# Response of Turkey Muscle Satellite Cells to Thermal Challenge. II. Transcriptome Effects in Differentiating Cells

**DOI:** 10.3389/fphys.2017.00948

**Published:** 2017-11-30

**Authors:** Kent M. Reed, Kristelle M. Mendoza, Gale M. Strasburg, Sandra G. Velleman

**Affiliations:** ^1^Department of Veterinary and Biomedical Sciences, University of Minnesota, St. Paul, MN, United States; ^2^Department of Food Science and Human Nutrition, Michigan State University, East Lansing, MI, United States; ^3^Department of Animal Sciences, Ohio Agricultural Research and Development Center, Ohio State University, Wooster, OH, United States

**Keywords:** satellite cell, skeletal muscle, growth-selected, turkey, differentiation

## Abstract

**Background:** Exposure of poultry to extreme temperatures during the critical period of post-hatch growth can seriously affect muscle development and thus compromise subsequent meat quality. This study was designed to characterize transcriptional changes induced in turkey muscle satellite cells by thermal challenge during differentiation. Our goal is to better define how thermal stress alters breast muscle ultrastructure and subsequent development.

**Results:** Skeletal muscle satellite cells previously isolated from the *Pectoralis major* muscle of 7-wk-old male turkeys (*Meleagris gallopavo*) from two breeding lines: the F-line (16 wk body weight-selected) and RBC2 (randombred control line) were used in this study. Cultured cells were induced to differentiate at 38°C (control) or thermal challenge temperatures of 33 or 43°C. After 48 h of differentiation, cells were harvested and total RNA was isolated for RNAseq analysis. Analysis of 39.9 Gb of sequence found 89% mapped to the turkey genome (UMD5.0, annotation 101) with average expression of 18,917 genes per library. In the cultured satellite cells, slow/cardiac muscle isoforms are generally present in greater abundance than fast skeletal isoforms. Statistically significant differences in gene expression were observed among treatments and between turkey lines, with a greater number of genes affected in the F-line cells following cold treatment whereas more differentially expressed (DE) genes were observed in the RBC2 cells following heat treatment. Many of the most significant pathways involved signaling, consistent with ongoing cellular differentiation. Regulation of Ca^2+^ homeostasis appears to be significantly affected by temperature treatment, particularly cold treatment.

**Conclusions:** Satellite cell differentiation is directly influenced by temperature at the level of gene transcription with greater effects attributed to selection for fast growth. At lower temperature, muscle-associated genes in the satellite cells were among the genes with the greatest down regulation consistent with slower differentiation and smaller myotubes. Fewer expression differences were observed in the differentiating cells than previously observed for proliferating cells. This suggests the impact of temperature on satellite cells occurs primarily at early points in satellite cell activation.

## Introduction

Skeletal muscle hypertrophy is a multifaceted process. During embryonic development, undifferentiated cells in the mesoderm (myoblasts) proliferate, differentiate, and fuse to form multinucleated myotubes that further differentiate into muscle fibers. Subsequent muscle growth is dependent upon satellite cells; stem cells located between the basement membrane and sarcolemma of skeletal muscle fibers (Mauro, 1961; Moss and Leblond, 1971). Satellite cells are self-renewing mesenchymal cells that enable further hypertrophy, maintenance, and repair of damaged skeletal muscle. After initial muscle growth and development, satellite cells become quiescent unless activated by antagonized Notch and Wnt signaling (reviewed in Fujimaki et al., 2013). Evidence from cell culture studies suggest that satellite cells are multi-potential and can be induced to follow osteogenic or adipogenic cellular pathways in addition to myogenesis (Asakura et al., 2001; Powell et al., 2016).

In the early post-hatch period, avian satellite cells are highly active (Halevy et al., 2000; Mozdziak et al., 2002). This activity can be directly affected by environmental stimuli with potential long-lasting effects on skeletal muscle growth (Piestun et al., 2013; Loyau et al., 2014). Thermal challenge is especially impactful on poults. Newly-hatched birds have immature thermoregulatory systems and are more susceptible to the effects of extreme ambient temperature (Myhre, 1978; Modrey and Nichelmann, 1992; Shinder et al., 2007). Given the need to transfer poults from hatch to grow facilities, this is also the period where birds are often exposed to acute thermal conditions, either hot or cold. The timing of thermal stress can differentially affect production performance. For example, broilers exposed early posthatch to elevated temperatures show increased body weight and yield at market age (Yahav and Plavnik, 1999; Yahav and McMurtry, 2001). Birds reared in elevated temperatures during later posthatch growth had reduced feed intake, weight gain, and meat yield (Ain Baziz et al., 1996; Halevy et al., 2001; Zhang et al., 2012).

Genetic selection has resulted in poultry with greater breast muscle weights and shorter growth periods (Havenstein et al., 2007). Satellite cells are in part responsible for the increase degree of muscle growth as the rates of satellite cell proliferation and differentiation are higher in birds selected for increased growth (Velleman et al., 2000). Extreme growth however, can be accompanied by physiological muscle defects that affect yield (Siller and Wight, 1979; Wilson et al., 1990). For example, increase in muscle fiber size displaces capillaries causing reduced blood supply, reduced heat dissipation and the accumulation of metabolic waste products (Sosnicki et al., 1991a,b; Kurnoth et al., 1994). Growth-selected birds respond differently to elevated environmental temperatures. The effects of long-term thermal stress on skeletal muscle growth and muscle damage is greater in fast-growing meat-type broilers as compared to slow-growing broilers (Ain Baziz et al., 1996; Lu et al., 2007).

As satellite cells are the only posthatch myonuclear source, they may directly modify skeletal muscle growth if functionally altered by temperature. Clark et al. (2016a) demonstrated *in vitro* that turkey satellite cell function is sensitive to both hot and cold temperatures with expression of key myogenic regulatory factors (myogenic differentiation factor, *MYOD1* and myogenin, *MYOG*) increasing with temperature. In addition, proliferation of satellite cells from growth-selected turkeys was increased compared to that of cells from a non-selected line when incubated at higher temperatures. The differential response of fast- and slow-growing turkeys to temperature may be due to differences in satellite cell thermal sensitivity. Understanding the interaction between temperature and muscle growth and its impact on yield is significant to the poultry industry.

Previously, we utilized cultured turkey satellite cells to study the effects of thermal challenge on the transcriptome of proliferating satellite cells (Reed et al., 2017). Significant gene expression differences were observed between cells incubated at both hot and cold temperatures. Enrichment analysis indicated a shift in cold-treated cells toward cell signaling whereas heat-treated cells had expression profiles shifted toward muscle development. Markers of cell proliferation such as *MYOD1* and several interacting genes were significantly upregulated in the heat-exposed cells and differential expression of chief myogenic regulators and pathways activated by Wnt ligands were observed. Greater differences in gene expression were observed for satellite cells from growth-selected birds as compared to random bred controls. The present study uses RNAseq transcriptome analysis to characterize gene expression in differentiating satellite cells under the same thermal challenge model. Understanding how temperature affects satellite cell differentiation can potentially be used to develop thermal management strategies to improve skeletal muscle growth.

## Methods

### Turkey myogenic satellite cells

Satellite cells used in this study were previously isolated from the *pectoralis major* (*p*. *major*) muscle of 7 wk old males from two turkey lines; the random bred control 2 (RBC2) and body weight-selected (F) (Velleman et al., 2000). The RBC2 line is maintained without conscious selection for any trait and the F line is derived from the RBC2 line and selected for 16 wk body weight (Nestor, 1977, 1984).

Turkey *p. major* satellite cells were replicate plated and proliferated as described in Reed et al. (2017). After 72 h of proliferation at 38°C, the growth medium was removed and the cells were fed a lower-serum medium containing DMEM, 3% horse serum (Gemini BioProducts), 1% antibiotics-antimycotics (Gemini BioProducts), 0.1% gentamicin (Gemini BioProducts), and 1 mg/mL bovine serum albumin (BSA, Sigma Aldrich) to induce differentiation. Cells were cultured in 95% air/5%CO_2_ incubators at 38°C (control) or at an experimental temperature (33 or 43°C). Medium was changed at 24 h. The control temperature of 38°C is approximately equal to that measured in newly hatched poults (38.0–38.5°C; Strasburg, unpublished). Initiation of differentiation was characterized by the visual observance of multinucleated myotubules. At the conclusion of the 48 h treatment, cell medium was removed, cells were rinsed with PBS and the plates were held at −80°C until RNA isolation.

### RNA isolation and sequencing

Total RNA was isolated from each culture by TRIzol extraction (Ambion, Inc.), DNase-treated (Turbo DNA-freeTM Kit, Ambion, Inc.), and stored at −80°C. Initial RNA concentration and quality was measured (Nanodrop 1000) and samples were submitted for library preparation and sequencing at the University of Minnesota Genomics Center (UMGC). Samples were quantified by RiboGreen Assay and RNA integrity was measured on a 2100 Bioanalyzer (Aligent Technologies). All sample had clear peak separation (18S and 28S) and RNA Integrity Numbers (RIN) ranged between 5.1 and 8.8. Indexed libraries were constructed with the TruSeq RNA Sample Preparation Kit version 2 (Illumina, Inc.) from 1 μg of total RNA/sample. Libraries were multiplexed, pooled and sequenced (101-bp paired-end reads) on the HiSeq 2000 using v3 chemistry (Illumina, Inc.). Replicate samples were sequenced from each treatment group (*n* = 12 libraries).

### RNAseq data analyses

Trimmomatic (Bolger et al., 2014) was used to remove sequence adapters and low quality bases and quality control checks were performed with FastQC (Andrews, 2010). Groomed reads were subsequently mapped to the turkey genome (UMD 5.0, NCBI Annotation 101) with Bowtie (v2.2.4.0). Read counts were normalized in CLC Genomics Workbench (CLCGWB v. 8.0.2, CLC Bio) as described in Reed et al. (2017). Using normalized read counts, samples were hierarchically clustered based on Euclidean sample distances with single linkage and principal component analysis (PCA) was performed in CLCGWB. Differential gene expression and ANOVA was performed in CLCGWB (Bonferroni and FDR corrected) and pair-wise comparisons between treatment groups were made in the Bioconductor (3.2) R package DESeq2 (Love et al., 2014) under the standard workflow. For all comparisons, *p* < 0.05 were considered significant. Affected gene pathways were investigated with Ingenuity Pathway Analysis (IPA, Qiagen Bioinformatics). For analysis, the turkey annotated gene set, was mapped to the IPA database (11,615 IDs) and Log_2_FC and FDR-corrected *p*-values were imported. Core and comparative analyses of gene expression were run with FDR *p*-val cutoff set at 0.05. The PANTHER Overrepresentation Test (release 20150430, Mi et al., 2013; http://geneontology.org/) was used to test for gene enrichment. Data are deposited in the NCBI's Gene Expression Omnibus (GEO) repository as part of SRA BioProject 341399.

## Results

Total RNA isolated from satellite cell cultures was used to construct 12 individual barcoded libraries. Sequencing of the libraries produced over 394 M combined reads with the number of reads per library ranging from 12.4 to 18.5 M (average 16.4 M). After read trimming and filtering, median read quality was consistently high and ranged from 36.8 to 37.3. Replicate libraries for each treatment produced comparable results with the number of reads per treatment group ranging from 29 to 36.4 M (average 32.9 ± 1.25 M reads). Cumulatively, corrected reads comprised 39.9 Gb of sequence for transcriptome analysis. Mean library insert was estimated from mapping results as 213.4 bp (Table 1).

**Table 1:** Summary of RNA-seq data.

**Line**	**Temp °C**	**Replicate**	**PE reads**	**Median read quality R1**	**Median read quality R2**	**Trimmed PE reads**	**% mapped**	**% concordant**	**Estimated insert mean (bp)**	**Total observed genes**	**Mean expressed genes**	**% expressed genes**
**RBC2**	**33**	A	17836372	37.1	36.9	16326998	89.9	82.3	216	16544	15351	73.1
		B	18039271	37.1	37.0	16541921	89.3	82.5	218	16622		
	**38**	A	16252079	37.2	36.9	14822122	89.1	82.8	213	16365	15193	72.3
		B	16698643	37.1	37.0	15361039	89.2	83.3	198	16532		
	**43**	A	15602450	37.2	37.0	14274919	89.7	83.4	226	16244	15399	73.3
		B	15303273	37.1	36.9	13986262	89.3	82.5	227	16397		
**F-Line**	**33**	A	16169884	37.2	37.0	14865209	88.9	82.2	215	16557	15314	72.9
		B	17084981	37.2	37.0	15702722	89.5	82.8	215	16355		
	**38**	A	18370285	37.2	37.0	16846899	89.9	83.5	217	16630	15270	72.7
		B	18085832	37.1	36.8	16419915	90.2	84.1	213	16575		
	**43**	A	14634877	37.2	36.9	13373887	89.2	83.0	226	16498	15364	73.1
		B	15356625	37.2	36.9	14035364	89.2	82.9	226	16583		
**Mean**			16619547.7	37.15	36.94	15213104.7	89.44	82.94	213.4	16491.8	15315.2	72.9

*For each library the total number of concatenated reads, median read qualities (R1 and R2), estimated mean insert length (bp), number of and percentage of aligned reads, percentage of concordant reads, and the number and percentage of observed genes (mapped reads > 1.0) and expressed genes (mean group normalized read count > 3.0) are given*.

### Gene expression

Approximately 89% of the quality trimmed fragments mapped uniquely to the annotated turkey gene set, and on average, 82.9% mapped concordantly (Table 1). Evidence for expression (at least one mapped read) in at least one treatment group was observed for 18,917 genes (average 16,491 per group, 78.5% of the turkey gene set) with a mean depth of 464 ± 28.9 (SE) reads/gene (Table S1). Using an expression cutoff for the average number of mapped reads = 3.0 resulted in 16,582 experiment-wise expressed genes (tRNAs excluded) with 14,038 shared in common among the six treatment groups. Within treatment groups, the mean number of expressed genes (average # of mapped reads ≥ 3.0) ranged from 15,193 to 15,399 and included on average 72.9% of the turkey gene set (Table 1).

**Figure 1:**
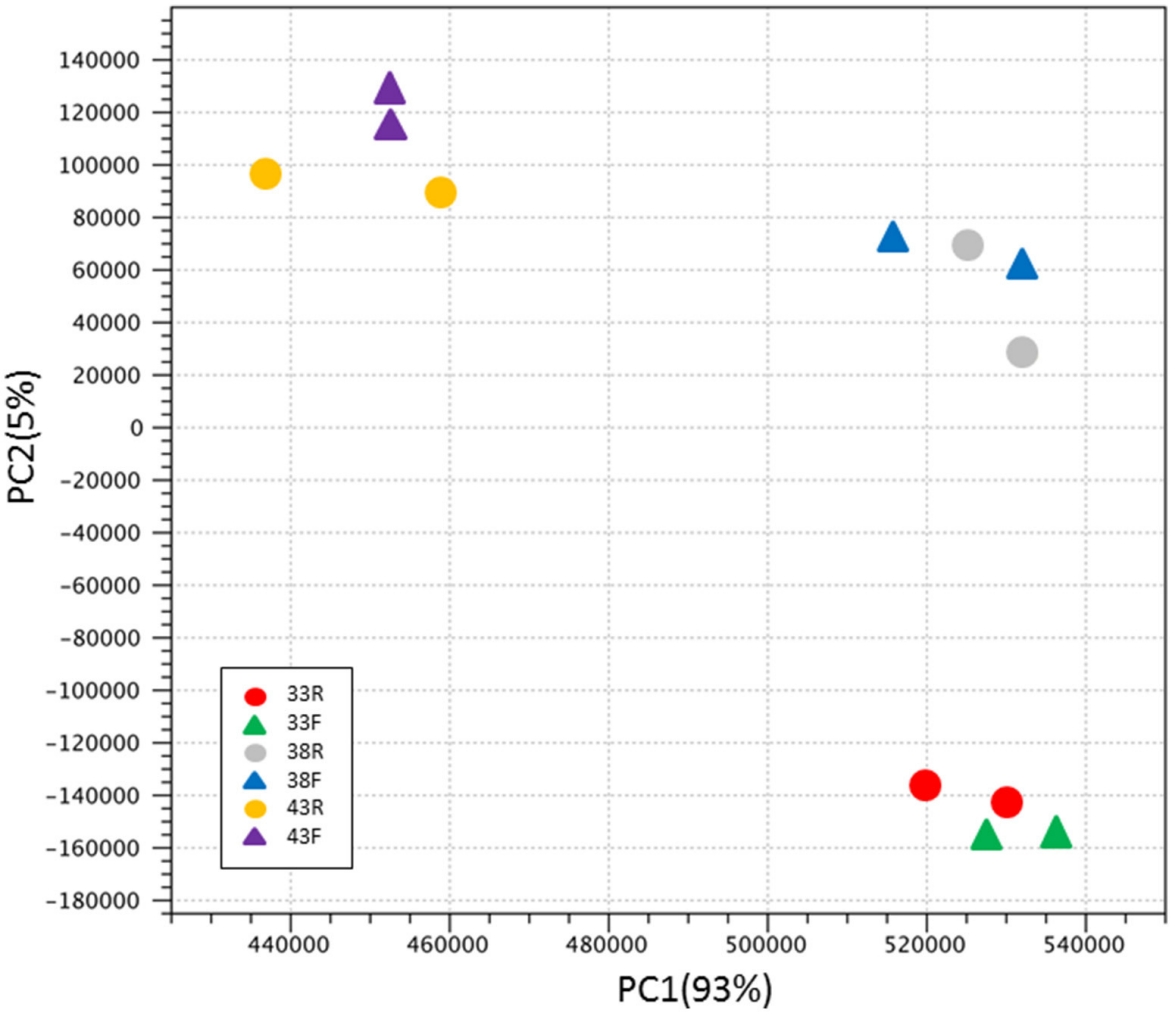
Principal component analysis (PCA) of normalized RNAseq read counts. Sample to sample distances (within- and between-treatments) are illustrated for each dataset on the first two principle components comprising ~98% of the variation. Samples are plotted according to treatment.

Variation among groups was evaluated by principal component analysis (PCA) of normalized read counts (Figure 1). Treatment groups clustered distinctly by Temp/Time within the first two principal components explaining ~98% of the observed variation. The relationships among treatment groups shown by PCA, with clustering by incubation temperature then turkey type, were reiterated in the hierarchical clustering of groups by Euclidean distance (Figure S1). Replicate treatment pairs clustered together as nearest neighbors, supporting the pooling of replicates for expression analyses.

Distribution of expressed genes (unique and shared) among treatment groups is summarized in Table 2. The number of uniquely expressed genes in the satellite cells was higher at both cold and hot temperatures as compared to control (38°C) in both lines. In paired comparisons between temperature treatments, the number of uniquely expressed genes was higher in both the cold and hot treatments than the control temperature (38°C). Within temperature, 14,903 expressed genes were shared on average among the lines (Table 2). Numbers of uniquely expressed genes were comparable being higher for the RBC2 line cells at 33 and 43°C (414 and 437, respectively) but higher for the F-line cells at 38°C (459).

**Table 2:** Summary of gene expression and significant differential expression (DE) in pair-wise comparisons of differentiating cells.

**Comparison**	**Groups**	**Expressed genes**	**Shared genes**	**Unique genes /group**	**FDR < 0.05**	**|Log_2_FC| > 1.0**	**|Log_2_FC| > 2.0**
**Cold**	33R vs. 38R	15,885	14,659	692/534	5,963	2,608	852 (0.337)
	33F vs. 38F	15,901	14,683	631/587	8,678	3,027	969 (0.372)
**Hot**	43R vs. 38R	15,933	14,695	704/498	7,160	3,355	845 (0.788)
	43F vs. 38F	15,951	14,757	607/513	9,039	2,905	703 (0.752)
**LINE**							
	33F vs. 33R	15,728	14,937	414/377	873	116	23 (0.130)
	38F vs. 38R	15,652	14,811	382/459	1	1	1 (0.000)
	43F vs. 43R	15,801	14,962	437/402	1,818	318	52 (0.500)

*For each comparison of the treatment groups (Temperature, 33, 38, or 43°C; Line, RBC2 or F), the total number of expressed, shared, and uniquely expressed genes, the number of genes with significant FDR p-value, and the numbers of significant genes also with |Log_2_ fold change| > 1.0 and > 2.0 are given. Only those genes with treatment group mean normalized read counts > 3.0 are included as expressed. Numbers in parentheses equal the proportion of up-regulated genes*.

Ordering and classification of the genes expressed at the control incubation temperature (38°C, Table S2) defines the baseline cellular processes of the satellite cells after 48 h of differentiation. The majority of gene products (68%) expressed by the RBC2 and F-line satellite cells is characterized as nuclear or cytoplasmic proteins (Figure S2). The largest represented functional class of gene products (49%, designated as other) included primarily structural proteins with enzymes (20%) and transcriptional regulators (9%) comprising the next two major groups. A Comparison Analysis between the RBC2 and F-line groups at 38°C was conducted using the normalized read counts for the 8,000 highest expressed genes from the 11,615 gene IDs mapped in the IPA database. The top *Metabolic* pathways included the tRNA charging pathway (33 of 39 pathway genes associated) and D-myo inositol pathway (Table 3). Myo-inositol is the structural basis for a number of secondary messengers in eukaryotic cells. The top two *Signaling* pathways included protein ubiquitination (177 of 255 genes associated) and EIF2 signaling (142 of 194 genes). Ubiquitination pathways primarily include the tagging of proteins for degradation by the proteasome whereas the eIF2 initiation complex regulates both global and specific mRNA translation in response to stress-related signals.

**Table 3:** Twenty most significant canonical pathways expressed in satellite cell cultures at 38°C after 48 h of differentiation.

	**RBC2**		**F-Line**	
**Metabolic Pathways**	**−log (*p*-value)**	**Ratio**	**−log (*p*-value)**	**Ratio**
tRNA charging	8.920	0.846	8.950	0.846
Superpathway of inositol phosphate compounds	6.870	0.540	7.260	0.545
3-phosphoinositide degradation	6.860	0.582	6.540	0.575
Colanic acid building blocks biosynthesis	6.050	1.000	6.070	1.000
3-phosphoinositide biosynthesis	5.960	0.543	6.020	0.543
D-myo-inositol (1,4,5,6)-tetrakisphosphate biosynthesis	5.720	0.574	5.770	0.574
D-myo-inositol (3,4,5,6)-tetrakisphosphate biosynthesis	5.720	0.574	5.770	0.574
D-myo-inositol-5-phosphate metabolism	4.930	0.544	4.980	0.544
Superpathway of D-myo-inositol (1,4,5)-trisphosphate metabolism	4.860	0.800	5.750	0.840
Fatty Acid β-oxidation I	4.200	0.719	3.600	0.688
Valine degradation I	4.130	0.833	4.140	0.833
Pyridoxal 5′-phosphate salvage pathway	3.660	0.594	3.680	0.594
Superpathway of cholesterol biosynthesis	3.650	0.714	3.670	0.714
Isoleucine degradation I	3.590	0.857	3.600	0.857
TCA Cycle II (Eukaryotic)	3.460	0.739	3.470	0.739
D-myo-inositol (1,4,5)-trisphosphate degradation	3.300	0.778	4.140	0.833
D-myo-inositol (1,3,4)-trisphosphate biosynthesis	3.210	0.750	3.990	0.800
Phosphatidylglycerol biosynthesis II (Non-plastidic)	3.080	0.692	3.090	0.692
Salvage pathways of pyrimidine ribonucleotides	2.850	0.527	2.880	0.527
GDP-mannose biosynthesis	2.590	1.000	2.600	1.000
**SIGNALING PATHWAYS**				
Protein ubiquitination pathway	25.400	0.694	25.500	0.694
EIF2 signaling	24.100	0.732	24.200	0.732
Regulation of eIF4 and p70S6K signaling	18.400	0.720	18.500	0.720
mTOR signaling	16.000	0.658	16.600	0.663
NRF2-mediated oxidative stress response	15.500	0.658	15.100	0.653
Estrogen receptor signaling	12.600	0.688	12.700	0.688
Molecular mechanisms of cancer	12.600	0.553	12.700	0.553
Hereditary breast cancer signaling	12.400	0.669	12.500	0.669
Aldosterone signaling in epithelial cells	12.300	0.645	12.400	0.645
Huntington's disease signaling	11.500	0.589	11.600	0.589
Role of BRCA1 in DNA damage response	10.700	0.744	10.800	0.744
PI3K/AKT signaling	10.400	0.661	10.500	0.661
Death receptor signaling	9.090	0.685	9.730	0.696
Integrin signaling	9.000	0.571	9.830	0.580
AMPK	8.660	0.582	8.730	0.582
Protein kinase A signaling	8.630	0.515	8.460	0.513
Mitotic roles of polo-like kinase	8.440	0.727	8.480	0.727
Glioma signaling	8.420	0.645	8.470	0.645
HIPPO signaling	8.040	0.674	8.090	0.674
Apidogenesis pathway	8.000	0.612	8.060	0.612

RNA-Seq affords the opportunity to gain new insights into temporal gene expression patterns of differentiating satellite cells, particularly with respect to muscle fiber-type-specific proteins. Although, the adult turkey *p*. *major* muscle comprises almost exclusively Type IIa (fast-contracting) muscle fibers, the satellite cells in this study which had undergone differentiation for 48 h, primarily expressed mRNAs corresponding to cardiac or slow-contracting sarcomeric and sarcoplasmic reticulum protein isoforms (Table S1). For example, the dominant myosin heavy chain based on number of reads were *MYH7B* (slow/tonic, cardiac isoform) and the non-muscle isoforms *MYH9* and *MHY10*. Likewise, the cardiac or slow-contracting muscle isoforms of actin (*ACTC1*), troponin C (*TNNC1*), troponin T (*TNNT2*), the sarcoplasmic/endoplasmic reticulum calcium ATPase (*ATP2A2*), and calsequestrin (*CASQ2*) predominate relative to their respective fast-contracting muscle isoforms. The adult isoforms of the myosin heavy chains associated with the myofibril (e.g., *MYH1, MYH2*), were not observed although it is possible that there are reads associated with gene loci that have not yet been annotated. Nevertheless, it is readily apparent that in direct comparisons between fast skeletal muscle isoforms and slow or cardiac isoforms, e.g., actin and the troponin subunits, the slow/cardiac isoforms are generally present in greater abundance.

### Differential expression

Gaussian-based ANOVA found 12,395 genes with significant (FDR *p*-val < 0.05) experiment-wise differential expression (Figure S3). Seven two-way contrasts were generated based on temperature treatment (cold and hot) and line (RBC2 and F). More significant differentially expressed (DE) genes were identified in the temperature contrasts than between genetic lines within temperature (Table 2, Table S4) and the majority of DE genes were unique to treatment groups (temperature/line; Figure S4).

**Figure 2:**
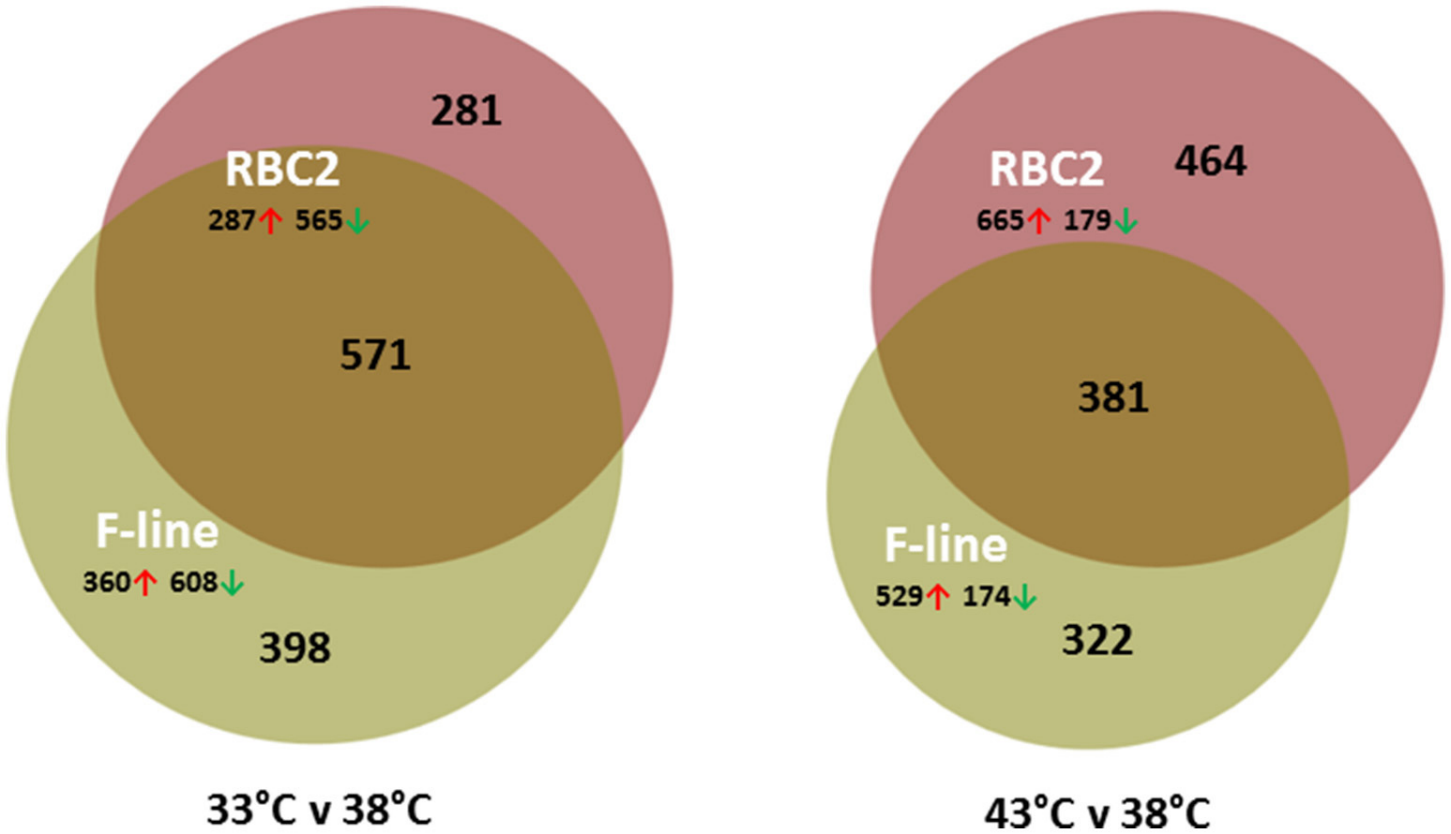
Distribution of differentially expressed genes during differentiation of cultured turkey *p. major* satellite cells. For each temperature comparison, the number of genes with FDR *p* < 0.05 and |Log_2_FC| > 2.0 that were shared or unique to each line (RBC2 and F) are indicated in the Venn diagram. Circle size is proportional to the number of genes.

On average, more genes were significantly affected by cold (33°C) treatment than by heat (43°C; Table 2). Comparison of the cold treated (33°C) to controls (38°C) found a greater number of DE genes in the F-line cells compared to the RBC2 cells (Figure 2, Figure S4). However, the opposite was observed in the 43 vs. 38°C comparison where a greater number of genes were significantly different in the RBC2 cells. Interestingly, the proportion of upregulated genes was ~2-fold higher in the heat-treated cells (0.77 vs. 0.36; Table 2). In the 33 vs. 38°C comparison (Figure 2), 571 (45.7%) of the DE genes were common to the two genetic lines and the number of unique DE genes was higher in the F-line cells compared to the RBC2 (398 [31.8%] and 281 [22.5%], respectively). Fewer DE genes (381, 32.6%) were shared between lines in the 43 vs. 38°C comparison, and the number of unique DE genes was higher in the RBC2 cells compared to the F-line (464 [39.8%] and 281 [27.6%], respectively).

The majority of DE genes identified in the treatment comparisons were unique to treatment groups (temperature/line; Figure S4). The 50 significant DE genes with the greatest expression change in each comparison are listed in Table S4. Prominent in the 33 vs. 38°C comparisons are the number of myosin-related genes that were significantly down regulated in the satellite cells. The gene with the greatest expression change was neuropeptide Y (*NPY*) with an average Log_2_FC = −10.63. In mammals, *NPY* is produced mainly by neurons in the brain and autonomic nervous system and can stimulate growth of adipose tissue (Wang et al., 2007).

Statistical overrepresentation tests (PANTHER) of genes differentially expressed between the 33 and 38°C found greatest enrichment for the GO *Biological Processes* of myofibril assembly, regulation of calcium ion import, muscle contraction and muscle development (Table 4). Greatest enrichment of *Cellular Components* included the troponin complex, muscle thin filament and myofilament, whereas actin and cytoskeletal protein binding were included in the top significantly enriched *Molecular Functions*. Myofibril assembly (over 13-fold enrichment), tropomyosin binding (28.97 x) and troponin complex (41.39 x) were the top overrepresented categories among the 457 DE genes shared among the F and RBC2 lines in the 33 vs. 38°C comparison. In contrast, overrepresentation tests of genes differentially expressed between the 43 and 38°C found very few significantly enriched categories and low enrichment values (Table 5).

**Table 4:** Summary of PANTHER Overrepresentation test of the 939 genes differentially expressed in *p. major* satellite cell cultures after 48 h of differentiation at 33 vs. 38°C.

**Biological process**	***Gallus gallus* (15,789)**	**Turkey DEGs (395 of 400)**	**Expected**	**over /under**	**Fold Enrichment**	***P*-value**
Myofibril assembly (GO:0030239)	38	8	0.96	+	8.31	4.46E-02
Regulation of calcium ion import (GO:0090279)	56	10	1.42	+	7.05	1.40E-02
Muscle contraction (GO:0006936)	106	16	2.69	+	5.96	1.38E-04
Striated muscle cell development (GO:0055002)	83	12	2.1	+	5.71	1.22E-02
Muscle system process (GO:0003012)	131	18	3.32	+	5.42	7.58E-05
Muscle cell development (GO:0055001)	93	12	2.36	+	5.09	3.81E-02
Striated muscle cell differentiation (GO:0051146)	123	14	3.12	+	4.49	2.73E-02
Regulation of metal ion transport (GO:0010959)	167	16	4.23	+	3.78	4.95E-02
Striated muscle tissue development (GO:0014706)	186	17	4.71	+	3.61	4.76E-02
Muscle tissue development (GO:0060537)	199	18	5.04	+	3.57	2.97E-02
Muscle structure development (GO:0061061)	278	24	7.04	+	3.41	1.82E-03
Regulation of ion transport (GO:0043269)	303	24	7.68	+	3.13	8.12E-03
Anatomical structure formation in morphogenesis (GO:0048646)	659	41	16.7	+	2.46	1.03E-03
System process (GO:0003008)	789	49	19.99	+	2.45	6.09E-05
Circulatory system development (GO:0072359)	529	32	13.4	+	2.39	4.33E-02
Cardiovascular system development (GO:0072358)	529	32	13.4	+	2.39	4.33E-02
Tissue development (GO:0009888)	961	51	24.35	+	2.09	3.76E-03
regulation of multicellular organismal development (GO:2000026)	1,017	52	25.76	+	2.02	8.59E-03
Regulation of transport (GO:0051049)	1,056	52	26.75	+	1.94	2.50E-02
Regulation of multicellular organismal process (GO:0051239)	1,555	76	39.39	+	1.93	1.32E-04
Anatomical structure morphogenesis (GO:0009653)	1,390	66	35.21	+	1.87	3.73E-03
Regulation of developmental process (GO:0050793)	1,369	64	34.68	+	1.85	9.56E-03
Animal organ development (GO:0048513)	1,697	79	42.99	+	1.84	5.10E-04
System development (GO:0048731)	2,360	108	59.79	+	1.81	2.23E-06
Regulation of localization (GO:0032879)	1,497	68	37.93	+	1.79	1.15E-02
Multicellular organismal process (GO:0032501)	3,429	153	86.87	+	1.76	3.04E-10
Single-multicellular organism process (GO:0044707)	3,050	136	77.27	+	1.76	2.03E-08
Multicellular organism development (GO:0007275)	2,614	116	66.22	+	1.75	2.42E-06
Anatomical structure development (GO:0048856)	2,936	129	74.38	+	1.73	2.81E-07
Single-organism developmental process (GO:0044767)	3,091	131	78.31	+	1.67	2.11E-06
Developmental process (GO:0032502)	3,122	131	79.09	+	1.66	4.10E-06
Cell communication (GO:0007154)	2,933	114	74.3	+	1.53	5.40E-03
Single organism signaling (GO:0044700)	2,872	111	72.76	+	1.53	1.06E-02
Signaling (GO:0023052)	2,875	111	72.84	+	1.52	1.12E-02
Signal transduction (GO:0007165)	2,680	103	67.9	+	1.52	3.77E-02
Response to stimulus (GO:0050896)	4,290	150	108.68	+	1.38	2.62E-02
Single-organism process (GO:0044699)	7,871	264	199.4	+	1.32	3.09E-07
Regulation of biological process (GO:0050789)	6,727	224	170.42	+	1.31	2.97E-04
Single-organism cellular process (GO:0044763)	6,748	221	170.95	+	1.29	1.97E-03
Regulation of cellular process (GO:0050794)	6,374	207	161.48	+	1.28	1.67E-02
Biological regulation (GO:0065007)	7,227	231	183.09	+	1.26	6.16E-03
Biological_process (GO:0008150)	11,618	333	294.33	+	1.13	1.81E-02
Unclassified (UNCLASSIFIED)	4,171	67	105.67	−	0.63	0.00E+00
**CELLULAR COMPONENT**						
Troponin complex (GO:0005861)	7	4	0.18	+	22.56	3.62E-02
Striated muscle thin filament (GO:0005865)	17	6	0.43	+	13.93	6.09E-03
Myofilament (GO:0036379)	18	6	0.46	+	13.16	8.40E-03
A band (GO:0031672)	21	6	0.53	+	11.28	1.99E-02
Sarcomere (GO:0030017)	110	25	2.79	+	8.97	3.33E-13
Contractile fiber part (GO:0044449)	120	25	3.04	+	8.22	2.33E-12
Myofibril (GO:0030016)	131	26	3.32	+	7.83	1.95E-12
Contractile fiber (GO:0043292)	138	26	3.5	+	7.44	6.43E-12
I band (GO:0031674)	77	14	1.95	+	7.18	1.87E-05
Receptor complex (GO:0043235)	236	20	5.98	+	3.35	3.97E-03
Proteinaceous extracellular matrix (GO:0005578)	238	20	6.03	+	3.32	4.49E-03
Extracellular matrix (GO:0031012)	305	24	7.73	+	3.11	1.54E-03
Plasma membrane part (GO:0044459)	1,442	67	36.53	+	1.83	1.09E-03
Plasma membrane (GO:0005886)	2,561	97	64.88	+	1.5	2.40E-02
Cell periphery (GO:0071944)	2,651	99	67.16	+	1.47	3.39E-02
Intrinsic component of membrane (GO:0031224)	3,877	134	98.22	+	1.36	3.72E-02
Membrane part (GO:0044425)	4,536	154	114.92	+	1.34	1.69E-02
Cellular component (GO:0005575)	12,385	347	313.76	+	1.11	1.41E-02
Intracellular membrane-bounded organelle (GO:0043231)	6,617	119	167.64	−	0.71	3.46E-04
Unclassified (UNCLASSIFIED)	3,404	53	86.24	−	0.61	0.00E+00
Nucleus (GO:0005634)	4,190	64	106.15	−	0.6	3.67E-04
Organelle lumen (GO:0043233)	2,350	28	59.54	−	0.47	9.99E-04
Intracellular organelle lumen (GO:0070013)	2,350	28	59.54	−	0.47	9.99E-04
Membrane-enclosed lumen (GO:0031974)	2,350	28	59.54	−	0.47	9.99E-04
Nuclear lumen (GO:0031981)	2,129	25	53.94	−	0.46	2.68E-03
Nuclear part (GO:0044428)	2,465	26	62.45	−	0.42	2.26E-05
Ribonucleoprotein complex (GO:1990904)	518	1	13.12	−	<0.2	2.39E-02
Intracellular ribonucleoprotein complex (GO:0030529)	518	1	13.12	−	<0.2	2.39E-02
**MOLECULAR FUNCTION**						
Actin binding (GO:0003779)	285	22	7.22	+	3.05	1.07E-02
Cytoskeletal protein binding (GO:0008092)	600	42	15.2	+	2.76	9.00E-06
Calcium ion binding (GO:0005509)	493	30	12.49	+	2.4	2.41E-02
Transmembrane receptor activity (GO:0099600)	690	38	17.48	+	2.17	1.60E-02
Receptor activity (GO:0004872)	886	45	22.45	+	2	1.77E-02
Molecular transducer activity (GO:0060089)	886	45	22.45	+	2	1.77E-02
Protein binding (GO:0005515)	4,019	148	101.82	+	1.45	4.40E-04
Unclassified (UNCLASSIFIED)	4,679	87	118.54	−	0.73	0.00E+00
Nucleic acid binding (GO:0003676)	2,522	27	63.89	−	0.42	4.12E-05
poly(A) RNA binding (GO:0044822)	835	5	21.15	−	0.24	4.04E-02
RNA binding (GO:0003723)	1,108	6	28.07	−	0.21	5.56E-04

*Turkey DEGs were matched to the chicken gene reference list IDs for analysis in PANTHER. For each Gene Ontology category, the number of genes in the reference list and those differentially expressed in the turkey are given. Fold enrichment is the number of DE genes divided by Expected. P-values are as determined by the binomial statistic*.

**Table 5:** Summary of PANTHER Overrepresentation test of the 856 genes differentially expressed in *p. major* satellite cell cultures after 48 h of differentiation at 43 vs. 38°C.

**Biological process**	***Gallus gallus*—(15789)**	**Turkey DEGs (248 of 249)**	**Expected**	**over /under**	**Fold Enrichment**	***P*-value**
Animal organ development (GO:0048513)	1,697	52	26.76	+	1.94	1.37E-02
Single-multicellular organism process (GO:0044707)	3,050	88	48.10	+	1.83	1.38E-05
System development (GO:0048731)	2,360	67	37.22	+	1.80	4.76E-03
Multicellular organism development (GO:0007275)	2,614	72	41.22	+	1.75	4.90E-03
Multicellular organismal process (GO:0032501)	3,429	91	54.08	+	1.68	4.14E-04
Negative regulation of cellular process (GO:0048523)	2,565	68	40.45	+	1.68	4.28E-02
Negative regulation of biological process (GO:0048519)	2,756	73	43.46	+	1.68	1.73E-02
Single-organism developmental process (GO:0044767)	3,091	79	48.75	+	1.62	2.24E-02
Developmental process (GO:0032502)	3,122	79	49.24	+	1.60	3.29E-02
Biological regulation (GO:0065007)	7,227	151	113.97	+	1.32	1.04E-02
Single-organism process (GO:0044699)	7,871	160	124.13	+	1.29	1.94E-02
Unclassified (UNCLASSIFIED)	4,171	46	65.78	−	0.70	0.00E+00
**CELLULAR COMPONENT**						
Unclassified (UNCLASSIFIED)	3,404	30	53.68	−	0.56	0.00E+00
**MOLECULAR FUNCTION**						
Unclassified (UNCLASSIFIED)	4,679	58	73.79	−	0.79	0.00E00

*Turkey DEGs were matched to the chicken gene reference list IDs for analysis in PANTHER. For each Gene Ontology category, the number of genes in the reference list and those differentially expressed in the turkey are given. Fold enrichment is the number of DE genes divided by Expected. P-values are as determined by the binomial statistic*.

**Figure 3:**
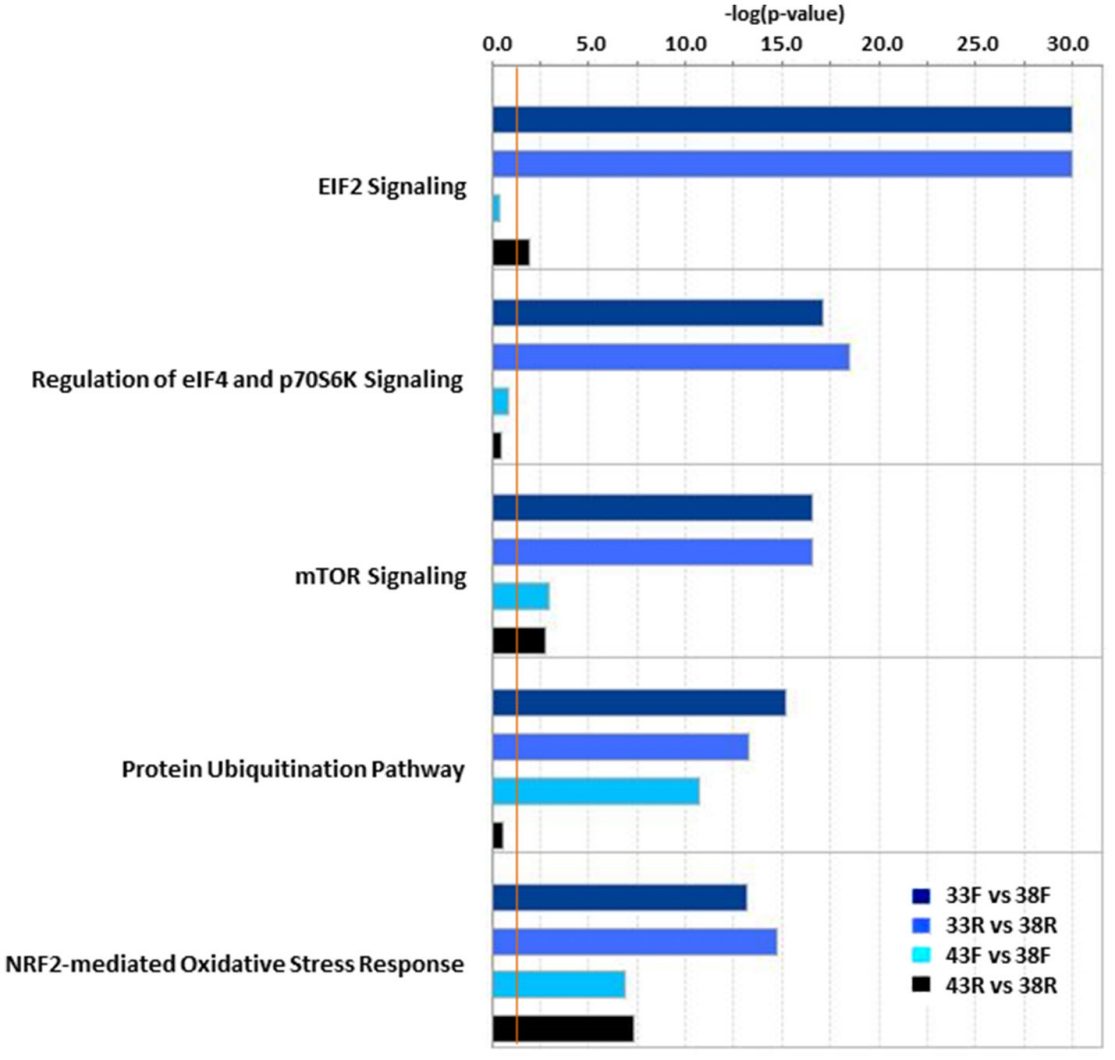
Significant pathway associations identified in IPA Comparison Analysis of thermal challenge vs. control temperature cells. In each pair-wise comparison, *P*-values are assigned to canonical pathways based on differential expression (DE). Bar plot provides the 5 most variable pathways with significance. Pathway associations must have a −log(*p*-value) > 1.3 (threshold, vertical orange line).

Analysis of DE genes in IPA outlined several temperature-induced shifts in the satellite cell transcriptomes. The top 10 canonical pathways for each temperature comparison are given in Table S5. Consistent with ongoing cellular development, many of the most significant pathways were signaling pathways. Comparison analysis across treatments identified EIF2 signaling, regulation of eIF4 and p70S6K signaling, mTOR signaling, protein ubiquitination and NRF2-mediated oxidative stress response as the most divergently altered pathways (Figure 3). In general, the first four pathways were most significantly altered in the cold treated cells (primarily by down regulation) whereas the differences seen in the NRF2-mediated OSR pathway were the result of greater bidirectional expression differences.

A total of 2,926 significant DE genes (|Log_2_FC| > 1.0) was shared among all treatment comparisons, but only 70 had |Log_2_FC| > 2.0 (Figure S4). The majority of these 70 genes (48) were up regulated in cells from both lines incubated at either higher (43°C) or lower temperatures (33°C) as compared to control (Table 6). In all but two genes, *CD36* and *GUCA1B*, the directionality of the expression changes was the same for all comparisons. Expression of *CD36* (thrombospondin receptor) was up regulated at 33°C (average Log_2_FC = 2.39) but down regulated at 43°C (average Log_2_FC = −2.37) compared to control. Conversely, expression of *GUCA1B* (guanylate cyclase activator 1B) was down regulated at 33°C (average Log_2_FC = −4.75) but up regulated at 43°C (average Log_2_FC = 3.15) compared to control. *CD36* is a member of the class B scavenger receptor family of cell surface proteins and in mammals, acts to import fatty acids inside cells, whereas *GUCA1B* is a calcium-binding protein that activates guanylate cyclases. These two genes are also represented in the two most significant scoring gene networks identified in IPA analysis of the 70 DE genes shared among the treatment comparisons (Figure S5).

**Table 6:** Common significant DE genes [FDR *p*-values (< 0.05), |Log_2_FC| >1] across treatments.

**Gene**	**Description**	**33R vs. 38R**		**33F vs. 38F**		**43R vs. 38R**		**43F vs. 38F**	
		**Log_2_FC**	**FDR**	**Log_2_FC**	**FDR**	**Log_2_FC**	**FDR**	**Log_2_FC**	**FDR**
ACTA1	Actin, alpha 1, skeletal muscle	−3.205	2.2E-154	−3.347	0	−2.861	0	−3.193	0
APOA1	Apolipoprotein A-I	−3.110	2.87E-36	−3.113	1.19E-45	−4.200	2.2E-53	−2.404	1.29E-28
BCAN	Brevican	−4.061	1.12E-67	−3.839	1.93E-40	−2.376	4.27E-27	−2.095	9.01E-45
C1QL1	Complement component 1, q subcomponent-like 1	−2.133	2.99E-40	−2.465	4.61E-89	−2.942	8.8E-80	−2.162	1.15E-32
CCDC69	Coiled-coil domain containing 69	−5.348	8.31E-17	−4.015	9.4E-110	−3.700	7.16E-11	−2.138	3.22E-95
CD36	CD36 molecule (thrombospondin receptor)	2.300	2.05E-46	2.479	9.79E-46	−2.606	3.59E-17	−2.131	3.52E-27
DRC7	Dynein regulatory complex subunit 7	−4.210	2.88E-16	−3.981	4.15E-24	−2.259	7.2E-09	−2.073	3.98E-12
EVC	Ellis van Creveld syndrome	3.026	0.005323	2.570	0.000106	3.119	0.000946	2.024	0.007842
FABP3	Fatty acid binding protein 3, muscle and heart	−5.284	7.79E-35	−4.858	7.79E-41	−3.020	1.97E-18	−3.959	2.29E-36
GUCA1B	Guanylate cyclase activator 1B (retina)	−3.228	2.28E-05	−6.281	2.68E-05	2.990	1.44E-23	3.316	1.18E-25
LNPEP	Leucyl/cystinyl aminopeptidase	2.589	1.2E-07	2.319	4.07E-13	3.110	3.88E-16	2.267	1.53E-12
LOC100539445	Tensin-3 (TNS3)	2.269	0.00123	2.321	6.04E-08	2.791	1.56E-09	3.026	9.17E-16
LOC100545344	Myosin-7-like	−4.975	2.4E-142	−4.716	1.5E-45	−2.988	8.58E-75	−2.169	5.61E-42
LOC100547876	Polypeptide N-acetylgalactosaminyltransferase 12-like	2.823	0.001667	2.930	9.26E-07	2.582	0.002754	2.215	0.001923
LOC100548077	Disheveled-associated activator of morphogenesis 2 (DAAM2)	2.703	0.000521	2.031	0.009045	3.218	3.7E-06	2.329	0.000755
LOC100548792	Collagen alpha-1(XII) chain-like	2.061	1.94E-06	2.720	2.64E-79	3.333	1.46E-33	2.652	4.26E-87
LOC100549353	Activating signal cointegrator 1 complex subunit 3 (ASCC3)	2.127	3.37E-06	2.379	4.43E-16	2.699	1.16E-13	2.276	1.47E-16
LOC100550192	HLA class II histocompatibility antigen, DM beta chain-like (DMB2)	2.263	0.003633	2.648	6.59E-05	3.586	4.06E-10	3.266	2.81E-09
LOC104909264	Multidrug resistance-associated protein 4-like	2.217	5.61E-06	3.408	1.25E-16	2.207	1.27E-05	2.329	4.25E-06
LOC104909289	Limbin-like	2.589	1.35E-06	3.524	3.22E-09	2.058	0.000628	2.740	0.000108
LOC104909397	Dolichyl pyrophosphate Glc1Man9GlcNAc2 alpha-1,3-glucosyltransferase (probable)	2.464	0.002752	2.186	1.95E-06	3.644	1.56E-10	2.282	1.87E-07
LOC104909635	Latent-transforming growth factor beta-binding protein 1-like	2.875	3.12E-11	3.039	2.66E-58	4.077	2.86E-66	3.716	3.9E-113
LOC104909708	Uncharacterized LOC104909708 (ncRNA)	−3.356	0.012361	−3.268	0.000646	−2.688	0.03465	−3.090	0.000878
LOC104909779	Uncharacterized LOC104909779 (ncRNA)	2.223	2.66E-05	2.640	7.28E-09	2.120	6.87E-05	2.361	2.74E-06
LOC104909819	Uncharacterized LOC104909819 (ncRNA)	−3.276	0.004612	−4.031	0.000265	−2.214	0.027631	−2.483	0.005267
LOC104909922	Unconventional myosin-VI-like	3.620	0.002294	4.227	1.24E-05	5.139	2.25E-08	4.453	1.39E-06
LOC104909972	Uncharacterized LOC104909972 (ncRNA)	2.761	0.025565	3.318	7.03E-05	3.046	0.001915	2.946	0.002409
LOC104910277	Uncharacterized LOC104910277 (ncRNA)	2.047	0.000265	2.011	8.08E-06	3.469	1.52E-14	3.345	6.5E-30
LOC104910488	Protein EFR3 homolog A-like	2.207	0.001044	2.478	2.77E-08	3.242	1.65E-13	2.702	1.86E-11
LOC104911116	Tetraspanin-18-like	2.542	1.11E-08	2.454	2.04E-16	3.174	1.45E-16	2.371	2.01E-15
LOC104911697	Lymphocyte antigen 75-like	2.320	3.64E-06	2.737	2.24E-27	3.010	3.28E-17	2.467	1.3E-17
LOC104912545	Serine/threonine-protein kinase ATR-like	2.556	0.008755	3.134	0.000424	2.518	0.004279	2.665	0.019456
LOC104913390	Multidrug resistance-associated protein 1-like	3.731	2.77E-06	3.871	2.66E-12	3.832	1.01E-06	3.542	1.1E-09
LOC104913470	Periplakin-like	6.430	2.91E-05	6.732	2.38E-07	6.104	0.000567	4.863	0.040654
LOC104913553	Uncharacterized LOC104913553 (ncRNA)	−2.071	7.56E-10	−2.542	1.04E-24	−2.187	8.27E-16	−3.085	1.65E-42
LOC104913838	Uncharacterized LOC104913838 (ncRNA)	−6.959	4.68E-07	−3.967	4.63E-07	−2.318	0.000899	−2.566	6.44E-05
LOC104914081	Regulator of G-protein signaling 9-binding protein-like	−3.633	1.72E-96	−3.782	1.47E-36	−4.970	1.7E-110	−4.224	3.51E-39
LOC104914095	Probable phospholipid-transporting ATPase IIA	3.083	0.02281	3.349	0.006467	3.225	0.018069	3.794	0.000706
LOC104914454	Importin-9-like	2.093	2E-07	2.484	6.1E-18	2.003	1.92E-09	2.319	3.02E-15
LOC104914932	Protein LAP2-like	2.253	1.33E-07	2.614	1.59E-18	2.978	1.19E-13	2.505	7.31E-17
LOC104914983	Rho guanine nucleotide exchange factor 28-like	2.756	0.021236	2.875	0.032997	3.290	0.000905	4.226	2.95E-05
LOC104915185	Peptidyl-glycine alpha-amidating monooxygenase-like	2.029	2.1E-13	2.194	1.66E-84	3.124	5.61E-93	2.504	3.61E-96
LOC104915209	Cyclin-G-associated kinase-like	2.718	0.003993	2.098	0.002392	2.970	0.000509	2.078	0.002028
LOC104915239	Solute carrier family 12 member 2-like	2.423	0.004607	4.386	1.2E-06	3.172	3.58E-07	4.979	8.76E-10
LOC104915240	Solute carrier family 12 member 2-like	2.331	0.008301	3.373	4.27E-05	2.581	0.003403	3.312	0.000191
LOC104915275	Versican core protein-like	2.117	3.28E-07	2.508	9.4E-19	4.283	6.06E-45	3.322	3.36E-41
LOC104915303	Structural maintenance of chromosomes protein 2-like	2.372	1.45E-09	3.005	1.51E-47	3.395	5.32E-41	2.418	2.86E-19
LOC104915418	Uncharacterized LOC104915418 (ncRNA)	2.830	0.014401	2.592	0.013781	3.176	0.000745	2.861	0.004775
LOC104916051	Uncharacterized LOC104916051	2.211	0.010683	4.739	1.72E-07	3.274	8.48E-08	5.184	2.35E-11
LOC104916792	Uncharacterized LOC104916792 (ncRNA)	2.430	0.004417	2.450	0.000331	3.118	8.43E-06	2.330	0.000822
LOC104916797	Kinesin-like protein KIF20B	5.546	0.012424	3.131	0.019325	5.632	0.003275	3.042	0.030237
LOC104916915	ETS translocation variant 3-like protein	2.198	7.61E-05	2.887	1.53E-07	2.179	6.09E-05	2.863	1.6E-07
LOC104916992	Uncharacterized LOC104916992 (ncRNA)	−6.565	2.46E-78	−6.353	4E-112	−2.320	9.47E-22	−2.022	2.86E-73
LOC104917145	Uncharacterized LOC104917145 (ncRNA)	2.147	5.47E-05	2.680	2.39E-09	2.443	1.16E-06	2.382	1.55E-10
LOC104917155	Alpha-mannosidase 2-like	3.184	8.61E-06	3.626	9.74E-23	4.006	1.55E-14	3.543	1.35E-21
LOC104917232	Alpha-mannosidase 2-like	3.317	1.09E-05	3.119	3.95E-11	3.585	5.11E-08	2.802	1.73E-07
LOC104917363	Extended synaptotagmin-2-A-like	2.174	0.01489	3.069	3.92E-12	2.452	0.000574	2.336	8.58E-05
LOC104917414	Uncharacterized LOC104917414 (ncRNA)	3.090	2.57E-06	3.525	2.34E-09	2.547	0.000728	2.029	0.020654
LOC104917580	Sister chromatid cohesion protein PDS5 homolog B-like	2.780	5.84E-05	2.052	2.19E-06	3.603	2.16E-11	2.307	2.47E-08
MKI67	Marker of proliferation Ki-67	2.867	6.91E-09	3.127	1.48E-31	4.181	6.74E-22	2.829	1.9E-24
MSTN	Myostatin	−3.824	4.36E-08	−2.382	1.94E-12	−3.424	5.62E-07	−2.480	5.71E-13
MTMR8	Myotubularin related protein 8	2.148	1.6E-07	2.456	1.51E-14	2.262	3.14E-10	2.143	3.91E-11
MYL3	Myosin, light chain 3, alkali; ventricular, skeletal, slow	−6.283	6.6E-130	−7.377	0	−3.687	3.26E-82	−3.705	0
PTGFRN	Prostaglandin F2 receptor inhibitor	3.291	4.18E-06	2.740	1.19E-06	3.536	1.27E-07	3.300	3.07E-11
REEP3	Receptor accessory protein 3	2.363	5.13E-14	2.201	9.59E-31	2.646	6.37E-23	2.124	4.64E-30
SLN	Sarcolipin	−5.226	1.34E-54	−6.440	7.03E-80	−3.335	2.34E-30	−2.338	1.23E-38
TEAD1	TEA domain family member 1 (SV40 transcriptional enhancer factor)	2.357	1.73E-12	2.259	9.1E-12	3.078	1.95E-19	2.654	5.9E-32
TNNC2	Troponin C type 2 (fast)	−3.312	5.9E-126	−3.349	0	−4.650	7.4E-152	−4.099	0
TPM2	Tropomyosin 2 (beta)	−2.732	1.51E-36	−2.541	1.5E-107	−2.289	1.47E-22	−2.163	9.04E-96
TSPAN10	Tetraspanin 10	−2.955	4.44E-18	−2.432	3.11E-72	−2.771	3.98E-16	−3.485	3.72E-88

*Comparisons highlighted red are up-regulated in the comparison whereas genes highlighted in green are down-regulated*.

To further examine differences between the temperature treatments, the 2,926 significant DE genes (|Log_2_FC| > 1.0) shared among all treatment comparisons were further investigated. These represent 23.3% of the total number of DE genes identified across treatments. Of the 2,926 DE genes, 637 showed consistent directional expression change, with the 33 vs. 38°C comparisons and the 43 vs. 38°C comparisons having the same directional response in both lines. Up regulation across all treatment comparisons was observed for 407 genes whereas 141 were down regulated. Of the remaining 89 genes, 53 were down regulated by cold treatment in both lines and 36 were down regulated by heat treatment (Table 7). These include *CD36* and *GUCA1B* discussed above. IPA analysis of these 89 genes suggests upstream effects attributed to the AHR (Aryl Hydrocarbon Receptor) transcription factor, and the transforming growth factors TGFB1 and TGFB3 (Figure S6).

**Table 7:** Common DE genes [FDR *p*-values (< 0.05), |Log_2_FC| >1.0] showing consistent directional change by treatment.

		**33R vs. 38R**		**33F vs. 38F**		**43R vs. 38R**		**43F vs. 38F**	
**Gene**	**Description**	**Log_2_FC**	**FDR pval**	**Log_2_FC**	**FDR pval**	**Log_2_FC**	**FDR pval**	**Log_2_FC**	**FDR pval**
ABLIM2	Actin binding LIM protein family, member 2	−2.054	0.001493	−1.390	0.004024	1.353	0.001549	1.418	4.88E-07
LOC104912064	Disintegrin and metalloproteinase domain-containing protein 12-like	−1.231	0.035684	−1.447	0.000151	1.739	2.65E-07	1.573	9.79E-13
APBA2	Amyloid beta (A4) precursor protein-binding, family A, member 2	−1.319	0.001841	−1.274	7.45E-09	2.041	1.52E-20	1.135	1.5E-14
ARHGAP28	Rho GTPase activating protein 28	−1.852	1.38E-05	−1.690	2.9E-07	1.581	3.62E-06	1.145	6.23E-07
ASAP3	ArfGAP with SH3 domain, ankyrin repeat and PH domain 3	−1.356	0.001483	−1.633	0.000236	1.193	0.000193	1.931	7.36E-16
BCL6	B-cell CLL/lymphoma 6	−1.024	1.31E-06	−1.079	8.27E-30	1.171	1.37E-09	1.177	1.3E-65
C8H10orf71	Chromosome 8 open reading frame, human C10orf71	−2.257	2.07E-27	−2.531	1.83E-83	1.786	3.24E-26	1.430	1.85E-50
CDK18	Cyclin-dependent kinase 18	−2.219	3.25E-10	−1.751	3.86E-11	1.300	4.65E-08	1.053	6.42E-09
CHST1	Carbohydrate (keratan sulfate Gal-6) sulfotransferase 1	−3.057	3.89E-06	−3.571	7.87E-08	1.440	1.37E-05	1.722	1.83E-08
COL3A1	Collagen, type III, alpha 1	−2.903	0	−2.382	0	1.659	2.98E-12	1.923	1.18E-27
COL4A1	Collagen, type IV, alpha 1	−1.606	5.48E-14	−1.224	0	1.217	1.83E-08	1.685	1.24E-40
DCX	Doublecortin	−2.199	4.6E-09	−1.987	2.44E-19	1.815	3.08E-11	1.073	1.75E-14
LOC104911002	Dickkopf-related protein 3-like	−2.513	4.9E-08	−1.496	9.78E-05	1.626	6.64E-10	1.797	3.55E-19
LOC100540803	Delta and Notch-like epidermal growth factor-related receptor	−2.602	0.003208	−2.278	0.009568	1.750	6.59E-05	1.973	6.31E-07
LOC100545932	Dystrobrevin alpha	−1.700	0.004161	−2.497	4.6E-06	1.703	1.34E-06	1.535	5.69E-08
LOC104910320	Dystrobrevin alpha-like	−2.452	0.000109	−2.072	1.99E-05	1.320	0.00051	1.007	0.001196
LOC104910337	Dystrobrevin alpha-like	−2.896	0.028765	−2.277	0.010759	2.334	1.68E-05	1.380	0.001723
LOC100539830	Dysferlin-like	−1.673	0.000158	−1.487	0.001369	1.095	0.000961	1.553	3.96E-07
FRAS1	Fraser extracellular matrix complex subunit 1	−1.772	0.001852	−2.548	1.79E-08	2.296	6.27E-12	1.137	7.91E-06
GNG2	Guanine nucleotide binding protein (G protein), gamma 2	−1.704	1.86E-06	−1.474	2.75E-06	1.126	9.24E-06	1.158	4.56E-08
GRIN2A	Glutamate receptor, ionotropic, N-methyl D-aspartate 2A	−2.471	4.6E-12	−1.899	1.35E-14	1.115	1.98E-07	1.354	4.38E-22
GUCA1B	Guanylate cyclase activator 1B (retina)	−3.228	2.28E-05	−6.281	2.68E-05	2.990	1.44E-23	3.316	1.18E-25
LOC104909294	Hypermethylated in cancer 1 protein	−2.281	2.93E-05	−3.489	6.51E-10	1.876	2.64E-09	2.233	1.28E-29
LOC100541783	Hypermethylated in cancer 1 protein-like	−1.977	0.027443	−3.105	0.002499	2.380	3.83E-09	2.684	2.86E-15
INHA	Inhibin, alpha	−5.346	1.21E-15	−6.115	4.21E-20	1.205	2.72E-05	1.180	2.92E-07
LOC100547979	Junctophilin-1	−4.175	1.97E-17	−3.904	7.22E-23	2.198	2.21E-19	1.723	2.29E-23
LOC104917153	Small conductance calcium-activated potassium channel protein 3	−1.103	0.027791	−1.368	0.000591	1.241	0.000199	1.234	1.4E-06
KLB	Klotho beta	−2.008	4.52E-14	−2.114	1.07E-43	1.060	3.28E-07	1.049	1.72E-16
KLHL31	Kelch-like family member 31	−2.298	4.77E-08	−3.752	1.06E-19	2.070	1.16E-12	1.284	5.06E-08
LEF1	Lymphoid enhancer-binding factor 1	−5.804	0.002693	−4.107	0.00012	1.684	0.003038	1.957	1.61E-06
LHFPL3	Lipoma HMGIC fusion partner-like 3	−1.892	0.001752	−1.144	0.005348	1.391	0.000141	1.788	6.76E-14
LOC104912085	Uncharacterized LOC	−1.805	6.64E-11	−1.791	3.01E-07	1.795	4.69E-15	1.270	5.13E-06
LOC104914708	Uncharacterized LOC	−3.041	4.19E-08	−2.491	1.47E-16	1.414	8.5E-05	1.543	3.96E-23
LTBP2	Latent transforming growth factor beta binding protein 2	−2.355	4.6E-44	−2.465	4.81E-43	1.535	2.35E-24	1.854	5.97E-21
LOC104909252	Myosin-3-like	−6.600	1.56E-05	−6.693	6.28E-07	1.071	0.039632	1.195	0.003104
LOC100543020	Myosin-7-like	−3.828	1.32E-05	−3.409	0.000241	1.192	0.007426	1.221	0.004462
LOC100549331	Myosin-7-like	−5.467	4.06E-71	−6.412	0	1.275	3.75E-06	1.390	6.35E-31
LOC100544354	Uncharacterized LOC	−5.397	0.014658	−5.702	0.002833	1.954	0.002783	1.448	0.009527
LOC104914128	Uncharacterized LOC	−2.861	0.00076	−3.030	1.8E-05	1.456	0.001195	1.698	7.65E-09
NFATC2	Nuclear factor of activated T-cells, cytoplasmic, calcineurin-dependent 2	−2.476	0.020677	−2.123	0.003688	1.779	0.000711	1.675	2.65E-05
OASL	2′–5′-Oligoadenylate synthetase-like	−1.909	1.5E-08	−1.554	4.62E-09	2.351	1.3E-29	2.333	7.81E-66
OPCML	Opioid binding protein/cell adhesion molecule-like	−2.642	1.09E-11	−1.680	1.43E-21	1.270	2.36E-06	2.338	5.65E-21
P2RY1	Purinergic receptor P2Y, G-protein coupled, 1	−1.507	0.000988	−1.140	0.013083	1.259	6.78E-05	1.932	6.03E-16
PALMD	Palmdelphin	−2.571	3.06E-05	−4.140	5.6E-09	1.568	5.2E-06	1.613	2.51E-08
LOC104914006	Receptor-type tyrosine-protein phosphatase T-like	−2.385	1.73E-12	−2.643	2E-28	2.389	1.61E-33	1.880	5.84E-89
LOC104914794	Iporin-like	−1.551	3.48E-05	−1.618	2.51E-10	1.509	9.48E-07	1.310	1.39E-12
RYR1	Ryanodine receptor 1 (skeletal)	−2.016	9.81E-05	−2.523	0.000488	1.361	2.2E-05	1.894	1.47E-09
LOC104916851	Ryanodine receptor 1-like	−1.297	0.01007	−2.003	7.49E-06	1.469	9.89E-06	1.875	1.34E-17
LOC104917345	Ryanodine receptor 1-like	−1.549	0.003033	−1.727	0.00029	1.208	0.001046	1.777	3.21E-13
LOC104913331	Putative E3 ubiquitin-protein ligase SH3RF2	−5.398	0.013363	−6.023	0.000157	2.091	0.000434	1.132	0.035376
SHROOM1	Shroom family member 1	−2.387	7.34E-07	−2.096	3.88E-07	1.263	4.57E-05	1.482	7.73E-11
LOC100546217	protein TENP	−5.045	0.038689	−4.947	0.043268	1.767	0.021551	3.304	1.16E-12
TIMP3	TIMP metallopeptidase inhibitor 3	−1.582	0	−1.683	0	1.690	5.37E-33	1.422	8.26E-31
LOC100542775	Alpha-2-macroglobulin-like protein 1	2.407	7.18E-09	2.252	2.22E-11	−1.990	0.040101	−2.381	0.00191
C1QTNF4	C1q and tumor necrosis factor related protein 4	1.106	1.2E-15	1.168	8.66E-38	−1.107	2.2E-12	−1.070	0
CD36	CD36 molecule (thrombospondin receptor)	2.300	2.05E-46	2.479	9.79E-46	−2.606	3.59E-17	−2.131	3.52E-27
CNDP1	Carnosine dipeptidase 1 (metallopeptidase M20 family)	1.117	0.007658	1.413	1.9E-05	−3.750	1.16E-07	−1.882	0.000797
LOC100550279	HLA class II histocompatibility antigen, DM beta chain-like	1.627	4.24E-29	1.928	6.54E-50	−1.339	0	−2.694	2.97E-91
ENTPD3	Ectonucleoside triphosphate diphosphohydrolase 3	1.002	0.012146	1.079	3.4E-05	−1.395	0.005746	−1.241	0.001609
ESAM	Endothelial cell adhesion molecule	1.958	1.42E-07	2.116	5.48E-25	−1.339	0.012003	−2.291	9.08E-08
ETV7	ets variant 7	2.160	2.67E-06	1.664	5.7E-08	−2.342	0.003252	−2.101	0.000492
LOC104917139	Germin-like protein subfamily 2 member 2	1.627	0.000198	1.141	6.34E-05	−5.294	4.91E-09	−3.698	1.06E-09
LOC100539100	Guanylate cyclase soluble subunit beta-2-like	2.063	6.72E-21	1.708	2.8E-25	−1.209	0.000197	−1.081	4.92E-05
LOC100543128	Histone H3-like	1.487	1.79E-07	1.169	1.34E-10	−2.755	5.02E-09	−2.480	2.18E-18
IL18BP	Interleukin 18 binding protein	1.513	0.000535	1.325	4.57E-06	−2.087	0.000679	−2.409	3.58E-06
LOC104916207	Kallikrein-8-like	1.284	0.000119	1.215	6.35E-06	−1.304	0.006723	−1.537	0.000479
LRAT	Lecithin retinol acyltransferase (phosphatidylcholine–retinol O-acyltransferase)	1.219	1.27E-05	1.178	4.02E-10	−1.302	0.000548	−1.028	0.000202
LOC100549167	Mannose-binding protein A-like	1.670	0.002909	1.232	0.010338	−3.692	0.004571	−3.695	0.00115
MTNR1A	Melatonin receptor 1A	1.515	0.000877	1.153	0.0135	−2.098	0.009624	−1.611	0.023993
LOC104909506	Uncharacterized LOC	1.567	0.006758	1.695	5.46E-05	−2.746	0.021177	−2.932	0.002457
LOC104910409	Uncharacterized LOC	1.067	0.003554	1.193	7.65E-05	−2.385	7.52E-06	−1.160	0.010109
LOC104911590	Uncharacterized LOC	1.319	2.43E-06	1.103	9.54E-09	−1.940	3.03E-07	−2.072	8.46E-11
LOC104912270	Uncharacterized LOC	1.400	3.32E-11	1.230	2.85E-15	−1.335	6.18E-05	−1.116	4.73E-08
LOC104912544	Uncharacterized LOC	1.332	2.14E-05	1.304	8.93E-10	−1.550	0.000542	−1.963	2.5E-08
LOC104913567	Uncharacterized LOC	1.240	1.61E-14	1.029	6.62E-35	−1.328	2.57E-11	−1.137	3.08E-26
LOC104913826	Uncharacterized LOC	1.515	1.98E-22	1.564	4.58E-37	−1.109	9.44E-07	−1.104	5.72E-11
LOC104914493	uncharacterized LOC	1.418	0.001313	1.516	5E-05	−1.869	0.007214	−2.320	0.002089
NOXO1	NADPH oxidase organizer 1	1.653	1.97E-10	1.357	7.28E-17	−2.124	1.47E-09	−1.591	6.04E-09
NTSR1	Neurotensin receptor 1 (high affinity)	1.209	0.029619	1.309	0.001145	−2.517	0.002856	−2.140	0.004664
PCBP3	Poly(rC) binding protein 3	1.527	8.42E-09	1.406	8.33E-16	−1.491	7.04E-05	−1.521	4.59E-08
PIGM	Phosphatidylinositol glycan anchor biosynthesis, class M	1.107	0.000363	1.095	4.43E-07	−1.324	0.000604	−1.023	0.000899
PLS1	Plastin 1	1.251	2.14E-09	1.473	1.37E-32	−2.179	3.22E-15	−1.691	3.69E-20
PTGR1	Prostaglandin reductase 1	2.046	7.33E-09	2.261	2.27E-14	−1.937	0.001524	−2.169	0.00077
RANBP17	RAN binding protein 17	1.835	6.16E-08	1.038	5.8E-05	−1.391	0.017844	−2.861	8.69E-10
SERPINF2	Serpin peptidase inhibitor, clade F (alpha-2 antiplasmin, pigment epithelium derived factor), member 2	1.395	2.6E-16	1.163	1.15E-27	−1.207	1.55E-08	−1.096	1.28E-14
SH2D4A	SH2 domain containing 4A	1.314	6.2E-07	1.371	8.49E-13	−1.828	2.37E-07	−2.367	1.25E-11
SH2D5	SH2 domain containing 5	1.108	0.019073	1.063	0.00494	−2.319	0.000812	−1.171	0.030757
SH3GLB2	SH3-domain GRB2-like endophilin B2	1.380	5.63E-21	1.065	1.78E-49	−1.095	7.41E-11	−1.345	3.32E-40
LOC104915044	spErm-associated antigen 4 protein-like	2.185	2.89E-06	1.644	1.1E–05	−3.967	0.000862	−2.010	0.007625

*Comparisons highlighted red are up regulated in the comparison whereas genes highlighted in green are down regulated*.

### Effects of selection (line differences)

**Figure 4:**
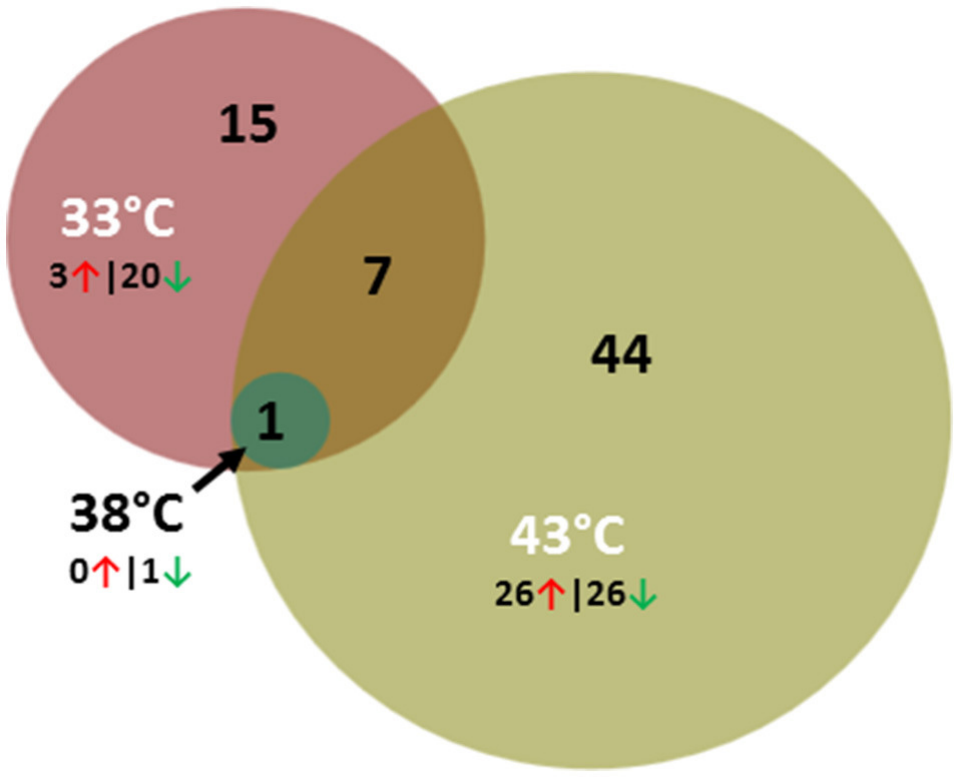
Distribution of differentially expressed genes between lines (F-line vs. RBC2) during p. major satellite cell differentiation. For each temperature comparison, the number of genes with FDR *p* < 0.05 and |Log_2_FC| > 2.0 that were shared or unique to each incubation temperature are indicated. The number and direction of expression change (↑ or ↓) for the genes included in each temperature group are listed outside the Venn diagram. Circle size is proportional to the number of genes.

Comparisons between lines within temperature treatment found relatively few DE genes at the three incubation temperatures (Table 2, Figure 4) and the majority were down regulated in the RBC2 cells. Interestingly at the control temperature (38°C), only a single gene, Trans-2,3-Enoyl-CoA Reductase-Like (*TECRL*), showed significant DE between lines. This gene is a protein coding regulatory gene that catalyzes an oxidation-reduction (redox) reaction and is thought to be involved in the fatty acid biosynthesis pathway. This gene also had significant DE between lines at 33 and 43°C (Table 7). In all cases *TECRL* was down regulated in the F line vs. RBC2 (average Log_2_FC = −6.96).

In contrast to the control temperature, the numbers of genes where the cold (33°C) and hot (43°C) treatments significantly affected expression were considerably higher (Table 2). At 33°C, 873 genes showed significant FDR *p*-values with 116 also having |Log_2_FC| > 1.0. Of these 23 had |Log_2_FC| > 2.0 with the majority (87%) being down regulated in the F line compared to the RBC2 cells. Fifteen of the 23 genes were unique to the 33°C temperature comparison and 7 were shared with the 43°C comparison (Figure 4, Table 8). Included in the group of 15 unique genes were; arf-GAP with coiled-coil, ANK repeat and PH domain-containing protein 1-like (*LOC104916358*), Rho guanine nucleotide exchange factor (GEF) 16 (*ARHGEF16*), carbamoyl-phosphate synthase 1, mitochondrial (*CPS1*), neutrophil cytosolic factor 1 (*NCF1*), tocopherol (alpha) transfer protein (*TTPA*), histamine N-methyltransferase-like (*LOC100542432*), receptor tyrosine-protein kinase erbB-3-like (*LOC100546071*), fibrocystin-like (*LOC104910058*), semaphorin-6C-like (*LOC104916327*), and 6 uncharacterized loci (*LOC104909501, LOC104910046, LOC104910133, LOC104911073, LOC104912259, LOC104916656*). With the exception of *LOC104911073*, all were down regulated in the F-line cells. Although, the specific function of many of these genes are not known, they may have roles in such diverse processes as protein-protein and protein-lipid interactions, the removal of excess urea from cells, the regulation of vitamin E levels and secretion of vitamin E from hepatocytes to circulating lipoproteins. In regards to muscle biology, semaphorin-6C-like has been found to be concentrated at neuromuscular junctions suggesting a role in neuromuscular communication (Svensson et al., 2008).

**Table 8:** Significant DE genes in between-line comparisons [FDR *p*-values (< 0.05), |Log_2_FC| >2.0] that were shared within temperature (see Figure 4).

		**33°C**		**38°C**		**43°C**	
**DEGs in “F vs. RBC2” at all temps**		**Log_2_FC**	**FDR *p*-val**	**Log_2_FC**	**FDR *p*-val**	**Log_2_FC**	**FDR *p*-val**
TECRL	Trans-2,3-enoyl-CoA reductase-like	−4.141	1E-07	−10.569	0.0037668	−6.170	6.44*E*−114
**DEGs IN “F vs. RBC2” AT 33° AND 43°C BUT NOT AT 38°C**							
CNGA3	Cyclic nucleotide gated channel alpha 3	2.740	0.0030845	1.741	1.000	4.090	0.0007882
COL24A1	Collagen, type XXIV, alpha 1	−2.815	1.983E-06	−2.710	1.000	−2.851	8.81*E*−10
LOC100539697	Integrin beta-like protein 1	2.998	0.0318933	1.024	1.000	3.096	0.0088252
LOC104915513	Histone deacetylase 7-like	−3.813	0.0051096	−5.804	1.000	−4.703	4.932*E*−07
LOC104917072	Zinc finger protein 502-like	−3.963	2.294E-06	−2.846	1.000	−5.982	0.0010235
MUC3A	Mucin 3A, cell surface associated	−6.218	0.0065859	−4.082	1.000	−6.338	1.051*E*−23
ROBO2	Roundabout, axon guidance receptor, homolog 2 (Drosophila)	−3.456	3.467E-05	−1.795	1.000	−2.654	0.0011681

*Comparisons highlighted red are up-regulated in the comparison whereas genes highlighted in green are down-regulated*.

Greater differential expression between the F and RBC2 cells was observed at 43°C (Table 2). Here the total number of DE genes (1818) was more than twice that observed at 33°C. In total 318 genes had |Log_2_FC| > 1.0 and of 52 had |Log_2_FC| > 2.0. Of these 52 genes, 44 were unique to this temperature comparison (Figure 4) and the directionality of expression changes was balanced with half of the genes being up regulated and half down regulated. Ten of these genes are annotated as uncharacterized loci. However, 14 (*ALDH1A3, B4GALT6, CD55, CYGB, ENPP2, GREM1, NPY, NRTN, PKHD1, RAC2, RARB, RSAD2, SLIT3*, and *WASF3*) co-occur in an IPA gene network corresponding to the processes of cell morphology, cellular assembly, organization, and development and 8 genes (*ANKS4B, CAPN9, CYP26B1, GALNT3, PXDN, RASD2, RPS4X, SPON1*, and *SLC7A14*) co-occur in a gene network corresponding to the processes of cellular movement, cell cycle and morphology. Interesting loci in these groups include genes involved in glycolipid biosynthesis (*B4GALT6*, Takizawa et al., 1999) down regulated in F-line cells, genes involved in regulating organogenesis, body patterning, cell and tissue differentiation [*GREM1* (Michos et al., 2004) and *RARB* (Hauksdottir et al., 2003)], and *NPY* a neurotransmitter with regulatory functions in bone homeostasis (Gu et al., 2016). The latter 3 genes were all upregulated in the F-line cells.

## Discussion

Muscle satellite cells are self-renewing and give rise to differentiated cell types, thus being true stem cells. Satellite cells and myofibers are of the same origin, being derived from somites (Armand et al., 1983). In mice, satellite cells are first identified morphologically toward the end of fetal development lying underneath the forming basement membrane (Ontell and Kozeka, 1984). They are the only undifferentiated cells present in the muscle at birth and during early postnatal growth, and are the primary source of myonuclei for skeletal muscle growth (Sherwood et al., 2004). During posthatch development in the chicken, satellite cells diversify in cell fate, with some cells entering quiescence (Halevy et al., 2004). In adult muscle, quiescent cells continue to provide nuclei for muscle hypertrophy and repair (Zammit et al., 2006; Zammit, 2008).

The function of satellite cells is modulated by the cellular microenvironment and quiescent satellite cells are identifiable and being both PAX7 and MYF5 positive. The state of satellite cells is defined by antagonistic Notch and Wnt signaling, with Notch maintaining PAX7 and Wnt signaling driving *MYOD* expression through beta-catenin (Fujimaki et al., 2013). Activation of satellite cells is thus marked by the onset of *MYOD* expression, downregulation of *PAX7* and the subsequent increase in myogenin that marks the commitment of the activated cell to differentiation (Zammit, 2008).

Thermal challenge of the turkey satellite cells had no significant effect on expression of *PAX7* in either the cold or heat-treated cells. Likewise, no significant expression differences were observed for *MYOD1*, and *MYF5* was only slightly downregulated in the heat-treated RBC2 cells. However, myogenin (*MYOG, LOC100303673*), was significantly down regulated in the cold treated cells (33°C) of both the RBC2 and F-line compared to controls (38°C) (Log_2_FC = −2.17 and −2.30, respectively). In addition, myocyte enhancer factors *MEF2B, MEF2C, and MEF2D* were significantly downregulated in cold treated cells (Log_2_FC = −3.04, −2.43, and −0.95, respectively, for RBC2 line and −2.75, −4.48, and −0.60 for F line, respectively. This suggests that decreased temperature can directly influence satellite cell differentiation by acting at the level of gene transcription. In the heat-treated cells (43°C), *MYOG* was slightly downregulated (Log_2_FC = −1.11 and −1.06). This result is counter to a previous study using the same dissociated cell culture system that found increased *MYOG* expression, as measured by qPCR, in cells incubated at temperatures above 38°C (Clark et al., 2016a). However, expression of *MYOG* in that study was conducted after 72 h of differentiation as opposed to 48 h as in the present study. Satellite cells from both the RBC2 and F lines differentiate faster and produce larger myotubes at higher temperatures with slower growth at lower temperatures (Clark et al., 2016a). This is consistent with the RNAseq results in that many muscle-associated genes were among the genes with the greatest down regulation in cold-treated cells (33°C).

The gene *MEF2B* was also down regulated in heat-treated cells (Log_2_FC = −1.52 and −1.54 for RBC2 and F lines, respectively), whereas *MEF2D* was modestly upregulated (Log_2_FC = 0.92 and 1.15). Myocyte enhancer factor 2 proteins play key roles as transcriptional regulators of skeletal muscle development as well as myofibrillar gene expression, fiber type control, and glucose regulation (Richter and Hargreaves, 2013). Myocyte enhancer factor 2c binds to myogenin to activate skeletal muscle differentiation (Molketin et al., 1995). Moreover, recent work by Anderson et al. (2015) demonstrated that *MEF2C* knockout mice had significantly reduced body weight compared to controls beginning at day 10 and persisting to day 52. Reduced *MEF2C* expression observed at cold temperatures could be a factor in the extensive down regulation of other genes associated with sarcomeric gene expression as well as genes associated with carbohydrate metabolism and reduced rate of differentiation observed by Clark et al. (2016a).

In addition to maintaining PAX7, Notch may also signal satellite cells to stop proliferating (Conboy and Rando, 2002). The transcription factor prospero homeobox 1 (PROX1) appears to be essential for myoblast differentiation in that has a bidirectional interaction with Notch1. High Notch activity inhibits PROX1, which in turn represses Notch1 signaling (Kivelä et al., 2016). PROX1 is thought to regulate muscle phenotype via NFAT (nuclear factor of activated T cells). *PROX1* was up regulated in the heat-treated turkey cells (Log_2_FC = 1.82 and 1.32 in the RBC2 and F line, respectively) consistent with enhanced differentiation.

The suggested upstream effects on AHR (Aryl Hydrocarbon Receptor) transcription factor, and the transforming growth factors TGFB1 and TGFB3 observed in comparison of the challenged cells are of interest because of the consistent directionality of expression changes and shared significance observed across treatment comparisons in the turkey satellite cells. Of particular interest is the effect of TGFB1 on genes like *NFATC2*. Both NFATC2 and the related NFATC3 are important transcription factors involved in muscle growth and differentiation. In the cold treated cells, *NFATC2* was downregulated but modestly upregulated in the heat comparisons. These genes may also be affected by temporal/spatial calcium-signaling. In differentiating satellite cell, calcium enters the cytoplasm via TRPC channels. *TRPC1* was modestly upregulated in cold but not hot-treated cells. Calcium increases translocation of both NFATC2 and NFATC3 to the nucleus. Increased translocation of NFATC2 and NFATC3 leads to increased expression of *MYOD* (Liu and Schneider, 2014).

In addition to TRPC channels, differentiating satellite cells beginning to express proteins responsible for regulation of Ca^2+^ homeostasis, including channel proteins, Ca^2+^-pumps, Ca^2+^-storage proteins, and proteins the regulate the activity of channels and pumps. These proteins are localized to the sarcoplasmic reticulum (SR) and the sarcolemma. The SR calcium release channels or ryanodine receptors RYR1 and RYR3 serve as conduits for Ca^2+^ to enter the sarcoplasm from the lumen of the sarcoplasmic reticulum (Rossi and Dirksen, 2006). In adult avian skeletal muscle, Ca^2+^ release via the ryanodine receptors is activated by voltage depolarization across the sarcolemma. This results in a conformational change in the α_1s_ subunit of the calcium voltage-gated calcium channel. The ryanodine receptor RYR1, which interacts with the α_1s_ subunit, responds by undergoing a conformational change causing the pore to open, thereby enabling calcium release from the SR. Increased cytosolic [Ca^2+^] activates RYR3 via a Ca^2+^ -induced Ca^2+^-release mechanism thereby amplifying Ca^2+^ release from the SR. The structure of the Ca^2+^-release complex includes associated proteins that modulate Ca^2+^-release activity. These include the Ca^2+^ storage protein calsequestrin (CASQ), the structural proteins junctophilin-2 (JPH2) and triadin (TRDN), and the Ca^2+^ pump protein (ATP2A1 and ATP2A2) and its associated inhibitory protein sarcolipin (SLN) (Rossi and Dirksen, 2006).

Interestingly, most of these genes associated with regulation of Ca^2+^ homeostasis were significantly affected by temperature treatment, particularly cold treatment. The *RYR1* isoform was downregulated >2-fold in cold treated cells from both lines, but modestly increased in the heat-treated cells. (Table S3). The α_1s_ subunit of the calcium voltage-gated calcium channel (*CACNA1S*) was significantly downregulated in both lines (Log_2_FC = −3.64 and −3.91 for RBC2 and F line, respectively), but was modestly upregulated by ~0.4-fold in heat-treated cells from both lines. Other Ca^2+^ regulatory, pump, and storage genes associated with the SR including, *TRDN, ATP2A2, SLN, CASQ2* were also significantly downregulated. Log_2_FC ranged from −0.71 for *ATP2A2* in the cold treated cells from RBC2 to −7.2 for *TRDN* from the same treatment (Table S3). Taken in total, the small increase in *TRPC1* expression in cold treated cells may be offset by decreased of expression of these other genes. This has important implications for the ability of these cells to maintain normal Ca^2+^ homeostasis in response to cold temperatures. Heat stress can effect an increase in intracellular [Ca^2+^] (Mikkelsen et al., 1991) and there is some evidence that elevated temperatures result in decreased ability of the SR to sequester Ca^2+^ in muscle (van der Poel and Stephenson, 2007). The intracellular Ca^2+^ concentration regulates a plethora of metabolic and transcriptional activities, so exposure to hot or cold temperatures early in development may have long-term implications for the fate of the developing muscle.

Among the most significantly altered gene pathways in the turkey satellite cells based on activation score were those corresponding to the eIF2, eIF4, and p70S6K, and mTOR signaling. The eIF2 imitation complex integrates a diverse array of stress-related signals to regulate mRNA translation, especially in response to stress. Likewise, eIF4 and p70S6K signaling play critical roles in translation regulation (Sonenberg and Hinnebusch, 2009). Genes of the mTOR signaling pathway play a critical role in the regulation of skeletal muscle hypertrophy (Bodine et al., 2001; Ohanna et al., 2005; Rommel et al., 2013) this pathway is one of the main signaling pathways controlling protein synthesis and cell proliferation in myogenic satellite cells (Han et al., 2008). Muscle growth after hatch occurs through hypertrophic growth of existing muscle fibers. Thus, the accretion of breast muscle mass would involve mTOR signaling. Down regulation of genes in these pathways by cold treatment would significantly affect downstream gene action through altered translation.

The mTOR protein is central to two multi-protein complexes with distinct cellular functions that integrate cellular signals to regulate metabolism, proliferation and autophagy (Laplante and Sabatini, 2009). mTORC1 positively regulates cell growth, protein synthesis, and the activity of sterol regulatory element binding protein 1 (SREBP1) and peroxisome proliferator-activated receptor gamma (PPARG) which are key genes involved in lipid homeostasis. Less is known about the mTORC2 complex although it too plays key roles in cell survival, metabolism, proliferation and cytoskeleton organization (Laplante and Sabatini, 2009). The expanded mTOR pathway also overlaps with aspects of eIF4 translational regulation. Thus cellular stress can produce a temporal reduction in protein synthesis. In the present study a greater number of mTOR pathway genes were significantly affected by cold treatment, primarily through down regulation.

Temperature can also stimulate the transdifferentiation of satellite cells to an adipogenic phenotype. Harding et al. (2015) demonstrated that elevated temperatures *in vitro* increased lipid accumulation in both broiler *p. major* and *b. femoris* satellite cells, and decreased temperatures reduced lipid accumulation in both cell types. Clark et al. (2016b) showed that elevated temperatures in the F and RBC2 turkey *p. major* satellite cells impacted the expression of adipogenic genes and increased lipid deposition. In the current study, expression of key adipogenic genes *CEBPB* [CCAAT/enhancer binding protein (C/EBP), beta] and *PPARG* in the differentiating turkey cells was not affected by thermal treatment. However, *SCD* [stearoyl-CoA desaturase (delta-9-desaturase), an enzyme responsible for complex lipid production] was significantly up regulated in cold-treated cells (Log_2_FC = 1.403 and 1.338 in the RBC2 and F line, respectively), but also slightly up regulated in the RBC2 cells at 43°C (Log_2_FC = 1.13).

Further support for the conversion of breast muscle satellite cells to an adipogenic cell fate, was the change in expression of *NPY* in the 33–38°C comparison. NPY is a neuropeptide that is widely expressed in the central nervous system (Wang et al., 2007). The peptide is thought to function through G protein-coupled receptors to activate mitogen-activated protein kinase (MAPK), regulate intracellular calcium levels, and activate potassium channels (RefSeq, Oct 2014). Recently, Zhang et al. (2015) found that NPY plays a role in promoting adipogenesis in chickens. Although, there was no effect on proliferation, supplementation of stromal-vascular fraction cells with NPY during differentiation was associated with greater glycerol-3-phosphate dehydrogenase activity, increased expression of fatty acid binding protein 4 and lipoprotein lipase, indicative of increased lipid accumulation. Additional research on the role of NPY in satellite cells and more broadly in muscle is clearly warranted.

The post-hatch time-frame is critical and exposure of poultry to extreme temperatures, can seriously compromise the quality of meat. This study demonstrates significant alterations in gene expression and supports the hypothesis that satellite cell differentiation is directly altered in turkeys in response to ambient temperature. Numerous expression differences were observed between cells incubated at both lower (33°C) and higher (43°C) temperatures as compared to control (38°C). Greater expression differences were seen in the cold treatments where a greater number of the DE genes were down regulated. Fewer expression differences in the differentiating cells were observed between the genetic lines than observed for proliferating cells in the same experimental system (Reed et al., 2017). This suggests that the impact of temperature on satellite cells attributed to selection for fast growth may occur primarily at early points in satellite cell activation. Studies are currently underway to examine the effects of thermal challenge on *in vivo* gene expression and early muscle development of poults.

## Author contributions

KR, SV, and GS conceived and designed the experiments. SV and KM performed the experiments. KR and KM analyzed the data. KR, KM, SV, and GS drafted the manuscript. All authors read and approved the final manuscript.

### Conflict of interest statement

The authors declare that the research was conducted in the absence of any commercial or financial relationships that could be construed as a potential conflict of interest.

## Supplementary material

Figure S1 Hierarchical clustering of samples based on Euclidean distance reiterated relationships shown by PCA. Global gene expression differences among groups are illustrated in the heat map constructed from the co-expressed genes with the greatest experiment-wise differences in average normalized expression.

Figure S2 GO classification of genes expressed in cultured turkey *p. major* satellite cells after 48 h differentiation at 38°C.

Figure S3 Volcano plot showing the relationship between the ANOVA *p*-values and experiment-wise Log_2_ fold change for gene expression in *p. major* satellite cell transcriptomes during differentiation.

Figure S4 Distribution of differentially expressed genes for cold (33 vs. 38°C) and hot (43 vs. 38°C) comparisons of each line (RBC2 and F) during *p. major* satellite cell differentiation. For each temperature comparison, the number of genes with FDR *p* < 0.05 and |Log_2_FC| > 2.0 that were shared or unique to each group are indicated. Numbers in parentheses are the number of DE genes in the group that were also identified as DE in proliferating satellite cells (Reed et al., 2017).

Figure S5 Example gene networks identified from differentially expressed genes using QIAGEN's Ingenuity® Pathway Analysis (IPA®, QIAGEN Bioinformatics) software showing differential response of *p. major* satellite cell transcriptome to temperature. Depicted are the two most significant scoring gene networks identified in IPA analysis of the 70 DE genes shared among the treatment comparisons. In each panel the direction (red = up regulated, green = down regulated) and magnitude (color intensity) of expression changes are indicated. Shapes within the networks correspond to genes, gene products, or small molecules.

Figure S6 Predicted upstream effects for three DE genes (*AHR, TGFB1*, and *TGFB3*) shared among treatments that showed directional by-temperature differences in expression in IPA analysis (Ingenuity Pathway Analysis, Qiagen Bioinformatics).
