# Supplementary figures and images for: Response of Turkey Muscle Satellite Cells to Thermal Challenge. II. Transcriptome Effects in Differentiating Cells
Source: Front Physiol. 2017 Nov 30;8:948. doi: 10.3389/fphys.2017.00948 (PMC5714890; doi:10.3389/fphys.2017.00948)

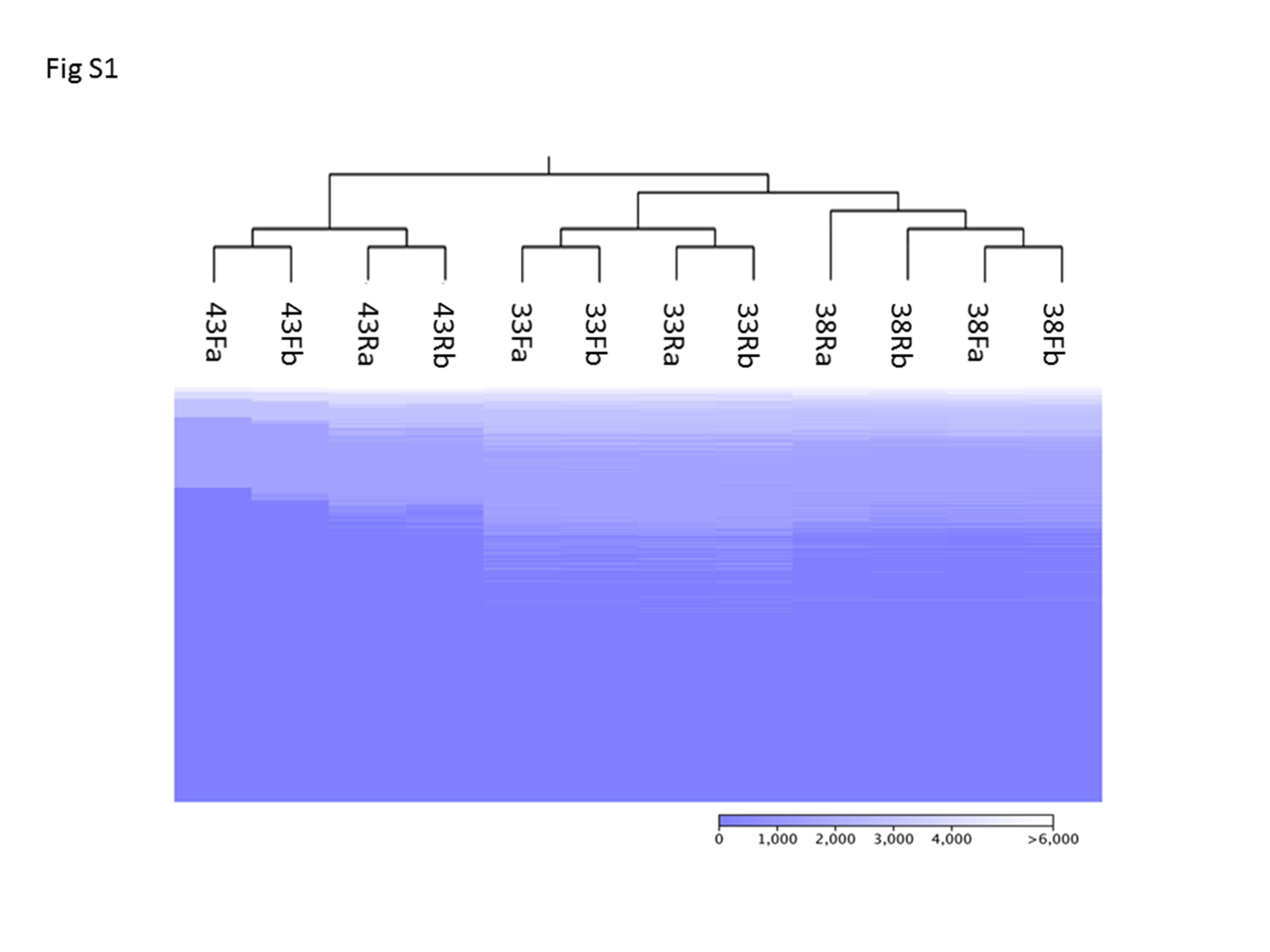

Supplement: Figure S1 — Hierarchical clustering of samples based on Euclidean distance reiterated relationships shown by PCA. Global gene expression differences among groups are illustrated in the heat map constructed from the co-expressed genes with the greatest experiment-wise differences in average normalized expression. [file Image1.TIF]

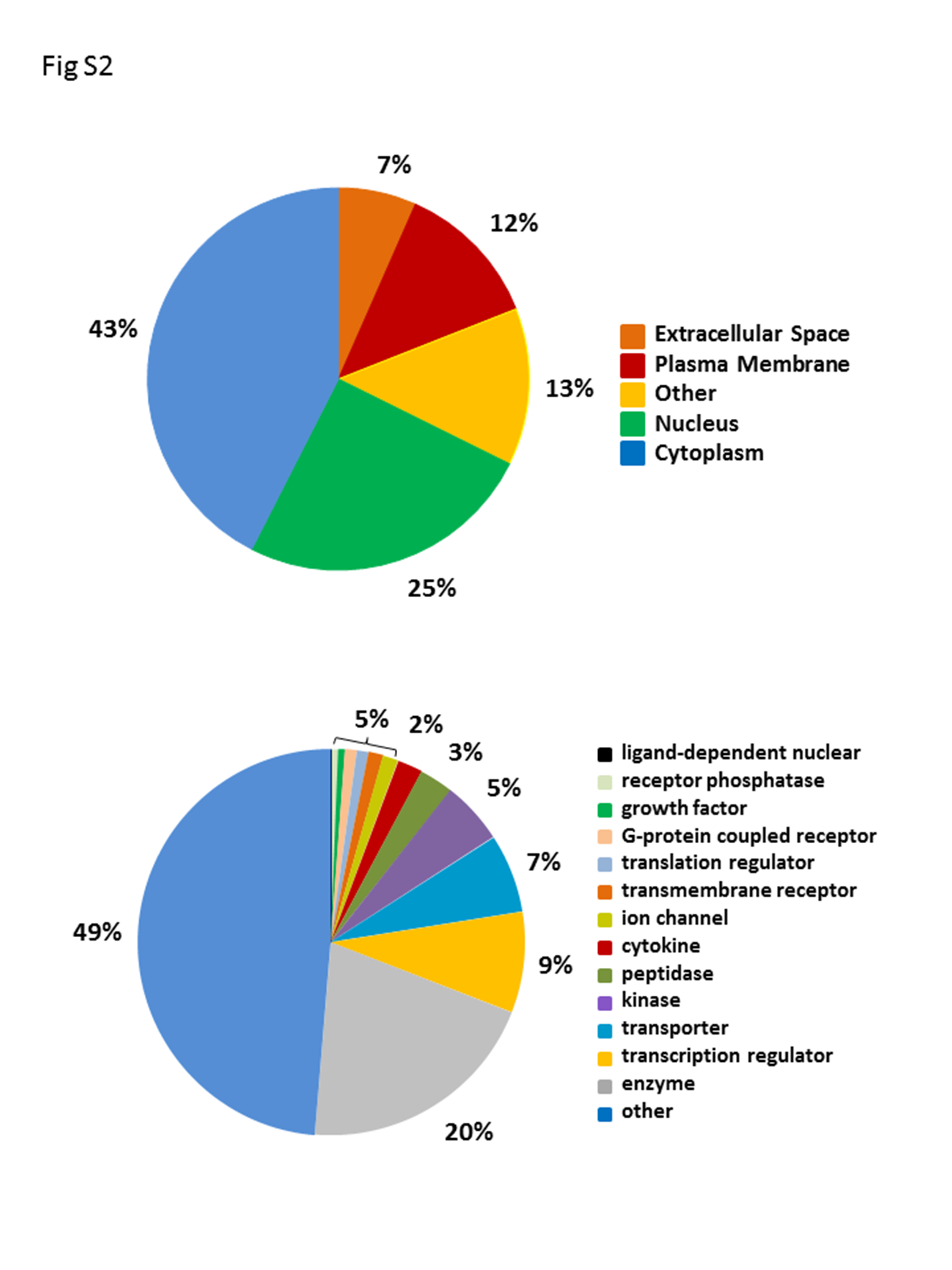

Supplement: Figure S2 — GO classification of genes expressed in cultured turkey p. major satellite cells after 48 h differentiation at 38°C. [file Image2.TIF]

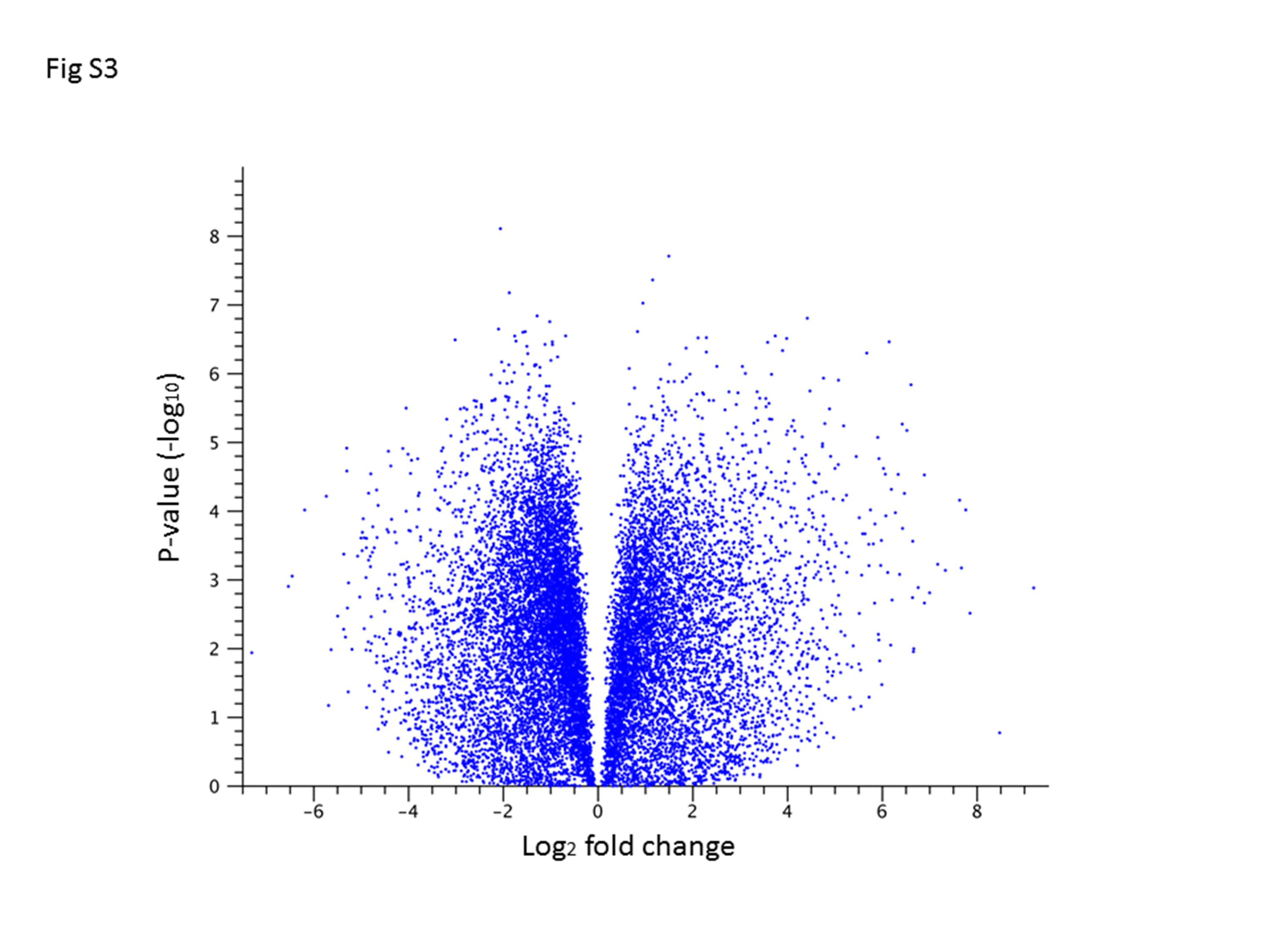

Supplement: Figure S3 — Volcano plot showing the relationship between the ANOVA p-values and experiment-wise Log2 fold change for gene expression in p. major satellite cell transcriptomes during differentiation. [file Image3.TIF]

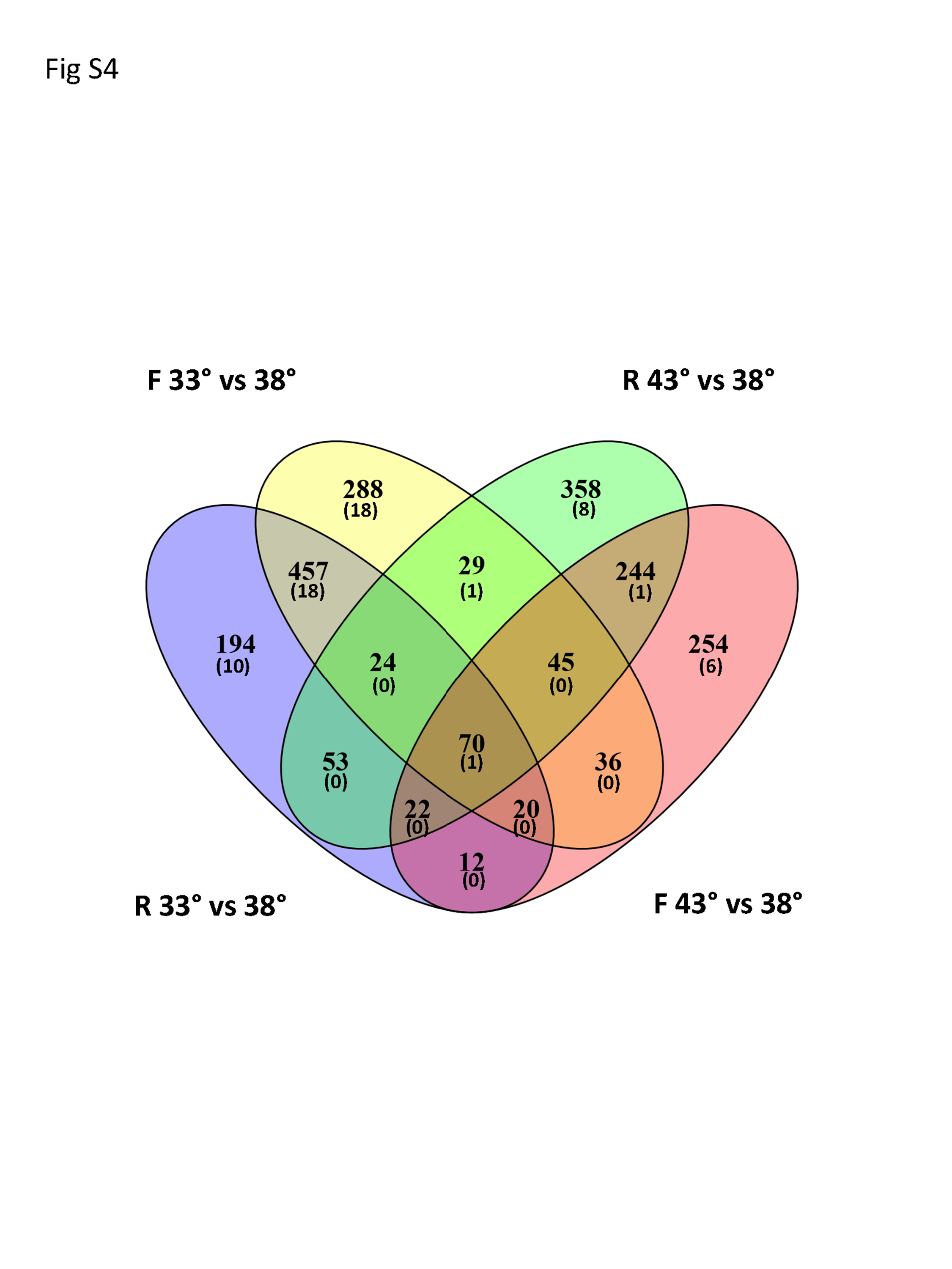

Supplement: Figure S4 — Distribution of differentially expressed genes for cold (33 vs. 38°C) and hot (43 vs. 38°C) comparisons of each line (RBC2 and F) during p. major satellite cell differentiation. For each temperature comparison, the number of genes with FDR p < 0.05 and |Log2FC| > 2.0 that were shared or unique to each group are indicated. Numbers in parentheses are the number of DE genes in the group that were also identified as DE in proliferating satellite cells (Reed et al., 2017). [file Image4.TIFF]

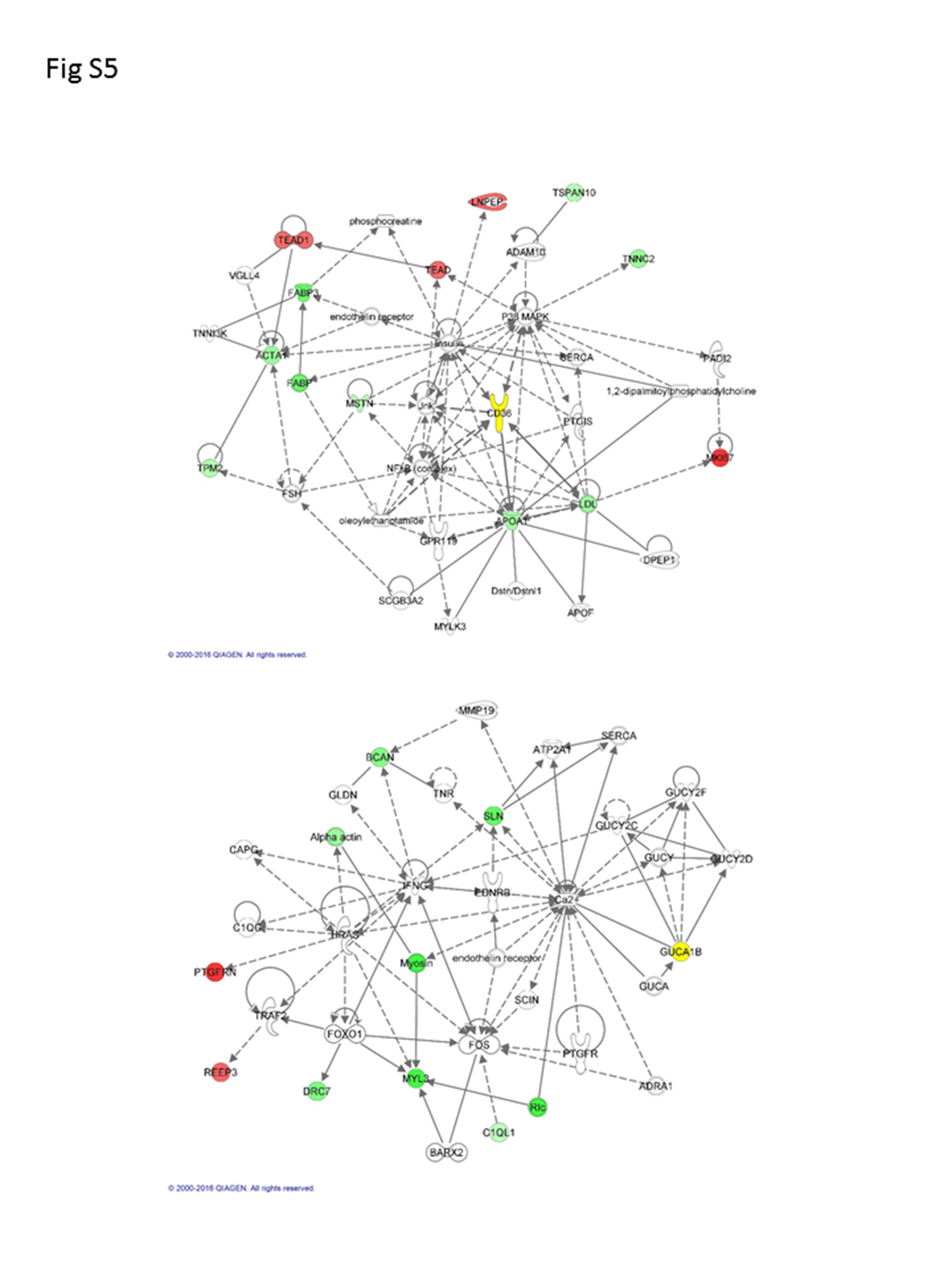

Supplement: Figure S5 — Example gene networks identified from differentially expressed genes using QIAGEN's Ingenuity® Pathway Analysis (IPA®, QIAGEN Bioinformatics) software showing differential response of p. major satellite cell transcriptome to temperature. Depicted are the two most significant scoring gene networks identified in IPA analysis of the 70 DE genes shared among the treatment comparisons. In each panel the direction (red = up regulated, green = down regulated) and magnitude (color intensity) of expression changes are indicated. Shapes within the networks correspond to genes, gene products, or small molecules. [file Image5.TIF]

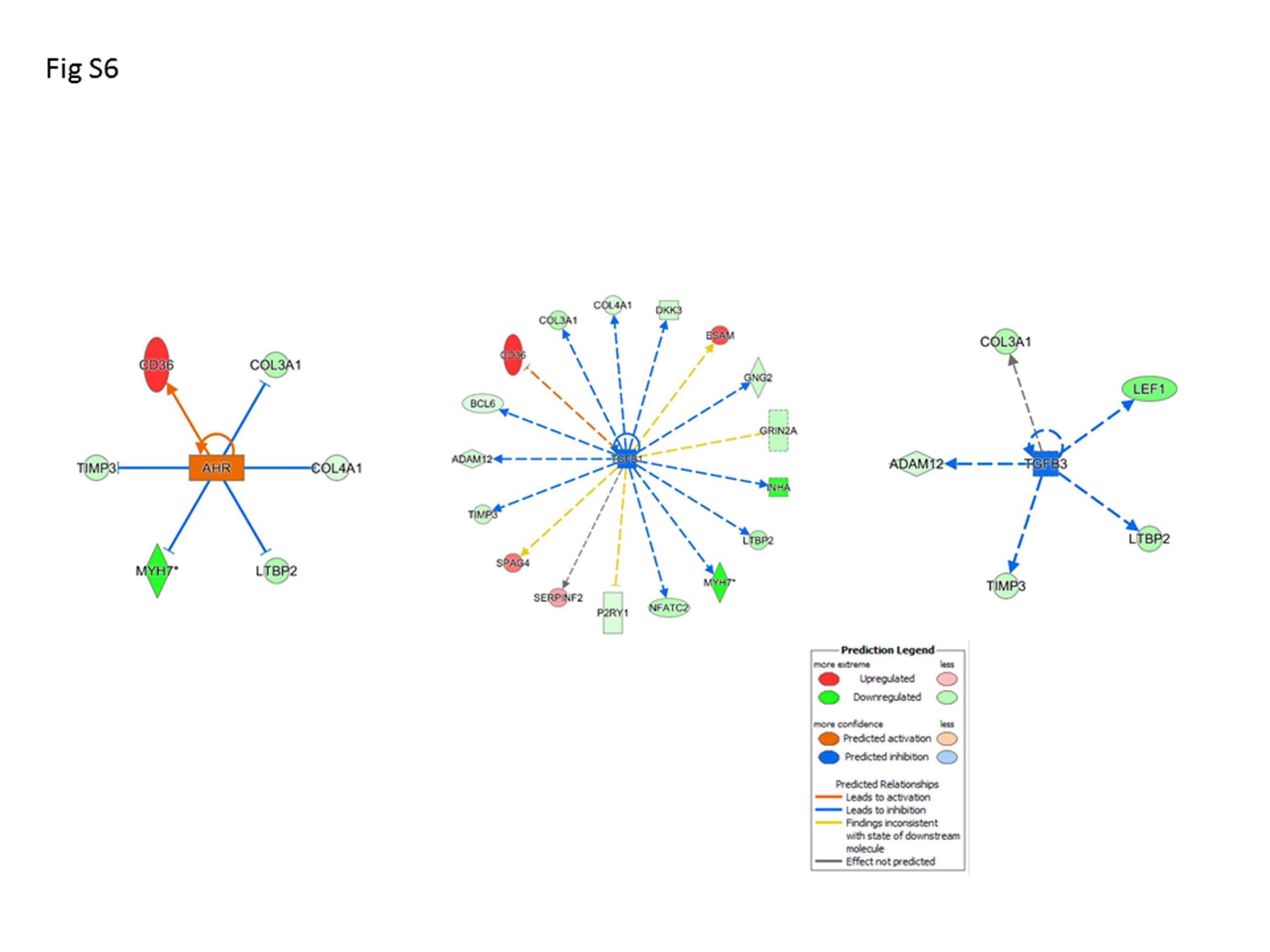

Supplement: Figure S6 — Predicted upstream effects for three DE genes (AHR, TGFB1, and TGFB3) shared among treatments that showed directional by-temperature differences in expression in IPA analysis (Ingenuity Pathway Analysis, Qiagen Bioinformatics). [file Image6.TIF]
