# Supplementary material for: Response of Turkey Muscle Satellite Cells to Thermal Challenge. II. Transcriptome Effects in Differentiating Cells
Source: Front Physiol. 2017 Nov 30;8:948. doi: 10.3389/fphys.2017.00948 (PMC5714890; doi:10.3389/fphys.2017.00948)
Supplement: Supplementary file 7 [file Table1.PDF]

Table S1. Mean quality-trimmed RNAseq read counts for turkey *p. major* muscle satellite cells from two genetic lines (RBC2 and F) after 48 hr differentiation. Cells were cultured at 33°, 38° or 43° C. For each gene, the average and standard deviation are given.

	RBC2			F-Line				
**Gene**	**33**	**38**	**43**	**33**	**38**	**43**	**Average**	**SD**
A1CF	2.0	0.5	1.5	1.5	0.5	2.0	1.3	0.68
A2M	10.5	11.0	14.0	10.5	9.0	15.0	11.7	2.32
A4GALT	155.5	77.0	116.0	155.5	107.0	128.5	123.3	30.21
A4GNT	0.0	0.0	0.0	0.0	0.0	0.0	0.0	0.00
AAAS	1221.0	946.0	500.5	972.5	810.5	471.5	820.3	291.05
AACS	1701.5	711.0	1383.5	1534.0	720.5	953.5	1167.3	428.98
AADAC	58.0	39.0	62.0	60.5	43.5	43.5	51.1	10.17
AADAT	362.0	266.0	374.0	304.5	287.5	340.0	322.3	43.03
AAED1	165.5	74.5	71.0	147.0	66.5	76.0	100.1	44.02
AAGAB	287.5	175.0	336.5	284.0	179.0	283.0	257.5	65.52
AAK1	101.5	78.0	197.0	122.5	88.0	206.0	132.2	55.81
AAMDC	206.5	173.0	223.5	157.0	194.0	202.5	192.8	24.08
AAMP	991.5	1110.5	798.5	981.0	1137.5	904.5	987.3	126.73
AANAT	1.0	3.5	0.5	2.5	5.0	2.0	2.4	1.66
AAR2	39.5	23.5	49.0	38.5	27.0	35.0	35.4	9.21
AARS	3533.5	2670.0	3021.0	3130.0	2461.5	2323.5	2856.6	454.99
AARS2	407.5	333.5	241.0	400.5	302.0	262.5	324.5	69.38
AARSD1	863.5	808.0	472.0	787.5	900.5	487.5	719.8	190.28
AASDH	173.5	76.0	117.5	160.0	90.0	123.0	123.3	38.07
AASDHPPT	143.0	128.0	97.0	142.5	157.5	108.0	129.3	23.05
AASS	87.5	52.0	45.0	88.5	68.0	51.5	65.4	19.07
AATF	104.5	79.0	70.0	84.5	89.5	66.5	82.3	13.87
ABAT	908.0	809.5	761.5	1016.0	1035.5	1084.0	935.8	130.72
ABCA1	430.0	658.5	347.5	516.5	857.5	709.0	586.5	189.81
ABCA12	25.0	11.0	7.5	22.0	14.0	8.5	14.7	7.26
ABCA13	33.0	26.5	16.5	25.0	16.5	15.0	22.1	7.21
ABCA2	170.5	227.5	554.5	146.5	212.0	524.5	305.9	183.46
ABCA3	2364.5	923.0	1447.0	1976.0	798.0	1340.5	1474.8	604.01
ABCA4	4.5	6.0	11.5	3.5	6.5	7.5	6.6	2.80
ABCA5	253.5	218.5	341.5	301.5	278.0	443.5	306.1	79.23
ABCA7	618.5	665.0	200.5	485.5	651.5	179.5	466.8	223.70
ABCB10	226.0	104.5	122.5	198.5	102.0	103.5	142.8	54.98
ABCB11	154.0	148.0	96.0	142.0	183.5	122.5	141.0	29.64
ABCB4	0.0	0.0	0.5	0.0	0.5	0.0	0.2	0.26
ABCB5	0.5	1.0	1.0	1.5	0.5	0.5	0.8	0.41
ABCB6	526.0	236.5	177.5	527.5	276.0	206.5	325.0	159.65
ABCB7	455.0	224.5	496.0	435.0	291.0	404.5	384.3	104.59
ABCB9	31.5	16.5	23.0	26.0	20.5	26.0	23.9	5.17
ABCC1	1362.0	509.0	596.5	1326.5	602.0	670.0	844.3	390.76
ABCC10	325.0	190.0	134.0	313.5	188.0	121.5	212.0	87.64
ABCC2	54.5	69.5	54.5	51.0	69.0	66.0	60.8	8.31
ABCC3	0.5	1.0	1.0	0.5	1.0	1.5	0.9	0.38
ABCC5	873.5	501.0	700.5	759.5	568.5	650.5	675.6	133.72
ABCC6	446.5	230.5	174.0	368.5	259.0	191.5	278.3	107.24
ABCC8	7.5	8.0	9.5	2.5	8.5	11.0	7.8	2.89
ABCC9	3.5	52.5	71.0	2.5	51.5	63.0	40.7	30.05
ABCD4	316.5	263.0	229.0	276.5	322.5	259.0	277.8	35.92
ABCE1	2184.5	1728.5	1663.0	2100.0	1864.0	1512.5	1842.1	259.94

ABCF2	5210.0	3851.5	2251.0	5232.5	4052.5	2179.5	3796.2	1351.44
ABCF3	1341.0	1268.0	862.5	1250.5	1188.0	870.0	1130.0	210.05
ABCG1	0.5	0.5	0.0	1.5	2.0	1.0	0.9	0.74
ABCG2	85.0	22.5	45.0	38.5	26.5	14.0	38.6	25.32
ABCG4	14.0	2.5	3.5	8.0	3.0	2.0	5.5	4.69
ABCG5	52.5	39.0	32.5	54.0	43.5	34.0	42.6	9.14
ABCG8	0.0	0.0	0.0	0.0	0.0	0.0	0.0	0.00
ABHD1	270.5	206.5	141.0	299.5	215.0	178.5	218.5	58.36
ABHD10	421.5	266.5	198.0	401.0	273.0	177.5	289.6	101.56
ABHD11	117.5	170.5	147.5	118.5	221.5	158.5	155.7	38.64
ABHD12	350.5	276.5	348.0	418.5	311.5	397.5	350.4	52.60
ABHD12B	366.5	274.5	323.0	366.0	254.5	280.0	310.8	48.44
ABHD13	387.5	276.5	293.5	402.0	302.0	260.0	320.3	59.66
ABHD17A	553.0	804.0	769.5	512.0	796.0	754.0	698.1	130.16
ABHD17B	280.5	299.0	258.5	309.5	392.0	231.5	295.2	55.15
ABHD17C	159.5	143.0	230.5	177.0	182.5	237.0	188.3	37.94
ABHD2	1989.5	558.0	757.0	1828.0	682.5	638.0	1075.5	650.65
ABHD3	12.5	19.0	12.0	17.5	34.5	32.5	21.3	9.83
ABHD5	336.5	325.5	513.5	323.0	361.0	439.5	383.2	77.17
ABHD6	243.0	94.5	48.5	238.5	80.5	47.5	125.4	91.19
ABHD8	12.0	6.0	3.0	8.5	2.5	1.5	5.6	4.07
ABI1	526.5	318.0	726.5	569.0	355.5	678.5	529.0	165.88
ABI2	1192.0	1225.0	1356.5	1167.0	1443.5	1313.0	1282.8	106.92
ABI3	3.5	2.5	1.0	6.5	2.0	1.0	2.8	2.07
ABI3BP	67.5	137.0	184.5	62.5	112.0	167.0	121.8	50.53
ABL1	1799.5	1402.0	2206.5	1859.0	1551.0	2001.5	1803.3	293.14
ABL2	44.0	39.0	128.0	49.5	52.5	128.0	73.5	42.47
ABLIM1	152.5	116.0	123.0	255.5	145.0	168.5	160.1	50.55
ABLIM2	5.0	16.5	48.5	9.0	21.0	56.0	26.0	21.22
ABLIM3	58.5	1013.0	720.0	60.5	1083.5	760.5	616.0	453.27
ABR	1394.0	1408.0	1296.0	1314.5	1603.0	1350.5	1394.3	111.08
ABRA	503.5	561.0	578.0	367.0	655.5	632.0	549.5	104.32
ABRACL	147.0	178.0	208.0	157.5	200.5	206.0	182.8	26.20
ABTB1	1523.5	1211.0	1640.5	1430.5	1482.0	1679.0	1494.4	167.82
ABTB2	52.5	22.0	70.0	55.0	32.0	47.0	46.4	17.15
ACAA1	627.5	311.5	332.0	536.5	335.5	237.5	396.8	150.56
ACAA2	5915.5	7146.0	3521.5	5719.0	7761.5	3046.0	5518.3	1896.25
ACACB	89.0	23.5	5.5	52.5	25.5	2.5	33.1	32.71
ACAD10	100.5	42.5	24.0	79.0	30.5	23.5	50.0	32.27
ACAD11	299.5	215.5	269.0	274.0	254.5	300.5	268.8	31.69
ACAD8	317.0	287.5	212.5	277.5	275.0	198.0	261.3	46.10
ACAD9	3483.5	2573.0	1989.5	3036.0	2857.0	1905.0	2640.7	613.72
ACADL	1937.5	1725.0	1498.5	1742.5	1735.0	1136.5	1629.2	278.60
ACADM	623.5	960.5	782.0	595.5	1129.0	719.5	801.7	206.77
ACADS	475.5	715.5	649.0	457.5	777.5	618.5	615.6	128.02
ACADSB	869.5	531.5	629.5	801.0	599.5	549.0	663.3	139.38
ACAN	0.0	0.0	0.0	0.0	0.0	0.0	0.0	0.00
ACAP2	3393.5	1993.5	1971.5	3270.0	2303.0	2116.0	2507.9	650.07
ACAP3	39.5	80.0	148.5	57.5	110.5	153.0	98.2	47.15
ACAT1	770.5	548.5	525.5	745.5	540.5	413.5	590.7	138.74
ACAT2	2422.0	1093.0	1287.5	2271.0	1102.5	866.5	1507.1	665.49
ACBD3	751.5	840.5	821.0	730.5	940.5	849.5	822.3	75.48
ACBD5	415.0	345.5	428.0	422.5	354.0	380.5	390.9	36.02
ACBD6	420.0	332.5	209.0	403.5	383.0	222.5	328.4	92.20

ACBD7	89.5	30.5	23.5	66.5	43.5	22.5	46.0	26.90
ACCS	247.5	201.5	221.0	190.5	221.0	224.0	217.6	19.76
ACE	16.5	195.0	215.0	18.0	197.0	183.5	137.5	93.69
ACE2	1.0	0.0	0.5	2.5	0.5	0.0	0.8	0.94
ACER1	0.0	0.0	0.0	0.0	0.0	0.0	0.0	0.00
ACER2	140.5	84.5	83.0	149.0	89.5	90.5	106.2	30.14
ACKR2	0.5	0.5	0.5	1.5	0.5	1.0	0.8	0.42
ACKR3	8.0	3.0	2.5	5.5	2.5	3.0	4.1	2.22
ACKR4	1.5	8.5	6.5	3.0	7.5	2.0	4.8	3.03
ACLY	3009.5	1502.0	2985.5	2854.5	1654.5	2368.0	2395.7	675.86
ACMSD	2.5	14.5	5.0	1.5	10.5	3.5	6.3	5.14
ACO1	919.0	607.0	847.0	889.0	649.5	709.5	770.2	131.94
ACO2	982.0	2231.0	3210.5	1053.0	2361.0	2822.0	2109.9	914.91
ACOT11	696.5	1131.0	1110.0	538.5	1195.0	951.5	937.1	264.95
ACOT12	2.0	1.5	2.5	2.0	1.0	2.5	1.9	0.58
ACOT13	305.5	349.5	304.0	280.0	374.0	265.5	313.1	41.32
ACOT7	308.0	204.5	200.5	330.0	193.0	202.5	239.8	61.90
ACOT8	362.0	503.0	243.5	265.0	502.0	218.5	349.0	128.46
ACOT9	2146.0	1630.0	1936.0	1901.0	1917.5	1763.0	1882.3	174.30
ACOX1	1714.0	993.5	896.0	1475.0	1057.5	883.5	1169.9	343.49
ACOX2	29.5	32.5	72.0	48.5	33.0	39.5	42.5	15.97
ACOX3	211.0	130.5	154.5	240.0	166.0	165.0	177.8	40.14
ACP1	643.0	682.5	551.5	588.0	761.0	474.5	616.8	101.13
ACP2	601.5	405.5	525.5	640.5	455.0	503.0	521.8	88.09
ACP5	108.5	56.0	21.5	89.0	51.5	12.5	56.5	37.27
ACP6	274.5	238.0	152.0	222.5	284.5	147.5	219.8	58.89
ACR	7.5	11.5	22.0	7.5	15.0	16.5	13.3	5.65
ACRBP	0.0	0.0	0.0	0.0	0.0	0.0	0.0	0.00
ACRC	108.5	47.0	71.0	118.0	46.0	42.5	72.2	33.53
ACRV1	44.5	132.0	140.5	42.5	109.0	173.5	107.0	53.37
ACSBG1	57.0	45.5	25.5	41.5	47.5	29.5	41.1	11.76
ACSBG2	866.5	350.0	783.5	804.0	315.0	641.0	626.7	239.77
ACSF2	586.5	426.5	321.0	556.5	478.5	431.5	466.8	96.58
ACSL1	252.0	104.5	193.0	264.0	100.5	150.0	177.3	71.12
ACSL3	282.0	272.5	200.5	312.0	312.5	223.0	267.1	46.28
ACSL4	466.0	394.0	670.5	409.5	526.0	612.5	513.1	111.32
ACSL5	100.0	90.5	67.5	81.0	83.0	49.5	78.6	17.86
ACSL6	1.0	1.5	2.5	1.5	1.5	1.5	1.6	0.49
ACSM3	6.0	2.0	0.5	4.0	2.0	0.0	2.4	2.25
ACSS1	200.0	100.5	131.5	206.0	104.5	135.0	146.3	46.13
ACSS2	1107.5	937.0	634.5	1005.0	966.5	632.5	880.5	199.82
ACSS3	25.0	39.0	32.0	21.5	47.0	44.0	34.8	10.31
ACTA1	855.5	6147.5	971.5	715.5	6395.0	700.0	2630.8	2822.68
ACTA2	1942.5	994.0	1632.5	2504.0	1174.0	1073.5	1553.4	591.35
ACTB	193818.5	137666.5	101394.5	188589.5	150500.5	95563.0	144588.8	41741.51
ACTC1	6618.5	74186.0	59715.0	5652.5	86005.5	54892.5	47845.0	34118.81
ACTL6A	522.0	203.0	364.0	595.5	237.0	295.5	369.5	158.19
ACTL9	0.0	0.0	0.0	0.0	0.0	0.0	0.0	0.00
ACTN1	6551.0	3904.0	4507.5	7317.0	4424.5	4541.0	5207.5	1378.51
ACTN2	4093.5	8765.0	10127.5	3335.0	10676.0	8473.0	7578.3	3112.95
ACTR1A	4946.5	6111.0	5624.5	4967.5	6687.0	5400.0	5622.8	679.31
ACTR2	1335.0	722.0	870.0	1449.5	746.5	693.5	969.4	334.98
ACTR3	3454.5	2282.5	2685.0	3793.5	2569.0	2474.5	2876.5	603.43
ACTR3B	1417.0	867.0	591.5	1354.0	949.5	554.0	955.5	366.97

ACTR5	178.5	146.0	116.5	191.5	139.0	101.0	145.4	34.84
ACTR6	964.0	1073.0	847.0	958.5	1174.5	764.0	963.5	148.26
ACTR8	429.5	369.0	374.0	455.0	440.0	413.5	413.5	35.27
ACTRT2	0.0	0.0	0.0	0.0	0.0	0.0	0.0	0.00
ACVR1	1830.5	1286.5	1794.5	1844.5	1488.5	1937.5	1697.0	252.55
ACVR1B	41.0	31.0	99.0	50.0	36.0	97.5	59.1	30.98
ACVR1C	0.5	0.0	0.5	0.0	0.0	0.5	0.3	0.27
ACVR2A	358.5	155.0	362.5	340.5	170.5	300.5	281.3	94.50
ACVR2B	23.0	14.5	15.0	10.5	15.0	9.0	14.5	4.88
ACVRL1	1.5	2.0	4.5	1.0	2.5	3.5	2.5	1.30
ACY1	494.0	358.0	254.0	441.5	358.5	261.5	361.3	95.43
ACYP1	86.5	112.0	71.5	98.0	102.5	95.0	94.3	13.97
ACYP2	478.5	576.0	317.0	398.5	669.0	298.5	456.3	146.77
ADA	16.5	26.5	51.5	22.5	37.5	61.0	35.9	17.44
ADAD1	1.0	2.0	0.5	1.5	1.5	2.0	1.4	0.58
ADAL	217.5	305.0	273.0	184.0	385.0	260.0	270.8	70.30
ADAM11	2.5	5.5	2.0	3.5	3.0	3.5	3.3	1.21
ADAM17	1219.0	753.5	1069.0	1244.5	870.5	1033.0	1031.6	192.43
ADAM19	0.0	0.0	1.0	0.5	0.5	0.5	0.4	0.38
ADAM22	8.0	6.0	16.0	11.0	5.5	15.5	10.3	4.62
ADAM23	1030.5	603.0	1074.0	775.5	697.0	970.5	858.4	193.30
ADAM33	395.0	985.0	873.0	359.5	1055.0	845.0	752.1	300.30
ADAM8	1572.5	1239.5	1765.5	1842.0	1410.5	1817.0	1607.8	244.66
ADAM9	813.5	555.5	966.0	710.5	602.0	835.5	747.2	154.43
ADAMTS1	180.0	124.5	220.5	85.5	107.0	107.0	137.4	51.82
ADAMTS10	106.5	513.5	410.5	98.0	567.0	750.0	407.6	260.91
ADAMTS13	165.0	64.0	97.5	121.5	85.5	100.5	105.7	34.66
ADAMTS14	716.5	1127.5	2572.0	755.5	1225.5	2776.5	1528.9	911.69
ADAMTS15	20.0	7.0	3.0	20.5	12.0	2.0	10.8	8.16
ADAMTS17	180.5	78.5	157.0	165.5	87.0	137.0	134.3	42.38
ADAMTS19	5.5	3.0	14.5	4.0	4.5	6.5	6.3	4.18
ADAMTS2	465.5	677.0	2103.0	643.0	866.0	2368.0	1187.1	826.25
ADAMTS20	181.5	132.5	243.0	167.0	147.0	218.0	181.5	42.23
ADAMTS3	38.0	8.0	34.5	29.5	9.0	15.0	22.3	13.28
ADAMTS4	44.0	51.0	58.0	30.0	37.5	59.0	46.6	11.56
ADAMTS5	29.0	17.5	83.0	30.5	23.5	72.0	42.6	27.65
ADAMTS6	190.0	125.0	203.0	216.0	158.5	250.5	190.5	44.09
ADAMTS7	18.5	36.5	64.5	17.0	44.0	116.5	49.5	37.23
ADAMTS8	16.5	21.0	31.5	16.0	27.5	31.5	24.0	7.13
ADAMTS9	38.5	101.0	60.5	57.0	100.0	104.0	76.8	28.24
ADAMTSL1	73.5	35.5	78.0	37.0	34.0	44.0	50.3	20.03
ADAMTSL2	16253.5	12683.5	10030.0	17116.0	15575.5	10627.5	13714.3	3021.53
ADAMTSL3	211.0	109.5	296.0	235.0	122.5	248.0	203.7	73.46
ADAMTSL5	3.5	15.0	13.0	1.0	17.0	26.0	12.6	9.19
ADAP2	56.5	39.0	33.0	64.5	41.0	41.5	45.9	11.96
ADAR	490.5	437.0	519.5	508.0	467.5	551.5	495.7	40.25
ADARB1	148.5	158.5	306.0	157.0	191.0	327.0	214.7	80.47
ADARB2	0.0	4.5	0.0	0.0	5.5	3.0	2.2	2.50
ADAT1	45.5	31.0	31.5	55.5	33.0	30.5	37.8	10.34
ADCK1	107.0	71.0	74.5	110.5	73.0	67.5	83.9	19.41
ADCK2	214.0	82.0	51.5	168.5	83.0	52.0	108.5	67.12
ADCK3	1244.5	1278.0	1797.5	1022.0	1449.5	1685.5	1412.8	290.84
ADCY2	220.0	427.5	493.0	154.5	533.0	514.5	390.4	162.68
ADCY3	0.5	0.0	5.5	0.5	1.0	3.0	1.8	2.12

ADCY5	373.5	248.0	346.0	375.0	314.0	300.0	326.1	48.91
ADCY8	0.0	12.0	20.0	0.0	5.5	15.0	8.8	8.24
ADCYAP1	1805.5	1511.5	1441.0	2037.5	1680.5	1434.5	1651.8	238.56
ADCYAP1R1	6.5	1.5	4.5	3.5	0.5	1.5	3.0	2.26
ADD1	2998.0	2390.0	3380.5	2990.0	2650.0	3174.0	2930.4	357.97
ADD2	3.0	1.0	4.0	2.0	2.0	1.0	2.2	1.17
ADD3	256.5	173.0	258.0	256.0	204.5	268.0	236.0	38.18
ADGB	0.0	0.5	0.0	0.5	0.0	0.0	0.2	0.26
ADHFE1	76.5	67.0	75.5	75.0	66.0	58.5	69.8	7.13
ADI1	321.5	215.0	253.0	317.0	205.5	210.5	253.8	53.46
ADIPOQ	237.0	146.5	114.0	117.0	162.5	99.0	146.0	50.25
ADIPOR1	1062.5	918.0	913.0	991.5	1058.5	838.5	963.7	89.28
ADIPOR2	2584.5	1416.5	1723.0	2585.5	1599.0	1590.5	1916.5	526.94
ADIRF	2.0	2.5	0.5	4.0	1.0	0.0	1.7	1.47
ADK	3264.0	3634.0	2729.0	3492.0	4060.0	2468.5	3274.6	590.06
ADNP	2329.5	2281.5	3037.5	2259.5	2638.5	2909.0	2575.9	339.25
ADNP2	275.5	247.5	475.5	276.0	310.0	444.0	338.1	96.82
ADORA2A	351.0	140.5	390.0	319.5	140.5	303.5	274.2	107.68
ADORA2B	4.5	10.5	34.5	3.5	9.0	18.5	13.4	11.63
ADPGK	462.5	317.0	537.5	508.5	333.5	512.5	445.3	96.19
ADPRH	2159.0	1197.5	1582.5	2040.0	1339.0	1519.5	1639.6	383.04
ADPRHL1	0.0	2.5	1.0	0.5	2.5	2.5	1.5	1.14
ADPRHL2	1007.5	696.5	1133.0	931.0	783.0	1230.0	963.5	203.28
ADPRM	402.0	396.5	241.5	380.5	409.5	216.0	341.0	87.84
ADRA1A	0.0	0.0	1.0	0.5	0.0	1.5	0.5	0.63
ADRA1B	1.5	1.5	1.0	4.0	3.0	0.0	1.8	1.44
ADRA1D	26.5	27.5	34.5	17.0	22.0	16.5	24.0	6.90
ADRA2A	58.0	106.5	31.0	99.0	113.0	84.0	81.9	31.72
ADRA2B	19.0	11.0	9.0	10.0	8.5	14.5	12.0	4.04
ADRA2C	1.5	0.5	1.0	0.0	0.0	0.5	0.6	0.58
ADRB1	0.0	0.0	0.0	0.5	0.0	0.0	0.1	0.20
ADRB2	1.5	6.5	18.0	1.0	5.5	6.5	6.5	6.14
ADRB3	0.0	0.0	0.0	0.0	0.0	0.0	0.0	0.00
ADRBK1	845.0	731.5	587.0	754.5	690.0	600.0	701.3	97.82
ADSL	1552.5	1243.0	1020.5	1343.0	1352.0	1022.5	1255.6	207.29
ADSSL1	1174.0	947.5	869.0	1505.5	1147.5	890.5	1089.0	241.64
ADTRP	3.0	4.5	2.5	1.0	3.0	0.5	2.4	1.46
AEBP1	852.5	1539.5	1820.5	898.5	1598.0	2314.0	1503.8	558.11
AEBP2	323.0	199.0	292.0	343.0	229.0	259.0	274.2	55.43
AEN	277.5	312.0	318.5	277.0	320.5	343.5	308.2	26.21
AFAP1	856.5	1239.0	3431.0	981.5	1471.0	3460.5	1906.6	1210.98
AFAP1L1	1237.0	1528.5	1303.5	997.0	1560.0	1223.5	1308.3	210.26
AFAP1L2	1302.0	1816.0	2526.0	1345.0	1933.0	2386.5	1884.8	510.09
AFF1	2284.5	1284.5	2259.0	1992.5	1284.0	1870.0	1829.1	450.40
AFF4	447.0	201.0	783.0	534.5	254.0	689.0	484.8	231.66
AFG3L2	1478.0	1027.0	1106.5	1339.0	1191.5	1056.0	1199.7	176.68
AFMID	190.0	246.0	281.5	166.0	226.5	227.5	222.9	40.80
AFTPH	333.0	256.0	388.0	383.5	301.5	347.5	334.9	50.37
AGAP1	540.5	396.5	773.5	467.5	419.5	790.5	564.7	175.49
AGAP3	448.5	502.5	387.0	427.5	449.0	438.5	442.2	37.41
AGBL1	16.0	115.5	69.0	13.5	104.5	28.0	57.8	45.24
AGBL2	0.5	0.0	0.5	1.0	0.5	0.0	0.4	0.38
AGBL3	4.5	2.5	2.0	4.5	1.5	2.0	2.8	1.33
AGBL4	1.0	0.5	0.0	0.0	0.5	1.0	0.5	0.45

AGBL5	167.5	163.5	109.5	176.0	172.5	138.0	154.5	25.82
AGFG1	843.5	795.0	885.5	879.0	908.0	830.0	856.8	41.60
AGGF1	415.5	301.0	326.5	318.5	304.5	300.5	327.8	44.24
AGK	86.0	40.5	43.0	91.5	36.0	36.5	55.6	25.88
AGMAT	0.0	0.5	4.0	1.5	4.5	2.5	2.2	1.83
AGMO	0.5	0.0	1.5	0.0	2.5	1.5	1.0	1.00
AGO1	371.5	229.5	868.0	412.0	255.0	755.0	481.8	266.80
AGO2	24.0	19.0	55.5	24.5	22.0	50.5	32.6	16.01
AGO3	105.5	55.5	145.5	107.0	58.0	118.5	98.3	35.27
AGO4	457.5	449.5	705.0	413.5	496.0	673.0	532.4	124.50
AGPAT2	336.5	320.0	184.5	276.0	290.0	172.5	263.3	69.14
AGPAT3	301.0	187.0	255.0	296.0	205.5	201.0	240.9	50.19
AGPAT4	359.0	120.0	139.0	339.5	151.5	123.5	205.4	112.15
AGPAT5	61.5	17.0	63.0	64.5	23.0	55.5	47.4	21.54
AGPAT6	1092.5	804.5	748.5	1049.0	879.5	727.0	883.5	154.95
AGPAT9	287.0	194.5	172.5	232.5	205.5	188.5	213.4	41.22
AGPS	713.5	629.5	718.0	701.5	678.0	667.0	684.6	33.54
AGR2	0.0	0.0	0.0	0.0	0.0	0.0	0.0	0.00
AGR3	0.5	0.0	0.0	1.5	0.5	0.0	0.4	0.58
AGRN	178.5	445.0	618.0	208.0	466.5	894.0	468.3	266.80
AGRP	6.5	3.0	4.0	3.0	3.0	2.5	3.7	1.47
AGT	4.0	1.5	1.5	4.0	2.0	3.0	2.7	1.17
AGTPBP1	1.5	0.0	2.0	2.5	0.0	0.0	1.0	1.14
AGTR1	45.0	33.5	61.5	56.5	42.5	39.0	46.3	10.66
AGTR2	0.0	0.0	1.0	0.5	0.0	0.0	0.3	0.42
AGTRAP	42.5	17.0	19.0	31.0	26.0	15.0	25.1	10.42
AGXT	0.0	0.0	0.0	0.0	0.0	0.0	0.0	0.00
AGXT2	3.5	1.0	0.5	9.0	0.5	0.0	2.4	3.46
AHCTF1	716.5	453.0	678.0	737.5	509.0	621.0	619.2	115.49
AHCY	11673.5	17872.0	12565.0	9666.0	19782.0	11326.0	13814.1	4040.35
AHCYL1	303.0	239.5	291.5	274.0	262.5	308.5	279.8	26.29
AHCYL2	533.5	914.0	1242.5	625.0	1066.0	1102.0	913.8	280.95
AHI1	116.0	49.0	78.0	98.5	70.5	58.5	78.4	25.06
AHNAK2	1266.0	346.5	1092.0	982.5	394.0	792.0	812.2	375.61
AHR	306.5	180.0	423.0	387.0	223.5	549.0	344.8	136.33
AHRR	1385.5	1202.0	1282.5	1575.5	1460.5	1640.5	1424.4	168.49
AHSA1	957.0	1444.5	1296.0	964.0	1633.0	1460.0	1292.4	278.42
AHSA2	572.5	412.5	613.0	533.5	459.5	604.5	532.6	81.23
AHSG	0.5	0.0	0.0	0.0	0.5	0.5	0.3	0.27
AICDA	10.0	4.5	0.5	11.5	4.5	1.0	5.3	4.55
AIDA	543.5	628.5	617.0	538.0	710.0	547.5	597.4	67.76
AIF1L	1961.0	4332.0	4579.5	2459.0	4685.5	3298.0	3552.5	1160.98
AIFM1	1564.0	1327.0	1054.5	1518.5	1434.5	1035.5	1322.3	229.56
AIFM2	209.0	114.5	79.5	228.5	143.0	77.0	141.9	64.59
AIFM3	4.5	1.0	1.5	1.5	0.5	1.0	1.7	1.44
AIG1	422.5	292.0	221.5	344.5	322.0	179.5	297.0	87.36
AIM1	66.0	11.0	54.5	46.0	10.5	17.0	34.2	24.32
AIM1L	0.0	0.0	1.0	0.5	0.0	0.5	0.3	0.41
AIMP1	589.5	663.5	642.5	577.0	763.5	580.5	636.1	71.80
AIMP2	1018.5	845.0	661.0	979.0	908.5	631.0	840.5	162.25
AIP	432.5	356.0	289.0	454.5	370.0	290.5	365.4	69.28
AIRE	15.0	7.0	7.5	16.5	9.0	8.5	10.6	4.09
AJAP1	19.5	54.0	84.0	54.5	67.0	162.5	73.6	48.44
AK1	423.0	555.5	308.5	418.5	670.5	455.0	471.8	125.43

AK2	728.5	626.0	478.5	779.5	605.5	518.5	622.8	116.39
AK3	313.0	198.5	160.5	328.0	229.5	144.5	229.0	76.97
AK4	534.5	414.5	552.0	528.5	468.5	649.5	524.6	79.67
AK5	2.0	1.5	1.0	6.5	0.5	1.5	2.2	2.18
AK6	522.5	555.0	412.5	570.5	621.5	370.0	508.7	97.33
AK8	12.5	19.0	26.0	6.5	25.5	20.5	18.3	7.61
AKAP1	1181.5	976.5	1421.5	1251.5	998.5	1129.0	1159.8	166.09
AKAP10	185.0	130.0	344.0	213.5	157.5	351.0	230.2	95.07
AKAP11	178.0	75.5	207.0	202.0	71.5	197.0	155.2	64.03
AKAP12	1708.5	1429.0	3674.5	1094.5	1594.0	2410.5	1985.2	934.55
AKAP13	266.5	414.0	1834.0	276.5	466.5	1431.5	781.5	675.99
AKAP14	1.0	4.0	3.0	3.5	5.5	5.5	3.8	1.70
AKAP17A	604.5	519.5	605.0	533.5	648.5	547.0	576.3	50.49
AKAP5	15.5	31.5	29.5	19.5	37.0	29.0	27.0	7.99
AKAP6	250.5	472.5	737.0	229.0	589.5	566.0	474.1	200.48
AKAP8	864.5	924.0	737.0	735.0	879.0	769.0	818.1	81.20
AKAP8L	783.5	660.0	598.0	812.0	732.0	602.5	698.0	91.68
AKAP9	1116.0	947.5	1771.5	1067.0	1094.0	1827.0	1303.8	388.53
AKIP1	184.0	193.0	165.0	181.5	197.0	185.0	184.3	11.12
AKIRIN2	1170.0	1274.0	996.0	1055.0	1474.0	988.5	1159.6	189.22
AKNAD1	34.0	31.0	13.0	26.0	31.0	8.5	23.9	10.61
AKR1A1	4862.5	3007.0	2145.5	4203.0	3031.0	1850.5	3183.3	1163.37
AKR1D1	0.0	3.0	0.5	1.0	3.0	2.5	1.7	1.33
AKT1	1290.0	916.0	1284.5	1176.0	1123.5	1145.5	1155.9	136.85
AKT3	434.0	429.5	692.0	397.5	485.0	693.5	521.9	135.26
AKTIP	591.0	501.5	372.5	566.5	600.5	319.5	491.9	119.37
ALAD	279.0	209.0	264.5	229.0	179.5	205.5	227.8	37.82
ALAS1	7393.0	7585.5	9589.0	8810.5	9232.5	8159.5	8461.7	893.06
ALB	7.5	4.5	4.5	8.5	6.0	6.5	6.3	1.60
ALCAM	1.5	4.5	3.0	1.5	2.0	4.5	2.8	1.40
ALDH18A1	3383.0	2341.0	4762.5	3400.0	2440.5	3548.5	3312.6	880.34
ALDH1A1	0.5	0.5	0.0	0.5	0.0	0.0	0.3	0.27
ALDH1A2	83.5	149.5	120.0	79.0	197.0	174.0	133.8	48.13
ALDH1A3	6.0	14.5	9.5	7.5	26.5	54.0	19.7	18.39
ALDH1L2	110.5	129.0	142.5	65.5	115.0	62.5	104.2	33.09
ALDH2	1725.5	1565.5	1505.0	1609.5	1644.5	1531.0	1596.8	80.92
ALDH3A2	1778.0	1013.5	1331.5	1451.5	999.0	1085.5	1276.5	305.88
ALDH4A1	1017.5	1009.5	1125.0	958.0	1051.5	994.0	1025.9	57.35
ALDH5A1	168.5	52.5	165.0	163.5	62.0	87.5	116.5	55.09
ALDH6A1	134.0	151.0	318.0	111.5	157.5	268.5	190.1	82.97
ALDH7A1	2503.5	1684.5	1855.5	2262.0	1793.0	1688.0	1964.4	339.17
ALDH8A1	1.5	2.0	1.0	1.5	1.5	0.0	1.3	0.69
ALDH9A1	1121.0	1061.0	1119.5	1111.5	1159.5	1040.5	1102.2	43.65
ALDOB	1.0	0.5	0.0	1.0	0.5	0.5	0.6	0.38
ALDOC	11786.5	8810.5	7596.5	11287.5	8109.5	7566.0	9192.8	1877.73
ALG10	533.0	258.0	362.5	547.5	302.0	287.5	381.8	127.50
ALG11	312.5	143.0	140.5	302.5	162.0	141.0	200.3	83.52
ALG12	946.0	649.5	1348.0	971.5	721.5	1115.5	958.7	256.26
ALG13	262.0	221.5	136.0	258.0	251.5	148.0	212.8	56.81
ALG14	110.5	64.0	46.0	79.5	61.0	43.0	67.3	24.94
ALG2	262.5	180.0	144.5	277.5	220.0	154.0	206.4	55.97
ALG3	388.5	371.5	300.0	337.5	386.0	280.5	344.0	45.85
ALG5	502.5	185.0	187.0	445.5	202.0	156.5	279.8	152.25
ALG6	704.5	395.5	385.0	644.0	468.0	401.5	499.8	139.59

ALG8	162.0	83.5	218.0	162.5	79.0	153.0	143.0	53.11
ALG9	916.5	611.5	574.0	856.0	661.0	560.5	696.6	152.20
ALK	191.0	342.5	212.0	229.5	344.0	164.0	247.2	77.57
ALKBH1	264.5	238.0	175.5	282.5	266.0	194.0	236.8	43.13
ALKBH2	102.0	67.0	85.0	80.5	86.5	68.0	81.5	13.05
ALKBH3	458.0	271.0	242.5	386.5	326.0	281.5	327.6	81.30
ALKBH4	242.5	211.0	203.5	202.0	231.5	202.5	215.5	17.32
ALKBH5	383.0	321.5	480.0	386.5	370.5	475.0	402.8	62.42
ALKBH8	167.0	123.5	110.0	144.0	130.5	93.0	128.0	25.89
ALMS1	429.0	278.5	470.0	470.0	311.0	495.0	408.9	91.51
ALOX5	3.5	6.5	13.5	3.0	3.0	12.0	6.9	4.73
ALOX5AP	0.0	0.0	0.0	0.5	0.0	0.0	0.1	0.20
ALPK1	165.5	62.5	54.0	138.5	73.5	52.5	91.1	48.53
ALPK2	139.5	134.5	168.5	125.0	145.5	92.0	134.2	25.29
ALPK3	980.0	759.0	1082.0	839.0	732.0	1124.0	919.3	166.90
ALS2	598.0	509.0	527.0	558.5	635.5	540.0	561.3	47.38
ALS2CL	145.5	36.5	71.5	161.5	41.0	52.5	84.8	54.84
ALX1	0.0	0.0	0.0	0.0	0.0	0.0	0.0	0.00
ALX3	0.0	1.0	1.0	0.0	0.0	0.5	0.4	0.49
ALX4	1344.0	661.0	1178.0	1223.0	766.0	1270.5	1073.8	286.34
ALYREF	1815.0	1686.5	1044.5	1825.0	1754.0	948.0	1512.2	403.84
AMACR	569.0	407.5	304.0	523.5	417.0	256.5	412.9	120.80
AMBP	0.5	0.0	0.0	0.0	0.0	0.0	0.1	0.20
AMD1	2620.5	1791.5	2066.5	2699.5	1884.5	1903.5	2161.0	397.33
AMDHD1	65.0	22.0	11.0	53.5	33.5	16.5	33.6	21.54
AMDHD2	456.5	432.0	332.5	481.5	460.5	363.0	421.0	59.66
AMER1	107.5	71.5	163.5	123.0	77.0	119.0	110.3	33.73
AMER2	0.0	0.5	0.0	0.0	0.0	0.5	0.2	0.26
AMER3	0.0	0.0	0.0	0.0	0.0	0.0	0.0	0.00
AMFR	1373.0	814.5	805.0	1366.5	881.5	837.5	1013.0	277.60
AMH	1.0	1.5	1.5	1.0	1.0	0.0	1.0	0.55
AMICA1	0.5	0.0	0.0	1.0	0.0	0.5	0.3	0.41
AMIGO1	81.5	49.5	71.0	57.5	50.5	76.0	64.3	13.66
AMIGO2	16.0	95.0	144.5	10.0	112.0	144.0	86.9	60.36
AMIGO3	25.5	14.0	22.5	22.5	11.5	29.0	20.8	6.75
AMMECR1	74.0	19.0	102.5	81.0	29.0	104.0	68.3	36.37
AMMECR1L	672.5	640.0	543.5	606.0	742.5	604.5	634.8	68.02
AMN	0.0	0.5	0.5	1.5	0.0	0.5	0.5	0.55
AMN1	11.0	7.0	13.5	14.0	6.0	15.5	11.2	3.91
AMOT	970.0	483.0	615.5	988.0	551.5	505.5	685.6	231.82
AMOTL1	49.5	45.0	139.0	69.0	42.0	122.0	77.8	42.27
AMOTL2	987.5	1199.0	1474.0	1152.0	1354.5	1679.0	1307.7	247.51
AMPD1	1.0	21.0	35.5	0.5	18.5	32.5	18.2	14.97
AMPD2	139.0	99.0	196.5	124.5	114.5	214.5	148.0	46.76
AMPD3	480.5	694.5	1692.5	449.0	902.5	1389.5	934.8	506.37
AMPH	0.0	0.0	0.5	0.0	0.0	0.0	0.1	0.20
AMT	179.0	181.5	118.5	178.5	194.5	132.0	164.0	30.87
AMZ1	1.0	4.5	2.0	0.0	6.5	2.5	2.8	2.38
ANAPC1	1110.5	730.0	863.0	1092.5	805.0	752.5	892.3	168.56
ANAPC10	188.5	182.0	150.5	188.5	186.5	138.5	172.4	22.08
ANAPC13	158.5	172.0	104.0	169.5	176.5	106.5	147.8	33.52
ANAPC15	406.5	526.5	300.5	334.0	527.0	301.5	399.3	105.95
ANAPC16	776.0	946.0	615.5	706.5	1002.0	674.5	786.8	155.03
ANAPC2	343.0	220.0	246.5	319.0	204.0	228.5	260.2	57.06

ANAPC4	778.0	566.0	608.0	751.5	647.0	501.0	641.9	107.07
ANAPC5	381.0	154.0	242.0	435.0	161.5	180.0	258.9	120.77
ANAPC7	461.0	269.5	331.0	449.0	317.5	285.0	352.2	82.72
ANGEL1	1140.5	958.0	1741.0	1221.0	1147.5	1852.0	1343.3	363.27
ANGEL2	750.0	1559.0	785.0	666.0	1785.0	893.0	1073.0	475.07
ANGPT1	0.0	0.5	0.5	1.0	0.0	0.5	0.4	0.38
ANGPT2	104.5	143.5	79.0	118.0	185.5	156.5	131.2	38.35
ANGPT4	1.0	5.5	3.5	2.5	2.5	1.5	2.8	1.60
ANGPTL1	72.0	101.5	40.0	70.0	128.0	53.5	77.5	32.23
ANGPTL2	432.5	336.0	1517.0	454.0	448.0	1451.5	773.2	552.85
ANGPTL3	1.0	0.0	0.0	0.0	0.0	0.0	0.2	0.41
ANGPTL4	1588.5	646.5	711.5	1464.5	744.5	940.5	1016.0	409.29
ANGPTL5	16.0	56.5	40.0	17.0	83.5	55.0	44.7	25.94
ANGPTL7	3.5	2.5	1.0	0.5	0.5	1.5	1.6	1.20
ANK1	33.0	248.0	179.5	23.5	292.0	239.5	169.3	114.99
ANK2	145.0	48.0	44.5	102.5	49.0	29.0	69.7	44.58
ANK3	73.5	43.5	204.5	61.0	56.0	148.5	97.8	64.18
ANKDD1A	38.0	91.5	91.5	50.0	108.5	75.0	75.8	27.05
ANKDD1B	1.0	2.0	1.5	0.5	3.0	1.5	1.6	0.86
ANKEF1	0.0	0.0	0.5	0.0	0.0	0.0	0.1	0.20
ANKFN1	1.5	0.0	0.0	1.0	1.0	0.0	0.6	0.66
ANKFY1	1016.5	811.0	1325.5	1017.0	814.5	1206.5	1031.8	206.50
ANKH	408.5	355.0	514.0	398.0	381.5	414.0	411.8	54.40
ANKIB1	319.0	139.0	491.0	400.0	179.5	487.5	336.0	151.44
ANKK1	1.0	0.5	4.0	0.0	4.5	2.5	2.1	1.88
ANKLE1	100.5	54.5	81.0	98.5	58.0	53.0	74.3	22.04
ANKLE2	549.5	333.0	806.0	594.0	413.0	738.5	572.3	182.14
ANKMY1	15.0	4.5	3.0	13.0	4.5	5.0	7.5	5.12
ANKMY2	1299.0	1032.5	863.0	1283.0	1290.0	815.0	1097.1	224.09
ANKRA2	870.0	595.0	714.5	820.0	677.0	681.5	726.3	101.24
ANKRD1	3139.5	9391.5	6622.0	2914.0	11034.0	6165.0	6544.3	3261.46
ANKRD10	826.5	1048.0	1459.0	726.5	1202.5	1360.0	1103.8	291.17
ANKRD11	1226.5	943.5	2072.0	1396.5	1034.5	1992.0	1444.2	482.17
ANKRD12	248.0	193.0	206.5	243.5	248.5	241.0	230.1	24.04
ANKRD13A	2230.5	1600.0	1669.5	2206.0	1806.0	1605.5	1852.9	292.66
ANKRD13B	67.0	127.5	128.5	50.5	91.0	116.0	96.8	32.81
ANKRD13C	744.5	773.0	1242.0	725.5	837.0	1178.5	916.8	231.33
ANKRD16	202.5	205.0	206.5	204.0	222.0	183.0	203.8	12.44
ANKRD17	1671.0	1364.0	2114.0	1563.5	1594.0	1983.0	1714.9	280.63
ANKRD2	219.0	1956.5	889.0	206.5	1993.0	946.5	1035.1	793.52
ANKRD22	0.5	0.0	0.0	1.5	0.5	0.0	0.4	0.58
ANKRD24	25.5	9.5	1.5	27.5	7.0	3.0	12.3	11.35
ANKRD27	214.5	116.0	159.0	198.0	181.0	127.5	166.0	39.07
ANKRD28	336.0	243.5	335.5	338.0	334.0	308.0	315.8	37.17
ANKRD29	442.5	170.5	304.5	481.0	185.5	253.5	306.3	130.30
ANKRD31	43.5	18.0	17.0	35.5	19.5	31.0	27.4	10.92
ANKRD32	422.0	216.0	197.5	424.0	230.5	192.5	280.4	111.27
ANKRD33	1.0	14.0	10.5	2.0	20.5	14.5	10.4	7.63
ANKRD33B	1.5	0.5	0.0	0.5	0.0	0.5	0.5	0.55
ANKRD34B	5.0	1.5	2.0	2.0	0.5	3.0	2.3	1.54
ANKRD34C	111.0	74.5	162.5	123.5	57.5	167.5	116.1	44.81
ANKRD40	135.5	93.5	67.5	113.5	93.5	73.5	96.2	25.29
ANKRD42	61.0	45.5	17.5	59.0	47.0	20.5	41.8	18.70
ANKRD44	567.0	155.5	376.5	500.5	189.5	290.0	346.5	165.87

ANKRD46	795.5	721.0	788.0	710.5	761.5	704.0	746.8	40.25
ANKRD49	214.0	208.5	205.5	223.0	265.5	163.5	213.3	32.81
ANKRD50	298.0	391.0	800.5	331.0	439.5	793.0	508.8	228.29
ANKRD52	149.0	62.0	209.0	173.0	81.0	183.5	142.9	58.88
ANKRD54	520.5	360.5	471.0	458.0	360.0	433.0	433.8	63.73
ANKRD55	3.0	3.5	2.0	1.5	1.0	1.5	2.1	0.97
ANKRD6	20.0	18.0	26.0	19.5	17.0	29.5	21.7	4.96
ANKRD60	1.0	1.0	6.0	4.0	2.5	4.0	3.1	1.96
ANKRD61	16.5	3.5	6.0	12.0	3.5	6.0	7.9	5.23
ANKRD66	99.5	78.0	56.0	93.0	89.0	65.0	80.1	16.94
ANKRD9	26.0	31.0	19.5	21.0	25.5	24.0	24.5	4.07
ANKS1A	21.5	16.5	46.5	20.0	14.5	49.5	28.1	15.65
ANKS1B	68.5	31.5	80.0	72.0	30.5	53.5	56.0	21.19
ANKS3	308.5	274.0	302.5	283.5	280.5	267.5	286.1	16.13
ANKS4B	5.5	2.5	12.0	0.5	0.5	2.0	3.8	4.40
ANKZF1	240.5	213.0	179.0	224.5	216.5	172.5	207.7	26.56
ANLN	3006.5	886.5	1777.5	3319.0	803.5	941.5	1789.1	1124.93
ANO1	29.0	208.0	357.0	19.0	179.5	123.5	152.7	126.13
ANO10	425.0	250.0	345.5	441.0	293.0	299.5	342.3	76.65
ANO3	99.5	147.5	234.0	90.5	210.5	386.5	194.8	110.21
ANO4	1.5	0.5	0.5	1.0	0.5	0.5	0.8	0.42
ANO5	721.0	246.5	1055.0	912.0	332.0	930.0	699.4	336.27
ANO8	273.5	469.5	733.0	242.0	441.0	693.5	475.4	205.13
ANO9	0.0	4.5	1.5	0.0	3.0	0.5	1.6	1.83
ANP32A	543.5	392.0	429.5	527.5	456.5	458.5	467.9	57.83
ANP32E	4919.5	2672.5	3153.0	4763.0	2983.5	2885.0	3562.8	1003.66
ANPEP	107.0	76.0	74.0	104.0	69.5	64.0	82.4	18.38
ANTXRL	303.0	264.5	475.5	255.0	230.0	474.0	333.7	111.78
ANXA10	2.0	0.0	1.0	1.0	1.0	1.0	1.0	0.63
ANXA11	1128.0	892.0	1164.0	1066.5	1082.5	1283.5	1102.8	129.00
ANXA13	2.5	1.5	0.5	0.5	2.0	1.0	1.3	0.82
ANXA2	16443.0	16985.0	21773.0	17333.5	19167.0	19707.0	18568.1	2023.54
ANXA4	992.5	1048.0	1181.5	994.5	1257.5	1217.5	1115.3	117.69
ANXA5	7145.5	8873.5	13843.0	6833.5	10155.0	11381.5	9705.3	2669.24
ANXA6	10423.0	11474.0	11489.5	10092.5	12449.5	10755.0	11113.9	860.70
ANXA7	1837.5	1287.5	839.5	1556.0	1450.5	789.5	1293.4	412.23
AOAH	19.5	1.5	5.5	13.5	3.0	8.0	8.5	6.85
AOX1	702.0	336.5	351.0	680.0	350.5	328.0	458.0	180.82
AP1B1	973.0	761.5	1015.5	900.0	734.0	935.0	886.5	114.49
AP1G1	683.0	367.5	776.0	676.5	373.5	724.5	600.2	181.43
AP1M1	2083.5	1628.5	1684.5	2195.5	1813.0	1561.5	1827.8	257.67
AP1S2	492.0	175.0	384.0	445.0	185.0	316.0	332.8	132.35
AP1S3	1.5	1.0	0.5	0.0	1.5	0.0	0.8	0.69
AP2A2	2077.5	1636.5	2298.0	2145.0	1684.0	2072.5	1985.6	265.27
AP2B1	1654.5	1510.5	2362.0	1635.0	1489.0	2125.0	1796.0	360.64
AP2M1	4594.5	2813.5	4461.0	4448.0	2870.0	3884.0	3845.2	814.98
AP3B1	1210.5	844.5	1179.0	1197.0	968.0	1028.0	1071.2	148.84
AP3B2	86.0	78.0	90.5	89.0	91.0	118.0	92.1	13.57
AP3D1	1472.0	1395.5	1891.0	1428.5	1519.5	1862.5	1594.8	222.50
AP3M1	5912.5	7907.0	8303.0	6268.0	9220.5	8108.5	7619.9	1272.21
AP3M2	731.0	346.5	295.0	712.0	395.5	283.5	460.6	206.09
AP3S1	356.0	189.5	248.0	371.0	233.0	230.0	271.3	74.19
AP4B1	422.0	342.5	334.0	444.0	391.5	330.0	377.3	48.93
AP4S1	138.5	144.5	91.5	130.0	142.5	56.5	117.3	35.63

AP5M1	100.5	93.5	79.0	121.5	87.0	63.0	90.8	19.85
AP5S1	217.0	257.0	137.5	188.5	250.0	166.0	202.7	47.31
AP5Z1	380.5	301.0	228.5	336.5	305.5	233.0	297.5	59.00
APAF1	436.0	292.0	301.0	396.5	366.5	272.0	344.0	65.52
APBA1	25.0	22.0	54.0	36.5	22.0	48.0	34.6	13.92
APBA2	35.0	67.5	320.0	40.0	85.5	187.0	122.5	111.38
APBA3	386.5	615.5	505.5	362.0	573.5	586.0	504.8	107.66
APBB2	295.0	383.5	456.5	346.5	480.0	400.0	393.6	68.56
APC	1237.5	1968.5	2972.5	1262.0	2407.5	2741.5	2098.3	739.02
APC2	0.5	0.5	1.0	0.5	0.5	1.5	0.8	0.42
APCDD1	4.0	6.5	6.5	5.0	9.5	18.5	8.3	5.32
APCDD1L	70.5	19.5	52.0	48.5	22.5	16.0	38.2	22.04
APEH	659.0	658.0	542.0	615.0	638.0	496.5	601.4	67.20
APEX1	14.0	17.5	94.5	21.5	30.5	90.0	44.7	37.29
APH1A	716.0	545.5	481.5	634.0	581.5	463.0	570.3	95.32
API5	1844.0	1281.0	1924.5	1949.0	1417.0	1759.0	1695.8	280.06
APIP	303.5	244.0	256.5	307.0	291.5	234.5	272.8	31.70
APITD1	288.5	134.5	103.5	303.0	143.5	73.0	174.3	97.38
APLF	108.0	52.5	65.5	86.5	61.5	50.0	70.7	22.42
APLN	6.0	12.5	5.0	3.5	18.5	10.0	9.3	5.63
APLNR	1.0	0.0	0.0	0.0	0.0	0.0	0.2	0.41
APLP2	9894.5	6545.5	10695.0	10499.5	7791.0	11498.5	9487.3	1909.21
APMAP	452.0	346.5	503.5	486.0	394.0	544.5	454.4	73.24
APOA1	206.5	1397.0	86.5	224.5	1710.0	322.0	657.8	704.86
APOA4	26.0	9.5	7.0	25.0	17.5	8.0	15.5	8.59
APOA5	29.0	34.0	26.5	29.0	36.0	18.5	28.8	6.17
APOB	0.0	0.5	2.5	0.0	1.0	3.5	1.3	1.44
APOBEC2	775.0	1799.5	1743.5	552.5	2041.0	1556.5	1411.3	603.50
APOBEC4	0.0	0.5	0.0	0.0	0.0	0.0	0.1	0.20
APOC3	10.0	13.5	2.5	15.0	18.0	5.0	10.7	6.00
APOD	4.0	24.0	28.5	7.0	30.0	45.5	23.2	15.51
APOH	0.0	0.0	0.0	0.0	0.5	1.0	0.3	0.42
APOLD1	26.0	56.5	60.5	24.5	65.5	57.0	48.3	18.17
APOO	477.5	572.0	511.0	434.5	627.0	458.5	513.4	73.31
APOOL	224.5	113.5	203.0	247.5	126.5	233.5	191.4	57.32
APOPT1	32.5	67.5	66.0	42.5	73.0	71.5	58.8	17.02
APP	3873.0	3421.0	7056.0	4060.0	4224.5	7792.5	5071.2	1857.05
APPBP2	873.5	700.0	816.5	849.0	820.0	702.5	793.6	74.48
APPL1	80.5	17.5	107.0	84.5	22.0	81.0	65.4	36.73
APPL2	302.5	105.5	364.5	352.5	147.0	294.0	261.0	108.69
APRT	231.0	324.5	275.0	179.0	291.0	286.0	264.4	51.59
AQP1	1.0	1.5	9.0	4.5	1.5	10.0	4.6	4.02
AQP10	9.0	1.0	1.0	6.5	2.5	1.5	3.6	3.37
AQP11	21.5	13.0	13.0	17.5	18.0	10.0	15.5	4.22
AQP2	0.0	0.0	0.0	0.0	0.0	0.0	0.0	0.00
AQP3	14.0	55.0	59.5	7.0	67.0	51.0	42.3	25.26
AQP4	0.0	0.0	0.0	0.0	0.0	0.5	0.1	0.20
AQP8	1.0	3.5	0.5	0.5	4.0	0.5	1.7	1.63
AQP9	31.5	11.0	12.0	38.0	10.0	6.5	18.2	13.14
AQR	369.5	201.0	504.0	371.0	215.0	406.5	344.5	116.60
AR	0.5	2.5	2.5	2.5	4.0	5.0	2.8	1.54
ARAP2	101.5	27.0	45.0	55.5	17.5	15.0	43.6	32.47
ARAP3	1.0	2.0	2.0	0.5	2.5	0.0	1.3	0.98
ARC	4.0	68.5	63.5	1.5	82.0	99.5	53.2	41.00

ARCN1	3293.0	2780.0	2703.0	3348.5	2986.0	2894.5	3000.8	266.55
AREG	0.5	0.0	0.0	0.0	0.0	0.0	0.1	0.20
AREL1	515.5	471.0	936.0	488.0	482.0	870.5	627.2	215.35
ARF1	4316.5	2533.0	4101.5	4359.0	2896.5	3943.0	3691.6	779.96
ARF4	3144.0	2854.5	2863.0	3162.0	3450.0	3053.0	3087.8	221.82
ARF6	638.0	578.0	697.5	664.5	620.5	664.0	643.8	41.54
ARFGAP2	908.0	866.5	704.0	944.5	871.5	713.0	834.6	101.67
ARFGAP3	767.5	545.5	709.5	817.5	663.0	724.0	704.5	93.96
ARFGEF1	62.5	16.5	110.0	72.0	23.5	76.5	60.2	35.06
ARFGEF2	604.5	506.5	734.0	572.0	580.0	679.5	612.8	81.54
ARFIP1	1207.0	855.0	907.5	1231.0	1011.5	875.0	1014.5	167.50
ARFIP2	647.5	609.5	449.5	590.5	546.0	514.0	559.5	71.49
ARFRP1	342.5	303.5	248.5	311.0	384.0	249.0	306.4	52.92
ARG2	73.0	64.0	74.5	59.0	79.0	69.0	69.8	7.32
ARGLU1	681.0	601.0	619.0	614.5	727.0	644.5	647.8	47.99
ARHGAP1	174.5	37.0	151.0	179.5	36.5	96.5	112.5	65.65
ARHGAP10	1023.5	659.5	570.0	927.0	841.5	462.5	747.3	218.04
ARHGAP11A	471.0	167.0	250.5	492.0	168.0	150.5	283.2	157.68
ARHGAP12	410.0	150.0	363.5	392.0	192.0	348.0	309.3	110.05
ARHGAP17	2157.5	2219.5	2144.0	2129.0	2522.0	2121.0	2215.5	154.17
ARHGAP18	50.0	62.0	167.5	72.5	75.0	113.5	90.1	43.53
ARHGAP19	956.0	262.5	440.0	948.0	251.5	277.5	522.6	339.66
ARHGAP20	35.5	67.0	59.5	45.5	94.0	93.0	65.8	24.11
ARHGAP21	853.0	834.5	1296.5	823.0	983.0	1254.0	1007.3	215.74
ARHGAP22	43.5	23.5	31.0	32.5	19.0	41.5	31.8	9.64
ARHGAP23	123.0	209.5	312.5	112.0	153.5	285.0	199.3	84.62
ARHGAP24	701.0	749.0	791.0	677.0	963.0	886.5	794.6	110.89
ARHGAP25	2.0	14.0	4.0	2.5	22.0	7.5	8.7	7.90
ARHGAP26	88.5	43.0	60.0	75.0	53.0	64.5	64.0	16.12
ARHGAP27	1.0	0.0	0.0	0.0	0.0	0.0	0.2	0.41
ARHGAP28	22.5	63.5	220.0	32.5	93.5	204.5	106.1	86.06
ARHGAP29	146.0	70.0	162.0	142.5	88.5	135.0	124.0	36.24
ARHGAP31	421.5	786.5	1417.0	286.5	761.0	728.0	733.4	391.90
ARHGAP32	146.0	186.5	373.0	193.5	239.0	464.0	267.0	124.38
ARHGAP35	91.5	89.0	330.0	104.0	68.5	284.5	161.3	114.57
ARHGAP39	29.0	49.0	109.5	27.0	29.0	91.5	55.8	35.97
ARHGAP40	67.5	659.0	1027.0	41.0	813.0	794.0	566.9	414.32
ARHGAP44	1.5	19.5	25.5	3.0	27.0	22.0	16.4	11.29
ARHGAP5	652.5	327.5	649.0	659.5	388.0	536.5	535.5	146.27
ARHGAP6	7.0	13.0	26.5	9.5	17.5	18.5	15.3	7.05
ARHGAP8	4.0	4.0	3.0	13.0	4.0	5.5	5.6	3.72
ARHGDIA	6743.0	6070.5	5593.0	6430.5	6177.0	5435.5	6074.9	494.84
ARHGDIB	3068.5	2422.0	1442.5	2518.0	2429.0	1447.0	2221.2	647.34
ARHGDIG	298.5	190.0	140.5	284.5	183.0	167.5	210.7	65.03
ARHGEF10	54.5	27.0	126.0	65.0	35.5	135.5	73.9	46.13
ARHGEF10L	317.0	318.0	328.0	268.5	365.5	332.0	321.5	31.40
ARHGEF11	944.0	700.5	758.0	903.0	674.5	790.0	795.0	108.36
ARHGEF12	1123.5	710.0	1469.0	1190.0	848.5	1452.0	1132.2	309.15
ARHGEF16	19.0	24.0	29.5	4.0	17.0	32.5	21.0	10.22
ARHGEF17	805.5	839.0	1180.0	788.5	767.5	1151.0	921.9	190.35
ARHGEF18	700.0	525.5	859.0	827.5	627.5	784.0	720.6	127.87
ARHGEF26	302.5	350.0	426.5	260.5	387.5	374.5	350.3	60.21
ARHGEF3	151.5	106.0	189.5	140.0	133.5	226.0	157.8	43.10
ARHGEF33	9.5	17.5	11.5	12.0	20.0	9.5	13.3	4.39

ARHGEF37	94.5	105.0	105.5	87.0	100.0	73.5	94.3	12.32
ARHGEF5	0.0	0.0	0.0	0.0	0.0	0.0	0.0	0.00
ARHGEF6	1476.0	1197.0	1714.0	1257.0	1197.0	1418.5	1376.6	202.13
ARHGEF7	567.0	691.5	765.5	540.5	769.5	686.0	670.0	97.08
ARHGEF9	5.5	2.0	4.5	3.5	4.0	7.0	4.4	1.72
ARID1B	416.0	333.0	668.0	426.0	361.5	687.5	482.0	155.59
ARID2	369.0	309.5	485.0	369.5	401.0	464.0	399.7	65.43
ARID3A	11.0	13.5	17.5	6.5	18.0	20.5	14.5	5.19
ARID3B	99.5	84.0	125.0	90.5	71.5	124.5	99.2	21.82
ARID3C	0.0	1.0	1.5	0.0	1.5	1.0	0.8	0.68
ARID4A	351.0	215.0	342.0	387.5	237.0	343.5	312.7	69.49
ARID4B	547.5	418.5	528.5	499.0	522.0	487.5	500.5	45.50
ARID5A	538.5	401.5	301.0	539.5	390.5	291.5	410.4	109.23
ARID5B	409.0	318.5	502.0	428.0	339.0	496.5	415.5	76.82
ARIH1	874.0	739.0	1122.0	843.5	839.5	1053.5	911.9	145.22
ARIH2	882.5	789.0	762.0	822.5	919.5	806.0	830.3	59.50
ARL1	1466.5	1130.5	816.0	1387.5	1384.5	798.5	1163.9	298.63
ARL10	4.0	5.0	3.5	10.5	8.5	5.0	6.1	2.78
ARL11	97.5	96.0	47.0	99.5	89.5	34.0	77.3	28.96
ARL13A	0.0	1.5	0.5	0.0	2.0	0.5	0.8	0.82
ARL13B	116.5	68.5	137.0	124.0	83.0	129.0	109.7	27.49
ARL14	0.0	0.0	0.5	1.5	0.5	0.0	0.4	0.58
ARL14EP	352.0	290.5	371.5	358.0	304.5	364.0	340.1	33.90
ARL14EPL	6.0	5.0	1.0	5.5	3.5	0.5	3.6	2.35
ARL16	112.5	76.0	42.0	107.0	68.0	62.5	78.0	27.10
ARL2BP	275.5	245.0	428.5	240.5	282.5	392.0	310.7	79.71
ARL3	152.5	173.5	268.0	155.0	170.5	254.5	195.7	51.64
ARL4A	286.0	161.5	213.0	262.5	170.0	177.5	211.8	52.02
ARL4C	704.5	1756.5	2032.0	865.0	2065.0	2638.5	1676.9	750.00
ARL5A	424.5	420.5	478.5	406.5	510.5	516.5	459.5	48.51
ARL5B	204.0	137.0	170.0	169.0	164.5	160.5	167.5	21.58
ARL6	38.5	16.5	40.5	49.5	26.0	39.0	35.0	11.76
ARL6IP1	3767.0	2145.5	2335.5	3509.5	2352.0	1918.5	2671.3	769.55
ARL6IP4	512.5	338.0	330.0	428.0	363.0	320.0	381.9	74.82
ARL6IP5	2149.5	1484.5	2200.0	2029.0	1738.0	1981.5	1930.4	271.61
ARL8A	299.0	513.5	620.5	322.5	530.0	604.5	481.7	138.86
ARL8B	1351.0	1420.5	1708.0	1424.0	1597.0	1507.5	1501.3	131.92
ARL9	745.0	787.0	484.0	722.5	1055.5	512.5	717.8	208.04
ARMC1	452.5	405.0	619.0	466.0	454.5	529.0	487.7	75.63
ARMC10	154.5	112.0	108.5	134.5	119.0	108.5	122.8	18.34
ARMC2	3.0	1.5	5.0	2.0	1.5	5.5	3.1	1.77
ARMC3	4.0	3.0	2.0	4.0	0.5	1.5	2.5	1.41
ARMC4	0.5	0.0	0.0	0.0	0.0	0.0	0.1	0.20
ARMC6	838.0	820.5	710.5	897.5	936.0	739.5	823.7	87.38
ARMC7	410.0	832.0	301.5	366.0	907.5	364.5	530.3	266.31
ARMC8	644.0	619.0	857.5	655.5	715.5	906.5	733.0	120.69
ARMC9	391.0	219.0	339.5	346.5	210.5	328.5	305.8	73.73
ARNT	895.0	926.5	919.5	848.5	888.0	968.0	907.6	40.45
ARNT2	109.0	46.5	171.5	136.5	59.5	115.5	106.4	46.95
ARNTL	293.5	135.5	159.0	261.5	172.0	140.0	193.6	67.09
ARNTL2	1031.0	869.0	714.0	994.5	1085.0	726.0	903.3	158.80
ARPC1A	3949.0	2715.0	2390.5	3227.0	2709.5	1944.0	2822.5	695.03
ARPC1B	1352.0	1042.5	1374.5	1283.0	974.0	1301.5	1221.3	169.66
ARPC2	4034.0	3176.0	3089.5	4150.5	3478.0	3141.5	3511.6	471.17

ARPC3	1564.0	1387.0	1183.5	1426.5	1539.0	1130.5	1371.8	179.89
ARPC4	2089.0	2653.0	1583.0	2089.5	2776.0	1556.5	2124.5	514.22
ARPC5	3531.5	3705.5	3087.0	3496.5	4295.0	3122.0	3539.6	442.56
ARPC5L	518.0	588.0	352.5	463.5	621.0	378.0	486.8	109.22
ARPP19	3741.5	3849.0	4209.0	3363.0	4391.5	4107.5	3943.6	370.08
ARPP21	262.0	440.5	348.5	217.5	522.5	354.0	357.5	112.19
ARR3	2.0	4.0	3.5	3.5	3.0	2.0	3.0	0.84
ARRDC1	60.5	179.5	257.0	57.0	179.0	162.0	149.2	77.41
ARRDC2	287.0	468.5	551.5	326.5	579.0	654.5	477.8	145.79
ARRDC4	182.5	68.5	122.0	149.0	79.5	94.0	115.9	43.86
ARRDC5	2.5	1.0	0.0	3.5	3.0	1.0	1.8	1.37
ARSA	445.0	464.0	594.0	511.0	485.5	659.5	526.5	83.32
ARSB	245.5	78.5	94.0	265.0	105.5	90.0	146.4	84.97
ARSG	17.0	24.0	42.5	16.0	23.5	39.0	27.0	11.19
ARSI	728.0	268.0	400.5	778.5	295.0	459.5	488.3	217.31
ARSJ	895.0	851.5	1254.5	1015.5	1028.0	1337.0	1063.6	194.00
ARSK	988.5	374.5	294.0	1031.5	382.0	285.5	559.3	351.60
ART4	0.0	0.0	0.0	0.0	0.0	0.0	0.0	0.00
ARTN	6.0	7.0	5.5	4.5	7.5	4.0	5.8	1.37
ARV1	280.5	206.5	293.5	254.5	250.0	290.0	262.5	32.86
ARVCF	308.0	148.5	468.0	330.0	167.0	404.0	304.3	126.91
ARX	1.0	1.0	0.0	0.0	0.0	0.0	0.3	0.52
AS3MT	119.5	188.0	177.5	100.0	187.5	169.5	157.0	37.74
ASAH1	1011.0	772.0	1005.0	1111.0	997.5	1057.5	992.3	116.11
ASAP1	234.0	154.0	231.5	222.5	191.5	266.0	216.6	38.86
ASAP2	77.0	77.0	237.0	80.0	86.0	180.0	122.8	68.84
ASAP3	16.0	32.0	84.0	8.5	23.5	89.5	42.3	35.38
ASB1	546.5	467.0	581.0	570.5	523.5	534.5	537.2	40.59
ASB10	0.0	2.0	0.0	1.0	1.0	0.5	0.8	0.76
ASB11	0.0	1.5	2.0	0.0	1.5	3.5	1.4	1.32
ASB12	37.5	45.0	61.5	34.5	51.5	54.5	47.4	10.36
ASB13	47.5	38.5	49.5	43.5	36.5	49.5	44.2	5.65
ASB14	68.5	25.0	72.0	67.0	26.0	66.5	54.2	22.29
ASB15	13.0	126.0	115.5	9.0	141.0	73.5	79.7	57.74
ASB18	1.5	62.0	42.5	1.0	49.5	61.5	36.3	28.16
ASB2	144.0	285.5	175.0	139.5	360.0	133.0	206.2	94.44
ASB3	191.5	193.0	201.0	221.0	219.5	165.5	198.6	20.60
ASB4	0.0	0.0	0.5	0.5	0.5	0.0	0.3	0.27
ASB5	1161.0	2381.0	2126.0	810.5	2633.5	1795.0	1817.8	710.51
ASB6	414.0	421.0	476.0	348.0	474.5	497.5	438.5	55.28
ASB7	46.5	23.5	60.5	41.5	28.0	59.5	43.3	15.48
ASB8	428.5	400.5	293.5	452.0	404.5	282.5	376.9	71.40
ASB9	12.5	10.5	14.5	27.5	11.5	34.5	18.5	10.02
ASCC1	853.0	933.0	571.5	794.5	943.0	561.0	776.0	171.38
ASCC2	1250.0	1386.0	1359.5	1323.0	1528.5	1524.0	1395.2	111.37
ASCL1	0.0	0.0	0.0	0.0	0.0	0.0	0.0	0.00
ASCL3	54.0	30.5	37.0	39.5	36.5	27.5	37.5	9.24
ASCL4	0.5	1.0	1.0	1.5	1.0	1.5	1.1	0.38
ASH1L	1177.5	759.5	1718.5	1109.0	766.0	1583.0	1185.6	401.36
ASH2L	911.5	753.0	796.5	845.0	807.5	767.5	813.5	57.79
ASIC1	7.0	18.0	26.5	6.5	14.5	35.0	17.9	11.20
ASIC2	0.0	0.0	0.0	0.0	1.0	1.5	0.4	0.66
ASIC4	6.5	88.0	129.5	6.5	88.0	94.0	68.8	50.63
ASIC5	0.0	0.0	0.0	0.0	0.0	0.0	0.0	0.00

ASIP	0.5	4.0	5.5	0.0	4.0	7.0	3.5	2.76
ASMTL	65.0	29.0	34.5	96.0	30.0	42.0	49.4	26.40
ASNA1	451.0	382.5	350.0	404.0	399.0	327.5	385.7	43.47
ASNS	1498.0	1128.5	1133.5	1381.5	1159.0	786.0	1181.1	245.85
ASNSD1	299.5	245.0	341.5	294.0	261.5	316.0	292.9	35.27
ASPA	12.5	11.5	4.0	13.0	11.5	4.0	9.4	4.24
ASPDH	3.5	3.5	1.5	1.0	1.0	0.5	1.8	1.33
ASPG	59.0	25.0	29.5	74.0	31.5	24.0	40.5	20.88
ASPH	1837.0	904.0	1485.0	1761.0	985.5	1177.5	1358.3	396.34
ASPHD2	18.5	17.5	9.5	13.5	10.0	14.5	13.9	3.72
ASPM	678.0	269.5	690.5	757.5	255.0	420.5	511.8	224.91
ASPN	2039.0	1140.5	1699.5	2244.5	1446.5	3246.5	1969.4	740.93
ASPSCR1	767.5	675.0	661.5	670.0	772.5	658.5	700.8	53.92
ASRGL1	159.5	110.5	48.5	163.5	112.0	67.0	110.2	46.79
ASS1	33.0	31.0	45.0	21.5	28.5	29.5	31.4	7.72
ASTE1	58.5	54.0	53.0	54.5	58.0	58.5	56.1	2.52
ASTN2	1.0	3.5	5.5	4.5	3.0	6.0	3.9	1.83
ASUN	4051.5	3322.0	4244.5	3841.0	3553.5	3779.0	3798.6	332.32
ASXL1	379.5	255.0	340.0	380.0	214.0	304.5	312.2	67.64
ASXL2	430.5	273.0	460.0	441.5	296.0	429.5	388.4	81.56
ASXL3	543.5	455.0	609.0	499.0	553.0	708.5	561.3	88.89
ASZ1	0.0	0.0	0.5	0.0	0.0	0.0	0.1	0.20
ATAD1	1828.0	948.0	885.0	1847.5	975.5	822.5	1217.8	483.19
ATAD2	412.0	152.0	336.5	474.5	155.0	265.5	299.3	133.00
ATAD2B	318.5	207.0	392.5	321.0	265.0	334.0	306.3	63.45
ATAD3A	823.0	657.0	466.0	758.5	652.5	424.0	630.2	157.69
ATAD5	331.0	120.5	241.5	312.0	133.0	188.5	221.1	89.11
ATCAY	0.0	0.5	0.5	1.0	0.0	0.0	0.3	0.41
ATE1	114.0	39.5	130.0	124.0	39.0	112.0	93.1	42.21
ATF1	474.0	264.5	565.5	505.5	309.0	493.0	435.3	119.86
ATF2	697.0	599.0	712.0	633.0	695.0	723.0	676.5	49.16
ATF3	382.5	287.5	521.0	393.0	324.0	482.0	398.3	89.59
ATF4	7199.5	10612.0	9626.0	7211.0	12052.5	9772.5	9412.3	1914.55
ATF6	703.0	693.0	804.5	708.0	685.0	746.5	723.3	45.08
ATF7	122.5	133.5	227.5	138.0	130.5	215.5	161.3	47.10
ATF7IP	223.0	135.5	294.5	240.5	152.5	236.5	213.8	59.52
ATG12	452.5	362.0	236.5	446.0	382.0	250.5	354.9	93.30
ATG13	2700.5	1854.5	1997.5	2352.0	2028.5	1823.5	2126.1	338.34
ATG14	299.5	320.0	374.5	271.0	346.0	342.5	325.6	36.84
ATG16L1	548.5	655.5	643.5	545.0	720.5	613.5	621.1	67.36
ATG2B	193.0	84.5	320.0	209.5	119.0	257.5	197.3	86.83
ATG3	823.0	627.0	561.5	766.0	684.0	474.0	655.9	129.36
ATG4A	652.5	667.0	623.5	627.0	762.0	578.5	651.8	61.91
ATG4B	423.0	319.0	580.0	439.5	320.5	522.5	434.1	105.27
ATG4C	456.5	283.5	361.0	405.5	324.5	307.0	356.3	65.15
ATG5	126.0	58.0	138.5	128.5	57.0	116.5	104.1	36.76
ATG7	310.5	213.0	232.5	290.5	259.0	224.0	254.9	38.96
ATG9A	767.5	412.0	533.0	739.0	371.5	489.5	552.1	166.05
ATHL1	315.5	173.0	109.5	286.0	157.5	119.0	193.4	86.91
ATIC	1586.0	1879.5	1309.0	1513.5	1866.0	1155.0	1551.5	291.60
ATL1	411.5	307.5	512.5	416.5	344.0	508.5	416.8	83.48
ATL2	738.0	571.5	581.5	635.0	617.0	543.0	614.3	68.89
ATMIN	106.0	53.5	87.5	109.0	65.5	82.0	83.9	21.90
ATOH7	3.0	3.0	2.5	3.0	1.5	2.0	2.5	0.63

ATOH8	951.0	674.5	1359.5	811.5	811.0	1295.0	983.8	280.82
ATOX1	1021.5	1015.0	502.0	878.0	1057.0	463.0	822.8	270.81
ATP10A	446.0	258.5	621.0	474.0	306.5	501.5	434.6	132.91
ATP10B	0.0	0.0	0.0	0.0	0.0	0.0	0.0	0.00
ATP10D	7.5	2.5	10.5	7.5	2.5	5.0	5.9	3.17
ATP11A	728.0	1278.5	1260.5	638.5	1622.5	1023.0	1091.8	370.82
ATP11B	1362.0	749.5	1608.5	1308.0	811.0	1355.5	1199.1	341.53
ATP11C	1204.0	817.5	1184.0	1319.0	933.5	1205.5	1110.6	191.74
ATP12A	50.5	13.0	5.5	57.0	20.5	10.5	26.2	22.00
ATP13A1	669.0	593.0	711.5	679.5	596.5	663.0	652.1	47.47
ATP13A2	142.5	114.0	98.0	126.5	113.5	95.5	115.0	17.67
ATP13A3	211.5	60.0	417.5	240.5	84.5	304.0	219.7	134.55
ATP13A5	1.0	1.0	0.5	1.0	2.0	1.5	1.2	0.52
ATP1A1	6851.5	6028.5	7937.5	6120.5	6655.5	5807.5	6566.8	778.87
ATP1B1	1087.5	329.0	829.5	631.5	322.0	392.0	598.6	311.54
ATP1B3	5631.0	3308.5	4411.0	6186.0	3844.5	4192.0	4595.5	1097.19
ATP1B4	120.0	51.5	71.5	91.0	60.5	53.5	74.7	26.52
ATP2A2	3533.0	4503.0	6002.5	3303.0	5178.0	4997.5	4586.2	1028.40
ATP2A3	0.5	7.0	25.0	1.0	3.0	53.5	15.0	20.96
ATP2B1	920.5	1210.5	1446.5	1200.5	1437.0	1305.0	1253.3	194.48
ATP2B2	53.0	17.5	8.0	48.0	21.5	14.0	27.0	18.80
ATP2B4	802.5	315.5	1098.0	821.5	335.0	945.5	719.7	323.32
ATP2C1	1444.5	974.0	1823.0	1529.5	1135.5	1583.5	1415.0	310.30
ATP2C2	52.0	22.5	16.0	59.5	31.0	15.0	32.7	18.92
ATP4B	0.5	0.0	0.0	0.0	0.0	0.0	0.1	0.20
ATP5A1	11143.5	8417.0	8350.0	10697.5	8582.5	6981.0	9028.6	1580.14
ATP5B	11117.0	12120.0	8072.0	9513.0	11655.0	7938.0	10069.2	1825.30
ATP5C1	2999.5	2893.5	2461.5	2762.5	3047.0	2069.0	2705.5	375.85
ATP5D	1895.5	2580.0	1494.0	1712.5	2654.5	1438.0	1962.4	533.25
ATP5E	625.5	1030.5	549.5	573.0	1042.0	508.5	721.5	246.75
ATP5F1	3365.5	3354.0	2298.5	3169.5	3526.0	2026.0	2956.6	631.47
ATP5G1	1690.0	1869.0	1126.0	1534.0	1860.0	947.5	1504.4	386.79
ATP5G3	4560.5	4837.5	2904.5	4220.0	5314.5	2637.5	4079.1	1078.04
ATP5H	2658.5	4461.5	2033.0	2336.0	5195.0	2202.0	3147.7	1338.10
ATP5I	2921.0	3505.5	1434.5	2640.0	3343.0	1330.0	2529.0	939.96
ATP5J	2120.0	2229.0	1331.5	1854.0	2277.0	1245.0	1842.8	454.63
ATP5J2	1153.5	1259.0	787.5	1078.5	1326.0	769.0	1062.3	235.94
ATP5O	1982.5	1440.5	924.0	1680.5	1454.5	802.5	1380.8	448.10
ATP5S	519.0	260.0	104.0	463.5	267.5	89.5	283.9	178.06
ATP6AP1	3964.0	1630.0	2163.0	3894.5	1886.0	1737.5	2545.8	1086.66
ATP6AP1L	9.0	5.0	3.5	9.0	3.5	4.5	5.8	2.58
ATP6AP2	824.5	606.0	693.0	832.0	733.0	594.5	713.8	102.84
ATP6V0A1	2706.0	1535.5	1339.0	2292.0	1693.5	1490.5	1842.8	536.94
ATP6V0A2	673.5	353.0	535.0	699.0	411.5	503.0	529.2	138.04
ATP6V0A4	7.0	1.5	2.5	1.0	3.0	4.0	3.2	2.16
ATP6V0B	620.5	588.0	450.0	557.5	672.5	440.0	554.8	93.21
ATP6V0C	2308.5	2198.5	2225.5	2158.0	2478.0	2086.5	2242.5	136.76
ATP6V0D1	911.0	1126.5	861.5	881.5	1261.5	894.0	989.3	164.97
ATP6V0E1	2186.5	4137.5	2699.0	2173.5	4717.0	2797.5	3118.5	1061.53
ATP6V1A	1009.5	830.5	1139.0	986.0	936.0	977.0	979.7	100.48
ATP6V1B2	2002.0	1436.5	1368.0	2053.5	1666.0	1270.0	1632.7	333.07
ATP6V1C1	642.0	778.0	734.5	630.5	868.5	621.0	712.4	99.17
ATP6V1C2	17.5	17.5	22.5	17.5	24.0	34.0	22.2	6.46
ATP6V1D	1864.5	1979.5	1101.0	1786.0	2210.5	937.0	1646.4	509.30

ATP6V1E1	879.0	858.0	936.0	885.5	1005.0	837.0	900.1	61.16
ATP6V1G1	2233.5	2230.5	1458.0	2142.0	2479.5	1338.0	1980.3	466.35
ATP6V1G3	0.0	0.0	0.0	0.5	0.0	0.0	0.1	0.20
ATP6V1H	621.0	562.0	644.0	678.5	659.0	557.0	620.3	50.70
ATP7A	193.5	41.5	277.0	199.5	59.5	202.5	162.3	91.97
ATP7B	2.5	2.5	6.0	3.5	2.5	2.5	3.3	1.41
ATP8A1	126.5	26.0	82.5	139.0	26.5	76.5	79.5	47.83
ATP8B1	9.5	4.5	3.0	13.0	3.0	2.0	5.8	4.41
ATP8B3	0.0	3.0	3.0	0.5	1.0	7.5	2.5	2.76
ATP9B	299.0	213.5	236.5	344.5	220.5	201.0	252.5	56.71
ATPAF1	424.0	282.0	172.5	340.0	305.0	158.0	280.3	101.40
ATPAF2	258.5	311.0	157.5	225.0	269.0	145.0	227.7	65.37
ATPIF1	2811.5	3072.0	1450.0	2599.0	2919.0	1450.5	2383.7	739.23
ATRAID	617.0	915.0	510.5	538.5	815.5	573.0	661.6	164.75
ATRIP	704.0	379.5	308.0	655.0	375.5	282.0	450.7	181.90
ATRNL1	3.0	1.5	7.0	2.5	1.0	10.0	4.2	3.56
ATRX	458.5	183.0	594.5	483.0	225.0	502.0	407.7	164.87
ATXN1	92.5	46.0	211.0	93.5	51.5	170.0	110.8	66.17
ATXN1L	521.5	351.0	484.5	499.5	360.0	503.0	453.3	76.68
ATXN2	511.0	453.0	751.0	490.5	503.5	727.0	572.7	130.60
ATXN3	401.5	299.0	438.5	433.0	324.0	418.5	385.8	59.45
ATXN7	241.0	126.0	159.0	208.0	132.5	160.5	171.2	44.79
ATXN7L1	28.5	10.0	45.0	20.5	21.0	47.0	28.7	14.68
AUH	162.5	216.5	210.5	160.5	256.0	191.0	199.5	36.23
AUP1	692.0	620.0	606.0	677.5	648.0	616.0	643.3	35.34
AURKA	669.0	286.5	427.0	762.0	245.0	264.0	442.3	223.08
AURKAIP1	459.0	550.0	361.0	436.0	532.5	350.5	448.2	83.53
AUTS2	451.5	369.0	726.0	436.0	411.5	667.0	510.2	148.18
AVEN	289.0	305.0	166.5	277.5	326.0	170.0	255.7	69.65
AVL9	386.0	188.0	192.5	359.0	232.0	209.5	261.2	88.03
AVPR1A	0.5	0.0	0.5	0.0	0.5	0.0	0.3	0.27
AVPR1B	0.5	0.5	0.0	0.5	0.0	0.0	0.3	0.27
AXIN1	292.5	386.0	372.0	298.5	433.0	402.0	364.0	56.84
AXIN2	89.5	101.0	118.0	88.5	121.5	168.5	114.5	29.87
AZI2	393.0	350.0	419.0	392.0	400.5	394.5	391.5	22.68
AZIN1	1989.0	1649.0	2167.5	1849.5	1725.0	1875.0	1875.8	185.89
AZIN2	24.5	21.0	7.0	20.0	14.0	8.0	15.8	7.24
B2M	3345.0	3125.5	3565.5	3166.5	3234.0	4090.5	3421.2	363.85
B3GALNT1	31.0	19.0	50.0	37.0	22.5	30.5	31.7	11.05
B3GALNT2	145.0	63.5	115.0	125.0	81.5	105.5	105.9	29.56
B3GALT1	32.5	11.5	12.5	16.5	4.5	6.5	14.0	10.03
B3GALT2	45.5	149.0	116.0	46.0	171.0	83.5	101.8	52.60
B3GALT4	100.0	92.0	76.5	79.0	79.5	77.0	84.0	9.70
B3GALT5	8.0	14.5	37.0	5.0	17.0	38.5	20.0	14.42
B3GALT6	356.0	151.0	178.5	405.0	193.5	160.5	240.8	110.33
B3GALTL	155.5	74.5	157.0	143.5	113.0	155.0	133.1	33.16
B3GAT1	9.5	7.0	3.5	4.0	9.5	9.0	7.1	2.75
B3GAT2	35.0	30.5	28.5	29.0	44.5	42.5	35.0	7.00
B3GNT1	957.0	644.5	530.5	776.5	464.5	526.5	649.9	186.67
B3GNT2	135.5	103.0	100.5	151.0	110.0	103.0	117.2	21.03
B3GNT3	0.5	1.0	0.0	0.0	1.0	0.0	0.4	0.49
B3GNT4	147.5	147.5	132.5	138.5	191.0	103.0	143.3	28.55
B3GNT5	102.5	78.0	160.0	138.0	100.0	151.5	121.7	32.77
B3GNT7	6.5	4.0	5.5	3.5	5.5	5.5	5.1	1.11

B3GNT9	78.0	76.5	87.0	87.0	69.0	73.0	78.4	7.34
B3GNTL1	29.0	32.0	43.0	23.0	43.0	33.5	33.9	7.90
B4GALNT3	320.5	185.5	285.0	293.5	181.5	166.0	238.7	68.15
B4GALNT4	76.0	77.5	148.5	73.5	55.0	107.0	89.6	33.35
B4GALT1	49.0	24.0	67.0	61.5	22.0	55.5	46.5	19.18
B4GALT2	5937.5	6425.5	4796.5	5820.0	6493.0	4849.0	5720.3	743.51
B4GALT3	115.0	142.5	188.5	126.5	123.5	202.0	149.7	36.66
B4GALT4	377.0	162.5	216.5	313.5	179.0	187.5	239.3	86.26
B4GALT5	1098.0	1179.0	1265.0	1236.0	1376.5	1411.5	1261.0	118.26
B4GALT6	3.5	2.0	10.0	1.0	2.5	1.5	3.4	3.34
B4GALT7	497.0	512.0	265.5	502.5	583.5	280.0	440.1	133.40
B9D1	54.5	15.5	40.0	50.0	30.0	35.0	37.5	14.13
BAAT	6.0	12.5	17.0	10.5	11.5	17.0	12.4	4.19
BABAM1	850.0	988.5	799.0	776.5	1038.5	838.5	881.8	106.55
BACE1	2437.0	3255.5	3617.5	2544.5	3698.0	4027.0	3263.3	647.64
BACE2	150.5	107.5	172.5	162.0	113.5	161.0	144.5	27.31
BACH1	654.0	739.5	1091.5	699.0	908.5	1137.5	871.7	207.37
BACH2	102.5	64.0	277.0	102.0	80.5	223.5	141.6	87.05
BAG1	397.0	311.0	210.5	350.0	366.5	206.0	306.8	81.26
BAG2	3036.0	3853.0	2774.5	2979.5	4176.0	3071.0	3315.0	560.86
BAG3	1486.0	1799.0	2105.0	1460.0	2113.5	2362.5	1887.7	367.64
BAG4	500.5	517.0	435.0	554.0	607.5	478.5	515.4	60.04
BAG5	360.0	334.5	433.0	376.5	448.0	472.5	404.1	54.76
BAHCC1	1039.5	1196.0	1524.5	1024.5	1168.5	1876.5	1304.9	333.01
BAHD1	783.5	534.5	690.0	807.5	592.5	667.5	679.3	105.88
BAI2	8.5	8.0	0.0	10.0	5.0	0.5	5.3	4.26
BAI3	1.0	1.5	2.0	3.0	3.0	2.0	2.1	0.80
BAIAP2	5.5	16.0	17.0	8.0	21.5	18.5	14.4	6.27
BAIAP2L1	116.0	57.0	48.5	108.5	72.5	52.5	75.8	29.45
BAIAP2L2	10.0	15.0	13.0	13.5	14.0	10.5	12.7	1.99
BAK1	165.5	294.5	428.0	200.5	253.5	360.5	283.8	98.63
BAMBI	46.0	42.0	25.5	49.0	53.0	37.5	42.2	9.78
BANK1	2.5	1.5	0.5	4.5	1.5	0.0	1.8	1.60
BAP1	1085.5	981.0	898.0	958.5	948.5	899.5	961.8	69.01
BARD1	295.5	84.0	205.5	331.0	75.5	111.0	183.8	111.00
BARHL1	0.0	0.0	0.0	0.0	0.0	0.0	0.0	0.00
BARHL2	0.0	0.0	0.0	0.0	0.0	0.0	0.0	0.00
BARX2	23.5	341.0	204.0	37.5	458.5	275.5	223.3	171.30
BASP1	3310.5	2455.0	3200.0	3628.0	2697.5	2854.5	3024.3	432.89
BATF	144.5	129.0	48.0	143.5	128.0	52.0	107.5	45.10
BATF3	6.0	2.5	2.0	4.5	1.0	3.5	3.3	1.81
BAZ1A	892.5	580.0	840.0	904.0	620.0	660.0	749.4	145.62
BAZ1B	268.0	144.0	480.5	306.5	139.0	394.5	288.8	135.70
BAZ2B	738.0	448.0	578.5	631.5	525.5	556.0	579.6	98.58
BBIP1	105.5	123.0	174.5	91.0	147.0	180.5	136.9	36.61
BBOX1	534.5	339.0	230.5	564.5	344.0	233.5	374.3	144.57
BBS10	102.0	66.5	66.0	104.5	71.0	79.0	81.5	17.50
BBS12	53.0	23.0	36.0	35.5	32.0	40.0	36.6	9.88
BBS2	165.5	154.5	165.0	165.0	171.5	193.0	169.1	12.94
BBS4	381.5	247.5	290.5	361.0	288.5	341.5	318.4	51.05
BBS5	453.0	226.5	316.0	402.5	266.0	265.5	321.6	88.46
BBS7	163.5	67.5	88.0	127.5	72.0	65.5	97.3	39.80
BBS9	219.0	73.5	101.0	198.5	86.5	88.5	127.8	63.61
BBX	195.5	91.5	324.5	203.5	103.0	290.5	201.4	94.75

BCAN	55.0	720.5	159.0	62.5	789.5	184.0	328.4	335.07
BCAP29	1402.0	513.5	980.0	1333.0	628.5	867.5	954.1	361.25
BCAR3	489.0	383.5	352.0	503.5	431.0	346.5	417.6	68.08
BCAS1	5.5	4.0	3.0	3.5	3.0	3.5	3.8	0.94
BCAS2	530.0	625.5	404.5	465.5	674.5	428.5	521.4	109.34
BCAT1	228.0	66.5	288.5	263.0	67.5	244.5	193.0	99.65
BCDIN3D	53.5	53.5	55.5	68.5	53.0	48.5	55.4	6.81
BCHE	12.0	16.0	73.5	8.5	14.0	75.5	33.3	32.05
BCKDHB	261.0	176.5	127.5	230.0	200.5	113.0	184.8	57.63
BCL10	184.5	179.0	220.5	173.0	188.0	194.5	189.9	16.70
BCL11A	2.5	0.5	8.0	1.0	2.5	3.5	3.0	2.68
BCL11B	0.0	0.0	0.0	0.0	0.0	0.0	0.0	0.00
BCL2	764.0	959.5	689.0	768.0	1212.0	867.5	876.7	189.27
BCL2A1	6.0	1.5	2.5	4.5	3.5	2.5	3.4	1.63
BCL2L1	791.5	781.0	533.5	1004.0	827.0	599.0	756.0	168.87
BCL2L13	14.5	8.0	26.0	14.0	6.0	16.0	14.1	7.05
BCL2L14	1.5	0.0	0.0	0.0	0.0	0.0	0.3	0.61
BCL2L15	1.0	0.0	0.5	0.5	1.0	0.0	0.5	0.45
BCL6	356.0	564.5	1461.0	379.5	707.5	1590.0	843.1	545.50
BCL7A	70.0	82.5	326.0	99.5	91.5	230.0	149.9	104.22
BCL7B	517.5	502.5	429.5	491.0	459.0	414.0	468.9	41.58
BCL9	168.5	191.0	407.0	162.0	203.0	450.0	263.6	129.32
BCL9L	126.5	61.5	311.0	167.5	63.0	261.5	165.2	103.18
BCLAF1	1925.0	1134.0	1115.0	1832.5	1232.0	997.5	1372.7	400.10
BCO1	2.0	3.0	6.5	6.5	6.0	1.5	4.3	2.34
BCO2	1.0	1.5	2.5	2.0	2.0	2.0	1.8	0.52
BCOR	261.5	73.5	175.0	187.0	88.0	145.5	155.1	69.26
BCR	287.5	339.0	524.5	269.0	439.0	531.0	398.3	116.36
BDH1	587.5	1534.0	2148.0	690.5	1810.0	1612.5	1397.1	625.17
BDH2	403.0	294.5	297.0	408.0	383.5	382.0	361.3	51.84
BDKRB1	29.0	13.0	7.5	45.5	10.0	11.5	19.4	14.88
BDKRB2	5.0	8.0	4.0	8.0	5.0	4.0	5.7	1.86
BDNF	27.0	45.5	25.5	29.0	55.5	39.5	37.0	11.97
BEGAIN	0.0	3.0	0.0	0.0	4.0	0.5	1.3	1.78
BEND3	209.0	167.0	200.0	229.5	197.0	219.5	203.7	21.67
BEND4	42.0	16.0	17.5	24.5	12.0	17.0	21.5	10.83
BEND5	158.0	246.5	155.0	140.0	261.5	154.5	185.9	53.32
BEND6	311.0	181.0	149.5	278.0	250.0	176.5	224.3	64.52
BEND7	176.0	98.0	130.0	132.5	109.0	133.5	129.8	26.83
BEST1	16.5	12.5	7.0	12.5	14.0	9.0	11.9	3.43
BEST3	1.0	7.5	10.5	1.0	1.0	3.0	4.0	4.06
BEST4	0.0	0.0	0.5	0.0	0.0	0.5	0.2	0.26
BET1	282.5	445.5	501.0	265.5	483.0	403.0	396.8	101.00
BET1L	966.5	567.0	518.0	897.5	580.5	551.0	680.1	197.46
BFAR	567.0	266.0	498.5	543.0	320.5	428.0	437.2	122.32
BFSP1	1.0	0.5	1.0	0.5	0.5	0.0	0.6	0.38
BFSP2	1.0	0.0	0.0	0.0	0.0	0.5	0.3	0.42
BHLHA15	147.0	227.0	152.0	166.5	261.0	134.5	181.3	50.76
BHLHA9	0.5	0.0	0.0	0.0	0.0	0.0	0.1	0.20
BHLHE23	0.0	0.0	1.0	1.0	0.0	0.5	0.4	0.49
BHLHE40	1134.0	1262.0	1359.0	1239.5	1537.0	1401.0	1322.1	141.13
BHLHE41	52.5	49.5	33.0	72.5	60.0	37.5	50.8	14.52
BICC1	726.5	442.5	820.0	710.5	508.5	770.0	663.0	151.58
BICD2	2017.0	1576.5	3406.0	2406.5	2034.5	3318.5	2459.8	747.35

BID	500.0	383.5	530.0	485.0	399.0	432.5	455.0	58.83
BIN2	1.5	0.0	0.0	0.5	0.0	0.0	0.3	0.61
BIRC2	1162.0	721.5	778.0	1132.0	754.0	873.0	903.4	195.54
BIRC6	105.5	26.0	173.0	93.0	37.5	156.0	98.5	59.85
BIRC7	8.0	8.5	9.5	3.0	6.0	6.0	6.8	2.34
BLCAP	3395.5	2990.5	1863.5	3141.0	3204.0	2007.0	2766.9	658.74
BLK	0.0	0.0	0.0	0.0	0.0	0.0	0.0	0.00
BLM	365.5	151.5	235.5	354.0	147.5	150.0	234.0	102.99
BLMH	1927.0	1506.5	1392.5	1961.0	1556.5	1196.5	1590.0	301.05
BLNK	0.0	0.5	0.0	0.0	0.0	0.0	0.1	0.20
BLOC1S1	226.0	360.0	205.5	245.5	353.0	218.0	268.0	69.81
BLOC1S2	812.5	590.5	423.5	718.5	635.5	342.5	587.2	177.21
BLOC1S4	520.0	661.0	461.0	443.0	690.5	430.0	534.3	114.23
BLOC1S5	1639.0	1404.5	1760.5	1350.5	1417.5	1319.0	1481.8	176.77
BLOC1S6	200.0	172.0	160.5	194.5	190.5	144.5	177.0	21.78
BLVRA	57.0	250.0	101.5	55.0	266.5	75.0	134.2	97.69
BLZF1	372.0	382.0	470.0	325.5	384.0	427.0	393.4	49.56
BMF	1062.5	619.5	719.0	887.5	789.0	947.5	837.5	160.71
BMP10	0.0	0.0	0.0	0.0	0.0	0.0	0.0	0.00
BMP15	1.5	1.0	2.5	3.0	3.5	0.0	1.9	1.32
BMP2	2.5	1.5	7.5	2.5	3.5	3.0	3.4	2.11
BMP2K	840.5	1411.5	1672.0	986.0	1641.0	1517.5	1344.8	349.89
BMP3	16.5	3.0	14.5	50.5	6.5	10.5	16.9	17.19
BMP4	179.5	282.0	555.0	100.0	251.5	392.5	293.4	161.57
BMP5	0.0	0.0	0.0	0.0	0.0	0.0	0.0	0.00
BMP6	74.0	42.0	38.0	51.0	49.0	52.0	51.0	12.52
BMP7	108.5	318.5	315.5	190.5	453.0	614.5	333.4	181.58
BMPER	32.5	16.5	16.0	87.0	36.0	19.5	34.6	27.03
BMPR1A	632.5	431.5	681.5	572.5	509.5	689.5	586.2	101.88
BMPR1B	34.0	104.0	44.0	49.5	141.5	75.5	74.8	41.37
BMPR2	980.5	669.0	1055.5	927.0	763.0	1042.5	906.3	157.22
BMS1	724.0	440.0	555.0	677.0	452.0	533.5	563.6	116.02
BNC1	13.0	6.0	10.0	10.5	8.0	12.0	9.9	2.58
BNC2	60.0	63.0	101.5	55.5	56.0	101.0	72.8	22.18
BNIP1	210.5	209.0	193.5	201.0	229.0	206.0	208.2	11.93
BNIP2	592.0	494.0	690.5	629.5	591.0	661.5	609.8	68.78
BNIP3	566.5	424.0	349.5	542.0	447.0	339.5	444.8	94.73
BNIP3L	411.0	275.0	635.0	429.5	323.0	595.5	444.8	144.17
BOC	733.0	414.0	444.5	603.0	468.0	427.5	515.0	126.73
BOD1	645.5	590.5	433.0	634.0	691.0	383.5	562.9	124.99
BOD1L1	257.0	100.5	459.0	266.0	125.5	344.0	258.7	134.33
BOK	29.5	105.0	92.5	20.0	97.5	74.0	69.8	36.45
BOLA3	427.5	625.0	330.5	419.0	564.0	347.0	452.2	118.28
BOLL	27.0	20.5	12.0	14.5	16.5	13.0	17.3	5.65
BORA	134.5	65.0	104.5	144.0	68.0	76.5	98.8	34.46
BPGM	882.5	545.5	782.5	840.5	559.5	638.0	708.1	146.23
BPHL	46.0	27.5	17.5	39.5	32.5	18.5	30.3	11.37
BPIFB6	0.0	0.0	0.0	0.0	0.0	0.0	0.0	0.00
BPNT1	234.0	164.5	201.5	194.0	187.5	166.5	191.3	25.62
BPTF	334.0	253.5	701.0	335.0	306.5	647.0	429.5	192.44
BRAP	1149.0	582.5	727.0	1025.5	695.5	666.5	807.7	225.24
BRAT1	251.5	253.0	259.5	248.0	307.5	307.0	271.1	28.26
BRCA1	208.5	154.0	190.5	185.5	137.5	125.5	166.9	32.80
BRCA2	232.0	74.5	127.5	220.0	101.5	92.0	141.3	67.95

BRCC3	461.0	419.5	305.5	509.5	502.0	291.0	414.8	95.92
BRD1	535.5	367.5	568.0	518.0	451.5	603.5	507.3	85.45
BRD3	142.0	104.5	226.0	137.0	115.5	197.5	153.8	47.84
BRD4	307.0	255.0	586.0	301.0	249.5	486.5	364.2	138.92
BRD7	1357.5	1290.0	1159.0	1346.0	1519.0	1210.5	1313.7	126.69
BRD8	788.5	666.0	736.5	701.5	710.0	635.5	706.3	53.56
BRD9	337.5	251.5	183.0	255.0	280.0	159.0	244.3	65.06
BRF1	164.0	99.0	151.5	153.5	100.5	154.0	137.1	29.25
BRF2	165.5	95.5	65.0	156.0	113.5	73.5	111.5	41.86
BRI3	539.5	712.5	504.0	473.5	804.0	530.0	593.9	132.54
BRI3BP	1877.0	890.0	1328.0	1823.0	932.0	1209.0	1343.2	426.11
BRICD5	0.0	1.0	1.0	0.5	3.0	1.0	1.1	1.02
BRINP1	11.5	1.0	1.0	6.5	0.5	1.5	3.7	4.43
BRINP2	0.0	3.0	1.5	2.0	2.5	1.5	1.8	1.04
BRINP3	0.0	0.0	0.0	0.0	0.0	0.0	0.0	0.00
BRIP1	199.5	132.5	205.5	208.5	133.5	165.0	174.1	35.43
BRIX1	1032.5	908.0	677.0	961.5	994.0	646.5	869.9	166.61
BRMS1L	464.5	298.0	300.5	409.5	350.5	284.5	351.3	72.23
BROX	445.0	342.0	387.0	409.5	403.0	328.0	385.8	43.87
BRPF1	281.5	288.0	306.0	231.0	256.0	266.0	271.4	26.36
BRPF3	551.5	505.5	392.5	535.5	605.5	461.0	508.6	74.44
BRS3	0.0	0.0	0.0	0.0	0.0	0.0	0.0	0.00
BRSK2	0.5	0.0	1.0	0.5	1.5	1.0	0.8	0.52
BRWD1	573.5	436.0	520.0	588.5	521.5	544.5	530.7	53.91
BRWD3	255.5	177.0	453.5	230.5	247.0	377.5	290.2	103.67
BSDC1	330.0	284.5	339.5	274.0	285.5	344.0	309.6	31.53
BSG	4247.0	2488.5	3987.0	4571.0	2670.0	4153.0	3686.1	880.10
BSN	3.5	7.0	14.5	8.0	3.5	19.0	9.3	6.25
BSND	0.0	0.0	0.0	0.0	0.0	0.0	0.0	0.00
BSPRY	5.5	4.5	2.5	2.0	4.0	6.0	4.1	1.59
BST1	345.0	209.0	191.5	321.5	228.0	148.0	240.5	76.92
BSX	0.0	0.0	0.0	0.0	0.0	0.0	0.0	0.00
BTAF1	586.0	517.0	711.5	600.0	614.5	601.5	605.1	62.57
BTBD1	1206.0	1214.5	1071.0	1097.0	1418.5	1094.5	1183.6	130.19
BTBD10	377.0	252.5	381.0	410.0	304.5	350.5	345.9	57.91
BTBD11	162.0	310.0	117.5	170.5	397.0	91.0	208.0	119.60
BTBD17	0.5	1.5	0.0	0.0	1.5	0.0	0.6	0.74
BTBD19	26.0	35.5	54.5	25.0	35.5	62.5	39.8	15.35
BTBD2	200.0	185.5	204.0	193.5	134.0	183.5	183.4	25.48
BTBD3	1224.0	956.5	1567.5	1109.0	974.5	1542.0	1228.9	270.56
BTBD6	177.5	250.0	178.0	129.5	215.5	120.0	178.4	49.61
BTBD7	99.5	43.5	181.0	119.0	37.5	148.5	104.8	56.98
BTBD8	14.5	5.0	9.0	8.0	6.5	11.5	9.1	3.46
BTBD9	91.5	27.0	139.5	78.0	38.5	99.5	79.0	41.43
BTC	704.5	480.5	162.0	583.0	446.0	192.5	428.1	214.37
BTD	146.5	98.5	129.0	160.5	86.0	127.5	124.7	28.17
BTF3	5169.0	3713.5	3205.5	4680.5	4065.5	2738.5	3928.8	906.44
BTF3L4	1922.5	2185.5	1788.5	1845.5	2502.0	1550.0	1965.7	333.60
BTG1	11165.0	5808.5	6036.5	9723.5	6432.5	5303.0	7411.5	2420.68
BTG2	1231.5	1090.0	1463.5	1174.5	1144.5	1396.0	1250.0	148.10
BTG4	0.0	0.0	0.0	0.0	0.0	0.5	0.1	0.20
BTK	32.5	25.5	14.0	23.5	16.5	12.5	20.8	7.74
BTRC	210.5	228.0	511.0	201.5	233.0	434.5	303.1	134.12
BUB1	852.5	281.0	489.5	1105.5	314.0	384.0	571.1	333.67

BUB3	1497.5	842.5	1032.5	1539.0	849.0	908.5	1111.5	322.64
BUD13	313.0	282.5	294.0	281.0	322.0	279.0	295.3	18.23
BUD31	1239.5	1340.0	960.0	1150.5	1453.0	983.0	1187.7	195.65
BVES	369.0	644.0	433.5	474.0	818.0	536.0	545.8	163.01
BYSL	434.0	613.5	420.5	397.0	649.5	441.5	492.7	109.19
BZRAP1	284.0	241.5	480.5	245.5	295.5	552.5	349.9	132.71
BZW1	3305.5	3425.5	3212.5	3275.5	3690.5	3118.5	3338.0	200.40
BZW2	4026.5	4408.5	2984.5	3794.0	4688.5	2583.0	3747.5	817.39
C10H14orf80	219.0	175.5	110.5	209.5	157.5	91.5	160.6	51.61
C10H1orf111	7.5	7.0	10.5	5.5	10.5	23.5	10.8	6.56
C10H1orf112	197.5	74.0	116.5	180.0	70.0	84.0	120.3	55.73
C10H1orf123	472.0	316.5	193.0	381.5	346.5	208.5	319.7	105.98
C10H1orf146	1.5	2.0	0.5	3.0	0.5	2.0	1.6	0.97
C10H1orf168	0.0	0.0	0.0	0.5	0.0	0.0	0.1	0.20
C10H1orf177	1.0	0.0	0.0	0.5	0.0	0.0	0.3	0.42
C10H1orf21	162.0	134.5	198.5	159.0	163.5	161.0	163.1	20.46
C10H1orf210	0.0	0.5	0.0	0.0	0.0	0.0	0.1	0.20
C10H1orf226	27.5	41.5	37.0	35.5	60.5	53.5	42.6	12.25
C10H1orf228	4.5	3.5	6.0	6.0	3.5	5.0	4.8	1.13
C10H1orf27	624.0	435.5	370.5	619.5	508.5	346.5	484.1	120.58
C10H1orf52	213.0	173.5	151.0	163.5	203.0	135.0	173.2	30.06
C11H18orf32	638.5	658.5	578.5	691.0	808.5	603.5	663.1	81.55
C11H21orf2	740.5	689.5	492.0	689.5	712.0	471.0	632.4	118.58
C11H2orf54	0.0	0.0	0.5	0.0	0.0	0.0	0.1	0.20
C11H2orf82	6.0	11.0	6.0	8.5	14.5	5.0	8.5	3.66
C11H3orf33	288.0	367.5	274.0	255.5	408.5	349.0	323.8	60.24
C11H3orf58	106.0	73.5	145.0	151.0	96.0	148.0	119.9	32.57
C11H3orf70	334.5	218.0	318.5	339.5	240.5	330.0	296.8	53.29
C12H15orf26	4.0	6.0	6.5	4.0	7.0	4.5	5.3	1.33
C12H15orf39	43.5	42.0	52.0	40.5	51.0	45.5	45.8	4.76
C12H15orf40	539.0	491.5	283.5	477.0	522.0	309.5	437.1	111.37
C12H15orf43	0.0	0.0	0.5	0.0	0.0	0.5	0.2	0.26
C12H15orf48	10.0	6.0	8.5	17.0	5.0	13.5	10.0	4.57
C12H15orf59	0.5	0.0	1.0	1.0	1.0	1.0	0.8	0.42
C12H15orf61	108.0	48.5	34.0	80.5	46.5	30.5	58.0	30.20
C12H15orf65	0.5	0.0	0.5	0.0	0.5	0.0	0.3	0.27
C13H16orf70	674.5	612.0	624.5	554.5	783.0	625.5	645.7	77.45
C13H16orf74	1.5	0.5	0.5	0.5	0.5	0.0	0.6	0.49
C13H16orf87	200.0	131.5	96.0	186.5	141.0	93.5	141.4	44.55
C13H19orf40	127.0	104.5	123.0	161.5	79.0	102.0	116.2	28.06
C14H3orf14	255.0	226.0	300.5	259.0	231.5	279.0	258.5	28.22
C14H3orf18	716.5	366.5	518.5	793.5	380.0	528.0	550.5	173.82
C14H3orf67	5.0	0.5	1.0	7.5	1.5	0.5	2.7	2.91
C14H9orf89	2013.0	1267.5	1419.0	2056.5	1427.5	1627.5	1635.2	330.26
C15H5orf15	2510.0	1690.5	2044.5	2516.0	1915.5	2234.0	2151.8	331.01
C15H5orf24	120.0	39.5	196.5	142.5	47.5	154.0	116.7	61.94
C15H5orf45	542.5	344.5	404.0	399.0	317.0	240.0	374.5	101.97
C15H5orf58	0.0	0.0	0.0	0.0	0.0	0.0	0.0	0.00
C16H16orf45	43.5	28.0	33.5	37.5	40.0	42.5	37.5	5.89
C16H16orf52	117.5	104.0	146.5	96.0	110.0	155.0	121.5	23.88
C16H16orf59	86.0	39.5	36.5	61.0	39.0	36.5	49.8	20.06
C16H16orf72	2749.5	2126.0	2090.0	2629.5	2398.0	1956.0	2324.8	319.14
C16H16orf96	37.5	18.5	6.0	27.0	12.5	10.0	18.6	11.81
C16H7orf26	204.5	187.0	205.0	209.0	216.5	177.0	199.8	14.81

C16H7orf50	219.5	171.0	144.5	218.0	163.5	100.0	169.4	45.49
C16H7orf62	1.0	0.5	0.5	1.5	1.5	1.0	1.0	0.45
C16H8orf33	228.5	126.5	145.5	213.5	127.0	133.5	162.4	46.14
C17H11orf85	0.0	0.0	3.0	0.0	1.0	0.5	0.8	1.17
C17H12orf43	55.5	47.0	35.5	52.5	44.0	34.0	44.8	8.74
C17H12orf49	331.0	166.0	183.0	343.5	157.5	163.0	224.0	88.22
C17H12orf65	388.5	401.5	362.0	382.0	434.0	324.0	382.0	37.12
C19H16orf93	116.5	108.0	77.5	91.5	113.5	73.0	96.7	18.76
C19H9orf114	138.5	168.0	120.5	124.5	162.5	130.0	140.7	20.05
C19H9orf142	77.5	109.0	80.5	66.0	109.5	102.0	90.8	18.46
C19H9orf16	574.0	432.5	321.0	543.5	418.5	309.5	433.2	109.63
C19H9orf171	0.0	0.0	0.0	0.0	0.0	0.0	0.0	0.00
C19H9orf172	7.0	4.5	4.5	11.0	8.0	9.5	7.4	2.63
C19H9orf173	6.5	5.5	1.5	9.5	4.0	3.0	5.0	2.83
C19H9orf69	215.0	233.0	200.5	216.0	264.5	229.0	226.3	21.96
C19H9orf78	338.0	293.0	345.5	300.0	333.5	364.0	329.0	27.33
C19H9orf9	13.0	9.5	31.5	9.5	9.5	25.5	16.4	9.65
C19H9orf91	2.0	3.0	0.5	1.0	3.5	0.5	1.8	1.29
C1D	642.0	518.0	366.5	586.5	576.0	324.0	502.2	128.47
C1GALT1	664.5	429.5	653.0	645.5	505.5	615.5	585.6	95.95
C1GALT1C1	1032.0	659.0	773.0	1073.0	720.5	665.0	820.4	184.94
C1H11orf30	220.0	199.0	238.0	199.0	220.5	227.0	217.3	15.56
C1H11orf54	402.5	209.0	185.5	365.5	255.5	203.0	270.2	91.91
C1H11orf70	35.5	11.5	16.5	32.0	18.0	15.5	21.5	9.79
C1H11orf73	657.0	577.0	450.5	600.5	593.0	360.5	539.8	111.14
C1H11orf87	1567.5	899.0	759.0	1299.0	992.0	1313.0	1138.3	304.33
C1H11orf97	0.5	1.5	0.0	2.5	1.5	2.0	1.3	0.93
C1H12orf29	387.5	260.0	256.0	388.5	275.0	194.0	293.5	78.27
C1H12orf4	447.5	263.0	252.0	419.5	266.5	198.0	307.8	100.87
C1H12orf40	0.5	0.0	0.0	1.0	0.0	0.0	0.3	0.42
C1H12orf45	547.0	644.0	395.5	527.5	712.5	366.5	532.2	135.20
C1H12orf5	437.0	348.5	231.0	377.0	413.0	207.5	335.7	95.40
C1H12orf50	7.0	2.5	1.5	12.0	3.0	1.5	4.6	4.16
C1H12orf57	667.5	1219.0	624.5	710.5	1323.5	725.0	878.3	308.15
C1H12orf66	414.5	535.0	348.5	413.0	637.0	359.0	451.2	112.60
C1H12orf73	699.0	1045.0	923.5	715.0	1228.5	970.0	930.2	201.77
C1H12orf75	63.0	36.0	76.0	59.0	58.5	71.0	60.6	13.88
C1H21orf140	0.0	0.0	0.0	0.0	0.0	0.0	0.0	0.00
C1H21orf33	696.5	545.5	421.5	582.5	497.0	344.5	514.6	123.75
C1H21orf59	453.5	356.0	354.5	400.0	368.5	305.5	373.0	49.83
C1H21orf62	0.5	0.5	0.0	3.0	0.5	0.0	0.8	1.13
C1H21orf91	225.5	205.0	231.0	203.5	206.0	256.0	221.2	20.65
C1H22orf23	322.5	155.5	102.5	258.5	159.5	109.0	184.6	87.65
C1H2orf40	17.5	16.5	33.0	14.0	16.5	23.5	20.2	7.04
C1H2orf49	457.5	558.0	540.5	484.0	608.0	522.0	528.3	53.66
C1H3orf17	273.0	430.0	309.0	310.0	484.5	331.0	356.3	82.25
C1H3orf30	0.0	0.5	1.0	1.5	0.5	0.0	0.6	0.58
C1H3orf38	336.5	206.5	166.0	343.0	227.0	162.0	240.2	80.96
C1H3orf52	0.0	0.0	1.5	0.0	1.5	1.0	0.7	0.75
C1H7orf49	108.5	75.0	54.5	103.0	90.5	55.0	81.1	23.43
C1H7orf55	410.0	305.5	149.0	410.0	292.0	104.5	278.5	128.49
C1H7orf60	140.5	89.0	219.0	145.5	87.0	203.5	147.4	55.47
C1HXorf22	1.0	0.5	1.0	0.0	0.5	0.0	0.5	0.45
C1HXorf23	659.0	405.0	433.0	580.5	454.0	376.0	484.6	110.76

C1HXorf30	0.5	0.0	1.0	1.5	0.0	0.0	0.5	0.63
C1HXorf36	4.0	2.0	0.0	4.5	1.5	0.0	2.0	1.92
C1QA	0.0	0.0	0.0	0.0	0.0	0.0	0.0	0.00
C1QB	0.0	0.0	0.0	0.0	0.0	0.0	0.0	0.00
C1QBP	3598.5	3743.0	2309.0	3298.0	3856.0	2250.5	3175.8	719.10
C1QC	0.0	0.0	0.0	0.0	0.0	0.0	0.0	0.00
C1QL1	158.5	542.5	81.0	109.0	530.5	118.0	256.6	218.27
C1QL4	0.5	0.0	0.5	0.0	0.0	0.0	0.2	0.26
C1QTNF1	20.0	11.5	10.5	15.5	9.0	7.5	12.3	4.63
C1QTNF2	0.0	0.0	0.0	1.0	0.0	1.0	0.3	0.52
C1QTNF3	556.5	823.0	4148.0	851.0	951.0	3628.5	1826.3	1610.87
C1QTNF4	3206.0	1156.5	615.5	3425.0	1343.0	638.0	1730.7	1262.13
C1QTNF5	0.5	0.5	0.0	0.0	0.0	0.0	0.2	0.26
C1QTNF6	1.0	0.0	1.5	1.0	0.5	0.5	0.8	0.52
C1QTNF7	0.0	0.0	0.0	0.5	0.0	0.0	0.1	0.20
C1QTNF8	0.0	0.0	0.0	0.0	0.0	1.0	0.2	0.41
C1R	11.0	17.0	5.0	15.5	21.0	5.5	12.5	6.47
C1S	23.0	19.0	6.0	21.5	19.0	9.0	16.3	7.01
C20H17orf58	89.0	299.5	288.5	132.0	389.5	535.0	288.9	164.55
C20H17orf62	420.0	333.0	214.0	479.5	425.5	202.5	345.8	116.45
C20H17orf67	6.0	2.5	0.5	4.0	3.0	0.0	2.7	2.23
C20H17orf70	581.5	547.5	684.0	423.0	530.0	643.5	568.3	91.90
C20H17orf75	1331.0	701.0	875.0	932.5	753.0	742.0	889.1	233.62
C20H17orf80	307.5	198.5	164.5	253.5	212.5	187.5	220.7	51.83
C20H17orf89	278.5	378.5	133.5	317.0	327.5	156.5	265.3	98.73
C21H17orf64	0.0	0.0	0.0	0.0	0.0	0.0	0.0	0.00
C21H17orf85	831.5	745.0	659.0	763.0	835.0	576.0	734.9	101.25
C21H17orf97	3.5	2.5	5.0	4.0	2.5	3.5	3.5	0.95
C22H20orf195	0.0	0.0	0.0	0.5	0.0	0.0	0.1	0.20
C22H20orf24	1394.0	1314.5	1158.0	1411.0	1431.5	1050.0	1293.2	155.59
C23H1orf158	13.0	9.0	8.0	18.0	9.5	5.0	10.4	4.52
C23H1orf159	14.5	6.5	11.0	13.0	10.5	5.0	10.1	3.68
C23H1orf167	6.0	2.5	3.0	2.5	4.5	0.5	3.2	1.89
C23H1orf174	68.0	27.0	44.0	75.5	39.5	37.5	48.6	18.94
C23H1orf50	186.5	182.5	129.0	160.0	176.0	99.0	155.5	34.71
C23H1orf86	80.5	41.5	33.0	56.0	31.0	23.0	44.2	21.04
C24H2orf42	164.5	170.5	123.5	164.0	195.5	129.5	157.9	26.99
C24H8orf4	1.0	5.5	5.5	0.0	0.5	1.5	2.3	2.50
C25H1orf216	2.5	1.0	3.0	0.5	1.5	3.5	2.0	1.18
C26H11orf1	0.0	0.5	2.0	0.0	0.0	2.5	0.8	1.13
C26H11orf52	1295.5	2453.0	2131.5	1018.5	2779.0	2012.5	1948.3	674.26
C26H11orf57	378.0	370.5	293.0	330.5	386.0	328.0	347.7	36.28
C26H11orf63	1.0	0.5	3.5	2.0	1.0	1.5	1.6	1.07
C26H11orf88	0.0	0.0	0.0	0.0	0.0	0.0	0.0	0.00
C28H1orf116	10.5	1.0	2.5	4.0	2.0	0.5	3.4	3.68
C28H1orf186	0.0	0.0	0.0	0.0	0.0	0.0	0.0	0.00
C28H1orf74	130.0	119.0	68.0	138.5	122.5	77.0	109.2	29.32
C28H6orf106	548.0	434.0	467.0	489.5	445.5	470.0	475.7	40.44
C28H6orf132	30.0	51.5	22.5	33.5	48.5	21.5	34.6	12.80
C28H6orf89	396.5	295.5	465.5	411.5	317.5	447.0	388.9	68.75
C29H17orf104	0.0	0.0	0.0	0.0	0.0	1.5	0.3	0.61
C2CD2	338.0	122.0	331.0	357.0	151.5	273.0	262.1	101.48
C2CD2L	305.5	217.5	250.5	271.5	248.5	256.5	258.3	29.08
C2CD3	23.5	4.5	32.5	22.0	8.5	21.0	18.7	10.35

C2CD4C	0.0	0.0	0.0	0.0	0.0	0.5	0.1	0.20
C2CD5	661.5	442.0	568.0	612.0	550.5	518.5	558.8	75.89
C2H1orf101	16.0	6.0	7.0	13.0	6.0	4.5	8.8	4.62
C2H1orf115	14.0	4.5	2.0	13.5	7.0	2.0	7.2	5.43
C2H1orf131	110.0	81.5	78.0	102.0	100.5	84.5	92.8	13.08
C2H1orf198	2153.5	1324.5	1865.0	1803.0	1396.0	1563.5	1684.3	314.34
C2H2orf43	222.0	152.5	231.5	241.5	152.5	210.0	201.7	39.48
C2H2orf50	1.0	0.0	0.0	0.5	0.0	0.0	0.3	0.42
C2H2orf70	0.0	0.0	0.0	0.0	0.0	0.0	0.0	0.00
C2H2orf71	1.0	0.5	0.0	1.5	1.5	0.0	0.8	0.69
C2H6orf120	166.0	119.5	180.0	167.0	153.5	191.5	162.9	24.93
C2H6orf163	0.5	6.0	6.5	1.5	9.5	4.0	4.7	3.36
C2H6orf165	10.5	23.0	11.5	10.5	36.0	37.5	21.5	12.73
C2H6orf203	581.5	511.0	320.5	544.5	513.0	326.0	466.1	113.58
C2H6orf211	276.5	219.5	349.0	348.0	261.0	282.5	289.4	50.78
C2H6orf57	277.5	503.5	345.0	274.0	623.0	434.5	409.6	137.83
C2HXorf21	0.0	0.0	0.0	0.0	0.0	0.0	0.0	0.00
C3	0.5	2.0	0.0	1.0	2.0	0.0	0.9	0.92
C30H19orf10	1540.0	1697.0	1457.5	1534.5	1872.5	1655.5	1626.2	148.89
C30H19orf35	0.0	0.0	0.5	0.0	0.0	0.5	0.2	0.26
C30H19orf44	336.0	388.0	290.5	375.5	472.5	342.0	367.4	61.77
C30H19orf45	13.0	7.0	6.0	16.5	7.5	3.5	8.9	4.85
C30H19orf60	295.5	410.5	348.5	281.5	398.5	360.5	349.2	52.50
C30H19orf70	454.5	614.5	336.0	386.5	586.0	286.5	444.0	133.51
C30H19orf71	2.0	0.0	0.5	0.0	0.5	0.0	0.5	0.77
C3AR1	0.0	0.5	0.0	0.0	0.0	0.0	0.1	0.20
C3H18orf21	488.0	360.5	178.0	458.0	358.0	172.0	335.8	134.86
C3H18orf42	67.5	70.0	76.0	60.5	102.0	93.5	78.3	16.13
C3H18orf63	0.0	0.0	0.0	0.0	0.0	0.0	0.0	0.00
C3H18orf8	417.0	324.0	341.5	419.0	380.0	327.0	368.1	43.51
C3H5orf22	255.0	143.0	155.5	242.5	163.5	119.5	179.8	55.56
C3H5orf49	3.0	9.5	14.5	1.0	11.5	18.5	9.7	6.70
C3H6orf52	683.5	479.5	485.5	677.5	517.0	477.0	553.3	99.56
C3H6orf62	4903.5	3486.0	3022.5	4641.0	3995.0	2715.0	3793.8	876.71
C3H7orf57	0.5	0.0	0.0	0.5	0.5	0.0	0.3	0.27
C3H7orf72	0.0	0.0	0.0	0.0	0.0	0.0	0.0	0.00
C3H8orf22	4.5	1.5	0.0	3.0	2.0	3.0	2.3	1.54
C3H8orf37	22.0	6.5	23.5	17.0	8.5	24.0	16.9	7.73
C3H8orf46	0.0	0.0	0.0	0.0	0.0	0.0	0.0	0.00
C3H8orf76	1414.0	814.0	579.0	1165.5	862.0	524.0	893.1	342.83
C4H20orf194	2425.5	1545.0	1774.5	1942.0	1641.0	1518.5	1807.8	340.96
C4H4orf17	6.5	1.0	4.0	1.0	3.0	1.5	2.8	2.16
C4H4orf19	0.0	0.0	0.0	0.0	0.0	0.0	0.0	0.00
C4H4orf27	624.5	369.5	299.5	535.5	343.0	237.5	401.6	147.94
C4H4orf29	476.0	396.0	438.5	430.0	443.5	420.5	434.1	26.52
C4H4orf33	175.5	92.5	193.0	191.5	107.0	186.5	157.7	45.51
C4H4orf46	196.0	176.5	160.5	206.0	169.0	178.0	181.0	16.99
C4H4orf47	274.0	95.0	61.0	234.0	97.0	62.5	137.3	92.59
C4H4orf48	77.0	100.0	48.0	106.5	165.0	62.0	93.1	41.61
C4H4orf50	0.0	0.0	0.0	0.0	0.0	0.0	0.0	0.00
C5	2.5	0.5	1.0	0.5	1.0	0.5	1.0	0.77
C5H11orf16	39.5	16.5	28.5	46.5	19.5	22.0	28.8	11.93
C5H11orf24	645.5	519.5	441.0	775.5	571.0	501.5	575.7	119.68
C5H11orf31	565.5	608.0	376.5	594.5	497.5	357.5	499.9	109.95

C5H11orf49	21.5	10.0	21.0	27.0	13.5	20.5	18.9	6.13
C5H11orf58	2921.5	2525.0	1415.5	2542.0	2561.0	1273.0	2206.3	685.27
C5H11orf74	10.5	13.0	6.0	18.0	11.0	6.0	10.8	4.54
C5H11orf94	23.5	15.5	6.5	18.5	20.5	5.5	15.0	7.45
C5H14orf105	0.0	0.0	0.0	0.5	0.0	0.0	0.1	0.20
C5H14orf132	3.0	0.5	0.0	1.5	0.0	0.0	0.8	1.21
C5H14orf159	233.5	193.0	109.0	175.0	182.0	48.5	156.8	66.59
C5H14orf166	2091.0	1942.5	1274.0	1893.0	2031.5	1216.5	1741.4	390.84
C5H14orf169	231.0	194.0	152.5	201.5	192.0	181.5	192.1	25.63
C5H14orf180	0.0	0.5	4.0	0.0	1.0	1.0	1.1	1.50
C5H14orf2	1106.5	1441.0	857.0	991.5	1388.5	748.0	1088.8	280.54
C5H14orf37	4.5	1.0	7.0	5.5	2.5	4.0	4.1	2.13
C5H14orf39	0.0	0.0	0.0	0.5	0.0	0.5	0.2	0.26
C5H14orf79	9.5	13.5	11.5	9.5	11.0	11.0	11.0	1.48
C5H15orf52	152.5	62.0	34.0	128.0	92.0	52.0	86.8	46.16
C5H15orf57	274.0	206.5	200.0	262.0	226.0	195.0	227.3	33.49
C5H15orf62	4.0	4.5	6.0	2.5	5.0	3.5	4.3	1.21
C6	0.0	0.0	0.0	0.0	0.0	0.0	0.0	0.00
C6H1orf35	571.5	446.0	282.0	520.5	398.5	250.5	411.5	127.70
C6H7orf25	234.0	199.0	228.5	203.5	210.0	234.0	218.2	15.86
C6H7orf31	2.5	1.0	0.0	0.5	1.0	0.5	0.9	0.86
C6H9orf152	0.0	1.5	0.5	1.0	2.0	0.0	0.8	0.82
C7	9.0	6.0	5.5	5.0	3.5	2.5	5.3	2.25
C7H21orf58	1.5	5.0	1.5	0.0	7.5	1.5	2.8	2.82
C7H2orf47	890.5	591.0	318.5	800.5	603.5	243.0	574.5	255.92
C7H2orf66	43.0	24.0	28.5	40.0	27.5	20.0	30.5	9.08
C7H2orf69	136.0	129.5	123.5	128.5	113.5	131.0	127.0	7.75
C7H2orf76	149.0	130.0	97.0	160.0	135.0	75.5	124.4	32.12
C7H2orf88	86.5	105.5	23.5	113.5	136.0	33.5	83.1	45.27
C8A	0.0	0.0	0.0	0.0	0.0	0.0	0.0	0.00
C8B	0.0	0.0	0.0	0.0	0.0	0.0	0.0	0.00
C8G	241.0	148.0	70.0	189.5	161.0	77.5	147.8	65.72
C8H10orf11	205.5	103.0	89.5	195.5	108.5	65.0	127.8	58.34
C8H10orf12	1277.0	1323.5	1057.5	1006.0	1380.5	1949.5	1332.3	337.16
C8H10orf128	0.0	0.0	0.0	0.0	1.0	0.5	0.3	0.42
C8H10orf2	572.0	689.5	465.5	609.0	657.5	462.0	575.9	95.75
C8H10orf32	213.5	160.5	263.0	205.5	235.5	210.5	214.8	34.08
C8H10orf54	194.0	136.5	276.0	191.0	139.0	236.5	195.5	54.51
C8H10orf71	83.0	309.0	1228.5	74.0	378.0	1011.5	514.0	489.47
C8H10orf76	609.5	660.5	644.0	622.5	651.5	663.5	641.9	21.61
C8H10orf88	422.0	487.5	435.5	390.5	496.0	369.5	433.5	50.80
C8H10orf90	0.0	1.5	2.0	0.0	1.5	2.5	1.3	1.04
C9H8orf48	102.5	63.0	82.5	92.5	81.5	78.0	83.3	13.40
C9HXorf56	513.0	581.0	633.0	497.0	614.0	662.5	583.4	66.45
C9HXorf57	338.0	151.0	496.5	332.5	173.5	363.5	309.2	128.72
C9HXorf65	0.0	0.0	0.0	0.0	0.0	0.5	0.1	0.20
CA10	0.5	0.0	1.0	0.0	0.0	0.5	0.3	0.41
CA12	5.5	4.5	31.5	8.0	7.5	19.0	12.7	10.60
CA14	9.5	9.0	2.0	6.5	9.5	1.5	6.3	3.72
CA4	0.0	0.0	0.0	0.0	0.0	0.0	0.0	0.00
CA5A	85.5	23.0	143.0	87.0	30.5	131.5	83.4	49.66
CA6	9.0	8.5	51.5	5.0	6.5	18.0	16.4	17.77
CA7	4.5	3.0	2.0	2.0	1.0	0.5	2.2	1.44
CA8	602.0	1014.5	929.0	564.0	1103.0	934.0	857.8	222.40

CAAP1	492.5	384.5	190.0	444.0	376.0	158.0	340.8	136.36
CAB39L	4.0	6.0	5.5	1.0	2.5	6.0	4.2	2.07
CABIN1	66.5	38.0	166.5	73.0	35.5	130.5	85.0	52.65
CABLES1	293.0	303.0	317.5	294.5	322.0	357.0	314.5	23.94
CABLES2	8.5	6.5	15.5	12.5	5.0	13.0	10.2	4.12
CABP1	0.5	0.0	0.0	0.5	0.0	0.5	0.3	0.27
CABP2	8.0	5.5	10.5	11.5	8.0	5.5	8.2	2.48
CABP4	1.5	1.0	0.0	0.5	0.5	1.0	0.8	0.52
CABP7	0.0	0.0	0.0	0.0	0.0	0.5	0.1	0.20
CABYR	15.5	7.0	8.0	10.5	5.5	10.0	9.4	3.51
CACFD1	39.5	32.5	218.0	52.0	44.5	205.0	98.6	87.79
CACHD1	370.0	290.5	418.5	398.0	340.0	462.0	379.8	60.40
CACNA1B	0.0	0.0	0.0	1.0	0.0	1.0	0.3	0.52
CACNA1D	5.5	0.5	2.5	6.5	1.0	2.0	3.0	2.45
CACNA1E	73.0	40.5	82.5	46.0	40.5	49.0	55.3	17.99
CACNA1G	0.5	1.0	1.0	0.5	2.0	1.5	1.1	0.58
CACNA1I	0.0	0.0	0.0	0.0	0.0	0.0	0.0	0.00
CACNA1S	91.5	887.5	1358.0	72.5	962.0	1282.5	775.7	566.70
CACNA2D2	962.5	376.0	360.5	953.5	359.0	314.5	554.3	313.36
CACNA2D3	2.0	1.5	1.0	1.5	6.0	0.5	2.1	1.99
CACNB2	3.0	2.0	2.0	2.0	0.5	1.0	1.8	0.88
CACNB4	2.0	0.0	2.0	1.0	0.0	1.5	1.1	0.92
CACNG1	70.0	1033.0	949.5	36.5	1042.5	719.0	641.8	470.65
CACNG2	2.5	4.5	7.0	3.0	6.0	2.5	4.3	1.92
CACNG3	706.5	873.0	855.5	774.5	1037.0	1319.5	927.7	221.84
CACNG4	21.5	265.0	531.5	23.0	250.0	417.5	251.4	205.55
CACNG5	248.0	169.0	133.5	251.5	204.5	139.5	191.0	52.03
CACTIN	851.5	740.0	606.5	834.5	813.5	644.5	748.4	103.23
CACUL1	424.5	368.0	459.0	373.0	386.0	438.5	408.2	37.72
CACYBP	391.5	306.0	432.5	367.5	357.5	345.0	366.7	42.86
CAD	435.5	339.0	390.5	381.5	302.5	383.5	372.1	45.86
CADM1	52.0	386.0	694.5	26.0	403.5	419.0	330.2	252.59
CADPS	2.0	2.0	3.5	1.5	4.0	2.5	2.6	0.97
CADPS2	25.5	19.0	41.0	11.5	16.0	23.0	22.7	10.26
CALB1	3.5	0.5	1.0	1.0	1.0	0.5	1.3	1.13
CALB2	0.5	1.0	0.0	0.0	0.0	0.0	0.3	0.42
CALCA	0.5	1.0	3.0	0.0	0.0	4.5	1.5	1.84
CALCOCO2	1066.0	628.0	763.5	1030.0	675.5	682.5	807.6	191.59
CALCRL	23.5	24.5	29.5	28.5	25.5	34.5	27.7	4.07
CALD1	739.5	618.5	2241.0	950.0	726.5	2322.0	1266.3	794.12
CALHM1	0.0	0.0	0.0	0.0	0.0	0.5	0.1	0.20
CALHM2	164.0	132.0	284.5	197.0	162.5	243.0	197.2	57.05
CALHM3	1.0	0.5	0.5	1.0	1.0	1.0	0.8	0.26
CALM1	4108.5	3241.5	3147.5	3908.0	3721.0	3032.0	3526.4	445.31
CALM2	11824.5	9461.5	9226.0	12149.0	10693.0	8543.0	10316.2	1472.37
CALML4	579.5	834.5	569.0	583.5	912.5	594.0	678.8	153.00
CALN1	1.0	1.5	0.0	3.0	4.5	0.0	1.7	1.78
CALR	9489.5	10249.0	9986.0	9472.5	10804.0	11084.5	10180.9	667.42
CALR3	4.0	4.0	5.5	3.5	4.5	5.5	4.5	0.84
CAMK1	116.5	172.5	164.5	86.0	147.5	170.0	142.8	34.74
CAMK1D	5.0	11.0	24.0	8.5	14.5	16.0	13.2	6.64
CAMK1G	15.5	6.0	3.0	21.0	5.0	5.5	9.3	7.19
CAMK2A	1886.0	9870.5	11831.0	1891.5	12209.5	11636.0	8220.8	4970.27
CAMK2B	37.0	59.5	56.5	32.0	65.0	71.0	53.5	15.61

CAMK2D	989.0	1351.5	2450.0	1166.5	1929.5	2813.0	1783.3	738.10
CAMK2G	184.0	151.0	298.0	186.5	158.5	264.5	207.1	60.04
CAMK4	65.5	52.0	35.5	103.0	69.5	67.5	65.5	22.40
CAMKK1	7.0	3.5	8.0	8.5	4.0	9.5	6.8	2.46
CAMKK2	225.0	160.0	505.0	194.0	180.5	506.5	295.2	164.48
CAMKMT	63.0	48.5	57.0	55.0	41.0	37.5	50.3	9.82
CAMKV	6.5	49.5	32.0	5.5	54.0	49.5	32.8	22.11
CAMLG	93.5	38.5	108.0	94.5	43.5	104.0	80.3	31.00
CAMSAP1	454.5	282.5	481.0	411.5	306.5	401.5	389.6	79.45
CAMSAP2	198.0	79.5	418.5	252.0	127.0	358.0	238.8	131.31
CAMTA1	759.0	536.0	565.0	748.0	661.0	565.5	639.1	98.24
CAND1	2075.0	1365.5	1806.0	2090.5	1470.5	1588.0	1732.6	308.25
CANT1	383.0	304.5	291.0	310.5	327.0	342.0	326.3	32.96
CANX	3634.0	2037.0	5844.0	4151.0	2497.0	4437.0	3766.7	1380.85
CAP1	8426.5	4973.5	4407.5	7810.5	5629.5	3765.0	5835.4	1882.85
CAP2	1425.5	2247.0	1608.5	1553.5	2705.5	1871.0	1901.8	489.95
CAPG	443.0	668.5	667.0	466.0	735.0	723.0	617.1	129.14
CAPN10	267.5	177.0	176.0	219.5	203.0	141.5	197.4	43.43
CAPN11	12230.0	10963.0	10791.5	11654.5	11408.0	10184.5	11205.3	716.22
CAPN13	9.5	20.5	36.0	9.5	23.5	36.0	22.5	11.90
CAPN14	13.0	9.0	10.5	14.5	9.0	4.5	10.1	3.51
CAPN15	532.5	570.5	1045.0	450.5	490.5	1033.0	687.0	275.63
CAPN2	3827.0	2726.0	2871.5	3678.0	2984.0	2752.5	3139.8	485.67
CAPN3	77.0	797.5	417.5	52.5	861.0	402.0	434.6	343.15
CAPN5	0.0	0.5	0.0	1.0	1.0	0.0	0.4	0.49
CAPN6	8.0	41.0	5.0	19.0	65.5	10.5	24.8	23.80
CAPN7	261.5	191.5	266.5	238.5	244.5	263.5	244.3	28.21
CAPN8	6.0	3.0	2.0	20.0	7.0	2.0	6.7	6.86
CAPN9	17.5	5.5	19.5	8.0	5.0	3.5	9.8	6.90
CAPNS2	44.0	24.0	33.5	43.0	28.0	22.0	32.4	9.45
CAPRIN1	4194.0	3662.0	4746.0	3982.5	4057.0	4268.5	4151.7	359.45
CAPS2	51.5	41.5	55.5	68.5	59.0	42.5	53.1	10.27
CAPSL	8.0	3.5	1.0	8.5	1.0	1.5	3.9	3.48
CAPZA1	2064.5	1970.5	1816.5	2038.0	2203.0	1583.0	1945.9	218.03
CAPZA2	739.5	493.0	831.0	797.5	563.5	681.5	684.3	133.10
CAPZA3	0.0	0.0	0.0	0.5	0.0	0.0	0.1	0.20
CAPZB	3834.0	3883.0	2916.5	3520.0	4476.5	2807.0	3572.8	632.63
CARD10	0.5	0.5	1.0	0.0	0.0	0.5	0.4	0.38
CARF	223.5	104.5	128.0	195.5	129.0	128.5	151.5	46.73
CARHSP1	637.0	530.5	415.0	647.5	596.5	438.5	544.2	100.07
CARKD	614.0	463.5	364.0	572.5	482.5	321.0	469.6	113.91
CARNS1	183.5	655.0	428.0	149.5	685.5	484.0	430.9	227.27
CARS	1400.5	1418.0	1732.0	1386.0	1495.0	1449.5	1480.2	129.35
CARS2	497.0	448.0	243.5	452.5	554.0	272.0	411.2	125.15
CARTPT	9.0	16.0	3.5	10.0	22.0	6.0	11.1	6.81
CASC1	44.0	65.5	57.0	45.0	53.0	41.0	50.9	9.33
CASC3	659.0	633.5	762.5	624.5	574.5	681.5	655.9	63.47
CASC4	2950.0	2178.0	2466.0	2929.5	2492.5	2618.0	2605.7	296.24
CASC5	530.5	148.0	394.5	629.5	150.5	208.5	343.6	206.43
CASK	582.0	399.5	642.0	536.5	496.0	653.5	551.6	95.85
CASKIN2	163.0	457.5	869.0	169.5	482.0	692.5	472.3	280.54
CASP10	305.5	206.0	278.0	308.5	237.5	214.0	258.3	45.31
CASP2	257.0	118.0	156.0	254.0	124.0	122.0	171.8	66.22
CASP3	181.5	150.5	293.0	194.0	177.0	265.0	210.2	55.88

CASP6	195.0	196.0	215.5	214.5	240.0	228.0	214.8	17.64
CASP7	116.0	56.5	75.0	100.0	52.0	76.0	79.3	24.78
CASP8AP2	294.0	166.0	292.5	269.5	194.5	260.5	246.2	53.44
CASP9	199.0	298.0	237.5	158.0	282.0	241.0	235.9	51.85
CASQ2	906.0	3202.0	4065.0	710.0	3519.0	2914.0	2552.7	1405.71
CASR	0.0	1.5	1.5	0.5	1.0	1.0	0.9	0.58
CAST	4599.0	3144.0	3062.5	4269.5	3620.5	2857.5	3592.2	706.32
CAT	350.0	177.5	225.0	275.5	230.0	264.0	253.7	58.39
CATIP	19.5	13.5	24.5	22.0	18.0	24.0	20.3	4.16
CAV1	6180.5	3204.0	5189.0	6403.5	3489.5	4048.0	4752.4	1374.40
CAV2	1131.0	843.0	1098.0	1084.5	994.0	1072.5	1037.2	105.41
CAV3	835.5	5546.5	2415.0	829.5	6310.0	2519.0	3075.9	2339.91
CBFA2T2	210.0	136.5	144.0	205.0	134.0	158.0	164.6	34.31
CBFB	403.0	527.0	599.5	437.5	546.5	482.0	499.3	72.70
CBL	1231.5	1094.0	1155.5	1101.5	1276.5	1100.0	1159.8	77.57
CBLB	150.5	133.0	261.5	163.5	137.0	211.5	176.2	50.49
CBLL1	411.5	369.0	378.0	409.0	388.0	394.5	391.7	16.83
CBLN1	62.5	109.5	136.5	37.5	105.5	182.5	105.7	51.73
CBLN2	0.0	0.0	0.0	1.0	0.0	0.0	0.2	0.41
CBLN4	0.0	0.0	0.0	0.5	0.5	0.5	0.3	0.27
CBR1	712.5	381.5	340.0	843.5	494.0	363.5	522.5	209.01
CBR4	279.0	179.5	94.0	242.5	186.5	93.0	179.1	75.78
CBX1	366.0	329.5	710.5	399.0	367.5	677.0	474.9	171.26
CBX2	0.5	2.0	15.0	1.5	3.0	12.0	5.7	6.19
CBX3	2620.5	2126.0	2361.0	2420.0	2396.5	2214.0	2356.3	172.50
CBX4	108.0	84.5	77.5	91.0	96.0	90.5	91.3	10.38
CBX7	15.5	6.5	10.5	4.0	10.5	6.0	8.8	4.17
CBY1	675.0	451.5	444.0	695.0	428.5	302.0	499.3	153.92
CC2D1B	847.0	569.5	530.0	818.0	652.0	580.0	666.1	135.10
CC2D2A	203.5	207.0	215.5	181.5	195.0	183.5	197.7	13.48
CCAR1	1469.5	1122.0	1072.0	1340.5	1266.0	982.0	1208.7	182.36
CCBL1	291.0	175.5	154.5	263.5	184.5	131.0	200.0	63.22
CCBL2	467.5	188.0	187.0	384.0	188.5	130.0	257.5	134.85
CCDC101	810.5	663.5	338.5	707.5	695.5	396.0	601.9	189.21
CCDC102A	330.5	321.0	208.0	315.0	316.0	262.5	292.2	47.65
CCDC102B	0.0	0.0	0.0	0.0	0.0	0.0	0.0	0.00
CCDC103	15.0	25.0	13.5	20.0	19.0	10.5	17.2	5.20
CCDC107	483.0	330.5	402.0	510.0	395.0	395.0	419.3	65.80
CCDC108	0.0	0.0	0.0	0.0	0.0	0.0	0.0	0.00
CCDC109B	210.5	221.5	245.0	206.5	284.0	225.5	232.2	28.77
CCDC110	25.5	11.5	27.0	24.0	10.5	18.0	19.4	7.21
CCDC112	16.0	8.0	2.5	14.5	6.5	3.0	8.4	5.70
CCDC113	95.0	41.0	31.5	75.5	48.5	37.0	54.8	25.04
CCDC117	488.0	397.0	472.0	490.5	467.5	498.5	468.9	37.12
CCDC12	404.0	430.5	339.5	389.5	472.5	313.0	391.5	58.50
CCDC122	0.0	0.0	0.0	0.0	0.0	0.0	0.0	0.00
CCDC124	941.0	1211.5	1132.0	860.0	1322.0	1198.0	1110.8	175.80
CCDC125	384.0	186.0	144.0	344.0	191.5	105.0	225.8	112.29
CCDC126	321.5	208.5	251.5	294.0	243.5	201.5	253.4	47.11
CCDC127	354.5	144.5	212.5	346.5	186.5	190.5	239.2	89.04
CCDC129	0.0	0.0	0.0	0.0	0.0	0.0	0.0	0.00
CCDC13	49.5	7.5	12.0	31.0	11.0	4.0	19.2	17.57
CCDC134	836.0	564.5	328.0	693.0	494.0	330.5	541.0	201.28
CCDC137	204.0	172.0	87.5	186.0	181.0	102.5	155.5	48.25

CCDC138	2.0	1.5	4.0	0.0	0.0	0.0	1.3	1.60
CCDC14	127.5	61.5	76.0	129.0	70.5	60.5	87.5	32.09
CCDC141	131.0	452.5	854.5	124.5	555.0	651.5	461.5	290.58
CCDC142	46.0	52.0	41.0	52.0	57.5	36.5	47.5	7.82
CCDC148	13.5	16.0	18.0	7.5	14.5	20.5	15.0	4.45
CCDC149	160.5	57.5	130.0	172.0	77.5	136.0	122.3	45.57
CCDC15	882.0	493.0	387.5	779.0	464.5	290.0	549.3	231.18
CCDC153	19.5	135.0	109.0	20.0	150.5	132.5	94.4	59.34
CCDC157	18.5	17.0	21.5	12.0	27.0	23.0	19.8	5.20
CCDC166	0.0	0.0	0.0	0.5	0.0	0.0	0.1	0.20
CCDC167	651.5	488.0	423.0	592.0	521.0	328.5	500.7	116.05
CCDC169	216.5	1720.5	1091.5	204.5	2114.5	937.5	1047.5	775.46
CCDC17	0.0	0.0	0.5	0.5	0.0	0.0	0.2	0.26
CCDC170	197.5	107.5	173.0	132.5	128.5	150.0	148.2	32.68
CCDC171	78.0	51.5	125.5	82.5	63.0	98.0	83.1	26.26
CCDC172	0.0	0.0	0.0	0.0	0.0	0.0	0.0	0.00
CCDC173	79.0	93.5	74.0	86.0	114.0	76.5	87.2	14.94
CCDC174	311.5	230.5	280.0	331.5	242.5	258.5	275.8	39.64
CCDC176	29.5	10.5	4.5	21.0	9.5	3.5	13.1	10.17
CCDC177	28.0	12.0	26.0	14.5	5.5	19.5	17.6	8.60
CCDC18	215.0	116.5	112.0	145.0	116.5	116.0	136.8	40.14
CCDC181	32.5	11.0	18.0	26.5	13.5	12.5	19.0	8.67
CCDC183	2.0	2.0	7.0	1.0	1.0	6.0	3.2	2.64
CCDC186	272.5	162.0	243.5	280.5	183.0	260.0	233.6	49.39
CCDC25	1172.5	1164.0	773.5	1059.0	1200.5	712.5	1013.7	215.95
CCDC27	117.5	77.5	45.5	105.0	87.0	45.5	79.7	29.89
CCDC28A	330.0	404.5	306.5	321.0	408.5	279.0	341.6	53.18
CCDC28B	256.0	240.5	94.0	195.5	202.0	111.0	183.2	66.72
CCDC3	0.0	1.5	2.5	0.0	2.0	2.5	1.4	1.16
CCDC30	80.5	89.5	115.5	64.5	81.5	130.5	93.7	24.59
CCDC33	0.0	0.0	0.0	0.0	0.0	0.0	0.0	0.00
CCDC34	204.0	124.5	129.5	193.5	154.5	99.0	150.8	41.22
CCDC36	0.0	6.0	4.0	1.5	6.0	2.0	3.3	2.48
CCDC37	0.0	0.5	0.0	0.5	0.0	0.0	0.2	0.26
CCDC39	53.0	25.5	35.0	66.0	34.0	36.5	41.7	14.91
CCDC40	95.5	100.0	111.5	107.5	103.0	108.0	104.3	5.89
CCDC42	0.0	0.0	0.0	0.0	0.0	0.5	0.1	0.20
CCDC42B	33.5	12.0	10.0	24.0	11.5	10.0	16.8	9.74
CCDC43	759.0	1060.0	690.0	723.0	1228.0	765.5	870.9	219.78
CCDC47	994.5	625.5	734.5	1015.0	707.0	587.0	777.3	184.23
CCDC50	1357.5	402.5	1608.0	1264.0	398.5	1458.5	1081.5	539.69
CCDC51	341.0	296.5	252.5	335.5	329.0	248.5	300.5	41.72
CCDC57	9.5	19.5	19.0	11.0	20.5	24.5	17.3	5.84
CCDC58	211.5	232.0	215.0	199.0	235.0	172.0	210.8	23.23
CCDC59	302.5	268.0	218.5	281.5	289.5	184.5	257.4	46.01
CCDC6	1280.0	937.5	1000.0	1282.0	1060.5	989.0	1091.5	151.91
CCDC60	0.0	0.0	0.0	0.0	0.0	0.0	0.0	0.00
CCDC61	495.0	394.5	236.5	393.5	388.0	220.5	354.7	105.70
CCDC63	0.0	0.0	0.5	0.0	0.0	0.0	0.1	0.20
CCDC64	309.0	406.0	241.0	319.0	434.5	245.0	325.8	80.37
CCDC65	2.5	0.5	2.5	0.5	1.0	2.0	1.5	0.95
CCDC66	156.0	113.0	119.0	163.0	137.5	114.0	133.8	21.92
CCDC67	0.5	0.0	0.0	0.5	1.0	1.0	0.5	0.45
CCDC68	2.0	3.0	0.5	5.0	3.0	0.0	2.3	1.84

CCDC69	10.0	324.5	28.5	32.5	464.0	105.0	160.8	188.97
CCDC71	708.5	578.0	690.0	669.0	671.5	706.5	670.6	48.33
CCDC71L	20.0	9.5	19.5	18.0	14.5	15.0	16.1	3.94
CCDC73	21.0	10.0	15.5	17.5	16.0	25.0	17.5	5.12
CCDC77	120.5	66.0	73.5	101.0	63.5	43.5	78.0	27.93
CCDC78	1.5	1.0	1.0	0.0	1.5	0.5	0.9	0.58
CCDC79	19.0	16.0	39.0	19.0	18.5	36.0	24.6	10.11
CCDC80	2294.0	1433.0	2496.5	2215.0	1718.5	3202.0	2226.5	619.18
CCDC81	0.0	0.0	0.0	0.0	0.5	0.5	0.2	0.26
CCDC82	113.5	86.5	78.0	111.5	73.0	72.5	89.2	18.77
CCDC83	5.0	5.5	4.0	2.5	2.5	2.5	3.7	1.37
CCDC84	77.5	64.5	44.0	64.0	71.0	60.0	63.5	11.37
CCDC85A	0.5	0.0	0.0	0.5	0.0	0.0	0.2	0.26
CCDC85C	0.5	2.0	6.0	4.0	0.5	6.0	3.2	2.54
CCDC86	806.0	934.0	476.5	765.0	940.0	418.5	723.3	225.28
CCDC88A	280.0	140.0	465.5	279.0	129.0	428.0	286.9	140.31
CCDC88C	1263.5	3947.5	4659.0	1566.0	5153.5	4891.0	3580.1	1727.21
CCDC89	24.0	7.5	8.0	25.0	14.5	14.5	15.6	7.55
CCDC90B	840.5	806.5	860.5	842.0	906.0	797.5	842.2	39.19
CCDC91	4.5	8.5	31.0	7.5	12.0	15.5	13.2	9.53
CCDC92	44.0	41.5	43.0	36.0	39.0	61.0	44.1	8.78
CCDC93	829.0	596.5	611.5	777.0	637.5	572.0	670.6	106.03
CCDC94	413.5	465.0	294.0	376.0	433.5	309.0	381.8	68.76
CCDC96	2.5	2.5	1.5	3.5	1.5	1.5	2.2	0.82
CCDC97	214.0	175.0	206.5	182.0	132.0	204.0	185.6	30.27
CCK	27.5	44.0	37.5	20.0	48.5	26.0	33.9	11.18
CCKAR	0.5	0.0	0.0	0.0	0.0	0.0	0.1	0.20
CCKBR	4.0	20.0	9.0	3.5	18.0	7.0	10.3	7.10
CCL1	0.0	0.0	0.0	0.0	0.0	0.0	0.0	0.00
CCL13	0.0	0.0	0.0	0.0	0.0	0.0	0.0	0.00
CCL17	0.0	0.0	0.0	0.0	0.0	0.0	0.0	0.00
CCL19	0.0	0.0	0.0	0.0	0.5	0.0	0.1	0.20
CCL20	2.0	2.5	4.0	2.0	1.0	1.0	2.1	1.11
CCL21	0.5	0.0	0.0	2.0	0.0	0.5	0.5	0.77
CCL24	24.0	6.5	1.5	24.5	2.5	1.5	10.1	11.13
CCL26	0.5	0.0	0.0	2.0	0.0	0.0	0.4	0.80
CCL5	0.0	0.0	0.0	0.0	0.0	0.0	0.0	0.00
CCM2	752.0	536.0	866.0	813.0	569.0	754.0	715.0	133.16
CCM2L	182.5	238.5	247.5	168.5	202.0	301.0	223.3	48.94
CCNA1	0.5	1.0	1.0	1.5	1.0	0.0	0.8	0.52
CCNA2	1177.5	498.0	628.5	1263.5	500.5	423.5	748.6	372.44
CCNB2	2391.5	1235.0	1362.5	2578.5	1359.0	1015.5	1657.0	656.32
CCNB3	967.0	492.0	506.0	1070.5	464.0	309.0	634.8	307.41
CCND1	411.0	519.5	675.0	365.5	627.5	588.5	531.2	122.70
CCND2	91.5	31.5	66.0	90.5	35.0	53.0	61.3	26.21
CCND3	751.0	286.5	287.5	676.5	269.5	301.0	428.7	222.30
CCNDBP1	948.5	890.0	667.0	834.5	974.5	563.5	813.0	163.95
CCNE2	40.5	19.5	32.5	36.5	12.0	21.5	27.1	11.08
CCNF	541.5	258.5	413.5	452.5	224.0	259.5	358.3	129.03
CCNG1	3595.5	2750.0	3336.0	3355.5	3144.0	2830.5	3168.6	327.21
CCNG2	1090.0	641.5	1188.5	985.5	739.5	998.0	940.5	209.28
CCNH	1313.5	909.5	835.5	1337.0	1084.0	895.5	1062.5	219.84
CCNI	2455.5	3251.0	3723.5	2111.5	3774.0	4193.0	3251.4	814.30
CCNJ	82.5	91.0	124.0	73.0	101.0	130.5	100.3	22.90

CCNJL	0.5	0.0	0.0	0.0	0.0	0.0	0.1	0.20
CCNK	611.0	541.0	707.0	624.0	664.0	682.5	638.3	59.58
CCNL1	3255.0	2328.5	1266.0	2556.0	2650.0	1484.0	2256.6	751.61
CCNL2	1456.0	2024.0	1615.5	1298.0	2515.5	1802.5	1785.3	439.36
CCNO	9.5	4.5	5.5	5.5	4.5	5.0	5.8	1.89
CCNT1	273.5	264.0	258.5	306.0	290.5	272.5	277.5	17.69
CCNT2	313.0	361.0	577.0	305.5	433.5	582.5	428.8	125.55
CCNYL1	3009.5	2346.5	2486.0	3163.5	2456.5	2466.5	2654.8	341.44
CCP110	199.5	108.0	242.0	193.0	135.0	236.5	185.7	54.04
CCPG1	3328.0	1991.5	4205.0	3343.5	2090.5	3351.0	3051.6	851.95
CCR10	0.0	0.0	0.5	0.0	0.0	1.0	0.3	0.42
CCR4	87.5	35.0	41.5	84.5	49.0	37.0	55.8	23.94
CCR5	0.0	0.0	1.0	0.0	0.5	0.0	0.3	0.42
CCR6	0.0	0.0	0.0	0.0	0.0	0.0	0.0	0.00
CCR7	86.0	46.0	67.0	64.0	54.0	118.0	72.5	26.07
CCR8	0.5	2.5	0.5	0.0	1.5	1.0	1.0	0.89
CCR9	0.5	0.0	0.5	2.0	1.0	0.0	0.7	0.75
CCRN4L	193.0	266.5	292.5	185.0	306.0	319.5	260.4	58.08
CCSAP	31.5	21.0	43.0	40.5	15.5	35.0	31.1	10.87
CCSER1	6.0	3.5	22.0	1.5	3.0	12.0	8.0	7.79
CCT2	2669.5	2914.5	2941.0	2680.5	3163.5	2750.5	2853.3	190.72
CCT3	3669.5	3376.5	2700.0	3379.5	2893.5	2496.0	3085.8	457.20
CCT4	3261.5	2924.5	2658.5	3133.5	3030.5	2438.0	2907.8	307.97
CCT5	3078.5	2803.5	3023.0	3034.0	2900.5	2727.5	2927.8	140.97
CCT6A	3528.5	3730.5	3600.0	3656.0	3980.5	3409.0	3650.8	195.49
CCT7	2611.5	2373.0	2257.5	2372.5	2378.0	2082.5	2345.8	173.33
CCT8	3609.5	3169.0	3847.0	3501.0	3367.5	3405.5	3483.3	230.98
CCZ1	1290.5	762.0	818.0	1105.0	874.5	740.0	931.7	219.32
CD109	1214.5	323.0	381.5	1251.5	358.5	324.5	642.3	458.27
CD151	2950.0	2488.0	2496.5	2574.5	2773.0	2798.5	2680.1	188.29
CD163	0.5	0.0	0.0	1.5	1.0	2.5	0.9	0.97
CD164	4374.0	3057.0	3261.5	4452.0	3459.5	3306.5	3651.8	604.02
CD164L2	1.0	1.0	0.0	0.5	0.5	0.5	0.6	0.38
CD180	7.0	3.0	1.5	11.0	3.5	3.5	4.9	3.48
CD2	0.0	0.5	0.0	1.0	0.5	0.0	0.3	0.41
CD200R1	0.5	0.0	0.5	0.0	0.0	0.0	0.2	0.26
CD24	142.0	208.0	300.0	197.0	253.5	324.0	237.4	68.23
CD247	67.0	70.5	56.0	55.5	58.0	58.0	60.8	6.31
CD274	4.0	1.0	0.5	4.0	2.0	1.0	2.1	1.56
CD276	707.0	681.0	1122.5	696.0	703.5	973.0	813.8	187.47
CD28	0.5	1.0	0.0	0.0	0.0	0.0	0.3	0.42
CD320	180.5	230.5	167.5	195.5	254.5	147.0	195.9	40.17
CD34	1.0	1.0	0.0	1.0	3.0	1.5	1.3	0.99
CD36	761.5	120.0	22.5	951.0	150.0	34.0	339.8	407.41
CD38	44.0	39.5	13.0	45.5	41.5	21.0	34.1	13.63
CD3E	2.5	0.0	0.5	3.5	0.0	0.5	1.2	1.47
CD4	0.0	0.0	0.0	0.0	0.0	0.0	0.0	0.00
CD40	715.5	196.5	210.0	500.5	147.5	162.0	322.0	232.83
CD40LG	0.0	0.0	0.0	0.0	0.0	0.0	0.0	0.00
CD44	2628.0	3507.5	6648.0	3727.0	3966.5	6028.0	4417.5	1567.13
CD46	502.5	332.5	964.5	585.5	373.0	837.0	599.2	253.70
CD47	2006.0	2529.0	4143.5	2158.0	3260.0	3914.5	3001.8	908.88
CD5	1.0	0.0	0.0	1.5	0.0	0.0	0.4	0.66
CD55	28.0	5.5	10.0	17.5	3.0	1.0	10.8	10.26

CD59	2473.0	1760.0	2985.5	2330.0	2029.0	2970.5	2424.7	494.39
CD6	1.5	0.0	0.0	0.0	0.0	0.0	0.3	0.61
CD63	8830.5	8326.0	5873.5	8684.0	8866.5	6577.0	7859.6	1299.49
CD7	0.5	3.5	3.5	1.0	10.0	4.5	3.8	3.40
CD74	333.5	265.5	248.0	244.0	253.5	191.0	255.9	45.90
CD79A	6.0	8.0	17.0	14.5	7.5	20.0	12.2	5.79
CD79B	1.5	2.0	1.0	0.0	3.5	2.0	1.7	1.17
CD80	1.0	0.0	2.0	0.5	2.0	0.5	1.0	0.84
CD81	1698.5	1230.5	1488.5	1632.0	1273.5	1442.0	1460.8	187.11
CD82	224.5	90.0	167.0	157.5	102.0	93.5	139.1	53.44
CD83	0.0	0.0	0.0	0.0	0.0	0.0	0.0	0.00
CD84	4.5	2.5	3.0	4.0	3.5	4.5	3.7	0.82
CD86	0.0	0.0	0.0	0.0	0.0	0.0	0.0	0.00
CD8B	0.5	0.0	0.5	0.5	1.0	0.0	0.4	0.38
CD9	0.0	0.0	0.0	0.0	0.5	0.0	0.1	0.20
CD93	28.0	13.0	8.0	23.0	22.5	7.5	17.0	8.65
CD96	0.0	0.0	1.0	0.5	0.0	0.5	0.3	0.41
CD99	2551.5	1880.5	1688.0	3555.0	2340.0	1585.0	2266.7	734.16
CD99L2	161.0	62.0	214.0	205.5	66.5	208.5	152.9	71.25
CDADC1	148.0	150.5	256.5	153.0	172.5	301.5	197.0	65.67
CDAN1	499.5	337.5	413.5	488.0	423.0	476.5	439.7	61.11
CDC123	775.5	309.5	338.5	643.0	387.0	302.5	459.3	200.29
CDC14B	105.5	35.0	47.0	103.0	36.5	55.5	63.8	32.25
CDC16	498.0	343.5	346.0	486.0	375.0	277.0	387.6	87.11
CDC20	1186.5	1185.5	797.0	1327.5	1351.5	842.5	1115.1	239.40
CDC20B	17.0	4.0	2.5	11.5	1.5	0.5	6.2	6.60
CDC23	778.0	418.5	348.0	715.5	456.5	306.0	503.8	196.43
CDC25A	246.5	146.5	169.0	247.5	127.0	134.5	178.5	54.93
CDC25B	3738.5	4334.5	2907.0	2946.5	3894.0	2594.0	3402.4	682.67
CDC26	183.0	190.5	76.5	176.0	192.5	63.5	147.0	60.07
CDC27	521.5	278.0	576.0	528.5	329.5	498.0	455.3	121.16
CDC34	284.0	229.0	272.0	245.0	266.5	288.5	264.2	23.05
CDC37	474.0	580.5	376.0	442.0	539.5	367.5	463.3	85.92
CDC37L1	122.5	134.0	146.0	107.0	154.0	122.0	130.9	17.28
CDC40	251.0	102.5	262.5	272.0	126.0	229.5	207.3	73.79
CDC42	5490.5	5071.0	5686.5	5418.0	5422.0	5325.0	5402.2	202.44
CDC42BPB	2965.5	1933.0	3032.0	3120.0	2235.5	2716.0	2667.0	480.60
CDC42EP1	636.0	509.0	833.5	574.5	477.5	671.0	616.9	128.90
CDC42EP2	36.0	71.0	118.0	45.5	89.5	115.5	79.3	34.64
CDC42EP3	832.0	794.5	925.5	764.5	853.5	891.5	843.6	59.84
CDC42EP4	499.0	650.5	465.5	555.0	661.5	472.5	550.7	87.52
CDC42SE1	1630.5	1844.0	1580.0	1484.0	1848.5	1578.0	1660.8	151.23
CDC42SE2	455.0	286.5	219.0	441.0	335.0	206.5	323.8	106.97
CDC45	517.5	208.5	338.5	558.0	212.5	194.5	338.3	163.54
CDC6	462.0	416.0	352.5	380.0	375.0	298.5	380.7	55.57
CDCA2	71.0	23.5	76.0	89.5	19.0	44.5	53.9	29.25
CDCA3	548.5	286.0	317.5	561.5	269.5	206.5	364.9	151.67
CDCA4	1656.0	1071.5	1067.5	1592.5	1198.0	989.5	1262.5	288.77
CDCA7	717.0	311.0	442.0	667.0	302.0	336.0	462.5	185.32
CDCA7L	1804.5	1130.0	891.5	1701.5	1330.0	899.5	1292.8	393.06
CDCA8	957.0	734.0	846.5	751.5	599.0	587.5	745.9	142.50
CDCP1	186.0	99.5	142.0	213.5	119.0	151.0	151.8	42.15
CDCP2	0.5	0.0	0.0	0.0	0.5	0.0	0.2	0.26
CDH1	1.0	0.5	1.5	0.0	1.0	5.0	1.5	1.79

CDH11	3.5	1.5	1.0	2.0	1.0	0.0	1.5	1.18
CDH13	2958.5	1550.0	4410.5	3530.5	1929.5	4379.5	3126.4	1210.64
CDH17	9.0	2.5	1.5	4.5	1.5	0.5	3.3	3.13
CDH18	0.5	1.0	1.0	0.0	1.0	1.0	0.8	0.42
CDH2	2238.5	2531.5	4901.0	1978.5	2963.5	4727.5	3223.4	1276.15
CDH20	0.0	0.0	0.0	0.0	0.0	0.0	0.0	0.00
CDH22	477.5	48.5	45.0	215.0	29.0	30.5	140.9	179.57
CDH23	558.5	410.0	532.5	554.0	394.5	433.5	480.5	75.85
CDH5	0.0	0.5	0.5	0.0	0.0	0.0	0.2	0.26
CDH6	251.0	74.0	97.0	63.0	28.5	32.0	90.9	82.58
CDHR1	0.0	0.0	0.0	0.0	0.0	0.0	0.0	0.00
CDHR2	28.5	29.5	7.0	19.0	32.0	12.5	21.4	10.20
CDHR3	10.0	1.5	0.0	5.0	1.0	1.5	3.2	3.75
CDHR4	2.5	1.5	5.0	3.5	3.0	3.5	3.2	1.17
CDHR5	0.0	0.0	0.0	0.0	0.0	0.0	0.0	0.00
CDIP1	1311.5	1068.5	703.0	1119.0	984.5	694.5	980.2	243.02
CDK1	1977.0	908.0	1123.5	2001.0	811.5	710.0	1255.2	584.67
CDK10	135.0	144.0	134.0	101.5	153.0	127.0	132.4	17.62
CDK12	376.5	256.5	470.0	377.0	250.0	411.5	356.9	87.26
CDK13	772.0	815.0	1113.5	759.5	955.0	1112.5	921.3	163.95
CDK14	13.0	19.5	39.5	17.5	9.0	28.0	21.1	11.08
CDK15	4.0	11.0	6.0	5.5	11.5	5.0	7.2	3.24
CDK17	834.5	689.0	889.5	787.0	775.5	843.0	803.1	69.40
CDK18	18.5	67.5	191.5	23.0	68.5	141.5	85.1	68.38
CDK19	623.0	318.0	565.0	530.0	363.5	518.0	486.3	119.33
CDK2	849.5	634.5	597.5	874.5	577.0	421.5	659.1	173.35
CDK2AP1	1431.0	1109.5	1421.0	1283.0	1309.0	1676.5	1371.7	189.42
CDK3	2.0	0.0	0.0	5.5	1.0	0.5	1.5	2.10
CDK5	200.5	264.0	253.5	191.5	255.0	278.5	240.5	35.71
CDK5R1	45.5	141.5	93.5	32.0	138.0	103.0	92.3	45.72
CDK5R2	0.0	0.0	0.0	0.0	0.0	0.0	0.0	0.00
CDK5RAP1	299.0	276.0	261.5	239.5	247.0	185.5	251.4	38.67
CDK5RAP2	402.5	184.5	281.0	433.5	203.0	245.5	291.7	103.92
CDK5RAP3	765.0	561.0	432.0	719.0	572.5	411.5	576.8	144.36
CDK6	203.0	151.5	245.5	229.0	169.5	194.5	198.8	35.28
CDK8	280.0	333.0	196.5	301.5	378.0	260.5	291.6	62.33
CDK9	226.5	160.5	210.0	219.5	160.5	213.5	198.4	29.90
CDKAL1	189.0	202.0	152.0	171.0	207.0	132.5	175.6	29.32
CDKL1	88.0	26.0	35.0	66.0	34.0	36.0	47.5	24.15
CDKL2	129.5	103.5	52.0	151.0	137.5	60.0	105.6	41.49
CDKL5	197.0	176.0	256.5	193.5	213.5	213.0	208.3	27.43
CDKN1A	878.5	650.5	1027.5	901.0	728.5	917.5	850.6	137.14
CDKN1B	2932.5	5773.5	3423.0	2835.0	6616.5	3351.5	4155.3	1618.45
CDKN2AIP	1240.5	967.0	1090.0	1118.0	1012.5	953.5	1063.6	108.64
CDKN2C	131.0	102.0	145.0	135.0	95.0	155.0	127.2	23.82
CDKN3	204.0	84.5	206.0	168.5	81.5	113.5	143.0	57.30
CDO1	13.0	10.5	23.5	11.5	13.0	33.0	17.4	8.96
CDON	1289.5	606.0	811.5	1078.5	548.0	712.0	840.9	288.49
CDPF1	395.5	384.0	370.5	411.0	439.0	321.5	386.9	39.81
CDR2	584.5	568.5	609.5	594.0	621.0	612.0	598.3	19.62
CDR2L	17.5	10.0	20.5	12.0	14.0	22.0	16.0	4.79
CDRT1	0.0	0.5	0.0	0.5	1.0	0.0	0.3	0.41
CDS1	34.0	9.0	4.5	36.5	6.5	5.5	16.0	15.01
CDS2	854.0	510.5	808.0	820.5	559.0	768.0	720.0	146.91

CDT1	227.5	140.0	153.5	183.5	111.0	106.5	153.7	45.99
CDV3	1391.0	1373.5	2005.5	1483.5	1416.0	1766.0	1572.6	256.86
CDX1	0.5	1.0	0.0	0.0	0.0	0.5	0.3	0.41
CDYL	170.5	92.0	151.0	163.0	116.0	152.0	140.8	30.33
CDYL2	233.0	132.5	210.5	214.0	153.5	169.0	185.4	39.49
CEBPB	363.5	479.5	623.5	395.0	483.0	727.5	512.0	138.93
CEBPG	1080.5	1381.0	1270.5	1226.5	1538.0	1286.0	1297.1	153.49
CEBPZ	970.5	669.0	1144.5	936.5	770.5	1033.5	920.8	174.04
CECR1	139.5	74.0	55.0	204.5	90.5	52.5	102.7	59.14
CECR5	392.0	294.0	333.5	356.5	365.5	309.5	341.8	36.59
CECR6	0.0	0.5	4.0	2.0	1.0	1.0	1.4	1.43
CEL	0.0	0.0	0.0	0.0	0.0	0.0	0.0	0.00
CELA1	0.0	0.0	0.0	0.0	0.0	0.0	0.0	0.00
CELF1	32.5	10.0	27.0	28.0	16.0	24.0	22.9	8.37
CELF2	170.5	86.5	360.5	189.5	97.5	305.0	201.6	110.58
CELF4	0.0	0.5	0.0	0.0	0.5	0.0	0.2	0.26
CELF5	19.5	21.5	16.5	20.5	19.5	34.0	21.9	6.15
CELF6	84.0	63.0	91.0	83.5	81.5	91.0	82.3	10.28
CELSR1	1455.5	1137.0	1430.5	1300.0	1364.0	1343.0	1338.3	113.94
CELSR3	191.5	262.0	321.5	207.0	265.5	323.5	261.8	55.37
CEMIP	9.5	41.5	120.0	28.5	72.5	210.5	80.4	74.55
CEND1	0.0	0.0	1.5	0.5	0.0	0.5	0.4	0.58
CENPA	12.5	18.0	21.5	24.5	15.5	16.5	18.1	4.32
CENPC	193.0	73.0	146.5	224.5	67.5	85.0	131.6	66.84
CENPE	1025.5	281.0	721.5	1035.5	264.5	393.5	620.3	357.76
CENPF	428.5	127.0	338.0	425.5	117.5	197.5	272.3	143.43
CENPH	927.5	430.0	651.5	894.5	439.0	431.5	629.0	234.44
CENPI	151.5	32.0	122.0	185.0	35.0	90.5	102.7	62.06
CENPK	280.0	85.0	181.5	304.0	80.5	103.0	172.3	99.88
CENPL	53.5	13.5	44.0	76.5	12.5	22.5	37.1	25.47
CENPM	882.0	461.5	532.5	996.5	481.5	400.0	625.7	249.22
CENPN	371.0	138.5	188.5	367.0	117.0	109.0	215.2	122.34
CENPO	51.5	19.0	90.0	51.0	22.5	37.5	45.3	25.85
CENPP	244.5	116.0	117.5	226.0	104.5	91.5	150.0	66.95
CENPQ	312.5	150.0	196.0	285.5	150.0	145.5	206.6	74.42
CENPT	354.5	143.0	171.5	399.0	147.0	147.5	227.1	117.21
CENPU	170.5	67.0	94.0	182.0	53.0	46.0	102.1	59.86
CENPW	56.0	27.5	58.0	86.0	27.0	46.5	50.2	22.11
CEP104	388.5	204.0	432.0	352.0	202.5	368.0	324.5	97.68
CEP120	162.0	82.0	289.0	187.0	102.0	242.0	177.3	79.68
CEP128	54.0	10.5	30.0	40.5	14.0	13.5	27.1	17.57
CEP131	788.5	637.0	456.5	683.5	632.0	467.5	610.8	128.33
CEP135	257.5	101.5	146.0	283.0	91.0	92.0	161.8	86.74
CEP152	136.0	73.0	123.0	117.5	77.0	106.5	105.5	25.50
CEP162	229.0	151.5	242.5	186.5	166.0	221.0	199.4	36.81
CEP164	84.5	51.0	103.0	76.0	58.5	93.0	77.7	20.02
CEP170	494.5	242.0	895.5	545.5	307.0	790.5	545.8	258.50
CEP170B	1490.5	1080.0	1123.5	1474.0	1302.5	1236.0	1284.4	172.49
CEP19	176.0	147.0	99.0	160.5	156.5	138.0	146.2	26.44
CEP192	43.5	15.0	72.5	48.5	7.0	37.0	37.3	23.74
CEP250	358.0	237.0	339.0	337.5	268.5	331.0	311.8	47.69
CEP290	200.0	114.5	150.0	188.0	93.5	122.0	144.7	42.45
CEP350	378.0	312.0	834.0	419.5	335.5	711.0	498.3	219.01
CEP41	127.0	118.5	78.5	125.5	116.0	87.0	108.8	20.73

CEP44	235.5	101.0	106.5	223.5	93.0	83.5	140.5	69.48
CEP55	290.0	111.5	176.0	302.0	107.0	108.5	182.5	91.76
CEP57	227.5	208.5	223.0	229.5	248.0	189.5	221.0	19.98
CEP57L1	73.5	54.5	35.0	74.0	62.5	45.5	57.5	15.57
CEP68	213.5	254.0	194.0	196.5	248.5	208.0	219.1	25.99
CEP70	251.0	112.0	102.5	227.5	102.5	76.5	145.3	74.07
CEP76	333.5	214.5	146.0	315.5	254.5	150.0	235.7	80.16
CEP78	436.0	287.0	221.0	387.0	355.0	173.5	309.9	100.91
CEP83	315.5	150.0	136.0	237.5	153.5	107.0	183.3	78.06
CEP85	289.5	168.0	149.5	291.5	146.5	131.5	196.1	74.05
CEP89	132.5	102.0	151.5	136.5	126.0	161.5	135.0	20.75
CEP95	399.0	127.5	114.5	359.5	112.0	92.0	200.8	139.29
CEP97	108.0	63.5	82.0	105.0	74.0	80.0	85.4	17.58
CEPT1	581.5	444.5	489.5	498.0	497.5	472.0	497.2	46.00
CER1	0.0	0.0	0.5	0.0	0.0	0.5	0.2	0.26
CERCAM	1384.5	1453.5	1515.0	1348.5	1663.0	2670.0	1672.4	501.16
CERK	73.0	27.5	106.0	69.0	34.0	87.5	66.2	30.42
CERKL	0.5	0.0	0.0	0.5	0.0	0.0	0.2	0.26
CERS1	25.0	10.5	54.5	21.0	6.5	38.5	26.0	17.97
CERS2	423.0	279.5	542.5	473.5	257.0	560.0	422.6	129.42
CERS3	15.0	12.5	12.0	11.5	12.0	11.5	12.4	1.32
CERS5	1158.5	671.5	684.0	1108.0	699.5	695.0	836.1	230.94
CERS6	9.0	4.5	16.5	4.0	5.5	8.0	7.9	4.64
CETN2	970.5	506.0	500.5	867.5	551.0	419.5	635.8	225.76
CETN3	696.0	502.5	346.5	626.5	512.5	322.0	501.0	148.20
CETP	0.0	0.0	1.0	0.0	0.0	0.5	0.3	0.42
CFAP20	800.5	665.0	499.0	752.5	669.5	477.0	643.9	131.35
CFAP221	0.0	0.0	0.0	0.0	0.0	0.0	0.0	0.00
CFAP36	1181.0	1307.0	1067.0	1092.0	1541.0	1140.0	1221.3	177.94
CFAP44	11.0	9.5	12.5	10.5	9.0	8.5	10.2	1.47
CFAP45	1.0	4.5	1.5	1.0	3.5	3.5	2.5	1.52
CFAP52	0.0	0.0	0.0	0.0	0.0	0.0	0.0	0.00
CFAP54	1126.0	547.0	745.0	1039.0	586.0	569.0	768.7	254.44
CFAP57	2.0	1.5	4.0	3.0	3.5	4.5	3.1	1.16
CFAP58	2.5	0.0	1.5	3.5	0.5	0.0	1.3	1.44
CFAP61	2.5	1.0	1.5	2.5	1.5	0.5	1.6	0.80
CFAP69	2.5	1.0	1.0	4.0	1.5	2.5	2.1	1.16
CFAP74	8.5	5.5	7.5	3.0	2.0	1.5	4.7	2.94
CFAP97	416.0	330.5	386.5	353.0	324.5	354.5	360.8	34.77
CFAP99	11.5	7.5	14.5	13.0	5.0	13.0	10.8	3.70
CFD	47.5	30.5	0.5	28.5	36.5	4.0	24.6	18.55
CFDP1	352.5	432.0	356.0	321.0	415.5	388.5	377.6	41.99
CFH	0.0	0.5	0.0	2.0	2.5	0.0	0.8	1.13
CFI	47.0	27.5	21.0	50.5	26.0	18.0	31.7	13.71
CFL2	5950.0	5914.5	5020.0	5455.0	6701.5	4300.0	5556.8	833.18
CFLAR	420.5	380.0	485.5	384.0	377.0	556.0	433.8	72.64
CFTR	0.0	0.0	0.0	0.0	0.0	0.0	0.0	0.00
CGA	3.5	0.5	0.5	2.5	1.5	0.0	1.4	1.36
CGGBP1	609.0	347.5	472.0	583.0	407.0	484.0	483.8	100.13
CGN	141.5	133.5	50.0	139.5	125.0	43.0	105.4	46.05
CGNL1	208.5	116.5	194.5	190.0	117.5	176.0	167.2	40.22
CGREF1	17.0	13.5	6.5	11.0	23.5	6.5	13.0	6.56
CGRRF1	353.5	227.5	242.0	365.5	296.5	255.5	290.1	58.61
CH25H	5.0	1.0	0.0	3.0	2.5	1.5	2.2	1.75

CHAC1	766.5	653.5	418.0	834.5	602.5	418.5	615.6	173.31
CHAD	346.5	76.5	113.0	273.5	61.0	82.5	158.8	120.54
CHADL	5.0	3.0	4.5	8.0	6.0	2.5	4.8	2.02
CHAF1A	597.0	294.5	328.0	634.5	306.5	253.0	402.3	167.59
CHAF1B	289.5	207.5	255.0	359.5	267.5	239.0	269.7	51.94
CHAMP1	607.0	460.5	521.0	629.5	478.5	474.0	528.4	72.82
CHAT	0.0	0.0	0.0	0.0	0.0	0.0	0.0	0.00
CHCHD1	450.0	420.0	276.5	393.5	471.5	249.0	376.8	92.59
CHCHD2	6014.0	6833.5	3616.0	5640.0	7035.0	3794.0	5488.8	1474.95
CHCHD3	893.0	666.5	573.5	885.0	747.0	498.0	710.5	161.83
CHCHD4	465.0	376.0	271.5	433.5	384.5	215.5	357.7	95.88
CHCHD6	545.5	563.0	382.0	540.0	585.0	371.0	497.8	95.29
CHCHD7	902.5	1312.0	624.0	855.0	1565.0	647.5	984.3	377.16
CHD1L	214.5	81.0	72.5	241.5	73.0	56.0	123.1	82.12
CHD2	918.0	993.0	1413.5	851.0	1157.0	1171.0	1083.9	205.72
CHD4	2825.5	2351.0	2670.0	2694.0	2466.0	2776.5	2630.5	184.45
CHD5	306.0	190.0	258.0	241.0	171.0	246.0	235.3	48.68
CHD7	628.0	765.5	958.5	569.0	955.0	955.5	805.3	177.37
CHD9	37.0	11.0	71.5	34.5	11.0	66.5	38.6	26.09
CHDC2	0.0	0.0	0.0	0.0	0.0	0.0	0.0	0.00
CHDH	187.0	119.0	60.5	209.0	158.5	69.5	133.9	61.35
CHEK1	348.0	116.0	92.5	320.0	103.0	68.0	174.6	124.80
CHEK2	130.0	82.0	75.5	141.0	109.0	80.0	102.9	28.05
CHERP	468.0	344.0	543.0	557.0	396.0	608.5	486.1	101.87
CHFR	479.5	290.5	464.5	465.0	363.0	376.5	406.5	75.32
CHGA	20.5	15.0	8.0	16.5	20.0	4.0	14.0	6.66
CHGB	83.0	25.0	63.5	68.0	32.0	34.0	50.9	23.64
CHIC1	178.5	122.0	171.0	174.0	148.0	160.5	159.0	21.17
CHIC2	89.0	88.5	111.5	100.0	99.0	110.0	99.7	9.86
CHID1	470.0	369.5	252.0	454.5	448.0	244.5	373.1	102.80
CHKA	1774.0	2674.5	1773.5	1713.0	3224.5	1634.5	2132.3	658.42
CHL1	37.5	209.5	185.5	25.5	214.5	235.5	151.3	94.25
CHM	278.5	159.0	311.0	280.0	195.0	283.0	251.1	59.70
CHMP1A	1049.5	858.5	678.5	868.5	826.0	629.5	818.4	150.23
CHMP1B	1301.0	1338.0	1320.0	1335.0	1495.5	1196.0	1330.9	96.30
CHMP2A	196.0	180.5	301.0	196.0	174.0	278.0	220.9	54.31
CHMP2B	737.5	386.5	373.5	698.5	423.5	306.5	487.7	182.80
CHMP3	1265.5	945.0	940.0	1120.5	1043.5	889.0	1033.9	140.67
CHMP4B	1932.5	1864.5	1433.0	1723.5	2051.0	1384.0	1731.4	272.06
CHMP4C	30.0	6.5	5.0	22.5	8.0	2.5	12.4	11.12
CHMP5	1289.5	1102.0	1093.5	1287.0	1190.5	1013.5	1162.7	112.28
CHMP6	1306.0	975.0	812.0	1304.0	1076.0	1005.0	1079.7	194.80
CHMP7	1325.5	1189.5	1081.5	1182.5	1201.0	1103.0	1180.5	86.41
CHN2	1902.5	1092.5	1484.5	1462.0	1157.0	1086.5	1364.2	318.65
CHODL	1.0	0.0	0.5	0.0	0.0	0.0	0.3	0.42
CHORDC1	504.5	413.0	563.0	494.5	442.0	504.5	486.9	52.81
CHP1	1148.5	966.5	1156.5	1232.5	1160.0	1298.5	1160.4	111.38
CHPF	522.0	632.0	843.5	590.5	707.5	946.0	706.9	160.73
CHPF2	2378.0	1921.5	1536.5	2535.5	2246.5	1711.5	2054.9	394.09
CHRAC1	229.0	148.5	183.5	210.5	153.0	135.5	176.7	37.36
CHRD	194.5	127.0	177.0	224.5	121.5	210.0	175.8	42.95
CHRDL1	323.0	305.5	477.5	223.0	297.0	519.5	357.6	115.14
CHRDL2	17.5	11.0	2.5	8.0	9.0	3.0	8.5	5.55
CHRFAM7A	5.0	2.0	2.5	5.5	3.5	1.0	3.3	1.75

CHRM2	0.0	0.0	0.0	0.0	0.0	0.0	0.0	0.00
CHRM3	1.0	0.0	2.5	0.0	0.5	1.5	0.9	0.97
CHRM4	4.0	24.0	8.5	9.5	24.0	14.5	14.1	8.38
CHRM5	177.5	46.0	26.0	122.5	61.0	29.5	77.1	60.41
CHRNA1	12262.0	19237.0	20897.5	11771.0	23107.0	17123.5	17399.7	4611.68
CHRNA10	0.0	0.0	0.0	0.0	0.5	0.0	0.1	0.20
CHRNA2	46.5	5.5	44.5	22.5	13.5	11.0	23.9	17.61
CHRNA3	214.5	224.5	129.0	196.0	311.5	138.0	202.3	66.50
CHRNA4	0.0	0.5	1.0	0.0	1.0	0.0	0.4	0.49
CHRNA5	46.5	16.5	28.0	29.5	19.5	23.5	27.3	10.64
CHRNA6	0.0	1.0	0.0	0.0	0.0	0.0	0.2	0.41
CHRNA9	0.0	0.0	1.0	0.0	0.5	0.0	0.3	0.42
CHRNB2	55.0	90.0	67.5	54.0	104.5	100.5	78.6	22.65
CHRNB3	0.0	0.0	0.5	0.0	1.5	0.0	0.3	0.61
CHRNB4	30.5	14.5	13.5	25.5	13.5	13.0	18.4	7.61
CHRND	729.0	1317.0	1359.5	726.0	1382.5	1113.0	1104.5	307.18
CHRNG	1275.5	2169.0	942.5	1034.0	2283.5	821.0	1420.9	642.39
CHST1	3.5	23.5	73.5	1.5	17.0	56.0	29.2	29.29
CHST10	239.5	80.5	124.0	224.5	91.5	111.0	145.2	69.09
CHST11	12.5	8.5	22.5	16.0	5.0	15.0	13.3	6.12
CHST12	124.0	69.0	114.0	143.5	78.5	114.5	107.3	28.22
CHST13	29.5	54.5	103.5	13.0	54.0	148.5	67.2	50.24
CHST14	1240.5	805.5	770.5	1217.5	955.5	877.5	977.8	204.79
CHST15	9.0	13.0	29.5	11.0	28.0	43.5	22.3	13.60
CHST2	214.5	166.0	131.0	189.0	162.0	123.5	164.3	34.39
CHST3	551.0	568.0	847.5	568.0	587.5	764.0	647.7	125.80
CHST4	3.5	4.0	1.5	0.5	1.5	2.0	2.2	1.33
CHST8	0.5	0.0	0.0	0.0	0.0	0.0	0.1	0.20
CHST9	9.5	7.5	12.0	7.5	10.0	20.5	11.2	4.88
CHSY1	504.0	517.0	672.0	565.5	616.5	692.5	594.6	78.91
CHTF18	345.5	234.0	274.0	345.5	212.0	275.0	281.0	55.45
CHTF8	273.5	127.0	140.0	263.5	133.5	74.0	168.6	80.92
CHTOP	1595.5	1539.0	1105.0	1426.5	1543.0	1117.5	1387.8	221.19
CHUK	1515.5	1489.5	1066.5	1358.5	1638.0	1139.0	1367.8	224.88
CHURC1	1101.5	1609.0	905.0	1003.0	1607.5	1029.0	1209.2	315.47
CIAO1	595.0	607.5	388.0	540.0	605.5	367.0	517.2	111.15
CIAPIN1	1127.5	1170.0	999.5	1086.0	1247.0	985.5	1102.6	100.63
CIART	43.5	152.5	84.5	49.0	194.5	154.5	113.1	62.71
CIB1	1427.5	1482.5	1184.5	1343.0	1542.5	1250.0	1371.7	138.02
CIB2	95.5	49.5	28.0	83.0	54.0	33.5	57.3	26.89
CIB3	6.0	6.5	4.0	4.0	6.5	3.5	5.1	1.39
CIDEA	906.5	963.0	809.0	842.0	1256.0	784.5	926.8	174.02
CIDEC	3.5	1.5	19.0	1.5	7.0	16.0	8.1	7.63
CIITA	1.5	0.5	1.0	2.0	1.0	2.0	1.3	0.61
CILP	80.0	58.5	165.5	46.5	67.0	125.0	90.4	45.69
CILP2	21.0	9.5	31.0	14.5	8.0	24.0	18.0	8.93
CINP	213.0	212.0	170.5	201.0	221.5	139.5	192.9	31.64
CIPC	164.5	88.5	162.0	150.0	83.0	141.5	131.6	36.50
CIRBP	12643.0	12376.0	7919.0	11520.5	13202.5	7886.0	10924.5	2402.92
CIRH1A	718.0	547.0	315.5	726.5	499.5	337.5	524.0	177.78
CISD1	1222.5	1321.0	1459.5	1202.0	1387.5	1442.5	1339.2	109.77
CISD2	676.0	399.0	410.0	644.0	457.0	422.0	501.3	124.85
CISD3	313.5	394.5	181.0	338.0	390.0	229.5	307.8	86.52
CISH	2743.0	2563.5	1747.0	2973.0	2731.0	1868.0	2437.6	506.60

CIT	110.0	24.0	150.0	168.0	21.5	82.5	92.7	61.88
CITED2	126.5	56.5	79.5	106.5	61.5	73.5	84.0	27.22
CITED4	57.5	68.0	46.0	56.0	75.0	50.5	58.8	10.86
CIZ1	1055.0	678.0	529.0	950.5	696.5	535.5	740.8	217.08
CKAP2	2187.0	975.5	1566.5	2237.5	866.5	868.0	1450.2	645.17
CKAP2L	518.5	242.0	362.0	586.0	242.0	271.0	370.3	149.23
CKAP4	1684.0	910.5	2590.0	1942.0	1083.5	2588.0	1799.7	718.78
CKB	6699.5	20141.0	10711.5	7557.0	26520.0	10874.0	13750.5	7867.46
CKLF	1170.5	1297.5	1123.5	1221.5	1408.0	1191.0	1235.3	102.51
CKMT2	0.5	3.0	0.5	0.0	3.5	1.0	1.4	1.46
CKS1B	1006.0	867.0	639.0	959.0	723.5	498.0	782.1	196.60
CKS2	355.0	181.0	195.0	297.0	160.0	120.0	218.0	89.30
CLASP1	404.5	251.0	540.5	412.0	273.5	486.0	394.6	114.30
CLASP2	999.5	630.5	909.0	989.5	812.0	935.5	879.3	139.33
CLCA2	12.5	7.0	9.5	8.0	5.5	8.5	8.5	2.39
CLCC1	371.5	223.5	259.0	340.5	218.5	214.5	271.3	68.23
CLCF1	8.5	11.5	6.0	4.0	10.5	11.0	8.6	3.02
CLCN1	28.0	6.5	30.0	28.0	10.0	19.5	20.3	10.10
CLCN2	3.5	40.5	49.5	7.0	38.5	37.0	29.3	19.19
CLCN3	1455.0	1018.5	1242.5	1469.5	1217.0	1353.5	1292.7	170.14
CLCN4	19.5	12.5	30.5	13.0	6.5	17.0	16.5	8.17
CLCN5	507.5	315.0	197.5	466.0	356.5	224.0	344.4	125.28
CLCN6	358.5	176.0	287.0	339.0	185.5	260.5	267.8	76.05
CLCN7	928.0	645.0	650.5	852.5	655.5	610.0	723.6	132.26
CLDN1	0.0	1.5	0.0	0.0	1.0	1.0	0.6	0.66
CLDN10	26.0	6.0	3.5	11.0	9.5	4.0	10.0	8.38
CLDN11	0.5	4.0	8.0	1.5	5.5	11.0	5.1	3.97
CLDN12	387.0	190.5	325.5	314.5	209.5	277.0	284.0	74.30
CLDN14	0.0	0.0	0.0	0.5	0.0	0.0	0.1	0.20
CLDN16	0.0	0.0	0.0	0.0	0.0	0.0	0.0	0.00
CLDN18	0.0	0.5	0.0	0.0	0.0	0.0	0.1	0.20
CLDN19	0.0	0.0	0.0	0.0	0.0	0.0	0.0	0.00
CLDN2	0.5	2.0	0.0	1.0	1.0	0.5	0.8	0.68
CLDN20	3.5	2.5	1.0	4.5	1.5	1.0	2.3	1.44
CLDN23	0.0	0.0	0.5	0.0	0.5	0.0	0.2	0.26
CLDN25	2.0	4.5	16.0	4.0	8.0	13.5	8.0	5.63
CLDN3	0.5	0.5	0.5	0.0	0.5	0.0	0.3	0.26
CLDN5	1.5	0.5	1.5	1.0	0.5	2.0	1.2	0.61
CLDND1	1278.5	1285.0	1612.0	1315.0	1490.5	1616.5	1432.9	160.45
CLEC16A	46.5	23.5	55.5	54.0	26.5	36.5	40.4	13.74
CLEC19A	11.0	20.5	13.5	15.0	17.5	8.0	14.3	4.48
CLEC3A	0.5	0.0	0.0	0.5	0.0	0.5	0.3	0.27
CLEC3B	93.5	167.0	91.5	94.0	234.0	167.5	141.3	58.21
CLGN	0.5	1.5	1.0	0.5	0.0	1.0	0.8	0.52
CLIC2	3047.0	2288.0	1592.5	2970.0	2478.5	1725.5	2350.3	608.99
CLIC3	116.5	84.0	55.0	81.5	88.5	39.5	77.5	27.02
CLIC4	7061.0	7697.5	7391.5	7250.5	8271.0	7227.0	7483.1	441.14
CLIC5	0.0	10.0	9.0	2.5	5.5	5.0	5.3	3.79
CLIC6	0.5	1.0	0.5	0.5	1.0	1.0	0.8	0.27
CLINT1	1990.0	1558.5	2461.0	1973.5	1776.0	2013.0	1962.0	299.98
CLIP1	920.0	786.5	946.0	861.5	889.5	833.5	872.8	58.28
CLIP2	540.0	464.0	839.5	605.5	467.5	966.0	647.1	208.66
CLIP4	197.0	115.5	117.5	205.5	135.0	158.5	154.8	39.22
CLK1	660.5	691.0	595.5	594.0	761.5	666.0	661.4	62.93

CLK2	318.5	301.0	282.0	299.0	331.5	275.0	301.2	21.34
CLK3	763.5	716.0	676.5	694.5	759.0	691.5	716.8	36.67
CLK4	429.0	530.5	489.5	384.0	595.5	553.0	496.9	79.26
CLMN	327.5	131.5	215.5	210.5	113.0	135.5	188.9	80.36
CLMP	185.5	43.5	135.5	224.0	45.0	161.0	132.4	74.27
CLN5	210.5	88.0	154.0	200.5	106.5	125.0	147.4	50.10
CLN6	419.0	335.5	262.5	387.0	318.0	276.0	333.0	61.26
CLN8	541.5	382.0	558.5	562.0	405.0	497.5	491.1	79.33
CLNS1A	906.0	636.5	578.5	904.5	699.5	569.0	715.7	154.10
CLOCK	269.5	216.5	337.5	233.0	253.0	343.5	275.5	53.47
CLP1	231.0	209.5	165.5	236.5	217.5	177.0	206.2	28.92
CLPP	0.0	0.0	0.0	0.0	0.0	0.0	0.0	0.00
CLPS	0.5	0.5	0.5	2.0	1.5	0.0	0.8	0.75
CLPTM1	422.0	240.5	437.0	423.5	208.5	372.0	350.6	100.65
CLPTM1L	747.5	549.5	641.5	757.5	597.0	634.5	654.6	82.65
CLPX	1682.0	1123.0	1142.5	1565.0	1343.5	1041.5	1316.3	260.54
CLRN1	18.5	21.0	19.5	10.5	20.5	28.0	19.7	5.61
CLRN2	12.0	12.0	6.5	11.0	17.0	7.5	11.0	3.75
CLRN3	4.0	2.0	1.5	3.0	1.5	0.0	2.0	1.38
CLSPN	127.5	55.0	120.0	137.5	47.5	73.0	93.4	39.53
CLSTN3	37.0	19.0	22.0	36.0	23.5	19.0	26.1	8.26
CLTA	837.0	535.5	420.0	839.5	578.0	371.5	596.9	201.36
CLTB	2829.5	3497.0	2639.5	2749.5	3642.0	2737.0	3015.8	435.58
CLTCL1	1499.0	1228.0	1532.5	1375.5	1246.5	955.0	1306.1	212.70
CLU	34608.5	32187.0	21415.0	38974.5	37319.0	35196.0	33283.3	6263.38
CLUAP1	178.0	100.0	124.5	160.0	97.5	98.5	126.4	34.94
CLUH	831.0	639.5	815.5	905.5	697.0	661.0	758.3	107.30
CLUL1	11.5	6.0	1.5	11.0	1.0	0.0	5.2	5.14
CLVS1	2.0	1.0	4.0	6.0	2.0	1.5	2.8	1.89
CLVS2	0.5	0.0	1.5	0.0	0.0	1.0	0.5	0.63
CLYBL	19.5	21.5	27.0	16.0	24.5	16.5	20.8	4.38
CMAS	209.0	141.0	201.0	214.0	141.0	169.5	179.3	33.43
CMC1	242.0	258.0	251.0	230.5	290.0	244.5	252.7	20.48
CMC2	216.5	174.5	152.5	216.0	170.5	136.5	177.8	32.76
CMC4	300.0	315.5	225.0	273.5	369.5	279.5	293.8	48.15
CMIP	167.0	96.0	143.5	153.5	83.0	124.5	127.9	33.08
CMKLR1	155.0	691.5	1160.0	176.5	637.5	716.0	589.4	377.77
CMPK1	344.5	190.0	400.0	374.5	221.0	355.0	314.2	86.82
CMPK2	41.5	32.5	96.5	41.5	37.0	100.5	58.3	31.38
CMSS1	209.0	167.0	185.5	215.5	169.5	206.5	192.2	21.09
CMTM3	168.5	68.5	157.5	195.0	77.5	119.0	131.0	51.22
CMTM4	367.0	117.5	421.5	427.5	156.0	370.5	310.0	137.05
CMTM7	3178.0	1327.0	1419.5	2608.5	1598.5	1723.0	1975.8	745.96
CMTM8	6.5	10.5	13.5	3.5	11.0	12.0	9.5	3.75
CMTR1	645.0	428.5	555.0	619.5	437.0	565.0	541.7	90.80
CMTR2	255.5	173.0	252.0	253.0	203.0	234.5	228.5	33.61
CMYA5	54.5	307.5	315.5	47.0	382.5	296.0	233.8	145.00
CNBP	16152.5	10543.5	6261.5	14718.5	11477.5	6138.0	10881.9	4167.03
CNDP1	53.0	19.0	1.5	51.5	17.0	4.5	24.4	22.61
CNDP2	733.5	563.5	476.5	664.0	567.0	395.0	566.6	122.41
CNEP1R1	100.0	45.5	90.5	83.0	66.5	78.0	77.3	19.24
CNGA1	0.0	0.0	0.0	0.0	0.0	0.0	0.0	0.00
CNGA2	277.0	316.0	153.5	267.5	366.0	187.0	261.2	79.22
CNGA3	2.0	4.0	0.5	13.5	15.0	10.0	7.5	6.16

CNGA4	18.5	155.0	78.5	12.5	133.5	63.0	76.8	58.41
CNGB1	1.0	1.0	2.0	1.0	0.5	2.0	1.3	0.61
CNGB3	2.0	0.5	1.0	0.0	0.5	3.0	1.2	1.13
CNIH1	1581.5	1365.0	1186.5	1565.0	1405.0	1139.0	1373.7	184.91
CNIH3	0.5	0.0	0.0	1.0	0.0	0.5	0.3	0.41
CNIH4	475.5	296.5	260.5	460.0	346.0	241.0	346.6	100.55
CNKSR1	6.0	6.0	4.5	4.5	4.0	6.5	5.3	1.04
CNKSR3	2208.5	1023.0	1501.0	1955.0	1151.0	1307.5	1524.3	467.30
CNN2	2011.5	4306.0	3009.0	1864.0	4505.5	3170.5	3144.4	1108.50
CNN3	6543.0	4227.5	6235.5	6382.5	4631.0	6489.0	5751.4	1037.39
CNNM1	60.5	46.5	42.0	35.5	42.0	58.5	47.5	9.95
CNNM2	116.5	85.0	226.5	117.5	74.0	189.0	134.8	60.26
CNNM4	222.5	145.5	242.5	260.0	154.0	264.0	214.8	52.52
CNOT1	1892.0	1284.0	1895.5	1867.0	1350.5	1655.5	1657.4	279.03
CNOT10	557.0	411.0	386.5	553.5	480.5	372.0	460.1	82.61
CNOT11	540.5	485.5	490.0	555.5	501.5	441.5	502.4	41.04
CNOT2	488.5	508.5	480.5	495.0	520.5	491.5	497.4	14.58
CNOT4	264.5	288.5	381.5	261.5	332.5	345.0	312.3	48.37
CNOT6	657.0	564.5	896.5	679.0	711.0	801.5	718.3	116.35
CNOT7	322.0	291.0	464.5	333.0	311.5	407.0	354.8	66.72
CNOT8	832.0	652.0	590.5	812.0	710.5	570.5	694.6	110.42
CNP	6081.5	5306.0	5402.0	4551.0	5290.5	4317.5	5158.1	636.67
CNPPD1	1719.0	930.0	564.5	1550.0	1005.5	559.5	1054.8	487.87
CNPY3	484.0	772.5	586.5	528.5	812.0	753.5	656.2	140.06
CNR1	876.5	299.0	529.0	699.0	319.0	415.5	523.0	227.74
CNR2	12.5	4.5	2.5	7.5	1.5	2.5	5.2	4.18
CNRIP1	18.0	50.5	84.0	11.0	52.0	69.5	47.5	28.45
CNST	115.5	109.5	136.0	103.5	127.5	152.5	124.1	18.28
CNTD1	42.5	106.5	119.0	52.0	95.0	132.5	91.3	36.43
CNTF	65.5	84.0	72.5	54.5	96.0	54.0	71.1	16.66
CNTN1	2.0	8.5	20.5	7.0	15.0	16.5	11.6	6.90
CNTN2	1.0	2.5	1.0	0.5	0.5	2.5	1.3	0.93
CNTN3	8.0	9.0	5.0	12.5	13.5	6.5	9.1	3.34
CNTN4	0.0	1.5	5.5	1.0	4.0	9.5	3.6	3.54
CNTN5	0.0	0.0	0.0	0.0	0.0	0.0	0.0	0.00
CNTN6	0.0	0.5	0.0	0.0	0.0	0.0	0.1	0.20
CNTNAP1	18.5	10.0	25.0	22.5	11.5	25.0	18.8	6.65
CNTNAP2	11.0	5.0	16.0	11.5	8.0	20.0	11.9	5.41
CNTRL	790.5	398.5	460.0	706.5	420.5	353.0	521.5	181.16
COASY	199.0	326.5	154.0	188.5	327.5	187.5	230.5	76.27
COCH	2.5	1.0	6.0	1.5	1.5	4.0	2.8	1.92
COG1	617.5	401.5	400.0	547.5	444.5	412.0	470.5	90.96
COG2	373.0	268.0	257.0	394.0	302.5	269.5	310.7	58.81
COG3	112.5	70.5	223.0	118.5	85.5	154.5	127.4	55.08
COG4	1380.0	983.5	701.0	1374.5	1031.0	704.0	1029.0	302.61
COG5	257.5	163.0	345.0	245.0	174.5	282.5	244.6	68.20
COG6	303.0	309.0	450.5	319.0	337.0	374.0	348.8	56.01
COG7	620.5	510.0	483.0	646.0	541.5	485.0	547.7	70.06
COG8	223.0	258.0	208.5	207.5	250.0	206.5	225.6	22.96
COIL	266.5	240.5	235.0	286.0	255.5	244.5	254.7	19.04
COL10A1	0.0	0.0	0.5	0.0	0.0	0.0	0.1	0.20
COL13A1	23.5	412.5	277.5	50.0	538.0	317.5	269.8	201.79
COL14A1	5.0	4.0	8.5	6.0	5.5	6.5	5.9	1.53
COL15A1	81.0	100.0	268.0	85.5	108.0	208.5	141.8	77.63

COL16A1	3.0	48.5	35.0	3.5	24.5	41.5	26.0	19.31
COL17A1	913.0	1091.5	579.0	943.5	1245.5	725.0	916.3	240.91
COL18A1	2757.5	4067.5	7963.5	3226.0	4531.0	10487.0	5505.4	3053.60
COL19A1	48.0	36.5	56.0	22.5	25.0	39.0	37.8	12.93
COL1A2	15364.5	24122.0	34732.5	16772.0	31037.0	48364.5	28398.8	12402.19
COL20A1	1.0	2.5	11.0	0.0	5.0	10.0	4.9	4.65
COL23A1	334.0	537.5	753.0	300.0	565.5	683.5	528.9	182.16
COL24A1	26.5	12.5	39.0	3.5	2.0	5.0	14.8	14.94
COL25A1	0.0	0.0	0.0	0.0	0.0	0.5	0.1	0.20
COL26A1	29.0	15.5	22.5	32.0	22.0	16.5	22.9	6.58
COL27A1	201.5	182.5	149.0	270.0	198.0	206.5	201.3	39.61
COL28A1	9.0	9.5	7.0	12.5	7.0	4.0	8.2	2.88
COL2A1	5.0	4.5	11.0	2.5	2.5	8.5	5.7	3.42
COL3A1	1108.5	6472.0	23445.0	2024.0	9325.5	35102.5	12912.9	13531.96
COL4A1	1047.5	2486.5	6637.5	1701.0	3516.0	11203.0	4431.9	3853.52
COL4A2	810.0	1522.0	4259.0	1345.5	1990.5	7187.5	2852.4	2438.30
COL4A3	214.5	166.5	174.0	203.5	170.5	143.0	178.7	26.11
COL4A3BP	243.5	225.0	262.5	268.5	249.5	251.0	250.0	15.28
COL4A4	13.0	12.0	13.0	27.0	8.5	14.5	14.7	6.37
COL4A5	24.0	38.5	42.0	26.5	34.0	132.5	49.6	41.20
COL4A6	9.0	24.5	24.0	7.5	27.5	62.5	25.8	19.86
COL5A1	2068.0	2308.5	6314.0	2815.0	3198.0	7402.5	4017.7	2261.46
COL5A2	8367.5	7384.0	15324.0	8764.5	8662.5	15847.5	10725.0	3800.22
COL6A1	12464.5	20640.0	36377.5	17643.5	25682.5	44587.0	26232.5	12122.01
COL6A2	11677.0	16610.5	27101.0	17909.0	20481.0	32650.0	21071.4	7601.63
COL6A3	3070.0	10775.0	12576.5	5594.0	14685.5	21135.0	11306.0	6486.68
COL6A6	4.0	2.5	6.0	2.0	2.5	3.0	3.3	1.47
COL7A1	858.5	1805.5	1579.0	1301.0	1995.5	3051.5	1765.2	745.85
COL8A1	4.5	17.0	36.0	3.0	27.5	30.5	19.8	13.86
COL8A2	6.5	7.5	160.5	3.5	7.5	131.5	52.8	72.76
COL9A1	1.0	0.0	2.0	0.0	0.0	2.5	0.9	1.11
COL9A2	1.0	1.0	6.0	1.0	1.0	3.0	2.2	2.04
COL9A3	36.0	20.0	26.0	25.0	25.0	27.5	26.6	5.26
COLCA2	3.5	0.0	0.0	2.5	0.5	0.0	1.1	1.53
COLEC10	86.0	32.5	13.5	85.0	29.0	15.5	43.6	33.30
COLEC11	2.0	2.5	2.5	1.0	1.0	2.0	1.8	0.68
COLEC12	3027.5	1745.5	2063.0	2870.5	2032.5	1931.5	2278.4	533.46
COLGALT2	49.0	68.0	211.0	60.0	71.0	173.0	105.3	68.62
COLQ	0.5	1.5	6.5	3.0	4.0	6.5	3.7	2.50
COMMD1	337.5	293.0	177.0	294.0	307.0	165.5	262.3	72.45
COMMD2	576.0	681.0	619.5	523.5	716.5	537.5	609.0	77.93
COMMD4	483.5	705.5	415.0	462.0	691.5	430.5	531.3	131.74
COMMD6	1060.0	681.5	791.0	1050.5	755.5	652.5	831.8	180.07
COMMD7	1029.0	979.0	777.5	810.0	994.0	708.5	883.0	133.99
COMMD8	298.5	247.5	265.0	287.5	264.0	230.0	265.4	25.14
COMMD9	513.5	330.0	259.0	476.0	355.5	245.5	363.3	110.62
COMP	450.5	171.0	183.0	255.0	151.0	108.0	219.8	122.82
COMT	2167.0	1832.0	2376.5	1845.0	2217.0	2178.5	2102.7	217.99
COMTD1	1327.0	858.5	690.5	1055.0	873.0	584.0	898.0	265.53
COPA	3650.0	2235.0	2907.0	3916.5	2404.0	2874.0	2997.8	667.44
COPB1	3302.0	2018.0	2636.0	3396.5	2210.0	2362.0	2654.1	575.92
COPB2	3459.0	2580.5	3347.0	3639.5	3062.5	3053.5	3190.3	375.62
COPE	3269.5	2562.5	1947.5	3389.0	2788.0	1876.5	2638.8	639.95
COPG1	2690.5	2211.5	2443.0	2845.5	2448.5	2594.5	2538.9	221.20

COPG2	1145.0	783.0	919.5	1179.0	848.0	777.0	941.9	178.45
COPRS	0.5	2.0	4.0	1.0	1.0	4.5	2.2	1.69
COPS2	1495.0	1032.5	1085.0	1293.0	1138.0	968.5	1168.7	194.17
COPS3	1343.5	1276.0	1043.0	1300.0	1295.0	939.5	1199.5	166.07
COPS4	1412.5	1134.0	999.5	1321.5	1197.0	873.0	1156.3	199.96
COPS5	834.5	669.0	566.5	825.0	732.5	524.0	691.9	129.72
COPS7A	1430.5	1227.0	938.5	1354.5	1336.5	968.0	1209.2	208.84
COPS7B	525.5	354.5	311.0	456.5	306.5	278.0	372.0	97.83
COPS8	1845.0	1977.0	1514.0	1669.5	2117.0	1368.0	1748.4	284.02
COQ10A	124.0	126.5	131.0	110.0	142.0	132.0	127.6	10.60
COQ10B	415.0	441.5	302.5	338.5	535.5	275.0	384.7	97.79
COQ2	97.0	106.5	81.5	103.0	101.0	92.5	96.9	8.97
COQ3	509.5	449.0	246.0	474.0	462.5	237.5	396.4	121.51
COQ4	399.5	358.5	225.0	350.5	362.0	229.0	320.8	74.56
COQ5	743.0	604.0	475.0	684.0	616.5	384.0	584.4	133.13
COQ6	350.5	358.0	215.5	403.0	371.0	196.5	315.8	87.10
COQ7	379.5	289.0	241.5	317.5	280.0	199.5	284.5	62.01
COQ9	679.0	960.5	546.0	666.5	982.5	598.5	738.8	186.65
CORO1B	1425.5	1154.0	1143.0	1436.5	1083.5	1155.5	1233.0	155.67
CORO1C	2313.5	1929.0	2091.0	2439.5	2024.5	1943.0	2123.4	208.43
CORO2A	3.0	1.0	1.0	3.0	1.0	3.0	2.0	1.10
CORO2B	43.0	19.5	9.5	30.0	17.0	6.0	20.8	13.71
CORO6	311.5	564.0	270.0	269.0	608.0	248.0	378.4	162.70
CORO7	81.5	33.5	65.5	59.0	34.5	64.5	56.4	18.92
CORT	3.0	0.0	0.5	2.0	0.0	1.0	1.1	1.20
COTL1	7430.0	4468.0	4515.5	7177.0	5207.0	4826.5	5604.0	1345.07
COX3	0.0	0.0	0.0	0.0	0.0	0.0	0.0	0.00
CP	365.0	413.0	328.5	310.5	559.5	361.5	389.7	90.33
CPA2	2.5	0.5	1.0	1.5	3.0	1.0	1.6	0.97
CPAMD8	10.5	20.0	65.5	18.5	30.0	39.5	30.7	19.80
CPB2	0.0	0.0	0.0	0.0	0.0	0.0	0.0	0.00
CPD	858.5	378.5	1263.5	932.0	400.5	1355.0	864.7	413.73
CPE	637.5	300.0	478.5	629.0	380.5	458.0	480.6	134.01
CPEB1	180.0	348.5	183.0	202.5	437.5	203.5	259.2	107.85
CPEB2	245.0	148.0	377.5	269.5	163.0	245.0	241.3	82.71
CPEB3	80.0	32.0	110.0	79.0	69.0	108.5	79.8	28.77
CPEB4	306.5	459.0	759.5	287.0	534.0	556.0	483.7	175.80
CPED1	4.5	0.5	6.5	3.5	1.5	1.5	3.0	2.26
CPLX2	2.5	0.5	2.5	0.5	2.0	2.0	1.7	0.93
CPLX3	0.0	0.0	0.0	0.0	0.0	0.0	0.0	0.00
CPLX4	0.0	0.0	0.0	0.0	0.0	0.5	0.1	0.20
CPM	2228.0	2000.5	3088.0	2551.5	2415.0	2723.0	2501.0	381.79
CPN1	8.5	6.0	4.5	3.5	6.5	5.0	5.7	1.75
CPNE1	1632.5	991.0	1115.5	1416.0	1166.5	1114.5	1239.3	238.19
CPNE2	78.0	30.0	31.0	57.0	43.5	40.5	46.7	18.22
CPNE4	28.0	21.0	18.5	15.5	24.0	20.5	21.3	4.34
CPNE7	3.5	8.0	5.0	2.0	7.0	6.0	5.3	2.23
CPNE8	96.0	37.5	125.5	119.5	35.0	109.5	87.2	40.69
CPNE9	17.0	23.0	11.0	14.5	26.0	13.5	17.5	5.83
CPO	0.0	0.5	0.0	0.5	0.0	0.0	0.2	0.26
CPOX	461.0	362.0	252.0	407.5	366.5	255.5	350.8	83.16
CPPED1	83.0	95.0	180.0	73.5	101.5	169.0	117.0	45.71
CPQ	978.5	677.5	515.5	1038.0	758.0	548.0	752.6	217.36
CPS1	15.5	10.5	20.5	3.5	8.0	5.0	10.5	6.49

CPSF1	31.0	15.0	48.5	21.5	8.5	46.0	28.4	16.39
CPSF2	691.5	529.5	699.5	686.0	629.0	598.0	638.9	66.93
CPSF3	1217.0	1055.0	1021.5	1168.0	1079.0	929.0	1078.3	103.29
CPSF3L	710.5	690.5	636.0	744.5	773.0	629.0	697.3	57.62
CPSF4	717.0	559.0	586.0	718.0	606.0	505.0	615.2	86.22
CPSF6	1763.0	1029.0	963.5	1672.0	1087.0	905.0	1236.6	378.60
CPSF7	67.0	49.0	80.0	67.5	36.5	66.0	61.0	15.55
CPT1A	1723.5	1206.0	1366.5	1704.5	1482.0	1394.0	1479.4	202.51
CPT2	502.5	339.0	340.5	434.5	317.5	258.5	365.4	87.88
CPTP	230.5	178.0	226.0	262.5	182.5	224.0	217.3	31.92
CPXM2	3.0	0.0	3.5	0.5	0.0	2.0	1.5	1.55
CR1	1.0	0.0	0.0	0.0	0.5	0.0	0.3	0.42
CRABP1	25.0	59.0	66.5	30.5	79.0	131.5	65.3	38.59
CRABP2	7.0	7.0	8.0	3.0	10.0	10.0	7.5	2.59
CRACR2B	299.0	187.5	314.5	204.5	152.5	304.5	243.8	70.40
CRADD	253.5	200.0	107.5	224.5	185.5	82.5	175.6	67.02
CRAMP1L	85.0	46.5	141.5	68.0	51.5	122.0	85.8	38.60
CRAT	226.5	133.5	215.5	236.0	135.5	216.0	193.8	46.58
CRB1	419.5	289.0	306.5	296.0	279.5	372.5	327.2	56.05
CRB2	12.5	21.0	15.5	20.5	28.5	17.5	19.3	5.53
CRBN	343.0	221.5	286.5	348.0	276.0	256.5	288.6	49.36
CRCP	1044.5	739.0	460.5	960.0	789.5	411.0	734.1	256.80
CREB1	126.0	48.0	217.0	117.5	56.5	173.0	123.0	65.46
CREB3	2180.0	1950.5	1819.0	2292.5	2041.0	1757.5	2006.8	206.58
CREB3L1	949.0	861.5	1333.5	1047.5	1020.5	1668.5	1146.8	301.12
CREB3L2	1523.0	691.0	2273.0	1681.5	764.0	2534.5	1577.8	756.44
CREB3L3	11.5	5.0	0.5	7.5	2.5	1.0	4.7	4.25
CREB5	21.0	14.5	38.0	22.5	12.0	44.5	25.4	13.04
CREBL2	1021.5	541.0	483.5	933.5	622.0	491.0	682.1	235.74
CREBRF	48.0	22.0	61.0	46.0	41.5	59.0	46.3	14.10
CREG1	160.0	26.5	59.0	200.5	33.5	33.5	85.5	75.32
CREG2	72.5	41.0	62.0	63.5	45.5	48.5	55.5	12.28
CRELD1	39.0	249.5	194.5	28.5	210.0	288.0	168.3	109.19
CRELD2	794.5	435.5	623.5	792.0	531.5	576.5	625.6	143.96
CREM	165.0	109.5	141.5	187.0	127.5	164.5	149.2	28.35
CRH	117.0	156.5	62.5	118.0	190.0	102.0	124.3	44.19
CRHBP	1.5	2.0	0.0	1.0	2.5	1.0	1.3	0.88
CRHR1	0.0	0.0	0.0	0.0	0.0	0.0	0.0	0.00
CRHR2	15.0	13.0	15.5	4.5	11.0	13.0	12.0	4.01
CRIM1	207.5	109.0	446.0	213.5	141.5	422.0	256.6	143.20
CRIP1	231.0	1083.5	327.0	225.0	1058.5	468.0	565.5	401.40
CRIPT	481.5	481.5	695.0	432.5	534.5	610.0	539.2	97.32
CRISPLD1	17.0	5.0	16.5	17.5	3.5	6.5	11.0	6.65
CRISPLD2	37.0	48.0	106.0	48.5	69.5	174.0	80.5	51.93
CRK	1652.0	1504.0	2099.0	1662.0	1696.0	1818.5	1738.6	203.22
CRKL	1188.5	1123.5	1340.0	1147.5	1191.0	1093.5	1180.7	86.63
CRLF1	252.5	320.0	1025.0	144.5	201.0	698.0	440.2	347.21
CRLF2	0.0	0.0	0.5	0.0	0.0	0.0	0.1	0.20
CRLF3	156.0	91.0	298.5	162.0	87.0	248.5	173.8	84.82
CRLS1	537.5	487.5	471.5	487.5	561.5	467.5	502.2	38.32
CRMP1	4.5	8.0	9.0	5.0	9.5	10.5	7.8	2.46
CRNKL1	791.0	507.5	573.0	767.5	582.5	532.5	625.7	122.26
CROCC	317.0	247.0	176.5	307.5	219.0	199.5	244.4	57.50
CROT	813.5	384.5	571.5	845.0	485.5	507.5	601.3	186.82

CRTAC1	143.5	340.5	577.5	121.0	298.5	453.0	322.3	176.41
CRTAM	0.0	0.0	0.0	0.0	0.0	0.0	0.0	0.00
CRTAP	1673.0	1168.0	1090.0	1681.0	1489.0	1210.5	1385.3	263.04
CRTC1	207.0	123.5	211.5	180.5	124.0	187.0	172.3	39.34
CRY1	1376.0	1050.5	963.0	1162.0	1193.0	838.5	1097.2	188.80
CRY2	311.0	460.5	1273.0	344.5	536.5	1640.5	761.0	557.20
CRYAA	21.0	69.5	45.0	22.5	117.5	62.0	56.3	35.97
CRYAB	92.5	1684.0	2555.5	47.0	1893.5	2780.5	1508.8	1186.11
CRYBA1	23.0	26.0	17.5	18.0	29.0	16.0	21.6	5.24
CRYBA2	0.0	0.0	0.0	0.0	0.0	0.0	0.0	0.00
CRYBA4	23.0	17.0	3.0	24.0	20.5	4.0	15.3	9.42
CRYBB1	37.0	17.5	10.0	41.0	31.5	9.5	24.4	13.87
CRYBB2	0.0	0.0	0.0	0.0	0.0	0.0	0.0	0.00
CRYBB3	0.5	0.5	1.0	0.0	0.5	0.5	0.5	0.32
CRYBG3	81.0	35.5	90.0	70.0	30.5	94.0	66.8	27.52
CRYGN	77.0	70.5	50.0	63.5	73.5	43.0	62.9	13.65
CRYGS	17.5	10.5	7.5	17.5	14.5	6.5	12.3	4.88
CRYM	36.5	18.5	54.5	28.5	15.5	28.0	30.3	14.08
CRYZ	865.5	622.5	550.5	859.5	662.5	579.5	690.0	138.93
CRYZL1	413.5	284.5	218.5	378.0	310.0	197.0	300.3	85.51
CS	3060.5	3588.5	3016.5	2970.5	3459.0	2872.5	3161.3	290.54
CSAD	4.5	4.5	1.0	1.5	4.5	1.0	2.8	1.83
CSDC2	0.5	0.0	0.5	0.0	0.0	0.0	0.2	0.26
CSDE1	7186.5	6372.0	7539.5	7063.0	7332.0	7077.5	7095.1	396.53
CSF1	134.5	119.0	172.0	116.0	100.5	182.0	137.3	32.72
CSF1R	0.5	4.5	1.5	1.5	4.0	1.5	2.3	1.60
CSF3	14.0	4.5	0.0	11.0	2.0	0.5	5.3	5.85
CSF3R	1.0	0.0	0.0	0.0	0.0	1.0	0.3	0.52
CSGALNACT1	31.5	15.5	31.0	27.5	22.5	41.5	28.3	8.83
CSGALNACT2	593.0	657.0	574.0	647.0	743.5	644.0	643.1	59.29
CSK	481.0	321.0	573.5	485.0	350.0	586.0	466.1	110.47
CSMD1	0.5	0.0	0.0	1.0	0.5	0.0	0.3	0.41
CSMD2	0.0	1.0	4.0	0.0	1.0	1.0	1.2	1.47
CSNK1A1	3501.0	2429.5	3458.5	3450.0	2777.5	2964.5	3096.8	443.55
CSNK1D	1256.5	977.0	1293.0	1163.5	1129.0	1282.0	1183.5	120.86
CSNK1E	295.5	290.0	777.5	347.0	357.5	612.5	446.7	201.11
CSNK1G1	734.5	572.5	621.5	709.0	612.0	650.5	650.0	61.46
CSNK1G2	302.0	304.5	383.0	306.5	260.0	437.0	332.2	64.99
CSNK2A1	1149.5	949.5	1094.0	1176.0	1110.0	1030.5	1084.9	83.04
CSNK2A2	1258.0	819.0	1160.0	1274.0	838.0	1092.5	1073.6	201.15
CSPG4	225.5	268.0	763.5	265.0	237.5	513.5	378.8	216.72
CSPG5	230.5	129.0	86.5	219.5	104.5	100.0	145.0	63.57
CSPP1	406.0	326.0	297.0	359.0	325.0	268.5	330.3	48.02
CSRNP1	284.0	197.0	462.5	357.0	233.0	413.5	324.5	104.20
CSRNP2	62.5	74.5	93.0	76.5	73.0	104.5	80.7	15.26
CSRNP3	47.0	15.5	37.0	40.0	15.5	29.5	30.8	13.08
CSRP1	6538.0	8905.5	4851.0	6009.0	9388.5	4520.5	6702.1	2037.89
CSRP2	7190.0	2224.5	1413.0	8715.5	2879.5	2257.5	4113.3	3048.66
CSRP2BP	342.5	297.5	382.5	312.5	285.0	358.0	329.7	37.64
CSRP3	667.0	2541.5	917.0	642.5	2994.0	879.0	1440.2	1044.03
CST7	161.5	84.0	124.0	178.5	89.0	100.0	122.8	39.42
CSTA	572.5	1008.0	1007.5	584.5	1193.5	868.0	872.3	250.04
CSTB	463.5	617.0	702.5	471.5	765.0	613.5	605.5	120.97
CSTF1	448.5	416.5	489.5	413.5	443.5	467.5	446.5	29.30

CSTF2	1003.5	872.0	530.5	948.5	885.5	548.5	798.1	205.82
CSTF3	917.0	639.0	611.5	802.5	744.5	516.5	705.2	144.67
CTAGE5	489.0	268.0	335.0	548.0	282.0	278.5	366.8	121.27
CTBP1	2238.5	2519.0	2758.5	2205.0	2954.0	2751.5	2571.1	303.86
CTBP2	859.5	610.5	908.5	731.5	626.5	790.0	754.4	121.37
CTBS	705.5	416.5	429.0	733.5	508.5	515.0	551.3	136.57
CTC1	397.0	379.0	483.5	384.5	404.5	379.5	404.7	39.93
CTCF	670.5	365.5	720.0	655.0	399.0	623.0	572.2	150.78
CTCFL	6.0	2.5	5.0	4.5	5.0	6.0	4.8	1.29
CTDP1	452.0	278.0	321.5	409.5	333.0	336.0	355.0	63.68
CTDSP1	249.5	202.5	299.0	226.0	186.0	334.0	249.5	57.24
CTDSPL	1129.5	674.5	813.5	953.5	769.0	868.5	868.1	158.69
CTDSPL2	826.5	545.0	751.5	790.5	586.5	652.5	692.1	114.57
CTH	874.5	1187.0	1063.0	833.0	1273.5	892.5	1020.6	182.42
CTHRC1	1142.0	781.5	925.5	1359.0	843.5	832.5	980.7	224.79
CTIF	153.0	116.5	344.0	170.5	97.0	268.5	191.6	95.57
CTLA4	0.0	0.0	0.0	0.0	0.0	0.0	0.0	0.00
CTNNA1	3460.0	1828.0	3922.5	3694.0	2011.0	3435.0	3058.4	901.63
CTNNAL1	27.0	25.0	39.0	25.5	19.5	37.0	28.8	7.57
CTNNB1	7596.5	5625.5	6013.0	7786.5	6401.5	5266.5	6448.3	1036.77
CTNNBIP1	776.0	823.5	560.0	764.0	897.5	558.0	729.8	140.40
CTNNBL1	1701.5	1677.5	1389.0	1594.5	1834.5	1457.5	1609.1	164.72
CTNND1	3657.0	3638.0	3691.0	3642.0	4071.0	3301.5	3666.8	244.52
CTNND2	2.0	2.5	5.0	0.0	1.5	0.5	1.9	1.77
CTNS	108.0	91.5	124.5	112.5	92.5	128.0	109.5	15.44
CTPS1	863.5	753.0	584.5	855.0	687.0	551.5	715.8	132.39
CTPS2	312.0	114.0	124.5	322.5	126.5	119.5	186.5	101.43
CTRC	0.0	0.0	0.0	0.5	0.0	0.0	0.1	0.20
CTRL	0.0	0.5	0.0	0.5	0.0	0.5	0.3	0.27
CTSA	3824.0	7338.0	5410.0	4122.5	7803.0	5764.5	5710.3	1624.88
CTSB	1462.5	821.5	1432.0	1428.0	958.5	1327.0	1238.3	277.00
CTSC	1932.0	1269.0	1354.0	2078.5	1550.5	1415.5	1599.9	330.38
CTSD	13748.0	13436.5	14544.5	14076.0	15562.0	11540.0	13817.8	1339.82
CTSE	0.0	0.0	0.0	0.0	0.0	0.0	0.0	0.00
CTSH	940.0	1122.0	771.0	1031.0	1267.0	1361.5	1082.1	216.07
CTSK	148.5	968.0	398.5	129.5	1153.5	602.0	566.7	424.49
CTSL	4673.0	1937.0	2479.5	4777.0	2529.5	2107.0	3083.8	1291.06
CTSO	396.0	269.5	456.5	336.5	299.5	448.0	367.7	77.98
CTSS	283.0	187.0	233.0	269.0	172.5	177.5	220.3	48.35
CTSZ	1630.5	1357.5	1228.0	1557.0	1673.0	1318.5	1460.8	183.41
CTTN	3068.0	2210.5	2809.5	3146.5	2580.0	2759.0	2762.3	340.89
CTTNBP2	50.5	16.0	48.5	25.5	16.0	29.0	30.9	15.30
CTTNBP2NL	319.5	245.0	355.5	315.5	285.0	377.0	316.3	47.53
CTU2	234.5	219.5	158.0	225.0	198.5	141.0	196.1	38.34
CTXN1	0.5	0.0	0.0	0.0	0.0	0.5	0.2	0.26
CTXN2	3.5	0.0	0.0	2.0	0.0	0.0	0.9	1.50
CTXN3	3.0	4.5	2.0	5.0	1.0	1.5	2.8	1.63
CUBN	11.0	10.0	11.5	12.0	15.5	11.5	11.9	1.88
CUEDC1	864.0	646.0	907.0	778.0	629.5	898.5	787.2	124.52
CUEDC2	956.5	687.5	668.0	843.0	683.0	554.0	732.0	143.45
CUL1	1095.5	1040.0	1142.0	1136.5	1177.5	1073.5	1110.8	50.44
CUL3	1632.0	1344.5	1206.5	1518.5	1494.0	1104.0	1383.3	201.57
CUL4A	585.5	395.5	294.5	689.5	508.0	340.5	468.9	152.37
CUL4B	1026.5	640.5	1143.5	1028.0	731.0	1035.5	934.2	199.47

CUL5	520.0	279.5	637.5	491.0	351.0	526.0	467.5	130.04
CUTA	2741.0	2314.0	1514.0	2228.5	2682.5	1588.5	2178.1	525.54
CUTC	396.0	356.0	237.0	360.5	423.5	253.5	337.8	75.95
CUX1	419.0	474.0	597.0	325.0	455.0	483.5	458.9	88.82
CUX2	1.5	3.5	6.0	1.0	3.0	4.0	3.2	1.81
CUZD1	0.0	1.0	1.5	0.0	1.5	2.0	1.0	0.84
CWC15	1192.5	1161.0	1134.5	1104.5	1274.5	1138.5	1167.6	60.03
CWC22	724.0	464.0	493.5	630.0	543.0	491.0	557.6	100.45
CWC25	284.0	249.5	248.0	275.0	272.0	229.0	259.6	20.78
CWC27	502.5	432.0	281.5	442.5	418.0	295.5	395.3	87.74
CWF19L1	775.5	520.5	360.0	732.0	487.5	333.5	534.8	184.58
CWF19L2	168.5	117.5	89.5	134.0	128.5	80.5	119.8	31.99
CX3CL1	55.5	34.5	12.5	46.0	21.5	9.0	29.8	18.69
CX3CR1	0.0	0.5	0.0	0.0	0.5	0.0	0.2	0.26
CXADR	2.5	1.0	2.5	1.5	1.5	1.0	1.7	0.68
CXCL12	120.0	70.5	568.5	100.5	84.5	201.0	190.8	190.63
CXCL14	0.5	0.0	0.0	0.0	0.5	0.0	0.2	0.26
CXCR4	73.5	65.0	46.5	44.0	74.0	70.0	62.2	13.52
CXCR5	598.0	457.0	579.0	531.5	451.0	525.5	523.7	60.62
CXXC5	905.0	1176.0	1409.0	890.0	1352.5	1310.0	1173.8	227.42
CYC	2056.5	2745.5	2083.5	1875.5	2909.0	1783.5	2242.3	469.57
CYFIP2	19.5	39.0	50.0	15.5	38.5	35.5	33.0	13.04
CYGB	77.5	338.0	166.0	81.5	481.5	835.5	330.0	293.58
CYHR1	49.0	15.5	51.0	45.5	12.5	42.5	36.0	17.31
CYLD	331.0	383.0	397.5	317.5	439.5	441.5	385.0	52.53
CYP1A5	1.0	0.0	1.0	0.0	0.0	0.5	0.4	0.49
CYP3A37	0.5	0.0	0.0	0.5	0.0	0.0	0.2	0.26
CYP3A80	2.5	0.0	0.5	1.0	3.0	0.0	1.2	1.29
CYR61	2117.5	1192.0	1835.5	2663.0	1198.0	2060.0	1844.3	571.79
CYSLTR1	4.5	6.0	7.5	6.0	2.0	4.0	5.0	1.92
CYSLTR2	0.0	0.0	0.0	0.0	0.0	0.0	0.0	0.00
CYTH1	353.0	374.0	286.0	330.5	345.0	334.5	337.2	29.46
CYTH4	1.5	1.5	3.5	1.5	5.5	0.5	2.3	1.83
CYTIP	2.0	1.5	1.0	1.0	0.5	1.5	1.3	0.52
CYYR1	63.0	52.5	86.0	41.0	46.5	61.0	58.3	15.93
CZH18orf25	291.5	160.0	270.0	338.0	234.5	261.5	259.3	59.71
CZH5orf28	310.5	193.5	323.5	342.0	194.0	272.5	272.7	65.24
CZH5orf30	84.5	70.5	125.5	58.5	71.5	103.0	85.6	24.73
CZH5orf34	218.0	124.5	113.0	211.5	137.5	117.5	153.7	48.08
CZH5orf42	437.5	247.5	368.5	312.5	253.0	261.5	313.4	76.32
CZH5orf51	247.5	129.5	81.0	223.5	140.0	91.0	152.1	68.77
CZH5orf63	111.5	68.5	29.5	106.0	78.0	17.5	68.5	38.65
CZH9orf41	146.0	129.5	177.0	149.5	127.0	137.5	144.4	18.24
CZH9orf64	473.5	445.5	377.0	489.5	509.5	341.0	439.3	66.63
CZH9orf72	612.5	391.5	601.5	573.0	452.0	460.0	515.1	92.30
CZH9orf84	0.0	0.0	0.0	0.5	1.0	0.0	0.3	0.42
D2HGDH	216.5	100.5	101.0	187.5	92.5	75.0	128.8	58.18
DAAM1	150.5	65.0	100.5	120.5	66.0	112.5	102.5	33.08
DAB1	0.0	0.0	0.0	0.0	0.0	0.0	0.0	0.00
DAB2	56.5	25.5	45.0	67.0	33.0	59.0	47.7	16.09
DAB2IP	568.0	461.5	962.0	641.0	511.5	1037.0	696.8	243.09
DACH1	15.0	6.5	7.0	7.5	2.0	5.5	7.3	4.27
DACH2	10.5	3.5	6.0	3.5	2.5	3.0	4.8	3.03
DACT1	413.5	605.5	1183.0	419.0	557.0	796.5	662.4	291.39

DACT2	1.0	0.0	0.5	0.5	0.0	0.5	0.4	0.38
DAD1	1697.5	1706.0	1030.0	1652.0	1892.5	1145.5	1520.6	347.17
DAG1	3647.0	2263.0	5137.0	3945.0	2482.0	4559.5	3672.3	1132.29
DAGLA	106.0	94.5	109.0	99.0	96.5	100.5	100.9	5.58
DAGLB	399.5	286.0	408.5	358.0	318.0	419.0	364.8	53.85
DAK	321.5	403.0	345.0	258.5	380.0	362.5	345.1	50.85
DALRD3	206.0	293.5	226.5	220.0	270.5	226.5	240.5	33.80
DAO	28.0	32.5	22.5	23.5	40.5	23.5	28.4	7.02
DAP	4803.0	3135.0	2797.5	4673.5	3711.5	3047.0	3694.6	863.07
DAP3	1848.5	1398.0	898.5	1575.5	1349.0	804.5	1312.3	398.64
DAPK1	500.0	419.5	358.0	437.0	493.5	467.0	445.8	53.18
DAPK2	612.5	2126.0	1560.0	533.0	2570.5	1716.5	1519.8	813.48
DAPK3	582.0	377.5	720.0	538.5	271.5	630.5	520.0	166.41
DAPP1	357.5	286.5	189.5	294.5	298.5	157.5	264.0	75.18
DARS	3302.0	2062.5	1497.5	3247.0	2276.5	1397.5	2297.2	826.59
DAW1	0.0	0.0	0.0	0.5	0.5	0.0	0.2	0.26
DAZAP1	1623.0	1271.0	981.0	1576.5	1449.0	954.0	1309.1	291.48
DAZAP2	78.0	69.5	111.5	116.5	99.5	135.5	101.8	24.74
DAZL	0.0	0.0	0.0	0.0	0.0	0.5	0.1	0.20
DBF4	439.5	292.0	314.0	430.5	307.0	243.0	337.7	79.42
DBF4B	150.0	103.0	113.5	136.0	108.0	80.0	115.1	24.84
DBH	1.0	4.0	1.0	1.0	2.0	5.5	2.4	1.91
DBI	3058.0	2362.0	1370.5	2786.0	2346.5	1150.0	2178.8	763.56
DBN1	8376.0	8102.0	6365.0	7625.5	7464.5	5767.5	7283.4	1015.39
DBNDD1	102.5	150.0	124.0	76.5	163.0	113.0	121.5	31.62
DBNDD2	1487.0	1710.0	739.0	1307.0	1917.5	780.5	1323.5	482.85
DBNL	669.5	868.0	725.5	564.5	727.5	741.5	716.1	99.09
DBR1	706.0	409.5	460.5	663.0	446.5	425.5	518.5	130.47
DBT	175.5	157.0	132.0	189.0	167.0	120.5	156.8	26.17
DBX1	939.0	1168.5	1045.5	630.0	1257.5	2189.0	1204.9	528.99
DBX2	1.5	0.0	1.0	4.0	1.5	1.0	1.5	1.34
DCAF10	949.0	622.0	764.0	882.0	699.5	753.5	778.3	119.46
DCAF12	63.5	36.0	110.0	76.5	44.5	78.5	68.2	26.62
DCAF13	2018.0	1401.0	1535.0	2040.0	1575.5	1341.5	1651.8	304.45
DCAF17	401.5	408.0	308.5	384.5	460.0	349.5	385.3	52.04
DCAF4	560.0	413.5	339.5	479.5	485.0	343.0	436.8	87.33
DCAF5	467.5	328.0	303.0	463.5	342.0	291.0	365.8	79.27
DCAF7	960.0	643.0	830.5	910.5	735.5	780.0	809.9	116.07
DCAF8	300.5	281.0	470.5	323.5	287.0	417.0	346.6	78.51
DCAKD	25.5	34.0	22.5	17.5	33.0	21.5	25.7	6.59
DCBLD1	72.5	41.5	49.5	72.0	52.0	48.5	56.0	13.06
DCBLD2	885.5	515.0	793.0	785.0	566.0	766.0	718.4	144.77
DCDC1	0.0	0.0	0.0	0.0	0.0	0.0	0.0	0.00
DCDC2	1.0	0.0	1.0	0.5	0.0	0.0	0.4	0.49
DCDC2B	154.5	139.5	132.0	125.0	163.0	147.5	143.6	14.20
DCHS1	346.0	290.5	862.5	371.0	308.0	868.5	507.8	278.55
DCHS2	0.0	0.5	0.0	0.0	0.5	1.5	0.4	0.58
DCK	76.5	32.5	97.0	99.0	29.0	66.5	66.8	30.49
DCLK1	80.5	227.5	474.0	107.5	278.5	368.5	256.1	151.23
DCLK2	875.5	684.0	501.0	948.5	777.5	468.0	709.1	195.83
DCLK3	1.0	1.5	2.0	0.5	0.5	2.0	1.3	0.69
DCLRE1A	501.5	234.0	259.0	458.0	264.0	230.0	324.4	121.84
DCLRE1B	154.5	104.5	174.5	160.0	109.0	116.0	136.4	30.07
DCLRE1C	186.5	112.5	109.0	152.5	113.0	109.0	130.4	32.17

DCN	171.0	642.5	659.5	262.5	1054.0	1733.0	753.8	574.68
DCP1A	84.0	34.5	128.5	100.0	47.5	107.5	83.7	36.25
DCP1B	67.5	47.5	63.0	73.5	49.0	69.5	61.7	10.94
DCP2	15.5	6.0	17.5	21.0	10.5	11.0	13.6	5.44
DCPS	275.0	194.5	161.5	265.0	200.5	132.0	204.8	56.31
DCST1	0.0	1.0	0.0	0.0	0.0	0.0	0.2	0.41
DCSTAMP	0.0	0.0	0.0	0.0	0.0	0.0	0.0	0.00
DCT	0.0	0.0	0.0	0.0	0.0	0.0	0.0	0.00
DCTD	47.5	12.5	27.5	59.0	16.0	13.5	29.3	19.62
DCTN3	1519.0	1442.5	1231.0	1400.5	1479.5	1117.0	1364.9	157.20
DCTN4	1933.5	1323.0	1176.5	1831.0	1356.5	1111.0	1455.3	344.49
DCTN5	709.5	440.5	360.5	626.5	449.5	295.5	480.3	158.08
DCTN6	616.5	610.0	440.5	607.5	565.5	440.0	546.7	84.37
DCUN1D1	385.0	301.0	449.0	360.0	328.5	420.5	374.0	55.68
DCUN1D2	347.0	368.5	264.0	353.0	445.5	245.5	337.3	73.24
DCUN1D3	188.0	128.0	171.5	193.0	158.5	153.0	165.3	24.12
DCUN1D4	478.0	346.0	281.5	426.5	361.0	258.5	358.6	83.66
DCUN1D5	495.0	605.5	543.5	427.5	642.0	454.0	527.9	84.74
DCX	24.0	86.5	352.0	39.0	137.0	286.5	154.2	135.45
DCXR	455.0	416.0	207.0	418.0	504.0	272.0	378.7	114.30
DDA1	329.0	259.0	315.5	346.0	303.0	272.5	304.2	33.28
DDAH1	184.0	56.5	335.0	274.0	61.5	251.5	193.8	115.02
DDB1	4496.0	4773.5	4270.5	4423.0	5259.5	4218.5	4573.5	389.12
DDB2	567.0	576.5	357.5	557.0	754.0	443.5	542.6	134.70
DDC	0.0	0.0	0.5	0.0	0.5	0.5	0.3	0.27
DDHD1	115.5	121.5	224.5	101.0	145.0	230.0	156.3	56.82
DDHD2	249.0	348.0	286.0	238.0	405.5	363.5	315.0	67.46
DDIAS	32.5	17.5	29.5	31.0	20.5	29.0	26.7	6.14
DDO	994.0	706.0	507.5	1004.0	728.5	406.5	724.4	244.67
DDOST	3726.5	2443.0	2735.0	3733.5	2651.5	2678.0	2994.6	578.15
DDR2	1029.0	866.5	1028.0	1021.5	762.0	939.0	941.0	108.98
DDRGK1	362.5	354.5	412.5	305.5	386.5	347.5	361.5	36.35
DDX1	2333.5	1569.5	1899.5	2283.5	1727.0	1648.5	1910.3	327.65
DDX11	187.5	112.5	149.0	165.5	102.5	109.5	137.8	34.78
DDX17	1365.5	867.5	2027.5	1424.0	1010.0	2004.0	1449.8	486.03
DDX18	871.5	611.5	577.0	919.0	652.5	616.0	707.9	147.83
DDX20	545.0	310.5	377.5	526.5	359.5	355.0	412.3	98.28
DDX23	615.0	989.0	1053.5	705.0	819.5	1053.0	872.5	187.66
DDX24	807.0	645.0	691.5	858.5	772.0	661.0	739.2	86.22
DDX28	134.0	164.5	151.5	147.5	171.5	145.5	152.4	13.58
DDX31	413.0	252.0	286.0	387.5	244.0	238.5	303.5	77.16
DDX3X	6798.0	5821.0	10942.0	6430.0	6929.0	10492.5	7902.1	2218.79
DDX4	0.0	0.0	0.0	0.0	0.0	0.0	0.0	0.00
DDX41	1023.5	732.0	616.0	965.5	736.5	639.5	785.5	169.91
DDX42	655.5	623.5	870.0	601.0	711.5	827.0	714.8	110.90
DDX43	0.0	0.0	0.0	0.0	2.0	0.0	0.3	0.82
DDX46	1019.0	669.0	907.5	942.0	747.0	828.5	852.2	129.72
DDX47	455.0	306.5	344.5	440.0	297.0	309.0	358.7	70.83
DDX49	590.0	515.0	379.5	645.0	490.5	381.5	500.3	107.71
DDX5	26742.5	12645.5	9721.5	21864.5	14387.0	9771.0	15855.3	6953.31
DDX51	487.5	367.0	337.0	464.0	393.5	321.5	395.1	67.63
DDX52	490.5	324.5	356.0	461.0	340.5	312.5	380.8	75.55
DDX54	583.5	607.0	511.0	544.5	582.5	490.0	553.1	45.87
DDX55	157.0	152.0	138.0	155.5	145.5	123.0	145.2	12.93

DDX59	217.0	299.5	267.0	238.5	335.0	182.5	256.6	55.64
DDX6	376.0	140.5	476.0	362.0	172.0	405.5	322.0	134.65
DEAF1	171.5	178.0	178.0	160.0	207.5	179.5	179.1	15.70
DECR1	844.0	600.0	727.0	656.5	486.5	417.5	621.9	156.31
DECR2	669.5	462.0	389.5	523.0	496.0	414.5	492.4	99.89
DEDD	450.5	389.0	438.0	416.0	367.5	410.5	411.9	30.61
DEF6	2.5	0.5	0.0	1.5	0.5	0.5	0.9	0.92
DEF8	131.5	173.0	137.5	104.5	145.0	122.0	135.6	23.06
DEGS1	1481.0	695.5	1490.0	1476.5	828.0	1217.5	1198.1	355.73
DEGS2	0.5	1.0	1.0	0.0	0.0	0.0	0.4	0.49
DEK	1004.5	339.0	987.5	1017.0	350.0	695.0	732.2	323.27
DENND1A	638.0	493.0	557.5	589.5	552.5	542.5	562.2	48.55
DENND1B	6.0	2.5	4.0	4.5	2.0	5.5	4.1	1.59
DENND2A	44.0	19.5	30.5	50.5	21.0	30.5	32.7	12.37
DENND2C	716.5	733.5	770.0	746.0	856.5	804.5	771.2	51.84
DENND2D	17.5	9.0	27.0	16.5	7.0	30.5	17.9	9.40
DENND3	279.0	178.0	206.0	251.0	209.5	218.0	223.6	35.89
DENND4A	192.5	99.5	221.0	198.0	109.5	210.5	171.8	53.18
DENND4C	408.0	232.0	431.5	391.0	267.5	391.0	353.5	82.49
DENND5A	1289.0	991.0	1185.0	1150.0	1159.5	1088.0	1143.8	99.53
DENND6A	163.0	78.5	185.5	163.5	93.5	173.0	142.8	45.03
DENND6B	212.0	212.0	222.5	190.0	231.0	233.0	216.8	15.88
DENR	1176.0	860.5	483.0	1047.0	850.0	448.5	810.8	293.90
DEPDC1	78.0	19.0	65.5	70.0	18.0	36.0	47.8	26.74
DEPDC1B	103.0	43.0	99.0	116.0	33.0	56.5	75.1	35.13
DEPDC5	172.5	110.5	192.5	165.0	139.0	158.0	156.3	28.45
DEPDC7	74.5	25.5	46.0	83.0	29.0	43.0	50.2	23.64
DEPTOR	160.0	114.0	213.5	83.5	166.0	268.0	167.5	66.62
DERA	201.5	113.5	86.0	214.0	147.5	92.0	142.4	55.14
DERL1	637.0	369.5	661.0	579.5	391.0	593.5	538.6	126.28
DERL2	663.0	453.0	378.0	596.0	474.0	354.5	486.4	121.34
DES	12337.0	32758.0	14704.0	10554.0	30786.5	17008.5	19691.3	9627.97
DESI1	454.0	314.5	618.5	391.0	347.0	592.0	452.8	127.26
DESI2	877.0	796.5	961.0	895.0	873.0	909.5	885.3	53.88
DET1	337.0	177.0	402.0	344.5	222.0	353.0	305.9	86.68
DEXI	0.5	0.0	0.5	1.5	0.5	0.5	0.6	0.49
DFFA	175.0	143.0	116.0	182.0	169.0	137.0	153.7	25.70
DFFB	77.0	42.5	63.0	63.5	49.0	53.5	58.1	12.31
DFNA5	355.0	244.5	146.5	356.5	234.0	153.0	248.3	92.47
DFNB31	0.0	0.0	1.0	0.0	0.0	1.0	0.3	0.52
DFNB59	221.5	130.0	148.5	144.5	146.5	137.5	154.8	33.40
DGAT2	2.5	1.0	2.0	5.0	0.5	4.5	2.6	1.83
DGCR14	434.0	357.5	354.0	351.5	405.0	310.5	368.8	43.83
DGCR2	1090.0	858.0	992.0	909.0	911.0	1003.5	960.6	83.93
DGCR6L	761.0	787.0	718.5	680.5	812.0	673.0	738.7	57.12
DGCR8	423.0	235.0	635.0	418.5	243.0	537.0	415.3	158.33
DGKB	21.5	24.5	20.0	36.5	46.5	19.5	28.1	11.00
DGKD	893.0	993.0	1094.0	786.5	1168.0	976.5	985.2	136.65
DGKE	839.5	451.0	697.5	874.5	501.5	615.5	663.3	173.41
DGKG	0.0	0.5	0.5	0.0	0.0	1.5	0.4	0.58
DGKH	8.5	20.5	13.5	8.5	24.0	13.0	14.7	6.35
DGKI	4.0	3.5	4.0	2.0	7.5	6.5	4.6	2.04
DGKK	2.5	6.0	4.5	1.0	1.0	2.5	2.9	1.99
DGKQ	84.0	69.0	96.0	115.0	76.0	97.5	89.6	16.67

DGKZ	1120.5	1407.0	912.5	1080.5	1588.0	1209.5	1219.7	242.66
DGUOK	250.0	244.5	133.5	197.5	239.0	160.5	204.2	48.76
DHCR24	1401.0	578.5	815.5	1348.5	603.0	578.0	887.4	388.23
DHDDS	852.5	703.5	673.0	792.0	724.5	693.5	739.8	68.68
DHDH	145.0	116.5	56.5	147.0	127.5	73.5	111.0	37.77
DHFR	334.0	326.0	443.0	350.0	355.0	407.5	369.3	46.02
DHH	0.0	1.5	1.0	1.0	1.5	0.5	0.9	0.58
DHODH	859.0	559.0	553.5	795.5	551.5	489.0	634.6	152.74
DHRS11	55.0	62.0	50.0	62.0	58.0	48.0	55.8	5.95
DHRS12	33.5	17.5	46.5	21.5	15.0	41.0	29.2	13.08
DHRS13	3.0	2.5	4.0	5.0	3.5	5.5	3.9	1.16
DHRS3	117.0	132.0	100.0	70.0	122.0	290.0	138.5	77.33
DHRS7	757.0	765.5	565.5	741.0	882.0	636.0	724.5	110.39
DHRS7B	342.0	294.0	330.5	366.0	319.5	326.0	329.7	23.92
DHRS7C	78.0	480.5	370.0	51.0	534.0	320.0	305.6	201.86
DHRS9	443.0	279.5	166.0	335.5	320.5	202.5	291.2	99.54
DHTKD1	32.5	42.5	127.5	35.5	44.5	110.0	65.4	41.91
DHX15	1433.5	1187.0	1898.0	1476.0	1394.0	1671.0	1509.9	245.46
DHX30	736.5	661.0	959.5	703.5	702.5	988.5	791.9	143.35
DHX32	8.0	13.5	25.5	3.0	8.5	8.5	11.2	7.77
DHX33	202.5	101.5	136.0	200.0	91.5	108.0	139.9	49.76
DHX34	30.5	26.0	46.5	32.5	11.0	63.5	35.0	18.04
DHX35	197.0	69.0	84.0	202.0	82.0	81.0	119.2	62.47
DHX36	1118.0	829.5	826.0	1087.0	800.0	717.5	896.3	165.02
DHX37	960.0	616.0	581.0	993.5	687.0	532.0	728.3	199.28
DHX38	1587.5	1198.0	1207.0	1576.0	1184.0	1116.5	1311.5	211.76
DHX40	439.0	278.0	248.0	363.5	301.0	222.5	308.7	80.12
DHX57	225.0	121.0	227.5	241.0	121.5	197.0	188.8	54.27
DHX58	63.5	73.0	67.0	58.5	82.0	81.0	70.8	9.52
DHX8	249.0	133.0	307.0	256.0	165.0	273.5	230.6	67.07
DHX9	6.5	5.0	6.5	4.5	6.0	8.5	6.2	1.40
DIABLO	710.5	567.0	528.5	569.5	532.5	409.0	552.8	96.99
DIAPH2	1583.0	1176.5	1756.5	1439.0	1360.5	1568.0	1480.6	201.43
DIAPH3	605.5	250.5	604.0	595.0	225.0	302.0	430.3	189.17
DICER1	74.5	18.5	93.5	67.0	23.0	73.0	58.3	30.41
DIDO1	590.0	445.5	790.0	617.5	508.5	667.0	603.1	120.98
DIEXF	462.0	396.5	436.5	502.0	445.0	400.5	440.4	39.54
DIMT1	296.0	208.0	283.5	349.0	227.0	259.5	270.5	50.80
DIO1	17.0	11.5	5.0	20.0	10.0	5.5	11.5	6.05
DIO2	28.0	24.0	16.0	24.5	28.0	25.5	24.3	4.42
DIO3	21.5	37.0	33.5	40.5	48.5	49.5	38.4	10.40
DIP2A	379.5	153.0	385.0	407.5	216.5	340.5	313.7	104.12
DIP2B	1515.0	1127.0	2397.5	1508.0	1364.5	1939.0	1641.8	455.04
DIP2C	764.5	1475.5	1718.0	649.5	1736.0	1533.5	1312.8	481.47
DIRAS1	28.0	147.5	121.5	28.0	128.5	143.0	99.4	56.12
DIRC2	181.0	122.5	210.5	191.0	131.5	178.0	169.1	34.64
DIS3	297.5	215.5	207.0	268.0	269.0	209.0	244.3	38.65
DIS3L	664.0	373.0	407.0	580.5	428.0	370.0	470.4	122.46
DIS3L2	552.5	368.0	266.5	526.5	362.0	290.5	394.3	119.45
DISC1	641.5	539.5	587.0	570.5	613.5	625.5	596.3	37.87
DISP1	1413.0	1081.0	897.5	1440.0	1234.0	1297.5	1227.2	207.30
DISP2	0.0	0.0	0.0	0.0	0.0	0.0	0.0	0.00
DIXDC1	127.0	70.0	29.0	96.0	86.5	40.0	74.8	36.45
DKC1	2362.0	1602.5	1496.0	2139.0	1659.0	1295.0	1758.9	406.69

DKK2	13.0	3.0	16.5	17.5	7.5	6.0	10.6	5.94
DKK3	1.5	6.0	64.5	6.0	7.5	44.0	21.6	26.20
DLAT	544.0	320.5	501.0	591.5	321.0	422.5	450.1	114.57
DLD	1830.0	1211.0	1266.5	1659.5	1373.0	1194.0	1422.3	263.04
DLEC1	2.0	1.5	1.0	2.5	1.0	1.0	1.5	0.63
DLEU7	0.0	0.0	0.0	0.0	0.0	0.0	0.0	0.00
DLG1	130.0	63.0	376.5	140.5	76.0	323.5	184.9	132.38
DLG2	263.5	1029.5	1367.0	150.0	1085.0	905.5	800.1	485.15
DLG5	395.0	254.0	587.0	320.5	311.0	427.0	382.4	117.83
DLGAP1	3.5	2.5	2.5	2.5	3.0	2.5	2.8	0.42
DLGAP2	18.0	13.0	25.0	16.5	14.0	19.0	17.6	4.29
DLGAP3	19.5	87.5	23.0	35.5	96.5	42.5	50.8	33.14
DLGAP4	2852.0	4631.0	5140.5	2617.5	4846.5	4818.5	4151.0	1111.56
DLGAP5	828.5	318.5	565.5	918.5	289.0	358.5	546.4	272.73
DLK1	4.0	3.5	1.0	2.5	2.0	0.5	2.3	1.37
DLK2	11.5	172.0	237.0	11.0	198.5	294.0	154.0	117.94
DLL1	365.0	118.5	127.0	272.0	118.5	102.5	183.9	108.61
DLL4	0.0	1.5	2.0	1.0	3.5	4.5	2.1	1.66
DLST	812.0	744.5	1085.5	832.5	752.5	903.0	855.0	126.89
DLX1	0.0	0.0	0.5	0.0	1.0	0.0	0.3	0.42
DLX5	0.5	1.0	2.0	3.0	3.0	4.0	2.3	1.33
DLX6	2.0	6.0	2.5	2.5	4.5	3.0	3.4	1.53
DMAP1	310.5	231.5	278.5	314.0	236.0	265.0	272.6	35.42
DMBX1	0.0	0.0	0.0	0.0	0.0	0.0	0.0	0.00
DMC1	29.5	10.0	23.5	31.0	18.5	14.5	21.2	8.34
DMD	2181.5	2656.5	3736.0	1946.0	3199.0	3468.5	2864.6	719.67
DMGDH	68.0	56.0	209.5	64.5	56.5	135.5	98.3	62.20
DMP1	183.0	18.0	21.5	155.0	15.5	10.0	67.2	79.46
DMRT1	0.5	0.0	3.5	0.0	1.0	2.5	1.3	1.44
DMRT2	24.5	259.0	140.0	35.5	361.5	319.0	189.9	144.62
DMRT3	0.0	0.0	0.0	0.0	0.0	0.0	0.0	0.00
DMRTA2	1.5	0.5	2.5	0.0	0.5	2.5	1.3	1.08
DMRTB1	0.0	2.0	0.5	0.0	0.5	0.5	0.6	0.74
DMTF1	153.5	92.5	161.5	130.5	94.5	130.0	127.1	28.85
DMXL2	13.0	6.0	48.0	14.0	12.5	29.5	20.5	15.56
DNA2	194.5	46.5	100.0	181.0	48.0	55.0	104.2	67.79
DNAAF1	208.5	106.0	78.0	221.5	127.0	111.5	142.1	58.80
DNAAF5	479.0	311.5	471.5	428.0	368.5	424.5	413.8	63.90
DNAH1	3.0	3.0	2.0	4.0	6.0	2.0	3.3	1.51
DNAH10	20.5	4.0	6.0	18.0	11.0	2.0	10.3	7.63
DNAH12	57.5	24.0	24.5	34.5	25.0	15.5	30.2	14.68
DNAH14	0.0	0.0	0.0	0.0	0.0	0.0	0.0	0.00
DNAH17	116.0	126.0	126.5	105.0	141.5	134.5	124.9	13.01
DNAH3	160.5	71.5	45.5	125.5	74.0	38.0	85.8	47.77
DNAH5	9.5	13.5	19.5	7.0	22.5	14.0	14.3	5.85
DNAI2	1.0	3.0	0.5	0.5	3.5	1.0	1.6	1.32
DNAJA1	509.5	1187.5	2693.0	353.0	1286.0	2659.5	1448.1	1018.99
DNAJA2	1777.5	1867.5	1477.0	1617.0	2187.0	1347.0	1712.2	300.52
DNAJA3	828.0	750.5	830.0	885.5	834.0	801.0	821.5	44.34
DNAJA4	280.0	298.0	401.5	265.5	342.0	402.5	331.6	60.30
DNAJB1	435.0	506.5	706.0	427.0	547.0	686.5	551.3	121.01
DNAJB11	1289.0	1145.5	947.0	1364.5	1215.5	1049.5	1168.5	154.21
DNAJB12	1252.0	1206.0	1221.0	1342.0	1362.0	1441.5	1304.1	92.64
DNAJB13	48.0	50.5	32.0	28.5	45.5	17.5	37.0	13.06

DNAJB14	1203.5	1214.5	1348.5	1156.5	1349.0	1218.5	1248.4	80.80
DNAJB2	1453.5	1842.0	1301.5	1419.0	1614.5	1289.0	1486.6	210.54
DNAJB4	1196.0	1129.5	1520.5	1237.0	1382.0	1703.5	1361.4	218.81
DNAJB5	299.5	1084.0	1725.0	346.0	1239.5	1660.0	1059.0	620.19
DNAJB6	3444.0	2339.0	3005.5	3323.5	2680.5	2527.5	2886.7	444.30
DNAJB8	1.5	0.5	0.0	2.0	0.0	1.5	0.9	0.86
DNAJB9	457.0	355.5	556.5	447.5	483.5	492.5	465.4	66.08
DNAJC1	649.5	640.5	415.5	608.0	722.5	462.5	583.1	118.65
DNAJC10	1796.0	1253.5	1720.5	1790.5	1416.0	1596.0	1595.4	220.47
DNAJC11	840.5	662.0	643.5	753.0	699.5	551.5	691.7	98.81
DNAJC12	415.5	179.0	120.0	356.5	256.5	150.5	246.3	118.77
DNAJC13	932.0	927.0	1237.5	829.5	1009.0	1080.0	1002.5	142.74
DNAJC14	488.0	497.5	436.0	406.5	492.0	454.5	462.4	36.46
DNAJC15	129.5	137.0	120.5	122.5	176.0	103.0	131.4	24.61
DNAJC16	482.0	321.5	328.5	470.5	356.5	307.5	377.8	78.03
DNAJC17	294.5	127.5	90.5	260.5	138.5	87.5	166.5	88.93
DNAJC18	1192.5	1024.5	770.0	1074.5	1122.5	738.5	987.1	188.94
DNAJC19	369.0	241.5	189.0	337.5	263.0	156.5	259.4	82.45
DNAJC2	968.0	864.0	575.5	969.0	925.0	548.5	808.3	194.80
DNAJC21	376.0	213.0	248.5	354.0	232.0	229.5	275.5	70.58
DNAJC22	0.0	0.0	0.0	0.0	0.0	0.5	0.1	0.20
DNAJC24	61.5	37.0	31.0	51.0	42.0	31.5	42.3	11.97
DNAJC25	236.5	193.0	266.0	228.5	224.5	253.5	233.7	25.34
DNAJC27	107.5	105.0	83.5	102.0	105.0	81.0	97.3	11.84
DNAJC28	43.5	55.0	67.5	45.5	60.0	47.5	53.2	9.38
DNAJC3	2190.5	911.0	1509.5	2094.0	1110.0	1482.5	1549.6	512.70
DNAJC5	658.0	425.0	545.5	724.5	448.5	534.5	556.0	116.69
DNAJC5B	87.5	102.0	62.0	90.0	93.5	64.0	83.2	16.38
DNAJC6	270.0	126.5	135.5	236.5	150.0	101.0	169.9	67.33
DNAJC7	3484.0	5237.5	3330.5	3380.0	6166.5	3265.0	4143.9	1244.18
DNAJC8	1464.5	1573.0	1262.5	1391.5	1659.0	1197.5	1424.7	177.48
DNAJC9	518.0	322.0	332.0	503.5	313.0	274.0	377.1	105.49
DNAL1	270.5	232.0	405.5	247.0	254.5	367.5	296.2	72.07
DNAL4	491.5	396.5	355.0	437.5	405.5	336.0	403.7	56.32
DNALI1	89.5	86.5	83.0	59.5	101.5	73.5	82.3	14.39
DNASE1L2	1.0	0.0	0.0	1.0	1.5	1.0	0.8	0.61
DNASE1L3	3.0	1.5	0.5	3.5	0.5	0.0	1.5	1.45
DNASE2B	19.5	56.5	75.5	20.5	74.0	52.5	49.8	24.80
DND1	0.0	0.5	0.0	0.0	0.0	0.0	0.1	0.20
DNM1	92.0	45.5	47.5	90.0	49.0	43.5	61.3	23.13
DNM1L	1292.0	1178.5	1662.5	1243.5	1402.5	1504.5	1380.6	180.44
DNMBP	429.5	304.0	306.5	417.0	305.0	369.0	355.2	58.38
DNMT1	509.5	423.0	639.5	538.5	403.5	580.5	515.8	90.85
DNMT3A	359.5	553.0	635.0	367.5	556.5	633.5	517.5	124.51
DNMT3B	0.5	8.0	2.5	0.5	7.0	5.5	4.0	3.29
DNTT	0.5	0.0	0.0	0.0	0.5	0.0	0.2	0.26
DNTTIP1	1063.5	955.0	537.5	861.0	926.0	514.5	809.6	229.31
DNTTIP2	1627.5	1241.0	1244.0	1635.5	1342.5	1149.0	1373.3	209.21
DOC2B	34.0	56.5	80.5	23.0	63.0	81.0	56.3	23.86
DOCK11	272.0	188.0	329.5	241.5	191.0	341.5	260.6	66.18
DOCK2	59.0	48.5	73.5	47.0	41.5	43.0	52.1	12.16
DOCK3	1.0	0.5	6.0	0.0	0.5	8.5	2.8	3.59
DOCK4	133.5	147.0	233.0	115.5	213.5	226.0	178.1	51.84
DOCK5	5.0	0.5	3.5	7.5	1.5	8.0	4.3	3.08

DOCK8	0.0	0.0	0.5	0.5	0.0	0.5	0.3	0.27
DOCK9	554.0	359.0	639.0	609.5	428.0	511.0	516.8	107.45
DOHH	706.0	1029.0	618.0	706.0	1022.5	665.5	791.2	184.59
DOK1	94.5	66.0	61.5	93.0	75.5	86.0	79.4	13.94
DOK2	67.0	159.0	180.5	106.5	197.0	231.5	156.9	60.59
DOK3	5.5	2.0	2.5	3.0	5.5	2.0	3.4	1.66
DOK4	61.0	244.5	132.5	75.0	217.5	163.0	148.9	74.16
DOK5	3.0	40.0	34.0	4.5	48.0	46.5	29.3	20.44
DOK7	102.0	35.0	122.0	95.0	37.5	105.5	82.8	37.17
DOLK	231.0	140.0	145.5	248.0	191.0	162.0	186.3	45.22
DOLPP1	346.5	293.0	279.0	318.5	337.0	230.0	300.7	43.04
DONSON	295.5	213.5	151.5	295.5	209.5	117.5	213.8	72.83
DOPEY1	233.0	150.0	196.5	261.5	170.0	207.5	203.1	40.70
DOPEY2	28.0	10.5	42.0	31.0	8.5	34.5	25.8	13.44
DOT1L	326.5	300.5	513.0	376.5	349.5	466.5	388.8	83.47
DPAGT1	670.0	580.5	604.5	638.0	642.0	592.5	621.3	34.22
DPCD	323.0	390.5	211.0	409.0	461.5	295.0	348.3	90.12
DPEP1	2.0	2.5	0.5	0.5	1.5	0.5	1.3	0.88
DPF3	3.0	1.0	3.0	5.5	2.0	0.5	2.5	1.79
DPH1	126.5	91.5	123.5	139.5	94.0	154.5	121.6	24.89
DPH2	180.0	158.0	110.5	191.5	147.0	119.0	151.0	32.28
DPH3	274.5	363.5	391.0	254.5	445.0	355.5	347.3	71.69
DPH5	398.5	197.5	142.5	423.5	238.5	150.5	258.5	123.34
DPH7	387.5	211.0	312.5	356.0	218.5	358.5	307.3	75.65
DPM2	541.0	579.0	386.0	514.0	598.0	375.5	498.9	96.14
DPM3	396.5	347.5	165.5	354.0	331.0	140.0	289.1	108.09
DPP6	2.0	12.0	10.5	2.0	7.0	3.0	6.1	4.43
DPP7	781.5	622.5	458.5	795.0	705.0	541.5	650.7	134.57
DPP8	616.5	476.0	595.0	606.0	539.0	498.0	555.1	59.55
DPP9	1103.0	502.5	575.5	1011.5	549.5	533.0	712.5	269.65
DPT	3.0	3.5	1.5	2.5	1.0	0.5	2.0	1.18
DPY19L1	1098.5	811.5	1225.5	1056.5	903.0	1155.0	1041.7	156.39
DPY19L4	457.0	320.5	433.0	464.5	336.5	441.0	408.8	63.36
DPY30	305.0	331.5	299.0	307.0	389.5	294.0	321.0	35.97
DPYS	0.0	0.0	0.0	0.0	0.0	0.0	0.0	0.00
DPYSL4	3.0	6.5	7.5	8.5	11.0	17.5	9.0	4.92
DPYSL5	120.0	605.0	441.5	95.0	604.5	422.5	381.4	226.02
DQX1	238.5	188.0	211.0	209.5	178.0	179.0	200.7	23.49
DR1	503.0	378.5	479.0	523.0	442.5	476.5	467.1	51.18
DRAM1	12.5	5.5	3.0	10.0	4.0	2.5	6.3	4.08
DRAM2	343.0	284.0	372.5	324.5	323.5	327.5	329.2	28.86
DRAXIN	11.5	1.0	4.5	4.0	0.5	1.5	3.8	4.09
DRC1	67.5	46.5	55.5	51.0	46.0	43.5	51.7	8.85
DRC7	3.5	52.5	12.5	4.0	57.5	13.5	23.9	24.48
DRD1	1.5	0.5	1.0	6.0	0.5	1.5	1.8	2.09
DRD2	0.0	0.0	0.0	0.0	0.5	0.0	0.1	0.20
DRD3	3.0	1.0	1.5	2.5	0.5	2.0	1.8	0.94
DRD4	2.0	0.5	0.0	1.0	4.0	1.0	1.4	1.43
DRD5	78.5	104.0	140.0	71.0	120.5	145.0	109.8	30.93
DRG1	1186.5	920.5	758.0	1109.0	946.0	628.5	924.8	209.05
DRG2	1274.0	1208.0	813.0	1128.5	1279.0	786.0	1081.4	225.25
DRGX	0.0	0.5	0.5	0.0	0.0	0.0	0.2	0.26
DROSHA	300.0	187.0	262.5	291.0	233.5	213.5	247.9	44.46
DRP2	9.5	0.5	3.0	13.5	3.5	4.0	5.7	4.84

DSCAM	0.0	0.5	0.0	0.0	0.0	0.0	0.1	0.20
DSCAML1	2.0	0.0	1.0	1.0	0.0	1.0	0.8	0.75
DSCC1	382.5	144.0	151.0	408.5	140.0	104.5	221.8	135.79
DSCR3	471.5	460.5	405.5	423.0	464.5	350.5	429.3	46.49
DSE	249.0	205.5	306.5	253.0	235.0	301.0	258.3	38.97
DSEL	16.5	8.5	14.0	18.0	9.5	10.0	12.8	3.98
DSG2	243.0	43.5	64.0	297.5	49.0	48.5	124.3	114.60
DSN1	590.0	459.5	354.0	565.0	471.0	319.5	459.8	108.65
DSP	3.0	3.5	9.5	6.0	5.5	9.5	6.2	2.82
DSTN	27341.0	15702.5	12677.5	26584.5	17458.0	11529.0	18548.8	6854.43
DSTYK	78.5	44.0	122.0	81.5	41.0	96.0	77.2	30.96
DTD1	501.5	347.0	263.0	439.0	422.0	218.0	365.1	109.27
DTD2	246.0	188.5	78.5	177.5	166.5	49.0	151.0	73.53
DTHD1	0.0	0.0	0.0	0.0	0.0	0.0	0.0	0.00
DTL	124.5	69.0	83.5	121.0	68.0	44.0	85.0	31.89
DTNB	78.5	57.0	74.5	74.5	64.5	70.5	69.9	7.90
DTNBP1	345.5	390.0	355.5	347.5	399.0	346.5	364.0	24.06
DTWD1	390.5	179.0	168.5	348.0	222.0	172.0	246.7	97.80
DTWD2	2.5	0.5	2.0	1.0	1.5	2.0	1.6	0.74
DTX1	0.0	0.5	0.0	0.0	0.0	0.0	0.1	0.20
DTX2	1556.5	1824.5	1366.0	1580.0	1841.5	1420.0	1598.1	199.07
DTX3L	240.0	128.5	234.5	211.5	159.0	193.5	194.5	43.76
DTX4	617.0	408.5	613.0	646.5	425.5	683.0	565.6	117.92
DTYMK	825.0	638.5	512.0	753.5	626.0	469.5	637.4	136.18
DUOX2	11.5	11.5	7.5	13.5	13.5	4.5	10.3	3.60
DUOXA1	0.0	0.0	0.0	0.0	0.0	0.0	0.0	0.00
DUOXA2	0.0	0.5	0.0	0.5	1.0	0.0	0.3	0.41
DUPD1	0.0	0.5	0.0	0.0	0.5	0.5	0.3	0.27
DUS1L	178.5	96.5	92.0	177.0	97.0	89.0	121.7	43.54
DUS2	150.5	110.5	96.0	148.0	120.0	78.0	117.2	28.62
DUS3L	269.5	199.5	184.0	240.0	204.5	172.5	211.7	36.46
DUS4L	401.0	289.0	270.0	336.5	353.0	293.0	323.8	49.05
DUSP1	115.5	664.5	650.0	84.0	782.5	692.5	498.2	312.19
DUSP10	407.5	317.5	585.0	403.0	386.0	553.0	442.0	104.04
DUSP11	309.5	308.0	214.0	323.0	304.0	214.5	278.8	50.43
DUSP12	376.0	281.0	180.5	332.5	341.5	176.5	281.3	85.27
DUSP14	466.0	2125.5	1256.0	601.0	2767.0	2454.5	1611.7	976.70
DUSP15	38.5	84.5	62.0	35.0	88.0	28.5	56.1	25.98
DUSP16	507.0	348.0	256.5	467.5	419.5	262.0	376.8	105.34
DUSP18	0.0	0.0	0.0	0.0	0.0	0.0	0.0	0.00
DUSP19	405.0	262.0	267.5	361.0	283.0	224.0	300.4	68.28
DUSP22	533.5	189.0	342.0	607.0	246.5	311.5	371.6	164.38
DUSP23	104.0	197.5	121.0	72.0	172.0	123.5	131.7	45.75
DUSP26	301.5	153.0	184.5	225.5	150.0	144.5	193.2	61.16
DUSP27	284.0	446.0	523.0	247.0	547.5	400.5	408.0	122.87
DUSP28	158.5	292.0	135.5	137.0	278.0	134.0	189.2	74.90
DUSP3	256.0	518.0	447.0	260.0	629.5	438.5	424.8	146.23
DUSP4	21.5	34.0	30.5	34.0	34.5	27.5	30.3	5.11
DUSP5	115.5	204.5	447.0	163.5	221.0	404.5	259.3	134.64
DUSP6	92.0	320.5	306.0	127.0	377.0	299.0	253.6	115.46
DUSP7	93.0	60.5	152.5	127.5	73.5	153.0	110.0	40.08
DUSP8	482.5	511.0	631.0	484.5	527.0	709.0	557.5	92.09
DUT	1190.5	742.0	679.5	1214.5	725.0	531.5	847.2	285.15
DVL1	1232.5	910.0	1449.0	1028.5	979.5	1142.0	1123.6	196.73

DVL3	708.0	464.5	690.0	664.0	474.5	663.5	610.8	110.73
DYDC1	55.5	26.0	3.0	53.5	22.5	5.5	27.7	22.68
DYNC1H1	9461.5	6964.0	11944.5	9543.0	7501.5	10377.0	9298.6	1840.10
DYNC1I2	2220.0	2295.0	1947.5	2076.0	2663.0	1969.0	2195.1	266.89
DYNC1LI1	908.5	851.0	790.0	907.0	966.0	808.0	871.8	67.31
DYNC2H1	20.0	12.5	49.5	29.5	9.0	37.5	26.3	15.51
DYNC2LI1	137.5	58.5	93.5	105.0	53.0	70.5	86.3	32.11
DYNLL1	52.0	74.5	40.0	73.5	82.5	55.5	63.0	16.30
DYNLL2	1762.5	1108.0	854.0	1695.0	1113.5	740.0	1212.2	426.10
DYNLRB1	1625.0	1590.5	1259.0	1404.0	1649.5	1122.5	1441.8	217.08
DYNLRB2	17.5	19.5	16.0	15.0	21.5	13.5	17.2	2.96
DYNLT1	330.0	307.5	370.5	336.5	344.0	314.5	333.8	22.52
DYNLT3	588.0	432.0	439.0	583.0	484.5	396.0	487.1	81.28
DYRK1A	725.0	884.5	753.5	667.0	1042.5	693.5	794.3	143.23
DYRK2	657.0	1010.5	995.5	601.0	1280.5	960.0	917.4	251.53
DYRK3	1058.0	2671.0	2191.5	1033.0	3134.5	1900.5	1998.1	849.27
DYTN	1.5	0.5	0.0	0.5	0.5	0.5	0.6	0.49
DYX1C1	120.0	51.5	41.0	106.0	60.0	39.5	69.7	34.67
DZANK1	42.5	24.5	21.5	59.5	36.0	35.5	36.6	13.68
DZIP1	166.0	79.0	88.0	129.0	76.5	93.0	105.3	35.26
DZIP1L	89.5	79.5	86.5	75.5	78.0	87.0	82.7	5.72
E2F1	1261.0	580.0	746.0	1196.0	497.5	568.0	808.1	336.33
E2F2	192.5	113.0	179.0	140.5	83.5	104.5	135.5	43.21
E2F4	413.5	459.5	362.0	440.0	582.0	389.0	441.0	77.36
E2F5	80.5	39.5	60.0	59.0	47.5	48.5	55.8	14.32
E2F6	266.0	175.0	147.5	250.0	198.5	143.0	196.7	51.81
E2F7	160.0	57.0	119.0	154.0	54.5	83.5	104.7	46.77
E2F8	534.0	147.0	237.0	491.0	118.5	131.5	276.5	187.96
E4F1	549.5	337.5	370.0	497.0	376.5	365.0	415.9	85.82
EAF1	875.5	565.0	629.0	865.0	653.5	561.5	691.6	142.98
EAF2	69.5	80.0	117.0	81.0	90.5	139.5	96.3	26.63
EAPP	504.5	468.0	349.0	542.5	516.0	331.0	451.8	90.05
EARS2	249.5	154.0	99.5	210.0	170.0	103.0	164.3	59.06
EBAG9	548.5	668.0	459.0	570.5	695.5	478.5	570.0	96.45
EBF1	665.0	287.0	296.0	681.0	329.5	272.5	421.8	195.52
EBF2	1.5	4.0	1.5	1.5	1.5	7.0	2.8	2.27
EBF3	75.0	45.5	41.0	62.5	52.5	28.5	50.8	16.41
EBNA1BP2	1066.0	1191.5	872.5	1070.0	1306.0	831.0	1056.2	181.91
ECD	819.5	486.5	464.5	788.0	547.0	435.5	590.2	169.74
ECE1	304.5	165.5	332.5	269.5	155.5	341.5	261.5	82.23
ECE2	12.0	9.0	7.0	13.0	5.5	10.5	9.5	2.90
ECEL1	0.5	0.0	0.0	0.0	0.0	0.5	0.2	0.26
ECH1	549.5	506.0	298.5	481.0	470.5	320.0	437.6	103.29
ECHDC2	1486.5	977.0	511.0	1246.5	1059.0	416.0	949.3	416.23
ECHDC3	147.0	148.5	98.5	130.0	142.5	94.5	126.8	24.41
ECHS1	653.5	732.5	458.5	599.5	722.0	445.0	601.8	125.96
ECI1	638.0	526.0	386.5	557.0	484.0	370.0	493.6	102.72
ECI2	769.0	426.5	365.0	708.0	489.5	308.0	511.0	187.35
ECM1	8781.5	12961.0	17887.0	9598.0	15189.0	18084.5	13750.2	4013.00
ECM2	1831.5	1673.0	1720.5	2048.5	2172.0	2158.5	1934.0	221.07
ECSCR	1.0	0.0	0.0	0.5	0.5	1.0	0.5	0.45
ECT2	490.0	230.0	418.5	544.5	242.0	328.5	375.6	130.17
EDA	428.5	344.0	362.0	458.0	426.5	363.5	397.1	46.35
EDA2R	531.5	681.0	1122.0	635.0	688.0	1153.5	801.8	266.33

EDAR	18.5	19.0	12.0	14.0	16.0	10.5	15.0	3.45
EDARADD	51.0	25.5	18.0	52.5	20.5	16.0	30.6	16.71
EDC3	183.0	129.5	205.0	184.5	149.5	179.5	171.8	27.33
EDEM1	1485.5	1345.0	1589.5	1557.0	1546.0	1819.5	1557.1	155.01
EDEM2	294.0	248.5	218.0	271.5	265.5	244.0	256.9	26.13
EDEM3	1505.0	934.5	1184.0	1381.0	941.5	1072.0	1169.7	234.19
EDF1	2667.5	2989.0	2499.5	2506.5	3311.0	2172.0	2690.9	403.44
EDIL3	0.0	0.0	0.5	0.0	0.0	0.5	0.2	0.26
EDN1	8.5	3.5	13.0	7.5	2.0	10.0	7.4	4.09
EDN2	0.5	0.0	4.0	0.5	1.0	4.0	1.7	1.83
EDNRA	33.5	13.0	22.5	37.0	17.0	22.0	24.2	9.33
EDNRB	1.0	1.0	7.0	0.0	1.0	4.5	2.4	2.73
EEA1	1195.5	774.5	1010.5	1103.0	804.0	968.5	976.0	164.77
EED	324.5	173.5	197.5	322.5	187.5	168.5	229.0	73.92
EEF1A1	151113.0	102181.0	80615.0	136525.5	103990.0	76844.5	108544.8	29803.35
EEF1A2	62.5	2331.0	1572.0	28.0	2050.0	1052.5	1182.7	982.60
EEF1B2	19967.5	12360.5	8421.0	16015.0	12657.5	7744.0	12860.9	4618.21
EEF1D	15255.0	17226.5	11262.5	12688.5	17487.5	10561.0	14080.2	3005.00
EEF1E1	730.0	700.0	546.5	684.5	729.0	509.5	649.9	96.73
EEF2	34108.0	19099.5	22815.5	30035.5	18978.0	21509.0	24424.3	6234.78
EEF2K	521.0	263.5	303.5	459.0	307.5	301.5	359.3	104.31
EEF2KMT	418.5	374.0	261.0	320.5	451.5	243.0	344.8	84.43
EEFSEC	268.5	221.5	227.0	281.0	232.0	222.5	242.1	25.88
EEPD1	362.5	241.5	261.0	264.5	283.5	229.5	273.8	47.37
EFCAB1	109.0	48.5	45.5	107.5	56.5	26.0	65.5	34.60
EFCAB11	22.0	21.0	14.5	34.5	36.0	30.0	26.3	8.50
EFCAB12	13.5	5.5	16.0	15.0	15.5	10.5	12.7	4.03
EFCAB14	1474.0	1121.0	1585.5	1311.0	1177.0	1370.5	1339.8	175.80
EFCAB2	2.0	2.0	0.0	1.0	0.5	0.0	0.9	0.92
EFCAB3	0.5	0.5	0.0	0.0	0.5	0.0	0.3	0.27
EFCAB5	19.0	9.0	4.0	8.5	9.0	11.0	10.1	4.94
EFCAB7	301.5	130.5	110.0	294.5	139.5	99.5	179.3	93.10
EFCC1	0.5	3.0	1.5	2.5	8.0	2.5	3.0	2.61
EFEMP1	28.0	2.5	5.5	20.5	3.0	2.0	10.3	11.17
EFHB	0.5	0.0	0.5	1.5	1.0	1.0	0.8	0.52
EFHC1	664.0	961.0	686.5	651.0	1109.0	763.0	805.8	187.70
EFHD1	3978.0	3465.0	4069.0	3972.0	3873.0	3292.0	3774.8	317.95
EFHD2	87.5	46.0	99.0	92.0	46.0	97.5	78.0	25.12
EFNA5	0.0	0.0	0.5	0.0	0.0	0.5	0.2	0.26
EFNB2	139.0	174.5	556.0	165.5	198.5	580.0	302.3	206.87
EFR3A	558.0	378.0	442.5	501.5	399.0	382.0	443.5	72.84
EFR3B	908.0	1081.5	905.0	812.5	1181.5	836.5	954.2	145.85
EFTUD1	624.0	481.5	543.5	530.0	544.5	560.5	547.3	46.28
EFTUD2	2003.5	1355.0	1432.5	1850.5	1270.5	1273.5	1530.9	316.29
EGF	3.5	216.5	211.5	5.0	272.5	169.0	146.3	114.87
EGFL6	11.0	18.0	21.5	10.0	31.5	39.5	21.9	11.64
EGFL7	10.0	18.0	15.0	14.5	15.0	12.5	14.2	2.70
EGFLAM	7.0	6.5	12.0	9.5	4.5	8.5	8.0	2.61
EGFR	531.0	507.5	947.5	672.5	702.0	1036.5	732.8	216.52
EGLN1	200.5	187.5	290.0	191.0	224.5	238.5	222.0	38.77
EGLN3	20.0	36.0	103.5	28.0	36.5	97.5	53.6	36.89
EGR1	311.0	1141.5	1666.0	341.0	1172.5	2175.5	1134.6	731.50
EGR4	0.0	3.0	7.0	0.5	4.0	17.0	5.3	6.29
EHBP1	995.5	676.5	993.0	820.0	695.5	833.0	835.6	138.23

EHD4	352.0	359.5	433.0	367.5	423.5	338.0	378.9	39.54
EHF	0.0	0.0	0.0	0.0	0.0	0.0	0.0	0.00
EHHADH	362.5	170.0	156.0	344.5	184.0	125.0	223.7	102.61
EHMT1	493.5	414.0	630.5	545.5	487.5	576.5	524.6	76.05
EI24	1348.5	1604.5	1555.5	1259.5	1651.0	1725.0	1524.0	181.53
EIF1	2974.5	4193.0	2592.5	2822.5	4504.0	2802.0	3314.8	815.88
EIF1AX	3313.0	2665.0	2228.5	3117.5	3097.5	2111.0	2755.4	502.02
EIF1B	929.0	1175.5	1219.0	938.5	1207.0	1132.0	1100.2	132.41
EIF2A	658.0	651.5	734.5	646.5	705.5	644.5	673.4	37.52
EIF2AK1	489.0	436.0	395.0	417.0	514.5	422.0	445.6	46.17
EIF2AK3	421.5	172.0	360.5	508.0	192.0	383.0	339.5	132.11
EIF2AK4	1192.0	1003.5	1018.5	1223.0	1158.5	935.0	1088.4	117.80
EIF2B1	594.5	442.0	389.5	559.5	485.5	343.5	469.1	96.97
EIF2B2	970.0	913.5	752.0	928.0	1035.0	771.5	895.0	111.68
EIF2B3	663.5	582.0	379.0	614.5	616.5	338.5	532.3	137.54
EIF2B4	604.5	601.5	451.0	563.0	578.0	447.5	540.9	72.65
EIF2B5	943.5	833.5	882.5	930.5	885.0	781.5	876.1	60.64
EIF2D	602.5	663.5	555.5	504.5	717.0	515.0	593.0	84.57
EIF2S1	2111.5	2108.5	1722.0	2056.5	2211.5	1608.0	1969.7	243.95
EIF2S2	2730.5	2808.5	2174.5	2782.0	2971.0	1902.5	2561.5	421.88
EIF2S3	3020.0	3101.5	3350.0	2701.0	3317.0	2918.0	3067.9	245.82
EIF3A	4104.5	3312.5	4363.5	3817.0	3527.5	3860.5	3830.9	379.57
EIF3B	3262.0	2393.5	3318.5	3169.0	2736.0	2914.0	2965.5	356.77
EIF3D	3787.5	2521.5	1864.5	3315.5	2584.0	1673.0	2624.3	815.51
EIF3E	8518.5	5604.0	4596.0	7983.0	6114.0	4346.5	6193.7	1727.73
EIF3F	4234.5	3309.0	2481.0	3426.5	3425.5	2404.0	3213.4	683.19
EIF3H	4389.0	3561.5	2645.0	3767.0	3817.0	2408.5	3431.3	756.31
EIF3I	4967.0	4761.5	3539.0	4354.5	5113.5	3206.5	4323.7	786.65
EIF3J	1068.5	1088.5	977.5	1046.5	1232.0	948.5	1060.3	99.89
EIF3K	2155.5	2006.0	970.0	1843.5	2060.5	1043.5	1679.8	531.62
EIF3L	4073.0	3511.0	2993.0	3511.5	3612.5	2634.0	3389.2	504.88
EIF3M	6244.5	3326.5	2472.5	5577.0	3532.5	2194.5	3891.3	1656.44
EIF4A2	10182.0	11219.5	16236.5	9741.0	12958.0	14596.5	12488.9	2576.46
EIF4A3	2086.5	2043.5	1707.0	2022.0	2077.0	1681.5	1936.3	189.05
EIF4B	1460.5	1304.0	676.5	1228.5	1305.5	690.0	1110.8	339.73
EIF4E	1538.0	1592.0	1464.5	1502.0	1621.5	1360.0	1513.0	94.36
EIF4E1B	2.5	1.0	0.0	2.0	2.0	0.0	1.3	1.08
EIF4E2	736.0	1022.0	572.0	679.5	1073.5	599.0	780.3	215.76
EIF4E3	226.0	310.0	308.0	248.0	399.0	340.5	305.3	62.64
EIF4EBP1	2399.5	2969.0	1888.0	2208.0	3195.0	1761.0	2403.4	576.72
EIF4EBP3	234.0	233.5	122.5	195.0	260.5	133.0	196.4	57.24
EIF4ENIF1	476.0	324.0	427.0	440.5	382.0	445.0	415.8	54.37
EIF4G1	4206.0	3920.0	4497.5	4109.5	3643.0	4164.5	4090.1	287.80
EIF4G2	18943.5	16845.0	25021.0	18381.5	19121.5	22188.5	20083.5	2980.59
EIF4G3	430.0	363.5	866.0	441.0	376.5	721.0	533.0	209.06
EIF4H	3236.0	2554.5	3500.5	3483.5	2823.5	3380.5	3163.1	388.53
EIF5	4129.0	3888.5	3697.5	3756.5	4296.0	3590.0	3892.9	270.85
EIF5A2	7941.5	5899.5	4785.5	6933.5	5746.5	3988.5	5882.5	1425.03
EIF5B	4054.5	3254.0	2625.0	3603.5	3500.0	2145.5	3197.1	696.91
EIF6	963.5	1088.5	1011.5	986.0	1157.5	912.0	1019.8	89.09
ELAC2	661.0	638.0	571.0	591.0	691.0	528.0	613.3	60.80
ELAVL1	1631.5	1866.5	2215.0	1617.5	2143.0	2044.0	1919.6	256.80
ELAVL2	0.0	0.0	0.0	0.0	0.0	0.0	0.0	0.00
ELAVL4	2.0	2.0	0.5	0.0	1.0	1.5	1.2	0.82

ELF1	618.0	500.5	474.0	629.5	571.5	490.0	547.3	68.10
ELF2	762.0	628.0	683.0	761.5	717.5	667.5	703.3	53.66
ELF3	1.5	0.5	0.5	1.0	0.5	1.0	0.8	0.41
ELF5	0.0	0.0	0.0	0.0	0.0	0.0	0.0	0.00
ELFN1	24.5	37.5	19.5	33.0	35.0	55.5	34.2	12.46
ELFN2	4.0	25.0	44.0	3.5	27.0	56.0	26.6	21.04
ELK3	126.0	95.5	174.5	147.0	99.0	199.0	140.2	41.42
ELK4	39.5	18.5	66.0	37.0	14.0	50.5	37.6	19.49
ELL	208.5	129.5	219.5	219.0	140.5	216.0	188.8	42.03
ELL2	160.5	96.0	119.0	179.5	137.5	133.0	137.6	29.59
ELMO1	174.0	54.0	37.0	205.0	51.0	44.0	94.2	74.72
ELMO2	3156.5	2737.0	2298.5	2959.0	2708.5	2252.0	2685.3	357.08
ELMO3	133.0	87.0	128.5	105.0	106.0	96.0	109.3	18.08
ELMOD1	15.5	0.5	4.5	14.5	5.5	3.5	7.3	6.18
ELMOD2	949.0	460.0	612.0	874.5	515.5	517.5	654.8	206.33
ELMOD3	304.5	344.0	288.5	247.0	361.5	309.0	309.1	40.66
ELMSAN1	43.0	15.0	122.0	47.0	17.0	101.5	57.6	44.41
ELOF1	396.5	636.0	341.5	405.5	570.0	320.5	445.0	128.24
ELOVL1	568.5	247.0	398.5	681.5	237.5	360.5	415.6	177.51
ELOVL2	0.0	0.0	1.0	0.5	0.0	0.5	0.3	0.41
ELOVL3	2.5	2.5	0.5	2.0	3.0	0.0	1.8	1.21
ELOVL4	0.0	0.0	0.0	0.0	0.5	0.0	0.1	0.20
ELOVL5	1410.5	889.0	1535.5	1336.0	902.0	1199.0	1212.0	268.34
ELOVL6	1030.5	756.0	1270.0	1036.0	754.5	1080.0	987.8	200.22
ELOVL7	5.0	24.5	34.5	5.5	36.0	25.0	21.8	13.63
ELP2	98.5	66.0	145.0	88.5	61.0	117.5	96.1	31.78
ELP3	509.5	415.0	289.0	484.0	402.5	276.0	396.0	96.83
ELP4	130.0	52.5	66.0	119.5	67.0	52.5	81.3	34.43
ELP6	627.5	413.5	280.5	550.0	485.5	295.0	442.0	138.90
ELTD1	4.5	0.0	1.0	1.5	0.0	0.0	1.2	1.75
EMB	4.0	0.5	1.0	4.0	0.0	0.5	1.7	1.83
EMC1	3210.0	1629.0	2073.5	3457.5	1839.0	2068.0	2379.5	761.30
EMC2	495.5	406.5	322.5	402.5	439.0	254.0	386.7	85.96
EMC3	1729.5	956.5	679.5	1541.5	1054.5	716.0	1112.9	432.93
EMC4	867.5	866.0	457.5	764.0	779.5	469.0	700.6	188.78
EMC6	630.0	802.5	514.5	585.5	819.5	537.0	648.2	132.41
EMC7	538.5	425.0	436.0	526.5	480.0	404.5	468.4	55.57
EMC8	473.5	477.0	414.0	420.5	472.5	347.0	434.1	51.07
EMCN	19.5	1.5	3.0	11.0	3.5	1.5	6.7	7.22
EME1	144.0	55.0	77.0	125.0	47.0	46.5	82.4	42.26
EME2	145.0	183.0	130.0	131.5	206.0	140.0	155.9	31.25
EMG1	356.5	301.0	225.0	383.0	286.0	205.0	292.8	70.16
EMID1	0.0	0.5	0.0	0.0	0.0	0.5	0.2	0.26
EMILIN1	815.5	750.0	475.5	660.5	712.5	565.5	663.3	124.86
EMILIN2	1445.5	1117.5	1694.0	1717.5	1262.0	1807.5	1507.3	277.52
EMILIN3	0.5	1.5	13.5	0.5	3.5	10.5	5.0	5.61
EML1	198.0	114.0	166.5	264.5	160.0	198.5	183.6	50.33
EML4	1293.5	1088.0	1912.0	1297.5	1205.0	1173.5	1328.3	296.57
EML5	24.5	14.5	44.0	31.0	14.5	62.0	31.8	18.51
EML6	2424.0	3860.0	2404.5	1778.0	4253.0	2270.5	2831.7	985.03
EMP1	3109.0	5515.5	7287.5	3630.0	6789.5	7300.0	5605.3	1857.18
EMP2	541.0	421.5	609.5	514.5	465.5	631.0	530.5	81.01
EMX1	0.0	0.0	0.0	0.0	0.0	0.0	0.0	0.00
EMX2	283.5	282.5	382.5	285.5	269.5	339.5	307.2	44.22

EN1	0.5	0.0	0.5	0.0	0.5	1.0	0.4	0.38
EN2	0.0	0.0	0.0	0.5	0.0	0.0	0.1	0.20
ENAH	3094.5	2774.5	3266.0	2937.5	3148.0	2989.0	3034.9	172.72
ENC1	208.0	459.0	741.0	183.0	472.5	641.5	450.8	224.41
ENDOD1	511.0	344.0	579.5	455.0	332.5	397.5	436.6	97.20
ENDOG	393.5	509.0	393.5	358.5	470.5	331.5	409.4	67.55
ENDOU	0.0	0.0	0.0	0.0	0.0	0.0	0.0	0.00
ENDOV	417.5	374.5	243.0	324.5	379.0	237.0	329.3	75.21
ENG	48.5	45.0	66.5	59.5	49.5	95.0	60.7	18.61
ENGASE	77.5	40.0	23.0	75.5	34.5	21.0	45.3	25.22
ENHO	174.0	43.5	38.0	191.5	39.0	34.5	86.8	74.62
ENKD1	205.0	169.0	126.5	174.0	175.0	129.0	163.1	30.18
ENKUR	47.0	19.5	9.0	46.0	20.0	4.0	24.3	18.30
ENO1	40938.0	22138.0	27462.0	38129.5	24229.5	21323.5	29036.8	8449.78
ENO2	109.5	54.5	216.0	84.0	75.0	181.5	120.1	64.37
ENO4	20.0	8.5	1.5	20.0	2.0	1.0	8.8	9.07
ENOPH1	2089.5	1841.5	1501.0	2134.5	2042.5	1400.0	1834.8	315.71
ENOSF1	0.0	0.0	0.0	0.0	0.0	0.0	0.0	0.00
ENOX2	784.0	914.0	1170.0	909.5	1078.0	1180.5	1006.0	160.99
ENPEP	0.5	0.5	0.0	0.5	0.0	1.0	0.4	0.38
ENPP2	1.5	5.0	3.0	1.0	4.5	19.0	5.7	6.72
ENPP3	1.0	2.5	4.0	3.0	2.5	6.0	3.2	1.69
ENPP4	91.5	41.5	80.5	70.0	46.5	52.0	63.7	20.10
ENPP7	0.0	0.0	1.0	0.0	0.0	1.0	0.3	0.52
ENSA	854.0	901.0	791.0	780.0	911.0	806.0	840.5	56.77
ENTHD2	386.0	365.0	270.0	336.5	328.0	339.5	337.5	39.40
ENTPD1	0.5	2.5	0.5	1.5	1.5	2.0	1.4	0.80
ENTPD2	28.5	20.5	12.0	34.0	21.0	11.5	21.3	8.90
ENTPD3	68.5	26.5	11.5	66.0	27.5	11.5	35.3	25.75
ENTPD4	210.0	93.5	192.0	182.5	108.5	154.5	156.8	47.06
ENTPD5	225.5	119.0	220.0	260.5	113.0	175.0	185.5	60.34
ENTPD6	520.0	321.5	381.5	586.0	318.0	321.5	408.1	116.61
ENTPD7	221.0	205.5	198.5	198.5	218.0	209.5	208.5	9.56
ENY2	438.0	334.0	246.0	406.5	364.5	216.0	334.2	87.94
EOGT	195.5	83.0	81.0	226.5	86.5	71.0	123.9	68.36
EOMES	0.0	1.0	0.0	0.0	0.5	0.0	0.3	0.42
EP300	555.0	431.0	1035.5	634.5	463.5	1030.5	691.7	273.85
EP400	516.0	427.5	649.5	509.0	478.0	643.5	537.3	90.22
EPAS1	3.5	0.0	4.5	2.0	1.0	1.5	2.1	1.66
EPB41	213.0	56.5	139.5	188.0	66.5	121.0	130.8	63.02
EPB41L1	1014.0	461.5	1093.0	981.0	518.5	1002.0	845.0	278.17
EPB41L2	136.0	29.0	131.0	112.0	23.0	91.0	87.0	49.88
EPB41L4A	3.0	4.5	5.5	2.0	6.0	6.5	4.6	1.77
EPB41L4B	3.5	9.5	15.0	2.0	21.0	36.5	14.6	12.87
EPB42	30.0	7.5	14.5	21.0	8.0	14.5	15.9	8.51
EPC1	477.0	364.0	387.0	437.0	396.0	376.5	406.3	42.65
EPC2	317.5	211.5	315.5	306.0	249.0	300.0	283.3	43.20
EPCAM	15.5	4.0	0.5	12.0	3.0	0.5	5.9	6.32
EPDR1	1214.0	881.0	1219.5	1229.5	1202.0	1448.5	1199.1	181.64
EPGN	88.0	70.0	81.0	83.0	84.5	80.5	81.2	6.10
EPHA1	25.0	8.0	1.0	27.0	7.5	1.5	11.7	11.50
EPHA2	481.0	375.5	768.5	616.0	377.0	699.5	552.9	167.06
EPHA4	2.5	0.5	5.0	3.5	1.0	3.0	2.6	1.66
EPHA5	380.0	358.0	435.5	377.5	370.0	475.5	399.4	45.89

EPHA7	24.0	11.0	28.5	7.0	3.5	7.0	13.5	10.26
EPHA8	1.0	0.0	0.0	0.5	0.5	0.0	0.3	0.41
EPHB1	273.0	295.0	379.5	323.5	375.5	546.0	365.4	98.15
EPHB2	46.5	39.5	112.0	53.0	48.5	104.5	67.3	32.08
EPHB3	193.0	226.5	331.5	204.0	242.0	337.5	255.8	63.37
EPHB6	44.5	19.5	47.0	54.0	15.0	67.5	41.3	20.28
EPHX1	87.5	41.5	78.5	86.5	46.5	78.0	69.8	20.39
EPHX2	2395.0	754.0	677.0	1724.0	654.0	581.5	1130.9	751.94
EPHX4	230.5	132.5	154.0	259.5	157.5	141.5	179.3	52.51
EPM2A	104.5	150.5	136.5	108.5	173.5	127.5	133.5	26.08
EPN2	1229.0	1270.0	2127.5	1245.0	1456.0	2017.5	1557.5	408.66
EPN3	418.0	242.5	226.0	369.5	205.5	165.0	271.1	99.67
EPRS	2863.0	2238.5	3005.0	2864.0	2487.0	2612.0	2678.3	286.27
EPS15	479.5	364.5	897.5	467.5	419.0	924.5	592.1	250.49
EPS15L1	489.0	292.5	454.0	489.5	348.0	422.5	415.9	80.12
EPS8L2	17.0	3.5	12.0	20.5	10.0	13.5	12.8	5.87
EPS8L3	1.5	1.5	0.5	0.5	1.5	0.5	1.0	0.55
EPT1	146.0	42.0	179.5	180.5	59.0	144.5	125.3	60.19
EPYC	0.0	0.0	0.0	0.0	0.0	0.0	0.0	0.00
ERAL1	558.0	519.0	508.5	522.0	567.0	552.0	537.8	24.18
ERAP1	1696.5	1024.5	1798.5	1644.0	1199.0	1880.0	1540.4	346.34
ERBB2	1055.5	964.0	828.0	832.5	897.0	829.5	901.1	92.72
ERBB4	3.0	15.0	43.5	5.0	20.0	21.0	17.9	14.60
ERC2	3.0	3.5	3.5	4.0	3.0	0.5	2.9	1.24
ERCC3	739.0	582.5	812.0	675.0	653.0	681.5	690.5	78.11
ERCC4	233.0	172.0	282.5	273.0	184.5	237.0	230.3	44.94
ERCC5	470.5	333.5	219.5	365.5	338.0	210.0	322.8	97.34
ERCC6L	395.5	144.5	295.0	462.0	147.5	176.0	270.1	136.26
ERCC8	67.0	36.0	47.5	74.5	25.0	45.5	49.3	18.62
EREG	11.5	10.0	10.5	10.5	4.5	13.5	10.1	3.01
ERG	156.5	59.0	104.5	168.0	67.5	76.0	105.3	46.87
ERGIC1	1523.5	1197.5	2211.5	1624.5	1389.0	2034.5	1663.4	387.70
ERGIC3	927.0	647.0	881.0	913.5	684.5	828.5	813.6	120.02
ERH	1976.5	1851.0	1321.0	1795.5	1945.0	1162.5	1675.3	345.62
ERI1	233.5	115.0	163.5	273.5	144.5	155.5	180.9	59.93
ERI2	44.0	12.0	35.5	46.0	14.0	19.0	28.4	15.29
ERI3	214.0	192.5	306.5	225.5	202.0	293.0	238.9	48.60
ERICH1	142.5	97.0	97.5	134.5	107.5	118.5	116.3	19.11
ERICH3	3.0	1.5	2.5	2.0	2.0	5.5	2.8	1.44
ERICH6	27.0	14.5	6.5	18.5	12.5	6.5	14.3	7.80
ERICH6B	5.5	4.5	5.5	4.5	13.5	7.5	6.8	3.44
ERLEC1	2298.0	1897.0	1462.5	2743.5	2293.0	1628.0	2053.7	479.18
ERLIN1	322.5	219.5	348.5	309.5	229.0	310.5	289.9	52.86
ERLIN2	4074.5	2956.5	3248.5	4116.5	3201.5	3062.0	3443.3	515.83
ERMARD	275.0	114.0	65.5	257.5	157.5	79.5	158.2	89.71
ERMN	0.5	1.0	2.0	1.0	0.5	0.0	0.8	0.68
ERMP1	427.0	154.0	265.5	401.5	153.5	198.0	266.6	121.74
ERN1	758.5	340.5	451.0	741.5	400.0	449.0	523.4	180.16
ERN2	305.5	306.0	415.5	265.5	292.0	375.5	326.7	56.74
ERO1L	1888.0	791.0	827.0	2150.0	965.0	798.5	1236.6	614.92
ERO1LB	313.0	107.0	260.0	335.0	107.5	216.5	223.2	98.84
ERP27	0.5	3.0	1.0	0.0	1.5	1.0	1.2	1.03
ERP29	904.0	1103.0	1648.0	984.0	1328.0	1796.0	1293.8	364.19
ERP44	1484.0	1105.0	1430.5	1654.0	1293.5	1457.5	1404.1	186.59

ERRFI1	438.5	288.0	311.0	408.0	350.5	293.5	348.3	62.83
ESAM	189.0	37.5	17.0	148.0	30.0	6.0	71.3	77.20
ESCO1	390.0	272.0	283.5	400.0	324.0	273.5	323.8	58.35
ESCO2	372.0	235.5	255.0	368.5	226.5	216.0	278.9	71.91
ESD	2210.5	1777.0	1603.5	1926.5	1879.5	1618.5	1835.9	225.91
ESF1	98.5	47.0	96.5	137.5	42.5	91.5	85.6	35.67
ESM1	156.5	12.5	30.5	94.5	10.0	9.5	52.3	60.53
ESPL1	445.0	210.5	428.0	456.5	171.5	287.5	333.2	126.48
ESPN	1.0	0.0	1.0	0.5	0.5	0.0	0.5	0.45
ESPNL	0.0	0.0	0.0	0.5	0.5	0.5	0.3	0.27
ESR1	29.5	17.5	93.0	16.5	22.0	70.0	41.4	32.22
ESR2	13.5	13.5	16.5	9.5	16.0	12.5	13.6	2.54
ESRP1	1.0	0.5	0.0	0.0	0.5	1.0	0.5	0.45
ESRP2	78.0	6.0	2.5	63.0	11.0	4.5	27.5	33.76
ESRRB	21.0	11.0	20.5	14.0	10.5	8.5	14.3	5.34
ESRRG	24.5	17.5	49.0	18.5	28.5	41.0	29.8	12.67
ESYT2	2851.5	1198.0	1690.0	3203.5	1430.0	1682.5	2009.3	817.06
ESYT3	0.0	0.0	0.0	2.0	0.0	1.5	0.6	0.92
ETAA1	341.5	262.0	275.5	335.5	269.0	273.0	292.8	35.78
ETF1	2653.5	4102.0	3207.5	2720.0	4800.0	3097.0	3430.0	848.05
ETFA	2335.0	1425.5	1207.0	2110.0	1406.5	989.5	1578.9	527.77
ETFDH	541.0	304.0	573.0	604.5	340.0	426.5	464.8	126.43
ETNK1	136.5	72.0	149.5	128.5	72.5	116.5	112.6	33.04
ETNK2	3.5	4.0	5.5	2.0	3.0	7.5	4.3	1.97
ETNPPL	1.0	3.5	9.5	2.5	7.0	16.0	6.6	5.56
ETS1	54.5	18.0	46.5	39.5	18.0	33.5	35.0	14.92
ETS2	239.5	180.0	291.5	302.5	232.5	313.0	259.8	51.35
ETV1	155.0	174.0	333.0	237.0	181.0	315.5	232.6	76.28
ETV4	206.0	311.0	635.0	242.0	323.5	672.0	398.3	202.76
ETV6	367.5	600.0	574.5	399.0	644.0	651.0	539.3	124.55
ETV7	67.0	11.5	2.5	56.0	15.5	3.5	26.0	28.14
EVA1A	23.0	30.0	11.0	31.5	52.5	27.0	29.2	13.59
EVA1B	25.0	32.5	58.0	23.5	30.0	45.0	35.7	13.34
EVA1C	36.5	19.5	41.5	26.5	19.5	44.5	31.3	11.02
EVC	11.5	1.0	11.0	17.5	2.5	10.5	9.0	6.18
EVI2A	0.0	0.0	0.0	0.5	1.0	0.0	0.3	0.42
EVI2B	0.0	0.0	0.0	1.5	0.0	0.0	0.3	0.61
EVL	905.0	628.0	861.5	874.5	707.0	974.0	825.0	130.47
EVPL	148.5	45.0	22.5	95.0	50.0	20.0	63.5	49.63
EVX1	0.0	0.0	0.0	0.0	0.0	0.5	0.1	0.20
EVX2	0.0	0.0	0.0	0.0	0.0	0.0	0.0	0.00
EWSR1	1007.0	820.5	628.0	950.0	833.5	661.5	816.8	150.98
EXD1	251.5	117.5	58.0	268.5	136.5	52.0	147.3	93.39
EXD2	210.5	114.0	125.0	177.0	130.0	142.0	149.8	36.81
EXD3	299.0	209.0	176.0	251.0	208.5	135.5	213.2	57.01
EXO1	201.0	47.5	89.5	252.0	49.5	53.0	115.4	88.89
EXOC1	107.0	29.0	129.5	115.0	28.5	116.5	87.6	46.14
EXOC2	803.0	504.0	545.5	782.5	591.0	500.0	621.0	137.21
EXOC3	1029.5	691.5	548.0	980.5	766.0	502.0	752.9	217.80
EXOC3L1	48.5	44.5	38.5	33.5	39.0	27.0	38.5	7.66
EXOC3L4	3.0	1.5	1.5	2.5	1.5	1.5	1.9	0.66
EXOC4	600.0	511.0	705.0	529.5	519.0	628.0	582.1	76.60
EXOC5	1498.5	1368.5	1504.0	1654.0	1543.0	1398.5	1494.4	102.95
EXOC6	459.0	533.5	625.0	388.5	634.5	557.0	532.9	95.60

EXOC7	922.5	751.0	892.0	966.5	774.5	813.5	853.3	86.44
EXOC8	194.5	150.5	202.0	195.0	153.0	201.5	182.8	24.23
EXOG	150.0	110.0	95.0	154.0	112.0	83.0	117.3	28.88
EXOSC1	801.5	714.0	368.5	672.0	719.5	383.5	609.8	185.98
EXOSC10	808.0	595.0	632.0	847.5	645.5	578.5	684.4	114.32
EXOSC2	758.5	600.5	423.0	768.5	643.5	372.5	594.4	166.32
EXOSC3	178.5	170.0	127.5	148.5	160.5	123.0	151.3	22.58
EXOSC4	204.0	179.0	93.0	198.0	152.5	110.0	156.1	46.25
EXOSC7	411.0	323.0	232.0	413.0	372.0	227.5	329.8	84.11
EXOSC8	396.5	331.0	228.0	391.5	336.5	213.5	316.2	78.84
EXOSC9	586.5	409.0	390.0	553.5	397.5	377.5	452.3	92.31
EXPH5	5.0	1.0	7.5	1.5	0.0	9.0	4.0	3.73
EXT1	471.0	302.5	545.0	530.5	331.5	549.5	455.0	110.90
EXT2	893.0	284.5	920.5	1099.5	351.5	854.0	733.8	333.59
EXTL1	411.0	405.0	370.5	278.5	326.0	274.0	344.2	60.71
EXTL2	548.0	213.5	141.5	564.5	239.5	133.5	306.8	197.57
EXTL3	1916.0	2173.0	2545.0	2160.0	2258.5	2399.5	2242.0	216.63
EYA2	100.0	1408.0	521.5	98.0	1895.5	536.5	759.9	733.48
EYA3	945.0	860.0	895.5	963.0	915.5	868.5	907.9	41.16
EZH1	521.0	438.5	446.5	413.5	486.5	545.5	475.3	51.27
EZH2	985.5	506.5	617.5	918.0	567.0	451.0	674.3	223.16
EZR	2109.0	1793.5	2105.0	2211.0	2122.5	1941.5	2047.1	151.79
F10	426.0	476.0	449.5	649.5	531.5	746.0	546.4	126.12
F11R	2.0	0.5	0.0	1.5	0.5	0.0	0.8	0.82
F13A1	70.5	78.0	79.5	114.5	89.0	58.5	81.7	19.03
F2	37.5	26.0	14.0	41.5	29.5	20.0	28.1	10.38
F2R	832.0	688.0	1554.0	862.0	802.0	1502.5	1040.1	383.04
F2RL1	863.5	659.0	1808.5	852.5	741.5	1706.5	1105.3	511.83
F2RL2	120.0	176.0	297.5	127.0	209.0	287.0	202.8	76.70
F2RL3	0.0	0.5	0.0	0.0	0.0	0.0	0.1	0.20
F3	4.0	6.5	4.0	7.0	4.5	7.5	5.6	1.59
F5	22.5	16.5	27.0	28.5	28.5	42.5	27.6	8.64
F7	0.0	0.0	0.0	0.5	0.0	0.0	0.1	0.20
F8	3.0	0.5	0.5	3.0	1.0	0.0	1.3	1.33
F9	1.0	12.0	6.5	0.5	15.5	10.5	7.7	6.09
FA2H	6.5	0.5	3.5	3.0	1.0	0.5	2.5	2.35
FAAH	622.0	577.0	439.5	511.0	590.5	384.0	520.7	93.46
FAAH2	195.5	194.5	128.5	210.5	191.0	120.0	173.3	38.70
FABP1	5.5	18.0	25.5	3.5	21.0	40.5	19.0	13.65
FABP2	0.0	2.0	0.5	1.0	1.0	0.5	0.8	0.68
FABP3	3.5	110.0	15.5	3.0	80.0	5.0	36.2	46.77
FABP6	2.0	2.0	1.0	1.5	0.0	0.0	1.1	0.92
FABP7	13.5	9.5	9.5	10.0	6.0	4.5	8.8	3.19
FADD	256.0	129.0	371.0	289.5	143.5	309.5	249.8	95.67
FADS1	1555.5	645.5	860.5	1586.0	646.0	729.5	1003.8	446.21
FADS2	270.5	133.5	752.0	353.0	109.0	416.0	339.0	235.17
FADS6	9.5	4.0	7.5	9.0	9.5	8.5	8.0	2.10
FAF1	252.0	166.0	291.0	252.5	182.5	268.0	235.3	49.67
FAF2	629.5	492.0	483.0	600.5	571.5	481.0	542.9	65.80
FAH	2.0	3.5	1.5	1.5	6.0	0.5	2.5	1.97
FAHD1	171.5	174.5	124.5	172.5	175.0	125.5	157.3	25.02
FAHD2A	565.0	643.5	284.0	504.5	630.5	297.5	487.5	160.37
FAIM	256.5	140.0	146.5	227.5	181.5	154.5	184.4	47.68
FAIM2	3.0	6.5	8.0	2.5	7.0	6.0	5.5	2.24

FAM101B	10037.5	14037.0	12195.5	9626.5	16327.5	13321.5	12590.9	2531.84
FAM102A	58.5	25.5	49.0	34.5	17.5	49.0	39.0	15.78
FAM102B	264.5	222.5	227.0	263.0	261.5	208.0	241.1	24.83
FAM103A1	816.0	676.0	613.5	775.5	704.0	503.0	681.3	113.09
FAM104A	256.5	256.5	265.0	257.0	276.0	246.5	259.6	9.96
FAM105A	262.0	118.5	159.0	216.0	112.5	131.5	166.6	60.12
FAM107A	0.0	0.5	0.5	0.5	0.5	1.5	0.6	0.49
FAM107B	314.0	172.5	209.0	288.5	233.0	160.5	229.6	61.75
FAM109A	9.5	11.0	10.5	8.5	15.0	13.5	11.3	2.46
FAM109B	2330.5	2548.0	2764.0	2333.5	3144.5	3182.5	2717.2	381.23
FAM110B	245.5	210.0	187.0	258.5	227.0	203.0	221.8	26.98
FAM110C	14.5	4.5	3.5	9.0	5.5	2.5	6.6	4.48
FAM110D	99.0	101.5	193.0	103.5	113.5	170.5	130.2	40.88
FAM114A1	394.5	398.5	543.5	471.5	505.0	645.0	493.0	94.76
FAM114A2	866.5	598.5	506.0	801.0	617.0	504.0	648.8	151.96
FAM117A	935.0	557.0	482.0	814.5	515.0	541.0	640.8	186.95
FAM117B	16.0	6.0	23.0	21.0	7.0	23.5	16.1	7.89
FAM118B	182.5	144.5	160.5	192.5	173.0	173.5	171.1	16.84
FAM120A	2278.0	1267.0	3339.0	2305.0	1652.5	3242.5	2347.3	829.61
FAM120B	235.0	147.0	160.5	229.0	135.5	137.0	174.0	45.84
FAM122B	325.0	227.5	532.5	360.5	242.5	471.5	359.9	122.29
FAM124A	0.0	0.0	0.0	0.0	0.5	0.0	0.1	0.20
FAM124B	20.0	12.0	15.0	14.5	10.0	7.5	13.2	4.37
FAM126A	1634.0	683.0	1097.5	1672.0	735.5	923.5	1124.3	435.14
FAM126B	24.5	13.0	39.0	30.0	17.0	34.5	26.3	10.09
FAM129A	85.0	146.0	110.5	97.5	187.0	81.0	117.8	41.18
FAM129B	4144.0	3547.0	3200.5	4819.0	3643.0	3475.5	3804.8	584.79
FAM131A	2.0	4.0	5.0	3.0	1.0	4.5	3.3	1.54
FAM131B	28.0	16.5	54.5	28.0	15.5	55.5	33.0	17.87
FAM132A	147.0	82.5	150.0	139.5	120.5	113.5	125.5	25.60
FAM133B	1820.5	1693.5	1271.0	1761.0	1834.5	1195.5	1596.0	286.37
FAM134A	1450.5	1099.0	952.5	1289.0	1135.5	974.5	1150.2	190.91
FAM134B	61.5	156.0	155.5	56.0	140.0	186.0	125.8	54.09
FAM134C	512.5	421.5	422.0	510.0	462.0	419.0	457.8	44.36
FAM135A	1721.5	1213.0	2504.0	1642.0	1284.5	1831.5	1699.4	464.19
FAM136A	524.5	314.5	111.0	445.5	277.0	95.5	294.7	173.02
FAM13A	1302.5	1211.5	2589.5	1148.5	1399.0	2417.5	1678.1	647.23
FAM149A	18.0	4.5	11.0	7.5	5.0	10.0	9.3	4.98
FAM149B1	927.5	488.0	561.5	875.0	569.5	523.0	657.4	191.83
FAM150B	65.0	90.0	66.0	40.5	92.0	61.5	69.2	19.30
FAM151B	237.0	115.0	238.0	238.0	120.5	239.5	198.0	62.19
FAM154A	24.0	10.0	16.5	16.0	8.5	14.0	14.8	5.52
FAM154B	2.5	3.0	2.0	2.0	1.0	1.5	2.0	0.71
FAM155A	0.0	0.0	0.5	0.0	0.0	0.5	0.2	0.26
FAM155B	2.5	2.0	8.5	3.0	3.5	4.5	4.0	2.37
FAM159A	2.5	6.0	0.5	4.5	5.0	2.5	3.5	2.02
FAM160A1	149.5	166.5	125.0	165.5	186.0	120.0	152.1	25.72
FAM160A2	244.0	614.0	654.0	226.5	555.0	685.5	496.5	207.08
FAM160B1	620.5	435.5	480.0	639.0	492.5	501.0	528.1	82.13
FAM161A	132.5	84.0	103.0	124.0	102.0	93.5	106.5	18.38
FAM161B	49.5	36.5	52.0	43.5	42.5	46.5	45.1	5.52
FAM162A	745.5	542.5	380.0	700.0	625.0	331.0	554.0	169.21
FAM162B	2.0	3.0	2.5	1.5	2.5	1.5	2.2	0.61
FAM163A	0.0	3.5	3.0	0.5	1.0	0.5	1.4	1.46

FAM163B	159.0	360.5	157.5	119.0	365.0	115.5	212.8	117.64
FAM166A	1.0	0.5	0.5	1.5	0.0	0.0	0.6	0.58
FAM167A	0.0	0.0	0.0	0.0	0.0	0.0	0.0	0.00
FAM167B	34.0	55.0	25.0	22.0	37.5	24.0	32.9	12.42
FAM168A	84.0	61.5	113.0	74.5	66.5	119.0	86.4	24.22
FAM168B	28.5	16.0	64.5	29.5	24.0	47.0	34.9	17.71
FAM169A	0.0	0.5	1.5	0.0	0.5	1.5	0.7	0.68
FAM169B	100.5	110.0	78.0	82.5	114.5	66.0	91.9	19.31
FAM171B	418.0	158.0	183.0	370.5	160.5	180.5	245.1	116.96
FAM172A	488.5	272.5	400.5	438.0	283.0	321.5	367.3	88.30
FAM173A	191.5	185.0	119.0	186.0	200.0	119.0	166.8	37.37
FAM173B	94.5	66.0	55.5	100.0	84.0	75.0	79.2	16.99
FAM175A	316.0	123.0	126.0	293.5	136.5	119.0	185.7	92.70
FAM175B	534.0	558.0	542.5	501.5	642.5	487.0	544.3	54.87
FAM177A1	1074.5	521.0	478.5	865.0	530.5	337.0	634.4	276.86
FAM178A	354.5	340.5	410.5	382.5	334.0	412.5	372.4	34.57
FAM178B	13.0	5.5	3.5	7.5	6.5	1.5	6.3	3.95
FAM179A	3.5	3.5	4.0	5.0	6.5	5.5	4.7	1.21
FAM179B	369.0	442.0	524.5	340.5	458.5	561.5	449.3	85.64
FAM180A	98.5	85.0	104.0	138.5	117.5	64.0	101.3	25.76
FAM180B	5.5	2.5	1.5	7.0	3.5	1.0	3.5	2.35
FAM181A	1.0	13.5	7.0	1.5	14.0	5.5	7.1	5.65
FAM183A	2.5	2.5	1.0	3.0	4.0	0.5	2.3	1.29
FAM184B	39.0	22.0	17.5	31.0	19.5	10.5	23.3	10.19
FAM185A	70.0	23.5	49.5	43.0	18.5	37.0	40.3	18.67
FAM188A	616.5	386.5	307.5	637.5	414.0	293.0	442.5	150.22
FAM188B	140.0	74.5	85.5	168.5	79.0	95.0	107.1	38.26
FAM189A1	180.0	82.5	119.5	125.5	60.0	94.5	110.3	41.78
FAM189A2	568.5	383.5	392.5	633.5	428.0	429.0	472.5	103.28
FAM192A	1008.0	857.0	593.0	874.0	834.5	542.0	784.8	179.55
FAM193A	299.0	199.0	445.0	296.0	232.5	379.5	308.5	91.31
FAM193B	311.0	394.0	351.5	300.5	396.5	394.0	357.9	43.89
FAM195A	56.0	85.5	158.0	36.0	84.0	144.0	93.9	48.12
FAM198A	0.0	0.0	0.0	0.0	0.0	0.5	0.1	0.20
FAM198B	790.0	980.0	774.0	985.0	1188.5	1081.5	966.5	162.01
FAM199X	933.5	660.5	864.0	917.0	747.0	780.5	817.1	106.31
FAM19A1	9.0	5.0	0.5	7.5	6.5	0.0	4.8	3.72
FAM19A2	0.0	0.0	0.5	0.0	0.0	0.0	0.1	0.20
FAM19A3	4.5	10.0	5.0	10.0	21.0	8.5	9.8	5.97
FAM19A4	0.0	0.5	1.0	0.0	0.0	0.0	0.3	0.42
FAM204A	388.0	292.5	140.5	337.0	294.5	151.0	267.3	100.40
FAM206A	218.0	237.5	150.0	221.0	223.5	156.0	201.0	37.82
FAM207A	1580.0	1205.0	670.0	1413.5	1302.0	729.5	1150.0	370.86
FAM208A	1002.0	658.0	751.5	924.5	784.5	718.0	806.4	130.80
FAM208B	317.5	175.5	410.0	310.5	172.0	326.5	285.3	93.61
FAM20A	19.0	45.0	64.5	15.0	38.0	59.5	40.2	20.37
FAM20B	368.0	260.5	334.5	352.5	297.5	388.5	333.6	47.33
FAM20C	967.0	693.0	773.5	877.5	841.0	877.5	838.3	94.82
FAM210A	519.0	342.5	516.5	446.0	372.5	436.5	438.8	72.38
FAM210B	185.0	89.0	218.5	236.5	106.5	238.0	178.9	65.94
FAM212B	376.5	410.5	299.0	307.0	417.0	311.5	353.6	54.23
FAM213A	139.5	91.5	34.0	151.0	86.5	59.5	93.7	45.10
FAM214A	818.5	686.5	1102.5	851.0	859.5	1075.0	898.8	159.93
FAM214B	96.0	102.0	90.0	90.0	124.0	103.5	100.9	12.67

FAM217B	117.0	63.0	225.0	99.5	82.0	179.0	127.6	62.10
FAM219A	6279.0	3624.0	4567.0	5748.0	4414.0	4428.0	4843.3	979.75
FAM219B	198.0	225.5	157.5	180.5	267.0	176.0	200.8	39.76
FAM21C	1309.5	928.5	1099.5	1307.0	1065.5	1038.5	1124.8	153.26
FAM221A	1.0	1.0	0.0	1.0	0.0	0.0	0.5	0.55
FAM222A	0.0	0.0	0.0	0.0	0.0	0.0	0.0	0.00
FAM222B	205.5	196.5	225.5	192.0	211.0	232.5	210.5	15.95
FAM228B	26.5	14.0	20.0	12.5	7.5	9.5	15.0	7.09
FAM26D	0.5	0.0	0.0	0.0	0.0	0.0	0.1	0.20
FAM26E	20.5	26.5	28.5	28.0	29.5	24.0	26.2	3.37
FAM26F	0.0	0.0	0.5	0.5	0.0	2.0	0.5	0.77
FAM32A	1950.0	2145.0	1428.0	1806.0	2236.5	1385.5	1825.2	357.35
FAM35A	242.5	263.5	338.0	208.0	293.0	257.0	267.0	44.52
FAM3B	1.5	1.0	2.5	0.5	1.0	1.5	1.3	0.68
FAM3C	855.0	492.5	551.5	794.5	557.5	534.5	630.9	153.05
FAM3D	0.5	0.0	0.0	0.5	0.0	0.0	0.2	0.26
FAM43A	214.5	251.5	551.5	208.0	255.0	742.5	370.5	223.33
FAM43B	0.5	1.0	3.0	1.5	0.5	2.0	1.4	0.97
FAM45A	435.5	321.5	334.5	396.0	363.0	324.0	362.4	45.60
FAM46A	154.5	205.5	552.5	230.5	272.5	687.0	350.4	216.32
FAM46C	227.5	116.0	243.5	161.0	137.5	305.5	198.5	72.45
FAM46D	1.0	0.0	3.0	1.5	0.0	7.0	2.1	2.65
FAM49A	2244.0	3149.5	3584.0	2237.0	3652.0	3239.5	3017.7	632.09
FAM49B	733.5	724.5	660.5	708.5	766.0	604.0	699.5	58.18
FAM53A	165.0	103.5	133.0	134.5	94.0	100.5	121.8	27.26
FAM57A	4337.5	3067.5	3035.0	4462.0	3440.0	3060.5	3567.1	663.31
FAM60A	244.0	207.0	334.5	329.5	273.5	374.0	293.8	62.91
FAM63A	683.0	736.0	651.5	560.0	674.5	685.0	665.0	58.42
FAM63B	87.5	39.0	115.5	92.5	42.5	98.0	79.2	31.24
FAM64A	453.5	170.5	363.5	433.5	145.0	178.5	290.8	141.75
FAM65A	897.0	698.0	855.0	910.0	749.5	814.5	820.7	83.83
FAM65B	1719.5	6172.5	6827.0	1455.0	7244.5	5484.0	4817.1	2573.43
FAM69A	85.5	88.5	137.5	74.0	101.0	140.5	104.5	28.09
FAM69B	343.5	311.5	243.0	311.0	343.0	283.5	305.9	38.22
FAM69C	0.5	0.0	0.0	0.5	0.0	0.0	0.2	0.26
FAM71D	0.0	0.0	0.0	0.0	0.0	0.0	0.0	0.00
FAM72A	264.5	99.0	218.0	295.0	102.5	173.5	192.1	81.91
FAM73B	303.5	166.0	263.0	233.0	208.0	253.0	237.8	47.43
FAM76A	237.0	150.5	241.5	248.0	163.5	242.0	213.8	44.29
FAM76B	246.5	169.5	204.0	249.0	207.5	204.0	213.4	30.01
FAM78A	68.5	225.5	406.0	50.0	233.5	299.0	213.8	136.14
FAM78B	231.5	328.0	239.0	223.5	371.5	274.0	277.9	59.85
FAM81A	1.0	1.0	1.5	1.0	1.5	0.5	1.1	0.38
FAM81B	2.5	0.0	0.0	2.0	0.5	0.5	0.9	1.07
FAM83A	1.5	0.5	1.5	2.0	1.5	0.0	1.2	0.75
FAM83B	0.0	0.0	0.0	0.0	0.0	0.0	0.0	0.00
FAM83C	8.5	4.0	5.0	7.5	9.5	3.5	6.3	2.50
FAM83F	0.0	0.0	0.0	0.5	0.0	0.0	0.1	0.20
FAM83G	0.0	0.0	0.0	0.0	0.0	0.0	0.0	0.00
FAM83H	12.5	11.0	2.5	14.5	16.5	6.5	10.6	5.22
FAM84A	2.0	5.5	13.0	9.5	4.0	10.0	7.3	4.17
FAM84B	11.5	7.5	26.0	13.5	10.0	29.5	16.3	9.13
FAM8A1	882.0	746.5	658.5	813.5	882.0	678.5	776.8	98.04
FAM91A1	571.0	432.0	615.5	564.0	469.5	555.5	534.6	69.19

FAM92A1	213.5	131.0	206.0	185.5	154.0	167.5	176.3	31.55
FAM96A	236.5	213.5	180.0	249.5	241.0	171.5	215.3	33.01
FAM96B	1031.0	828.0	782.5	938.5	924.0	753.5	876.3	106.10
FAM98A	1724.5	1414.0	1444.5	1557.5	1728.0	1420.0	1548.1	147.44
FAM98B	333.0	184.5	227.5	326.5	186.0	195.0	242.1	69.69
FAN1	137.0	108.0	143.5	133.0	120.5	136.0	129.7	13.04
FANCA	38.5	7.0	23.5	37.0	8.5	13.0	21.3	14.03
FANCB	111.5	53.5	130.5	102.5	62.5	70.0	88.4	30.76
FANCC	308.0	291.5	200.5	294.5	335.5	181.5	268.6	62.37
FANCD2	272.5	118.0	295.5	285.0	116.5	182.0	211.6	83.45
FANCE	316.5	178.5	128.5	285.5	137.5	96.0	190.4	90.14
FANCF	419.0	129.0	89.0	360.5	118.0	73.5	198.2	150.86
FANCG	112.5	78.5	121.0	102.5	85.5	91.0	98.5	16.39
FANCI	225.0	74.5	210.0	260.0	60.0	119.0	158.1	84.49
FANCL	124.0	68.0	86.5	143.0	63.0	50.0	89.1	36.84
FANCM	646.0	805.5	712.0	599.0	856.0	868.5	747.8	112.60
FAR2	100.5	35.5	128.5	123.5	47.0	90.0	87.5	38.72
FARP1	322.0	239.0	420.5	335.0	273.5	400.5	331.8	70.29
FARP2	282.5	185.5	353.0	287.5	209.0	527.5	307.5	123.35
FARS2	282.0	132.5	134.0	215.0	147.5	121.0	172.0	63.49
FARSA	702.0	725.0	550.5	680.5	738.5	517.0	652.3	94.50
FARSB	1379.5	972.5	612.0	1093.5	920.0	506.5	914.0	319.26
FAS	421.5	659.5	390.5	427.5	854.0	402.5	525.9	189.55
FASLG	15.0	6.5	14.5	11.5	10.0	11.0	11.4	3.12
FASN	511.0	281.0	850.5	564.0	320.0	911.5	573.0	262.60
FASTK	270.0	322.5	461.0	263.5	258.5	438.5	335.7	91.58
FASTKD1	262.5	247.0	260.5	246.5	254.0	244.5	252.5	7.70
FASTKD2	312.0	206.0	209.5	303.0	207.0	192.0	238.3	54.06
FASTKD3	200.5	153.5	199.0	220.5	145.0	166.0	180.8	30.14
FASTKD5	97.0	119.5	120.0	90.0	130.0	111.0	111.3	15.17
FAT1	2337.0	2014.5	4716.0	2459.0	2423.0	3831.5	2963.5	1064.37
FAT2	0.0	0.0	0.5	0.5	0.0	0.0	0.2	0.26
FAT4	79.0	15.0	29.5	78.5	20.0	33.5	42.6	28.78
FAU	6740.0	6002.0	3008.5	5523.5	6059.5	3001.0	5055.8	1635.34
FAXC	52.5	16.5	64.5	66.5	20.5	65.5	47.7	23.19
FAXDC2	16.0	24.5	12.5	19.0	21.0	16.5	18.3	4.20
FBF1	442.5	365.5	343.5	380.5	359.0	327.0	369.7	40.15
FBLIM1	249.0	217.5	219.5	245.5	230.0	217.5	229.8	14.31
FBLN2	473.0	1099.5	2141.5	1185.5	1708.0	3923.0	1755.1	1204.11
FBLN5	97.5	102.0	177.5	119.5	99.5	177.5	128.9	38.43
FBLN7	116.0	289.5	138.0	125.0	339.5	258.0	211.0	96.57
FBN1	578.0	298.0	705.0	605.5	390.0	788.0	560.8	185.98
FBN2	0.0	3.0	3.0	0.0	8.0	9.5	3.9	4.01
FBN3	0.0	0.0	0.5	0.0	0.0	0.5	0.2	0.26
FBRSL1	342.0	391.0	459.0	296.0	423.0	455.0	394.3	64.97
FBXL12	104.0	97.5	91.0	80.5	100.5	100.0	95.6	8.56
FBXL13	265.0	163.0	158.5	217.5	160.5	133.5	183.0	48.73
FBXL14	137.0	138.0	176.0	143.0	150.5	184.0	154.8	20.29
FBXL15	84.0	44.5	110.0	69.5	51.5	97.0	76.1	25.67
FBXL16	0.0	0.5	0.5	0.0	0.5	0.0	0.3	0.27
FBXL17	58.5	24.5	61.5	55.5	24.0	54.5	46.4	17.34
FBXL18	263.5	184.0	245.0	256.0	197.0	248.0	232.3	33.23
FBXL2	43.0	37.5	51.0	34.5	37.0	43.5	41.1	6.01
FBXL20	821.5	608.5	729.0	679.0	634.5	710.0	697.1	75.84

FBXL21	533.0	285.5	233.5	472.0	331.5	247.0	350.4	124.14
FBXL22	802.0	1305.5	1044.5	812.5	1513.0	1044.5	1087.0	279.07
FBXL3	524.5	270.5	369.0	465.0	349.0	325.5	383.9	93.87
FBXL4	8.0	1.5	20.5	12.5	4.5	24.5	11.9	9.07
FBXL5	650.5	482.5	479.5	626.5	547.0	440.0	537.7	85.65
FBXL7	249.0	322.5	262.0	260.5	362.0	241.0	282.8	48.31
FBXL8	348.5	183.5	156.0	330.5	198.0	147.5	227.3	88.96
FBXO11	810.0	575.5	735.5	750.5	630.0	607.0	684.8	93.34
FBXO16	4.5	2.5	5.5	4.5	2.5	5.0	4.1	1.28
FBXO18	293.0	300.0	524.5	260.0	321.5	472.0	361.8	108.77
FBXO2	186.0	92.5	54.0	141.0	87.5	49.0	101.7	52.90
FBXO21	323.5	274.0	224.0	299.5	310.5	213.5	274.2	46.03
FBXO22	552.5	458.5	570.5	600.5	514.0	580.5	546.1	51.93
FBXO25	389.0	198.0	221.5	394.0	254.0	209.5	277.7	90.15
FBXO28	327.0	387.5	336.0	318.5	418.5	311.5	349.8	43.12
FBXO3	554.0	575.0	680.0	539.5	632.0	654.0	605.8	57.52
FBXO30	735.0	745.0	1144.0	799.5	933.0	1058.0	902.4	171.41
FBXO31	764.5	755.5	853.5	588.5	772.0	796.5	755.1	88.91
FBXO32	449.5	157.0	748.5	495.0	179.5	567.0	432.8	228.93
FBXO33	451.0	316.5	427.5	475.5	358.5	360.5	398.3	62.12
FBXO34	153.5	151.5	179.0	153.5	120.0	172.0	154.9	20.53
FBXO38	399.0	305.5	385.5	420.5	327.5	337.5	362.6	45.44
FBXO39	21.0	11.0	14.0	26.5	10.0	10.0	15.4	6.84
FBXO4	90.5	46.5	67.5	87.0	40.0	67.0	66.4	20.49
FBXO40	60.5	49.0	48.5	51.5	47.5	37.0	49.0	7.55
FBXO41	0.0	0.0	0.5	0.0	0.5	0.0	0.2	0.26
FBXO42	380.0	411.0	393.0	401.0	422.0	389.0	399.3	15.32
FBXO43	6.0	1.0	3.0	3.0	2.0	1.0	2.7	1.86
FBXO45	1093.0	611.5	682.0	966.0	709.5	687.0	791.5	191.51
FBXO47	7.0	5.0	4.0	7.5	9.0	2.0	5.8	2.56
FBXO48	50.5	25.0	25.5	42.0	32.5	22.0	32.9	11.21
FBXO5	151.5	73.0	132.0	173.0	70.0	95.5	115.8	42.78
FBXO8	160.0	128.0	156.5	158.5	147.0	164.0	152.3	13.20
FBXO9	1704.0	1527.5	1509.5	1565.5	1660.5	1590.5	1592.9	76.03
FBXW11	1409.0	1190.0	1319.0	1381.0	1280.0	1186.0	1294.2	93.91
FBXW2	556.0	405.0	541.5	504.0	499.0	534.0	506.6	54.40
FBXW4	530.0	476.0	432.5	428.5	477.0	412.0	459.3	43.58
FBXW5	1050.5	1045.5	911.5	1025.5	1158.0	868.0	1009.8	104.80
FBXW7	187.0	161.5	164.0	205.5	200.0	160.5	179.8	20.38
FBXW8	274.5	227.5	185.0	239.5	212.0	195.5	222.3	32.44
FCF1	659.5	649.5	439.0	689.5	721.0	400.0	593.1	137.31
FCHO1	25.0	17.0	22.5	20.5	22.5	26.5	22.3	3.36
FCHO2	441.5	327.0	372.0	437.0	392.5	363.0	388.8	44.46
FCHSD1	112.0	67.5	63.0	87.0	46.5	54.0	71.7	24.08
FCHSD2	62.5	52.0	83.0	66.0	62.5	95.5	70.3	15.95
FDFT1	5793.5	4032.0	5263.5	4902.5	4113.5	4517.0	4770.3	685.32
FDPS	2170.0	1743.5	2200.5	1943.5	1559.5	1791.5	1901.4	251.91
FDX1	506.0	343.5	283.0	456.5	341.5	300.0	371.8	89.38
FDXACB1	121.5	75.5	74.5	95.5	87.0	78.0	88.7	17.97
FDXR	601.0	419.5	308.0	520.5	447.5	372.5	444.8	104.61
FECH	65.0	9.5	30.0	75.5	10.5	25.0	35.9	27.97
FEM1A	781.5	546.0	1407.0	855.0	588.0	1223.5	900.2	346.53
FEM1B	655.5	560.5	847.5	670.5	591.0	788.0	685.5	111.74
FEM1C	162.0	117.5	325.5	176.0	131.0	257.0	194.8	80.53

FEN1	1031.0	474.0	643.5	1045.5	464.0	456.0	685.7	281.85
FER	89.0	36.0	112.5	78.0	64.0	93.5	78.8	26.49
FER1L6	3.5	0.5	0.5	1.5	0.0	0.5	1.1	1.28
FERD3L	0.0	0.0	0.0	0.0	0.0	0.0	0.0	0.00
FERMT1	81.0	23.0	11.5	54.5	23.5	12.0	34.3	27.75
FERMT2	5580.5	7129.5	6793.0	5783.5	8515.0	6646.5	6741.3	1055.77
FES	0.0	2.0	1.0	0.0	1.0	0.5	0.8	0.76
FETUB	1.0	0.0	0.5	0.0	0.0	0.0	0.3	0.42
FEV	0.0	0.0	0.0	0.0	0.0	0.0	0.0	0.00
FEZ1	47.5	65.5	45.5	31.5	55.0	39.0	47.3	11.94
FEZF1	0.0	0.0	0.5	0.0	0.0	0.0	0.1	0.20
FEZF2	0.0	0.0	0.0	0.0	0.0	0.0	0.0	0.00
FFAR4	1.5	3.0	2.5	1.5	2.5	1.5	2.1	0.66
FGA	0.0	0.0	0.5	0.0	0.5	0.5	0.3	0.27
FGB	0.0	0.0	0.0	0.0	0.0	0.0	0.0	0.00
FGD3	253.5	144.5	261.5	275.0	154.0	211.5	216.7	56.45
FGD5	10.5	6.0	5.0	7.0	5.5	4.5	6.4	2.18
FGD6	128.0	104.5	139.0	136.5	109.0	92.0	118.2	19.09
FGF10	48.0	22.5	47.0	36.0	33.5	53.0	40.0	11.37
FGF12	8.0	44.5	31.0	9.0	43.0	56.0	31.9	19.79
FGF13	34.0	604.5	488.5	36.5	787.0	557.5	418.0	312.53
FGF14	2.0	0.5	1.5	0.5	0.5	2.0	1.2	0.75
FGF16	30.0	7.5	6.0	30.0	9.5	9.0	15.3	11.43
FGF18	1.0	0.0	0.5	0.5	1.5	0.0	0.6	0.58
FGF2	159.0	62.5	49.5	118.0	63.5	44.5	82.8	45.62
FGF20	0.5	0.0	0.0	0.5	1.0	0.0	0.3	0.41
FGF22	12.0	20.0	29.0	8.5	14.0	38.0	20.3	11.29
FGF23	18.5	10.5	8.5	14.5	15.0	15.0	13.7	3.59
FGF3	0.0	0.0	0.0	0.0	0.0	0.0	0.0	0.00
FGF4	0.5	2.0	1.5	0.0	1.5	3.0	1.4	1.07
FGF5	0.5	4.0	4.0	4.0	5.0	3.0	3.4	1.56
FGF6	1.0	0.0	0.5	0.0	0.0	0.0	0.3	0.42
FGF7	0.0	4.5	0.5	0.0	4.0	1.0	1.7	2.04
FGF8	0.0	0.5	0.0	0.5	0.0	1.0	0.3	0.41
FGFBP1	1.0	0.0	0.0	0.5	0.5	0.0	0.3	0.41
FGFBP2	1.0	1.0	0.5	2.5	1.5	2.5	1.5	0.84
FGFR1	683.5	665.5	1036.0	672.0	629.5	1241.0	821.3	254.79
FGFR1OP	290.0	684.0	569.5	287.5	821.5	627.5	546.7	216.56
FGFR1OP2	705.5	462.5	497.0	729.0	544.5	474.0	568.8	118.65
FGFR3	5.5	0.5	3.5	5.5	2.0	1.0	3.0	2.19
FGFR4	5886.5	3532.5	3341.0	4921.5	3414.5	4186.5	4213.8	1016.30
FGFRL1	12.0	5.5	27.5	12.5	12.5	15.5	14.3	7.28
FGG	0.0	1.0	0.5	1.0	2.5	0.0	0.8	0.93
FGGY	129.5	83.0	42.0	129.5	80.0	33.0	82.8	41.27
FGL1	12.5	16.5	34.0	10.5	18.5	47.5	23.3	14.49
FGL2	2.5	0.0	1.5	6.5	2.0	1.0	2.3	2.25
FH	1073.5	985.0	959.5	1014.0	1099.0	926.5	1009.6	66.51
FHAD1	0.0	0.0	0.5	0.0	0.0	1.0	0.3	0.42
FHDC1	194.0	78.0	108.5	175.0	87.5	87.5	121.8	49.98
FHIT	126.0	125.0	94.0	128.5	147.0	90.0	118.4	22.01
FHL1	8234.5	8889.5	7404.0	6284.5	10119.5	5624.0	7759.3	1669.40
FHL2	16.5	31.5	9.5	14.0	44.5	13.0	21.5	13.61
FHL3	7295.5	3784.5	4925.0	6716.0	4068.0	4513.0	5217.0	1450.54
FHL5	2.5	2.5	5.0	3.0	1.0	3.0	2.8	1.29

FHOD1	797.0	1283.5	769.0	645.5	1188.0	815.0	916.3	256.20
FHOD3	1427.0	1485.0	2984.0	1493.5	1791.0	2201.5	1897.0	606.46
FIBCD1	0.0	1.0	1.0	1.0	3.0	1.0	1.2	0.98
FIBIN	65.5	98.5	81.5	67.5	95.5	121.5	88.3	21.25
FICD	238.0	196.5	223.0	249.5	201.5	210.5	219.8	20.92
FIG4	64.0	27.5	94.5	87.0	27.5	100.5	66.8	32.89
FIGF	0.0	0.0	0.0	0.0	0.0	0.0	0.0	0.00
FIGN	40.5	15.5	62.0	37.0	19.0	56.5	38.4	18.93
FIGNL1	146.5	64.0	82.0	120.0	61.0	72.5	91.0	34.56
FIGNL2	39.5	72.5	134.5	43.5	86.0	118.0	82.3	38.59
FILIP1	1.5	4.0	16.0	1.5	4.0	10.5	6.3	5.80
FILIP1L	16.5	18.0	24.0	20.0	17.0	34.5	21.7	6.85
FIP1L1	407.5	288.0	330.0	369.0	354.0	316.5	344.2	42.06
FITM2	2002.0	1120.5	1007.0	2295.5	1408.5	988.0	1470.3	554.45
FKBP10	1641.0	1422.0	1967.5	1833.0	1610.0	2607.5	1846.8	417.69
FKBP14	650.5	668.5	947.0	623.5	795.0	981.5	777.7	156.45
FKBP15	681.5	490.5	510.5	580.0	533.0	493.0	548.1	73.22
FKBP1A	2240.5	2991.0	3094.0	2064.5	3326.0	3099.5	2802.6	518.32
FKBP1B	348.5	782.5	778.0	315.5	936.0	845.5	667.7	266.41
FKBP3	1681.0	3149.5	1822.0	1637.0	3511.0	2014.5	2302.5	814.97
FKBP4	1008.5	646.0	1085.0	840.0	680.0	871.0	855.1	173.92
FKBP5	1761.5	563.0	1187.5	1320.5	451.5	878.0	1027.0	493.98
FKBP6	13.0	8.0	7.5	18.0	10.5	7.0	10.7	4.24
FKBP7	1616.0	1040.5	1065.5	1474.5	1209.5	1257.5	1277.3	227.83
FKBP8	1165.0	1224.0	894.0	1111.5	1160.5	923.5	1079.8	137.49
FKBP9	2877.5	1511.5	1675.0	2753.5	1639.5	1479.0	1989.3	645.41
FKRP	421.0	619.0	382.5	364.0	621.0	384.0	465.3	121.29
FKTN	1506.5	886.0	779.5	1086.5	972.0	643.5	979.0	300.29
FLAD1	229.0	203.0	168.0	214.0	227.0	196.0	206.2	22.75
FLCN	876.0	547.5	669.0	861.5	574.5	603.0	688.6	145.36
FLI1	11.5	9.5	31.0	17.0	11.0	27.0	17.8	9.10
FLII	1799.0	1739.5	1672.5	1613.5	1575.0	1544.5	1657.3	98.47
FLNB	11146.5	5295.5	8308.5	12310.0	5951.5	8744.5	8626.1	2767.12
FLOT2	697.5	616.5	513.5	646.0	653.0	419.0	590.9	104.36
FLRT2	100.5	113.5	554.0	90.0	115.5	343.0	219.4	189.80
FLRT3	63.0	44.5	63.0	65.5	36.5	90.0	60.4	18.65
FLT3	6.0	2.0	6.0	5.0	2.5	9.0	5.1	2.58
FLT4	0.0	0.0	0.5	1.0	1.0	1.5	0.7	0.61
FLVCR1	331.5	261.5	263.5	337.5	297.5	277.5	294.8	33.36
FLVCR2	1374.0	1381.0	895.0	1276.5	1386.5	854.0	1194.5	251.49
FMN1	152.0	122.0	181.0	142.0	145.5	165.0	151.3	20.24
FMNL1	1.5	0.5	1.0	0.5	0.0	1.5	0.8	0.61
FMNL2	47.0	27.0	76.5	54.0	44.5	92.5	56.9	23.69
FMO4	2.5	2.5	1.5	1.0	1.5	0.5	1.6	0.80
FMOD	2.0	4.0	3.5	8.0	2.5	1.5	3.6	2.35
FMR1	452.0	289.5	477.0	453.5	334.0	435.5	406.9	76.20
FMR1NB	1.5	1.5	1.5	5.0	2.5	0.0	2.0	1.67
FN1	4154.0	2266.5	6995.5	7122.0	3007.0	9282.5	5471.3	2744.16
FN3K	64.5	32.0	32.5	52.5	47.0	39.0	44.6	12.65
FN3KRP	663.5	504.0	644.5	638.5	600.5	597.5	608.1	57.14
FNBP1	882.0	493.5	1087.5	828.0	548.0	1128.5	827.9	264.89
FNBP4	1602.5	1914.0	1177.0	1414.0	2009.5	1364.5	1580.3	326.48
FNDC1	1.5	28.5	41.5	2.5	51.5	80.5	34.3	30.34
FNDC3A	710.5	408.0	506.5	640.0	444.0	525.0	539.0	115.80

FNDC3B	946.0	561.5	1755.0	1336.0	751.0	1846.5	1199.3	532.59
FNDC4	86.0	66.0	44.5	80.5	72.5	66.5	69.3	14.48
FNDC5	4722.5	8045.0	6344.0	4355.5	8672.5	6678.5	6469.7	1727.19
FNDC7	54.0	21.0	21.5	43.0	25.5	27.5	32.1	13.41
FNDC9	0.0	1.0	0.0	0.0	0.0	0.0	0.2	0.41
FNIP1	407.5	263.0	968.0	442.5	327.0	928.0	556.0	310.27
FNIP2	303.5	307.5	463.0	312.0	402.5	423.5	368.7	69.64
FNTA	835.0	612.0	664.0	722.0	595.0	525.5	658.9	108.72
FOPNL	578.5	332.0	267.0	475.0	349.0	210.5	368.7	135.96
FOSL2	2481.0	1238.0	2718.0	2720.5	1424.5	2663.0	2207.5	686.89
FOXA1	0.0	0.0	0.0	0.0	0.0	0.0	0.0	0.00
FOXA2	0.5	0.0	0.0	0.0	0.0	0.0	0.1	0.20
FOXB1	0.0	0.0	0.0	0.0	0.0	0.0	0.0	0.00
FOXC1	14.5	1.5	14.5	11.5	6.0	13.5	10.3	5.35
FOXE3	13.0	8.5	17.5	9.0	10.5	14.5	12.2	3.49
FOXF1	1.0	0.0	0.0	1.0	0.0	0.5	0.4	0.49
FOXF2	0.0	0.0	0.0	0.0	0.0	0.0	0.0	0.00
FOXG1	0.0	0.0	0.5	0.0	0.0	0.0	0.1	0.20
FOXI1	2.5	1.0	2.0	1.0	1.5	1.5	1.6	0.58
FOXI2	0.0	0.0	0.0	0.0	0.0	0.0	0.0	0.00
FOXI3	0.0	0.0	1.0	0.0	0.0	0.0	0.2	0.41
FOXJ1	0.0	0.0	0.0	1.0	1.5	0.5	0.5	0.63
FOXJ2	1208.0	889.0	885.0	1078.0	998.5	854.0	985.4	137.79
FOXK1	109.0	75.0	106.0	104.5	85.0	119.0	99.8	16.42
FOXK2	195.5	124.0	291.0	172.5	130.5	254.5	194.7	67.03
FOXL1	0.0	0.0	0.5	0.0	0.0	0.0	0.1	0.20
FOXL2	8.0	5.5	12.5	14.0	5.5	4.5	8.3	4.01
FOXM1	532.5	233.0	522.0	547.5	241.5	303.5	396.7	152.61
FOXN1	0.0	1.5	0.5	0.0	0.5	0.0	0.4	0.58
FOXN2	35.0	18.5	61.0	50.0	21.5	49.5	39.3	17.07
FOXN3	40.0	15.5	45.5	27.5	17.0	38.5	30.7	12.61
FOXN4	1.5	0.0	0.0	0.5	0.0	0.0	0.3	0.61
FOXO1	623.0	443.0	663.0	606.5	497.5	686.0	586.5	95.91
FOXO3	425.0	260.5	423.0	373.0	304.5	381.0	361.2	65.99
FOXO4	739.0	539.0	1091.5	731.0	539.5	1162.0	800.3	268.51
FOXO6	121.5	188.5	566.0	113.5	174.0	648.0	301.9	239.50
FOXP1	323.0	246.5	294.0	303.0	299.0	358.5	304.0	36.78
FOXP2	74.0	25.0	114.5	55.5	28.0	87.0	64.0	34.84
FOXP4	708.0	689.5	979.5	785.0	649.5	912.5	787.3	132.26
FOXRED1	167.0	237.0	207.5	142.5	223.5	202.0	196.6	35.52
FOXRED2	636.0	447.0	396.5	630.0	536.5	422.0	511.3	105.42
FOXS1	7.5	6.5	6.5	12.0	6.5	7.5	7.8	2.14
FPGS	553.5	383.0	246.0	469.0	319.0	259.5	371.7	121.50
FPGT	336.5	255.5	223.5	294.0	281.0	228.0	269.8	43.02
FRA10AC1	205.5	130.0	252.5	145.5	139.5	191.5	177.4	47.60
FRAS1	9.5	25.5	145.0	6.0	31.5	69.0	47.8	52.68
FRG1	565.0	362.0	364.5	531.5	403.0	343.5	428.3	95.53
FRK	0.0	0.0	0.0	0.5	0.0	0.0	0.1	0.20
FRMD1	46.5	28.0	28.0	22.5	29.5	21.5	29.3	9.01
FRMD3	285.5	1054.0	632.0	226.0	1282.5	546.0	671.0	420.57
FRMD4A	535.5	548.0	762.5	525.5	660.0	805.5	639.5	122.71
FRMD4B	571.0	1061.5	892.0	581.5	1327.5	1139.5	928.8	306.91
FRMD5	287.0	285.0	481.0	191.0	250.5	378.0	312.1	102.66
FRMD6	488.0	546.5	767.5	439.0	533.0	871.0	607.5	171.45

FRMD7	8.0	7.5	11.0	11.0	10.5	12.5	10.1	1.93
FRMPD1	6.0	7.5	15.0	6.0	7.5	33.5	12.6	10.79
FRMPD2	16.5	15.0	36.5	14.5	10.5	24.5	19.6	9.48
FRMPD3	0.0	1.0	1.0	0.0	0.0	1.5	0.6	0.66
FRMPD4	47.0	20.5	10.0	34.5	32.5	13.0	26.3	14.22
FRRS1L	20.5	25.0	25.0	15.0	21.0	20.5	21.2	3.70
FRS2	188.5	81.0	274.5	199.5	92.5	227.0	177.2	76.16
FRS3	354.5	315.0	358.5	342.0	354.5	311.5	339.3	20.98
FRY	98.0	69.5	22.0	89.5	86.5	45.0	68.4	29.50
FRZB	28.0	31.0	57.0	15.0	34.0	60.5	37.6	17.66
FSBP	161.0	168.5	256.0	141.5	226.5	223.0	196.1	45.19
FSCN2	0.0	4.5	2.0	0.0	2.5	3.0	2.0	1.76
FSD1	81.5	143.5	151.0	78.5	137.0	140.0	121.9	32.82
FSD1L	123.5	109.0	112.0	110.0	126.5	106.0	114.5	8.41
FSD2	61.0	119.0	85.5	42.5	130.0	87.0	87.5	33.26
FSHB	0.0	0.0	0.0	0.0	0.0	0.0	0.0	0.00
FST	40.0	10.5	98.0	43.5	15.5	70.5	46.3	33.28
FSTL1	7105.0	4014.0	6891.5	8163.5	4500.0	6536.0	6201.7	1608.35
FSTL3	65.5	55.0	115.0	82.0	76.0	267.5	110.2	79.72
FSTL5	1.5	1.0	0.0	1.0	2.5	1.0	1.2	0.82
FTCD	7.0	3.5	7.5	8.0	4.5	2.5	5.5	2.30
FTH1	36831.5	27836.0	16306.5	28366.5	27587.0	14040.5	25161.3	8503.94
FTO	2.0	0.5	5.5	4.0	0.0	1.5	2.3	2.12
FTSJ2	38.0	24.0	21.0	39.0	30.0	18.5	28.4	8.71
FTSJ3	840.0	748.0	648.5	841.5	708.0	684.5	745.1	80.86
FUBP1	1169.5	632.0	999.5	1188.5	713.5	915.0	936.3	230.08
FUBP3	926.0	477.5	613.5	851.5	554.5	536.0	659.8	184.07
FUCA1	521.0	397.0	279.0	541.5	462.5	389.0	431.7	97.29
FUCA2	154.5	88.0	204.5	134.0	114.5	185.0	146.8	43.62
FUK	212.5	198.5	181.0	200.5	155.0	204.0	191.9	20.83
FUNDC1	1031.0	679.0	512.5	971.5	739.0	508.5	740.3	222.42
FUNDC2	6127.5	6617.0	6350.0	5830.0	7220.0	6319.5	6410.7	474.75
FUT10	50.0	31.5	44.5	34.5	32.5	52.5	40.9	9.28
FUT11	1091.0	691.0	1052.0	990.5	763.0	999.0	931.1	163.85
FUT4	93.0	45.0	68.0	75.5	56.0	58.0	65.9	16.90
FUT7	0.5	2.0	2.5	2.0	3.0	1.5	1.9	0.86
FUT8	142.0	45.5	77.5	154.0	47.0	66.5	88.8	47.60
FUT9	0.0	0.0	0.5	0.0	0.0	0.5	0.2	0.26
FXN	839.0	548.5	825.0	916.0	653.0	712.5	749.0	136.02
FXR1	1791.0	1529.5	2116.0	1749.5	1778.5	1872.5	1806.2	190.34
FXYD2	14.5	3.5	2.5	3.5	1.5	1.0	4.4	5.04
FXYD6	18366.0	12926.0	7742.5	14771.0	12690.5	8562.5	12509.8	3947.72
FYB	0.5	0.0	0.0	0.0	0.5	0.5	0.3	0.27
FYCO1	729.5	382.5	1052.0	750.5	420.5	941.5	712.8	269.67
FYTTD1	1410.5	1000.5	1023.0	1379.0	1148.5	999.5	1160.2	190.16
FZD1	459.0	344.0	1091.5	464.0	372.5	1062.5	632.3	347.82
FZD10	4.0	0.5	4.5	2.0	1.0	1.0	2.2	1.69
FZD3	42.5	29.5	68.0	42.0	34.5	62.5	46.5	15.41
FZD4	444.0	336.5	626.0	438.0	355.0	704.0	483.9	148.78
FZD5	9.5	5.5	1.0	11.5	5.5	2.5	5.9	4.01
FZD6	1215.5	881.5	1474.5	1169.5	1023.5	1189.5	1159.0	199.57
FZD7	128.5	106.5	199.5	122.5	90.5	143.0	131.8	37.82
FZD8	45.0	17.0	20.0	28.0	11.5	15.5	22.8	12.18
FZD9	772.5	1158.5	1125.5	765.5	881.0	1365.0	1011.3	242.58

FZR1	266.5	150.0	282.0	286.0	150.0	254.5	231.5	64.12
G0S2	1247.5	770.5	717.0	1747.5	905.0	650.0	1006.3	420.49
G2E3	409.5	159.0	439.5	472.0	186.0	311.0	329.5	133.27
G3BP1	2303.0	2440.0	3213.0	2231.0	2505.5	2852.5	2590.8	373.61
G6PC	33.0	18.0	10.5	15.0	15.5	9.5	16.9	8.51
G6PC2	7.0	2.0	3.5	8.0	1.5	4.0	4.3	2.64
G6PC3	193.0	195.0	294.0	205.0	172.5	296.0	225.9	54.55
GAA	441.5	375.5	311.5	429.0	398.0	382.0	389.6	46.20
GAB1	367.0	380.0	399.5	397.5	474.5	420.5	406.5	37.98
GAB2	224.5	53.5	145.0	304.5	60.0	134.0	153.6	96.99
GAB3	20.5	24.0	30.0	32.0	30.0	41.5	29.7	7.24
GABARAPL1	1962.5	1278.0	1289.5	1820.5	1389.0	1226.0	1494.3	315.41
GABARAPL2	1464.0	1171.5	880.0	1243.0	1322.0	883.0	1160.6	236.98
GABPA	1891.0	1124.5	1265.5	1771.5	1220.0	1166.0	1406.4	334.66
GABPB1	237.5	131.0	248.0	225.0	170.0	207.0	203.1	44.69
GABPB2	238.5	227.0	226.5	249.5	231.0	216.0	231.4	11.48
GABRA1	21.0	5.0	15.5	58.0	11.5	26.0	22.8	18.71
GABRA2	4.5	1.0	2.5	1.0	0.5	3.0	2.1	1.53
GABRA3	1.0	2.5	1.0	1.5	2.0	6.0	2.3	1.89
GABRA4	5.0	5.0	9.5	4.0	2.0	7.0	5.4	2.58
GABRA5	0.0	0.0	0.0	0.0	0.0	0.0	0.0	0.00
GABRA6	0.0	0.5	0.5	0.0	0.0	0.0	0.2	0.26
GABRB1	129.0	120.0	80.0	83.0	144.0	90.0	107.7	26.88
GABRB2	0.0	0.5	1.5	0.5	0.5	1.0	0.7	0.52
GABRB3	0.0	0.0	0.0	0.0	0.0	0.5	0.1	0.20
GABRD	32.5	13.5	7.0	29.0	15.0	7.0	17.3	10.95
GABRE	0.0	0.0	0.0	0.0	0.0	0.0	0.0	0.00
GABRG1	3.5	0.5	0.5	0.0	0.0	0.0	0.8	1.37
GABRG2	1.5	0.0	0.5	0.0	0.0	0.5	0.4	0.58
GABRG3	0.0	0.5	0.5	0.0	0.0	0.0	0.2	0.26
GABRP	35.5	10.0	16.0	23.0	10.5	10.5	17.6	10.11
GABRR1	15.0	17.0	21.0	12.0	15.0	8.0	14.7	4.41
GABRR2	21.5	29.5	15.0	36.5	31.5	18.0	25.3	8.43
GABRR3	7.0	0.5	0.5	4.0	3.5	1.0	2.8	2.58
GAD2	0.0	0.0	0.0	0.0	0.0	0.0	0.0	0.00
GADD45A	4781.5	4954.0	2715.0	4619.5	5389.5	2553.0	4168.8	1217.34
GADD45G	335.5	566.0	476.5	271.5	657.5	349.5	442.8	149.65
GADD45GIP1	203.0	286.5	205.5	181.0	223.0	174.0	212.2	40.50
GADL1	9.0	1.0	3.5	3.0	2.0	2.5	3.5	2.83
GAL	5.0	4.0	5.5	4.5	2.0	3.5	4.1	1.24
GAL3ST1	0.5	0.0	0.5	0.0	0.0	0.0	0.2	0.26
GAL3ST2	2.5	0.5	1.0	0.0	0.5	0.0	0.8	0.94
GAL3ST4	90.5	159.0	293.5	108.5	165.5	415.5	205.4	125.09
GALC	52.5	38.0	164.5	60.0	43.0	123.5	80.3	51.62
GALE	425.0	447.0	422.0	439.5	414.5	447.0	432.5	13.86
GALK1	376.0	314.5	101.5	339.0	324.5	93.5	258.2	126.22
GALK2	652.0	392.5	364.0	595.5	435.0	286.0	454.2	141.20
GALM	381.0	394.5	365.5	383.0	514.5	401.5	406.7	54.25
GALNS	143.5	67.0	198.5	143.5	89.0	169.5	135.2	49.21
GALNT1	717.0	422.5	1033.5	773.5	484.0	864.0	715.8	230.64
GALNT10	78.0	16.5	154.0	77.5	14.5	99.5	73.3	52.77
GALNT11	355.0	266.5	302.5	290.5	260.0	263.0	289.6	36.23
GALNT14	557.0	461.5	464.5	394.5	485.5	334.5	449.6	76.77
GALNT15	4.5	1.5	1.5	4.5	3.5	2.5	3.0	1.38

GALNT16	176.0	80.0	98.0	164.5	89.5	93.0	116.8	41.95
GALNT18	32.5	38.0	54.5	33.5	43.5	38.5	40.1	8.09
GALNT2	681.0	376.5	911.0	742.5	460.0	886.5	676.3	219.24
GALNT3	17.5	17.0	21.5	10.0	12.5	2.0	13.4	6.90
GALNT4	564.5	347.5	397.5	624.0	395.0	413.5	457.0	110.19
GALNT5	5.0	14.0	18.5	7.0	19.5	28.5	15.4	8.70
GALNT6	0.0	0.0	0.0	0.0	0.5	0.0	0.1	0.20
GALNT7	159.5	60.5	129.0	158.0	64.5	95.0	111.1	44.39
GALNT9	28.5	54.0	51.5	11.0	49.0	69.0	43.8	20.67
GALR1	0.0	0.5	0.0	0.0	0.5	3.5	0.8	1.37
GALR2	8.0	4.0	6.0	9.0	11.0	5.0	7.2	2.64
GALR3	1.0	0.5	0.0	0.0	0.0	1.0	0.4	0.49
GALT	168.0	123.5	82.5	154.5	119.5	89.0	122.8	34.13
GAMT	194.0	122.5	53.5	182.0	114.5	45.0	118.6	62.28
GAN	43.5	24.5	29.5	42.5	30.0	34.0	34.0	7.60
GANC	266.5	134.5	210.0	228.0	157.5	202.0	199.8	47.76
GAP43	0.0	0.5	0.5	0.0	0.0	0.5	0.3	0.27
GAPDH	61980.0	53631.5	48242.5	54808.5	54212.0	44894.0	52961.4	5901.03
GAPVD1	1170.0	904.5	1036.5	1185.0	1117.0	1103.5	1086.1	103.46
GAR1	873.5	520.0	293.0	763.5	539.5	258.0	541.3	245.80
GAREM	54.5	23.5	108.5	92.5	32.5	139.0	75.1	45.61
GAREML	0.0	0.0	0.0	0.0	0.0	0.5	0.1	0.20
GARNL3	296.5	128.5	236.0	253.5	163.0	224.5	217.0	61.35
GARS	3042.0	2739.5	3244.5	3024.5	2734.5	2450.5	2872.6	284.62
GART	1046.0	628.5	361.0	928.0	694.5	371.0	671.5	281.12
GAS1	450.0	180.5	270.0	429.5	179.5	297.0	301.1	117.45
GAS2	291.5	175.0	162.5	281.5	211.5	159.0	213.5	59.61
GAS2L1	82.5	74.0	104.5	76.0	42.5	83.5	77.2	20.14
GAS2L2	0.5	0.5	1.0	0.0	0.0	0.5	0.4	0.38
GAS2L3	67.5	18.0	122.5	90.5	27.5	65.0	65.2	38.93
GAS6	11.5	7.5	33.5	16.0	9.0	24.5	17.0	10.14
GAS8	162.5	145.0	160.0	124.0	135.5	152.5	146.6	14.86
GATA2	155.0	64.0	165.5	129.0	77.0	96.5	114.5	41.79
GATA3	0.0	0.0	0.0	0.0	0.0	0.5	0.1	0.20
GATA5	0.0	0.0	0.0	0.0	0.0	0.0	0.0	0.00
GATA6	7.0	5.0	5.5	1.5	1.5	0.5	3.5	2.66
GATAD1	246.0	170.5	133.5	241.5	202.5	160.5	192.4	45.50
GATAD2A	437.0	228.0	365.0	424.5	245.5	310.0	335.0	88.77
GATAD2B	89.5	81.5	188.0	79.5	87.0	138.5	110.7	43.78
GATB	228.0	170.0	195.0	208.0	179.0	187.5	194.6	20.95
GATM	3.0	5.5	13.0	3.5	7.5	8.0	6.8	3.67
GATSL2	496.5	615.5	531.5	420.5	645.0	452.5	526.9	88.99
GATSL3	112.0	144.5	156.0	114.5	127.5	182.5	139.5	27.10
GBA2	1008.0	925.0	588.5	905.5	898.0	621.5	824.4	174.72
GBAS	1171.5	1179.5	1065.0	1034.0	1301.5	965.0	1119.4	121.31
GBF1	2148.0	1821.0	2321.0	2471.5	2055.5	2685.0	2250.3	308.42
GBGT1	134.5	97.5	154.0	148.5	102.0	147.5	130.7	24.83
GBX1	0.0	0.5	0.0	0.0	0.0	0.5	0.2	0.26
GC	0.0	0.5	0.0	0.0	0.5	0.0	0.2	0.26
GCAT	260.0	170.0	172.5	192.5	116.5	109.5	170.2	55.02
GCC1	272.0	273.0	250.5	298.5	279.0	244.0	269.5	19.80
GCC2	472.0	247.5	291.0	469.5	273.0	267.0	336.7	104.79
GCFC2	1068.5	834.5	629.0	933.5	1014.5	623.5	850.6	190.87
GCG	494.0	1010.0	417.5	454.0	1331.5	416.5	687.3	389.11

GCGR	12.0	2.5	3.0	5.0	1.0	2.0	4.3	4.02
GCH1	130.0	131.5	105.0	137.5	144.0	75.0	120.5	25.93
GCHFR	4.5	3.0	6.0	5.5	8.0	9.0	6.0	2.21
GCK	29.5	24.5	12.0	17.5	22.0	11.0	19.4	7.26
GCLC	219.0	129.0	311.0	247.5	163.0	265.0	222.4	67.17
GCLM	265.0	112.5	243.5	281.0	143.5	219.5	210.8	68.12
GCM1	1.0	0.0	0.0	0.0	1.0	0.0	0.3	0.52
GCM2	0.5	0.0	1.0	0.5	0.0	0.5	0.4	0.38
GCN1L1	1291.0	1280.0	1785.5	1342.0	1366.5	1506.0	1428.5	192.74
GCNT1	1.5	0.5	1.0	0.5	0.5	0.5	0.8	0.42
GCNT3	0.0	1.0	0.5	0.0	1.0	1.0	0.6	0.49
GCNT4	2.5	2.5	4.0	2.5	2.5	6.0	3.3	1.44
GCNT7	335.0	96.0	122.5	235.5	120.0	123.0	172.0	93.74
GCSH	279.5	206.0	128.5	267.5	194.0	94.0	194.9	73.68
GDAP1	2.5	5.0	6.0	1.5	4.0	4.5	3.9	1.66
GDAP2	414.0	385.0	483.0	413.0	437.0	396.5	421.4	34.95
GDE1	608.0	257.5	340.0	635.0	292.5	330.0	410.5	166.25
GDF1	167.5	128.5	206.0	108.0	94.0	164.0	144.7	42.02
GDF10	0.0	0.0	0.0	0.5	0.5	0.0	0.2	0.26
GDF11	0.0	0.5	4.5	0.0	0.0	5.0	1.7	2.40
GDF15	26.0	19.5	37.0	45.0	18.0	53.0	33.1	14.26
GDF2	0.0	0.0	0.0	0.0	0.5	0.0	0.1	0.20
GDF5	0.0	1.5	0.5	1.0	0.5	0.5	0.7	0.52
GDF7	0.0	0.0	0.5	0.0	0.0	0.0	0.1	0.20
GDF9	0.5	0.0	1.0	0.0	2.5	2.0	1.0	1.05
GDI2	4047.0	3848.0	4492.5	3846.0	4335.5	4204.0	4128.8	263.12
GDNF	10.0	16.0	24.5	11.0	18.0	23.0	17.1	5.99
GDPD1	371.5	407.0	430.0	364.0	451.5	400.0	404.0	33.51
GDPD2	0.0	0.0	0.5	0.0	0.0	0.0	0.1	0.20
GDPD5	13.0	21.0	14.0	26.5	31.0	30.0	22.6	7.86
GDPGP1	32.5	16.0	8.5	19.0	8.0	7.0	15.2	9.78
GEM	24.0	20.0	64.0	28.5	32.5	43.0	35.3	16.12
GEMIN2	403.0	337.0	350.5	382.0	383.0	331.0	364.4	29.00
GEMIN4	334.0	297.5	336.5	353.5	280.5	311.5	318.9	27.28
GEMIN5	166.0	52.0	283.5	207.0	47.0	190.5	157.7	92.55
GEMIN6	268.5	304.5	282.0	326.5	305.5	257.0	290.7	26.06
GEN1	257.5	146.5	176.5	224.0	163.5	150.0	186.3	44.74
GET4	1020.5	815.0	775.5	1038.0	914.0	725.5	881.4	130.28
GFAP	17.5	15.5	25.5	14.0	10.5	19.5	17.1	5.14
GFER	16.0	20.5	32.5	20.5	18.5	31.5	23.3	6.98
GFI1	0.0	0.0	0.0	0.0	0.0	0.0	0.0	0.00
GFI1B	0.0	0.0	0.0	0.0	0.0	0.0	0.0	0.00
GFM1	543.0	503.0	586.5	508.5	535.0	484.5	526.8	36.29
GFOD1	314.0	233.0	446.5	323.0	270.5	449.0	339.3	89.96
GFOD2	154.0	109.5	135.0	159.5	111.0	142.5	135.3	21.18
GFPT1	892.5	522.5	1283.5	875.5	496.5	1061.5	855.3	305.67
GFPT2	4.5	2.5	0.5	5.5	1.0	0.0	2.3	2.25
GFRA2	0.0	0.0	1.0	0.5	2.0	3.0	1.1	1.20
GFRA3	13.5	4.5	17.0	18.5	4.0	8.0	10.9	6.30
GFRA4	0.0	0.5	0.0	0.0	0.0	0.0	0.1	0.20
GFRAL	0.0	0.0	0.0	0.0	0.0	0.0	0.0	0.00
GGA1	819.5	712.5	872.5	748.0	782.0	832.5	794.5	58.64
GGA2	238.5	207.5	211.0	226.5	209.0	228.0	220.1	12.70
GGA3	613.5	501.5	530.5	479.0	543.0	538.5	534.3	45.86

GGACT	32.0	21.5	16.0	37.0	19.5	12.0	23.0	9.61
GGCT	118.0	72.0	73.5	147.0	80.5	65.0	92.7	32.56
GGCX	80.5	75.5	133.5	78.0	66.0	156.5	98.3	37.20
GGH	938.5	879.5	1281.5	842.5	1103.0	1356.0	1066.8	215.82
GGNBP2	542.0	438.5	437.5	562.5	504.5	426.5	485.3	59.12
GGPS1	86.0	62.5	172.5	97.0	85.5	156.0	109.9	43.87
GGT1	6.0	6.0	5.5	7.0	8.0	6.0	6.4	0.92
GGT5	3.0	2.5	1.5	5.0	0.5	2.5	2.5	1.52
GGT7	635.0	308.0	109.5	657.0	387.5	155.5	375.4	232.57
GHDC	233.0	135.0	98.5	193.5	122.5	112.5	149.2	52.57
GHITM	4601.5	3144.5	2806.5	4287.0	3401.5	2550.5	3465.3	817.80
GHRH	0.5	0.5	0.5	1.0	0.5	0.0	0.5	0.32
GHRHR	0.0	0.0	0.5	0.0	0.0	0.0	0.1	0.20
GHRL	7.0	3.5	1.0	5.5	5.0	2.5	4.1	2.18
GHSR	4.0	1.5	2.5	6.5	6.5	4.0	4.2	2.04
GID4	415.0	388.5	355.5	393.0	406.5	342.0	383.4	28.78
GID8	619.0	560.0	762.5	609.5	628.5	694.0	645.6	71.61
GIF	5.0	4.0	0.0	8.5	2.0	1.5	3.5	3.03
GIMAP8	5.0	1.0	2.0	1.0	3.0	1.0	2.2	1.60
GIMD1	47.5	124.0	112.0	39.0	151.0	126.0	99.9	45.77
GIN1	91.5	82.0	80.0	87.5	108.5	73.5	87.2	12.16
GINM1	727.0	489.5	596.0	718.5	567.5	691.0	631.6	95.66
GINS1	165.5	88.5	65.5	147.5	76.5	42.5	97.7	48.36
GINS2	328.0	254.5	232.0	296.5	230.0	165.0	251.0	56.94
GINS3	639.5	654.5	581.5	593.0	701.5	603.5	628.9	45.21
GINS4	505.5	390.5	455.5	488.0	377.0	389.5	434.3	55.88
GIP	0.0	0.0	0.0	0.0	0.0	0.0	0.0	0.00
GIPC1	156.0	198.0	200.0	144.5	165.0	211.0	179.1	27.35
GIPC2	392.5	298.5	294.0	433.5	357.5	261.5	339.6	66.04
GIPC3	15.5	9.5	3.5	4.0	5.0	2.0	6.6	5.05
GIT1	259.5	343.0	232.5	220.0	304.0	259.0	269.7	46.09
GIT2	482.0	230.5	676.5	546.0	281.5	572.0	464.8	174.18
GJA1	408.5	548.5	960.0	550.5	659.0	1217.5	724.0	304.60
GJA10	0.0	0.0	0.0	0.0	0.0	1.0	0.2	0.41
GJA3	0.0	0.0	0.0	0.0	0.0	0.0	0.0	0.00
GJA4	0.0	1.5	0.0	0.0	0.0	0.0	0.3	0.61
GJA5	2.5	67.5	62.0	3.5	88.5	55.5	46.6	35.53
GJA8	1.0	0.5	0.0	0.0	1.5	1.5	0.8	0.69
GJA9	26.0	87.5	61.5	38.0	128.0	78.5	69.9	36.81
GJB1	0.0	0.0	0.0	0.0	0.0	0.5	0.1	0.20
GJB2	0.0	0.5	0.0	0.0	0.0	0.0	0.1	0.20
GJB5	0.0	0.0	0.0	0.5	0.5	0.0	0.2	0.26
GJC1	1262.0	1380.5	1044.0	1291.5	1621.0	1253.5	1308.8	188.83
GJC2	380.5	159.5	345.5	438.0	166.0	254.0	290.6	115.65
GJD2	1.5	42.0	38.5	4.0	62.0	62.0	35.0	26.84
GJD4	68.0	475.0	1120.0	49.5	636.5	995.5	557.4	451.44
GK	106.5	57.0	39.0	122.5	50.5	27.5	67.2	38.36
GKAP1	382.5	155.0	187.5	334.0	189.5	160.5	234.8	97.81
GKN2	0.5	0.0	0.0	1.0	0.5	0.0	0.3	0.41
GLA	407.0	373.0	291.5	378.0	482.0	294.0	370.9	72.00
GLB1	1112.0	705.0	907.5	1079.5	830.5	807.5	907.0	160.21
GLB1L	536.5	368.5	180.0	509.5	428.0	260.0	380.4	140.07
GLCCI1	238.5	156.5	317.0	228.5	172.0	303.0	235.9	65.62
GLCE	569.0	390.5	632.5	615.0	462.0	802.0	578.5	143.67

GLDC	0.5	1.0	2.5	2.5	1.0	0.5	1.3	0.93
GLDN	1.0	2.0	5.5	3.0	1.0	1.0	2.3	1.78
GLE1	274.0	194.5	258.5	271.5	231.0	237.0	244.4	30.11
GLG1	1006.5	331.5	1221.5	1117.0	337.5	978.5	832.1	394.94
GLI1	0.0	1.0	0.5	0.0	1.0	3.0	0.9	1.11
GLI2	85.5	59.0	226.5	111.5	67.0	265.5	135.8	88.09
GLI3	467.0	469.0	593.5	519.0	526.0	608.5	530.5	60.03
GLIPR2	1746.0	1255.0	1387.5	1360.0	1243.5	929.0	1320.2	264.80
GLIS1	414.0	414.0	621.5	435.0	516.5	633.0	505.7	101.54
GLMN	139.5	87.0	91.5	146.0	92.0	97.0	108.8	26.54
GLO1	3327.5	2109.5	2544.0	2749.5	2241.0	2073.5	2507.5	479.60
GLOD4	1605.0	1555.5	1224.0	1536.5	1788.5	1411.0	1520.1	189.92
GLOD5	0.0	0.0	0.0	0.0	0.0	0.0	0.0	0.00
GLP1R	524.5	374.0	194.5	550.0	410.5	198.0	375.3	153.72
GLP2R	15.0	12.5	18.5	21.5	13.0	16.5	16.2	3.43
GLRA1	0.0	0.5	0.0	0.0	1.0	0.5	0.3	0.41
GLRA2	0.0	0.0	0.0	0.0	0.5	0.0	0.1	0.20
GLRA4	0.0	0.5	1.0	1.5	0.0	0.0	0.5	0.63
GLRB	0.5	0.0	2.0	1.0	0.5	1.0	0.8	0.68
GLRX	22.5	35.0	21.5	38.5	58.5	17.5	32.3	15.26
GLRX2	717.0	792.0	648.0	620.0	865.0	597.0	706.5	105.30
GLRX3	1263.5	1275.0	1135.0	1180.5	1304.0	895.5	1175.6	151.14
GLRX5	960.0	802.5	736.5	858.0	862.0	611.0	805.0	120.35
GLS	331.5	175.5	509.5	354.0	207.5	510.0	348.0	142.92
GLT1D1	40.5	29.0	27.0	45.0	40.5	23.5	34.3	8.82
GLT8D1	764.5	720.5	831.0	733.0	793.0	908.0	791.7	69.75
GLT8D2	643.5	772.5	418.0	672.5	888.5	465.5	643.4	178.91
GLTP	529.5	368.5	186.5	424.0	336.5	134.5	329.9	147.63
GLTSCR1L	487.0	259.0	184.0	407.0	303.0	174.0	302.3	124.42
GLUD1	4168.0	2113.0	2226.5	3824.0	2466.5	1903.5	2783.6	962.72
GLUL	59.0	15.0	9.0	32.0	13.5	12.0	23.4	19.22
GLYATL3	9.0	16.5	4.0	12.0	15.5	7.5	10.8	4.82
GLYCTK	30.0	45.0	46.5	24.0	41.5	50.5	39.6	10.34
GLYR1	2155.0	1800.5	2523.0	2028.0	1979.5	2075.5	2093.6	241.54
GM2A	707.0	1229.0	428.0	1165.0	1927.0	886.5	1057.1	518.72
GMCL1	284.5	266.5	238.5	237.0	242.5	242.0	251.8	19.31
GMDS	949.5	766.5	646.0	904.0	783.5	595.5	774.2	138.63
GMEB1	88.0	83.5	111.0	84.0	71.0	103.5	90.2	14.60
GMEB2	446.5	348.5	226.0	401.5	320.5	236.0	329.8	88.06
GMFB	2500.5	1664.0	2332.0	2297.0	1850.5	1828.5	2078.8	339.52
GMIP	0.0	0.5	0.0	0.0	1.0	0.0	0.3	0.42
GMNC	0.0	0.0	0.0	0.0	0.0	0.0	0.0	0.00
GMNN	423.0	249.5	254.5	383.0	275.0	198.5	297.3	86.63
GMPPA	659.5	777.5	706.5	626.0	814.5	775.5	726.6	74.48
GMPPB	770.5	841.5	512.0	720.5	929.0	614.5	731.3	151.40
GMPR	111.0	136.0	249.5	116.0	148.0	166.5	154.5	50.85
GMPS	1053.5	705.0	857.0	1083.5	779.0	861.5	889.9	150.08
GNA11	472.5	491.5	340.5	448.0	540.0	324.5	436.2	85.94
GNA12	1445.0	1630.0	2203.5	1482.0	1941.0	2274.0	1829.3	362.85
GNA13	135.5	62.0	220.5	143.5	70.0	169.0	133.4	60.13
GNA14	4.5	12.5	14.0	6.0	7.5	9.0	8.9	3.71
GNAI1	401.5	293.5	373.5	368.5	330.5	350.5	353.0	37.59
GNAI2	1582.0	1059.0	1204.0	1541.5	1045.5	1265.0	1282.8	232.11
GNAO1	31.0	24.0	43.0	27.5	24.5	42.5	32.1	8.63

GNAQ	38.0	10.0	44.5	35.5	11.0	34.0	28.8	14.65
GNAS	471.0	371.0	309.5	442.5	431.0	297.0	387.0	72.71
GNAT1	0.0	0.0	0.0	0.5	0.0	0.0	0.1	0.20
GNAT3	0.0	0.0	0.0	0.0	0.0	0.0	0.0	0.00
GNAZ	6.0	2.5	5.5	4.5	2.0	9.5	5.0	2.72
GNB1	5109.0	3564.5	5917.0	5263.5	4131.0	5279.5	4877.4	863.25
GNB1L	256.0	249.5	258.5	198.5	254.5	170.0	231.2	37.56
GNB2L1	18086.5	15579.0	10789.0	15569.0	15504.5	10333.0	14310.2	3069.14
GNB3	6.0	0.5	1.0	4.0	0.5	1.0	2.2	2.29
GNB4	179.5	56.5	382.5	208.5	75.0	302.5	200.8	126.90
GNB5	384.0	327.5	391.5	308.0	359.0	388.0	359.7	34.97
GNE	584.0	362.5	566.0	550.5	396.5	538.5	499.7	94.93
GNG11	191.5	534.5	246.0	153.5	504.5	189.5	303.3	170.36
GNG12	985.0	688.5	969.5	840.5	722.5	952.0	859.7	130.21
GNG13	1.5	1.5	0.5	0.5	4.0	1.5	1.6	1.28
GNG2	21.0	53.5	134.5	19.5	48.0	106.5	63.8	46.84
GNG4	37.0	15.0	9.0	33.0	17.5	10.0	20.3	11.92
GNG5	1007.0	1109.0	885.5	1016.0	1198.0	871.5	1014.5	126.29
GNGT2	16.5	3.5	4.0	8.5	5.0	1.5	6.5	5.41
GNL2	916.5	644.5	673.5	914.5	693.0	637.0	746.5	132.45
GNL3	1534.0	1076.0	930.5	1427.0	1153.5	882.0	1167.2	263.78
GNMT	2495.5	3900.0	1680.0	2689.5	4069.0	1845.5	2779.9	1008.80
GNPAT	1099.0	1058.0	870.0	1078.5	1255.5	849.5	1035.1	152.82
GNPDA2	660.5	320.0	325.5	530.0	365.0	294.5	415.9	146.66
GNPNAT1	728.0	713.0	661.0	755.0	796.0	662.0	719.2	52.83
GNPTAB	410.0	142.5	501.5	348.5	149.5	348.0	316.7	143.63
GNPTG	689.0	535.5	426.0	605.5	542.0	396.0	532.3	109.45
GNS	3931.5	3360.0	5387.5	3963.5	3999.5	4749.0	4231.8	718.23
GOLGA1	596.0	420.0	575.0	568.0	520.5	551.0	538.4	63.29
GOLGA2	2197.5	1835.0	1522.5	2143.5	2117.0	1673.0	1914.8	279.94
GOLGA3	1017.0	1039.0	1422.0	1066.5	1256.5	1401.5	1200.4	184.56
GOLGA4	602.0	384.0	577.5	556.5	481.0	542.0	523.8	79.72
GOLGA5	479.5	347.0	415.0	419.5	373.5	404.5	406.5	45.17
GOLGA7	1534.0	1081.5	1113.5	1410.0	1108.0	1014.0	1210.2	209.58
GOLGA7B	19.0	118.0	74.5	32.5	113.5	69.0	71.1	40.54
GOLGB1	324.0	188.0	667.5	360.0	222.5	656.5	403.1	210.29
GOLIM4	626.0	355.5	1082.5	666.5	444.0	1249.0	737.3	355.02
GOLM1	474.5	373.5	483.0	461.5	446.0	400.5	439.8	43.63
GOLPH3	1063.5	839.0	1016.0	949.5	1018.0	941.0	971.2	79.47
GOLPH3L	1.5	6.0	1.5	1.0	8.0	0.0	3.0	3.21
GOLT1A	0.0	0.0	0.5	0.0	0.0	0.0	0.1	0.20
GOLT1B	986.5	642.0	747.0	963.0	737.5	779.0	809.2	136.36
GON4L	1113.0	825.0	1128.0	875.0	871.5	884.5	949.5	134.13
GOPC	1241.0	827.5	947.5	1194.5	937.0	891.0	1006.4	169.72
GORAB	390.5	517.5	456.5	405.0	620.0	477.0	477.8	83.87
GORASP1	542.0	471.0	638.5	527.0	484.0	608.0	545.1	66.70
GORASP2	1976.5	2128.0	2454.5	1992.0	2431.5	2353.5	2222.7	217.83
GOSR1	158.5	63.5	183.0	167.0	87.5	166.5	137.7	49.39
GOSR2	574.0	536.5	576.0	599.5	593.0	588.5	577.9	22.54
GOT1	1150.0	731.0	552.5	991.0	757.0	459.5	773.5	260.22
GOT2	2923.0	3240.0	2053.5	3039.5	3198.5	1929.0	2730.6	585.13
GP1BB	102.5	68.5	72.0	96.0	92.0	70.5	83.6	14.94
GP5	1587.5	1052.5	1495.0	1590.5	1262.0	1371.0	1393.1	210.16
GP9	0.0	0.0	0.0	0.0	0.0	0.0	0.0	0.00

GPA33	0.0	0.0	0.0	0.5	0.5	0.0	0.2	0.26
GPALPP1	340.0	266.0	223.5	271.5	321.0	185.0	267.8	58.10
GPAM	128.0	91.5	117.5	128.5	112.0	110.5	114.7	13.70
GPAT2	6.5	10.0	2.5	4.0	13.5	9.5	7.7	4.11
GPATCH1	49.0	18.0	58.5	49.0	22.0	42.0	39.8	16.22
GPATCH11	326.5	252.5	219.0	275.0	277.0	187.0	256.2	48.81
GPATCH2	65.0	30.0	115.5	69.0	43.5	108.0	71.8	34.12
GPATCH2L	477.5	319.0	548.0	426.5	378.0	503.0	442.0	84.48
GPATCH3	115.0	100.5	77.0	102.0	121.0	74.0	98.3	19.27
GPATCH8	365.0	395.0	484.0	370.0	300.5	599.0	418.9	106.36
GPBP1	495.5	387.5	409.5	457.0	481.5	465.0	449.3	42.14
GPC1	2815.5	1677.5	3335.5	3385.5	1938.5	2727.0	2646.6	706.56
GPC3	5.0	5.5	3.0	9.0	9.0	9.5	6.8	2.70
GPC4	3533.5	2946.5	3541.0	4311.5	3443.5	2981.5	3459.6	495.97
GPC5	1.5	0.5	1.5	0.5	2.0	2.0	1.3	0.68
GPCPD1	1155.0	1291.0	1167.0	1264.0	1624.0	1400.0	1316.8	175.14
GPD1	37.0	52.0	49.5	29.0	48.5	66.0	47.0	12.79
GPD1L	2498.5	2855.5	2725.5	2314.5	3305.5	2177.0	2646.1	409.09
GPER1	2.0	2.5	3.0	0.5	0.5	2.5	1.8	1.08
GPHB5	0.5	0.5	0.0	0.0	0.0	0.0	0.2	0.26
GPHN	336.0	234.5	323.5	310.0	248.5	249.0	283.6	44.44
GPI	9342.5	3804.5	5932.5	8976.0	4164.0	5250.0	6244.9	2384.41
GPKOW	120.0	143.0	79.0	130.5	133.0	83.0	114.8	27.18
GPLD1	1.5	1.0	0.5	0.0	0.0	0.0	0.5	0.63
GPM6B	1707.5	1072.5	2242.0	1827.0	1262.5	1986.5	1683.0	441.69
GPN1	790.0	636.5	459.5	686.0	584.5	422.0	596.4	138.86
GPN2	310.5	272.0	225.0	267.5	303.0	215.0	265.5	39.16
GPN3	558.0	419.5	315.0	538.0	517.0	291.0	439.8	116.36
GPNMB	7.5	9.5	12.0	4.0	8.0	7.0	8.0	2.66
GPR1	2722.5	2534.5	3674.0	2758.5	2894.0	3571.0	3025.8	477.41
GPR107	1344.5	954.0	1313.0	1392.0	1083.5	1157.5	1207.4	170.93
GPR112	0.0	0.0	0.5	1.0	1.5	0.0	0.5	0.63
GPR114	3.5	4.5	1.0	3.5	3.5	2.5	3.1	1.20
GPR115	0.5	1.0	2.0	0.5	1.5	3.0	1.4	0.97
GPR116	0.5	0.5	1.0	0.5	1.5	1.5	0.9	0.49
GPR119	4.0	1.5	3.0	4.0	1.0	2.0	2.6	1.28
GPR12	0.0	0.0	0.0	0.0	0.5	0.5	0.2	0.26
GPR123	223.5	59.0	47.0	198.0	55.0	50.0	105.4	82.09
GPR124	29.0	13.0	44.0	25.5	15.0	48.0	29.1	14.49
GPR125	545.5	545.0	953.5	561.0	664.5	790.5	676.7	165.93
GPR126	7.0	10.5	53.5	9.0	10.5	57.5	24.7	23.95
GPR128	0.0	0.5	2.5	0.0	0.5	4.0	1.3	1.64
GPR132	0.5	0.5	1.5	0.5	0.0	0.5	0.6	0.49
GPR133	2.5	2.0	1.5	1.5	0.5	0.0	1.3	0.93
GPR135	8.5	8.0	4.0	12.0	8.0	11.0	8.6	2.80
GPR137B	336.5	286.0	227.0	309.0	306.0	245.0	284.9	41.56
GPR137C	104.5	39.0	55.5	77.5	45.0	54.0	62.6	24.37
GPR139	0.0	1.0	2.0	0.5	2.5	1.0	1.2	0.93
GPR141	0.0	0.0	0.0	0.0	0.0	0.0	0.0	0.00
GPR142	0.0	0.0	0.5	0.0	0.0	0.5	0.2	0.26
GPR143	1.0	0.5	0.5	1.5	0.0	0.0	0.6	0.58
GPR144	1781.0	1055.5	1089.0	1535.0	1103.5	1021.5	1264.3	316.04
GPR146	4.0	0.5	1.0	2.0	1.5	0.0	1.5	1.41
GPR148	0.0	0.0	0.0	0.0	0.0	0.0	0.0	0.00

GPR149	0.0	0.0	1.0	0.0	1.0	0.5	0.4	0.49
GPR15	0.0	0.0	0.0	0.0	0.0	0.0	0.0	0.00
GPR156	25.0	29.5	21.5	25.0	28.5	39.5	28.2	6.24
GPR157	148.0	226.5	240.5	128.0	219.5	246.0	201.4	50.43
GPR158	193.0	128.5	238.0	138.0	140.5	146.5	164.1	42.69
GPR160	51.0	39.0	37.5	59.5	50.5	35.0	45.4	9.67
GPR161	266.0	140.0	226.0	292.5	160.5	219.0	217.3	58.83
GPR162	285.0	135.5	138.5	271.5	127.0	133.0	181.8	74.97
GPR17	5.5	18.5	7.0	9.0	22.5	2.5	10.8	7.88
GPR171	1.5	3.5	2.5	6.5	9.0	1.5	4.1	3.04
GPR174	0.0	0.0	0.0	0.0	0.0	0.5	0.1	0.20
GPR176	424.0	479.5	432.5	465.0	639.5	470.5	485.2	78.72
GPR18	7.0	5.0	3.5	4.0	8.0	3.0	5.1	2.01
GPR182	8.5	10.5	3.0	16.5	18.0	10.5	11.2	5.47
GPR19	8.5	0.5	0.0	7.5	0.0	0.0	2.8	4.08
GPR20	6.5	20.0	29.5	10.0	26.0	36.0	21.3	11.44
GPR21	51.0	32.0	30.5	46.5	36.0	12.5	34.8	13.60
GPR22	12.0	23.5	29.0	9.5	23.0	25.0	20.3	7.76
GPR26	0.0	0.0	0.0	0.0	0.0	0.0	0.0	0.00
GPR27	295.0	430.5	785.5	283.0	416.0	582.0	465.3	190.80
GPR34	0.0	0.0	1.0	0.0	0.0	0.5	0.3	0.42
GPR37	15.0	2.5	15.0	18.5	3.0	9.5	10.6	6.72
GPR37L1	3.0	8.0	12.0	2.5	10.5	8.5	7.4	3.89
GPR39	0.5	0.0	0.0	0.0	0.0	0.0	0.1	0.20
GPR52	9.5	6.5	2.5	13.0	10.5	5.0	7.8	3.87
GPR55	0.0	0.0	0.0	0.5	0.0	0.0	0.1	0.20
GPR56	2572.5	3736.5	2379.5	2406.0	4106.5	2479.5	2946.8	766.98
GPR6	0.5	0.0	0.0	0.0	0.5	0.5	0.3	0.27
GPR61	6.5	2.5	1.0	4.0	2.5	0.5	2.8	2.18
GPR62	21.5	26.0	18.5	12.0	29.5	17.5	20.8	6.27
GPR63	27.5	2.0	21.5	22.5	6.5	23.5	17.3	10.37
GPR64	15.5	3.0	11.0	26.5	5.5	4.0	10.9	8.99
GPR65	0.0	0.5	0.0	0.0	0.0	0.0	0.1	0.20
GPR68	3.5	22.0	21.5	6.0	43.0	19.5	19.3	14.14
GPR75	167.5	59.5	80.0	145.0	63.0	86.0	100.2	45.14
GPR78	0.0	0.0	0.0	0.0	0.0	0.0	0.0	0.00
GPR82	1.5	2.0	5.5	2.5	2.5	5.5	3.3	1.78
GPR83	14.0	8.5	11.0	7.0	4.0	5.5	8.3	3.68
GPR85	3.5	1.0	3.5	1.5	1.5	0.5	1.9	1.28
GPR87	6.0	7.0	3.5	5.5	6.0	2.5	5.1	1.72
GPR89B	314.5	155.5	152.5	331.5	202.0	128.0	214.0	87.92
GPR97	0.5	0.5	1.0	0.0	1.0	0.0	0.5	0.45
GPRC5B	276.0	210.0	718.0	215.5	241.0	513.5	362.3	207.99
GPRC5C	0.5	2.5	2.5	0.5	1.5	0.5	1.3	0.98
GPRC6A	0.0	0.0	0.0	0.0	0.0	0.0	0.0	0.00
GPRIN1	61.5	88.0	73.0	111.0	158.5	100.0	98.7	34.32
GPRIN2	5.0	1.0	1.5	3.0	1.0	0.5	2.0	1.70
GPRIN3	300.5	328.0	500.0	175.5	319.0	418.0	340.2	110.35
GPS1	536.5	630.0	764.0	526.0	667.0	703.5	637.8	93.72
GPSM1	728.0	208.0	341.0	563.0	227.0	337.5	400.8	204.12
GPSM2	581.5	362.0	545.0	561.0	381.5	417.5	474.8	98.44
GPT2	31.5	37.5	147.0	41.0	39.5	128.5	70.8	52.26
GPX1	3222.0	3410.5	1820.5	3057.5	3323.0	2086.0	2819.9	686.65
GPX2	0.5	0.0	0.0	0.0	0.0	0.0	0.1	0.20

GPX3	3562.5	5225.0	1306.5	3335.0	5882.5	2492.5	3634.0	1697.16
GPX4	4040.5	6033.0	2733.5	2844.0	5533.5	2601.5	3964.3	1508.30
GPX7	275.0	270.0	188.5	344.0	390.5	306.5	295.8	69.31
GPX8	753.5	435.5	473.5	631.5	543.5	448.5	547.7	124.36
GRAMD1B	57.0	57.5	137.5	61.0	73.0	136.0	87.0	38.97
GRAMD1C	191.5	101.5	86.5	182.5	94.5	63.0	119.9	53.63
GRAMD2	121.5	84.5	78.0	121.5	80.0	76.5	93.7	21.73
GRAP	0.0	0.0	0.5	0.0	0.0	0.0	0.1	0.20
GRAP2	11.0	10.5	2.0	12.0	10.0	0.5	7.7	5.04
GRB10	1552.0	2861.0	3349.0	1680.5	3187.5	2963.5	2598.9	781.05
GRB2	3165.5	2463.5	3067.0	2956.5	2855.5	2921.5	2904.9	242.65
GRB7	0.0	0.5	0.0	0.0	0.5	0.0	0.2	0.26
GREB1L	5.5	11.0	12.0	6.5	13.5	11.5	10.0	3.22
GREM1	12.0	7.5	10.5	18.0	15.0	40.5	17.3	11.95
GREM2	4.5	2.5	10.0	1.0	1.5	3.5	3.8	3.28
GRHL2	2.5	28.5	23.5	5.5	31.5	17.5	18.2	11.99
GRHL3	47.5	105.0	158.5	39.5	117.0	133.5	100.2	47.49
GRHPR	264.0	117.5	76.5	187.0	119.5	61.0	137.6	75.83
GRIA2	0.0	0.5	0.5	0.0	0.5	0.0	0.3	0.27
GRIA3	148.5	309.5	218.0	141.0	368.5	164.5	225.0	94.18
GRID2IP	0.0	0.0	0.0	0.0	0.0	0.0	0.0	0.00
GRIK1	1.5	1.0	2.0	1.5	1.5	1.5	1.5	0.32
GRIK3	1.0	0.0	1.5	0.5	1.0	1.5	0.9	0.58
GRIK4	0.0	0.0	0.5	0.0	0.0	0.0	0.1	0.20
GRIN1	0.0	0.0	0.0	0.0	0.0	0.5	0.1	0.20
GRIN2A	21.0	91.5	228.0	27.0	89.0	226.5	113.8	92.74
GRIN2B	1.0	1.0	1.5	1.0	1.5	1.5	1.3	0.27
GRIN2C	10.0	4.5	8.5	7.5	6.0	9.5	7.7	2.11
GRIN3A	5.5	2.5	5.5	3.0	5.0	6.5	4.7	1.57
GRIP1	9.5	3.5	6.0	6.0	6.5	3.0	5.8	2.34
GRIP2	113.5	136.0	144.5	85.5	164.0	155.5	133.2	29.14
GRK6	183.5	107.5	102.5	164.5	117.5	117.5	132.2	33.46
GRK7	24.5	21.0	14.5	21.5	19.0	8.5	18.2	5.78
GRM1	0.0	0.0	0.0	0.0	0.0	0.0	0.0	0.00
GRM2	0.0	0.5	1.0	0.0	0.0	0.0	0.3	0.42
GRM3	0.0	0.0	0.0	0.0	0.0	0.0	0.0	0.00
GRM7	0.5	1.5	6.0	1.0	3.5	5.0	2.9	2.27
GRM8	2.5	4.5	5.5	2.5	3.0	6.0	4.0	1.55
GRP	18.0	38.5	19.5	16.5	48.5	37.5	29.8	13.47
GRPEL1	1279.0	1304.5	844.0	1264.0	1317.5	747.5	1126.1	258.37
GRPEL2	811.5	717.0	965.5	864.5	805.0	855.5	836.5	82.08
GRPR	0.0	0.0	0.5	0.0	0.0	0.0	0.1	0.20
GRSF1	635.5	541.5	513.0	586.0	613.5	455.5	557.5	67.34
GRTP1	2.5	3.0	2.0	0.5	1.5	3.5	2.2	1.08
GRXCR1	0.0	0.0	0.0	0.0	0.0	0.0	0.0	0.00
GRXCR2	0.0	5.5	13.0	0.5	7.5	9.0	5.9	5.03
GSC	43.5	34.5	28.0	60.0	32.5	30.0	38.1	12.01
GSC2	0.0	0.0	0.0	0.0	0.5	0.0	0.1	0.20
GSE1	506.5	681.0	1001.5	513.5	729.5	1025.0	742.8	227.59
GSG1	29.5	109.0	124.0	31.5	97.0	103.5	82.4	41.20
GSG1L	0.0	0.0	2.5	0.0	0.5	0.0	0.5	1.00
GSG2	199.0	103.0	169.0	224.0	101.0	114.0	151.7	53.16
GSK3B	710.5	796.0	1123.5	736.5	898.5	1060.0	887.5	172.09
GSKIP	381.0	310.5	404.5	349.5	318.5	408.0	362.0	42.39

GSN	7893.0	7295.0	5152.0	8156.0	8359.5	6380.5	7206.0	1233.96
GSPT1	1244.5	943.5	988.5	1238.0	1098.0	907.5	1070.0	147.32
GSR	1041.5	628.5	412.0	812.5	628.0	341.5	644.0	257.77
GSS	623.5	1521.0	706.5	539.0	1838.5	1093.0	1053.6	530.35
GSTA1.1	13.0	37.0	16.5	14.5	39.0	10.5	21.8	12.75
GSTA1.3	1556.0	1202.5	1011.0	1469.5	1223.0	747.5	1201.6	296.77
GSTA2	727.0	582.5	735.0	713.0	726.5	725.5	701.6	58.77
GSTA3	4.0	3.5	8.0	4.0	9.5	6.5	5.9	2.48
GSTA4	313.0	228.0	212.5	276.5	256.0	213.0	249.8	39.92
GSTK1	53.5	72.5	112.5	57.0	77.5	116.5	81.6	27.08
GSTZ1	166.0	160.5	158.0	142.5	166.0	146.5	156.6	9.95
GSX1	0.0	0.0	0.5	0.0	0.0	0.0	0.1	0.20
GSX2	0.0	0.0	0.0	0.0	0.0	0.0	0.0	0.00
GTF2A1	1353.0	696.0	912.5	1290.5	875.5	815.0	990.4	267.67
GTF2A2	777.0	908.0	644.0	718.0	976.0	540.0	760.5	162.77
GTF2B	1160.5	1027.0	878.5	1103.0	1202.0	808.5	1029.9	157.46
GTF2E1	782.0	549.0	544.5	811.0	616.5	498.5	633.6	132.01
GTF2E2	617.5	532.0	415.0	555.0	580.0	364.5	510.7	99.15
GTF2F2	470.0	612.0	527.0	445.0	672.5	499.5	537.7	87.68
GTF2H1	735.0	724.5	630.0	695.5	819.0	586.5	698.4	82.19
GTF2H3	457.0	286.5	284.0	425.0	291.0	235.0	329.8	89.12
GTF2H5	397.0	453.0	351.5	360.5	469.0	386.5	402.9	48.21
GTF3C1	973.5	883.5	854.5	970.5	985.5	826.5	915.7	69.22
GTF3C2	564.5	565.0	440.5	479.5	504.5	454.5	501.4	53.71
GTF3C3	641.0	399.0	366.0	567.0	428.0	370.0	461.8	114.78
GTF3C4	310.5	199.0	293.0	338.0	216.5	277.0	272.3	54.25
GTF3C5	211.5	159.0	129.5	198.5	183.5	138.0	170.0	33.17
GTF3C6	205.0	173.5	88.0	199.5	178.0	94.5	156.4	51.94
GTPBP1	1101.5	845.0	849.5	1013.0	823.5	835.5	911.3	116.78
GTPBP2	883.0	728.0	653.0	816.5	759.5	703.0	757.2	82.46
GTPBP3	139.0	158.5	53.0	128.5	152.5	64.5	116.0	45.71
GTPBP4	1728.5	1186.5	985.5	1538.5	1152.5	923.5	1252.5	316.94
GTPBP6	94.0	90.0	107.0	80.5	106.5	99.5	96.3	10.23
GTPBP8	440.5	423.5	502.5	410.0	523.0	528.0	471.3	52.64
GTSE1	273.5	95.0	157.0	355.0	93.5	93.5	177.9	111.41
GTSF1	150.5	95.5	75.5	103.0	87.0	88.5	100.0	26.39
GUCA1A	0.0	0.0	0.0	0.0	0.0	0.0	0.0	0.00
GUCA1B	2.0	15.5	142.5	0.0	9.0	90.0	43.2	59.25
GUCA1C	1.5	7.5	9.5	0.0	15.0	21.5	9.2	8.15
GUCD1	419.5	264.0	312.5	368.0	268.5	281.5	319.0	62.49
GUCY1A3	55.5	30.0	49.0	35.5	28.5	34.5	38.8	10.92
GUCY1B3	379.0	236.0	289.0	361.5	237.5	265.0	294.7	61.97
GUCY2C	2.5	3.0	1.5	6.5	4.5	0.5	3.1	2.15
GUCY2F	0.0	0.0	0.0	0.0	0.0	0.0	0.0	0.00
GUF1	289.0	244.0	263.0	278.5	253.5	217.0	257.5	25.70
GUK1	423.5	250.0	307.5	330.0	258.0	288.0	309.5	63.36
GULP1	700.0	485.5	773.5	767.0	651.0	738.0	685.8	108.21
GUSB	2440.5	1367.5	947.5	2535.0	1572.0	937.5	1633.3	706.19
GXYLT1	484.5	296.5	517.5	421.0	328.0	370.0	402.9	87.33
GXYLT2	4709.0	5549.5	5717.0	5211.5	6557.5	7578.5	5887.2	1029.32
GYG1	1040.5	718.0	985.0	925.5	858.0	896.0	903.8	111.74
GYG2	4.5	5.0	3.5	7.0	6.5	3.5	5.0	1.48
GYLTL1B	110.5	114.5	156.0	133.0	128.0	167.5	134.9	22.67
GYPC	94.5	41.5	121.0	90.0	45.5	69.0	76.9	30.75

GYS2	0.0	0.0	0.0	0.0	0.0	0.0	0.0	0.00
GZF1	528.5	333.0	495.5	493.0	343.0	435.0	438.0	83.16
H3F3A	3528.5	3905.5	3404.0	3030.5	4696.5	3580.5	3690.9	568.21
H3F3B	7119.5	5723.5	4076.5	7021.5	6213.5	4456.5	5768.5	1279.02
H6PD	107.0	34.5	112.0	103.0	41.0	84.0	80.3	34.32
HAAO	0.0	0.0	0.5	0.5	0.0	0.0	0.2	0.26
HABP4	314.0	202.0	249.5	267.0	220.0	212.0	244.1	42.01
HACE1	944.5	493.5	662.5	953.5	569.5	563.5	697.8	201.85
HACL1	71.0	52.0	76.5	70.5	57.5	58.0	64.3	9.69
HADH	1765.0	1498.5	1166.0	1605.0	1669.5	1061.5	1460.9	284.48
HADHA	4413.0	3080.5	2410.5	4147.5	3113.0	2125.5	3215.0	912.96
HADHB	4680.0	4006.5	2294.0	4334.5	4504.0	2322.0	3690.2	1093.48
HAGH	1778.5	1419.0	1608.0	1632.5	1511.5	1702.5	1608.7	129.33
HAL	53.0	24.5	25.5	47.5	31.0	18.5	33.3	13.80
HAND1	0.0	0.0	0.0	0.0	0.0	0.0	0.0	0.00
HAND2	1.0	5.0	2.5	2.0	4.0	3.5	3.0	1.45
HAO2	0.0	0.0	0.5	0.0	0.0	0.5	0.2	0.26
HAP1	21.0	6.0	8.0	21.5	12.0	5.5	12.3	7.28
HAPLN1	2.0	0.0	1.0	2.0	0.0	0.5	0.9	0.92
HAPLN2	1.5	1.5	2.0	0.5	2.0	0.5	1.3	0.68
HAPLN3	37.5	22.5	22.5	36.0	24.0	20.5	27.2	7.52
HAPLN4	0.0	0.0	0.0	0.0	0.0	0.0	0.0	0.00
HARBI1	270.0	216.5	120.0	271.0	199.5	118.0	199.2	68.31
HARS	1109.5	1092.0	1073.0	1020.0	1153.5	985.5	1072.3	61.05
HAS2	78.5	67.0	97.5	100.5	84.0	136.5	94.0	24.21
HAS3	6.0	0.5	0.0	3.5	0.5	0.0	1.8	2.46
HAT1	1015.0	418.5	463.0	967.5	482.0	385.0	621.8	288.54
HAUS1	861.5	361.0	466.0	913.0	307.5	264.5	528.9	286.09
HAUS2	387.5	263.0	256.5	374.5	278.0	236.0	299.3	64.88
HAUS3	211.5	114.0	264.5	226.0	104.0	208.5	188.1	64.50
HAUS6	1119.0	499.0	761.5	1149.0	501.5	527.5	759.6	306.33
HAUS8	359.0	152.0	228.5	339.5	172.0	175.5	237.8	90.21
HAVCR2	5.5	2.5	1.0	6.0	1.5	1.5	3.0	2.19
HBEGF	124.0	129.0	98.5	115.0	127.5	111.0	117.5	11.70
HBS1L	952.0	821.0	798.0	962.5	935.5	736.0	867.5	94.95
HCK	15.0	36.0	9.0	19.0	30.0	10.5	19.9	10.89
HCN1	0.0	0.0	0.0	0.0	0.0	0.0	0.0	0.00
HCN2	33.5	28.5	61.0	28.5	26.0	75.5	42.2	20.86
HCN4	0.5	0.5	0.5	1.0	0.5	1.0	0.7	0.26
HCRT	3.5	4.0	2.0	1.0	1.5	3.5	2.6	1.24
HDAC1	1513.5	750.5	1326.5	1522.0	905.0	1407.0	1237.4	329.09
HDAC10	84.5	25.5	68.5	81.0	35.0	64.5	59.8	24.29
HDAC11	95.5	61.0	60.0	65.5	76.0	54.0	68.7	15.05
HDAC2	3703.5	3070.0	3118.5	3381.5	3477.0	2963.5	3285.7	282.36
HDAC3	536.0	414.0	380.0	519.0	466.0	390.5	450.9	66.55
HDAC4	213.5	158.5	195.5	173.5	168.5	183.5	182.2	19.92
HDAC7	1614.5	1783.0	1974.5	1785.5	1871.0	2193.5	1870.3	197.77
HDAC8	833.0	735.0	596.0	804.5	774.0	594.5	722.8	104.05
HDAC9	270.0	261.5	380.5	253.5	311.0	310.0	297.8	47.36
HDC	21.5	23.5	32.0	23.5	35.0	39.0	29.1	7.23
HDDC2	782.5	807.0	424.5	761.5	895.0	455.0	687.6	197.52
HDGF	2754.0	3065.5	2006.5	2403.5	3085.5	1932.5	2541.3	508.29
HDHD1	54.5	27.0	67.0	50.5	33.5	56.5	48.2	15.05
HDHD2	308.0	221.0	259.5	258.0	254.5	247.0	258.0	28.30

HDHD3	1417.0	1361.5	631.0	1285.5	1380.5	652.0	1121.3	374.14
HDLBP	9993.0	10235.5	12663.5	10251.0	10494.0	11825.0	10910.3	1078.44
HDX	41.0	31.0	82.0	39.0	29.0	67.5	48.3	21.52
HEATR1	1097.5	725.0	838.0	1075.5	692.0	709.5	856.3	185.66
HEATR5A	724.5	494.0	943.5	785.0	648.0	1155.0	791.7	231.89
HEATR6	434.0	309.0	332.0	419.0	341.0	316.5	358.6	54.01
HEBP1	263.5	206.0	355.5	305.5	238.5	364.5	288.9	64.01
HECA	1385.0	972.5	1268.0	1303.5	1037.0	1100.0	1177.7	164.25
HECTD1	2089.5	1636.0	2296.0	2084.5	1998.5	2068.5	2028.8	216.78
HECTD2	349.0	330.5	394.5	269.0	396.0	390.0	354.8	50.00
HECTD4	1425.5	1096.0	1901.0	1135.0	1199.0	1659.5	1402.7	323.11
HECW1	1096.5	558.0	857.5	1141.0	616.0	817.5	847.8	239.39
HECW2	4889.0	2640.0	4302.0	4699.0	2678.0	4014.5	3870.4	986.60
HEG1	135.0	95.5	260.5	129.0	115.0	217.0	158.7	64.98
HELB	132.5	116.5	123.5	150.5	123.0	96.5	123.8	17.82
HELLS	1423.5	934.0	1057.5	1333.0	1008.0	835.0	1098.5	231.08
HELQ	302.5	225.5	216.5	273.5	260.0	260.5	256.4	31.60
HELZ	311.5	246.5	494.0	354.0	287.5	452.5	357.7	96.96
HELZ2	37.0	21.5	87.5	41.0	28.5	103.0	53.1	33.71
HEMGN	0.0	0.0	0.5	0.0	0.5	0.5	0.3	0.27
HEMK1	664.0	383.0	240.0	611.0	404.5	256.5	426.5	176.92
HENMT1	204.5	160.5	84.0	201.0	172.5	72.5	149.2	57.53
HEP21	0.0	0.0	0.0	0.0	0.0	0.0	0.0	0.00
HEPACAM	0.0	0.0	0.0	0.0	0.0	0.0	0.0	0.00
HEPACAM2	0.0	0.0	0.0	0.0	0.0	0.0	0.0	0.00
HEPH	523.5	480.5	392.0	538.5	590.5	505.0	505.0	66.55
HEPHL1	6.5	3.0	0.5	8.0	2.0	0.0	3.3	3.25
HERC3	405.5	387.5	416.0	348.0	518.5	426.0	416.9	56.83
HERC4	989.0	694.0	1471.5	887.0	788.0	1284.5	1019.0	300.85
HERPUD1	275.0	364.5	386.0	314.0	385.5	401.5	354.4	49.44
HERPUD2	1475.0	1421.5	1229.5	1389.0	1627.0	1234.5	1396.1	151.07
HES1	559.0	860.0	534.5	562.5	865.5	509.5	648.5	167.06
HES4	33.5	44.0	58.0	22.0	46.0	48.0	41.9	12.52
HES6	1036.0	537.5	487.0	826.0	527.0	453.5	644.5	233.56
HESX1	160.0	61.0	57.5	122.0	48.5	39.0	81.3	48.36
HEXA	1211.5	1033.5	1006.5	1230.5	1116.5	1174.5	1128.8	93.23
HEXB	894.5	404.5	298.0	970.5	510.5	365.0	573.8	287.25
HEXDC	299.5	278.0	171.0	290.5	368.5	181.5	264.8	75.54
HEY1	1230.0	758.0	683.0	1275.5	878.0	647.0	911.9	275.97
HEY2	169.0	225.0	162.5	217.5	306.0	164.5	207.4	55.61
HEYL	36.0	101.0	121.0	50.0	84.0	194.5	97.8	56.91
HGD	0.0	0.5	0.0	0.0	1.5	0.0	0.3	0.61
HGF	0.5	0.0	0.5	1.5	0.0	0.0	0.4	0.58
HGH1	281.0	312.0	137.5	217.0	308.5	114.5	228.4	86.65
HGS	1102.0	988.0	1047.0	1037.5	996.5	1006.0	1029.5	42.40
HGSNAT	1051.0	476.0	330.5	1186.5	590.0	391.5	670.9	360.22
HHAT	235.5	341.0	302.5	262.5	393.5	309.5	307.4	56.14
HHATL	17.5	28.5	59.0	7.5	23.0	73.5	34.8	25.72
HHEX	175.0	105.0	84.0	194.5	112.0	102.0	128.8	44.78
HHIP	3.5	4.0	4.5	3.5	1.5	1.5	3.1	1.28
HHIPL1	274.5	402.0	715.5	459.5	493.0	1011.5	559.3	264.23
HHIPL2	27.0	22.0	18.5	36.0	30.5	29.0	27.2	6.23
HHLA1	0.0	0.0	0.0	0.0	0.0	0.0	0.0	0.00
HHLA2	55.0	30.5	98.5	48.0	41.5	80.0	58.9	25.52

HIAT1	939.5	572.0	796.5	953.0	626.5	762.5	775.0	156.60
HIBADH	1087.0	940.5	580.5	898.0	1067.0	478.0	841.8	254.70
HIBCH	800.5	970.0	1003.5	770.0	1078.5	834.5	909.5	124.92
HIC2	18.0	2.5	22.0	12.5	6.5	19.5	13.5	7.74
HID1	402.5	268.5	118.5	405.0	311.0	175.5	280.2	117.24
HIF1A	318.5	100.0	381.0	358.0	135.5	385.0	279.7	128.12
HIF1AN	738.5	786.5	705.5	761.5	891.5	688.0	761.9	72.93
HIGD1A	2153.0	979.0	830.5	1636.0	953.5	687.5	1206.6	566.89
HIGD2A	524.5	764.0	321.0	524.0	736.5	372.5	540.4	181.82
HINFP	214.0	106.0	111.0	172.5	117.0	98.5	136.5	46.28
HINT1	1894.5	2114.0	1242.0	1638.5	2057.5	1144.0	1681.8	414.18
HINT3	324.5	303.5	322.0	338.0	329.0	275.0	315.3	22.79
HIP1	921.0	517.5	692.5	929.0	429.0	656.5	690.9	204.69
HIP1R	248.5	151.0	129.0	231.5	162.0	135.5	176.3	51.00
HIPK1	207.0	126.5	689.0	276.5	190.0	615.5	350.8	239.50
HIPK2	703.5	743.5	820.5	775.5	894.5	1002.5	823.3	109.72
HIPK3	140.0	58.0	277.5	158.0	85.0	230.0	158.1	83.81
HIVEP1	254.5	223.5	465.5	252.5	274.5	482.0	325.4	116.16
HIVEP2	624.0	480.5	460.5	617.0	487.0	413.0	513.7	86.74
HIVEP3	7.5	10.0	38.5	5.5	5.5	39.5	17.8	16.55
HJURP	301.5	104.0	215.5	335.0	97.5	131.5	197.5	103.10
HK1	1691.5	1080.0	1831.0	1819.5	1256.0	1777.0	1575.8	324.49
HK2	786.5	659.5	1184.5	740.0	690.0	1237.5	883.0	258.28
HK3	34.0	39.5	46.0	37.0	54.0	56.0	44.4	9.12
HKDC1	70.0	26.0	41.5	66.0	19.5	29.5	42.1	21.35
HLCS	159.5	85.0	137.5	162.0	123.5	122.5	131.7	28.49
HLF	42.0	17.5	22.0	48.0	19.5	22.5	28.6	12.98
HM13	4384.5	2637.5	2019.0	4503.0	2917.0	2021.5	3080.4	1113.12
HMBOX1	137.5	129.5	111.0	115.5	160.5	85.0	123.2	25.70
HMBS	714.0	615.5	622.0	679.0	639.0	614.0	647.3	40.70
HMCES	458.0	489.0	361.5	500.0	518.5	448.5	462.6	55.97
HMCN1	3180.5	1909.0	2091.0	3528.5	2271.0	2759.5	2623.3	642.82
HMG20A	913.0	674.5	590.5	873.0	727.5	505.5	714.0	158.33
HMG20B	910.0	691.0	592.5	897.0	677.5	553.5	720.3	151.03
HMGB1	4095.5	1901.0	2110.0	3575.5	1948.5	1729.5	2560.0	1008.90
HMGB2	1628.0	759.5	601.0	1610.5	699.0	474.5	962.1	518.12
HMGB3	2305.5	1569.0	1721.5	2325.5	1640.5	1430.0	1832.0	386.63
HMGCL	383.5	388.5	312.0	333.0	402.0	292.5	351.9	45.44
HMGCLL1	1.0	3.5	1.0	1.5	2.5	1.0	1.8	1.04
HMGCR	2873.5	1262.0	2648.0	2646.0	1284.5	1795.5	2084.9	728.75
HMGCS1	2617.5	623.0	1669.5	2628.5	623.0	1094.5	1542.7	921.17
HMGCS2	8.5	9.0	2.5	8.0	6.5	3.0	6.3	2.84
HMGN1	2847.5	1690.0	1555.5	2676.5	1636.0	1249.0	1942.4	655.16
HMGN2	3778.0	3176.5	2781.0	3605.0	3418.0	2503.5	3210.3	491.17
HMGN3	2853.5	2254.0	1747.5	2265.5	2346.5	1523.5	2165.1	471.34
HMGXB3	555.5	405.0	634.0	512.0	427.5	632.5	527.8	98.38
HMGXB4	634.0	581.0	478.0	624.0	647.0	461.0	570.8	81.73
HMMR	573.5	208.5	354.0	568.5	207.0	205.5	352.8	178.33
HMOX1	1073.5	887.5	748.5	1200.0	913.5	473.5	882.8	254.37
HMOX2	1473.0	902.0	655.0	1098.0	855.5	657.5	940.2	309.41
HMX1	0.0	0.0	0.0	0.0	0.0	0.0	0.0	0.00
HMX2	0.0	0.0	0.0	0.0	0.0	0.0	0.0	0.00
HMX3	0.0	0.0	0.5	0.5	0.0	0.0	0.2	0.26
HN1	4440.5	11020.0	5584.0	4452.0	13701.0	5887.5	7514.2	3892.65

HN1L	1620.5	1414.5	1378.5	1876.0	1618.0	1459.0	1561.1	185.03
HNF1A	48.5	52.0	41.5	50.5	54.5	35.0	47.0	7.35
HNF1B	0.0	1.5	0.0	0.0	0.0	0.0	0.3	0.61
HNF4A	5.5	4.5	3.0	4.5	2.0	5.5	4.2	1.40
HNF4G	0.0	0.0	0.0	0.0	0.0	0.0	0.0	0.00
HNRNPA0	555.5	347.0	439.0	603.5	292.5	435.0	445.4	118.60
HNRNPA2B1	5298.0	5150.0	4452.5	5081.5	5572.0	4357.0	4985.2	481.04
HNRNPA3	4165.5	3804.5	4232.0	4333.0	3878.5	3496.5	3985.0	315.36
HNRNPAB	2549.5	2294.0	2263.5	2527.5	2543.5	2027.0	2367.5	210.65
HNRNPD	1933.5	1517.5	905.0	1784.0	1625.0	875.5	1440.1	448.77
HNRNPDL	2034.0	2451.0	2640.5	2021.0	2637.5	2655.0	2406.5	303.06
HNRNPH1	7401.5	5366.0	6234.5	7150.0	5764.5	5938.5	6309.2	803.66
HNRNPH3	4821.5	3356.5	3292.5	4583.5	3807.0	3096.5	3826.3	721.44
HNRNPK	1783.5	1046.5	1589.0	1880.0	1159.5	1329.5	1464.7	339.37
HNRNPL	956.5	836.5	900.5	854.0	842.5	808.5	866.4	53.39
HNRNPM	2984.5	2316.0	2580.0	2799.0	2406.5	2266.0	2558.7	285.39
HNRNPR	1314.5	1446.5	1244.5	1257.5	1614.5	1177.0	1342.4	161.12
HNRNPU	4174.5	2810.0	3284.0	4034.0	3109.0	3114.5	3421.0	552.61
HOGA1	19.5	285.5	161.0	14.0	269.5	207.0	159.4	119.17
HOMER1	31.0	17.5	44.0	32.0	15.0	36.5	29.3	11.15
HOMER2	41.0	30.5	20.5	21.5	21.0	17.0	25.3	8.93
HOMER3	1801.0	2078.0	1904.0	1735.0	2331.5	1878.5	1954.7	217.92
HOOK1	33.0	4.5	30.0	45.5	17.5	18.5	24.8	14.34
HOOK3	1756.5	1540.5	1218.5	1604.0	1658.0	1314.5	1515.3	207.58
HOPX	1568.5	6502.5	6576.5	1138.5	7227.5	6419.5	4905.5	2769.56
HORMAD1	15.5	3.5	12.5	15.5	9.0	4.5	10.1	5.30
HORMAD2	16.5	17.0	15.0	16.0	16.5	10.5	15.3	2.42
HOXA10	312.5	235.0	244.0	306.5	261.0	214.5	262.3	39.60
HOXA11	11.5	12.5	34.5	18.0	13.0	31.0	20.1	10.13
HOXA13	0.5	0.0	2.0	1.5	1.0	1.5	1.1	0.74
HOXA2	7.5	16.5	9.0	9.0	18.0	14.5	12.4	4.47
HOXA3	71.0	58.0	60.0	49.0	51.0	71.5	60.1	9.58
HOXA9	140.0	125.0	89.0	97.5	171.5	97.0	120.0	31.85
HOXB1	0.0	0.0	0.0	0.0	0.0	0.0	0.0	0.00
HOXB13	0.0	0.0	0.0	0.0	0.0	0.0	0.0	0.00
HOXB2	3.0	5.0	3.5	4.5	2.0	10.5	4.8	3.01
HOXB3	6.5	3.0	3.5	4.5	3.5	5.0	4.3	1.29
HOXB5	0.0	0.0	0.0	0.0	0.0	0.0	0.0	0.00
HOXB8	0.0	0.0	0.0	0.0	0.0	0.0	0.0	0.00
HOXB9	0.0	0.0	0.0	0.0	0.0	0.0	0.0	0.00
HOXC10	0.0	0.0	0.0	0.0	0.0	0.0	0.0	0.00
HOXC11	0.0	0.0	0.0	0.0	0.0	0.0	0.0	0.00
HOXC12	0.0	0.0	0.0	0.0	0.0	0.0	0.0	0.00
HOXC5	0.0	0.0	0.0	0.0	0.0	0.0	0.0	0.00
HOXC6	0.0	0.0	0.0	0.0	0.0	0.0	0.0	0.00
HOXC8	0.0	0.0	0.0	0.0	0.0	0.0	0.0	0.00
HOXD10	0.5	0.0	0.5	1.0	0.5	0.5	0.5	0.32
HOXD11	0.0	0.0	0.0	0.0	0.0	0.0	0.0	0.00
HOXD12	0.0	0.0	0.0	0.0	0.0	0.0	0.0	0.00
HOXD4	0.0	0.5	0.0	0.0	0.0	0.0	0.1	0.20
HOXD8	10.0	5.0	3.5	14.5	6.0	7.0	7.7	4.00
HP1BP3	4203.5	3453.0	3113.5	3809.0	3745.5	2882.5	3534.5	485.06
HPCA	15.0	12.5	2.5	11.5	15.0	5.5	10.3	5.18
HPCAL1	627.0	786.5	454.0	661.0	915.0	475.5	653.2	177.94

HPCAL4	19.5	48.0	51.0	19.5	53.0	57.0	41.3	17.16
HPD	0.5	3.0	2.0	0.0	2.0	0.0	1.3	1.25
HPDL	105.5	65.5	45.0	114.5	73.5	37.0	73.5	31.35
HPGD	0.0	0.0	0.0	0.0	0.0	0.0	0.0	0.00
HPGDS	12.0	1.0	1.0	3.5	1.0	0.0	3.1	4.52
HPRT1	656.5	996.5	812.0	608.0	977.5	613.5	777.3	178.56
HPS1	575.5	672.0	491.0	527.5	642.5	457.5	561.0	84.74
HPS3	167.5	159.0	189.5	145.5	205.0	172.5	173.2	21.35
HPS4	329.0	199.0	222.0	334.5	203.0	209.0	249.4	64.27
HPS5	474.0	376.0	493.5	450.0	411.5	318.5	420.6	65.62
HPS6	503.0	556.0	608.5	516.0	553.5	668.0	567.5	61.52
HPSE	13.0	9.5	26.5	19.0	6.5	10.0	14.1	7.41
HPSE2	0.0	0.5	0.0	0.5	1.0	0.5	0.4	0.38
HPX	3.5	2.0	3.0	2.0	2.0	1.5	2.3	0.75
HRAS	353.5	752.5	521.5	359.5	768.0	589.5	557.4	181.92
HRASLS	364.5	342.0	377.0	322.0	368.5	368.5	357.1	20.84
HRG	0.5	0.0	0.0	0.0	0.0	0.0	0.1	0.20
HRH1	38.5	22.0	56.0	34.0	26.5	70.0	41.2	18.40
HRH2	0.5	0.0	0.0	0.0	0.0	0.0	0.1	0.20
HRH3	12.0	12.5	5.0	13.5	12.5	6.5	10.3	3.61
HRSP12	233.0	197.5	182.5	193.5	225.5	161.0	198.8	26.86
HS1BP3	946.5	697.0	810.5	827.0	753.0	661.0	782.5	102.57
HS2ST1	1221.0	769.0	966.0	1289.5	846.0	842.5	989.0	216.78
HS3ST1	1.0	0.5	0.0	0.5	0.0	0.0	0.3	0.41
HS3ST2	2.5	20.5	48.0	4.0	17.0	22.0	19.0	16.45
HS3ST3B1	44.5	32.5	31.0	30.5	28.5	25.0	32.0	6.65
HS3ST5	60.5	162.0	170.5	85.5	211.5	255.0	157.5	73.76
HS3ST6	0.0	0.0	0.0	0.0	0.0	0.0	0.0	0.00
HS6ST1	544.5	610.5	856.5	573.0	692.0	1037.0	718.9	191.99
HS6ST2	332.0	369.5	292.0	383.5	497.5	350.0	370.8	69.80
HS6ST3	41.0	148.5	285.0	29.0	201.0	234.0	156.4	104.08
HSBP1	2863.0	2987.0	2157.5	3019.0	3372.5	2260.5	2776.6	472.28
HSBP1L1	21.5	19.0	12.0	13.0	16.5	12.5	15.8	3.91
HSCB	304.0	345.5	253.5	238.0	343.5	252.0	289.4	48.20
HSD11B2	21.5	138.0	72.0	14.0	143.0	73.0	76.9	55.08
HSD17B10	554.0	471.0	365.0	463.5	429.0	300.5	430.5	88.51
HSD17B12	1477.0	2310.5	1923.0	1519.0	2541.0	1828.5	1933.2	424.86
HSD17B2	0.0	0.0	0.0	0.0	0.0	0.0	0.0	0.00
HSD17B4	304.5	94.0	281.0	295.0	87.0	234.0	215.9	100.15
HSD17B7	978.0	453.0	364.5	864.5	405.5	320.0	564.3	282.30
HSDL1	739.5	468.5	433.0	961.0	612.5	483.0	616.3	203.58
HSF2	2556.0	3177.0	2573.0	2410.5	3618.0	2615.5	2825.0	469.96
HSF4	46.0	34.5	42.5	39.5	28.0	40.5	38.5	6.38
HSF5	35.0	20.0	9.5	26.0	33.0	10.5	22.3	10.93
HSP90AA1	2962.0	4896.5	12277.0	3045.0	5140.5	11854.5	6695.9	4258.83
HSP90AB1	4629.0	3307.5	1794.5	3948.0	3298.5	1505.5	3080.5	1215.23
HSP90B1	4728.0	5104.0	10908.0	5243.0	5747.0	10578.0	7051.3	2880.02
HSPA12A	6.0	3.5	11.5	9.5	1.5	8.5	6.8	3.79
HSPA13	401.0	174.0	201.0	383.5	182.0	194.5	256.0	106.10
HSPA14	637.0	439.5	304.5	636.5	512.5	282.5	468.8	155.49
HSPA2	3084.5	2250.0	2552.0	3217.0	2420.5	2961.5	2747.6	393.05
HSPA4	1336.0	1539.0	1934.0	1232.0	1585.5	1674.5	1550.2	249.50
HSPA4L	1517.5	1130.5	1990.0	1603.5	1255.5	1766.0	1543.8	318.33
HSPA5	6505.5	4668.0	6802.0	7373.0	5293.5	6048.0	6115.0	998.02

HSPA8	17520.0	15502.0	15168.0	18084.5	16269.0	14265.5	16134.8	1453.79
HSPA9	4838.0	3395.0	3088.5	4917.0	3514.0	2800.0	3758.8	901.88
HSPB1	5001.0	10537.5	8293.0	4564.5	11620.5	8170.0	8031.1	2844.38
HSPB11	330.0	185.0	129.5	277.0	236.0	118.5	212.7	83.67
HSPB2	145.0	1964.0	1593.0	107.0	2160.5	1631.5	1266.8	908.60
HSPB3	7.0	14.5	6.0	3.0	17.5	10.0	9.7	5.47
HSPB7	624.5	702.5	142.0	606.0	731.5	120.0	487.8	280.36
HSPB8	1179.0	6409.0	2741.5	1006.0	6966.5	2666.0	3494.7	2582.89
HSPBAP1	263.0	213.0	154.0	260.0	246.5	163.0	216.6	48.45
HSPBP1	786.5	791.5	539.5	766.0	686.5	604.5	695.8	105.04
HSPD1	3830.0	4006.0	5840.5	3952.0	4313.0	5186.5	4521.3	811.63
HSPE1	2042.5	2149.5	1717.0	1767.5	2170.5	1589.5	1906.1	246.15
HSPG2	3890.5	3047.0	12362.5	4068.0	2248.0	14814.0	6738.3	5401.44
HTATIP2	106.5	72.5	48.0	98.0	68.0	55.0	74.7	23.26
HTATSF1	720.0	491.5	613.0	720.0	561.5	541.0	607.8	95.22
HTR1A	0.0	0.0	0.0	0.0	0.0	0.0	0.0	0.00
HTR1B	6.0	5.0	7.0	6.0	2.5	5.0	5.3	1.54
HTR1D	189.0	106.0	121.0	196.0	125.0	143.0	146.7	37.47
HTR1E	0.5	0.0	0.0	0.0	0.0	0.0	0.1	0.20
HTR1F	0.0	0.0	0.0	0.0	0.0	0.0	0.0	0.00
HTR2B	350.0	195.0	217.0	355.0	187.0	234.5	256.4	76.29
HTR2C	5.5	8.5	3.0	7.0	10.0	3.5	6.3	2.77
HTR3A	3.0	3.0	8.0	2.5	5.0	17.5	6.5	5.76
HTR4	178.5	239.5	148.0	251.0	233.0	167.5	202.9	43.41
HTR5A	0.0	0.5	0.5	0.0	0.5	0.5	0.3	0.26
HTR6	8.0	11.5	11.0	4.5	13.0	22.0	11.7	5.90
HTR7	356.5	145.0	217.5	350.0	188.0	195.5	242.1	89.29
HTRA1	26297.0	27744.5	17315.5	30206.0	33390.0	28282.0	27205.8	5428.40
HTRA2	1091.0	996.0	643.0	944.0	885.5	629.0	864.8	189.67
HTRA3	158.0	141.0	206.5	203.0	191.5	217.0	186.2	30.03
HUNK	0.0	0.0	0.0	0.0	0.0	0.0	0.0	0.00
HUS1	237.0	94.5	70.0	260.0	91.5	63.5	136.1	88.19
HVCN1	1.5	5.5	5.5	2.0	3.5	2.5	3.4	1.74
HYAL1	45.0	106.5	95.5	57.0	118.0	108.5	88.4	30.09
HYAL2	170.5	244.5	184.5	175.5	257.0	194.0	204.3	37.05
HYAL3	57.5	68.5	83.5	46.5	66.5	94.5	69.5	17.34
HYDIN	53.5	44.0	38.5	52.0	45.5	48.5	47.0	5.53
HYI	200.0	122.5	60.5	158.5	119.5	65.5	121.1	53.65
HYKK	61.0	36.0	22.5	48.5	35.5	20.0	37.3	15.56
HYLS1	171.0	96.5	88.5	198.0	90.0	63.0	117.8	53.57
HYOU1	2336.5	1419.0	2119.5	2375.0	1551.5	2207.5	2001.5	412.28
HYPK	606.5	922.5	467.5	605.5	988.0	503.5	682.3	219.50
IAH1	225.0	266.0	230.0	142.5	236.5	173.0	212.2	45.52
IAPP	0.0	0.0	0.0	0.0	0.0	0.0	0.0	0.00
IARS	1791.5	1665.0	2585.0	1831.5	1633.5	1866.0	1895.4	350.13
IARS2	1237.5	610.5	734.0	1151.0	627.5	644.5	834.2	283.48
IBA57	249.0	244.5	379.0	285.0	324.5	377.0	309.8	60.16
IBSP	24.0	14.5	11.0	16.5	19.0	5.5	15.1	6.41
IBTK	1178.0	1254.5	1500.0	1235.0	1470.0	1484.5	1353.7	146.18
ICA1	243.5	160.5	105.5	197.5	181.5	97.0	164.3	55.99
ICK	47.0	29.5	60.5	55.5	19.0	63.0	45.8	17.84
ICOS	2.0	2.0	2.0	1.0	0.5	0.0	1.3	0.88
ICOSLG	146.5	74.5	171.0	181.0	69.0	121.0	127.2	47.72
ICT1	427.5	447.0	222.5	399.0	441.5	213.0	358.4	110.26

ID1	909.5	1267.0	703.0	939.5	1210.5	663.5	948.8	250.27
ID2	115.0	94.0	172.5	111.5	97.0	141.5	121.9	30.00
ID3	743.5	457.5	1052.0	612.5	476.0	949.0	715.1	246.20
ID4	93.0	74.0	184.5	145.0	81.5	179.0	126.2	49.73
IDE	1978.5	1419.5	1862.0	1895.5	1681.0	1579.5	1736.0	213.45
IDH1	3752.0	3092.0	2147.0	3688.5	3459.0	1846.5	2997.5	814.47
IDH2	3364.5	2683.0	2131.0	2855.0	2479.0	2091.0	2600.6	479.59
IDH3A	3147.5	2880.5	2078.0	2920.5	3324.0	1868.0	2703.1	591.59
IDH3B	1599.0	2178.0	1566.0	1466.0	2003.0	1463.5	1712.6	302.72
IDNK	65.5	62.0	41.5	55.0	73.5	48.0	57.6	11.77
IDO2	0.0	0.0	0.0	0.0	0.0	0.0	0.0	0.00
IDS	435.0	205.0	146.5	429.0	256.5	142.0	269.0	133.07
IDUA	176.0	99.5	135.0	219.0	127.5	122.0	146.5	43.43
IER3IP1	475.5	568.0	414.5	473.0	633.0	419.0	497.2	86.56
IER5	556.0	649.0	478.5	475.0	613.5	547.5	553.3	70.07
IFFO1	787.0	982.5	639.5	615.0	1024.5	618.0	777.8	186.56
IFFO2	273.0	312.5	221.5	270.0	363.0	178.5	269.8	65.10
IFI30	148.0	119.5	52.0	150.0	149.0	54.5	112.2	47.06
IFI35	712.0	498.5	471.0	597.0	516.5	454.0	541.5	97.21
IFIH1	152.0	84.0	123.5	167.0	89.5	131.0	124.5	33.08
IFITM10	43.5	33.5	19.5	35.5	38.0	24.5	32.4	8.88
IFITM5	3.5	1.5	3.5	3.5	1.5	1.5	2.5	1.10
IFNAR1	195.0	123.0	213.5	226.0	158.0	211.0	187.8	39.49
IFNAR2	725.0	330.5	288.0	654.0	366.5	276.0	440.0	197.19
IFNG	0.0	0.0	0.0	0.0	0.0	0.0	0.0	0.00
IFNGR1	580.0	510.0	596.0	586.5	543.0	531.0	557.8	34.63
IFNGR2	1607.0	1041.0	942.0	1519.0	1193.0	857.5	1193.3	308.65
IFNLR1	34.0	15.0	33.5	17.0	22.5	35.0	26.2	9.11
IFRD1	2235.0	1805.0	1324.0	2186.5	1990.5	1264.0	1800.8	421.61
IFT122	400.5	305.5	491.0	255.0	267.0	336.0	342.5	89.65
IFT140	161.5	104.0	132.5	123.0	105.5	131.0	126.3	21.17
IFT172	402.0	253.5	367.5	271.5	233.5	298.5	304.4	66.70
IFT20	728.5	563.0	489.0	646.5	530.5	491.5	574.8	95.04
IFT22	358.5	279.0	364.5	313.0	297.0	328.0	323.3	33.82
IFT27	97.5	78.0	142.0	94.0	84.5	136.0	105.3	27.04
IFT43	181.0	126.5	137.0	137.0	145.0	128.5	142.5	20.00
IFT46	320.0	279.0	280.0	259.5	326.0	291.5	292.7	25.72
IFT52	1283.0	809.0	896.0	1240.0	908.5	862.0	999.8	206.11
IFT57	366.0	296.5	308.5	325.0	339.0	278.0	318.8	31.44
IFT80	279.0	242.0	307.0	275.0	259.0	273.0	272.5	21.69
IFT81	145.5	120.5	205.0	131.0	135.5	189.5	154.5	34.43
IFT88	119.0	80.5	169.0	120.5	67.5	140.5	116.2	37.55
IGBP1	871.0	740.5	492.5	818.0	774.5	495.0	698.6	164.58
IGDCC3	3.0	8.5	19.0	8.0	9.5	16.0	10.7	5.83
IGDCC4	34.5	49.5	61.0	37.5	65.5	126.0	62.3	33.53
IGF1R	293.5	318.5	299.0	259.0	367.0	247.5	297.4	43.06
IGF2	30.5	94.5	143.0	41.5	102.0	157.5	94.8	51.54
IGF2BP1	45.0	35.5	29.5	29.5	20.0	20.5	30.0	9.44
IGF2BP2	289.0	385.0	461.5	237.0	371.5	472.5	369.4	93.12
IGF2BP3	126.5	60.5	120.0	110.5	54.5	74.0	91.0	31.73
IGF2R	792.5	678.0	1100.0	843.5	780.0	983.0	862.8	152.99
IGFALS	1.5	8.5	8.5	1.5	6.5	8.5	5.8	3.44
IGFBP1	0.0	0.0	0.5	0.0	0.0	0.0	0.1	0.20
IGFBP2	908.0	191.0	532.5	793.0	161.0	342.5	488.0	312.32

IGFBP3	560.5	375.0	393.5	562.0	419.0	381.0	448.5	88.63
IGFBP4	554.0	313.0	629.5	496.5	272.0	892.0	526.2	226.37
IGFBP5	53.0	36.5	28.5	61.5	44.0	16.5	40.0	16.40
IGFBP7	1335.5	1152.5	1219.0	1387.5	1346.5	1352.0	1298.8	91.75
IGFN1	26.0	106.0	235.5	21.5	122.5	252.5	127.3	99.31
IGHMBP2	101.5	55.5	106.5	119.0	70.5	92.5	90.9	23.74
IGJ	1322.5	3093.0	4149.0	1356.5	3525.0	3139.0	2764.2	1166.64
IGLL1	0.0	0.5	1.5	0.0	0.0	0.0	0.3	0.61
IGSF10	20.5	10.0	25.5	15.0	11.0	24.5	17.8	6.73
IGSF11	2.0	46.0	40.0	1.5	57.5	20.5	27.9	23.55
IGSF21	10.0	4.0	2.5	8.5	2.0	2.0	4.8	3.53
IGSF3	883.5	233.5	1501.5	842.5	228.5	1197.5	814.5	510.91
IGSF5	13.5	21.0	13.0	11.0	20.0	11.0	14.9	4.45
IGSF6	2.5	1.5	5.0	2.5	2.0	1.0	2.4	1.39
IGSF9	99.0	156.5	245.0	108.0	139.0	232.5	163.3	62.12
IGSF9B	4.5	3.5	16.5	2.5	2.5	17.0	7.8	7.01
IK	1563.0	1379.5	1092.5	1466.5	1322.0	969.5	1298.8	226.26
IKBIP	245.5	203.5	220.0	362.0	271.0	285.0	264.5	56.62
IKBKAP	775.0	862.5	1166.5	892.0	923.0	1019.5	939.8	136.71
IKBKB	643.5	390.5	459.0	658.0	465.0	402.0	503.0	118.32
IKBKE	68.0	232.0	200.0	102.0	263.5	170.5	172.7	75.46
IKZF1	0.0	0.0	0.0	0.0	0.0	0.0	0.0	0.00
IKZF2	29.0	16.5	17.0	29.0	17.5	14.0	20.5	6.69
IKZF3	2.0	5.5	0.0	1.0	6.5	2.5	2.9	2.56
IKZF4	37.0	39.0	68.0	44.5	44.5	68.0	50.2	14.13
IKZF5	533.0	437.0	867.0	556.0	527.0	779.5	616.6	167.44
IL10	0.0	2.0	4.5	1.5	2.5	3.5	2.3	1.57
IL10RA	8.0	5.5	3.0	6.0	4.0	6.0	5.4	1.74
IL10RB	426.5	174.0	116.5	423.5	208.5	113.5	243.8	144.90
IL11RA	56.5	57.0	66.5	49.5	58.0	75.0	60.4	8.96
IL12A	0.0	0.5	1.0	0.0	0.0	0.0	0.3	0.42
IL12B	0.0	0.0	0.0	0.0	0.0	0.0	0.0	0.00
IL13RA1	23.5	11.0	9.5	12.5	11.5	14.5	13.8	5.06
IL13RA2	9.5	34.0	35.5	31.5	46.5	84.5	40.3	24.82
IL15	0.0	1.0	1.0	0.5	0.5	1.0	0.7	0.41
IL16	39.5	12.5	14.5	48.5	23.0	11.5	24.9	15.59
IL17B	0.0	0.5	0.0	0.5	0.0	0.0	0.2	0.26
IL17C	7.5	7.5	3.5	2.5	6.5	1.5	4.8	2.66
IL17RA	182.5	49.5	157.0	214.0	61.5	145.0	134.9	66.02
IL17RC	507.0	499.0	342.0	511.0	423.0	413.0	449.2	68.01
IL17RD	96.5	54.0	140.0	117.0	89.0	173.0	111.6	41.62
IL17RE	73.0	54.5	36.0	72.0	42.0	34.0	51.9	17.48
IL17REL	1.5	3.0	5.5	1.5	11.0	21.0	7.3	7.62
IL18	476.5	511.0	599.0	438.5	478.5	433.5	489.5	60.79
IL18BP	63.0	17.0	4.5	70.0	24.5	4.5	30.6	28.94
IL18R1	16.0	14.5	26.0	11.0	17.5	25.5	18.4	6.08
IL18RAP	4.0	1.0	0.5	3.0	0.5	0.0	1.5	1.61
IL1R2	4.5	7.5	15.0	3.5	14.5	12.5	9.6	5.08
IL1RAP	168.0	96.5	231.5	173.5	139.5	281.5	181.8	65.93
IL1RAPL1	0.0	0.0	0.0	0.0	0.0	0.0	0.0	0.00
IL1RAPL2	388.5	319.5	345.5	363.0	353.0	293.0	343.8	33.54
IL1RL1	7.5	5.0	4.5	4.0	4.5	14.5	6.7	4.03
IL1RN	0.0	0.0	0.5	0.0	0.0	0.0	0.1	0.20
IL20RA	6.5	13.5	9.5	13.0	22.0	9.0	12.3	5.45

IL20RB	2.5	0.5	1.5	3.5	1.0	1.5	1.8	1.08
IL21	0.0	0.0	0.0	0.0	0.0	0.5	0.1	0.20
IL21R	1.5	0.5	3.0	0.0	2.0	0.5	1.3	1.13
IL22	0.0	0.0	0.0	0.0	0.0	0.0	0.0	0.00
IL22RA1	8.0	26.0	22.0	5.0	34.5	13.0	18.1	11.37
IL22RA2	1.0	0.0	1.0	2.0	1.5	1.5	1.2	0.68
IL23A	3.0	1.0	1.0	1.0	3.0	3.0	2.0	1.10
IL26	0.0	0.0	0.0	0.0	0.0	0.0	0.0	0.00
IL2RB	0.5	0.0	0.0	0.0	1.0	0.5	0.3	0.41
IL2RG	1.0	0.5	3.0	2.0	0.5	2.0	1.5	1.00
IL31RA	6.5	0.0	3.0	2.0	0.0	1.0	2.1	2.46
IL34	192.5	284.0	145.0	163.0	240.5	127.0	192.0	60.15
IL4I1	0.0	0.0	0.0	0.0	0.0	0.0	0.0	0.00
IL4R	630.5	573.0	702.0	600.0	577.0	729.5	635.3	66.14
IL5RA	2.0	1.5	7.5	2.5	2.0	4.0	3.3	2.25
IL6	35.0	19.0	7.5	52.5	26.0	15.5	25.9	16.03
IL6R	46.5	47.0	13.5	35.5	44.5	30.5	36.3	12.96
IL7R	2.0	0.5	0.0	1.5	0.5	0.0	0.8	0.82
IL8	296.0	152.0	110.0	375.5	178.5	129.5	206.9	105.42
ILDR1	0.5	0.0	0.5	1.0	0.0	0.0	0.3	0.41
ILDR2	3.5	2.5	9.0	5.5	1.5	11.0	5.5	3.78
ILF2	2937.0	3198.0	2367.5	2733.0	3530.5	2322.0	2848.0	472.65
ILF3	708.5	492.5	699.0	728.0	439.5	630.5	616.3	122.15
ILK	4403.5	4551.0	3471.0	4041.0	4636.5	3575.0	4113.0	501.41
ILKAP	827.0	818.5	540.5	729.5	852.0	561.0	721.4	138.68
IMMP1L	164.5	140.5	120.5	165.0	134.5	102.0	137.8	24.71
IMMP2L	27.5	39.5	23.0	26.0	38.0	19.0	28.8	8.23
IMMT	947.0	1020.5	901.0	1018.0	1136.5	859.0	980.3	99.55
IMP3	843.5	1152.5	629.5	880.0	1039.0	759.5	884.0	188.70
IMP4	1311.0	1425.0	1087.5	1296.5	1399.0	1101.5	1270.1	144.74
IMPA1	1306.5	543.5	496.5	1223.0	645.0	434.5	774.8	386.57
IMPA2	1340.0	1027.0	721.0	1159.0	1168.5	746.0	1026.9	248.20
IMPACT	160.0	127.5	123.5	124.5	150.0	114.5	133.3	17.62
IMPAD1	692.5	474.0	379.0	593.5	507.5	364.5	501.8	126.10
IMPDH2	2028.0	2983.5	2140.0	1943.5	2891.0	1934.0	2320.0	484.70
IMPG1	0.0	1.0	1.0	1.0	0.0	0.0	0.5	0.55
IMPG2	56.0	84.0	71.5	40.5	92.5	73.0	69.6	18.86
INA	36.5	173.5	143.0	45.5	183.0	122.5	117.3	63.00
INCENP	813.5	304.5	468.5	869.0	283.0	287.5	504.3	270.48
INF2	260.0	245.5	574.0	401.5	322.5	555.5	393.2	143.98
ING1	355.0	286.5	226.0	328.0	350.0	233.0	296.4	57.23
ING2	116.0	67.5	74.5	113.5	93.5	56.5	86.9	24.71
ING3	311.0	234.5	233.0	280.0	272.0	237.5	261.3	31.69
ING4	489.5	482.0	513.5	506.0	513.5	475.0	496.6	16.67
ING5	528.5	225.0	387.5	501.5	264.0	394.0	383.4	122.05
INHA	1.0	36.0	95.5	0.5	38.5	87.0	43.1	40.82
INHBA	187.5	165.5	78.5	264.5	213.5	151.0	176.8	62.62
INHBB	1.0	1.0	2.0	0.5	1.0	1.0	1.1	0.49
INHBC	0.0	0.5	0.0	0.0	0.0	0.0	0.1	0.20
INIP	676.5	547.5	480.0	617.5	561.5	403.0	547.7	97.13
INO80	573.0	405.0	535.0	560.0	473.0	581.0	521.2	68.96
INO80B	215.5	301.5	177.0	233.5	278.0	164.0	228.3	54.38
INO80C	177.0	187.5	213.0	182.5	238.5	229.5	204.7	26.01
INO80D	43.5	39.0	70.0	40.5	47.5	65.0	50.9	13.26

INPP1	61.5	84.5	64.5	54.0	60.0	94.0	69.8	15.78
INPP4A	863.5	525.0	677.0	748.5	695.0	1012.0	753.5	167.59
INPP4B	25.5	45.5	50.5	13.5	40.0	19.0	32.3	15.11
INPP5A	84.0	43.5	105.5	98.5	55.5	86.0	78.8	24.37
INPP5B	692.0	383.5	458.0	635.0	494.0	454.5	519.5	118.53
INPP5D	0.0	1.0	2.0	0.0	1.0	1.0	0.8	0.75
INPP5E	676.5	510.0	705.0	700.5	571.5	603.5	627.8	79.06
INPP5F	695.5	504.5	761.0	650.0	589.0	700.0	650.0	91.41
INPP5J	16.5	42.5	23.0	17.5	34.0	31.5	27.5	10.24
INPP5K	664.5	307.5	383.5	632.0	341.0	348.0	446.1	158.78
INS	0.0	0.0	0.0	0.0	0.0	0.0	0.0	0.00
INSC	0.0	0.0	0.0	0.0	0.0	0.0	0.0	0.00
INSIG1	3582.5	1652.5	3332.5	3146.0	1716.5	2529.0	2659.8	832.09
INSIG2	505.0	359.5	601.0	498.5	451.0	594.5	501.6	90.87
INSL5	82.0	53.5	20.0	68.5	61.5	26.0	51.9	24.35
INSR	298.5	140.5	420.0	313.5	140.0	317.0	271.6	110.52
INSRR	52.5	31.0	9.0	32.5	21.5	12.0	26.4	15.96
INTS1	1003.5	650.0	927.0	903.0	693.5	863.0	840.0	138.82
INTS10	336.5	245.0	167.5	336.0	274.5	168.0	254.6	76.04
INTS12	287.0	158.0	155.5	294.0	212.0	148.5	209.2	66.99
INTS2	1523.0	1093.5	1306.0	1258.5	1004.5	993.5	1196.5	205.48
INTS4	479.0	345.0	477.5	491.5	395.5	452.0	440.1	57.84
INTS5	89.5	63.0	132.0	93.0	36.5	109.0	87.2	33.70
INTS6	831.0	380.0	400.0	771.5	508.0	436.0	554.4	197.01
INTS7	401.0	289.5	308.5	453.5	325.0	254.0	338.6	74.50
INTS8	690.0	506.0	566.5	626.0	565.5	533.0	581.2	66.76
INTS9	604.5	489.5	506.5	537.5	555.5	487.0	530.1	45.38
INTU	116.0	60.5	109.0	96.0	73.5	118.5	95.6	23.84
IP6K1	813.0	436.0	446.5	728.5	437.0	550.0	568.5	164.59
IP6K2	839.0	472.5	641.5	842.5	589.0	599.0	663.9	148.00
IP6K3	139.5	1351.0	1128.0	119.5	1429.0	841.0	834.7	583.20
IPCEF1	65.0	18.0	16.0	49.0	34.0	8.0	31.7	21.95
IPMK	354.0	249.0	331.5	355.0	281.0	346.5	319.5	44.22
IPO13	431.5	308.0	373.5	337.0	315.0	319.0	347.3	47.48
IPO5	2913.5	1747.5	1988.0	2526.0	1707.5	1580.5	2077.2	529.31
IPO8	576.5	419.5	588.0	554.5	475.5	479.0	515.5	67.30
IPP	195.5	137.0	219.5	198.0	150.5	205.0	184.3	32.74
IPPK	46.5	11.0	65.0	58.5	26.0	53.5	43.4	20.78
IQCA1	0.5	0.0	1.0	0.5	0.0	0.5	0.4	0.38
IQCB1	433.0	302.0	172.0	406.0	376.0	216.0	317.5	106.12
IQCC	166.5	153.5	122.0	116.5	154.5	114.0	137.8	22.89
IQCD	3.0	2.5	2.0	1.0	0.5	2.0	1.8	0.93
IQCE	34.0	7.0	27.0	32.0	14.0	26.0	23.3	10.61
IQCG	10.0	8.5	19.0	7.5	13.0	12.5	11.8	4.16
IQCH	37.5	27.0	24.5	36.0	19.5	26.0	28.4	6.97
IQCJ	0.0	0.0	0.0	0.0	0.0	0.0	0.0	0.00
IQCK	158.5	106.0	45.5	122.5	116.5	34.0	97.2	47.99
IQGAP1	3419.0	1507.5	2901.5	3623.0	1709.5	2514.0	2612.4	871.92
IQGAP2	433.5	463.5	746.0	531.0	628.5	1214.5	669.5	290.47
IQSEC1	751.0	634.5	691.0	663.0	753.5	775.5	711.4	56.78
IQSEC3	1.5	0.0	1.0	2.5	0.5	0.0	0.9	0.97
IQUB	111.0	216.5	145.0	82.0	195.5	91.0	140.2	55.79
IRAK1BP1	109.0	107.5	70.5	100.5	142.5	58.5	98.1	30.05
IRAK2	1085.5	602.5	680.5	1117.0	698.5	629.5	802.3	234.36

IRAK4	547.0	280.0	254.5	471.5	336.5	232.5	353.7	127.67
IREB2	762.0	692.5	945.0	825.0	776.0	841.0	806.9	85.58
IRF1	109.5	95.5	145.0	99.0	158.5	160.0	127.9	29.94
IRF2	537.0	452.5	438.0	472.0	463.5	397.5	460.1	45.89
IRF2BP2	872.0	1142.5	612.5	806.5	1276.5	744.5	909.1	251.57
IRF2BPL	824.0	570.0	704.5	818.0	607.0	662.0	697.6	106.09
IRF4	1.5	0.0	0.5	1.5	3.5	1.0	1.3	1.21
IRF5	8.5	9.0	9.0	8.5	13.0	10.0	9.7	1.72
IRF6	2.5	2.5	2.5	0.5	1.5	3.0	2.1	0.92
IRF7	89.0	68.0	60.5	91.5	77.0	77.5	77.3	11.89
IRF8	262.5	156.5	174.5	322.0	164.0	172.0	208.6	67.78
IRG1	3.0	9.0	4.5	3.0	6.5	4.5	5.1	2.31
IRS1	55.0	30.0	84.0	69.5	31.5	59.5	54.9	21.21
IRS2	7.0	16.5	13.0	7.5	15.5	12.0	11.9	3.97
IRS4	1203.5	526.5	910.5	1170.5	619.0	864.0	882.3	276.87
IRX1	0.0	0.0	0.0	0.5	1.0	0.0	0.3	0.42
IRX4	3.0	2.0	0.0	1.5	0.0	0.5	1.2	1.21
ISCA1	28.0	19.0	20.5	21.5	21.5	12.0	20.4	5.15
ISCA2	582.5	602.0	433.5	551.5	741.0	513.5	570.7	102.55
ISCU	1634.0	1084.0	769.0	1460.5	1079.5	704.0	1121.8	368.59
ISG20L2	188.0	181.5	158.5	188.0	177.5	152.5	174.3	15.25
ISL1	0.0	0.0	0.5	0.0	0.0	0.0	0.1	0.20
ISM1	5.5	2.0	3.5	0.5	6.0	4.5	3.7	2.11
ISM2	100.5	177.0	255.0	112.5	217.0	313.5	195.9	82.68
ISPD	287.5	156.0	134.0	284.5	177.0	131.0	195.0	72.43
IST1	2610.5	2379.0	2040.0	2393.5	2448.5	2022.5	2315.7	235.19
ISX	1.0	0.0	0.0	0.0	0.0	0.0	0.2	0.41
ISY1	1126.0	838.5	737.5	1061.5	935.5	665.5	894.1	180.77
ITCH	431.0	338.0	480.0	426.5	403.5	426.0	417.5	46.39
ITFG2	456.0	381.5	372.5	418.0	434.5	351.0	402.3	40.30
ITFG3	1005.5	962.0	1456.5	1213.0	1085.5	1492.0	1202.4	227.50
ITGA11	0.0	0.0	0.5	0.0	1.0	2.5	0.7	0.98
ITGA4	542.5	294.5	328.5	591.5	389.5	483.0	438.3	119.53
ITGA6	26643.0	16736.5	34012.5	30939.0	19343.5	28218.0	25982.1	6693.97
ITGA7	135.0	176.0	69.0	116.0	178.0	42.0	119.3	55.54
ITGA9	10.5	4.5	49.5	10.5	4.5	27.5	17.8	17.66
ITGAV	3309.0	817.0	5365.5	3859.0	1092.5	3908.0	3058.5	1768.44
ITGB1	8534.5	5665.5	11562.0	9032.0	6497.5	10818.5	8685.0	2318.39
ITGB1BP1	361.0	325.5	235.5	297.0	325.5	222.5	294.5	54.80
ITGB1BP2	466.5	881.5	293.5	360.0	929.0	304.0	539.1	290.59
ITGB2	1.0	15.0	11.0	3.5	22.5	9.5	10.4	7.81
ITGB3	2.0	176.0	78.5	2.0	146.5	68.0	78.8	72.03
ITGB3BP	209.0	91.0	118.5	206.0	89.0	68.0	130.3	61.96
ITGB4	0.0	0.0	0.5	0.0	1.0	0.0	0.3	0.42
ITGB5	154.5	104.5	225.0	197.0	120.5	337.5	189.8	85.36
ITGB6	0.0	2.5	15.5	0.0	1.5	8.5	4.7	6.17
ITGB8	11.5	18.0	101.0	4.0	13.5	88.0	39.3	43.17
ITIH2	107.0	52.0	36.5	161.0	43.5	39.5	73.3	50.33
ITIH5	62.0	15.0	8.0	34.5	12.5	10.5	23.8	21.00
ITK	15.0	3.5	0.5	24.5	3.0	1.5	8.0	9.64
ITM2A	3106.5	2840.5	3913.0	3191.0	3522.0	4394.0	3494.5	575.39
ITM2B	671.5	290.0	426.5	792.0	352.0	522.5	509.1	192.92
ITM2C	229.5	198.0	209.5	231.5	218.5	227.5	219.1	13.19
ITPA	365.0	425.0	231.5	355.0	398.5	222.0	332.8	85.89

ITPK1	276.5	904.0	447.0	242.0	991.0	413.5	545.7	322.05
ITPKA	938.0	778.0	1193.5	937.0	859.0	1033.0	956.4	144.30
ITPKB	257.5	344.0	375.5	223.5	357.0	382.0	323.3	66.37
ITPR1	747.5	373.5	684.0	778.0	402.0	584.0	594.8	173.84
ITPR2	107.5	47.0	89.0	124.5	61.5	79.0	84.8	28.69
ITPR3	726.5	2336.0	2827.0	669.5	2703.5	1855.5	1853.0	956.56
ITPRIP	295.5	166.0	418.0	295.0	188.5	388.5	291.9	101.78
ITSN1	303.5	147.0	467.0	330.0	178.5	483.5	318.3	140.46
ITSN2	311.5	151.5	299.5	304.0	163.0	284.0	252.3	74.22
IVD	144.0	264.5	289.0	139.0	282.5	307.0	237.7	75.74
IVNS1ABP	1391.0	1063.5	1241.0	1346.0	1147.0	1184.5	1228.8	123.43
IWS1	1213.0	958.5	924.0	1146.0	1052.5	866.0	1026.7	134.63
IYD	28.5	65.0	98.5	16.0	51.5	70.5	55.0	29.89
JADE1	732.0	257.0	334.5	664.0	277.0	307.5	428.7	211.38
JADE3	660.5	567.5	767.0	709.0	626.5	736.5	677.8	74.13
JAG1	73.0	20.5	98.5	85.5	43.0	114.5	72.5	35.17
JAG2	1733.0	1133.5	1477.5	1900.0	1321.5	1141.5	1451.2	314.70
JAGN1	473.0	433.0	271.0	480.5	451.5	322.5	405.3	87.22
JAK2	257.0	166.5	292.0	230.0	177.5	287.5	235.1	53.88
JAK3	4.0	1.0	1.5	2.0	2.0	2.0	2.1	1.02
JAKMIP2	113.5	108.5	349.0	95.0	121.0	325.5	185.4	118.15
JAKMIP3	0.5	0.5	0.0	0.5	0.0	0.5	0.3	0.26
JAM2	337.5	194.5	267.0	298.0	214.5	274.5	264.3	52.83
JAM3	14440.5	12904.0	15905.5	14076.5	14061.0	12667.0	14009.1	1167.23
JARID2	545.5	110.0	205.0	499.5	133.5	191.5	280.8	191.04
JDP2	228.0	270.0	359.0	237.5	283.5	337.0	285.8	52.74
JKAMP	426.0	267.0	329.0	397.5	256.0	261.0	322.8	74.38
JMJD1C	654.0	536.5	1089.0	606.5	589.5	1058.5	755.7	249.42
JMJD4	616.0	496.0	636.5	576.5	547.0	586.0	576.3	50.20
JMJD6	325.5	409.0	558.5	328.5	440.5	456.5	419.8	87.59
JMJD7	169.0	144.0	75.0	222.0	165.5	82.5	143.0	56.05
JMJD8	370.0	389.5	274.5	336.0	430.0	347.0	357.8	52.72
JMY	76.0	42.0	77.5	72.0	30.0	62.5	60.0	19.68
JOSD1	755.5	475.0	599.0	675.0	512.0	581.5	599.7	103.46
JPH2	38.0	140.5	268.5	40.0	140.0	211.0	139.7	91.60
JUN	1021.5	900.0	1238.0	1035.0	988.0	1342.0	1087.4	167.09
JUND	2574.5	3342.5	1622.0	2154.0	3341.5	1839.0	2478.9	741.39
JUP	6595.0	4722.0	6478.5	6222.5	5142.5	6184.5	5890.8	769.89
KAL1	0.5	0.0	6.0	2.0	0.5	8.0	2.8	3.36
KANK1	80.0	39.0	110.0	81.0	52.0	104.5	77.8	28.06
KANK3	1727.0	1922.0	1785.5	1769.5	2018.0	1863.0	1847.5	108.91
KANK4	184.5	435.5	433.5	232.5	547.5	826.5	443.3	232.07
KANSL1	213.0	141.0	218.0	206.5	136.0	235.5	191.7	42.32
KANSL1L	428.5	401.0	513.0	427.5	477.0	548.0	465.8	56.86
KANSL3	928.5	780.0	625.5	858.0	779.5	616.5	764.7	124.36
KARS	3113.5	1955.5	1673.0	2935.5	2030.5	1545.0	2208.8	658.82
KAT2A	384.0	360.0	426.5	348.5	314.5	401.5	372.5	39.96
KAT2B	488.5	253.0	426.5	492.0	310.5	473.0	407.3	101.62
KAT6A	235.0	89.0	421.0	228.5	97.0	366.0	239.4	135.68
KAT6B	146.5	70.0	130.0	149.5	90.0	127.0	118.8	31.98
KAT7	1028.5	722.0	795.5	1068.0	853.0	716.5	863.9	151.93
KATNA1	368.5	212.0	344.0	371.0	217.0	238.0	291.8	77.12
KATNAL1	867.5	479.5	570.5	744.0	582.0	642.0	647.6	138.70
KATNAL2	146.5	132.5	97.5	124.0	141.0	87.5	121.5	23.94

KATNB1	348.0	267.5	259.0	349.5	287.5	314.0	304.3	39.33
KATNBL1	461.0	400.5	487.5	484.0	446.0	457.5	456.1	31.57
KAZALD1	130.0	649.0	1381.5	173.5	623.0	1052.0	668.2	488.45
KAZN	7.0	3.0	15.0	12.5	5.0	10.5	8.8	4.61
KBTBD11	56.0	65.0	62.5	52.0	67.0	97.0	66.6	15.93
KBTBD12	0.0	8.0	1.0	0.5	4.0	3.0	2.8	3.00
KBTBD13	0.0	1.0	3.5	0.5	0.5	0.5	1.0	1.26
KBTBD2	626.5	659.5	967.0	604.5	746.5	947.0	758.5	161.30
KBTBD3	12.0	6.0	9.0	12.5	6.0	10.0	9.3	2.82
KBTBD4	470.0	455.5	356.0	480.0	483.0	356.0	433.4	60.73
KBTBD8	212.5	194.0	279.5	220.5	208.5	223.0	223.0	29.53
KCMF1	0.0	0.0	0.0	0.0	0.0	0.0	0.0	0.00
KCNA1	0.0	1.0	0.5	0.5	0.0	1.0	0.5	0.45
KCNA10	0.5	0.0	0.0	0.0	0.5	0.0	0.2	0.26
KCNA2	0.0	0.5	0.5	0.5	0.0	1.0	0.4	0.38
KCNA4	2.5	0.5	2.5	1.5	2.5	4.5	2.3	1.33
KCNA5	134.0	59.5	149.5	123.0	69.5	103.5	106.5	35.96
KCNA6	1.0	0.5	1.5	0.0	0.5	2.0	0.9	0.74
KCNAB1	64.0	120.5	189.5	66.0	147.0	228.0	135.8	65.99
KCNAB2	215.0	285.0	219.0	204.0	313.0	231.5	244.6	43.96
KCNB1	0.5	1.0	2.5	2.0	0.5	0.5	1.2	0.88
KCNB2	3.0	2.5	2.5	1.0	0.5	3.5	2.2	1.17
KCNC1	20.0	28.0	20.5	17.0	31.5	17.0	22.3	6.03
KCNC2	49.0	18.5	48.0	50.5	27.5	50.5	40.7	14.01
KCNC4	0.0	0.0	0.0	0.0	0.5	0.0	0.1	0.20
KCNE1	0.5	3.5	2.5	1.5	2.5	1.0	1.9	1.11
KCNE2	6.5	4.0	3.0	3.0	7.0	1.5	4.2	2.16
KCNE3	2.5	0.5	0.0	1.5	0.5	0.0	0.8	0.98
KCNE4	6.0	6.0	0.5	2.5	13.5	2.0	5.1	4.68
KCNF1	0.0	0.0	0.5	0.0	0.5	0.5	0.3	0.27
KCNG1	0.5	0.0	0.5	0.0	0.0	0.0	0.2	0.26
KCNG2	0.0	0.0	0.0	1.0	0.0	0.0	0.2	0.41
KCNG3	0.5	0.0	0.0	0.0	0.5	0.0	0.2	0.26
KCNG4	1.5	0.5	0.0	1.0	0.0	0.0	0.5	0.63
KCNH1	1.0	0.5	2.0	3.0	1.5	1.0	1.5	0.89
KCNH2	16.0	40.0	31.5	15.5	37.5	28.5	28.2	10.46
KCNH4	0.5	0.0	1.5	0.0	0.0	0.0	0.3	0.61
KCNH5	1.0	3.0	2.5	5.0	8.5	3.5	3.9	2.60
KCNH6	245.5	410.5	475.0	200.5	442.0	343.0	352.8	110.47
KCNH8	0.0	0.0	0.0	0.0	0.0	0.0	0.0	0.00
KCNIP1	425.0	463.0	155.0	317.0	396.0	56.0	302.0	162.63
KCNIP2	1.5	0.0	1.0	3.5	1.0	1.0	1.3	1.17
KCNIP4	5.5	3.0	5.0	4.5	2.0	2.0	3.7	1.54
KCNJ1	0.0	0.0	0.0	0.0	0.0	0.0	0.0	0.00
KCNJ10	0.0	0.0	0.5	0.0	0.5	0.0	0.2	0.26
KCNJ11	2.0	23.0	13.5	4.5	14.0	11.5	11.4	7.51
KCNJ12	4.5	2.0	11.5	4.5	5.0	6.0	5.6	3.18
KCNJ13	1.0	0.0	0.0	0.0	0.0	0.0	0.2	0.41
KCNJ15	0.0	0.0	2.0	0.5	1.0	2.0	0.9	0.92
KCNJ16	0.5	1.5	1.5	0.0	0.5	1.0	0.8	0.61
KCNJ2	45.0	42.0	194.0	56.5	42.0	154.5	89.0	67.42
KCNJ3	0.0	2.0	9.5	0.0	2.5	6.5	3.4	3.81
KCNJ4	0.5	0.5	3.0	0.5	2.5	0.5	1.3	1.17
KCNJ5	7.5	241.0	290.0	7.0	313.0	268.5	187.8	141.89

KCNJ6	0.0	0.5	0.0	0.0	1.5	0.5	0.4	0.58
KCNJ8	18.0	19.0	91.5	7.0	18.5	60.5	35.8	32.99
KCNK1	196.5	63.5	139.5	139.5	62.0	64.5	110.9	56.13
KCNK10	1.0	1.0	2.0	0.0	0.0	1.5	0.9	0.80
KCNK12	0.5	0.0	0.5	0.0	0.0	0.0	0.2	0.26
KCNK13	9.0	11.0	19.0	8.0	13.5	13.5	12.3	3.97
KCNK16	0.0	0.0	0.0	0.0	0.0	0.0	0.0	0.00
KCNK18	0.0	0.0	0.0	0.0	0.0	0.0	0.0	0.00
KCNK2	0.0	1.0	0.5	0.5	0.0	0.5	0.4	0.38
KCNK3	0.0	0.0	0.0	0.0	0.0	0.0	0.0	0.00
KCNK5	26.0	6.0	36.0	21.5	7.0	26.0	20.4	11.78
KCNK9	0.0	0.5	0.5	0.0	0.0	0.0	0.2	0.26
KCNMA1	233.0	183.0	627.5	258.0	257.5	698.5	376.3	224.91
KCNMB1	50.5	44.5	61.0	43.5	53.5	53.0	51.0	6.47
KCNMB2	89.5	43.5	56.5	96.5	58.5	50.5	65.8	21.80
KCNMB4	2.5	3.0	1.0	2.0	5.5	1.0	2.5	1.67
KCNN2	26.5	19.0	24.0	26.0	23.5	17.5	22.8	3.70
KCNQ1	0.0	0.0	0.0	0.0	0.5	0.5	0.2	0.26
KCNQ2	0.0	0.0	0.0	0.0	0.0	0.0	0.0	0.00
KCNQ3	0.0	0.0	0.0	0.5	0.0	0.0	0.1	0.20
KCNQ5	6.5	4.0	11.0	12.0	4.0	10.5	8.0	3.62
KCNRG	25.0	22.5	22.5	21.5	27.5	20.5	23.3	2.56
KCNS1	0.0	0.0	0.0	0.5	0.5	0.5	0.3	0.27
KCNS2	0.5	0.5	2.0	0.0	2.5	0.5	1.0	1.00
KCNS3	0.0	0.0	0.0	0.0	0.0	0.0	0.0	0.00
KCNT1	618.5	904.5	798.0	513.0	924.5	720.5	746.5	161.79
KCNU1	0.5	0.0	1.0	0.0	0.0	1.0	0.4	0.49
KCNV1	0.0	0.0	0.0	0.5	0.0	1.0	0.3	0.42
KCNV2	1.0	1.0	0.5	0.0	0.5	1.0	0.7	0.41
KCTD10	370.0	161.0	538.5	498.0	158.5	468.5	365.8	168.99
KCTD14	0.0	0.0	0.0	0.0	0.0	0.0	0.0	0.00
KCTD15	451.5	262.5	312.0	373.0	307.0	326.5	338.8	65.69
KCTD16	0.5	0.0	3.0	0.5	0.0	1.5	0.9	1.16
KCTD17	0.5	0.0	3.5	1.0	1.0	7.0	2.2	2.66
KCTD18	171.5	101.5	68.5	153.5	101.5	69.0	110.9	42.94
KCTD19	0.5	0.0	1.5	2.0	0.0	2.0	1.0	0.95
KCTD2	3071.0	2517.5	2442.5	3155.0	2713.5	2296.0	2699.3	348.62
KCTD20	1815.0	1302.5	1304.5	1720.5	1399.5	1204.0	1457.7	249.81
KCTD21	49.5	49.0	115.0	50.5	50.5	105.0	69.9	31.21
KCTD3	1265.5	1150.0	1107.0	1121.5	1323.0	1123.5	1181.8	90.09
KCTD4	0.5	0.0	0.0	0.0	0.5	0.0	0.2	0.26
KCTD6	293.5	265.0	202.5	285.0	307.0	223.5	262.8	41.41
KCTD7	93.0	90.0	110.5	107.5	82.5	103.5	97.8	11.01
KCTD8	0.5	0.0	0.0	0.0	0.0	0.0	0.1	0.20
KCTD9	717.0	417.0	373.0	689.5	464.0	399.0	509.9	152.92
KDELC1	134.0	58.5	213.5	143.5	67.0	185.5	133.7	62.05
KDELC2	988.5	533.5	730.5	1006.5	528.0	578.0	727.5	221.70
KDELR2	3301.0	3476.5	3947.5	3494.5	3908.0	4041.5	3694.8	307.36
KDELR3	452.5	267.0	546.0	417.0	312.5	524.0	419.8	112.00
KDF1	5.0	1.5	2.5	2.5	1.5	1.5	2.4	1.36
KDM1A	1467.0	1285.5	1175.0	1485.0	1379.5	1129.5	1320.3	148.92
KDM1B	170.5	125.5	186.0	152.5	156.0	191.0	163.6	24.22
KDM2A	852.5	708.0	798.0	850.5	759.5	796.5	794.2	55.18
KDM2B	441.0	376.5	530.5	446.5	400.5	513.0	451.3	60.64

KDM3A	1129.5	909.5	1546.5	1084.0	1073.5	1353.0	1182.7	228.25
KDM4A	724.5	483.0	594.5	674.5	546.0	592.5	602.5	86.84
KDM4B	655.0	389.5	553.5	663.5	480.5	568.0	551.7	104.71
KDM5B	835.0	841.5	987.5	811.0	961.5	1176.0	935.4	138.32
KDM7A	25.0	20.0	93.5	21.0	25.0	72.5	42.8	31.88
KDM8	75.5	66.5	53.5	56.0	75.0	55.0	63.6	10.14
KDSR	503.0	299.5	369.0	580.0	367.5	364.0	413.8	105.14
KEAP1	186.0	284.5	340.0	204.5	257.0	397.5	278.3	80.59
KEL	0.0	0.5	1.0	0.0	0.5	1.0	0.5	0.45
KERA	0.0	0.5	0.0	0.0	0.0	1.5	0.3	0.61
KHDRBS1	275.5	207.5	364.5	299.0	228.0	328.0	283.8	59.50
KHDRBS3	1452.5	1047.5	792.5	1253.0	1133.5	880.5	1093.3	242.49
KHK	2.0	0.5	1.0	1.0	0.0	0.0	0.8	0.76
KIAA0040	70.5	57.5	54.5	69.0	55.5	42.0	58.2	10.51
KIAA0100	704.0	575.0	591.5	634.0	517.0	631.0	608.8	63.34
KIAA0141	59.5	27.5	34.5	59.0	28.0	39.5	41.3	14.57
KIAA0195	885.0	1103.0	1134.5	844.5	1276.0	1019.5	1043.8	161.97
KIAA0196	604.0	515.5	652.5	638.5	580.5	580.5	595.3	49.03
KIAA0226	122.5	83.0	97.0	142.0	94.5	124.0	110.5	22.42
KIAA0226L	6.5	1.0	4.0	5.5	3.5	3.5	4.0	1.90
KIAA0232	453.5	259.5	826.5	449.5	284.0	630.5	483.9	215.08
KIAA0319	452.5	256.5	183.5	362.5	295.0	154.0	284.0	111.74
KIAA0319L	1057.0	686.0	867.0	1066.5	805.0	736.0	869.6	161.01
KIAA0368	1210.0	858.5	1138.5	1118.0	926.5	1001.0	1042.1	135.65
KIAA0391	181.0	216.0	304.0	191.0	221.5	274.0	231.3	48.15
KIAA0408	82.0	98.0	128.0	88.5	104.5	130.5	105.3	20.15
KIAA0430	863.5	482.5	840.0	806.0	580.5	747.5	720.0	154.28
KIAA0513	27.5	48.5	53.5	20.5	54.0	74.5	46.4	19.65
KIAA0556	118.0	46.5	123.0	110.5	61.5	104.0	93.9	31.94
KIAA0586	137.5	164.5	206.0	276.0	221.5	298.5	217.3	62.19
KIAA0753	83.5	27.0	43.5	93.0	36.0	45.0	54.7	26.95
KIAA0895	131.0	56.5	99.5	102.0	44.5	77.0	85.1	32.04
KIAA0895L	58.0	74.5	63.0	46.0	72.5	89.5	67.3	15.04
KIAA0907	381.5	199.5	420.5	398.5	226.0	400.0	337.7	97.91
KIAA0922	555.0	436.0	431.0	522.0	493.5	477.0	485.8	48.41
KIAA0930	103.5	61.5	118.5	117.5	54.5	88.0	90.6	27.66
KIAA1024	0.0	0.5	1.0	0.0	0.0	0.0	0.3	0.42
KIAA1024L	0.0	0.0	0.0	0.0	0.0	0.0	0.0	0.00
KIAA1033	29.5	12.5	103.5	45.0	11.5	72.5	45.8	36.31
KIAA1045	0.0	0.0	0.5	0.0	0.0	0.0	0.1	0.20
KIAA1107	305.0	306.5	368.0	338.0	409.0	375.0	350.3	41.21
KIAA1109	764.0	495.5	724.0	759.5	571.5	667.5	663.7	109.25
KIAA1143	1332.0	634.5	441.0	1322.0	818.0	428.0	829.3	411.18
KIAA1147	413.5	222.5	198.0	315.5	280.0	218.5	274.7	80.90
KIAA1161	1045.5	662.5	578.5	1059.5	766.0	567.0	779.8	222.97
KIAA1191	290.0	266.0	244.5	342.0	360.0	278.0	296.8	44.99
KIAA1210	78.0	77.5	238.5	74.5	110.5	250.0	138.2	83.30
KIAA1211	149.0	115.0	97.5	132.0	122.0	120.5	122.7	17.19
KIAA1211L	1.0	0.0	0.5	1.0	1.5	0.0	0.7	0.61
KIAA1217	245.5	322.0	784.5	231.0	395.5	627.5	434.3	224.08
KIAA1279	1199.5	765.5	728.5	1274.5	975.5	741.5	947.5	242.79
KIAA1324	11.5	13.0	12.0	10.5	6.0	15.5	11.4	3.15
KIAA1324L	6.0	8.5	24.5	6.0	5.5	21.5	12.0	8.64
KIAA1328	113.0	44.0	121.0	130.5	47.0	110.0	94.3	38.44

KIAA1377	5.0	3.5	5.0	7.0	6.5	6.5	5.6	1.32
KIAA1407	90.0	66.0	142.5	75.0	73.0	115.0	93.6	29.65
KIAA1429	851.5	580.5	735.5	781.5	735.5	697.5	730.3	90.42
KIAA1456	1.5	0.5	1.0	1.0	1.5	0.5	1.0	0.45
KIAA1462	1116.0	532.0	660.5	1285.5	632.0	621.5	807.9	311.95
KIAA1467	192.5	157.5	197.0	177.0	169.0	159.5	175.4	16.59
KIAA1468	601.0	381.5	448.5	578.5	456.0	389.0	475.8	93.58
KIAA1522	72.0	52.0	87.0	64.5	49.0	68.5	65.5	13.92
KIAA1524	789.5	263.5	528.5	833.5	256.0	295.5	494.4	265.69
KIAA1549L	13.0	8.0	9.0	10.0	9.0	6.5	9.3	2.19
KIAA1551	324.5	190.0	303.5	278.5	214.5	281.5	265.4	52.25
KIAA1644	1.5	0.0	0.0	0.5	1.0	0.0	0.5	0.63
KIAA1671	111.0	168.5	410.5	108.5	212.0	382.0	232.1	133.18
KIAA1715	183.5	96.5	204.0	205.5	114.0	165.0	161.4	46.30
KIAA1755	23.5	23.0	14.0	21.5	22.5	34.5	23.2	6.57
KIAA1919	508.0	273.5	346.0	521.0	300.5	292.5	373.6	111.80
KIAA2013	2205.0	1264.0	1415.5	2175.0	1475.5	1330.0	1644.2	429.02
KIAA2018	34.5	15.0	80.0	35.5	15.5	71.5	42.0	27.73
KIAA2022	33.0	22.0	48.5	28.0	27.0	39.0	32.9	9.57
KIAA2026	383.0	340.5	391.5	341.0	402.5	416.0	379.1	31.69
KIDINS220	128.0	32.5	368.5	153.0	45.0	250.0	162.8	128.12
KIF11	577.5	174.5	350.5	717.0	149.0	220.0	364.8	233.77
KIF12	3.0	1.0	1.0	1.5	1.5	1.5	1.6	0.74
KIF13A	1385.0	1297.5	1716.5	1416.5	1471.0	1522.0	1468.1	143.73
KIF13B	367.5	1080.0	1859.0	341.0	1093.0	1446.0	1031.1	596.72
KIF14	699.0	260.0	352.0	817.0	242.5	239.0	434.9	256.36
KIF15	751.5	208.5	652.5	836.0	196.0	337.5	497.0	283.95
KIF16B	563.0	421.5	470.5	551.0	432.5	405.5	474.0	67.87
KIF17	5.0	19.0	24.5	6.5	21.5	30.5	17.8	10.13
KIF18A	117.0	54.0	86.5	125.0	43.5	58.0	80.7	34.43
KIF18B	374.0	270.5	308.0	361.0	243.5	261.5	303.1	54.31
KIF19	0.5	5.5	3.5	0.5	4.5	3.5	3.0	2.07
KIF1B	740.0	561.5	2425.5	752.0	665.5	1746.0	1148.4	760.22
KIF20A	763.5	233.5	708.5	835.5	211.0	399.5	525.3	277.95
KIF21A	220.5	57.0	105.5	230.5	73.0	79.0	127.6	77.50
KIF21B	12.0	9.0	29.0	7.5	17.0	41.5	19.3	13.35
KIF23	783.0	297.0	761.0	749.0	308.0	479.5	562.9	230.20
KIF24	1018.5	2377.5	2038.0	1127.5	2889.5	1639.5	1848.4	728.27
KIF25	195.0	91.5	32.0	166.5	86.5	25.0	99.4	69.21
KIF26A	3.0	219.5	244.5	8.0	346.0	531.5	225.4	202.67
KIF26B	3.0	2.0	2.5	6.0	4.0	3.0	3.4	1.43
KIF27	17.0	7.0	35.5	14.0	11.5	33.5	19.8	11.90
KIF2B	0.0	0.0	0.0	0.0	0.0	0.0	0.0	0.00
KIF2C	850.0	364.0	553.0	919.0	352.0	359.5	566.3	258.72
KIF3A	793.5	475.5	580.5	807.5	547.0	525.0	621.5	142.86
KIF3B	337.5	278.5	378.0	330.0	298.5	360.0	330.4	37.16
KIF3C	88.0	69.0	124.5	70.5	74.5	92.0	86.4	20.89
KIF4A	1219.5	455.5	675.5	1332.0	387.5	413.0	747.2	423.41
KIF5C	101.5	26.0	69.5	97.5	35.0	62.5	65.3	31.10
KIF7	682.5	491.0	705.5	683.0	460.5	658.0	613.4	108.12
KIF9	129.0	232.0	187.0	131.0	235.5	175.5	181.7	46.56
KIFAP3	1025.0	1401.5	1935.0	1180.0	1667.5	2288.5	1582.9	476.55
KIFC3	2958.5	2027.5	1753.0	2033.0	1769.5	1473.0	2002.4	512.33
KIN	159.0	120.0	190.5	155.5	152.5	168.5	157.7	23.01

KIRREL	3058.5	1938.5	2757.0	2908.5	2013.5	3011.0	2614.5	505.82
KIRREL3	1.0	0.0	1.0	0.0	0.0	1.0	0.5	0.55
KIT	8.5	14.5	3.0	3.5	23.0	8.5	10.2	7.55
KITLG	8.0	12.5	5.0	9.0	7.0	5.0	7.8	2.82
KL	1.5	1.5	2.0	1.0	0.5	1.0	1.3	0.52
KLB	66.0	206.5	496.0	55.5	212.0	436.5	245.4	184.50
KLC1	819.5	402.0	1021.5	792.5	460.0	918.5	735.7	250.08
KLC4	4772.5	5347.0	4977.0	3264.5	5048.0	3616.5	4504.3	851.64
KLF10	677.5	352.0	440.0	730.5	299.0	390.5	481.6	179.16
KLF11	667.0	480.0	544.5	660.0	526.5	594.0	578.7	75.20
KLF12	170.0	96.5	168.0	77.0	67.5	130.0	118.2	44.81
KLF15	4.0	21.0	52.0	2.5	30.0	45.5	25.8	20.66
KLF2	345.5	679.5	648.0	339.0	706.5	707.5	571.0	178.53
KLF3	367.5	320.0	432.0	324.0	355.0	395.0	365.6	42.89
KLF4	109.0	94.0	117.0	106.5	102.0	96.0	104.1	8.58
KLF5	733.5	1312.5	1122.5	689.5	1604.0	1027.5	1081.6	348.06
KLF6	1645.5	1836.0	2585.5	1814.0	2028.5	2428.5	2056.3	372.92
KLF7	684.5	281.0	649.0	739.5	351.5	636.0	556.9	191.14
KLF8	277.0	394.0	251.0	307.0	405.5	272.5	317.8	66.02
KLHDC1	441.0	386.5	343.0	391.0	423.0	354.0	389.8	37.97
KLHDC10	329.5	344.0	336.0	312.5	376.0	326.5	337.4	21.62
KLHDC2	1476.5	1478.5	1251.5	1303.0	1450.5	1136.0	1349.3	141.65
KLHDC4	831.0	615.0	588.5	671.0	575.5	544.0	637.5	103.98
KLHDC7A	20.0	2.0	0.0	17.5	1.0	0.0	6.8	9.36
KLHDC8A	74.5	260.0	260.5	64.0	244.5	201.5	184.2	91.63
KLHDC8B	6.0	5.0	4.0	9.0	8.5	2.5	5.8	2.54
KLHL10	50.5	40.0	35.5	36.5	49.5	47.5	43.3	6.72
KLHL11	829.0	352.5	463.0	795.0	405.5	504.0	558.2	203.49
KLHL12	368.5	375.5	321.0	335.5	389.5	372.5	360.4	26.30
KLHL13	633.0	309.0	284.5	621.5	389.0	239.0	412.7	173.24
KLHL14	4.5	3.0	1.0	2.5	1.0	2.0	2.3	1.33
KLHL15	554.0	354.0	477.5	552.0	353.0	386.5	446.2	94.39
KLHL17	42.0	31.5	37.5	38.5	31.5	44.0	37.5	5.21
KLHL18	355.0	369.5	355.5	386.5	365.0	423.5	375.8	26.04
KLHL2	761.0	514.5	534.5	761.5	527.0	468.5	594.5	131.18
KLHL20	490.5	290.0	415.0	483.0	303.0	384.0	394.3	85.87
KLHL21	158.0	105.5	252.5	162.0	109.0	216.5	167.3	58.33
KLHL22	284.0	187.5	300.5	269.0	225.0	303.0	261.5	46.07
KLHL23	21.0	15.0	10.5	22.0	14.5	11.5	15.8	4.78
KLHL24	1244.0	1158.5	1442.0	1050.0	1208.0	1292.0	1232.4	131.87
KLHL25	255.5	155.5	225.5	220.0	165.5	156.5	196.4	42.70
KLHL26	95.0	91.0	138.0	98.5	99.5	159.0	113.5	28.07
KLHL28	352.0	390.0	509.0	369.0	403.5	503.0	421.1	68.12
KLHL29	197.5	288.0	251.0	183.0	337.0	271.0	254.6	57.57
KLHL3	52.0	50.5	67.0	55.0	54.0	51.0	54.9	6.17
KLHL30	51.0	118.0	112.0	36.5	97.5	78.5	82.3	33.10
KLHL31	11.5	44.5	216.5	4.0	49.0	118.5	74.0	80.73
KLHL32	0.0	3.5	0.5	0.0	1.5	1.0	1.1	1.32
KLHL33	197.5	342.0	194.5	211.5	355.5	201.0	250.3	76.57
KLHL34	9.0	102.5	34.5	13.0	147.5	45.5	58.7	55.00
KLHL35	0.0	0.0	0.0	0.0	0.0	0.0	0.0	0.00
KLHL36	173.5	62.0	308.5	207.5	70.0	311.5	188.8	109.69
KLHL38	2.0	5.0	17.0	1.5	5.0	8.5	6.5	5.73
KLHL4	1.5	0.0	0.5	0.0	0.0	0.5	0.4	0.58

KLHL40	407.5	1025.5	777.0	310.5	1135.0	758.5	735.7	326.85
KLHL41	924.0	825.0	1283.5	780.0	940.0	1037.5	965.0	180.46
KLHL5	638.0	312.0	341.0	626.0	362.0	337.5	436.1	152.63
KLHL6	1.0	0.0	0.0	0.0	0.0	1.0	0.3	0.52
KLHL7	339.5	287.0	394.5	351.0	311.5	333.5	336.2	36.52
KLHL8	125.5	89.0	93.5	110.0	98.5	68.0	97.4	19.49
KMO	1.5	4.0	1.0	0.0	8.0	3.0	2.9	2.87
KMT2A	219.0	160.0	363.0	195.5	177.5	384.5	249.9	98.13
KMT2C	664.0	602.5	1217.0	619.0	721.0	1303.0	854.4	318.00
KMT2E	606.0	629.5	1376.0	527.0	786.0	1366.0	881.8	388.19
KNDC1	2.5	0.0	0.0	0.0	0.0	0.0	0.4	1.02
KNG1	3.5	1.5	0.5	2.0	0.0	0.0	1.3	1.37
KNOP1	913.0	1056.5	860.5	783.0	1224.0	802.0	939.8	170.28
KNSTRN	536.0	256.0	238.0	484.5	220.0	181.5	319.3	150.81
KNTC1	356.0	102.0	344.0	383.0	100.0	182.5	244.6	131.56
KPNA2	2813.0	1277.5	1646.5	3399.5	1208.0	1039.5	1897.3	975.00
KPNA3	170.5	83.5	282.5	181.0	117.5	251.0	181.0	75.96
KPNA4	2507.5	2421.0	2167.0	2356.5	2588.5	1924.5	2327.5	244.24
KPNA5	69.5	20.0	65.0	80.5	18.5	45.5	49.8	26.26
KPNA6	873.5	1160.5	1206.0	858.0	1276.5	1177.0	1091.9	179.70
KPNA7	0.0	0.5	0.0	0.0	2.5	0.5	0.6	0.97
KPTN	75.5	57.5	68.5	56.5	51.0	60.5	61.6	8.91
KRAS	1675.5	1296.0	1578.0	1621.0	1504.0	1510.5	1530.8	132.34
KRBA1	2.0	0.0	0.5	2.0	0.5	0.5	0.9	0.86
KREMEN1	3897.0	2694.5	3947.0	3644.5	3036.5	3594.5	3469.0	499.02
KRIT1	869.0	686.5	1249.5	818.5	747.0	1086.0	909.4	215.78
KRR1	477.0	453.0	566.0	444.0	519.5	539.0	499.8	49.26
KRT18	0.0	0.5	0.0	0.0	0.0	0.0	0.1	0.20
KRT20	4.5	1.5	0.5	1.0	2.0	0.5	1.7	1.51
KRT222	11.0	6.0	7.5	4.5	5.0	8.0	7.0	2.39
KRT23	32.0	11.5	27.5	51.5	9.5	33.5	27.6	15.57
KRT8	1.5	0.0	1.0	0.5	0.5	1.0	0.8	0.52
KRT80	116.0	67.0	37.5	113.5	76.5	33.5	74.0	35.64
KRTCAP3	17.5	7.5	6.5	20.0	9.5	6.0	11.2	6.05
KSR1	669.0	408.5	672.0	717.0	524.0	614.0	600.8	115.13
KSR2	0.0	0.5	0.0	0.0	0.0	0.0	0.1	0.20
KTN1	1593.5	1091.5	1376.0	1552.5	1221.0	1290.0	1354.1	193.88
KXD1	460.5	450.5	266.5	444.5	467.5	257.0	391.1	100.54
KY	0.0	0.0	0.0	0.0	0.0	0.0	0.0	0.00
L2HGDH	457.0	220.0	182.5	355.0	244.5	178.0	272.8	110.83
L3HYPDH	220.0	155.5	62.5	191.0	144.0	74.0	141.2	62.63
L3MBTL1	100.0	55.5	76.0	89.0	72.5	76.5	78.3	15.15
L3MBTL2	439.0	332.0	407.0	454.0	341.5	386.0	393.3	49.91
LACC1	0.0	0.0	0.0	1.0	0.0	0.5	0.3	0.42
LACE1	88.5	85.5	113.0	95.5	107.5	88.0	96.3	11.41
LACTB	309.5	297.0	314.0	299.0	327.5	298.5	307.6	11.91
LACTB2	461.0	292.0	406.5	470.5	315.0	310.0	375.8	80.27
LACTBL1	0.0	0.0	1.5	0.5	0.0	0.0	0.3	0.61
LAD1	3.5	15.5	9.5	2.5	19.0	19.5	11.6	7.55
LAG3	0.0	0.0	0.0	0.0	0.0	0.0	0.0	0.00
LAMA1	156.0	67.0	140.5	216.0	74.5	133.0	131.2	55.19
LAMA3	104.0	55.0	112.0	136.0	79.0	133.5	103.3	31.56
LAMA4	1465.0	959.0	836.5	1448.0	1112.5	1312.5	1188.9	261.35
LAMA5	9856.0	6679.5	13901.5	10924.0	7605.5	16085.0	10841.9	3622.98

LAMB1	873.0	420.5	821.0	758.5	508.5	1370.5	792.0	335.13
LAMB2	7234.5	8916.0	6875.0	7173.5	8786.0	8812.5	7966.3	963.84
LAMB3	16.5	8.5	8.0	26.5	3.5	4.0	11.2	8.84
LAMC1	4538.5	2580.5	5891.0	4831.0	2917.0	7636.0	4732.3	1883.98
LAMC2	1369.5	410.5	169.0	1295.5	424.5	213.0	647.0	541.26
LAMC3	31.5	20.5	10.5	33.5	27.5	14.5	23.0	9.35
LAMP1	5257.5	4171.5	5086.0	5674.5	5021.0	5495.0	5117.6	524.90
LAMP2	354.0	158.0	579.0	457.5	182.5	483.5	369.1	170.04
LAMP3	2.0	0.0	1.0	1.0	1.5	0.5	1.0	0.71
LAMP5	498.5	250.0	652.5	581.5	257.5	628.0	478.0	181.49
LAMTOR1	353.0	508.0	440.5	334.5	492.5	438.0	427.8	70.97
LAMTOR2	991.0	1254.0	670.5	935.0	1237.5	677.5	960.9	256.37
LAMTOR3	854.0	987.5	829.5	785.5	1132.0	844.5	905.5	130.06
LAMTOR4	323.0	450.0	251.5	281.0	505.5	277.0	348.0	104.57
LAMTOR5	661.5	863.0	565.5	632.5	913.0	536.5	695.3	156.63
LANCL1	576.5	387.0	423.5	621.5	394.5	366.5	461.6	108.93
LANCL2	417.5	185.5	327.5	377.5	141.0	279.0	288.0	108.20
LANCL3	0.0	0.0	0.0	0.5	0.0	0.0	0.1	0.20
LAP3	1099.0	796.5	529.0	1125.5	899.0	486.5	822.6	273.43
LAPTM4A	1138.5	942.5	1454.5	1189.5	1065.0	1449.5	1206.6	207.45
LAPTM4B	37.0	24.0	9.5	45.5	21.5	15.0	25.4	13.54
LAPTM5	6.5	1.0	0.0	6.5	1.5	1.0	2.8	2.95
LARP1	282.0	147.5	573.0	344.5	161.0	479.0	331.2	170.66
LARP1B	416.0	228.5	286.5	394.0	268.0	270.5	310.6	75.91
LARP4	193.5	73.0	315.5	242.0	70.0	208.5	183.8	96.61
LARP4B	1267.0	1490.5	1675.0	1139.0	1677.0	1455.0	1450.6	216.35
LARP6	910.0	580.5	879.0	811.5	623.5	854.5	776.5	139.62
LARP7	427.0	354.0	367.0	450.0	422.0	350.0	395.0	43.05
LARS	1148.0	916.0	954.0	1172.5	955.5	825.0	995.2	136.60
LARS2	413.5	279.5	276.5	423.5	334.0	266.0	332.2	71.01
LASP1	5712.0	2655.0	3362.5	5630.0	2692.5	3327.5	3896.6	1407.19
LAT2	16.0	31.0	28.0	27.0	32.0	29.0	27.2	5.78
LATS1	367.0	170.0	420.5	363.5	188.0	359.5	311.4	105.11
LATS2	188.5	146.5	513.0	217.5	193.0	449.5	284.7	155.28
LBH	3798.5	849.0	2094.5	4422.5	820.0	2331.5	2386.0	1486.28
LBR	1995.0	774.5	1125.5	1969.5	796.5	891.5	1258.8	574.11
LBX1	31.5	35.5	44.0	34.5	23.0	44.5	35.5	8.08
LCA5	176.5	113.5	155.0	135.0	86.5	104.5	128.5	33.51
LCA5L	362.5	228.5	191.5	300.0	246.5	165.0	249.0	72.49
LCAT	182.5	72.0	20.5	102.5	65.0	23.0	77.6	60.09
LCK	2.0	2.0	0.5	0.0	1.0	5.5	1.8	1.97
LCMT1	339.0	273.5	197.5	313.0	266.5	198.5	264.7	58.02
LCMT2	381.5	446.5	357.5	327.5	476.0	352.5	390.3	58.36
LCOR	18.0	3.0	25.5	22.0	5.0	30.0	17.3	11.02
LCORL	189.0	111.0	209.5	192.0	124.0	180.5	167.7	40.20
LCP1	12.0	4.0	1.0	7.5	2.5	2.5	4.9	4.12
LCP2	0.0	0.0	0.0	0.0	0.0	0.0	0.0	0.00
LCT	184.5	246.0	165.5	216.5	286.5	132.0	205.2	56.15
LDB1	210.0	189.5	343.0	233.5	172.0	361.0	251.5	80.71
LDB2	69.5	32.0	36.0	90.0	34.5	19.5	46.9	26.88
LDB3	10.0	585.5	515.0	12.0	714.5	535.5	395.4	305.75
LDHB	8371.0	5778.0	4598.0	7779.5	5736.5	3938.5	6033.6	1738.71
LDHC	1435.0	970.5	2846.5	1540.0	1343.5	2664.0	1799.9	766.67
LDHD	43.5	60.5	70.5	52.0	67.5	90.0	64.0	16.17

LDLRAD1	330.0	249.5	190.0	307.0	251.0	191.0	253.1	57.75
LDLRAD3	16.0	1.5	15.0	12.0	4.5	12.0	10.2	5.85
LDLRAD4	369.0	452.5	566.5	360.0	526.0	527.5	466.9	87.53
LDLRAP1	1145.5	703.0	756.0	954.0	769.0	677.5	834.2	180.77
LECT1	93.0	103.0	349.0	75.5	99.0	195.5	152.5	105.11
LECT2	46.5	15.0	6.0	32.5	8.0	3.5	18.6	17.23
LEF1	0.0	6.0	22.5	0.5	9.5	37.0	12.6	14.50
LEFTY1	17.0	4.0	6.0	12.0	10.0	6.5	9.3	4.77
LEKR1	133.5	169.5	310.5	155.5	175.0	354.5	216.4	92.11
LEMD2	1148.0	902.0	1204.0	1153.5	1029.5	1177.0	1102.3	114.94
LEMD3	513.0	217.5	398.5	542.5	247.5	355.5	379.1	133.41
LENG8	34.0	17.0	26.0	24.0	13.0	38.0	25.3	9.58
LEO1	429.0	387.5	527.5	421.5	449.0	505.5	453.3	53.25
LEPR	27.0	15.5	16.5	25.5	13.0	19.0	19.4	5.65
LEPREL2	605.0	423.0	366.5	564.5	485.0	394.5	473.1	95.86
LEPROT	1897.5	1221.0	2252.0	1840.0	1438.0	2283.0	1821.9	427.11
LEPROTL1	942.0	752.5	876.5	938.5	851.5	878.0	873.2	69.38
LETM1	406.5	212.5	451.5	366.5	252.0	334.0	337.2	91.15
LETM2	143.0	88.5	85.5	132.0	96.5	71.5	102.8	28.26
LETMD1	10.5	5.5	22.0	12.5	5.0	17.5	12.2	6.69
LFNG	493.5	529.0	997.0	508.5	491.5	794.5	635.7	211.82
LGALS1	6205.5	9160.0	5919.5	6653.0	10058.0	6152.5	7358.1	1782.41
LGALS2	2.5	2.5	2.0	5.0	8.5	2.5	3.8	2.52
LGALS3	1967.5	1923.5	1572.0	1825.0	2394.5	1712.0	1899.1	282.20
LGALS8	1312.0	1027.0	1198.5	1393.5	1303.0	1316.5	1258.4	129.32
LGALSL	122.5	57.5	48.5	123.0	65.5	52.0	78.2	35.01
LGI1	23.0	11.5	11.5	17.0	7.0	7.5	12.9	6.11
LGI2	211.5	166.5	203.5	244.5	203.5	231.0	210.1	26.87
LGI3	6.0	13.5	6.0	3.0	8.5	11.5	8.1	3.89
LGMN	1729.0	1169.0	1681.5	2107.0	1428.5	2061.5	1696.1	361.41
LGR4	204.0	98.0	98.5	212.0	130.5	100.0	140.5	53.77
LGR5	730.0	498.0	736.0	823.0	629.0	407.0	637.2	158.32
LGR6	0.0	0.5	0.5	0.0	0.5	0.0	0.3	0.27
LGSN	0.0	0.0	0.0	0.0	0.0	0.0	0.0	0.00
LH-BETA	12.5	35.0	14.5	10.5	36.5	17.0	21.0	11.64
LHCGR	0.0	0.0	0.0	0.0	0.0	0.0	0.0	0.00
LHFP	1166.5	347.0	625.0	841.0	319.0	306.0	600.8	349.01
LHFPL2	65.0	29.5	104.5	69.0	26.5	66.5	60.2	28.92
LHFPL3	7.0	20.5	62.0	12.5	24.5	84.5	35.2	30.95
LHFPL4	17.0	12.5	17.5	18.5	10.5	18.5	15.8	3.40
LHFPL5	15.0	4.0	11.0	14.5	5.0	11.0	10.1	4.65
LHPP	46.0	46.5	51.5	51.0	51.5	43.0	48.3	3.59
LHX1	0.0	0.0	0.0	0.5	0.0	0.0	0.1	0.20
LHX2	25.5	5.5	7.5	9.5	5.0	4.5	9.6	8.02
LHX3	0.0	0.0	0.0	0.0	0.0	0.0	0.0	0.00
LHX4	1.5	0.0	0.0	0.0	0.0	0.0	0.3	0.61
LHX5	0.5	0.0	0.0	0.5	0.0	0.0	0.2	0.26
LHX6	8.5	5.5	2.0	7.0	6.0	2.0	5.2	2.66
LHX8	0.5	0.0	0.5	0.5	0.0	0.0	0.3	0.27
LHX9	0.5	0.0	0.0	0.0	0.0	0.0	0.1	0.20
LIAS	840.5	674.5	620.5	725.0	738.5	555.0	692.3	99.49
LIF	6.5	22.5	63.5	14.5	19.5	50.5	29.5	22.36
LIFR	374.5	143.0	186.0	351.0	169.0	191.5	235.8	100.03
LIG1	428.5	482.0	456.0	376.0	413.5	410.0	427.7	37.25

LIG3	993.0	851.5	849.5	966.5	866.0	816.5	890.5	71.50
LIG4	142.0	129.0	217.5	142.0	138.5	208.5	162.9	39.19
LIM2	16.5	56.0	46.5	11.5	37.5	49.0	36.2	18.23
LIMA1	3319.5	2455.0	2657.0	3402.5	2722.0	2485.0	2840.2	416.65
LIMCH1	7.5	187.5	566.0	6.0	208.0	357.5	222.1	214.82
LIMD1	2355.0	2773.5	2971.5	2411.0	3130.0	3261.0	2817.0	373.94
LIMD2	1.0	1.0	0.5	1.5	0.0	0.0	0.7	0.61
LIME1	4.0	10.5	6.5	5.0	6.0	6.5	6.4	2.22
LIMK1	56.5	67.5	55.5	56.0	59.0	60.5	59.2	4.51
LIMK2	289.5	216.5	322.5	308.5	269.5	346.5	292.2	45.57
LIMS1	778.5	503.5	564.0	782.0	532.5	509.5	611.7	132.31
LIN28A	0.0	0.0	0.5	0.0	0.0	0.0	0.1	0.20
LIN28B	0.5	0.5	0.5	0.0	0.5	1.0	0.5	0.32
LIN52	217.5	183.5	242.0	231.0	202.0	215.0	215.2	20.75
LIN54	132.0	41.0	101.0	118.5	35.5	79.0	84.5	40.02
LIN7A	203.5	160.5	187.5	220.0	185.0	213.5	195.0	21.84
LIN7C	702.0	457.5	804.5	607.0	468.5	653.0	615.4	135.10
LIN9	153.5	71.5	123.0	169.0	86.0	95.5	116.4	38.89
LINGO1	79.0	88.5	163.5	63.0	75.5	119.0	98.1	37.19
LINGO2	8.0	3.5	3.5	5.0	2.5	1.5	4.0	2.28
LINGO3	0.0	0.5	1.0	0.0	1.0	1.0	0.6	0.49
LINS	50.5	40.0	45.0	31.5	32.0	26.0	37.5	9.27
LIPA	3122.5	1285.5	1945.0	3315.5	1598.0	1634.5	2150.2	856.03
LIPC	2.5	1.0	1.0	1.5	1.0	0.0	1.2	0.82
LIPG	45.0	58.5	77.5	41.5	36.5	50.5	51.6	14.79
LIPH	5.5	7.0	13.0	1.0	7.0	16.5	8.3	5.55
LIPI	0.0	0.0	0.0	0.0	0.0	0.0	0.0	0.00
LIPT1	150.0	128.5	106.0	129.5	128.5	116.5	126.5	14.76
LITAF	24.5	22.5	36.0	28.5	38.5	29.5	29.9	6.28
LIX1	24.0	7.0	24.0	37.5	10.5	16.5	19.9	11.04
LLGL1	685.5	491.5	495.0	608.0	450.0	507.0	539.5	88.72
LLGL2	200.5	107.0	110.5	202.5	118.5	101.5	140.1	47.90
LLPH	446.0	735.5	412.0	437.0	798.5	437.0	544.3	173.99
LMAN1	2543.0	1429.5	1499.5	2757.0	1629.5	1492.0	1891.8	594.79
LMAN2	1603.5	1215.5	1113.0	1620.0	1361.0	1264.5	1362.9	208.74
LMAN2L	1044.0	950.5	772.0	956.5	971.0	733.5	904.6	122.88
LMBR1	146.5	55.0	132.0	159.0	69.5	123.5	114.3	42.32
LMBR1L	11.0	10.0	14.5	11.5	8.0	8.5	10.6	2.35
LMBRD1	433.5	327.5	517.5	389.0	381.5	378.0	404.5	64.82
LMBRD2	447.5	278.0	450.5	445.0	307.0	380.5	384.8	76.62
LMCD1	5571.0	6491.0	3509.5	6699.0	7898.0	4229.0	5732.9	1638.73
LMF1	270.0	190.0	239.0	272.5	193.0	208.0	228.8	37.23
LMF2	4941.5	3527.0	4173.5	5222.5	3306.0	4345.5	4252.7	755.34
LMLN	253.5	119.5	147.0	211.5	119.0	121.5	162.0	57.17
LMNB2	4050.5	2906.5	3927.5	4136.0	2655.0	3085.5	3460.2	650.98
LMO2	275.5	105.5	74.5	191.5	91.5	48.5	131.2	85.73
LMO3	0.0	0.5	0.0	1.0	0.0	0.5	0.3	0.41
LMO4	1551.5	775.5	1087.5	1235.5	734.5	866.5	1041.8	314.80
LMO7	2053.5	3201.5	4753.5	1736.5	3591.5	3490.0	3137.8	1103.07
LMOD1	14.0	10.5	17.0	15.0	8.5	20.5	14.3	4.34
LMOD2	134.0	2291.0	1889.5	118.0	2645.0	1956.5	1505.7	1102.04
LMOD3	3.0	81.0	73.5	2.0	84.0	85.5	54.8	40.75
LMTK2	649.0	399.0	751.0	680.5	518.0	675.5	612.2	129.32
LMX1A	0.0	0.0	0.5	0.0	0.0	0.0	0.1	0.20

LMX1B	1.0	0.0	1.0	2.0	1.0	0.5	0.9	0.66
LNP1	1.0	0.5	0.5	3.0	1.0	0.0	1.0	1.05
LNPEP	63.0	8.0	80.5	65.5	11.5	55.5	47.3	30.24
LNX1	0.5	0.5	0.0	1.5	1.5	0.5	0.8	0.61
LNX2	215.0	214.0	367.0	180.5	215.5	347.0	256.5	79.23
LOC100126556	1.0	0.0	0.0	0.5	0.0	0.0	0.3	0.42
LOC100303663	42.0	275.5	139.0	32.0	246.0	99.5	139.0	102.47
LOC100303669	0.0	0.0	0.0	0.0	0.0	0.0	0.0	0.00
LOC100303670	17.5	12.5	2.5	12.5	8.0	3.5	9.4	5.82
LOC100303673	3640.0	12766.0	6790.0	3382.0	14710.5	7017.5	8051.0	4700.96
LOC100303679	0.0	0.0	0.0	0.0	0.0	0.0	0.0	0.00
LOC100303681	39.5	54.5	21.5	28.5	45.0	18.0	34.5	14.23
LOC100303683	0.0	0.0	0.0	0.0	0.0	0.0	0.0	0.00
LOC100303687	0.0	0.0	0.0	0.0	0.0	0.0	0.0	0.00
LOC100303688	0.0	0.0	0.0	0.0	0.0	0.5	0.1	0.20
LOC100303695	13.5	9.5	10.5	12.5	15.0	12.0	12.2	1.99
LOC100303701	5.0	1.0	5.5	3.5	1.5	4.0	3.4	1.83
LOC100303705	0.5	0.0	0.0	0.0	0.0	0.0	0.1	0.20
LOC100303707	0.0	0.0	0.0	0.5	0.0	0.5	0.2	0.26
LOC100303709	2.0	6.5	32.5	0.5	6.5	11.0	9.8	11.71
LOC100538357	1047.0	1143.0	900.0	973.5	1315.0	923.0	1050.3	157.08
LOC100538358	117.0	114.5	114.5	133.5	122.0	102.0	117.3	10.34
LOC100538367	0.0	0.0	0.5	0.0	0.5	2.5	0.6	0.97
LOC100538371	447.5	393.5	414.0	458.5	476.5	427.5	436.3	30.47
LOC100538374	0.0	0.5	0.0	0.0	0.0	0.0	0.1	0.20
LOC100538375	1.0	1.0	3.5	1.0	4.5	4.5	2.6	1.77
LOC100538380	0.5	2.0	1.0	0.0	4.5	2.5	1.8	1.64
LOC100538403	0.0	0.0	0.0	0.0	0.0	0.0	0.0	0.00
LOC100538406	4.0	2.5	0.0	7.5	2.0	1.0	2.8	2.66
LOC100538407	0.0	0.0	0.0	0.5	0.5	0.0	0.2	0.26
LOC100538412	3.0	1.5	2.5	0.5	3.0	3.0	2.3	1.04
LOC100538416	32.5	8.5	10.0	36.5	10.5	13.5	18.6	12.50
LOC100538424	0.0	0.0	0.0	0.0	0.0	0.0	0.0	0.00
LOC100538433	8.5	1.5	4.5	7.5	3.0	4.0	4.8	2.68
LOC100538434	2.5	3.5	16.5	2.5	5.5	13.5	7.3	6.11
LOC100538436	0.0	0.0	0.0	0.0	1.0	0.0	0.2	0.41
LOC100538440	825.0	992.0	890.0	804.5	1148.5	1090.5	958.4	142.04
LOC100538445	2.5	0.0	1.0	1.0	0.0	0.5	0.8	0.93
LOC100538446	9.0	3.5	9.0	9.5	2.5	6.0	6.6	3.06
LOC100538447	2.5	1.5	7.5	3.5	2.5	3.5	3.5	2.10
LOC100538449	5.5	2.0	2.5	0.5	0.0	0.0	1.8	2.12
LOC100538453	1.0	0.0	2.0	0.0	0.5	0.0	0.6	0.80
LOC100538454	4.5	34.0	10.5	10.5	38.0	9.0	17.8	14.36
LOC100538455	232.5	194.5	252.5	196.5	146.0	172.5	199.1	38.79
LOC100538468	132.0	105.5	123.5	132.0	133.5	119.0	124.3	10.82
LOC100538484	47.5	9.0	64.0	39.5	10.5	44.5	35.8	21.82
LOC100538487	366.5	181.0	320.5	383.0	208.0	335.0	299.0	84.35
LOC100538504	29.0	21.0	21.0	19.5	12.5	16.5	19.9	5.51
LOC100538505	0.0	0.0	0.0	0.0	0.0	0.0	0.0	0.00
LOC100538506	1.0	1.0	2.0	1.0	3.0	2.0	1.7	0.82
LOC100538510	27.5	9.0	21.0	40.5	16.0	14.0	21.3	11.31
LOC100538523	0.0	0.0	1.0	0.0	0.5	0.5	0.3	0.41
LOC100538525	132.5	73.5	60.5	110.5	80.5	37.5	82.5	34.29
LOC100538528	0.0	0.5	0.0	0.0	0.0	0.0	0.1	0.20

LOC100538538	3.0	8.5	6.5	4.5	3.5	3.0	4.8	2.23
LOC100538539	1585.5	1673.0	1282.0	1528.5	1719.5	1182.5	1495.2	216.53
LOC100538554	7.5	31.5	14.0	6.0	32.5	16.0	17.9	11.55
LOC100538555	128.0	190.0	177.0	146.5	233.0	184.0	176.4	36.56
LOC100538558	377.5	659.5	442.5	356.0	687.5	432.0	492.5	144.17
LOC100538559	11.0	6.5	3.5	15.0	2.5	2.0	6.8	5.24
LOC100538560	2379.5	1727.5	2141.0	1847.5	1857.5	2091.0	2007.3	240.70
LOC100538570	0.0	0.0	0.0	0.0	0.0	0.0	0.0	0.00
LOC100538580	3972.5	2140.0	3770.5	3576.5	2116.0	2924.0	3083.3	819.39
LOC100538582	1.0	1.0	0.5	1.5	2.0	0.0	1.0	0.71
LOC100538586	506.0	387.5	625.0	497.0	428.5	518.5	493.8	81.91
LOC100538587	139.0	137.0	213.5	115.0	157.5	165.5	154.6	33.82
LOC100538588	0.5	10.0	6.0	0.5	7.0	4.5	4.8	3.75
LOC100538591	0.0	0.0	0.0	0.0	0.0	0.0	0.0	0.00
LOC100538595	3219.0	1564.0	1389.0	2750.5	1803.5	1096.0	1970.3	832.25
LOC100538605	37.0	67.5	56.0	44.0	77.5	60.5	57.1	14.92
LOC100538613	0.0	0.0	0.0	0.0	0.0	0.0	0.0	0.00
LOC100538614	1.5	1.5	2.0	0.5	1.5	1.5	1.4	0.49
LOC100538629	2623.5	2491.5	3189.5	2613.0	2781.0	2775.5	2745.7	243.41
LOC100538632	46.0	30.0	20.5	38.0	28.0	19.0	30.3	10.34
LOC100538652	0.0	0.0	0.0	0.0	0.0	0.0	0.0	0.00
LOC100538662	5.0	3.0	1.0	4.5	5.0	2.0	3.4	1.69
LOC100538666	118.5	46.5	70.0	113.5	38.0	45.5	72.0	35.77
LOC100538679	70.0	63.5	43.5	67.0	58.5	30.5	55.5	15.39
LOC100538692	54.0	25.5	97.0	49.5	25.0	97.5	58.1	32.60
LOC100538693	130.5	79.5	118.0	115.0	100.5	96.0	106.6	18.20
LOC100538694	3487.5	3036.0	2056.0	3084.5	3248.0	1910.0	2803.7	656.59
LOC100538708	813.5	532.0	865.0	774.0	580.0	801.0	727.6	137.00
LOC100538712	0.0	0.5	0.0	0.0	0.5	0.0	0.2	0.26
LOC100538719	306.5	112.5	575.0	366.5	143.0	419.5	320.5	174.16
LOC100538725	1.0	2.5	3.0	1.5	3.0	8.5	3.3	2.70
LOC100538736	0.5	0.0	0.5	0.0	0.0	0.0	0.2	0.26
LOC100538756	68.0	22.0	21.5	54.0	17.5	11.5	32.4	22.89
LOC100538764	1.0	1.5	2.5	0.0	1.5	3.5	1.7	1.21
LOC100538773	4.0	1.5	16.0	5.5	4.0	5.0	6.0	5.09
LOC100538779	314.0	199.0	296.0	288.5	220.5	242.0	260.0	46.11
LOC100538780	248.0	100.0	187.5	266.5	97.0	173.0	178.7	71.40
LOC100538782	6.0	3.0	16.5	10.0	3.0	10.0	8.1	5.18
LOC100538789	0.5	0.0	0.0	0.0	0.0	0.0	0.1	0.20
LOC100538796	979.5	1147.5	1409.0	964.0	1322.5	1456.0	1213.1	214.60
LOC100538805	1173.0	1309.0	1007.5	1137.5	1396.5	998.5	1170.3	159.69
LOC100538808	11.5	18.5	28.0	15.0	16.0	22.0	18.5	5.83
LOC100538811	6.5	1.0	3.5	3.5	0.5	12.0	4.5	4.25
LOC100538813	487.0	442.5	569.0	456.0	468.5	577.0	500.0	58.47
LOC100538821	57.5	38.5	60.5	42.5	54.0	70.0	53.8	11.69
LOC100538825	17.0	5.0	18.0	19.5	3.5	16.0	13.2	7.02
LOC100538829	0.5	1.0	0.5	2.0	0.0	2.0	1.0	0.84
LOC100538839	508.0	345.5	630.5	528.0	349.5	517.5	479.8	111.59
LOC100538841	23.0	31.5	81.5	34.0	55.0	200.5	70.9	66.89
LOC100538845	334.0	200.0	217.5	328.0	245.0	226.0	258.4	58.09
LOC100538849	926.5	734.0	579.0	897.5	827.5	589.0	758.9	150.90
LOC100538852	274.5	214.0	202.0	214.0	236.0	154.5	215.8	39.52
LOC100538860	307.0	292.5	432.5	348.0	250.5	357.0	331.3	62.94
LOC100538862	16.0	13.0	7.0	20.5	22.0	19.5	16.3	5.62

LOC100538867	28.0	13.0	30.5	24.0	19.0	43.0	26.3	10.34
LOC100538868	2.0	0.5	1.5	1.0	2.0	1.5	1.4	0.58
LOC100538873	31.5	5.5	13.5	40.0	3.0	9.5	17.2	15.07
LOC100538883	155.0	67.5	234.0	129.0	69.0	205.0	143.3	68.76
LOC100538884	0.0	0.0	0.0	0.0	0.0	0.0	0.0	0.00
LOC100538887	0.0	0.0	0.0	0.0	0.0	0.0	0.0	0.00
LOC100538893	1371.5	2136.0	710.5	1285.0	2210.5	765.0	1413.1	646.57
LOC100538897	90.0	8.0	12.5	76.5	5.5	11.5	34.0	38.47
LOC100538901	2.0	4.0	4.5	3.5	1.0	2.0	2.8	1.37
LOC100538902	1584.5	1889.5	722.0	1255.5	1873.0	878.0	1367.1	498.78
LOC100538920	1631.5	1738.0	1125.5	1552.5	1863.0	1004.0	1485.8	344.53
LOC100538921	4101.5	2992.0	3272.0	3635.5	2637.0	3066.0	3284.0	518.34
LOC100538926	0.0	0.5	0.0	0.5	0.5	0.0	0.3	0.27
LOC100538928	0.0	0.0	0.5	0.0	0.0	0.5	0.2	0.26
LOC100538933	32.5	20.5	36.5	25.5	27.0	34.5	29.4	6.10
LOC100538944	33.0	11.5	31.5	34.5	16.0	25.0	25.3	9.59
LOC100538956	4.5	6.5	6.5	4.5	7.0	4.0	5.5	1.30
LOC100538957	6.5	5.0	1.0	8.0	3.5	3.0	4.5	2.53
LOC100538959	1751.5	1943.5	2268.0	1644.0	2288.5	1974.0	1978.3	262.52
LOC100538963	26.0	19.5	19.5	22.0	11.0	18.0	19.3	4.96
LOC100538965	1.0	0.5	4.0	3.5	1.0	1.0	1.8	1.51
LOC100538966	1265.5	756.0	1501.5	1165.5	832.5	1220.0	1123.5	280.66
LOC100538971	1958.5	3139.5	1482.0	1673.0	3517.0	1159.5	2154.9	952.80
LOC100538974	395.5	342.5	355.5	459.5	366.5	299.5	369.8	54.05
LOC100538975	19.5	17.5	10.5	17.5	17.5	10.5	15.5	3.95
LOC100538976	14.5	15.5	7.5	11.0	28.0	19.5	16.0	7.16
LOC100538984	0.0	0.0	0.0	0.0	0.0	0.0	0.0	0.00
LOC100538986	0.0	0.0	0.0	0.0	0.0	0.0	0.0	0.00
LOC100539001	176.0	181.0	160.0	168.5	243.5	186.0	185.8	29.71
LOC100539002	384.0	515.0	761.0	387.0	589.0	759.5	565.9	169.57
LOC100539006	15.0	4.5	0.5	14.5	8.5	0.5	7.3	6.52
LOC100539008	7.0	3.5	2.5	13.5	6.5	1.5	5.8	4.38
LOC100539010	202.5	201.5	105.0	201.0	222.5	124.0	176.1	48.75
LOC100539020	0.0	0.0	0.0	0.0	0.0	0.0	0.0	0.00
LOC100539021	0.5	1.0	0.0	0.0	1.0	0.5	0.5	0.45
LOC100539027	783.0	630.0	822.0	754.0	772.5	854.0	769.3	77.19
LOC100539032	3.0	0.5	0.5	3.5	1.0	1.5	1.7	1.29
LOC100539035	1.0	0.0	1.0	0.5	0.5	0.5	0.6	0.38
LOC100539037	0.0	0.0	0.0	0.0	0.0	0.0	0.0	0.00
LOC100539040	1.5	0.0	0.0	0.5	0.0	0.0	0.3	0.61
LOC100539041	0.0	0.0	0.0	0.0	0.0	0.0	0.0	0.00
LOC100539048	6.0	75.5	41.5	4.0	71.0	30.5	38.1	30.80
LOC100539056	0.0	0.0	0.0	1.0	1.0	0.0	0.3	0.52
LOC100539065	11.5	5.5	6.0	13.0	6.0	8.5	8.4	3.18
LOC100539073	0.0	0.0	0.0	0.0	0.0	0.0	0.0	0.00
LOC100539075	213.0	129.5	154.5	200.0	128.0	183.5	168.1	36.20
LOC100539082	10.5	21.0	9.5	12.0	18.0	9.5	13.4	4.89
LOC100539085	140.0	51.0	130.5	152.5	47.0	73.0	99.0	47.37
LOC100539092	1.0	0.0	0.5	0.0	0.0	0.0	0.3	0.42
LOC100539100	358.0	66.5	33.0	285.5	77.0	36.0	142.7	141.62
LOC100539104	252.0	115.5	371.0	296.5	141.5	302.0	246.4	99.29
LOC100539108	0.5	0.0	0.0	0.0	0.5	0.5	0.3	0.27
LOC100539112	3042.5	3204.5	3301.0	2786.5	3410.0	4234.0	3329.8	493.61
LOC100539124	15.0	20.0	15.5	10.5	22.0	15.0	16.3	4.09

LOC100539126	2.0	3.0	0.5	2.0	0.5	0.5	1.4	1.07
LOC100539130	53.5	13.5	3.0	33.0	17.0	5.0	20.8	19.25
LOC100539136	0.5	0.0	0.0	1.0	0.0	0.5	0.3	0.41
LOC100539139	32.0	18.0	42.0	37.0	18.5	32.5	30.0	9.79
LOC100539142	500.0	290.0	335.5	406.5	329.5	388.0	374.9	74.35
LOC100539152	1057.0	862.0	1383.5	1166.0	914.0	1489.5	1145.3	251.87
LOC100539157	47.0	17.0	38.0	47.0	27.0	42.5	36.4	12.08
LOC100539161	25.5	29.5	45.5	45.5	39.0	49.5	39.1	9.67
LOC100539169	856.0	674.5	901.0	798.5	842.0	1031.0	850.5	117.40
LOC100539172	353.5	271.0	383.5	309.5	256.5	341.5	319.3	49.31
LOC100539174	0.0	0.0	0.5	0.0	0.5	0.0	0.2	0.26
LOC100539176	0.0	0.0	0.0	0.0	0.0	0.0	0.0	0.00
LOC100539183	57.0	102.5	794.0	148.5	107.5	750.0	326.6	346.51
LOC100539194	1.0	0.5	0.5	0.5	0.0	0.0	0.4	0.38
LOC100539204	19.5	33.5	33.5	14.0	34.0	37.5	28.7	9.51
LOC100539216	1233.5	619.0	1041.5	1010.0	656.5	1049.0	934.9	243.51
LOC100539227	2.5	0.5	0.0	1.5	0.0	0.0	0.8	1.04
LOC100539228	0.0	0.0	0.0	0.0	0.0	0.0	0.0	0.00
LOC100539236	5.5	1.0	2.5	7.0	1.0	1.5	3.1	2.56
LOC100539239	0.0	0.0	0.5	0.0	0.0	0.5	0.2	0.26
LOC100539246	681.0	638.0	595.5	613.0	650.5	586.5	627.4	35.82
LOC100539247	411.0	313.5	221.0	385.5	310.5	211.5	308.8	81.89
LOC100539248	13.0	2.5	2.0	7.5	1.5	1.5	4.7	4.68
LOC100539273	0.0	0.0	0.0	0.0	0.0	0.0	0.0	0.00
LOC100539278	81.5	151.0	206.5	81.5	172.0	163.0	142.6	50.80
LOC100539280	1905.0	1975.0	1188.0	1697.5	2177.5	1148.5	1681.9	426.61
LOC100539289	12.0	6.0	25.5	14.5	5.5	14.5	13.0	7.31
LOC100539290	197.0	68.5	233.0	212.5	78.0	176.0	160.8	70.43
LOC100539298	60.5	22.5	71.5	76.0	34.5	52.5	52.9	20.98
LOC100539302	423.5	1519.0	883.0	480.5	1472.5	1269.0	1007.9	485.91
LOC100539311	335.0	285.5	199.0	317.5	330.0	202.0	278.2	62.59
LOC100539329	4.0	5.0	7.5	5.0	2.0	6.5	5.0	1.92
LOC100539332	50.0	39.5	34.0	40.5	53.0	28.5	40.9	9.30
LOC100539335	189.0	42.0	118.0	194.5	34.0	72.5	108.3	71.04
LOC100539357	142.5	75.5	171.0	126.5	77.5	139.5	122.1	38.18
LOC100539361	2.0	1.5	7.0	0.5	1.0	1.0	2.2	2.42
LOC100539364	221.5	143.5	91.0	231.5	168.5	105.0	160.2	58.35
LOC100539365	195.0	230.0	155.0	187.0	260.5	154.0	196.9	42.04
LOC100539368	0.0	0.0	0.0	0.0	0.0	0.0	0.0	0.00
LOC100539376	815.0	687.5	779.0	820.0	920.5	901.0	820.5	84.76
LOC100539381	0.5	1.0	0.0	0.0	0.5	0.0	0.3	0.41
LOC100539382	985.5	2341.5	1887.0	909.0	2654.0	1789.0	1761.0	704.04
LOC100539384	37.5	55.0	35.5	35.5	55.0	37.0	42.6	9.65
LOC100539388	5.0	3.0	1.0	9.0	3.0	2.0	3.8	2.86
LOC100539391	16.5	14.5	14.0	20.0	9.0	17.0	15.2	3.70
LOC100539397	0.0	0.0	0.0	0.0	0.0	0.5	0.1	0.20
LOC100539427	41.0	18.0	97.5	54.5	33.5	70.5	52.5	28.41
LOC100539435	2.0	2.0	3.5	2.0	4.0	4.0	2.9	1.02
LOC100539441	12.0	38.5	35.5	9.0	42.0	47.5	30.8	16.21
LOC100539445	35.0	5.5	44.5	34.5	6.0	49.5	29.2	19.01
LOC100539447	17.0	18.5	27.5	8.5	22.0	27.5	20.2	7.21
LOC100539460	167.0	127.0	99.0	150.5	130.0	111.5	130.8	24.89
LOC100539461	0.0	0.0	0.0	0.0	0.5	0.0	0.1	0.20
LOC100539476	0.0	0.0	0.0	0.0	0.0	0.0	0.0	0.00

LOC100539477	565.0	496.5	387.5	509.0	497.5	368.5	470.7	76.30
LOC100539483	37.0	66.0	54.0	36.5	83.0	55.0	55.3	17.73
LOC100539484	1553.0	1635.0	1678.0	1752.0	1778.5	1549.5	1657.7	97.04
LOC100539486	6.0	2.5	11.5	4.5	2.0	20.0	7.8	6.90
LOC100539487	304.0	299.5	141.0	230.5	294.5	153.0	237.1	74.85
LOC100539489	0.5	0.0	0.0	0.0	0.0	0.0	0.1	0.20
LOC100539493	142.0	71.0	84.0	145.5	71.0	76.5	98.3	35.52
LOC100539497	0.5	0.0	0.0	0.0	0.0	0.0	0.1	0.20
LOC100539498	53.5	21.5	49.0	53.0	22.0	34.0	38.8	15.01
LOC100539521	0.0	0.5	0.0	0.0	1.5	0.0	0.3	0.61
LOC100539530	1046.5	967.5	1034.0	941.0	1196.5	1126.0	1051.9	96.10
LOC100539535	12.5	17.0	5.5	11.5	18.5	7.0	12.0	5.20
LOC100539550	213.5	151.5	420.5	282.5	183.5	420.5	278.7	118.09
LOC100539553	382.5	235.5	1297.0	362.0	301.5	1023.0	600.3	445.12
LOC100539565	237.5	148.5	468.0	233.0	174.0	369.5	271.8	122.87
LOC100539575	6.5	3.5	60.5	16.5	6.0	70.0	27.2	29.98
LOC100539580	0.0	0.0	0.0	0.0	0.0	0.0	0.0	0.00
LOC100539591	96.0	69.5	58.0	105.0	66.0	50.0	74.1	21.73
LOC100539600	0.5	0.5	0.0	1.0	1.0	0.0	0.5	0.45
LOC100539601	603.0	527.5	729.5	583.5	642.0	743.0	638.1	84.64
LOC100539603	2.0	2.0	2.5	1.0	0.0	2.0	1.6	0.92
LOC100539604	0.0	0.0	0.0	0.0	0.0	0.0	0.0	0.00
LOC100539605	77.0	71.0	49.5	141.5	91.5	91.5	87.0	30.90
LOC100539619	283.0	183.0	341.0	303.0	218.0	337.5	277.6	64.47
LOC100539623	2147.0	1962.5	1725.5	1712.0	2061.0	1586.5	1865.8	222.65
LOC100539630	799.0	545.5	1042.0	730.0	622.0	932.0	778.4	186.84
LOC100539632	210.5	628.0	418.5	171.5	752.0	431.5	435.3	227.09
LOC100539633	0.5	3.0	0.0	1.5	0.5	0.5	1.0	1.10
LOC100539637	2.0	1.5	3.5	5.0	2.5	3.5	3.0	1.26
LOC100539639	13.0	7.5	6.5	13.0	9.0	10.0	9.8	2.73
LOC100539640	131.5	49.5	119.5	139.0	56.0	107.5	100.5	38.56
LOC100539641	817.5	574.5	494.0	823.5	671.0	483.0	643.9	152.51
LOC100539643	1.0	0.5	0.5	2.5	1.0	0.0	0.9	0.86
LOC100539644	0.0	0.5	0.5	0.0	0.0	0.0	0.2	0.26
LOC100539646	6.5	1.0	0.5	4.5	0.0	1.5	2.3	2.58
LOC100539648	8.5	6.5	9.0	7.0	4.5	2.5	6.3	2.46
LOC100539649	0.0	0.0	0.0	0.0	0.0	0.0	0.0	0.00
LOC100539654	77.5	31.0	100.0	109.0	46.5	85.5	74.9	30.48
LOC100539660	1.5	1.0	1.0	1.0	0.5	4.5	1.6	1.46
LOC100539663	256.5	84.0	121.5	266.0	82.0	104.0	152.3	85.64
LOC100539666	2.0	0.0	3.5	2.5	0.5	1.0	1.6	1.32
LOC100539678	0.0	0.0	0.0	0.0	0.0	0.0	0.0	0.00
LOC100539680	0.0	0.0	0.0	0.5	0.0	0.0	0.1	0.20
LOC100539684	4.0	18.5	20.5	5.5	16.0	24.5	14.8	8.30
LOC100539689	3569.5	2641.5	4625.5	3604.0	2530.0	3955.0	3487.6	795.79
LOC100539697	1.0	1.5	1.0	8.5	3.5	9.0	4.1	3.73
LOC100539699	0.0	0.0	1.0	0.0	0.5	0.0	0.3	0.42
LOC100539703	36.5	24.5	26.5	34.0	23.5	24.0	28.2	5.64
LOC100539718	19.0	10.0	3.5	9.5	8.5	5.0	9.3	5.43
LOC100539719	285.5	190.5	256.0	263.5	237.0	251.5	247.3	32.09
LOC100539727	1727.5	632.5	759.0	1190.0	619.0	581.0	918.2	455.86
LOC100539729	19.5	16.0	8.5	10.0	10.0	8.5	12.1	4.58
LOC100539736	3.5	4.0	5.5	4.5	6.5	9.5	5.6	2.20
LOC100539738	43.5	48.0	22.0	47.5	58.0	30.5	41.6	13.08

LOC100539745	138.0	46.5	110.0	165.0	45.5	92.5	99.6	48.28
LOC100539746	17.5	8.5	18.5	19.5	15.0	9.0	14.7	4.82
LOC100539753	11.0	6.5	46.0	10.5	7.5	22.5	17.3	15.16
LOC100539757	0.0	0.5	0.0	0.0	0.0	0.0	0.1	0.20
LOC100539767	2.5	1.5	10.5	0.0	0.5	7.0	3.7	4.18
LOC100539772	580.5	698.0	487.0	544.5	647.5	501.0	576.4	83.13
LOC100539773	6.0	3.5	18.5	6.0	5.0	26.0	10.8	9.20
LOC100539788	4.0	5.0	13.0	8.5	5.5	10.0	7.7	3.46
LOC100539791	0.0	0.0	0.0	0.0	0.0	0.0	0.0	0.00
LOC100539800	16.0	4.5	12.0	15.0	4.0	12.5	10.7	5.19
LOC100539804	0.5	1.5	6.5	0.5	4.5	7.5	3.5	3.10
LOC100539811	896.5	767.5	649.5	851.0	863.0	674.0	783.6	103.78
LOC100539812	835.0	270.0	962.5	818.5	298.0	770.0	659.0	297.49
LOC100539818	3.5	0.0	0.5	2.0	0.5	1.5	1.3	1.29
LOC100539819	1344.0	1260.0	735.5	1375.0	1553.0	1734.5	1333.7	338.77
LOC100539820	109.0	170.5	181.5	116.5	181.0	163.5	153.7	32.49
LOC100539821	30.5	11.5	23.5	20.5	11.5	22.5	20.0	7.40
LOC100539825	24.0	9.0	12.5	25.0	7.0	13.0	15.1	7.63
LOC100539826	0.0	1.5	8.0	1.0	1.5	5.5	2.9	3.12
LOC100539830	24.5	61.0	150.0	22.5	56.5	163.5	79.7	61.92
LOC100539836	277.5	279.5	358.0	272.5	346.5	313.0	307.8	37.44
LOC100539842	0.0	0.0	0.0	0.0	0.0	0.0	0.0	0.00
LOC100539846	0.5	0.0	0.0	1.5	0.0	0.0	0.3	0.61
LOC100539847	5.5	7.0	6.5	6.5	8.0	5.0	6.4	1.07
LOC100539849	458.5	578.5	702.5	428.0	516.0	649.5	555.5	107.89
LOC100539852	2.5	5.0	1.0	1.5	5.5	2.0	2.9	1.88
LOC100539860	1528.5	917.0	941.5	1546.0	881.0	710.5	1087.4	357.73
LOC100539868	120.5	175.0	339.5	155.5	226.5	302.5	219.9	86.27
LOC100539883	358.5	356.5	472.0	420.0	410.5	558.0	429.3	76.34
LOC100539892	69.5	109.5	118.5	48.0	101.5	134.5	96.9	32.26
LOC100539893	0.0	0.0	0.0	0.0	0.0	0.0	0.0	0.00
LOC100539895	4.0	2.0	1.0	3.0	1.5	1.5	2.2	1.13
LOC100539898	225.5	118.0	299.5	212.0	126.5	292.5	212.3	78.06
LOC100539904	0.0	0.0	0.0	0.0	0.0	0.0	0.0	0.00
LOC100539909	4.0	1.0	12.5	10.0	4.0	7.0	6.4	4.27
LOC100539913	0.0	0.0	0.0	0.0	0.5	0.0	0.1	0.20
LOC100539914	393.5	531.5	256.0	460.0	507.5	252.0	400.1	122.58
LOC100539922	0.5	0.0	0.0	0.0	0.0	0.0	0.1	0.20
LOC100539929	92.0	82.5	117.0	106.0	96.5	122.0	102.7	15.16
LOC100539934	350.0	242.0	718.5	354.0	300.5	652.5	436.3	198.39
LOC100539940	1511.5	1291.0	1295.5	1415.5	1485.5	1286.0	1380.8	103.51
LOC100539942	72.0	66.5	59.0	64.5	71.0	76.5	68.3	6.20
LOC100539943	676.0	183.0	803.0	826.0	228.0	636.5	558.8	283.36
LOC100539971	37.0	15.0	53.0	41.5	24.5	43.0	35.7	13.72
LOC100539974	17773.0	18211.5	11350.5	18011.5	17133.5	10567.5	15507.9	3550.84
LOC100539980	3.5	0.0	1.5	2.5	0.0	0.5	1.3	1.44
LOC100539992	730.0	566.0	439.0	648.0	586.5	384.5	559.0	128.70
LOC100540000	2435.5	6548.0	3108.5	2459.0	6956.5	2674.5	4030.3	2126.11
LOC100540001	0.0	0.0	0.0	0.0	0.0	0.0	0.0	0.00
LOC100540007	0.0	1.0	0.0	0.5	0.5	0.0	0.3	0.41
LOC100540008	0.0	1.0	2.5	0.0	0.5	5.0	1.5	1.95
LOC100540009	7.5	2.0	1.5	2.5	3.0	4.0	3.4	2.18
LOC100540017	166.5	100.5	189.0	163.0	100.0	173.0	148.7	38.55
LOC100540025	147.5	63.0	41.5	98.0	53.5	36.5	73.3	42.38

LOC100540035	0.0	0.0	0.0	0.0	0.0	0.0	0.0	0.00
LOC100540040	155.5	90.5	62.5	117.5	86.5	56.5	94.8	36.87
LOC100540041	1028.0	550.0	955.0	1028.5	543.5	810.5	819.3	225.57
LOC100540047	118.0	117.0	157.5	118.0	134.5	119.0	127.3	16.20
LOC100540049	23.0	232.0	127.0	21.0	321.5	181.0	150.9	118.65
LOC100540050	107.5	82.0	35.5	102.5	90.0	51.5	78.2	28.77
LOC100540051	152.5	49.0	71.5	104.5	51.0	48.5	79.5	41.76
LOC100540054	10.5	4.5	1.5	9.0	1.0	1.0	4.6	4.24
LOC100540056	321.5	325.0	459.0	337.5	386.0	442.5	378.6	60.70
LOC100540064	0.5	0.0	0.0	0.0	0.0	0.0	0.1	0.20
LOC100540081	755.0	823.0	263.0	778.0	828.5	422.5	645.0	241.07
LOC100540082	205.5	63.5	107.0	189.5	62.0	84.5	118.7	63.43
LOC100540083	1.5	0.5	1.0	0.0	0.0	0.0	0.5	0.63
LOC100540086	271.5	229.5	333.0	320.0	209.0	243.5	267.8	50.01
LOC100540096	791.0	670.5	604.5	708.0	746.0	616.0	689.3	73.25
LOC100540100	0.0	0.0	0.0	0.0	0.0	0.0	0.0	0.00
LOC100540103	0.5	1.0	1.0	0.0	0.0	0.0	0.4	0.49
LOC100540105	14.0	7.0	5.0	12.0	12.5	5.5	9.3	3.95
LOC100540107	229.5	117.5	280.0	270.0	127.5	255.5	213.3	72.45
LOC100540115	6.0	1.5	4.0	7.5	3.0	4.5	4.4	2.13
LOC100540124	184.0	256.5	135.5	220.5	384.0	166.5	224.5	88.78
LOC100540132	18.5	6.0	7.0	9.0	9.0	3.0	8.8	5.27
LOC100540135	848.5	725.0	711.5	827.0	705.0	693.0	751.7	67.82
LOC100540140	192.0	104.5	322.5	186.5	96.5	307.0	201.5	96.46
LOC100540154	76.0	496.5	250.5	196.5	760.0	576.5	392.7	260.18
LOC100540167	0.0	0.0	0.0	0.0	0.0	0.0	0.0	0.00
LOC100540170	0.5	0.0	0.0	0.0	0.5	0.5	0.3	0.27
LOC100540172	1.0	0.0	0.5	1.0	0.5	0.0	0.5	0.45
LOC100540176	47.5	70.5	63.0	60.0	94.5	128.0	77.3	29.34
LOC100540178	56.0	27.0	20.0	33.5	21.5	19.0	29.5	14.06
LOC100540180	0.0	1.5	0.0	0.5	1.5	0.0	0.6	0.74
LOC100540188	3.5	2.0	0.5	0.0	0.0	2.0	1.3	1.40
LOC100540196	342.0	188.5	209.0	337.0	211.5	186.5	245.8	73.35
LOC100540203	0.5	0.0	0.0	0.0	0.5	0.0	0.2	0.26
LOC100540209	70.5	19.0	76.5	86.5	15.5	54.0	53.7	30.14
LOC100540211	13.0	4.0	4.5	16.0	7.0	8.0	8.8	4.79
LOC100540213	159.5	37.0	57.0	164.5	41.0	45.5	84.1	60.74
LOC100540222	0.5	0.0	0.0	0.0	0.5	0.0	0.2	0.26
LOC100540231	548.5	1825.0	1567.0	452.0	2004.0	1314.5	1285.2	651.96
LOC100540238	54.5	13.0	69.0	57.5	22.5	71.0	47.9	24.40
LOC100540242	67.0	37.0	128.0	84.0	34.5	99.5	75.0	36.44
LOC100540249	3.5	5.0	5.0	2.0	3.5	5.5	4.1	1.32
LOC100540256	0.0	0.0	0.0	0.0	0.0	0.0	0.0	0.00
LOC100540261	143.5	47.0	122.5	181.5	62.0	102.5	109.8	50.44
LOC100540262	45.5	26.0	35.5	50.5	16.0	43.0	36.1	13.04
LOC100540273	49.5	15.5	72.0	83.0	16.0	62.0	49.7	28.51
LOC100540274	2.0	2.0	1.5	2.0	1.5	2.0	1.8	0.26
LOC100540275	0.0	0.0	0.5	0.5	0.0	0.0	0.2	0.26
LOC100540278	0.0	0.0	0.0	0.0	0.0	1.0	0.2	0.41
LOC100540282	0.0	0.0	0.0	1.0	0.0	0.0	0.2	0.41
LOC100540289	0.0	1.0	2.5	0.5	2.0	6.0	2.0	2.17
LOC100540290	12.0	5.5	9.5	7.5	2.0	4.5	6.8	3.60
LOC100540301	3.5	3.0	3.5	5.5	2.0	7.5	4.2	1.99
LOC100540302	301.0	203.5	176.5	283.0	232.5	219.0	235.9	47.61

LOC100540306	1.0	0.5	1.0	2.0	1.0	0.0	0.9	0.66
LOC100540309	86.0	25.0	40.0	78.5	22.0	20.0	45.3	29.60
LOC100540318	386.0	273.0	435.5	354.5	303.0	445.5	366.3	69.71
LOC100540331	134.5	302.0	320.0	80.0	263.0	289.0	231.4	99.46
LOC100540334	2679.0	1836.0	2129.0	2064.0	1929.5	2238.5	2146.0	297.53
LOC100540339	56.0	25.0	44.0	52.0	27.5	47.5	42.0	12.88
LOC100540340	485.0	348.0	512.0	432.0	330.0	507.5	435.8	80.36
LOC100540358	1423.5	1221.5	1173.5	1322.0	1355.0	1180.0	1279.3	102.71
LOC100540364	0.0	0.0	0.0	0.0	0.0	0.0	0.0	0.00
LOC100540365	3.0	5.0	4.0	1.5	3.0	6.5	3.8	1.75
LOC100540368	53.0	14.5	133.0	51.0	19.0	100.5	61.8	46.54
LOC100540378	151.5	133.0	93.0	128.5	151.5	112.5	128.3	22.78
LOC100540379	310.5	95.0	381.0	338.0	103.5	331.5	259.9	126.57
LOC100540386	1.0	3.5	0.5	0.5	4.0	1.5	1.8	1.54
LOC100540389	196.0	134.0	171.5	211.5	172.0	117.0	167.0	35.93
LOC100540394	125.5	126.0	184.5	149.5	127.0	175.5	148.0	26.54
LOC100540396	5.5	17.0	5.5	8.0	28.5	7.5	12.0	9.14
LOC100540406	1.5	0.5	0.5	1.0	0.5	0.5	0.8	0.42
LOC100540409	8.0	10.0	7.5	4.5	3.0	7.0	6.7	2.52
LOC100540410	2.5	1.0	2.5	3.0	2.0	5.0	2.7	1.33
LOC100540413	0.0	0.0	0.0	0.0	0.0	0.0	0.0	0.00
LOC100540415	0.0	0.0	0.0	0.0	0.5	0.5	0.2	0.26
LOC100540418	0.0	0.5	0.0	0.0	0.0	0.5	0.2	0.26
LOC100540428	319.0	140.0	279.5	304.5	210.0	250.5	250.6	66.78
LOC100540432	39.0	51.0	47.5	34.0	52.5	53.0	46.2	7.89
LOC100540438	1.0	0.5	1.0	0.5	1.0	0.0	0.7	0.41
LOC100540439	10.5	10.5	9.5	8.5	9.5	9.5	9.7	0.75
LOC100540450	4.0	2.0	0.5	5.0	1.5	1.5	2.4	1.72
LOC100540451	345.0	443.0	937.5	394.5	457.0	969.0	591.0	283.53
LOC100540453	151.5	79.5	148.5	167.5	93.5	108.5	124.8	35.76
LOC100540457	1100.0	961.5	1560.5	1085.0	1164.5	1482.0	1225.6	239.55
LOC100540462	861.5	734.5	729.5	842.5	947.0	756.5	811.9	86.60
LOC100540465	51.5	22.0	52.0	65.0	20.5	37.5	41.4	17.89
LOC100540472	22.5	7.5	37.5	25.5	11.5	37.0	23.6	12.52
LOC100540476	1.0	0.5	4.0	3.5	0.0	3.5	2.1	1.77
LOC100540496	176.5	172.0	186.0	155.0	176.5	200.5	177.8	15.10
LOC100540502	0.0	0.5	0.0	0.0	0.0	0.0	0.1	0.20
LOC100540503	104.0	28.5	49.0	127.5	31.5	47.0	64.6	41.14
LOC100540511	80.0	20.5	96.5	93.0	34.0	69.0	65.5	31.49
LOC100540515	1.0	0.0	0.0	0.0	0.0	0.0	0.2	0.41
LOC100540527	0.0	0.0	0.0	0.0	0.0	0.0	0.0	0.00
LOC100540528	10.0	1.5	1.0	9.0	1.0	0.0	3.8	4.49
LOC100540532	477.5	290.0	504.5	436.5	362.0	492.0	427.1	84.69
LOC100540561	49.0	36.0	33.0	44.0	40.5	41.0	40.6	5.68
LOC100540564	721.0	628.0	555.0	608.5	701.0	543.0	626.1	73.34
LOC100540568	0.0	0.0	0.0	0.0	0.0	0.0	0.0	0.00
LOC100540575	189.0	83.5	196.5	199.0	109.0	180.0	159.5	50.09
LOC100540586	0.0	0.5	3.5	0.5	0.0	1.5	1.0	1.34
LOC100540589	47.5	12.5	70.0	40.0	16.5	69.0	42.6	24.76
LOC100540591	198.5	35.0	66.0	174.0	38.5	60.5	95.4	71.80
LOC100540595	2.5	2.5	2.5	1.0	2.0	1.0	1.9	0.74
LOC100540607	87.5	47.0	95.5	91.5	52.5	94.5	78.1	22.19
LOC100540610	34.5	48.5	58.5	41.5	40.5	105.0	54.8	25.95
LOC100540612	565.0	550.5	932.5	490.0	676.5	789.5	667.3	167.86

LOC100540620	1595.5	997.5	806.0	1799.0	1289.5	690.0	1196.3	442.60
LOC100540622	246.5	339.5	466.0	343.5	463.0	719.5	429.7	164.64
LOC100540623	422.0	260.0	186.0	320.0	295.5	163.0	274.4	94.60
LOC100540635	629.0	567.0	512.0	577.0	590.5	474.5	558.3	55.90
LOC100540641	10.5	30.5	15.5	12.5	34.5	16.0	19.9	10.03
LOC100540643	0.0	0.0	0.0	0.0	0.5	1.0	0.3	0.42
LOC100540665	169.0	103.0	225.0	176.0	111.0	162.0	157.7	45.13
LOC100540667	218.5	100.0	114.0	140.5	94.0	73.0	123.3	51.72
LOC100540668	509.5	229.5	1202.5	542.5	272.0	944.0	616.7	383.67
LOC100540670	0.0	0.0	0.0	0.0	0.0	0.0	0.0	0.00
LOC100540676	329.5	127.5	201.0	275.5	179.5	199.5	218.8	72.16
LOC100540688	67.0	18.5	29.5	50.5	27.0	34.0	37.8	17.81
LOC100540701	0.0	0.5	0.0	1.0	0.0	1.0	0.4	0.49
LOC100540703	6.0	8.5	3.0	7.5	7.5	5.0	6.3	2.02
LOC100540713	307.5	154.5	186.5	265.0	161.0	144.5	203.2	67.19
LOC100540723	0.0	0.0	0.0	0.0	0.0	1.0	0.2	0.41
LOC100540739	1174.0	855.0	591.5	1138.0	1029.0	567.5	892.5	266.75
LOC100540742	0.0	0.0	0.0	0.0	0.0	0.0	0.0	0.00
LOC100540743	80.0	31.0	153.5	80.5	32.5	126.0	83.9	49.16
LOC100540751	0.5	7.5	7.5	0.0	9.5	4.0	4.8	3.97
LOC100540753	53.5	16.5	71.5	67.5	23.0	55.0	47.8	22.93
LOC100540763	5.0	3.5	2.0	4.0	2.0	4.0	3.4	1.20
LOC100540766	5.0	0.5	2.5	2.5	3.0	0.5	2.3	1.69
LOC100540769	100.0	57.5	232.5	86.5	81.5	162.5	120.1	65.38
LOC100540781	0.0	0.0	0.0	0.0	0.0	0.0	0.0	0.00
LOC100540786	1.0	0.5	1.0	1.0	0.0	1.0	0.8	0.42
LOC100540787	3.5	0.5	0.5	1.0	0.5	3.0	1.5	1.38
LOC100540799	0.0	0.0	0.5	1.5	0.0	0.5	0.4	0.58
LOC100540803	2.0	10.0	39.0	2.0	9.0	35.5	16.3	16.65
LOC100540806	111.5	139.0	155.5	111.0	176.5	152.5	141.0	25.99
LOC100540816	2380.5	2864.0	1447.5	2134.5	2878.0	1514.5	2203.2	628.26
LOC100540821	4318.5	3669.5	2788.5	4108.0	3732.0	2685.0	3550.3	674.90
LOC100540823	0.0	0.0	0.0	0.0	0.5	0.0	0.1	0.20
LOC100540824	0.0	0.0	0.0	0.0	0.0	0.0	0.0	0.00
LOC100540834	5.5	7.0	5.0	3.5	5.5	6.0	5.4	1.16
LOC100540839	155.0	80.5	117.5	136.5	111.0	106.0	117.8	25.73
LOC100540841	2.5	2.0	9.0	2.5	2.0	7.5	4.3	3.14
LOC100540846	1675.5	699.0	907.0	1447.0	714.5	665.0	1018.0	435.24
LOC100540850	0.0	0.0	0.0	0.0	0.0	0.0	0.0	0.00
LOC100540851	109.0	33.0	194.5	146.5	35.0	135.5	108.9	64.30
LOC100540856	1.5	2.0	1.0	0.5	2.0	1.5	1.4	0.58
LOC100540871	1.0	2.5	5.0	1.0	3.0	3.0	2.6	1.50
LOC100540872	105.0	81.0	101.0	109.5	82.0	102.0	96.8	12.18
LOC100540873	473.0	261.5	281.0	451.0	270.5	245.5	330.4	102.82
LOC100540876	0.0	0.0	0.0	0.0	0.0	0.0	0.0	0.00
LOC100540882	0.0	0.0	0.5	0.0	0.0	0.0	0.1	0.20
LOC100540888	167.0	132.0	86.0	147.0	124.5	73.0	121.6	35.92
LOC100540893	0.0	0.0	0.0	0.0	0.0	0.0	0.0	0.00
LOC100540894	15.5	59.5	57.0	19.5	67.5	165.5	64.1	54.25
LOC100540904	0.0	0.5	0.0	0.5	0.0	1.0	0.3	0.41
LOC100540905	5.0	5.0	8.0	4.0	4.0	8.0	5.7	1.86
LOC100540906	214.0	77.0	369.0	224.0	92.0	343.0	219.8	121.83
LOC100540910	60.0	18.5	11.0	49.0	16.0	9.0	27.3	21.66
LOC100540915	441.0	276.5	455.0	397.0	326.5	420.5	386.1	70.18

LOC100540918	0.0	0.0	0.0	0.0	0.0	0.0	0.0	0.00
LOC100540920	96.5	63.0	112.0	73.5	55.0	79.5	79.9	21.23
LOC100540921	233.5	258.0	358.5	302.0	311.0	218.0	280.2	53.10
LOC100540933	0.0	0.0	0.0	0.0	0.0	0.0	0.0	0.00
LOC100540939	16.5	17.0	11.5	12.5	28.0	9.5	15.8	6.63
LOC100540942	1311.5	1072.5	229.5	1076.5	962.5	294.0	824.4	450.93
LOC100540945	0.0	0.0	0.0	0.0	0.0	0.0	0.0	0.00
LOC100540961	2.0	0.0	9.0	0.5	0.0	3.0	2.4	3.44
LOC100540962	505.5	332.0	298.0	472.5	360.5	284.5	375.5	92.43
LOC100540965	326.0	255.5	208.5	287.5	283.0	240.5	266.8	41.02
LOC100540970	0.0	0.0	0.0	0.0	0.0	0.0	0.0	0.00
LOC100540973	3.0	0.5	5.5	4.0	2.0	3.5	3.1	1.72
LOC100540974	2.5	0.5	0.5	1.0	1.5	0.5	1.1	0.80
LOC100540978	31.0	7.0	21.5	38.5	10.5	17.5	21.0	12.04
LOC100540985	105.5	72.0	95.5	96.0	64.5	126.0	93.3	22.42
LOC100540989	669.0	549.0	866.0	664.5	570.5	723.5	673.8	114.69
LOC100541022	116.0	756.0	722.5	95.0	899.0	577.5	527.7	342.68
LOC100541035	138.5	46.5	116.0	144.0	50.5	91.0	97.8	42.51
LOC100541041	2.5	4.0	11.0	8.0	6.0	9.0	6.8	3.19
LOC100541042	82.0	27.0	36.0	83.0	31.0	31.0	48.3	26.62
LOC100541044	7.0	11.0	3.0	12.5	10.0	10.0	8.9	3.41
LOC100541052	56.0	30.0	98.0	62.5	27.5	97.0	61.8	30.89
LOC100541057	36.0	23.5	24.0	46.5	23.5	34.5	31.3	9.36
LOC100541067	0.0	0.0	0.0	1.0	0.5	0.0	0.3	0.42
LOC100541069	0.5	1.0	1.0	0.0	0.5	0.5	0.6	0.38
LOC100541072	0.0	0.0	0.0	0.0	0.0	0.0	0.0	0.00
LOC100541074	0.5	0.0	0.5	1.5	2.0	1.0	0.9	0.74
LOC100541078	4.0	1.0	2.5	1.5	2.0	3.5	2.4	1.16
LOC100541081	17735.5	15587.5	7633.0	15091.5	14879.0	7510.5	13072.8	4380.24
LOC100541083	0.0	0.0	0.0	0.0	0.0	0.0	0.0	0.00
LOC100541086	0.5	0.5	0.0	0.0	0.0	0.0	0.2	0.26
LOC100541106	191.5	86.0	272.5	214.5	101.0	230.5	182.7	74.11
LOC100541107	513.5	831.5	504.0	493.5	854.5	353.5	591.8	203.35
LOC100541117	18.0	9.0	39.5	20.0	14.0	27.5	21.3	10.83
LOC100541118	359.5	164.5	498.0	343.0	144.5	405.0	319.1	138.55
LOC100541119	27.5	23.5	25.0	32.0	27.0	34.0	28.2	4.06
LOC100541121	5.5	39.5	36.0	7.0	53.5	49.5	31.8	20.82
LOC100541131	0.5	0.5	0.0	0.0	0.5	0.5	0.3	0.26
LOC100541134	1891.5	1157.0	2120.5	1748.5	1277.5	1872.0	1677.8	378.37
LOC100541135	0.0	0.0	0.0	0.0	0.0	0.0	0.0	0.00
LOC100541138	0.0	0.0	0.0	0.0	0.0	0.0	0.0	0.00
LOC100541145	4.5	4.5	2.0	2.5	6.5	1.5	3.6	1.91
LOC100541147	11.5	2.0	4.0	7.0	8.5	5.0	6.3	3.40
LOC100541155	71.0	711.5	1515.5	102.5	804.5	2089.0	882.3	794.81
LOC100541159	1.0	0.0	5.0	1.5	2.5	1.5	1.9	1.72
LOC100541160	4.5	11.0	7.5	3.5	6.0	11.0	7.3	3.21
LOC100541166	0.5	0.0	0.5	0.5	0.0	0.0	0.3	0.27
LOC100541174	3.0	2.0	2.0	0.5	2.5	0.5	1.8	1.04
LOC100541175	0.0	0.0	0.0	0.0	1.0	0.0	0.2	0.41
LOC100541177	239.0	83.0	73.5	167.5	69.5	43.5	112.7	74.87
LOC100541180	1.5	2.0	3.5	1.0	2.0	1.0	1.8	0.93
LOC100541187	7.0	1.0	4.0	10.5	2.0	5.5	5.0	3.48
LOC100541195	187.5	148.0	290.5	214.5	152.5	261.5	209.1	57.97
LOC100541197	0.0	0.5	0.0	0.0	0.0	0.5	0.2	0.26

LOC100541200	12.0	19.0	31.0	11.0	24.5	27.0	20.8	8.16
LOC100541203	0.0	0.0	0.0	0.0	0.0	0.0	0.0	0.00
LOC100541205	0.0	0.0	0.0	0.0	0.0	0.0	0.0	0.00
LOC100541206	0.0	0.0	0.0	0.0	0.5	0.0	0.1	0.20
LOC100541207	4690.0	7159.5	4608.0	3948.0	8332.5	4870.0	5601.3	1730.48
LOC100541211	150.0	60.0	134.5	137.5	71.5	140.5	115.7	39.18
LOC100541213	233.0	188.5	147.5	207.0	195.0	161.0	188.7	30.99
LOC100541214	1460.5	510.0	425.5	1745.5	574.0	444.0	859.9	584.95
LOC100541229	2925.5	2678.5	4292.5	3002.5	3100.5	4584.0	3430.6	798.28
LOC100541237	135.5	76.5	108.5	117.0	88.5	68.5	99.1	25.66
LOC100541254	62.5	19.0	14.0	58.5	27.5	15.0	32.8	22.05
LOC100541257	0.0	0.0	0.0	0.0	0.0	0.0	0.0	0.00
LOC100541261	0.0	0.5	0.0	0.0	0.0	0.0	0.1	0.20
LOC100541270	18.0	9.5	20.0	17.5	13.5	13.5	15.3	3.86
LOC100541274	42.5	29.5	41.5	43.5	28.0	57.0	40.3	10.61
LOC100541286	0.0	0.0	0.0	0.0	0.0	0.0	0.0	0.00
LOC100541290	393.0	398.0	590.5	370.0	415.0	406.0	428.8	80.68
LOC100541291	0.5	0.0	0.0	0.0	0.0	0.5	0.2	0.26
LOC100541293	0.0	0.0	0.0	0.0	0.0	0.0	0.0	0.00
LOC100541297	17.0	9.5	6.5	12.5	15.5	8.5	11.6	4.13
LOC100541314	0.0	2.0	0.0	0.0	0.5	0.0	0.4	0.80
LOC100541319	2.5	5.5	0.5	4.5	3.5	3.0	3.3	1.72
LOC100541326	0.0	0.0	0.0	0.0	0.0	0.0	0.0	0.00
LOC100541332	0.5	0.0	0.0	0.0	0.0	0.0	0.1	0.20
LOC100541340	0.0	0.0	0.0	0.0	0.0	0.0	0.0	0.00
LOC100541343	752.0	339.5	793.5	838.5	492.5	1058.0	712.3	257.25
LOC100541356	52.0	20.5	24.0	39.0	20.0	15.5	28.5	14.06
LOC100541366	38.0	7.0	20.0	43.0	4.5	12.5	20.8	16.21
LOC100541369	0.5	0.5	1.0	0.0	0.0	1.0	0.5	0.45
LOC100541374	92.5	62.0	73.0	78.5	62.0	87.5	75.9	12.74
LOC100541378	60.5	55.0	30.0	39.0	72.5	31.0	48.0	17.32
LOC100541387	0.0	0.0	0.0	0.0	0.0	0.0	0.0	0.00
LOC100541391	321.0	349.5	470.5	290.0	366.5	379.0	362.8	61.81
LOC100541395	0.5	0.0	0.0	0.5	0.5	0.0	0.3	0.27
LOC100541400	139.5	39.0	128.0	101.5	37.0	58.0	83.8	45.24
LOC100541411	47.0	20.5	91.0	67.5	27.5	63.0	52.8	26.44
LOC100541413	0.5	0.0	9.5	0.0	0.5	9.5	3.3	4.78
LOC100541423	46.5	9.5	40.0	49.0	13.5	38.5	32.8	17.03
LOC100541429	13.0	2.5	1.0	10.5	2.0	0.5	4.9	5.40
LOC100541434	8.0	9.0	4.5	13.5	10.0	6.0	8.5	3.16
LOC100541445	0.0	0.0	0.0	0.0	0.0	0.0	0.0	0.00
LOC100541447	0.0	0.0	0.0	0.0	0.5	0.0	0.1	0.20
LOC100541450	8.5	9.5	32.0	9.5	8.0	22.0	14.9	9.90
LOC100541451	0.5	0.0	0.0	0.0	0.0	0.0	0.1	0.20
LOC100541460	28.5	27.0	87.5	15.0	44.0	73.5	45.9	28.67
LOC100541463	287.0	1063.5	1187.5	372.0	1205.5	1525.5	940.2	497.76
LOC100541464	233.0	156.0	164.0	222.0	168.5	196.5	190.0	32.28
LOC100541465	0.0	0.5	0.0	0.0	4.5	0.0	0.8	1.81
LOC100541468	1734.5	848.5	1269.5	1628.5	749.5	900.5	1188.5	421.79
LOC100541469	0.5	0.0	0.0	0.0	0.0	0.0	0.1	0.20
LOC100541471	5.0	2.0	5.5	6.0	3.0	5.5	4.5	1.61
LOC100541480	19.0	9.0	3.5	17.5	6.5	5.5	10.2	6.52
LOC100541485	11.5	2.5	0.5	9.0	4.0	0.5	4.7	4.59
LOC100541489	0.0	0.0	0.0	0.0	0.0	0.0	0.0	0.00

LOC100541494	27.5	34.0	35.5	28.5	28.0	25.0	29.8	4.08
LOC100541498	288.0	176.5	185.0	304.5	182.0	175.5	218.6	60.49
LOC100541500	300.0	138.0	258.0	247.0	187.5	234.0	227.4	56.95
LOC100541513	66.5	15.0	21.0	39.0	20.0	13.0	29.1	20.51
LOC100541518	6.0	27.0	28.0	6.0	38.0	55.0	26.7	18.91
LOC100541519	83.5	44.0	114.0	77.5	51.5	85.0	75.9	25.33
LOC100541523	972.0	941.0	634.5	849.5	1075.5	652.0	854.1	178.64
LOC100541529	22.0	22.5	36.0	21.5	20.0	44.0	27.7	9.92
LOC100541538	0.0	0.0	0.0	0.0	0.0	0.0	0.0	0.00
LOC100541541	5.0	5.0	42.5	2.5	6.5	24.0	14.3	15.89
LOC100541557	1.0	0.5	0.5	1.5	0.0	0.5	0.7	0.52
LOC100541562	9.5	13.0	18.0	13.5	18.0	20.5	15.4	4.09
LOC100541564	1.0	0.0	0.0	0.5	1.0	0.5	0.5	0.45
LOC100541567	0.0	0.5	0.0	0.0	0.5	0.0	0.2	0.26
LOC100541582	75.0	28.5	18.5	72.5	42.0	20.5	42.8	25.34
LOC100541592	47.5	70.0	134.0	51.0	75.0	100.5	79.7	32.73
LOC100541596	213.0	220.0	194.0	320.0	264.5	323.0	255.8	55.94
LOC100541598	0.0	0.0	0.0	0.0	0.0	0.0	0.0	0.00
LOC100541600	0.0	0.0	0.0	0.0	0.0	0.0	0.0	0.00
LOC100541603	0.0	0.0	0.0	0.0	0.0	0.0	0.0	0.00
LOC100541604	0.0	0.0	0.0	0.0	0.0	0.0	0.0	0.00
LOC100541605	595.5	364.0	435.5	492.5	383.5	390.0	443.5	87.58
LOC100541607	1.5	1.5	0.0	4.0	3.5	1.5	2.0	1.48
LOC100541608	2726.0	1835.0	2061.5	2531.0	1873.5	1688.5	2119.3	416.59
LOC100541609	741.5	718.0	759.0	663.5	813.5	669.0	727.4	56.91
LOC100541617	0.5	0.0	0.5	0.0	0.0	0.0	0.2	0.26
LOC100541624	1158.0	756.0	623.5	1059.5	674.0	543.5	802.4	249.09
LOC100541632	0.0	0.5	0.5	0.0	0.0	1.0	0.3	0.41
LOC100541641	2663.0	2588.0	2130.5	2123.5	2604.5	1765.0	2312.4	361.28
LOC100541644	0.0	1.0	1.5	0.0	1.5	1.0	0.8	0.68
LOC100541646	2008.5	1741.5	1619.0	1847.5	2045.5	1484.5	1791.1	219.66
LOC100541650	107.0	72.5	127.5	149.5	82.5	108.5	107.9	28.33
LOC100541658	3.5	1.5	0.5	4.0	1.0	1.5	2.0	1.41
LOC100541666	255.5	202.0	249.0	234.0	206.0	186.5	222.2	27.97
LOC100541679	165.5	227.5	126.0	205.5	335.5	237.5	216.3	71.60
LOC100541687	0.0	0.0	0.0	0.0	0.0	0.0	0.0	0.00
LOC100541689	0.0	0.0	0.0	0.0	0.0	0.0	0.0	0.00
LOC100541693	107.5	50.0	105.5	109.5	53.0	108.5	89.0	29.09
LOC100541694	0.0	0.0	0.0	0.0	0.0	0.0	0.0	0.00
LOC100541702	1.0	0.0	0.0	0.0	0.0	0.0	0.2	0.41
LOC100541707	90.0	14.0	103.0	114.0	23.5	57.0	66.9	42.04
LOC100541714	396.0	279.5	149.5	337.0	275.5	182.5	270.0	92.37
LOC100541737	47.5	31.5	89.0	45.5	29.5	121.0	60.7	36.52
LOC100541738	42.5	55.0	24.0	30.0	63.5	37.5	42.1	14.96
LOC100541742	53.0	42.5	57.0	51.0	62.5	51.5	52.9	6.67
LOC100541746	302.0	276.5	145.5	310.0	306.0	194.0	255.7	69.29
LOC100541750	0.0	0.5	0.5	0.0	0.5	0.0	0.3	0.27
LOC100541754	58.5	14.0	62.0	62.0	19.0	45.5	43.5	21.84
LOC100541755	0.0	0.0	0.5	0.5	0.5	0.5	0.3	0.26
LOC100541756	0.0	0.0	0.0	0.0	0.0	0.0	0.0	0.00
LOC100541770	0.0	1.0	0.0	0.5	1.0	0.5	0.5	0.45
LOC100541773	34.0	25.5	40.5	30.5	25.5	34.5	31.8	5.81
LOC100541776	619.0	450.0	1068.5	635.5	533.5	990.0	716.1	252.70
LOC100541781	189.5	126.5	64.5	184.0	120.5	67.0	125.3	54.20

LOC100541783	3.0	9.5	57.5	1.0	8.5	55.0	22.4	26.41
LOC100541789	1.5	11.0	17.5	5.5	12.0	22.0	11.6	7.52
LOC100541791	23.5	41.5	158.5	37.5	45.0	165.5	78.6	65.06
LOC100541793	0.0	0.0	0.0	0.0	0.5	0.5	0.2	0.26
LOC100541794	134.0	148.5	367.5	131.0	160.5	255.5	199.5	94.30
LOC100541803	14.0	10.5	29.0	11.5	12.5	29.0	17.8	8.79
LOC100541825	10.0	7.5	3.0	16.0	14.0	7.5	9.7	4.75
LOC100541835	41.0	15.0	6.5	25.0	15.5	17.0	20.0	11.85
LOC100541844	6.0	3.5	14.0	2.5	2.5	14.5	7.2	5.64
LOC100541845	0.0	0.0	0.0	0.0	0.0	0.0	0.0	0.00
LOC100541846	490.0	305.0	674.5	477.5	368.5	603.0	486.4	138.47
LOC100541852	40.5	19.0	29.0	39.5	17.0	26.0	28.5	9.94
LOC100541858	1.5	1.0	2.0	0.5	0.5	2.0	1.3	0.69
LOC100541860	0.0	0.0	0.0	0.0	0.5	0.0	0.1	0.20
LOC100541862	588.5	502.0	494.5	500.5	508.5	448.0	507.0	45.50
LOC100541865	0.0	0.0	0.0	0.0	0.0	0.5	0.1	0.20
LOC100541871	0.0	0.5	0.5	1.0	0.0	0.0	0.3	0.41
LOC100541880	134.0	48.0	157.0	128.0	48.5	125.0	106.8	46.68
LOC100541881	155.0	86.0	175.5	86.5	58.0	88.5	108.3	46.02
LOC100541890	1.0	0.0	0.0	0.0	1.0	0.0	0.3	0.52
LOC100541896	98.5	69.0	33.5	88.5	82.0	57.0	71.4	23.62
LOC100541903	12.5	7.5	27.5	20.5	10.5	21.0	16.6	7.62
LOC100541905	120.0	36.5	57.0	113.0	36.5	37.5	66.8	39.38
LOC100541906	1.0	0.5	1.5	1.5	0.5	0.0	0.8	0.61
LOC100541913	0.0	0.0	0.0	0.0	0.0	0.0	0.0	0.00
LOC100541927	0.0	0.0	1.0	0.0	1.0	2.0	0.7	0.82
LOC100541930	317.5	394.0	318.0	293.5	379.5	302.5	334.2	42.03
LOC100541931	178.5	100.0	71.0	155.5	101.5	64.5	111.8	45.84
LOC100541941	10.5	4.5	8.5	17.5	8.5	8.5	9.7	4.31
LOC100541947	0.0	0.0	0.0	0.0	0.5	0.0	0.1	0.20
LOC100541954	0.0	0.0	0.0	0.0	0.0	0.5	0.1	0.20
LOC100541957	7.5	9.5	28.0	6.5	14.5	21.5	14.6	8.59
LOC100541961	57.0	32.5	9.0	67.0	26.5	6.0	33.0	24.82
LOC100541966	371.0	216.0	192.0	341.0	244.5	174.0	256.4	81.24
LOC100541969	98.0	141.0	237.5	111.5	160.0	221.0	161.5	57.05
LOC100541987	19.0	18.5	35.5	10.5	19.5	27.0	21.7	8.56
LOC100541990	10.0	3.5	18.5	13.5	4.5	19.5	11.6	6.81
LOC100541993	25.5	58.5	105.0	28.5	68.5	125.0	68.5	40.18
LOC100541995	0.0	0.5	0.0	0.0	0.0	0.0	0.1	0.20
LOC100541996	0.0	0.0	0.0	0.0	0.0	0.0	0.0	0.00
LOC100542004	96.0	41.0	80.0	89.5	58.5	60.5	70.9	21.05
LOC100542006	169.0	114.5	215.0	192.5	123.0	212.5	171.1	43.87
LOC100542016	0.0	0.0	0.0	0.0	0.0	0.0	0.0	0.00
LOC100542017	870.0	435.5	544.5	992.5	494.5	494.5	638.6	232.56
LOC100542020	58.0	17.0	102.5	47.5	28.5	77.0	55.1	31.47
LOC100542032	3208.0	3146.5	2117.0	2697.5	2943.5	1689.5	2633.7	608.49
LOC100542038	58.5	21.5	111.0	71.5	32.5	108.0	67.2	37.34
LOC100542043	0.0	0.0	0.0	0.0	0.0	0.0	0.0	0.00
LOC100542046	2.5	1.5	3.5	3.0	2.0	5.0	2.9	1.24
LOC100542048	5.0	3.5	2.5	6.0	3.0	4.5	4.1	1.32
LOC100542049	0.0	0.0	0.0	0.0	0.0	0.0	0.0	0.00
LOC100542067	0.0	0.0	1.0	0.0	0.0	0.5	0.3	0.42
LOC100542070	0.0	0.0	0.0	0.0	0.0	0.0	0.0	0.00
LOC100542090	1.0	0.0	4.0	0.0	1.5	3.0	1.6	1.63

LOC100542094	409.5	129.5	416.0	469.0	145.5	340.0	318.3	145.98
LOC100542101	2.0	0.0	0.5	0.5	0.0	0.0	0.5	0.77
LOC100542106	0.0	0.0	0.0	0.0	0.0	0.0	0.0	0.00
LOC100542110	18.0	8.5	20.0	24.5	8.5	13.5	15.5	6.47
LOC100542113	1743.0	435.0	2510.0	2382.0	623.0	2981.5	1779.1	1047.66
LOC100542122	976.0	522.0	447.0	939.5	565.0	395.0	640.8	252.75
LOC100542130	1.0	1.5	3.0	1.0	1.5	2.5	1.8	0.82
LOC100542155	0.0	0.0	0.0	0.5	0.0	0.5	0.2	0.26
LOC100542157	6.0	3.5	8.5	2.5	6.5	4.0	5.2	2.23
LOC100542159	0.5	0.5	1.0	0.0	0.0	1.0	0.5	0.45
LOC100542160	1.0	1.0	1.5	2.0	0.5	2.5	1.4	0.74
LOC100542161	1551.5	1483.0	1061.5	1393.0	1602.0	1015.0	1351.0	252.62
LOC100542164	134.5	39.0	110.5	190.0	43.0	111.5	104.8	57.21
LOC100542170	173.5	93.5	75.0	161.0	96.5	91.5	115.2	41.22
LOC100542173	1170.0	998.0	671.0	1157.0	963.5	602.0	926.9	240.62
LOC100542179	94.5	40.0	63.0	99.5	61.5	69.5	71.3	22.28
LOC100542183	0.0	1.0	1.5	0.0	0.0	0.0	0.4	0.66
LOC100542184	198.0	171.5	142.0	216.5	190.0	140.5	176.4	30.84
LOC100542185	0.0	0.0	1.0	0.0	0.0	0.5	0.3	0.42
LOC100542192	1.0	0.0	1.5	2.0	0.5	2.0	1.2	0.82
LOC100542196	4.0	3.5	5.5	1.0	2.0	4.5	3.4	1.66
LOC100542198	162.5	61.0	169.5	147.5	64.0	137.0	123.6	48.67
LOC100542202	4.5	5.5	2.5	4.5	1.0	3.0	3.5	1.64
LOC100542208	594.0	955.0	1681.5	555.5	1072.0	1247.5	1017.6	422.72
LOC100542210	6773.0	7127.5	7082.0	6909.5	8481.0	6881.5	7209.1	636.84
LOC100542217	37.0	190.5	98.0	20.0	202.5	87.0	105.8	76.20
LOC100542219	122.0	80.0	71.5	121.5	110.0	72.5	96.3	24.21
LOC100542220	0.5	3.5	6.5	2.5	7.0	6.5	4.4	2.65
LOC100542221	171.5	93.5	102.5	173.5	85.0	99.0	120.8	40.46
LOC100542222	0.0	0.0	0.0	0.0	0.0	0.0	0.0	0.00
LOC100542224	1.5	1.0	0.0	0.5	0.5	0.0	0.6	0.58
LOC100542230	75.0	28.0	146.0	87.0	40.5	126.0	83.8	46.32
LOC100542236	77.5	24.5	62.0	59.0	28.5	46.5	49.7	20.52
LOC100542241	837.5	569.5	1159.0	802.0	647.0	1189.5	867.4	257.40
LOC100542243	5.5	7.0	2.5	11.0	5.0	3.5	5.8	3.01
LOC100542248	26.5	10.5	21.0	15.0	12.0	11.5	16.1	6.37
LOC100542256	0.0	0.0	0.0	0.0	0.0	0.0	0.0	0.00
LOC100542258	2676.0	3718.0	2178.0	2505.0	3959.5	2415.5	2908.7	742.08
LOC100542269	0.0	0.0	0.0	0.0	0.0	0.0	0.0	0.00
LOC100542272	742.5	531.0	800.0	719.5	535.5	734.5	677.2	114.78
LOC100542279	1.5	2.0	0.5	0.5	0.0	1.5	1.0	0.77
LOC100542292	111.0	168.0	101.0	117.5	191.5	131.0	136.7	35.55
LOC100542299	1.5	9.0	8.0	1.0	15.5	10.0	7.5	5.50
LOC100542304	0.0	0.0	0.0	0.0	0.0	0.0	0.0	0.00
LOC100542306	0.5	0.0	0.5	0.0	0.0	0.0	0.2	0.26
LOC100542307	1015.5	308.0	445.5	926.0	404.0	419.0	586.3	302.69
LOC100542320	64.5	24.5	25.5	56.0	38.0	21.5	38.3	18.09
LOC100542349	176.0	52.5	178.5	189.0	57.5	154.5	134.7	62.74
LOC100542357	55.0	42.5	17.5	36.0	33.5	13.0	32.9	15.64
LOC100542364	145.0	36.0	207.5	118.5	48.0	210.0	127.5	75.21
LOC100542367	9.0	15.5	21.0	13.5	14.5	22.0	15.9	4.87
LOC100542387	17.5	7.0	20.5	18.5	4.5	18.0	14.3	6.77
LOC100542388	258.5	209.0	240.0	263.5	248.5	204.5	237.3	25.08
LOC100542393	1319.5	611.5	1989.5	1457.5	678.5	1614.5	1278.5	539.82

LOC100542400	1.5	0.0	2.5	1.5	0.5	1.0	1.2	0.88
LOC100542403	0.5	0.0	0.5	0.5	0.5	0.0	0.3	0.26
LOC100542409	0.0	0.0	0.0	0.0	0.0	0.0	0.0	0.00
LOC100542421	230.5	64.0	89.5	205.5	78.0	56.5	120.7	76.66
LOC100542427	50.5	13.0	94.0	69.5	12.5	58.5	49.7	32.14
LOC100542428	31.5	10.5	10.0	43.0	15.0	9.0	19.8	14.12
LOC100542432	6.5	8.5	85.0	0.0	6.0	47.5	25.6	33.78
LOC100542433	0.0	0.0	0.0	0.0	0.0	0.0	0.0	0.00
LOC100542437	3.0	4.0	5.5	3.5	9.0	3.0	4.7	2.32
LOC100542440	3.0	2.0	3.0	2.0	5.0	1.0	2.7	1.37
LOC100542442	182.5	85.5	80.0	170.0	95.5	95.5	118.2	45.56
LOC100542445	1241.0	771.5	904.5	1153.5	819.0	830.5	953.3	195.65
LOC100542446	1310.0	1851.5	1714.0	1222.0	1925.5	1600.0	1603.8	286.04
LOC100542457	106.0	49.5	154.5	135.0	67.5	141.0	108.9	42.52
LOC100542468	92.0	35.5	60.5	73.0	50.0	50.0	60.2	19.97
LOC100542473	0.0	0.0	0.0	0.0	0.0	0.0	0.0	0.00
LOC100542475	0.0	0.0	0.0	0.0	0.0	0.0	0.0	0.00
LOC100542484	298.5	64.0	186.0	314.0	56.0	122.0	173.4	113.11
LOC100542486	4.0	1.0	2.0	1.5	3.5	1.0	2.2	1.29
LOC100542495	0.5	0.5	0.0	0.0	0.0	0.0	0.2	0.26
LOC100542503	0.0	0.0	0.5	0.5	0.0	0.0	0.2	0.26
LOC100542515	2.5	2.0	3.0	7.0	3.5	3.5	3.6	1.77
LOC100542519	3523.0	3577.5	4497.5	4401.5	4036.5	5886.5	4320.4	867.06
LOC100542520	225.0	232.5	261.0	201.0	250.5	264.0	239.0	24.18
LOC100542525	0.0	0.0	0.0	0.0	0.0	0.0	0.0	0.00
LOC100542534	92.5	109.5	93.5	76.5	135.5	102.5	101.7	19.95
LOC100542537	2385.5	1826.5	1214.5	2104.5	1919.0	1045.5	1749.3	519.04
LOC100542547	13.0	4.0	1.5	4.5	0.0	3.0	4.3	4.56
LOC100542552	0.0	24.0	14.0	0.0	13.5	16.0	11.3	9.50
LOC100542556	33.5	20.0	16.0	36.0	20.0	20.0	24.3	8.32
LOC100542562	0.0	4.0	0.0	0.5	2.0	0.5	1.2	1.57
LOC100542571	2.5	3.0	1.5	4.0	4.0	3.5	3.1	0.97
LOC100542574	59.5	20.0	150.0	64.0	31.0	122.5	74.5	51.38
LOC100542589	19.5	12.0	5.0	7.5	16.0	5.5	10.9	5.94
LOC100542602	116.5	102.0	105.5	107.0	101.5	96.5	104.8	6.78
LOC100542612	676.0	784.5	1081.0	605.5	695.5	1135.5	829.7	223.88
LOC100542617	24.0	7.5	37.0	20.0	5.0	36.0	21.6	13.62
LOC100542618	12.5	5.5	29.5	10.5	9.0	25.0	15.3	9.62
LOC100542621	0.0	0.0	0.0	0.0	0.0	0.0	0.0	0.00
LOC100542624	633.0	556.5	510.5	647.0	653.5	508.5	584.8	67.90
LOC100542626	0.0	0.0	3.0	0.0	0.0	0.0	0.5	1.22
LOC100542630	0.0	0.0	0.0	0.5	0.0	0.0	0.1	0.20
LOC100542633	20.0	23.5	24.5	25.0	23.5	22.0	23.1	1.83
LOC100542634	63.0	15.0	16.5	39.5	16.5	5.5	26.0	21.32
LOC100542638	69.0	85.0	128.0	77.0	109.5	175.0	107.3	39.75
LOC100542660	1.0	0.0	0.0	0.0	0.0	0.0	0.2	0.41
LOC100542665	784.0	776.0	832.5	812.0	816.0	865.0	814.3	32.54
LOC100542676	1106.5	424.5	374.0	1136.0	497.5	316.0	642.4	375.79
LOC100542678	0.5	0.5	2.5	3.5	2.5	1.0	1.8	1.25
LOC100542684	0.0	0.0	0.0	0.0	0.0	0.0	0.0	0.00
LOC100542686	884.0	742.5	640.0	824.5	776.0	639.0	751.0	98.64
LOC100542689	0.0	0.0	0.0	0.0	0.0	0.0	0.0	0.00
LOC100542691	122.5	94.0	207.5	113.5	84.5	152.0	129.0	45.12
LOC100542699	23.0	18.5	56.0	27.5	21.0	53.0	33.2	16.81

LOC100542702	95.5	40.0	146.5	109.0	38.0	161.0	98.3	51.79
LOC100542710	856.5	488.0	840.0	821.0	524.0	814.5	724.0	169.89
LOC100542725	315.0	418.0	294.0	267.0	428.5	287.5	335.0	70.13
LOC100542733	73.0	39.5	42.5	66.5	44.5	35.5	50.3	15.54
LOC100542744	4.5	2.5	9.0	8.0	4.0	5.5	5.6	2.48
LOC100542745	45.0	32.0	102.5	42.5	33.0	136.5	65.3	43.67
LOC100542752	937.0	850.0	1871.5	923.0	933.5	1543.0	1176.3	425.35
LOC100542756	3.0	10.5	19.5	2.0	12.0	15.5	10.4	6.88
LOC100542757	1087.5	1327.0	1325.0	1092.5	1424.0	1278.5	1255.8	136.86
LOC100542763	3.5	1.5	0.0	8.5	1.5	2.5	2.9	2.97
LOC100542770	0.0	0.0	0.0	0.0	0.0	0.0	0.0	0.00
LOC100542772	2.5	0.0	3.5	0.5	0.0	1.5	1.3	1.44
LOC100542775	62.0	9.0	2.5	60.0	11.0	2.0	24.4	28.56
LOC100542776	11.0	7.5	6.5	7.5	8.5	14.0	9.2	2.82
LOC100542779	0.0	0.0	0.0	0.0	0.0	0.0	0.0	0.00
LOC100542789	0.0	0.0	0.0	0.0	0.0	0.5	0.1	0.20
LOC100542796	0.0	0.0	0.0	0.0	0.0	2.5	0.4	1.02
LOC100542800	3543.0	4228.5	3414.5	3758.0	4640.0	3445.5	3838.3	494.47
LOC100542816	2128.5	591.5	1510.5	2408.5	682.0	1150.0	1411.8	747.11
LOC100542821	151.5	43.0	40.0	139.5	44.5	32.5	75.2	54.77
LOC100542822	328.0	129.5	229.0	474.0	174.0	436.0	295.1	141.00
LOC100542828	270.0	102.0	89.5	179.0	120.5	68.0	138.2	74.78
LOC100542830	737.0	788.0	571.5	646.0	953.5	509.0	700.8	160.80
LOC100542834	1.0	1.5	0.5	3.5	2.0	2.0	1.8	1.04
LOC100542849	31.0	6.5	25.5	31.0	6.5	29.5	21.7	11.92
LOC100542853	13.0	1.5	5.5	8.0	2.5	4.0	5.8	4.23
LOC100542860	214.0	328.5	185.0	230.0	316.0	206.5	246.7	60.44
LOC100542863	125.0	103.5	127.0	124.5	109.5	138.5	121.3	12.71
LOC100542866	0.0	1.0	2.5	0.0	0.0	0.5	0.7	0.98
LOC100542871	6.5	7.0	27.0	5.5	5.0	30.0	13.5	11.68
LOC100542873	521.0	338.0	382.0	470.0	412.0	318.0	406.8	77.84
LOC100542874	0.5	0.5	2.5	0.5	0.5	3.5	1.3	1.33
LOC100542876	0.5	7.5	1.0	0.0	7.0	2.5	3.1	3.34
LOC100542877	0.5	2.5	3.0	0.0	2.0	1.0	1.5	1.18
LOC100542888	0.0	0.0	1.0	0.0	0.0	0.0	0.2	0.41
LOC100542889	0.5	0.0	0.5	2.0	0.0	0.5	0.6	0.74
LOC100542901	0.0	0.0	0.0	0.0	0.0	0.0	0.0	0.00
LOC100542902	28.5	19.0	28.5	28.0	22.5	27.0	25.6	3.94
LOC100542906	0.0	0.0	0.5	1.0	1.5	0.0	0.5	0.63
LOC100542915	218.5	168.5	222.0	209.0	155.5	228.5	200.3	30.63
LOC100542919	0.0	0.0	0.0	0.5	0.0	0.0	0.1	0.20
LOC100542926	0.0	0.0	0.0	0.0	0.0	0.0	0.0	0.00
LOC100542931	0.0	0.0	2.0	0.0	2.5	1.0	0.9	1.11
LOC100542970	7941.5	4353.0	4565.5	8498.0	4937.5	3742.5	5673.0	2018.09
LOC100542972	0.0	1.5	0.5	0.0	0.0	0.0	0.3	0.61
LOC100542975	9.0	16.0	15.0	14.0	19.0	15.0	14.7	3.27
LOC100542976	86.5	35.5	33.5	80.0	35.5	25.0	49.3	26.64
LOC100542989	13.0	5.5	1.0	4.5	5.0	0.5	4.9	4.49
LOC100542991	20235.5	16023.0	11033.5	18272.0	15958.5	10805.5	15388.0	3808.03
LOC100543002	0.0	0.0	2.5	0.0	0.0	0.5	0.5	1.00
LOC100543003	14.0	4.5	7.0	11.0	5.5	6.0	8.0	3.70
LOC100543005	2253.5	4269.0	2314.0	1722.0	5030.5	2150.0	2956.5	1349.49
LOC100543007	0.0	0.0	0.0	0.0	0.0	0.0	0.0	0.00
LOC100543009	114.5	99.0	216.0	91.5	108.5	159.5	131.5	47.75

LOC100543013	486.0	520.5	483.5	426.0	511.5	475.5	483.8	33.25
LOC100543018	354.0	287.5	203.0	314.0	223.0	167.0	258.1	71.77
LOC100543020	1.0	12.5	33.0	1.0	10.5	24.5	13.8	12.83
LOC100543023	228.5	234.5	265.5	227.5	228.0	302.0	247.7	30.35
LOC100543030	0.0	0.0	0.5	0.0	0.0	0.0	0.1	0.20
LOC100543035	17.5	10.0	6.0	16.0	8.0	6.5	10.7	4.94
LOC100543038	0.0	1.0	0.0	0.0	1.0	0.0	0.3	0.52
LOC100543050	50.5	51.0	68.5	49.5	58.0	56.5	55.7	7.17
LOC100543055	0.0	0.0	0.5	0.0	0.0	1.0	0.3	0.42
LOC100543059	30.5	5.0	19.0	32.5	4.5	12.0	17.3	12.26
LOC100543064	0.5	0.5	0.0	0.5	0.5	3.0	0.8	1.08
LOC100543069	0.5	1.0	2.5	0.0	1.5	2.0	1.3	0.94
LOC100543078	92.5	122.0	111.5	84.5	144.5	105.5	110.1	21.51
LOC100543080	1.5	4.5	5.0	3.5	5.5	5.5	4.3	1.54
LOC100543103	136.0	230.5	228.5	137.0	253.5	251.5	206.2	54.94
LOC100543106	1087.0	865.5	943.5	888.5	922.0	821.0	921.3	91.87
LOC100543114	156.5	56.5	58.5	133.0	65.0	53.5	87.2	45.38
LOC100543118	76.0	44.0	100.5	77.5	27.0	116.0	73.5	33.44
LOC100543119	0.0	0.0	0.0	0.0	0.0	0.0	0.0	0.00
LOC100543123	16.0	20.0	4.5	9.0	19.5	6.5	12.6	6.78
LOC100543124	124.0	49.0	47.5	121.5	52.0	35.0	71.5	40.13
LOC100543128	247.5	68.5	11.5	201.5	79.0	14.0	103.7	98.63
LOC100543133	168.5	280.0	314.5	150.0	309.0	364.5	264.4	86.08
LOC100543145	36.5	33.0	140.5	40.5	51.5	110.5	68.8	45.40
LOC100543147	36.0	11.5	59.0	45.0	14.5	44.0	35.0	18.60
LOC100543156	10.0	4.5	2.0	5.0	8.0	2.0	5.3	3.22
LOC100543157	0.0	0.5	0.0	0.0	0.0	0.0	0.1	0.20
LOC100543159	1777.0	1331.0	1239.0	1720.0	1452.0	1169.5	1448.1	251.86
LOC100543166	654.5	480.0	375.0	643.0	452.0	346.0	491.8	131.12
LOC100543167	14.5	14.0	21.5	19.0	11.5	24.5	17.5	4.99
LOC100543177	12.0	7.5	2.0	14.0	7.0	2.0	7.4	4.96
LOC100543179	1.5	5.5	30.5	1.5	2.5	12.0	8.9	11.30
LOC100543185	0.0	2.5	1.0	0.0	2.0	1.0	1.1	1.02
LOC100543188	142.5	133.5	283.0	147.5	156.0	263.5	187.7	66.98
LOC100543189	0.5	0.0	0.0	0.5	0.0	0.0	0.2	0.26
LOC100543196	25.0	6.5	27.0	27.0	6.5	19.0	18.5	9.75
LOC100543199	0.0	0.0	0.0	0.0	0.0	0.0	0.0	0.00
LOC100543202	0.0	0.0	0.0	0.0	0.0	0.0	0.0	0.00
LOC100543206	485.5	226.0	368.5	466.5	232.0	342.5	353.5	110.96
LOC100543214	21.0	27.5	27.5	23.0	29.5	10.0	23.1	7.15
LOC100543226	821.5	571.5	553.0	773.5	655.5	648.0	670.5	107.47
LOC100543235	211.0	279.0	424.5	131.5	281.5	426.5	292.3	116.83
LOC100543241	0.5	0.0	1.5	0.0	1.5	2.0	0.9	0.86
LOC100543242	12.5	7.0	10.5	8.5	5.5	7.0	8.5	2.59
LOC100543251	820.5	345.5	316.5	777.5	392.5	251.0	483.9	248.71
LOC100543260	0.0	0.0	0.0	0.0	0.0	0.0	0.0	0.00
LOC100543264	771.0	737.5	508.0	703.5	765.5	565.0	675.1	111.46
LOC100543274	0.0	0.0	0.5	0.0	0.0	0.5	0.2	0.26
LOC100543277	0.0	0.5	0.0	1.0	0.0	0.0	0.3	0.42
LOC100543282	62.5	34.5	47.0	50.0	42.5	44.0	46.8	9.32
LOC100543283	0.0	0.0	0.5	0.0	0.0	0.0	0.1	0.20
LOC100543285	148.5	93.5	131.0	103.5	72.5	71.0	103.3	31.30
LOC100543297	1063.0	873.0	1008.0	1283.5	929.5	913.0	1011.7	149.84
LOC100543299	0.0	1.0	0.5	1.0	0.5	1.0	0.7	0.41

LOC100543301	459.5	186.5	468.5	510.5	207.0	416.0	374.7	141.19
LOC100543303	496.0	378.0	379.5	460.5	466.5	321.0	416.9	67.42
LOC100543305	525.0	146.5	310.5	534.5	123.5	185.5	304.3	186.25
LOC100543307	0.0	0.0	0.0	0.0	0.0	0.0	0.0	0.00
LOC100543316	37.0	17.5	43.0	44.0	25.0	35.5	33.7	10.44
LOC100543320	1048.5	494.0	344.5	959.0	479.5	309.5	605.8	317.89
LOC100543329	0.0	0.0	0.0	0.0	0.0	0.0	0.0	0.00
LOC100543330	0.5	166.0	178.0	0.0	148.5	116.5	101.6	81.18
LOC100543336	296.0	427.5	192.5	237.0	443.0	228.0	304.0	107.08
LOC100543342	3.0	1.0	1.5	6.5	2.5	3.5	3.0	1.95
LOC100543343	1124.0	1311.0	723.0	1041.0	1330.5	711.0	1040.1	273.36
LOC100543349	566.5	446.5	667.0	567.0	575.0	614.5	572.8	73.00
LOC100543356	0.0	0.0	0.0	0.0	0.0	0.5	0.1	0.20
LOC100543359	0.0	0.0	0.0	0.0	0.0	0.0	0.0	0.00
LOC100543367	0.0	0.0	0.0	0.0	0.0	0.0	0.0	0.00
LOC100543379	162.0	158.5	127.0	163.0	194.0	182.0	164.4	22.92
LOC100543381	62.0	57.5	56.0	50.5	66.0	57.5	58.3	5.30
LOC100543391	280.0	124.0	365.0	328.0	154.5	331.5	263.8	100.70
LOC100543395	123.5	34.0	40.0	103.5	35.5	37.0	62.3	40.25
LOC100543400	0.0	80.0	64.0	5.0	126.5	107.5	63.8	52.21
LOC100543401	0.0	0.0	0.0	0.0	0.0	0.0	0.0	0.00
LOC100543404	4.0	19.5	18.5	2.0	24.5	15.5	14.0	9.02
LOC100543408	0.5	0.0	0.0	0.0	0.0	1.0	0.3	0.42
LOC100543412	997.5	997.5	767.5	913.0	968.0	755.5	899.8	111.57
LOC100543417	0.5	0.0	0.0	0.0	0.0	0.0	0.1	0.20
LOC100543419	113.0	156.5	174.5	158.0	177.0	276.0	175.8	54.18
LOC100543433	0.0	0.0	0.0	0.0	0.0	0.0	0.0	0.00
LOC100543439	0.0	0.0	0.0	0.0	0.0	0.0	0.0	0.00
LOC100543441	10.5	11.5	5.0	11.0	19.5	4.5	10.3	5.45
LOC100543444	0.0	0.0	0.0	0.0	0.0	0.0	0.0	0.00
LOC100543458	0.0	0.0	0.0	2.0	0.0	0.0	0.3	0.82
LOC100543464	127.5	835.5	1283.5	174.5	1019.5	1206.0	774.4	507.47
LOC100543469	0.5	0.0	0.0	0.0	0.0	0.0	0.1	0.20
LOC100543473	0.0	0.0	0.5	0.0	0.0	0.5	0.2	0.26
LOC100543474	339.5	87.5	75.0	232.5	64.5	35.5	139.1	119.96
LOC100543476	2.5	0.5	0.0	1.0	0.0	0.0	0.7	0.98
LOC100543487	0.0	0.0	0.0	0.0	0.0	0.0	0.0	0.00
LOC100543496	3.0	9.0	9.5	1.0	10.5	12.0	7.5	4.43
LOC100543498	0.0	0.0	0.0	0.0	0.0	0.0	0.0	0.00
LOC100543500	185.5	206.0	139.0	173.0	217.0	170.5	181.8	27.84
LOC100543508	2.0	3.0	2.0	2.0	4.5	0.5	2.3	1.33
LOC100543510	0.0	0.0	0.0	0.0	1.0	0.0	0.2	0.41
LOC100543523	0.0	0.0	0.0	0.0	0.0	0.0	0.0	0.00
LOC100543527	1484.5	1084.5	1016.5	1486.0	1170.0	845.5	1181.2	258.53
LOC100543530	75.0	29.5	113.5	73.0	52.5	65.0	68.1	27.84
LOC100543537	2.0	0.5	0.0	2.5	0.5	0.0	0.9	1.07
LOC100543540	591.5	514.5	733.0	581.5	631.0	717.0	628.1	84.06
LOC100543544	1.0	0.0	0.0	0.0	0.5	1.0	0.4	0.49
LOC100543546	259.5	269.5	250.5	220.0	245.5	259.0	250.7	17.14
LOC100543548	0.0	0.0	0.0	0.0	0.0	0.0	0.0	0.00
LOC100543549	1.5	2.5	0.0	3.0	0.0	0.5	1.3	1.29
LOC100543552	1160.0	453.0	396.5	1267.5	484.0	429.0	698.3	401.71
LOC100543556	25.0	414.0	282.0	29.0	504.0	310.0	260.7	197.39
LOC100543562	6.0	1.0	2.0	4.5	1.5	2.0	2.8	1.97

LOC100543567	691.0	448.0	255.0	673.5	509.0	260.5	472.8	190.96
LOC100543573	0.5	0.0	0.0	0.0	1.5	0.0	0.3	0.61
LOC100543582	0.0	0.0	0.0	0.0	0.0	0.0	0.0	0.00
LOC100543589	6.0	16.5	8.0	8.5	12.0	6.5	9.6	3.99
LOC100543590	0.5	1.5	1.5	2.5	0.0	3.0	1.5	1.14
LOC100543595	25.5	22.5	20.0	19.5	32.0	14.0	22.3	6.11
LOC100543599	49.5	14.5	94.5	67.5	16.5	84.0	54.4	33.79
LOC100543611	28.0	126.0	98.5	26.5	157.0	85.5	86.9	52.34
LOC100543612	1.0	0.0	2.0	0.0	0.0	0.5	0.6	0.80
LOC100543615	2.0	1.5	1.0	1.5	1.0	0.5	1.3	0.52
LOC100543617	2155.0	1811.0	1837.5	2040.0	1996.0	1645.0	1914.1	184.12
LOC100543622	0.5	2.0	0.5	2.0	2.0	0.5	1.3	0.82
LOC100543634	0.0	0.0	0.0	0.0	0.0	0.0	0.0	0.00
LOC100543636	0.0	0.0	0.0	0.0	0.0	0.0	0.0	0.00
LOC100543637	0.0	0.5	0.5	0.0	1.0	0.0	0.3	0.41
LOC100543639	0.5	5.0	12.0	1.5	10.0	4.0	5.5	4.60
LOC100543646	0.0	0.0	0.0	0.0	0.5	0.0	0.1	0.20
LOC100543661	365.0	326.5	559.5	398.0	445.0	546.0	440.0	95.72
LOC100543673	0.0	0.0	0.0	0.0	0.0	0.0	0.0	0.00
LOC100543675	27.5	20.0	17.0	20.0	21.5	18.5	20.8	3.64
LOC100543679	185.5	102.0	307.5	196.5	106.5	265.5	193.9	82.70
LOC100543691	3.0	1.5	2.0	2.5	2.0	1.5	2.1	0.58
LOC100543701	0.0	0.0	0.0	0.0	0.0	0.0	0.0	0.00
LOC100543707	28.5	10.0	45.0	30.0	24.0	37.0	29.1	11.89
LOC100543736	267.0	224.5	117.0	280.5	245.5	135.0	211.6	69.21
LOC100543737	0.0	0.0	0.0	0.0	0.0	0.0	0.0	0.00
LOC100543743	10384.0	8820.0	5519.0	8467.5	9190.0	6000.0	8063.4	1903.77
LOC100543744	18.0	5.0	17.5	24.5	3.5	16.0	14.1	8.17
LOC100543747	0.0	0.0	0.0	0.0	0.0	0.0	0.0	0.00
LOC100543749	0.0	0.5	0.0	0.5	1.0	0.5	0.4	0.38
LOC100543750	140.5	68.5	166.0	131.5	65.5	148.0	120.0	42.60
LOC100543754	0.0	1.0	0.5	0.0	0.5	0.5	0.4	0.38
LOC100543777	36.5	29.0	71.5	41.5	36.5	52.5	44.6	15.30
LOC100543785	2542.0	2539.5	3063.0	2625.0	3073.5	3437.0	2880.0	367.80
LOC100543786	0.5	4.5	0.0	0.5	3.5	0.0	1.5	1.97
LOC100543796	993.5	475.5	648.5	848.5	347.0	455.5	628.1	250.84
LOC100543818	52.0	41.0	115.5	50.0	50.0	92.5	66.8	29.94
LOC100543828	2.0	1.0	2.5	1.5	1.0	5.5	2.3	1.70
LOC100543834	475.0	292.5	530.5	421.0	327.5	500.0	424.4	96.26
LOC100543835	20.0	3.5	36.5	22.5	10.5	33.0	21.0	12.68
LOC100543837	60.5	23.0	21.0	60.5	37.0	27.0	38.2	18.16
LOC100543839	16.0	4.0	54.0	13.0	3.0	42.5	22.1	21.20
LOC100543851	1.0	0.5	0.0	0.0	1.5	1.0	0.7	0.61
LOC100543857	0.0	0.0	0.0	0.0	0.0	0.0	0.0	0.00
LOC100543858	0.0	0.0	0.0	0.0	0.0	0.0	0.0	0.00
LOC100543865	8.5	0.5	10.5	9.0	2.0	6.5	6.2	4.05
LOC100543880	0.0	0.0	0.0	0.0	0.0	0.0	0.0	0.00
LOC100543887	2.5	1.5	5.5	1.5	1.5	2.5	2.5	1.55
LOC100543889	0.0	1.0	0.0	0.0	0.5	0.0	0.3	0.42
LOC100543893	0.0	1.5	3.0	1.0	3.0	2.0	1.8	1.17
LOC100543903	0.0	0.0	0.0	0.0	0.0	0.0	0.0	0.00
LOC100543904	6.5	1.5	0.5	7.5	3.5	1.5	3.5	2.90
LOC100543905	108.5	107.5	48.0	103.5	122.5	59.5	91.6	30.22
LOC100543914	364.0	144.0	222.5	371.0	182.0	244.5	254.7	93.97

LOC100543920	2.0	1.0	0.5	0.5	4.0	1.0	1.5	1.34
LOC100543921	0.0	1.0	1.5	0.5	0.5	1.5	0.8	0.61
LOC100543930	0.0	0.0	2.0	1.5	0.5	0.5	0.8	0.82
LOC100543947	26.0	14.5	7.5	16.5	14.0	5.0	13.9	7.40
LOC100543953	0.0	0.0	0.0	0.0	0.0	0.0	0.0	0.00
LOC100543957	429.5	237.0	359.5	357.5	271.0	315.5	328.3	69.07
LOC100543967	0.0	0.0	0.0	0.0	0.0	0.0	0.0	0.00
LOC100543969	5157.0	4152.0	6325.5	7023.5	4973.0	9560.5	6198.6	1937.76
LOC100543979	109.0	65.0	125.5	116.0	68.0	127.5	101.8	28.19
LOC100543986	2.0	0.0	4.0	1.0	0.5	1.5	1.5	1.41
LOC100544007	42.5	17.0	58.0	42.0	14.0	43.0	36.1	17.07
LOC100544020	48.5	29.0	76.0	49.0	21.5	45.5	44.9	18.96
LOC100544022	0.5	0.5	0.0	0.0	0.0	0.0	0.2	0.26
LOC100544043	0.0	0.5	4.5	0.0	3.0	0.5	1.4	1.88
LOC100544046	1.0	2.0	1.5	0.5	0.5	1.5	1.2	0.61
LOC100544056	21.0	8.5	40.5	24.5	10.0	45.5	25.0	15.32
LOC100544060	0.0	0.0	0.0	0.0	0.0	0.0	0.0	0.00
LOC100544062	40.0	21.5	11.0	29.5	13.0	7.0	20.3	12.58
LOC100544066	516.5	481.5	386.5	461.0	544.5	408.5	466.4	61.00
LOC100544071	0.0	0.0	0.0	0.0	0.0	0.0	0.0	0.00
LOC100544072	212.5	227.0	350.0	221.5	283.0	373.5	277.9	69.88
LOC100544079	0.0	0.0	0.0	0.0	0.0	0.0	0.0	0.00
LOC100544081	122.0	70.5	56.5	135.5	58.5	41.5	80.8	38.54
LOC100544088	176.0	71.0	122.5	168.5	64.5	72.0	112.4	50.86
LOC100544098	2.5	1.0	0.5	2.0	1.0	3.0	1.7	0.98
LOC100544105	765.5	268.5	949.0	877.5	327.5	710.0	649.7	285.53
LOC100544107	1.0	0.0	0.0	0.5	0.0	0.5	0.3	0.41
LOC100544113	2.5	7.5	1.0	3.5	6.0	2.5	3.8	2.44
LOC100544119	47.5	28.5	34.0	73.5	36.0	22.5	40.3	18.27
LOC100544123	189.5	179.0	144.0	224.0	272.5	244.0	208.8	46.88
LOC100544124	510.0	491.0	340.0	477.0	549.0	295.5	443.8	101.54
LOC100544125	6.0	0.0	2.0	9.0	0.0	4.0	3.5	3.56
LOC100544134	6.0	0.0	10.0	6.0	1.5	7.0	5.1	3.69
LOC100544136	170.5	119.5	574.0	215.0	150.0	464.0	282.2	189.28
LOC100544139	8.0	12.0	0.0	10.5	15.0	3.5	8.2	5.57
LOC100544143	786.0	817.5	585.5	663.0	879.5	604.5	722.7	121.61
LOC100544156	28.0	12.0	15.0	29.0	20.0	14.5	19.8	7.26
LOC100544159	0.0	0.0	0.0	0.0	0.0	0.0	0.0	0.00
LOC100544162	0.0	0.0	0.0	0.0	0.0	0.0	0.0	0.00
LOC100544165	30.5	15.0	26.0	36.0	17.5	18.5	23.9	8.29
LOC100544169	0.0	0.0	0.0	0.0	0.0	0.0	0.0	0.00
LOC100544170	18.5	47.0	14.5	12.5	50.0	22.5	27.5	16.65
LOC100544185	0.0	0.0	0.0	0.0	0.0	0.0	0.0	0.00
LOC100544196	208.5	153.5	193.0	214.0	162.5	198.0	188.3	24.74
LOC100544198	2.0	297.0	758.0	0.0	279.5	480.5	302.8	290.35
LOC100544199	269.5	279.0	351.0	268.5	337.0	311.0	302.7	35.81
LOC100544203	169.5	146.5	137.0	188.5	177.0	105.5	154.0	30.52
LOC100544221	466.0	357.0	312.5	509.0	430.0	297.0	395.3	86.16
LOC100544224	0.0	0.0	0.0	0.0	0.0	0.0	0.0	0.00
LOC100544231	6.0	5.5	1.5	1.5	3.5	4.5	3.8	1.94
LOC100544232	0.0	0.0	0.5	0.0	0.0	0.0	0.1	0.20
LOC100544235	3.5	1.0	14.0	6.0	2.0	8.0	5.8	4.79
LOC100544245	52.5	25.5	117.5	13.0	61.5	65.0	55.8	36.56
LOC100544252	727.0	484.5	982.0	740.0	589.0	1046.5	761.5	218.17

LOC100544253	75.5	64.5	78.0	71.0	65.0	89.0	73.8	9.20
LOC100544274	0.5	5.0	0.5	0.0	4.5	5.5	2.7	2.58
LOC100544277	0.0	1.5	1.5	0.0	2.0	1.0	1.0	0.84
LOC100544286	0.0	1.0	0.0	0.0	1.5	0.0	0.4	0.66
LOC100544288	13.5	22.5	24.5	8.0	12.5	13.0	15.7	6.41
LOC100544290	0.0	0.5	0.5	0.0	0.5	0.0	0.3	0.27
LOC100544291	198.5	176.5	165.0	221.5	208.0	202.0	195.3	20.85
LOC100544294	66.0	22.0	129.5	76.0	28.0	113.5	72.5	43.62
LOC100544297	6.5	1.0	1.5	4.0	7.0	1.5	3.6	2.67
LOC100544298	3.0	2.0	0.5	5.5	1.0	0.5	2.1	1.93
LOC100544306	990.0	871.0	1080.5	930.5	886.0	1030.5	964.8	83.09
LOC100544311	0.0	0.5	0.0	0.0	1.5	0.0	0.3	0.61
LOC100544321	202.0	147.5	110.0	190.5	175.5	172.5	166.3	33.19
LOC100544322	0.0	0.0	0.0	0.0	0.0	0.0	0.0	0.00
LOC100544327	153.0	87.0	91.5	153.5	102.5	74.5	110.3	34.43
LOC100544335	276.0	152.0	371.5	261.5	214.0	383.5	276.4	89.56
LOC100544338	8.0	4.5	9.0	9.5	1.5	9.0	6.9	3.22
LOC100544340	91.0	63.5	94.0	76.5	85.5	95.0	84.3	12.23
LOC100544344	20.0	33.5	22.0	14.0	29.5	23.5	23.8	6.93
LOC100544354	0.0	4.5	20.5	0.0	6.0	16.5	7.9	8.63
LOC100544355	457.5	367.0	214.5	427.0	406.0	213.0	347.5	107.71
LOC100544367	0.5	4.5	0.5	0.5	4.5	1.0	1.9	2.01
LOC100544369	88.0	53.0	178.5	110.0	61.5	161.5	108.8	51.81
LOC100544371	10.5	1.0	17.0	9.0	3.0	13.5	9.0	6.11
LOC100544372	333.0	325.0	202.5	307.5	332.0	279.5	296.6	50.30
LOC100544385	1.0	0.5	0.5	2.0	0.0	0.0	0.7	0.75
LOC100544389	5.0	2.5	4.0	8.5	2.5	2.5	4.2	2.36
LOC100544391	0.0	0.0	0.0	0.0	0.0	0.0	0.0	0.00
LOC100544408	363.5	315.5	280.5	320.5	310.5	250.0	306.8	38.50
LOC100544421	416.0	240.5	503.5	367.5	198.0	445.0	361.8	119.60
LOC100544424	0.0	0.5	0.0	0.0	1.0	0.5	0.3	0.41
LOC100544425	120.0	65.0	175.0	127.0	74.0	164.5	120.9	45.14
LOC100544433	6.0	2.5	12.5	7.0	3.0	19.0	8.3	6.34
LOC100544434	727.0	448.5	340.0	712.0	418.0	309.0	492.4	183.08
LOC100544435	16.0	8.5	16.5	21.5	4.5	11.5	13.1	6.14
LOC100544436	33.0	13.5	13.0	37.0	30.0	17.0	23.9	10.64
LOC100544441	18.0	5.5	4.0	10.5	7.0	5.5	8.4	5.19
LOC100544443	1023.5	389.0	1462.0	1105.0	463.5	1113.0	926.0	416.19
LOC100544448	5.5	8.5	9.5	5.5	12.5	27.0	11.4	8.08
LOC100544450	0.5	2.0	41.5	0.0	4.5	40.0	14.8	20.21
LOC100544458	28.0	22.0	17.0	24.5	17.5	15.5	20.8	4.91
LOC100544459	66.5	32.5	76.5	63.5	19.0	99.5	59.6	29.40
LOC100544461	134.5	108.5	252.5	125.5	98.5	181.5	150.2	57.82
LOC100544468	0.5	0.0	0.0	0.0	0.0	0.5	0.2	0.26
LOC100544471	873.5	619.5	1060.0	852.5	667.0	1008.5	846.8	176.83
LOC100544478	0.0	0.5	0.0	0.5	0.0	0.0	0.2	0.26
LOC100544485	1.5	3.0	0.0	0.0	2.5	0.0	1.2	1.37
LOC100544490	36.5	24.0	56.5	25.0	37.0	70.0	41.5	18.23
LOC100544491	113.0	66.0	59.5	121.0	71.0	76.0	84.4	25.95
LOC100544502	972.5	1138.0	1291.5	926.5	1159.0	1232.5	1120.0	143.61
LOC100544508	7.5	66.5	69.5	25.5	115.5	132.0	69.4	48.60
LOC100544511	653.5	615.0	391.5	621.0	708.5	440.0	571.6	126.12
LOC100544513	500.0	449.0	391.5	511.5	497.5	415.0	460.8	49.99
LOC100544524	82.0	106.0	94.5	58.5	117.0	65.0	87.2	22.97

LOC100544528	0.0	0.0	1.0	0.0	0.0	0.0	0.2	0.41
LOC100544530	0.0	0.0	0.0	0.0	1.0	0.5	0.3	0.42
LOC100544531	0.0	0.0	0.0	0.0	0.0	0.0	0.0	0.00
LOC100544543	44.5	38.0	53.0	46.5	37.5	39.5	43.2	6.03
LOC100544544	2.0	1.5	0.5	1.0	2.0	1.0	1.3	0.61
LOC100544547	28.0	21.5	14.5	45.0	30.5	17.0	26.1	11.12
LOC100544550	0.0	0.0	0.0	0.0	0.0	0.0	0.0	0.00
LOC100544552	0.0	0.0	0.0	0.0	0.0	0.0	0.0	0.00
LOC100544555	0.0	0.0	0.0	0.0	0.0	0.0	0.0	0.00
LOC100544563	0.0	0.0	0.0	0.0	0.0	0.0	0.0	0.00
LOC100544568	630.0	384.5	1450.0	720.0	436.0	1353.5	829.0	461.36
LOC100544577	172.0	143.0	471.5	161.0	186.5	407.0	256.8	143.47
LOC100544580	9044.0	11360.0	15528.0	9695.5	13572.0	19018.5	13036.3	3799.78
LOC100544583	48035.5	32074.0	38567.0	44039.5	33516.5	37160.0	38898.8	6141.44
LOC100544584	0.0	0.0	0.0	0.5	0.0	0.5	0.2	0.26
LOC100544585	253.0	179.0	190.0	230.5	230.0	215.0	216.3	27.64
LOC100544587	672.0	568.5	635.5	645.5	734.0	632.5	648.0	54.21
LOC100544588	0.0	0.0	0.0	0.0	0.0	0.5	0.1	0.20
LOC100544592	60.0	29.0	50.5	69.0	22.0	41.5	45.3	18.04
LOC100544598	0.0	0.0	0.0	0.0	0.0	0.0	0.0	0.00
LOC100544614	22.0	11.5	5.5	11.0	6.5	6.5	10.5	6.17
LOC100544619	1849.5	1208.5	1985.0	1911.0	1251.5	1751.5	1659.5	341.65
LOC100544620	6280.5	5745.0	4834.0	6122.5	6363.0	4482.0	5637.8	795.91
LOC100544625	0.0	0.5	0.0	1.5	0.0	0.0	0.3	0.61
LOC100544628	0.0	0.0	0.0	0.0	0.0	0.0	0.0	0.00
LOC100544636	0.0	0.5	0.0	0.5	0.0	0.0	0.2	0.26
LOC100544640	0.0	0.0	0.5	1.0	0.0	0.0	0.3	0.42
LOC100544642	0.0	0.0	0.0	0.0	0.0	0.0	0.0	0.00
LOC100544649	600.5	358.5	325.0	542.0	377.0	318.0	420.2	120.44
LOC100544657	2.0	0.0	1.0	0.5	0.0	0.5	0.7	0.75
LOC100544679	918.5	644.5	610.5	944.0	749.0	584.5	741.8	157.23
LOC100544684	0.5	0.0	0.0	1.0	0.5	0.0	0.3	0.41
LOC100544690	1.5	0.0	0.0	0.0	0.0	0.0	0.3	0.61
LOC100544693	2.5	2.5	0.5	0.0	1.5	1.5	1.4	1.02
LOC100544702	57.0	59.5	37.5	59.5	47.0	42.5	50.5	9.48
LOC100544708	2085.5	1557.5	1506.0	2291.0	1830.0	1444.5	1785.8	344.64
LOC100544709	0.0	0.0	0.0	0.0	0.0	0.0	0.0	0.00
LOC100544713	0.0	0.0	0.0	0.0	0.0	0.0	0.0	0.00
LOC100544724	1350.5	2347.5	3066.5	948.0	1880.0	3103.5	2116.0	887.48
LOC100544731	50.0	25.5	11.0	58.0	26.5	24.0	32.5	17.75
LOC100544738	231.5	147.0	98.5	176.5	173.5	86.5	152.3	53.96
LOC100544740	10.0	12.5	9.0	6.5	8.0	6.0	8.7	2.40
LOC100544745	69.0	101.0	57.0	87.5	176.0	44.0	89.1	47.25
LOC100544750	0.0	0.0	0.0	0.0	0.0	0.0	0.0	0.00
LOC100544756	199.0	56.0	290.0	213.5	72.5	201.0	172.0	90.08
LOC100544767	144.0	64.5	166.0	132.0	85.5	136.0	121.3	38.35
LOC100544777	2.0	1.0	1.0	2.0	1.0	1.0	1.3	0.52
LOC100544780	1.0	1.0	0.5	0.5	0.0	0.5	0.6	0.38
LOC100544787	0.0	0.0	0.0	0.0	0.0	0.0	0.0	0.00
LOC100544792	0.0	0.0	0.0	0.0	0.0	0.0	0.0	0.00
LOC100544802	0.0	0.5	0.0	1.0	0.0	1.0	0.4	0.49
LOC100544805	0.0	0.0	0.0	0.0	0.0	0.0	0.0	0.00
LOC100544806	504.0	2525.0	1295.0	528.0	2702.5	1161.5	1452.7	956.72
LOC100544815	8.5	4.5	2.5	4.0	2.0	0.5	3.7	2.77

LOC100544818	139.5	105.0	89.0	146.5	119.5	77.5	112.8	27.45
LOC100544821	0.5	0.5	0.5	2.0	0.5	0.5	0.8	0.61
LOC100544825	0.0	1.0	0.0	0.5	0.0	0.0	0.3	0.42
LOC100544832	76.0	28.5	171.5	88.5	26.5	183.0	95.7	67.99
LOC100544843	350.5	139.5	208.0	323.0	145.0	221.0	231.2	88.48
LOC100544848	1.5	2.0	0.5	1.0	1.5	0.0	1.1	0.74
LOC100544862	1.5	0.0	0.0	0.0	0.0	0.0	0.3	0.61
LOC100544866	1.5	0.0	0.0	3.5	0.5	0.5	1.0	1.34
LOC100544870	1.5	0.0	0.0	1.5	0.0	0.0	0.5	0.77
LOC100544871	7.0	4.0	4.5	8.0	3.5	5.5	5.4	1.77
LOC100544877	237.0	242.0	174.0	241.5	268.0	183.5	224.3	37.08
LOC100544881	0.5	0.0	0.5	2.5	0.0	1.0	0.8	0.94
LOC100544896	5.5	12.0	9.0	3.0	15.5	13.5	9.8	4.82
LOC100544897	0.0	0.0	0.0	0.0	0.0	0.0	0.0	0.00
LOC100544899	3157.5	3308.0	5487.0	3172.5	3553.0	4797.5	3912.6	987.38
LOC100544913	359.5	275.5	348.0	269.5	276.0	274.5	300.5	41.47
LOC100544917	0.5	0.5	0.0	0.0	0.5	1.5	0.5	0.55
LOC100544920	9.0	0.5	6.0	4.5	2.5	6.0	4.8	2.98
LOC100544929	1.0	2.0	4.5	1.5	1.0	7.0	2.8	2.42
LOC100544938	10.0	1.5	0.5	21.0	4.5	8.0	7.6	7.52
LOC100544939	0.0	0.0	0.0	0.0	0.0	0.0	0.0	0.00
LOC100544943	0.0	0.0	0.0	0.0	0.0	0.0	0.0	0.00
LOC100544944	9.0	10.0	10.0	4.5	4.0	6.5	7.3	2.71
LOC100544946	0.0	0.0	0.5	0.0	0.0	0.0	0.1	0.20
LOC100544965	0.0	0.0	0.0	0.0	0.0	0.0	0.0	0.00
LOC100544972	110.5	240.5	157.5	104.5	220.5	167.5	166.8	55.59
LOC100544973	1.0	0.0	0.0	0.0	0.5	0.0	0.3	0.42
LOC100544984	0.0	0.0	0.0	0.0	0.0	0.0	0.0	0.00
LOC100544999	6.5	2.0	2.5	3.0	1.5	1.0	2.8	1.97
LOC100545010	0.0	0.5	0.5	0.0	0.5	0.5	0.3	0.26
LOC100545013	8.0	1.5	2.0	2.0	0.5	0.0	2.3	2.89
LOC100545035	79.0	40.0	19.5	99.5	53.5	19.5	51.8	32.38
LOC100545042	23.0	4.5	24.5	16.5	3.0	18.5	15.0	9.20
LOC100545050	8.0	0.5	3.5	3.5	3.5	2.5	3.6	2.46
LOC100545053	554.0	320.5	332.5	452.0	331.0	323.0	385.5	96.67
LOC100545069	389.0	108.5	611.0	438.0	143.0	529.5	369.8	204.17
LOC100545074	1068.0	1043.5	1066.5	980.0	1137.5	1016.0	1051.9	53.53
LOC100545080	501.5	231.5	65.0	226.5	283.5	109.0	236.2	153.77
LOC100545091	2.5	0.0	2.5	1.0	1.5	2.0	1.6	0.97
LOC100545094	0.0	0.0	0.0	0.0	0.0	0.0	0.0	0.00
LOC100545095	229.0	66.0	118.0	267.0	85.0	118.0	147.2	81.50
LOC100545101	369.0	536.5	347.0	325.0	528.0	406.0	418.6	92.06
LOC100545105	4.5	2.0	9.5	3.5	1.5	7.5	4.8	3.16
LOC100545106	0.5	0.5	1.5	1.5	0.0	0.5	0.8	0.61
LOC100545114	0.0	0.0	0.0	0.0	0.0	0.0	0.0	0.00
LOC100545120	0.0	1.0	2.0	1.0	2.5	2.0	1.4	0.92
LOC100545121	226.5	190.5	325.0	238.5	201.0	300.0	246.9	54.21
LOC100545122	3.0	2.0	0.0	2.5	2.0	0.5	1.7	1.17
LOC100545125	47.0	18.5	16.0	28.5	27.0	10.5	24.6	12.91
LOC100545133	7.5	14.0	52.5	11.5	15.5	33.5	22.4	17.25
LOC100545134	1413.0	1367.0	775.5	1298.0	1432.0	784.0	1178.3	312.12
LOC100545141	2749.0	1779.5	1197.5	2560.0	1779.0	1095.5	1860.1	680.70
LOC100545142	0.0	0.0	0.0	0.0	0.0	0.0	0.0	0.00
LOC100545149	7.0	2.0	1.5	8.0	1.5	0.0	3.3	3.31

LOC100545163	24.0	15.0	8.0	8.5	10.0	2.5	11.3	7.39
LOC100545164	556.5	242.0	89.0	878.0	284.0	92.0	356.9	307.20
LOC100545183	14.5	4.0	3.5	19.5	1.5	2.5	7.6	7.51
LOC100545184	0.0	0.0	0.0	0.0	0.0	0.0	0.0	0.00
LOC100545191	1.0	0.0	1.0	0.0	0.0	0.5	0.4	0.49
LOC100545203	10.0	2.5	24.0	12.5	3.5	52.0	17.4	18.63
LOC100545207	11.5	5.5	9.0	12.0	7.5	6.0	8.6	2.75
LOC100545208	748.5	793.0	1232.5	806.0	929.5	1251.0	960.1	226.36
LOC100545215	12.0	3.5	23.0	21.0	5.0	17.0	13.6	8.16
LOC100545217	1087.5	842.5	546.0	849.0	817.0	484.5	771.1	221.90
LOC100545218	232.0	216.5	357.0	262.0	230.5	327.5	270.9	57.97
LOC100545219	469.0	372.0	275.0	445.5	353.5	276.5	365.3	81.74
LOC100545225	148.0	96.0	23.0	120.0	125.5	22.5	89.2	54.04
LOC100545228	0.0	0.0	0.0	0.0	0.5	0.5	0.2	0.26
LOC100545232	0.0	0.0	0.0	0.0	0.0	0.0	0.0	0.00
LOC100545236	0.0	0.0	0.0	0.0	0.0	0.0	0.0	0.00
LOC100545241	273.0	142.5	432.5	249.0	151.5	358.5	267.8	113.98
LOC100545242	0.0	0.0	0.0	0.0	0.0	0.0	0.0	0.00
LOC100545251	0.0	0.5	0.0	0.0	0.0	0.0	0.1	0.20
LOC100545254	0.0	0.0	0.0	0.0	0.0	0.0	0.0	0.00
LOC100545259	158.5	168.5	151.0	141.0	196.5	175.5	165.2	19.64
LOC100545266	17.5	5.5	7.0	18.0	9.0	6.5	10.6	5.67
LOC100545269	0.0	0.0	0.0	0.0	0.0	0.0	0.0	0.00
LOC100545276	0.0	0.0	0.0	0.0	0.0	0.0	0.0	0.00
LOC100545280	0.0	0.5	1.0	0.0	0.5	1.0	0.5	0.45
LOC100545285	9.0	8.0	9.5	11.0	6.5	6.0	8.3	1.89
LOC100545300	0.0	1.0	0.5	0.5	0.5	0.0	0.4	0.38
LOC100545311	51.0	23.5	36.0	46.0	16.0	41.5	35.7	13.50
LOC100545312	2885.0	2213.0	2333.0	2875.5	2225.5	2077.5	2434.9	354.38
LOC100545313	0.0	1.5	0.5	0.0	3.0	0.0	0.8	1.21
LOC100545318	70.5	32.5	18.5	62.5	30.0	18.0	38.7	22.49
LOC100545319	169.5	164.5	295.0	146.5	152.0	237.5	194.2	59.30
LOC100545322	622.5	178.5	162.5	317.0	161.5	47.0	248.2	202.50
LOC100545337	2.5	7.0	2.0	1.5	4.0	5.0	3.7	2.09
LOC100545338	40.0	24.5	41.5	68.0	27.5	58.5	43.3	17.08
LOC100545341	2.0	8.0	8.5	0.5	12.5	19.5	8.5	6.98
LOC100545343	436.5	220.0	704.5	463.0	218.5	548.5	431.8	189.39
LOC100545344	46.5	1142.5	164.5	43.0	997.0	221.0	435.8	497.99
LOC100545353	0.0	0.0	0.0	0.0	1.5	0.5	0.3	0.61
LOC100545360	108.0	45.0	275.0	125.5	50.0	223.5	137.8	93.30
LOC100545362	0.5	0.0	0.0	1.0	0.0	0.0	0.3	0.42
LOC100545365	2.5	0.5	1.0	1.0	0.0	0.0	0.8	0.93
LOC100545373	108.5	51.5	148.5	110.0	63.5	160.0	107.0	43.62
LOC100545386	66.5	21.5	84.5	61.5	24.0	53.0	51.8	24.79
LOC100545389	0.5	0.0	0.0	0.5	0.0	0.0	0.2	0.26
LOC100545392	0.5	0.0	0.0	0.0	0.0	0.5	0.2	0.26
LOC100545397	62.0	79.0	52.0	66.0	70.0	69.0	66.3	9.00
LOC100545399	0.5	0.0	0.5	0.0	0.0	0.0	0.2	0.26
LOC100545410	53.0	20.5	121.5	63.5	22.5	107.0	64.7	42.16
LOC100545415	517.0	340.5	325.0	530.5	377.5	291.0	396.9	102.19
LOC100545419	5.0	1.0	0.5	2.5	1.0	0.0	1.7	1.83
LOC100545420	2.0	0.0	1.0	4.0	1.0	0.0	1.3	1.51
LOC100545421	144.0	53.0	218.0	162.0	72.5	190.0	139.9	65.13
LOC100545422	540.0	541.5	597.5	487.0	586.0	525.5	546.3	40.52

LOC100545423	37.5	18.5	31.5	45.0	21.0	24.5	29.7	10.26
LOC100545427	1.0	0.0	0.0	1.0	0.5	1.0	0.6	0.49
LOC100545429	0.0	0.0	0.5	0.0	0.0	0.5	0.2	0.26
LOC100545430	0.0	0.0	0.0	0.0	0.5	0.0	0.1	0.20
LOC100545434	739.5	993.5	690.0	706.5	1099.5	641.0	811.7	187.67
LOC100545437	7.5	21.0	23.5	13.5	26.0	24.5	19.3	7.28
LOC100545443	32.0	43.5	21.5	31.0	45.0	22.0	32.5	10.11
LOC100545452	55.0	35.0	88.5	68.5	33.0	63.0	57.2	21.09
LOC100545453	7.0	5.0	42.5	14.0	3.5	32.0	17.3	16.19
LOC100545461	5.5	5.5	2.0	3.0	5.0	3.5	4.1	1.46
LOC100545463	2.0	2.5	1.0	2.0	2.5	1.0	1.8	0.68
LOC100545464	18.5	5.5	23.5	18.0	5.0	23.5	15.7	8.41
LOC100545469	0.5	0.0	0.0	0.5	2.0	0.0	0.5	0.77
LOC100545470	113.5	31.5	136.5	115.0	35.5	127.5	93.3	47.06
LOC100545471	0.0	0.0	0.5	0.0	1.0	0.5	0.3	0.41
LOC100545477	3608.5	2724.0	1971.5	3612.0	2765.5	1629.0	2718.4	817.03
LOC100545478	2.5	0.5	0.0	0.5	1.0	0.0	0.8	0.94
LOC100545481	3.0	1.0	0.5	1.5	1.0	2.0	1.5	0.89
LOC100545485	0.5	0.0	2.0	0.0	0.0	0.0	0.4	0.80
LOC100545500	1.0	0.5	0.0	0.5	1.5	0.5	0.7	0.52
LOC100545512	39.5	23.0	17.0	34.0	24.5	16.0	25.7	9.36
LOC100545524	90.0	32.0	115.0	98.0	25.5	105.0	77.6	38.76
LOC100545545	55.0	29.5	25.0	48.5	29.0	30.0	36.2	12.37
LOC100545547	172.5	69.0	172.5	163.5	57.5	153.0	131.3	53.35
LOC100545558	0.0	0.0	0.0	0.0	0.0	0.0	0.0	0.00
LOC100545577	590.0	455.0	520.5	505.0	486.5	436.5	498.9	54.36
LOC100545581	203.5	91.0	327.0	207.5	115.5	264.5	201.5	88.73
LOC100545583	1.0	0.0	0.0	0.0	0.0	0.0	0.2	0.41
LOC100545586	6.0	4.0	7.0	5.0	2.0	2.0	4.3	2.07
LOC100545589	0.0	0.0	0.0	0.0	0.0	0.0	0.0	0.00
LOC100545599	2.0	4.5	2.5	0.5	4.5	10.0	4.0	3.32
LOC100545600	0.5	0.0	0.0	0.0	0.0	0.0	0.1	0.20
LOC100545607	0.0	0.0	0.0	0.0	0.0	0.0	0.0	0.00
LOC100545609	0.0	0.0	0.5	0.5	0.5	0.5	0.3	0.26
LOC100545615	83.5	87.5	125.0	86.0	76.5	113.0	95.3	19.16
LOC100545617	7.0	8.0	7.0	6.0	7.5	5.0	6.8	1.08
LOC100545624	42.5	6.0	46.5	26.5	11.5	34.5	27.9	16.45
LOC100545629	218.0	136.0	72.0	210.5	163.0	97.5	149.5	59.14
LOC100545648	9.0	6.5	9.5	7.0	5.0	9.0	7.7	1.78
LOC100545656	12.0	7.0	9.0	11.0	6.0	9.5	9.1	2.29
LOC100545658	59.5	23.0	91.5	74.5	26.0	102.0	62.8	33.00
LOC100545666	22.5	15.0	25.0	22.5	21.5	24.0	21.8	3.53
LOC100545668	2305.5	1544.5	1194.5	1924.5	1618.5	1035.5	1603.8	466.79
LOC100545675	508.5	415.0	343.0	458.0	427.0	312.5	410.7	72.54
LOC100545676	4.0	3.5	2.0	9.0	5.5	3.0	4.5	2.49
LOC100545683	0.5	0.0	0.0	0.0	0.0	0.0	0.1	0.20
LOC100545689	395.0	140.5	387.5	385.5	188.0	299.0	299.3	111.33
LOC100545705	112.5	48.5	84.0	99.5	55.5	73.0	78.8	24.84
LOC100545707	135.5	139.5	162.0	132.0	130.5	203.0	150.4	28.20
LOC100545720	1.0	0.0	0.0	0.0	0.0	0.0	0.2	0.41
LOC100545726	125.0	53.0	152.0	118.5	66.5	132.0	107.8	39.14
LOC100545729	2.5	2.5	1.0	0.0	1.5	1.0	1.4	0.97
LOC100545731	1.0	2.0	1.5	0.0	2.0	1.5	1.3	0.75
LOC100545735	916.5	686.5	540.0	974.0	659.0	611.0	731.2	174.07

LOC100545741	1060.0	1650.0	1361.0	836.5	1763.5	1155.0	1304.3	356.20
LOC100545742	0.5	0.5	0.0	0.0	0.0	0.0	0.2	0.26
LOC100545745	2381.5	2564.0	1546.0	2247.0	2850.0	1470.5	2176.5	556.19
LOC100545752	1.0	3.0	0.5	0.5	1.0	2.5	1.4	1.07
LOC100545757	10034.5	7755.0	3430.0	9360.0	8115.0	6651.0	7557.6	2349.14
LOC100545760	62.0	31.0	31.5	44.5	32.5	35.0	39.4	12.14
LOC100545769	1.0	0.0	2.0	1.0	0.0	2.5	1.1	1.02
LOC100545776	2.0	5.5	3.5	3.5	7.5	0.0	3.7	2.62
LOC100545780	0.0	0.0	0.5	0.5	0.0	0.0	0.2	0.26
LOC100545797	286.5	258.5	125.0	270.0	294.0	111.5	224.3	83.15
LOC100545800	351.5	278.0	281.5	344.0	323.5	372.0	325.1	38.41
LOC100545806	2.0	0.0	0.5	1.0	0.0	0.5	0.7	0.75
LOC100545814	308.0	338.5	364.0	309.5	373.5	349.5	340.5	27.36
LOC100545816	23.0	17.5	47.0	19.0	22.0	39.0	27.9	12.12
LOC100545817	1067.5	597.5	1046.5	1049.0	590.5	1100.5	908.6	244.45
LOC100545820	86.0	45.0	160.0	88.0	61.5	131.5	95.3	43.15
LOC100545821	434.0	321.0	264.5	394.0	351.5	268.0	338.8	68.00
LOC100545842	14.0	2.0	0.0	15.5	4.5	2.5	6.4	6.63
LOC100545856	10.5	1.0	14.5	11.0	2.5	8.0	7.9	5.23
LOC100545863	0.0	0.0	0.0	0.0	0.5	0.0	0.1	0.20
LOC100545870	1.0	0.5	0.5	0.5	1.0	0.0	0.6	0.38
LOC100545877	8.0	1.0	23.0	3.5	2.0	10.5	8.0	8.20
LOC100545878	8.0	4.5	1.0	10.5	6.5	4.0	5.8	3.33
LOC100545885	0.0	0.0	0.0	0.5	0.0	0.0	0.1	0.20
LOC100545886	123.5	49.5	48.5	115.0	49.5	26.0	68.7	40.29
LOC100545893	41.5	25.5	25.0	24.0	28.5	23.5	28.0	6.84
LOC100545895	133.5	201.5	89.0	141.5	259.5	84.0	151.5	67.90
LOC100545905	36.0	27.0	33.5	36.0	26.0	27.5	31.0	4.68
LOC100545914	18.5	6.0	12.5	12.0	6.5	9.0	10.8	4.66
LOC100545924	1906.0	1195.0	1829.0	2142.0	1440.0	1643.5	1692.6	340.57
LOC100545925	63.0	25.5	14.0	72.5	44.0	22.0	40.2	23.71
LOC100545931	4.5	0.0	2.0	2.0	0.5	2.0	1.8	1.57
LOC100545932	9.0	23.0	86.5	4.5	23.0	66.5	35.4	33.28
LOC100545943	26.0	9.5	33.0	22.5	8.0	35.0	22.3	11.47
LOC100545944	51.5	36.0	53.5	39.5	47.0	45.0	45.4	6.76
LOC100545946	441.5	430.5	507.5	420.0	483.5	435.5	453.1	34.45
LOC100545947	191.5	37.0	410.0	257.5	42.5	274.5	202.2	144.48
LOC100545950	4134.5	2363.5	7925.0	4260.0	2788.5	7867.5	4889.8	2443.11
LOC100545957	517.5	321.5	375.5	490.0	382.0	369.0	409.3	76.73
LOC100545963	111.5	58.5	239.5	133.0	73.5	200.0	136.0	71.18
LOC100545973	442.0	294.0	235.5	337.5	316.0	172.0	299.5	92.09
LOC100545977	396.0	341.0	708.0	427.0	415.5	668.0	492.6	154.74
LOC100545985	195.0	81.0	405.5	187.5	97.0	353.0	219.8	132.84
LOC100546006	145.5	39.0	65.0	108.0	43.5	42.5	73.9	43.58
LOC100546012	33.0	69.0	89.5	50.0	74.0	153.0	78.1	41.63
LOC100546025	25.5	19.5	42.5	35.0	20.0	35.0	29.6	9.33
LOC100546034	186.5	47.5	253.5	261.0	48.5	164.5	160.3	94.61
LOC100546043	1.0	0.0	0.0	0.0	0.0	0.0	0.2	0.41
LOC100546061	1.5	1.5	1.0	1.0	0.5	1.0	1.1	0.38
LOC100546064	0.5	0.0	0.0	0.0	0.0	0.0	0.1	0.20
LOC100546066	3.5	2.0	1.0	2.5	3.0	1.5	2.3	0.94
LOC100546071	8.5	41.0	54.0	1.0	26.5	35.5	27.8	20.06
LOC100546086	563.0	514.0	420.0	907.0	879.0	588.0	645.2	200.58
LOC100546087	215.0	1327.5	1086.0	180.5	1545.5	1096.0	908.4	576.01

LOC100546100	597.0	484.0	411.0	545.5	536.0	391.0	494.1	80.78
LOC100546110	120.0	46.0	25.0	142.5	63.0	42.0	73.1	47.19
LOC100546113	11.5	2.0	5.0	7.0	0.5	4.5	5.1	3.89
LOC100546114	1887.0	2969.5	1827.5	1761.5	3181.5	1986.5	2268.9	632.70
LOC100546118	0.0	0.0	0.0	0.0	0.0	0.0	0.0	0.00
LOC100546123	20.0	52.0	30.0	12.0	46.5	22.5	30.5	15.72
LOC100546124	143.0	61.5	90.5	142.5	75.0	71.0	97.3	36.46
LOC100546134	67.0	20.0	115.0	70.0	18.5	96.5	64.5	39.26
LOC100546136	0.0	0.0	0.0	0.0	0.0	0.0	0.0	0.00
LOC100546141	366.5	668.5	708.0	376.0	766.0	808.0	615.5	195.16
LOC100546146	74.5	89.0	101.0	76.5	77.0	106.5	87.4	13.75
LOC100546153	11.0	4.0	2.0	10.0	8.0	2.0	6.2	4.02
LOC100546173	584.0	1463.5	949.0	521.0	1688.5	894.0	1016.7	469.79
LOC100546176	9.0	26.0	50.0	12.5	18.0	40.0	25.9	16.17
LOC100546177	0.0	0.0	0.0	0.0	0.0	0.0	0.0	0.00
LOC100546179	1.5	1.5	0.0	0.5	0.5	1.0	0.8	0.61
LOC100546181	234.0	155.0	420.0	254.0	175.0	355.0	265.5	103.35
LOC100546187	8.5	2.5	2.5	3.5	0.5	1.5	3.2	2.80
LOC100546195	454.5	405.0	523.0	425.0	414.5	496.5	453.1	47.68
LOC100546203	163.0	174.5	137.5	164.5	188.0	120.0	157.9	24.91
LOC100546217	0.0	3.5	14.0	0.0	3.5	35.5	9.4	13.77
LOC100546220	3.5	4.5	6.0	1.5	4.5	6.5	4.4	1.80
LOC100546225	6.0	1.0	2.0	1.5	4.0	1.0	2.6	2.01
LOC100546239	2.5	1.5	0.5	3.0	1.5	0.0	1.5	1.14
LOC100546242	0.0	0.0	0.0	0.0	0.0	0.5	0.1	0.20
LOC100546243	86.0	71.0	45.5	94.0	77.0	49.5	70.5	19.50
LOC100546254	562.0	538.0	370.0	573.5	520.5	316.5	480.1	108.91
LOC100546258	208.0	162.5	223.0	194.0	187.0	196.5	195.2	20.38
LOC100546272	601.0	581.0	683.5	548.5	673.5	679.0	627.8	58.33
LOC100546305	196.0	118.0	101.5	179.0	140.5	138.0	145.5	35.91
LOC100546312	14.0	3.0	19.5	19.5	8.5	26.0	15.1	8.35
LOC100546313	0.0	2.0	1.5	0.0	2.5	0.5	1.1	1.07
LOC100546322	2.0	4.5	2.5	2.5	3.5	7.5	3.8	2.04
LOC100546323	1499.0	1368.5	1879.5	1390.5	1391.0	1969.0	1582.9	269.78
LOC100546324	25.5	6.5	56.5	23.0	9.5	42.0	27.2	19.18
LOC100546326	132.0	106.5	67.0	113.0	95.0	62.0	95.9	27.18
LOC100546330	3.5	1.0	1.5	3.5	1.0	2.0	2.1	1.16
LOC100546335	0.0	1.5	0.0	0.0	3.0	1.0	0.9	1.20
LOC100546341	302.5	197.0	300.0	290.5	193.0	269.0	258.7	50.72
LOC100546345	0.5	0.0	0.0	0.0	0.0	0.0	0.1	0.20
LOC100546347	12.0	2.0	11.5	14.5	4.0	4.5	8.1	5.19
LOC100546349	0.0	0.0	0.0	0.0	0.0	0.5	0.1	0.20
LOC100546350	1030.0	1009.0	568.5	899.0	1053.0	620.5	863.3	215.47
LOC100546353	851.0	753.5	561.5	751.5	951.5	1171.5	840.1	207.52
LOC100546354	0.0	0.0	0.0	0.5	0.0	0.0	0.1	0.20
LOC100546355	83.5	69.0	59.0	72.0	80.0	62.5	71.0	9.58
LOC100546358	168.5	101.0	146.0	152.5	102.0	123.5	132.3	27.86
LOC100546360	4.5	2.0	2.0	6.0	1.5	3.5	3.3	1.75
LOC100546369	97.5	151.5	113.5	111.0	167.0	89.5	121.7	30.80
LOC100546371	0.0	0.0	0.0	0.0	0.0	0.0	0.0	0.00
LOC100546372	0.5	7.0	5.0	0.5	3.0	11.5	4.6	4.24
LOC100546378	1527.0	1823.0	1427.5	1329.5	1826.5	1422.0	1559.3	214.95
LOC100546381	1506.5	1048.5	494.0	1217.0	1131.0	512.5	984.9	403.85
LOC100546382	36.0	16.0	37.0	51.5	20.5	30.0	31.8	12.75

LOC100546398	40.5	13.0	33.0	46.0	13.5	22.0	28.0	13.97
LOC100546401	431.5	279.5	349.0	386.5	328.5	351.0	354.3	51.57
LOC100546403	381.0	323.5	219.0	322.5	356.0	261.0	310.5	60.29
LOC100546408	257.0	123.5	216.5	277.5	123.5	225.0	203.8	65.98
LOC100546418	187.5	197.0	195.5	171.5	202.0	231.5	197.5	19.78
LOC100546426	10.5	9.0	7.0	10.5	5.0	8.5	8.4	2.13
LOC100546433	213.5	334.5	230.0	178.5	443.0	202.0	266.9	101.80
LOC100546434	0.0	0.5	0.5	0.0	0.0	0.0	0.2	0.26
LOC100546435	2632.5	2823.0	10649.0	3270.0	2773.5	11948.0	5682.7	4374.57
LOC100546438	0.0	0.0	1.0	0.0	1.0	0.0	0.3	0.52
LOC100546440	1.0	2.5	7.0	1.0	9.0	9.0	4.9	3.85
LOC100546441	93.0	88.5	92.5	102.5	112.5	89.5	96.4	9.31
LOC100546443	589.5	483.5	841.5	543.0	595.5	721.5	629.1	130.35
LOC100546458	592.0	448.5	569.5	546.5	447.5	559.0	527.2	63.12
LOC100546462	0.5	0.0	0.0	0.5	0.0	0.0	0.2	0.26
LOC100546469	1.5	0.0	0.5	0.0	1.0	3.0	1.0	1.14
LOC100546474	158.0	60.0	189.5	188.0	71.5	158.0	137.5	57.37
LOC100546477	0.0	0.0	0.0	0.0	0.0	0.0	0.0	0.00
LOC100546484	34.0	71.5	44.5	47.0	76.0	81.5	59.1	19.65
LOC100546489	13.0	9.5	36.0	10.5	13.0	42.5	20.8	14.54
LOC100546493	15.5	3.0	34.5	25.0	8.5	41.5	21.3	15.02
LOC100546494	50.0	40.0	58.0	39.5	41.0	51.5	46.7	7.63
LOC100546503	32.5	14.0	128.5	17.0	6.5	66.0	44.1	46.46
LOC100546506	1.5	1.5	0.5	0.5	1.0	0.0	0.8	0.61
LOC100546507	0.0	0.0	0.5	0.5	0.5	0.0	0.3	0.27
LOC100546512	83.0	25.0	53.0	90.5	18.0	51.5	53.5	29.38
LOC100546519	0.0	0.0	0.0	0.0	0.0	0.0	0.0	0.00
LOC100546521	7.0	7.0	7.0	9.5	14.5	7.0	8.7	3.03
LOC100546529	2.5	1.0	0.0	0.5	2.5	0.5	1.2	1.08
LOC100546534	1322.5	1429.0	1733.0	1329.5	1513.5	1730.0	1509.6	185.71
LOC100546535	0.0	0.0	0.0	0.0	0.0	0.0	0.0	0.00
LOC100546538	112.0	56.0	83.5	118.5	66.0	71.0	84.5	25.50
LOC100546539	7111.5	4850.5	3533.0	8173.0	5554.5	4235.0	5576.3	1766.42
LOC100546546	124.0	87.5	125.0	105.5	111.0	128.5	113.6	15.58
LOC100546547	204.0	216.5	134.0	165.0	209.5	157.0	181.0	33.59
LOC100546553	1828.0	971.5	893.0	1782.0	1014.0	696.0	1197.4	483.35
LOC100546568	278.0	136.0	134.5	184.5	134.0	108.5	162.6	61.71
LOC100546571	5.0	11.5	18.0	6.0	10.5	12.5	10.6	4.73
LOC100546575	5.5	2.5	2.0	4.0	3.5	1.0	3.1	1.59
LOC100546578	208.0	118.0	323.0	192.0	136.0	273.5	208.4	78.81
LOC100546590	28.5	27.0	111.0	34.0	27.0	127.5	59.2	46.90
LOC100546606	0.0	0.0	0.0	3.0	0.5	0.0	0.6	1.20
LOC100546609	0.0	0.0	0.0	0.0	0.5	0.0	0.1	0.20
LOC100546611	0.0	0.0	0.0	0.0	0.0	0.5	0.1	0.20
LOC100546620	0.0	0.0	0.0	0.0	0.5	0.0	0.1	0.20
LOC100546621	361.5	253.5	168.5	337.0	299.5	148.5	261.4	87.89
LOC100546622	1363.0	1164.0	1075.0	1269.5	1247.5	964.0	1180.5	144.22
LOC100546634	44.0	33.5	91.5	54.5	34.5	128.5	64.4	37.96
LOC100546637	0.0	0.0	0.0	0.0	0.0	0.0	0.0	0.00
LOC100546639	24.5	6.0	35.0	32.5	3.0	27.5	21.4	13.64
LOC100546640	1790.5	1390.0	1229.5	1686.0	1581.5	1246.5	1487.3	234.18
LOC100546647	4.5	3.5	8.5	5.0	4.0	12.0	6.3	3.33
LOC100546652	31.0	8.5	33.0	21.5	11.0	33.5	23.1	11.23
LOC100546653	225.5	169.5	92.0	164.0	225.0	126.5	167.1	53.04

LOC100546655	0.0	0.0	1.5	0.0	0.0	0.5	0.3	0.61
LOC100546656	31.0	12.5	76.5	37.0	23.0	57.0	39.5	23.47
LOC100546659	9.0	5.5	2.5	6.5	4.0	4.0	5.3	2.30
LOC100546662	0.5	0.0	4.5	0.5	2.5	1.5	1.6	1.69
LOC100546675	11.5	11.0	8.0	9.0	16.0	8.5	10.7	2.96
LOC100546688	141.0	232.0	185.0	145.5	243.5	242.0	198.2	47.62
LOC100546696	107.0	60.5	126.0	133.5	55.0	105.5	97.9	32.97
LOC100546708	219.0	167.5	130.0	177.0	155.0	126.0	162.4	34.27
LOC100546724	0.0	1.5	1.5	0.5	0.0	2.5	1.0	1.00
LOC100546728	1.0	0.0	0.0	0.0	1.0	0.0	0.3	0.52
LOC100546734	0.0	4.5	2.0	0.5	12.0	4.5	3.9	4.40
LOC100546746	5.5	3.5	0.5	3.5	3.0	1.0	2.8	1.83
LOC100546747	231.0	130.5	209.5	244.0	148.0	225.5	198.1	47.22
LOC100546750	182.0	79.5	211.5	178.5	98.5	197.5	157.9	54.99
LOC100546753	0.0	2.5	6.5	0.5	0.0	2.5	2.0	2.49
LOC100546764	17.0	14.0	30.0	13.5	11.0	11.5	16.2	7.10
LOC100546766	24.0	24.0	21.0	22.0	28.5	24.0	23.9	2.58
LOC100546777	169.0	114.0	53.0	161.5	112.0	50.5	110.0	50.88
LOC100546791	4.5	3.5	4.0	10.0	13.5	5.0	6.8	4.06
LOC100546792	19.0	5.0	14.0	14.5	7.5	10.0	11.7	5.13
LOC100546802	4.0	2.5	1.0	3.5	3.0	0.5	2.4	1.39
LOC100546803	0.0	0.5	0.5	0.0	0.5	1.0	0.4	0.38
LOC100546808	17.5	5.0	47.5	11.5	3.0	26.5	18.5	16.60
LOC100546809	1.0	0.0	0.0	1.5	1.5	0.0	0.7	0.75
LOC100546811	0.5	0.5	0.0	0.0	0.0	0.0	0.2	0.26
LOC100546813	0.5	0.0	0.0	0.0	0.0	0.0	0.1	0.20
LOC100546815	11.0	5.5	20.5	18.5	5.0	23.0	13.9	7.82
LOC100546824	950.0	1410.0	1667.0	928.0	1627.5	1601.5	1364.0	340.96
LOC100546836	4.5	10.0	9.5	3.5	7.0	5.0	6.6	2.71
LOC100546837	20.5	2.0	33.0	18.5	5.5	20.0	16.6	11.28
LOC100546839	11.5	4.0	3.0	3.0	1.5	10.0	5.5	4.17
LOC100546840	0.0	0.0	0.0	0.0	0.0	0.0	0.0	0.00
LOC100546851	183.5	254.0	205.5	169.0	251.5	259.0	220.4	39.53
LOC100546859	0.0	1.5	5.5	0.5	1.5	4.5	2.3	2.23
LOC100546870	30.5	19.0	4.0	33.0	16.5	2.0	17.5	12.92
LOC100546874	0.0	0.0	0.5	0.5	0.5	0.5	0.3	0.26
LOC100546884	0.5	0.0	0.0	0.0	0.5	0.0	0.2	0.26
LOC100546887	168.5	174.5	342.0	135.0	206.5	313.0	223.3	84.38
LOC100546891	364.5	166.5	211.0	299.0	184.0	192.5	236.3	78.14
LOC100546893	265.0	284.0	626.5	318.5	289.5	619.0	400.4	173.09
LOC100546894	0.0	0.0	0.5	2.0	0.0	0.0	0.4	0.80
LOC100546895	1.0	0.5	0.5	1.0	0.0	0.5	0.6	0.38
LOC100546896	9.5	3.0	11.5	6.5	4.0	10.0	7.4	3.46
LOC100546899	0.0	1.0	1.0	0.0	0.0	0.5	0.4	0.49
LOC100546910	45.0	9.0	51.0	43.0	7.0	23.0	29.7	19.25
LOC100546913	4.0	2.5	1.5	4.5	2.0	0.5	2.5	1.52
LOC100546924	547.5	426.0	434.5	558.5	459.5	472.5	483.1	56.79
LOC100546929	2615.5	708.5	1177.5	1808.5	711.5	819.5	1306.8	765.63
LOC100546931	72.0	77.5	101.0	81.5	87.0	144.5	93.9	26.69
LOC100546932	0.0	0.0	0.0	0.0	0.0	0.0	0.0	0.00
LOC100546933	292.5	159.5	175.0	297.5	224.5	142.5	215.3	67.59
LOC100546941	0.0	0.0	0.0	0.0	0.5	0.0	0.1	0.20
LOC100546946	0.0	1.0	0.5	0.0	1.0	0.5	0.5	0.45
LOC100546961	0.0	0.0	0.0	0.0	0.0	0.5	0.1	0.20

LOC100546964	0.0	0.0	0.5	0.5	0.0	0.0	0.2	0.26
LOC100546965	7.5	6.5	6.5	5.0	4.0	3.0	5.4	1.72
LOC100546971	190.0	260.5	277.5	189.0	269.0	229.5	235.9	39.44
LOC100546974	161.5	119.5	148.0	162.0	130.5	140.5	143.7	16.97
LOC100546979	8.5	2.0	3.0	7.5	1.0	2.5	4.1	3.12
LOC100546986	2343.0	2898.5	2406.5	2307.5	2771.5	2790.5	2586.3	261.81
LOC100546988	10.0	13.5	17.0	5.0	12.5	18.0	12.7	4.77
LOC100546989	48.0	23.0	10.0	23.0	23.5	17.0	24.1	12.83
LOC100546992	1632.5	1986.0	2072.0	1393.5	2166.0	1889.5	1856.6	291.42
LOC100546995	166.0	104.5	62.5	138.5	134.5	62.0	111.3	42.73
LOC100547028	0.0	0.0	0.0	0.0	0.0	0.0	0.0	0.00
LOC100547030	0.0	0.5	0.0	0.5	0.0	0.5	0.3	0.27
LOC100547042	165.5	67.0	396.5	172.0	64.5	297.5	193.8	131.08
LOC100547060	0.5	0.0	0.0	0.0	0.0	0.0	0.1	0.20
LOC100547061	471.5	891.0	1079.5	406.5	1038.5	1043.0	821.7	304.06
LOC100547088	5.5	4.0	0.5	6.0	6.0	4.0	4.3	2.09
LOC100547089	1.5	1.5	2.0	2.0	1.5	3.0	1.9	0.58
LOC100547091	190.5	85.0	270.0	181.0	111.5	280.5	186.4	79.73
LOC100547096	0.0	0.0	0.0	0.0	0.0	0.0	0.0	0.00
LOC100547097	271.0	281.5	255.0	367.5	371.5	314.5	310.2	49.93
LOC100547098	1672.0	1050.5	662.0	1505.0	1120.5	618.5	1104.8	428.40
LOC100547103	0.0	0.0	0.0	0.0	0.0	0.0	0.0	0.00
LOC100547109	2.5	4.5	12.0	7.0	7.0	14.5	7.9	4.53
LOC100547124	1056.5	1149.0	1232.0	1001.5	1320.5	1187.5	1157.8	116.30
LOC100547129	0.0	0.0	0.0	0.5	0.0	0.0	0.1	0.20
LOC100547145	40.0	12.0	35.5	31.0	13.0	34.0	27.6	12.04
LOC100547151	34.5	14.0	8.0	30.5	21.0	13.5	20.3	10.42
LOC100547156	359.0	158.0	242.5	343.0	162.5	201.5	244.4	88.17
LOC100547159	18.5	10.5	14.5	5.5	6.0	2.0	9.5	6.19
LOC100547196	307.5	145.0	614.0	322.0	153.0	522.5	344.0	191.13
LOC100547197	0.0	0.0	2.5	0.0	0.0	1.5	0.7	1.08
LOC100547210	0.0	0.0	0.0	0.0	0.0	0.0	0.0	0.00
LOC100547218	207.5	156.5	146.5	189.5	153.0	161.5	169.1	23.99
LOC100547219	1056.5	469.5	640.5	1112.5	567.0	656.0	750.3	267.71
LOC100547230	551.5	471.0	258.5	448.5	453.0	264.0	407.8	119.43
LOC100547239	276.0	533.0	796.0	342.0	592.0	871.5	568.4	237.59
LOC100547241	331.5	234.5	309.0	332.5	242.5	315.0	294.2	44.15
LOC100547242	47.5	46.0	21.0	44.0	38.0	30.5	37.8	10.35
LOC100547244	115.5	44.5	50.0	131.0	40.5	27.5	68.2	43.58
LOC100547251	10.0	6.5	10.5	14.5	12.5	9.5	10.6	2.73
LOC100547252	0.0	0.0	2.0	0.0	0.0	2.0	0.7	1.03
LOC100547253	0.0	0.0	1.5	0.0	0.0	0.0	0.3	0.61
LOC100547273	1221.0	1599.0	1658.5	1476.5	2188.0	1588.0	1621.8	317.91
LOC100547274	47.5	13.5	10.5	50.0	16.5	12.0	25.0	18.52
LOC100547276	0.0	0.0	0.0	0.0	0.0	0.0	0.0	0.00
LOC100547278	0.0	0.0	0.0	0.0	0.0	0.0	0.0	0.00
LOC100547295	0.0	0.0	0.0	0.0	0.0	0.0	0.0	0.00
LOC100547297	36.0	39.0	70.5	38.0	36.0	74.5	49.0	18.28
LOC100547303	198.0	142.0	204.0	142.0	132.5	166.5	164.2	30.73
LOC100547331	1041.5	479.5	800.5	1019.5	521.0	696.0	759.7	240.05
LOC100547334	1.0	1.0	0.5	0.5	0.0	1.5	0.8	0.52
LOC100547338	117.0	84.5	113.0	109.5	106.0	100.0	105.0	11.61
LOC100547348	384.5	269.0	591.0	396.5	266.0	540.0	407.8	134.97
LOC100547350	338.0	653.0	385.5	322.5	743.0	408.0	475.0	177.78

LOC100547354	1456.0	1273.5	1211.5	1253.5	1387.0	1073.0	1275.8	134.61
LOC100547356	0.0	0.0	0.0	0.0	0.0	0.0	0.0	0.00
LOC100547361	0.5	0.5	0.0	0.0	0.0	0.0	0.2	0.26
LOC100547363	135.5	48.5	153.0	131.0	49.0	154.0	111.8	49.72
LOC100547373	92.5	101.5	118.0	80.5	132.5	125.0	108.3	20.14
LOC100547382	32.5	6.5	21.5	35.5	12.5	20.0	21.4	11.18
LOC100547383	82.0	132.5	86.0	82.0	117.0	85.0	97.4	21.79
LOC100547387	86.0	41.5	59.0	90.5	57.0	55.5	64.9	19.15
LOC100547397	762.0	542.5	677.5	688.5	535.5	750.0	659.3	98.92
LOC100547402	409.5	254.5	284.0	379.0	277.0	204.0	301.3	77.84
LOC100547403	107.0	61.5	35.5	95.5	72.5	53.0	70.8	26.73
LOC100547404	0.0	0.0	0.0	0.0	0.0	0.0	0.0	0.00
LOC100547415	44.0	17.5	47.0	40.5	32.0	44.5	37.6	11.14
LOC100547424	3.0	3.5	5.0	3.5	1.0	5.5	3.6	1.59
LOC100547425	12.5	1.5	3.0	3.0	2.0	2.0	4.0	4.21
LOC100547427	2.5	8.5	8.0	4.0	8.0	8.0	6.5	2.57
LOC100547434	0.5	0.0	1.5	0.5	1.0	0.0	0.6	0.58
LOC100547435	221.5	166.5	178.0	194.0	198.0	147.5	184.3	25.99
LOC100547436	134.0	84.5	108.5	121.0	72.5	89.5	101.7	23.49
LOC100547437	74.5	46.5	30.0	93.5	60.5	42.0	57.8	23.28
LOC100547441	400.0	405.5	532.0	405.0	428.0	466.5	439.5	51.62
LOC100547442	515.0	444.5	314.5	587.5	425.0	240.5	421.2	127.32
LOC100547447	0.0	0.0	0.0	0.0	0.0	0.0	0.0	0.00
LOC100547451	24.5	15.0	23.5	30.0	15.5	18.0	21.1	5.91
LOC100547453	2006.0	2024.5	1377.5	1579.0	1675.5	1363.0	1670.9	292.12
LOC100547459	0.0	0.0	0.5	0.0	0.0	0.5	0.2	0.26
LOC100547461	2.0	1.0	2.0	2.5	3.0	5.0	2.6	1.36
LOC100547471	1638.5	1009.0	1618.5	1396.5	1215.5	1470.0	1391.3	243.31
LOC100547473	0.0	1.0	0.0	0.0	2.0	0.0	0.5	0.84
LOC100547482	91.5	43.0	151.5	110.5	61.5	137.0	99.2	42.26
LOC100547485	40.5	16.5	63.5	52.0	21.5	54.0	41.3	18.85
LOC100547495	232.0	125.5	291.0	236.5	120.0	278.0	213.8	74.19
LOC100547499	0.0	0.0	0.0	0.0	0.0	1.0	0.2	0.41
LOC100547506	0.0	0.0	0.0	0.0	0.0	0.0	0.0	0.00
LOC100547507	279.5	263.0	752.0	351.5	415.0	894.5	492.6	265.66
LOC100547518	184.0	55.0	35.0	165.0	46.0	23.5	84.8	70.57
LOC100547524	13.5	8.0	12.0	11.5	4.5	15.5	10.8	3.97
LOC100547530	2013.5	1559.5	1392.5	2019.5	1682.5	1470.0	1689.6	271.00
LOC100547559	95.0	214.5	238.5	94.5	259.5	253.0	192.5	77.28
LOC100547566	21.5	71.0	59.0	24.5	95.5	154.0	70.9	49.49
LOC100547576	0.5	1.0	0.0	0.0	0.0	0.0	0.3	0.42
LOC100547584	3.0	40.0	49.0	2.0	32.5	31.0	26.3	19.48
LOC100547592	4.0	1.0	0.5	1.5	2.0	1.0	1.7	1.25
LOC100547598	615.5	1056.5	419.5	565.0	1096.0	451.0	700.6	299.98
LOC100547599	794.5	703.5	693.0	782.0	727.0	684.0	730.7	47.03
LOC100547603	0.0	0.5	0.0	0.0	0.0	0.5	0.2	0.26
LOC100547610	231.5	208.5	92.0	249.5	182.5	98.0	177.0	67.40
LOC100547611	91.0	103.5	139.5	86.5	119.5	133.5	112.3	22.08
LOC100547618	622.0	611.0	681.0	544.0	609.5	635.5	617.2	44.46
LOC100547620	41.0	11.0	34.5	40.5	7.0	19.0	25.5	15.11
LOC100547627	1.5	2.0	0.5	3.0	1.0	0.5	1.4	0.97
LOC100547642	0.0	0.0	0.0	0.0	0.0	0.0	0.0	0.00
LOC100547653	0.0	0.0	0.5	0.0	0.0	0.0	0.1	0.20
LOC100547657	0.5	0.0	6.0	0.5	0.0	5.5	2.1	2.85

LOC100547664	54.5	21.5	72.0	36.0	39.0	55.5	46.4	17.81
LOC100547665	0.0	0.0	0.0	1.0	2.5	1.5	0.8	1.03
LOC100547676	157.0	56.0	215.5	171.5	63.5	151.0	135.8	63.09
LOC100547680	10.5	11.0	7.0	23.5	14.0	15.0	13.5	5.66
LOC100547687	416.0	372.5	400.0	432.5	462.0	439.5	420.4	31.53
LOC100547689	2.0	1.5	2.0	3.5	1.0	0.0	1.7	1.17
LOC100547693	3.5	2.5	1.5	5.0	6.5	4.0	3.8	1.78
LOC100547702	38.0	56.5	31.0	28.5	67.5	45.0	44.4	15.19
LOC100547703	7.0	3.5	4.0	4.0	3.0	11.0	5.4	3.07
LOC100547709	221.5	140.5	348.5	209.0	166.5	283.5	228.3	76.74
LOC100547714	7.0	5.0	10.0	6.5	6.0	2.5	6.2	2.46
LOC100547716	0.5	0.0	0.0	0.0	0.0	0.0	0.1	0.20
LOC100547719	26.5	11.0	42.5	26.5	6.5	23.0	22.7	12.81
LOC100547721	141.5	52.0	94.5	143.0	64.5	114.0	101.6	38.33
LOC100547722	0.5	1.0	0.5	0.5	0.0	0.5	0.5	0.32
LOC100547726	1.5	0.5	2.0	1.0	0.5	1.0	1.1	0.58
LOC100547732	1773.0	1699.5	1381.0	1638.0	1979.5	1308.5	1629.9	250.14
LOC100547734	506.5	322.0	376.5	550.5	351.5	318.0	404.2	99.60
LOC100547760	745.5	605.0	661.0	639.5	658.0	524.0	638.8	72.89
LOC100547762	1.5	0.5	0.0	0.5	0.5	0.5	0.6	0.49
LOC100547765	53.0	44.5	64.0	46.0	43.0	56.0	51.1	8.11
LOC100547767	695.0	391.0	367.5	570.5	326.0	369.5	453.3	146.03
LOC100547769	1.0	2.0	0.5	0.5	2.0	3.0	1.5	1.00
LOC100547773	169.5	114.5	112.5	136.0	136.5	150.5	136.6	21.66
LOC100547793	7.0	6.0	15.5	5.0	10.5	23.5	11.3	7.12
LOC100547794	226.5	320.5	291.5	180.5	289.5	187.0	249.3	59.33
LOC100547801	0.0	0.5	0.0	0.0	0.0	0.0	0.1	0.20
LOC100547805	410.0	117.0	80.5	330.5	111.0	79.0	188.0	144.22
LOC100547818	0.5	0.0	2.0	0.5	0.5	0.5	0.7	0.68
LOC100547821	0.0	0.0	0.0	0.0	0.0	0.0	0.0	0.00
LOC100547826	161.0	93.0	62.0	180.5	80.0	47.0	103.9	54.43
LOC100547828	27.0	38.5	79.5	32.0	43.0	85.5	50.9	25.14
LOC100547835	4.5	3.5	2.5	6.0	4.0	3.5	4.0	1.18
LOC100547843	446.5	323.0	257.0	464.5	389.5	259.5	356.7	90.87
LOC100547855	0.0	0.0	0.0	0.0	0.0	0.0	0.0	0.00
LOC100547860	2.0	0.5	1.0	0.5	0.0	0.0	0.7	0.75
LOC100547871	0.0	0.0	0.0	0.0	0.0	0.0	0.0	0.00
LOC100547876	14.5	1.5	11.0	22.5	2.5	12.0	10.7	7.84
LOC100547877	121.0	165.0	119.5	125.5	195.0	125.0	141.8	31.13
LOC100547883	3696.5	1443.0	1665.5	2935.0	1362.5	1495.5	2099.7	977.32
LOC100547885	39.5	23.5	16.5	44.5	33.0	30.0	31.2	10.26
LOC100547893	85.5	38.5	61.0	75.0	33.0	58.0	58.5	20.29
LOC100547897	230.5	360.5	425.0	223.5	384.0	495.5	353.2	107.97
LOC100547911	468.5	3975.0	1286.0	364.0	4211.5	1808.0	2018.8	1694.57
LOC100547916	645.5	464.0	1034.5	668.5	409.5	1042.5	710.8	272.97
LOC100547917	1580.5	1697.5	1351.5	1305.0	1339.5	1259.0	1422.2	174.96
LOC100547920	35.0	24.0	66.0	49.0	19.0	53.5	41.1	18.19
LOC100547923	298.5	263.0	303.0	245.0	255.0	335.5	283.3	34.71
LOC100547927	247.5	288.0	313.0	229.5	272.5	289.0	273.3	30.41
LOC100547929	0.0	0.0	0.0	0.0	0.0	0.0	0.0	0.00
LOC100547940	1721.0	4730.0	5328.5	1057.5	5414.5	4772.5	3837.3	1928.15
LOC100547944	0.0	2.0	0.0	0.5	4.5	0.0	1.2	1.81
LOC100547963	0.0	3.5	2.5	0.0	4.5	3.5	2.3	1.91
LOC100547971	210.5	271.5	201.5	232.5	270.5	206.5	232.2	31.89

LOC100547972	68.5	33.5	108.0	87.5	35.0	69.0	66.9	29.17
LOC100547979	3.5	51.0	270.5	4.0	54.5	179.0	93.8	107.77
LOC100548001	3.5	4.5	13.0	6.5	4.5	18.5	8.4	6.02
LOC100548015	42.5	28.0	74.0	47.0	25.0	63.0	46.6	19.23
LOC100548019	82.5	66.5	87.0	77.0	59.0	89.5	76.9	12.01
LOC100548022	0.0	0.0	0.0	0.0	0.5	0.0	0.1	0.20
LOC100548025	317.5	162.0	233.0	293.0	177.5	199.5	230.4	63.16
LOC100548026	0.0	0.0	0.0	0.0	0.0	0.0	0.0	0.00
LOC100548027	32.5	11.0	15.0	19.5	13.5	24.5	19.3	8.03
LOC100548043	2.5	0.0	0.5	2.0	0.5	0.0	0.9	1.07
LOC100548047	86.5	80.5	101.0	66.5	79.0	88.5	83.7	11.47
LOC100548053	359.0	608.5	376.0	325.5	576.0	290.5	422.6	135.04
LOC100548064	709.0	325.5	285.0	487.0	296.5	284.5	397.9	170.79
LOC100548067	10.5	6.5	11.5	6.0	5.0	4.5	7.3	2.94
LOC100548069	329.5	589.5	495.5	266.0	519.0	556.5	459.3	130.76
LOC100548070	59.0	53.5	36.0	49.5	49.5	31.0	46.4	10.71
LOC100548071	9.0	7.5	4.0	7.5	7.5	6.0	6.9	1.72
LOC100548072	2512.5	823.0	547.5	2300.5	908.5	524.0	1269.3	896.07
LOC100548077	17.5	2.0	22.5	12.0	2.5	13.0	11.6	8.13
LOC100548078	0.5	1.5	3.0	4.5	1.0	0.5	1.8	1.60
LOC100548083	0.0	0.0	1.0	0.0	0.0	0.0	0.2	0.41
LOC100548086	375.5	213.5	264.5	313.5	257.5	256.5	280.2	56.50
LOC100548097	180.0	253.0	282.0	179.5	308.5	316.5	253.3	61.13
LOC100548098	1621.0	2038.0	1222.0	1449.0	2016.5	1150.5	1582.8	382.55
LOC100548105	1365.5	1030.5	1085.5	1019.5	1002.5	1049.0	1092.1	136.92
LOC100548106	0.0	0.0	0.5	1.5	0.5	0.0	0.4	0.58
LOC100548108	0.0	0.5	0.0	0.0	0.0	1.0	0.3	0.42
LOC100548109	57.0	35.5	38.0	54.5	25.5	41.0	41.9	11.94
LOC100548115	211.0	90.5	208.0	210.0	110.0	151.5	163.5	54.28
LOC100548116	0.0	0.0	0.5	0.0	0.0	0.0	0.1	0.20
LOC100548118	0.0	0.0	0.0	0.0	0.0	0.0	0.0	0.00
LOC100548121	22.0	22.0	27.5	16.0	17.5	14.0	19.8	4.95
LOC100548127	43.5	19.0	58.0	50.5	19.5	61.0	41.9	18.58
LOC100548128	38.5	18.5	79.5	29.5	26.0	77.5	44.9	26.80
LOC100548130	16.5	12.0	29.5	24.0	7.5	29.0	19.8	9.16
LOC100548137	684.5	570.5	650.5	627.5	621.0	606.0	626.7	38.80
LOC100548146	3.0	1.0	0.0	2.5	1.5	1.0	1.5	1.10
LOC100548147	284.5	212.0	126.0	272.5	207.5	123.0	204.3	69.13
LOC100548148	0.0	0.0	0.0	0.0	0.0	0.0	0.0	0.00
LOC100548153	0.0	0.0	0.0	0.0	0.0	0.0	0.0	0.00
LOC100548169	29.0	30.5	37.0	29.5	39.0	31.0	32.7	4.24
LOC100548173	0.5	2.5	3.5	0.5	4.0	8.0	3.2	2.79
LOC100548193	302.0	168.5	450.0	345.5	228.5	418.0	318.8	108.44
LOC100548196	0.0	0.0	0.0	0.0	0.0	0.0	0.0	0.00
LOC100548199	4.0	5.0	8.0	3.0	7.0	8.5	5.9	2.25
LOC100548214	1440.0	1864.0	1671.5	1191.5	2071.0	1577.0	1635.8	310.47
LOC100548218	0.5	0.5	1.5	0.5	0.5	0.5	0.7	0.41
LOC100548226	23.0	20.5	44.0	26.0	13.5	35.0	27.0	10.90
LOC100548228	1.0	16.5	21.0	0.5	15.5	9.0	10.6	8.53
LOC100548247	133.5	88.0	67.5	132.0	91.0	60.0	95.3	31.29
LOC100548251	1314.5	1122.0	1009.5	1221.0	1137.5	900.0	1117.4	147.59
LOC100548257	1.0	0.0	0.0	0.0	1.0	0.0	0.3	0.52
LOC100548260	0.5	2.0	1.5	2.0	1.0	2.0	1.5	0.63
LOC100548266	5.0	11.5	3.5	3.0	8.5	6.0	6.3	3.24

LOC100548269	0.5	4.0	2.5	2.5	1.5	1.5	2.1	1.20
LOC100548270	468.5	443.0	374.5	486.5	517.0	419.5	451.5	50.65
LOC100548272	1763.5	1433.5	1901.0	1950.0	1546.5	1812.5	1734.5	203.49
LOC100548274	490.5	299.5	303.5	479.0	345.0	247.5	360.8	100.92
LOC100548275	3.5	2.0	2.5	3.5	2.5	1.0	2.5	0.95
LOC100548279	0.0	0.0	0.0	0.0	0.0	0.0	0.0	0.00
LOC100548280	34.0	73.0	29.5	22.0	89.0	69.5	52.8	27.72
LOC100548286	4499.0	3187.0	2721.5	4877.5	3764.0	2916.5	3660.9	877.99
LOC100548287	689.5	1335.5	1269.0	802.5	1502.0	1395.5	1165.7	335.88
LOC100548288	3.5	6.5	1.0	1.5	6.5	1.0	3.3	2.62
LOC100548295	94.5	68.0	81.0	96.5	76.5	76.5	82.2	11.17
LOC100548298	0.0	0.0	0.0	0.0	0.0	0.0	0.0	0.00
LOC100548306	1058.5	1284.0	546.0	949.5	1269.0	567.5	945.8	326.96
LOC100548307	56.5	16.0	110.0	62.5	27.0	74.0	57.7	33.78
LOC100548320	0.0	0.0	0.0	0.0	0.0	0.0	0.0	0.00
LOC100548321	1.5	0.0	0.5	0.5	0.0	1.5	0.7	0.68
LOC100548322	27.0	32.5	27.0	23.0	30.5	29.5	28.3	3.33
LOC100548327	0.0	0.0	0.0	0.0	0.0	0.0	0.0	0.00
LOC100548330	1372.0	1610.5	854.0	1225.0	1637.5	782.5	1246.9	366.32
LOC100548331	11113.0	9352.0	9220.5	10053.0	10418.5	8309.5	9744.4	990.97
LOC100548332	175.0	206.0	167.0	170.5	225.5	156.5	183.4	26.51
LOC100548334	122.5	124.0	185.5	112.5	150.5	211.0	151.0	39.53
LOC100548337	1232.5	1450.0	789.0	1127.5	1441.0	835.0	1145.8	286.86
LOC100548350	309.5	230.5	386.5	273.5	318.0	403.5	320.3	65.85
LOC100548360	266.5	338.0	527.0	309.0	407.0	443.0	381.8	95.89
LOC100548372	0.0	0.0	0.0	0.0	0.0	0.0	0.0	0.00
LOC100548375	0.0	0.0	0.0	0.0	0.0	0.0	0.0	0.00
LOC100548376	13.5	8.0	12.5	11.5	8.5	9.0	10.5	2.30
LOC100548381	359.5	388.0	475.5	313.0	391.0	502.5	404.9	71.41
LOC100548382	0.0	0.0	0.0	0.0	0.0	0.0	0.0	0.00
LOC100548407	240.5	105.5	204.5	236.5	114.0	144.5	174.3	60.70
LOC100548422	1.5	0.0	1.5	1.0	0.5	1.0	0.9	0.58
LOC100548429	2.0	1.0	2.0	3.0	0.5	2.5	1.8	0.93
LOC100548433	0.0	0.0	0.0	0.0	0.0	0.0	0.0	0.00
LOC100548437	116.0	22.5	117.5	107.5	27.0	89.0	79.9	43.94
LOC100548454	468.5	242.0	355.5	393.5	272.0	353.0	347.4	82.08
LOC100548465	759.0	614.5	731.0	723.5	665.5	629.5	687.2	59.12
LOC100548468	151.0	90.0	202.5	144.5	68.0	188.0	140.7	52.98
LOC100548480	35.0	71.5	199.5	18.5	98.5	151.5	95.8	69.46
LOC100548498	1162.0	1144.5	1402.0	1030.5	1200.5	1256.5	1199.3	124.30
LOC100548520	9.5	19.0	19.0	10.5	17.0	34.0	18.2	8.80
LOC100548521	141.5	53.5	264.0	148.5	68.0	135.5	135.2	74.83
LOC100548527	0.0	0.0	0.0	0.0	0.0	0.0	0.0	0.00
LOC100548529	6.5	1.5	3.0	5.0	3.0	2.5	3.6	1.83
LOC100548534	0.0	0.5	1.0	0.5	2.5	1.0	0.9	0.86
LOC100548539	0.0	0.0	0.0	0.0	0.0	0.0	0.0	0.00
LOC100548555	902.0	620.0	707.5	917.5	616.5	657.0	736.8	138.05
LOC100548556	17.5	2.5	3.5	7.5	4.5	3.0	6.4	5.71
LOC100548557	3.0	9.0	9.5	3.0	10.0	14.5	8.2	4.46
LOC100548568	13.0	7.5	1.5	16.5	3.5	2.5	7.4	6.14
LOC100548577	0.0	0.0	0.0	0.0	0.5	0.0	0.1	0.20
LOC100548578	37.5	15.5	43.5	44.0	25.5	45.0	35.2	12.08
LOC100548579	440.0	1024.0	3101.5	416.0	1146.5	1858.0	1331.0	1017.03
LOC100548582	55.5	43.0	117.5	97.0	54.5	98.5	77.7	30.41

LOC100548583	77.0	42.0	101.0	71.5	49.0	77.5	69.7	21.41
LOC100548588	1.0	1.5	1.0	4.0	4.5	2.5	2.4	1.53
LOC100548591	795.0	591.5	631.5	821.0	612.5	540.5	665.3	114.91
LOC100548603	100.5	39.5	150.5	109.5	52.0	118.0	95.0	41.90
LOC100548608	20.5	13.0	7.5	22.0	18.5	11.0	15.4	5.77
LOC100548610	1.5	6.5	8.0	3.0	10.0	2.5	5.3	3.42
LOC100548634	0.0	0.0	1.5	0.0	0.0	0.0	0.3	0.61
LOC100548643	0.0	0.0	0.0	0.0	0.5	0.0	0.1	0.20
LOC100548653	15.0	9.5	19.0	13.5	16.5	14.0	14.6	3.18
LOC100548654	209.5	74.0	42.5	156.0	77.5	48.5	101.3	66.72
LOC100548663	22.5	11.0	8.5	15.5	17.5	8.5	13.9	5.59
LOC100548666	1.5	4.5	0.5	2.5	4.5	2.0	2.6	1.63
LOC100548675	0.0	0.0	0.0	0.0	0.0	0.0	0.0	0.00
LOC100548678	209.5	212.5	530.5	227.5	263.5	481.5	320.8	145.54
LOC100548679	3.0	2.5	11.5	4.5	0.0	8.0	4.9	4.16
LOC100548686	494.0	452.5	368.0	567.0	450.5	546.5	479.8	72.60
LOC100548691	191.0	8.0	12.0	107.5	9.0	9.0	56.1	76.86
LOC100548692	208.0	104.5	202.0	222.0	146.0	186.0	178.1	44.50
LOC100548693	0.0	0.0	0.0	0.0	0.0	0.0	0.0	0.00
LOC100548696	12.0	11.0	15.0	11.0	12.0	21.5	13.8	4.07
LOC100548705	333.0	222.0	364.5	339.0	242.0	379.5	313.3	65.53
LOC100548706	512.0	306.5	496.5	540.0	354.5	481.0	448.4	94.62
LOC100548707	2.5	2.0	24.5	2.5	2.0	8.0	6.9	8.92
LOC100548708	12.5	5.5	5.5	7.5	2.5	0.5	5.7	4.17
LOC100548709	3450.5	3674.0	4855.5	3442.0	4075.0	4232.5	3954.9	547.53
LOC100548710	628.0	333.5	470.5	640.0	390.0	400.0	477.0	129.23
LOC100548711	1064.5	1184.0	1061.5	1098.5	1198.5	987.0	1099.0	80.33
LOC100548716	109.5	47.5	102.5	74.0	52.5	62.5	74.8	25.95
LOC100548731	2.5	1.0	1.5	1.5	1.0	2.5	1.7	0.68
LOC100548732	0.0	1.0	0.5	1.0	0.0	0.0	0.4	0.49
LOC100548734	277.5	301.5	117.0	222.0	339.5	122.5	230.0	93.54
LOC100548741	2544.5	3282.5	2700.5	2179.5	3599.0	1971.0	2712.8	628.18
LOC100548742	147.5	113.0	301.5	145.5	147.0	318.5	195.5	89.81
LOC100548747	0.0	0.0	0.0	0.0	0.0	0.0	0.0	0.00
LOC100548761	12.0	25.0	31.5	7.0	26.0	26.5	21.3	9.57
LOC100548773	2750.5	1802.5	2455.5	2650.5	1924.5	2175.5	2293.2	388.25
LOC100548778	308.0	351.5	299.0	396.0	370.5	382.0	351.2	39.80
LOC100548787	315.0	279.0	450.5	257.0	292.0	332.5	321.0	68.76
LOC100548792	271.0	50.0	582.5	400.0	53.5	334.5	281.9	206.46
LOC100548793	0.0	0.0	2.0	0.0	0.5	0.0	0.4	0.80
LOC100548799	87.0	89.0	79.0	95.5	97.5	66.0	85.7	11.67
LOC100548805	31.0	13.0	39.0	50.5	18.0	30.0	30.3	13.67
LOC100548808	1039.0	724.0	906.5	975.0	788.0	721.5	859.0	134.38
LOC100548817	2.0	2.5	3.0	1.5	1.0	1.0	1.8	0.82
LOC100548822	47.5	14.5	15.5	36.0	11.5	7.5	22.1	15.91
LOC100548827	2513.0	1213.5	1145.0	2508.5	1190.0	831.5	1566.9	743.97
LOC100548828	8.0	4.0	4.5	8.0	6.5	4.5	5.9	1.83
LOC100548829	0.0	0.0	0.0	0.0	0.0	0.0	0.0	0.00
LOC100548833	0.0	0.0	0.0	0.0	0.0	0.0	0.0	0.00
LOC100548836	11.5	7.5	3.0	14.0	6.5	5.0	7.9	4.12
LOC100548837	0.5	0.0	0.0	1.0	0.5	0.0	0.3	0.41
LOC100548843	0.5	1.0	0.0	0.5	0.0	0.5	0.4	0.38
LOC100548849	41.5	16.5	23.0	41.0	28.5	27.5	29.7	9.92
LOC100548859	669.5	468.5	456.0	593.5	555.0	425.5	528.0	94.10

LOC100548864	0.5	0.0	1.0	0.0	0.0	1.0	0.4	0.49
LOC100548894	5547.0	4970.5	4685.5	5114.0	5984.0	4010.5	5051.9	684.57
LOC100548900	19.0	22.0	15.5	15.5	16.0	20.0	18.0	2.74
LOC100548902	299.0	74.0	460.5	352.0	77.5	304.5	261.3	154.96
LOC100548914	3.5	3.5	10.0	5.5	1.5	7.5	5.3	3.09
LOC100548920	1.5	0.5	0.0	0.5	0.0	0.5	0.5	0.55
LOC100548925	26.0	7.0	3.5	18.0	9.5	4.0	11.3	8.91
LOC100548929	16.0	8.0	14.5	11.0	8.5	21.5	13.3	5.15
LOC100548930	0.0	0.5	0.0	0.0	0.0	0.0	0.1	0.20
LOC100548938	363.5	357.0	378.0	373.5	404.0	402.0	379.7	19.53
LOC100548941	0.5	0.0	0.5	1.0	0.5	0.0	0.4	0.38
LOC100548946	2565.0	1200.5	2840.0	3016.5	1357.5	2429.5	2234.8	769.90
LOC100548949	253.5	310.5	578.5	282.0	412.5	499.5	389.4	130.11
LOC100548954	82.0	52.5	93.0	70.5	48.0	75.0	70.2	17.25
LOC100548957	8.5	5.5	16.0	12.0	7.5	11.5	10.2	3.76
LOC100548962	0.0	0.0	0.0	0.0	0.0	0.0	0.0	0.00
LOC100548963	2.0	10.5	5.0	2.0	7.5	1.5	4.8	3.64
LOC100548964	25.5	11.0	15.0	27.0	11.0	14.0	17.3	7.17
LOC100548965	63.5	87.0	68.5	58.5	92.0	44.0	68.9	17.99
LOC100548974	100.5	45.5	12.5	95.5	56.5	26.0	56.1	35.90
LOC100548984	0.0	0.0	0.0	0.0	0.0	0.0	0.0	0.00
LOC100548989	306.0	1225.0	776.0	316.0	1348.0	936.0	817.8	441.90
LOC100548991	15.0	7.5	18.0	13.5	6.5	11.0	11.9	4.44
LOC100548996	2.5	2.0	0.0	1.5	2.5	1.0	1.6	0.97
LOC100549000	203.0	117.0	98.0	179.0	128.0	72.5	132.9	49.36
LOC100549006	0.0	0.0	0.0	0.0	0.0	0.0	0.0	0.00
LOC100549016	5.0	15.5	14.5	5.0	13.5	20.0	12.3	6.04
LOC100549021	264.5	338.5	319.0	237.5	342.5	357.0	309.8	47.93
LOC100549028	634.0	778.5	621.5	510.5	741.0	616.5	650.3	96.38
LOC100549029	6.5	3.0	1.5	5.0	2.0	0.5	3.1	2.27
LOC100549030	6.5	14.5	15.0	11.0	19.0	12.5	13.1	4.21
LOC100549042	0.0	0.5	0.0	0.0	0.0	1.5	0.3	0.61
LOC100549046	0.0	0.0	0.0	0.0	0.0	0.0	0.0	0.00
LOC100549047	0.0	0.0	0.0	0.0	0.0	0.0	0.0	0.00
LOC100549054	0.0	0.0	0.5	0.0	0.5	0.0	0.2	0.26
LOC100549055	1848.5	1726.0	1904.0	4079.5	2856.5	5243.0	2942.9	1438.59
LOC100549062	21.5	8.5	72.5	32.0	13.5	72.0	36.7	28.69
LOC100549070	45.0	27.0	51.5	43.5	28.5	47.5	40.5	10.25
LOC100549073	409.5	666.0	327.0	381.0	619.5	340.5	457.3	147.37
LOC100549076	0.5	1.0	10.0	0.5	0.5	3.5	2.7	3.78
LOC100549082	103.5	318.0	116.0	97.0	383.0	175.5	198.8	122.46
LOC100549086	546.5	283.0	103.0	456.5	299.5	96.0	297.4	182.17
LOC100549091	0.0	0.0	0.0	0.0	0.0	0.0	0.0	0.00
LOC100549093	5.5	0.5	16.0	5.0	1.5	13.5	7.0	6.36
LOC100549101	1685.0	994.0	1104.0	1549.5	1169.0	1210.5	1285.3	270.71
LOC100549107	3677.5	3867.5	2836.5	3167.5	3736.0	2081.0	3227.7	684.34
LOC100549110	92.5	112.5	62.5	108.5	111.5	68.0	92.6	22.44
LOC100549111	188.0	134.5	74.5	181.0	138.0	75.0	131.8	49.26
LOC100549117	0.0	0.0	0.0	0.0	0.0	0.0	0.0	0.00
LOC100549118	144.5	67.0	95.0	123.0	72.5	83.0	97.5	30.43
LOC100549121	0.5	0.0	0.0	0.0	0.0	0.0	0.1	0.20
LOC100549134	1.5	5.0	7.0	4.0	10.5	9.5	6.3	3.42
LOC100549138	1368.0	855.0	830.0	1229.0	867.0	815.0	994.0	240.62
LOC100549140	0.0	0.0	0.0	0.0	0.0	0.0	0.0	0.00

LOC100549141	193.5	274.0	123.0	173.5	256.0	198.0	203.0	55.20
LOC100549146	725.0	738.5	403.0	574.0	737.0	448.0	604.3	152.35
LOC100549159	1.5	1.0	2.5	0.5	3.0	1.0	1.6	0.97
LOC100549160	52.5	56.5	115.0	27.5	49.0	83.0	63.9	30.68
LOC100549167	29.0	7.0	0.5	21.5	8.0	0.5	11.1	11.66
LOC100549172	178.0	89.0	254.0	234.0	89.5	264.0	184.8	79.73
LOC100549177	293.5	192.5	285.5	276.0	208.5	211.5	244.6	45.08
LOC100549178	1.5	0.0	4.0	2.5	1.0	0.5	1.6	1.46
LOC100549188	0.5	0.0	0.0	0.0	0.0	0.5	0.2	0.26
LOC100549193	3.0	2.5	1.5	1.0	0.0	1.5	1.6	1.07
LOC100549197	91.0	35.0	168.0	119.0	35.5	101.5	91.7	51.06
LOC100549201	0.0	0.0	0.0	0.0	0.0	0.0	0.0	0.00
LOC100549202	0.0	0.0	0.5	0.0	0.0	0.0	0.1	0.20
LOC100549208	11.5	2.5	12.0	12.5	0.0	2.0	6.8	5.82
LOC100549217	243.0	292.0	178.5	193.0	342.0	209.5	243.0	63.22
LOC100549224	285.5	179.5	300.0	263.0	196.5	263.5	248.0	48.83
LOC100549245	4.5	0.0	0.5	0.0	0.0	0.5	0.9	1.77
LOC100549247	10.0	2.5	23.5	14.0	4.5	17.0	11.9	7.90
LOC100549259	0.0	0.0	0.5	0.0	0.0	0.5	0.2	0.26
LOC100549268	4.5	5.5	18.0	5.5	6.0	19.0	9.8	6.80
LOC100549271	0.0	0.0	0.0	0.0	0.0	0.0	0.0	0.00
LOC100549275	15.5	3.0	10.0	10.0	6.0	7.0	8.6	4.29
LOC100549281	120.5	94.5	62.0	122.0	86.0	49.5	89.1	29.70
LOC100549283	0.0	0.0	0.0	0.0	0.0	0.0	0.0	0.00
LOC100549289	299.5	352.5	325.0	212.5	335.0	298.0	303.8	49.36
LOC100549299	127.5	109.0	150.5	151.5	124.5	143.5	134.4	16.88
LOC100549312	1.5	3.0	0.5	0.5	0.0	1.5	1.2	1.08
LOC100549326	457.5	485.0	249.5	470.0	549.0	244.0	409.2	129.70
LOC100549331	36.5	1264.5	3509.0	21.0	1586.0	4130.0	1757.8	1728.94
LOC100549337	4.5	3.0	7.5	4.0	3.0	8.5	5.1	2.35
LOC100549340	102.5	65.5	115.5	69.0	37.0	71.0	76.8	28.16
LOC100549350	0.0	0.0	0.0	1.0	0.0	0.0	0.2	0.41
LOC100549351	53.0	22.5	94.5	32.0	21.0	80.0	50.5	31.01
LOC100549353	82.5	14.5	109.5	98.0	16.5	80.0	66.8	41.19
LOC100549354	618.5	565.0	698.0	656.5	619.0	719.0	646.0	56.90
LOC100549359	200.0	89.0	119.0	194.5	102.5	113.5	136.4	48.26
LOC100549361	19.5	13.0	16.5	23.5	21.5	18.0	18.7	3.72
LOC100549369	1200.0	1034.0	1127.5	1248.0	1282.5	1098.0	1165.0	94.84
LOC100549370	3.5	0.5	0.0	6.0	0.0	0.0	1.7	2.52
LOC100549374	20.0	17.5	12.0	22.5	11.5	19.0	17.1	4.44
LOC100549389	759.0	754.5	778.0	712.0	789.0	737.5	755.0	27.77
LOC100549393	381.5	156.0	187.5	404.0	155.0	120.0	234.0	125.01
LOC100549411	62.5	110.5	245.5	46.5	107.0	145.5	119.6	71.23
LOC100549421	265.5	204.5	244.0	288.5	207.5	278.0	248.0	35.76
LOC100549425	1188.5	913.0	1315.0	1235.5	1029.0	1333.5	1169.1	166.32
LOC100549427	0.0	0.0	0.0	0.0	0.0	0.0	0.0	0.00
LOC100549440	2.5	1.5	1.0	5.0	0.5	1.0	1.9	1.66
LOC100549445	0.0	0.0	0.0	0.0	0.0	0.0	0.0	0.00
LOC100549446	55.0	98.0	105.5	40.0	71.0	96.0	77.6	26.46
LOC100549451	1.0	0.0	0.0	0.0	0.0	0.0	0.2	0.41
LOC100549452	128.5	87.5	90.5	113.0	83.5	74.0	96.2	20.43
LOC100549453	0.5	0.0	0.5	0.5	0.5	0.0	0.3	0.26
LOC100549460	2.0	0.5	1.0	0.5	0.5	0.0	0.8	0.69
LOC100549461	0.0	0.0	0.0	0.0	0.0	0.0	0.0	0.00

LOC100549463	279.5	168.0	141.0	296.5	163.0	204.0	208.7	64.92
LOC100549470	2447.5	1352.5	2038.0	2263.0	1351.0	1490.5	1823.8	486.73
LOC100549479	24.0	15.0	22.0	24.5	12.5	21.5	19.9	4.97
LOC100549485	1.5	10.5	24.5	2.0	18.0	22.0	13.1	9.98
LOC100549494	1.5	0.0	1.5	0.5	0.5	0.5	0.8	0.61
LOC100549505	0.5	0.0	0.5	1.0	0.5	0.5	0.5	0.32
LOC100549507	1.0	0.5	0.5	0.5	0.0	1.0	0.6	0.38
LOC100549511	0.0	0.0	0.0	0.0	0.0	0.0	0.0	0.00
LOC100549513	77.0	18.0	99.5	88.5	23.5	81.5	64.7	34.90
LOC100549516	3.5	0.0	0.0	1.0	1.0	0.0	0.9	1.36
LOC100549522	216.5	111.0	251.5	211.0	105.5	200.0	182.6	60.13
LOC100549525	75.5	74.5	223.5	68.0	97.0	183.0	120.3	66.28
LOC100549531	171.0	146.0	217.0	174.5	184.0	208.0	183.4	25.95
LOC100549535	1263.5	1395.0	1479.0	1159.5	1668.5	1425.5	1398.5	176.33
LOC100549541	13.5	22.0	80.0	24.0	27.5	92.5	43.3	33.86
LOC100549545	2.5	0.5	0.5	0.5	1.5	2.0	1.3	0.88
LOC100549552	795.5	346.0	301.0	789.0	383.5	328.0	490.5	235.28
LOC100549557	207.5	84.0	180.5	201.0	106.0	164.0	157.2	51.02
LOC100549568	0.0	0.0	0.0	0.0	0.0	0.0	0.0	0.00
LOC100549590	7.5	2.0	0.5	4.5	3.5	2.0	3.3	2.46
LOC100549601	89.5	86.5	105.0	89.5	74.0	84.5	88.2	10.04
LOC100549614	0.0	0.5	1.0	1.5	1.0	1.0	0.8	0.52
LOC100549616	0.0	0.0	0.0	0.0	0.0	0.0	0.0	0.00
LOC100549617	4.0	5.0	12.0	3.0	0.5	4.5	4.8	3.86
LOC100549626	1.0	3.5	3.0	2.5	6.0	5.0	3.5	1.79
LOC100549632	15.0	15.5	12.0	24.5	9.0	15.5	15.3	5.20
LOC100549639	13.5	9.0	1.0	10.5	9.5	5.5	8.2	4.36
LOC100549646	80.0	106.0	102.0	57.5	105.5	123.0	95.7	23.21
LOC100549648	265.5	72.0	52.5	248.0	67.0	49.0	125.7	102.05
LOC100549666	0.0	0.5	0.0	0.5	0.5	0.0	0.3	0.27
LOC100549668	1.0	3.0	7.0	5.5	4.5	3.0	4.0	2.12
LOC100549671	39.0	1.0	0.5	38.0	3.0	0.5	13.7	19.26
LOC100549678	35.5	75.5	62.5	36.5	100.0	78.0	64.7	25.27
LOC100549687	4.0	3.5	2.5	3.5	3.5	1.5	3.1	0.92
LOC100549689	0.0	0.0	0.0	0.0	0.0	0.0	0.0	0.00
LOC100549696	4.5	12.0	4.5	5.5	14.0	39.5	13.3	13.45
LOC100549706	6.0	0.0	8.5	2.0	0.5	12.0	4.8	4.82
LOC100549707	121.5	52.5	86.0	132.5	82.0	87.5	93.7	29.02
LOC100549708	3.5	3.5	2.5	3.5	5.5	4.0	3.8	0.99
LOC100549719	1.0	4.5	2.5	2.5	1.5	2.5	2.4	1.20
LOC100549720	7.5	1.0	0.5	8.5	1.0	0.0	3.1	3.84
LOC100549722	0.0	0.0	0.5	0.0	0.0	2.0	0.4	0.80
LOC100549728	1041.5	902.5	811.0	872.5	1028.5	794.5	908.4	105.76
LOC100549732	37.5	17.5	40.0	43.0	19.0	32.0	31.5	10.89
LOC100549733	801.0	784.0	1134.0	757.0	818.0	929.0	870.5	141.99
LOC100549735	247.5	104.5	218.0	259.5	113.0	216.0	193.1	67.50
LOC100549737	456.5	223.5	225.0	452.5	264.0	214.0	305.9	116.37
LOC100549755	77.0	44.5	55.5	55.0	37.0	57.0	54.3	13.56
LOC100549758	57.5	42.0	85.0	52.5	52.5	82.0	61.9	17.49
LOC100549762	54.0	28.0	31.5	63.0	26.0	24.5	37.8	16.43
LOC100549764	8.0	9.0	7.5	11.5	6.5	3.0	7.6	2.82
LOC100549766	45.0	10.5	1.5	49.0	24.5	7.5	23.0	20.10
LOC100549788	0.5	0.0	0.5	0.5	0.0	0.0	0.3	0.27
LOC100549795	15.0	6.0	49.5	19.5	6.0	37.0	22.2	17.60

LOC100549801	648.5	1185.5	914.0	624.0	1297.0	884.5	925.6	273.88
LOC100549803	436.0	359.0	376.5	397.5	326.0	355.5	375.1	38.12
LOC100549810	234.0	61.5	318.0	204.0	64.0	267.0	191.4	106.60
LOC100549814	0.0	0.5	0.5	0.0	1.0	0.0	0.3	0.41
LOC100549822	1615.5	1240.0	1451.5	1857.0	1542.5	1459.5	1527.7	204.74
LOC100549823	402.0	245.5	472.5	473.5	258.5	432.5	380.8	103.33
LOC100549824	761.5	543.0	480.5	789.0	637.5	474.0	614.3	138.13
LOC100549826	4174.5	3385.0	2747.0	3880.0	3762.5	2667.0	3436.0	619.32
LOC100549829	844.5	286.5	137.0	773.0	299.0	184.5	420.8	307.51
LOC100549832	90.5	52.5	133.5	85.5	61.0	153.0	96.0	39.78
LOC100549837	727.5	417.0	399.0	644.0	475.5	423.0	514.3	137.75
LOC100549851	28.5	17.0	87.0	31.5	13.5	82.5	43.3	32.82
LOC100549854	876.0	783.5	639.5	901.5	837.0	664.0	783.6	109.87
LOC100549855	55.0	11.5	63.5	65.5	12.5	56.5	44.1	25.17
LOC100549857	0.0	0.0	0.0	0.0	0.0	0.5	0.1	0.20
LOC100549863	40.0	30.5	26.0	24.0	24.5	24.5	28.3	6.23
LOC100549869	57.0	69.5	61.5	62.0	82.0	69.5	66.9	8.86
LOC100549877	0.0	0.0	0.0	0.0	0.0	0.0	0.0	0.00
LOC100549885	0.0	0.0	4.0	0.5	0.0	1.5	1.0	1.58
LOC100549893	3110.5	3062.0	2371.0	3041.0	3270.5	2167.0	2837.0	451.89
LOC100549894	1201.0	747.0	1266.5	1210.5	845.5	1139.5	1068.3	216.82
LOC100549896	1.0	0.5	0.5	1.0	0.5	1.0	0.8	0.27
LOC100549911	63.0	68.0	76.0	60.0	79.5	56.5	67.2	9.09
LOC100549918	339.5	205.5	499.5	365.0	193.0	481.5	347.3	130.74
LOC100549920	17.5	3.5	8.0	11.0	1.5	4.5	7.7	5.89
LOC100549940	296.0	136.5	281.0	337.0	142.5	262.0	242.5	83.54
LOC100549945	23.5	13.0	10.5	21.0	9.5	6.0	13.9	6.88
LOC100549955	456.5	651.0	375.0	395.0	537.5	416.0	471.8	104.92
LOC100549962	0.0	0.5	0.0	0.0	1.5	2.5	0.8	1.04
LOC100549969	702.5	393.0	273.5	670.5	451.0	301.5	465.3	182.99
LOC100549986	0.0	2.5	4.0	0.0	1.0	5.5	2.2	2.25
LOC100549990	1085.5	1036.5	1241.5	998.0	1128.5	1057.5	1091.3	85.82
LOC100549991	0.0	0.0	0.0	0.0	1.0	0.0	0.2	0.41
LOC100549995	0.0	0.0	0.0	0.0	0.0	0.0	0.0	0.00
LOC100549996	14.0	11.0	16.0	13.5	24.0	14.5	15.5	4.47
LOC100550004	0.0	0.0	0.0	0.0	0.0	0.0	0.0	0.00
LOC100550010	0.5	0.5	1.5	0.0	0.0	0.0	0.4	0.58
LOC100550016	1.5	1.0	0.5	1.5	1.0	0.0	0.9	0.58
LOC100550020	9.5	5.5	11.5	4.0	2.5	3.0	6.0	3.69
LOC100550023	0.5	4.0	5.0	0.0	6.5	3.5	3.3	2.54
LOC100550029	0.5	0.0	1.0	2.5	1.0	0.0	0.8	0.93
LOC100550035	277.5	74.0	175.0	215.5	81.5	107.0	155.1	81.65
LOC100550042	0.0	0.0	1.5	0.0	1.0	0.5	0.5	0.63
LOC100550044	277.0	213.5	252.5	262.0	249.5	192.5	241.2	31.78
LOC100550045	1.5	3.5	8.0	0.5	1.0	1.0	2.6	2.85
LOC100550050	10.5	5.0	0.5	4.5	4.5	1.0	4.3	3.59
LOC100550056	73.5	62.0	56.5	64.0	87.5	64.0	67.9	11.05
LOC100550062	2308.0	1672.5	2682.0	2725.0	2053.5	2365.5	2301.1	396.34
LOC100550063	348.5	206.5	457.5	340.0	164.0	369.0	314.3	109.12
LOC100550074	128.0	37.5	40.5	125.0	42.5	29.0	67.1	46.26
LOC100550081	130.0	79.5	168.5	114.0	86.5	110.5	114.8	32.19
LOC100550096	0.0	0.0	0.0	0.0	0.0	0.0	0.0	0.00
LOC100550104	0.0	0.0	0.0	0.0	0.0	0.0	0.0	0.00
LOC100550119	626.0	297.5	1126.0	619.5	367.0	1134.0	695.0	361.81

LOC100550121	29.0	18.0	56.0	29.5	24.0	44.0	33.4	14.02
LOC100550127	66.5	15.5	47.0	42.5	16.0	37.5	37.5	19.51
LOC100550128	696.0	255.0	221.5	1090.5	311.0	86.0	443.3	377.50
LOC100550133	2.5	2.0	1.0	4.0	4.5	2.5	2.8	1.29
LOC100550136	0.0	0.5	0.0	0.0	0.0	0.0	0.1	0.20
LOC100550137	32.5	13.5	10.5	41.0	9.5	4.5	18.6	14.62
LOC100550153	2212.0	2082.0	1250.0	2207.0	2104.0	1264.0	1853.2	464.79
LOC100550164	18.0	19.0	57.0	15.5	17.0	24.5	25.2	15.90
LOC100550169	4.0	0.0	1.0	3.5	0.5	0.0	1.5	1.79
LOC100550170	992.5	1115.5	1007.0	1088.5	1287.5	1030.5	1086.9	109.12
LOC100550171	0.0	0.0	0.0	0.0	0.0	0.0	0.0	0.00
LOC100550173	82.5	30.5	105.0	81.0	47.5	87.5	72.3	27.72
LOC100550174	1.5	0.5	1.0	2.0	0.0	1.0	1.0	0.71
LOC100550180	85.0	71.0	170.0	98.0	77.0	161.5	110.4	43.89
LOC100550182	671.0	555.0	506.0	650.0	671.0	484.5	589.6	85.01
LOC100550192	16.0	2.5	36.0	18.5	2.5	25.0	16.8	13.03
LOC100550202	402.5	258.5	275.5	352.5	290.0	243.5	303.8	61.31
LOC100550208	95.0	21.5	63.0	100.0	22.0	59.5	60.2	33.94
LOC100550216	855.0	260.0	2163.5	1317.5	302.5	1473.0	1061.9	736.26
LOC100550217	34.0	55.0	54.0	25.5	65.0	54.5	48.0	14.96
LOC100550226	0.0	0.0	0.0	0.0	0.0	0.5	0.1	0.20
LOC100550229	656.0	351.0	441.0	596.5	385.5	364.0	465.7	129.51
LOC100550231	502.0	306.0	476.0	576.5	342.5	376.0	429.8	104.60
LOC100550234	40.5	31.0	115.0	37.5	33.0	118.5	62.6	42.10
LOC100550249	0.0	0.0	0.0	0.0	0.0	0.0	0.0	0.00
LOC100550253	12.5	3.0	11.5	11.0	6.0	4.0	8.0	4.16
LOC100550262	1032.0	742.0	729.0	1029.0	728.5	668.0	821.4	163.98
LOC100550263	5.0	3.5	9.0	8.0	2.0	5.5	5.5	2.65
LOC100550264	0.0	0.0	0.0	0.0	0.0	0.0	0.0	0.00
LOC100550265	2.0	0.0	0.0	0.0	0.0	1.0	0.5	0.84
LOC100550268	34.0	14.5	27.5	32.5	15.5	36.5	26.8	9.57
LOC100550270	0.0	0.5	0.5	0.5	0.0	0.0	0.3	0.27
LOC100550272	1032.0	1007.5	863.5	882.5	1176.0	1002.5	994.0	113.39
LOC100550279	8084.0	2030.0	919.5	10213.0	2367.0	366.5	3996.7	4111.44
LOC100550280	1.5	3.5	2.0	1.0	2.5	2.5	2.2	0.88
LOC100550282	990.5	426.0	2330.5	1100.5	565.0	2077.0	1248.3	786.04
LOC100550289	9.0	35.0	34.0	7.0	42.0	21.0	24.7	14.60
LOC100550291	600.5	494.5	494.5	653.5	615.5	656.5	585.8	73.96
LOC100550296	181.0	147.0	329.5	208.5	162.5	325.0	225.6	81.38
LOC100550313	1721.0	887.0	1230.0	1688.5	877.5	1063.5	1244.6	379.38
LOC100550314	705.5	420.5	423.5	770.5	488.5	363.0	528.6	168.27
LOC100550316	0.0	0.0	0.0	0.0	0.0	0.0	0.0	0.00
LOC100550324	11.0	35.0	19.0	7.5	35.5	14.0	20.3	12.16
LOC100550328	8.0	3.5	25.5	6.0	3.5	16.5	10.5	8.78
LOC100550331	116.5	90.0	144.5	121.5	124.5	154.5	125.3	22.63
LOC100550333	1.0	0.5	3.5	0.5	0.0	2.0	1.3	1.29
LOC100550341	92.5	64.5	83.0	87.5	68.5	67.0	77.2	11.96
LOC100550348	129.0	111.5	89.5	121.0	113.5	70.0	105.8	21.95
LOC100550358	0.5	0.5	5.0	1.0	1.0	5.5	2.3	2.34
LOC100550363	1.5	1.5	4.5	1.5	1.0	3.0	2.2	1.33
LOC100550364	0.5	0.0	1.0	1.5	0.5	0.5	0.7	0.52
LOC100550375	1.0	1.0	0.5	4.0	2.0	2.5	1.8	1.29
LOC100550376	147.0	166.5	153.0	125.0	177.5	146.0	152.5	18.17
LOC100550377	145.0	83.0	123.5	71.0	67.0	64.5	92.3	33.78

LOC100550389	118.5	62.0	43.5	128.0	78.5	46.0	79.4	36.33
LOC100550402	0.0	0.0	0.0	0.0	0.0	0.0	0.0	0.00
LOC100550412	109.0	22.5	20.5	75.0	25.5	13.5	44.3	38.66
LOC100550418	48.5	29.0	62.5	54.5	40.0	69.0	50.6	14.69
LOC100550420	299.5	415.5	411.0	263.0	349.0	615.0	392.2	124.65
LOC100550421	667.5	453.5	1244.5	750.0	488.0	988.0	765.3	304.35
LOC100550423	429.5	384.0	174.5	385.0	385.0	193.5	325.3	110.94
LOC100550424	10.5	2.5	1.0	14.0	1.0	0.0	4.8	5.90
LOC100550425	0.0	0.0	0.0	0.0	0.0	0.0	0.0	0.00
LOC100550430	0.0	1.0	2.0	0.5	1.0	0.5	0.8	0.68
LOC100550431	30.5	52.0	94.0	17.0	37.5	31.5	43.8	27.10
LOC100550436	0.0	0.0	0.5	0.0	0.5	0.5	0.3	0.27
LOC100550445	191.0	122.5	111.0	182.5	127.5	58.0	132.1	49.11
LOC100550448	22.5	30.0	22.0	23.5	30.5	17.0	24.3	5.16
LOC100550455	3.0	0.5	1.5	4.5	0.5	1.5	1.9	1.56
LOC100550460	0.5	1.5	2.0	1.0	2.0	1.0	1.3	0.61
LOC100550466	456.5	451.5	453.5	452.5	500.5	371.5	447.7	41.82
LOC100550472	0.0	0.0	0.0	0.0	0.0	0.0	0.0	0.00
LOC100550475	0.0	0.0	0.0	0.0	0.0	0.0	0.0	0.00
LOC100550490	65.0	404.5	162.5	79.5	512.0	280.0	250.6	181.31
LOC100550494	694.5	245.5	289.5	535.0	256.5	217.5	373.1	195.24
LOC100550496	344.5	305.5	286.0	361.5	324.0	247.5	311.5	41.30
LOC100550503	430.5	278.5	804.0	504.0	302.5	670.0	498.3	206.98
LOC100550505	64.5	25.5	171.0	71.0	19.5	134.0	80.9	60.24
LOC100550511	0.5	1.0	2.5	1.0	1.5	1.0	1.3	0.69
LOC100550516	975.5	1190.0	1415.0	870.5	1463.0	1250.5	1194.1	235.29
LOC100550517	0.0	0.5	0.0	0.0	0.0	0.0	0.1	0.20
LOC100550520	2755.5	1357.0	2693.5	2900.5	1458.0	2200.5	2227.5	678.16
LOC100550523	0.0	0.0	0.0	0.0	0.0	0.0	0.0	0.00
LOC100550524	61.0	50.0	42.0	51.5	28.0	68.5	50.2	14.24
LOC100550526	0.0	0.0	0.0	1.0	0.5	0.5	0.3	0.41
LOC100550530	15.0	10.0	21.0	7.5	15.0	15.5	14.0	4.72
LOC100550532	548.0	280.0	336.5	516.0	286.5	200.0	361.2	139.73
LOC100550551	0.0	0.0	0.0	0.5	0.0	0.0	0.1	0.20
LOC100550557	0.0	0.0	1.5	0.0	0.0	3.5	0.8	1.44
LOC100550565	0.0	0.0	0.0	0.0	0.0	0.0	0.0	0.00
LOC100550569	38.0	18.5	80.0	45.5	28.0	63.5	45.6	22.83
LOC100550576	7.0	1.5	9.5	7.0	1.0	9.5	5.9	3.79
LOC100550580	0.0	8.0	3.5	0.0	10.0	4.0	4.3	4.10
LOC100550583	0.0	0.0	0.0	0.0	0.0	0.0	0.0	0.00
LOC100550586	1.0	2.0	3.0	0.5	0.0	6.0	2.1	2.20
LOC100550587	0.0	0.0	0.0	0.0	0.0	0.0	0.0	0.00
LOC100550588	1705.5	537.0	680.5	1631.0	560.0	500.5	935.8	571.08
LOC100550589	268.5	160.0	266.0	288.0	179.0	233.5	232.5	52.19
LOC100550598	0.0	0.0	0.0	0.0	0.0	0.0	0.0	0.00
LOC100550599	1.0	0.5	2.0	3.0	1.0	4.0	1.9	1.36
LOC100550605	208.5	107.5	419.0	208.0	119.0	360.5	237.1	127.06
LOC100550606	545.0	277.5	533.0	578.0	292.0	450.0	445.9	131.85
LOC100550610	0.0	0.0	0.0	0.5	0.0	0.5	0.2	0.26
LOC100550618	0.0	0.5	2.0	0.0	1.5	0.5	0.8	0.82
LOC100550620	0.0	0.0	0.0	0.0	0.0	0.0	0.0	0.00
LOC100550621	589.0	479.0	853.5	631.5	522.0	766.0	640.2	144.21
LOC100550622	45.5	38.5	253.0	47.5	45.5	211.0	106.8	97.91
LOC100550623	96.5	53.5	136.0	101.0	67.5	126.0	96.8	32.06

LOC100550625	0.0	0.0	0.0	0.0	0.0	0.0	0.0	0.00
LOC100550626	0.0	0.0	0.0	0.0	0.0	0.0	0.0	0.00
LOC100550636	6.5	2.0	10.0	4.0	2.5	5.5	5.1	2.96
LOC100550642	284.5	242.5	412.5	287.0	341.5	548.5	352.8	112.38
LOC100550646	0.0	0.0	0.0	0.0	0.0	0.0	0.0	0.00
LOC100550649	186.5	117.0	345.5	222.0	135.5	336.5	223.8	98.09
LOC100550655	5.5	5.5	5.0	3.0	6.0	10.0	5.8	2.29
LOC100550661	105.5	63.0	35.5	90.0	77.5	45.5	69.5	26.66
LOC100550667	1014.0	917.5	889.5	1061.0	1050.5	896.0	971.4	79.24
LOC100550673	61.0	54.5	92.5	66.5	45.0	90.0	68.3	19.22
LOC100550679	9631.0	7431.5	6812.5	10444.5	9090.0	6835.5	8374.2	1553.91
LOC100550690	90.0	78.5	77.5	76.5	88.5	94.5	84.3	7.68
LOC100550693	0.0	0.0	0.0	0.0	0.0	0.0	0.0	0.00
LOC100550697	44.5	32.0	82.5	42.5	35.5	98.5	55.9	27.64
LOC100550702	10.5	11.5	8.0	6.5	10.0	7.0	8.9	2.04
LOC100550711	3.0	3.0	5.5	5.5	5.0	10.0	5.3	2.56
LOC100550717	598.5	449.0	342.5	420.0	450.0	271.0	421.8	111.17
LOC100550719	0.0	0.0	0.0	0.0	0.0	0.0	0.0	0.00
LOC100550721	35.0	34.5	5.5	29.0	28.0	12.5	24.1	12.22
LOC100550735	152.0	120.5	157.0	177.0	157.5	175.0	156.5	20.40
LOC100550736	457.0	292.0	316.5	423.5	329.5	295.5	352.3	70.28
LOC100550739	2.5	4.5	7.0	5.5	5.5	23.0	8.0	7.50
LOC100550740	316.0	174.0	259.0	272.5	141.0	97.0	209.9	85.29
LOC100550742	0.0	0.0	0.5	0.0	0.0	0.0	0.1	0.20
LOC100550744	310.0	199.0	162.0	312.0	253.5	166.0	233.8	68.22
LOC100550749	625.5	598.0	545.0	588.0	684.5	558.0	599.8	50.45
LOC100550754	3.0	0.5	1.5	5.0	1.5	0.0	1.9	1.83
LOC100550755	2.5	6.0	3.0	3.5	3.5	7.0	4.3	1.81
LOC100550763	1.5	1.5	10.5	2.0	2.5	6.5	4.1	3.67
LOC100550767	650.5	738.0	871.5	619.0	844.0	792.5	752.6	102.54
LOC100550777	7.0	5.0	5.5	6.0	6.0	11.5	6.8	2.38
LOC100550780	0.0	0.0	0.0	0.0	0.0	0.0	0.0	0.00
LOC100550794	981.0	1084.5	783.5	1012.0	1288.0	945.0	1015.7	166.74
LOC100550801	666.5	537.5	432.0	597.0	591.0	419.5	540.6	98.02
LOC100550809	2.5	3.5	6.0	6.0	5.0	13.0	6.0	3.70
LOC100550812	5.0	2.0	1.5	6.0	3.0	1.5	3.2	1.91
LOC100550814	47.0	18.0	43.5	48.0	17.5	39.5	35.6	14.13
LOC100550818	35.0	34.0	20.5	24.0	43.5	45.5	33.8	10.05
LOC100550820	0.0	0.0	0.0	0.0	0.0	0.0	0.0	0.00
LOC100550826	360.5	311.5	381.0	425.5	360.0	468.0	384.4	55.08
LOC100550829	397.0	498.5	304.5	351.0	528.5	280.0	393.3	101.85
LOC100550830	0.5	0.5	0.5	1.5	0.0	0.0	0.5	0.55
LOC100550832	4063.5	3710.5	3818.5	3893.0	4241.0	4012.0	3956.4	189.24
LOC100550836	57.0	16.0	58.5	36.0	12.5	31.0	35.2	19.60
LOC100550847	0.0	0.0	0.0	0.0	0.0	0.0	0.0	0.00
LOC100550849	15.0	5.0	22.5	17.5	8.0	16.5	14.1	6.46
LOC100550859	1364.0	1137.0	780.5	1243.5	1171.5	813.0	1084.9	236.58
LOC100550869	1.5	1.0	3.0	0.5	1.5	2.0	1.6	0.86
LOC100550877	0.0	0.5	0.0	0.0	0.0	0.0	0.1	0.20
LOC100550884	0.0	0.0	0.0	0.0	0.0	0.0	0.0	0.00
LOC100550885	1.5	0.5	13.5	6.5	2.0	20.0	7.3	7.85
LOC100550886	28.0	32.5	15.5	11.5	38.0	9.0	22.4	12.02
LOC100550893	15.0	4.0	1.5	12.0	9.0	5.0	7.8	5.16
LOC100550906	1416.0	938.0	569.0	1370.0	868.5	584.5	957.7	368.44

LOC100550908	95.0	108.0	56.0	77.5	125.0	59.0	86.8	27.51
LOC100550909	0.5	0.0	0.0	0.0	0.0	0.0	0.1	0.20
LOC100550910	26.0	12.5	7.5	12.0	6.0	9.5	12.3	7.19
LOC100550934	0.0	0.0	0.0	0.0	0.0	0.0	0.0	0.00
LOC100550942	0.0	0.0	0.0	0.0	0.0	0.0	0.0	0.00
LOC100550948	4.0	2.5	5.5	1.0	4.0	3.5	3.4	1.53
LOC100550949	0.0	0.0	0.0	0.0	0.0	0.0	0.0	0.00
LOC100550954	1295.0	1283.5	1440.5	1083.5	1302.0	1638.5	1340.5	185.29
LOC100550956	67.0	32.5	109.0	81.0	46.5	83.0	69.8	27.49
LOC100550971	1.5	2.5	7.0	3.5	6.5	6.5	4.6	2.38
LOC100550975	1.0	2.0	0.0	3.5	2.0	1.0	1.6	1.20
LOC100550977	39.0	37.0	66.0	49.0	41.0	64.0	49.3	12.82
LOC100550978	246.0	1018.5	981.5	211.0	1084.0	935.0	746.0	403.96
LOC100550986	262.0	123.0	453.0	289.5	111.5	392.5	271.9	138.31
LOC100550995	5.5	5.5	3.0	8.5	5.0	4.0	5.3	1.86
LOC100551000	150.0	116.0	92.0	136.5	148.5	82.0	120.8	29.07
LOC100551004	59.5	19.5	36.0	71.0	19.0	29.0	39.0	21.60
LOC100551007	0.0	0.0	0.0	0.0	0.0	0.0	0.0	0.00
LOC100551010	3.0	5.5	9.0	0.5	5.0	3.0	4.3	2.89
LOC100551022	4221.0	2870.5	3893.5	4380.0	3186.5	3744.0	3715.9	587.10
LOC100551023	28.0	8.5	1.5	16.5	6.0	2.0	10.4	10.20
LOC100551027	1087.0	905.5	989.5	1056.5	891.0	865.0	965.8	92.60
LOC100551033	3.0	6.5	18.5	4.0	4.5	18.0	9.1	7.19
LOC100551038	407.0	426.5	723.5	372.5	426.0	501.0	476.1	128.30
LOC100551040	1266.5	605.0	368.5	1207.0	609.5	375.5	738.7	400.35
LOC100551042	0.0	0.0	0.0	0.0	0.0	0.0	0.0	0.00
LOC100551043	171.0	66.0	93.5	172.0	80.0	94.5	112.8	46.62
LOC100551044	68.5	31.0	96.5	87.5	43.0	70.0	66.1	25.17
LOC100551049	14.0	16.0	8.0	22.5	23.0	13.0	16.1	5.80
LOC100551066	310.5	220.0	427.5	348.5	194.0	446.0	324.4	104.01
LOC100551070	1.0	5.0	1.5	1.5	1.5	2.5	2.2	1.47
LOC100551071	54.5	16.5	65.5	47.0	21.0	61.5	44.3	20.84
LOC100551072	0.0	0.0	0.0	0.0	0.0	0.0	0.0	0.00
LOC100551074	409.0	322.0	596.0	395.5	392.5	638.5	458.9	127.05
LOC100551087	47.5	93.5	159.5	68.5	100.0	175.5	107.4	50.40
LOC100551088	3253.0	3351.5	2889.0	3107.0	3671.0	3179.5	3241.8	261.87
LOC100551109	0.0	0.0	0.0	0.5	1.0	0.5	0.3	0.41
LOC100551117	569.5	779.0	607.0	497.5	706.0	704.5	643.9	104.03
LOC100551119	108.5	39.5	120.0	96.0	38.0	105.5	84.6	36.32
LOC100551121	0.0	0.0	0.0	0.0	0.0	0.0	0.0	0.00
LOC100551127	22.0	12.0	9.0	16.0	15.0	5.5	13.3	5.78
LOC100551128	142.0	105.0	58.5	127.5	129.0	38.0	100.0	42.31
LOC100551131	1.0	2.5	4.0	2.0	2.5	1.0	2.2	1.13
LOC100551134	106.5	94.5	138.0	105.5	84.0	135.5	110.7	21.82
LOC100551145	1013.5	838.5	1717.5	1056.0	1020.5	1683.0	1221.5	378.61
LOC100551155	144.5	215.5	140.0	122.0	251.5	149.5	170.5	50.96
LOC100551159	0.0	0.0	0.0	0.0	0.0	0.0	0.0	0.00
LOC100551160	873.5	991.0	1347.0	828.5	1021.5	1379.0	1073.4	235.66
LOC100551166	0.0	0.0	0.0	0.0	0.0	0.0	0.0	0.00
LOC100551187	282.0	148.5	295.0	285.0	151.0	205.5	227.8	68.42
LOC100551190	6.0	0.5	1.5	4.5	1.0	4.0	2.9	2.22
LOC100551192	2.5	2.0	1.5	2.5	2.5	4.0	2.5	0.84
LOC100551205	0.0	0.0	0.0	0.0	0.0	0.0	0.0	0.00
LOC100551216	4.0	3.0	9.0	8.5	6.0	34.0	10.8	11.64

LOC100551225	758.0	431.5	397.5	745.0	393.5	288.0	502.3	199.02
LOC100551228	6.5	7.0	10.0	8.5	7.5	11.5	8.5	1.92
LOC100551229	1662.5	1964.0	4092.0	1979.5	1727.0	3297.0	2453.7	1001.39
LOC100551231	0.5	4.0	5.0	0.5	4.0	7.0	3.5	2.57
LOC100551250	189.5	65.5	121.5	180.0	61.5	109.5	121.3	54.64
LOC100551262	294.0	197.0	653.0	340.5	253.0	636.5	395.7	198.71
LOC100551267	166.5	110.0	403.0	160.5	138.5	280.5	209.8	111.06
LOC100551269	3109.0	2158.0	3350.5	3099.5	2361.5	3092.5	2861.8	480.69
LOC100551278	536.5	443.5	598.0	532.0	465.5	483.0	509.8	56.68
LOC100551279	0.5	0.0	1.0	0.0	0.0	1.0	0.4	0.49
LOC100551282	23.5	65.5	24.5	19.5	72.5	32.0	39.6	23.25
LOC100551284	1.5	0.0	2.0	2.5	0.5	3.0	1.6	1.16
LOC100551287	0.0	0.0	0.0	0.0	0.0	0.0	0.0	0.00
LOC100551292	188.5	103.5	258.5	211.5	134.5	244.0	190.1	61.05
LOC100551294	163.5	44.0	124.0	142.5	60.0	96.5	105.1	46.92
LOC100551296	4918.0	3811.0	2719.0	4439.5	4509.0	3262.0	3943.1	836.28
LOC100551326	72.5	16.5	11.0	62.5	10.5	4.5	29.6	29.78
LOC100551338	2.0	1.5	5.0	0.5	2.0	5.5	2.8	2.02
LOC100551343	0.0	0.0	0.0	0.0	0.0	0.0	0.0	0.00
LOC100551355	175.5	67.5	230.0	174.0	82.0	185.5	152.4	63.69
LOC100551358	0.5	0.0	0.0	0.0	0.0	0.0	0.1	0.20
LOC100551363	2.0	1.0	0.0	5.5	0.0	0.0	1.4	2.15
LOC100551372	535.0	709.0	1253.5	632.5	701.5	1337.0	861.4	342.83
LOC100551374	418.0	278.5	151.5	398.0	280.5	157.0	280.6	113.67
LOC100551377	38.5	42.0	74.5	31.0	50.5	83.5	53.3	21.04
LOC100551379	284.0	176.5	139.5	232.5	162.5	107.5	183.8	64.35
LOC100551380	235.0	190.0	153.0	193.0	197.0	123.0	181.8	38.84
LOC100551381	0.0	0.0	0.0	0.0	0.0	0.0	0.0	0.00
LOC100551394	0.0	0.0	0.0	0.0	0.0	0.0	0.0	0.00
LOC100551399	468.5	418.0	589.0	534.5	516.5	587.5	519.0	67.22
LOC100551401	85.0	32.0	52.5	69.0	29.5	47.0	52.5	21.48
LOC100551403	107.5	61.5	69.0	100.0	55.5	59.0	75.4	22.52
LOC100551412	674.5	542.5	414.5	649.5	578.5	454.5	552.3	103.65
LOC100551413	0.0	0.0	0.0	0.0	0.0	0.0	0.0	0.00
LOC100551422	117.5	53.0	52.0	99.0	58.5	31.5	68.6	32.60
LOC100551424	0.0	0.5	0.0	0.5	0.0	0.0	0.2	0.26
LOC100551434	699.0	738.0	649.0	688.0	762.5	621.5	693.0	52.85
LOC100551438	0.5	0.0	0.0	0.0	0.0	0.0	0.1	0.20
LOC100551442	53.5	81.0	22.5	40.5	61.0	28.0	47.8	21.88
LOC100551461	401.0	302.5	315.5	464.0	335.0	302.0	353.3	65.58
LOC100551463	88.5	7.5	118.0	71.5	18.0	57.0	60.1	42.04
LOC100551492	5.5	1.5	2.0	3.0	6.0	5.0	3.8	1.91
LOC104909199	2083.5	2174.0	2734.0	2084.5	2607.5	3615.5	2549.8	591.29
LOC104909200	14.5	11.0	15.0	9.5	5.5	14.0	11.6	3.68
LOC104909202	0.0	0.0	1.0	0.0	0.0	0.0	0.2	0.41
LOC104909203	0.0	0.0	0.0	0.0	0.0	0.0	0.0	0.00
LOC104909204	105.5	104.5	71.5	109.0	87.5	61.0	89.8	20.00
LOC104909205	447.0	182.0	365.5	421.5	189.0	362.5	327.9	115.01
LOC104909207	0.0	0.0	0.0	0.0	0.0	0.0	0.0	0.00
LOC104909208	0.0	0.0	0.0	0.0	0.0	0.0	0.0	0.00
LOC104909209	10.5	4.5	24.5	5.5	6.0	19.5	11.8	8.35
LOC104909210	546.5	551.5	468.0	537.0	652.5	453.0	534.8	71.31
LOC104909211	0.0	0.0	0.0	0.0	0.0	1.0	0.2	0.41
LOC104909212	0.0	0.0	0.0	0.0	0.0	0.0	0.0	0.00

LOC104909213	387.0	223.5	203.5	326.5	194.0	205.0	256.6	80.48
LOC104909215	0.5	2.0	8.5	1.0	2.0	6.0	3.3	3.19
LOC104909216	0.0	0.0	0.0	0.0	0.0	0.0	0.0	0.00
LOC104909218	103.0	41.5	94.5	124.5	49.5	90.5	83.9	32.09
LOC104909219	159.0	120.0	84.0	157.0	138.5	89.5	124.7	32.64
LOC104909220	0.0	0.0	0.0	0.0	0.0	0.0	0.0	0.00
LOC104909221	0.0	0.0	0.0	0.0	0.0	0.0	0.0	0.00
LOC104909224	68.0	93.0	189.5	63.5	93.5	186.5	115.7	57.39
LOC104909225	68.0	98.0	110.5	81.5	104.0	168.0	105.0	34.56
LOC104909228	2.0	1.0	2.0	0.5	0.5	0.5	1.1	0.74
LOC104909229	0.5	0.0	1.0	0.0	0.5	0.0	0.3	0.41
LOC104909230	97.0	78.0	114.5	95.5	103.5	142.5	105.2	21.83
LOC104909231	438.5	561.0	303.0	372.5	556.5	288.0	419.9	120.19
LOC104909233	614.5	404.5	446.5	532.0	426.0	408.5	472.0	83.98
LOC104909235	0.0	0.0	0.5	0.0	0.0	0.5	0.2	0.26
LOC104909236	783.5	673.0	552.0	662.5	741.5	541.0	658.9	97.86
LOC104909237	181.0	131.5	75.0	107.0	132.5	60.0	114.5	43.87
LOC104909238	0.0	0.0	0.5	0.0	0.0	0.0	0.1	0.20
LOC104909239	0.0	0.0	0.0	0.0	0.5	0.0	0.1	0.20
LOC104909240	0.0	0.0	0.0	0.0	0.0	0.0	0.0	0.00
LOC104909241	0.0	0.0	0.0	0.0	0.0	0.0	0.0	0.00
LOC104909245	275.0	172.0	417.0	314.0	181.0	380.5	289.9	100.92
LOC104909246	8.0	4.5	9.5	7.0	3.5	8.5	6.8	2.36
LOC104909248	14.0	2.0	9.0	16.5	3.0	12.5	9.5	5.95
LOC104909250	218.0	323.5	241.0	240.0	357.0	214.5	265.7	59.74
LOC104909252	0.0	10.5	25.5	0.0	12.0	27.5	12.6	11.92
LOC104909254	35.5	122.5	71.5	44.0	134.0	70.0	79.6	40.42
LOC104909256	21.5	14.5	40.0	24.0	15.0	30.5	24.3	9.75
LOC104909257	640.5	570.5	484.0	556.5	534.5	513.5	549.9	54.00
LOC104909259	0.0	0.0	0.5	0.0	0.5	0.0	0.2	0.26
LOC104909260	7.5	2.0	1.5	2.5	1.5	1.5	2.8	2.36
LOC104909261	21.5	27.5	31.0	39.5	28.0	44.0	31.9	8.34
LOC104909263	0.5	2.0	2.0	1.5	6.5	3.5	2.7	2.11
LOC104909264	45.5	7.5	40.5	55.5	4.5	23.0	29.4	21.00
LOC104909265	0.5	0.0	0.0	0.0	0.0	0.0	0.1	0.20
LOC104909266	0.5	0.5	2.5	0.0	0.5	8.0	2.0	3.07
LOC104909267	410.5	310.0	271.5	403.0	314.5	277.0	331.1	61.11
LOC104909268	91.5	49.5	72.5	112.0	43.0	65.5	72.3	25.96
LOC104909269	74.5	38.0	46.5	65.0	41.5	43.5	51.5	14.71
LOC104909270	51.5	79.0	66.5	59.5	87.0	73.5	69.5	13.00
LOC104909271	43.5	7.5	22.0	57.0	13.5	14.5	26.3	19.56
LOC104909272	66.5	36.5	133.5	68.5	38.5	102.5	74.3	37.70
LOC104909274	0.0	0.0	0.0	0.0	0.0	0.0	0.0	0.00
LOC104909277	13.5	6.0	7.5	14.5	3.0	4.0	8.1	4.85
LOC104909278	0.5	0.0	1.0	0.0	0.0	0.5	0.3	0.41
LOC104909279	172.0	71.5	198.0	164.0	91.0	147.5	140.7	49.22
LOC104909281	37.5	21.0	26.0	38.0	21.0	28.5	28.7	7.61
LOC104909282	0.5	0.0	0.0	0.0	0.0	0.0	0.1	0.20
LOC104909283	516.0	540.5	648.0	397.0	635.0	569.0	550.9	91.48
LOC104909284	15.5	15.5	17.5	16.5	12.0	12.5	14.9	2.20
LOC104909285	1.0	4.5	16.5	7.5	5.0	12.5	7.8	5.71
LOC104909286	10.0	5.0	54.0	17.0	15.0	48.0	24.8	20.78
LOC104909287	0.0	0.0	0.0	0.0	0.0	0.0	0.0	0.00
LOC104909288	156.5	127.5	129.5	134.5	148.5	117.5	135.7	14.39

LOC104909289	35.5	4.5	22.0	27.5	2.0	14.0	17.6	13.16
LOC104909290	18.5	6.5	25.5	16.5	7.0	22.0	16.0	7.80
LOC104909291	0.5	2.0	6.0	0.0	7.5	6.0	3.7	3.22
LOC104909292	1.0	10.5	4.5	4.0	10.5	8.0	6.4	3.87
LOC104909293	1097.0	1268.5	1325.5	970.5	1498.0	1223.5	1230.5	183.03
LOC104909294	6.5	25.0	105.5	2.5	26.0	122.0	47.9	52.13
LOC104909297	0.0	0.5	0.5	0.0	0.5	0.0	0.3	0.27
LOC104909298	5.5	5.5	4.0	3.5	4.5	2.5	4.3	1.17
LOC104909299	0.0	0.0	0.0	0.0	0.0	0.0	0.0	0.00
LOC104909300	0.0	0.5	0.5	0.0	0.0	0.5	0.3	0.27
LOC104909301	0.5	5.0	0.5	0.0	5.5	4.0	2.6	2.52
LOC104909304	213.0	155.5	298.5	227.0	168.5	251.0	218.9	52.97
LOC104909305	5.5	1.0	5.5	6.0	0.0	1.0	3.2	2.77
LOC104909306	1.5	4.0	5.0	0.5	2.0	3.0	2.7	1.66
LOC104909307	98.0	58.5	116.5	112.0	60.0	90.5	89.3	25.06
LOC104909308	0.0	0.0	0.0	0.0	0.0	0.0	0.0	0.00
LOC104909309	69.0	49.0	100.0	63.0	53.5	92.0	71.1	20.69
LOC104909310	208.0	100.5	98.0	191.5	112.0	86.5	132.8	52.79
LOC104909311	0.0	1.0	0.5	0.5	0.5	0.5	0.5	0.32
LOC104909312	143.0	70.0	36.5	132.0	97.0	50.0	88.1	43.50
LOC104909314	19.5	7.0	28.0	19.5	11.5	23.5	18.2	7.72
LOC104909316	0.0	0.5	0.0	0.0	0.0	0.0	0.1	0.20
LOC104909317	109.5	84.5	49.5	110.5	78.5	48.5	80.2	27.37
LOC104909318	0.0	2.0	5.0	0.5	2.0	19.0	4.8	7.20
LOC104909319	26.0	3.5	37.5	28.5	7.5	27.0	21.7	13.23
LOC104909320	6.5	4.0	4.5	9.0	2.0	3.0	4.8	2.54
LOC104909321	4.5	0.0	0.0	0.5	0.0	1.0	1.0	1.76
LOC104909322	0.0	0.0	5.5	0.0	0.0	4.5	1.7	2.60
LOC104909324	0.0	0.5	1.5	3.0	0.0	2.0	1.2	1.21
LOC104909325	0.0	0.0	1.0	0.0	0.0	0.0	0.2	0.41
LOC104909326	231.0	248.5	509.0	144.0	219.0	517.5	311.5	160.31
LOC104909327	0.0	0.0	0.0	0.0	0.0	0.0	0.0	0.00
LOC104909328	1.5	2.5	7.5	1.0	3.5	3.0	3.2	2.32
LOC104909329	30.5	37.0	13.5	39.0	38.5	24.0	30.4	10.10
LOC104909330	128.5	65.0	104.0	116.5	62.5	63.5	90.0	29.88
LOC104909332	22.0	64.0	47.0	19.5	78.5	32.0	43.8	23.78
LOC104909334	0.0	0.0	0.0	0.0	0.0	0.0	0.0	0.00
LOC104909335	57.5	29.5	103.0	68.0	34.5	97.0	64.9	30.74
LOC104909336	37.5	18.0	18.5	18.5	19.0	16.5	21.3	7.97
LOC104909337	119.5	96.5	90.5	93.0	94.5	113.5	101.3	12.12
LOC104909339	2.0	9.0	20.0	2.0	9.5	22.0	10.8	8.60
LOC104909340	191.5	104.0	190.5	198.5	107.0	189.5	163.5	45.05
LOC104909342	0.0	0.0	0.0	0.0	0.0	0.0	0.0	0.00
LOC104909344	0.0	0.0	0.0	0.0	0.0	0.0	0.0	0.00
LOC104909345	0.0	5.0	13.5	0.0	1.5	12.5	5.4	6.16
LOC104909346	48.5	8.0	12.5	46.0	14.0	9.0	23.0	18.93
LOC104909348	0.0	0.0	0.0	0.0	0.0	0.0	0.0	0.00
LOC104909349	0.0	0.0	0.0	0.0	0.0	0.0	0.0	0.00
LOC104909350	216.5	109.0	240.0	221.5	125.5	214.5	187.8	55.66
LOC104909351	62.5	33.0	63.0	76.5	32.0	67.0	55.7	18.64
LOC104909352	0.5	0.5	1.0	0.0	0.0	0.0	0.3	0.41
LOC104909353	0.0	0.0	0.0	0.0	0.0	0.0	0.0	0.00
LOC104909356	33.0	32.0	36.5	25.5	31.0	42.5	33.4	5.70
LOC104909357	65.0	22.5	118.0	72.5	26.0	109.5	68.9	40.20

LOC104909358	0.0	0.0	0.0	0.0	0.0	0.0	0.0	0.00
LOC104909359	3.5	2.5	2.0	1.5	1.0	1.0	1.9	0.97
LOC104909362	356.0	144.5	275.0	346.5	162.0	225.5	251.6	90.15
LOC104909364	94.5	34.5	40.0	79.5	37.0	29.0	52.4	27.44
LOC104909368	0.5	0.0	0.0	0.0	0.0	0.0	0.1	0.20
LOC104909369	820.5	1325.5	833.0	705.0	1400.0	840.0	987.3	295.88
LOC104909370	80.5	51.5	112.0	61.0	49.0	84.0	73.0	24.00
LOC104909371	10.5	6.0	17.0	14.5	8.0	9.5	10.9	4.12
LOC104909372	13.0	3.5	26.0	16.0	2.5	12.0	12.2	8.66
LOC104909373	292.0	227.5	204.0	263.0	230.0	188.5	234.2	38.04
LOC104909374	1.5	0.5	1.5	0.0	0.0	1.0	0.8	0.69
LOC104909376	48.5	21.0	78.5	62.5	16.5	52.0	46.5	23.94
LOC104909378	16.5	9.5	3.5	21.0	13.5	2.5	11.1	7.31
LOC104909379	0.5	0.0	1.5	1.5	1.0	1.5	1.0	0.63
LOC104909380	0.0	0.0	0.0	0.0	0.5	0.0	0.1	0.20
LOC104909381	0.5	0.5	0.0	0.0	0.0	1.0	0.3	0.41
LOC104909382	275.0	154.5	292.0	151.0	152.0	220.5	207.5	64.73
LOC104909383	17.0	22.0	58.0	8.5	17.5	47.0	28.3	19.53
LOC104909384	0.5	0.0	0.0	0.0	0.0	0.0	0.1	0.20
LOC104909385	0.0	1.5	0.5	0.0	3.0	1.0	1.0	1.14
LOC104909386	0.5	0.5	0.5	0.5	0.5	2.0	0.8	0.61
LOC104909387	10.0	7.5	12.5	5.5	7.0	12.5	9.2	2.96
LOC104909388	10.0	4.5	9.5	9.0	1.5	8.5	7.2	3.40
LOC104909389	0.5	6.5	18.0	0.5	4.5	5.5	5.9	6.44
LOC104909390	2.5	1.0	9.5	4.5	1.0	6.0	4.1	3.31
LOC104909392	0.0	1.0	0.5	0.0	0.5	0.0	0.3	0.41
LOC104909393	0.5	0.0	0.0	0.0	0.0	0.0	0.1	0.20
LOC104909394	3.5	2.0	14.5	5.0	2.5	5.0	5.4	4.62
LOC104909395	53.0	44.5	52.5	44.0	47.0	29.0	45.0	8.73
LOC104909396	0.5	0.0	0.5	0.0	0.0	0.5	0.3	0.27
LOC104909397	18.5	2.5	37.5	31.5	6.0	29.5	20.9	14.34
LOC104909398	4.0	1.0	2.5	1.5	0.5	2.0	1.9	1.24
LOC104909399	43.0	19.5	32.0	42.0	19.5	22.5	29.8	10.89
LOC104909400	0.0	1.0	0.0	0.0	0.5	0.0	0.3	0.42
LOC104909401	0.0	0.0	0.0	0.0	0.0	0.0	0.0	0.00
LOC104909404	1.5	4.5	4.5	3.0	4.0	3.5	3.5	1.14
LOC104909405	251.0	146.5	150.5	198.5	163.5	145.0	175.8	41.88
LOC104909406	27.0	19.0	52.5	27.0	19.0	55.5	33.3	16.43
LOC104909408	2.0	0.5	0.0	2.5	1.0	1.0	1.2	0.93
LOC104909409	9.5	5.0	6.5	9.5	9.5	4.0	7.3	2.50
LOC104909410	9.0	10.0	137.5	4.5	11.5	58.5	38.5	52.48
LOC104909411	451.0	308.5	560.0	394.0	378.5	575.0	444.5	105.66
LOC104909412	0.0	0.0	0.0	0.0	1.0	0.0	0.2	0.41
LOC104909413	3.0	4.0	7.5	4.5	1.0	4.0	4.0	2.12
LOC104909414	0.0	0.0	0.0	0.0	0.0	0.0	0.0	0.00
LOC104909415	0.0	0.5	0.5	0.5	0.0	0.0	0.3	0.27
LOC104909416	0.0	0.0	0.0	0.0	0.0	0.0	0.0	0.00
LOC104909417	0.0	0.0	0.0	0.0	0.0	0.0	0.0	0.00
LOC104909419	0.0	0.5	1.0	1.0	0.5	0.5	0.6	0.38
LOC104909420	26.5	8.0	26.5	29.5	12.0	24.0	21.1	8.85
LOC104909421	0.0	0.0	0.5	0.0	0.0	0.5	0.2	0.26
LOC104909422	0.0	0.5	0.0	0.0	0.0	0.0	0.1	0.20
LOC104909425	0.0	0.0	0.0	0.0	0.0	0.0	0.0	0.00
LOC104909426	0.0	0.0	0.0	0.0	0.0	0.0	0.0	0.00

LOC104909427	0.0	0.0	0.0	0.0	0.0	0.0	0.0	0.00
LOC104909429	0.0	0.0	0.0	0.0	0.0	0.0	0.0	0.00
LOC104909430	0.5	2.0	0.0	0.0	0.5	0.0	0.5	0.77
LOC104909432	31.0	46.0	48.0	25.0	37.0	41.5	38.1	8.89
LOC104909433	17.5	13.5	24.0	17.5	15.0	15.0	17.1	3.73
LOC104909434	0.0	0.0	0.0	0.0	0.0	0.0	0.0	0.00
LOC104909436	0.0	0.0	0.0	0.0	0.0	0.0	0.0	0.00
LOC104909438	69.0	27.5	33.5	55.5	16.0	22.5	37.3	20.59
LOC104909440	162.5	105.5	39.5	155.5	114.5	44.5	103.7	52.70
LOC104909441	5.0	3.0	5.5	7.0	5.5	7.5	5.6	1.59
LOC104909445	34.5	19.5	33.0	33.0	18.0	36.0	29.0	8.03
LOC104909448	75.5	95.5	106.5	60.0	88.0	100.0	87.6	17.21
LOC104909449	33.5	39.5	29.5	23.0	25.0	32.0	30.4	6.00
LOC104909451	390.0	240.5	191.5	375.0	254.0	198.0	274.8	86.90
LOC104909452	49.5	25.0	38.0	37.5	32.0	27.5	34.9	8.84
LOC104909453	305.0	210.0	202.0	277.5	222.0	197.0	235.6	44.79
LOC104909454	0.0	0.0	0.0	0.0	0.0	0.0	0.0	0.00
LOC104909455	1.5	3.0	1.5	4.0	2.5	2.5	2.5	0.95
LOC104909456	18.0	10.0	13.0	28.0	11.0	8.0	14.7	7.37
LOC104909457	91.0	45.5	110.0	89.5	44.5	97.5	79.7	27.81
LOC104909458	173.5	167.5	113.0	209.0	202.0	126.5	165.3	38.91
LOC104909459	9.0	3.0	15.5	11.0	0.5	11.5	8.4	5.63
LOC104909460	1.5	4.0	1.5	0.5	4.0	2.5	2.3	1.44
LOC104909461	6.5	1.5	3.5	3.0	5.5	1.0	3.5	2.17
LOC104909462	0.5	0.0	0.5	1.5	1.0	0.5	0.7	0.52
LOC104909463	33.5	60.5	38.5	27.5	60.0	36.0	42.7	14.10
LOC104909464	810.5	817.0	2716.0	802.5	1037.0	1927.5	1351.8	796.53
LOC104909465	3.5	1.0	3.0	3.0	3.0	4.5	3.0	1.14
LOC104909466	1.5	0.0	1.0	0.0	0.0	0.0	0.4	0.66
LOC104909467	78.5	24.5	56.0	66.5	25.5	61.5	52.1	22.26
LOC104909468	2.5	1.0	3.5	2.5	1.0	0.5	1.8	1.17
LOC104909469	0.0	0.0	0.0	0.0	0.0	0.0	0.0	0.00
LOC104909470	0.0	0.0	0.0	0.0	0.0	0.0	0.0	0.00
LOC104909471	14.5	8.5	6.0	15.0	13.5	12.0	11.6	3.60
LOC104909472	1.0	0.0	0.0	0.0	0.0	0.0	0.2	0.41
LOC104909473	0.0	0.0	0.5	0.0	0.0	0.0	0.1	0.20
LOC104909474	0.0	0.0	0.0	0.0	0.5	0.0	0.1	0.20
LOC104909475	0.0	0.0	0.0	0.0	0.0	0.0	0.0	0.00
LOC104909476	3.0	3.0	5.0	0.0	2.5	3.0	2.8	1.60
LOC104909477	8.5	5.5	18.0	18.0	3.5	15.0	11.4	6.41
LOC104909478	55.5	57.5	57.0	50.5	51.0	43.0	52.4	5.49
LOC104909479	41.5	5.0	23.0	38.0	9.5	19.5	22.8	14.73
LOC104909481	1592.5	1496.5	2016.5	1503.0	1624.0	1708.0	1656.8	193.17
LOC104909482	95.0	60.5	58.5	94.5	75.5	56.5	73.4	17.83
LOC104909483	50.0	45.0	43.0	66.5	53.5	71.0	54.8	11.48
LOC104909484	50.0	21.5	19.0	41.0	18.5	19.5	28.3	13.70
LOC104909488	3.0	1.5	3.5	10.0	1.5	3.0	3.8	3.17
LOC104909489	0.5	0.0	0.0	0.5	0.0	0.0	0.2	0.26
LOC104909490	52.0	17.0	7.0	34.0	17.5	5.5	22.2	17.81
LOC104909491	72.0	22.5	103.5	80.5	24.0	65.0	61.3	32.17
LOC104909493	64.5	129.5	67.0	62.5	120.5	70.0	85.7	30.70
LOC104909494	125.5	118.5	77.0	133.0	163.0	81.0	116.3	32.68
LOC104909495	0.0	0.0	0.0	0.0	0.0	0.0	0.0	0.00
LOC104909497	0.0	0.0	0.0	0.0	0.0	1.0	0.2	0.41

LOC104909499	0.0	0.0	0.0	0.0	0.0	0.0	0.0	0.00
LOC104909500	1.5	1.5	0.5	1.0	0.5	0.0	0.8	0.61
LOC104909501	26.0	12.5	8.5	3.5	3.0	2.0	9.3	9.13
LOC104909502	35.5	10.5	24.5	9.0	6.0	3.0	14.8	12.58
LOC104909504	80.0	43.5	29.0	60.0	40.5	24.5	46.3	20.69
LOC104909505	0.5	0.5	0.0	0.0	0.0	0.0	0.2	0.26
LOC104909506	25.0	6.5	1.0	31.5	8.5	1.0	12.3	12.91
LOC104909507	0.0	0.0	0.0	0.0	0.0	0.0	0.0	0.00
LOC104909508	2.5	3.5	3.0	2.0	10.0	1.5	3.8	3.14
LOC104909510	0.0	0.0	0.0	0.0	0.0	0.0	0.0	0.00
LOC104909512	0.0	0.5	1.0	0.0	0.0	0.0	0.3	0.42
LOC104909513	7.5	7.0	8.0	5.5	7.5	8.0	7.3	0.94
LOC104909514	414.0	127.5	290.0	443.0	178.0	296.5	291.5	124.71
LOC104909515	45.0	19.5	59.0	54.5	21.5	57.0	42.8	17.90
LOC104909517	236.0	219.0	144.5	205.0	215.5	141.5	193.6	40.44
LOC104909518	338.0	388.0	378.0	357.5	438.5	426.5	387.8	38.89
LOC104909519	50.5	18.5	51.5	53.5	18.5	49.0	40.3	16.91
LOC104909520	64.0	44.0	46.5	79.0	42.0	46.5	53.7	14.70
LOC104909521	165.5	147.0	71.5	163.5	181.5	88.5	136.3	45.24
LOC104909522	0.0	0.0	0.5	0.0	0.0	0.0	0.1	0.20
LOC104909523	0.0	0.0	0.0	0.0	0.0	0.0	0.0	0.00
LOC104909525	214.0	101.5	470.0	258.0	133.5	467.0	274.0	160.63
LOC104909526	7.5	0.0	5.0	4.0	1.0	4.0	3.6	2.73
LOC104909527	1.0	1.0	0.0	0.0	1.5	0.5	0.7	0.61
LOC104909528	31.5	37.0	42.0	34.5	33.5	33.0	35.3	3.78
LOC104909529	0.0	0.5	0.5	0.0	0.0	0.0	0.2	0.26
LOC104909530	0.0	0.0	0.0	0.0	0.0	0.0	0.0	0.00
LOC104909531	1.0	0.5	0.0	0.0	0.5	0.0	0.3	0.41
LOC104909532	3.5	2.0	6.5	5.5	3.0	6.0	4.4	1.83
LOC104909533	0.5	0.5	0.0	1.0	0.0	0.0	0.3	0.41
LOC104909534	1.5	0.5	0.5	3.0	0.0	1.0	1.1	1.07
LOC104909535	0.0	0.0	0.0	1.0	1.0	0.0	0.3	0.52
LOC104909536	0.0	0.0	0.0	0.0	0.0	0.0	0.0	0.00
LOC104909537	7.5	1.0	7.0	5.5	2.5	3.0	4.4	2.63
LOC104909538	1.5	1.0	1.0	3.5	0.5	2.0	1.6	1.07
LOC104909539	6.5	3.5	10.5	7.0	5.0	10.5	7.2	2.86
LOC104909540	1.0	0.0	0.0	0.0	0.0	0.0	0.2	0.41
LOC104909541	0.5	0.0	0.0	1.5	0.5	0.0	0.4	0.58
LOC104909542	0.5	0.0	3.0	0.0	1.0	5.5	1.7	2.18
LOC104909543	38.0	28.5	32.5	27.5	37.5	42.0	34.3	5.77
LOC104909544	8.0	3.0	11.0	8.0	2.0	3.0	5.8	3.66
LOC104909545	0.5	0.5	1.5	0.0	0.0	0.0	0.4	0.58
LOC104909546	0.0	0.0	0.0	0.0	0.0	0.0	0.0	0.00
LOC104909547	0.0	1.0	0.0	3.0	0.5	0.5	0.8	1.13
LOC104909548	5.5	13.0	0.5	20.0	27.5	30.0	16.1	11.86
LOC104909549	0.0	0.0	0.0	0.0	0.0	0.0	0.0	0.00
LOC104909550	0.0	0.0	0.0	0.0	0.0	0.0	0.0	0.00
LOC104909551	0.5	0.0	0.0	0.0	0.0	0.0	0.1	0.20
LOC104909552	0.0	0.0	0.0	0.0	0.0	0.0	0.0	0.00
LOC104909553	0.5	0.5	2.5	0.5	0.0	0.5	0.8	0.88
LOC104909554	20.0	14.5	46.0	32.0	9.5	38.0	26.7	14.27
LOC104909555	0.5	2.0	1.5	1.0	0.5	1.5	1.2	0.61
LOC104909557	6.5	6.0	4.5	8.0	2.0	6.5	5.6	2.08
LOC104909558	7.5	6.0	9.0	5.0	4.5	8.5	6.8	1.86

LOC104909559	26.5	10.0	37.0	30.0	20.0	34.0	26.3	9.93
LOC104909560	19.0	4.0	26.0	26.5	5.0	48.5	21.5	16.49
LOC104909561	0.0	1.5	0.0	0.0	0.0	0.0	0.3	0.61
LOC104909562	0.0	0.5	0.0	0.5	0.0	0.0	0.2	0.26
LOC104909563	84.0	72.5	99.0	100.0	76.5	105.0	89.5	13.63
LOC104909564	34.5	15.5	64.0	41.0	15.5	61.5	38.7	21.26
LOC104909565	22.5	51.5	46.0	13.0	58.0	38.5	38.3	17.39
LOC104909566	0.0	0.0	0.0	0.0	0.0	0.0	0.0	0.00
LOC104909567	0.5	1.0	0.5	0.0	0.0	0.0	0.3	0.41
LOC104909569	0.0	0.0	0.0	0.0	0.0	0.0	0.0	0.00
LOC104909570	0.0	0.0	0.0	0.0	0.0	0.0	0.0	0.00
LOC104909571	0.0	0.0	1.0	0.0	0.0	0.0	0.2	0.41
LOC104909572	1.5	1.0	6.0	0.5	1.0	4.0	2.3	2.18
LOC104909573	0.0	0.0	0.0	0.5	0.0	1.0	0.3	0.42
LOC104909574	5.0	3.0	1.0	2.5	2.0	2.5	2.7	1.33
LOC104909575	0.0	0.5	0.0	0.0	0.0	0.5	0.2	0.26
LOC104909576	0.0	0.5	0.0	0.0	1.0	0.0	0.3	0.42
LOC104909579	4.0	0.5	2.0	1.0	1.0	1.5	1.7	1.25
LOC104909580	299.0	245.5	181.5	278.5	299.5	155.0	243.2	61.84
LOC104909581	15.5	3.0	2.0	12.0	6.5	2.5	6.9	5.63
LOC104909582	0.0	0.0	0.0	0.0	0.0	0.0	0.0	0.00
LOC104909583	0.0	0.0	0.0	0.0	0.0	0.0	0.0	0.00
LOC104909584	0.0	0.0	0.0	0.5	0.0	0.0	0.1	0.20
LOC104909585	0.0	0.0	0.0	0.0	0.0	0.0	0.0	0.00
LOC104909586	0.0	0.0	0.0	0.0	0.0	0.0	0.0	0.00
LOC104909587	26.5	24.5	17.5	14.0	24.0	32.0	23.1	6.45
LOC104909588	1.0	1.0	0.0	0.5	1.5	1.0	0.8	0.52
LOC104909589	1.5	3.5	1.0	2.0	2.5	0.5	1.8	1.08
LOC104909590	0.0	0.0	0.0	0.0	0.0	0.0	0.0	0.00
LOC104909591	966.0	417.5	1725.5	1098.5	467.5	1358.0	1005.5	507.55
LOC104909592	0.0	0.5	0.5	0.0	0.0	0.0	0.2	0.26
LOC104909593	1.0	0.0	1.0	1.0	0.5	0.0	0.6	0.49
LOC104909594	46.5	28.0	33.5	46.5	38.5	30.0	37.2	8.06
LOC104909595	450.0	151.5	115.0	354.5	149.5	94.5	219.2	146.58
LOC104909596	0.0	0.0	0.0	0.0	0.0	0.0	0.0	0.00
LOC104909597	1.0	0.0	0.5	1.5	0.0	1.0	0.7	0.61
LOC104909599	13.5	6.5	2.0	12.5	5.5	1.5	6.9	5.10
LOC104909601	3.5	9.0	12.5	7.0	12.5	7.0	8.6	3.51
LOC104909602	1.5	5.0	3.0	0.0	6.5	1.5	2.9	2.44
LOC104909604	1.0	2.5	3.5	0.5	4.5	2.5	2.4	1.50
LOC104909608	0.0	0.0	0.0	0.0	0.0	0.0	0.0	0.00
LOC104909609	0.0	0.0	0.0	0.0	0.0	0.0	0.0	0.00
LOC104909610	0.0	0.0	0.0	0.0	0.0	0.0	0.0	0.00
LOC104909611	0.0	0.0	0.5	0.0	0.0	0.0	0.1	0.20
LOC104909612	322.5	228.0	235.5	293.5	299.5	246.0	270.8	39.26
LOC104909613	143.5	58.5	53.0	153.5	69.5	39.5	86.3	49.28
LOC104909614	5.0	2.0	3.0	8.0	4.0	2.0	4.0	2.28
LOC104909616	0.0	0.0	0.0	0.0	0.0	0.0	0.0	0.00
LOC104909618	0.0	0.5	1.5	0.0	1.0	1.0	0.7	0.61
LOC104909620	37.5	18.5	83.5	34.5	19.5	66.5	43.3	26.26
LOC104909621	450.0	340.0	409.0	465.0	392.5	436.5	415.5	45.50
LOC104909622	10.5	3.0	9.5	11.0	6.0	4.0	7.3	3.46
LOC104909623	0.0	0.0	0.0	0.0	0.0	0.0	0.0	0.00
LOC104909624	0.0	0.0	0.0	0.0	0.0	0.5	0.1	0.20

LOC104909625	19.5	6.0	52.0	21.0	3.5	49.5	25.3	20.97
LOC104909626	285.5	225.0	304.0	310.0	280.5	298.0	283.8	30.88
LOC104909627	0.5	0.0	0.5	0.5	0.0	0.0	0.3	0.27
LOC104909628	1.0	3.0	1.0	0.5	1.0	2.0	1.4	0.92
LOC104909629	8.0	6.0	17.5	13.0	6.5	13.5	10.8	4.61
LOC104909630	0.0	0.0	0.0	0.0	0.0	0.0	0.0	0.00
LOC104909631	233.0	102.0	344.5	270.0	139.5	279.0	228.0	91.29
LOC104909632	868.5	712.5	642.5	806.0	796.0	640.5	744.3	93.87
LOC104909633	313.0	300.0	146.5	287.5	314.5	126.5	248.0	87.15
LOC104909634	5.0	1.5	4.5	1.5	3.5	3.5	3.3	1.47
LOC104909635	163.0	17.0	332.5	224.5	24.0	315.0	179.3	137.59
LOC104909636	3.0	10.0	5.5	3.5	12.0	4.0	6.3	3.76
LOC104909637	130.0	49.5	159.5	160.5	59.5	138.0	116.2	49.33
LOC104909638	104.0	95.5	52.0	105.0	90.5	45.5	82.1	26.46
LOC104909639	0.5	0.0	0.5	0.5	1.0	1.5	0.7	0.52
LOC104909640	0.0	0.0	1.5	0.0	0.0	0.0	0.3	0.61
LOC104909641	4.0	3.5	5.5	3.0	2.0	6.0	4.0	1.52
LOC104909642	2245.5	2212.0	1630.0	1935.5	2384.5	1766.5	2029.0	297.86
LOC104909644	15653.0	16279.5	13916.0	13128.5	14008.5	12395.5	14230.2	1479.98
LOC104909646	4.0	6.0	4.0	5.0	4.5	4.0	4.6	0.80
LOC104909647	3.0	1.0	4.5	1.5	1.5	4.0	2.6	1.46
LOC104909648	609.5	698.0	1005.5	559.0	816.5	900.0	764.8	173.10
LOC104909649	2.5	4.5	5.0	0.5	4.5	0.0	2.8	2.18
LOC104909650	2.0	0.0	0.5	3.5	1.5	0.0	1.3	1.37
LOC104909651	0.0	0.0	0.0	0.0	0.0	0.0	0.0	0.00
LOC104909652	22.0	4.5	1.0	7.5	6.0	0.5	6.9	7.88
LOC104909653	18.5	0.5	26.0	14.5	3.5	13.0	12.7	9.46
LOC104909654	2.5	1.0	1.0	1.5	1.5	0.5	1.3	0.68
LOC104909655	0.0	0.0	0.5	0.0	0.0	0.0	0.1	0.20
LOC104909656	9.5	6.0	5.5	6.0	6.0	4.0	6.2	1.81
LOC104909657	536.0	634.0	717.0	563.0	743.0	653.5	641.1	81.85
LOC104909658	0.0	0.0	0.0	0.0	0.0	0.0	0.0	0.00
LOC104909659	0.5	0.0	0.0	0.0	0.0	0.0	0.1	0.20
LOC104909661	0.0	0.0	0.5	0.5	0.0	0.0	0.2	0.26
LOC104909662	0.0	0.0	0.0	0.5	0.5	0.0	0.2	0.26
LOC104909663	6.0	3.5	3.5	1.0	1.0	1.0	2.7	2.04
LOC104909664	5.5	0.5	1.0	2.5	3.0	1.5	2.3	1.81
LOC104909665	0.0	0.0	0.0	0.5	0.0	0.0	0.1	0.20
LOC104909666	208.5	63.0	137.5	210.0	64.0	119.0	133.7	65.58
LOC104909667	0.0	0.0	0.5	0.0	0.0	0.0	0.1	0.20
LOC104909668	7.0	7.5	7.0	9.0	6.5	4.0	6.8	1.63
LOC104909669	7.0	4.0	4.5	10.0	5.5	5.5	6.1	2.18
LOC104909670	204.5	181.0	247.0	191.5	189.5	241.0	209.1	28.14
LOC104909671	147.5	97.0	75.5	147.5	105.0	91.5	110.7	30.12
LOC104909672	154.5	54.0	295.0	164.5	58.0	231.5	159.6	94.90
LOC104909673	39.0	17.0	49.5	47.5	20.0	33.5	34.4	13.65
LOC104909674	0.0	0.0	0.0	0.0	0.0	0.0	0.0	0.00
LOC104909675	7.5	9.0	5.5	7.5	11.5	5.0	7.7	2.38
LOC104909676	124.5	64.5	71.5	135.5	84.0	77.0	92.8	29.70
LOC104909677	51.0	33.0	48.5	65.5	40.0	39.0	46.2	11.54
LOC104909678	0.0	0.0	0.0	0.0	0.0	0.0	0.0	0.00
LOC104909679	0.0	0.5	0.0	0.0	0.0	0.0	0.1	0.20
LOC104909680	0.0	0.0	0.0	0.0	0.0	0.0	0.0	0.00
LOC104909681	2.0	0.5	0.0	3.5	1.5	0.5	1.3	1.29

LOC104909682	33.0	17.0	8.0	26.5	15.5	12.0	18.7	9.36
LOC104909683	22.0	9.5	19.5	19.5	7.5	16.5	15.8	5.91
LOC104909684	2.5	0.5	2.0	1.0	0.0	1.5	1.3	0.94
LOC104909685	0.0	0.0	0.0	0.0	0.0	0.0	0.0	0.00
LOC104909686	1.5	0.5	3.5	0.0	1.0	4.0	1.8	1.64
LOC104909687	2.0	5.0	1.0	1.0	6.5	3.0	3.1	2.25
LOC104909688	322.0	216.0	112.5	293.0	218.5	115.0	212.8	87.21
LOC104909690	2.5	2.0	4.0	2.0	4.0	3.0	2.9	0.92
LOC104909691	16.5	0.5	1.5	10.5	1.0	4.0	5.7	6.47
LOC104909693	21.5	12.5	14.5	22.0	18.5	35.5	20.8	8.15
LOC104909695	113.5	85.5	49.5	96.5	103.0	51.0	83.2	27.07
LOC104909696	1.0	0.0	0.5	0.0	1.0	0.5	0.5	0.45
LOC104909699	9.0	2.5	0.5	4.5	1.5	0.5	3.1	3.26
LOC104909700	0.0	0.0	0.0	0.0	0.0	0.0	0.0	0.00
LOC104909701	0.0	0.0	0.0	0.0	0.0	0.0	0.0	0.00
LOC104909702	0.0	0.0	0.5	0.0	0.0	1.0	0.3	0.42
LOC104909705	0.0	0.0	0.0	0.5	0.0	0.0	0.1	0.20
LOC104909707	1.5	19.0	22.0	6.5	21.0	26.0	16.0	9.70
LOC104909708	1.0	9.0	1.5	1.0	9.5	1.0	3.8	4.20
LOC104909710	0.0	0.0	0.0	1.5	0.0	0.0	0.3	0.61
LOC104909711	35.0	9.0	26.5	30.5	6.0	16.5	20.6	11.87
LOC104909712	2.5	0.5	0.0	0.5	0.0	0.0	0.6	0.97
LOC104909713	12.5	4.5	0.5	9.5	0.5	1.5	4.8	5.09
LOC104909714	0.0	0.0	0.0	0.0	0.0	0.0	0.0	0.00
LOC104909715	1.0	0.0	3.0	0.0	0.5	0.5	0.8	1.13
LOC104909716	0.0	0.0	0.0	0.5	0.0	0.0	0.1	0.20
LOC104909717	379.0	375.5	395.0	327.5	328.5	348.0	358.9	28.33
LOC104909718	2.0	2.0	6.5	0.0	7.5	6.0	4.0	3.05
LOC104909719	1.0	1.0	0.5	1.0	0.5	0.0	0.7	0.41
LOC104909721	2.0	0.5	0.0	0.5	0.0	0.0	0.5	0.77
LOC104909723	0.0	0.0	0.5	0.0	0.0	0.5	0.2	0.26
LOC104909724	25.0	5.0	57.5	24.5	13.5	45.0	28.4	19.59
LOC104909725	617.5	838.5	370.5	469.0	868.5	561.0	620.8	199.01
LOC104909726	193.5	229.5	202.0	178.5	237.5	245.5	214.4	26.86
LOC104909727	1.0	1.0	0.5	2.0	0.5	0.0	0.8	0.68
LOC104909728	7.0	5.5	1.5	10.5	4.0	1.5	5.0	3.46
LOC104909729	85.5	44.0	17.0	84.5	43.0	19.5	48.9	30.16
LOC104909730	497.0	225.5	233.5	491.5	240.5	232.5	320.1	135.00
LOC104909731	66.5	25.0	30.0	72.0	30.0	36.0	43.3	20.51
LOC104909732	118.0	26.0	88.5	122.5	29.5	72.0	76.1	41.86
LOC104909734	0.0	1.0	0.0	0.5	0.5	0.0	0.3	0.41
LOC104909735	14.0	6.0	61.0	18.5	17.0	42.0	26.4	20.79
LOC104909736	19.5	15.5	9.5	24.0	14.0	9.5	15.3	5.70
LOC104909737	0.0	0.0	0.0	0.0	0.0	0.0	0.0	0.00
LOC104909738	1.0	0.5	0.0	1.5	0.5	0.0	0.6	0.58
LOC104909739	12.0	8.0	5.5	7.5	11.0	1.5	7.6	3.81
LOC104909740	159.5	133.5	134.5	132.5	151.5	156.0	144.6	12.42
LOC104909742	3.0	0.5	1.5	2.0	1.0	0.0	1.3	1.08
LOC104909743	2.0	1.5	0.0	4.5	4.0	0.5	2.1	1.83
LOC104909744	0.5	0.0	1.5	0.5	0.0	0.5	0.5	0.55
LOC104909745	23.0	1.0	20.5	25.0	5.5	7.5	13.8	10.27
LOC104909746	45.0	39.0	96.5	54.0	50.0	87.0	61.9	23.84
LOC104909747	264.5	225.5	358.5	250.0	255.0	329.0	280.4	51.57
LOC104909748	1.0	5.0	7.5	7.5	7.0	4.5	5.4	2.52

LOC104909749	10.0	7.0	3.0	10.5	7.0	4.0	6.9	3.04
LOC104909750	0.0	0.0	0.5	0.0	0.0	0.0	0.1	0.20
LOC104909751	23.5	10.5	55.0	23.0	11.0	49.0	28.7	19.01
LOC104909752	18.0	6.5	2.5	16.0	6.5	2.0	8.6	6.82
LOC104909753	0.0	0.0	0.0	0.5	0.0	0.5	0.2	0.26
LOC104909754	1.0	0.0	0.0	0.5	2.5	1.0	0.8	0.93
LOC104909756	10.5	0.5	2.5	11.0	3.0	3.0	5.1	4.49
LOC104909758	3.5	1.0	15.5	2.0	0.5	8.0	5.1	5.77
LOC104909759	1.0	0.0	0.0	0.0	0.0	0.5	0.3	0.42
LOC104909760	510.0	285.0	361.0	552.5	381.5	350.0	406.7	102.64
LOC104909761	154.0	167.5	311.0	154.0	210.0	298.5	215.8	71.98
LOC104909763	3.5	0.0	0.5	0.0	3.0	2.0	1.5	1.55
LOC104909764	0.0	1.0	0.0	1.0	1.0	0.0	0.5	0.55
LOC104909765	1.5	0.0	0.0	0.5	0.0	0.0	0.3	0.61
LOC104909766	1.5	2.0	0.5	1.0	1.5	1.0	1.3	0.52
LOC104909767	4.0	6.5	2.0	4.0	2.0	0.5	3.2	2.11
LOC104909768	576.5	681.0	399.0	694.5	767.5	406.5	587.5	155.57
LOC104909771	1.0	0.5	1.0	2.0	0.0	0.0	0.8	0.76
LOC104909772	0.0	0.0	0.0	0.0	1.0	0.0	0.2	0.41
LOC104909773	0.0	0.0	0.0	0.0	0.0	0.0	0.0	0.00
LOC104909774	3.5	0.5	6.5	4.0	2.5	5.0	3.7	2.07
LOC104909775	391.0	856.0	765.0	423.0	1061.5	652.5	691.5	258.18
LOC104909776	13.5	12.5	42.0	14.0	16.0	40.0	23.0	14.00
LOC104909777	21.5	11.0	13.5	15.0	14.5	11.0	14.4	3.87
LOC104909778	0.0	0.0	0.0	0.0	1.0	0.5	0.3	0.42
LOC104909779	33.5	5.5	28.0	36.0	5.0	26.0	22.3	13.72
LOC104909781	0.0	0.0	0.0	0.0	0.0	0.5	0.1	0.20
LOC104909782	32.0	12.5	46.5	32.5	11.0	29.5	27.3	13.47
LOC104909783	0.5	0.5	0.5	5.0	1.5	1.0	1.5	1.76
LOC104909784	0.5	0.5	1.5	0.0	0.5	1.5	0.8	0.61
LOC104909786	11.5	29.0	63.5	15.5	30.5	113.5	43.9	38.69
LOC104909787	0.0	0.0	1.0	0.0	0.0	0.5	0.3	0.42
LOC104909788	0.0	0.0	0.5	0.0	0.0	3.0	0.6	1.20
LOC104909789	2.5	0.5	0.0	5.5	1.0	1.0	1.8	2.02
LOC104909791	85.0	74.5	33.5	82.0	99.0	47.0	70.2	24.86
LOC104909792	10.0	2.0	17.0	11.5	4.0	15.0	9.9	5.94
LOC104909793	114.5	48.0	103.5	116.0	47.5	85.5	85.8	31.45
LOC104909794	908.0	732.5	339.5	656.0	679.0	367.0	613.7	220.38
LOC104909795	24.0	22.5	64.5	19.0	30.0	42.0	33.7	17.13
LOC104909796	45.5	30.5	118.0	40.0	35.0	88.0	59.5	35.36
LOC104909797	6.5	12.0	11.0	7.5	19.5	12.0	11.4	4.60
LOC104909798	108.5	60.5	69.5	125.5	88.5	70.0	87.1	25.47
LOC104909799	5.0	46.0	65.0	8.0	66.0	78.0	44.7	31.30
LOC104909800	0.0	0.5	0.0	0.0	0.0	0.0	0.1	0.20
LOC104909802	0.0	0.0	0.0	0.0	0.0	0.0	0.0	0.00
LOC104909803	0.0	0.0	0.0	0.0	0.0	0.0	0.0	0.00
LOC104909804	0.0	1.0	0.5	0.0	1.0	1.5	0.7	0.61
LOC104909805	0.0	10.0	6.5	0.0	11.0	7.5	5.8	4.80
LOC104909807	256.5	296.5	342.0	232.5	362.0	489.5	329.8	92.35
LOC104909808	92.0	516.5	230.0	101.5	560.0	195.5	282.6	205.49
LOC104909809	0.0	0.0	0.0	0.0	0.0	0.0	0.0	0.00
LOC104909810	0.0	0.0	0.0	0.0	0.0	0.0	0.0	0.00
LOC104909811	0.0	0.0	0.0	0.0	0.0	0.0	0.0	0.00
LOC104909812	0.5	0.0	0.5	0.0	0.0	0.5	0.3	0.27

LOC104909813	0.0	0.0	0.0	0.5	0.0	0.0	0.1	0.20
LOC104909814	0.0	0.5	0.5	0.0	1.5	0.5	0.5	0.55
LOC104909815	0.0	1.5	0.0	0.0	4.0	0.0	0.9	1.63
LOC104909816	2.5	3.5	9.0	4.5	8.5	11.0	6.5	3.45
LOC104909817	0.0	1.0	1.5	0.0	3.0	2.0	1.3	1.17
LOC104909818	1.0	5.0	0.5	2.5	4.0	1.5	2.4	1.77
LOC104909819	1.0	8.5	2.0	0.5	9.0	1.5	3.8	3.91
LOC104909821	56.0	15.0	15.5	30.5	12.0	7.5	22.8	18.04
LOC104909822	13.5	1.0	4.5	8.5	1.5	1.0	5.0	5.08
LOC104909823	1246.0	1170.5	1127.0	1235.0	1288.5	1097.5	1194.1	74.39
LOC104909824	1.5	0.5	3.5	1.5	2.0	0.5	1.6	1.11
LOC104909825	1.5	0.0	0.5	1.5	1.5	0.5	0.9	0.66
LOC104909826	769.5	483.0	560.0	678.0	544.5	487.0	587.0	113.96
LOC104909827	0.5	3.5	1.0	0.0	5.0	3.5	2.3	2.02
LOC104909828	0.0	0.5	0.5	0.0	0.5	1.0	0.4	0.38
LOC104909829	6.0	4.0	8.5	10.0	5.5	12.5	7.8	3.17
LOC104909830	16.0	1.5	5.5	7.0	0.5	2.0	5.4	5.76
LOC104909831	0.0	0.0	0.0	0.0	0.0	0.0	0.0	0.00
LOC104909832	2.0	0.5	0.5	1.0	2.0	0.5	1.1	0.74
LOC104909834	474.0	424.0	390.5	434.0	473.0	337.5	422.2	52.13
LOC104909835	0.0	0.0	0.0	0.0	0.0	0.5	0.1	0.20
LOC104909836	9.5	1.5	3.0	6.0	1.5	3.0	4.1	3.12
LOC104909837	728.0	455.5	435.5	632.0	496.5	498.0	540.9	114.47
LOC104909838	2.0	4.5	3.0	1.5	3.0	3.0	2.8	1.03
LOC104909839	254.5	114.0	88.5	220.5	124.5	66.5	144.8	75.40
LOC104909840	62.5	21.0	61.0	50.5	19.5	65.5	46.7	21.08
LOC104909841	1.0	0.0	0.5	0.0	0.5	0.0	0.3	0.41
LOC104909842	0.0	1.0	0.0	0.0	0.0	1.0	0.3	0.52
LOC104909843	0.0	0.0	0.0	0.0	0.0	0.0	0.0	0.00
LOC104909844	82.5	28.0	24.5	79.0	25.0	22.0	43.5	28.94
LOC104909845	0.0	0.0	0.5	0.0	0.0	0.0	0.1	0.20
LOC104909846	3.0	3.5	12.5	3.5	4.0	11.0	6.3	4.30
LOC104909847	11.5	7.5	27.0	13.5	7.0	16.5	13.8	7.39
LOC104909848	9.0	3.0	18.0	12.5	3.0	12.5	9.7	5.91
LOC104909849	134.0	23.5	45.5	163.5	23.0	33.0	70.4	61.93
LOC104909850	55.5	15.5	55.5	67.5	20.5	62.5	46.2	22.34
LOC104909851	27.0	10.5	22.0	21.0	11.0	23.5	19.2	6.83
LOC104909852	0.5	1.5	2.0	2.5	1.5	2.5	1.8	0.76
LOC104909853	13.5	9.5	10.5	7.0	11.0	8.5	10.0	2.24
LOC104909855	7.0	6.0	9.0	6.0	10.5	13.5	8.7	2.96
LOC104909856	21.5	14.0	19.5	19.5	26.5	26.0	21.2	4.67
LOC104909857	1.5	1.0	1.0	2.0	1.5	1.0	1.3	0.41
LOC104909858	251.5	199.5	79.0	216.5	193.0	61.5	166.8	77.71
LOC104909859	119.0	48.0	44.5	104.5	43.5	33.0	65.4	36.53
LOC104909861	5.0	10.0	4.5	2.5	10.0	8.0	6.7	3.13
LOC104909862	0.0	0.0	0.0	0.0	0.0	0.0	0.0	0.00
LOC104909863	20.5	7.5	23.5	11.5	5.5	22.5	15.2	7.97
LOC104909864	1.0	0.0	1.5	0.5	0.5	1.5	0.8	0.61
LOC104909865	0.0	0.0	0.0	0.0	0.0	0.0	0.0	0.00
LOC104909866	36.0	17.5	68.0	25.5	30.5	62.0	39.9	20.45
LOC104909867	50.5	75.5	66.0	64.0	87.0	61.0	67.3	12.57
LOC104909868	0.0	0.5	1.0	0.5	0.0	1.0	0.5	0.45
LOC104909869	27.0	12.5	26.5	33.0	14.0	16.0	21.5	8.43
LOC104909870	10.0	3.0	10.5	10.5	0.5	6.0	6.8	4.30

LOC104909871	0.0	0.0	0.0	0.0	0.0	0.0	0.0	0.00
LOC104909872	0.5	0.0	0.0	0.0	0.0	0.5	0.2	0.26
LOC104909873	74.5	54.0	75.0	97.0	66.0	92.0	76.4	16.02
LOC104909874	1.0	0.5	1.5	0.0	0.0	1.5	0.8	0.69
LOC104909875	333.5	226.5	169.0	318.0	260.5	166.5	245.7	71.66
LOC104909876	85.5	30.0	63.0	79.0	30.5	66.5	59.1	23.78
LOC104909877	0.0	0.0	0.0	0.0	0.0	0.0	0.0	0.00
LOC104909878	0.0	0.0	0.0	0.0	0.0	0.0	0.0	0.00
LOC104909879	15.0	4.5	18.5	11.0	2.5	7.0	9.8	6.22
LOC104909881	86.5	36.0	87.5	87.5	32.0	69.5	66.5	26.12
LOC104909883	43.5	15.0	45.5	47.5	16.0	41.0	34.8	15.07
LOC104909884	20.0	4.5	37.5	23.0	5.0	23.5	18.9	12.54
LOC104909885	0.0	0.0	0.0	0.0	0.0	0.0	0.0	0.00
LOC104909886	0.0	0.0	0.0	0.0	0.0	0.0	0.0	0.00
LOC104909887	0.0	0.0	0.0	0.0	0.0	0.0	0.0	0.00
LOC104909888	0.0	0.0	0.0	0.0	0.0	0.0	0.0	0.00
LOC104909889	0.0	0.0	0.0	0.0	0.0	0.0	0.0	0.00
LOC104909890	0.0	0.0	0.0	0.0	0.0	0.0	0.0	0.00
LOC104909891	1.0	3.0	1.5	1.0	5.5	1.0	2.2	1.81
LOC104909892	0.5	27.0	60.0	1.5	22.0	34.0	24.2	22.21
LOC104909893	2.0	0.0	2.5	0.5	2.5	0.5	1.3	1.13
LOC104909895	14.0	12.0	34.5	18.0	18.0	27.0	20.6	8.55
LOC104909896	607.5	511.5	467.5	617.5	567.0	421.0	532.0	78.91
LOC104909897	0.0	0.0	0.0	0.0	0.0	0.0	0.0	0.00
LOC104909898	10.0	9.0	10.0	9.5	12.0	2.5	8.8	3.27
LOC104909900	6.0	2.5	6.5	10.5	5.0	7.5	6.3	2.66
LOC104909901	0.0	0.0	0.0	0.0	0.0	0.0	0.0	0.00
LOC104909903	2.0	1.0	0.5	2.5	2.5	0.5	1.5	0.95
LOC104909904	3.0	9.0	11.5	5.5	15.0	18.0	10.3	5.67
LOC104909905	0.5	0.5	0.0	0.5	0.5	0.0	0.3	0.26
LOC104909906	1.0	1.0	0.5	1.5	4.0	3.5	1.9	1.46
LOC104909907	0.0	0.0	0.0	0.0	0.0	0.0	0.0	0.00
LOC104909908	0.0	0.0	0.0	0.0	0.0	0.0	0.0	0.00
LOC104909910	0.5	0.5	1.0	1.5	2.5	0.0	1.0	0.89
LOC104909911	0.5	0.0	0.5	0.5	0.5	0.0	0.3	0.26
LOC104909912	0.0	0.0	0.0	0.0	0.0	0.0	0.0	0.00
LOC104909913	0.0	0.0	0.0	0.0	0.0	0.0	0.0	0.00
LOC104909914	0.0	0.0	0.0	0.0	0.0	0.0	0.0	0.00
LOC104909916	50.0	183.5	140.0	32.5	204.5	109.0	119.9	69.64
LOC104909917	151.5	50.0	189.5	148.5	63.0	145.5	124.7	55.32
LOC104909918	641.5	455.5	504.5	501.0	516.5	363.0	497.0	90.57
LOC104909919	13.0	4.5	29.0	21.5	1.0	21.5	15.1	10.87
LOC104909920	336.5	641.0	284.0	316.5	608.0	251.0	406.2	171.92
LOC104909922	9.5	0.5	25.0	13.0	0.5	13.5	10.3	9.23
LOC104909923	56.0	23.5	16.5	56.5	24.5	11.5	31.4	19.82
LOC104909924	6.5	3.5	5.0	7.5	5.5	3.5	5.3	1.60
LOC104909925	1.0	0.0	0.5	0.0	0.0	0.0	0.3	0.42
LOC104909926	8.0	3.0	0.0	8.0	4.0	2.0	4.2	3.25
LOC104909927	45.0	30.0	44.0	32.5	42.0	51.5	40.8	8.12
LOC104909928	89.0	37.0	48.5	57.5	57.0	46.5	55.9	17.89
LOC104909930	0.5	0.0	0.0	0.0	0.0	0.0	0.1	0.20
LOC104909931	3.0	0.0	3.5	0.0	1.5	1.0	1.5	1.48
LOC104909932	1.0	1.0	1.0	0.5	0.0	1.0	0.8	0.42
LOC104909933	1.5	1.0	0.5	4.5	0.0	1.0	1.4	1.59

LOC104909935	19.5	5.5	31.5	11.0	7.5	37.5	18.8	13.24
LOC104909937	28.5	21.0	58.0	29.0	23.5	66.0	37.7	19.26
LOC104909938	3.0	9.0	29.0	0.5	9.0	8.5	9.8	10.04
LOC104909939	16.0	207.0	65.5	6.5	170.5	15.5	80.2	87.39
LOC104909940	0.5	0.5	1.0	0.0	0.0	0.5	0.4	0.38
LOC104909941	17.0	9.0	11.5	21.0	8.0	7.0	12.3	5.58
LOC104909942	558.5	405.5	479.5	564.5	429.0	411.5	474.8	72.08
LOC104909943	430.5	272.0	250.0	418.0	321.0	278.0	328.3	77.94
LOC104909944	9.5	4.5	1.5	8.0	3.0	2.0	4.8	3.30
LOC104909945	0.0	0.0	0.0	0.0	0.0	0.0	0.0	0.00
LOC104909946	0.5	0.0	1.5	0.0	0.0	0.5	0.4	0.58
LOC104909947	93.0	49.5	362.0	96.5	63.0	328.0	165.3	140.71
LOC104909948	40.5	33.5	106.0	35.5	45.0	100.5	60.2	33.66
LOC104909949	184.5	82.5	137.0	227.5	89.5	136.5	142.9	55.61
LOC104909950	0.0	0.0	0.0	0.0	0.0	0.0	0.0	0.00
LOC104909951	0.0	0.5	0.0	0.0	0.0	0.0	0.1	0.20
LOC104909952	0.5	2.0	3.0	0.0	2.0	1.0	1.4	1.11
LOC104909953	0.0	0.0	0.0	0.0	0.0	0.0	0.0	0.00
LOC104909954	0.0	0.0	0.0	0.0	1.5	0.5	0.3	0.61
LOC104909955	9.0	3.5	10.5	6.0	1.5	7.0	6.3	3.36
LOC104909956	14.0	3.5	10.5	14.0	5.0	6.0	8.8	4.63
LOC104909958	2.0	0.0	2.5	0.5	0.5	1.5	1.2	0.98
LOC104909959	9.5	5.0	7.5	19.0	3.5	16.0	10.1	6.18
LOC104909960	11.0	5.0	2.0	9.0	3.5	1.0	5.3	3.97
LOC104909961	1689.5	2172.0	2532.0	1689.0	2641.0	2906.0	2271.6	508.90
LOC104909962	78.0	58.0	234.0	76.0	71.5	234.5	125.3	84.65
LOC104909963	81.0	49.5	260.0	91.0	63.5	275.5	136.8	102.59
LOC104909964	61.5	39.0	271.0	66.0	46.0	254.5	123.0	108.82
LOC104909965	10.5	2.0	1.5	9.0	4.5	3.5	5.2	3.74
LOC104909966	5.0	2.5	2.5	4.0	6.5	4.0	4.1	1.53
LOC104909967	0.0	0.0	0.0	0.0	0.0	0.0	0.0	0.00
LOC104909969	0.0	0.0	0.0	0.0	0.0	0.0	0.0	0.00
LOC104909971	0.5	0.5	0.0	0.0	0.0	0.0	0.2	0.26
LOC104909972	9.5	1.0	10.5	12.5	1.0	8.5	7.2	4.96
LOC104909974	0.0	0.5	0.0	0.0	0.0	0.0	0.1	0.20
LOC104909975	635.0	527.0	792.5	543.5	573.0	727.0	633.0	106.89
LOC104909976	0.0	0.0	1.0	1.5	0.0	1.0	0.6	0.66
LOC104909977	200.5	178.0	239.5	195.0	189.5	185.5	198.0	21.75
LOC104909978	13.0	7.0	24.0	14.5	7.0	15.0	13.4	6.30
LOC104909979	5.0	1.5	14.0	4.5	0.5	3.5	4.8	4.81
LOC104909980	166.0	165.5	230.0	165.0	191.0	201.5	186.5	26.31
LOC104909981	0.0	0.0	0.0	0.0	0.0	0.5	0.1	0.20
LOC104909982	283.0	249.0	270.5	266.0	284.0	250.0	267.1	15.30
LOC104909983	0.5	0.0	0.5	0.5	0.0	0.0	0.3	0.27
LOC104909984	1398.5	733.0	1676.5	1520.5	794.5	1575.0	1283.0	412.55
LOC104909986	15.0	12.5	10.0	9.5	12.0	7.5	11.1	2.63
LOC104909987	1.5	0.5	4.0	2.0	1.0	2.0	1.8	1.21
LOC104909988	28.0	11.0	71.5	27.5	9.0	49.5	32.8	23.96
LOC104909989	83.5	41.5	211.5	104.5	45.0	187.5	112.3	72.01
LOC104909990	1148.5	1433.0	1590.5	1069.0	1568.0	1510.5	1386.6	223.40
LOC104909991	1.0	0.0	0.0	0.5	1.0	0.0	0.4	0.49
LOC104909992	0.5	0.0	0.0	0.0	0.5	0.5	0.3	0.27
LOC104909993	18.0	7.5	26.5	16.5	6.0	23.5	16.3	8.27
LOC104909994	15.0	6.0	0.5	9.5	4.0	3.0	6.3	5.21

LOC104909995	4.0	0.0	6.5	4.5	1.0	3.0	3.2	2.38
LOC104909996	0.0	0.0	0.0	0.0	0.0	0.0	0.0	0.00
LOC104909997	8.0	5.5	17.0	2.5	5.5	7.5	7.7	4.97
LOC104909998	34.5	5.0	79.5	30.0	9.0	55.5	35.6	28.28
LOC104910000	0.0	0.0	0.0	0.0	0.0	0.0	0.0	0.00
LOC104910001	0.0	0.0	0.0	0.0	0.0	0.0	0.0	0.00
LOC104910002	5.0	3.0	1.5	2.0	2.5	3.5	2.9	1.24
LOC104910003	1.5	0.0	1.5	1.0	0.5	1.5	1.0	0.63
LOC104910004	0.0	0.0	0.5	0.0	0.0	0.0	0.1	0.20
LOC104910005	0.0	0.5	1.5	0.0	0.0	0.0	0.3	0.61
LOC104910007	0.0	0.0	0.0	0.0	0.0	0.0	0.0	0.00
LOC104910008	0.0	0.0	0.0	0.0	0.0	0.0	0.0	0.00
LOC104910009	16.5	1.0	4.0	13.0	0.5	2.5	6.3	6.79
LOC104910010	0.5	0.5	0.0	0.5	1.5	0.5	0.6	0.49
LOC104910011	5.0	0.5	1.0	3.5	0.5	0.0	1.8	2.02
LOC104910012	0.0	0.0	0.0	0.0	0.0	0.0	0.0	0.00
LOC104910013	0.0	0.0	0.0	0.0	0.0	0.0	0.0	0.00
LOC104910014	0.0	0.0	0.0	0.0	0.0	0.0	0.0	0.00
LOC104910015	0.0	0.0	0.0	0.0	0.0	0.0	0.0	0.00
LOC104910019	2930.5	2555.5	756.0	2654.0	2823.5	857.0	2096.1	1007.86
LOC104910020	1.0	1.0	0.0	1.0	0.5	0.0	0.6	0.49
LOC104910021	168.5	77.5	233.5	179.5	74.0	237.0	161.7	72.07
LOC104910022	0.0	1.0	1.0	0.0	1.5	0.0	0.6	0.66
LOC104910023	1.0	11.0	4.0	0.5	6.5	6.0	4.8	3.91
LOC104910024	2.5	2.5	5.0	6.0	3.5	3.5	3.8	1.40
LOC104910026	11.5	5.0	1.0	8.0	4.0	0.5	5.0	4.21
LOC104910027	171.0	200.5	153.0	185.5	227.0	177.0	185.7	25.64
LOC104910030	0.0	0.0	0.0	0.0	0.0	0.5	0.1	0.20
LOC104910032	6.5	4.0	7.0	8.5	4.5	5.5	6.0	1.67
LOC104910034	0.0	0.0	0.0	0.0	0.0	0.0	0.0	0.00
LOC104910037	198.0	146.0	215.0	190.5	136.5	194.5	180.1	31.36
LOC104910038	626.5	428.0	553.5	522.5	486.0	544.5	526.8	66.95
LOC104910040	1.5	0.5	6.5	3.0	2.5	2.5	2.8	2.04
LOC104910041	12.0	1.5	6.0	8.0	5.0	8.5	6.8	3.56
LOC104910042	4.5	6.5	2.5	3.0	7.5	2.5	4.4	2.15
LOC104910043	388.0	547.5	351.0	360.0	631.0	363.5	440.2	119.09
LOC104910044	362.5	206.5	302.5	345.5	232.5	306.5	292.7	61.65
LOC104910045	0.0	0.0	0.0	0.0	0.0	0.0	0.0	0.00
LOC104910046	49.0	11.5	4.5	11.0	3.5	2.0	13.6	17.80
LOC104910047	2.0	1.0	1.0	1.5	3.0	0.0	1.4	1.02
LOC104910049	3.0	5.0	0.5	7.0	5.5	0.5	3.6	2.71
LOC104910050	0.0	0.0	0.0	0.0	0.0	0.0	0.0	0.00
LOC104910051	0.0	0.0	0.0	0.0	0.0	0.0	0.0	0.00
LOC104910052	0.0	0.0	0.0	0.0	0.0	0.0	0.0	0.00
LOC104910053	0.0	0.0	0.0	0.0	0.0	0.0	0.0	0.00
LOC104910054	102.5	36.0	132.5	99.5	42.0	118.0	88.4	40.10
LOC104910057	0.0	0.0	0.0	0.0	0.0	0.0	0.0	0.00
LOC104910058	25.0	1.5	5.0	2.5	0.5	0.5	5.8	9.54
LOC104910059	23.5	4.5	3.5	8.5	2.0	1.0	7.2	8.41
LOC104910060	38.0	9.5	5.5	11.0	11.0	1.0	12.7	13.00
LOC104910061	0.0	0.0	0.0	0.0	0.0	0.0	0.0	0.00
LOC104910062	0.0	0.0	0.0	0.0	0.0	0.0	0.0	0.00
LOC104910063	0.0	0.0	0.0	0.0	0.5	0.5	0.2	0.26
LOC104910064	0.0	6.0	1.5	0.0	5.5	3.5	2.8	2.66

LOC104910065	0.0	0.0	0.0	0.0	0.0	0.0	0.0	0.00
LOC104910066	0.0	0.0	0.0	0.0	0.0	0.0	0.0	0.00
LOC104910067	0.0	0.0	0.0	1.5	0.5	0.0	0.3	0.61
LOC104910068	0.0	0.0	0.0	0.0	0.0	0.0	0.0	0.00
LOC104910069	0.5	0.0	1.0	0.0	0.0	0.5	0.3	0.41
LOC104910070	0.0	0.0	0.0	0.0	0.0	0.0	0.0	0.00
LOC104910071	0.5	0.0	0.5	0.5	0.0	0.0	0.3	0.27
LOC104910072	0.0	0.0	0.0	0.0	0.0	0.0	0.0	0.00
LOC104910073	0.0	0.5	0.0	0.0	0.0	1.0	0.3	0.42
LOC104910074	1.5	0.5	1.0	0.0	0.0	3.0	1.0	1.14
LOC104910075	170.0	102.0	57.5	197.5	108.0	52.0	114.5	58.86
LOC104910076	140.0	120.0	82.5	126.5	139.5	83.0	115.3	26.31
LOC104910077	0.0	0.0	0.0	0.0	0.0	0.0	0.0	0.00
LOC104910078	361.0	163.5	247.5	333.5	204.0	249.5	259.8	75.26
LOC104910081	0.5	0.0	1.0	1.5	0.0	0.0	0.5	0.63
LOC104910083	0.5	0.0	0.0	0.0	0.0	0.5	0.2	0.26
LOC104910085	0.5	0.0	0.0	0.0	0.0	0.5	0.2	0.26
LOC104910088	0.0	0.0	0.0	0.0	0.0	0.0	0.0	0.00
LOC104910089	1.5	0.0	0.0	0.0	0.5	0.0	0.3	0.61
LOC104910090	0.5	0.0	0.0	0.0	0.0	0.0	0.1	0.20
LOC104910091	2.5	3.5	2.5	3.0	3.5	5.0	3.3	0.93
LOC104910092	0.0	0.0	0.0	0.0	1.0	0.5	0.3	0.42
LOC104910094	0.0	0.5	0.5	0.0	0.0	0.0	0.2	0.26
LOC104910095	0.0	0.0	0.0	0.0	0.0	0.0	0.0	0.00
LOC104910097	41.5	44.5	27.5	37.5	68.0	66.0	47.5	16.17
LOC104910098	4.5	4.5	10.0	7.0	10.0	9.0	7.5	2.57
LOC104910099	5.5	8.5	10.5	6.0	11.0	11.0	8.8	2.50
LOC104910100	0.0	0.0	0.0	0.0	0.0	0.0	0.0	0.00
LOC104910102	0.5	0.0	0.5	0.0	0.5	0.5	0.3	0.26
LOC104910104	6.5	1.5	0.5	2.0	1.0	1.5	2.2	2.18
LOC104910105	3.0	0.5	1.5	1.0	0.5	0.0	1.1	1.07
LOC104910106	342.5	408.0	387.5	304.0	407.5	272.5	353.7	56.88
LOC104910107	5.5	1.0	0.0	5.5	0.0	0.0	2.0	2.74
LOC104910108	0.0	0.0	0.0	0.0	0.0	0.0	0.0	0.00
LOC104910109	0.0	0.0	0.0	0.0	0.0	0.0	0.0	0.00
LOC104910110	0.0	0.0	0.0	0.0	0.0	0.0	0.0	0.00
LOC104910111	0.0	0.5	0.0	0.0	0.5	0.5	0.3	0.27
LOC104910112	3.0	5.5	9.5	1.5	3.0	13.5	6.0	4.63
LOC104910113	2.5	1.5	2.5	1.0	3.5	1.0	2.0	1.00
LOC104910114	2.0	2.0	1.0	1.0	1.0	1.5	1.4	0.49
LOC104910115	0.0	0.0	0.0	0.0	0.0	0.0	0.0	0.00
LOC104910116	0.5	0.5	1.0	1.0	1.0	0.0	0.7	0.41
LOC104910117	1.0	0.0	0.5	0.0	0.0	0.5	0.3	0.41
LOC104910118	17.5	7.5	3.5	17.0	10.5	4.0	10.0	6.16
LOC104910119	1.0	0.5	2.5	1.0	3.0	1.5	1.6	0.97
LOC104910121	21117.5	30955.0	21174.0	20989.5	34554.0	19948.5	24789.8	6289.51
LOC104910123	2.5	2.5	2.5	5.0	2.0	2.0	2.8	1.13
LOC104910124	0.0	0.0	0.0	0.0	0.0	0.0	0.0	0.00
LOC104910125	0.5	0.5	0.0	1.0	0.0	0.0	0.3	0.41
LOC104910126	3.0	2.0	0.5	8.5	2.5	1.0	2.9	2.89
LOC104910128	2.0	0.0	0.5	1.5	2.0	0.0	1.0	0.95
LOC104910129	23.5	12.5	15.0	26.0	12.5	18.0	17.9	5.72
LOC104910130	0.5	0.5	1.0	0.5	1.5	0.5	0.8	0.42
LOC104910131	142.5	124.5	245.0	126.0	146.5	226.0	168.4	53.03

LOC104910132	0.0	0.0	0.0	0.0	0.0	0.0	0.0	0.00
LOC104910133	42.5	14.5	23.0	7.0	7.0	5.5	16.6	14.31
LOC104910134	53.5	19.0	13.5	33.0	19.0	23.5	26.9	14.57
LOC104910135	0.0	0.0	0.0	0.0	0.0	0.0	0.0	0.00
LOC104910136	0.0	0.0	0.0	0.0	0.0	0.0	0.0	0.00
LOC104910137	0.0	0.0	0.0	0.0	0.0	0.0	0.0	0.00
LOC104910138	96.5	86.0	96.0	85.0	117.0	114.5	99.2	13.74
LOC104910139	24.0	10.0	10.0	13.0	10.5	15.0	13.8	5.40
LOC104910145	1210.0	2776.5	1318.0	1116.0	3039.0	1322.0	1796.9	867.80
LOC104910147	155.0	118.0	149.5	121.5	133.0	148.0	137.5	15.60
LOC104910148	0.0	0.0	0.0	0.0	0.0	0.0	0.0	0.00
LOC104910149	0.0	0.0	0.0	0.0	0.0	0.0	0.0	0.00
LOC104910150	0.0	0.0	0.0	0.5	0.0	0.0	0.1	0.20
LOC104910151	0.0	0.0	0.0	0.0	0.0	0.0	0.0	0.00
LOC104910152	0.5	0.0	0.5	0.5	0.5	0.0	0.3	0.26
LOC104910153	0.0	0.0	0.0	0.0	0.0	0.0	0.0	0.00
LOC104910154	0.0	0.0	0.5	0.0	0.0	0.0	0.1	0.20
LOC104910155	0.0	0.0	0.0	0.0	0.0	0.0	0.0	0.00
LOC104910156	1.5	1.0	1.0	2.5	2.0	1.0	1.5	0.63
LOC104910157	0.0	0.0	0.0	0.0	0.0	0.0	0.0	0.00
LOC104910158	0.0	0.0	0.0	0.0	0.0	0.0	0.0	0.00
LOC104910159	1.0	0.5	0.5	0.0	1.0	0.0	0.5	0.45
LOC104910160	1.5	0.5	1.0	1.0	2.5	0.5	1.2	0.75
LOC104910161	0.0	0.0	0.0	0.0	0.0	0.0	0.0	0.00
LOC104910162	0.0	0.0	0.0	0.0	0.0	0.5	0.1	0.20
LOC104910163	0.0	0.0	0.0	0.0	0.0	0.0	0.0	0.00
LOC104910164	0.0	0.5	0.0	0.0	0.5	0.0	0.2	0.26
LOC104910166	1.0	0.5	0.0	0.5	0.0	0.0	0.3	0.41
LOC104910167	0.0	0.0	0.0	0.0	0.0	0.0	0.0	0.00
LOC104910168	0.0	0.0	0.0	0.0	0.0	0.0	0.0	0.00
LOC104910169	0.0	0.0	0.0	0.0	0.0	0.0	0.0	0.00
LOC104910170	0.0	0.0	0.0	0.0	0.0	0.0	0.0	0.00
LOC104910171	0.0	0.0	0.5	0.0	0.0	0.0	0.1	0.20
LOC104910172	10.5	14.5	9.5	13.5	20.0	12.5	13.4	3.72
LOC104910173	0.0	0.0	0.0	0.0	0.0	0.0	0.0	0.00
LOC104910174	0.0	0.0	0.0	0.0	0.0	0.0	0.0	0.00
LOC104910175	1.0	0.0	0.0	0.0	0.0	0.5	0.3	0.42
LOC104910176	0.5	0.0	0.0	0.0	0.0	0.0	0.1	0.20
LOC104910177	0.5	0.0	0.0	0.0	0.0	0.0	0.1	0.20
LOC104910178	40.0	123.5	185.5	33.5	169.5	169.5	120.3	67.95
LOC104910179	550.5	569.5	782.0	476.5	700.5	873.0	658.7	152.14
LOC104910180	4.5	2.0	2.0	3.5	2.0	2.5	2.8	1.04
LOC104910181	5.0	7.5	7.5	8.5	12.5	5.0	7.7	2.77
LOC104910182	0.0	0.0	0.0	0.5	0.0	0.0	0.1	0.20
LOC104910183	2.5	5.5	0.5	0.5	3.0	0.5	2.1	2.01
LOC104910184	0.0	0.0	0.0	0.0	0.0	0.0	0.0	0.00
LOC104910185	46.0	45.0	20.5	35.5	56.0	38.5	40.3	11.99
LOC104910186	0.0	0.0	0.0	0.0	0.0	0.0	0.0	0.00
LOC104910187	0.0	0.0	0.0	0.0	0.0	0.0	0.0	0.00
LOC104910188	0.0	0.0	0.0	0.0	0.0	0.0	0.0	0.00
LOC104910189	2.5	4.5	0.0	4.5	4.5	0.5	2.8	2.09
LOC104910190	13.0	4.0	1.0	9.0	6.5	1.0	5.8	4.73
LOC104910191	0.0	0.0	0.0	0.0	0.0	0.0	0.0	0.00
LOC104910193	0.0	0.0	0.0	0.0	0.0	0.0	0.0	0.00

LOC104910194	1.0	0.0	4.5	2.5	0.5	3.0	1.9	1.72
LOC104910195	9.0	59.0	71.0	17.0	73.5	139.0	61.4	46.87
LOC104910196	311.5	371.5	292.0	297.0	421.5	297.0	331.8	52.99
LOC104910197	0.5	2.5	2.5	3.5	0.0	2.5	1.9	1.36
LOC104910198	25.0	39.0	78.5	27.5	26.0	70.0	44.3	23.87
LOC104910199	2.0	2.0	2.0	0.5	1.5	2.0	1.7	0.61
LOC104910200	11.0	20.5	9.0	13.5	24.0	7.0	14.2	6.71
LOC104910201	33.0	17.5	16.0	36.5	14.0	21.5	23.1	9.43
LOC104910202	0.0	0.0	0.0	0.0	0.0	0.0	0.0	0.00
LOC104910204	45.5	15.5	60.0	46.0	19.0	41.5	37.9	17.23
LOC104910205	1.5	2.0	2.5	0.0	0.0	0.5	1.1	1.07
LOC104910206	0.5	1.5	2.5	2.0	2.0	2.0	1.8	0.69
LOC104910207	0.5	0.5	0.5	0.5	0.0	0.5	0.4	0.20
LOC104910208	0.5	4.0	1.5	2.5	0.0	0.5	1.5	1.52
LOC104910211	3.0	0.0	2.5	3.5	1.0	5.0	2.5	1.79
LOC104910212	5.0	0.0	4.0	5.0	0.0	2.0	2.7	2.34
LOC104910213	92.5	54.5	47.0	91.0	76.0	48.5	68.3	20.96
LOC104910214	51.5	15.5	69.5	68.5	21.5	88.5	52.5	28.89
LOC104910215	0.5	0.0	1.0	0.0	1.5	0.0	0.5	0.63
LOC104910216	4.0	0.5	4.5	7.5	2.0	4.5	3.8	2.40
LOC104910217	226.5	146.5	146.0	200.5	149.0	115.5	164.0	41.10
LOC104910218	7.0	1.0	2.5	6.0	3.5	3.0	3.8	2.25
LOC104910219	1.0	1.5	0.5	2.0	1.5	0.5	1.2	0.61
LOC104910220	0.0	0.5	1.0	0.5	1.0	2.0	0.8	0.68
LOC104910221	6.0	8.5	17.0	11.0	11.0	18.5	12.0	4.85
LOC104910222	58.5	118.0	179.5	77.5	150.0	261.5	140.8	74.10
LOC104910223	93.5	198.0	488.5	90.0	265.5	327.0	243.8	152.06
LOC104910224	17.5	2.5	17.0	20.5	5.0	11.0	12.3	7.31
LOC104910225	1.0	0.0	0.0	1.0	0.0	0.0	0.3	0.52
LOC104910228	0.0	0.0	0.0	0.0	0.0	0.0	0.0	0.00
LOC104910229	34.0	5.0	3.5	31.5	7.5	5.0	14.4	14.28
LOC104910231	0.0	0.0	1.0	0.0	0.0	0.0	0.2	0.41
LOC104910232	129.0	147.0	154.0	132.0	133.5	151.5	141.2	10.92
LOC104910234	21.0	8.0	2.0	15.0	5.0	1.0	8.7	7.87
LOC104910235	0.0	0.0	0.0	0.0	0.0	0.0	0.0	0.00
LOC104910236	0.0	0.0	0.0	0.0	0.0	0.0	0.0	0.00
LOC104910237	0.0	0.0	0.0	0.5	0.0	0.0	0.1	0.20
LOC104910238	0.0	0.0	0.0	0.0	0.0	0.0	0.0	0.00
LOC104910239	0.0	0.0	0.0	0.0	0.0	0.0	0.0	0.00
LOC104910240	0.0	0.0	0.0	0.0	0.0	0.0	0.0	0.00
LOC104910241	6.5	2.0	8.0	7.0	2.0	2.5	4.7	2.79
LOC104910242	10.5	0.5	5.0	14.5	0.5	3.5	5.8	5.65
LOC104910243	12.0	1.5	3.0	19.5	0.5	3.5	6.7	7.50
LOC104910244	0.0	0.0	0.0	0.5	0.5	0.0	0.2	0.26
LOC104910245	0.0	0.0	0.0	0.0	0.0	0.0	0.0	0.00
LOC104910246	397.0	244.5	375.5	418.0	271.0	239.5	324.3	81.35
LOC104910247	38.5	8.0	58.5	55.5	6.5	42.0	34.8	22.69
LOC104910248	26.5	53.0	273.5	37.5	60.5	224.5	112.6	107.45
LOC104910249	1682.0	2448.0	1423.0	1451.0	2795.5	1265.0	1844.1	626.63
LOC104910250	26.5	27.0	20.0	36.0	41.0	25.0	29.3	7.74
LOC104910251	1.5	7.5	2.0	2.0	6.5	5.0	4.1	2.60
LOC104910252	0.0	0.0	0.5	0.0	0.0	0.0	0.1	0.20
LOC104910256	13.5	3.5	14.0	10.0	2.0	10.5	8.9	5.05
LOC104910257	30.5	8.5	33.5	36.5	6.5	28.0	23.9	13.05

LOC104910258	20.0	8.5	16.0	21.5	11.0	13.0	15.0	5.11
LOC104910259	0.0	0.0	0.0	0.0	0.0	0.0	0.0	0.00
LOC104910260	84.5	121.0	45.5	83.5	126.0	56.0	86.1	32.78
LOC104910261	667.5	720.5	946.5	737.0	832.5	1056.5	826.8	149.57
LOC104910262	0.0	0.0	0.0	0.0	0.0	0.0	0.0	0.00
LOC104910263	0.0	0.0	0.0	0.0	0.0	0.0	0.0	0.00
LOC104910264	0.0	1.0	6.0	1.0	1.0	5.0	2.3	2.50
LOC104910265	0.0	0.0	0.5	0.0	0.0	0.0	0.1	0.20
LOC104910266	61.0	35.0	72.0	40.5	40.0	70.0	53.1	16.51
LOC104910267	346.5	228.5	188.0	369.5	265.0	178.5	262.7	80.34
LOC104910268	166.0	69.5	145.0	165.0	74.0	111.0	121.8	43.58
LOC104910269	3.0	1.0	1.0	4.5	4.0	0.0	2.3	1.84
LOC104910270	0.5	1.0	1.5	0.0	0.0	1.0	0.7	0.61
LOC104910271	1.5	1.0	3.0	3.5	3.0	3.0	2.5	1.00
LOC104910272	0.0	0.0	0.0	0.0	0.0	0.0	0.0	0.00
LOC104910273	41.0	86.0	86.0	32.0	94.0	77.5	69.4	26.18
LOC104910274	361.5	235.5	384.0	403.5	283.0	396.5	344.0	68.84
LOC104910275	0.0	0.0	0.0	0.0	0.0	0.0	0.0	0.00
LOC104910276	34.0	55.5	71.5	37.0	57.0	76.5	55.3	17.34
LOC104910277	40.5	7.5	97.5	37.0	8.0	82.0	45.4	37.37
LOC104910279	0.0	0.0	0.0	0.0	0.0	0.0	0.0	0.00
LOC104910280	170.0	93.0	81.0	123.0	79.5	75.0	103.6	36.90
LOC104910281	22.5	22.5	42.5	21.5	30.5	34.0	28.9	8.37
LOC104910282	146.5	60.5	203.0	148.5	72.5	194.0	137.5	59.73
LOC104910283	0.5	5.0	11.0	1.5	6.0	9.0	5.5	4.10
LOC104910284	43.0	13.5	37.0	39.0	18.5	41.0	32.0	12.65
LOC104910285	0.5	0.5	0.5	0.0	0.0	0.0	0.3	0.27
LOC104910286	7.5	43.0	50.0	6.5	42.5	39.5	31.5	19.29
LOC104910287	0.0	0.0	0.0	0.0	0.0	0.0	0.0	0.00
LOC104910288	0.0	0.0	0.0	0.0	0.0	0.0	0.0	0.00
LOC104910289	0.0	0.5	0.0	0.0	0.0	0.0	0.1	0.20
LOC104910291	4.0	2.5	1.0	4.0	2.5	0.5	2.4	1.46
LOC104910292	1.0	0.0	0.0	2.5	0.5	0.0	0.7	0.98
LOC104910294	0.5	0.0	0.5	0.5	0.0	0.5	0.3	0.26
LOC104910295	0.0	0.0	1.0	0.0	0.0	0.0	0.2	0.41
LOC104910296	0.0	0.0	0.0	0.0	0.0	0.0	0.0	0.00
LOC104910297	0.0	0.0	0.0	0.0	0.0	0.0	0.0	0.00
LOC104910298	8.5	1.5	1.0	10.5	6.5	4.0	5.3	3.83
LOC104910299	22.0	8.0	6.5	27.5	21.0	6.5	15.3	9.32
LOC104910300	0.0	0.0	0.0	0.0	0.0	0.0	0.0	0.00
LOC104910301	16.0	30.0	33.0	11.5	49.5	22.5	27.1	13.66
LOC104910302	4.0	14.5	19.0	2.5	13.5	15.0	11.4	6.61
LOC104910303	0.0	0.0	0.0	0.0	0.0	0.0	0.0	0.00
LOC104910304	0.0	0.0	0.0	0.0	0.0	0.0	0.0	0.00
LOC104910305	0.5	0.0	0.0	0.0	0.0	0.0	0.1	0.20
LOC104910306	2.5	0.0	0.5	0.0	0.0	0.0	0.5	1.00
LOC104910307	14.5	10.5	18.0	11.0	8.5	19.0	13.6	4.28
LOC104910308	0.0	0.0	0.0	0.0	0.0	0.0	0.0	0.00
LOC104910309	0.0	0.0	0.0	0.0	0.0	0.0	0.0	0.00
LOC104910310	0.0	0.0	0.0	0.0	0.0	0.0	0.0	0.00
LOC104910311	0.0	0.0	0.0	0.0	0.0	0.0	0.0	0.00
LOC104910313	0.0	0.0	0.0	0.0	0.0	0.0	0.0	0.00
LOC104910314	1.0	2.0	0.0	2.0	1.5	0.0	1.1	0.92
LOC104910315	1.5	2.0	1.0	2.0	1.5	3.0	1.8	0.68

LOC104910316	94.5	46.0	148.5	68.0	47.0	109.0	85.5	39.88
LOC104910318	34.0	22.0	16.5	34.0	15.5	21.5	23.9	8.23
LOC104910319	0.5	0.0	0.5	0.0	0.0	0.0	0.2	0.26
LOC104910320	4.0	17.5	50.5	6.5	24.5	49.0	25.3	20.32
LOC104910321	3.5	4.0	8.0	3.5	1.5	2.0	3.8	2.30
LOC104910322	92.5	58.5	29.0	73.0	66.0	34.5	58.9	23.95
LOC104910323	0.0	0.0	0.0	0.0	0.0	0.5	0.1	0.20
LOC104910324	1.0	0.0	0.5	0.0	0.0	2.0	0.6	0.80
LOC104910325	0.0	0.0	0.5	0.0	0.0	1.0	0.3	0.42
LOC104910326	0.0	0.0	0.5	0.0	0.0	0.5	0.2	0.26
LOC104910327	71.0	13.5	35.0	71.0	22.0	30.0	40.4	24.79
LOC104910328	32.0	12.0	9.5	32.0	15.5	7.5	18.1	11.11
LOC104910329	0.0	0.0	0.0	0.0	0.0	0.0	0.0	0.00
LOC104910330	0.5	0.0	0.0	0.0	0.0	0.0	0.1	0.20
LOC104910331	0.0	0.0	0.0	0.0	0.0	0.0	0.0	0.00
LOC104910332	0.5	0.0	0.0	0.0	0.0	0.0	0.1	0.20
LOC104910333	0.0	0.0	0.0	0.0	0.5	0.5	0.2	0.26
LOC104910334	0.0	0.0	0.5	0.0	0.0	0.0	0.1	0.20
LOC104910335	274.0	197.0	251.0	180.0	202.5	190.0	215.8	37.69
LOC104910336	50.0	30.5	41.5	59.0	20.0	28.5	38.3	14.61
LOC104910337	1.0	6.5	38.5	2.0	9.0	23.5	13.4	14.72
LOC104910338	1.0	0.5	3.0	2.0	1.0	1.5	1.5	0.89
LOC104910339	5.0	4.0	4.5	4.0	2.0	4.5	4.0	1.05
LOC104910340	0.0	0.0	0.0	0.0	0.0	0.0	0.0	0.00
LOC104910341	9.5	5.0	1.0	10.0	4.5	1.0	5.2	3.93
LOC104910342	1.0	1.5	0.0	0.0	0.0	0.0	0.4	0.66
LOC104910343	0.0	0.0	0.0	0.0	0.0	0.0	0.0	0.00
LOC104910344	42.5	21.0	15.5	35.0	27.0	12.0	25.5	11.70
LOC104910345	5.0	3.5	9.5	8.0	14.5	8.5	8.2	3.84
LOC104910346	5.0	1.0	2.5	0.5	4.0	2.5	2.6	1.72
LOC104910347	0.5	0.0	0.0	0.0	0.5	0.0	0.2	0.26
LOC104910348	0.0	0.0	0.0	0.0	0.0	0.0	0.0	0.00
LOC104910349	0.0	0.0	0.0	0.0	0.0	0.0	0.0	0.00
LOC104910350	34.0	10.0	29.5	25.5	12.0	24.5	22.6	9.60
LOC104910351	40.5	17.0	20.0	53.0	29.5	16.0	29.3	14.84
LOC104910352	64.0	54.0	40.5	53.0	42.5	24.5	46.4	13.72
LOC104910353	14.0	3.5	15.0	11.0	4.5	9.5	9.6	4.77
LOC104910354	0.0	0.0	0.0	0.0	0.0	0.0	0.0	0.00
LOC104910355	46.5	26.0	105.5	67.0	23.0	78.5	57.8	32.06
LOC104910356	0.0	0.5	0.0	1.5	0.5	0.0	0.4	0.58
LOC104910357	53.5	46.5	23.0	87.0	62.0	40.0	52.0	21.64
LOC104910358	0.0	0.0	0.0	0.0	0.0	0.0	0.0	0.00
LOC104910359	2.0	1.0	5.5	2.0	0.5	6.5	2.9	2.48
LOC104910360	1.5	0.5	4.5	2.0	0.0	3.5	2.0	1.73
LOC104910361	461.5	344.5	357.0	409.0	382.0	351.5	384.3	44.68
LOC104910362	0.5	0.0	1.5	0.0	0.0	0.5	0.4	0.58
LOC104910363	0.0	0.0	0.0	0.0	0.0	0.0	0.0	0.00
LOC104910364	1.0	1.0	0.5	0.0	1.0	0.0	0.6	0.49
LOC104910365	1.0	0.0	7.0	3.0	1.0	7.0	3.2	3.13
LOC104910366	0.0	1.0	0.5	0.5	0.0	0.0	0.3	0.41
LOC104910367	0.0	0.0	0.0	0.0	0.0	0.0	0.0	0.00
LOC104910368	25.0	28.0	47.0	31.0	41.0	80.0	42.0	20.38
LOC104910369	0.0	0.0	1.0	0.5	0.0	1.0	0.4	0.49
LOC104910370	0.0	0.0	0.0	0.0	0.5	0.5	0.2	0.26

LOC104910371	43.5	31.5	73.5	33.5	32.0	62.5	46.1	17.87
LOC104910372	0.0	0.0	0.0	0.0	0.0	0.0	0.0	0.00
LOC104910373	8.5	6.0	21.5	6.5	4.5	18.5	10.9	7.21
LOC104910374	2.0	4.5	1.5	3.0	2.5	4.5	3.0	1.26
LOC104910375	0.0	0.0	0.0	0.0	0.5	0.0	0.1	0.20
LOC104910377	0.0	0.5	0.5	0.0	0.0	1.0	0.3	0.41
LOC104910378	0.5	0.0	0.0	0.0	0.0	0.5	0.2	0.26
LOC104910379	0.0	3.0	42.5	0.5	1.5	22.0	11.6	17.30
LOC104910381	0.0	0.0	1.0	2.0	1.0	0.5	0.8	0.76
LOC104910382	36.0	41.5	46.0	29.0	37.0	33.5	37.2	5.97
LOC104910383	0.0	0.0	0.0	0.0	0.0	0.0	0.0	0.00
LOC104910384	11.0	1.0	2.0	3.0	1.0	3.0	3.5	3.78
LOC104910385	1.5	6.0	27.0	0.5	8.5	11.5	9.2	9.67
LOC104910387	71.5	27.5	114.5	82.5	21.0	97.5	69.1	37.68
LOC104910388	43.5	20.5	54.0	42.0	17.0	42.0	36.5	14.51
LOC104910389	0.0	0.0	0.0	0.0	0.0	0.0	0.0	0.00
LOC104910390	0.0	0.0	0.0	0.0	0.0	0.0	0.0	0.00
LOC104910391	4.5	1.5	3.5	4.5	0.5	1.5	2.7	1.72
LOC104910392	0.0	0.0	0.0	0.0	0.0	0.0	0.0	0.00
LOC104910393	10.5	3.5	14.5	11.0	4.5	13.0	9.5	4.51
LOC104910394	3.0	2.0	9.5	3.0	1.5	7.0	4.3	3.19
LOC104910396	0.0	0.0	0.0	0.0	0.0	0.0	0.0	0.00
LOC104910397	191.5	84.5	176.5	192.5	106.0	150.5	150.3	45.73
LOC104910398	0.0	0.0	0.0	0.0	0.0	0.0	0.0	0.00
LOC104910399	102.5	37.5	120.0	132.5	46.5	95.5	89.1	38.82
LOC104910400	0.5	0.0	0.5	0.0	0.0	0.0	0.2	0.26
LOC104910401	0.0	1.0	2.5	1.0	4.0	3.0	1.9	1.50
LOC104910402	0.0	0.0	0.0	0.0	0.5	0.5	0.2	0.26
LOC104910403	0.5	1.5	0.0	0.5	1.5	0.0	0.7	0.68
LOC104910404	1.5	0.0	3.0	0.0	1.0	1.5	1.2	1.13
LOC104910405	1.0	0.0	0.5	2.0	0.0	0.0	0.6	0.80
LOC104910406	72.0	57.0	79.0	75.5	53.0	65.5	67.0	10.39
LOC104910407	0.0	1.0	0.5	0.5	2.0	0.0	0.7	0.75
LOC104910408	1616.5	1635.0	1118.0	1378.5	1708.5	904.5	1393.5	323.27
LOC104910409	69.0	25.5	5.5	56.0	21.5	9.5	31.2	25.70
LOC104910410	35.0	32.0	33.5	31.0	47.5	39.5	36.4	6.19
LOC104910411	0.0	0.0	0.5	0.5	0.0	0.0	0.2	0.26
LOC104910415	0.0	0.0	0.0	0.0	0.0	0.0	0.0	0.00
LOC104910416	0.0	0.0	0.0	0.5	0.5	0.0	0.2	0.26
LOC104910419	0.0	0.0	0.0	0.5	0.0	0.0	0.1	0.20
LOC104910420	0.0	0.0	0.0	0.0	0.0	0.0	0.0	0.00
LOC104910421	0.0	0.0	0.0	0.0	0.0	0.0	0.0	0.00
LOC104910422	0.0	0.0	0.0	0.5	0.0	0.0	0.1	0.20
LOC104910423	2.5	2.0	1.0	4.5	1.0	3.5	2.4	1.39
LOC104910424	0.0	0.0	0.0	0.0	0.0	0.5	0.1	0.20
LOC104910425	100.5	58.5	93.5	92.0	57.5	70.5	78.8	18.95
LOC104910426	26.0	5.5	12.0	6.5	2.0	2.0	9.0	9.10
LOC104910427	10.0	4.0	14.5	9.5	3.5	12.5	9.0	4.45
LOC104910428	0.5	0.0	0.5	0.5	0.0	0.5	0.3	0.26
LOC104910429	0.0	0.0	0.0	0.0	0.0	0.0	0.0	0.00
LOC104910430	0.0	0.0	0.0	0.0	0.0	0.0	0.0	0.00
LOC104910431	6.5	5.5	22.0	3.0	4.5	3.5	7.5	7.22
LOC104910432	0.0	0.0	0.0	0.0	0.0	0.0	0.0	0.00
LOC104910433	0.0	0.0	0.0	0.0	0.0	0.0	0.0	0.00

LOC104910434	0.0	0.0	0.0	0.0	0.0	0.0	0.0	0.00
LOC104910436	0.5	0.0	0.0	0.0	0.0	0.5	0.2	0.26
LOC104910437	0.0	0.0	0.0	1.0	0.0	0.0	0.2	0.41
LOC104910438	0.0	0.0	0.0	0.0	0.0	0.0	0.0	0.00
LOC104910439	0.0	0.0	0.0	0.0	0.0	0.0	0.0	0.00
LOC104910440	0.0	0.0	0.0	0.0	0.0	0.0	0.0	0.00
LOC104910441	0.0	0.0	0.0	0.0	0.0	0.0	0.0	0.00
LOC104910442	0.0	0.0	0.0	0.0	0.0	0.0	0.0	0.00
LOC104910443	3.0	0.0	0.5	3.0	0.0	0.5	1.2	1.44
LOC104910444	0.0	1.0	1.0	0.0	2.0	1.0	0.8	0.75
LOC104910445	45.0	21.5	22.0	36.5	22.5	10.0	26.3	12.45
LOC104910446	3.5	0.5	0.5	2.0	0.5	0.0	1.2	1.33
LOC104910447	0.0	0.0	0.0	0.0	0.0	0.0	0.0	0.00
LOC104910448	6.5	3.0	1.0	5.0	4.5	3.5	3.9	1.88
LOC104910449	0.0	0.5	0.5	0.0	1.0	0.5	0.4	0.38
LOC104910450	72.0	28.5	134.0	70.5	26.5	86.0	69.6	39.92
LOC104910451	100.5	97.0	130.5	92.5	111.0	122.0	108.9	14.99
LOC104910453	0.0	0.0	0.0	0.0	0.0	0.0	0.0	0.00
LOC104910454	0.0	0.0	0.0	0.0	0.0	0.0	0.0	0.00
LOC104910455	0.0	0.0	0.0	0.0	0.0	0.0	0.0	0.00
LOC104910456	81.5	87.5	175.5	82.0	101.0	183.0	118.4	47.70
LOC104910457	5.0	3.0	10.5	3.0	2.0	9.0	5.4	3.53
LOC104910458	0.0	0.0	0.0	0.0	0.0	0.0	0.0	0.00
LOC104910459	42.0	18.0	67.0	38.5	23.0	59.5	41.3	19.38
LOC104910460	0.0	0.0	3.5	0.5	1.5	2.5	1.3	1.44
LOC104910461	0.0	0.0	0.0	0.0	0.0	0.5	0.1	0.20
LOC104910463	0.5	4.0	2.5	1.0	5.5	9.5	3.8	3.34
LOC104910464	0.5	1.0	1.0	1.0	0.0	0.5	0.7	0.41
LOC104910465	2.5	2.5	1.5	1.0	3.0	2.0	2.1	0.74
LOC104910466	13.0	2.5	13.0	8.0	5.0	14.5	9.3	4.92
LOC104910467	26.5	16.5	72.5	26.5	19.0	48.0	34.8	21.53
LOC104910468	0.0	0.0	0.0	0.0	0.0	0.0	0.0	0.00
LOC104910469	238.0	113.0	186.5	266.0	112.5	193.0	184.8	63.05
LOC104910470	18.0	5.5	12.5	11.0	7.0	11.0	10.8	4.41
LOC104910471	0.5	0.0	0.0	0.0	0.0	0.0	0.1	0.20
LOC104910472	56.0	33.0	118.5	79.0	42.0	156.0	80.8	47.93
LOC104910473	3.0	3.5	8.5	4.0	8.5	11.5	6.5	3.48
LOC104910474	65.5	29.5	88.5	81.5	39.0	86.0	65.0	25.31
LOC104910476	1.0	2.5	27.5	0.5	3.0	8.0	7.1	10.35
LOC104910477	0.0	0.0	0.0	0.0	0.0	0.0	0.0	0.00
LOC104910479	9.0	1.0	10.0	7.0	3.0	9.5	6.6	3.75
LOC104910481	31.5	11.0	27.5	28.5	8.5	31.0	23.0	10.40
LOC104910482	487.5	374.5	411.0	491.5	410.0	389.5	427.3	50.05
LOC104910483	0.0	0.0	0.0	0.0	0.0	0.0	0.0	0.00
LOC104910484	1.5	0.0	0.0	0.5	0.0	1.0	0.5	0.63
LOC104910485	0.0	0.0	0.0	0.0	0.0	0.0	0.0	0.00
LOC104910488	33.5	5.5	61.0	38.5	6.0	39.5	30.7	21.49
LOC104910489	0.0	0.0	0.0	0.0	0.0	0.0	0.0	0.00
LOC104910490	0.0	0.0	0.0	0.0	0.0	0.0	0.0	0.00
LOC104910491	0.0	0.0	0.0	0.0	0.0	0.0	0.0	0.00
LOC104910492	0.0	0.0	0.0	0.0	0.0	0.0	0.0	0.00
LOC104910493	44.0	34.0	92.0	35.0	15.0	75.5	49.3	28.82
LOC104910494	84.0	71.0	162.5	86.5	50.5	174.5	104.8	51.08
LOC104910495	0.0	0.0	0.5	0.5	0.0	0.0	0.2	0.26

LOC104910496	1.5	3.0	1.5	8.5	1.5	4.5	3.4	2.76
LOC104910497	0.0	7.0	5.5	0.5	6.0	3.5	3.8	2.95
LOC104910498	0.5	0.0	0.5	4.5	1.0	2.0	1.4	1.66
LOC104910500	0.0	0.0	0.0	0.0	0.0	0.0	0.0	0.00
LOC104910501	0.0	0.0	0.0	1.0	0.0	0.0	0.2	0.41
LOC104910503	9.0	10.5	7.0	6.0	8.5	7.5	8.1	1.59
LOC104910506	0.0	0.0	0.0	0.0	0.0	0.0	0.0	0.00
LOC104910507	0.0	0.0	0.0	0.0	0.0	0.0	0.0	0.00
LOC104910508	1.0	0.0	0.5	0.5	0.0	0.5	0.4	0.38
LOC104910509	0.0	0.0	0.0	0.0	0.0	0.0	0.0	0.00
LOC104910510	0.0	0.0	0.0	0.0	0.0	0.0	0.0	0.00
LOC104910511	12.0	2.0	5.0	5.0	4.5	10.0	6.4	3.77
LOC104910512	0.0	0.0	0.0	0.0	0.0	0.0	0.0	0.00
LOC104910513	0.0	0.0	0.0	0.0	0.0	0.0	0.0	0.00
LOC104910514	0.0	0.0	0.0	0.0	0.0	0.0	0.0	0.00
LOC104910515	1.5	0.0	0.0	2.0	0.0	0.0	0.6	0.92
LOC104910516	0.0	0.0	0.0	0.0	0.0	0.0	0.0	0.00
LOC104910517	0.0	0.0	0.0	0.0	0.0	0.0	0.0	0.00
LOC104910518	0.0	0.0	0.0	0.0	0.0	0.0	0.0	0.00
LOC104910519	0.0	0.0	0.0	0.0	0.0	0.0	0.0	0.00
LOC104910520	0.0	0.0	0.0	0.0	0.0	0.0	0.0	0.00
LOC104910521	0.0	0.0	0.5	0.5	0.0	0.0	0.2	0.26
LOC104910522	0.5	0.5	0.5	0.0	0.0	0.0	0.3	0.27
LOC104910523	0.0	0.0	0.0	0.0	0.0	0.0	0.0	0.00
LOC104910524	0.0	0.0	0.0	0.0	0.0	0.0	0.0	0.00
LOC104910525	3.5	17.0	9.5	4.5	14.5	9.5	9.8	5.33
LOC104910526	2.0	1.0	0.5	1.5	1.5	1.0	1.3	0.52
LOC104910527	0.0	0.0	0.0	0.0	0.0	0.0	0.0	0.00
LOC104910529	5.0	7.0	11.5	2.5	3.0	5.0	5.7	3.28
LOC104910530	3.5	7.0	9.5	1.5	6.0	5.5	5.5	2.77
LOC104910531	0.5	0.5	0.0	0.5	0.5	0.5	0.4	0.20
LOC104910532	0.5	2.5	1.5	0.0	1.5	0.0	1.0	1.00
LOC104910533	2.0	8.5	6.5	1.5	8.5	5.5	5.4	3.07
LOC104910534	2.5	2.5	16.5	3.0	5.5	15.5	7.6	6.62
LOC104910535	0.0	0.0	0.5	0.0	2.5	0.5	0.6	0.97
LOC104910536	0.0	0.0	0.0	0.0	0.5	0.0	0.1	0.20
LOC104910537	7.5	8.5	8.0	5.5	9.0	5.0	7.3	1.64
LOC104910538	195.0	159.0	165.5	153.5	185.5	188.5	174.5	17.32
LOC104910539	1.0	0.5	0.0	2.5	1.5	0.5	1.0	0.89
LOC104910540	1.0	0.0	0.0	0.0	1.0	0.0	0.3	0.52
LOC104910541	1.5	2.0	0.0	1.5	2.5	0.0	1.3	1.04
LOC104910542	3.0	2.5	3.0	1.5	2.0	2.0	2.3	0.61
LOC104910543	0.5	0.0	0.0	1.5	0.0	0.5	0.4	0.58
LOC104910544	0.0	0.0	0.5	0.0	0.0	0.0	0.1	0.20
LOC104910545	0.0	0.0	0.5	0.5	0.0	0.0	0.2	0.26
LOC104910546	0.0	0.0	0.0	0.0	0.0	0.0	0.0	0.00
LOC104910547	192.5	227.0	125.0	173.0	279.5	131.0	188.0	58.92
LOC104910548	0.0	4.5	0.0	2.0	3.0	1.0	1.8	1.78
LOC104910550	2.0	0.5	0.5	0.0	0.5	0.0	0.6	0.74
LOC104910551	134.0	191.5	153.5	129.5	222.5	194.0	170.8	37.41
LOC104910552	23.5	145.0	184.0	28.0	127.0	106.5	102.3	64.58
LOC104910553	14.5	13.0	7.5	19.5	15.5	8.0	13.0	4.60
LOC104910555	0.0	1.5	0.5	0.5	0.0	0.0	0.4	0.58
LOC104910556	0.0	0.0	0.0	0.5	0.0	2.5	0.5	1.00

LOC104910557	0.0	0.5	0.0	0.5	0.5	0.0	0.3	0.27
LOC104910558	124.5	141.5	61.0	114.5	126.5	56.5	104.1	36.19
LOC104910559	1.0	0.5	2.5	0.5	1.0	6.0	1.9	2.13
LOC104910560	2.5	0.5	2.5	4.5	1.5	5.0	2.8	1.72
LOC104910561	16.0	6.5	4.5	19.0	13.5	2.5	10.3	6.74
LOC104910563	10.5	2.0	4.5	15.0	2.5	3.5	6.3	5.24
LOC104910564	1.5	2.0	1.5	0.5	0.5	1.0	1.2	0.61
LOC104910565	29.0	9.5	3.5	28.0	11.0	4.0	14.2	11.49
LOC104910566	42.0	16.5	11.5	45.5	18.5	8.5	23.8	15.93
LOC104910568	44.0	26.5	14.0	46.5	28.5	16.0	29.3	13.65
LOC104910571	22.5	9.0	17.5	29.0	12.5	9.5	16.7	7.93
LOC104910573	54.0	27.0	28.0	37.0	28.5	22.0	32.8	11.48
LOC104910574	69.5	69.5	57.0	53.5	57.5	61.5	61.4	6.76
LOC104910575	0.0	1.0	0.5	0.0	0.0	0.0	0.3	0.42
LOC104910576	15.5	6.5	39.0	20.5	6.0	30.0	19.6	13.11
LOC104910577	1.0	4.0	13.5	0.5	1.5	8.5	4.8	5.17
LOC104910578	0.0	0.5	3.5	0.0	0.0	0.0	0.7	1.40
LOC104910579	0.5	2.0	5.5	0.5	2.5	2.5	2.3	1.84
LOC104910580	0.5	0.0	0.5	0.5	0.5	0.0	0.3	0.26
LOC104910581	0.0	0.0	0.5	3.0	0.0	4.0	1.3	1.78
LOC104910582	0.5	0.0	0.0	0.0	0.0	0.0	0.1	0.20
LOC104910583	0.0	0.5	0.0	0.0	0.0	0.5	0.2	0.26
LOC104910584	12.5	16.5	14.0	5.0	21.5	14.0	13.9	5.40
LOC104910585	0.0	0.0	0.0	0.0	0.0	0.0	0.0	0.00
LOC104910586	0.0	0.0	0.0	0.0	0.0	0.0	0.0	0.00
LOC104910587	0.0	0.0	1.0	0.5	1.0	1.5	0.7	0.61
LOC104910588	396.0	256.5	317.5	450.0	288.0	240.5	324.8	82.37
LOC104910590	154.5	595.5	394.0	123.0	601.5	327.5	366.0	206.97
LOC104910593	0.0	0.5	0.5	0.0	1.0	2.0	0.7	0.75
LOC104910594	17.0	13.0	4.0	12.0	16.0	3.5	10.9	5.85
LOC104910596	2.0	1.0	7.0	0.0	1.5	5.0	2.8	2.68
LOC104910597	0.0	0.0	0.0	0.0	0.0	0.0	0.0	0.00
LOC104910598	0.0	0.0	0.0	0.0	0.0	0.0	0.0	0.00
LOC104910599	2.0	0.5	1.5	2.0	0.0	1.0	1.2	0.82
LOC104910600	0.0	0.5	0.0	0.0	0.0	0.0	0.1	0.20
LOC104910601	860.5	228.0	301.0	537.5	262.0	242.0	405.2	250.68
LOC104910602	2.0	0.0	6.0	2.5	0.0	3.0	2.3	2.23
LOC104910603	0.0	0.0	0.0	0.0	0.0	0.0	0.0	0.00
LOC104910604	8.5	16.0	26.5	6.0	20.0	18.5	15.9	7.60
LOC104910605	1.0	0.5	5.0	2.0	1.5	1.0	1.8	1.63
LOC104910606	106.0	187.0	398.5	117.0	180.0	345.5	222.3	121.55
LOC104910607	132.5	101.0	265.5	145.0	107.5	244.5	166.0	71.10
LOC104910608	41.5	11.0	131.5	40.0	26.5	99.5	58.3	46.74
LOC104910609	34.5	9.0	7.0	30.0	13.0	5.5	16.5	12.54
LOC104910610	2.5	1.0	0.5	3.5	0.5	0.0	1.3	1.37
LOC104910611	4.5	5.0	1.5	2.0	4.5	2.5	3.3	1.51
LOC104910612	0.0	0.0	0.5	0.0	0.0	0.0	0.1	0.20
LOC104910613	0.0	0.0	0.0	0.5	0.0	0.0	0.1	0.20
LOC104910615	0.5	1.0	2.0	0.5	3.5	0.5	1.3	1.21
LOC104910616	7.0	18.0	4.5	10.5	13.0	7.5	10.1	4.87
LOC104910617	14.5	4.5	59.0	19.5	6.0	44.5	24.7	22.17
LOC104910618	2.0	0.5	0.5	0.0	0.5	0.5	0.7	0.68
LOC104910619	1.5	1.5	3.0	1.5	1.0	4.0	2.1	1.16
LOC104910620	24.5	14.5	65.0	25.5	12.0	45.5	31.2	20.36

LOC104910621	187.0	95.0	150.0	179.5	112.5	151.5	145.9	36.26
LOC104910622	1.5	0.0	0.0	0.0	0.0	0.0	0.3	0.61
LOC104910623	1102.0	808.0	600.0	962.5	822.5	530.5	804.3	214.80
LOC104910624	0.0	0.0	0.0	0.0	0.0	0.0	0.0	0.00
LOC104910625	124.0	83.5	54.0	97.0	67.0	33.5	76.5	32.16
LOC104910626	0.0	0.0	0.0	0.0	0.0	0.0	0.0	0.00
LOC104910627	0.0	0.0	0.0	0.0	0.0	0.0	0.0	0.00
LOC104910628	0.0	0.0	0.0	0.0	0.0	0.0	0.0	0.00
LOC104910629	0.0	0.0	0.0	0.0	0.0	0.0	0.0	0.00
LOC104910630	0.0	0.5	0.0	0.0	0.0	0.0	0.1	0.20
LOC104910631	0.0	0.0	0.0	0.0	0.0	0.0	0.0	0.00
LOC104910632	0.0	0.0	0.0	0.0	1.0	0.0	0.2	0.41
LOC104910633	0.0	4.0	7.0	0.0	1.5	6.0	3.1	3.04
LOC104910634	0.5	0.0	0.0	2.0	0.0	0.0	0.4	0.80
LOC104910635	0.0	0.0	0.0	0.0	0.0	0.0	0.0	0.00
LOC104910636	19.0	9.5	45.0	24.0	13.0	40.5	25.2	14.57
LOC104910637	0.0	0.0	0.0	0.0	0.0	0.0	0.0	0.00
LOC104910638	0.0	0.0	0.0	0.0	0.0	0.0	0.0	0.00
LOC104910639	28.5	7.0	62.0	34.0	11.0	76.0	36.4	27.57
LOC104910640	0.5	0.0	1.0	0.5	0.5	0.5	0.5	0.32
LOC104910641	15.0	6.0	19.0	20.5	9.5	10.0	13.3	5.76
LOC104910642	14.5	3.0	5.5	18.0	4.0	2.5	7.9	6.63
LOC104910643	9.5	1.0	5.0	3.0	2.5	7.0	4.7	3.16
LOC104910644	78.5	57.5	136.0	31.5	34.0	90.5	71.3	39.43
LOC104910646	20.0	8.0	34.0	21.5	10.5	28.5	20.4	10.04
LOC104910647	11.5	2.0	12.5	10.0	3.0	6.0	7.5	4.47
LOC104910649	0.0	0.5	0.0	0.0	0.0	0.0	0.1	0.20
LOC104910650	12.0	1.5	1.0	10.5	2.5	4.0	5.3	4.78
LOC104910651	2.0	0.0	0.0	2.5	1.0	0.0	0.9	1.11
LOC104910652	19.0	7.5	12.5	15.0	9.5	7.0	11.8	4.68
LOC104910653	39.5	10.5	39.5	39.5	16.0	31.0	29.3	13.00
LOC104910654	40.0	15.0	14.0	33.5	12.5	10.5	20.9	12.53
LOC104910655	44.0	9.5	9.5	25.5	17.0	6.5	18.7	14.19
LOC104910656	15.5	2.5	4.5	8.5	1.5	4.5	6.2	5.16
LOC104910657	55.5	36.0	51.0	44.5	45.5	31.5	44.0	8.99
LOC104910658	0.0	1.5	1.5	0.5	1.0	1.5	1.0	0.63
LOC104910659	5.0	3.5	1.0	3.0	7.0	4.0	3.9	2.01
LOC104910660	570.5	417.5	415.0	537.0	475.0	417.5	472.1	68.01
LOC104910661	0.0	0.0	0.0	0.0	0.0	0.0	0.0	0.00
LOC104910662	0.0	0.5	0.0	0.0	0.0	0.0	0.1	0.20
LOC104910663	4.5	34.5	61.5	3.0	45.5	74.0	37.2	29.19
LOC104910664	0.0	0.0	0.0	0.0	0.0	0.0	0.0	0.00
LOC104910665	2.0	5.5	6.0	3.5	10.5	16.5	7.3	5.34
LOC104910666	0.0	0.0	0.0	0.0	0.0	0.0	0.0	0.00
LOC104910668	36.0	13.5	63.5	43.0	16.0	48.5	36.8	19.31
LOC104910669	0.0	0.0	0.0	0.0	0.0	0.0	0.0	0.00
LOC104910671	11.0	3.0	2.5	5.5	2.0	0.5	4.1	3.76
LOC104910672	0.0	0.0	0.0	0.0	0.0	0.0	0.0	0.00
LOC104910673	50.0	70.0	201.5	53.5	85.5	156.0	102.8	61.91
LOC104910674	1.0	1.0	2.0	0.0	0.5	1.5	1.0	0.71
LOC104910675	52.5	65.0	10.5	73.0	131.0	23.5	59.3	42.59
LOC104910677	0.5	3.0	2.5	1.0	5.0	5.5	2.9	2.04
LOC104910678	11.0	2.5	13.5	16.0	4.0	16.5	10.6	6.03
LOC104910679	15.0	3.0	27.0	14.0	3.5	21.0	13.9	9.49

LOC104910680	15.5	10.0	48.0	16.5	7.5	48.5	24.3	18.83
LOC104910681	5.0	5.5	13.5	4.5	3.5	6.5	6.4	3.61
LOC104910682	240.5	160.5	272.5	199.5	162.5	239.5	212.5	45.80
LOC104910683	0.0	0.5	0.0	0.5	0.0	0.5	0.3	0.27
LOC104910685	0.5	0.0	0.0	0.0	0.5	0.0	0.2	0.26
LOC104910686	474.0	200.0	481.0	430.5	237.0	455.5	379.7	126.60
LOC104910688	0.0	0.0	0.0	0.0	0.0	0.0	0.0	0.00
LOC104910689	0.5	0.0	0.0	0.0	0.0	0.0	0.1	0.20
LOC104910690	4.5	0.5	2.0	0.5	1.0	1.5	1.7	1.51
LOC104910691	2.0	0.5	3.0	0.0	0.5	3.0	1.5	1.34
LOC104910692	5.5	0.0	6.0	2.5	1.0	8.0	3.8	3.14
LOC104910693	260.0	166.5	365.0	299.5	211.0	425.5	287.9	96.38
LOC104910694	126.5	25.5	145.5	161.5	34.0	113.0	101.0	57.66
LOC104910695	0.0	0.0	0.0	0.0	0.0	0.0	0.0	0.00
LOC104910696	0.0	0.0	0.5	0.0	0.0	0.0	0.1	0.20
LOC104910697	0.0	0.5	0.0	0.0	0.0	0.0	0.1	0.20
LOC104910698	0.5	0.5	0.0	0.0	0.0	0.0	0.2	0.26
LOC104910699	4.0	2.0	0.0	7.0	1.5	0.5	2.5	2.61
LOC104910700	0.0	0.0	1.0	0.0	0.0	0.0	0.2	0.41
LOC104910701	3.5	2.5	1.5	0.0	1.0	1.5	1.7	1.21
LOC104910702	13.0	11.0	24.0	3.0	7.0	1.0	9.8	8.30
LOC104910703	0.0	0.5	0.0	0.0	0.0	0.0	0.1	0.20
LOC104910704	0.0	0.5	0.0	1.0	0.0	0.0	0.3	0.42
LOC104910705	35.0	13.5	22.0	22.0	16.5	26.0	22.5	7.56
LOC104910706	54.0	25.5	10.5	50.0	23.0	15.5	29.8	18.09
LOC104910708	13.0	9.0	6.0	10.5	11.0	7.5	9.5	2.53
LOC104910709	14.5	11.0	13.0	11.0	7.0	10.5	11.2	2.54
LOC104910710	127.5	105.0	68.5	107.5	119.0	100.0	104.6	20.32
LOC104910711	1285.5	1155.0	635.0	1128.0	1295.5	622.0	1020.2	310.78
LOC104910712	0.0	0.0	0.0	0.0	0.0	0.0	0.0	0.00
LOC104910713	0.0	0.0	0.0	0.0	0.0	0.0	0.0	0.00
LOC104910714	0.0	0.0	0.0	0.0	0.0	0.0	0.0	0.00
LOC104910715	0.0	0.0	0.0	0.0	0.0	0.0	0.0	0.00
LOC104910716	0.0	0.0	0.0	0.0	0.0	0.0	0.0	0.00
LOC104910717	0.0	0.0	0.0	0.0	0.0	0.0	0.0	0.00
LOC104910718	0.0	0.0	0.0	0.0	0.0	0.0	0.0	0.00
LOC104910719	145.5	65.0	42.0	177.0	72.0	56.5	93.0	54.72
LOC104910720	0.0	0.0	1.5	0.0	0.5	0.0	0.3	0.61
LOC104910721	0.0	0.0	0.0	1.0	0.0	0.0	0.2	0.41
LOC104910722	1.0	0.5	1.0	0.0	0.0	0.5	0.5	0.45
LOC104910723	2.5	0.0	1.5	0.0	0.0	0.5	0.8	1.04
LOC104910724	1.0	1.5	0.0	3.0	1.5	0.5	1.3	1.04
LOC104910725	13.0	2.0	1.5	15.0	4.5	0.5	6.1	6.30
LOC104910726	4.0	0.5	1.5	2.5	1.5	0.5	1.8	1.33
LOC104910727	1.0	0.0	0.5	0.5	0.0	1.0	0.5	0.45
LOC104910728	0.0	0.5	1.5	1.0	2.5	0.5	1.0	0.89
LOC104910729	12.5	5.5	9.5	8.0	11.5	12.0	9.8	2.71
LOC104910731	7.0	3.5	0.5	2.5	5.5	1.0	3.3	2.54
LOC104910733	1.5	0.0	0.5	0.5	0.0	0.0	0.4	0.58
LOC104910734	103.5	41.0	72.0	96.5	39.5	50.5	67.2	28.04
LOC104910736	0.0	0.0	0.0	0.0	0.0	0.0	0.0	0.00
LOC104910737	12.0	12.0	46.5	8.0	18.5	30.0	21.2	14.62
LOC104910738	44.5	20.5	26.0	19.0	17.0	16.5	23.9	10.65
LOC104910740	0.0	1.0	0.5	1.0	0.5	1.5	0.8	0.52

LOC104910741	2.0	1.5	0.0	1.0	0.5	1.0	1.0	0.71
LOC104910742	1.5	1.0	1.5	1.0	0.0	0.0	0.8	0.68
LOC104910743	3.0	3.0	0.5	2.5	2.0	1.5	2.1	0.97
LOC104910744	12.0	5.0	11.5	6.0	5.5	10.0	8.3	3.19
LOC104910745	7.5	1.5	5.5	5.5	2.0	2.0	4.0	2.49
LOC104910746	0.5	0.0	0.0	0.0	1.0	0.0	0.3	0.42
LOC104910747	0.0	0.5	1.5	0.0	0.0	1.0	0.5	0.63
LOC104910748	0.0	0.0	0.0	0.0	0.0	0.0	0.0	0.00
LOC104910749	13.0	5.0	24.5	8.0	6.0	18.5	12.5	7.73
LOC104910752	8.5	4.0	4.5	7.5	4.5	6.0	5.8	1.83
LOC104910753	67.0	54.5	51.0	63.5	70.0	51.5	59.6	8.29
LOC104910754	0.0	0.0	0.0	0.0	0.0	0.5	0.1	0.20
LOC104910755	0.5	0.0	1.5	0.0	0.0	0.0	0.3	0.61
LOC104910756	7.5	0.5	0.0	9.5	3.5	0.0	3.5	4.14
LOC104910757	0.0	0.0	0.0	0.0	0.0	0.0	0.0	0.00
LOC104910758	1043.0	982.0	1064.5	1014.5	1026.5	997.5	1021.3	30.07
LOC104910759	3.5	2.5	1.5	2.5	1.5	2.0	2.3	0.76
LOC104910760	3.0	2.0	6.5	4.0	1.0	3.0	3.3	1.89
LOC104910761	26.5	6.5	23.0	23.0	19.0	17.5	19.3	7.02
LOC104910762	6.0	4.0	7.0	7.0	2.5	4.0	5.1	1.86
LOC104910763	0.0	0.0	0.0	0.0	0.0	0.0	0.0	0.00
LOC104910765	7.0	4.0	1.0	7.5	3.0	1.0	3.9	2.84
LOC104910766	6.5	1.5	2.5	8.0	0.5	1.5	3.4	3.07
LOC104910767	20.5	4.0	33.5	22.0	4.0	23.0	17.8	11.66
LOC104910768	36.5	16.5	49.5	43.5	9.5	35.5	31.8	15.60
LOC104910769	43.5	41.5	78.5	39.5	40.5	46.0	48.3	15.00
LOC104910770	1.0	0.5	1.0	0.0	0.0	1.5	0.7	0.61
LOC104910771	28.0	9.5	14.0	12.5	8.0	6.0	13.0	7.91
LOC104910772	0.0	0.0	0.0	0.0	0.0	0.0	0.0	0.00
LOC104910773	277.5	309.0	254.0	250.5	377.5	241.5	285.0	51.44
LOC104910774	4.0	2.5	4.0	1.5	1.5	3.5	2.8	1.17
LOC104910775	11.5	2.0	34.5	12.0	4.0	23.0	14.5	12.28
LOC104910776	1.0	0.0	0.5	0.0	0.5	0.0	0.3	0.41
LOC104910777	50.0	14.0	19.5	32.0	13.5	19.0	24.7	14.09
LOC104910780	191.0	102.0	123.5	215.5	115.5	128.5	146.0	45.90
LOC104910781	782.0	471.0	185.5	660.5	494.5	183.5	462.8	243.70
LOC104910782	14.0	10.0	23.5	11.0	12.0	20.0	15.1	5.44
LOC104910783	33.5	8.0	34.0	32.0	12.5	25.0	24.2	11.34
LOC104910784	0.0	0.0	0.0	0.0	0.0	0.0	0.0	0.00
LOC104910785	9.5	2.0	21.5	9.0	6.5	16.5	10.8	7.04
LOC104910786	133.5	62.5	50.5	102.5	82.5	68.5	83.3	30.39
LOC104910787	0.0	0.0	0.0	0.0	0.0	0.0	0.0	0.00
LOC104910788	0.0	0.0	0.0	0.0	0.0	0.0	0.0	0.00
LOC104910789	0.0	0.0	0.0	0.0	0.0	0.0	0.0	0.00
LOC104910790	0.0	0.0	0.0	0.5	0.0	0.0	0.1	0.20
LOC104910792	0.5	0.0	0.0	0.0	0.0	0.0	0.1	0.20
LOC104910793	2.0	2.0	1.5	1.5	0.5	0.5	1.3	0.68
LOC104910794	9.0	1.5	0.5	8.0	1.5	0.0	3.4	3.99
LOC104910796	0.5	0.5	0.0	1.5	1.0	0.0	0.6	0.58
LOC104910797	0.0	0.0	0.0	0.0	0.0	0.0	0.0	0.00
LOC104910798	0.0	0.0	0.0	0.0	0.0	0.0	0.0	0.00
LOC104910799	34.5	25.5	31.5	26.0	28.0	26.5	28.7	3.59
LOC104910800	0.0	0.0	0.0	0.0	0.0	0.0	0.0	0.00
LOC104910801	0.5	0.5	0.0	0.0	0.0	0.0	0.2	0.26

LOC104910802	0.0	0.0	0.0	0.5	0.0	0.5	0.2	0.26
LOC104910803	1.0	0.0	0.5	0.0	0.5	0.0	0.3	0.41
LOC104910804	0.0	0.0	0.5	0.0	0.0	0.0	0.1	0.20
LOC104910805	0.0	0.0	0.0	0.0	0.0	0.0	0.0	0.00
LOC104910806	0.0	0.0	0.0	0.0	0.5	0.0	0.1	0.20
LOC104910807	2.0	0.5	4.5	0.0	0.5	0.5	1.3	1.69
LOC104910808	3.0	0.5	0.0	2.5	1.0	0.0	1.2	1.29
LOC104910809	41.0	11.5	19.5	30.5	13.5	14.5	21.8	11.64
LOC104910810	0.0	0.0	0.5	0.5	0.0	1.0	0.3	0.41
LOC104910811	0.0	0.0	1.0	1.5	1.5	0.0	0.7	0.75
LOC104910812	0.5	0.5	1.0	0.5	0.0	0.0	0.4	0.38
LOC104910813	2.0	5.5	0.5	3.0	3.0	2.5	2.8	1.64
LOC104910814	27.0	16.0	22.0	24.5	25.0	25.0	23.3	3.90
LOC104910815	2.0	0.5	1.5	6.5	3.0	2.5	2.7	2.07
LOC104910816	1.0	0.0	0.0	0.0	0.0	0.0	0.2	0.41
LOC104910818	40.5	59.0	54.0	24.5	54.0	37.0	44.8	13.13
LOC104910819	1.5	8.5	4.0	2.0	6.0	2.0	4.0	2.77
LOC104910820	0.0	1.0	5.0	0.0	3.5	4.0	2.3	2.19
LOC104910821	0.0	0.0	0.0	0.0	0.0	0.0	0.0	0.00
LOC104910823	95.0	21.0	57.0	73.0	24.0	56.5	54.4	28.44
LOC104910824	2.0	0.0	0.5	1.5	0.0	0.0	0.7	0.88
LOC104910825	5.0	5.0	2.5	8.5	3.5	1.5	4.3	2.46
LOC104910826	237.5	73.5	353.5	335.5	91.0	339.0	238.3	127.84
LOC104910827	33.0	57.0	39.5	34.0	76.5	50.0	48.3	16.67
LOC104910828	0.0	0.0	0.0	0.0	0.0	0.0	0.0	0.00
LOC104910829	0.0	0.0	0.0	0.0	0.0	0.0	0.0	0.00
LOC104910831	0.0	0.0	0.0	0.0	0.0	0.0	0.0	0.00
LOC104910832	0.0	0.0	0.0	0.0	0.0	0.0	0.0	0.00
LOC104910833	721.0	261.5	807.5	838.0	315.0	659.0	600.3	250.45
LOC104910834	4.5	0.5	7.0	6.0	2.5	5.0	4.3	2.38
LOC104910835	0.5	0.0	1.0	1.0	0.0	0.0	0.4	0.49
LOC104910836	0.0	0.0	0.0	0.0	0.0	0.0	0.0	0.00
LOC104910837	0.0	0.0	0.0	0.0	0.0	0.0	0.0	0.00
LOC104910838	0.0	0.0	0.0	0.0	0.0	0.0	0.0	0.00
LOC104910839	0.0	0.5	0.0	2.0	0.0	0.0	0.4	0.80
LOC104910840	2.0	3.0	6.0	1.0	4.0	4.0	3.3	1.75
LOC104910841	154.0	100.5	137.0	126.0	116.0	138.5	128.7	18.81
LOC104910842	186.5	130.5	92.0	203.0	139.0	94.5	140.9	46.03
LOC104910843	137.5	67.5	93.5	134.0	79.5	82.0	99.0	29.66
LOC104910844	0.0	0.0	0.0	0.0	0.0	0.0	0.0	0.00
LOC104910846	0.0	0.0	0.0	0.0	0.0	0.0	0.0	0.00
LOC104910848	158.5	63.0	63.5	101.5	49.5	53.0	81.5	42.03
LOC104910849	0.0	0.0	0.0	0.0	0.0	0.0	0.0	0.00
LOC104910850	24.0	9.0	22.0	31.0	12.5	19.5	19.7	7.97
LOC104910851	84.5	45.5	106.0	88.5	49.0	93.5	77.8	24.79
LOC104910852	16.5	9.0	25.5	25.0	9.0	22.5	17.9	7.61
LOC104910853	1.0	2.5	1.0	0.0	1.5	2.5	1.4	0.97
LOC104910855	26.5	23.5	34.0	26.5	44.5	49.5	34.1	10.71
LOC104910857	256.5	150.5	142.0	232.0	198.5	138.0	186.3	50.48
LOC104910858	254.0	237.0	149.5	228.5	278.5	141.5	214.8	56.41
LOC104910859	108.5	41.0	41.0	88.0	64.0	45.0	64.6	28.19
LOC104910860	1.5	1.0	2.0	6.0	1.0	2.5	2.3	1.89
LOC104910861	63.5	48.0	74.0	49.0	40.0	56.5	55.2	12.21
LOC104910862	849.0	724.5	492.0	860.0	856.0	547.0	721.4	165.32

LOC104910863	0.0	0.0	0.0	0.0	0.0	0.0	0.0	0.00
LOC104910864	58.0	73.5	96.5	45.5	102.0	123.5	83.2	29.34
LOC104910865	209.5	129.5	92.5	211.0	145.5	81.0	144.8	55.87
LOC104910866	1.0	4.0	3.5	0.5	5.5	6.0	3.4	2.27
LOC104910867	11.5	2.0	19.5	19.5	5.5	18.0	12.7	7.59
LOC104910868	20.5	9.5	45.0	31.5	10.0	40.0	26.1	15.14
LOC104910869	22.0	5.5	18.5	10.0	12.0	17.0	14.2	6.09
LOC104910870	210.5	101.0	252.0	209.0	92.0	205.5	178.3	65.69
LOC104910871	0.0	0.0	0.0	0.0	0.0	0.5	0.1	0.20
LOC104910872	69.0	9.5	32.5	61.0	19.5	24.5	36.0	23.80
LOC104910873	14.5	6.0	36.0	24.5	8.5	36.5	21.0	13.42
LOC104910874	0.0	0.5	2.5	0.0	2.0	6.5	1.9	2.48
LOC104910875	54.5	21.5	342.0	65.5	28.0	278.0	131.6	140.62
LOC104910876	11.0	4.5	47.0	23.5	16.0	46.5	24.8	18.14
LOC104910877	1.5	2.0	0.0	2.0	1.0	0.0	1.1	0.92
LOC104910878	0.0	0.0	0.0	0.0	0.0	0.0	0.0	0.00
LOC104910879	8.0	4.5	5.0	5.0	5.5	5.0	5.5	1.26
LOC104910880	0.0	0.0	0.0	0.0	0.0	0.0	0.0	0.00
LOC104910882	24.5	93.5	135.0	27.5	90.5	170.5	90.3	57.82
LOC104910883	0.0	0.0	0.5	0.0	0.0	0.0	0.1	0.20
LOC104910884	2.0	0.0	0.5	0.0	0.0	0.5	0.5	0.77
LOC104910885	2.0	1.5	3.0	1.5	1.5	0.5	1.7	0.82
LOC104910887	0.5	0.5	0.0	1.5	0.5	1.0	0.7	0.52
LOC104910888	7.0	3.0	10.5	4.0	1.0	5.5	5.2	3.33
LOC104910889	14.0	14.0	28.5	16.5	10.0	36.0	19.8	10.12
LOC104910890	1.0	0.0	0.5	1.5	0.0	2.5	0.9	0.97
LOC104910891	0.0	0.0	0.0	0.0	0.0	0.0	0.0	0.00
LOC104910892	0.0	0.0	0.0	0.0	0.0	0.0	0.0	0.00
LOC104910893	0.0	0.0	0.0	0.0	0.0	0.0	0.0	0.00
LOC104910894	0.0	0.0	0.0	0.0	0.0	0.0	0.0	0.00
LOC104910895	0.5	1.5	1.5	2.5	2.0	5.0	2.2	1.54
LOC104910896	13.0	3.5	1.5	11.0	6.5	0.0	5.9	5.23
LOC104910897	0.0	0.0	0.0	0.0	0.0	0.0	0.0	0.00
LOC104910898	0.0	0.0	0.0	0.0	0.0	0.0	0.0	0.00
LOC104910899	0.0	0.0	0.0	0.0	0.0	0.0	0.0	0.00
LOC104910900	0.0	0.0	0.0	0.0	0.0	0.0	0.0	0.00
LOC104910901	0.0	0.0	0.0	0.0	0.0	0.0	0.0	0.00
LOC104910902	0.5	0.5	0.5	0.0	2.0	2.0	0.9	0.86
LOC104910903	0.0	0.5	0.5	0.0	0.0	1.5	0.4	0.58
LOC104910904	3.5	1.0	1.0	1.0	0.5	0.5	1.3	1.13
LOC104910905	0.0	0.0	0.0	0.0	0.0	0.0	0.0	0.00
LOC104910906	5.0	3.5	2.0	3.5	0.5	1.5	2.7	1.63
LOC104910907	2.0	2.0	5.0	3.0	4.0	4.0	3.3	1.21
LOC104910908	551.0	146.0	426.5	604.5	146.5	250.0	354.1	201.97
LOC104910909	4.5	8.5	34.5	6.5	15.0	29.0	16.3	12.57
LOC104910910	3.0	2.0	9.0	2.5	1.5	5.5	3.9	2.85
LOC104910911	0.0	0.0	0.0	0.0	0.0	0.0	0.0	0.00
LOC104910912	0.0	0.0	0.0	0.0	0.0	0.0	0.0	0.00
LOC104910913	0.0	0.0	1.0	0.0	0.0	0.5	0.3	0.42
LOC104910914	0.0	0.0	0.5	0.0	0.5	0.5	0.3	0.27
LOC104910915	0.0	0.0	0.0	0.0	0.0	0.0	0.0	0.00
LOC104910916	0.0	0.0	0.0	0.0	0.0	0.0	0.0	0.00
LOC104910917	52.5	16.5	86.5	52.0	22.0	92.5	53.7	31.54
LOC104910921	9.0	6.0	6.0	9.5	7.0	10.0	7.9	1.80

LOC104910922	6.5	6.0	6.5	5.5	6.0	6.5	6.2	0.41
LOC104910926	0.0	0.0	0.0	0.0	0.0	0.0	0.0	0.00
LOC104910927	0.0	0.0	0.5	0.0	0.5	1.0	0.3	0.41
LOC104910928	2.5	0.5	2.0	5.5	4.0	2.5	2.8	1.72
LOC104910929	0.5	0.0	0.5	0.0	1.5	0.5	0.5	0.55
LOC104910930	2.5	2.0	2.0	2.5	1.0	0.5	1.8	0.82
LOC104910931	0.0	0.0	2.0	1.0	0.0	0.0	0.5	0.84
LOC104910932	13.0	2.5	8.5	9.5	6.5	10.0	8.3	3.56
LOC104910934	0.0	0.0	0.0	0.0	0.0	0.0	0.0	0.00
LOC104910936	0.0	0.0	0.0	0.0	0.0	0.0	0.0	0.00
LOC104910938	494.5	220.5	127.5	463.5	209.0	100.5	269.3	169.14
LOC104910939	267.5	59.0	85.0	289.5	56.5	64.0	136.9	110.35
LOC104910940	8.5	4.5	1.5	14.0	5.5	0.5	5.8	4.96
LOC104910942	1.0	0.5	0.0	0.5	2.0	0.0	0.7	0.75
LOC104910946	3.0	8.0	21.0	2.5	19.0	29.0	13.8	10.84
LOC104910947	3.5	4.0	10.5	5.5	3.0	2.5	4.8	2.96
LOC104910950	13.5	12.0	6.0	13.5	18.5	8.0	11.9	4.44
LOC104910953	664.0	279.0	659.0	646.5	343.0	557.0	524.8	171.29
LOC104910954	102.5	9.5	80.5	129.5	19.0	44.0	64.2	47.83
LOC104910956	62.5	13.0	47.0	77.5	7.5	29.0	39.4	27.81
LOC104910957	92.5	57.5	65.0	78.5	61.5	53.0	68.0	14.81
LOC104910961	0.0	5.5	0.5	0.0	1.5	0.0	1.3	2.16
LOC104910962	365.0	183.5	88.0	281.5	178.0	82.0	196.3	110.46
LOC104910963	55.5	20.0	17.0	56.5	16.0	9.5	29.1	21.13
LOC104910964	363.5	336.0	268.0	326.0	360.5	245.5	316.6	49.01
LOC104910965	1.5	0.5	1.0	1.5	0.0	1.0	0.9	0.58
LOC104910966	17.5	14.0	12.5	18.0	13.5	7.5	13.8	3.82
LOC104910968	0.0	0.0	0.0	0.0	0.0	0.0	0.0	0.00
LOC104910969	0.0	0.0	0.0	0.0	0.0	0.0	0.0	0.00
LOC104910970	294.5	239.5	168.0	253.5	266.0	174.5	232.7	50.95
LOC104910971	0.0	0.0	0.0	0.0	0.0	0.0	0.0	0.00
LOC104910972	0.5	0.0	0.0	0.5	0.0	0.0	0.2	0.26
LOC104910973	0.0	0.5	0.0	0.0	0.0	0.0	0.1	0.20
LOC104910974	89.5	42.0	32.0	74.5	35.5	23.5	49.5	26.30
LOC104910975	1163.0	1304.5	1815.0	1052.0	1371.0	1679.5	1397.5	295.82
LOC104910976	1.5	0.5	5.5	1.0	1.0	2.5	2.0	1.84
LOC104910980	119.5	28.5	195.0	151.5	40.0	145.5	113.3	65.99
LOC104910981	4581.5	4404.5	3682.5	4170.0	5027.5	3503.0	4228.2	569.54
LOC104910982	64.5	7.0	14.0	52.0	9.0	15.0	26.9	24.77
LOC104910983	713.5	463.5	502.5	686.5	490.0	475.5	555.3	113.21
LOC104910984	30.5	18.5	44.0	36.5	17.0	33.5	30.0	10.51
LOC104910985	15.0	3.0	15.0	19.5	5.0	11.5	11.5	6.37
LOC104910986	24.0	6.5	1.5	14.0	7.5	1.5	9.2	8.61
LOC104910987	76.0	51.0	189.5	62.0	60.5	143.0	97.0	56.19
LOC104910989	2.0	2.0	1.5	3.0	0.5	2.0	1.8	0.82
LOC104910990	7.0	0.5	10.0	6.0	2.0	11.5	6.2	4.32
LOC104910991	0.0	0.0	1.0	1.0	0.5	0.0	0.4	0.49
LOC104910992	3.5	2.5	5.5	2.0	6.0	3.5	3.8	1.60
LOC104910993	5.0	2.0	5.0	2.5	4.0	5.5	4.0	1.45
LOC104910994	0.0	0.0	0.0	0.0	0.0	0.0	0.0	0.00
LOC104910995	4.0	3.5	5.5	0.0	3.5	1.0	2.9	2.04
LOC104910996	2.5	1.0	0.0	0.0	0.5	0.0	0.7	0.98
LOC104910997	0.0	0.0	0.0	0.0	0.0	0.0	0.0	0.00
LOC104910998	0.0	0.0	0.0	0.0	0.0	0.0	0.0	0.00

LOC104910999	62.0	26.0	34.5	60.0	38.0	35.5	42.7	14.78
LOC104911001	369.5	360.0	203.0	322.0	369.5	203.5	304.6	80.42
LOC104911002	8.0	36.0	128.0	16.5	41.5	143.5	62.3	58.45
LOC104911003	63.0	55.0	246.5	86.0	67.5	236.5	125.8	90.29
LOC104911005	774.0	688.0	569.0	702.5	794.5	576.5	684.1	95.34
LOC104911006	2.5	1.5	1.5	3.0	1.5	5.5	2.6	1.56
LOC104911008	586.5	620.5	315.0	525.5	660.0	283.0	498.4	160.97
LOC104911009	0.0	3.0	2.5	2.5	4.5	3.5	2.7	1.51
LOC104911010	18.0	16.0	12.5	19.0	26.0	15.5	17.8	4.59
LOC104911011	419.0	227.0	391.5	416.5	203.0	330.5	331.3	95.83
LOC104911012	25.0	18.0	8.5	13.5	20.0	5.0	15.0	7.46
LOC104911013	6.0	3.5	3.5	5.0	7.0	3.0	4.7	1.60
LOC104911014	1718.5	1505.5	1364.5	2050.0	1870.5	1687.5	1699.4	245.95
LOC104911015	3.0	1.0	3.5	3.5	2.0	5.5	3.1	1.53
LOC104911016	0.0	0.0	0.0	0.0	0.0	0.0	0.0	0.00
LOC104911017	32.5	21.5	54.5	21.0	25.0	80.5	39.2	23.79
LOC104911019	55.5	21.5	91.5	62.5	23.0	55.0	51.5	26.31
LOC104911020	4.0	2.5	0.0	3.0	1.5	0.5	1.9	1.53
LOC104911021	100.5	39.5	78.0	114.5	27.0	67.5	71.2	33.92
LOC104911022	22.5	44.0	56.5	25.0	46.5	54.5	41.5	14.55
LOC104911023	52.0	91.5	48.0	47.5	91.0	57.5	64.6	20.97
LOC104911024	827.0	1533.0	1677.0	664.5	1634.0	1503.0	1306.4	441.93
LOC104911025	0.0	0.0	0.0	0.0	0.0	0.0	0.0	0.00
LOC104911026	0.0	0.0	0.0	0.0	0.0	0.0	0.0	0.00
LOC104911027	0.5	0.0	0.0	0.0	0.0	0.0	0.1	0.20
LOC104911028	169.0	54.0	209.5	247.5	64.0	216.0	160.0	82.19
LOC104911029	40.5	10.5	109.5	46.5	15.0	86.0	51.3	39.28
LOC104911030	74.0	21.5	138.5	78.0	37.0	112.0	76.8	44.04
LOC104911031	0.0	0.0	0.0	0.0	0.0	0.0	0.0	0.00
LOC104911033	0.0	0.0	0.0	1.5	0.0	1.0	0.4	0.66
LOC104911034	0.0	0.0	0.5	0.0	0.0	0.0	0.1	0.20
LOC104911035	0.0	0.0	0.0	0.0	0.0	0.0	0.0	0.00
LOC104911036	0.0	0.0	0.0	0.0	0.0	0.0	0.0	0.00
LOC104911037	0.0	0.0	0.5	0.5	0.5	0.0	0.3	0.27
LOC104911040	0.0	0.0	0.0	0.0	0.0	0.0	0.0	0.00
LOC104911041	0.0	0.0	0.0	0.0	0.0	0.0	0.0	0.00
LOC104911042	0.0	0.0	0.0	0.0	0.0	0.0	0.0	0.00
LOC104911043	0.0	0.0	0.5	0.0	1.0	0.5	0.3	0.41
LOC104911044	0.0	0.0	1.0	0.0	0.5	0.5	0.3	0.41
LOC104911045	0.0	0.5	0.5	0.5	0.5	1.0	0.5	0.32
LOC104911046	0.5	0.0	1.5	0.0	0.5	0.0	0.4	0.58
LOC104911047	16.5	9.5	10.5	12.5	14.5	9.5	12.2	2.88
LOC104911048	1.0	1.0	1.5	1.5	1.5	0.5	1.2	0.41
LOC104911049	4.0	1.5	3.0	3.0	3.0	4.0	3.1	0.92
LOC104911050	1.0	0.0	1.5	2.5	2.0	1.5	1.4	0.86
LOC104911052	2.5	2.5	1.0	3.0	0.0	0.5	1.6	1.24
LOC104911053	3.5	2.5	3.0	2.5	1.0	0.5	2.2	1.17
LOC104911054	242.5	174.0	180.5	234.0	193.0	158.0	197.0	33.99
LOC104911055	0.0	0.0	0.0	0.0	0.0	0.0	0.0	0.00
LOC104911056	0.0	0.0	0.0	0.0	0.0	0.0	0.0	0.00
LOC104911060	0.0	0.0	0.0	0.0	0.0	0.0	0.0	0.00
LOC104911061	2.0	2.0	1.0	2.5	1.0	2.0	1.8	0.61
LOC104911062	0.0	0.0	0.0	0.0	0.0	0.0	0.0	0.00
LOC104911063	0.5	0.0	0.0	0.0	0.0	0.0	0.1	0.20

LOC104911064	0.0	0.0	0.0	0.0	0.0	0.0	0.0	0.00
LOC104911065	180.0	676.5	449.0	218.5	878.5	356.0	459.8	271.91
LOC104911066	0.0	0.0	0.0	0.0	0.0	0.0	0.0	0.00
LOC104911067	0.0	0.0	0.0	0.0	0.0	0.0	0.0	0.00
LOC104911068	0.0	1.0	0.0	0.0	0.0	1.0	0.3	0.52
LOC104911069	0.0	0.0	0.0	0.0	0.0	0.0	0.0	0.00
LOC104911070	8.0	6.0	4.5	6.0	5.5	2.0	5.3	1.99
LOC104911071	7.5	3.5	14.0	2.0	6.0	7.0	6.7	4.17
LOC104911072	0.0	0.0	0.0	0.0	0.0	0.0	0.0	0.00
LOC104911073	4.0	10.5	5.5	17.0	11.5	11.0	9.9	4.66
LOC104911074	0.5	0.5	0.0	0.0	0.0	0.0	0.2	0.26
LOC104911075	12.0	5.5	10.0	12.5	10.0	7.0	9.5	2.76
LOC104911076	0.0	3.5	2.0	0.5	3.5	3.0	2.1	1.53
LOC104911077	306.5	308.0	443.0	302.0	355.0	415.0	354.9	61.18
LOC104911079	0.0	0.0	0.0	0.5	0.0	0.0	0.1	0.20
LOC104911080	0.5	4.5	2.5	0.5	4.0	1.0	2.2	1.78
LOC104911081	0.5	1.0	1.5	2.0	0.5	1.0	1.1	0.58
LOC104911082	102.0	103.5	61.5	89.0	110.0	81.0	91.2	17.96
LOC104911083	1378.0	1011.5	932.5	1253.5	1158.0	883.0	1102.8	193.46
LOC104911084	0.0	0.0	0.0	0.0	0.0	0.0	0.0	0.00
LOC104911085	0.0	0.0	0.0	0.0	0.0	0.0	0.0	0.00
LOC104911086	13.5	17.0	18.5	10.5	11.5	13.0	14.0	3.13
LOC104911087	2.5	0.5	2.0	1.0	2.0	1.5	1.6	0.74
LOC104911089	0.0	0.0	0.0	1.0	0.0	0.0	0.2	0.41
LOC104911090	205.5	171.0	355.5	208.0	184.0	376.0	250.0	90.94
LOC104911091	43.0	23.5	138.5	45.0	27.5	106.0	63.9	47.09
LOC104911092	18.5	3.0	31.5	21.0	6.5	35.0	19.3	12.87
LOC104911093	1.5	0.5	1.5	3.5	1.5	0.5	1.5	1.10
LOC104911094	1577.0	1134.5	1071.0	1410.5	1237.5	951.5	1230.3	230.22
LOC104911095	204.5	80.0	712.0	218.0	80.5	537.0	305.3	260.06
LOC104911096	0.0	1.0	0.0	0.0	0.5	0.0	0.3	0.42
LOC104911097	0.0	0.0	5.0	0.0	1.0	3.5	1.6	2.15
LOC104911099	27.5	10.5	22.0	29.0	11.5	25.0	20.9	8.05
LOC104911100	1.0	0.0	2.0	2.0	1.0	1.0	1.2	0.75
LOC104911101	112.0	54.0	172.5	125.5	53.5	145.0	110.4	48.38
LOC104911102	1040.5	596.0	1059.5	1168.0	672.0	836.0	895.3	230.38
LOC104911103	0.5	2.0	3.0	1.5	1.0	2.0	1.7	0.88
LOC104911104	433.0	176.0	388.5	435.5	151.5	402.0	331.1	131.09
LOC104911105	35.0	8.0	30.5	28.0	7.5	22.5	21.9	11.69
LOC104911106	597.0	250.5	408.5	386.0	257.0	269.0	361.3	134.21
LOC104911107	4.5	70.0	53.0	8.0	105.5	62.0	50.5	38.64
LOC104911108	4.5	46.0	37.5	7.0	56.5	37.0	31.4	21.12
LOC104911110	15.5	33.0	17.5	21.0	52.5	19.5	26.5	14.14
LOC104911111	49.5	40.0	63.5	52.5	50.5	46.0	50.3	7.80
LOC104911112	0.5	0.0	0.0	0.0	1.0	0.0	0.3	0.42
LOC104911114	91.0	24.0	66.5	144.5	25.0	69.0	70.0	45.05
LOC104911116	64.5	8.5	89.5	84.5	13.5	70.0	55.1	35.38
LOC104911118	756.5	608.5	505.5	711.0	707.5	530.0	636.5	104.19
LOC104911120	288.5	104.5	100.0	229.0	122.5	84.0	154.8	83.63
LOC104911121	466.0	328.5	463.5	445.0	446.5	423.5	428.8	51.48
LOC104911122	0.0	0.0	0.0	0.0	0.0	0.0	0.0	0.00
LOC104911123	2.5	1.0	9.5	2.0	1.5	5.0	3.6	3.22
LOC104911124	0.5	0.0	1.0	0.5	0.0	0.0	0.3	0.41
LOC104911126	0.0	0.5	0.0	0.0	0.0	1.0	0.3	0.42

LOC104911127	1.0	0.0	0.0	1.0	0.5	0.0	0.4	0.49
LOC104911128	0.0	0.0	0.0	0.0	0.5	0.0	0.1	0.20
LOC104911129	113.0	82.0	244.5	152.0	99.5	205.0	149.3	64.03
LOC104911130	2.0	0.0	0.0	0.0	0.5	0.0	0.4	0.80
LOC104911131	262.0	284.5	208.0	237.0	284.5	217.5	248.9	33.19
LOC104911132	2.0	1.0	4.0	1.0	0.5	0.0	1.4	1.43
LOC104911133	313.5	209.0	333.0	271.0	249.0	331.0	284.4	50.00
LOC104911135	40.5	18.5	11.0	35.0	25.5	8.5	23.2	12.89
LOC104911136	0.0	0.0	0.0	0.0	0.0	0.0	0.0	0.00
LOC104911138	25.0	47.0	37.0	47.5	62.0	38.5	42.8	12.47
LOC104911140	0.0	0.0	0.0	0.5	0.0	0.0	0.1	0.20
LOC104911143	336.5	153.5	178.5	216.0	136.5	153.0	195.7	74.35
LOC104911144	20.5	10.5	25.5	25.5	14.5	15.5	18.7	6.18
LOC104911145	0.0	0.0	0.0	0.0	0.5	0.0	0.1	0.20
LOC104911146	14.0	4.0	15.5	11.0	2.5	21.5	11.4	7.21
LOC104911147	6.0	0.5	4.0	0.5	1.5	2.0	2.4	2.18
LOC104911148	23.5	5.0	34.5	19.5	4.0	24.0	18.4	11.87
LOC104911149	13.0	4.0	4.0	16.5	6.0	4.0	7.9	5.46
LOC104911150	14.5	4.0	7.5	19.0	7.0	7.5	9.9	5.63
LOC104911151	0.0	0.0	0.0	0.0	0.0	0.0	0.0	0.00
LOC104911152	6.0	3.5	1.0	7.0	2.0	3.0	3.8	2.32
LOC104911153	1.5	0.5	0.5	1.5	0.0	1.5	0.9	0.66
LOC104911154	2.5	1.0	0.0	0.5	3.0	1.5	1.4	1.16
LOC104911155	3.0	2.0	6.0	5.0	2.5	2.5	3.5	1.61
LOC104911156	0.0	0.0	0.0	0.0	0.0	0.0	0.0	0.00
LOC104911157	0.0	0.0	0.0	0.0	0.0	0.0	0.0	0.00
LOC104911158	131.5	72.5	52.0	115.0	78.5	62.5	85.3	31.16
LOC104911159	57.5	14.5	15.5	48.5	14.5	11.5	27.0	20.38
LOC104911160	2.5	2.5	9.5	4.0	2.5	4.5	4.3	2.72
LOC104911161	35.5	11.5	52.0	34.5	16.5	51.0	33.5	16.89
LOC104911162	1.0	1.0	12.5	3.0	2.5	9.0	4.8	4.78
LOC104911163	5.5	1.0	4.5	6.5	5.5	4.5	4.6	1.91
LOC104911164	19.0	8.5	19.0	14.0	8.5	22.0	15.2	5.77
LOC104911165	1.0	1.5	5.5	4.0	2.5	8.5	3.8	2.82
LOC104911166	24.5	15.0	7.5	23.5	10.5	9.0	15.0	7.42
LOC104911167	351.0	113.0	85.5	296.0	136.0	83.0	177.4	116.12
LOC104911168	98.0	106.5	104.5	107.5	113.5	124.5	109.1	9.06
LOC104911169	3.5	1.0	2.5	7.0	2.5	3.5	3.3	2.02
LOC104911170	0.0	0.0	0.0	0.0	1.0	1.0	0.3	0.52
LOC104911171	29.5	46.0	36.0	35.0	53.0	26.0	37.6	10.17
LOC104911172	20.5	5.5	13.0	22.0	4.5	7.0	12.1	7.70
LOC104911173	5.0	6.5	4.0	6.0	0.0	5.0	4.4	2.33
LOC104911174	45.0	15.5	78.5	35.0	18.5	80.0	45.4	28.35
LOC104911175	0.0	1.0	0.0	0.5	1.5	0.0	0.5	0.63
LOC104911176	1.0	1.0	0.0	0.5	0.0	0.0	0.4	0.49
LOC104911177	0.0	0.0	0.0	0.0	0.0	0.0	0.0	0.00
LOC104911178	0.0	0.0	0.0	0.0	0.0	0.0	0.0	0.00
LOC104911179	6.0	2.5	10.5	3.0	2.5	1.5	4.3	3.39
LOC104911181	0.0	0.0	0.0	0.0	0.0	0.0	0.0	0.00
LOC104911182	2.0	2.0	1.5	0.5	1.0	1.0	1.3	0.61
LOC104911183	0.0	0.0	0.5	0.0	0.0	0.0	0.1	0.20
LOC104911184	0.0	0.0	0.0	0.0	0.0	1.0	0.2	0.41
LOC104911185	1.0	3.0	0.0	1.5	3.0	0.0	1.4	1.36
LOC104911186	0.0	0.0	0.5	0.0	0.0	1.0	0.3	0.42

LOC104911187	0.0	1.0	7.5	0.5	1.0	9.5	3.3	4.13
LOC104911188	0.0	0.0	0.0	0.0	0.0	0.0	0.0	0.00
LOC104911190	0.0	0.0	0.0	0.0	0.0	0.0	0.0	0.00
LOC104911191	0.0	0.0	0.0	0.0	0.0	0.0	0.0	0.00
LOC104911194	0.0	0.0	0.0	0.0	0.0	0.0	0.0	0.00
LOC104911195	0.0	0.0	0.0	0.5	0.0	0.0	0.1	0.20
LOC104911196	0.0	0.0	0.0	0.0	0.0	0.0	0.0	0.00
LOC104911197	0.0	0.0	0.0	0.5	0.0	0.0	0.1	0.20
LOC104911201	1503.5	1677.5	808.0	1429.0	1969.0	817.5	1367.4	468.13
LOC104911202	0.5	1.0	0.0	0.5	0.0	0.0	0.3	0.41
LOC104911204	11.0	7.0	2.0	16.5	7.5	1.5	7.6	5.65
LOC104911205	35.0	21.0	8.0	43.0	28.0	16.0	25.2	12.80
LOC104911207	10.5	2.5	4.0	13.0	1.5	4.0	5.9	4.68
LOC104911209	2.0	0.0	1.5	0.0	0.0	0.5	0.7	0.88
LOC104911210	0.5	0.0	0.0	0.0	0.5	0.0	0.2	0.26
LOC104911211	0.5	0.0	0.0	0.0	0.0	0.0	0.1	0.20
LOC104911212	0.0	0.0	0.0	0.0	0.0	0.0	0.0	0.00
LOC104911213	0.0	0.0	0.0	0.0	0.5	0.0	0.1	0.20
LOC104911215	3.0	1.5	1.5	1.5	4.0	1.0	2.1	1.16
LOC104911217	5.0	2.5	5.5	4.5	2.0	3.0	3.8	1.44
LOC104911218	0.0	0.0	0.0	0.0	0.0	0.0	0.0	0.00
LOC104911219	0.0	0.0	2.0	0.0	0.5	0.0	0.4	0.80
LOC104911221	3.5	2.5	2.5	3.5	2.5	6.0	3.4	1.36
LOC104911222	0.0	0.0	0.0	0.0	0.0	0.0	0.0	0.00
LOC104911223	2.0	0.5	0.0	0.5	2.0	0.5	0.9	0.86
LOC104911224	0.5	0.5	0.5	2.0	1.0	2.0	1.1	0.74
LOC104911225	19.0	9.5	7.5	19.0	9.0	9.5	12.3	5.28
LOC104911227	993.0	967.0	612.0	855.5	1151.5	739.0	886.3	192.93
LOC104911228	0.0	0.5	0.0	1.5	0.5	0.0	0.4	0.58
LOC104911229	2.5	4.0	7.0	1.5	1.5	4.0	3.4	2.08
LOC104911230	0.0	0.5	0.0	0.5	0.0	0.5	0.3	0.27
LOC104911232	41.5	15.5	43.0	39.0	29.0	28.0	32.7	10.53
LOC104911233	44.0	32.0	57.5	48.5	37.5	42.5	43.7	8.84
LOC104911235	12.5	2.0	4.0	19.0	3.5	1.5	7.1	7.08
LOC104911236	327.5	368.0	356.5	341.5	431.0	357.5	363.7	35.89
LOC104911237	0.0	0.0	0.0	0.0	0.0	0.0	0.0	0.00
LOC104911238	0.0	0.0	0.0	0.0	0.0	0.0	0.0	0.00
LOC104911239	0.0	0.0	0.0	0.0	0.0	0.0	0.0	0.00
LOC104911240	33.5	18.5	9.0	19.5	15.5	7.0	17.2	9.45
LOC104911241	10.0	16.0	6.0	20.0	25.0	16.0	15.5	6.80
LOC104911242	11.5	55.5	81.5	7.5	52.0	84.5	48.8	33.15
LOC104911243	10.0	2.0	1.5	8.5	3.5	3.0	4.8	3.59
LOC104911245	350.5	242.5	277.5	405.0	297.5	229.5	300.4	66.88
LOC104911246	0.5	0.5	0.0	1.0	1.0	0.0	0.5	0.45
LOC104911248	2.5	1.0	0.5	0.5	1.5	0.0	1.0	0.89
LOC104911249	0.0	0.5	0.0	0.5	1.5	0.0	0.4	0.58
LOC104911250	29.5	28.5	41.0	30.0	45.0	59.5	38.9	12.18
LOC104911252	4.5	4.5	2.0	9.5	8.5	4.0	5.5	2.88
LOC104911253	6.5	6.5	2.5	7.5	11.0	3.0	6.2	3.13
LOC104911254	5.5	1.5	4.5	5.5	1.0	1.0	3.2	2.23
LOC104911256	68.0	41.0	64.0	57.0	31.5	37.0	49.8	15.24
LOC104911257	1.0	0.5	0.0	0.0	0.0	0.0	0.3	0.42
LOC104911258	0.0	0.0	0.0	0.0	0.0	0.0	0.0	0.00
LOC104911259	0.0	0.0	0.5	0.0	0.0	0.0	0.1	0.20

LOC104911263	80.5	73.0	68.0	74.0	76.0	73.5	74.2	4.08
LOC104911264	5.5	2.0	1.5	7.5	2.5	0.5	3.3	2.68
LOC104911265	0.0	0.0	0.0	0.5	0.0	0.5	0.2	0.26
LOC104911267	3.5	5.5	2.0	2.5	1.0	2.0	2.8	1.57
LOC104911269	1.0	0.5	1.5	3.0	0.0	0.5	1.1	1.07
LOC104911270	5.5	2.0	21.0	5.5	6.5	26.5	11.2	10.02
LOC104911271	25.0	13.0	11.5	18.5	13.0	11.0	15.3	5.44
LOC104911272	1430.0	1530.0	829.5	1201.5	1700.5	833.0	1254.1	365.16
LOC104911273	0.0	0.0	0.0	0.0	0.0	0.0	0.0	0.00
LOC104911274	0.5	0.5	4.5	0.5	1.0	3.0	1.7	1.69
LOC104911275	1.0	0.5	0.5	0.5	0.0	0.0	0.4	0.38
LOC104911276	103.5	122.5	84.5	121.0	129.0	88.5	108.2	18.83
LOC104911277	88.0	48.5	65.0	82.5	40.5	55.0	63.3	18.92
LOC104911278	138.0	102.0	66.5	118.5	96.0	50.0	95.2	32.52
LOC104911281	0.0	0.0	0.0	0.0	0.0	0.0	0.0	0.00
LOC104911282	0.0	0.0	0.0	0.0	0.0	0.5	0.1	0.20
LOC104911284	9.5	7.0	6.5	5.0	3.5	1.5	5.5	2.81
LOC104911285	3.0	2.0	2.5	0.0	4.5	2.0	2.3	1.47
LOC104911288	0.0	0.0	0.0	0.0	0.0	0.0	0.0	0.00
LOC104911290	0.0	0.0	0.0	0.0	0.0	0.0	0.0	0.00
LOC104911291	222.5	478.0	315.0	178.0	446.0	317.0	326.1	118.59
LOC104911292	157.5	25.5	19.0	91.5	25.0	7.0	54.3	58.66
LOC104911293	13.0	26.5	69.0	8.0	29.0	54.5	33.3	23.84
LOC104911296	16.5	6.5	11.5	16.5	6.5	8.0	10.9	4.69
LOC104911297	4.5	3.5	2.5	12.0	1.0	2.0	4.3	3.98
LOC104911299	0.0	0.0	0.0	0.0	0.0	0.0	0.0	0.00
LOC104911300	0.0	0.0	0.0	0.0	0.0	0.0	0.0	0.00
LOC104911303	0.0	0.0	0.0	0.0	0.0	0.0	0.0	0.00
LOC104911304	0.0	0.0	0.0	0.0	0.0	0.0	0.0	0.00
LOC104911305	133.0	111.5	135.5	110.0	118.0	132.5	123.4	11.59
LOC104911306	87.0	61.0	43.0	81.5	62.5	47.5	63.8	17.66
LOC104911307	0.0	0.0	0.0	0.0	0.0	0.0	0.0	0.00
LOC104911308	178.0	195.0	215.0	156.0	220.5	230.5	199.2	28.34
LOC104911310	586.0	190.0	285.5	480.5	163.5	257.0	327.1	168.98
LOC104911311	0.0	0.0	0.0	0.0	0.0	0.0	0.0	0.00
LOC104911312	0.0	0.0	0.0	0.0	0.0	0.0	0.0	0.00
LOC104911313	3.5	2.0	2.0	3.5	5.5	4.0	3.4	1.32
LOC104911316	3.0	1.0	0.0	2.5	1.0	0.0	1.3	1.25
LOC104911318	15.5	4.5	59.5	18.5	3.0	29.0	21.7	20.86
LOC104911319	0.0	0.5	0.0	0.0	0.5	0.0	0.2	0.26
LOC104911320	0.0	0.0	0.0	0.0	0.0	0.0	0.0	0.00
LOC104911321	20.5	32.5	40.5	16.5	36.0	61.0	34.5	15.90
LOC104911322	0.0	0.0	0.0	0.0	0.5	0.0	0.1	0.20
LOC104911323	0.0	0.0	0.0	0.0	0.0	0.0	0.0	0.00
LOC104911324	0.0	1.5	0.5	0.0	0.5	1.5	0.7	0.68
LOC104911326	1.0	3.5	0.5	0.5	2.0	1.0	1.4	1.16
LOC104911327	448.5	428.5	238.0	478.0	533.0	312.5	406.4	110.08
LOC104911328	0.0	0.0	0.0	0.0	0.0	0.0	0.0	0.00
LOC104911329	2.5	0.0	1.5	0.5	1.0	0.0	0.9	0.97
LOC104911330	5.5	1.5	2.0	1.5	2.0	2.5	2.5	1.52
LOC104911331	22.5	54.5	36.0	34.5	72.5	25.5	40.9	19.10
LOC104911332	0.0	0.0	0.0	0.0	0.5	0.0	0.1	0.20
LOC104911333	0.5	0.0	0.0	0.0	0.0	1.0	0.3	0.42
LOC104911334	20.0	4.5	6.5	21.0	7.5	3.5	10.5	7.88

LOC104911335	0.0	0.0	0.0	0.0	0.0	0.0	0.0	0.00
LOC104911336	0.5	0.0	0.0	0.5	0.0	0.0	0.2	0.26
LOC104911338	6.0	2.0	14.5	6.5	2.0	21.0	8.7	7.57
LOC104911339	63.0	87.0	145.5	83.5	114.0	118.5	101.9	29.65
LOC104911340	69.0	41.5	32.5	39.5	42.0	25.0	41.6	14.92
LOC104911341	2.0	2.0	7.0	3.5	2.0	6.0	3.8	2.23
LOC104911342	0.5	0.0	0.0	1.5	0.0	0.0	0.3	0.61
LOC104911344	14.0	9.5	8.0	13.0	5.5	5.5	9.3	3.64
LOC104911346	0.0	0.0	0.0	0.0	0.0	0.0	0.0	0.00
LOC104911347	0.0	0.0	0.0	0.0	0.0	0.0	0.0	0.00
LOC104911348	808.5	666.0	736.0	822.5	788.5	676.5	749.7	67.55
LOC104911349	0.0	0.0	0.0	0.0	0.0	0.0	0.0	0.00
LOC104911350	0.0	0.5	0.0	0.0	0.0	1.0	0.3	0.42
LOC104911351	42.0	14.0	20.5	45.0	8.0	17.0	24.4	15.37
LOC104911352	12.5	10.0	19.0	9.5	9.5	6.0	11.1	4.40
LOC104911353	0.0	0.0	0.0	0.0	0.5	0.5	0.2	0.26
LOC104911354	11.0	9.5	4.5	7.5	4.5	7.5	7.4	2.62
LOC104911355	144.0	89.5	68.0	119.0	110.5	75.0	101.0	28.85
LOC104911356	36.0	15.0	32.0	33.5	18.0	24.5	26.5	8.69
LOC104911357	2.0	4.5	5.0	6.0	8.0	3.0	4.8	2.14
LOC104911358	280.5	121.0	180.0	291.0	132.5	109.5	185.8	81.15
LOC104911359	0.0	0.0	0.0	0.0	0.0	0.0	0.0	0.00
LOC104911360	0.0	0.0	0.5	0.0	0.0	0.0	0.1	0.20
LOC104911361	0.0	0.0	0.0	0.0	0.0	0.0	0.0	0.00
LOC104911362	4.5	0.5	2.0	1.0	1.5	2.5	2.0	1.41
LOC104911363	343.5	359.5	355.0	335.0	420.5	335.0	358.1	32.20
LOC104911364	0.5	0.0	0.0	0.0	0.5	0.0	0.2	0.26
LOC104911365	1.0	2.5	2.0	5.0	1.0	1.5	2.2	1.51
LOC104911366	253.0	336.5	352.5	236.0	374.0	331.0	313.8	56.00
LOC104911367	1653.0	2730.0	1912.5	1278.5	3180.0	1868.5	2103.8	710.86
LOC104911368	0.5	0.5	2.5	0.0	1.0	1.0	0.9	0.86
LOC104911369	0.5	1.0	0.5	0.0	1.0	2.0	0.8	0.68
LOC104911370	24.0	17.5	17.0	26.0	22.0	16.0	20.4	4.15
LOC104911371	63.5	26.0	24.0	54.0	38.0	22.5	38.0	17.24
LOC104911372	49.0	31.5	45.5	34.0	39.5	28.0	37.9	8.22
LOC104911373	542.5	359.0	331.5	506.5	430.5	301.0	411.8	97.89
LOC104911374	32.0	12.5	54.0	37.5	23.0	55.0	35.7	16.88
LOC104911375	603.0	490.5	325.0	571.0	535.5	274.5	466.6	135.48
LOC104911377	2.5	0.5	4.0	2.0	1.0	3.5	2.3	1.37
LOC104911378	3.5	3.0	2.5	3.5	3.5	3.0	3.2	0.41
LOC104911379	2.5	0.5	1.0	3.0	0.0	1.0	1.3	1.17
LOC104911380	3.0	0.5	0.5	2.5	1.0	0.0	1.3	1.21
LOC104911381	4.5	7.0	5.5	8.0	5.5	5.0	5.9	1.32
LOC104911382	1.5	1.5	1.0	1.0	3.0	2.0	1.7	0.75
LOC104911384	3.0	2.0	18.5	8.0	1.5	16.5	8.3	7.55
LOC104911385	6.0	3.5	12.0	4.5	4.5	14.0	7.4	4.44
LOC104911387	0.0	0.0	0.0	0.0	0.0	0.0	0.0	0.00
LOC104911388	1.0	0.5	1.0	1.0	0.0	2.0	0.9	0.66
LOC104911389	2.5	28.0	10.0	4.0	22.5	12.5	13.3	10.15
LOC104911391	0.0	0.5	0.0	0.0	0.0	0.0	0.1	0.20
LOC104911392	39.0	19.0	19.5	37.5	12.0	17.0	24.0	11.36
LOC104911393	0.5	0.0	0.0	0.0	0.0	0.0	0.1	0.20
LOC104911394	286.5	103.5	609.5	333.5	131.0	425.5	314.9	189.04
LOC104911395	36.0	20.5	58.0	46.5	21.0	56.0	39.7	16.61

LOC104911396	20.0	13.0	42.0	25.0	11.0	23.5	22.4	11.10
LOC104911397	100.0	70.5	133.5	107.5	69.0	121.0	100.3	26.27
LOC104911398	1296.0	891.5	1454.0	1247.5	1020.5	1352.0	1210.3	212.53
LOC104911401	23.5	29.5	23.5	25.5	36.5	18.0	26.1	6.31
LOC104911402	1.0	0.0	0.0	0.0	0.0	0.0	0.2	0.41
LOC104911403	70.0	26.0	39.0	60.5	26.0	33.5	42.5	18.54
LOC104911404	16.5	9.0	34.0	23.0	9.0	17.0	18.1	9.45
LOC104911405	1003.5	776.5	594.0	926.5	827.0	554.0	780.3	178.50
LOC104911406	79.0	30.5	87.0	85.5	38.0	77.0	66.2	25.12
LOC104911407	28.5	13.5	28.0	25.0	11.5	17.5	20.7	7.47
LOC104911408	108.5	29.5	38.0	101.0	49.0	30.5	59.4	35.88
LOC104911409	21.5	16.5	15.0	15.5	15.5	11.5	15.9	3.23
LOC104911410	8.5	14.5	9.5	11.0	17.5	16.0	12.8	3.68
LOC104911411	1.0	0.0	1.0	0.5	0.5	0.0	0.5	0.45
LOC104911412	290.0	279.0	266.5	272.5	349.5	230.5	281.3	39.01
LOC104911413	62.0	45.0	91.0	64.5	53.0	72.5	64.7	16.03
LOC104911414	49.5	24.5	71.0	51.5	30.0	51.5	46.3	16.82
LOC104911415	2.5	1.5	1.5	1.5	0.5	1.5	1.5	0.63
LOC104911416	0.0	0.0	0.0	0.0	0.0	0.0	0.0	0.00
LOC104911418	31.5	19.0	30.0	20.5	16.5	22.0	23.3	6.11
LOC104911419	1.0	0.5	0.0	0.0	0.0	0.5	0.3	0.41
LOC104911420	4.5	5.5	18.0	5.5	5.5	8.5	7.9	5.12
LOC104911421	1754.5	1090.0	1327.5	1670.5	1183.0	1179.0	1367.4	279.20
LOC104911422	207.5	95.5	157.5	196.5	108.0	132.0	149.5	46.00
LOC104911423	0.0	0.0	0.0	0.0	0.0	0.5	0.1	0.20
LOC104911424	3.5	1.0	3.0	2.5	1.0	2.0	2.2	1.03
LOC104911425	82.0	125.5	121.0	103.0	167.5	98.5	116.3	29.66
LOC104911426	0.0	0.5	0.5	1.0	1.0	0.5	0.6	0.38
LOC104911427	0.5	0.0	0.0	0.5	0.0	0.5	0.3	0.27
LOC104911428	1.0	2.0	1.5	0.5	3.0	3.0	1.8	1.03
LOC104911429	361.5	218.0	328.5	321.0	212.0	275.0	286.0	61.57
LOC104911430	104.5	94.0	263.5	42.0	39.0	122.5	110.9	82.05
LOC104911431	2.0	1.0	9.0	0.0	0.0	1.5	2.3	3.40
LOC104911432	7.5	1.5	41.0	4.0	1.0	17.0	12.0	15.37
LOC104911433	0.0	0.0	0.0	0.0	0.0	0.0	0.0	0.00
LOC104911434	1.5	0.0	1.0	1.0	0.0	0.0	0.6	0.66
LOC104911435	1.0	2.5	0.5	1.5	0.5	1.5	1.3	0.76
LOC104911436	0.0	2.5	0.5	0.0	2.0	1.0	1.0	1.05
LOC104911437	0.0	0.0	0.0	0.0	0.0	0.5	0.1	0.20
LOC104911438	0.5	0.0	1.5	0.5	0.0	1.5	0.7	0.68
LOC104911439	17.5	5.5	19.5	15.5	3.5	18.5	13.3	7.00
LOC104911440	0.0	0.0	0.0	0.0	0.0	0.0	0.0	0.00
LOC104911441	0.5	0.0	0.0	0.0	0.0	0.0	0.1	0.20
LOC104911442	1.0	0.0	0.0	0.0	0.0	0.0	0.2	0.41
LOC104911443	74.0	21.5	55.0	85.0	23.0	46.0	50.8	26.00
LOC104911444	0.0	0.0	0.0	0.0	0.0	0.0	0.0	0.00
LOC104911445	0.0	0.5	0.0	0.0	0.0	0.0	0.1	0.20
LOC104911446	0.0	0.0	0.0	0.0	0.0	0.0	0.0	0.00
LOC104911447	0.0	0.0	0.0	0.0	0.0	0.0	0.0	0.00
LOC104911448	8.5	0.5	2.5	8.5	0.0	1.0	3.5	3.96
LOC104911449	5.5	0.0	0.0	2.5	1.0	0.0	1.5	2.19
LOC104911450	5.0	0.0	0.5	2.5	0.5	0.0	1.4	1.99
LOC104911451	5.0	4.5	4.0	6.5	2.5	4.0	4.4	1.32
LOC104911452	198.0	122.5	123.0	166.5	154.5	153.5	153.0	28.43

LOC104911453	100.5	60.0	99.0	120.0	87.5	90.0	92.8	19.75
LOC104911454	43.0	25.5	48.0	45.5	22.5	42.0	37.8	10.89
LOC104911455	407.0	406.0	214.0	345.0	420.0	225.0	336.2	94.11
LOC104911456	16.0	5.0	5.5	9.5	6.5	7.0	8.3	4.11
LOC104911457	0.0	0.5	0.0	0.5	0.0	2.0	0.5	0.77
LOC104911458	54.5	23.0	58.0	34.0	18.5	48.0	39.3	16.63
LOC104911459	19.5	6.5	50.0	17.5	5.5	38.5	22.9	17.83
LOC104911460	219.5	182.5	210.5	257.0	172.0	203.0	207.4	30.03
LOC104911462	11.0	1.0	5.0	4.5	2.5	3.0	4.5	3.49
LOC104911463	13.5	8.5	6.5	21.0	9.5	7.5	11.1	5.43
LOC104911464	2933.5	1757.5	9111.5	3549.0	1985.5	11178.5	5085.9	4025.19
LOC104911466	9.0	33.5	13.5	8.0	51.5	10.5	21.0	17.70
LOC104911468	17.0	13.5	33.0	14.5	14.0	23.0	19.2	7.63
LOC104911469	1.0	1.5	0.0	2.5	0.5	2.0	1.3	0.94
LOC104911470	101.0	55.5	56.5	108.5	74.5	51.0	74.5	24.88
LOC104911471	1.5	0.0	0.5	0.5	0.0	0.5	0.5	0.55
LOC104911472	4.0	1.5	2.0	1.0	0.5	0.5	1.6	1.32
LOC104911473	0.5	0.5	0.0	0.5	0.5	0.5	0.4	0.20
LOC104911474	0.0	0.0	0.0	0.0	1.0	0.0	0.2	0.41
LOC104911475	0.0	0.0	0.0	0.5	0.0	0.5	0.2	0.26
LOC104911476	1.0	2.5	0.5	0.5	0.0	0.0	0.8	0.94
LOC104911477	1.0	0.0	0.0	1.0	0.0	0.0	0.3	0.52
LOC104911478	3.0	0.5	1.0	1.0	1.0	2.0	1.4	0.92
LOC104911479	0.0	0.0	0.0	0.0	0.0	0.0	0.0	0.00
LOC104911480	0.0	0.0	0.0	0.0	0.0	0.0	0.0	0.00
LOC104911481	0.0	0.0	0.0	0.0	0.0	0.0	0.0	0.00
LOC104911482	0.0	0.0	0.0	0.0	0.0	0.0	0.0	0.00
LOC104911483	2.5	1.5	0.5	1.5	4.5	3.0	2.3	1.41
LOC104911484	1437.5	2270.5	1096.0	1422.0	2466.5	1100.0	1632.1	592.68
LOC104911485	138.5	146.0	85.5	169.0	196.5	116.5	142.0	38.91
LOC104911486	158.0	57.5	95.5	147.5	84.5	98.5	106.9	38.48
LOC104911487	0.0	0.0	0.0	0.0	0.5	0.0	0.1	0.20
LOC104911488	4.5	4.5	3.5	8.5	1.5	2.5	4.2	2.42
LOC104911490	1.0	0.5	0.0	2.5	0.0	1.0	0.8	0.93
LOC104911491	0.0	0.0	0.0	0.0	1.0	0.0	0.2	0.41
LOC104911494	0.5	0.0	0.0	0.5	1.0	0.0	0.3	0.41
LOC104911495	0.0	0.0	0.0	0.0	0.0	0.0	0.0	0.00
LOC104911497	14.0	2.0	5.0	12.5	5.0	7.5	7.7	4.69
LOC104911498	83.5	35.5	46.0	78.5	49.0	38.0	55.1	20.74
LOC104911499	3.0	3.5	2.5	1.5	2.0	1.5	2.3	0.82
LOC104911500	0.0	1.0	1.5	0.5	1.0	3.0	1.2	1.03
LOC104911501	9.5	1.0	17.0	10.5	5.0	8.5	8.6	5.40
LOC104911502	16.0	5.0	23.0	11.0	4.0	15.5	12.4	7.24
LOC104911503	778.5	603.0	625.0	726.5	746.5	552.5	672.0	90.68
LOC104911504	21.0	13.0	44.0	24.5	19.5	37.5	26.6	11.77
LOC104911505	5.5	19.0	5.0	10.5	18.0	5.5	10.6	6.46
LOC104911506	0.0	1.0	1.0	0.0	2.0	3.0	1.2	1.17
LOC104911507	988.0	588.0	936.5	941.5	623.5	709.0	797.8	177.96
LOC104911508	12.5	4.0	8.0	8.5	2.5	6.0	6.9	3.57
LOC104911509	1.5	0.0	0.5	1.0	0.0	0.0	0.5	0.63
LOC104911510	0.0	1.0	0.0	0.5	0.5	0.0	0.3	0.41
LOC104911511	0.0	0.0	0.0	0.0	0.0	0.0	0.0	0.00
LOC104911512	0.0	0.0	0.0	0.5	0.0	0.0	0.1	0.20
LOC104911513	21.5	14.0	15.5	21.5	14.0	13.5	16.7	3.80

LOC104911514	3.0	0.5	0.0	1.5	0.5	0.5	1.0	1.10
LOC104911515	112.5	59.5	63.0	111.5	64.0	54.5	77.5	26.93
LOC104911516	0.0	0.5	0.5	0.0	1.0	1.0	0.5	0.45
LOC104911517	0.0	0.0	0.0	0.5	0.0	0.0	0.1	0.20
LOC104911518	0.0	0.0	0.0	0.0	0.0	0.0	0.0	0.00
LOC104911519	21.0	38.5	53.5	17.0	51.5	45.0	37.8	15.50
LOC104911520	9.5	5.5	39.0	11.5	7.5	26.0	16.5	13.21
LOC104911521	24.5	6.0	41.5	28.0	11.5	38.0	24.9	14.10
LOC104911522	3.0	1.0	1.0	0.0	1.0	3.0	1.5	1.22
LOC104911525	0.0	0.0	0.0	0.0	0.0	0.0	0.0	0.00
LOC104911526	0.0	0.0	0.0	0.0	0.0	0.0	0.0	0.00
LOC104911527	0.0	0.0	0.0	0.0	0.0	0.0	0.0	0.00
LOC104911528	0.0	0.0	0.0	0.0	0.0	0.0	0.0	0.00
LOC104911529	0.0	1.0	2.0	0.0	0.5	0.0	0.6	0.80
LOC104911530	1.0	0.5	1.5	0.5	0.0	0.0	0.6	0.58
LOC104911532	0.0	0.5	0.5	1.0	0.0	0.5	0.4	0.38
LOC104911533	0.0	0.0	0.0	0.0	0.0	0.0	0.0	0.00
LOC104911534	0.0	0.0	0.0	0.0	0.0	0.0	0.0	0.00
LOC104911535	0.0	0.0	0.0	0.0	0.0	0.0	0.0	0.00
LOC104911536	2.5	0.5	2.5	2.5	0.0	0.5	1.4	1.20
LOC104911537	66.0	51.0	20.5	42.5	39.5	23.5	40.5	17.06
LOC104911538	73.0	53.0	28.0	87.0	80.0	41.0	60.3	23.37
LOC104911540	0.0	0.0	0.0	0.0	0.0	0.0	0.0	0.00
LOC104911541	0.5	0.0	0.5	1.0	0.0	0.5	0.4	0.38
LOC104911542	0.0	1.0	0.0	0.0	0.0	1.0	0.3	0.52
LOC104911543	0.5	0.0	1.0	0.0	0.5	0.5	0.4	0.38
LOC104911545	0.0	0.0	0.0	0.0	0.0	0.0	0.0	0.00
LOC104911546	4.0	3.0	5.0	6.5	3.0	3.0	4.1	1.43
LOC104911547	54.0	14.5	3.0	41.5	17.0	9.0	23.2	20.04
LOC104911548	77.0	60.0	71.5	88.5	73.0	66.0	72.7	9.76
LOC104911549	0.5	0.0	0.0	0.0	0.5	0.0	0.2	0.26
LOC104911554	4.0	0.5	0.0	0.0	1.0	0.5	1.0	1.52
LOC104911555	0.0	0.0	0.0	0.0	0.0	0.0	0.0	0.00
LOC104911556	0.0	0.0	0.0	0.0	0.0	0.0	0.0	0.00
LOC104911557	0.0	0.0	0.0	0.0	0.0	0.0	0.0	0.00
LOC104911558	1.0	2.0	4.5	0.5	5.5	7.5	3.5	2.77
LOC104911559	3.5	6.5	1.5	3.0	2.5	2.0	3.2	1.78
LOC104911560	2.5	5.0	3.0	6.5	6.5	4.5	4.7	1.69
LOC104911561	20.5	12.5	4.0	17.5	13.0	6.5	12.3	6.28
LOC104911562	1.0	0.5	0.0	0.0	1.5	1.0	0.7	0.61
LOC104911564	0.0	0.0	0.0	0.5	0.0	0.0	0.1	0.20
LOC104911565	0.0	0.0	0.0	0.0	0.0	0.0	0.0	0.00
LOC104911566	0.5	0.0	0.5	0.0	0.0	0.5	0.3	0.27
LOC104911567	0.0	0.0	0.0	0.0	0.0	0.0	0.0	0.00
LOC104911568	0.0	0.0	0.0	0.0	0.0	0.5	0.1	0.20
LOC104911569	18.0	7.0	9.5	12.5	3.5	8.0	9.8	5.01
LOC104911570	0.5	0.0	0.5	0.0	0.0	0.0	0.2	0.26
LOC104911571	4.0	3.5	1.5	3.0	4.5	2.0	3.1	1.16
LOC104911572	0.0	0.0	0.0	0.0	0.0	0.0	0.0	0.00
LOC104911573	0.0	0.0	0.0	0.0	0.0	0.0	0.0	0.00
LOC104911574	0.0	0.5	0.0	0.0	0.0	0.0	0.1	0.20
LOC104911575	0.0	0.0	0.0	0.0	0.0	0.0	0.0	0.00
LOC104911576	0.0	0.0	0.0	0.0	0.0	0.0	0.0	0.00
LOC104911578	0.0	0.5	1.0	0.0	0.5	1.5	0.6	0.58

LOC104911579	6.5	4.5	4.0	4.5	7.0	4.5	5.2	1.25
LOC104911580	4.0	4.5	6.0	9.0	2.0	9.0	5.8	2.82
LOC104911581	177.0	133.0	113.0	164.0	134.0	93.5	135.8	31.03
LOC104911584	662.0	529.5	542.0	489.0	554.0	435.0	535.3	75.74
LOC104911585	0.0	0.0	0.0	0.0	0.0	3.5	0.6	1.43
LOC104911586	17.0	6.0	3.5	25.5	15.0	5.5	12.1	8.56
LOC104911589	7.5	7.5	2.5	17.0	12.0	5.5	8.7	5.13
LOC104911590	166.5	52.0	15.5	181.0	74.0	17.5	84.4	72.74
LOC104911591	12.0	5.5	1.0	9.0	5.0	1.5	5.7	4.26
LOC104911592	3.0	1.5	1.5	3.5	3.0	1.5	2.3	0.93
LOC104911593	1.0	0.0	0.5	2.5	2.0	0.5	1.1	0.97
LOC104911594	0.5	0.5	1.0	0.5	0.5	0.0	0.5	0.32
LOC104911595	0.0	0.0	0.0	0.0	0.0	0.5	0.1	0.20
LOC104911600	32.5	14.0	47.5	47.5	20.0	46.0	34.6	14.86
LOC104911601	4.5	3.0	12.0	4.0	2.0	15.5	6.8	5.54
LOC104911602	12.5	3.0	17.0	18.0	3.5	9.5	10.6	6.46
LOC104911603	0.5	0.0	0.0	1.0	0.0	0.0	0.3	0.42
LOC104911604	0.0	0.0	0.5	0.5	0.0	0.0	0.2	0.26
LOC104911605	4.0	0.0	1.0	2.5	0.0	0.0	1.3	1.67
LOC104911606	2.0	4.0	0.5	1.5	2.5	2.5	2.2	1.17
LOC104911607	0.0	0.0	0.0	0.0	0.0	0.0	0.0	0.00
LOC104911609	0.0	0.0	0.0	0.0	0.0	0.0	0.0	0.00
LOC104911610	70.5	38.5	34.0	83.5	51.0	36.0	52.3	20.48
LOC104911611	0.0	8.0	9.5	2.5	12.5	10.0	7.1	4.81
LOC104911613	4.5	3.0	0.5	6.0	2.0	1.0	2.8	2.11
LOC104911615	4.5	39.0	46.0	8.0	37.5	50.5	30.9	19.71
LOC104911616	1.0	0.5	0.0	0.0	0.5	0.0	0.3	0.41
LOC104911617	0.0	0.0	0.0	0.0	0.0	0.0	0.0	0.00
LOC104911619	0.0	0.0	0.0	0.5	0.5	1.5	0.4	0.58
LOC104911620	0.0	0.0	0.0	0.0	0.0	0.5	0.1	0.20
LOC104911621	0.0	0.0	0.5	0.0	0.0	0.0	0.1	0.20
LOC104911622	0.0	0.0	0.0	0.0	0.0	0.0	0.0	0.00
LOC104911623	0.0	0.0	0.0	0.0	0.0	0.0	0.0	0.00
LOC104911624	0.0	0.0	0.0	0.0	0.0	0.0	0.0	0.00
LOC104911625	1.0	0.5	0.5	1.0	1.0	1.0	0.8	0.26
LOC104911626	4.0	2.5	4.5	5.5	1.0	3.5	3.5	1.58
LOC104911627	0.5	0.5	0.5	0.5	2.0	0.5	0.8	0.61
LOC104911628	1.5	0.0	0.0	1.5	1.5	0.5	0.8	0.75
LOC104911629	2.0	1.0	4.5	3.0	2.0	3.0	2.6	1.20
LOC104911630	30.0	17.5	37.0	36.5	18.5	31.5	28.5	8.58
LOC104911632	202.5	165.5	123.0	204.0	186.5	114.0	165.9	39.37
LOC104911633	3.0	4.0	2.5	4.0	2.5	2.0	3.0	0.84
LOC104911635	9.0	3.5	4.5	6.0	6.0	2.0	5.2	2.42
LOC104911636	48.0	29.5	70.0	51.0	26.5	75.5	50.1	20.14
LOC104911637	17.0	8.0	13.0	14.0	7.0	14.0	12.2	3.87
LOC104911638	16.0	6.0	25.5	15.5	9.5	20.5	15.5	7.09
LOC104911639	1.5	0.5	3.0	0.5	0.5	2.0	1.3	1.03
LOC104911640	1.0	0.5	1.0	0.5	0.5	0.0	0.6	0.38
LOC104911641	3.0	1.0	3.5	3.5	1.5	1.5	2.3	1.13
LOC104911642	0.0	0.0	0.0	0.0	0.5	0.5	0.2	0.26
LOC104911644	15.5	4.0	5.5	15.0	10.5	9.5	10.0	4.73
LOC104911645	60.5	24.0	9.0	57.0	17.5	16.0	30.7	22.30
LOC104911646	2.5	3.0	1.5	1.0	1.0	1.5	1.8	0.82
LOC104911647	510.0	588.0	486.0	488.5	739.5	584.5	566.1	96.41

LOC104911649	180.0	181.0	101.0	158.5	192.5	94.5	151.3	42.92
LOC104911650	55.0	11.0	109.5	57.5	12.0	66.0	51.8	36.94
LOC104911651	212.0	117.5	156.0	230.5	131.5	134.5	163.7	46.64
LOC104911652	1.5	1.5	0.0	3.5	1.0	0.5	1.3	1.21
LOC104911655	2.5	3.5	1.5	1.5	0.0	1.5	1.8	1.17
LOC104911656	1.5	0.0	0.0	0.5	0.0	0.0	0.3	0.61
LOC104911657	467.0	333.0	300.5	441.0	412.0	288.0	373.6	76.24
LOC104911658	6.0	4.5	2.5	5.0	5.5	1.0	4.1	1.93
LOC104911659	1.0	1.0	0.0	0.0	0.5	0.5	0.5	0.45
LOC104911660	0.0	0.0	0.0	0.0	0.0	0.0	0.0	0.00
LOC104911661	0.0	2.5	5.0	1.0	9.0	3.0	3.4	3.23
LOC104911662	8.0	7.0	3.0	8.5	5.5	0.5	5.4	3.12
LOC104911663	0.0	0.0	0.5	0.0	0.0	0.5	0.2	0.26
LOC104911664	0.0	0.0	0.5	0.5	0.0	0.0	0.2	0.26
LOC104911665	236.0	132.5	123.0	206.0	190.0	127.5	169.2	47.89
LOC104911666	0.0	0.0	0.0	0.0	0.0	0.0	0.0	0.00
LOC104911667	1627.5	1950.0	2106.0	1564.0	2309.5	1987.0	1924.0	284.22
LOC104911668	0.0	0.0	0.0	0.0	0.0	0.0	0.0	0.00
LOC104911670	0.0	0.0	0.0	0.0	0.5	0.0	0.1	0.20
LOC104911671	525.5	429.0	1280.0	581.5	579.5	1261.5	776.2	387.12
LOC104911672	0.0	0.0	0.0	0.0	0.0	0.0	0.0	0.00
LOC104911673	0.0	0.0	0.0	0.0	0.0	0.0	0.0	0.00
LOC104911675	0.0	0.0	0.0	0.0	0.0	0.0	0.0	0.00
LOC104911676	3.5	1.5	0.5	1.0	0.5	0.0	1.2	1.25
LOC104911677	1.5	6.0	4.0	1.0	1.0	5.0	3.1	2.20
LOC104911679	84.0	108.5	85.0	95.5	102.5	101.0	96.1	9.89
LOC104911681	234.0	248.5	182.0	197.0	237.0	163.5	210.3	34.36
LOC104911682	0.0	0.0	0.0	0.0	0.5	0.0	0.1	0.20
LOC104911683	35.5	30.0	34.0	23.0	33.5	31.0	31.2	4.48
LOC104911685	6.0	2.0	0.5	3.0	1.5	1.0	2.3	1.99
LOC104911686	0.0	0.0	0.0	0.0	0.0	0.0	0.0	0.00
LOC104911687	269.5	257.5	100.0	211.0	263.5	149.5	208.5	69.87
LOC104911688	201.5	207.0	154.5	156.5	185.0	131.5	172.7	29.83
LOC104911689	0.0	0.0	0.0	0.0	0.0	0.0	0.0	0.00
LOC104911690	561.5	998.5	790.0	451.5	933.0	866.5	766.8	216.03
LOC104911691	0.0	14.5	10.5	0.5	12.5	8.0	7.7	6.14
LOC104911692	0.5	4.5	0.0	0.0	5.0	1.5	1.9	2.27
LOC104911693	13.0	7.0	43.5	14.5	14.0	42.0	22.3	16.05
LOC104911694	52.5	22.5	84.0	70.0	23.0	69.5	53.6	25.89
LOC104911695	101.0	60.5	133.5	117.0	73.5	121.0	101.1	28.67
LOC104911696	471.5	317.0	321.0	536.5	393.5	345.0	397.4	89.33
LOC104911697	68.5	10.5	98.0	99.0	13.0	72.0	60.2	39.60
LOC104911698	0.0	0.5	0.0	1.0	0.5	0.0	0.3	0.41
LOC104911699	259.0	310.5	311.5	222.0	367.0	334.0	300.7	52.25
LOC104911700	19.0	3.0	27.0	14.0	4.0	16.0	13.8	9.15
LOC104911701	23.0	5.5	42.5	42.5	4.5	36.5	25.8	17.58
LOC104911702	11.0	15.0	5.5	12.5	16.5	12.5	12.2	3.82
LOC104911703	68.5	60.5	61.5	75.0	59.0	41.0	60.9	11.46
LOC104911704	11.0	3.5	17.5	20.5	5.0	10.0	11.3	6.71
LOC104911705	0.5	0.0	0.0	0.5	0.0	0.0	0.2	0.26
LOC104911706	0.0	0.0	0.5	0.0	0.0	0.0	0.1	0.20
LOC104911707	105.0	54.5	194.5	98.5	59.0	110.5	103.7	50.47
LOC104911708	0.0	0.0	0.0	0.0	0.0	0.0	0.0	0.00
LOC104911709	0.0	0.5	0.0	0.0	0.0	0.0	0.1	0.20

LOC104911710	1.5	0.5	1.0	0.0	0.5	0.0	0.6	0.58
LOC104911711	3.5	1.0	1.0	0.5	2.0	0.5	1.4	1.16
LOC104911712	0.5	0.0	0.0	0.0	0.0	0.0	0.1	0.20
LOC104911713	4.5	4.5	4.5	2.0	4.5	2.0	3.7	1.29
LOC104911714	0.0	0.0	0.0	0.0	0.0	0.0	0.0	0.00
LOC104911715	50.0	92.5	14.0	54.0	126.5	56.5	65.6	38.87
LOC104911716	0.5	1.0	1.5	2.5	2.0	1.0	1.4	0.74
LOC104911718	0.0	0.0	0.0	0.0	0.0	0.0	0.0	0.00
LOC104911719	0.0	0.0	0.0	0.0	0.0	0.0	0.0	0.00
LOC104911720	19.0	4.0	33.0	11.0	3.5	23.5	15.7	11.65
LOC104911721	83.0	57.5	128.5	86.0	68.5	107.5	88.5	25.92
LOC104911722	0.0	0.0	0.0	0.5	0.5	0.0	0.2	0.26
LOC104911723	487.5	648.5	662.0	430.0	796.5	639.0	610.6	132.07
LOC104911724	1.0	0.0	0.5	0.0	0.0	0.0	0.3	0.42
LOC104911725	0.5	0.0	0.5	1.0	0.0	1.0	0.5	0.45
LOC104911726	0.0	0.0	0.0	0.0	0.0	0.0	0.0	0.00
LOC104911727	0.5	0.5	1.5	2.0	1.0	0.5	1.0	0.63
LOC104911728	5.5	2.5	1.5	8.0	3.0	1.0	3.6	2.67
LOC104911729	582.0	657.5	527.5	534.0	711.0	549.5	593.6	74.65
LOC104911730	92.5	22.5	132.0	94.5	40.0	81.5	77.2	39.83
LOC104911731	525.5	289.0	204.0	461.5	317.5	179.0	329.4	138.58
LOC104911732	287.0	192.5	98.0	279.0	213.0	108.0	196.3	81.02
LOC104911733	0.5	0.0	0.0	0.0	1.0	0.5	0.3	0.41
LOC104911734	0.0	0.0	0.0	0.0	1.0	0.5	0.3	0.42
LOC104911735	0.0	0.0	0.0	0.0	0.0	0.0	0.0	0.00
LOC104911737	46.5	25.0	58.5	60.0	26.5	60.0	46.1	16.55
LOC104911738	3.5	3.0	2.5	3.5	2.5	5.0	3.3	0.93
LOC104911739	0.0	0.0	0.0	0.0	0.0	0.0	0.0	0.00
LOC104911740	5.5	4.5	14.0	6.0	7.0	15.0	8.7	4.60
LOC104911741	0.0	0.0	1.0	0.0	0.0	0.0	0.2	0.41
LOC104911742	0.0	0.0	0.0	0.0	0.0	0.0	0.0	0.00
LOC104911743	0.5	1.0	0.5	0.0	1.0	0.5	0.6	0.38
LOC104911744	1.0	0.5	1.0	0.0	0.0	0.0	0.4	0.49
LOC104911745	0.5	1.0	0.5	0.0	0.0	0.5	0.4	0.38
LOC104911746	243.5	170.5	253.0	254.0	186.0	261.0	228.0	39.24
LOC104911747	13.5	10.5	1.5	10.5	17.5	4.5	9.7	5.85
LOC104911748	525.5	936.5	436.0	534.5	992.5	465.0	648.3	248.29
LOC104911749	242.0	266.5	150.5	231.0	257.5	164.0	218.6	49.25
LOC104911750	268.5	159.5	220.5	261.0	166.5	193.0	211.5	46.59
LOC104911751	97.0	15.5	59.5	83.5	17.0	53.0	54.3	33.47
LOC104911752	2456.5	1350.5	1483.5	2131.5	1424.5	1280.5	1687.8	485.49
LOC104911753	0.0	0.0	0.0	0.0	0.0	0.0	0.0	0.00
LOC104911754	3.5	5.0	13.0	10.5	4.5	6.5	7.2	3.76
LOC104911756	0.0	0.0	0.0	0.0	0.0	0.5	0.1	0.20
LOC104911757	0.0	0.5	0.0	0.0	0.0	0.0	0.1	0.20
LOC104911758	0.0	0.0	0.0	0.0	0.0	0.0	0.0	0.00
LOC104911759	16.5	14.5	22.5	16.0	11.5	20.5	16.9	4.01
LOC104911761	762.0	365.0	170.5	779.0	347.0	182.5	434.3	272.63
LOC104911762	17.5	51.0	52.5	13.5	39.0	64.0	39.6	20.31
LOC104911763	0.0	5.0	7.5	0.0	3.5	5.5	3.6	3.06
LOC104911764	506.5	419.5	465.5	570.5	501.5	504.5	494.7	50.07
LOC104911765	273.5	86.0	253.0	372.0	116.0	249.0	224.9	106.32
LOC104911766	1.0	2.0	6.5	2.0	2.5	2.5	2.8	1.92
LOC104911767	899.0	992.5	474.5	856.0	1061.0	505.5	798.1	249.31

LOC104911768	1.0	1.0	1.0	1.5	0.0	0.5	0.8	0.52
LOC104911769	0.0	0.0	0.0	0.0	0.0	0.0	0.0	0.00
LOC104911770	297.0	211.5	498.0	399.5	321.0	402.0	354.8	99.75
LOC104911771	0.0	0.5	0.0	0.0	0.0	0.0	0.1	0.20
LOC104911772	0.5	0.0	0.0	0.5	0.0	0.0	0.2	0.26
LOC104911774	2.5	3.0	3.0	4.0	4.5	1.5	3.1	1.07
LOC104911775	1.0	1.0	0.0	2.5	2.5	0.0	1.2	1.13
LOC104911776	106.5	54.0	136.5	95.0	62.5	121.5	96.0	32.52
LOC104911777	0.0	0.0	0.5	0.0	0.0	0.0	0.1	0.20
LOC104911778	49.5	38.0	47.0	40.0	46.5	42.0	43.8	4.50
LOC104911780	428.0	396.5	318.5	382.0	443.5	306.0	379.1	56.33
LOC104911781	26.0	9.0	34.0	19.5	13.0	29.0	21.8	9.64
LOC104911785	14.0	14.0	4.0	20.0	18.5	7.0	12.9	6.30
LOC104911786	0.0	0.5	0.0	0.5	0.0	0.0	0.2	0.26
LOC104911787	260.0	366.5	300.5	295.0	468.0	445.0	355.8	85.55
LOC104911788	0.0	0.0	0.0	0.0	0.0	0.0	0.0	0.00
LOC104911789	19.5	13.5	23.0	21.5	20.5	18.5	19.4	3.29
LOC104911790	1.5	1.5	0.5	0.5	0.5	1.0	0.9	0.49
LOC104911791	0.0	0.0	0.5	0.5	0.5	1.5	0.5	0.55
LOC104911793	1.0	0.5	0.5	0.5	0.0	0.0	0.4	0.38
LOC104911794	1.0	0.0	0.5	0.5	0.0	1.5	0.6	0.58
LOC104911795	34.0	25.5	27.5	29.5	26.5	26.5	28.3	3.13
LOC104911796	163.0	86.0	123.0	147.0	85.0	113.0	119.5	31.67
LOC104911797	237.5	439.5	204.0	196.5	485.0	188.0	291.8	133.91
LOC104911798	11.0	14.0	16.5	12.0	14.5	14.0	13.7	1.94
LOC104911799	459.0	751.5	1272.0	425.5	847.5	853.5	768.2	310.07
LOC104911800	0.0	0.0	0.0	0.0	0.0	0.0	0.0	0.00
LOC104911801	35.0	13.0	137.5	41.0	13.5	98.5	56.4	50.53
LOC104911803	2.5	0.0	7.5	1.0	3.0	7.5	3.6	3.22
LOC104911804	440.0	126.5	283.0	520.5	159.0	259.0	298.0	155.02
LOC104911805	4.0	1.0	6.0	2.5	0.0	4.5	3.0	2.26
LOC104911806	16.0	2.5	26.0	11.0	5.5	19.5	13.4	8.83
LOC104911807	48.0	86.0	72.0	55.0	101.0	64.5	71.1	19.76
LOC104911808	15.0	13.5	30.0	18.5	17.5	31.5	21.0	7.77
LOC104911809	0.5	0.0	0.0	0.5	0.5	0.0	0.3	0.27
LOC104911810	3.0	0.0	0.5	0.0	1.0	1.0	0.9	1.11
LOC104911811	0.5	2.5	0.0	0.0	0.5	0.0	0.6	0.97
LOC104911812	66.0	21.0	100.5	64.5	29.5	94.5	62.7	32.54
LOC104911813	20.5	9.0	14.5	22.5	7.0	10.5	14.0	6.34
LOC104911815	0.0	0.0	0.0	0.0	0.0	0.0	0.0	0.00
LOC104911816	125.5	33.5	96.5	129.0	28.5	83.5	82.8	43.65
LOC104911817	2.0	0.0	0.0	0.5	2.5	0.0	0.8	1.13
LOC104911818	2.5	1.5	2.0	2.0	2.0	3.5	2.3	0.69
LOC104911819	3.0	1.0	7.5	4.5	3.0	8.0	4.5	2.76
LOC104911820	0.5	0.5	0.0	0.0	0.0	0.5	0.3	0.27
LOC104911821	1021.0	2770.0	1315.5	689.0	3050.0	1303.0	1691.4	975.25
LOC104911823	8.5	9.5	11.0	5.0	14.0	9.0	9.5	2.97
LOC104911824	1.5	1.0	1.0	2.0	2.5	1.5	1.6	0.58
LOC104911825	0.0	0.0	0.0	0.0	0.0	0.0	0.0	0.00
LOC104911826	45.0	15.0	19.0	37.5	16.0	19.5	25.3	12.67
LOC104911827	0.0	0.0	0.0	0.0	0.0	0.0	0.0	0.00
LOC104911828	0.0	0.0	0.0	0.5	0.0	0.5	0.2	0.26
LOC104911829	1.5	5.5	2.0	3.5	2.5	1.0	2.7	1.63
LOC104911830	3.0	4.0	1.5	3.5	5.0	1.0	3.0	1.52

LOC104911831	0.0	1.0	0.0	0.5	0.0	0.0	0.3	0.42
LOC104911832	10.5	4.0	3.5	9.5	4.5	5.5	6.3	3.00
LOC104911833	0.0	0.0	0.0	0.0	0.0	0.0	0.0	0.00
LOC104911834	0.0	0.0	0.5	0.5	0.5	0.0	0.3	0.27
LOC104911835	864.0	543.0	1043.5	900.0	614.0	842.0	801.1	187.50
LOC104911836	0.0	0.0	0.0	0.0	0.0	0.0	0.0	0.00
LOC104911837	125.5	61.0	100.0	87.5	58.0	77.5	84.9	25.43
LOC104911838	112.0	79.0	102.0	83.0	86.5	197.0	109.9	44.45
LOC104911839	0.5	0.5	22.5	0.0	0.5	24.5	8.1	11.96
LOC104911840	0.0	0.0	0.5	0.0	0.0	0.5	0.2	0.26
LOC104911841	0.5	0.0	0.0	0.5	0.0	0.0	0.2	0.26
LOC104911842	117.0	105.0	53.5	103.0	124.5	47.0	91.7	33.10
LOC104911843	74.0	49.0	81.5	77.5	49.5	65.5	66.2	14.13
LOC104911844	13.0	5.5	6.5	13.5	11.5	3.5	8.9	4.27
LOC104911845	55.5	14.5	33.0	54.0	13.0	22.0	32.0	19.00
LOC104911846	22.5	9.5	14.5	26.0	11.5	10.0	15.7	6.96
LOC104911848	8.5	1.5	3.5	6.5	1.5	2.5	4.0	2.88
LOC104911849	0.5	0.0	0.0	1.0	0.0	0.5	0.3	0.41
LOC104911850	38.5	51.0	41.0	38.0	67.0	36.5	45.3	11.82
LOC104911851	2.5	2.0	11.5	2.0	1.5	17.5	6.2	6.74
LOC104911852	0.0	0.0	0.5	0.0	0.5	0.0	0.2	0.26
LOC104911853	0.5	2.5	0.0	1.0	1.0	1.0	1.0	0.84
LOC104911854	0.5	2.0	1.0	2.0	0.5	1.0	1.2	0.68
LOC104911855	0.0	0.0	0.0	0.0	0.0	0.5	0.1	0.20
LOC104911857	0.0	0.0	1.5	0.5	0.0	1.0	0.5	0.63
LOC104911858	0.0	0.0	0.5	0.5	0.5	0.0	0.3	0.27
LOC104911859	51.5	171.0	123.0	64.5	227.5	311.0	158.1	99.72
LOC104911860	0.0	0.0	0.5	1.0	0.0	1.0	0.4	0.49
LOC104911862	49.5	28.0	43.5	50.5	37.0	46.0	42.4	8.56
LOC104911863	0.0	0.0	0.0	0.0	0.0	0.0	0.0	0.00
LOC104911864	239.0	210.5	275.5	189.5	264.0	255.0	238.9	33.09
LOC104911865	1.0	3.0	4.0	0.0	7.0	6.5	3.6	2.84
LOC104911866	0.0	0.0	0.0	0.0	0.0	0.0	0.0	0.00
LOC104911867	0.0	0.0	0.5	0.0	0.0	0.0	0.1	0.20
LOC104911868	0.0	0.0	0.0	0.0	0.0	0.0	0.0	0.00
LOC104911870	0.5	0.5	0.0	0.5	0.5	0.0	0.3	0.26
LOC104911871	2.5	10.5	16.0	1.0	8.5	11.5	8.3	5.68
LOC104911872	10.0	0.0	0.0	11.5	0.5	0.0	3.7	5.51
LOC104911873	1.5	0.0	0.0	0.5	0.0	0.0	0.3	0.61
LOC104911874	0.0	0.0	0.0	0.0	0.0	1.0	0.2	0.41
LOC104911875	0.0	0.0	0.0	0.0	0.0	0.0	0.0	0.00
LOC104911876	0.0	0.0	0.0	0.0	0.0	0.0	0.0	0.00
LOC104911877	0.0	0.0	0.0	0.0	0.0	0.0	0.0	0.00
LOC104911878	0.5	1.0	4.0	1.5	4.5	4.5	2.7	1.86
LOC104911879	1.0	0.0	0.0	0.5	0.5	0.0	0.3	0.41
LOC104911880	1.5	1.0	1.0	0.5	1.0	0.0	0.8	0.52
LOC104911881	0.5	0.5	0.0	0.0	0.5	0.0	0.3	0.27
LOC104911882	0.0	0.0	0.0	0.0	0.0	0.0	0.0	0.00
LOC104911885	0.0	1.0	0.0	0.5	0.5	0.0	0.3	0.41
LOC104911886	0.5	0.5	0.0	0.5	0.0	0.5	0.3	0.26
LOC104911887	26.0	10.5	32.0	19.5	12.0	22.0	20.3	8.22
LOC104911891	4.5	0.5	6.5	8.0	1.5	5.0	4.3	2.88
LOC104911892	0.0	0.0	0.0	0.0	0.0	0.0	0.0	0.00
LOC104911893	0.5	0.0	0.0	0.0	0.0	0.0	0.1	0.20

LOC104911894	0.0	1.0	0.0	0.5	0.5	0.5	0.4	0.38
LOC104911895	0.0	0.0	0.0	0.0	0.0	0.0	0.0	0.00
LOC104911896	0.0	0.5	0.0	0.0	0.5	0.0	0.2	0.26
LOC104911897	0.5	0.0	2.0	1.0	1.0	1.5	1.0	0.71
LOC104911898	32.5	6.5	38.0	40.5	7.0	29.0	25.6	15.14
LOC104911899	198.0	143.5	303.0	174.5	178.0	270.5	211.3	61.89
LOC104911900	1.0	1.0	0.5	1.0	0.0	1.0	0.8	0.42
LOC104911901	0.0	0.0	3.0	0.0	1.0	1.0	0.8	1.17
LOC104911902	11.5	14.0	40.5	5.5	16.0	37.0	20.8	14.42
LOC104911903	0.0	0.0	0.0	0.0	0.0	0.0	0.0	0.00
LOC104911904	0.0	0.0	0.5	0.0	0.0	0.5	0.2	0.26
LOC104911905	1.0	0.5	3.5	0.5	0.0	1.5	1.2	1.25
LOC104911906	4.0	9.0	2.0	2.5	8.5	1.0	4.5	3.44
LOC104911907	21.0	21.0	14.5	20.0	20.5	23.5	20.1	2.99
LOC104911908	0.5	0.5	1.5	3.0	1.5	1.5	1.4	0.92
LOC104911909	180.5	71.0	84.0	150.0	72.5	75.0	105.5	47.49
LOC104911910	238.5	138.0	147.0	207.0	162.0	123.5	169.3	44.36
LOC104911911	5.5	3.0	20.5	9.5	2.5	18.5	9.9	7.85
LOC104911912	3.0	0.5	0.5	2.5	2.5	0.5	1.6	1.20
LOC104911914	4.5	2.5	4.0	5.0	5.0	4.0	4.2	0.93
LOC104911915	3.0	7.5	35.5	6.0	8.0	20.5	13.4	12.38
LOC104911916	14.5	15.0	39.5	6.0	14.0	33.0	20.3	12.93
LOC104911917	1.0	0.0	0.0	0.0	0.0	0.0	0.2	0.41
LOC104911918	15.0	17.0	52.5	22.5	11.0	43.0	26.8	16.89
LOC104911919	168.0	200.5	329.5	109.0	208.5	301.0	219.4	82.57
LOC104911920	402.0	865.0	623.0	297.0	978.5	565.0	621.8	262.07
LOC104911921	0.5	3.0	5.0	1.0	1.0	10.5	3.5	3.82
LOC104911922	0.0	0.0	0.0	0.0	0.0	0.0	0.0	0.00
LOC104911923	0.0	0.0	0.0	0.0	0.0	0.0	0.0	0.00
LOC104911924	0.0	0.0	0.0	0.5	0.0	0.0	0.1	0.20
LOC104911925	2.0	1.0	0.0	1.0	0.5	0.0	0.8	0.76
LOC104911926	43.5	32.5	64.0	47.5	38.0	79.5	50.8	17.67
LOC104911927	2.0	4.5	22.5	2.0	4.0	11.0	7.7	7.99
LOC104911928	7.0	0.5	6.5	10.5	0.0	2.5	4.5	4.16
LOC104911933	2.0	1.5	3.5	1.5	1.5	2.5	2.1	0.80
LOC104911934	1.0	1.5	2.5	1.0	0.5	1.5	1.3	0.68
LOC104911937	1.5	2.0	3.5	0.5	3.0	4.0	2.4	1.32
LOC104911938	49.0	41.5	60.5	29.5	40.5	51.0	45.3	10.62
LOC104911939	23.0	110.0	69.5	19.0	141.0	82.0	74.1	47.95
LOC104911940	4.0	11.5	4.0	6.0	13.0	13.0	8.6	4.39
LOC104911941	2.0	1.0	0.5	2.0	0.5	0.5	1.1	0.74
LOC104911942	11.5	3.5	2.0	10.0	3.0	2.0	5.3	4.26
LOC104911943	7.0	2.5	1.5	3.0	4.0	1.5	3.3	2.07
LOC104911944	1.5	0.0	0.5	0.0	0.0	0.0	0.3	0.61
LOC104911945	9.0	2.0	4.5	5.0	1.0	2.0	3.9	2.94
LOC104911946	1.0	0.5	0.0	1.0	1.0	1.0	0.8	0.42
LOC104911947	0.0	0.0	0.0	0.0	0.0	0.5	0.1	0.20
LOC104911948	15.5	12.0	10.0	15.5	11.5	8.0	12.1	2.99
LOC104911949	11.5	8.5	17.5	5.5	6.5	19.0	11.4	5.70
LOC104911950	5.0	16.5	25.5	5.0	23.0	17.0	15.3	8.72
LOC104911951	6.0	25.5	22.0	7.0	22.5	13.5	16.1	8.43
LOC104911952	3.5	14.5	14.0	7.0	27.5	21.5	14.7	8.89
LOC104911953	88.0	60.5	69.0	92.5	63.0	64.0	72.8	13.84
LOC104911954	20.0	25.0	44.0	15.5	19.5	25.0	24.8	10.06

LOC104911955	5.5	2.0	2.0	1.5	1.5	1.5	2.3	1.57
LOC104911956	1.0	2.0	2.0	2.5	0.5	0.0	1.3	0.98
LOC104911957	10.5	4.0	9.0	4.0	4.0	7.0	6.4	2.87
LOC104911958	1.5	11.0	9.0	0.5	13.5	12.0	7.9	5.56
LOC104911959	0.0	0.0	0.0	0.0	0.0	0.0	0.0	0.00
LOC104911963	4.0	0.5	12.5	3.0	2.0	3.0	4.2	4.25
LOC104911964	17.5	20.5	50.5	30.5	32.5	37.5	31.5	11.97
LOC104911965	0.5	0.0	1.0	0.0	0.0	0.0	0.3	0.42
LOC104911966	0.0	0.0	0.0	0.0	0.0	0.0	0.0	0.00
LOC104911967	0.0	0.0	0.0	0.0	0.0	0.0	0.0	0.00
LOC104911968	0.0	0.0	0.0	0.0	0.0	0.0	0.0	0.00
LOC104911969	6.5	3.0	1.5	4.5	1.0	0.5	2.8	2.32
LOC104911970	1.0	0.0	1.0	0.0	0.0	0.0	0.3	0.52
LOC104911971	108.0	65.0	55.5	106.0	63.0	40.5	73.0	27.71
LOC104911972	0.0	0.5	0.5	0.0	0.0	0.0	0.2	0.26
LOC104911974	0.0	0.0	0.0	0.0	0.0	0.0	0.0	0.00
LOC104911975	0.0	0.0	0.0	0.0	0.0	0.0	0.0	0.00
LOC104911976	2.5	0.5	0.5	1.5	0.5	0.0	0.9	0.92
LOC104911977	0.5	0.5	0.0	0.5	0.5	0.0	0.3	0.26
LOC104911978	1.0	0.0	0.0	1.0	0.0	0.0	0.3	0.52
LOC104911979	300.0	177.0	158.0	242.5	185.5	144.5	201.3	58.96
LOC104911981	0.0	0.0	0.0	0.0	0.0	0.0	0.0	0.00
LOC104911982	0.0	0.0	0.0	0.0	0.0	0.0	0.0	0.00
LOC104911983	0.5	0.5	0.0	0.5	0.5	0.0	0.3	0.26
LOC104911984	4.0	1.5	0.0	1.5	0.5	0.5	1.3	1.44
LOC104911985	0.5	0.0	0.0	0.0	0.0	0.0	0.1	0.20
LOC104911986	262.0	232.0	139.5	249.5	265.0	181.0	221.5	50.58
LOC104911987	17.0	17.0	10.5	8.5	17.0	10.0	13.3	4.07
LOC104911988	0.0	0.0	0.0	0.0	0.0	0.0	0.0	0.00
LOC104911990	3.0	1.0	1.0	4.5	2.5	2.5	2.4	1.32
LOC104911993	1.0	1.0	0.0	0.0	1.0	0.5	0.6	0.49
LOC104911994	0.5	0.0	0.0	1.5	0.0	0.0	0.3	0.61
LOC104911995	153.5	431.0	401.0	215.0	454.0	410.5	344.2	126.71
LOC104911996	5.5	4.5	2.5	9.5	5.0	1.0	4.7	2.91
LOC104911997	1.0	25.0	12.5	4.0	36.0	17.0	15.9	13.14
LOC104911999	0.0	1.5	1.0	0.0	1.0	1.5	0.8	0.68
LOC104912000	38.5	31.0	32.0	34.0	33.5	24.0	32.2	4.76
LOC104912001	17.5	12.0	10.0	13.0	12.0	6.5	11.8	3.61
LOC104912002	1.5	1.5	4.5	2.0	1.0	1.5	2.0	1.26
LOC104912003	0.5	0.0	0.5	0.0	0.0	0.0	0.2	0.26
LOC104912004	0.0	0.5	0.5	0.0	2.0	0.0	0.5	0.77
LOC104912005	46.5	29.0	34.0	49.5	48.0	21.5	38.1	11.61
LOC104912006	8.5	22.0	26.5	14.5	29.0	32.0	22.1	9.03
LOC104912007	0.0	0.5	1.0	1.0	0.5	0.5	0.6	0.38
LOC104912008	2.0	1.5	1.5	4.0	0.5	2.0	1.9	1.16
LOC104912009	8.0	8.5	6.5	9.5	10.5	10.5	8.9	1.56
LOC104912011	0.0	0.0	0.0	0.5	0.0	0.0	0.1	0.20
LOC104912012	0.0	1.0	0.0	1.0	0.0	0.5	0.4	0.49
LOC104912013	0.0	0.0	0.0	0.0	0.0	0.0	0.0	0.00
LOC104912014	0.0	0.0	0.0	0.0	0.0	0.0	0.0	0.00
LOC104912015	57.5	16.5	50.0	68.0	12.5	41.5	41.0	22.34
LOC104912017	0.0	0.0	0.0	0.0	0.0	0.0	0.0	0.00
LOC104912020	0.0	0.0	0.0	0.0	0.0	0.0	0.0	0.00
LOC104912022	0.0	0.0	0.0	0.0	0.0	0.0	0.0	0.00

LOC104912027	43.5	27.0	39.0	37.5	36.0	48.5	38.6	7.28
LOC104912028	3.0	1.0	1.5	6.0	0.5	2.0	2.3	1.99
LOC104912029	230.5	191.0	161.0	218.5	240.5	188.0	204.9	30.05
LOC104912030	5.0	1.5	6.5	2.0	1.5	6.0	3.8	2.34
LOC104912031	0.0	0.0	0.0	0.0	0.0	0.0	0.0	0.00
LOC104912032	3.0	1.0	0.0	4.5	1.0	1.0	1.8	1.67
LOC104912034	0.5	0.0	0.0	0.5	1.0	1.0	0.5	0.45
LOC104912035	0.0	0.0	0.0	0.0	0.0	0.0	0.0	0.00
LOC104912037	0.0	0.5	4.0	0.5	0.5	1.5	1.2	1.47
LOC104912038	0.0	0.0	0.0	0.5	0.0	0.5	0.2	0.26
LOC104912039	1.0	1.0	1.0	1.0	3.0	1.5	1.4	0.80
LOC104912040	2.5	1.5	4.0	4.0	0.0	2.5	2.4	1.53
LOC104912041	11.0	4.0	12.5	13.5	2.5	6.5	8.3	4.63
LOC104912042	0.5	0.5	0.5	0.0	1.5	1.0	0.7	0.52
LOC104912044	19.5	8.0	17.5	12.5	5.5	9.5	12.1	5.50
LOC104912045	0.5	2.5	3.0	1.5	2.0	2.5	2.0	0.89
LOC104912046	0.0	0.0	0.0	0.0	0.0	0.0	0.0	0.00
LOC104912047	0.0	0.0	0.0	0.0	0.0	0.0	0.0	0.00
LOC104912048	169.5	78.5	224.0	174.0	95.5	199.5	156.8	57.77
LOC104912049	0.0	0.0	0.0	0.0	0.0	0.0	0.0	0.00
LOC104912050	0.0	0.0	0.0	0.0	0.0	0.0	0.0	0.00
LOC104912051	0.5	0.5	3.0	0.0	0.0	1.5	0.9	1.16
LOC104912052	2.0	6.5	6.5	2.0	4.0	19.0	6.7	6.37
LOC104912053	1077.5	324.0	938.5	1169.5	310.0	532.5	725.3	384.06
LOC104912055	0.0	0.5	2.0	0.5	0.5	1.5	0.8	0.75
LOC104912056	1.5	0.0	0.0	0.5	0.0	0.0	0.3	0.61
LOC104912058	0.0	0.0	0.0	0.0	0.0	0.5	0.1	0.20
LOC104912059	81.5	28.5	128.5	79.5	34.5	123.0	79.3	42.24
LOC104912060	1.0	4.5	15.5	3.5	6.0	14.5	7.5	6.04
LOC104912061	23.0	13.5	52.5	28.0	8.0	46.5	28.6	17.75
LOC104912063	0.0	0.0	0.0	0.5	0.0	1.0	0.3	0.42
LOC104912064	12.0	22.0	84.5	13.0	31.5	93.5	42.8	36.62
LOC104912065	139.5	74.0	63.5	99.5	69.5	56.0	83.7	31.09
LOC104912067	73.5	31.5	79.5	69.5	31.0	60.0	57.5	21.30
LOC104912068	339.5	367.0	655.5	363.0	450.5	784.5	493.3	184.13
LOC104912069	0.0	0.0	0.0	0.0	0.0	0.0	0.0	0.00
LOC104912070	0.0	0.0	0.0	0.0	0.0	0.5	0.1	0.20
LOC104912072	0.0	0.0	0.0	0.0	0.0	0.0	0.0	0.00
LOC104912073	49.0	50.5	149.0	54.5	58.5	114.5	79.3	42.17
LOC104912074	0.5	0.5	1.5	0.0	1.0	1.0	0.8	0.52
LOC104912075	9.5	18.5	12.0	16.5	17.5	14.0	14.7	3.47
LOC104912076	114.5	88.5	100.5	85.5	138.5	130.5	109.7	21.93
LOC104912077	3.0	2.0	2.0	1.5	2.5	1.0	2.0	0.71
LOC104912078	44.0	17.0	31.0	35.0	13.5	20.5	26.8	11.78
LOC104912079	0.0	0.0	0.0	0.0	0.5	0.5	0.2	0.26
LOC104912080	8.5	5.5	3.5	6.0	5.5	4.5	5.6	1.69
LOC104912081	140.0	89.5	170.0	143.5	110.0	192.0	140.8	37.58
LOC104912082	3.5	3.0	19.0	3.0	3.0	20.0	8.6	8.46
LOC104912083	22.0	8.0	47.5	23.0	10.5	43.0	25.7	16.37
LOC104912084	1635.5	1679.5	2788.0	1607.0	1983.0	2629.5	2053.8	527.29
LOC104912085	75.5	205.0	822.5	69.5	214.5	508.5	315.9	294.90
LOC104912086	74.0	31.5	107.5	75.0	29.5	112.5	71.7	35.66
LOC104912087	262.5	123.5	197.0	243.0	151.5	194.5	195.3	52.67
LOC104912088	0.0	0.0	0.0	0.0	0.0	0.0	0.0	0.00

LOC104912089	0.0	0.0	0.0	0.0	0.0	0.0	0.0	0.00
LOC104912090	0.0	0.0	0.0	0.0	0.0	0.0	0.0	0.00
LOC104912091	1148.0	1142.5	1028.5	1153.0	1356.0	943.5	1128.6	139.40
LOC104912092	2.0	0.5	0.5	1.5	2.0	3.0	1.6	0.97
LOC104912093	46.5	37.5	51.0	45.0	39.0	59.0	46.3	7.95
LOC104912094	0.5	1.0	0.0	0.0	0.0	0.5	0.3	0.41
LOC104912095	2.5	0.0	0.5	0.0	0.5	0.0	0.6	0.97
LOC104912096	0.0	0.5	1.0	1.5	1.0	2.5	1.1	0.86
LOC104912097	2714.0	946.5	1032.5	2157.0	908.5	989.0	1457.9	778.55
LOC104912098	11.0	4.0	2.5	4.0	2.0	1.5	4.2	3.50
LOC104912099	1.0	0.5	0.0	1.0	0.5	0.0	0.5	0.45
LOC104912101	7.0	10.0	11.5	6.0	13.0	11.5	9.8	2.77
LOC104912102	0.0	0.0	0.0	0.0	0.0	0.0	0.0	0.00
LOC104912103	107.5	95.0	110.5	95.0	95.0	112.5	102.6	8.46
LOC104912106	1.0	1.5	0.0	1.0	1.0	1.0	0.9	0.49
LOC104912108	187.5	82.0	214.0	184.5	102.5	195.5	161.0	54.62
LOC104912109	41.5	5.0	1.5	34.0	8.5	2.0	15.4	17.64
LOC104912110	0.0	1.0	0.5	0.0	2.0	1.0	0.8	0.76
LOC104912112	2.0	3.5	2.5	1.5	1.0	1.5	2.0	0.89
LOC104912114	312.0	482.5	444.5	294.5	512.5	533.0	429.8	102.63
LOC104912115	14.0	6.0	5.0	17.0	8.5	7.0	9.6	4.82
LOC104912116	16.5	2.5	36.0	10.0	4.5	30.5	16.7	13.84
LOC104912117	388.0	307.0	284.0	384.5	307.0	283.5	325.7	48.08
LOC104912118	2.0	2.0	8.0	9.0	4.0	15.0	6.7	5.05
LOC104912120	43.0	66.5	61.5	36.0	75.0	51.0	55.5	14.79
LOC104912121	13.5	15.5	41.0	18.5	10.5	49.5	24.8	16.31
LOC104912123	0.0	0.0	0.0	0.0	0.0	0.5	0.1	0.20
LOC104912124	0.0	0.0	0.0	0.0	0.0	0.5	0.1	0.20
LOC104912125	15.0	4.5	31.0	18.5	3.0	24.0	16.0	10.93
LOC104912126	1.0	2.5	2.0	1.5	2.5	3.0	2.1	0.74
LOC104912127	40.0	18.5	85.0	42.0	14.5	61.5	43.6	26.56
LOC104912128	38.0	37.0	105.0	43.5	45.5	101.0	61.7	32.20
LOC104912129	0.0	0.0	0.0	0.0	0.5	0.0	0.1	0.20
LOC104912130	0.0	0.0	0.0	0.0	0.0	0.0	0.0	0.00
LOC104912131	8.0	8.0	19.5	5.0	5.5	10.0	9.3	5.31
LOC104912132	10.0	6.5	24.0	15.5	12.5	25.5	15.7	7.65
LOC104912133	0.0	0.5	0.5	1.0	0.5	0.0	0.4	0.38
LOC104912134	42.0	30.5	20.0	39.0	23.0	15.0	28.3	10.78
LOC104912135	5.5	4.5	6.0	2.5	8.0	11.0	6.3	2.95
LOC104912136	0.0	0.0	0.0	0.0	0.0	0.0	0.0	0.00
LOC104912137	1.0	0.5	2.5	0.0	0.0	0.5	0.8	0.94
LOC104912138	39.0	36.5	24.5	27.0	33.5	21.5	30.3	7.02
LOC104912139	8625.5	8261.0	3768.5	7542.0	8389.0	3723.0	6718.2	2330.67
LOC104912140	67.5	68.0	138.5	81.0	34.5	125.5	85.8	39.14
LOC104912141	317.0	346.5	463.0	299.5	310.0	448.0	364.0	72.73
LOC104912142	6.5	9.0	6.0	4.5	6.0	11.5	7.3	2.54
LOC104912145	1.5	2.0	2.5	3.5	2.0	4.5	2.7	1.13
LOC104912147	0.0	0.0	0.0	0.0	0.0	0.0	0.0	0.00
LOC104912148	0.0	0.0	0.5	0.0	0.0	0.0	0.1	0.20
LOC104912150	169.0	65.0	300.0	181.0	66.5	249.0	171.8	94.85
LOC104912151	115.5	76.5	132.0	112.0	80.5	118.5	105.8	22.27
LOC104912152	7.0	4.0	3.5	8.0	4.0	2.0	4.8	2.27
LOC104912153	0.0	0.0	0.0	0.0	0.0	0.0	0.0	0.00
LOC104912154	3.5	2.0	7.0	4.0	0.5	3.0	3.3	2.18

LOC104912155	0.0	1.0	0.0	1.0	0.0	1.0	0.5	0.55
LOC104912156	18.0	4.5	1.5	26.5	5.5	3.5	9.9	10.01
LOC104912157	107.5	42.5	41.5	98.0	46.5	40.0	62.7	31.27
LOC104912158	30.5	11.5	12.0	26.0	15.5	17.0	18.8	7.78
LOC104912159	33.5	11.0	42.0	15.0	7.5	12.0	20.2	14.09
LOC104912161	0.5	0.0	0.0	0.0	0.0	0.0	0.1	0.20
LOC104912162	0.0	0.0	0.0	0.0	0.0	0.0	0.0	0.00
LOC104912163	2.0	0.0	1.5	1.5	0.0	1.0	1.0	0.84
LOC104912164	0.0	0.0	0.5	0.0	0.0	0.0	0.1	0.20
LOC104912165	2.5	2.0	3.0	0.5	2.0	1.0	1.8	0.93
LOC104912166	0.0	0.0	0.0	0.0	0.5	0.0	0.1	0.20
LOC104912167	19.0	13.0	10.0	22.5	10.0	11.0	14.3	5.27
LOC104912168	0.0	0.0	0.0	0.0	0.0	0.0	0.0	0.00
LOC104912169	2.0	5.5	0.0	1.5	6.5	0.0	2.6	2.78
LOC104912170	0.0	0.0	0.0	0.0	0.0	0.0	0.0	0.00
LOC104912171	0.0	0.0	0.0	0.0	0.0	0.0	0.0	0.00
LOC104912172	0.0	0.0	0.0	1.5	0.0	0.0	0.3	0.61
LOC104912173	0.5	0.5	1.5	0.5	0.5	1.5	0.8	0.52
LOC104912174	2.5	0.5	0.0	0.5	0.5	1.5	0.9	0.92
LOC104912175	50.5	43.0	28.5	47.0	41.0	35.5	40.9	7.96
LOC104912176	1.5	0.5	0.0	1.5	0.0	0.0	0.6	0.74
LOC104912177	0.0	0.0	0.0	0.0	0.5	0.0	0.1	0.20
LOC104912178	0.0	0.0	0.0	0.0	0.0	0.0	0.0	0.00
LOC104912179	361.5	834.0	482.5	210.0	874.5	458.0	536.8	264.22
LOC104912180	25.0	28.0	28.5	23.0	33.0	20.5	26.3	4.45
LOC104912181	6.0	0.5	1.0	9.0	6.5	3.0	4.3	3.37
LOC104912182	0.0	0.5	1.5	0.0	1.5	2.0	0.9	0.86
LOC104912183	0.5	1.0	1.0	0.5	0.0	0.0	0.5	0.45
LOC104912184	5.0	33.0	46.0	11.0	43.0	66.0	34.0	22.89
LOC104912185	0.0	0.5	2.0	0.0	1.5	6.0	1.7	2.27
LOC104912186	246.5	182.0	151.5	245.5	181.5	214.0	203.5	38.40
LOC104912188	1254.5	1241.0	1743.0	1518.0	1605.5	2294.5	1609.4	389.17
LOC104912189	0.5	0.5	0.0	0.5	0.0	0.0	0.3	0.27
LOC104912190	71.0	85.0	34.0	62.0	110.0	28.0	65.0	30.98
LOC104912191	0.5	1.0	0.0	3.5	4.5	2.0	1.9	1.77
LOC104912192	0.0	0.0	0.0	0.0	0.0	0.0	0.0	0.00
LOC104912194	0.5	0.5	0.5	0.0	0.0	0.0	0.3	0.27
LOC104912195	8.0	27.0	29.0	6.0	21.5	23.5	19.2	9.80
LOC104912196	0.0	0.5	0.5	0.0	0.0	0.5	0.3	0.27
LOC104912198	2.5	1.5	3.5	0.5	1.0	5.0	2.3	1.69
LOC104912200	0.5	1.0	1.0	0.0	1.5	1.5	0.9	0.58
LOC104912201	0.0	0.0	0.0	0.0	0.5	0.0	0.1	0.20
LOC104912202	1.0	2.5	1.5	3.5	0.5	1.0	1.7	1.13
LOC104912203	0.0	0.0	1.0	0.0	0.0	1.5	0.4	0.66
LOC104912204	0.0	0.0	0.0	0.0	0.0	0.0	0.0	0.00
LOC104912205	0.0	0.0	0.0	0.0	0.0	0.0	0.0	0.00
LOC104912208	0.0	0.0	0.0	0.0	0.0	0.0	0.0	0.00
LOC104912209	0.0	0.0	0.5	0.0	0.0	0.5	0.2	0.26
LOC104912210	1.0	0.5	0.0	0.5	0.5	0.5	0.5	0.32
LOC104912211	1.0	0.5	0.5	2.5	2.0	1.0	1.3	0.82
LOC104912212	3.5	5.5	3.0	5.5	3.5	3.0	4.0	1.18
LOC104912214	297.5	164.0	271.0	263.5	179.5	263.5	239.8	54.42
LOC104912217	137.0	47.5	132.0	130.0	55.5	97.0	99.8	40.10
LOC104912219	1.0	3.5	1.5	1.0	2.0	0.5	1.6	1.07

LOC104912220	0.0	0.5	2.5	2.5	1.0	1.5	1.3	1.03
LOC104912221	1.5	2.5	0.0	0.5	1.5	0.5	1.1	0.92
LOC104912222	2.0	1.5	6.5	3.5	3.5	9.5	4.4	3.04
LOC104912223	345.5	366.0	387.5	342.0	504.5	425.5	395.2	61.77
LOC104912224	248.0	108.0	308.5	324.0	128.5	327.0	240.7	99.23
LOC104912226	0.0	0.0	0.5	0.5	0.0	0.5	0.3	0.27
LOC104912227	52.5	25.0	45.5	51.0	37.5	38.5	41.7	10.24
LOC104912228	34.5	24.0	129.0	38.0	33.0	108.0	61.1	45.20
LOC104912229	0.0	0.0	0.0	0.0	0.5	0.0	0.1	0.20
LOC104912230	0.5	0.0	0.0	0.0	0.0	0.0	0.1	0.20
LOC104912231	1.0	0.0	0.0	0.5	0.0	0.0	0.3	0.42
LOC104912233	5.5	5.0	4.0	5.0	5.0	4.5	4.8	0.52
LOC104912234	1.5	0.0	0.0	0.0	0.0	0.0	0.3	0.61
LOC104912236	2.0	0.5	0.5	1.0	0.5	2.0	1.1	0.74
LOC104912238	0.0	0.0	0.0	0.0	0.0	0.0	0.0	0.00
LOC104912240	0.0	0.0	0.0	0.0	0.0	0.0	0.0	0.00
LOC104912241	0.5	0.0	0.5	0.0	0.0	0.0	0.2	0.26
LOC104912242	2.5	1.5	0.5	4.0	2.0	0.0	1.8	1.44
LOC104912244	0.0	0.0	0.5	0.0	0.0	0.0	0.1	0.20
LOC104912245	2.5	4.5	6.5	3.5	5.0	3.5	4.3	1.41
LOC104912246	38.5	13.5	24.0	30.0	16.5	7.0	21.6	11.55
LOC104912247	40.0	15.5	61.0	48.0	12.5	21.5	33.1	19.58
LOC104912248	228.5	146.5	219.0	241.5	193.0	231.5	210.0	35.21
LOC104912249	3.0	0.0	0.0	0.5	1.0	0.5	0.8	1.13
LOC104912250	2.0	0.5	0.5	1.5	1.0	0.0	0.9	0.74
LOC104912252	16.0	6.0	15.5	14.0	7.0	12.5	11.8	4.32
LOC104912253	154.0	77.5	155.0	130.0	110.5	168.0	132.5	33.89
LOC104912254	686.5	639.0	654.0	653.5	696.5	562.5	648.7	47.52
LOC104912255	29.5	16.5	17.5	18.5	9.5	18.5	18.3	6.43
LOC104912256	62.0	70.5	68.0	71.0	69.0	63.5	67.3	3.74
LOC104912257	24.0	10.5	18.0	28.5	18.5	20.0	19.9	6.08
LOC104912258	422.5	534.5	329.0	415.0	533.5	307.0	423.6	96.95
LOC104912259	17.0	6.5	6.5	4.0	6.0	3.5	7.3	4.95
LOC104912260	5.5	2.0	12.0	13.0	5.5	7.0	7.5	4.22
LOC104912261	8.0	3.0	4.0	6.5	4.5	3.0	4.8	2.02
LOC104912262	4.5	1.0	0.5	3.0	1.0	1.5	1.9	1.53
LOC104912263	0.5	0.0	1.5	0.5	0.0	0.5	0.5	0.55
LOC104912264	0.0	0.0	0.0	0.0	0.0	0.0	0.0	0.00
LOC104912265	0.0	0.0	0.0	0.0	0.0	0.0	0.0	0.00
LOC104912266	0.0	0.0	0.0	0.0	0.0	0.0	0.0	0.00
LOC104912267	0.0	0.5	0.0	0.5	0.0	0.0	0.2	0.26
LOC104912268	0.5	0.5	0.5	0.0	1.5	2.0	0.8	0.75
LOC104912269	2.5	2.5	1.0	2.0	2.0	0.0	1.7	0.98
LOC104912270	338.0	99.5	45.0	273.5	102.5	47.0	150.9	124.12
LOC104912271	1.5	1.0	2.5	0.0	3.0	1.0	1.5	1.10
LOC104912272	0.5	0.0	0.0	0.0	0.0	2.0	0.4	0.80
LOC104912273	13.5	19.0	26.0	11.5	19.0	32.5	20.3	7.85
LOC104912274	29.5	27.0	31.0	31.5	38.5	40.0	32.9	5.17
LOC104912275	1.5	0.0	2.5	0.0	0.0	0.5	0.8	1.04
LOC104912276	477.0	816.0	644.0	403.5	1009.5	553.0	650.5	226.69
LOC104912277	0.0	0.0	0.0	0.0	0.0	0.0	0.0	0.00
LOC104912278	0.0	0.0	0.0	0.0	1.0	0.0	0.2	0.41
LOC104912279	2.0	3.0	0.5	1.5	3.5	0.5	1.8	1.25
LOC104912280	29.5	10.5	25.5	34.5	12.5	14.5	21.2	9.99

LOC104912282	228.0	161.5	151.5	202.5	190.0	154.0	181.3	30.76
LOC104912283	1200.0	972.0	1057.5	1297.0	1125.5	1112.5	1127.4	112.51
LOC104912284	206.5	85.0	75.0	180.5	86.0	60.5	115.6	61.60
LOC104912285	0.0	0.0	2.0	0.0	0.0	0.5	0.4	0.80
LOC104912286	0.0	0.0	0.0	0.0	0.5	0.0	0.1	0.20
LOC104912287	124.5	140.0	118.5	122.5	152.5	94.5	125.4	19.79
LOC104912288	1.0	0.0	0.0	0.0	0.0	0.0	0.2	0.41
LOC104912289	0.0	0.0	0.0	0.0	0.0	0.0	0.0	0.00
LOC104912290	0.0	1.5	0.5	0.5	1.0	1.0	0.8	0.52
LOC104912291	0.0	0.0	0.0	0.0	0.0	0.0	0.0	0.00
LOC104912292	0.0	0.0	0.0	0.0	0.0	0.0	0.0	0.00
LOC104912293	1.0	0.5	0.5	0.5	0.0	1.0	0.6	0.38
LOC104912294	1.0	0.5	1.0	0.5	0.0	1.5	0.8	0.52
LOC104912295	0.5	2.0	1.0	0.0	2.0	1.5	1.2	0.82
LOC104912297	0.0	0.0	0.5	0.5	0.0	0.0	0.2	0.26
LOC104912298	0.0	0.5	0.0	0.0	0.0	0.0	0.1	0.20
LOC104912299	39.0	19.0	7.5	32.5	22.0	14.5	22.4	11.61
LOC104912301	0.0	0.0	0.0	0.0	1.5	0.0	0.3	0.61
LOC104912302	3.0	1.5	3.5	2.0	1.0	2.5	2.3	0.94
LOC104912303	0.5	0.0	0.5	0.0	0.0	2.0	0.5	0.77
LOC104912304	15.5	3.0	2.5	26.0	5.0	1.5	8.9	9.82
LOC104912305	5.5	2.0	3.0	4.5	2.5	1.5	3.2	1.54
LOC104912306	0.0	0.0	0.0	0.0	0.0	0.0	0.0	0.00
LOC104912307	0.5	0.5	0.0	0.0	0.0	0.0	0.2	0.26
LOC104912308	3.5	14.5	20.5	2.5	13.5	13.5	11.3	6.97
LOC104912309	25.5	8.5	16.5	15.0	16.0	5.5	14.5	6.99
LOC104912310	7.0	0.5	7.0	3.5	1.5	4.0	3.9	2.71
LOC104912312	0.0	0.0	0.0	0.0	0.0	0.0	0.0	0.00
LOC104912313	2.5	0.5	2.0	0.5	1.5	1.0	1.3	0.82
LOC104912314	171.0	88.5	411.0	224.0	106.5	404.0	234.2	142.64
LOC104912315	43.0	37.5	32.5	46.5	42.5	65.5	44.6	11.35
LOC104912316	14.5	45.5	55.0	21.0	53.0	118.0	51.2	36.80
LOC104912317	0.0	0.0	0.0	0.0	0.0	0.0	0.0	0.00
LOC104912318	0.0	0.0	0.0	0.0	0.0	0.5	0.1	0.20
LOC104912319	0.0	2.5	0.5	0.5	0.0	1.0	0.8	0.94
LOC104912320	517.5	512.5	463.5	484.5	652.0	473.5	517.3	69.36
LOC104912321	1.5	1.5	0.5	0.5	2.0	2.5	1.4	0.80
LOC104912322	1.0	3.0	4.5	3.0	4.5	4.5	3.4	1.39
LOC104912323	3.0	4.5	2.0	5.5	5.0	5.0	4.2	1.37
LOC104912324	1.5	5.0	1.5	0.5	4.5	1.5	2.4	1.86
LOC104912327	22.0	133.5	136.5	31.0	165.0	258.5	124.4	88.40
LOC104912328	79.5	45.0	35.5	63.0	30.5	28.5	47.0	20.31
LOC104912329	1600.0	2610.0	1666.0	1438.0	3028.5	1733.5	2012.7	646.12
LOC104912330	396.5	307.0	461.0	326.5	311.0	356.0	359.7	59.80
LOC104912332	230.0	135.5	262.5	171.0	105.5	271.0	195.9	68.82
LOC104912334	0.0	0.0	0.0	0.0	0.0	0.0	0.0	0.00
LOC104912335	118.0	88.5	110.0	120.0	89.0	96.5	103.7	14.20
LOC104912336	1906.5	1642.5	2176.0	1973.5	1917.5	2197.0	1968.8	203.97
LOC104912337	22.5	11.0	59.5	33.5	12.5	39.0	29.7	18.36
LOC104912338	4.0	3.0	1.0	2.0	3.5	1.5	2.5	1.18
LOC104912339	1.0	0.5	0.0	0.0	0.5	0.0	0.3	0.41
LOC104912341	0.0	0.0	0.0	0.0	0.0	1.0	0.2	0.41
LOC104912342	0.0	0.0	1.0	0.5	0.5	0.0	0.3	0.41
LOC104912343	172.5	127.5	95.0	143.5	135.5	93.0	127.8	30.30

LOC104912346	93.5	54.0	73.0	62.0	47.0	77.5	67.8	16.96
LOC104912348	10.0	4.0	23.0	11.5	3.5	13.0	10.8	7.13
LOC104912349	0.5	6.5	4.0	0.5	6.5	7.0	4.2	3.03
LOC104912350	48.5	39.0	43.5	56.5	49.5	62.5	49.9	8.53
LOC104912351	0.0	0.0	0.5	0.0	0.0	0.0	0.1	0.20
LOC104912352	0.5	0.0	0.5	1.0	0.0	0.5	0.4	0.38
LOC104912353	12.0	23.5	42.5	13.5	20.5	46.0	26.3	14.56
LOC104912354	0.0	1.5	0.0	0.0	1.0	1.0	0.6	0.66
LOC104912355	44.0	21.0	41.5	56.0	28.5	37.5	38.1	12.26
LOC104912356	1427.5	982.5	1265.5	1206.5	1141.0	1167.5	1198.4	146.91
LOC104912358	84.0	127.0	112.0	77.5	161.0	163.0	120.8	36.72
LOC104912359	56.0	19.0	23.0	45.5	21.5	19.5	30.8	15.91
LOC104912360	4.5	2.5	3.0	3.5	2.5	4.5	3.4	0.92
LOC104912361	126.0	58.0	53.0	147.0	53.0	33.5	78.4	46.25
LOC104912362	277.0	263.5	247.5	262.5	327.0	219.0	266.1	35.81
LOC104912363	922.0	630.0	1129.5	928.0	763.5	1168.5	923.6	206.99
LOC104912364	45.5	7.0	7.0	44.0	10.5	5.5	19.9	19.31
LOC104912365	26.5	5.0	3.5	24.0	2.5	4.0	10.9	11.16
LOC104912366	0.5	0.5	1.0	0.5	0.0	0.0	0.4	0.38
LOC104912367	36.5	10.5	10.0	36.5	5.5	7.5	17.8	14.63
LOC104912368	11.0	11.0	50.5	12.0	20.5	40.5	24.3	17.14
LOC104912369	3.5	0.0	10.5	3.5	0.5	7.5	4.3	4.07
LOC104912370	0.0	0.0	6.5	1.0	0.0	4.0	1.9	2.73
LOC104912371	10.5	7.0	4.5	5.0	5.5	4.0	6.1	2.40
LOC104912372	28.5	8.5	57.5	28.0	16.0	47.5	31.0	18.55
LOC104912373	0.0	0.0	0.0	0.0	0.0	0.0	0.0	0.00
LOC104912374	40.0	18.5	16.0	28.0	15.5	15.5	22.3	9.92
LOC104912375	110.0	57.5	71.5	106.5	66.0	144.5	92.7	33.39
LOC104912376	0.0	0.0	0.0	0.0	0.0	0.0	0.0	0.00
LOC104912377	25.5	16.0	31.0	25.5	12.5	23.5	22.3	6.83
LOC104912381	1606.5	2726.0	2485.0	1054.0	2726.0	2843.5	2240.2	735.47
LOC104912382	27.5	13.0	15.0	24.0	13.0	14.5	17.8	6.28
LOC104912384	25.0	30.5	31.5	15.5	25.5	24.5	25.4	5.70
LOC104912385	0.0	0.0	0.0	0.5	0.0	0.0	0.1	0.20
LOC104912386	0.0	0.0	0.0	1.5	0.0	0.0	0.3	0.61
LOC104912388	0.0	1.0	0.5	0.5	1.0	0.5	0.6	0.38
LOC104912389	37.5	15.0	29.5	40.0	18.5	36.5	29.5	10.53
LOC104912390	0.0	0.0	0.0	0.0	0.0	0.5	0.1	0.20
LOC104912391	91.5	44.0	145.5	96.0	50.5	141.0	94.8	43.04
LOC104912393	0.5	0.0	0.0	0.0	0.0	0.0	0.1	0.20
LOC104912394	0.5	0.5	0.0	0.5	1.5	0.0	0.5	0.55
LOC104912395	0.5	0.5	0.0	0.5	1.0	0.5	0.5	0.32
LOC104912396	0.0	0.0	0.0	0.0	0.0	0.0	0.0	0.00
LOC104912397	0.0	0.5	0.5	0.0	0.0	0.5	0.3	0.27
LOC104912398	3.0	2.0	2.0	3.0	2.5	1.5	2.3	0.61
LOC104912399	0.0	0.0	0.0	0.0	0.0	2.0	0.3	0.82
LOC104912401	73.5	44.5	77.5	67.5	61.5	69.0	65.6	11.67
LOC104912402	0.5	2.0	5.5	0.5	3.0	2.5	2.3	1.86
LOC104912403	1.0	0.0	0.0	0.0	0.0	0.0	0.2	0.41
LOC104912404	1.0	0.0	0.0	0.0	1.0	0.0	0.3	0.52
LOC104912405	0.0	0.0	0.0	0.0	0.0	0.0	0.0	0.00
LOC104912406	1.0	0.0	0.0	0.5	0.0	0.0	0.3	0.42
LOC104912410	0.0	0.0	0.5	0.0	0.0	0.5	0.2	0.26
LOC104912411	0.0	0.0	0.0	0.0	0.0	0.0	0.0	0.00

LOC104912412	6425.0	14505.0	16312.5	6201.0	15154.0	15163.0	12293.4	4669.42
LOC104912413	4.0	5.5	6.5	3.0	1.5	7.0	4.6	2.13
LOC104912415	15.0	42.0	63.0	6.0	55.0	87.0	44.7	30.39
LOC104912416	0.0	0.0	0.0	0.0	0.0	0.5	0.1	0.20
LOC104912417	53.0	11.0	134.0	70.0	18.0	100.0	64.3	47.49
LOC104912418	1.5	0.5	0.5	0.0	3.0	1.5	1.2	1.08
LOC104912421	471.0	529.0	512.0	467.5	539.5	518.5	506.3	30.17
LOC104912422	28.5	35.0	26.5	29.5	48.5	57.0	37.5	12.44
LOC104912423	3.0	1.0	3.0	2.5	1.5	4.0	2.5	1.10
LOC104912424	0.0	0.0	0.0	0.0	0.0	0.0	0.0	0.00
LOC104912425	0.0	1.0	0.5	0.0	0.5	0.0	0.3	0.41
LOC104912426	0.0	0.5	0.0	0.0	0.0	1.0	0.3	0.42
LOC104912427	295.5	530.0	420.0	249.5	552.5	259.5	384.5	135.96
LOC104912428	442.5	560.5	337.5	275.0	556.5	217.0	398.2	144.95
LOC104912429	0.5	1.0	0.0	0.5	0.0	2.5	0.8	0.94
LOC104912430	44.0	37.0	17.0	39.5	38.5	15.5	31.9	12.37
LOC104912431	5.5	5.0	4.5	5.5	6.0	5.5	5.3	0.52
LOC104912432	1.0	0.0	0.5	0.0	0.0	0.5	0.3	0.41
LOC104912434	2.0	0.5	0.0	1.0	2.0	0.0	0.9	0.92
LOC104912435	1.0	0.0	0.0	1.0	1.0	1.0	0.7	0.52
LOC104912436	1052.0	592.0	852.5	967.0	700.0	803.0	827.8	169.00
LOC104912440	98.5	86.0	91.0	92.0	80.0	82.0	88.3	6.91
LOC104912442	28.5	15.5	5.0	25.0	10.0	3.5	14.6	10.38
LOC104912443	15.5	17.0	8.5	8.5	21.5	11.0	13.7	5.22
LOC104912445	0.0	0.0	0.0	0.0	0.0	0.0	0.0	0.00
LOC104912446	74.5	29.5	178.5	102.5	37.5	149.0	95.3	59.93
LOC104912447	166.5	150.0	99.0	163.5	115.0	71.0	127.5	38.69
LOC104912448	655.5	544.0	651.0	623.0	607.5	610.5	615.3	40.29
LOC104912449	22.0	4.0	28.0	18.0	6.0	19.5	16.3	9.38
LOC104912450	345.0	224.5	132.5	337.5	254.0	126.5	236.7	95.23
LOC104912451	39.5	16.5	64.5	39.5	17.5	54.5	38.7	19.28
LOC104912452	60.0	20.5	79.0	57.0	28.0	65.0	51.6	22.60
LOC104912453	1.5	0.5	0.0	1.0	0.0	0.0	0.5	0.63
LOC104912454	0.0	0.0	0.0	0.0	0.0	0.0	0.0	0.00
LOC104912455	0.0	5.0	2.0	0.0	7.0	3.5	2.9	2.80
LOC104912457	2.5	0.5	0.0	2.5	0.0	0.0	0.9	1.24
LOC104912458	9.5	4.0	6.0	9.5	3.0	5.0	6.2	2.77
LOC104912459	11.0	25.5	52.5	10.5	33.5	88.0	36.8	29.54
LOC104912460	92.0	52.5	67.5	96.5	61.0	56.0	70.9	18.82
LOC104912462	0.5	0.5	0.0	1.0	0.5	1.0	0.6	0.38
LOC104912464	0.0	0.0	0.0	1.0	0.5	0.0	0.3	0.42
LOC104912465	23.5	25.5	31.0	25.0	25.0	24.0	25.7	2.71
LOC104912467	44.5	156.0	100.5	39.0	203.0	145.5	114.8	65.29
LOC104912468	87.0	39.5	146.0	90.5	44.5	123.0	88.4	42.05
LOC104912469	444.0	420.0	396.5	390.5	470.5	400.0	420.3	31.46
LOC104912471	121.5	55.0	84.0	131.5	58.0	66.5	86.1	33.04
LOC104912473	16.5	45.5	39.5	22.0	58.0	59.5	40.2	17.95
LOC104912474	94.0	58.0	72.0	101.0	69.5	63.5	76.3	17.24
LOC104912475	0.0	0.0	0.0	0.0	0.0	0.0	0.0	0.00
LOC104912476	0.5	5.0	8.5	0.0	5.5	7.0	4.4	3.46
LOC104912477	413.0	507.5	553.5	334.5	445.0	459.5	452.2	75.91
LOC104912481	2.0	4.0	6.0	1.5	5.0	4.5	3.8	1.75
LOC104912482	134.5	169.0	183.0	102.5	186.5	181.5	159.5	33.85
LOC104912483	1543.0	965.5	1011.0	1347.0	1052.0	918.0	1139.4	248.75

LOC104912484	919.0	3745.0	3049.5	815.5	4523.0	2727.0	2629.8	1498.80
LOC104912485	14.5	5.5	17.0	13.0	10.0	11.0	11.8	3.98
LOC104912486	121.5	131.0	68.5	125.0	134.0	76.5	109.4	29.04
LOC104912488	1.5	1.0	2.5	1.5	0.5	2.0	1.5	0.71
LOC104912491	75.0	14.5	217.0	106.5	24.0	155.5	98.8	78.12
LOC104912492	1.5	0.0	0.0	1.0	0.0	0.0	0.4	0.66
LOC104912493	0.0	0.0	0.0	0.0	0.0	0.0	0.0	0.00
LOC104912494	168.0	256.5	193.5	136.0	331.5	110.5	199.3	82.04
LOC104912495	43.0	23.5	78.0	47.5	22.0	71.5	47.6	23.46
LOC104912496	0.5	0.0	0.0	0.0	0.0	0.0	0.1	0.20
LOC104912497	121.0	104.5	318.0	146.5	103.5	328.0	186.9	106.60
LOC104912498	232.0	180.0	226.5	232.5	190.0	239.5	216.8	25.14
LOC104912499	41.0	26.0	24.5	52.5	23.5	15.0	30.4	13.71
LOC104912500	53.0	33.5	35.5	41.5	41.0	37.5	40.3	6.93
LOC104912501	5.5	9.5	6.5	7.0	11.5	12.5	8.8	2.86
LOC104912504	1.5	1.0	6.5	1.5	1.0	2.0	2.3	2.12
LOC104912505	16.5	6.0	13.5	16.5	10.0	9.0	11.9	4.28
LOC104912507	33.0	39.0	58.5	22.0	35.5	43.0	38.5	12.10
LOC104912508	2.5	1.5	2.0	1.0	1.5	2.5	1.8	0.61
LOC104912509	29.0	48.5	34.0	24.0	45.0	37.0	36.3	9.33
LOC104912510	3.5	4.0	6.5	2.5	2.0	2.0	3.4	1.72
LOC104912511	465.0	440.0	392.0	402.0	391.0	409.0	416.5	29.74
LOC104912512	537.5	700.0	578.0	530.5	848.5	654.0	641.4	121.32
LOC104912514	23.0	16.5	43.5	22.0	13.0	30.5	24.8	10.97
LOC104912515	1.5	0.5	0.5	2.0	0.5	1.0	1.0	0.63
LOC104912516	9.0	3.5	7.0	7.0	7.0	8.0	6.9	1.86
LOC104912517	151.5	164.0	121.5	154.0	196.0	135.0	153.7	25.64
LOC104912520	52.0	38.5	11.0	31.0	27.5	5.5	27.6	17.26
LOC104912521	0.5	0.0	0.0	0.0	0.0	0.5	0.2	0.26
LOC104912522	0.0	0.0	0.0	0.0	0.0	0.0	0.0	0.00
LOC104912523	0.0	0.0	0.0	0.0	0.0	0.0	0.0	0.00
LOC104912524	2.5	2.5	1.0	1.5	0.0	1.5	1.5	0.95
LOC104912525	5.5	3.0	2.5	13.0	4.5	3.0	5.3	3.96
LOC104912526	18.5	61.5	48.0	16.0	65.0	54.0	43.8	21.44
LOC104912527	0.0	0.0	1.0	0.0	1.0	1.5	0.6	0.66
LOC104912528	1.5	6.5	5.5	1.0	3.5	1.5	3.3	2.32
LOC104912529	1.0	0.5	3.0	0.0	1.5	2.0	1.3	1.08
LOC104912530	439.5	485.0	358.5	403.0	525.5	333.0	424.1	73.86
LOC104912531	0.0	0.0	0.5	0.0	0.0	0.0	0.1	0.20
LOC104912532	0.0	0.5	0.5	0.0	1.0	0.5	0.4	0.38
LOC104912533	73.5	25.5	25.5	78.5	22.5	28.0	42.3	26.25
LOC104912534	1.0	0.5	0.5	1.0	1.5	1.0	0.9	0.38
LOC104912536	0.5	0.0	0.5	1.0	0.0	0.0	0.3	0.41
LOC104912537	0.0	0.0	0.5	1.0	0.0	0.0	0.3	0.42
LOC104912538	0.0	0.0	0.0	0.0	0.5	0.5	0.2	0.26
LOC104912539	0.0	1.0	0.0	0.0	0.5	0.5	0.3	0.41
LOC104912540	0.0	0.5	0.5	0.0	0.5	0.5	0.3	0.26
LOC104912541	1.0	0.0	0.0	0.0	0.0	0.0	0.2	0.41
LOC104912542	0.0	0.0	0.0	0.5	0.0	0.0	0.1	0.20
LOC104912543	25.5	7.0	16.5	30.5	13.5	11.0	17.3	8.97
LOC104912544	104.0	32.0	12.5	116.5	41.5	10.5	52.8	46.16
LOC104912545	12.0	1.5	10.5	11.0	1.0	7.0	7.2	4.89
LOC104912546	216.5	174.5	123.5	207.5	210.5	124.5	176.2	42.97
LOC104912547	32.5	40.5	27.5	25.5	35.0	19.0	30.0	7.60

LOC104912548	148.5	174.0	169.0	153.5	167.5	124.5	156.2	18.33
LOC104912549	10.0	15.0	13.0	12.5	18.5	22.5	15.3	4.55
LOC104912550	154.5	128.0	129.5	169.0	124.5	135.0	140.1	17.72
LOC104912551	18.0	16.5	22.5	35.0	31.0	19.5	23.8	7.54
LOC104912553	8.5	4.5	4.0	7.5	4.0	4.5	5.5	1.97
LOC104912554	0.5	0.0	0.0	0.0	0.5	0.0	0.2	0.26
LOC104912555	48.0	28.0	86.0	46.0	37.5	57.5	50.5	20.05
LOC104912556	1749.0	1920.5	1579.5	1594.0	2242.0	1635.5	1786.8	256.73
LOC104912557	29.0	5.5	12.5	39.5	10.5	12.5	18.3	13.08
LOC104912558	2.0	0.0	0.5	0.0	0.5	1.0	0.7	0.75
LOC104912559	0.0	0.0	0.0	0.0	0.0	0.5	0.1	0.20
LOC104912560	0.0	0.5	0.0	1.5	0.0	0.5	0.4	0.58
LOC104912561	12.5	58.0	60.5	20.5	62.5	64.0	46.3	23.33
LOC104912562	558.0	495.0	329.0	484.0	571.5	318.5	459.3	110.47
LOC104912563	0.0	0.5	0.0	0.5	0.0	0.0	0.2	0.26
LOC104912564	2.0	0.5	1.0	0.5	1.0	0.5	0.9	0.58
LOC104912565	2.5	1.0	0.5	3.0	0.0	0.5	1.3	1.21
LOC104912566	5.5	0.5	4.5	7.5	3.5	4.0	4.3	2.32
LOC104912567	3.0	0.0	1.0	2.0	0.5	0.0	1.1	1.20
LOC104912568	20.5	6.5	6.0	15.5	9.0	6.5	10.7	5.99
LOC104912569	0.0	0.0	0.0	0.0	0.0	0.0	0.0	0.00
LOC104912570	0.5	1.0	0.5	0.5	0.0	0.0	0.4	0.38
LOC104912571	319.0	157.0	225.0	294.0	178.0	202.0	229.2	64.58
LOC104912572	3.5	2.5	2.0	6.0	7.0	0.5	3.6	2.48
LOC104912573	278.5	110.5	271.5	289.5	126.0	242.0	219.7	80.27
LOC104912574	2.5	2.0	3.0	0.5	0.0	0.5	1.4	1.24
LOC104912575	0.0	0.0	0.5	0.0	0.0	0.0	0.1	0.20
LOC104912576	2.0	0.5	0.0	3.5	1.0	3.5	1.8	1.51
LOC104912577	1.5	0.5	0.0	0.0	0.0	0.0	0.3	0.61
LOC104912578	0.0	0.0	0.0	0.0	0.0	0.0	0.0	0.00
LOC104912579	0.0	0.0	0.0	0.0	0.0	0.0	0.0	0.00
LOC104912581	133.5	42.0	199.5	172.5	46.0	222.5	136.0	77.19
LOC104912582	6.0	1.5	1.5	3.0	2.0	3.0	2.8	1.69
LOC104912583	1008.0	817.0	837.5	1062.5	1013.0	1020.0	959.7	104.57
LOC104912584	248.5	120.0	273.0	243.0	148.0	236.0	211.4	61.88
LOC104912585	372.5	440.0	229.0	321.5	556.5	311.0	371.8	114.35
LOC104912586	62.5	25.5	69.5	78.0	23.5	54.5	52.3	22.86
LOC104912587	0.0	0.0	0.0	0.0	0.0	0.0	0.0	0.00
LOC104912588	0.0	0.0	0.0	0.5	0.0	0.0	0.1	0.20
LOC104912589	0.5	0.0	1.0	0.5	0.0	0.0	0.3	0.41
LOC104912590	78.5	26.0	97.5	134.0	20.5	131.5	81.3	49.64
LOC104912591	5.5	2.5	1.0	8.5	1.0	1.5	3.3	3.04
LOC104912593	6.5	5.5	19.0	5.5	4.0	26.0	11.1	9.15
LOC104912594	0.0	0.0	0.0	0.0	0.0	0.0	0.0	0.00
LOC104912596	2266.0	1265.0	1565.0	2137.5	1290.0	1310.5	1639.0	450.93
LOC104912598	0.5	0.0	1.5	0.5	2.0	0.0	0.8	0.82
LOC104912599	0.0	0.0	0.0	0.0	0.5	0.0	0.1	0.20
LOC104912600	0.0	0.0	0.0	0.0	0.0	0.0	0.0	0.00
LOC104912601	0.5	0.0	0.5	0.0	1.0	1.0	0.5	0.45
LOC104912602	5.0	4.5	5.0	4.5	6.0	10.5	5.9	2.31
LOC104912603	0.0	0.0	0.0	0.0	0.0	0.0	0.0	0.00
LOC104912605	80.0	32.0	39.5	66.5	35.0	27.0	46.7	21.42
LOC104912607	1.5	2.0	9.0	4.5	2.0	2.5	3.6	2.85
LOC104912609	254.0	63.5	151.5	260.0	85.5	142.0	159.4	82.57

LOC104912610	3372.5	3298.5	4833.0	3532.0	3905.5	4612.0	3925.6	655.59
LOC104912612	29.5	11.0	40.5	23.5	12.5	36.0	25.5	12.12
LOC104912614	0.0	0.0	0.0	1.0	0.0	0.0	0.2	0.41
LOC104912615	0.0	3.0	2.5	0.5	1.5	3.5	1.8	1.40
LOC104912616	75.5	96.0	38.5	65.5	89.5	52.5	69.6	21.93
LOC104912617	112.5	44.5	307.5	134.5	41.0	225.0	144.2	104.77
LOC104912619	1.5	0.5	0.0	0.5	0.5	0.0	0.5	0.55
LOC104912620	1.5	0.0	1.0	0.0	0.0	0.0	0.4	0.66
LOC104912621	0.0	0.0	0.5	0.5	1.0	0.5	0.4	0.38
LOC104912622	0.0	0.0	0.5	0.0	0.0	0.0	0.1	0.20
LOC104912625	332.5	321.5	552.5	325.0	398.0	548.5	413.0	110.13
LOC104912627	0.5	0.0	0.0	1.5	0.0	0.0	0.3	0.61
LOC104912628	31.0	4.0	11.5	30.0	5.5	12.5	15.8	11.89
LOC104912630	0.0	0.0	0.5	0.5	0.0	0.0	0.2	0.26
LOC104912631	1.0	0.0	0.5	0.0	1.0	0.0	0.4	0.49
LOC104912632	53.0	14.0	79.5	57.0	19.0	84.0	51.1	29.44
LOC104912634	0.0	1.5	2.0	2.0	0.0	2.0	1.3	0.99
LOC104912635	2.5	1.5	3.5	3.5	0.5	3.5	2.5	1.26
LOC104912637	158.0	101.5	153.5	151.0	111.0	159.5	139.1	25.79
LOC104912638	0.5	0.0	0.0	1.0	0.0	1.0	0.4	0.49
LOC104912639	0.0	0.0	0.5	1.0	0.0	0.5	0.3	0.41
LOC104912647	45.5	35.5	84.0	69.0	36.5	108.5	63.2	29.37
LOC104912648	53.0	23.5	43.5	66.5	24.0	33.5	40.7	17.04
LOC104912649	118.0	42.0	130.5	127.5	35.0	142.0	99.2	47.66
LOC104912650	36.0	15.0	12.5	37.5	17.0	15.5	22.3	11.33
LOC104912651	46.5	30.0	91.5	69.5	34.0	90.0	60.3	27.35
LOC104912652	57.5	42.0	74.5	48.5	52.0	77.5	58.7	14.37
LOC104912653	0.5	0.0	0.5	0.5	0.0	0.0	0.3	0.27
LOC104912655	0.0	0.0	0.0	0.0	0.0	0.5	0.1	0.20
LOC104912656	4.5	5.5	22.0	6.0	10.5	19.0	11.3	7.51
LOC104912657	9.5	17.0	47.5	6.5	17.5	34.5	22.1	15.80
LOC104912658	21.5	8.5	5.5	19.0	17.0	5.0	12.8	7.27
LOC104912660	0.0	0.0	0.0	0.0	0.0	0.0	0.0	0.00
LOC104912662	2.0	0.5	0.0	3.0	2.0	2.5	1.7	1.17
LOC104912664	3.5	13.5	8.0	3.0	9.0	15.5	8.8	5.09
LOC104912665	957.0	495.5	645.0	811.5	505.0	689.5	683.9	178.83
LOC104912666	1.5	1.5	1.5	1.0	5.0	2.5	2.2	1.47
LOC104912668	45.5	97.5	79.0	45.5	103.5	93.5	77.4	26.01
LOC104912669	16.0	17.5	18.5	15.0	19.5	16.0	17.1	1.72
LOC104912671	0.0	0.0	0.0	0.0	0.0	0.0	0.0	0.00
LOC104912672	2.0	0.0	0.5	0.5	1.0	0.5	0.8	0.69
LOC104912674	0.0	0.0	0.0	1.0	0.5	0.0	0.3	0.42
LOC104912675	1.0	1.5	0.0	3.5	0.0	0.5	1.1	1.32
LOC104912676	0.0	0.0	0.0	0.0	0.0	0.0	0.0	0.00
LOC104912679	0.0	0.0	0.0	0.0	1.5	0.0	0.3	0.61
LOC104912680	0.0	0.5	0.0	0.0	0.0	0.0	0.1	0.20
LOC104912681	6.0	1.5	2.0	10.5	0.5	4.5	4.2	3.71
LOC104912684	0.0	0.0	0.0	0.0	0.0	0.0	0.0	0.00
LOC104912685	0.0	0.0	0.0	0.0	0.0	0.0	0.0	0.00
LOC104912686	0.5	0.5	0.0	0.5	0.0	0.0	0.3	0.27
LOC104912687	5.0	3.5	5.0	3.0	2.0	2.5	3.5	1.26
LOC104912688	1.5	1.0	2.5	0.0	1.5	3.5	1.7	1.21
LOC104912689	6.5	9.0	7.5	2.5	9.0	7.5	7.0	2.41
LOC104912690	0.0	0.0	0.0	0.5	0.0	0.0	0.1	0.20

LOC104912691	386.5	514.5	670.5	415.5	621.0	667.5	545.9	125.98
LOC104912692	2.0	1.0	1.5	2.5	0.5	2.0	1.6	0.74
LOC104912693	0.0	0.0	0.0	0.0	0.0	0.0	0.0	0.00
LOC104912694	1.0	0.5	0.0	0.5	0.0	0.0	0.3	0.41
LOC104912695	391.5	207.5	325.0	409.0	274.0	298.5	317.6	75.18
LOC104912696	22.0	15.0	41.5	23.5	16.0	33.5	25.3	10.36
LOC104912699	5.0	11.0	10.0	3.0	5.5	31.0	10.9	10.31
LOC104912700	46.5	6.5	17.0	47.5	13.0	26.5	26.2	17.39
LOC104912701	386.0	339.5	345.0	409.0	386.0	387.5	375.5	27.25
LOC104912702	0.0	0.5	1.5	0.0	0.5	0.5	0.5	0.55
LOC104912703	0.0	0.0	0.0	0.0	0.0	0.0	0.0	0.00
LOC104912704	0.5	0.0	0.0	0.0	0.0	0.5	0.2	0.26
LOC104912705	0.0	0.0	0.0	0.0	0.0	0.0	0.0	0.00
LOC104912707	0.0	0.0	0.0	0.0	0.0	0.0	0.0	0.00
LOC104912708	0.0	0.0	0.0	0.0	0.0	0.0	0.0	0.00
LOC104912709	0.0	0.5	2.0	0.0	0.0	1.0	0.6	0.80
LOC104912710	0.5	0.0	0.0	0.0	0.0	0.0	0.1	0.20
LOC104912711	0.0	0.0	1.0	0.0	0.0	0.0	0.2	0.41
LOC104912712	0.0	0.5	0.5	0.0	0.0	0.0	0.2	0.26
LOC104912713	0.0	0.0	0.0	0.0	0.0	0.0	0.0	0.00
LOC104912714	0.0	0.0	0.0	0.0	0.0	0.0	0.0	0.00
LOC104912715	0.5	0.0	1.5	0.0	0.0	2.0	0.7	0.88
LOC104912716	0.0	0.0	0.0	0.0	0.0	0.5	0.1	0.20
LOC104912717	0.0	0.0	0.0	0.0	0.0	0.0	0.0	0.00
LOC104912718	0.5	0.0	0.5	0.0	0.5	0.0	0.3	0.27
LOC104912719	32.0	14.5	82.5	23.5	26.0	41.5	36.7	24.18
LOC104912720	39.0	18.0	36.0	34.0	21.0	32.5	30.1	8.53
LOC104912721	0.0	0.0	1.5	0.0	0.5	0.5	0.4	0.58
LOC104912722	75.5	81.5	250.0	80.5	103.0	148.0	123.1	67.73
LOC104912723	0.0	0.0	0.0	0.5	0.0	0.0	0.1	0.20
LOC104912724	128.0	188.5	117.0	124.0	195.5	164.0	152.8	34.49
LOC104912726	0.0	0.0	0.0	0.0	0.5	0.0	0.1	0.20
LOC104912727	0.0	0.0	0.0	0.0	0.0	0.0	0.0	0.00
LOC104912728	2.0	3.5	1.5	1.5	2.0	1.0	1.9	0.86
LOC104912729	0.0	0.0	0.0	0.0	0.0	0.0	0.0	0.00
LOC104912730	0.0	0.0	0.0	0.0	0.0	0.0	0.0	0.00
LOC104912731	661.0	1040.5	452.0	634.0	1182.0	461.0	738.4	304.60
LOC104912732	0.5	1.0	0.0	0.5	0.0	0.0	0.3	0.41
LOC104912738	5.0	3.0	0.5	12.0	4.5	1.5	4.4	4.09
LOC104912739	1.5	2.5	1.5	2.0	2.5	1.5	1.9	0.49
LOC104912740	0.5	0.0	0.0	0.0	0.0	0.0	0.1	0.20
LOC104912741	47.5	71.5	53.5	48.0	100.0	63.0	63.9	19.96
LOC104912746	0.0	0.0	0.0	0.0	0.0	0.0	0.0	0.00
LOC104912747	879.0	509.0	439.0	787.5	559.0	432.0	600.9	188.20
LOC104912748	2.0	3.5	6.5	3.5	2.0	1.5	3.2	1.83
LOC104912749	248.0	327.5	231.0	221.0	375.5	206.5	268.3	67.61
LOC104912750	0.0	0.0	0.0	0.0	0.0	0.0	0.0	0.00
LOC104912752	5.0	1.0	1.5	9.5	2.0	1.0	3.3	3.37
LOC104912760	44.5	39.5	30.5	37.5	38.0	42.5	38.8	4.86
LOC104912761	161.0	103.5	105.0	150.0	100.0	111.5	121.8	26.57
LOC104912762	1.0	0.0	0.5	0.5	0.0	0.5	0.4	0.38
LOC104912763	1032.0	1052.0	1184.0	1013.0	1170.0	1415.0	1144.3	150.92
LOC104912764	16.0	8.0	30.0	36.0	7.0	31.0	21.3	12.61
LOC104912766	270.0	195.5	178.0	263.5	227.0	176.5	218.4	41.67

LOC104912768	20.5	5.0	1.5	22.5	2.5	0.5	8.8	10.01
LOC104912769	19.5	7.5	47.0	19.5	11.5	44.5	24.9	16.81
LOC104912770	55.0	29.5	159.5	62.0	36.0	143.5	80.9	56.18
LOC104912771	19.5	14.0	33.0	19.5	21.0	34.5	23.6	8.24
LOC104912772	0.0	0.0	0.0	0.0	0.0	0.0	0.0	0.00
LOC104912773	2.0	2.5	3.5	1.0	4.5	1.0	2.4	1.39
LOC104912774	1.0	2.0	2.5	2.5	1.5	0.5	1.7	0.82
LOC104912775	0.5	0.0	0.0	0.5	0.0	0.0	0.2	0.26
LOC104912776	1730.5	1255.5	1511.5	1514.5	1407.5	1434.0	1475.6	156.58
LOC104912777	0.0	0.0	0.0	0.0	0.0	0.0	0.0	0.00
LOC104912779	11.0	5.0	3.0	6.5	8.5	2.5	6.1	3.28
LOC104912780	393.0	176.0	661.0	395.0	251.5	621.0	416.3	193.74
LOC104912781	48.0	28.5	18.0	58.5	30.0	29.0	35.3	14.92
LOC104912782	557.5	628.0	400.0	538.5	783.0	427.5	555.8	139.91
LOC104912783	10.0	6.5	4.0	13.0	11.0	3.5	8.0	3.91
LOC104912784	1.0	0.0	3.0	0.0	0.0	2.0	1.0	1.26
LOC104912785	92.5	58.0	52.0	83.5	70.5	46.5	67.2	18.23
LOC104912786	208.5	151.5	144.0	218.5	187.0	138.5	174.7	34.65
LOC104912787	351.5	302.5	367.5	336.0	375.5	357.0	348.3	26.24
LOC104912788	0.5	1.0	2.0	1.0	0.5	1.5	1.1	0.58
LOC104912791	38.0	16.0	132.5	43.5	22.0	110.0	60.3	48.77
LOC104912792	20.5	4.5	86.5	20.5	6.5	58.0	32.8	32.60
LOC104912793	4.0	22.5	30.0	4.5	28.5	39.0	21.4	14.31
LOC104912794	10.5	4.0	14.0	12.5	3.0	10.5	9.1	4.53
LOC104912795	2.5	0.5	0.5	2.5	1.5	0.5	1.3	0.98
LOC104912796	0.0	0.0	0.0	0.0	0.0	0.0	0.0	0.00
LOC104912797	88.5	103.0	34.5	92.0	119.5	38.0	79.3	35.03
LOC104912799	4.5	1.5	1.5	7.0	0.5	1.0	2.7	2.54
LOC104912800	0.0	0.5	1.5	1.0	0.5	0.5	0.7	0.52
LOC104912801	1.0	1.0	0.0	1.0	1.5	2.0	1.1	0.66
LOC104912802	220.0	92.5	88.5	195.0	86.0	91.5	128.9	61.42
LOC104912803	2059.0	1733.5	859.5	1595.0	1879.5	783.5	1485.0	537.09
LOC104912804	0.0	0.0	0.0	0.0	0.0	0.5	0.1	0.20
LOC104912805	0.0	0.0	0.0	0.5	0.0	0.0	0.1	0.20
LOC104912806	9.0	9.0	10.0	11.0	10.5	13.0	10.4	1.50
LOC104912807	11.0	6.5	23.5	17.5	2.5	19.0	13.3	8.03
LOC104912808	2.5	0.5	4.0	1.0	1.5	2.5	2.0	1.26
LOC104912809	0.0	0.0	0.0	0.0	0.0	0.0	0.0	0.00
LOC104912811	3.5	1.5	2.5	2.0	0.5	0.5	1.8	1.17
LOC104912812	0.5	0.0	7.0	4.5	1.5	3.5	2.8	2.68
LOC104912815	25.0	11.5	98.0	36.5	15.0	71.5	42.9	34.58
LOC104912816	6.0	0.0	31.0	10.0	3.0	17.0	11.2	11.37
LOC104912818	0.0	0.0	0.0	0.5	0.0	0.0	0.1	0.20
LOC104912820	8.0	7.0	5.5	7.0	5.5	6.0	6.5	1.00
LOC104912821	0.0	0.0	0.0	0.0	0.5	0.5	0.2	0.26
LOC104912822	112.0	60.0	68.5	95.0	79.0	66.5	80.2	19.82
LOC104912823	19.0	12.5	30.5	32.5	7.5	24.0	21.0	9.90
LOC104912824	67.0	97.0	59.0	56.0	125.0	56.0	76.7	28.32
LOC104912825	1.5	0.5	3.0	1.0	0.5	2.5	1.5	1.05
LOC104912826	0.0	0.0	0.0	0.5	0.5	0.0	0.2	0.26
LOC104912827	4.5	0.5	0.5	0.5	0.5	0.0	1.1	1.69
LOC104912828	1.0	0.0	0.5	1.0	0.0	0.0	0.4	0.49
LOC104912829	0.5	0.5	0.0	2.0	0.0	0.0	0.5	0.77
LOC104912830	0.0	0.5	0.0	0.5	0.5	0.0	0.3	0.27

LOC104912831	1.0	0.0	0.0	1.0	0.0	0.5	0.4	0.49
LOC104912832	0.0	0.0	0.0	0.0	0.0	1.0	0.2	0.41
LOC104912833	2.5	1.0	3.5	5.0	2.0	3.5	2.9	1.39
LOC104912834	10442.5	8097.0	5450.5	8610.5	8418.0	5404.0	7737.1	1967.08
LOC104912835	166.5	63.5	43.0	143.5	62.5	73.0	92.0	50.29
LOC104912836	18.0	3.5	28.0	18.0	2.5	27.0	16.2	11.06
LOC104912838	0.5	0.0	0.5	0.5	0.5	2.5	0.8	0.88
LOC104912839	201.0	139.0	136.0	164.5	146.5	115.5	150.4	29.43
LOC104912841	7.5	4.5	6.5	3.5	5.5	3.0	5.1	1.74
LOC104912842	10.0	3.5	8.0	10.5	3.0	4.5	6.6	3.34
LOC104912843	0.0	0.0	0.0	0.5	0.0	0.0	0.1	0.20
LOC104912844	0.0	0.0	0.0	0.0	0.0	0.0	0.0	0.00
LOC104912845	0.5	0.0	0.5	0.0	0.0	0.0	0.2	0.26
LOC104912846	0.5	0.0	0.0	0.0	0.0	0.0	0.1	0.20
LOC104912847	0.0	2.0	0.0	0.5	1.5	0.0	0.7	0.88
LOC104912848	0.0	0.0	0.5	0.0	0.5	0.0	0.2	0.26
LOC104912849	0.0	0.0	0.0	0.0	0.0	0.0	0.0	0.00
LOC104912851	22.0	15.0	19.5	20.0	16.5	17.5	18.4	2.56
LOC104912852	0.5	0.5	0.0	1.5	4.5	0.5	1.3	1.67
LOC104912853	93.5	54.5	44.0	79.5	71.0	35.0	62.9	22.29
LOC104912854	1.0	0.0	0.5	0.0	0.0	0.0	0.3	0.42
LOC104912855	113.5	48.0	78.0	140.5	60.5	100.0	90.1	34.59
LOC104912856	1254.5	934.0	488.5	1095.5	1059.0	425.0	876.1	341.13
LOC104912857	0.5	0.0	0.0	1.0	1.0	0.0	0.4	0.49
LOC104912858	20.0	53.5	52.0	21.5	48.5	74.0	44.9	20.74
LOC104912859	12.5	31.5	42.0	13.5	41.5	43.5	30.8	14.39
LOC104912860	0.0	0.0	0.0	0.0	0.0	0.0	0.0	0.00
LOC104912861	0.0	0.5	0.0	1.0	0.5	0.5	0.4	0.38
LOC104912862	0.0	0.0	0.0	0.0	0.0	0.0	0.0	0.00
LOC104912863	1.0	0.0	0.0	2.5	1.0	0.0	0.8	0.99
LOC104912864	0.0	0.0	0.0	0.0	0.0	0.0	0.0	0.00
LOC104912865	3.0	1.5	0.0	2.0	3.0	0.5	1.7	1.25
LOC104912866	49.0	18.5	25.5	32.0	14.5	14.0	25.6	13.39
LOC104912867	0.0	0.0	0.0	0.0	0.0	0.0	0.0	0.00
LOC104912869	0.0	0.5	0.5	0.0	0.5	0.5	0.3	0.26
LOC104912871	63.5	59.5	73.5	74.5	73.0	65.5	68.3	6.26
LOC104912872	2.0	0.5	2.0	2.0	0.0	1.5	1.3	0.88
LOC104912873	1.5	2.0	2.0	2.0	1.5	1.5	1.8	0.27
LOC104912874	0.0	0.0	1.5	0.0	0.5	0.5	0.4	0.58
LOC104912875	0.5	0.0	0.0	0.5	0.0	0.0	0.2	0.26
LOC104912876	201.5	55.5	64.0	92.0	38.0	45.5	82.8	61.11
LOC104912877	5.5	0.5	1.0	2.5	1.0	0.5	1.8	1.94
LOC104912879	1.5	4.0	15.5	0.5	5.0	8.5	5.8	5.51
LOC104912881	0.0	0.0	0.5	0.0	0.0	0.0	0.1	0.20
LOC104912882	0.0	0.0	0.0	0.0	0.5	0.0	0.1	0.20
LOC104912883	811.5	588.5	463.5	668.0	657.5	504.0	615.5	125.86
LOC104912884	62.0	42.5	53.0	69.0	51.0	49.0	54.4	9.54
LOC104912885	8.5	3.0	12.5	8.5	3.5	8.5	7.4	3.58
LOC104912886	8.5	14.5	14.5	7.0	17.5	11.0	12.2	4.02
LOC104912887	0.0	0.0	0.0	0.0	0.0	0.0	0.0	0.00
LOC104912888	145.5	83.0	137.0	102.0	89.5	126.5	113.9	26.01
LOC104912892	67.5	50.5	107.5	76.0	57.0	116.0	79.1	26.90
LOC104912895	554.0	590.0	599.0	596.5	553.0	680.5	595.5	46.47
LOC104912896	12.0	10.5	15.0	9.5	10.5	15.0	12.1	2.40

LOC104912897	947.0	1236.5	783.0	846.0	1444.5	781.5	1006.4	273.72
LOC104912898	0.5	0.0	0.0	0.5	0.0	0.0	0.2	0.26
LOC104912900	57.0	12.5	63.0	61.5	15.5	48.5	43.0	23.04
LOC104912904	5.5	8.0	1.5	4.0	7.5	1.5	4.7	2.84
LOC104912905	5.5	10.0	6.0	2.5	4.0	6.5	5.8	2.54
LOC104912906	3.5	0.0	0.5	4.0	1.0	0.0	1.5	1.79
LOC104912907	0.5	0.0	0.5	0.0	0.0	0.0	0.2	0.26
LOC104912908	0.0	0.0	0.0	0.0	0.0	0.0	0.0	0.00
LOC104912909	19.0	17.5	14.0	15.5	12.5	5.5	14.0	4.77
LOC104912910	7.0	0.5	1.5	4.5	0.0	1.0	2.4	2.75
LOC104912911	30.5	24.0	35.0	30.5	22.5	36.0	29.8	5.54
LOC104912912	184.5	63.0	225.5	182.0	61.0	192.5	151.4	70.99
LOC104912913	2.5	4.5	3.0	2.5	4.5	5.5	3.8	1.25
LOC104912914	0.0	0.5	0.0	0.0	0.0	0.0	0.1	0.20
LOC104912915	8.0	4.0	2.0	7.5	0.5	1.5	3.9	3.18
LOC104912916	17.5	8.0	4.0	15.0	3.0	5.5	8.8	6.04
LOC104912917	161.0	60.0	212.5	190.5	68.0	185.5	146.3	65.83
LOC104912918	95.5	56.5	126.0	114.0	77.0	144.5	102.3	32.44
LOC104912919	1393.0	1073.5	1004.5	1281.5	1179.5	936.0	1144.7	173.12
LOC104912920	6.5	1.5	5.5	6.0	1.5	8.0	4.8	2.71
LOC104912921	5.0	2.0	9.0	5.0	3.0	7.5	5.3	2.64
LOC104912922	3.5	3.0	5.0	1.0	5.0	8.0	4.3	2.36
LOC104912923	12.5	20.5	17.0	6.5	27.5	27.0	18.5	8.24
LOC104912924	8.5	13.0	30.0	13.0	12.0	10.5	14.5	7.78
LOC104912925	0.0	0.0	0.0	0.0	0.5	0.0	0.1	0.20
LOC104912926	572.0	528.5	586.5	599.5	683.0	541.5	585.2	54.90
LOC104912929	17.5	5.0	16.0	16.5	2.0	11.0	11.3	6.54
LOC104912930	1.5	1.0	1.5	2.0	4.0	0.5	1.8	1.21
LOC104912931	800.0	529.0	514.5	760.5	618.0	585.5	634.6	119.56
LOC104912932	19.0	8.0	16.0	17.0	11.5	16.5	14.7	4.09
LOC104912933	0.0	0.0	0.0	0.0	0.0	0.0	0.0	0.00
LOC104912934	0.5	0.0	0.0	1.0	0.5	0.0	0.3	0.41
LOC104912935	0.5	0.0	0.0	0.0	1.0	1.0	0.4	0.49
LOC104912936	2.5	2.5	1.5	1.5	1.5	1.5	1.8	0.52
LOC104912937	0.0	0.0	0.0	0.0	0.0	0.0	0.0	0.00
LOC104912938	0.5	0.0	3.5	0.5	1.0	4.0	1.6	1.72
LOC104912939	272.0	199.5	184.5	275.5	223.0	209.5	227.3	38.11
LOC104912940	382.5	460.5	504.0	365.0	549.5	538.0	466.6	78.49
LOC104912941	21.0	14.5	27.5	26.0	13.0	31.0	22.2	7.28
LOC104912942	90.5	45.0	179.0	98.0	55.0	153.0	103.4	53.15
LOC104912943	84.0	22.5	124.0	81.0	25.5	97.5	72.4	40.47
LOC104912944	0.0	0.0	0.0	0.0	0.0	0.0	0.0	0.00
LOC104912945	95.5	19.5	86.5	103.5	24.0	64.0	65.5	36.40
LOC104912946	8.0	13.0	7.0	7.0	6.5	9.5	8.5	2.45
LOC104912947	0.0	0.0	0.0	0.0	0.0	0.0	0.0	0.00
LOC104912948	2.0	1.5	0.5	1.5	0.5	1.0	1.2	0.61
LOC104912949	0.0	0.5	0.5	0.0	0.0	0.0	0.2	0.26
LOC104912950	1.0	1.0	0.0	0.0	0.0	0.5	0.4	0.49
LOC104912951	529.0	620.0	416.0	490.0	657.5	384.0	516.1	108.73
LOC104912952	139.5	181.5	114.5	128.5	244.5	126.0	155.8	49.26
LOC104912953	3.0	0.5	6.5	1.0	0.0	1.5	2.1	2.40
LOC104912954	12.5	3.0	28.0	14.0	7.5	23.5	14.8	9.47
LOC104912955	0.5	0.5	0.0	1.0	0.0	1.0	0.5	0.45
LOC104912956	14.0	4.5	15.5	7.5	3.5	11.0	9.3	4.97

LOC104912957	57.0	18.0	99.0	59.0	26.0	86.5	57.6	31.99
LOC104912958	223.5	155.5	125.0	171.0	156.0	124.5	159.3	36.53
LOC104912959	1.5	1.0	2.0	0.5	0.5	4.5	1.7	1.51
LOC104912960	0.0	0.0	0.0	0.0	0.0	0.0	0.0	0.00
LOC104912961	0.0	0.0	0.0	0.0	0.0	0.0	0.0	0.00
LOC104912962	174.5	70.0	64.0	150.5	85.5	66.0	101.8	48.26
LOC104912963	1423.5	3268.0	4091.5	1785.0	3977.5	6059.5	3434.2	1697.63
LOC104912964	10.5	4.5	13.5	12.5	6.0	13.0	10.0	3.85
LOC104912965	4.5	0.5	0.0	7.5	0.5	0.0	2.2	3.13
LOC104912966	1.5	5.5	9.5	1.5	3.0	6.5	4.6	3.17
LOC104912967	9.5	8.5	2.0	6.5	8.5	2.5	6.3	3.25
LOC104912968	2.0	1.5	0.0	1.0	2.5	0.5	1.3	0.94
LOC104912971	0.0	0.0	0.0	0.0	0.0	0.0	0.0	0.00
LOC104912973	0.5	0.0	0.0	0.5	0.0	0.0	0.2	0.26
LOC104912974	2.5	0.0	0.0	0.5	0.0	0.5	0.6	0.97
LOC104912976	8.0	10.5	7.0	16.0	9.0	7.0	9.6	3.41
LOC104912977	3.5	2.5	5.5	4.0	1.5	1.5	3.1	1.56
LOC104912978	52.0	16.5	64.5	56.5	12.5	47.5	41.6	21.75
LOC104912979	9.5	14.5	13.5	12.5	17.0	14.0	13.5	2.47
LOC104912981	0.5	0.0	1.5	0.0	0.0	1.0	0.5	0.63
LOC104912982	607.5	518.5	402.0	593.0	581.5	389.5	515.3	97.56
LOC104912983	88.5	60.0	84.5	39.5	63.0	57.0	65.4	18.30
LOC104912984	305.0	329.5	214.5	293.0	325.5	228.5	282.7	49.43
LOC104912985	22.5	13.5	41.0	22.0	9.0	34.5	23.8	12.17
LOC104912986	1.0	0.0	1.0	1.0	0.0	0.5	0.6	0.49
LOC104912988	86.0	30.0	205.0	99.5	24.5	158.0	100.5	70.97
LOC104912989	3.5	1.0	2.5	1.0	1.5	1.5	1.8	0.98
LOC104912990	11.0	13.5	3.0	7.5	23.5	10.5	11.5	6.89
LOC104912991	102.0	125.5	67.5	79.5	146.5	74.5	99.3	31.43
LOC104912992	27.5	13.5	49.0	38.5	19.0	36.0	30.6	13.17
LOC104912993	683.5	488.5	269.5	574.5	518.5	286.5	470.2	163.14
LOC104912994	121.5	42.0	99.0	160.5	42.0	118.0	97.2	47.17
LOC104912995	38.5	7.5	6.5	20.0	2.5	2.5	12.9	14.09
LOC104912996	0.0	0.0	0.0	0.0	0.0	0.5	0.1	0.20
LOC104912998	45.0	17.5	52.0	35.5	19.0	46.0	35.8	14.62
LOC104912999	136.0	79.5	41.0	145.0	105.0	59.5	94.3	41.70
LOC104913001	0.0	0.0	0.0	0.0	0.0	0.0	0.0	0.00
LOC104913002	0.0	0.0	0.0	0.0	1.0	2.0	0.5	0.84
LOC104913003	385.5	242.0	278.0	357.5	230.5	237.0	288.4	67.02
LOC104913005	13.5	4.0	1.5	15.5	3.0	1.5	6.5	6.30
LOC104913007	1.0	1.0	1.0	0.0	0.5	1.0	0.8	0.42
LOC104913009	741.0	647.5	858.0	679.0	706.0	820.0	741.9	82.14
LOC104913010	140.5	112.5	127.0	116.0	135.0	120.0	125.2	11.01
LOC104913011	154.0	64.5	265.0	155.5	61.0	225.0	154.2	82.49
LOC104913014	104.5	54.0	86.5	56.5	59.0	51.5	68.7	21.69
LOC104913015	322.0	179.5	311.5	310.5	229.5	280.5	272.3	56.53
LOC104913016	21.5	18.0	21.5	17.5	12.5	28.5	19.9	5.35
LOC104913017	0.5	0.0	0.5	1.5	0.0	0.0	0.4	0.58
LOC104913018	1.5	0.0	0.0	1.5	0.0	0.0	0.5	0.77
LOC104913019	1385.0	898.0	1455.0	1229.5	1101.0	1299.0	1227.9	203.10
LOC104913021	11.5	1.5	0.5	9.0	1.5	0.5	4.1	4.86
LOC104913023	0.0	0.0	0.0	0.0	1.0	0.0	0.2	0.41
LOC104913024	3.5	0.0	1.0	1.5	0.5	0.5	1.2	1.25
LOC104913025	19.0	8.0	18.5	8.0	8.5	20.5	13.8	6.15

LOC104913026	54.5	23.5	81.5	66.5	18.0	73.0	52.8	26.43
LOC104913028	300.0	210.0	137.5	285.5	199.0	109.0	206.8	76.57
LOC104913029	0.0	0.0	0.0	0.0	0.0	0.0	0.0	0.00
LOC104913030	591.0	1356.5	645.5	508.0	1413.5	672.0	864.4	407.52
LOC104913032	1313.5	1715.0	1083.0	1228.0	1774.5	1172.5	1381.1	292.11
LOC104913033	568.5	511.0	709.0	568.0	451.5	711.0	586.5	104.97
LOC104913034	382.5	159.0	205.5	354.0	149.0	153.0	233.8	106.47
LOC104913035	68.5	20.0	49.5	76.0	14.0	24.5	42.1	26.41
LOC104913036	2.5	3.0	4.5	2.5	5.5	2.0	3.3	1.37
LOC104913037	438.0	574.5	404.5	452.5	698.0	437.5	500.8	113.03
LOC104913038	0.0	0.0	0.0	1.0	0.0	0.5	0.3	0.42
LOC104913039	20.0	23.5	36.0	19.0	18.5	53.5	28.4	13.92
LOC104913040	0.0	0.0	0.0	0.0	0.0	0.0	0.0	0.00
LOC104913041	0.0	0.5	1.0	0.0	1.0	5.5	1.3	2.09
LOC104913042	0.0	0.0	0.0	0.0	0.0	0.0	0.0	0.00
LOC104913043	0.0	0.0	0.0	0.0	0.0	0.0	0.0	0.00
LOC104913045	43.5	13.0	29.5	53.5	16.5	32.0	31.3	15.49
LOC104913046	0.0	0.0	0.0	0.0	0.0	0.0	0.0	0.00
LOC104913047	0.0	0.0	1.0	0.0	0.0	0.5	0.3	0.42
LOC104913048	42.5	37.0	42.5	78.5	48.5	58.0	51.2	15.18
LOC104913049	37.0	15.0	8.0	39.0	17.5	14.0	21.8	12.98
LOC104913050	0.0	0.0	0.0	0.0	0.0	0.0	0.0	0.00
LOC104913051	5.5	4.0	8.5	4.0	7.5	7.5	6.2	1.94
LOC104913054	2.5	2.0	1.5	2.0	1.5	1.5	1.8	0.41
LOC104913055	17.0	5.5	26.5	28.0	6.5	24.5	18.0	10.04
LOC104913056	0.0	0.0	0.0	0.0	0.0	0.0	0.0	0.00
LOC104913057	1350.0	1238.5	980.5	1247.0	1365.5	1027.0	1201.4	162.28
LOC104913059	4.5	0.0	0.5	3.0	0.5	0.5	1.5	1.82
LOC104913060	71.0	34.0	105.5	74.5	47.5	101.0	72.3	28.34
LOC104913062	0.5	0.0	0.5	0.0	0.0	0.0	0.2	0.26
LOC104913063	62.5	19.5	32.5	56.5	31.5	17.0	36.6	18.90
LOC104913064	94.5	62.0	47.0	80.0	44.5	34.0	60.3	23.15
LOC104913065	0.0	0.0	0.5	0.0	0.0	0.0	0.1	0.20
LOC104913067	289.5	236.0	291.5	272.0	237.5	246.0	262.1	25.53
LOC104913068	186.5	172.0	102.5	181.5	152.5	95.0	148.3	40.20
LOC104913069	148.0	89.5	226.5	140.0	107.0	174.5	147.6	49.05
LOC104913071	33.0	15.5	46.0	34.0	19.0	38.0	30.9	11.59
LOC104913072	570.0	104.0	221.0	554.0	113.5	143.0	284.3	219.10
LOC104913073	0.0	1.5	0.5	0.0	1.0	0.0	0.5	0.63
LOC104913074	7.5	1.0	0.5	2.0	1.0	0.5	2.1	2.71
LOC104913075	1.5	9.0	8.5	0.5	16.5	7.0	7.2	5.81
LOC104913076	0.0	0.0	0.0	0.0	0.0	0.0	0.0	0.00
LOC104913077	0.0	0.0	0.0	0.0	0.0	0.0	0.0	0.00
LOC104913078	0.0	0.0	0.0	0.0	0.0	0.0	0.0	0.00
LOC104913080	0.0	0.0	0.0	0.0	0.0	0.0	0.0	0.00
LOC104913082	0.0	0.0	0.0	0.0	0.0	0.5	0.1	0.20
LOC104913084	56.5	105.5	136.5	61.5	105.0	181.0	107.7	46.84
LOC104913085	104.0	42.5	167.0	112.5	26.5	133.0	97.6	53.71
LOC104913086	0.0	0.0	0.0	0.0	0.0	0.0	0.0	0.00
LOC104913088	11.0	11.0	20.5	11.0	13.0	24.0	15.1	5.71
LOC104913089	0.0	0.5	0.0	0.0	0.0	0.0	0.1	0.20
LOC104913090	0.0	0.0	0.0	0.0	0.5	0.0	0.1	0.20
LOC104913091	0.0	1.5	1.5	0.0	1.5	2.0	1.1	0.86
LOC104913092	0.0	0.0	0.0	1.0	0.0	0.5	0.3	0.42

LOC104913093	0.0	0.5	0.5	1.0	0.5	0.0	0.4	0.38
LOC104913094	0.5	0.0	0.0	0.0	0.0	0.0	0.1	0.20
LOC104913095	0.5	0.5	0.5	1.0	0.5	0.0	0.5	0.32
LOC104913096	1.5	4.5	1.5	3.0	3.5	3.5	2.9	1.20
LOC104913097	0.5	0.0	1.0	0.5	0.5	1.0	0.6	0.38
LOC104913098	42.5	15.5	59.5	37.5	17.0	54.5	37.8	18.45
LOC104913099	0.0	0.0	0.0	0.0	0.0	0.0	0.0	0.00
LOC104913100	1.0	0.5	1.0	1.5	0.5	0.5	0.8	0.41
LOC104913101	27.0	16.5	22.0	28.5	18.5	21.5	22.3	4.68
LOC104913102	1024.5	801.5	404.5	1003.5	889.0	452.0	762.5	271.60
LOC104913104	180.5	183.5	217.5	169.5	228.0	235.0	202.3	27.80
LOC104913105	13.0	16.5	6.5	9.5	18.5	10.0	12.3	4.55
LOC104913106	20.5	7.5	13.0	24.0	11.0	11.0	14.5	6.36
LOC104913107	7.0	4.5	1.0	7.5	4.0	2.5	4.4	2.52
LOC104913108	1.5	2.0	10.5	0.5	3.5	15.5	5.6	6.04
LOC104913109	0.5	0.0	3.5	1.0	3.0	5.0	2.2	1.97
LOC104913110	11.5	8.0	8.0	9.5	5.5	7.5	8.3	2.02
LOC104913111	2.0	0.5	0.5	1.5	0.5	0.5	0.9	0.66
LOC104913112	14.5	3.0	11.5	17.5	5.5	17.0	11.5	6.06
LOC104913113	288.0	208.5	129.0	318.0	292.0	221.0	242.8	70.31
LOC104913115	67.5	46.0	152.5	67.5	44.0	142.0	86.6	48.18
LOC104913116	0.0	0.0	0.0	0.0	0.5	0.0	0.1	0.20
LOC104913117	6.0	11.5	14.5	6.0	3.5	9.5	8.5	4.09
LOC104913119	31.0	52.0	24.5	24.5	56.5	31.5	36.7	14.02
LOC104913121	156.5	230.5	124.0	135.0	199.5	151.5	166.2	40.76
LOC104913122	4.5	5.5	4.5	9.5	3.0	6.5	5.6	2.25
LOC104913123	2.5	4.0	2.5	3.0	3.0	3.0	3.0	0.55
LOC104913124	18.5	9.0	9.5	22.5	11.5	15.5	14.4	5.39
LOC104913126	224.5	248.5	188.5	212.5	255.5	199.5	221.5	26.64
LOC104913127	1.5	2.0	0.0	1.0	2.0	0.5	1.2	0.82
LOC104913129	2.0	1.0	0.0	1.5	0.0	0.5	0.8	0.82
LOC104913130	133.5	28.0	79.5	136.5	20.0	52.0	74.9	50.97
LOC104913132	0.0	0.0	0.0	0.0	0.0	0.0	0.0	0.00
LOC104913134	3.5	1.0	5.0	3.0	2.0	4.5	3.2	1.51
LOC104913135	0.0	0.0	0.0	0.0	0.0	0.0	0.0	0.00
LOC104913136	20.5	4.0	40.0	24.0	7.0	38.5	22.3	15.17
LOC104913137	0.0	0.0	0.0	0.0	0.0	0.0	0.0	0.00
LOC104913138	627.0	614.5	328.0	559.5	582.0	385.5	516.1	127.00
LOC104913139	0.0	0.0	0.0	0.0	0.0	0.0	0.0	0.00
LOC104913140	185.0	209.5	250.0	155.0	202.0	252.0	208.9	37.61
LOC104913142	83.0	81.0	83.5	82.0	80.5	79.5	81.6	1.53
LOC104913144	1.5	3.5	5.0	1.0	4.5	3.0	3.1	1.59
LOC104913145	0.0	0.0	0.0	0.0	0.0	0.0	0.0	0.00
LOC104913146	0.0	0.0	0.0	0.0	0.0	0.0	0.0	0.00
LOC104913147	0.0	0.0	0.0	0.0	0.0	0.0	0.0	0.00
LOC104913148	0.0	0.0	0.0	0.0	0.5	0.0	0.1	0.20
LOC104913149	1.5	0.5	3.0	0.0	0.5	0.0	0.9	1.16
LOC104913150	185.5	140.0	156.5	166.5	210.0	136.0	165.8	28.22
LOC104913151	0.5	0.0	0.5	0.0	0.0	0.0	0.2	0.26
LOC104913154	1.0	1.0	1.5	2.0	4.5	1.5	1.9	1.32
LOC104913156	0.0	0.0	0.0	0.0	0.0	0.0	0.0	0.00
LOC104913158	0.0	0.0	0.5	0.5	0.5	0.5	0.3	0.26
LOC104913159	1.5	0.5	0.0	0.0	0.0	0.5	0.4	0.58
LOC104913160	0.0	0.5	0.0	1.0	0.5	0.5	0.4	0.38

LOC104913161	8.0	6.5	1.5	7.5	8.5	3.5	5.9	2.80
LOC104913162	3.0	2.5	2.5	1.0	3.5	1.5	2.3	0.93
LOC104913163	0.0	0.0	0.0	0.0	0.0	0.0	0.0	0.00
LOC104913164	0.0	0.0	0.5	0.0	0.0	0.0	0.1	0.20
LOC104913165	4.5	2.5	0.0	3.0	0.5	2.5	2.2	1.66
LOC104913166	10.5	53.0	33.0	10.5	44.0	30.0	30.2	17.29
LOC104913167	829.5	1214.0	753.0	729.5	1239.5	683.0	908.1	251.48
LOC104913168	110.5	97.5	127.0	114.5	104.5	127.0	113.5	11.93
LOC104913169	2.5	2.5	7.0	1.0	3.5	8.0	4.1	2.78
LOC104913175	0.5	0.0	0.0	0.0	0.0	0.0	0.1	0.20
LOC104913176	0.0	0.0	0.0	0.0	0.0	0.0	0.0	0.00
LOC104913177	1.0	1.0	0.5	2.0	0.5	0.0	0.8	0.68
LOC104913178	7.0	8.0	10.0	11.5	3.5	11.0	8.5	3.00
LOC104913181	0.0	0.0	0.0	0.0	0.0	0.0	0.0	0.00
LOC104913184	0.0	1.0	0.5	2.0	2.0	2.0	1.3	0.88
LOC104913189	0.0	0.0	0.0	0.0	0.0	0.0	0.0	0.00
LOC104913190	6.5	1.0	0.5	2.0	3.0	2.0	2.5	2.14
LOC104913191	0.0	0.5	0.5	0.5	1.5	1.5	0.8	0.61
LOC104913192	1.5	0.0	0.0	0.5	2.0	0.0	0.7	0.88
LOC104913193	0.0	0.5	0.0	0.0	0.0	0.5	0.2	0.26
LOC104913194	0.0	0.0	0.0	0.0	0.0	0.0	0.0	0.00
LOC104913195	0.0	0.0	0.0	0.0	0.0	0.0	0.0	0.00
LOC104913196	6.5	1.5	5.5	5.5	6.0	6.5	5.3	1.89
LOC104913197	0.5	0.0	0.0	0.5	0.5	1.5	0.5	0.55
LOC104913198	2.5	1.5	3.0	0.5	2.0	1.5	1.8	0.88
LOC104913199	11.5	5.0	1.0	14.0	9.0	1.5	7.0	5.36
LOC104913200	0.0	0.0	0.0	0.0	0.0	0.0	0.0	0.00
LOC104913201	0.0	0.5	0.0	0.0	0.0	0.0	0.1	0.20
LOC104913202	0.0	0.0	0.0	0.0	0.0	0.0	0.0	0.00
LOC104913204	0.0	0.0	0.0	0.0	0.0	0.0	0.0	0.00
LOC104913205	1.5	0.0	0.5	0.0	0.5	0.0	0.4	0.58
LOC104913206	0.0	0.0	0.0	0.5	0.5	0.0	0.2	0.26
LOC104913207	2.0	1.0	1.5	0.0	0.0	0.5	0.8	0.82
LOC104913209	214.5	129.5	193.0	177.0	133.0	162.0	168.2	33.50
LOC104913210	0.0	0.0	0.5	2.5	0.0	0.0	0.5	1.00
LOC104913211	0.5	0.5	1.5	0.0	0.5	2.0	0.8	0.75
LOC104913214	35.0	32.5	7.0	33.0	28.5	25.0	26.8	10.36
LOC104913215	1.5	1.0	5.0	3.0	1.0	2.0	2.3	1.54
LOC104913216	0.0	0.0	0.0	0.0	0.0	1.0	0.2	0.41
LOC104913217	13.0	3.0	27.0	19.5	4.0	19.5	14.3	9.50
LOC104913218	87.5	76.0	210.5	87.0	86.0	218.5	127.6	67.51
LOC104913219	0.5	1.0	1.0	0.0	0.0	0.0	0.4	0.49
LOC104913220	0.0	0.0	0.0	0.0	0.0	0.0	0.0	0.00
LOC104913221	0.5	0.0	1.0	0.0	0.0	0.0	0.3	0.42
LOC104913222	0.0	1.0	3.0	0.5	0.5	0.5	0.9	1.07
LOC104913223	16.0	9.5	37.0	29.0	8.5	38.0	23.0	13.41
LOC104913227	751.0	653.0	840.0	741.0	670.5	799.0	742.4	72.07
LOC104913229	26.0	13.0	66.5	27.5	7.5	53.5	32.3	23.11
LOC104913230	7.0	6.5	2.5	8.0	6.5	4.5	5.8	1.99
LOC104913231	1.0	0.0	0.0	0.0	1.0	0.0	0.3	0.52
LOC104913232	0.5	3.5	4.0	1.5	4.0	4.5	3.0	1.61
LOC104913233	0.0	0.0	0.0	0.0	0.0	0.0	0.0	0.00
LOC104913234	3.5	6.5	6.5	8.0	13.0	11.0	8.1	3.43
LOC104913240	0.5	0.0	0.0	0.0	0.0	0.0	0.1	0.20

LOC104913241	0.0	0.5	0.0	0.0	1.0	0.0	0.3	0.42
LOC104913242	0.5	0.0	0.0	0.0	0.0	0.0	0.1	0.20
LOC104913243	0.0	0.0	0.0	0.0	0.0	0.0	0.0	0.00
LOC104913244	0.0	0.0	0.0	0.0	0.0	0.0	0.0	0.00
LOC104913246	0.0	0.0	0.0	0.0	0.0	0.0	0.0	0.00
LOC104913247	0.0	0.0	0.5	0.0	0.0	0.0	0.1	0.20
LOC104913248	0.0	0.0	0.0	0.0	0.0	0.0	0.0	0.00
LOC104913250	0.0	0.0	0.0	0.0	0.0	0.0	0.0	0.00
LOC104913252	1.5	1.0	1.0	2.5	1.5	0.0	1.3	0.82
LOC104913253	100.0	92.0	121.5	85.5	84.5	120.0	100.6	16.58
LOC104913254	11.0	14.0	8.5	11.0	22.5	8.5	12.6	5.27
LOC104913255	0.0	0.0	0.5	0.0	1.0	0.0	0.3	0.42
LOC104913256	92.0	78.5	36.5	62.0	82.5	32.0	63.9	24.98
LOC104913257	0.5	4.5	1.0	0.5	4.0	0.0	1.8	1.97
LOC104913258	0.0	0.0	0.0	0.0	0.0	0.0	0.0	0.00
LOC104913259	0.0	0.0	0.0	1.0	0.0	0.0	0.2	0.41
LOC104913261	0.0	0.0	0.0	0.0	0.0	0.0	0.0	0.00
LOC104913262	0.0	0.0	0.0	0.0	0.0	0.0	0.0	0.00
LOC104913263	0.5	2.0	1.5	2.5	3.5	1.0	1.8	1.08
LOC104913264	13.0	14.0	11.0	16.0	12.5	9.5	12.7	2.27
LOC104913265	0.5	0.5	0.0	0.0	2.0	1.5	0.8	0.82
LOC104913266	1.5	0.5	0.0	0.5	0.0	0.0	0.4	0.58
LOC104913268	2.0	0.0	1.5	1.0	0.5	1.5	1.1	0.74
LOC104913269	17.0	20.5	14.5	13.0	18.5	11.5	15.8	3.43
LOC104913271	3.5	3.5	6.0	3.5	2.5	8.5	4.6	2.25
LOC104913274	0.0	0.0	0.0	0.0	0.0	0.0	0.0	0.00
LOC104913275	1.0	0.0	2.0	1.5	1.5	3.0	1.5	1.00
LOC104913276	0.0	0.0	0.0	0.0	0.0	0.0	0.0	0.00
LOC104913277	3.5	1.0	1.0	2.5	1.0	1.0	1.7	1.08
LOC104913278	8.5	6.0	1.5	14.0	3.5	3.0	6.1	4.60
LOC104913279	0.5	1.0	3.0	0.5	1.0	0.5	1.1	0.97
LOC104913280	0.0	0.0	0.0	0.0	0.0	0.0	0.0	0.00
LOC104913281	6.5	64.0	48.0	5.0	74.5	53.0	41.8	29.42
LOC104913282	0.0	0.0	1.0	0.0	3.5	1.0	0.9	1.36
LOC104913283	54.0	26.0	145.0	69.0	35.5	123.5	75.5	48.35
LOC104913284	51.0	22.0	185.5	60.0	21.0	135.0	79.1	66.69
LOC104913285	0.0	0.0	0.0	0.5	0.0	0.0	0.1	0.20
LOC104913287	0.0	1.5	1.0	1.5	4.0	3.0	1.8	1.44
LOC104913288	37.0	42.0	26.0	68.0	48.0	32.0	42.2	14.78
LOC104913289	0.5	7.0	2.0	1.0	8.5	3.0	3.7	3.31
LOC104913290	11.5	11.5	24.5	12.0	11.0	15.0	14.3	5.22
LOC104913291	3.5	6.5	4.0	2.5	7.0	5.5	4.8	1.78
LOC104913293	1.5	0.0	0.5	2.5	0.5	0.0	0.8	0.98
LOC104913294	1.5	0.0	3.5	2.5	1.0	3.5	2.0	1.41
LOC104913296	0.0	0.0	0.5	0.0	0.0	0.0	0.1	0.20
LOC104913297	0.0	0.0	0.0	0.0	0.5	0.0	0.1	0.20
LOC104913298	4.0	7.5	6.5	5.0	10.5	7.5	6.8	2.27
LOC104913299	0.5	0.0	0.0	0.0	0.0	0.0	0.1	0.20
LOC104913300	180.0	111.5	73.5	160.0	107.5	70.0	117.1	44.81
LOC104913301	0.5	2.0	4.0	0.0	1.0	1.5	1.5	1.41
LOC104913302	0.0	0.0	0.0	0.0	0.0	0.0	0.0	0.00
LOC104913303	0.0	0.0	0.5	0.0	0.0	0.0	0.1	0.20
LOC104913304	700.5	444.0	544.0	701.0	474.5	564.5	571.4	109.44
LOC104913305	168.0	175.5	117.0	157.0	191.0	156.0	160.8	25.04

LOC104913308	0.0	0.0	0.5	3.0	0.5	1.0	0.8	1.13
LOC104913309	15.0	5.5	5.5	14.0	8.5	3.0	8.6	4.91
LOC104913310	223.0	162.5	227.0	200.5	170.5	229.5	202.2	29.59
LOC104913311	85.5	24.5	128.0	78.5	29.5	114.0	76.7	42.56
LOC104913312	1.0	23.0	57.0	4.5	27.0	47.5	26.7	22.44
LOC104913313	0.0	0.0	0.0	0.0	0.0	1.0	0.2	0.41
LOC104913314	5.5	5.5	0.5	5.0	9.5	2.0	4.7	3.14
LOC104913315	1.0	1.0	1.0	0.0	0.5	0.5	0.7	0.41
LOC104913316	44.5	18.0	54.5	41.5	17.5	50.0	37.7	16.06
LOC104913317	0.0	2.5	1.5	0.0	0.5	2.5	1.2	1.17
LOC104913318	48.0	35.5	27.0	32.0	38.5	31.0	35.3	7.35
LOC104913321	1.0	2.0	5.5	1.5	1.0	3.5	2.4	1.77
LOC104913322	0.0	0.0	0.0	0.0	0.0	0.0	0.0	0.00
LOC104913323	0.0	0.0	0.0	0.0	0.0	0.0	0.0	0.00
LOC104913324	1928.0	1349.0	823.5	1607.5	1498.0	854.5	1343.4	434.69
LOC104913325	7.0	2.0	6.0	6.0	2.5	4.0	4.6	2.06
LOC104913326	1.0	0.0	0.0	0.0	0.0	0.0	0.2	0.41
LOC104913327	0.0	0.0	0.0	0.0	0.0	0.5	0.1	0.20
LOC104913328	549.0	332.0	574.5	465.5	336.5	504.0	460.3	104.53
LOC104913329	120.5	36.0	129.0	119.0	42.5	119.0	94.3	42.88
LOC104913330	0.5	5.0	21.0	0.0	14.0	21.0	10.3	9.72
LOC104913331	0.0	4.5	22.5	0.0	7.5	16.5	8.5	9.18
LOC104913333	2.5	0.0	0.5	2.5	0.0	1.5	1.2	1.17
LOC104913334	0.0	0.0	0.0	0.0	0.0	0.5	0.1	0.20
LOC104913336	102.5	40.0	41.5	88.5	56.0	35.0	60.6	28.28
LOC104913337	0.5	0.0	0.0	1.0	0.0	0.5	0.3	0.41
LOC104913338	0.0	0.0	0.0	0.0	0.0	0.0	0.0	0.00
LOC104913339	36.0	12.0	26.5	34.0	13.5	48.0	28.3	13.91
LOC104913340	49.0	33.5	17.0	71.0	37.0	21.5	38.2	19.72
LOC104913341	1.5	1.0	0.5	3.0	3.5	0.5	1.7	1.29
LOC104913344	37.0	32.0	65.5	31.0	22.5	76.0	44.0	21.50
LOC104913345	51.0	44.0	41.0	59.5	41.0	32.0	44.8	9.46
LOC104913346	0.5	0.5	0.0	0.0	0.0	0.0	0.2	0.26
LOC104913349	25.0	19.5	49.5	39.0	24.5	47.5	34.2	12.88
LOC104913350	27.5	13.5	22.0	24.0	21.5	12.5	20.2	5.95
LOC104913351	12.0	9.0	6.5	13.0	5.5	4.0	8.3	3.63
LOC104913352	0.0	0.0	0.0	0.0	0.0	0.0	0.0	0.00
LOC104913353	131.5	118.0	79.0	94.0	124.5	67.5	102.4	26.13
LOC104913356	4668.0	3714.0	3980.5	4458.5	4080.0	3863.5	4127.4	365.03
LOC104913357	16.5	9.5	67.5	33.0	9.0	52.5	31.3	24.31
LOC104913358	16.5	26.0	13.0	17.0	28.5	18.0	19.8	6.04
LOC104913359	90.0	55.5	34.5	77.0	64.5	26.0	57.9	24.53
LOC104913360	14.0	15.0	17.0	12.5	15.5	22.5	16.1	3.48
LOC104913361	389.5	487.0	269.0	352.5	556.0	318.5	395.4	107.66
LOC104913362	0.0	0.0	0.0	0.0	0.0	0.0	0.0	0.00
LOC104913363	0.5	0.0	0.0	0.0	1.5	1.5	0.6	0.74
LOC104913364	0.0	0.0	0.0	0.0	0.0	0.0	0.0	0.00
LOC104913365	3.0	15.5	8.5	2.0	7.5	7.5	7.3	4.80
LOC104913366	30.5	11.0	26.5	33.0	13.5	42.0	26.1	11.89
LOC104913367	2.0	1.0	0.0	0.5	1.0	0.0	0.8	0.76
LOC104913368	0.0	0.5	0.5	0.0	0.5	1.0	0.4	0.38
LOC104913369	235.0	118.5	531.5	261.5	145.0	460.5	292.0	168.31
LOC104913373	11.5	4.0	20.0	10.5	5.5	14.5	11.0	5.88
LOC104913374	326.5	253.5	321.5	307.5	291.5	303.0	300.6	26.31

LOC104913375	11.0	6.0	4.5	14.0	3.0	4.0	7.1	4.41
LOC104913376	288.5	441.0	182.5	273.5	477.0	165.0	304.6	129.58
LOC104913377	35.0	18.5	65.0	42.0	16.5	58.5	39.3	20.04
LOC104913378	0.0	0.0	1.5	0.0	0.0	0.0	0.3	0.61
LOC104913379	38.5	36.5	40.5	39.5	41.5	51.5	41.3	5.27
LOC104913381	1.5	0.5	2.5	0.5	0.0	2.0	1.2	0.98
LOC104913382	0.0	0.0	0.5	0.5	0.0	0.0	0.2	0.26
LOC104913383	0.0	0.0	0.0	0.0	0.0	0.0	0.0	0.00
LOC104913384	49.0	12.5	87.0	43.0	12.5	65.5	44.9	29.38
LOC104913385	195.5	177.0	170.5	189.5	186.5	156.0	179.2	14.45
LOC104913386	5.5	2.5	5.5	6.5	3.5	7.5	5.2	1.86
LOC104913387	4.5	2.0	3.0	8.0	2.0	1.0	3.4	2.54
LOC104913389	0.0	1.5	4.0	1.0	2.0	2.5	1.8	1.37
LOC104913390	27.5	1.5	26.5	35.0	2.0	24.5	19.5	14.20
LOC104913391	292.0	339.0	435.5	265.5	414.0	367.0	352.2	66.77
LOC104913392	0.5	0.5	0.0	0.5	0.0	0.5	0.3	0.26
LOC104913393	806.5	1001.0	762.5	673.0	1153.5	715.0	851.9	186.54
LOC104913394	3.0	0.0	3.5	3.5	3.0	3.0	2.7	1.33
LOC104913397	10.5	1.5	5.5	19.0	4.5	8.0	8.2	6.13
LOC104913398	173.5	167.5	273.5	171.5	195.5	246.0	204.6	44.68
LOC104913399	11680.5	6612.5	4592.0	10076.5	6442.0	4031.0	7239.1	3036.57
LOC104913401	0.0	0.0	0.0	0.0	0.0	0.0	0.0	0.00
LOC104913402	13.5	14.0	4.5	6.0	11.5	4.0	8.9	4.60
LOC104913403	0.0	0.0	0.5	0.0	0.0	0.0	0.1	0.20
LOC104913404	0.0	0.0	0.0	0.0	0.0	0.0	0.0	0.00
LOC104913405	0.0	0.0	0.0	0.0	0.0	0.0	0.0	0.00
LOC104913406	0.0	0.5	0.0	0.0	1.0	0.5	0.3	0.41
LOC104913409	4.0	6.0	8.5	5.0	6.0	2.0	5.3	2.19
LOC104913410	0.0	0.0	0.0	0.0	0.5	0.0	0.1	0.20
LOC104913411	885.5	1020.5	1188.0	806.5	1105.0	1123.0	1021.4	148.13
LOC104913412	139.5	119.5	120.0	130.0	113.5	123.5	124.3	9.19
LOC104913413	48.0	55.0	68.0	58.0	61.0	70.0	60.0	8.22
LOC104913414	80.0	62.0	153.5	92.5	74.0	144.5	101.1	38.50
LOC104913417	13.5	5.0	29.0	14.5	2.0	30.0	15.7	11.75
LOC104913418	100.5	144.5	120.0	109.0	148.5	105.0	121.3	20.64
LOC104913419	53.5	72.0	51.0	60.5	73.5	52.5	60.5	10.04
LOC104913420	1.0	0.0	0.0	1.0	0.0	0.5	0.4	0.49
LOC104913421	0.0	0.0	0.0	0.5	0.0	0.5	0.2	0.26
LOC104913422	15.5	3.5	6.5	18.0	3.0	8.5	9.2	6.26
LOC104913423	2.0	0.0	0.5	0.0	0.0	0.0	0.4	0.80
LOC104913424	8.5	2.5	1.0	3.0	0.5	0.0	2.6	3.12
LOC104913425	9.5	3.0	1.5	7.5	3.5	2.0	4.5	3.24
LOC104913426	17.0	7.5	2.0	12.0	6.5	2.0	7.8	5.85
LOC104913427	0.0	1.5	1.0	0.0	0.5	0.5	0.6	0.58
LOC104913428	1.5	0.0	2.5	1.0	0.5	1.5	1.2	0.88
LOC104913429	0.0	0.0	0.0	0.0	0.0	0.0	0.0	0.00
LOC104913430	0.5	1.5	1.5	2.5	1.0	0.5	1.3	0.76
LOC104913431	14.5	7.0	23.5	11.5	7.0	25.0	14.8	7.90
LOC104913432	79.0	39.0	12.0	60.5	27.0	15.0	38.8	26.50
LOC104913433	5.5	1.5	3.0	9.0	2.0	3.5	4.1	2.78
LOC104913435	2.0	1.0	4.5	1.0	1.5	5.0	2.5	1.79
LOC104913437	0.0	0.0	0.5	0.0	0.0	0.0	0.1	0.20
LOC104913439	185.5	43.5	215.0	183.5	35.5	134.0	132.8	76.87
LOC104913440	6.0	4.5	11.5	3.0	4.5	19.0	8.1	6.11

LOC104913441	811.5	569.0	442.5	767.5	676.5	493.5	626.8	149.30
LOC104913442	12.5	6.5	11.0	13.0	17.5	6.0	11.1	4.33
LOC104913443	3.0	3.5	3.0	5.0	6.0	3.5	4.0	1.22
LOC104913444	0.0	0.0	0.0	0.0	0.0	0.0	0.0	0.00
LOC104913445	3.5	1.0	1.5	3.5	3.0	1.5	2.3	1.13
LOC104913446	4.0	19.5	18.0	5.0	21.5	22.0	15.0	8.26
LOC104913447	0.5	3.0	8.0	1.0	4.5	8.0	4.2	3.30
LOC104913449	16.5	15.0	11.0	19.0	15.0	25.5	17.0	4.91
LOC104913452	115.5	40.5	133.5	122.5	39.5	101.5	92.2	41.72
LOC104913453	69.5	65.5	30.5	55.0	81.0	37.0	56.4	19.54
LOC104913454	0.0	0.0	0.0	0.0	0.0	0.0	0.0	0.00
LOC104913455	0.5	0.0	1.0	0.0	0.0	0.0	0.3	0.42
LOC104913456	0.0	0.0	0.0	0.0	0.0	0.0	0.0	0.00
LOC104913457	0.5	1.0	0.0	1.0	0.0	0.0	0.4	0.49
LOC104913458	11.0	13.5	18.0	9.0	10.5	21.0	13.8	4.72
LOC104913459	1.5	0.5	3.0	3.0	1.0	5.0	2.3	1.66
LOC104913460	11.5	1.5	11.0	13.0	6.5	9.5	8.8	4.22
LOC104913461	0.0	0.0	0.5	0.0	0.0	0.0	0.1	0.20
LOC104913462	2.0	21.5	22.0	2.0	10.0	52.5	18.3	18.94
LOC104913463	0.5	1.0	6.0	0.0	0.5	9.5	2.9	3.92
LOC104913466	258.5	197.5	168.0	180.0	196.5	148.5	191.5	37.64
LOC104913467	0.0	0.0	0.0	0.0	0.0	0.0	0.0	0.00
LOC104913468	0.0	0.0	0.0	0.0	0.0	0.0	0.0	0.00
LOC104913469	20.5	2.5	9.5	21.0	3.0	5.5	10.3	8.44
LOC104913470	12.0	0.0	9.0	14.0	0.0	3.5	6.4	6.10
LOC104913472	7.0	2.5	1.0	2.0	1.5	2.0	2.7	2.18
LOC104913473	1.5	1.5	0.0	0.5	1.0	0.5	0.8	0.61
LOC104913477	322.5	313.0	132.5	298.0	291.0	148.0	250.8	86.51
LOC104913478	0.5	0.5	1.0	0.0	1.0	0.0	0.5	0.45
LOC104913482	1.5	1.0	0.5	1.5	0.5	0.5	0.9	0.49
LOC104913486	50.0	26.0	35.5	45.5	23.5	26.0	34.4	11.20
LOC104913488	0.5	0.5	2.5	0.0	0.0	1.0	0.8	0.94
LOC104913489	7.5	2.5	3.5	7.0	4.0	1.5	4.3	2.42
LOC104913491	6.0	5.0	2.0	5.0	2.0	3.0	3.8	1.72
LOC104913492	0.0	0.5	0.0	0.0	2.0	1.5	0.7	0.88
LOC104913493	4.0	5.5	3.5	3.5	9.0	3.0	4.8	2.25
LOC104913496	74.0	76.5	250.0	107.0	57.0	268.0	138.8	94.70
LOC104913497	0.0	0.0	0.0	0.0	0.0	0.0	0.0	0.00
LOC104913498	275.0	123.0	152.0	238.5	147.5	159.0	182.5	59.90
LOC104913499	9.5	1.0	9.5	9.0	2.5	9.5	6.8	3.97
LOC104913500	0.0	0.0	0.0	0.5	0.0	0.0	0.1	0.20
LOC104913501	5.0	1.5	7.5	1.5	2.0	5.5	3.8	2.52
LOC104913502	35.0	19.5	32.5	42.0	15.5	23.5	28.0	10.14
LOC104913504	920.5	457.0	277.5	999.0	564.5	272.0	581.8	314.08
LOC104913505	0.0	0.0	0.0	0.0	0.0	0.0	0.0	0.00
LOC104913507	0.0	0.0	0.0	0.0	0.0	0.0	0.0	0.00
LOC104913508	0.0	0.0	0.0	0.5	0.0	0.5	0.2	0.26
LOC104913510	63.5	125.0	124.5	81.5	135.5	134.5	110.8	30.52
LOC104913512	61.5	92.5	23.0	83.0	140.0	58.0	76.3	39.40
LOC104913513	11.0	20.5	10.5	27.5	29.0	24.5	20.5	8.09
LOC104913514	0.0	2.0	4.0	1.0	1.5	4.0	2.1	1.63
LOC104913515	1.0	1.0	2.5	2.0	0.5	1.0	1.3	0.75
LOC104913516	0.0	0.5	2.5	1.0	0.5	0.0	0.8	0.94
LOC104913517	1.0	1.5	9.0	1.5	1.0	6.5	3.4	3.46

LOC104913519	5.0	1.0	2.5	5.0	4.0	0.5	3.0	1.97
LOC104913520	102.0	70.0	20.0	151.0	74.0	26.0	73.8	48.89
LOC104913521	76.5	58.0	83.0	67.0	72.5	83.0	73.3	9.72
LOC104913523	126.0	109.0	44.5	276.5	163.0	73.5	132.1	81.83
LOC104913524	1.5	1.0	0.5	0.0	1.0	0.5	0.8	0.52
LOC104913525	39.5	41.0	27.0	37.0	44.5	35.0	37.3	6.03
LOC104913526	1.5	0.5	0.5	0.5	0.0	0.0	0.5	0.55
LOC104913527	4.5	21.5	32.0	4.0	18.0	33.5	18.9	12.82
LOC104913528	2.0	4.0	4.5	0.5	4.0	6.0	3.5	1.95
LOC104913529	3.0	2.5	1.0	4.0	4.0	1.5	2.7	1.25
LOC104913531	6.5	2.0	3.0	6.5	2.5	5.0	4.3	2.02
LOC104913532	0.0	0.0	0.0	0.0	0.0	0.0	0.0	0.00
LOC104913533	1.0	0.5	6.0	0.5	1.0	7.0	2.7	2.99
LOC104913535	13.5	19.5	27.0	15.0	14.5	25.0	19.1	5.77
LOC104913536	8.0	5.0	18.0	9.5	4.5	15.5	10.1	5.54
LOC104913537	94.5	64.0	66.5	82.5	73.5	70.0	75.2	11.46
LOC104913538	36.5	20.5	29.0	30.5	18.5	16.0	25.2	8.02
LOC104913539	0.0	0.0	0.5	0.5	0.0	0.5	0.3	0.27
LOC104913543	7.5	4.5	2.0	6.5	1.0	2.5	4.0	2.61
LOC104913544	1.5	0.5	1.0	3.5	2.5	0.0	1.5	1.30
LOC104913548	14.0	8.5	7.5	13.5	4.5	7.0	9.2	3.79
LOC104913549	16.5	16.5	34.5	12.5	16.0	34.0	21.7	9.86
LOC104913550	270.0	317.0	256.5	225.5	354.0	274.5	282.9	45.71
LOC104913551	13.5	0.0	6.0	15.0	4.5	5.0	7.3	5.76
LOC104913552	428.0	882.0	397.0	405.0	1062.0	442.5	602.8	292.08
LOC104913553	38.5	127.0	32.0	30.0	155.0	18.0	66.8	58.57
LOC104913554	4.5	2.5	29.5	6.0	3.0	23.0	11.4	11.74
LOC104913555	0.5	0.0	0.5	1.0	1.5	1.0	0.8	0.52
LOC104913556	24.5	56.5	9.5	46.5	80.5	14.0	38.6	27.53
LOC104913558	4.5	2.0	5.5	4.0	1.5	4.5	3.7	1.57
LOC104913559	350.0	226.5	331.5	308.0	283.5	287.5	297.8	43.26
LOC104913560	2.0	0.0	0.0	0.0	1.0	0.5	0.6	0.80
LOC104913561	0.5	0.0	0.0	1.0	0.0	0.0	0.3	0.42
LOC104913562	20.5	6.5	8.5	15.5	6.5	4.0	10.3	6.37
LOC104913563	1.0	0.0	0.0	0.5	0.0	0.0	0.3	0.42
LOC104913564	66.5	32.0	33.5	42.0	37.0	21.5	38.8	15.19
LOC104913565	30.0	8.5	9.5	22.5	14.5	9.5	15.8	8.73
LOC104913566	0.0	0.0	0.5	0.0	0.0	0.0	0.1	0.20
LOC104913567	1274.0	420.0	192.5	1053.5	455.5	206.0	600.3	454.86
LOC104913568	10.0	4.5	12.5	12.0	2.0	12.0	8.8	4.48
LOC104913569	85.0	17.5	25.0	69.5	16.0	11.0	37.3	31.63
LOC104913570	440.0	370.0	377.5	369.5	307.0	350.5	369.1	43.13
LOC104913571	5.0	0.0	1.5	6.5	1.0	2.5	2.8	2.50
LOC104913572	45.0	86.0	149.0	25.0	67.5	137.5	85.0	49.72
LOC104913574	11.5	10.0	16.0	10.5	10.0	12.0	11.7	2.27
LOC104913575	10.0	4.0	5.5	8.5	6.0	4.5	6.4	2.35
LOC104913578	0.0	0.0	0.5	0.0	0.5	0.0	0.2	0.26
LOC104913579	1.0	1.0	1.0	2.5	3.5	3.0	2.0	1.14
LOC104913580	335.5	216.0	342.5	308.5	233.5	314.0	291.7	53.66
LOC104913581	34.5	11.5	51.5	30.0	15.5	44.0	31.2	15.64
LOC104913585	607.0	483.0	612.5	568.0	509.0	518.5	549.7	54.11
LOC104913586	0.0	0.0	0.5	0.5	0.0	0.0	0.2	0.26
LOC104913587	2171.0	2316.5	1528.5	2104.0	2453.5	1527.5	2016.8	397.43
LOC104913588	119.5	71.0	202.5	112.5	74.0	155.5	122.5	50.20

LOC104913589	2.0	1.0	2.5	2.0	1.5	0.5	1.6	0.74
LOC104913591	0.0	0.0	0.0	0.5	0.0	2.5	0.5	1.00
LOC104913592	10.0	8.0	18.5	10.5	2.5	23.5	12.2	7.57
LOC104913593	0.5	0.0	0.5	0.0	0.5	0.0	0.3	0.27
LOC104913595	1702.0	1493.0	1663.5	1594.0	1529.0	1593.0	1595.8	78.62
LOC104913597	47.0	11.5	101.5	59.5	13.5	71.0	50.7	34.65
LOC104913598	39.5	11.0	28.0	37.0	9.5	22.0	24.5	12.70
LOC104913600	112.5	105.0	59.5	89.0	141.0	67.0	95.7	30.33
LOC104913601	27.5	14.5	8.5	32.0	13.5	6.5	17.1	10.36
LOC104913602	0.0	0.0	0.0	0.0	0.0	0.0	0.0	0.00
LOC104913604	0.5	1.5	0.0	0.5	0.5	0.0	0.5	0.55
LOC104913605	0.5	0.5	0.0	0.5	0.5	0.0	0.3	0.26
LOC104913606	123.5	259.0	119.5	165.0	282.0	140.5	181.6	71.09
LOC104913607	0.0	0.0	0.0	0.0	0.0	0.0	0.0	0.00
LOC104913608	0.0	0.0	0.0	0.0	0.0	0.0	0.0	0.00
LOC104913609	31.0	24.0	73.5	38.0	32.0	57.5	42.7	18.93
LOC104913610	72.5	41.0	29.5	58.5	33.0	30.5	44.2	17.58
LOC104913611	0.0	0.0	0.0	0.0	0.0	0.0	0.0	0.00
LOC104913612	37.0	20.5	22.5	38.5	17.0	26.5	27.0	8.89
LOC104913614	0.0	0.0	0.0	0.0	0.0	0.0	0.0	0.00
LOC104913617	69.5	15.5	18.0	34.0	10.5	9.0	26.1	23.06
LOC104913620	108.5	93.0	154.5	103.5	77.0	179.0	119.3	39.12
LOC104913621	106.5	43.5	60.0	101.5	37.0	85.5	72.3	29.74
LOC104913622	9.0	3.0	3.5	6.5	2.0	4.0	4.7	2.60
LOC104913623	19.5	14.0	10.5	25.0	19.0	29.0	19.5	6.81
LOC104913624	0.0	11.0	9.0	0.5	12.0	10.5	7.2	5.45
LOC104913625	103.5	112.0	113.0	105.5	110.5	104.5	108.2	4.14
LOC104913627	11.0	4.5	5.0	12.5	3.0	1.5	6.3	4.46
LOC104913628	12.5	48.5	35.0	15.5	64.5	53.0	38.2	20.99
LOC104913629	1.0	7.0	8.5	2.5	7.0	8.5	5.8	3.21
LOC104913630	0.0	0.0	0.5	0.0	0.0	0.0	0.1	0.20
LOC104913631	225.5	86.0	229.5	171.0	86.0	200.5	166.4	65.71
LOC104913632	9.0	7.0	9.0	6.0	6.0	9.5	7.8	1.60
LOC104913633	4.5	5.0	4.0	4.5	9.5	2.5	5.0	2.37
LOC104913634	252.5	110.0	219.5	293.0	84.0	169.5	188.1	81.76
LOC104913635	35.5	37.5	13.0	34.5	31.5	15.5	27.9	10.79
LOC104913636	716.0	850.5	1054.0	844.5	885.0	1307.5	942.9	208.97
LOC104913637	0.0	0.5	0.0	0.0	0.0	0.5	0.2	0.26
LOC104913638	0.0	0.0	0.5	0.0	0.0	0.0	0.1	0.20
LOC104913639	68.5	45.0	88.5	70.5	35.0	86.5	65.7	21.70
LOC104913643	30.0	8.0	7.5	31.5	9.5	4.5	15.2	12.19
LOC104913644	52.0	40.5	47.0	49.0	40.5	53.0	47.0	5.47
LOC104913648	0.0	0.0	0.0	0.0	0.0	0.5	0.1	0.20
LOC104913651	15.5	18.0	13.5	19.5	18.5	9.5	15.8	3.76
LOC104913653	37.5	65.0	58.5	38.5	67.5	57.0	54.0	13.00
LOC104913658	98.0	160.5	98.5	148.0	217.5	126.5	141.5	45.05
LOC104913659	19.5	3.0	1.5	11.5	4.5	1.5	6.9	7.20
LOC104913661	0.0	0.0	0.0	0.5	0.0	0.0	0.1	0.20
LOC104913662	0.0	2.5	0.0	0.0	0.5	0.5	0.6	0.97
LOC104913664	14.5	7.0	10.0	20.0	3.5	3.5	9.8	6.53
LOC104913665	151.5	90.0	86.5	133.0	112.5	71.0	107.4	30.63
LOC104913666	0.0	0.0	0.0	0.0	0.0	0.0	0.0	0.00
LOC104913668	0.0	0.0	0.0	0.0	0.0	0.0	0.0	0.00
LOC104913670	24.5	33.0	111.0	46.0	55.0	136.0	67.6	45.26

LOC104913673	0.5	0.0	2.0	0.5	1.5	2.5	1.2	0.98
LOC104913674	41.5	19.5	20.5	46.0	16.5	15.0	26.5	13.58
LOC104913675	9.5	3.5	1.5	4.0	1.5	3.0	3.8	2.96
LOC104913676	28.0	21.0	25.0	22.0	26.5	27.5	25.0	2.92
LOC104913678	11.5	2.5	9.5	12.5	4.0	6.0	7.7	4.11
LOC104913681	0.0	0.5	0.5	0.0	0.0	0.5	0.3	0.27
LOC104913683	13.0	18.5	42.5	15.0	21.5	57.5	28.0	17.93
LOC104913684	1.0	0.5	0.0	0.5	0.0	1.0	0.5	0.45
LOC104913685	0.0	0.0	0.0	1.0	0.0	0.0	0.2	0.41
LOC104913686	1.0	1.5	2.5	2.0	2.0	1.5	1.8	0.52
LOC104913687	1.5	2.0	2.5	3.5	1.5	5.0	2.7	1.37
LOC104913688	0.0	0.0	0.0	0.0	0.5	1.5	0.3	0.61
LOC104913690	176.5	162.5	68.5	164.0	160.0	64.5	132.7	51.58
LOC104913691	0.5	0.5	0.0	1.5	0.5	1.0	0.7	0.52
LOC104913692	27.0	15.0	7.0	23.5	21.5	10.0	17.3	7.94
LOC104913693	14.0	11.0	9.0	16.0	12.5	12.5	12.5	2.41
LOC104913694	1434.0	2772.0	2525.0	1740.5	3714.5	3165.5	2558.6	858.50
LOC104913695	20.5	15.0	42.0	19.5	19.5	55.0	28.6	16.08
LOC104913696	24.5	30.0	6.0	43.5	35.5	13.0	25.4	14.01
LOC104913697	0.0	0.0	0.0	0.0	0.0	0.5	0.1	0.20
LOC104913698	0.0	0.0	0.0	0.0	0.0	0.5	0.1	0.20
LOC104913700	17.5	25.5	19.5	21.0	14.0	21.0	19.8	3.86
LOC104913701	98.5	62.0	418.0	154.5	70.5	436.0	206.6	173.87
LOC104913702	1.0	1.0	1.0	2.5	0.5	2.0	1.3	0.75
LOC104913703	0.0	0.5	0.5	0.5	0.5	0.5	0.4	0.20
LOC104913704	2.5	0.5	1.0	3.0	1.0	1.5	1.6	0.97
LOC104913705	0.0	0.0	1.0	0.0	0.5	1.0	0.4	0.49
LOC104913706	0.0	0.5	0.0	0.0	0.0	0.0	0.1	0.20
LOC104913707	0.0	0.0	0.0	0.0	0.0	0.0	0.0	0.00
LOC104913708	0.0	0.0	0.0	0.0	0.0	0.0	0.0	0.00
LOC104913710	0.0	0.0	0.0	0.0	0.0	0.0	0.0	0.00
LOC104913712	3.0	6.5	4.5	6.5	10.5	4.5	5.9	2.62
LOC104913713	0.0	0.0	0.0	0.0	0.0	0.0	0.0	0.00
LOC104913717	0.0	0.0	0.0	0.0	0.0	0.0	0.0	0.00
LOC104913718	18.0	3.5	22.5	26.0	4.0	8.0	13.7	9.78
LOC104913722	4.0	2.0	5.5	11.5	2.0	5.0	5.0	3.51
LOC104913723	15.5	3.0	20.0	11.5	5.0	11.0	11.0	6.35
LOC104913724	3.5	1.0	2.0	1.0	1.0	7.0	2.6	2.38
LOC104913725	154.0	514.0	1044.0	134.5	590.0	653.5	515.0	340.51
LOC104913726	1.0	42.0	70.0	0.0	33.0	61.5	34.6	29.53
LOC104913727	0.5	31.5	21.5	0.0	31.5	15.5	16.8	14.17
LOC104913729	0.0	4.0	2.0	0.0	2.5	0.0	1.4	1.69
LOC104913730	3206.0	25982.0	15243.0	2895.0	29008.5	12002.0	14722.8	11054.93
LOC104913731	0.0	2.0	15.0	0.0	1.5	5.5	4.0	5.75
LOC104913732	4.0	5.5	9.0	3.5	6.0	12.0	6.7	3.25
LOC104913733	0.0	0.0	1.0	0.0	0.0	0.5	0.3	0.42
LOC104913734	0.0	1.0	4.5	0.5	1.0	2.0	1.5	1.61
LOC104913735	70.5	15.5	23.5	82.5	18.5	28.0	39.8	29.03
LOC104913736	78.0	93.5	91.5	61.0	78.5	95.0	82.9	13.08
LOC104913737	0.0	2.0	2.5	1.5	0.5	2.0	1.4	0.97
LOC104913738	0.0	0.0	0.0	0.0	0.0	0.0	0.0	0.00
LOC104913740	3.0	0.5	2.5	3.5	0.5	2.0	2.0	1.26
LOC104913741	573.5	274.5	171.0	512.5	342.5	154.5	338.1	174.08
LOC104913742	0.5	43.0	54.0	0.5	41.5	52.5	32.0	24.90

LOC104913743	109.0	28.0	99.5	108.5	25.5	72.5	73.8	38.82
LOC104913745	1.0	3.0	5.5	2.5	3.0	4.5	3.3	1.57
LOC104913746	3.5	2.0	1.5	1.5	3.0	0.5	2.0	1.10
LOC104913747	3.5	1.5	0.5	3.0	0.0	0.0	1.4	1.53
LOC104913748	3.0	1.0	0.5	0.0	0.0	0.0	0.8	1.17
LOC104913749	27.0	5.5	13.5	18.0	9.0	13.0	14.3	7.52
LOC104913751	28.5	24.5	29.5	20.5	31.0	20.0	25.7	4.72
LOC104913752	63.0	39.5	21.5	44.0	31.5	29.0	38.1	14.55
LOC104913753	35.5	176.5	75.5	30.5	254.5	86.5	109.8	88.22
LOC104913755	3.5	1.0	20.5	6.5	2.5	21.0	9.2	9.15
LOC104913756	16.5	11.5	7.5	15.0	10.0	11.0	11.9	3.31
LOC104913758	0.0	0.0	0.0	0.0	0.0	0.0	0.0	0.00
LOC104913759	12.0	22.0	33.0	18.0	26.0	40.5	25.3	10.31
LOC104913760	40.5	23.0	41.5	42.0	24.5	33.0	34.1	8.66
LOC104913763	13.5	4.5	13.5	6.0	3.0	4.5	7.5	4.74
LOC104913764	4.5	5.0	2.0	2.0	6.0	2.5	3.7	1.72
LOC104913766	6.0	2.0	7.0	1.5	4.0	11.0	5.3	3.55
LOC104913767	1.0	1.5	2.0	1.0	0.5	1.0	1.2	0.52
LOC104913769	80.5	52.0	283.0	113.5	59.5	297.5	147.7	112.58
LOC104913771	812.5	732.5	408.0	747.0	811.5	406.0	652.9	193.26
LOC104913773	229.0	210.0	223.0	189.0	231.5	252.5	222.5	21.46
LOC104913775	616.5	666.5	468.5	535.0	728.5	569.0	597.3	93.43
LOC104913777	39.0	100.5	50.5	31.5	120.5	71.5	68.9	35.49
LOC104913778	25.5	16.0	64.0	24.5	13.0	42.5	30.9	19.20
LOC104913779	92.0	122.0	45.0	81.0	113.0	48.0	83.5	32.17
LOC104913781	0.0	0.0	0.0	1.0	0.0	0.5	0.3	0.42
LOC104913782	1.5	1.0	1.5	1.0	1.0	1.5	1.3	0.27
LOC104913783	3.5	0.0	2.5	2.5	1.0	2.5	2.0	1.26
LOC104913784	148.0	38.0	158.0	179.5	35.0	111.5	111.7	62.25
LOC104913785	9.5	6.5	20.0	13.5	4.0	13.0	11.1	5.70
LOC104913786	7.0	5.5	16.0	11.0	5.5	13.0	9.7	4.36
LOC104913789	1.5	0.5	0.0	0.0	0.5	0.0	0.4	0.58
LOC104913791	368.0	126.5	154.5	191.5	73.0	50.0	160.6	114.08
LOC104913792	0.0	0.0	0.0	0.0	0.0	0.0	0.0	0.00
LOC104913793	3.5	1.5	1.5	1.5	1.5	1.0	1.8	0.88
LOC104913795	12.5	8.5	12.0	8.0	8.5	10.0	9.9	1.93
LOC104913796	0.5	0.5	0.0	0.0	5.0	0.5	1.1	1.93
LOC104913797	19.5	56.0	44.0	19.0	72.0	65.0	45.9	22.68
LOC104913798	2.0	0.5	0.5	2.0	0.5	2.0	1.3	0.82
LOC104913799	0.0	0.5	0.0	0.0	0.0	0.0	0.1	0.20
LOC104913802	11.0	10.0	13.5	11.5	5.5	12.0	10.6	2.75
LOC104913803	23.0	15.0	26.0	28.5	15.5	18.5	21.1	5.62
LOC104913804	55.5	34.5	44.5	54.0	41.5	34.5	44.1	9.16
LOC104913806	0.0	0.0	0.0	1.5	0.5	0.0	0.3	0.61
LOC104913808	1.5	1.0	1.5	3.0	1.0	1.0	1.5	0.77
LOC104913809	12.5	4.0	7.5	6.5	9.0	20.5	10.0	5.87
LOC104913810	1.5	0.0	0.0	1.0	0.5	1.5	0.8	0.69
LOC104913812	100.5	98.0	73.5	79.5	119.0	80.5	91.8	17.13
LOC104913813	289.5	271.5	173.0	282.0	329.0	185.0	255.0	62.12
LOC104913814	16.5	5.5	25.0	14.0	5.5	14.5	13.5	7.36
LOC104913816	76.5	58.5	2.5	108.0	62.5	8.5	52.8	40.57
LOC104913817	714.5	717.5	591.5	716.0	791.0	573.0	683.9	84.15
LOC104913818	0.0	0.0	0.0	0.0	0.0	0.0	0.0	0.00
LOC104913819	57.5	26.0	8.0	43.0	31.0	7.5	28.8	19.62

LOC104913820	6.0	0.0	2.5	9.0	0.5	0.0	3.0	3.73
LOC104913821	802.5	828.5	863.5	771.0	976.5	929.0	861.8	78.16
LOC104913822	3.0	2.5	2.5	2.0	4.0	6.5	3.4	1.66
LOC104913824	135.0	52.5	58.0	121.0	74.5	67.0	84.7	34.68
LOC104913826	999.0	271.5	144.0	840.0	250.0	116.0	436.8	381.97
LOC104913827	19.0	20.0	22.0	20.0	18.0	23.5	20.4	2.01
LOC104913829	0.0	0.0	0.0	0.0	0.0	0.5	0.1	0.20
LOC104913830	0.0	0.0	0.0	0.0	0.0	0.0	0.0	0.00
LOC104913831	79.0	198.5	88.0	72.5	232.0	110.5	130.1	68.04
LOC104913832	0.0	0.0	0.0	0.0	0.0	0.0	0.0	0.00
LOC104913833	290.5	267.5	361.5	290.5	270.5	363.5	307.3	43.82
LOC104913836	0.0	0.0	0.0	0.0	0.0	0.0	0.0	0.00
LOC104913837	14.5	10.0	2.5	14.0	7.5	2.5	8.5	5.32
LOC104913838	0.0	13.5	3.0	1.0	15.5	2.5	5.9	6.76
LOC104913839	0.0	0.0	0.0	0.0	0.0	0.0	0.0	0.00
LOC104913840	0.0	0.0	0.0	0.0	0.0	0.0	0.0	0.00
LOC104913841	0.0	0.0	0.0	0.0	0.0	0.0	0.0	0.00
LOC104913842	0.0	13.0	7.0	2.5	10.5	9.5	7.1	4.97
LOC104913844	0.0	0.5	1.0	0.0	0.5	0.0	0.3	0.41
LOC104913846	0.5	0.5	0.5	0.0	0.0	1.0	0.4	0.38
LOC104913847	2.5	2.0	3.5	1.5	1.5	2.5	2.3	0.76
LOC104913848	319.0	281.0	120.5	244.0	293.0	141.5	233.2	83.01
LOC104913849	8.0	6.0	18.5	7.5	5.5	14.0	9.9	5.19
LOC104913850	1.0	0.0	0.5	0.5	1.5	0.0	0.6	0.58
LOC104913851	21.0	21.5	33.0	18.5	18.5	21.0	22.3	5.43
LOC104913853	0.0	0.0	0.0	0.0	0.0	0.0	0.0	0.00
LOC104913856	1.5	6.0	6.0	1.5	4.0	4.0	3.8	2.02
LOC104913857	452.5	754.0	544.5	370.0	716.5	490.5	554.7	151.45
LOC104913858	0.0	0.0	0.0	0.0	0.0	0.0	0.0	0.00
LOC104913859	0.0	0.0	0.5	0.0	0.0	0.5	0.2	0.26
LOC104913860	0.0	1.5	0.0	2.5	6.0	0.5	1.8	2.30
LOC104913861	0.0	3.5	1.5	0.5	8.5	1.0	2.5	3.18
LOC104913862	91.5	72.5	109.0	112.5	67.0	122.0	95.8	22.50
LOC104913863	116.0	81.0	129.0	105.5	106.5	114.0	108.7	15.97
LOC104913864	3.5	1.0	3.0	4.5	2.5	2.5	2.8	1.17
LOC104913865	10.0	3.0	3.0	7.0	3.5	1.5	4.7	3.19
LOC104913866	226.0	154.0	190.5	198.0	184.5	188.0	190.2	23.20
LOC104913867	0.0	0.0	0.0	0.0	0.0	0.0	0.0	0.00
LOC104913868	0.0	0.0	0.0	0.0	0.0	0.0	0.0	0.00
LOC104913869	1.0	0.0	0.0	0.0	0.0	0.0	0.2	0.41
LOC104913870	0.0	0.0	0.0	0.0	0.0	0.0	0.0	0.00
LOC104913871	0.0	0.0	0.0	0.0	0.0	0.0	0.0	0.00
LOC104913872	14.5	12.0	18.5	8.0	16.5	31.5	16.8	8.06
LOC104913873	381.5	445.5	418.0	308.5	486.0	367.0	401.1	62.59
LOC104913875	0.0	0.0	0.0	0.0	0.0	0.0	0.0	0.00
LOC104913877	478.0	466.5	416.5	468.0	508.5	402.0	456.6	39.92
LOC104913878	45.0	10.5	75.0	56.0	12.5	87.0	47.7	31.59
LOC104913879	944.5	860.5	1035.5	970.5	966.0	936.0	952.2	56.91
LOC104913880	25.5	11.5	9.5	31.5	23.0	5.0	17.7	10.45
LOC104913882	734.5	1632.5	1152.0	611.0	1686.0	1208.5	1170.8	443.65
LOC104913884	114.0	58.0	201.5	128.5	46.0	142.0	115.0	57.28
LOC104913885	0.0	1.5	4.5	1.0	1.5	2.0	1.8	1.51
LOC104913886	0.5	0.0	0.0	0.0	0.0	0.0	0.1	0.20
LOC104913887	0.0	0.0	0.5	0.0	0.0	0.0	0.1	0.20

LOC104913889	0.0	0.0	0.0	0.0	0.0	0.0	0.0	0.00
LOC104913890	211.5	38.0	124.0	133.0	27.0	91.0	104.1	68.19
LOC104913891	64.5	9.0	37.5	55.0	6.0	34.0	34.3	23.63
LOC104913892	0.0	0.0	1.5	0.5	0.0	0.0	0.3	0.61
LOC104913893	82.0	41.0	71.0	81.0	47.5	60.0	63.8	17.20
LOC104913894	34.0	16.5	37.0	33.0	17.5	32.5	28.4	8.99
LOC104913895	2.5	0.5	0.0	2.0	0.5	0.5	1.0	1.00
LOC104913898	318.0	176.0	317.5	276.5	183.0	261.0	255.3	62.93
LOC104913899	0.0	0.0	0.5	0.0	0.0	0.0	0.1	0.20
LOC104913900	0.5	0.0	0.5	0.5	0.0	0.0	0.3	0.27
LOC104913901	0.0	0.0	0.0	0.0	0.0	0.0	0.0	0.00
LOC104913902	0.0	0.0	0.0	0.0	0.0	0.0	0.0	0.00
LOC104913903	268.0	185.5	241.5	280.0	208.5	209.5	232.2	37.18
LOC104913904	8.5	1.0	3.5	8.0	0.5	3.5	4.2	3.40
LOC104913905	1.0	1.0	0.5	5.5	0.5	1.5	1.7	1.91
LOC104913906	20.5	22.5	14.5	18.5	27.5	12.5	19.3	5.46
LOC104913907	284.5	135.0	241.5	268.5	165.0	223.0	219.6	58.71
LOC104913908	842.5	793.0	548.5	833.0	946.5	555.0	753.1	164.00
LOC104913909	32.0	29.0	30.0	27.0	29.5	29.5	29.5	1.61
LOC104913910	4332.5	3589.5	3176.0	4115.0	4614.0	2718.5	3757.6	726.35
LOC104913913	16.5	6.5	13.0	11.5	7.5	6.0	10.2	4.19
LOC104913914	142.0	128.5	160.5	121.5	147.0	145.0	140.8	13.92
LOC104913915	0.0	0.0	1.0	0.0	0.0	0.0	0.2	0.41
LOC104913917	0.0	0.5	0.5	0.0	0.5	0.0	0.3	0.27
LOC104913918	1.0	1.5	2.5	0.5	1.0	5.5	2.0	1.84
LOC104913919	21.5	13.5	46.5	32.0	11.5	66.5	31.9	21.32
LOC104913920	57.0	63.0	34.5	49.5	44.5	32.0	46.8	12.24
LOC104913921	27.0	7.0	8.5	10.0	10.5	3.0	11.0	8.29
LOC104913922	0.5	0.0	0.5	0.5	1.0	1.5	0.7	0.52
LOC104913924	12.5	2.0	16.5	13.5	3.5	16.5	10.8	6.42
LOC104913925	49.5	41.0	98.0	57.0	49.0	103.5	66.3	27.19
LOC104913926	31.5	9.5	30.0	23.0	19.0	37.5	25.1	10.04
LOC104913927	18.0	8.0	29.0	20.5	6.5	36.5	19.8	11.69
LOC104913928	1.0	0.0	0.0	0.0	0.0	0.0	0.2	0.41
LOC104913929	193.0	169.5	113.5	145.0	178.0	134.0	155.5	29.82
LOC104913930	66.0	29.5	202.0	68.5	28.5	146.0	90.1	69.51
LOC104913931	233.0	235.5	286.5	252.5	233.0	263.0	250.6	21.42
LOC104913932	100.0	106.0	58.0	107.5	140.5	87.5	99.9	27.03
LOC104913933	13.5	7.5	12.0	11.5	9.0	14.5	11.3	2.66
LOC104913934	0.0	0.0	0.0	0.0	0.5	0.0	0.1	0.20
LOC104913935	0.0	0.0	1.0	0.0	0.0	0.5	0.3	0.42
LOC104913936	161.5	281.5	246.0	135.0	356.5	261.0	240.3	81.19
LOC104913937	0.0	6.5	15.5	1.0	4.5	10.5	6.3	5.89
LOC104913940	444.0	408.0	475.0	358.0	481.5	447.0	435.6	46.14
LOC104913942	60.5	38.5	29.0	56.0	33.0	29.0	41.0	13.88
LOC104913943	9.0	7.5	15.0	16.5	3.0	20.5	11.9	6.51
LOC104913944	698.5	2158.0	1761.5	694.5	2357.5	1498.0	1528.0	710.38
LOC104913945	3.5	3.0	10.5	0.5	3.5	9.5	5.1	3.98
LOC104913946	4.0	7.5	5.0	6.0	6.5	7.5	6.1	1.39
LOC104913948	2.0	2.0	6.0	2.0	1.5	0.5	2.3	1.89
LOC104913950	142.0	127.5	98.5	144.5	143.0	92.5	124.7	23.48
LOC104913952	0.0	0.5	0.0	0.5	0.0	0.0	0.2	0.26
LOC104913953	0.5	0.5	2.5	0.0	3.0	1.0	1.3	1.21
LOC104913956	70.0	60.5	55.0	64.5	54.5	70.5	62.5	7.05

LOC104913957	107.5	68.0	175.5	135.5	53.0	208.0	124.6	60.48
LOC104913958	118.0	69.5	102.0	114.0	63.0	96.5	93.8	22.84
LOC104913960	0.0	0.0	1.0	0.0	0.0	0.0	0.2	0.41
LOC104913961	1913.5	2060.0	1273.5	1778.0	2574.5	1273.5	1812.2	496.86
LOC104913962	208.0	41.0	405.0	259.0	32.0	337.0	213.7	152.79
LOC104913964	15.5	6.5	7.0	7.0	8.5	10.5	9.2	3.43
LOC104913965	344.5	591.5	261.0	303.0	671.0	289.0	410.0	175.30
LOC104913967	43.5	64.0	34.5	23.5	51.0	30.0	41.1	14.86
LOC104913969	1420.0	1658.5	1178.0	1313.5	1894.0	1263.5	1454.6	271.58
LOC104913970	62.0	74.5	73.0	59.5	84.5	81.5	72.5	10.08
LOC104913973	1.5	7.0	5.5	1.0	5.0	10.0	5.0	3.39
LOC104913974	0.0	0.0	0.0	0.0	1.0	0.0	0.2	0.41
LOC104913976	0.0	0.0	0.0	0.0	0.0	0.0	0.0	0.00
LOC104913979	64.0	41.0	82.0	69.5	43.5	64.5	60.8	15.75
LOC104913980	1.5	1.5	1.5	1.0	0.5	0.0	1.0	0.63
LOC104913981	1.0	0.0	0.0	0.0	0.0	0.5	0.3	0.42
LOC104913982	0.0	0.0	0.0	0.0	0.0	0.0	0.0	0.00
LOC104913984	1.0	27.0	39.5	0.5	28.0	46.5	23.8	19.25
LOC104913985	0.0	0.0	0.5	1.0	0.0	1.0	0.4	0.49
LOC104913986	0.0	0.0	0.0	0.0	0.0	0.5	0.1	0.20
LOC104913987	66.5	25.0	11.5	52.5	18.5	10.5	30.8	23.30
LOC104913988	1074.0	635.0	603.5	1136.0	692.0	574.0	785.8	251.13
LOC104913990	0.0	0.5	0.0	0.0	0.0	0.0	0.1	0.20
LOC104913992	0.0	0.5	0.5	0.5	1.0	2.5	0.8	0.88
LOC104913993	216.0	243.5	289.5	190.0	261.5	318.0	253.1	47.03
LOC104913994	70.0	27.0	124.5	69.5	28.0	107.0	71.0	39.87
LOC104913995	0.0	0.0	0.0	0.0	0.0	0.0	0.0	0.00
LOC104913996	0.0	0.0	0.0	0.0	0.0	0.0	0.0	0.00
LOC104913997	0.0	0.0	0.0	0.0	0.5	0.0	0.1	0.20
LOC104913998	0.0	0.0	0.0	0.0	0.0	0.0	0.0	0.00
LOC104913999	0.5	0.0	0.5	1.0	1.0	0.0	0.5	0.45
LOC104914000	0.0	0.0	0.0	0.0	0.0	0.0	0.0	0.00
LOC104914002	35.5	18.0	17.0	32.0	16.5	14.0	22.2	9.14
LOC104914003	0.0	0.0	6.0	0.0	0.5	5.5	2.0	2.92
LOC104914004	0.5	2.5	5.0	1.0	2.5	8.0	3.3	2.81
LOC104914005	0.0	1.0	4.0	2.5	4.0	5.5	2.8	2.07
LOC104914006	25.5	104.0	628.5	22.0	122.0	447.0	224.8	252.33
LOC104914007	0.5	4.0	7.0	0.5	9.0	13.0	5.7	4.96
LOC104914008	5.0	2.5	4.0	8.0	2.0	2.5	4.0	2.26
LOC104914009	798.0	306.5	491.0	811.0	271.0	286.0	493.9	253.40
LOC104914010	0.0	0.0	0.0	0.5	0.0	0.0	0.1	0.20
LOC104914011	0.0	0.0	0.0	0.0	0.0	0.0	0.0	0.00
LOC104914012	0.5	2.0	19.0	0.0	1.0	13.0	5.9	8.06
LOC104914013	0.5	4.5	32.5	0.0	5.0	16.5	9.8	12.60
LOC104914014	98.0	106.5	82.5	85.0	88.0	86.5	91.1	9.24
LOC104914016	0.0	0.0	0.0	0.0	0.0	0.0	0.0	0.00
LOC104914020	0.0	0.0	0.0	0.0	0.0	0.0	0.0	0.00
LOC104914021	0.0	0.0	0.0	0.0	0.0	0.0	0.0	0.00
LOC104914022	0.0	0.0	0.0	0.0	0.0	0.0	0.0	0.00
LOC104914023	0.0	0.0	0.0	0.0	0.0	0.0	0.0	0.00
LOC104914024	0.0	0.0	0.0	0.0	0.0	0.0	0.0	0.00
LOC104914025	0.0	0.0	0.0	0.0	0.0	0.0	0.0	0.00
LOC104914026	0.0	0.0	0.0	0.0	0.0	0.0	0.0	0.00
LOC104914027	0.0	0.5	1.0	0.0	1.5	0.5	0.6	0.58

LOC104914028	0.5	14.5	15.0	0.0	16.5	11.0	9.6	7.45
LOC104914031	2.0	0.5	0.0	0.0	1.5	0.5	0.8	0.82
LOC104914032	4.5	64.0	26.5	5.0	84.0	30.0	35.7	32.15
LOC104914033	0.0	0.0	0.0	0.0	0.0	0.0	0.0	0.00
LOC104914035	723.5	459.5	487.5	752.0	510.0	415.5	558.0	143.04
LOC104914036	8.5	8.0	23.5	5.5	9.5	19.0	12.3	7.17
LOC104914038	7.5	1.5	1.5	2.5	1.0	1.0	2.5	2.51
LOC104914039	1030.0	592.0	324.5	904.5	630.5	323.0	634.1	291.30
LOC104914040	0.0	0.0	0.0	0.0	0.0	0.0	0.0	0.00
LOC104914041	1544.0	1145.5	1135.0	1523.0	1171.5	978.0	1249.5	230.33
LOC104914042	0.0	0.5	0.0	4.0	0.5	0.5	0.9	1.53
LOC104914043	0.0	0.0	0.0	0.0	0.0	0.0	0.0	0.00
LOC104914044	26.5	16.5	56.5	42.0	18.0	33.5	32.2	15.29
LOC104914045	234.0	130.5	395.0	203.5	178.5	389.5	255.2	111.48
LOC104914046	11.0	1.0	3.5	8.0	1.0	1.5	4.3	4.22
LOC104914047	0.0	0.0	0.0	0.0	0.0	0.0	0.0	0.00
LOC104914048	26.5	19.5	47.5	21.0	21.0	33.0	28.1	10.75
LOC104914049	13.5	37.0	51.5	12.5	43.5	63.0	36.8	20.40
LOC104914050	1.0	0.0	0.0	0.5	0.0	0.0	0.3	0.42
LOC104914052	275.5	2108.5	1459.0	352.0	2432.0	1204.5	1305.3	885.17
LOC104914053	4.0	3.5	1.0	4.0	3.0	0.5	2.7	1.54
LOC104914055	0.0	0.0	0.0	0.0	0.0	0.0	0.0	0.00
LOC104914056	78.5	55.0	132.5	86.0	56.5	84.5	82.2	28.16
LOC104914057	41.0	20.0	65.0	54.0	18.0	34.5	38.8	18.58
LOC104914059	580.5	460.5	729.0	641.0	619.5	804.5	639.2	119.30
LOC104914060	14.0	8.5	46.0	31.0	6.5	30.0	22.7	15.52
LOC104914061	35.5	21.5	77.5	25.5	22.0	52.0	39.0	22.10
LOC104914063	18.5	8.0	17.5	12.0	13.5	10.0	13.3	4.13
LOC104914064	59.0	13.5	40.0	61.0	14.0	34.0	36.9	20.78
LOC104914065	2.5	3.0	0.5	2.0	0.5	1.5	1.7	1.03
LOC104914066	0.0	0.0	0.0	0.0	0.0	0.0	0.0	0.00
LOC104914067	1.0	0.5	6.5	1.5	0.5	2.5	2.1	2.29
LOC104914068	71.0	216.0	71.5	74.5	248.5	95.5	129.5	80.76
LOC104914069	0.5	0.0	0.0	0.5	0.0	0.0	0.2	0.26
LOC104914071	0.5	0.0	0.0	0.5	0.0	0.0	0.2	0.26
LOC104914072	0.0	0.0	1.0	1.0	0.0	0.0	0.3	0.52
LOC104914073	0.5	0.0	1.5	0.5	1.0	1.0	0.8	0.52
LOC104914076	0.0	0.0	0.0	0.0	0.0	0.0	0.0	0.00
LOC104914077	3.0	1.5	0.5	1.5	1.5	0.5	1.4	0.92
LOC104914079	9.0	2.0	1.0	8.5	2.0	0.0	3.8	3.95
LOC104914081	83.5	807.5	29.5	70.5	855.5	45.5	315.3	400.55
LOC104914082	0.0	0.0	0.0	0.5	0.0	0.0	0.1	0.20
LOC104914083	62.5	76.5	43.0	57.5	92.0	64.5	66.0	16.75
LOC104914085	601.0	397.5	554.0	605.5	486.0	503.0	524.5	79.19
LOC104914086	0.5	0.0	0.5	0.5	0.0	0.0	0.3	0.27
LOC104914088	1.5	0.0	0.5	0.0	0.5	0.5	0.5	0.55
LOC104914089	0.0	0.0	1.5	0.5	0.0	0.0	0.3	0.61
LOC104914090	201.5	56.0	51.5	179.5	38.0	89.0	102.6	70.48
LOC104914091	13.5	23.0	14.0	18.0	21.5	17.0	17.8	3.86
LOC104914093	75.0	48.5	61.5	75.5	31.5	47.5	56.6	17.31
LOC104914095	6.5	0.5	6.5	7.0	0.5	8.5	4.9	3.50
LOC104914102	0.0	0.0	0.5	0.0	0.5	1.5	0.4	0.58
LOC104914104	4.0	13.0	6.0	6.5	22.5	9.0	10.2	6.79
LOC104914106	0.0	0.0	0.0	0.0	0.0	0.0	0.0	0.00

LOC104914108	43.0	34.5	48.5	52.5	37.5	46.5	43.8	6.81
LOC104914110	0.0	0.5	0.0	1.0	2.0	1.5	0.8	0.82
LOC104914112	68.5	58.5	70.0	73.5	63.0	69.5	67.2	5.44
LOC104914114	109.0	54.0	34.5	96.5	61.5	50.5	67.7	28.84
LOC104914115	0.0	0.0	0.0	0.0	0.0	0.0	0.0	0.00
LOC104914119	12.5	16.0	15.0	15.5	8.0	15.5	13.8	3.08
LOC104914121	76.5	38.0	151.5	86.5	46.0	85.0	80.6	40.27
LOC104914122	0.0	0.0	0.0	0.0	0.0	0.0	0.0	0.00
LOC104914124	138.5	68.0	172.0	121.5	75.0	165.0	123.3	44.13
LOC104914125	0.0	0.0	0.0	0.0	0.0	0.0	0.0	0.00
LOC104914126	0.0	0.0	0.0	0.0	0.0	0.0	0.0	0.00
LOC104914127	0.5	1.5	2.5	0.0	3.5	3.0	1.8	1.40
LOC104914128	2.0	12.0	38.0	2.5	19.0	61.5	22.5	23.26
LOC104914131	283.5	84.0	117.0	226.0	79.5	98.5	148.1	85.61
LOC104914133	0.5	0.5	0.5	0.0	0.0	0.0	0.3	0.27
LOC104914134	1.5	1.0	0.5	1.5	0.0	0.5	0.8	0.61
LOC104914136	0.0	0.0	0.0	0.0	0.0	0.0	0.0	0.00
LOC104914141	1.0	0.0	5.5	0.0	1.0	3.5	1.8	2.21
LOC104914142	1.0	1.5	0.5	1.0	2.0	0.5	1.1	0.58
LOC104914144	2.0	3.5	2.5	0.5	1.0	5.0	2.4	1.66
LOC104914145	5.0	1.0	6.0	3.0	6.0	9.5	5.1	2.91
LOC104914146	563.0	530.0	170.0	657.5	532.0	130.0	430.4	222.46
LOC104914147	235.5	216.0	236.5	188.0	239.5	217.5	222.2	19.55
LOC104914148	40.5	19.5	29.5	44.5	13.5	43.0	31.8	13.07
LOC104914149	0.5	2.0	0.0	2.5	3.5	0.0	1.4	1.46
LOC104914150	5.5	3.0	6.0	2.5	2.0	1.5	3.4	1.88
LOC104914152	1.5	0.5	0.0	0.0	0.0	0.0	0.3	0.61
LOC104914153	19.5	3.0	1.5	11.5	3.0	0.0	6.4	7.56
LOC104914155	254.0	191.5	296.5	215.0	195.0	235.5	231.3	39.88
LOC104914156	9.0	9.5	8.0	9.5	7.0	6.5	8.3	1.29
LOC104914157	5.0	4.5	1.0	5.0	2.5	6.0	4.0	1.87
LOC104914158	1.0	3.0	2.5	1.5	1.0	0.5	1.6	0.97
LOC104914159	19.5	9.0	72.5	26.0	11.5	46.5	30.8	24.43
LOC104914161	685.5	672.0	782.0	605.5	793.0	713.0	708.5	70.76
LOC104914162	4.5	0.0	0.0	2.5	0.5	0.5	1.3	1.81
LOC104914163	350.0	192.5	310.0	300.5	245.0	246.0	274.0	56.64
LOC104914164	9.0	4.5	17.5	7.0	4.0	13.5	9.3	5.32
LOC104914165	8.5	1.5	24.5	7.5	6.0	24.0	12.0	9.79
LOC104914166	11.5	5.0	51.0	10.5	7.0	27.0	18.7	17.64
LOC104914167	0.0	0.0	6.0	0.5	1.0	12.0	3.3	4.86
LOC104914168	0.0	0.0	0.0	0.0	0.5	0.0	0.1	0.20
LOC104914170	1.0	0.0	1.0	0.0	0.0	0.5	0.4	0.49
LOC104914171	36.0	20.5	117.0	41.0	20.5	85.0	53.3	39.15
LOC104914173	692.0	1074.0	672.0	572.0	1220.0	845.5	845.9	253.35
LOC104914174	1554.5	1359.5	1110.0	1357.0	1480.5	1233.5	1349.2	161.34
LOC104914175	195.0	208.5	274.0	169.0	204.0	282.0	222.1	45.49
LOC104914176	691.5	802.5	773.5	626.0	996.5	888.5	796.4	133.51
LOC104914177	7.5	18.5	21.5	7.5	18.0	14.5	14.6	5.92
LOC104914178	9.0	6.5	6.5	6.0	6.5	4.5	6.5	1.45
LOC104914179	2.0	0.0	0.0	0.5	0.5	0.0	0.5	0.77
LOC104914181	0.0	0.0	0.0	2.5	0.0	0.0	0.4	1.02
LOC104914182	156.5	123.0	51.5	103.0	147.0	56.0	106.2	44.72
LOC104914184	21.5	5.0	48.0	20.0	13.5	33.5	23.6	15.22
LOC104914185	175.0	126.0	199.5	183.0	183.0	239.5	184.3	36.79

LOC104914186	20.0	10.5	2.5	14.5	16.0	14.5	13.0	5.98
LOC104914187	0.5	0.0	0.5	1.0	0.0	0.0	0.3	0.41
LOC104914188	268.0	249.0	499.0	327.5	311.5	532.5	364.6	120.95
LOC104914191	0.0	0.0	0.0	0.0	0.5	0.0	0.1	0.20
LOC104914193	5.0	8.0	5.0	5.5	2.0	2.0	4.6	2.29
LOC104914194	3.0	2.5	0.0	2.0	1.0	2.0	1.8	1.08
LOC104914197	16.0	12.0	14.5	18.5	10.0	16.0	14.5	3.07
LOC104914200	92.5	19.0	20.5	82.5	23.5	27.0	44.2	33.83
LOC104914201	10.5	19.5	44.5	12.5	25.5	54.0	27.8	17.74
LOC104914202	2.5	4.0	7.5	2.5	2.5	9.5	4.8	3.03
LOC104914203	13329.0	18508.5	17901.0	13021.0	18699.0	24019.5	17579.7	4063.00
LOC104914204	187.0	235.5	135.0	161.5	228.0	165.0	185.3	39.64
LOC104914206	358.5	483.0	451.0	308.0	467.5	475.5	423.9	72.80
LOC104914207	22.5	22.0	38.0	27.0	20.5	54.0	30.7	13.09
LOC104914208	1.5	4.0	11.5	6.5	1.5	7.5	5.4	3.88
LOC104914209	0.0	0.0	0.0	0.0	0.0	0.0	0.0	0.00
LOC104914210	133.0	63.5	170.0	122.5	80.5	138.0	117.9	39.31
LOC104914211	2.0	0.5	0.0	2.5	0.0	0.0	0.8	1.13
LOC104914212	657.0	617.5	694.0	614.5	732.0	681.5	666.1	45.75
LOC104914214	4.5	0.5	6.0	1.5	2.5	3.5	3.1	2.01
LOC104914215	67.5	38.5	98.5	66.0	37.5	93.0	66.8	25.89
LOC104914218	14.5	10.5	13.0	28.5	13.0	24.0	17.3	7.23
LOC104914219	4.0	1.0	2.0	5.0	1.0	1.5	2.4	1.69
LOC104914220	0.0	0.0	0.0	0.0	0.0	0.0	0.0	0.00
LOC104914221	0.0	0.0	0.0	0.0	0.0	0.0	0.0	0.00
LOC104914223	3.0	2.5	18.5	6.5	3.5	16.0	8.3	7.09
LOC104914225	1095.0	1115.0	399.5	970.0	1086.0	403.0	844.8	347.26
LOC104914226	0.0	0.0	0.0	0.0	0.0	0.0	0.0	0.00
LOC104914227	12.0	2.5	3.5	7.5	6.5	1.5	5.6	3.90
LOC104914228	0.0	0.0	0.5	0.0	0.0	1.0	0.3	0.42
LOC104914230	6.5	3.0	26.5	4.5	5.0	16.0	10.3	9.21
LOC104914232	0.5	6.0	12.5	2.0	8.0	9.5	6.4	4.55
LOC104914233	0.0	1.0	1.0	0.5	2.5	1.0	1.0	0.84
LOC104914234	0.0	0.0	0.0	0.0	0.0	0.0	0.0	0.00
LOC104914235	12.0	33.0	37.5	10.0	43.5	33.0	28.2	13.86
LOC104914236	0.0	0.0	0.0	0.0	0.5	0.0	0.1	0.20
LOC104914237	1.5	1.0	1.5	1.0	1.5	1.0	1.3	0.27
LOC104914238	2.5	4.5	2.0	4.0	2.0	4.0	3.2	1.13
LOC104914239	2.0	1.0	2.0	1.0	1.0	0.5	1.3	0.61
LOC104914240	0.5	1.5	0.5	1.0	2.5	1.0	1.2	0.75
LOC104914241	63.0	11.0	72.0	76.5	18.0	49.0	48.3	27.87
LOC104914242	151.5	76.0	65.0	132.5	81.0	67.5	95.6	36.90
LOC104914243	46.0	31.5	28.0	40.0	32.5	27.5	34.3	7.30
LOC104914244	14.5	37.5	41.0	11.0	39.0	49.5	32.1	15.58
LOC104914245	0.0	0.0	0.0	0.0	0.0	0.0	0.0	0.00
LOC104914246	0.0	0.0	0.0	0.0	0.0	0.0	0.0	0.00
LOC104914247	0.0	0.0	0.0	0.0	0.0	0.0	0.0	0.00
LOC104914248	0.0	0.0	0.0	0.0	0.0	0.0	0.0	0.00
LOC104914250	0.0	0.5	0.0	0.0	0.5	0.0	0.2	0.26
LOC104914251	0.0	0.0	0.0	0.0	0.0	0.0	0.0	0.00
LOC104914253	14.5	12.0	8.0	22.0	7.5	13.0	12.8	5.28
LOC104914254	2.0	3.5	2.5	2.5	2.0	5.0	2.9	1.16
LOC104914255	31.0	32.0	17.5	39.5	44.0	26.0	31.7	9.45
LOC104914256	15.5	5.5	15.0	10.5	5.0	9.5	10.2	4.49

LOC104914260	0.0	0.0	0.5	0.0	0.0	0.0	0.1	0.20
LOC104914261	2388.5	1696.0	2199.5	2414.0	1868.0	2004.0	2095.0	289.08
LOC104914262	163.5	125.0	172.5	177.0	121.0	160.5	153.3	24.21
LOC104914263	111.5	24.0	104.0	125.5	27.5	91.0	80.6	43.93
LOC104914264	337.0	190.0	439.0	414.0	200.5	397.5	329.7	109.46
LOC104914265	8.0	4.5	12.0	14.0	3.5	13.0	9.2	4.50
LOC104914266	0.5	0.5	0.5	1.5	0.5	0.5	0.7	0.41
LOC104914267	0.0	0.0	0.0	0.0	0.0	0.0	0.0	0.00
LOC104914268	0.0	0.0	0.0	0.0	0.0	0.0	0.0	0.00
LOC104914270	16.0	7.5	30.5	15.5	10.0	30.0	18.3	9.84
LOC104914271	0.0	4.0	2.5	0.0	5.5	2.0	2.3	2.18
LOC104914272	147.0	57.0	276.5	179.0	55.5	213.0	154.7	87.49
LOC104914273	266.0	429.0	294.5	281.0	425.5	267.5	327.3	78.15
LOC104914277	125.0	118.5	109.5	102.5	138.5	174.0	128.0	25.76
LOC104914280	25.5	15.0	29.5	19.5	8.5	34.5	22.1	9.61
LOC104914281	365.5	202.0	173.0	241.5	158.0	151.5	215.3	80.70
LOC104914283	25.0	18.5	22.5	30.5	23.0	22.0	23.6	3.99
LOC104914284	1.5	2.5	15.5	2.5	4.0	14.5	6.8	6.45
LOC104914285	0.0	0.5	3.5	0.5	2.5	6.5	2.3	2.48
LOC104914286	1.0	1.0	6.0	1.0	4.0	4.5	2.9	2.20
LOC104914287	4.0	0.0	0.0	1.0	0.5	0.0	0.9	1.56
LOC104914288	138.0	165.5	45.0	121.0	166.5	40.0	112.7	57.03
LOC104914289	0.0	0.0	0.0	0.0	0.0	0.0	0.0	0.00
LOC104914290	297.0	243.5	378.5	278.0	284.5	409.5	315.2	64.34
LOC104914291	697.0	764.5	709.0	602.0	684.5	694.5	691.9	52.41
LOC104914292	62.0	48.5	32.5	43.5	50.5	25.0	43.7	13.26
LOC104914294	214.5	272.0	137.0	173.0	323.5	154.5	212.4	72.79
LOC104914295	4.0	0.0	2.0	0.5	0.0	0.5	1.2	1.57
LOC104914297	0.5	0.0	0.0	0.5	0.0	1.0	0.3	0.41
LOC104914298	10.5	12.0	7.0	10.5	5.5	6.5	8.7	2.66
LOC104914299	0.0	0.0	0.0	0.0	0.0	0.0	0.0	0.00
LOC104914300	123.0	49.5	137.5	115.0	61.0	117.5	100.6	36.15
LOC104914301	40.0	69.0	52.5	46.5	104.0	66.0	63.0	22.97
LOC104914302	49.0	31.0	11.0	32.5	28.5	16.0	28.0	13.44
LOC104914304	0.0	0.0	0.0	0.0	0.0	0.0	0.0	0.00
LOC104914305	0.0	0.0	0.0	0.0	0.0	0.0	0.0	0.00
LOC104914307	420.5	283.5	209.5	324.0	297.5	197.5	288.8	81.62
LOC104914309	6.0	2.5	5.0	10.5	7.5	10.5	7.0	3.16
LOC104914310	149.5	168.5	176.0	123.0	173.5	193.0	163.9	24.44
LOC104914311	0.5	0.0	0.5	1.5	0.5	0.0	0.5	0.55
LOC104914312	18.0	13.5	34.0	21.5	10.5	20.5	19.7	8.18
LOC104914314	28.5	9.5	28.0	32.5	8.0	27.0	22.3	10.63
LOC104914316	0.0	0.0	0.0	0.0	0.0	0.0	0.0	0.00
LOC104914317	8.5	2.0	8.5	8.0	3.5	4.0	5.8	2.91
LOC104914319	171.5	172.5	104.5	111.0	181.5	79.0	136.7	43.65
LOC104914320	594.0	223.5	762.5	757.0	251.5	640.5	538.2	242.07
LOC104914321	255.5	257.0	220.5	221.5	228.0	248.0	238.4	17.00
LOC104914325	8.5	7.5	4.0	5.5	11.0	5.0	6.9	2.60
LOC104914326	23.0	14.0	3.5	27.5	15.0	2.5	14.3	10.06
LOC104914327	0.0	0.5	0.5	0.0	0.5	1.0	0.4	0.38
LOC104914332	1017.5	1700.0	3281.0	1036.5	1681.0	3034.0	1958.3	978.27
LOC104914333	355.0	456.5	1673.0	406.0	409.5	1499.0	799.8	612.29
LOC104914335	2.0	1.0	3.0	3.0	1.5	4.0	2.4	1.11
LOC104914336	0.0	0.0	0.0	0.0	0.0	0.5	0.1	0.20

LOC104914337	177.0	349.0	435.0	191.5	369.0	421.0	323.8	112.73
LOC104914338	0.0	0.0	0.0	0.0	0.0	0.0	0.0	0.00
LOC104914340	0.0	0.0	0.0	0.0	0.0	0.0	0.0	0.00
LOC104914341	139.0	83.5	82.0	97.0	90.0	71.5	93.8	23.71
LOC104914342	5.0	3.5	0.0	7.5	1.5	1.5	3.2	2.75
LOC104914343	0.0	0.5	2.0	0.0	1.5	0.0	0.7	0.88
LOC104914344	35.5	101.0	13.0	38.5	138.0	57.0	63.8	46.81
LOC104914345	1.0	0.0	0.0	0.5	0.5	0.0	0.3	0.41
LOC104914346	0.0	0.0	0.0	0.0	0.0	0.0	0.0	0.00
LOC104914347	53.0	15.5	20.5	40.0	21.0	19.0	28.2	14.91
LOC104914348	517.0	98.5	63.0	441.5	114.5	72.5	217.8	204.71
LOC104914349	0.0	0.0	0.0	0.0	0.0	0.0	0.0	0.00
LOC104914350	0.0	0.0	0.0	0.0	0.5	0.5	0.2	0.26
LOC104914351	0.5	1.0	2.5	2.0	0.5	2.5	1.5	0.95
LOC104914353	0.0	0.0	0.0	0.0	0.0	0.0	0.0	0.00
LOC104914355	0.0	0.5	0.0	0.5	0.0	0.0	0.2	0.26
LOC104914357	5.0	1.5	0.0	1.5	0.0	0.0	1.3	1.94
LOC104914359	13.5	22.5	17.0	14.0	27.5	18.5	18.8	5.36
LOC104914360	0.0	3.5	2.5	0.0	5.0	1.5	2.1	1.99
LOC104914363	83.0	80.0	70.0	87.0	97.5	81.5	83.2	9.01
LOC104914367	20.5	3.0	2.5	11.5	3.5	0.5	6.9	7.66
LOC104914368	14.5	9.0	4.0	11.0	4.5	7.5	8.4	3.99
LOC104914370	0.0	0.0	0.0	1.0	0.0	0.5	0.3	0.42
LOC104914371	1.5	0.5	0.5	1.0	1.5	2.0	1.2	0.61
LOC104914373	0.0	0.0	0.0	0.0	1.0	0.0	0.2	0.41
LOC104914376	93.5	42.5	43.0	81.0	45.0	39.5	57.4	23.51
LOC104914377	1.0	12.0	8.0	1.5	7.5	7.0	6.2	4.20
LOC104914378	4.5	7.0	24.0	2.0	6.0	18.0	10.3	8.70
LOC104914384	0.0	0.5	0.0	0.0	0.0	0.0	0.1	0.20
LOC104914390	0.0	0.0	0.5	0.0	1.5	1.5	0.6	0.74
LOC104914392	38.0	61.5	54.5	34.0	55.5	65.5	51.5	12.72
LOC104914393	9.0	7.0	22.5	9.0	7.0	19.0	12.3	6.74
LOC104914394	23.0	8.0	18.0	20.0	15.0	20.5	17.4	5.33
LOC104914395	3.0	2.0	3.0	1.0	0.5	2.5	2.0	1.05
LOC104914396	1.0	0.0	2.5	0.5	0.0	1.0	0.8	0.93
LOC104914397	1.0	2.0	3.5	0.0	0.5	5.0	2.0	1.92
LOC104914398	10.5	2.0	5.5	5.0	3.5	6.5	5.5	2.92
LOC104914402	140.5	91.0	173.0	148.0	125.0	166.0	140.6	29.84
LOC104914404	0.0	0.0	0.0	0.0	0.0	0.5	0.1	0.20
LOC104914405	1.5	1.0	0.0	1.5	0.5	0.5	0.8	0.61
LOC104914407	0.0	0.0	0.0	0.0	0.0	0.0	0.0	0.00
LOC104914408	0.0	0.0	0.0	0.0	0.0	0.0	0.0	0.00
LOC104914409	0.0	0.0	0.0	0.0	0.0	0.0	0.0	0.00
LOC104914410	8.0	1.0	1.5	5.0	2.5	4.0	3.7	2.60
LOC104914411	25.5	10.0	7.5	18.0	14.5	7.5	13.8	7.05
LOC104914412	0.0	0.0	0.0	0.0	0.0	0.0	0.0	0.00
LOC104914413	0.0	0.0	0.0	0.0	0.0	0.0	0.0	0.00
LOC104914414	0.0	0.0	0.0	0.0	0.0	0.0	0.0	0.00
LOC104914415	0.0	0.0	0.0	0.0	0.0	0.0	0.0	0.00
LOC104914416	0.0	0.0	0.0	0.0	0.0	0.0	0.0	0.00
LOC104914417	0.0	0.0	0.5	0.0	0.0	0.0	0.1	0.20
LOC104914418	0.0	0.0	0.0	0.0	0.0	0.0	0.0	0.00
LOC104914419	0.0	0.0	0.0	0.0	0.0	0.0	0.0	0.00
LOC104914420	0.0	0.5	0.0	0.0	0.0	0.0	0.1	0.20

LOC104914422	0.0	0.0	0.0	0.0	0.0	0.0	0.0	0.00
LOC104914423	36.0	28.5	18.0	29.0	30.0	25.0	27.8	5.96
LOC104914425	33.0	29.0	10.0	24.5	24.5	19.0	23.3	8.06
LOC104914426	66.5	65.0	72.0	63.0	65.5	65.5	66.3	3.05
LOC104914427	0.0	0.5	0.0	0.5	1.0	0.0	0.3	0.41
LOC104914428	35.5	20.0	8.5	23.5	19.5	12.5	19.9	9.39
LOC104914429	0.0	0.0	0.0	0.0	0.0	0.0	0.0	0.00
LOC104914431	2.0	0.5	1.0	0.0	0.5	0.0	0.7	0.75
LOC104914439	0.5	0.0	0.0	0.0	0.0	0.0	0.1	0.20
LOC104914441	0.0	0.0	0.0	0.0	0.0	0.0	0.0	0.00
LOC104914447	1.0	0.0	1.0	0.0	0.0	1.0	0.5	0.55
LOC104914448	0.0	0.0	0.0	0.0	0.0	0.0	0.0	0.00
LOC104914449	0.0	0.0	0.5	0.0	0.0	0.0	0.1	0.20
LOC104914452	233.0	307.5	348.0	285.5	317.0	299.5	298.4	38.30
LOC104914453	4.0	2.5	11.5	1.0	2.5	8.0	4.9	4.02
LOC104914454	91.5	16.5	76.5	83.0	13.0	65.0	57.6	34.31
LOC104914455	115.0	160.5	84.5	117.0	145.0	103.0	120.8	27.73
LOC104914456	183.0	108.0	416.0	161.0	127.5	414.0	234.9	141.89
LOC104914458	1.0	0.5	0.0	0.0	0.5	0.0	0.3	0.41
LOC104914459	0.0	0.5	1.5	0.5	0.0	0.0	0.4	0.58
LOC104914461	3035.0	2406.0	2037.5	2672.5	2812.5	2071.0	2505.8	405.02
LOC104914462	1195.5	1091.0	974.0	1188.5	1212.0	926.0	1097.8	123.05
LOC104914463	17.5	7.5	16.5	10.5	7.0	13.0	12.0	4.45
LOC104914464	0.5	0.5	0.0	0.0	0.0	0.5	0.3	0.27
LOC104914465	2683.5	7113.5	6282.5	2584.5	7663.0	5779.5	5351.1	2203.28
LOC104914466	43.0	31.5	49.0	35.5	21.0	33.5	35.6	9.68
LOC104914467	4.0	6.0	11.5	15.5	5.0	7.0	8.2	4.43
LOC104914470	24.5	98.0	75.5	29.5	109.5	86.5	70.6	35.65
LOC104914473	2.5	7.5	8.0	3.5	7.0	11.5	6.7	3.27
LOC104914474	0.0	0.0	0.0	0.0	0.0	0.0	0.0	0.00
LOC104914475	0.0	0.0	0.5	0.5	0.0	0.0	0.2	0.26
LOC104914478	0.0	0.0	0.0	0.0	0.0	0.0	0.0	0.00
LOC104914479	102.0	79.0	98.0	55.5	74.5	51.5	76.8	20.92
LOC104914480	0.5	0.5	0.5	0.0	0.0	0.0	0.3	0.27
LOC104914481	0.0	0.0	0.0	0.0	0.0	0.0	0.0	0.00
LOC104914482	3.5	0.5	1.5	3.0	0.5	0.0	1.5	1.45
LOC104914483	0.0	0.0	0.0	0.5	0.5	0.0	0.2	0.26
LOC104914484	6.5	21.5	15.5	5.0	24.0	17.0	14.9	7.74
LOC104914487	10.0	1.5	3.5	13.0	5.5	1.5	5.8	4.73
LOC104914488	0.0	0.5	0.0	0.0	0.0	0.0	0.1	0.20
LOC104914489	0.0	0.0	0.0	0.0	0.0	0.0	0.0	0.00
LOC104914490	0.0	1.0	1.0	0.0	1.0	0.0	0.5	0.55
LOC104914491	15.0	15.5	10.0	27.5	21.5	19.5	18.2	6.06
LOC104914493	45.0	13.0	4.0	42.5	13.0	2.5	20.0	18.93
LOC104914494	1.0	0.0	1.5	1.5	0.0	0.5	0.8	0.69
LOC104914495	12.5	5.0	11.0	11.0	4.0	11.0	9.1	3.61
LOC104914496	1.0	4.0	9.0	3.0	3.5	12.0	5.4	4.18
LOC104914497	0.0	1.5	13.0	0.0	2.0	9.5	4.3	5.53
LOC104914498	0.5	0.5	1.5	1.0	0.5	0.0	0.7	0.52
LOC104914499	1.0	1.5	2.0	1.5	3.0	1.0	1.7	0.75
LOC104914500	0.0	0.5	0.0	0.0	0.0	0.0	0.1	0.20
LOC104914501	0.5	0.0	1.5	0.0	0.5	1.5	0.7	0.68
LOC104914502	0.0	0.0	1.0	0.0	1.5	0.5	0.5	0.63
LOC104914503	0.0	0.0	0.0	0.0	0.0	0.0	0.0	0.00

LOC104914507	12.0	1.0	1.0	6.5	1.5	1.0	3.8	4.55
LOC104914508	0.5	0.0	0.0	0.0	0.0	0.0	0.1	0.20
LOC104914509	0.5	0.0	0.0	0.0	0.0	0.0	0.1	0.20
LOC104914510	37.0	9.0	9.5	36.0	8.5	14.5	19.1	13.67
LOC104914513	0.0	0.0	0.0	0.0	0.0	0.0	0.0	0.00
LOC104914514	0.0	1.0	3.0	0.0	0.0	9.5	2.3	3.74
LOC104914515	8.0	2.5	11.5	8.5	4.5	13.0	8.0	4.00
LOC104914519	0.0	0.0	0.0	0.0	0.0	0.0	0.0	0.00
LOC104914521	247.0	359.5	212.5	236.0	350.0	217.5	270.4	66.57
LOC104914523	0.0	3.0	14.0	0.5	1.5	7.5	4.4	5.42
LOC104914524	208.0	314.0	216.5	226.0	299.5	208.0	245.3	48.25
LOC104914527	166.5	125.5	208.0	162.0	150.5	202.5	169.2	31.41
LOC104914528	1116.5	560.5	1093.5	780.0	561.5	936.0	841.3	248.68
LOC104914530	11.5	23.5	47.0	14.5	31.5	118.5	41.1	40.03
LOC104914531	4.5	2.0	3.0	1.5	2.5	1.0	2.4	1.24
LOC104914533	413.5	244.5	190.0	368.0	260.5	167.5	274.0	97.72
LOC104914535	0.5	0.0	0.0	0.0	0.0	0.0	0.1	0.20
LOC104914536	30.5	48.5	154.5	34.5	51.5	86.5	67.7	46.91
LOC104914538	0.0	0.0	10.0	0.5	2.0	6.5	3.2	4.16
LOC104914539	0.0	0.0	0.0	0.0	0.0	0.0	0.0	0.00
LOC104914540	2.5	0.0	0.0	3.5	0.0	0.5	1.1	1.53
LOC104914541	1.5	3.0	0.0	1.0	1.0	0.5	1.2	1.03
LOC104914543	0.0	0.0	0.5	1.5	0.5	0.0	0.4	0.58
LOC104914544	0.0	0.5	0.5	0.5	1.5	0.0	0.5	0.55
LOC104914545	0.0	0.0	0.0	0.0	0.0	0.0	0.0	0.00
LOC104914549	8.0	3.5	3.0	9.5	2.0	2.0	4.7	3.25
LOC104914551	0.0	0.0	0.0	0.0	0.0	0.0	0.0	0.00
LOC104914552	44.0	29.5	63.0	36.5	23.5	58.0	42.4	15.68
LOC104914553	0.0	0.0	0.0	0.0	0.5	0.0	0.1	0.20
LOC104914555	0.0	0.0	0.0	0.0	0.0	0.0	0.0	0.00
LOC104914556	48.0	60.0	45.5	56.0	50.0	47.5	51.2	5.63
LOC104914557	3.0	4.5	2.0	1.5	2.5	0.5	2.3	1.37
LOC104914561	1585.5	2130.0	1293.5	1442.0	1986.0	1512.0	1658.2	327.55
LOC104914563	104.0	49.0	143.5	109.0	52.5	123.5	96.9	38.32
LOC104914564	0.0	0.0	1.0	1.0	0.0	0.5	0.4	0.49
LOC104914565	16.0	3.0	25.0	14.5	5.5	26.5	15.1	9.67
LOC104914567	1.5	2.0	4.0	0.5	3.5	4.0	2.6	1.46
LOC104914568	15.5	15.0	33.0	15.0	10.0	29.0	19.6	9.16
LOC104914570	68.0	56.0	56.5	44.0	71.0	64.5	60.0	9.89
LOC104914571	0.0	0.5	0.0	0.0	0.0	0.5	0.2	0.26
LOC104914572	0.0	0.0	0.0	0.0	0.0	0.0	0.0	0.00
LOC104914574	72.0	15.0	64.0	96.0	18.5	58.5	54.0	31.59
LOC104914577	39.5	39.5	26.5	49.0	32.0	23.5	35.0	9.49
LOC104914579	0.0	1.0	2.5	0.5	0.5	1.0	0.9	0.86
LOC104914580	0.0	0.5	0.0	0.0	0.0	0.0	0.1	0.20
LOC104914581	9.5	2.0	0.0	4.0	0.5	1.0	2.8	3.56
LOC104914582	9.0	10.0	10.0	3.5	7.0	6.0	7.6	2.58
LOC104914583	0.0	0.0	0.0	0.0	0.5	0.0	0.1	0.20
LOC104914585	3.5	2.5	11.5	1.5	2.5	5.0	4.4	3.67
LOC104914586	0.0	0.0	0.0	0.0	0.0	0.0	0.0	0.00
LOC104914587	0.5	0.0	0.5	0.0	0.0	2.5	0.6	0.97
LOC104914588	127.5	161.0	170.5	116.5	169.0	188.0	155.4	27.56
LOC104914589	157.5	133.0	136.5	135.5	98.5	154.0	135.8	20.98
LOC104914590	85.5	66.0	91.5	71.5	68.0	78.0	76.8	10.15

LOC104914591	0.0	0.0	0.0	0.0	0.0	0.0	0.0	0.00
LOC104914592	0.0	0.0	0.0	0.0	0.0	0.0	0.0	0.00
LOC104914596	0.0	0.0	0.0	0.0	0.0	0.0	0.0	0.00
LOC104914598	134.5	174.0	89.0	112.5	139.0	114.5	127.3	29.05
LOC104914600	54.0	107.5	221.0	50.5	89.5	208.5	121.8	75.21
LOC104914601	121.0	241.5	408.5	109.0	238.5	394.0	252.1	128.50
LOC104914602	12.0	11.5	15.0	7.0	9.5	19.5	12.4	4.38
LOC104914604	513.5	291.5	883.5	449.0	293.5	688.5	519.9	232.00
LOC104914605	523.0	239.0	715.5	613.5	201.0	530.0	470.3	206.39
LOC104914606	2.5	2.5	1.0	4.0	1.5	2.0	2.3	1.04
LOC104914608	0.0	0.0	0.0	0.0	0.0	0.0	0.0	0.00
LOC104914610	0.0	0.0	0.0	0.0	0.0	0.0	0.0	0.00
LOC104914612	181.5	180.0	194.0	140.0	203.5	199.0	183.0	23.05
LOC104914614	377.0	749.0	324.5	354.5	688.0	418.5	485.3	184.27
LOC104914617	330.0	364.5	364.0	341.5	382.5	329.5	352.0	21.59
LOC104914618	3.5	1.5	0.0	1.5	1.0	1.5	1.5	1.14
LOC104914619	117.0	90.5	71.5	101.0	77.0	69.0	87.7	18.81
LOC104914622	0.0	8.5	1.5	1.5	5.0	3.5	3.3	3.08
LOC104914623	0.5	0.0	0.5	0.5	0.0	0.0	0.3	0.27
LOC104914624	40.0	23.0	35.5	41.0	23.0	32.5	32.5	7.97
LOC104914628	0.5	1.5	2.0	0.0	0.5	0.5	0.8	0.75
LOC104914629	0.5	1.0	1.0	1.5	0.0	0.5	0.8	0.52
LOC104914631	86.5	62.0	166.0	111.5	87.5	209.0	120.4	55.95
LOC104914633	657.5	627.5	945.0	593.5	563.0	971.5	726.3	182.62
LOC104914634	136.0	68.5	52.5	92.5	55.0	48.0	75.4	33.77
LOC104914635	0.5	0.0	0.0	0.0	0.0	0.0	0.1	0.20
LOC104914636	0.0	0.0	0.0	0.0	0.0	0.0	0.0	0.00
LOC104914637	0.0	0.0	0.0	0.0	0.0	0.0	0.0	0.00
LOC104914639	25.5	9.5	37.5	29.5	8.0	29.0	23.2	11.85
LOC104914640	0.0	0.0	0.5	0.0	0.0	0.0	0.1	0.20
LOC104914641	0.0	0.0	0.0	0.0	0.5	0.0	0.1	0.20
LOC104914642	0.5	2.5	6.5	0.0	1.0	3.5	2.3	2.42
LOC104914643	4.5	2.0	2.5	2.5	4.0	1.5	2.8	1.17
LOC104914644	0.0	0.0	1.0	0.0	0.0	0.0	0.2	0.41
LOC104914645	4.5	2.5	1.5	1.5	1.0	0.5	1.9	1.43
LOC104914647	20.0	30.5	31.0	18.0	31.5	30.5	26.9	6.18
LOC104914649	191.0	148.5	126.5	181.0	118.0	120.0	147.5	31.88
LOC104914651	1.5	0.0	1.0	0.5	0.5	0.0	0.6	0.58
LOC104914652	1.5	6.5	10.5	0.0	4.5	9.5	5.4	4.22
LOC104914654	0.0	0.0	0.0	0.5	0.0	0.0	0.1	0.20
LOC104914656	41.5	20.0	8.0	40.5	33.5	9.5	25.5	15.08
LOC104914657	0.0	0.0	0.0	0.0	0.5	0.5	0.2	0.26
LOC104914658	1.0	0.5	0.0	0.0	0.5	0.5	0.4	0.38
LOC104914659	2.5	0.5	1.0	1.5	1.0	0.5	1.2	0.75
LOC104914660	23.5	18.0	3.0	15.5	8.0	6.5	12.4	7.83
LOC104914661	0.0	0.0	0.0	0.0	0.0	0.5	0.1	0.20
LOC104914662	1.5	0.5	0.0	1.0	0.5	0.0	0.6	0.58
LOC104914663	25.5	23.5	12.5	21.5	15.0	23.5	20.3	5.25
LOC104914664	1012.0	1155.5	823.0	893.5	1073.0	871.5	971.4	129.56
LOC104914665	48.5	65.0	43.0	49.5	44.0	45.5	49.3	8.12
LOC104914666	20.0	11.0	29.5	13.5	8.0	36.0	19.7	11.07
LOC104914670	125.0	363.0	347.0	132.5	361.5	346.5	279.3	116.81
LOC104914671	0.0	0.0	0.0	0.0	0.0	0.0	0.0	0.00
LOC104914673	0.0	0.0	0.0	0.0	0.0	0.0	0.0	0.00

LOC104914674	0.0	0.5	0.0	0.0	0.5	0.5	0.3	0.27
LOC104914676	158.5	177.0	114.0	139.0	188.0	101.0	146.3	34.58
LOC104914677	1228.0	963.5	822.0	1074.0	965.0	766.0	969.8	167.91
LOC104914678	3.0	0.0	0.0	0.0	0.5	0.5	0.7	1.17
LOC104914681	0.0	0.0	0.0	0.0	1.0	0.0	0.2	0.41
LOC104914682	188.0	417.0	341.0	141.5	408.5	405.0	316.8	121.76
LOC104914683	0.0	0.0	0.0	0.0	0.0	0.0	0.0	0.00
LOC104914684	11.5	5.0	20.5	17.0	5.0	14.5	12.3	6.35
LOC104914686	201.5	392.5	251.5	220.0	458.0	251.0	295.8	104.18
LOC104914687	14.0	72.5	35.5	8.0	102.5	43.0	45.9	36.00
LOC104914688	1.5	4.0	2.5	1.0	4.5	5.5	3.2	1.78
LOC104914690	61.0	16.5	76.0	77.0	17.5	60.5	51.4	27.58
LOC104914691	1.5	0.5	2.0	2.0	3.5	1.5	1.8	0.98
LOC104914697	16.5	18.5	61.0	18.0	11.0	48.0	28.8	20.48
LOC104914698	6.0	5.5	9.5	6.0	3.0	8.0	6.3	2.23
LOC104914699	115.5	78.0	188.5	115.0	83.5	185.5	127.7	48.52
LOC104914701	45.0	40.0	57.5	45.0	44.0	57.5	48.2	7.46
LOC104914702	0.0	0.0	0.0	0.0	0.0	0.0	0.0	0.00
LOC104914703	0.0	1.0	0.0	0.0	0.5	0.5	0.3	0.41
LOC104914705	0.0	0.0	1.5	0.5	0.5	0.5	0.5	0.55
LOC104914706	1143.0	908.0	786.0	1097.5	903.5	795.0	938.8	150.40
LOC104914707	62.0	90.5	43.0	50.0	96.0	50.0	65.3	22.60
LOC104914708	9.5	61.5	189.0	14.0	70.0	203.0	91.2	84.89
LOC104914710	11.0	9.0	17.0	15.5	15.0	24.0	15.3	5.23
LOC104914711	370.5	147.0	118.5	283.5	162.0	114.5	199.3	104.16
LOC104914713	140.5	165.0	79.0	112.0	139.0	71.5	117.8	37.08
LOC104914716	0.0	0.5	0.0	0.0	0.5	1.0	0.3	0.41
LOC104914717	0.0	0.0	0.0	0.0	0.0	0.5	0.1	0.20
LOC104914718	5.5	0.5	0.5	6.0	1.0	0.0	2.3	2.73
LOC104914720	0.0	0.0	0.0	0.0	0.0	0.0	0.0	0.00
LOC104914722	55.5	51.5	134.0	58.0	43.5	90.0	72.1	34.26
LOC104914728	89.0	30.0	10.0	60.5	34.0	9.0	38.8	31.01
LOC104914729	148.5	39.0	175.5	164.5	49.0	150.5	121.2	60.66
LOC104914731	3.0	1.5	2.0	3.0	1.0	2.0	2.1	0.80
LOC104914732	4.5	0.5	7.0	7.0	2.0	7.0	4.7	2.86
LOC104914733	25.0	6.0	3.5	21.5	7.0	5.0	11.3	9.37
LOC104914734	181.5	62.0	244.0	218.0	79.5	244.5	171.6	81.61
LOC104914735	0.0	0.0	0.0	0.0	0.0	0.0	0.0	0.00
LOC104914736	0.0	0.0	0.0	0.0	0.0	0.0	0.0	0.00
LOC104914738	0.5	2.0	0.0	0.0	2.0	0.5	0.8	0.93
LOC104914742	40.5	23.0	9.0	71.0	18.5	5.0	27.8	24.54
LOC104914743	50.5	32.0	62.5	44.5	36.0	67.0	48.8	14.05
LOC104914744	53.5	32.0	85.0	53.5	39.0	82.0	57.5	21.82
LOC104914746	314.0	238.0	151.0	303.0	250.5	175.0	238.6	65.87
LOC104914750	108.5	33.5	186.0	125.5	46.0	171.5	111.8	62.82
LOC104914751	48.0	21.5	53.5	51.0	33.0	58.0	44.2	13.99
LOC104914752	17.0	9.0	29.5	27.0	9.5	22.0	19.0	8.69
LOC104914753	151.0	91.0	141.5	161.5	149.0	150.5	140.8	25.20
LOC104914755	0.0	0.0	0.0	0.0	0.0	0.0	0.0	0.00
LOC104914759	0.0	0.0	0.0	0.0	0.0	0.0	0.0	0.00
LOC104914760	45.0	43.0	45.5	52.0	41.5	40.5	44.6	4.12
LOC104914761	62.5	54.0	75.5	78.5	48.0	63.5	63.7	11.83
LOC104914762	201.0	317.5	307.0	180.5	347.0	337.5	281.8	72.19
LOC104914763	0.5	0.0	0.5	0.5	0.0	0.5	0.3	0.26

LOC104914767	17.5	8.5	1.5	15.5	6.5	4.0	8.9	6.36
LOC104914768	736.0	544.0	783.0	734.5	617.5	651.0	677.7	89.36
LOC104914769	35.5	16.0	59.5	29.0	30.5	49.5	36.7	15.56
LOC104914770	64.5	78.5	61.5	68.5	98.0	47.5	69.8	17.13
LOC104914772	58.5	27.0	10.0	38.5	19.0	9.5	27.1	18.89
LOC104914773	6.0	2.0	3.0	11.5	3.0	0.5	4.3	3.95
LOC104914775	2.5	2.0	2.0	1.5	3.5	2.0	2.3	0.69
LOC104914776	0.0	0.0	0.0	0.0	0.0	0.0	0.0	0.00
LOC104914777	0.0	0.0	0.0	0.0	0.0	0.0	0.0	0.00
LOC104914779	7.5	5.0	18.0	7.0	2.5	12.0	8.7	5.55
LOC104914780	162.5	316.5	242.0	186.5	325.0	192.5	237.5	69.52
LOC104914781	2.5	2.5	3.5	1.0	4.0	1.5	2.5	1.14
LOC104914782	8.5	13.5	22.0	10.5	16.0	20.5	15.2	5.38
LOC104914783	127.5	102.5	150.5	120.0	106.0	121.5	121.3	17.21
LOC104914786	31.0	10.0	27.0	22.0	8.0	22.0	20.0	9.19
LOC104914787	3131.0	4140.0	2004.5	2798.5	4529.5	2085.0	3114.8	1043.64
LOC104914788	10.0	13.0	46.0	8.5	12.0	29.5	19.8	14.91
LOC104914789	1.0	2.5	13.5	1.0	3.5	8.0	4.9	4.93
LOC104914790	0.0	0.0	0.0	0.0	0.0	0.0	0.0	0.00
LOC104914791	0.5	0.5	4.5	0.5	2.0	2.5	1.8	1.60
LOC104914793	1831.5	1557.0	1492.5	1804.0	1655.5	1333.5	1612.3	190.68
LOC104914794	95.5	218.0	713.5	87.0	237.0	580.0	321.8	262.45
LOC104914795	0.5	0.0	0.0	0.0	0.0	0.0	0.1	0.20
LOC104914796	0.0	0.5	0.0	0.0	2.5	0.5	0.6	0.97
LOC104914797	0.0	0.0	0.0	0.0	0.0	0.0	0.0	0.00
LOC104914799	0.0	0.0	0.0	0.0	0.0	0.0	0.0	0.00
LOC104914800	45.5	42.0	48.5	49.0	52.0	51.0	48.0	3.70
LOC104914801	19.0	11.0	10.0	13.5	9.0	9.5	12.0	3.78
LOC104914802	64.5	45.5	237.0	63.5	68.0	163.0	106.9	76.21
LOC104914803	4.0	9.0	15.0	7.0	5.5	16.0	9.4	5.00
LOC104914804	35.0	33.0	49.5	46.5	32.5	50.5	41.2	8.54
LOC104914806	35.0	14.0	74.5	34.0	16.0	55.0	38.1	23.26
LOC104914807	245.0	93.0	615.0	274.0	77.5	477.5	297.0	212.95
LOC104914810	263.5	528.5	398.0	278.0	517.5	462.0	407.9	116.02
LOC104914811	65.0	106.0	132.5	80.5	86.0	135.5	100.9	28.81
LOC104914812	28.0	26.0	14.5	20.0	23.5	9.0	20.2	7.26
LOC104914813	55.0	34.5	56.5	80.0	37.0	40.0	50.5	17.18
LOC104914814	89.5	59.0	30.5	69.5	41.5	19.0	51.5	26.16
LOC104914815	162.5	179.5	136.0	223.0	225.5	159.0	180.9	36.33
LOC104914816	269.5	252.0	157.5	271.0	243.0	138.0	221.8	58.68
LOC104914818	3.0	8.0	13.0	7.0	13.0	14.0	9.7	4.37
LOC104914821	8.5	36.0	17.5	16.5	37.0	15.5	21.8	11.80
LOC104914822	0.0	0.0	0.0	0.0	0.5	0.0	0.1	0.20
LOC104914825	197.5	285.5	103.5	225.0	432.5	179.5	237.3	112.61
LOC104914826	1.0	1.5	3.0	2.5	0.5	4.0	2.1	1.32
LOC104914828	177.0	105.0	265.5	186.5	119.0	245.5	183.1	64.72
LOC104914829	92.5	38.5	150.5	120.0	44.5	125.0	95.2	45.51
LOC104914830	168.0	54.0	221.5	185.5	42.0	184.5	142.6	75.41
LOC104914834	0.0	0.0	0.0	0.5	0.0	0.0	0.1	0.20
LOC104914835	15.0	7.5	30.5	18.0	6.0	30.5	17.9	10.73
LOC104914838	1.0	0.0	3.0	1.5	1.0	2.5	1.5	1.10
LOC104914839	0.0	0.0	0.0	0.0	0.0	0.0	0.0	0.00
LOC104914840	0.5	0.0	0.0	0.0	0.5	0.0	0.2	0.26
LOC104914841	52.5	40.0	40.5	56.0	51.5	35.5	46.0	8.35

LOC104914842	46.0	13.5	103.5	47.5	12.5	67.5	48.4	34.39
LOC104914843	0.0	0.0	0.0	0.0	0.0	0.0	0.0	0.00
LOC104914844	0.0	0.0	0.0	0.0	0.0	0.0	0.0	0.00
LOC104914847	1.0	1.5	2.0	1.0	3.0	1.5	1.7	0.75
LOC104914849	0.5	1.5	0.0	0.0	0.0	0.0	0.3	0.61
LOC104914851	0.0	0.0	0.0	0.0	0.0	0.0	0.0	0.00
LOC104914852	0.0	0.0	0.0	0.0	0.0	0.0	0.0	0.00
LOC104914853	49.0	32.0	52.0	36.5	37.5	50.0	42.8	8.48
LOC104914856	78.0	39.0	101.0	81.0	40.5	95.0	72.4	26.71
LOC104914857	39.0	26.0	54.0	49.5	31.0	45.0	40.8	10.82
LOC104914859	6.5	14.5	11.5	9.0	21.0	18.5	13.5	5.58
LOC104914861	18.0	6.0	14.0	16.0	7.0	15.0	12.7	4.97
LOC104914865	384.5	255.5	227.0	367.0	285.0	266.5	297.6	63.64
LOC104914866	168.0	36.0	125.0	124.0	26.5	89.5	94.8	55.27
LOC104914870	4.5	3.5	6.5	4.0	3.5	6.5	4.8	1.41
LOC104914872	0.0	0.0	0.0	0.0	0.0	0.0	0.0	0.00
LOC104914873	1.5	3.5	0.5	2.0	3.5	1.0	2.0	1.26
LOC104914875	79.5	31.5	20.0	77.0	37.5	15.5	43.5	28.05
LOC104914876	3.5	1.5	1.5	2.5	1.0	3.5	2.3	1.08
LOC104914877	1.5	0.0	1.0	0.5	0.0	0.0	0.5	0.63
LOC104914878	1.5	0.5	2.0	0.5	0.0	1.0	0.9	0.74
LOC104914879	0.0	0.0	0.0	0.0	0.0	0.0	0.0	0.00
LOC104914880	5.5	2.0	6.0	6.0	4.5	5.0	4.8	1.51
LOC104914881	0.0	2.0	2.0	1.5	1.0	4.5	1.8	1.51
LOC104914883	7.0	2.0	2.5	7.0	1.0	1.5	3.5	2.76
LOC104914886	1848.5	581.5	1084.0	1610.5	594.0	801.5	1086.7	535.54
LOC104914888	230.0	352.5	216.0	209.5	468.5	217.0	282.3	106.11
LOC104914889	17.0	5.0	36.0	19.5	3.5	26.5	17.9	12.48
LOC104914890	130.5	42.0	257.0	124.5	51.5	183.5	131.5	81.14
LOC104914891	261.5	83.0	302.0	309.0	95.5	228.0	213.2	100.42
LOC104914894	3.0	0.0	1.5	1.5	1.0	0.5	1.3	1.04
LOC104914895	2.5	0.0	0.5	4.0	0.0	1.5	1.4	1.59
LOC104914896	16.5	3.5	9.5	19.5	5.0	5.5	9.9	6.64
LOC104914898	3.5	0.0	9.5	4.0	1.5	3.0	3.6	3.25
LOC104914900	84.5	86.5	53.0	95.5	93.0	58.0	78.4	18.27
LOC104914902	86.5	49.0	89.0	112.0	57.0	65.0	76.4	23.60
LOC104914903	17.5	40.5	63.5	19.0	47.0	54.5	40.3	18.75
LOC104914905	0.0	0.0	0.0	0.0	0.0	0.0	0.0	0.00
LOC104914906	2.5	4.5	16.0	5.0	5.0	13.0	7.7	5.46
LOC104914909	501.5	312.0	216.0	478.0	385.5	200.0	348.8	128.44
LOC104914911	3.0	1.0	14.0	5.0	2.5	18.0	7.3	7.01
LOC104914913	0.0	0.0	0.0	0.0	0.0	0.0	0.0	0.00
LOC104914914	0.0	0.0	0.0	0.0	0.0	0.0	0.0	0.00
LOC104914915	0.0	0.0	0.0	0.0	0.0	0.0	0.0	0.00
LOC104914916	14.5	5.0	19.5	15.5	5.5	18.0	13.0	6.26
LOC104914917	12.5	5.5	21.0	5.5	7.5	14.0	11.0	6.07
LOC104914919	65.5	27.0	74.5	88.0	22.0	61.0	56.3	26.37
LOC104914920	101.5	37.0	153.0	102.5	36.5	100.0	88.4	44.75
LOC104914921	617.5	444.0	405.0	648.0	487.5	388.0	498.3	110.05
LOC104914926	42.0	17.0	55.0	40.5	20.5	38.5	35.6	14.31
LOC104914928	715.0	399.0	370.0	669.0	396.0	230.0	463.2	188.39
LOC104914929	2.5	0.0	5.5	6.5	1.0	6.5	3.7	2.88
LOC104914930	8.5	1.5	1.0	4.0	4.0	1.5	3.4	2.82
LOC104914931	4.5	0.5	4.0	4.0	1.0	4.5	3.1	1.83

LOC104914932	62.0	10.0	92.0	80.5	11.5	65.5	53.6	34.88
LOC104914933	423.5	180.5	318.5	447.0	237.0	303.5	318.3	103.39
LOC104914934	0.0	0.0	0.0	0.5	0.0	0.0	0.1	0.20
LOC104914935	0.0	0.0	0.0	0.0	0.0	0.0	0.0	0.00
LOC104914937	0.0	0.0	0.0	0.0	0.0	0.0	0.0	0.00
LOC104914938	0.0	0.0	0.0	0.0	0.0	0.0	0.0	0.00
LOC104914939	0.0	0.0	0.0	0.0	0.0	0.0	0.0	0.00
LOC104914940	0.0	0.0	0.0	0.0	0.0	0.0	0.0	0.00
LOC104914941	0.0	0.0	0.0	0.0	1.0	0.0	0.2	0.41
LOC104914942	8.0	4.0	23.0	9.5	5.5	14.5	10.8	7.02
LOC104914943	439.0	377.0	217.0	413.5	373.0	217.0	339.4	97.90
LOC104914944	208.0	83.0	175.0	201.5	91.5	161.0	153.3	54.05
LOC104914945	193.0	88.5	68.5	192.5	111.5	91.5	124.3	54.78
LOC104914946	79.0	135.5	224.0	94.0	149.0	161.0	140.4	51.83
LOC104914947	8.0	1.0	4.5	6.0	4.0	6.5	5.0	2.43
LOC104914948	6.5	0.5	7.0	8.0	2.0	3.5	4.6	3.02
LOC104914949	0.0	0.0	0.0	0.0	0.0	0.0	0.0	0.00
LOC104914952	1.0	0.0	0.5	0.5	1.0	0.0	0.5	0.45
LOC104914955	236.5	163.5	73.0	226.5	189.0	116.0	167.4	63.72
LOC104914956	250.0	594.5	559.5	205.0	580.5	443.5	438.8	172.75
LOC104914959	170.5	74.0	345.0	151.5	92.0	288.5	186.9	108.25
LOC104914961	23.5	3.5	33.0	30.0	7.5	32.5	21.7	13.03
LOC104914962	0.0	0.0	0.0	0.0	0.0	0.0	0.0	0.00
LOC104914965	8.0	5.0	11.0	8.5	4.0	12.0	8.1	3.17
LOC104914968	68.0	38.0	72.5	69.5	43.0	69.5	60.1	15.32
LOC104914969	0.0	0.5	0.0	1.0	0.0	1.0	0.4	0.49
LOC104914972	59.5	17.0	53.5	56.5	25.5	51.5	43.9	17.97
LOC104914973	6.5	2.0	5.5	6.5	3.5	6.5	5.1	1.91
LOC104914976	35.0	14.0	15.0	29.5	17.0	19.5	21.7	8.59
LOC104914977	111.5	63.5	73.0	90.0	65.5	55.0	76.4	20.84
LOC104914979	12.0	3.5	7.5	14.0	5.0	6.5	8.1	4.09
LOC104914980	32.5	20.5	27.5	22.5	16.5	34.5	25.7	7.05
LOC104914981	2.5	1.5	4.0	2.0	0.5	3.0	2.3	1.21
LOC104914982	2.5	0.5	3.0	2.5	0.0	4.0	2.1	1.53
LOC104914983	9.5	1.0	12.5	5.0	0.5	11.5	6.7	5.26
LOC104914985	1.0	0.0	0.0	0.0	0.0	0.0	0.2	0.41
LOC104914986	39.0	17.0	42.5	56.0	17.0	30.5	33.7	15.30
LOC104914987	8.5	13.0	9.0	6.0	11.0	8.0	9.3	2.44
LOC104914990	65.5	47.0	41.5	57.0	55.0	33.0	49.8	11.70
LOC104914991	0.0	0.0	0.0	0.0	0.0	0.0	0.0	0.00
LOC104914992	2.5	1.0	1.0	2.0	0.0	1.0	1.3	0.88
LOC104914993	0.0	0.0	0.0	0.0	0.0	0.0	0.0	0.00
LOC104914994	12.5	3.5	1.0	8.5	4.5	3.0	5.5	4.23
LOC104914995	101.0	40.5	100.5	66.5	32.5	50.5	65.3	29.75
LOC104914997	458.0	413.0	259.5	425.0	445.0	237.5	373.0	97.93
LOC104914998	62.5	52.5	65.5	60.5	66.0	48.0	59.2	7.33
LOC104915000	23.0	9.0	75.5	22.0	8.0	46.5	30.7	25.99
LOC104915001	0.5	0.0	1.5	0.5	0.0	0.5	0.5	0.55
LOC104915002	12.0	4.0	3.5	4.0	3.0	3.5	5.0	3.45
LOC104915003	476.0	470.5	312.0	447.5	534.0	287.0	421.2	98.78
LOC104915004	38.5	12.0	35.5	35.0	14.5	23.0	26.4	11.52
LOC104915005	0.5	0.0	1.0	2.5	0.5	1.5	1.0	0.89
LOC104915006	8.0	3.0	6.5	8.0	1.0	3.0	4.9	2.97
LOC104915007	822.0	639.0	985.0	756.0	776.0	952.0	821.7	129.17

LOC104915009	0.0	0.0	0.0	1.0	0.0	1.0	0.3	0.52
LOC104915011	4.0	1.0	0.0	7.0	1.0	0.0	2.2	2.79
LOC104915012	0.0	0.0	0.5	0.5	0.0	0.5	0.3	0.27
LOC104915014	0.5	0.5	0.0	0.5	0.0	1.0	0.4	0.38
LOC104915015	0.0	0.0	1.0	0.0	0.0	0.0	0.2	0.41
LOC104915016	9.5	3.5	6.0	8.0	2.0	3.5	5.4	2.92
LOC104915017	10.5	2.5	10.0	9.0	5.0	2.5	6.6	3.71
LOC104915018	15.0	5.0	4.5	10.0	3.5	5.0	7.2	4.46
LOC104915019	22.5	20.5	23.0	22.5	22.0	30.0	23.4	3.34
LOC104915020	19.0	7.5	23.0	21.0	10.0	27.0	17.9	7.62
LOC104915021	3.0	2.5	5.5	2.0	3.5	3.5	3.3	1.21
LOC104915022	1.5	1.5	1.0	0.0	1.0	1.0	1.0	0.55
LOC104915023	1.0	1.0	2.5	2.0	1.5	2.0	1.7	0.61
LOC104915024	8.0	3.0	3.5	6.0	4.0	7.5	5.3	2.14
LOC104915025	1.5	0.5	4.5	2.5	3.5	2.5	2.5	1.41
LOC104915026	253.0	201.5	184.5	226.0	225.0	197.5	214.6	24.83
LOC104915027	1.5	0.0	0.5	2.0	0.5	5.0	1.6	1.83
LOC104915028	1.0	0.0	1.0	1.0	0.5	1.0	0.8	0.42
LOC104915034	3.5	2.0	0.5	1.0	0.5	0.5	1.3	1.21
LOC104915035	13.5	7.0	7.0	11.5	3.0	3.0	7.5	4.31
LOC104915036	7.5	4.5	4.0	4.5	3.0	1.0	4.1	2.13
LOC104915037	23.0	19.5	8.5	27.5	13.5	5.0	16.2	8.68
LOC104915038	1.0	0.0	0.0	0.0	0.0	1.0	0.3	0.52
LOC104915039	13.5	3.0	5.5	8.0	6.5	1.5	6.3	4.23
LOC104915040	0.0	0.0	0.0	0.0	0.0	0.0	0.0	0.00
LOC104915043	238.5	208.5	269.5	220.5	220.0	245.5	233.8	22.09
LOC104915044	50.0	8.5	0.5	37.5	10.5	2.5	18.3	20.48
LOC104915045	0.5	6.5	1.0	3.0	4.0	1.0	2.7	2.32
LOC104915046	0.0	0.5	0.5	0.0	0.0	0.0	0.2	0.26
LOC104915047	10.5	7.0	1.0	8.0	2.5	5.5	5.8	3.53
LOC104915049	557.5	488.5	448.5	498.5	576.0	384.0	492.2	70.65
LOC104915050	27.5	9.5	59.5	32.0	11.0	37.5	29.5	18.53
LOC104915051	6.5	1.5	12.0	4.0	1.0	15.5	6.8	5.87
LOC104915052	4.0	6.0	2.0	2.0	4.0	2.5	3.4	1.56
LOC104915053	40.0	9.0	66.5	36.5	11.0	39.5	33.8	21.37
LOC104915054	45.0	14.0	98.0	50.0	13.5	68.5	48.2	32.49
LOC104915057	43.0	8.0	35.5	47.0	9.0	26.0	28.1	16.78
LOC104915058	7030.0	6190.0	4849.0	6791.0	7571.0	5606.0	6339.5	997.92
LOC104915059	4.0	2.5	4.0	5.5	5.0	3.5	4.1	1.07
LOC104915061	0.0	0.0	0.0	0.0	0.0	0.0	0.0	0.00
LOC104915062	415.0	204.5	106.5	359.0	247.5	90.5	237.2	131.32
LOC104915063	0.0	0.0	0.0	0.0	0.0	0.0	0.0	0.00
LOC104915064	0.0	0.0	0.5	0.0	0.0	0.0	0.1	0.20
LOC104915065	0.0	0.0	0.0	0.0	0.0	0.0	0.0	0.00
LOC104915066	1035.5	1375.5	2143.5	1080.5	1604.5	1600.0	1473.3	409.38
LOC104915067	3.5	0.5	7.0	4.5	3.5	4.5	3.9	2.11
LOC104915068	13.5	5.5	64.0	29.0	11.5	37.5	26.8	21.76
LOC104915069	76.5	26.5	218.5	70.5	35.0	122.0	91.5	70.91
LOC104915070	47.5	11.5	167.0	53.0	12.5	76.5	61.3	57.49
LOC104915071	72.0	24.0	276.0	97.0	30.0	157.5	109.4	95.10
LOC104915072	11.0	5.0	7.5	6.0	8.5	3.0	6.8	2.80
LOC104915073	0.0	1.0	0.0	0.5	1.5	0.5	0.6	0.58
LOC104915074	8.0	3.0	3.5	8.0	2.5	2.0	4.5	2.76
LOC104915076	19.5	16.0	6.5	18.0	13.5	7.5	13.5	5.43

LOC104915077	0.0	1.0	0.0	0.0	0.0	0.0	0.2	0.41
LOC104915078	44.0	16.0	34.0	35.5	12.0	35.5	29.5	12.58
LOC104915080	79.0	72.5	57.5	79.0	78.5	50.5	69.5	12.45
LOC104915082	226.5	278.0	431.5	217.0	325.5	383.5	310.3	86.08
LOC104915083	6.0	1.5	1.0	4.0	2.0	2.5	2.8	1.86
LOC104915084	0.0	0.0	0.0	0.0	0.0	0.0	0.0	0.00
LOC104915085	0.5	0.0	0.0	0.0	0.0	0.0	0.1	0.20
LOC104915088	719.5	395.0	742.5	662.0	461.5	748.0	621.4	154.14
LOC104915089	33.5	15.0	78.5	36.5	25.0	72.5	43.5	25.96
LOC104915091	13.5	4.0	19.0	15.0	5.5	11.0	11.3	5.74
LOC104915092	67.5	74.0	83.0	90.0	75.0	66.5	76.0	9.08
LOC104915093	20.5	3.5	22.5	22.0	11.0	19.5	16.5	7.62
LOC104915094	306.5	303.5	298.5	282.5	367.5	340.5	316.5	31.39
LOC104915095	0.0	0.0	0.0	0.5	0.0	0.0	0.1	0.20
LOC104915100	0.0	0.5	0.0	0.5	0.0	2.0	0.5	0.77
LOC104915101	0.0	0.0	0.0	0.0	0.0	0.0	0.0	0.00
LOC104915102	0.0	0.0	0.5	0.0	0.5	0.5	0.3	0.27
LOC104915104	5.5	5.0	7.5	3.5	0.5	3.0	4.2	2.40
LOC104915106	14.5	13.0	18.0	12.0	12.5	28.5	16.4	6.30
LOC104915109	2.0	1.0	2.0	2.5	0.0	1.0	1.4	0.92
LOC104915110	9.0	5.0	21.0	8.0	6.5	17.0	11.1	6.41
LOC104915116	28.0	9.5	29.5	29.0	10.5	18.0	20.8	9.34
LOC104915117	302.0	263.5	131.5	283.5	306.0	163.0	241.6	75.27
LOC104915118	26.5	13.0	20.0	20.0	18.0	17.0	19.1	4.45
LOC104915119	6.5	5.0	4.0	9.5	4.5	3.0	5.4	2.31
LOC104915120	5.0	0.0	3.5	3.0	0.5	1.5	2.3	1.92
LOC104915121	6.0	0.5	4.0	7.5	4.0	5.0	4.5	2.37
LOC104915122	21.5	2.0	24.5	33.0	7.5	13.5	17.0	11.49
LOC104915123	5.5	5.0	3.0	6.0	5.5	2.0	4.5	1.61
LOC104915124	275.5	115.5	109.5	264.0	132.5	94.5	165.3	81.94
LOC104915125	0.0	0.0	0.5	0.5	0.0	0.0	0.2	0.26
LOC104915127	1.0	0.0	0.0	1.0	0.0	1.0	0.5	0.55
LOC104915129	0.0	0.0	0.0	0.0	0.0	0.0	0.0	0.00
LOC104915130	1.0	6.0	0.5	1.5	7.5	1.5	3.0	2.97
LOC104915131	0.0	0.0	0.0	0.0	0.0	0.0	0.0	0.00
LOC104915132	0.0	0.0	0.0	0.0	0.0	0.0	0.0	0.00
LOC104915134	101.0	45.5	57.5	93.5	49.5	52.0	66.5	24.25
LOC104915138	189.0	222.0	190.0	192.0	256.5	182.0	205.3	28.70
LOC104915139	0.0	0.0	0.0	0.0	0.0	0.0	0.0	0.00
LOC104915140	40.5	57.0	112.0	40.0	64.0	88.0	66.9	28.31
LOC104915141	28.5	9.0	66.0	38.0	6.0	46.5	32.3	22.89
LOC104915142	55.0	16.0	73.0	67.5	24.5	52.5	48.1	23.02
LOC104915143	65.0	26.0	63.5	57.0	31.5	54.5	49.6	16.70
LOC104915145	0.0	0.0	0.0	0.0	0.0	0.0	0.0	0.00
LOC104915146	0.0	0.0	0.0	0.0	0.0	0.0	0.0	0.00
LOC104915148	22.5	19.0	45.5	12.5	16.0	23.5	23.2	11.68
LOC104915150	1.5	0.0	2.5	0.5	0.0	0.0	0.8	1.04
LOC104915152	118.5	56.5	169.5	131.5	61.0	144.0	113.5	45.65
LOC104915153	15.5	4.0	34.0	14.5	2.0	24.5	15.8	12.15
LOC104915154	12.5	5.0	29.5	9.0	5.5	27.5	14.8	10.94
LOC104915155	8.5	1.5	42.0	15.0	5.0	34.5	17.8	16.66
LOC104915156	36.0	12.0	92.0	41.5	22.0	82.5	47.7	32.51
LOC104915157	0.0	0.0	0.0	0.0	0.0	0.5	0.1	0.20
LOC104915158	1.0	4.5	0.0	0.0	0.0	1.0	1.1	1.74

LOC104915160	622.0	485.5	559.5	597.0	549.5	510.0	553.9	51.29
LOC104915161	97.5	55.5	149.0	111.0	63.5	139.5	102.7	38.36
LOC104915162	9.5	3.5	17.0	9.5	2.0	17.0	9.8	6.39
LOC104915163	18.0	20.5	34.0	24.5	34.5	34.0	27.6	7.51
LOC104915164	11.0	9.5	22.0	12.0	13.5	17.0	14.2	4.61
LOC104915166	31.5	6.0	53.0	23.5	13.0	47.5	29.1	18.65
LOC104915167	2.5	2.0	4.5	4.5	8.0	3.5	4.2	2.14
LOC104915169	0.5	0.0	1.0	0.5	0.0	1.0	0.5	0.45
LOC104915170	8.5	23.5	17.5	9.5	21.0	12.0	15.3	6.25
LOC104915171	90.0	73.0	219.5	116.0	82.0	221.0	133.6	68.65
LOC104915172	0.0	0.0	0.0	0.0	0.0	0.0	0.0	0.00
LOC104915176	0.0	0.0	0.0	0.0	0.0	0.0	0.0	0.00
LOC104915177	1.0	0.5	0.5	0.5	0.0	1.0	0.6	0.38
LOC104915179	1.0	1.0	1.0	1.5	0.5	0.0	0.8	0.52
LOC104915180	135.5	129.5	167.5	128.5	152.5	177.0	148.4	20.59
LOC104915181	118.0	43.0	171.0	118.5	61.0	142.5	109.0	48.57
LOC104915182	132.5	39.0	189.5	123.5	37.5	138.0	110.0	60.15
LOC104915183	2.5	0.5	1.0	1.0	4.0	1.5	1.8	1.29
LOC104915184	32.0	8.0	73.0	51.5	12.0	58.5	39.2	26.20
LOC104915185	439.0	83.0	832.5	500.0	96.5	543.0	415.7	286.41
LOC104915187	14.0	11.5	3.5	17.0	15.0	2.5	10.6	6.14
LOC104915188	0.0	0.0	0.0	0.0	0.0	0.0	0.0	0.00
LOC104915189	13.5	11.0	45.0	18.5	9.5	32.5	21.7	14.14
LOC104915190	0.0	0.0	0.0	0.0	0.0	0.0	0.0	0.00
LOC104915191	439.0	457.0	412.5	382.0	575.0	401.0	444.4	69.34
LOC104915195	4.0	3.5	5.0	4.0	1.5	5.5	3.9	1.39
LOC104915196	2.5	1.0	0.5	2.0	0.0	1.0	1.2	0.93
LOC104915197	410.0	412.5	257.0	332.5	449.5	250.0	351.9	85.25
LOC104915198	31.5	16.0	83.0	45.0	26.0	81.0	47.1	28.63
LOC104915199	59.0	7.5	8.0	33.5	7.0	2.0	19.5	22.34
LOC104915200	0.0	0.0	0.0	0.0	0.0	0.0	0.0	0.00
LOC104915201	0.0	0.0	0.0	0.0	0.0	0.0	0.0	0.00
LOC104915202	36.5	4.0	27.0	41.0	5.0	17.0	21.8	15.71
LOC104915204	0.0	0.0	0.5	0.5	0.0	0.0	0.2	0.26
LOC104915205	6.0	0.0	4.0	5.5	1.0	5.0	3.6	2.50
LOC104915207	63.5	54.0	59.5	54.0	54.0	53.0	56.3	4.22
LOC104915208	53.0	17.0	42.5	63.0	14.5	24.5	35.8	20.08
LOC104915209	13.5	1.5	14.5	15.0	3.0	13.0	10.1	6.13
LOC104915210	0.0	0.0	0.0	0.0	0.0	0.0	0.0	0.00
LOC104915211	0.0	0.0	0.0	0.0	0.0	0.0	0.0	0.00
LOC104915212	0.0	0.0	0.0	0.0	0.0	0.0	0.0	0.00
LOC104915213	0.0	0.0	0.0	0.0	0.0	0.0	0.0	0.00
LOC104915214	147.0	124.5	151.0	166.0	131.5	158.5	146.4	15.83
LOC104915216	24.5	13.0	28.5	21.0	16.5	20.0	20.6	5.53
LOC104915218	0.0	0.0	0.0	0.0	0.0	0.0	0.0	0.00
LOC104915219	0.5	0.0	3.0	0.0	1.0	1.0	0.9	1.11
LOC104915220	0.0	0.0	0.0	0.0	0.0	0.0	0.0	0.00
LOC104915221	12.0	15.0	22.0	9.0	16.0	24.0	16.3	5.75
LOC104915223	1.5	0.0	0.5	1.5	0.0	0.0	0.6	0.74
LOC104915224	1.5	1.5	4.0	0.0	2.0	2.0	1.8	1.29
LOC104915225	363.0	180.5	251.5	274.5	204.0	245.5	253.2	63.68
LOC104915226	0.0	0.0	0.0	0.0	0.0	0.0	0.0	0.00
LOC104915227	281.5	122.5	266.0	229.0	121.5	256.5	212.8	72.40
LOC104915229	48.5	31.0	111.5	58.0	41.0	74.0	60.7	28.93

LOC104915231	0.5	1.5	0.5	0.0	2.0	1.0	0.9	0.74
LOC104915232	0.5	0.5	0.5	0.0	0.5	0.5	0.4	0.20
LOC104915233	96.0	86.0	79.5	109.0	109.5	72.5	92.1	15.39
LOC104915234	7.5	2.5	13.5	19.5	3.0	9.0	9.2	6.49
LOC104915235	22.0	11.0	4.5	13.0	9.5	2.5	10.4	6.92
LOC104915237	0.5	0.0	0.5	0.5	0.0	0.0	0.3	0.27
LOC104915238	0.0	0.0	0.0	0.5	0.0	0.0	0.1	0.20
LOC104915239	18.0	2.5	27.0	14.5	0.5	19.5	13.7	10.29
LOC104915240	13.5	2.0	14.5	13.0	1.0	11.0	9.2	6.06
LOC104915241	133.0	87.0	124.0	100.5	97.0	109.5	108.5	17.32
LOC104915242	0.0	0.0	0.0	0.0	0.0	0.5	0.1	0.20
LOC104915243	0.0	0.0	0.0	0.0	0.0	0.0	0.0	0.00
LOC104915244	30.5	18.0	52.5	32.5	15.0	36.0	30.8	13.52
LOC104915247	0.0	0.0	0.0	0.0	0.0	0.0	0.0	0.00
LOC104915248	0.0	0.0	3.5	0.0	0.5	1.5	0.9	1.39
LOC104915249	0.0	0.5	3.5	0.5	0.5	1.5	1.1	1.28
LOC104915251	1.5	27.0	29.0	2.5	29.0	26.0	19.2	13.35
LOC104915253	28.5	8.5	54.5	30.5	13.5	50.0	30.9	18.61
LOC104915255	34.0	11.5	4.0	23.0	10.0	4.0	14.4	11.85
LOC104915256	60.0	34.5	118.0	50.0	64.5	104.0	71.8	32.34
LOC104915257	0.0	0.0	0.0	0.0	0.0	0.0	0.0	0.00
LOC104915258	0.0	0.0	0.0	0.0	0.0	0.0	0.0	0.00
LOC104915259	0.0	0.0	0.0	0.0	0.0	0.0	0.0	0.00
LOC104915260	116.0	85.5	147.5	108.0	129.5	157.0	123.9	26.36
LOC104915261	0.0	0.0	0.0	0.0	0.0	0.0	0.0	0.00
LOC104915262	0.0	0.0	0.0	0.0	0.0	0.0	0.0	0.00
LOC104915264	9.5	39.5	42.0	15.0	51.5	44.5	33.7	17.15
LOC104915265	2.5	7.5	24.0	2.5	10.0	20.0	11.1	9.03
LOC104915266	0.0	0.0	0.0	0.0	0.0	0.0	0.0	0.00
LOC104915267	0.0	0.0	0.0	0.0	0.0	0.0	0.0	0.00
LOC104915269	38.5	21.0	106.0	36.0	24.0	107.0	55.4	40.14
LOC104915270	1.0	0.5	0.5	0.5	1.5	0.5	0.8	0.42
LOC104915271	0.0	0.0	0.0	0.0	0.0	0.0	0.0	0.00
LOC104915274	0.0	0.0	0.0	0.5	0.0	0.0	0.1	0.20
LOC104915275	62.0	11.0	250.0	91.0	14.0	140.0	94.7	90.30
LOC104915277	47.5	21.5	17.5	42.0	28.0	17.5	29.0	12.91
LOC104915278	222.5	134.0	110.5	190.0	134.0	92.5	147.3	49.37
LOC104915286	0.0	0.0	0.0	0.0	0.0	1.0	0.2	0.41
LOC104915287	3.0	2.0	5.0	4.0	3.0	3.5	3.4	1.02
LOC104915288	1.0	0.0	0.0	0.0	1.0	0.0	0.3	0.52
LOC104915289	15.0	9.5	21.5	15.5	9.0	14.5	14.2	4.58
LOC104915290	377.0	181.0	217.5	393.5	204.5	197.5	261.8	96.46
LOC104915292	572.5	469.0	247.5	526.5	518.5	240.0	429.0	147.22
LOC104915295	0.0	0.0	0.0	0.0	0.0	0.0	0.0	0.00
LOC104915298	0.5	0.0	0.5	0.0	2.0	0.0	0.5	0.77
LOC104915299	84.0	70.0	88.0	67.0	64.0	73.5	74.4	9.59
LOC104915300	7.0	2.5	9.5	11.0	4.5	7.0	6.9	3.12
LOC104915303	114.5	17.0	207.5	142.0	15.5	83.0	96.6	74.54
LOC104915305	0.5	0.0	0.0	0.0	0.0	0.0	0.1	0.20
LOC104915307	208.0	188.0	221.5	195.5	214.5	200.0	204.6	12.45
LOC104915308	0.0	0.0	0.0	0.0	0.0	0.0	0.0	0.00
LOC104915310	0.0	0.0	0.0	0.0	0.0	0.0	0.0	0.00
LOC104915311	0.0	0.0	0.0	0.0	0.0	0.0	0.0	0.00
LOC104915312	673.0	624.5	437.5	598.0	693.0	401.0	571.2	122.96

LOC104915313	52.0	33.5	72.0	42.0	34.0	35.0	44.8	15.10
LOC104915314	1.0	1.5	2.5	0.5	1.0	1.0	1.3	0.69
LOC104915316	19.0	6.0	14.5	18.5	10.0	13.0	13.5	5.00
LOC104915317	74.5	29.0	38.5	71.5	35.0	34.0	47.1	20.33
LOC104915318	13.0	6.0	15.5	17.5	8.5	6.0	11.1	4.95
LOC104915319	131.5	96.0	84.5	137.0	105.5	58.5	102.2	29.47
LOC104915320	3.0	1.0	1.0	1.5	1.0	1.0	1.4	0.80
LOC104915321	0.0	0.0	0.0	0.0	0.0	0.0	0.0	0.00
LOC104915322	0.0	0.0	0.0	0.0	0.0	0.0	0.0	0.00
LOC104915323	0.0	0.0	0.0	0.0	0.0	0.0	0.0	0.00
LOC104915324	0.0	0.0	0.0	0.0	0.0	0.0	0.0	0.00
LOC104915325	0.0	0.0	0.0	0.0	0.0	0.0	0.0	0.00
LOC104915326	54.0	53.0	61.5	41.5	46.0	50.5	51.1	6.91
LOC104915327	12.0	2.5	33.0	16.0	6.0	16.5	14.3	10.68
LOC104915330	125.0	76.5	80.0	102.5	87.5	82.0	92.3	18.46
LOC104915331	0.0	0.5	5.5	0.0	0.0	4.0	1.7	2.44
LOC104915335	0.5	0.0	0.0	0.0	0.5	0.0	0.2	0.26
LOC104915336	62.5	32.5	41.5	55.5	35.0	44.5	45.3	11.70
LOC104915338	288.5	247.5	191.0	246.5	247.5	139.5	226.8	52.83
LOC104915340	44.5	7.0	62.5	49.5	13.5	48.0	37.5	22.07
LOC104915341	325.0	251.5	249.0	320.5	278.5	263.5	281.3	33.77
LOC104915342	17.5	7.5	28.0	15.5	12.5	18.5	16.6	6.86
LOC104915343	525.0	664.5	862.0	403.0	698.0	652.5	634.2	156.59
LOC104915344	17.0	3.5	11.5	20.5	3.5	8.5	10.8	7.00
LOC104915345	907.0	769.5	567.0	851.0	853.0	558.0	750.9	152.44
LOC104915347	13.5	2.0	4.0	7.0	5.0	2.5	5.7	4.24
LOC104915348	303.5	216.5	152.0	266.5	200.0	125.5	210.7	67.20
LOC104915349	176.5	111.0	99.5	170.5	110.0	90.5	126.3	37.35
LOC104915350	101.0	33.5	54.0	92.0	40.5	52.5	62.3	27.75
LOC104915351	112.5	96.5	114.0	123.0	88.5	95.5	105.0	13.39
LOC104915352	66.0	26.5	100.5	65.5	27.0	77.5	60.5	29.06
LOC104915354	3.0	2.5	10.0	11.0	3.5	10.5	6.8	4.13
LOC104915355	2.5	2.5	1.5	0.5	1.5	1.5	1.7	0.75
LOC104915356	13.0	3.0	6.0	21.5	6.0	6.5	9.3	6.81
LOC104915357	4.0	8.0	7.5	5.5	5.5	10.5	6.8	2.32
LOC104915358	19.5	15.5	18.5	18.5	26.0	22.0	20.0	3.61
LOC104915359	115.5	62.0	121.0	126.5	67.5	112.5	100.8	28.41
LOC104915360	0.5	0.5	0.0	0.0	0.0	0.0	0.2	0.26
LOC104915361	6.5	3.0	17.5	8.0	5.0	5.0	7.5	5.18
LOC104915362	5.5	4.5	12.5	6.5	4.5	8.5	7.0	3.08
LOC104915365	73.0	17.5	66.0	75.5	30.5	68.0	55.1	24.66
LOC104915366	5.0	6.0	8.0	5.5	9.5	6.5	6.8	1.70
LOC104915367	0.0	1.0	2.0	0.0	1.5	3.5	1.3	1.33
LOC104915368	187.5	185.5	198.5	201.5	230.5	193.0	199.4	16.42
LOC104915371	0.0	0.0	0.0	0.0	0.0	0.0	0.0	0.00
LOC104915373	0.0	0.0	0.5	0.5	0.0	0.0	0.2	0.26
LOC104915374	0.0	0.0	0.0	0.0	0.0	0.0	0.0	0.00
LOC104915375	0.0	0.0	0.0	0.0	0.0	0.0	0.0	0.00
LOC104915377	758.5	432.0	198.5	664.0	409.5	190.5	442.2	233.73
LOC104915382	9.0	1.0	1.0	9.5	3.0	3.5	4.5	3.82
LOC104915384	0.5	0.0	1.0	1.0	0.0	1.0	0.6	0.49
LOC104915385	0.0	0.0	0.0	0.0	0.0	0.0	0.0	0.00
LOC104915386	0.0	0.0	0.0	0.0	0.0	0.0	0.0	0.00
LOC104915387	0.0	0.0	0.0	0.0	0.0	0.0	0.0	0.00

LOC104915388	6912.0	4690.5	3827.5	6112.5	4707.5	3470.0	4953.3	1324.94
LOC104915389	0.0	0.0	0.0	0.0	0.0	0.0	0.0	0.00
LOC104915390	0.0	0.0	0.0	0.0	0.0	0.0	0.0	0.00
LOC104915391	0.0	0.0	0.0	0.0	0.0	0.0	0.0	0.00
LOC104915392	60.0	17.0	69.0	61.0	23.5	57.0	47.9	21.89
LOC104915393	1.0	0.5	1.5	1.0	0.0	1.5	0.9	0.58
LOC104915394	0.0	0.0	0.0	0.0	0.0	0.0	0.0	0.00
LOC104915395	0.0	0.0	0.0	0.0	0.5	0.5	0.2	0.26
LOC104915397	7.0	12.0	5.5	15.0	18.5	7.5	10.9	5.13
LOC104915398	15.5	12.0	2.0	21.0	26.5	15.5	15.4	8.32
LOC104915399	1.5	0.0	2.0	3.5	0.0	1.0	1.3	1.33
LOC104915400	8.5	3.0	12.0	12.5	2.0	11.0	8.2	4.61
LOC104915401	28.5	18.5	12.5	17.5	19.0	8.5	17.4	6.79
LOC104915402	1.0	0.5	0.0	0.5	0.0	0.0	0.3	0.41
LOC104915403	0.0	0.0	0.0	0.5	0.0	0.0	0.1	0.20
LOC104915404	0.0	0.5	1.5	0.5	0.0	0.0	0.4	0.58
LOC104915405	0.0	0.0	0.0	0.0	0.0	0.0	0.0	0.00
LOC104915406	74.5	99.5	43.0	84.5	97.0	70.0	78.1	20.82
LOC104915407	2.0	2.0	12.0	1.5	3.5	7.0	4.7	4.12
LOC104915408	0.0	0.0	0.0	0.0	0.0	0.0	0.0	0.00
LOC104915409	0.0	0.5	0.5	1.5	0.0	0.0	0.4	0.58
LOC104915410	0.0	0.0	0.0	0.0	0.0	0.5	0.1	0.20
LOC104915411	24.0	8.5	18.5	20.5	8.5	10.0	15.0	6.83
LOC104915412	44.0	22.5	24.0	38.0	28.5	20.0	29.5	9.52
LOC104915413	433.0	1013.5	551.5	369.5	1157.0	683.5	701.3	319.36
LOC104915414	0.0	0.0	0.0	0.0	0.0	0.0	0.0	0.00
LOC104915415	0.0	0.0	0.5	0.0	1.0	0.0	0.3	0.42
LOC104915416	9.0	4.5	25.5	9.5	9.0	17.5	12.5	7.64
LOC104915417	0.0	0.0	0.0	0.0	0.0	0.0	0.0	0.00
LOC104915418	10.0	1.0	11.5	7.5	1.0	8.0	6.5	4.49
LOC104915419	895.5	1033.0	908.5	941.5	1178.0	903.5	976.7	110.94
LOC104915420	3.0	1.0	2.0	1.5	0.0	0.5	1.3	1.08
LOC104915421	0.0	0.0	0.0	0.5	1.0	0.0	0.3	0.42
LOC104915422	1.0	0.0	0.0	0.0	0.5	0.0	0.3	0.42
LOC104915423	252.0	143.5	163.5	246.0	187.5	149.0	190.3	48.02
LOC104915425	53.5	23.0	42.5	72.5	26.5	42.0	43.3	18.18
LOC104915426	0.0	0.0	0.0	0.0	0.0	0.0	0.0	0.00
LOC104915427	18.0	20.5	44.0	19.0	25.5	43.0	28.3	12.03
LOC104915428	12.5	1.5	6.5	5.5	2.5	2.0	5.1	4.15
LOC104915429	0.0	0.0	0.0	0.0	0.0	0.0	0.0	0.00
LOC104915430	56.5	43.0	26.5	47.5	52.0	25.5	41.8	13.07
LOC104915431	515.5	951.5	693.0	374.0	898.0	647.0	679.8	220.54
LOC104915432	0.0	0.0	0.0	0.0	0.0	0.0	0.0	0.00
LOC104915433	0.0	0.0	0.0	0.0	0.0	0.0	0.0	0.00
LOC104915434	107.5	119.0	107.5	113.0	114.5	118.5	113.3	5.07
LOC104915435	0.0	0.0	0.0	0.0	0.0	0.0	0.0	0.00
LOC104915436	0.0	0.0	0.0	0.0	0.0	0.0	0.0	0.00
LOC104915438	0.0	0.0	0.0	0.0	0.0	0.0	0.0	0.00
LOC104915439	68.5	48.5	55.0	46.0	34.0	42.5	49.1	11.77
LOC104915440	36.5	29.0	29.0	40.0	36.0	33.5	34.0	4.39
LOC104915441	0.0	0.0	0.0	0.0	0.0	0.0	0.0	0.00
LOC104915442	0.0	0.0	0.5	0.0	0.0	0.0	0.1	0.20
LOC104915443	80.0	35.0	49.0	78.0	42.0	43.0	54.5	19.50
LOC104915444	22.5	16.5	5.0	17.5	9.5	8.0	13.2	6.69

LOC104915445	0.0	0.0	0.5	0.0	0.0	0.0	0.1	0.20
LOC104915446	1.5	0.0	0.0	0.5	0.0	0.0	0.3	0.61
LOC104915448	0.0	0.0	0.0	0.0	0.0	0.0	0.0	0.00
LOC104915449	0.0	0.0	0.0	0.0	0.0	0.0	0.0	0.00
LOC104915450	0.0	0.0	0.0	0.0	0.0	0.0	0.0	0.00
LOC104915451	0.0	0.0	1.0	0.0	0.5	0.0	0.3	0.42
LOC104915452	0.0	0.0	0.0	0.0	0.0	0.0	0.0	0.00
LOC104915453	0.0	0.0	0.0	0.0	0.0	0.0	0.0	0.00
LOC104915454	0.0	0.0	0.0	0.0	0.0	0.0	0.0	0.00
LOC104915455	0.0	0.0	0.0	0.0	0.0	0.0	0.0	0.00
LOC104915456	0.0	0.0	0.0	0.0	0.0	0.0	0.0	0.00
LOC104915457	20.5	11.5	16.5	23.5	10.5	11.5	15.7	5.42
LOC104915458	0.5	0.0	0.0	0.0	0.5	0.0	0.2	0.26
LOC104915459	0.0	0.0	0.0	0.0	0.0	0.0	0.0	0.00
LOC104915461	1.5	0.0	0.0	1.0	1.0	0.5	0.7	0.61
LOC104915462	11.5	6.5	86.0	8.0	4.5	67.5	30.7	36.24
LOC104915463	445.5	821.0	806.5	554.0	884.0	857.0	728.0	182.14
LOC104915464	0.0	0.0	0.0	0.0	0.0	0.0	0.0	0.00
LOC104915465	0.0	0.0	0.0	0.0	0.0	0.0	0.0	0.00
LOC104915466	0.0	0.0	0.0	1.0	0.5	1.0	0.4	0.49
LOC104915467	0.0	0.0	0.0	0.0	0.0	0.0	0.0	0.00
LOC104915468	0.0	0.0	0.0	0.0	0.0	0.0	0.0	0.00
LOC104915469	1.0	1.0	0.0	0.5	0.0	0.0	0.4	0.49
LOC104915470	0.0	0.0	1.0	0.0	0.5	0.0	0.3	0.42
LOC104915471	0.5	0.0	1.0	0.0	0.0	0.0	0.3	0.42
LOC104915472	0.0	0.0	0.0	0.0	0.0	0.0	0.0	0.00
LOC104915473	0.5	0.0	0.0	0.0	0.0	0.5	0.2	0.26
LOC104915474	0.0	0.0	0.5	0.5	0.0	0.0	0.2	0.26
LOC104915475	1.5	0.0	0.0	0.0	0.0	0.0	0.3	0.61
LOC104915476	0.0	0.0	0.0	0.0	0.0	0.0	0.0	0.00
LOC104915477	0.0	0.0	0.0	0.0	0.5	0.0	0.1	0.20
LOC104915478	0.0	0.0	0.0	0.0	0.0	0.0	0.0	0.00
LOC104915479	0.0	0.0	0.0	0.0	0.0	0.0	0.0	0.00
LOC104915480	0.0	0.0	0.0	0.0	0.0	0.0	0.0	0.00
LOC104915481	0.0	0.0	0.0	0.0	0.0	0.0	0.0	0.00
LOC104915482	0.0	0.0	0.0	0.0	0.0	0.0	0.0	0.00
LOC104915483	0.0	0.0	0.0	0.0	0.0	0.0	0.0	0.00
LOC104915484	0.0	0.0	0.0	0.0	0.0	0.0	0.0	0.00
LOC104915485	1.0	1.5	5.0	0.0	1.5	13.0	3.7	4.88
LOC104915486	162.0	89.5	78.5	123.0	113.5	74.0	106.8	33.26
LOC104915487	71.0	46.0	105.5	56.5	37.0	114.5	71.8	31.84
LOC104915488	0.0	0.0	0.0	0.0	0.0	0.0	0.0	0.00
LOC104915489	4.0	2.0	7.5	2.0	2.0	6.5	4.0	2.47
LOC104915490	1.5	2.0	6.5	3.0	5.0	5.5	3.9	2.04
LOC104915491	1.0	0.0	0.0	0.5	0.5	0.5	0.4	0.38
LOC104915493	54.5	32.5	10.5	40.5	41.0	11.5	31.8	17.56
LOC104915494	10.0	7.0	1.5	7.5	6.5	1.5	5.7	3.44
LOC104915495	26.5	9.0	8.0	17.0	9.0	1.5	11.8	8.71
LOC104915496	4.0	1.0	3.5	5.0	1.0	1.5	2.7	1.72
LOC104915497	129.0	53.0	18.0	141.5	83.5	20.0	74.2	53.22
LOC104915498	69.0	32.0	102.5	72.5	36.5	79.5	65.3	26.79
LOC104915499	49.5	53.5	58.5	34.0	50.5	59.5	50.9	9.23
LOC104915500	1.5	0.5	1.0	2.5	2.0	1.0	1.4	0.74
LOC104915501	3595.0	3978.0	2493.5	3405.0	4489.5	2543.5	3417.4	789.01

LOC104915502	92.5	115.5	58.5	87.0	137.0	61.0	91.9	30.61
LOC104915504	0.5	1.0	0.0	0.0	0.0	0.5	0.3	0.41
LOC104915505	0.0	0.5	0.5	0.5	0.5	0.5	0.4	0.20
LOC104915506	17.5	8.5	49.0	24.0	9.5	69.5	29.7	24.48
LOC104915507	0.0	0.5	0.0	0.0	0.0	0.0	0.1	0.20
LOC104915508	0.0	0.0	0.0	0.5	0.0	0.5	0.2	0.26
LOC104915509	0.5	0.5	5.0	0.0	0.5	0.5	1.2	1.89
LOC104915510	652.5	594.0	638.5	668.0	630.5	616.5	633.3	26.21
LOC104915511	83.5	51.0	110.0	70.5	59.5	71.5	74.3	20.70
LOC104915512	0.0	0.0	1.0	0.0	0.0	1.0	0.3	0.52
LOC104915513	9.0	6.5	17.0	0.5	0.0	0.5	5.6	6.72
LOC104915514	1.5	0.5	0.5	1.0	0.5	0.0	0.7	0.52
LOC104915515	23.0	10.5	10.0	7.0	10.0	17.0	12.9	5.94
LOC104915516	0.0	0.0	0.0	0.0	0.0	0.0	0.0	0.00
LOC104915517	0.0	0.0	0.5	0.5	0.5	0.0	0.3	0.27
LOC104915518	1.0	2.0	2.0	2.0	4.5	3.0	2.4	1.20
LOC104915519	17.5	13.5	17.0	11.5	8.0	20.0	14.6	4.42
LOC104915520	1.0	2.5	1.0	1.0	1.0	7.0	2.3	2.40
LOC104915521	1.0	0.0	0.0	0.0	0.5	1.5	0.5	0.63
LOC104915522	21.0	3.0	2.5	6.5	6.5	1.0	6.8	7.33
LOC104915523	0.0	0.0	0.0	0.0	0.5	0.0	0.1	0.20
LOC104915524	35.0	24.5	18.0	38.5	32.0	20.5	28.1	8.29
LOC104915525	0.0	0.0	0.0	0.0	0.0	0.0	0.0	0.00
LOC104915526	1.0	64.0	76.5	0.5	58.5	75.0	45.9	35.63
LOC104915527	1.5	218.5	113.5	1.0	226.5	101.5	110.4	99.08
LOC104915528	0.0	0.0	0.0	0.0	0.0	0.0	0.0	0.00
LOC104915529	0.5	0.0	0.0	0.5	0.0	0.0	0.2	0.26
LOC104915530	0.0	0.0	0.0	0.0	0.0	0.0	0.0	0.00
LOC104915532	0.0	0.0	0.0	0.0	0.0	0.0	0.0	0.00
LOC104915533	1255.0	1227.0	999.0	1308.0	1335.0	942.0	1177.7	165.90
LOC104915534	0.0	0.0	0.0	0.0	0.0	0.0	0.0	0.00
LOC104915535	0.0	0.0	0.0	0.0	0.0	0.0	0.0	0.00
LOC104915536	0.0	0.0	0.0	0.0	0.0	0.0	0.0	0.00
LOC104915537	0.0	0.0	0.0	0.0	0.0	0.0	0.0	0.00
LOC104915538	51.0	43.5	27.5	48.5	46.5	16.5	38.9	13.78
LOC104915539	0.5	0.0	0.5	0.0	0.0	0.0	0.2	0.26
LOC104915540	0.0	0.0	0.0	0.0	0.0	0.0	0.0	0.00
LOC104915541	25.5	16.5	15.0	26.5	25.5	13.5	20.4	6.02
LOC104915542	127.5	74.0	71.5	118.5	85.0	62.0	89.8	26.93
LOC104915543	0.0	0.0	0.0	0.0	0.0	0.0	0.0	0.00
LOC104915544	15.0	9.5	22.5	12.0	19.5	17.5	16.0	4.82
LOC104915545	80.5	64.5	97.0	79.0	53.0	79.0	75.5	15.09
LOC104915546	9.5	3.5	19.0	12.0	3.5	15.0	10.4	6.22
LOC104915547	6.5	3.0	0.0	4.0	2.5	1.5	2.9	2.22
LOC104915548	157.5	98.0	114.5	140.5	97.5	144.5	125.4	25.58
LOC104915549	2.0	0.0	0.0	0.0	0.0	0.0	0.3	0.82
LOC104915550	170.0	178.5	135.0	150.5	180.0	144.0	159.7	19.04
LOC104915551	0.0	0.0	0.0	0.0	0.0	0.0	0.0	0.00
LOC104915552	0.5	1.0	0.0	0.0	1.0	0.0	0.4	0.49
LOC104915553	0.5	1.5	0.5	0.0	0.5	1.5	0.8	0.61
LOC104915554	88.5	43.0	56.0	88.0	52.5	65.5	65.6	18.98
LOC104915555	14.0	7.5	3.0	11.5	6.0	5.0	7.8	4.16
LOC104915556	38.0	38.0	42.5	48.5	47.0	39.0	42.2	4.65
LOC104915557	0.0	0.0	0.0	0.0	0.0	0.0	0.0	0.00

LOC104915558	149.5	90.5	180.0	157.5	87.0	160.5	137.5	39.09
LOC104915559	0.0	0.0	0.0	0.0	0.0	0.0	0.0	0.00
LOC104915560	167.5	197.5	197.0	161.0	177.0	205.0	184.2	18.12
LOC104915561	56.0	37.0	24.0	36.5	26.0	15.5	32.5	14.09
LOC104915562	0.0	0.0	0.0	0.0	0.0	0.0	0.0	0.00
LOC104915563	0.0	0.0	0.0	0.0	0.0	0.0	0.0	0.00
LOC104915564	0.0	0.0	0.0	0.0	0.0	0.0	0.0	0.00
LOC104915566	280.5	216.0	221.5	281.5	235.0	169.0	233.9	42.72
LOC104915567	96.0	25.0	111.0	73.5	41.0	78.5	70.8	32.57
LOC104915568	0.0	0.0	0.0	0.0	0.5	0.0	0.1	0.20
LOC104915569	7.5	6.0	7.5	7.0	2.5	6.5	6.2	1.89
LOC104915570	0.0	0.0	0.0	0.0	0.0	0.0	0.0	0.00
LOC104915571	0.0	0.0	0.5	0.0	0.0	0.0	0.1	0.20
LOC104915572	149.0	116.5	122.0	165.0	112.5	113.0	129.7	22.03
LOC104915573	0.0	0.0	0.0	0.0	0.0	0.0	0.0	0.00
LOC104915575	0.0	0.0	0.0	0.0	0.0	0.0	0.0	0.00
LOC104915578	0.0	0.0	0.0	0.0	0.0	0.0	0.0	0.00
LOC104915579	1.0	0.5	0.0	1.0	1.0	0.0	0.6	0.49
LOC104915580	1.5	0.0	0.0	0.0	0.5	0.5	0.4	0.58
LOC104915581	0.0	0.0	0.0	0.0	0.0	0.0	0.0	0.00
LOC104915582	0.0	0.0	0.0	0.0	0.0	0.0	0.0	0.00
LOC104915583	0.0	0.0	0.0	0.0	0.0	0.0	0.0	0.00
LOC104915584	0.0	0.0	0.0	0.0	0.0	0.0	0.0	0.00
LOC104915585	50.5	23.0	15.0	38.0	31.0	10.5	28.0	14.94
LOC104915586	0.0	0.0	0.0	0.0	0.0	0.0	0.0	0.00
LOC104915588	0.0	0.0	0.0	0.0	0.0	0.0	0.0	0.00
LOC104915589	0.0	0.0	0.0	0.0	0.0	0.0	0.0	0.00
LOC104915590	0.0	0.0	0.0	0.0	0.0	2.0	0.3	0.82
LOC104915591	0.0	0.0	0.0	0.0	0.0	0.0	0.0	0.00
LOC104915593	0.0	0.5	0.0	0.0	0.0	0.0	0.1	0.20
LOC104915594	12.5	4.0	4.5	9.5	3.5	8.0	7.0	3.61
LOC104915595	1.0	0.0	0.0	0.0	0.0	0.0	0.2	0.41
LOC104915596	0.0	0.0	0.0	0.0	0.0	0.0	0.0	0.00
LOC104915597	0.5	1.5	0.5	1.5	0.5	2.5	1.2	0.82
LOC104915598	471.0	472.0	464.0	412.5	525.0	437.5	463.7	37.92
LOC104915599	341.0	323.0	205.5	304.0	308.5	194.5	279.4	62.96
LOC104915600	0.0	0.0	0.0	0.0	0.0	0.0	0.0	0.00
LOC104915601	0.0	0.0	0.0	0.0	0.0	0.0	0.0	0.00
LOC104915602	0.0	0.0	0.0	0.0	0.0	0.0	0.0	0.00
LOC104915603	0.0	0.0	0.0	0.0	0.0	0.0	0.0	0.00
LOC104915604	0.0	0.0	0.0	0.0	0.0	0.0	0.0	0.00
LOC104915605	0.0	0.0	0.0	0.0	0.0	0.0	0.0	0.00
LOC104915606	0.0	0.0	0.0	0.0	0.0	0.0	0.0	0.00
LOC104915607	0.0	0.0	0.0	0.0	0.0	0.0	0.0	0.00
LOC104915608	0.0	0.0	0.0	0.0	0.0	0.0	0.0	0.00
LOC104915609	0.0	0.0	0.0	0.0	0.0	0.0	0.0	0.00
LOC104915610	0.5	5.5	1.0	2.0	1.5	5.5	2.7	2.25
LOC104915611	0.0	0.0	0.0	0.0	0.0	0.0	0.0	0.00
LOC104915613	0.0	0.0	0.0	0.0	0.0	0.0	0.0	0.00
LOC104915614	0.0	2.0	0.0	0.5	0.5	0.0	0.5	0.77
LOC104915615	0.0	0.0	0.0	0.0	0.0	0.0	0.0	0.00
LOC104915616	0.5	1.0	0.0	1.5	2.0	1.0	1.0	0.71
LOC104915617	0.0	0.0	0.0	0.0	0.0	0.0	0.0	0.00
LOC104915618	0.0	0.0	0.0	0.0	0.0	0.0	0.0	0.00

LOC104915619	0.0	0.0	0.0	0.0	0.5	0.5	0.2	0.26
LOC104915620	0.0	0.0	4.5	0.0	1.0	3.5	1.5	2.00
LOC104915621	130.5	35.5	85.0	125.5	39.0	69.5	80.8	41.02
LOC104915622	0.0	0.0	0.0	0.0	0.0	0.0	0.0	0.00
LOC104915623	0.0	0.0	0.0	0.0	0.0	0.0	0.0	0.00
LOC104915624	0.0	0.0	0.0	0.0	0.0	0.0	0.0	0.00
LOC104915625	0.0	0.0	0.0	0.0	0.0	0.0	0.0	0.00
LOC104915626	0.0	0.0	0.0	0.0	0.5	0.0	0.1	0.20
LOC104915627	5.0	1.0	1.5	5.0	1.0	3.0	2.8	1.89
LOC104915628	0.0	0.0	0.5	0.0	0.0	0.0	0.1	0.20
LOC104915629	0.0	0.0	0.0	0.0	0.5	0.5	0.2	0.26
LOC104915630	0.0	0.0	0.0	0.5	0.0	0.0	0.1	0.20
LOC104915631	0.0	0.0	1.0	0.0	0.0	0.5	0.3	0.42
LOC104915632	0.0	0.0	0.0	0.0	0.0	0.0	0.0	0.00
LOC104915633	52.5	42.0	39.5	55.0	47.5	29.5	44.3	9.37
LOC104915634	0.0	0.0	0.0	0.5	0.0	0.0	0.1	0.20
LOC104915635	0.0	0.0	0.0	0.0	0.0	0.0	0.0	0.00
LOC104915637	0.5	0.5	1.0	0.5	1.5	2.0	1.0	0.63
LOC104915638	0.0	0.5	0.5	0.0	0.0	0.5	0.3	0.27
LOC104915639	0.0	0.0	0.0	0.0	0.0	0.0	0.0	0.00
LOC104915640	0.0	1.5	0.0	0.0	2.5	1.0	0.8	1.03
LOC104915641	0.0	0.0	0.0	1.0	0.5	1.0	0.4	0.49
LOC104915642	0.0	0.0	0.0	0.0	0.0	0.0	0.0	0.00
LOC104915643	0.5	0.5	0.5	0.0	0.0	0.0	0.3	0.27
LOC104915644	0.0	0.0	0.0	0.0	0.0	0.0	0.0	0.00
LOC104915647	2.0	1.0	7.0	2.0	2.5	9.5	4.0	3.42
LOC104915648	0.0	1.0	3.0	1.5	0.0	2.0	1.3	1.17
LOC104915649	0.0	0.0	0.0	0.0	0.0	0.0	0.0	0.00
LOC104915650	1.5	1.0	0.5	2.0	1.5	3.5	1.7	1.03
LOC104915651	0.0	0.0	0.0	0.0	0.0	1.0	0.2	0.41
LOC104915652	0.0	0.0	0.0	0.0	0.0	0.0	0.0	0.00
LOC104915653	0.5	0.0	0.0	0.0	0.0	0.0	0.1	0.20
LOC104915654	0.0	0.0	0.0	0.0	0.0	0.0	0.0	0.00
LOC104915655	0.0	0.0	0.0	0.0	0.0	0.0	0.0	0.00
LOC104915656	0.5	1.5	3.0	2.0	5.0	3.5	2.6	1.59
LOC104915657	4.5	2.5	0.0	2.0	0.0	0.0	1.5	1.84
LOC104915658	3.0	6.0	2.0	3.0	7.0	0.5	3.6	2.46
LOC104915659	0.0	0.5	0.0	0.5	0.5	0.0	0.3	0.27
LOC104915660	0.0	1.5	1.0	0.5	0.5	0.5	0.7	0.52
LOC104915661	65.0	17.0	70.5	22.5	12.0	45.5	38.8	25.28
LOC104915662	0.0	0.0	0.0	0.0	0.0	0.0	0.0	0.00
LOC104915663	0.0	0.0	0.0	0.0	0.0	0.0	0.0	0.00
LOC104915664	69.0	69.0	45.5	78.0	59.5	40.0	60.2	14.81
LOC104915665	0.0	0.0	0.0	0.0	0.0	0.0	0.0	0.00
LOC104915666	3.0	7.0	7.0	3.0	7.5	10.0	6.3	2.75
LOC104915667	0.0	0.0	0.0	0.0	0.0	0.0	0.0	0.00
LOC104915668	6.0	2.0	2.5	3.0	0.0	1.5	2.5	2.00
LOC104915669	1.5	0.5	0.0	0.0	0.0	0.0	0.3	0.61
LOC104915670	175.5	312.0	155.5	171.0	298.0	140.0	208.7	75.79
LOC104915672	5.5	3.5	4.5	4.5	3.0	0.5	3.6	1.74
LOC104915673	1.5	0.5	0.5	0.0	0.0	0.0	0.4	0.58
LOC104915674	0.0	0.0	0.0	0.0	0.0	0.0	0.0	0.00
LOC104915675	0.0	0.0	0.0	0.0	0.0	0.0	0.0	0.00
LOC104915676	3.0	3.5	1.5	6.0	5.5	1.5	3.5	1.92

LOC104915677	0.0	0.5	0.0	0.5	0.0	0.0	0.2	0.26
LOC104915678	45.0	27.0	59.0	21.5	24.5	36.5	35.6	14.37
LOC104915679	0.0	0.0	0.0	0.0	0.0	0.0	0.0	0.00
LOC104915680	0.0	0.0	0.0	0.0	0.0	0.0	0.0	0.00
LOC104915681	62.0	26.5	69.5	49.0	32.0	60.0	49.8	17.33
LOC104915682	0.0	0.0	1.0	0.0	0.0	0.0	0.2	0.41
LOC104915683	0.0	0.5	0.0	0.0	1.0	0.0	0.3	0.42
LOC104915684	0.0	0.0	0.0	0.0	0.0	0.0	0.0	0.00
LOC104915685	0.0	0.0	0.0	0.0	0.0	0.0	0.0	0.00
LOC104915686	7.0	3.5	31.0	6.5	4.5	25.0	12.9	11.91
LOC104915687	0.5	1.0	8.5	0.5	1.0	1.5	2.2	3.13
LOC104915688	0.0	0.0	0.0	0.0	0.0	0.5	0.1	0.20
LOC104915689	0.0	1.5	0.5	0.0	0.5	0.0	0.4	0.58
LOC104915690	0.0	0.0	0.0	0.0	0.0	0.0	0.0	0.00
LOC104915692	0.0	0.0	0.0	0.0	0.0	0.0	0.0	0.00
LOC104915693	250.0	174.5	253.5	243.5	124.0	260.5	217.7	55.62
LOC104915694	0.0	0.0	0.0	0.0	0.0	0.0	0.0	0.00
LOC104915695	2.0	0.0	0.5	1.0	1.0	0.5	0.8	0.68
LOC104915697	5.5	2.0	9.5	5.5	0.5	7.5	5.1	3.35
LOC104915698	1.0	0.5	0.5	1.0	0.5	0.0	0.6	0.38
LOC104915699	288.0	193.0	268.0	248.5	153.5	203.5	225.8	50.92
LOC104915701	0.0	0.0	0.0	0.0	0.0	0.0	0.0	0.00
LOC104915702	6.5	67.5	45.0	7.5	107.0	38.0	45.3	38.18
LOC104915703	16.5	8.0	4.0	14.5	7.0	4.5	9.1	5.23
LOC104915704	0.0	0.0	0.5	0.0	0.0	0.0	0.1	0.20
LOC104915705	0.0	0.0	0.0	0.0	0.0	0.0	0.0	0.00
LOC104915706	19.0	8.0	15.5	29.5	18.0	20.0	18.3	6.97
LOC104915707	95.5	78.5	61.5	78.5	88.0	63.5	77.6	13.33
LOC104915708	9.0	7.5	19.0	8.5	2.5	21.5	11.3	7.33
LOC104915709	0.0	1.0	0.0	0.0	1.0	0.0	0.3	0.52
LOC104915710	0.0	0.0	0.0	0.0	0.0	0.0	0.0	0.00
LOC104915712	10.0	2.5	2.0	3.0	1.0	1.0	3.3	3.40
LOC104915714	0.0	0.0	0.0	0.0	0.5	0.0	0.1	0.20
LOC104915716	180.0	260.5	177.5	150.5	235.5	162.5	194.4	43.58
LOC104915717	0.0	0.0	0.0	0.0	0.0	0.0	0.0	0.00
LOC104915718	0.0	0.0	0.0	0.0	0.0	0.0	0.0	0.00
LOC104915721	13.0	15.0	6.0	15.0	27.5	11.0	14.6	7.16
LOC104915722	728.0	837.5	488.0	643.5	865.5	513.0	679.3	159.75
LOC104915724	0.0	0.0	0.0	0.0	0.0	0.0	0.0	0.00
LOC104915725	6.5	5.5	1.5	10.5	9.0	5.5	6.4	3.14
LOC104915726	0.5	0.0	0.5	0.0	0.5	0.5	0.3	0.26
LOC104915727	11.5	1.5	7.5	7.5	1.5	6.5	6.0	3.89
LOC104915728	0.0	0.0	0.0	0.0	0.0	0.0	0.0	0.00
LOC104915729	12.5	8.5	36.0	11.0	6.5	36.5	18.5	13.90
LOC104915730	0.0	0.0	0.0	0.0	0.0	0.0	0.0	0.00
LOC104915731	0.0	0.0	0.0	0.0	0.0	0.0	0.0	0.00
LOC104915732	0.0	0.0	0.0	0.0	0.0	0.0	0.0	0.00
LOC104915733	0.0	0.0	0.0	0.0	0.0	0.0	0.0	0.00
LOC104915734	3.0	1.0	2.5	5.5	0.0	2.0	2.3	1.89
LOC104915735	0.0	0.0	0.5	0.0	0.0	0.0	0.1	0.20
LOC104915736	14.5	5.5	11.0	9.0	7.5	11.5	9.8	3.19
LOC104915737	0.0	0.0	0.0	0.0	0.0	0.0	0.0	0.00
LOC104915738	1.5	0.5	0.0	0.0	0.0	1.5	0.6	0.74
LOC104915739	26.0	20.0	10.5	20.0	21.5	4.0	17.0	8.13

LOC104915740	1.5	0.5	1.0	2.0	1.0	0.0	1.0	0.71
LOC104915742	0.0	0.0	0.0	0.0	0.0	0.0	0.0	0.00
LOC104915743	162.5	135.5	285.0	178.5	107.5	267.0	189.3	71.58
LOC104915744	0.0	3.0	0.5	0.0	0.5	0.0	0.7	1.17
LOC104915745	0.0	0.0	0.0	0.0	0.0	0.0	0.0	0.00
LOC104915746	0.0	0.0	0.0	0.0	0.0	0.0	0.0	0.00
LOC104915747	0.0	0.0	0.0	0.0	0.0	0.0	0.0	0.00
LOC104915748	83.0	71.0	53.5	87.0	70.0	71.5	72.7	11.75
LOC104915749	0.0	0.0	0.0	0.0	0.0	0.0	0.0	0.00
LOC104915750	0.0	0.0	0.0	0.0	0.0	0.0	0.0	0.00
LOC104915751	0.0	0.0	0.0	0.0	0.5	0.0	0.1	0.20
LOC104915752	0.0	0.0	0.0	0.0	0.0	0.0	0.0	0.00
LOC104915753	0.0	0.0	0.0	0.0	0.0	0.0	0.0	0.00
LOC104915754	0.0	0.0	0.0	0.0	0.0	0.0	0.0	0.00
LOC104915755	0.0	0.0	0.0	0.0	0.0	0.0	0.0	0.00
LOC104915756	0.0	0.0	0.0	0.0	0.0	0.0	0.0	0.00
LOC104915757	0.0	0.0	0.0	0.0	0.0	0.0	0.0	0.00
LOC104915759	34.0	47.0	32.0	35.0	46.0	30.5	37.4	7.21
LOC104915760	36.5	24.5	15.0	21.5	34.0	19.5	25.2	8.44
LOC104915761	623.0	544.0	269.5	484.5	513.0	340.5	462.4	132.34
LOC104915762	0.5	2.0	0.0	2.5	1.5	1.0	1.3	0.94
LOC104915763	225.0	287.0	193.5	166.0	296.5	235.0	233.8	51.11
LOC104915764	3.5	8.5	3.0	0.5	4.5	2.0	3.7	2.73
LOC104915765	1.0	6.5	4.5	0.0	2.0	11.0	4.2	4.11
LOC104915766	0.0	0.0	0.0	0.0	0.0	0.0	0.0	0.00
LOC104915767	0.0	0.0	0.0	0.0	0.0	0.0	0.0	0.00
LOC104915769	0.5	0.5	0.0	0.5	1.0	0.5	0.5	0.32
LOC104915770	68.5	49.0	91.0	70.5	47.0	96.5	70.4	20.57
LOC104915771	0.0	0.5	0.0	0.0	0.0	0.0	0.1	0.20
LOC104915772	0.0	0.0	0.0	0.0	0.0	2.0	0.3	0.82
LOC104915773	0.0	0.0	0.0	0.0	0.0	0.0	0.0	0.00
LOC104915774	689.5	619.5	889.5	699.5	600.0	572.5	678.4	114.86
LOC104915776	738.5	621.0	421.0	708.0	602.5	383.5	579.1	146.68
LOC104915777	0.0	0.0	0.0	0.0	0.0	0.0	0.0	0.00
LOC104915778	0.5	0.0	0.5	0.5	0.5	0.0	0.3	0.26
LOC104915779	15.0	7.5	23.5	15.5	8.0	18.0	14.6	6.09
LOC104915780	0.0	0.0	0.0	0.0	0.0	0.0	0.0	0.00
LOC104915781	111.0	65.0	90.5	193.0	68.0	137.0	110.8	48.55
LOC104915782	139.0	149.5	205.0	136.0	160.5	211.5	166.9	33.22
LOC104915783	7.5	7.0	13.5	6.5	10.5	15.0	10.0	3.61
LOC104915785	0.0	0.0	0.0	0.0	0.0	0.0	0.0	0.00
LOC104915786	20.0	28.5	37.0	26.5	28.0	36.5	29.4	6.45
LOC104915787	77.0	93.0	44.5	66.5	72.5	60.0	68.9	16.37
LOC104915788	45.5	15.0	40.5	34.5	15.0	37.0	31.3	13.12
LOC104915789	1.0	0.5	0.0	2.5	0.5	0.0	0.8	0.94
LOC104915790	0.5	4.5	3.5	0.0	3.5	6.5	3.1	2.46
LOC104915791	19.5	9.5	36.5	14.0	8.5	17.0	17.5	10.22
LOC104915794	2.5	5.0	5.0	2.5	2.5	3.0	3.4	1.24
LOC104915795	362.5	317.5	319.0	313.0	318.0	333.5	327.3	18.62
LOC104915796	0.0	0.0	0.0	0.0	0.0	0.5	0.1	0.20
LOC104915797	74.5	122.0	85.0	80.0	96.5	101.0	93.2	17.28
LOC104915798	43.0	38.5	142.0	40.0	25.0	140.0	71.4	54.26
LOC104915799	0.0	18.5	12.0	1.0	24.0	16.0	11.9	9.67
LOC104915800	173.5	143.5	202.0	174.5	113.5	193.0	166.7	32.90

LOC104915801	60.0	43.0	37.0	48.0	41.5	40.5	45.0	8.18
LOC104915802	0.0	0.0	0.0	0.0	0.0	0.0	0.0	0.00
LOC104915803	0.0	0.0	0.0	0.0	0.0	0.0	0.0	0.00
LOC104915804	0.0	0.0	0.0	0.0	0.0	0.0	0.0	0.00
LOC104915805	0.0	0.0	0.0	0.0	0.0	0.0	0.0	0.00
LOC104915806	0.0	0.0	0.0	0.0	0.0	0.0	0.0	0.00
LOC104915807	415.0	440.5	281.0	380.0	386.5	270.0	362.2	70.60
LOC104915808	0.0	1.0	1.0	0.5	1.0	2.5	1.0	0.84
LOC104915809	66.5	48.0	63.0	68.5	45.5	60.0	58.6	9.65
LOC104915810	320.5	247.0	452.5	275.0	212.5	406.0	318.9	93.65
LOC104915811	537.0	474.5	271.0	454.5	504.0	296.0	422.8	111.74
LOC104915813	0.5	1.5	9.0	2.5	1.5	12.0	4.5	4.79
LOC104915814	403.0	223.5	175.5	396.5	214.5	148.5	260.3	111.41
LOC104915815	0.5	0.0	1.5	0.0	0.5	1.0	0.6	0.58
LOC104915816	0.0	0.0	0.0	0.0	0.0	0.0	0.0	0.00
LOC104915817	0.0	0.0	0.0	0.0	0.0	0.0	0.0	0.00
LOC104915818	1.0	0.0	0.0	0.0	0.5	0.0	0.3	0.42
LOC104915820	0.0	0.0	0.0	0.5	0.0	0.0	0.1	0.20
LOC104915821	0.0	0.0	0.0	0.0	0.0	0.0	0.0	0.00
LOC104915822	0.0	0.0	0.0	0.0	0.0	0.0	0.0	0.00
LOC104915823	1812.5	1804.0	835.5	1637.0	1592.5	814.5	1416.0	466.16
LOC104915824	0.0	0.0	0.5	0.0	0.0	0.0	0.1	0.20
LOC104915825	0.0	0.0	0.0	0.0	0.0	0.0	0.0	0.00
LOC104915827	774.0	816.5	2372.0	746.0	504.5	2155.5	1228.1	812.43
LOC104915828	7.0	9.0	20.0	4.5	3.0	10.5	9.0	6.06
LOC104915830	14.0	7.0	3.5	16.0	8.0	1.5	8.3	5.71
LOC104915831	186.0	198.0	255.5	255.5	243.5	325.5	244.0	49.77
LOC104915832	66.5	29.0	75.0	67.0	19.0	85.0	56.9	26.56
LOC104915833	4.0	1.5	1.5	1.5	1.5	0.0	1.7	1.29
LOC104915834	0.0	0.0	0.0	0.0	0.0	0.0	0.0	0.00
LOC104915835	299.5	242.0	161.0	254.0	255.0	125.5	222.8	65.65
LOC104915836	0.0	0.0	0.0	0.0	0.0	0.0	0.0	0.00
LOC104915838	6.5	15.5	6.0	8.0	13.0	7.5	9.4	3.89
LOC104915840	367.0	257.5	293.0	340.0	260.0	273.0	298.4	45.30
LOC104915841	66.0	65.0	61.0	67.0	54.5	59.0	62.1	4.82
LOC104915842	126.0	175.0	71.5	114.0	160.0	69.0	119.3	43.92
LOC104915843	0.0	0.5	2.0	1.0	0.0	0.5	0.7	0.75
LOC104915844	6.5	7.5	27.5	11.5	5.0	23.5	13.6	9.56
LOC104915845	1.5	2.5	1.0	1.0	1.5	1.0	1.4	0.58
LOC104915846	18.5	12.0	30.0	17.5	10.5	33.0	20.3	9.29
LOC104915848	176.0	240.5	144.5	161.5	263.5	167.0	192.2	48.02
LOC104915850	0.0	0.0	0.0	0.0	0.0	0.0	0.0	0.00
LOC104915851	0.0	0.0	0.0	0.0	0.0	0.0	0.0	0.00
LOC104915852	0.0	0.0	0.0	0.0	0.0	0.0	0.0	0.00
LOC104915853	333.5	344.5	171.5	324.5	299.5	166.0	273.3	82.31
LOC104915856	281.5	360.5	124.5	260.5	326.0	169.0	253.7	90.91
LOC104915857	156.0	84.5	227.5	167.5	72.5	228.0	156.0	67.10
LOC104915858	7.5	14.5	20.5	12.0	19.0	23.5	16.2	5.93
LOC104915860	0.0	0.0	0.0	0.0	0.0	0.0	0.0	0.00
LOC104915861	294.5	330.5	311.0	305.0	326.0	428.5	332.6	48.84
LOC104915862	1296.0	1433.0	744.0	962.0	1167.0	749.5	1058.6	287.10
LOC104915863	117.5	126.0	197.0	107.5	122.5	214.0	147.4	45.74
LOC104915864	0.0	0.0	0.0	0.0	0.0	0.0	0.0	0.00
LOC104915867	11.0	11.0	16.5	6.0	17.0	20.0	13.6	5.14

LOC104915868	28.0	22.0	51.0	38.0	35.0	58.0	38.7	13.65
LOC104915869	382.0	501.0	278.0	376.5	470.0	302.5	385.0	88.34
LOC104915870	0.5	0.0	0.5	0.0	0.0	1.0	0.3	0.41
LOC104915871	335.5	270.5	211.5	237.0	256.5	177.0	248.0	54.28
LOC104915872	358.0	420.5	183.0	276.5	405.0	177.5	303.4	107.78
LOC104915873	655.5	840.0	479.0	612.0	959.0	599.0	690.8	176.03
LOC104915874	0.0	0.5	0.0	0.0	0.0	0.0	0.1	0.20
LOC104915875	1367.5	1687.5	1201.5	1080.5	1637.0	1210.5	1364.1	248.81
LOC104915876	0.0	0.0	0.0	0.0	0.0	0.0	0.0	0.00
LOC104915877	130.5	135.5	236.0	144.5	159.0	214.0	169.9	44.30
LOC104915878	2.5	1.0	0.5	1.0	2.5	1.0	1.4	0.86
LOC104915879	0.0	0.0	0.0	0.0	0.0	0.0	0.0	0.00
LOC104915880	0.0	0.0	0.0	0.0	0.0	0.0	0.0	0.00
LOC104915881	0.0	0.0	0.0	0.0	0.0	0.0	0.0	0.00
LOC104915882	0.0	0.0	0.0	0.0	0.0	0.0	0.0	0.00
LOC104915883	0.0	0.0	0.0	0.0	0.0	0.0	0.0	0.00
LOC104915884	2.0	0.5	3.0	2.5	0.0	0.5	1.4	1.24
LOC104915885	1.0	1.5	1.5	0.0	0.0	0.0	0.7	0.75
LOC104915888	0.0	0.0	0.0	0.0	0.0	0.0	0.0	0.00
LOC104915889	0.5	0.0	0.0	0.0	0.0	0.5	0.2	0.26
LOC104915890	0.0	0.0	0.0	0.5	0.0	0.0	0.1	0.20
LOC104915891	0.0	0.0	0.5	0.0	0.0	0.0	0.1	0.20
LOC104915892	620.0	628.5	587.5	528.5	528.5	623.5	586.1	46.87
LOC104915894	11.0	17.5	33.0	6.0	18.5	48.0	22.3	15.53
LOC104915895	0.0	1.0	0.0	0.0	2.5	0.5	0.7	0.98
LOC104915897	201.5	216.0	314.0	183.0	194.5	307.5	236.1	58.85
LOC104915898	48.0	84.0	40.5	60.5	77.0	61.5	61.9	16.55
LOC104915899	5.0	18.5	40.5	3.5	8.5	45.0	20.2	18.31
LOC104915901	0.0	0.0	0.0	0.0	0.0	0.0	0.0	0.00
LOC104915902	3.5	2.5	1.0	4.5	4.5	1.0	2.8	1.60
LOC104915905	35.0	23.0	46.0	33.5	22.5	42.0	33.7	9.61
LOC104915908	203.5	307.0	158.5	212.5	294.0	166.0	223.6	63.24
LOC104915909	7.0	22.0	23.5	3.0	17.5	31.0	17.3	10.57
LOC104915912	59.0	61.0	45.5	54.5	57.0	46.0	53.8	6.62
LOC104915913	0.0	0.0	0.0	0.0	0.0	0.0	0.0	0.00
LOC104915914	0.0	0.0	0.0	0.0	0.0	0.0	0.0	0.00
LOC104915915	0.0	0.0	0.0	0.0	0.0	0.0	0.0	0.00
LOC104915916	0.0	0.0	0.0	0.0	0.0	0.5	0.1	0.20
LOC104915917	0.0	0.0	0.0	0.0	0.0	0.0	0.0	0.00
LOC104915918	0.0	0.5	1.5	0.0	1.0	2.0	0.8	0.82
LOC104915919	0.5	0.0	0.0	0.0	0.5	0.5	0.3	0.27
LOC104915920	0.0	0.0	0.0	0.0	0.0	0.0	0.0	0.00
LOC104915921	498.0	233.5	884.0	565.5	188.0	639.5	501.4	260.60
LOC104915922	0.0	0.0	0.0	0.0	0.0	0.0	0.0	0.00
LOC104915923	2.5	6.0	0.0	3.0	2.0	2.0	2.6	1.96
LOC104915924	123.5	159.5	218.5	134.5	153.0	169.5	159.8	33.30
LOC104915925	0.0	0.0	0.0	0.0	0.0	0.0	0.0	0.00
LOC104915926	198.0	323.5	254.0	387.0	386.0	501.5	341.7	107.91
LOC104915931	110.5	83.5	47.0	93.0	83.0	33.5	75.1	29.07
LOC104915932	391.0	145.0	122.0	393.0	140.5	96.5	214.7	138.42
LOC104915935	0.0	0.0	0.0	0.5	0.5	1.5	0.4	0.58
LOC104915936	298.0	415.5	148.5	261.0	391.0	215.5	288.3	102.38
LOC104915937	0.0	0.0	0.0	1.0	0.0	0.0	0.2	0.41
LOC104915938	2.0	3.5	0.5	2.0	2.5	1.0	1.9	1.07

LOC104915939	0.0	0.0	0.0	0.0	0.0	0.0	0.0	0.00
LOC104915940	0.0	0.0	0.0	0.0	0.0	0.0	0.0	0.00
LOC104915941	263.5	278.5	151.5	219.0	281.5	137.0	221.8	64.28
LOC104915944	334.0	276.0	199.5	309.0	240.5	150.0	251.5	69.05
LOC104915946	6.0	10.5	44.0	5.5	9.5	36.0	18.6	16.89
LOC104915947	3.5	2.0	1.0	4.0	2.5	2.0	2.5	1.10
LOC104915949	0.0	0.0	0.0	0.0	0.0	0.0	0.0	0.00
LOC104915950	4.0	40.0	27.0	5.0	27.5	39.0	23.8	15.89
LOC104915951	75.0	37.5	47.5	80.5	41.0	51.0	55.4	18.02
LOC104915952	0.0	0.0	0.0	0.0	0.0	0.0	0.0	0.00
LOC104915953	2473.5	1506.0	5932.5	3066.5	1621.5	6632.0	3538.7	2211.96
LOC104915954	331.5	363.0	174.5	197.5	306.5	151.0	254.0	90.29
LOC104915955	0.0	0.0	0.0	0.0	0.0	0.0	0.0	0.00
LOC104915956	0.0	0.0	0.0	0.0	0.0	0.0	0.0	0.00
LOC104915957	0.0	0.0	0.0	0.0	0.0	0.0	0.0	0.00
LOC104915958	0.5	1.5	1.5	1.0	0.5	2.5	1.3	0.76
LOC104915959	0.0	0.0	0.0	0.0	0.0	0.0	0.0	0.00
LOC104915960	121.0	147.0	86.0	116.5	125.5	112.5	118.1	19.81
LOC104915965	513.0	249.5	252.5	467.5	286.0	290.0	343.1	116.10
LOC104915967	21.0	33.5	43.0	28.0	44.0	61.0	38.4	14.13
LOC104915970	897.5	807.5	914.0	848.5	751.5	980.5	866.6	81.54
LOC104915971	254.5	240.5	265.5	226.0	190.5	314.0	248.5	41.35
LOC104915972	1.0	0.0	0.5	0.5	2.0	1.0	0.8	0.68
LOC104915974	0.0	0.0	0.0	0.0	0.0	0.0	0.0	0.00
LOC104915975	36.0	38.0	42.0	29.0	31.5	51.0	37.9	7.90
LOC104915976	53.5	44.5	50.0	55.0	39.5	60.0	50.4	7.44
LOC104915977	32.5	16.0	25.5	28.0	12.5	23.0	22.9	7.49
LOC104915978	354.5	337.0	163.0	266.5	298.0	134.5	258.9	91.10
LOC104915979	3.5	10.0	5.0	6.0	7.0	9.5	6.8	2.54
LOC104915980	53.0	34.0	76.5	39.0	18.5	63.0	47.3	21.01
LOC104915981	121.5	83.0	131.5	118.5	85.5	140.0	113.3	23.79
LOC104915983	0.0	1.5	1.5	0.0	0.0	0.0	0.5	0.77
LOC104915984	0.0	0.0	1.5	0.5	0.0	0.0	0.3	0.61
LOC104915985	17.0	20.0	6.0	18.0	23.0	10.5	15.8	6.32
LOC104915986	2.5	5.0	2.5	2.0	6.0	9.5	4.6	2.89
LOC104915987	44.0	35.5	102.0	35.0	29.5	85.5	55.3	30.63
LOC104915988	706.0	923.5	540.5	620.5	881.5	479.5	691.9	180.51
LOC104915989	0.0	0.0	0.0	0.0	0.0	0.0	0.0	0.00
LOC104915990	0.0	0.0	0.0	0.0	0.0	0.0	0.0	0.00
LOC104915991	4.5	0.5	4.5	3.5	2.0	4.0	3.2	1.60
LOC104915992	148.5	179.5	172.0	179.5	203.5	165.0	174.7	18.24
LOC104915993	180.5	308.5	289.0	165.0	258.0	329.0	255.0	68.05
LOC104915995	16.5	14.5	22.5	20.5	9.0	27.0	18.3	6.36
LOC104915996	0.0	0.0	1.0	0.0	0.0	0.5	0.3	0.42
LOC104915997	0.0	0.0	1.5	0.0	0.0	0.0	0.3	0.61
LOC104915999	0.0	0.0	0.0	0.0	0.0	0.0	0.0	0.00
LOC104916000	0.0	0.0	0.0	0.0	0.0	0.0	0.0	0.00
LOC104916001	0.0	0.0	0.0	0.0	0.0	0.0	0.0	0.00
LOC104916002	0.0	0.0	0.0	0.0	0.0	0.0	0.0	0.00
LOC104916003	1.0	2.5	2.0	1.0	2.0	10.0	3.1	3.44
LOC104916004	0.0	0.0	0.5	1.0	0.0	0.0	0.3	0.42
LOC104916005	0.0	0.0	0.0	0.0	0.0	0.0	0.0	0.00
LOC104916006	0.0	0.0	0.0	0.0	0.0	0.0	0.0	0.00
LOC104916007	0.0	0.0	0.0	0.0	0.0	0.0	0.0	0.00

LOC104916008	2.0	0.0	0.0	0.5	0.0	0.0	0.4	0.80
LOC104916009	0.0	0.0	0.0	0.0	0.0	0.0	0.0	0.00
LOC104916010	0.0	0.0	0.0	0.0	0.0	0.0	0.0	0.00
LOC104916013	0.5	3.5	5.0	0.5	4.0	4.0	2.9	1.93
LOC104916014	0.0	0.0	0.0	0.0	0.0	0.0	0.0	0.00
LOC104916015	584.0	238.5	606.0	697.5	252.0	547.0	487.5	194.14
LOC104916016	148.5	178.0	73.5	109.5	187.5	109.0	134.3	44.48
LOC104916018	138.5	177.0	89.0	125.0	193.5	96.5	136.6	42.15
LOC104916019	110.0	128.0	83.5	117.5	118.5	103.0	110.1	15.52
LOC104916022	97.5	157.0	71.0	73.0	153.0	86.5	106.3	38.93
LOC104916023	45.5	66.5	39.5	48.0	64.0	45.5	51.5	11.04
LOC104916024	0.5	1.5	1.0	3.0	2.0	3.0	1.8	1.03
LOC104916025	377.5	418.0	642.5	146.0	356.0	421.5	393.6	158.94
LOC104916026	11.5	29.0	13.5	6.0	20.0	10.0	15.0	8.26
LOC104916027	0.0	0.0	0.0	0.0	0.5	0.0	0.1	0.20
LOC104916028	0.0	1.0	0.0	0.0	0.0	0.0	0.2	0.41
LOC104916029	44.5	44.0	26.0	44.0	38.5	32.0	38.2	7.67
LOC104916030	0.0	0.0	3.5	0.5	2.5	7.0	2.3	2.73
LOC104916031	42.5	33.5	43.0	44.5	26.0	59.0	41.4	11.16
LOC104916032	0.0	4.5	12.0	0.5	4.0	9.0	5.0	4.72
LOC104916034	132.0	112.5	108.5	124.5	120.0	103.0	116.8	10.76
LOC104916035	183.0	157.5	179.5	148.0	153.0	177.0	166.3	15.21
LOC104916036	2.0	0.0	11.0	4.0	2.0	8.0	4.5	4.18
LOC104916038	1.5	3.0	3.0	0.5	1.5	2.5	2.0	1.00
LOC104916039	18.5	20.5	32.5	23.5	22.0	35.0	25.3	6.77
LOC104916040	935.5	621.0	561.5	832.0	593.0	501.0	674.0	170.47
LOC104916041	458.5	526.5	444.0	439.5	505.5	535.0	484.8	42.65
LOC104916042	148.5	94.5	73.0	120.0	80.0	62.5	96.4	32.38
LOC104916043	5163.5	4131.5	2141.5	4890.5	3841.5	2216.5	3730.8	1295.32
LOC104916044	1524.0	1818.5	1200.5	1356.0	1929.5	1209.0	1506.3	310.28
LOC104916045	0.0	0.0	0.0	0.5	0.0	0.0	0.1	0.20
LOC104916047	326.5	448.5	186.5	307.0	342.0	169.5	296.7	104.32
LOC104916048	0.0	0.0	0.0	0.0	0.0	0.0	0.0	0.00
LOC104916049	0.0	0.0	0.0	0.0	0.0	0.0	0.0	0.00
LOC104916050	0.0	0.0	0.0	0.0	0.0	0.0	0.0	0.00
LOC104916051	15.5	2.5	29.0	18.5	0.5	22.5	14.8	11.23
LOC104916052	0.0	0.0	0.0	0.0	0.0	0.0	0.0	0.00
LOC104916053	0.0	0.0	0.0	0.0	0.0	0.0	0.0	0.00
LOC104916054	0.0	0.0	0.0	0.0	0.0	0.0	0.0	0.00
LOC104916055	0.0	1.0	0.0	0.0	1.0	0.0	0.3	0.52
LOC104916056	0.0	0.0	0.0	0.0	0.0	0.0	0.0	0.00
LOC104916057	1.0	0.0	0.0	0.0	0.0	0.0	0.2	0.41
LOC104916058	20.0	28.5	24.0	18.5	20.5	20.0	21.9	3.71
LOC104916059	0.0	0.0	0.0	0.0	0.0	0.0	0.0	0.00
LOC104916060	1.5	2.5	1.5	4.0	2.0	2.0	2.3	0.94
LOC104916061	0.0	0.0	0.0	0.0	0.0	0.0	0.0	0.00
LOC104916062	0.0	0.0	0.0	0.0	0.0	0.0	0.0	0.00
LOC104916063	0.0	0.0	0.0	0.0	0.0	0.0	0.0	0.00
LOC104916064	0.0	0.0	0.0	0.0	0.0	0.0	0.0	0.00
LOC104916066	27.5	20.0	39.0	40.5	29.5	43.5	33.3	9.09
LOC104916067	3.5	3.0	9.5	4.0	1.5	6.5	4.7	2.88
LOC104916068	134.5	114.0	170.5	152.5	104.5	183.0	143.2	31.12
LOC104916069	0.0	0.5	0.0	0.5	0.0	0.0	0.2	0.26
LOC104916070	483.5	450.5	206.5	352.0	373.5	170.0	339.3	127.11

LOC104916071	375.5	181.5	1108.0	511.5	212.5	1125.5	585.8	428.14
LOC104916072	8.5	20.0	43.5	14.5	18.0	34.0	23.1	13.10
LOC104916073	0.0	0.0	0.0	0.0	0.0	0.0	0.0	0.00
LOC104916074	44.0	13.0	33.5	46.0	12.0	27.5	29.3	14.70
LOC104916079	5.0	9.5	4.5	8.0	13.5	4.5	7.5	3.59
LOC104916080	0.5	4.0	10.5	0.5	4.5	8.5	4.8	4.10
LOC104916081	34.5	36.5	40.0	38.0	31.0	26.5	34.4	4.95
LOC104916082	0.0	0.0	0.0	0.0	0.5	0.0	0.1	0.20
LOC104916083	40.0	60.0	68.5	33.0	52.5	115.5	61.6	29.40
LOC104916084	0.0	2.0	1.0	1.0	3.0	2.0	1.5	1.05
LOC104916085	0.0	0.0	0.0	0.0	0.0	0.0	0.0	0.00
LOC104916086	26.5	31.0	36.0	31.0	24.0	47.0	32.6	8.19
LOC104916088	116.5	119.5	53.0	110.5	127.0	46.0	95.4	36.03
LOC104916090	159.5	160.5	294.0	153.0	128.5	261.5	192.8	67.57
LOC104916092	0.0	0.0	0.0	0.0	0.0	0.0	0.0	0.00
LOC104916093	54.5	47.5	55.0	55.0	31.0	60.0	50.5	10.35
LOC104916095	18.5	21.5	11.5	15.0	18.5	15.5	16.8	3.49
LOC104916099	0.0	0.0	0.0	0.0	0.0	0.0	0.0	0.00
LOC104916100	2.5	1.5	3.0	3.0	2.5	3.0	2.6	0.58
LOC104916101	35.0	17.5	59.5	42.5	15.0	39.0	34.8	16.60
LOC104916103	1.0	3.5	1.0	1.5	1.5	1.5	1.7	0.93
LOC104916105	0.0	0.0	0.0	0.0	0.0	0.0	0.0	0.00
LOC104916107	103.5	98.5	44.0	92.5	98.0	43.5	80.0	28.29
LOC104916108	118.5	115.0	62.5	114.5	149.0	68.0	104.6	33.11
LOC104916109	10.5	8.5	2.5	6.0	6.0	4.5	6.3	2.84
LOC104916110	0.0	0.0	0.5	0.0	0.0	0.0	0.1	0.20
LOC104916111	0.0	0.0	0.0	0.0	0.0	0.0	0.0	0.00
LOC104916112	43.0	50.0	148.0	63.0	68.0	113.5	80.9	41.09
LOC104916113	159.5	303.0	530.0	56.5	279.5	374.0	283.8	165.08
LOC104916114	0.5	1.5	1.5	1.0	2.0	3.5	1.7	1.03
LOC104916115	483.5	348.5	471.5	462.5	319.5	498.5	430.7	76.40
LOC104916116	288.5	444.0	355.5	262.0	375.5	339.5	344.2	64.73
LOC104916117	0.0	0.0	0.0	0.0	0.0	0.0	0.0	0.00
LOC104916118	13.0	17.5	11.5	10.5	12.0	8.0	12.1	3.15
LOC104916119	0.0	0.0	0.0	0.0	0.0	0.0	0.0	0.00
LOC104916121	0.0	0.5	1.0	0.0	1.5	0.0	0.5	0.63
LOC104916122	2.5	3.5	3.0	3.0	2.5	4.0	3.1	0.58
LOC104916123	0.0	0.0	0.5	0.0	0.0	0.0	0.1	0.20
LOC104916126	0.0	0.0	0.5	0.0	0.0	0.0	0.1	0.20
LOC104916127	0.0	0.0	0.0	0.0	0.0	0.0	0.0	0.00
LOC104916128	0.0	0.0	0.0	0.0	0.0	0.0	0.0	0.00
LOC104916129	0.0	0.0	0.0	0.0	0.0	0.0	0.0	0.00
LOC104916130	0.0	0.0	0.0	0.0	0.0	0.0	0.0	0.00
LOC104916131	0.0	0.0	0.0	0.0	0.0	0.0	0.0	0.00
LOC104916132	1.5	2.0	5.5	2.0	4.0	3.5	3.1	1.53
LOC104916133	0.0	0.0	0.0	0.0	0.0	0.0	0.0	0.00
LOC104916134	4.5	7.5	8.5	5.0	7.5	8.5	6.9	1.74
LOC104916135	0.0	0.0	0.0	0.0	0.0	0.0	0.0	0.00
LOC104916136	0.0	0.0	0.0	0.0	0.0	0.0	0.0	0.00
LOC104916137	0.0	0.0	0.0	0.0	0.0	0.0	0.0	0.00
LOC104916138	0.0	0.0	0.0	0.0	0.0	0.0	0.0	0.00
LOC104916139	0.0	0.0	0.0	0.0	0.0	0.0	0.0	0.00
LOC104916140	0.0	0.0	0.0	0.5	0.0	0.0	0.1	0.20
LOC104916142	1.0	0.5	0.0	0.5	0.0	0.0	0.3	0.41

LOC104916146	127.0	67.5	90.5	156.0	48.0	105.5	99.1	39.35
LOC104916148	0.0	0.0	0.0	0.0	0.0	0.0	0.0	0.00
LOC104916149	190.5	110.0	188.5	182.5	115.0	145.0	155.3	37.05
LOC104916151	0.0	0.0	0.0	0.0	0.0	0.0	0.0	0.00
LOC104916152	8.5	12.0	11.5	5.5	8.0	7.0	8.8	2.54
LOC104916153	56.0	23.0	46.5	51.0	23.5	47.5	41.3	14.33
LOC104916154	27.5	16.5	55.5	24.0	9.0	48.5	30.2	18.21
LOC104916155	831.5	1214.5	596.0	874.5	1138.5	621.5	879.4	256.35
LOC104916157	85.0	62.5	85.0	86.0	53.0	77.5	74.8	13.91
LOC104916158	2.0	5.5	5.5	6.5	6.5	2.5	4.8	1.99
LOC104916159	92.0	118.0	75.0	92.0	120.5	79.0	96.1	19.22
LOC104916160	667.0	774.0	685.0	584.5	894.5	696.0	716.8	106.09
LOC104916161	3.0	2.0	4.0	1.0	2.5	2.0	2.4	1.02
LOC104916163	107.5	58.0	43.0	118.5	82.0	55.5	77.4	30.51
LOC104916164	8.5	2.0	0.5	3.0	1.0	0.5	2.6	3.06
LOC104916165	44.5	19.0	25.0	15.0	8.0	6.0	19.6	14.07
LOC104916166	1.5	0.0	0.0	1.0	0.5	0.0	0.5	0.63
LOC104916167	12.5	10.5	20.0	4.0	1.5	1.0	8.3	7.45
LOC104916168	0.0	0.0	0.0	0.0	0.0	0.0	0.0	0.00
LOC104916169	0.0	0.0	0.0	0.0	0.0	0.0	0.0	0.00
LOC104916170	0.0	0.0	0.0	0.0	0.0	0.0	0.0	0.00
LOC104916171	2.0	1.5	6.5	0.0	1.5	2.0	2.3	2.21
LOC104916172	0.0	0.0	0.0	0.0	0.5	0.0	0.1	0.20
LOC104916173	0.0	0.0	0.0	0.0	0.0	0.0	0.0	0.00
LOC104916174	0.0	0.0	0.0	0.0	0.0	0.0	0.0	0.00
LOC104916175	96.0	44.0	25.0	86.0	61.0	34.5	57.8	28.54
LOC104916179	0.0	0.0	0.0	0.0	0.0	0.0	0.0	0.00
LOC104916180	163.0	82.0	148.0	158.0	67.5	111.0	121.6	40.85
LOC104916181	553.0	304.0	785.5	576.0	267.0	590.5	512.7	194.95
LOC104916182	156.0	152.0	81.0	151.0	143.0	91.0	129.0	33.72
LOC104916183	20.5	5.0	10.0	19.5	5.0	10.0	11.7	6.84
LOC104916184	0.0	0.0	0.0	0.0	0.0	0.0	0.0	0.00
LOC104916185	0.0	0.0	0.0	0.0	0.0	0.0	0.0	0.00
LOC104916187	155.0	175.5	86.5	141.0	188.0	65.5	135.3	49.13
LOC104916188	114.0	157.5	219.0	123.5	161.0	235.5	168.4	49.42
LOC104916191	2.0	0.5	1.5	5.5	0.5	2.5	2.1	1.86
LOC104916192	0.5	0.0	0.5	0.0	0.0	1.0	0.3	0.41
LOC104916195	51.5	42.5	70.0	69.0	44.0	68.5	57.6	13.06
LOC104916196	148.0	725.5	473.0	96.5	773.0	491.5	451.3	282.36
LOC104916197	65.5	330.5	97.0	59.0	333.0	58.5	157.3	135.91
LOC104916198	50.5	51.5	84.5	58.0	72.5	93.0	68.3	17.86
LOC104916199	0.0	0.0	1.0	0.0	0.0	0.0	0.2	0.41
LOC104916200	11.5	13.5	20.5	15.0	18.5	27.5	17.8	5.79
LOC104916203	3.5	1.5	5.5	5.5	2.0	4.0	3.7	1.69
LOC104916204	216.0	243.5	135.5	208.0	211.0	145.5	193.3	42.87
LOC104916205	4319.5	4377.0	1915.5	3382.0	4038.5	1745.0	3296.3	1190.48
LOC104916207	88.0	28.0	13.0	66.0	25.0	8.5	38.1	31.77
LOC104916208	0.0	0.0	0.0	0.0	0.0	0.5	0.1	0.20
LOC104916209	0.5	0.0	0.0	1.0	1.0	0.5	0.5	0.45
LOC104916210	154.0	179.5	121.0	148.0	170.0	147.0	153.3	20.38
LOC104916211	16.0	7.5	17.5	9.5	9.0	18.5	13.0	4.86
LOC104916212	434.0	501.5	343.0	326.0	455.5	305.5	394.3	79.99
LOC104916214	0.5	1.5	5.5	1.0	0.5	1.0	1.7	1.91
LOC104916215	143.5	142.0	152.5	122.5	132.5	179.0	145.3	19.42

LOC104916217	0.0	0.0	0.0	0.0	0.0	0.0	0.0	0.00
LOC104916218	0.5	0.0	0.0	1.0	0.5	0.0	0.3	0.41
LOC104916219	0.0	1.0	0.0	0.5	2.5	1.0	0.8	0.93
LOC104916221	10.0	23.5	7.5	13.5	29.5	15.0	16.5	8.40
LOC104916222	21.0	33.5	17.0	26.5	27.5	20.0	24.3	6.04
LOC104916223	4.5	3.0	6.5	4.5	4.0	10.0	5.4	2.52
LOC104916224	0.0	0.0	0.0	0.0	0.0	0.0	0.0	0.00
LOC104916225	7.5	3.0	5.0	3.0	1.5	8.0	4.7	2.64
LOC104916228	153.0	172.0	270.0	167.0	138.0	259.0	193.2	56.62
LOC104916229	14.0	9.0	9.5	16.5	5.5	10.0	10.8	3.91
LOC104916231	0.0	0.0	0.0	0.0	0.0	0.0	0.0	0.00
LOC104916232	0.5	2.0	0.0	0.5	0.0	0.5	0.6	0.74
LOC104916233	0.0	0.0	0.0	0.0	0.0	0.0	0.0	0.00
LOC104916234	0.0	0.0	0.0	0.0	0.0	0.0	0.0	0.00
LOC104916235	0.0	0.0	0.0	0.0	0.0	0.0	0.0	0.00
LOC104916237	56.0	60.0	47.0	51.0	68.0	50.0	55.3	7.74
LOC104916241	1760.0	1556.0	982.0	1515.5	1566.5	1005.0	1397.5	324.26
LOC104916242	0.0	0.0	0.0	0.0	0.0	0.0	0.0	0.00
LOC104916243	0.0	1.0	0.0	0.0	0.0	0.0	0.2	0.41
LOC104916244	0.0	2.0	1.5	0.0	2.0	4.5	1.7	1.66
LOC104916245	0.0	0.5	0.0	0.0	0.5	0.5	0.3	0.27
LOC104916246	183.0	275.0	148.5	237.5	310.0	164.5	219.8	64.80
LOC104916247	563.0	657.5	558.5	525.5	655.5	600.5	593.4	54.35
LOC104916248	10.5	6.5	6.0	8.5	4.5	2.5	6.4	2.84
LOC104916249	0.0	0.0	0.0	0.0	0.0	0.0	0.0	0.00
LOC104916251	4.0	3.5	2.5	2.0	2.5	2.5	2.8	0.75
LOC104916253	0.0	0.0	1.0	0.0	0.0	0.0	0.2	0.41
LOC104916254	89.5	107.0	66.5	81.0	104.5	71.5	86.7	16.78
LOC104916255	193.0	197.0	130.5	175.5	155.5	131.5	163.8	29.37
LOC104916258	466.0	264.5	375.5	505.0	245.5	373.0	371.6	104.02
LOC104916259	143.0	142.0	260.5	144.0	137.0	219.5	174.3	52.55
LOC104916260	121.5	82.0	155.0	127.5	58.5	128.0	112.1	35.21
LOC104916261	0.0	0.0	0.0	0.0	0.0	0.0	0.0	0.00
LOC104916263	288.0	327.0	176.5	255.5	342.0	199.0	264.7	67.20
LOC104916264	0.0	0.0	0.0	0.0	0.0	0.0	0.0	0.00
LOC104916265	0.5	0.0	0.5	0.0	0.5	0.5	0.3	0.26
LOC104916266	102.5	40.5	95.0	104.5	39.5	83.5	77.6	30.03
LOC104916267	19.5	20.0	37.5	23.5	20.5	44.0	27.5	10.56
LOC104916268	104.5	117.0	90.5	109.0	118.0	102.0	106.8	10.28
LOC104916269	0.0	0.0	0.0	0.0	0.0	0.0	0.0	0.00
LOC104916270	7.5	4.5	7.5	6.5	3.0	2.0	5.2	2.36
LOC104916271	1.0	3.5	6.0	2.0	5.5	3.0	3.5	1.95
LOC104916272	8.5	3.5	0.5	6.5	0.0	0.0	3.2	3.66
LOC104916274	4.0	9.0	3.5	0.5	9.0	5.5	5.3	3.33
LOC104916275	36.5	13.5	26.0	26.0	10.0	27.0	23.2	9.75
LOC104916276	373.0	213.5	452.5	346.5	195.0	397.0	329.6	103.36
LOC104916277	1.0	8.0	16.0	1.5	7.0	21.5	9.2	8.13
LOC104916278	0.0	0.0	0.5	0.5	1.0	0.0	0.3	0.41
LOC104916280	20.5	18.0	21.5	14.5	14.0	20.0	18.1	3.18
LOC104916281	0.0	0.0	0.0	0.0	0.0	0.0	0.0	0.00
LOC104916282	1.5	0.5	1.0	0.5	0.0	0.5	0.7	0.52
LOC104916283	12.0	4.0	5.0	8.0	4.0	3.0	6.0	3.41
LOC104916284	0.0	0.0	0.0	0.0	0.0	0.0	0.0	0.00
LOC104916285	17.0	18.0	22.5	18.0	19.0	16.5	18.5	2.14

LOC104916287	28.5	46.5	72.5	41.0	70.0	78.0	56.1	20.12
LOC104916289	181.0	315.0	170.0	154.5	305.0	196.0	220.3	70.90
LOC104916290	22.5	6.5	117.5	24.5	7.5	129.0	51.3	56.38
LOC104916291	3.0	4.0	7.0	2.5	2.5	3.0	3.7	1.72
LOC104916292	46.5	163.0	112.0	40.0	171.5	104.0	106.2	55.63
LOC104916293	1.0	2.5	2.0	0.5	0.5	3.5	1.7	1.21
LOC104916294	136.0	106.0	82.0	163.0	93.0	99.0	113.2	30.45
LOC104916295	2.0	0.5	11.5	1.0	1.5	8.0	4.1	4.55
LOC104916296	0.0	0.0	0.0	0.0	0.0	0.0	0.0	0.00
LOC104916298	55.0	35.0	79.0	53.5	44.0	115.0	63.6	29.18
LOC104916299	26.5	7.0	23.0	28.0	11.0	16.5	18.7	8.57
LOC104916300	0.0	0.0	0.0	0.0	0.0	0.0	0.0	0.00
LOC104916301	89.0	145.5	63.5	82.5	131.0	78.5	98.3	32.36
LOC104916302	706.5	449.0	1664.5	929.0	482.5	1885.5	1019.5	614.16
LOC104916303	101.0	60.0	122.5	77.5	45.5	127.0	88.9	33.39
LOC104916305	5.5	6.5	18.0	9.0	5.5	19.0	10.6	6.27
LOC104916307	1466.0	1952.0	1211.5	1430.5	1761.5	1294.0	1519.3	283.59
LOC104916308	250.5	294.0	211.5	238.0	309.0	225.5	254.8	38.75
LOC104916309	113.0	126.0	175.0	127.5	112.5	191.5	140.9	33.79
LOC104916310	0.0	0.0	0.0	0.0	0.0	0.5	0.1	0.20
LOC104916311	0.0	0.0	0.5	0.0	0.5	0.5	0.3	0.27
LOC104916312	2.0	0.5	0.5	0.0	0.0	0.0	0.5	0.77
LOC104916313	209.0	270.0	121.0	142.5	243.5	122.0	184.7	64.95
LOC104916314	79.5	50.5	50.5	58.5	48.5	40.0	54.6	13.56
LOC104916315	0.0	0.0	0.0	0.0	0.0	0.0	0.0	0.00
LOC104916316	0.0	0.0	0.0	0.0	0.0	0.0	0.0	0.00
LOC104916317	152.5	116.5	64.0	123.5	91.0	68.5	102.7	34.37
LOC104916319	0.0	0.0	0.0	0.0	0.0	0.0	0.0	0.00
LOC104916320	0.0	0.0	0.0	0.0	0.0	0.0	0.0	0.00
LOC104916321	0.0	0.0	0.0	0.0	0.0	0.0	0.0	0.00
LOC104916322	0.0	0.0	0.0	0.0	0.0	0.0	0.0	0.00
LOC104916323	0.0	0.0	0.0	0.0	0.0	0.0	0.0	0.00
LOC104916324	2.5	2.0	9.5	1.5	6.0	25.0	7.8	8.98
LOC104916326	0.0	0.0	0.0	0.0	0.0	0.0	0.0	0.00
LOC104916327	8.0	2.0	7.5	1.0	5.0	4.5	4.7	2.82
LOC104916328	1110.0	751.5	858.0	1173.5	815.5	857.0	927.6	171.55
LOC104916329	0.0	0.0	0.0	0.0	0.0	0.0	0.0	0.00
LOC104916330	0.0	0.0	0.0	0.0	0.0	0.0	0.0	0.00
LOC104916331	0.0	0.0	0.0	0.0	0.0	0.0	0.0	0.00
LOC104916332	0.0	0.0	0.0	0.0	0.0	0.0	0.0	0.00
LOC104916334	0.0	0.0	0.0	0.0	0.0	0.0	0.0	0.00
LOC104916335	153.0	107.5	97.0	141.5	90.0	106.5	115.9	25.37
LOC104916336	948.0	1383.0	2501.5	869.5	1404.0	2304.0	1568.3	685.06
LOC104916337	137.0	143.5	72.0	142.0	136.0	100.0	121.8	29.21
LOC104916338	61.0	47.0	91.5	64.0	37.5	103.0	67.3	25.34
LOC104916339	32.0	56.0	70.5	22.5	45.0	76.5	50.4	21.27
LOC104916340	24.0	33.0	52.5	24.5	25.0	34.0	32.2	10.90
LOC104916343	5.5	0.5	3.0	2.5	1.5	0.5	2.3	1.89
LOC104916345	0.0	0.0	0.5	0.5	0.0	0.0	0.2	0.26
LOC104916346	84.5	55.0	102.0	65.0	48.5	105.5	76.8	24.22
LOC104916348	0.5	0.0	0.5	0.5	0.0	0.0	0.3	0.27
LOC104916349	0.5	0.0	0.5	0.5	0.5	1.0	0.5	0.32
LOC104916350	5.5	24.5	51.0	5.0	24.0	58.0	28.0	22.33
LOC104916351	16.5	6.0	12.5	12.5	2.5	12.5	10.4	5.14

LOC104916352	1.0	0.5	0.0	0.5	0.0	0.5	0.4	0.38
LOC104916353	10.0	7.0	8.5	8.5	7.0	10.5	8.6	1.46
LOC104916356	107.0	94.5	205.0	116.5	79.0	210.5	135.4	57.45
LOC104916357	8.0	18.5	23.5	12.0	24.0	34.5	20.1	9.47
LOC104916358	24.0	15.5	8.5	5.5	7.5	5.0	11.0	7.40
LOC104916359	0.0	0.0	0.5	0.0	0.0	0.0	0.1	0.20
LOC104916360	0.0	0.5	0.5	0.0	0.0	0.5	0.3	0.27
LOC104916362	0.0	0.0	0.0	0.0	0.0	0.0	0.0	0.00
LOC104916363	7.5	9.5	22.5	9.5	6.5	19.0	12.4	6.65
LOC104916364	0.0	0.0	0.5	0.0	0.0	0.0	0.1	0.20
LOC104916365	13.0	6.0	6.0	15.0	7.5	6.0	8.9	4.03
LOC104916367	302.0	497.5	223.0	258.0	476.0	253.5	335.0	120.41
LOC104916369	84.0	59.0	106.5	81.5	41.0	87.5	76.6	23.10
LOC104916371	160.5	50.5	277.0	189.0	53.5	232.0	160.4	92.79
LOC104916372	28.0	15.0	81.0	45.5	12.5	89.0	45.2	33.10
LOC104916374	100.0	145.5	71.0	99.0	125.5	63.5	100.8	31.28
LOC104916375	1.0	1.5	1.0	0.0	0.0	0.0	0.6	0.66
LOC104916377	50.5	126.5	40.5	58.5	85.5	52.0	68.9	32.04
LOC104916378	108.0	90.5	105.5	79.0	88.5	82.5	92.3	11.93
LOC104916379	15.5	5.0	14.5	12.5	3.5	4.5	9.3	5.49
LOC104916380	4.5	2.5	1.0	4.0	1.5	0.5	2.3	1.63
LOC104916381	849.5	727.0	446.5	813.5	697.5	446.0	663.3	177.02
LOC104916382	39.5	107.5	94.0	58.5	101.0	113.0	85.6	29.66
LOC104916383	572.0	406.0	843.0	546.5	368.0	765.5	583.5	189.69
LOC104916384	90.5	64.0	157.5	112.5	69.5	138.0	105.3	37.56
LOC104916385	10.5	7.5	7.5	17.0	3.5	9.5	9.3	4.49
LOC104916386	836.5	587.5	964.0	850.0	521.0	773.5	755.4	168.82
LOC104916387	0.0	1.5	1.0	0.0	0.5	2.5	0.9	0.97
LOC104916388	3.0	2.5	11.5	4.5	4.0	8.5	5.7	3.56
LOC104916390	42.0	56.0	151.0	39.5	40.5	151.5	80.1	55.45
LOC104916391	0.0	0.0	0.0	0.0	0.0	0.0	0.0	0.00
LOC104916392	0.0	0.0	0.0	0.0	0.0	0.0	0.0	0.00
LOC104916393	47.5	35.5	32.5	30.5	31.0	47.5	37.4	8.00
LOC104916394	4.0	14.5	18.5	2.0	13.0	15.5	11.3	6.67
LOC104916395	39.5	21.5	31.0	45.5	31.0	25.5	32.3	8.86
LOC104916396	13.5	12.5	8.0	9.5	11.0	11.5	11.0	2.00
LOC104916399	0.0	0.0	0.0	0.0	0.0	0.0	0.0	0.00
LOC104916400	18.0	18.5	49.5	10.5	19.5	28.0	24.0	13.67
LOC104916401	19.0	15.5	12.5	27.5	18.0	14.0	17.8	5.35
LOC104916402	27.5	17.0	46.5	22.5	16.0	46.5	29.3	13.92
LOC104916403	1.0	0.0	0.5	0.0	0.5	0.0	0.3	0.41
LOC104916404	186.5	177.0	212.5	168.0	164.5	240.0	191.4	29.36
LOC104916405	99.0	83.5	80.5	100.5	52.5	82.5	83.1	17.32
LOC104916408	32.5	25.0	52.0	34.0	13.0	40.5	32.8	13.28
LOC104916409	407.0	403.5	463.5	394.5	368.0	462.5	416.5	38.52
LOC104916410	3.0	2.5	1.5	1.0	1.5	0.0	1.6	1.07
LOC104916411	0.0	0.0	0.0	0.0	0.0	0.0	0.0	0.00
LOC104916413	0.0	0.0	0.0	0.0	0.0	0.0	0.0	0.00
LOC104916414	1.5	0.5	4.0	1.0	1.5	7.0	2.6	2.48
LOC104916416	0.5	0.0	0.0	0.0	0.0	0.0	0.1	0.20
LOC104916417	0.5	0.5	0.0	0.0	0.0	0.0	0.2	0.26
LOC104916418	19.0	19.0	37.5	17.5	9.5	32.0	22.4	10.33
LOC104916420	0.0	1.5	3.0	1.5	2.5	3.0	1.9	1.16
LOC104916421	48.0	18.0	46.0	36.5	14.5	42.0	34.2	14.47

LOC104916422	0.0	0.0	0.0	0.5	0.0	0.5	0.2	0.26
LOC104916423	0.0	0.0	0.0	0.0	0.0	0.0	0.0	0.00
LOC104916424	2.0	1.5	2.5	4.0	3.0	3.5	2.8	0.94
LOC104916425	15.0	14.5	26.5	15.5	8.0	22.0	16.9	6.46
LOC104916426	2.5	0.0	6.5	1.0	1.0	3.0	2.3	2.32
LOC104916427	0.0	0.0	0.0	0.0	0.0	0.0	0.0	0.00
LOC104916428	0.0	0.0	0.0	0.0	0.0	0.0	0.0	0.00
LOC104916429	0.0	0.0	0.0	0.0	0.0	0.0	0.0	0.00
LOC104916430	0.0	0.0	0.0	0.0	0.0	0.0	0.0	0.00
LOC104916431	0.0	0.0	0.0	0.0	0.0	0.0	0.0	0.00
LOC104916432	0.0	0.0	0.0	0.0	0.0	0.0	0.0	0.00
LOC104916434	0.5	0.0	0.0	0.0	0.0	0.0	0.1	0.20
LOC104916435	232.5	325.5	601.0	242.0	261.0	648.5	385.1	189.06
LOC104916438	466.5	711.5	312.5	377.5	708.0	294.0	478.3	189.16
LOC104916440	172.5	185.0	205.0	155.0	225.5	182.5	187.6	24.75
LOC104916443	2.0	2.5	4.0	1.0	3.0	4.5	2.8	1.29
LOC104916444	0.0	0.0	0.5	0.5	0.0	0.0	0.2	0.26
LOC104916445	0.0	0.0	0.0	0.0	0.0	0.0	0.0	0.00
LOC104916447	0.0	0.0	0.0	0.0	0.0	0.0	0.0	0.00
LOC104916448	96.0	92.0	52.5	84.0	80.5	47.0	75.3	20.64
LOC104916449	0.0	0.0	0.5	0.0	0.0	0.5	0.2	0.26
LOC104916451	49.0	49.0	56.0	58.0	45.0	65.0	53.7	7.37
LOC104916452	1123.0	1332.0	942.0	1029.5	1268.0	914.0	1101.4	171.52
LOC104916453	15.0	23.5	37.5	22.5	30.5	50.5	29.9	12.65
LOC104916454	1.0	0.5	0.5	0.0	1.0	0.0	0.5	0.45
LOC104916455	43.0	57.0	107.0	57.5	79.0	137.5	80.2	35.89
LOC104916456	0.0	0.0	0.0	0.0	0.0	0.0	0.0	0.00
LOC104916458	4.0	0.5	4.5	3.5	0.0	2.5	2.5	1.87
LOC104916459	84.5	48.5	46.5	49.5	36.5	65.0	55.1	17.07
LOC104916460	18.0	11.0	9.5	13.5	9.0	5.0	11.0	4.42
LOC104916462	2.5	10.0	19.0	0.0	9.5	19.5	10.1	8.10
LOC104916463	42.0	152.5	199.0	36.0	122.5	207.5	126.6	74.62
LOC104916465	45.0	35.0	31.0	65.0	39.5	36.5	42.0	12.20
LOC104916466	6.5	4.5	10.0	9.0	5.0	9.0	7.3	2.32
LOC104916468	15.5	7.5	14.5	13.5	6.5	14.0	11.9	3.88
LOC104916469	0.0	0.0	0.0	0.0	0.0	0.0	0.0	0.00
LOC104916470	6.0	0.0	4.0	3.5	0.5	1.5	2.6	2.31
LOC104916471	0.0	0.0	0.0	0.5	0.0	0.0	0.1	0.20
LOC104916472	29.0	15.0	27.0	26.0	13.5	25.5	22.7	6.65
LOC104916474	16.0	15.5	3.5	14.0	13.0	12.0	12.3	4.58
LOC104916475	179.5	233.0	158.5	170.5	204.0	143.5	181.5	32.42
LOC104916478	0.0	0.0	0.0	0.5	0.0	0.0	0.1	0.20
LOC104916481	65.0	33.5	169.0	63.0	34.0	152.5	86.2	59.57
LOC104916482	0.5	0.0	0.5	0.5	0.5	0.0	0.3	0.26
LOC104916483	0.5	1.5	1.0	0.0	0.0	0.5	0.6	0.58
LOC104916484	0.0	0.0	0.0	0.0	0.0	0.0	0.0	0.00
LOC104916485	2.0	0.5	2.0	1.0	1.0	2.5	1.5	0.77
LOC104916486	88.5	147.0	137.0	70.5	124.5	141.5	118.2	31.38
LOC104916487	0.0	0.0	0.0	0.0	0.0	0.0	0.0	0.00
LOC104916488	0.0	0.0	0.0	0.0	0.0	0.0	0.0	0.00
LOC104916489	0.0	0.0	0.0	0.0	0.0	0.0	0.0	0.00
LOC104916490	10.0	3.0	12.0	13.0	5.0	8.5	8.6	3.93
LOC104916491	0.0	0.5	0.5	0.0	0.0	0.5	0.3	0.27
LOC104916492	0.0	0.0	0.0	0.0	0.0	0.0	0.0	0.00

LOC104916493	1.0	0.0	0.0	0.0	0.0	0.0	0.2	0.41
LOC104916494	0.0	0.0	0.0	0.0	0.0	0.0	0.0	0.00
LOC104916496	6.0	2.0	2.5	1.5	1.0	1.5	2.4	1.83
LOC104916497	0.0	0.0	0.0	0.0	0.0	0.0	0.0	0.00
LOC104916498	1.5	3.0	10.0	1.0	8.0	10.0	5.6	4.22
LOC104916499	313.5	286.5	461.0	360.0	293.5	511.0	370.9	94.10
LOC104916500	30.0	40.0	46.0	23.5	25.5	36.0	33.5	8.73
LOC104916501	0.0	0.0	0.0	0.0	0.0	0.0	0.0	0.00
LOC104916502	85.0	70.5	68.5	67.0	63.0	74.5	71.4	7.66
LOC104916504	0.0	0.0	0.0	0.0	0.0	0.0	0.0	0.00
LOC104916505	59.0	34.5	40.0	64.5	28.5	40.5	44.5	14.16
LOC104916506	1.5	1.5	4.5	2.0	2.0	7.5	3.2	2.40
LOC104916507	0.0	0.0	0.0	0.0	0.0	0.0	0.0	0.00
LOC104916508	65.0	117.0	102.0	82.5	94.5	100.5	93.6	17.93
LOC104916510	0.0	0.0	0.0	0.0	0.0	0.0	0.0	0.00
LOC104916511	546.5	499.0	207.0	440.5	492.0	194.0	396.5	155.55
LOC104916512	0.5	0.0	2.0	0.0	0.5	1.0	0.7	0.75
LOC104916513	129.5	97.0	124.5	110.0	91.5	110.5	110.5	14.84
LOC104916514	0.0	0.0	0.0	0.0	0.0	0.0	0.0	0.00
LOC104916515	25.0	12.0	16.0	27.0	12.5	12.5	17.5	6.77
LOC104916516	62.5	36.5	32.5	59.5	39.0	19.0	41.5	16.63
LOC104916517	19.5	6.0	26.0	29.0	11.5	14.5	17.8	8.78
LOC104916518	9.0	5.5	15.0	6.0	3.5	9.5	8.1	4.07
LOC104916519	0.0	0.0	0.0	0.0	0.0	0.0	0.0	0.00
LOC104916520	0.0	0.0	0.5	0.0	0.0	1.5	0.3	0.61
LOC104916521	43.0	64.5	52.5	31.5	52.0	51.0	49.1	11.02
LOC104916522	0.0	0.0	0.0	0.0	0.0	0.0	0.0	0.00
LOC104916523	2142.5	2589.5	1952.0	2071.5	2855.0	2194.5	2300.8	346.72
LOC104916524	104.5	82.5	185.5	98.5	76.0	172.0	119.8	46.99
LOC104916525	414.0	330.0	543.0	383.5	401.0	472.5	424.0	74.32
LOC104916526	0.0	0.0	0.0	0.0	0.0	0.0	0.0	0.00
LOC104916527	51.5	74.0	52.5	46.0	59.0	47.5	55.1	10.32
LOC104916528	1065.0	702.0	676.0	1144.5	626.5	800.5	835.8	217.40
LOC104916529	0.5	0.5	0.5	1.0	0.0	0.5	0.5	0.32
LOC104916532	14.0	9.0	34.0	14.5	3.5	25.5	16.8	11.16
LOC104916533	1.5	0.0	0.0	0.5	0.5	0.5	0.5	0.55
LOC104916534	45.0	19.5	30.5	43.5	18.5	42.0	33.2	12.11
LOC104916535	54.5	24.5	87.5	48.5	27.0	74.0	52.7	25.07
LOC104916536	9.5	12.0	22.0	14.0	7.5	11.5	12.8	5.05
LOC104916537	0.0	0.0	0.0	0.0	0.0	0.0	0.0	0.00
LOC104916538	60.0	26.0	61.5	65.5	26.0	66.0	50.8	19.37
LOC104916540	10.0	1.5	10.0	13.5	3.5	12.0	8.4	4.81
LOC104916541	0.0	0.0	0.0	0.0	0.0	0.0	0.0	0.00
LOC104916542	0.0	0.0	0.0	0.0	0.0	0.0	0.0	0.00
LOC104916543	3.0	1.0	14.0	1.0	3.0	9.0	5.2	5.23
LOC104916544	0.0	0.0	0.0	0.0	0.0	0.0	0.0	0.00
LOC104916545	2.5	0.0	0.0	1.0	0.5	0.5	0.8	0.94
LOC104916546	0.0	0.0	0.0	0.0	0.0	0.0	0.0	0.00
LOC104916547	9.0	64.0	90.5	7.5	61.5	83.0	52.6	36.07
LOC104916548	0.0	0.0	0.0	0.0	0.0	0.0	0.0	0.00
LOC104916549	5.0	0.5	6.5	4.5	0.5	1.5	3.1	2.58
LOC104916550	0.0	0.0	0.0	0.0	0.0	0.5	0.1	0.20
LOC104916551	0.0	0.0	0.0	0.0	0.0	0.0	0.0	0.00
LOC104916552	0.0	0.0	0.0	0.0	0.0	0.0	0.0	0.00

LOC104916553	0.0	0.0	0.0	0.0	0.0	0.0	0.0	0.00
LOC104916554	0.0	0.0	0.0	0.0	0.0	0.0	0.0	0.00
LOC104916555	0.0	0.0	0.0	0.0	0.0	0.0	0.0	0.00
LOC104916556	0.0	0.0	0.0	0.0	0.0	0.0	0.0	0.00
LOC104916557	0.0	0.5	0.5	0.0	0.0	0.0	0.2	0.26
LOC104916558	49.5	23.5	44.0	24.0	6.5	19.0	27.8	16.11
LOC104916559	0.0	0.0	0.0	0.0	0.0	0.0	0.0	0.00
LOC104916560	0.0	0.0	0.0	0.0	0.0	0.0	0.0	0.00
LOC104916561	0.0	0.0	0.0	0.0	0.0	0.0	0.0	0.00
LOC104916562	0.0	0.0	0.0	1.0	0.0	0.0	0.2	0.41
LOC104916563	49.5	57.5	29.5	51.0	66.0	37.0	48.4	13.32
LOC104916564	0.0	0.0	0.0	0.0	0.0	0.0	0.0	0.00
LOC104916565	0.0	0.0	0.0	0.0	0.0	0.0	0.0	0.00
LOC104916566	0.0	0.0	0.0	0.0	0.0	0.0	0.0	0.00
LOC104916567	0.0	0.0	0.0	0.0	0.0	0.0	0.0	0.00
LOC104916568	0.0	0.0	0.0	0.0	0.0	0.0	0.0	0.00
LOC104916569	0.0	1.0	0.0	0.0	0.0	0.5	0.3	0.42
LOC104916570	0.5	0.0	0.0	0.0	0.0	0.0	0.1	0.20
LOC104916571	0.0	0.0	0.0	0.0	0.0	0.0	0.0	0.00
LOC104916572	3.0	2.5	1.5	6.0	1.0	0.0	2.3	2.09
LOC104916573	0.0	0.0	0.0	0.0	0.5	0.0	0.1	0.20
LOC104916574	0.0	0.0	0.0	0.0	0.0	0.0	0.0	0.00
LOC104916575	0.0	0.0	0.0	0.0	0.0	0.0	0.0	0.00
LOC104916576	9.5	9.5	12.5	7.5	10.0	9.5	9.8	1.60
LOC104916577	0.5	0.0	0.5	0.0	0.0	0.0	0.2	0.26
LOC104916578	18.0	6.0	1.5	15.0	6.0	7.5	9.0	6.22
LOC104916579	0.0	0.0	0.0	0.0	0.0	0.0	0.0	0.00
LOC104916580	3.0	0.0	0.5	0.5	0.0	2.0	1.0	1.22
LOC104916581	37.5	24.0	15.5	62.5	30.0	35.0	34.1	16.02
LOC104916582	0.0	0.0	0.0	0.0	0.0	0.0	0.0	0.00
LOC104916584	0.0	0.0	0.0	0.0	0.0	0.0	0.0	0.00
LOC104916585	0.0	0.0	0.0	0.0	0.0	0.0	0.0	0.00
LOC104916586	0.0	0.0	0.0	0.0	0.0	0.0	0.0	0.00
LOC104916587	0.0	0.0	0.0	0.0	0.0	0.0	0.0	0.00
LOC104916588	0.0	1.0	0.0	0.0	0.0	0.0	0.2	0.41
LOC104916589	0.0	0.0	0.0	0.0	0.0	0.0	0.0	0.00
LOC104916590	0.0	0.0	0.0	0.0	0.0	0.0	0.0	0.00
LOC104916591	0.0	0.0	0.0	0.0	0.0	0.0	0.0	0.00
LOC104916592	0.0	0.0	0.0	0.0	0.0	0.0	0.0	0.00
LOC104916593	0.0	0.0	0.0	0.0	0.0	0.0	0.0	0.00
LOC104916594	0.0	0.0	0.0	0.0	0.0	0.0	0.0	0.00
LOC104916595	0.0	0.0	0.0	0.0	0.5	0.0	0.1	0.20
LOC104916596	0.0	0.0	0.0	0.0	0.0	0.0	0.0	0.00
LOC104916597	0.0	0.0	0.0	0.0	0.0	0.0	0.0	0.00
LOC104916598	0.0	0.0	0.0	0.0	0.0	0.0	0.0	0.00
LOC104916599	0.0	0.0	0.0	0.0	0.0	0.0	0.0	0.00
LOC104916600	0.0	0.0	0.0	0.0	0.0	0.0	0.0	0.00
LOC104916601	0.5	0.0	0.0	0.0	0.0	0.0	0.1	0.20
LOC104916602	64.5	33.5	93.5	66.0	28.5	84.0	61.7	26.20
LOC104916603	1.5	0.0	0.5	1.0	0.0	0.0	0.5	0.63
LOC104916604	0.0	0.0	0.0	0.0	0.0	0.0	0.0	0.00
LOC104916606	2.0	1.0	0.0	1.5	1.0	2.0	1.3	0.76
LOC104916607	2.5	3.0	1.0	6.0	4.5	2.0	3.2	1.81
LOC104916608	61.0	24.0	47.0	58.5	23.0	35.0	41.4	16.67

LOC104916609	0.0	0.5	4.0	0.0	1.0	2.5	1.3	1.60
LOC104916610	0.0	0.0	0.0	0.5	0.0	0.0	0.1	0.20
LOC104916611	0.0	0.0	0.0	0.0	0.0	0.0	0.0	0.00
LOC104916612	0.0	0.0	0.0	0.0	0.5	0.0	0.1	0.20
LOC104916614	0.0	0.0	0.0	0.0	0.0	0.0	0.0	0.00
LOC104916615	0.0	0.0	2.0	0.0	1.0	0.0	0.5	0.84
LOC104916616	0.5	0.0	0.5	0.0	0.0	0.0	0.2	0.26
LOC104916617	0.0	0.0	0.0	0.0	0.0	0.0	0.0	0.00
LOC104916618	0.0	6.0	10.0	2.0	1.5	9.5	4.8	4.30
LOC104916619	2.0	467.0	22.0	4.5	697.5	303.5	249.4	291.19
LOC104916620	0.0	0.0	0.0	0.0	0.0	0.0	0.0	0.00
LOC104916621	6.5	3.5	1.5	8.5	1.0	1.0	3.7	3.17
LOC104916622	0.0	0.5	1.0	0.0	0.0	0.0	0.3	0.42
LOC104916624	1.0	0.5	2.0	0.0	1.5	5.5	1.8	1.97
LOC104916625	0.0	0.0	0.0	0.0	0.0	0.0	0.0	0.00
LOC104916626	0.0	0.5	0.0	0.0	0.0	0.0	0.1	0.20
LOC104916627	0.0	0.5	0.5	0.5	0.0	1.0	0.4	0.38
LOC104916629	2.0	2.0	6.0	1.0	0.5	3.5	2.5	2.00
LOC104916630	0.0	0.0	0.0	0.0	0.0	0.0	0.0	0.00
LOC104916631	0.5	0.0	0.0	0.0	0.0	0.0	0.1	0.20
LOC104916632	0.0	0.0	0.0	0.0	0.0	0.0	0.0	0.00
LOC104916633	0.0	0.5	0.0	0.5	0.0	0.0	0.2	0.26
LOC104916634	0.0	0.0	0.0	0.0	0.0	0.0	0.0	0.00
LOC104916635	0.0	0.0	0.0	0.0	0.0	0.0	0.0	0.00
LOC104916636	0.0	0.0	0.0	0.0	0.0	0.0	0.0	0.00
LOC104916637	15.5	3.0	0.0	9.5	2.5	2.0	5.4	5.89
LOC104916638	0.5	0.0	0.0	0.0	0.0	0.0	0.1	0.20
LOC104916639	0.0	0.0	0.0	0.0	0.0	0.0	0.0	0.00
LOC104916640	0.0	0.0	0.0	0.0	0.0	0.0	0.0	0.00
LOC104916641	0.0	0.0	0.0	0.0	0.0	0.0	0.0	0.00
LOC104916642	569.0	737.5	355.5	530.5	702.0	344.5	539.8	166.40
LOC104916643	0.0	0.0	0.0	0.0	0.0	0.0	0.0	0.00
LOC104916644	0.0	0.0	1.5	0.0	0.0	0.0	0.3	0.61
LOC104916645	0.0	0.0	0.0	0.0	0.5	0.0	0.1	0.20
LOC104916646	2.5	4.0	6.0	5.5	7.0	7.0	5.3	1.78
LOC104916647	8.5	8.5	1.0	6.0	12.5	5.5	7.0	3.85
LOC104916648	0.0	0.0	0.5	1.0	1.0	1.0	0.6	0.49
LOC104916649	1.0	1.0	1.0	2.0	0.0	2.0	1.2	0.75
LOC104916650	5.0	0.0	1.0	3.5	3.5	1.0	2.3	1.94
LOC104916651	40.5	16.5	55.0	35.5	14.0	33.5	32.5	15.35
LOC104916652	0.0	0.0	0.0	0.0	0.0	0.0	0.0	0.00
LOC104916653	0.0	0.0	0.0	1.0	0.0	0.0	0.2	0.41
LOC104916654	6.5	4.5	4.5	4.5	2.0	5.0	4.5	1.45
LOC104916655	10.0	18.5	25.5	14.5	25.0	45.5	23.2	12.47
LOC104916656	10.0	6.0	4.0	1.0	1.5	0.0	3.8	3.76
LOC104916657	0.0	0.0	0.0	0.0	0.0	0.0	0.0	0.00
LOC104916658	2.5	1.0	1.0	1.5	0.5	0.0	1.1	0.86
LOC104916659	1.0	0.0	0.0	0.0	0.0	0.0	0.2	0.41
LOC104916660	0.0	0.0	0.0	0.5	0.0	0.0	0.1	0.20
LOC104916661	19.5	5.5	7.5	13.5	6.0	11.5	10.6	5.39
LOC104916662	3.0	0.0	2.0	2.0	1.0	2.0	1.7	1.03
LOC104916663	0.0	0.0	0.5	0.0	0.0	0.0	0.1	0.20
LOC104916664	0.0	0.0	0.0	0.0	0.0	0.0	0.0	0.00
LOC104916665	1.0	0.5	0.0	0.0	0.0	0.0	0.3	0.42

LOC104916666	1.0	0.5	0.5	1.0	0.0	0.0	0.5	0.45
LOC104916667	0.0	0.0	0.0	0.0	0.0	0.0	0.0	0.00
LOC104916668	51.5	23.0	9.0	59.5	27.0	17.5	31.3	19.89
LOC104916670	0.0	0.0	0.0	0.0	0.0	0.0	0.0	0.00
LOC104916671	6366.0	9889.0	8722.5	6197.0	9956.0	8010.0	8190.1	1650.47
LOC104916672	150.0	77.5	45.5	139.5	80.5	58.5	91.9	43.01
LOC104916673	0.0	0.0	0.0	0.0	0.0	0.0	0.0	0.00
LOC104916674	0.0	0.5	0.5	0.0	0.0	0.0	0.2	0.26
LOC104916675	0.0	0.0	0.0	0.0	0.0	0.0	0.0	0.00
LOC104916676	0.0	0.0	0.0	0.0	0.0	0.0	0.0	0.00
LOC104916677	43.0	29.0	26.5	44.0	24.0	35.0	33.6	8.51
LOC104916678	45.5	47.5	42.5	42.0	58.5	43.0	46.5	6.24
LOC104916679	0.0	0.0	0.0	0.0	0.0	0.0	0.0	0.00
LOC104916680	10.5	4.0	9.0	19.0	9.5	11.5	10.6	4.87
LOC104916681	2.5	0.5	0.0	1.0	0.0	0.0	0.7	0.98
LOC104916682	1.5	1.0	1.0	3.0	0.5	1.5	1.4	0.86
LOC104916683	2.0	1.0	1.5	2.5	0.5	0.5	1.3	0.82
LOC104916685	199.0	260.5	220.0	190.0	230.5	232.5	222.1	25.38
LOC104916686	135.5	129.0	165.5	126.0	110.5	140.0	134.4	18.28
LOC104916687	0.0	0.0	0.0	0.0	0.0	0.0	0.0	0.00
LOC104916688	0.0	0.0	0.0	0.0	0.0	0.0	0.0	0.00
LOC104916689	0.0	0.0	0.0	0.0	0.0	0.0	0.0	0.00
LOC104916690	0.0	0.0	0.0	0.0	0.0	0.0	0.0	0.00
LOC104916691	0.0	0.0	0.0	0.0	0.0	0.5	0.1	0.20
LOC104916692	173.0	273.0	364.5	200.5	369.5	431.0	301.9	102.85
LOC104916693	23.0	13.5	21.0	28.0	12.0	22.5	20.0	6.11
LOC104916694	0.0	0.0	0.0	0.0	0.0	0.0	0.0	0.00
LOC104916695	0.0	0.0	0.0	0.0	0.0	0.0	0.0	0.00
LOC104916696	0.0	0.0	0.0	0.0	0.0	0.0	0.0	0.00
LOC104916697	0.0	0.0	0.0	0.0	0.0	0.5	0.1	0.20
LOC104916698	0.0	0.0	0.0	0.0	0.0	0.0	0.0	0.00
LOC104916699	0.0	0.0	0.0	0.0	0.0	0.0	0.0	0.00
LOC104916700	3125.0	3930.5	4049.5	3286.0	4241.5	3623.5	3709.3	441.78
LOC104916701	627.0	533.5	622.5	638.0	544.5	630.5	599.3	47.14
LOC104916702	12.0	11.5	42.5	19.5	10.0	37.0	22.1	14.18
LOC104916703	0.0	0.0	0.0	0.0	0.0	0.0	0.0	0.00
LOC104916704	0.0	0.0	0.0	0.0	0.0	0.0	0.0	0.00
LOC104916705	0.0	0.0	0.0	0.0	0.0	0.0	0.0	0.00
LOC104916706	0.0	0.0	0.0	0.0	0.0	0.0	0.0	0.00
LOC104916707	98.5	102.5	52.5	89.5	102.5	53.5	83.2	23.85
LOC104916708	0.0	0.0	0.0	0.0	0.0	0.0	0.0	0.00
LOC104916709	89.0	63.0	30.5	68.0	49.0	21.0	53.4	25.16
LOC104916710	0.0	0.0	0.0	0.0	0.0	0.0	0.0	0.00
LOC104916711	1.0	1.0	2.5	0.5	1.0	0.0	1.0	0.84
LOC104916712	258.5	176.0	55.0	228.0	165.5	66.5	158.3	82.90
LOC104916713	0.0	0.0	0.0	0.0	0.0	0.0	0.0	0.00
LOC104916714	0.0	0.0	0.0	0.5	0.0	0.0	0.1	0.20
LOC104916715	800.5	991.0	453.5	774.5	910.0	497.0	737.8	218.11
LOC104916716	105.0	83.0	178.5	76.5	74.0	160.0	112.8	45.42
LOC104916717	95.0	125.5	170.5	93.5	113.5	176.0	129.0	36.33
LOC104916718	0.0	0.0	0.0	0.0	0.0	0.0	0.0	0.00
LOC104916720	681.5	599.0	289.0	723.0	739.0	333.0	560.8	199.93
LOC104916721	119.5	76.0	126.0	132.0	75.0	123.0	108.6	25.95
LOC104916722	0.0	0.0	0.0	0.0	0.0	0.0	0.0	0.00

LOC104916725	0.0	0.0	0.0	0.0	0.0	0.0	0.0	0.00
LOC104916726	77.5	40.0	57.5	63.5	34.5	46.0	53.2	16.07
LOC104916727	0.0	0.0	0.0	0.0	0.0	0.0	0.0	0.00
LOC104916728	0.0	0.0	0.0	0.0	0.0	0.0	0.0	0.00
LOC104916729	0.0	0.0	0.0	0.0	0.0	0.0	0.0	0.00
LOC104916730	247.5	330.5	174.5	242.0	346.5	180.5	253.6	72.55
LOC104916732	57.5	86.5	49.5	54.5	77.5	54.0	63.3	15.02
LOC104916733	591.5	886.5	458.0	665.5	887.0	582.0	678.4	174.59
LOC104916734	24.0	18.5	5.0	26.5	18.0	6.5	16.4	8.88
LOC104916735	0.0	0.0	0.0	0.0	0.0	0.0	0.0	0.00
LOC104916736	0.5	1.0	0.5	0.5	2.5	1.0	1.0	0.77
LOC104916737	3.0	3.0	0.0	1.0	4.5	1.5	2.2	1.63
LOC104916738	0.0	0.5	0.0	0.0	0.0	0.0	0.1	0.20
LOC104916739	0.0	0.0	0.0	0.0	0.0	0.0	0.0	0.00
LOC104916740	0.0	0.0	0.0	0.0	0.0	0.0	0.0	0.00
LOC104916741	7.5	9.0	0.5	6.5	10.0	4.0	6.3	3.50
LOC104916742	0.0	0.0	0.0	0.0	0.0	0.0	0.0	0.00
LOC104916744	0.0	1.0	0.0	0.0	0.0	0.0	0.2	0.41
LOC104916745	0.0	0.0	0.0	0.0	0.0	0.0	0.0	0.00
LOC104916746	0.0	0.0	0.0	0.0	0.0	0.0	0.0	0.00
LOC104916748	3.0	1.5	5.5	3.5	0.0	3.0	2.8	1.86
LOC104916749	132.0	115.0	155.0	108.5	112.0	146.5	128.2	19.45
LOC104916751	126.0	173.5	256.5	123.0	145.0	215.0	173.2	53.35
LOC104916752	0.0	0.0	0.0	0.0	0.0	0.0	0.0	0.00
LOC104916753	0.0	0.0	0.0	0.0	0.0	0.0	0.0	0.00
LOC104916754	0.0	0.0	0.0	0.0	0.0	0.0	0.0	0.00
LOC104916755	0.0	0.0	0.0	0.0	0.0	0.0	0.0	0.00
LOC104916756	0.0	0.0	0.0	0.0	0.0	0.0	0.0	0.00
LOC104916757	0.0	0.0	0.5	0.0	0.0	0.0	0.1	0.20
LOC104916758	32.0	34.0	26.5	31.0	40.5	31.5	32.6	4.60
LOC104916759	44.5	51.0	98.5	35.0	47.0	108.5	64.1	31.14
LOC104916760	0.0	0.0	0.0	0.0	0.0	0.0	0.0	0.00
LOC104916763	1.0	0.0	0.0	0.0	0.5	0.5	0.3	0.41
LOC104916764	0.0	0.0	0.0	0.0	0.0	0.5	0.1	0.20
LOC104916765	0.0	0.0	0.0	0.0	0.0	0.0	0.0	0.00
LOC104916766	0.0	0.0	0.0	0.0	0.0	0.0	0.0	0.00
LOC104916767	0.5	1.5	0.0	0.0	0.0	0.5	0.4	0.58
LOC104916768	221.0	143.5	131.5	172.0	129.0	111.5	151.4	39.55
LOC104916769	93.0	91.0	53.0	82.0	64.0	48.0	71.8	19.51
LOC104916770	13.0	22.0	23.0	14.5	23.0	47.0	23.8	12.21
LOC104916771	9.0	8.5	13.5	7.5	9.0	8.5	9.3	2.11
LOC104916772	0.0	0.0	0.0	0.0	0.0	0.0	0.0	0.00
LOC104916773	0.0	0.0	0.0	0.0	0.0	0.0	0.0	0.00
LOC104916774	47.5	33.5	60.5	57.5	31.0	71.5	50.3	15.92
LOC104916775	0.0	0.0	0.0	0.0	0.0	0.0	0.0	0.00
LOC104916776	0.0	0.0	0.0	0.0	0.0	0.0	0.0	0.00
LOC104916777	1.5	0.0	0.0	0.0	0.0	0.0	0.3	0.61
LOC104916778	0.0	0.0	0.0	0.0	0.0	0.0	0.0	0.00
LOC104916779	0.0	0.0	0.0	0.5	1.0	1.0	0.4	0.49
LOC104916780	3.5	0.5	2.5	3.5	2.0	1.5	2.3	1.17
LOC104916781	11.5	17.0	8.0	5.0	28.0	9.0	13.1	8.35
LOC104916782	22.5	64.5	21.0	26.0	73.0	24.0	38.5	23.64
LOC104916783	21.0	17.0	28.5	23.0	22.0	29.5	23.5	4.73
LOC104916784	2.5	2.0	0.0	1.5	1.5	0.5	1.3	0.93

LOC104916785	44.5	49.0	63.5	65.0	49.0	109.0	63.3	23.89
LOC104916786	9.0	10.0	22.5	9.0	13.5	28.5	15.4	8.21
LOC104916787	15.5	11.5	18.5	11.0	12.5	19.5	14.8	3.66
LOC104916788	0.0	0.0	0.0	0.0	0.0	0.0	0.0	0.00
LOC104916789	0.0	0.0	0.0	0.0	0.0	0.0	0.0	0.00
LOC104916790	39.5	47.0	50.0	41.5	33.5	51.0	43.8	6.79
LOC104916791	7.5	21.0	21.5	11.0	12.5	24.0	16.3	6.76
LOC104916792	14.5	2.0	21.0	16.0	2.5	13.0	11.5	7.66
LOC104916794	0.0	0.0	0.0	0.0	0.0	0.0	0.0	0.00
LOC104916795	1.0	1.5	1.0	0.5	1.5	0.5	1.0	0.45
LOC104916796	0.0	0.0	0.0	0.0	0.0	0.0	0.0	0.00
LOC104916797	6.5	0.0	6.5	6.0	0.5	5.0	4.1	3.02
LOC104916798	0.5	0.0	0.0	0.0	0.0	0.0	0.1	0.20
LOC104916799	0.0	0.0	0.0	0.0	0.0	0.0	0.0	0.00
LOC104916800	0.0	0.0	0.0	0.0	0.0	0.0	0.0	0.00
LOC104916802	0.0	0.0	0.0	0.0	0.0	0.0	0.0	0.00
LOC104916803	0.5	1.0	2.5	0.0	1.0	1.5	1.1	0.86
LOC104916804	1548.5	1274.0	941.5	1271.0	1117.0	945.5	1182.9	231.82
LOC104916806	1.5	1.0	4.5	3.5	1.0	7.0	3.1	2.40
LOC104916807	0.0	0.0	0.0	0.0	0.0	0.0	0.0	0.00
LOC104916808	0.5	0.0	0.0	0.0	0.0	0.0	0.1	0.20
LOC104916809	0.0	0.5	0.5	0.5	0.0	1.0	0.4	0.38
LOC104916811	39.0	41.5	37.5	34.0	46.0	30.5	38.1	5.47
LOC104916812	0.0	0.0	0.0	0.5	0.0	0.0	0.1	0.20
LOC104916813	0.0	0.0	0.0	0.0	0.0	0.5	0.1	0.20
LOC104916814	0.0	0.0	0.0	0.0	0.0	0.0	0.0	0.00
LOC104916815	4.0	4.0	7.5	6.0	2.0	5.0	4.8	1.89
LOC104916816	1.5	0.5	2.5	1.5	2.0	0.5	1.4	0.80
LOC104916817	0.0	0.0	0.0	0.0	0.0	0.0	0.0	0.00
LOC104916819	0.0	0.0	0.0	0.0	0.0	0.0	0.0	0.00
LOC104916820	0.0	0.0	0.0	0.0	0.0	0.0	0.0	0.00
LOC104916821	21.0	12.5	8.5	30.0	12.5	10.5	15.8	8.15
LOC104916822	2.5	1.5	6.0	2.5	0.5	9.5	3.8	3.37
LOC104916823	119.5	98.0	123.0	120.0	87.5	110.0	109.7	14.20
LOC104916825	0.0	0.0	0.0	0.0	0.0	0.0	0.0	0.00
LOC104916826	175.0	410.5	224.0	138.5	367.5	231.5	257.8	107.96
LOC104916827	4.0	2.0	5.0	2.0	0.5	9.5	3.8	3.20
LOC104916829	0.0	0.0	0.0	0.0	0.0	0.0	0.0	0.00
LOC104916830	1.0	1.0	1.5	0.0	0.0	0.0	0.6	0.66
LOC104916832	0.5	1.0	1.0	0.0	0.5	1.5	0.8	0.52
LOC104916834	7.0	2.5	0.5	6.0	5.0	4.0	4.2	2.38
LOC104916835	6.5	7.5	1.0	7.0	8.5	3.0	5.6	2.92
LOC104916836	111.0	100.5	45.5	92.5	109.0	59.5	86.3	27.38
LOC104916837	52.0	35.0	73.0	62.0	21.5	60.0	50.6	19.04
LOC104916838	0.0	0.0	0.0	0.0	0.0	0.0	0.0	0.00
LOC104916839	0.0	0.0	0.0	0.0	0.0	0.0	0.0	0.00
LOC104916841	216.0	179.5	227.0	222.0	199.5	241.5	214.3	21.88
LOC104916842	112.5	58.0	85.5	123.5	48.0	65.5	82.2	30.56
LOC104916843	0.0	0.0	0.0	0.0	0.0	0.0	0.0	0.00
LOC104916846	0.0	0.0	0.0	0.0	0.0	0.0	0.0	0.00
LOC104916848	5.0	11.0	15.0	10.0	11.5	23.0	12.6	6.04
LOC104916850	10.5	20.0	31.5	11.5	5.5	32.0	18.5	11.27
LOC104916851	19.0	36.5	116.5	8.5	30.5	111.5	53.8	47.68
LOC104916852	0.0	0.0	0.5	0.0	0.5	0.5	0.3	0.27

LOC104916853	24.5	17.0	31.5	29.5	11.5	38.5	25.4	9.90
LOC104916855	0.0	0.0	0.0	0.0	0.0	1.0	0.2	0.41
LOC104916857	7.0	7.5	11.0	8.5	7.5	8.5	8.3	1.44
LOC104916858	0.0	0.0	0.0	0.0	0.0	0.0	0.0	0.00
LOC104916859	162.5	104.0	154.5	170.0	130.5	127.5	141.5	25.08
LOC104916861	24.0	14.5	17.5	15.5	8.5	19.0	16.5	5.15
LOC104916862	44.5	40.5	33.0	45.0	34.5	43.0	40.1	5.17
LOC104916863	80.5	74.0	276.5	85.5	46.5	270.5	138.9	105.13
LOC104916864	1016.0	734.0	1040.0	727.0	559.0	743.5	803.3	187.16
LOC104916865	130.5	133.0	113.5	143.5	145.5	106.5	128.8	15.79
LOC104916866	113.0	73.5	201.0	103.5	65.5	156.5	118.8	51.65
LOC104916867	68.0	52.0	133.0	56.0	44.5	118.5	78.7	37.54
LOC104916868	48.5	36.0	38.0	48.5	28.0	30.5	38.3	8.72
LOC104916869	69.0	69.5	74.0	66.5	55.0	83.5	69.6	9.33
LOC104916870	12.5	28.0	34.0	10.5	26.0	63.0	29.0	19.00
LOC104916871	19.5	26.0	17.0	16.5	20.0	14.5	18.9	4.02
LOC104916872	0.0	0.0	0.0	0.0	0.0	0.0	0.0	0.00
LOC104916874	89.0	67.0	156.0	63.5	70.5	103.0	91.5	35.00
LOC104916875	0.0	0.0	0.0	0.0	0.0	0.0	0.0	0.00
LOC104916876	0.0	0.0	0.0	0.0	0.0	0.0	0.0	0.00
LOC104916877	0.0	0.0	0.0	0.0	0.0	0.0	0.0	0.00
LOC104916878	0.0	0.0	0.5	0.0	0.0	0.5	0.2	0.26
LOC104916879	0.0	0.0	0.0	0.0	0.0	0.5	0.1	0.20
LOC104916880	89.0	23.5	163.0	60.0	15.0	113.0	77.3	56.27
LOC104916883	0.0	0.0	0.0	0.0	0.0	0.0	0.0	0.00
LOC104916884	67.0	34.5	122.0	59.5	26.0	118.0	71.2	40.78
LOC104916885	18.0	10.0	28.5	23.5	4.5	18.0	17.1	8.74
LOC104916886	547.0	432.5	435.0	738.5	523.0	383.0	509.8	127.68
LOC104916887	0.0	0.0	0.0	0.0	0.0	0.0	0.0	0.00
LOC104916888	199.0	201.0	196.5	186.0	188.0	179.0	191.6	8.60
LOC104916889	6.5	3.0	14.0	5.0	6.0	13.5	8.0	4.62
LOC104916890	168.0	107.0	264.0	183.5	97.0	244.5	177.3	68.63
LOC104916891	1.0	1.0	2.0	0.0	0.5	0.0	0.8	0.76
LOC104916893	14.5	13.5	22.0	10.0	16.0	15.5	15.3	3.93
LOC104916894	17.5	10.0	10.5	15.0	13.5	15.0	13.6	2.89
LOC104916895	368.5	683.5	201.0	299.5	656.5	212.5	403.6	215.38
LOC104916896	61.5	82.5	73.0	64.5	82.0	67.0	71.8	8.97
LOC104916897	0.5	0.0	1.5	6.0	3.5	2.5	2.3	2.21
LOC104916898	0.0	0.0	0.0	0.0	0.0	0.0	0.0	0.00
LOC104916901	5.0	1.0	14.5	5.0	3.0	10.0	6.4	4.96
LOC104916902	0.0	0.0	0.0	0.0	0.0	0.0	0.0	0.00
LOC104916903	0.0	0.0	0.0	0.0	0.0	0.0	0.0	0.00
LOC104916904	0.0	0.5	0.0	0.0	0.0	0.0	0.1	0.20
LOC104916905	0.0	0.0	0.0	0.5	0.0	0.0	0.1	0.20
LOC104916906	123.0	100.5	133.5	126.0	87.0	121.5	115.3	17.68
LOC104916908	13.5	9.0	10.5	16.5	10.0	8.0	11.3	3.17
LOC104916909	3.5	0.0	1.0	0.5	1.5	0.0	1.1	1.32
LOC104916910	118.5	184.5	254.5	115.0	173.0	260.5	184.3	63.24
LOC104916914	30.5	30.5	57.5	32.5	44.5	53.0	41.4	12.00
LOC104916915	30.0	5.0	26.5	26.0	3.0	22.5	18.8	11.75
LOC104916917	1.5	0.5	0.5	2.0	0.0	0.0	0.8	0.82
LOC104916918	370.0	532.0	438.0	328.0	497.5	456.0	436.9	76.70
LOC104916919	36.0	20.0	39.0	42.5	14.0	29.5	30.2	11.22
LOC104916922	1.0	0.0	0.5	0.5	0.0	0.5	0.4	0.38

LOC104916923	0.0	0.5	1.0	0.0	0.0	1.5	0.5	0.63
LOC104916924	314.0	313.5	267.5	328.5	306.0	254.0	297.3	29.51
LOC104916925	44.5	24.5	30.5	61.0	26.0	34.0	36.8	13.86
LOC104916927	146.0	95.5	276.0	165.5	97.0	238.5	169.8	74.03
LOC104916929	209.5	315.5	112.5	216.0	366.5	153.5	228.9	96.14
LOC104916930	16.0	9.0	6.0	19.0	5.0	6.0	10.2	5.91
LOC104916931	62.0	88.0	89.0	55.5	88.5	76.0	76.5	14.72
LOC104916932	54.0	32.0	34.0	50.5	35.0	29.0	39.1	10.46
LOC104916933	0.0	0.0	0.0	0.0	0.0	0.0	0.0	0.00
LOC104916934	2.0	2.0	0.5	5.5	2.5	1.0	2.3	1.75
LOC104916935	540.5	552.5	168.0	509.0	608.0	248.5	437.8	182.41
LOC104916936	8.5	20.0	5.0	7.0	14.5	1.5	9.4	6.73
LOC104916937	149.0	218.0	179.5	152.0	206.5	182.5	181.3	27.89
LOC104916938	0.0	0.0	0.0	0.0	0.5	0.0	0.1	0.20
LOC104916939	45.0	55.5	84.5	33.5	46.5	81.5	57.8	20.79
LOC104916940	59.5	82.0	69.5	68.0	91.0	97.0	77.8	14.56
LOC104916941	39.5	47.5	44.5	35.5	46.5	37.5	41.8	5.01
LOC104916943	3.5	16.5	16.0	6.5	17.5	21.5	13.6	6.99
LOC104916944	0.5	0.0	0.0	0.0	0.0	0.0	0.1	0.20
LOC104916946	73.0	79.5	62.5	67.0	58.0	59.5	66.6	8.35
LOC104916947	15.0	9.5	49.0	10.5	4.0	31.0	19.8	16.99
LOC104916948	66.5	41.0	170.0	55.0	44.0	107.0	80.6	49.90
LOC104916949	0.0	0.0	0.0	1.0	0.0	0.0	0.2	0.41
LOC104916950	47.5	39.0	46.5	76.5	40.5	41.5	48.6	14.09
LOC104916952	12.5	16.5	17.5	11.0	13.0	15.5	14.3	2.54
LOC104916953	159.0	89.5	223.0	184.0	76.5	221.5	158.9	63.66
LOC104916955	0.0	0.0	0.0	0.0	0.0	0.0	0.0	0.00
LOC104916956	0.0	0.0	0.0	0.0	0.0	0.0	0.0	0.00
LOC104916957	83.5	98.5	158.5	62.0	83.5	148.5	105.8	38.90
LOC104916958	96.5	114.5	176.5	125.5	80.0	181.0	129.0	41.57
LOC104916959	52.5	57.5	52.0	37.5	43.0	45.0	47.9	7.36
LOC104916960	1.5	1.0	1.0	0.5	0.5	0.5	0.8	0.41
LOC104916961	73.5	48.0	57.0	62.0	47.0	76.5	60.7	12.47
LOC104916962	127.5	77.5	95.5	127.5	71.5	112.0	101.9	24.39
LOC104916963	1.0	0.0	0.0	0.5	0.0	0.5	0.3	0.41
LOC104916964	85.0	65.5	91.0	95.5	49.0	98.5	80.8	19.47
LOC104916965	22.0	15.0	23.0	13.0	11.5	12.5	16.2	5.05
LOC104916966	48.0	54.5	35.0	42.0	54.5	32.5	44.4	9.52
LOC104916967	357.0	116.0	608.0	378.5	95.5	453.5	334.8	198.13
LOC104916969	0.0	0.5	0.5	0.0	0.0	0.0	0.2	0.26
LOC104916970	2.5	2.0	0.5	5.5	2.0	2.0	2.4	1.66
LOC104916972	15.0	9.5	16.0	13.0	15.0	21.5	15.0	3.94
LOC104916973	0.0	0.5	0.5	0.5	1.0	0.5	0.5	0.32
LOC104916974	0.0	0.0	0.0	0.0	0.0	0.0	0.0	0.00
LOC104916975	31.5	32.0	42.5	34.5	23.0	45.5	34.8	8.15
LOC104916976	612.0	497.5	515.0	576.0	551.5	480.5	538.8	50.15
LOC104916977	0.0	0.0	0.0	0.0	0.0	0.0	0.0	0.00
LOC104916979	0.0	0.0	0.0	0.0	0.0	0.0	0.0	0.00
LOC104916980	12.0	5.5	0.5	12.5	2.5	0.5	5.6	5.48
LOC104916981	0.0	2.0	0.0	0.0	0.0	0.0	0.3	0.82
LOC104916982	0.0	0.0	0.0	0.0	0.0	0.0	0.0	0.00
LOC104916983	0.5	1.0	2.5	0.0	1.5	1.0	1.1	0.86
LOC104916984	18.0	30.5	39.0	14.0	22.0	38.0	26.9	10.51
LOC104916985	0.0	0.5	0.0	0.0	0.0	0.5	0.2	0.26

LOC104916986	435.5	282.0	350.0	430.0	250.5	356.0	350.7	75.19
LOC104916987	0.0	0.0	2.0	0.5	0.0	1.0	0.6	0.80
LOC104916988	0.0	0.0	0.0	0.0	0.0	0.0	0.0	0.00
LOC104916989	34.5	34.0	47.0	46.5	41.0	53.0	42.7	7.55
LOC104916990	2.0	3.0	10.5	1.5	2.5	12.0	5.3	4.70
LOC104916991	0.5	0.5	8.5	0.0	0.5	10.0	3.3	4.61
LOC104916992	4.5	342.5	78.5	5.0	369.0	90.5	148.3	164.83
LOC104916993	110.5	114.0	493.0	63.0	74.5	313.5	194.8	172.33
LOC104916994	0.0	0.0	0.0	0.0	0.0	0.0	0.0	0.00
LOC104916995	0.0	0.0	0.0	0.0	0.0	0.0	0.0	0.00
LOC104916996	468.5	415.0	375.0	388.0	445.0	350.5	407.0	44.42
LOC104916997	5.0	4.5	1.5	0.5	2.0	0.5	2.3	1.97
LOC104916998	3.5	11.0	36.5	2.0	3.5	31.5	14.7	15.38
LOC104916999	129.0	349.0	360.5	124.0	396.5	422.5	296.9	134.57
LOC104917000	5.5	6.0	5.5	8.0	6.5	6.5	6.3	0.93
LOC104917001	3.5	3.0	4.0	2.5	3.5	7.0	3.9	1.59
LOC104917002	1678.0	2125.5	1061.5	1450.5	2035.0	1086.5	1572.8	456.74
LOC104917003	14.5	1.0	0.5	5.5	0.5	1.5	3.9	5.52
LOC104917004	4.5	0.5	0.0	3.0	0.5	0.5	1.5	1.82
LOC104917005	107.0	275.5	88.0	71.5	230.0	132.5	150.8	82.84
LOC104917006	0.0	0.0	0.0	0.0	0.0	0.0	0.0	0.00
LOC104917007	3.0	2.5	1.0	3.5	1.0	4.5	2.6	1.39
LOC104917008	0.0	0.5	0.0	0.0	0.0	0.0	0.1	0.20
LOC104917009	54.0	49.0	75.0	57.5	44.0	72.5	58.7	12.57
LOC104917010	0.0	0.0	0.0	0.0	0.0	0.0	0.0	0.00
LOC104917011	0.0	1.0	1.0	0.5	0.5	1.0	0.7	0.41
LOC104917012	5258.5	7795.5	9507.5	4894.5	6844.0	9463.5	7293.9	1998.06
LOC104917013	0.0	0.0	0.0	0.0	0.0	0.0	0.0	0.00
LOC104917014	5.0	3.5	22.5	3.0	1.5	15.5	8.5	8.50
LOC104917015	326.0	359.5	310.0	338.0	291.0	321.5	324.3	23.46
LOC104917016	0.5	0.5	0.0	0.5	0.0	0.5	0.3	0.26
LOC104917017	1.0	1.0	0.5	0.0	1.0	4.5	1.3	1.60
LOC104917018	70.5	79.5	116.5	89.0	76.5	118.0	91.7	20.70
LOC104917019	327.5	182.0	98.5	316.0	168.5	64.0	192.8	109.10
LOC104917020	11.5	13.5	16.5	13.0	10.0	20.5	14.2	3.79
LOC104917021	8.0	4.0	8.5	4.0	2.5	7.5	5.8	2.54
LOC104917022	0.0	0.5	0.5	0.5	0.5	0.0	0.3	0.26
LOC104917024	658.5	540.0	292.0	512.0	492.5	306.5	466.9	142.17
LOC104917025	4.0	3.0	2.0	3.0	2.5	2.0	2.8	0.76
LOC104917027	40.0	56.5	25.0	31.5	45.0	35.5	38.9	11.02
LOC104917028	289.0	211.0	136.5	298.5	218.0	177.0	221.7	62.95
LOC104917029	741.5	9731.5	3829.5	613.5	10378.0	5287.5	5096.9	4244.59
LOC104917030	0.0	0.0	0.0	0.0	0.0	0.0	0.0	0.00
LOC104917033	0.0	0.0	1.0	0.0	2.0	0.0	0.5	0.84
LOC104917034	0.0	0.0	0.0	0.0	0.0	0.0	0.0	0.00
LOC104917035	0.0	0.0	0.0	0.0	0.0	0.0	0.0	0.00
LOC104917036	0.0	0.0	0.0	0.0	0.0	0.0	0.0	0.00
LOC104917037	0.0	1.0	1.5	0.5	1.5	1.5	1.0	0.63
LOC104917038	33.0	17.5	73.0	32.0	18.0	65.0	39.8	23.74
LOC104917039	0.0	0.0	3.5	0.0	0.5	12.0	2.7	4.77
LOC104917040	14.0	13.0	35.0	14.0	11.5	33.5	20.2	10.96
LOC104917041	0.0	0.0	0.0	0.0	0.0	0.0	0.0	0.00
LOC104917042	0.0	0.0	1.0	0.0	0.5	0.0	0.3	0.42
LOC104917043	2.0	0.0	1.5	1.5	0.0	1.0	1.0	0.84

LOC104917044	0.0	0.0	0.0	0.0	0.0	0.0	0.0	0.00
LOC104917045	0.0	0.0	0.0	0.0	0.0	0.0	0.0	0.00
LOC104917046	0.0	0.0	0.0	0.0	0.0	0.0	0.0	0.00
LOC104917048	0.0	0.5	0.0	1.0	0.0	0.0	0.3	0.42
LOC104917049	39.5	18.0	75.5	49.0	20.5	48.0	41.8	21.22
LOC104917050	489.5	619.5	539.0	467.0	540.5	666.0	553.6	76.12
LOC104917051	27.5	36.5	28.0	16.5	23.0	17.5	24.8	7.48
LOC104917052	4.5	0.5	4.0	2.0	0.0	0.0	1.8	2.02
LOC104917053	515.0	571.5	402.0	492.5	622.0	413.5	502.8	86.39
LOC104917054	0.0	0.0	0.0	0.0	0.5	0.5	0.2	0.26
LOC104917055	0.0	0.0	0.0	0.0	0.0	0.0	0.0	0.00
LOC104917056	82.5	96.5	245.0	86.0	66.5	217.5	132.3	77.71
LOC104917057	8.0	3.5	10.0	10.5	8.5	16.0	9.4	4.07
LOC104917058	137.5	75.5	126.5	136.0	57.5	125.5	109.8	34.32
LOC104917059	17.0	11.5	36.0	17.0	8.0	35.5	20.8	12.05
LOC104917060	0.5	0.0	0.0	0.0	0.0	0.5	0.2	0.26
LOC104917061	2.0	8.5	10.0	2.0	7.0	9.5	6.5	3.63
LOC104917062	53.0	54.0	220.5	33.0	36.5	170.5	94.6	80.20
LOC104917063	692.5	737.5	661.0	641.5	675.5	726.5	689.1	37.40
LOC104917065	4.5	4.0	9.0	2.0	1.5	19.5	6.8	6.79
LOC104917066	318.0	231.0	203.5	301.5	262.5	251.5	261.3	42.87
LOC104917067	10.5	9.5	23.5	16.5	6.0	21.5	14.6	7.03
LOC104917068	0.0	0.0	0.0	0.0	0.0	0.0	0.0	0.00
LOC104917071	0.0	0.0	0.0	0.0	0.0	0.0	0.0	0.00
LOC104917072	26.0	4.0	8.0	1.5	0.5	0.0	6.7	9.92
LOC104917073	12.0	55.5	48.0	17.0	48.0	130.5	51.8	42.50
LOC104917074	7.0	2.5	2.0	2.0	2.0	2.0	2.9	2.01
LOC104917075	54.0	20.5	19.0	31.5	19.5	15.0	26.6	14.52
LOC104917076	1.0	0.5	0.0	0.0	0.0	1.0	0.4	0.49
LOC104917077	0.0	0.0	0.0	0.0	0.0	0.0	0.0	0.00
LOC104917078	273.0	301.0	152.0	266.5	350.0	182.5	254.2	74.11
LOC104917079	0.0	0.0	0.0	0.0	0.0	0.0	0.0	0.00
LOC104917080	2.0	5.0	1.5	9.0	13.5	12.0	7.2	5.11
LOC104917081	0.0	0.0	0.0	0.5	0.0	0.0	0.1	0.20
LOC104917082	74.0	44.0	130.0	73.5	34.0	125.0	80.1	40.04
LOC104917086	0.0	1.0	0.0	1.0	0.0	0.0	0.3	0.52
LOC104917089	50.5	102.0	119.0	69.5	119.0	142.0	100.3	34.26
LOC104917090	82.5	42.5	41.5	75.0	39.0	41.0	53.6	19.67
LOC104917094	17.5	23.0	35.5	15.0	21.0	37.5	24.9	9.41
LOC104917097	0.0	0.0	0.0	1.0	0.0	0.5	0.3	0.42
LOC104917099	65.5	75.5	145.0	78.0	62.0	156.5	97.1	42.15
LOC104917100	2.0	0.5	4.5	2.0	0.5	3.0	2.1	1.53
LOC104917101	8.0	15.0	41.5	3.0	6.5	34.5	18.1	16.07
LOC104917102	1109.0	1410.0	695.5	839.5	1427.0	774.0	1042.5	322.81
LOC104917103	56.5	52.5	65.5	70.5	51.0	83.5	63.3	12.48
LOC104917104	0.0	0.0	0.0	0.0	0.0	0.5	0.1	0.20
LOC104917105	3.5	3.5	10.0	4.5	2.5	9.0	5.5	3.18
LOC104917110	480.0	402.5	407.5	444.5	414.0	414.0	427.1	29.78
LOC104917111	131.5	130.5	172.5	121.5	98.0	163.5	136.3	27.54
LOC104917112	102.0	79.0	95.0	93.5	80.0	119.0	94.8	14.89
LOC104917113	0.5	2.0	2.0	1.0	1.5	3.5	1.8	1.04
LOC104917114	129.5	137.0	211.5	131.0	134.5	198.5	157.0	37.50
LOC104917115	0.0	0.5	1.5	0.5	0.0	2.5	0.8	0.98
LOC104917116	0.0	0.0	0.0	0.0	0.0	0.0	0.0	0.00

LOC104917117	0.0	0.0	0.0	0.0	0.0	0.0	0.0	0.00
LOC104917118	19.5	39.0	67.0	14.0	37.5	170.5	57.9	58.20
LOC104917119	0.0	0.5	1.0	0.0	0.0	2.0	0.6	0.80
LOC104917120	90.5	29.0	192.5	79.0	25.5	161.5	96.3	68.38
LOC104917121	0.0	0.0	0.0	0.0	0.0	0.0	0.0	0.00
LOC104917122	0.0	0.5	0.0	0.0	0.0	0.0	0.1	0.20
LOC104917123	0.0	0.0	0.0	0.0	0.0	0.0	0.0	0.00
LOC104917125	0.0	0.0	0.0	0.0	0.0	0.0	0.0	0.00
LOC104917126	0.5	0.0	0.5	0.0	0.0	0.0	0.2	0.26
LOC104917127	0.0	1.0	0.0	0.0	0.5	0.0	0.3	0.42
LOC104917128	296.5	252.0	172.5	262.5	236.5	184.0	234.0	47.60
LOC104917129	15.5	85.0	180.5	10.5	81.5	119.0	82.0	64.21
LOC104917130	1.0	0.5	1.5	0.5	1.5	0.5	0.9	0.49
LOC104917131	0.0	0.0	0.0	0.0	0.0	0.0	0.0	0.00
LOC104917132	4.5	6.5	3.0	4.5	3.0	5.0	4.4	1.32
LOC104917133	15.0	5.0	0.5	15.5	4.5	2.0	7.1	6.54
LOC104917134	472.0	411.0	570.5	413.0	441.5	599.5	484.6	81.41
LOC104917135	68.5	41.0	56.5	70.5	46.5	61.0	57.3	11.80
LOC104917138	0.0	0.0	0.0	0.0	0.0	0.0	0.0	0.00
LOC104917139	85.0	21.5	0.5	69.0	27.5	2.0	34.3	35.13
LOC104917140	0.0	0.0	0.0	0.0	0.0	0.0	0.0	0.00
LOC104917141	0.0	0.0	0.0	1.0	0.0	0.0	0.2	0.41
LOC104917142	52.5	24.0	11.5	38.5	29.5	11.5	27.9	15.96
LOC104917144	0.0	0.0	0.5	0.5	0.0	0.0	0.2	0.26
LOC104917145	55.0	9.5	60.5	59.0	8.0	42.0	39.0	24.32
LOC104917146	33.5	9.5	17.0	48.5	8.0	19.5	22.7	15.59
LOC104917147	2.0	0.0	0.0	0.5	0.0	0.5	0.5	0.77
LOC104917148	0.0	0.0	0.0	0.0	0.0	0.5	0.1	0.20
LOC104917149	120.5	45.5	53.0	91.5	48.5	47.0	67.7	31.18
LOC104917152	354.5	715.0	254.0	300.0	701.5	261.5	431.1	217.67
LOC104917153	14.0	23.5	64.0	12.0	27.5	64.5	34.3	23.94
LOC104917154	0.5	0.0	0.0	0.0	0.0	0.0	0.1	0.20
LOC104917155	42.5	3.5	67.0	64.5	4.5	53.5	39.3	28.66
LOC104917158	78.0	37.5	66.5	71.0	29.5	67.0	58.3	19.77
LOC104917160	250.0	142.0	272.5	273.0	131.0	279.0	224.6	69.03
LOC104917161	105.5	85.5	90.0	88.0	64.0	97.0	88.3	13.95
LOC104917163	13.5	2.0	5.5	15.5	3.5	2.0	7.0	5.98
LOC104917166	3896.5	4550.5	5787.5	3597.0	6226.5	5694.5	4958.8	1094.05
LOC104917167	1757.5	1453.5	3749.5	1962.0	1623.0	3763.5	2384.8	1075.47
LOC104917168	282.5	262.0	235.0	278.0	276.5	224.5	259.8	24.46
LOC104917171	3.0	3.5	1.0	3.5	4.0	0.0	2.5	1.61
LOC104917173	0.0	0.0	0.0	0.0	0.0	0.0	0.0	0.00
LOC104917174	0.0	0.0	0.0	0.5	0.5	0.0	0.2	0.26
LOC104917175	0.5	0.0	0.0	0.0	0.0	0.0	0.1	0.20
LOC104917176	0.0	0.5	0.0	0.0	0.0	0.5	0.2	0.26
LOC104917177	0.0	0.0	0.0	0.0	0.5	0.0	0.1	0.20
LOC104917178	0.0	0.0	0.0	0.0	0.0	0.0	0.0	0.00
LOC104917179	13.5	10.0	2.5	15.0	10.5	5.0	9.4	4.83
LOC104917180	99.5	52.0	52.5	82.0	60.5	50.0	66.1	20.21
LOC104917181	0.0	0.5	0.0	0.0	1.0	0.5	0.3	0.41
LOC104917182	0.0	0.0	0.0	0.0	0.0	0.0	0.0	0.00
LOC104917183	11.0	12.0	17.5	9.5	16.0	15.5	13.6	3.18
LOC104917184	85.5	56.0	79.0	73.0	61.0	69.0	70.6	11.01
LOC104917185	0.0	0.0	0.0	0.0	0.0	0.0	0.0	0.00

LOC104917186	16.5	19.5	50.5	20.0	23.0	46.5	29.3	15.04
LOC104917187	0.0	2.0	0.5	0.0	0.0	1.5	0.7	0.88
LOC104917188	9.5	18.0	23.0	4.5	20.0	22.0	16.2	7.47
LOC104917189	912.5	980.0	486.5	725.5	884.0	443.0	738.6	228.37
LOC104917195	0.0	0.0	0.0	0.0	0.0	0.0	0.0	0.00
LOC104917196	0.0	0.0	0.0	0.0	0.0	0.0	0.0	0.00
LOC104917197	282.0	122.0	210.0	273.0	145.0	183.5	202.6	65.57
LOC104917199	24.5	18.5	13.5	35.0	23.5	8.0	20.5	9.43
LOC104917200	28.0	26.5	7.5	41.5	45.5	9.0	26.3	15.85
LOC104917201	1.5	0.0	2.5	1.5	1.5	0.5	1.3	0.88
LOC104917203	76.0	43.5	151.5	76.0	47.5	133.5	88.0	44.75
LOC104917204	338.5	265.5	384.5	361.5	239.5	375.5	327.5	60.69
LOC104917205	27.0	44.5	72.0	44.5	42.5	65.5	49.3	16.54
LOC104917206	105.5	92.5	206.5	99.5	85.0	186.0	129.2	52.81
LOC104917208	339.0	215.0	273.5	360.5	236.5	297.0	286.9	56.79
LOC104917209	0.0	0.0	0.0	0.0	0.0	0.0	0.0	0.00
LOC104917210	50.5	32.0	75.0	46.5	24.0	65.0	48.8	19.26
LOC104917211	17.0	20.5	7.5	22.0	18.5	9.5	15.8	5.96
LOC104917212	66.0	51.0	40.0	48.5	56.5	31.0	48.8	12.28
LOC104917213	2.0	0.5	0.0	0.0	0.0	0.0	0.4	0.80
LOC104917214	0.0	0.0	0.0	0.0	0.0	0.0	0.0	0.00
LOC104917216	0.0	0.0	0.0	0.0	0.0	0.0	0.0	0.00
LOC104917217	0.0	0.0	0.0	0.0	0.0	0.5	0.1	0.20
LOC104917218	132.0	208.5	509.0	138.5	198.0	455.0	273.5	165.27
LOC104917219	0.5	105.5	182.5	1.0	130.5	245.5	110.9	97.90
LOC104917220	554.5	315.5	307.0	524.0	328.5	313.5	390.5	115.83
LOC104917221	327.0	224.0	121.5	325.5	231.0	131.5	226.8	89.42
LOC104917222	188.0	150.0	117.5	153.0	149.0	94.5	142.0	32.27
LOC104917223	0.0	0.0	0.0	0.0	0.0	0.0	0.0	0.00
LOC104917224	0.0	0.0	0.0	0.0	0.0	0.0	0.0	0.00
LOC104917226	40.0	90.0	106.0	40.0	71.5	119.5	77.8	33.41
LOC104917227	0.0	0.0	0.0	0.0	0.0	0.0	0.0	0.00
LOC104917228	0.0	0.0	0.0	0.0	0.0	0.0	0.0	0.00
LOC104917229	0.0	0.0	0.0	0.0	0.0	0.0	0.0	0.00
LOC104917230	0.0	0.0	0.0	0.0	0.0	0.0	0.0	0.00
LOC104917231	0.0	0.0	0.0	0.0	0.0	0.0	0.0	0.00
LOC104917232	27.0	2.0	29.0	35.5	3.5	25.0	20.3	14.08
LOC104917234	0.0	0.0	0.0	0.5	0.0	0.5	0.2	0.26
LOC104917235	1.5	4.5	3.0	2.5	3.5	6.0	3.5	1.58
LOC104917237	44.5	54.5	60.5	41.5	50.0	55.0	51.0	7.10
LOC104917238	0.0	0.0	0.0	0.0	0.0	0.0	0.0	0.00
LOC104917239	2.0	0.0	0.0	0.5	0.0	2.0	0.8	0.99
LOC104917240	450.5	302.0	419.0	497.5	270.0	383.0	387.0	87.40
LOC104917241	2.0	0.0	0.0	1.0	0.0	0.0	0.5	0.84
LOC104917242	2.5	0.0	0.5	2.5	0.0	1.5	1.2	1.17
LOC104917243	0.0	0.0	0.0	0.0	0.0	0.0	0.0	0.00
LOC104917244	0.0	0.0	0.0	0.0	0.0	0.0	0.0	0.00
LOC104917245	0.0	0.0	0.0	0.0	0.0	0.0	0.0	0.00
LOC104917246	0.0	0.0	0.0	0.0	0.0	0.0	0.0	0.00
LOC104917247	8.5	19.0	37.5	7.5	14.5	48.5	22.6	16.72
LOC104917248	38.5	52.5	62.0	31.5	49.5	89.0	53.8	20.29
LOC104917249	0.5	1.0	2.0	0.5	0.0	0.0	0.7	0.75
LOC104917250	96.5	72.5	142.0	106.5	82.0	107.0	101.1	24.25
LOC104917251	55.0	58.5	32.5	45.5	66.0	34.5	48.7	13.49

LOC104917252	0.0	0.0	0.0	0.0	0.0	0.0	0.0	0.00
LOC104917253	0.0	0.0	0.0	0.0	0.0	0.0	0.0	0.00
LOC104917254	4.5	3.0	1.0	3.5	2.5	3.0	2.9	1.16
LOC104917255	0.0	0.0	0.0	0.0	0.0	0.0	0.0	0.00
LOC104917256	0.0	0.0	0.0	0.0	0.0	0.0	0.0	0.00
LOC104917257	0.0	0.0	0.0	0.0	0.0	0.0	0.0	0.00
LOC104917258	0.0	0.0	0.0	0.0	0.0	0.0	0.0	0.00
LOC104917259	0.0	0.0	0.0	0.0	0.0	0.0	0.0	0.00
LOC104917260	0.0	0.0	0.0	0.0	0.0	0.0	0.0	0.00
LOC104917261	0.0	0.0	0.0	0.0	0.0	0.0	0.0	0.00
LOC104917262	0.0	0.0	0.0	0.0	0.0	0.0	0.0	0.00
LOC104917263	0.0	0.0	0.0	0.0	0.0	0.0	0.0	0.00
LOC104917264	0.0	0.0	0.0	0.0	0.0	0.0	0.0	0.00
LOC104917265	0.0	0.0	1.0	0.0	0.0	0.0	0.2	0.41
LOC104917266	0.0	0.0	0.0	0.0	0.0	0.0	0.0	0.00
LOC104917267	0.0	0.0	0.0	0.0	0.0	0.0	0.0	0.00
LOC104917268	0.0	0.0	0.0	0.0	0.0	0.0	0.0	0.00
LOC104917270	0.0	0.0	0.0	0.0	0.0	0.0	0.0	0.00
LOC104917271	0.0	0.0	0.0	0.0	0.0	0.0	0.0	0.00
LOC104917272	0.0	0.0	0.0	0.0	0.0	0.0	0.0	0.00
LOC104917273	0.0	0.0	0.0	0.0	0.0	0.0	0.0	0.00
LOC104917274	0.0	0.0	0.0	0.0	0.0	0.0	0.0	0.00
LOC104917275	0.0	0.0	0.0	0.0	0.0	0.0	0.0	0.00
LOC104917276	0.0	0.0	0.0	0.0	0.0	0.0	0.0	0.00
LOC104917277	0.0	0.0	0.0	0.0	0.0	0.0	0.0	0.00
LOC104917278	0.0	0.0	0.0	0.0	0.0	0.0	0.0	0.00
LOC104917279	0.0	0.0	2.0	0.0	1.0	3.0	1.0	1.26
LOC104917280	1.0	0.5	0.0	0.5	0.5	0.0	0.4	0.38
LOC104917281	0.0	0.0	0.0	0.0	0.0	0.0	0.0	0.00
LOC104917282	0.0	0.0	0.0	0.0	0.0	0.0	0.0	0.00
LOC104917284	0.0	0.0	0.0	0.0	0.0	0.0	0.0	0.00
LOC104917285	0.0	0.0	0.0	0.0	0.0	0.0	0.0	0.00
LOC104917286	1.0	0.5	1.5	0.5	3.5	0.5	1.3	1.17
LOC104917287	0.5	2.0	0.0	1.0	1.5	3.5	1.4	1.24
LOC104917288	0.0	0.0	0.0	0.0	0.0	0.0	0.0	0.00
LOC104917289	12.0	3.5	7.0	8.5	3.0	2.5	6.1	3.76
LOC104917290	0.0	0.0	0.0	0.0	0.0	0.0	0.0	0.00
LOC104917291	0.0	0.5	0.0	0.0	1.0	0.5	0.3	0.41
LOC104917292	0.0	0.0	0.0	0.0	0.0	0.0	0.0	0.00
LOC104917293	0.0	0.0	0.0	0.0	0.0	0.0	0.0	0.00
LOC104917294	0.0	0.0	0.0	0.0	0.0	0.0	0.0	0.00
LOC104917295	0.0	0.0	0.0	0.0	0.0	0.0	0.0	0.00
LOC104917296	0.0	0.0	0.0	0.0	0.0	0.0	0.0	0.00
LOC104917297	1.0	1.5	5.0	2.0	1.5	5.0	2.7	1.83
LOC104917298	0.0	0.0	0.0	0.0	0.0	0.0	0.0	0.00
LOC104917299	0.0	0.0	0.0	0.0	0.0	0.0	0.0	0.00
LOC104917300	2.0	1.5	2.0	0.0	2.0	2.5	1.7	0.88
LOC104917301	0.0	0.0	2.0	0.0	0.0	0.5	0.4	0.80
LOC104917302	0.0	1.5	0.0	0.5	0.0	1.0	0.5	0.63
LOC104917303	0.0	0.0	0.0	0.0	0.0	0.0	0.0	0.00
LOC104917304	0.0	0.0	0.0	0.0	0.0	0.0	0.0	0.00
LOC104917305	30.0	39.5	33.5	45.0	45.0	79.0	45.3	17.57
LOC104917306	5.0	2.5	8.0	2.0	3.0	13.0	5.6	4.25
LOC104917307	0.0	1.5	1.0	1.5	0.0	1.0	0.8	0.68

LOC104917308	0.0	0.0	0.0	0.0	0.0	0.0	0.0	0.00
LOC104917309	29.0	92.0	31.0	34.5	113.5	66.0	61.0	35.69
LOC104917310	25.5	63.5	24.0	28.0	67.0	38.5	41.1	19.43
LOC104917311	0.0	0.0	0.0	0.0	0.0	0.0	0.0	0.00
LOC104917312	0.0	0.0	0.5	0.5	0.0	0.0	0.2	0.26
LOC104917313	0.0	0.0	0.0	0.0	0.0	0.0	0.0	0.00
LOC104917314	32.0	37.5	47.0	51.5	49.5	60.0	46.3	10.07
LOC104917315	13.0	40.0	15.5	12.5	38.0	35.5	25.8	13.35
LOC104917316	96.5	60.0	87.0	81.0	48.5	68.5	73.6	17.89
LOC104917317	7.0	1.5	6.5	4.0	6.5	7.5	5.5	2.30
LOC104917318	0.0	0.0	0.0	0.0	0.0	0.0	0.0	0.00
LOC104917319	0.0	1.0	1.0	0.0	1.5	0.5	0.7	0.61
LOC104917321	102.0	174.5	117.0	110.5	171.0	140.0	135.8	31.27
LOC104917322	194.5	225.5	146.5	167.0	225.0	158.0	186.1	34.23
LOC104917323	6.0	2.0	6.0	4.5	4.5	6.0	4.8	1.57
LOC104917324	116.0	266.0	240.5	142.5	299.5	224.5	214.8	71.47
LOC104917325	0.0	0.0	0.5	0.0	0.0	0.0	0.1	0.20
LOC104917326	14.5	4.5	13.5	18.5	9.5	11.5	12.0	4.76
LOC104917328	22.5	9.5	18.0	35.5	10.5	21.0	19.5	9.49
LOC104917329	11.5	9.5	21.0	9.5	12.5	15.0	13.2	4.36
LOC104917330	0.0	0.0	0.0	0.0	0.0	0.0	0.0	0.00
LOC104917331	0.0	0.0	0.0	0.0	0.0	0.0	0.0	0.00
LOC104917332	1.0	0.0	0.0	0.0	0.5	0.0	0.3	0.42
LOC104917333	0.0	0.0	0.0	0.0	0.0	0.0	0.0	0.00
LOC104917334	504.5	348.5	404.0	462.0	383.0	516.0	436.3	68.20
LOC104917335	11.0	1.5	8.5	11.0	1.5	5.5	6.5	4.37
LOC104917336	0.0	0.0	0.0	0.5	0.0	0.5	0.2	0.26
LOC104917337	9.5	14.0	6.5	4.5	11.0	10.0	9.3	3.36
LOC104917338	9.0	3.5	6.0	7.5	2.0	7.0	5.8	2.62
LOC104917339	238.5	56.0	71.0	204.5	51.0	62.5	113.9	84.29
LOC104917340	5.0	5.5	7.0	3.5	5.0	5.5	5.3	1.13
LOC104917341	146.0	44.5	105.5	156.0	52.0	82.0	97.7	46.81
LOC104917342	111.5	80.0	71.5	110.5	59.0	57.5	81.7	24.19
LOC104917343	0.0	0.0	0.0	0.5	0.0	0.0	0.1	0.20
LOC104917344	4.5	2.5	4.5	7.5	3.5	5.5	4.7	1.72
LOC104917345	19.0	43.5	115.5	12.0	35.5	121.0	57.8	48.23
LOC104917346	0.5	3.5	1.5	2.0	1.5	0.0	1.5	1.22
LOC104917347	1.0	3.0	7.0	0.5	5.5	1.0	3.0	2.70
LOC104917348	1.0	0.0	2.5	2.0	0.0	0.5	1.0	1.05
LOC104917350	0.0	0.0	0.0	0.0	0.0	0.0	0.0	0.00
LOC104917351	0.0	0.0	0.0	0.0	0.5	0.0	0.1	0.20
LOC104917352	20.5	23.5	13.5	14.0	24.0	18.5	19.0	4.54
LOC104917353	0.0	0.0	0.0	0.0	0.0	0.0	0.0	0.00
LOC104917354	0.0	0.0	0.0	0.0	0.0	0.0	0.0	0.00
LOC104917355	0.0	0.0	0.0	0.5	0.0	0.5	0.2	0.26
LOC104917357	1.0	2.5	1.0	0.0	1.0	5.0	1.8	1.78
LOC104917358	85.5	80.0	48.5	74.5	87.0	61.5	72.8	15.07
LOC104917359	672.0	777.5	287.5	620.0	534.0	294.5	530.9	201.89
LOC104917361	70.5	159.5	96.0	69.0	168.0	135.0	116.3	43.90
LOC104917362	0.0	0.0	0.0	0.0	0.0	0.0	0.0	0.00
LOC104917363	18.0	3.0	19.5	39.0	4.0	20.5	17.3	13.17
LOC104917364	0.5	0.0	0.0	0.0	0.0	0.0	0.1	0.20
LOC104917365	23.0	10.0	18.0	29.0	9.5	10.5	16.7	8.10
LOC104917366	24.0	8.5	5.5	29.5	4.5	6.0	13.0	10.87

LOC104917367	396.0	532.5	599.0	279.0	503.5	516.5	471.1	114.70
LOC104917369	0.0	0.0	6.0	0.0	0.0	6.0	2.0	3.10
LOC104917370	16.0	24.0	6.5	26.0	20.5	12.0	17.5	7.44
LOC104917371	0.0	0.0	0.0	0.0	0.0	0.0	0.0	0.00
LOC104917372	0.0	0.0	0.0	0.0	0.0	0.0	0.0	0.00
LOC104917373	11.5	15.5	21.0	12.5	10.5	22.5	15.6	5.08
LOC104917374	0.0	0.0	0.0	0.0	0.0	0.0	0.0	0.00
LOC104917375	0.0	0.0	0.0	0.0	0.0	0.0	0.0	0.00
LOC104917376	0.0	0.0	0.0	0.0	0.0	0.0	0.0	0.00
LOC104917377	0.0	0.0	0.0	0.0	0.0	0.0	0.0	0.00
LOC104917378	0.0	0.0	0.0	0.0	0.0	0.0	0.0	0.00
LOC104917379	0.0	0.0	0.0	0.0	0.0	0.0	0.0	0.00
LOC104917380	0.0	0.0	0.0	0.0	0.0	0.0	0.0	0.00
LOC104917381	0.0	0.0	0.5	0.0	0.0	0.0	0.1	0.20
LOC104917382	0.0	0.0	0.0	0.0	0.0	0.0	0.0	0.00
LOC104917383	1.0	1.0	1.0	0.0	1.0	0.0	0.7	0.52
LOC104917384	5.0	5.5	2.5	6.0	4.0	0.5	3.9	2.08
LOC104917385	0.0	0.0	0.0	0.0	0.0	0.0	0.0	0.00
LOC104917386	0.0	0.0	0.0	0.0	0.0	0.0	0.0	0.00
LOC104917387	0.0	0.0	0.0	0.0	0.0	0.0	0.0	0.00
LOC104917388	91.0	92.5	237.0	73.5	87.0	250.0	138.5	81.71
LOC104917389	0.0	0.0	0.0	0.0	0.0	0.0	0.0	0.00
LOC104917390	0.0	0.0	0.0	0.0	0.0	0.0	0.0	0.00
LOC104917391	0.0	0.0	0.0	0.0	0.0	0.0	0.0	0.00
LOC104917392	40.0	29.0	28.0	44.0	36.0	25.5	33.8	7.39
LOC104917393	1.0	1.0	0.0	0.0	0.0	1.0	0.5	0.55
LOC104917394	0.5	0.5	0.0	0.0	0.5	0.5	0.3	0.26
LOC104917395	2.5	0.0	0.0	2.5	1.0	0.5	1.1	1.16
LOC104917396	0.0	0.0	0.0	0.0	0.0	0.0	0.0	0.00
LOC104917397	1.0	0.0	0.0	1.0	0.0	0.0	0.3	0.52
LOC104917398	0.0	0.0	0.0	0.0	0.0	0.0	0.0	0.00
LOC104917399	0.0	0.0	0.0	0.0	0.0	0.0	0.0	0.00
LOC104917400	0.0	0.0	0.0	0.0	0.0	0.0	0.0	0.00
LOC104917401	0.0	0.0	0.0	0.0	0.0	0.0	0.0	0.00
LOC104917402	36.5	20.5	18.0	30.0	35.5	21.5	27.0	8.06
LOC104917403	7.5	20.5	21.5	7.5	24.5	34.0	19.3	10.27
LOC104917404	0.0	0.0	0.0	0.0	0.5	0.0	0.1	0.20
LOC104917405	29.0	7.0	14.0	27.5	10.5	15.0	17.2	9.05
LOC104917406	0.5	0.0	0.0	0.0	0.0	0.0	0.1	0.20
LOC104917408	39.5	12.0	88.0	30.5	20.5	71.0	43.6	29.81
LOC104917409	21.0	18.0	23.5	31.0	18.5	15.0	21.2	5.61
LOC104917410	68.5	50.5	47.5	64.5	49.0	33.5	52.3	12.66
LOC104917411	2.5	1.5	2.0	1.5	2.0	0.5	1.7	0.68
LOC104917412	112.0	177.5	83.0	101.0	194.0	54.0	120.3	54.65
LOC104917413	396.0	202.0	313.5	360.5	171.0	279.5	287.1	88.00
LOC104917414	28.5	2.5	17.5	27.5	2.0	8.5	14.4	11.92
LOC104917415	1.5	1.0	1.0	3.5	3.0	4.0	2.3	1.33
LOC104917416	0.5	0.0	0.0	0.0	0.0	0.0	0.1	0.20
LOC104917417	83.5	25.0	47.0	81.5	30.5	44.5	52.0	25.04
LOC104917418	22.0	8.5	22.0	28.0	4.5	24.5	18.3	9.45
LOC104917419	528.5	328.5	190.5	445.0	322.5	235.0	341.7	126.77
LOC104917420	3.5	1.5	2.5	2.5	2.5	1.0	2.3	0.88
LOC104917421	1.0	1.5	1.0	1.5	3.0	0.5	1.4	0.86
LOC104917422	94.0	131.0	67.5	82.5	120.5	59.5	92.5	28.57

LOC104917423	1.5	0.5	0.5	1.5	0.0	2.0	1.0	0.77
LOC104917424	0.0	0.5	1.5	3.0	3.5	3.5	2.0	1.55
LOC104917425	13.5	3.5	24.0	9.0	3.5	22.0	12.6	8.92
LOC104917426	76.0	16.5	119.0	78.0	15.0	97.5	67.0	42.64
LOC104917428	32.0	19.5	18.5	29.0	21.5	14.0	22.4	6.79
LOC104917430	0.0	0.5	1.0	1.0	0.5	2.0	0.8	0.68
LOC104917431	28.0	18.5	29.0	29.5	19.0	33.0	26.2	5.99
LOC104917432	150.5	136.5	112.0	145.5	181.5	121.5	141.3	24.46
LOC104917433	187.0	35.0	109.0	216.5	33.5	92.5	112.3	76.19
LOC104917434	50.5	51.5	71.0	70.5	52.5	64.0	60.0	9.65
LOC104917435	56.5	27.5	67.5	61.5	32.5	64.0	51.6	17.17
LOC104917436	2.5	0.0	2.5	3.5	0.5	2.5	1.9	1.36
LOC104917437	0.0	0.0	0.0	0.0	0.0	0.0	0.0	0.00
LOC104917438	0.0	0.0	0.0	0.0	0.0	0.0	0.0	0.00
LOC104917440	76.0	30.5	130.5	85.5	31.0	88.5	73.7	38.16
LOC104917441	41.0	24.5	20.0	41.0	30.5	11.0	28.0	11.91
LOC104917442	9.0	10.0	1.5	10.0	9.5	1.0	6.8	4.34
LOC104917443	0.0	0.0	0.0	0.0	0.0	0.0	0.0	0.00
LOC104917444	0.0	0.0	0.0	0.0	0.0	0.0	0.0	0.00
LOC104917445	0.0	0.0	0.0	0.0	0.0	0.0	0.0	0.00
LOC104917446	5.0	1.0	1.5	1.5	1.5	1.0	1.9	1.53
LOC104917447	0.0	0.0	0.0	0.0	0.0	0.0	0.0	0.00
LOC104917449	0.0	0.0	0.0	0.0	0.0	0.0	0.0	0.00
LOC104917450	0.0	0.0	0.0	0.0	0.0	0.0	0.0	0.00
LOC104917451	0.0	0.0	0.0	0.0	0.0	0.0	0.0	0.00
LOC104917452	0.0	0.0	0.0	0.0	0.0	0.0	0.0	0.00
LOC104917453	0.0	0.0	0.0	0.0	0.0	0.0	0.0	0.00
LOC104917454	1.0	0.5	0.5	0.5	2.0	0.0	0.8	0.69
LOC104917455	3.0	0.5	2.5	3.0	2.5	1.0	2.1	1.07
LOC104917456	3.0	17.0	7.0	2.5	14.0	6.0	8.3	5.95
LOC104917457	0.5	0.5	0.0	0.5	0.5	0.0	0.3	0.26
LOC104917458	30.0	13.5	11.0	14.0	13.0	14.5	16.0	6.96
LOC104917459	1.0	0.5	1.0	1.0	0.5	1.5	0.9	0.38
LOC104917461	0.0	0.0	0.0	0.0	0.0	0.0	0.0	0.00
LOC104917462	58.0	20.5	86.5	62.5	25.0	67.5	53.3	25.64
LOC104917463	7.5	13.5	27.5	13.0	15.5	17.5	15.8	6.66
LOC104917464	0.0	0.0	0.0	0.5	0.5	0.0	0.2	0.26
LOC104917465	1.0	0.5	14.0	2.0	2.5	5.5	4.3	5.09
LOC104917466	701.0	508.5	662.0	672.0	614.0	608.0	627.6	68.26
LOC104917467	8.5	2.0	1.5	7.5	2.5	0.0	3.7	3.47
LOC104917468	6.5	9.0	22.5	3.5	10.0	15.0	11.1	6.78
LOC104917469	0.0	0.0	0.0	0.0	0.0	0.0	0.0	0.00
LOC104917470	0.0	0.0	0.0	0.0	0.0	0.0	0.0	0.00
LOC104917471	1.5	0.0	0.0	0.0	0.0	0.5	0.3	0.61
LOC104917472	32.0	18.5	49.5	32.5	23.5	31.0	31.2	10.56
LOC104917473	18.0	7.0	13.5	9.5	7.5	7.5	10.5	4.39
LOC104917474	9.5	2.5	9.5	5.5	2.0	4.5	5.6	3.29
LOC104917475	6.5	1.0	12.5	3.5	2.0	3.5	4.8	4.19
LOC104917477	21.0	25.5	24.0	22.5	22.5	10.0	20.9	5.56
LOC104917479	10.0	3.5	11.5	11.0	5.0	5.5	7.8	3.47
LOC104917480	0.0	0.0	0.0	0.0	0.0	0.0	0.0	0.00
LOC104917481	0.0	0.0	0.0	0.0	0.0	0.0	0.0	0.00
LOC104917482	0.0	0.0	0.0	0.0	0.5	0.0	0.1	0.20
LOC104917483	0.0	0.0	0.0	0.0	0.0	0.0	0.0	0.00

LOC104917484	286.5	211.0	119.0	253.0	222.0	127.5	203.2	67.31
LOC104917485	0.0	0.0	0.0	0.0	0.0	0.0	0.0	0.00
LOC104917486	0.0	0.0	0.0	0.0	0.0	1.0	0.2	0.41
LOC104917487	35.0	13.0	51.5	32.0	15.0	39.5	31.0	14.76
LOC104917488	0.0	0.0	0.0	0.0	0.0	0.5	0.1	0.20
LOC104917489	0.0	0.0	0.0	0.0	0.5	0.0	0.1	0.20
LOC104917490	0.0	0.5	0.0	0.0	0.0	0.5	0.2	0.26
LOC104917491	0.0	0.0	0.5	0.0	0.0	0.0	0.1	0.20
LOC104917492	48.5	29.5	21.5	51.0	27.0	24.5	33.7	12.76
LOC104917493	8.0	3.5	7.0	18.5	4.5	10.0	8.6	5.40
LOC104917494	15.5	4.0	3.5	9.5	3.0	1.5	6.2	5.33
LOC104917495	426.0	78.0	70.0	371.0	81.0	50.0	179.3	171.00
LOC104917496	2.5	1.5	0.5	4.0	2.5	0.5	1.9	1.36
LOC104917498	3.0	6.0	6.0	3.5	6.0	3.5	4.7	1.47
LOC104917499	12.5	4.5	3.5	12.0	6.0	2.5	6.8	4.36
LOC104917500	5.5	5.0	9.5	2.5	4.0	8.5	5.8	2.68
LOC104917501	297.5	361.0	264.5	208.0	438.5	270.5	306.7	81.53
LOC104917502	0.0	0.0	0.0	0.0	0.0	0.0	0.0	0.00
LOC104917503	410.0	421.5	396.0	373.5	515.5	385.0	416.9	51.24
LOC104917505	3.0	10.0	20.0	6.5	21.5	25.0	14.3	9.01
LOC104917506	0.0	0.5	2.5	0.0	0.5	2.5	1.0	1.18
LOC104917507	0.0	1.5	6.5	0.5	2.0	9.0	3.3	3.64
LOC104917508	0.0	0.0	0.5	1.5	0.0	3.0	0.8	1.21
LOC104917509	6.5	5.0	7.5	5.0	3.0	11.0	6.3	2.75
LOC104917510	0.0	0.0	0.0	0.0	0.0	0.0	0.0	0.00
LOC104917512	34.0	9.5	59.5	48.5	12.0	46.0	34.9	20.41
LOC104917513	16.5	4.0	23.0	19.5	6.0	25.0	15.7	8.78
LOC104917514	0.0	0.0	0.0	0.0	0.0	0.0	0.0	0.00
LOC104917517	372.0	306.5	418.5	391.5	291.5	342.5	353.8	49.37
LOC104917519	0.0	0.0	1.0	1.0	0.0	0.0	0.3	0.52
LOC104917520	78.0	80.0	84.5	80.5	89.0	84.0	82.7	3.97
LOC104917521	0.0	2.0	3.5	0.0	2.5	5.5	2.3	2.12
LOC104917522	2.5	0.5	3.0	0.5	0.0	0.5	1.2	1.25
LOC104917523	0.0	0.0	0.0	0.0	0.0	0.0	0.0	0.00
LOC104917525	6.5	5.0	10.5	12.0	4.5	6.5	7.5	3.05
LOC104917526	3.5	1.5	1.0	2.0	1.0	1.5	1.8	0.94
LOC104917527	201.0	115.0	127.0	175.5	136.0	82.0	139.4	42.79
LOC104917528	0.0	0.0	0.0	0.0	0.0	0.0	0.0	0.00
LOC104917530	0.5	2.0	1.5	0.0	0.5	1.5	1.0	0.77
LOC104917532	3.0	4.0	9.5	7.0	7.5	8.5	6.6	2.56
LOC104917533	28.0	71.5	35.5	37.0	92.0	65.0	54.8	25.20
LOC104917534	208.5	229.0	91.5	199.5	239.5	91.5	176.6	67.42
LOC104917535	0.0	0.0	0.0	0.0	0.0	0.0	0.0	0.00
LOC104917537	139.0	76.5	137.5	166.5	93.0	111.5	120.7	33.23
LOC104917538	2.5	2.5	30.5	1.5	7.0	13.5	9.6	11.18
LOC104917539	8.0	5.0	0.5	6.0	5.0	3.5	4.7	2.52
LOC104917540	7.5	14.0	20.0	3.5	10.0	14.5	11.6	5.83
LOC104917541	0.5	0.5	10.0	0.5	0.5	3.5	2.6	3.83
LOC104917542	0.0	0.0	0.0	0.0	0.0	0.0	0.0	0.00
LOC104917543	2.5	0.0	0.0	1.5	1.0	0.0	0.8	1.03
LOC104917544	0.0	0.0	0.0	0.0	0.0	0.0	0.0	0.00
LOC104917545	5.5	2.5	6.0	11.5	4.0	3.5	5.5	3.21
LOC104917546	100.0	53.5	47.0	105.5	56.5	63.0	70.9	25.25
LOC104917547	4.5	3.5	2.5	2.0	4.0	2.5	3.2	0.98

LOC104917548	63.5	34.0	35.5	60.5	46.5	31.5	45.3	13.98
LOC104917549	1.0	1.5	5.0	1.0	1.0	3.0	2.1	1.63
LOC104917550	1.5	0.0	0.5	0.0	0.0	0.0	0.3	0.61
LOC104917551	0.5	0.0	1.0	0.5	0.5	0.5	0.5	0.32
LOC104917553	59.0	22.0	87.5	75.5	40.0	78.0	60.3	25.16
LOC104917554	3.5	1.5	3.0	4.0	3.5	3.0	3.1	0.86
LOC104917555	1.0	0.0	0.0	1.5	0.0	0.5	0.5	0.63
LOC104917556	0.5	0.5	1.0	1.0	0.5	0.0	0.6	0.38
LOC104917557	10.5	4.0	8.0	16.0	2.5	10.5	8.6	4.91
LOC104917558	10.5	3.5	5.5	11.0	5.5	5.0	6.8	3.13
LOC104917559	5633.5	12044.5	9603.5	5947.5	15033.0	7690.0	9325.3	3681.37
LOC104917561	1.0	1.5	0.5	2.0	3.5	0.5	1.5	1.14
LOC104917562	331.0	318.5	355.5	264.0	366.5	329.0	327.4	35.87
LOC104917563	0.0	0.5	0.0	0.0	0.0	0.0	0.1	0.20
LOC104917564	258.5	1501.0	1244.5	479.0	1970.5	1494.0	1157.9	658.53
LOC104917565	36.0	91.0	313.0	90.0	98.0	335.5	160.6	128.91
LOC104917566	0.0	0.0	0.0	0.0	0.0	0.0	0.0	0.00
LOC104917567	0.0	0.0	0.0	0.0	0.0	0.0	0.0	0.00
LOC104917568	158.0	85.5	86.5	142.5	96.5	99.0	111.3	31.00
LOC104917569	0.0	0.0	0.0	0.0	0.5	0.0	0.1	0.20
LOC104917571	0.0	0.0	0.0	0.0	0.0	0.0	0.0	0.00
LOC104917572	0.0	0.0	0.0	0.0	0.0	0.0	0.0	0.00
LOC104917573	0.0	0.5	0.0	0.0	0.0	0.0	0.1	0.20
LOC104917574	4.0	21.5	8.0	5.0	22.0	11.0	11.9	8.00
LOC104917576	0.0	0.0	0.0	0.0	0.0	0.0	0.0	0.00
LOC104917577	50.0	32.0	33.0	36.5	37.5	39.0	38.0	6.46
LOC104917578	0.5	0.0	1.0	0.5	0.0	1.0	0.5	0.45
LOC104917579	0.5	0.0	0.0	0.0	0.0	0.5	0.2	0.26
LOC104917580	27.5	3.0	43.5	31.0	6.5	32.5	24.0	15.88
LOC104917581	2.0	0.0	2.0	2.5	2.0	2.0	1.8	0.88
LOC104917582	9.5	4.0	3.0	8.0	6.0	5.0	5.9	2.46
LOC104917583	10.0	1.5	7.5	7.5	3.0	6.5	6.0	3.16
LOC104917584	163.5	87.5	59.0	163.0	100.5	69.0	107.1	45.82
LOC104917585	263.0	215.5	263.0	260.0	240.5	235.0	246.2	19.26
LOC104917587	57.5	24.5	129.0	65.0	27.0	141.0	74.0	50.05
LOC104917588	0.0	0.0	0.0	0.0	0.0	0.0	0.0	0.00
LOC104917589	0.0	0.0	0.0	0.0	0.0	0.0	0.0	0.00
LOC104917590	0.0	1.0	2.0	0.0	2.0	0.5	0.9	0.92
LOC104917591	0.0	0.0	0.0	0.0	0.0	0.5	0.1	0.20
LOC104917592	0.0	0.5	0.0	0.0	0.0	0.0	0.1	0.20
LOC104917593	1.0	0.0	0.0	0.0	0.5	0.0	0.3	0.42
LOC104917594	157.0	69.5	89.0	174.0	75.0	90.0	109.1	44.74
LOC104917595	2.0	7.0	3.0	4.0	8.0	3.0	4.5	2.43
LOC104917596	5.0	4.5	1.0	2.0	1.5	0.5	2.4	1.88
LOC104917597	0.0	0.0	0.0	0.0	0.0	0.0	0.0	0.00
LOC104917598	102.0	35.0	57.5	82.5	28.0	48.0	58.8	28.50
LOC104917599	0.0	0.0	0.0	0.0	0.0	0.0	0.0	0.00
LOC104917600	120.0	72.0	140.5	136.5	104.5	127.5	116.8	25.43
LOC104917602	891.0	637.5	326.5	777.0	720.5	330.0	613.8	236.01
LOC104917603	174.5	33.5	39.0	203.0	38.5	18.5	84.5	81.59
LOC104917604	216.0	219.0	148.0	181.0	238.5	155.0	192.9	37.12
LOC104917605	14.5	4.5	38.0	12.0	10.0	36.5	19.3	14.33
LOC104917606	0.5	0.5	1.0	0.0	0.5	1.0	0.6	0.38
LOC104917607	1.0	1.0	0.0	1.0	1.0	0.0	0.7	0.52

LOC104917608	15.0	3.0	21.0	16.0	6.0	23.0	14.0	8.00
LOC104917609	0.0	0.0	0.0	0.0	0.0	0.0	0.0	0.00
LOC104917610	0.5	0.5	0.0	0.5	0.0	0.0	0.3	0.27
LOC104917611	5.5	3.0	8.5	7.0	5.0	7.5	6.1	1.99
LOC104917612	55.5	39.0	57.0	47.0	59.5	65.0	53.8	9.34
LOC104917613	84.0	155.5	298.0	100.0	174.0	308.0	186.6	96.23
LOC104917614	0.0	0.0	0.0	0.0	0.0	0.0	0.0	0.00
LOC104917615	0.0	0.0	0.0	0.0	0.0	0.0	0.0	0.00
LOC104917616	1.5	0.5	0.5	0.0	0.5	1.5	0.8	0.61
LOC104917618	7.5	3.5	27.0	13.0	6.0	16.5	12.3	8.65
LOC104917620	54.5	27.0	15.0	37.5	25.5	15.0	29.1	15.04
LOC104917621	16.0	15.0	60.5	25.5	10.5	39.0	27.8	18.99
LOC104917622	0.0	0.0	0.0	0.0	0.0	0.0	0.0	0.00
LOC654833	6287.5	11177.0	5362.5	6042.0	11508.0	5023.5	7566.8	2961.61
LOC678666	0.0	0.0	0.0	0.0	0.0	0.0	0.0	0.00
LOC723978	1607.5	1531.5	831.5	1358.0	1644.0	745.5	1286.3	398.92
LOC723980	64.0	111.0	135.0	98.5	140.0	230.0	129.8	56.27
LOC723981	3021.0	3686.5	2012.5	2742.0	3745.5	1797.0	2834.1	818.69
LOC723983	3182.0	3373.5	2071.5	2710.5	3209.0	1857.0	2733.9	639.36
LOH12CR1	247.0	177.0	246.0	235.5	174.0	198.0	212.9	34.04
LONP1	788.0	527.5	937.5	754.0	552.0	776.0	722.5	155.86
LONRF2	854.0	738.5	1072.0	879.0	946.0	1099.0	931.4	137.13
LONRF3	128.5	146.5	134.5	100.0	156.5	129.0	132.5	19.29
LOXHD1	9.0	83.0	37.5	18.5	110.5	40.0	49.8	39.19
LOXL1	1039.5	1165.5	1266.0	1023.0	1294.5	1827.0	1269.3	295.27
LOXL2	227.0	118.0	762.0	269.5	122.0	536.5	339.2	257.39
LOXL3	1703.5	2244.5	2487.0	2013.0	2325.5	3342.5	2352.7	556.25
LOXL4	6.5	73.5	134.5	6.5	62.0	100.0	63.8	50.94
LPAR1	6.5	9.0	28.0	11.5	9.0	30.5	15.8	10.61
LPAR2	91.0	79.5	42.5	92.0	74.5	60.0	73.3	19.12
LPAR3	0.0	0.0	0.0	0.0	0.0	0.0	0.0	0.00
LPAR4	18.0	30.5	44.0	22.0	45.5	92.5	42.1	27.11
LPAR5	0.0	0.0	0.0	0.0	1.0	0.0	0.2	0.41
LPAR6	3.5	4.0	3.0	2.0	3.5	3.5	3.3	0.69
LPCAT1	168.0	84.0	76.5	145.0	90.0	69.5	105.5	40.76
LPCAT2	1067.5	356.5	817.0	1093.5	409.5	643.0	731.2	317.30
LPCAT3	621.0	331.5	483.0	638.5	365.5	444.5	480.7	127.62
LPGAT1	583.5	312.5	399.5	544.0	346.5	367.5	425.6	111.41
LPHN2	429.5	175.0	317.5	376.5	192.0	257.5	291.3	101.56
LPIN2	202.5	80.0	238.0	244.0	90.5	186.0	173.5	71.78
LPL	1815.0	2493.5	2716.5	1653.5	3113.5	2349.0	2356.8	549.35
LPO	0.0	0.0	0.5	0.0	0.0	0.0	0.1	0.20
LPXN	112.0	198.0	69.5	106.0	215.5	73.5	129.1	62.74
LRAT	126.0	42.0	19.5	153.0	59.5	29.0	71.5	55.02
LRBA	208.5	87.0	165.5	178.5	82.0	136.0	142.9	50.92
LRCH2	93.5	77.0	204.0	127.0	84.0	174.5	126.7	52.26
LRCH3	1102.0	489.5	974.0	998.5	485.0	926.5	829.3	271.07
LRFN1	38.5	38.0	60.0	52.0	29.0	65.5	47.2	14.24
LRFN2	0.5	0.5	0.5	0.5	1.0	0.5	0.6	0.20
LRFN5	107.5	68.5	219.0	136.5	75.5	215.5	137.1	66.68
LRG1	3.0	4.5	5.0	3.5	10.0	7.5	5.6	2.67
LRGUK	10.0	5.0	1.0	9.5	4.5	2.5	5.4	3.65
LRIF1	404.0	252.0	282.5	392.5	309.5	297.0	322.9	61.53
LRIG1	688.0	557.5	587.5	734.5	694.0	571.0	638.8	75.45

LRIG2	836.0	570.0	634.0	819.5	708.0	799.0	727.8	108.88
LRIT1	0.0	0.0	0.0	0.0	0.0	0.0	0.0	0.00
LRIT2	0.0	0.0	0.5	0.0	0.0	0.0	0.1	0.20
LRIT3	2.0	4.0	6.5	2.0	5.5	10.0	5.0	3.05
LRMP	163.0	246.0	225.5	167.5	228.5	154.5	197.5	40.09
LRP11	354.0	124.0	133.5	420.0	125.0	169.5	221.0	131.32
LRP12	907.0	832.5	1392.5	875.0	951.5	1284.0	1040.4	236.47
LRP2	1.0	0.0	1.0	0.0	1.5	1.0	0.8	0.61
LRP2BP	238.0	180.5	116.5	197.5	204.5	153.5	181.8	42.40
LRP3	117.0	64.5	201.5	135.5	67.5	128.0	119.0	50.57
LRP4	267.5	164.5	542.0	260.5	187.5	560.5	330.4	175.79
LRP5	306.5	144.5	205.0	264.5	137.0	201.5	209.8	66.33
LRP6	39.0	15.5	94.5	49.5	19.5	102.0	53.3	37.04
LRP8	143.5	78.0	151.0	150.0	86.0	132.5	123.5	32.91
LRPAP1	375.0	173.5	296.0	430.5	225.5	318.5	303.2	94.35
LRPPRC	1883.5	1208.5	1457.0	1901.0	1269.0	1147.5	1477.8	337.44
LRR1	206.0	121.0	130.0	208.0	118.5	103.0	147.8	46.72
LRRC1	35.5	18.0	31.0	24.0	18.0	19.0	24.3	7.45
LRRC10	0.0	0.0	0.0	0.0	0.0	0.0	0.0	0.00
LRRC10B	37.0	82.5	60.0	31.0	76.5	61.5	58.1	20.63
LRRC14	103.5	106.0	118.0	95.0	121.5	146.0	115.0	18.03
LRRC14B	1.0	37.5	16.0	0.5	37.5	16.5	18.2	16.50
LRRC15	181.5	288.5	552.5	229.5	294.5	391.0	322.9	132.71
LRRC17	346.5	207.5	331.0	199.5	171.5	391.0	274.5	92.38
LRRC18	0.0	0.0	0.0	0.0	0.0	0.0	0.0	0.00
LRRC19	9.0	8.5	4.0	5.0	8.5	2.5	6.3	2.77
LRRC20	683.5	564.0	423.0	675.5	597.0	431.5	562.4	114.21
LRRC23	178.0	97.0	69.5	157.5	112.5	70.5	114.2	45.06
LRRC26	0.0	0.0	0.5	0.0	0.5	1.0	0.3	0.41
LRRC28	417.5	280.5	348.0	388.5	316.5	308.0	343.2	51.92
LRRC30	0.0	1.0	2.0	0.0	0.0	2.0	0.8	0.98
LRRC31	8.0	5.5	7.5	14.0	6.5	4.5	7.7	3.36
LRRC32	37.0	158.0	174.5	51.5	221.0	247.0	148.2	86.68
LRRC34	7.5	3.5	5.5	5.5	4.0	4.5	5.1	1.43
LRRC37A3	408.5	350.5	217.0	384.0	424.0	242.5	337.8	87.63
LRRC38	278.5	264.5	325.0	261.5	277.5	299.0	284.3	23.93
LRRC39	94.0	113.0	94.5	77.0	126.0	76.0	96.8	19.76
LRRC3B	0.0	0.0	0.0	0.0	0.0	0.0	0.0	0.00
LRRC3C	0.5	0.5	0.0	0.0	1.0	0.0	0.3	0.41
LRRC4	20.0	14.5	66.0	17.0	10.0	40.5	28.0	21.41
LRRC42	458.5	353.0	303.5	434.5	416.0	331.5	382.8	62.15
LRRC43	23.5	16.0	15.0	20.0	19.0	12.5	17.7	3.95
LRRC45	380.5	317.0	300.5	338.0	407.0	373.0	352.7	40.86
LRRC46	30.0	30.0	29.5	26.5	31.0	31.0	29.7	1.66
LRRC47	491.5	317.5	287.5	449.0	338.0	269.0	358.8	90.59
LRRC48	3.5	1.0	3.5	3.5	3.0	2.0	2.8	1.04
LRRC49	220.0	136.0	199.5	220.5	178.0	204.0	193.0	32.01
LRRC4B	9.0	4.5	25.5	4.0	4.0	11.5	9.8	8.31
LRRC4C	10.5	9.5	7.5	17.0	17.0	6.0	11.3	4.72
LRRC52	111.0	62.0	49.0	100.0	51.0	36.0	68.2	30.27
LRRC55	0.0	0.5	0.0	0.0	0.0	0.0	0.1	0.20
LRRC56	8.5	11.0	2.5	7.0	8.5	3.0	6.8	3.36
LRRC57	399.5	268.0	284.0	397.5	315.5	268.5	322.2	61.60
LRRC58	244.5	94.5	251.5	272.0	114.0	273.0	208.3	81.56

LRRC59	1397.0	1084.5	706.0	1372.0	1210.5	792.0	1093.7	291.38
LRRC6	0.0	0.5	0.0	0.5	0.0	0.0	0.2	0.26
LRRC61	85.0	115.0	68.0	70.5	104.0	70.0	85.4	19.92
LRRC66	2972.5	2643.0	2944.5	2957.0	3176.0	3021.5	2952.4	173.77
LRRC7	57.0	59.5	34.0	59.0	58.5	59.0	54.5	10.08
LRRC70	23.5	13.5	8.0	27.5	18.0	8.5	16.5	7.97
LRRC71	145.5	109.0	64.5	136.5	116.5	69.0	106.8	33.75
LRRC72	32.5	11.5	9.0	24.0	20.0	7.0	17.3	9.92
LRRC73	125.5	159.0	143.0	121.0	169.5	166.5	147.4	20.90
LRRC74A	10.0	4.0	1.0	17.5	6.0	5.0	7.3	5.81
LRRC75B	184.5	327.5	173.5	222.5	331.0	214.0	242.2	69.85
LRRC8A	483.0	279.0	517.5	455.0	291.0	488.0	418.9	105.68
LRRC8B	301.0	135.0	199.0	299.0	144.5	124.0	200.4	81.34
LRRC8C	163.0	58.0	116.5	204.5	86.5	123.5	125.3	52.55
LRRC8D	804.0	452.0	472.5	832.0	452.0	373.0	564.3	199.68
LRRCC1	172.0	83.5	128.5	154.5	81.5	87.5	117.9	39.53
LRRD1	6.0	1.5	3.0	4.0	0.5	3.0	3.0	1.92
LRRFIP1	1098.0	1057.5	1331.5	1144.5	1266.0	1231.0	1188.1	105.40
LRRFIP2	1639.5	1897.0	1801.0	1628.0	2357.5	2071.0	1899.0	279.35
LRRIQ1	0.0	0.5	0.0	0.5	0.0	0.0	0.2	0.26
LRRIQ4	2.5	1.0	0.0	1.0	0.5	1.5	1.1	0.86
LRRK1	111.0	80.5	104.0	116.5	86.0	84.0	97.0	15.41
LRRK2	416.5	217.5	237.0	356.0	215.5	196.5	273.2	90.57
LRRN1	12.0	4.0	8.5	13.0	3.5	9.5	8.4	3.97
LRRN2	0.5	2.0	2.0	0.5	0.5	0.5	1.0	0.77
LRRN3	1.5	3.5	4.0	0.5	3.0	10.0	3.8	3.33
LRRN4	67.5	28.5	128.5	67.5	32.0	87.5	68.6	37.14
LRRTM1	0.0	0.0	0.0	0.0	0.0	0.0	0.0	0.00
LRRTM2	3.5	4.0	2.5	0.5	1.0	1.0	2.1	1.46
LRRTM3	9.0	3.5	0.5	5.5	2.0	2.0	3.8	3.08
LRRTM4	0.5	0.0	0.0	0.5	0.0	0.0	0.2	0.26
LRSAM1	545.5	405.5	334.5	486.5	428.5	310.5	418.5	89.15
LRTM1	0.0	0.0	0.0	0.0	0.0	0.0	0.0	0.00
LRTM2	5.0	0.5	0.5	2.0	0.0	1.0	1.5	1.84
LRWD1	796.0	589.0	448.5	800.0	590.0	446.0	611.6	157.80
LSAMP	1.5	11.5	35.0	1.5	16.5	29.0	15.8	13.93
LSG1	1041.0	791.5	757.5	934.0	761.0	741.0	837.7	121.99
LSM1	232.0	299.0	191.0	229.0	318.0	194.5	243.9	53.16
LSM10	57.5	89.0	60.0	52.5	87.0	80.0	71.0	16.16
LSM11	44.5	40.0	95.5	63.0	54.0	103.5	66.8	26.70
LSM14A	326.0	114.5	341.0	314.0	136.0	309.5	256.8	102.73
LSM14B	326.5	183.0	258.0	293.0	209.5	256.5	254.4	52.57
LSM3	350.5	254.0	170.5	347.0	261.0	140.5	253.9	87.02
LSM4	708.0	501.5	346.5	650.0	519.0	326.5	508.6	154.50
LSM5	372.0	263.5	217.0	287.5	280.0	184.0	267.3	64.83
LSM6	347.5	261.5	190.0	294.5	296.5	172.0	260.3	67.56
LSM7	567.5	400.0	336.0	631.5	434.0	277.5	441.1	135.48
LSM8	225.5	207.5	149.5	214.5	212.0	142.5	191.9	36.13
LSMEM1	135.0	303.5	177.5	114.0	303.5	109.5	190.5	90.77
LSMEM2	25.5	66.0	47.0	18.0	60.5	55.0	45.3	19.46
LSP1	1122.0	2678.5	2061.0	1220.0	3046.0	2053.0	2030.1	766.14
LSS	2905.0	1631.0	2151.5	2464.5	1565.0	1931.5	2108.1	513.37
LTA4H	1141.0	718.0	767.5	1081.0	822.0	733.0	877.1	185.66
LTB4R	0.0	0.0	0.0	0.0	0.5	0.0	0.1	0.20

LTBP2	165.5	656.5	2184.5	109.0	531.0	1910.0	926.1	897.39
LTK	9.5	7.0	25.0	4.0	7.0	24.0	12.8	9.27
LTN1	597.0	387.5	507.0	570.0	463.5	439.0	494.0	79.81
LTV1	408.0	309.0	244.0	367.0	331.5	240.5	316.7	66.69
LUC7L	1418.0	1465.0	968.5	1228.0	1710.0	1066.0	1309.3	275.16
LUC7L3	1143.5	812.5	974.5	1022.0	899.5	872.5	954.1	118.82
LUM	4.0	14.5	12.0	5.5	19.0	42.0	16.2	13.84
LURAP1	85.0	56.0	44.0	77.5	60.5	50.5	62.3	15.90
LURAP1L	43.0	17.0	10.0	35.5	13.5	2.5	20.3	15.66
LUZP1	283.0	199.5	476.5	306.0	211.5	446.0	320.4	116.81
LUZP2	1.0	0.0	0.0	1.0	1.0	0.0	0.5	0.55
LXN	153.5	89.5	37.5	166.5	96.0	37.5	96.8	55.06
LY86	7.0	2.0	0.5	4.0	1.5	0.0	2.5	2.61
LY96	200.5	235.0	123.5	205.0	303.0	159.5	204.4	62.02
LYAR	811.5	495.0	479.0	751.0	579.5	461.5	596.3	150.14
LYN	175.5	100.0	138.5	167.0	89.5	111.5	130.3	35.76
LYPD1	3.5	8.0	75.0	2.5	13.5	54.0	26.1	30.74
LYPD6	7.0	5.0	16.0	5.5	3.0	18.0	9.1	6.30
LYPD6B	7.5	3.5	5.5	19.0	4.0	4.0	7.3	5.94
LYPLA1	1595.0	1220.5	1415.0	1467.0	1300.5	1198.5	1366.1	153.99
LYPLA2	980.5	690.0	847.5	984.5	669.5	761.0	822.2	138.98
LYPLAL1	86.0	60.0	45.0	78.5	60.0	38.5	61.3	18.41
LYRM1	539.5	546.5	417.5	565.5	580.5	443.0	515.4	68.00
LYRM2	257.0	261.0	146.0	242.5	307.5	149.5	227.3	65.34
LYRM4	196.5	143.5	91.5	160.0	194.5	84.0	145.0	48.81
LYRM5	128.0	90.5	65.5	125.5	102.5	55.5	94.6	30.08
LYRM7	490.0	475.0	326.0	369.0	508.5	260.5	404.8	101.25
LYRM9	25.5	23.0	12.5	20.5	33.5	9.5	20.8	8.77
LYSMD2	696.5	797.0	623.5	815.0	954.5	731.0	769.6	114.17
LYSMD3	311.5	209.0	202.0	327.0	245.0	242.0	256.1	52.08
LYSMD4	475.5	383.0	668.5	407.5	337.5	474.5	457.8	116.26
LYST	196.5	118.0	304.0	164.5	150.0	340.0	212.2	89.47
LYVE1	10.0	4.0	6.0	10.0	7.0	4.5	6.9	2.62
LYZ	3.5	1.0	0.5	6.5	0.0	0.5	2.0	2.53
LZIC	558.5	354.5	488.0	558.5	369.0	386.0	452.4	94.58
LZTFL1	320.5	211.0	295.0	292.5	202.5	213.5	255.8	52.36
LZTR1	683.0	482.0	433.5	594.5	536.5	437.5	527.8	97.73
LZTS1	300.5	613.5	751.5	334.0	778.5	656.5	572.4	206.92
LZTS2	409.5	459.5	410.5	391.5	483.5	543.5	449.7	57.58
M1AP	155.5	95.0	75.5	121.0	90.5	82.5	103.3	29.92
M6PR	3847.5	3357.5	4260.5	3703.5	3863.0	3566.5	3766.4	306.97
MAATS1	24.5	40.0	29.5	20.5	50.5	34.5	33.3	10.94
MAB21L1	4.0	4.5	0.5	3.5	6.0	2.0	3.4	1.93
MAB21L2	2.5	3.5	14.0	3.0	3.0	5.5	5.3	4.41
MAB21L3	1.5	4.0	7.5	3.0	7.5	3.5	4.5	2.47
MACC1	2.0	3.0	1.5	0.5	2.5	1.0	1.8	0.94
MACF1	155.0	165.5	811.5	157.5	127.5	742.0	359.8	323.94
MACROD2	386.5	217.5	264.5	347.5	220.5	242.5	279.8	70.70
MAD1L1	71.5	26.0	56.0	81.5	32.0	62.5	54.9	21.92
MAD2L1	725.5	444.0	545.5	712.0	463.5	434.0	554.1	133.52
MAD2L1BP	164.5	58.5	113.5	215.0	64.0	77.0	115.4	62.75
MAD2L2	355.0	319.0	243.5	308.0	318.5	240.0	297.3	45.92
MADCAM1	7.0	8.0	6.5	3.0	7.5	5.5	6.3	1.81
MAEA	2168.0	1870.0	2238.5	2209.5	2166.5	2195.5	2141.3	135.64

MAEL	0.0	0.0	0.0	0.0	0.0	0.0	0.0	0.00
MAF	23.0	18.5	81.0	25.5	18.5	104.0	45.1	37.54
MAF1	133.0	214.5	432.0	155.0	158.5	443.0	256.0	143.18
MAFA	69.5	162.0	303.5	60.0	165.5	281.5	173.7	102.43
MAFB	44.0	142.5	84.5	65.0	149.5	118.0	100.6	42.85
MAFF	1359.0	1816.0	1217.0	1366.0	1988.5	1550.0	1549.4	298.08
MAFG	350.0	387.0	390.0	335.5	429.0	439.5	388.5	41.32
MAFK	325.5	445.0	237.5	263.5	493.5	265.5	338.4	106.48
MAGI1	281.5	487.5	369.0	288.0	588.0	386.5	400.1	118.94
MAGI3	269.0	142.5	349.5	270.5	151.0	336.5	253.2	88.84
MAGOH	1472.0	1189.0	750.0	1352.5	1214.5	658.0	1106.0	328.88
MAGT1	2201.0	1784.5	2487.5	2225.5	1973.0	2328.5	2166.7	251.92
MAK	26.5	18.5	24.5	19.0	19.5	20.5	21.4	3.29
MAK16	742.0	891.0	765.5	686.5	978.5	723.5	797.8	112.61
MAL2	0.0	0.0	0.0	0.0	0.0	0.0	0.0	0.00
MALL	66.5	23.0	19.0	70.0	17.0	18.5	35.7	25.34
MALRD1	0.0	0.0	0.0	0.0	0.0	0.0	0.0	0.00
MALSU1	504.0	433.5	345.5	459.5	464.5	326.5	422.3	70.77
MALT1	824.5	386.5	449.5	737.5	430.5	419.5	541.3	188.78
MAMDC2	2399.5	1007.0	3359.5	2981.0	1262.5	3351.0	2393.4	1038.91
MAMDC4	4.5	6.5	2.0	3.5	3.0	6.0	4.3	1.75
MAML1	500.0	348.0	704.5	508.0	419.0	656.0	522.6	136.25
MAML2	337.0	216.0	494.5	396.5	228.0	534.5	367.8	132.82
MAML3	288.0	236.5	246.0	260.5	267.0	249.0	257.8	18.30
MAN1A1	706.0	291.0	738.0	936.0	407.0	777.5	642.6	243.53
MAN1A2	1831.5	1234.5	1724.0	1902.5	1367.0	1604.5	1610.7	263.70
MAN1B1	715.0	376.0	813.5	787.0	408.0	719.0	636.4	193.40
MAN1C1	476.5	356.0	596.0	416.5	418.5	628.5	482.0	108.34
MAN2A1	1161.0	531.0	904.5	1270.0	632.0	826.0	887.4	288.98
MAN2A2	788.5	754.5	817.5	750.0	775.0	963.5	808.2	79.96
MAN2C1	796.5	639.5	514.0	775.5	634.0	518.0	646.3	121.17
MANBA	273.0	262.0	262.5	329.0	313.0	291.0	288.4	27.80
MANEA	201.5	95.0	196.0	190.5	114.5	128.5	154.3	47.00
MANEAL	11.5	7.0	9.0	9.0	8.0	20.0	10.8	4.77
MANF	874.5	952.5	711.5	852.0	1032.5	846.5	878.3	108.46
MANSC1	130.5	84.5	140.0	207.5	100.0	177.0	139.9	46.25
MANSC4	1.5	0.5	0.0	0.5	1.0	1.5	0.8	0.61
MAP10	114.5	43.0	32.5	116.0	60.0	38.5	67.4	38.17
MAP1A	3345.5	2688.5	4859.0	3208.0	3293.0	4320.0	3619.0	805.58
MAP1LC3A	690.5	916.5	806.0	723.5	1052.0	851.5	840.0	132.67
MAP1LC3C	1991.5	687.0	337.5	1357.0	698.5	344.5	902.7	649.79
MAP1S	1192.5	816.0	1326.5	1295.0	692.0	1315.5	1106.3	279.72
MAP2	1.0	0.5	1.0	0.5	1.5	3.5	1.3	1.13
MAP2K1	3709.5	3563.0	4220.0	4023.0	4059.0	3688.5	3877.2	258.56
MAP2K2	788.0	609.5	466.0	757.5	632.5	479.5	622.2	134.78
MAP2K3	255.5	155.5	251.0	268.5	173.5	248.5	225.4	48.03
MAP2K4	655.5	466.5	332.0	586.0	517.0	324.0	480.2	134.04
MAP2K5	655.0	408.0	433.0	586.5	433.0	396.0	485.3	108.13
MAP2K6	97.0	112.0	325.5	105.0	141.5	300.0	180.2	104.11
MAP3K13	37.5	35.0	121.5	36.0	44.0	84.5	59.8	35.62
MAP3K14	792.0	924.0	1001.0	762.5	1034.0	1252.0	960.9	179.41
MAP3K19	10.5	2.5	3.0	5.0	3.0	0.5	4.1	3.46
MAP3K2	271.0	185.5	269.0	232.5	204.0	232.5	232.4	34.16
MAP3K3	697.0	526.5	615.0	671.0	579.5	617.0	617.7	61.50

MAP3K4	438.5	285.5	391.5	420.5	330.5	339.0	367.6	58.87
MAP3K6	5.5	17.0	15.0	6.0	15.0	12.5	11.8	4.93
MAP3K7	2370.5	2150.5	2941.5	2164.5	2458.0	2682.5	2461.3	307.40
MAP3K7CL	26.0	290.0	222.0	16.0	276.0	219.5	174.9	122.55
MAP3K8	22.5	32.0	32.0	18.0	43.5	21.5	28.3	9.43
MAP3K9	63.0	19.0	44.0	59.5	14.0	30.5	38.3	20.57
MAP4	4482.5	1926.5	5063.0	4300.5	1594.5	4555.0	3653.7	1491.79
MAP4K3	648.0	468.0	621.0	613.0	528.0	599.5	579.6	67.85
MAP4K4	1279.0	872.0	2210.0	1194.5	887.5	1692.0	1355.8	515.36
MAP4K5	641.0	502.5	678.0	672.5	563.5	663.5	620.2	71.35
MAP6	0.0	0.0	0.5	0.0	0.0	0.5	0.2	0.26
MAP7D1	1431.0	2018.0	2313.0	1284.0	1869.0	2075.0	1831.7	396.86
MAP7D3	1435.5	1131.5	1320.5	1252.0	1346.0	1204.0	1281.6	108.42
MAP9	117.0	105.0	106.5	92.0	112.0	72.5	100.8	16.23
MAPK1	1489.0	1091.5	1686.5	1622.5	1318.0	1411.0	1436.4	216.13
MAPK10	10.5	8.0	4.0	7.0	7.0	6.0	7.1	2.15
MAPK11	251.0	184.5	152.5	271.5	260.0	193.0	218.8	48.48
MAPK12	135.5	164.5	194.0	123.5	173.0	214.5	167.5	34.38
MAPK13	447.5	536.0	355.5	461.0	706.5	418.5	487.5	122.31
MAPK14	296.5	125.5	311.0	310.0	145.0	249.0	239.5	84.08
MAPK15	122.0	93.0	64.5	104.5	104.5	74.0	93.8	21.33
MAPK1IP1L	2228.5	1690.5	1918.5	2148.5	1852.5	1788.0	1937.8	209.83
MAPK6	1442.5	761.5	2014.0	1616.0	979.5	1773.0	1431.1	478.18
MAPK8	699.5	608.5	726.5	640.5	738.0	697.0	685.0	50.43
MAPK8IP1	63.5	172.5	118.0	68.5	199.5	111.5	122.3	54.71
MAPK8IP2	48.0	31.5	15.5	37.0	29.0	16.5	29.6	12.39
MAPK8IP3	1279.0	994.0	949.0	1054.0	1084.5	1094.0	1075.8	113.97
MAPK9	39.0	24.5	87.5	36.5	27.5	67.5	47.1	24.98
MAPKAP1	631.5	525.5	413.0	731.5	600.5	427.0	554.8	123.69
MAPKAPK2	524.0	519.0	1034.5	511.5	649.5	882.0	686.8	221.71
MAPKAPK5	494.0	284.5	347.5	500.5	332.5	325.5	380.8	92.65
MAPKBP1	159.5	70.0	208.5	143.0	84.0	176.0	140.2	53.69
MAPRE1	2857.0	3892.0	4185.0	2695.5	4134.5	3479.0	3540.5	644.48
MAPRE2	93.0	54.5	159.0	139.0	76.5	173.0	115.8	47.95
MAPRE3	963.5	943.0	610.5	1044.0	957.5	644.0	860.4	184.32
MAPT	13.0	15.0	20.5	9.5	12.0	42.5	18.8	12.21
MAR1	11.5	18.5	12.5	11.0	20.5	11.0	14.2	4.22
MAR2	418.0	344.0	310.5	379.5	361.5	300.0	352.3	44.03
MAR4	0.0	0.0	0.0	0.0	0.0	0.0	0.0	0.00
MAR5	689.0	439.5	467.0	646.5	491.0	452.0	530.8	108.27
MAR6	695.5	572.5	734.0	668.0	655.5	644.0	661.6	54.25
MAR7	1092.5	847.0	907.5	1074.5	973.5	910.5	967.6	98.47
MAR8	491.5	231.0	245.0	415.0	257.5	188.5	304.8	119.82
MARCH11	0.5	1.0	0.5	0.5	2.0	1.0	0.9	0.58
MARCO	0.5	0.5	0.5	0.0	0.0	0.0	0.3	0.27
MARK1	1103.0	822.0	1161.5	1096.5	1013.0	1086.0	1047.0	120.00
MARK3	1007.5	1169.5	1225.5	1051.0	1374.0	1212.5	1173.3	131.91
MARS2	150.5	181.5	182.0	154.5	186.5	192.5	174.6	17.60
MARVELD1	548.5	728.5	1054.0	554.5	783.0	1296.5	827.5	295.10
MARVELD3	1091.0	553.0	516.5	996.0	615.5	566.0	723.0	252.07
MAS1	0.0	0.0	0.0	0.0	0.0	0.0	0.0	0.00
MASP1	0.5	11.0	7.0	1.0	13.5	6.5	6.6	5.21
MASP2	191.5	89.0	81.5	153.5	102.0	89.5	117.8	44.51
MAST3	115.5	135.0	163.0	126.5	130.0	174.5	140.8	22.91

MAST4	1110.0	533.0	697.0	1209.5	666.5	676.0	815.3	274.76
MAT1A	125.5	136.5	214.0	90.0	109.0	125.5	133.4	42.68
MAT2B	2271.0	1464.0	1561.5	2372.5	1700.0	1577.5	1824.4	393.78
MATK	15.0	3.5	4.0	13.5	6.0	3.5	7.6	5.27
MATN1	0.5	1.5	0.5	0.5	0.5	0.0	0.6	0.49
MATN2	28.0	48.0	45.5	30.0	53.5	57.5	43.8	12.19
MATN3	45.5	16.5	20.5	61.5	20.0	15.5	29.9	19.05
MATN4	0.0	0.0	0.5	0.0	0.5	0.0	0.2	0.26
MATR3	3952.5	3187.5	3445.5	3588.0	3552.0	3231.0	3492.8	278.52
MAU2	211.0	106.5	244.5	231.5	122.5	241.5	192.9	62.07
MAVS	25.5	15.0	84.0	38.5	18.0	52.5	38.9	26.10
MAX	362.5	458.5	390.0	338.5	477.0	404.5	405.2	53.85
MAZ	118.0	106.0	167.5	106.5	88.0	191.5	129.6	40.56
MB	12.0	20.0	30.0	10.5	30.5	46.5	24.9	13.58
MB21D1	91.5	34.0	42.0	92.0	37.0	32.0	54.8	28.86
MB21D2	70.0	406.5	275.0	69.5	445.0	313.5	263.3	161.93
MBD3	755.0	407.0	360.5	638.0	406.0	346.0	485.4	169.38
MBD4	570.5	503.0	400.0	466.0	477.0	349.5	461.0	77.70
MBD5	450.5	285.5	651.5	447.5	341.5	621.5	466.3	146.46
MBIP	349.0	216.5	169.0	310.0	250.5	170.5	244.3	73.76
MBLAC1	60.5	51.0	58.0	64.0	36.5	52.0	53.7	9.77
MBNL1	913.5	423.5	1751.5	1103.0	509.0	1494.0	1032.4	528.02
MBNL2	899.0	432.0	678.5	759.0	500.5	575.5	640.8	172.98
MBNL3	2854.0	1867.5	2116.5	2733.0	2012.0	1770.5	2225.6	457.21
MBOAT1	26.5	98.5	65.5	20.0	107.0	108.5	71.0	40.17
MBOAT4	0.0	0.0	0.5	0.0	0.0	0.0	0.1	0.20
MBTD1	308.0	217.5	300.0	304.5	294.5	348.5	295.5	42.78
MBTPS1	1883.0	1053.5	1948.0	1934.5	1264.0	1941.0	1670.7	402.73
MC1R	2.0	2.5	3.5	1.0	1.5	1.0	1.9	0.97
MC2R	0.0	0.0	0.0	0.0	0.0	0.0	0.0	0.00
MC3R	0.0	0.0	0.0	0.0	0.0	0.0	0.0	0.00
MC4R	0.0	0.0	0.0	0.0	1.0	1.0	0.3	0.52
MC5R	7.0	1.5	2.0	7.0	2.5	0.0	3.3	2.96
MCAM	1750.5	1586.0	3817.5	1818.5	1645.5	3021.5	2273.3	926.39
MCAT	284.5	236.0	249.0	241.0	247.0	209.5	244.5	24.23
MCCC1	343.5	242.0	307.0	282.0	238.5	275.0	281.3	39.86
MCEE	380.0	200.5	287.0	337.0	233.5	218.0	276.0	71.45
MCF2	782.5	565.0	930.5	865.5	608.5	870.0	770.3	150.43
MCF2L	20.0	12.5	17.0	28.0	18.5	36.5	22.1	8.69
MCF2L2	47.5	30.5	19.5	54.5	38.5	14.0	34.1	15.78
MCFD2	1211.5	749.5	850.0	1191.0	811.5	701.0	919.1	224.55
MCHR1	0.0	0.0	0.0	0.0	0.5	0.0	0.1	0.20
MCL1	1590.5	2877.0	2127.0	1648.0	2866.5	2067.0	2196.0	565.98
MCM10	301.5	133.0	220.0	296.5	128.0	126.0	200.8	83.86
MCM2	1290.0	709.0	654.5	1353.0	658.5	486.0	858.5	367.00
MCM3	1159.0	618.5	601.5	1090.0	529.5	348.0	724.4	325.12
MCM3AP	913.5	714.5	870.5	914.5	838.5	886.0	856.3	75.06
MCM4	1083.0	537.5	785.5	1091.0	541.5	531.5	761.7	269.81
MCM5	1577.0	764.0	661.5	1557.0	653.0	426.0	939.8	498.31
MCM6	1741.0	847.5	1114.5	1966.5	950.5	826.0	1241.0	490.68
MCM8	442.0	275.5	201.5	404.5	349.0	189.5	310.3	105.22
MCM9	19.0	1.5	10.0	21.5	8.0	11.0	11.8	7.35
MCMBP	1467.0	1033.0	1057.0	1493.5	1083.0	967.0	1183.4	233.28
MCMDC2	26.5	7.0	21.5	32.5	16.5	25.0	21.5	8.87

MCOLN1	666.0	440.5	469.5	636.0	444.5	486.5	523.8	100.38
MCOLN2	0.0	0.0	0.0	0.0	0.0	0.0	0.0	0.00
MCOLN3	1.5	0.5	1.5	1.0	0.0	2.0	1.1	0.74
MCPH1	41.5	9.5	28.5	43.0	12.0	15.0	24.9	14.95
MCRS1	1372.5	1575.5	1159.0	1284.0	1526.5	1095.0	1335.4	193.44
MCTP1	1.0	1.5	9.5	0.0	3.5	4.0	3.3	3.42
MCTP2	3.0	9.0	21.0	2.5	8.5	17.0	10.2	7.46
MCTS1	1353.5	1169.5	831.0	1217.5	1272.5	767.0	1101.8	243.26
MCU	1663.0	2413.0	2864.5	1516.5	2710.5	2582.5	2291.7	565.50
MCUR1	744.5	618.0	560.0	741.5	701.5	564.0	654.9	85.24
MDFI	199.5	69.5	78.5	165.0	63.0	86.5	110.3	57.32
MDFIC	1336.5	880.5	845.0	1312.0	1118.0	1283.0	1129.2	220.36
MDGA1	3.5	5.5	11.0	2.5	3.0	9.0	5.8	3.50
MDGA2	0.0	0.0	0.0	0.0	0.0	0.0	0.0	0.00
MDH1	4186.0	3616.0	3546.5	3874.5	3787.5	2940.5	3658.5	417.48
MDH1B	8.5	6.0	12.5	10.0	11.5	5.0	8.9	2.99
MDH2	2580.5	3505.5	2939.0	2622.5	3655.5	2525.0	2971.3	495.60
MDK	210.0	471.5	278.0	155.5	588.0	294.5	332.9	164.53
MDM1	221.0	144.5	182.5	215.0	167.5	153.5	180.7	31.71
MDM2	338.5	161.5	647.0	379.0	198.5	473.0	366.3	179.56
MDM4	1167.5	591.5	1420.0	1131.5	648.5	1126.5	1014.3	324.64
MDN1	959.0	776.5	1238.0	895.5	883.5	1190.5	990.5	183.59
ME1	329.5	252.5	273.0	276.0	251.0	252.5	272.4	30.08
ME3	253.0	344.5	383.0	258.0	377.0	339.0	325.8	57.12
MEAF6	732.0	664.0	585.5	661.5	739.0	572.0	659.0	70.33
MECOM	41.5	25.0	26.5	71.0	31.0	53.0	41.3	17.94
MECR	269.0	175.0	142.5	253.0	203.0	136.5	196.5	55.62
MED1	1036.5	659.5	961.5	1073.5	745.0	920.0	899.3	164.18
MED10	1059.5	933.5	664.0	1167.0	1059.5	709.0	932.1	204.59
MED11	201.0	281.0	151.5	187.0	245.0	136.5	200.3	55.03
MED12	635.5	509.0	768.5	670.5	545.5	778.5	651.3	111.33
MED12L	365.0	447.5	789.0	302.5	529.0	963.0	566.0	257.91
MED13	745.5	563.5	953.0	701.0	718.5	888.0	761.6	139.75
MED13L	796.5	502.5	1235.0	819.0	609.5	1253.0	869.3	313.31
MED14	525.0	356.5	718.5	506.5	412.0	607.5	521.0	130.89
MED15	471.0	470.0	420.5	498.0	581.0	423.5	477.3	58.98
MED16	387.0	341.0	358.5	423.5	378.5	396.5	380.8	28.96
MED17	711.0	484.0	494.0	719.0	553.0	445.0	567.7	119.28
MED18	642.0	356.5	716.5	660.0	428.5	657.0	576.8	146.73
MED19	317.5	272.5	190.0	294.0	283.5	186.5	257.3	55.55
MED20	519.5	515.5	381.5	512.0	548.5	400.5	479.6	70.08
MED21	256.0	329.0	373.5	245.0	350.0	357.0	318.4	54.62
MED22	441.5	310.0	462.5	436.0	317.5	375.5	390.5	66.17
MED23	426.5	243.5	453.0	426.5	286.0	379.5	369.2	85.34
MED24	309.5	263.5	333.5	262.5	220.5	303.0	282.1	40.88
MED26	222.0	238.5	239.0	205.0	237.5	227.0	228.2	13.32
MED27	362.0	398.5	416.5	373.0	455.5	381.0	397.8	34.23
MED28	369.0	312.0	262.0	393.0	359.0	252.0	324.5	58.62
MED29	914.5	1094.5	566.0	874.0	1115.5	549.0	852.3	247.48
MED30	555.5	557.0	452.0	561.5	604.5	457.0	531.3	62.17
MED31	915.5	1037.5	708.5	965.0	1255.0	776.5	943.0	195.05
MED4	269.0	204.5	164.5	255.0	233.0	162.0	214.7	45.41
MED6	716.5	846.0	670.5	660.0	920.0	622.5	739.3	117.66
MED7	376.0	369.0	239.5	363.0	416.0	276.0	339.9	67.29

MED8	293.5	318.0	277.5	291.0	348.0	277.0	300.8	27.50
MED9	753.0	742.5	523.5	714.5	811.5	548.5	682.3	117.87
MEF2A	4858.5	2849.5	3971.5	4608.0	3404.0	3347.0	3839.8	782.14
MEF2B	12.0	77.5	31.0	11.0	66.0	22.5	36.7	28.38
MEF2BNB	306.0	388.5	276.0	306.5	414.0	272.5	327.3	59.64
MEF2C	7.5	32.0	63.0	2.0	42.0	52.0	33.1	24.31
MEF2D	240.5	363.5	789.0	271.0	363.5	804.0	471.9	256.22
MEGF10	115.0	57.5	136.5	186.5	57.5	128.0	113.5	49.68
MEGF11	4.5	9.0	12.0	4.5	11.0	16.0	9.5	4.49
MEGF6	3.5	4.0	17.0	4.0	8.5	8.0	7.5	5.14
MEGF9	213.0	90.5	123.5	228.5	100.0	141.5	149.5	58.21
MEI1	40.5	9.5	7.0	38.5	15.5	6.0	19.5	15.85
MEI4	0.0	0.0	0.0	0.0	0.0	0.0	0.0	0.00
MEIG1	0.5	0.0	0.0	0.5	0.5	0.5	0.3	0.26
MEIOB	2.5	4.0	8.5	4.0	4.5	6.0	4.9	2.08
MEIS1	359.5	301.5	296.5	284.5	322.0	307.5	311.9	26.39
MEIS2	1.5	1.5	2.0	2.0	2.0	1.5	1.8	0.27
MELK	8.5	4.5	8.0	11.5	4.5	7.0	7.3	2.66
MEMO1	1525.5	1022.5	785.5	1274.0	1137.5	716.5	1076.9	303.82
MEOX1	2.0	5.0	9.0	0.5	6.0	10.5	5.5	3.87
MEOX2	3.5	6.0	3.5	1.5	1.5	0.0	2.7	2.11
MEP1A	2.0	3.0	4.5	0.5	1.5	3.5	2.5	1.45
MEPCE	117.5	87.0	119.5	108.5	58.5	108.0	99.8	23.30
MERTK	896.0	539.0	355.5	751.5	589.5	343.5	579.2	217.88
MESDC1	262.5	320.5	330.5	294.0	386.5	373.0	327.8	46.81
MESDC2	561.0	285.0	723.0	558.0	367.0	665.5	526.6	169.63
MEST	40.0	25.5	56.5	50.0	28.5	59.0	43.3	14.23
MET	3504.0	1564.5	2020.5	3503.5	1842.5	1889.5	2387.4	877.39
METAP1	634.0	519.5	711.5	700.5	606.0	648.5	636.7	69.99
METAP1D	51.0	24.0	24.5	45.0	34.5	19.5	33.1	12.69
METAP2	2027.5	1782.5	1458.0	1908.5	1914.0	1352.5	1740.5	273.06
METRN	33.0	128.5	132.5	10.5	99.5	173.0	96.2	62.62
METRNL	1030.5	880.5	1032.0	929.0	914.5	1014.0	966.8	66.56
METTL11B	8.0	64.0	131.5	6.0	83.0	127.5	70.0	55.20
METTL13	348.0	286.0	193.0	313.5	288.5	222.5	275.3	57.61
METTL14	1018.5	683.5	566.5	1031.5	724.0	548.5	762.1	214.40
METTL15	35.5	27.0	46.0	39.0	28.0	33.5	34.8	7.10
METTL16	793.0	515.0	543.5	668.0	578.5	457.0	592.5	120.72
METTL17	80.0	132.0	57.0	73.5	106.5	76.0	87.5	27.04
METTL18	199.0	102.0	100.5	232.5	111.5	81.0	137.8	62.14
METTL20	20.5	24.0	9.5	12.5	17.5	9.5	15.6	6.04
METTL21A	728.5	555.0	588.5	715.5	616.5	557.5	626.9	77.12
METTL21C	168.5	250.0	92.5	142.0	310.5	117.0	180.1	83.82
METTL22	208.5	159.0	153.5	198.0	161.0	138.0	169.7	27.44
METTL23	2196.5	2082.0	1580.5	1953.0	2354.0	1497.5	1943.9	341.31
METTL24	203.0	236.0	407.0	209.5	262.5	324.0	273.7	78.70
METTL25	139.0	105.5	76.0	159.0	98.5	72.0	108.3	34.58
METTL2A	488.5	369.5	343.0	466.5	418.0	327.5	402.2	66.31
METTL3	37.0	69.0	78.5	57.0	70.5	67.5	63.3	14.59
METTL5	1105.0	1086.0	746.5	1056.0	1254.0	716.5	994.0	214.71
METTL6	263.0	155.0	171.0	237.5	185.0	172.5	197.3	42.85
METTL7A	8.5	16.5	10.5	13.0	17.0	11.5	12.8	3.37
METTL8	351.0	187.5	112.0	308.5	205.5	106.5	211.8	100.41
METTL9	646.5	365.5	219.5	601.0	404.0	199.0	405.9	187.16

MEX3A	26.5	42.0	62.5	25.5	40.5	81.0	46.3	21.65
MEX3B	163.0	385.0	327.5	147.5	407.5	396.5	304.5	118.96
MEX3D	144.0	181.0	330.5	143.5	195.5	315.0	218.3	83.63
MFAP1	1124.0	938.5	848.5	1130.5	1013.5	810.0	977.5	135.93
MFAP2	121.0	86.5	33.5	149.0	84.0	60.5	89.1	41.35
MFAP3	1217.0	872.5	1576.0	1124.5	1014.0	1341.5	1190.9	248.49
MFAP3L	2.5	3.0	2.5	6.5	1.5	2.0	3.0	1.79
MFAP5	22.5	7.0	44.0	26.5	6.5	33.0	23.3	14.71
MFF	193.0	127.5	148.5	180.5	130.5	136.0	152.7	27.65
MFGE8	21994.5	9261.5	9518.0	18268.5	9541.0	9528.5	13018.7	5635.12
MFHAS1	135.0	68.5	287.5	133.0	59.5	237.5	153.5	91.47
MFI2	22.5	26.5	71.5	18.5	45.0	79.5	43.9	26.22
MFN1	1428.0	1336.0	1430.5	1253.5	1536.5	1322.5	1384.5	100.47
MFN2	579.0	284.5	700.0	564.5	335.0	558.5	503.6	159.63
MFNG	0.0	1.0	0.0	0.0	0.0	1.0	0.3	0.52
MFRP	0.0	0.0	0.0	0.0	0.0	0.0	0.0	0.00
MFSD1	817.0	373.5	484.0	769.5	459.5	444.5	558.0	186.50
MFSD10	511.5	375.0	368.5	495.0	437.0	362.0	424.8	66.61
MFSD11	895.0	572.0	656.0	797.0	608.0	562.0	681.7	135.09
MFSD12	59.5	68.0	47.0	68.5	55.5	29.0	54.6	14.90
MFSD2A	301.5	727.0	558.0	313.0	708.0	566.5	529.0	185.42
MFSD2B	1.5	1.0	0.0	1.5	0.0	0.0	0.7	0.75
MFSD4	8.0	7.0	10.0	9.0	12.0	15.0	10.2	2.93
MFSD5	442.5	539.5	426.5	369.5	511.0	443.5	455.4	61.13
MFSD6	707.5	532.5	437.0	815.0	628.0	433.5	592.3	153.00
MFSD7	107.0	52.5	81.5	114.5	61.0	92.5	84.8	24.72
MFSD8	204.0	74.0	106.5	208.5	100.0	98.0	131.8	58.70
MFSD9	240.0	189.5	180.0	223.5	191.0	156.0	196.7	30.37
MGARP	9.0	5.5	2.0	12.5	7.5	3.0	6.6	3.92
MGAT1	537.5	588.5	476.5	553.5	536.5	504.0	532.8	38.90
MGAT2	554.5	437.5	425.0	585.0	423.5	449.5	479.2	71.45
MGAT3	248.5	162.0	465.5	207.0	206.5	490.0	296.6	143.18
MGAT4A	36.5	21.0	29.0	41.5	18.0	29.5	29.3	8.91
MGAT4B	2555.0	1538.5	1530.5	2006.0	1576.0	1395.5	1766.9	438.34
MGAT4C	0.0	0.0	0.0	0.5	0.0	0.0	0.1	0.20
MGAT4D	2.0	1.0	0.5	1.5	2.0	2.0	1.5	0.63
MGAT5	67.0	12.5	169.0	79.5	21.5	126.0	79.3	60.30
MGAT5B	0.5	0.5	0.0	0.0	0.0	0.0	0.2	0.26
MGEA5	2589.0	2313.0	2441.0	2241.5	2737.0	2537.5	2476.5	183.01
MGLL	3.5	3.0	16.5	2.5	11.5	22.0	9.8	8.20
MGME1	222.5	159.5	196.0	230.5	173.5	156.5	189.8	31.80
MGMT	49.5	22.0	31.5	41.5	23.0	23.5	31.8	11.40
MGP	731.0	5673.0	2426.0	196.0	3797.5	1915.5	2456.5	2026.08
MGST1	837.0	363.0	345.0	843.5	398.0	314.5	516.8	251.98
MGST2	17.0	16.5	7.0	16.5	9.0	6.0	12.0	5.21
MGST3	936.5	722.5	493.5	875.5	832.5	433.0	715.6	208.46
MIA3	1123.5	613.5	995.5	1056.5	736.5	925.5	908.5	196.26
MIB1	1178.0	415.5	787.0	1358.5	528.5	751.0	836.4	366.33
MIB2	205.0	93.5	233.0	203.5	93.5	231.0	176.6	65.55
MICAL1	3393.5	3464.5	2993.0	2905.5	3496.0	2791.5	3174.0	312.22
MICALL1	6912.5	7044.0	7501.0	6732.0	7872.0	6848.5	7151.7	442.21
MICALL2	1478.0	709.5	677.5	1390.0	700.5	629.0	930.8	391.80
MICU1	1313.0	2225.5	1188.5	1228.5	2540.5	1281.5	1629.6	593.58
MICU2	560.0	902.0	1080.0	561.0	1088.0	891.0	847.0	237.27

MID1	166.5	115.5	181.5	148.0	108.0	148.0	144.6	28.46
MID1IP1	116.5	207.0	152.5	133.0	196.5	195.5	166.8	37.94
MIDN	146.0	243.5	303.5	150.0	237.5	333.5	235.7	76.96
MIEF1	733.0	543.0	817.0	718.5	635.0	783.5	705.0	100.81
MIEF2	235.5	391.5	316.5	194.0	396.5	249.0	297.2	84.74
MIEN1	36.0	60.0	80.0	44.5	56.5	76.0	58.8	17.18
MIER1	520.0	379.0	492.0	475.5	459.0	508.5	472.3	50.73
MIER2	337.0	277.5	297.0	314.0	336.5	276.0	306.3	27.38
MIER3	210.5	168.5	217.0	211.5	212.0	198.5	203.0	17.98
MIF	3674.0	1880.0	2899.0	3343.5	2212.5	2460.5	2744.9	686.68
MIF4GD	168.0	123.5	181.5	191.0	146.0	194.5	167.4	27.84
MIIP	230.5	138.5	143.0	217.5	148.0	151.5	171.5	41.11
MILR1	77.0	52.5	28.5	65.5	54.5	26.0	50.7	20.16
MINA	895.0	533.5	473.0	886.5	596.0	428.0	635.3	205.80
MINOS1	959.0	1266.5	855.5	856.5	1364.0	806.5	1018.0	237.56
MINPP1	1592.5	1063.5	1711.5	1768.0	1208.5	1149.0	1415.5	310.15
MIOS	256.0	141.5	199.5	260.0	146.5	193.0	199.4	51.12
MIOX	21.0	3.5	2.0	12.0	2.0	1.5	7.0	7.92
MIP	0.0	0.0	0.5	0.0	1.0	1.0	0.4	0.49
MIPOL1	87.0	73.5	75.5	68.5	67.5	74.0	74.3	6.98
MIS12	162.5	76.0	142.0	183.0	94.0	120.5	129.7	40.77
MIS18A	187.0	101.5	122.5	204.0	100.0	98.0	135.5	47.62
MIS18BP1	279.5	125.0	195.5	328.5	141.5	150.0	203.3	82.87
MISP	0.0	0.5	0.0	0.0	0.0	0.5	0.2	0.26
MITD1	106.0	51.5	72.5	80.0	44.5	49.0	67.3	23.62
MITF	69.5	20.5	94.0	63.5	27.0	96.5	61.8	32.31
MKI67	67.0	7.0	149.5	105.0	10.5	75.0	69.0	54.92
MKKS	1180.5	945.0	1138.5	1070.0	1063.0	1013.5	1068.4	84.56
MKL1	938.5	1027.5	1542.5	868.5	1075.0	1402.5	1142.4	269.10
MKL2	445.5	323.0	481.0	432.0	420.0	491.5	432.2	60.25
MKLN1	368.0	192.0	265.5	357.0	239.5	242.5	277.4	70.19
MKNK1	179.5	87.0	68.5	161.5	87.0	79.5	110.5	47.31
MKRN1	871.5	389.5	641.5	845.5	448.0	583.0	629.8	199.03
MKRN2	531.0	322.0	286.0	522.5	345.0	292.5	383.2	113.25
MKRN2OS	119.5	110.5	50.0	107.0	115.0	44.0	91.0	34.39
MKS1	213.5	193.5	231.5	203.0	190.5	274.5	217.8	31.55
MKX	1.5	0.5	4.0	0.0	0.0	3.0	1.5	1.67
MLANA	0.0	10.0	20.0	0.5	8.5	49.5	14.8	18.53
MLC1	0.0	0.0	0.0	0.0	0.0	0.0	0.0	0.00
MLEC	993.0	690.0	697.5	1025.5	728.0	589.0	787.2	178.56
MLF1	626.0	557.5	480.0	543.0	645.0	420.5	545.3	85.38
MLF2	1612.5	1271.5	1210.5	1569.5	1360.0	1123.0	1357.8	196.96
MLH1	149.0	71.5	132.0	173.5	85.0	124.0	122.5	38.48
MLH3	279.5	178.0	211.5	256.5	178.5	229.5	222.3	41.21
MLKL	0.5	0.5	0.0	0.5	0.5	0.0	0.3	0.26
MLLT1	140.0	58.5	145.0	125.5	62.0	145.5	112.8	41.32
MLLT10	356.0	316.0	345.0	348.0	384.5	333.0	347.1	23.02
MLLT11	157.0	296.0	248.5	154.0	371.5	252.5	246.6	83.28
MLLT3	155.0	108.5	192.0	161.5	134.5	175.0	154.4	29.64
MLLT4	1173.0	1389.5	2167.5	1107.5	1746.0	2057.5	1606.8	452.02
MLN	0.5	4.5	5.5	0.0	6.5	5.0	3.7	2.73
MLNR	1.5	2.5	1.0	4.5	5.5	1.5	2.8	1.84
MLPH	4.5	11.0	4.0	7.0	8.5	3.5	6.4	2.96
MLST8	959.5	1088.5	795.0	867.0	1141.5	778.0	938.3	152.06

MLX	731.0	698.0	681.0	645.0	789.5	674.5	703.2	50.89
MLXIP	415.5	329.5	924.5	515.5	400.5	752.0	556.3	232.84
MLXIPL	5.0	2.0	6.0	5.5	3.5	7.5	4.9	1.93
MLYCD	418.5	246.0	218.5	335.0	279.0	196.5	282.3	82.57
MMAA	379.0	299.0	337.0	368.5	333.5	319.0	339.3	30.01
MMAB	477.5	376.0	347.5	346.0	365.0	280.5	365.4	64.16
MMACHC	52.0	41.5	74.5	36.0	46.0	67.5	52.9	15.12
MMADHC	748.5	465.0	524.5	649.5	567.0	475.5	571.7	109.73
MMD	1426.5	771.5	904.0	1446.0	836.0	1039.5	1070.6	296.92
MMD2	7.5	11.0	6.0	5.5	11.0	6.0	7.8	2.54
MME	5.0	10.5	27.0	2.5	6.0	17.5	11.4	9.28
MMEL1	0.0	0.5	0.0	0.0	0.0	0.0	0.1	0.20
MMGT1	831.0	619.5	585.5	797.0	635.0	625.5	682.3	103.97
MMP11	339.5	366.0	283.5	242.0	460.0	640.5	388.6	144.23
MMP13	0.5	0.5	1.5	0.0	1.0	2.5	1.0	0.89
MMP15	1443.0	1228.5	999.0	1409.5	1194.0	1161.0	1239.2	165.23
MMP17	10.0	10.5	5.5	10.0	10.0	13.0	9.8	2.42
MMP2	479.0	415.5	1859.5	684.0	475.5	2450.0	1060.6	872.61
MMP23B	163.0	154.5	122.5	136.0	177.0	111.0	144.0	25.20
MMP24	462.5	375.0	315.5	429.0	389.0	311.5	380.4	60.27
MMP28	197.0	690.5	700.5	229.0	810.0	1103.5	621.8	350.18
MMP7	0.0	25.5	11.5	1.0	27.0	14.5	13.3	11.57
MMP9	419.5	691.0	408.0	726.5	825.5	639.0	618.3	169.76
MMRN1	1.5	3.5	1.0	2.5	8.5	1.5	3.1	2.80
MMRN2	0.5	0.5	0.5	0.5	0.0	0.0	0.3	0.26
MMS19	1307.5	1794.5	1936.5	1370.0	1841.0	1894.0	1690.6	277.42
MNAT1	850.5	687.0	511.0	669.5	675.5	447.5	640.2	143.07
MND1	323.5	136.0	121.0	272.0	142.5	83.0	179.7	95.17
MNS1	87.5	80.0	94.5	78.0	89.5	92.0	86.9	6.60
MNT	411.0	489.0	537.5	388.5	538.0	548.0	485.3	69.77
MNX1	3.5	106.0	40.0	1.5	159.5	56.0	61.1	61.71
MOB1A	1335.5	1678.5	1290.5	1266.0	1629.5	1295.0	1415.8	186.47
MOB1B	1726.5	1299.5	1251.0	1499.5	1421.0	1145.0	1390.4	206.75
MOB2	1708.5	1572.5	1113.5	1694.5	1831.0	1044.5	1494.1	332.50
MOB3A	525.5	408.5	267.0	476.0	378.5	265.0	386.8	106.70
MOB3B	1.0	0.5	1.0	2.5	0.0	1.5	1.1	0.86
MOB3C	68.5	43.0	59.0	58.5	41.5	61.5	55.3	10.75
MOCOS	21.0	7.5	20.0	17.5	7.0	16.5	14.9	6.16
MOCS1	1203.5	827.0	694.0	1057.5	877.0	556.5	869.3	235.60
MOCS3	185.5	102.5	74.5	132.0	97.5	85.5	112.9	40.51
MOGAT1	12.0	4.5	17.5	12.5	4.0	21.0	11.9	6.81
MOGAT2	0.5	0.0	0.0	0.0	0.0	0.0	0.1	0.20
MOGS	99.0	122.0	108.5	95.0	120.0	146.5	115.2	18.78
MOK	70.0	65.0	67.5	62.0	71.0	61.0	66.1	4.13
MON1A	258.5	287.5	265.5	236.0	288.0	312.5	274.7	26.89
MON2	679.0	406.5	619.0	670.5	446.0	597.0	569.7	115.95
MORC2	1371.0	1574.0	1908.5	1318.0	1867.0	1853.0	1648.6	264.18
MORC4	806.0	511.5	773.5	770.5	549.5	703.5	685.8	125.37
MORF4L1	1316.0	1179.5	1016.5	1403.5	1337.0	1137.5	1231.7	145.34
MORN2	6.0	1.5	0.5	9.5	5.5	2.5	4.3	3.37
MORN3	0.5	1.5	3.0	1.5	1.5	4.5	2.1	1.43
MORN4	538.0	602.5	536.5	529.0	557.0	609.5	562.1	35.32
MOS	0.0	0.5	0.5	0.5	1.0	1.0	0.6	0.38
MOSPD1	678.5	671.5	596.0	635.0	756.5	582.0	653.3	63.75

MOV10	77.5	58.0	58.0	76.0	41.5	53.5	60.8	13.79
MOV10L1	0.0	0.0	0.0	0.0	0.0	0.0	0.0	0.00
MOXD1	106.0	368.5	662.5	217.0	534.0	1943.0	638.5	670.42
MPC1	1766.0	3768.0	2860.5	1974.0	4483.0	2658.0	2918.3	1045.07
MPC2	974.5	846.0	507.0	952.0	806.5	410.5	749.4	235.76
MPDZ	173.5	125.0	364.0	190.0	153.0	321.0	221.1	97.47
MPEG1	36.0	34.5	22.5	36.0	34.0	28.5	31.9	5.38
MPG	351.5	450.0	265.5	340.5	461.5	273.0	357.0	84.02
MPHOSPH10	633.5	521.0	552.0	658.5	546.5	444.5	559.3	77.73
MPHOSPH6	425.5	451.5	461.0	365.5	473.0	433.0	434.9	38.25
MPHOSPH8	599.0	409.5	396.5	535.0	469.0	420.5	471.6	80.45
MPHOSPH9	192.0	97.0	99.5	182.0	103.5	87.0	126.8	47.03
MPI	437.0	504.0	425.5	454.0	518.5	406.5	457.6	44.59
MPL	61.5	31.5	23.0	54.0	29.5	23.5	37.2	16.45
MPLKIP	108.0	56.5	52.0	84.0	71.5	58.5	71.8	21.27
MPND	11.5	15.0	6.5	13.5	16.0	7.5	11.7	3.93
MPP1	346.5	195.0	142.5	243.0	201.0	92.0	203.3	87.45
MPP2	16.5	12.5	9.0	12.5	9.5	9.5	11.6	2.87
MPP3	661.5	670.5	478.5	523.5	736.0	399.5	578.3	130.56
MPP4	135.0	128.0	87.0	116.5	152.0	86.5	117.5	26.45
MPP5	158.5	70.0	204.0	161.0	73.5	171.0	139.7	55.07
MPP6	4.5	10.5	21.5	9.5	12.5	34.5	15.5	10.84
MPP7	1.5	0.5	2.5	2.0	2.5	2.0	1.8	0.75
MPPE1	404.5	355.5	398.0	370.5	433.0	403.0	394.1	27.43
MPPED2	695.0	353.5	678.0	610.0	405.0	638.0	563.3	146.52
MPRIP	2141.0	3459.5	4122.5	2010.0	3851.0	4438.5	3337.1	1029.65
MPST	904.0	742.0	601.5	821.0	787.5	487.0	723.8	153.19
MPV17	521.5	411.5	191.0	487.5	392.5	201.0	367.5	141.10
MPV17L2	761.5	816.5	535.0	654.0	811.5	590.5	694.8	119.13
MPZL1	68.5	29.5	49.5	52.5	28.0	43.0	45.2	15.24
MPZL2	1.0	0.0	0.5	3.0	0.0	0.0	0.8	1.17
MPZL3	0.0	0.0	0.0	0.0	0.0	0.0	0.0	0.00
MRAP	0.0	0.0	1.0	0.0	0.0	0.0	0.2	0.41
MRAP2	0.5	0.5	0.5	1.0	0.5	0.0	0.5	0.32
MRAS	3826.5	10381.5	7381.0	3457.5	12024.5	6353.0	7237.3	3450.40
MRC2	376.5	635.5	1864.5	592.5	647.5	2271.5	1064.7	793.84
MRE11A	731.5	368.0	352.5	705.5	426.5	310.5	482.4	186.79
MREG	57.0	113.5	77.0	53.5	143.5	102.5	91.2	35.08
MRGBP	720.5	591.0	373.5	726.0	641.5	371.0	570.6	161.71
MRM1	172.0	191.0	150.5	134.0	163.0	141.0	158.6	21.11
MRPL1	531.0	350.5	293.5	558.5	396.5	256.0	397.7	123.93
MRPL10	535.0	855.0	520.0	466.0	793.0	537.0	617.7	163.07
MRPL12	778.0	992.0	500.0	693.0	973.5	474.0	735.1	223.56
MRPL13	616.5	477.5	308.0	588.5	484.0	266.5	456.8	143.09
MRPL14	865.5	832.5	616.0	815.0	966.5	621.0	786.1	140.02
MRPL15	382.0	402.5	394.0	405.5	483.5	407.5	412.5	36.02
MRPL16	984.0	1011.5	578.0	859.0	958.0	552.5	823.8	206.96
MRPL17	1593.0	1912.0	1057.5	1664.0	1976.5	1222.5	1570.9	367.42
MRPL18	1466.5	1707.0	1052.0	1378.5	1810.5	1051.0	1410.9	319.32
MRPL19	361.0	350.5	311.5	353.5	380.5	287.0	340.7	34.62
MRPL2	517.5	506.0	265.5	358.5	408.5	264.5	386.8	111.57
MRPL20	1652.0	1682.0	998.0	1480.0	1844.0	971.0	1437.8	369.76
MRPL21	583.0	508.5	361.0	547.0	574.5	316.0	481.7	114.79
MRPL22	596.5	568.5	510.0	559.0	584.5	474.0	548.8	47.21

MRPL23	353.5	347.0	227.0	345.0	330.0	227.0	304.9	60.84
MRPL24	627.0	780.0	496.0	621.5	802.5	578.5	650.9	118.59
MRPL27	700.0	839.5	586.5	646.5	834.0	508.0	685.8	133.27
MRPL28	1653.0	1637.0	892.5	1549.0	1776.0	892.0	1399.9	399.84
MRPL3	806.5	716.0	568.5	807.5	781.5	456.5	689.4	145.40
MRPL30	370.5	388.5	263.5	312.0	413.5	216.5	327.4	76.97
MRPL32	582.0	476.5	404.5	530.5	446.5	320.5	460.1	92.57
MRPL33	1465.0	1933.0	1112.0	1529.0	2164.5	1137.0	1556.8	422.87
MRPL34	871.0	1182.0	733.5	841.5	1272.0	701.0	933.5	237.81
MRPL35	516.0	469.0	259.5	509.0	533.0	242.0	421.4	133.97
MRPL37	1422.5	1009.5	587.0	1312.0	1096.0	627.5	1009.1	344.67
MRPL38	751.5	699.5	605.5	651.0	663.5	485.5	642.8	91.28
MRPL39	505.0	384.5	335.5	466.5	410.0	255.5	392.8	90.02
MRPL40	1204.5	1089.0	567.0	1038.5	1111.0	563.5	928.9	286.79
MRPL41	1065.0	1083.0	369.0	1076.0	1060.0	367.0	836.7	363.12
MRPL42	506.0	550.0	409.0	480.0	590.5	373.0	484.8	82.63
MRPL43	337.5	373.0	158.0	282.0	340.5	163.0	275.7	93.89
MRPL44	625.0	581.0	370.0	552.0	612.5	361.5	517.0	119.91
MRPL45	1144.5	1274.5	889.5	1013.5	1282.5	857.0	1076.9	186.22
MRPL46	451.0	473.5	334.5	412.5	462.5	296.0	405.0	73.51
MRPL47	501.0	553.5	375.0	459.0	550.5	340.5	463.3	89.51
MRPL48	720.0	419.5	341.5	632.0	453.0	287.0	475.5	168.17
MRPL50	383.0	451.5	283.5	352.5	438.5	277.0	364.3	74.50
MRPL51	1176.5	728.5	334.0	1063.0	728.5	328.0	726.4	354.57
MRPL52	207.0	262.0	173.0	194.0	258.0	173.5	211.3	39.91
MRPL53	204.0	159.5	136.5	186.5	173.5	119.5	163.3	31.46
MRPL54	710.5	656.5	245.5	637.5	627.5	226.5	517.3	219.88
MRPL55	527.0	923.0	585.0	444.5	903.5	632.5	669.3	199.23
MRPL57	721.0	815.5	477.5	655.5	803.5	468.5	656.9	153.91
MRPL9	864.0	1221.5	580.5	795.0	1253.0	604.5	886.4	292.77
MRPS10	473.0	306.5	239.5	407.0	331.0	208.5	327.6	99.85
MRPS11	1626.0	1837.0	922.0	1466.5	1842.0	934.5	1438.0	419.13
MRPS12	1759.5	1272.5	529.0	1591.0	1158.5	487.5	1133.0	529.88
MRPS14	249.5	609.5	736.0	224.5	719.5	767.0	551.0	249.08
MRPS15	1191.5	1340.0	725.5	1080.0	1358.5	684.0	1063.3	296.11
MRPS16	655.0	884.0	459.0	614.5	845.5	498.5	659.4	175.02
MRPS17	1119.5	1227.5	898.5	956.0	1250.0	853.5	1050.8	171.31
MRPS18A	329.5	312.5	278.0	296.0	345.0	231.0	298.7	40.75
MRPS18B	360.0	459.5	293.5	328.5	476.0	278.5	366.0	83.90
MRPS18C	611.5	396.5	206.0	577.0	437.5	190.0	403.1	178.41
MRPS2	1096.5	935.0	816.5	944.0	940.5	818.5	925.2	103.18
MRPS21	699.5	917.0	470.0	639.0	889.0	446.5	676.8	200.24
MRPS22	361.5	312.0	238.0	359.5	335.5	208.5	302.5	64.66
MRPS23	750.5	763.5	400.0	689.0	840.0	373.0	636.0	199.33
MRPS24	223.0	269.0	130.5	213.5	251.5	145.5	205.5	56.11
MRPS25	742.5	924.5	579.5	695.0	999.5	509.5	741.8	190.94
MRPS26	835.0	880.5	708.5	695.5	852.0	592.5	760.7	112.67
MRPS27	488.0	348.0	332.5	410.5	359.5	271.0	368.3	73.96
MRPS28	402.0	528.0	249.0	402.5	612.5	294.0	414.7	137.33
MRPS30	491.5	207.0	269.5	416.5	241.5	234.5	310.1	115.69
MRPS31	1492.5	935.5	545.5	1298.5	944.5	412.0	938.1	416.70
MRPS33	1316.0	1095.0	662.5	1176.5	1230.0	603.5	1013.9	304.27
MRPS34	902.0	981.5	844.0	821.0	1012.0	790.5	891.8	89.62
MRPS35	377.0	323.5	329.0	343.0	341.0	281.5	332.5	31.17

MRPS5	570.0	498.5	392.5	520.5	513.0	385.5	480.0	74.51
MRPS6	1173.5	868.0	511.0	1266.0	1025.0	550.0	898.9	315.98
MRPS7	924.5	1079.5	938.0	835.5	1118.0	889.5	964.2	110.77
MRPS9	631.0	429.0	295.5	563.0	433.5	253.5	434.3	146.45
MRRF	296.0	410.0	291.5	271.5	421.5	275.0	327.6	69.03
MRS2	126.0	125.5	139.5	106.5	139.5	138.5	129.3	12.95
MRTO4	575.5	625.5	448.0	549.5	619.5	444.5	543.8	80.60
MRVI1	18.5	3.5	10.5	22.0	7.0	8.0	11.6	7.17
MSANTD1	2.0	0.5	0.0	0.0	1.0	0.0	0.6	0.80
MSANTD2	171.0	199.0	147.0	150.5	216.5	141.5	170.9	30.73
MSANTD3	74.5	77.5	126.0	71.5	91.0	107.0	91.3	21.52
MSANTD4	260.5	279.0	282.0	215.5	286.0	302.5	270.9	30.31
MSGN1	0.5	0.0	0.0	1.0	0.0	0.5	0.3	0.41
MSH2	448.5	206.5	285.0	445.0	208.0	234.0	304.5	113.79
MSH3	200.0	112.0	127.0	213.0	118.0	94.5	144.1	49.67
MSH4	359.5	185.5	118.5	292.0	221.0	112.0	214.8	97.61
MSH5	0.0	0.0	1.0	0.0	0.0	0.0	0.2	0.41
MSH6	532.0	400.5	400.5	539.5	406.5	323.0	433.7	84.92
MSI1	266.0	103.0	165.0	257.0	121.0	155.0	177.8	68.64
MSL1	851.0	676.0	470.0	768.0	685.5	587.0	672.9	133.78
MSL2	294.5	243.0	333.0	271.0	259.0	341.5	290.3	40.13
MSL3	469.5	431.0	477.5	479.0	501.0	432.5	465.1	27.86
MSMO1	5688.0	2211.0	3689.0	6140.0	2430.5	3265.0	3903.9	1653.74
MSMP	136.0	176.0	159.5	129.0	211.0	178.0	164.9	30.24
MSN	8515.0	7910.0	11078.5	8789.0	9671.5	9687.0	9275.2	1118.62
MSRA	169.0	142.0	81.0	162.5	131.0	73.0	126.4	40.73
MSRB1	694.5	460.5	350.0	640.5	483.0	343.0	495.3	145.90
MSRB3	3541.0	2094.5	1877.5	3915.5	2618.5	1926.5	2662.3	874.56
MSS51	2.5	1.0	1.0	2.5	0.0	1.0	1.3	0.98
MST1	44.5	61.0	31.0	50.5	65.0	69.5	53.6	14.43
MST1R	59.0	70.5	76.5	67.0	56.0	101.5	71.8	16.39
MSTN	2.5	29.0	3.0	11.0	51.0	9.0	17.6	19.00
MSTO1	1087.5	1052.5	671.5	991.0	1074.0	773.0	941.6	176.03
MSX1	115.0	169.5	141.5	126.5	200.0	224.0	162.8	42.97
MSX2	8.0	3.0	6.0	3.0	4.0	2.0	4.3	2.25
MT2A	199.5	115.5	84.5	141.0	112.0	68.5	120.2	46.38
MTA1	1241.0	1145.0	1151.5	1167.5	1305.0	1288.0	1216.3	71.11
MTA3	499.5	472.0	301.5	479.0	531.0	248.5	421.9	116.84
MTBP	82.5	19.0	62.5	79.0	24.5	28.5	49.3	28.72
MTCH1	405.0	302.0	227.0	357.5	329.0	221.0	306.9	72.75
MTCH2	1824.0	1631.0	1057.5	1627.5	1650.5	977.5	1461.3	352.39
MTCL1	357.0	397.0	751.0	364.0	422.5	680.0	495.3	173.68
MTCP1	93.5	69.5	94.0	108.5	77.5	99.0	90.3	14.33
MTDH	1647.0	1073.5	1573.0	1732.0	1264.0	1610.5	1483.3	256.48
MTERF2	270.5	141.0	189.0	229.0	171.5	171.0	195.3	46.77
MTERF3	299.5	266.0	224.5	258.5	265.0	191.0	250.8	37.77
MTF1	146.0	67.5	258.0	147.5	84.5	195.5	149.8	70.50
MTFMT	414.5	275.0	203.0	398.0	291.5	221.5	300.6	88.30
MTFP1	92.0	169.5	109.0	97.0	175.0	88.5	121.8	39.70
MTFR1	1140.5	1339.5	1258.5	1082.5	1455.5	1142.5	1236.5	142.00
MTFR1L	1745.5	2419.0	2066.0	1391.5	2596.0	1655.5	1978.9	466.09
MTFR2	406.5	145.5	191.5	451.5	149.0	119.5	243.9	145.91
MTG1	1251.5	947.5	430.5	1103.0	897.5	443.5	845.6	340.02
MTG2	216.5	171.0	160.0	215.0	196.5	133.5	182.1	33.01

MTHFD1	606.0	516.5	665.5	613.5	567.0	467.5	572.7	71.61
MTHFD1L	695.5	659.5	1096.0	652.0	669.5	749.0	753.6	171.40
MTHFD2	668.5	925.5	1069.0	710.5	907.5	972.5	875.6	155.21
MTHFD2L	14.0	8.5	28.0	14.0	11.0	23.0	16.4	7.50
MTHFR	453.0	166.5	118.5	364.5	189.0	112.0	233.9	141.28
MTHFS	66.0	63.0	58.0	63.0	85.0	55.0	65.0	10.56
MTHFSD	287.5	245.5	184.5	280.5	268.5	199.5	244.3	43.24
MTIF2	378.5	251.5	279.5	327.0	272.0	263.0	295.3	48.34
MTIF3	261.0	230.0	208.0	232.5	243.0	189.0	227.3	25.52
MTL5	1.0	1.0	1.5	1.0	3.5	5.0	2.2	1.69
MTMR1	840.0	539.0	569.0	757.5	583.5	551.5	640.1	126.55
MTMR10	283.5	132.0	413.0	276.5	143.5	350.5	266.5	111.47
MTMR12	343.0	278.0	299.0	283.5	340.5	289.0	305.5	28.93
MTMR14	382.0	336.5	281.5	360.5	341.0	245.5	324.5	51.20
MTMR2	1259.5	750.5	803.0	1211.5	886.0	751.0	943.6	231.96
MTMR3	540.0	340.5	769.5	533.5	349.0	722.5	542.5	180.15
MTMR4	416.5	376.5	483.0	412.5	432.5	471.0	432.0	39.56
MTMR6	531.5	415.0	472.5	539.5	490.0	440.0	481.4	49.32
MTMR8	72.0	12.5	69.5	81.5	13.0	57.5	51.0	30.60
MTMR9	1090.5	549.5	597.0	916.5	576.0	506.0	705.9	238.92
MTNR1A	42.5	11.5	3.0	28.0	11.0	3.5	16.6	15.59
MTO1	848.5	547.5	395.0	779.5	589.0	347.0	584.4	200.71
MTOR	743.5	535.0	622.0	672.5	574.0	539.0	614.3	82.12
MTPN	3433.5	3000.5	3298.5	3220.5	3408.0	2806.0	3194.5	246.07
MTR	175.0	92.5	159.5	182.5	115.0	140.5	144.2	35.18
MTRF1	388.0	286.5	263.0	256.0	288.0	190.0	278.6	64.38
MTRF1L	143.5	104.0	111.5	163.5	109.0	95.0	121.1	26.50
MTRR	536.5	323.5	375.5	495.0	323.0	317.0	395.1	96.73
MTSS1L	809.5	1194.5	1548.5	709.5	1181.0	1412.5	1142.6	328.72
MTTP	1.0	2.5	1.5	1.5	1.5	0.0	1.3	0.82
MTURN	1847.5	842.0	624.0	1723.5	997.0	597.5	1105.3	548.38
MTUS1	322.5	247.0	193.0	257.5	285.5	175.5	246.8	55.35
MTX2	1583.5	1659.5	1212.5	1391.0	1713.5	1103.5	1443.9	249.35
MTX3	179.0	83.5	143.0	180.5	88.0	123.0	132.8	42.54
MUC	15.5	12.0	18.5	16.0	15.5	22.5	16.7	3.53
MUC13	0.5	0.0	1.0	0.0	0.0	0.5	0.3	0.41
MUC2	10.0	2.5	4.5	3.0	3.0	2.0	4.2	2.98
MUC3A	9.5	9.5	53.0	0.0	0.5	0.5	12.2	20.50
MUC4	114.0	179.5	51.0	97.5	172.0	73.0	114.5	52.11
MUC6	1.0	0.5	1.0	0.5	0.0	0.0	0.5	0.45
MUL1	187.0	220.0	154.0	169.0	202.5	169.0	183.6	24.48
MURC	83.5	1241.0	1443.5	59.0	1139.5	736.5	783.8	598.12
MUSK	409.5	731.0	937.5	260.0	776.5	888.5	667.2	272.13
MUT	306.5	186.5	309.0	291.5	202.0	265.5	260.2	53.58
MUTYH	83.5	62.5	37.0	91.0	56.0	33.5	60.6	23.51
MVB12A	1038.0	1146.0	839.5	902.0	1158.5	845.5	988.3	145.80
MVB12B	173.5	121.5	337.0	180.5	121.0	291.0	204.1	89.93
MVD	760.5	563.5	461.5	636.5	458.0	347.5	537.9	147.24
MVK	1070.0	559.0	736.5	947.5	578.5	616.0	751.3	212.33
MVP	1425.0	1134.5	1095.0	1259.0	1040.0	1140.0	1182.3	139.09
MX1	3.0	3.5	5.0	0.5	4.0	6.5	3.8	2.02
MXD1	194.5	145.5	241.5	202.5	160.0	216.0	193.3	35.55
MXD4	311.0	212.0	415.5	297.0	227.5	432.5	315.9	92.20
MXI1	469.0	373.0	425.5	428.5	422.0	451.5	428.3	32.56

MXRA5	1508.5	1022.0	3773.0	1226.0	883.0	3023.5	1906.0	1198.57
MXRA7	1941.0	1625.5	2248.5	1840.5	1968.0	2023.0	1941.1	205.74
MXRA8	10766.0	9380.5	10628.0	11725.5	10979.0	12866.0	11057.5	1166.60
MYADM	0.0	0.0	0.5	0.0	0.0	0.5	0.2	0.26
MYADML2	11.0	207.5	191.0	11.5	249.5	186.5	142.8	104.32
MYB	42.0	20.5	37.0	41.0	21.0	24.0	30.9	10.16
MYBBP1A	588.0	457.0	468.0	644.0	446.5	395.5	499.8	95.01
MYBL1	494.0	191.0	311.0	531.5	237.0	193.5	326.3	151.25
MYBL2	488.0	287.5	355.5	488.0	315.5	312.0	374.4	90.64
MYBPC1	0.5	2.5	3.5	0.0	4.5	10.0	3.5	3.62
MYBPC3	35.5	432.0	338.5	35.0	506.5	351.0	283.1	201.30
MYBPH	381.0	3289.5	2892.0	261.5	3398.0	2634.5	2142.8	1437.83
MYC	498.5	544.5	533.5	497.0	566.5	566.0	534.3	31.05
MYCBP	458.0	473.5	259.0	364.5	517.5	250.5	387.2	114.08
MYCBP2	205.5	86.0	415.0	246.0	97.0	367.5	236.2	135.78
MYCBPAP	306.5	111.5	106.0	202.0	96.0	82.5	150.8	87.27
MYCL	343.0	702.0	534.0	497.5	829.0	759.0	610.8	183.49
MYCN	54.5	33.0	89.5	52.0	41.5	83.5	59.0	22.72
MYCT1	0.0	0.0	0.0	0.0	0.0	0.0	0.0	0.00
MYD88	295.0	194.5	264.0	302.0	234.0	275.0	260.8	40.48
MYEF2	300.5	257.5	388.0	273.5	286.5	328.0	305.7	46.96
MYEOV2	337.0	564.5	376.5	294.5	598.0	373.5	424.0	125.83
MYF5	1196.5	1010.0	575.0	1224.5	946.0	840.0	965.3	241.26
MYF6	833.5	518.5	320.5	845.5	517.5	370.5	567.7	224.84
MYH10	3773.0	2097.5	4264.0	4013.5	2284.5	3374.5	3301.2	910.48
MYH11	178.5	37.0	59.5	167.5	55.0	51.5	91.5	63.67
MYH7B	42.0	3238.0	1284.5	53.0	4118.5	2299.0	1839.2	1678.77
MYH9	9016.5	3385.0	8553.5	9622.0	3592.5	7451.5	6936.8	2764.55
MYL1	171.5	12856.0	6592.5	149.0	13838.5	6102.5	6618.3	5911.34
MYL10	456.0	14822.5	9700.0	337.0	17613.0	8112.0	8506.8	7154.74
MYL2	0.0	1.0	0.5	0.5	1.0	0.5	0.6	0.38
MYL3	36.0	2198.0	196.0	23.0	3387.5	258.5	1016.5	1429.25
MYL4	69.0	230.5	131.5	62.0	209.5	104.5	134.5	71.13
MYL9	6089.5	9770.5	6922.0	5650.5	10163.0	6377.0	7495.4	1962.24
MYLIP	349.0	250.0	360.0	298.5	294.0	358.0	318.3	44.51
MYLK	16.0	9.0	36.0	18.0	17.5	27.0	20.6	9.49
MYLK2	540.0	263.0	117.0	454.0	260.0	153.5	297.9	166.86
MYLK3	103.0	59.0	106.0	94.5	62.5	87.0	85.3	20.20
MYLK4	25.5	34.5	42.5	24.0	43.5	35.0	34.2	8.20
MYNN	464.0	269.0	354.5	437.0	308.5	330.5	360.6	75.60
MYO10	630.5	1641.5	1518.0	789.5	1880.5	1121.5	1263.6	496.76
MYO15A	5.0	0.5	0.0	3.0	1.5	2.0	2.0	1.82
MYO16	43.5	630.0	1024.0	32.5	686.5	715.5	522.0	399.18
MYO18A	2135.5	1508.5	1843.5	1949.0	1462.0	1601.0	1749.9	268.48
MYO18B	338.5	1284.0	716.0	232.0	1238.5	804.0	768.8	438.95
MYO19	177.5	139.5	225.5	196.0	158.5	227.5	187.4	35.67
MYO1A	6.0	2.5	3.0	5.0	4.5	5.5	4.4	1.39
MYO1B	1189.5	807.5	1170.5	2004.0	1150.0	1530.0	1308.6	410.39
MYO1C	3637.5	3004.5	4376.5	4134.5	3435.5	3879.0	3744.6	494.80
MYO1D	324.0	346.5	525.5	320.5	347.5	683.0	424.5	148.29
MYO1E	270.0	312.0	524.5	281.0	395.0	519.5	383.7	115.77
MYO1F	2.5	13.5	2.5	3.0	9.0	8.0	6.4	4.51
MYO1G	1.0	1.0	1.5	2.0	1.5	1.0	1.3	0.41
MYO1H	2782.0	2342.0	2392.0	2940.0	2461.0	2330.0	2541.2	256.91

MYO3A	4.0	1.5	0.5	1.5	2.0	1.0	1.8	1.21
MYO3B	1.5	0.5	1.5	1.0	0.5	1.0	1.0	0.45
MYO5C	2.5	0.0	1.5	2.5	0.0	0.5	1.2	1.17
MYO7A	0.0	0.5	0.0	0.0	0.5	0.0	0.2	0.26
MYO7B	20.5	64.0	54.0	18.0	76.0	40.0	45.4	23.48
MYO9A	470.0	546.5	856.0	440.0	657.0	777.0	624.4	168.46
MYO9B	1088.5	656.0	1182.0	1091.0	755.5	1098.5	978.6	216.47
MYOC	80.0	57.5	67.5	81.5	57.5	66.5	68.4	10.47
MYOCD	815.5	244.0	194.5	922.0	282.0	246.0	450.7	326.78
MYOD1	4323.0	6608.5	7396.5	4036.0	7131.0	7627.5	6187.1	1594.24
MYOF	2316.0	1297.0	2269.0	2127.0	1493.0	2125.5	1937.9	431.79
MYOM1	673.5	2672.0	2683.5	612.0	3087.0	2539.0	2044.5	1101.37
MYOM2	15.0	96.0	86.5	3.5	81.0	21.5	50.6	41.49
MYOM3	2669.0	4079.5	5909.5	1796.0	4780.5	4375.0	3934.9	1483.65
MYOT	8.0	39.5	61.0	5.5	57.0	91.0	43.7	33.06
MYOZ1	73.0	492.0	598.5	52.5	607.5	574.5	399.7	264.22
MYOZ2	941.0	5344.5	3503.5	926.0	6274.0	3277.0	3377.7	2201.42
MYOZ3	88.0	44.5	24.5	98.0	51.0	51.0	59.5	27.89
MYPN	186.0	922.5	899.5	138.5	1232.0	870.5	708.2	442.83
MYRF	53.0	32.0	87.0	62.0	23.5	55.0	52.1	22.57
MYRFL	0.0	1.0	0.0	0.0	0.0	0.5	0.3	0.42
MYRIP	3.0	2.5	4.0	4.0	3.5	5.0	3.7	0.88
MYZAP	5.5	16.0	25.5	5.5	8.0	16.0	12.8	7.89
MZB1	0.5	0.0	0.0	0.0	0.5	0.5	0.3	0.27
MZT1	201.0	178.0	182.5	169.0	195.0	153.5	179.8	17.32
N4BP1	699.5	507.5	676.0	658.0	623.5	655.0	636.6	68.03
N4BP2	85.5	26.5	203.5	73.0	29.5	169.0	97.8	73.15
N4BP2L1	46.5	15.5	19.0	55.5	19.5	17.0	28.8	17.46
N4BP3	185.5	149.0	135.0	158.5	111.0	117.0	142.7	27.73
N6AMT1	374.5	448.0	256.0	359.0	486.0	187.5	351.8	113.14
N6AMT2	494.0	493.0	478.0	489.5	533.0	496.5	497.3	18.64
NAA15	1948.5	1589.5	1846.0	2043.0	1805.0	1558.0	1798.3	192.92
NAA16	603.5	601.5	514.5	566.0	662.5	488.0	572.7	63.95
NAA20	959.5	1127.5	662.5	859.0	1270.5	598.0	912.8	261.10
NAA25	682.0	438.5	508.0	647.0	450.0	456.0	530.3	107.26
NAA30	294.5	372.0	404.0	268.0	385.5	387.5	351.9	56.30
NAA35	548.0	203.0	295.0	511.5	236.0	279.0	345.4	146.85
NAA38	1364.5	1764.0	1579.5	1202.5	1780.0	1580.0	1545.1	226.05
NAA40	306.5	383.5	306.0	319.5	378.5	291.0	330.8	39.92
NAA50	2647.5	2366.0	2749.0	2717.0	2515.5	2518.0	2585.5	145.26
NAA60	392.0	389.0	532.0	408.0	364.5	469.5	425.8	62.86
NAAA	154.5	147.5	142.5	155.0	178.5	135.5	152.3	14.83
NAALADL2	134.0	14.5	21.5	132.5	20.5	17.0	56.7	59.38
NAB1	544.0	507.0	787.0	532.0	578.0	814.5	627.1	136.73
NAB2	102.0	115.0	217.0	90.0	102.0	246.5	145.4	67.98
NABP1	607.5	538.0	815.0	450.5	536.0	607.5	592.4	123.52
NABP2	182.0	196.5	91.0	198.5	195.0	108.5	161.9	48.82
NACA	11406.5	8196.0	3783.0	9954.0	8626.5	3746.5	7618.8	3189.25
NACAD	309.5	199.5	270.5	359.0	243.0	291.5	278.8	55.09
NACC1	21.0	13.0	70.5	28.5	15.5	47.0	32.6	22.23
NACC2	256.0	133.5	145.5	288.0	135.5	138.0	182.8	69.99
NADK	254.0	159.5	557.5	270.0	178.0	501.5	320.1	168.60
NADK2	403.5	307.5	301.5	397.5	364.5	331.5	351.0	44.32
NADSYN1	202.0	52.0	57.5	162.5	42.5	47.5	94.0	69.67

NAE1	693.0	493.0	429.0	672.0	543.5	351.0	530.3	134.56
NAF1	492.5	301.0	385.0	370.0	305.5	278.0	355.3	79.15
NAGA	887.5	1132.0	1275.5	960.0	1403.5	1601.5	1210.0	271.14
NAGLU	370.5	365.5	362.0	346.0	351.5	372.0	361.3	10.46
NAGPA	173.5	147.5	160.5	176.5	155.5	165.5	163.2	10.97
NAIF1	652.5	632.0	575.0	644.5	650.0	620.5	629.1	29.09
NALCN	64.0	22.5	30.5	77.5	31.0	37.0	43.8	21.84
NAMPT	119.0	60.0	227.5	135.0	61.0	208.5	135.2	71.16
NANP	50.0	15.0	45.0	58.5	20.5	38.0	37.8	17.02
NANS	1128.5	754.5	725.5	1154.5	749.0	600.5	852.1	231.23
NAP1L1	6425.5	4255.5	4281.0	5397.0	4205.5	3570.0	4689.1	1035.09
NAP1L4	2822.0	2835.5	2666.0	2624.0	2993.0	2443.0	2730.6	193.11
NAPB	145.0	95.5	120.0	127.5	128.5	149.5	127.7	19.35
NAPEPLD	7.5	7.0	11.5	14.0	13.5	15.0	11.4	3.43
NAPG	349.5	197.5	396.5	307.0	221.5	278.0	291.7	75.56
NAPRT	384.5	250.0	209.5	299.0	240.0	197.0	263.3	69.24
NARF	951.0	1142.0	906.0	938.5	1359.5	834.5	1021.9	194.39
NARFL	443.0	315.0	488.0	329.0	322.5	491.0	398.1	85.00
NARS	1053.0	871.0	1003.0	982.5	1002.0	895.5	967.8	69.97
NARS2	244.5	172.5	102.5	217.5	175.0	115.0	171.2	55.52
NASP	1283.0	930.5	699.0	1262.5	954.5	612.0	956.9	277.81
NAT10	969.5	669.5	799.5	1115.5	747.0	699.5	833.4	174.07
NAT6	25.5	31.5	20.0	24.5	16.5	23.0	23.5	5.11
NAT9	716.0	490.5	315.5	534.5	434.5	273.5	460.8	160.20
NATD1	40.0	18.5	54.5	40.5	19.0	51.0	37.3	15.42
NAV1	285.0	177.5	404.0	245.5	183.0	394.0	281.5	99.49
NAV2	308.0	307.0	911.5	317.5	446.5	968.5	543.2	312.39
NAV3	187.5	155.5	542.5	302.0	170.0	504.0	310.3	173.34
NBAS	599.0	399.5	583.5	585.0	471.5	602.0	540.1	84.47
NBEAL1	583.0	431.0	810.5	469.5	512.5	722.5	588.2	149.67
NBEAL2	182.5	490.0	391.0	127.0	501.0	454.0	357.6	162.69
NBN	123.5	60.5	138.5	100.0	55.5	97.5	95.9	33.11
NBR1	1041.0	641.5	937.5	956.0	724.5	859.5	860.0	151.05
NCALD	302.5	3063.0	4011.0	756.0	3900.5	3370.0	2567.2	1622.46
NCAM1	948.5	2931.0	7080.0	1171.5	3370.5	6277.0	3629.8	2557.33
NCAM2	0.0	0.0	0.0	0.5	0.0	0.0	0.1	0.20
NCAN	0.0	1.0	0.5	0.0	1.0	0.0	0.4	0.49
NCAPD3	738.5	568.5	475.0	674.5	540.0	348.0	557.4	139.70
NCAPG	399.0	132.5	299.0	436.0	141.0	185.0	265.4	132.43
NCAPG2	210.5	81.0	252.0	215.5	75.5	121.0	159.3	76.15
NCAPH	989.0	388.5	617.5	981.0	347.0	361.0	614.0	303.80
NCAPH2	516.0	229.0	288.0	463.5	214.0	188.0	316.4	139.21
NCBP1	1320.0	943.5	851.5	1270.5	1025.5	801.5	1035.4	216.03
NCBP2	1579.0	1198.5	992.0	1547.5	1266.5	980.0	1260.6	260.18
NCDN	428.5	461.0	437.5	444.5	467.0	386.0	437.4	29.00
NCEH1	733.5	334.5	782.5	786.0	412.0	713.0	626.9	199.99
NCF1	36.5	19.0	14.0	7.0	8.5	7.5	15.4	11.32
NCF2	71.0	37.5	32.5	63.5	34.5	30.5	44.9	17.61
NCF4	2.0	5.5	4.5	5.0	4.0	4.5	4.3	1.21
NCK2	608.5	847.5	1103.0	665.0	939.0	946.5	851.6	186.43
NCKAP1	3975.5	2284.0	2992.5	3991.5	2455.5	2816.0	3085.8	739.39
NCKAP1L	24.0	22.5	25.0	17.0	17.5	24.5	21.8	3.59
NCKAP5	23.5	17.0	50.5	19.5	31.5	31.0	28.8	12.14
NCKIPSD	351.5	456.0	441.0	324.5	514.0	527.0	435.7	82.90

NCL	13320.0	6702.0	6632.0	12074.0	7049.5	5935.5	8618.8	3203.87
NCLN	1110.5	655.0	783.0	1095.0	743.0	776.0	860.4	193.22
NCMAP	74.0	56.5	33.0	69.0	53.0	20.0	50.9	20.84
NCOA1	304.5	205.5	368.5	245.0	213.0	373.5	285.0	75.22
NCOA2	72.5	37.5	165.5	69.5	38.5	157.5	90.2	57.26
NCOA3	43.5	9.5	128.5	58.5	13.0	115.0	61.3	50.48
NCOA4	4627.0	2853.0	3020.5	3763.0	3285.0	2889.0	3406.3	686.24
NCOA5	766.0	537.5	449.0	729.0	558.0	447.5	581.2	136.96
NCOA6	535.0	494.5	873.0	552.5	588.5	835.0	646.4	164.06
NCOA7	189.5	188.5	285.0	193.5	219.0	307.5	230.5	52.62
NCOR1	205.0	171.5	280.5	205.5	225.0	269.5	226.2	41.70
NCOR2	1087.5	841.5	2555.0	1277.0	845.0	2712.5	1553.1	854.26
NCS1	270.0	390.0	548.0	294.5	422.5	473.5	399.8	105.78
NCSTN	593.0	357.0	671.5	616.5	379.5	610.0	537.9	134.22
NDC1	1111.0	499.5	657.0	1096.0	481.0	470.5	719.2	305.36
NDC80	124.5	28.5	185.5	150.5	42.5	87.0	103.1	61.63
NDE1	736.5	420.0	682.0	709.5	428.0	499.5	579.3	146.18
NDEL1	1035.0	844.5	937.0	998.5	846.5	996.5	943.0	81.80
NDFIP1	4373.0	3589.0	2737.0	4181.0	4056.5	2628.0	3594.1	752.72
NDFIP2	1723.5	1109.5	1309.5	1671.5	1212.0	1286.0	1385.3	252.19
NDNF	0.0	0.5	0.5	2.5	1.5	2.0	1.2	0.98
NDOR1	238.5	229.5	190.0	242.0	232.5	192.5	220.8	23.34
NDP	608.0	748.0	438.5	743.5	848.5	389.0	629.3	184.27
NDRG3	2433.0	2062.5	2083.0	2155.0	2261.0	2954.5	2324.8	337.16
NDRG4	266.0	284.0	779.5	246.5	310.0	712.0	433.0	244.09
NDST1	3312.5	1324.0	1326.0	2844.0	1285.5	1613.5	1950.9	893.50
NDST2	248.5	218.5	395.0	235.0	261.5	331.5	281.7	67.83
NDST3	129.5	48.0	83.5	121.0	48.0	64.0	82.3	35.82
NDST4	0.0	0.0	0.0	0.0	0.0	0.0	0.0	0.00
NDUFA1	1061.5	1199.5	556.0	912.5	1161.5	562.5	908.9	288.47
NDUFA10	2964.0	2193.5	1671.5	2598.0	2245.5	1377.5	2175.0	582.46
NDUFA12	765.0	678.0	384.0	686.5	700.0	338.5	592.0	181.90
NDUFA13	587.0	712.5	567.0	508.0	642.0	575.5	598.7	70.33
NDUFA2	469.0	733.0	321.0	423.0	673.5	351.5	495.2	170.43
NDUFA4	3255.5	3499.0	2134.5	2715.0	3588.0	1841.0	2838.8	731.82
NDUFA5	1021.5	2020.0	1564.5	956.5	2186.0	1386.5	1522.5	505.84
NDUFA6	617.5	764.0	283.5	569.5	881.5	307.5	570.6	239.96
NDUFA7	495.5	629.0	339.5	480.5	660.0	372.5	496.2	130.05
NDUFA8	886.5	913.0	566.0	815.5	914.5	513.0	768.1	181.45
NDUFA9	771.5	626.0	778.5	673.0	632.5	643.0	687.4	69.76
NDUFAF1	322.5	294.5	282.5	331.5	346.0	292.0	311.5	25.38
NDUFAF2	406.5	467.0	361.5	446.0	554.5	361.0	432.8	73.60
NDUFAF3	267.0	555.0	212.5	271.0	541.5	260.0	351.2	154.16
NDUFAF4	105.5	66.5	89.5	97.5	61.0	54.0	79.0	21.26
NDUFAF5	356.0	238.0	155.0	324.5	269.5	164.5	251.3	82.02
NDUFAF6	125.0	181.0	93.5	134.5	212.0	107.5	142.3	45.41
NDUFAF7	131.0	146.5	129.5	106.5	152.5	124.5	131.8	16.39
NDUFB1	3190.0	4477.0	1947.5	2777.0	4699.5	2001.0	3182.0	1188.50
NDUFB10	1703.0	1373.0	886.5	1542.5	1516.0	934.0	1325.8	338.86
NDUFB2	698.5	868.0	358.0	639.5	818.0	426.5	634.8	205.93
NDUFB3	1080.0	1325.0	733.5	989.0	1391.0	628.5	1024.5	306.74
NDUFB4	1665.0	1568.0	1252.5	1508.5	1690.0	1159.0	1473.8	219.75
NDUFB5	1229.0	1400.5	971.0	1083.0	1537.0	977.0	1199.6	232.58
NDUFB6	2278.5	2970.5	1460.5	2064.5	3062.0	1429.0	2210.8	707.37

NDUFB7	1074.5	1398.0	706.0	987.5	1425.0	772.0	1060.5	303.71
NDUFB8	1209.5	1401.0	1046.5	1180.0	1403.5	1035.5	1212.7	162.46
NDUFB9	1208.0	1511.0	733.0	1133.5	1461.0	679.0	1120.9	352.51
NDUFC1	609.0	840.0	445.5	547.0	832.5	470.0	624.0	174.28
NDUFC2	717.5	732.0	393.0	620.5	781.5	400.0	607.4	171.53
NDUFS1	1295.0	1084.0	1440.0	1362.0	1141.0	1137.5	1243.3	143.17
NDUFS2	1343.0	1400.5	884.0	1231.5	1387.5	851.5	1183.0	251.54
NDUFS3	1454.0	1628.5	1078.5	1268.5	1644.0	993.5	1344.5	276.43
NDUFS4	1037.0	1063.5	681.0	947.0	1237.5	622.5	931.4	236.92
NDUFS6	388.5	661.5	402.5	360.5	719.0	373.5	484.3	161.22
NDUFS7	1229.0	1113.5	617.0	1144.0	1090.5	533.0	954.5	298.87
NDUFS8	671.0	759.5	524.0	609.0	781.5	521.5	644.4	112.79
NDUFV1	2056.5	1933.0	1355.0	1799.0	1973.0	1368.5	1747.5	310.19
NDUFV2	1368.5	1042.5	835.0	1274.0	1147.0	784.5	1075.3	234.08
NDUFV3	1089.0	1087.0	814.5	987.0	1230.0	857.0	1010.8	156.67
NECAB1	6.0	3.0	3.5	2.5	4.0	3.5	3.8	1.21
NECAB2	48.0	22.5	7.5	44.5	26.5	13.0	27.0	16.40
NECAB3	843.0	597.0	462.0	718.5	553.5	449.5	603.9	152.88
NECAP1	1009.5	682.5	544.5	1015.0	790.0	461.0	750.4	232.15
NECAP2	523.5	399.0	398.5	463.0	480.0	414.0	446.3	50.87
NEDD4	33.5	15.5	40.0	43.0	14.0	31.5	29.6	12.24
NEDD8	287.5	405.0	194.5	288.0	386.5	208.5	295.0	87.36
NEDD9	1264.5	737.5	1065.5	1416.0	729.0	920.5	1022.2	280.37
NEFH	1.0	1.0	3.5	1.5	1.5	2.0	1.8	0.94
NEFL	0.5	0.0	1.5	0.0	0.0	0.5	0.4	0.58
NEFM	0.0	0.0	0.0	0.5	0.0	0.0	0.1	0.20
NEGR1	0.0	0.0	0.0	0.0	0.0	0.0	0.0	0.00
NEIL2	7.0	2.5	3.0	3.0	3.0	0.5	3.2	2.11
NEIL3	42.0	13.5	46.5	30.5	18.0	20.0	28.4	13.55
NEK1	744.5	508.0	641.0	694.0	584.0	610.0	630.3	83.34
NEK10	0.5	0.5	1.0	0.5	2.0	2.0	1.1	0.74
NEK11	70.5	41.5	82.5	68.5	56.0	77.0	66.0	14.97
NEK2	467.5	183.5	200.5	554.5	172.0	143.0	286.8	176.80
NEK3	54.5	34.5	20.0	46.0	39.5	26.5	36.8	12.64
NEK4	860.0	640.0	500.5	708.0	666.5	474.5	641.6	141.82
NEK5	2.5	3.0	1.5	2.5	4.5	2.5	2.8	0.99
NEK6	6089.0	3025.5	2452.5	5903.5	3601.0	2181.0	3875.4	1715.02
NEK7	630.5	484.5	438.5	626.5	504.0	378.0	510.3	101.31
NEK8	199.5	122.0	176.5	193.0	113.0	191.5	165.9	38.36
NEK9	1168.0	2256.0	2103.0	1262.5	2445.0	2051.0	1880.9	534.38
NELFA	251.5	219.5	376.5	243.0	276.0	405.0	295.3	76.68
NELFB	617.5	500.0	478.0	611.0	531.5	465.5	533.9	66.17
NELFCD	812.0	392.5	679.0	787.0	454.5	598.5	620.6	171.95
NELFE	477.5	483.5	275.0	394.5	478.0	281.5	398.3	98.74
NELL1	5.0	2.0	9.0	1.0	2.5	6.5	4.3	3.06
NELL2	1645.0	1765.5	2138.5	1466.0	1666.5	1610.5	1715.3	228.94
NEMF	798.0	651.0	666.5	804.0	658.0	591.0	694.8	86.51
NENF	557.5	285.5	386.5	532.5	306.0	330.5	399.8	117.75
NEO1	299.5	91.5	390.5	345.0	125.5	304.5	259.4	121.88
NES	46597.5	48515.0	37110.5	36469.0	44120.5	30390.0	40533.8	6992.43
NET1	866.0	1160.5	1386.5	662.0	1346.5	1182.0	1100.6	282.78
NETO1	1.0	0.5	0.5	1.0	0.0	0.0	0.5	0.45
NETO2	75.5	35.0	80.5	71.5	54.0	101.0	69.6	22.73
NEU2	0.0	2.5	0.5	0.0	3.0	1.0	1.2	1.29

NEU3	117.5	104.0	82.5	89.0	117.5	93.0	100.6	14.85
NEU4	0.0	0.0	0.0	0.0	0.0	0.0	0.0	0.00
NEURL1	163.0	137.5	418.5	194.0	156.0	376.0	240.8	123.26
NEURL1B	203.5	592.5	1184.0	147.0	595.0	824.5	591.1	388.20
NEURL2	18.0	57.5	27.0	21.0	49.5	46.5	36.6	16.63
NEUROD2	0.0	0.0	0.0	0.0	0.0	0.0	0.0	0.00
NEUROD4	0.0	0.0	0.0	0.0	0.0	0.0	0.0	0.00
NEUROD6	0.0	0.0	0.0	0.0	0.0	0.0	0.0	0.00
NEXN	580.5	1159.5	2484.0	549.0	1508.0	1992.5	1378.9	773.14
NF1	375.5	173.0	640.5	398.0	183.0	493.5	377.3	180.39
NF2	1771.0	1936.0	1734.0	1652.5	1989.5	1599.0	1780.3	154.56
NFAM1	24.0	22.5	25.0	18.5	26.5	40.5	26.2	7.53
NFAT5	1539.5	1180.5	1829.0	1560.5	1417.5	1796.5	1553.9	242.00
NFATC1	255.5	215.5	650.0	281.5	310.0	678.0	398.4	208.25
NFATC2	1.5	7.0	28.0	2.5	10.0	32.0	13.5	13.21
NFATC3	519.5	240.5	1417.5	582.5	329.5	1035.5	687.5	451.93
NFE2L1	2380.0	1928.0	3386.5	2179.0	1893.0	2846.5	2435.5	582.08
NFE2L2	1465.0	1054.0	1051.0	1446.5	1142.5	991.5	1191.8	210.18
NFIA	27.0	9.0	16.0	25.5	18.0	17.5	18.8	6.61
NFIC	231.5	233.0	382.0	223.0	248.0	360.0	279.6	71.61
NFIL3	1067.0	751.0	938.0	1011.0	834.0	718.5	886.6	141.47
NFIX	477.0	317.0	604.0	368.5	245.5	489.0	416.8	130.78
NFKB1	371.0	243.5	400.0	402.5	292.0	437.5	357.8	74.43
NFKB2	517.5	450.5	429.0	503.0	456.5	444.0	466.8	35.22
NFKBIA	170.0	162.5	156.5	192.0	188.0	169.5	173.1	14.07
NFKBIB	709.0	933.5	545.0	657.0	982.5	597.0	737.3	180.29
NFKBID	5.5	1.0	0.5	4.0	1.5	0.0	2.1	2.18
NFKBIE	254.0	191.0	143.5	243.0	187.0	170.0	198.1	42.63
NFKBIZ	241.5	127.5	85.5	166.5	137.5	106.0	144.1	55.12
NFRKB	509.0	386.5	435.0	465.0	399.5	428.0	437.2	44.74
NFS1	940.5	631.0	838.5	917.0	776.0	729.0	805.3	117.40
NFU1	477.5	512.5	505.5	426.5	561.5	421.0	484.1	54.03
NFX1	443.5	334.5	535.5	422.0	404.5	498.0	439.7	71.01
NFXL1	89.0	23.5	68.5	92.5	18.5	46.5	56.4	32.03
NFYA	207.0	104.0	221.5	180.5	115.5	213.5	173.7	51.52
NFYB	500.0	407.0	298.5	469.5	476.0	316.5	411.3	86.22
NFYC	1626.5	1262.5	886.5	1513.5	1356.0	930.5	1262.6	301.94
NGB	0.5	0.0	0.0	2.0	0.0	0.0	0.4	0.80
NGEF	1.0	0.5	0.0	0.5	0.0	0.0	0.3	0.41
NGF	2.0	1.5	0.5	0.5	1.5	3.5	1.6	1.11
NGFR	65.5	128.0	62.0	77.5	147.0	33.5	85.6	43.15
NGLY1	527.5	381.5	427.5	444.0	443.0	340.5	427.3	63.56
NHLH1	119.5	45.5	34.0	97.5	33.0	26.5	59.3	39.20
NHLRC1	321.0	225.0	183.5	313.0	206.5	186.5	239.3	62.11
NHLRC2	267.5	110.0	363.0	245.5	152.5	346.5	247.5	101.47
NHLRC3	389.0	226.0	259.0	372.5	263.5	274.5	297.4	66.75
NHLRC4	0.0	0.0	0.0	0.0	0.0	0.0	0.0	0.00
NHP2	412.5	387.5	221.5	372.0	363.0	220.0	329.4	85.83
NHP2L1	1963.5	1528.5	1073.5	1798.5	1548.0	982.0	1482.3	388.69
NHS	148.5	106.0	304.0	173.5	148.5	254.5	189.2	74.73
NHSL1	463.0	940.0	1151.5	527.0	1141.5	1205.5	904.8	330.60
NHSL2	40.5	113.5	189.0	23.5	109.0	171.5	107.8	66.81
NICN1	61.0	71.0	22.5	88.5	65.0	43.5	58.6	22.92
NID1	660.5	175.5	299.0	685.0	236.0	473.5	421.6	218.72

NID2	9317.5	7004.5	12113.5	8885.5	7496.0	13958.5	9795.9	2714.32
NIF3L1	605.5	451.0	255.0	544.0	471.5	242.0	428.2	149.61
NIFK	1137.0	1112.0	901.5	1089.5	1123.0	847.5	1035.1	126.51
NIM1K	66.5	41.0	65.0	64.0	42.5	43.0	53.7	12.64
NIN	319.0	169.5	334.5	272.0	175.5	288.5	259.8	71.16
NINJ1	694.0	335.5	858.5	709.5	369.0	738.0	617.4	213.63
NINJ2	2.0	3.5	1.5	1.0	3.0	0.5	1.9	1.16
NIP7	690.5	889.5	438.0	685.0	852.5	435.5	665.2	195.35
NIPA1	628.0	314.0	533.0	560.0	344.5	457.0	472.8	124.29
NIPA2	1000.5	831.5	798.0	888.5	1004.5	860.0	897.2	86.95
NIPAL1	2.0	0.0	1.5	0.0	0.0	2.0	0.9	1.02
NIPAL2	0.0	1.0	0.5	0.5	1.5	0.5	0.7	0.52
NIPAL3	69.0	100.0	323.5	92.0	143.0	228.0	159.3	98.13
NIPAL4	0.0	0.0	0.0	0.0	0.0	0.0	0.0	0.00
NIPSNAP1	488.0	508.0	255.0	495.5	547.0	248.5	423.7	134.72
NIPSNAP3A	840.0	612.5	650.5	826.0	628.5	608.0	694.3	108.59
NISCH	1022.0	1280.5	1647.5	953.0	1561.5	1495.5	1326.7	290.23
NIT1	110.0	241.5	177.0	108.5	234.0	199.5	178.4	58.45
NIT2	780.5	707.5	431.0	847.0	868.0	449.5	680.6	194.53
NKAIN1	0.5	5.5	12.0	2.0	3.5	14.5	6.3	5.66
NKAIN2	0.5	0.0	0.5	0.5	0.5	0.5	0.4	0.20
NKAIN3	4.0	3.5	1.5	7.0	2.0	2.5	3.4	1.99
NKAIN4	61.0	184.0	199.5	34.5	212.0	165.0	142.7	75.65
NKAP	606.5	510.0	360.0	574.0	617.5	386.0	509.0	112.10
NKD1	10.0	71.0	82.5	17.5	113.0	181.5	79.3	63.69
NKIRAS1	43.0	29.5	16.0	20.0	29.5	24.5	27.1	9.43
NKIRAS2	944.0	1102.0	847.5	820.5	1025.0	886.5	937.6	108.76
NKPD1	1.5	2.5	6.0	0.5	0.5	11.0	3.7	4.13
NKRF	367.0	322.0	278.0	372.5	349.5	276.0	327.5	42.90
NKTR	436.0	324.0	779.0	441.0	385.0	810.0	529.2	210.05
NKX2-1	0.0	0.0	0.0	0.0	0.0	0.0	0.0	0.00
NKX2-3	0.5	0.0	0.0	0.5	0.0	0.0	0.2	0.26
NKX2-6	0.0	0.0	0.0	0.0	0.0	0.0	0.0	0.00
NKX2-8	0.0	0.0	0.0	0.0	0.0	0.0	0.0	0.00
NKX3-1	0.0	0.0	0.0	1.0	0.0	0.5	0.3	0.42
NKX6-2	0.0	0.5	0.0	0.5	1.0	0.0	0.3	0.41
NKX6-3	0.5	18.0	11.5	2.5	15.5	18.0	11.0	7.76
NLE1	219.0	224.0	155.5	205.5	239.0	144.0	197.8	38.93
NLGN1	4.0	3.5	3.0	5.5	4.5	0.5	3.5	1.70
NLGN3	1.0	0.0	3.5	0.0	1.5	2.0	1.3	1.33
NLGN4X	16.0	3.5	3.0	13.5	0.5	4.0	6.8	6.36
NLK	227.0	155.0	226.5	214.0	201.0	196.5	203.3	26.83
NLRC3	1.0	0.5	0.0	0.0	0.0	0.5	0.3	0.41
NLRX1	211.0	137.5	209.5	169.0	124.0	195.0	174.3	37.22
NMB	0.5	0.0	0.5	1.0	0.5	0.5	0.5	0.32
NMBR	0.0	0.0	0.0	0.0	0.0	0.0	0.0	0.00
NMD3	673.5	413.5	448.0	766.5	480.5	403.5	530.9	151.83
NME2	9283.5	8519.0	4287.5	8024.5	8814.0	4285.5	7202.3	2295.32
NME3	400.0	431.0	409.0	391.0	439.5	383.5	409.0	22.22
NME4	1.0	0.0	0.0	0.0	1.0	0.5	0.4	0.49
NME5	32.0	21.0	40.0	33.5	21.5	26.5	29.1	7.44
NME7	755.5	634.0	537.5	466.5	607.0	362.5	560.5	137.23
NME8	0.0	0.0	0.0	0.0	0.0	0.0	0.0	0.00
NME9	2.0	1.0	3.0	2.0	0.5	2.0	1.8	0.88

NMI	623.5	529.0	710.5	668.0	589.0	704.5	637.4	70.81
NMNAT1	86.0	57.5	90.5	103.0	56.0	64.5	76.3	19.56
NMNAT2	1.5	3.0	7.0	2.0	8.0	5.5	4.5	2.72
NMNAT3	122.0	57.5	53.5	99.5	54.5	47.0	72.3	30.79
NMRAL1	1571.0	834.5	858.5	1461.0	856.5	826.0	1067.9	349.05
NMRK1	14.5	8.0	8.5	13.0	11.5	12.0	11.3	2.54
NMRK2	433.0	442.0	379.0	399.5	415.5	267.5	389.4	63.90
NMT1	861.0	1173.0	931.0	787.0	1168.5	817.5	956.3	173.00
NMT2	722.5	546.5	666.0	768.0	608.5	584.5	649.3	85.04
NMU	178.5	348.5	358.5	140.5	420.5	348.5	299.2	112.11
NMUR1	8.0	3.5	1.0	6.5	3.0	0.5	3.8	2.98
NMUR2	0.5	0.0	0.0	0.0	0.0	0.0	0.1	0.20
NNT	317.5	99.5	453.0	318.0	111.0	346.0	274.2	140.05
NOA1	719.0	443.0	405.5	647.5	469.5	381.5	511.0	138.67
NOB1	509.5	531.5	295.5	432.5	491.5	314.0	429.1	101.93
NOBOX	0.0	0.0	0.0	0.0	0.0	0.0	0.0	0.00
NOC2L	814.5	796.5	711.0	807.0	864.5	680.0	778.9	69.41
NOC3L	689.0	527.0	560.0	666.0	573.5	520.5	589.3	71.48
NOC4L	555.0	361.5	413.5	554.0	370.5	385.5	440.0	90.43
NOD1	343.0	216.5	291.5	383.0	270.0	312.0	302.7	57.94
NODAL	0.0	0.0	0.0	0.0	0.5	0.0	0.1	0.20
NOG	0.0	0.0	0.5	1.5	0.0	0.0	0.3	0.61
NOL11	790.5	555.5	521.5	769.0	585.0	458.0	613.3	135.89
NOL12	752.5	769.0	516.5	751.0	830.0	501.5	686.8	140.74
NOL4	0.0	0.0	0.0	0.5	0.0	0.0	0.1	0.20
NOL4L	72.5	70.5	75.5	71.5	82.5	89.5	77.0	7.50
NOL6	288.0	119.0	378.0	306.0	131.0	328.5	258.4	107.72
NOL7	965.5	994.5	586.0	844.5	1050.5	633.5	845.8	195.40
NOL8	500.5	231.0	393.0	495.0	237.0	318.5	362.5	120.42
NOL9	1564.5	1195.5	762.5	1463.5	1280.5	789.0	1175.9	336.35
NOLC1	2631.5	1763.0	1869.0	2599.0	1872.0	1730.5	2077.5	420.45
NOM1	264.0	246.0	317.5	265.0	270.5	289.0	275.3	24.83
NONO	3492.5	3026.0	3026.0	3366.0	3217.0	2762.0	3148.3	264.47
NOP10	384.0	377.5	117.5	376.5	346.5	107.0	284.8	134.35
NOP14	348.0	197.5	297.0	372.0	225.5	265.5	284.3	68.20
NOP16	513.0	749.0	366.0	414.0	695.0	310.0	507.8	179.60
NOP2	1258.0	872.0	821.5	1226.0	891.5	797.0	977.7	207.79
NOP56	7479.5	7981.5	4953.5	7111.0	7499.0	5204.5	6704.8	1291.81
NOP58	2199.5	1872.5	1184.5	2226.5	1982.0	1121.0	1764.3	492.37
NOP9	380.0	476.0	257.5	288.0	463.5	273.5	356.4	97.64
NOS1	0.0	0.5	0.0	0.0	0.0	0.0	0.1	0.20
NOS1AP	48.5	53.0	64.0	51.5	44.5	97.0	59.8	19.38
NOS2	195.0	81.0	63.5	176.5	108.0	54.0	113.0	59.56
NOS3	0.5	3.5	10.0	0.0	4.0	12.5	5.1	5.09
NOTCH1	454.5	227.0	981.5	435.5	230.5	866.5	532.6	320.37
NOTCH2	502.0	282.5	1040.5	679.5	311.0	1207.0	670.4	382.88
NOTCH2NL	20.5	3.5	40.0	28.5	6.5	44.5	23.9	16.94
NOTO	0.0	0.0	0.0	0.0	0.0	0.0	0.0	0.00
NOTUM	196.5	198.0	296.0	180.0	245.5	304.5	236.8	53.89
NOV	3.0	1.0	3.5	4.0	3.0	2.0	2.8	1.08
NOX1	6.5	37.5	17.0	5.5	40.5	26.5	22.3	15.10
NOX3	28.5	5.5	6.0	26.0	6.0	1.0	12.2	11.86
NOX4	0.5	0.0	6.5	0.5	1.0	3.5	2.0	2.53
NOX5	0.0	0.0	0.0	0.0	0.0	0.5	0.1	0.20

NOXA1	20.0	14.5	4.5	7.5	15.0	10.0	11.9	5.65
NOXO1	248.0	61.0	16.0	212.5	73.0	24.0	105.8	99.44
NOXRED1	0.5	0.5	0.5	0.0	1.0	0.0	0.4	0.38
NPAS2	294.5	135.0	188.0	245.5	144.0	172.5	196.6	61.95
NPAS3	66.0	15.0	44.0	43.5	19.5	34.5	37.1	18.59
NPAT	191.0	150.0	270.0	191.0	168.0	249.0	203.2	46.75
NPB	368.0	353.5	157.0	315.0	302.0	130.5	271.0	101.83
NPBWR1	0.0	0.5	0.0	0.0	0.0	0.0	0.1	0.20
NPBWR2	0.0	0.0	0.0	0.0	0.0	0.0	0.0	0.00
NPC1	1098.0	447.5	654.5	1117.0	504.5	557.0	729.8	300.49
NPC2	1205.0	1127.0	990.0	1208.0	1265.5	1132.0	1154.6	95.98
NPDC1	615.5	452.0	440.0	461.5	430.0	356.0	459.2	85.27
NPEPL1	785.5	625.5	593.5	678.0	688.5	542.5	652.3	84.79
NPEPPS	2448.0	2726.0	3287.0	2163.0	2847.5	2848.5	2720.0	384.32
NPFFR1	1.5	2.0	3.0	0.5	2.5	5.0	2.4	1.53
NPFFR2	0.0	0.0	0.0	0.0	0.0	0.0	0.0	0.00
NPHP1	326.5	189.0	207.5	352.0	225.5	233.0	255.6	67.06
NPHP3	408.5	356.5	476.5	382.5	416.5	419.5	410.0	40.44
NPHP4	339.5	298.5	338.0	304.0	274.5	321.5	312.7	25.19
NPHS2	0.0	0.0	0.0	0.0	0.0	0.0	0.0	0.00
NPL	52.5	35.5	18.0	43.0	38.5	26.0	35.6	12.25
NPLOC4	4763.0	3863.0	2323.5	4133.5	3628.0	2202.5	3485.6	1020.89
NPM1	22208.0	12173.0	8988.5	19133.5	12358.0	7833.5	13782.4	5702.89
NPM3	1593.5	1810.5	585.0	1435.5	1682.5	650.0	1292.8	537.56
NPNT	126.0	50.5	189.0	133.5	71.0	203.0	128.8	61.04
NPR2	2840.5	4358.0	3881.0	2466.0	4487.0	4477.5	3751.7	887.14
NPRL2	195.5	297.5	252.0	197.5	298.5	264.0	250.8	45.89
NPRL3	759.0	567.0	379.0	737.0	581.5	345.0	561.4	173.44
NPS	1.5	7.0	5.5	2.5	5.0	1.5	3.8	2.32
NPSR1	0.0	0.0	0.0	0.0	0.0	0.0	0.0	0.00
NPTN	702.0	336.5	1185.0	739.0	425.0	970.0	726.3	320.40
NPTX1	10.5	7.0	8.5	8.0	9.5	18.0	10.3	3.98
NPTX2	39.0	6.0	14.0	38.0	10.0	9.0	19.3	15.07
NPVF	0.0	0.0	0.0	0.0	0.0	0.0	0.0	0.00
NPY	0.0	115.5	30.5	1.0	184.0	179.0	85.0	85.81
NPY1R	0.5	0.0	0.0	0.0	0.0	0.0	0.1	0.20
NPY2R	190.5	104.5	119.5	213.5	130.5	114.5	145.5	45.15
NPY5R	0.0	0.0	0.0	0.0	0.0	0.0	0.0	0.00
NQO1	796.5	643.0	448.5	733.0	641.5	450.5	618.8	143.56
NQO2	85.0	31.5	39.0	74.0	29.5	37.5	49.4	23.83
NR0B1	2.5	0.5	3.0	0.5	0.0	0.5	1.2	1.25
NR0B2	8.0	3.5	1.5	10.5	8.5	3.5	5.9	3.56
NR1D2	377.5	419.0	668.0	364.5	500.0	894.0	537.2	207.41
NR1H3	3388.0	1898.5	2157.0	3183.0	2073.5	2224.0	2487.3	631.13
NR1H4	0.0	0.5	0.0	0.0	0.0	0.0	0.1	0.20
NR1I3	1.0	0.0	0.0	0.0	0.0	0.0	0.2	0.41
NR2C1	545.5	562.0	633.0	444.5	626.0	619.5	571.8	72.04
NR2C2	950.0	673.5	1106.0	882.5	790.5	1053.0	909.3	162.13
NR2C2AP	156.0	263.5	158.0	141.5	287.0	158.0	194.0	63.67
NR2E1	43.5	51.0	42.0	38.0	58.5	42.5	45.9	7.48
NR2E3	0.0	0.0	0.0	0.0	1.0	0.0	0.2	0.41
NR3C2	101.0	59.0	173.5	101.5	83.5	107.5	104.3	38.22
NR4A1	20.0	101.0	131.5	27.5	79.0	162.0	86.8	56.38
NR4A3	76.0	206.0	529.0	138.5	221.0	713.5	314.0	250.43

NR5A1	4.0	3.5	2.0	1.5	1.5	1.0	2.3	1.21
NR6A1	14.5	6.5	22.0	13.0	11.5	16.0	13.9	5.13
NRAP	76.0	478.5	192.0	58.0	565.0	357.0	287.8	212.06
NRARP	14.5	10.0	21.5	20.0	15.0	27.5	18.1	6.19
NRAS	163.0	77.5	96.5	145.0	70.0	65.5	102.9	41.36
NRBF2	641.0	666.0	515.5	598.0	789.0	506.5	619.3	105.26
NRBP1	1944.5	1750.0	1283.5	2021.0	1880.0	1325.0	1700.7	319.94
NRBP2	59.5	167.0	199.0	50.5	129.0	193.0	133.0	65.32
NRCAM	3.0	25.5	16.5	0.5	25.0	19.0	14.9	10.80
NRD1	2348.5	1752.0	1673.0	1973.0	1887.0	1464.5	1849.7	301.51
NRDE2	197.5	121.5	156.0	223.5	153.5	155.5	167.9	36.40
NRF1	56.0	30.5	96.0	64.5	33.0	75.0	59.2	25.11
NRG1	0.0	0.0	0.0	0.0	0.0	0.0	0.0	0.00
NRG2	0.5	5.0	5.5	1.0	6.5	8.5	4.5	3.15
NRG3	0.0	0.0	0.5	0.0	0.0	0.0	0.1	0.20
NRG4	355.5	379.5	218.5	331.5	407.5	221.0	318.9	80.85
NRGN	0.0	0.0	0.0	0.0	0.0	0.0	0.0	0.00
NRIP1	119.0	49.0	181.0	106.0	52.5	131.0	106.4	50.06
NRIP3	0.5	0.5	1.5	1.0	1.0	2.0	1.1	0.58
NRK	530.5	510.5	1041.0	507.0	554.0	777.5	653.4	215.63
NRN1	0.0	0.0	0.0	0.0	0.0	0.0	0.0	0.00
NRN1L	39.0	180.5	85.5	60.0	180.0	166.0	118.5	64.36
NRP1	3423.0	1546.5	3540.5	4046.5	1706.5	3327.5	2931.8	1042.24
NRP2	1874.0	1118.5	2511.5	2246.5	1348.5	2274.0	1895.5	556.65
NRROS	7.5	15.0	17.0	10.0	15.0	21.5	14.3	5.00
NRSN1	148.0	60.0	44.5	132.5	74.5	76.0	89.3	41.41
NRSN2	99.5	80.5	43.5	98.5	68.0	38.5	71.4	26.38
NRTN	1.0	19.5	7.0	1.0	33.5	32.0	15.7	14.86
NRXN3	8.5	44.0	30.0	7.5	45.0	27.0	27.0	16.40
NSA2	3296.0	2114.0	1776.5	2819.5	2219.5	1600.5	2304.3	642.84
NSD1	1100.0	794.5	1298.0	954.5	811.0	1246.0	1034.0	215.59
NSDHL	895.5	408.0	754.5	805.5	413.5	568.0	640.8	207.89
NSF	1229.5	935.5	1096.5	1271.0	1033.5	1196.5	1127.1	128.39
NSFL1C	1184.5	1174.0	786.5	1085.5	1293.0	758.5	1047.0	222.77
NSL1	312.0	225.5	308.5	306.5	214.0	265.5	272.0	44.02
NSMAF	1103.0	1038.0	1010.5	994.5	1172.0	944.0	1043.7	81.82
NSMCE1	445.0	343.0	319.5	403.5	369.0	297.5	362.9	54.74
NSMCE2	446.5	336.5	288.0	419.0	386.5	233.0	351.6	81.37
NSMCE4A	346.5	599.5	545.0	305.5	619.5	606.0	503.7	140.54
NSMF	102.5	202.5	242.0	99.5	222.5	334.5	200.6	89.41
NSRP1	311.0	315.5	278.5	299.5	365.0	265.0	305.8	34.86
NSUN2	642.5	462.0	487.0	627.5	532.5	454.5	534.3	82.74
NSUN3	63.0	56.5	51.0	91.5	63.0	68.0	65.5	14.04
NSUN4	270.0	224.5	213.0	235.5	248.0	186.0	229.5	29.01
NSUN5	817.5	694.5	541.0	746.5	721.0	539.5	676.7	113.33
NSUN6	143.5	97.0	110.0	120.5	92.0	102.0	110.8	18.90
NSUN7	36.5	62.5	27.5	33.0	73.5	38.5	45.3	18.35
NT5C1A	38.0	668.0	398.5	73.5	759.0	501.0	406.3	299.44
NT5C2	1094.0	1316.5	1837.5	1044.0	1442.0	1513.0	1374.5	293.05
NT5C3A	444.5	259.0	221.5	402.0	273.5	239.0	306.6	93.05
NT5C3B	505.5	648.0	657.5	463.5	620.5	602.5	582.9	79.81
NT5DC1	188.0	84.5	67.5	152.5	75.0	80.5	108.0	49.84
NT5DC2	1499.0	1542.0	882.0	1406.0	1629.0	833.0	1298.5	349.41
NT5DC3	166.0	75.0	77.5	247.0	114.5	150.5	138.4	64.81

NT5E	0.5	1.0	2.0	1.5	2.0	4.5	1.9	1.39
NT5M	217.5	211.5	280.5	231.5	250.5	236.0	237.9	25.02
NTAN1	445.0	449.0	314.0	433.5	518.5	300.5	410.1	85.18
NTF3	0.0	1.0	1.5	0.5	0.5	0.0	0.6	0.58
NTHL1	187.5	133.0	126.5	180.5	135.5	117.0	146.7	29.70
NTM	118.5	55.5	22.0	108.5	48.0	15.0	61.3	43.36
NTMT1	1161.5	1707.5	1073.0	1093.5	1841.0	1227.5	1350.7	335.24
NTN1	12.0	15.0	11.0	23.0	17.0	23.0	16.8	5.23
NTN3	1.5	0.0	1.0	3.5	0.0	0.5	1.1	1.32
NTN4	2840.5	2460.5	2780.0	3147.5	2808.5	3337.0	2895.7	307.11
NTNG1	2.5	2.0	1.0	3.0	2.5	2.0	2.2	0.68
NTNG2	9.5	21.0	29.5	12.0	20.5	32.0	20.8	9.02
NTPCR	410.5	259.5	232.5	363.5	263.5	149.0	279.8	93.93
NTRK1	10.0	0.0	0.0	2.0	2.0	1.0	2.5	3.78
NTRK2	1.5	1.0	3.0	2.0	0.0	1.5	1.5	1.00
NTRK3	0.5	0.5	0.5	1.0	0.5	0.0	0.5	0.32
NTS	0.0	0.0	0.5	0.0	1.5	0.0	0.3	0.61
NTSR1	31.5	10.5	2.0	32.5	11.5	2.5	15.1	13.68
NUAK1	1012.0	633.5	1490.5	1160.5	773.5	1541.0	1101.8	369.48
NUAK2	50.5	157.5	259.5	79.5	165.0	273.5	164.3	90.75
NUB1	825.5	563.0	697.0	878.5	566.0	595.5	687.6	137.36
NUBP1	802.5	621.0	488.0	769.5	655.5	504.0	640.1	130.67
NUBP2	613.5	654.5	452.5	605.0	649.5	469.0	574.0	89.99
NUBPL	710.5	398.0	344.5	606.5	474.5	368.0	483.7	146.09
NUCKS1	936.5	981.5	464.0	861.5	1070.5	409.0	787.2	280.47
NUDC	1894.5	1772.5	2006.0	1940.0	1876.0	1672.0	1860.2	120.14
NUDCD2	782.0	782.0	687.5	821.5	824.5	696.0	765.6	60.13
NUDCD3	427.0	551.0	646.0	366.5	540.5	669.0	533.3	118.83
NUDT12	478.0	343.5	236.0	498.5	411.0	271.5	373.1	107.85
NUDT13	187.5	112.5	94.5	169.5	146.0	99.5	134.9	38.66
NUDT14	420.0	551.0	310.0	411.5	623.5	235.5	425.3	144.65
NUDT15	381.5	333.0	258.0	363.0	346.0	243.0	320.8	57.00
NUDT16L1	3340.0	3742.0	1683.5	3070.0	3868.0	1702.5	2901.0	977.99
NUDT19	176.0	123.5	147.0	167.5	136.5	119.5	145.0	23.05
NUDT2	492.5	451.0	463.0	458.0	500.5	393.0	459.7	38.15
NUDT21	1217.0	1216.0	1055.5	1144.0	1320.5	1003.5	1159.4	116.46
NUDT3	173.5	266.5	249.0	169.5	275.0	252.5	231.0	47.06
NUDT4	1241.0	1195.5	1399.5	1259.5	1328.0	1284.0	1284.6	71.49
NUDT5	485.0	172.5	167.0	402.5	177.5	130.5	255.8	148.80
NUDT6	165.5	68.0	104.5	140.0	74.5	95.5	108.0	38.00
NUDT7	554.5	401.0	427.0	405.0	407.5	338.0	422.2	71.52
NUDT8	70.0	70.0	51.5	55.5	93.5	53.5	65.7	15.90
NUDT9	873.5	507.0	455.0	767.5	510.0	431.5	590.8	183.58
NUF2	323.5	75.5	239.0	333.5	54.5	133.5	193.3	122.81
NUFIP1	435.5	324.5	308.0	459.0	319.5	288.5	355.8	72.27
NUFIP2	337.0	192.0	236.0	353.0	235.0	222.0	262.5	66.04
NUMA1	1548.0	1256.5	1639.0	1446.5	1230.5	1652.0	1462.1	184.87
NUMB	1855.5	1841.0	1443.5	1784.5	2332.5	2041.0	1883.0	294.07
NUP107	1091.5	590.5	780.0	1096.0	639.5	673.0	811.8	227.13
NUP133	905.0	462.5	559.0	898.0	500.0	475.5	633.3	210.36
NUP153	909.0	582.0	931.0	895.5	690.0	907.5	819.2	146.38
NUP155	1417.5	652.0	1154.5	1525.5	637.0	880.5	1044.5	381.99
NUP160	1144.5	737.5	907.5	1191.0	699.5	787.0	911.2	211.26
NUP188	1352.5	1044.5	1440.0	1314.5	1116.5	1414.0	1280.3	162.60

NUP205	1530.0	775.0	1147.0	1541.5	875.5	957.0	1137.7	331.66
NUP210	213.5	83.5	166.0	158.0	67.0	85.0	128.8	58.65
NUP210L	16.5	17.5	7.0	12.0	20.5	15.5	14.8	4.73
NUP214	722.5	622.5	924.5	817.0	634.0	816.0	756.1	117.93
NUP35	404.0	288.0	318.5	309.0	259.0	234.5	302.2	58.87
NUP37	331.5	259.5	201.5	321.0	295.0	204.5	268.8	56.73
NUP43	531.5	245.5	277.0	514.0	255.0	198.0	336.8	146.41
NUP50	1251.0	800.5	918.0	1218.0	897.0	875.0	993.3	191.32
NUP54	540.5	540.0	584.5	561.5	572.5	581.0	563.3	19.56
NUP62CL	784.0	808.5	596.5	730.5	812.5	596.5	721.4	101.08
NUP85	2180.0	1613.0	1277.5	2102.5	1740.0	1309.0	1703.7	382.85
NUP88	1018.0	966.5	924.0	943.5	1097.5	911.5	976.8	70.05
NUP98	1108.0	702.5	886.5	1109.5	702.5	802.5	885.3	186.31
NUPL2	113.0	83.5	56.0	126.5	92.0	69.0	90.0	26.47
NUS1	720.5	703.5	574.5	711.5	744.0	557.0	668.5	80.93
NUSAP1	599.0	270.5	502.0	531.0	256.0	291.0	408.3	152.41
NUTF2	1535.0	1455.5	1045.0	1475.5	1547.0	1013.0	1345.2	247.54
NVL	492.5	336.0	475.0	530.0	388.0	409.0	438.4	72.87
NWD2	1.5	1.0	4.5	1.0	0.0	4.0	2.0	1.82
NXN	35.0	41.0	127.0	36.0	52.0	145.0	72.7	49.75
NXNL1	1.0	0.0	0.5	0.5	0.5	0.0	0.4	0.38
NXNL2	22.0	6.5	10.5	17.0	2.5	3.5	10.3	7.79
NXPE3	243.0	127.0	128.5	216.0	135.0	113.0	160.4	54.66
NXPH1	0.0	0.0	0.0	0.0	0.0	0.0	0.0	0.00
NXPH2	30.0	15.0	4.0	25.0	9.5	9.0	15.4	10.11
NXPH3	3.0	7.0	5.5	3.0	6.5	11.0	6.0	2.98
NXPH4	646.0	994.5	1171.0	738.5	1182.5	1638.5	1061.8	358.06
NXT2	1498.0	811.5	613.0	1493.5	899.0	602.0	986.2	410.93
NYAP2	0.0	0.0	0.0	0.0	0.0	0.0	0.0	0.00
NYX	4.0	3.0	4.5	2.0	7.5	7.5	4.8	2.30
OAF	368.5	521.0	400.5	341.5	539.0	560.5	455.2	95.79
OARD1	878.5	921.0	846.5	828.5	1054.0	842.0	895.1	84.63
OASL	25.5	75.0	439.5	34.0	88.5	444.0	184.4	200.75
OAT	3242.5	2401.0	2253.5	3333.0	2830.5	2319.5	2730.0	477.53
OAZ1	21323.0	17682.5	12518.0	19477.5	20153.0	13223.0	17396.2	3705.29
OAZ2	799.0	1083.0	791.5	722.5	1146.5	866.5	901.5	172.53
OBFC1	191.5	105.5	144.5	209.0	86.5	111.5	141.4	49.57
OBSCN	261.5	817.5	1252.0	240.5	975.0	1090.5	772.8	428.54
OBSL1	72.5	966.0	1702.5	67.0	953.5	1447.5	868.2	681.70
OC90	0.0	0.0	0.0	0.0	1.0	0.0	0.2	0.41
OCIAD1	876.5	696.5	475.0	801.5	784.5	433.0	677.8	183.07
OCRL	644.0	481.5	523.5	511.5	516.5	531.0	534.7	56.19
OCSTAMP	7.0	48.0	61.0	7.0	35.5	41.0	33.3	22.05
ODC1	972.0	1564.0	817.5	1051.5	1950.0	992.0	1224.5	437.04
ODF2	355.0	187.0	344.5	374.0	201.0	279.5	290.2	81.11
ODF2L	90.5	26.0	16.5	75.5	28.0	13.5	41.7	32.83
OFCC1	0.0	0.0	0.0	0.0	0.0	0.0	0.0	0.00
OFD1	922.0	831.5	662.0	885.5	961.5	638.0	816.8	136.29
OGDH	682.5	640.5	761.0	657.0	610.0	709.0	676.7	53.54
OGDHL	0.5	0.0	0.0	0.0	0.0	0.0	0.1	0.20
OGFOD1	688.0	659.0	545.5	583.5	703.5	539.0	619.8	72.88
OGFOD2	183.0	121.5	54.0	192.0	126.0	60.0	122.8	58.48
OGFOD3	880.5	751.5	616.5	818.5	825.5	623.5	752.7	110.64
OGFR	246.0	231.0	232.5	234.5	262.0	215.5	236.9	15.69

OGFRL1	207.0	102.0	88.5	254.0	127.0	65.0	140.6	73.99
OGG1	101.0	95.0	77.5	101.0	92.5	77.0	90.7	10.92
OGN	7.0	9.0	14.5	7.5	16.5	12.5	11.2	3.92
OGT	5871.0	5822.5	5386.5	5087.0	7113.5	6478.0	5959.8	737.12
OIP5	1134.0	808.5	808.5	868.5	828.5	620.5	844.8	165.78
OIT3	466.0	648.5	300.0	399.5	798.5	319.5	488.7	197.09
OLAH	0.0	0.0	0.0	1.5	0.0	0.0	0.3	0.61
OLFM1	5.0	4.0	35.5	4.5	5.0	8.5	10.4	12.39
OLFM3	3.5	2.0	5.5	5.0	4.0	3.0	3.8	1.29
OLFM4	0.0	0.5	0.0	0.0	0.0	0.0	0.1	0.20
OLFML1	0.0	0.0	0.0	0.0	0.0	0.0	0.0	0.00
OLFML2A	2298.5	6607.0	6336.0	2204.0	7305.0	8845.0	5599.3	2735.89
OLFML2B	2811.5	3928.0	3855.0	2102.5	3807.5	5085.0	3598.3	1028.12
OLFML3	1337.5	1253.5	576.5	1508.0	1430.5	904.5	1168.4	357.73
OLIG3	1.0	2.5	3.5	0.0	0.0	0.0	1.2	1.51
OMA1	184.5	84.0	79.5	181.0	77.5	72.0	113.1	54.11
OMD	4.5	7.0	20.5	5.0	7.0	20.5	10.8	7.62
OMG	0.5	2.5	2.0	2.0	3.5	1.5	2.0	1.00
OMP	0.0	0.0	0.0	0.0	0.0	0.0	0.0	0.00
ONECUT3	0.0	0.0	0.0	0.0	0.5	0.0	0.1	0.20
OPA1	213.0	94.0	292.0	266.5	114.0	225.5	200.8	80.42
OPA3	69.0	52.0	34.0	69.0	44.5	32.5	50.2	16.24
OPCML	28.5	140.0	386.5	56.5	160.0	805.0	262.8	294.07
OPHN1	175.5	376.5	422.5	196.0	388.0	520.0	346.4	134.46
OPN3	99.0	62.5	109.5	58.0	45.0	51.0	70.8	26.77
OPN4	1.0	6.0	2.5	1.0	12.0	3.0	4.3	4.22
OPN5	0.0	0.0	0.0	0.0	0.0	0.0	0.0	0.00
OPRD1	55.5	114.0	212.5	39.0	147.0	378.0	157.7	124.98
OPRK1	0.0	0.0	0.0	0.0	0.0	0.0	0.0	0.00
OPRL1	0.0	0.0	0.0	0.5	0.0	0.0	0.1	0.20
OPRM1	17.0	5.5	3.5	17.5	1.5	5.0	8.3	7.05
OPTC	186.5	547.0	147.0	361.5	810.5	475.5	421.3	246.64
OPTN	740.0	631.0	590.0	684.0	693.5	527.5	644.3	77.36
ORAI1	221.0	117.5	204.0	216.0	120.5	194.5	178.9	47.33
ORAI2	109.0	46.5	105.0	106.5	46.0	97.5	85.1	30.32
ORAOV1	172.0	133.5	102.0	124.0	134.0	92.0	126.3	28.18
ORC2	79.5	44.0	89.0	84.0	39.5	71.0	67.8	21.10
ORC3	578.5	579.5	418.0	530.5	589.5	387.5	513.9	89.04
ORC4	1427.5	947.0	984.5	1398.0	1048.5	904.5	1118.3	233.09
ORC5	114.0	59.0	126.5	108.5	64.0	95.0	94.5	27.54
ORC6	344.5	234.0	297.0	332.0	245.0	245.0	282.9	48.31
ORMDL1	323.5	187.5	110.5	287.5	190.0	126.5	204.3	85.40
ORMDL2	947.5	838.0	686.0	819.5	876.5	720.5	814.7	97.43
ORMDL3	401.5	240.0	342.0	381.0	250.5	373.0	331.3	69.45
OSBP	999.0	963.0	996.5	946.5	794.5	1045.0	957.4	86.74
OSBP2	70.5	33.5	71.5	70.5	41.5	86.0	62.3	20.21
OSBPL10	346.5	162.0	197.0	270.0	145.0	208.0	221.4	75.04
OSBPL11	993.5	988.0	711.0	1081.5	1112.0	676.0	927.0	187.56
OSBPL1A	503.5	283.0	215.5	448.0	317.0	235.5	333.8	116.93
OSBPL2	1067.5	773.0	946.5	995.5	889.0	799.0	911.8	113.98
OSBPL3	831.5	598.0	776.0	1101.5	721.5	942.0	828.4	175.93
OSBPL5	583.0	284.0	753.0	775.0	363.0	780.0	589.7	220.05
OSBPL6	94.0	68.0	131.0	69.0	79.0	123.0	94.0	27.33
OSBPL7	69.5	50.0	63.0	69.5	40.5	71.5	60.7	12.64

OSBPL8	549.0	161.0	897.5	493.0	180.0	655.5	489.3	283.29
OSCP1	108.5	78.5	78.5	99.5	67.5	63.0	82.6	17.90
OSER1	822.5	734.5	680.5	710.0	825.0	734.5	751.2	59.64
OSGEP	232.0	252.5	106.5	176.0	203.5	100.0	178.4	63.74
OSGEPL1	274.0	175.0	159.5	288.5	186.0	150.5	205.6	60.05
OSGIN1	387.0	103.0	35.0	332.0	113.5	80.0	175.1	146.41
OSGIN2	96.0	36.0	51.0	93.0	33.5	28.5	56.3	30.52
OSMR	1251.0	1549.0	2274.0	1479.5	1879.5	2498.0	1821.8	486.30
OSR1	45.5	67.0	41.5	66.5	66.0	57.5	57.3	11.34
OSR2	79.0	83.5	163.5	53.0	73.0	146.5	99.8	44.37
OST4	672.0	1279.5	782.5	596.0	1138.0	675.0	857.2	282.31
OSTC	1824.5	1407.5	1424.0	1795.5	1651.0	1627.5	1621.7	177.31
OSTF1	930.5	548.0	555.5	768.0	597.5	509.5	651.5	163.96
OSTM1	552.5	232.0	433.0	520.5	243.0	358.0	389.8	136.30
OSTN	1.5	0.5	2.0	0.5	0.0	0.5	0.8	0.75
OTC	0.0	0.0	0.0	0.0	0.0	0.0	0.0	0.00
OTOA	9.0	15.5	16.0	17.5	16.5	23.5	16.3	4.63
OTOF	0.0	0.0	0.0	0.5	0.0	0.0	0.1	0.20
OTOGL	1.0	0.0	0.0	1.5	1.5	0.0	0.7	0.75
OTOL1	0.0	1.5	0.5	0.0	2.0	0.5	0.8	0.82
OTOP1	0.0	0.0	0.0	0.0	0.5	0.0	0.1	0.20
OTOP2	0.5	0.0	0.5	0.0	0.0	0.5	0.3	0.27
OTOP3	0.0	0.0	0.0	0.0	0.0	1.0	0.2	0.41
OTOR	28.0	56.5	39.0	36.0	66.0	30.0	42.6	15.29
OTOS	1.0	0.5	0.5	0.0	0.0	0.5	0.4	0.38
OTUD1	139.0	194.5	245.0	148.0	234.5	272.0	205.5	54.17
OTUD3	143.0	137.5	118.5	138.0	154.0	120.5	135.3	13.58
OTUD4	641.5	504.0	1045.0	621.0	633.0	948.5	732.2	213.14
OTUD5	268.0	254.0	233.5	242.5	237.5	222.5	243.0	16.04
OTUD6B	2616.5	1939.0	1674.0	2666.5	2223.0	1611.5	2121.8	457.39
OTUD7A	35.5	39.5	66.5	21.0	41.5	52.0	42.7	15.41
OTUD7B	294.5	268.0	267.0	262.5	265.5	280.5	273.0	12.21
OTULIN	42.5	32.0	23.5	43.0	30.5	22.0	32.3	9.00
OTX2	0.0	0.0	0.0	0.0	0.0	0.0	0.0	0.00
OVCA2	156.5	121.0	101.0	155.5	123.5	125.5	130.5	21.61
OVCH2	65.5	70.0	81.5	59.5	76.5	92.0	74.2	11.70
OVOL2	50.0	26.0	25.5	27.5	24.5	16.0	28.3	11.41
OXCT1	46.0	18.5	89.5	25.5	20.0	43.5	40.5	26.72
OXGR1	0.0	0.0	0.0	0.0	0.5	0.0	0.1	0.20
OXNAD1	153.5	176.0	165.5	157.5	202.0	161.5	169.3	17.77
OXR1	122.0	89.5	129.5	103.5	80.5	141.5	111.1	23.84
OXSM	43.0	24.0	47.5	34.5	22.5	36.0	34.6	9.98
OXSR1	958.0	909.5	994.5	794.0	1017.5	779.5	908.8	101.47
OXTR	48.0	60.0	30.5	43.5	75.5	39.0	49.4	16.10
P2RX1	0.0	0.5	0.0	0.0	0.5	0.0	0.2	0.26
P2RX2	4.0	1.0	1.5	1.0	2.5	0.0	1.7	1.40
P2RX3	75.5	50.5	21.5	77.0	36.0	32.0	48.8	23.25
P2RX4	543.5	338.5	420.5	497.0	383.0	392.0	429.1	76.79
P2RX5	38.5	103.0	157.5	58.0	130.0	133.5	103.4	46.51
P2RX6	3.0	1.0	0.5	0.5	1.0	0.5	1.1	0.97
P2RX7	56.5	19.5	30.5	59.5	26.5	17.0	34.9	18.54
P2RY1	13.5	30.0	82.5	12.0	23.5	89.5	41.8	34.91
P2RY12	1.0	0.5	0.0	0.0	0.0	0.0	0.3	0.42
P2RY13	1.0	0.5	0.5	0.0	0.5	1.0	0.6	0.38

P2RY14	3.0	2.0	0.5	3.0	2.0	3.5	2.3	1.08
P2RY2	0.0	0.0	0.5	1.0	0.5	0.5	0.4	0.38
P2RY4	0.0	0.0	0.0	0.0	0.0	0.0	0.0	0.00
P2RY6	0.5	0.0	0.0	0.0	0.0	0.0	0.1	0.20
P2RY8	2.0	0.0	0.0	1.5	1.0	0.0	0.8	0.88
P3H1	975.0	1435.0	1207.5	956.5	1649.5	1946.5	1361.7	391.84
P3H2	197.0	555.5	269.5	277.0	677.0	434.0	401.7	187.31
P4HA1	4232.5	2598.5	4945.0	4591.0	3021.0	5056.0	4074.0	1029.69
P4HA2	513.5	574.0	1817.5	581.5	696.5	2270.0	1075.5	765.82
P4HA3	292.5	149.5	138.0	239.5	159.5	124.0	183.8	66.87
P4HB	9878.0	6980.5	7024.5	10891.5	8310.5	7443.5	8421.4	1625.99
P4HTM	90.5	97.5	115.5	74.5	99.0	128.0	100.8	18.80
PA2G4	1387.0	2513.0	2467.0	1414.0	2833.5	2225.0	2139.9	604.70
PAAF1	540.0	346.5	255.0	504.0	320.5	258.0	370.7	122.98
PABPC1	24648.0	23382.5	27786.5	20771.0	26838.0	26143.5	24928.3	2570.77
PABPC1L	7.0	6.0	8.5	7.0	9.0	7.0	7.4	1.11
PABPC4	222.5	304.5	217.5	242.0	295.0	240.5	253.7	37.10
PABPN1L	1.5	2.0	1.0	2.0	5.5	4.5	2.8	1.81
PACRG	10.0	3.0	2.5	7.0	3.0	1.0	4.4	3.38
PACRGL	8.0	4.0	12.5	3.0	4.5	3.5	5.9	3.68
PACS2	1189.5	1058.5	1930.0	969.5	1173.5	1552.5	1312.3	362.08
PACSIN1	2.0	0.0	0.5	0.0	1.0	0.5	0.7	0.75
PACSIN2	777.0	387.5	581.0	797.0	417.5	504.0	577.3	176.12
PACSIN3	2438.0	3629.5	2490.5	2304.5	3914.0	2424.0	2866.8	709.37
PADI1	1216.0	1355.5	674.0	1133.5	1560.5	889.5	1138.2	319.05
PADI2	4.0	3.0	0.0	0.5	1.5	2.5	1.9	1.53
PAF1	860.0	815.5	676.0	836.0	816.5	696.0	783.3	77.37
PAFAH1B1	3623.0	3476.5	3697.5	3642.0	3911.5	3513.0	3643.9	154.97
PAFAH1B2	1818.0	1390.5	1527.5	1914.0	1581.5	1452.0	1613.9	208.01
PAFAH2	237.5	250.5	92.5	302.0	265.0	117.0	210.8	85.25
PAG1	107.0	36.5	105.0	71.0	31.5	46.0	66.2	33.73
PAH	0.0	0.0	0.0	0.0	0.0	0.0	0.0	0.00
PAICS	0.5	0.0	1.5	0.5	0.5	1.5	0.8	0.61
PAIP1	1138.5	904.0	949.5	1105.0	957.5	906.0	993.4	102.33
PAIP2	1002.0	945.5	1084.5	1029.5	1003.0	1019.5	1014.0	45.16
PAIP2B	266.5	289.5	122.5	228.0	307.0	148.0	226.9	76.20
PAK1IP1	641.5	512.0	373.5	739.0	602.0	412.0	546.7	140.26
PAK2	690.0	500.5	901.0	711.0	576.5	875.0	709.0	158.63
PAK3	174.5	122.5	506.0	173.0	123.5	382.0	246.9	159.24
PAK4	357.0	302.0	369.0	344.0	321.0	391.5	347.4	32.48
PAK6	2.5	0.5	0.0	1.0	0.0	0.0	0.7	0.98
PAK7	8.5	6.0	12.5	1.5	5.5	11.0	7.5	4.01
PALB2	365.0	232.0	251.5	323.5	248.0	228.0	274.7	56.20
PALD1	3107.5	3249.5	2445.5	3159.0	3282.5	2194.5	2906.4	465.33
PALLD	5367.5	5749.5	7487.5	5161.5	6772.5	6538.5	6179.5	902.44
PALM	1.0	0.5	0.0	1.0	0.5	2.5	0.9	0.86
PALM2	67.0	38.0	136.0	57.0	43.5	85.0	71.1	36.00
PALMD	4.0	19.0	65.0	1.0	17.5	53.5	26.7	26.48
PAM16	270.0	388.5	239.0	265.5	393.0	221.5	296.3	75.33
PAMR1	3.5	2.5	0.0	1.5	1.5	2.5	1.9	1.20
PAN2	1096.0	1394.0	845.0	985.0	1444.0	1018.0	1130.3	238.43
PAN3	1106.0	797.5	1178.0	987.0	967.0	1168.5	1034.0	146.04
PANK1	267.5	140.0	138.0	246.0	152.0	119.5	177.2	62.88
PANK2	595.0	695.0	337.5	531.5	839.5	373.5	562.0	191.13

PANK3	3307.0	2667.0	6890.5	3168.0	2844.5	5207.0	4014.0	1679.00
PANK4	253.5	125.0	178.0	189.5	165.0	149.0	176.7	43.91
PANX2	51.5	25.0	40.0	55.0	30.5	49.0	41.8	12.11
PANX3	36.5	54.5	42.5	33.0	64.0	61.5	48.7	13.16
PAOX	159.0	136.5	60.0	166.5	154.0	114.0	131.7	39.83
PAPD4	863.0	458.0	347.5	831.5	525.5	374.0	566.6	226.54
PAPD7	572.5	545.5	506.0	589.0	552.0	481.5	541.1	40.50
PAPLN	918.0	825.5	718.0	853.0	996.5	748.0	843.2	104.16
PAPOLA	973.0	630.0	1177.0	981.0	715.5	1018.0	915.8	203.96
PAPOLG	2.0	0.5	1.5	1.0	0.0	0.0	0.8	0.82
PAPPA	4.0	0.0	2.0	3.0	1.5	0.5	1.8	1.51
PAPPA2	0.0	0.0	0.5	0.0	0.0	0.5	0.2	0.26
PAPSS2	376.0	378.5	357.5	358.5	446.5	400.5	386.3	33.45
PAQR3	106.0	77.5	142.0	110.5	80.0	144.5	110.1	28.94
PAQR5	79.5	52.5	67.5	76.5	51.5	89.5	69.5	15.27
PAQR7	171.5	336.5	321.0	189.5	367.0	287.5	278.8	80.56
PAQR8	40.5	35.0	72.0	34.5	39.0	59.0	46.7	15.33
PAQR9	12.5	2.0	1.5	20.5	5.0	3.5	7.5	7.52
PARD3	139.0	78.5	148.5	162.5	83.5	141.0	125.5	35.48
PARD3B	1348.5	1366.0	1291.5	1509.5	1654.5	1481.0	1441.8	132.97
PARD6A	8.5	13.0	11.0	9.5	10.0	8.5	10.1	1.72
PARD6B	123.5	52.0	75.0	133.5	49.0	60.5	82.3	37.08
PARD6G	110.5	74.5	46.5	125.5	94.0	57.0	84.7	30.81
PARG	1379.0	709.0	1620.5	1409.0	780.0	1317.5	1202.5	369.81
PARK2	59.0	38.5	44.5	48.5	49.5	46.0	47.7	6.77
PARK7	1800.5	1706.5	1067.0	1581.0	1812.5	1013.5	1496.8	363.67
PARL	1857.0	1507.5	677.0	1705.5	1554.0	589.5	1315.1	542.92
PARM1	917.0	287.0	196.0	709.5	263.0	261.5	439.0	298.76
PARN	972.0	690.0	629.5	1002.5	822.0	650.5	794.4	163.96
PARP1	1816.0	1418.0	1844.5	1863.0	1517.0	1549.0	1667.9	195.21
PARP11	238.0	131.5	145.5	233.5	152.5	136.5	172.9	49.23
PARP12	332.0	211.0	331.5	316.0	219.5	278.5	281.4	54.89
PARP14	9.0	2.5	6.5	8.5	5.5	8.0	6.7	2.42
PARP15	62.0	56.5	158.0	68.5	62.5	159.5	94.5	49.91
PARP16	222.5	259.0	234.0	209.0	261.5	235.0	236.8	20.45
PARP3	1549.5	719.0	396.0	1190.5	661.5	328.0	807.4	474.48
PARP4	196.5	119.5	233.5	182.5	153.0	222.5	184.6	42.92
PARP6	167.0	174.0	197.0	146.0	146.0	198.0	171.3	23.15
PARP8	123.5	143.5	117.0	100.5	165.0	140.5	131.7	22.73
PARP9	86.0	79.5	145.0	88.0	106.0	158.5	110.5	33.41
PARS2	346.0	288.0	167.5	340.5	293.0	189.0	270.7	75.72
PARVA	351.5	157.5	317.0	305.5	180.5	278.0	265.0	78.35
PARVG	1393.0	1810.5	3038.5	1421.5	2100.0	2547.5	2051.8	649.79
PASD1	169.0	250.0	183.0	191.5	263.5	303.5	226.8	53.43
PASK	77.5	28.5	74.0	79.5	42.5	38.0	56.7	22.80
PATL1	658.5	600.5	673.5	711.0	621.0	675.5	656.7	40.05
PATL2	49.0	30.5	64.5	48.0	34.0	52.0	46.3	12.45
PATZ1	84.0	45.5	66.0	73.0	46.5	59.0	62.3	15.11
PAWR	5.5	2.0	8.5	7.5	6.0	7.5	6.2	2.32
PAX1	0.0	0.0	0.5	0.0	0.0	0.0	0.1	0.20
PAX2	0.0	0.0	0.5	0.0	0.5	0.0	0.2	0.26
PAX5	0.0	0.5	0.0	0.0	0.0	0.0	0.1	0.20
PAX6	1.0	0.0	0.5	0.5	0.5	0.5	0.5	0.32
PAX7	238.5	298.0	399.5	212.0	338.5	509.5	332.7	109.91

PAX9	3.0	4.0	2.5	1.0	1.0	2.0	2.3	1.17
PAXBP1	774.0	806.5	875.5	697.5	896.5	844.0	815.7	73.08
PAXIP1	252.5	178.0	222.0	251.0	175.5	232.0	218.5	34.34
PBK	562.5	228.5	340.0	588.5	230.0	192.0	356.9	176.62
PBLD	708.5	260.5	157.5	590.5	288.0	125.5	355.1	238.96
PBRM1	1448.0	843.5	1247.0	1348.5	885.5	1101.5	1145.7	246.48
PBX1	35.0	19.5	62.5	22.5	22.5	65.5	37.9	20.92
PBX3	285.5	227.5	344.5	232.0	244.5	242.0	262.7	45.06
PBX4	172.5	159.5	135.0	169.5	203.0	134.0	162.3	25.95
PBXIP1	505.5	466.5	476.5	454.0	328.5	405.5	439.4	63.47
PCBD1	1280.5	1177.5	1314.0	1333.0	1219.0	1266.0	1265.0	58.38
PCBD2	336.5	355.0	239.5	312.5	347.5	236.5	304.6	53.55
PCBP2	2384.0	2790.0	2909.5	2496.5	3413.0	2929.0	2820.3	365.29
PCBP3	168.5	45.5	18.5	170.0	56.5	19.5	79.8	70.88
PCBP4	1823.5	1297.0	1153.0	1383.0	1199.5	914.0	1295.0	303.81
PCCB	686.5	615.0	468.0	627.5	582.0	409.5	564.8	104.93
PCDH1	56.5	37.5	53.5	21.5	41.0	38.0	41.3	12.63
PCDH10	0.5	0.0	0.0	0.0	0.0	1.0	0.3	0.42
PCDH11X	279.5	199.0	340.5	360.5	246.5	368.5	299.1	68.51
PCDH12	35.0	14.0	15.0	43.5	21.5	24.5	25.6	11.61
PCDH15	3.0	4.5	2.0	1.5	4.0	5.0	3.3	1.40
PCDH17	1.0	0.0	0.5	3.0	0.0	4.0	1.4	1.69
PCDH18	193.0	211.5	359.0	186.0	212.5	333.0	249.2	76.16
PCDH19	37.5	9.0	46.5	38.0	5.0	35.5	28.6	17.19
PCDH20	44.0	15.0	19.0	44.0	16.0	32.5	28.4	13.60
PCDH7	2.5	0.0	1.5	2.0	1.0	0.5	1.3	0.94
PCDH8	1.0	0.5	4.5	1.5	1.0	3.5	2.0	1.61
PCDH9	0.0	0.0	0.5	1.0	1.0	0.0	0.4	0.49
PCDHAC1	1.0	2.0	1.0	0.5	1.0	1.5	1.2	0.52
PCF11	1148.5	964.0	987.0	1054.5	1083.0	993.0	1038.3	70.16
PCGF1	107.5	102.5	111.5	88.0	102.0	103.0	102.4	7.96
PCGF2	34.0	25.5	18.5	37.0	29.5	16.5	26.8	8.24
PCGF3	954.5	753.5	659.0	831.0	962.5	660.5	803.5	136.14
PCGF5	2344.5	1351.0	2235.5	2105.5	1536.0	2053.0	1937.6	400.30
PCGF6	547.0	448.5	380.5	519.0	501.0	364.0	460.0	75.37
PCID2	1295.0	1024.5	1132.5	1266.0	1146.5	964.0	1138.1	129.84
PCIF1	1142.0	800.5	989.0	1107.5	777.5	947.5	960.7	151.41
PCK1	0.0	0.0	0.0	0.0	0.0	0.0	0.0	0.00
PCLO	0.0	0.0	0.0	0.0	0.0	0.0	0.0	0.00
PCM1	745.5	443.5	741.5	739.5	484.0	609.5	627.3	137.29
PCMT1	1552.0	1361.5	1070.0	1455.5	1377.0	925.0	1290.2	240.97
PCMTD1	1549.5	1153.0	1510.5	1411.5	1364.5	1466.0	1409.2	142.00
PCMTD2	125.5	46.0	135.0	164.0	58.0	110.0	106.4	45.83
PCNA	2575.5	1126.0	978.0	2616.5	1079.0	756.0	1521.8	841.85
PCNP	1179.0	1019.5	1225.0	1092.5	1179.5	1146.5	1140.3	73.65
PCNT	509.0	401.5	561.5	472.0	446.5	478.5	478.2	54.43
PCNX	1012.0	566.5	1098.0	1010.0	639.5	918.5	874.1	218.74
PCNXL2	9.0	3.5	10.0	12.0	4.5	6.0	7.5	3.35
PCNXL4	334.5	320.0	242.0	298.0	380.0	299.0	312.3	45.74
PCOLCE2	2562.5	533.5	809.5	2437.5	539.5	593.0	1245.9	977.40
PCP4	0.0	0.0	0.0	0.0	0.0	0.0	0.0	0.00
PCP4L1	0.0	0.0	0.0	0.0	0.0	0.0	0.0	0.00
PCSK1	357.0	253.0	225.0	394.5	383.0	346.0	326.4	70.47
PCSK2	0.0	0.0	0.5	0.0	0.0	0.0	0.1	0.20

PCSK4	2.5	0.5	0.0	1.0	1.5	1.0	1.1	0.86
PCSK6	789.5	418.0	735.5	699.5	414.5	581.0	606.3	162.37
PCSK7	1389.0	1230.0	845.0	1490.5	1274.0	921.5	1191.7	256.77
PCSK9	48.5	40.0	32.0	33.5	33.5	21.5	34.8	8.98
PCTP	211.5	125.0	65.5	176.0	133.0	63.5	129.1	58.94
PCYOX1	824.0	758.5	1029.5	925.0	888.0	1094.5	919.9	125.53
PCYOX1L	272.0	153.0	124.0	242.0	178.5	133.5	183.8	60.41
PCYT1A	3384.5	2146.5	2062.5	3125.5	2220.0	2036.0	2495.8	597.25
PCYT1B	32.0	13.5	14.0	25.5	13.0	13.0	18.5	8.21
PCYT2	1154.5	1296.0	1003.5	1175.5	1501.5	1028.0	1193.2	184.77
PDAP1	1753.0	1827.5	1379.0	1706.5	1904.0	1380.0	1658.3	226.16
PDC	1.0	0.0	0.0	1.0	0.0	0.0	0.3	0.52
PDCD1	1.0	3.0	1.5	1.0	2.5	3.0	2.0	0.95
PDCD10	790.0	651.0	590.5	774.5	753.0	544.5	683.9	103.42
PDCD11	704.5	378.0	506.5	671.5	394.0	370.0	504.1	151.13
PDCD1LG2	0.0	1.0	0.0	0.0	0.5	0.0	0.3	0.42
PDCD2	496.0	499.0	272.5	451.0	525.0	269.5	418.8	116.96
PDCD2L	457.0	323.0	177.0	359.0	368.5	168.5	308.8	114.28
PDCD4	2056.5	979.0	814.5	1813.0	1087.5	945.0	1282.6	518.38
PDCD5	1113.0	915.0	780.5	920.0	1037.0	670.0	905.9	162.32
PDCD6	1470.5	1397.0	1197.5	1374.5	1483.0	1069.0	1331.9	164.48
PDCD6IP	1162.5	673.0	1316.5	1257.0	741.5	1077.0	1037.9	269.70
PDCD7	379.0	327.0	248.5	355.5	364.0	225.0	316.5	64.48
PDCL	1562.0	1357.5	2115.5	1571.0	1474.0	1862.0	1657.0	279.95
PDCL2	31.5	25.0	22.0	39.0	28.5	20.0	27.7	6.95
PDCL3	1863.0	2358.5	1172.5	1845.0	2705.0	1133.5	1846.3	626.43
PDDC1	401.5	295.0	296.5	384.5	295.0	233.5	317.7	63.33
PDE10A	19.5	9.0	14.0	15.0	6.0	7.5	11.8	5.18
PDE11A	3.0	3.5	0.0	2.0	4.5	1.0	2.3	1.66
PDE12	443.5	480.5	335.0	466.5	477.0	340.0	423.8	68.07
PDE1A	8.5	1.0	3.0	2.0	1.0	2.0	2.9	2.84
PDE1B	1.0	0.0	0.0	0.5	0.0	0.0	0.3	0.42
PDE1C	1.5	1.5	1.5	1.5	0.0	2.5	1.4	0.80
PDE2A	3.0	11.5	21.5	3.0	15.5	16.5	11.8	7.55
PDE3A	0.0	0.0	0.0	0.0	0.0	0.0	0.0	0.00
PDE3B	597.0	304.5	748.0	511.0	317.5	464.5	490.4	169.27
PDE4B	236.0	569.0	539.5	207.5	655.0	598.0	467.5	194.35
PDE4C	25.0	32.0	35.5	14.5	37.5	30.5	29.2	8.39
PDE4D	639.5	566.5	372.0	578.5	654.5	383.5	532.4	124.55
PDE5A	716.0	616.5	1130.5	829.0	789.5	1343.5	904.2	276.12
PDE6B	35.0	15.5	9.5	30.5	12.0	6.0	18.1	11.86
PDE6C	33.0	32.5	26.0	27.0	31.0	15.5	27.5	6.54
PDE6D	1072.5	1068.5	1013.0	1069.5	1136.5	1074.5	1072.4	39.15
PDE6G	9.0	25.5	11.0	10.5	21.5	15.0	15.4	6.69
PDE6H	5.0	5.0	2.0	1.0	1.5	0.5	2.5	2.00
PDE7B	33.5	33.0	118.5	23.0	42.5	157.5	68.0	55.95
PDE8A	247.0	240.5	266.0	212.0	312.5	223.0	250.2	35.87
PDE8B	91.5	55.5	47.5	72.0	88.0	58.5	68.8	18.06
PDF	115.5	181.0	107.0	111.0	177.5	113.5	134.3	34.99
PDGFA	397.5	198.5	197.5	378.5	269.5	227.5	278.2	89.22
PDGFB	282.0	249.5	429.0	362.5	341.5	576.0	373.4	117.46
PDGFC	0.0	0.0	0.0	0.0	0.0	0.0	0.0	0.00
PDGFD	263.0	687.5	633.5	274.5	823.0	661.0	557.1	232.68
PDGFRA	0.0	0.0	0.0	0.0	0.0	0.0	0.0	0.00

PDGFRB	1489.5	713.5	1085.0	1595.0	792.0	1139.5	1135.8	356.40
PDGFRL	243.0	162.0	132.5	248.5	212.5	132.5	188.5	53.14
PDHA1	2000.0	1313.0	976.5	1963.0	1467.0	892.0	1435.3	473.07
PDHB	2161.0	2080.0	1587.0	1959.0	2289.5	1477.5	1925.7	325.03
PDHX	502.5	457.0	506.5	543.5	526.0	422.0	492.9	45.27
PDIA2	0.5	0.0	0.0	0.0	0.0	1.5	0.3	0.61
PDIA3	9940.5	7525.5	9806.0	10923.0	8677.0	10988.0	9643.3	1340.11
PDIA4	3153.5	1879.5	3589.0	3555.0	2166.5	4124.5	3078.0	878.26
PDIA6	2932.5	2285.5	4314.5	3083.5	2619.0	4614.0	3308.2	941.31
PDIK1L	303.5	227.0	272.0	259.0	280.0	271.5	268.8	25.26
PDILT	0.0	0.0	0.5	0.0	0.0	0.0	0.1	0.20
PDK1	4574.5	6008.0	6312.5	4349.0	6977.0	4994.0	5535.8	1051.61
PDK3	54.0	30.0	99.5	54.5	25.5	83.5	57.8	29.12
PDK4	412.5	50.5	39.5	250.0	59.0	38.5	141.7	155.76
PDLIM1	5343.5	7856.5	6655.5	4525.0	8233.5	5341.5	6325.9	1501.18
PDLIM3	1690.5	5611.0	4935.5	1042.5	6624.0	4483.5	4064.5	2220.15
PDLIM4	1180.5	1979.0	1774.5	1623.0	2179.5	3199.0	1989.3	683.25
PDLIM5	5178.5	2922.5	5606.5	6164.0	3466.0	5184.0	4753.6	1272.27
PDLIM7	8009.5	6925.5	4256.0	6137.0	6405.5	3631.5	5894.2	1653.00
PDP1	402.0	277.5	414.0	356.0	395.5	465.0	385.0	63.30
PDP2	92.0	61.0	137.5	95.5	69.0	139.5	99.1	33.25
PDPK1	181.0	121.0	318.0	199.0	130.5	266.5	202.7	77.12
PDPN	953.0	610.0	725.0	854.5	650.0	562.0	725.8	151.11
PDPR	784.0	454.0	441.0	724.0	499.5	414.0	552.8	159.46
PDS5A	392.0	218.0	563.0	411.0	274.5	549.0	401.3	139.87
PDS5B	281.5	82.5	333.0	288.5	103.0	257.5	224.3	105.01
PDSS1	158.5	94.5	89.5	154.5	112.0	73.5	113.8	35.34
PDSS2	625.5	400.5	441.0	574.0	449.5	409.5	483.3	93.47
PDX1	0.0	0.0	0.0	0.0	0.0	0.0	0.0	0.00
PDXDC1	241.5	143.5	234.0	257.5	178.0	206.0	210.1	43.10
PDXK	405.5	234.5	482.5	393.0	244.5	376.5	356.1	97.40
PDXP	230.5	175.0	216.0	238.0	219.0	208.5	214.5	22.03
PDYN	0.0	0.0	0.0	0.0	0.0	0.0	0.0	0.00
PDZD11	405.0	362.0	410.5	404.5	389.5	399.5	395.2	17.72
PDZD2	470.5	909.0	790.0	559.0	1207.5	861.5	799.6	264.00
PDZD3	9.0	50.0	37.0	13.0	52.5	34.0	32.6	18.23
PDZD7	62.5	65.5	86.5	75.0	88.5	103.0	80.2	15.39
PDZK1IP1	0.0	0.0	0.0	0.0	0.0	0.5	0.1	0.20
PDZRN3	605.0	639.5	1098.0	746.5	841.0	1090.0	836.7	215.95
PDZRN4	0.0	1.0	2.0	0.5	1.5	1.5	1.1	0.74
PEA15	83.0	140.5	317.5	89.0	118.5	266.0	169.1	98.61
PEAK1	1340.0	1764.5	2639.5	1373.5	2252.5	2681.5	2008.6	603.37
PEAR1	0.5	0.5	0.0	0.0	0.0	0.0	0.2	0.26
PEBP1	2784.0	3200.5	2142.0	2659.5	3446.0	2189.0	2736.8	525.37
PEBP4	8.5	23.5	13.0	14.0	22.5	8.5	15.0	6.60
PECAM1	53.0	28.0	24.0	44.5	29.0	23.0	33.6	12.27
PECR	268.0	144.5	178.5	237.0	155.5	165.5	191.5	49.54
PEF1	1197.0	979.5	664.0	1070.5	1010.0	602.0	920.5	235.66
PELI1	188.0	180.5	246.0	176.5	180.0	262.0	205.5	38.09
PELI2	27.5	14.5	11.0	15.5	14.5	15.5	16.4	5.68
PELO	492.0	470.5	539.5	528.0	542.5	510.5	513.8	28.45
PENK	4736.5	10068.0	9547.0	5106.0	13372.0	24706.5	11256.0	7349.17
PEPD	747.5	468.5	290.0	664.5	493.5	247.0	485.2	198.06
PER2	65.5	44.0	78.0	59.5	69.0	121.5	72.9	26.35

PER3	456.0	750.0	392.5	381.5	1075.5	1058.0	685.6	324.33
PES1	1225.0	1100.0	965.0	1216.0	1085.5	825.5	1069.5	153.16
PET117	5.5	2.5	10.5	9.5	5.0	5.0	6.3	3.04
PEX1	460.0	309.5	494.5	430.0	322.0	393.5	401.6	74.47
PEX10	419.5	329.0	320.0	383.5	326.0	278.5	342.8	50.33
PEX11A	64.5	46.5	52.5	48.0	55.0	37.0	50.6	9.22
PEX12	68.5	61.0	64.5	85.5	56.0	55.0	65.1	11.22
PEX13	662.0	391.0	482.0	678.0	400.0	463.5	512.8	126.88
PEX14	338.5	246.0	293.5	329.0	278.5	288.0	295.6	33.97
PEX16	528.0	445.0	273.5	468.0	465.5	330.0	418.3	96.13
PEX19	729.5	1090.0	1034.5	568.0	950.5	913.5	881.0	197.00
PEX2	221.5	156.0	168.5	193.5	163.0	153.0	175.9	26.57
PEX26	185.5	88.0	206.0	199.5	82.0	172.5	155.6	55.92
PEX3	469.5	398.0	336.0	448.0	425.5	358.5	405.9	51.76
PEX5	330.0	243.0	207.0	290.5	273.0	203.0	257.8	49.63
PEX5L	4.5	4.5	9.0	4.0	8.0	8.0	6.3	2.23
PEX6	658.0	850.0	754.0	584.0	865.0	824.0	755.8	113.61
PEX7	150.5	65.0	65.0	133.0	80.5	50.5	90.8	41.00
PFDN1	526.5	740.0	370.0	516.0	689.5	455.0	549.5	140.49
PFDN2	899.5	1025.0	488.5	800.0	1048.5	502.5	794.0	248.03
PFDN4	767.0	830.0	781.0	662.5	902.0	713.5	776.0	84.48
PFDN5	1217.5	2146.0	1512.0	1070.0	2242.0	1575.5	1627.2	477.79
PFKFB2	19.5	4.0	22.5	25.0	6.5	17.5	15.8	8.62
PFKFB3	3.5	2.0	10.5	6.5	3.5	6.0	5.3	3.04
PFKL	1567.5	1030.0	733.0	1531.5	1073.0	760.0	1115.8	363.09
PFKM	287.0	3201.5	1783.5	195.0	3315.0	2006.5	1798.1	1354.07
PFKP	666.5	213.5	993.0	725.0	248.5	884.5	621.8	324.19
PFN2	6932.0	5566.0	4994.0	6851.5	6202.5	4744.5	5881.8	930.32
PFN3	0.0	0.5	0.0	0.0	0.0	0.0	0.1	0.20
PFN4	26.0	19.0	12.5	21.5	21.5	20.5	20.2	4.42
PGAM1	16066.0	14689.0	16181.5	16677.5	16962.5	17575.0	16358.6	984.94
PGAM5	1402.5	1220.5	1044.0	1393.0	1393.0	1060.0	1252.2	169.43
PGAP1	119.5	68.0	161.0	134.5	73.0	144.5	116.8	38.32
PGAP2	236.5	194.5	199.0	221.5	205.5	241.0	216.3	19.68
PGAP3	323.5	250.5	178.0	314.5	249.5	157.0	245.5	68.23
PGBD5	20.0	27.0	55.5	17.0	25.5	29.5	29.1	13.74
PGD	1753.0	851.0	902.5	1721.5	877.5	790.5	1149.3	457.03
PGF	18.0	61.0	55.0	26.0	75.5	114.0	58.3	34.88
PGGT1B	268.0	205.0	207.5	236.5	235.5	207.5	226.7	24.85
PGK1	12631.0	7262.0	8947.5	12203.0	8139.0	7711.5	9482.3	2344.03
PGLS	851.0	1036.0	564.0	804.5	1094.5	577.5	821.3	222.50
PGLYRP2	0.0	0.0	0.0	0.0	0.0	0.0	0.0	0.00
PGM1	1185.5	780.5	575.0	1090.5	874.0	608.0	852.3	248.98
PGM2	862.0	307.0	311.5	941.5	406.5	347.5	529.3	291.75
PGM2L1	667.5	293.0	332.5	498.0	312.0	288.0	398.5	153.26
PGM3	610.0	329.0	506.5	731.0	375.5	442.5	499.1	150.70
PGM5	76.0	20.0	94.0	61.5	22.0	44.5	53.0	29.68
PGP	1249.0	843.5	743.5	1147.5	934.5	651.0	928.2	232.02
PGPEP1	496.5	405.0	439.5	412.5	479.5	495.0	454.7	41.15
PGPEP1L	959.5	913.0	1025.5	911.5	985.0	976.5	961.8	44.11
PGR	11.5	52.5	22.0	10.0	83.0	43.0	37.0	28.27
PGRMC2	1425.5	894.5	894.0	1444.0	1038.0	846.5	1090.4	274.41
PGS1	104.5	63.0	168.5	110.5	72.0	157.0	112.6	43.07
PHACTR1	1.5	0.5	1.0	0.5	0.0	0.0	0.6	0.58

PHACTR2	837.5	824.0	773.5	941.5	1098.0	1015.5	915.0	125.38
PHACTR4	264.5	281.0	254.0	240.5	319.0	274.5	272.3	27.09
PHAX	899.0	845.5	925.0	871.5	946.5	871.0	893.1	37.75
PHB	1871.5	2043.0	1394.0	1676.0	2093.5	1205.0	1713.8	357.77
PHB2	3413.5	3009.5	2199.0	3182.5	3091.5	1996.0	2815.3	575.81
PHC1	245.0	240.5	320.5	245.5	236.0	322.5	268.3	41.33
PHC3	92.0	51.0	55.0	111.5	75.5	59.0	74.0	23.86
PHEX	0.0	0.0	0.0	0.0	0.0	0.0	0.0	0.00
PHF10	228.5	202.5	300.5	235.0	230.0	294.0	248.4	39.53
PHF11	288.0	233.0	204.5	275.5	303.0	213.0	252.8	41.43
PHF12	877.0	772.0	1164.5	879.5	926.5	1144.5	960.7	158.55
PHF13	329.5	270.5	253.5	267.0	294.5	293.5	284.8	27.09
PHF19	311.5	164.5	247.5	281.0	136.0	225.0	227.6	67.33
PHF2	203.0	158.5	329.0	199.0	187.5	320.5	232.9	72.87
PHF20	1922.0	1295.5	1604.0	1864.5	1408.5	1620.0	1619.1	245.47
PHF21A	1421.5	974.0	907.5	1168.0	1128.5	931.5	1088.5	194.59
PHF21B	1.0	0.0	0.0	1.0	0.0	0.0	0.3	0.52
PHF3	1155.5	1007.5	1486.5	1259.0	1283.0	1525.0	1286.1	196.29
PHF5A	712.5	657.5	555.0	597.5	660.5	490.0	612.2	81.08
PHF6	799.0	648.5	895.5	780.5	729.5	818.5	778.6	83.68
PHGDH	2529.0	1961.0	1901.0	1805.5	1525.5	1383.5	1850.9	400.22
PHIP	292.5	152.5	425.5	285.0	157.5	329.0	273.7	104.67
PHKA1	1663.0	1334.5	926.0	1570.0	1419.0	1120.5	1338.8	276.79
PHKG1	1213.5	1358.0	1084.5	1021.5	1403.5	1055.5	1189.4	162.46
PHLDA1	6202.5	7072.5	17642.0	6759.0	7599.5	18971.0	10707.8	5918.30
PHLDA2	620.0	678.5	430.0	680.0	616.0	415.0	573.3	120.04
PHLDA3	90.0	304.5	103.5	112.5	260.5	119.0	165.0	92.59
PHLDB1	1414.5	1494.5	1514.5	1188.5	1445.5	1362.5	1403.3	118.65
PHLPP1	145.5	80.0	313.5	147.0	68.0	301.0	175.8	106.94
PHLPP2	544.5	430.5	538.5	492.0	497.0	489.5	498.7	41.15
PHOSPHO1	655.5	639.5	359.0	567.5	665.0	329.5	536.0	152.71
PHOSPHO2	184.0	138.0	137.5	203.5	162.0	139.5	160.8	27.85
PHOX2B	0.0	0.0	0.0	0.0	0.0	0.0	0.0	0.00
PHPT1	685.0	868.0	502.5	600.0	871.5	519.0	674.3	164.73
PHRF1	866.0	649.0	989.0	794.0	768.0	851.0	819.5	113.36
PHTF1	362.0	227.0	311.5	362.5	270.5	284.0	302.9	53.45
PHYH	216.0	151.0	281.5	168.5	180.0	268.5	210.9	54.16
PHYHD1	181.5	245.0	66.0	145.0	292.5	87.0	169.5	88.42
PHYHIPL	3.5	5.5	9.0	2.0	14.0	15.0	8.2	5.45
PHYKPL	59.0	20.0	35.5	49.5	30.0	22.0	36.0	15.49
PI15	0.0	1.0	0.5	0.5	1.5	3.5	1.2	1.25
PI16	6.5	13.5	47.0	10.0	13.5	47.0	22.9	18.83
PI3	0.5	0.0	0.0	0.0	0.0	0.0	0.1	0.20
PI4K2A	413.0	425.0	462.0	393.5	477.0	493.0	443.9	39.21
PI4K2B	194.5	56.0	109.0	199.5	59.5	85.5	117.3	64.65
PI4KA	861.0	695.5	1081.0	849.5	873.5	1187.5	924.7	177.97
PI4KB	980.5	1122.0	1381.0	997.0	1000.5	1478.0	1159.8	217.09
PIANP	29.0	3.5	2.0	28.5	5.0	1.5	11.6	13.35
PIAS1	248.5	158.5	239.5	204.0	187.5	233.5	211.9	34.86
PIAS2	128.0	72.0	162.0	128.0	100.0	132.5	120.4	30.83
PIAS4	104.0	68.0	131.5	92.5	72.5	100.5	94.8	23.17
PIBF1	81.5	113.0	117.0	70.0	114.5	80.5	96.1	20.97
PICALM	1061.5	828.5	1023.0	1156.0	942.5	1003.5	1002.5	110.76
PICK1	247.5	215.0	227.5	246.0	215.5	199.0	225.1	19.08

PID1	233.5	373.0	376.0	294.5	459.5	460.5	366.2	89.99
PIDD1	94.0	54.5	55.5	78.5	54.0	59.0	65.9	16.59
PIEZO1	3081.5	2227.5	2419.0	3080.5	2193.5	2523.0	2587.5	401.12
PIF1	38.0	25.0	24.5	47.0	23.5	17.0	29.2	11.09
PIFO	0.0	0.0	0.0	0.0	0.0	0.0	0.0	0.00
PIGA	165.0	52.5	81.0	163.5	79.0	71.0	102.0	49.26
PIGB	446.0	255.0	334.0	405.0	289.0	311.5	340.1	72.30
PIGC	266.0	272.5	158.0	250.0	263.0	140.5	225.0	59.39
PIGF	247.5	182.0	421.5	237.5	219.5	357.0	277.5	91.70
PIGG	144.5	12.5	94.0	158.5	25.5	79.0	85.7	59.76
PIGH	78.5	43.0	76.0	58.5	43.5	63.5	60.5	15.31
PIGK	191.5	95.5	279.0	225.5	137.0	275.5	200.7	74.23
PIGL	115.5	112.0	60.0	130.5	124.0	59.0	100.2	32.16
PIGM	115.5	41.5	19.0	102.0	42.0	20.5	56.8	41.69
PIGN	193.5	103.0	186.5	194.0	109.0	147.5	155.6	42.12
PIGO	142.5	110.5	291.0	162.0	89.5	253.0	174.8	80.29
PIGP	195.5	146.0	132.0	192.0	172.0	116.5	159.0	32.54
PIGQ	184.0	63.5	103.5	155.0	65.0	82.0	108.8	50.01
PIGR	0.0	0.0	0.0	0.0	0.0	0.0	0.0	0.00
PIGS	620.5	537.5	498.5	637.5	541.0	561.0	566.0	53.11
PIGT	1715.5	1140.5	1361.0	1620.0	1174.0	1265.5	1379.4	238.09
PIGU	470.5	385.0	373.0	439.5	402.5	343.0	402.3	46.28
PIGV	266.5	259.0	213.0	263.0	246.0	243.5	248.5	19.68
PIGW	240.5	158.0	120.5	237.0	160.5	139.0	175.9	50.78
PIGX	1350.5	1257.5	1165.0	1234.0	1321.0	1323.0	1275.2	69.58
PIGY	996.5	749.0	509.5	874.5	798.5	458.5	731.1	209.42
PIGZ	14.5	17.5	19.0	21.0	19.0	18.5	18.3	2.16
PIH1D2	72.5	58.5	42.5	55.5	72.5	26.5	54.7	17.85
PIH1D3	7.5	8.0	17.0	7.0	13.0	13.0	10.9	4.03
PIK3AP1	24.0	24.5	19.0	25.5	33.5	21.0	24.6	4.99
PIK3C2B	187.5	344.5	563.5	191.0	385.5	592.5	377.4	174.85
PIK3C3	325.0	130.5	322.5	370.5	124.0	239.0	251.9	105.51
PIK3CA	271.0	145.0	342.5	235.0	170.0	280.5	240.7	73.56
PIK3CD	603.5	632.0	403.5	612.5	760.0	417.0	571.4	137.11
PIK3IP1	618.0	489.5	171.0	513.0	599.0	449.0	473.3	161.59
PIK3R1	14.5	1.0	11.5	7.5	2.5	8.5	7.6	5.16
PIK3R2	1622.5	982.0	1741.0	1379.0	919.5	1575.5	1369.9	345.60
PIK3R3	499.0	340.5	583.0	422.0	413.5	380.0	439.7	87.70
PIK3R4	606.5	412.0	494.5	647.0	447.0	443.0	508.3	96.30
PIK3R5	2.0	2.0	1.0	1.5	2.0	1.0	1.6	0.49
PIK3R6	109.0	408.0	189.0	83.0	441.0	207.0	239.5	151.08
PIKFYVE	158.5	98.0	281.0	188.0	113.5	252.5	181.9	73.63
PIM1	434.5	665.0	622.5	529.0	709.0	754.0	619.0	118.82
PIM3	491.0	892.0	621.0	463.5	1039.0	614.5	686.8	229.74
PIN4	406.5	464.5	204.5	367.5	505.0	224.0	362.0	123.95
PINK1	992.5	816.0	889.5	804.0	846.5	814.0	860.4	71.83
PINX1	127.0	68.0	79.5	100.5	76.5	85.0	89.4	21.35
PIP4K2A	1203.5	729.0	1001.0	1103.0	763.5	924.5	954.1	186.79
PIP4K2B	417.5	184.5	495.5	474.0	189.5	394.5	359.3	138.36
PIP5K1A	246.5	117.5	264.0	255.0	118.5	278.5	213.3	74.60
PIP5K1B	607.5	326.0	223.5	595.0	372.0	212.5	389.4	174.92
PIP5K1C	108.5	99.5	98.5	111.5	99.5	90.5	101.3	7.57
PIPOX	9.5	11.0	2.0	8.5	12.5	2.0	7.6	4.53
PIRT	0.0	0.0	0.0	0.5	0.0	0.0	0.1	0.20

PISD	550.5	323.5	432.0	539.0	322.0	358.5	420.9	103.95
PITHD1	1251.0	1210.0	646.0	1181.0	1219.5	722.0	1038.3	276.36
PITPNA	1296.5	895.0	1030.5	1178.5	984.5	919.5	1050.8	156.84
PITPNC1	84.5	29.0	171.5	105.0	40.5	161.5	98.7	59.55
PITPNM1	417.0	319.0	452.5	372.0	312.5	381.5	375.8	54.52
PITPNM2	233.0	194.0	357.0	226.5	221.5	401.5	272.3	85.11
PITPNM3	124.0	48.5	56.0	117.5	54.0	58.0	76.3	34.61
PITRM1	1040.5	651.0	842.5	963.0	775.0	724.0	832.7	147.25
PITX1	0.5	0.5	1.5	0.5	2.0	1.5	1.1	0.66
PITX2	295.0	195.0	204.0	218.5	165.0	164.5	207.0	48.17
PITX3	906.5	1342.0	1042.0	993.0	1318.0	1375.0	1162.8	205.11
PIWIL1	52.5	27.5	11.5	35.5	42.5	13.0	30.4	16.30
PJA2	1064.5	614.0	707.5	938.5	702.0	689.5	786.0	174.89
PKD1	117.0	72.5	328.0	102.0	73.0	333.0	170.9	124.80
PKD1L2	1.0	2.5	14.5	1.0	0.5	14.0	5.6	6.75
PKD2	221.5	74.0	295.0	251.0	74.5	234.0	191.7	94.29
PKD2L1	0.5	1.0	2.0	0.0	0.5	0.0	0.7	0.75
PKD2L2	252.5	293.0	212.0	217.0	328.0	247.5	258.3	44.86
PKDCC	33.5	24.5	44.5	18.0	15.5	18.5	25.8	11.23
PKDREJ	2.5	2.0	0.5	1.5	1.5	0.5	1.4	0.80
PKHD1	48.5	6.5	10.0	17.5	5.5	0.0	14.7	17.55
PKIA	1171.5	1507.5	2147.0	983.5	1891.0	1769.5	1578.3	443.18
PKIB	1.0	0.0	4.5	0.0	1.0	6.5	2.2	2.70
PKIG	9031.5	12860.5	7975.0	9205.5	15897.0	8967.5	10656.2	3069.52
PKM	33042.5	13873.5	15073.5	31641.5	13217.0	13590.0	20073.0	9534.16
PKN2	404.0	271.0	625.0	435.5	322.5	536.0	432.3	131.74
PKN3	111.0	42.5	117.5	92.5	68.0	121.0	92.1	31.24
PKNOX1	74.5	69.5	164.5	91.0	82.0	108.5	98.3	35.22
PKNOX2	153.0	310.0	264.5	153.5	362.0	261.5	250.8	83.91
PKP1	0.5	2.0	2.5	0.0	2.0	2.5	1.6	1.07
PKP2	196.0	155.5	225.5	86.5	146.5	97.5	151.3	54.11
PKP3	4.5	9.5	6.0	4.0	8.0	3.5	5.9	2.40
PKP4	1301.0	1029.0	1605.0	1169.0	1126.5	1552.5	1297.2	235.56
PLA1A	6.0	128.0	72.0	9.0	152.5	59.5	71.2	60.16
PLA2G10	331.5	309.0	212.0	273.0	300.0	174.0	266.6	61.19
PLA2G12A	779.0	631.5	863.0	781.5	712.5	930.5	783.0	105.92
PLA2G12B	2.0	0.0	2.0	3.0	0.5	1.0	1.4	1.11
PLA2G15	63.5	12.5	90.0	82.5	20.0	65.5	55.7	32.23
PLA2G4A	257.5	169.5	199.0	229.0	179.0	242.0	212.7	35.56
PLA2G6	219.5	162.5	180.0	219.5	170.5	157.0	184.8	27.95
PLA2G7	233.5	278.0	324.5	200.0	329.0	320.5	280.9	54.01
PLA2R1	492.0	281.0	470.5	571.0	321.5	521.0	442.8	115.44
PLAA	216.0	78.0	239.0	242.0	98.5	190.0	177.3	71.72
PLAC8	58.0	10.0	3.5	40.5	8.5	5.5	21.0	22.68
PLAC9	262.5	111.5	51.0	216.0	132.0	83.0	142.7	80.98
PLAG1	109.0	49.0	109.0	114.0	78.5	107.5	94.5	25.67
PLAGL1	60.5	26.5	44.5	52.0	23.0	35.5	40.3	14.67
PLAGL2	194.0	160.5	252.0	184.5	171.5	199.5	193.7	31.98
PLAT	67.0	54.0	93.0	57.0	73.5	104.0	74.8	19.98
PLAU	65.0	32.5	58.0	84.0	46.5	55.0	56.8	17.37
PLB1	26.0	18.5	9.0	24.5	27.5	11.5	19.5	7.83
PLBD1	1786.5	611.5	483.5	1204.5	616.0	463.0	860.8	528.72
PLBD2	342.5	571.0	610.5	330.5	575.5	919.0	558.2	215.29
PLCB2	28.5	9.0	20.5	19.5	12.0	21.0	18.4	6.98

PLCB4	141.0	210.0	260.0	110.5	245.0	165.5	188.7	59.39
PLCD1	73.5	16.5	18.5	61.5	22.0	18.5	35.1	25.46
PLCD3	178.5	393.0	461.0	161.5	409.5	556.5	360.0	157.92
PLCD4	199.0	226.5	80.5	179.0	273.5	114.5	178.8	71.34
PLCE1	133.0	109.0	276.0	121.0	148.5	212.0	166.6	64.63
PLCG1	188.0	388.0	398.5	216.0	450.5	499.5	356.8	126.63
PLCG2	12.5	11.0	13.0	12.0	12.5	25.0	14.3	5.27
PLCH1	9.5	6.0	10.5	8.0	8.0	9.5	8.6	1.59
PLCL1	141.5	76.0	53.5	122.0	97.0	75.0	94.2	32.79
PLCL2	79.5	37.0	107.5	49.0	58.0	86.5	69.6	26.24
PLCXD1	11.0	13.5	10.0	6.5	12.5	17.5	11.8	3.68
PLCXD2	23.5	27.0	73.0	21.0	26.5	82.5	42.3	27.75
PLCXD3	0.5	0.0	0.0	0.0	0.0	0.0	0.1	0.20
PLD1	956.0	598.5	1061.0	848.5	695.5	1002.5	860.3	181.93
PLD4	7.5	2.0	3.0	3.5	1.5	2.5	3.3	2.16
PLD5	2.5	0.0	0.0	1.0	0.0	0.5	0.7	0.98
PLEK	71.5	49.5	54.0	46.0	48.0	56.0	54.2	9.28
PLEK2	5.0	4.5	7.5	9.0	3.5	6.5	6.0	2.05
PLEKHA1	1218.0	1762.0	1221.5	1454.0	2134.0	1817.5	1601.2	365.84
PLEKHA2	85.5	45.0	56.0	75.0	36.5	42.5	56.8	19.55
PLEKHA3	1034.5	514.0	418.5	961.0	582.5	425.5	656.0	272.54
PLEKHA4	0.0	0.0	0.0	0.0	0.0	0.5	0.1	0.20
PLEKHA5	25.0	13.0	34.0	27.0	18.0	33.5	25.1	8.37
PLEKHA6	33.0	17.5	45.5	35.0	19.5	54.0	34.1	14.26
PLEKHA8	669.0	431.0	955.5	585.0	494.5	785.5	653.4	194.09
PLEKHB1	0.5	1.0	1.5	0.0	0.5	2.0	0.9	0.74
PLEKHB2	525.5	253.0	347.5	532.5	271.5	283.5	368.9	128.03
PLEKHD1	7.0	4.5	9.5	10.5	4.5	6.0	7.0	2.53
PLEKHF1	1328.0	1039.0	1295.5	1219.0	1166.5	1160.5	1201.4	104.36
PLEKHF2	262.0	177.5	200.5	233.0	207.5	189.0	211.6	31.02
PLEKHG1	40.0	79.5	90.5	31.0	84.5	101.0	71.1	28.62
PLEKHG3	274.5	221.5	269.0	279.5	207.5	265.0	252.8	30.42
PLEKHG4	178.0	121.0	220.0	204.5	139.5	189.5	175.4	38.18
PLEKHG5	149.5	65.0	28.5	116.5	51.0	13.5	70.7	52.50
PLEKHG7	4.5	0.5	1.5	2.5	0.5	1.0	1.8	1.54
PLEKHH1	281.5	228.0	202.0	228.0	240.0	173.5	225.5	36.37
PLEKHH2	81.0	41.5	49.5	74.5	46.0	39.5	55.3	17.83
PLEKHH3	165.5	75.5	124.0	167.5	50.5	118.5	116.9	47.11
PLEKHJ1	580.5	411.0	509.0	570.5	427.5	504.5	500.5	70.32
PLEKHM1	225.5	147.0	138.0	203.5	104.0	150.0	161.3	44.86
PLEKHM2	1581.0	1321.5	1626.0	1453.0	1142.5	1516.5	1440.1	180.58
PLEKHM3	316.5	195.0	205.5	310.5	190.5	174.5	232.1	63.88
PLEKHN1	86.5	20.0	65.0	89.5	16.0	52.5	54.9	31.73
PLEKHO1	3009.5	2552.0	3019.5	2983.0	2938.0	2494.5	2832.8	242.08
PLEKHO2	545.5	261.5	426.0	546.5	309.5	383.0	412.0	118.41
PLEKHS1	3.5	2.0	1.0	4.5	2.0	1.5	2.4	1.32
PLIN1	4.0	0.0	1.0	3.5	1.0	0.5	1.7	1.66
PLIN2	2652.0	846.0	1622.5	2869.0	994.5	1260.0	1707.3	860.14
PLK1	946.5	419.5	571.5	1085.0	408.0	408.0	639.8	300.97
PLK2	745.5	680.5	976.0	1022.5	792.5	754.5	828.6	137.81
PLK3	85.5	147.5	123.0	87.5	141.0	153.5	123.0	30.07
PLK4	281.5	102.5	259.0	350.5	103.0	147.0	207.3	104.10
PLLP	87.0	96.0	132.5	61.5	105.0	129.0	101.8	26.72
PLN	328.0	1429.0	977.0	208.0	1392.0	647.0	830.2	523.19

PLOD1	1616.5	1183.5	5419.5	1947.0	1603.0	6325.0	3015.8	2244.20
PLOD2	6643.5	4359.5	5129.0	7347.0	5144.0	5399.5	5670.4	1106.34
PLP1	2.0	5.5	4.0	1.0	4.0	4.5	3.5	1.67
PLRG1	1215.0	761.5	1011.5	1169.0	835.5	900.0	982.1	182.72
PLS1	388.0	126.5	32.0	465.5	148.0	45.5	200.9	182.22
PLS3	10182.0	16978.0	17794.5	10054.0	20517.5	15001.5	15087.9	4237.79
PLSCR1	1.5	2.5	3.5	3.5	3.0	4.5	3.1	1.02
PLSCR5	0.0	5.0	1.0	0.5	5.0	0.0	1.9	2.42
PLTP	549.5	1749.5	760.0	772.5	2288.5	1412.0	1255.3	680.13
PLVAP	2.0	1.5	0.5	0.5	1.5	3.0	1.5	0.95
PLXDC1	8.0	14.0	16.5	12.5	16.0	10.0	12.8	3.36
PLXDC2	93.0	84.5	123.0	85.5	96.0	130.5	102.1	19.74
PLXNA1	3365.0	2100.5	5324.0	3584.0	2269.0	4759.0	3566.9	1294.95
PLXNA2	12.0	20.0	40.0	8.0	30.5	95.5	34.3	32.21
PLXNA4	2.5	1.0	1.5	2.0	4.0	5.0	2.7	1.54
PLXNB1	2.0	0.5	8.0	0.5	0.0	2.0	2.2	2.98
PLXNB2	836.0	825.5	1642.5	858.5	993.5	1405.0	1093.5	346.93
PLXNC1	16.0	9.0	30.5	16.0	14.0	21.0	17.8	7.35
PLXND1	26.5	113.5	189.5	21.0	140.0	219.0	118.3	81.97
PM20D1	115.0	84.5	68.5	128.0	102.5	82.5	96.8	22.33
PM20D2	1198.0	1662.0	1622.5	984.0	1985.5	1387.0	1473.2	358.57
PMAIP1	11.0	56.0	84.5	10.5	55.5	97.5	52.5	36.21
PMCH	3.5	0.5	1.0	2.0	0.0	1.5	1.4	1.24
PMEL	7.0	11.5	9.5	12.5	10.0	20.0	11.8	4.46
PMEPA1	173.5	230.5	300.5	191.0	279.5	325.0	250.0	61.25
PMFBP1	7.5	12.0	14.0	4.0	12.0	10.0	9.9	3.64
PML	23.5	12.0	27.0	28.0	14.5	18.5	20.6	6.63
PMM1	477.0	291.5	274.5	404.5	314.5	263.5	337.6	84.90
PMM2	454.0	574.5	645.0	452.5	655.0	591.0	562.0	89.65
PMP22	133.5	261.0	220.0	162.0	282.5	273.0	222.0	62.00
PMPCA	1497.5	1458.5	1203.0	1448.5	1593.0	1220.0	1403.4	157.27
PMPCB	406.0	841.5	900.0	388.0	906.0	813.0	709.1	244.34
PMS1	172.5	73.0	86.5	149.5	79.0	94.5	109.2	41.44
PMS2	541.5	351.5	435.0	632.5	459.5	449.0	478.2	96.89
PMVK	183.5	220.5	184.5	153.5	217.5	217.0	196.1	26.83
PNISR	2312.5	975.0	1018.0	1989.0	1184.5	1094.0	1428.8	572.92
PNLDC1	6.5	7.5	15.0	6.0	9.0	19.5	10.6	5.45
PNMT	0.5	0.0	0.0	0.0	0.0	0.0	0.1	0.20
PNN	1304.0	1031.5	1067.0	1276.0	1167.0	1007.0	1142.1	127.19
PNO1	964.0	919.5	439.5	922.5	962.0	467.5	779.2	253.12
PNOC	0.0	0.0	0.0	0.0	0.0	0.0	0.0	0.00
PNP	0.0	0.5	0.0	0.0	0.0	0.0	0.1	0.20
PNPLA1	0.5	0.5	0.5	0.5	0.0	1.0	0.5	0.32
PNPLA2	976.0	488.5	683.5	969.5	557.0	558.0	705.4	216.45
PNPLA4	51.5	33.0	32.0	46.0	36.5	34.0	38.8	8.02
PNPLA6	359.0	410.0	454.0	258.5	416.5	517.5	402.6	88.04
PNPLA7	841.5	477.0	557.0	791.5	482.0	547.0	616.0	159.48
PNPLA8	645.0	528.0	661.5	579.0	591.0	629.5	605.7	49.38
PNPO	96.0	96.5	59.5	91.0	90.0	64.5	82.9	16.48
PNPT1	508.5	279.0	267.0	528.5	279.5	226.5	348.2	133.50
PNRC1	1847.5	1359.0	1793.0	1539.0	1618.0	1659.0	1635.9	176.85
PNRC2	1215.0	945.0	1590.0	1211.0	1081.0	1431.5	1245.6	233.56
POC1A	159.0	108.0	99.0	133.5	108.5	84.5	115.4	26.66
POC1B	232.0	169.0	234.5	229.5	180.5	204.5	208.3	28.39

POC5	313.5	179.0	219.0	296.0	173.5	152.0	222.2	67.76
PODN	318.5	142.0	75.5	241.0	180.0	91.5	174.8	92.67
PODXL	0.5	1.0	1.5	0.5	0.0	1.0	0.8	0.52
PODXL2	248.5	136.0	204.0	153.0	124.0	237.0	183.8	53.36
POF1B	160.5	88.5	134.0	150.5	103.5	119.5	126.1	27.60
POFUT1	646.5	462.0	528.0	759.0	541.5	614.0	591.8	104.78
POFUT2	1304.5	1020.0	1465.0	1379.0	1129.5	1416.0	1285.7	175.03
POGLUT1	984.5	476.0	428.5	1121.0	530.0	444.5	664.1	306.11
POGZ	491.0	437.5	540.5	459.5	475.0	533.5	489.5	40.87
POLA1	66.0	14.5	44.0	68.0	7.0	32.0	38.6	25.56
POLB	1028.0	661.0	449.0	993.5	747.0	441.5	720.0	254.90
POLD2	710.5	731.0	625.5	609.0	712.0	537.5	654.3	76.02
POLD3	30.0	12.5	31.5	38.5	18.0	31.5	27.0	9.73
POLDIP2	1199.5	1704.5	1339.5	1109.5	1822.5	1253.0	1404.8	290.16
POLDIP3	801.0	605.5	737.0	750.0	641.5	680.0	702.5	73.19
POLE	277.5	121.5	184.0	235.5	100.5	123.5	173.8	71.14
POLE2	452.0	211.5	226.5	415.0	240.0	214.5	293.3	109.73
POLE3	302.0	214.0	265.0	271.5	241.0	228.0	253.6	32.17
POLE4	113.0	110.0	99.0	104.5	127.5	105.5	109.9	9.87
POLG	346.5	294.5	318.5	326.0	317.5	301.0	317.3	18.52
POLH	286.5	218.5	169.0	256.5	221.0	169.5	220.2	46.71
POLL	222.0	221.0	200.5	184.0	210.0	197.0	205.8	14.78
POLN	57.0	18.5	51.5	51.0	28.5	50.5	42.8	15.48
POLQ	94.0	31.0	46.0	96.5	32.0	24.5	54.0	32.72
POLR1A	613.0	496.5	707.5	595.5	580.5	700.5	615.6	79.35
POLR1B	701.0	545.0	648.0	679.0	553.5	597.5	620.7	65.39
POLR1C	459.5	454.5	333.5	455.0	440.5	318.0	410.2	65.88
POLR1D	420.0	523.0	419.0	376.5	598.0	463.5	466.7	81.25
POLR1E	883.5	1019.0	657.0	927.5	1242.0	744.5	912.3	207.11
POLR2B	1003.0	428.0	857.0	1084.0	507.0	753.5	772.1	263.38
POLR2C	1253.5	1221.0	863.0	1109.5	1206.0	769.5	1070.4	204.78
POLR2D	605.5	539.5	508.0	553.0	600.0	467.5	545.6	53.20
POLR2E	931.5	1100.5	941.5	817.5	1087.5	939.0	969.6	107.10
POLR2F	902.0	768.0	521.5	757.5	724.0	488.5	693.6	158.53
POLR2H	577.0	605.5	419.5	508.5	663.5	372.0	524.3	112.41
POLR2I	594.5	637.5	446.5	527.0	671.5	455.5	555.4	94.23
POLR2J	396.0	345.0	220.5	353.5	355.0	206.0	312.7	79.15
POLR2K	218.0	300.5	221.5	208.5	301.5	193.0	240.5	47.89
POLR2L	860.5	1008.5	491.5	835.5	1067.0	522.5	797.6	241.59
POLR2M	289.0	296.5	185.0	277.5	313.0	180.0	256.8	58.74
POLR3A	168.5	96.5	214.5	180.0	114.5	195.5	161.6	46.45
POLR3B	323.0	247.5	359.5	332.5	260.5	306.5	304.9	43.22
POLR3C	26.5	26.5	43.5	33.5	27.0	56.5	35.6	12.19
POLR3D	104.5	128.0	75.5	104.5	113.0	72.5	99.7	21.68
POLR3E	438.5	327.0	466.5	502.0	368.5	417.5	420.0	64.09
POLR3F	199.0	189.0	208.0	217.0	192.0	197.5	200.4	10.43
POLR3G	18.5	9.0	7.5	10.5	15.5	8.5	11.6	4.41
POLR3H	773.5	675.0	423.5	674.0	694.0	374.0	602.3	162.63
POLR3K	138.5	159.0	87.0	124.0	140.5	108.0	126.2	25.71
POLRMT	362.0	345.5	436.5	358.5	305.5	437.0	374.2	52.47
POMC	0.0	1.0	0.0	0.0	1.5	0.0	0.4	0.66
POMGNT1	1011.5	726.0	885.0	974.5	729.5	761.0	847.9	126.96
POMGNT2	138.0	82.5	84.0	122.5	98.5	99.5	104.2	21.96
POMK	194.0	145.0	141.0	189.5	146.0	89.5	150.8	38.14

POMP	2230.0	2173.0	1557.5	2019.0	2295.0	1424.5	1949.8	369.36
POMT1	676.0	524.0	647.0	678.5	632.0	607.5	627.5	57.39
POMT2	614.0	399.0	378.5	617.5	460.0	406.0	479.2	109.18
POP1	239.0	151.0	149.5	208.5	176.0	117.5	173.6	44.14
POPDC2	23.0	669.0	461.0	23.0	737.5	427.0	390.1	308.02
POPDC3	492.0	1088.5	428.5	869.0	1593.5	922.5	899.0	425.84
POR	769.5	578.0	610.5	728.0	613.5	612.0	651.9	77.28
PORCN	213.0	175.0	133.0	194.5	161.5	137.0	169.0	31.62
POT1	425.0	183.0	224.5	431.0	227.0	192.0	280.4	115.64
POU1F1	0.0	0.0	0.0	0.0	0.5	0.0	0.1	0.20
POU2AF1	18.5	1.0	3.0	9.5	2.0	4.0	6.3	6.66
POU2F1	52.0	69.5	53.0	52.5	86.0	56.5	61.6	13.66
POU2F3	0.0	0.0	0.0	0.0	0.5	0.5	0.2	0.26
POU3F1	2.0	1.0	3.0	2.0	0.5	0.5	1.5	1.00
POU3F2	0.5	0.5	0.0	0.0	0.0	0.5	0.3	0.27
POU4F3	0.0	0.0	0.0	0.0	0.5	0.0	0.1	0.20
POU6F1	27.0	36.5	15.5	33.5	67.0	27.0	34.4	17.52
POU6F2	0.0	0.5	0.0	1.0	0.0	0.0	0.3	0.42
PP2D1	1292.5	1298.0	1047.0	1177.0	1464.0	1062.0	1223.4	159.72
PPA1	1780.5	1667.5	1172.5	1678.5	1770.0	1123.0	1532.0	301.58
PPA2	623.0	531.5	283.0	509.5	581.0	279.5	467.9	149.87
PPAP2A	127.5	141.0	152.0	151.0	168.0	160.0	149.9	14.26
PPAP2B	217.5	177.0	130.5	244.0	214.5	178.0	193.6	40.12
PPAP2C	765.0	574.0	771.0	749.5	588.5	859.0	717.8	112.60
PPAPDC1A	0.5	0.0	0.0	1.0	0.0	0.0	0.3	0.42
PPAPDC1B	113.5	115.0	90.5	131.5	98.5	112.0	110.2	14.25
PPAPDC2	523.0	357.5	192.5	485.0	352.0	176.5	347.8	143.59
PPAPDC3	145.0	236.0	361.5	112.5	256.0	333.5	240.8	99.05
PPARA	153.5	115.5	151.5	158.5	135.0	136.5	141.8	15.97
PPARD	348.5	283.0	337.0	320.0	286.5	281.5	309.4	29.67
PPARG	310.0	239.5	209.0	337.0	247.0	154.0	249.4	66.61
PPARGC1A	8.5	33.5	134.0	9.5	46.5	92.5	54.1	49.82
PPARGC1B	30.0	23.0	51.5	23.5	21.5	40.5	31.7	11.99
PPAT	719.0	341.0	248.0	623.5	319.5	208.0	409.8	210.27
PPCDC	88.5	127.0	100.5	69.0	118.5	127.0	105.1	23.40
PPCS	274.5	246.0	168.5	254.5	264.5	148.0	226.0	53.73
PPDPF	857.0	2963.0	2070.0	831.5	2910.0	2117.5	1958.2	941.86
PPEF1	78.5	128.0	58.0	88.0	132.5	60.5	90.9	32.48
PPEF2	11.5	20.0	17.5	14.5	26.5	20.5	18.4	5.22
PPFIA1	707.0	521.0	733.5	727.0	589.5	696.5	662.4	86.82
PPFIA2	9.5	14.5	13.0	4.5	12.5	13.5	11.3	3.71
PPFIBP2	5.5	10.5	15.0	4.0	9.5	16.0	10.1	4.85
PPHLN1	376.5	254.0	242.0	342.0	287.0	235.0	289.4	58.00
PPIA	13390.5	13917.0	10746.5	12052.5	14455.5	10545.5	12517.9	1656.38
PPIB	12691.5	10671.0	7566.0	12870.0	11858.0	9968.0	10937.4	2001.82
PPIC	2444.5	1591.0	1008.5	2492.5	1961.0	1138.5	1772.7	636.26
PPID	1141.0	1244.0	736.5	1044.5	1262.5	607.5	1006.0	273.34
PPIE	684.5	776.0	536.0	659.5	722.0	544.0	653.7	96.43
PPIF	670.5	532.5	477.0	647.0	546.5	435.0	551.4	92.51
PPIG	1653.5	1305.5	1069.5	1529.5	1448.0	1034.5	1340.1	250.42
PPIH	756.0	657.5	568.5	747.5	709.5	515.5	659.1	98.57
PPIL1	298.5	250.5	97.0	270.0	248.5	102.0	211.1	88.30
PPIL2	801.5	636.5	738.5	623.5	579.5	601.5	663.5	87.06
PPIL3	688.5	474.0	307.5	553.0	527.5	304.0	475.8	149.48

PPIL4	639.5	520.5	443.0	622.5	602.5	380.0	534.7	105.67
PPIL6	40.0	7.5	5.5	35.0	9.5	5.5	17.2	15.90
PPL	491.5	77.0	159.5	385.0	71.5	109.0	215.6	178.50
PPM1A	692.0	623.0	738.0	651.0	750.0	705.0	693.2	49.16
PPM1B	1677.5	1490.0	1508.0	1622.5	1759.0	1466.5	1587.3	117.65
PPM1D	329.5	387.5	450.0	334.5	440.0	435.0	396.1	54.13
PPM1E	704.5	714.0	1959.0	654.0	817.0	1859.5	1118.0	615.99
PPM1F	740.0	709.5	766.0	687.5	821.5	667.5	732.0	56.35
PPM1G	730.5	743.5	847.5	754.5	798.0	808.5	780.4	45.00
PPM1H	8.0	4.0	9.5	6.5	6.0	11.5	7.6	2.67
PPM1J	111.5	134.0	48.5	103.5	112.0	50.5	93.3	35.44
PPM1L	325.5	549.0	627.5	350.5	662.5	862.5	562.9	202.74
PPM1M	878.0	1159.0	869.5	866.5	1313.0	956.0	1007.0	186.88
PPME1	1068.0	724.5	852.5	1005.0	728.0	756.0	855.7	148.88
PPP1CA	4042.5	4152.0	2726.0	3637.5	3999.5	2638.5	3532.7	681.57
PPP1CB	7017.5	5942.5	6154.0	6493.5	6606.5	5473.5	6281.3	543.38
PPP1CC	1244.0	1051.5	789.0	1185.0	1113.5	756.0	1023.2	205.00
PPP1R10	8.0	5.5	15.0	12.0	5.0	14.5	10.0	4.44
PPP1R12A	909.0	844.5	1120.0	906.5	1014.5	1063.0	976.3	106.21
PPP1R13B	193.5	161.5	186.0	202.0	180.5	181.5	184.2	13.73
PPP1R14D	422.0	319.0	192.0	375.5	381.0	157.0	307.8	108.87
PPP1R15B	765.5	1126.0	1560.5	746.0	1239.0	1403.5	1140.1	332.19
PPP1R16B	198.5	115.0	123.0	171.5	182.0	138.0	154.7	34.08
PPP1R17	0.0	0.0	0.0	0.0	0.0	1.0	0.2	0.41
PPP1R1B	106.0	68.0	83.5	103.0	68.5	65.5	82.4	18.27
PPP1R1C	4.5	4.5	6.5	2.5	5.5	7.5	5.2	1.75
PPP1R2	725.0	447.0	641.5	730.0	488.5	475.5	584.6	129.74
PPP1R21	372.5	310.0	342.0	392.5	359.5	314.5	348.5	32.60
PPP1R26	155.5	132.0	275.0	181.0	150.0	268.5	193.7	62.52
PPP1R27	2.5	7.0	0.5	7.0	3.0	1.5	3.6	2.78
PPP1R36	2.0	0.5	0.5	4.0	1.5	0.0	1.4	1.46
PPP1R3A	9.0	17.5	8.0	4.5	19.0	8.5	11.1	5.79
PPP1R3B	9.5	8.0	13.5	12.0	9.5	13.0	10.9	2.22
PPP1R3C	18.0	10.5	6.5	22.0	15.5	4.5	12.8	6.82
PPP1R3D	105.5	81.0	213.0	93.5	107.0	160.0	126.7	50.17
PPP1R42	6.5	6.0	4.0	7.0	8.5	5.5	6.3	1.51
PPP1R7	1349.0	1422.0	1101.5	1211.0	1507.0	1058.5	1274.8	179.99
PPP1R8	260.0	219.5	271.5	253.5	204.0	210.5	236.5	28.59
PPP1R9A	33.5	27.5	33.0	36.0	31.5	28.0	31.6	3.31
PPP2CA	1989.5	1821.0	1805.5	1881.0	2162.0	1705.5	1894.1	161.13
PPP2CB	2755.5	2269.5	1813.0	2508.5	2403.5	1779.0	2254.8	389.61
PPP2R2A	2009.5	1451.5	1445.5	1979.5	1622.0	1301.5	1634.9	296.62
PPP2R2B	13.5	3.5	12.5	11.5	11.0	12.5	10.8	3.66
PPP2R2D	1086.5	953.5	1112.0	1057.5	1105.5	1139.0	1075.7	65.72
PPP2R3B	971.5	1932.0	1019.5	798.5	2334.0	920.0	1329.3	639.68
PPP2R3C	342.5	210.0	178.0	320.0	229.0	172.0	241.9	72.63
PPP2R4	1453.0	1187.5	1069.0	1364.5	1157.5	923.0	1192.4	193.17
PPP2R5A	733.5	635.5	585.0	605.0	751.0	570.5	646.8	77.32
PPP2R5C	886.5	516.5	700.5	791.5	566.5	640.0	683.6	139.02
PPP2R5D	1412.5	1212.0	1169.5	1354.0	1236.0	1249.0	1272.2	92.08
PPP2R5E	587.0	458.0	562.5	564.5	539.5	525.5	539.5	45.27
PPP3CA	485.0	222.0	577.5	457.0	253.0	650.0	440.8	171.94
PPP3CB	1008.5	585.0	857.5	995.5	693.5	809.0	824.8	166.70
PPP3R1	1724.5	1728.0	1991.0	1755.0	2046.5	1991.0	1872.7	151.63

PPP4R1	709.5	362.5	723.5	760.5	419.0	715.5	615.1	175.58
PPP4R2	1154.0	1165.0	1576.5	1055.5	1310.0	1531.0	1298.7	214.11
PPP4R4	13.5	23.5	19.5	26.0	39.5	24.0	24.3	8.65
PPP6C	763.5	624.0	475.0	765.5	596.5	486.0	618.4	127.51
PPP6R2	471.5	433.5	486.0	483.5	428.5	431.0	455.7	27.51
PPP6R3	374.5	246.5	551.0	417.5	270.5	538.5	399.8	129.05
PPRC1	261.0	266.5	325.5	266.0	262.5	350.0	288.6	38.92
PPT1	3223.5	3328.5	3420.5	2995.5	3665.0	3320.0	3325.5	220.74
PPT2	105.0	137.5	109.5	108.5	124.0	125.5	118.3	12.68
PPTC7	244.0	154.0	376.0	272.5	184.5	353.0	264.0	88.72
PPWD1	592.5	457.0	459.0	569.0	433.0	396.5	484.5	78.24
PQLC1	546.0	264.0	259.0	581.0	286.0	270.0	367.7	152.37
PQLC2	140.5	119.5	86.0	145.0	120.5	93.5	117.5	23.94
PQLC2L	0.0	0.0	0.0	0.0	0.0	0.0	0.0	0.00
PQLC3	1006.5	488.0	464.0	882.5	522.5	399.0	627.1	252.23
PRADC1	282.0	197.5	156.0	266.0	232.0	180.5	219.0	49.52
PRC1	997.0	476.5	617.0	1001.5	409.0	458.0	659.8	271.85
PRCC	533.5	466.5	595.5	560.5	531.5	604.5	548.7	50.45
PRCP	591.5	293.0	349.5	639.0	281.5	235.5	398.3	172.56
PRDM10	916.0	665.0	788.5	837.5	751.0	839.0	799.5	86.19
PRDM11	200.5	88.5	77.0	158.5	76.5	62.5	110.6	55.62
PRDM12	0.0	0.0	0.0	0.0	0.0	0.0	0.0	0.00
PRDM13	0.0	0.0	0.0	0.0	0.0	0.0	0.0	0.00
PRDM15	203.0	174.5	197.0	207.0	208.5	213.0	200.5	13.83
PRDM16	0.0	0.0	1.5	0.0	0.0	1.5	0.5	0.77
PRDM4	169.5	215.0	228.0	191.5	232.0	213.5	208.3	23.70
PRDM5	184.0	104.5	72.0	139.5	123.0	57.0	113.3	46.33
PRDM8	8.0	13.0	13.0	5.5	11.5	4.5	9.3	3.78
PRDX1	13756.5	9492.0	5142.5	12872.0	10380.0	4777.5	9403.4	3781.44
PRDX3	1559.5	1451.5	1436.0	1509.0	1565.0	1264.0	1464.2	111.61
PRDX4	3298.0	2328.5	2729.5	3319.5	2626.5	3005.0	2884.5	393.56
PRDX6	1847.0	2621.5	2397.5	1719.0	2671.5	2311.0	2261.3	396.17
PRELID1	1653.0	2275.5	1523.0	1515.0	2494.0	1581.5	1840.3	430.18
PRELP	4211.0	2307.0	5725.5	5212.5	2506.0	5770.5	4288.8	1563.63
PREP	1288.0	827.0	944.0	1255.5	891.0	756.5	993.7	224.60
PREPL	68.0	23.5	30.5	65.5	34.5	24.0	41.0	20.38
PREX1	162.5	65.5	388.0	204.0	85.0	381.0	214.3	141.15
PREX2	18.0	8.0	60.5	26.5	19.5	61.5	32.3	22.98
PRF1	10.5	7.5	4.5	7.5	7.5	9.5	7.8	2.07
PRG4	106.0	149.5	138.5	101.0	189.5	106.0	131.8	34.55
PRICKLE1	330.0	252.0	374.0	283.0	269.5	237.5	291.0	51.61
PRICKLE2	75.0	60.0	119.0	100.5	52.5	77.5	80.8	25.00
PRICKLE4	86.5	68.5	47.0	85.5	63.0	63.5	69.0	15.03
PRIM1	1205.0	1230.0	1144.0	1207.5	1312.0	1076.0	1195.8	79.91
PRIMA1	384.0	479.5	531.5	382.5	571.5	482.5	471.9	76.63
PRIMPOL	233.0	114.0	110.0	230.5	98.0	75.0	143.4	69.76
PRKAA1	261.5	160.5	309.5	306.5	173.5	297.5	251.5	67.78
PRKAA2	0.0	0.5	0.0	0.0	0.5	0.0	0.2	0.26
PRKAB1	244.0	119.5	312.5	247.5	126.0	221.0	211.8	75.39
PRKAB2	558.5	491.0	341.5	526.5	443.5	353.0	452.3	90.00
PRKACB	2518.5	1863.5	2318.5	2445.0	2141.0	2152.5	2239.8	238.73
PRKAG2	1182.5	1397.0	1298.0	1052.5	1525.0	1056.5	1251.9	190.01
PRKAG3	16.0	19.5	25.0	9.5	16.5	24.5	18.5	5.84
PRKAR1A	795.0	229.0	907.5	912.0	300.0	742.5	647.7	304.71

PRKAR1B	372.5	245.0	325.5	359.0	277.5	296.0	312.6	48.96
PRKAR2A	177.5	102.5	315.5	205.5	101.5	219.0	186.9	80.52
PRKAR2B	3.5	1.5	8.0	3.5	1.0	4.5	3.7	2.50
PRKCA	852.5	645.0	865.5	910.5	629.0	763.5	777.7	119.05
PRKCB	109.0	309.0	329.5	112.0	414.0	433.0	284.4	142.83
PRKCE	160.5	82.0	174.5	157.0	82.0	144.0	133.3	40.93
PRKCH	76.0	45.5	36.0	93.0	56.0	44.0	58.4	21.86
PRKCI	935.5	627.0	969.5	905.0	742.5	892.5	845.3	132.35
PRKCQ	44.0	560.0	664.5	57.0	734.5	580.5	440.1	308.15
PRKD1	343.5	147.5	258.0	261.5	164.0	204.5	229.8	72.76
PRKDC	69.0	20.0	104.5	48.0	10.0	54.5	51.0	34.20
PRKG1	114.5	152.0	272.0	135.5	151.0	348.5	195.6	93.05
PRKG2	337.5	215.0	184.5	276.5	237.5	187.0	239.7	58.92
PRKRIP1	426.0	453.0	392.5	356.0	463.0	346.5	406.2	49.15
PRKRIR	205.0	142.5	221.5	234.5	169.0	204.0	196.1	34.26
PRKX	59.5	14.5	74.5	66.5	21.5	66.0	50.4	25.65
PRL	0.0	1.0	0.5	0.0	0.0	0.0	0.3	0.42
PRLH	1.5	9.5	9.5	1.5	14.0	10.5	7.8	5.12
PRLHR	0.5	0.0	0.0	0.0	0.0	0.5	0.2	0.26
PRLR	20.5	3.5	5.5	11.0	6.0	5.0	8.6	6.37
PRMT3	765.0	507.0	387.0	707.0	539.5	539.0	574.1	138.60
PRMT5	434.5	411.5	341.5	423.5	360.5	279.5	375.2	59.46
PRMT7	325.5	135.0	145.0	350.5	165.5	104.0	204.3	105.78
PRMT8	45.0	31.5	43.0	35.0	38.0	20.5	35.5	8.88
PRMT9	923.5	738.0	774.5	899.0	816.5	714.5	811.0	85.33
PRNP	1022.0	1542.0	1723.0	991.0	1552.0	1710.5	1423.4	331.93
PROB1	304.0	484.0	397.5	259.5	473.0	378.5	382.8	89.47
PROCA1	32.0	11.5	5.5	23.0	10.5	4.0	14.4	10.91
PROCR	458.5	212.0	69.0	389.5	241.5	111.5	247.0	152.54
PROK1	0.0	0.0	0.5	1.5	0.5	0.5	0.5	0.55
PROK2	19.0	8.5	13.0	21.5	11.5	11.5	14.2	5.00
PROKR1	0.5	0.0	0.5	0.0	1.5	0.0	0.4	0.58
PROM1	153.5	114.5	105.5	194.0	111.5	88.0	127.8	38.90
PROM2	0.0	0.0	1.0	0.0	0.0	0.5	0.3	0.42
PROS1	1157.0	809.5	599.0	1119.0	863.0	832.5	896.7	209.12
PROSC	654.0	534.0	549.0	581.0	529.0	501.5	558.1	53.73
PROSER1	401.5	362.5	326.5	401.0	416.0	330.0	372.9	38.90
PROSER2	9.5	6.5	26.0	20.5	11.5	23.0	16.2	8.02
PROX1	45.0	29.0	118.0	41.5	44.0	110.0	64.6	38.79
PROX2	0.0	0.0	1.0	0.0	2.0	2.5	0.9	1.11
PROZ	21.5	20.5	15.0	21.5	43.5	15.5	22.9	10.50
PRPF18	1040.0	762.0	478.0	892.0	806.5	489.5	744.7	223.28
PRPF19	3021.5	2441.0	2201.0	3173.5	2417.5	2271.5	2587.7	407.76
PRPF3	691.5	452.0	462.0	666.0	472.5	489.5	538.9	109.32
PRPF38A	546.5	442.5	362.5	537.5	465.5	349.0	450.6	83.81
PRPF38B	956.5	941.5	856.0	896.0	1059.0	861.5	928.4	75.87
PRPF39	1759.0	1351.0	1425.0	1674.0	1574.0	1303.0	1514.3	183.43
PRPF4	735.5	545.5	432.5	712.5	615.5	430.0	578.6	132.99
PRPF40A	1081.0	935.0	1155.5	1076.5	1050.5	1177.0	1079.3	86.04
PRPF4B	1056.0	644.0	897.0	1010.5	752.5	837.0	866.2	155.53
PRPF6	1105.5	532.5	886.5	1141.0	574.5	834.5	845.8	256.18
PRPF8	2838.0	1809.5	3381.5	2850.5	1958.0	2996.5	2639.0	618.91
PRPH	14.5	4.0	3.0	9.5	9.0	5.0	7.5	4.34
PRPH2	0.0	0.5	0.0	0.0	2.0	0.0	0.4	0.80

PRPS1	2099.0	1176.0	1315.5	2118.0	1247.5	1238.5	1532.4	448.46
PRPS2	37.0	5.5	14.5	20.5	3.5	10.0	15.2	12.34
PRPSAP1	379.0	215.0	230.0	357.5	241.0	217.5	273.3	74.42
PRPSAP2	1027.0	530.5	584.0	954.0	635.0	551.5	713.7	218.54
PRR11	364.0	180.0	257.0	318.5	171.0	155.5	241.0	86.40
PRR14L	135.5	56.0	207.0	143.0	62.0	169.5	128.8	59.63
PRR15L	0.0	0.0	0.5	1.0	0.5	0.0	0.3	0.41
PRR16	5.0	4.5	1.5	3.0	4.5	0.5	3.2	1.83
PRR22	2.5	7.5	9.0	4.0	5.0	7.5	5.9	2.48
PRR29	0.0	0.5	0.0	0.5	0.5	0.0	0.3	0.27
PRR33	3.0	28.0	28.0	6.0	42.0	26.5	22.3	14.89
PRR35	0.0	0.0	0.0	0.0	0.0	0.0	0.0	0.00
PRR5	158.0	248.0	258.0	138.5	273.5	307.5	230.6	67.17
PRR5L	220.0	279.0	495.5	216.5	347.0	533.5	348.6	137.55
PRR7	402.0	563.5	387.0	395.0	563.0	436.5	457.8	83.38
PRRC1	1093.5	728.5	937.0	1124.0	814.5	939.0	939.4	153.56
PRRC2B	192.5	94.0	460.0	202.5	119.0	363.5	238.6	143.71
PRRC2C	774.0	780.5	1312.5	882.5	940.5	1176.0	977.7	220.11
PRRG1	117.0	59.0	136.0	93.5	92.5	145.5	107.3	32.00
PRRG3	385.5	358.0	244.0	335.5	405.5	273.0	333.6	63.50
PRRG4	54.0	19.0	19.0	49.0	29.5	15.0	30.9	16.73
PRRT1	19.5	58.0	48.0	18.0	53.5	38.5	39.3	17.17
PRRT3	0.0	3.5	1.0	0.0	2.5	1.0	1.3	1.40
PRRX1	102.0	230.5	265.0	148.5	317.5	469.0	255.4	130.50
PRRX2	13.5	14.5	27.5	19.5	17.0	43.0	22.5	11.22
PRSS12	197.5	192.0	328.5	215.0	238.5	340.5	252.0	66.04
PRSS23	1609.0	2277.0	3645.5	2252.0	2998.5	4663.5	2907.6	1109.03
PRSS35	922.0	2295.0	3512.5	1365.5	3302.0	5140.0	2756.2	1553.28
PRSS55	6.5	2.0	0.5	5.5	3.5	1.5	3.3	2.36
PRSS56	0.0	1.0	0.5	1.0	0.5	0.0	0.5	0.45
PRSS57	0.0	0.0	0.0	0.0	0.0	0.0	0.0	0.00
PRUNE	730.5	736.0	510.0	639.0	714.0	592.0	653.6	90.53
PRUNE2	90.5	22.0	296.0	97.0	28.0	222.5	126.0	110.21
PSAP	4153.5	1391.5	5681.0	4411.0	1555.5	4329.0	3586.9	1725.25
PSAT1	233.0	86.5	151.0	207.5	60.5	74.0	135.4	73.13
PSCA	0.0	1.0	0.5	0.0	0.5	1.5	0.6	0.58
PSD	310.0	267.5	142.5	345.5	269.5	198.0	255.5	74.14
PSD3	561.5	463.5	917.0	515.5	525.5	846.5	638.3	192.50
PSEN1	165.0	94.0	313.5	181.5	120.0	294.5	194.8	90.39
PSEN2	757.0	432.5	589.0	649.0	540.5	489.0	576.2	116.34
PSIP1	344.0	590.0	606.5	343.5	639.0	554.0	512.8	133.81
PSKH1	113.5	74.0	242.5	114.5	93.0	204.5	140.3	67.21
PSMA1	2806.5	2268.0	1351.5	2637.5	2404.5	1282.5	2125.1	653.27
PSMA2	3131.0	2368.0	1622.0	3037.5	2584.0	1384.5	2354.5	721.08
PSMA3	3027.5	2594.0	1831.0	2687.5	2858.0	1644.5	2440.4	567.20
PSMA4	5158.5	3963.5	2510.5	4811.5	4324.0	2257.0	3837.5	1200.43
PSMA5	2422.5	2851.5	1542.0	2075.0	2801.0	1360.5	2175.4	630.06
PSMA6	2751.5	1936.0	1290.0	2484.5	2163.5	1109.0	1955.8	650.62
PSMA7	4051.5	3811.0	2292.0	3484.5	4098.0	2070.0	3301.2	897.29
PSMB1	2036.5	1748.5	1310.5	1901.5	1777.5	1092.5	1644.5	364.55
PSMB2	3092.5	3209.5	1770.5	2793.0	3245.5	1641.5	2625.4	730.87
PSMB3	2589.5	1827.5	1009.5	2259.0	1804.5	828.0	1719.7	687.85
PSMB4	3427.5	2784.5	1703.5	3054.5	2659.0	1576.0	2534.2	742.31
PSMB5	1232.5	1495.5	906.0	1127.0	1523.0	921.0	1200.8	269.19

PSMB7	3599.0	3276.0	1866.0	3339.0	3457.0	1714.0	2875.2	849.12
PSMC1	2794.5	2293.5	1431.0	2462.0	2538.0	1367.5	2147.8	602.18
PSMC2	3355.0	3130.0	2171.5	3074.0	3331.0	1970.5	2838.7	607.96
PSMC3	3999.5	4218.0	2558.0	3822.5	4701.0	2652.5	3658.6	867.89
PSMC3IP	146.5	70.5	60.5	131.5	79.5	43.0	88.6	41.16
PSMC5	2737.5	2458.0	1341.5	2417.5	2401.5	1208.5	2094.1	647.48
PSMC6	2706.5	2578.5	2028.0	2592.0	2909.5	2046.5	2476.8	360.61
PSMD1	5456.0	2893.5	3214.5	5634.5	3121.0	2827.5	3857.8	1315.98
PSMD10	704.0	722.5	471.5	668.5	820.5	484.0	645.2	139.19
PSMD11	1561.0	1792.0	1369.5	1525.0	1778.0	1372.0	1566.3	186.53
PSMD12	1567.5	1539.0	1176.5	1616.5	1783.0	1014.5	1449.5	291.48
PSMD13	3008.0	3352.0	2097.0	2824.5	3403.5	1827.0	2752.0	654.20
PSMD2	4211.5	3792.0	3021.0	4011.5	3794.5	2791.5	3603.7	566.88
PSMD3	3428.5	3098.5	1927.0	3100.5	2974.5	1712.0	2706.8	706.92
PSMD4	2265.5	2253.5	1130.0	1874.0	2018.5	1089.0	1771.8	533.89
PSMD5	1285.0	884.5	901.5	1163.0	906.0	815.0	992.5	186.31
PSMD6	1326.0	1017.0	1052.5	1249.0	1151.5	897.0	1115.5	158.01
PSMD7	2598.0	2332.0	1392.0	2485.5	2426.0	1460.0	2115.6	541.50
PSMD9	793.5	1043.0	809.5	701.5	1121.5	716.5	864.3	175.76
PSME3	2104.5	1657.0	1822.5	2062.5	1747.5	1570.5	1827.4	216.10
PSME4	937.0	698.5	906.5	974.0	857.5	884.5	876.3	96.10
PSMF1	1233.5	931.5	569.0	1087.0	894.0	509.0	870.7	284.43
PSMG1	392.0	308.5	277.5	349.5	365.5	276.0	328.2	48.16
PSMG2	565.5	422.5	282.5	540.5	415.5	253.0	413.3	128.25
PSMG3	352.0	288.5	116.0	305.0	306.0	112.5	246.7	104.73
PSMG4	369.5	282.5	153.5	328.0	330.0	197.0	276.8	84.44
PSPH	316.5	646.0	642.0	278.5	583.5	426.5	482.2	164.12
PSTK	254.0	210.0	110.5	253.5	228.5	125.5	197.0	63.56
PSTPIP2	50.0	163.0	59.5	100.0	277.5	72.5	120.4	87.03
PTAFR	2.5	1.0	0.0	2.5	1.5	2.0	1.6	0.97
PTBP1	861.5	347.0	749.0	868.5	371.5	693.0	648.4	233.87
PTBP2	643.5	433.5	521.0	555.0	515.5	453.5	520.3	75.37
PTBP3	210.0	104.5	136.0	209.0	131.5	121.5	152.1	45.77
PTCD1	336.0	255.5	278.5	323.0	267.0	235.5	282.6	39.23
PTCD2	32.5	11.0	17.5	21.5	18.5	18.0	19.8	7.10
PTCD3	764.5	1070.5	910.0	747.5	1196.5	868.5	926.3	176.38
PTCH2	1.5	2.0	4.0	0.5	5.0	5.5	3.1	2.04
PTCHD1	69.0	92.5	157.0	64.5	118.5	174.0	112.6	45.60
PTCHD2	18.5	8.5	9.5	11.0	8.0	13.5	11.5	3.96
PTCHD3	263.0	159.0	253.5	264.5	186.0	228.5	225.8	44.05
PTCRA	28.0	9.5	5.0	30.0	7.5	5.0	14.2	11.63
PTDSS1	305.0	195.5	397.5	279.5	213.0	349.0	289.9	77.76
PTDSS2	204.5	70.5	172.5	199.0	59.5	171.5	146.3	64.45
PTEN	1550.0	1900.0	2612.0	1538.0	2082.5	2495.5	2029.7	457.72
PTER	201.5	160.0	210.5	178.0	160.5	199.0	184.9	21.88
PTGDR	10.5	15.0	16.5	9.5	14.5	20.5	14.4	4.03
PTGER2	7.0	20.0	12.5	10.5	20.0	31.0	16.8	8.68
PTGER4	97.0	240.0	238.5	86.0	280.0	244.5	197.7	83.71
PTGES	171.0	34.0	28.0	61.0	21.0	15.5	55.1	58.95
PTGES2	846.5	655.5	365.5	807.5	665.5	334.0	612.4	217.26
PTGES3	3612.5	3676.0	3010.5	3360.5	3700.0	3032.0	3398.6	316.23
PTGES3L	693.0	752.0	376.0	591.5	757.5	358.0	588.0	181.39
PTGFR	26.5	16.0	55.0	39.5	19.0	51.0	34.5	16.52
PTGFRN	26.5	2.0	28.0	23.5	3.0	30.5	18.9	12.92

PTGR1	91.0	17.0	5.0	76.5	14.0	3.0	34.4	38.85
PTGR2	189.0	153.0	289.5	276.0	181.0	284.5	228.8	61.04
PTGS1	337.0	317.0	283.0	282.0	331.0	273.5	303.9	27.72
PTGS2	29.5	7.0	16.5	25.0	3.0	14.5	15.9	10.16
PTH	0.0	0.0	0.0	0.0	0.0	0.0	0.0	0.00
PTH1R	1.0	0.0	0.5	1.0	2.0	0.5	0.8	0.68
PTHLH	22.5	68.5	97.5	19.0	65.5	93.5	61.1	33.80
PTK2	102.5	65.5	131.5	93.5	74.5	114.0	96.9	24.58
PTK2B	33.0	4.5	3.0	15.0	2.0	2.5	10.0	12.28
PTK7	3346.0	3143.5	3991.0	3266.5	3034.5	4185.0	3494.4	475.84
PTMA	5951.5	3462.5	2998.5	5372.0	3607.0	2785.0	4029.4	1312.11
PTMS	1455.5	763.5	1422.0	1507.0	880.5	1285.0	1218.9	318.28
PTN	22.5	42.5	118.5	10.0	15.0	43.5	42.0	39.97
PTP4A1	4349.5	4671.5	5735.0	4133.5	5491.0	4706.0	4847.8	634.12
PTP4A2	1411.0	1522.5	1280.5	1293.0	1654.0	1356.0	1419.5	144.93
PTP4A3	304.0	523.0	653.5	283.5	499.5	549.0	468.8	145.55
PTPDC1	546.5	379.0	178.0	528.5	393.0	234.5	376.6	149.57
PTPLA	1170.0	2231.5	1562.0	1210.5	2543.5	1307.0	1670.8	580.15
PTPLAD1	783.0	538.0	787.0	880.0	577.0	721.0	714.3	132.24
PTPLAD2	71.5	33.0	26.0	50.0	40.0	38.5	43.2	16.00
PTPLB	811.5	389.0	463.5	780.0	414.5	389.5	541.3	199.18
PTPMT1	189.0	332.5	213.0	169.5	342.0	242.5	248.1	73.31
PTPN1	133.5	52.0	190.0	130.0	67.0	168.0	123.4	54.50
PTPN11	1194.5	951.0	1162.5	1130.0	1003.0	975.0	1069.3	105.19
PTPN12	764.5	707.0	868.0	773.0	743.5	748.0	767.3	54.32
PTPN14	1516.5	754.5	1721.0	1395.0	873.0	1381.5	1273.6	378.28
PTPN21	145.5	73.0	448.0	133.0	85.0	348.0	205.4	154.95
PTPN22	37.0	30.0	29.5	31.5	35.5	30.0	32.3	3.21
PTPN23	377.5	352.5	405.0	368.0	352.0	368.0	370.5	19.59
PTPN3	239.0	165.0	255.0	241.0	177.0	234.5	218.6	37.68
PTPN4	124.5	151.5	245.5	142.0	199.5	197.5	176.8	45.28
PTPN5	444.0	246.0	223.5	418.0	297.5	318.0	324.5	89.62
PTPN6	46.0	8.5	4.5	43.0	8.5	6.5	19.5	19.44
PTPN7	0.5	5.0	4.5	0.0	6.0	4.0	3.3	2.48
PTPN9	597.5	907.0	915.5	585.0	969.5	1054.0	838.1	198.27
PTPRA	831.5	896.5	1014.5	745.0	985.0	1088.0	926.8	126.58
PTPRB	2.0	0.0	3.0	2.5	0.5	1.0	1.5	1.18
PTPRC	129.0	44.5	66.5	82.5	54.5	65.5	73.8	29.92
PTPRE	84.5	40.5	53.5	87.0	39.5	54.5	59.9	20.99
PTPRF	1962.0	1438.5	2445.0	1866.5	1589.5	2516.5	1969.7	438.71
PTPRG	5221.0	2522.0	4403.0	6129.0	3014.5	3862.5	4192.0	1351.98
PTPRJ	0.0	1.0	1.5	3.5	0.0	2.5	1.4	1.39
PTPRK	98.5	52.0	73.0	74.5	51.0	96.0	74.2	20.49
PTPRN	2047.5	1950.5	1088.5	1840.0	1944.0	1201.5	1678.7	420.08
PTPRN2	1.5	1.5	3.0	1.5	1.0	3.0	1.9	0.86
PTPRQ	244.5	207.5	426.5	274.5	223.0	392.5	294.8	92.31
PTPRR	17.0	6.5	5.0	25.0	9.0	6.0	11.4	7.95
PTPRS	1669.5	1607.5	2505.0	1575.0	1759.0	2828.5	1990.8	537.20
PTPRU	11.0	73.5	77.0	16.0	119.0	205.0	83.6	72.08
PTPRZ1	0.0	0.0	0.0	0.0	0.0	0.5	0.1	0.20
PTRF	11229.5	6644.5	8176.5	10847.5	6152.5	6829.5	8313.3	2218.14
PTRH2	484.0	432.5	284.0	523.5	459.5	316.5	416.7	95.57
PTS	237.5	238.0	116.0	218.0	236.0	107.0	192.1	62.93
PTTG1	391.0	164.0	261.5	388.5	168.5	168.5	257.0	109.16

PTTG1IP	559.5	426.0	331.5	550.0	482.0	365.5	452.4	94.59
PTX3	2000.0	831.5	1219.5	2317.0	834.0	882.0	1347.3	652.49
PTX4	0.0	0.0	0.0	0.0	0.0	0.0	0.0	0.00
PUF60	1714.5	1611.5	1063.0	1675.0	1659.0	1003.5	1454.4	328.44
PUM2	1459.0	1459.0	2185.0	1430.0	1743.0	2118.0	1732.3	344.84
PURA	80.5	77.5	71.0	111.5	79.0	79.5	83.2	14.29
PURG	184.0	142.0	126.5	151.5	143.5	134.0	146.9	20.08
PUS1	516.0	390.5	433.5	449.0	418.5	365.5	428.8	52.18
PUS3	304.5	315.5	285.5	316.5	323.5	310.0	309.3	13.28
PUS7	165.0	68.5	110.0	190.5	77.5	92.0	117.3	49.58
PUS7L	171.5	102.5	115.5	154.0	114.0	96.0	125.6	30.20
PUSL1	107.5	72.0	93.5	111.0	107.5	98.0	98.3	14.46
PVALB	1.0	1.0	2.0	2.0	1.0	1.0	1.3	0.52
PVRL1	196.0	111.5	175.0	219.0	122.0	158.5	163.7	41.76
PVRL3	308.5	215.5	247.0	294.5	245.0	255.0	260.9	34.46
PVRL4	8.5	4.0	1.5	5.5	7.5	4.0	5.2	2.56
PWP1	349.5	277.5	380.5	363.5	333.0	335.0	339.8	35.39
PWWP2A	159.5	108.0	139.5	131.5	103.0	134.5	129.3	20.94
PWWP2B	158.0	157.5	186.5	149.5	174.0	197.5	170.5	18.78
PXDC1	395.0	259.0	272.0	421.0	283.5	250.0	313.4	74.60
PXDN	281.0	76.0	606.5	324.5	106.5	458.5	308.8	203.56
PXK	132.5	65.5	77.5	118.5	70.5	97.5	93.7	27.32
PXMP4	2411.0	1745.0	1193.5	2234.5	1805.5	1238.0	1771.3	498.78
PXN	2461.5	2437.5	3629.5	2654.5	2613.0	3645.5	2906.9	572.10
PYCR1	1553.0	1964.5	1966.0	1397.5	1944.0	1761.5	1764.4	241.77
PYCRL	465.5	456.5	323.0	437.5	492.0	291.0	410.9	83.00
PYGL	1549.0	1228.0	1118.5	1476.0	1227.0	1142.0	1290.1	179.34
PYGO1	64.5	29.0	175.0	59.0	35.0	108.0	78.4	54.96
PYGO2	115.0	74.5	82.0	109.0	68.0	89.5	89.7	18.84
PYROXD1	234.0	137.5	136.5	261.5	154.0	126.0	174.9	57.78
PYROXD2	24.5	31.5	28.5	23.5	19.0	31.5	26.4	4.96
QARS	1539.0	2029.5	1258.5	1502.5	1892.0	1252.5	1579.0	321.77
QDPR	607.0	311.0	381.5	553.5	358.5	299.0	418.4	130.05
QKI	170.5	103.5	416.0	198.0	136.0	312.5	222.8	118.73
QPCT	375.0	252.0	196.5	404.5	356.5	224.5	301.5	87.68
QPRT	0.0	0.0	0.0	0.0	0.0	0.0	0.0	0.00
QRFP	0.0	0.0	0.0	0.0	0.5	0.0	0.1	0.20
QRFPR	22.5	30.0	32.0	31.5	39.0	42.0	32.8	6.92
QRICH1	1229.5	1214.5	1601.0	1260.5	1345.0	1437.5	1348.0	149.36
QRSL1	353.5	208.0	226.5	303.0	215.0	191.0	249.5	64.10
QSER1	663.5	483.0	883.0	628.0	540.5	853.0	675.2	162.63
QSOX1	643.5	577.5	862.0	696.0	584.5	872.5	706.0	132.17
QSOX2	474.5	170.5	316.5	475.0	175.5	253.5	310.9	137.89
QTRT1	92.5	64.0	38.0	76.0	55.5	39.0	60.8	21.30
QTRTD1	237.0	221.5	242.5	246.0	229.5	242.5	236.5	9.34
R3HCC1	953.0	788.5	1014.5	902.5	808.0	985.5	908.7	93.47
R3HCC1L	98.5	136.0	162.0	103.0	131.5	176.0	134.5	30.91
R3HDM1	533.5	486.5	939.0	565.5	562.0	874.0	660.1	194.05
R3HDM2	292.0	308.0	340.0	286.0	336.5	387.5	325.0	37.85
R3HDM4	163.5	180.0	162.0	155.5	185.5	159.0	167.6	12.19
R3HDML	7.5	46.5	26.5	5.5	52.5	51.0	31.6	21.56
RAB10	3212.5	3205.5	4522.0	3248.5	3854.0	4191.0	3705.6	570.32
RAB11A	1903.5	2002.0	1889.0	1878.0	2231.5	1912.5	1969.4	135.76
RAB11B	2295.5	2539.5	2307.5	2158.5	2744.0	2075.0	2353.3	248.26

RAB11FIP1	797.5	581.5	725.0	793.0	722.0	681.0	716.7	80.00
RAB11FIP2	1789.0	1147.5	1339.5	1687.5	1250.0	1138.5	1392.0	279.96
RAB11FIP3	565.0	764.5	664.0	577.5	851.5	872.5	715.8	134.08
RAB11FIP4	6.5	7.5	21.0	7.5	8.5	11.0	10.3	5.45
RAB11FIP5	12.0	6.0	11.0	11.0	10.5	11.0	10.3	2.14
RAB12	810.0	768.5	877.0	697.5	938.0	909.0	833.3	91.42
RAB14	774.5	471.0	983.0	809.0	577.0	827.0	740.3	185.20
RAB17	0.5	1.5	0.5	1.5	0.0	0.0	0.7	0.68
RAB18	2170.0	1581.0	1587.5	2032.5	1725.5	1488.5	1764.2	275.30
RAB19	36.5	32.5	44.0	35.0	31.5	31.5	35.2	4.77
RAB1A	5273.5	5195.0	4370.0	5150.5	5806.0	4239.5	5005.8	593.33
RAB20	122.5	87.0	45.5	112.5	91.5	47.5	84.4	32.17
RAB21	1024.0	1229.5	1222.0	915.5	1400.5	1145.5	1156.2	170.24
RAB22A	148.5	211.5	289.5	128.0	255.5	248.5	213.6	63.72
RAB23	897.5	1195.0	2144.0	1323.5	1491.5	2775.5	1637.8	694.97
RAB24	1096.5	1741.0	909.5	969.5	1695.5	836.5	1208.1	404.52
RAB25	1.0	1.5	2.5	0.0	0.0	2.5	1.3	1.13
RAB26	104.5	77.5	157.0	65.0	68.5	122.0	99.1	35.98
RAB27A	1004.0	682.5	903.0	983.5	768.0	760.0	850.2	132.08
RAB28	117.0	85.5	136.0	111.0	94.5	139.5	113.9	21.66
RAB29	437.0	356.5	421.5	398.0	423.0	417.0	408.8	28.56
RAB2A	3394.5	2771.5	2438.0	3162.5	3134.5	2372.5	2878.9	418.08
RAB30	1022.5	592.5	754.0	858.0	692.0	791.5	785.1	147.27
RAB31	30.0	21.5	39.5	39.5	19.5	26.0	29.3	8.68
RAB32	42.5	28.0	30.5	34.0	31.5	7.5	29.0	11.65
RAB33A	18.0	30.5	29.5	18.0	37.0	39.0	28.7	9.03
RAB33B	1123.0	864.5	624.5	1159.5	929.5	627.0	888.0	231.85
RAB34	733.5	782.5	660.5	635.0	828.0	688.0	721.3	74.21
RAB35	378.5	262.0	494.0	432.0	328.5	434.0	388.2	83.42
RAB36	0.0	0.5	0.0	0.0	0.5	0.0	0.2	0.26
RAB37	3.0	0.5	0.5	1.5	0.0	1.5	1.2	1.08
RAB38	1.0	21.5	12.0	0.0	32.5	4.5	11.9	12.90
RAB39A	84.0	35.0	97.5	104.0	41.5	86.5	74.8	29.27
RAB39B	121.5	63.0	120.0	117.5	66.0	108.0	99.3	27.40
RAB3A	66.5	36.0	62.0	62.0	37.5	51.5	52.6	13.23
RAB3C	3.0	1.5	1.5	4.0	2.0	1.5	2.3	1.04
RAB3GAP1	838.0	491.0	708.5	827.0	603.5	615.5	680.6	136.47
RAB3GAP2	3499.5	2285.5	2579.5	3131.5	2549.5	2582.5	2771.3	451.14
RAB3IL1	1069.0	637.5	753.0	699.5	603.0	608.0	728.3	176.60
RAB3IP	50.0	37.5	43.5	35.0	40.0	32.0	39.7	6.43
RAB40B	6.5	4.5	5.5	7.0	2.5	3.0	4.8	1.83
RAB40C	74.0	57.0	66.5	78.5	52.5	80.5	68.2	11.54
RAB41	1427.5	727.5	634.5	1186.0	744.0	584.5	884.0	341.36
RAB43	116.5	45.5	121.5	142.0	56.5	107.5	98.3	38.47
RAB44	3.0	1.0	7.0	1.5	3.5	9.5	4.3	3.33
RAB4A	349.0	205.0	257.0	309.0	232.0	232.5	264.1	54.40
RAB5A	206.0	128.0	221.0	223.5	149.5	226.0	192.3	42.63
RAB5B	1191.5	1196.0	801.5	1009.5	1177.5	801.5	1029.6	189.86
RAB5C	2421.0	1715.0	1564.5	2234.0	1809.5	1609.0	1892.2	352.78
RAB6A	516.0	364.0	351.0	493.0	461.0	358.0	423.8	74.67
RAB7A	4588.0	4650.5	3389.0	4559.5	5190.5	3427.0	4300.8	729.13
RAB7B	43.5	80.0	96.5	43.0	84.5	124.0	78.6	31.36
RAB8A	661.0	422.5	553.5	643.0	469.0	498.0	541.2	95.95
RAB8B	1105.5	899.0	1119.5	1130.5	1015.5	1072.0	1057.0	87.83

RAB9A	1012.5	1014.0	1101.0	928.0	1160.5	1050.0	1044.3	80.26
RAB9B	391.0	531.5	482.0	354.5	597.5	441.0	466.3	90.02
RABEP1	989.0	855.0	1463.5	920.5	984.0	1321.5	1088.9	244.33
RABEPK	903.0	973.0	534.5	775.0	1014.5	531.0	788.5	214.12
RABGAP1	466.5	339.0	667.0	539.0	444.0	599.5	509.2	117.46
RABGEF1	956.0	751.0	980.0	977.0	903.0	1005.0	928.7	93.56
RABGGTB	1032.5	770.5	1210.0	1057.0	866.5	1128.0	1010.8	164.07
RABIF	593.5	724.0	469.5	529.5	730.0	541.0	597.9	107.49
RABL3	579.5	429.0	427.0	539.5	525.0	380.0	480.0	78.58
RABL6	1865.5	2019.0	1907.5	1609.5	2114.5	1756.5	1878.8	180.94
RAC1	6946.0	4750.0	6535.5	6570.5	5301.0	5898.0	6000.2	844.83
RAC2	9.0	14.5	14.0	5.0	14.0	2.5	9.8	5.18
RAC3	731.0	1002.5	761.0	687.0	1087.5	824.0	848.8	160.61
RACGAP1	685.5	235.0	406.0	726.5	205.0	272.0	421.7	231.07
RAD1	279.5	243.5	215.0	280.0	295.0	202.5	252.6	38.15
RAD17	390.5	229.0	226.5	353.0	243.0	227.0	278.2	73.70
RAD18	481.0	227.5	272.5	441.0	201.0	209.5	305.4	123.67
RAD21	2739.5	1823.0	2463.5	2696.0	2280.0	2222.0	2370.7	340.81
RAD21L1	10.5	3.5	4.5	8.0	6.0	2.0	5.8	3.11
RAD23B	1164.5	1167.5	1270.5	1123.0	1374.5	1162.5	1210.4	94.26
RAD50	671.0	337.0	485.5	650.0	368.0	500.0	501.9	138.51
RAD51	416.5	165.0	227.5	368.5	158.5	118.5	242.4	122.32
RAD51AP1	331.0	134.5	104.5	285.5	123.0	85.5	177.3	103.77
RAD51AP2	0.0	0.0	0.0	0.5	0.0	0.0	0.1	0.20
RAD51B	321.5	279.5	258.0	310.5	321.5	251.5	290.4	31.69
RAD51C	283.0	193.5	123.0	279.5	207.0	146.5	205.4	66.21
RAD51D	242.0	288.0	198.5	203.0	287.5	204.0	237.2	42.20
RAD52	515.0	471.0	238.5	407.0	528.0	208.5	394.7	139.49
RAD54B	200.0	75.0	117.5	188.0	95.5	89.0	127.5	53.44
RAD54L	47.5	13.5	32.5	49.0	8.5	27.0	29.7	16.83
RAD54L2	329.5	213.0	420.0	331.0	179.0	407.5	313.3	98.93
RAD9A	307.0	246.5	189.0	265.0	258.5	218.5	247.4	40.58
RAD9B	152.5	163.0	89.5	129.5	205.0	92.0	138.6	44.41
RADIL	4.0	3.0	1.0	1.0	5.5	4.5	3.2	1.86
RAE1	1682.5	986.5	1046.5	1534.5	1061.0	929.5	1206.8	318.14
RAF1	963.0	945.5	937.5	970.0	1079.5	865.5	960.2	69.33
RAG1	0.5	0.0	0.0	0.5	0.5	0.0	0.3	0.27
RAG2	3.5	1.0	0.0	3.0	0.0	0.5	1.3	1.54
RAI1	178.0	97.5	294.5	245.5	74.5	277.0	194.5	93.26
RAI14	826.5	608.0	1063.0	763.5	681.0	961.5	817.3	171.19
RAI2	496.5	508.0	422.5	463.0	505.0	467.0	477.0	32.84
RALA	1081.0	924.0	910.0	983.0	994.5	923.5	969.3	64.75
RALB	1046.0	711.0	1080.5	1168.0	787.5	1039.5	972.1	180.21
RALBP1	1895.0	1900.5	2338.5	2008.5	2163.0	2421.0	2121.1	224.24
RALGAPA1	557.5	449.0	815.5	627.0	525.5	815.5	631.7	153.46
RALGAPA2	129.5	93.5	179.0	134.0	112.0	162.5	135.1	31.54
RALGAPB	895.0	785.0	1092.5	906.5	845.5	1136.0	943.4	139.79
RALGDS	1749.5	1306.0	1507.0	1570.5	1429.5	1509.5	1512.0	147.72
RALGPS1	223.5	124.5	123.5	211.5	146.0	112.0	156.8	48.41
RALGPS2	323.0	549.5	756.5	368.0	691.5	655.5	557.3	177.83
RALY	717.0	696.5	686.5	762.0	759.0	631.0	708.7	49.23
RALYL	4.5	4.0	6.5	3.5	0.5	2.5	3.6	2.01
RAMP1	172.5	208.0	161.0	99.5	241.0	162.5	174.1	47.96
RAMP2	5.0	3.5	3.0	6.5	5.5	2.0	4.3	1.70

RAMP3	0.0	0.0	0.5	1.0	0.0	0.0	0.3	0.42
RAN	5439.5	4863.5	3395.0	5398.5	5158.0	3121.0	4562.6	1034.82
RANBP1	2.0	2.0	0.0	4.5	0.5	1.5	1.8	1.57
RANBP10	407.0	373.5	353.5	403.0	375.0	322.0	372.3	31.75
RANBP17	131.0	28.5	12.5	70.0	30.0	4.0	46.0	47.43
RANBP3	1259.5	1347.5	1298.5	1208.0	1517.5	1239.0	1311.7	111.87
RANBP3L	5.0	1.0	0.0	5.5	1.5	2.0	2.5	2.24
RANBP9	1012.5	753.5	830.0	869.0	824.0	765.5	842.4	93.76
RANGAP1	807.5	322.5	547.0	814.0	307.0	387.5	530.9	232.83
RAP1A	1118.5	910.0	1004.0	982.0	1110.5	1017.0	1023.7	79.52
RAP1B	2222.0	2386.5	2396.0	2202.0	2659.5	2079.5	2324.3	203.33
RAP1GAP	6.5	13.0	11.0	7.5	12.0	21.0	11.8	5.16
RAP1GAP2	39.5	79.5	81.5	26.5	79.5	87.5	65.7	25.80
RAP1GDS1	3406.5	2426.0	2834.0	3544.5	2752.0	2872.5	2972.6	422.38
RAP2B	39.5	23.0	35.0	42.5	20.5	30.0	31.8	8.86
RAP2C	2744.0	1608.5	2009.5	2567.0	1742.5	1686.0	2059.6	484.15
RAPGEF1	1921.5	3009.0	3749.5	2269.5	3435.0	3272.5	2942.8	707.46
RAPGEF2	838.0	766.5	957.5	863.5	914.5	840.5	863.4	66.37
RAPGEF3	0.0	1.0	3.0	0.5	1.5	5.0	1.8	1.86
RAPGEF5	206.5	325.5	190.0	205.5	373.0	241.5	257.0	74.94
RAPGEF6	116.5	97.0	384.5	110.5	108.5	362.5	196.6	137.36
RAPGEFL1	6.0	7.5	9.5	5.0	3.5	9.0	6.8	2.34
RAPH1	436.5	374.0	582.0	417.5	399.0	480.0	448.2	74.73
RAPSN	1462.5	1944.5	1226.5	1067.0	2020.5	997.5	1453.1	440.79
RARB	4.5	4.5	2.0	2.0	9.5	9.5	5.3	3.42
RARRES1	32.0	45.5	61.5	28.5	43.0	42.0	42.1	11.63
RARRES2	670.5	571.5	419.5	513.0	558.0	458.5	531.8	89.26
RARS	1817.0	1314.5	1398.0	1745.0	1388.0	1281.5	1490.7	230.26
RARS2	288.5	205.0	206.5	293.0	200.0	192.5	230.9	46.63
RASA1	936.5	868.0	1436.0	826.5	997.0	1378.5	1073.8	265.44
RASA2	431.5	404.5	411.5	416.0	539.0	449.5	442.0	50.18
RASA3	341.0	410.0	435.5	224.5	471.0	379.0	376.8	87.07
RASAL1	0.5	5.5	1.5	1.0	5.5	1.5	2.6	2.29
RASAL2	209.0	152.5	575.0	219.5	159.5	486.5	300.3	182.57
RASAL3	1.0	0.0	0.5	0.0	0.0	0.5	0.3	0.41
RASD1	232.5	104.0	41.0	237.0	89.0	44.0	124.6	88.83
RASD2	2.5	26.5	4.0	9.0	33.5	53.0	21.4	19.92
RASEF	20.0	16.0	21.5	13.0	8.5	5.5	14.1	6.32
RASGEF1A	39.0	27.0	45.0	32.0	35.0	41.5	36.6	6.58
RASGEF1B	522.5	1077.5	1007.5	669.0	1202.0	1061.0	923.3	265.64
RASGEF1C	70.5	143.5	401.5	56.0	179.0	349.5	200.0	144.29
RASGRF1	96.0	85.5	137.0	112.0	86.5	169.5	114.4	33.18
RASGRF2	0.0	0.0	0.0	0.5	0.0	0.0	0.1	0.20
RASGRP1	216.5	101.5	250.0	221.0	111.0	220.0	186.7	63.51
RASGRP3	91.5	590.5	517.5	97.5	708.5	523.0	421.4	262.42
RASL10B	26.5	9.0	3.5	30.5	9.5	2.5	13.6	11.96
RASL11A	2.5	2.5	3.0	2.5	4.5	1.5	2.8	0.99
RASL11B	441.0	337.0	171.0	395.0	323.0	187.5	309.1	109.18
RASL12	2.5	5.5	2.0	4.5	6.0	5.0	4.3	1.64
RASSF1	96.5	152.0	103.0	101.0	114.0	130.5	116.2	21.37
RASSF10	1.0	0.0	1.0	0.0	0.0	0.5	0.4	0.49
RASSF2	3.0	27.0	39.5	4.5	29.5	70.5	29.0	24.95
RASSF3	6172.0	6099.5	8737.0	5818.0	7413.5	7350.5	6931.8	1111.46
RASSF5	116.5	73.5	53.0	125.5	92.5	75.5	89.4	27.63

RASSF6	260.0	159.5	244.5	270.0	173.0	177.0	214.0	49.40
RASSF7	8.0	8.5	7.0	1.5	8.0	13.0	7.7	3.68
RASSF8	44.0	69.0	103.0	53.5	76.0	101.5	74.5	24.27
RASSF9	0.0	0.0	0.0	0.0	0.0	0.0	0.0	0.00
RAVER2	239.5	153.5	212.0	258.5	172.0	213.0	208.1	39.62
RAX2	0.0	0.0	0.0	0.0	0.0	0.0	0.0	0.00
RB1CC1	896.0	786.0	1259.5	830.5	952.0	1051.5	962.6	172.76
RBBP5	324.0	222.0	477.5	340.0	262.5	443.5	344.9	99.66
RBBP6	764.0	659.0	832.5	669.5	794.0	768.0	747.8	69.28
RBBP7	141.0	66.0	70.0	146.5	54.0	37.0	85.8	46.40
RBBP8	597.0	257.0	394.5	538.0	279.0	271.5	389.5	147.53
RBBP8NL	0.0	0.0	0.0	0.0	0.0	0.5	0.1	0.20
RBFA	438.0	329.0	207.0	388.0	329.5	197.5	314.8	96.26
RBFOX2	417.0	524.0	653.0	330.0	573.0	565.0	510.3	117.21
RBFOX3	1.5	4.0	5.0	0.0	1.5	1.5	2.3	1.86
RBKS	148.0	88.5	112.5	179.0	96.5	90.0	119.1	36.77
RBL1	556.0	327.5	346.0	533.5	361.5	298.5	403.8	111.38
RBL2	475.5	258.0	423.0	496.0	312.0	360.5	387.5	93.78
RBM11	5.0	3.5	0.5	6.0	0.5	1.0	2.8	2.42
RBM12	672.5	531.5	713.0	638.5	591.5	657.5	634.1	64.20
RBM12B	485.5	296.5	458.5	436.0	366.5	430.5	412.3	69.13
RBM15	29.0	8.0	55.5	20.0	7.5	39.5	26.6	18.77
RBM15B	397.5	403.5	379.0	398.5	403.5	361.0	390.5	17.05
RBM17	707.0	559.0	543.0	644.5	611.5	526.5	598.6	69.11
RBM18	380.5	445.5	401.0	393.0	502.0	394.5	419.4	46.20
RBM19	315.0	386.0	482.5	386.0	404.5	500.5	412.4	68.73
RBM20	707.0	506.5	973.0	732.0	599.5	987.0	750.8	194.94
RBM22	1287.5	965.0	1010.0	1223.5	998.5	916.0	1066.8	151.17
RBM24	7540.5	10094.5	7131.0	7743.5	12761.5	8569.0	8973.3	2132.15
RBM25	508.5	323.5	587.5	539.0	413.5	493.5	477.6	94.77
RBM26	854.0	740.0	930.0	874.5	815.5	860.0	845.7	63.71
RBM27	878.0	697.0	736.0	847.0	732.5	722.5	768.8	74.47
RBM33	247.0	124.5	321.0	233.0	140.0	280.0	224.3	77.60
RBM34	1169.5	762.0	515.5	838.0	719.0	464.5	744.8	253.44
RBM39	2030.0	1932.5	1935.5	1842.5	2161.5	1875.5	1962.9	116.41
RBM41	157.0	125.5	93.5	143.0	143.0	86.5	124.8	28.80
RBM43	182.5	158.0	170.0	163.5	165.0	168.0	167.8	8.29
RBM44	36.5	46.0	48.0	41.0	39.0	38.0	41.4	4.61
RBM45	893.5	597.0	522.5	759.5	690.0	491.5	659.0	152.70
RBM46	7.5	3.5	1.5	12.0	2.5	2.0	4.8	4.12
RBM47	0.5	1.5	0.0	0.0	0.0	0.5	0.4	0.58
RBM48	351.5	311.0	237.0	294.0	329.0	213.0	289.3	53.84
RBM5	4126.0	3549.5	2767.0	3252.5	4094.5	3032.0	3470.3	558.57
RBM7	518.5	547.0	302.5	519.5	557.5	313.0	459.7	118.70
RBM8A	588.5	602.0	426.0	555.0	543.0	405.0	519.9	83.94
RBMS2	158.0	128.5	221.5	139.5	108.0	204.0	159.9	44.36
RBMS3	38.5	18.5	54.0	39.0	24.5	59.0	38.9	15.85
RBMX	1078.5	987.0	946.5	1047.5	989.5	870.0	986.5	73.98
RBMX2	698.0	640.0	518.5	551.5	581.5	423.5	568.8	95.78
RBP1	137.0	84.5	56.5	128.5	90.5	45.0	90.3	37.06
RBP2	24.0	15.5	7.5	13.0	10.5	7.0	12.9	6.32
RBP3	0.0	0.0	0.5	0.0	0.5	0.0	0.2	0.26
RBP4	25.5	12.5	0.0	19.5	15.0	0.0	12.1	10.35
RBP5	10.5	13.0	6.0	9.5	11.5	3.0	8.9	3.73

RBP7	0.5	2.0	0.5	3.0	1.5	0.5	1.3	1.03
RBPJ	114.5	40.0	141.5	130.5	47.5	118.0	98.7	43.66
RBPJL	0.0	0.0	0.5	0.0	0.0	0.0	0.1	0.20
RBPMS	383.5	326.0	427.5	361.5	320.0	434.5	375.5	48.94
RBPMS2	1.5	3.5	4.0	0.5	3.5	0.5	2.3	1.60
RBSN	818.0	549.5	659.0	799.0	654.0	615.0	682.4	105.40
RBX1	883.5	963.0	452.5	800.5	925.0	463.0	747.9	231.18
RC3H2	156.5	66.0	228.5	175.0	77.5	175.0	146.4	62.75
RCAN1	398.5	75.0	385.5	438.5	85.0	274.0	276.1	161.42
RCAN2	2.5	1.0	7.5	4.0	3.0	7.0	4.2	2.58
RCAN3	616.5	581.5	526.0	626.5	701.0	587.5	606.5	58.13
RCBTB1	381.5	263.5	564.0	377.0	318.0	433.0	389.5	103.49
RCBTB2	364.0	333.0	393.0	329.0	333.0	397.5	358.3	31.34
RCC1	627.0	565.5	787.5	529.0	520.5	690.0	619.9	104.07
RCC2	1860.0	1613.0	2146.0	1898.0	1896.5	2223.0	1939.4	218.86
RCCD1	133.0	172.0	106.5	128.0	173.5	125.0	139.7	27.15
RCHY1	1644.0	1042.5	567.0	1390.5	1094.0	608.5	1057.8	423.94
RCL1	162.5	97.5	132.0	154.0	110.5	103.0	126.6	27.32
RCN1	3548.0	3020.5	3339.5	3708.0	3528.5	3702.5	3474.5	260.45
RCN2	1660.0	1226.5	1328.5	1562.5	1384.5	1321.0	1413.8	164.05
RCOR1	810.5	508.0	881.5	861.5	526.5	801.5	731.6	168.83
RCOR3	611.0	557.5	713.5	619.0	659.5	739.0	649.9	67.94
RCSD1	15.0	67.5	88.0	6.0	78.5	66.0	53.5	34.37
RD3	10.5	5.5	16.5	9.0	8.5	9.5	9.9	3.64
RD3L	0.0	0.0	0.0	0.0	0.0	0.0	0.0	0.00
RDH10	97.0	94.0	94.0	74.0	109.5	75.5	90.7	13.60
RDH16	144.0	103.5	38.5	189.5	126.5	63.5	110.9	54.88
RDH5	13.5	12.5	9.5	12.0	8.5	19.0	12.5	3.70
RDH8	0.0	0.0	0.0	0.0	0.0	0.0	0.0	0.00
RDX	4043.5	3478.0	3683.0	3745.0	3945.5	3777.5	3778.8	199.33
REC114	10.5	3.0	7.5	6.5	2.0	3.0	5.4	3.31
REC8	2.0	1.5	0.5	1.0	6.5	0.0	1.9	2.35
RECK	498.5	224.0	445.0	464.5	248.5	420.0	383.4	117.10
RECQL	709.0	513.0	426.0	700.0	552.5	416.0	552.8	128.41
REEP1	197.0	2182.5	2488.0	169.5	2641.5	2008.5	1614.5	1130.75
REEP2	370.0	273.0	276.0	354.0	283.0	316.5	312.1	41.97
REEP3	170.0	25.5	184.5	175.0	33.5	145.5	122.3	73.10
REEP5	3163.0	2535.0	1662.5	2824.5	2965.0	1563.5	2452.3	682.21
REEP6	3.5	2.0	1.5	4.5	3.0	0.0	2.4	1.59
REG4	2.5	2.5	1.0	8.5	1.0	2.5	3.0	2.79
REL	79.5	42.0	102.5	89.5	60.5	95.0	78.2	22.92
RELL1	130.0	33.5	94.5	155.5	39.5	97.5	91.8	48.35
RELL2	0.5	1.0	0.0	0.0	0.5	0.0	0.3	0.41
RELT	30.5	38.0	24.5	42.5	36.0	25.5	32.8	7.19
REM1	148.0	96.5	46.5	153.5	70.0	46.5	93.5	48.06
REN	0.5	0.0	0.5	0.0	0.0	1.0	0.3	0.41
REP15	0.0	0.5	1.0	0.0	1.0	0.5	0.5	0.45
REPS1	326.5	176.5	346.5	340.0	232.0	317.5	289.8	69.32
REPS2	2.0	0.5	1.0	0.0	0.0	0.5	0.7	0.75
RER1	1367.5	1019.0	929.5	1327.5	1212.0	887.5	1123.8	206.51
RERG	24.0	13.0	27.5	27.0	7.5	25.5	20.8	8.41
RERGL	52.0	44.5	163.0	56.0	47.5	184.5	91.3	64.38
REST	376.5	192.5	640.5	350.5	234.0	672.5	411.1	202.47
RET	289.0	57.5	107.0	194.0	29.5	40.5	119.6	102.59

RETSAT	969.5	937.5	399.5	1374.5	1194.5	720.5	932.7	344.80
REV1	329.0	228.5	256.5	311.0	280.0	261.5	277.8	37.09
REXO1	527.0	431.5	568.5	517.0	440.0	565.5	508.3	59.81
REXO2	1279.5	1273.5	668.0	1250.5	1389.0	714.0	1095.8	317.49
REXO4	475.5	292.0	402.0	421.0	257.5	270.5	353.1	91.30
RFC1	677.5	227.5	328.0	637.0	277.5	269.0	402.8	200.12
RFC2	834.5	497.5	466.0	758.5	501.0	333.5	565.2	190.85
RFC3	930.0	420.5	492.5	845.5	459.5	333.0	580.2	245.61
RFC4	1268.5	883.5	540.0	1258.5	849.0	444.0	873.9	346.60
RFC5	569.5	871.0	612.5	568.0	954.0	560.0	689.2	175.93
RFESD	2.0	1.0	2.0	1.5	1.0	4.0	1.9	1.11
RFFL	28.0	11.0	43.5	36.0	17.5	32.0	28.0	12.00
RFK	29.0	13.0	12.0	23.0	18.5	19.0	19.1	6.34
RFNG	735.5	475.5	510.0	725.5	539.5	489.5	579.3	119.16
RFT1	1014.5	700.5	518.0	915.0	790.5	547.0	747.6	198.13
RFTN2	895.0	434.0	441.0	831.0	486.0	384.0	578.5	223.65
RFWD2	536.5	432.5	493.5	456.5	382.5	434.0	455.9	53.53
RFWD3	177.0	102.0	156.5	150.5	88.0	108.5	130.4	35.61
RFX2	130.0	70.5	102.5	105.0	68.0	72.5	91.4	25.06
RFX4	1.5	0.0	0.0	1.0	1.0	0.0	0.6	0.66
RFX5	202.0	132.0	134.0	174.5	118.0	134.5	149.2	32.08
RFX6	0.5	0.0	0.0	0.0	0.0	0.0	0.1	0.20
RFX7	546.5	266.5	942.0	579.5	328.0	794.0	576.1	260.64
RFX8	0.0	0.0	0.0	0.0	0.0	0.0	0.0	0.00
RFXANK	604.0	667.0	406.0	563.5	601.5	399.5	540.3	111.58
RGCC	0.5	6.0	2.0	1.5	8.0	2.5	3.4	2.92
RGL1	21.0	16.0	37.5	24.0	17.0	38.0	25.6	9.85
RGMA	772.5	669.5	1042.5	815.0	759.0	1016.5	845.8	150.18
RGMB	104.5	129.5	187.0	91.5	164.0	208.0	147.4	46.48
RGN	266.5	432.5	87.5	249.0	446.5	133.5	269.3	148.29
RGP1	282.0	365.5	266.5	273.0	370.0	296.5	308.9	46.69
RGR	0.0	0.0	0.0	0.0	0.5	0.0	0.1	0.20
RGS1	0.0	0.5	0.5	0.0	0.5	0.5	0.3	0.26
RGS10	463.0	369.5	690.0	493.0	443.5	888.5	557.9	194.19
RGS11	20.5	13.0	9.0	14.0	16.5	21.0	15.7	4.62
RGS12	3.5	1.5	9.5	6.0	0.5	7.0	4.7	3.44
RGS13	0.0	0.0	0.0	0.0	0.0	1.0	0.2	0.41
RGS14	364.5	347.5	266.5	313.0	294.5	265.5	308.6	41.20
RGS16	0.0	0.5	0.0	0.0	0.5	0.5	0.3	0.27
RGS17	0.5	0.0	4.0	2.0	1.5	3.5	1.9	1.59
RGS18	0.0	0.0	0.0	0.0	0.0	0.0	0.0	0.00
RGS19	2.5	2.0	2.5	3.0	1.0	3.0	2.3	0.75
RGS2	347.0	355.5	266.0	236.0	410.5	312.0	321.2	63.57
RGS20	53.5	129.5	144.0	46.5	135.5	100.5	101.6	42.61
RGS21	0.0	1.0	0.0	0.0	0.0	0.0	0.2	0.41
RGS22	7.0	32.0	44.0	1.0	28.0	24.0	22.7	16.05
RGS3	238.0	397.5	341.5	303.0	468.0	331.5	346.6	79.09
RGS4	23.0	22.5	20.5	18.0	20.5	11.5	19.3	4.23
RGS5	21.5	73.5	50.0	19.0	93.5	56.5	52.3	29.08
RGS6	1.5	1.5	0.5	0.0	1.0	1.5	1.0	0.63
RGS7	8.5	3.0	1.5	4.5	1.5	0.5	3.3	2.93
RGS8	0.0	0.0	0.0	0.0	0.0	0.0	0.0	0.00
RGS9	325.0	537.0	544.0	355.5	585.0	460.5	467.8	107.13
RGS9BP	294.0	167.5	306.5	311.0	167.0	233.0	246.5	67.47

RGSL1	1.5	1.0	1.0	1.0	0.0	0.5	0.8	0.52
RHAG	0.0	0.0	0.0	0.0	0.0	0.0	0.0	0.00
RHBDD1	72.0	15.0	49.0	73.0	28.5	48.5	47.7	23.11
RHBDD2	343.0	266.5	302.5	303.0	287.0	288.0	298.3	25.62
RHBDF1	381.0	517.0	737.5	400.5	539.0	665.5	540.1	141.44
RHBDF2	4.5	6.5	2.0	7.5	7.5	9.0	6.2	2.52
RHBDL1	1.0	0.5	2.0	0.0	0.5	1.0	0.8	0.68
RHBDL3	122.0	25.5	33.5	72.5	24.5	52.0	55.0	37.60
RHBG	131.5	159.5	107.5	113.0	139.0	127.0	129.6	18.74
RHCE	412.0	411.0	351.0	389.0	535.0	364.5	410.4	65.76
RHCG	1.0	9.5	1.5	1.0	13.5	6.0	5.4	5.23
RHEB	1981.5	1930.5	1550.5	1711.0	2215.5	1389.0	1796.3	303.85
RHNO1	12.5	4.0	7.5	11.5	6.0	4.0	7.6	3.68
RHO	5.5	18.0	31.5	9.5	21.0	33.0	19.8	11.19
RHOA	14709.5	12320.0	11655.0	14084.5	13147.5	11291.5	12868.0	1356.92
RHOBTB2	185.5	202.5	142.0	132.5	169.5	113.5	157.6	33.92
RHOBTB3	153.0	214.5	233.5	193.5	302.5	265.5	227.1	52.88
RHOC	1985.0	2861.5	1600.5	1886.0	2954.5	1756.5	2174.0	583.73
RHOF	9.5	3.0	8.0	12.0	10.0	3.5	7.7	3.66
RHOG	3.0	1.0	6.5	0.5	2.5	3.0	2.8	2.12
RHOH	2.5	3.5	6.0	3.5	1.0	3.5	3.3	1.63
RHOJ	222.0	195.5	301.0	261.0	212.0	192.0	230.6	42.48
RHOQ	94.0	85.5	288.5	112.5	84.0	226.5	148.5	87.27
RHOT1	496.5	311.5	578.0	499.0	343.5	503.5	455.3	104.08
RHOT2	754.0	552.0	562.5	785.0	571.0	562.0	631.1	107.83
RHOU	207.5	96.0	191.5	123.5	97.0	184.0	149.9	50.22
RHOV	1.0	1.0	0.5	0.5	1.5	1.0	0.9	0.38
RHPN1	32.0	23.0	23.0	28.0	32.0	40.0	29.7	6.47
RIBC2	0.0	0.0	0.0	0.0	0.0	0.0	0.0	0.00
RIC1	230.5	115.5	349.0	263.5	148.5	318.0	237.5	92.13
RIC3	64.5	40.5	75.0	69.0	47.0	59.5	59.3	13.21
RIC8A	960.0	606.5	645.5	880.0	671.5	659.0	737.1	145.57
RIC8B	734.5	602.5	437.0	763.5	668.5	455.0	610.2	138.92
RICTOR	74.5	75.5	166.0	79.5	75.0	141.5	102.0	40.87
RIF1	382.5	134.0	396.5	429.0	117.5	337.0	299.4	137.83
RILP	128.5	69.5	65.0	83.5	70.5	68.5	80.9	24.15
RIMBP2	100.5	107.0	79.0	78.0	131.0	89.5	97.5	20.04
RIMS1	24.0	15.5	39.5	28.5	18.5	25.0	25.2	8.44
RIMS3	1.0	0.5	1.5	0.5	0.0	2.0	0.9	0.74
RIMS4	4.0	2.0	5.0	3.5	2.5	6.5	3.9	1.66
RIN2	792.0	628.5	872.5	713.0	670.0	949.5	770.9	123.67
RIN3	4.5	2.0	4.0	6.5	3.0	1.0	3.5	1.95
RINT1	250.0	173.5	221.0	265.5	181.0	184.0	212.5	39.02
RIOK1	680.5	425.5	346.0	575.5	523.5	345.0	482.7	134.40
RIOK2	737.0	481.0	551.0	782.0	512.5	508.0	595.3	129.95
RIOK3	1831.5	1241.0	957.5	1741.0	1436.0	869.5	1346.1	397.20
RIPK1	428.5	287.0	341.0	484.5	325.0	319.5	364.3	75.68
RIPK2	85.5	44.0	51.5	82.0	44.0	44.5	58.6	19.73
RIPK4	0.0	0.0	0.5	0.0	0.0	0.0	0.1	0.20
RIPPLY1	639.5	434.5	144.0	541.5	482.0	176.0	402.9	200.48
RIPPLY2	3.0	0.0	0.0	2.5	0.0	0.5	1.0	1.38
RIPPLY3	0.0	0.0	0.0	0.0	0.0	0.0	0.0	0.00
RIT1	713.0	548.5	643.0	701.0	618.5	584.5	634.8	64.51
RLBP1	1.0	1.5	0.0	0.0	0.0	0.5	0.5	0.63

RLF	301.0	271.5	397.0	312.0	317.5	385.5	330.8	49.62
RLIM	991.5	703.5	797.5	970.5	865.5	737.5	844.3	119.55
RLTPR	411.0	391.5	249.5	339.5	401.0	235.5	338.0	78.10
RMDN1	201.0	125.0	62.5	192.0	134.0	67.5	130.3	58.97
RMDN2	62.5	25.0	31.5	49.5	24.5	24.5	36.3	16.06
RMDN3	315.0	225.0	256.5	302.5	223.5	243.0	260.9	39.20
RMI1	296.0	151.0	210.5	313.5	167.5	141.0	213.3	74.96
RMND1	612.0	522.0	508.5	510.5	574.5	445.0	528.8	58.01
RMND5A	327.0	176.5	280.5	319.0	224.5	257.0	264.1	57.50
RNASEH1	837.0	960.5	599.0	730.0	1017.5	601.5	790.9	178.13
RNASEH2B	561.5	256.5	338.0	488.5	256.0	272.0	362.1	131.79
RNASEK	817.5	1447.5	864.5	771.5	1541.5	898.5	1056.8	343.01
RNASEL	182.5	140.5	164.0	180.5	162.0	191.0	170.1	18.32
RNASET2	640.0	1423.5	973.5	590.5	1730.0	844.0	1033.6	453.39
RND1	14.0	7.5	7.0	12.0	5.0	4.0	8.3	3.95
RND2	5.0	29.0	53.0	3.5	33.0	18.5	23.7	18.75
RND3	283.0	396.5	398.0	292.0	446.5	336.0	358.7	65.39
RNF10	2913.5	3313.0	3134.5	2922.5	3741.0	3129.5	3192.3	307.72
RNF103	387.0	287.5	292.0	329.0	328.0	276.5	316.7	40.74
RNF11	860.5	900.5	647.0	770.0	1046.0	674.5	816.4	150.15
RNF111	274.5	220.0	397.5	274.0	253.0	428.5	307.9	84.35
RNF113A	356.0	316.5	194.5	316.5	312.0	182.5	279.7	72.50
RNF114	1002.0	823.0	896.5	996.0	895.0	892.5	917.5	69.00
RNF121	386.0	321.5	265.5	318.5	344.5	268.5	317.4	45.92
RNF122	3570.5	3573.5	2812.5	3253.5	3811.5	2781.5	3300.5	428.55
RNF123	243.5	262.5	242.0	235.0	238.0	265.0	247.7	12.84
RNF126	659.0	555.5	503.5	609.0	575.5	470.0	562.1	68.89
RNF128	864.5	256.5	165.5	641.0	325.5	188.0	406.8	282.71
RNF13	3016.5	1778.0	1742.0	3006.0	1907.5	1708.5	2193.1	637.34
RNF138	405.5	203.0	226.5	391.0	239.5	187.0	275.4	96.98
RNF139	996.5	577.5	632.5	928.0	653.5	608.0	732.7	180.93
RNF14	233.0	235.0	240.0	260.5	237.0	231.0	239.4	10.79
RNF141	461.0	451.5	745.5	470.0	442.0	612.5	530.4	122.89
RNF144A	521.5	420.5	594.5	585.5	523.0	681.0	554.3	87.86
RNF144B	15.0	7.5	37.5	16.0	7.0	19.5	17.1	11.15
RNF145	333.5	358.5	474.5	374.0	405.0	570.0	419.3	88.40
RNF146	637.5	890.5	574.0	567.5	1025.0	611.0	717.6	192.52
RNF149	469.0	268.0	293.0	436.0	304.0	303.5	345.6	84.49
RNF150	46.0	63.5	136.0	60.5	66.0	117.5	81.6	36.14
RNF152	0.5	0.5	3.0	0.0	2.5	4.0	1.8	1.64
RNF157	3.0	41.5	36.0	3.5	35.0	32.5	25.3	17.29
RNF165	133.5	86.5	257.0	116.5	131.5	229.0	159.0	67.78
RNF166	126.0	119.0	188.0	137.0	115.0	167.0	142.0	29.26
RNF168	428.5	266.0	382.5	431.5	288.0	331.5	354.7	70.67
RNF169	72.5	34.0	61.0	68.0	36.0	69.5	56.8	17.34
RNF17	2.5	0.5	0.5	1.0	2.0	0.5	1.2	0.88
RNF170	182.5	86.0	171.0	187.0	98.0	146.0	145.1	43.67
RNF180	469.0	319.0	722.0	486.0	359.0	885.0	540.0	219.87
RNF182	1.5	1.0	0.0	2.5	8.5	2.5	2.7	3.01
RNF185	502.0	469.5	417.0	488.5	523.0	409.0	468.2	46.23
RNF186	0.5	0.0	1.0	0.5	0.0	1.0	0.5	0.45
RNF19A	1366.5	619.0	810.0	1253.5	600.0	620.5	878.3	344.94
RNF19B	740.0	444.5	350.5	713.0	463.5	382.5	515.7	168.56
RNF2	67.5	33.0	37.5	66.0	40.0	37.5	46.9	15.54

RNF20	1238.5	1191.5	1943.5	1117.5	1257.0	1775.0	1420.5	347.36
RNF207	116.0	119.0	53.5	95.5	121.0	50.0	92.5	32.87
RNF208	226.0	253.5	344.5	180.5	211.0	323.0	256.4	64.73
RNF212	0.0	0.0	0.0	0.0	0.0	0.0	0.0	0.00
RNF212B	87.5	42.5	24.5	97.0	43.0	29.5	54.0	30.64
RNF213	235.5	123.0	127.5	232.5	127.5	180.5	171.1	53.14
RNF214	597.5	531.0	475.5	592.0	583.5	524.0	550.6	48.43
RNF215	704.0	489.5	357.0	598.5	466.5	423.5	506.5	125.38
RNF217	141.0	130.0	98.0	151.0	173.5	89.5	130.5	31.99
RNF219	576.5	425.5	404.0	540.0	508.5	415.5	478.3	72.96
RNF222	0.5	1.0	0.5	0.0	0.0	3.5	0.9	1.32
RNF223	0.5	3.5	0.5	1.0	1.5	0.0	1.2	1.25
RNF224	1.5	3.0	1.5	2.0	2.5	3.0	2.3	0.69
RNF24	74.5	81.5	162.0	78.0	80.5	192.5	111.5	51.89
RNF25	872.0	635.0	424.0	781.0	647.0	438.0	632.8	179.41
RNF26	731.5	372.0	539.5	754.5	414.0	508.0	553.3	159.22
RNF32	11.5	11.5	4.5	8.0	8.5	5.5	8.3	2.93
RNF34	1002.5	751.0	728.5	938.5	855.5	698.0	829.0	123.40
RNF38	751.5	671.0	893.5	743.0	818.0	995.5	812.1	117.18
RNF4	182.5	93.0	147.0	142.0	95.0	113.0	128.8	34.82
RNF41	726.0	858.5	820.5	637.0	839.5	816.0	782.9	84.80
RNF43	23.5	1.5	6.5	14.5	2.5	6.5	9.2	8.38
RNF44	88.5	64.5	153.0	88.0	54.5	142.5	98.5	40.52
RNF6	449.5	379.0	446.5	436.0	464.5	465.0	440.1	31.92
RNF8	395.5	319.0	392.5	411.0	353.5	358.0	371.6	34.18
RNFT1	693.0	717.5	751.0	676.0	819.5	723.0	730.0	50.88
RNFT2	194.5	129.0	109.0	177.0	151.0	112.5	145.5	34.98
RNGTT	701.5	403.0	573.5	715.5	492.0	506.5	565.3	123.57
RNH1	7320.5	14087.0	10270.0	8567.0	16554.5	10075.5	11145.8	3495.72
RNLS	123.0	74.0	56.0	115.0	68.0	50.0	81.0	30.74
RNMT	73.5	40.0	113.5	104.5	54.5	98.0	80.7	29.45
RNMTL1	282.5	256.5	189.5	261.0	280.5	196.0	244.3	41.31
RNPC3	186.5	93.5	139.0	178.0	100.5	143.0	140.1	38.31
RNPEP	497.0	323.5	273.0	430.0	320.0	302.5	357.7	86.54
RNPEPL1	481.0	531.5	608.5	423.5	598.0	566.0	534.8	71.68
RNPS1	1268.0	1310.5	1232.5	1223.0	1409.5	1197.0	1273.4	77.43
ROBO1	15.0	20.5	104.0	10.5	32.5	129.0	51.9	51.18
ROBO2	18.5	5.0	14.0	1.5	1.5	2.0	7.1	7.36
ROBO3	0.0	0.0	0.0	0.0	0.0	0.0	0.0	0.00
ROBO4	0.5	0.0	0.0	0.0	0.5	0.0	0.2	0.26
ROCK1	990.0	689.0	1244.0	962.0	802.5	1180.0	977.9	212.74
ROCK2	432.5	218.0	707.0	429.5	261.0	578.0	437.7	185.36
ROGDI	410.0	223.5	157.5	326.5	204.5	136.0	243.0	105.34
ROMO1	733.5	1072.5	797.0	768.5	1114.5	770.5	876.1	170.13
ROPN1L	43.0	42.5	13.0	37.0	52.5	11.5	33.3	17.02
ROR1	64.5	27.0	44.0	53.0	21.5	21.0	38.5	18.13
ROR2	892.0	575.0	939.0	1008.0	700.0	886.5	833.4	162.76
RORA	8.5	2.5	0.5	3.5	2.0	2.0	3.2	2.79
RORB	0.0	0.0	1.0	0.0	0.0	0.0	0.2	0.41
RP1L1	7.5	1.5	0.0	3.0	1.0	0.0	2.2	2.84
RP2	1062.0	455.5	611.5	1324.5	582.0	678.0	785.6	334.40
RP9	188.0	226.0	193.5	133.5	201.0	204.5	191.1	31.08
RPA1	1488.0	729.0	1202.0	1455.0	809.0	901.0	1097.3	331.32
RPA2	1338.5	1272.0	1356.5	1329.0	1341.0	1070.0	1284.5	109.03

RPA3	116.5	57.0	93.5	117.0	71.0	64.0	86.5	26.44
RPAIN	148.5	144.0	131.0	127.0	159.0	130.5	140.0	12.57
RPAP1	548.0	373.5	443.0	531.0	511.0	487.0	482.3	64.68
RPAP2	176.0	138.5	145.0	186.0	147.0	139.5	155.3	20.39
RPAP3	566.5	385.5	424.0	569.0	450.5	344.0	456.6	93.32
RPE	569.5	691.5	438.5	519.0	724.0	427.0	561.6	125.22
RPE65	99.0	56.0	48.0	103.0	49.5	43.0	66.4	27.14
RPF1	556.5	442.0	400.0	474.0	440.0	383.0	449.3	61.75
RPF2	1147.0	873.0	841.5	1058.5	924.0	724.5	928.1	152.92
RPGRIP1L	15.0	7.0	15.0	16.0	13.0	14.0	13.3	3.27
RPH3A	8.0	2.5	3.5	4.0	7.0	5.0	5.0	2.12
RPH3AL	53.5	55.0	88.0	66.0	68.0	105.0	72.6	20.13
RPIA	2658.0	1113.5	502.0	2352.5	1195.5	607.5	1404.8	899.78
RPL10	14878.0	10068.5	7114.0	12402.5	9757.5	6839.5	10176.7	3090.66
RPL10A	15575.5	10350.0	5716.0	12597.0	10298.5	5026.5	9927.3	4027.38
RPL11	14052.5	11064.5	5961.0	12094.0	10999.5	5717.0	9981.4	3393.97
RPL12	8407.5	6904.5	4305.5	7042.0	7057.0	4015.5	6288.7	1739.36
RPL13	17863.0	13169.5	7575.0	14632.0	12639.5	7051.0	12155.0	4171.52
RPL14	11576.5	9746.5	5234.0	9630.5	9745.5	4902.5	8472.6	2736.87
RPL15	13736.5	11104.5	6197.0	12043.5	11027.5	6215.5	10054.1	3136.26
RPL18A	10133.5	9041.0	4643.0	9181.5	8816.5	4395.5	7701.8	2507.05
RPL19	13046.5	12440.5	6516.5	12031.0	12562.5	6756.0	10558.8	3056.57
RPL21	17602.5	14398.0	8433.5	14129.0	15080.0	7865.5	12918.1	3896.52
RPL22	4510.5	4064.5	2136.0	3937.0	3949.0	2055.0	3442.0	1064.02
RPL22L1	3984.5	3315.5	1652.0	2963.0	3187.5	1483.5	2764.3	988.83
RPL23	13047.0	9782.5	5214.5	11324.5	9966.0	5164.5	9083.2	3235.01
RPL23A	10360.5	8906.5	4854.0	8794.0	8958.0	4552.0	7737.5	2421.34
RPL24	10309.0	9572.0	4437.5	9029.5	10077.0	4321.5	7957.8	2806.72
RPL26L1	14414.0	14208.5	6307.0	11792.0	13973.5	6228.0	11153.8	3900.59
RPL27	12039.5	9760.0	5490.5	10351.0	9713.0	5192.5	8757.8	2778.89
RPL27A	13568.0	10826.0	7204.5	11401.5	11042.5	6638.0	10113.4	2664.75
RPL29	8885.5	8605.5	3403.0	7364.0	8157.5	3198.5	6602.3	2609.45
RPL3	24757.0	15550.0	13080.5	21970.5	15493.0	12063.0	17152.3	5076.37
RPL30	10653.5	8654.5	3975.5	9257.0	8959.5	3766.0	7544.3	2927.38
RPL31	7591.0	6929.0	3145.0	6325.5	7016.0	3036.5	5673.8	2040.96
RPL32	13737.0	11206.5	5892.5	12013.5	11427.5	5772.5	10008.3	3354.27
RPL34	4411.5	4334.5	2255.5	3611.0	4326.0	2211.5	3525.0	1041.83
RPL35	8076.5	8106.5	4005.5	7268.5	8014.5	4024.5	6582.7	2012.96
RPL35A	8728.0	7437.0	3792.5	7478.0	7607.5	3662.5	6450.9	2162.98
RPL36	6197.0	5748.0	2533.0	5493.5	5580.5	2472.5	4670.8	1696.86
RPL37	9027.5	8963.0	4942.5	8012.5	8855.0	4401.5	7367.0	2126.39
RPL37A	12392.0	12261.5	5559.0	10793.0	11845.5	5217.0	9678.0	3371.89
RPL38	3330.5	3629.0	1692.5	2982.5	3632.5	1584.5	2808.6	937.93
RPL39	12553.0	10914.5	5427.5	10939.0	11543.5	5016.5	9399.0	3292.23
RPL3L	24.0	17.0	3.0	13.0	12.5	5.5	12.5	7.64
RPL4	35512.0	23250.0	14908.0	30201.0	22414.0	13607.0	23315.3	8507.91
RPL5	26471.0	19839.5	12920.5	23601.5	20643.0	12223.5	19283.2	5706.03
RPL6	19537.5	15702.0	10913.5	17439.5	16279.5	9731.5	14933.9	3823.62
RPL7	20985.5	15686.0	9709.0	17922.5	16048.0	9012.5	14893.9	4684.30
RPL7A	18497.0	15665.0	9774.0	16776.0	15753.5	9486.5	14325.3	3778.14
RPL7L1	2168.5	1623.5	1390.5	2333.0	1782.5	1298.0	1766.0	415.72
RPL8	19836.0	12944.5	7744.0	16642.0	12720.5	7500.0	12897.8	4855.70
RPL9	15536.0	13665.0	8554.5	13417.5	14144.5	8311.5	12271.5	3063.26
RPLP0	26532.0	20236.5	13727.0	21944.0	20027.5	13298.5	19294.3	5055.19

RPLP1	18813.0	17968.0	8225.0	16123.5	17833.5	8534.5	14582.9	4884.64
RPLP2	12257.5	9890.0	5053.0	10504.5	10336.0	4994.5	8839.3	3063.32
RPN1	7433.5	4898.5	5686.5	7890.0	5515.0	6252.0	6279.3	1163.57
RPN2	5115.5	3016.5	4686.5	5979.5	3678.5	4805.0	4546.9	1054.32
RPP14	771.0	768.0	416.5	721.0	817.0	411.5	650.8	185.95
RPP25	0.5	0.0	0.0	0.5	0.0	0.0	0.2	0.26
RPP25L	731.5	634.5	502.5	662.0	601.5	444.0	596.0	105.90
RPP30	336.5	315.0	296.5	336.5	344.5	291.5	320.1	22.51
RPP38	357.0	156.5	93.5	298.0	169.5	92.5	194.5	109.35
RPP40	281.5	219.0	123.0	253.5	205.0	122.5	200.8	66.06
RPRD1A	130.5	119.5	157.0	125.0	163.5	157.0	142.1	19.18
RPRD1B	120.5	80.5	143.5	136.0	67.0	122.5	111.7	30.88
RPRD2	478.5	259.5	573.5	483.5	299.0	503.5	432.9	124.41
RPRML	0.0	0.0	0.0	0.0	0.0	0.0	0.0	0.00
RPS10	9678.5	9554.0	5410.5	8665.5	9396.0	5111.0	7969.3	2129.33
RPS11	9913.0	10411.5	5282.0	9144.5	10984.0	5246.0	8496.8	2576.04
RPS12	20581.0	16003.0	8368.5	17565.0	15830.0	7835.5	14363.8	5143.93
RPS13	6214.0	5502.0	3768.0	5292.0	5882.0	3508.0	5027.7	1125.01
RPS14	10406.0	8507.0	4598.5	9298.5	8701.0	4581.0	7682.0	2485.01
RPS15	11438.0	11780.5	5154.5	9630.0	12110.5	5410.5	9254.0	3194.76
RPS15A	15368.5	14479.5	7086.5	13992.5	14422.5	6723.5	12012.2	3982.87
RPS16	11272.0	8983.0	4895.5	9860.0	9421.0	4596.5	8171.3	2763.94
RPS17	9367.0	10039.0	4605.0	8626.0	9970.0	4706.5	7885.6	2553.18
RPS19BP1	415.0	414.5	294.5	361.5	395.5	293.5	362.4	56.45
RPS2	29630.0	20089.5	10321.5	25051.5	19739.0	9515.0	19057.8	7957.54
RPS20	16474.5	13632.0	7087.5	14342.0	14604.0	6885.0	12170.8	4124.83
RPS21	4810.5	4693.5	1907.0	4132.5	4797.5	1878.0	3703.2	1424.52
RPS23	15090.0	13353.0	6302.0	13727.5	13398.0	6262.0	11355.4	3980.36
RPS24	18836.5	15656.0	8922.5	15002.5	16044.5	8053.0	13752.5	4291.84
RPS25	9558.0	8662.0	4374.5	8589.5	9137.5	4352.5	7445.7	2412.96
RPS27	6025.5	6460.5	3123.5	5506.0	6739.5	3533.5	5231.4	1537.35
RPS27A	10547.0	8621.0	5140.5	8807.0	8859.0	4745.0	7786.6	2313.46
RPS27L	312.0	301.0	193.5	267.0	318.0	172.5	260.7	63.05
RPS28	5204.0	9112.5	2872.5	4846.0	7325.0	3016.0	5396.0	2445.62
RPS29	5092.0	4720.0	1808.5	4744.0	4554.5	1934.5	3808.9	1511.41
RPS3	25086.5	16909.0	8958.0	21015.5	16493.0	8509.5	16161.9	6547.20
RPS3A	8637.0	7755.5	4514.0	7513.5	8174.0	4173.5	6794.6	1939.52
RPS4X	702.5	477.5	181.0	334.0	555.0	39.0	381.5	245.81
RPS6	30861.5	24327.5	13249.5	27364.5	23792.0	12534.0	22021.5	7511.41
RPS6KA1	1612.5	1172.0	1127.0	1753.0	1239.0	1163.5	1344.5	268.20
RPS6KA2	10.5	5.0	6.5	5.5	7.0	3.0	6.3	2.50
RPS6KA3	938.0	845.0	1262.0	924.0	946.5	1126.0	1006.9	155.40
RPS6KA5	511.5	288.0	363.5	461.5	345.0	370.0	389.9	81.74
RPS6KA6	532.5	533.5	631.5	437.0	633.0	643.0	568.4	81.84
RPS6KB1	94.5	64.5	155.0	101.0	58.0	129.0	100.3	37.20
RPS6KB2	450.0	740.5	493.0	423.5	705.0	581.0	565.5	133.48
RPS6KC1	338.5	335.5	217.5	323.5	340.5	201.0	292.8	65.16
RPS6KL1	173.0	184.5	157.0	180.0	223.0	233.5	191.8	29.90
RPS7	16523.5	13653.5	6734.5	13961.5	13547.0	6410.5	11805.1	4199.45
RPS8	17150.5	14597.0	7427.0	15527.5	14461.5	7560.0	12787.3	4211.45
RPSA	28920.5	23418.5	15270.0	25330.5	22473.0	13789.0	21533.6	5875.88
RPTOR	674.0	571.5	662.0	700.5	556.5	624.5	631.5	57.92
RPUSD1	112.0	58.5	63.0	95.0	44.0	58.5	71.8	25.92
RPUSD2	219.5	135.5	109.0	196.0	151.0	104.0	152.5	46.73

RPUSD3	278.0	381.5	217.0	303.0	370.5	226.5	296.1	69.72
RRAD	724.0	353.0	454.5	675.0	359.0	300.0	477.6	179.64
RRAGC	319.5	162.5	334.0	303.0	200.0	311.5	271.8	71.83
RRAGD	1328.5	1487.0	1474.5	1209.5	1579.5	1161.5	1373.4	166.96
RRAS2	2072.0	1774.5	2615.5	2033.5	1954.5	2403.5	2142.3	309.67
RRBP1	867.5	798.0	1305.5	951.0	944.5	1425.0	1048.6	254.41
RREB1	387.0	215.5	604.0	388.5	244.0	451.5	381.8	142.09
RRH	162.0	135.0	60.5	132.0	156.0	53.5	116.5	47.58
RRM1	1358.5	517.5	850.5	1363.5	498.0	532.0	853.3	414.19
RRM2	2211.0	786.0	1261.5	2178.0	723.0	682.5	1307.0	718.39
RRM2B	590.5	549.0	526.5	580.0	636.0	434.0	552.7	69.14
RRN3	431.0	267.0	449.5	371.5	286.0	309.5	352.4	76.83
RRNAD1	201.0	130.0	108.5	271.5	126.0	74.5	151.9	71.75
RRP12	1494.0	1339.0	1307.0	1523.0	1318.0	1303.5	1380.8	100.15
RRP15	362.5	347.0	301.0	365.5	347.0	250.0	328.8	45.01
RRP1B	905.0	611.0	766.5	938.0	639.0	745.0	767.4	133.78
RRP7A	224.5	257.0	216.0	209.0	236.0	195.5	223.0	21.58
RRP9	1087.0	957.0	507.0	983.0	920.0	541.5	832.6	245.44
RS1	0.0	4.5	0.5	0.0	0.5	0.0	0.9	1.77
RSAD1	55.5	19.5	29.5	38.0	18.0	24.5	30.8	14.11
RSAD2	2.5	1.5	2.5	4.5	1.5	14.0	4.4	4.82
RSBN1	133.0	92.5	148.5	152.5	102.5	153.5	130.4	26.73
RSBN1L	488.5	386.0	443.0	440.5	381.0	397.0	422.7	41.96
RSF1	417.5	336.5	540.5	425.0	411.5	535.0	444.3	79.08
RSG1	324.0	371.0	222.0	295.5	330.5	259.5	300.4	53.46
RSL1D1	926.5	769.0	673.0	1001.0	761.5	586.5	786.3	154.55
RSL24D1	1554.0	1400.5	1056.0	1520.0	1578.0	1009.5	1353.0	255.88
RSPH1	0.5	0.0	0.0	0.0	0.0	0.0	0.1	0.20
RSPH10B	9.0	5.0	22.5	10.0	9.0	19.5	12.5	6.87
RSPH14	0.0	0.0	0.0	0.5	0.0	0.0	0.1	0.20
RSPH3	99.0	47.0	75.0	55.5	50.0	51.5	63.0	20.27
RSPH9	21.0	21.0	18.5	18.5	31.0	16.0	21.0	5.24
RSPO1	0.0	0.0	0.0	0.0	0.0	0.0	0.0	0.00
RSPO2	0.5	0.0	1.0	0.5	0.0	0.0	0.3	0.41
RSPO3	16.5	6.5	4.5	13.0	7.5	5.0	8.8	4.83
RSPO4	142.5	194.5	91.0	272.0	277.5	191.5	194.8	72.55
RSPRY1	181.5	153.5	209.5	201.5	177.5	201.0	187.4	20.76
RSRC1	385.0	376.0	333.0	357.5	416.5	300.5	361.4	40.83
RSRC2	1061.5	1041.5	888.0	1022.5	1228.5	923.5	1027.6	120.05
RSRP1	883.0	871.5	1756.5	767.5	1108.0	2214.0	1266.8	585.68
RSU1	1050.5	947.0	1347.0	1162.5	1101.0	1198.5	1134.4	136.65
RTCA	637.0	433.0	341.0	684.5	504.0	368.0	494.6	141.31
RTCB	314.0	120.5	244.0	369.0	120.0	215.5	230.5	100.87
RTEL1	119.5	116.5	86.5	128.5	150.0	70.0	111.8	29.01
RTFDC1	1608.5	1099.5	910.5	1422.5	1176.0	911.0	1188.0	280.54
RTKN2	17.5	3.0	28.0	19.5	7.5	17.5	15.5	8.95
RTN1	15.5	3.5	3.5	17.0	4.5	3.5	7.9	6.48
RTN4R	0.5	0.0	0.0	0.0	0.0	0.0	0.1	0.20
RTN4RL1	2.0	1.5	4.5	0.5	1.5	6.0	2.7	2.11
RTN4RL2	214.0	175.0	165.5	169.5	180.0	252.0	192.7	33.83
RUFY1	167.0	78.5	142.0	170.0	97.0	125.0	129.9	37.09
RUFY2	243.5	144.0	172.5	215.0	151.5	162.0	181.4	39.33
RUFY3	1379.5	1344.0	1339.0	1207.0	1574.5	1212.5	1342.8	134.51
RUFY4	0.0	0.5	0.0	1.5	0.0	0.0	0.3	0.61

RUNDC1	997.0	762.0	719.5	967.5	802.0	669.0	819.5	133.92
RUNDC3A	0.0	0.0	0.0	0.0	0.5	0.0	0.1	0.20
RUNX1	56.0	26.5	176.5	57.5	28.5	150.5	82.6	64.56
RUNX2	7.0	6.5	6.0	6.0	6.0	10.5	7.0	1.76
RUNX3	0.0	0.0	0.0	0.5	0.0	0.0	0.1	0.20
RUSC1	839.0	964.5	913.0	732.0	961.0	939.5	891.5	90.69
RUVBL1	1299.0	906.5	912.5	1223.5	943.0	798.5	1013.8	199.23
RWDD1	435.5	361.5	256.0	409.5	367.0	226.5	342.7	83.71
RWDD2A	81.5	70.0	74.0	87.5	81.0	59.0	75.5	10.15
RWDD2B	58.5	46.0	34.5	52.5	56.0	25.5	45.5	13.03
RWDD3	114.5	74.5	67.0	123.5	97.0	54.5	88.5	27.52
RWDD4	967.5	723.0	433.0	895.0	832.5	379.5	705.1	245.57
RXFP1	195.5	193.5	137.0	194.5	200.0	116.5	172.8	36.35
RXFP3	5.5	3.0	3.5	4.0	1.0	2.0	3.2	1.57
RXRA	91.0	118.0	217.0	97.5	129.5	216.5	144.9	57.34
RXRG	2.5	0.0	0.0	0.5	0.0	0.0	0.5	1.00
RYBP	173.5	184.5	239.5	203.0	226.5	209.5	206.1	24.83
RYK	521.5	442.5	479.5	594.5	482.0	524.5	507.4	52.37
RYR1	8.0	25.5	75.5	3.5	18.5	68.5	33.3	31.07
RYR2	4.0	3.0	2.5	1.0	1.0	5.0	2.8	1.60
RYR3	585.0	505.5	566.5	474.0	634.0	620.5	564.3	63.34
S100A1	870.5	1585.5	785.5	961.5	1796.0	1065.5	1177.4	413.79
S100A10	11861.0	13276.5	7294.0	10347.5	13775.0	7423.0	10662.8	2824.80
S100A11	12190.0	6942.5	4951.5	11234.5	7447.0	4617.5	7897.2	3165.86
S100A12	0.0	0.0	0.0	0.0	1.5	0.0	0.3	0.61
S100A13	5.0	3.5	1.5	4.0	3.5	2.5	3.3	1.21
S100A14	0.0	0.0	0.0	0.5	1.0	0.0	0.3	0.42
S100A16	12.0	8.0	6.5	12.0	5.0	4.5	8.0	3.33
S100A4	5317.0	5350.5	2828.0	3955.5	4947.0	2221.5	4103.3	1336.40
S100B	8.5	3.5	6.5	8.0	6.0	4.0	6.1	2.04
S100Z	47.5	99.5	175.5	30.5	135.0	185.5	112.3	64.69
S1PR1	77.0	17.0	49.5	90.0	19.0	57.0	51.6	29.71
S1PR2	20.0	18.5	63.5	31.5	17.0	54.5	34.2	20.11
S1PR3	244.5	100.5	97.0	228.5	101.0	78.5	141.7	74.09
S1PR4	70.5	67.0	66.0	67.5	60.0	49.5	63.4	7.64
SACM1L	1086.5	794.5	899.0	1040.0	900.5	862.5	930.5	110.76
SACS	1262.5	1424.5	3387.5	1209.5	2020.5	3505.5	2135.0	1056.67
SAG	0.5	0.0	3.0	0.0	1.5	3.5	1.4	1.53
SALL1	111.5	104.5	195.0	57.0	82.5	99.0	108.3	46.74
SALL3	0.5	0.5	0.0	0.0	0.5	0.0	0.3	0.27
SALL4	1127.5	855.0	774.5	1005.5	877.0	800.0	906.6	134.86
SAMD10	0.0	0.5	3.5	0.0	1.0	3.0	1.3	1.54
SAMD11	21.5	168.0	231.0	31.5	211.5	378.5	173.7	134.22
SAMD12	72.5	15.0	36.5	74.5	31.5	30.0	43.3	24.45
SAMD13	90.5	55.5	72.0	94.5	59.5	66.0	73.0	16.17
SAMD15	50.0	24.5	36.5	47.0	23.0	33.5	35.8	11.17
SAMD3	3.5	0.5	2.5	2.5	2.5	3.0	2.4	1.02
SAMD4A	497.0	1112.5	1678.5	490.5	1159.0	1594.5	1088.7	513.26
SAMD4B	97.5	85.0	132.0	105.5	84.0	120.0	104.0	19.21
SAMD7	2.5	3.5	1.0	2.5	2.5	1.0	2.2	0.98
SAMD8	163.5	56.0	259.0	141.0	80.5	180.0	146.7	73.00
SAMHD1	108.0	139.5	176.0	101.5	164.0	299.0	164.7	72.12
SAMM50	1256.5	984.0	828.5	1091.0	1051.0	757.5	994.8	181.65
SAMSN1	5.0	0.5	0.0	1.0	0.0	0.0	1.1	1.96

SAP130	505.5	445.5	486.0	509.5	507.5	428.0	480.3	35.23
SAP18	2301.5	2808.0	2327.0	2265.5	2877.0	2236.0	2469.2	291.65
SAP30	1113.5	637.5	392.5	1058.5	720.5	359.0	713.6	320.49
SAP30BP	671.5	459.5	473.5	607.5	576.5	482.0	545.1	86.36
SAP30L	114.5	72.5	186.0	148.0	91.0	160.0	128.7	43.40
SAPCD2	306.5	175.5	197.5	306.5	179.5	128.5	215.7	73.96
SAR1A	1595.0	1854.5	1253.0	1578.5	1942.0	1449.0	1612.0	254.78
SAR1B	2438.5	2245.5	2398.0	2332.0	2672.5	2524.5	2435.2	149.88
SARAF	682.5	319.0	568.0	688.5	361.5	546.5	527.7	156.83
SARDH	781.5	3069.0	2258.0	666.5	3285.5	1962.5	2003.8	1106.90
SARM1	16.5	60.0	68.5	13.5	73.5	91.5	53.9	31.87
SARNP	298.5	175.0	283.5	289.5	199.0	281.0	254.4	53.11
SARS	1830.0	1804.0	1313.0	1640.5	1615.0	1167.0	1561.6	267.35
SARS2	215.5	211.0	181.5	199.5	216.0	161.5	197.5	21.93
SART3	544.0	271.5	393.0	533.0	308.5	375.0	404.2	113.03
SASH1	639.5	602.0	1109.5	670.0	795.0	1098.0	819.0	229.92
SASH3	1.0	0.5	0.0	1.5	0.5	0.0	0.6	0.58
SASS6	663.5	271.5	458.0	677.0	285.5	334.0	448.3	184.14
SAT1	4508.5	5380.5	2952.0	3934.0	6173.0	4181.5	4521.6	1130.45
SATB2	75.5	28.0	46.0	71.5	34.0	42.0	49.5	19.65
SAV1	834.5	558.0	630.5	872.0	573.0	669.5	689.6	133.47
SBDS	1766.5	2288.0	2246.0	1828.0	2729.0	2212.0	2178.3	350.23
SBF1	1218.5	1074.5	1645.5	1378.5	1019.5	1676.0	1335.4	281.27
SBK1	0.5	0.5	0.5	1.5	0.5	2.5	1.0	0.84
SBK2	3.5	4.0	3.0	1.0	4.0	12.5	4.7	4.00
SBK3	5.0	3.5	1.0	2.5	5.0	2.5	3.3	1.57
SBNO1	2664.5	1608.5	2187.0	2491.0	1828.5	1965.0	2124.1	402.28
SBNO2	591.5	1551.0	1819.0	540.5	1832.5	1783.0	1352.9	618.26
SBSPON	154.5	111.5	117.5	137.0	115.5	80.0	119.3	25.20
SC5D	591.0	368.0	689.0	547.0	383.5	589.0	527.9	126.85
SCAF4	370.0	341.0	407.0	356.0	389.0	364.5	371.3	23.61
SCAF8	490.0	409.0	518.0	467.0	486.0	482.0	475.3	36.51
SCAI	275.5	204.0	205.0	259.5	222.5	242.0	234.8	29.35
SCAMP1	210.0	88.0	163.5	217.5	131.5	166.5	162.8	48.60
SCAMP2	1303.5	1138.5	1143.0	1277.5	1292.0	1107.5	1210.3	89.59
SCAMP4	800.5	688.5	890.5	833.5	712.0	817.5	790.4	76.48
SCAMP5	749.5	646.5	412.0	735.0	690.5	487.0	620.1	139.01
SCAP	1257.0	475.5	404.5	1210.5	412.0	498.5	709.7	407.81
SCAPER	72.0	21.5	69.0	76.0	19.5	49.5	51.3	25.51
SCARA3	0.0	0.0	0.0	0.0	0.5	0.5	0.2	0.26
SCARA5	8.0	12.5	16.0	6.0	7.5	8.5	9.8	3.75
SCARB1	639.5	621.5	768.5	591.5	723.0	826.5	695.1	92.38
SCARB2	1360.5	520.0	580.5	1360.0	641.0	611.0	845.5	400.73
SCARF1	19.5	8.0	10.0	13.5	9.0	11.5	11.9	4.19
SCARF2	98.5	103.0	141.0	98.0	92.5	176.0	118.2	33.31
SCCPDH	1444.5	1053.0	1278.0	1594.0	1196.0	1209.5	1295.8	193.76
SCD	5975.0	1757.5	4396.0	5552.0	1938.5	3099.5	3786.4	1804.55
SCD5	471.0	184.0	298.0	451.0	217.0	295.5	319.4	118.45
SCEL	1.5	0.0	1.5	1.0	0.0	1.5	0.9	0.74
SCFD1	669.5	443.0	528.5	688.5	447.0	471.0	541.3	111.14
SCFD2	132.5	45.0	95.0	129.5	51.5	89.0	90.4	37.14
SCG2	437.0	149.5	121.5	893.0	202.5	109.5	318.8	306.14
SCG3	260.5	184.0	289.5	286.0	202.0	295.0	252.8	48.17
SCG5	5.5	2.0	7.0	5.0	5.0	9.0	5.6	2.33

SCGN	0.0	0.0	0.0	0.0	2.0	0.0	0.3	0.82
SCLT1	339.5	189.5	211.5	297.5	235.0	212.5	247.6	58.32
SCLY	374.0	470.5	523.5	375.0	487.0	483.5	452.3	62.74
SCMH1	262.0	220.0	293.5	231.0	214.0	284.5	250.8	34.00
SCML2	71.0	29.0	28.5	51.0	38.5	18.0	39.3	19.07
SCN2B	55.0	23.0	33.5	36.5	10.5	41.5	33.3	15.33
SCN3B	706.0	656.5	298.0	620.5	762.0	414.5	576.3	180.73
SCN4A	32.0	177.0	140.0	31.0	175.5	132.0	114.6	66.88
SCN4B	452.5	286.0	215.0	344.0	266.0	342.0	317.6	82.07
SCN8A	27.0	48.5	10.5	36.0	79.5	12.5	35.7	25.81
SCNN1A	10.5	30.5	48.5	12.5	46.0	33.0	30.2	16.09
SCNN1B	5.5	4.0	6.0	4.5	6.0	6.0	5.3	0.88
SCNN1D	0.0	0.0	0.0	0.0	0.5	0.0	0.1	0.20
SCNN1G	57.5	33.0	29.5	54.0	36.0	26.0	39.3	13.20
SCOC	885.0	822.0	1085.5	887.0	898.5	993.0	928.5	94.52
SCP2	1866.5	1511.0	1615.5	1524.5	1812.0	1392.0	1620.3	184.73
SCP2D1	208.5	124.5	150.5	167.0	105.5	117.5	145.6	38.16
SCPEP1	588.5	310.5	256.5	575.5	353.5	299.0	397.3	146.46
SCRG1	1.0	0.0	0.5	0.5	0.0	0.5	0.4	0.38
SCRIB	655.5	789.5	1096.0	555.5	771.5	1008.5	812.8	205.76
SCRN1	57.5	50.0	57.0	51.0	56.5	53.0	54.2	3.27
SCRN2	241.0	272.5	207.5	217.0	280.5	227.0	240.9	29.82
SCRN3	1201.0	768.5	693.5	908.5	769.5	612.5	825.6	208.36
SCRT2	0.5	1.5	1.0	0.5	1.5	2.5	1.3	0.76
SCT	0.5	0.0	0.0	0.0	0.0	0.5	0.2	0.26
SCTR	0.0	0.0	0.0	0.0	0.0	0.0	0.0	0.00
SCUBE1	0.5	0.0	0.5	0.5	0.5	0.0	0.3	0.26
SCUBE2	419.0	148.0	236.0	373.0	184.0	348.0	284.7	110.49
SCX	8.0	6.5	5.5	2.0	2.0	2.0	4.3	2.68
SCYL2	924.5	550.0	727.5	817.0	630.5	650.5	716.7	136.35
SCYL3	626.5	469.5	494.0	582.5	530.5	463.5	527.8	65.45
SDAD1	703.0	510.5	492.5	740.5	514.0	424.0	564.1	126.92
SDC1	287.0	111.0	168.0	502.5	149.5	176.0	232.3	144.83
SDC2	3885.5	1902.0	2277.0	3966.0	2047.5	1734.5	2635.4	1015.56
SDC3	0.5	0.0	0.5	0.0	0.0	0.5	0.3	0.27
SDC4	381.0	212.0	223.5	387.5	266.5	269.0	289.9	76.53
SDCBP	2964.0	1945.0	2212.5	2754.5	2173.0	1697.5	2291.1	481.62
SDCBP2	0.5	1.0	1.5	0.5	1.0	2.5	1.2	0.75
SDCCAG3	116.0	207.5	94.0	124.0	222.5	118.5	147.1	53.80
SDCCAG8	189.0	163.0	138.0	134.0	177.0	147.0	158.0	22.11
SDE2	888.5	924.0	866.0	928.5	1059.5	844.5	918.5	76.35
SDF2	964.0	1213.0	733.0	967.5	1409.0	776.0	1010.4	259.16
SDF2L1	1699.5	731.0	712.0	1958.5	851.0	731.0	1113.8	562.16
SDF4	682.0	320.0	623.0	758.0	375.5	632.0	565.1	175.91
SDHA	2730.0	2024.5	1907.5	2595.0	2137.5	1575.5	2161.7	433.21
SDHAF2	643.5	598.5	284.0	616.0	605.0	266.0	502.2	176.73
SDHB	2597.5	2713.5	1756.0	2350.0	2779.0	1721.0	2319.5	473.36
SDHC	2136.5	2375.0	1731.5	2039.5	2654.0	1621.0	2092.9	387.93
SDHD	1864.0	2135.0	1580.0	1592.0	2255.0	1525.5	1825.3	311.94
SDK2	1106.5	2549.5	3736.0	1123.0	2548.0	3497.0	2426.7	1125.36
SDPR	5906.5	3875.0	5486.5	4355.0	3549.0	3365.5	4422.9	1050.61
SDR42E1	98.5	43.0	46.0	92.0	45.0	38.5	60.5	27.12
SEBOX	0.0	2.5	1.5	1.5	1.0	2.5	1.5	0.95
SEC11A	1195.0	874.0	812.5	1184.0	992.5	818.5	979.4	175.14

SEC11C	454.0	352.0	305.0	429.5	373.5	303.5	369.6	62.58
SEC13	1614.5	1745.0	1491.5	1687.0	1832.5	1517.5	1648.0	132.37
SEC14L1	510.0	293.0	1091.5	542.5	311.5	832.0	596.8	311.20
SEC14L2	324.0	151.0	67.0	217.0	136.5	69.0	160.8	97.68
SEC14L5	1.5	3.0	2.5	1.0	3.5	1.5	2.2	0.98
SEC16A	1005.5	867.5	1733.5	966.0	997.5	1521.0	1181.8	354.92
SEC16B	16.0	13.0	9.0	13.0	11.5	8.5	11.8	2.80
SEC22A	296.5	147.0	302.5	281.5	133.5	237.0	233.0	75.53
SEC22B	1450.5	1107.5	866.5	1395.5	1167.5	859.0	1141.1	251.90
SEC22C	139.5	79.5	116.0	145.5	86.0	127.5	115.7	27.52
SEC23A	2920.5	2402.0	3675.0	2814.0	2743.5	3056.5	2935.3	423.79
SEC23B	964.5	493.5	434.5	1015.0	493.0	411.0	635.3	276.96
SEC23IP	676.0	382.0	632.5	641.0	443.0	511.5	547.7	120.07
SEC24A	2616.5	1745.0	2108.5	2344.0	1926.5	2173.0	2152.3	307.07
SEC24B	354.0	295.0	363.5	334.5	342.5	324.5	335.7	24.24
SEC24C	1759.5	1214.5	1716.0	1864.0	1414.5	1642.5	1601.8	242.24
SEC24D	1000.5	827.5	1681.0	1100.5	982.5	1766.0	1226.3	395.81
SEC31A	1896.0	1498.5	2587.0	2024.0	1811.5	2630.5	2074.6	448.81
SEC31B	911.0	813.5	977.5	895.0	836.0	838.5	878.6	61.25
SEC61A1	1128.0	293.0	981.0	1307.0	310.5	826.5	807.7	422.94
SEC61A2	829.0	476.5	656.5	782.5	537.5	554.5	639.4	142.02
SEC61B	1565.5	1696.0	979.0	1689.5	1938.0	1158.5	1504.4	362.88
SEC61G	1418.5	1526.0	906.5	1238.0	1573.5	868.5	1255.2	307.49
SEC62	1354.0	1035.5	1316.0	1325.5	1276.0	1311.0	1269.7	117.44
SEC63	366.0	107.5	369.0	448.0	132.0	309.0	288.6	138.28
SECISBP2	321.0	237.0	347.0	290.0	278.5	368.0	306.9	48.00
SECISBP2L	1281.5	1213.5	1492.0	1237.0	1322.0	1388.0	1322.3	103.82
SEH1L	509.0	568.5	554.5	509.5	679.5	511.0	555.3	66.04
SEL1L	1828.5	983.0	1896.5	1879.5	1157.0	1642.0	1564.4	397.33
SEL1L3	496.5	369.5	504.5	521.0	415.5	471.5	463.1	58.79
SELENBP1	791.0	332.5	587.5	533.0	341.0	504.5	514.9	170.56
SELP	0.5	0.0	0.0	0.0	0.0	0.0	0.1	0.20
SELPLG	3.0	2.0	1.5	1.5	1.0	1.5	1.8	0.69
SEMA3B	5.5	12.0	46.5	7.0	14.0	45.5	21.8	19.04
SEMA3C	321.0	89.0	646.0	426.5	106.5	551.0	356.7	228.85
SEMA3D	199.0	120.0	330.0	277.5	158.0	286.5	228.5	82.07
SEMA3E	1.0	0.0	0.5	0.5	0.5	0.0	0.4	0.38
SEMA3F	78.0	78.0	135.0	88.0	87.0	173.0	106.5	38.94
SEMA3G	8.5	6.5	11.0	7.5	8.5	8.5	8.4	1.50
SEMA4B	623.0	322.5	292.5	586.5	266.5	303.5	399.1	160.75
SEMA4C	143.5	139.5	187.0	145.0	107.0	234.0	159.3	44.56
SEMA4D	139.5	101.0	145.5	104.5	105.0	123.0	119.8	19.32
SEMA4G	186.0	237.5	228.5	192.0	268.5	218.0	221.8	30.52
SEMA5A	10.5	28.0	40.0	3.5	28.5	10.5	20.2	14.06
SEMA6A	181.5	112.0	206.5	188.0	131.0	251.5	178.4	50.78
SEMA6B	2076.5	2553.0	1040.5	1751.0	2506.0	1274.5	1866.9	627.76
SEMA6C	19.0	8.5	38.5	11.5	11.0	26.0	19.1	11.49
SEMA6D	48.0	46.5	101.5	33.5	48.5	80.0	59.7	25.62
SEMA7A	176.5	246.5	297.0	125.5	224.0	394.0	243.9	94.14
SENP1	123.5	70.5	160.0	117.5	72.5	135.5	113.3	35.47
SENP2	165.0	152.0	129.0	151.0	152.5	115.5	144.2	18.24
SENP5	979.5	895.0	841.0	924.5	966.0	869.5	912.6	54.34
SENP7	84.5	57.5	104.0	100.0	66.5	105.5	86.3	20.47
SENP8	156.5	81.5	127.0	141.5	89.0	106.0	116.9	29.75

SEPHS1	795.0	476.0	599.5	859.5	536.0	653.5	653.3	148.79
SEPN1	3144.0	2753.5	2585.5	3180.0	2997.5	2467.5	2854.7	297.50
SEPP1	65.0	15.5	54.5	63.5	30.5	62.0	48.5	20.63
SEPSECS	104.5	45.0	114.5	113.0	54.0	78.5	84.9	30.46
SEPT10	120.5	77.5	77.5	107.0	85.5	71.5	89.9	19.46
SEPT11	6199.5	5080.0	7063.0	6257.5	5391.5	5891.5	5980.5	701.75
SEPT12	0.0	0.0	0.0	0.0	0.0	0.5	0.1	0.20
SEPT2	412.0	98.5	394.5	413.5	102.0	303.0	287.3	150.46
SEPT3	0.0	0.0	0.0	0.0	0.0	0.0	0.0	0.00
SEPT4	1.5	11.0	13.5	2.5	8.0	8.0	7.4	4.68
SEPT5	1829.0	982.5	1917.5	1955.0	1049.5	1682.0	1569.3	439.24
SEPT6	2869.5	3325.5	2773.5	2765.5	3731.5	2804.5	3045.0	397.60
SEPT7	4849.0	3956.0	4188.0	4645.0	4681.0	3873.0	4365.3	413.08
SEPT8	1299.0	1289.0	1518.5	1499.0	1500.5	1730.0	1472.7	163.51
SEPT9	3752.0	2859.5	3354.0	3491.5	3073.5	3449.0	3329.9	318.14
SERAC1	317.5	234.0	283.0	294.5	255.5	261.0	274.3	30.01
SERBP1	9044.0	7316.0	5926.0	7964.5	7600.5	5511.5	7227.1	1313.65
SERGEF	14.0	5.5	25.0	14.0	4.0	25.0	14.6	9.08
SERHL2	445.0	237.5	194.5	426.5	269.5	240.0	302.2	106.36
SERINC1	5376.0	4327.0	5732.0	4978.0	5228.0	5564.0	5200.8	501.73
SERINC2	16.0	39.5	55.5	12.5	44.5	54.5	37.1	18.72
SERINC3	6578.0	4298.0	6016.5	7399.5	5451.5	6085.0	5971.4	1048.14
SERINC4	54.0	35.0	33.0	41.5	45.5	33.0	40.3	8.38
SERINC5	104.5	55.5	208.5	133.0	63.0	174.0	123.1	60.84
SERPINA10	0.0	0.5	0.0	0.5	0.5	0.0	0.3	0.27
SERPINB10	5.5	2.5	2.5	6.0	3.5	2.0	3.7	1.69
SERPINB12	0.0	0.0	0.0	0.0	0.0	0.0	0.0	0.00
SERPINB14	0.5	0.0	0.0	0.5	0.0	0.0	0.2	0.26
SERPINB5	5.5	0.5	2.0	5.5	1.5	4.0	3.2	2.14
SERPINC1	23.5	13.5	13.0	19.0	12.5	10.0	15.3	5.01
SERPIND1	1.5	0.5	0.5	0.5	2.5	0.0	0.9	0.92
SERPINE2	10119.5	5515.5	7825.0	10764.5	6362.5	7897.5	8080.8	2048.95
SERPINE3	0.0	0.0	0.0	0.0	0.0	0.0	0.0	0.00
SERPINF1	3247.0	5073.0	8608.0	3413.0	5283.0	7251.5	5479.3	2115.93
SERPINF2	691.5	204.0	101.5	559.5	220.5	102.5	313.3	250.40
SERPING1	151.0	204.0	245.5	156.0	191.5	353.5	216.9	75.29
SERPINH1	4638.5	7404.0	27213.0	5685.0	8753.5	30476.0	14028.3	11609.02
SERPINI1	58.0	36.0	66.0	59.0	45.5	56.0	53.4	10.80
SERTAD2	52.0	28.0	134.5	57.5	35.0	104.0	68.5	41.89
SERTAD4	249.5	220.0	460.5	283.5	280.0	450.0	323.9	104.36
SERTM1	13.5	12.5	5.5	16.5	9.5	8.5	11.0	3.94
SESN1	1248.0	588.0	962.5	1032.5	646.0	777.0	875.7	251.40
SESN2	105.0	93.5	133.5	122.0	62.5	100.5	102.8	24.62
SESN3	191.5	87.0	207.5	167.5	84.5	189.5	154.6	54.82
SESTD1	1970.0	1201.0	1318.0	1768.5	1318.5	1179.0	1459.2	329.09
SET	3096.0	2496.5	2417.5	2911.0	2746.0	2300.0	2661.2	308.42
SETBP1	59.5	62.5	93.0	62.0	91.0	88.0	76.0	16.18
SETD1B	161.5	174.0	132.0	137.5	171.5	148.5	154.2	17.60
SETD2	1597.0	1347.5	2256.5	1610.5	1437.0	2201.0	1741.6	390.48
SETD3	1245.0	1154.5	1119.0	1175.0	1294.5	1100.5	1181.4	74.89
SETD4	52.5	23.0	35.0	27.0	22.5	30.0	31.7	11.21
SETD5	577.0	401.5	733.0	603.0	421.0	705.0	573.4	138.89
SETD6	678.0	556.5	315.5	649.0	529.0	329.5	509.6	155.23
SETD7	3307.5	5779.0	5092.0	3093.5	6944.5	4792.5	4834.8	1468.00

SETD8	276.0	298.5	355.5	314.5	330.5	357.0	322.0	32.07
SETD9	76.5	47.0	34.5	91.0	50.0	40.5	56.6	22.19
SETDB1	600.5	610.0	596.5	593.0	605.5	716.5	620.3	47.50
SETDB2	500.0	375.0	266.0	464.0	372.0	209.5	364.4	111.49
SETX	366.5	192.5	536.5	350.5	248.0	504.0	366.3	135.93
SEZ6	0.0	0.0	0.0	0.0	0.0	0.0	0.0	0.00
SEZ6L	0.0	0.0	0.0	0.0	0.0	0.0	0.0	0.00
SF3A1	1634.5	1038.0	1369.0	1585.0	1144.5	1217.0	1331.3	241.54
SF3A2	594.5	391.0	458.5	612.0	444.0	423.5	487.3	92.83
SF3A3	1541.0	1009.5	1092.5	1412.0	1077.5	970.0	1183.8	234.69
SF3B1	3975.0	3183.0	3606.5	3996.5	3695.0	3426.5	3647.1	315.47
SF3B3	3555.0	2156.5	2451.5	2976.5	2056.0	2127.5	2553.8	596.48
SF3B4	752.5	653.0	809.5	787.0	698.5	757.5	743.0	57.86
SF3B5	923.5	603.0	382.5	897.5	559.0	373.0	623.1	241.03
SF3B6	1050.0	853.5	693.5	885.5	879.5	672.5	839.1	139.59
SFMBT1	143.0	103.5	183.0	127.0	96.0	205.5	143.0	43.66
SFMBT2	60.0	18.5	88.5	80.0	20.0	68.0	55.8	29.98
SFN	0.0	0.0	0.0	0.0	0.5	0.0	0.1	0.20
SFPQ	1749.0	1245.0	1226.0	1666.5	1317.0	1158.0	1393.6	249.93
SFR1	77.5	22.0	39.5	71.0	25.0	24.0	43.2	24.95
SFRP1	0.5	0.5	6.0	1.0	0.0	0.5	1.4	2.27
SFRP2	9.0	326.0	554.5	15.0	593.0	856.0	392.3	339.20
SFRP4	140.0	140.5	123.5	165.5	200.0	296.5	177.7	64.03
SFRP5	57.5	201.5	194.0	73.0	270.0	363.5	193.3	116.48
SFSWAP	1129.0	1081.0	930.0	940.0	1225.5	920.0	1037.6	126.87
SFT2D1	824.5	549.5	637.5	892.5	614.0	585.0	683.8	140.12
SFT2D2	243.0	64.5	91.0	253.0	64.5	56.5	128.8	93.16
SFXN1	1468.0	968.0	728.0	1390.0	1103.5	714.0	1061.9	321.13
SFXN2	127.5	103.0	126.0	116.5	107.0	156.5	122.8	19.23
SFXN4	385.0	303.5	139.0	374.5	345.5	140.5	281.3	113.25
SFXN5	58.0	28.0	29.0	63.0	42.5	26.0	41.1	16.21
SGCB	661.0	327.5	1041.0	822.0	369.0	975.0	699.3	302.20
SGCD	3691.0	2898.0	2509.5	3310.0	3329.5	2857.0	3099.2	422.99
SGCE	2185.5	1173.5	1614.0	2300.0	1407.5	1689.5	1728.3	438.44
SGCG	1742.0	2776.0	3668.5	1781.0	3446.5	3059.5	2745.6	822.21
SGCZ	0.0	2.5	2.5	0.5	0.0	1.5	1.2	1.17
SGK3	765.0	375.5	542.0	778.5	425.0	469.0	559.2	173.57
SGMS1	404.0	312.5	429.0	464.5	354.5	376.5	390.2	54.25
SGMS2	232.5	68.0	145.5	294.5	65.5	117.5	153.9	92.23
SGOL1	482.5	179.5	260.0	517.0	168.0	155.0	293.7	164.15
SGOL2	19.5	11.5	8.5	16.5	10.0	6.0	12.0	5.08
SGPL1	5685.5	2853.5	3221.0	5805.0	3266.5	3069.5	3983.5	1372.77
SGPP1	2795.5	2156.5	2939.0	2904.0	2790.0	2870.0	2742.5	293.06
SGPP2	6.5	8.0	7.0	9.5	13.0	47.0	15.2	15.77
SGSH	287.0	289.0	306.5	281.0	305.0	313.5	297.0	13.01
SGSM1	16.0	29.5	64.0	13.0	40.0	67.5	38.3	23.37
SGSM2	397.0	223.5	264.0	374.0	246.5	270.5	295.9	71.65
SGSM3	184.0	236.0	186.5	202.5	227.0	226.0	210.3	22.38
SGTA	1860.0	2786.5	2297.5	1754.0	3111.0	2071.5	2313.4	536.15
SGTB	379.0	240.0	208.0	401.5	271.0	259.5	293.2	78.50
SH2B2	91.0	200.5	279.0	85.5	261.0	266.0	197.2	88.61
SH2B3	19.0	23.0	16.5	18.0	23.5	17.0	19.5	3.03
SH2D1A	0.0	0.0	0.0	0.0	0.0	0.0	0.0	0.00
SH2D2A	418.0	420.0	248.5	333.5	490.5	230.0	356.8	103.89

SH2D3C	1559.5	1569.5	1133.0	1298.0	1496.5	1061.5	1353.0	222.04
SH2D4A	184.5	57.5	18.5	184.0	62.5	12.0	86.5	78.36
SH2D4B	2.5	3.0	10.5	1.0	2.5	4.0	3.9	3.37
SH2D5	37.5	13.5	3.0	38.0	16.0	7.0	19.2	15.12
SH3BGR	88.5	1172.0	1182.0	51.0	1406.0	859.5	793.2	586.86
SH3BGRL	3653.5	3810.5	5668.0	3183.0	4592.0	4968.5	4312.6	928.21
SH3BGRL2	130.5	52.5	195.0	183.0	79.0	217.0	142.8	66.67
SH3BGRL3	2351.5	1909.5	1782.5	2237.5	2023.0	1648.5	1992.1	268.02
SH3BP1	0.0	0.5	0.0	0.0	0.5	0.0	0.2	0.26
SH3BP2	48.5	93.5	82.0	58.0	137.0	125.0	90.7	35.37
SH3BP4	458.0	438.5	870.5	520.0	479.5	558.0	554.1	160.90
SH3BP5	333.0	225.0	178.5	311.5	269.5	170.0	247.9	68.06
SH3D19	528.5	927.5	1207.5	600.0	1118.5	1091.0	912.2	285.19
SH3GL1	320.0	192.5	475.5	318.0	197.0	419.0	320.3	114.34
SH3GL3	99.0	99.5	160.0	86.0	115.0	147.5	117.8	29.56
SH3GLB1	2374.0	2452.0	1625.0	2267.5	2671.5	1543.0	2155.5	462.83
SH3GLB2	1921.5	575.0	309.0	1631.5	687.5	269.5	899.0	703.72
SH3KBP1	453.0	323.0	233.5	406.0	318.5	226.0	326.7	90.72
SH3PXD2A	917.0	1035.5	1606.0	829.5	1181.5	1381.5	1158.5	294.01
SH3PXD2B	280.5	506.5	494.5	279.5	616.5	482.0	443.3	135.24
SH3RF1	325.0	396.5	345.5	316.0	454.0	344.0	363.5	52.39
SH3RF3	1121.0	510.5	590.0	956.5	554.5	535.5	711.3	260.19
SH3TC1	0.5	0.5	0.0	0.5	0.5	1.0	0.5	0.32
SH3TC2	0.0	2.5	3.0	0.0	3.0	3.5	2.0	1.58
SH3YL1	18.0	9.0	5.5	13.0	6.5	3.0	9.2	5.50
SHANK2	0.0	0.5	0.5	0.0	0.0	0.5	0.3	0.27
SHANK3	0.5	1.0	0.0	0.5	1.0	0.0	0.5	0.45
SHB	60.0	56.5	89.0	72.5	64.5	78.5	70.2	12.26
SHC1	1146.0	1044.5	928.0	1034.5	942.0	927.5	1003.8	87.41
SHC2	66.5	79.5	77.0	50.5	79.5	79.0	72.0	11.65
SHC3	6.0	3.5	1.0	6.0	1.0	0.5	3.0	2.55
SHC4	10.0	5.0	15.5	14.5	3.5	7.0	9.3	4.97
SHCBP1	256.0	71.0	342.0	271.5	84.5	206.5	205.3	107.95
SHE	0.0	0.0	0.0	0.0	0.0	0.0	0.0	0.00
SHF	1451.0	440.0	346.5	1014.5	385.0	351.5	664.8	462.32
SHFM1	1082.5	1606.0	1071.0	855.5	1670.0	875.0	1193.3	357.83
SHH	1.5	0.5	0.5	0.0	0.0	1.0	0.6	0.58
SHISA2	473.0	5221.5	4906.0	582.0	5926.5	4523.5	3605.4	2428.44
SHISA4	233.0	258.5	231.0	216.5	245.5	216.0	233.4	16.56
SHISA5	48.0	28.5	62.5	63.0	31.5	55.5	48.2	15.13
SHISA6	14.0	7.5	6.5	6.0	6.5	4.0	7.4	3.43
SHISA8	2198.0	1352.5	1222.0	1928.5	1405.0	1175.5	1546.9	417.37
SHISA9	0.0	0.5	0.5	0.0	0.0	0.0	0.2	0.26
SHMT1	148.5	81.0	72.5	135.0	87.0	57.0	96.8	36.48
SHOC2	454.0	446.0	424.0	432.5	512.5	401.5	445.1	37.76
SHOX	0.0	0.0	0.0	0.0	0.0	0.0	0.0	0.00
SHOX2	0.0	0.0	0.0	0.0	0.0	0.0	0.0	0.00
SHPK	229.0	201.5	126.5	243.0	241.0	120.0	193.5	56.43
SHPRH	243.5	157.0	220.5	203.5	190.0	158.0	195.4	34.39
SHQ1	135.0	84.5	65.0	124.0	90.5	78.0	96.2	27.39
SHROOM1	7.5	31.0	85.5	8.0	30.5	85.0	41.3	35.60
SHROOM2	42.0	9.0	9.5	36.5	4.5	3.5	17.5	17.10
SHROOM3	369.5	614.0	783.0	366.0	762.0	688.5	597.2	187.40
SI	0.5	2.0	0.0	0.0	0.5	0.0	0.5	0.77

SIAH1	636.5	748.5	892.5	602.0	860.0	867.5	767.8	125.77
SIAH2	275.5	253.5	246.5	300.0	234.0	259.5	261.5	23.36
SIAH3	1.0	0.0	0.0	1.0	0.0	0.5	0.4	0.49
SIDT1	6.5	18.0	7.5	7.5	29.0	10.5	13.2	8.83
SIDT2	699.0	870.0	1154.0	683.0	851.5	1266.0	920.6	239.45
SIGIRR	0.0	0.0	0.5	0.0	1.0	0.0	0.3	0.42
SIGLEC15	2.5	8.0	15.5	3.5	10.5	5.5	7.6	4.86
SIGMAR1	531.0	545.0	482.5	517.0	508.0	430.5	502.3	41.09
SIK1	1491.5	674.0	1411.0	1499.5	831.5	1339.0	1207.8	360.73
SIK2	161.5	110.5	302.0	147.5	117.5	245.5	180.8	76.56
SIK3	256.5	185.5	276.0	245.0	170.0	278.0	235.2	46.41
SIL1	155.5	121.0	107.0	153.5	119.5	109.0	127.6	21.58
SIM2	1283.0	897.5	1004.5	1338.5	1230.0	1426.5	1196.7	203.93
SIMC1	81.0	87.0	83.5	81.0	123.5	93.5	91.6	16.33
SIN3A	559.5	436.5	591.0	570.0	469.5	593.0	536.6	66.79
SIN3B	831.0	490.5	635.0	805.5	510.0	624.0	649.3	143.44
SIPA1L1	761.0	502.0	784.0	628.5	518.0	736.0	654.9	124.35
SIPA1L2	603.0	557.0	808.5	649.0	618.5	754.5	665.1	96.47
SIRT1	789.0	642.5	810.0	671.0	741.5	785.5	739.9	68.76
SIRT3	254.0	280.0	288.5	224.0	305.0	332.5	280.7	38.11
SIRT4	129.5	55.0	67.5	79.5	71.0	56.5	76.5	27.54
SIRT5	426.5	348.5	431.0	416.0	346.5	408.5	396.2	38.52
SIRT6	787.5	712.5	643.5	784.0	776.0	645.5	724.8	67.98
SIRT7	311.0	240.5	220.5	283.0	231.5	216.5	250.5	38.04
SIX2	103.5	51.5	111.5	101.5	58.5	83.5	85.0	25.07
SIX4	50.0	20.5	112.5	37.5	21.5	81.5	53.9	36.46
SKA2	1217.5	1144.5	824.0	1126.5	1258.0	812.0	1063.8	196.31
SKA3	279.0	156.5	246.5	285.0	153.5	173.5	215.7	61.50
SKAP1	0.0	0.0	0.0	0.0	0.0	0.0	0.0	0.00
SKAP2	682.5	340.5	400.0	585.5	374.5	344.0	454.5	144.03
SKI	120.5	54.0	190.5	112.5	61.5	145.5	114.1	51.47
SKIDA1	37.0	32.0	32.5	33.0	38.5	30.0	33.8	3.24
SKIL	350.0	186.0	505.5	394.5	230.5	478.0	357.4	129.14
SKIV2L	241.5	218.5	243.5	226.5	153.5	238.5	220.3	34.13
SKOR2	0.0	0.0	0.0	0.0	0.0	0.0	0.0	0.00
SKP1	3163.0	2497.5	2930.0	3149.5	2938.5	2923.5	2933.7	240.65
SKP2	825.0	621.0	722.5	821.5	665.5	690.0	724.3	83.54
SLA	0.0	0.0	0.0	0.0	0.0	0.0	0.0	0.00
SLA2	174.0	123.0	74.5	135.5	138.0	164.5	134.9	35.23
SLAIN1	1.0	0.0	0.5	0.5	0.0	1.0	0.5	0.45
SLAIN2	824.0	862.0	1042.5	824.5	1008.5	1043.0	934.1	108.15
SLBP	143.5	74.0	94.5	142.0	74.0	54.0	97.0	37.68
SLC10A2	0.0	0.0	0.0	0.0	0.0	0.0	0.0	0.00
SLC10A4	8.0	36.5	38.0	8.5	53.5	44.5	31.5	18.98
SLC10A7	824.0	414.0	520.5	827.0	443.0	537.5	594.3	184.92
SLC11A1	21.0	11.0	8.5	25.5	8.5	8.5	13.8	7.49
SLC12A1	1.5	0.0	1.0	1.0	0.5	0.5	0.8	0.52
SLC12A3	5.0	5.0	2.0	4.5	4.5	2.5	3.9	1.32
SLC12A4	697.5	251.5	1099.5	721.0	324.0	877.5	661.8	324.14
SLC12A5	49.0	43.0	86.0	49.0	39.5	90.5	59.5	22.61
SLC12A7	340.5	118.0	155.0	250.0	120.0	83.5	177.8	97.99
SLC12A8	2.0	2.0	2.0	1.5	4.5	2.0	2.3	1.08
SLC12A9	376.5	283.0	282.0	325.5	307.5	302.0	312.8	35.21
SLC13A1	5.5	0.5	1.0	13.0	0.0	0.0	3.3	5.17

SLC13A2	0.0	2.5	1.0	0.0	4.5	1.0	1.5	1.73
SLC13A3	50.0	489.5	216.0	54.0	498.5	116.0	237.3	207.69
SLC13A4	3.0	2.0	3.5	2.0	5.0	2.5	3.0	1.14
SLC13A5	0.0	0.0	1.0	0.0	0.0	0.0	0.2	0.41
SLC14A2	0.0	0.0	2.0	1.0	4.0	1.5	1.4	1.50
SLC15A1	3.0	3.0	0.0	2.0	2.0	0.5	1.8	1.25
SLC15A2	10.5	5.5	11.0	11.0	8.0	17.5	10.6	4.02
SLC15A4	670.0	395.0	484.5	572.0	475.0	456.5	508.8	97.36
SLC16A1	2895.0	1349.0	1574.0	2957.0	1364.5	1800.5	1990.0	743.67
SLC16A12	5.0	3.5	2.5	9.5	3.0	1.5	4.2	2.86
SLC16A14	9.0	6.0	5.0	17.5	11.0	6.5	9.2	4.63
SLC16A3	572.0	414.0	1533.5	583.0	621.0	1643.5	894.5	543.33
SLC16A4	35.0	15.0	12.5	40.5	18.0	11.0	22.0	12.55
SLC16A5	17.5	57.0	41.0	24.0	56.5	44.0	40.0	16.37
SLC16A6	18.0	19.0	17.5	22.0	18.0	25.0	19.9	2.97
SLC16A7	1.5	0.0	1.0	0.5	0.5	0.0	0.6	0.58
SLC16A8	1.0	1.0	0.5	1.0	1.5	0.5	0.9	0.38
SLC16A9	76.0	23.0	19.5	52.5	18.5	21.0	35.1	23.83
SLC17A5	491.5	242.0	216.5	511.0	289.5	206.5	326.2	138.76
SLC17A6	1.0	0.5	0.0	0.5	0.5	1.0	0.6	0.38
SLC17A8	4.0	0.5	0.5	1.0	1.0	0.5	1.3	1.37
SLC17A9	36.0	13.0	10.5	39.5	19.0	9.0	21.2	13.34
SLC18A1	10.0	5.0	2.5	15.5	4.0	2.5	6.6	5.17
SLC18A2	0.5	0.5	0.0	0.0	0.0	0.0	0.2	0.26
SLC18A3	0.5	0.0	0.5	0.0	0.0	0.0	0.2	0.26
SLC18B1	35.5	17.5	25.0	33.0	24.0	12.5	24.6	8.79
SLC19A1	561.0	324.0	240.5	556.5	307.5	207.0	366.1	155.26
SLC19A3	59.0	107.5	44.0	62.5	104.0	57.5	72.4	26.60
SLC1A1	0.0	0.0	0.0	0.0	0.0	0.0	0.0	0.00
SLC1A2	71.0	91.0	327.5	105.5	119.5	456.5	195.2	158.64
SLC1A3	0.0	0.0	0.0	0.0	0.0	0.5	0.1	0.20
SLC1A4	656.5	394.5	773.5	791.5	406.0	538.0	593.3	175.16
SLC1A7	44.0	21.5	28.5	43.0	39.0	22.0	33.0	10.30
SLC20A1	859.5	535.0	461.0	819.5	542.5	427.5	607.5	185.36
SLC20A2	523.0	503.0	503.0	639.5	550.0	444.0	527.1	65.18
SLC22A15	67.0	20.0	63.0	67.0	25.5	40.5	47.2	21.40
SLC22A16	2257.5	918.5	798.0	2382.5	980.5	574.0	1318.5	789.08
SLC22A23	77.5	79.5	217.5	43.5	64.0	98.5	96.8	61.90
SLC22A3	68.5	64.0	165.0	126.0	76.0	139.0	106.4	42.52
SLC22A4	483.5	297.0	334.5	558.0	404.5	401.0	413.1	95.74
SLC22A5	37.5	14.5	80.5	57.0	19.5	81.0	48.3	29.22
SLC22A7	2.0	2.0	1.5	4.5	1.5	1.5	2.2	1.17
SLC23A1	111.0	172.5	96.5	94.0	201.5	104.0	129.9	45.55
SLC23A2	87.5	31.0	164.5	88.0	31.5	118.5	86.8	51.41
SLC23A3	0.0	0.0	0.0	0.0	0.0	0.0	0.0	0.00
SLC24A1	126.5	111.0	69.0	127.0	117.5	85.0	106.0	23.78
SLC24A2	1.0	1.5	0.5	0.0	1.0	0.5	0.8	0.52
SLC24A3	138.5	117.0	110.0	130.0	114.0	115.5	120.8	10.98
SLC24A4	3.5	11.0	7.5	4.5	9.5	4.5	6.8	3.06
SLC24A5	192.5	174.0	175.5	172.0	215.0	157.0	181.0	20.12
SLC25A1	1375.0	1354.5	1428.5	1459.5	1455.0	1357.5	1405.0	48.43
SLC25A10	343.0	286.0	175.0	349.5	260.5	165.0	263.2	79.71
SLC25A13	336.5	321.5	345.5	442.5	412.0	508.0	394.3	72.92
SLC25A14	453.0	664.5	508.5	469.5	762.5	551.5	568.3	121.56

SLC25A15	447.0	241.5	176.0	394.0	238.0	157.5	275.7	118.19
SLC25A16	258.5	197.0	214.5	288.5	239.0	213.0	235.1	34.00
SLC25A17	334.0	198.0	188.0	333.5	201.5	183.0	239.7	73.18
SLC25A19	140.0	108.0	208.5	128.0	99.0	146.0	138.3	38.88
SLC25A20	964.5	1216.5	940.5	1002.5	1385.0	877.5	1064.4	194.94
SLC25A21	57.0	20.5	11.5	58.5	24.5	15.0	31.2	21.08
SLC25A22	170.0	91.0	156.0	171.5	78.0	146.5	135.5	40.78
SLC25A24	2623.0	1238.5	1131.5	2426.5	1301.0	995.0	1619.3	711.76
SLC25A25	175.5	77.5	197.0	196.5	100.0	181.0	154.6	52.18
SLC25A26	163.5	218.0	172.5	166.0	240.5	167.5	188.0	32.87
SLC25A28	876.5	856.5	450.0	720.0	927.5	508.0	723.1	201.97
SLC25A29	481.0	251.0	160.0	406.5	280.0	173.0	291.9	128.25
SLC25A3	6983.5	5268.0	3945.0	6480.5	5948.0	3255.0	5313.3	1460.52
SLC25A30	25.0	49.5	170.0	21.5	59.0	99.0	70.7	56.14
SLC25A32	336.5	245.5	287.0	298.0	242.5	269.0	279.8	35.45
SLC25A33	320.0	255.0	249.0	261.5	243.5	196.5	254.3	39.63
SLC25A34	10.5	15.0	8.0	16.0	18.0	12.5	13.3	3.71
SLC25A36	349.5	218.5	612.0	335.0	266.5	557.0	389.8	159.06
SLC25A37	64.5	40.0	56.0	59.0	31.0	58.5	51.5	13.02
SLC25A38	360.0	439.0	554.0	387.5	572.0	622.5	489.2	108.05
SLC25A39	809.5	789.5	614.0	767.5	776.0	655.5	735.3	80.27
SLC25A4	252.0	548.5	462.0	218.0	561.5	324.5	394.4	150.01
SLC25A40	1.5	1.5	1.0	2.0	2.5	0.5	1.5	0.71
SLC25A42	180.5	135.5	152.5	145.5	163.5	175.0	158.8	17.41
SLC25A43	525.0	439.0	605.5	504.5	494.5	666.0	539.1	82.36
SLC25A44	104.5	104.5	103.0	91.5	111.0	120.0	105.8	9.43
SLC25A46	849.0	703.5	559.5	809.5	803.0	543.5	711.3	132.87
SLC25A47	73.0	20.5	20.5	84.5	20.0	8.5	37.8	32.23
SLC25A48	0.0	7.5	5.5	0.0	11.5	5.0	4.9	4.44
SLC25A5	1686.0	1765.5	1166.5	1711.5	1988.5	1243.0	1593.5	320.40
SLC25A51	412.0	340.5	411.0	394.0	379.5	396.0	388.8	26.57
SLC25A6	22987.0	15344.0	11258.5	20201.0	15677.5	10050.0	15919.7	4996.93
SLC26A1	22.5	29.5	29.5	12.5	34.0	23.0	25.2	7.59
SLC26A11	284.5	158.5	145.0	260.0	166.0	132.5	191.1	64.38
SLC26A2	75.0	21.5	44.0	72.5	27.0	33.0	45.5	23.13
SLC26A3	4.0	3.0	6.0	5.5	5.0	4.5	4.7	1.08
SLC26A5	951.5	349.0	308.0	1030.0	353.5	282.5	545.8	346.59
SLC26A6	2.5	3.0	0.5	2.5	1.5	1.0	1.8	0.98
SLC26A7	0.0	0.0	0.0	0.5	0.5	0.0	0.2	0.26
SLC26A8	202.5	176.5	115.0	176.0	204.0	127.0	166.8	37.69
SLC26A9	0.0	0.0	0.0	0.0	0.0	0.5	0.1	0.20
SLC27A1	169.0	90.0	67.0	120.0	103.5	76.5	104.3	36.88
SLC27A4	996.5	616.0	645.5	1007.0	622.0	651.5	756.4	190.54
SLC28A2	57.5	31.0	14.5	42.5	27.0	18.5	31.8	15.97
SLC28A3	2.0	0.0	0.5	0.0	0.0	0.5	0.5	0.77
SLC29A1	519.5	419.0	323.5	499.5	493.5	327.0	430.3	88.25
SLC29A3	21.5	12.0	18.5	24.5	10.0	15.0	16.9	5.60
SLC29A4	14.0	8.5	8.5	7.0	10.5	7.5	9.3	2.58
SLC2A1	542.0	380.0	708.5	605.5	446.0	738.0	570.0	142.07
SLC2A10	301.5	369.0	271.5	299.0	388.5	263.0	315.4	51.68
SLC2A12	9.5	24.5	77.5	11.0	29.5	56.5	34.8	26.96
SLC2A2	1.0	0.0	3.0	0.0	0.5	1.0	0.9	1.11
SLC2A4RG	48.5	25.5	28.0	48.0	29.0	35.5	35.8	10.23
SLC2A5	7.0	9.5	15.0	7.0	6.5	13.5	9.8	3.67

SLC2A6	102.5	179.5	456.0	128.5	205.0	541.0	268.8	183.59
SLC2A8	1029.5	735.0	778.5	952.0	859.0	664.5	836.4	137.36
SLC30A1	173.5	89.0	150.0	185.0	101.0	130.5	138.2	38.57
SLC30A10	15.5	7.0	5.5	11.0	7.5	10.5	9.5	3.62
SLC30A2	56.5	17.5	15.0	23.5	16.5	9.5	23.1	16.98
SLC30A3	1.5	1.0	1.5	0.5	0.0	1.0	0.9	0.58
SLC30A4	661.5	439.5	717.0	715.5	551.5	642.5	621.3	107.72
SLC30A5	1363.5	985.5	912.5	1367.0	1190.0	871.0	1114.9	222.80
SLC30A6	322.0	272.0	347.0	332.5	309.0	317.5	316.7	25.51
SLC30A7	216.5	92.0	125.5	249.0	97.0	113.0	148.8	66.87
SLC30A8	0.0	2.0	3.0	0.0	3.5	0.5	1.5	1.55
SLC30A9	1071.0	921.5	903.0	993.0	1061.0	910.0	976.6	76.41
SLC31A1	658.5	402.0	348.5	654.0	407.0	375.0	474.2	142.59
SLC31A2	51.5	58.0	53.5	59.5	67.0	68.5	59.7	6.92
SLC32A1	0.0	0.0	0.5	0.0	0.0	1.0	0.3	0.42
SLC33A1	759.5	536.5	503.0	751.5	550.5	501.5	600.4	121.64
SLC34A1	1.0	0.5	0.5	1.0	0.5	0.0	0.6	0.38
SLC34A2	0.0	0.5	0.0	1.0	1.5	0.0	0.5	0.63
SLC35A1	240.0	155.0	143.5	234.5	168.5	144.0	180.9	44.61
SLC35A3	187.5	102.5	139.5	207.5	100.5	104.0	140.3	47.06
SLC35A4	235.0	321.5	278.5	226.5	307.5	276.0	274.2	37.89
SLC35A5	652.0	382.0	317.5	595.5	441.5	352.5	456.8	136.71
SLC35B1	831.0	672.0	681.0	826.0	766.5	723.0	749.9	69.55
SLC35B2	533.5	285.0	421.0	521.0	285.0	368.0	402.3	109.86
SLC35B3	367.0	159.0	183.5	330.0	187.5	164.0	231.8	91.78
SLC35B4	881.5	542.0	590.0	752.5	560.0	499.5	637.6	147.78
SLC35C1	363.5	296.0	232.0	361.5	307.0	255.0	302.5	53.83
SLC35C2	1116.0	397.0	234.0	887.0	447.0	270.5	558.6	359.30
SLC35D1	272.5	143.0	180.5	215.0	152.0	122.0	180.8	55.31
SLC35D2	23.5	11.5	9.0	30.0	16.0	14.0	17.3	7.94
SLC35D3	0.0	0.5	1.5	0.5	1.0	3.5	1.2	1.25
SLC35E1	589.5	393.0	457.5	624.0	436.0	414.0	485.7	96.85
SLC35E3	1741.0	1852.5	2185.0	2224.0	2345.5	2355.0	2117.2	259.33
SLC35E4	70.0	92.5	95.0	65.0	74.5	120.0	86.2	20.53
SLC35F1	0.5	0.5	0.0	0.0	0.5	2.5	0.7	0.93
SLC35F2	165.0	45.5	27.5	154.5	38.5	24.5	75.9	65.46
SLC35F3	8.0	10.0	7.5	6.0	13.0	19.0	10.6	4.78
SLC35F4	0.0	0.0	0.0	0.0	0.5	0.0	0.1	0.20
SLC35F5	199.0	267.5	389.5	193.0	335.5	291.0	279.3	76.78
SLC35F6	986.0	647.0	406.0	928.5	582.0	422.0	661.9	247.29
SLC35G1	75.5	18.0	119.5	84.0	30.0	84.0	68.5	37.85
SLC35G2	337.0	503.0	696.0	322.0	585.5	818.5	543.7	196.97
SLC36A4	56.0	83.0	112.0	58.0	106.5	89.5	84.2	23.59
SLC37A1	298.0	126.5	145.5	271.5	123.0	109.0	178.9	83.22
SLC37A2	16.5	26.0	17.0	11.5	30.0	23.5	20.8	6.90
SLC37A3	396.5	395.5	642.5	443.5	442.0	553.0	478.8	98.61
SLC37A4	333.5	208.0	223.5	319.0	246.0	196.5	254.4	58.24
SLC38A1	681.5	358.0	902.5	648.5	434.5	807.5	638.8	210.00
SLC38A10	4802.0	2162.0	3031.0	5062.5	2178.5	2708.5	3324.1	1291.04
SLC38A11	0.0	0.5	0.5	0.5	0.0	0.0	0.3	0.27
SLC38A3	6.5	6.0	21.5	4.0	2.0	25.0	10.8	9.81
SLC38A6	1954.5	915.0	1112.0	1588.5	985.0	883.0	1239.7	435.07
SLC38A7	738.0	526.0	509.0	915.0	559.0	593.0	640.0	157.58
SLC38A8	11.5	3.0	3.0	13.0	4.5	3.0	6.3	4.64

SLC38A9	138.0	45.5	91.0	135.0	54.5	86.5	91.8	38.90
SLC39A10	882.0	498.0	774.0	734.0	573.0	761.0	703.7	141.65
SLC39A12	2.5	1.5	0.0	0.5	0.0	0.0	0.8	1.04
SLC39A13	2377.5	2456.0	2977.0	2674.0	2843.0	3030.5	2726.3	270.73
SLC39A14	202.0	238.0	302.0	173.5	239.0	383.5	256.3	75.77
SLC39A3	117.5	255.5	256.0	124.0	235.0	267.5	209.3	69.38
SLC39A6	357.5	260.5	548.5	384.0	263.5	510.5	387.4	121.22
SLC39A8	13.5	8.5	17.5	10.5	7.0	20.5	12.9	5.28
SLC39A9	69.5	17.0	147.0	83.0	26.5	124.5	77.9	51.76
SLC3A1	111.0	64.5	67.5	116.5	87.0	68.0	85.8	23.17
SLC40A1	5347.0	924.5	1194.0	3665.0	855.0	1094.5	2180.0	1882.42
SLC41A1	45.0	33.5	93.0	65.5	38.5	114.5	65.0	32.65
SLC41A2	290.0	126.5	442.5	358.0	140.0	388.0	290.8	131.69
SLC41A3	69.5	167.5	155.0	74.5	172.5	192.5	138.6	52.99
SLC43A2	403.5	88.0	94.0	304.5	115.0	90.0	182.5	136.82
SLC43A3	816.5	258.5	173.0	634.0	226.0	191.0	383.2	272.78
SLC44A1	629.5	224.0	498.5	668.5	243.0	504.0	461.3	188.89
SLC44A3	5.5	6.5	10.5	7.0	4.0	7.0	6.8	2.16
SLC44A5	23.5	6.5	34.0	26.5	8.5	18.0	19.5	10.65
SLC45A1	592.0	413.0	490.0	531.5	462.5	484.0	495.5	61.10
SLC45A2	2.5	2.5	0.5	3.0	2.0	2.5	2.2	0.88
SLC45A3	16.0	47.5	62.0	14.0	59.0	99.0	49.6	31.88
SLC45A4	315.5	118.0	246.5	296.5	118.0	220.5	219.2	85.42
SLC46A1	27.5	20.0	35.0	22.5	26.0	37.5	28.1	6.90
SLC46A2	20.0	24.5	19.5	30.0	33.5	26.0	25.6	5.51
SLC46A3	33.0	10.5	27.5	24.0	15.0	20.0	21.7	8.24
SLC48A1	366.5	308.5	388.5	322.5	329.0	358.5	345.6	30.43
SLC4A10	12.0	6.5	17.0	19.0	5.0	15.0	12.4	5.68
SLC4A11	109.0	24.5	108.5	74.0	16.5	61.0	65.6	39.79
SLC4A1AP	396.5	276.5	262.5	368.0	333.5	249.5	314.4	60.53
SLC4A2	914.5	1044.5	1014.5	840.5	821.0	1074.0	951.5	108.02
SLC4A3	120.5	159.0	181.5	103.5	136.0	180.5	146.8	32.16
SLC4A5	2.0	2.5	1.5	2.0	2.5	2.5	2.2	0.41
SLC4A7	419.5	207.5	445.5	423.5	234.0	321.5	341.9	103.47
SLC4A8	17.0	36.5	41.5	7.5	30.5	49.0	30.3	15.56
SLC4A9	1.0	0.5	0.0	1.5	0.0	0.0	0.5	0.63
SLC51A	1.0	0.5	0.5	0.5	0.5	0.0	0.5	0.32
SLC51B	0.5	0.0	0.5	1.5	0.0	0.5	0.5	0.55
SLC52A3	108.5	89.0	73.0	115.5	76.0	65.5	87.9	20.26
SLC5A1	12.0	10.0	1.5	6.0	8.5	2.0	6.7	4.29
SLC5A10	0.0	0.0	0.0	0.0	0.0	0.0	0.0	0.00
SLC5A11	0.0	0.0	0.5	0.5	0.0	0.0	0.2	0.26
SLC5A12	0.0	0.0	0.0	0.0	0.0	0.0	0.0	0.00
SLC5A3	20.0	4.0	45.0	28.5	5.5	29.0	22.0	15.62
SLC5A5	0.5	0.0	1.0	0.5	0.5	1.0	0.6	0.38
SLC5A6	531.5	493.5	374.0	519.0	416.5	348.0	447.1	78.16
SLC5A7	433.0	223.5	368.0	461.5	233.0	300.5	336.6	100.70
SLC5A8	1.5	2.5	1.0	1.5	2.0	2.0	1.8	0.52
SLC5A9	371.5	154.0	145.5	281.0	177.0	139.5	211.4	94.30
SLC6A1	6.0	1.5	4.0	4.5	2.5	0.5	3.2	2.04
SLC6A11	0.0	0.0	0.5	0.0	1.0	0.0	0.3	0.42
SLC6A12	0.0	0.0	0.0	0.0	0.0	0.5	0.1	0.20
SLC6A14	0.0	0.0	0.0	0.0	0.0	0.0	0.0	0.00
SLC6A15	0.5	0.5	2.0	0.0	1.0	4.0	1.3	1.47

SLC6A17	0.0	5.0	18.0	0.5	1.0	17.5	7.0	8.51
SLC6A18	0.0	0.0	0.0	0.0	0.0	0.0	0.0	0.00
SLC6A19	0.0	0.0	0.0	0.0	0.5	0.0	0.1	0.20
SLC6A2	3.5	0.5	0.5	1.5	0.5	0.0	1.1	1.28
SLC6A20	0.0	0.5	1.0	0.0	0.0	0.5	0.3	0.41
SLC6A4	1.0	0.5	1.0	2.0	2.0	1.5	1.3	0.61
SLC6A5	4.5	2.0	1.5	1.5	1.0	1.0	1.9	1.32
SLC6A6	173.5	54.0	224.0	164.0	74.0	287.0	162.8	88.33
SLC6A7	117.5	657.5	346.5	97.5	778.5	307.0	384.1	279.60
SLC6A8	1978.0	2544.0	2553.5	1778.0	2340.0	2057.0	2208.4	319.47
SLC6A9	1387.0	1023.5	1168.0	1263.0	926.5	857.0	1104.2	204.19
SLC7A1	655.0	537.5	799.5	707.0	635.5	793.0	687.9	100.31
SLC7A10	7.0	9.5	25.5	4.5	12.5	14.5	12.3	7.43
SLC7A11	154.0	84.5	106.5	166.0	83.5	51.5	107.7	44.33
SLC7A14	0.0	1.5	0.5	0.0	2.5	6.5	1.8	2.48
SLC7A3	350.5	364.0	358.0	376.5	328.0	329.5	351.1	19.29
SLC7A4	23.5	10.0	4.0	27.5	13.0	7.0	14.2	9.36
SLC7A6	1059.0	633.5	431.0	1031.0	698.0	387.0	706.6	287.38
SLC7A6OS	210.5	143.5	189.5	219.0	153.0	192.5	184.7	30.42
SLC7A9	0.5	0.5	0.0	0.5	0.0	1.0	0.4	0.38
SLC8A2	1.0	0.0	0.5	0.0	0.0	0.5	0.3	0.41
SLC8A3	104.0	66.5	88.0	87.0	63.0	105.5	85.7	17.99
SLC8B1	106.5	91.5	94.5	63.5	83.0	76.0	85.8	15.09
SLC9A1	1193.5	790.5	877.5	1102.5	752.0	795.0	918.5	184.67
SLC9A2	4.5	26.5	11.5	4.0	27.5	6.5	13.4	10.86
SLC9A3	0.0	0.0	0.0	0.0	0.0	0.5	0.1	0.20
SLC9A3R1	71.0	52.0	63.0	53.5	47.0	41.0	54.6	10.86
SLC9A3R2	371.5	94.5	92.0	306.0	98.5	98.0	176.8	127.20
SLC9A4	0.0	0.0	0.0	0.0	0.0	0.0	0.0	0.00
SLC9A5	7.0	13.5	9.5	8.5	15.5	14.0	11.3	3.44
SLC9A6	1099.0	634.5	734.5	981.5	693.5	630.5	795.6	196.97
SLC9A7	187.0	122.0	128.5	206.5	147.0	115.5	151.1	37.42
SLC9A8	745.5	479.5	436.5	704.5	573.0	416.0	559.2	139.95
SLC9B2	747.0	476.5	763.0	768.5	509.5	789.5	675.7	142.53
SLCO1A2	0.0	0.0	0.0	0.5	0.0	1.0	0.3	0.42
SLCO2A1	1.5	1.0	1.0	0.5	1.0	1.5	1.1	0.38
SLCO2B1	0.0	1.5	0.5	0.0	0.0	0.5	0.4	0.58
SLCO3A1	1.0	0.5	1.0	2.5	0.5	2.5	1.3	0.93
SLCO4A1	23.5	36.5	42.0	18.0	41.0	67.0	38.0	17.20
SLCO4C1	0.0	0.0	0.0	0.0	0.0	0.0	0.0	0.00
SLCO5A1	102.5	78.5	127.0	73.0	51.0	50.0	80.3	30.02
SLIRP	990.0	1015.0	458.0	928.5	1084.5	435.5	818.6	292.41
SLIT1	1893.5	1448.5	2754.0	1472.0	1499.0	4641.5	2284.8	1257.36
SLIT2	158.5	35.5	219.5	83.5	30.5	93.5	103.5	73.39
SLIT3	2144.5	351.0	1252.5	755.0	156.0	252.0	818.5	765.38
SLITRK1	0.0	0.0	0.0	0.0	0.0	0.0	0.0	0.00
SLITRK2	323.0	312.0	428.0	281.5	345.5	407.5	349.6	57.05
SLITRK3	0.0	0.0	0.0	0.0	0.0	0.0	0.0	0.00
SLITRK4	0.0	1.0	3.0	0.0	1.0	3.0	1.3	1.37
SLITRK5	0.0	0.0	0.0	0.0	0.0	0.5	0.1	0.20
SLITRK6	0.5	0.0	0.0	0.5	0.5	0.0	0.3	0.27
SLK	255.0	101.5	495.5	272.0	140.5	411.5	279.3	152.15
SLMAP	1166.0	931.5	943.0	1048.5	1085.5	877.5	1008.7	109.28
SLMO1	837.0	886.0	680.0	736.0	917.5	631.5	781.3	116.08

SLMO2	3113.5	2180.5	1896.0	3228.0	2382.5	1754.0	2425.8	618.14
SLN	5.5	164.0	18.5	2.0	163.0	32.0	64.2	77.67
SLTM	1985.0	1684.5	1638.5	1668.0	1884.5	1591.5	1742.0	155.88
SLU7	503.0	373.0	542.5	495.0	443.5	511.0	478.0	60.63
SLX4	461.5	303.5	440.0	482.5	356.0	408.0	408.6	67.91
SLX4IP	86.0	39.5	43.0	81.0	39.5	39.5	54.8	22.37
SMAD1	601.0	634.0	854.0	607.0	804.0	977.5	746.3	155.89
SMAD3	1699.0	1008.5	2042.0	1330.5	982.5	1685.5	1458.0	423.15
SMAD5	1370.5	1165.0	1468.0	1499.5	1407.5	1534.0	1407.4	132.89
SMAD6	29.0	44.5	49.0	30.0	48.0	53.0	42.3	10.25
SMAD7	14.5	10.0	17.0	9.5	13.5	14.5	13.2	2.89
SMAD9	381.5	502.5	656.0	493.0	605.5	775.5	569.0	139.21
SMAGP	0.0	0.0	0.0	0.0	0.0	0.0	0.0	0.00
SMAP1	1368.5	1044.0	765.0	1408.0	1216.5	815.0	1102.8	274.61
SMAP2	433.5	431.5	391.5	417.0	524.0	450.5	441.3	45.06
SMARCA1	540.5	321.0	478.5	541.0	356.5	438.5	446.0	92.42
SMARCA5	1356.5	889.5	1110.0	1265.5	1018.5	905.0	1090.8	190.66
SMARCAD1	483.0	244.0	338.5	455.5	292.5	302.0	352.6	95.66
SMARCAL1	225.0	83.5	138.5	215.0	93.5	117.5	145.5	60.88
SMARCB1	915.5	807.0	734.5	947.5	913.0	729.0	841.1	97.08
SMARCD1	862.0	759.5	683.5	808.5	789.0	722.5	770.8	63.49
SMARCD2	438.0	325.0	570.5	431.0	322.5	567.5	442.4	109.88
SMARCD3	3216.5	3150.5	2320.0	2719.5	3672.5	2205.5	2880.8	567.23
SMARCE1	2161.0	1970.5	2104.5	2041.5	1991.0	2009.5	2046.3	73.11
SMC2	1845.5	770.5	1936.0	1849.5	704.0	1005.0	1351.8	584.90
SMC3	1324.0	791.0	1198.0	1273.0	841.5	959.0	1064.4	229.91
SMC4	899.0	491.0	1173.0	890.5	495.5	850.5	799.9	263.77
SMC5	629.5	364.5	322.0	568.0	425.0	311.0	436.7	133.20
SMC6	519.0	175.0	194.5	466.5	193.0	138.0	281.0	166.11
SMCHD1	456.0	282.0	579.0	395.0	325.5	518.0	425.9	113.64
SMCO3	3.5	0.5	1.5	3.0	1.5	1.0	1.8	1.17
SMCO4	120.5	161.0	159.5	134.5	189.5	219.0	164.0	35.96
SMCR8	253.0	166.5	253.0	239.0	153.5	226.0	215.2	44.09
SMDT1	264.0	495.5	360.0	267.5	555.5	354.0	382.8	119.40
SMEK1	476.5	473.5	520.0	429.0	537.0	459.5	482.6	39.71
SMEK2	1106.5	988.0	1223.0	1100.0	1063.0	1165.0	1107.6	81.20
SMG1	1068.0	792.5	1234.5	1044.0	928.0	1182.0	1041.5	162.84
SMG5	177.0	167.0	268.5	161.5	117.0	237.0	188.0	55.11
SMG6	259.5	315.5	637.0	255.0	314.0	582.5	393.9	170.03
SMG7	1100.0	936.0	1290.5	1062.0	1072.5	1167.5	1104.8	118.16
SMG8	448.5	366.5	325.5	427.0	360.0	301.0	371.4	57.01
SMIM11	919.0	716.5	651.0	668.5	680.0	505.0	690.0	133.75
SMIM12	282.0	270.0	138.0	293.5	268.5	109.0	226.8	81.07
SMIM13	891.0	545.0	580.5	828.5	568.5	481.5	649.2	167.83
SMIM14	2159.5	1875.5	1811.0	2086.0	2117.5	1805.0	1975.8	162.70
SMIM15	1368.5	947.0	923.0	1306.0	1043.5	847.0	1072.5	215.37
SMIM18	46.5	41.5	51.5	33.0	37.0	43.5	42.2	6.62
SMIM19	458.5	733.5	406.0	491.5	808.5	469.0	561.2	166.64
SMIM20	501.0	431.0	227.5	439.5	450.5	230.0	379.9	119.59
SMIM24	0.5	1.0	0.5	0.5	1.5	0.0	0.7	0.52
SMIM3	244.5	289.0	150.0	274.5	365.5	196.0	253.3	75.29
SMIM4	292.5	314.5	199.5	249.0	307.0	181.5	257.3	56.81
SMIM5	61.0	56.0	56.0	43.0	63.5	60.5	56.7	7.32
SMIM8	195.5	210.5	96.5	172.5	228.0	103.5	167.8	55.60

SMNDC1	811.0	629.0	743.0	865.5	784.5	685.5	753.1	86.08
SMO	1594.0	1178.5	1597.5	1595.5	1172.5	1515.5	1442.3	208.95
SMOC1	63.0	19.0	17.5	60.0	24.0	10.0	32.3	23.12
SMOC2	91.0	11.0	14.5	86.5	13.0	11.0	37.8	39.49
SMOX	369.0	286.0	519.0	319.5	363.5	612.5	411.6	126.71
SMPD1	494.5	470.5	494.0	532.0	457.0	561.0	501.5	38.77
SMPD2	290.5	212.5	146.0	254.5	203.0	148.0	209.1	57.36
SMPD3	18.0	9.0	7.5	20.0	13.5	3.0	11.8	6.52
SMPD4	427.0	257.5	294.5	392.5	283.0	261.5	319.3	72.18
SMPDL3A	65.0	45.5	77.5	112.5	40.0	82.5	70.5	26.62
SMPDL3B	226.5	203.0	141.0	220.0	228.0	171.5	198.3	35.13
SMPX	409.0	430.0	311.0	354.0	468.5	232.5	367.5	86.52
SMS	144.5	82.0	197.0	180.5	110.5	164.0	146.4	43.53
SMTN	854.5	2360.0	1721.5	811.0	2416.5	1840.5	1667.3	702.56
SMTNL1	20.5	22.5	18.5	13.5	22.0	26.5	20.6	4.36
SMURF2	435.0	484.0	876.5	467.0	587.5	894.0	624.0	208.79
SMYD1	3893.5	2557.5	2764.0	3594.0	2927.0	2667.0	3067.2	546.23
SMYD2	978.0	826.0	416.5	847.5	931.0	405.5	734.1	256.28
SMYD4	395.5	308.5	252.5	397.5	307.5	225.5	314.5	71.13
SMYD5	552.0	312.5	606.5	570.0	308.0	506.5	475.9	132.31
SNAI1	1598.5	825.0	1038.0	1337.0	947.5	868.5	1102.4	303.63
SNAI2	765.5	1089.0	1105.5	917.5	1402.0	1317.5	1099.5	238.27
SNAP23	1193.0	818.5	1117.5	1214.0	1004.0	1098.0	1074.2	145.88
SNAP25	1.0	0.5	0.5	0.0	0.0	1.5	0.6	0.58
SNAP29	140.5	50.5	232.5	158.5	60.0	158.5	133.4	68.40
SNAP47	386.0	242.0	256.0	284.5	282.0	249.0	283.3	53.25
SNAP91	8.5	15.0	6.5	11.5	22.5	7.0	11.8	6.11
SNAPC1	338.0	286.5	261.0	310.0	315.5	237.5	291.4	37.29
SNAPC3	2.5	1.0	2.0	0.5	1.0	2.0	1.5	0.77
SNAPC5	1238.5	1842.0	1307.0	1174.5	2023.0	1272.5	1476.3	360.68
SNAPIN	324.0	494.5	233.5	301.0	516.5	244.5	352.3	123.57
SNCA	0.5	0.0	0.0	0.0	0.0	0.0	0.1	0.20
SNCAIP	51.0	167.0	237.0	85.0	196.0	251.5	164.6	81.27
SNCB	319.0	229.0	82.0	446.0	327.5	167.0	261.8	129.54
SNCG	76.5	68.0	32.5	84.5	89.0	58.0	68.1	20.72
SNED1	190.0	118.5	104.5	173.0	127.0	147.5	143.4	33.07
SNF8	662.5	659.0	412.5	626.0	688.0	375.5	570.6	138.69
SNIP1	364.5	341.5	370.5	372.5	367.0	334.5	358.4	16.21
SNN	33.0	19.5	28.5	28.5	23.5	16.0	24.8	6.35
SNPH	0.0	0.0	0.0	0.0	0.0	0.0	0.0	0.00
SNRK	389.0	259.5	366.5	361.5	308.0	309.5	332.3	48.26
SNRNP200	2551.0	2550.5	2723.5	2321.5	2475.5	2993.0	2602.5	231.33
SNRNP27	359.0	440.5	286.5	325.0	415.0	285.0	351.8	65.36
SNRNP35	1084.0	1140.5	1152.0	979.0	1357.0	1112.5	1137.5	124.10
SNRNP40	688.0	494.5	397.0	632.0	483.0	353.0	507.9	130.33
SNRNP48	126.0	113.5	79.5	134.0	92.5	59.0	100.8	28.87
SNRPA1	598.5	493.0	362.0	569.0	512.5	357.5	482.1	102.07
SNRPB	1344.5	1030.5	754.5	1204.5	954.5	675.5	994.0	256.46
SNRPB2	777.0	740.0	554.0	783.5	798.5	509.5	693.8	127.73
SNRPC	1919.0	1901.0	1135.5	1746.5	1982.0	1072.5	1626.1	412.23
SNRPD1	1261.5	1247.5	768.0	1138.5	1177.0	687.5	1046.7	252.41
SNRPD3	2377.5	2322.5	1702.0	2386.5	2577.5	1644.0	2168.3	393.68
SNRPE	1242.0	1365.5	836.0	1240.0	1445.5	817.5	1157.8	268.00
SNRPF	1557.5	1429.0	527.0	1451.5	1414.0	532.0	1151.8	484.67

SNRPG	748.5	972.5	595.0	704.0	981.5	553.0	759.1	183.06
SNTA1	152.5	203.5	274.5	185.0	190.0	254.5	210.0	45.86
SNTB1	46.5	19.5	25.0	30.0	15.0	12.5	24.8	12.44
SNTB2	106.0	116.0	86.0	105.5	113.0	111.0	106.3	10.71
SNTG2	0.0	0.0	0.0	0.5	0.0	0.0	0.1	0.20
SNTN	0.0	0.0	0.0	0.0	0.0	0.0	0.0	0.00
SNUPN	285.0	218.5	154.0	270.5	208.0	123.0	209.8	63.35
SNW1	964.5	535.0	667.0	893.5	625.0	661.5	724.4	166.88
SNX1	1912.0	1946.0	2397.5	1838.5	2113.0	2285.5	2082.1	222.98
SNX10	288.0	127.5	76.0	303.5	144.5	87.5	171.2	99.84
SNX11	876.0	1299.0	647.5	729.0	1230.5	639.0	903.5	293.28
SNX12	1109.5	1667.5	1540.5	1025.5	1735.5	1517.0	1432.6	295.23
SNX13	278.0	107.0	365.5	261.0	133.0	325.5	245.0	103.86
SNX14	589.0	320.5	410.0	579.5	364.0	324.0	431.2	122.97
SNX16	127.5	98.5	93.0	126.5	110.5	77.5	105.6	19.69
SNX17	1395.5	1108.0	1196.0	1367.0	1072.5	1210.5	1224.9	132.08
SNX18	141.0	96.0	150.5	127.5	110.5	129.5	125.8	19.90
SNX19	307.5	412.0	342.5	310.0	502.0	349.5	370.6	74.66
SNX2	826.0	798.0	1046.0	710.0	929.0	912.0	870.2	117.42
SNX20	0.5	3.5	0.5	0.5	2.0	1.0	1.3	1.21
SNX22	73.5	34.0	13.5	61.0	43.5	19.0	40.8	23.46
SNX24	97.5	68.0	86.0	95.5	85.0	68.5	83.4	12.76
SNX25	984.5	455.5	493.0	846.5	534.5	507.5	636.9	221.63
SNX27	558.5	665.0	705.5	519.5	722.0	678.0	641.4	82.75
SNX3	2084.0	2087.0	2143.0	1888.0	2274.0	1861.5	2056.3	156.77
SNX30	643.0	680.0	546.0	577.5	814.0	554.5	635.8	101.82
SNX33	349.0	414.0	441.5	349.0	411.5	461.0	404.3	46.61
SNX4	698.0	429.5	404.5	752.5	470.5	354.0	518.2	165.68
SNX5	278.0	176.5	167.5	294.0	193.0	148.0	209.5	61.22
SNX6	826.5	539.0	870.0	790.0	565.0	737.5	721.3	138.44
SNX7	1006.0	797.5	900.5	987.0	942.0	907.5	923.4	74.62
SNX8	580.5	524.5	556.5	641.5	630.0	605.0	589.7	44.68
SOAT1	311.5	147.0	294.5	352.0	183.5	281.0	261.6	79.19
SOCS1	58.5	68.5	25.0	50.5	85.0	38.0	54.3	21.45
SOCS2	23.0	20.0	8.0	19.0	13.0	7.0	15.0	6.66
SOCS3	118.5	92.5	84.0	119.0	88.5	80.5	97.2	17.20
SOCS4	167.0	113.5	215.5	157.5	112.5	184.5	158.4	40.35
SOCS5	240.5	217.5	310.5	283.0	230.0	310.5	265.3	41.34
SOCS6	128.5	97.5	191.5	110.0	116.5	171.5	135.9	37.24
SOCS7	114.0	106.0	129.0	96.5	100.5	115.0	110.2	11.75
SOD1	2873.5	2934.0	2052.5	2679.5	2991.5	1879.0	2568.3	481.57
SOD2	1777.5	1655.5	1415.0	1678.0	1795.0	1232.0	1592.2	222.70
SOD3	509.0	475.0	449.5	544.0	559.0	543.0	513.3	43.52
SOGA1	1044.5	1349.5	2049.5	997.0	1119.0	2046.0	1434.3	490.39
SOGA3	13.0	16.0	17.5	9.5	12.0	24.5	15.4	5.29
SON	875.0	510.5	1515.0	972.5	572.5	1241.0	947.8	386.18
SORBS1	477.0	599.5	899.5	471.0	650.5	716.5	635.7	161.22
SORBS2	102.5	106.5	164.5	66.0	100.0	104.5	107.3	31.82
SORCS1	22.5	34.5	63.5	42.0	47.0	62.0	45.3	15.88
SORCS2	447.0	319.5	1089.5	570.0	355.5	1142.0	653.9	368.43
SORCS3	0.5	0.0	0.0	1.0	0.0	0.0	0.3	0.42
SORD	228.0	198.5	161.5	230.5	197.0	152.5	194.7	32.54
SORL1	8.5	5.0	5.5	8.0	6.5	8.0	6.9	1.46
SOS2	579.5	434.0	636.5	492.0	457.0	542.5	523.6	77.06

SOST	19.0	16.0	10.5	13.0	19.0	7.5	14.2	4.68
SOSTDC1	8.0	4.5	4.5	8.5	9.5	2.0	6.2	2.93
SOWAHA	0.0	0.5	0.0	0.5	0.0	0.0	0.2	0.26
SOWAHB	9.5	11.5	3.5	11.0	13.5	8.0	9.5	3.48
SOWAHC	294.0	168.0	219.0	358.0	179.0	181.0	233.2	76.66
SOX10	13.5	1.0	2.5	3.5	1.5	2.0	4.0	4.73
SOX11	119.5	295.0	682.5	142.0	303.0	570.0	352.0	228.28
SOX13	52.0	78.0	68.0	42.0	66.0	55.0	60.2	12.94
SOX14	0.0	0.5	0.5	0.0	0.0	0.0	0.2	0.26
SOX18	0.0	0.0	0.0	0.5	0.0	0.0	0.1	0.20
SOX2	3.0	2.5	8.5	5.0	2.5	3.0	4.1	2.35
SOX30	0.5	0.0	0.5	0.0	0.0	0.5	0.3	0.27
SOX4	202.0	169.0	257.0	160.5	135.0	338.0	210.3	75.38
SOX7	63.5	43.5	25.0	53.0	55.5	33.0	45.6	14.57
SOX8	400.0	195.0	473.0	339.5	225.5	460.0	348.8	117.78
SOX9	27.5	25.0	63.0	33.0	21.5	29.0	33.2	15.12
SP1	645.5	554.5	1111.0	655.5	513.5	1150.5	771.8	283.51
SP2	337.5	264.0	248.5	321.0	319.5	253.5	290.7	39.54
SP4	72.5	38.5	111.0	71.0	51.5	116.0	76.8	31.19
SP9	0.0	0.0	0.0	0.0	0.0	0.0	0.0	0.00
SPACA1	1.5	10.5	1.5	0.5	4.5	3.0	3.6	3.67
SPAG1	102.5	119.0	150.5	93.0	144.0	113.5	120.4	22.73
SPAG16	2.0	1.0	1.5	1.0	0.5	1.5	1.3	0.52
SPAG17	0.0	2.0	1.0	1.5	1.5	1.5	1.3	0.69
SPAG5	252.0	151.5	137.0	203.5	160.5	93.5	166.3	55.03
SPAG6	9.5	10.5	15.5	8.5	11.5	16.0	11.9	3.14
SPAG8	3.0	1.0	1.5	6.0	2.0	2.0	2.6	1.80
SPAG9	421.5	188.5	711.0	472.0	215.5	628.0	439.4	211.57
SPAM1	19.5	17.5	16.5	19.0	26.0	19.0	19.6	3.34
SPARC	19513.5	30916.0	39167.0	24431.0	39418.5	58507.5	35325.6	13835.23
SPARCL1	5.5	8.5	4.5	8.0	11.5	4.5	7.1	2.76
SPATA1	50.0	26.0	26.5	42.0	30.5	29.0	34.0	9.77
SPATA13	87.0	130.0	160.5	90.0	146.5	173.0	131.2	36.03
SPATA16	0.0	0.0	0.0	0.0	0.0	0.0	0.0	0.00
SPATA17	12.5	11.5	13.5	9.0	14.5	9.0	11.7	2.29
SPATA18	35.5	14.5	12.5	27.0	18.0	10.5	19.7	9.69
SPATA2	142.0	94.5	137.0	135.5	122.0	125.5	126.1	17.18
SPATA20	373.0	230.5	220.5	323.0	200.0	189.0	256.0	74.45
SPATA22	0.0	0.0	0.0	0.5	0.0	0.0	0.1	0.20
SPATA2L	357.5	358.5	242.0	323.5	344.5	264.0	315.0	50.14
SPATA4	24.0	7.0	2.0	19.5	7.0	4.5	10.7	8.90
SPATA5	363.0	243.5	253.0	328.5	236.5	245.5	278.3	53.61
SPATA5L1	216.0	152.0	117.5	233.5	178.0	132.5	171.6	46.22
SPATA7	112.5	114.0	111.5	112.0	143.5	145.5	123.2	16.56
SPATS2	612.5	522.5	679.0	560.0	585.0	663.5	603.8	60.29
SPATS2L	98.5	49.5	164.0	115.0	69.5	180.0	112.8	51.44
SPC25	756.0	230.5	425.0	693.0	249.0	271.5	437.5	233.59
SPCS1	1015.5	856.5	527.0	918.5	882.0	518.0	786.3	211.34
SPCS2	655.5	302.5	313.5	664.5	357.0	288.5	430.3	179.45
SPCS3	2359.0	1839.0	1828.5	2132.5	2010.5	1728.5	1983.0	234.03
SPDEF	0.0	0.0	0.5	0.5	0.0	1.0	0.3	0.41
SPDL1	697.0	446.0	510.5	653.0	470.0	525.0	550.3	101.61
SPDYA	25.0	29.5	33.0	41.0	35.5	32.0	32.7	5.42
SPECC1	666.0	1833.0	2469.0	611.5	1830.0	1711.5	1520.2	732.94

SPECC1L	114.0	43.0	133.0	122.5	45.0	88.0	90.9	39.28
SPEF1	68.5	80.5	37.0	53.0	61.0	37.0	56.2	17.39
SPEF2	0.0	0.0	0.0	0.0	0.0	0.0	0.0	0.00
SPEG	191.5	296.0	381.5	160.5	242.5	382.0	275.7	94.20
SPEN	699.5	652.5	1142.5	654.5	755.5	1055.0	826.6	215.92
SPERT	0.5	0.0	0.0	0.5	0.0	0.0	0.2	0.26
SPG11	690.5	611.0	733.0	648.5	647.5	711.5	673.7	45.78
SPG20	2176.0	4247.5	3717.0	2569.5	5314.5	3537.0	3593.6	1137.18
SPG21	1041.0	1012.5	851.0	1012.5	1122.0	742.0	963.5	139.75
SPG7	3377.0	3092.0	2751.0	3265.5	3351.5	2853.0	3115.0	264.16
SPHK1	290.5	397.5	363.0	333.0	390.0	517.0	381.8	77.02
SPHKAP	0.5	1.0	1.0	0.5	2.5	7.0	2.1	2.52
SPI1	16.5	18.0	5.5	9.5	30.5	3.0	13.8	10.08
SPICE1	343.5	335.0	354.0	319.5	341.5	347.0	340.1	11.87
SPIDR	113.0	101.5	71.0	113.0	89.0	71.5	93.2	19.15
SPIN1	2323.5	1098.0	1792.5	2081.0	1219.0	1603.5	1686.3	478.42
SPINK2	17.5	16.5	14.5	17.0	17.0	17.5	16.7	1.13
SPINK4	1.0	0.0	2.5	0.5	0.0	1.0	0.8	0.93
SPINT1	814.0	637.5	979.5	793.0	693.0	843.5	793.4	119.96
SPINT4	0.0	0.0	0.0	0.0	0.0	0.0	0.0	0.00
SPIRE1	392.0	312.5	425.5	428.5	298.5	324.0	363.5	58.77
SPIRE2	7.5	4.0	7.0	9.5	4.0	8.5	6.8	2.30
SPNS2	0.0	0.0	0.0	1.0	0.0	0.0	0.2	0.41
SPNS3	1.0	2.5	2.0	2.0	1.5	1.0	1.7	0.61
SPO11	78.5	37.5	15.0	53.0	22.0	7.5	35.6	26.63
SPOCK1	0.0	0.5	1.0	0.0	0.5	4.5	1.1	1.72
SPOCK2	263.5	291.5	209.5	258.5	277.5	239.0	256.6	29.10
SPOCK3	1.5	2.0	6.0	2.5	2.0	6.5	3.4	2.22
SPON1	44.0	74.0	109.0	164.5	138.5	484.5	169.1	160.46
SPON2	1.0	2.5	1.0	0.5	4.0	2.5	1.9	1.32
SPOP	995.0	944.5	889.5	937.5	973.0	906.5	941.0	39.51
SPOPL	1648.5	950.5	977.0	1601.0	1178.5	974.5	1221.7	323.24
SPP1	181.5	158.5	380.5	219.0	135.5	206.0	213.5	87.30
SPP2	0.0	1.5	0.0	0.0	0.0	0.0	0.3	0.61
SPPL2A	2385.5	1749.0	2784.5	2470.5	2070.5	2553.0	2335.5	369.82
SPPL2B	356.5	248.0	701.0	360.0	270.0	579.0	419.1	181.08
SPPL3	599.0	449.0	547.0	579.0	505.0	569.0	541.3	55.47
SPR	211.5	159.0	114.0	215.0	173.0	130.5	167.2	41.29
SPRED1	392.0	445.0	547.5	474.5	486.0	596.0	490.2	72.70
SPRED2	130.5	112.0	361.5	149.5	123.0	324.0	200.1	111.81
SPRTN	818.5	610.5	447.5	660.0	678.0	425.0	606.6	149.10
SPRY1	93.0	85.5	221.5	92.0	96.0	263.5	141.9	79.11
SPRY2	1025.5	730.0	591.0	955.0	768.5	733.0	800.5	160.58
SPRY3	25.0	20.0	14.0	42.0	17.0	19.0	22.8	10.07
SPRY4	6.5	9.0	19.5	9.5	6.0	14.0	10.8	5.15
SPRYD7	542.5	474.0	583.0	500.0	558.5	549.0	534.5	40.13
SPSB1	24.5	17.5	41.5	37.5	19.0	51.5	31.9	13.68
SPSB3	472.5	421.5	399.0	423.5	473.5	427.0	436.2	30.18
SPSB4	41.0	32.5	58.5	49.0	28.5	54.5	44.0	12.06
SPTAN1	5860.0	5004.5	6294.0	5617.5	5693.5	5648.0	5686.3	417.27
SPTB	852.0	1206.0	1081.5	633.0	1174.0	980.5	987.8	217.00
SPTBN1	6021.0	4063.5	10242.0	6080.0	4961.5	8935.5	6717.3	2381.61
SPTBN5	0.0	1.5	1.0	1.0	0.5	0.5	0.8	0.52
SPTLC2	82.5	36.0	90.0	94.5	47.5	86.0	72.8	24.61

SPTSSB	8.0	11.5	9.0	7.5	11.0	6.5	8.9	1.99
SPTY2D1	906.5	696.0	748.5	891.5	719.0	710.0	778.6	94.96
SPX	0.5	0.0	0.0	0.0	0.0	0.0	0.1	0.20
SQLE	1902.5	613.0	1475.0	1935.0	660.5	1083.5	1278.3	586.83
SQRDL	718.5	297.5	151.0	766.5	412.0	379.5	454.2	241.34
SQSTM1	1960.0	832.0	421.0	1614.5	929.5	438.5	1032.6	628.74
SRA1	390.5	479.0	244.0	365.0	501.5	278.5	376.4	103.56
SRBD1	1548.0	905.0	743.0	1483.5	952.5	645.0	1046.2	380.69
SRC	72.0	62.0	160.0	70.0	59.0	150.5	95.6	46.57
SRD5A1	320.5	165.0	168.5	280.5	186.0	116.5	206.2	77.66
SRD5A2	0.5	0.5	0.0	1.0	0.0	0.5	0.4	0.38
SRD5A3	395.0	245.5	237.0	348.0	252.5	224.5	283.8	70.20
SREBF1	928.5	747.5	1525.0	867.0	654.5	1411.0	1022.3	359.86
SREBF2	3469.5	1316.5	3351.5	3392.5	1253.5	2883.5	2611.2	1047.75
SREK1	243.0	214.0	446.0	224.5	266.0	425.0	303.1	104.29
SREK1IP1	220.0	275.5	189.0	215.0	259.5	168.0	221.2	40.80
SRF	938.5	764.5	1045.5	1064.0	649.5	1046.0	918.0	173.22
SRFBP1	436.5	996.5	1688.0	526.0	1359.5	2347.5	1225.7	728.70
SRGAP1	1093.0	973.5	1277.0	1019.0	1132.5	1529.0	1170.7	204.49
SRGAP2	511.0	312.5	533.5	457.0	373.5	537.5	454.2	92.79
SRGN	6.5	1.0	1.0	4.0	2.0	0.0	2.4	2.42
SRI	1953.5	1576.0	1213.0	1791.5	1730.0	1209.0	1578.8	309.54
SRL	1833.5	5695.5	5323.0	1396.5	5417.0	3820.0	3914.3	1902.27
SRM	643.5	355.5	231.5	607.5	369.5	250.5	409.7	176.32
SRMS	0.0	0.5	0.0	0.5	0.0	0.0	0.2	0.26
SRP14	1510.0	1244.5	792.0	1364.5	1312.5	825.5	1174.8	296.89
SRP19	1384.5	1270.0	917.5	1336.0	1391.0	867.0	1194.3	238.50
SRP54	1612.5	1136.0	1175.0	1440.0	1250.5	1094.0	1284.7	201.62
SRP68	1108.5	908.0	954.5	1033.0	979.5	967.0	991.8	69.99
SRP72	2709.5	2172.0	1440.0	2663.5	2257.0	1369.5	2101.9	581.04
SRP9	1066.5	1258.5	1166.5	1098.5	1397.5	1136.0	1187.3	122.31
SRPK1	261.5	110.5	348.5	299.5	138.0	302.0	243.3	96.67
SRPK2	1393.5	1431.0	1160.5	1261.0	1754.0	1210.0	1368.3	215.91
SRPR	2096.0	1971.5	3250.0	2295.5	2043.0	3083.5	2456.6	563.00
SRPRB	1864.0	1296.0	1559.5	2009.5	1459.0	1575.0	1627.2	263.64
SRPX	39.0	57.0	115.5	49.5	60.5	118.0	73.3	34.50
SRPX2	317.0	217.5	265.0	375.5	245.5	266.0	281.1	56.57
SRR	196.0	275.5	173.0	170.5	317.5	164.5	216.2	64.52
SRRD	498.0	392.0	232.0	417.5	403.5	248.5	365.3	103.84
SRRM1	2350.0	2385.5	1944.0	2257.5	2755.0	2068.0	2293.3	282.47
SRRM3	0.0	0.0	0.0	0.5	0.0	0.0	0.1	0.20
SRRM4	13.5	17.5	11.5	9.0	18.0	15.0	14.1	3.48
SRSF1	5405.5	4822.5	3729.0	4894.0	5175.5	3544.5	4595.2	773.12
SRSF10	1254.0	1440.0	1361.0	1188.5	1486.5	1299.0	1338.2	113.06
SRSF11	2371.5	2214.5	1896.5	2225.5	2626.5	2044.0	2229.8	254.29
SRSF2	987.5	511.5	931.5	1036.5	569.5	840.0	812.8	221.53
SRSF3	2633.5	2778.5	1836.5	2546.5	2871.5	1715.5	2397.0	495.50
SRSF4	84.0	113.5	191.0	73.5	131.0	173.5	127.8	47.25
SRSF5	3236.5	2051.5	1449.0	2871.5	2290.5	1522.0	2236.8	717.08
SRSF6	1247.0	1542.5	2659.0	1307.5	1866.0	2845.5	1911.3	689.31
SRXN1	359.5	460.5	425.0	364.5	427.0	421.0	409.6	39.51
SS18	1393.0	1066.5	896.0	1270.5	1197.5	849.0	1112.1	214.14
SS18L1	12.5	5.5	9.5	11.5	11.0	8.0	9.7	2.58
SS18L2	861.0	927.5	494.0	818.5	956.0	474.0	755.2	215.64

SSB	1418.0	909.0	917.0	1482.5	947.0	823.0	1082.8	288.36
SSBP1	484.5	525.0	403.0	447.0	565.0	380.5	467.5	71.06
SSBP2	74.5	38.0	67.0	55.5	39.5	67.0	56.9	15.33
SSC5D	0.0	0.0	3.0	0.5	0.5	3.5	1.3	1.57
SSFA2	407.5	209.5	469.0	385.5	222.0	353.5	341.2	104.30
SSH1	270.5	212.0	281.0	243.5	233.5	302.5	257.2	33.43
SSH2	147.0	225.0	267.0	151.0	217.5	266.0	212.3	53.08
SSH3	121.0	242.5	203.0	92.0	209.0	274.5	190.3	70.41
SSNA1	29.5	24.5	14.0	22.5	23.0	14.5	21.3	6.02
SSPN	1043.0	1851.5	1576.5	1043.5	2125.0	1595.5	1539.2	432.94
SSPO	0.5	1.0	4.0	0.0	2.0	0.5	1.3	1.47
SSR1	5147.5	3859.5	4673.5	5947.0	4505.0	4477.0	4768.3	710.24
SSR2	3795.5	3330.0	2153.5	3692.5	3750.0	2596.5	3219.7	689.00
SSR3	2531.5	1884.0	1467.5	2508.5	2077.0	1461.5	1988.3	476.06
SSR4	1241.0	982.5	695.0	1145.5	940.5	754.0	959.8	213.03
SSRP1	1626.5	1144.5	1115.5	1534.5	1057.0	1040.0	1253.0	258.15
SST	0.0	3.0	3.5	0.0	5.0	3.5	2.5	2.05
SSTR1	0.0	0.0	0.0	0.0	0.0	0.0	0.0	0.00
SSTR2	463.0	117.0	150.5	370.5	118.0	121.0	223.3	153.15
SSTR3	0.0	0.0	0.0	0.0	0.0	0.0	0.0	0.00
SSTR4	753.0	479.0	239.5	738.0	591.0	295.5	516.0	217.95
SSTR5	0.5	0.0	0.0	0.0	2.0	0.0	0.4	0.80
SSU72	1897.5	1554.5	1226.5	1829.5	1800.5	1199.0	1584.6	310.59
SSX2IP	28.5	14.5	12.0	18.0	10.5	7.0	15.1	7.55
ST13	779.5	501.5	1150.5	758.5	632.0	1114.0	822.7	259.94
ST14	206.5	98.5	220.5	142.0	71.5	126.5	144.3	58.98
ST3GAL1	35.5	13.0	37.0	46.5	17.5	52.0	33.6	15.52
ST3GAL2	1491.0	940.5	1032.0	1506.0	1065.5	1087.5	1187.1	246.43
ST3GAL4	124.5	154.0	247.0	159.5	196.0	281.5	193.8	60.15
ST3GAL5	200.5	252.5	216.5	187.0	326.0	433.5	269.3	94.61
ST3GAL6	388.0	295.5	225.5	357.0	323.5	189.5	296.5	76.49
ST5	1266.0	1009.0	940.5	1407.5	1107.0	909.5	1106.6	195.91
ST6GAL1	16.0	8.0	4.0	29.5	11.0	3.5	12.0	9.75
ST6GAL2	102.0	232.0	544.0	41.5	138.5	245.5	217.3	177.85
ST6GALNAC1	22.0	24.0	14.0	17.5	24.5	9.0	18.5	6.16
ST6GALNAC2	0.5	2.0	1.0	0.5	0.0	1.0	0.8	0.68
ST6GALNAC3	232.0	166.0	369.0	246.5	190.5	295.0	249.8	73.65
ST6GALNAC4	496.5	581.0	402.0	426.5	656.5	525.5	514.7	95.32
ST6GALNAC5	191.5	158.0	253.0	202.0	198.5	321.5	220.8	58.02
ST6GALNAC6	2593.5	2271.0	1918.0	2487.5	2395.5	2761.0	2404.4	291.30
ST7	251.0	118.5	81.0	213.0	128.5	74.5	144.4	71.97
ST7L	604.5	364.0	320.5	584.5	404.5	355.0	438.8	123.67
ST8SIA1	2.5	5.0	9.0	10.0	4.5	9.5	6.8	3.14
ST8SIA2	0.5	4.0	2.5	2.0	3.5	8.5	3.5	2.74
ST8SIA3	0.0	0.0	0.0	0.0	0.0	0.0	0.0	0.00
ST8SIA4	796.0	411.5	845.5	926.0	489.0	837.5	717.6	212.73
ST8SIA5	9.0	14.5	26.0	13.0	19.5	19.0	16.8	5.96
ST8SIA6	21.5	12.0	57.5	21.5	12.0	37.5	27.0	17.61
STAB1	7.0	8.5	3.5	9.5	5.5	2.0	6.0	2.90
STAB2	0.0	0.0	0.5	0.5	0.5	2.5	0.7	0.93
STAC	1.0	4.0	5.0	0.0	7.0	7.5	4.1	3.07
STAG2	487.5	263.5	617.0	513.0	281.0	532.5	449.1	143.81
STAM2	974.0	742.0	778.5	975.5	884.0	731.0	847.5	112.41
STAMBP	269.0	283.5	361.5	282.5	281.0	333.0	301.8	36.75

STAMBPL1	669.5	264.5	226.0	567.5	294.0	205.5	371.2	196.67
STAP1	112.0	85.5	66.0	110.5	101.0	64.5	89.9	21.32
STAR	2.5	5.0	8.5	4.0	8.0	10.0	6.3	2.93
STARD13	494.5	117.5	510.5	427.5	120.5	468.0	356.4	186.04
STARD3	496.0	326.0	296.0	371.0	295.0	273.5	342.9	82.25
STARD3NL	288.5	247.5	209.5	265.0	296.5	204.0	251.8	39.02
STARD4	925.0	404.0	1245.5	925.5	419.0	979.0	816.3	335.34
STARD5	121.5	160.5	121.5	154.5	208.0	166.5	155.4	32.30
STARD8	661.5	389.0	726.5	641.5	394.0	717.5	588.3	155.85
STARD9	225.0	147.5	336.0	183.0	194.5	353.0	239.8	84.95
STAT1	1142.0	501.0	681.0	1462.0	584.5	688.5	843.2	375.89
STAT2	125.0	139.5	133.0	122.5	124.0	148.5	132.1	10.32
STAT3	1902.0	1666.0	2529.0	2004.0	1597.0	2896.5	2099.1	511.51
STAT4	3.5	2.0	1.0	6.5	1.5	1.0	2.6	2.13
STAU1	2128.0	2065.0	2406.5	1900.5	2149.5	2121.0	2128.4	163.58
STBD1	330.0	134.5	56.5	341.0	127.5	70.0	176.6	126.91
STC1	62.0	157.0	156.5	132.5	175.0	269.5	158.8	67.21
STC2	946.5	774.0	2580.5	1037.0	819.5	2471.0	1438.1	848.32
STEAP3	39.0	192.0	188.5	33.0	188.5	123.5	127.4	75.34
STEAP4	9.0	8.5	14.0	11.5	7.0	11.5	10.3	2.54
STIL	107.0	43.5	77.5	94.0	41.0	40.5	67.3	29.56
STIM1	1604.5	849.0	821.5	1498.5	844.0	888.0	1084.3	364.11
STIM2	633.0	317.5	402.0	649.5	363.5	402.5	461.3	142.91
STK10	46.5	58.0	103.5	52.5	63.5	61.5	64.3	20.20
STK11	262.0	248.5	407.0	256.0	284.0	384.0	306.9	70.01
STK11IP	266.5	129.5	214.0	314.5	133.5	178.0	206.0	74.01
STK16	185.0	155.0	106.5	173.0	148.5	110.5	146.4	32.11
STK17A	4405.5	6024.5	4792.5	3916.5	6547.0	3859.0	4924.2	1120.77
STK17B	2273.0	1143.0	1397.0	2349.5	1400.0	1897.5	1743.3	503.98
STK19	234.0	403.5	188.5	220.0	336.5	232.5	269.2	82.56
STK24	130.0	79.5	82.5	129.0	104.0	81.5	101.1	23.74
STK25	1201.0	999.0	1395.5	1176.0	1132.0	1231.0	1189.1	129.67
STK26	315.0	126.5	364.5	323.0	169.5	305.5	267.3	95.58
STK3	1300.5	1192.5	1274.0	1198.5	1385.5	1165.5	1252.8	83.11
STK31	0.0	0.0	0.0	0.0	0.0	0.0	0.0	0.00
STK32A	1.0	0.0	0.0	0.0	0.0	0.5	0.3	0.42
STK32B	0.0	0.0	0.0	0.5	0.0	0.5	0.2	0.26
STK32C	3.5	19.5	34.0	8.0	27.5	96.0	31.4	33.65
STK35	73.0	29.0	90.5	64.0	36.0	71.5	60.7	23.59
STK36	96.0	103.0	125.5	94.0	91.5	154.5	110.8	24.74
STK38	27.0	13.5	33.5	22.5	10.5	30.5	22.9	9.27
STK38L	757.5	450.0	993.5	657.5	499.0	832.0	698.3	205.70
STK39	29.5	7.0	27.0	14.5	8.0	8.5	15.8	10.06
STK40	146.5	103.5	209.5	137.5	97.0	189.0	147.2	45.06
STKLD1	50.5	34.0	38.5	50.0	26.5	23.5	37.2	11.44
STMN1	15440.5	21157.0	13118.0	11992.5	21474.0	10168.0	15558.3	4776.61
STMN2	0.0	0.0	1.5	0.0	0.5	0.0	0.3	0.61
STMN3	0.0	2.0	2.0	1.0	6.0	1.0	2.0	2.10
STMN4	1.0	60.5	11.5	1.5	51.0	22.0	24.6	25.51
STMND1	0.0	0.0	0.0	0.0	0.0	0.0	0.0	0.00
STOM	2320.5	2272.0	1504.0	2296.0	2392.0	1724.0	2084.8	373.38
STOML1	197.0	125.0	145.0	188.5	133.0	134.5	153.8	30.93
STOML2	466.5	527.0	453.0	449.5	556.0	414.0	477.7	53.20
STOML3	0.5	0.5	0.5	0.5	1.5	0.5	0.7	0.41

STON1	21.5	18.0	41.5	32.0	13.5	43.5	28.3	12.57
STON2	120.5	100.5	90.5	130.5	127.0	167.5	122.8	26.91
STOX1	15.5	7.0	8.0	10.5	6.0	12.5	9.9	3.63
STOX2	94.5	30.0	86.0	106.0	43.0	71.5	71.8	29.88
STRA6	0.0	0.5	0.0	0.5	0.5	0.5	0.3	0.26
STRA8	7.5	2.0	9.0	10.5	4.5	6.5	6.7	3.08
STRADA	1072.0	897.5	909.0	947.5	949.0	848.0	937.2	75.82
STRADB	243.0	132.5	232.0	267.0	145.5	170.0	198.3	56.16
STRAP	1858.0	1306.0	1143.5	1706.0	1445.5	1101.0	1426.7	305.04
STRBP	28.0	18.0	19.5	20.0	12.5	16.0	19.0	5.19
STRIP1	1192.0	1252.0	1061.5	1095.5	1273.5	1041.0	1152.6	100.06
STRIP2	5.0	12.5	11.0	7.0	20.0	10.5	11.0	5.21
STRN	66.5	28.0	55.0	58.0	27.5	46.0	46.8	16.17
STRN3	557.5	391.0	934.0	590.5	486.0	908.5	644.6	225.11
STS	75.5	24.0	29.0	48.0	26.5	31.0	39.0	19.79
STT3A	2408.0	2323.5	3149.0	2623.5	2613.0	3413.0	2755.0	431.80
STT3B	1278.5	1071.5	1446.5	1357.0	1235.5	1363.5	1292.1	130.54
STUB1	1582.0	1259.0	909.5	1424.0	1223.5	906.0	1217.3	271.72
STX11	0.0	0.0	4.5	0.5	0.5	2.5	1.3	1.81
STX12	637.0	533.0	770.5	671.5	615.5	720.5	658.0	83.21
STX16	598.0	356.0	585.0	605.5	387.0	497.0	504.8	110.76
STX17	26.5	13.5	27.5	20.5	18.5	32.5	23.2	6.92
STX18	381.5	238.0	223.0	347.5	264.0	248.0	283.7	64.92
STX19	2.0	0.0	1.0	0.5	0.0	0.5	0.7	0.75
STX1A	477.5	630.0	711.0	385.0	640.0	770.0	602.3	144.86
STX2	1350.0	929.5	1010.5	1188.0	1053.5	961.0	1082.1	159.30
STX3	45.5	87.5	67.5	40.0	63.5	69.0	62.2	17.25
STX6	188.0	185.5	299.5	192.0	176.0	296.0	222.8	58.28
STX7	639.5	526.5	424.0	604.0	584.5	475.5	542.3	82.15
STX8	705.5	650.0	462.5	622.5	679.0	442.0	593.6	113.14
STXBP1	497.5	426.5	766.0	442.5	453.0	735.0	553.4	154.78
STXBP3	195.5	128.0	172.5	224.5	150.0	171.0	173.6	33.81
STXBP4	313.5	166.0	413.5	362.5	194.5	364.5	302.4	100.18
STXBP5	291.5	130.5	248.0	268.0	172.0	228.5	223.1	60.88
STXBP5L	20.0	54.0	55.5	10.0	65.0	91.0	49.3	29.83
STXBP6	1193.5	781.5	771.0	1061.5	907.5	762.0	912.8	179.45
STYK1	29.5	18.0	9.5	32.0	16.0	11.0	19.3	9.41
STYX	535.0	548.5	476.0	543.0	627.5	429.0	526.5	67.95
STYXL1	56.0	15.5	12.5	51.0	21.5	10.0	27.8	20.37
SUB1	2586.5	2310.0	2500.5	2460.0	2592.0	2272.5	2453.6	136.00
SUCLA2	1560.0	1543.5	1355.5	1651.5	1770.0	1287.5	1528.0	180.38
SUCLG1	1379.0	1140.0	1207.0	1250.5	1142.0	1012.5	1188.5	123.26
SUCLG2	847.0	600.0	606.0	842.0	655.0	552.0	683.7	128.79
SUCNR1	3.5	4.0	8.5	6.0	7.5	4.5	5.7	2.02
SUCO	965.5	670.5	1062.5	872.0	832.5	806.5	868.3	135.20
SUDS3	898.5	745.5	732.5	765.0	891.5	689.0	787.0	87.33
SUFU	1667.5	1128.0	1602.5	1589.0	1214.5	1626.5	1471.3	235.56
SUGCT	49.5	25.0	10.5	71.5	38.5	14.0	34.8	23.22
SUGP1	337.5	266.5	276.0	301.5	298.0	259.0	289.8	28.85
SUGP2	728.5	439.0	799.0	665.0	507.0	753.5	648.7	144.40
SUGT1	700.0	769.0	808.0	655.5	865.5	803.5	766.9	76.99
SULT4A1	231.0	128.0	170.0	188.0	161.5	170.0	174.8	33.90
SULT6B1	44.5	13.5	11.0	50.0	16.0	4.5	23.3	19.06
SUMF1	591.0	331.0	362.0	588.0	392.5	380.5	440.8	117.01

SUMF2	122.0	142.5	130.5	111.5	155.0	121.5	130.5	15.87
SUMO1	2007.0	2198.0	1702.5	1856.5	2465.0	1677.5	1984.4	305.66
SUMO2	2481.5	2749.0	1859.0	2204.0	3064.5	1758.0	2352.7	509.87
SUN1	2601.5	1409.0	1931.5	2260.5	1600.0	1729.5	1922.0	442.79
SUN2	661.5	241.5	682.0	569.5	217.5	563.0	489.2	206.85
SUN3	351.0	327.0	151.5	234.5	327.0	124.5	252.6	97.68
SUOX	148.5	113.5	111.5	131.5	105.5	133.5	124.0	16.47
SUPT3H	68.5	20.0	35.0	61.0	17.5	40.0	40.3	20.90
SUPT4H1	626.0	855.5	503.0	581.0	874.5	514.0	659.0	165.88
SUPT5H	1227.0	1083.0	1038.0	1075.5	1075.5	1021.5	1086.8	72.90
SUPT6H	985.0	808.5	1334.5	944.5	816.0	1209.5	1016.3	213.55
SUPT7L	144.0	59.0	220.5	167.5	65.5	178.5	139.2	64.56
SUPV3L1	603.0	484.5	399.5	588.5	466.5	368.5	485.1	95.77
SURF1	456.0	294.5	256.5	344.5	276.0	185.0	302.1	91.67
SURF2	515.0	366.5	188.0	439.0	336.0	187.0	338.6	132.40
SURF4	3140.0	2425.5	3494.5	3077.0	2756.0	3351.0	3040.7	393.17
SURF6	813.0	888.0	555.5	787.5	882.5	558.0	747.4	152.73
SUSD1	20.0	11.5	27.5	20.0	10.0	22.0	18.5	6.62
SUSD2	0.0	0.0	0.0	0.0	0.0	0.0	0.0	0.00
SUSD3	9.0	25.5	8.5	6.5	19.5	8.5	12.9	7.71
SUSD4	74.5	19.0	7.5	62.5	29.5	18.0	35.2	27.01
SUSD5	0.0	3.0	0.5	1.0	1.5	2.5	1.4	1.16
SUSD6	1568.5	828.5	1403.0	1463.5	981.5	1641.0	1314.3	331.13
SUV39H2	267.0	174.0	231.5	265.0	162.5	207.5	217.9	44.54
SUV420H1	215.0	205.0	206.5	201.0	261.5	190.0	213.2	25.04
SUZ12	805.5	771.0	826.5	797.5	809.0	717.0	787.8	39.11
SV2B	0.0	19.0	16.0	0.5	34.5	32.5	17.1	14.92
SV2C	0.0	1.0	3.5	2.0	1.5	8.0	2.7	2.86
SVEP1	81.5	200.5	236.0	104.5	253.0	488.0	227.3	145.43
SVIL	1095.0	1040.5	2104.0	958.5	1242.0	1928.0	1394.7	493.23
SVOP	1.0	0.0	0.5	1.5	0.5	1.0	0.8	0.52
SVOPL	19.5	33.0	42.5	19.5	49.5	58.0	37.0	15.85
SWAP70	186.5	50.5	192.0	199.5	45.5	186.5	143.4	74.08
SWI5	777.0	735.5	455.5	713.0	807.0	512.0	666.7	146.47
SWT1	276.0	136.0	139.0	219.0	142.0	127.5	173.3	60.47
SYAP1	392.5	343.5	274.0	325.0	376.5	280.5	332.0	48.66
SYBU	30.0	26.0	32.0	43.5	34.5	54.5	36.8	10.48
SYCE3	1.0	0.5	0.0	2.0	0.5	0.0	0.7	0.75
SYCN	12.5	6.0	10.0	5.5	10.5	8.0	8.8	2.73
SYCP1	31.0	14.5	9.0	19.5	9.5	4.5	14.7	9.51
SYCP2	82.0	67.0	52.5	70.0	74.5	38.5	64.1	15.89
SYCP3	248.0	245.0	316.0	201.5	269.5	287.5	261.3	39.41
SYDE2	588.5	562.5	687.5	593.0	646.0	769.5	641.2	77.36
SYF2	854.5	723.0	562.5	777.5	768.5	476.0	693.7	144.18
SYK	0.0	0.0	0.0	0.5	0.0	0.0	0.1	0.20
SYN2	50.5	37.0	34.0	47.0	40.5	59.5	44.8	9.48
SYNC	1951.0	2361.0	1795.5	1903.5	2376.5	1879.5	2044.5	256.22
SYNCRIP	1619.5	1392.0	1974.5	1750.0	1586.0	1843.0	1694.2	206.12
SYNDIG1	0.0	0.0	0.5	1.0	0.0	0.5	0.3	0.41
SYNDIG1L	5.0	11.5	20.5	7.0	16.5	29.0	14.9	9.00
SYNE1	1606.0	1188.0	1650.5	1255.0	1393.5	1413.0	1417.7	184.12
SYNE2	1416.0	1346.5	2882.5	1250.5	1509.0	2501.5	1817.7	693.08
SYNE3	130.5	60.5	170.5	115.0	67.5	109.0	108.8	40.88
SYNGR1	1243.5	609.5	756.5	1213.0	677.0	696.5	866.0	284.66

SYNGR2	1119.0	1127.5	784.0	989.5	1110.0	846.0	996.0	150.27
SYNGR3	103.5	42.5	19.0	80.5	47.0	17.0	51.6	34.33
SYNJ1	430.5	393.0	489.0	417.5	543.5	511.5	464.2	59.21
SYNJ2	844.0	1547.0	1532.0	1005.5	1780.5	1528.5	1372.9	363.51
SYNM	679.5	965.0	1770.0	814.5	1295.0	1550.0	1179.0	430.94
SYNPO	1651.0	1226.0	1105.5	1927.5	1376.5	1190.5	1412.8	316.77
SYNPO2	305.0	578.5	1419.0	296.5	729.0	1198.5	754.4	465.22
SYNPO2L	6807.0	13725.0	9104.5	6742.5	15516.5	8871.5	10127.8	3663.10
SYNPR	0.0	0.0	0.5	0.0	0.0	0.0	0.1	0.20
SYNRG	1368.5	1187.5	1861.0	1315.0	1240.5	1659.5	1438.7	264.46
SYPL1	0.0	0.5	0.0	0.0	0.0	0.0	0.1	0.20
SYPL2	77.0	209.0	149.0	62.5	213.5	152.5	143.9	63.69
SYS1	290.0	267.5	234.5	283.5	317.0	225.5	269.7	34.75
SYT1	7.0	11.0	13.0	5.5	11.0	12.0	9.9	2.97
SYT11	3698.5	3603.5	5095.0	3695.5	4095.0	4877.5	4177.5	652.66
SYT12	8.5	7.0	5.0	4.5	8.5	11.0	7.4	2.44
SYT13	19.5	6.0	19.0	21.0	4.5	9.5	13.3	7.42
SYT14	16.0	10.0	33.5	13.5	14.0	23.0	18.3	8.59
SYT15	315.5	98.0	60.0	235.0	88.5	150.0	157.8	98.83
SYT16	11.5	5.0	16.5	21.0	8.0	14.0	12.7	5.79
SYT17	167.0	80.0	131.5	157.0	87.5	127.0	125.0	35.40
SYT2	1.0	1.5	1.5	0.5	0.0	1.0	0.9	0.58
SYT4	0.0	0.0	0.0	0.0	0.0	0.0	0.0	0.00
SYT6	0.5	1.0	0.0	0.0	1.0	2.5	0.8	0.93
SYT8	0.5	0.0	0.0	0.0	0.5	0.0	0.2	0.26
SYTL2	41.0	59.5	41.0	22.5	69.0	39.5	45.4	16.45
SYTL4	68.0	92.0	71.5	72.0	111.0	100.0	85.8	17.82
SYTL5	3.5	6.0	1.0	9.0	9.5	3.5	5.4	3.37
SZRD1	246.0	232.0	333.0	212.0	243.5	303.5	261.7	46.39
SZT2	82.5	51.5	173.5	87.5	48.0	124.0	94.5	47.57
T	1.0	1.0	1.0	0.5	1.0	2.0	1.1	0.49
TAAR5	0.0	0.0	0.0	0.0	0.0	0.0	0.0	0.00
TAB1	296.0	191.5	220.5	266.0	208.5	172.5	225.8	46.67
TAB2	930.0	1048.0	1658.5	926.5	1198.5	1576.0	1222.9	322.26
TAB3	422.5	240.0	366.5	398.5	287.5	332.5	341.3	68.90
TAC1	0.0	0.5	0.5	0.0	0.5	0.0	0.3	0.27
TACC1	2120.0	2554.5	3449.5	2058.5	2667.0	3141.5	2665.2	551.28
TACR1	0.0	2.0	57.5	0.0	2.0	20.0	13.6	22.84
TACR2	37.0	20.5	19.0	19.5	25.0	24.5	24.3	6.74
TACSTD2	121.0	54.5	32.5	113.5	80.0	26.5	71.3	40.30
TADA1	260.0	293.5	235.0	221.0	299.5	251.5	260.1	31.31
TADA2A	214.0	198.0	204.5	227.5	215.0	232.0	215.2	13.00
TADA2B	58.0	28.5	53.5	57.5	31.5	52.5	46.9	13.31
TADA3	484.0	586.5	405.5	416.0	481.5	416.5	465.0	68.83
TAF1	694.5	900.5	1122.0	700.5	937.0	1029.5	897.3	172.86
TAF11	353.0	407.0	290.5	363.0	393.5	253.5	343.4	59.85
TAF12	956.5	926.0	677.0	896.5	1011.5	686.5	859.0	142.50
TAF13	1001.0	1077.0	833.5	902.0	1136.0	746.5	949.3	148.74
TAF15	709.0	721.0	558.5	635.0	824.0	545.0	665.4	106.77
TAF1A	611.5	543.0	612.5	552.5	594.0	559.5	578.8	30.93
TAF1B	309.5	226.0	268.0	263.0	205.5	220.0	248.7	38.68
TAF1D	427.0	286.0	312.5	371.0	308.0	299.5	334.0	54.13
TAF2	1143.5	731.5	963.5	1126.0	872.5	848.0	947.5	162.92
TAF3	893.0	730.0	709.0	861.5	848.5	654.5	782.8	97.31

TAF4	128.0	92.0	146.5	132.5	123.5	141.0	127.3	19.21
TAF4B	76.5	40.0	52.5	85.0	32.0	47.0	55.5	20.90
TAF5	167.0	119.0	232.0	225.5	133.5	208.0	180.8	48.18
TAF5L	409.5	296.5	383.0	389.0	331.5	325.5	355.8	44.16
TAF6	98.0	71.0	54.5	90.0	52.0	46.0	68.6	21.51
TAF7L	1351.0	1550.0	832.0	1320.5	1627.0	791.0	1245.3	355.69
TAF8	222.5	204.5	194.0	208.5	201.0	183.0	202.3	13.37
TAF9B	459.5	452.0	424.0	418.0	497.5	411.5	443.8	32.55
TAGAP	1.0	4.5	1.0	5.0	5.5	1.5	3.1	2.13
TAGLN	2037.5	1237.5	1238.0	2593.0	1267.0	1312.0	1614.2	571.41
TAGLN2	5371.5	5263.5	6374.0	5006.0	5109.0	6305.0	5571.5	608.37
TAGLN3	60.0	32.5	14.5	64.5	37.5	22.5	38.6	20.04
TAL1	0.0	0.5	0.5	0.0	1.5	1.0	0.6	0.58
TAL2	109.0	154.0	81.0	48.0	75.0	24.5	81.9	45.66
TAMM41	290.0	189.0	240.0	291.0	228.0	305.0	257.2	45.39
TANC1	866.5	504.5	623.0	869.0	601.0	624.0	681.3	150.94
TANGO2	429.5	491.0	436.5	372.5	516.5	275.5	420.3	87.01
TANGO6	827.0	521.5	207.0	630.0	431.5	209.0	471.0	242.68
TANK	633.5	496.5	501.0	573.5	561.5	429.0	532.5	71.73
TAOK1	315.0	252.5	773.0	376.5	253.0	738.5	451.4	240.41
TAPBP	434.0	418.0	291.5	561.5	491.0	513.0	451.5	94.31
TAPBPL	93.5	70.0	97.0	75.5	66.0	102.0	84.0	15.33
TAPT1	488.0	469.0	520.5	482.5	575.5	476.0	501.9	40.21
TARBP1	737.0	559.0	495.0	621.0	647.0	470.0	588.2	100.18
TARBP2	150.0	224.0	163.0	135.0	198.5	159.0	171.6	33.19
TARDBP	1745.5	1094.0	1501.0	1532.0	1174.0	1399.0	1407.6	241.40
TARS	1399.5	875.0	1377.0	1293.5	919.0	1080.0	1157.3	231.49
TARS2	374.5	360.5	277.5	340.0	348.0	281.5	330.3	41.09
TARSL2	483.5	449.5	429.0	453.5	490.0	364.5	445.0	45.47
TASP1	29.0	17.0	41.0	31.5	15.5	31.5	27.6	9.70
TAT	2.0	1.0	0.5	0.5	1.0	1.0	1.0	0.55
TATDN1	360.0	331.5	280.0	313.0	340.0	225.5	308.3	48.77
TATDN3	624.5	419.5	307.5	594.0	477.5	331.5	459.1	131.70
TAX1BP1	1906.0	1599.5	2050.0	1887.5	1911.0	1805.5	1859.9	149.94
TAX1BP3	2998.0	2733.5	3177.0	3020.0	3222.0	3153.5	3050.7	179.00
TAZ	273.0	420.0	240.0	247.5	398.0	235.5	302.3	83.92
TBC1D1	233.0	235.0	363.5	276.0	278.5	361.5	291.3	58.49
TBC1D10A	690.0	811.5	573.0	678.5	776.5	666.5	699.3	84.97
TBC1D12	369.5	132.0	194.5	337.5	153.5	164.5	225.3	101.87
TBC1D13	525.0	364.5	440.5	497.0	365.5	457.5	441.7	66.35
TBC1D14	2014.0	1638.0	1744.0	1662.5	1944.5	1763.0	1794.3	152.41
TBC1D15	1332.0	896.0	1046.0	1309.5	985.0	904.0	1078.8	195.54
TBC1D16	136.5	308.0	282.0	130.5	354.5	300.5	252.0	94.87
TBC1D19	353.5	137.0	172.5	309.0	146.5	146.5	210.8	95.07
TBC1D2	516.0	346.0	296.5	449.5	368.0	258.5	372.4	95.93
TBC1D20	554.5	523.0	638.0	562.5	525.0	617.0	570.0	47.67
TBC1D22A	117.0	39.5	116.0	111.5	43.5	99.0	87.8	36.42
TBC1D22B	213.5	138.5	216.0	174.5	151.0	214.5	184.7	34.85
TBC1D23	1267.5	943.5	1044.0	1213.5	1086.0	1041.5	1099.3	120.15
TBC1D24	216.5	115.0	202.0	204.0	134.0	186.5	176.3	41.70
TBC1D25	248.0	218.5	278.0	244.0	196.5	309.0	249.0	40.38
TBC1D30	42.5	29.0	19.0	34.5	36.5	23.0	30.8	8.79
TBC1D31	322.0	166.0	210.5	288.0	190.0	195.0	228.6	61.83
TBC1D32	143.0	106.5	177.0	118.5	128.0	140.0	135.5	24.43

TBC1D4	351.5	359.5	297.5	326.5	442.0	325.0	350.3	49.98
TBC1D7	220.0	232.0	167.5	210.0	236.5	167.5	205.6	30.92
TBC1D8	483.5	731.5	704.5	336.0	877.0	658.5	631.8	192.64
TBC1D8B	856.5	524.5	593.5	797.0	609.0	555.5	656.0	136.81
TBC1D9	588.0	419.5	549.0	551.0	438.0	489.0	505.8	67.81
TBC1D9B	1777.5	929.0	1332.5	1767.0	1086.0	1257.0	1358.2	349.90
TBCA	1332.5	1281.5	992.0	1106.0	1360.5	791.5	1144.0	223.66
TBCC	68.5	57.5	73.5	78.5	57.5	82.0	69.6	10.41
TBCCD1	387.0	232.0	146.0	337.5	222.0	148.5	245.5	98.52
TBCD	799.5	440.5	517.0	834.5	495.0	461.0	591.3	177.20
TBCE	705.5	379.0	343.5	592.0	434.5	285.0	456.6	160.64
TBCEL	344.5	288.5	429.0	313.5	309.0	382.5	344.5	52.80
TBK1	354.5	212.5	425.0	349.0	242.0	388.0	328.5	83.53
TBL1X	627.5	546.0	717.0	614.5	595.5	651.0	625.3	57.21
TBL1XR1	187.5	208.0	327.5	203.5	219.0	282.0	237.9	54.68
TBL2	778.5	616.5	364.0	733.5	678.5	412.5	597.3	171.42
TBL3	671.0	496.0	540.0	725.0	502.0	486.5	570.1	102.17
TBP	474.0	419.5	486.5	513.5	478.5	445.5	469.6	32.87
TBPL1	269.0	293.0	329.0	271.0	310.5	325.0	299.6	26.18
TBPL2	1.5	1.5	1.5	0.5	3.0	2.0	1.7	0.82
TBRG4	1113.5	915.0	701.5	1036.0	835.0	639.5	873.4	185.23
TBX10	0.0	0.0	0.0	0.0	0.0	0.0	0.0	0.00
TBX15	57.5	197.5	269.0	51.5	209.5	169.0	159.0	87.28
TBX18	6.0	2.0	7.0	1.5	0.0	2.0	3.1	2.76
TBX19	1.0	0.0	1.0	0.0	1.5	1.0	0.8	0.61
TBX2	0.0	0.5	1.0	0.0	0.0	0.0	0.3	0.42
TBX20	0.0	0.0	1.5	0.0	0.0	0.0	0.3	0.61
TBX22	0.0	0.0	0.0	0.0	0.0	0.5	0.1	0.20
TBX4	156.5	143.5	75.5	145.5	163.0	93.0	129.5	36.20
TBX5	0.0	0.0	0.0	0.0	0.0	0.0	0.0	0.00
TBXA2R	76.5	18.0	19.0	46.5	10.5	9.0	29.9	26.54
TC2N	5.5	10.5	11.0	6.0	10.5	13.0	9.4	2.99
TCAIM	449.0	335.0	362.5	419.0	363.5	407.0	389.3	42.64
TCAP	3.0	2.0	0.5	0.5	1.0	2.5	1.6	1.07
TCEA1	712.0	610.0	601.0	630.0	674.0	556.0	630.5	55.45
TCEA2	456.0	257.0	129.5	501.0	293.0	147.0	297.3	154.32
TCEA3	85.5	105.0	163.5	51.5	85.5	154.0	107.5	43.38
TCEANC	115.5	99.5	111.5	105.0	109.0	93.5	105.7	8.10
TCEANC2	117.0	108.0	113.0	128.0	139.0	117.5	120.4	11.24
TCEB1	1136.5	1548.5	1481.5	1109.0	1756.0	1490.5	1420.3	251.10
TCEB3	652.0	531.0	622.5	586.0	571.5	540.0	583.8	46.93
TCERG1	1421.0	1042.5	1023.5	1380.5	1156.0	1051.5	1179.2	178.19
TCERG1L	0.0	0.0	0.0	0.5	0.5	0.0	0.2	0.26
TCF15	298.0	370.0	311.5	233.5	342.5	411.0	327.8	61.59
TCF20	709.5	500.5	786.5	641.5	571.5	807.0	669.4	120.95
TCF21	0.0	0.0	0.0	0.0	0.0	0.0	0.0	0.00
TCF24	72.0	44.5	82.0	42.5	45.0	62.0	58.0	16.61
TCF25	1036.0	863.5	656.0	873.0	802.5	603.5	805.8	157.62
TCF3	107.5	65.5	231.0	105.5	68.5	196.5	129.1	68.80
TCF7	9.5	14.5	29.5	14.5	16.0	21.5	17.6	6.99
TCF7L1	250.5	215.0	224.5	262.0	221.0	238.5	235.3	18.37
TCFL5	29.0	12.0	35.0	26.5	14.0	23.5	23.3	8.88
TCHH	3.0	8.0	7.0	1.0	11.0	6.0	6.0	3.58
TCHP	1672.0	1640.0	1084.0	1474.0	1779.0	1142.5	1465.3	290.28

TCIRG1	590.0	560.0	561.0	523.0	498.0	582.0	552.3	35.34
TCN2	1297.5	741.5	487.5	1206.0	829.5	588.0	858.3	328.27
TCOF1	3106.0	3210.5	3043.5	3036.5	3696.0	3286.5	3229.8	248.33
TCP1	3328.5	2945.5	3259.0	3543.0	3200.0	2849.5	3187.6	254.69
TCP11	16.5	3.0	2.0	11.0	2.5	1.0	6.0	6.28
TCP11L1	84.0	51.5	125.5	97.0	61.0	114.0	88.8	29.11
TCP11L2	1173.0	677.5	874.0	1115.5	846.5	820.0	917.8	188.96
TCTE1	1.5	3.0	5.5	1.0	1.5	2.5	2.5	1.64
TCTE3	38.0	21.5	9.5	30.5	20.5	4.5	20.8	12.52
TCTEX1D1	27.5	11.0	8.0	26.5	11.0	11.5	15.9	8.68
TCTEX1D2	95.0	88.5	73.0	98.5	84.0	66.0	84.2	12.62
TCTEX1D4	0.0	0.5	1.0	0.0	0.5	1.0	0.5	0.45
TCTN1	261.0	133.5	177.0	225.0	136.5	170.0	183.8	50.32
TCTN2	308.0	206.5	259.0	326.5	201.0	254.5	259.3	51.18
TCTN3	774.0	580.5	707.5	702.5	654.5	658.0	679.5	65.01
TDG	152.0	77.0	146.0	162.5	102.5	139.5	129.9	33.02
TDO2	2.5	2.0	3.0	0.5	2.0	4.0	2.3	1.17
TDP1	202.5	161.5	166.0	213.0	170.5	147.0	176.8	25.49
TDP2	339.5	242.5	203.0	309.0	274.5	154.0	253.8	68.54
TDRD1	2.0	1.5	4.0	1.0	1.5	2.5	2.1	1.07
TDRD12	11.0	2.5	5.5	11.5	5.5	9.0	7.5	3.56
TDRD3	675.5	581.0	554.0	611.5	638.5	519.0	596.6	57.07
TDRD5	0.0	0.5	0.0	1.0	0.0	0.0	0.3	0.42
TDRD6	3.0	2.0	7.5	3.0	3.0	3.5	3.7	1.94
TDRD7	585.0	395.0	328.0	547.5	493.5	347.0	449.3	107.83
TDRD9	8.0	3.0	2.5	3.0	6.5	1.5	4.1	2.56
TDRP	8.5	0.0	4.5	4.5	1.5	3.5	3.8	2.93
TEAD1	129.5	19.5	191.5	152.0	28.0	175.5	116.0	74.53
TEAD3	480.0	395.5	889.0	476.0	394.0	745.0	563.3	205.17
TEC	19.5	5.5	13.5	12.5	8.0	13.0	12.0	4.86
TECPR1	397.5	289.5	358.5	319.0	349.0	333.5	341.2	36.76
TECPR2	433.5	407.5	511.5	452.0	480.0	467.5	458.7	36.39
TECR	132.5	76.5	118.5	117.5	88.0	93.5	104.4	21.57
TECRL	507.0	179.5	389.5	28.0	0.0	5.0	184.8	217.63
TECTA	14.0	20.0	21.5	8.5	20.0	26.0	18.3	6.16
TECTB	1.0	0.0	0.5	0.0	0.5	0.5	0.4	0.38
TEF	2571.5	1589.0	1873.5	2473.0	1916.5	2209.5	2105.5	379.30
TEK	0.0	0.0	0.0	0.0	0.0	0.0	0.0	0.00
TEKT1	439.0	226.5	249.0	435.5	215.5	186.0	291.9	114.39
TEKT2	2.5	7.5	3.5	2.0	8.5	8.5	5.4	3.07
TEKT3	0.0	0.5	0.0	0.0	0.0	0.0	0.1	0.20
TEKT4	1.0	0.0	0.5	0.0	0.0	0.0	0.3	0.42
TEKT5	0.0	1.0	0.0	0.0	0.5	0.5	0.3	0.41
TELO2	191.0	138.5	201.0	190.0	120.5	163.0	167.3	32.41
TEN1	16.5	6.5	4.0	19.5	10.0	2.5	9.8	6.88
TENM1	0.5	0.0	0.0	0.0	0.0	0.5	0.2	0.26
TENM2	5.0	3.0	3.5	3.0	4.0	13.0	5.3	3.87
TERF1	290.5	92.5	144.5	268.0	100.5	84.0	163.3	92.46
TERF2	479.5	309.5	425.5	486.0	339.5	390.0	405.0	72.34
TERF2IP	389.5	295.5	270.0	360.5	328.0	259.5	317.2	51.47
TERT	1.0	0.0	0.0	1.0	0.5	0.5	0.5	0.45
TES	924.5	577.5	841.5	898.0	608.0	677.0	754.4	152.22
TESC	5.5	2.0	0.5	6.5	2.5	1.0	3.0	2.45
TESK1	837.0	2047.5	1020.5	923.5	2386.0	1196.0	1401.8	651.32

TESPA1	0.0	0.0	0.0	0.0	0.0	0.0	0.0	0.00
TET1	112.0	74.0	157.5	95.0	91.0	140.0	111.6	31.71
TET2	61.5	30.5	127.5	57.0	34.0	137.0	74.6	46.40
TET3	84.5	81.0	113.5	69.0	69.0	106.0	87.2	18.73
TEX10	234.0	185.5	336.0	216.5	209.5	332.0	252.3	65.22
TEX11	106.5	61.5	65.5	111.0	70.5	62.5	79.6	22.85
TEX12	1.0	0.5	0.5	1.5	2.5	0.0	1.0	0.89
TEX14	1190.5	644.5	621.5	985.5	828.5	874.0	857.4	214.22
TEX2	1725.5	731.0	1015.5	1560.0	821.5	914.0	1127.9	413.19
TEX264	872.5	631.0	503.5	824.5	610.5	567.0	668.2	147.14
TEX30	82.0	43.5	23.0	87.0	47.5	28.0	51.8	26.96
TEX33	2.5	2.5	1.0	2.0	2.5	3.5	2.3	0.82
TEX36	1.5	1.0	0.0	1.0	0.5	0.0	0.7	0.61
TEX9	90.0	56.0	39.0	94.5	58.5	43.0	63.5	23.52
TF	190.0	73.0	60.0	162.5	82.0	73.5	106.8	54.92
TFAM	816.5	871.0	718.5	768.5	828.0	678.5	780.2	72.30
TFAP2A	1.0	0.0	0.0	0.0	0.0	0.0	0.2	0.41
TFAP2C	0.5	3.5	2.5	2.5	1.0	1.0	1.8	1.17
TFAP2D	0.0	0.0	0.0	0.0	0.0	0.0	0.0	0.00
TFAP2E	0.0	0.0	0.5	1.0	1.5	0.0	0.5	0.63
TFAP4	110.5	95.5	91.5	100.5	82.5	108.0	98.1	10.50
TFB1M	259.5	165.5	219.0	212.5	198.0	192.5	207.8	31.43
TFB2M	450.5	317.0	359.0	400.5	348.0	289.0	360.7	58.07
TFCP2	842.5	569.0	626.0	687.5	570.5	538.0	638.9	112.90
TFCP2L1	252.5	644.5	451.0	245.5	695.5	728.0	502.8	218.84
TFDP1	1152.0	761.0	852.0	1101.0	802.5	655.5	887.3	196.90
TFEB	1013.5	673.0	821.5	975.0	648.0	908.0	839.8	153.65
TFEC	0.0	0.0	0.0	0.0	0.0	0.0	0.0	0.00
TFG	1765.0	1504.5	1279.5	1673.5	1511.0	1201.5	1489.2	217.94
TFIP11	615.5	241.5	506.5	576.0	294.0	447.5	446.8	151.14
TFPI	294.0	206.0	88.0	259.5	248.0	129.5	204.2	80.16
TFPI2	2456.0	2018.0	2586.0	2202.5	2141.0	1636.0	2173.3	336.14
TFRC	5394.0	3758.0	6180.0	5210.5	4202.0	5415.0	5026.6	887.61
TG	0.5	0.0	0.0	1.0	0.0	0.0	0.3	0.42
TGDS	39.5	17.5	42.0	35.0	22.0	46.5	33.8	11.55
TGFA	2.0	2.5	10.0	3.5	4.0	8.5	5.1	3.34
TGFB2	92.5	148.5	146.0	107.0	161.0	174.0	138.2	31.72
TGFB3	6492.5	13301.0	11717.5	6791.5	15108.5	14352.5	11293.9	3779.92
TGFBI	1008.5	924.0	712.5	1024.0	1224.0	955.5	974.8	165.78
TGFBR1	674.0	532.0	787.0	709.0	595.0	772.5	678.3	99.99
TGFBR2	403.5	164.0	349.5	443.0	204.0	357.0	320.2	111.47
TGFBRAP1	337.0	258.5	243.5	316.5	302.0	259.0	286.1	37.63
TGIF1	221.5	329.0	336.5	245.0	359.0	346.5	306.3	57.91
TGIF2	134.5	148.0	240.5	151.5	134.5	217.5	171.1	45.97
TGM2	71.0	98.0	32.0	36.5	138.5	85.0	76.8	39.97
TGM4	223.5	181.5	119.5	218.0	185.0	163.0	181.8	38.20
TGOLN2	1207.5	619.5	1013.0	1326.5	710.0	942.0	969.8	274.44
TGS1	432.0	268.5	413.5	416.0	274.0	373.0	362.8	73.58
TH	0.0	0.5	0.0	0.0	0.0	0.0	0.1	0.20
THADA	373.0	280.0	315.0	307.0	283.5	307.5	311.0	33.48
THAP11	68.0	43.0	74.5	76.0	45.5	74.0	63.5	15.18
THAP4	315.0	282.5	350.0	302.5	343.5	335.0	321.4	26.09
THAP5	255.5	228.0	226.5	247.5	240.5	249.0	241.2	11.80
THAP7	459.5	643.5	428.0	439.0	638.5	439.5	508.0	103.53

THAP9	793.5	611.5	393.5	750.5	704.0	389.5	607.1	177.59
THBD	3386.5	1290.5	1511.0	3508.5	1565.0	1898.5	2193.3	991.54
THBS1	13397.00	8742.00	23196.00	20307.50	10689.00	26284.00	17102.6	7161.90
THBS2	3.5	4.5	22	1	5	5.5	6.9	7.56
THBS4	51.5	9.5	21	48	6.5	6	23.8	20.89
THEMIS	0	0	0	0	0	0	0.0	0.00
THEMIS2	4	9	0.5	4.5	7.5	2	4.6	3.22
THG1L	273.5	248	220	258	240	198.5	239.7	26.95
THNSL1	261	112	143	247.5	123.5	122	168.2	67.57
THNSL2	163.5	111.5	128	176	130.5	164	145.6	25.63
THOC1	589.5	413	457	601.5	472	400	488.8	86.91
THOC2	1233.5	1159.5	1331	1201	1327.5	1281	1255.6	69.58
THOC3	298	265.5	248	295	271	238	269.3	24.23
THOC5	1275.5	1229	1058	1212.5	1158.5	951.5	1147.5	121.49
THOC6	79	80	102	79	86	109.5	89.3	13.26
THOC7	1420.5	1199.5	935.5	1205.5	1319.5	817.5	1149.7	229.78
THOP1	793	808.5	917.5	777	828	851.5	829.3	50.52
THP2	0.5	0	0	0	0	0	0.1	0.20
THPO	18	21.5	26.5	9	13.5	21	18.3	6.23
THRA	54	80	92.5	37	66.5	106	72.7	25.36
THRAP3	2218.5	2055.5	2271.5	2197	2306.5	2332	2230.2	99.65
THRB	421.5	348.5	710	466.5	427.5	755	521.5	168.43
THSD1	137.5	46.5	57.5	138.5	50.5	56	81.1	44.26
THSD7A	20.5	12	13	22.5	13	15.5	16.1	4.40
THUMPD1	625	407	308.5	489.5	381.5	261	412.1	131.03
THUMPD2	290	164	156	255.5	186.5	142	199.0	59.92
THUMPD3	1017.5	921	813.5	952.5	945.5	773	903.8	92.30
THY1	181.5	331.5	587.5	232.5	412.5	903	441.4	267.78
THYN1	2743	2228	1985.5	2455.5	2280	1717	2234.8	357.58
TIA1	656	565.5	655.5	644.5	643	647	635.3	34.61
TIAL1	1119	793	756.5	1048	895	713	887.4	164.89
TIAM1	199.5	206	182.5	218	234	302.5	223.8	42.30
TIAM2	181.5	116.5	226.5	158.5	146	220	174.8	43.02
TICAM1	564.5	435.5	376	556	492	446	478.3	73.48
TICRR	419.5	154.5	314	431.5	148	207.5	279.2	128.06
TIE1	1.5	2.5	4.5	2.5	0.5	4	2.6	1.50
TIFA	60.5	27	28	46.5	26.5	29	36.3	14.10
TIGD3	195.5	195	199	171	180	213.5	192.3	14.95
TIGD5	56	76.5	99	57.5	89	92	78.3	18.24
TIMELESS	172	99.5	90	163	79.5	82	114.3	41.87
TIMM10	392	394	143	384	351.5	142.5	301.2	123.66
TIMM13	185	177.5	84.5	202.5	213	98	160.1	54.94
TIMM17A	1368	1436.5	802	1186	1497	792	1180.3	314.67
TIMM21	638	399	378	466.5	412	295.5	431.5	115.47
TIMM22	606	612.5	424	532	682.5	448.5	550.9	101.09
TIMM44	762.5	648.5	410	713	669.5	376	596.6	162.83
TIMM50	883	813.5	390.5	788	846.5	387.5	684.8	231.36
TIMM8A	1039.5	1551	742	1059	1523.5	713.5	1104.8	364.83
TIMM9	394.5	398	262	292	412	239.5	333.0	77.09
TIMMDC1	639.5	520.5	283	629	540.5	263.5	479.3	166.51
TIMP2	10831	5578.5	5535.5	10511	5195	7093.5	7457.4	2576.06
TIMP3	995.5	2316	8574	1018	2885.5	7668.5	3909.6	3356.42
TIMP4	133	92	60	128	112.5	67	98.8	30.90
TINAG	0	0	0	0	0	0	0.0	0.00

TINAGL1	80	28.5	88.5	77	28	66.5	61.4	26.64
TIPARP	1882	1660	1356	1877	1746.5	1684.5	1701.0	193.28
TIPIN	912.5	722	651.5	993	815.5	606.5	783.5	151.06
TIPRL	449.5	303.5	319	421	336.5	297.5	354.5	64.62
TIRAP	205	239	200	180.5	255	260.5	223.3	32.70
TJAP1	45.5	41.5	132	64.5	47	128	76.4	42.27
TJP1	44	22	189	54.5	23.5	133	77.7	68.06
TJP2	958.5	382.5	664	874.5	382	445.5	617.8	254.86
TJP3	172	201	161	106.5	196	216.5	175.5	39.33
TK1	940.5	525.5	650.5	837.5	494	403.5	641.9	209.64
TK2	549.5	418.5	715	575	487.5	676.5	570.3	111.87
TKT	1149.5	412	241	1116	430.5	213	593.7	426.78
TLCD1	1080.5	811	379	1017	764.5	397	741.5	298.75
TLCD2	31	34.5	13.5	25	26	16.5	24.4	8.12
TLDC1	461	246	282	477	245.5	219.5	321.8	115.83
TLDC2	0.5	1	1	2.5	1	0.5	1.1	0.74
TLE1	170.5	202	260	174.5	237.5	283.5	221.3	46.42
TLE2	8	7.5	7	8	4.5	5	6.7	1.54
TLE3	188.5	288.5	318	194	328	293	268.3	61.55
TLK2	580	694	679.5	568	733	690.5	657.5	67.26
TLL1	128.5	774	1272	157	970	1672.5	829.0	612.03
TLL2	73.5	140	133	71.5	143.5	146	117.9	35.46
TLR3	139	66.5	156	135	73.5	119	114.8	36.74
TLR4	0	0	0	0	0	0	0.0	0.00
TLR5	17	12	5.5	23.5	10	6	12.3	6.91
TLR7	4	0	1.5	0.5	1	3	1.7	1.54
TLX1	0	0.5	0	0	0	0	0.1	0.20
TLX2	0	0	2	0	0.5	5	1.3	1.99
TM2D1	882	872	867	836	972	892	886.8	45.83
TM2D2	546	484.5	328	500.5	476.5	336	445.3	91.00
TM2D3	728	435.5	357.5	802	471	341.5	522.6	195.22
TM4SF19	0.5	0.5	0	0	1.5	0	0.4	0.58
TM4SF4	0.5	0	0	0.5	0	1	0.3	0.41
TM6SF1	611	622.5	853.5	558	615.5	640.5	650.2	103.38
TM6SF2	0	0	0	0	0	0	0.0	0.00
TM7SF3	414.5	158	291.5	434.5	206	259.5	294.0	111.08
TM9SF2	2182	1158	2286	2269.5	1455	2031	1896.9	475.55
TM9SF3	3679	2319	3064.5	4101.5	2656	2944.5	3127.4	658.22
TM9SF4	1005	746.5	1253.5	992.5	820.5	1167	997.5	194.26
TMA16	807.5	599.5	454.5	702	643	391.5	599.7	154.89
TMBIM1	2398.5	1435.5	1362	2143.5	1453	1489	1713.6	441.19
TMBIM4	1026.5	603	702.5	967.5	706	627.5	772.2	179.79
TMC2	0.5	1.5	0.5	0.5	0.5	0.5	0.7	0.41
TMC3	1.5	1	2	0	6.5	2	2.2	2.25
TMC5	6	2	0	7.5	4	2	3.6	2.80
TMC6	645.5	507	489.5	630.5	519.5	542	555.7	66.20
TMCC1	378	284	456	379	331	408.5	372.8	59.82
TMCC2	192	291.5	428.5	160	322	395.5	298.3	107.18
TMCC3	84.5	61	109	25	20.5	40	56.7	34.99
TMCO1	667	585	457	664	685	474	588.7	101.58
TMCO3	280	168.5	175	258.5	172	189.5	207.3	49.03
TMCO4	122	85	116.5	110.5	63	86	97.2	22.84
TMCO6	389	468.5	313	387	452.5	359	394.8	58.01
TMED10	3287.5	1963.5	2472	3338.5	2146	2404.5	2602.0	580.32

TMED2	2241	1325	2053	2159	1519	2095	1898.7	379.62
TMED3	331.5	556	490.5	356.5	648	598.5	496.8	129.43
TMED5	3359.5	3306	3480	3425	3591.5	2941.5	3350.6	223.49
TMED6	6	23	4.5	8	22.5	9.5	12.3	8.31
TMED7	420	228.5	438	465	295.5	370.5	369.6	91.35
TMED8	150	149	276	146.5	173	243.5	189.7	56.07
TMEFF1	7.5	11	54.5	6.5	15.5	29.5	20.8	18.53
TMEFF2	122.5	197.5	216	89.5	234	183.5	173.8	56.21
TMEM100	14.5	62	48	13	72.5	116.5	54.4	38.96
TMEM101	350.5	355	270.5	340	390.5	271.5	329.7	48.51
TMEM104	368	209.5	238.5	421	257	274	294.7	81.97
TMEM106B	1774	1193.5	1694.5	1795.5	1335.5	1455.5	1541.4	250.17
TMEM106C	36	20	40	29	22.5	33	30.1	7.77
TMEM108	8	5	2.5	8	6	3	5.4	2.38
TMEM109	69.5	156	129	64.5	155.5	157	121.9	43.85
TMEM11	302	410	324.5	323	455.5	337.5	358.8	60.18
TMEM114	2	6	2	1.5	5.5	5	3.7	2.04
TMEM115	1676	1260.5	1136	1555	1322	1122.5	1345.3	225.62
TMEM116	195	169.5	173	197	213	174.5	187.0	17.31
TMEM117	420	425	321.5	607.5	734	579	514.5	151.78
TMEM119	38.5	24.5	23.5	49.5	26	17.5	29.9	11.81
TMEM120A	480	315.5	211.5	432.5	335	244.5	336.5	104.25
TMEM120B	253	119	89.5	243	119.5	77.5	150.3	77.54
TMEM123	1439.5	1013	810	1453	1231.5	910.5	1142.9	273.38
TMEM125	4	15.5	6.5	3	10	10.5	8.3	4.68
TMEM126A	391	318.5	219	382	329.5	196.5	306.1	81.57
TMEM127	603.5	506	395	584.5	509.5	415.5	502.3	84.98
TMEM128	185.5	69	76.5	161.5	82	81	109.3	50.55
TMEM129	205	116	184.5	209.5	120.5	155.5	165.2	41.07
TMEM130	340	147	138.5	381	158	172.5	222.8	108.02
TMEM131	1222	813	1486	1146.5	960	1469.5	1182.8	269.58
TMEM132A	796.5	611	839	819.5	584	889	756.5	127.17
TMEM132B	4.5	3	4.5	2.5	1.5	4.5	3.4	1.28
TMEM132C	1	2.5	11	2	2.5	10.5	4.9	4.55
TMEM132D	1	5.5	7	1.5	6.5	12	5.6	4.04
TMEM132E	1	1.5	1.5	1	0.5	0	0.9	0.58
TMEM136	49.5	28	12	46.5	27.5	13	29.4	15.96
TMEM138	627.5	657	487	589	627	486	578.9	74.77
TMEM139	0.5	0.5	0	0	0	0.5	0.3	0.27
TMEM140	241.5	147.5	236.5	252.5	170.5	196.5	207.5	42.69
TMEM141	160.5	149	70	146	168.5	67.5	126.9	45.78
TMEM144	67	33.5	48	55	35	47.5	47.7	12.56
TMEM14A	200.5	198	130.5	177.5	266.5	151.5	187.4	47.26
TMEM150A	211	178	128	194	181	150.5	173.8	30.01
TMEM150C	118.5	96	174.5	161	128	192	145.0	36.68
TMEM151B	0	0	0.5	0	0	0	0.1	0.20
TMEM154	0.5	0	0	0	0.5	0	0.2	0.26
TMEM156	0	1	0	0	0.5	0.5	0.3	0.41
TMEM159	1817.5	1168.5	1163.5	1764.5	1264	1253	1405.2	302.20
TMEM161A	183	158.5	172.5	163	152.5	157.5	164.5	11.29
TMEM161B	467.5	345.5	375	500.5	403	330	403.6	67.95
TMEM164	605	316.5	510.5	596	367	372	461.2	125.74
TMEM165	1379.5	1229.5	1177.5	1333	1466.5	1158	1290.7	122.27
TMEM168	737	443.5	757	728	529.5	627	637.0	127.89

TMEM169	380	283.5	312	395.5	315.5	386.5	345.5	47.41
TMEM17	51.5	43.5	47	57	63	48	51.7	7.19
TMEM170A	335.5	295.5	285	308.5	309.5	295.5	304.9	17.57
TMEM170B	408	246	270	388.5	290	200.5	300.5	81.62
TMEM171	4.5	1.5	15	2.5	2.5	10	6.0	5.37
TMEM173	55.5	29.5	20	46.5	42	23.5	36.2	14.01
TMEM174	0	0	0	0	0.5	0	0.1	0.20
TMEM175	317.5	110.5	299.5	291	120.5	269	234.7	93.67
TMEM177	316	311	203.5	349.5	354	217	291.8	65.64
TMEM178B	2	1.5	6	1.5	2	3	2.7	1.72
TMEM179	0	0	0	0	0	0	0.0	0.00
TMEM18	148.5	69.5	130	132	93.5	91.5	110.8	30.39
TMEM180	1193.5	1514.5	1464	1365	1725.5	1496.5	1459.8	175.99
TMEM182	68	251.5	182.5	42	288	153	164.2	97.57
TMEM184A	892.5	735	804.5	857	855.5	923.5	844.7	66.95
TMEM184B	1177.5	561	1230	1134.5	639.5	1142	980.8	297.70
TMEM184C	648.5	349.5	720.5	620.5	361.5	642.5	557.2	159.83
TMEM185A	354	356.5	444.5	358.5	414.5	428	392.7	40.94
TMEM186	478.5	371	475	516.5	388.5	457	447.8	56.39
TMEM19	472	334.5	403	398	339.5	277	370.7	67.99
TMEM192	253	143	144	220.5	145.5	130	172.7	50.99
TMEM194A	225.5	121.5	178.5	258	135	164.5	180.5	52.62
TMEM194B	1109.5	526.5	538.5	1125.5	569	498.5	727.9	302.66
TMEM196	1.5	2.5	2.5	0.5	1	4.5	2.1	1.43
TMEM198	69.5	236	271.5	63.5	234	266	190.1	96.95
TMEM199	387	425.5	208.5	370.5	450	209	341.8	106.76
TMEM200A	1175.5	1656.5	2132	1229	1909.5	1798.5	1650.2	380.57
TMEM200B	298.5	181.5	92.5	214.5	192.5	95	179.1	77.80
TMEM200C	0.5	0.5	1.5	0.5	2.5	3.5	1.5	1.26
TMEM201	1041.5	657.5	1353.5	932	731	1234	991.6	273.97
TMEM203	320	376.5	210	300	382.5	222	301.8	73.79
TMEM204	6.5	22.5	19	9.5	28.5	42	21.3	13.00
TMEM206	245.5	208	207	251	235.5	211.5	226.4	19.95
TMEM207	0	0	0	0	0	0	0.0	0.00
TMEM209	165.5	72.5	203	180	93.5	163.5	146.3	51.48
TMEM213	8	10.5	2	4.5	5	2.5	5.4	3.28
TMEM214	1351.5	997.5	594.5	1431	1003	713.5	1015.2	333.00
TMEM215	0	1.5	2.5	1	0.5	0.5	1.0	0.89
TMEM216	179	173.5	147.5	171	176.5	158.5	167.7	12.18
TMEM220	125.5	73.5	49	128	84.5	49.5	85.0	35.15
TMEM221	1.5	0.5	1	0.5	0.5	2	1.0	0.63
TMEM222	555.5	488	422	554	444.5	453.5	486.3	57.15
TMEM223	178	256.5	215	211	276	249	230.9	35.96
TMEM229B	21	7.5	35	19	12.5	40	22.5	12.66
TMEM230	852.5	897	711	809.5	988	699.5	826.3	110.89
TMEM231	1549	465	386	1521.5	508.5	355	797.5	574.13
TMEM233	15.5	174	240	18	215	227.5	148.3	104.31
TMEM234	195.5	152.5	112.5	170	180	98	151.4	38.66
TMEM237	776.5	649	770	730	697.5	733	726.0	47.52
TMEM240	0.5	0	0	0	1	0.5	0.3	0.41
TMEM241	289	217.5	155.5	238	209	150.5	209.9	52.14
TMEM242	612.5	404.5	262.5	514	411.5	240	407.5	143.16
TMEM243	186	155	105.5	138.5	175	118	146.3	31.63
TMEM244	0.5	0	1	0	0.5	0	0.3	0.41

TMEM245	626	239.5	557	575.5	256.5	453	451.3	167.29
TMEM246	49.5	41	79.5	66	40.5	77	58.9	17.61
TMEM247	2	0.5	1	3	3.5	0	1.7	1.40
TMEM248	1028	603	958	1054.5	648.5	909.5	866.9	194.22
TMEM252	0	0	0	0	0	0	0.0	0.00
TMEM254	71.5	32.5	75	67	36.5	85	61.3	21.59
TMEM255A	492.5	384	404	394	407	383.5	410.8	41.19
TMEM255B	1.5	0	1	0.5	0.5	0.5	0.7	0.52
TMEM256	415	556.5	213	444	555	242	404.3	148.66
TMEM258	1086	1258	919	1037	1358	924.5	1097.1	178.44
TMEM259	493.5	338	573.5	494	308	542.5	458.3	109.49
TMEM26	0	0	1.5	2	0.5	0.5	0.8	0.82
TMEM260	471	276	307.5	441.5	275	275.5	341.1	90.55
TMEM263	1514.5	1184.5	1652	1378.5	1378.5	1523	1438.5	161.38
TMEM27	0	0.5	0	0	0	0	0.1	0.20
TMEM30A	583	270.5	812.5	628	348.5	701.5	557.3	208.53
TMEM33	1495.5	975.5	1104	1468.5	1022.5	936	1167.0	250.48
TMEM35	20.5	8	13	9.5	5	5.5	10.3	5.80
TMEM37	2	1	3	2	0.5	2.5	1.8	0.93
TMEM38A	962	1323	1175.5	923.5	1540	1036	1160.0	237.37
TMEM38B	331.5	232	499.5	253.5	287	357.5	326.8	96.68
TMEM39A	649	471	646	686.5	551.5	676.5	613.4	84.58
TMEM39B	173	142.5	116	151	133	135.5	141.8	19.19
TMEM40	2.5	6	4.5	5.5	5	6.5	5.0	1.41
TMEM41A	228.5	209.5	143	210.5	243.5	172	201.2	37.23
TMEM41B	101	28	134	104	41	107	85.8	41.67
TMEM43	2882.5	1248.5	1630	2868	1444.5	1519.5	1932.2	741.04
TMEM45A	1023.5	650	1119.5	1130	781	1316.5	1003.4	245.88
TMEM47	6477	1486.5	2986.5	6384	1699.5	2421.5	3575.8	2274.57
TMEM5	221	113	123	206.5	139.5	116.5	153.3	47.96
TMEM50A	735	439	771	830	543.5	663.5	663.7	147.78
TMEM50B	680	572.5	622	603	614	629	620.1	35.40
TMEM51	3.5	2	2	2.5	4	2.5	2.8	0.82
TMEM52	253	230	177	218	321	201.5	233.4	50.01
TMEM52B	0	0	0	0	0	0	0.0	0.00
TMEM53	506	283.5	252.5	472.5	286	234	339.1	118.41
TMEM55A	47	16	50	52.5	22	51	39.8	16.28
TMEM57	891	1072.5	844	943	1330	891.5	995.3	181.80
TMEM59	2067	1312.5	1812	2059.5	1599	1799	1774.8	287.41
TMEM59L	22	15	19	13	10.5	15	15.8	4.14
TMEM60	376.5	352.5	257.5	318.5	374	245.5	320.8	57.65
TMEM61	4	5.5	3.5	2.5	1.5	7	4.0	2.00
TMEM62	300.5	187.5	247.5	266.5	205.5	205	235.4	43.51
TMEM63A	127.5	76	89.5	116.5	83	88.5	96.8	20.38
TMEM63C	147.5	62.5	47	120	55.5	71.5	84.0	40.32
TMEM64	278	381	548	313	477	521.5	419.8	112.31
TMEM65	269	198	279.5	251	224.5	238	243.3	29.88
TMEM67	46	11.5	38.5	40	16.5	38.5	31.8	14.18
TMEM69	218.5	150.5	142.5	211.5	197.5	148.5	178.2	34.73
TMEM70	620.5	598.5	548.5	527.5	607	420	553.7	74.70
TMEM71	5.5	1.5	0.5	2	1.5	1.5	2.1	1.74
TMEM72	2.5	0	0.5	1.5	0	0.5	0.8	0.98
TMEM74	20	8.5	17	14.5	12	13	14.2	4.01
TMEM79	257.5	153	64	245	137.5	54.5	151.9	86.30

TMEM80	339.5	166.5	145.5	329	201	176	226.3	85.60
TMEM81	266.5	292.5	232	245.5	323.5	257.5	269.6	33.41
TMEM82	23.5	20	10	17.5	20	14.5	17.6	4.77
TMEM86A	197.5	44.5	27.5	168.5	52.5	38	88.1	74.54
TMEM87A	715	183.5	460	774.5	226.5	381	456.8	245.39
TMEM88B	1.5	3.5	4.5	2	5	2	3.1	1.46
TMEM8A	672.5	399.5	520.5	637.5	381	451	510.3	122.56
TMEM8C	3413.5	6493.5	5001.5	3800.5	7578.5	5388.5	5279.3	1582.26
TMEM9	76.5	159	260.5	63.5	163.5	179.5	150.4	72.43
TMEM97	612.5	415	321.5	571.5	484.5	365	461.7	115.33
TMEM98	13.5	39.5	6	16	36.5	8.5	20.0	14.42
TMEM9B	1786	1114	1167.5	1636.5	1307	1054.5	1344.3	300.05
TMF1	673	386	630.5	693	466	605.5	575.7	122.59
TMIE	0	0	0	0.5	0	0	0.1	0.20
TMIGD1	14	4.5	1.5	7.5	5	3	5.9	4.44
TMLHE	820	799	766.5	856.5	997.5	767	834.4	86.84
TMOD1	1196	3796.5	2772.5	908	4199	2761	2605.5	1332.52
TMOD2	36.5	15.5	34.5	56.5	18	32	32.2	14.80
TMOD3	731	308	741.5	738.5	372.5	671	593.8	199.09
TMOD4	93.5	75.5	70.5	58.5	60.5	89	74.6	14.42
TMPO	2354.5	1454	1608	2196	1546.5	1249	1734.7	438.89
TMPPE	40.5	11.5	63	35.5	18	48.5	36.2	19.13
TMPRSS12	1735	1923	1420.5	1551	2061.5	1443.5	1689.1	263.45
TMPRSS13	241.5	118.5	53	187	109	75	130.7	71.00
TMPRSS2	0	0	0	0	0	0	0.0	0.00
TMPRSS3	0	0	0	0	0	0	0.0	0.00
TMPRSS4	0	0.5	0	1	1	0	0.4	0.49
TMPRSS5	6	0.5	1	5.5	3.5	1	2.9	2.44
TMPRSS6	0.5	0	0	0	0	0	0.1	0.20
TMPRSS7	0	1	0	0.5	1	1.5	0.7	0.61
TMPRSS9	0.5	1	0	1.5	1.5	1	0.9	0.58
TMSB15B	43641	68278.5	34770	36091.5	67451	30322.5	46759.1	16903.61
TMSB4X	5	1.5	0.5	2.5	0.5	0	1.7	1.86
TMTC1	173	147	356.5	185	201	339.5	233.7	90.46
TMTC2	16.5	4.5	11.5	27	6	12	12.9	8.16
TMTC3	940	670	1096	944.5	788.5	936	895.8	147.32
TMTC4	385.5	69	134	365.5	67.5	78.5	183.3	150.98
TMUB1	1041.5	1073	570.5	972.5	992.5	623.5	878.9	221.86
TMX2	465.5	499.5	457.5	439	552.5	423	472.8	46.85
TMX3	1098.5	691	919.5	1062.5	753	761.5	881.0	172.33
TMX4	1624	647	820	1677.5	741	742	1041.9	475.08
TNC	14034	8001.5	28369	20999	8803.5	27203	17901.7	8960.16
TNFAIP1	281.5	161.5	460	323	191.5	372	298.3	111.89
TNFAIP2	1099	1725	1565.5	1405	1983	1560	1556.3	297.54
TNFAIP3	152.5	258	201	151.5	295.5	167.5	204.3	60.06
TNFAIP6	638	1070.5	1861	1251	1405	2173	1399.8	551.63
TNFAIP8	88	44.5	106.5	86	47	74	74.3	24.47
TNFAIP8L1	349.5	149	156.5	349.5	172	129.5	217.7	103.03
TNFAIP8L3	530.5	906	1966	432.5	885.5	1299	1003.3	563.37
TNFRSF10B	347.5	258.5	363.5	318.5	273	301.5	310.4	41.07
TNFRSF11A	439	257.5	435	433	297	376	372.9	78.65
TNFRSF11B	368	109	79	347.5	99.5	44	174.5	143.83
TNFRSF13B	1.5	2	0	1	1.5	1	1.2	0.68
TNFRSF13C	0.5	0	0	0	0	0	0.1	0.20

TNFRSF18	0	0	1	0	1	1.5	0.6	0.66
TNFRSF19	200	556	643	292.5	659	747.5	516.3	219.81
TNFRSF1A	1248	1088	1082	1151.5	1267	1078	1152.4	85.92
TNFRSF1B	2	0	0	2	0.5	0	0.8	0.99
TNFRSF21	3684.5	2689.5	2183.5	3532.5	2622	2385	2849.5	616.50
TNFRSF25	2.5	0	1.5	1.5	1	0	1.1	0.97
TNFRSF4	595.5	398.5	354.5	623	474	359.5	467.5	118.15
TNFRSF6B	82	82.5	28.5	166	105.5	68.5	88.8	45.55
TNFRSF8	0.5	0	0	0	0.5	0	0.2	0.26
TNFRSF9	204	104	96.5	193	141	95.5	139.0	49.13
TNFSF10	1518.5	404.5	694.5	1566.5	486	793	910.5	509.25
TNFSF11	0	0	0.5	0	0	1.5	0.3	0.61
TNFSF13B	1.5	1	8.5	1	2	22.5	6.1	8.54
TNFSF15	0	0	0.5	0	0.5	0	0.2	0.26
TNFSF4	17	25	44	22	27.5	23.5	26.5	9.26
TNFSF8	2	0	0	0.5	0.5	1.5	0.8	0.82
TNIK	1350.5	927.5	1339.5	1123.5	1048	1190.5	1163.3	165.72
TNIP1	1677.5	1517.5	1137	1393.5	1541.5	1282	1424.8	195.07
TNIP2	24.5	15	13.5	29	15.5	14	18.6	6.52
TNIP3	0	0	0	0	1	0	0.2	0.41
TNK2	3360.5	3321	2424.5	3256	3400.5	2783.5	3091.0	396.82
TNKS	1415	1001.5	1147	1304	1085.5	1026.5	1163.3	163.93
TNKS1BP1	615	405.5	357	507.5	423.5	431.5	456.7	91.58
TNMD	3	1.5	5	2.5	3.5	4	3.3	1.21
TNN	2.5	2.5	2.5	2	3	2	2.4	0.38
TNNC1	10732	22949.5	9602	7983.5	25337	8375.5	14163.3	7827.09
TNNC2	651.5	5051.5	230.5	615	5512	320.5	2063.5	2502.40
TNNI1	784.5	19455.5	7946	707.5	22626.5	8131	9941.8	9249.89
TNNI2	3909.5	22375	7422	3909	23161	6273.5	11175.0	9086.21
TNNI3K	10.5	8	4	12.5	5.5	6.5	7.8	3.19
TNNT2	5600	46390	22296.5	4231	46767.5	20214.5	24249.9	18793.09
TNNT3	1794.5	26863	11836	1445.5	29592	9727.5	13543.1	12139.53
TNPO1	176	82.5	248.5	165.5	85	178	155.9	63.20
TNPO3	901	882	926	833.5	887	849	879.8	33.86
TNR	6.5	1	3.5	7	6.5	1	4.3	2.81
TNRC6A	533.5	359	978.5	508	455	797.5	605.3	234.13
TNRC6B	204.5	127.5	200.5	181	157	168.5	173.2	28.84
TNRC6C	206	115	258	211	115	229	189.0	60.14
TNS1	772.5	511	1130	733.5	491	1085	787.2	273.25
TNS4	233.5	131	228	191.5	123	226	188.8	50.19
TNXB	6.5	3.5	6.5	6.5	4	10	6.2	2.32
TOB1	1087	634.5	740	948	690	679.5	796.5	179.89
TOB2	805	408	865	788	418.5	763	674.6	205.23
TOE1	614	553.5	378	594	550.5	369.5	509.9	108.23
TOLLIP	372.5	252	494	399	299	434	375.1	88.46
TOM1	517.5	1511	788	488.5	1707.5	855.5	978.0	513.62
TOM1L1	17.5	12.5	11.5	29	16.5	17.5	17.4	6.23
TOMM20	2493.5	2355	1416	2266.5	2666	1298.5	2082.6	579.03
TOMM22	1598.5	1785.5	1004	1483.5	1872	986.5	1455.0	381.42
TOMM34	380	337	240	411	374.5	266.5	334.8	67.94
TOMM40L	663.5	776.5	434	539.5	728	405	591.1	155.13
TOMM5	651	474	215.5	569	412.5	237.5	426.6	175.16
TOMM70A	1349	847.5	964.5	1424	1014	851.5	1075.1	250.81
TOP1	733.5	567.5	1131.5	783.5	653	1035	817.3	220.76

TOP1MT	363.5	451.5	437.5	351.5	424	359	397.8	44.66
TOP2A	3220	951.5	2331.5	3281.5	820	1296.5	1983.5	1115.85
TOP2B	1814	1515.5	2006	1595.5	1762	1917	1768.3	186.82
TOP3A	194	122.5	182.5	167.5	133.5	173	162.2	28.17
TOP3B	452.5	300.5	379	443	339.5	291	367.6	69.55
TOPAZ1	0	0	0.5	0	0	0	0.1	0.20
TOPBP1	4268	3089.5	2836	3999.5	3359.5	2540.5	3348.8	671.12
TOR1A	783.5	672.5	732.5	758.5	695.5	723	727.6	40.48
TOR1AIP1	1296.5	588.5	814.5	1373	718	674.5	910.8	337.21
TOR1B	716.5	576.5	684.5	645	630.5	677.5	655.1	49.03
TOR2A	177.5	145.5	143.5	182	161.5	136.5	157.8	18.96
TOR3A	1722.5	1130.5	1157.5	2034.5	1367	1423	1472.5	348.74
TOR4A	326.5	307.5	270.5	357	324.5	264	308.3	35.66
TOX	22.5	9.5	54	9.5	15.5	32.5	23.9	17.13
TOX3	4.5	3	1	4	3	2	2.9	1.28
TP53BP1	912	1036.5	910.5	823.5	1149	905.5	956.2	116.48
TP53BP2	1116	734	936.5	1103.5	816	927.5	938.9	152.11
TP53I11	900.5	502.5	760.5	977	582.5	789.5	752.1	181.73
TP53I3	145	82.5	83.5	95.5	73.5	74	92.3	27.02
TP53INP1	483	421.5	496	437	479.5	447.5	460.8	29.56
TP53INP2	1028.5	1326.5	729	984.5	1449.5	733	1041.8	297.97
TP53RK	339.5	371	179	309.5	399	194.5	298.8	91.93
TP53TG5	11	2	0.5	7	5	1.5	4.5	4.00
TP63	26.5	95.5	210.5	69.5	119	200.5	120.3	72.89
TP73	24.5	9	27	17.5	14	14.5	17.8	6.82
TPBG	96.5	83	130.5	94	91.5	131	104.4	20.90
TPBGL	0.5	0.5	0.5	0.5	1	0	0.5	0.32
TPCN1	513.5	239.5	222.5	456.5	244.5	205	313.6	134.71
TPCN2	69	35	56.5	92	46.5	48.5	57.9	20.15
TPD52	2495.5	3125.5	2448.5	2436	3488	2318	2718.6	473.42
TPD52L1	3	2	0.5	2	4	1	2.1	1.28
TPD52L2	756	571.5	671	745	598.5	598	656.7	79.94
TPGS1	257	110	63	251	107.5	73.5	143.7	87.45
TPGS2	365.5	366	431.5	349.5	378	391	380.3	28.68
TPH1	2.5	0	0.5	0	0	0.5	0.6	0.97
TPH2	12.5	28.5	57.5	11.5	29	83.5	37.1	28.18
TPI1	7376.5	5476	4641	6878	5987	4239	5766.3	1229.68
TPK1	323	340.5	248.5	322	347	250	305.2	44.39
TPL	0	0.5	0	0	0	0	0.1	0.20
TPM1	4385.5	9102.5	7730.5	4188.5	9914	6232	6925.5	2397.59
TPM2	66	342.5	80.5	71	365	81	167.7	144.43
TPM3	10151	12307.5	7449.5	9875.5	13499.5	7780.5	10177.3	2402.17
TPM4	3606.5	3074	1796	3471.5	3178.5	1771.5	2816.3	822.67
TPMT	195	129.5	106	175	154.5	110	145.0	35.99
TPP1	656	665	519.5	617	695.5	642	632.5	61.12
TPP2	460.5	303.5	499	432.5	340.5	425.5	410.3	74.01
TPPP	101.5	70.5	221.5	71.5	76	116.5	109.6	57.87
TPPP2	13	3.5	1.5	11	1.5	0.5	5.2	5.42
TPPP3	2971	1764	3888	3214.5	1570	2170	2596.3	907.61
TPR	781	581	1155.5	826	589	1000.5	822.2	226.78
TPRA1	431	320.5	391.5	432.5	347.5	405.5	388.1	45.48
TPRG1L	1829	1713	2448	1456	1667	2009	1853.7	343.73
TPRKB	274.5	212	101.5	229	231	107	192.5	71.43
TPRN	204.5	232.5	247	228	227.5	280	236.6	25.28

TPST1	615	664	1385	660	671	1279	879.0	353.04
TPST2	898.5	444	462	829	514	439.5	597.8	208.83
TPT1	25365	37642	21434.5	22937.5	42061.5	20761.5	28367.0	9142.86
TPX2	2066.5	794	1157	2311.5	782.5	801.5	1318.8	693.01
TRA2A	1133	853	1374	1045	1074	1230.5	1118.3	176.58
TRA2B	2008.5	1626.5	946	1871	1704.5	869.5	1504.3	481.33
TRABD	427	187	271.5	411	230.5	231	293.0	101.32
TRADD	444	266	366.5	371	304.5	335	347.8	61.44
TRAF1	37.5	21	13.5	40.5	19	19	25.1	11.11
TRAF2	357	246	415	348.5	215.5	415	332.8	84.43
TRAF3	81.5	29.5	85	99	39	68	67.0	27.38
TRAF3IP1	180	117.5	121.5	159	117.5	99	132.4	30.50
TRAF3IP2	577	256	240	479	297.5	271.5	353.5	139.96
TRAF4	156	158	91	129	165	86.5	130.9	34.91
TRAF5	144	86.5	64	88	77	49	84.8	32.54
TRAF6	60.5	33	121	68.5	45	109	72.8	35.10
TRAF7	1569.5	1161.5	1557	1386.5	1179.5	1522	1396.0	186.47
TRAFD1	648.5	387.5	453.5	661.5	431	420	500.3	121.75
TRAIP	686.5	252	286.5	665	261.5	180	388.6	225.34
TRAK1	1527.5	1620	1933.5	1418.5	1798.5	1573	1645.2	188.53
TRAK2	94.5	49.5	139	99	48	150.5	96.8	43.11
TRAM1	2146.5	1433.5	1395.5	2257.5	1586.5	1348	1694.6	402.62
TRAM2	384.5	371.5	783.5	385	375	725	504.1	194.73
TRANK1	137	64	134.5	134.5	81	129	113.3	32.19
TRAP1	1671	1138.5	1019	1588.5	1123.5	908.5	1241.5	312.99
TRAPPC10	341	225	464	363	291.5	398.5	347.2	83.18
TRAPPC11	857.5	523	684	775.5	594	584.5	669.8	127.42
TRAPPC12	345	244.5	294	315.5	255	282	289.3	37.54
TRAPPC13	108.5	54.5	105	108.5	61.5	86.5	87.4	24.31
TRAPPC2	751	609	599	706.5	757.5	574	666.2	81.78
TRAPPC2L	194.5	213.5	173	153.5	208	148.5	181.8	27.71
TRAPPC3	1192.5	1303.5	1047.5	1072	1428.5	1057	1183.5	155.84
TRAPPC3L	1	2	1.5	0.5	2	2	1.5	0.63
TRAPPC4	677	675	482.5	628	755	493.5	618.5	109.05
TRAPPC5	612.5	547.5	460.5	545.5	544.5	462	528.8	58.32
TRAPPC6B	401.5	364.5	334	300	359.5	269	338.1	47.79
TRAPPC8	619.5	471.5	655.5	560.5	600	645	592.0	68.08
TRAPPC9	69	18	114.5	91	24	91.5	68.0	39.19
TRDMT1	355	256.5	174.5	324.5	277.5	165	258.8	77.24
TRDN	0	16	23	0.5	16.5	16.5	12.1	9.53
TRERF1	707.5	484.5	517.5	625.5	508	389.5	538.8	111.91
TRH	0	0.5	0	0	1	0	0.3	0.42
TRHDE	44.5	17	47	34	16	45	33.9	14.24
TRHR	0	0	0	0	0	0	0.0	0.00
TRIAP1	397.5	574	372	417.5	625.5	371.5	459.7	111.06
TRIB1	94	351.5	276.5	106	399	225.5	242.1	125.30
TRIB2	143	305.5	505	158	355.5	541.5	334.8	167.94
TRIL	0.5	2.5	4	0.5	3.5	12	3.8	4.26
TRIM13	311	175	190.5	297	193	181	224.6	62.01
TRIM14	51	20.5	27	41.5	28	16.5	30.8	13.08
TRIM2	1	0	6.5	1	0.5	1.5	1.8	2.38
TRIM23	343	227.5	394.5	363	280.5	358.5	327.8	61.90
TRIM25	82	21.5	65	73	17.5	78.5	56.3	29.07
TRIM29	0.5	2	1	1.5	1	2	1.3	0.61

TRIM32	172.5	136	191.5	170.5	132.5	168	161.8	22.96
TRIM35	529	317	342.5	442	341	382.5	392.3	80.04
TRIM37	261.5	136.5	285.5	247	146	232	218.1	62.15
TRIM41	311.5	256.5	293	316.5	263	277.5	286.3	24.90
TRIM42	0	0	0	0	0	0	0.0	0.00
TRIM45	619	720	534.5	547.5	786.5	439	607.8	128.18
TRIM47	673.5	660	984	732.5	703	938.5	781.9	141.88
TRIM50	6	2	1	9.5	3.5	2.5	4.1	3.15
TRIM54	3315.5	4129	1711.5	2962.5	4195	1775.5	3014.8	1091.96
TRIM55	1005	3864.5	3339.5	719	4027.5	2723	2613.1	1433.87
TRIM59	636	259.5	399	721.5	263	361.5	440.1	194.63
TRIM62	183.5	177	166.5	139.5	154	182.5	167.2	17.54
TRIM63	152	582.5	516	113	537	482	397.1	207.88
TRIM65	117	96.5	84	99	88.5	79	94.0	13.53
TRIM66	0	4.5	15.5	1.5	5	15.5	7.0	6.84
TRIM67	11.5	4.5	2.5	9.5	7	5	6.7	3.36
TRIM7	30.5	54	69.5	21.5	32	64.5	45.3	19.96
TRIM71	4	5.5	5	4	5.5	6.5	5.1	0.97
TRIM8	1395	1954	1602.5	1372.5	2243	1790	1726.2	338.57
TRIM9	76	91	165	61.5	82	157	105.4	44.18
TRIO	1827	1957	3404	1727	2345	3066	2387.7	697.31
TRIOBP	1448	1097.5	1261.5	1348	1140.5	1180	1245.9	133.41
TRIP11	397	312.5	599	392	410	575.5	447.7	113.66
TRIP12	718.5	323	1130.5	751.5	452.5	1002	729.7	309.07
TRIP13	133.5	73	107	186	57.5	64	103.5	49.65
TRIP4	379	181	113.5	337	169.5	102	213.7	116.68
TRIQK	179	85	82.5	191.5	123	107	128.0	46.94
TRIT1	491.5	494	444	443.5	542	452.5	477.9	38.81
TRMT10A	130	115	156.5	113	139	140.5	132.3	16.58
TRMT10B	488.5	414.5	313	371.5	419.5	345	392.0	62.36
TRMT10C	412	300	271	431.5	348.5	274	339.5	69.78
TRMT11	231	223.5	251	245.5	270	230	241.8	17.23
TRMT112	12.5	8	26.5	5	4.5	11.5	11.3	8.12
TRMT12	149.5	133	128.5	127.5	148.5	147	139.0	10.42
TRMT13	496.5	414	281.5	437	419.5	208	376.1	108.38
TRMT1L	1233.5	994	915	1157	1126.5	1019	1074.2	118.15
TRMT2A	364.5	383.5	414.5	363.5	444	382	392.0	31.47
TRMT2B	336	215.5	257	282	220.5	220	255.2	47.47
TRMT5	203	149	150	182.5	150.5	137	162.0	25.19
TRMT6	213	183	221.5	176.5	212.5	228.5	205.8	21.15
TRMT61A	711	937.5	783.5	777.5	1180.5	875.5	877.6	168.51
TRMT61B	243	177.5	140	309	225	156.5	208.5	63.12
TRMU	333	265	243.5	296.5	293	249.5	280.1	33.88
TRNAU1AP	245.5	256	150.5	223.5	261	130	211.1	56.73
TRNT1	1378.5	905	757	1218.5	934.5	650	973.9	276.33
TROVE2	58.5	31	112	56.5	36	96	65.0	32.50
TRPA1	2	3	1	1.5	1	3	1.9	0.92
TRPC1	1313	744	903	1149.5	842.5	786.5	956.4	225.50
TRPC3	11.5	5.5	6.5	9	8.5	10	8.5	2.21
TRPC4AP	621	349	629	633.5	429	556	536.3	120.16
TRPC5	0.5	1	0.5	2	0.5	2	1.1	0.74
TRPC6	0.5	0	1.5	0	1	1.5	0.8	0.69
TRPC7	0	0	2.5	0	0	0	0.4	1.02
TRPM1	3	0.5	0.5	0.5	2.5	0.5	1.3	1.17

TRPM2	14	8.5	2.5	8	8.5	7	8.1	3.68
TRPM5	0	0	0.5	0	0	0	0.1	0.20
TRPM7	586	372	462	526	494.5	535.5	496.0	73.61
TRPM8	0.5	1	3.5	1	3	1	1.7	1.25
TRPS1	25.5	16	37	22	17	34.5	25.3	8.81
TRPT1	37.5	53.5	15.5	37	52.5	28.5	37.4	14.47
TRPV1	6	5	10.5	7	8.5	6.5	7.3	1.97
TRPV2	3190	3809	5339	3498	4478.5	4984	4216.4	854.83
TRPV3	4.5	6.5	6	3	3	5	4.7	1.47
TRPV4	0	0	0.5	0.5	0	0.5	0.3	0.27
TRRAP	801	429.5	1095.5	851	465.5	897	756.6	259.69
TRUB1	80.5	77.5	80	95	103.5	79	85.9	10.72
TRUB2	463	427.5	282	435.5	415.5	274	382.9	82.79
TSC1	791	715.5	1012.5	673.5	783.5	802.5	796.4	117.12
TSC2	1011.5	848.5	1152	873.5	929	1177	998.6	140.39
TSC22D2	624.5	583.5	826.5	566	665	758.5	670.7	102.68
TSEN2	205.5	142.5	152.5	188	136.5	134.5	159.9	29.73
TSEN34	402.5	311	167.5	314	239.5	175.5	268.3	91.13
TSEN54	547	389.5	362	524.5	402.5	383	434.8	79.64
TSG101	1006	872	738.5	940	1018	676.5	875.2	141.34
TSGA10	0	0.5	0	0.5	0.5	0.5	0.3	0.26
TSHB	0	0	0.5	0	0	0	0.1	0.20
TSHR	0	0	0	0	0	0	0.0	0.00
TSHZ1	114.5	52	159.5	121	61.5	165.5	112.3	47.66
TSHZ2	149.5	122	131.5	172	129.5	136.5	140.2	18.07
TSHZ3	166.5	64.5	92	112.5	66.5	94	99.3	37.57
TSKU	123.5	395.5	960.5	158	435	676.5	458.2	318.30
TSN	1983	1752	1436	1951.5	1940.5	1420	1747.2	260.24
TSNARE1	1.5	0	1.5	0.5	0.5	0	0.7	0.68
TSNAX	631.5	464.5	444.5	574.5	515	404.5	505.8	85.24
TSNAXIP1	20.5	6	13.5	19	7.5	7.5	12.3	6.31
TSPAN1	0.5	0	1	0	0	0	0.3	0.42
TSPAN10	29	176	29.5	51	243	21.5	91.7	94.22
TSPAN12	1446.5	1697	1986.5	1118	2038.5	1583.5	1645.0	344.99
TSPAN13	33	40	43.5	30.5	32.5	41.5	36.8	5.47
TSPAN14	427.5	196.5	425	468.5	262.5	405	364.2	108.36
TSPAN15	12.5	13	5	18.5	18	12.5	13.3	4.89
TSPAN19	0	1.5	1	0	1	0	0.6	0.66
TSPAN2	106.5	64	72	101	73	108.5	87.5	19.93
TSPAN3	4832	2648	2835	4860	3163	2499.5	3472.9	1086.47
TSPAN32	2	1.5	1	2	3	1	1.8	0.76
TSPAN4	988	786.5	1442	904	900	1110.5	1021.8	232.23
TSPAN5	136	107	192.5	118	122	159	139.1	31.70
TSPAN6	546.5	207.5	354	586.5	211	304	368.3	163.89
TSPAN7	165.5	730	1251	140.5	830	995	685.3	448.52
TSPAN8	171	61	65	181	83.5	29.5	98.5	62.57
TSPAN9	411.5	310	451.5	431.5	424.5	413.5	407.1	49.72
TSPEAR	47	44	39.5	29	50	32.5	40.3	8.27
TSPO	389	299	183	381.5	308.5	214.5	295.9	84.30
TSPO2	9	3	1.5	7.5	4	1.5	4.4	3.15
TSR1	818.5	905.5	851.5	767	907	827.5	846.2	54.09
TSR3	487	454	327.5	435	454	291	408.1	79.21
TSSC1	330.5	233	183	285.5	294	177.5	250.6	62.78
TSSC4	358	258.5	218.5	376	347.5	208	294.4	74.88

TSSK3	0.5	0	0	0	0	0	0.1	0.20
TST	1016.5	684	360	842.5	671.5	352.5	654.5	262.86
TSTA3	392	344	278.5	349.5	346	268	329.7	47.27
TSTD2	1097	1405	1061.5	956.5	1606.5	1000	1187.8	259.02
TSTD3	111	67.5	29.5	117.5	69.5	41.5	72.8	35.64
TTBK2	22	11	42.5	13	16.5	29.5	22.4	11.90
TTC1	744.5	650.5	626.5	674.5	729.5	552	662.9	70.72
TTC12	31.5	15.5	19.5	23.5	17.5	12	19.9	6.86
TTC13	247.5	129	164	204.5	150.5	154.5	175.0	43.32
TTC14	862	341	409.5	646.5	428	364.5	508.6	204.49
TTC16	29	13	18	54.5	12.5	22.5	24.9	15.76
TTC19	703.5	487.5	221	625	569	243	474.8	201.02
TTC21B	388	188	260.5	361	223	229.5	275.0	80.89
TTC25	129	146	136	102	146	156.5	135.9	19.11
TTC26	225	102.5	116.5	198	133	113	148.0	50.88
TTC28	1869.5	1260	1776	1803	1447	1573	1621.4	237.40
TTC29	1.5	0	0	2	0.5	0	0.7	0.88
TTC3	682	643.5	892	678.5	702	788	731.0	92.55
TTC30B	176.5	110.5	83.5	153.5	108	67.5	116.6	41.39
TTC32	248	216	134.5	235.5	231	129	199.0	53.12
TTC33	27	8.5	37	33.5	14	27	24.5	11.10
TTC34	0	0	1.5	0.5	0	0.5	0.4	0.58
TTC36	0	0	0	0.5	0.5	0	0.2	0.26
TTC37	1097	724.5	884	1060.5	811	807.5	897.4	149.71
TTC38	1155	609	530.5	1092	666	546	766.4	281.46
TTC39A	231	791	439.5	243.5	945.5	587	539.6	290.91
TTC39B	4.5	2.5	6.5	3.5	3	7.5	4.6	2.01
TTC39C	192.5	176	170.5	160	194	164.5	176.3	14.24
TTC4	679.5	780	539.5	625.5	813.5	509.5	657.9	123.84
TTC7A	213.5	128.5	125	205.5	132.5	125.5	155.1	42.31
TTC7B	275	190	195	274	189	171.5	215.8	46.20
TTC8	258	178.5	212	231.5	177	235.5	215.4	32.64
TTC9	1200	3115.5	2058	1259	3727.5	2035.5	2232.6	1009.73
TTF1	334.5	273.5	322.5	373	341	347	331.9	33.18
TTF2	368.5	189.5	204	313	207	154.5	239.4	82.56
TTI1	148	122	182.5	139	149.5	167.5	151.4	21.26
TTI2	271.5	231.5	179	270	262	182.5	232.8	42.80
TTK	526.5	205	414.5	566.5	203	245	360.1	164.46
TTL	5102.5	4634.5	3342.5	5071.5	4875.5	3095.5	4353.7	898.07
TTLL1	223	158	275.5	212.5	148	233.5	208.4	48.06
TTLL10	0	0	0	0	0	0	0.0	0.00
TTLL11	540.5	322	320	463	300	305.5	375.2	101.41
TTLL12	932	441.5	492	1011	467.5	416.5	626.8	269.39
TTLL2	5	5	9.5	8	14	3.5	7.5	3.87
TTLL5	889	974	1085	860.5	1046.5	1056.5	985.3	93.52
TTLL6	111.5	105	84	96	109	74.5	96.7	14.79
TTLL7	456.5	444.5	462	325	456.5	414	426.4	52.62
TTLL9	574.5	747.5	313.5	520.5	725	322	533.8	188.39
TTN	3788.5	11877	14445	3078	15049	12174.5	10068.7	5290.95
TTPA	23.5	11.5	19	5.5	7	23.5	15.0	8.09
TTPAL	716.5	449.5	740.5	794	581	774	675.9	133.91
TTR	0	0	0	0	0	0	0.0	0.00
TTYH2	860	311.5	379.5	604	305.5	345.5	467.7	221.76
TUB	174	164.5	491.5	202	192	440.5	277.4	147.55

TUBAL3	1796.5	11113.5	7070.5	1522.5	11379.5	5887	6461.6	4305.45
TUBB1	59.5	49.5	39	74	48.5	40.5	51.8	13.11
TUBB3	28	21.5	8.5	36	27	7.5	21.4	11.38
TUBB4B	4483	3801	3217	4470.5	3886	2898.5	3792.7	644.32
TUBB6	7588	15823.5	10887.5	8290	19119.5	11236.5	12157.5	4476.79
TUBD1	216	199	318	200	219.5	230	230.4	44.52
TUBG1	810.5	746.5	661.5	829	753.5	650.5	741.9	73.85
TUBGCP2	697.5	454	584	718	510	527.5	581.8	106.19
TUBGCP3	416.5	231.5	260.5	371.5	301	237.5	303.1	75.88
TUBGCP4	573	330	761	579.5	422	643.5	551.5	154.55
TUBGCP5	555	274.5	370.5	431.5	304	291	371.1	107.44
TUBGCP6	383	279	322	371	313.5	319.5	331.3	38.80
TUFM	0	0	0	0	0	0	0.0	0.00
TUFT1	1354.5	2030	1151.5	1248	2146	1319	1541.5	430.53
TULP1	9.5	2.5	6.5	3	2	4	4.6	2.89
TULP3	191	77.5	139.5	156.5	89.5	142.5	132.8	42.48
TUSC2	274.5	251.5	241.5	253.5	233	248.5	250.4	13.98
TUSC3	12	2.5	1	6.5	3	4.5	4.9	3.94
TVP23A	24	41	60.5	17.5	60.5	48.5	42.0	18.18
TVP23B	790	480	619	844.5	549.5	659	657.0	139.41
TWF1	1607.5	1099.5	1080.5	1666	1318.5	1008.5	1296.8	283.60
TWF2	3846	5405.5	4558.5	3764.5	5717.5	4821.5	4685.6	796.37
TWIST2	3	13.5	11.5	10	18	18	12.3	5.64
TWISTNB	460	298.5	218.5	390.5	317.5	210.5	315.9	97.19
TWSG1	1843.5	1589	2108	1982.5	1770	2379.5	1945.4	277.28
TXK	0	0	0.5	0	0.5	0	0.2	0.26
TXLNA	793.5	674.5	621	761.5	659	597	684.4	77.78
TXLNB	14	110.5	58.5	13	132.5	27	59.3	51.42
TXLNG	387.5	315	324.5	371.5	395	321.5	352.5	36.18
TXN	6511	10285	7445	5576.5	10462	7182.5	7910.3	2015.01
TXN2	1285	1395	990	1143	1421	928	1193.7	207.45
TXNDC11	798.5	587.5	490	821	709	502	651.3	145.72
TXNDC12	420	450.5	563	374.5	513	706	504.5	119.21
TXNDC15	322	219	176	307.5	284.5	179.5	248.1	64.91
TXNDC16	219	175	222.5	202.5	201	209	204.8	16.97
TXNDC17	969.5	1147.5	921.5	984.5	1265	846.5	1022.4	154.83
TXNDC5	8507.5	3311	2938.5	7588	3624	2905	4812.3	2536.69
TXNDC9	980	796	733	971.5	855.5	657.5	832.3	129.20
TXNIP	2364.5	1088.5	979	1878.5	1064	1231.5	1434.3	559.95
TXNL1	1640	1426.5	1071	1421	1455	902.5	1319.3	275.13
TXNL4A	495.5	437	253	416	472.5	231	384.2	113.73
TXNL4B	64.5	40.5	22	39	44	26.5	39.4	14.98
TXNRD1	170.5	49.5	63	179	58	40	93.3	63.60
TXNRD2	478.5	238	312	360	261.5	269	319.8	88.92
TXNRD3	1184	876.5	1103	1132.5	926	987	1034.8	122.99
TYK2	215.5	171.5	290.5	192.5	190	283	223.8	50.75
TYMS	806.5	326	322	788	302	237	463.6	260.49
TYR	0	0	0.5	0	0	0	0.1	0.20
TYRO3	589	643.5	765	592.5	732.5	856.5	696.5	106.55
TYRP1	0	0	0	0	0	0	0.0	0.00
TYSND1	143.5	135.5	132	158.5	145.5	141.5	142.8	9.23
TYW1	81.5	33.5	98.5	106	29.5	77	71.0	32.42
TYW3	106	107	102.5	101	120	107	107.3	6.72
TYW5	868	625.5	464.5	714	734	491	649.5	154.25

U2AF1	2264.5	1637	1497.5	2191	1810	1364.5	1794.1	367.77
U2SURP	2053	1303.5	1121	1811	1439	1065.5	1465.5	392.47
UACA	1069	352	608	1018	456.5	742	707.6	292.43
UAP1	250.5	337	260.5	269.5	366	329	302.1	47.92
UAP1L1	193.5	118	109.5	245	149.5	142	159.6	51.18
UBA2	1194.5	1009.5	1121.5	1220	1081.5	1004.5	1105.3	90.86
UBA3	1034.5	988.5	1020	976	1127.5	970	1019.4	58.64
UBA5	353.5	297	612.5	395	339.5	585	430.4	134.38
UBA52	6221.5	7231	3775.5	5836.5	7291	3809.5	5694.2	1577.55
UBA6	274.5	165	242	334	176	235	237.8	62.84
UBAC1	231.5	118	322.5	188	124.5	243.5	204.7	77.88
UBAC2	589.5	466.5	437	532.5	530	354.5	485.0	83.52
UBALD1	1682.5	1891	899.5	1419.5	1962	1050	1484.1	439.93
UBAP1	332.5	213	230	278.5	234	193.5	246.9	50.55
UBAP1L	12.5	23	36	10	23	33	22.9	10.47
UBAP2L	790.5	761	1120	855	851.5	1089	911.2	154.31
UBASH3A	0	0	0.5	0	0	0	0.1	0.20
UBASH3B	1028.5	494.5	874	986	537.5	930	808.4	232.81
UBB	22866.5	25458	22965	22511.5	26546	21688.5	23672.6	1891.00
UBE2A	1588	1609.5	1423	1455	1880	1493	1574.8	166.50
UBE2B	1349.5	1352	1185.5	1201.5	1592.5	1328	1334.8	146.31
UBE2D1	2113.5	1728.5	1348	2000	2002.5	1283.5	1746.0	357.21
UBE2D3	3183	3163	2794	3004	3456.5	2467.5	3011.3	344.58
UBE2E1	773	666.5	482.5	730.5	723.5	422	633.0	145.32
UBE2E2	202	223.5	216	186	289.5	231	224.7	35.58
UBE2E3	576.5	388.5	357.5	545	454	317.5	439.8	104.23
UBE2F	914	952	650	744.5	1000	624	814.1	162.15
UBE2G1	1451.5	1591.5	1488	1313	1864.5	1426	1522.4	190.35
UBE2G2	2233	1479	1138	1784.5	1472	908	1502.4	469.24
UBE2H	1807.5	1481	1545.5	1615	1746	1403.5	1599.8	155.14
UBE2I	1731	1492.5	1428	1686	1650.5	1329	1552.8	160.21
UBE2J1	631.5	618	676.5	635	748.5	669.5	663.2	47.61
UBE2J2	1897	1552.5	1254.5	1888.5	1699	1198	1581.6	304.12
UBE2K	640	576.5	439.5	535.5	629.5	408.5	538.3	96.68
UBE2L3	2087.5	1620.5	1733	1938.5	1677	1369.5	1737.7	251.27
UBE2M	604.5	826.5	608.5	527.5	873.5	615	675.9	139.35
UBE2N	2011.5	2387.5	2361.5	2040	2791.5	2178	2295.0	289.34
UBE2O	371.5	300.5	430	365	305	458.5	371.8	64.03
UBE2Q1	1362.5	1557	1253.5	1310.5	1624.5	1263	1395.2	157.83
UBE2Q2	1108.5	1335.5	1042.5	1090	1653.5	1073	1217.2	238.19
UBE2QL1	287.5	260.5	260.5	353.5	274.5	264	283.4	35.88
UBE2R2	798	710.5	942	748	759.5	870.5	804.8	86.44
UBE2T	161	101.5	180.5	144.5	107	110.5	134.2	32.68
UBE2V1	625.5	503.5	602.5	634.5	529	544.5	573.3	54.75
UBE2V2	946	856	666.5	878.5	943.5	639	821.6	135.76
UBE2W	195.5	126.5	177	176.5	156.5	156.5	164.8	23.80
UBE2Z	139	63.5	140	165.5	70.5	122	116.8	41.02
UBE3A	1046.5	904.5	956.5	1040.5	937	885.5	961.8	67.99
UBE3B	1072.5	903.5	883.5	955.5	928	819.5	927.1	84.83
UBE3C	572.5	210.5	476.5	574	277	422.5	422.2	151.32
UBE3D	91.5	51.5	39	90	49	33	59.0	25.49
UBE4A	1355.5	1202	1485.5	1310	1301.5	1324	1329.8	92.15
UBFD1	1492.5	1247.5	932.5	1392.5	1335.5	858.5	1209.8	257.23
UBIAD1	1425	981.5	1155	1418.5	1162.5	1102	1207.4	178.22

UBL3	1042	766.5	789	889	874	744.5	850.8	110.25
UBL7	1043.5	858.5	450	985.5	868	399	767.4	275.20
UBLCP1	544	409.5	469.5	507	436	410	462.7	54.66
UBN1	219.5	122	189	210.5	153	177	178.5	36.50
UBN2	72.5	63.5	142.5	78	74	138	94.8	35.59
UBOX5	246.5	261	203	235	253	207	234.3	24.24
UBP1	1472	1599	2224.5	1482	1834	1989	1766.8	303.17
UBQLN1	409.5	278.5	701	484	293.5	618.5	464.2	171.40
UBQLN4	1483.5	1264.5	1247	1421	1470.5	1162.5	1341.5	134.18
UBR1	239	135	407	294	150	396	270.2	117.31
UBR2	549.5	272.5	531.5	528	311.5	473.5	444.4	121.39
UBR4	2207	2093	2482	2083.5	2262	2495.5	2270.5	182.17
UBR5	1574.5	988.5	1474	1505	1134	1354	1338.3	230.80
UBTD1	558	1429.5	825	571.5	1680	1024.5	1014.8	459.51
UBTD2	982	986.5	1105.5	927.5	1072.5	1101	1029.2	73.82
UBTF	1162.5	1149.5	1698	1263	1285.5	1809	1394.6	285.29
UBXN10	768	786.5	464.5	638.5	903	480.5	673.5	176.93
UBXN11	998.5	1010	554.5	874.5	1026	562.5	837.7	222.84
UBXN2A	899	664	919.5	795	756.5	744	796.3	97.52
UBXN2B	173.5	71	98.5	148	121	78	115.0	40.31
UBXN4	3123	2901.5	2959.5	3174	3254	2688.5	3016.8	208.14
UBXN6	504.5	356.5	435.5	459	405	385.5	424.3	53.36
UBXN7	423.5	294.5	530	434	336	475	415.5	87.20
UCHL1	25	34.5	9.5	21	34.5	10.5	22.5	11.04
UCHL3	1024	994.5	805.5	1012	1096.5	691	937.3	154.76
UCHL5	1222.5	801	735.5	1253	898	667.5	929.6	250.71
UCK1	349	699.5	1137	375.5	723	882.5	694.4	301.04
UCK2	416	375.5	317	432	335.5	286.5	360.4	57.29
UCKL1	231.5	309.5	197	255	357.5	244.5	265.8	57.96
UCN3	1.5	0	2	0	1	4	1.4	1.50
UFC1	834	783	344	762	783	328	639.0	235.95
UFD1L	1270.5	1083.5	762.5	1048	1035.5	733.5	988.9	205.13
UFL1	667.5	439.5	478.5	668	491	549	548.9	98.52
UFM1	490	270	380	474	277.5	339	371.8	94.67
UFSP2	591.5	343.5	391	623	402.5	320.5	445.3	129.36
UGCG	28.5	15	24.5	21	18.5	26.5	22.3	5.11
UGDH	1867.5	1691.5	1496	1933.5	1886	1972.5	1807.8	180.75
UGGT1	1422	1126.5	1675	1404	1222	1318.5	1361.3	189.89
UGGT2	57	12	138.5	76	11	104.5	66.5	50.71
UGP2	2899	1715.5	2075.5	2090.5	1727.5	1544.5	2008.8	486.68
UGT8	0.5	0	0	0	0	0	0.1	0.20
UHMK1	1282	1053.5	1122.5	1197.5	1076	1030	1126.9	96.36
UHRF1	958.5	212	597.5	1156.5	241.5	335	583.5	396.45
UHRF1BP1	97	55	223.5	87.5	68	183.5	119.1	68.20
UHRF1BP1L	548	328.5	538	553.5	377.5	458.5	467.3	96.28
UHRF2	114	76.5	129	96.5	82.5	122	103.4	21.55
UIMC1	322.5	223.5	225.5	279	214	197.5	243.7	47.35
ULK1	287.5	225.5	426.5	247.5	266	377.5	305.1	79.34
ULK2	2317	1441.5	1727.5	2089	1636.5	1695	1817.8	322.63
ULK3	221.5	279.5	232.5	183.5	254.5	240	235.3	32.33
ULK4	39	23.5	39.5	33	18	22	29.2	9.23
UMODL1	0.5	2	0	2.5	3	1.5	1.6	1.16
UMPS	921.5	535.5	353	897.5	575	336	603.1	255.85
UNC119	7.5	4	7	8	6	6	6.4	1.43

UNC119B	529	419	637	518	458.5	635	532.8	89.45
UNC13A	0	0.5	0	0.5	0.5	0	0.3	0.27
UNC13C	1	0	1	0.5	0	1	0.6	0.49
UNC13D	4.5	1.5	1.5	3	1	2	2.3	1.29
UNC45A	534	516.5	717.5	541.5	419	707	572.6	116.84
UNC45B	947	2935.5	2262	706	3392.5	1928.5	2028.6	1064.55
UNC50	595	399	498.5	553	427	538	501.8	75.88
UNC5A	230	361	394.5	255.5	356	479.5	346.1	91.81
UNC5B	51	20	94.5	52.5	24.5	93	55.9	32.18
UNC5C	4	12	28	5.5	15	28.5	15.5	10.68
UNC5D	2.5	3.5	21.5	1.5	8	21	9.7	9.24
UNC79	3.5	6	4	4.5	7	6.5	5.3	1.44
UNC80	49	33	16.5	36	28.5	18.5	30.3	12.02
UNC93A	1	1	0	0.5	0	0	0.4	0.49
UNC93B1	107	138	217.5	113	145.5	220.5	156.9	50.24
UNG	288.5	196	212	306.5	179.5	188	228.4	54.87
UNK	52	37.5	61	70.5	38	54.5	52.3	12.93
UNKL	427.5	298.5	271	407.5	340	275.5	336.7	67.51
UPB1	16.5	15	28	14	14.5	20	18.0	5.36
UPF1	2487.5	1672	2683	2311.5	1791	2341	2214.3	398.27
UPF2	719	562	807.5	749	615.5	748.5	700.3	92.58
UPF3A	26.5	7.5	23	15	7.5	20.5	16.7	8.03
UPF3B	1036.5	946.5	780.5	918	1084	714	913.3	143.39
UPK1B	113	32	17	51.5	15	8.5	39.5	39.17
UPK3A	0	0	0	0	0.5	0	0.1	0.20
UPK3B	1	2	3.5	0.5	2	1.5	1.8	1.04
UPP1	20.5	4	9	16.5	6	4	10.0	6.95
UPP2	68.5	79.5	112	66.5	88	135.5	91.7	27.08
UPRT	440	355.5	288	444.5	387.5	297	368.8	67.84
URB2	237.5	195.5	338.5	252.5	196	300.5	253.4	57.20
URGCP	154.5	85.5	34	182	107.5	38.5	100.3	60.16
URM1	1741.5	1498.5	706	1509.5	1575.5	676.5	1284.6	467.82
UROC1	26	13.5	6	17.5	10	8.5	13.6	7.29
UROD	503.5	570	366	437	551	398	470.9	83.39
UROS	217.5	149	155	242	185	205.5	192.3	36.33
USB1	152	118.5	65	139.5	126	92.5	115.6	31.96
USE1	598	464	440.5	519	483	365.5	478.3	77.93
USF1	521.5	552.5	615.5	518	533	573.5	552.3	37.26
USH1G	0	0	0.5	0	1	0	0.3	0.42
USH2A	24.5	29	22	22.5	40	25.5	27.3	6.73
USHBP1	1.5	2	2.5	1	1.5	3.5	2.0	0.89
USMG5	1345.5	1536.5	989	1250.5	1695	884.5	1283.5	311.19
USO1	1906	2080.5	2279	1849.5	2213.5	2406	2122.4	217.40
USP1	1551.5	1101	1234.5	1610	1201.5	1141.5	1306.7	218.08
USP10	1288	805	1402.5	1339	938.5	1215.5	1164.8	238.86
USP12	101	39.5	48	101.5	48	45.5	63.9	29.09
USP13	252.5	221.5	670	209	272	504.5	354.9	188.74
USP14	1284	1244	1098	1161	1323	923.5	1172.3	146.89
USP15	929.5	765.5	946	917	848.5	813	869.9	72.33
USP16	729	688	788.5	659	762	702.5	721.5	48.11
USP18	78.5	41	61	81	40.5	47.5	58.3	18.24
USP19	1040.5	1005.5	1053	997.5	905.5	964	994.3	53.89
USP2	22.5	63	47	10.5	49	27.5	36.6	19.60
USP20	2009	1735	1873.5	1801	1981.5	1979.5	1896.6	111.80

USP24	174.5	37.5	267.5	152	44	224.5	150.0	93.65
USP25	1905.5	1199	1140	1651	1375	1108.5	1396.5	320.02
USP28	535.5	785.5	952.5	482.5	952	978.5	781.1	222.28
USP3	213.5	113.5	273.5	242	142.5	198.5	197.3	60.16
USP30	124	48.5	82.5	115.5	52	87	84.9	31.25
USP31	39	14	53.5	37	12	57	35.4	19.05
USP32	344	214.5	379	292	246	334	301.6	62.62
USP33	1360.5	805.5	881.5	1187.5	952.5	813.5	1000.2	225.40
USP36	866.5	879	746	877.5	849	804	837.0	52.51
USP37	255.5	161	278	268	201	261	237.4	46.11
USP38	589	539	541	493.5	657.5	503	553.8	60.98
USP39	232.5	175.5	204.5	236	185	195.5	204.8	24.81
USP4	903	707	900	899.5	778	851	839.8	80.95
USP40	233.5	142	299.5	209.5	157.5	240.5	213.8	57.95
USP42	52.5	37.5	51.5	43.5	44	53	47.0	6.29
USP43	2	12.5	25.5	1	10	27	13.0	11.19
USP44	240	188	194	213.5	212	202	208.3	18.45
USP46	225.5	119.5	130.5	198	133.5	120	154.5	45.53
USP47	1537.5	1201	1238	1407.5	1343	1202	1321.5	134.38
USP48	1409	1072.5	1148.5	1368.5	1152.5	1208.5	1226.6	133.47
USP49	1247	1395	824.5	1240	1521.5	826.5	1175.8	290.58
USP5	2378	1522	1408.5	2220	1228	1211.5	1661.3	509.74
USP50	1	3	7.5	1	2.5	4	3.2	2.42
USP53	89.5	27	169	97	34	161.5	96.3	60.44
USP54	727.5	655	1010.5	697.5	760.5	924	795.8	139.95
USP6NL	565	517.5	712.5	609.5	655	699.5	626.5	76.80
USP8	713.5	399.5	725.5	762	526	728.5	642.5	145.76
USP9X	2338.5	2095.5	3025.5	2244.5	2437	2720.5	2476.9	340.74
USPL1	1087.5	973.5	1128.5	925	1190.5	1281.5	1097.8	133.19
UST	373	235	532	365	287	478.5	378.4	112.02
UTP11L	1531.5	1371.5	1000	1466	1409.5	854.5	1272.2	276.43
UTP14A	686.5	683.5	577	705	710.5	565.5	654.7	65.54
UTP15	860	702.5	800.5	904.5	802	740.5	801.7	74.24
UTP18	910.5	644	581	829.5	656	534	692.5	146.65
UTP20	425	317	529	446	337	441.5	415.9	78.03
UTP23	117	148	90.5	134	164.5	100	125.7	28.45
UTP3	528	507.5	454.5	517.5	507	418	488.8	42.97
UTP6	462.5	313.5	326.5	454	330.5	264	358.5	80.87
UTS2	1	0.5	3.5	2.5	3	5	2.6	1.66
UTS2B	0	0.5	0.5	0	0.5	1	0.4	0.38
UVRAG	415	282	291.5	405	346.5	266	334.3	64.64
UVSSA	117	68.5	96.5	120	80.5	97	96.6	20.06
UXS1	1771	792.5	758	2045	926	672	1160.8	590.95
VAMP1	4	3	11.5	8	8.5	7.5	7.1	3.12
VAMP3	1606	2017.5	1632.5	1434.5	1948.5	1779	1736.3	221.25
VAMP4	421.5	371.5	341	422	391.5	308.5	376.0	45.23
VAMP7	479.5	431.5	474	502	469.5	413	461.6	32.97
VANGL1	165.5	68	204	142.5	65	146.5	131.9	55.16
VANGL2	259	191.5	350	336.5	224	378.5	289.9	75.65
VAPB	2682	2274.5	1750.5	2236.5	2492.5	1687	2187.2	397.24
VASH1	2771	2272	4450.5	2367	2476.5	3823.5	3026.8	898.33
VASH2	1422.5	1721.5	3604	1445.5	2168.5	3133	2249.2	919.88
VASN	1491.5	1350.5	1556	1680.5	1341	1837	1542.8	192.89
VAT1	257	89	334	234	90	232.5	206.1	97.59

VAT1L	274	134	387	264.5	164.5	266	248.3	90.07
VAV2	2617.5	2565	3126.5	2880	2724	3557	2911.7	375.64
VAV3	15	20	24	15	22	13	18.2	4.45
VBP1	333	234.5	319.5	291.5	262.5	326	294.5	39.33
VCAM1	0	0	0	0.5	0	0	0.1	0.20
VCL	1342.5	627.5	2849.5	2014	812.5	2484.5	1688.4	905.57
VCPIP1	136	62.5	209.5	139.5	76	203	137.8	61.46
VCPKMT	162	161.5	111.5	121.5	164	108.5	138.2	27.01
VDAC1	551	257	308	539	255	326.5	372.8	136.39
VDAC2	7852.5	7731.5	5924.5	7393	8647.5	5106.5	7109.3	1326.72
VDAC3	2491.5	2317.5	1449	2227.5	2480	1372.5	2056.3	510.46
VDR	59	80.5	40.5	52.5	82.5	67.5	63.8	16.35
VEGFA	47.5	39.5	179	67	56	140.5	88.3	57.44
VEGFC	107	137	209.5	115.5	164	231.5	160.8	50.78
VENTX	0	0	0	0	0	0	0.0	0.00
VEPH1	1754.5	1814	1752.5	2193	1994	1699	1867.8	189.31
VEZF1	525.5	367	818.5	508.5	449	786.5	575.8	184.41
VEZT	405.5	221.5	296.5	383	231.5	219	292.8	83.83
VGLL1	25	48.5	40	34.5	58	32	39.7	11.96
VGLL2	0.5	12	6	0	20	8.5	7.8	7.54
VGLL3	1259	1385.5	2073.5	1379.5	1519.5	1888	1584.2	323.43
VGLL4	117	61	272.5	109.5	74.5	244.5	146.5	89.68
VIL1	45.5	10	2.5	17	8	1	14.0	16.46
VILL	1	2	1.5	3.5	0.5	1	1.6	1.07
VIM	44517	50421	50741.5	40145	56781.5	44204.5	47801.8	5974.85
VIMP	863.5	620.5	495.5	830.5	761	597	694.7	145.65
VIP	2.5	6	4.5	0.5	5.5	4	3.8	2.04
VIPAS39	556.5	631.5	560	477	679.5	601.5	584.3	69.95
VIPR2	2	1	6	2.5	3	5.5	3.3	1.99
VIT	2	0.5	0.5	1	1	1.5	1.1	0.58
VKORC1L1	86	54.5	85	78	60.5	84.5	74.8	13.79
VLDLR	917	827	1172	883	1019.5	1278.5	1016.2	176.90
VMA21	1120	828.5	900.5	1110.5	891.5	883.5	955.8	126.11
VMO1	0	0	0	0.5	0.5	0	0.2	0.26
VOPP1	2139.5	1911.5	1175	1818	2139.5	1235.5	1736.5	430.90
VPRBP	526	322.5	533	522	383	503.5	465.0	89.56
VPREB3	0	0	0	0.5	0	0	0.1	0.20
VPS11	786	760.5	698.5	740	827	653.5	744.3	61.97
VPS13A	311	123.5	564	307	148.5	465	319.8	172.53
VPS13B	448	284.5	594.5	378.5	351.5	512	428.2	113.20
VPS13C	413	158	395	388	185	323	310.3	112.09
VPS16	1595	1598	850	1392.5	1477.5	847	1293.3	353.08
VPS18	403	319	389.5	419	345	358.5	372.3	37.94
VPS26A	540	450.5	601.5	551	506.5	522.5	528.7	50.14
VPS26B	864.5	828	799	923	825	845	847.4	42.97
VPS29	1868.5	1478.5	1378.5	1829	1619.5	1179	1558.8	266.76
VPS33A	633	545	425.5	590	576.5	378.5	524.8	100.30
VPS35	2217	1669	2123.5	2253	1805	1942.5	2001.7	235.47
VPS36	615.5	390.5	428	641	431	395.5	483.6	113.54
VPS37A	414.5	397.5	357	362	452	348	388.5	40.31
VPS37B	441.5	321	879.5	420	380.5	686.5	521.5	215.49
VPS37C	262.5	177	160	226	193	171	198.3	38.94
VPS39	418.5	375.5	365.5	387.5	442.5	317.5	384.5	43.50
VPS41	864	515	562.5	852	560	596.5	658.3	156.86

VPS45	848	659.5	530	754	657	525.5	662.3	125.81
VPS4A	2089	2208	1488.5	1980.5	2167.5	1547	1913.4	316.70
VPS4B	1741.5	1208.5	1121.5	1699	1287	1007	1344.1	306.18
VPS51	528	414	479	461	426	452.5	460.1	40.78
VPS53	1247.5	1508	1807.5	1228	1659	1712.5	1527.1	244.29
VPS54	485.5	349	369	511	346.5	322.5	397.3	80.02
VPS72	752	825.5	653	683.5	783.5	650	724.6	73.19
VPS9D1	121	61.5	96.5	109	52.5	110.5	91.8	28.22
VRK1	330.5	106.5	88.5	350.5	124	58	176.3	129.18
VRK2	675.5	434.5	340.5	598	481	305.5	472.5	144.08
VRK3	593.5	520.5	369	447	574	414	486.3	90.43
VRTN	0	1	1	0.5	1	1	0.8	0.42
VSIG1	21.5	24.5	28.5	18	35	20.5	24.7	6.22
VSIG10	817	842	444	847.5	1036	577.5	760.7	213.06
VSIG4	0	0	1	1	0	1	0.5	0.55
VSIG8	4	6.5	6.5	0.5	4.5	2	4.0	2.41
VSNL1	7	16	67	10	17	27	24.0	22.16
VSTM2A	0	0	0	0	0	0	0.0	0.00
VSTM2L	13.5	81	71.5	12	65.5	65.5	51.5	30.55
VSTM4	174	178.5	264	187	233.5	349	231.0	67.77
VSTM5	6	0.5	5.5	6	1	2	3.5	2.61
VSX1	0.5	1	1	0.5	1.5	2	1.1	0.58
VSX2	0	1	0	0	0	0	0.2	0.41
VTA1	1337.5	1311.5	1040	1298.5	1453	911	1225.3	205.11
VTCN1	16.5	18	31.5	23.5	27.5	22	23.2	5.67
VTI1A	50	31.5	49.5	54.5	39	42.5	44.5	8.47
VTI1B	373.5	271	252.5	338	297.5	225	292.9	55.27
VTN	3	13	11.5	2	16.5	10	9.3	5.72
VWA1	2974	2379.5	4797.5	3492.5	2841.5	5198.5	3613.9	1136.35
VWA2	1.5	19	25	6.5	13	20.5	14.3	8.96
VWA3A	20.5	17	33.5	22.5	13.5	29	22.7	7.46
VWA3B	0	1.5	0	0.5	0.5	0.5	0.5	0.55
VWA5B1	23	19.5	11.5	15.5	28	18.5	19.3	5.75
VWA5B2	90.5	87	51	82.5	73.5	60.5	74.2	15.65
VWA8	167.5	58	114	144.5	61.5	69	102.4	46.70
VWA9	424.5	384.5	381	420	429.5	352.5	398.7	30.72
VWC2	0	0	0	0	0	0	0.0	0.00
VWC2L	0.5	1	1	0	0	1	0.6	0.49
VWCE	13.5	10.5	6.5	17.5	7.5	5.5	10.2	4.63
VWDE	0.5	0	0.5	0	0	0	0.2	0.26
VWF	0	0.5	0.5	0.5	0	0.5	0.3	0.26
WAC	522	364	757	533.5	471	700	557.9	146.20
WAPAL	972	587.5	1086.5	949.5	598	961	859.1	212.11
WARS	598	472	688	569	460	489	546.0	88.90
WARS2	171.5	124	210.5	168.5	168	165.5	168.0	27.43
WAS	1	3	2	1	2	1	1.7	0.82
WASF1	21.5	32.5	55	29	43	49.5	38.4	12.87
WASF2	234	89.5	350	265.5	76	316.5	221.9	115.08
WASF3	0.5	2.5	1	0.5	6	10	3.4	3.84
WASH1	466	399	362	430.5	402	328	397.9	48.77
WASL	521	472.5	575.5	497	533.5	546	524.3	36.38
WBP1	584	642.5	475.5	543.5	601	473	553.3	68.95
WBP11	1547.5	1231.5	1272.5	1511.5	1328.5	1162.5	1342.3	155.18
WBP1L	215.5	180.5	357.5	177.5	199.5	282	235.4	70.90

WBP2	443.5	409	327.5	432.5	470	327.5	401.7	60.70
WBP2NL	3737.5	2493.5	1955	3543.5	2661	1744	2689.1	812.44
WBP4	385	383.5	280.5	331	418.5	270.5	344.8	60.65
WBSCR16	150	79	162.5	146.5	78.5	127.5	124.0	36.80
WBSCR22	422	525.5	343.5	433	524	357.5	434.3	78.31
WDFY1	1376.5	1168.5	1696	1471	1407	1756	1479.2	217.25
WDFY2	232	152.5	165	256.5	189	176.5	195.3	40.58
WDFY4	2.5	3.5	2	1	0.5	1.5	1.8	1.08
WDHD1	269	114.5	225	283.5	130	135.5	192.9	75.40
WDPCP	40.5	24	40.5	37	32	27.5	33.6	6.91
WDR1	6094	5417.5	5751.5	6489.5	6007	5143.5	5817.2	486.33
WDR12	1508.5	857.5	1078	1521	954.5	952	1145.3	294.68
WDR13	195	196	207	182	185	192	192.8	8.89
WDR17	2	3	2	3	1.5	1	2.1	0.80
WDR18	67	52.5	106	78.5	74.5	103.5	80.3	20.91
WDR19	193	81	153	196.5	105.5	138	144.5	46.31
WDR20	366	268	361	355	298	316.5	327.4	39.73
WDR24	771.5	785.5	554.5	743	771.5	546.5	695.4	113.13
WDR25	60	45	57	55	39	58.5	52.4	8.45
WDR26	1750.5	1556	1961.5	1708.5	2035.5	1847	1809.8	175.23
WDR27	0	1	1	1.5	1	1	0.9	0.49
WDR3	722.5	480.5	557.5	742.5	505.5	461.5	578.3	123.85
WDR31	3	6	4	3.5	5.5	6.5	4.8	1.44
WDR34	72.5	105.5	77	69	94	89	84.5	14.09
WDR35	198	143	201.5	184.5	149.5	175.5	175.3	24.48
WDR37	82.5	55.5	91.5	73.5	68	83.5	75.8	12.88
WDR4	647.5	433.5	346	586.5	515	338	477.8	127.18
WDR41	280.5	237.5	281.5	242.5	283	245	261.7	22.06
WDR43	1283	928.5	855	1215.5	985	763.5	1005.1	204.29
WDR44	399	334	540.5	418	368	536.5	432.7	86.80
WDR45B	581.5	314	440.5	551.5	307.5	391.5	431.1	116.41
WDR47	469.5	414.5	485.5	481	498	485	472.3	29.73
WDR48	441	336	527	412	343.5	500.5	426.7	78.84
WDR5	716	463	649	734	533	575.5	611.8	106.62
WDR53	477.5	406.5	384.5	485	428.5	378.5	426.8	45.81
WDR54	1066.5	617	736	827.5	468	743	743.0	201.66
WDR55	425.5	578.5	333	399	520	357.5	435.6	95.51
WDR59	281.5	118	207.5	237.5	118.5	194.5	192.9	65.12
WDR6	216	158.5	118	143.5	159	119.5	152.4	35.97
WDR60	177	76.5	110.5	152.5	79.5	81.5	112.9	42.68
WDR61	1040	655.5	646.5	976.5	691	589	766.4	191.22
WDR63	0	0	0	0	0	0	0.0	0.00
WDR66	35.5	75.5	150.5	38.5	107	93	83.3	43.64
WDR7	141.5	89.5	204	156.5	110.5	185	147.8	43.47
WDR70	658.5	444.5	532	552.5	501.5	540.5	538.3	70.49
WDR72	3	0	2	0	0	1	1.0	1.26
WDR73	156.5	129.5	109	165.5	98.5	106.5	127.6	27.98
WDR75	860	519.5	510.5	821	477.5	449.5	606.3	183.49
WDR76	379	182	133.5	357.5	187.5	142	230.3	109.20
WDR77	899	815.5	578	893.5	859	550	765.8	159.40
WDR78	5	0.5	1	3	2.5	1.5	2.3	1.64
WDR82	1258.5	1109	1341.5	1344	1255.5	1269	1262.9	85.50
WDR83	49.5	61	33	35.5	47.5	31	42.9	11.72
WDR83OS	884	1167	505.5	764.5	1058	521	816.7	272.94

WDR86	469	198	230.5	374.5	231	210	285.5	110.40
WDR88	0.5	0	0.5	0.5	1	0.5	0.5	0.32
WDR89	529.5	309	219.5	480	334	254.5	354.4	124.17
WDR90	184	80	100.5	159.5	65.5	77	111.1	48.95
WDR91	783.5	608.5	500	714	636	484.5	621.1	117.23
WDR92	298	316.5	339.5	265.5	295.5	298	302.2	24.59
WDR93	0.5	1	0.5	1.5	0	0.5	0.7	0.52
WDSUB1	241.5	128	126	234	133	121	163.9	57.37
WDTC1	2131	1244.5	1224.5	1983	1368.5	1154.5	1517.7	426.01
WDYHV1	95	71	74	96.5	78	58.5	78.8	14.64
WEE1	533	224.5	366.5	574	219.5	268.5	364.3	156.29
WEE2	7.5	22	10	11	25.5	15.5	15.3	7.16
WFDC1	1497	901	1607.5	1280.5	1045	1611	1323.7	300.44
WFDC3	1.5	0.5	0	0	0	0	0.3	0.61
WFIKKN2	0	0	0	0.5	0	0.5	0.2	0.26
WFS1	744.5	549.5	716.5	722.5	679.5	637	674.9	72.14
WHAMM	290	214.5	145	230.5	273	161.5	219.1	58.12
WHSC1L1	416	181.5	455.5	436.5	217	399	350.9	119.54
WIBG	227	315	201	229.5	280.5	210	243.8	44.45
WIF1	294.5	126.5	81	388.5	177.5	90.5	193.1	123.48
WIPF1	113.5	55.5	151.5	118	53.5	137.5	104.9	41.38
WIPF2	212.5	191	189	183.5	165	207.5	191.4	17.14
WIPF3	1722	3569	3159.5	1173.5	4320	3174.5	2853.1	1179.99
WIPI1	2466.5	3058.5	3196	2235	3397	3053.5	2901.1	450.21
WIPI2	1086.5	872	879.5	981.5	942.5	828.5	931.8	93.27
WISP1	2.5	1	1	1.5	1.5	1.5	1.5	0.55
WISP2	8.5	26.5	45	12	27.5	85	34.1	28.12
WISP3	41.5	22.5	10.5	41	22.5	5	23.8	15.12
WIZ	562.5	470.5	793	565	461.5	788.5	606.8	149.05
WLS	1824	1737	7233	2010	1731	5695	3371.7	2446.27
WNK1	1353	920.5	1531.5	1169.5	932.5	1365	1212.0	249.11
WNK2	5.5	32.5	32.5	6.5	51.5	36.5	27.5	18.07
WNK4	6.5	4.5	2.5	3	6	3.5	4.3	1.63
WNT10A	96.5	117.5	225.5	101.5	112	228	146.8	62.35
WNT11	0	0	0.5	0	0	0.5	0.2	0.26
WNT16	0	0	1	0	0	2	0.5	0.84
WNT2	0	0	0.5	0.5	1	1.5	0.6	0.58
WNT2B	3.5	1	1	5	1	3	2.4	1.69
WNT3	0	0	0	0	0	0	0.0	0.00
WNT3A	0	0	0	0	0	0	0.0	0.00
WNT4	87.5	111	63	108	146.5	128.5	107.4	29.51
WNT5A	19	21	8	12.5	20.5	7.5	14.8	6.22
WNT5B	39	77	89	79.5	67	160	85.3	40.43
WNT6	5	29	32.5	2.5	30	85	30.7	29.70
WNT7A	78	361	312	94.5	454.5	408	284.7	160.94
WNT7B	0	0	0	0	0	0	0.0	0.00
WNT8A	0	0	0	1	0	0	0.2	0.41
WNT8B	31	21	35	44	26.5	43	33.4	9.10
WNT9A	0	0.5	0	0.5	0	0	0.2	0.26
WNT9B	0	0	0	0	0	0	0.0	0.00
WRB	210.5	86	156.5	197.5	97	129	146.1	51.41
WRN	739	413	354.5	598.5	398	362	477.5	156.19
WRNIP1	62	21	8	40.5	17.5	9	26.3	21.05
WSB2	1070.5	1884	1396.5	1037.5	1906	1446	1456.8	377.64

WSCD1	2.5	8.5	3.5	4	5.5	8	5.3	2.46
WSCD2	4.5	29	42.5	6.5	29	39	25.1	16.10
WT1	65	23.5	13	54	26.5	13	32.5	21.89
WTAP	1159	865	890	1095.5	1018	900.5	988.0	121.73
WTIP	246.5	182.5	286	232.5	206	253.5	234.5	36.54
WWC1	202.5	203.5	209	174.5	201	183	195.6	13.58
WWC2	192	138.5	422.5	213	154	354.5	245.8	115.71
WWC3	196	90	317.5	208.5	93.5	318.5	204.0	101.27
WWTR1	2979.5	6824.5	7115	2786.5	7577	7071	5725.6	2216.09
XAF1	1	0	0	0	0	0	0.2	0.41
XBP1	2035	1687.5	2005.5	2157	1759.5	1921	1927.6	176.74
XCR1	1.5	1	0.5	2.5	0	2.5	1.3	1.03
XDH	3.5	0.5	1	1.5	0	2.5	1.5	1.30
XG	67	39.5	20	45	50.5	12	39.0	20.21
XIAP	160	166	323.5	176.5	198	288	218.7	69.60
XIRP1	344	2901	4461.5	412.5	3627	3581.5	2554.6	1757.05
XIRP2	0	0	0	0	0	0	0.0	0.00
XK	0.5	1.5	0	0	0.5	0	0.4	0.58
XKR4	60	38.5	53.5	70.5	42.5	32.5	49.6	14.33
XKR5	13	11	17	5.5	5.5	9.5	10.3	4.46
XKR6	5	1.5	5	3.5	3	1.5	3.3	1.57
XKR7	54	30.5	76.5	59.5	22	95	56.3	27.43
XKR8	279	369.5	225	282.5	399	304.5	309.9	63.93
XKR9	0.5	0.5	0.5	0	0.5	1	0.5	0.32
XKRX	136	806.5	366	140	861	395.5	450.8	316.46
XPA	1749	1523	1143	1254.5	1557	942	1361.4	299.88
XPC	301.5	212.5	317	278.5	255.5	326.5	281.9	42.73
XPNPEP1	710	692.5	546.5	610.5	750.5	516.5	637.8	94.55
XPNPEP2	37	37	61	39	35.5	59.5	44.8	12.00
XPNPEP3	861	561.5	425.5	737	550.5	330.5	577.7	195.40
XPO4	384	630.5	865.5	394.5	772.5	696.5	623.9	197.96
XPO5	1151	1105	1263	1161.5	1151.5	1207	1173.2	54.70
XPO6	1480	1103.5	1144.5	1472	1219.5	1206	1270.9	164.33
XPO7	868.5	886.5	1147.5	912.5	883.5	1135.5	972.3	131.85
XPOT	1836	1314.5	1689.5	1833	1477	1575	1620.8	206.23
XPR1	3914	2284	3106	2983	2220.5	2848	2892.6	619.85
XRCC1	105	79.5	80	94.5	77	61.5	82.9	15.07
XRCC2	224.5	149.5	105	210.5	181	105.5	162.7	51.41
XRCC3	273.5	140.5	184	283.5	159	145	197.6	64.56
XRCC4	227	148.5	134.5	211	160.5	113.5	165.8	44.34
XRCC5	899.5	365	549	795	398.5	428.5	572.6	224.08
XRCC6	664	468.5	356	677	529	360	509.1	141.35
XRCC6BP1	535	448	270.5	484	495	288.5	420.2	112.58
XRN1	1025.5	629	911.5	1051	795	741	858.8	166.29
XRN2	605.5	256	496.5	633	280.5	423.5	449.2	159.31
XRRA1	208	155	91	182	169.5	103.5	151.5	45.66
XXYLT1	1291	997.5	1083	1266.5	1059	1194.5	1148.6	119.54
XYLB	71.5	39.5	25.5	58.5	44.5	40	46.6	16.16
XYLT1	24.5	32	46.5	36	36	43	36.3	7.83
XYLT2	355	187.5	286	367.5	170	297	277.2	82.72
YAE1D1	272	167.5	171	220	194.5	189.5	202.4	38.95
YAF2	268	267	268.5	235	305	271.5	269.2	22.20
YAP1	768	480	1312.5	762.5	635	1264.5	870.4	340.80
YARS	2475	3036.5	2289	2403.5	2898	2082	2530.7	366.00

YARS2	279.5	276	252	255	282	244	264.8	16.31
YBEY	16.5	8.5	31	22	6.5	36	20.1	11.90
YBX1	22382.5	19691	13955.5	20117	21238.5	12854	18373.1	3975.92
YBX3	2911	3645	2138	2752	3834	2187	2911.2	712.41
YDJC	2922.5	3673.5	2863	2380.5	3962	2462.5	3044.0	642.63
YEATS2	796	722	658.5	829	818	694.5	753.0	70.94
YEATS4	671.5	460	287.5	586.5	488	310.5	467.3	150.53
YES1	1794.5	1444	1846	1682	1694	1781.5	1707.0	143.17
YIPF1	1885.5	963.5	742.5	1708.5	1022	763.5	1180.9	492.70
YIPF3	2043	1485.5	952.5	1979.5	1566.5	991	1503.0	466.52
YIPF4	141.5	59	127.5	153.5	72	124	112.9	38.42
YIPF5	1735.5	1868.5	1712.5	2112	2344	2148	1986.8	253.63
YIPF6	994.5	803.5	738	882.5	898.5	689.5	834.4	112.52
YIPF7	0	2	0.5	0	0	2	0.8	0.99
YJEFN3	108	114	64.5	83	117.5	46.5	88.9	29.12
YKT6	411.5	428	366	427	402	334.5	394.8	37.24
YLPM1	452	409.5	694.5	413	431.5	606	501.1	119.85
YME1L1	4077.5	2342.5	2677	3810	2636.5	2270.5	2969.0	776.18
YOD1	350.5	333.5	326.5	320	360	293.5	330.7	23.56
YPEL1	896	1113.5	1148.5	854	1403	1167	1097.0	200.45
YPEL2	198	263	486.5	196	279	555.5	329.7	153.50
YPEL4	43	42.5	34	38.5	51.5	44	42.3	5.85
YPEL5	581.5	355	665.5	517.5	400.5	599.5	519.9	120.65
YRDC	335	323	231.5	328	383.5	242	307.2	58.75
YTHDC1	925.5	609.5	725.5	822.5	752.5	695.5	755.2	108.82
YTHDC2	700.5	719	898.5	581.5	888	933.5	786.8	140.31
YTHDF1	928.5	765	973.5	929.5	870	844	885.1	74.85
YTHDF2	833	641	794.5	833.5	685	796.5	763.9	81.18
YTHDF3	447	629.5	845	535.5	788.5	778.5	670.7	158.68
YWHAB	2166	1200.5	1923	2235	1273	1674	1745.3	441.21
YWHAE	8677	9764	9259.5	7978.5	11113	8301.5	9182.3	1144.75
YWHAG	951	724.5	1494.5	825.5	776.5	1052	970.7	283.12
YWHAH	1792.5	3428	1882.5	2051.5	3868	1849.5	2478.7	920.44
YWHAZ	8566	10370	9051	8569.5	12784.5	9093.5	9739.1	1631.37
YY1	867	791	880.5	812.5	916.5	864	855.3	45.96
ZADH2	638.5	316	373	520.5	349	337	422.3	128.66
ZAR1L	20	16	2	13	19.5	4.5	12.5	7.64
ZBBX	5.5	1	0.5	6.5	4	3	3.4	2.40
ZBED1	223.5	232.5	277	294	296.5	288.5	268.7	32.33
ZBED4	532.5	508	656	538	598.5	620.5	575.6	58.04
ZBTB1	971.5	907.5	1129	1030.5	1085.5	1148	1045.3	93.62
ZBTB10	610.5	474.5	653.5	624.5	583	605	591.8	62.03
ZBTB11	43	20	121.5	55.5	21.5	98	59.9	41.52
ZBTB14	34.5	31	64	33.5	37	59	43.2	14.42
ZBTB16	147.5	40	99.5	132	38.5	89	91.1	45.40
ZBTB17	615	676	657	606.5	668	585.5	634.7	37.19
ZBTB18	447.5	443.5	560	494	514	503	493.7	43.70
ZBTB2	557	570.5	662	558.5	649	610.5	601.3	46.45
ZBTB20	13	6.5	19.5	9	6	17.5	11.9	5.70
ZBTB21	150	67.5	225	142.5	79.5	188	142.1	60.87
ZBTB24	132	77.5	143.5	154.5	107.5	148.5	127.3	29.48
ZBTB25	209	259	242	226.5	284.5	256	246.2	26.52
ZBTB26	190	136	194	204	163.5	186.5	179.0	24.97
ZBTB33	1298.5	777.5	940.5	1153.5	865	930.5	994.3	194.20

ZBTB34	31.5	18.5	56.5	29.5	12	46.5	32.4	16.72
ZBTB37	297.5	121.5	372.5	311	129	334.5	261.0	108.21
ZBTB38	186.5	122	129	197	135	126.5	149.3	33.29
ZBTB39	213	335	361	236	420	522.5	347.9	115.53
ZBTB4	110	56.5	186.5	107.5	36.5	177	112.3	60.96
ZBTB40	176	160.5	226	159.5	190.5	243	192.6	34.82
ZBTB42	66.5	24.5	96	66	26	88	61.2	30.22
ZBTB43	267.5	200	355	258	234	344.5	276.5	61.43
ZBTB44	408	199	397.5	349	240	318.5	318.7	84.41
ZBTB45	118	126	115.5	112	116.5	113	116.8	5.01
ZBTB46	290	210	330.5	249.5	233.5	349	277.1	55.41
ZBTB47	197	235	181.5	173	236	157	196.6	32.80
ZBTB48	296.5	281	260	278.5	341	253	285.0	31.55
ZBTB49	336	221.5	255.5	201.5	232.5	212.5	243.3	49.05
ZBTB5	92.5	55.5	238	123.5	68.5	232.5	135.1	80.99
ZBTB6	165.5	137	152.5	142	132	129.5	143.1	13.69
ZBTB7A	204.5	221.5	256.5	208.5	212	270	228.8	27.58
ZBTB7C	21.5	16	40.5	28.5	18	51	29.3	13.87
ZBTB8A	261.5	291	296.5	261.5	307.5	375.5	298.9	41.97
ZBTB8B	354.5	350	344.5	345.5	361	378.5	355.7	12.74
ZBTB8OS	742.5	494.5	418.5	695	598.5	422.5	561.9	138.72
ZC2HC1A	48	17.5	114	55.5	26.5	77.5	56.5	35.33
ZC2HC1B	0	0	0	0	0	0.5	0.1	0.20
ZC2HC1C	21.5	16.5	58.5	24	25	59.5	34.2	19.46
ZC3H10	131.5	171	119	134.5	146.5	113	135.9	20.86
ZC3H11A	985.5	1074.5	1235.5	874.5	1258.5	1332	1126.8	177.55
ZC3H12A	390.5	290	326.5	412	298.5	263	330.1	59.12
ZC3H12B	150.5	270	460	150.5	311	422.5	294.1	131.24
ZC3H12C	186.5	178.5	261.5	219	192	260	216.3	37.06
ZC3H12D	1.5	1.5	3	2	1.5	3.5	2.2	0.88
ZC3H13	1829.5	1493.5	1494.5	1718.5	1642.5	1407.5	1597.7	160.01
ZC3H14	623	450.5	523.5	600.5	477.5	487	527.0	70.05
ZC3H15	1329.5	1029.5	1108	1168	1083	952.5	1111.8	129.26
ZC3H3	353	319	281.5	277	329	273	305.4	32.97
ZC3H6	721.5	445	693	706	518.5	616.5	616.8	113.05
ZC3H7A	1287.5	1003.5	845	1199.5	1158.5	833.5	1054.6	190.51
ZC3H7B	554.5	288	844.5	542	274.5	742.5	541.0	231.49
ZC3HC1	618.5	515	447	538	423.5	396	489.7	83.12
ZC4H2	18	14.5	45	19	12.5	41.5	25.1	14.31
ZCCHC10	315	276.5	272	319.5	306.5	265.5	292.5	23.82
ZCCHC17	248.5	288.5	199.5	231.5	258	184.5	235.1	38.46
ZCCHC2	293.5	232.5	334.5	297.5	266.5	355.5	296.7	44.57
ZCCHC24	858	395	654.5	774	462	559	617.1	179.34
ZCCHC4	357.5	191.5	152.5	328	173.5	122.5	220.9	97.57
ZCCHC6	425	230.5	395	379	278.5	362	345.0	74.67
ZCCHC7	307.5	204	211	327	223.5	208	246.8	55.28
ZCCHC8	648.5	408	448.5	568	499.5	445.5	503.0	90.23
ZCCHC9	415.5	349	333	381	339	321	356.4	35.37
ZCRB1	838.5	869	614.5	814.5	939	596.5	778.7	140.62
ZDHHC1	80.5	32	75	72	34	62.5	59.3	21.23
ZDHHC12	418	222.5	196	368	224	190	269.8	97.73
ZDHHC14	351	293	310	364.5	384	327.5	338.3	34.37
ZDHHC15	181	61.5	148.5	168.5	59	116	122.4	52.93
ZDHHC16	291.5	260	214	244	261.5	186	242.8	37.61

ZDHHC17	344	190.5	336.5	325.5	220.5	280	282.8	64.59
ZDHHC18	31.5	26.5	53	38	32.5	49	38.4	10.49
ZDHHC2	79.5	32.5	32	53.5	40	30.5	44.7	19.09
ZDHHC20	574	435.5	588.5	574.5	503	577.5	542.2	60.61
ZDHHC21	309	153	177.5	297	180.5	173.5	215.1	68.88
ZDHHC22	0	0	1	0.5	0.5	2	0.7	0.75
ZDHHC23	83	78.5	60	70	71	63.5	71.0	8.70
ZDHHC3	430.5	297	385	371	317.5	351	358.7	48.09
ZDHHC4	60	26	33.5	78.5	25.5	27	41.8	22.28
ZDHHC5	660	498.5	906.5	712.5	617	963.5	726.3	177.29
ZDHHC6	864	556	609.5	819.5	579.5	532.5	660.2	143.64
ZDHHC7	179	113	292.5	216.5	112.5	241.5	192.5	71.94
ZDHHC8	302	259.5	407	297.5	349.5	382	332.9	56.17
ZDHHC9	1123.5	1252.5	1446.5	960	1223.5	1467.5	1245.6	193.19
ZEB1	848	965.5	1528.5	854.5	1156	1448.5	1133.5	297.77
ZEB2	537.5	404	1275.5	448	515	1130	718.3	381.01
ZER1	890	584.5	564	872.5	683.5	577.5	695.3	150.23
ZFAND1	713	503.5	329.5	674	586.5	355.5	527.0	160.45
ZFAND2A	588	683	239.5	585	699	246.5	506.8	209.71
ZFAND2B	207.5	236	176.5	176.5	261.5	158.5	202.8	39.79
ZFAND3	2199.5	1900.5	2159.5	2049	2072.5	1974	2059.2	111.78
ZFAND4	420	389	476.5	402.5	509	428.5	437.6	46.04
ZFAND5	86.5	46	126.5	101	68	112	90.0	29.56
ZFAND6	1062	900.5	1542.5	1158	1129.5	1424.5	1202.8	237.97
ZFAT	33	11	47	31.5	7.5	37	27.8	15.42
ZFC3H1	1440.5	732	1123	1271.5	796.5	1082.5	1074.3	271.95
ZFHX3	38	25.5	108	63	21.5	104.5	60.1	38.60
ZFP36L1	573	378	1121	682	392	946	682.0	300.27
ZFP91	305.5	235	361	297	244	356	299.8	53.40
ZFPM1	123	222	328.5	147.5	216	339.5	229.4	89.67
ZFPM2	0	0	0.5	0	0	0	0.1	0.20
ZFR	1085.5	777	970	1058	936	917.5	957.3	110.71
ZFYVE1	578.5	461.5	533.5	531	530.5	529	527.3	37.46
ZFYVE16	1001.5	737.5	973.5	890.5	819.5	950	895.4	101.05
ZFYVE19	1054	611.5	456	922	587	373	667.3	266.54
ZFYVE21	297.5	222	218.5	273	255	237	250.5	30.84
ZFYVE26	256.5	118	205	235	121.5	184	186.7	57.48
ZFYVE27	616.5	421.5	338.5	687.5	540.5	376	496.8	139.95
ZFYVE9	30.5	20	29.5	27	17.5	20	24.1	5.58
ZGPAT	388	371	368.5	373	360	349	368.3	13.11
ZGRF1	182	97	165.5	217	93.5	117	145.3	50.42
ZHX1	965.5	838	1587	898	961.5	1626	1146.0	359.96
ZHX2	642	506	515	555	541.5	565	554.1	48.68
ZHX3	229.5	375.5	522.5	280.5	448.5	561.5	403.0	132.16
ZIC1	0	0	0	1.5	0	0	0.3	0.61
ZMAT2	1232	1297	884.5	1153	1493	821	1146.8	254.82
ZMAT3	114	47	134	132	51	136	102.3	42.07
ZMAT4	1	1	1.5	1.5	1.5	0.5	1.2	0.41
ZMAT5	192.5	145.5	89	169.5	145.5	70.5	135.4	46.89
ZMIZ1	481.5	311.5	449	538	357.5	443.5	430.2	82.65
ZMIZ2	102.5	132.5	167.5	81	102	192.5	129.7	43.03
ZMYM2	280	266	451	248.5	303	384.5	322.2	79.01
ZMYM3	292	322	345.5	251	319.5	347	312.8	36.39
ZMYM4	529.5	419.5	543	489.5	463.5	493	489.7	44.83

ZMYM5	277.5	131	241	285	153	225	218.8	63.87
ZMYM6NB	539.5	516	224	585.5	562	312.5	456.6	150.33
ZMYND10	16.5	9.5	7	9.5	9	5	9.4	3.89
ZMYND11	789	401.5	576	682	487	568	583.9	137.60
ZMYND12	68	63	88.5	61.5	82.5	81	74.1	11.36
ZMYND19	279.5	250	213.5	278.5	237.5	241	250.0	25.50
ZNF106	334	843	2414.5	292.5	1036	1638.5	1093.1	815.67
ZNF131	312.5	222	108	279	254	122.5	216.3	83.87
ZNF142	140.5	128	147	117.5	118.5	128.5	130.0	11.78
ZNF143	290.5	279.5	302	283.5	298	291	290.8	8.47
ZNF148	145.5	107	139.5	135	101	133	126.8	18.30
ZNF185	62.5	23.5	41.5	52.5	17.5	43	40.1	17.04
ZNF207	2036.5	1707.5	1711.5	1976.5	1906.5	1468.5	1801.2	211.99
ZNF217	532.5	223	850	609	276.5	746.5	539.6	250.39
ZNF236	141	71.5	175	140.5	86.5	181	132.6	45.01
ZNF276	371.5	260.5	200	343.5	280.5	224.5	280.1	66.73
ZNF277	277	188.5	175.5	241.5	235	165.5	213.8	43.93
ZNF280D	546.5	409.5	360.5	498	486	335	439.3	83.78
ZNF281	384.5	264.5	377.5	392	276.5	314.5	334.9	57.13
ZNF292	330	250.5	537	313	271	458.5	360.0	113.18
ZNF318	657.5	348.5	381.5	604	362	342.5	449.3	142.17
ZNF319	248.5	233	248.5	234.5	222.5	268.5	242.6	16.14
ZNF326	735.5	563	665.5	705	634	596.5	649.9	65.21
ZNF330	758	366	255.5	647.5	426.5	195	441.4	220.86
ZNF335	1275	1242	1141	1309.5	1270	1263.5	1250.2	57.78
ZNF341	247	147	126.5	186	165.5	118	165.0	47.29
ZNF346	372.5	193	305.5	312.5	185	236	267.4	74.62
ZNF362	243.5	228.5	352	228	198.5	361.5	268.7	69.84
ZNF365	391.5	510.5	570.5	420.5	610.5	598	516.9	93.03
ZNF366	0.5	0	0	0	0.5	0.5	0.3	0.27
ZNF367	761.5	306	677.5	727.5	321.5	399	532.2	212.20
ZNF384	45.5	58	85	60.5	50	72	61.8	14.58
ZNF385B	318.5	628	549.5	256.5	734.5	434.5	486.9	184.02
ZNF385C	12.5	5	11.5	8.5	7	6	8.4	3.02
ZNF385D	10.5	19	10	15	38	26	19.8	10.73
ZNF395	104	65	270.5	105	68	234.5	141.2	88.64
ZNF407	48	16.5	109.5	54.5	22	89	56.6	36.69
ZNF410	551.5	511	571.5	544	572.5	560	551.8	22.84
ZNF414	108	108	115	109.5	97	100.5	106.3	6.51
ZNF423	102	44.5	87	112	36.5	91	78.8	31.06
ZNF438	177.5	85	109.5	154.5	101	106	122.3	35.66
ZNF451	117	47.5	153.5	127	55	113.5	102.3	41.98
ZNF462	328.5	83.5	579	336	97.5	542.5	327.8	210.71
ZNF467	67	58	63.5	49.5	53	58.5	58.3	6.46
ZNF469	21.5	30	84.5	39.5	29.5	105.5	51.8	34.63
ZNF488	59	93	55	43	68.5	51.5	61.7	17.50
ZNF507	178.5	106	216.5	175.5	108.5	184	161.5	44.51
ZNF511	266.5	219	148.5	197	216.5	133.5	196.8	49.15
ZNF512	816.5	525.5	770	808.5	589.5	715	704.2	120.85
ZNF512B	290	170.5	211	300.5	185	228	230.8	53.84
ZNF513	416.5	384	293	420	383.5	307	367.3	54.58
ZNF518A	136.5	74	133.5	116.5	91.5	121.5	112.3	24.64
ZNF518B	167	152.5	246	169	161.5	244.5	190.1	43.12
ZNF521	530	345	595.5	499	444	543	492.8	88.00

ZNF532	261.5	181.5	318	247	230	331.5	261.6	56.03
ZNF536	4	1	18	4.5	2	18	7.9	7.91
ZNF574	25.5	28.5	49	29.5	19	33.5	30.8	10.13
ZNF592	280.5	221	395	264	245.5	374.5	296.8	71.27
ZNF593	1180.5	1507	798.5	1101.5	1506.5	791	1147.5	319.44
ZNF598	1064	679.5	749	1030	738	755	835.9	166.04
ZNF608	254.5	282.5	453.5	242	328.5	468	338.2	99.60
ZNF609	722.5	536	839	790	496	823	701.1	149.37
ZNF618	43.5	13	55	45.5	14.5	51.5	37.2	18.61
ZNF622	897.5	676.5	659.5	929.5	775	622.5	760.1	129.49
ZNF628	595	566.5	993	588.5	558.5	998	716.6	216.47
ZNF639	456.5	442	329	398.5	473	299	399.7	71.45
ZNF644	1150.5	905	1209.5	1210	1109.5	1068	1108.8	114.30
ZNF648	502.5	548.5	446	432.5	566.5	369.5	477.6	75.19
ZNF652	529.5	476.5	584.5	497	589	588.5	544.2	50.24
ZNF653	83.5	53	65	71	51.5	66	65.0	11.88
ZNF654	52	30.5	107	72	43.5	92	66.2	29.51
ZNF687	586.5	394	386.5	562.5	388	374	448.6	98.05
ZNF703	400	641.5	574.5	428.5	717.5	603.5	560.9	123.64
ZNF704	435.5	433.5	422	426.5	475.5	448.5	440.3	19.50
ZNF706	1423.5	3566	3779	1345	4156.5	3735.5	3000.9	1267.28
ZNF710	65.5	57	96.5	71.5	54.5	90	72.5	17.30
ZNF711	789	580	594	703.5	673.5	611	658.5	79.82
ZNF750	0	0	0.5	0	0	0	0.1	0.20
ZNF76	186	158.5	96.5	190.5	157	108.5	149.5	39.09
ZNF770	241	125	185.5	197	161	175.5	180.8	38.58
ZNF800	73	60	77.5	66.5	58.5	45	63.4	11.62
ZNF804A	0	0.5	0	0	0	0.5	0.2	0.26
ZNF804B	0	0	0	0	0	0	0.0	0.00
ZNF821	645.5	790	800	586.5	855.5	844	753.6	111.03
ZNF827	56	31.5	134	54.5	27.5	169	78.8	58.59
ZNF830	444	311.5	252	396	348.5	264	336.0	75.18
ZNF831	0.5	0	0	1	0.5	0.5	0.4	0.38
ZNF839	700.5	489.5	407	614.5	564	385.5	526.8	122.44
ZNF865	149	169	172	162	137.5	207	166.1	23.85
ZNFX1	270.5	196.5	466.5	262.5	211	342	291.5	99.96
ZNHIT2	193	263.5	193	171	259.5	208.5	214.8	38.15
ZNHIT3	327	358.5	196	304	395	214	299.1	79.25
ZNHIT6	359	303	273	355.5	325.5	274	315.0	38.15
ZNRF1	321.5	477.5	414.5	294.5	528	457	415.5	91.32
ZNRF3	239	232	218.5	247	264	244	240.8	15.26
ZP1	1.5	1.5	3.5	0.5	1.5	4.5	2.2	1.51
ZP2	2	1.5	3	1.5	2	3	2.2	0.68
ZP4	3	2.5	3	0.5	0	10	3.2	3.59
ZPAX	0	0	0	0	0	0	0.0	0.00
ZPBP	0	0	0	0	0	0	0.0	0.00
ZPBP2	0	0	0	0	0	0	0.0	0.00
ZPLD1	1.5	0	1	2	0	0	0.8	0.88
ZPR1	712	832	502.5	730	885	540	700.3	153.17
ZRANB1	1392	1326.5	1492	1254.5	1432	1339	1372.7	84.10
ZRANB2	207.5	106.5	217	197.5	119.5	207.5	175.9	49.30
ZRSR2	380.5	393	348.5	371	431.5	331.5	376.0	35.07
ZSCAN21	0	0	0	0	0	0	0.0	0.00
ZSWIM1	204	213.5	122	207.5	262	161.5	195.1	48.00

ZSWIM5	289.5	376.5	667	328.5	405	651.5	453.0	164.67
ZSWIM7	176.5	178	120	152.5	190	86	150.5	40.22
ZSWIM8	482	385.5	448.5	409	434.5	428.5	431.3	33.10
ZUFSP	210.5	98.5	127.5	188.5	121	115	143.5	44.97
ZW10	765	560.5	689.5	751.5	620.5	643	671.7	78.99
ZWILCH	398.5	311	300.5	406	287.5	261	327.4	60.38
ZXDC	528	346.5	404	476.5	449.5	373.5	429.7	67.80
ZYX	4413.5	5304.5	6326.5	4132	5623.5	6344.5	5357.4	935.91
ZZZ3	430.5	308.5	420	473	355	431	403.0	59.94
	518.23	468.36	466.13	494.78	508.29	451.43	484.54	131.16
