# Supplementary material for: Response of Turkey Muscle Satellite Cells to Thermal Challenge. II. Transcriptome Effects in Differentiating Cells
Source: Front Physiol. 2017 Nov 30;8:948. doi: 10.3389/fphys.2017.00948 (PMC5714890; doi:10.3389/fphys.2017.00948)
Supplement: Supplementary file 8 [file Table2.PDF]

Table S2. Normalized mean RNAseq read counts observed in cultured *p. major* satellite cells from RBC2 and F line turkeys after 48 hr differentiation at 38° C. Genes are sorted in descending order by average number of reads.

Feature ID	Description	38R - Normalized means	38F - Normalized means	Average
ACTB	actin, beta	232528.9	234482.0	233505.49
EEF1A1	eukaryotic translation elongation factor 1 alpha 1	172530.1	161859.1	167194.59
ACTC1	actin, alpha, cardiac muscle 1	125315.8	133863.3	129589.56
TMSB15B	thymosin beta 15B	115296.8	104990.5	110143.68
GAPDH	glyceraldehyde-3-phosphate dehydrogenase	90600.4	84397.9	87499.17
VIM	vimentin	85151.7	88340.5	86746.10
TNNT2	troponin T type 2 (cardiac)	78365.2	72775.4	75570.29
NES	nestin	81945.3	68668.6	75306.92
TPT1	tumor protein, translationally-controlled 1	63554.2	65510.1	64532.14
SPARC	secreted protein, acidic, cysteine-rich (osteonectin)	52256.2	61423.2	56839.68
CLU	clusterin	54404.1	58164.5	56284.28
LOC100544583	actin, cytoplasmic 2-like	54173.3	52191.1	53182.23
LOC104910121	tubulin beta-2 chain	52282.5	53814.5	53048.55
DES	desmin	55346.8	47864.5	51605.66
HTRA1	HtrA serine peptidase 1	46857.8	52001.9	49429.85
TNNT3	troponin T type 3 (skeletal, fast)	45361.4	46067.0	45714.17
FTH1	ferritin, heavy polypeptide 1	46996.5	42995.0	44995.76
COL1A2	collagen, type I, alpha 2	40756.5	48309.6	44533.03
LOC104913730	myosin heavy chain, cardiac muscle isoform	43892.4	45147.7	44520.04
UBB	ubiquitin B	43001.4	41354.9	42178.13
PABPC1	poly(A) binding protein, cytoplasmic 1	39489.8	41771.9	40630.87
TNNC1	troponin C type 1 (slow)	38765.5	39468.6	39117.06
RPS6	ribosomal protein S6	41079.2	37090.7	39084.92
CKB	creatine kinase, brain	34031.8	41274.0	37652.90
ENO1	enolase 1, (alpha)	37397.9	37722.1	37559.98
COL6A1	collagen, type VI, alpha 1	34884.6	39960.1	37422.37
RPSA	ribosomal protein SA	39551.9	35024.0	37287.97
RPL4	ribosomal protein L4	39256.6	34902.0	37079.30
TNNI2	troponin I type 2 (skeletal, fast)	37784.0	36073.4	36928.73
STMN1	stathmin 1	35715.3	33431.5	34573.41
CHRNA1	cholinergic receptor, nicotinic, alpha 1 (muscle)	32499.0	35951.9	34225.45
TNNI1	troponin I type 1 (skeletal, slow)	32871.9	35214.8	34043.34
YBX1	Y box binding protein 1	33255.4	33080.2	33167.79
RPL5	ribosomal protein L5	33502.4	32139.2	32820.83
RPLP0	ribosomal protein, large, P0	34177.6	31201.8	32689.75
RPS2	ribosomal protein S2	33926.2	30769.1	32347.63
EEF2	eukaryotic translation elongation factor 2	32263.5	29531.2	30897.35
OAZ1	ornithine decarboxylase antizyme 1	29858.6	31369.0	30613.79
AHCY	adenosylhomocysteinase	30184.5	30790.3	30487.41
PLS3	plastin 3	28670.4	31896.5	30283.45
LOC104914203	basement membrane-specific heparan sulfate proteoglycan core protein-like	31274.4	29111.9	30193.12
COL6A2	collagen, type VI, alpha 2	28075.3	31878.3	29976.79
ANXA2	annexin A2	28687.7	29857.9	29272.76
ITGA6	integrin, alpha 6	28269.7	30113.0	29191.37
EIF4G2	eukaryotic translation initiation factor 4 gamma, 2	28451.9	29748.6	29100.22
RPLP1	ribosomal protein, large, P1	30342.3	27744.8	29043.58
LOC100539974	tubulin alpha-1B chain	30759.3	26689.2	28724.27
TUBB6	tubulin, beta 6 class V	26727.2	29732.0	28229.60
EEF1D	eukaryotic translation elongation factor 1 delta (guanine nucleotide exchange protein)	29089.6	27227.3	28158.44
RPS3	ribosomal protein S3	28552.7	25684.3	27118.54
DSTN	destrin (actin depolymerizing factor)	26516.6	27183.7	26850.14

MYL10	myosin, light chain 10, regulatory	25033.9	27401.1	26217.51
LOC100542991	40S ribosomal protein S4-like	27062.1	24850.9	25956.53
RPL6	ribosomal protein L6	26511.8	25353.6	25932.70
RPS12	ribosomal protein S12	27015.7	24659.0	25837.32
HSPA8	heat shock 70kDa protein 8	26182.3	25332.1	25757.19
RPL7	ribosomal protein L7	26488.0	24993.9	25740.93
RPS24	ribosomal protein S24	26434.8	24988.4	25711.59
PGAM1	phosphoglycerate mutase 1 (brain)	24812.8	26388.3	25600.55
RPL7A	ribosomal protein L7a	26456.1	24539.9	25498.03
GNB2L1	guanine nucleotide binding protein (G protein), beta polypeptide 2-like 1	26309.3	24149.3	25229.29
RPL3	ribosomal protein L3	26257.3	24124.7	25190.98
SLC25A6	solute carrier family 25 (mitochondrial carrier; adenine nucleotide translocator), member 6	25906.6	24400.1	25153.38
RNH1	ribonuclease/angiogenin inhibitor 1	23797.9	25762.3	24780.14
LOC100541081	protein S100-A6	26331.9	23180.8	24756.36
LOC104909644	spectrin beta chain, non-erythrocytic 5-like	27511.3	21786.4	24648.82
FAM101B	family with sequence similarity 101, member B	23712.0	25425.2	24568.60
LOC104912412	protein wntless homolog	24496.7	23591.3	24044.00
RPL21	ribosomal protein L21	24309.0	23483.8	23896.41
SYNPO2L	synaptopodin 2-like	23186.0	24131.4	23658.71
RPS8	ribosomal protein S8	24650.6	22527.7	23589.12
CTSD	cathepsin D	22699.7	24224.1	23461.88
RPS15A	ribosomal protein S15a	24447.2	22432.9	23440.03
PKIG	protein kinase (cAMP-dependent, catalytic) inhibitor gamma	21725.3	24747.4	23236.32
TGFB3	transforming growth factor, beta 3	22470.8	23535.2	23003.00
PPIA	peptidylprolyl isomerase A (cyclophilin A)	23503.9	22501.1	23002.50
RPS20	ribosomal protein S20	23015.9	22748.0	22881.92
RPL26L1	ribosomal protein L26-like 1	23990.3	21755.8	22873.08
ADAMTSL2	ADAMTS-like 2	21440.2	24211.5	22825.85
ECM1	extracellular matrix protein 1	21901.5	23679.7	22790.62
RPL9	ribosomal protein L9	23073.5	22018.2	22545.82
LOC100303673	myogenin	21563.1	22904.9	22233.99
RPS7	ribosomal protein S7	23053.3	21087.8	22070.54
PKM	pyruvate kinase, muscle	23433.8	20582.5	22008.17
S100A10	S100 calcium binding protein A10	22417.8	21448.1	21932.94
DDX5	DEAD (Asp-Glu-Ala-Asp) box helicase 5	21356.8	22383.5	21870.17
LOC104917559	serine/threonine-protein kinase DCLK1-like	20342.6	23355.4	21848.99
JAM3	junctional adhesion molecule 3	21793.6	21890.0	21841.82
TTN	titin	20066.7	23410.1	21738.41
RPS23	ribosomal protein S23	22544.5	20856.8	21700.64
MYL1	myosin, light chain 1, alkali; skeletal, fast	21710.8	21549.0	21629.86
RPL13	ribosomal protein L13	22236.9	19686.7	20961.76
TPM3	tropomyosin 3	20789.6	21018.9	20904.29
RPL8	ribosomal protein L8	21859.1	19810.7	20834.89
FXYD6	FXYD domain containing ion transport regulator 6	21829.6	19759.5	20794.55
CIRBP	cold inducible RNA binding protein	20897.8	20553.4	20725.61
RHOA	ras homolog family member A	20802.3	20457.8	20630.08
COL6A3	collagen, type VI, alpha 3	18217.1	22818.4	20517.72
RPL19	ribosomal protein L19	21012.1	19570.8	20291.47
EEF1B2	eukaryotic translation elongation factor 1 beta 2	20872.9	19708.8	20290.84
LOC100544580	collagen alpha-1(I) chain-like	19196.8	21161.6	20179.21
HN1	hematological and neurological expressed 1	18617.1	21311.5	19964.30
NPM1	nucleophosmin (nucleolar phosphoprotein B23, numatrin)	20553.1	19236.3	19894.66
RPL37A	ribosomal protein L37a	20707.4	18436.1	19571.77
EIF4A2	eukaryotic translation initiation factor 4A2	18945.0	20154.9	19549.93
ANXA6	annexin A6	19387.8	19379.7	19383.74
RPS15	ribosomal protein S15	19896.1	18852.6	19374.34

ATP5B	ATP synthase, H+ transporting, mitochondrial F1 complex, beta polypeptide	20472.6	18160.4	19316.51
PENK	proenkephalin	17013.7	20855.1	18934.39
YWHAZ	tyrosine 3-monooxygenase/tryptophan 5-monooxygenase activation protein, zeta	17511.5	19892.1	18701.76
RBM24	RNA binding motif protein 24	17053.8	19838.4	18446.10
LOC654833	myosin light polypeptide 6	18877.6	17915.7	18396.66
RPL32	ribosomal protein L32	18924.2	17805.5	18364.87
ATF4	activating transcription factor 4	17924.0	18748.0	18335.99
PPIB	peptidylprolyl isomerase B (cyclophilin B)	18026.2	18502.4	18264.30
TUBAL3	tubulin, alpha-like 3	18769.5	17706.5	18238.03
RPL39	ribosomal protein L39	18434.9	17987.2	18211.02
CAPN11	calpain 11	18523.2	17754.9	18139.05
MRAS	muscle RAS oncogene homolog	17530.5	18699.8	18115.19
RPL15	ribosomal protein L15	18750.4	17178.2	17964.31
HSPB1	heat shock 27kDa protein 1	17797.4	18104.1	17950.77
RPL11	ribosomal protein L11	18682.8	17134.8	17908.83
CAMK2A	calcium/calmodulin-dependent protein kinase II alpha	16681.6	18987.4	17834.48
CNBP	CCHC-type zinc finger, nucleic acid binding protein	17802.0	17866.6	17834.27
RPL27A	ribosomal protein L27a	18279.2	17195.6	17737.40
RPS11	ribosomal protein S11	17583.2	17104.2	17343.67
CALR	calreticulin	17316.8	16838.1	17077.45
YWHAE	tyrosine 3-monooxygenase/tryptophan 5-monooxygenase activation protein, epsilon	16487.3	17297.6	16892.41
TXN	thioredoxin	17363.2	16295.8	16829.47
HDLBP	high density lipoprotein binding protein	17292.9	16337.2	16815.05
RPL10A	ribosomal protein L10a	17475.9	16043.0	16759.45
ANKRD1	ankyrin repeat domain 1 (cardiac muscle)	15859.0	17167.7	16513.36
MXRA8	matrix-remodelling associated 8	15845.4	17105.1	16475.25
RPLP2	ribosomal protein, large, P2	16695.8	16097.6	16396.70
LOC104917029	troponin T, slow skeletal muscle-like	16440.7	16172.4	16306.53
CALM2	calmodulin 2 (phosphorylase kinase, delta)	15976.9	16632.9	16304.89
RPS17	ribosomal protein S17	16952.4	15531.8	16242.11
MYL9	myosin, light chain 9, regulatory	16505.0	15839.4	16172.22
PRDX1	peroxiredoxin 1	16029.0	16169.4	16099.19
RPL10	ribosomal protein L10	17001.1	15187.4	16094.24
LOC104916671	tubulin alpha-1A chain-like	16703.9	15482.7	16093.29
RPL23	ribosomal protein L23	16521.2	15524.2	16022.65
LOC100548331	14-3-3 protein theta	15794.8	16215.1	16004.97
RPL24	ribosomal protein L24	16161.2	15685.9	15923.55
RPL14	ribosomal protein L14	16455.9	15167.6	15811.76
RPL27	ribosomal protein L27	16482.4	15125.2	15803.82
ACTN2	actinin, alpha 2	14803.7	16582.4	15693.04
THBS1	thrombospondin 1	14767.8	16617.4	15692.62
LGALS1	lectin, galactoside-binding, soluble, 1	15473.8	15672.3	15573.03
TPM1	tropomyosin 1 (alpha)	15371.2	15419.2	15395.17
ANXA5	annexin A5	14983.7	15799.1	15391.41
RPS10	ribosomal protein S10	16133.3	14639.5	15386.38
FHL1	four and a half LIM domains 1	15013.6	15747.9	15380.74
MFGE8	milk fat globule-EGF factor 8 protein	15645.9	14869.0	15257.43
RPS16	ribosomal protein S16	15167.1	14666.0	14916.55
CSRP1	cysteine and glycine-rich protein 1	15042.3	14611.9	14827.11
LOC100543743	60S ribosomal protein L17-like	14897.6	14298.7	14598.16
RPL23A	ribosomal protein L23a	15040.6	13959.6	14500.09
RPL18A	ribosomal protein L18a	15265.1	13717.5	14491.29
RPL37	ribosomal protein L37	15134.7	13789.9	14462.31
RPS25	ribosomal protein S25	14626.8	14225.3	14426.05
LAMB2	laminin, beta 2 (laminin S)	15065.3	13672.4	14368.84

RPL30	ribosomal protein L30	14609.1	13938.3	14273.73
MSN	moesin	13359.0	15035.3	14197.19
RPS27A	ribosomal protein S27a	14555.7	13791.6	14173.69
NME2	NME/NM23 nucleoside diphosphate kinase 2	14386.6	13734.3	14060.45
RPS14	ribosomal protein S14	14366.0	13548.9	13957.47
CD63	CD63 molecule	14066.6	13810.3	13938.43
AP3M1	adaptor-related protein complex 3, mu 1 subunit	13354.7	14347.0	13850.88
ATP5A1	ATP synthase, H+ transporting, mitochondrial F1 complex, alpha subunit 1, cardiac muscle	14217.1	13375.3	13796.19
ALDOC	aldolase C, fructose-bisphosphate	14882.0	12617.3	13749.67
NACA	nascent polypeptide-associated complex alpha subunit	13841.8	13442.3	13642.05
RPL29	ribosomal protein L29	14533.4	12702.8	13618.09
TNC	tenascin C	13517.4	13696.5	13606.98
ALAS1	5'-aminolevulinate synthase 1	12814.0	14351.8	13582.94
FNDC5	fibronectin type III domain containing 5	13594.0	13505.7	13549.85
LOC104912139	60S ribosomal protein L36a	13950.2	13076.9	13513.58
LOC104912834	nicotinamide riboside kinase 2-like	13670.8	13111.6	13391.23
RPS28	ribosomal protein S28	15393.4	11374.1	13383.76
LOC100550679	NADH-cytochrome b5 reductase 3	12553.1	14139.3	13346.17
VDAC2	voltage-dependent anion channel 2	13056.3	13455.5	13255.92
PDIA3	protein disulfide isomerase family A, member 3	12712.5	13520.5	13116.48
RPL35	ribosomal protein L35	13686.7	12471.0	13078.85
SERPINH1	serpin peptidase inhibitor, clade H (heat shock protein 47), member 1, (collagen binding protein 1)	12511.7	13629.6	13070.67
PDLIM1	PDZ and LIM domain 1	13268.9	12813.5	13041.16
COL5A2	collagen, type V, alpha 2	12471.9	13472.6	12972.24
CLIC4	chloride intracellular channel 4	13002.3	12877.7	12939.99
RPS3A	ribosomal protein S3A	13096.2	12730.6	12913.39
LOC100545757	protein-arginine deiminase type-1-like	13102.6	12620.6	12861.58
COL3A1	collagen, type III, alpha 1	10940.8	14498.2	12719.50
GSN	gelsolin	12321.8	13009.8	12665.77
DBN1	drebrin 1	13688.2	11601.1	12644.65
FERMT2	fermitin family member 2	12043.3	13245.0	12644.12
LOC100542210	reticulon-4-like	12038.9	13197.4	12618.15
NOP56	NOP56 ribonucleoprotein	13485.7	11673.3	12579.49
LOC100541207	transforming acidic coiled-coil-containing protein 2-like	12090.9	12959.7	12525.31
PGK1	phosphoglycerate kinase 1	12263.6	12664.0	12463.80
P4HB	prolyl 4-hydroxylase, beta polypeptide	11792.2	12940.9	12366.54
CTSA	cathepsin A	12397.3	12149.8	12273.58
RPL35A	ribosomal protein L35a	12556.7	11845.7	12201.19
SERBP1	SERPINE1 mRNA binding protein 1	12354.9	11831.9	12093.42
MICALL1	MICAL-like 1	11901.4	12240.8	12071.14
ACAA2	acetyl-CoA acyltransferase 2	12068.3	12072.7	12070.48
LOC104914465	myogenin pseudogene	12018.3	11914.6	11966.41
LOC104917012	plectin-like	13168.8	10648.8	11908.76
PHLDA1	pleckstrin homology-like domain, family A, member 1	11945.7	11814.1	11879.90
UBA52	ubiquitin A-52 residue ribosomal protein fusion product 1	12211.1	11367.3	11789.22
NID2	nidogen 2 (osteonidogen)	11832.6	11666.2	11749.38
DYNC1H1	dynein, cytoplasmic 1, heavy chain 1	11760.9	11675.8	11718.34
S100A11	S100 calcium binding protein A11	11726.4	11608.2	11667.32
WWTR1	WW domain containing transcription regulator 1	11526.9	11780.7	11653.80
LMCD1	LIM and cysteine-rich domains 1	10964.9	12285.3	11625.10
APLP2	amyloid beta (A4) precursor-like protein 2	11057.6	12129.7	11593.63
LAMA5	laminin, alpha 5	11286.5	11838.6	11562.54
TMEM8C	transmembrane protein 8C	10971.2	11807.7	11389.45
RPL12	ribosomal protein L12	11657.2	10994.7	11325.94
RPL31	ribosomal protein L31	11700.6	10928.3	11314.49
OLFML2A	olfactomedin-like 2A	11166.1	11371.8	11268.92

CHCHD2	coiled-coil-helix-coiled-coil-helix domain containing 2	11539.0	10956.1	11247.57
FUNDC2	FUN14 domain containing 2	11177.5	11227.6	11202.56
NCL	nucleolin	11318.5	10983.2	11150.86
MYOD1	myogenic differentiation 1	11160.1	11097.7	11128.89
LOC104915058	uncharacterized LOC104915058	10456.3	11787.4	11121.86
HOPX	HOP homeobox	10981.4	11257.4	11119.42
CSDE1	cold shock domain containing E1, RNA-binding	10761.0	11400.7	11080.88
LOC100540000	tropomyosin beta chain	11061.0	10849.7	10955.33
RASSF3	Ras association (RalGDS/AF-6) domain family member 3	10301.3	11521.9	10911.60
FAM65B	family with sequence similarity 65, member B	10425.2	11252.1	10838.65
HSPB8	heat shock 22kDa protein 8	10822.8	10851.0	10836.93
PDLIM7	PDZ and LIM domain 7 (enigma)	11697.8	9974.4	10836.11
RPS27	ribosomal protein S27	10909.4	10488.7	10699.06
LOC104913399	uncharacterized LOC104913399	11166.1	10031.0	10598.56
PDK1	pyruvate dehydrogenase kinase, isozyme 1	10147.4	10845.8	10496.63
B4GALT2	UDP-Gal:betaGlcNAc beta 1,4- galactosyltransferase, polypeptide 2	10855.7	10106.4	10481.08
OGT	O-linked N-acetylglucosamine (GlcNAc) transferase	9838.0	11063.6	10450.82
PTRF	polymerase I and transcript release factor	11224.5	9569.0	10396.74
ACTR1A	ARP1 actin-related protein 1 homolog A, centractin alpha (yeast)	10322.2	10409.0	10365.63
DDX3X	DEAD (Asp-Glu-Ala-Asp) box helicase 3, X-linked	9829.9	10770.8	10300.37
SETD7	SET domain containing (lysine methyltransferase) 7	9762.2	10815.1	10288.66
ATP1A1	ATPase, Na+/K+ transporting, alpha 1 polypeptide	10183.0	10361.3	10272.14
CFL2	cofilin 2 (muscle)	9989.0	10420.6	10204.78
ACTA1	actin, alpha 1, skeletal muscle	10382.4	9978.1	10180.26
STK17A	serine/threonine kinase 17a	10173.3	10182.4	10177.85
PPP1CB	protein phosphatase 1, catalytic subunit, beta isozyme	10033.4	10279.4	10156.41
PALLD	palladin, cytoskeletal associated protein	9710.9	10520.2	10115.55
CDKN1B	cyclin-dependent kinase inhibitor 1B (p27, Kip1)	9750.6	10288.9	10019.77
EMP1	epithelial membrane protein 1	9316.6	10570.2	9943.43
ARHGDIA	Rho GDP dissociation inhibitor (GDI) alpha	10253.5	9615.6	9934.56
BTG1	B-cell translocation gene 1, anti-proliferative	9807.3	10009.7	9908.51
PDLIM3	PDZ and LIM domain 3	9476.2	10295.5	9885.82
ITGB1	integrin, beta 1 (fibronectin receptor, beta polypeptide, antigen CD29 includes MDF2, MSK12)	9568.7	10110.3	9839.46
LOC100544620	septin-2	9701.2	9900.4	9800.82
FAU	Finkel-Biskis-Reilly murine sarcoma virus (FBR-MuSV) ubiquitously expressed	10134.8	9440.4	9787.60
GXYLT2	glucoside xylosyltransferase 2	9373.2	10197.4	9785.30
CTNNB1	catenin (cadherin-associated protein), beta 1, 88kDa	9500.5	9956.1	9728.30
H3F3B	H3 histone, family 3B (H3.3B)	9666.9	9674.6	9670.73
SERPINE2	serpin peptidase inhibitor, clade E (nexin, plasminogen activator inhibitor type 1), member 2	9314.4	9899.6	9607.03
CAV3	caveolin 3	9370.5	9815.3	9592.92
PFN2	profilin 2	9400.1	9654.5	9527.28
EIF3E	eukaryotic translation initiation factor 3, subunit E	9463.4	9513.6	9488.53
EIF5A2	eukaryotic translation initiation factor 5A2	9961.5	8944.1	9452.79
GPX4	glutathione peroxidase 4	10186.3	8614.6	9400.48
MYOZ2	myozenin 2	9026.9	9764.7	9395.79
SAT1	spermidine/spermine N1-acetyltransferase 1	9087.5	9609.0	9348.25
LDHB	lactate dehydrogenase B	9756.3	8935.7	9345.99
TPI1	triosephosphate isomerase 1	9248.0	9326.0	9286.99
WDR1	WD repeat domain 1	9148.6	9349.7	9249.12
RPS13	ribosomal protein S13	9288.9	9166.1	9227.48
DNAJC7	DnaJ (Hsp40) homolog, subfamily C, member 7	8846.2	9592.8	9219.50
RPL36	ribosomal protein L36	9706.6	8694.8	9200.70
FLNB	filamin B, beta	8944.2	9256.3	9100.22

SLC25A3	solute carrier family 25 (mitochondrial carrier; phosphate carrier), member 3	8896.0	9264.7	9080.38
SRL	sarcalumenin	9620.4	8431.7	9026.05
SHISA2	shisa family member 2	8820.8	9215.0	9017.89
HNRNPH1	heterogeneous nuclear ribonucleoprotein H1 (H)	9062.5	8969.8	9016.18
TWF2	twinfilin actin-binding protein 2	9131.6	8894.3	9012.91
GPX3	glutathione peroxidase 3 (plasma)	8828.5	9155.9	8992.20
RAB1A	RAB1A, member RAS oncogene family	8772.9	9031.7	8902.31
ZYX	zyxin	8960.7	8756.5	8858.60
LOC100548894	myosin regulatory light chain 2, smooth muscle minor isoform	8395.5	9312.7	8854.10
HSP90B1	heat shock protein 90kDa beta (Grp94), member 1	8621.8	8949.9	8785.87
TIMP2	TIMP metallopeptidase inhibitor 2	9423.3	8076.1	8749.73
HNRNPA2B1	heterogeneous nuclear ribonucleoprotein A2/B1	8698.3	8680.8	8689.56
LOC104917166	uncharacterized LOC104917166	7683.8	9686.2	8685.01
SPTAN1	spectrin, alpha, non-erythrocytic 1	8455.7	8865.4	8660.54
CNP	2',3'-cyclic nucleotide 3' phosphodiesterase	8961.0	8230.6	8595.80
CAP1	CAP, adenylate cyclase-associated protein 1 (yeast)	8401.7	8763.2	8582.43
TNNC2	troponin C type 2 (fast)	8535.3	8591.2	8563.24
CDC42	cell division cycle 42	8565.2	8444.8	8504.97
SEPT11	septin 11	8578.4	8390.5	8484.45
KLC4	kinesin light chain 4	9030.4	7851.5	8440.91
RPN1	ribophorin I	8275.0	8590.5	8432.73
TAGLN2	transgelin 2	8889.6	7954.7	8422.16
LOC100546539	aldose reductase-related protein 2-like	8191.6	8645.2	8418.36
SERPINF1	serpin peptidase inhibitor, clade F (alpha-2 antiplasmin, pigment epithelium derived factor), member 1	8568.0	8233.2	8400.61
GADD45A	growth arrest and DNA-damage-inducible, alpha	8367.7	8396.1	8381.91
S100A4	S100 calcium binding protein A4	9036.7	7714.8	8375.78
ATP5G3	ATP synthase, H+ transporting, mitochondrial Fo complex, subunit C3 (subunit 9)	8168.1	8276.0	8222.07
PTP4A1	protein tyrosine phosphatase type IVA, member 1	7888.0	8539.6	8213.76
LOC100547940	nebulin-like	7992.7	8408.8	8200.78
HSP90AA1	heat shock protein 90kDa alpha (cytosolic), class A member 1	8268.7	8002.0	8135.37
RAC1	ras-related C3 botulinum toxin substrate 1 (rho family, small GTP binding protein Rac1)	8021.8	8242.7	8132.24
DDB1	damage-specific DNA binding protein 1, 127kDa	8064.2	8187.7	8125.92
RAN	RAN, member RAS oncogene family	8212.3	8031.3	8121.79
SRSF1	serine/arginine-rich splicing factor 1	8144.7	8060.1	8102.39
HSPA5	heat shock 70kDa protein 5 (glucose-regulated protein, 78kDa)	7884.3	8250.7	8067.50
EIF3I	eukaryotic translation initiation factor 3, subunit I	8041.9	7953.4	7997.67
JUP	junction plakoglobin	7975.7	7998.8	7987.25
RAB7A	RAB7A, member RAS oncogene family	7853.0	8072.8	7962.92
SERINC3	serine incorporator 3	7261.1	8482.7	7871.92
ATP2A2	ATPase, Ca++ transporting, cardiac muscle, slow twitch 2	7605.6	8062.0	7833.79
COTL1	coactosin-like F-actin binding protein 1	7547.3	8104.1	7825.74
ATP5H	ATP synthase, H+ transporting, mitochondrial Fo complex, subunit d	7535.9	8085.8	7810.83
MGP	matrix Gla protein	9570.3	5915.2	7742.76
SERINC1	serine incorporator 1	7306.5	8134.7	7720.64
SPG20	spastic paraplegia 20 (Troyer syndrome)	7174.6	8258.4	7716.48
TTL	tubulin tyrosine ligase	7828.5	7588.9	7708.71
RPS21	ribosomal protein S21	7925.7	7466.5	7696.07
DLGAP4	discs, large (Drosophila) homolog-associated protein 4	7821.6	7544.2	7682.90
PLOD2	procollagen-lysine, 2-oxoglutarate 5-dioxygenase 2	7363.0	8002.5	7682.73
LOC104910981	importin-7-like	7438.5	7817.1	7627.78
LOC104915388	40S ribosomal protein S3a pseudogene	7919.6	7329.0	7624.29
RPS29	ribosomal protein S29	7971.0	7097.9	7534.45
LOC100542970	annexin A1 isoform p37-like	7351.8	7687.6	7519.69

LOC100543005	kelch-like protein 41	7208.0	7825.6	7516.82
ILK	integrin-linked kinase	7687.9	7214.4	7451.13
NDUFB1	NADH dehydrogenase (ubiquinone) 1 beta subcomplex, 1, 7kDa	7559.5	7316.4	7437.96
LAMP1	lysosomal-associated membrane protein 1	7045.5	7817.6	7431.52
LOC100543969	connective tissue growth factor-like	7012.0	7730.9	7371.43
BZW2	basic leucine zipper and W2 domains 2	7443.0	7295.3	7369.15
CCDC88C	coiled-coil domain containing 88C	6670.8	8005.5	7338.18
AIF1L	allograft inflammatory factor 1-like	7315.8	7295.6	7305.74
SPTBN1	spectrin, beta, non-erythrocytic 1	6866.4	7707.3	7286.87
PSMC3	proteasome (prosome, macropain) 26S subunit, ATPase, 3	7124.3	7321.4	7222.86
ETF1	eukaryotic translation termination factor 1	6928.1	7469.5	7198.79
LOC100542800	actin, cytoplasmic type 5	7142.6	7217.6	7180.08
NPR2	natriuretic peptide receptor 2	7363.6	6979.8	7171.71
CNN3	calponin 3, acidic	7138.5	7199.5	7169.01
ATP6V0E1	ATPase, H+ transporting, lysosomal 9kDa, V0 subunit e1	6987.4	7347.8	7167.59
MYOM3	myomesin 3	6891.0	7428.9	7159.95
CNN2	calponin 2	7272.5	7013.9	7143.22
EIF1	eukaryotic translation initiation factor 1	7081.5	7011.5	7046.48
RPL34	ribosomal protein L34	7318.1	6737.0	7027.56
LOC104914787	transitional endoplasmic reticulum ATPase-like	6992.1	7053.2	7022.64
SEPT7	septin 7	6681.0	7274.0	6977.50
COL18A1	collagen, type XVIII, alpha 1	6875.1	7040.4	6957.76
H3F3A	H3 histone, family 3A	6595.0	7310.2	6952.57
FSTL1	follistatin-like 1	6781.0	6998.8	6889.89
HADHB	hydroxyacyl-CoA dehydrogenase/3-ketoacyl-CoA thiolase/enoyl-CoA hydratase (trifunctional protein), beta subunit	6767.4	7006.4	6886.91
NAP1L1	nucleosome assembly protein 1-like 1	7184.1	6545.4	6864.72
LOC104915501	catenin alpha-1 pseudogene	6718.6	6992.9	6855.74
LOC104916205	uncharacterized LOC104916205	7388.0	6286.4	6837.23
SH3BGRL	SH3 domain binding glutamate-rich protein like	6435.9	7138.2	6787.02
SSR1	signal sequence receptor, alpha	6519.6	7015.9	6767.71
CAPZB	capping protein (actin filament) muscle Z-line, beta	6557.6	6968.7	6763.17
TRIM54	tripartite motif containing 54	6975.7	6533.4	6754.57
ACTN1	actinin, alpha 1	6595.3	6885.1	6740.19
HSPD1	heat shock 60kDa protein 1 (chaperonin)	6765.4	6714.1	6739.74
LOC100551296	cystatin	6436.5	7018.8	6727.62
PSMA4	proteasome (prosome, macropain) subunit, alpha type, 4	6693.1	6734.8	6713.95
TRPV2	transient receptor potential cation channel, subfamily V, member 2	6434.5	6970.6	6702.52
CDC25B	cell division cycle 25B	7318.5	6069.2	6693.87
LOC104912484	nexilin-like	6326.0	7041.6	6683.76
MPC1	mitochondrial pyruvate carrier 1	6364.4	6979.2	6671.81
ARPP19	cAMP-regulated phosphoprotein, 19kDa	6499.6	6830.8	6665.17
LOC100547911	heat shock protein 30C-like	6714.5	6552.5	6633.48
EIF5	eukaryotic translation initiation factor 5	6566.9	6692.6	6629.74
LOC104916700	uncharacterized LOC104916700	6639.3	6616.9	6628.13
GDI2	GDP dissociation inhibitor 2	6498.8	6752.6	6625.66
LOC104913910	WD repeat and SOCS box-containing protein 1-like	6063.7	7173.6	6618.64
PPP1CA	protein phosphatase 1, catalytic subunit, alpha isozyme	7013.0	6223.2	6618.08
FDFT1	farnesyl-diphosphate farnesyltransferase 1	6809.7	6407.7	6608.70
EML6	echinoderm microtubule associated protein like 6	6517.3	6618.1	6567.68
RPL22	ribosomal protein L22	6865.1	6155.7	6510.37
BAG2	BCL2-associated athanogene 2	6508.1	6501.9	6504.99
MAPRE1	microtubule-associated protein, RP/EB family, member 1	6572.5	6432.6	6502.54
FAM219A	family with sequence similarity 219, member A	6123.4	6858.4	6490.91
LOC104916043	uncharacterized LOC104916043	6977.4	5978.3	6477.82
ARPC5	actin related protein 2/3 complex, subunit 5, 16kDa	6257.5	6687.3	6472.43
TMOD1	tropomodulin 1	6411.3	6529.3	6470.32

GNMT	glycine N-methyltransferase	6588.7	6336.8	6462.73
GPI	glucose-6-phosphate isomerase	6425.7	6481.1	6453.39
TFRC	transferrin receptor	6347.2	6538.9	6443.04
LOC100550832	liprin-beta-1	6267.7	6601.3	6434.50
PSMA7	proteasome (prosome, macropain) subunit, alpha type, 7	6434.8	6384.7	6409.75
ABCF2	ATP-binding cassette, sub-family F (GCN20), member 2	6504.6	6305.8	6405.23
TRIM55	tripartite motif containing 55	6527.2	6261.4	6394.30
WIPF3	WAS/WASL interacting protein family, member 3	6028.8	6718.9	6373.86
FHL3	four and a half LIM domains 3	6391.5	6333.6	6362.54
GPR56	G protein-coupled receptor 56	6313.9	6393.4	6353.66
LOC104913356	adenylate cyclase type 9	6274.5	6352.7	6313.60
BTF3	basic transcription factor 3	6270.5	6330.6	6300.55
OLFML2B	olfactomedin-like 2B	6634.4	5919.6	6277.00
LOC100548709	LIM and senescent cell antigen-like-containing domain protein 2	6204.9	6333.9	6269.42
CCT6A	chaperonin containing TCP1, subunit 6A (zeta 1)	6299.0	6197.1	6248.05
CAPRIN1	cell cycle associated protein 1	6184.2	6311.5	6247.86
ZNF706	zinc finger protein 706	6022.8	6467.8	6245.27
CTNND1	catenin (cadherin-associated protein), delta 1	6146.3	6328.5	6237.44
TUBB4B	tubulin, beta 4B class IVb	6420.0	6052.0	6236.01
SYT11	synaptotagmin XI	6088.5	6377.0	6232.75
HNRNPA3	heterogeneous nuclear ribonucleoprotein A3	6424.8	6035.6	6230.19
ADK	adenosine kinase	6137.0	6319.5	6228.24
GNB1	guanine nucleotide binding protein (G protein), beta polypeptide 1	6019.1	6423.8	6221.47
LOC100542258	TSC22 domain family protein 3-like	6280.5	6156.5	6218.46
NDFIP1	Nedd4 family interacting protein 1	6061.7	6316.1	6188.90
RBM5	RNA binding motif protein 5	5995.6	6372.9	6184.24
YDJC	YdjC homolog (bacterial)	6203.7	6164.5	6184.06
APP	amyloid beta (A4) precursor protein	5777.7	6573.0	6175.38
LOC100549107	cytochrome c oxidase subunit 6C	6528.9	5820.0	6174.44
NUDT16L1	nudix (nucleoside diphosphate linked moiety X)-type motif 16-like 1	6319.0	6024.3	6171.63
MAP2K1	mitogen-activated protein kinase kinase 1	6016.9	6316.0	6166.46
C1QBP	complement component 1, q subcomponent binding protein	6321.2	6001.5	6161.36
LOC100542519	rho-related GTP-binding protein RhoB	6044.3	6271.4	6157.81
PSMD2	proteasome (prosome, macropain) 26S subunit, non-ATPase, 2	6405.5	5908.7	6157.08
EIF4G1	eukaryotic translation initiation factor 4 gamma, 1	6624.5	5660.3	6142.44
PACSIN3	protein kinase C and casein kinase substrate in neurons 3	6130.7	6092.4	6111.53
NPLOC4	nuclear protein localization 4 homolog (S. cerevisiae)	6526.3	5644.9	6085.60
YBX3	Y box binding protein 3	6154.6	5968.7	6061.67
C3H6orf62	chromosome 3 open reading frame, human C6orf62	5886.9	6213.8	6050.38
CD44	CD44 molecule (Indian blood group)	5924.3	6172.9	6048.56
SDPR	serum deprivation response	6541.4	5519.8	6030.62
LOC723981	cytochrome c oxidase subunit VIIb precursor	6224.5	5835.5	6030.02
RDX	radixin	5874.2	6134.0	6004.10
LOC100540821	guanine nucleotide-binding protein G(k) subunit alpha-like	6197.6	5810.1	6003.83
MDH1	malate dehydrogenase 1, NAD (soluble)	6105.8	5895.1	6000.46
PTGES3	prostaglandin E synthase 3 (cytosolic)	6208.2	5761.4	5984.81
RNF122	ring finger protein 122	6036.0	5933.1	5984.56
KDELR2	KDEL (Lys-Asp-Glu-Leu) endoplasmic reticulum protein retention receptor 2	5871.8	6086.2	5978.98
EIF3H	eukaryotic translation initiation factor 3, subunit H	6013.4	5941.4	5977.39
PAFAH1B1	platelet-activating factor acetylhydrolase 1b, regulatory subunit 1 (45kDa)	5871.0	6079.7	5975.35
GNS	glucosamine (N-acetyl)-6-sulfatase	5675.2	6223.7	5949.49
MYH7B	myosin, heavy chain 7B, cardiac muscle, beta	5475.2	6411.4	5943.27
EFHD1	EF-hand domain family, member D1	5851.8	6027.4	5939.63
MPRIP	myosin phosphatase Rho interacting protein	5845.7	5988.3	5916.99

YWHAH	tyrosine 3-monooxygenase/tryptophan 5-monooxygenase activation protein, eta	5789.0	6019.7	5904.35
RPL38	ribosomal protein L38	6129.3	5660.6	5894.94
LOC104912963	72 kDa type IV collagenase-like	5519.6	6191.1	5855.32
M6PR	mannose-6-phosphate receptor (cation dependent)	5670.2	6008.1	5839.11
FAM129B	family with sequence similarity 129, member B	5993.4	5663.7	5828.57
HP1BP3	heterochromatin protein 1, binding protein 3	5831.0	5825.3	5828.14
LOC104912610	uncharacterized LOC104912610	5572.4	6071.6	5822.00
MDH2	malate dehydrogenase 2, NAD (mitochondrial)	5919.6	5695.9	5807.79
HNRNPH3	heterogeneous nuclear ribonucleoprotein H3 (2H9)	5669.1	5927.8	5798.45
CLTB	clathrin, light chain B	5908.0	5669.5	5788.75
ATP1B3	ATPase, Na+/K+ transporting, beta 3 polypeptide	5588.6	5981.6	5785.08
LOC100549826	serine/arginine-rich splicing factor 5-like	5716.4	5853.7	5785.04
EIF3L	eukaryotic translation initiation factor 3, subunit L	5929.6	5624.1	5776.88
BZW1	basic leucine zipper and W2 domains 1	5784.7	5742.2	5763.46
NDUFA4	NDUFA4, mitochondrial complex associated	5908.9	5589.2	5749.03
PTMA	prothymosin, alpha	5846.7	5623.9	5735.31
SSR2	signal sequence receptor, beta (translocon-associated protein beta)	5623.9	5841.5	5732.72
CS	citrate synthase	6061.3	5385.9	5723.59
SEPT6	septin 6	5617.3	5804.8	5711.01
RNF10	ring finger protein 10	5596.0	5822.8	5709.42
RAB10	RAB10, member RAS oncogene family	5414.0	5996.2	5705.12
LOC100551088	actin, gamma-enteric smooth muscle	5661.7	5732.4	5697.06
CCNI	cyclin I	5491.6	5871.7	5681.62
PPT1	palmitoyl-protein thioesterase 1	5622.8	5702.8	5662.80
MYH9	myosin, heavy chain 9, non-muscle	5719.2	5585.6	5652.40
MICAL1	microtubule associated monooxygenase, calponin and LIM domain containing 1	5853.7	5444.0	5648.85
FGFR4	fibroblast growth factor receptor 4	5967.1	5315.4	5641.25
CALM1	calmodulin 1 (phosphorylase kinase, delta)	5473.5	5788.0	5630.79
BACE1	beta-site APP-cleaving enzyme 1	5501.4	5755.4	5628.40
LOC100548286	nucleobindin-2-like	5384.1	5860.5	5622.28
NCALD	neurocalcin delta	5175.4	6068.6	5622.03
TXNDC5	thioredoxin domain containing 5 (endoplasmic reticulum)	5592.3	5638.9	5615.58
HSPA9	heat shock 70kDa protein 9 (mortalin)	5733.3	5465.9	5599.62
TCOF1	Treacher Collins-Franceschetti syndrome 1	5423.8	5754.6	5589.21
TMED5	transmembrane emp24 protein transport domain containing 5	5582.8	5593.2	5588.01
ATP5F1	ATP synthase, H+ transporting, mitochondrial Fo complex, subunit B1	5664.2	5495.0	5579.62
LOC100548741	myosin regulatory light chain 2, smooth muscle minor isoform-like	5542.5	5600.9	5571.71
ATP5I	ATP synthase, H+ transporting, mitochondrial Fo complex, subunit E	5921.1	5213.3	5567.18
ASUN	asunder spermatogenesis regulator	5610.0	5523.6	5566.84
SF3B1	splicing factor 3b, subunit 1, 155kDa	5376.7	5742.1	5559.39
EIF3M	eukaryotic translation initiation factor 3, subunit M	5617.3	5495.2	5556.21
LOC100544899	EH domain-containing protein 3	5586.6	5522.7	5554.64
LMF2	lipase maturation factor 2	5961.3	5146.9	5554.08
EIF3A	eukaryotic translation initiation factor 3, subunit A	5594.3	5486.1	5540.18
DAP	death-associated protein	5294.3	5776.5	5535.39
TTC9	tetratricopeptide repeat domain 9	5263.7	5803.9	5533.80
SMARCD3	SWI/SNF related, matrix associated, actin dependent regulator of chromatin, subfamily d, member 3	5321.8	5713.6	5517.72
LMO7	LIM domain 7	5407.5	5585.7	5496.59
HSF2	heat shock transcription factor 2	5364.8	5627.7	5496.25
FAM49A	family with sequence similarity 49, member A	5317.5	5674.2	5495.87
PSMD13	proteasome (prosome, macropain) 26S subunit, non-ATPase, 13	5661.1	5297.3	5479.19
EIF5B	eukaryotic translation initiation factor 5B	5494.6	5449.1	5471.84

CAST	calpastatin	5310.3	5630.3	5470.28
GPX1	glutathione peroxidase 1	5760.8	5171.7	5466.22
EIF3F	eukaryotic translation initiation factor 3, subunit F	5587.4	5334.0	5460.68
PSMB7	proteasome (prosome, macropain) subunit, beta type, 7	5532.3	5386.5	5459.42
MATR3	matrin 3	5383.0	5523.0	5453.02
TNK2	tyrosine kinase, non-receptor, 2	5610.7	5293.8	5452.26
ILF2	interleukin enhancer binding factor 2	5402.0	5500.9	5451.45
CASQ2	calsequestrin 2 (cardiac muscle)	5409.1	5479.3	5444.21
MYBPH	myosin binding protein H	5556.0	5290.3	5423.17
JUND	jun D proto-oncogene	5644.8	5199.7	5422.25
CAV1	caveolin 1, caveolae protein, 22kDa	5412.0	5428.7	5420.34
RPN2	ribophorin II	5096.3	5733.8	5415.04
FKBP3	FK506 binding protein 3, 25kDa	5319.5	5469.1	5394.31
ARPC2	actin related protein 2/3 complex, subunit 2, 34kDa	5363.3	5414.9	5389.09
LOC100538971	uncharacterized LOC100538971	5300.4	5472.6	5386.49
PEBP1	phosphatidylethanolamine binding protein 1	5405.2	5364.2	5384.70
LOC100539112	neuroblastoma suppressor of tumorigenicity 1	5414.4	5310.4	5362.41
HSP90AB1	heat shock protein 90kDa alpha (cytosolic), class B member 1	5585.9	5135.6	5360.73
UBE2D3	ubiquitin-conjugating enzyme E2D 3	5340.9	5378.2	5359.56
NEK6	NIMA-related kinase 6	5109.2	5600.6	5354.86
IGJ	immunoglobulin J polypeptide, linker protein for immunoglobulin alpha and mu polypeptides	5224.2	5481.2	5352.71
TPD52	tumor protein D52	5278.5	5425.1	5351.83
LOC723983	cytochrome c oxidase subunit VIa	5696.7	4995.5	5346.11
HMGN2	high mobility group nucleosomal binding domain 2	5363.9	5325.8	5344.87
GHITM	growth hormone inducible transmembrane protein	5310.3	5294.9	5302.59
IDH1	isocitrate dehydrogenase 1 (NADP+), soluble	5221.6	5381.4	5301.53
PALD1	phosphatase domain containing, paladin 1	5489.7	5108.4	5299.04
RCN1	reticulocalbin 1, EF-hand calcium binding domain	5102.4	5494.1	5298.24
HDAC2	histone deacetylase 2	5184.3	5411.1	5297.67
CCT8	chaperonin containing TCP1, subunit 8 (theta)	5351.2	5242.4	5296.81
PFKM	phosphofructokinase, muscle	5407.7	5154.4	5281.03
RPL22L1	ribosomal protein L22-like 1	5596.4	4965.2	5280.79
CD164	CD164 molecule, sialomucin	5163.1	5386.5	5274.82
FAM57A	family with sequence similarity 57, member A	5183.5	5357.9	5270.71
XIRP1	xin actin-binding repeat containing 1	4902.0	5638.6	5270.31
PSMB2	proteasome (prosome, macropain) subunit, beta type, 2	5420.2	5056.0	5238.09
PSMC2	proteasome (prosome, macropain) 26S subunit, ATPase, 2	5285.6	5183.4	5234.49
LOC104913694	pleckstrin homology-like domain family B member 2	4683.3	5773.0	5228.14
WIPI1	WD repeat domain, phosphoinositide interacting 1	5165.3	5290.7	5228.02
GOT2	glutamic-oxaloacetic transaminase 2, mitochondrial	5473.2	4981.0	5227.10
TOPBP1	topoisomerase (DNA) II binding protein 1	5217.8	5229.8	5223.80
SPG7	spastic paraplegia 7 (pure and complicated autosomal recessive)	5223.3	5218.2	5220.75
MYO1C	myosin IC	5076.2	5345.6	5210.92
RAPGEF1	Rap guanine nucleotide exchange factor (GEF) 1	5084.1	5336.1	5210.10
EIF2S3	eukaryotic translation initiation factor 2, subunit 3 gamma, 52kDa	5236.3	5161.4	5198.86
MTPN	myotrophin	5066.9	5298.4	5182.67
GPC4	glypican 4	4976.9	5364.5	5170.68
PDLIM5	PDZ and LIM domain 5	4935.4	5380.9	5158.12
B2M	beta-2-microglobulin	5278.5	5031.9	5155.15
SARDH	sarcosine dehydrogenase	5183.8	5113.8	5148.81
HSBP1	heat shock factor binding protein 1	5044.4	5252.6	5148.47
ITM2A	integral membrane protein 2A	4798.4	5480.0	5139.17
LOC100549893	cytochrome b-c1 complex subunit 1, mitochondrial	5172.8	5090.4	5131.62
FKBP1A	FK506 binding protein 1A, 12kDa	5051.9	5182.8	5117.31
UNC45B	unc-45 homolog B (C. elegans)	4958.5	5275.7	5117.08
CCT3	chaperonin containing TCP1, subunit 3 (gamma)	5703.8	4507.2	5105.53
EDF1	endothelial differentiation-related factor 1	5047.9	5152.3	5100.13

NCAM1	neural cell adhesion molecule 1	4952.9	5239.0	5095.98
ARF4	ADP-ribosylation factor 4	4821.2	5369.3	5095.27
LOC100538694	cytochrome c oxidase subunit 4 isoform 1, mitochondrial	5127.2	5060.8	5094.00
TPM4	tropomyosin 4	5191.5	4954.1	5072.79
NONO	non-POU domain containing, octamer-binding	5109.7	5004.4	5057.07
MEF2A	myocyte enhancer factor 2A	4812.9	5286.1	5049.51
SGCD	sarcoglycan, delta (35kDa dystrophin-associated glycoprotein)	4895.4	5176.1	5035.72
SGCG	sarcoglycan, gamma (35kDa dystrophin-associated glycoprotein)	4689.7	5362.1	5025.88
HADHA	hydroxyacyl-CoA dehydrogenase/3-ketoacyl-CoA thiolase/enoyl-CoA hydratase (trifunctional protein), alpha subunit	5203.2	4847.7	5025.43
BLCAP	bladder cancer associated protein	5051.4	4989.9	5020.64
PTK7	protein tyrosine kinase 7	5311.7	4726.3	5018.97
IDH3A	isocitrate dehydrogenase 3 (NAD+) alpha	4864.5	5172.9	5018.74
PCBP2	poly(rC) binding protein 2	4713.2	5317.8	5015.52
EIF4EBP1	eukaryotic translation initiation factor 4E binding protein 1	5013.9	4973.8	4993.86
HDGF	hepatoma-derived growth factor	5176.8	4810.3	4993.53
ERLIN2	ER lipid raft associated 2	4992.8	4983.8	4988.30
LOC100546114	selenoprotein M	5017.0	4956.8	4986.90
UBXN4	UBX domain protein 4	4899.9	5058.8	4979.35
GPD1L	glycerol-3-phosphate dehydrogenase 1-like	4821.8	5132.8	4977.26
TCP1	t-complex 1	4974.2	4979.3	4976.73
NCOA4	nuclear receptor coactivator 4	4818.2	5109.6	4963.93
SGPL1	sphingosine-1-phosphate lyase 1	4818.9	5081.7	4950.30
LOC100542032	cytochrome b-c1 complex subunit 2, mitochondrial	5313.3	4583.1	4948.25
PHB2	prohibitin 2	5082.2	4811.5	4946.84
PSMD3	proteasome (prosome, macropain) 26S subunit, non-ATPase, 3	5233.5	4632.3	4932.88
CCT2	chaperonin containing TCP1, subunit 2 (beta)	4921.5	4923.6	4922.52
LOC100551022	clathrin heavy chain 1	4848.3	4959.5	4903.91
AKR1A1	aldo-keto reductase family 1, member A1 (aldehyde reductase)	5077.4	4717.4	4897.38
NDUFB6	NADH dehydrogenase (ubiquinone) 1 beta subcomplex, 6, 17kDa	5017.1	4771.7	4894.40
GRB10	growth factor receptor-bound protein 10	4830.7	4952.4	4891.52
PSMD1	proteasome (prosome, macropain) 26S subunit, non-ATPase, 1	4886.9	4858.2	4872.57
ATPIF1	ATPase inhibitory factor 1	5187.9	4540.8	4864.36
COL4A1	collagen, type IV, alpha 1	4202.9	5457.7	4830.30
CCT4	chaperonin containing TCP1, subunit 4 (delta)	4938.9	4720.0	4829.46
MAP1A	microtubule-associated protein 1A	4541.8	5116.8	4829.28
YARS	tyrosyl-tRNA synthetase	5127.5	4503.9	4815.68
ATP5C1	ATP synthase, H+ transporting, mitochondrial F1 complex, gamma polypeptide 1	4886.0	4744.7	4815.36
TAX1BP3	Tax1 (human T-cell leukemia virus type I) binding protein 3	4615.4	5013.2	4814.31
SEPT9	septin 9	4831.0	4787.3	4809.15
SOD1	superoxide dismutase 1, soluble	4954.0	4656.7	4805.33
HNRNPU	heterogeneous nuclear ribonucleoprotein U (scaffold attachment factor A)	4745.8	4839.2	4792.50
ENAH	enabled homolog (Drosophila)	4685.0	4895.5	4790.27
LOC104911367	supervillin-like	4610.5	4952.4	4781.43
LIMD1	LIM domains containing 1	4685.3	4873.1	4779.19
RAB2A	RAB2A, member RAS oncogene family	4681.0	4877.3	4779.11
SGTA	small glutamine-rich tetratricopeptide repeat (TPR)-containing, alpha	4706.8	4841.7	4774.25
CCNG1	cyclin G1	4645.0	4899.0	4772.01
IMPDH2	IMP (inosine 5'-monophosphate) dehydrogenase 2	5038.6	4499.4	4769.03
PPDPF	pancreatic progenitor cell differentiation and proliferation factor	5003.8	4528.4	4766.10
CHKA	choline kinase alpha	4518.1	5013.8	4765.94
DMD	dystrophin	4486.4	4976.6	4731.49
NAP1L4	nucleosome assembly protein 1-like 4	4787.8	4653.8	4720.78
LOC104911821	uncharacterized LOC104911821	4679.8	4756.2	4718.00
RHOC	ras homolog family member C	4833.8	4599.9	4716.87

LOC104910145	tubulin beta chain	4688.7	4728.7	4708.70
SUMO2	small ubiquitin-like modifier 2	4642.6	4771.9	4707.25
LRRC66	leucine rich repeat containing 66	4463.9	4945.9	4704.92
TSPAN3	tetraspanin 3	4472.2	4923.9	4698.08
DYRK3	dual-specificity tyrosine-(Y)-phosphorylation regulated kinase 3	4511.7	4878.4	4695.05
EIF2S2	eukaryotic translation initiation factor 2, subunit 2 beta, 38kDa	4742.0	4626.0	4684.03
LOC100541229	A disintegrin and metalloproteinase with thrombospondin motifs 12-like	4524.6	4822.7	4673.67
ARCN1	archain 1	4695.7	4646.5	4671.10
CD47	CD47 molecule	4272.1	5068.3	4670.23
EIF1AX	eukaryotic translation initiation factor 1A, X-linked	4500.1	4821.3	4660.69
MCL1	myeloid cell leukemia 1	4859.4	4460.5	4659.93
LOC100540816	ATP synthase subunit g, mitochondrial-like	4837.5	4482.2	4659.87
SEPN1	selenoprotein N, 1	4651.4	4667.5	4659.45
MYOM1	myomesin 1	4514.0	4796.6	4655.32
KREMEN1	kringle containing transmembrane protein 1	4551.8	4725.6	4638.73
LSP1	lymphocyte-specific protein 1	4526.0	4736.1	4631.05
CAPN2	calpain 2, (m/II) large subunit	4604.0	4646.8	4625.39
CCT5	chaperonin containing TCP1, subunit 5 (epsilon)	4734.8	4513.6	4624.25
SAP18	Sin3A-associated protein, 18kDa	4741.6	4479.4	4610.50
AP2M1	adaptor-related protein complex 2, mu 1 subunit	4752.1	4461.2	4606.64
LOC100546986	signal transducer and activator of transcription 5B	4897.7	4307.8	4602.78
FAM109B	family with sequence similarity 109, member B	4304.8	4889.3	4597.05
PSMA5	proteasome (prosome, macropain) subunit, alpha type, 5	4815.6	4365.0	4590.32
SRSF3	serine/arginine-rich splicing factor 3	4691.7	4473.5	4582.61
CYC	cytochrome c	4635.7	4528.8	4582.24
ANP32E	acidic (leucine-rich) nuclear phosphoprotein 32 family, member E	4513.7	4645.6	4579.68
LOC100538921	heterogeneous nuclear ribonucleoprotein A1-like	5053.9	4100.3	4577.09
COPB2	coatomer protein complex, subunit beta 2 (beta prime)	4358.5	4766.6	4562.54
LOC104912329	uncharacterized LOC104912329	4409.0	4715.3	4562.15
P4HA1	prolyl 4-hydroxylase, alpha polypeptide I	4390.6	4700.9	4545.71
LOC100546435	low-density lipoprotein receptor-related protein 1-like	4773.5	4300.8	4537.14
LOC100543785	zinc finger and BTB domain-containing protein 41	4289.1	4780.0	4534.54
LPL	lipoprotein lipase	4212.1	4844.9	4528.49
LMNB2	lamin B2	4906.8	4138.0	4522.43
NPEPPS	aminopeptidase puromycin sensitive	4604.5	4433.2	4518.85
PRSS35	protease, serine, 35	3879.1	5135.4	4507.27
HM13	histocompatibility (minor) 13	4455.5	4555.0	4505.23
MYL3	myosin, light chain 3, alkali; ventricular, skeletal, slow	3715.1	5275.4	4495.26
CSRP3	cysteine and glycine-rich protein 3 (cardiac LIM protein)	4293.2	4659.3	4476.28
PTPRG	protein tyrosine phosphatase, receptor type, G	4260.2	4692.1	4476.17
PANK3	pantothenate kinase 3	4503.7	4423.0	4463.34
SDHB	succinate dehydrogenase complex, subunit B, iron sulfur (Ip)	4582.1	4326.4	4454.23
LOC100545477	histone H2A.Z	4600.0	4307.5	4453.73
REEP5	receptor accessory protein 5	4281.3	4615.9	4448.64
LAMC1	laminin, gamma 1 (formerly LAMB2)	4360.3	4535.5	4447.90
PSMC6	proteasome (prosome, macropain) 26S subunit, ATPase, 6	4354.3	4530.5	4442.41
PLEKHO1	pleckstrin homology domain containing, family O member 1	4311.7	4572.3	4442.04
GARS	glycyl-tRNA synthetase	4626.8	4256.1	4441.43
CDH2	cadherin 2, type 1, N-cadherin (neuronal)	4275.9	4604.2	4440.04
COL5A1	collagen, type V, alpha 1	3901.1	4976.3	4438.70
SMYD1	SET and MYND domain containing 1	4320.1	4554.7	4437.36
CTBP1	C-terminal binding protein 1	4254.1	4595.4	4424.73
LOC104912381	myomegalin-like	4607.0	4241.7	4424.37
PSMB4	proteasome (prosome, macropain) subunit, beta type, 4	4702.8	4140.8	4421.80
ELMO2	engulfment and cell motility 2	4622.4	4215.7	4419.08
PSMA3	proteasome (prosome, macropain) subunit, alpha type, 3	4380.3	4449.7	4415.01
LOC104916523	mucin-2-like	4374.6	4444.9	4409.73

ARPC4	actin related protein 2/3 complex, subunit 4, 20kDa	4480.3	4325.2	4402.72
ARPC1A	actin related protein 2/3 complex, subunit 1A, 41kDa	4584.0	4220.9	4402.44
ACAD9	acyl-CoA dehydrogenase family, member 9	4345.4	4448.5	4396.93
SKP1	S-phase kinase-associated protein 1	4217.2	4569.5	4393.35
GPR1	G protein-coupled receptor 1	4280.6	4504.0	4392.31
ARF1	ADP-ribosylation factor 1	4277.1	4502.8	4389.96
LOC100545745	cytochrome c oxidase subunit 5A, mitochondrial	4330.0	4438.3	4384.15
LOC104910019	sterile alpha motif domain-containing protein 12-like	4318.1	4393.2	4355.64
EIF4H	eukaryotic translation initiation factor 4H	4314.6	4385.2	4349.89
LASP1	LIM and SH3 protein 1	4484.8	4194.0	4339.40
COPE	coatomer protein complex, subunit epsilon	4327.6	4339.1	4333.31
PA2G4	proliferation-associated 2G4, 38kDa	4245.1	4407.1	4326.11
HSPG2	heparan sulfate proteoglycan 2	5153.0	3477.5	4315.24
TNFRSF21	tumor necrosis factor receptor superfamily, member 21	4542.8	4082.1	4312.47
HECW2	HECT, C2 and WW domain containing E3 ubiquitin protein ligase 2	4457.6	4159.6	4308.61
GRB2	growth factor receptor-bound protein 2	4161.0	4445.4	4303.18
PRDX6	peroxiredoxin 6	4428.1	4159.7	4293.90
CISH	cytokine inducible SH2-containing protein	4331.0	4252.4	4291.69
SLC39A13	solute carrier family 39 (zinc transporter), member 13	4148.7	4424.3	4286.51
VAV2	vav 2 guanine nucleotide exchange factor	4334.3	4235.1	4284.69
RAB11B	RAB11B, member RAS oncogene family	4288.6	4272.5	4280.56
NTN4	netrin 4	4157.5	4371.2	4264.32
LOC100538629	kinesin-1 heavy chain	4207.1	4318.3	4262.69
CD151	CD151 molecule (Raph blood group)	4202.4	4317.6	4259.98
PRSS23	protease, serine, 23	3847.6	4668.9	4258.25
FN1	fibronectin 1	3832.4	4681.4	4256.87
KIF24	kinesin family member 24	4016.2	4490.7	4253.41
SET	SET nuclear proto-oncogene	4215.2	4277.0	4246.11
ATP5D	ATP synthase, H+ transporting, mitochondrial F1 complex, delta subunit	4357.2	4133.3	4245.22
LOC104910249	uncharacterized LOC104910249	4134.1	4355.4	4244.75
LOC100544806	16 kDa beta-galactoside-binding lectin	4264.1	4212.2	4238.12
KCTD2	potassium channel tetramerization domain containing 2	4251.9	4220.1	4236.04
C26H11orf52	chromosome 26 open reading frame, human C11orf52	4142.8	4322.9	4232.87
TACC1	transforming, acidic coiled-coil containing protein 1	4316.1	4148.4	4232.24
OAT	ornithine aminotransferase	4054.6	4405.7	4230.16
VWA1	von Willebrand factor A domain containing 1	4020.5	4428.7	4224.59
LOC104914461	mitogen-activated protein kinase 14-like	4063.6	4372.6	4218.13
LOC100541641	betaine--homocysteine S-methyltransferase 1	4369.4	4058.0	4213.71
CSNK1A1	casein kinase 1, alpha 1	4102.8	4320.1	4211.47
TIMP3	TIMP metallopeptidase inhibitor 3	3911.8	4485.4	4198.62
LOC100539689	talin-1	4463.9	3930.5	4197.18
IDH2	isocitrate dehydrogenase 2 (NADP+), mitochondrial	4531.3	3860.3	4195.79
SURF4	surfeit 4	4096.6	4291.6	4194.08
RAP1GDS1	RAP1, GTP-GDP dissociation stimulator 1	4097.6	4284.7	4191.12
LIMA1	LIM domain and actin binding 1	4146.4	4235.0	4190.73
UBE2N	ubiquitin-conjugating enzyme E2N	4031.8	4343.2	4187.49
BSG	basigin (Ok blood group)	4203.4	4157.9	4180.65
WBP2NL	WBP2 N-terminal like	4211.2	4139.8	4175.47
AARS	alanyl-tRNA synthetase	4509.3	3832.3	4170.85
BASP1	brain abundant, membrane attached signal protein 1	4146.7	4193.4	4170.04
SEC23A	Sec23 homolog A (S. cerevisiae)	4055.1	4266.7	4160.93
LOC100545950	transcription factor 12	3992.4	4325.2	4158.77
SRRM1	serine/arginine repetitive matrix 1	4029.8	4283.7	4156.71
EIF3B	eukaryotic translation initiation factor 3, subunit B	4042.4	4257.1	4149.74
SH3GLB1	SH3-domain GRB2-like endophilin B1	4140.6	4156.5	4148.57
MCU	mitochondrial calcium uniporter	4075.5	4215.0	4145.23
EIF3D	eukaryotic translation initiation factor 3, subunit D	4257.8	4025.1	4141.44

SDK2	sidekick cell adhesion molecule 2	4308.8	3963.1	4135.97
DDOST	dolichyl-diphosphooligosaccharide--protein glycosyltransferase subunit (non-catalytic)	4126.6	4132.2	4129.41
C5H11orf58	chromosome 5 open reading frame, human C11orf58	4263.5	3986.2	4124.87
HNRNPDL	heterogeneous nuclear ribonucleoprotein D-like	4138.7	4107.6	4123.13
CSRP2	cysteine and glycine-rich protein 2	3758.4	4481.6	4120.01
SEMA6B	sema domain, transmembrane domain (TM), and cytoplasmic domain, (semaphorin) 6B	4314.0	3902.9	4108.45
PDCL3	phosducin-like 3	3983.4	4209.7	4096.59
PXN	paxillin	4117.9	4063.0	4090.45
RAP1B	RAP1B, member of RAS oncogene family	4029.7	4134.9	4082.28
MGEA5	meningioma expressed antigen 5 (hyaluronidase)	3906.9	4253.2	4080.05
SNRNP200	small nuclear ribonucleoprotein 200kDa (U5)	4309.3	3850.4	4079.88
ADD1	adducin 1 (alpha)	4036.0	4119.4	4077.71
ITPR3	inositol 1,4,5-trisphosphate receptor, type 3	3946.6	4204.2	4075.43
SDHC	succinate dehydrogenase complex, subunit C, integral membrane protein, 15kDa	4010.7	4133.2	4071.96
TOMM20	translocase of outer mitochondrial membrane 20 homolog (yeast)	3976.9	4154.1	4065.45
MTFR1L	mitochondrial fission regulator 1-like	4084.4	4045.3	4064.84
DNAJB6	DnaJ (Hsp40) homolog, subfamily B, member 6	3950.9	4166.7	4058.79
ASB5	ankyrin repeat and SOCS box containing 5	4019.8	4097.2	4058.51
KPNA4	karyopherin alpha 4 (importin alpha 3)	4088.3	4027.7	4057.99
SBDS	Shwachman-Bodian-Diamond syndrome	3864.6	4247.5	4056.07
LOC100539382	A-kinase anchor protein 2-like	3956.3	4131.3	4043.79
CUTA	cutA divalent cation tolerance homolog (E. coli)	3908.7	4177.4	4043.05
YME1L1	YME1-like 1 ATPase	3956.3	4101.7	4029.02
CCNL1	cyclin L1	3932.7	4125.1	4028.93
TM9SF3	transmembrane 9 superfamily member 3	3916.6	4130.0	4023.28
PRDX4	peroxiredoxin 4	3933.7	4089.4	4011.53
PSMA2	proteasome (prosome, macropain) subunit, alpha type, 2	3999.0	4021.0	4010.01
G3BP1	GTPase activating protein (SH3 domain) binding protein 1	4119.8	3899.1	4009.42
DYNC1I2	dynein, cytoplasmic 1, intermediate chain 2	3876.1	4140.5	4008.27
CAP2	CAP, adenylate cyclase-associated protein, 2 (yeast)	3796.7	4208.0	4002.32
STT3A	STT3A, subunit of the oligosaccharyltransferase complex (catalytic)	3925.9	4069.1	3997.49
SGPP1	sphingosine-1-phosphate phosphatase 1	3642.7	4339.6	3991.12
LMOD2	leiomodin 2 (cardiac)	3869.3	4109.8	3989.56
ADNP	activity-dependent neuroprotector homeobox	3853.8	4098.1	3975.95
SAR1B	secretion associated, Ras related GTPase 1B	3792.4	4159.5	3975.94
SLC6A8	solute carrier family 6 (neurotransmitter transporter), member 8	4297.3	3643.5	3970.37
PDIA6	protein disulfide isomerase family A, member 6	3860.7	4079.3	3969.98
SNRPD3	small nuclear ribonucleoprotein D3 polypeptide 18kDa	3921.8	4014.7	3968.26
SUB1	SUB1 homolog (S. cerevisiae)	3900.2	4033.4	3966.79
NAA50	N(alpha)-acetyltransferase 50, NatE catalytic subunit	3995.5	3914.6	3955.03
DUSP14	dual specificity phosphatase 14	3591.9	4307.7	3949.82
PSMC5	proteasome (prosome, macropain) 26S subunit, ATPase, 5	4151.0	3740.2	3945.58
PRPF19	pre-mRNA processing factor 19	4123.8	3764.2	3944.00
ARHGDIB	Rho GDP dissociation inhibitor (GDI) beta	4090.9	3782.9	3936.92
HSD17B12	hydroxysteroid (17-beta) dehydrogenase 12	3902.0	3953.4	3927.66
ACTR3	ARP3 actin-related protein 3 homolog (yeast)	3855.0	3996.2	3925.60
HNRNPAB	heterogeneous nuclear ribonucleoprotein A/B	3873.9	3962.3	3918.14
IST1	increased sodium tolerance 1 homolog (yeast)	4018.7	3814.6	3916.64
SRSF11	serine/arginine-rich splicing factor 11	3740.9	4087.9	3914.41
RAB3GAP2	RAB3 GTPase activating protein subunit 2 (non-catalytic)	3861.0	3966.4	3913.70
PSMC1	proteasome (prosome, macropain) 26S subunit, ATPase, 1	3873.1	3949.0	3911.08
CHD4	chromodomain helicase DNA binding protein 4	3971.7	3834.9	3903.28
PRELP	proline/arginine-rich end leucine-rich repeat protein	3899.1	3898.1	3898.61
REEP1	receptor accessory protein 1	3687.3	4109.7	3898.52

CCNYL1	cyclin Y-like 1	3963.4	3823.5	3893.46
MYO1H	myosin IH	3955.9	3830.1	3893.01
VDAC3	voltage-dependent anion channel 3	3914.2	3863.4	3888.82
LOC104909961	dystonin-like	3669.0	4106.2	3887.62
ALDH18A1	aldehyde dehydrogenase 18 family, member A1	3952.7	3797.8	3875.27
SMTN	smoothelin	3987.3	3760.8	3874.07
CTTN	cortactin	3733.3	4013.0	3873.18
LOC104913587	dynein light chain 1, cytoplasmic-like	3911.7	3825.7	3868.74
PRELID1	PRELI domain containing 1	3843.6	3885.0	3864.30
PTPLA	protein tyrosine phosphatase-like (proline instead of catalytic arginine), member A	3768.8	3958.1	3863.43
VAPB	VAMP (vesicle-associated membrane protein)-associated protein B and C	3841.4	3882.5	3861.95
CLIC2	chloride intracellular channel 2	3864.9	3857.8	3861.33
PSMD7	proteasome (prosome, macropain) 26S subunit, non-ATPase, 7	3938.8	3783.3	3861.04
LOC104909199	paired box protein Pax-7-like	3674.6	4045.6	3860.07
MICU1	mitochondrial calcium uptake 1	3760.3	3957.1	3858.71
CCT7	chaperonin containing TCP1, subunit 7 (eta)	4007.5	3701.4	3854.47
VASH1	vasohibin 1	3837.0	3853.7	3845.31
SYNC	syncoilin, intermediate filament protein	3988.9	3700.6	3844.77
DAG1	dystroglycan 1 (dystrophin-associated glycoprotein 1)	3824.5	3862.8	3843.65
LOC104911484	uncharacterized LOC104911484	3833.9	3843.9	3838.88
NCKAP1	NCK-associated protein 1	3857.4	3816.2	3836.77
ARHGAP17	Rho GTPase activating protein 17	3749.0	3921.6	3835.33
HNRNPM	heterogeneous nuclear ribonucleoprotein M	3911.6	3743.8	3827.70
EPRS	glutamyl-prolyl-tRNA synthetase	3780.6	3867.0	3823.81
DBI	diazepam binding inhibitor (GABA receptor modulator, acyl-CoA binding protein)	3987.7	3654.9	3821.31
ATP6V1G1	ATPase, H+ transporting, lysosomal 13kDa, V1 subunit G1	3766.7	3861.8	3814.25
NEK9	NIMA-related kinase 9	3811.1	3802.7	3806.88
MSRB3	methionine sulfoxide reductase B3	3538.0	4069.7	3803.84
DAPK2	death-associated protein kinase 2	3590.7	3997.5	3794.10
BTF3L4	basic transcription factor 3-like 4	3690.4	3890.1	3790.22
PSMA1	proteasome (prosome, macropain) subunit, alpha type, 1	3829.7	3747.3	3788.47
PPP2CB	protein phosphatase 2, catalytic subunit, beta isozyme	3833.0	3741.0	3786.99
ST6GALNAC6	ST6 (alpha-N-acetyl-neuraminyl-2,3-beta-galactosyl-1,3)-N-acetylgalactosaminide alpha-2,6-sialyltransferase 6	3837.7	3731.0	3784.35
ATP6V0C	ATPase, H+ transporting, lysosomal 16kDa, V0 subunit c	3712.3	3854.7	3783.51
HSPA2	heat shock 70kDa protein 2	3799.6	3766.1	3782.85
STOM	stomatin	3837.2	3725.8	3781.48
CASC4	cancer susceptibility candidate 4	3678.4	3880.4	3779.42
SUMO1	small ubiquitin-like modifier 1	3711.5	3837.5	3774.50
COPG1	coatomer protein complex, subunit gamma 1	3736.0	3812.0	3774.03
MSMO1	methylsterol monooxygenase 1	3733.8	3785.9	3759.87
COPA	coatomer protein complex, subunit alpha	3776.3	3743.4	3759.82
LOC104913961	vacuole membrane protein 1-like	3479.5	4009.7	3744.60
HMGN3	high mobility group nucleosomal binding domain 3	3804.6	3651.6	3728.10
MAP3K7	mitogen-activated protein kinase kinase kinase 7	3632.1	3820.4	3726.26
LOC104909642	CSC1-like protein 2	3737.5	3713.8	3725.63
ACO2	aconitase 2, mitochondrial	3768.5	3674.9	3721.70
LOXL3	lysyl oxidase-like 3	3792.9	3621.4	3707.16
GLUD1	glutamate dehydrogenase 1	3568.4	3836.1	3702.29
SLMO2	slowmo homolog 2 (Drosophila)	3682.0	3709.3	3695.64
GORASP2	golgi reassembly stacking protein 2, 55kDa	3594.0	3780.8	3687.38
LOC100549055	ferritin light chain-like	2918.2	4448.2	3683.20
LOC104914052	junctophilin-2-like	3561.7	3786.4	3674.03
CCNL2	cyclin L2	3419.2	3913.6	3666.39
USP9X	ubiquitin specific peptidase 9, X-linked	3539.0	3790.2	3664.63

CANX	calnexin	3441.1	3880.0	3660.52
C16H16orf72	chromosome 16 open reading frame, human C16orf72	3590.1	3729.6	3659.86
CBX3	chromobox homolog 3	3590.0	3729.3	3659.69
LOC100551269	tyrosine-protein kinase JAK1-like	3644.9	3672.1	3658.49
XPR1	xenotropic and polytropic retrovirus receptor 1	3857.5	3458.1	3657.79
THYN1	thymocyte nuclear protein 1	3762.6	3550.8	3656.68
LOC104913944	uncharacterized LOC104913944	3644.0	3667.4	3655.70
ATP5J	ATP synthase, H+ transporting, mitochondrial Fo complex, subunit F6	3763.8	3546.1	3654.98
ARL6IP1	ADP-ribosylation factor-like 6 interacting protein 1	3623.8	3657.5	3640.66
POMP	proteasome maturation protein	3669.0	3573.5	3621.24
CHRNG	cholinergic receptor, nicotinic, gamma (muscle)	3664.0	3559.8	3611.87
CTSL	cathepsin L	3272.4	3938.4	3605.43
NDUFA10	NADH dehydrogenase (ubiquinone) 1 alpha subcomplex, 10, 42kDa	3704.1	3500.3	3602.19
LOC100545312	uncharacterized LOC100545312	3737.7	3466.4	3602.08
EXTL3	exostosin-like glycosyltransferase 3	3671.9	3515.1	3593.51
METTL23	methyltransferase like 23	3516.2	3668.1	3592.14
SRP72	signal recognition particle 72kDa	3668.3	3512.5	3590.43
TESK1	testis-specific kinase 1	3460.4	3717.6	3589.00
PIEZO1	piezo-type mechanosensitive ion channel component 1	3763.9	3410.2	3587.05
FAT1	FAT atypical cadherin 1	3402.9	3764.6	3583.75
CPM	carboxypeptidase M	3380.0	3760.4	3570.22
HOMER3	homer homolog 3 (Drosophila)	3510.6	3627.6	3569.12
EEF1A2	eukaryotic translation elongation factor 1 alpha 2	3936.1	3192.3	3564.19
LOC100550279	fatty acid-binding protein, adipocyte-like	3429.2	3689.1	3559.16
PFDN5	prefoldin subunit 5	3624.1	3493.1	3558.60
SDHD	succinate dehydrogenase complex, subunit D, integral membrane protein	3604.9	3508.2	3556.58
FAM32A	family with sequence similarity 32, member A	3622.2	3483.4	3552.80
VPS4A	vacuolar protein sorting 4 homolog A (S. cerevisiae)	3728.9	3372.5	3550.70
MYH10	myosin, heavy chain 10, non-muscle	3543.0	3556.3	3549.66
PCYT1A	phosphate cytidylyltransferase 1, choline, alpha	3625.8	3453.5	3539.69
PDHB	pyruvate dehydrogenase (lipoamide) beta	3512.2	3561.9	3537.06
PLXNA1	plexin A1	3549.5	3521.4	3535.47
APC	adenomatous polyposis coli	3325.7	3742.6	3534.13
SNX3	sorting nexin 3	3524.2	3538.1	3531.14
THRAP3	thyroid hormone receptor associated protein 3	3471.7	3586.4	3529.04
UBR4	ubiquitin protein ligase E3 component n-recognin 4	3536.1	3521.3	3528.68
LOC100538893	tubulin alpha-5 chain	3608.0	3442.1	3525.06
GLO1	glyoxalase I	3561.7	3485.5	3523.59
SLC38A10	solute carrier family 38, member 10	3652.9	3392.4	3522.67
SRSF5	serine/arginine-rich splicing factor 5	3465.5	3562.2	3513.86
NSA2	NSA2 ribosome biogenesis homolog (S. cerevisiae)	3570.0	3457.7	3513.84
DARS	aspartyl-tRNA synthetase	3483.1	3542.9	3512.97
HSPE1	heat shock 10kDa protein 1	3629.6	3380.7	3505.14
EIF2S1	eukaryotic translation initiation factor 2, subunit 1 alpha, 35kDa	3560.6	3444.7	3502.64
NDRG3	NDRG family member 3	3484.3	3519.0	3501.65
LGALS3	lectin, galactoside-binding, soluble, 3	3250.2	3728.1	3489.16
USO1	USO1 vesicle transport factor	3515.1	3444.3	3479.68
ACAP2	ArfGAP with coiled-coil, ankyrin repeat and PH domains 2	3366.9	3583.3	3475.06
TRIO	trio Rho guanine nucleotide exchange factor	3305.9	3643.2	3474.56
PSMD4	proteasome (prosome, macropain) 26S subunit, non-ATPase, 4	3806.0	3141.1	3473.51
LOC100538580	lanosterol 14-alpha demethylase	3613.4	3295.5	3454.45
LOC100544724	cadherin EGF LAG seven-pass G-type receptor 3-like	3967.0	2925.0	3446.02
PPP2R3B	protein phosphatase 2, regulatory subunit B'', beta	3263.0	3628.4	3445.72
LOC104911667	ras-associated and pleckstrin homology domains-containing protein 1-like	3293.5	3590.4	3442.00

LRRFIP2	leucine rich repeat (in FLII) interacting protein 2	3204.5	3667.3	3435.88
RAB11A	RAB11A, member RAS oncogene family	3380.9	3474.3	3427.60
COPB1	coatomer protein complex, subunit beta 1	3408.8	3436.9	3422.84
LOC100538959	dihydropyrimidinase-related protein 2	3282.2	3558.7	3420.47
SF3B3	splicing factor 3b, subunit 3, 130kDa	3642.1	3198.0	3420.05
STAU1	staufen double-stranded RNA binding protein 1	3486.6	3343.0	3414.83
CD99	CD99 molecule	3176.6	3641.2	3408.92
NDUFA5	NADH dehydrogenase (ubiquinone) 1 alpha subcomplex, 5	3411.3	3401.8	3406.57
YIPF5	Yip1 domain family, member 5	3157.7	3649.5	3403.56
TRIM8	tripartite motif containing 8	3301.3	3493.4	3397.34
IDH3B	isocitrate dehydrogenase 3 (NAD+) beta	3679.0	3115.0	3397.00
LOC100550153	polyubiquitin-B	3515.8	3273.9	3394.82
ATP6V1D	ATPase, H+ transporting, lysosomal 34kDa, V1 subunit D	3342.8	3441.3	3392.02
SLC35E3	solute carrier family 35, member E3	3130.6	3651.1	3390.81
ERLEC1	endoplasmic reticulum lectin 1	3205.1	3570.6	3387.84
HINT1	histidine triad nucleotide binding protein 1	3569.4	3203.9	3386.61
TUFT1	tuftelin 1	3429.4	3339.3	3384.37
HMCN1	hemicentin 1	3226.9	3536.4	3381.66
LOC104917002	cytochrome c oxidase subunit 6B1	3590.0	3171.2	3380.63
CAPZA1	capping protein (actin filament) muscle Z-line, alpha 1	3327.4	3427.1	3377.22
CHPF2	chondroitin polymerizing factor 2	3246.2	3500.0	3373.11
SDHA	succinate dehydrogenase complex, subunit A, flavoprotein (Fp)	3419.0	3324.4	3371.73
CDC42BPB	CDC42 binding protein kinase beta (DMPK-like)	3265.4	3476.2	3370.81
PDLIM4	PDZ and LIM domain 4	3343.5	3392.2	3367.85
TFPI2	tissue factor pathway inhibitor 2	3407.5	3327.1	3367.33
LOC104912556	E3 ubiquitin-protein ligase TRIP12-like	3243.8	3489.7	3366.76
NUMB	numb homolog (Drosophila)	3110.5	3622.5	3366.50
OTUD6B	OTU domain containing 6B	3274.0	3458.4	3366.18
LOC100546992	cysteine-rich protein 2-like	3355.5	3373.5	3364.51
LOC100539280	DDB1- and CUL4-associated factor 6-like	3335.3	3389.9	3362.56
PHB	prohibitin	3449.9	3262.5	3356.18
RHEB	Ras homolog enriched in brain	3259.7	3446.9	3353.29
RABL6	RAB, member RAS oncogene family-like 6	3410.5	3290.1	3350.27
LOC104914561	uncharacterized LOC104914561	3597.3	3094.8	3346.06
EIF4A3	eukaryotic translation initiation factor 4A3	3451.8	3234.8	3343.30
HSPB2	heat shock 27kDa protein 2	3317.8	3359.9	3338.82
SDCBP	syndecan binding protein (syntenin)	3284.2	3379.8	3331.99
TMED10	transmembrane emp24-like trafficking protein 10 (yeast)	3316.4	3340.4	3328.40
PSMA6	proteasome (prosome, macropain) subunit, alpha type, 6	3269.5	3370.8	3320.16
MRPL33	mitochondrial ribosomal protein L33	3264.2	3372.4	3318.29
COPS8	COP9 signalosome subunit 8	3337.8	3296.5	3317.16
RBM39	RNA binding motif protein 39	3263.9	3364.8	3314.33
MIF	macrophage migration inhibitory factor (glycosylation-inhibiting factor)	3175.5	3447.8	3311.66
RAD21	RAD21 homolog (S. pombe)	3078.3	3541.8	3310.04
CCPG1	cell cycle progression 1	3363.0	3250.6	3306.81
EIF3K	eukaryotic translation initiation factor 3, subunit K	3387.9	3210.0	3298.92
LOC100548098	cytochrome b-c1 complex subunit 6, mitochondrial	3441.9	3140.8	3291.36
SNX1	sorting nexin 1	3287.0	3289.7	3288.33
RALBP1	ralA binding protein 1	3209.6	3364.9	3287.24
VOPP1	vesicular, overexpressed in cancer, prosurvival protein 1	3228.1	3332.1	3280.08
DNAJA2	DnaJ (Hsp40) homolog, subfamily A, member 2	3153.8	3403.6	3278.67
PDIA4	protein disulfide isomerase family A, member 4	3175.1	3375.1	3275.12
COMT	catechol-O-methyltransferase	3094.2	3450.9	3272.58
MAEA	macrophage erythroblast attacher	3157.6	3367.1	3262.36
LOC100539623	dihydropyrimidinase-related protein 3	3313.9	3209.7	3261.80
PLTP	phospholipid transfer protein	2957.8	3562.5	3260.16
SRPR	signal recognition particle receptor (docking protein)	3330.4	3181.5	3255.93

ELAVL1	ELAV like RNA binding protein 1	3151.8	3333.4	3242.59
PEAK1	pseudopodium-enriched atypical kinase 1	2981.1	3497.9	3239.49
PRKACB	protein kinase, cAMP-dependent, catalytic, beta	3147.1	3328.6	3237.87
CREB3	cAMP responsive element binding protein 3	3294.4	3179.2	3236.82
KARS	lysyl-tRNA synthetase	3302.4	3161.8	3232.10
SMIM14	small integral membrane protein 14	3167.5	3292.0	3229.78
TMPRSS12	transmembrane (C-terminal) protease, serine 12	3247.6	3209.3	3228.45
PTEN	phosphatase and tensin homolog	3208.8	3240.3	3224.59
C5H14orf166	chromosome 5 open reading frame, human C14orf166	3280.0	3165.7	3222.86
PPP2CA	protein phosphatase 2, catalytic subunit, alpha isozyme	3075.3	3366.1	3220.70
VAMP3	vesicle-associated membrane protein 3	3407.4	3031.5	3219.48
SSPN	sarcospan	3128.6	3307.4	3218.03
ZFAND3	zinc finger, AN1-type domain 3	3210.1	3224.4	3217.25
NR1H3	nuclear receptor subfamily 1, group H, member 3	3206.4	3225.5	3215.99
RAPSN	receptor-associated protein of the synapse	3284.4	3145.2	3214.78
SMARCE1	SWI/SNF related, matrix associated, actin dependent regulator of chromatin, subfamily e, member 1	3328.3	3096.8	3212.52
SSR3	signal sequence receptor, gamma (translocon-associated protein gamma)	3182.0	3232.1	3207.08
CORO1C	coronin, actin binding protein, 1C	3258.1	3151.9	3205.01
ANKRD2	ankyrin repeat domain 2 (stretch responsive muscle)	3304.5	3105.5	3204.99
KIRREL	kin of IRRE like (Drosophila)	3275.2	3132.9	3204.02
SDC2	syndecan 2	3211.8	3187.0	3199.42
GOLGA2	golgin A2	3100.5	3293.6	3197.09
KANK3	KN motif and ankyrin repeat domains 3	3247.6	3139.3	3193.43
QARS	glutaminyl-tRNA synthetase	3428.7	2948.0	3188.37
LOC100548214	receptor-type tyrosine-protein phosphatase V-like	3150.0	3224.3	3187.15
SH3BGRL3	SH3 domain binding glutamate-rich protein like 3	3224.9	3147.3	3186.06
NF2	neurofibromin 2 (merlin)	3269.7	3097.4	3183.57
FNBP4	formin binding protein 4	3234.0	3130.9	3182.47
PYCR1	pyrroline-5-carboxylate reductase 1	3317.5	3027.9	3172.71
CHMP4B	charged multivesicular body protein 4B	3148.2	3191.7	3169.96
NDUFV1	NADH dehydrogenase (ubiquinone) flavoprotein 1, 51kDa	3264.6	3073.3	3168.96
EZR	ezrin	3029.4	3299.5	3164.47
BAG3	BCL2-associated athanogene 3	3038.6	3287.3	3162.96
PTPRN	protein tyrosine phosphatase, receptor type, N	3295.0	3029.0	3161.97
PARVG	parvin, gamma	3057.8	3261.2	3159.50
MAP7D1	MAP7 domain containing 1	3407.9	2908.9	3158.39
MRPL17	mitochondrial ribosomal protein L17	3229.7	3073.0	3151.36
SNRPC	small nuclear ribonucleoprotein polypeptide C	3210.6	3089.0	3149.78
PLEKHA1	pleckstrin homology domain containing, family A (phosphoinositide binding specific) member 1	2976.8	3317.9	3147.37
ENOPH1	enolase-phosphatase 1	3110.6	3180.6	3145.59
ATG13	autophagy related 13	3132.1	3155.3	3143.68
MBNL3	muscleblind-like splicing regulator 3	3153.8	3129.1	3141.47
GBF1	golgi brefeldin A resistant guanine nucleotide exchange factor 1	3077.2	3197.7	3137.41
VASH2	vasohibin 2	2908.8	3362.2	3135.50
SNAPC5	small nuclear RNA activating complex, polypeptide 5, 19kDa	3110.8	3146.8	3128.79
KLF6	Kruppel-like factor 6	3099.7	3152.2	3125.93
NOP58	NOP58 ribonucleoprotein	3162.4	3089.3	3125.88
UBALD1	UBA-like domain containing 1	3194.2	3054.5	3124.32
HMGB1	high mobility group box 1	3209.7	3033.2	3121.41
SPCS3	signal peptidase complex subunit 3 homolog (S. cerevisiae)	3105.4	3132.4	3118.86
APOBEC2	apolipoprotein B mRNA editing enzyme, catalytic polypeptide-like 2	3038.8	3178.5	3108.66
CTNNA1	catenin (cadherin-associated protein), alpha 1, 102kDa	3088.2	3122.0	3105.10
ECM2	extracellular matrix protein 2, female organ and adipocyte specific	2826.8	3378.3	3102.57
LOC100540231	GTPase IMAP family member 6-like	3082.1	3118.1	3100.09

NFE2L1	nuclear factor, erythroid 2-like 1	3257.8	2941.2	3099.54
CCDC169	coiled-coil domain containing 169	2906.1	3286.6	3096.39
ARL4C	ADP-ribosylation factor-like 4C	2967.9	3215.4	3091.63
SPPL2A	signal peptide peptidase like 2A	2954.3	3221.6	3087.98
KIFC3	kinesin family member C3	3423.7	2751.2	3087.42
VEPH1	ventricular zone expressed PH domain-containing 1	3064.5	3105.9	3085.20
LOC100543617	transaldolase-like	3058.5	3111.5	3085.00
MAFF	v-maf avian musculoaponeurotic fibrosarcoma oncogene homolog F	3067.7	3094.9	3081.30
COL7A1	collagen, type VII, alpha 1	3053.1	3109.1	3081.10
SAR1A	secretion associated, Ras related GTPase 1A	3132.0	3022.4	3077.19
ERH	enhancer of rudimentary homolog (Drosophila)	3125.3	3028.3	3076.79
IFRD1	interferon-related developmental regulator 1	3047.4	3101.2	3074.30
WSB2	WD repeat and SOCS box containing 2	3182.2	2966.2	3074.19
CD59	CD59 molecule, complement regulatory protein	2972.4	3160.4	3066.40
LOC100541646	ADP-ribosylation factor-like protein 8A	2940.7	3186.8	3063.72
LOC100542446	tripartite motif-containing protein 7-like	3127.4	2995.7	3061.54
GLYR1	glyoxylate reductase 1 homolog (Arabidopsis)	3041.1	3079.9	3060.48
COLEC12	collectin sub-family member 12	2948.3	3163.2	3055.74
PRPF8	pre-mRNA processing factor 8	3057.4	3049.9	3053.63
LOC100538902	transmembrane protein 110-like	3189.3	2913.4	3051.35
LOC100540334	myomegalin	3102.3	2999.5	3050.87
LOC100547273	prolyl endopeptidase FAP	2702.0	3397.3	3049.68
PPP3R1	protein phosphatase 3, regulatory subunit B, alpha	2917.9	3180.7	3049.29
MAGT1	magnesium transporter 1	3013.9	3070.5	3042.22
AFAP1L2	actin filament associated protein 1-like 2	3068.2	3011.8	3039.98
LOC104916044	heterogeneous nuclear ribonucleoprotein C-like	3070.6	3009.4	3039.97
ATIC	5-aminoimidazole-4-carboxamide ribonucleotide formyltransferase/IMP cyclohydrolase	3174.8	2903.4	3039.08
SLC25A5	solute carrier family 25 (mitochondrial carrier; adenine nucleotide translocator), member 5	2982.5	3095.2	3038.83
LOC100542537	coiled-coil domain-containing protein 173-like	3084.1	2988.9	3036.50
ATP5G1	ATP synthase, H+ transporting, mitochondrial Fo complex, subunit C1 (subunit 9)	3155.7	2895.7	3025.73
PDAP1	PDGFA associated protein 1	3086.4	2962.8	3024.57
LOC104911464	collagen alpha-2(I) chain-like	2970.0	3076.0	3023.02
LOC104916307	eukaryotic translation initiation factor 5A-1-like	3298.0	2743.8	3020.94
LOC100548773	histone-binding protein RBBP4	3043.6	2996.4	3020.02
RRAS2	related RAS viral (r-ras) oncogene homolog 2	2996.5	3040.4	3018.45
TSPAN12	tetraspanin 12	2865.5	3171.1	3018.31
UBE2D1	ubiquitin-conjugating enzyme E2D 1	2919.3	3115.7	3017.48
LOC100547453	methionine--tRNA ligase, cytoplasmic-like	3419.6	2605.2	3012.40
LOC100550062	uncharacterized LOC100550062	2824.5	3195.9	3010.20
LOC100541608	putative E3 ubiquitin-protein ligase UBR7	3098.2	2916.4	3007.30
USP20	ubiquitin specific peptidase 20	2931.0	3082.5	3006.71
LOC100551229	tubulin beta chain-like	3318.8	2682.1	3000.48
TNFAIP2	tumor necrosis factor, alpha-induced protein 2	2914.4	3082.7	2998.56
CDC42SE1	CDC42 small effector 1	3114.5	2879.8	2997.15
METAP2	methionyl aminopeptidase 2	3010.1	2977.4	2993.77
SCD	stearoyl-CoA desaturase (delta-9-desaturase)	2969.0	3015.8	2992.39
TSN	translin	2958.4	3020.6	2989.46
RNF13	ring finger protein 13	3002.5	2965.9	2984.21
MRPS11	mitochondrial ribosomal protein S11	3101.6	2865.9	2983.75
AMD1	adenosylmethionine decarboxylase 1	3025.3	2935.0	2980.19
LOC100547732	MOB-like protein phocein	2870.1	3082.0	2976.07
DTX2	deltex 2, E3 ubiquitin ligase	3082.3	2866.2	2974.25
SEC24A	SEC24 family member A	2947.4	2996.6	2972.00
SPECC1	sperm antigen with calponin homology and coiled-coil domains 1	3095.0	2846.8	2970.86

TOMM22	translocase of outer mitochondrial membrane 22 homolog (yeast)	3015.8	2914.9	2965.31
ESD	esterase D	3000.9	2925.8	2963.33
HDAC7	histone deacetylase 7	3012.9	2910.7	2961.80
LOC100546378	AN1-type zinc finger protein 5-like	3079.0	2844.3	2961.63
NUDC	nudC nuclear distribution protein	2993.7	2919.6	2956.65
LOC104912084	transforming acidic coiled-coil-containing protein 2-like	2837.7	3071.3	2954.48
LOC104911201	uncharacterized LOC104911201	2832.6	3069.9	2951.22
PSMB3	proteasome (prosome, macropain) subunit, beta type, 3	3085.8	2814.1	2949.93
MMS19	MMS19 nucleotide excision repair homolog (S. cerevisiae)	3032.2	2867.0	2949.60
PM20D2	peptidase M20 domain containing 2	2806.9	3090.7	2948.81
NRD1	nardilysin (N-arginine dibasic convertase)	2959.7	2937.8	2948.79
NOLC1	nucleolar and coiled-body phosphoprotein 1	2977.4	2914.5	2945.95
NRBP1	nuclear receptor binding protein 1	2956.6	2927.2	2941.93
SEC61B	Sec61 beta subunit	2865.0	3013.0	2938.99
DBNDD2	dysbindin (dystrobrevin binding protein 1) domain containing 2	2888.2	2986.3	2937.25
HECTD1	HECT domain containing E3 ubiquitin protein ligase 1	2763.5	3109.0	2936.25
LOC104912803	uncharacterized LOC104912803	2927.5	2925.6	2926.53
ZNF207	zinc finger protein 207	2884.3	2967.4	2925.84
GPC1	glypican 1	2835.1	3016.4	2925.73
LOC100538920	ATP synthase F(0) complex subunit C2, mitochondrial-like	2935.5	2901.9	2918.69
C15H5orf15	chromosome 15 open reading frame, human C5orf15	2854.8	2980.3	2917.57
DAD1	defender against cell death 1	2881.5	2948.6	2915.07
BICD2	bicaudal D homolog 2 (Drosophila)	2663.8	3160.5	2912.13
ABCE1	ATP-binding cassette, sub-family E (OABP), member 1	2919.0	2901.1	2910.03
LOC100538560	C-type lectin domain family 2 member D-like	2918.2	2894.0	2906.12
MXRA7	matrix-remodelling associated 7	2745.7	3061.3	2903.50
SEC13	SEC13 homolog (S. cerevisiae)	2946.8	2849.7	2898.25
WDR26	WD repeat domain 26	2628.0	3166.3	2897.11
UGDH	UDP-glucose 6-dehydrogenase	2857.0	2936.4	2896.66
PSMD11	proteasome (prosome, macropain) 26S subunit, non-ATPase, 11	3026.7	2765.9	2896.30
CRYAB	crystallin, alpha B	2844.4	2947.1	2895.77
TBC1D14	TBC1 domain family, member 14	2767.0	3022.9	2894.94
C30H19orf10	chromosome 30 open reading frame, human C19orf10	2865.9	2914.2	2890.03
SLTM	SAFB-like, transcription modulator	2845.1	2933.1	2889.09
LOC100545141	acidic leucine-rich nuclear phosphoprotein 32 family member B	3004.9	2773.1	2889.01
GNA12	guanine nucleotide binding protein (G protein) alpha 12	2753.1	3019.9	2886.49
LOC104914261	uncharacterized LOC104914261	2864.6	2906.8	2885.70
TMEM200A	transmembrane protein 200A	2797.4	2970.7	2884.05
LOC104912336	protein PRRC2C-like	2774.5	2985.2	2879.86
PXMP4	peroxisomal membrane protein 4, 24kDa	2947.6	2811.0	2879.28
NTMT1	N-terminal Xaa-Pro-Lys N-methyltransferase 1	2884.1	2870.2	2877.13
NAA38	N(alpha)-acetyltransferase 38, NatC auxiliary subunit	2979.0	2768.5	2873.74
LOC104913969	uncharacterized LOC104913969	2801.1	2946.3	2873.70
PPIC	peptidylprolyl isomerase C (cyclophilin C)	2687.1	3055.0	2871.01
ACOT9	acyl-CoA thioesterase 9	2753.0	2986.1	2869.56
MAPK1IP1L	mitogen-activated protein kinase 1 interacting protein 1-like	2855.4	2883.0	2869.21
MAP4	microtubule-associated protein 4	3255.1	2468.6	2861.84
PSMB1	proteasome (prosome, macropain) subunit, beta type, 1	2952.6	2766.5	2859.51
FAM133B	family with sequence similarity 133, member B	2859.8	2858.0	2858.89
POLDIP2	polymerase (DNA-directed), delta interacting protein 2	2878.8	2836.4	2857.62
RAB5C	RAB5C, member RAS oncogene family	2896.5	2817.4	2856.95
MRPL20	mitochondrial ribosomal protein L20	2840.7	2872.1	2856.39
PARK7	parkinson protein 7	2881.6	2821.8	2851.70
MRPL18	mitochondrial ribosomal protein L18	2882.8	2818.9	2850.86
MYO10	myosin X	2772.4	2924.8	2848.62
ATP6AP1	ATPase, H+ transporting, lysosomal accessory protein 1	2752.8	2941.0	2846.94
CTNNBL1	catenin, beta like 1	2833.3	2855.4	2844.34
GMFB	glia maturation factor, beta	2809.9	2877.3	2843.58

PHGDH	phosphoglycerate dehydrogenase	3310.2	2371.9	2841.07
NPM3	nucleophosmin/nucleoplasmin 3	3057.7	2624.2	2840.96
RCC2	regulator of chromosome condensation 2	2724.9	2948.3	2836.64
TAX1BP1	Tax1 (human T-cell leukemia virus type I) binding protein 1	2701.3	2971.5	2836.38
ODC1	ornithine decarboxylase 1	2641.4	3030.8	2836.09
COL4A2	collagen, type IV, alpha 2	2573.9	3091.7	2832.77
TEF	thyrotrophic embryonic factor	2684.2	2978.6	2831.42
LOC104913032	breast cancer anti-estrogen resistance protein 1-like	2897.4	2764.3	2830.82
UBE2A	ubiquitin-conjugating enzyme E2A	2718.0	2927.3	2822.69
ALDH7A1	aldehyde dehydrogenase 7 family, member A1	2844.4	2789.1	2816.76
DNAJB2	DnaJ (Hsp40) homolog, subfamily B, member 2	3111.7	2513.8	2812.79
VPS35	vacuolar protein sorting 35 homolog (S. cerevisiae)	2818.4	2806.4	2812.39
WLS	wntless Wnt ligand secretion mediator	2935.0	2686.8	2810.87
CDH13	cadherin 13	2619.2	2999.5	2809.32
EMC1	ER membrane protein complex subunit 1	2752.4	2864.5	2808.42
UPF1	UPF1 regulator of nonsense transcripts homolog (yeast)	2824.2	2788.8	2806.50
ACADL	acyl-CoA dehydrogenase, long chain	2912.1	2699.2	2805.62
IPO5	importin 5	2950.9	2658.6	2804.78
LOC104917564	uncharacterized LOC104917564	2535.7	3070.2	2802.93
SOD2	superoxide dismutase 2, mitochondrial	2795.4	2794.9	2795.18
UBE2G1	ubiquitin-conjugating enzyme E2G 1	2687.7	2900.5	2794.07
XBP1	X-box binding protein 1	2849.2	2737.9	2793.56
UGP2	UDP-glucose pyrophosphorylase 2	2896.9	2688.9	2792.93
U2AF1	U2 small nuclear RNA auxiliary factor 1	2764.4	2820.3	2792.35
ALYREF	Aly/REF export factor	2848.0	2734.6	2791.31
RAB24	RAB24, member RAS oncogene family	2940.5	2640.4	2790.44
NELL2	NEL-like 2 (chicken)	2980.6	2594.8	2787.70
PPA1	pyrophosphatase (inorganic) 1	2816.5	2758.3	2787.39
AP1M1	adaptor-related protein complex 1, mu 1 subunit	2749.9	2819.7	2784.81
MORC2	MORC family CW-type zinc finger 2	2659.2	2904.6	2781.91
SARS	seryl-tRNA synthetase	3047.4	2513.1	2780.27
SBNO1	strawberry notch homolog 1 (Drosophila)	2716.4	2842.7	2779.55
UBP1	upstream binding protein 1 (LBP-1a)	2700.7	2849.2	2774.93
SACS	sacsin molecular chaperone	2407.1	3135.2	2771.17
TCHP	trichoplein, keratin filament binding	2770.5	2771.2	2770.84
OSMR	oncostatin M receptor	2616.3	2924.1	2770.19
TRAK1	trafficking protein, kinesin binding 1	2736.0	2797.4	2766.70
MRPL28	mitochondrial ribosomal protein L28	2764.6	2767.7	2766.13
LOC100545741	UAP56-interacting factor-like	2787.0	2745.3	2766.12
LOC100539484	uncharacterized LOC100539484	2762.3	2769.2	2765.79
LOC104915823	histone H2A.V-like	3046.7	2480.8	2763.74
TIPARP	TCDD-inducible poly(ADP-ribose) polymerase	2803.0	2718.0	2760.51
SNX12	sorting nexin 12	2816.4	2704.6	2760.47
RPL7L1	ribosomal protein L7-like 1	2742.2	2777.1	2759.62
PSME3	proteasome (prosome, macropain) activator subunit 3 (PA28 gamma; Ki)	2798.5	2718.8	2758.68
ANKRD13A	ankyrin repeat domain 13A	2702.4	2810.9	2756.69
MET	MET proto-oncogene, receptor tyrosine kinase	2642.2	2867.6	2754.88
MOB2	MOB kinase activator 2	2655.5	2850.9	2753.19
LOC100538539	cytochrome b-c1 complex subunit Rieske, mitochondrial	2825.5	2680.3	2752.87
SRSF6	serine/arginine-rich splicing factor 6	2606.3	2898.7	2752.49
NAA15	N(alpha)-acetyltransferase 15, NatA auxiliary subunit	2684.6	2809.8	2747.19
TPRG1L	tumor protein p63 regulated 1-like	2892.1	2593.3	2742.70
PKIA	protein kinase (cAMP-dependent, catalytic) inhibitor alpha	2545.9	2937.1	2741.53
LOC104914332	microtubule-actin cross-linking factor 1-like	2873.1	2608.4	2740.74
LOC100544708	alcohol dehydrogenase class-3	2630.9	2849.1	2739.99
S100A1	S100 calcium binding protein A1	2678.9	2797.3	2738.09
AZIN1	antizyme inhibitor 1	2784.4	2686.0	2735.23

SBNO2	strawberry notch homolog 2 (Drosophila)	2620.8	2847.7	2734.22
MTX2	metaxin 2	2801.8	2664.1	2732.92
INSIG1	insulin induced gene 1	2790.1	2674.9	2732.51
LOC104911014	oxysterol-binding protein-related protein 5-like	2543.6	2910.3	2726.96
LOC100538595	glutathione S-transferase theta-1	2642.0	2807.7	2724.86
PTPRS	protein tyrosine phosphatase, receptor type, S	2715.2	2734.3	2724.73
TWSG1	twisted gastrulation BMP signaling modulator 1	2683.6	2755.6	2719.60
NUP85	nucleoporin 85kDa	2724.5	2709.2	2716.82
GSS	glutathione synthetase	2570.6	2861.2	2715.90
SSU72	SSU72 RNA polymerase II CTD phosphatase homolog (S. cerevisiae)	2625.1	2803.7	2714.41
TPPP3	tubulin polymerization-promoting protein family member 3	2979.7	2448.4	2714.05
RAP2C	RAP2C, member of RAS oncogene family	2716.2	2708.4	2712.28
LOC104910408	cytochrome b-c1 complex subunit 7	2760.6	2659.6	2710.11
ANGEL2	angel homolog 2 (Drosophila)	2633.2	2778.5	2705.82
GLOD4	glyoxalase domain containing 4	2627.6	2783.4	2705.50
BDH1	3-hydroxybutyrate dehydrogenase, type 1	2591.3	2818.0	2704.64
TRA2B	transformer 2 beta homolog (Drosophila)	2746.7	2652.9	2699.82
HMGN1	high mobility group nucleosome binding domain 1	2852.8	2546.5	2699.65
LOC104915875	dynactin subunit 2-like	2849.6	2548.9	2699.24
CLINT1	clathrin interactor 1	2631.6	2760.6	2696.07
FLII	flightless I homolog (Drosophila)	2938.3	2453.2	2695.76
SYNJ2	synaptojanin 2	2614.0	2769.8	2691.87
LOC104913882	telomerase-binding protein EST1A-like	2757.8	2625.0	2691.42
AP2A2	adaptor-related protein complex 2, alpha 2 subunit	2763.6	2618.9	2691.22
FDPS	farnesyl diphosphate synthase	2944.4	2431.8	2688.11
PSMD12	proteasome (prosome, macropain) 26S subunit, non-ATPase, 12	2598.7	2773.5	2686.09
MOB1A	MOB kinase activator 1A	2834.6	2537.0	2685.81
SCP2	sterol carrier protein 2	2551.5	2818.3	2684.91
SUCLA2	succinate-CoA ligase, ADP-forming, beta subunit	2606.0	2755.1	2680.55
SRI	sorcin	2661.1	2693.3	2677.22
RAB18	RAB18, member RAS oncogene family	2669.8	2683.9	2676.84
IARS	isoleucyl-tRNA synthetase	2811.7	2541.1	2676.42
SEC31A	SEC31 homolog A (S. cerevisiae)	2531.9	2820.6	2676.26
TCEB1	transcription elongation factor B (SIII), polypeptide 1 (15kDa, elongin C)	2615.1	2733.2	2674.12
FXR1	fragile X mental retardation, autosomal homolog 1	2583.2	2764.6	2673.87
UBE2L3	ubiquitin-conjugating enzyme E2L 3	2736.5	2607.4	2671.95
DDX1	DEAD (Asp-Glu-Ala-Asp) box helicase 1	2651.0	2687.2	2669.10
MTCH2	mitochondrial carrier 2	2754.7	2571.6	2663.17
EYA2	EYA transcriptional coactivator and phosphatase 2	2379.5	2944.1	2661.82
SHFM1	split hand/foot malformation (ectrodactyly) type 1	2710.9	2601.4	2656.17
NDUFS3	NADH dehydrogenase (ubiquinone) Fe-S protein 3, 30kDa (NADH-coenzyme Q reductase)	2749.8	2559.8	2654.80
PUF60	poly-U binding splicing factor 60KDa	2721.5	2583.3	2652.43
TOP2B	topoisomerase (DNA) II beta 180kDa	2559.2	2741.0	2650.10
STAT3	signal transducer and activator of transcription 3 (acute-phase response factor)	2815.2	2481.8	2648.48
DKC1	dyskeratosis congenita 1, dyskerin	2706.1	2583.2	2644.63
FHOD3	formin homology 2 domain containing 3	2508.6	2779.1	2643.83
NDUFB4	NADH dehydrogenase (ubiquinone) 1 beta subcomplex, 4, 15kDa	2648.2	2632.1	2640.14
EI24	etoposide induced 2.4	2710.5	2569.5	2639.99
CAMK2D	calcium/calmodulin-dependent protein kinase II delta	2283.5	2996.2	2639.82
LOC100548330	cytochrome c oxidase subunit 7A2, mitochondrial	2719.2	2552.1	2635.62
NRP1	neuropilin 1	2612.4	2655.6	2633.97
UBE2J2	ubiquitin-conjugating enzyme E2, J2	2621.9	2641.8	2631.89
DYNLRB1	dynein, light chain, roadblock-type 1	2686.0	2568.9	2627.43
LOC100547530	15 kDa selenoprotein	2633.8	2620.0	2626.89

PPM1B	protein phosphatase, Mg2+/Mn2+ dependent, 1B	2515.8	2733.4	2624.60
TMEM180	transmembrane protein 180	2558.8	2685.5	2622.16
MCAM	melanoma cell adhesion molecule	2680.0	2560.3	2620.12
DNAJC8	DnaJ (Hsp40) homolog, subfamily C, member 8	2656.3	2581.6	2618.96
LOC104911272	uncharacterized LOC104911272	2584.5	2649.1	2616.80
USMG5	up-regulated during skeletal muscle growth 5 homolog (mouse)	2594.3	2637.4	2615.81
ATP6V0A1	ATPase, H+ transporting, lysosomal V0 subunit a1	2594.4	2635.7	2615.01
CHURC1	churchill domain containing 1	2717.7	2506.2	2611.98
UBE2H	ubiquitin-conjugating enzyme E2H	2501.3	2714.7	2607.99
TOM1	target of myb1 (chicken)	2552.6	2658.9	2605.71
EIF4E	eukaryotic translation initiation factor 4E	2688.0	2523.1	2605.55
ARL6IP5	ADP-ribosylation factor-like 6 interacting protein 5	2507.1	2700.3	2603.70
ALDH2	aldehyde dehydrogenase 2 family (mitochondrial)	2644.2	2561.8	2603.03
IQGAP1	IQ motif containing GTPase activating protein 1	2545.9	2658.9	2602.36
LOC104914793	transitional endoplasmic reticulum ATPase-like	2630.0	2573.5	2601.72
HMGB3	high mobility group box 3	2649.1	2554.2	2601.61
LSS	lanosterol synthase (2,3-oxidosqualene-lanosterol cyclase)	2754.5	2438.3	2596.42
DIP2C	DIP2 disco-interacting protein 2 homolog C (Drosophila)	2492.0	2697.4	2594.69
HOOK3	hook microtubule-tethering protein 3	2601.8	2578.3	2590.07
CRK	v-crk avian sarcoma virus CT10 oncogene homolog	2539.7	2636.8	2588.26
PUM2	pumilio RNA-binding family member 2	2463.9	2709.0	2586.42
ADCYAP1	adenylate cyclase activating polypeptide 1 (pituitary)	2553.2	2615.7	2584.44
FBXO9	F-box protein 9	2579.8	2583.2	2581.49
UBE2Q1	ubiquitin-conjugating enzyme E2Q family member 1	2629.5	2530.1	2579.79
C4H20orf194	chromosome 4 open reading frame, human C20orf194	2609.4	2548.9	2579.15
TMEM47	transmembrane protein 47	2510.8	2641.3	2576.08
LOC723978	cytochrome c oxidase subunit VIIc precursor	2586.8	2562.6	2574.68
TAF7L	TAF7-like RNA polymerase II, TATA box binding protein (TBP)-associated factor, 50kDa	2617.5	2531.8	2574.66
PTP4A2	protein tyrosine phosphatase type IVA, member 2	2571.2	2574.7	2572.98
NT5DC2	5'-nucleotidase domain containing 2	2604.7	2540.0	2572.34
SRPK2	SRSF protein kinase 2	2416.7	2727.2	2571.92
LUC7L	LUC7-like (S. cerevisiae)	2474.7	2663.5	2569.11
LOC104911024	uncharacterized LOC104911024	2588.8	2543.6	2566.19
HADH	hydroxyacyl-CoA dehydrogenase	2530.0	2601.0	2565.46
VPS53	vacuolar protein sorting 53 homolog (S. cerevisiae)	2547.2	2580.6	2563.91
LOC100545668	aldose reductase-like	2607.8	2517.7	2562.75
LARP4B	La ribonucleoprotein domain family, member 4B	2517.1	2605.1	2561.08
MAT2B	methionine adenosyltransferase II, beta	2472.6	2642.9	2557.74
ACLY	ATP citrate lyase	2536.5	2576.0	2556.28
FKBP9	FK506 binding protein 9, 63 kDa	2552.6	2553.3	2552.95
LOC100546173	cytochrome c oxidase copper chaperone	2471.3	2627.1	2549.18
RNASET2	ribonuclease T2	2404.2	2691.2	2547.74
CCDC80	coiled-coil domain containing 80	2420.5	2673.1	2546.81
HNRNPD	heterogeneous nuclear ribonucleoprotein D (AU-rich element RNA binding protein 1, 37kDa)	2562.7	2530.2	2546.43
AEBP1	AE binding protein 1	2601.6	2489.9	2545.74
UBE2I	ubiquitin-conjugating enzyme E2I	2519.8	2568.1	2543.98
GM2A	GM2 ganglioside activator	2078.9	3001.8	2540.35
ZC3H13	zinc finger CCCH-type containing 13	2522.8	2555.6	2539.18
FAM98A	family with sequence similarity 98, member A	2388.1	2687.2	2537.65
YES1	YES proto-oncogene 1, Src family tyrosine kinase	2438.6	2631.9	2535.25
LOC104916241	B-cell receptor-associated protein 31-like	2628.2	2440.2	2534.17
CHUK	conserved helix-loop-helix ubiquitous kinase	2516.2	2550.1	2533.11
HSPA4	heat shock 70kDa protein 4	2598.6	2467.4	2533.00
LOC104915953	collagen alpha-1(I) chain-like	2545.0	2517.7	2531.38
MLLT4	myeloid/lymphoid or mixed-lineage leukemia (trithorax homolog, Drosophila); translocated to, 4	2347.5	2715.3	2531.37

LOC104909481	exportin-1	2526.9	2525.2	2526.05
MGAT4B	mannosyl (alpha-1,3-)-glycoprotein beta-1,4-N-acetylglucosaminyltransferase, isozyme B	2598.6	2451.1	2524.83
CERCAM	cerebral endothelial cell adhesion molecule	2456.4	2591.2	2523.78
MCRS1	microspherule protein 1	2661.2	2374.4	2517.80
SEC61G	Sec61 gamma subunit	2576.5	2452.1	2514.30
UBTD1	ubiquitin domain containing 1	2415.4	2612.8	2514.08
APOA1	apolipoprotein A-I	2361.7	2664.0	2512.84
PRNP	prion protein	2603.7	2418.5	2511.09
ATP6V1B2	ATPase, H+ transporting, lysosomal 56/58kDa, V1 subunit B2	2426.4	2592.5	2509.42
VPS29	vacuolar protein sorting 29 homolog (S. cerevisiae)	2496.5	2519.1	2507.77
IDE	insulin-degrading enzyme	2397.0	2611.7	2504.35
AFAP1L1	actin filament associated protein 1-like 1	2582.6	2421.3	2501.96
CHTOP	chromatin target of PRMT1	2599.4	2402.7	2501.01
VPS16	vacuolar protein sorting 16 homolog (S. cerevisiae)	2699.3	2300.9	2500.10
LOC100542161	serine/arginine-rich splicing factor 7-like	2504.0	2494.8	2499.38
XPA	xeroderma pigmentosum, complementation group A	2571.8	2423.6	2497.68
P3H1	prolyl 3-hydroxylase 1	2425.0	2569.3	2497.18
TIMM8A	translocase of inner mitochondrial membrane 8 homolog A (yeast)	2619.7	2372.4	2496.04
NHP2L1	NHP2 non-histone chromosome protein 2-like 1 (S. cerevisiae)	2581.5	2408.7	2495.10
URM1	ubiquitin related modifier 1	2530.2	2455.4	2492.80
AHSA1	AHA1, activator of heat shock 90kDa protein ATPase homolog 1 (yeast)	2440.0	2543.2	2491.60
SH2D3C	SH2 domain containing 3C	2651.5	2327.3	2489.41
ULK2	unc-51 like autophagy activating kinase 2	2434.5	2543.8	2489.17
PPP2R2A	protein phosphatase 2, regulatory subunit B, alpha	2451.1	2524.5	2487.82
LOC104917167	neuroblast differentiation-associated protein AHNAK-like	2456.5	2518.0	2487.25
RRAGD	Ras-related GTP binding D	2511.1	2456.6	2483.86
BLMH	bleomycin hydrolase	2544.1	2423.5	2483.82
PARL	presenilin associated, rhomboid-like	2545.4	2421.8	2483.61
TNIP1	TNFAIP3 interacting protein 1	2563.4	2400.2	2481.82
KIFAP3	kinesin-associated protein 3	2367.6	2595.7	2481.63
HNRNPR	heterogeneous nuclear ribonucleoprotein R	2442.8	2513.8	2478.31
LMAN1	lectin, mannose-binding, 1	2414.8	2540.0	2477.39
LOC100549535	ubiquitin carboxyl-terminal hydrolase 7-like	2355.5	2598.6	2477.03
LOC100547917	pre-B-cell leukemia transcription factor-interacting protein 1-like	2868.2	2083.9	2476.06
YIPF3	Yip1 domain family, member 3	2509.1	2440.2	2474.67
OSTC	oligosaccharyltransferase complex subunit (non-catalytic)	2377.4	2568.8	2473.09
PMPCA	peptidase (mitochondrial processing) alpha	2463.6	2478.9	2471.26
BMP2K	BMP2 inducible kinase	2384.7	2548.4	2466.54
HERPUD2	HERPUD family member 2	2400.6	2531.3	2465.96
LOC100546824	uncharacterized LOC100546824	2381.5	2534.0	2457.72
FKBP10	FK506 binding protein 10, 65 kDa	2403.7	2506.5	2455.12
HN1L	hematological and neurological expressed 1-like	2389.1	2518.8	2453.91
CIB1	calcium and integrin binding 1 (calmyrin)	2504.6	2403.0	2453.81
PTPRF	protein tyrosine phosphatase, receptor type, F	2430.3	2472.3	2451.29
LOC104913595	serine/threonine-protein kinase TAO3-like	2521.8	2379.6	2450.69
CTSZ	cathepsin Z	2292.9	2603.2	2448.06
PSMB5	proteasome (prosome, macropain) subunit, beta type, 5	2525.6	2369.0	2447.30
TFG	TRK-fused gene	2541.2	2352.6	2446.93
AKAP12	A kinase (PRKA) anchor protein 12	2413.7	2478.7	2446.22
TRAM1	translocation associated membrane protein 1	2421.2	2466.3	2443.72
ZNF593	zinc finger protein 593	2544.8	2342.6	2443.68
PRDX3	peroxiredoxin 3	2451.0	2436.0	2443.49
ARL8B	ADP-ribosylation factor-like 8B	2399.3	2484.2	2441.74
ADIPOR2	adiponectin receptor 2	2392.6	2490.7	2441.66
PARD3B	par-3 family cell polarity regulator beta	2308.1	2574.3	2441.20
ABR	active BCR-related	2379.0	2496.7	2437.84

TSTD2	thiosulfate sulfurtransferase (rhodanese)-like domain containing 2	2373.7	2500.8	2437.23
AP2B1	adaptor-related protein complex 2, beta 1 subunit	2551.5	2316.7	2434.10
NUTF2	nuclear transport factor 2	2458.1	2410.0	2434.03
SUN1	Sad1 and UNC84 domain containing 1	2379.7	2487.9	2433.84
TMPO	thymopoietin	2454.8	2407.1	2430.96
LOC104909990	kinase D-interacting substrate of 220 kDa-like	2420.0	2438.0	2428.97
LOC100539302	tumor necrosis factor receptor superfamily member 16-like	2566.3	2289.8	2428.08
GJC1	gap junction protein, gamma 1, 45kDa	2332.6	2522.1	2427.34
DGKZ	diacylglycerol kinase, zeta	2378.0	2469.6	2423.80
RNASEK	ribonuclease, RNase K	2444.7	2398.8	2421.75
UBE2Q2	ubiquitin-conjugating enzyme E2Q family member 2	2255.9	2573.9	2414.89
LOC100548272	cytoplasmic FMR1-interacting protein 1-like	2421.6	2407.1	2414.36
NDUFB9	NADH dehydrogenase (ubiquinone) 1 beta subcomplex, 9, 22kDa	2551.3	2275.3	2413.31
MYO18A	myosin XVIIIA	2548.9	2272.1	2410.53
LOC104915066	transmembrane protein 2-like	2323.0	2496.7	2409.84
SYNCRIP	synaptotagmin binding, cytoplasmic RNA interacting protein	2350.8	2467.0	2408.93
RSL24D1	ribosomal L24 domain containing 1	2364.9	2452.9	2408.90
DCAF13	DDB1 and CUL4 associated factor 13	2366.0	2451.4	2408.68
HYOU1	hypoxia up-regulated 1	2397.2	2416.0	2406.60
PNRC1	proline-rich nuclear receptor coactivator 1	2295.0	2517.7	2406.39
LOC100546640	translocation protein SEC63 homolog	2347.4	2464.7	2406.04
PAFAH1B2	platelet-activating factor acetylhydrolase 1b, catalytic subunit 2 (30kDa)	2348.4	2459.7	2404.05
UBE2G2	ubiquitin-conjugating enzyme E2G 2	2497.2	2289.0	2393.10
ABL1	ABL proto-oncogene 1, non-receptor tyrosine kinase	2368.8	2413.7	2391.23
ANKRD17	ankyrin repeat domain 17	2304.4	2476.4	2390.39
SLIT1	slit homolog 1 (Drosophila)	2448.4	2330.0	2389.19
PHLDB1	pleckstrin homology-like domain, family B, member 1	2524.7	2250.3	2387.48
LOC100546534	procollagen galactosyltransferase 1-like	2414.3	2360.2	2387.25
PSAP	prosaposin	2351.1	2412.7	2381.92
UBE2B	ubiquitin-conjugating enzyme E2B	2283.4	2478.0	2380.70
TIMM17A	translocase of inner mitochondrial membrane 17 homolog A (yeast)	2425.6	2333.2	2379.44
NDUFB5	NADH dehydrogenase (ubiquinone) 1 beta subcomplex, 5, 16kDa	2365.1	2393.4	2379.22
GUSB	glucuronidase, beta	2310.1	2447.4	2378.78
PARP1	poly (ADP-ribose) polymerase 1	2394.7	2361.6	2378.17
KLHDC2	kelch domain containing 2	2497.0	2254.8	2375.91
HAGH	hydroxyacylglutathione hydrolase	2396.5	2355.2	2375.83
PPP1R7	protein phosphatase 1, regulatory subunit 7	2401.2	2346.0	2373.58
SRSF10	serine/arginine-rich splicing factor 10	2431.5	2311.4	2371.49
ARPC3	actin related protein 2/3 complex, subunit 3, 21kDa	2341.8	2398.7	2370.23
DCTN3	dynactin 3 (p22)	2436.1	2304.0	2370.09
PRKAG2	protein kinase, AMP-activated, gamma 2 non-catalytic subunit	2359.0	2374.9	2366.95
CMTM7	CKLF-like MARVEL transmembrane domain containing 7	2241.8	2490.0	2365.87
PRPF39	pre-mRNA processing factor 39	2282.0	2449.3	2365.66
USP49	ubiquitin specific peptidase 49	2356.1	2368.9	2362.53
CEBPG	CCAAT/enhancer binding protein (C/EBP), gamma	2332.1	2392.1	2362.11
AP3D1	adaptor-related protein complex 3, delta 1 subunit	2357.3	2366.0	2361.65
CARS	cysteinyl-tRNA synthetase	2394.3	2326.1	2360.21
RUFY3	RUN and FYVE domain containing 3	2270.0	2450.3	2360.16
ASCC2	activating signal cointegrator 1 complex subunit 2	2341.8	2378.3	2360.07
PADI1	peptidyl arginine deiminase, type I	2289.5	2428.4	2358.96
EXOC5	exocyst complex component 5	2310.9	2400.1	2355.51
KLF5	Kruppel-like factor 5 (intestinal)	2216.3	2493.5	2354.94
FAM120A	family with sequence similarity 120A	2140.6	2567.9	2354.26
TMEM59	transmembrane protein 59	2216.6	2487.5	2352.05

GPCPD1	glycerophosphocholine phosphodiesterase GDE1 homolog (S. cerevisiae)	2180.8	2523.2	2352.02
VGLL3	vestigial-like family member 3	2340.0	2363.0	2351.51
ATP5O	ATP synthase, H+ transporting, mitochondrial F1 complex, O subunit	2433.0	2266.2	2349.58
LOC100548337	protein farnesyltransferase subunit beta-like	2449.6	2243.4	2346.50
TMBIM1	transmembrane BAX inhibitor motif containing 1	2425.0	2262.9	2343.97
ATP11A	ATPase, class VI, type 11A	2159.4	2523.6	2341.50
NDUFB10	NADH dehydrogenase (ubiquinone) 1 beta subcomplex, 10, 22kDa	2318.6	2361.8	2340.20
EDEM1	ER degradation enhancer, mannosidase alpha-like 1	2272.4	2405.8	2339.12
TXNL1	thioredoxin-like 1	2408.4	2267.7	2338.05
PCGF5	polycomb group ring finger 5	2282.0	2390.5	2336.22
PDCD6	programmed cell death 6	2358.9	2307.4	2333.14
LIPA	lipase A, lysosomal acid, cholesterol esterase	2171.3	2488.8	2330.06
CHCHD7	coiled-coil-helix-coiled-coil-helix domain containing 7	2216.4	2436.5	2326.48
MFN1	mitofusin 1	2256.6	2391.4	2323.99
LOC100546087	dystrobrevin alpha-like	2242.5	2404.2	2323.31
RANBP3	RAN binding protein 3	2276.3	2361.4	2318.86
TMEM38A	transmembrane protein 38A	2234.8	2399.1	2316.94
SYNE2	spectrin repeat containing, nuclear envelope 2	2274.3	2345.7	2310.03
THBD	thrombomodulin	2181.1	2435.7	2308.40
SNRPF	small nuclear ribonucleoprotein polypeptide F	2412.6	2202.2	2307.44
LOC104913324	uncharacterized LOC104913324	2278.0	2332.3	2305.19
CFAP36	cilia and flagella associated protein 36	2207.7	2398.9	2303.29
LOC100549331	myosin-7-like	2138.0	2468.4	2303.19
LOC104917102	eukaryotic translation initiation factor 3 subunit C-like	2381.8	2222.0	2301.88
PAN2	PAN2 poly(A) specific ribonuclease subunit	2354.7	2248.5	2301.58
TMED2	transmembrane emp24 domain trafficking protein 2	2237.9	2362.4	2300.17
C5H14orf2	chromosome 5 open reading frame, human C14orf2	2433.4	2162.2	2297.83
ETFA	electron-transfer-flavoprotein, alpha polypeptide	2407.4	2188.2	2297.83
CUL3	cullin 3	2270.7	2324.8	2297.76
LOC104914174	uncharacterized LOC104914174	2295.9	2299.2	2297.57
CAND1	cullin-associated and neddylation-dissociated 1	2306.3	2286.7	2296.51
LOC104912188	uncharacterized LOC104912188	2097.3	2495.5	2296.42
LOC100548287	USP6 N-terminal-like protein	2257.0	2335.4	2296.19
NISCH	nischarin	2163.1	2425.8	2294.43
CHMP1B	charged multivesicular body protein 1B	2259.4	2327.5	2293.44
IMP4	IMP4, U3 small nucleolar ribonucleoprotein	2406.9	2176.6	2291.78
PDCL	phosducin-like	2292.4	2290.1	2291.23
NDUFB7	NADH dehydrogenase (ubiquinone) 1 beta subcomplex, 7, 18kDa	2361.2	2215.5	2288.37
PLN	phospholamban	2412.5	2163.8	2288.16
BLOC1S5	biogenesis of lysosomal organelles complex-1, subunit 5, muted	2371.4	2204.5	2287.96
TXN2	thioredoxin 2	2355.6	2210.9	2283.27
FBXL22	F-box and leucine-rich repeat protein 22	2205.1	2355.8	2280.46
LOC100547883	SWI/SNF complex subunit SMARCC1-like	2437.1	2121.3	2279.21
SNRPE	small nuclear ribonucleoprotein polypeptide E	2305.6	2252.2	2278.87
CTSC	cathepsin C	2143.3	2413.8	2278.55
LOC100550520	transitional endoplasmic reticulum ATPase-like	2292.3	2262.9	2277.61
SLC25A1	solute carrier family 25 (mitochondrial carrier; citrate transporter), member 1	2288.1	2265.2	2276.64
NDUFB8	NADH dehydrogenase (ubiquinone) 1 beta subcomplex, 8, 19kDa	2365.9	2186.3	2276.14
LOC100539819	cytochrome b reductase 1	2128.7	2416.1	2272.39
BRD7	bromodomain containing 7	2178.6	2364.1	2271.33
LOC100545134	proteasomal ubiquitin receptor ADRM1-like	2309.2	2232.8	2270.99
MTFR1	mitochondrial fission regulator 1	2262.3	2266.5	2264.37
KRAS	Kirsten rat sarcoma viral oncogene homolog	2188.6	2338.4	2263.52
BUD31	BUD31 homolog (S. cerevisiae)	2263.2	2263.5	2263.36
PCYT2	phosphate cytidylyltransferase 2, ethanolamine	2190.1	2336.6	2263.36

NDUFS2	NADH dehydrogenase (ubiquinone) Fe-S protein 2, 49kDa (NADH-coenzyme Q reductase)	2365.4	2161.1	2263.22
LOC104916336	EH domain-containing protein 3 pseudogene	2336.8	2187.4	2262.11
BHLHE40	basic helix-loop-helix family, member e40	2131.7	2392.1	2261.92
FBLN2	fibulin 2	1859.4	2662.0	2260.68
CDV3	CDV3 homolog (mouse)	2319.3	2200.6	2259.97
ZMAT2	zinc finger, matrin-type 2	2190.4	2327.6	2259.00
XPOT	exportin, tRNA	2219.9	2297.6	2258.75
MYOF	myoferlin	2190.8	2322.7	2256.73
SEPT8	septin 8	2177.5	2335.4	2256.44
UTP11L	UTP11-like, U3 small nucleolar ribonucleoprotein (yeast)	2315.8	2196.6	2256.18
LOC100543159	cytochrome P450 20A1	2247.7	2261.9	2254.80
SETD2	SET domain containing 2	2276.6	2232.2	2254.45
IP6K3	inositol hexakisphosphate kinase 3	2282.1	2222.4	2252.21
PDHA1	pyruvate dehydrogenase (lipoamide) alpha 1	2217.6	2284.8	2251.17
LOC100549822	epididymis-specific alpha-mannosidase	2095.0	2404.2	2249.62
LOC104911752	uncharacterized LOC104911752	2280.9	2218.4	2249.61
TP53INP2	tumor protein p53 inducible nuclear protein 2	2240.1	2255.8	2247.96
LOC104913030	protein VAC14 homolog	2291.9	2203.2	2247.54
FLVCR2	feline leukemia virus subgroup C cellular receptor family, member 2	2332.1	2160.3	2246.20
CNIH1	cornichon family AMPA receptor auxiliary protein 1	2305.3	2186.8	2246.03
CLDND1	claudin domain containing 1	2170.1	2320.5	2245.33
LOC100539940	pumilio homolog 1	2180.2	2310.0	2245.09
PLOD1	procollagen-lysine, 2-oxoglutarate 5-dioxygenase 1	2000.1	2489.5	2244.79
ACVR1	activin A receptor, type I	2173.2	2314.2	2243.69
KIF13A	kinesin family member 13A	2191.6	2288.2	2239.88
PHKG1	phosphorylase kinase, gamma 1 (muscle)	2293.7	2185.6	2239.62
USP5	ubiquitin specific peptidase 5 (isopeptidase T)	2570.8	1907.2	2239.01
AIFM1	apoptosis-inducing factor, mitochondrion-associated, 1	2241.3	2234.3	2237.81
SHISA8	shisa family member 8	2284.5	2190.7	2237.61
LOC100546323	bromodomain containing 2	2312.1	2161.8	2236.98
VTA1	vesicle (multivesicular body) trafficking 1	2214.3	2258.4	2236.35
LOC104909293	eyes absent homolog 1	2142.3	2330.3	2236.32
PP2D1	protein phosphatase 2C-like domain containing 1	2191.6	2279.0	2235.30
NT5C2	5'-nucleotidase, cytosolic II	2223.9	2244.5	2234.18
ZRANB1	zinc finger, RAN-binding domain containing 1	2240.6	2227.5	2234.04
DAP3	death associated protein 3	2361.4	2103.0	2232.22
PHKA1	phosphorylase kinase, alpha 1 (muscle)	2254.8	2207.2	2231.00
SRPRB	signal recognition particle receptor, B subunit	2188.8	2270.3	2229.53
PPIG	peptidylprolyl isomerase G (cyclophilin G)	2204.9	2254.0	2229.48
LOC100542757	ubiquitin carboxyl-terminal hydrolase 22-A	2241.4	2216.9	2229.18
STRAP	serine/threonine kinase receptor associated protein	2205.3	2249.9	2227.58
C22H20orf24	chromosome 22 open reading frame, human C20orf24	2219.8	2230.5	2225.17
HDHD3	haloacid dehalogenase-like hydrolase domain containing 3	2299.5	2149.4	2224.46
AKIRIN2	akirin 2	2151.5	2293.2	2222.34
PCMT1	protein-L-isoaspartate (D-aspartate) O-methyltransferase	2299.4	2142.9	2221.12
U2SURP	U2 snRNP-associated SURP domain containing	2201.7	2239.6	2220.68
KIAA2013	KIAA2013 ortholog	2135.0	2297.2	2216.08
ANXA7	annexin A7	2174.1	2258.0	2216.04
RALGDS	ral guanine nucleotide dissociation stimulator	2206.6	2222.4	2214.46
UBQLN4	ubiquilin 4	2136.5	2289.3	2212.94
TRAPPC3	trafficking protein particle complex 3	2201.4	2223.7	2212.58
LOC104909369	uncharacterized LOC104909369	2238.5	2178.1	2208.32
ADCK3	aarF domain containing kinase 3	2158.5	2251.6	2205.08
C2H1orf198	chromosome 2 open reading frame, human C1orf198	2236.6	2171.5	2204.05
MOB1B	MOB kinase activator 1B	2194.8	2212.7	2203.74
EPN2	epsin 2	2144.8	2262.6	2203.72

NDUFB3	NADH dehydrogenase (ubiquinone) 1 beta subcomplex, 3, 12kDa	2237.4	2168.3	2202.86
RNPS1	RNA binding protein S1, serine-rich domain	2213.1	2192.4	2202.73
SLC16A1	solute carrier family 16 (monocarboxylate transporter), member 1	2278.6	2125.8	2202.19
IBTK	inhibitor of Bruton agammaglobulinemia tyrosine kinase	2118.9	2284.6	2201.73
DAZAP1	DAZ associated protein 1	2146.6	2254.6	2200.58
CISD1	CDGSH iron sulfur domain 1	2230.5	2158.6	2194.52
LOC100549470	isopentenyl-diphosphate Delta-isomerase 1-like	2283.2	2105.8	2194.47
LOC100538805	aflatoxin B1 aldehyde reductase member 2-like	2211.0	2177.1	2194.04
IK	IK cytokine, down-regulator of HLA II	2330.2	2056.7	2193.47
CKLF	chemokine-like factor	2191.8	2193.1	2192.45
C8H10orf12	chromosome 8 open reading frame, human C10orf12	2236.1	2148.0	2192.03
RARS	arginyl-tRNA synthetase	2220.0	2161.4	2190.74
MRPS15	mitochondrial ribosomal protein S15	2262.6	2117.5	2190.08
CHRND	cholinergic receptor, nicotinic, delta (muscle)	2224.4	2155.4	2189.90
KCTD20	potassium channel tetramerization domain containing 20	2200.0	2178.7	2189.38
PHF20	PHD finger protein 20	2188.3	2190.0	2189.14
AFAP1	actin filament associated protein 1	2092.8	2285.4	2189.07
TTC28	tetratricopeptide repeat domain 28	2128.6	2249.2	2188.87
VASN	vasorin	2281.7	2086.1	2183.92
API5	apoptosis inhibitor 5	2163.3	2204.4	2183.86
C14H9orf89	chromosome 14 open reading frame, human C9orf89	2141.1	2221.2	2181.15
TMEM165	transmembrane protein 165	2076.1	2285.3	2180.74
TMEM43	transmembrane protein 43	2108.8	2247.8	2178.30
ABTB1	ankyrin repeat and BTB (POZ) domain containing 1	2045.8	2302.6	2174.18
OLFML3	olfactomedin-like 3	2118.0	2229.8	2173.91
DCTN4	dynactin 4 (p62)	2234.7	2111.6	2173.17
LOC104912897	inositol hexakisphosphate and diphosphoinositol-pentakisphosphate kinase 1-like	2088.8	2251.0	2169.89
RAB23	RAB23, member RAS oncogene family	2019.4	2320.2	2169.81
CPT1A	carnitine palmitoyltransferase 1A (liver)	2037.1	2301.6	2169.35
LOC104910975	uncharacterized LOC104910975	2202.7	2131.8	2167.23
RIOK3	RIO kinase 3	2095.7	2231.8	2163.75
DNAJC10	DnaJ (Hsp40) homolog, subfamily C, member 10	2117.4	2204.0	2160.68
GABARAPL1	GABA(A) receptor-associated protein like 1	2158.3	2162.0	2160.14
PITX3	paired-like homeodomain 3	2267.5	2052.6	2160.07
POPDC3	popeye domain containing 3	1840.7	2479.1	2159.87
KDM1A	lysine (K)-specific demethylase 1A	2171.7	2147.0	2159.37
RRP12	ribosomal RNA processing 12 homolog (S. cerevisiae)	2262.0	2052.2	2157.13
REXO2	RNA exonuclease 2	2150.4	2162.8	2156.56
ABI2	abl-interactor 2	2069.1	2243.0	2156.03
LOC104912776	disintegrin and metalloproteinase domain-containing protein 10-like	2120.2	2190.3	2155.23
LOC100547354	uncharacterized LOC100547354	2150.3	2158.2	2154.27
SRP19	signal recognition particle 19kDa	2144.5	2163.3	2153.90
FOSL2	FOS-like antigen 2	2091.2	2214.8	2153.01
AHRR	aryl-hydrocarbon receptor repressor	2031.0	2270.6	2150.80
SRP9	signal recognition particle 9kDa	2125.4	2175.7	2150.57
NEXN	nexilin (F actin binding protein)	1959.0	2340.4	2149.70
LEPROT	leptin receptor overlapping transcript	2061.7	2237.6	2149.67
ADAM8	ADAM metallopeptidase domain 8	2094.6	2196.8	2145.74
CRTAP	cartilage associated protein	1972.7	2316.8	2144.74
LOC100550516	adenosine receptor A1	2010.4	2278.6	2144.51
LOC100543343	NAD-dependent protein deacetylase sirtuin-2-like	2214.3	2072.9	2143.60
GCN1L1	GCN1 general control of amino-acid synthesis 1-like 1 (yeast)	2161.6	2124.7	2143.17
TBCA	tubulin folding cofactor A	2163.6	2118.3	2140.96
ATP2B1	ATPase, Ca++ transporting, plasma membrane 1	2044.5	2235.8	2140.15
CNOT1	CCR4-NOT transcription complex, subunit 1	2169.0	2100.8	2134.86
EFTUD2	elongation factor Tu GTP binding domain containing 2	2288.6	1979.5	2134.02

MLF2	myeloid leukemia factor 2	2147.6	2117.0	2132.28
MINOS1	mitochondrial inner membrane organizing system 1	2138.5	2122.7	2130.58
LOC100545924	E3 SUMO-protein ligase RanBP2-like	2018.3	2241.0	2129.69
BTBD1	BTB (POZ) domain containing 1	2051.1	2207.7	2129.37
RAB21	RAB21, member RAS oncogene family	2076.1	2178.6	2127.36
SEC24C	SEC24 family member C	2051.8	2201.4	2126.58
GRPEL1	GrpE-like 1, mitochondrial (E. coli)	2202.5	2048.7	2125.57
ESYT2	extended synaptotagmin-like protein 2	2023.8	2223.8	2123.79
SDF2	stromal cell-derived factor 2	2049.2	2194.7	2121.94
NFYC	nuclear transcription factor Y, gamma	2132.2	2111.0	2121.63
TMEM258	transmembrane protein 258	2124.9	2117.5	2121.20
EIF4B	eukaryotic translation initiation factor 4B	2202.5	2034.3	2118.40
LOC104915862	methionine--tRNA ligase, cytoplasmic-like	2420.2	1815.6	2117.91
NDST1	N-deacetylase/N-sulfotransferase (heparan glucosaminyl) 1	2237.1	1998.1	2117.56
RPA2	replication protein A2, 32kDa	2147.9	2086.3	2117.09
WDTC1	WD and tetratricopeptide repeats 1	2101.9	2130.6	2116.24
PGAM5	phosphoglycerate mutase family member 5	2061.2	2170.5	2115.85
RCN2	reticulocalbin 2, EF-hand calcium binding domain	2071.3	2155.3	2113.26
LOC104916452	spliceosome RNA helicase DDX39B-like	2249.8	1973.4	2111.61
FAM13A	family with sequence similarity 13, member A	2046.2	2174.1	2110.11
TM9SF2	transmembrane 9 superfamily member 2	1955.9	2264.1	2109.99
LOC104913057	uncharacterized LOC104913057	2092.1	2127.3	2109.69
SYNPO	synaptopodin	2072.1	2145.7	2108.90
MAN1A2	mannosidase, alpha, class 1A, member 2	2085.0	2127.3	2106.16
SLC25A20	solute carrier family 25 (carnitine/acylcarnitine translocase), member 20	2054.6	2156.2	2105.38
ADSL	adenylosuccinate lyase	2099.5	2105.3	2102.40
CCNB2	cyclin B2	2085.8	2117.3	2101.53
NFAT5	nuclear factor of activated T-cells 5, tonicity-responsive	1994.6	2204.7	2099.67
LGMN	legumain	1975.0	2223.5	2099.25
LOC100550954	frizzled-2	2168.6	2027.9	2098.27
ATP5J2	ATP synthase, H+ transporting, mitochondrial Fo complex, subunit F2	2126.6	2067.4	2096.97
TMEM115	transmembrane protein 115	2129.3	2061.9	2095.57
DNTTIP2	deoxynucleotidyltransferase, terminal, interacting protein 2	2095.7	2090.3	2093.00
UBFD1	ubiquitin family domain containing 1	2106.7	2078.8	2092.77
GLRX3	glutaredoxin 3	2152.5	2032.2	2092.33
DLD	dihydrolipoamide dehydrogenase	2045.0	2136.8	2090.88
ERGIC1	endoplasmic reticulum-golgi intermediate compartment (ERGIC) 1	2022.6	2158.7	2090.68
PIGX	phosphatidylinositol glycan anchor biosynthesis, class X	2124.0	2057.1	2090.51
ASPN	asporin	1926.9	2251.0	2088.96
SREBF2	sterol regulatory element binding transcription factor 2	2224.5	1950.6	2087.55
SGCE	sarcoglycan, epsilon	1982.2	2191.8	2086.99
LMAN2	lectin, mannose-binding 2	2052.9	2120.1	2086.52
COPS3	COP9 signalosome subunit 3	2155.1	2017.9	2086.51
DNM1L	dynamin 1-like	1990.7	2180.7	2085.72
DHX15	DEAH (Asp-Glu-Ala-His) box helicase 15	2004.5	2166.7	2085.64
LOC100540358	protein SCAF11	2063.1	2106.9	2085.00
SYNE1	spectrin repeat containing, nuclear envelope 1	2006.5	2162.9	2084.71
LOC100548989	dysferlin-like	2070.1	2099.2	2084.67
SH3BGR	SH3 domain binding glutamate-rich protein	1979.4	2189.1	2084.26
AFF1	AF4/FMR2 family, member 1	2170.0	1998.0	2083.97
STK3	serine/threonine kinase 3	2014.1	2153.4	2083.73
USP25	ubiquitin specific peptidase 25	2024.5	2137.3	2080.93
WDFY1	WD repeat and FYVE domain containing 1	1973.5	2186.9	2080.19
USP14	ubiquitin specific peptidase 14 (tRNA-guanine transglycosylase)	2100.4	2058.5	2079.47
DNAJB12	DnaJ (Hsp40) homolog, subfamily B, member 12	2037.5	2118.8	2078.11
SMAD5	SMAD family member 5	1967.6	2188.3	2077.96

SFPQ	splicing factor proline/glutamine-rich	2103.0	2050.6	2076.80
DNAJB14	DnaJ (Hsp40) homolog, subfamily B, member 14	2051.4	2100.1	2075.75
LOC104915533	protein MANBAL-like	2071.6	2079.1	2075.37
COPS7A	COP9 signalosome subunit 7A	2072.5	2077.5	2074.99
WBP11	WW domain binding protein 11	2080.1	2068.4	2074.26
MRPL45	mitochondrial ribosomal protein L45	2152.5	1995.4	2073.97
LOC100548306	cytochrome b-c1 complex subunit 8	2168.6	1978.0	2073.31
SRP14	signal recognition particle 14kDa (homologous Alu RNA binding protein)	2101.5	2044.5	2072.98
TMEM263	transmembrane protein 263	2000.8	2144.2	2072.47
QRICH1	glutamine-rich 1	2051.5	2092.4	2071.95
LOC104912596	uncharacterized LOC104912596	2135.7	2006.8	2071.25
LOC100548946	collagen alpha-1(XII) chain	2028.3	2113.4	2070.84
AMOTL2	angiomotin like 2	2026.1	2107.9	2066.98
B4GALT5	UDP-Gal:betaGlcNAc beta 1,4- galactosyltransferase, polypeptide 5	1992.1	2140.7	2066.39
HMGCR	3-hydroxy-3-methylglutaryl-CoA reductase	2130.6	1999.0	2064.80
C1H12orf57	chromosome 1 open reading frame, human C12orf57	2058.8	2059.8	2059.31
PRIM1	primase, DNA, polypeptide 1 (49kDa)	2078.0	2039.9	2058.92
SLC25A24	solute carrier family 25 (mitochondrial carrier; phosphate carrier), member 24	2091.5	2025.7	2058.64
USP47	ubiquitin specific peptidase 47	2028.3	2087.4	2057.87
MARK3	MAP/microtubule affinity-regulating kinase 3	1975.8	2138.5	2057.16
NUDT21	nudix (nucleoside diphosphate linked moiety X)-type motif 21	2053.5	2057.7	2055.60
RAD23B	RAD23 homolog B (S. cerevisiae)	1971.8	2138.6	2055.20
SNX11	sorting nexin 11	2193.6	1916.3	2054.99
ADPRH	ADP-ribosylarginine hydrolase	2022.9	2085.2	2054.04
STK17B	serine/threonine kinase 17b	1930.8	2177.0	2053.90
CDC20	cell division cycle 20	2003.2	2103.6	2053.41
CCDC124	coiled-coil domain containing 124	2046.8	2058.0	2052.38
SECISBP2L	SECIS binding protein 2-like	2049.1	2054.7	2051.85
DIAPH2	diaphanous-related formin 2	1986.4	2115.5	2050.93
STRIP1	striatin interacting protein 1	2115.3	1982.0	2048.63
NAGA	N-acetylgalactosaminidase, alpha-	1912.8	2184.4	2048.61
MYO18B	myosin XVIIIB	2169.8	1924.0	2046.90
TMEM106B	transmembrane protein 106B	2015.4	2076.3	2045.87
CXXC5	CXXC finger protein 5	1985.8	2104.2	2045.01
FITM2	fat storage-inducing transmembrane protein 2	1893.1	2192.5	2042.80
LYPLA1	lysophospholipase I	2060.6	2021.9	2041.28
NUDT4	nudix (nucleoside diphosphate linked moiety X)-type motif 4	2018.9	2062.8	2040.82
SESTD1	SEC14 and spectrin domains 1	2028.0	2049.7	2038.87
THOC7	THO complex 7 homolog (Drosophila)	2025.3	2052.3	2038.83
ZNF335	zinc finger protein 335	2098.9	1977.5	2038.18
MORF4L1	mortality factor 4 like 1	1992.1	2080.1	2036.15
PCMTD1	protein-L-isoaspartate (D-aspartate) O-methyltransferase domain containing 1	1947.2	2121.1	2034.16
PPID	peptidylprolyl isomerase D	2100.4	1966.0	2033.19
TAGLN	transgelin	2090.9	1974.7	2032.84
ARL1	ADP-ribosylation factor-like 1	1909.3	2155.5	2032.37
FAM207A	family with sequence similarity 207, member A	2034.5	2029.8	2032.10
PCSK7	proprotein convertase subtilisin/kexin type 7	2078.3	1985.4	2031.84
CD81	CD81 molecule	2078.5	1984.3	2031.39
YPEL1	yippee-like 1 (Drosophila)	1881.3	2179.9	2030.61
PCBP4	poly(rC) binding protein 4	2190.6	1867.8	2029.22
UBE4A	ubiquitination factor E4A	2030.5	2027.0	2028.77
NET1	neuroepithelial cell transforming 1	1960.3	2095.5	2027.90
DNAJB4	DnaJ (Hsp40) homolog, subfamily B, member 4	1907.5	2148.1	2027.76
GLIPR2	GLI pathogenesis-related 2	2118.5	1933.6	2026.08

C1QTNF4	C1q and tumor necrosis factor related protein 4	1953.6	2094.2	2023.91
FAM135A	family with sequence similarity 135, member A	2048.4	1998.9	2023.66
EBNA1BP2	EBNA1 binding protein 2	2012.1	2033.1	2022.63
LAMTOR2	late endosomal/lysosomal adaptor, MAPK and MTOR activator 2	2117.6	1926.9	2022.27
VPS4B	vacuolar protein sorting 4 homolog B (S. cerevisiae)	2040.9	2002.4	2021.63
CELSR1	cadherin, EGF LAG seven-pass G-type receptor 1	1920.6	2122.5	2021.56
IGFBP7	insulin-like growth factor binding protein 7	1946.7	2096.2	2021.45
NARF	nuclear prelamin A recognition factor	1928.8	2114.0	2021.42
KPNA2	karyopherin alpha 2 (RAG cohort 1, importin alpha 1)	2157.0	1883.7	2020.35
SNRNP35	small nuclear ribonucleoprotein 35kDa (U11/U12)	1926.0	2113.9	2019.94
LOC104912091	type I inositol 1,4,5-trisphosphate 5-phosphatase-like	1929.6	2109.9	2019.76
TOR3A	torsin family 3, member A	1910.3	2128.8	2019.54
GABARAPL2	GABA(A) receptor-associated protein-like 2	1978.0	2058.9	2018.43
NUMA1	nuclear mitotic apparatus protein 1	2123.5	1913.0	2018.21
DRG2	developmentally regulated GTP binding protein 2	2040.3	1993.4	2016.86
STUB1	STIP1 homology and U-box containing protein 1, E3 ubiquitin protein ligase	2126.4	1903.9	2015.16
DIP2B	DIP2 disco-interacting protein 2 homolog B (Drosophila)	1903.7	2122.1	2012.89
THOC2	THO complex 2	1958.4	2066.2	2012.30
ID1	inhibitor of DNA binding 1, dominant negative helix-loop-helix protein	2139.4	1884.0	2011.73
SNAI2	snail family zinc finger 2	1839.8	2183.6	2011.66
LOC100549801	cytochrome b-c1 complex subunit 10	2002.1	2019.2	2010.69
ZDHHC9	zinc finger, DHHC-type containing 9	2115.7	1905.0	2010.34
MRPS17	mitochondrial ribosomal protein S17	2072.2	1946.8	2009.50
FHOD1	formin homology 2 domain containing 1	2168.6	1849.5	2009.01
SOGA1	suppressor of glucose, autophagy associated 1	2279.8	1737.2	2008.48
GBAS	glioblastoma amplified sequence	1991.8	2024.1	2007.93
LRPPRC	leucine-rich pentatricopeptide repeat containing	2040.6	1975.1	2007.86
MRPL9	mitochondrial ribosomal protein L9	2063.1	1949.7	2006.39
NOL9	nucleolar protein 9	2019.4	1993.2	2006.31
CLTCL1	clathrin, heavy chain-like 1	2073.8	1938.4	2006.10
PLEKHM2	pleckstrin homology domain containing, family M (with RUN domain) member 2	2233.2	1775.7	2004.47
DNAJA1	DnaJ (Hsp40) homolog, subfamily A, member 1	2005.7	2000.7	2003.21
MAP7D3	MAP7 domain containing 3	1911.4	2094.8	2003.10
YWHAB	tyrosine 3-monooxygenase/tryptophan 5-monooxygenase activation protein, beta	2027.9	1976.9	2002.43
PPP4R2	protein phosphatase 4, regulatory subunit 2	1967.3	2036.4	2001.86
PPM1M	protein phosphatase, Mg2+/Mn2+ dependent, 1M	1958.1	2043.4	2000.75
KCTD3	potassium channel tetramerization domain containing 3	1942.6	2058.2	2000.38
FBXW11	F-box and WD repeat domain containing 11	2009.9	1990.5	2000.22
NCBP2	nuclear cap binding protein subunit 2, 20kDa	2024.2	1973.5	1998.84
LOC100547124	PERQ amino acid-rich with GYF domain-containing protein 2	1941.1	2055.9	1998.50
NSFL1C	NSFL1 (p97) cofactor (p47)	1982.7	2014.0	1998.36
LOC100538796	probable E3 ubiquitin-protein ligase HERC1	1938.4	2056.9	1997.65
TNFAIP6	tumor necrosis factor, alpha-induced protein 6	1808.5	2186.0	1997.29
ABCF3	ATP-binding cassette, sub-family F (GCN20), member 3	2142.1	1850.2	1996.15
NRP2	neuropilin 2	1889.9	2099.5	1994.67
LOC100544619	nodal modulator 1	2040.9	1947.0	1993.94
CLPX	caseinolytic mitochondrial matrix peptidase chaperone subunit	1896.3	2090.9	1993.63
CTH	cystathionine gamma-lyase	2004.2	1982.3	1993.29
PYGL	phosphorylase, glycogen, liver	2074.5	1911.6	1993.02
LOC104913167	uncharacterized LOC104913167	2050.0	1934.5	1992.26
LOXL1	lysyl oxidase-like 1	1969.0	2013.4	1991.22
LOC104909823	WD40 repeat-containing protein SMU1-like	1976.9	2003.5	1990.17
CDCA7L	cell division cycle associated 7-like	1908.1	2070.8	1989.42
LOC100538357	cytochrome c oxidase assembly factor 5	1930.0	2048.2	1989.10

MRPL34	mitochondrial ribosomal protein L34	1995.9	1980.5	1988.20
PPP2R5D	protein phosphatase 2, regulatory subunit B', delta	2047.4	1923.1	1985.21
LOC104910711	uncharacterized LOC104910711	1950.6	2019.0	1984.79
RNF20	ring finger protein 20, E3 ubiquitin protein ligase	2012.1	1957.2	1984.68
JAG2	jagged 2	1914.4	2054.8	1984.62
MTA1	metastasis associated 1	1934.2	2032.1	1983.17
SETD3	SET domain containing 3	1949.5	2012.5	1981.01
MCTS1	malignant T cell amplified sequence 1	1974.6	1981.4	1978.00
MRPS12	mitochondrial ribosomal protein S12	2148.7	1803.3	1976.00
KPNA6	karyopherin alpha 6 (importin alpha 7)	1960.3	1986.6	1973.44
PITHD1	PITH (C-terminal proteasome-interacting domain of thioredoxin-like) domain containing 1	2044.2	1899.5	1971.84
UBTF	upstream binding transcription factor, RNA polymerase I	1942.4	2001.2	1971.75
CWC15	CWC15 spliceosome-associated protein	1960.7	1982.7	1971.70
TMEM159	transmembrane protein 159	1973.5	1967.5	1970.51
LOC100541134	uncharacterized LOC100541134	1954.1	1985.6	1969.87
POLR2C	polymerase (RNA) II (DNA directed) polypeptide C, 33kDa	2061.4	1878.2	1969.80
SNRPD1	small nuclear ribonucleoprotein D1 polypeptide 16kDa	2105.8	1832.5	1969.15
SCAMP2	secretory carrier membrane protein 2	1923.0	2011.1	1967.03
GSTA1.3	glutathione S-transferase alpha class A1.3	2030.2	1903.7	1966.97
MMP15	matrix metallopeptidase 15 (membrane-inserted)	2075.8	1858.0	1966.94
OST4	oligosaccharyltransferase 4 homolog (S. cerevisiae)	2160.7	1772.3	1966.49
ERP29	endoplasmic reticulum protein 29	1863.3	2069.7	1966.49
SYNRG	synergin, gamma	2005.9	1927.0	1966.42
DBX1	developing brain homeobox 1	1973.5	1957.4	1965.44
PRPS1	phosphoribosyl pyrophosphate synthetase 1	1986.0	1941.0	1963.51
CIAPIN1	cytokine induced apoptosis inhibitor 1	1976.1	1941.4	1958.73
IRF2BP2	interferon regulatory factor 2 binding protein 2	1929.5	1987.4	1958.47
TMEM9B	TMEM9 domain family, member B	1881.1	2035.1	1958.12
CDK2AP1	cyclin-dependent kinase 2 associated protein 1	1873.7	2038.0	1955.90
RPS6KA1	ribosomal protein S6 kinase, 90kDa, polypeptide 1	1980.7	1929.3	1955.02
TWF1	twinfilin actin-binding protein 1	1856.9	2052.6	1954.77
LOC100546622	survival of motor neuron protein-like	1965.7	1942.0	1953.83
TXNDC17	thioredoxin domain containing 17	1937.9	1967.0	1952.41
LOC100548827	tubulin alpha-8 chain	2049.1	1855.0	1952.05
MAGOH	mago-nashi homolog, proliferation-associated (Drosophila)	2007.9	1889.6	1948.71
MAPK1	mitogen-activated protein kinase 1	1843.6	2048.8	1946.18
LOC104916804	zinc finger protein 638-like	2152.5	1739.4	1945.94
SKA2	spindle and kinetochore associated complex subunit 2	1932.8	1959.0	1945.90
LOC100550170	putative RNA-binding protein Luc7-like 2	1884.0	2004.9	1944.46
PCBD1	pterin-4 alpha-carbinolamine dehydratase/dimerization cofactor of hepatocyte nuclear factor 1 alpha	1988.6	1897.5	1943.02
ERP44	endoplasmic reticulum protein 44	1866.5	2016.9	1941.72
NAA20	N(alpha)-acetyltransferase 20, NatB catalytic subunit	1904.0	1979.3	1941.63
TMEM57	transmembrane protein 57	1811.6	2069.6	1940.65
RAB11FIP2	RAB11 family interacting protein 2 (class I)	1937.9	1943.3	1940.58
ARHGEF6	Rac/Cdc42 guanine nucleotide exchange factor (GEF) 6	2021.9	1858.9	1940.39
THOC5	THO complex 5	2075.8	1804.7	1940.23
CYR61	cysteine-rich, angiogenic inducer, 61	2013.0	1866.1	1939.51
CHMP7	charged multivesicular body protein 7	2009.0	1867.5	1938.26
NPC2	Niemann-Pick disease, type C2	1904.2	1970.8	1937.48
FKBP8	FK506 binding protein 8, 38kDa	2067.6	1807.3	1937.45
CTSH	cathepsin H	1895.8	1973.4	1934.61
MURC	muscle-related coiled-coil protein	2094.8	1771.7	1933.24
DHX38	DEAH (Asp-Glu-Ala-His) box polypeptide 38	2023.7	1842.4	1933.00
ATP6V0D1	ATPase, H+ transporting, lysosomal 38kDa, V0 subunit d1	1903.0	1962.3	1932.68
CCAR1	cell division cycle and apoptosis regulator 1	1894.8	1970.5	1932.65
SPTB	spectrin, beta, erythrocytic	2038.1	1827.1	1932.61

HSPA4L	heat shock 70kDa protein 4-like	1909.1	1955.2	1932.13
SRP54	signal recognition particle 54kDa	1918.8	1945.2	1931.98
LOC100548711	selenoprotein T	1999.2	1864.3	1931.72
EIF1B	eukaryotic translation initiation factor 1B	1985.0	1876.6	1930.77
FRMD4B	FERM domain containing 4B	1793.4	2064.6	1929.00
RAB5B	RAB5B, member RAS oncogene family	2020.3	1835.3	1927.76
EMILIN2	elastin microfibril interfacer 2	1888.2	1964.9	1926.56
KIAA0195	KIAA0195 ortholog	1863.3	1988.7	1925.98
MTSS1L	metastasis suppressor 1-like	2018.2	1832.1	1925.14
CEP170B	centrosomal protein 170B	1824.7	2022.7	1923.67
LOC104911094	RNA polymerase-associated protein RTF1 homolog	1915.5	1926.9	1921.20
LOC100550794	transmembrane protein 14C-like	1832.1	2007.8	1919.96
NDUFA1	NADH dehydrogenase (ubiquinone) 1 alpha subcomplex, 1, 7.5kDa	2025.8	1810.4	1918.10
BAHCC1	BAH domain and coiled-coil containing 1	2021.2	1814.6	1917.90
KLHL24	kelch-like family member 24	1956.8	1879.0	1917.86
CBL	Cbl proto-oncogene, E3 ubiquitin protein ligase	1848.2	1986.4	1917.28
CCDC25	coiled-coil domain containing 25	1965.2	1868.2	1916.71
BCLAF1	BCL2-associated transcription factor 1	1915.1	1917.4	1916.25
PPP1R15B	protein phosphatase 1, regulatory subunit 15B	1901.8	1927.9	1914.82
DNAJB11	DnaJ (Hsp40) homolog, subfamily B, member 11	1935.0	1893.1	1914.06
WDR82	WD repeat domain 82	1873.2	1954.0	1913.60
LOC104916155	ubiquitin thioesterase OTUB1-like	2051.1	1769.5	1910.30
SMO	smoothened, frizzled class receptor	1990.8	1824.0	1907.40
ADAMTS14	ADAM metallopeptidase with thrombospondin type 1 motif, 14	1905.2	1906.6	1905.90
TNFRSF1A	tumor necrosis factor receptor superfamily, member 1A	1837.8	1972.1	1904.95
PPP2R4	protein phosphatase 2A activator, regulatory subunit 4	2005.9	1803.4	1904.66
UGGT1	UDP-glucose glycoprotein glucosyltransferase 1	1902.6	1901.8	1902.19
LOC100548498	pre-mRNA 3'-end-processing factor FIP1-like	1932.8	1868.5	1900.63
NMT1	N-myristoyltransferase 1	1981.2	1818.3	1899.78
GTPBP4	GTP binding protein 4	2003.8	1795.4	1899.60
SRFBP1	serum response factor binding protein 1	1683.9	2114.7	1899.28
TRAF7	TNF receptor-associated factor 7, E3 ubiquitin protein ligase	1961.9	1835.8	1898.86
GABPA	GA binding protein transcription factor, alpha subunit 60kDa	1898.9	1897.9	1898.42
SUFU	suppressor of fused homolog (Drosophila)	1905.5	1889.9	1897.70
METTL5	methyltransferase like 5	1833.9	1953.6	1893.72
COL17A1	collagen, type XVII, alpha 1	1843.8	1940.1	1891.95
MTDH	metadherin	1813.4	1966.6	1890.01
GCG	glucagon	1707.0	2072.0	1889.51
HK1	hexokinase 1	1825.1	1952.9	1889.01
COPS4	COP9 signalosome subunit 4	1914.8	1863.1	1888.96
GPM6B	glycoprotein M6B	1811.6	1965.8	1888.72
FRMD3	FERM domain containing 3	1780.0	1993.5	1886.74
ACOT11	acyl-CoA thioesterase 11	1910.8	1860.1	1885.47
ZC3H11A	zinc finger CCCH-type containing 11A	1815.2	1955.4	1885.29
MRPS33	mitochondrial ribosomal protein S33	1848.9	1915.2	1882.06
XPO6	exportin 6	1864.4	1897.5	1880.97
LGALS8	lectin, galactoside-binding, soluble, 8	1734.5	2027.4	1880.92
LOC104914041	exportin-2-like	1934.5	1825.9	1880.18
NDFIP2	Nedd4 family interacting protein 2	1874.0	1886.1	1880.03
EIF3J	eukaryotic translation initiation factor 3, subunit J	1838.3	1920.3	1879.32
PIGT	phosphatidylinositol glycan anchor biosynthesis, class T	1926.7	1829.1	1877.92
DNAJB5	DnaJ (Hsp40) homolog, subfamily B, member 5	1831.9	1922.8	1877.34
LRRFIP1	leucine rich repeat (in FLII) interacting protein 1	1786.4	1966.4	1876.40
NDUFV3	NADH dehydrogenase (ubiquinone) flavoprotein 3, 10kDa	1835.8	1915.3	1875.57
EGR1	early growth response 1	1927.2	1823.7	1875.48
CRKL	v-crk avian sarcoma virus CT10 oncogene homolog-like	1897.4	1853.1	1875.27
ANKMY2	ankyrin repeat and MYND domain containing 2	1743.7	2005.7	1874.72
SPIN1	spindlin 1	1853.8	1893.6	1873.69

DISP1	dispatched homolog 1 (Drosophila)	1826.3	1920.9	1873.59
MBTPS1	membrane-bound transcription factor peptidase, site 1	1779.5	1966.2	1872.86
LOC100550859	vacuolar protein sorting-associated protein 8 homolog	1920.6	1824.7	1872.63
RPIA	ribose 5-phosphate isomerase A	1880.2	1863.2	1871.74
KTN1	kinectin 1 (kinesin receptor)	1843.5	1900.0	1871.72
GNPAT	glyceronephosphate O-acyltransferase	1787.0	1954.4	1870.68
LOC100549369	ribosomal biogenesis protein LAS1L-like	1746.1	1994.9	1870.46
GP5	glycoprotein V (platelet)	1777.5	1962.7	1870.12
MVB12A	multivesicular body subunit 12A	1935.7	1803.0	1869.36
GNG5	guanine nucleotide binding protein (G protein), gamma 5	1872.7	1864.8	1868.74
SEC62	SEC62 homolog (S. cerevisiae)	1749.1	1987.2	1868.14
STT3B	STT3B, subunit of the oligosaccharyltransferase complex (catalytic)	1809.5	1923.9	1866.69
LOC104914462	uncharacterized LOC104914462	1843.0	1889.4	1866.24
SFSWAP	splicing factor, suppressor of white-apricot family	1826.8	1905.2	1865.99
USP1	ubiquitin specific peptidase 1	1859.1	1871.2	1865.14
ANXA4	annexin A4	1770.1	1957.8	1863.97
LDHC	lactate dehydrogenase C	1639.6	2086.8	1863.19
EFCAB14	EF-hand calcium binding domain 14	1893.3	1832.2	1862.73
LOC100544502	nuclear cap-binding protein subunit 2	1921.3	1803.9	1862.59
NDUFS4	NADH dehydrogenase (ubiquinone) Fe-S protein 4, 18kDa (NADH-coenzyme Q reductase)	1796.1	1928.9	1862.48
LRRC59	leucine rich repeat containing 59	1831.9	1885.0	1858.43
LOC104914173	CDP-diacylglycerol--serine O-phosphatidyltransferase	1814.7	1900.7	1857.69
HECTD4	HECT domain containing E3 ubiquitin protein ligase 4	1851.2	1863.8	1857.51
CHMP5	charged multivesicular body protein 5	1860.6	1850.2	1855.41
ASNS	asparagine synthetase (glutamine-hydrolyzing)	1905.1	1804.9	1854.99
EML4	echinoderm microtubule associated protein like 4	1837.3	1872.2	1854.73
GOLGA3	golgin A3	1755.4	1953.9	1854.61
MED31	mediator complex subunit 31	1752.1	1954.2	1853.15
SUCLG1	succinate-CoA ligase, alpha subunit	1925.2	1777.8	1851.49
CCDC43	coiled-coil domain containing 43	1790.3	1912.4	1851.33
PHF3	PHD finger protein 3	1702.2	1993.3	1847.73
DYRK2	dual-specificity tyrosine-(Y)-phosphorylation regulated kinase 2	1706.6	1988.1	1847.33
LOC100540620	uncharacterized LOC100540620	1685.1	2005.2	1845.14
KNOP1	lysine-rich nucleolar protein 1	1784.3	1904.4	1844.33
SEC22B	SEC22 vesicle trafficking protein homolog B (S. cerevisiae)	1870.3	1816.7	1843.47
RTFDC1	replication termination factor 2 domain containing 1	1856.9	1829.4	1843.12
RASGEF1B	RasGEF domain family, member 1B	1819.6	1865.9	1842.76
SVIL	supervillin	1758.0	1924.9	1841.42
SAMD4A	sterile alpha motif domain containing 4A	1879.3	1802.1	1840.73
LOC104911421	pituitary tumor-transforming gene 1 protein-interacting protein-like	1840.9	1840.4	1840.69
MINPP1	multiple inositol-polyphosphate phosphatase 1	1796.0	1881.4	1838.66
C1H12orf73	chromosome 1 open reading frame, human C12orf73	1765.0	1911.7	1838.35
LOC100541463	tumor necrosis factor ligand superfamily member 10-like	1797.6	1878.0	1837.77
TARDBP	TAR DNA binding protein	1847.2	1826.9	1837.06
CDCA4	cell division cycle associated 4	1809.5	1864.6	1837.05
TRAP1	TNF receptor-associated protein 1	1922.9	1749.9	1836.37
PTCD3	pentatricopeptide repeat domain 3	1807.8	1861.4	1834.58
RSRC2	arginine/serine-rich coiled-coil 2	1759.0	1910.0	1834.47
LOC100548251	cytochrome b5 type B	1894.8	1770.5	1832.68
SS18	synovial sarcoma translocation, chromosome 18	1801.0	1863.8	1832.40
EFR3B	EFR3 homolog B (S. cerevisiae)	1826.7	1837.4	1832.08
MAMDC2	MAM domain containing 2	1701.4	1959.9	1830.65
XPO5	exportin 5	1866.7	1792.8	1829.75
POLR1E	polymerase (RNA) I polypeptide E, 53kDa	1721.4	1934.9	1828.12
SMAP1	small ArfGAP 1	1763.5	1892.2	1827.83
LOC100543527	cytochrome c-type heme lyase	1831.3	1819.8	1825.53

LOC104912919	uncharacterized LOC104912919	1812.5	1835.2	1823.86
AAMP	angio-associated, migratory cell protein	1875.7	1770.8	1823.25
CHN2	chimerin 2	1844.3	1798.4	1821.34
EIF6	eukaryotic translation initiation factor 6	1838.4	1803.9	1821.13
SYNM	synemin, intermediate filament protein	1630.2	2012.0	1821.13
ACTR6	ARP6 actin-related protein 6 homolog (yeast)	1811.8	1828.4	1820.11
FKBP7	FK506 binding protein 7	1757.4	1882.8	1820.10
SCCPDH	saccharopine dehydrogenase (putative)	1778.5	1860.4	1819.46
HARS	histidyl-tRNA synthetase	1844.2	1793.7	1818.98
ANKRD10	ankyrin repeat domain 10	1769.6	1867.7	1818.65
CORO1B	coronin, actin binding protein, 1B	1949.4	1687.4	1818.43
SYNGR2	synaptogyrin 2	1904.4	1728.8	1816.59
TAB2	TGF-beta activated kinase 1/MAP3K7 binding protein 2	1770.0	1863.1	1816.54
TMEM123	transmembrane protein 123	1710.9	1918.1	1814.49
CRY1	cryptochrome circadian clock 1	1774.0	1854.3	1814.15
TRIOBP	TRIO and F-actin binding protein	1854.0	1774.1	1814.05
NIFK	nucleolar protein interacting with the FHA domain of MKI67	1877.7	1749.3	1813.50
FAM134A	family with sequence similarity 134, member A	1856.8	1768.9	1812.83
BTG2	BTG family, member 2	1841.1	1780.9	1811.01
WDR83OS	WD repeat domain 83 opposite strand	1970.7	1648.0	1809.36
LOC104914664	amino-terminal enhancer of split-like	1952.3	1665.8	1809.08
MLST8	MTOR associated protein, LST8 homolog (S. cerevisiae)	1838.5	1777.0	1807.75
OAZ2	ornithine decarboxylase antizyme 2	1828.9	1786.4	1807.64
IFNGR2	interferon gamma receptor 2 (interferon gamma transducer 1)	1758.4	1856.3	1807.34
CLCN3	chloride channel, voltage-sensitive 3	1720.8	1893.6	1807.18
GNL3	guanine nucleotide binding protein-like 3 (nucleolar)	1817.4	1794.8	1806.10
PACS2	phosphofurin acidic cluster sorting protein 2	1788.0	1823.4	1805.72
RER1	retention in endoplasmic reticulum sorting receptor 1	1720.9	1888.8	1804.84
NDUFS1	NADH dehydrogenase (ubiquinone) Fe-S protein 1, 75kDa (NADH-coenzyme Q reductase)	1830.7	1776.1	1803.39
GTF2B	general transcription factor IIB	1734.4	1871.6	1803.02
USP48	ubiquitin specific peptidase 48	1811.9	1793.7	1802.80
DYNLL2	dynein, light chain, LC8-type 2	1870.9	1734.0	1802.42
LOC104912731	uncharacterized LOC104912731	1757.4	1842.4	1799.87
LOC104912763	uncharacterized LOC104912763	1776.7	1822.3	1799.49
ALDH9A1	aldehyde dehydrogenase 9 family, member A1	1791.9	1805.0	1798.46
ERAP1	endoplasmic reticulum aminopeptidase 1	1730.4	1865.3	1797.85
LOC100547471	metastasis suppressor protein 1	1704.3	1887.2	1795.77
CUL1	cullin 1	1756.5	1832.5	1794.52
AFG3L2	AFG3-like AAA ATPase 2	1734.4	1854.1	1794.26
TAF13	TAF13 RNA polymerase II, TATA box binding protein (TBP)-associated factor, 18kDa	1818.5	1768.7	1793.58
MED29	mediator complex subunit 29	1848.6	1736.6	1792.60
PCNA	proliferating cell nuclear antigen	1900.9	1681.3	1791.13
SH3PXD2A	SH3 and PX domains 2A	1749.7	1832.3	1790.96
NDUFS7	NADH dehydrogenase (ubiquinone) Fe-S protein 7, 20kDa (NADH-coenzyme Q reductase)	1880.4	1700.4	1790.43
SSRP1	structure specific recognition protein 1	1932.9	1647.6	1790.24
IVNS1ABP	influenza virus NS1A binding protein	1796.1	1784.3	1790.20
CIDEA	cell death-inducing DFFA-like effector a	1627.0	1952.7	1789.82
LOC104915419	uncharacterized LOC104915419	1744.2	1832.5	1788.34
PDE6D	phosphodiesterase 6D, cGMP-specific, rod, delta	1804.6	1771.2	1787.87
LOC104914225	uncharacterized LOC104914225	1883.2	1692.2	1787.73
NSMAF	neutral sphingomyelinase (N-SMase) activation associated factor	1753.0	1822.4	1787.68
HNRNPK	heterogeneous nuclear ribonucleoprotein K	1767.2	1801.4	1784.29
FBXW5	F-box and WD repeat domain containing 5	1766.1	1802.0	1784.05
MRPL40	mitochondrial ribosomal protein L40	1838.5	1728.9	1783.67

PLEKHF1	pleckstrin homology domain containing, family F (with FYVE domain) member 1	1754.6	1812.4	1783.51
ACAT2	acetyl-CoA acetyltransferase 2	1845.4	1719.0	1782.17
MRPS7	mitochondrial ribosomal protein S7	1823.2	1740.4	1781.77
IMP3	IMP3, U3 small nucleolar ribonucleoprotein	1946.7	1615.6	1781.15
TCERG1	transcription elongation regulator 1	1760.5	1799.1	1779.82
PCNP	PEST proteolytic signal containing nuclear protein	1721.5	1837.9	1779.69
CSTA	cystatin A (stefin A)	1702.6	1856.6	1779.58
PNN	pinin, desmosome associated protein	1742.2	1816.0	1779.10
NFE2L2	nuclear factor, erythroid 2-like 2	1779.9	1776.4	1778.13
POLR2E	polymerase (RNA) II (DNA directed) polypeptide E, 25kDa	1858.2	1694.1	1776.17
GOLGA7	golgin A7	1826.2	1725.4	1775.78
IMPA2	inositol(myo)-1(or 4)-monophosphatase 2	1734.6	1815.7	1775.19
PES1	pescadillo ribosomal biogenesis factor 1	1857.6	1690.3	1773.95
NDUFV2	NADH dehydrogenase (ubiquinone) flavoprotein 2, 24kDa	1760.6	1785.6	1773.11
ROMO1	reactive oxygen species modulator 1	1811.6	1734.3	1772.95
SNX17	sorting nexin 17	1872.1	1669.3	1770.69
TP53BP1	tumor protein p53 binding protein 1	1750.5	1789.5	1770.02
LOC100545074	cullin-associated NEDD8-dissociated protein 1-like	1762.7	1771.8	1767.24
MVP	major vault protein	1916.4	1617.8	1767.12
SF3A1	splicing factor 3a, subunit 1, 120kDa	1753.0	1780.8	1766.91
KHDRBS3	KH domain containing, RNA binding, signal transduction associated 3	1768.4	1764.7	1766.57
LOC100550272	P protein	1702.0	1829.7	1765.84
LOC100546381	N-alpha-acetyltransferase 35, NatC auxiliary subunit	1771.0	1759.3	1765.11
KIF13B	kinesin family member 13B	1823.8	1699.3	1761.58
NXPH4	neurexophilin 4	1680.6	1840.0	1760.28
SLC30A5	solute carrier family 30 (zinc transporter), member 5	1664.9	1853.4	1759.15
CNKSR3	CNKSR family member 3	1727.5	1790.6	1759.07
RAB9A	RAB9A, member RAS oncogene family	1712.6	1805.4	1758.98
LOC100547098	ES1 protein homolog, mitochondrial-like	1774.1	1743.6	1758.82
LOC104915413	proline-rich protein 5-like	1711.9	1803.2	1757.54
PCID2	PCI domain containing 2	1730.0	1785.0	1757.45
COPS2	COP9 signalosome subunit 2	1743.5	1771.1	1757.29
LOC104911083	transmembrane protein 87A-like	1708.5	1804.2	1756.32
ISCU	iron-sulfur cluster assembly enzyme	1830.3	1680.8	1755.56
PSMD6	proteasome (prosome, macropain) 26S subunit, non-ATPase, 6	1717.3	1793.0	1755.16
PPP1CC	protein phosphatase 1, catalytic subunit, gamma isozyme	1775.8	1733.2	1754.50
ACTA2	actin, alpha 2, smooth muscle, aorta	1680.0	1828.5	1754.22
SUPT5H	suppressor of Ty 5 homolog (S. cerevisiae)	1829.7	1678.1	1753.94
FAM198B	family with sequence similarity 198, member B	1655.7	1850.4	1753.01
PSMD9	proteasome (prosome, macropain) 26S subunit, non-ATPase, 9	1761.0	1745.0	1752.98
LOC100548579	myosin-7B-like	1729.9	1774.7	1752.29
BCL2	B-cell CLL/lymphoma 2	1620.7	1883.9	1752.29
LOC100549990	phosphorylase b kinase regulatory subunit beta-like	1750.0	1754.6	1752.28
GPR144	G protein-coupled receptor 144	1783.2	1721.2	1752.19
NUP188	nucleoporin 188kDa	1764.3	1738.8	1751.58
KLHL40	kelch-like family member 40	1732.3	1767.0	1749.66
MEMO1	mediator of cell motility 1	1726.8	1771.2	1749.02
LOC100549101	mitochondrial amidoxime reducing component 2-like	1678.8	1818.6	1748.65
ZC3H7A	zinc finger CCCH-type containing 7A	1694.6	1802.4	1748.50
EIF2AK4	eukaryotic translation initiation factor 2 alpha kinase 4	1694.8	1801.9	1748.34
IMMT	inner membrane protein, mitochondrial	1723.7	1772.5	1748.11
LOC100539530	cyclin-dependent kinase 11B	1634.8	1860.8	1747.84
USPL1	ubiquitin specific peptidase like 1	1644.5	1849.6	1747.02
TXNIP	thioredoxin interacting protein	1838.3	1654.2	1746.27
LOC100547598	zinc finger SWIM domain-containing protein 3-like	1785.2	1707.3	1746.22
CPNE1	copine I	1673.8	1816.6	1745.20

DGKD	diacylglycerol kinase, delta 130kDa	1677.5	1812.1	1744.84
PKP4	plakophilin 4	1737.7	1752.0	1744.82
LOC104913393	uncharacterized LOC104913393	1690.6	1797.2	1743.88
PNISR	PNN-interacting serine/arginine-rich protein	1647.0	1840.5	1743.77
LOC100545344	myosin-7-like	1930.1	1554.4	1742.24
HEXA	hexosaminidase A (alpha polypeptide)	1745.8	1737.6	1741.72
MRPL41	mitochondrial ribosomal protein L41	1829.3	1650.5	1739.87
POFUT2	protein O-fucosyltransferase 2	1722.6	1757.1	1739.86
DNAJC18	DnaJ (Hsp40) homolog, subfamily C, member 18	1730.2	1748.6	1739.38
FYTTD1	forty-two-three domain containing 1	1689.6	1786.6	1738.12
CRIP1	cysteine-rich protein 1 (intestinal)	1829.5	1646.0	1737.75
DENND5A	DENN/MADD domain containing 5A	1673.5	1801.5	1737.49
CHD2	chromodomain helicase DNA binding protein 2	1677.7	1795.9	1736.81
MYPN	myopalladin	1558.4	1913.0	1735.72
UBIAD1	UbiA prenyltransferase domain containing 1	1657.7	1809.7	1733.70
LOC100538440	glutathione S-transferase theta-1-like	1675.9	1790.3	1733.10
TGFBI	transforming growth factor, beta-induced, 68kDa	1561.1	1902.9	1732.02
RCHY1	ring finger and CHY zinc finger domain containing 1, E3 ubiquitin protein ligase	1760.7	1703.3	1731.96
CPSF3	cleavage and polyadenylation specific factor 3, 73kDa	1781.9	1680.3	1731.07
SEL1L	sel-1 suppressor of lin-12-like (C. elegans)	1660.1	1801.6	1730.82
NKIRAS2	NFKB inhibitor interacting Ras-like 2	1861.1	1596.4	1728.74
UHMK1	U2AF homology motif (UHM) kinase 1	1779.9	1675.7	1727.78
PGLS	6-phosphogluconolactonase	1749.9	1705.2	1727.57
SPG21	spastic paraplegia 21 (autosomal recessive, Mast syndrome)	1709.8	1743.5	1726.64
MSTO1	misato 1, mitochondrial distribution and morphology regulator	1777.8	1674.1	1725.97
PI4KB	phosphatidylinositol 4-kinase, catalytic, beta	1895.7	1556.0	1725.88
STK25	serine/threonine kinase 25	1687.3	1761.1	1724.20
RETSAT	retinol saturase (all-trans-retinol 13,14-reductase)	1585.0	1860.9	1722.95
LOC104913411	TOM1-like protein 2	1724.1	1720.5	1722.32
UFD1L	ubiquitin fusion degradation 1 like (yeast)	1829.5	1612.1	1720.79
SPOPL	speckle-type POZ protein-like	1605.3	1832.7	1719.00
LOC104912356	roquin-1-like	1659.5	1776.2	1717.81
CHP1	calcineurin-like EF-hand protein 1	1632.2	1803.4	1717.80
LOC100540457	cas scaffolding protein family member 4-like	1624.5	1809.2	1716.84
TRMT1L	tRNA methyltransferase 1 homolog (S. cerevisiae)-like	1678.8	1754.2	1716.52
UBR5	ubiquitin protein ligase E3 component n-recognin 5	1669.7	1763.1	1716.36
MCMBP	minichromosome maintenance complex binding protein	1744.9	1687.2	1716.05
CTSK	cathepsin K	1635.9	1795.1	1715.49
LAMTOR3	late endosomal/lysosomal adaptor, MAPK and MTOR activator 3	1667.7	1763.1	1715.39
CPSF6	cleavage and polyadenylation specific factor 6, 68kDa	1737.7	1692.8	1715.23
SIM2	single-minded family bHLH transcription factor 2	1516.5	1912.3	1714.40
CA8	carbonic anhydrase VIII	1713.3	1715.1	1714.21
ST5	suppression of tumorigenicity 5	1704.3	1723.4	1713.85
DLG2	discs, large homolog 2 (Drosophila)	1738.5	1688.7	1713.58
ZEB1	zinc finger E-box binding homeobox 1	1631.0	1795.3	1713.14
LOC104911227	histone H1.01-like	1633.2	1791.7	1712.46
UBA3	ubiquitin-like modifier activating enzyme 3	1669.6	1753.2	1711.41
ZC3H15	zinc finger CCCH-type containing 15	1738.2	1683.7	1710.98
TRMT61A	tRNA methyltransferase 61A	1584.0	1836.6	1710.33
GNAI2	guanine nucleotide binding protein (G protein), alpha inhibiting activity polypeptide 2	1789.2	1628.3	1708.76
PDZD2	PDZ domain containing 2	1535.8	1877.1	1706.41
ATP2C1	ATPase, Ca++ transporting, type 2C, member 1	1644.8	1767.6	1706.21
MRPL37	mitochondrial ribosomal protein L37	1705.0	1707.1	1706.08
PFKL	phosphofructokinase, liver	1740.1	1670.4	1705.23
MKL1	megakaryoblastic leukemia (translocation) 1	1735.8	1672.5	1704.16
INTS2	integrator complex subunit 2	1845.6	1562.7	1704.15

SRGAP1	SLIT-ROBO Rho GTPase activating protein 1	1644.6	1762.0	1703.32
LOC100550978	iporin-like	1721.2	1685.3	1703.25
CSNK1D	casein kinase 1, delta	1649.9	1754.8	1702.36
SLIRP	SRA stem-loop interacting RNA binding protein	1713.6	1690.1	1701.89
SBF1	SET binding factor 1	1816.1	1585.5	1700.80
ANGEL1	angel homolog 1 (Drosophila)	1618.5	1783.0	1700.76
PHF21A	PHD finger protein 21A	1645.2	1755.2	1700.19
ABLIM3	actin binding LIM protein family, member 3	1711.7	1685.9	1698.82
EIF4E2	eukaryotic translation initiation factor 4E family member 2	1726.2	1670.8	1698.52
OSBPL11	oxysterol binding protein-like 11	1668.7	1727.9	1698.28
LOC104912283	uncharacterized LOC104912283	1641.8	1751.0	1696.42
LOC100545434	ras-related GTP-binding protein A	1678.0	1712.6	1695.30
UBA2	ubiquitin-like modifier activating enzyme 2	1704.5	1682.9	1693.70
RAC3	ras-related C3 botulinum toxin substrate 3 (rho family, small GTP binding protein Rac3)	1693.3	1692.6	1692.96
UCHL3	ubiquitin carboxyl-terminal esterase L3 (ubiquitin thiolesterase)	1679.2	1706.6	1692.91
ADSSL1	adenylosuccinate synthase like 1	1600.5	1784.9	1692.70
SF3A3	splicing factor 3a, subunit 3, 60kDa	1704.8	1677.7	1691.27
ACADM	acyl-CoA dehydrogenase, C-4 to C-12 straight chain	1622.7	1756.7	1689.71
TNKS	tankyrase, TRF1-interacting ankyrin-related ADP-ribose polymerase	1691.7	1687.4	1689.57
FH	fumarate hydratase	1663.7	1711.0	1687.38
CACNG1	calcium channel, voltage-dependent, gamma subunit 1	1744.7	1625.6	1685.19
PFDN2	prefoldin subunit 2	1731.0	1632.8	1681.90
MAPK8IP3	mitogen-activated protein kinase 8 interacting protein 3	1679.4	1684.4	1681.90
POLR2L	polymerase (RNA) II (DNA directed) polypeptide L, 7.6kDa	1702.8	1660.5	1681.66
ATOX1	antioxidant 1 copper chaperone	1714.2	1647.8	1680.98
NHSL1	NHS-like 1	1588.3	1773.3	1680.82
ATP5E	ATP synthase, H+ transporting, mitochondrial F1 complex, epsilon subunit	1739.9	1621.5	1680.70
EPDR1	ependymin related 1	1488.7	1870.8	1679.76
TMUB1	transmembrane and ubiquitin-like domain containing 1	1812.2	1546.5	1679.34
SFXN1	sideroflexin 1	1634.6	1717.6	1676.09
EFHC1	EF-hand domain (C-terminal) containing 1	1623.4	1728.6	1675.98
LAMA4	laminin, alpha 4	1620.2	1730.4	1675.32
PDCD4	programmed cell death 4 (neoplastic transformation inhibitor)	1653.2	1693.8	1673.52
LOC100546350	tripartite motif-containing protein 15-like	1704.7	1639.7	1672.21
ALDH4A1	aldehyde dehydrogenase 4 family, member A1	1705.2	1637.1	1671.13
NUP88	nucleoporin 88kDa	1632.4	1709.7	1671.08
CDIP1	cell death-inducing p53 target 1	1804.8	1531.7	1668.28
XXYLT1	xyloside xylosyltransferase 1	1685.1	1651.3	1668.19
UBTD2	ubiquitin domain containing 2	1666.2	1669.5	1667.83
DOHH	deoxyhypusine hydroxylase/monooxygenase	1738.1	1591.8	1665.00
PPP2R2D	protein phosphatase 2, regulatory subunit B, delta	1610.4	1719.4	1664.89
CSNK2A1	casein kinase 2, alpha 1 polypeptide	1604.1	1725.3	1664.69
NUCKS1	nuclear casein kinase and cyclin-dependent kinase substrate 1	1657.2	1671.7	1664.48
LOC104911767	T-box brain protein 1-like	1675.9	1651.8	1663.88
FZD9	frizzled class receptor 9	1958.1	1368.1	1663.10
ACOX1	acyl-CoA oxidase 1, palmitoyl	1677.9	1646.8	1662.39
SMEK2	SMEK homolog 2, suppressor of mek1 (Dictyostelium)	1668.7	1655.1	1661.94
CHMP6	charged multivesicular body protein 6	1647.3	1675.3	1661.27
PEX19	peroxisomal biogenesis factor 19	1841.0	1480.6	1660.78
RAE1	ribonucleic acid export 1	1666.1	1651.3	1658.69
ITFG3	integrin alpha FG-GAP repeat containing 3	1625.2	1690.6	1657.89
NOL7	nucleolar protein 7, 27kDa	1679.6	1636.1	1657.83
HIBCH	3-hydroxyisobutyryl-CoA hydrolase	1638.3	1676.2	1657.24
RSU1	Ras suppressor protein 1	1599.7	1713.0	1656.35
LOC100540942	death-associated protein kinase 2-like	1811.2	1500.9	1656.04

PCF11	PCF11 cleavage and polyadenylation factor subunit	1628.1	1683.2	1655.63
ADAM33	ADAM metallopeptidase domain 33	1664.5	1642.5	1653.52
SH3D19	SH3 domain containing 19	1567.2	1739.7	1653.43
GSPT1	G1 to S phase transition 1	1593.5	1710.6	1652.04
UBXN11	UBX domain protein 11	1705.7	1597.4	1651.53
AKAP9	A kinase (PRKA) anchor protein 9	1601.1	1699.6	1650.34
LOC100548105	mesothelin-like	1739.8	1558.0	1648.92
ECHDC2	enoyl CoA hydratase domain containing 2	1649.6	1647.2	1648.38
SAMM50	SAMM50 sorting and assembly machinery component	1661.6	1635.0	1648.29
GPR107	G protein-coupled receptor 107	1611.1	1685.5	1648.28
AKT1	v-akt murine thymoma viral oncogene homolog 1	1547.1	1749.1	1648.10
SEPT5	septin 5	1659.9	1632.3	1646.10
BABAM1	BRISC and BRCA1 A complex member 1	1669.7	1618.9	1644.30
UPF3B	UPF3 regulator of nonsense transcripts homolog B (yeast)	1598.5	1687.2	1642.84
TBC1D23	TBC1 domain family, member 23	1593.3	1688.3	1640.76
ARPC1B	actin related protein 2/3 complex, subunit 1B, 41kDa	1760.4	1517.2	1638.82
LOC100542208	motile sperm domain-containing protein 2	1612.7	1664.6	1638.68
RAF1	Raf-1 proto-oncogene, serine/threonine kinase	1596.8	1680.2	1638.50
ZFAND6	zinc finger, AN1-type domain 6	1520.9	1754.9	1637.88
PNRC2	proline-rich nuclear receptor coactivator 2	1595.7	1679.8	1637.76
TTLL5	tubulin tyrosine ligase-like family, member 5	1645.6	1628.6	1637.10
LOC104912483	uncharacterized LOC104912483	1630.4	1637.8	1634.07
DNAJC3	DnaJ (Hsp40) homolog, subfamily C, member 3	1538.8	1729.1	1633.95
GAPVD1	GTPase activating protein and VPS9 domains 1	1528.1	1739.1	1633.58
COG4	component of oligomeric golgi complex 4	1661.2	1604.2	1632.68
LOC100551160	sideroflexin-1-like	1674.3	1590.7	1632.48
ALDH3A2	aldehyde dehydrogenase 3 family, member A2	1711.5	1553.1	1632.27
RAP1A	RAP1A, member of RAS oncogene family	1536.7	1727.6	1632.15
LOC100541523	6-phosphofructo-2-kinase/fructose-2,6-bisphosphatase 4	1589.5	1671.8	1630.62
SLMAP	sarcolemma associated protein	1573.4	1687.6	1630.53
EMC3	ER membrane protein complex subunit 3	1615.5	1643.1	1629.27
TBC1D9B	TBC1 domain family, member 9B (with GRAM domain)	1569.5	1688.0	1628.75
IWS1	IWS1 homolog (S. cerevisiae)	1619.0	1638.2	1628.59
HECA	headcase homolog (Drosophila)	1642.1	1614.1	1628.13
IFFO1	intermediate filament family orphan 1	1659.8	1594.4	1627.08
LOC104910758	uncharacterized LOC104910758	1658.2	1594.4	1626.30
ZNF644	zinc finger protein 644	1528.2	1724.2	1626.19
LAPTM4A	lysosomal protein transmembrane 4 alpha	1592.0	1659.1	1625.53
SMG7	SMG7 nonsense mediated mRNA decay factor	1581.2	1669.7	1625.45
HIBADH	3-hydroxyisobutyrate dehydrogenase	1588.1	1660.6	1624.32
TMEM214	transmembrane protein 214	1685.5	1563.0	1624.25
MKKS	McKusick-Kaufman syndrome	1595.6	1652.8	1624.21
ST3GAL2	ST3 beta-galactoside alpha-2,3-sialyltransferase 2	1589.3	1658.1	1623.72
TMEM33	transmembrane protein 33	1647.3	1592.1	1619.73
PRPF38B	pre-mRNA processing factor 38B	1590.1	1645.6	1617.83
CCDC6	coiled-coil domain containing 6	1583.1	1649.2	1616.16
SMAD3	SMAD family member 3	1703.0	1529.3	1616.14
MRPS34	mitochondrial ribosomal protein S34	1657.5	1574.6	1616.07
ORC4	origin recognition complex, subunit 4	1599.4	1631.4	1615.42
SHC1	SHC (Src homology 2 domain containing) transforming protein 1	1764.3	1465.9	1615.15
LOC104913019	solute carrier family 12 member 4-like	1516.3	1713.4	1614.82
PEF1	penta-EF-hand domain containing 1	1654.1	1573.2	1613.66
FAM21C	family with sequence similarity 21, member C	1568.3	1657.6	1612.94
MED10	mediator complex subunit 10	1576.7	1648.6	1612.62
LOC104912856	uncharacterized LOC104912856	1577.4	1647.7	1612.59
SNRPB	small nuclear ribonucleoprotein polypeptides B and B1	1740.2	1484.6	1612.38
SMIM15	small integral membrane protein 15	1599.3	1623.8	1611.57
CCNH	cyclin H	1536.0	1686.8	1611.40

MDFIC	MyoD family inhibitor domain containing	1487.2	1735.5	1611.38
ZBTB1	zinc finger and BTB domain containing 1	1532.7	1689.2	1610.98
CHMP3	charged multivesicular body protein 3	1595.6	1626.0	1610.78
RABEPK	Rab9 effector protein with kelch motifs	1643.2	1578.4	1610.76
CKAP4	cytoskeleton-associated protein 4	1538.0	1682.7	1610.33
YIPF1	Yip1 domain family, member 1	1627.5	1593.1	1610.26
ENOX2	ecto-NOX disulfide-thiol exchanger 2	1543.6	1676.7	1610.16
HGS	hepatocyte growth factor-regulated tyrosine kinase substrate	1669.1	1550.7	1609.93
MANF	mesencephalic astrocyte-derived neurotrophic factor	1608.9	1610.8	1609.86
LOC100546553	transforming acidic coiled-coil-containing protein 3-like	1640.4	1578.1	1609.23
MICU2	mitochondrial calcium uptake 2	1523.4	1691.3	1607.38
PRPF40A	PRP40 pre-mRNA processing factor 40 homolog A (S. cerevisiae)	1579.3	1634.9	1607.08
SDE2	SDE2 telomere maintenance homolog (S. pombe)	1560.7	1647.8	1604.23
STX2	syntaxin 2	1569.7	1638.3	1603.98
CDKN2AIP	CDKN2A interacting protein	1633.0	1574.3	1603.63
KDM3A	lysine (K)-specific demethylase 3A	1536.3	1670.5	1603.39
RBMX	RNA binding motif protein, X-linked	1666.7	1540.0	1603.35
RNASEH1	ribonuclease H1	1621.8	1584.7	1603.27
SLC30A9	solute carrier family 30 (zinc transporter), member 9	1556.3	1649.8	1603.04
AHCYL2	adenosylhomocysteinase-like 2	1544.4	1658.8	1601.59
AKAP1	A kinase (PRKA) anchor protein 1	1649.1	1553.0	1601.04
COMMD7	COMM domain containing 7	1653.0	1548.2	1600.59
GGH	gamma-glutamyl hydrolase (conjugase, folylpolygammaglutamyl hydrolase)	1485.5	1715.7	1600.58
ANKRD11	ankyrin repeat domain 11	1594.6	1606.3	1600.49
HPRT1	hypoxanthine phosphoribosyltransferase 1	1682.0	1518.8	1600.43
MRPL16	mitochondrial ribosomal protein L16	1708.4	1491.9	1600.13
ADIPOR1	adiponectin receptor 1	1550.7	1648.6	1599.66
RSRP1	arginine/serine-rich protein 1	1472.6	1725.1	1598.83
TNIK	TRAF2 and NCK interacting kinase	1566.3	1629.4	1597.89
OARD1	O-acyl-ADP-ribose deacylase 1	1555.3	1638.6	1596.94
LOC100543412	mitochondrial import inner membrane translocase subunit Tim23	1684.9	1507.4	1596.13
MRPL12	mitochondrial ribosomal protein L12	1675.1	1515.9	1595.49
NSF	N-ethylmaleimide-sensitive factor	1580.3	1608.6	1594.45
ANXA11	annexin A11	1506.5	1682.0	1594.25
LOC100550667	uncharacterized LOC100550667	1549.9	1638.1	1593.97
NCBP1	nuclear cap binding protein subunit 1, 80kDa	1593.6	1593.9	1593.73
LOC100542173	nucleolar RNA helicase 2	1685.3	1501.4	1593.33
RBM22	RNA binding motif protein 22	1630.1	1554.8	1592.44
MAP1LC3A	microtubule-associated protein 1 light chain 3 alpha	1547.8	1636.6	1592.20
MYF5	myogenic factor 5	1706.2	1472.8	1589.53
SNRPG	small nuclear ribonucleoprotein polypeptide G	1641.8	1530.5	1586.14
SLC6A9	solute carrier family 6 (neurotransmitter transporter, glycine), member 9	1729.0	1443.3	1586.13
MAP3K14	mitogen-activated protein kinase kinase kinase 14	1561.1	1607.9	1584.50
PTPN11	protein tyrosine phosphatase, non-receptor type 11	1605.9	1561.5	1583.69
MFAP1	microfibrillar-associated protein 1	1585.1	1580.1	1582.63
UBE2F	ubiquitin-conjugating enzyme E2F (putative)	1607.1	1556.4	1581.78
ANAPC16	anaphase promoting complex subunit 16	1597.6	1559.9	1578.79
PDCD5	programmed cell death 5	1544.9	1612.6	1578.74
PAIP2	poly(A) binding protein interacting protein 2	1596.4	1559.5	1577.96
ARNTL2	aryl hydrocarbon receptor nuclear translocator-like 2	1467.6	1687.3	1577.47
EIF2B2	eukaryotic translation initiation factor 2B, subunit 2 beta, 39kDa	1543.2	1611.7	1577.46
COQ9	coenzyme Q9	1622.6	1531.8	1577.21
MYO1B	myosin IB	1364.8	1786.1	1575.45
WFDC1	WAP four-disulfide core domain 1	1522.1	1628.3	1575.18
RNF11	ring finger protein 11	1520.8	1627.6	1574.18
HELLS	helicase, lymphoid-specific	1577.3	1569.4	1573.33

LOC104913552	DEP domain-containing protein 5-like	1489.8	1654.1	1571.97
LOC100549425	vasculin-like protein 1	1542.2	1600.1	1571.13
LOC104911690	striated muscle-specific serine/threonine-protein kinase-like	1687.4	1451.8	1569.62
TAF12	TAF12 RNA polymerase II, TATA box binding protein (TBP)-associated factor, 20kDa	1563.6	1575.1	1569.36
HIGD1A	HIG1 hypoxia inducible domain family, member 1A	1652.5	1484.4	1568.43
DNAJC13	DnaJ (Hsp40) homolog, subfamily C, member 13	1565.8	1570.9	1568.34
CNPPD1	cyclin Pas1/PHO80 domain containing 1	1570.8	1564.5	1567.64
BAP1	BRCA1 associated protein-1 (ubiquitin carboxy-terminal hydrolase)	1657.3	1476.9	1567.11
LOC104914677	uncharacterized LOC104914677	1627.2	1503.8	1565.49
BTBD3	BTB (POZ) domain containing 3	1614.9	1515.6	1565.27
RUSC1	RUN and SH3 domain containing 1	1629.4	1498.8	1564.07
PGRMC2	progesterone receptor membrane component 2	1510.7	1617.3	1563.97
LOC104911748	uncharacterized LOC104911748	1581.3	1546.2	1563.78
LEMD2	LEM domain containing 2	1523.9	1603.1	1563.51
SSR4	signal sequence receptor, delta	1659.9	1466.2	1563.10
LOC100549728	N-chimaerin	1523.9	1601.2	1562.52
LOC100547061	neuron-specific protein family member 1	1504.8	1616.9	1560.85
PIM3	Pim-3 proto-oncogene, serine/threonine kinase	1506.6	1614.5	1560.52
ATAD1	ATPase family, AAA domain containing 1	1600.8	1518.5	1559.66
OXSR1	oxidative stress responsive 1	1536.2	1583.2	1559.66
MRPS25	mitochondrial ribosomal protein S25	1561.1	1557.9	1559.53
LMAN2L	lectin, mannose-binding 2-like	1606.1	1512.5	1559.31
OBSL1	obscurin-like 1	1632.7	1483.0	1557.87
DYRK1A	dual-specificity tyrosine-(Y)-phosphorylation regulated kinase 1A	1493.8	1621.5	1557.67
TRA2A	transformer 2 alpha homolog (Drosophila)	1440.9	1668.5	1554.72
SPOP	speckle-type POZ protein	1595.1	1513.6	1554.36
RALA	v-ral simian leukemia viral oncogene homolog A (ras related)	1560.0	1547.7	1553.85
USP19	ubiquitin specific peptidase 19	1698.6	1408.4	1553.49
NFKBIB	nuclear factor of kappa light polypeptide gene enhancer in B-cells inhibitor, beta	1576.5	1528.2	1552.31
PHACTR2	phosphatase and actin regulator 2	1392.2	1710.1	1551.14
WDR43	WD repeat domain 43	1567.6	1533.3	1550.46
HYPK	huntingtin interacting protein K	1558.1	1540.5	1549.30
MXRA5	matrix-remodelling associated 5	1725.2	1372.5	1548.87
RNF146	ring finger protein 146	1503.9	1593.0	1548.45
RAB8B	RAB8B, member RAS oncogene family	1518.1	1577.7	1547.93
LOC104911398	ATP-dependent 6-phosphofructokinase, platelet type-like	1505.7	1589.5	1547.62
LOC104909775	uncharacterized LOC104909775	1445.8	1649.0	1547.40
CACNG3	calcium channel, voltage-dependent, gamma subunit 3	1475.0	1616.3	1545.66
LOC104916715	RNA-binding protein FUS-like	1673.9	1416.7	1545.30
SMARCA5	SWI/SNF related, matrix associated, actin dependent regulator of chromatin, subfamily a, member 5	1502.2	1586.8	1544.50
PIK3R2	phosphoinositide-3-kinase, regulatory subunit 2 (beta)	1658.7	1428.4	1543.56
ACSS2	acyl-CoA synthetase short-chain family member 2	1582.6	1503.9	1543.27
MAPRE3	microtubule-associated protein, RP/EB family, member 3	1593.4	1490.9	1542.14
FZD6	frizzled class receptor 6	1488.7	1591.3	1540.00
SLC22A16	solute carrier family 22 (organic cation/carnitine transporter), member 16	1551.4	1528.3	1539.83
BRIX1	BRX1, biogenesis of ribosomes, homolog (S. cerevisiae)	1533.0	1546.3	1539.67
FARSB	phenylalanyl-tRNA synthetase, beta subunit	1642.5	1435.4	1538.99
SLC38A6	solute carrier family 38, member 6	1545.5	1532.4	1538.91
ITGAV	integrin, alpha V	1380.5	1695.5	1537.97
PGPEP1L	pyroglutamyl-peptidase I-like	1541.9	1532.6	1537.24
ITPK1	inositol-tetrakisphosphate 1-kinase	1527.3	1544.0	1535.65
RBX1	ring-box 1, E3 ubiquitin protein ligase	1626.2	1440.3	1533.25
C1H11orf87	chromosome 1 open reading frame, human C11orf87	1518.3	1544.6	1531.43

ASPH	aspartate beta-hydroxylase	1526.8	1535.5	1531.16
HTRA2	HtrA serine peptidase 2	1682.2	1379.8	1531.02
MRPS6	mitochondrial ribosomal protein S6	1466.2	1594.7	1530.45
TSG101	tumor susceptibility 101	1472.6	1587.0	1529.76
DVL1	dishevelled segment polarity protein 1	1536.7	1522.3	1529.52
SRP68	signal recognition particle 68kDa	1533.7	1524.7	1529.22
NASP	nuclear autoantigenic sperm protein (histone-binding)	1571.3	1486.9	1529.14
JUN	jun proto-oncogene	1520.3	1536.3	1528.28
DNTTIP1	deoxynucleotidyltransferase, terminal, interacting protein 1	1612.9	1442.2	1527.58
FOXJ2	forkhead box J2	1501.5	1552.7	1527.13
SS18L2	synovial sarcoma translocation gene on chromosome 18-like 2	1566.2	1487.2	1526.71
GTF2A2	general transcription factor IIA, 2, 12kDa	1533.2	1518.5	1525.87
TMEM230	transmembrane protein 230	1514.6	1536.3	1525.42
MFAP3	microfibrillar-associated protein 3	1473.8	1576.9	1525.36
PNO1	partner of NOB1 homolog (S. cerevisiae)	1553.0	1497.7	1525.34
RRP9	ribosomal RNA processing 9, small subunit (SSU) processome component, homolog (yeast)	1616.1	1433.9	1524.99
MRPS31	mitochondrial ribosomal protein S31	1579.5	1470.2	1524.86
PTPRA	protein tyrosine phosphatase, receptor type, A	1514.4	1534.1	1524.27
CREB3L1	cAMP responsive element binding protein 3-like 1	1455.9	1590.0	1522.95
ASCC1	activating signal cointegrator 1 complex subunit 1	1576.0	1469.5	1522.75
LOC100540739	cytochrome b5	1443.8	1600.7	1522.28
MRPS2	mitochondrial ribosomal protein S2	1579.2	1465.3	1522.25
PITPNA	phosphatidylinositol transfer protein, alpha	1511.6	1532.6	1522.10
TBC1D15	TBC1 domain family, member 15	1513.0	1531.1	1522.07
CCDC86	coiled-coil domain containing 86	1577.4	1466.6	1522.03
WTAP	Wilms tumor 1 associated protein	1460.9	1582.7	1521.77
EDEM3	ER degradation enhancer, mannosidase alpha-like 3	1578.1	1464.8	1521.47
PTPN9	protein tyrosine phosphatase, non-receptor type 9	1532.1	1509.9	1520.96
SLC4A2	solute carrier family 4 (anion exchanger), member 2	1764.8	1276.0	1520.40
ARSJ	arylsulfatase family, member J	1438.3	1598.6	1518.48
VSIG10	V-set and immunoglobulin domain containing 10	1423.1	1611.9	1517.50
LARS	leucyl-tRNA synthetase	1546.6	1487.2	1516.88
DDX17	DEAD (Asp-Glu-Ala-Asp) box helicase 17	1465.7	1567.7	1516.70
LOC104917189	uncharacterized LOC104917189	1655.3	1378.0	1516.66
NARS	asparaginyl-tRNA synthetase	1470.9	1560.9	1515.89
ZNF106	zinc finger protein 106	1423.9	1606.8	1515.37
MAK16	MAK16 homolog (S. cerevisiae)	1504.3	1524.5	1514.41
THUMPD3	THUMP domain containing 3	1555.2	1473.1	1514.18
DRG1	developmentally regulated GTP binding protein 1	1554.6	1473.4	1514.00
GTF3C1	general transcription factor IIIC, polypeptide 1, alpha 220kDa	1492.5	1533.5	1513.00
ERBB2	v-erb-b2 avian erythroblastic leukemia viral oncogene homolog 2	1628.3	1397.2	1512.76
SLAIN2	SLAIN motif family, member 2	1455.6	1567.5	1511.54
SEC11A	SEC11 homolog A (S. cerevisiae)	1476.2	1545.4	1510.82
CCNDBP1	cyclin D-type binding-protein 1	1503.1	1518.2	1510.63
SEC16A	SEC16 homolog A (S. cerevisiae)	1465.5	1553.7	1509.57
RASA1	RAS p21 protein activator (GTPase activating protein) 1	1466.3	1551.1	1508.68
PAIP1	poly(A) binding protein interacting protein 1	1526.9	1490.0	1508.42
ARFIP1	ADP-ribosylation factor interacting protein 1	1444.0	1572.4	1508.21
SENP5	SUMO1/sentrin specific peptidase 5	1511.8	1504.3	1508.03
LOC104912097	uncharacterized LOC104912097	1598.0	1414.0	1506.02
ATP6V1E1	ATPase, H+ transporting, lysosomal 31kDa, V1 subunit E1	1448.7	1562.4	1505.54
SRBD1	S1 RNA binding domain 1	1528.5	1481.8	1505.14
TOMM70A	translocase of outer mitochondrial membrane 70 homolog A (S. cerevisiae)	1431.7	1576.5	1504.14
SSB	Sjogren syndrome antigen B (autoantigen La)	1534.7	1472.6	1503.66
FKTN	fukutin	1496.1	1510.4	1503.23
LOC100551145	utrophin	1416.3	1588.4	1502.37

WNK1	WNK lysine deficient protein kinase 1	1555.1	1448.3	1501.70
PPP1R12A	protein phosphatase 1, regulatory subunit 12A	1426.3	1576.7	1501.50
LOC104915431	protein scribble homolog	1607.5	1393.7	1500.58
GOLPH3	golgi phosphoprotein 3 (coat-protein)	1416.8	1583.0	1499.91
RUVBL1	RuvB-like AAA ATPase 1	1530.7	1468.6	1499.66
MTG1	mitochondrial ribosome-associated GTPase 1	1600.6	1398.7	1499.65
CACNA1S	calcium channel, voltage-dependent, L type, alpha 1S subunit	1499.7	1498.6	1499.19
LOC100543464	cytosolic 5'-nucleotidase 1A-like	1411.8	1585.8	1498.83
CKAP2	cytoskeleton associated protein 2	1647.0	1349.1	1498.01
STRADA	STE20-related kinase adaptor alpha	1515.8	1477.1	1496.43
GCFC2	GC-rich sequence DNA-binding factor 2	1409.5	1577.4	1493.43
TM2D1	TM2 domain containing 1	1472.8	1512.7	1492.73
UBE3A	ubiquitin protein ligase E3A	1527.4	1457.4	1492.37
LOC104909464	reticulon-4-like	1379.8	1604.9	1492.37
LOC104911920	uncharacterized LOC104911920	1460.7	1523.0	1491.85
VLDLR	very low density lipoprotein receptor	1397.1	1585.6	1491.37
TRNT1	tRNA nucleotidyl transferase, CCA-adding, 1	1528.3	1453.7	1491.00
TAF1	TAF1 RNA polymerase II, TATA box binding protein (TBP)-associated factor, 250kDa	1521.1	1459.1	1490.09
ABAT	4-aminobutyrate aminotransferase	1367.7	1610.7	1489.17
RABEP1	rabaptin, RAB GTPase binding effector protein 1	1444.0	1530.3	1487.16
MTHFD2	methylenetetrahydrofolate dehydrogenase (NADP+ dependent) 2, methenyltetrahydrofolate cyclohydrolase	1562.6	1411.7	1487.16
PLIN2	perilipin 2	1429.4	1544.2	1486.76
MTURN	maturin, neural progenitor differentiation regulator homolog (Xenopus)	1422.1	1551.0	1486.58
ARL9	ADP-ribosylation factor-like 9	1329.4	1642.8	1486.11
CAV2	caveolin 2	1423.7	1547.0	1485.34
UBE3B	ubiquitin protein ligase E3B	1526.1	1444.2	1485.18
NDUFA8	NADH dehydrogenase (ubiquinone) 1 alpha subcomplex, 8, 19kDa	1541.5	1426.2	1483.83
NIPA2	non imprinted in Prader-Willi/Angelman syndrome 2	1404.3	1562.8	1483.54
KCNT1	potassium channel, subfamily T, member 1	1528.4	1437.9	1483.18
MRPL55	mitochondrial ribosomal protein L55	1558.5	1406.8	1482.67
PSMF1	proteasome (prosome, macropain) inhibitor subunit 1 (PI31)	1573.4	1391.0	1482.21
MARK1	MAP/microtubule affinity-regulating kinase 1	1388.3	1575.2	1481.76
GBA2	glucosidase, beta (bile acid) 2	1562.5	1398.1	1480.27
RFC5	replication factor C (activator 1) 5, 36.5kDa	1471.4	1489.0	1480.17
LOC104912583	phosphatidylinositol 4,5-bisphosphate 3-kinase catalytic subunit beta isoform-like	1379.8	1577.1	1478.45
LOC104913879	tyrosine-protein kinase BAZ1B-like	1453.6	1503.0	1478.32
BRI3BP	BRI3 binding protein	1503.1	1451.4	1477.24
LOC104912276	glycogen debranching enzyme-like	1378.1	1570.9	1474.48
ARNT	aryl hydrocarbon receptor nuclear translocator	1565.7	1381.5	1473.63
DDX23	DEAD (Asp-Glu-Ala-Asp) box polypeptide 23	1670.9	1275.3	1473.11
SNAP23	synaptosomal-associated protein, 23kDa	1382.4	1562.2	1472.31
PAPLN	papilin, proteoglycan-like sulfated glycoprotein	1394.0	1550.2	1472.14
MAR7	membrane-associated ring finger (C3HC4) 7, E3 ubiquitin protein ligase	1430.4	1513.6	1471.97
TSR1	TSR1, 20S rRNA accumulation, homolog (S. cerevisiae)	1529.5	1412.9	1471.19
ACTR3B	ARP3 actin-related protein 3 homolog B (yeast)	1464.1	1477.6	1470.88
PER3	period circadian clock 3	1267.4	1673.4	1470.41
LOC104914706	PWWP domain-containing protein MUM1	1533.5	1406.6	1470.03
PLXNB2	plexin B2	1394.2	1545.7	1469.95
DYNC1LI1	dynein, cytoplasmic 1, light intermediate chain 1	1437.1	1502.7	1469.91
ARHGAP21	Rho GTPase activating protein 21	1409.8	1529.9	1469.82
WIPI2	WD repeat domain, phosphoinositide interacting 2	1472.6	1466.0	1469.29
ENSA	endosulfine alpha	1521.8	1416.3	1469.09
LOC100550906	putative methyltransferase DDB_G0268948	1584.2	1352.9	1468.56

WDR12	WD repeat domain 12	1448.6	1487.3	1467.92
ITGB1BP2	integrin beta 1 binding protein (melusin) 2	1489.2	1446.6	1467.91
MRPS21	mitochondrial ribosomal protein S21	1548.8	1385.7	1467.28
LOC104915988	UV excision repair protein RAD23 homolog A-like	1560.1	1372.8	1466.42
AP3B1	adaptor-related protein complex 3, beta 1 subunit	1426.2	1505.6	1465.88
AKAP8	A kinase (PRKA) anchor protein 8	1561.0	1368.5	1464.75
ZCRB1	zinc finger CCHC-type and RNA binding motif 1	1467.4	1460.8	1464.12
SUSD6	sushi domain containing 6	1399.4	1527.6	1463.50
SEC24D	SEC24 family member D	1398.1	1526.4	1462.28
HMOX1	heme oxygenase (decycling) 1	1499.0	1424.5	1461.71
SLMO1	slowmo homolog 1 (Drosophila)	1496.1	1426.9	1461.51
TXNRD3	thioredoxin reductase 3	1480.0	1440.1	1460.04
LOC100539860	multifunctional protein ADE2	1547.9	1371.0	1459.49
LOC104913821	uncharacterized LOC104913821	1399.2	1518.8	1459.02
LOC100551027	importin subunit beta-1-like	1529.5	1388.3	1458.88
LOC100543297	tubulin beta-4B chain-like	1475.4	1442.1	1458.74
KDM5B	lysine (K)-specific demethylase 5B	1421.7	1494.8	1458.27
UBE2V2	ubiquitin-conjugating enzyme E2 variant 2	1445.2	1469.3	1457.28
LOC104915873	uncharacterized LOC104915873	1418.9	1494.3	1456.57
RPF2	ribosome production factor 2 homolog (S. cerevisiae)	1473.9	1438.4	1456.16
ZHX1	zinc fingers and homeoboxes 1	1415.5	1496.0	1455.76
MCM6	minichromosome maintenance complex component 6	1431.3	1479.7	1455.50
MRPL14	mitochondrial ribosomal protein L14	1405.9	1504.8	1455.38
METRNL	meteorin, glial cell differentiation regulator-like	1486.4	1423.7	1455.02
TNFAIP8L3	tumor necrosis factor, alpha-induced protein 8-like 3	1529.9	1378.7	1454.31
RAB33B	RAB33B, member RAS oncogene family	1460.5	1448.1	1454.28
LOC104914176	HMG box-containing protein 1-like	1355.5	1551.4	1453.45
TARS	threonyl-tRNA synthetase	1477.5	1428.1	1452.78
ELOVL5	ELOVL fatty acid elongase 5	1501.0	1404.2	1452.60
PSMD5	proteasome (prosome, macropain) 26S subunit, non-ATPase, 5	1493.8	1410.8	1452.29
OFD1	oral-facial-digital syndrome 1	1404.2	1497.6	1450.90
TMLHE	trimethyllysine hydroxylase, epsilon	1349.9	1550.3	1450.10
PHAX	phosphorylated adaptor for RNA export	1427.8	1471.6	1449.72
DNAJC2	DnaJ (Hsp40) homolog, subfamily C, member 2	1459.0	1440.4	1449.69
LOC100543106	schwannomin-interacting protein 1	1461.7	1436.6	1449.15
RPS6KA3	ribosomal protein S6 kinase, 90kDa, polypeptide 3	1427.1	1470.9	1448.99
OBSCN	obscurin, cytoskeletal calmodulin and titin-interacting RhoGEF	1381.6	1513.7	1447.65
IKBKAP	inhibitor of kappa light polypeptide gene enhancer in B-cells, kinase complex-associated protein	1456.9	1436.7	1446.79
NCK2	NCK adaptor protein 2	1431.5	1460.8	1446.13
SLC25A28	solute carrier family 25 (mitochondrial iron transporter), member 28	1446.7	1444.9	1445.80
SLC40A1	solute carrier family 40 (iron-regulated transporter), member 1	1560.7	1329.2	1444.93
KIAA0368	KIAA0368 ortholog	1450.1	1438.0	1444.04
LRP12	low density lipoprotein receptor-related protein 12	1406.2	1480.3	1443.28
LOC100542752	tight junction protein ZO-1-like	1435.5	1449.6	1442.53
TOP2A	topoisomerase (DNA) II alpha 170kDa	1606.8	1277.9	1442.31
ACBD3	acyl-CoA binding domain containing 3	1419.7	1462.8	1441.23
LAMTOR5	late endosomal/lysosomal adaptor, MAPK and MTOR activator 5	1457.4	1423.3	1440.34
CTSB	cathepsin B	1388.1	1492.3	1440.16
LOC104916733	uncharacterized LOC104916733	1498.0	1381.9	1439.93
PGP	phosphoglycolate phosphatase	1424.6	1455.2	1439.87
TSC2	tuberous sclerosis 2	1433.4	1444.2	1438.81
LOC100539152	fibronectin type-III domain-containing protein 3a-like	1456.1	1421.2	1438.67
EYA3	EYA transcriptional coactivator and phosphatase 3	1453.0	1424.1	1438.59
XPO7	exportin 7	1497.9	1375.1	1436.51
SURF6	surfeit 6	1499.7	1373.2	1436.48
ISY1	ISY1 splicing factor homolog (S. cerevisiae)	1416.1	1456.0	1436.03

SNAI1	snail family zinc finger 1	1393.4	1476.4	1434.87
TNPO3	transportin 3	1490.1	1378.6	1434.36
GMPPB	GDP-mannose pyrophosphorylase B	1421.6	1446.3	1433.94
DGCR2	DiGeorge syndrome critical region gene 2	1449.0	1417.4	1433.19
C1QTNF3	C1q and tumor necrosis factor related protein 3	1390.0	1476.2	1433.10
TMEM131	transmembrane protein 131	1373.7	1492.3	1433.03
PICALM	phosphatidylinositol binding clathrin assembly protein	1399.4	1465.6	1432.52
CDK13	cyclin-dependent kinase 13	1376.7	1486.1	1431.41
OSBP	oxysterol binding protein	1627.1	1235.3	1431.19
LOC100550313	interleukin-6 receptor subunit beta-like	1497.9	1363.3	1430.64
AAAS	achalasia, adrenocortical insufficiency, alacrimia	1597.7	1263.2	1430.44
MED6	mediator complex subunit 6	1428.7	1432.0	1430.31
NOP2	NOP2 nucleolar protein	1472.5	1388.0	1430.26
ATP6V1A	ATPase, H+ transporting, lysosomal 70kDa, V1 subunit A	1402.8	1456.4	1429.61
ASAH1	N-acylsphingosine amidohydrolase (acid ceramidase) 1	1304.1	1554.5	1429.30
HMOX2	heme oxygenase (decycling) 2	1523.1	1333.5	1428.32
GOPC	golgi-associated PDZ and coiled-coil motif containing	1397.3	1456.7	1426.99
CSTF2	cleavage stimulation factor, 3' pre-RNA, subunit 2, 64kDa	1473.0	1379.9	1426.44
SQSTM1	sequestosome 1	1405.6	1447.1	1426.33
MAP4K4	mitogen-activated protein kinase kinase kinase kinase 4	1472.8	1378.8	1425.78
PAN3	PAN3 poly(A) specific ribonuclease subunit	1346.9	1503.6	1425.25
LOC100544306	protein HIRA	1471.4	1378.6	1424.97
CHST14	carbohydrate (N-acetylgalactosamine 4-0) sulfotransferase 14	1360.2	1487.7	1423.93
KLHL41	kelch-like family member 41	1393.1	1453.2	1423.17
TBRG4	transforming growth factor beta regulator 4	1545.5	1300.6	1423.02
ARMC6	armadillo repeat containing 6	1385.9	1459.0	1422.41
SMYD2	SET and MYND domain containing 2	1394.9	1448.6	1421.73
AIMP2	aminoacyl tRNA synthetase complex-interacting multifunctional protein 2	1427.1	1415.7	1421.41
HBS1L	HBS1-like translational GTPase	1386.4	1456.0	1421.23
ERO1L	ERO1-like (S. cerevisiae)	1336.4	1504.6	1420.52
USP33	ubiquitin specific peptidase 33	1360.5	1480.1	1420.28
FAM96B	family with sequence similarity 96, member B	1398.2	1438.5	1418.35
ATP11C	ATPase, class VI, type 11C	1381.0	1453.2	1417.08
PMPCB	peptidase (mitochondrial processing) beta	1421.1	1412.3	1416.70
LYSMD2	LysM, putative peptidoglycan-binding, domain containing 2	1346.0	1484.9	1415.48
NIP7	NIP7, nucleolar pre-rRNA processing protein	1501.8	1328.2	1415.00
CALML4	calmodulin-like 4	1409.6	1420.3	1414.99
PHPT1	phosphohistidine phosphatase 1	1466.0	1358.2	1412.10
SPCS1	signal peptidase complex subunit 1 homolog (S. cerevisiae)	1446.8	1374.5	1410.64
ARFGAP2	ADP-ribosylation factor GTPase activating protein 2	1463.2	1356.9	1410.04
TLL1	tolloid-like 1	1307.8	1510.6	1409.20
RRBP1	ribosome binding protein 1	1348.2	1469.8	1408.97
ARMC7	armadillo repeat containing 7	1405.3	1411.6	1408.45
USP10	ubiquitin specific peptidase 10	1359.5	1456.8	1408.13
ATRAID	all-trans retinoic acid-induced differentiation factor	1545.2	1270.3	1407.75
LOC104913636	intercellular adhesion molecule 1-like	1437.2	1377.8	1407.52
RFC4	replication factor C (activator 1) 4, 37kDa	1492.1	1322.9	1407.48
MRPS26	mitochondrial ribosomal protein S26	1486.9	1327.0	1406.91
LOC100542830	cytochrome c oxidase subunit 7A-related protein, mitochondrial	1330.6	1483.1	1406.85
LOC104913908	uncharacterized LOC104913908	1339.2	1473.5	1406.32
SNX7	sorting nexin 7	1346.8	1464.8	1405.80
SF3B6	splicing factor 3b, subunit 6, 14kDa	1441.3	1369.0	1405.14
KIAA1279	KIAA1279 ortholog	1292.9	1517.1	1404.98
USP28	ubiquitin specific peptidase 28	1326.9	1482.7	1404.81
SALL4	spalt-like transcription factor 4	1444.2	1364.3	1404.25
USP36	ubiquitin specific peptidase 36	1485.5	1321.9	1403.71
COMTD1	catechol-O-methyltransferase domain containing 1	1449.4	1357.7	1403.51

SUPT4H1	suppressor of Ty 4 homolog 1 (S. cerevisiae)	1444.6	1362.2	1403.37
MAPK6	mitogen-activated protein kinase 6	1286.4	1520.0	1403.22
TTC39A	tetratricopeptide repeat domain 39A	1335.9	1470.4	1403.14
PFDN4	prefoldin subunit 4	1401.5	1404.8	1403.13
MRPS16	mitochondrial ribosomal protein S16	1492.7	1313.2	1402.93
RB1CC1	RB1-inducible coiled-coil 1	1327.5	1477.7	1402.58
PBRM1	polybromo 1	1424.8	1380.1	1402.44
LOC100548072	transmembrane protein 110-like	1389.9	1414.4	1402.13
PGD	phosphogluconate dehydrogenase	1436.9	1365.0	1400.93
UBL7	ubiquitin-like 7	1450.1	1351.5	1400.79
GET4	golgi to ER traffic protein 4 homolog (S. cerevisiae)	1376.6	1423.2	1399.92
ABCA3	ATP-binding cassette, sub-family A (ABC1), member 3	1559.4	1240.0	1399.69
HES1	hes family bHLH transcription factor 1	1452.1	1346.9	1399.49
CDK1	cyclin-dependent kinase 1	1532.7	1265.7	1399.21
SIDT2	SID1 transmembrane family, member 2	1470.0	1327.0	1398.52
LOC100549138	importin-9	1444.0	1350.3	1397.14
SNX2	sorting nexin 2	1347.4	1445.4	1396.41
CTNNBIP1	catenin, beta interacting protein 1	1390.8	1398.1	1394.43
SCOC	short coiled-coil protein	1388.6	1399.8	1394.21
LIG3	ligase III, DNA, ATP-dependent	1438.3	1348.7	1393.49
EIF2B5	eukaryotic translation initiation factor 2B, subunit 5 epsilon, 82kDa	1407.7	1378.5	1393.09
VMA21	VMA21 vacuolar H+-ATPase homolog (S. cerevisiae)	1398.9	1387.1	1393.01
SMARCB1	SWI/SNF related, matrix associated, actin dependent regulator of chromatin, subfamily b, member 1	1363.2	1422.4	1392.77
ZPR1	ZPR1 zinc finger	1405.6	1379.6	1392.57
PREP	prolyl endopeptidase	1396.7	1386.7	1391.71
PEX6	peroxisomal biogenesis factor 6	1436.3	1346.9	1391.58
SMG1	SMG1 phosphatidylinositol 3-kinase-related kinase	1338.6	1444.4	1391.52
RNF114	ring finger protein 114	1389.9	1392.8	1391.34
PRRC2C	proline-rich coiled-coil 2C	1318.5	1464.1	1391.32
UXS1	UDP-glucuronate decarboxylase 1	1338.7	1443.2	1390.99
LOC100545208	RING finger protein 151-like	1339.9	1442.0	1390.95
ORMDL2	ORMDL sphingolipid biosynthesis regulator 2	1415.6	1365.5	1390.53
IFT52	intraflagellar transport 52	1366.2	1414.7	1390.44
CHD7	chromodomain helicase DNA binding protein 7	1293.5	1485.2	1389.38
FKBP1B	FK506 binding protein 1B, 12.6 kDa	1321.9	1455.7	1388.78
DENR	density-regulated protein	1452.9	1323.9	1388.38
DPY19L1	dpy-19-like 1 (C. elegans)	1370.4	1404.9	1387.64
LUC7L3	LUC7-like 3 (S. cerevisiae)	1372.4	1400.5	1386.44
CCDC90B	coiled-coil domain containing 90B	1362.0	1410.7	1386.35
LOC104912179	transmembrane protein 182-like	1408.3	1362.9	1385.57
NXT2	nuclear transport factor 2-like export factor 2	1370.3	1399.5	1384.90
LOC104909725	uncharacterized LOC104909725	1416.7	1351.9	1384.28
PCGF3	polycomb group ring finger 3	1273.1	1494.7	1383.89
ZFR	zinc finger RNA binding protein	1312.3	1454.8	1383.53
AARSD1	alanyl-tRNA synthetase domain containing 1	1364.9	1402.0	1383.45
LOC104915722	chromosome unknown open reading frame, human C19orf43	1414.6	1348.9	1381.74
ARHGAP24	Rho GTPase activating protein 24	1265.2	1497.7	1381.44
ARIH2	ariadne RBR E3 ubiquitin protein ligase 2	1332.5	1429.5	1381.02
YY1	YY1 transcription factor	1335.7	1425.3	1380.46
LOC104915463	dual specificity testis-specific protein kinase 1-like	1387.8	1372.8	1380.31
MOCS1	molybdenum cofactor synthesis 1	1396.5	1364.0	1380.25
TFAM	transcription factor A, mitochondrial	1470.6	1289.8	1380.18
HDDC2	HD domain containing 2	1363.2	1395.3	1379.25
UBE2M	ubiquitin-conjugating enzyme E2M	1396.2	1361.1	1378.65
AGFG1	ArfGAP with FG repeats 1	1343.1	1413.8	1378.41
YIPF6	Yip1 domain family, member 6	1357.0	1399.0	1378.03
PAXBP1	PAX3 and PAX7 binding protein 1	1362.0	1393.9	1377.94

RNF41	ring finger protein 41, E3 ubiquitin protein ligase	1450.5	1305.1	1377.80
RAB12	RAB12, member RAS oncogene family	1297.7	1457.3	1377.50
LOC100546353	dynein heavy chain 7, axonemal	1273.4	1481.0	1377.22
HPCAL1	hippocalcin-like 1	1328.3	1423.8	1376.06
LOC100544143	single-stranded DNA-binding protein 3	1380.8	1370.5	1375.67
ANLN	anillin, actin binding protein	1496.8	1252.4	1374.60
UCHL5	ubiquitin carboxyl-terminal hydrolase L5	1352.2	1396.8	1374.48
NUP50	nucleoporin 50kDa	1351.9	1396.7	1374.32
GON4L	gon-4-like (C. elegans)	1393.7	1354.9	1374.31
AMFR	autocrine motility factor receptor, E3 ubiquitin protein ligase	1375.8	1372.3	1374.05
STIM1	stromal interaction molecule 1	1434.2	1312.5	1373.35
LAP3	leucine aminopeptidase 3	1345.3	1401.4	1373.34
FAM192A	family with sequence similarity 192, member A	1446.9	1299.5	1373.20
SACM1L	SAC1 suppressor of actin mutations 1-like (yeast)	1342.0	1403.2	1372.57
PHF12	PHD finger protein 12	1304.2	1440.2	1372.20
GSK3B	glycogen synthase kinase 3 beta	1344.3	1399.9	1372.14
BUB3	BUB3 mitotic checkpoint protein	1422.3	1320.3	1371.30
NDEL1	nudE neurodevelopment protein 1-like 1	1426.6	1315.7	1371.15
NMRAL1	NmrA-like family domain containing 1	1410.2	1331.9	1371.06
NDUFB2	NADH dehydrogenase (ubiquinone) 1 beta subcomplex, 2, 8kDa	1465.7	1275.1	1370.43
LOC104913102	high mobility group protein HMGI-C-like	1353.8	1385.3	1369.54
UBXN10	UBX domain protein 10	1328.1	1407.7	1367.95
CHMP1A	charged multivesicular body protein 1A	1450.1	1285.8	1367.94
LOC100541107	transcription factor Dp-2	1403.8	1330.9	1367.36
NCOR2	nuclear receptor corepressor 2	1422.1	1310.4	1366.25
TSPAN4	tetraspanin 4	1328.8	1403.2	1366.01
TIAL1	TIA1 cytotoxic granule-associated RNA binding protein-like 1	1339.2	1391.4	1365.30
AGPAT6	1-acylglycerol-3-phosphate O-acyltransferase 6	1358.9	1369.4	1364.13
STXBP6	syntaxin binding protein 6 (amisyn)	1319.8	1408.0	1363.87
DCN	decorin	1085.9	1639.9	1362.92
HNRNPL	heterogeneous nuclear ribonucleoprotein L	1413.4	1312.4	1362.90
LONRF2	LON peptidase N-terminal domain and ring finger 2	1248.0	1473.1	1360.56
MRPL27	mitochondrial ribosomal protein L27	1417.9	1300.4	1359.15
C3H8orf76	chromosome 3 open reading frame, human C8orf76	1374.7	1343.2	1358.98
HIF1AN	hypoxia inducible factor 1, alpha subunit inhibitor	1328.4	1388.0	1358.24
RAPGEF2	Rap guanine nucleotide exchange factor (GEF) 2	1294.8	1421.3	1358.04
WDR77	WD repeat domain 77	1377.2	1338.0	1357.58
NDUFC1	NADH dehydrogenase (ubiquinone) 1, subcomplex unknown, 1, 6kDa	1418.4	1295.7	1357.06
LOC100540462	uncharacterized LOC100540462	1240.3	1473.3	1356.84
G0S2	G0/G1 switch 2	1301.5	1410.0	1355.73
PROS1	protein S (alpha)	1367.5	1343.5	1355.47
CLIP1	CAP-GLY domain containing linker protein 1	1328.3	1382.2	1355.28
GTPBP1	GTP binding protein 1	1427.5	1282.0	1354.74
FBXO30	F-box protein 30	1258.3	1451.1	1354.72
ILKAP	integrin-linked kinase-associated serine/threonine phosphatase	1382.5	1326.7	1354.58
TCF25	transcription factor 25 (basic helix-loop-helix)	1458.5	1249.6	1354.04
LBH	limb bud and heart development	1434.2	1271.8	1352.99
PTX3	pentraxin 3, long	1404.6	1299.3	1351.95
DESI2	desumoylating isopeptidase 2	1345.1	1358.3	1351.69
XKRX	XK, Kell blood group complex subunit-related, X-linked	1362.8	1340.5	1351.65
LOC104916160	natterin-3-like	1307.2	1392.6	1349.87
LOC104914081	regulator of G-protein signaling 9-binding protein-like	1364.5	1333.4	1348.95
GLRX5	glutaredoxin 5	1354.8	1341.7	1348.24
LOC100545217	BRCA2 and CDKN1A-interacting protein-like	1422.2	1273.5	1347.85
PINK1	PTEN induced putative kinase 1	1378.1	1317.4	1347.75
FANCM	Fanconi anemia, complementation group M	1361.0	1332.2	1346.59

TIMM50	translocase of inner mitochondrial membrane 50 homolog (S. cerevisiae)	1373.8	1318.0	1345.92
NOC2L	nucleolar complex associated 2 homolog (S. cerevisiae)	1345.5	1345.2	1345.31
CAPN3	calpain 3, (p94)	1347.0	1341.5	1344.26
CSNK2A2	casein kinase 2, alpha prime polypeptide	1383.4	1304.5	1343.96
GLT8D2	glycosyltransferase 8 domain containing 2	1305.0	1382.4	1343.73
OSBPL2	oxysterol binding protein-like 2	1305.6	1381.7	1343.63
MDN1	MDN1, midasin homolog (yeast)	1311.6	1374.5	1343.08
MPC2	mitochondrial pyruvate carrier 2	1428.3	1256.1	1342.20
VPS26B	vacuolar protein sorting 26 homolog B (S. pombe)	1398.6	1285.8	1342.18
EWSR1	EWS RNA-binding protein 1	1386.0	1297.9	1341.94
GLRX2	glutaredoxin 2	1337.2	1344.8	1340.97
LOC100540081	AP-3 complex subunit sigma-2	1390.5	1290.2	1340.35
MRPL10	mitochondrial ribosomal protein L10	1444.0	1235.1	1339.57
PGM1	phosphoglucomutase 1	1318.5	1360.4	1339.47
LOC100541022	keratin, type I cytoskeletal 19-like	1277.3	1399.6	1338.45
HDAC1	histone deacetylase 1	1267.9	1408.8	1338.35
EMC4	ER membrane protein complex subunit 4	1462.6	1213.4	1338.03
SEC31B	SEC31 homolog B (S. cerevisiae)	1374.3	1301.3	1337.76
TXNDC9	thioredoxin domain containing 9	1344.0	1330.5	1337.25
NUP205	nucleoporin 205kDa	1309.1	1363.5	1336.29
RABGEF1	RAB guanine nucleotide exchange factor (GEF) 1	1268.3	1403.1	1335.71
CDC42EP3	CDC42 effector protein (Rho GTPase binding) 3	1341.7	1329.5	1335.61
ZNF821	zinc finger protein 821	1334.3	1332.8	1333.53
DHRS7	dehydrogenase/reductase (SDR family) member 7	1293.0	1374.0	1333.53
PCYOX1	prenylcysteine oxidase 1	1281.8	1384.3	1333.02
ATP6V1C1	ATPase, H+ transporting, lysosomal 42kDa, V1 subunit C1	1313.7	1350.0	1331.86
NDUFA6	NADH dehydrogenase (ubiquinone) 1 alpha subcomplex, 6, 14kDa	1290.3	1372.5	1331.41
PTMS	parathymosin	1289.6	1372.8	1331.22
ZBTB33	zinc finger and BTB domain containing 33	1313.0	1346.3	1329.65
BACH1	BTB and CNC homology 1, basic leucine zipper transcription factor 1	1248.7	1409.6	1329.14
OIP5	Opa interacting protein 5	1365.1	1291.7	1328.42
THOP1	thimet oligopeptidase 1	1365.7	1288.1	1326.94
PAF1	Paf1, RNA polymerase II associated factor, homolog (S. cerevisiae)	1377.6	1273.8	1325.71
HIPK2	homeodomain interacting protein kinase 2	1256.2	1394.8	1325.51
UBL3	ubiquitin-like 3	1294.2	1356.8	1325.50
ITPKA	inositol-trisphosphate 3-kinase A	1314.1	1335.5	1324.81
SUDS3	suppressor of defective silencing 3 homolog (S. cerevisiae)	1259.2	1390.1	1324.68
DDR2	discoidin domain receptor tyrosine kinase 2	1463.9	1185.4	1324.67
RABGGTB	Rab geranylgeranyltransferase, beta subunit	1301.5	1347.1	1324.32
CCZ1	CCZ1 vacuolar protein trafficking and biogenesis associated homolog (S. cerevisiae)	1286.8	1361.5	1324.17
HEY1	hes-related family bHLH transcription factor with YRPW motif 1	1280.7	1367.3	1323.97
SUGT1	SGT1, suppressor of G2 allele of SKP1 (S. cerevisiae)	1298.7	1346.9	1322.81
SMC3	structural maintenance of chromosomes 3	1335.8	1309.8	1322.78
DHX36	DEAH (Asp-Glu-Ala-His) box polypeptide 36	1401.1	1244.3	1322.68
YTHDF1	YTH N(6)-methyladenosine RNA binding protein 1	1291.9	1353.3	1322.62
LOC104910623	ras GTPase-activating protein-binding protein 2	1364.6	1280.3	1322.46
MPV17L2	MPV17 mitochondrial membrane protein-like 2	1379.4	1265.4	1322.39
MTMR2	myotubularin related protein 2	1267.8	1376.6	1322.20
RALGAPB	Ral GTPase activating protein, beta subunit (non-catalytic)	1326.1	1316.3	1321.22
COPG2	coatomer protein complex, subunit gamma 2	1322.4	1319.8	1321.10
LOC100539811	focal adhesion kinase 1-like	1296.4	1343.7	1320.06
ANKFY1	ankyrin repeat and FYVE domain containing 1	1369.6	1266.9	1318.20
FAM8A1	family with sequence similarity 8, member A1	1261.1	1375.0	1318.01
SUPT6H	suppressor of Ty 6 homolog (S. cerevisiae)	1366.0	1268.9	1317.43
NUP62CL	nucleoporin 62kDa C-terminal like	1365.5	1266.8	1316.14

CTHRC1	collagen triple helix repeat containing 1	1320.0	1312.3	1316.14
EMC6	ER membrane protein complex subunit 6	1355.2	1276.5	1315.85
PTPN14	protein tyrosine phosphatase, non-receptor type 14	1274.4	1355.8	1315.12
MRPL57	mitochondrial ribosomal protein L57	1377.2	1250.9	1314.05
LOC104915345	uncharacterized LOC104915345	1299.3	1328.3	1313.82
STAM2	signal transducing adaptor molecule (SH3 domain and ITAM motif) 2	1253.2	1374.2	1313.73
ADAM17	ADAM metallopeptidase domain 17	1272.7	1354.6	1313.64
LOC100549854	superkiller viralicidic activity 2-like 2	1322.9	1303.1	1313.02
RAB11FIP3	RAB11 family interacting protein 3 (class II)	1291.8	1325.2	1308.51
HS2ST1	heparan sulfate 2-O-sulfotransferase 1	1298.4	1317.8	1308.09
VPS72	vacuolar protein sorting 72 homolog (S. cerevisiae)	1394.2	1221.1	1307.64
USP15	ubiquitin specific peptidase 15	1293.0	1319.3	1306.18
RAB34	RAB34, member RAS oncogene family	1321.5	1289.1	1305.30
UBAP2L	ubiquitin associated protein 2-like	1285.9	1324.6	1305.28
ARHGEF17	Rho guanine nucleotide exchange factor (GEF) 17	1418.0	1192.2	1305.08
BCL2L1	BCL2-like 1	1319.7	1289.0	1304.33
ANKRD13C	ankyrin repeat domain 13C	1305.6	1302.3	1303.93
MMD	monocyte to macrophage differentiation-associated	1303.0	1300.2	1301.59
NUDCD2	NudC domain containing 2	1320.5	1282.1	1301.28
NSD1	nuclear receptor binding SET domain protein 1	1342.4	1260.0	1301.18
SIAH1	siah E3 ubiquitin protein ligase 1	1263.9	1337.3	1300.63
RNF34	ring finger protein 34, E3 ubiquitin protein ligase	1268.4	1331.8	1300.10
TBC1D8	TBC1 domain family, member 8 (with GRAM domain)	1235.7	1363.8	1299.76
LOC100541468	large neutral amino acids transporter small subunit 1-like	1432.7	1166.8	1299.72
DBNL	drebrin-like	1466.1	1132.4	1299.23
LOC104911405	tRNA-splicing ligase RtcB homolog	1311.3	1286.2	1298.77
ABHD17A	abhydrolase domain containing 17A	1358.0	1239.1	1298.56
MRPS23	mitochondrial ribosomal protein S23	1289.4	1307.6	1298.49
LEPROTL1	leptin receptor overlapping transcript-like 1	1270.6	1325.4	1297.99
LOC100549733	microtubule-associated serine/threonine-protein kinase 2	1324.5	1270.1	1297.32
YTHDC2	YTH domain containing 2	1214.5	1380.0	1297.25
DGCR6L	DiGeorge syndrome critical region gene 6-like	1329.1	1265.3	1297.23
TAF2	TAF2 RNA polymerase II, TATA box binding protein (TBP)-associated factor, 150kDa	1235.7	1357.4	1296.58
CKS1B	CDC28 protein kinase regulatory subunit 1B	1464.2	1128.4	1296.29
LOC100539376	solute carrier family 35 member E2-like	1161.2	1430.9	1296.04
NOL12	nucleolar protein 12	1298.7	1292.0	1295.35
R3HCC1	R3H domain and coiled-coil containing 1	1332.2	1257.9	1295.03
LOC104911799	uncharacterized LOC104911799	1269.2	1319.0	1294.08
PLRG1	pleiotropic regulator 1	1286.0	1299.8	1292.92
NDP	Norrie disease (pseudoglioma)	1263.6	1319.9	1291.75
STC2	stanniocalcin 2	1307.8	1275.4	1291.59
TTC4	tetratricopeptide repeat domain 4	1317.3	1265.8	1291.56
GMPPA	GDP-mannose pyrophosphorylase A	1313.8	1269.0	1291.38
LOC100542665	histone H2A type 2-B	1311.1	1269.4	1290.22
TBC1D10A	TBC1 domain family, member 10A	1371.1	1208.0	1289.56
SLC2A8	solute carrier family 2 (facilitated glucose transporter), member 8	1241.5	1336.7	1289.10
LOC100549894	protein tweety homolog 3-like	1262.0	1314.4	1288.17
LOC100542445	probable tumor suppressor protein MN1	1302.8	1272.1	1287.48
DENND2C	DENN/MADD domain containing 2C	1239.7	1333.4	1286.53
TMEM184A	transmembrane protein 184A	1241.7	1330.9	1286.31
RPP14	ribonuclease P/MRP 14kDa subunit	1296.8	1275.4	1286.11
AMPD3	adenosine monophosphate deaminase 3	1173.6	1398.4	1286.02
VPS11	vacuolar protein sorting 11 homolog (S. cerevisiae)	1284.5	1286.9	1285.69
LOC100538966	peptidyl-glycine alpha-amidating monooxygenase-like	1276.3	1293.9	1285.12
MRPL24	mitochondrial ribosomal protein L24	1317.6	1251.4	1284.51
CNPY3	canopy FGF signaling regulator 3	1305.1	1263.8	1284.44

TRPC1	transient receptor potential cation channel, subfamily C, member 1	1256.6	1310.5	1283.56
DNAJA3	DnaJ (Hsp40) homolog, subfamily A, member 3	1267.9	1296.1	1281.98
NFIL3	nuclear factor, interleukin 3 regulated	1267.9	1295.7	1281.84
TLCD1	TLC domain containing 1	1369.9	1192.3	1281.10
PCIF1	PDX1 C-terminal inhibiting factor 1	1352.3	1209.8	1281.05
SUZ12	SUZ12 polycomb repressive complex 2 subunit	1302.1	1259.6	1280.84
TPX2	TPX2, microtubule-associated	1341.1	1219.6	1280.36
LOC100550767	glucosamine-6-phosphate isomerase 1	1246.4	1312.2	1279.27
EEA1	early endosome antigen 1	1308.1	1250.4	1279.25
SDF2L1	stromal cell-derived factor 2-like 1	1234.9	1323.4	1279.11
LOC104910862	uncharacterized LOC104910862	1223.5	1333.4	1278.46
C21H17orf85	chromosome 21 open reading frame, human C17orf85	1258.3	1298.0	1278.14
RANBP9	RAN binding protein 9	1272.6	1282.7	1277.61
TAF3	TAF3 RNA polymerase II, TATA box binding protein (TBP)-associated factor, 140kDa	1232.7	1322.1	1277.38
OGFOD3	2-oxoglutarate and iron-dependent oxygenase domain containing 3	1269.4	1285.0	1277.20
ARIH1	ariadne RBR E3 ubiquitin protein ligase 1	1248.3	1305.6	1276.99
GYG1	glycogenin 1	1212.5	1335.0	1273.73
LBR	lamin B receptor	1307.7	1239.0	1273.36
NIT2	nitrilase family, member 2	1195.0	1350.5	1272.73
KAT7	K(lysine) acetyltransferase 7	1219.6	1325.3	1272.47
TCN2	transcobalamin II	1252.9	1291.5	1272.21
PRPF18	pre-mRNA processing factor 18	1287.2	1256.6	1271.88
SLC25A39	solute carrier family 25, member 39	1333.9	1208.3	1271.10
UFC1	ubiquitin-fold modifier conjugating enzyme 1	1322.5	1218.5	1270.53
TM9SF4	transmembrane 9 superfamily protein member 4	1261.0	1278.7	1269.86
LOC104916328	uncharacterized LOC104916328	1269.1	1269.5	1269.29
GTF2A1	general transcription factor IIA, 1, 19/37kDa	1175.5	1361.1	1268.31
RUNDC1	RUN domain containing 1	1287.1	1249.2	1268.13
RLIM	ring finger protein, LIM domain interacting	1187.9	1346.1	1267.01
TFDP1	transcription factor Dp-1	1284.8	1248.8	1266.83
LOC104915970	signal transducer and activator of transcription 6-like	1364.8	1168.7	1266.71
PI4KA	phosphatidylinositol 4-kinase, catalytic, alpha	1175.0	1358.1	1266.56
KANSL3	KAT8 regulatory NSL complex subunit 3	1317.7	1213.5	1265.62
SCRIB	scribbled planar cell polarity protein	1334.2	1196.9	1265.56
WDR24	WD repeat domain 24	1327.0	1203.4	1265.17
MCFD2	multiple coagulation factor deficiency 2	1265.6	1263.4	1264.52
ASH2L	ash2 (absent, small, or homeotic)-like (Drosophila)	1271.8	1256.6	1264.21
LOC100538849	protein C19orf12 homolog	1239.5	1288.7	1264.09
ATP11B	ATPase, class VI, type 11B	1266.0	1260.9	1263.48
TSPAN7	tetraspanin 7	1232.9	1293.9	1263.39
OSER1	oxidative stress responsive serine-rich 1	1240.6	1284.8	1262.70
ZFYVE16	zinc finger, FYVE domain containing 16	1245.9	1276.5	1261.16
LSG1	large 60S subunit nuclear export GTPase 1	1336.6	1183.6	1260.12
PRMT9	protein arginine methyltransferase 9	1246.3	1272.6	1259.44
MED9	mediator complex subunit 9	1253.8	1264.6	1259.18
ARHGEF12	Rho guanine nucleotide exchange factor (GEF) 12	1199.8	1318.4	1259.11
MGST3	microsomal glutathione S-transferase 3	1220.2	1297.3	1258.73
RBM26	RNA binding motif protein 26	1249.8	1267.7	1258.73
RWDD4	RWD domain containing 4	1220.9	1296.3	1258.63
CACTIN	cactin, spliceosome C complex subunit	1250.2	1267.0	1258.61
GMDS	GDP-mannose 4,6-dehydratase	1294.8	1219.7	1257.23
LOC104910261	uncharacterized LOC104910261	1216.9	1296.9	1256.90
TEX2	testis expressed 2	1234.6	1279.0	1256.78
PSME4	proteasome (prosome, macropain) activator subunit 4	1179.8	1333.1	1256.47
ARHGAP31	Rho GTPase activating protein 31	1328.0	1184.7	1256.33

SMARCD1	SWI/SNF related, matrix associated, actin dependent regulator of chromatin, subfamily d, member 1	1282.8	1228.3	1255.52
MCM3AP	minichromosome maintenance complex component 3 associated protein	1207.0	1302.9	1254.95
LOC104911065	uncharacterized LOC104911065	1143.2	1366.1	1254.65
TP53BP2	tumor protein p53 binding protein 2	1239.7	1269.6	1254.63
PIGY	phosphatidylinositol glycan anchor biosynthesis, class Y	1264.7	1243.9	1254.28
SLC9A1	solute carrier family 9, subfamily A (NHE1, cation proton antiporter 1), member 1	1335.5	1172.1	1253.80
LOC104912512	GPI-anchor transamidase-like	1182.4	1321.6	1252.03
LOC100549389	hepatoma-derived growth factor-related protein 2	1274.4	1228.4	1251.39
TAF15	TAF15 RNA polymerase II, TATA box binding protein (TBP)-associated factor, 68kDa	1217.8	1284.6	1251.17
LOC104913771	uncharacterized LOC104913771	1237.1	1264.4	1250.78
NDUFS8	NADH dehydrogenase (ubiquinone) Fe-S protein 8, 23kDa (NADH-coenzyme Q reductase)	1282.8	1218.3	1250.58
SWI5	SWI5 recombination repair homolog (yeast)	1242.4	1257.4	1249.90
PSMD10	proteasome (prosome, macropain) 26S subunit, non-ATPase, 10	1220.4	1279.0	1249.66
GTF2H1	general transcription factor IIH, polypeptide 1, 62kDa	1223.6	1275.0	1249.28
PPM1G	protein phosphatase, Mg2+/Mn2+ dependent, 1G	1255.9	1242.4	1249.13
FAM214A	family with sequence similarity 214, member A	1159.8	1338.2	1248.97
PRRC1	proline-rich coiled-coil 1	1230.4	1267.2	1248.80
SMIM19	small integral membrane protein 19	1238.8	1258.6	1248.71
SCRN3	secernin 3	1297.8	1197.5	1247.66
YEATS2	YEATS domain containing 2	1219.6	1272.5	1246.05
SNRPB2	small nuclear ribonucleoprotein polypeptide B	1249.5	1242.4	1245.96
LTA4H	leukotriene A4 hydrolase	1212.5	1279.3	1245.95
ADAMTS2	ADAM metallopeptidase with thrombospondin type 1 motif, 2	1144.3	1347.2	1245.75
TIPIN	TIMELESS interacting protein	1219.4	1270.3	1244.88
RPA1	replication protein A1, 70kDa	1231.0	1257.4	1244.20
ANAPC1	anaphase promoting complex subunit 1	1233.2	1254.5	1243.82
RNFT1	ring finger protein, transmembrane 1	1211.6	1275.4	1243.54
LLPH	LLP homolog, long-term synaptic facilitation (Aplysia)	1241.9	1244.8	1243.36
TTC37	tetratricopeptide repeat domain 37	1223.7	1261.3	1242.48
RSL1D1	ribosomal L1 domain containing 1	1298.5	1185.4	1241.95
GLB1	galactosidase, beta 1	1191.0	1292.2	1241.60
PANK2	pantothenate kinase 2	1174.0	1307.6	1240.81
MAN2A2	mannosidase, alpha, class 2A, member 2	1274.9	1205.8	1240.35
FAM20C	family with sequence similarity 20, member C	1170.4	1310.0	1240.20
MYCL	v-myc avian myelocytomatosis viral oncogene lung carcinoma derived homolog	1186.5	1292.5	1239.53
FBXO31	F-box protein 31	1276.1	1202.7	1239.38
PPM1F	protein phosphatase, Mg2+/Mn2+ dependent, 1F	1198.4	1279.7	1239.06
LOC100541609	SERTA domain-containing protein 2-like	1212.5	1265.5	1238.99
PPM1E	protein phosphatase, Mg2+/Mn2+ dependent, 1E	1206.1	1271.5	1238.83
MPST	mercaptopyruvate sulfurtransferase	1252.8	1223.6	1238.21
CRCP	CGRP receptor component	1247.9	1228.0	1237.91
ZFC3H1	zinc finger, C3H1-type containing	1236.6	1238.7	1237.64
EXOC7	exocyst complex component 7	1268.5	1205.9	1237.20
ASH1L	ash1 (absent, small, or homeotic)-like (Drosophila)	1283.1	1190.5	1236.78
LOC104909984	14-3-3 protein theta pseudogene	1238.3	1232.6	1235.46
LOC100549028	protein phosphatase 1 regulatory subunit 3C-B-like	1315.0	1154.2	1234.61
HRAS	Harvey rat sarcoma viral oncogene homolog	1271.2	1195.8	1233.50
GRPEL2	GrpE-like 2, mitochondrial (E. coli)	1211.1	1252.3	1231.71
DEGS1	delta(4)-desaturase, sphingolipid 1	1174.7	1287.4	1231.05
LOC100542686	protein-L-isoaspartate(D-aspartate) O-methyltransferase-like	1253.8	1208.3	1231.05
TCP11L2	t-complex 11, testis-specific-like 2	1143.9	1316.3	1230.11
APPBP2	amyloid beta precursor protein (cytoplasmic tail) binding protein 2	1182.1	1276.2	1229.14

RRM2	ribonucleotide reductase M2	1327.0	1128.7	1227.86
BRI3	brain protein I3	1203.4	1252.3	1227.83
IGBP1	immunoglobulin (CD79A) binding protein 1	1250.6	1204.3	1227.45
ELOVL6	ELOVL fatty acid elongase 6	1276.7	1176.8	1226.77
MMP9	matrix metallopeptidase 9 (gelatinase B, 92kDa gelatinase, 92kDa type IV collagenase)	1168.3	1284.7	1226.51
LOC100541155	regulator of cell cycle RGCC-like	1201.9	1250.7	1226.31
MARVELD1	MARVEL domain containing 1	1230.8	1221.8	1226.29
LMO4	LIM domain only 4	1309.2	1143.3	1226.25
GLT8D1	glycosyltransferase 8 domain containing 1	1217.0	1235.2	1226.12
NDUFC2	NADH dehydrogenase (ubiquinone) 1, subcomplex unknown, 2, 14.5kDa	1235.9	1216.3	1226.05
MAP1S	microtubule-associated protein 1S	1378.9	1071.8	1225.37
PTGES3L	prostaglandin E synthase 3 (cytosolic)-like	1270.0	1180.1	1225.04
LOC100539169	chromodomain-helicase-DNA-binding protein 6	1139.6	1310.4	1225.02
LOC104909648	uncharacterized LOC104909648	1178.7	1269.2	1223.98
BCAN	brevican	1217.7	1229.6	1223.64
ABCA1	ATP-binding cassette, sub-family A (ABC1), member 1	1112.9	1333.9	1223.41
HDAC8	histone deacetylase 8	1241.4	1205.3	1223.34
LOC100548808	ubiquitin carboxyl-terminal hydrolase 12-like	1222.6	1223.9	1223.26
TOMM40L	translocase of outer mitochondrial membrane 40 homolog (yeast)-like	1311.5	1134.6	1223.07
PARN	poly(A)-specific ribonuclease	1165.5	1279.8	1222.64
FAS	Fas cell surface death receptor	1113.8	1330.5	1222.13
GNPNAT1	glucosamine-phosphate N-acetyltransferase 1	1204.2	1239.7	1221.95
LOC104909632	baculoviral IAP repeat-containing protein 6-like	1203.3	1239.7	1221.49
PDGFD	platelet derived growth factor D	1161.3	1281.5	1221.39
LOC104913817	cytochrome c oxidase assembly protein COX11, mitochondrial	1211.7	1230.9	1221.33
MUSK	muscle, skeletal, receptor tyrosine kinase	1234.7	1207.4	1221.07
NANS	N-acetylneuraminic acid synthase	1274.0	1166.7	1220.32
TRIM45	tripartite motif containing 45	1216.0	1224.0	1220.02
PDGFRB	platelet-derived growth factor receptor, beta polypeptide	1205.4	1234.4	1219.91
LOC100543264	E3 ubiquitin-protein ligase RNF130-like	1245.8	1192.7	1219.24
SLC25A46	solute carrier family 25, member 46	1188.0	1249.1	1218.59
LOC100551434	transmembrane protein 183A-like	1246.4	1189.2	1217.82
TUBG1	tubulin, gamma 1	1261.3	1174.0	1217.65
HIGD2A	HIG1 hypoxia inducible domain family, member 2A	1290.6	1143.8	1217.21
UTP15	UTP15, U3 small nucleolar ribonucleoprotein, homolog (S. cerevisiae)	1186.2	1247.9	1217.05
PPIE	peptidylprolyl isomerase E (cyclophilin E)	1310.6	1123.4	1216.99
FGFR1OP	FGFR1 oncogene partner	1155.3	1276.8	1216.06
SIK1	salt-inducible kinase 1	1139.0	1292.2	1215.59
LOC104916196	ryanodine receptor 1-like	1225.6	1205.4	1215.52
AP1B1	adaptor-related protein complex 1, beta 1 subunit	1286.5	1143.4	1214.95
MMP28	matrix metallopeptidase 28	1166.8	1262.7	1214.73
YWHAG	tyrosine 3-monooxygenase/tryptophan 5-monooxygenase activation protein, gamma	1223.5	1205.5	1214.52
SUCO	SUN domain containing ossification factor	1132.4	1295.4	1213.92
SPRY2	sprouty homolog 2 (Drosophila)	1232.4	1195.0	1213.70
RALB	v-ral simian leukemia viral oncogene homolog B	1200.9	1224.8	1212.81
DLST	dihydrolipoamide S-succinyltransferase (E2 component of 2-oxo-glutarate complex)	1257.5	1168.0	1212.79
MRPL3	mitochondrial ribosomal protein L3	1208.9	1216.4	1212.68
POLR2F	polymerase (RNA) II (DNA directed) polypeptide F	1296.9	1128.2	1212.56
TSC1	tuberous sclerosis 1	1208.2	1216.2	1212.24
ARHGAP10	Rho GTPase activating protein 10	1113.7	1309.9	1211.82
GGA1	golgi-associated, gamma adaptin ear containing, ARF binding protein 1	1203.6	1217.5	1210.56

ACADS	acyl-CoA dehydrogenase, C-2 to C-3 short chain	1208.7	1211.2	1209.98
ALPK3	alpha-kinase 3	1282.6	1136.8	1209.68
PIP4K2A	phosphatidylinositol-5-phosphate 4-kinase, type II, alpha	1230.8	1188.1	1209.47
SYF2	SYF2 pre-mRNA-splicing factor	1220.8	1198.1	1209.46
SNX30	sorting nexin family member 30	1148.7	1266.7	1207.69
FAM49B	family with sequence similarity 49, member B	1223.2	1191.8	1207.51
LOC100551117	ankyrin repeat domain-containing protein 13D-like	1316.3	1097.3	1206.80
RFT1	RFT1 homolog (S. cerevisiae)	1183.0	1229.6	1206.31
GOT1	glutamic-oxaloacetic transaminase 1, soluble	1234.6	1177.6	1206.10
KIAA0319L	KIAA0319-like ortholog	1158.8	1252.8	1205.79
ROCK1	Rho-associated, coiled-coil containing protein kinase 1	1163.4	1248.0	1205.74
SIRT6	sirtuin 6	1203.2	1208.1	1205.68
GTPBP2	GTP binding protein 2	1229.5	1181.3	1205.43
PARG	poly (ADP-ribose) glycohydrolase	1197.3	1211.4	1204.37
MLX	MLX, MAX dimerization protein	1179.1	1229.5	1204.28
F2R	coagulation factor II (thrombin) receptor	1161.8	1246.5	1204.14
LOC100542612	phospholipid-transporting ATPase ID-like	1325.7	1080.5	1203.12
HSPBP1	HSPA (heat shock 70kDa) binding protein, cytoplasmic cochaperone 1	1336.7	1069.3	1203.00
RNF38	ring finger protein 38	1133.6	1271.4	1202.49
RBM34	RNA binding motif protein 34	1286.2	1118.3	1202.25
USP4	ubiquitin specific peptidase 4 (proto-oncogene)	1194.4	1209.8	1202.08
GMPS	guanine monophosphate synthase	1190.8	1211.6	1201.20
ANKRD46	ankyrin repeat domain 46	1217.6	1183.4	1200.53
ATOH8	atonal homolog 8 (Drosophila)	1139.0	1259.8	1199.41
SMC2	structural maintenance of chromosomes 2	1300.7	1097.2	1198.99
HERC4	HECT and RLD domain containing E3 ubiquitin protein ligase 4	1171.8	1224.8	1198.28
LOC104911005	fatty acyl-CoA reductase 1-like	1161.8	1234.5	1198.17
LOC100549146	glutaminyl-peptide cyclotransferase-like protein	1247.3	1147.7	1197.50
OCIAD1	OCIA domain containing 1	1175.8	1219.1	1197.45
ADPRHL2	ADP-ribosylhydrolase like 2	1176.2	1216.9	1196.54
TTLL9	tubulin tyrosine ligase-like family, member 9	1262.3	1130.5	1196.40
BIRC2	baculoviral IAP repeat containing 2	1218.8	1173.0	1195.89
CLK3	CDC-like kinase 3	1209.6	1179.7	1194.67
PDZRN3	PDZ domain containing ring finger 3	1080.3	1308.5	1194.39
LOC104913857	protein CASP-like	1273.4	1115.3	1194.35
LOC100550262	hippocampus abundant transcript 1 protein-like	1253.2	1134.7	1193.95
RAB41	RAB41, member RAS oncogene family	1228.6	1157.9	1193.26
LDLRAP1	low density lipoprotein receptor adaptor protein 1	1187.0	1198.3	1192.69
PLK2	polo-like kinase 2	1149.8	1233.7	1191.75
DDX41	DEAD (Asp-Glu-Ala-Asp) box polypeptide 41	1236.7	1146.5	1191.61
DUT	deoxyuridine triphosphatase	1252.9	1129.1	1190.99
UBE2R2	ubiquitin-conjugating enzyme E2R 2	1200.1	1181.4	1190.78
NECAP1	NECAP endocytosis associated 1	1152.6	1228.3	1190.45
ACTR2	ARP2 actin-related protein 2 homolog (yeast)	1219.2	1161.6	1190.38
NEDD9	neural precursor cell expressed, developmentally down-regulated 9	1245.1	1134.4	1189.74
IREB2	iron-responsive element binding protein 2	1169.6	1207.4	1188.51
FARSA	phenylalanyl-tRNA synthetase, alpha subunit	1224.7	1152.1	1188.41
ARHGAP40	Rho GTPase activating protein 40	1113.3	1263.0	1188.16
TEX14	testis expressed 14	1088.9	1287.1	1188.01
EMILIN1	elastin microfibril interfacer 1	1266.4	1108.6	1187.53
KDM2A	lysine (K)-specific demethylase 2A	1196.3	1178.7	1187.49
HMGB2	high mobility group box 2	1282.4	1089.7	1186.05
C11H18orf32	chromosome 11 open reading frame, human C18orf32	1112.3	1258.8	1185.54
CPSF3L	cleavage and polyadenylation specific factor 3-like	1166.3	1203.5	1184.90
LOC104914161	kinesin-like protein KIF1B	1134.9	1233.2	1184.04
CDK17	cyclin-dependent kinase 17	1163.6	1204.1	1183.84

FTSJ3	FtsJ homolog 3 (E. coli)	1263.5	1103.2	1183.33
NR2C2	nuclear receptor subfamily 2, group C, member 2	1137.5	1228.9	1183.22
MRPL51	mitochondrial ribosomal protein L51	1230.3	1135.8	1183.03
FKBP14	FK506 binding protein 14, 22 kDa	1128.7	1237.0	1182.85
DCLK2	doublecortin-like kinase 2	1155.6	1209.6	1182.64
POMGNT1	protein O-linked mannose N-acetylglucosaminyltransferase 1 (beta 1,2-)	1226.5	1137.2	1181.89
ARHGEF7	Rho guanine nucleotide exchange factor (GEF) 7	1167.6	1195.6	1181.59
BVES	blood vessel epicardial substance	1088.2	1273.7	1180.94
RALY	RALY heterogeneous nuclear ribonucleoprotein	1176.7	1183.3	1180.00
RABIF	RAB interacting factor	1222.7	1137.2	1179.94
EXOC3	exocyst complex component 3	1167.8	1191.9	1179.82
ECHS1	enoyl CoA hydratase, short chain, 1, mitochondrial	1237.0	1121.3	1179.14
TMTC3	transmembrane and tetratricopeptide repeat containing 3	1131.8	1225.5	1178.64
IGF2R	insulin-like growth factor 2 receptor	1145.1	1212.0	1178.56
PPME1	protein phosphatase methylesterase 1	1223.7	1133.2	1178.46
LOC104914291	probable beta-D-xylosidase 2	1291.3	1065.4	1178.37
PRUNE	prune exopolyphosphatase	1243.5	1112.1	1177.80
FUT11	fucosyltransferase 11 (alpha (1,3) fucosyltransferase)	1167.1	1187.9	1177.52
CREB3L2	cAMP responsive element binding protein 3-like 2	1167.3	1186.8	1177.06
C20H17orf75	chromosome 20 open reading frame, human C17orf75	1183.6	1169.8	1176.72
LOC104911348	WASH complex subunit 7-like	1124.9	1227.7	1176.31
CLK1	CDC-like kinase 1	1167.1	1185.3	1176.25
NRBF2	nuclear receptor binding factor 2	1124.7	1226.5	1175.58
RBBP6	retinoblastoma binding protein 6	1113.3	1237.8	1175.50
PTPN12	protein tyrosine phosphatase, non-receptor type 12	1194.1	1156.5	1175.32
ZNF622	zinc finger protein 622	1142.4	1208.2	1175.32
RPS6KB2	ribosomal protein S6 kinase, 70kDa, polypeptide 2	1251.2	1098.2	1174.70
USP16	ubiquitin specific peptidase 16	1162.0	1186.8	1174.43
HS1BP3	HCLS1 binding protein 3	1177.0	1171.2	1174.07
NOP16	NOP16 nucleolar protein	1264.5	1082.7	1173.59
NUS1	nuclear undecaprenyl pyrophosphate synthase 1 homolog (S. cerevisiae)	1188.1	1158.5	1173.29
LOC104909768	uncharacterized LOC104909768	1150.9	1194.6	1172.74
RAB27A	RAB27A, member RAS oncogene family	1152.4	1193.0	1172.72
KIAA1143	KIAA1143 ortholog	1071.5	1272.7	1172.08
FAM65A	family with sequence similarity 65, member A	1179.3	1164.8	1172.01
POLD2	polymerase (DNA directed), delta 2, accessory subunit	1234.6	1109.4	1172.00
CTPS1	CTP synthase 1	1272.0	1070.6	1171.33
DUSP1	dual specificity phosphatase 1	1122.3	1219.4	1170.86
ASPSCR1	alveolar soft part sarcoma chromosome region, candidate 1	1139.9	1201.1	1170.51
XPNPEP1	X-prolyl aminopeptidase (aminopeptidase P) 1, soluble	1169.6	1170.1	1169.81
OIT3	oncoprotein induced transcript 3	1095.9	1243.2	1169.56
LOC104916642	paladin-like	1245.8	1092.7	1169.25
TMX3	thioredoxin-related transmembrane protein 3	1166.9	1170.9	1168.88
ACP1	acid phosphatase 1, soluble	1152.2	1183.9	1168.04
CTDSPL	CTD (carboxy-terminal domain, RNA polymerase II, polypeptide A) small phosphatase-like	1139.2	1195.8	1167.51
NUP160	nucleoporin 160kDa	1245.9	1088.6	1167.26
LOC104914614	cytochrome c oxidase assembly factor 3 homolog, mitochondrial	1265.0	1068.8	1166.91
LOC104911723	E3 ubiquitin-protein ligase UBR3-like	1095.3	1237.9	1166.64
FAM208A	family with sequence similarity 208, member A	1111.3	1221.2	1166.26
MFSD2A	major facilitator superfamily domain containing 2A	1228.2	1102.4	1165.31
SLC35B1	solute carrier family 35, member B1	1134.7	1195.7	1165.17
EXOSC1	exosome component 1	1205.9	1122.3	1164.10
CEBPZ	CCAAT/enhancer binding protein (C/EBP), zeta	1130.0	1198.1	1164.00
DDO	D-aspartate oxidase	1192.4	1135.5	1163.91
HSPB7	heat shock 27kDa protein family, member 7 (cardiovascular)	1187.0	1140.2	1163.62

CLASP2	cytoplasmic linker associated protein 2	1065.0	1261.4	1163.20
COMMD6	COMM domain containing 6	1150.8	1175.5	1163.13
SLC6A7	solute carrier family 6 (neurotransmitter transporter), member 7	1110.6	1215.6	1163.12
CPQ	carboxypeptidase Q	1144.3	1181.0	1162.68
LOC100541624	E3 ubiquitin-protein ligase MGRN1	1277.0	1048.3	1162.67
PFDN1	prefoldin subunit 1	1250.2	1072.8	1161.49
LOC100546141	protein phosphatase 1K, mitochondrial	1129.3	1193.5	1161.40
AACS	acetoacetyl-CoA synthetase	1200.4	1122.3	1161.34
LOC100540135	enhancer of mRNA-decapping protein 4-like	1224.9	1097.7	1161.33
KRIT1	KRIT1, ankyrin repeat containing	1159.4	1161.9	1160.69
SMAD1	SMAD family member 1	1071.0	1249.8	1160.42
LOC100547599	probable G-protein coupled receptor 153	1188.4	1132.3	1160.33
VCL	vinculin	1060.3	1259.2	1159.75
BMPR2	bone morphogenetic protein receptor, type II (serine/threonine kinase)	1129.9	1186.8	1158.38
TRAPPC4	trafficking protein particle complex 4	1140.0	1176.5	1158.30
RBM27	RNA binding motif protein 27	1177.3	1138.8	1158.06
DHDDS	dehydrodolichyl diphosphate synthase	1188.1	1127.8	1157.97
EEF1E1	eukaryotic translation elongation factor 1 epsilon 1	1181.8	1133.4	1157.63
TMEM45A	transmembrane protein 45A	1098.1	1216.3	1157.18
LOC104916381	protein syndesmos pseudogene	1227.8	1086.5	1157.14
ATG4A	autophagy related 4A, cysteine peptidase	1126.7	1186.4	1156.52
KIAA1161	KIAA1161 ortholog	1119.2	1193.3	1156.28
TLK2	tousled-like kinase 2	1172.1	1139.8	1155.99
MOSPD1	motile sperm domain containing 1	1134.1	1177.4	1155.77
ADRBK1	adrenergic, beta, receptor kinase 1	1235.9	1074.1	1155.00
SLC25A14	solute carrier family 25 (mitochondrial carrier, brain), member 14	1122.6	1187.3	1154.94
NT5C1A	5'-nucleotidase, cytosolic IA	1129.1	1180.8	1154.91
RGMA	repulsive guidance molecule family member a	1131.6	1177.6	1154.59
MCM5	minichromosome maintenance complex component 5	1290.2	1018.5	1154.34
ALX4	ALX homeobox 4	1116.8	1191.6	1154.19
AIMP1	aminoacyl tRNA synthetase complex-interacting multifunctional protein 1	1120.3	1187.8	1154.06
LOC104916438	UV excision repair protein RAD23 homolog A-like	1201.7	1104.9	1153.32
UCK1	uridine-cytidine kinase 1	1181.6	1124.0	1152.77
PITRM1	pitrilysin metallopeptidase 1	1099.5	1205.7	1152.57
LOC100546929	growth hormone receptor	1196.2	1106.7	1151.45
HEATR1	HEAT repeat containing 1	1224.7	1077.5	1151.09
LOC104917152	uncharacterized LOC104917152	1208.0	1093.0	1150.55
SRF	serum response factor (c-fos serum response element-binding transcription factor)	1291.9	1008.7	1150.28
MLEC	malectin	1165.2	1135.2	1150.25
UBXN2A	UBX domain protein 2A	1121.2	1177.0	1149.07
LOC104917063	nuclear receptor subfamily 2 group F member 6-like	1246.2	1051.9	1149.05
XRN1	5'-3' exoribonuclease 1	1062.2	1235.8	1149.02
NSUN5	NOP2/Sun domain family, member 5	1173.0	1123.7	1148.34
FUNDC1	FUN14 domain containing 1	1146.6	1149.5	1148.06
SCN3B	sodium channel, voltage-gated, type III, beta subunit	1109.2	1186.5	1147.81
ZNF598	zinc finger protein 598	1147.6	1147.9	1147.77
FAM126A	family with sequence similarity 126, member A	1153.2	1142.3	1147.75
LOC104909794	laminin subunit alpha-2-like	1237.2	1058.1	1147.66
LOC100540096	scaffold attachment factor B1-like	1132.7	1161.9	1147.26
RPE	ribulose-5-phosphate-3-epimerase	1168.1	1126.4	1147.23
NAT10	N-acetyltransferase 10 (GCN5-related)	1130.9	1163.3	1147.10
SPTY2D1	SPT2, Suppressor of Ty, domain containing 1 (S. cerevisiae)	1175.4	1118.7	1147.05
LOC100540846	retinol dehydrogenase 12-like	1180.3	1113.0	1146.67
FAM63A	family with sequence similarity 63, member A	1243.6	1049.4	1146.51
PHRF1	PHD and ring finger domains 1	1096.1	1195.8	1145.94

EPHX2	epoxide hydrolase 2, cytoplasmic	1273.3	1018.3	1145.78
PRDM10	PR domain containing 10	1123.4	1168.1	1145.76
DDX24	DEAD (Asp-Glu-Ala-Asp) box helicase 24	1089.2	1201.9	1145.53
DDX46	DEAD (Asp-Glu-Ala-Asp) box polypeptide 46	1130.0	1161.0	1145.46
LOC104909236	lysine-specific demethylase 6A-like	1136.7	1153.0	1144.87
LOC100551372	ephrin-B1	1198.0	1091.6	1144.79
USP54	ubiquitin specific peptidase 54	1106.7	1182.8	1144.75
MICALL2	MICAL-like 2	1198.5	1090.9	1144.70
YTHDF3	YTH N(6)-methyladenosine RNA binding protein 3	1063.3	1225.5	1144.43
PDCD6IP	programmed cell death 6 interacting protein	1136.7	1151.8	1144.28
CHCHD3	coiled-coil-helix-coiled-coil-helix domain containing 3	1125.5	1162.5	1144.01
LIAS	lipoic acid synthetase	1138.9	1148.5	1143.68
GNG12	guanine nucleotide binding protein (G protein), gamma 12	1162.6	1124.3	1143.48
LOC104915007	probable global transcription activator SNF2L2	1079.1	1207.2	1143.13
NDUFA2	NADH dehydrogenase (ubiquinone) 1 alpha subcomplex, 2, 8kDa	1237.7	1048.3	1143.00
KMT2E	lysine (K)-specific methyltransferase 2E	1063.7	1221.0	1142.38
GSE1	Gse1 coiled-coil protein	1150.6	1133.4	1142.02
SMNDC1	survival motor neuron domain containing 1	1062.2	1220.1	1141.13
MYO9B	myosin IXB	1108.0	1173.9	1140.97
METTL14	methyltransferase like 14	1154.1	1127.8	1140.92
LOC104912782	fanconi-associated nuclease 1-like	1060.9	1220.2	1140.54
SREBF1	sterol regulatory element binding transcription factor 1	1262.8	1017.9	1140.37
POPDC2	popeye domain containing 2	1130.3	1150.1	1140.20
NUP98	nucleoporin 98kDa	1186.7	1093.2	1139.92
POLB	polymerase (DNA directed), beta	1116.2	1163.1	1139.68
SPEN	spen family transcriptional repressor	1102.4	1175.9	1139.13
MPP3	membrane protein, palmitoylated 3 (MAGUK p55 subfamily member 3)	1132.5	1145.6	1139.04
FAM199X	family with sequence similarity 199, X-linked	1115.5	1162.3	1138.92
CAPG	capping protein (actin filament), gelsolin-like	1129.7	1144.5	1137.07
NFS1	NFS1 cysteine desulfurase	1065.6	1208.3	1136.95
KBTBD2	kelch repeat and BTB (POZ) domain containing 2	1113.9	1159.9	1136.94
MED1	mediator complex subunit 1	1114.5	1159.3	1136.90
BMF	Bcl2 modifying factor	1046.4	1227.2	1136.80
SCAMP4	secretory carrier membrane protein 4	1163.2	1109.9	1136.59
MEAF6	MYST/Esa1-associated factor 6	1121.2	1151.3	1136.26
C11H21orf2	chromosome 11 open reading frame, human C21orf2	1164.5	1106.9	1135.72
PDCD10	programmed cell death 10	1099.3	1171.5	1135.39
NUAK1	NUAK family, SNF1-like kinase, 1	1070.1	1200.5	1135.29
COPS5	COP9 signalosome subunit 5	1129.5	1140.8	1135.13
HMG20A	high mobility group 20A	1138.9	1130.9	1134.94
COMMD4	COMM domain containing 4	1191.7	1077.0	1134.34
SMIM11	small integral membrane protein 11	1209.1	1058.6	1133.83
F2RL1	coagulation factor II (thrombin) receptor-like 1	1112.8	1154.7	1133.78
PDE5A	phosphodiesterase 5A, cGMP-specific	1041.6	1225.1	1133.32
XPO4	exportin 4	1065.1	1200.4	1132.77
COMMD2	COMM domain containing 2	1149.8	1115.6	1132.70
CSGALNACT2	chondroitin sulfate N-acetylgalactosaminyltransferase 2	1109.7	1155.1	1132.41
LOC100539027	ubiquitin carboxyl-terminal hydrolase 34	1064.2	1200.1	1132.19
NCLN	nicalin	1106.6	1156.9	1131.75
LOC100547350	GTPase HRas pseudogene	1103.2	1158.1	1130.61
UTP14A	UTP14, U3 small nucleolar ribonucleoprotein, homolog A (yeast)	1154.7	1106.5	1130.58
LOC104913775	uncharacterized LOC104913775	1125.7	1134.8	1130.25
PRPF4B	pre-mRNA processing factor 4B	1087.6	1169.8	1128.69
LOC100544679	CAAX prenyl protease 1 homolog	1088.4	1167.4	1127.90
AKAP8L	A kinase (PRKA) anchor protein 8-like	1115.1	1139.4	1127.26
SASH1	SAM and SH3 domain containing 1	1017.1	1235.6	1126.37
LOC104912363	formin-binding protein 1-like	1064.0	1187.9	1125.95

UBE2E1	ubiquitin-conjugating enzyme E2E 1	1125.5	1125.6	1125.56
C13H16orf70	chromosome 13 open reading frame, human C16orf70	1033.7	1217.3	1125.54
PIK3CD	phosphatidylinositol-4,5-bisphosphate 3-kinase, catalytic subunit delta	1067.2	1183.6	1125.43
ANGPTL4	angiopoietin-like 4	1092.0	1157.5	1124.76
TMEM201	transmembrane protein 201	1110.5	1138.2	1124.35
MAP1LC3C	microtubule-associated protein 1 light chain 3 gamma	1159.8	1088.1	1123.97
LZTS1	leucine zipper, putative tumor suppressor 1	1036.9	1210.2	1123.59
TMX4	thioredoxin-related transmembrane protein 4	1092.5	1154.1	1123.30
SNX27	sorting nexin family member 27	1123.1	1123.3	1123.24
CD276	CD276 molecule	1150.2	1095.9	1123.07
KLF2	Kruppel-like factor 2	1147.4	1098.3	1122.86
PARP3	poly (ADP-ribose) polymerase family, member 3	1214.5	1031.0	1122.78
FGF13	fibroblast growth factor 13	1021.5	1223.0	1122.27
IQSEC1	IQ motif and Sec7 domain 1	1071.9	1171.9	1121.87
ZFAND2A	zinc finger, AN1-type domain 2A	1153.2	1089.3	1121.28
PPP2R5A	protein phosphatase 2, regulatory subunit B', alpha	1073.3	1169.2	1121.28
SIRT1	sirtuin 1	1085.1	1153.5	1119.31
NDUFS6	NADH dehydrogenase (ubiquinone) Fe-S protein 6, 13kDa (NADH-coenzyme Q reductase)	1117.3	1121.0	1119.14
CSTF3	cleavage stimulation factor, 3' pre-RNA, subunit 3, 77kDa	1079.2	1158.5	1118.86
ATF6	activating transcription factor 6	1170.6	1065.9	1118.28
EIF2D	eukaryotic translation initiation factor 2D	1120.4	1115.7	1118.05
AMMECR1L	AMMECR1-like	1081.1	1154.4	1117.74
LOC100546086	serpin B6-like	868.9	1366.5	1117.68
C1GALT1C1	C1GALT1-specific chaperone 1	1113.0	1122.0	1117.51
FAM103A1	family with sequence similarity 103, member A1	1141.3	1093.7	1117.49
NDUFA12	NADH dehydrogenase (ubiquinone) 1 alpha subcomplex, 12	1144.7	1090.1	1117.39
TTC1	tetratricopeptide repeat domain 1	1098.7	1135.9	1117.31
CDKN1A	cyclin-dependent kinase inhibitor 1A (p21, Cip1)	1099.1	1134.4	1116.79
ARHGEF11	Rho guanine nucleotide exchange factor (GEF) 11	1183.8	1049.6	1116.72
VIMP	VCP-interacting membrane protein	1048.1	1185.3	1116.71
CCNG2	cyclin G2	1083.4	1149.9	1116.69
CSTB	cystatin B (stefin B)	1042.1	1189.8	1115.96
LOC100539632	dickkopf-related protein 3-like	1060.8	1171.0	1115.90
GOLT1B	golgi transport 1B	1083.9	1147.3	1115.58
PHF6	PHD finger protein 6	1095.3	1135.5	1115.40
DCAF7	DDB1 and CUL4 associated factor 7	1086.1	1143.9	1114.98
BRD8	bromodomain containing 8	1124.9	1104.5	1114.69
CIZ1	CDKN1A interacting zinc finger protein 1	1145.3	1083.2	1114.26
TYRO3	TYRO3 protein tyrosine kinase	1087.2	1141.0	1114.09
ATG16L1	autophagy related 16-like 1 (S. cerevisiae)	1107.1	1120.6	1113.86
LOC104909657	uncharacterized LOC104909657	1070.8	1155.8	1113.29
EHBP1	EH domain binding protein 1	1143.0	1083.3	1113.15
PIM1	Pim-1 proto-oncogene, serine/threonine kinase	1123.1	1102.9	1113.01
CERS5	ceramide synthase 5	1134.3	1090.4	1112.32
CUEDC2	CUE domain containing 2	1161.0	1063.6	1112.29
MCM2	minichromosome maintenance complex component 2	1197.0	1026.5	1111.77
HMG20B	high mobility group 20B	1167.3	1055.4	1111.36
SHROOM3	shroom family member 3	1037.4	1185.0	1111.22
POLR3H	polymerase (RNA) III (DNA directed) polypeptide H (22.9kD)	1139.9	1080.6	1110.23
EDA2R	ectodysplasin A2 receptor	1150.1	1069.3	1109.73
CUL4B	cullin 4B	1081.9	1137.5	1109.72
FCF1	FCF1 rRNA-processing protein	1096.8	1122.3	1109.50
TOR1A	torsin family 1, member A (torsin A)	1135.7	1082.8	1109.20
ALG12	ALG12, alpha-1,6-mannosyltransferase	1096.8	1121.5	1109.13
PPM1A	protein phosphatase, Mg2+/Mn2+ dependent, 1A	1052.0	1165.1	1108.53
LOC104911729	serine/threonine-protein kinase tousled-like 1	1110.4	1106.1	1108.25

MRPL38	mitochondrial ribosomal protein L38	1181.4	1033.4	1107.40
PRKCI	protein kinase C, iota	1058.8	1155.9	1107.35
PPIH	peptidylprolyl isomerase H (cyclophilin H)	1110.1	1104.2	1107.16
MNAT1	MNAT CDK-activating kinase assembly factor 1	1159.9	1052.3	1106.11
NBR1	neighbor of BRCA1 gene 1	1083.4	1127.9	1105.65
EBAG9	estrogen receptor binding site associated, antigen, 9	1128.1	1082.2	1105.13
MSL1	male-specific lethal 1 homolog (Drosophila)	1141.9	1067.5	1104.69
TRIM47	tripartite motif containing 47	1114.8	1094.0	1104.41
DHX30	DEAH (Asp-Glu-Ala-His) box helicase 30	1116.7	1092.0	1104.38
LOC104915343	small EDRK-rich factor 1-like	1121.9	1086.6	1104.24
DNAJC1	DnaJ (Hsp40) homolog, subfamily C, member 1	1081.9	1126.0	1103.95
DNAJC11	DnaJ (Hsp40) homolog, subfamily C, member 11	1118.1	1089.5	1103.80
OGFOD1	2-oxoglutarate and iron-dependent oxygenase domain containing 1	1112.8	1094.2	1103.51
TPP1	tripeptidyl peptidase I	1123.7	1082.8	1103.24
UBE2J1	ubiquitin-conjugating enzyme E2, J1	1043.8	1162.5	1103.17
LYPLA2	lysophospholipase II	1165.6	1040.5	1103.06
TRAPPC2	trafficking protein particle complex 2	1028.5	1177.5	1102.99
CNOT8	CCR4-NOT transcription complex, subunit 8	1101.1	1104.8	1102.94
CCDC101	coiled-coil domain containing 101	1121.0	1082.6	1101.80
TST	thiosulfate sulfurtransferase (rhodanese)	1155.1	1046.6	1100.86
ZNF385B	zinc finger protein 385B	1060.5	1140.9	1100.72
ZNF703	zinc finger protein 703	1083.7	1117.7	1100.70
YTHDC1	YTH domain containing 1	1029.5	1171.8	1100.65
NDUFA13	NADH dehydrogenase (ubiquinone) 1 alpha subcomplex, 13	1203.1	997.7	1100.42
SAP30	Sin3A-associated protein, 30kDa	1076.6	1123.5	1100.07
LOC104917602	zinc finger MYM-type protein 2-like	1076.5	1123.0	1099.71
TYW5	tRNA-yW synthesizing protein 5	1056.5	1142.2	1099.35
EIF2A	eukaryotic translation initiation factor 2A, 65kDa	1100.1	1096.7	1098.43
GINS3	GINS complex subunit 3 (Psf3 homolog)	1105.3	1091.1	1098.22
C1H12orf45	chromosome 1 open reading frame, human C12orf45	1087.4	1107.8	1097.56
LOC104913009	unconventional prefoldin RPB5 interactor 1-like	1093.6	1098.6	1096.08
SF3B4	splicing factor 3b, subunit 4, 49kDa	1103.2	1088.1	1095.66
BLOC1S4	biogenesis of lysosomal organelles complex-1, subunit 4, cappuccino	1116.2	1074.3	1095.24
OPTC	opticin	925.4	1263.6	1094.52
C8H10orf2	chromosome 8 open reading frame, human C10orf2	1164.9	1023.0	1093.93
HK2	hexokinase 2	1114.3	1072.7	1093.51
LOC100539772	vacuolar protein sorting-associated protein 33B-like	1179.4	1006.7	1093.03
LOC100545735	uncharacterized LOC100545735	1160.0	1025.8	1092.88
SNF8	SNF8, ESCRT-II complex subunit	1112.8	1072.6	1092.71
LOC100538558	autophagy-related protein 101-like	1114.2	1071.1	1092.66
MIA3	melanoma inhibitory activity family, member 3	1036.5	1148.0	1092.26
WDR61	WD repeat domain 61	1106.6	1076.1	1091.38
LOC104914212	protein SON-like	1043.1	1139.6	1091.32
ZBTB17	zinc finger and BTB domain containing 17	1142.0	1038.8	1090.40
TMEM237	transmembrane protein 237	1096.0	1083.8	1089.90
INTS1	integrator complex subunit 1	1097.8	1080.4	1089.14
LOC104911503	TBC1 domain family member 5-like	1018.3	1159.9	1089.11
TTC3	tetratricopeptide repeat domain 3	1087.1	1090.8	1088.93
PAPOLA	poly(A) polymerase alpha	1063.9	1113.3	1088.60
LOC104916895	glutaryl-CoA dehydrogenase, mitochondrial-like	1154.3	1022.6	1088.47
ELF2	E74-like factor 2 (ets domain transcription factor)	1060.7	1116.1	1088.43
FUBP1	far upstream element (FUSE) binding protein 1	1067.3	1108.4	1087.87
FOXP4	forkhead box P4	1165.0	1010.7	1087.86
PLA2G12A	phospholipase A2, group XIIA	1066.8	1108.0	1087.41
SCARB1	scavenger receptor class B, member 1	1049.9	1124.7	1087.32
MAPK8	mitogen-activated protein kinase 8	1027.7	1146.8	1087.22

CARNS1	carnosine synthase 1	1107.0	1067.4	1087.21
CDCA8	cell division cycle associated 8	1239.1	933.2	1086.18
CALD1	caldesmon 1	1044.9	1126.6	1085.71
CHPF	chondroitin polymerizing factor	1068.0	1102.0	1084.99
TFCP2L1	transcription factor CP2-like 1	1088.6	1080.8	1084.70
ISCA2	iron-sulfur cluster assembly 2	1016.9	1151.4	1084.15
GNL2	guanine nucleotide binding protein-like 2 (nucleolar)	1088.5	1079.4	1083.96
AIDA	axin interactor, dorsalization associated	1061.6	1105.3	1083.45
CFAP20	cilia and flagella associated protein 20	1123.1	1043.5	1083.29
TXLNA	taxilin alpha	1139.3	1026.2	1082.76
SCAMP5	secretory carrier membrane protein 5	1092.0	1073.3	1082.67
ATP6AP2	ATPase, H+ transporting, lysosomal accessory protein 2	1023.4	1141.1	1082.29
CLUH	clustered mitochondria (cluA/CLU1) homolog	1080.3	1084.2	1082.23
TPST1	tyrosylprotein sulfotransferase 1	1121.7	1042.2	1081.99
LOC104916720	keratin-associated protein 6-2-like	1012.2	1151.4	1081.77
LOC104912254	uncharacterized LOC104912254	1079.0	1083.6	1081.32
CLNS1A	chloride channel, nucleotide-sensitive, 1A	1074.9	1087.4	1081.15
LOC104915827	plectin-like	1380.2	781.8	1081.01
CMKLR1	chemokine-like receptor 1	1167.7	994.0	1080.86
EVL	Enah/Vasp-like	1060.5	1099.7	1080.14
LOC104916528	histone H1x	1185.6	974.3	1079.94
ERGIC3	ERGIC and golgi 3	1092.6	1066.7	1079.66
DDX42	DEAD (Asp-Glu-Ala-Asp) box helicase 42	1053.2	1105.6	1079.41
CCDC47	coiled-coil domain containing 47	1056.5	1101.8	1079.14
ARMC8	armadillo repeat containing 8	1045.2	1112.0	1078.62
STX8	syntaxin 8	1097.8	1058.8	1078.29
SLC7A6	solute carrier family 7 (amino acid transporter light chain, y+L system), member 6	1070.0	1085.6	1077.79
SPINT1	serine peptidase inhibitor, Kunitz type 1	1076.7	1078.1	1077.38
MTHFD1L	methylenetetrahydrofolate dehydrogenase (NADP+ dependent) 1-like	1113.6	1040.9	1077.22
ELAC2	elaC ribonuclease Z 2	1077.7	1075.4	1076.55
TGOLN2	trans-golgi network protein 2	1046.5	1106.0	1076.21
SLC9A6	solute carrier family 9, subfamily A (NHE6, cation proton antiporter 6), member 6	1071.6	1080.4	1076.03
LOC100540564	scaffold attachment factor B2-like	1061.1	1090.2	1075.61
DPP7	dipeptidyl-peptidase 7	1051.8	1098.9	1075.36
MRPS14	mitochondrial ribosomal protein S14	1029.5	1120.3	1074.92
FKBP4	FK506 binding protein 4, 59kDa	1090.9	1058.4	1074.66
DDB2	damage-specific DNA binding protein 2, 48kDa	974.1	1175.0	1074.55
LOC104913988	serine/threonine-protein kinase 4	1072.5	1076.5	1074.47
YTHDF2	YTH N(6)-methyladenosine RNA binding protein 2	1083.0	1065.3	1074.14
LOC104917359	nucleoside diphosphate kinase pseudogene	1313.9	833.6	1073.75
LOC104913227	ankyrin repeat and KH domain-containing protein 1-like	1103.5	1043.0	1073.27
ARGLU1	arginine and glutamate rich 1	1015.0	1131.1	1073.02
TOB1	transducer of ERBB2, 1	1071.5	1073.9	1072.67
TFEB	transcription factor EB	1137.0	1007.5	1072.22
LOC104911647	SPATS2-like protein	993.1	1150.5	1071.82
OPTN	optineurin	1065.5	1078.1	1071.78
PTGES2	prostaglandin E synthase 2	1107.0	1036.4	1071.68
LOC100544511	selenoprotein K	1038.6	1103.6	1071.11
GART	phosphoribosylglycinamide formyltransferase, phosphoribosylglycinamide synthetase, phosphoribosylaminoimidazole synthetase	1061.4	1080.3	1070.88
PHF5A	PHD finger protein 5A	1110.2	1028.6	1069.42
DCAF10	DDB1 and CUL4 associated factor 10	1050.6	1087.8	1069.20
KMT2C	lysine (K)-specific methyltransferase 2C	1017.7	1120.5	1069.10
ABCA7	ATP-binding cassette, sub-family A (ABC1), member 7	1123.7	1014.2	1068.96

TIMM44	translocase of inner mitochondrial membrane 44 homolog (yeast)	1095.2	1042.7	1068.95
VPS45	vacuolar protein sorting 45 homolog (S. cerevisiae)	1114.0	1023.9	1068.94
FBXO45	F-box protein 45	1032.8	1104.1	1068.45
HPS1	Hermansky-Pudlak syndrome 1	1135.1	1001.4	1068.27
LOC104915312	phospholipase A-2-activating protein-like	1054.4	1080.7	1067.58
MCUR1	mitochondrial calcium uniporter regulator 1	1043.8	1091.2	1067.49
OSBPL3	oxysterol binding protein-like 3	1010.3	1121.9	1066.12
MYO16	myosin XVI	1064.0	1067.0	1065.52
LOC104916247	chromosome unknown open reading frame, human C1orf43	1110.7	1019.9	1065.31
LOC104911118	exostosin-2-like	1027.8	1102.0	1064.89
THAP9	THAP domain containing 9	1032.9	1096.1	1064.53
C8H10orf76	chromosome 8 open reading frame, human C10orf76	1115.7	1012.3	1063.99
CDC42EP4	CDC42 effector protein (Rho GTPase binding) 4	1098.6	1029.4	1063.99
PJA2	praja ring finger 2, E3 ubiquitin protein ligase	1036.9	1090.4	1063.62
NPEPL1	aminopeptidase-like 1	1056.3	1070.2	1063.24
ATAD3A	ATPase family, AAA domain containing 3A	1109.6	1016.9	1063.24
KIAA1429	KIAA1429 ortholog	980.5	1144.4	1062.44
VIPAS39	VPS33B interacting protein, apical-basolateral polarity regulator, spe-39 homolog	1066.8	1058.0	1062.35
POLR2I	polymerase (RNA) II (DNA directed) polypeptide I, 14.5kDa	1076.8	1047.4	1062.10
NEMF	nuclear export mediator factor	1099.7	1023.2	1061.44
ATG3	autophagy related 3	1058.8	1063.8	1061.30
TMBIM4	transmembrane BAX inhibitor motif containing 4	1018.4	1100.9	1059.66
NEK4	NIMA-related kinase 4	1080.6	1036.4	1058.51
AGPS	alkylglycerone phosphate synthase	1063.1	1053.9	1058.48
NUBP2	nucleotide binding protein 2	1105.4	1011.4	1058.40
GSTA2	glutathione S-transferase	983.9	1132.1	1058.00
PHOSPHO1	phosphatase, orphan 1	1080.1	1034.4	1057.23
FNDC3B	fibronectin type III domain containing 3B	948.7	1165.5	1057.09
TOR1AIP1	torsin A interacting protein 1	994.0	1118.1	1056.02
CALCOCO2	calcium binding and coiled-coil domain 2	1060.4	1051.1	1055.75
CLCN7	chloride channel, voltage-sensitive 7	1089.7	1020.8	1055.27
LOC104916864	serine hydroxymethyltransferase, mitochondrial-like	1239.4	871.1	1055.22
DHX37	DEAH (Asp-Glu-Ala-His) box polypeptide 37	1040.4	1069.4	1054.90
SYNPO2	synaptopodin 2	977.5	1132.3	1054.87
HS6ST1	heparan sulfate 6-O-sulfotransferase 1	1031.3	1077.5	1054.43
BCAS2	breast carcinoma amplified sequence 2	1056.8	1052.0	1054.41
UTP18	UTP18 small subunit (SSU) processome component homolog (yeast)	1087.3	1021.4	1054.36
PHLDA2	pleckstrin homology-like domain, family A, member 2	1145.9	961.2	1053.57
APEH	acylaminoacyl-peptide hydrolase	1111.5	994.1	1052.82
RAB11FIP1	RAB11 family interacting protein 1 (class I)	982.4	1123.2	1052.81
IRAK2	interleukin-1 receptor-associated kinase 2	1017.8	1087.3	1052.54
FGFR1	fibroblast growth factor receptor 1	1124.1	979.5	1051.81
RIN2	Ras and Rab interactor 2	1061.3	1041.2	1051.26
GPS1	G protein pathway suppressor 1	1064.1	1038.0	1051.07
LDB3	LIM domain binding 3	989.2	1110.9	1050.07
ADAM23	ADAM metallopeptidase domain 23	1018.5	1081.4	1049.95
LOC100544587	WASH complex subunit CCDC53-like	960.2	1139.6	1049.92
RASGRP3	RAS guanyl releasing protein 3 (calcium and DAG-regulated)	997.7	1101.6	1049.62
TBL2	transducin (beta)-like 2	1041.5	1057.0	1049.29
TIMM22	translocase of inner mitochondrial membrane 22 homolog (yeast)	1034.7	1062.5	1048.59
TXNDC11	thioredoxin domain containing 11	992.4	1103.6	1048.02
FADS1	fatty acid desaturase 1	1090.2	1005.4	1047.77
NUP155	nucleoporin 155kDa	1100.6	991.8	1046.20
PLD1	phospholipase D1, phosphatidylcholine-specific	1011.1	1081.2	1046.13
ATF2	activating transcription factor 2	1011.6	1079.5	1045.56
LOC100549073	uncharacterized LOC100549073	1124.9	964.9	1044.89

NDUFA7	NADH dehydrogenase (ubiquinone) 1 alpha subcomplex, 7, 14.5kDa	1062.0	1026.8	1044.42
LOC100539246	activating molecule in BECN1-regulated autophagy protein 1-like	1077.6	1010.8	1044.19
LOC104912436	ubiquitin carboxyl-terminal hydrolase 24-like	999.9	1088.1	1044.00
PRKCQ	protein kinase C, theta	945.9	1141.0	1043.43
TMEM138	transmembrane protein 138	1109.7	976.7	1043.23
LOC100542393	adenylate cyclase type 9-like	1033.5	1052.7	1043.13
MRPL54	mitochondrial ribosomal protein L54	1108.6	977.0	1042.80
RAI14	retinoic acid induced 14	1026.9	1058.4	1042.68
SPRTN	SprT-like N-terminal domain	1031.0	1054.0	1042.47
SYNGR1	synaptogyrin 1	1029.9	1054.8	1042.35
SKP2	S-phase kinase-associated protein 2, E3 ubiquitin protein ligase	1048.8	1035.5	1042.17
NQO1	NAD(P)H dehydrogenase, quinone 1	1086.0	998.1	1042.08
RBM45	RNA binding motif protein 45	1008.1	1074.9	1041.49
LOC100544471	nipped-B-like protein	1046.2	1036.2	1041.21
THAP7	THAP domain containing 7	1086.8	994.7	1040.75
CRYZ	crystallin, zeta (quinone reductase)	1051.1	1029.8	1040.45
GTF2F2	general transcription factor IIF, polypeptide 2, 30kDa	1033.5	1047.1	1040.27
MRC2	mannose receptor, C type 2	1074.1	1005.5	1039.85
NAIF1	nuclear apoptosis inducing factor 1	1067.5	1011.8	1039.64
RNF25	ring finger protein 25	1072.7	1005.8	1039.26
TCEA1	transcription elongation factor A (SII), 1	1030.0	1048.4	1039.17
LOC104911008	39S ribosomal protein L23, mitochondrial-like	1048.0	1029.1	1038.54
RAB30	RAB30, member RAS oncogene family	1000.7	1075.8	1038.28
LOC100550749	cullin-2	1010.0	1065.0	1037.46
BOD1	biorientation of chromosomes in cell division 1	997.0	1077.3	1037.13
RCAN3	RCAN family member 3	982.1	1091.3	1036.74
LOC100548465	NAD(P) transhydrogenase, mitochondrial-like	1037.8	1035.0	1036.43
LOC104912951	HEAT repeat-containing protein 3-like	1046.8	1023.9	1035.38
NUBP1	nucleotide binding protein 1	1048.8	1021.1	1034.96
CUEDC1	CUE domain containing 1	1091.1	978.3	1034.74
MED13	mediator complex subunit 13	952.1	1117.1	1034.61
RIC8A	RIC8 guanine nucleotide exchange factor A	1024.5	1044.7	1034.58
FAHD2A	fumarylacetoacetate hydrolase domain containing 2A	1086.7	981.7	1034.22
KAZALD1	Kazal-type serine peptidase inhibitor domain 1	1096.4	971.3	1033.84
MAN2C1	mannosidase, alpha, class 2C, member 1	1080.5	987.0	1033.75
PRKCA	protein kinase C, alpha	1089.3	978.0	1033.67
LOC100539216	trinucleotide repeat-containing gene 18 protein-like	1045.9	1021.0	1033.45
TTC38	tetratricopeptide repeat domain 38	1028.7	1037.5	1033.12
BRAP	BRCA1 associated protein	983.9	1081.8	1032.85
LRIG2	leucine-rich repeats and immunoglobulin-like domains 2	963.0	1101.0	1032.00
SQLE	squalene epoxidase	1035.2	1028.6	1031.88
PPP3CB	protein phosphatase 3, catalytic subunit, beta isozyme	988.1	1075.6	1031.83
ALG9	ALG9, alpha-1,2-mannosyltransferase	1032.9	1030.3	1031.61
RFXANK	regulatory factor X-associated ankyrin-containing protein	1126.5	936.3	1031.39
NT5C3B	5'-nucleotidase, cytosolic IIIB	1094.3	966.7	1030.49
CEP131	centrosomal protein 131kDa	1076.4	983.9	1030.14
ROR2	receptor tyrosine kinase-like orphan receptor 2	971.2	1088.7	1029.98
STX1A	syntaxin 1A (brain)	1064.3	995.3	1029.79
FLOT2	flotillin 2	1041.6	1017.9	1029.75
ANKRA2	ankyrin repeat, family A (RFXANK-like), 2	1004.8	1054.4	1029.60
LOC100542816	annexin A1	999.0	1060.1	1029.54
CNOT6	CCR4-NOT transcription complex, subunit 6	953.5	1105.3	1029.38
RIC8B	RIC8 guanine nucleotide exchange factor B	1017.3	1040.4	1028.84
AUP1	ancient ubiquitous protein 1	1047.2	1010.4	1028.82
LOC104913037	uncharacterized LOC104913037	970.2	1086.4	1028.29
POLR2H	polymerase (RNA) II (DNA directed) polypeptide H	1022.5	1033.4	1027.91
TMCO1	transmembrane and coiled-coil domains 1	988.0	1067.7	1027.81

NUP153	nucleoporin 153kDa	983.1	1072.2	1027.62
BCL6	B-cell CLL/lymphoma 6	954.0	1099.5	1026.78
DTYMK	deoxythymidylate kinase (thymidylate kinase)	1078.1	975.3	1026.70
P4HA2	prolyl 4-hydroxylase, alpha polypeptide II	969.7	1082.2	1025.95
LOC104910179	protein cordon-bleu-like	961.9	1088.9	1025.38
LOC104911102	cytoskeleton-associated protein 5-like	1006.7	1043.8	1025.26
ZER1	zyg-11 related, cell cycle regulator	987.6	1062.5	1025.06
DDX18	DEAD (Asp-Glu-Ala-Asp) box polypeptide 18	1032.8	1016.3	1024.53
BYSL	bystin-like	1036.3	1012.6	1024.48
IER5	immediate early response 5	1095.9	952.4	1024.17
NAA16	N(alpha)-acetyltransferase 16, NatA auxiliary subunit	1015.8	1030.0	1022.89
LOC104913965	NF-kappa-B-activating protein-like	999.3	1046.1	1022.69
LOC100547760	beclin-1	1021.7	1022.6	1022.15
GATSL2	GATS protein-like 2	1039.6	1004.4	1021.96
NDUFA9	NADH dehydrogenase (ubiquinone) 1 alpha subcomplex, 9, 39kDa	1057.1	985.1	1021.09
SH3GLB2	SH3-domain GRB2-like endophilin B2	971.4	1070.5	1020.90
ATP6V0B	ATPase, H+ transporting, lysosomal 21kDa, V0 subunit b	993.2	1047.7	1020.43
CHAC1	ChaC glutathione-specific gamma-glutamylcyclotransferase 1	1103.2	936.6	1019.93
GSR	glutathione reductase	1061.3	978.2	1019.74
SPG11	spastic paraplegia 11 (autosomal recessive)	1032.1	1007.3	1019.71
PDPN	podoplanin	1030.1	1009.0	1019.57
NUP214	nucleoporin 214kDa	1051.6	986.5	1019.05
HMBS	hydroxymethylbilane synthase	1039.6	997.0	1018.26
MMGT1	membrane magnesium transporter 1	1046.4	989.3	1017.83
ACO1	aconitase 1, soluble	1025.1	1009.5	1017.30
SUCLG2	succinate-CoA ligase, GDP-forming, beta subunit	1013.2	1018.8	1015.97
LOC100539727	general transcription factor II-I	1067.6	963.2	1015.39
LOC104909920	collagen alpha-1(XII) chain-like	1082.8	947.7	1015.22
OGDH	oxoglutarate (alpha-ketoglutarate) dehydrogenase (lipoamide)	1082.2	948.2	1015.19
LOC100546272	nuclear receptor corepressor 1-like	981.6	1048.6	1015.12
RRP1B	ribosomal RNA processing 1B	1031.9	994.9	1013.40
ZNF711	zinc finger protein 711	979.6	1046.8	1013.17
TMEM248	transmembrane protein 248	1018.3	1007.9	1013.10
LOC100540154	carbonic anhydrase 9-like	839.8	1185.1	1012.43
SORBS1	sorbin and SH3 domain containing 1	1012.6	1011.3	1011.92
LRIG1	leucine-rich repeats and immunoglobulin-like domains 1	942.0	1081.6	1011.79
MRTO4	mRNA turnover 4 homolog (S. cerevisiae)	1056.3	965.6	1010.95
DCUN1D5	DCN1, defective in cullin neddylation 1, domain containing 5	1022.4	998.5	1010.44
CCDC71	coiled-coil domain containing 71	976.6	1043.8	1010.18
WBP1	WW domain binding protein 1	1085.5	934.8	1010.16
HMGCS1	3-hydroxy-3-methylglutaryl-CoA synthase 1 (soluble)	1051.8	968.0	1009.91
POLDIP3	polymerase (DNA-directed), delta interacting protein 3	1022.7	997.0	1009.84
TSC22D2	TSC22 domain family, member 2	985.6	1033.4	1009.48
WDR91	WD repeat domain 91	1027.7	990.7	1009.17
SEH1L	SEH1-like (S. cerevisiae)	959.9	1057.3	1008.62
LOC104912883	uncharacterized LOC104912883	994.1	1022.7	1008.39
LOC100539641	glycosyltransferase-like protein LARGE1	970.5	1045.9	1008.22
FBXL20	F-box and leucine-rich repeat protein 20	1027.9	988.4	1008.15
TEX264	testis expressed 264	1066.0	950.1	1008.07
LOC104913441	uncharacterized LOC104913441	961.0	1054.4	1007.72
ETV6	ets variant 6	1013.3	1002.0	1007.68
EXOSC2	exosome component 2	1014.2	1001.0	1007.62
RAB3IL1	RAB3A interacting protein (rabin3)-like 1	1076.3	938.5	1007.42
NME7	NME/NM23 family member 7	1069.9	944.9	1007.40
MAP2K2	mitogen-activated protein kinase kinase 2	1029.6	985.0	1007.33
FKRP	fukutin related protein	1045.5	968.7	1007.09
TMA16	translation machinery associated 16 homolog (S. cerevisiae)	1012.2	1001.1	1006.67
ACYP2	acylphosphatase 2, muscle type	972.4	1039.9	1006.14

NIPSNAP3A	nipsnap homolog 3A (C. elegans)	1034.3	977.7	1006.02
SLC27A4	solute carrier family 27 (fatty acid transporter), member 4	1040.7	970.8	1005.75
EXOSC10	exosome component 10	1004.9	1005.7	1005.28
TM6SF1	transmembrane 6 superfamily member 1	1051.0	958.5	1004.75
LOC100548555	pre-mRNA 3' end processing protein WDR33	1047.2	961.0	1004.13
IARS2	isoleucyl-tRNA synthetase 2, mitochondrial	1031.0	976.7	1003.87
RPP25L	ribonuclease P/MRP 25kDa subunit-like	1071.3	936.0	1003.65
ABHD2	abhydrolase domain containing 2	942.8	1064.2	1003.49
CTBP2	C-terminal binding protein 2	1031.0	975.3	1003.16
ST6GALNAC4	ST6 (alpha-N-acetyl-neuraminyl-2,3-beta-galactosyl-1,3)-N-acetylgalactosaminide alpha-2,6-sialyltransferase 4	981.9	1022.3	1002.09
MAPK13	mitogen-activated protein kinase 13	905.6	1098.5	1002.05
MDM4	MDM4, p53 regulator	999.6	1004.3	1001.93
RALGPS2	Ral GEF with PH domain and SH3 binding motif 2	928.6	1073.2	1000.89
SLC35F6	solute carrier family 35, member F6	1092.9	907.2	1000.03
AK2	adenylate kinase 2	1057.6	942.2	999.92
CCDC93	coiled-coil domain containing 93	1007.5	992.2	999.85
SESN1	sestrin 1	993.2	1006.4	999.84
TCTN3	tectonic family member 3	980.6	1018.2	999.43
ERCC3	excision repair cross-complementation group 3	983.6	1015.2	999.40
PSPH	phosphoserine phosphatase	1090.6	906.8	998.68
NUP107	nucleoporin 107kDa	997.4	997.4	997.39
MRGBP	MRG/MORF4L binding protein	997.8	996.8	997.31
P3H2	prolyl 3-hydroxylase 2	938.7	1055.6	997.17
RNF139	ring finger protein 139	975.5	1017.2	996.35
PLBD1	phospholipase B domain containing 1	1032.8	957.5	995.16
PSIP1	PC4 and SFRS1 interacting protein 1	996.6	993.2	994.88
HMGXB4	HMG box domain containing 4	981.2	1007.9	994.55
PMM2	phosphomannomutase 2	970.0	1017.2	993.63
LOC104915776	mitochondrial inner membrane protein OXA1L	1048.9	937.8	993.35
RBMX2	RNA binding motif protein, X-linked 2	1080.7	905.9	993.31
BLOC1S2	biogenesis of lysosomal organelles complex-1, subunit 2	997.1	988.7	992.89
WFS1	Wolfram syndrome 1 (wolframin)	928.3	1057.2	992.74
MAR6	membrane-associated ring finger (C3HC4) 6, E3 ubiquitin protein ligase	966.9	1018.2	992.56
LOC100543226	protein FAM65C-like	965.3	1019.1	992.20
GPN1	GPN-loop GTPase 1	1074.7	909.5	992.13
LOC104915510	gem-associated protein 5-like	1003.3	980.9	992.07
AK1	adenylate kinase 1	938.5	1044.1	991.29
LOC100550182	thyroglobulin	937.2	1044.7	990.97
PPP6C	protein phosphatase 6, catalytic subunit	1053.7	928.1	990.88
LOC100540612	protein kinase C-binding protein 1-like	929.8	1051.9	990.86
PATL1	protein associated with topoisomerase II homolog 1 (yeast)	1014.9	966.0	990.44
COQ5	coenzyme Q5 homolog, methyltransferase (S. cerevisiae)	1019.9	960.7	990.26
LOC104915774	uncharacterized LOC104915774	1046.1	934.3	990.19
LOC104914039	uncharacterized LOC104914039	999.4	981.0	990.18
GADD45G	growth arrest and DNA-damage-inducible, gamma	955.9	1024.2	990.09
LOC100547618	uncharacterized LOC100547618	1031.9	948.2	990.05
DPAGT1	dolichyl-phosphate (UDP-N-acetylglucosamine) N-acetylglucosaminephosphotransferase 1 (GlcNAc-1-P transferase)	980.6	999.4	990.03
PDE4B	phosphodiesterase 4B, cAMP-specific	961.1	1017.1	989.11
RNF19A	ring finger protein 19A, RBR E3 ubiquitin protein ligase	1045.3	932.2	988.73
KIF1B	kinesin family member 1B	948.7	1028.7	988.70
PPIL2	peptidylprolyl isomerase (cyclophilin)-like 2	1074.7	902.6	988.64
NSMCE4A	non-SMC element 4 homolog A (S. cerevisiae)	1012.2	963.8	988.01
PDE4D	phosphodiesterase 4D, cAMP-specific	956.6	1018.1	987.36
CC2D1B	coiled-coil and C2 domain containing 1B	961.9	1012.8	987.34
ATP6V1H	ATPase, H+ transporting, lysosomal 50/57kDa, V1 subunit H	949.0	1025.3	987.18

TDRD3	tudor domain containing 3	981.1	992.9	987.02
TOP1	topoisomerase (DNA) I	958.7	1015.1	986.87
LOC104916040	3-beta-hydroxysteroid-Delta(8),Delta(7)-isomerase-like	1048.9	924.1	986.52
ZBTB2	zinc finger and BTB domain containing 2	963.7	1009.3	986.48
CDK2	cyclin-dependent kinase 2	1071.7	901.0	986.34
SETDB1	SET domain, bifurcated 1	1030.6	941.9	986.29
FAM114A2	family with sequence similarity 114, member A2	1010.8	960.9	985.87
EAF1	ELL associated factor 1	954.1	1016.9	985.49
LOC100551040	lambda-crystallin-like	1021.6	949.1	985.38
RCOR3	REST corepressor 3	941.6	1025.3	983.45
ABRA	actin-binding Rho activating protein	947.5	1019.3	983.42
CIAO1	cytosolic iron-sulfur assembly component 1	1025.8	940.7	983.26
INPP4A	inositol polyphosphate-4-phosphatase, type I, 107kDa	887.1	1079.1	983.07
LOC100542241	CREB-binding protein-like	962.3	1003.8	983.06
CASC3	cancer susceptibility candidate 3	1070.2	894.8	982.52
LOC104911507	raftlin-like	993.1	970.7	981.93
TNFRSF19	tumor necrosis factor receptor superfamily, member 19	939.2	1024.5	981.82
DPYSL5	dihydropyrimidinase-like 5	1022.4	939.7	981.04
ANAPC4	anaphase promoting complex subunit 4	955.8	1005.7	980.79
FNTA	farnesyltransferase, CAAX box, alpha	1033.3	927.3	980.32
ARPC5L	actin related protein 2/3 complex, subunit 5-like	993.0	967.5	980.27
ELOF1	elongation factor 1 homolog (S. cerevisiae)	1074.0	886.0	980.02
PPM1L	protein phosphatase, Mg2+/Mn2+ dependent, 1L	927.6	1032.2	979.90
LOC100542624	son of sevenless homolog 1-like	940.1	1017.5	978.83
TMEM70	transmembrane protein 70	1010.6	945.7	978.13
TIA1	TIA1 cytotoxic granule-associated RNA binding protein	955.3	1001.0	978.12
LOC104912926	low-density lipoprotein receptor-related protein 3-like	892.8	1062.9	977.85
TOR1B	torsin family 1, member B (torsin B)	973.5	982.2	977.85
GPR125	G protein-coupled receptor 125	920.4	1033.6	977.03
SYDE2	synapse defective 1, Rho GTPase, homolog 2 (C. elegans)	950.1	1003.9	976.99
SDHAF2	succinate dehydrogenase complex assembly factor 2	1010.6	942.9	976.73
UBE2K	ubiquitin-conjugating enzyme E2K	973.4	979.9	976.67
FBXO3	F-box protein 3	970.9	982.3	976.61
GJA1	gap junction protein, alpha 1, 43kDa	926.6	1026.6	976.59
FRMD4A	FERM domain containing 4A	925.8	1026.7	976.23
FBXO11	F-box protein 11	971.9	980.1	976.03
LOC100548591	peptidyl-prolyl cis-trans isomerase D-like	998.7	953.3	975.97
PCNX	pecanex homolog (Drosophila)	957.0	994.2	975.61
ARFGAP3	ADP-ribosylation factor GTPase activating protein 3	921.3	1029.5	975.39
TARBP1	TAR (HIV-1) RNA binding protein 1	944.2	1006.5	975.31
C30H19orf70	chromosome 30 open reading frame, human C19orf70	1037.6	911.5	974.55
LARP6	La ribonucleoprotein domain family, member 6	980.3	968.6	974.48
TMED3	transmembrane emp24 protein transport domain containing 3	939.3	1008.5	973.91
LOC104909210	E3 ubiquitin-protein ligase KCMF1-like	931.3	1015.7	973.48
EGFR	epidermal growth factor receptor	857.7	1089.1	973.42
CCNK	cyclin K	913.8	1032.7	973.26
ZFYVE19	zinc finger, FYVE domain containing 19	1032.7	913.1	972.91
IER3IP1	immediate early response 3 interacting protein 1	959.4	986.2	972.82
PCCB	propionyl CoA carboxylase, beta polypeptide	1038.3	906.5	972.39
RBSN	rabenosyn, RAB effector	928.1	1016.6	972.35
TRIAP1	TP53 regulated inhibitor of apoptosis 1	969.3	975.0	972.13
BAZ1A	bromodomain adjacent to zinc finger domain, 1A	979.8	964.3	972.05
MLF1	myeloid leukemia factor 1	941.4	1002.7	972.05
EIF2B3	eukaryotic translation initiation factor 2B, subunit 3 gamma, 58kDa	982.8	961.0	971.90
LOC104913138	alpha/beta hydrolase domain-containing protein 14B-like	1037.9	905.6	971.75
MYO9A	myosin IXA	923.7	1019.8	971.73
FXN	frataxin	926.5	1016.4	971.43
APOO	apolipoprotein O	966.0	976.7	971.34

ARF6	ADP-ribosylation factor 6	976.2	966.0	971.11
TMEM184B	transmembrane protein 184B	947.8	994.4	971.08
FAM175B	family with sequence similarity 175, member B	942.3	999.5	970.86
TMEM132A	transmembrane protein 132A	1032.5	908.9	970.71
HIAT1	hippocampus abundant transcript 1	966.1	974.9	970.50
LOC104910590	uncharacterized LOC104910590	1005.7	934.9	970.27
C9HXorf56	chromosome 9 open reading frame, human CXorf56	981.3	956.0	968.66
C7H2orf47	chromosome 7 open reading frame, human C2orf47	997.9	939.2	968.56
ZNF326	zinc finger protein 326	950.8	986.0	968.41
LOC104917053	E3 ubiquitin-protein ligase HUWE1	965.4	971.3	968.35
LOC100549955	perilipin-3-like	1099.9	836.0	967.94
MAST4	microtubule associated serine/threonine kinase family member 4	900.2	1035.3	967.76
MRPL44	mitochondrial ribosomal protein L44	981.2	954.4	967.76
KLHDC4	kelch domain containing 4	1038.4	897.1	967.75
LTBP2	latent transforming growth factor beta binding protein 2	1108.6	825.9	967.27
LOC104914633	retinoic acid receptor alpha	1060.4	873.9	967.17
CAMTA1	calmodulin binding transcription activator 1	905.0	1029.3	967.14
APBA3	amyloid beta (A4) precursor protein-binding, family A, member 3	1039.8	893.4	966.59
DDX54	DEAD (Asp-Glu-Ala-Asp) box polypeptide 54	1025.3	907.8	966.56
BOLA3	bolA family member 3	1055.8	877.1	966.47
USP38	ubiquitin specific peptidase 38	910.2	1022.5	966.36
JADE3	jade family PHD finger 3	958.5	974.0	966.29
LOC100548137	phosphatidylinositol 4-phosphate 3-kinase C2 domain-containing subunit alpha	963.6	967.3	965.45
POR	P450 (cytochrome) oxidoreductase	976.6	954.3	965.42
ATP13A1	ATPase type 13A1	1001.9	928.0	964.96
LOC100545817	class I histocompatibility antigen, F10 alpha chain-like	1009.5	918.8	964.11
CDR2	cerebellar degeneration-related protein 2, 62kDa	960.1	967.1	963.59
ATL2	atlastin GTPase 2	965.2	961.4	963.29
LOC100548053	proline dehydrogenase 1, mitochondrial-like	1028.0	897.7	962.85
NEURL1B	neuralized E3 ubiquitin protein ligase 1B	1001.1	922.5	961.82
NR2C1	nuclear receptor subfamily 2, group C, member 1	949.2	974.1	961.63
GID8	GID complex subunit 8	945.7	977.4	961.57
TMEM50B	transmembrane protein 50B	966.7	955.9	961.26
TES	testis derived transcript (3 LIM domains)	975.2	946.3	960.79
WAPAL	wings apart-like homolog (Drosophila)	992.2	929.1	960.62
IMPA1	inositol(myo)-1(or 4)-monophosphatase 1	917.9	1003.1	960.46
CSNK1G1	casein kinase 1, gamma 1	967.1	952.3	959.74
DHCR24	24-dehydrocholesterol reductase	976.8	940.2	958.52
EIF2B4	eukaryotic translation initiation factor 2B, subunit 4 delta, 67kDa	1016.1	900.8	958.43
LOC100549354	cell division cycle 5-like protein	954.3	962.6	958.43
RRM2B	ribonucleotide reductase M2 B (TP53 inducible)	927.2	988.6	957.89
LRWD1	leucine-rich repeats and WD repeat domain containing 1	994.8	919.3	957.01
ZW10	zw10 kinetochore protein	946.8	965.6	956.18
MFSD11	major facilitator superfamily domain containing 11	966.1	945.8	955.94
DCTN6	dynactin 6	1030.3	881.2	955.73
LOC100549824	protein SAAL1-like	917.0	992.2	954.56
DPM2	dolichyl-phosphate mannosyltransferase polypeptide 2, regulatory subunit	978.1	931.0	954.52
LOC104910043	cell division cycle 5-like protein	924.6	984.4	954.49
SCYL2	SCY1-like 2 (S. cerevisiae)	928.7	980.0	954.37
IRF2BPL	interferon regulatory factor 2 binding protein-like	962.8	944.5	953.65
LOC104914886	chondroitin sulfate proteoglycan 4-like	982.1	924.6	953.32
LOC104914956	uncharacterized LOC104914956	1003.8	902.6	953.17
UPF2	UPF2 regulator of nonsense transcripts homolog (yeast)	949.2	956.0	952.64
MIEF1	mitochondrial elongation factor 1	917.3	987.7	952.51
AK6	adenylate kinase 6	937.1	967.2	952.11
HECW1	HECT, C2 and WW domain containing E3 ubiquitin protein ligase 1	942.7	960.1	951.39

SIPA1L2	signal-induced proliferation-associated 1 like 2	940.9	961.8	951.36
STYX	serine/threonine/tyrosine interacting protein	926.3	975.2	950.74
NUDT14	nudix (nucleoside diphosphate linked moiety X)-type motif 14	930.6	970.7	950.65
MCF2	MCF.2 cell line derived transforming sequence	954.5	946.6	950.52
LOC104909283	lethal(3)malignant brain tumor-like protein 3	912.7	987.5	950.07
CORO6	coronin 6	952.8	947.0	949.92
FAM160A2	family with sequence similarity 160, member A2	1037.6	860.6	949.10
TPR	translocated promoter region, nuclear basket protein	981.5	915.8	948.67
METTL21A	methyltransferase like 21A	937.3	959.6	948.41
C1H11orf73	chromosome 1 open reading frame, human C11orf73	974.3	922.5	948.40
TPD52L2	tumor protein D52-like 2	965.1	931.4	948.27
C1H12orf66	chromosome 1 open reading frame, human C12orf66	903.7	992.7	948.20
SLC7A1	solute carrier family 7 (cationic amino acid transporter, y+ system), member 1	907.9	987.9	947.93
ORC3	origin recognition complex, subunit 3	978.7	916.9	947.79
RBM17	RNA binding motif protein 17	944.0	951.1	947.55
FAAH	fatty acid amide hydrolase	974.4	918.6	946.50
MARVELD3	MARVEL domain containing 3	934.0	958.6	946.31
USP6NL	USP6 N-terminal like	874.2	1017.1	945.67
LOC100539601	CCR4-NOT transcription complex subunit 6-like	891.0	999.0	945.02
FAM162A	family with sequence similarity 162, member A	916.1	973.4	944.74
LOC100539630	rho GTPase-activating protein 39-like	921.8	967.6	944.66
CPSF4	cleavage and polyadenylation specific factor 4, 30kDa	944.2	944.9	944.57
SF3B5	splicing factor 3b, subunit 5, 10kDa	1018.4	870.6	944.53
LOC104917050	tripartite motif-containing protein 3-like	1046.8	841.4	944.10
EXOC6	exocyst complex component 6	901.0	987.1	944.07
C1H2orf49	chromosome 1 open reading frame, human C2orf49	942.4	945.6	944.02
DACT1	dishevelled-binding antagonist of beta-catenin 1	1022.5	865.0	943.78
PPAP2C	phosphatidic acid phosphatase type 2C	969.8	917.0	943.40
RIT1	Ras-like without CAAX 1	926.5	960.2	943.32
GTF2E1	general transcription factor IIE, polypeptide 1, alpha 56kDa	927.3	958.7	943.00
RPS6KA6	ribosomal protein S6 kinase, 90kDa, polypeptide 6	900.8	985.1	942.95
MAPKAPK2	mitogen-activated protein kinase-activated protein kinase 2	876.8	1009.0	942.92
AKAP17A	A kinase (PRKA) anchor protein 17A	877.4	1008.3	942.86
MYEOV2	myeloma overexpressed 2	953.2	932.0	942.63
QSOX1	quiescin Q6 sulfhydryl oxidase 1	975.8	908.7	942.25
MORN4	MORN repeat containing 4	1018.0	866.0	942.00
PRPSAP2	phosphoribosyl pyrophosphate synthetase-associated protein 2	895.7	988.1	941.91
KIAA1462	KIAA1462 ortholog	899.0	984.6	941.80
SFT2D1	SFT2 domain containing 1	928.1	955.0	941.53
LRRC20	leucine rich repeat containing 20	952.5	930.1	941.29
MAN2A1	mannosidase, alpha, class 2A, member 1	896.8	985.3	941.04
LOC104915892	dynactin subunit 2-like	1061.6	820.1	940.83
CREBL2	cAMP responsive element binding protein-like 2	913.5	967.5	940.52
MED30	mediator complex subunit 30	940.5	940.0	940.25
LOC104914768	uncharacterized LOC104914768	918.7	961.5	940.10
LOC104912320	parafibromin	865.4	1014.2	939.82
ARFIP2	ADP-ribosylation factor interacting protein 2	1029.6	849.9	939.73
PRPF4	pre-mRNA processing factor 4	921.2	957.5	939.37
LOC104916619	myosin light chain 3, skeletal muscle isoform-like	790.9	1087.2	939.02
LOC104916935	histone H1.11R-like	933.3	944.3	938.80
SCARB2	scavenger receptor class B, member 2	878.6	998.5	938.51
MFSD6	major facilitator superfamily domain containing 6	899.6	977.3	938.44
CDON	cell adhesion associated, oncogene regulated	1023.6	852.5	938.05
SNW1	SNW domain containing 1	903.5	972.4	937.95
ADAM9	ADAM metallopeptidase domain 9	938.2	937.6	937.92
CHST3	carbohydrate (chondroitin 6) sulfotransferase 3	959.4	915.1	937.24
LOC100540635	exocyst complex component 1	957.4	916.4	936.90

CPSF2	cleavage and polyadenylation specific factor 2, 100kDa	894.1	978.7	936.42
LOC104911835	bcl-2-like protein 13	917.3	954.0	935.66
POMT1	protein-O-mannosyltransferase 1	885.2	985.7	935.41
MCM3	minichromosome maintenance complex component 3	1044.3	826.1	935.18
MRPL22	mitochondrial ribosomal protein L22	959.9	910.4	935.13
LOC100539992	dolichol-phosphate mannosyltransferase subunit 1	955.4	913.8	934.57
NECAB3	N-terminal EF-hand calcium binding protein 3	1008.3	860.7	934.51
GRSF1	G-rich RNA sequence binding factor 1	914.5	954.1	934.31
NMT2	N-myristoyltransferase 2	922.9	945.5	934.20
SNX8	sorting nexin 8	886.0	980.3	933.17
FEM1B	fem-1 homolog b (C. elegans)	946.8	919.5	933.14
IL4R	interleukin 4 receptor	967.7	898.4	933.04
LOC104912448	oxysterol-binding protein-related protein 9	918.9	945.5	932.20
DISC1	disrupted in schizophrenia 1	911.5	952.2	931.81
NPRL3	nitrogen permease regulator-like 3 (S. cerevisiae)	957.9	905.5	931.66
ARHGEF18	Rho/Rac guanine nucleotide exchange factor (GEF) 18	887.6	975.1	931.33
RBM8A	RNA binding motif protein 8A	1016.8	845.0	930.91
PLBD2	phospholipase B domain containing 2	965.0	896.5	930.77
BET1L	Bet1 golgi vesicular membrane trafficking protein-like	957.8	903.5	930.67
CHCHD6	coiled-coil-helix-coiled-coil-helix domain containing 6	950.8	910.1	930.43
TMEM117	transmembrane protein 117	718.5	1141.8	930.15
CLPTM1L	CLPTM1-like	928.2	930.8	929.53
LOC104912895	furin-like	997.0	860.7	928.82
LOC104912931	probable C-mannosyltransferase DPY19L3	893.5	962.0	927.78
OSTF1	osteoclast stimulating factor 1	925.1	929.9	927.52
STX12	syntaxin 12	900.2	954.6	927.40
DUSP3	dual specificity phosphatase 3	875.1	979.0	927.07
CCND1	cyclin D1	877.7	975.6	926.67
LOC100543540	transmembrane protein 189-like	869.0	983.5	926.25
IRS4	insulin receptor substrate 4	889.3	962.6	925.91
MRPL42	mitochondrial ribosomal protein L42	928.5	921.3	924.90
TFCP2	transcription factor CP2	961.2	888.0	924.61
TBL1X	transducin (beta)-like 1X-linked	922.2	926.8	924.49
NOL11	nucleolar protein 11	937.9	910.7	924.33
ALS2	amyotrophic lateral sclerosis 2 (juvenile)	859.6	988.8	924.19
MRPS28	mitochondrial ribosomal protein S28	891.7	954.1	922.88
POLR2D	polymerase (RNA) II (DNA directed) polypeptide D	910.9	934.2	922.53
BCAP29	B-cell receptor-associated protein 29	867.2	977.4	922.32
MVK	mevalonate kinase	944.1	900.5	922.31
TAF1A	TATA box binding protein (TBP)-associated factor, RNA polymerase I, A, 48kDa	917.0	925.4	921.19
MTO1	mitochondrial tRNA translation optimization 1	924.5	917.5	920.99
ZDHHC6	zinc finger, DHHC-type containing 6	939.0	902.4	920.69
HEATR5A	HEAT repeat containing 5A	834.6	1005.6	920.12
RYR3	ryanodine receptor 3	853.8	985.4	919.61
CDK5RAP3	CDK5 regulatory subunit associated protein 3	947.6	890.8	919.23
GORAB	golgin, RAB6-interacting	874.2	964.2	919.19
CFAP54	cilia and flagella associated 54	923.4	912.5	917.96
CNDP2	CNDP dipeptidase 2 (metallopeptidase M20 family)	951.6	884.1	917.88
LOC104912691	uncharacterized LOC104912691	868.9	966.3	917.62
OTUD4	OTU deubiquitinase 4	851.4	983.9	917.62
SAV1	salvador family WW domain containing protein 1	942.7	891.8	917.26
RARRES2	retinoic acid receptor responder (tazarotene induced) 2	965.2	869.1	917.14
FEM1A	fem-1 homolog a (C. elegans)	922.3	911.8	917.05
RNF126	ring finger protein 126	938.3	894.8	916.54
GULP1	GULP, engulfment adaptor PTB domain containing 1	820.3	1012.7	916.53
CHSY1	chondroitin sulfate synthase 1	873.4	959.4	916.44

CTDSPL2	CTD (carboxy-terminal domain, RNA polymerase II, polypeptide A) small phosphatase like 2	920.6	911.5	916.07
TBC1D8B	TBC1 domain family, member 8B (with GRAM domain)	886.1	945.7	915.90
NUB1	negative regulator of ubiquitin-like proteins 1	950.7	880.8	915.75
ACADSB	acyl-CoA dehydrogenase, short/branched chain	897.6	932.8	915.20
RPTOR	regulatory associated protein of MTOR, complex 1	965.6	864.5	915.05
BTAF1	BTAF1 RNA polymerase II, B-TFIID transcription factor-associated, 170kDa	873.6	956.1	914.86
MGAT1	mannosyl (alpha-1,3-)-glycoprotein beta-1,2-N-acetylglucosaminyltransferase	994.1	835.1	914.62
MERTK	MER proto-oncogene, tyrosine kinase	910.6	918.5	914.53
ST13	suppression of tumorigenicity 13 (colon carcinoma) (Hsp70 interacting protein)	846.9	981.9	914.43
MINA	MYC induced nuclear antigen	900.9	927.7	914.30
GOSR2	golgi SNAP receptor complex member 2	906.3	922.1	914.22
PRR7	proline rich 7 (synaptic)	952.1	876.0	914.02
RNPEPL1	arginyl aminopeptidase (aminopeptidase B)-like 1	897.6	930.3	913.97
LOC100550801	GTP-binding protein 10-like	907.6	919.9	913.74
N4BP1	NEDD4 binding protein 1	857.0	969.8	913.40
LYRM1	LYR motif containing 1	923.0	903.7	913.34
APH1A	APH1A gamma secretase subunit	921.5	904.8	913.12
LOC100545422	acyl-coenzyme A thioesterase 5-like	914.4	911.4	912.91
ZNF628	zinc finger protein 628	957.5	868.3	912.90
CARHSP1	calcium regulated heat stable protein 1, 24kDa	896.1	929.3	912.70
RAB9B	RAB9B, member RAS oncogene family	897.9	927.2	912.52
MTMR9	myotubularin related protein 9	928.2	895.8	911.99
BAHD1	bromo adjacent homology domain containing 1	902.7	920.7	911.71
NKAP	NFKB activating protein	861.2	961.6	911.39
TGFBR1	transforming growth factor, beta receptor 1	898.5	924.0	911.27
CLK4	CDC-like kinase 4	896.0	926.0	910.98
LOC104914528	high affinity cGMP-specific 3',5'-cyclic phosphodiesterase 9A-like	946.3	875.7	910.95
UROD	uroporphyrinogen decarboxylase	962.7	859.2	910.93
JMJD1C	jumonji domain containing 1C	906.0	915.8	910.91
RHOT2	ras homolog family member T2	932.6	889.1	910.87
LOC100547239	ectoderm-neural cortex protein 1-like	900.6	921.0	910.83
MAPKAP1	mitogen-activated protein kinase associated protein 1	887.7	933.9	910.81
C2H6orf57	chromosome 2 open reading frame, human C6orf57	850.7	969.8	910.26
CDC37	cell division cycle 37	980.5	840.0	910.26
TRIM63	tripartite motif containing 63, E3 ubiquitin protein ligase	983.8	836.1	909.98
LGR5	leucine-rich repeat containing G protein-coupled receptor 5	841.6	978.1	909.90
METAP1	methionyl aminopeptidase 1	877.4	942.3	909.86
RBM12	RNA binding motif protein 12	897.4	922.1	909.75
LOC100551412	cytochrome b ascorbate-dependent protein 3	916.4	902.4	909.40
MTMR1	myotubularin related protein 1	910.3	908.2	909.26
BAG4	BCL2-associated athanogene 4	873.3	945.1	909.21
VPS33A	vacuolar protein sorting 33 homolog A (S. cerevisiae)	920.4	897.7	909.05
RGS9	regulator of G-protein signaling 9	907.1	909.7	908.44
FLCN	folliculin	925.0	891.8	908.38
PPIL4	peptidylprolyl isomerase (cyclophilin)-like 4	878.9	937.1	908.02
LOC104917466	E3 ubiquitin-protein ligase MYCBP2-like	858.8	957.0	907.93
LOC104909231	uncharacterized LOC104909231	947.7	867.0	907.35
SH3PXD2B	SH3 and PX domains 2B	855.9	958.6	907.24
LOC104912428	cytochrome P450 2J2-like	946.5	867.7	907.13
LOC100540989	copine-3	927.1	886.6	906.86
POLR1D	polymerase (RNA) I polypeptide D, 16kDa	883.1	929.9	906.50
B3GNT1	UDP-GlcNAc:betaGal beta-1,3-N-acetylglucosaminyltransferase 1	1088.2	724.3	906.26
ZNF365	zinc finger protein 365	862.3	950.1	906.18
PNPLA8	patatin-like phospholipase domain containing 8	891.7	920.2	905.92

NMI	N-myc (and STAT) interactor	893.4	916.6	905.01
PTPLAD1	protein tyrosine phosphatase-like A domain containing 1	908.6	898.9	903.80
ZNF648	zinc finger protein 648	926.4	880.4	903.38
TRAPPC11	trafficking protein particle complex 11	883.1	922.8	902.96
ZNF512	zinc finger protein 512	887.7	917.4	902.52
XPNPEP3	X-prolyl aminopeptidase (aminopeptidase P) 3, putative	948.1	856.9	902.52
CLTA	clathrin, light chain A	904.1	900.8	902.45
SMIM13	small integral membrane protein 13	920.1	884.6	902.36
RNF214	ring finger protein 214	897.2	907.2	902.23
GPR176	G protein-coupled receptor 176	810.2	993.9	902.06
DHODH	dihydroorotate dehydrogenase (quinone)	944.2	859.8	901.99
LOC104916386	integrin alpha-3-like	992.6	810.8	901.70
TMEM256	transmembrane protein 256	939.9	863.1	901.54
NUP54	nucleoporin 54kDa	912.1	890.9	901.50
C5H11orf31	chromosome 5 open reading frame, human C11orf31	1026.8	775.9	901.34
PAK1IP1	PAK1 interacting protein 1	864.6	937.9	901.25
LOC100548069	dnaJ homolog subfamily A member 1-like	995.7	806.7	901.21
PPA2	pyrophosphatase (inorganic) 2	897.6	904.5	901.03
NCAPD3	non-SMC condensin II complex, subunit D3	960.0	842.0	901.02
ZDHHC5	zinc finger, DHHC-type containing 5	842.3	959.0	900.62
MYC	v-myc avian myelocytomatosis viral oncogene homolog	919.2	881.9	900.59
DNMT3A	DNA (cytosine-5-)-methyltransferase 3 alpha	934.2	866.5	900.35
GTF2E2	general transcription factor IIE, polypeptide 2, beta 34kDa	898.4	901.9	900.12
HPS6	Hermansky-Pudlak syndrome 6	939.1	860.9	900.04
LOC100538708	kinesin-like protein KIF2A	898.5	901.5	900.03
UMPS	uridine monophosphate synthetase	904.1	895.2	899.63
SLC25A4	solute carrier family 25 (mitochondrial carrier; adenine nucleotide translocator), member 4	926.1	873.1	899.59
INIP	INTS3 and NABP interacting protein	924.4	874.5	899.45
STX7	syntaxin 7	888.9	909.3	899.12
YAP1	Yes-associated protein 1	810.9	985.4	898.17
LOC104909257	glutamate receptor ionotropic, NMDA 3B-like	963.6	832.7	898.11
PPFIA1	protein tyrosine phosphatase, receptor type, f polypeptide (PTPRF), interacting protein (liprin), alpha 1	879.7	916.4	898.07
BRPF3	bromodomain and PHD finger containing, 3	853.9	942.0	897.95
MTOR	mechanistic target of rapamycin (serine/threonine kinase)	903.6	892.2	897.90
MED13L	mediator complex subunit 13-like	848.9	946.7	897.82
ABCC1	ATP-binding cassette, sub-family C (CFTR/MRP), member 1	859.7	935.9	897.80
GJD4	gap junction protein, delta 4, 40.1kDa	802.7	990.1	896.41
SPATS2	spermatogenesis associated, serine-rich 2	882.8	910.1	896.41
TOE1	target of EGR1, member 1 (nuclear)	935.0	857.8	896.38
LOC100550291	heat shock factor protein 3	835.6	957.0	896.31
SMAD9	SMAD family member 9	849.0	943.1	896.03
BPGM	2,3-bisphosphoglycerate mutase	921.1	870.8	895.98
RBM7	RNA binding motif protein 7	923.8	868.2	895.96
MRPL47	mitochondrial ribosomal protein L47	934.6	856.9	895.75
PRPF6	pre-mRNA processing factor 6	899.2	892.3	895.74
CCM2	cerebral cavernous malformation 2	905.5	885.5	895.49
MAP3K3	mitogen-activated protein kinase kinase kinase 3	889.5	901.1	895.31
ZBED4	zinc finger, BED-type containing 4	857.8	931.0	894.41
SNX6	sorting nexin 6	910.1	877.8	893.99
COL23A1	collagen, type XXIII, alpha 1	907.7	880.1	893.87
SLC35B4	solute carrier family 35 (UDP-xylose/UDP-N-acetylglucosamine transporter), member B4	915.3	872.0	893.63
WDR55	WD repeat domain 55	977.3	809.8	893.55
RBM20	RNA binding motif protein 20	855.6	930.7	893.14
DIABLO	diablo, IAP-binding mitochondrial protein	957.5	828.5	893.02

TCEB3	transcription elongation factor B (SIII), polypeptide 3 (110kDa, elongin A)	896.7	889.0	892.85
TANC1	tetratricopeptide repeat, ankyrin repeat and coiled-coil containing 1	852.1	933.2	892.63
FN3KRP	fructosamine 3 kinase related protein	851.0	934.2	892.62
LOC100539002	zinc finger CCHC domain-containing protein 14	869.8	914.8	892.32
NOC3L	nucleolar complex associated 3 homolog (S. cerevisiae)	890.2	893.2	891.66
POLR1B	polymerase (RNA) I polypeptide B, 128kDa	920.5	861.6	891.07
LOC104913725	myosin-7-like	868.6	913.5	891.02
LOC104912982	uncharacterized LOC104912982	875.9	905.8	890.84
LOC100539849	stress-induced-phosphoprotein 1-like	977.4	803.5	890.49
AKTIP	AKT interacting protein	846.8	933.9	890.38
PAPD7	PAP associated domain containing 7	921.1	859.5	890.30
NDUFAF3	NADH dehydrogenase (ubiquinone) complex I, assembly factor 3	937.4	842.5	889.96
LOC100550588	7-dehydrocholesterol reductase-like	906.6	873.0	889.81
LOC104909975	peroxidasin homolog	890.0	889.4	889.69
RBFOX2	RNA binding protein, fox-1 homolog (C. elegans) 2	885.2	892.2	888.69
KIAA0100	KIAA0100 ortholog	971.5	805.2	888.33
NCOA5	nuclear receptor coactivator 5	908.0	868.5	888.24
IFT20	intraflagellar transport 20	950.7	825.3	887.97
RMND1	required for meiotic nuclear division 1 homolog (S. cerevisiae)	881.3	894.1	887.71
MYOZ1	myozenin 1	831.1	944.0	887.56
PLEKHA3	pleckstrin homology domain containing, family A (phosphoinositide binding specific) member 3	867.8	907.3	887.55
B4GALT7	xylosylprotein beta 1,4-galactosyltransferase, polypeptide 7	864.9	909.7	887.30
TMEM194B	transmembrane protein 194B	889.3	884.7	886.97
LOC100540041	ATP-dependent RNA helicase DDX19B	928.8	844.2	886.51
KIAA0196	KIAA0196 ortholog	870.8	902.0	886.38
VRK3	vaccinia related kinase 3	879.3	893.4	886.35
TRAPPC5	trafficking protein particle complex 5	924.7	847.3	886.01
NUDCD3	NudC domain containing 3	930.8	840.9	885.84
C1D	C1D nuclear receptor corepressor	874.4	896.6	885.47
METTL16	methyltransferase like 16	869.5	900.9	885.19
EXOC2	exocyst complex component 2	850.8	918.7	884.79
WDR54	WD repeat domain 54	1042.3	726.7	884.51
DECR1	2,4-dienoyl CoA reductase 1, mitochondrial	1012.4	756.2	884.26
RAB3GAP1	RAB3 GTPase activating protein subunit 1 (catalytic)	829.2	938.3	883.73
INPP5F	inositol polyphosphate-5-phosphatase F	852.2	914.8	883.52
ACAT1	acetyl-CoA acetyltransferase 1	926.2	840.7	883.40
ZBTB8OS	zinc finger and BTB domain containing 8 opposite strand	835.1	931.4	883.28
SSBP1	single-stranded DNA binding protein 1, mitochondrial	886.5	880.0	883.26
C5H11orf24	chromosome 5 open reading frame, human C11orf24	877.7	888.8	883.25
RCC1	regulator of chromosome condensation 1	955.2	811.2	883.25
NEK1	NIMA-related kinase 1	858.0	906.8	882.42
SLC33A1	solute carrier family 33 (acetyl-CoA transporter), member 1	906.3	857.6	881.92
SETD6	SET domain containing 6	939.9	823.7	881.80
ZFAND1	zinc finger, AN1-type domain 1	850.5	913.0	881.77
CRNKL1	crooked neck pre-mRNA splicing factor 1	857.1	906.2	881.66
USF1	upstream transcription factor 1	933.3	829.5	881.43
SLC35G2	solute carrier family 35, member G2	849.6	911.1	880.35
ERAL1	Era-like 12S mitochondrial rRNA chaperone 1	876.7	882.6	879.65
SLC38A7	solute carrier family 38, member 7	889.2	870.0	879.60
IQGAP2	IQ motif containing GTPase activating protein 2	783.0	975.9	879.46
PIK3IP1	phosphoinositide-3-kinase interacting protein 1	827.1	931.5	879.34
ARFGEF2	ADP-ribosylation factor guanine nucleotide-exchange factor 2 (brefeldin A-inhibited)	855.5	902.8	879.14
LOC100542122	AP-1 complex-associated regulatory protein-like	881.6	876.4	878.97
AURKAIP1	aurora kinase A interacting protein 1	928.9	828.8	878.84

STAT1	signal transducer and activator of transcription 1, 91kDa	846.6	910.6	878.64
TP53I11	tumor protein p53 inducible protein 11	848.8	908.5	878.63
LOC104911584	N-acetylglucosamine-1-phosphotransferase subunits alpha/beta-like	893.8	862.1	877.97
NAB1	NGFI-A binding protein 1 (EGR1 binding protein 1)	856.3	899.2	877.77
STOML2	stomatin (EPB72)-like 2	890.1	865.0	877.53
E2F1	E2F transcription factor 1	979.6	775.2	877.38
MRPL21	mitochondrial ribosomal protein L21	858.6	895.7	877.16
LOC100541214	netrin-4	861.3	892.5	876.94
ZNF410	zinc finger protein 410	863.1	890.8	876.94
LOC104912427	cytochrome P450 2J2-like	895.0	858.8	876.90
MTHFD1	methylenetetrahydrofolate dehydrogenase (NADP+ dependent) 1, methenyltetrahydrofolate cyclohydrolase, formyltetrahydrofolate synthetase	871.9	881.3	876.61
INPP5E	inositol polyphosphate-5-phosphatase, 72 kDa	861.5	891.2	876.33
FRMD6	FERM domain containing 6	923.3	829.3	876.28
PPP2R5C	protein phosphatase 2, regulatory subunit B', gamma	872.2	880.2	876.22
BNIP2	BCL2/adenovirus E1B 19kDa interacting protein 2	834.1	917.8	875.94
SON	SON DNA binding protein	862.3	889.3	875.82
MCM4	minichromosome maintenance complex component 4	907.5	843.8	875.69
PIGS	phosphatidylinositol glycan anchor biosynthesis, class S	908.0	842.4	875.21
DCBLD2	discoidin, CUB and LCCL domain containing 2	869.7	880.5	875.09
LOC100547397	C-type lectin domain family 2 member B-like	916.6	833.5	875.02
NCOA6	nuclear receptor coactivator 6	835.4	914.7	875.01
SRSF2	serine/arginine-rich splicing factor 2	864.0	886.1	875.01
LONP1	lon peptidase 1, mitochondrial	890.9	858.6	874.77
C20H17orf70	chromosome 20 open reading frame, human C17orf70	924.8	824.7	874.76
ADAMTS10	ADAM metallopeptidase with thrombospondin type 1 motif, 10	867.7	881.5	874.63
PPIF	peptidylprolyl isomerase F	899.0	850.1	874.57
GNPTG	N-acetylglucosamine-1-phosphate transferase, gamma subunit	904.2	844.6	874.43
FOXO4	forkhead box O4	910.8	837.5	874.17
SLC20A1	solute carrier family 20 (phosphate transporter), member 1	903.7	844.6	874.14
LOC104916701	reticulon-3-like	901.6	845.8	873.68
LOC104909896	uncharacterized LOC104909896	863.9	882.4	873.14
ZMYM6NB	ZMYM6 neighbor	871.8	874.1	872.93
LOC104909808	uncharacterized LOC104909808	872.1	872.6	872.36
HSDL1	hydroxysteroid dehydrogenase like 1	791.5	953.1	872.31
NABP1	nucleic acid binding protein 1	908.4	835.6	872.02
C1QL1	complement component 1, q subcomponent-like 1	916.3	826.7	871.49
LOC100546443	spastin	816.4	925.4	870.87
FAM117A	family with sequence similarity 117, member A	940.7	800.7	870.73
PCOLCE2	procollagen C-endopeptidase enhancer 2	900.9	840.4	870.65
PAK2	p21 protein (Cdc42/Rac)-activated kinase 2	845.3	895.7	870.52
LOC104914059	phosphatidylinositol 3,4,5-trisphosphate-dependent Rac exchanger 1 protein-like	778.1	962.9	870.51
GTF3C2	general transcription factor IIIC, polypeptide 2, beta 110kDa	954.4	786.6	870.50
VPS41	vacuolar protein sorting 41 homolog (S. cerevisiae)	869.6	871.3	870.45
CBFB	core-binding factor, beta subunit	890.0	850.7	870.36
POLR1A	polymerase (RNA) I polypeptide A, 194kDa	838.7	901.6	870.14
NFU1	NFU1 iron-sulfur cluster scaffold	865.5	874.6	870.06
TADA3	transcriptional adaptor 3	990.6	748.9	869.75
MAGI1	membrane associated guanylate kinase, WW and PDZ domain containing 1	823.6	915.1	869.34
EZH2	enhancer of zeste 2 polycomb repressive complex 2 subunit	855.4	883.0	869.17
LYAR	Ly1 antibody reactive	835.8	900.9	868.34
TCF20	transcription factor 20 (AR1)	845.4	889.7	867.52
INTS8	integrator complex subunit 8	854.5	879.8	867.14
LOC100544252	protocadherin gamma-C5-like	818.7	915.3	867.00

LOC104912421	dedicator of cytokinesis protein 7-like	893.6	839.7	866.66
CDS2	CDP-diacylglycerol synthase (phosphatidate cytidylyltransferase) 2	862.3	870.9	866.60
SP1	Sp1 transcription factor	937.1	796.1	866.59
SLC39A10	solute carrier family 39 (zinc transporter), member 10	841.2	891.2	866.24
LOC104912258	rho GTPase-activating protein 29-like	902.5	829.9	866.19
ELF1	E74-like factor 1 (ets domain transcription factor)	845.2	887.0	866.09
SMURF2	SMAD specific E3 ubiquitin protein ligase 2	817.6	913.9	865.73
SSTR4	somatostatin receptor 4	809.6	921.8	865.66
ABCC5	ATP-binding cassette, sub-family C (CFTR/MRP), member 5	846.5	884.1	865.29
LOC104912747	mucosa-associated lymphoid tissue lymphoma translocation protein 1-like	859.8	870.6	865.24
HEPH	hephaestin	811.9	918.4	865.12
LOC100542272	1-phosphatidylinositol 4,5-bisphosphate phosphodiesterase gamma-1-like	896.9	833.3	865.10
MPHOSPH10	M-phase phosphoprotein 10 (U3 small nucleolar ribonucleoprotein)	879.7	850.0	864.87
TRAPPC8	trafficking protein particle complex 8	796.5	933.1	864.82
LOC100545101	F-box/WD repeat-containing protein 7-like	906.4	821.0	863.73
HES6	hes family bHLH transcription factor 6	907.7	819.3	863.49
PROSC	proline synthetase co-transcribed homolog (bacterial)	901.6	825.1	863.33
SH3RF3	SH3 domain containing ring finger 3	862.2	863.7	862.95
MAP4K5	mitogen-activated protein kinase kinase kinase kinase 5	848.6	877.0	862.83
RECQL	RecQ helicase-like	866.4	859.0	862.71
LOC104912562	uncharacterized LOC104912562	835.7	889.7	862.70
KIAA1109	KIAA1109 ortholog	837.1	888.2	862.63
LOC104914146	uncharacterized oxidoreductase C663.09c-like	895.1	829.3	862.20
KDELC2	KDEL (Lys-Asp-Glu-Leu) containing 2	901.0	823.2	862.09
MED20	mediator complex subunit 20	870.4	853.7	862.08
NRK	Nik related kinase	862.2	861.9	862.06
CAPN15	calpain 15	964.4	759.5	861.93
CCDC134	coiled-coil domain containing 134	953.3	770.2	861.76
HGSNAT	heparan-alpha-glucosaminide N-acetyltransferase	804.4	918.8	861.56
LOC104915049	focadhesin-like	825.1	897.8	861.46
ZNF652	zinc finger protein 652	804.8	916.9	860.85
TCIRG1	T-cell, immune regulator 1, ATPase, H+ transporting, lysosomal V0 subunit A3	946.3	775.4	860.84
FAF2	Fas associated factor family member 2	831.0	889.9	860.48
TIMMDC1	translocase of inner mitochondrial membrane domain containing 1	878.9	841.6	860.28
OAF	OAF homolog (Drosophila)	880.1	839.8	859.97
HACE1	HECT domain and ankyrin repeat containing E3 ubiquitin protein ligase 1	833.4	885.8	859.61
GAR1	GAR1 ribonucleoprotein	878.1	841.0	859.55
LOC100546254	cytochrome c oxidase assembly protein COX15 homolog	908.5	809.7	859.12
MORC4	MORC family CW-type zinc finger 4	863.9	853.9	858.89
KIAA0430	KIAA0430 ortholog	815.0	901.4	858.22
KATNAL1	katanin p60 subunit A-like 1	809.9	905.9	857.89
LOC104915761	josephin-2-like	918.7	797.1	857.89
IP6K2	inositol hexakisphosphate kinase 2	798.2	917.2	857.69
AKAP6	A kinase (PRKA) anchor protein 6	798.2	917.0	857.60
MDK	midkine (neurite growth-promoting factor 2)	796.8	916.8	856.78
RHBDF1	rhomboid 5 homolog 1 (Drosophila)	873.5	839.8	856.68
SIGMAR1	sigma non-opioid intracellular receptor 1	920.7	791.4	856.06
TANK	TRAF family member-associated NFKB activator	838.3	873.8	856.01
FAM149B1	family with sequence similarity 149, member B1	824.3	887.2	855.73
CETN2	centrin, EF-hand protein, 2	854.3	857.0	855.65
GINM1	glycoprotein integral membrane 1	826.8	883.7	855.21

TTC19	tetratricopeptide repeat domain 19	823.4	886.6	855.01
MED12	mediator complex subunit 12	860.1	849.7	854.91
ANAPC15	anaphase promoting complex subunit 15	889.2	820.4	854.82
CAPZA2	capping protein (actin filament) muscle Z-line, alpha 2	832.5	876.7	854.58
NIPSNAP1	nipsnap homolog 1 (C. elegans)	858.0	851.1	854.56
DNAJB1	DnaJ (Hsp40) homolog, subfamily B, member 1	855.8	852.2	853.99
IFNGR1	interferon gamma receptor 1	861.4	846.0	853.70
ZBTB10	zinc finger and BTB domain containing 10	801.4	906.0	853.70
MFSD5	major facilitator superfamily domain containing 5	911.2	795.1	853.16
SLC20A2	solute carrier family 20 (phosphate transporter), member 2	849.7	855.9	852.80
TMX2	thioredoxin-related transmembrane protein 2	843.8	861.2	852.49
ZNF839	zinc finger protein 839	826.7	877.9	852.29
FAM177A1	family with sequence similarity 177, member A1	879.4	825.0	852.23
DPP9	dipeptidyl-peptidase 9	848.9	854.6	851.79
HTATSF1	HIV-1 Tat specific factor 1	829.9	873.2	851.57
WBSCR22	Williams Beuren syndrome chromosome region 22	887.6	815.5	851.54
COG7	component of oligomeric golgi complex 7	861.3	841.1	851.19
CIRH1A	cirrhosis, autosomal recessive 1A (cirhin)	924.1	778.3	851.18
SMDT1	single-pass membrane protein with aspartate-rich tail 1	836.9	865.1	851.04
SLC9A8	solute carrier family 9, subfamily A (NHE8, cation proton antiporter 8), member 8	809.8	892.0	850.88
LOC100542113	latent-transforming growth factor beta-binding protein 1	735.0	966.4	850.71
PRIMA1	proline rich membrane anchor 1	810.2	890.7	850.46
TBC1D20	TBC1 domain family, member 20	883.4	817.0	850.22
LOC104914810	gamma-secretase subunit Aph-1b-like	892.8	806.3	849.57
FAM3C	family with sequence similarity 3, member C	831.8	866.7	849.26
ZHX2	zinc fingers and homeoboxes 2	854.5	843.3	848.92
CRLS1	cardiolipin synthase 1	823.0	874.3	848.66
MED15	mediator complex subunit 15	793.9	903.2	848.55
LOC104916976	homeobox protein six1-like	840.7	856.0	848.40
R3HDM1	R3H domain containing 1	822.2	873.9	848.05
PRMT3	protein arginine methyltransferase 3	856.0	838.8	847.43
C1H21orf33	chromosome 1 open reading frame, human C21orf33	920.7	773.5	847.09
RAB14	RAB14, member RAS oncogene family	795.4	896.8	846.08
DENND1A	DENN/MADD domain containing 1A	832.6	859.5	846.08
ARRDC2	arrestin domain containing 2	791.7	900.2	845.95
GGA3	golgi-associated, gamma adaptin ear containing, ARF binding protein 3	847.0	844.8	845.91
PNPLA2	patatin-like phospholipase domain containing 2	825.2	866.5	845.83
ARL8A	ADP-ribosylation factor-like 8A	867.4	823.9	845.65
INTS9	integrator complex subunit 9	826.8	864.4	845.59
KLHL2	kelch-like family member 2	869.0	819.5	844.26
LOC104913361	uncharacterized LOC104913361	822.5	865.5	844.01
JMJD4	jumonji domain containing 4	837.7	850.2	843.93
LOC100539914	solute carrier family 22 member 18-like	897.3	790.5	843.91
GNG11	guanine nucleotide binding protein (G protein), gamma 11	902.3	784.9	843.60
NRAP	nebulin-related anchoring protein	808.2	878.1	843.16
TAPT1	transmembrane anterior posterior transformation 1	792.0	894.3	843.16
FNBP1	formin binding protein 1	833.6	852.1	842.85
LOC104917367	cytochrome b-c1 complex subunit 9	899.1	785.3	842.22
DUSP8	dual specificity phosphatase 8	863.2	819.9	841.55
LOC100544124	uncharacterized LOC100544124	829.1	853.9	841.48
GFM1	G elongation factor, mitochondrial 1	849.5	833.2	841.32
E2F4	E2F transcription factor 4, p107/p130-binding	776.3	905.9	841.08
MYF6	myogenic factor 6 (herculin)	875.5	805.4	840.48
NAE1	NEDD8 activating enzyme E1 subunit 1	832.7	846.6	839.61
TRIT1	tRNA isopentenyltransferase 1	834.4	844.2	839.32
LOC104917024	5'-AMP-activated protein kinase subunit gamma-1-like	912.1	766.1	839.11

LIMS1	LIM and senescent cell antigen-like domains 1	850.3	827.6	838.94
MED17	mediator complex subunit 17	817.5	859.8	838.64
LOC104916041	immediate early response gene 5-like protein	889.7	786.5	838.11
LOC100547219	protein disulfide-isomerase A5-like	793.1	882.7	837.90
RCOR1	REST corepressor 1	858.3	817.1	837.70
ZNF609	zinc finger protein 609	906.0	769.4	837.68
AMOT	angiomotin	816.0	858.6	837.26
LOC100543013	NEDD4-like E3 ubiquitin-protein ligase WWP2	879.3	795.0	837.16
UBE2V1	ubiquitin-conjugating enzyme E2 variant 1	850.3	823.3	836.78
LOC104915160	uncharacterized LOC104915160	819.8	853.6	836.69
LOC100549326	transmembrane 4 L6 family member 1-like	819.4	853.7	836.55
RP2	retinitis pigmentosa 2 (X-linked recessive)	769.4	903.7	836.55
ECD	ecdysoneless homolog (Drosophila)	821.6	851.4	836.48
LOC104916918	NAD(P)H-hydrate epimerase-like	898.5	773.7	836.09
EXOC4	exocyst complex component 4	863.2	809.0	836.08
SAP30BP	SAP30 binding protein	776.0	896.0	836.00
GNA11	guanine nucleotide binding protein (G protein), alpha 11 (Gq class)	830.2	841.8	835.96
SOD3	superoxide dismutase 3, extracellular	802.4	869.4	835.94
UBASH3B	ubiquitin associated and SH3 domain containing B	835.3	836.3	835.82
NELFB	negative elongation factor complex member B	844.5	826.9	835.70
SPRYD7	SPRY domain containing 7	800.4	869.5	834.93
FUBP3	far upstream element (FUSE) binding protein 3	806.6	861.9	834.26
CNOT2	CCR4-NOT transcription complex, subunit 2	858.7	809.6	834.13
FBXL5	F-box and leucine-rich repeat protein 5	815.0	852.0	833.49
LOC104915191	chromodomain-helicase-DNA-binding protein 1-like	771.9	895.0	833.42
MMADHC	methylmalonic aciduria (cobalamin deficiency) cblD type, with homocystinuria	785.3	880.9	833.09
KDM4A	lysine (K)-specific demethylase 4A	815.9	850.2	833.06
TMC6	transmembrane channel-like 6	856.6	809.4	833.03
SLC16A3	solute carrier family 16 (monocarboxylate transporter), member 3	699.5	966.1	832.79
MVD	mevalonate (diphospho) decarboxylase	951.5	713.3	832.41
LOC100550829	chromosome unknown open reading frame, human C12orf10	842.0	822.4	832.22
TVP23B	Golgi apparatus membrane protein TVP23 homolog B	810.8	853.6	832.18
NOB1	NIN1/RPN12 binding protein 1 homolog (S. cerevisiae)	897.6	765.8	831.69
TTPAL	tocopherol (alpha) transfer protein-like	759.4	903.9	831.63
N6AMT2	N-6 adenine-specific DNA methyltransferase 2 (putative)	832.4	830.6	831.49
RPS4X	ribosomal protein S4, X-linked	805.7	856.9	831.30
SDAD1	SDA1 domain containing 1	862.2	800.1	831.15
LOC104909826	importin subunit alpha-6-like	815.5	846.5	831.04
C2H6orf203	chromosome 2 open reading frame, human C6orf203	862.8	799.2	831.00
MNT	MAX network transcriptional repressor	826.1	835.6	830.82
PTP4A3	protein tyrosine phosphatase type IVA, member 3	883.5	778.2	830.82
LOC104911375	uncharacterized LOC104911375	828.2	833.4	830.81
LOC100544066	uncharacterized LOC100544066	813.7	846.9	830.29
PDCD2	programmed cell death 2	842.6	818.0	830.29
EFTUD1	elongation factor Tu GTP binding domain containing 1	813.1	846.3	829.68
MPI	mannose phosphate isomerase	851.3	807.9	829.60
TK1	thymidine kinase 1, soluble	887.3	771.3	829.29
LFNG	LFNG O-fucosylpeptide 3-beta-N-acetylglucosaminyltransferase	893.1	765.1	829.10
FKBP15	FK506 binding protein 15, 133kDa	828.5	828.2	828.37
GFPT1	glutamine--fructose-6-phosphate transaminase 1	882.6	773.6	828.10
LSM4	LSM4 homolog, U6 small nuclear RNA associated (S. cerevisiae)	847.0	809.0	827.99
QSER1	glutamine and serine rich 1	815.8	839.7	827.73
TMEM39A	transmembrane protein 39A	795.8	859.6	827.71
LOC100548859	protein DDX26B-like	791.4	863.4	827.39
KIF3A	kinesin family member 3A	802.9	851.7	827.30
SIPA1L1	signal-induced proliferation-associated 1 like 1	848.1	805.9	827.01
FKBP5	FK506 binding protein 5	950.4	703.0	826.68

LOC104913504	uncharacterized LOC104913504	772.1	879.6	825.84
LOC100546100	COMM domain-containing protein 5-like	817.4	834.1	825.74
NDUFAF2	NADH dehydrogenase (ubiquinone) complex I, assembly factor 2	788.7	862.3	825.48
RRM1	ribonucleotide reductase M1	873.8	776.3	825.08
NUDT9	nudix (nucleoside diphosphate linked moiety X)-type motif 9	856.3	793.6	824.97
LZTR1	leucine-zipper-like transcription regulator 1	814.0	835.8	824.89
TMEM127	transmembrane protein 127	854.9	794.4	824.63
LOC100543349	lysine-specific demethylase 5A	753.9	894.1	824.00
IFI35	interferon-induced protein 35	842.1	805.3	823.66
UTP3	UTP3, small subunit (SSU) processome component, homolog (S. cerevisiae)	857.1	789.6	823.35
CETN3	centrin, EF-hand protein, 3	848.3	797.4	822.86
CRIPT	cysteine-rich PDZ-binding protein	812.9	831.2	822.05
RAI2	retinoic acid induced 2	858.1	785.9	821.99
DPP8	dipeptidyl-peptidase 8	804.0	839.2	821.59
SEC61A2	Sec61 alpha 2 subunit (S. cerevisiae)	804.8	837.8	821.28
DHRS7C	dehydrogenase/reductase (SDR family) member 7C	811.6	830.6	821.12
RFNG	RFNG O-fucosylpeptide 3-beta-N-acetylglucosaminyltransferase	803.1	839.1	821.10
ECI1	enoyl-CoA delta isomerase 1	888.2	753.9	821.06
C12H15orf40	chromosome 12 open reading frame, human C15orf40	829.9	812.1	821.01
MRPS5	mitochondrial ribosomal protein S5	841.9	798.1	820.01
SNAPIN	SNAP-associated protein	835.4	804.4	819.87
LOC100541862	RNA polymerase-associated protein RTF1 homolog	847.7	791.5	819.59
LOC100542710	growth arrest-specific protein 7-like	824.1	814.6	819.37
PELO	pelota homolog (Drosophila)	794.7	843.7	819.21
PQLC3	PQ loop repeat containing 3	824.1	813.3	818.67
SEPHS1	selenophosphate synthetase 1	804.0	833.1	818.53
CWF19L1	CWF19-like 1, cell cycle control (S. pombe)	879.2	757.8	818.50
LOC104912530	uncharacterized LOC104912530	818.8	817.2	817.99
CCDC167	coiled-coil domain containing 167	824.0	811.1	817.57
DDX49	DEAD (Asp-Glu-Ala-Asp) box polypeptide 49	869.8	765.0	817.38
SMEK1	SMEK homolog 1, suppressor of mek1 (Dictyostelium)	799.8	834.9	817.34
HMCES	5-hydroxymethylcytosine (hmC) binding, ES cell-specific	826.1	808.5	817.28
LOC104912993	WD repeat-containing protein 59-like	825.2	808.6	816.95
LOC104912940	uncharacterized LOC104912940	777.8	855.1	816.45
TANGO2	transport and golgi organization 2 homolog (Drosophila)	829.0	803.7	816.33
ACOT8	acyl-CoA thioesterase 8	849.5	782.3	815.93
F10	coagulation factor X	804.2	827.5	815.83
SLC25A38	solute carrier family 25, member 38	741.6	890.0	815.83
KLF11	Kruppel-like factor 11	810.7	820.6	815.65
SNRPA1	small nuclear ribonucleoprotein polypeptide A'	832.6	798.2	815.40
CWC22	CWC22 spliceosome-associated protein	783.8	846.3	815.08
POGLUT1	protein O-glucosyltransferase 1	804.0	825.3	814.63
TNS1	tensin 1	863.4	764.9	814.15
WASL	Wiskott-Aldrich syndrome-like	798.0	829.9	813.92
FGFR1OP2	FGFR1 oncogene partner 2	781.0	846.7	813.86
ZNRF1	zinc and ring finger 1, E3 ubiquitin protein ligase	806.6	821.0	813.80
ASXL3	additional sex combs like transcriptional regulator 3	768.7	858.3	813.52
LOC104915003	pumilio domain-containing protein KIAA0020 homolog	794.4	831.5	812.93
CCDC141	coiled-coil domain containing 141	764.3	861.2	812.77
LAMTOR1	late endosomal/lysosomal adaptor, MAPK and MTOR activator 1	858.0	767.1	812.54
POFUT1	protein O-fucosyltransferase 1	780.4	842.5	811.45
HAUS6	HAUS augmin-like complex, subunit 6	842.4	780.4	811.41
MTA3	metastasis associated 1 family, member 3	797.2	825.3	811.27
LOC104912665	cell wall protein DAN4-like	836.7	785.8	811.27
PPIL3	peptidylprolyl isomerase (cyclophilin)-like 3	800.4	821.8	811.10
SIN3B	SIN3 transcription regulator family member B	828.3	793.8	811.03
MRPL35	mitochondrial ribosomal protein L35	792.0	829.6	810.82

LOC104911671	myc box-dependent-interacting protein 1-like	724.8	896.8	810.81
LOC100550621	protein DDI1 homolog 2	808.9	811.9	810.40
RFC2	replication factor C (activator 1) 2, 40kDa	840.1	780.3	810.22
CCNA2	cyclin A2	840.8	779.4	810.08
WDR75	WD repeat domain 75	877.2	742.9	810.06
TBL3	transducin (beta)-like 3	837.6	782.4	810.01
CARS2	cysteinyl-tRNA synthetase 2, mitochondrial (putative)	756.8	863.0	809.93
LOC100547331	transmembrane 9 superfamily member 2-like	809.8	810.0	809.90
RAD52	RAD52 homolog (S. cerevisiae)	795.2	823.1	809.16
SCYL3	SCY1-like 3 (S. cerevisiae)	793.1	824.3	808.68
OCRL	oculocerebrorenal syndrome of Lowe	813.4	803.8	808.59
XRCC6	X-ray repair complementing defective repair in Chinese hamster cells 6	791.3	823.9	807.62
LOC104915598	putative homeodomain transcription factor 2	797.1	817.6	807.34
PRCC	papillary renal cell carcinoma (translocation-associated)	788.0	826.6	807.30
C3H6orf52	chromosome 3 open reading frame, human C6orf52	809.5	804.5	807.02
LOC104912114	protein eva-1 homolog C-like	815.4	798.3	806.85
ING4	inhibitor of growth family, member 4	814.1	798.8	806.41
UBAC2	UBA domain containing 2	787.8	824.8	806.28
WDR5	WD repeat domain 5	781.8	830.7	806.24
PPP2R5E	protein phosphatase 2, regulatory subunit B', epsilon isoform	773.5	838.9	806.20
LOC100539477	WD repeat-containing protein 81-like	838.6	773.5	806.04
CRY2	cryptochrome circadian clock 2	778.1	833.7	805.87
GLI3	GLI family zinc finger 3	792.1	819.4	805.77
TRERF1	transcriptional regulating factor 1	818.3	792.8	805.54
MAP4K3	mitogen-activated protein kinase kinase kinase kinase 3	790.3	820.5	805.39
RIOK2	RIO kinase 2	812.3	797.0	804.68
LOC104916511	uncharacterized LOC104916511	842.8	765.6	804.17
NSUN2	NOP2/Sun RNA methyltransferase family, member 2	780.2	827.9	804.02
IL18	interleukin 18	862.8	744.8	803.77
LOC104912585	period circadian protein homolog 2-like	743.4	863.7	803.54
NBEAL2	neurobeachin-like 2	828.3	778.8	803.53
DNAJC14	DnaJ (Hsp40) homolog, subfamily C, member 14	840.4	766.7	803.53
LOC104913585	uncharacterized LOC104913585	815.6	791.1	803.34
RNF185	ring finger protein 185	792.9	813.0	802.92
MYCBP	MYC binding protein	799.6	806.2	802.90
CPEB4	cytoplasmic polyadenylation element binding protein 4	775.3	830.4	802.84
SLC13A3	solute carrier family 13 (sodium-dependent dicarboxylate transporter), member 3	826.7	777.7	802.20
CDC42EP1	CDC42 effector protein (Rho GTPase binding) 1	859.8	743.7	801.75
DUSP27	dual specificity phosphatase 27 (putative)	753.4	849.8	801.60
C2CD5	C2 calcium-dependent domain containing 5	746.4	856.7	801.55
ASB1	ankyrin repeat and SOCS box containing 1	788.7	814.0	801.33
NEK7	NIMA-related kinase 7	818.1	784.0	801.06
ZFYVE1	zinc finger, FYVE domain containing 1	779.6	822.5	801.02
SMC4	structural maintenance of chromosomes 4	829.1	772.1	800.59
SEC23B	Sec23 homolog B (S. cerevisiae)	833.7	767.4	800.57
PSD3	pleckstrin and Sec7 domain containing 3	783.0	817.7	800.36
SNX25	sorting nexin 25	769.7	831.0	800.33
CNOT11	CCR4-NOT transcription complex, subunit 11	819.9	779.4	799.67
WDR3	WD repeat domain 3	811.5	787.5	799.51
SLC30A4	solute carrier family 30 (zinc transporter), member 4	742.6	856.2	799.36
LOC104915292	hydroxysteroid dehydrogenase-like protein 2	792.1	806.6	799.35
JOSD1	Josephin domain containing 1	802.5	795.9	799.20
SLC9B2	solute carrier family 9, subfamily B (NHA2, cation proton antiporter 2), member 2	805.0	793.0	798.97
LOC100550282	L-lactate dehydrogenase A chain-like	719.8	876.6	798.22
C8H10orf88	chromosome 8 open reading frame, human C10orf88	823.3	772.8	798.01

LYRM7	LYR motif containing 7	802.1	792.2	797.17
MBD4	methyl-CpG binding domain protein 4	849.4	744.9	797.16
EAPP	E2F-associated phosphoprotein	790.3	803.5	796.92
NDC1	NDC1 transmembrane nucleoporin	843.6	749.8	796.69
ENDOG	endonuclease G	859.4	733.0	796.18
MAP2K4	mitogen-activated protein kinase kinase 4	787.9	803.7	795.77
IMPAD1	inositol monophosphatase domain containing 1	800.7	790.8	795.75
PAPD4	PAP associated domain containing 4	773.4	817.7	795.58
PDHX	pyruvate dehydrogenase complex, component X	771.8	819.3	795.52
SRA1	steroid receptor RNA activator 1	809.2	781.3	795.25
FOXRED2	FAD-dependent oxidoreductase domain containing 2	755.2	834.9	795.03
LOC100541776	protein FAM13B-like	759.9	828.6	794.25
TMEM50A	transmembrane protein 50A	741.3	846.4	793.88
SNRNP40	small nuclear ribonucleoprotein 40kDa (U5)	835.1	752.4	793.74
ECH1	enoyl CoA hydratase 1, peroxisomal	854.5	732.8	793.65
LOC104915811	phosphoenolpyruvate carboxykinase [GTP], mitochondrial-like	801.9	784.9	793.40
KANK4	KN motif and ankyrin repeat domains 4	736.0	850.2	793.10
TSNAX	translin-associated factor X	784.4	800.3	792.31
EPB41L1	erythrocyte membrane protein band 4.1-like 1	779.9	804.5	792.19
LDLRAD4	low density lipoprotein receptor class A domain containing 4	764.3	818.9	791.61
LOC104916367	anion exchange protein 2-like	840.4	742.2	791.30
GPX8	glutathione peroxidase 8 (putative)	735.3	846.9	791.09
LRCH3	leucine-rich repeats and calponin homology (CH) domain containing 3	826.7	753.8	790.29
LOC104913877	uncharacterized LOC104913877	788.0	792.6	790.27
LOC100543320	mitochondrial ubiquitin ligase activator of nfkb 1-A-like	834.5	745.3	789.94
ELMOD2	ELMO/CED-12 domain containing 2	776.7	802.3	789.51
COQ10B	coenzyme Q10 homolog B (S. cerevisiae)	745.8	832.6	789.17
MED12L	mediator complex subunit 12-like	756.1	821.6	788.88
TMEM231	transmembrane protein 231	785.3	791.4	788.36
LOC104915869	uncharacterized LOC104915869	846.6	729.5	788.04
RALGAPA1	Ral GTPase activating protein, alpha subunit 1 (catalytic)	758.8	816.4	787.63
KRR1	KRR1, small subunit (SSU) processome component, homolog (yeast)	765.0	809.2	787.09
TMEM168	transmembrane protein 168	749.2	824.9	787.03
DAB2IP	DAB2 interacting protein	780.1	793.7	786.90
FBXO22	F-box protein 22	774.4	798.9	786.66
BAZ2B	bromodomain adjacent to zinc finger domain, 2B	756.6	816.4	786.48
LOC104909918	unconventional myosin-VI-like	768.9	802.1	785.50
NCKIPSD	NCK interacting protein with SH3 domain	770.5	800.2	785.35
PSEN2	presenilin 2	730.3	840.4	785.34
PIN4	protein (peptidylprolyl cis/trans isomerase) NIMA-interacting, 4 (parvulin)	784.4	785.4	784.92
LOC104914035	proteasomal ubiquitin receptor ADRM1	776.4	793.3	784.83
HIVEP2	human immunodeficiency virus type I enhancer binding protein 2	811.5	757.7	784.59
CP	ceruloplasmin (ferroxidase)	697.9	870.0	783.93
LOC104910781	uncharacterized LOC104910781	795.4	771.0	783.19
NTAN1	N-terminal asparagine amidase	758.3	807.1	782.71
LOC104913033	breast cancer anti-estrogen resistance protein 1-like	863.7	700.9	782.28
CRELD2	cysteine-rich with EGF-like domains 2	735.4	827.7	781.59
BCL7B	B-cell CLL/lymphoma 7B	848.8	713.9	781.37
TM2D2	TM2 domain containing 2	818.4	743.1	780.75
TUBGCP2	tubulin, gamma complex associated protein 2	767.1	794.1	780.60
CEBPB	CCAAT/enhancer binding protein (C/EBP), beta	809.9	750.9	780.40
NUP133	nucleoporin 133kDa	781.1	779.4	780.24
MRPL13	mitochondrial ribosomal protein L13	806.1	754.3	780.21
NELFE	negative elongation factor complex member E	816.6	743.7	780.18
TXNDC12	thioredoxin domain containing 12 (endoplasmic reticulum)	760.8	798.8	779.83

PEPD	peptidase D	790.9	768.4	779.68
IKZF5	IKAROS family zinc finger 5 (Pegasus)	738.0	819.8	778.88
ZC3H6	zinc finger CCCH-type containing 6	751.6	806.0	778.79
CCDC15	coiled-coil domain containing 15	832.5	724.9	778.72
PNPLA7	patatin-like phospholipase domain containing 7	805.7	751.4	778.56
ARPP21	cAMP-regulated phosphoprotein, 21kDa	744.1	812.6	778.36
LOC104916212	alpha-sarcoglycan-like	847.3	709.3	778.33
AHCTF1	AT hook containing transcription factor 1	765.0	791.2	778.09
ZFYVE27	zinc finger, FYVE domain containing 27	712.3	843.1	777.67
LOC104911327	uncharacterized LOC104911327	724.1	831.0	777.52
CCNB3	cyclin B3	830.8	723.8	777.33
PROB1	proline-rich basic protein 1	817.8	736.7	777.23
DECR2	2,4-dienoyl CoA reductase 2, peroxisomal	780.3	773.6	776.98
RNF215	ring finger protein 215	826.9	726.4	776.69
PDE12	phosphodiesterase 12	811.3	741.9	776.62
SCLY	selenocysteine lyase	794.6	758.1	776.35
TANGO6	transport and golgi organization 6 homolog (Drosophila)	881.0	671.5	776.22
LOC100548270	UPF0606 protein KIAA1549-like	748.4	804.0	776.20
SHOC2	soc-2 suppressor of clear homolog (C. elegans)	753.0	798.4	775.70
ADCY2	adenylate cyclase 2 (brain)	722.1	828.6	775.37
TPST2	tyrosylprotein sulfotransferase 2	749.8	800.0	774.90
ATXN2	ataxin 2	765.3	784.2	774.73
GORASP1	golgi reassembly stacking protein 1, 65kDa	795.3	754.1	774.73
LOC104912477	CMP-N-acetylneuraminate-beta-1,4-galactoside alpha-2,3-sialyltransferase-like	857.4	691.9	774.62
VPS26A	vacuolar protein sorting 26 homolog A (S. pombe)	760.8	788.1	774.41
LOC100543567	core histone macro-H2A.2	756.6	792.0	774.31
ZBTB18	zinc finger and BTB domain containing 18	749.2	797.9	773.54
FBXW4	F-box and WD repeat domain containing 4	803.9	742.9	773.38
AGAP3	ArfGAP with GTPase domain, ankyrin repeat and PH domain 3	848.9	697.5	773.20
KIF7	kinesin family member 7	829.4	716.8	773.11
BRWD1	bromodomain and WD repeat domain containing 1	736.6	809.6	773.10
CZH9orf64	chromosome Z open reading frame, human C9orf64	752.4	793.5	772.94
LAMTOR4	late endosomal/lysosomal adaptor, MAPK and MTOR activator 4	760.0	785.8	772.92
SPPL3	signal peptide peptidase like 3	758.3	786.6	772.46
LOC104910983	myotubularin-related protein 13-like	782.6	762.1	772.39
SUPV3L1	suppressor of var1, 3-like 1 (S. cerevisiae)	818.2	726.4	772.33
LOC104916886	tubulin alpha chain-like	732.2	812.3	772.25
AREL1	apoptosis resistant E3 ubiquitin protein ligase 1	795.6	748.7	772.16
PDPR	pyruvate dehydrogenase phosphatase regulatory subunit	766.8	777.4	772.10
LOC104914206	polyhomeotic-like protein 2	816.0	728.0	772.03
SMAP2	small ArfGAP2	729.0	815.0	772.03
DGKE	diacylglycerol kinase, epsilon 64kDa	761.7	781.1	771.41
NPC1	Niemann-Pick disease, type C1	756.0	786.2	771.09
SAP130	Sin3A-associated protein, 130kDa	752.6	789.5	771.06
DIDO1	death inducer-obliterator 1	752.4	789.8	771.06
HIP1	huntingtin interacting protein 1	874.6	666.7	770.67
EMC8	ER membrane protein complex subunit 8	805.4	735.8	770.61
RABL3	RAB, member of RAS oncogene family-like 3	724.5	816.7	770.57
LOC100550466	ubiquinol-cytochrome-c reductase complex assembly factor 1-like	762.4	778.7	770.52
LOC104909837	DNA polymerase zeta catalytic subunit-like	769.2	771.8	770.51
HSPA14	heat shock 70kDa protein 14	742.2	797.8	769.99
ARSA	arylsulfatase A	784.2	755.4	769.76
NUDT2	nudix (nucleoside diphosphate linked moiety X)-type motif 2	761.5	777.7	769.60
BICC1	BicC family RNA binding protein 1	747.3	790.8	769.08
TAF9B	TAF9B RNA polymerase II, TATA box binding protein (TBP)-associated factor, 31kDa	763.5	774.5	768.99
EIF2AK1	eukaryotic translation initiation factor 2-alpha kinase 1	736.6	801.4	768.96

PCGF6	polycomb group ring finger 6	757.3	779.0	768.14
YEATS4	YEATS domain containing 4	776.8	759.5	768.13
STK38L	serine/threonine kinase 38 like	760.2	775.9	768.04
PYCRL	pyrroline-5-carboxylate reductase-like	771.0	764.6	767.78
USE1	unconventional SNARE in the ER 1 homolog (S. cerevisiae)	783.5	751.0	767.28
COL13A1	collagen, type XIII, alpha 1	697.4	837.0	767.20
RIOK1	RIO kinase 1	718.8	815.4	767.07
RBM18	RNA binding motif protein 18	752.3	781.6	766.97
PTBP2	polypyrimidine tract binding protein 2	732.1	801.5	766.77
GALNT14	polypeptide N-acetylgalactosaminyltransferase 14	779.4	754.1	766.74
WDR4	WD repeat domain 4	731.9	801.2	766.55
CARKD	carbohydrate kinase domain containing	782.6	750.0	766.32
LOC100544513	protein SCO1 homolog, mitochondrial	758.2	774.3	766.26
MSRB1	methionine sulfoxide reductase B1	777.8	754.4	766.10
SUGP2	SURP and G patch domain containing 2	741.5	789.7	765.62
WDR70	WD repeat domain 70	750.5	780.2	765.33
LLGL1	lethal giant larvae homolog 1 (Drosophila)	830.3	700.3	765.26
AGO4	argonaute RISC catalytic component 4	759.3	769.9	764.62
GTPBP8	GTP-binding protein 8 (putative)	715.4	813.5	764.46
ZC3HC1	zinc finger, C3HC-type containing 1	870.1	658.6	764.34
CENPM	centromere protein M	779.2	749.4	764.31
RHCE	Rh blood group, CcEe antigens	694.0	834.4	764.20
XRCC6BP1	XRCC6 binding protein 1	756.7	771.3	763.97
LZTS2	leucine zipper, putative tumor suppressor 2	776.4	751.5	763.96
C10H1orf27	chromosome 10 open reading frame, human C1orf27	735.5	791.6	763.57
LOC100550421	glioma pathogenesis-related protein 1-like	766.0	760.5	763.26
LOC100545577	acyl-coenzyme A thioesterase 1-like	768.6	757.9	763.25
GGNBP2	gametogenetin binding protein 2	740.6	784.8	762.68
NOP9	NOP9 nucleolar protein	804.2	721.2	762.66
NBEAL1	neurobeachin-like 1	728.0	796.9	762.45
FEN1	flap structure-specific endonuclease 1	800.5	724.3	762.42
CEPT1	choline/ethanolamine phosphotransferase 1	750.6	774.1	762.38
UNC45A	unc-45 homolog A (C. elegans)	872.5	651.4	761.95
RNF144A	ring finger protein 144A	710.2	813.6	761.91
MIB1	mindbomb E3 ubiquitin protein ligase 1	701.9	820.7	761.30
FOXO1	forkhead box O1	748.2	774.2	761.21
CHAMP1	chromosome alignment maintaining phosphoprotein 1	777.6	744.1	760.86
KBTBD4	kelch repeat and BTB (POZ) domain containing 4	769.4	752.2	760.82
TARSL2	threonyl-tRNA synthetase-like 2	759.1	762.3	760.72
BMPR1A	bone morphogenetic protein receptor, type IA	728.9	792.2	760.58
RASA2	RAS p21 protein activator 2	683.2	837.9	760.55
DVL3	dishevelled segment polarity protein 3	784.9	736.1	760.53
MAFK	v-maf avian musculoaponeurotic fibrosarcoma oncogene homolog K	751.7	769.2	760.44
CASKIN2	CASK interacting protein 2	773.4	747.1	760.23
PRKAB2	protein kinase, AMP-activated, beta 2 non-catalytic subunit	829.5	690.7	760.08
MRPL46	mitochondrial ribosomal protein L46	799.6	720.1	759.84
GOLGA1	golgin A1	709.4	810.0	759.70
LOC100543552	multidrug resistance-associated protein 4	765.1	753.6	759.36
MYBPC3	myosin binding protein C, cardiac	729.8	788.8	759.32
ZDHHC20	zinc finger, DHHC-type containing 20	735.7	782.3	758.98
MAX	MYC associated factor X	774.7	742.3	758.49
MRPS18B	mitochondrial ribosomal protein S18B	776.0	741.0	758.49
ARID4B	AT rich interactive domain 4B (RBP1-like)	706.8	810.0	758.40
TMEM222	transmembrane protein 222	824.3	692.1	758.21
ILF3	interleukin enhancer binding factor 3, 90kDa	832.2	683.9	758.08
RTCA	RNA 3'-terminal phosphate cyclase	731.2	784.9	758.05
LOC100543166	nuclear pore complex protein Nup93-like	810.6	704.7	757.62

TBCD	tubulin folding cofactor D	743.9	771.0	757.49
GPN3	GPN-loop GTPase 3	708.5	805.2	756.85
ID3	inhibitor of DNA binding 3, dominant negative helix-loop-helix protein	772.5	740.6	756.58
LOC104917503	conserved oligomeric Golgi complex subunit 3-like	711.8	801.3	756.56
PPP1R2	protein phosphatase 1, regulatory (inhibitor) subunit 2	755.0	757.8	756.38
WARS	tryptophanyl-tRNA synthetase	796.8	715.7	756.25
WIZ	widely interspaced zinc finger motifs	795.2	717.3	756.23
C1GALT1	core 1 synthase, glycoprotein-N-acetylgalactosamine 3-beta-galactosyltransferase 1	725.2	786.8	756.01
DSN1	DSN1, MIS12 kinetochore complex component	776.1	735.2	755.68
SLC25A43	solute carrier family 25, member 43	741.6	769.7	755.66
POLR2B	polymerase (RNA) II (DNA directed) polypeptide B, 140kDa	723.1	788.1	755.58
N6AMT1	N-6 adenine-specific DNA methyltransferase 1 (putative)	756.3	754.9	755.58
CLIP2	CAP-GLY domain containing linker protein 2	783.9	726.6	755.28
LOC104913873	endoplasmic reticulum-Golgi intermediate compartment protein 2-like	752.3	756.9	754.63
ENC1	ectodermal-neural cortex 1 (with BTB domain)	775.2	734.1	754.62
LOC104916302	uncharacterized LOC104916302	758.8	750.3	754.57
LOC104914921	importin-11-like	749.9	759.1	754.52
SYNJ1	synaptojanin 1	663.9	845.1	754.52
RNF219	ring finger protein 219	718.6	790.2	754.41
CUX1	cut-like homeobox 1	800.7	707.3	753.98
LOC100551399	importin subunit alpha-5	706.2	801.8	753.97
MSL3	male-specific lethal 3 homolog (Drosophila)	727.8	779.3	753.58
SPRED1	sprouty-related, EVH1 domain containing 1	751.5	755.4	753.45
MBNL2	muscleblind-like splicing regulator 2	729.6	777.2	753.37
UFL1	UFM1-specific ligase 1	742.3	764.0	753.17
MAR5	membrane-associated ring finger (C3HC4) 5	742.3	763.7	753.00
LOC100542017	nucleolar RNA helicase 2-like	735.5	770.5	752.99
SMPD1	sphingomyelin phosphodiesterase 1, acid lysosomal	795.0	710.8	752.93
NAT9	N-acetyltransferase 9 (GCN5-related, putative)	828.1	677.7	752.91
ZC3H14	zinc finger CCCH-type containing 14	760.9	744.6	752.73
KIAA0922	KIAA0922 ortholog	736.5	768.7	752.64
BTC	betacellulin	811.5	693.6	752.53
NCDN	neurochondrin	778.7	726.1	752.41
GLIS1	GLIS family zinc finger 1	699.5	804.5	752.00
TICAM1	toll-like receptor adaptor molecule 1	735.9	768.1	751.99
DERL2	derlin 2	765.0	738.8	751.90
MBNL1	muscleblind-like splicing regulator 1	715.6	787.6	751.60
KSR1	kinase suppressor of ras 1	690.1	812.8	751.46
ARL5A	ADP-ribosylation factor-like 5A	710.1	792.2	751.17
LAMB1	laminin, beta 1	710.5	791.6	751.06
BET1	Bet1 golgi vesicular membrane trafficking protein	752.1	749.8	750.95
PCM1	pericentriolar material 1	749.1	752.6	750.87
LOC104911291	uncharacterized LOC104911291	807.4	694.3	750.85
FAM160B1	family with sequence similarity 160, member B1	735.5	765.7	750.59
LIN7C	lin-7 homolog C (C. elegans)	772.4	728.6	750.52
EIF2B1	eukaryotic translation initiation factor 2B, subunit 1 alpha, 26kDa	746.3	754.6	750.44
PHLPP2	PH domain and leucine rich repeat protein phosphatase 2	727.2	773.6	750.43
IL17RC	interleukin 17 receptor C	843.1	657.6	750.36
LOC100547230	histidine--tRNA ligase, cytoplasmic-like	795.5	705.0	750.27
DSCR3	Down syndrome critical region 3	777.5	723.0	750.26
MRPL32	mitochondrial ribosomal protein L32	804.5	695.1	749.78
LOC104911797	uncharacterized LOC104911797	742.4	756.9	749.67
MPHOSPH6	M-phase phosphoprotein 6	762.3	736.6	749.46
PRPF3	pre-mRNA processing factor 3	763.5	734.9	749.20
EZH1	enhancer of zeste 1 polycomb repressive complex 2 subunit	740.8	756.0	748.38

RYK	receptor-like tyrosine kinase	747.4	748.6	747.99
NIF3L1	NIF3 NGG1 interacting factor 3-like 1 (S. cerevisiae)	761.6	734.1	747.89
GTF2H5	general transcription factor IIH, polypeptide 5	764.9	730.6	747.77
TMCO6	transmembrane and coiled-coil domains 6	791.5	704.0	747.76
PLEKHA8	pleckstrin homology domain containing, family A (phosphoinositide binding specific) member 8	728.0	767.3	747.65
LCMT2	leucine carboxyl methyltransferase 2	754.2	741.0	747.60
CTBS	chitobiase, di-N-acetyl-	703.5	791.2	747.35
USP8	ubiquitin specific peptidase 8	675.1	817.0	746.04
LOC100542676	monocarboxylate transporter 10-like	717.1	774.0	745.56
MRPL2	mitochondrial ribosomal protein L2	854.4	636.6	745.49
BRCC3	BRCA1/BRCA2-containing complex, subunit 3	708.7	782.2	745.46
RFTN2	raftlin family member 2	732.9	757.4	745.16
KXD1	KxDL motif containing 1	760.9	728.7	744.82
LOC104913304	E3 ubiquitin-protein ligase RNF130-like	750.1	738.7	744.41
LOC104913376	uncharacterized LOC104913376	744.7	742.8	743.76
LOC104911764	unconventional myosin-X-like	708.9	778.6	743.74
SH3BP4	SH3-domain binding protein 4	740.9	746.1	743.45
DCXR	dicarbonyl/L-xylulose reductase	702.5	784.2	743.37
PRKRIP1	PRKR interacting protein 1 (IL11 inducible)	765.0	721.3	743.15
RIPPLY1	ripply transcriptional repressor 1	734.4	750.3	742.36
SPDL1	spindle apparatus coiled-coil protein 1	753.2	731.0	742.12
NR1D2	nuclear receptor subfamily 1, group D, member 2	707.9	776.1	742.01
IRF2	interferon regulatory factor 2	764.0	719.6	741.83
ZNF639	zinc finger protein 639	746.5	736.9	741.74
DYNLT3	dynein, light chain, Tctex-type 3	729.5	753.7	741.59
LOC100543556	uncharacterized LOC100543556	699.7	783.4	741.52
VRK2	vaccinia related kinase 2	733.9	748.8	741.38
ACSL4	acyl-CoA synthetase long-chain family member 4	665.3	817.4	741.36
SLC5A6	solute carrier family 5 (sodium/multivitamin and iodide cotransporter), member 6	833.8	648.9	741.34
ZEB2	zinc finger E-box binding homeobox 2	682.4	799.4	740.90
ECI2	enoyl-CoA delta isomerase 2	720.2	761.2	740.72
LOC100550490	synaptotagmin-7	683.8	797.2	740.50
C1H3orf17	chromosome 1 open reading frame, human C3orf17	726.3	753.8	740.09
HEXB	hexosaminidase B (beta polypeptide)	683.5	796.6	740.04
AKT3	v-akt murine thymoma viral oncogene homolog 3	725.5	754.5	740.02
LOC100545946	protein zyg-11 homolog B	727.1	752.8	739.94
LOC104910038	uncharacterized LOC104910038	722.9	756.6	739.77
MPG	N-methylpurine-DNA glycosylase	760.1	719.2	739.67
SNX19	sorting nexin 19	696.3	782.9	739.62
NOA1	nitric oxide associated 1	748.2	731.0	739.60
ANO8	anoctamin 8	793.4	685.4	739.38
LMTK2	lemur tyrosine kinase 2	674.0	804.5	739.24
COQ3	coenzyme Q3 methyltransferase	758.4	719.9	739.13
AGRN	agrin	751.8	725.8	738.80
POGZ	pogo transposable element with ZNF domain	738.9	737.9	738.41
SLC29A1	solute carrier family 29 (equilibrative nucleoside transporter), member 1	707.9	768.6	738.27
LOC100538813	polycomb complex protein BMI-1	747.4	728.8	738.12
DAPK1	death-associated protein kinase 1	708.5	766.9	737.72
PEX16	peroxisomal biogenesis factor 16	751.6	723.7	737.66
TXNL4A	thioredoxin-like 4A	738.0	737.0	737.48
WDR47	WD repeat domain 47	700.1	774.3	737.15
TSR3	TSR3, 20S rRNA accumulation, homolog (S. cerevisiae)	766.9	707.4	737.10
SFRP2	secreted frizzled-related protein 2	551.4	922.8	737.08
CXCR5	chemokine (C-X-C motif) receptor 5	771.9	702.2	737.01
ZNF704	zinc finger protein 704	732.3	741.2	736.77

TTLL12	tubulin tyrosine ligase-like family, member 12	745.7	727.3	736.49
SH2D2A	SH2 domain containing 2A	709.1	763.9	736.48
TAPBP	TAP binding protein (tapasin)	706.7	766.0	736.32
LOC100551278	serine/threonine-protein phosphatase 4 regulatory subunit 1-like	748.9	723.6	736.26
RNF19B	ring finger protein 19B	751.0	721.5	736.25
NFKB2	nuclear factor of kappa light polypeptide gene enhancer in B-cells 2 (p49/p100)	761.2	711.0	736.08
PRPF38A	pre-mRNA processing factor 38A	747.2	724.6	735.90
LOC100550314	isochorismatase domain-containing protein 1-like	710.1	760.8	735.43
MAD2L1	MAD2 mitotic arrest deficient-like 1 (yeast)	749.8	720.9	735.34
PTTG1IP	pituitary tumor-transforming 1 interacting protein	719.7	750.8	735.23
TM2D3	TM2 domain containing 3	735.5	734.0	734.75
LEPREL2	leprecan-like 2	714.7	754.6	734.69
SIN3A	SIN3 transcription regulator family member A	737.4	730.8	734.08
LRRC8D	leucine rich repeat containing 8 family, member D	763.5	704.3	733.89
MYBBP1A	MYB binding protein (P160) 1a	771.9	695.5	733.70
GALM	galactose mutarotase (aldose 1-epimerase)	666.5	800.6	733.54
ADAR	adenosine deaminase, RNA-specific	738.3	728.4	733.33
GALNT1	polypeptide N-acetylgalactosaminyltransferase 1	713.8	752.8	733.30
EP400	E1A binding protein p400	722.1	744.4	733.26
LOC100548686	cullin-9-like	764.9	701.2	733.04
ZCCHC8	zinc finger, CCHC domain containing 8	689.2	776.7	732.92
ACSF2	acyl-CoA synthetase family member 2	720.5	745.0	732.75
TK2	thymidine kinase 2, mitochondrial	706.9	758.4	732.61
CCDC12	coiled-coil domain containing 12	727.1	737.5	732.34
EMC7	ER membrane protein complex subunit 7	717.8	746.6	732.18
HSD17B10	hydroxysteroid (17-beta) dehydrogenase 10	795.7	668.5	732.10
MTMR6	myotubularin related protein 6	700.8	762.3	731.58
HERC3	HECT and RLD domain containing E3 ubiquitin protein ligase 3	654.5	808.2	731.36
TTLL7	tubulin tyrosine ligase-like family, member 7	750.7	711.1	730.92
CCDC94	coiled-coil domain containing 94	785.3	674.9	730.11
PI4K2A	phosphatidylinositol 4-kinase type 2 alpha	717.9	741.9	729.93
LOC100540451	fascin-like	748.6	711.3	729.92
FBXW2	F-box and WD repeat domain containing 2	683.9	775.2	729.52
LIG1	ligase I, DNA, ATP-dependent	814.0	645.0	729.51
VAMP7	vesicle-associated membrane protein 7	728.7	730.2	729.47
FAM179B	family with sequence similarity 179, member B	746.7	711.8	729.30
LOC100550717	lethal(3)malignant brain tumor-like protein 4	758.4	700.1	729.27
FAM91A1	family with sequence similarity 91, member A1	729.4	729.1	729.26
EHMT1	euchromatic histone-lysine N-methyltransferase 1	699.4	758.3	728.85
FAM114A1	family with sequence similarity 114, member A1	673.1	784.5	728.81
ST8SIA4	ST8 alpha-N-acetyl-neuraminide alpha-2,8-sialyltransferase 4	695.1	762.4	728.77
HAT1	histone acetyltransferase 1	706.6	750.6	728.62
SNX4	sorting nexin 4	725.4	731.7	728.52
CUL4A	cullin 4A	668.0	788.4	728.23
TP53INP1	tumor protein p53 inducible nuclear protein 1	711.7	744.6	728.15
ELP6	elongator acetyltransferase complex subunit 6	698.6	757.2	727.89
MALSU1	mitochondrial assembly of ribosomal large subunit 1	732.0	723.7	727.86
SMPX	small muscle protein, X-linked	726.1	729.5	727.77
TMEM97	transmembrane protein 97	701.1	754.4	727.73
DCAF4	DDB1 and CUL4 associated factor 4	698.6	756.3	727.41
MOXD1	monooxygenase, DBH-like 1	623.0	831.4	727.22
LOC100546458	E3 ubiquitin-protein ligase HECTD3-like	757.7	696.6	727.14
POLR1C	polymerase (RNA) I polypeptide C, 30kDa	767.5	686.1	726.80
TBP	TATA box binding protein	708.4	744.9	726.67
LOC104909834	pre-mRNA-processing factor 17-like	716.0	736.8	726.39
ASB6	ankyrin repeat and SOCS box containing 6	711.2	738.8	725.02
TATDN3	TatD DNase domain containing 3	708.3	741.3	724.81

RNF141	ring finger protein 141	762.5	686.8	724.69
ZFAND4	zinc finger, AN1-type domain 4	656.9	791.8	724.38
SPSB3	splA/ryanodine receptor domain and SOCS box containing 3	712.0	736.0	723.98
IPO8	importin 8	708.5	739.3	723.87
TRRAP	transformation/transcription domain-associated protein	725.6	722.0	723.81
EP300	E1A binding protein p300	728.4	719.1	723.75
MRPL50	mitochondrial ribosomal protein L50	762.5	684.3	723.40
BMS1	BMS1 ribosome biogenesis factor	743.1	703.6	723.37
SCAF8	SR-related CTD-associated factor 8	690.6	756.1	723.36
PTRH2	peptidyl-tRNA hydrolase 2	730.5	715.6	723.06
ZNF280D	zinc finger protein 280D	691.5	754.6	723.03
AMDHD2	amidohydrolase domain containing 2	729.8	716.0	722.93
PPWD1	peptidylprolyl isomerase domain and WD repeat containing 1	771.7	674.1	722.93
CASK	calcium/calmodulin-dependent serine protein kinase (MAGUK family)	674.7	771.0	722.87
HHIPL1	HHIP-like 1	679.5	766.2	722.85
NMD3	NMD3 ribosome export adaptor	698.2	747.5	722.82
RNGTT	RNA guanylyltransferase and 5'-phosphatase	680.5	764.7	722.60
SCAP	SREBF chaperone	803.6	640.8	722.24
LOC104910660	uncharacterized LOC104910660	705.0	739.2	722.13
SOS2	son of sevenless homolog 2 (Drosophila)	732.9	711.2	722.05
DCTN5	dynactin 5 (p25)	743.8	700.2	722.03
SCFD1	sec1 family domain containing 1	748.1	695.8	721.96
LOC100549837	lon protease homolog 2, peroxisomal	704.2	739.6	721.87
RAB8A	RAB8A, member RAS oncogene family	713.6	729.4	721.49
SRXN1	sulfiredoxin 1	778.2	664.5	721.36
CHCHD1	coiled-coil-helix-coiled-coil-helix domain containing 1	709.1	733.2	721.15
ICT1	immature colon carcinoma transcript 1	754.6	687.5	721.06
PRC1	protein regulator of cytokinesis 1	804.8	637.1	720.93
TOMM5	translocase of outer mitochondrial membrane 5 homolog (yeast)	800.4	641.4	720.90
CNOT10	CCR4-NOT transcription complex, subunit 10	694.2	747.5	720.83
LOC104912469	vasculin-like protein 1	709.3	731.4	720.38
NAA25	N(alpha)-acetyltransferase 25, NatB auxiliary subunit	740.5	698.9	719.70
TNFSF10	tumor necrosis factor (ligand) superfamily, member 10	683.4	755.7	719.58
MMP2	matrix metallopeptidase 2 (gelatinase A, 72kDa gelatinase, 72kDa type IV collagenase)	701.9	736.7	719.31
LOC104913940	uncharacterized LOC104913940	689.2	748.1	718.62
EMP2	epithelial membrane protein 2	711.8	725.1	718.47
MCOLN1	mucolipin 1	744.3	692.2	718.26
LOC100546924	caspase recruitment domain-containing protein 9-like	719.7	715.2	717.45
TDRD7	tudor domain containing 7	667.3	767.3	717.30
JAGN1	jagunal homolog 1 (Drosophila)	731.3	703.2	717.24
LOC104911995	uncharacterized LOC104911995	728.0	706.2	717.11
TECPR2	tectonin beta-propeller repeat containing 2	688.2	745.9	717.04
ZMYND11	zinc finger, MYND-type containing 11	677.9	755.8	716.86
NPDC1	neural proliferation, differentiation and control, 1	763.4	670.3	716.84
INTS6	integrator complex subunit 6	641.9	791.1	716.52
MRPL15	mitochondrial ribosomal protein L15	679.9	752.6	716.29
FAM134C	family with sequence similarity 134, member C	711.9	719.8	715.90
THOC1	THO complex 1	697.5	734.2	715.83
PGPEP1	pyroglutamyl-peptidase I	684.2	746.6	715.39
ZMYM4	zinc finger, MYM-type 4	708.6	721.8	715.16
RPF1	ribosome production factor 1 homolog (S. cerevisiae)	746.3	684.0	715.14
CBY1	chibby homolog 1 (Drosophila)	762.3	667.6	714.95
BOC	BOC cell adhesion associated, oncogene regulated	699.5	729.9	714.72
AK4	adenylate kinase 4	700.3	729.0	714.61
SMIM20	small integral membrane protein 20	727.8	700.8	714.26
LOC104909591	cold shock domain-containing protein E1	705.4	722.9	714.15

NFYB	nuclear transcription factor Y, beta	687.4	740.9	714.13
KCTD9	potassium channel tetramerization domain containing 9	704.4	723.2	713.79
RGN	regucalcin	730.6	696.4	713.50
RPAP1	RNA polymerase II associated protein 1	631.1	795.7	713.40
RFC3	replication factor C (activator 1) 3, 38kDa	710.1	716.4	713.27
C28H6orf106	chromosome 28 open reading frame, human C6orf106	732.9	693.4	713.15
RASA3	RAS p21 protein activator 3	692.3	734.0	713.14
LOC104914085	uncharacterized LOC104914085	671.1	755.1	713.14
HDAC3	histone deacetylase 3	699.4	725.9	712.62
STXBP1	syntaxin binding protein 1	720.7	702.2	711.46
TOP1MT	topoisomerase (DNA) I, mitochondrial	762.4	660.5	711.45
AKAP13	A kinase (PRKA) anchor protein 13	699.9	722.7	711.30
UNC119B	unc-119 homolog B (C. elegans)	707.6	714.9	711.24
WBP2	WW domain binding protein 2	690.8	731.6	711.23
NECAP2	NECAP endocytosis associated 2	674.0	747.6	710.79
MPHOSPH8	M-phase phosphoprotein 8	691.5	728.9	710.22
LOC100547916	alpha-actinin-4	784.4	635.6	710.03
INO80	INO80 complex subunit	684.1	735.8	709.93
TMEM199	transmembrane protein 199	718.6	700.9	709.78
KANSL1L	KAT8 regulatory NSL complex subunit 1-like	677.4	741.4	709.43
LOC104913567	uncharacterized LOC104913567	709.6	708.6	709.14
CDC23	cell division cycle 23	706.8	711.1	708.97
INPP5B	inositol polyphosphate-5-phosphatase, 75kDa	647.7	769.5	708.62
SLC45A1	solute carrier family 45, member 1	697.8	718.8	708.29
DNAJC5	DnaJ (Hsp40) homolog, subfamily C, member 5	718.0	698.3	708.14
IP6K1	inositol hexakisphosphate kinase 1	736.6	678.7	707.64
STRN3	striatin, calmodulin binding protein 3	660.2	753.7	706.95
CBR1	carbonyl reductase 1	644.5	769.3	706.91
LOC100547442	ubiquitin-conjugating enzyme E2 C-like	750.8	662.5	706.63
MRPL48	mitochondrial ribosomal protein L48	708.3	704.8	706.53
REXO1	REX1, RNA exonuclease 1 homolog (S. cerevisiae)	728.7	684.3	706.50
LOC100543336	D(1) dopamine receptor-like	722.1	690.7	706.40
NME3	NME/NM23 nucleoside diphosphate kinase 3	727.7	684.2	705.94
BNIP3	BCL2/adenovirus E1B 19kDa interacting protein 3	715.8	695.5	705.69
TNFRSF4	tumor necrosis factor receptor superfamily, member 4	673.0	738.1	705.54
AHSA2	AHA1, activator of heat shock 90kDa protein ATPase homolog 2 (yeast)	696.7	714.0	705.31
CENPH	centromere protein H	725.9	684.2	705.05
NUBPL	nucleotide binding protein-like	672.2	737.6	704.91
NBAS	neuroblastoma amplified sequence	674.9	733.6	704.24
LOC100544434	adenylosuccinate synthetase isozyme 2	757.0	650.3	703.64
LOC100538371	synaptojanin-2-binding protein-like	664.5	742.4	703.43
SLC15A4	solute carrier family 15 (oligopeptide transporter), member 4	667.1	738.6	702.89
KDM4B	lysine (K)-specific demethylase 4B	657.8	747.2	702.51
LOC104914333	uncharacterized LOC104914333	771.8	632.8	702.31
DCAF17	DDB1 and CUL4 associated factor 17	689.1	715.5	702.29
CROT	carnitine O-octanoyltransferase	649.4	755.0	702.23
FDXR	ferredoxin reductase	708.5	695.4	701.97
CMTR1	cap methyltransferase 1	723.6	680.2	701.87
GPBP1	GC-rich promoter binding protein 1	654.3	748.7	701.49
TMEM11	transmembrane protein 11	692.5	709.8	701.15
LOC104912223	myotubularin-like	618.1	783.9	700.97
GALE	UDP-galactose-4-epimerase	755.3	645.2	700.24
KCNIP1	Kv channel interacting protein 1	781.4	618.3	699.86
MRPS9	mitochondrial ribosomal protein S9	724.4	675.0	699.69
PPP6R2	protein phosphatase 6, regulatory subunit 2	732.3	667.0	699.66
TRPM7	transient receptor potential cation channel, subfamily M, member 7	628.5	770.4	699.45

RGS3	regulator of G-protein signaling 3	671.8	727.1	699.44
CCDC117	coiled-coil domain containing 117	670.4	727.8	699.12
LOC104915197	uncharacterized LOC104915197	696.6	701.6	699.07
HS6ST2	heparan sulfate 6-O-sulfotransferase 2	624.5	773.5	698.99
MGAT2	mannosyl (alpha-1,6-)-glycoprotein beta-1,2-N-acetylglucosaminyltransferase	738.8	658.7	698.73
ALG6	ALG6, alpha-1,3-glucosyltransferase	668.2	728.6	698.39
HSD17B7	hydroxysteroid (17-beta) dehydrogenase 7	765.1	631.7	698.39
GOLGA4	golgin A4	648.7	747.3	697.96
LOC104916996	maestro heat-like repeat-containing protein family member 1	701.1	693.4	697.24
APBB2	amyloid beta (A4) precursor protein-binding, family B, member 2	647.6	746.4	697.03
NMRK2	nicotinamide riboside kinase 2	746.2	647.8	697.03
CSTF1	cleavage stimulation factor, 3' pre-RNA, subunit 1, 50kDa	703.4	690.5	696.95
ACP2	acid phosphatase 2, lysosomal	685.1	707.9	696.50
KLC1	kinesin light chain 1	679.1	713.7	696.43
C30H19orf44	chromosome 30 open reading frame, human C19orf44	655.6	737.0	696.28
PIK3R4	phosphoinositide-3-kinase, regulatory subunit 4	695.8	696.5	696.13
FUCA1	fucosidase, alpha-L- 1, tissue	670.7	720.9	695.80
C1HXorf23	chromosome 1 open reading frame, human CXorf23	684.1	707.2	695.64
SNRNP27	small nuclear ribonucleoprotein 27kDa (U4/U6.U5)	743.9	647.0	695.47
POMT2	protein-O-mannosyltransferase 2	674.2	716.6	695.41
LOC104914997	uncharacterized LOC104914997	697.3	693.5	695.36
GDPD1	glycerophosphodiester phosphodiesterase domain containing 1	687.6	702.6	695.07
ARMC1	armadillo repeat containing 1	683.8	706.0	694.87
TBC1D9	TBC1 domain family, member 9 (with GRAM domain)	708.4	680.4	694.39
SLC10A7	solute carrier family 10, member 7	699.3	689.3	694.30
LOC104914273	tubulin-folding cofactor B-like	724.5	663.2	693.89
DBR1	debranching RNA lariats 1	691.6	695.1	693.36
CISD2	CDGSH iron sulfur domain 2	673.9	712.5	693.17
CHORDC1	cysteine and histidine-rich domain (CHORD) containing 1	697.5	687.7	692.59
ZCCHC24	zinc finger, CCHC domain containing 24	667.1	718.0	692.55
TMEM64	transmembrane protein 64	643.6	740.9	692.27
LOC104915088	vacuolar protein sorting-associated protein 13A-like	667.3	716.0	691.63
LOC100551038	cystathionine beta-synthase	720.1	663.0	691.54
KIF16B	kinesin family member 16B	711.7	670.7	691.23
KCNH6	potassium voltage-gated channel, subfamily H (eag-related), member 6	693.7	688.7	691.19
MED27	mediator complex subunit 27	673.3	709.1	691.17
C19H9orf16	chromosome 19 open reading frame, human C9orf16	730.5	651.0	690.76
IKBKB	inhibitor of kappa light polypeptide gene enhancer in B-cells, kinase beta	659.5	721.9	690.69
MB21D2	Mab-21 domain containing 2	686.8	694.0	690.41
GLA	galactosidase, alpha	630.3	750.4	690.34
LOC104917134	PH domain leucine-rich repeat-containing protein phosphatase 1-like	694.2	686.0	690.11
FNDC3A	fibronectin type III domain containing 3A	689.3	690.8	690.04
MON2	MON2 homolog (S. cerevisiae)	686.5	693.4	689.98
GAB1	GRB2-associated binding protein 1	642.0	737.9	689.95
CWC27	CWC27 spliceosome-associated protein homolog (S. cerevisiae)	729.3	650.5	689.88
DPCD	deleted in primary ciliary dyskinesia homolog (mouse)	659.7	718.1	688.91
CFDP1	craniofacial development protein 1	729.6	648.2	688.91
GLCE	glucuronic acid epimerase	659.7	718.0	688.87
PDSS2	prenyl (decaprenyl) diphosphate synthase, subunit 2	676.5	700.2	688.35
JMJD6	jumonji domain containing 6	691.0	685.5	688.27
TMF1	TATA element modulatory factor 1	652.0	724.3	688.15
LTN1	listerin E3 ubiquitin protein ligase 1	654.6	721.6	688.10
LOC100544443	importin-7	657.2	718.8	688.01
SH3RF1	SH3 domain containing ring finger 1	669.6	706.1	687.87

LOC104914686	serine/threonine-protein kinase STK11-like	662.9	712.9	687.86
PIK3R6	phosphoinositide-3-kinase, regulatory subunit 6	689.4	686.0	687.71
NDE1	nudE neurodevelopment protein 1	709.3	665.0	687.18
C6H1orf35	chromosome 6 open reading frame, human C1orf35	753.2	620.7	686.97
VPS37A	vacuolar protein sorting 37 homolog A (S. cerevisiae)	671.3	702.4	686.86
KIF4A	kinesin family member 4A	769.2	604.4	686.82
GPR27	G protein-coupled receptor 27	726.8	646.7	686.71
LOC100542725	ADP-ribosylation factor 5	706.0	667.2	686.61
ANP32A	acidic (leucine-rich) nuclear phosphoprotein 32 family, member A	662.2	710.8	686.49
PCNT	pericentrin	678.1	694.3	686.22
UBLCP1	ubiquitin-like domain containing CTD phosphatase 1	691.5	679.4	685.48
KATNBL1	katanin p80 subunit B-like 1	676.3	694.1	685.22
COG1	component of oligomeric golgi complex 1	678.2	691.9	685.01
NELFCD	negative elongation factor complex member C/D	663.0	707.0	684.97
TRUB2	TruB pseudouridine (psi) synthase family member 2	722.0	647.7	684.85
EMC2	ER membrane protein complex subunit 2	686.3	683.1	684.71
LOC104915377	uncharacterized LOC104915377	729.5	637.8	683.66
LOC100545675	tyrosine-protein phosphatase non-receptor type 2-like	700.7	665.7	683.20
SLC35C2	solute carrier family 35 (GDP-fucose transporter), member C2	670.5	695.8	683.16
LOC100549969	ubiquitin carboxyl-terminal hydrolase 45-like	663.9	702.1	682.99
TKT	transketolase	696.0	669.8	682.87
RND3	Rho family GTPase 3	669.6	694.8	682.19
LOC100543303	histone-lysine N-methyltransferase NSD2	638.4	725.2	681.81
UBE2E3	ubiquitin-conjugating enzyme E2E 3	656.1	707.5	681.80
CZH9orf72	chromosome Z open reading frame, human C9orf72	661.3	702.1	681.70
MAP2K5	mitogen-activated protein kinase kinase 5	688.9	674.1	681.49
YLPM1	YLP motif containing 1	691.6	671.3	681.42
VPS51	vacuolar protein sorting 51 homolog (S. cerevisiae)	699.4	662.6	680.97
CCDC64	coiled-coil domain containing 64	685.6	676.3	680.95
RNF6	ring finger protein (C3H2C3 type) 6	640.3	721.6	680.92
DIEXF	digestive organ expansion factor homolog (zebrafish)	669.5	692.2	680.85
PSMG2	proteasome (prosome, macropain) assembly chaperone 2	713.4	646.9	680.14
PLEKHJ1	pleckstrin homology domain containing, family J member 1	694.2	665.2	679.72
LOC104911780	corepressor interacting with RBPJ 1-like	669.5	689.9	679.70
C4H4orf29	chromosome 4 open reading frame, human C4orf29	668.8	689.6	679.20
SLC37A3	solute carrier family 37, member 3	668.1	689.8	678.94
PLCG1	phospholipase C, gamma 1	655.7	701.1	678.39
MKRN1	makorin ring finger protein 1	657.9	696.5	677.24
LAMC2	laminin, gamma 2	693.5	661.0	677.24
C17H12orf65	chromosome 17 open reading frame, human C12orf65	678.1	676.4	677.22
TRMT10B	tRNA methyltransferase 10 homolog B (S. cerevisiae)	700.1	653.8	676.91
LEO1	Leo1, Paf1/RNA polymerase II complex component, homolog (S. cerevisiae)	654.5	699.1	676.83
MIER1	mesoderm induction early response 1, transcriptional regulator	640.1	713.4	676.76
KIAA1468	KIAA1468 ortholog	644.2	709.2	676.70
WDR53	WD repeat domain 53	686.5	666.7	676.60
FBXO42	F-box protein 42	694.2	658.8	676.51
SF3A2	splicing factor 3a, subunit 2, 66kDa	660.4	692.5	676.47
LRSAM1	leucine rich repeat and sterile alpha motif containing 1	685.0	667.8	676.40
DNAJB9	DnaJ (Hsp40) homolog, subfamily B, member 9	600.4	752.4	676.40
LOC104909942	uncharacterized LOC104909942	684.9	667.7	676.31
LOC104912511	protein SZT2-like	743.3	609.0	676.15
TRMT13	tRNA methyltransferase 13 homolog (S. cerevisiae)	699.0	653.2	676.13
DTX4	deltex 4, E3 ubiquitin ligase	690.2	661.6	675.88
GALNT2	polypeptide N-acetylgalactosaminyltransferase 2	635.9	715.8	675.82
MRPS18C	mitochondrial ribosomal protein S18C	669.7	681.8	675.76
LSM7	LSM7 homolog, U6 small nuclear RNA associated (S. cerevisiae)	675.5	675.9	675.69
RPAP3	RNA polymerase II associated protein 3	651.0	699.9	675.48

LOC100547441	ADP-ribosylation factor GTPase-activating protein 1-like	684.8	665.8	675.29
PCSK6	proprotein convertase subtilisin/kexin type 6	706.1	644.0	675.04
HMGXB3	HMG box domain containing 3	684.4	664.4	674.40
LOC104911787	integrin beta-5-like	619.3	728.8	674.02
MRRF	mitochondrial ribosome recycling factor	692.5	655.0	673.75
YKT6	YKT6 v-SNARE homolog (S. cerevisiae)	722.9	624.4	673.67
LOC100547687	cohesin subunit SA-1	629.3	717.9	673.59
LOC104909233	ATP-binding cassette sub-family D member 2-like	683.1	663.3	673.20
PLK1	polo-like kinase 1	708.4	637.8	673.09
LOC104915807	valine--tRNA ligase, mitochondrial-like	744.2	601.5	672.84
WAC	WW domain containing adaptor with coiled-coil	614.8	730.8	672.81
MFSD1	major facilitator superfamily domain containing 1	630.9	714.2	672.56
TSKU	tsukushi, small leucine rich proteoglycan	668.1	676.9	672.47
PID1	phosphotyrosine interaction domain containing 1	630.2	714.3	672.22
TNKS1BP1	tankyrase 1 binding protein 1, 182kDa	684.9	659.4	672.18
LOC100543796	uncharacterized LOC100543796	803.1	539.9	671.50
SHF	Src homology 2 domain containing F	743.2	599.8	671.48
DNMT1	DNA (cytosine-5-)-methyltransferase 1	714.7	628.1	671.42
SLC35E1	solute carrier family 35, member E1	663.8	679.0	671.40
ANKRD50	ankyrin repeat domain 50	660.5	682.3	671.36
LOC104916070	programmed cell death protein 4-like	760.8	581.4	671.09
LOC100551225	deoxycytidine kinase-like	728.3	613.2	670.77
FAM212B	family with sequence similarity 212, member B	693.4	647.9	670.67
LOC104914682	ABC transporter F family member 4-like	704.4	636.7	670.53
LOC104915872	uncharacterized LOC104915872	710.1	630.8	670.47
PNPLA6	patatin-like phospholipase domain containing 6	692.9	648.0	670.44
GTF3C3	general transcription factor IIIC, polypeptide 3, 102kDa	673.9	666.7	670.33
TOB2	transducer of ERBB2, 2	689.1	651.5	670.29
LOC100541343	uncharacterized LOC100541343	573.7	766.1	669.90
TRMT2A	tRNA methyltransferase 2 homolog A (S. cerevisiae)	647.6	692.1	669.81
UNC50	unc-50 homolog (C. elegans)	673.9	665.6	669.75
GALK2	galactokinase 2	662.8	676.7	669.73
SNX33	sorting nexin 33	699.2	640.0	669.63
PPM1D	protein phosphatase, Mg2+/Mn2+ dependent, 1D	654.4	684.9	669.62
LOC104911455	uncharacterized LOC104911455	685.7	653.5	669.57
LOC104909518	heterogeneous nuclear ribonucleoprotein L-like	655.5	682.5	669.01
ITPA	inosine triphosphatase (nucleoside triphosphate pyrophosphatase)	717.5	620.5	668.98
LOC104916116	stress-induced-phosphoprotein 1-like	749.9	585.9	667.94
AMACR	alpha-methylacyl-CoA racemase	687.9	647.9	667.94
SLC2A1	solute carrier family 2 (facilitated glucose transporter), member 1	641.9	693.2	667.54
MMP11	matrix metallopeptidase 11 (stromelysin 3)	618.7	716.3	667.49
ZRSR2	zinc finger (CCCH type), RNA-binding motif and serine/arginine rich 2	663.7	671.2	667.44
PEX3	peroxisomal biogenesis factor 3	672.1	662.7	667.37
STARD4	StAR-related lipid transfer (START) domain containing 4	682.3	651.9	667.10
PAPSS2	3'-phosphoadenosine 5'-phosphosulfate synthase 2	639.5	694.6	667.06
EEF2KMT	eukaryotic elongation factor 2 lysine methyltransferase	631.6	702.4	667.01
NSDHL	NAD(P) dependent steroid dehydrogenase-like	689.0	644.5	666.76
RAB6A	RAB6A, member RAS oncogene family	614.8	718.6	666.72
SEC23IP	SEC23 interacting protein	645.2	688.0	666.61
SLC35A5	solute carrier family 35, member A5	645.2	687.8	666.51
NGLY1	N-glycanase 1	644.2	688.8	666.49
ZHX3	zinc fingers and homeoboxes 3	634.7	697.9	666.30
SETD5	SET domain containing 5	678.4	653.5	665.95
CDPF1	cysteine-rich, DPF motif domain containing 1	648.6	682.5	665.52
CYLD	cylindromatosis (turban tumor syndrome)	647.0	683.6	665.27
GDAP2	ganglioside induced differentiation associated protein 2	650.4	679.6	665.02

ZSWIM8	zinc finger, SWIM-type containing 8	651.1	677.6	664.37
TAZ	tafazzin	709.3	619.4	664.36
JMJD8	jumonji domain containing 8	658.1	670.4	664.25
VPS36	vacuolar protein sorting 36 homolog (S. cerevisiae)	659.5	668.9	664.22
ELP3	elongator acetyltransferase complex subunit 3	700.9	627.3	664.12
DR1	down-regulator of transcription 1, TBP-binding (negative cofactor 2)	639.2	688.7	663.97
CNTRL	centriolin	673.1	654.5	663.83
LOC100546195	zinc finger protein 777-like	684.2	643.1	663.65
RFWD2	ring finger and WD repeat domain 2, E3 ubiquitin protein ligase	730.3	596.0	663.17
MTCL1	microtubule crosslinking factor 1	670.7	655.3	662.99
GOLM1	golgi membrane protein 1	630.9	694.5	662.70
AXIN1	axin 1	652.0	673.3	662.66
LOC100544568	protocadherin gamma-C3-like	650.0	675.2	662.64
LOC104917110	eukaryotic translation initiation factor 3 subunit C-like	680.0	645.1	662.56
TRAFD1	TRAF-type zinc finger domain containing 1	654.5	670.6	662.53
LOC104914888	uncharacterized LOC104914888	595.3	728.9	662.07
LOC104910106	uncharacterized LOC104910106	688.9	634.2	661.59
TMEM242	transmembrane protein 242	682.7	640.1	661.41
CHID1	chitinase domain containing 1	624.1	698.6	661.34
VPS39	vacuolar protein sorting 39 homolog (S. cerevisiae)	634.2	688.5	661.33
BRD1	bromodomain containing 1	620.7	701.6	661.17
AGAP1	ArfGAP with GTPase domain, ankyrin repeat and PH domain 1	669.9	652.5	661.16
MALT1	MALT1 paracaspase	652.8	669.3	661.05
LOC100538586	phosphatidylinositol-binding clathrin assembly protein	654.5	667.2	660.87
FBRSL1	fibrosin-like 1	660.6	660.1	660.38
LOC104912068	protein FAM53B-like	620.3	700.3	660.26
ITFG2	integrin alpha FG-GAP repeat containing 2	644.2	675.9	660.07
SLU7	SLU7 splicing factor homolog (S. cerevisiae)	629.9	689.9	659.94
CYGB	cytoglobin	571.6	748.3	659.93
BCAR3	breast cancer anti-estrogen resistance 3	647.6	671.9	659.77
MBD3	methyl-CpG binding domain protein 3	687.5	632.0	659.76
MAFG	v-maf avian musculoaponeurotic fibrosarcoma oncogene homolog G	653.7	665.3	659.46
VEZF1	vascular endothelial zinc finger 1	619.9	698.4	659.16
CCDC28A	coiled-coil domain containing 28A	683.1	634.7	658.87
TBCE	tubulin folding cofactor E	640.1	677.4	658.73
VWA9	von Willebrand factor A domain containing 9	649.2	668.2	658.71
WRN	Werner syndrome, RecQ helicase-like	697.5	619.6	658.58
WNT7A	wingless-type MMTV integration site family, member 7A	610.1	706.8	658.43
LOC100541290	cytosolic 5'-nucleotidase 1B-like	672.0	644.5	658.24
RPS19BP1	ribosomal protein S19 binding protein 1	699.9	615.6	657.73
DCUN1D2	DCN1, defective in cullin neddylation 1, domain containing 2	622.4	692.2	657.32
RGS10	regulator of G-protein signaling 10	624.1	690.3	657.20
NCS1	neuronal calcium sensor 1	659.1	655.0	657.04
MFSD10	major facilitator superfamily domain containing 10	633.5	680.4	656.96
C30H19orf60	chromosome 30 open reading frame, human C19orf60	693.3	620.3	656.81
TIMM21	translocase of inner mitochondrial membrane 21 homolog (yeast)	673.6	639.9	656.76
TNRC6A	trinucleotide repeat containing 6A	606.6	706.8	656.72
FAM189A2	family with sequence similarity 189, member A2	647.9	665.3	656.57
TIMM9	translocase of inner mitochondrial membrane 9 homolog (yeast)	671.9	640.9	656.38
SLC31A1	solute carrier family 31 (copper transporter), member 1	679.0	633.4	656.17
CHMP2B	charged multivesicular body protein 2B	652.5	659.3	655.88
KLHDC1	kelch domain containing 1	652.8	658.6	655.72
PUS1	pseudouridylate synthase 1	659.5	651.7	655.61
NUDT7	nudix (nucleoside diphosphate linked moiety X)-type motif 7	677.0	634.2	655.57
EXOSC9	exosome component 9	690.6	619.2	654.91
INSIG2	insulin induced gene 2	607.3	702.4	654.86

RBM15B	RNA binding motif protein 15B	681.5	627.6	654.57
LOC104915936	uncharacterized LOC104915936	701.8	607.2	654.52
PMS2	PMS2 postmeiotic segregation increased 2 (S. cerevisiae)	593.6	714.7	654.13
MSH6	mutS homolog 6	676.4	631.6	654.01
MTMR4	myotubularin related protein 4	635.9	672.1	654.00
ACTR8	ARP8 actin-related protein 8 homolog (yeast)	623.3	684.5	653.88
CDH23	cadherin-related 23	692.9	614.3	653.60
ADPRM	ADP-ribose/CDP-alcohol diphosphatase, manganese-dependent	669.6	637.3	653.46
ASB8	ankyrin repeat and SOCS box containing 8	676.4	630.4	653.43
DPH3	diphthamide biosynthesis 3	613.7	692.5	653.08
MPV17	MpV17 mitochondrial inner membrane protein	695.0	610.9	652.96
FBXO28	F-box protein 28	654.4	650.7	652.57
UACA	uveal autoantigen with coiled-coil domains and ankyrin repeats	594.5	708.7	651.63
PACSIN2	protein kinase C and casein kinase substrate in neurons 2	654.4	647.8	651.06
MEF2BNB	MEF2B neighbor	656.2	645.3	650.75
PLCD3	phospholipase C, delta 3	663.9	637.1	650.53
PTPLB	protein tyrosine phosphatase-like (proline instead of catalytic arginine), member b	656.9	643.6	650.23
DNAL4	dynein, axonemal, light chain 4	669.5	631.0	650.21
WASH1	WAS protein family homolog 1	673.8	626.6	650.17
TAF11	TAF11 RNA polymerase II, TATA box binding protein (TBP)-associated factor, 28kDa	687.4	612.8	650.10
MRPL30	mitochondrial ribosomal protein L30	655.9	643.7	649.80
WBP4	WW domain binding protein 4	647.9	651.5	649.65
PBXIP1	pre-B-cell leukemia homeobox interacting protein 1	788.4	510.2	649.33
CCDC50	coiled-coil domain containing 50	680.2	618.3	649.25
GNAS	GNAS complex locus	626.7	671.6	649.15
FYCO1	FYVE and coiled-coil domain containing 1	646.2	651.9	649.05
SLC1A4	solute carrier family 1 (glutamate/neutral amino acid transporter), member 4	666.0	631.4	648.67
DIS3L	DIS3 like exosome 3'-5' exoribonuclease	630.2	667.0	648.57
FAM188A	family with sequence similarity 188, member A	652.6	644.3	648.49
LOC100540622	synaptonemal complex protein SC65-like	574.0	721.9	647.95
KLF8	Kruppel-like factor 8	665.6	629.7	647.68
SGK3	serum/glucocorticoid regulated kinase family, member 3	634.0	660.8	647.38
TBC1D4	TBC1 domain family, member 4	607.2	687.4	647.31
LOC104911236	autophagy-related protein 2 homolog B-like	621.6	669.9	645.78
LOC104917501	leucine-rich repeat and calponin homology domain-containing protein 1-like	609.8	681.7	645.76
LOC104916047	cytochrome c oxidase subunit 7C, mitochondrial pseudogene	757.9	532.8	645.37
GOLIM4	golgi integral membrane protein 4	600.6	689.6	645.10
SRRD	SRR1 domain containing	662.0	628.0	645.01
LOC104914928	uncharacterized LOC104914928	673.6	616.4	644.98
GLB1L	galactosidase, beta 1-like	622.7	666.5	644.63
GID4	GID complex subunit 4	656.3	633.0	644.62
BARX2	BARX homeobox 2	576.3	712.2	644.21
MRPL39	mitochondrial ribosomal protein L39	649.2	638.5	643.81
KLHL28	kelch-like family member 28	658.9	628.4	643.68
NEDD8	neural precursor cell expressed, developmentally down-regulated 8	684.2	602.6	643.42
ARID5A	AT rich interactive domain 5A (MRF1-like)	678.4	608.4	643.40
IGFBP3	insulin-like growth factor binding protein 3	633.3	653.3	643.32
CDC6	cell division cycle 6	702.7	583.8	643.27
MXI1	MAX interactor 1, dimerization protein	630.1	656.2	643.15
MRE11A	MRE11 meiotic recombination 11 homolog A (S. cerevisiae)	621.6	664.2	642.93
RLTPR	RGD motif, leucine rich repeats, tropomodulin domain and proline-rich containing	661.0	624.6	642.77
ZXDC	ZXD family zinc finger C	585.3	699.8	642.59

LOC104910196	testis-expressed sequence 10 protein homolog	627.4	657.5	642.48
MEX3B	mex-3 RNA binding family member B	650.4	633.6	642.01
FAM193B	family with sequence similarity 193, member B	665.7	617.9	641.79
TSEN54	TSEN54 tRNA splicing endonuclease subunit	658.2	625.2	641.70
PEX13	peroxisomal biogenesis factor 13	660.3	622.9	641.62
RSRC1	arginine/serine-rich coiled-coil 1	635.0	648.1	641.54
HMGCL	3-hydroxymethyl-3-methylglutaryl-CoA lyase	656.1	626.5	641.31
ZNF330	zinc finger protein 330	618.1	664.0	641.04
CCNT2	cyclin T2	609.7	672.4	641.04
TMEM255A	transmembrane protein 255A	648.7	633.0	640.86
THUMPD1	THUMP domain containing 1	687.1	594.5	640.78
RBM19	RNA binding motif protein 19	652.2	628.2	640.21
DTNBP1	dystrobrevin binding protein 1	658.6	621.3	639.94
MOB3A	MOB kinase activator 3A	689.8	590.0	639.91
SLC38A1	solute carrier family 38, member 1	604.7	675.1	639.90
CADM1	cell adhesion molecule 1	651.6	628.1	639.82
TEAD3	TEA domain family member 3	668.2	611.0	639.59
HEMK1	HemK methyltransferase family member 1	646.9	631.2	639.08
SPHK1	sphingosine kinase 1	671.6	606.3	638.93
MIEF2	mitochondrial elongation factor 2	661.1	616.6	638.82
SMC5	structural maintenance of chromosomes 5	615.6	660.2	637.92
CLN8	ceroid-lipofuscinosis, neuronal 8 (epilepsy, progressive with mental retardation)	645.2	630.1	637.67
PIGU	phosphatidylinositol glycan anchor biosynthesis, class U	650.4	624.5	637.45
NRG4	neuregulin 4	640.7	634.1	637.44
LOC104911373	partitioning defective 3 homolog	606.3	668.6	637.42
NFRKB	nuclear factor related to kappaB binding protein	652.8	622.0	637.40
HPS5	Hermansky-Pudlak syndrome 5	634.9	639.2	637.08
ATG9A	autophagy related 9A	696.2	577.8	636.98
METTL2A	methyltransferase like 2A	624.1	649.8	636.94
DAK	dihydroxyacetone kinase 2 homolog (S. cerevisiae)	680.9	592.8	636.82
RNF26	ring finger protein 26	628.6	645.1	636.81
MPPE1	metallophosphoesterase 1	600.6	672.8	636.68
ZNF521	zinc finger protein 521	582.7	690.4	636.55
CISD3	CDGSH iron sulfur domain 3	666.4	606.6	636.53
CCDC69	coiled-coil domain containing 69	549.1	723.4	636.25
LOC100544221	ESF1 homolog	603.0	669.0	635.99
LOC104910482	trafficking protein particle complex subunit 9-like	632.4	639.2	635.82
GLP1R	glucagon-like peptide 1 receptor	631.7	639.7	635.69
CCDC61	coiled-coil domain containing 61	666.2	604.8	635.51
DOCK9	dedicator of cytokinesis 9	606.3	664.7	635.48
MED7	mediator complex subunit 7	623.0	647.3	635.17
SEL1L3	sel-1 suppressor of lin-12-like 3 (C. elegans)	624.1	646.2	635.12
ZNF687	zinc finger protein 687	665.9	603.7	634.80
STARD8	StAR-related lipid transfer (START) domain containing 8	657.3	612.2	634.72
CPEB1	cytoplasmic polyadenylation element binding protein 1	589.0	680.4	634.69
MAN1B1	mannosidase, alpha, class 1B, member 1	635.2	634.0	634.58
CTC1	CTS telomere maintenance complex component 1	640.1	629.1	634.57
BID	BH3 interacting domain death agonist	647.5	620.8	634.18
LANCL1	LanC lantibiotic synthetase component C-like 1 (bacterial)	653.5	614.7	634.11
ASNA1	arsA arsenite transporter, ATP-binding, homolog 1 (bacterial)	646.1	621.0	633.54
PAM16	presequence translocase-associated motor 16 homolog (S. cerevisiae)	656.0	611.0	633.48
TMEM8A	transmembrane protein 8A	674.9	591.6	633.24
MED18	mediator complex subunit 18	602.1	664.3	633.20
ZSWIM5	zinc finger, SWIM-type containing 5	636.1	630.0	633.05
EPS15	epidermal growth factor receptor pathway substrate 15	615.9	649.1	632.50
EHD4	EH-domain containing 4	607.1	657.7	632.43

LOC104916826	bone morphogenetic protein 1-like	693.4	571.1	632.26
LOC100548381	heme-binding protein 1-like	655.5	609.0	632.25
ANGPTL2	angiopoietin-like 2	567.5	696.1	631.82
RABGAP1	RAB GTPase activating protein 1	572.6	690.7	631.67
AUTS2	autism susceptibility candidate 2	623.3	640.0	631.63
PPP4R1	protein phosphatase 4, regulatory subunit 1	612.3	650.8	631.55
BAG5	BCL2-associated athanogene 5	565.0	696.9	630.94
LOC104911363	poly(A) RNA polymerase, mitochondrial-like	607.1	654.5	630.81
CPD	carboxypeptidase D	639.7	621.8	630.76
CUTC	cutC copper transporter	601.3	658.7	630.00
RAB29	RAB29, member RAS oncogene family	602.1	657.7	629.91
LOC104916383	lamin-A-like	686.0	573.4	629.70
KDM2B	lysine (K)-specific demethylase 2B	635.9	623.5	629.66
LOC104916025	acetylcholinesterase-like	705.6	553.3	629.45
PROSER1	proline and serine rich 1	612.3	646.2	629.27
EFR3A	EFR3 homolog A (S. cerevisiae)	638.2	620.3	629.24
C11H3orf33	chromosome 11 open reading frame, human C3orf33	620.8	636.3	628.53
ITPR1	inositol 1,4,5-trisphosphate receptor, type 1	630.9	625.3	628.07
LOC100541930	zinc finger CCCH domain-containing protein 18-like	665.5	590.5	628.01
PRMT5	protein arginine methyltransferase 5	694.9	561.1	627.98
GAA	glucosidase, alpha; acid	634.5	621.1	627.79
BCR	breakpoint cluster region	572.7	682.8	627.72
TRPC4AP	transient receptor potential cation channel, subfamily C, member 4 associated protein	589.5	665.6	627.55
LOC100546433	ras-related protein Rab-18-B-like	565.1	689.9	627.47
LOC104916409	S-adenosylmethionine synthase isoform type-2	681.6	572.8	627.21
LARP7	La ribonucleoprotein domain family, member 7	597.8	656.2	627.01
THRB	thyroid hormone receptor, beta	588.7	663.8	626.28
LOC100544355	tRNA-dihydrouridine(16/17) synthase [NAD(P)(+)]-like	619.8	632.7	626.26
MAN1C1	mannosidase, alpha, class 1C, member 1	601.3	650.8	626.07
LRRC37A3	leucine rich repeat containing 37, member A3	591.9	660.2	626.06
RAPH1	Ras association (RalGDS/AF-6) and pleckstrin homology domains 1	631.6	620.4	626.02
PDCD11	programmed cell death 11	638.4	613.4	625.93
PTPDC1	protein tyrosine phosphatase domain containing 1	640.3	610.8	625.54
LOC104911121	tetratricopeptide repeat protein 17-like	554.9	695.2	625.02
NPHP3	nephronophthisis 3 (adolescent)	602.3	647.7	625.00
TP53RK	TP53 regulating kinase	626.7	621.6	624.16
LOC100550423	acylglycerol kinase, mitochondrial-like	648.5	599.7	624.09
TRIP12	thyroid hormone receptor interactor 12	545.8	701.8	623.78
ZFP36L1	ZFP36 ring finger protein-like 1	638.6	608.7	623.62
TMEM185A	transmembrane protein 185A	602.1	645.1	623.61
METTL9	methyltransferase like 9	617.1	629.8	623.45
GINS4	GINS complex subunit 4 (Sld5 homolog)	659.7	586.8	623.24
ZNF513	zinc finger protein 513	648.7	597.4	623.04
XKR8	XK, Kell blood group complex subunit-related family, member 8	624.4	621.3	622.86
EDA	ectodysplasin A	581.1	664.4	622.77
ST7L	suppression of tumorigenicity 7 like	614.8	630.0	622.39
RSBN1L	round spermatid basic protein 1-like	651.7	592.6	622.13
BMP7	bone morphogenetic protein 7	538.4	705.3	621.85
MED14	mediator complex subunit 14	602.1	641.5	621.83
LRRC42	leucine rich repeat containing 42	596.3	646.9	621.61
NMU	neuromedin U	588.5	654.2	621.38
LOC100550420	deleted in malignant brain tumors 1 protein-like	702.3	540.4	621.36
TTC14	tetratricopeptide repeat domain 14	576.0	666.5	621.27
DTD1	D-tyrosyl-tRNA deacylase 1	585.9	656.4	621.17
BLZF1	basic leucine zipper nuclear factor 1	645.1	596.8	620.94
LOC100543661	uncharacterized LOC100543661	551.5	690.4	620.94
KLHL12	kelch-like family member 12	634.5	607.0	620.73

DUSP16	dual specificity phosphatase 16	588.0	653.0	620.46
OPHN1	oligophrenin 1	636.2	603.8	619.99
LOC100539883	general transcription factor II-I repeat domain-containing protein 1-like	602.5	637.4	619.97
MMP24	matrix metallopeptidase 24 (membrane-inserted)	633.4	606.0	619.71
TJP2	tight junction protein 2	645.9	593.5	619.67
MAML1	mastermind-like 1 (Drosophila)	587.8	651.1	619.47
LPCAT2	lysophosphatidylcholine acyltransferase 2	602.1	636.8	619.47
RGS2	regulator of G-protein signaling 2	600.5	637.9	619.19
VAMP4	vesicle-associated membrane protein 4	627.4	610.8	619.10
NAA40	N(alpha)-acetyltransferase 40, NatD catalytic subunit	647.9	589.7	618.76
CTCF	CCCTC-binding factor (zinc finger protein)	617.3	620.0	618.68
FRG1	FSHD region gene 1	611.4	625.8	618.60
ATP6V0A2	ATPase, H+ transporting, lysosomal V0 subunit a2	596.3	640.5	618.41
XRCC5	X-ray repair complementing defective repair in Chinese hamster cells 5 (double-strand-break rejoining)	616.3	620.1	618.17
RNF145	ring finger protein 145	605.6	630.6	618.11
PRRG3	proline rich Gla (G-carboxyglutamic acid) 3 (transmembrane)	604.8	631.4	618.09
DGCR14	DiGeorge syndrome critical region gene 14	603.8	631.3	617.54
CAAP1	caspase activity and apoptosis inhibitor 1	649.3	585.5	617.43
UBXN6	UBX domain protein 6	602.0	631.6	616.76
CHCHD4	coiled-coil-helix-coiled-coil-helix domain containing 4	635.0	598.0	616.49
MGST1	microsomal glutathione S-transferase 1	613.1	619.5	616.30
DERL1	derlin 1	623.9	608.3	616.14
DDX51	DEAD (Asp-Glu-Ala-Asp) box polypeptide 51	619.8	612.2	615.96
TMEM203	transmembrane protein 203	636.0	595.7	615.82
LOC100548938	atrophin-1-like	603.1	628.4	615.74
TMEM186	transmembrane protein 186	626.6	604.2	615.38
C1H12orf5	chromosome 1 open reading frame, human C12orf5	588.4	642.3	615.36
EPC1	enhancer of polycomb homolog 1 (Drosophila)	614.8	615.3	615.06
DEDD	death effector domain containing	657.2	572.2	614.72
ALG3	ALG3, alpha-1,3- mannosyltransferase	627.6	601.7	614.67
GNE	glucosamine (UDP-N-acetyl)-2-epimerase/N-acetylmannosamine kinase	612.3	616.4	614.32
SLC2A10	solute carrier family 2 (facilitated glucose transporter), member 10	623.5	605.0	614.25
IGF2BP2	insulin-like growth factor 2 mRNA binding protein 2	650.3	577.9	614.13
NPTN	neuroplastin	568.3	659.7	614.03
NAA30	N(alpha)-acetyltransferase 30, NatC catalytic subunit	628.4	599.6	613.97
CDAN1	codanin 1	570.2	657.2	613.70
ARSK	arylsulfatase family, member K	632.5	594.8	613.67
MPPED2	metallophosphoesterase domain containing 2	596.9	630.1	613.50
C26H11orf57	chromosome 26 open reading frame, human C11orf57	625.7	601.2	613.45
CFLAR	CASP8 and FADD-like apoptosis regulator	641.8	585.0	613.39
CBLL1	Cbl proto-oncogene-like 1, E3 ubiquitin protein ligase	623.4	603.3	613.33
KLHL11	kelch-like family member 11	595.3	630.8	613.09
ATRIP	ATR interacting protein	641.0	584.3	612.65
NAA60	N(alpha)-acetyltransferase 60, NatF catalytic subunit	657.2	567.7	612.42
C20H17orf62	chromosome 20 open reading frame, human C17orf62	562.5	661.8	612.15
ENDOV	endonuclease V	632.4	590.9	611.68
RPUSD3	RNA pseudouridylate synthase domain containing 3	644.5	578.1	611.30
CACUL1	CDK2-associated, cullin domain 1	621.4	600.6	611.00
LOC100545977	serine/threonine-protein kinase MRCK alpha	576.3	645.7	610.99
YPEL5	yippee-like 5 (Drosophila)	599.6	622.1	610.87
EPHA2	EPH receptor A2	634.4	586.5	610.48
ZNHIT3	zinc finger, HIT-type containing 3	605.3	615.3	610.26
CGGBP1	CGG triplet repeat binding protein 1	586.9	633.2	610.06
NHP2	NHP2 ribonucleoprotein	654.6	565.5	610.05
ZBTB39	zinc finger and BTB domain containing 39	566.1	654.0	610.02

PIK3R3	phosphoinositide-3-kinase, regulatory subunit 3 (gamma)	575.2	644.5	609.85
SC5D	sterol-C5-desaturase	621.6	597.7	609.65
DDHD2	DDHD domain containing 2	588.1	631.1	609.60
PDZD11	PDZ domain containing 11	611.4	607.4	609.39
NUDT12	nudix (nucleoside diphosphate linked moiety X)-type motif 12	580.2	638.3	609.24
LOC104914943	cyclin-dependent kinase 7-like	636.6	580.5	608.54
LOC104913391	uncharacterized LOC104913391	572.5	643.4	607.99
RANBP10	RAN binding protein 10	631.0	584.2	607.59
HERPUD1	homocysteine-inducible, endoplasmic reticulum stress-inducible, ubiquitin-like domain member 1	615.5	599.6	607.54
TUBGCP4	tubulin, gamma complex associated protein 4	557.4	656.8	607.09
AZI2	5-azacytidine induced 2	591.0	622.6	606.81
TRIB1	tribbles pseudokinase 1	593.6	619.9	606.76
TIMM10	translocase of inner mitochondrial membrane 10 homolog (yeast)	665.3	547.4	606.38
LOC100541605	golgin subfamily B member 1-like	614.7	597.3	605.99
SETDB2	SET domain, bifurcated 2	633.2	578.2	605.73
TMEM161B	transmembrane protein 161B	583.6	627.8	605.70
NAV2	neuron navigator 2	518.9	692.3	605.63
TRAM2	translocation associated membrane protein 2	627.6	583.6	605.63
LOC100542873	serine/threonine-protein kinase 38	570.9	639.9	605.43
LOC104914617	V-type proton ATPase 116 kDa subunit a isoform 1-like	615.7	594.3	605.00
C14H3orf18	chromosome 14 open reading frame, human C3orf18	619.3	590.6	604.94
MRPL1	mitochondrial ribosomal protein L1	591.8	617.4	604.59
TMEM101	transmembrane protein 101	599.8	608.6	604.15
RSF1	remodeling and spacing factor 1	568.3	639.7	604.02
LOC100547897	lysosomal alpha-glucosidase-like	609.2	598.2	603.67
TPGS2	tubulin polyglutamylase complex subunit 2	618.2	588.8	603.52
UFSP2	UFM1-specific peptidase 2	580.2	626.7	603.43
LOC104916999	beta-enolase-like	589.6	617.0	603.28
ATG12	autophagy related 12	611.4	594.4	602.90
NCEH1	neutral cholesterol ester hydrolase 1	565.2	640.2	602.68
STK19	serine/threonine kinase 19	681.5	523.7	602.60
GPSM2	G-protein signaling modulator 2	611.2	593.7	602.42
LOC104911657	alpha-aspartyl dipeptidase-like	562.7	642.1	602.36
ANKLE2	ankyrin repeat and LEM domain containing 2	562.5	641.8	602.17
BROX	BRO1 domain and CAAX motif containing	577.5	626.8	602.13
UPRT	uracil phosphoribosyltransferase (FUR1) homolog (S. cerevisiae)	600.3	603.8	602.09
MMAB	methylmalonic aciduria (cobalamin deficiency) cblB type	634.9	569.1	601.98
LOC100548360	urotensin-2 receptor-like	571.2	631.8	601.53
THY1	Thy-1 cell surface antigen	560.6	642.3	601.48
GALNT4	polypeptide N-acetylgalactosaminyltransferase 4	586.8	615.0	600.89
STX16	syntaxin 16	601.2	600.3	600.76
AP1G1	adaptor-related protein complex 1, gamma 1 subunit	621.0	580.1	600.55
AP3M2	adaptor-related protein complex 3, mu 2 subunit	585.2	615.3	600.24
DDRGK1	DDRGK domain containing 1	598.7	601.8	600.24
KIAA2026	KIAA2026 ortholog	575.3	625.0	600.12
EIF4G3	eukaryotic translation initiation factor 4 gamma, 3	614.1	585.4	599.79
APMAP	adipocyte plasma membrane associated protein	585.4	613.7	599.58
ITCH	itchy E3 ubiquitin protein ligase	570.8	628.3	599.57
MKL2	MKL/myocardin-like 2	545.6	653.4	599.52
NCAPH	non-SMC condensin I complex, subunit H	655.9	541.6	598.77
AHNAK2	AHNAK nucleoprotein 2	585.3	612.2	598.76
INTS4	integrator complex subunit 4	582.7	614.7	598.70
CHERP	calcium homeostasis endoplasmic reticulum protein	581.4	615.9	598.64
ERN1	endoplasmic reticulum to nucleus signaling 1	575.2	621.6	598.39
ARHGEF26	Rho guanine nucleotide exchange factor (GEF) 26	591.2	603.6	597.39
RGP1	RGP1 retrograde golgi transport homolog (S. cerevisiae)	617.3	577.1	597.19
NCSTN	nicastrin	602.9	591.1	597.04

ANKH	ANKH inorganic pyrophosphate transport regulator	599.4	594.5	596.98
LOC100543251	phosphoribosyltransferase domain-containing protein 1-like	583.5	610.2	596.82
NFX1	nuclear transcription factor, X-box binding 1	565.0	628.6	596.80
CACNA2D2	calcium channel, voltage-dependent, alpha 2/delta subunit 2	635.3	558.0	596.67
LOC100550229	nuclear GTPase SLIP-GC-like	592.8	599.9	596.39
KLHL18	kelch-like family member 18	624.3	567.6	595.95
EXTL1	exostosin-like glycosyltransferase 1	684.1	507.4	595.74
LOC100544649	inorganic pyrophosphatase-like	605.4	585.7	595.53
RBM25	RNA binding motif protein 25	546.4	642.9	594.63
HHAT	hedgehog acyltransferase	576.1	611.9	594.01
NOC4L	nucleolar complex associated 4 homolog (S. cerevisiae)	610.5	577.5	593.99
AP4B1	adaptor-related protein complex 4, beta 1 subunit	578.6	608.1	593.31
ZGPAT	zinc finger, CCCH-type with G patch domain	626.6	559.7	593.15
DIS3L2	DIS3 like 3'-5' exoribonuclease 2	621.5	564.2	592.86
LOC104909621	serine/threonine-protein kinase D3-like	574.2	610.7	592.44
TSPAN9	tetraspanin 9	523.6	661.2	592.36
TBC1D13	TBC1 domain family, member 13	615.7	568.8	592.25
SLC25A13	solute carrier family 25 (aspartate/glutamate carrier), member 13	543.2	640.8	591.99
MRPL19	mitochondrial ribosomal protein L19	591.8	592.1	591.95
LOC104917334	MARCKS-related protein-like	588.6	594.9	591.73
LOC104911001	RNA polymerase-associated protein CTR9 homolog	607.9	574.8	591.36
RWDD1	RWD domain containing 1	610.4	571.9	591.16
LOC100549552	histone H2B 5-like	584.4	597.6	591.00
SCAF4	SR-related CTD-associated factor 4	576.0	606.0	591.00
CPOX	coproporphyrinogen oxidase	611.4	570.4	590.87
COQ6	coenzyme Q6 monooxygenase	604.7	576.8	590.75
PIK3C2B	phosphatidylinositol-4-phosphate 3-kinase, catalytic subunit type 2 beta	582.1	599.1	590.62
EPHA5	EPH receptor A5	604.8	575.9	590.33
LOC104916525	COUP transcription factor 2	557.3	622.9	590.13
LOC100545219	E3 ubiquitin-protein ligase RNF220-like	628.3	550.7	589.49
SMG8	SMG8 nonsense mediated mRNA decay factor	619.0	559.9	589.41
NOP10	NOP10 ribonucleoprotein	637.8	540.9	589.37
TMEM60	transmembrane protein 60	595.1	583.5	589.30
MEF2D	myocyte enhancer factor 2D	614.4	564.1	589.24
AIP	aryl hydrocarbon receptor interacting protein	601.2	577.1	589.17
MLXIP	MLX interacting protein	556.7	621.1	588.90
FAM163B	family with sequence similarity 163, member B	608.7	568.2	588.45
LOC104910361	brefeldin A-inhibited guanine nucleotide-exchange protein 1-like	581.8	594.9	588.37
FBF1	Fas (TNFRSF6) binding factor 1	617.6	559.1	588.32
SEC11C	SEC11 homolog C (S. cerevisiae)	594.5	582.0	588.26
SRM	spermidine synthase	600.4	575.3	587.85
TRAPPC6B	trafficking protein particle complex 6B	615.3	559.7	587.48
C1H21orf59	chromosome 1 open reading frame, human C21orf59	601.2	573.5	587.39
LOC104912701	PHD finger protein 13-like	573.6	600.4	587.00
LOC104914670	uncharacterized LOC104914670	613.4	560.1	586.76
HECTD2	HECT domain containing E3 ubiquitin protein ligase 2	558.4	614.8	586.56
CCDC107	coiled-coil domain containing 107	558.1	614.8	586.47
LYSMD4	LysM, putative peptidoglycan-binding, domain containing 4	646.7	526.2	586.46
ACOT13	acyl-CoA thioesterase 13	590.0	582.8	586.36
LZIC	leucine zipper and CTNNBIP1 domain containing	598.6	573.4	586.02
NVL	nuclear VCP-like	567.4	604.2	585.81
SUMF1	sulfatase modifying factor 1	559.1	611.5	585.30
LOC100548778	chromosome unknown open reading frame, human C1orf85	593.9	576.3	585.14
COQ4	coenzyme Q4	605.5	564.2	584.87
LRRC45	leucine rich repeat containing 45	535.6	634.1	584.85
ANKRD54	ankyrin repeat domain 54	608.8	560.8	584.80
LOC100548710	calcium-binding protein 39	563.3	606.3	584.79

NSMCE2	non-SMC element 2, MMS21 homolog (S. cerevisiae)	568.1	601.5	584.77
P2RX4	purinergic receptor P2X, ligand-gated ion channel, 4	571.7	597.3	584.50
ME3	malic enzyme 3, NADP(+)-dependent, mitochondrial	581.9	586.5	584.21
CYTH1	cytohesin 1	631.7	536.6	584.17
LOC100547767	eukaryotic translation initiation factor 4B-like	660.6	506.5	583.52
KLHDC10	kelch domain containing 10	581.1	586.0	583.52
LOC100548949	alpha-catulin	524.5	642.5	583.50
GOLGA5	golgin A5	586.0	580.2	583.09
GEMIN2	gem (nuclear organelle) associated protein 2	569.2	596.9	583.09
C3H18orf21	chromosome 3 open reading frame, human C18orf21	608.7	557.3	582.96
SLC25A51	solute carrier family 25, member 51	575.0	590.8	582.91
DPY30	dpy-30 homolog (C. elegans)	559.8	606.0	582.91
PRKCB	protein kinase C, beta	522.1	643.6	582.83
MED16	mediator complex subunit 16	576.2	589.0	582.60
SYAP1	synapse associated protein 1	580.0	584.8	582.40
NAGLU	N-acetylglucosaminidase, alpha	617.5	547.2	582.34
TRIP11	thyroid hormone receptor interactor 11	527.8	636.3	582.05
UNC5A	unc-5 homolog A (C. elegans)	610.0	553.8	581.93
ACY1	aminoacylase 1	604.7	558.9	581.79
KIF2C	kinesin family member 2C	614.7	548.6	581.64
PTBP1	polypyrimidine tract binding protein 1	586.2	577.1	581.61
CDC16	cell division cycle 16	579.9	582.7	581.33
FCHO2	FCH domain only 2	552.3	610.3	581.31
LOC104912625	disks large homolog 1-like	543.1	619.5	581.27
LOC104914337	microtubule-actin cross-linking factor 1-like	589.6	572.6	581.12
LOC100545415	cytochrome b5 reductase 4	575.1	586.6	580.84
LOC100541391	transcriptional enhancer factor TEF-3-like	590.4	570.0	580.20
MRPL43	mitochondrial ribosomal protein L43	629.8	530.0	579.91
FZD1	frizzled class receptor 1	581.1	578.0	579.51
KIAA1217	KIAA1217 ortholog	544.0	614.9	579.45
TCF15	transcription factor 15 (basic helix-loop-helix)	625.1	533.7	579.41
ACBD6	acyl-CoA binding domain containing 6	561.7	596.7	579.22
FAM210A	family with sequence similarity 210, member A	578.3	579.5	578.94
SKAP2	src kinase associated phosphoprotein 2	574.9	582.9	578.92
C4H4orf27	chromosome 4 open reading frame, human C4orf27	623.9	533.7	578.80
BLOC1S1	biogenesis of lysosomal organelles complex-1, subunit 1	608.0	549.5	578.75
TBC1D2	TBC1 domain family, member 2	584.3	573.1	578.66
LOC104911761	tubulin alpha-5 chain-like	616.6	540.5	578.56
UCK2	uridine-cytidine kinase 2	634.1	522.7	578.42
ARHGAP5	Rho GTPase activating protein 5	553.1	603.1	578.08
E4F1	E4F transcription factor 1	570.0	585.2	577.59
KIAA1107	KIAA1107 ortholog	517.8	637.3	577.55
LOC104916992	uncharacterized LOC104916992	578.8	575.5	577.11
LOC100551074	discoidin domain-containing receptor 2-like	543.9	610.2	577.10
LOC104914825	atrial natriuretic peptide receptor 1-like	482.3	671.5	576.86
RRAD	Ras-related associated with diabetes	595.9	557.4	576.65
ZBTB8B	zinc finger and BTB domain containing 8B	591.1	561.7	576.43
LOC104914521	ubiquinol-cytochrome-c reductase complex assembly factor 2	607.1	545.5	576.34
PPAPDC2	phosphatidic acid phosphatase type 2 domain containing 2	603.9	548.7	576.32
TOMM34	translocase of outer mitochondrial membrane 34	569.3	583.2	576.30
TMEM184C	transmembrane protein 184C	590.2	562.3	576.26
ATXN1L	ataxin 1-like	593.0	559.5	576.24
NSMCE1	non-SMC element 1 homolog (S. cerevisiae)	579.1	573.4	576.23
LOC100547843	serine/threonine-protein phosphatase 2A regulatory subunit B'' subunit alpha	545.6	606.6	576.10
LOC100545814	E3 ubiquitin-protein ligase TRIM33-like	571.5	580.5	575.99
HRASLS	HRAS-like suppressor	577.6	574.3	575.94
ZNF318	zinc finger protein 318	588.6	563.1	575.88

TARS2	threonyl-tRNA synthetase 2, mitochondrial (putative)	609.1	542.4	575.75
PGM2	phosphoglucomutase 2	518.6	632.8	575.67
CDK8	cyclin-dependent kinase 8	562.4	588.8	575.59
LOC104911366	uncharacterized LOC104911366	568.5	581.5	574.99
LOC100540056	serine-protein kinase ATM-like	548.9	601.1	574.98
LOC100538974	periodic tryptophan protein 2 homolog	578.3	570.9	574.58
LOC100542307	solute carrier family 2, facilitated glucose transporter member 3	520.3	628.6	574.45
LOC104911696	uncharacterized LOC104911696	535.3	613.5	574.42
SNIP1	Smad nuclear interacting protein 1	576.7	571.8	574.25
MRPS27	mitochondrial ribosomal protein S27	587.6	560.3	573.95
C20H17orf89	chromosome 20 open reading frame, human C17orf89	639.1	508.6	573.86
LOC104915926	protein-lysine methyltransferase METTL21B-like	547.1	600.3	573.73
LMBRD1	LMBR1 domain containing 1	553.1	594.2	573.63
KLHL15	kelch-like family member 15	597.7	548.7	573.21
TPM2	tropomyosin 2 (beta)	578.8	567.6	573.16
GLTP	glycolipid transfer protein	622.0	524.2	573.13
LOC104909717	zinc finger protein 70-like	634.3	511.9	573.12
FNIP2	folliculin interacting protein 2	519.2	626.7	572.97
TXLNG	taxilin gamma	532.0	613.8	572.92
ARID2	AT rich interactive domain 2 (ARID, RFX-like)	522.9	622.8	572.87
DCUN1D4	DCN1, defective in cullin neddylation 1, domain containing 4	584.3	561.3	572.76
ELMOD3	ELMO/CED-12 domain containing 3	581.0	563.4	572.16
EIF4E3	eukaryotic translation initiation factor 4E family member 3	523.4	620.9	572.15
SQRDL	sulfide quinone reductase-like (yeast)	502.8	641.3	572.03
MAR2	membrane-associated ring finger (C3HC4) 2, E3 ubiquitin protein ligase	581.1	562.9	571.98
FPGS	folylpolyglutamate synthase	647.0	496.9	571.91
MESDC1	mesoderm development candidate 1	541.2	602.4	571.79
NKTR	natural killer cell triggering receptor	547.3	596.3	571.79
RAD50	RAD50 homolog (S. cerevisiae)	569.3	573.4	571.35
BTBD11	BTB (POZ) domain containing 11	523.6	618.8	571.21
PCBD2	pterin-4 alpha-carbinolamine dehydratase/dimerization cofactor of hepatocyte nuclear factor 1 alpha (TCF1) 2	599.7	542.6	571.12
PTPN23	protein tyrosine phosphatase, non-receptor type 23	595.5	546.8	571.12
SURF2	surfeit 2	618.9	522.8	570.87
EIF4ENIF1	eukaryotic translation initiation factor 4E nuclear import factor 1	547.2	594.4	570.81
VPRBP	Vpr (HIV-1) binding protein	544.7	596.9	570.79
RSG1	REM2 and RAB-like small GTPase 1	626.7	514.8	570.75
CD46	CD46 molecule, complement regulatory protein	561.5	579.9	570.72
SPATA2L	spermatogenesis associated 2-like	605.8	535.7	570.70
UHRF1BP1L	UHRF1 binding protein 1-like	554.7	586.6	570.67
YRDC	yrdC N(6)-threonylcarbamoyltransferase domain containing	545.5	595.8	570.65
MYO1E	myosin IE	527.1	613.7	570.37
NINJ1	ninjurin 1	566.7	573.7	570.16
PAQR7	progestin and adipoQ receptor family member VII	568.7	571.5	570.10
SLC26A5	solute carrier family 26 (anion exchanger), member 5	589.5	550.5	569.97
C3H18orf8	chromosome 3 open reading frame, human C18orf8	547.3	592.3	569.80
UAP1	UDP-N-acetylglucosamine pyrophosphorylase 1	569.2	570.4	569.79
PGM3	phosphoglucomutase 3	555.8	583.4	569.60
LOC100545957	uncharacterized LOC100545957	543.2	594.5	568.85
ITPKB	inositol-trisphosphate 3-kinase B	581.2	556.5	568.85
LOC104912749	transcriptional enhancer factor TEF-3-like	553.0	584.4	568.69
WDR44	WD repeat domain 44	564.1	572.2	568.19
POLR2J	polymerase (RNA) II (DNA directed) polypeptide J, 13.3kDa	582.5	553.6	568.04
DUSP10	dual specificity phosphatase 10	536.1	599.9	568.03
ARL6IP4	ADP-ribosylation factor-like 6 interacting protein 4	571.0	564.4	567.69
LOC100549082	proproteinase E-like	537.4	596.4	566.89
ACBD5	acyl-CoA binding domain containing 5	583.5	550.1	566.79

GPATCH8	G patch domain containing 8	667.7	465.8	566.75
PCNXL4	pecanex-like 4 (Drosophila)	540.6	592.4	566.48
FAM78B	family with sequence similarity 78, member B	554.1	578.7	566.41
PPP1R14D	protein phosphatase 1, regulatory (inhibitor) subunit 14D	538.6	593.4	566.02
KLHL33	kelch-like family member 33	577.6	554.4	566.01
ENY2	enhancer of yellow 2 homolog (Drosophila)	564.0	567.7	565.82
VPS37B	vacuolar protein sorting 37 homolog B (S. cerevisiae)	542.4	589.1	565.72
MLLT10	myeloid/lymphoid or mixed-lineage leukemia (trithorax homolog, Drosophila); translocated to, 10	533.8	597.1	565.48
TCAIM	T cell activation inhibitor, mitochondrial	565.7	565.0	565.36
SLC22A4	solute carrier family 22 (organic cation/zwitterion transporter), member 4	501.8	628.5	565.18
DUSP6	dual specificity phosphatase 6	541.5	588.4	564.95
RAPGEF5	Rap guanine nucleotide exchange factor (GEF) 5	549.9	580.0	564.92
LPCAT3	lysophosphatidylcholine acyltransferase 3	559.9	569.9	564.87
LOC104910964	cleavage and polyadenylation specificity factor subunit 7-like	567.6	561.9	564.74
IFNAR2	interferon (alpha, beta and omega) receptor 2	558.1	571.3	564.72
PIP5K1B	phosphatidylinositol-4-phosphate 5-kinase, type I, beta	550.7	578.0	564.37
CBX1	chromobox homolog 1	556.6	571.7	564.18
VPS54	vacuolar protein sorting 54 homolog (S. cerevisiae)	589.4	538.8	564.12
ENTHD2	ENTH domain containing 2	616.8	511.3	564.08
LOC100538839	homeobox protein Hox-A7	583.6	542.8	563.24
EXOSC7	exosome component 7	545.5	580.8	563.17
KLHL13	kelch-like family member 13	522.0	604.3	563.16
SIRT5	sirtuin 5	588.7	537.6	563.15
RRP15	ribosomal RNA processing 15 homolog (S. cerevisiae)	585.9	540.2	563.06
LOC104914909	uncharacterized LOC104914909	526.9	599.1	563.03
SGCB	sarcoglycan, beta (43kDa dystrophin-associated glycoprotein)	553.2	572.7	562.94
POLR3E	polymerase (RNA) III (DNA directed) polypeptide E (80kD)	552.3	573.5	562.89
SLC7A3	solute carrier family 7 (cationic amino acid transporter, y+ system), member 3	615.0	510.5	562.73
GPATCH2L	G patch domain containing 2-like	538.8	586.5	562.66
MYO1D	myosin ID	585.7	539.4	562.54
SDF4	stromal cell derived factor 4	540.6	584.2	562.40
ARID1B	AT rich interactive domain 1B (SWI1-like)	562.7	561.8	562.26
CDC123	cell division cycle 123	522.6	601.7	562.15
YOD1	YOD1 deubiquitinase	563.2	560.7	561.95
MAN1A1	mannosidase, alpha, class 1A, member 1	491.6	632.0	561.79
GGT7	gamma-glutamyltransferase 7	520.5	601.9	561.19
LOC100540962	putative RNA exonuclease NEF-sp	560.8	560.4	560.60
ALKBH5	AlkB family member 5, RNA demethylase	543.0	577.6	560.28
FZD4	frizzled class receptor 4	568.4	551.7	560.04
TSTA3	tissue specific transplantation antigen P35B	581.1	538.8	559.95
PDCD7	programmed cell death 7	552.3	567.5	559.93
PDCD2L	programmed cell death 2-like	545.5	573.1	559.35
HSCB	HscB mitochondrial iron-sulfur cluster co-chaperone	583.5	535.0	559.28
LOC100549289	RNA-binding protein 38-like	595.4	521.9	558.65
LOC104915856	uncharacterized LOC104915856	609.0	508.0	558.51
ZCCHC9	zinc finger, CCHC domain containing 9	589.4	527.3	558.35
DEK	DEK proto-oncogene	572.4	544.1	558.24
MTMR3	myotubularin related protein 3	575.4	541.1	558.24
RBL1	retinoblastoma-like 1	553.0	562.7	557.88
ADAL	adenosine deaminase-like	515.1	600.4	557.76
TGIF1	TGFB-induced factor homeobox 1	555.8	559.2	557.49
TPK1	thiamin pyrophosphokinase 1	575.0	539.7	557.39
ALK	anaplastic lymphoma receptor tyrosine kinase	578.5	536.0	557.26
LOC100549803	RING finger protein 145-like	606.5	507.5	556.97
ABHD17B	abhydrolase domain containing 17B	504.8	609.0	556.90

AOX1	aldehyde oxidase 1	568.3	545.4	556.86
CMYA5	cardiomyopathy associated 5	519.4	593.5	556.46
C20H17orf58	chromosome 20 open reading frame, human C17orf58	506.0	606.8	556.40
GNB5	guanine nucleotide binding protein (G protein), beta 5	553.1	559.2	556.18
ARFRP1	ADP-ribosylation factor related protein 1	512.6	598.7	555.68
FDX1	ferredoxin 1	580.0	531.1	555.55
ABHD5	abhydrolase domain containing 5	549.6	561.3	555.45
COMMD9	COMM domain containing 9	557.3	553.5	555.40
IGF1R	insulin-like growth factor 1 receptor	538.0	572.2	555.08
FBN1	fibrillin 1	503.5	606.3	554.91
LOC104910773	uncharacterized LOC104910773	521.6	587.7	554.66
GNPDA2	glucosamine-6-phosphate deaminase 2	540.3	568.8	554.57
LOC104909411	alkaline ceramidase 3-like	521.2	587.7	554.45
C8H10orf71	chromosome 8 open reading frame, human C10orf71	522.0	586.8	554.40
SRGAP2	SLIT-ROBO Rho GTPase activating protein 2	527.9	580.6	554.22
BBOX1	butyrobetaine (gamma), 2-oxoglutarate dioxygenase (gamma-butyrobetaine hydroxylase) 1	572.5	535.7	554.10
LOC104917562	transcription factor SPT20 homolog	537.9	570.2	554.04
CMC4	C-x(9)-C motif containing 4	533.0	575.0	553.98
FBXL7	F-box and leucine-rich repeat protein 7	544.8	563.1	553.96
SNX14	sorting nexin 14	541.2	566.3	553.75
FAM45A	family with sequence similarity 45, member A	542.8	564.2	553.48
TMEM164	transmembrane protein 164	534.6	571.5	553.01
LOC104916929	uncharacterized LOC104916929	533.0	571.8	552.38
ARHGEF10L	Rho guanine nucleotide exchange factor (GEF) 10-like	537.4	567.1	552.26
LOC100549021	RNA-binding protein 6-like	571.8	532.1	551.93
CNGA2	cyclic nucleotide gated channel alpha 2	534.0	569.6	551.78
CD109	CD109 molecule	545.5	558.0	551.75
FOPNL	FGFR1OP N-terminal like	560.6	542.8	551.72
ITGA4	integrin, alpha 4 (antigen CD49D, alpha 4 subunit of VLA-4 receptor)	497.6	605.0	551.33
LOC104913570	thyroid adenoma-associated protein homolog	624.9	477.6	551.27
LOC104909250	paraspeckle component 1-like	546.3	556.2	551.25
DHFR	dihydrofolate reductase	550.3	551.8	551.09
CDK19	cyclin-dependent kinase 19	537.1	564.9	550.99
LOC100540340	RNA-binding protein 4B-like	588.1	513.7	550.92
DPM3	dolichyl-phosphate mannosyltransferase polypeptide 3	587.0	514.5	550.75
WDR48	WD repeat domain 48	567.4	534.0	550.70
SARAF	store-operated calcium entry-associated regulatory factor	538.8	562.4	550.62
LOC100546403	malignant fibrous histiocytoma-amplified sequence 1 homolog	546.3	554.8	550.55
NSRP1	nuclear speckle splicing regulatory protein 1	532.9	567.9	550.40
STIM2	stromal interaction molecule 2	536.2	564.5	550.39
NUDT15	nudix (nucleoside diphosphate linked moiety X)-type motif 15	562.3	537.7	549.97
MRPL23	mitochondrial ribosomal protein L23	586.1	513.8	549.92
MED21	mediator complex subunit 21	555.6	544.2	549.90
DNAAF5	dynein, axonemal, assembly factor 5	526.0	573.4	549.70
CPE	carboxypeptidase E	506.7	592.5	549.57
ENDOD1	endonuclease domain containing 1	581.0	518.1	549.51
MTMR14	myotubularin related protein 14	568.3	530.5	549.41
TMOD3	tropomodulin 3 (ubiquitous)	520.1	578.6	549.32
SPICE1	spindle and centriole associated protein 1	565.9	531.9	548.87
DNAJC16	DnaJ (Hsp40) homolog, subfamily C, member 16	543.2	554.1	548.68
GRIA3	glutamate receptor, ionotropic, AMPA 3	522.9	574.4	548.65
RPS6KC1	ribosomal protein S6 kinase, 52kDa, polypeptide 1	566.5	530.5	548.52
RNF180	ring finger protein 180	538.8	558.1	548.42
GZF1	GDNF-inducible zinc finger protein 1	562.4	534.4	548.42
LOC104916730	uncharacterized LOC104916730	558.1	538.7	548.41

SMARCA1	SWI/SNF related, matrix associated, actin dependent regulator of chromatin, subfamily a, member 1	542.1	554.4	548.28
LOC104911699	uncharacterized LOC104911699	524.6	571.7	548.16
KAT2A	K(lysine) acetyltransferase 2A	608.3	488.0	548.15
BAG1	BCL2-associated athanogene	525.0	570.5	547.79
IQCB1	IQ motif containing B1	510.2	585.0	547.63
FAM178A	family with sequence similarity 178, member A	574.9	520.1	547.51
LOC104912787	talin-2-like	510.9	583.7	547.31
PTPMT1	protein tyrosine phosphatase, mitochondrial 1	561.6	532.7	547.14
IFFO2	intermediate filament family orphan 2	527.8	566.4	547.10
BDH2	3-hydroxybutyrate dehydrogenase, type 2	497.4	596.3	546.84
TMEM19	transmembrane protein 19	564.7	528.3	546.48
KLF3	Kruppel-like factor 3 (basic)	540.5	551.9	546.20
ZDHHC14	zinc finger, DHHC-type containing 14	494.8	597.5	546.14
SELENBP1	selenium-binding protein 1	561.6	530.7	546.13
FBXO33	F-box protein 33	534.5	557.6	546.05
L3MBTL2	l(3)mbt-like 2 (Drosophila)	560.8	531.1	545.97
SORCS2	sortilin-related VPS10 domain containing receptor 2	539.8	552.1	545.94
LOC104915954	glutaryl-CoA dehydrogenase, mitochondrial-like	613.0	478.2	545.62
LOC100547734	26S proteasome non-ATPase regulatory subunit 14	544.0	546.9	545.43
KLHL5	kelch-like family member 5	526.9	563.4	545.15
HAUS1	HAUS augmin-like complex, subunit 1	609.3	481.0	545.14
ERCC5	excision repair cross-complementation group 5	563.2	526.8	544.98
PSMG1	proteasome (prosome, macropain) assembly chaperone 1	521.0	568.8	544.92
ABI1	abl-interactor 1	537.2	552.1	544.66
TATDN1	TatD DNase domain containing 1	559.6	529.6	544.60
LOC100545821	aldose reductase-like	542.0	546.8	544.38
LOC100547507	pleckstrin homology-like domain family B member 2	444.5	644.0	544.25
RNF8	ring finger protein 8, E3 ubiquitin protein ligase	538.6	549.8	544.19
LOC100541846	AT-rich interactive domain-containing protein 1A	515.3	572.8	544.02
CLCN5	chloride channel, voltage-sensitive 5	532.0	555.5	543.77
GMEB2	glucocorticoid modulatory element binding protein 2	588.8	498.6	543.70
IL1RAPL2	interleukin 1 receptor accessory protein-like 2	539.5	547.8	543.66
NKRF	NFKB repressing factor	543.9	543.4	543.63
LOC100550826	phospholipid-transporting ATPase ID-like	526.3	560.8	543.57
CSK	c-src tyrosine kinase	542.3	544.6	543.41
DCAF5	DDB1 and CUL4 associated factor 5	554.1	532.4	543.25
LOC104913550	protein SFI1 homolog	535.7	550.7	543.21
LOC104916115	ceramide synthase 2-like	588.7	497.7	543.21
NADK2	NAD kinase 2, mitochondrial	519.4	566.8	543.14
PKNOX2	PBX/knotted 1 homeobox 2	523.6	562.3	542.98
LOC104916263	F-box only protein 46-like	552.3	533.0	542.66
MED28	mediator complex subunit 28	526.8	558.4	542.62
LOC104915094	vacuolar protein sorting-associated protein 13A-like	512.6	572.1	542.36
GLG1	golgi glycoprotein 1	560.1	524.6	542.32
PAAF1	proteasomal ATPase-associated factor 1	585.2	499.2	542.21
DDX20	DEAD (Asp-Glu-Ala-Asp) box polypeptide 20	524.4	559.7	542.02
PDP1	pyruvate dehyrogenase phosphatase catalytic subunit 1	468.7	614.9	541.83
FRS3	fibroblast growth factor receptor substrate 3	532.1	551.5	541.77
LOC104913328	dihydropyrimidinase-related protein 3-like	560.7	522.6	541.66
QDPR	quinoid dihydropteridine reductase	525.2	557.5	541.32
EPHB1	EPH receptor B1	498.8	583.7	541.28
EXOSC8	exosome component 8	558.9	523.6	541.23
PPP1R21	protein phosphatase 1, regulatory subunit 21	523.5	559.0	541.22
ZMIZ1	zinc finger, MIZ-type containing 1	526.4	555.3	540.83
LHFP	lipoma HMGIC fusion partner	585.8	495.4	540.59
TPRA1	transmembrane protein, adipocyte asscociated 1	541.3	539.7	540.52
ACSBG2	acyl-CoA synthetase bubblegum family member 2	591.2	489.2	540.19

SGMS1	sphingomyelin synthase 1	527.9	552.3	540.11
MKRN2	makorin ring finger protein 2	543.9	536.3	540.08
RNF121	ring finger protein 121	543.0	536.8	539.89
RCBTB2	regulator of chromosome condensation (RCC1) and BTB (POZ) domain containing protein 2	562.6	517.1	539.81
ATG14	autophagy related 14	540.4	538.9	539.66
MED8	mediator complex subunit 8	537.1	542.2	539.63
UCKL1	uridine-cytidine kinase 1-like 1	522.9	556.3	539.62
MLLT11	myeloid/lymphoid or mixed-lineage leukemia (trithorax homolog, Drosophila); translocated to, 11	500.0	578.5	539.29
DDX52	DEAD (Asp-Glu-Ala-Asp) box polypeptide 52	548.0	530.3	539.15
KDSR	3-ketodihydrosphingosine reductase	505.9	571.6	538.74
MRPS35	mitochondrial ribosomal protein S35	546.1	531.4	538.74
LOC104916197	calponin-1-like	558.1	519.1	538.61
TMEM177	transmembrane protein 177	525.3	551.5	538.40
TFB2M	transcription factor B2, mitochondrial	535.2	541.4	538.26
COPS7B	COP9 signalosome subunit 7B	598.8	477.7	538.24
LOC104914762	ubiquitin-associated protein 2-like	536.5	539.5	538.02
ZADH2	zinc binding alcohol dehydrogenase domain containing 2	533.7	542.3	537.98
C15H5orf45	chromosome 15 open reading frame, human C5orf45	581.4	494.3	537.87
VPS18	vacuolar protein sorting 18 homolog (S. cerevisiae)	538.7	537.0	537.85
LOC104909760	transmembrane protein 181-like	481.3	593.5	537.42
SCPEP1	serine carboxypeptidase 1	524.3	549.9	537.14
C10H1orf123	chromosome 10 open reading frame, human C1orf123	534.6	539.5	537.07
PPAT	phosphoribosyl pyrophosphate amidotransferase	575.7	498.4	537.05
ZZZ3	zinc finger, ZZ-type containing 3	521.0	552.7	536.82
CACYBP	calcyclin binding protein	516.8	555.9	536.34
LOC104911077	uncharacterized LOC104911077	520.4	551.9	536.13
LOC100550119	E3 ubiquitin-protein ligase HERC2	503.0	569.0	535.98
TBC1D16	TBC1 domain family, member 16	520.5	551.1	535.80
RASL11B	RAS-like, family 11, member B	569.0	502.5	535.74
RBM12B	RNA binding motif protein 12B	500.7	570.7	535.69
DESI1	desumoylating isopeptidase 1	531.2	539.4	535.32
RBFA	ribosome binding factor A (putative)	555.4	513.9	534.65
LOC100548706	AF4/FMR2 family member 2-like	517.6	551.0	534.28
ZNF830	zinc finger protein 830	526.0	542.4	534.18
LRRC75B	leucine rich repeat containing 75B	553.1	514.6	533.89
TRIB2	tribbles pseudokinase 2	515.9	551.7	533.80
RPP30	ribonuclease P/MRP 30kDa subunit	531.9	535.5	533.70
SLX4	SLX4 structure-specific endonuclease subunit	512.7	554.7	533.67
SPCS2	signal peptidase complex subunit 2 homolog (S. cerevisiae)	510.8	556.4	533.63
NPB	neuropeptide B	596.8	470.0	533.40
CPT2	carnitine palmitoyltransferase 2	572.5	494.2	533.34
LOC104912141	protein Shroom4-like	585.3	481.2	533.27
LPGAT1	lysophosphatidylglycerol acyltransferase 1	527.8	538.5	533.19
NIPA1	non imprinted in Prader-Willi/Angelman syndrome 1	530.4	536.0	533.19
TRIM35	tripartite motif containing 35	535.6	530.6	533.08
LOC100544372	chloride channel protein ClC-Kb-like	549.1	516.8	532.97
MRPS18A	mitochondrial ribosomal protein S18A	527.6	537.4	532.53
SLITRK2	SLIT and NTRK-like family, member 2	526.8	538.0	532.42
ARID5B	AT rich interactive domain 5B (MRF1-like)	537.9	526.9	532.41
LOC104915861	vitamin K epoxide reductase complex subunit 1-like	558.6	506.2	532.39
GPX7	glutathione peroxidase 7	456.3	608.3	532.27
CECR5	cat eye syndrome chromosome region, candidate 5	496.5	568.0	532.22
DPY19L4	dpy-19-like 4 (C. elegans)	541.3	523.0	532.18
LOC104912984	RNA-binding Raly-like protein	556.6	507.0	531.80
CFAP97	cilia and flagella associated protein 97	558.2	505.1	531.64
LOC104909807	leucine-rich repeats and immunoglobulin-like domains protein 3	500.8	562.4	531.60

LRRC47	leucine rich repeat containing 47	536.3	526.9	531.57
CLN6	ceroid-lipofuscinosis, neuronal 6, late infantile, variant	566.8	496.1	531.49
PEX10	peroxisomal biogenesis factor 10	555.8	507.1	531.41
APAF1	apoptotic peptidase activating factor 1	493.1	569.2	531.16
COASY	CoA synthase	551.6	509.9	530.76
SUN3	Sad1 and UNC84 domain containing 3	552.2	509.2	530.72
GIPC2	GIPC PDZ domain containing family, member 2	504.2	556.9	530.55
LOC104917015	dynactin subunit 1-like	607.7	452.4	530.04
FAM69B	family with sequence similarity 69, member B	526.1	533.7	529.91
RHOT1	ras homolog family member T1	526.0	533.7	529.82
KLF10	Kruppel-like factor 10	594.5	465.0	529.77
UTP20	UTP20, small subunit (SSU) processome component, homolog (yeast)	535.3	523.9	529.61
DAPK3	death-associated protein kinase 3	637.8	420.9	529.34
POLRMT	polymerase (RNA) mitochondrial (DNA directed)	583.7	474.7	529.18
SMIM3	small integral membrane protein 3	488.2	570.1	529.15
ITGB1BP1	integrin beta 1 binding protein 1	549.7	507.7	528.68
ATP1B1	ATPase, Na+/K+ transporting, beta 1 polypeptide	555.4	501.5	528.43
LOC104917419	basic immunoglobulin-like variable motif-containing protein	554.6	501.9	528.28
CSPP1	centrosome and spindle pole associated protein 1	550.8	505.1	527.93
LOC100545053	laminin subunit alpha-2	541.5	514.0	527.77
ADPGK	ADP-dependent glucokinase	535.4	519.6	527.46
TMEM120A	transmembrane protein 120A	533.0	521.9	527.43
LOC100547097	dehydrogenase/reductase SDR family member on chromosome X-like	475.8	578.9	527.34
DYNLT1	dynein, light chain, Tctex-type 1	519.3	535.3	527.31
CHFR	checkpoint with forkhead and ring finger domains, E3 ubiquitin protein ligase	490.8	563.7	527.23
ATL1	atlastin GTPase 1	519.4	534.5	526.91
GIT1	G protein-coupled receptor kinase interacting ArfGAP 1	579.6	474.2	526.89
ATP2B4	ATPase, Ca++ transporting, plasma membrane 4	533.2	520.4	526.78
HEATR6	HEAT repeat containing 6	521.9	531.3	526.56
LOC104917078	zinc finger protein 774-like	508.4	544.6	526.52
LOC100540532	huntingtin	489.9	562.9	526.38
MESDC2	mesoderm development candidate 2	481.4	570.8	526.13
SFXN4	sideroflexin 4	512.6	539.3	525.96
DOT1L	DOT1-like histone H3K79 methyltransferase	507.8	543.7	525.78
PTGS1	prostaglandin-endoperoxide synthase 1 (prostaglandin G/H synthase and cyclooxygenase)	535.7	515.4	525.55
ZC3H3	zinc finger CCCH-type containing 3	538.7	512.2	525.49
TERF2	telomeric repeat binding factor 2	522.7	528.1	525.41
LOC100550231	transmembrane protein 68	516.9	533.8	525.32
TMEM126A	transmembrane protein 126A	537.8	512.8	525.29
INPP5K	inositol polyphosphate-5-phosphatase K	519.3	531.0	525.14
SMARCD2	SWI/SNF related, matrix associated, actin dependent regulator of chromatin, subfamily d, member 2	549.0	501.0	525.01
GPRIN3	GPRIN family member 3	553.7	496.1	524.89
MRPS22	mitochondrial ribosomal protein S22	526.8	522.7	524.74
TMEM259	transmembrane protein 259	571.1	478.2	524.65
MTRR	5-methyltetrahydrofolate-homocysteine methyltransferase reductase	546.3	502.8	524.52
ACAA1	acetyl-CoA acyltransferase 1	526.0	523.0	524.50
BRMS1L	breast cancer metastasis-suppressor 1-like	503.3	545.6	524.45
RBPMS	RNA binding protein with multiple splicing	550.6	498.1	524.36
LOC104915853	spore wall protein 2-like	582.0	466.6	524.33
TRMT10C	tRNA methyltransferase 10 homolog C (S. cerevisiae)	506.6	542.0	524.33
SMOX	spermine oxidase	483.0	564.7	523.88
LOC100542860	cytochrome b561 domain-containing protein 2	554.9	492.3	523.63

PISD	phosphatidylserine decarboxylase	546.3	500.8	523.58
CEP350	centrosomal protein 350kDa	527.3	519.8	523.57
RNPEP	arginyl aminopeptidase (aminopeptidase B)	546.4	499.1	522.74
COG6	component of oligomeric golgi complex 6	521.8	523.6	522.72
RGS14	regulator of G-protein signaling 14	586.9	458.5	522.71
CSNK1E	casein kinase 1, epsilon	489.8	555.2	522.51
UTP6	UTP6, small subunit (SSU) processome component, homolog (yeast)	529.5	515.3	522.39
NUFIP1	nuclear fragile X mental retardation protein interacting protein 1	548.0	496.3	522.17
R3HDM2	R3H domain containing 2	520.3	523.5	521.89
LOC104917220	zinc finger protein OZF-like	532.9	510.6	521.77
CAD	carbamoyl-phosphate synthetase 2, aspartate transcarbamylase, and dihydroorotase	572.6	470.3	521.46
ASB2	ankyrin repeat and SOCS box containing 2	482.3	560.6	521.44
OSBPL5	oxysterol binding protein-like 5	480.1	562.7	521.41
HEXDC	hexosaminidase (glycosyl hydrolase family 20, catalytic domain) containing	469.6	573.0	521.30
ETFDH	electron-transferring-flavoprotein dehydrogenase	513.4	529.0	521.23
LOC100548274	mitochondrial intermediate peptidase-like	505.9	536.2	521.06
TPP2	tripeptidyl peptidase II	512.5	529.5	520.98
SH3KBP1	SH3-domain kinase binding protein 1	545.4	496.5	520.94
WDR89	WD repeat domain 89	522.0	519.8	520.89
HNRNPA0	heterogeneous nuclear ribonucleoprotein A0	586.1	455.6	520.86
ZMYM3	zinc finger, MYM-type 3	544.1	496.4	520.21
CRTAC1	cartilage acidic protein 1	575.0	465.1	520.03
DLAT	dihydrolipoamide S-acetyltransferase	541.3	498.7	520.00
LOC104914780	protein unc-13 homolog B-like	534.7	505.1	519.89
CNIH4	cornichon family AMPA receptor auxiliary protein 4	500.7	538.9	519.79
LOC100548734	cohesin subunit SA-2-like	509.3	528.5	518.88
DUS4L	dihydrouridine synthase 4-like (S. cerevisiae)	488.1	549.5	518.82
RBM48	RNA binding motif protein 48	525.3	512.2	518.73
ENTPD6	ectonucleoside triphosphate diphosphohydrolase 6 (putative)	542.9	494.5	518.72
ITM2B	integral membrane protein 2B	490.0	547.4	518.72
PUS3	pseudouridylate synthase 3	532.9	504.3	518.58
LTV1	LTV1 ribosome biogenesis factor	521.7	515.4	518.54
ATG4B	autophagy related 4B, cysteine peptidase	538.9	498.1	518.53
FIP1L1	factor interacting with PAPOLA and CPSF1	486.4	550.2	518.28
CEP78	centrosomal protein 78kDa	484.7	551.7	518.23
NDUFAF1	NADH dehydrogenase (ubiquinone) complex I, assembly factor 1	497.5	538.9	518.22
TIPRL	TOR signaling pathway regulator	512.6	523.9	518.21
TOP3B	topoisomerase (DNA) III beta	507.4	528.5	517.95
MAD2L2	MAD2 mitotic arrest deficient-like 2 (yeast)	538.6	497.0	517.82
GALK1	galactokinase 1	531.4	504.2	517.78
LOC104916692	uncharacterized LOC104916692	461.3	573.8	517.58
DNAJA4	DnaJ (Hsp40) homolog, subfamily A, member 4	503.3	531.8	517.55
SLC48A1	solute carrier family 48 (heme transporter), member 1	521.1	513.9	517.49
CCDC102A	coiled-coil domain containing 102A	542.3	492.6	517.46
EPS15L1	epidermal growth factor receptor pathway substrate 15-like 1	494.1	540.6	517.32
NARFL	nuclear prelamin A recognition factor-like	531.9	502.2	517.04
AARS2	alanyl-tRNA synthetase 2, mitochondrial	563.5	469.8	516.62
MRPS10	mitochondrial ribosomal protein S10	517.6	515.6	516.58
LOC104915978	uncharacterized LOC104915978	568.6	464.4	516.54
UNKL	unkempt family zinc finger-like	504.2	528.8	516.51
LOC100551461	toll-like receptor 1	511.0	521.8	516.39
DNAJC9	DnaJ (Hsp40) homolog, subfamily C, member 9	543.8	487.8	515.78
TECPR1	tectonin beta-propeller repeat containing 1	489.1	542.1	515.60
LOC104915795	AT-hook DNA-binding motif-containing protein 1-like	536.2	495.0	515.60
SEC24B	SEC24 family member B	498.1	533.1	515.58

LOC104913936	uncharacterized LOC104913936	475.7	555.2	515.42
ERRFI1	ERBB receptor feedback inhibitor 1	486.4	544.4	515.41
PAX7	paired box protein Pax-7	503.6	526.7	515.18
LOC104912053	titin-like	547.2	482.9	515.08
LOC100540124	probable acyl-CoA dehydrogenase 6	433.4	596.7	515.05
ETV4	ets variant 4	525.3	503.8	514.56
IFT57	intraflagellar transport 57	500.9	528.1	514.51
UBA5	ubiquitin-like modifier activating enzyme 5	501.6	526.7	514.18
EXT1	exostosin glycosyltransferase 1	511.0	516.9	513.96
VPS13B	vacuolar protein sorting 13 homolog B (yeast)	480.5	547.3	513.95
EXT2	exostosin glycosyltransferase 2	480.8	547.1	513.92
ING1	inhibitor of growth family, member 1	483.7	543.9	513.81
DDX59	DEAD (Asp-Glu-Ala-Asp) box polypeptide 59	505.8	521.8	513.80
LOC104915599	uncharacterized LOC104915599	545.5	480.9	513.23
SLC19A1	solute carrier family 19 (folate transporter), member 1	547.2	479.1	513.16
AEN	apoptosis enhancing nuclease	527.2	499.1	513.11
LOC100549217	uncharacterized LOC100549217	493.1	533.0	513.09
FBXO38	F-box protein 38	516.1	509.4	512.74
CLK2	CDC-like kinase 2	508.4	516.7	512.54
PITPNM1	phosphatidylinositol transfer protein, membrane-associated 1	539.0	486.0	512.49
HINT3	histidine triad nucleotide binding protein 3	512.5	512.3	512.41
TOR4A	torsin family 4, member A	519.4	505.4	512.39
MMAA	methylmalonic aciduria (cobalamin deficiency) cblA type	504.9	519.6	512.27
PEX1	peroxisomal biogenesis factor 1	522.7	500.7	511.66
RANGAP1	Ran GTPase activating protein 1	544.6	478.6	511.57
CANT1	calcium activated nucleotidase 1	514.5	508.4	511.45
AVEN	apoptosis, caspase activation inhibitor	515.0	507.8	511.41
PCSK1	proprotein convertase subtilisin/kexin type 1	427.4	595.2	511.29
SLC35A4	solute carrier family 35, member A4	543.2	478.9	511.02
RPS6KA5	ribosomal protein S6 kinase, 90kDa, polypeptide 5	486.2	535.2	510.73
MTCH1	mitochondrial carrier 1	510.1	511.1	510.60
KLF7	Kruppel-like factor 7 (ubiquitous)	474.6	546.2	510.39
TYMS	thymidylate synthetase	550.5	469.9	510.21
RNF113A	ring finger protein 113A	534.5	485.9	510.20
UBXN7	UBX domain protein 7	497.5	522.7	510.08
LOC100550496	obg-like ATPase 1	516.0	503.9	509.99
SMG6	SMG6 nonsense mediated mRNA decay factor	533.2	486.7	509.98
DOLPP1	dolichyldiphosphatase 1	494.8	524.9	509.84
GSKIP	GSK3B interacting protein	524.3	495.0	509.68
CACHD1	cache domain containing 1	490.8	528.2	509.49
DCUN1D1	DCN1, defective in cullin neddylation 1, domain containing 1	508.4	510.3	509.34
ZNHIT6	zinc finger, HIT-type containing 6	511.6	506.1	508.85
MED22	mediator complex subunit 22	523.5	494.1	508.81
SETD8	SET domain containing (lysine methyltransferase) 8	504.1	513.4	508.75
ZNF367	zinc finger protein 367	516.7	500.7	508.70
TAF5L	TAF5-like RNA polymerase II, p300/CBP-associated factor (PCAF)-associated factor, 65kDa	500.7	516.0	508.36
CUL5	cullin 5	472.1	544.2	508.15
FBLN7	fibulin 7	489.2	526.8	508.01
LOC100544408	cytochrome b-245 light chain	533.0	483.0	507.99
LOC104911412	coiled-coil domain-containing protein 132	471.0	544.7	507.87
UVRAG	UV radiation resistance associated	476.2	538.7	507.46
FANCC	Fanconi anemia, complementation group C	492.1	521.8	506.93
C19H9orf78	chromosome 19 open reading frame, human C9orf78	494.9	518.9	506.87
LOC104909267	leucine-rich repeat-containing protein 40-like	523.5	489.8	506.65
LOC100539247	Sjoegren syndrome nuclear autoantigen 1 homolog	529.3	483.7	506.54
CCDC51	coiled-coil domain containing 51	500.7	512.2	506.48
LOC104914452	protein phosphatase 1 regulatory subunit 12B-like	519.7	492.9	506.32

MBD5	methyl-CpG binding domain protein 5	482.5	529.9	506.23
THAP4	THAP domain containing 4	477.3	535.0	506.15
CABLES1	Cdk5 and Abl enzyme substrate 1	511.7	500.0	505.85
MEIS1	Meis homeobox 1	509.2	501.9	505.56
TTLL11	tubulin tyrosine ligase-like family, member 11	543.8	467.2	505.51
LACTB	lactamase, beta	501.7	509.2	505.44
PRR5L	proline rich 5 like	471.4	539.4	505.41
GXYLT1	glucoside xylosyltransferase 1	500.6	510.1	505.34
KLHL29	kelch-like family member 29	486.6	523.8	505.17
LOC100548064	membrane primary amine oxidase-like	549.7	460.6	505.15
STARD3	StAR-related lipid transfer (START) domain containing 3	550.8	459.5	505.10
LOC100539836	vacuole membrane protein 1-like	472.3	537.8	505.03
IPO13	importin 13	520.4	489.4	504.91
PAK4	p21 protein (Cdc42/Rac)-activated kinase 4	510.2	499.5	504.81
KIAA1191	KIAA1191 ortholog	449.6	560.0	504.80
SMIM4	small integral membrane protein 4	531.0	478.0	504.51
TERF2IP	telomeric repeat binding factor 2, interacting protein	499.2	509.9	504.50
MCM8	minichromosome maintenance complex component 8	465.4	543.6	504.49
PDE3B	phosphodiesterase 3B, cGMP-inhibited	514.1	494.6	504.32
GNAI1	guanine nucleotide binding protein (G protein), alpha inhibiting activity polypeptide 1	495.6	512.7	504.14
WDR45B	WD repeat domain 45B	530.3	477.3	503.80
LOC104916289	amyloid beta A4 precursor protein-binding family B member 1-like	532.2	475.1	503.68
ATXN3	ataxin 3	505.1	502.2	503.66
FBXO18	F-box protein, helicase, 18	506.8	500.2	503.50
FMR1	fragile X mental retardation 1	488.9	518.0	503.45
HGH1	HGH1 homolog (S. cerevisiae)	526.8	479.9	503.38
DUSP12	dual specificity phosphatase 12	474.7	531.8	503.24
SMYD5	SMYD family member 5	527.9	478.5	503.22
ZBTB48	zinc finger and BTB domain containing 48	474.7	531.6	503.14
CNOT4	CCR4-NOT transcription complex, subunit 4	487.2	518.7	502.95
LOC104916924	interleukin enhancer-binding factor 3-like	529.6	476.3	502.94
PKD2L2	polycystic kidney disease 2-like 2	494.7	511.0	502.87
CDC42SE2	CDC42 small effector 2	483.8	521.8	502.80
LOC100550736	cyclin-C	493.1	512.3	502.72
LOC104910953	transaldolase-like	471.2	533.6	502.40
LOC100543834	methyltransferase-like protein 10	494.0	509.6	501.82
LOC100539142	dispanin subfamily A member 2b-like	489.9	513.4	501.62
ST3GAL6	ST3 beta-galactoside alpha-2,3-sialyltransferase 6	499.0	503.5	501.27
TTYH2	tweety family member 2	526.1	476.0	501.02
RPS27L	ribosomal protein S27-like	508.1	493.8	500.99
DMRT2	doublesex and mab-3 related transcription factor 2	437.7	564.1	500.89
LOC104912330	phosphofurin acidic cluster sorting protein 2-like	518.6	483.1	500.88
LSM1	LSM1, U6 small nuclear RNA associated	504.9	495.9	500.45
CNR1	cannabinoid receptor 1 (brain)	504.9	495.8	500.37
APRT	adenine phosphoribosyltransferase	547.9	452.5	500.18
MFN2	mitofusin 2	480.6	519.4	500.00
SMYD4	SET and MYND domain containing 4	521.0	478.9	499.92
FBXL3	F-box and leucine-rich repeat protein 3	456.8	542.8	499.80
TWISTNB	TWIST neighbor	503.8	495.6	499.72
TMEM30A	transmembrane protein 30A	456.9	542.3	499.61
MTMR12	myotubularin related protein 12	469.6	529.1	499.38
TMEM81	transmembrane protein 81	494.1	504.0	499.07
EBF1	early B-cell factor 1	484.8	513.3	499.06
FBXL21	F-box and leucine-rich repeat protein 21	482.2	515.6	498.89
MAPKAPK5	mitogen-activated protein kinase-activated protein kinase 5	480.5	517.3	498.89
TLE3	transducin-like enhancer of split 3	487.3	510.2	498.76
MAP3K4	mitogen-activated protein kinase kinase kinase 4	482.2	514.5	498.33

RNF103	ring finger protein 103	485.6	511.0	498.32
LOC104913741	kinesin-like protein KIF21A	463.7	532.9	498.30
LOC104909633	tetratricopeptide repeat protein 27-like	506.5	489.8	498.15
IRAK4	interleukin-1 receptor-associated kinase 4	473.0	523.2	498.11
LOC104914524	uncharacterized protein C3orf18 homolog	530.3	465.9	498.09
LOC100539311	ribonuclease P protein subunit p29-like	482.1	513.9	497.97
LOC100544199	ATP-dependent DNA helicase Q5	471.3	524.5	497.93
CDCA7	cell division cycle associated 7	525.0	470.7	497.87
ZDHHC3	zinc finger, DHHC-type containing 3	501.6	494.0	497.83
LOC104912117	TSC22 domain family protein 3-like	518.7	476.6	497.66
INTS7	integrator complex subunit 7	489.0	505.5	497.27
TMCC1	transmembrane and coiled-coil domain family 1	479.7	514.8	497.26
TMCC2	transmembrane and coiled-coil domain family 2	492.4	501.8	497.13
DHRS7B	dehydrogenase/reductase (SDR family) member 7B	496.7	497.3	496.99
WDR92	WD repeat domain 92	534.4	459.6	496.97
AIG1	androgen-induced 1	493.1	500.8	496.93
DUSP11	dual specificity phosphatase 11 (RNA/RNP complex 1-interacting)	520.2	473.6	496.92
POLG	polymerase (DNA directed), gamma	497.5	495.3	496.41
TTF1	transcription termination factor, RNA polymerase I	461.9	530.9	496.37
C28H6orf89	chromosome 28 open reading frame, human C6orf89	499.3	493.2	496.23
AGPAT2	1-acylglycerol-3-phosphate O-acyltransferase 2	540.5	451.9	496.23
LARS2	leucyl-tRNA synthetase 2, mitochondrial	472.1	519.9	496.02
LOC100547794	cytochrome P450 2J2-like	541.1	450.8	495.96
MIER2	mesoderm induction early response 1, family member 2	468.9	522.9	495.89
SPIRE1	spire-type actin nucleation factor 1	527.9	463.7	495.76
LOC104915670	tubulin alpha-4A chain-like	527.1	463.9	495.49
GEMIN6	gem (nuclear organelle) associated protein 6	514.3	476.6	495.46
PSMG4	proteasome (prosome, macropain) assembly chaperone 4	477.2	513.5	495.36
RIPK1	receptor (TNFRSF)-interacting serine-threonine kinase 1	484.7	504.9	494.77
DLGAP5	discs, large (Drosophila) homolog-associated protein 5	538.0	451.1	494.55
ATF3	activating transcription factor 3	485.4	503.6	494.48
PLA2G10	phospholipase A2, group X	521.8	467.1	494.46
LOC100545973	aldose reductase-like	496.3	491.3	493.82
DNMBP	dynamin binding protein	513.7	473.9	493.81
POLR2M	polymerase (RNA) II (DNA directed) polypeptide M	500.7	486.7	493.71
ZNF608	zinc finger protein 608	477.3	510.0	493.64
CTDP1	CTD (carboxy-terminal domain, RNA polymerase II, polypeptide A) phosphatase, subunit 1	469.4	517.4	493.41
SLC4A1AP	solute carrier family 4 (anion exchanger), member 1, adaptor protein	467.0	519.6	493.26
PWP1	PWP1 homolog (S. cerevisiae)	468.6	517.6	493.08
TSPO	translocator protein (18kDa)	505.0	480.4	492.72
LSMEM1	leucine-rich single-pass membrane protein 1	512.6	472.7	492.64
ATG4C	autophagy related 4C, cysteine peptidase	478.8	505.4	492.11
LOC100546401	histone RNA hairpin-binding protein-like	472.1	511.9	492.02
AP5Z1	adaptor-related protein complex 5, zeta 1 subunit	508.3	475.4	491.89
NAF1	nuclear assembly factor 1 ribonucleoprotein	508.1	475.0	491.58
DRAM2	DNA-damage regulated autophagy modulator 2	479.7	503.3	491.55
LACTB2	lactamase, beta 2	493.1	490.0	491.52
PLA2G7	phospholipase A2, group VII (platelet-activating factor acetylhydrolase, plasma)	469.5	513.3	491.39
PMM1	phosphomannomutase 1	492.3	490.2	491.28
LOC104913477	E3 ubiquitin-protein ligase MGRN1-like	528.7	453.7	491.22
LOC104911731	calcium-binding mitochondrial carrier protein Aralar1-like	488.0	494.3	491.16
SMCHD1	structural maintenance of chromosomes flexible hinge domain containing 1	476.2	505.9	491.06
KIF23	kinesin family member 23	501.4	480.5	490.98
AGGF1	angiogenic factor with G patch and FHA domains 1	508.3	473.6	490.97

UBE2O	ubiquitin-conjugating enzyme E2O	507.6	474.3	490.92
ZDHHC8	zinc finger, DHHC-type containing 8	438.4	543.0	490.69
QPCT	glutaminyl-peptide cyclotransferase	425.9	555.3	490.60
CDC27	cell division cycle 27	469.6	511.5	490.55
PGM2L1	phosphoglucomutase 2-like 1	494.8	486.2	490.49
TMEM170A	transmembrane protein 170A	499.1	481.7	490.41
DDX47	DEAD (Asp-Glu-Ala-Asp) box polypeptide 47	517.5	462.6	490.06
IFT46	intraflagellar transport 46	471.2	507.6	489.39
SEC14L1	SEC14-like 1 (S. cerevisiae)	495.1	483.5	489.26
BUD13	BUD13 homolog (S. cerevisiae)	477.2	501.3	489.25
DAGLB	diacylglycerol lipase, beta	483.1	494.4	488.75
TBPL1	TBP-like 1	494.8	482.6	488.71
SLC35C1	solute carrier family 35 (GDP-fucose transporter), member C1	500.0	477.4	488.70
TSSC4	tumor suppressing subtransferable candidate 4	436.6	540.7	488.65
LOC104916308	lysosomal alpha-mannosidase-like	496.8	480.4	488.61
MYBL2	v-myb avian myeloblastosis viral oncogene homolog-like 2	485.4	491.7	488.56
CHRDL1	chordin-like 1	515.8	461.1	488.47
POLR2K	polymerase (RNA) II (DNA directed) polypeptide K, 7.0kDa	507.4	469.1	488.24
LOC104915908	2-oxoglutarate dehydrogenase, mitochondrial-like	518.5	457.9	488.20
SEC61A1	Sec61 alpha 1 subunit (S. cerevisiae)	495.0	481.1	488.04
LOC104915082	UPF0586 protein C9orf41-like	469.3	506.3	487.80
SNAPC1	small nuclear RNA activating complex, polypeptide 1, 43kDa	483.7	491.6	487.62
CNOT7	CCR4-NOT transcription complex, subunit 7	491.4	483.7	487.58
PLA2R1	phospholipase A2 receptor 1, 180kDa	474.7	500.4	487.55
LOC100540915	E3 ubiquitin-protein ligase SMURF1	467.0	507.8	487.40
CHAF1A	chromatin assembly factor 1, subunit A (p150)	497.2	477.5	487.34
SLC35F5	solute carrier family 35, member F5	451.6	521.5	486.58
RAD51B	RAD51 paralog B	472.1	501.0	486.54
PABPC4	poly(A) binding protein, cytoplasmic 4 (inducible form)	514.3	458.7	486.49
LOC100545800	lysosomal amino acid transporter 1 homolog	469.6	502.9	486.25
LOC104917517	retinoblastoma-associated protein-like	517.7	454.7	486.21
ZWILCH	zwilch kinetochore protein	525.0	447.2	486.09
COMMD1	copper metabolism (Murr1) domain containing 1	494.6	477.0	485.82
LOC104913813	uncharacterized LOC104913813	458.8	512.9	485.82
ERN2	endoplasmic reticulum to nucleus signaling 2	517.0	454.6	485.79
FASN	fatty acid synthase	474.8	496.6	485.75
OSBPL1A	oxysterol binding protein-like 1A	478.0	493.0	485.51
DHRS9	dehydrogenase/reductase (SDR family) member 9	472.1	498.6	485.31
C1H7orf55	chromosome 1 open reading frame, human C7orf55	515.9	454.6	485.22
DBF4	DBF4 zinc finger	493.0	477.4	485.22
PHACTR4	phosphatase and actin regulator 4	474.8	495.5	485.17
DCHS1	dachsous cadherin-related 1	490.9	478.9	484.91
TMEM169	transmembrane protein 169	478.8	490.6	484.73
ZBTB8A	zinc finger and BTB domain containing 8A	491.8	477.3	484.52
WIBG	within bgcn homolog (Drosophila)	532.0	436.9	484.49
KLHL7	kelch-like family member 7	484.7	484.0	484.34
SOX11	SRY (sex determining region Y)-box 11	498.3	470.1	484.22
KCNAB2	potassium voltage-gated channel, shaker-related subfamily, beta member 2	481.5	486.7	484.09
TBCEL	tubulin folding cofactor E-like	487.3	480.0	483.66
NPRL2	nitrogen permease regulator-like 2 (S. cerevisiae)	502.5	464.5	483.51
LRRC28	leucine rich repeat containing 28	473.7	493.0	483.39
PAIP2B	poly(A) binding protein interacting protein 2B	489.0	477.4	483.19
BNIP3L	BCL2/adenovirus E1B 19kDa interacting protein 3-like	464.4	501.6	483.02
LOC100539487	rab-like protein 2A	505.8	459.0	482.39
SGSH	N-sulfoglucosamine sulfohydrolase	488.3	476.3	482.33
NDRG4	NDRG family member 4	480.0	484.6	482.28
ALKBH3	alkB, alkylation repair homolog 3 (E. coli)	457.8	506.5	482.14

BUB1	BUB1 mitotic checkpoint serine/threonine kinase	474.7	489.2	481.94
ARL14EP	ADP-ribosylation factor-like 14 effector protein	490.6	473.2	481.87
LOC104914294	uncharacterized LOC104914294	459.4	504.3	481.85
PSMG3	proteasome (prosome, macropain) assembly chaperone 3	487.1	476.5	481.80
CRYZL1	crystallin, zeta (quinone reductase)-like 1	480.5	483.1	481.77
TAF1D	TATA box binding protein (TBP)-associated factor, RNA polymerase I, D, 41kDa	483.1	480.0	481.54
ESCO1	establishment of sister chromatid cohesion N-acetyltransferase 1	459.2	503.7	481.47
FAM136A	family with sequence similarity 136, member A	531.1	431.2	481.15
TADA1	transcriptional adaptor 1	495.6	466.0	480.83
KLHL20	kelch-like family member 20	489.7	471.6	480.61
LOC100544105	WD repeat and SOCS box-containing protein 1	453.6	507.4	480.54
RFX7	regulatory factor X, 7	450.1	509.9	480.00
SRR	serine racemase	465.5	494.4	479.97
TUBGCP6	tubulin, gamma complex associated protein 6	471.3	488.6	479.93
NOTCH2	notch 2	477.7	482.1	479.90
GPR137B	G protein-coupled receptor 137B	483.3	475.9	479.58
NENF	neudesin neurotrophic factor	482.1	476.6	479.37
SEMA4B	sema domain, immunoglobulin domain (Ig), transmembrane domain (TM) and short cytoplasmic domain, (semaphorin) 4B	544.9	413.9	479.36
LOC104909943	uncharacterized LOC104909943	459.4	499.2	479.28
PKN2	protein kinase N2	457.8	500.6	479.22
PDDC1	Parkinson disease 7 domain containing 1	498.1	459.6	478.85
SLC12A9	solute carrier family 12, member 9	478.1	478.3	478.23
LOC104916435	epithelial discoidin domain-containing receptor 1-like	550.2	405.6	477.90
INCENP	inner centromere protein antigens 135/155kDa	514.2	441.3	477.76
EMG1	EMG1 N1-specific pseudouridine methyltransferase	508.3	446.6	477.45
LOC104912362	metal-response element-binding transcription factor 2-like	445.1	509.4	477.24
ZC3H12A	zinc finger CCCH-type containing 12A	489.7	464.6	477.17
LOC100543133	metallophosphoesterase domain-containing protein 1-like	473.0	480.9	476.97
RAB35	RAB35, member RAS oncogene family	442.5	510.7	476.63
ABHD12	abhydrolase domain containing 12	467.1	486.1	476.58
ALMS1	Alstrom syndrome 1	470.5	482.5	476.49
RLF	rearranged L-myc fusion	458.8	494.1	476.43
CAMSAP1	calmodulin regulated spectrin-associated protein 1	477.1	475.0	476.06
FAM204A	family with sequence similarity 204, member A	493.9	458.1	476.00
IGFBP4	insulin-like growth factor binding protein 4	528.6	423.2	475.94
PDGFB	platelet-derived growth factor beta polypeptide	421.6	530.1	475.82
FNIP1	folliculin interacting protein 1	444.6	506.3	475.43
ATPAF1	ATP synthase mitochondrial F1 complex assembly factor 1	476.1	473.8	474.97
ANAPC7	anaphase promoting complex subunit 7	455.1	494.6	474.86
LOC100549829	TGF-beta receptor type-2-like	483.9	465.7	474.78
DAPP1	dual adaptor of phosphotyrosine and 3-phosphoinositides	483.9	465.3	474.58
LOC104912494	cytochrome P450 2J2-like	433.3	515.5	474.44
GPALPP1	GPALPP motifs containing 1	449.2	499.1	474.15
LOC104916246	dehydrogenase/reductase SDR family member on chromosome X-like	464.7	482.5	473.60
SIRT3	sirtuin 3	473.0	474.2	473.58
LMBRD2	LMBR1 domain containing 2	469.5	477.6	473.56
ACSL3	acyl-CoA synthetase long-chain family member 3	460.3	486.7	473.53
LOC104914604	puromycin-sensitive aminopeptidase-like protein	492.5	454.5	473.51
ABCD4	ATP-binding cassette, sub-family D (ALD), member 4	444.3	502.6	473.44
FBXO21	F-box protein 21	462.8	484.0	473.44
LOC104915763	trichohyalin-like	484.9	461.7	473.26
LRRC15	leucine rich repeat containing 15	487.3	459.1	473.19
LOC104916113	acetylcholinesterase-like	511.9	434.3	473.08
FASTK	Fas-activated serine/threonine kinase	544.9	401.2	473.04
CSRP2BP	CSRP2 binding protein	502.5	443.6	473.03

SYS1	Sys1 golgi trafficking protein	451.7	494.1	472.93
BFAR	bifunctional apoptosis regulator	449.2	496.6	472.87
LOC100549086	molybdopterin synthase catalytic subunit-like	477.9	466.8	472.39
UBR2	ubiquitin protein ligase E3 component n-recognin 2	460.3	484.2	472.25
ATPAF2	ATP synthase mitochondrial F1 complex assembly factor 2	525.3	419.1	472.19
ZCCHC10	zinc finger, CCHC domain containing 10	466.9	476.9	471.91
LOC100541746	prolyl-tRNA synthetase associated domain-containing protein 1-like	467.0	476.4	471.73
SP2	Sp2 transcription factor	446.2	497.0	471.58
CASP9	caspase 9, apoptosis-related cysteine peptidase	503.3	439.5	471.37
PARS2	prolyl-tRNA synthetase 2, mitochondrial (putative)	486.4	456.3	471.37
LRRC57	leucine rich repeat containing 57	452.6	490.0	471.30
SLC30A6	solute carrier family 30 (zinc transporter), member 6	459.5	482.0	470.78
LOC104914307	uncharacterized LOC104914307	478.8	462.6	470.70
LOC100550642	protein NDRG1-like	409.9	531.3	470.60
INO80B	INO80 complex subunit B	509.1	432.0	470.56
LOC104914009	protein FAM83D-like	517.5	422.7	470.08
LOC100550503	dedicator of cytokinesis protein 7	470.6	469.5	470.04
GEMIN4	gem (nuclear organelle) associated protein 4	502.4	437.7	470.02
RNF128	ring finger protein 128, E3 ubiquitin protein ligase	433.1	506.8	469.95
ZC3H12B	zinc finger CCCH-type containing 12B	456.1	483.3	469.67
RCBTB1	regulator of chromosome condensation (RCC1) and BTB (POZ) domain containing protein 1	444.9	493.7	469.33
LOC104916499	RNA-binding protein 24-B-like	484.2	453.9	469.04
DHX40	DEAH (Asp-Glu-Ala-His) box polypeptide 40	469.5	468.6	469.03
SART3	squamous cell carcinoma antigen recognized by T cells 3	458.5	479.4	468.94
KDELR3	KDEL (Lys-Asp-Glu-Leu) endoplasmic reticulum protein retention receptor 3	451.1	486.7	468.92
HID1	HID1 domain containing	453.6	483.5	468.56
GTF2H3	general transcription factor IIH, polypeptide 3, 34kDa	483.8	453.2	468.52
ABHD13	abhydrolase domain containing 13	467.0	469.9	468.47
TUBGCP5	tubulin, gamma complex associated protein 5	463.4	472.9	468.15
LOC100544198	myosin heavy chain, skeletal muscle, adult	501.8	434.0	467.89
ZNF143	zinc finger protein 143	472.0	463.8	467.89
KIF3B	kinesin family member 3B	470.4	464.3	467.33
PRCP	prolylcarboxypeptidase (angiotensinase C)	494.7	439.2	466.96
ST3GAL5	ST3 beta-galactoside alpha-2,3-sialyltransferase 5	426.7	507.0	466.85
MON1A	MON1 secretory trafficking family member A	485.7	447.9	466.81
RAD51D	RAD51 paralog D	486.4	446.8	466.63
MYNN	myoneurin	454.4	478.8	466.57
MTRF1	mitochondrial translational release factor 1	483.7	449.3	466.48
LOC100540318	KDEL motif-containing protein 1-like	461.0	471.4	466.20
IFT22	intraflagellar transport 22	471.1	461.0	466.05
ANO5	anoctamin 5	416.6	515.5	466.03
LOC104913848	uncharacterized LOC104913848	474.6	457.1	465.87
METTL13	methyltransferase like 13	483.0	448.6	465.79
NPHP4	nephronophthisis 4	504.4	427.0	465.74
ANKRD28	ankyrin repeat domain 28	411.1	520.4	465.74
GPN2	GPN-loop GTPase 2	459.3	472.1	465.70
KIAA1919	KIAA1919 ortholog	462.0	469.0	465.49
IFT122	intraflagellar transport 122	515.7	415.1	465.42
LOC104910833	uncharacterized LOC104910833	441.7	488.7	465.16
LOC104916181	integrin alpha-3-like	513.7	415.7	464.73
LOC104917240	T-complex protein 1 subunit gamma	510.2	419.0	464.63
MANBA	mannosidase, beta A, lysosomal	442.6	486.6	464.62
SLC12A4	solute carrier family 12 (potassium/chloride transporter), member 4	424.8	503.4	464.12
LOC100546893	TSC22 domain family protein 1-like	479.9	448.3	464.08

CCRN4L	CCR4 carbon catabolite repression 4-like (S. cerevisiae)	450.2	477.0	463.57
SURF1	surfeit 1	497.1	429.7	463.39
KCTD6	potassium channel tetramerization domain containing 6	447.6	478.8	463.19
RNF149	ring finger protein 149	452.6	473.6	463.09
ATF1	activating transcription factor 1	446.9	478.8	462.88
SLC35B2	solute carrier family 35 (adenosine 3'-phospho 5'-phosphosulfate transporter), member B2	481.5	444.2	462.85
DUSP28	dual specificity phosphatase 28	493.2	432.5	462.84
LOC100548787	pyruvate dehydrogenase (acetyl-transferring) kinase isozyme 3, mitochondrial-like	471.1	454.0	462.56
UBQLN1	ubiquilin 1	470.3	454.6	462.48
YAF2	YY1 associated factor 2	450.8	474.1	462.47
HDAC9	histone deacetylase 9	441.6	483.1	462.38
TMEM53	transmembrane protein 53	478.9	445.7	462.29
SPOCK2	sparc/osteonectin, cwcv and kazal-like domains proteoglycan (testican) 2	492.3	432.0	462.16
TRADD	TNFRSF1A-associated via death domain	449.4	474.9	462.12
COQ7	coenzyme Q7 homolog, ubiquinone (yeast)	488.0	435.9	461.97
BSDC1	BSD domain containing 1	480.6	443.1	461.86
PLEKHO2	pleckstrin homology domain containing, family O member 2	441.8	481.7	461.73
COG2	component of oligomeric golgi complex 2	452.7	470.8	461.71
PPARD	peroxisome proliferator-activated receptor delta	478.0	445.3	461.67
LOC104911131	uncharacterized LOC104911131	480.3	443.0	461.63
LRRC8A	leucine rich repeat containing 8 family, member A	471.3	451.6	461.47
EEF2K	eukaryotic elongation factor 2 kinase	445.1	477.8	461.44
CRB1	crumbs family member 1, photoreceptor morphogenesis associated	488.0	434.5	461.25
LOC100550606	dnaJ homolog subfamily A member 2-like	468.8	453.3	461.02
ASXL2	additional sex combs like transcriptional regulator 2	461.2	460.7	460.96
RBL2	retinoblastoma-like 2	435.8	486.0	460.90
LOC104915993	ataxin-7-like protein 3	521.0	400.7	460.86
CTSO	cathepsin O	455.2	466.3	460.75
LOC100540921	extracellular sulfatase Sulf-2	435.9	485.4	460.65
VTI1B	vesicle transport through interaction with t-SNAREs 1B	457.6	463.3	460.44
ZMYM2	zinc finger, MYM-type 2	449.3	471.4	460.38
LOC104915117	aminopeptidase O-like	444.9	475.6	460.25
PHLDA3	pleckstrin homology-like domain, family A, member 3	514.5	405.9	460.20
DCAF8	DDB1 and CUL4 associated factor 8	474.9	445.2	460.04
LYRM2	LYR motif containing 2	440.8	479.0	459.93
KCTD15	potassium channel tetramerization domain containing 15	443.4	476.4	459.91
LOC100540331	steroid 17-alpha-hydroxylase/17,20 lyase	510.0	409.4	459.69
LOC100550532	baculoviral IAP repeat-containing protein 5-like	472.7	446.4	459.51
CSNK1G2	casein kinase 1, gamma 2	514.5	404.4	459.42
MTFMT	mitochondrial methionyl-tRNA formyltransferase	464.3	454.2	459.28
IBA57	IBA57, iron-sulfur cluster assembly homolog (S. cerevisiae)	413.1	505.3	459.22
MAP3K7CL	MAP3K7 C-terminal like	489.6	428.8	459.20
LOC100539812	anthrax toxin receptor 2-like	456.2	461.8	459.03
NFIX	nuclear factor I/X (CCAAT-binding transcription factor)	535.3	382.7	459.01
SNRK	SNF related kinase	438.1	479.7	458.93
DALRD3	DALR anticodon binding domain containing 3	495.8	421.7	458.72
STAMBP	STAM binding protein	478.9	437.8	458.37
LOC100550128	myelin P2 protein	430.7	486.0	458.33
NFATC3	nuclear factor of activated T-cells, cytoplasmic, calcineurin-dependent 3	406.5	509.7	458.11
MSANTD4	Myb/SANT-like DNA-binding domain containing 4 with coiled-coils	471.2	444.9	458.07
INF2	inverted formin, FH2 and WH2 domain containing	414.8	501.1	457.97
WDR20	WD repeat domain 20	452.7	463.2	457.94
THADA	thyroid adenoma associated	472.9	441.7	457.29

LOC104917324	tetratricopeptide repeat protein 9A-like	449.4	464.7	457.08
PHF13	PHD finger protein 13	456.9	457.2	457.06
ATP10A	ATPase, class V, type 10A	436.6	477.4	457.00
SUGP1	SURP and G patch domain containing 1	450.1	463.9	456.98
FOXO3	forkhead box O3	439.9	474.0	456.95
ACAD8	acyl-CoA dehydrogenase family, member 8	485.6	428.0	456.82
ARSI	arylsulfatase family, member I	452.8	459.7	456.25
ZC3H7B	zinc finger CCCH-type containing 7B	486.7	425.0	455.86
DLG5	discs, large homolog 5 (Drosophila)	429.0	482.5	455.74
KAT2B	K(lysine) acetyltransferase 2B	427.3	483.1	455.22
LOC100547927	uridine-cytidine kinase 1-like 1-like	486.4	423.4	454.92
LOC104915941	suppressor of SWI4 1 homolog	470.5	438.8	454.67
LOC100543235	endothelial zinc finger protein induced by tumor necrosis factor alpha-like	471.2	437.6	454.41
LOC100550216	versican core protein-like	439.3	469.4	454.33
KIF26A	kinesin family member 26A	371.2	537.2	454.22
DDA1	DET1 and DDB1 associated 1	437.5	470.7	454.11
LOC100548097	mastermind-like protein 2	427.4	480.7	454.05
LOC100541679	uncharacterized LOC100541679	384.6	523.3	453.97
LOC100551374	deoxyribodipyrimidine photo-lyase-like	470.6	437.2	453.87
GLTSCR1L	GLTSCR1-like	437.5	470.1	453.79
ADCY5	adenylate cyclase 5	418.9	488.1	453.52
LRIF1	ligand dependent nuclear receptor interacting factor 1	425.6	481.0	453.31
METTL21C	methyltransferase like 21C	422.2	484.1	453.14
BRAT1	BRCA1-associated ATM activator 1	427.4	478.6	453.02
ALG10	ALG10, alpha-1,2-glucosyltransferase	435.7	470.4	453.01
TGFBRAP1	transforming growth factor, beta receptor associated protein 1	436.6	469.3	452.99
PNPT1	polyribonucleotide nucleotidyltransferase 1	471.2	434.2	452.73
LOC104914188	PR domain zinc finger protein 2-like	420.8	484.1	452.48
STAMBPL1	STAM binding protein-like 1	446.7	458.1	452.41
YARS2	tyrosyl-tRNA synthetase 2, mitochondrial	466.0	438.7	452.34
BPTF	bromodomain PHD finger transcription factor	428.3	476.2	452.23
FLVCR1	feline leukemia virus subgroup C cellular receptor 1	441.6	462.4	452.01
RPRD2	regulation of nuclear pre-mRNA domain containing 2	438.6	465.3	451.94
CCND3	cyclin D3	483.9	419.8	451.88
TRMU	tRNA 5-methylaminomethyl-2-thiouridylate methyltransferase	447.5	455.7	451.60
LSM6	LSM6 homolog, U6 small nuclear RNA associated (S. cerevisiae)	441.5	461.5	451.49
FAM20B	family with sequence similarity 20, member B	439.9	462.9	451.43
CCDC59	coiled-coil domain containing 59	452.5	450.3	451.41
CDCA3	cell division cycle associated 3	482.8	419.9	451.34
HOGA1	4-hydroxy-2-oxoglutarate aldolase 1	482.4	420.2	451.30
SASS6	spindle assembly 6 homolog (C. elegans)	458.4	443.9	451.14
MED19	mediator complex subunit 19	460.2	441.6	450.92
REEP2	receptor accessory protein 2	461.0	440.5	450.77
AFTPH	aftiphilin	432.3	468.5	450.40
LOC100541714	heme-binding protein 2-like	472.0	428.7	450.34
KATNB1	katanin p80 (WD repeat containing) subunit B 1	452.0	448.2	450.11
FAM172A	family with sequence similarity 172, member A	460.2	440.0	450.09
FAM35A	family with sequence similarity 35, member A	445.2	454.8	450.02
BTBD10	BTB (POZ) domain containing 10	426.4	473.2	449.84
ADNP2	ADNP homeobox 2	417.9	481.7	449.81
ANKS3	ankyrin repeat and sterile alpha motif domain containing 3	462.8	436.7	449.76
CCNT1	cyclin T1	446.2	453.0	449.59
LOC100540623	retinol dehydrogenase 14-like	439.1	459.0	449.03
SNCB	synuclein, beta	387.2	510.5	448.86
NUP37	nucleoporin 37kDa	438.3	459.5	448.85
RNF168	ring finger protein 168, E3 ubiquitin protein ligase	449.3	448.4	448.83
SCN4B	sodium channel, voltage-gated, type IV, beta subunit	483.0	414.4	448.71

TSEN34	TSEN34 tRNA splicing endonuclease subunit	525.4	371.9	448.66
RHBDD2	rhomboid domain containing 2	450.1	447.0	448.54
SCRN2	secernin 2	460.3	436.8	448.54
TNFRSF11A	tumor necrosis factor receptor superfamily, member 11a, NFKB activator	434.9	461.8	448.33
AADAT	aminoadipate aminotransferase	449.3	447.3	448.30
EMX2	empty spiracles homeobox 2	477.2	418.9	448.00
JDP2	Jun dimerization protein 2	456.0	440.0	447.99
TRAF3IP2	TRAF3 interacting protein 2	432.4	462.9	447.69
RRN3	RRN3 RNA polymerase I transcription factor homolog (S. cerevisiae)	450.8	444.4	447.61
TMEM260	transmembrane protein 260	466.2	428.8	447.51
LOC100544913	erythroid differentiation-related factor 1-like	465.2	429.5	447.38
TNFAIP3	tumor necrosis factor, alpha-induced protein 3	435.9	458.9	447.37
GCC1	GRIP and coiled-coil domain containing 1	461.1	433.5	447.32
PARM1	prostate androgen-regulated mucin-like protein 1	484.7	409.9	447.29
LOC100545797	cytochrome c oxidase assembly factor 7	436.5	457.8	447.18
LOC100546621	cohesin subunit SA-2-like	428.2	465.2	446.69
KCNJ5	potassium inwardly-rectifying channel, subfamily J, member 5	407.2	486.2	446.69
REXO4	REX4, RNA exonuclease 4 homolog (S. cerevisiae)	493.0	399.6	446.29
KIAA0319	KIAA0319 ortholog	433.0	459.4	446.19
NR2C2AP	nuclear receptor 2C2-associated protein	445.0	447.3	446.14
PDE8A	phosphodiesterase 8A	406.2	486.1	446.12
LOC100540049	anoctamin-6-like	392.2	500.0	446.07
CTAGE5	CTAGE family, member 5	452.6	439.2	445.93
PQLC1	PQ loop repeat containing 1	446.0	445.4	445.71
BAK1	BCL2-antagonist/killer 1	497.7	393.7	445.68
TCEA2	transcription elongation factor A (SII), 2	434.1	456.7	445.43
TOLLIP	toll interacting protein	425.7	465.0	445.35
GDE1	glycerophosphodiester phosphodiesterase 1	435.1	455.4	445.23
ZCCHC17	zinc finger, CCHC domain containing 17	487.3	401.6	444.44
NUP35	nucleoporin 35kDa	486.2	402.6	444.39
SLC25A10	solute carrier family 25 (mitochondrial carrier; dicarboxylate transporter), member 10	483.2	405.5	444.34
PBLD	phenazine biosynthesis-like protein domain containing	439.8	448.8	444.31
TMEM52	transmembrane protein 52	388.5	499.9	444.22
LOC100550202	protein tweety homolog 3-like	436.6	451.4	444.02
UFM1	ubiquitin-fold modifier 1	455.8	431.7	443.76
IVD	isovaleryl-CoA dehydrogenase	446.7	440.6	443.67
CMC1	C-x(9)-C motif containing 1	435.7	451.6	443.62
CENPE	centromere protein E, 312kDa	474.5	411.3	442.92
BRPF1	bromodomain and PHD finger containing, 1	486.6	398.6	442.63
CEP170	centrosomal protein 170kDa	408.8	476.2	442.50
PRRX1	paired related homeobox 1	389.6	494.8	442.23
LOC100548350	histone H2A deubiquitinase MYSM1	389.4	494.0	441.67
DUSP19	dual specificity phosphatase 19	442.5	440.7	441.60
LOC100538860	leucine-rich repeat-containing protein 75A-like	494.1	389.0	441.55
FOXP1	forkhead box P1	416.3	465.9	441.08
STAG2	stromal antigen 2	445.1	436.6	440.83
LOC104910588	uncharacterized LOC104910588	433.0	448.4	440.69
ZBTB25	zinc finger and BTB domain containing 25	437.4	443.9	440.63
LOC104913374	uncharacterized LOC104913374	428.1	452.9	440.50
LOC104909580	corepressor interacting with RBPJ 1-like	414.5	466.5	440.50
PMP22	peripheral myelin protein 22	441.0	439.9	440.46
LSM5	LSM5 homolog, U6 small nuclear RNA associated (S. cerevisiae)	444.9	435.9	440.42
MYEF2	myelin expression factor 2	434.9	445.8	440.33
KEAP1	kelch-like ECH-associated protein 1	480.8	399.7	440.24
LOC100547971	5-hydroxytryptamine receptor 2A-like	458.5	421.9	440.20

PIGB	phosphatidylinositol glycan anchor biosynthesis, class B	430.6	449.3	439.94
STARD3NL	STARD3 N-terminal like	418.0	461.8	439.90
MYLIP	myosin regulatory light chain interacting protein	422.2	457.5	439.83
TGS1	trimethylguanosine synthase 1	453.5	425.8	439.62
PXDC1	PX domain containing 1	437.4	441.4	439.42
ANO10	anoctamin 10	422.2	456.6	439.41
NUDT3	nudix (nucleoside diphosphate linked moiety X)-type motif 3	450.2	428.2	439.23
LRRC38	leucine rich repeat containing 38	446.6	431.8	439.21
PTPN5	protein tyrosine phosphatase, non-receptor type 5 (striatum-enriched)	415.6	462.7	439.11
KIAA0232	KIAA0232 ortholog	438.4	439.7	439.06
YPEL2	yippee-like 2 (Drosophila)	444.4	433.3	438.84
ZNF281	zinc finger protein 281	446.6	430.9	438.74
SPEG	SPEG complex locus	500.3	376.7	438.48
LOC104913606	probable RNA-binding protein 19	437.6	439.1	438.32
ZNF276	zinc finger protein 276	440.0	436.3	438.15
LCMT1	leucine carboxyl methyltransferase 1	461.9	414.4	438.14
PPHLN1	periphilin 1	429.1	447.1	438.10
HAUS2	HAUS augmin-like complex, subunit 2	444.0	432.2	438.08
SMPD4	sphingomyelin phosphodiesterase 4, neutral membrane (neutral sphingomyelinase-3)	434.9	441.1	438.00
LOC104914865	selenoprotein P-like	431.5	444.4	437.94
LOC100539934	RILP-like protein 1	408.9	466.9	437.91
SMIM12	small integral membrane protein 12	456.0	418.6	437.33
ABHD10	abhydrolase domain containing 10	450.0	424.2	437.12
ANK1	ankyrin 1, erythrocytic	419.0	454.7	436.83
LOC104913833	uncharacterized LOC104913833	451.8	421.7	436.74
TMEM182	transmembrane protein 182	424.6	448.9	436.74
LOC104917168	cullin-9-like	442.6	430.5	436.57
LOC100540965	neuferricin	431.6	441.2	436.39
LOC104911245	protein LBH-like	409.8	462.5	436.17
PSD	pleckstrin and Sec7 domain containing	452.0	419.9	435.94
RAD1	RAD1 checkpoint DNA exonuclease	411.2	460.5	435.85
CERS2	ceramide synthase 2	472.3	399.4	435.83
FRMD5	FERM domain containing 5	481.3	390.3	435.82
THOC3	THO complex 3	448.5	421.6	435.06
RNMTL1	RNA methyltransferase like 1	433.4	436.5	434.96
PHYHD1	phytanoyl-CoA dioxygenase domain containing 1	413.9	455.8	434.87
PIGC	phosphatidylinositol glycan anchor biosynthesis, class C	460.2	409.5	434.83
SREK1IP1	SREK1-interacting protein 1	465.2	404.3	434.73
ULK3	unc-51 like kinase 3	472.2	396.3	434.26
FPGT	fucose-1-phosphate guanylyltransferase	431.6	436.9	434.23
RBBP8	retinoblastoma binding protein 8	433.9	434.5	434.21
LY96	lymphocyte antigen 96	397.0	470.9	433.95
BMP4	bone morphogenetic protein 4	476.1	391.6	433.84
UBE2QL1	ubiquitin-conjugating enzyme E2Q family-like 1	440.0	427.5	433.79
XRN2	5'-3' exoribonuclease 2	432.4	435.0	433.67
C1H12orf29	chromosome 1 open reading frame, human C12orf29	439.1	428.2	433.62
LOC104915965	uncharacterized LOC104915965	421.3	445.7	433.52
SMARCAD1	SWI/SNF-related, matrix-associated actin-dependent regulator of chromatin, subfamily a, containing DEAD/H box 1	412.1	454.9	433.49
LOC104916986	uncharacterized LOC104916986	476.6	390.3	433.44
BBS4	Bardet-Biedl syndrome 4	418.2	448.6	433.40
BZRAP1	benzodiazepine receptor (peripheral) associated protein 1	408.0	458.7	433.37
LOC100547348	probable global transcription activator SNF2L2	454.3	412.4	433.34
AURKA	aurora kinase A	483.8	382.3	433.05
PHF11	PHD finger protein 11	393.7	472.3	433.02

NFKB1	nuclear factor of kappa light polypeptide gene enhancer in B-cells 1	411.3	454.4	432.88
OTUD7B	OTU deubiquitinase 7B	452.7	412.9	432.80
APIP	APAF1 interacting protein	412.0	453.5	432.77
TFIP11	tuftelin interacting protein 11	408.0	456.9	432.45
TMEM170B	transmembrane protein 170B	415.3	449.5	432.39
JADE1	jade family PHD finger 1	434.0	430.4	432.19
HELZ	helicase with zinc finger	416.5	447.8	432.14
NT5C3A	5'-nucleotidase, cytosolic IIIA	437.3	426.9	432.09
LOC100539553	supervillin-like	398.0	465.8	431.91
TRDMT1	tRNA aspartic acid methyltransferase 1	433.1	430.7	431.91
CHRNA3	cholinergic receptor, nicotinic, alpha 3 (neuronal)	379.1	484.5	431.80
TMEM223	transmembrane protein 223	433.3	430.1	431.71
GUCD1	guanylyl cyclase domain containing 1	445.8	417.6	431.67
FAM104A	family with sequence similarity 104, member A	433.2	429.5	431.38
LOC104909982	nucleolar protein 10-like	420.5	442.2	431.36
MTERF3	mitochondrial transcription termination factor 3	449.1	413.1	431.12
TNFRSF10B	tumor necrosis factor receptor superfamily, member 10b	436.7	425.4	431.03
LOC100540873	cyclin-dependent kinase 7	441.5	420.3	430.92
MTUS1	microtubule associated tumor suppressor 1	417.1	444.5	430.78
ETAA1	Ewing tumor-associated antigen 1	442.4	418.7	430.56
LOC100547402	fatty acid amide hydrolase-like	429.9	431.1	430.54
BRD9	bromodomain containing 9	424.7	436.3	430.52
LCT	lactase	415.5	445.3	430.43
LOC100549141	heat shock protein beta-11-like	462.9	397.8	430.36
STK11	serine/threonine kinase 11	419.8	440.7	430.25
SLC25A29	solute carrier family 25 (mitochondrial carnitine/acylcarnitine carrier), member 29	423.8	436.5	430.18
ABHD12B	abhydrolase domain containing 12B	463.8	396.6	430.17
SLC17A5	solute carrier family 17 (acidic sugar transporter), member 5	408.8	451.1	429.95
TUBGCP3	tubulin, gamma complex associated protein 3	390.8	468.4	429.63
C1H12orf4	chromosome 1 open reading frame, human C12orf4	444.1	414.7	429.36
LOC104915341	transcription factor TFIIIB component B'' homolog	424.8	433.3	429.08
PPRC1	peroxisome proliferator-activated receptor gamma, coactivator-related 1	450.1	408.0	429.05
LOC100546971	protein VAC14 homolog	440.2	417.5	428.86
CTTNBP2NL	CTTNBP2 N-terminal like	413.9	443.6	428.73
GPATCH11	G patch domain containing 11	426.3	431.0	428.66
LOC104915871	geranylgeranyl transferase type-2 subunit alpha-like	456.8	400.5	428.65
IPMK	inositol polyphosphate multikinase	420.5	436.5	428.50
LOC100539172	butyrophilin subfamily 1 member A1-like	457.9	398.7	428.30
MED23	mediator complex subunit 23	411.3	445.0	428.13
HEY2	hes-related family bHLH transcription factor with YRPW motif 2	380.3	475.7	427.97
MED11	mediator complex subunit 11	474.7	381.1	427.90
CELSR3	cadherin, EGF LAG seven-pass G-type receptor 3	442.7	412.7	427.67
ATP5S	ATP synthase, H+ transporting, mitochondrial Fo complex, subunit s (factor B)	439.1	416.0	427.52
NUSAP1	nucleolar and spindle associated protein 1	456.6	398.4	427.49
LOC104911770	prolyl endopeptidase FAP-like	357.6	497.4	427.49
GALNT11	polypeptide N-acetylgalactosaminyltransferase 11	450.0	404.8	427.40
ZBED1	zinc finger, BED-type containing 1	392.8	461.4	427.07
LOC104914290	intersectin-1	411.3	442.8	427.06
ARL2BP	ADP-ribosylation factor-like 2 binding protein	413.8	439.9	426.83
CRLF1	cytokine receptor-like factor 1	540.0	312.7	426.34
ASPM	asp (abnormal spindle) homolog, microcephaly associated (Drosophila)	455.0	397.6	426.27
IL34	interleukin 34	479.6	372.7	426.17
TAB3	TGF-beta activated kinase 1/MAP3K7 binding protein 3	405.3	446.8	426.04

PRKAR1A	protein kinase, cAMP-dependent, regulatory, type I, alpha	386.9	464.7	425.81
LOC104909612	uncharacterized LOC104909612	385.3	466.4	425.81
TSSC1	tumor suppressing subtransferable candidate 1	393.5	457.9	425.71
LOC100545164	keratin, type II cytoskeletal 5-like	409.2	442.0	425.58
MYOCD	myocardin	412.4	438.7	425.54
LOC104911749	obg-like ATPase 1	450.1	400.4	425.24
GMNN	geminin, DNA replication inhibitor	421.2	429.1	425.15
ZNHIT2	zinc finger, HIT-type containing 2	445.1	404.6	424.85
JKAMP	JNK1/MAPK8-associated membrane protein	450.8	398.9	424.84
PLEKHB2	pleckstrin homology domain containing, family B (evectins) member 2	427.3	422.4	424.82
CDK5RAP1	CDK5 regulatory subunit associated protein 1	466.1	383.5	424.81
MYLK2	myosin light chain kinase 2	444.3	405.2	424.72
MTIF2	mitochondrial translational initiation factor 2	424.8	424.6	424.70
MLYCD	malonyl-CoA decarboxylase	415.4	433.9	424.62
EEPD1	endonuclease/exonuclease/phosphatase family domain containing 1	407.8	441.4	424.59
PEX14	peroxisomal biogenesis factor 14	415.5	433.5	424.50
LOC104913826	uncharacterized LOC104913826	458.5	390.4	424.47
SLC39A6	solute carrier family 39 (zinc transporter), member 6	440.0	408.9	424.43
LOC100303663	mitochondrial uncoupling protein 3	465.2	383.2	424.19
CLASP1	cytoplasmic linker associated protein 1	424.0	424.3	424.17
NTPCR	nucleoside-triphosphatase, cancer-related	438.1	409.5	423.82
SNAP47	synaptosomal-associated protein, 47kDa	408.8	438.8	423.78
TRIM59	tripartite motif containing 59	438.1	409.2	423.68
MRPS24	mitochondrial ribosomal protein S24	454.3	392.8	423.53
PTCD1	pentatricopeptide repeat domain 1	431.5	414.9	423.20
CGRRF1	cell growth regulator with ring finger domain 1	384.4	461.9	423.15
TMED7	transmembrane emp24 protein transport domain containing 7	386.1	459.9	422.99
ACP6	acid phosphatase 6, lysophosphatidic	402.0	443.8	422.91
ZDHHC16	zinc finger, DHHC-type containing 16	439.1	406.7	422.88
NFATC1	nuclear factor of activated T-cells, cytoplasmic, calcineurin-dependent 1	364.1	481.4	422.73
LOC104911687	serine/threonine-protein kinase 16-like	435.0	410.1	422.57
PRKAR1B	protein kinase, cAMP-dependent, regulatory, type I, beta	413.8	431.4	422.55
PRICKLE1	prickle homolog 1 (Drosophila)	425.6	419.2	422.39
PARP16	poly (ADP-ribose) polymerase family, member 16	437.3	406.8	422.07
PRR5	proline rich 5 (renal)	419.0	425.1	422.07
KIAA1524	KIAA1524 ortholog	444.9	399.2	422.05
CD74	CD74 molecule, major histocompatibility complex, class II invariant chain	448.2	395.4	421.82
CWC25	CWC25 spliceosome-associated protein homolog (S. cerevisiae)	421.3	422.2	421.78
MRPL52	mitochondrial ribosomal protein L52	442.3	400.9	421.60
ZNF292	zinc finger protein 292	423.1	420.0	421.57
UST	uronyl-2-sulfotransferase	396.8	446.3	421.57
GCC2	GRIP and coiled-coil domain containing 2	418.0	425.0	421.51
TRIM41	tripartite motif containing 41	433.4	409.4	421.38
CDK5	cyclin-dependent kinase 5	445.9	396.7	421.34
PTGER4	prostaglandin E receptor 4 (subtype EP4)	405.3	436.4	420.85
WDR41	WD repeat domain 41	401.2	440.4	420.77
LOC100547923	phosphofurin acidic cluster sorting protein 1-like	444.3	397.1	420.71
LOC104915944	polyamine-modulated factor 1-like	466.2	374.3	420.23
INTS10	integrator complex subunit 10	413.7	426.6	420.14
LOC104915840	serine/arginine-rich splicing factor 7	434.8	404.1	419.43
TRNAU1AP	tRNA selenocysteine 1 associated protein 1	432.5	406.3	419.37
SPPL2B	signal peptide peptidase like 2B	418.9	419.8	419.35
TMEM38B	transmembrane protein 38B	391.8	446.0	418.93
LOC104910274	uncharacterized LOC104910274	397.7	440.1	418.89

LOC100543546	zinc finger protein 710-like	455.2	382.5	418.89
TDP2	tyrosyl-DNA phosphodiesterase 2	409.6	427.2	418.40
KIF18B	kinesin family member 18B	456.9	379.1	417.98
CACNG4	calcium channel, voltage-dependent, gamma subunit 4	447.5	388.5	417.97
PPP6R3	protein phosphatase 6, regulatory subunit 3	416.5	419.1	417.81
LSM3	LSM3 homolog, U6 small nuclear RNA associated (S. cerevisiae)	428.8	406.7	417.77
NOM1	nucleolar protein with MIF4G domain 1	415.4	420.1	417.74
PAFAH2	platelet-activating factor acetylhydrolase 2, 40kDa	423.2	412.1	417.69
BLVRA	biliverdin reductase A	422.2	413.2	417.66
LOC104910246	uncharacterized LOC104910246	412.8	422.2	417.46
ARHGAP19	Rho GTPase activating protein 19	443.3	391.5	417.44
UBOX5	U-box domain containing 5	440.8	394.0	417.40
SLIT3	slit homolog 3 (Drosophila)	591.8	243.0	417.39
PEX5	peroxisomal biogenesis factor 5	410.3	424.2	417.26
LOC104916313	uncharacterized LOC104916313	456.0	378.4	417.18
LOC104910858	uncharacterized LOC104910858	400.2	433.8	417.02
LOC100543018	coronin-7-like	485.7	347.8	416.77
SYCP3	synaptonemal complex protein 3	413.7	419.8	416.75
TRAPPC10	trafficking protein particle complex 10	380.1	453.3	416.67
EDEM2	ER degradation enhancer, mannosidase alpha-like 2	419.8	413.3	416.53
SECISBP2	SECIS binding protein 2	400.3	432.6	416.45
TRAIP	TRAF interacting protein	425.6	407.1	416.37
MTHFSD	methenyltetrahydrofolate synthetase domain containing	414.6	418.1	416.35
RHOBTB3	Rho-related BTB domain containing 3	362.5	470.1	416.31
PASD1	PAS domain containing 1	422.5	410.1	416.31
LOC100545080	lipocalin-like	391.0	440.9	415.92
RNASEH2B	ribonuclease H2, subunit B	433.0	398.4	415.70
ABCB7	ATP-binding cassette, sub-family B (MDR/TAP), member 7	379.1	452.2	415.65
LOC100548454	ubiquitin-conjugating enzyme E2 D3-like	408.5	422.1	415.32
GADD45GIP1	growth arrest and DNA-damage-inducible, gamma interacting protein 1	483.7	346.9	415.30
VDAC1	voltage-dependent anion channel 1	434.0	395.5	414.73
TBC1D1	TBC1 (tre-2/USP6, BUB2, cdc16) domain family, member 1	397.0	432.5	414.71
ABCB6	ATP-binding cassette, sub-family B (MDR/TAP), member 6 (Langereis blood group)	399.7	429.2	414.45
COMMD8	COMM domain containing 8	417.9	410.8	414.35
FARP1	FERM, RhoGEF (ARHGEF) and pleckstrin domain protein 1 (chondrocyte-derived)	403.6	424.7	414.19
PPCS	phosphopantothenoylcysteine synthetase	415.5	412.4	413.95
GMCL1	germ cell-less, spermatogenesis associated 1	450.1	377.8	413.94
UBE2E2	ubiquitin-conjugating enzyme E2E 2	377.5	450.3	413.86
LOC104916258	DNA-directed RNA polymerase II subunit RPB1-like	446.9	380.6	413.78
SGTB	small glutamine-rich tetratricopeptide repeat (TPR)-containing, beta	405.3	421.8	413.55
LOC100544877	MAP kinase-activated protein kinase 3	408.6	417.3	412.94
GIT2	G protein-coupled receptor kinase interacting ArfGAP 2	389.5	435.9	412.67
COG8	component of oligomeric golgi complex 8	435.8	389.4	412.62
MBTD1	mbt domain containing 1	367.4	457.2	412.28
PMEPA1	prostate transmembrane protein, androgen induced 1	389.3	434.7	412.01
LOC104913559	calcineurin-binding protein cabin-1-like	382.5	441.1	411.79
LOC100544072	WD repeat-containing protein C2orf44 homolog	383.5	439.8	411.65
GUK1	guanylate kinase 1	422.2	401.0	411.62
BEND5	BEN domain containing 5	416.2	407.0	411.62
LOC104911106	MAP kinase-activating death domain protein-like	422.9	400.1	411.49
POLR3B	polymerase (RNA) III (DNA directed) polypeptide B	418.0	405.0	411.46
LOC104917005	microfibril-associated glycoprotein 4-like	465.2	357.6	411.44
AP5S1	adaptor-related protein complex 5, sigma 1 subunit	434.0	388.9	411.43
LAMP5	lysosomal-associated membrane protein family, member 5	422.3	400.5	411.39

CDK12	cyclin-dependent kinase 12	433.4	388.9	411.15
CSPG4	chondroitin sulfate proteoglycan 4	452.8	369.3	411.07
ZCCHC6	zinc finger, CCHC domain containing 6	389.3	432.6	410.96
REV1	REV1, polymerase (DNA directed)	386.0	435.8	410.86
LOC104917204	reticulon-3 pseudogene	448.7	372.6	410.64
FAM43A	family with sequence similarity 43, member A	425.3	396.0	410.64
NDUFAF5	NADH dehydrogenase (ubiquinone) complex I, assembly factor 5	402.0	419.2	410.60
ASNSD1	asparagine synthetase domain containing 1	413.7	407.1	410.44
LOC100543957	DNA polymerase kappa-like	400.2	420.6	410.39
TRIM23	tripartite motif containing 23	384.3	436.5	410.38
PIGV	phosphatidylinositol glycan anchor biosynthesis, class V	437.5	383.2	410.37
SERHL2	serine hydrolase-like 2	401.2	419.2	410.18
LOC100546851	E3 ubiquitin-protein ligase RNF19B-like	429.2	391.1	410.16
KLHDC8A	kelch domain containing 8A	439.0	380.9	409.99
LOC104910970	DNA-binding protein SMUBP-2-like	404.6	415.2	409.90
SEMA4G	sema domain, immunoglobulin domain (Ig), transmembrane domain (TM) and short cytoplasmic domain, (semaphorin) 4G	401.2	418.5	409.85
TAOK1	TAO kinase 1	426.5	393.1	409.80
PDIK1L	PDLIM1 interacting kinase 1 like	383.5	436.1	409.77
ING3	inhibitor of growth family, member 3	395.9	423.4	409.69
RAD9A	RAD9 homolog A (S. pombe)	416.5	402.8	409.60
LOC104913993	uncharacterized LOC104913993	411.5	407.5	409.48
SHISA4	shisa family member 4	436.8	381.8	409.29
CEP250	centrosomal protein 250kDa	400.4	418.1	409.28
LOC104910547	uncharacterized LOC104910547	383.4	435.1	409.27
BRD4	bromodomain containing 4	430.8	387.7	409.22
LOC104914533	uncharacterized LOC104914533	412.9	405.4	409.17
LOC104913126	zinc finger protein 384-like	419.8	397.7	408.74
IAH1	isoamyl acetate-hydrolyzing esterase 1 homolog (S. cerevisiae)	449.1	368.2	408.65
LOC100549823	MICOS complex subunit MIC60-like	414.8	402.4	408.59
DNAJC19	DnaJ (Hsp40) homolog, subfamily C, member 19	407.7	409.4	408.56
LOC104915848	chromosome unknown open reading frame, human C17orf53	406.2	410.6	408.39
DCLK1	doublecortin-like kinase 1	384.3	432.5	408.38
GNB1L	guanine nucleotide binding protein (G protein), beta polypeptide 1-like	421.3	395.3	408.27
ME1	malic enzyme 1, NADP(+)-dependent, cytosolic	426.4	390.1	408.26
KIF14	kinesin family member 14	438.9	377.4	408.19
ALKBH1	alkB, alkylation repair homolog 1 (E. coli)	402.1	414.3	408.17
LOC104909626	uncharacterized LOC104909626	380.0	436.1	408.08
CEP68	centrosomal protein 68kDa	429.1	386.9	408.01
CCDC109B	coiled-coil domain containing 109B	374.1	441.8	407.92
RFC1	replication factor C (activator 1) 1, 145kDa	384.2	431.4	407.77
MAML3	mastermind-like 3 (Drosophila)	399.6	414.9	407.24
AHCYL1	adenosylhomocysteinase-like 1	404.7	409.3	406.99
HYAL2	hyaluronoglucosaminidase 2	413.0	400.8	406.89
GFOD1	glucose-fructose oxidoreductase domain containing 1	393.5	420.3	406.88
LOC100550494	metalloreductase STEAP2	414.4	399.3	406.86
TXNDC15	thioredoxin domain containing 15	369.8	443.2	406.51
FASTKD1	FAST kinase domains 1	417.2	395.6	406.39
LDLRAD1	low density lipoprotein receptor class A domain containing 1	421.3	391.2	406.24
MSL2	male-specific lethal 2 homolog (Drosophila)	410.4	401.9	406.13
STX18	syntaxin 18	402.0	410.1	406.06
RNF123	ring finger protein 123	443.4	368.7	406.06
IFT80	intraflagellar transport 80	408.6	403.4	405.95
KIAA1147	KIAA1147 ortholog	375.9	435.9	405.91
NUP43	nucleoporin 43kDa	414.6	397.2	405.89
PGAP3	post-GPI attachment to proteins 3	423.1	388.2	405.68
CZH5orf42	chromosome Z open reading frame, human C5orf42	418.0	393.2	405.59

LOC100540668	sodium-coupled neutral amino acid transporter 2	387.8	421.9	404.85
TRAPPC12	trafficking protein particle complex 12	412.9	396.4	404.67
PPIL1	peptidylprolyl isomerase (cyclophilin)-like 1	422.9	386.3	404.64
SLC25A33	solute carrier family 25 (pyrimidine nucleotide carrier), member 33	430.5	378.3	404.41
PLCD4	phospholipase C, delta 4	382.9	425.2	404.04
TXNRD2	thioredoxin reductase 2	401.9	406.1	404.01
LOC104913113	E3 ubiquitin-protein ligase RNF123-like	352.5	455.2	403.83
METTL24	methyltransferase like 24	398.6	408.9	403.75
SRD5A3	steroid 5 alpha-reductase 3	414.6	392.9	403.75
DENND4C	DENN/MADD domain containing 4C	391.8	415.2	403.50
LOC104915835	NAD-dependent malic enzyme, mitochondrial-like	408.7	398.3	403.48
SERTAD4	SERTA domain containing 4	371.7	435.0	403.37
GUF1	GUF1 GTPase homolog (S. cerevisiae)	412.1	394.5	403.33
ZCCHC2	zinc finger, CCHC domain containing 2	392.7	413.9	403.31
LOC104915716	uncharacterized LOC104915716	439.9	366.6	403.22
C19H9orf69	chromosome 19 open reading frame, human C9orf69	393.5	412.8	403.14
ZNF217	zinc finger protein 217	376.6	429.6	403.11
DDX31	DEAD (Asp-Glu-Ala-Asp) box polypeptide 31	425.5	380.5	403.04
DCLRE1A	DNA cross-link repair 1A	395.0	410.6	402.82
ZFAND2B	zinc finger, AN1-type domain 2B	398.7	406.6	402.68
ANTXRL	anthrax toxin receptor-like	446.6	358.0	402.30
LOC104911986	V-type proton ATPase subunit e 2	392.0	412.5	402.23
PHTF1	putative homeodomain transcription factor 1	383.6	420.6	402.11
HIVEP1	human immunodeficiency virus type I enhancer binding protein 1	377.6	426.4	402.02
COIL	coilin	406.2	397.6	401.94
HOXA10	homeobox A10	396.8	407.1	401.94
LOC104915338	uncharacterized LOC104915338	417.9	385.7	401.84
VBP1	von Hippel-Lindau binding protein 1	396.0	407.7	401.83
LOC104914816	uncharacterized LOC104914816	425.7	377.9	401.79
CRBN	cereblon	374.1	429.2	401.66
TMEM245	transmembrane protein 245	404.7	398.0	401.36
LARP1B	La ribonucleoprotein domain family, member 1B	386.1	416.6	401.33
LOC104909451	uncharacterized LOC104909451	406.2	395.8	401.02
ZNRF3	zinc and ring finger 3	391.9	409.9	400.91
RRP7A	ribosomal RNA processing 7 homolog A (S. cerevisiae)	434.0	367.5	400.78
ABCA5	ATP-binding cassette, sub-family A (ABC1), member 5	369.2	432.1	400.70
IKBKE	inhibitor of kappa light polypeptide gene enhancer in B-cells, kinase epsilon	392.0	409.3	400.65
EIF4EBP3	eukaryotic translation initiation factor 4E binding protein 3	394.5	406.8	400.65
CDC34	cell division cycle 34	386.7	414.5	400.64
TIRAP	toll-interleukin 1 receptor (TIR) domain containing adaptor protein	403.9	397.2	400.54
LOC104917066	early activation antigen CD69-like	390.3	409.0	399.64
NELFA	negative elongation factor complex member A	370.8	428.2	399.52
LOC104916685	KAT8 regulatory NSL complex subunit 2-like	440.1	358.6	399.35
SH3BP5	SH3-domain binding protein 5 (BTK-associated)	380.0	418.6	399.30
TTI2	TELO2 interacting protein 2	391.0	407.5	399.27
OTUD5	OTU deubiquitinase 5	429.1	369.3	399.17
LOC104910267	centrosomal protein of 290 kDa-like	385.9	412.4	399.15
PPAPDC3	phosphatidic acid phosphatase type 2 domain containing 3	398.6	399.4	398.97
TRMT11	tRNA methyltransferase 11 homolog (S. cerevisiae)	377.5	420.2	398.90
SLC39A3	solute carrier family 39 (zinc transporter), member 3	431.5	366.2	398.88
TLDC1	TBC/LysM-associated domain containing 1	415.5	382.1	398.82
OGFR	opioid growth factor receptor	390.2	407.2	398.71
NAPRT	nicotinate phosphoribosyltransferase	422.4	374.7	398.53
FAM219B	family with sequence similarity 219, member B	380.8	416.2	398.49
USP13	ubiquitin specific peptidase 13 (isopeptidase T-3)	374.2	421.6	397.93

BBS5	Bardet-Biedl syndrome 5	382.5	413.1	397.82
LOC104916015	T-complex protein 1 subunit delta-like	402.9	392.7	397.80
ORMDL3	ORMDL sphingolipid biosynthesis regulator 3	405.4	389.8	397.61
PDS5A	PDS5 cohesin associated factor A	368.2	426.7	397.46
LOC104917128	sodium channel modifier 1-like	425.7	368.6	397.15
LOC100539365	uncharacterized LOC100539365	388.4	405.7	397.04
ULK1	unc-51 like autophagy activating kinase 1	380.8	413.2	397.00
LOC104910601	latrophilin-3-like	385.0	407.9	396.45
THG1L	tRNA-histidine guanylyltransferase 1-like (S. cerevisiae)	418.6	374.2	396.39
ABCC6	ATP-binding cassette, sub-family C (CFTR/MRP), member 6	389.6	403.0	396.30
SLC25A32	solute carrier family 25 (mitochondrial folate carrier), member 32	414.6	377.9	396.24
SIAH2	siah E3 ubiquitin protein ligase 2	428.1	364.2	396.15
SERAC1	serine active site containing 1	395.2	396.9	396.07
ZMYND19	zinc finger, MYND-type containing 19	422.1	369.9	396.02
NHLRC3	NHL repeat containing 3	381.7	410.2	395.94
LOC104914746	uncharacterized LOC104914746	402.0	389.8	395.90
MAR8	membrane-associated ring finger (C3HC4) 8, E3 ubiquitin protein ligase	390.1	401.7	395.88
IFT172	intraflagellar transport 172	428.1	363.3	395.68
ING5	inhibitor of growth family, member 5	380.0	410.7	395.37
BHLHA15	basic helix-loop-helix family, member a15	383.5	406.3	394.90
EGF	epidermal growth factor	365.8	424.0	394.89
LOC104911681	tubulin polyglutamylase TTLL4-like	419.8	369.6	394.71
LOC104914321	zinc finger and BTB domain-containing protein 8A-like	434.1	355.0	394.57
GINS2	GINS complex subunit 2 (Psf2 homolog)	429.8	359.3	394.54
LOC100549737	carbohydrate sulfotransferase 6-like	377.5	411.4	394.41
PPARG	peroxisome proliferator-activated receptor gamma	404.3	384.3	394.28
SLC43A3	solute carrier family 43, member 3	436.5	352.0	394.22
MED24	mediator complex subunit 24	445.2	342.9	394.03
LOC104909875	activating signal cointegrator 1 complex subunit 3-like	382.5	405.5	394.01
DNAL1	dynein, axonemal, light chain 1	391.7	396.0	393.87
TUSC2	tumor suppressor candidate 2	424.8	362.5	393.67
ELOVL1	ELOVL fatty acid elongase 1	417.4	369.7	393.53
WHAMM	WAS protein homolog associated with actin, golgi membranes and microtubules	362.3	424.3	393.29
CKAP2L	cytoskeleton associated protein 2-like	408.8	377.1	392.91
CD320	CD320 molecule	389.6	396.2	392.88
UBE3C	ubiquitin protein ligase E3C	355.6	430.1	392.85
TPCN1	two pore segment channel 1	404.5	381.1	392.80
HELQ	helicase, POLQ-like	380.9	404.6	392.77
NOD1	nucleotide-binding oligomerization domain containing 1	365.7	419.7	392.74
DGUOK	deoxyguanosine kinase	412.9	372.2	392.55
CCNF	cyclin F	436.5	348.6	392.55
LIMK2	LIM domain kinase 2	365.8	419.1	392.43
GSTA4	glutathione S-transferase 3	384.9	398.5	391.73
LOC100542520	glyoxylate reductase/hydroxypyruvate reductase-like	392.7	390.7	391.72
LOC100541596	interferon-induced guanylate-binding protein 1-like	371.9	411.5	391.71
LOC100543103	quinone oxidoreductase-like protein 2	389.4	393.9	391.67
DIS3	DIS3 exosome endoribonuclease and 3'-5' exoribonuclease	363.9	419.3	391.58
RBBP5	retinoblastoma binding protein 5	375.1	407.9	391.50
MCAT	malonyl CoA:ACP acyltransferase (mitochondrial)	398.5	384.3	391.42
AGO1	argonaute RISC catalytic component 1	387.8	394.7	391.26
GPHN	gephyrin	396.2	386.2	391.22
FAM102B	family with sequence similarity 102, member B	375.8	406.0	390.89
SLC25A36	solute carrier family 25 (pyrimidine nucleotide carrier), member 36	369.1	412.3	390.72
MCCC1	methylcrotonoyl-CoA carboxylase 1 (alpha)	408.6	372.2	390.37
MIDN	midnolin	411.5	369.2	390.33

SPATA5	spermatogenesis associated 5	411.2	368.3	389.74
NFIC	nuclear factor I/C (CCAAT-binding transcription factor)	393.7	385.5	389.60
SLC25A15	solute carrier family 25 (mitochondrial carrier; ornithine transporter) member 15	407.8	370.0	388.90
PALB2	partner and localizer of BRCA2	391.8	385.5	388.69
LOC104914601	voltage-dependent L-type calcium channel subunit beta-1-like	408.2	369.0	388.58
LOC104912695	uncharacterized LOC104912695	350.6	426.3	388.45
SPC25	SPC25, NDC80 kinetochore complex component	389.1	387.5	388.31
DFNA5	deafness, autosomal dominant 5	412.8	363.7	388.29
C2H6orf211	chromosome 2 open reading frame, human C6orf211	370.8	405.7	388.22
LOC104909747	sorting nexin-9-like	381.0	395.3	388.13
PDXK	pyridoxal (pyridoxine, vitamin B6) kinase	395.9	380.2	388.09
ORC6	origin recognition complex, subunit 6	395.1	381.0	388.06
ZFP91	ZFP91 zinc finger protein	397.1	378.7	387.93
LOC104912450	uncharacterized LOC104912450	379.3	396.3	387.81
KNSTRN	kinetochore-localized astrin/SPAG5 binding protein	432.1	343.3	387.71
SREK1	splicing regulatory glutamine/lysine-rich protein 1	361.4	413.4	387.42
FAM60A	family with sequence similarity 60, member A	349.7	425.0	387.31
NDST2	N-deacetylase/N-sulfotransferase (heparan glucosaminyl) 2	369.3	405.2	387.25
LOC104915138	uncharacterized LOC104915138	375.1	399.4	387.25
DGCR8	DGCR8 microprocessor complex subunit	397.0	376.9	386.96
PHC1	polyhomeotic homolog 1 (Drosophila)	406.2	367.4	386.79
ROCK2	Rho-associated, coiled-coil containing protein kinase 2	368.3	405.0	386.62
DIAPH3	diaphanous-related formin 3	422.9	350.2	386.55
SLC39A14	solute carrier family 39 (zinc transporter), member 14	402.1	371.0	386.54
SDC4	syndecan 4	358.0	414.7	386.37
LOC100547241	S-methyl-5'-thioadenosine phosphorylase-like	395.9	376.4	386.17
MED26	mediator complex subunit 26	402.9	369.3	386.08
NIT1	nitrilase 1	407.9	363.9	385.93
ZFYVE21	zinc finger, FYVE domain containing 21	374.9	396.6	385.76
LOC100546688	transducin-like enhancer protein 1	392.1	378.8	385.42
LRMP	lymphoid-restricted membrane protein	415.4	355.1	385.26
ZNF131	zinc finger protein 131	375.1	395.2	385.16
FAM46A	family with sequence similarity 46, member A	347.3	422.9	385.10
SZRD1	SUZ RNA binding domain containing 1	392.0	377.5	384.75
OSTM1	osteopetrosis associated transmembrane protein 1	391.9	377.4	384.66
PTS	6-pyruvoyltetrahydropterin synthase	401.8	367.4	384.60
LOC104913067	uncharacterized LOC104913067	398.7	370.5	384.60
FOXM1	forkhead box M1	393.5	375.6	384.56
LCA5L	Leber congenital amaurosis 5-like	385.7	383.1	384.43
HDHD2	haloacid dehalogenase-like hydrolase domain containing 2	373.2	395.5	384.38
AFMID	arylformamidase	415.5	352.7	384.11
PPP3CA	protein phosphatase 3, catalytic subunit, alpha isozyme	374.9	393.2	384.06
ZSWIM1	zinc finger, SWIM-type containing 1	360.8	407.2	384.03
COL4A3BP	collagen, type IV, alpha 3 (Goodpasture antigen) binding protein	379.9	388.1	384.00
GUCY1B3	guanylate cyclase 1, soluble, beta 3	398.5	369.5	383.97
LOC104914320	histone-binding protein RBBP4-like	377.4	390.5	383.96
LOC104913003	uncharacterized LOC104913003	408.6	358.9	383.80
LOC100548678	ubiquitin-associated protein 2-like	359.3	408.1	383.70
GATAD2A	GATA zinc finger domain containing 2A	385.0	382.3	383.70
HTR4	5-hydroxytryptamine (serotonin) receptor 4, G protein-coupled	404.4	362.7	383.57
MTIF3	mitochondrial translational initiation factor 3	388.3	378.0	383.16
CCDC174	coiled-coil domain containing 174	389.3	376.9	383.13
CHAF1B	chromatin assembly factor 1, subunit B (p60)	350.6	415.7	383.13
SIRT7	sirtuin 7	406.3	359.9	383.09
LOC104911864	uncharacterized LOC104911864	355.5	410.7	383.09
LOC100547559	zinc finger protein 664-like	362.5	403.6	383.06
RNF14	ring finger protein 14	396.9	369.2	383.06

ALG13	ALG13, UDP-N-acetylglucosaminyltransferase subunit	374.1	391.5	382.82
IKBIP	IKBKB interacting protein	343.8	421.7	382.78
NSUN4	NOP2/Sun domain family, member 4	379.2	386.2	382.67
SEMA7A	semaphorin 7A, GPI membrane anchor (John Milton Hagen blood group)	416.4	348.7	382.55
ANKRD49	ankyrin repeat domain 49	352.0	412.8	382.42
RAD17	RAD17 homolog (S. pombe)	386.6	378.2	382.41
RECK	reversion-inducing-cysteine-rich protein with kazal motifs	378.3	386.5	382.41
ECT2	epithelial cell transforming 2	388.4	376.2	382.32
ZBTB47	zinc finger and BTB domain containing 47	397.0	367.5	382.25
PBX3	pre-B-cell leukemia homeobox 3	384.1	380.3	382.22
ASXL1	additional sex combs like transcriptional regulator 1	430.8	333.5	382.13
RNF111	ring finger protein 111	371.7	392.3	381.97
TMEM198	transmembrane protein 198	398.7	364.8	381.74
AUH	AU RNA binding protein/enoyl-CoA hydratase	365.6	397.5	381.52
ATG7	autophagy related 7	359.7	402.6	381.19
SFRP5	secreted frizzled-related protein 5	340.5	421.4	380.96
LOC100543736	ras GTPase-activating protein 4-like	379.3	382.4	380.84
ATAD2B	ATPase family, AAA domain containing 2B	349.5	411.7	380.60
RSPO4	R-spondin 4	328.9	432.2	380.55
SGSM2	small G protein signaling modulator 2	377.6	383.5	380.54
FAM122B	family with sequence similarity 122B	384.3	376.6	380.43
LOC100548086	caltractin	360.6	400.1	380.33
LOC104917534	mitochondrial intermediate peptidase-like	386.8	373.7	380.22
LOC104909326	ras-related protein Rab-44-like	419.6	340.8	380.20
ACAD11	acyl-CoA dehydrogenase family, member 11	363.9	396.4	380.15
LOC104913931	uncharacterized LOC104913931	397.7	362.1	379.93
TBC1D7	TBC1 domain family, member 7	391.7	367.7	379.72
NOL8	nucleolar protein 8	390.1	369.2	379.61
EPHB3	EPH receptor B3	382.8	376.3	379.56
PLEKHH1	pleckstrin homology domain containing, family H (with MyTH4 domain) member 1	385.1	373.8	379.50
CLOCK	clock circadian regulator	365.7	393.3	379.49
KIF9	kinesin family member 9	391.9	366.9	379.38
THAP5	THAP domain containing 5	384.9	373.5	379.22
DMAP1	DNA methyltransferase 1 associated protein 1	391.0	367.3	379.19
CEP76	centrosomal protein 76kDa	362.2	395.9	379.07
LOC104909726	uncharacterized LOC104909726	387.8	370.1	378.99
CROCC	ciliary rootlet coiled-coil, rootletin	417.3	340.6	378.98
BTBD6	BTB (POZ) domain containing 6	422.1	335.8	378.97
CCDC58	coiled-coil domain containing 58	391.7	365.7	378.70
XPC	xeroderma pigmentosum, complementation group C	359.0	397.7	378.32
RNF208	ring finger protein 208	428.1	328.1	378.09
SLC44A1	solute carrier family 44 (choline transporter), member 1	378.3	377.7	378.00
ZNF592	zinc finger protein 592	373.2	382.6	377.92
LOC100551155	transmembrane protein 178B-like	364.0	391.8	377.89
PDGFA	platelet-derived growth factor alpha polypeptide	335.3	420.4	377.87
MBIP	MAP3K12 binding inhibitory protein 1	365.4	390.2	377.81
C2CD2L	C2CD2-like	367.5	387.5	377.48
TMEM104	transmembrane protein 104	354.1	400.9	377.46
RAB22A	RAB22A, member RAS oncogene family	357.2	397.6	377.39
LOC104909730	mitogen-activated protein kinase kinase kinase 5-like	380.8	373.3	377.06
LOC104914204	E3 ubiquitin-protein ligase TRIM62-like	397.9	355.0	376.42
SUV420H1	suppressor of variegation 4-20 homolog 1 (Drosophila)	346.2	406.3	376.26
LOC104914068	uncharacterized LOC104914068	365.0	387.2	376.10
SGSM3	small G protein signaling modulator 3	398.7	353.2	375.93
DOK4	docking protein 4	413.0	338.6	375.80
LEMD3	LEM domain containing 3	367.4	384.1	375.73

LOC100548705	protein FAM122A	375.0	376.4	375.68
LOC100543023	acetyl-CoA carboxylase-like	396.2	354.8	375.45
L2HGDH	L-2-hydroxyglutarate dehydrogenase	371.6	379.2	375.45
NLE1	notchless homolog 1 (Drosophila)	378.3	372.6	375.44
LETM1	leucine zipper-EF-hand containing transmembrane protein 1	358.9	391.5	375.21
TRAF2	TNF receptor-associated factor 2	415.7	334.6	375.17
TMEM14A	transmembrane protein 14A	334.4	415.5	374.97
ESCO2	establishment of sister chromatid cohesion N-acetyltransferase 2	397.8	352.0	374.88
C1H11orf54	chromosome 1 open reading frame, human C11orf54	352.9	396.8	374.88
SRPX2	sushi-repeat containing protein, X-linked 2	367.5	382.0	374.75
NDOR1	NADPH dependent diflavin oxidoreductase 1	387.6	361.7	374.69
IGSF3	immunoglobulin superfamily, member 3	394.5	354.8	374.68
LOC100544972	ADAMTS-like protein 2	406.2	342.9	374.56
FAM206A	family with sequence similarity 206, member A	401.0	347.2	374.13
LOC100550044	lamin-L(III)-like	360.6	387.6	374.09
CRELD1	cysteine-rich with EGF-like domains 1	421.4	326.6	374.01
NT5M	5',3'-nucleotidase, mitochondrial	357.3	390.4	373.85
FOXRED1	FAD-dependent oxidoreductase domain containing 1	400.3	347.3	373.76
BTRC	beta-transducin repeat containing E3 ubiquitin protein ligase	385.1	362.3	373.69
LOC104915810	SWI/SNF complex subunit SMARCC2-like	417.3	330.1	373.68
TPRN	taperin	392.7	354.6	373.64
PFKP	phosphofructokinase, platelet	360.7	386.0	373.32
SUN2	Sad1 and UNC84 domain containing 2	407.9	338.2	373.06
LOC104910223	protein FAM65B-like	334.7	410.8	372.76
CD24	CD24 molecule	351.3	394.1	372.70
IDS	iduronate 2-sulfatase	346.3	399.1	372.67
LOC100541966	ubiquitin carboxyl-terminal hydrolase CYLD-like	364.9	380.3	372.60
USP32	ubiquitin specific peptidase 32	362.2	382.9	372.55
SH2B2	SH2B adaptor protein 2	338.7	406.2	372.46
FAM78A	family with sequence similarity 78, member A	380.9	363.8	372.36
PBK	PDZ binding kinase	385.7	358.8	372.25
EPC2	enhancer of polycomb homolog 2 (Drosophila)	357.2	387.2	372.22
HSPBAP1	HSPB (heat shock 27kDa) associated protein 1	359.7	384.5	372.13
OSGEP	O-sialoglycoprotein endopeptidase	426.5	317.7	372.09
PVRL3	poliovirus receptor-related 3	363.9	380.2	372.07
LOC100545895	vesicle-associated membrane protein 2-like	340.5	403.5	371.99
RREB1	ras responsive element binding protein 1	364.0	379.5	371.78
SLC25A26	solute carrier family 25 (S-adenosylmethionine carrier), member 26	368.3	374.5	371.42
LOC100543206	RNA polymerase-associated protein CTR9 homolog	381.7	360.6	371.10
C11H3orf70	chromosome 11 open reading frame, human C3orf70	368.2	373.9	371.08
C14H3orf14	chromosome 14 open reading frame, human C3orf14	381.6	360.5	371.07
LOC104909373	centrosomal protein KIAA1731-like	384.2	357.9	371.03
GABPB2	GA binding protein transcription factor, beta subunit 2	383.5	358.6	371.02
LOC104917604	intraflagellar transport protein 88 homolog	369.9	371.7	370.81
NOTCH1	notch 1	383.6	357.6	370.63
LOC104911133	uncharacterized LOC104911133	353.2	387.4	370.27
TSPAN14	tetraspanin 14	331.8	408.6	370.21
SLC5A7	solute carrier family 5 (sodium/choline cotransporter), member 7	377.4	362.8	370.12
ZNF319	zinc finger protein 319	393.5	346.5	370.04
LOC104916204	histidine triad nucleotide-binding protein 2, mitochondrial-like	411.3	328.6	369.97
LOC100542388	G patch domain-containing protein 1-like	352.9	386.1	369.53
LOC104917221	probable germin-like protein subfamily 2 member 5	378.3	360.3	369.31
LOC104917585	transcription factor SPT20 homolog	363.9	374.7	369.30
MYADML2	myeloid-associated differentiation marker-like 2	350.6	387.9	369.24
CEP57	centrosomal protein 57kDa	352.3	386.1	369.21
PRPSAP1	phosphoribosyl pyrophosphate synthetase-associated protein 1	363.1	375.2	369.12
TBCCD1	TBCC domain containing 1	391.8	346.1	368.94

ARV1	ARV1 homolog (S. cerevisiae)	348.7	389.1	368.89
POLE3	polymerase (DNA directed), epsilon 3, accessory subunit	361.5	375.3	368.37
LOC104914147	conserved oligomeric Golgi complex subunit 5-like	364.8	371.9	368.31
FAM96A	family with sequence similarity 96, member A	360.4	375.7	368.06
EEFSEC	eukaryotic elongation factor, selenocysteine-tRNA-specific	374.0	361.4	367.70
ZBTB49	zinc finger and BTB domain containing 49	373.8	361.6	367.70
PLCB4	phospholipase C, beta 4	354.7	380.5	367.57
LOC104914794	iporin-like	368.6	366.3	367.46
SLC37A4	solute carrier family 37 (glucose-6-phosphate transporter), member 4	351.4	383.2	367.31
PROCR	protein C receptor, endothelial	358.0	376.6	367.28
TBK1	TANK-binding kinase 1	359.0	375.5	367.27
LYSMD3	LysM, putative peptidoglycan-binding, domain containing 3	353.0	381.4	367.17
UHRF1	ubiquitin-like with PHD and ring finger domains 1	358.0	376.2	367.10
SSH3	slingshot protein phosphatase 3	409.7	324.5	367.06
VEZT	vezatin, adherens junctions transmembrane protein	374.0	360.0	367.02
AFF4	AF4/FMR2 family, member 4	339.7	394.3	367.02
TFPI	tissue factor pathway inhibitor (lipoprotein-associated coagulation inhibitor)	347.9	385.9	366.89
TMEM41A	transmembrane protein 41A	353.9	379.7	366.77
EXTL2	exostosin-like glycosyltransferase 2	360.5	372.4	366.44
PRKG2	protein kinase, cGMP-dependent, type II	363.1	369.4	366.23
SVEP1	sushi, von Willebrand factor type A, EGF and pentraxin domain containing 1	338.8	393.3	366.06
ARID4A	AT rich interactive domain 4A (RBP1-like)	363.2	368.8	366.01
LOC104917208	uncharacterized LOC104917208	363.3	368.3	365.80
QTRTD1	queuine tRNA-ribosyltransferase domain containing 1	374.1	357.2	365.65
POLE2	polymerase (DNA directed), epsilon 2, accessory subunit	357.1	374.1	365.63
LOC100550744	ubiquitin-conjugating enzyme E2 variant 3-like	336.2	395.0	365.62
LOC104915062	gamma-secretase-activating protein-like	345.3	385.7	365.54
CCDC126	coiled-coil domain containing 126	352.0	379.0	365.48
LOC104917322	CCAAT/enhancer-binding protein delta-like	380.8	349.7	365.27
CLPTM1	cleft lip and palate associated transmembrane protein 1	406.5	323.8	365.15
LOC104915566	scaffold attachment factor B1-like	365.0	364.7	364.87
EPN3	epsin 3	409.8	319.9	364.82
FBXO25	F-box protein 25	334.3	394.9	364.58
GPRC5B	G protein-coupled receptor, class C, group 5, member B	354.8	373.4	364.07
RMDN3	regulator of microtubule dynamics 3	380.0	348.1	364.02
LOC104913580	uncharacterized LOC104913580	364.9	363.1	363.96
LOC100538852	5-phosphohydroxy-L-lysine phospho-lyase	361.3	366.3	363.82
RAMP1	receptor (G protein-coupled) activity modifying protein 1	351.3	375.8	363.55
ERLIN1	ER lipid raft associated 1	370.8	356.2	363.51
LOC100544123	uncharacterized LOC100544123	302.5	424.3	363.36
SOCS5	suppressor of cytokine signaling 5	367.5	358.8	363.14
MRPS30	mitochondrial ribosomal protein S30	349.6	376.0	362.83
CHTF18	CTF18, chromosome transmission fidelity factor 18 homolog (S. cerevisiae)	395.3	330.4	362.82
TTC32	tetratricopeptide repeat domain 32	364.9	360.0	362.42
FBLIM1	filamin binding LIM protein 1	367.5	357.3	362.37
ZDHHC12	zinc finger, DHHC-type containing 12	375.9	348.5	362.20
UBAP1	ubiquitin associated protein 1	359.8	364.5	362.16
PRDM4	PR domain containing 4	363.3	360.9	362.11
ANKRD32	ankyrin repeat domain 32	364.8	359.3	362.06
LOC100551262	neurabin-1-like	332.8	391.3	362.05
GPR157	G protein-coupled receptor 157	382.7	341.1	361.92
LOC100545218	ATP-binding cassette sub-family D member 3-like	365.6	358.1	361.88
LOC104915527	myosin-8-like	369.2	354.1	361.62
KIF20A	kinesin family member 20A	394.3	328.6	361.46

SSH1	slingshot protein phosphatase 1	358.1	363.5	360.80
DNAJC21	DnaJ (Hsp40) homolog, subfamily C, member 21	359.6	361.1	360.34
CCDC28B	coiled-coil domain containing 28B	406.3	314.3	360.31
WEE1	WEE1 G2 checkpoint kinase	379.1	341.5	360.27
PCDH11X	protocadherin 11 X-linked	336.2	384.0	360.10
NCAPH2	non-SMC condensin II complex, subunit H2	386.8	333.0	359.90
TPRKB	TP53RK binding protein	358.0	361.1	359.57
MAML2	mastermind-like 2 (Drosophila)	364.9	354.2	359.53
ZBTB46	zinc finger and BTB domain containing 46	354.8	364.1	359.47
LOC100538845	rab GTPase-activating protein 1-like, isoform 10	337.8	380.9	359.36
CLCC1	chloride channel CLIC-like 1	377.5	340.9	359.20
TMEM206	transmembrane protein 206	351.3	366.9	359.09
HEBP1	heme binding protein 1	347.9	370.2	359.04
SSH2	slingshot protein phosphatase 2	380.1	337.8	358.96
TEKT1	tektin 1	382.5	335.4	358.96
CCM2L	cerebral cavernous malformation 2-like	402.9	314.9	358.93
DENND6B	DENN/MADD domain containing 6B	358.1	359.4	358.71
MAPK11	mitogen-activated protein kinase 11	311.7	405.3	358.51
NOTUM	notum pectinacetylesterase homolog (Drosophila)	334.3	382.3	358.33
CASP10	caspase 10, apoptosis-related cysteine peptidase	347.9	368.7	358.33
RACGAP1	Rac GTPase activating protein 1	396.8	319.8	358.30
ALKBH4	alkB, alkylation repair homolog 4 (E. coli)	356.4	359.9	358.12
MYL4	myosin, light chain 4, alkali; atrial, embryonic	389.3	326.4	357.85
RNF138	ring finger protein 138, E3 ubiquitin protein ligase	342.9	372.7	357.80
LOC104914605	importin subunit beta-1	403.9	311.6	357.75
NACAD	NAC alpha domain containing	337.2	378.0	357.57
LOC104913773	uncharacterized LOC104913773	354.6	360.1	357.39
SHPK	sedoheptulokinase	340.4	374.2	357.31
NSL1	NSL1, MIS12 kinetochore complex component	380.8	333.5	357.15
ABCA2	ATP-binding cassette, sub-family A (ABC1), member 2	384.5	329.8	357.13
LOC104915831	choline transporter-like protein 2	334.7	379.1	356.92
SLC4A7	solute carrier family 4, sodium bicarbonate cotransporter, member 7	350.5	363.3	356.89
FBXW8	F-box and WD repeat domain containing 8	384.2	329.6	356.89
POLH	polymerase (DNA directed), eta	369.1	344.6	356.87
LOC100544421	zinc finger protein 84-like	406.4	307.0	356.68
LOC100540086	C-terminal-binding protein 2	387.6	325.4	356.50
DSE	dermatan sulfate epimerase	347.1	365.2	356.13
LOC104915814	chromobox protein homolog 5-like	377.6	334.6	356.13
TIAM1	T-cell lymphoma invasion and metastasis 1	348.1	363.8	355.95
PGGT1B	protein geranylgeranyltransferase type I, beta subunit	346.2	365.5	355.84
ANKRD12	ankyrin repeat domain 12	326.0	385.6	355.80
PMVK	phosphomevalonate kinase	372.3	339.2	355.76
SETX	senataxin	325.1	386.1	355.64
MXD4	MAX dimerization protein 4	358.1	353.2	355.63
MACROD2	MACRO domain containing 2	367.3	343.8	355.55
SMIM8	small integral membrane protein 8	355.5	355.5	355.52
UIMC1	ubiquitin interaction motif containing 1	377.5	333.5	355.48
LOC104910044	zinc finger protein 91-like	348.8	362.0	355.43
ACTL6A	actin-like 6A	342.9	367.8	355.34
PSTK	phosphoseryl-tRNA kinase	354.8	355.8	355.26
PPP2R3C	protein phosphatase 2, regulatory subunit B'', gamma	354.7	355.7	355.21
ZBTB44	zinc finger and BTB domain containing 44	335.9	374.5	355.20
ZFPM1	zinc finger protein, FOG family member 1	375.3	335.1	355.19
LOC104916475	nucleolar protein 16-like	393.4	316.9	355.15
TLE1	transducin-like enhancer of split 1 (E(sp1) homolog, Drosophila)	341.1	369.1	355.14
LOC100545343	serine/threonine-protein kinase TAO3	371.6	338.5	355.08
KIAA0391	KIAA0391 ortholog	364.7	345.0	354.86

BNIP1	BCL2/adenovirus E1B 19kDa interacting protein 1	353.0	356.6	354.80
KCNMA1	potassium large conductance calcium-activated channel, subfamily M, alpha member 1	309.1	400.1	354.62
NAA35	N(alpha)-acetyltransferase 35, NatC auxiliary subunit	343.0	365.6	354.31
MED4	mediator complex subunit 4	345.4	362.8	354.08
BST1	bone marrow stromal cell antigen 1	353.0	355.1	354.02
PSTPIP2	proline-serine-threonine phosphatase interacting protein 2	275.6	432.2	353.91
FAM110B	family with sequence similarity 110, member B	354.7	353.0	353.85
RAB4A	RAB4A, member RAS oncogene family	346.1	361.3	353.66
TCF7L1	transcription factor 7-like 1 (T-cell specific, HMG-box)	363.3	344.0	353.62
ZNF511	zinc finger protein 511	369.9	337.3	353.61
TRMT2B	tRNA methyltransferase 2 homolog B (S. cerevisiae)	364.0	342.8	353.40
LOC104914163	uncharacterized LOC104914163	325.2	381.4	353.28
LOC100540302	Golgi integral membrane protein 4-like	343.8	361.6	352.70
LOC104910686	anthrax toxin receptor 2-like	337.8	367.5	352.67
CASP6	caspase 6, apoptosis-related cysteine peptidase	331.1	374.1	352.59
KHDRBS1	KH domain containing, RNA binding, signal transduction associated 1	350.4	354.6	352.52
DPH5	diphthamide biosynthesis 5	333.6	371.2	352.40
LOC104909517	uncharacterized LOC104909517	369.8	335.0	352.36
LOC104909688	cytochrome c oxidase assembly factor 6 homolog	364.8	339.9	352.34
SCMH1	sex comb on midleg homolog 1 (Drosophila)	371.7	332.4	352.05
GPSM1	G-protein signaling modulator 1	351.4	352.6	352.01
DIMT1	DIM1 dimethyladenosine transferase 1 homolog (S. cerevisiae)	351.4	352.6	351.98
ZBTB7A	zinc finger and BTB domain containing 7A	374.2	329.6	351.90
ATP9B	ATPase, class II, type 9B	360.6	342.9	351.74
SLC25A16	solute carrier family 25 (mitochondrial carrier), member 16	332.7	370.6	351.66
CINP	cyclin-dependent kinase 2 interacting protein	358.0	345.1	351.55
LRRK2	leucine-rich repeat kinase 2	367.4	335.4	351.41
CAPN7	calpain 7	323.5	379.2	351.39
C1H3orf38	chromosome 1 open reading frame, human C3orf38	348.7	354.0	351.32
LOC104917484	pseudouridylate synthase 7 homolog	356.4	346.2	351.31
DNAJC12	DnaJ (Hsp40) homolog, subfamily C, member 12	302.5	400.1	351.31
LOC104915971	signal transducer and transcription activator 6-like	406.5	295.3	350.89
MCEE	methylmalonyl CoA epimerase	338.6	363.0	350.79
TAF1B	TATA box binding protein (TBP)-associated factor, RNA polymerase I, B, 63kDa	381.7	319.9	350.79
NHLRC1	NHL repeat containing E3 ubiquitin protein ligase 1	380.0	321.2	350.59
C5H15orf57	chromosome 5 open reading frame, human C15orf57	348.7	352.4	350.53
SPATA20	spermatogenesis associated 20	389.4	311.5	350.47
ZBTB43	zinc finger and BTB domain containing 43	337.7	362.9	350.30
DUSP22	dual specificity phosphatase 22	319.3	381.1	350.22
LOC104913121	E3 ubiquitin-protein ligase RNF123-like	389.4	310.9	350.15
LOC104911011	uncharacterized LOC104911011	383.4	316.8	350.10
LOC104909453	5'-3' exoribonuclease 2-like	354.8	344.8	349.82
LOC100548332	elongator complex protein 2-like	347.9	351.7	349.80
PHF10	PHD finger protein 10	342.0	357.6	349.80
LOC104910938	palmitoyltransferase ZDHHC13-like	372.3	327.2	349.77
POLL	polymerase (DNA directed), lambda	373.2	326.1	349.67
PICK1	protein interacting with PRKCA 1	363.2	335.7	349.44
SSFA2	sperm specific antigen 2	353.7	345.1	349.43
TMEFF2	transmembrane protein with EGF-like and two follistatin-like domains 2	333.7	364.9	349.30
LOC104911429	protein FAM171A1-like	368.1	330.1	349.10
SMPDL3B	sphingomyelin phosphodiesterase, acid-like 3B	342.9	355.3	349.07
ANKZF1	ankyrin repeat and zinc finger domain containing 1	359.8	337.7	348.76
FLAD1	flavin adenine dinucleotide synthetase 1	342.9	354.5	348.74
PARP12	poly (ADP-ribose) polymerase family, member 12	356.4	341.1	348.74

FAM193A	family with sequence similarity 193, member A	336.1	361.1	348.61
RAD18	RAD18 E3 ubiquitin protein ligase	384.1	313.0	348.52
KATNA1	katanin p60 (ATPase containing) subunit A 1	358.0	338.9	348.44
PTPRQ	protein tyrosine phosphatase, receptor type, Q	350.4	346.5	348.43
ARMC9	armadillo repeat containing 9	369.8	327.0	348.43
LOC104912029	uncharacterized LOC104912029	322.6	374.2	348.43
MKLN1	muskelin 1, intracellular mediator containing kelch motifs	324.4	372.4	348.38
SDCCAG3	serologically defined colon cancer antigen 3	350.6	346.0	348.30
PLEKHG3	pleckstrin homology domain containing, family G (with RhoGef domain) member 3	374.3	322.2	348.25
LNX2	ligand of numb-protein X 2	361.4	334.9	348.16
LOC104917028	3 beta-hydroxysteroid dehydrogenase type 7-like	356.3	339.9	348.12
DPH7	diphthamide biosynthesis 7	356.4	339.5	347.99
ROGDI	rogdi homolog (Drosophila)	377.4	318.6	347.98
PRSS12	protease, serine, 12 (neurotrypsin, motopsin)	324.4	371.4	347.88
LOC104913831	5-hydroxytryptamine receptor 3A-like	335.3	360.4	347.87
CSRNP1	cysteine-serine-rich nuclear protein 1	332.7	362.3	347.49
LOC104915043	uncharacterized LOC104915043	352.0	342.5	347.25
PRADC1	protease-associated domain containing 1	333.7	360.7	347.20
LOC104913753	uncharacterized LOC104913753	298.3	396.0	347.13
RP9	retinitis pigmentosa 9 (autosomal dominant)	381.5	312.7	347.07
ZNF362	zinc finger protein 362	386.1	308.0	347.05
BEND6	BEN domain containing 6	305.8	388.3	347.01
AK3	adenylate kinase 3	335.3	358.7	346.98
WDR86	WD repeat domain 86	334.5	359.4	346.93
OTUD1	OTU deubiquitinase 1	328.5	364.3	346.42
SNUPN	snurportin 1	368.9	323.8	346.39
SARS2	seryl-tRNA synthetase 2, mitochondrial	356.4	336.3	346.35
TMEM241	transmembrane protein 241	367.3	325.3	346.31
TBL1XR1	transducin (beta)-like 1 X-linked receptor 1	351.3	341.2	346.25
ZCCHC7	zinc finger, CCHC domain containing 7	344.6	347.8	346.22
LOC100546547	low density lipoprotein receptor adapter protein 1-like	365.6	326.8	346.19
MYD88	myeloid differentiation primary response 88	328.4	363.9	346.15
CLP1	cleavage and polyadenylation factor I subunit 1	353.8	338.3	346.10
NR4A3	nuclear receptor subfamily 4, group A, member 3	348.0	344.1	346.07
LOC104910027	uncharacterized LOC104910027	338.6	353.4	346.03
MYBL1	v-myb avian myeloblastosis viral oncogene homolog-like 1	322.6	369.1	345.84
AEBP2	AE binding protein 2	336.0	355.3	345.63
ANKRD16	ankyrin repeat domain 16	346.2	344.9	345.59
LOC104915026	uncharacterized LOC104915026	340.3	350.6	345.45
NUFIP2	nuclear fragile X mental retardation protein interacting protein 2	324.4	366.4	345.38
SCAI	suppressor of cancer cell invasion	344.6	345.7	345.11
LOC100539719	AP-4 complex subunit epsilon-1	321.9	367.7	344.81
LOC104916937	autophagy-related protein 101-like	368.2	321.3	344.74
ANAPC2	anaphase promoting complex subunit 2	371.5	317.9	344.70
DUSP5	dual specificity phosphatase 5	345.4	343.9	344.63
LOC104912780	thrombospondin type-1 domain-containing protein 4-like	297.3	391.5	344.43
RPP40	ribonuclease P/MRP 40kDa subunit	369.8	319.0	344.42
PCDH18	protocadherin 18	357.2	331.2	344.20
PPP1R8	protein phosphatase 1, regulatory subunit 8	370.7	317.7	344.19
KIAA0907	KIAA0907 ortholog	337.1	351.2	344.17
LOC104912952	non-canonical poly(A) RNA polymerase PAPD5-like	306.6	381.7	344.14
NSMF	NMDA receptor synaptonuclear signaling and neuronal migration factor	342.0	346.1	344.05
INO80C	INO80 complex subunit C	316.8	370.9	343.82
GLE1	GLE1 RNA export mediator	328.5	359.1	343.78
REST	RE1-silencing transcription factor	325.2	362.3	343.76

SERPINF2	serpin peptidase inhibitor, clade F (alpha-2 antiplasmin, pigment epithelium derived factor), member 2	344.5	342.8	343.67
DONSON	downstream neighbor of SON	360.5	326.7	343.60
CHL1	cell adhesion molecule L1-like	353.6	333.5	343.57
LOC104915921	integrin alpha-3-like	394.6	292.5	343.53
ENTPD7	ectonucleoside triphosphate diphosphohydrolase 7	347.2	339.8	343.47
LOC100539010	uncharacterized LOC100539010	340.3	346.5	343.40
TARBP2	TAR (HIV-1) RNA binding protein 2	378.4	308.4	343.39
ARHGAP32	Rho GTPase activating protein 32	315.2	371.5	343.34
QRSL1	glutaminyl-tRNA synthase (glutamine-hydrolyzing)-like 1	351.2	335.4	343.27
MUL1	mitochondrial E3 ubiquitin protein ligase 1	371.7	314.9	343.27
LOC100543500	uncharacterized protein C2orf54-like	348.0	337.6	342.81
SCLT1	sodium channel and clathrin linker 1	320.1	365.4	342.74
SYPL2	synaptophysin-like 2	352.9	332.5	342.69
HRSP12	heat-responsive protein 12	333.6	351.3	342.43
TRAPPC2L	trafficking protein particle complex 2-like	360.5	324.2	342.35
ZNF277	zinc finger protein 277	318.3	366.4	342.35
ACCS	1-aminocyclopropane-1-carboxylate synthase homolog (Arabidopsis)(non-functional)	340.4	344.3	342.35
LOC100539001	uncharacterized protein F13E9.13, mitochondrial-like	305.5	378.8	342.15
ABHD1	abhydrolase domain containing 1	348.9	335.2	342.04
NOP14	NOP14 nucleolar protein	333.6	350.2	341.92
HABP4	hyaluronan binding protein 4	341.1	342.5	341.79
TMEM65	transmembrane protein 65	334.3	349.1	341.72
LOC100538555	zinc finger SWIM domain-containing protein 6	320.9	362.4	341.65
LOC104912766	E3 ubiquitin-protein ligase NEDD4-like	330.1	353.1	341.63
LOC104912939	borealin-2	336.9	346.3	341.57
CDC45	cell division cycle 45	352.1	330.8	341.48
AP3S1	adaptor-related protein complex 3, sigma 1 subunit	320.1	362.4	341.24
BRWD3	bromodomain and WD repeat domain containing 3	299.1	383.3	341.21
ADI1	acireductone dioxygenase 1	363.0	319.2	341.12
LOC100548147	centrosomal protein of 63 kDa-like	358.0	323.6	340.80
HSPB11	heat shock protein family B (small), member 11	312.5	368.0	340.21
LSM8	LSM8 homolog, U6 small nuclear RNA associated (S. cerevisiae)	350.4	329.9	340.14
SOX8	SRY (sex determining region Y)-box 8	329.4	350.8	340.10
LOC100538779	uncharacterized LOC100538779	336.0	344.0	339.99
CTU2	cytosolic thiouridylase subunit 2 homolog (S. pombe)	370.7	309.2	339.97
DROSHA	drosha, ribonuclease type III	315.8	363.9	339.87
LOC104909213	delta(3,5)-Delta(2,4)-dienoyl-CoA isomerase, mitochondrial-like	377.4	302.2	339.80
TSPAN6	tetraspanin 6	350.5	328.9	339.71
C1H11orf30	chromosome 1 open reading frame, human C11orf30	336.1	343.2	339.65
KLB	klotho beta	348.6	330.4	339.52
AVL9	AVL9 homolog (S. cerevisiase)	317.6	360.8	339.23
TUBD1	tubulin, delta 1	336.1	341.9	338.97
NCOA1	nuclear receptor coactivator 1	347.3	330.6	338.96
EXD3	exonuclease 3'-5' domain containing 3	353.1	324.6	338.85
LOC104915348	uncharacterized LOC104915348	365.6	311.5	338.56
NAPG	N-ethylmaleimide-sensitive factor attachment protein, gamma	333.4	343.4	338.43
LOC104912085	uncharacterized LOC104912085	346.4	330.3	338.40
DNAJC25	DnaJ (Hsp40) homolog, subfamily C , member 25	326.0	350.5	338.24
GGA2	golgi-associated, gamma adaptin ear containing, ARF binding protein 2	350.6	325.6	338.06
TSPAN10	tetraspanin 10	297.5	378.6	338.03
HARBI1	harbinger transposase derived 1	365.7	309.8	337.72
SMPD2	sphingomyelin phosphodiesterase 2, neutral membrane (neutral sphingomyelinase)	359.0	316.2	337.61
ITM2C	integral membrane protein 2C	334.4	340.5	337.45
HMMR	hyaluronan-mediated motility receptor (RHAMM)	352.0	322.5	337.27

TBC1D25	TBC1 domain family, member 25	369.3	305.2	337.23
TRABD	TraB domain containing	315.8	358.4	337.09
LOC100551066	furin-like	371.9	301.7	336.82
TAMM41	TAM41, mitochondrial translocator assembly and maintenance protein, homolog (S. cerevisiae)	319.2	354.2	336.73
GTF3C4	general transcription factor IIIC, polypeptide 4, 90kDa	336.2	337.1	336.66
MSANTD2	Myb/SANT-like DNA-binding domain containing 2	336.1	337.0	336.57
AQR	aquarius intron-binding spliceosomal factor	339.4	333.4	336.43
SKIL	SKI-like proto-oncogene	314.2	358.4	336.27
PITPNM2	phosphatidylinositol transfer protein, membrane-associated 2	327.8	344.7	336.25
LOC104914933	protein LAP2-like	304.9	367.6	336.25
MSH2	mutS homolog 2	348.7	323.7	336.16
VANGL2	VANGL planar cell polarity protein 2	323.6	348.5	336.06
LOC104911308	gastrula zinc finger protein XlCGF67.1-like	329.5	342.6	336.04
LOC104915368	zinc finger X-chromosomal protein	313.3	357.6	335.44
LZTFL1	leucine zipper transcription factor-like 1	356.2	314.6	335.41
IQUB	IQ motif and ubiquitin domain containing	365.5	305.0	335.25
LPXN	leupaxin	334.6	335.8	335.20
FASTKD2	FAST kinase domains 2	347.8	322.4	335.09
LOC104910551	uncharacterized LOC104910551	323.7	346.1	334.88
NPHP1	nephronophthisis 1 (juvenile)	319.3	350.4	334.82
LOC104914175	uncharacterized LOC104914175	352.3	317.3	334.76
LOC100549421	gap junction gamma-1 protein-like	345.4	323.7	334.56
LOC104913140	alpha/beta hydrolase domain-containing protein 14A-like	353.9	314.8	334.38
TADA2A	transcriptional adaptor 2A	334.4	334.2	334.29
ASB3	ankyrin repeat and SOCS box containing 3	326.1	342.4	334.25
LOC104915897	LIX1-like protein	364.8	302.3	333.56
KLHL22	kelch-like family member 22	316.7	349.5	333.08
C1H21orf91	chromosome 1 open reading frame, human C21orf91	346.2	319.9	333.06
LUZP1	leucine zipper protein 1	337.2	328.8	333.01
EGLN1	egl-9 family hypoxia-inducible factor 1	316.7	349.2	332.97
C20H17orf80	chromosome 20 open reading frame, human C17orf80	335.3	330.6	332.96
LOC104913104	atrophin-1-like	310.1	355.8	332.94
ETS2	v-ets avian erythroblastosis virus E26 oncogene homolog 2	304.1	361.7	332.91
LOC104913028	condensin complex subunit 1-like	354.7	310.7	332.68
ZDHHC17	zinc finger, DHHC-type containing 17	321.7	343.0	332.36
VSTM4	V-set and transmembrane domain containing 4	301.6	362.7	332.17
TTC21B	tetratricopeptide repeat domain 21B	317.4	346.7	332.05
ZNF532	zinc finger protein 532	306.7	357.4	332.04
LOC104916440	nuclear factor 1 C-type-like	312.5	351.5	331.98
CMPK1	cytidine monophosphate (UMP-CMP) kinase 1, cytosolic	320.9	343.0	331.93
RYBP	RING1 and YY1 binding protein	311.7	352.1	331.90
GOLGB1	golgin B1	317.7	345.9	331.79
C6H7orf25	chromosome 6 open reading frame, human C7orf25	336.1	327.4	331.71
NID1	nidogen 1	296.3	367.0	331.67
LOC104916276	neurabin-2-like	360.8	302.4	331.59
CDKAL1	CDK5 regulatory subunit associated protein 1-like 1	341.0	322.1	331.54
LOC100539943	protein LAP2-like	309.2	353.8	331.52
JAM2	junctional adhesion molecule 2	328.4	334.4	331.40
TMEM87A	transmembrane protein 87A	310.0	352.4	331.19
POT1	protection of telomeres 1	309.0	353.1	331.04
TTK	TTK protein kinase	346.2	315.6	330.90
LOC104911919	ankyrin-3-like	338.7	323.1	330.88
LOC100541666	caspase-8-like	341.1	320.6	330.84
TCTN2	tectonic family member 2	348.8	312.4	330.62
PTDSS1	phosphatidylserine synthase 1	330.3	330.8	330.52
RHOJ	ras homolog family member J	330.3	330.2	330.23
FAM222B	family with sequence similarity 222, member B	332.0	328.3	330.16

LOC104917218	uncharacterized LOC104917218	352.2	307.9	330.05
CEP104	centrosomal protein 104kDa	344.6	315.4	329.99
RPS6KL1	ribosomal protein S6 kinase-like 1	311.7	348.1	329.89
TBX15	T-box 15	333.7	326.1	329.87
LOC100544585	ankyrin repeat domain-containing protein 26-like	302.5	357.2	329.84
ZNFX1	zinc finger, NFX1-type containing 1	331.8	327.7	329.76
LOC104913015	chromodomain-helicase-DNA-binding protein 9-like	303.1	356.2	329.66
ATRX	alpha thalassemia/mental retardation syndrome X-linked	309.1	350.1	329.64
REPS1	RALBP1 associated Eps domain containing 1	298.2	360.5	329.37
NCOA7	nuclear receptor coactivator 7	318.4	339.9	329.13
TAF8	TAF8 RNA polymerase II, TATA box binding protein (TBP)-associated factor, 43kDa	345.2	312.9	329.02
MSH4	mutS homolog 4	313.3	344.5	328.91
KIF15	kinesin family member 15	352.0	305.3	328.67
RARS2	arginyl-tRNA synthetase 2, mitochondrial	346.3	310.9	328.60
CAT	catalase	299.7	357.4	328.58
LOC104911732	uncharacterized LOC104911732	325.0	331.4	328.18
WWC1	WW and C2 domain containing 1	343.7	312.6	328.17
MID1IP1	MID1 interacting protein 1	349.6	305.6	327.59
DUS3L	dihydrouridine synthase 3-like (S. cerevisiae)	337.1	317.7	327.36
LOC104914815	SH2 domain-containing protein 2A-like	303.4	351.2	327.31
KIAA1551	KIAA1551 ortholog	320.9	333.4	327.14
FAM107B	family with sequence similarity 107, member B	291.4	362.9	327.14
C16H7orf26	chromosome 16 open reading frame, human C7orf26	315.9	337.5	326.69
CC2D2A	coiled-coil and C2 domain containing 2A	349.6	303.8	326.68
SPAG9	sperm associated antigen 9	318.4	334.0	326.22
HPS4	Hermansky-Pudlak syndrome 4	336.2	316.1	326.11
KBTBD8	kelch repeat and BTB (POZ) domain containing 8	327.6	324.3	325.93
TRMT61B	tRNA methyltransferase 61B	300.0	351.0	325.51
LOC104915307	E3 ubiquitin-protein ligase Topors-like	317.5	333.5	325.48
LOC100549177	UPF0505 protein C16orf62-like	325.2	325.0	325.09
AHR	aryl hydrocarbon receptor	303.9	346.0	324.96
GCSH	glycine cleavage system protein H (aminomethyl carrier)	347.8	302.1	324.93
RAD51C	RAD51 paralog C	326.8	322.4	324.58
SCD5	stearoyl-CoA desaturase 5	310.8	338.0	324.41
PIGF	phosphatidylinositol glycan anchor biosynthesis, class F	307.4	341.3	324.37
AGPAT9	1-acylglycerol-3-phosphate O-acyltransferase 9	328.5	320.0	324.25
LOC100540196	beta-galactosidase-1-like protein 2	318.4	329.9	324.17
PGAP2	post-GPI attachment to proteins 2	328.5	319.7	324.12
SLC25A17	solute carrier family 25 (mitochondrial carrier; peroxisomal membrane protein, 34kDa), member 17	334.4	313.7	324.04
LOC104910335	DNA-dependent protein kinase catalytic subunit-like	332.6	315.2	323.89
CLDN12	claudin 12	321.7	326.0	323.82
RMND5A	required for meiotic nuclear division 5 homolog A (S. cerevisiae)	298.1	349.5	323.77
CLEC3B	C-type lectin domain family 3, member B	282.2	365.3	323.77
TAB1	TGF-beta activated kinase 1/MAP3K7 binding protein 1	323.4	324.0	323.72
DTWD1	DTW domain containing 1	302.4	345.0	323.67
LOC100539868	ras-specific guanine nucleotide-releasing factor RalGPS1-like	295.7	351.3	323.49
USP44	ubiquitin specific peptidase 44	317.5	329.4	323.49
LOC100549918	CSC1-like protein 2	347.4	299.5	323.45
ALG2	ALG2, alpha-1,3/1,6-mannosyltransferase	304.0	342.9	323.44
LOC100546418	ubiquitin-like modifier-activating enzyme 1	333.0	313.7	323.35
LOC100539619	unconventional prefoldin RPB5 interactor-like	309.2	337.4	323.29
ACOT7	acyl-CoA thioesterase 7	345.4	300.5	322.95
FICD	FIC domain containing	331.9	313.6	322.74
TJP3	tight junction protein 3	339.7	304.5	322.14
LRPAP1	low density lipoprotein receptor-related protein associated protein 1	293.1	350.8	321.94

DET1	de-etiolated homolog 1 (Arabidopsis)	299.0	344.7	321.84
WHSC1L1	Wolf-Hirschhorn syndrome candidate 1-like 1	306.6	336.8	321.71
SERPING1	serpin peptidase inhibitor, clade G (C1 inhibitor), member 1	344.7	298.4	321.59
LOC104911859	inactive dipeptidyl peptidase 10-like	289.0	354.0	321.54
MPP1	membrane protein, palmitoylated 1, 55kDa	329.4	312.9	321.13
TTF2	transcription termination factor, RNA polymerase II	320.1	321.9	320.97
SORD	sorbitol dehydrogenase	335.2	306.6	320.94
NKAIN4	Na+/K+ transporting ATPase interacting 4	310.8	330.4	320.59
DCPS	decapping enzyme, scavenger	328.5	312.7	320.58
LIMCH1	LIM and calponin homology domains 1	316.8	323.7	320.25
NCOR1	nuclear receptor corepressor 1	289.7	350.6	320.14
TRMT6	tRNA methyltransferase 6	309.1	330.9	320.01
TEX10	testis expressed 10	313.3	325.9	319.60
LOC104913466	chitobiosyldiphosphodolichol beta-mannosyltransferase-like	333.4	305.6	319.51
SNTA1	syntrophin, alpha 1	343.9	295.1	319.49
LOC100548193	RNA-binding motif, single-stranded-interacting protein 1-like	284.6	354.1	319.39
ERI3	ERI1 exoribonuclease family member 3	325.2	313.6	319.38
LOC104911688	uncharacterized LOC104911688	349.6	289.1	319.34
RXFP1	relaxin/insulin-like family peptide receptor 1	326.7	311.9	319.30
FARP2	FERM, RhoGEF and pleckstrin domain protein 2	313.4	325.1	319.26
RAD54L2	RAD54-like 2 (S. cerevisiae)	360.0	278.3	319.13
LOC104913903	DDB1- and CUL4-associated factor 6-like	313.3	325.0	319.13
LOC104909858	reticulon-4-interacting protein 1, mitochondrial-like	336.9	301.4	319.12
BCL9	B-cell CLL/lymphoma 9	322.7	315.4	319.05
LOC100543301	bifunctional 3'-phosphoadenosine 5'-phosphosulfate synthase 1	315.1	321.9	318.53
FSBP	fibrinogen silencer binding protein	284.7	352.4	318.53
PDXP	pyridoxal (pyridoxine, vitamin B6) phosphatase	295.6	341.1	318.36
ACE	angiotensin I converting enzyme	329.4	307.3	318.36
METTL8	methyltransferase like 8	316.7	320.0	318.33
LOC100542217	cysteine string protein-like	321.8	314.9	318.33
NABP2	nucleic acid binding protein 2	332.2	304.4	318.27
C8H10orf32	chromosome 8 open reading frame, human C10orf32	271.1	365.4	318.24
LOC104916071	uncharacterized LOC104916071	306.6	329.8	318.21
LOC100546653	uncharacterized LOC100546653	286.2	350.0	318.07
AGPAT3	1-acylglycerol-3-phosphate O-acyltransferase 3	315.9	320.2	318.03
ISM2	isthmin 2	299.2	336.5	317.85
NDUFAF6	NADH dehydrogenase (ubiquinone) complex I, assembly factor 6	305.8	329.9	317.84
TMEM62	transmembrane protein 62	316.7	318.9	317.77
LOC100547610	peptidyl-prolyl cis-trans isomerase-like 1	352.1	283.4	317.76
URB2	URB2 ribosome biogenesis 2 homolog (S. cerevisiae)	330.2	304.7	317.42
CZH18orf25	chromosome Z open reading frame, human C18orf25	270.4	364.4	317.37
LOC100545121	torsin-1A-interacting protein 2-like	321.8	312.4	317.12
LSM14B	LSM14B, SCD6 homolog B (S. cerevisiae)	309.0	324.8	316.87
PPAP2B	phosphatidic acid phosphatase type 2B	299.0	334.3	316.65
ABHD11	abhydrolase domain containing 11	288.1	344.7	316.43
AKIP1	A kinase (PRKA) interacting protein 1	325.9	306.8	316.38
LOC100546341	double-stranded RNA-binding protein Staufen homolog 2-like	332.7	299.9	316.27
ALAD	aminolevulinate dehydratase	353.1	279.0	316.08
LOC104914264	uncharacterized LOC104914264	321.0	311.0	316.01
LOC104916888	ribosome biogenesis protein bop1-like	339.6	292.3	315.94
TIMM13	translocase of inner mitochondrial membrane 13 homolog (yeast)	299.8	332.0	315.87
MAP3K2	mitogen-activated protein kinase kinase kinase 2	313.3	318.1	315.68
SH3GL1	SH3-domain GRB2-like 1	325.3	305.5	315.36
CTDSP1	CTD (carboxy-terminal domain, RNA polymerase II, polypeptide A) small phosphatase 1	342.1	288.5	315.31
EME2	essential meiotic structure-specific endonuclease subunit 2	309.1	320.9	315.03
CDKL5	cyclin-dependent kinase-like 5	297.2	332.5	314.88
ANO1	anoctamin 1, calcium activated chloride channel	351.1	278.6	314.86

TMEM233	transmembrane protein 233	293.9	335.3	314.60
LOC100538487	TBC1 domain family member 2B-like	305.7	323.4	314.57
MUT	methylmalonyl CoA mutase	315.0	314.1	314.56
ODF2	outer dense fiber of sperm tails 2	316.0	313.1	314.54
WTIP	Wilms tumor 1 interacting protein	308.3	320.7	314.50
CZH5orf28	chromosome Z open reading frame, human C5orf28	326.7	301.9	314.31
SLC24A5	solute carrier family 24 (sodium/potassium/calcium exchanger), member 5	293.8	334.0	313.92
LOC104909916	uncharacterized LOC104909916	309.8	317.9	313.86
LOC100551380	RUN and FYVE domain-containing protein 1-like	320.8	306.9	313.82
CDK5RAP2	CDK5 regulatory subunit associated protein 2	311.6	315.3	313.47
C5H14orf169	chromosome 5 open reading frame, human C14orf169	327.6	298.9	313.25
ALG5	ALG5, dolichyl-phosphate beta-glucosyltransferase	312.4	314.1	313.23
LOC104914155	uncharacterized LOC104914155	323.4	302.9	313.11
CRADD	CASP2 and RIPK1 domain containing adaptor with death domain	337.6	288.6	313.11
DENND3	DENN/MADD domain containing 3	300.7	325.5	313.10
FAAH2	fatty acid amide hydrolase 2	328.6	297.3	312.98
SCG3	secretogranin III	310.7	314.9	312.80
PLEKHM3	pleckstrin homology domain containing, family M, member 3	329.2	296.2	312.73
MSMP	microseminoprotein, prostate associated	297.3	328.0	312.67
FAM173A	family with sequence similarity 173, member A	312.4	311.6	312.00
LOC104915290	hydroxysteroid dehydrogenase-like protein 2	305.7	318.1	311.92
GAS2	growth arrest-specific 2	295.7	328.1	311.89
LIN52	lin-52 DREAM MuvB core complex component	309.9	313.6	311.77
MKS1	Meckel syndrome, type 1	326.9	296.7	311.77
DEAF1	DEAF1 transcription factor	300.7	322.8	311.76
ESPL1	extra spindle pole bodies homolog 1 (S. cerevisiae)	355.6	267.5	311.58
PLEKHF2	pleckstrin homology domain containing, family F (with FYVE domain) member 2	299.7	323.1	311.38
LOC104912546	putative glycerol kinase 5	294.8	327.9	311.35
LRP2BP	LRP2 binding protein	304.9	317.7	311.30
KIAA0586	KIAA0586 ortholog	278.1	344.5	311.29
F2RL2	coagulation factor II (thrombin) receptor-like 2	297.3	325.2	311.26
LOC104915225	zinc finger protein 462-like	304.8	317.5	311.13
LOC104912724	uncharacterized LOC104912724	318.5	303.6	311.09
SLC2A6	solute carrier family 2 (facilitated glucose transporter), member 6	303.5	318.7	311.09
LMF1	lipase maturation factor 1	320.9	300.8	310.85
LOC100541213	interferon-induced guanylate-binding protein 1-like	318.5	303.2	310.83
LOC100544291	proactivator polypeptide-like	298.4	323.3	310.82
CDC26	cell division cycle 26	321.8	299.6	310.67
HTR2B	5-hydroxytryptamine (serotonin) receptor 2B, G protein-coupled	329.3	291.1	310.16
LOC104914612	uncharacterized LOC104914612	304.1	316.1	310.10
LOC104915992	DNA-directed RNA polymerase II subunit RPB1	303.4	316.3	309.87
WDR13	WD repeat domain 13	331.1	288.3	309.73
PRDM15	PR domain containing 15	294.8	324.2	309.50
LOC100546933	phosphatidylinositol 4,5-bisphosphate 3-kinase catalytic subunit gamma isoform-like	269.4	349.6	309.49
FBXL8	F-box and leucine-rich repeat protein 8	309.9	309.1	309.47
GLS	glutaminase	296.5	322.4	309.46
POLR3F	polymerase (RNA) III (DNA directed) polypeptide F, 39 kDa	319.3	299.2	309.26
IL10RB	interleukin 10 receptor, beta	293.9	324.4	309.18
STEAP3	STEAP family member 3, metalloreductase	324.4	293.8	309.09
TMEM116	transmembrane protein 116	286.4	331.4	308.88
FBXL18	F-box and leucine-rich repeat protein 18	310.9	306.8	308.85
LRRC17	leucine rich repeat containing 17	350.2	266.7	308.46
MFSD9	major facilitator superfamily domain containing 9	320.0	296.8	308.41
SLC26A8	solute carrier family 26 (anion exchanger), member 8	298.2	317.5	307.82
TTC7B	tetratricopeptide repeat domain 7B	320.9	294.7	307.80

COL27A1	collagen, type XXVII, alpha 1	308.5	307.0	307.75
LOC100546887	putative zinc finger protein 724	294.9	320.5	307.72
LOC104909939	M-protein, striated muscle-like	349.3	266.1	307.70
WBP1L	WW domain binding protein 1-like	304.9	310.4	307.68
ABCC10	ATP-binding cassette, sub-family C (CFTR/MRP), member 10	321.0	293.9	307.44
CAPN10	calpain 10	299.0	315.6	307.29
DOCK11	dedicator of cytokinesis 11	317.5	296.9	307.16
MIER3	mesoderm induction early response 1, family member 3	284.6	329.4	306.96
ZNF346	zinc finger protein 346	325.9	287.7	306.82
LOC104916841	RAC-beta serine/threonine-protein kinase-like	303.3	310.0	306.69
NFKBIE	nuclear factor of kappa light polypeptide gene enhancer in B-cells inhibitor, epsilon	322.6	290.6	306.61
KIAA1671	KIAA1671 ortholog	284.6	328.5	306.57
OXNAD1	oxidoreductase NAD-binding domain containing 1	297.3	315.6	306.48
ABRACL	ABRA C-terminal like	300.6	312.2	306.41
INHBA	inhibin, beta A	279.7	333.0	306.37
ORMDL1	ORMDL sphingolipid biosynthesis regulator 1	316.8	295.8	306.27
SAMD11	sterile alpha motif domain containing 11	284.0	328.3	306.15
MECR	mitochondrial trans-2-enoyl-CoA reductase	295.6	316.7	306.13
CCDC125	coiled-coil domain containing 125	314.2	297.8	305.99
UNG	uracil-DNA glycosylase	331.0	280.0	305.50
LRRC32	leucine rich repeat containing 32	267.0	343.6	305.31
BCKDHB	branched chain keto acid dehydrogenase E1, beta polypeptide	298.0	312.3	305.14
MEX3D	mex-3 RNA binding family member D	305.7	304.1	304.91
CCBL2	cysteine conjugate-beta lyase 2	317.4	292.4	304.90
LOC104913658	ectonucleoside triphosphate diphosphohydrolase 2-like	271.3	338.5	304.89
AS3MT	arsenite methyltransferase	317.5	292.3	304.88
LOC104909761	tubby-related protein 4-like	282.9	326.6	304.77
LOC104915560	protein SMG5-like	333.6	275.8	304.70
ADD3	adducin 3 (gamma)	292.1	317.3	304.69
AMT	aminomethyltransferase	306.5	302.8	304.68
LOC104910693	teneurin-3	281.3	328.0	304.66
LOC100549224	HEAT repeat-containing protein 3	303.2	305.7	304.46
TXNDC16	thioredoxin domain containing 16	295.7	313.2	304.44
C10H1orf52	chromosome 10 open reading frame, human C1orf52	292.9	315.9	304.40
TIGD3	tigger transposable element derived 3	329.4	279.2	304.28
C5H14orf159	chromosome 5 open reading frame, human C14orf159	325.9	282.6	304.22
FAM76B	family with sequence similarity 76, member B	286.3	321.9	304.08
CMTR2	cap methyltransferase 2	292.2	315.7	303.93
SKIV2L	superkiller viralicidic activity 2-like (S. cerevisiae)	369.2	238.4	303.84
MAF1	MAF1 homolog (S. cerevisiae)	362.6	245.0	303.79
ST6GAL2	ST6 beta-galactosamide alpha-2,6-sialyltranferase 2	391.3	215.1	303.18
LOC104917413	uncharacterized LOC104917413	341.3	265.0	303.17
TMEM200B	transmembrane protein 200B	306.6	299.6	303.08
RHOBTB2	Rho-related BTB domain containing 2	342.1	264.0	303.06
PIP4K2B	phosphatidylinositol-5-phosphate 4-kinase, type II, beta	311.8	294.1	302.97
ARHGDIG	Rho GDP dissociation inhibitor (GDI) gamma	320.9	285.0	302.96
LOC104911649	muskelin-like	305.6	299.8	302.73
SARNP	SAP domain containing ribonucleoprotein	295.5	309.4	302.46
CACNG5	calcium channel, voltage-dependent, gamma subunit 5	285.6	319.3	302.45
MZT1	mitotic spindle organizing protein 1	300.5	304.3	302.43
SLC23A1	solute carrier family 23 (ascorbic acid transporter), member 1	291.2	313.2	302.24
E2F6	E2F transcription factor 6	295.6	308.6	302.09
FAM73B	family with sequence similarity 73, member B	280.5	323.4	301.93
PDGFRL	platelet-derived growth factor receptor-like	273.6	330.2	301.88
HS3ST5	heparan sulfate (glucosamine) 3-O-sulfotransferase 5	273.8	329.6	301.71
EFNB2	ephrin-B2	294.9	308.5	301.67
LOC100550063	maestro heat-like repeat-containing protein family member 1	349.0	254.0	301.50

LOC104917412	uncharacterized LOC104917412	299.7	303.2	301.46
GATAD1	GATA zinc finger domain containing 1	288.0	314.9	301.42
MAPK8IP1	mitogen-activated protein kinase 8 interacting protein 1	291.5	310.9	301.18
LOC104909205	switch-associated protein 70	307.5	293.9	300.71
FAM98B	family with sequence similarity 98, member B	311.6	289.8	300.70
LOC104916018	uncharacterized LOC104916018	299.1	302.3	300.67
DUSP23	dual specificity phosphatase 23	333.5	267.5	300.51
LOC104909670	HEAT repeat-containing protein 5B-like	305.7	294.9	300.32
ZC3H12C	zinc finger CCCH-type containing 12C	301.6	298.8	300.17
WDR76	WD repeat domain 76	307.5	292.4	299.92
LOC104912498	terminal uridylyltransferase 4-like	304.1	295.3	299.68
VPS37C	vacuolar protein sorting 37 homolog C (S. cerevisiae)	299.1	300.3	299.68
DLK2	delta-like 2 homolog (Drosophila)	290.7	308.2	299.43
TTC39C	tetratricopeptide repeat domain 39C	297.1	301.5	299.34
SNX5	sorting nexin 5	298.0	300.2	299.13
G6PC3	glucose 6 phosphatase, catalytic, 3	329.4	268.6	298.99
ANAPC10	anaphase promoting complex subunit 10	307.3	290.6	298.98
MSX1	msh homeobox 1	286.5	311.0	298.75
LOC104909458	nephrocystin-1-like	282.9	314.4	298.68
LGI2	leucine-rich repeat LGI family, member 2	281.2	316.1	298.66
MARS2	methionyl-tRNA synthetase 2, mitochondrial	306.6	290.3	298.43
ZSWIM7	zinc finger, SWIM-type containing 7	300.6	296.1	298.33
SMC6	structural maintenance of chromosomes 6	295.6	300.7	298.12
B4GALNT3	beta-1,4-N-acetyl-galactosaminyl transferase 3	313.3	282.7	297.96
INTS12	integrator complex subunit 12	266.8	329.1	297.95
LOC104909977	uncharacterized LOC104909977	300.6	295.0	297.80
TRIM13	tripartite motif containing 13	295.5	300.0	297.76
MGAT3	mannosyl (beta-1,4-)-glycoprotein beta-1,4-N-acetylglucosaminyltransferase	273.7	321.6	297.65
DQX1	DEAQ box RNA-dependent ATPase 1	317.6	277.6	297.61
LOC104910606	protein phosphatase 1K, mitochondrial-like	315.9	279.2	297.55
TGM4	transglutaminase 4	306.7	288.3	297.49
STARD5	StAR-related lipid transfer (START) domain containing 5	271.3	323.5	297.43
BCL10	B-cell CLL/lymphoma 10	302.2	292.5	297.32
MTG2	mitochondrial ribosome-associated GTPase 2	288.7	305.9	297.31
LPHN2	latrophilin 2	295.6	298.8	297.18
LOC104911054	uncharacterized LOC104911054	293.8	300.4	297.15
DIP2A	DIP2 disco-interacting protein 2 homolog A (Drosophila)	258.4	335.8	297.14
RAD9B	RAD9 homolog B (S. pombe)	275.3	318.7	297.01
TGFBR2	transforming growth factor, beta receptor II (70/80kDa)	277.1	316.9	296.99
AAMDC	adipogenesis associated, Mth938 domain containing	292.0	301.8	296.93
LOC104910078	tensin-3-like	276.2	317.6	296.93
CBR4	carbonyl reductase 4	303.0	290.8	296.93
LOC104913305	zinc-binding protein A33-like	296.6	297.2	296.90
TMEM80	transmembrane protein 80	281.2	312.4	296.79
ZCCHC4	zinc finger, CCHC domain containing 4	323.4	270.1	296.76
LOC104916016	geranylgeranyl transferase type-2 subunit alpha-like	300.8	292.5	296.65
R3HDM4	R3H domain containing 4	304.0	288.4	296.24
LOC104914676	uncharacterized LOC104914676	299.0	293.4	296.20
ARHGAP23	Rho GTPase activating protein 23	354.2	238.0	296.08
C24H2orf42	chromosome 24 open reading frame, human C2orf42	288.0	304.1	296.05
GIPC1	GIPC PDZ domain containing family, member 1	334.6	256.5	295.57
LOC100545259	zinc finger protein 664-like	284.7	305.3	295.01
LOC104913385	transmembrane channel-like protein 7	299.0	291.0	294.99
LOC100547435	ectonucleoside triphosphate diphosphohydrolase 8-like	281.3	308.4	294.80
EIF2AK3	eukaryotic translation initiation factor 2-alpha kinase 3	290.5	298.8	294.68
LOC100544335	protein furry homolog-like	256.8	332.4	294.58
LOC104912186	mitochondrial fission factor homolog B-like	307.5	281.4	294.47

FOXO6	forkhead box O6	318.5	270.2	294.32
XIAP	X-linked inhibitor of apoptosis, E3 ubiquitin protein ligase	280.5	308.1	294.29
LOC104916187	uncharacterized LOC104916187	296.4	292.1	294.26
BEND3	BEN domain containing 3	282.0	306.6	294.26
LOC100546203	DNA-directed RNA polymerases I and III subunit RPAC2-like	294.6	293.3	293.98
TFB1M	transcription factor B1, mitochondrial	279.5	307.9	293.70
LOC104914281	uncharacterized LOC104914281	341.0	246.3	293.66
HPS3	Hermansky-Pudlak syndrome 3	268.6	318.5	293.57
LOC104913398	uncharacterized LOC104913398	282.9	304.2	293.55
LDB1	LIM domain binding 1	320.2	266.8	293.53
STX6	syntaxin 6	313.4	273.5	293.46
LOC104911979	uncharacterized LOC104911979	299.0	287.9	293.42
TM7SF3	transmembrane 7 superfamily member 3	266.9	319.9	293.40
SNCAIP	synuclein, alpha interacting protein	282.2	304.6	293.38
LOC104910962	glutamine-dependent NAD(+) synthetase	309.9	276.7	293.31
PBX4	pre-B-cell leukemia homeobox 4	269.4	317.0	293.20
BLOC1S6	biogenesis of lysosomal organelles complex-1, subunit 6, pallidin	290.5	295.8	293.14
PITX2	paired-like homeodomain 2	329.2	256.8	292.99
YAE1D1	Yae1 domain containing 1	282.9	303.1	292.98
CHD5	chromodomain helicase DNA binding protein 5	320.9	265.0	292.97
TYK2	tyrosine kinase 2	289.8	295.8	292.82
CLCN6	chloride channel, voltage-sensitive 6	297.2	288.4	292.81
OSGEPL1	O-sialoglycoprotein endopeptidase-like 1	295.5	290.1	292.79
LOC100542184	cat eye syndrome critical region protein 5 homolog	289.6	295.9	292.73
NAV1	neuron navigator 1	299.8	285.3	292.58
RRAGC	Ras-related GTP binding C	274.4	310.7	292.57
USP39	ubiquitin specific peptidase 39	296.6	288.4	292.51
CTSS	cathepsin S	315.8	269.1	292.48
NRN1L	neuritin 1-like	305.0	279.9	292.45
CPTP	ceramide-1-phosphate transfer protein	300.7	283.8	292.29
EED	embryonic ectoderm development	293.0	291.3	292.15
PELI1	pellino E3 ubiquitin protein ligase 1	304.9	279.3	292.11
USP37	ubiquitin specific peptidase 37	272.0	312.0	292.02
CCBL1	cysteine conjugate-beta lyase, cytoplasmic	296.4	287.6	292.01
FBXW7	F-box and WD repeat domain containing 7, E3 ubiquitin protein ligase	272.8	311.0	291.89
GAS1	growth arrest-specific 1	304.9	278.7	291.79
ISG20L2	interferon stimulated exonuclease gene 20kDa-like 2	306.6	276.3	291.46
TMEM150A	transmembrane protein 150A	300.7	282.0	291.31
AP1S2	adaptor-related protein complex 1, sigma 2 subunit	295.5	287.1	291.30
CASP8AP2	caspase 8 associated protein 2	280.4	302.2	291.30
LOC100547877	claudin-22-like	278.8	303.7	291.22
PDF	peptide deformylase (mitochondrial)	305.7	276.4	291.09
STXBP4	syntaxin binding protein 4	280.5	301.6	291.06
C23H1orf50	chromosome 23 open reading frame, human C1orf50	308.2	273.8	290.98
LOC100541498	tetratricopeptide repeat protein 27-like	298.1	283.6	290.86
LOC104912517	uncharacterized LOC104912517	277.0	304.7	290.86
LOC104916910	retinoic acid receptor RXR-beta-like	311.8	269.9	290.84
ARRDC1	arrestin domain containing 1	303.3	278.4	290.81
LOC104915550	mothers against decapentaplegic homolog 4-like	301.6	280.0	290.77
MDM2	MDM2 proto-oncogene, E3 ubiquitin protein ligase	272.8	308.4	290.62
XYLT2	xylosyltransferase II	316.9	264.3	290.57
LOC100542292	butyrophilin subfamily 1 member A1-like	283.9	297.1	290.50
LOC104913898	DDB1- and CUL4-associated factor 6-like	297.3	283.6	290.45
DOCK4	dedicator of cytokinesis 4	248.3	331.6	289.96
LOC104912467	cyclin-dependent kinase inhibitor 1-like	263.5	315.9	289.72
WIPF2	WAS/WASL interacting protein family, member 2	322.6	256.7	289.67
MLH3	mutL homolog 3	300.6	278.3	289.45

NEK2	NIMA-related kinase 2	309.8	268.9	289.31
LATS1	large tumor suppressor kinase 1	287.2	291.2	289.15
INA	internexin neuronal intermediate filament protein, alpha	293.2	284.5	288.82
SIK3	SIK family kinase 3	313.3	264.2	288.75
ANO3	anoctamin 3	249.3	327.9	288.57
ERCC4	excision repair cross-complementation group 4	290.5	286.4	288.48
ANKRD29	ankyrin repeat domain 29	288.0	288.9	288.48
DTD2	D-tyrosyl-tRNA deacylase 2 (putative)	318.0	258.7	288.36
FUK	fucokinase	335.3	241.4	288.35
TTC8	tetratricopeptide repeat domain 8	301.5	275.1	288.27
NF1	neurofibromin 1	292.3	284.2	288.26
ST6GALNAC3	ST6 (alpha-N-acetyl-neuraminyl-2,3-beta-galactosyl-1,3)-N-acetylgalactosaminide alpha-2,6-sialyltransferase 3	280.4	296.1	288.25
LOC104911746	transcription factor Sp3-like	288.0	288.5	288.24
TBC1D31	TBC1 domain family, member 31	280.4	296.0	288.20
HSPA13	heat shock protein 70kDa family, member 13	293.8	282.6	288.20
LOC104909980	uncharacterized LOC104909980	279.5	296.8	288.15
GPR89B	G protein-coupled receptor 89B	262.6	313.7	288.11
LOC104912482	uncharacterized LOC104912482	285.4	290.6	288.01
MRM1	mitochondrial rRNA methyltransferase 1 homolog (S. cerevisiae)	322.4	253.5	287.97
RTN4RL2	reticulon 4 receptor-like 2	295.5	280.4	287.97
LOC104911310	SWI/SNF complex subunit SMARCC1-like	320.9	254.9	287.91
MTHFR	methylenetetrahydrofolate reductase (NAD(P)H)	281.2	294.5	287.84
ST6GALNAC5	ST6 (alpha-N-acetyl-neuraminyl-2,3-beta-galactosyl-1,3)-N-acetylgalactosaminide alpha-2,6-sialyltransferase 5	267.0	308.6	287.82
ZNF512B	zinc finger protein 512B	288.2	287.4	287.82
SAPCD2	suppressor APC domain containing 2	296.4	279.2	287.81
LOC104911460	UBAP1-MVB12-associated (UMA)-domain containing protein 1-like	308.2	267.3	287.74
TUB	tubby bipartite transcription factor	277.8	297.5	287.66
ETV1	ets variant 1	293.8	281.5	287.65
DOK2	docking protein 2, 56kDa	268.6	306.1	287.38
CHMP2A	charged multivesicular body protein 2A	304.8	269.9	287.36
RINT1	RAD50 interactor 1	293.0	281.6	287.31
LOC104916255	evolutionarily conserved signaling intermediate in Toll pathway, mitochondrial-like	332.7	241.9	287.30
ITGA7	integrin, alpha 7	297.3	277.3	287.29
LOC104911090	MAX gene-associated protein-like	288.9	285.4	287.16
NLK	nemo-like kinase	261.8	312.4	287.09
LOC104914319	uncharacterized protein KIAA1522-like	291.3	282.6	286.95
PAXIP1	PAX interacting (with transcription-activation domain) protein 1	300.6	273.1	286.88
EHHADH	enoyl-CoA, hydratase/3-hydroxyacyl CoA dehydrogenase	287.2	286.6	286.86
AAGAB	alpha- and gamma-adaptin binding protein	295.5	278.1	286.81
IGFBP2	insulin-like growth factor binding protein 2, 36kDa	322.6	250.7	286.61
ITPRIP	inositol 1,4,5-trisphosphate receptor interacting protein	280.3	292.7	286.51
CCDC137	coiled-coil domain containing 137	290.5	281.8	286.17
LOC104909245	cell division cycle 5-like protein	290.5	281.4	285.96
POC5	POC5 centriolar protein	302.2	269.5	285.90
SCN4A	sodium channel, voltage-gated, type IV, alpha subunit	299.1	272.6	285.86
LOC104914955	arylsulfatase B-like	276.1	295.3	285.71
MUC4	mucin 4, cell surface associated	303.3	267.8	285.57
CHM	choroideremia (Rab escort protein 1)	268.6	302.4	285.51
PRR11	proline rich 11	304.0	266.8	285.37
GTF3C6	general transcription factor IIIC, polypeptide 6, alpha 35kDa	293.0	277.7	285.34
LOC100543379	phosphatidylethanolamine N-methyltransferase-like	267.8	302.6	285.23
LOC104911632	transcription intermediary factor 1-alpha-like	279.5	290.8	285.14
LOC104917019	uncharacterized LOC104917019	307.3	262.9	285.13
FAM160A1	family with sequence similarity 160, member A1	281.2	288.8	285.02
LOC100539820	mitochondrial chaperone BCS1	288.0	281.9	284.95

TNFAIP1	tumor necrosis factor, alpha-induced protein 1 (endothelial)	272.8	297.0	284.91
TRIP4	thyroid hormone receptor interactor 4	305.7	264.0	284.88
BPNT1	3'(2'), 5'-bisphosphate nucleotidase 1	277.8	291.7	284.71
LOH12CR1	loss of heterozygosity, 12, chromosomal region 1	298.9	270.4	284.64
LRP4	low density lipoprotein receptor-related protein 4	277.9	290.8	284.33
SRD5A1	steroid-5-alpha-reductase, alpha polypeptide 1 (3-oxo-5 alpha-steroid delta 4-dehydrogenase alpha 1)	278.7	289.9	284.27
LOC104916210	glucosylceramidase-like	303.2	265.3	284.27
TMEM216	transmembrane protein 216	293.0	275.0	283.99
FAHD1	fumarylacetoacetate hydrolase domain containing 1	294.7	273.0	283.85
SMCO4	single-pass membrane protein with coiled-coil domains 4	271.9	295.7	283.79
SCG2	secretogranin II	252.8	314.8	283.78
THUMPD2	THUMP domain containing 2	277.0	290.6	283.77
LOC100546891	cholinephosphotransferase 1-like	281.2	286.3	283.75
LOC104912282	dual specificity protein phosphatase CDC14A-like	272.7	294.7	283.71
ZBTB40	zinc finger and BTB domain containing 40	271.2	295.7	283.42
NFKBIA	nuclear factor of kappa light polypeptide gene enhancer in B-cells inhibitor, alpha	274.5	292.2	283.37
POC1B	POC1 centriolar protein B	285.4	281.1	283.28
NUDT5	nudix (nucleoside diphosphate linked moiety X)-type motif 5	291.2	275.3	283.25
LOC100540496	RNA-binding protein 14-like	290.5	275.4	282.94
LOC100546258	pre-mRNA 3' end processing protein WDR33-like	274.5	291.0	282.75
PTPN4	protein tyrosine phosphatase, non-receptor type 4 (megakaryocyte)	256.0	309.5	282.75
LOC104915699	WD repeat-containing protein 62-like	326.1	239.3	282.71
GATB	glutamyl-tRNA(Gln) amidotransferase, subunit B	287.2	278.1	282.64
ANAPC13	anaphase promoting complex subunit 13	290.5	274.5	282.48
SGOL1	shugoshin-like 1 (S. pombe)	303.0	261.5	282.27
PLA2G4A	phospholipase A2, group IVA (cytosolic, calcium-dependent)	286.4	278.1	282.23
ADARB1	adenosine deaminase, RNA-specific, B1	267.7	296.7	282.18
LOC104913929	breast carcinoma-amplified sequence 3-like	286.3	278.0	282.13
ST3GAL4	ST3 beta-galactoside alpha-2,3-sialyltransferase 4	260.3	303.9	282.06
NARS2	asparaginyl-tRNA synthetase 2, mitochondrial (putative)	291.3	272.7	282.01
FAM208B	family with sequence similarity 208, member B	296.6	267.0	281.80
FAF1	Fas (TNFRSF6) associated factor 1	280.4	283.0	281.70
LOC104910857	lateral signaling target protein 2 homolog	254.3	309.0	281.65
LOC100540428	zinc finger protein 516	236.6	326.4	281.51
LOC104913150	FYVE, RhoGEF and PH domain-containing protein 3-like	236.4	326.3	281.36
HS6ST3	heparan sulfate 6-O-sulfotransferase 3	250.9	311.4	281.19
TMEM69	transmembrane protein 69	254.3	308.0	281.13
SOWAHC	sosondowah ankyrin repeat domain family member C	283.7	278.2	280.97
QSOX2	quiescin Q6 sulfhydryl oxidase 2	288.2	273.5	280.80
C4H4orf46	chromosome 4 open reading frame, human C4orf46	298.1	263.2	280.66
CD40	CD40 molecule, TNF receptor superfamily member 5	332.0	229.1	280.56
TMTC1	transmembrane and tetratricopeptide repeat containing 1	248.4	312.6	280.52
SETD1B	SET domain containing 1B	293.9	267.1	280.47
LOC104917321	ATP-binding cassette sub-family B member 8, mitochondrial-like	294.7	266.0	280.39
SHPRH	SNF2 histone linker PHD RING helicase, E3 ubiquitin protein ligase	265.2	295.5	280.33
RCCD1	RCC1 domain containing 1	290.6	269.9	280.26
CIART	circadian associated repressor of transcription	257.7	302.8	280.25
SLC35B3	solute carrier family 35 (adenosine 3'-phospho 5'-phosphosulfate transporter), member B3	268.5	291.9	280.20
PIAS1	protein inhibitor of activated STAT, 1	267.7	292.2	279.94
CMC2	C-x(9)-C motif containing 2	294.6	265.0	279.77
CRH	corticotropin releasing hormone	264.3	295.1	279.72
MTFP1	mitochondrial fission process 1	286.3	272.5	279.39
PHF2	PHD finger protein 2	267.8	290.8	279.26
LIN7A	lin-7 homolog A (C. elegans)	271.0	287.5	279.26

NIN	ninein (GSK3B interacting protein)	286.2	272.3	279.25
PTCHD3	patched domain containing 3	268.5	289.8	279.14
ENKD1	enkurin domain containing 1	285.5	272.6	279.04
ASAP1	ArfGAP with SH3 domain, ankyrin repeat and PH domain 1	260.2	297.8	278.99
ARL3	ADP-ribosylation factor-like 3	293.0	265.0	278.98
ALDH1A2	aldehyde dehydrogenase 1 family, member A2	252.6	305.3	278.95
CCDC186	coiled-coil domain containing 186	273.6	284.2	278.87
LOC104910538	uncharacterized LOC104910538	268.7	289.0	278.84
LOC100550376	uncharacterized LOC100550376	281.1	276.5	278.79
LEKR1	leucine, glutamate and lysine rich 1	286.4	271.1	278.73
G2E3	G2/M-phase specific E3 ubiquitin protein ligase	268.5	288.8	278.67
ANKRD44	ankyrin repeat domain 44	262.5	294.4	278.48
JAK2	Janus kinase 2	281.1	275.7	278.43
B3GALT6	UDP-Gal:betaGal beta 1,3-galactosyltransferase polypeptide 6	255.1	301.5	278.30
GKAP1	G kinase anchoring protein 1	261.6	294.8	278.23
LOC100538455	steroid 21-hydroxylase	328.5	227.7	278.10
LOC104914310	discoidin, CUB and LCCL domain-containing protein 1-like	284.8	270.5	277.68
LOC104912214	A-kinase anchor protein 17B-like	277.1	278.2	277.63
CKS2	CDC28 protein kinase regulatory subunit 2	305.5	249.5	277.50
ICA1	islet cell autoantigen 1, 69kDa	271.1	283.8	277.42
LOC104916404	immediate early response gene 5-like protein	299.0	255.5	277.25
VPS13C	vacuolar protein sorting 13 homolog C (S. cerevisiae)	266.9	287.5	277.21
LOC104916712	putative N-acetyltransferase 8B	297.2	257.1	277.14
GTF3C5	general transcription factor IIIC, polypeptide 5, 63kDa	268.5	285.3	276.93
LOC104912548	transcription factor Dp-2	293.8	259.8	276.77
CRACR2B	calcium release activated channel regulator 2B	316.5	236.8	276.66
PPP1R13B	protein phosphatase 1, regulatory subunit 13B	272.9	280.3	276.59
B4GALT4	UDP-Gal:betaGlcNAc beta 1,4- galactosyltransferase, polypeptide 4	274.4	278.8	276.58
PTPN3	protein tyrosine phosphatase, non-receptor type 3	278.7	274.4	276.57
LOC104911485	uncharacterized LOC104911485	246.7	305.7	276.18
TMCO3	transmembrane and coiled-coil domains 3	284.5	267.8	276.16
LOC100545322	keratin, type II cytoskeletal cochleal	301.2	250.9	276.07
UBA6	ubiquitin-like modifier activating enzyme 6	278.8	273.3	276.04
ARHGAP12	Rho GTPase activating protein 12	253.4	298.6	275.98
SLC41A3	solute carrier family 41, member 3	283.1	268.8	275.95
WDFY2	WD repeat and FYVE domain containing 2	257.6	293.9	275.72
STARD9	StAR-related lipid transfer (START) domain containing 9	249.2	302.0	275.62
SDCCAG8	serologically defined colon cancer antigen 8	275.3	275.9	275.61
LOC100551379	lateral signaling target protein 2 homolog	298.2	252.8	275.53
OSBPL8	oxysterol binding protein-like 8	271.9	278.5	275.24
CAMKK2	calcium/calmodulin-dependent protein kinase kinase 2, beta	270.3	280.1	275.22
LAMP2	lysosomal-associated membrane protein 2	266.9	283.2	275.05
METTL6	methyltransferase like 6	261.8	288.1	274.94
SFRP4	secreted frizzled-related protein 4	237.6	311.6	274.59
LOC100548025	sentrin-specific protease 6-like	273.5	275.4	274.45
LOC100550589	tumor susceptibility gene 101 protein	270.2	278.3	274.23
PORCN	porcupine homolog (Drosophila)	295.7	252.1	273.88
MAPK12	mitogen-activated protein kinase 12	277.8	269.9	273.83
LOC104912248	transforming growth factor beta receptor type 3-like	247.5	300.1	273.82
PRG4	proteoglycan 4	252.4	294.9	273.69
LOC104913866	general transcription factor II-I-like	260.2	286.8	273.50
LOC104912786	uncharacterized LOC104912786	255.9	290.9	273.44
LATS2	large tumor suppressor kinase 2	247.5	299.3	273.42
SUV39H2	suppressor of variegation 3-9 homolog 2 (Drosophila)	294.0	252.7	273.35
COL4A3	collagen, type IV, alpha 3 (Goodpasture antigen)	281.2	265.5	273.35
COG5	component of oligomeric golgi complex 5	275.4	271.1	273.29
B3GNT4	UDP-GlcNAc:betaGal beta-1,3-N-acetylglucosaminyltransferase 4	249.1	297.2	273.15

PARVA	parvin, alpha	266.0	280.1	273.08
KMT2A	lysine (K)-specific methyltransferase 2A	270.4	275.6	272.99
NADK	NAD kinase	269.5	276.3	272.91
PPTC7	PTC7 protein phosphatase homolog (S. cerevisiae)	260.1	285.6	272.86
CMSS1	cms1 ribosomal small subunit homolog (yeast)	281.9	263.8	272.86
LOC104915842	gonadotropin-releasing hormone II receptor-like	295.6	250.0	272.80
LYRM4	LYR motif containing 4	242.3	302.9	272.60
DDX28	DEAD (Asp-Glu-Ala-Asp) box polypeptide 28	277.9	267.1	272.48
AGBL5	ATP/GTP binding protein-like 5	276.4	268.4	272.37
FBXO32	F-box protein 32	265.3	278.7	271.99
ARHGAP11A	Rho GTPase activating protein 11A	281.9	261.4	271.64
C16H7orf50	chromosome 16 open reading frame, human C7orf50	288.7	254.3	271.50
LOC104916292	hemojuvelin-like	275.4	267.5	271.45
RGS9BP	regulator of G protein signaling 9 binding protein	282.8	259.2	270.99
C10H14orf80	chromosome 10 open reading frame, human C14orf80	296.4	245.2	270.77
LOC100539550	rho GTPase-activating protein 7-like	255.9	285.4	270.64
LOC104912571	dedicator of cytokinesis protein 10-like	265.2	276.0	270.61
PRKAA1	protein kinase, AMP-activated, alpha 1 catalytic subunit	271.1	269.5	270.30
PTGR2	prostaglandin reductase 2	258.4	281.6	270.00
PLA2G6	phospholipase A2, group VI (cytosolic, calcium-independent)	274.6	265.1	269.84
PTTG1	pituitary tumor-transforming 1	276.7	262.9	269.83
UROS	uroporphyrinogen III synthase	251.8	287.9	269.82
MRPL53	mitochondrial ribosomal protein L53	269.3	270.2	269.77
MGME1	mitochondrial genome maintenance exonuclease 1	269.4	270.1	269.75
EXOSC4	exosome component 4	302.3	237.2	269.72
TRIM62	tripartite motif containing 62	299.1	240.2	269.69
LOC104913310	lysine-specific demethylase 3B-like	274.5	264.8	269.67
LOC100543419	immunoglobulin superfamily containing leucine-rich repeat protein 2-like	264.4	274.9	269.64
ZDHHC21	zinc finger, DHHC-type containing 21	258.4	280.8	269.59
TDP1	tyrosyl-DNA phosphodiesterase 1	272.8	266.0	269.39
ISPD	isoprenoid synthase domain containing	263.4	275.3	269.38
HENMT1	HEN1 methyltransferase homolog 1 (Arabidopsis)	271.0	267.5	269.27
TMEM234	transmembrane protein 234	257.6	280.8	269.18
LOC104914288	uncharacterized LOC104914288	279.6	258.7	269.16
RASSF6	Ras association (RalGDS/AF-6) domain family member 6	269.3	269.0	269.12
PLAGL2	pleiomorphic adenoma gene-like 2	271.1	267.1	269.10
LOC100549463	uncharacterized LOC100549463	283.8	254.2	269.02
ATHL1	ATH1, acid trehalase-like 1 (yeast)	292.3	245.7	268.99
STC1	stanniocalcin 1	265.4	272.3	268.88
EXOSC3	exosome component 3	287.0	250.5	268.78
SPR	sepiapterin reductase (7,8-dihydrobiopterin:NADP+ oxidoreductase)	268.5	268.8	268.69
FAM129A	family with sequence similarity 129, member A	246.7	290.6	268.68
HTR7	5-hydroxytryptamine (serotonin) receptor 7, adenylate cyclase-coupled	244.8	292.4	268.60
ARL4A	ADP-ribosylation factor-like 4A	272.6	264.5	268.56
HTRA3	HtrA serine peptidase 3	238.2	298.9	268.55
C19H9orf114	chromosome 19 open reading frame, human C9orf114	283.7	253.0	268.36
PCYOX1L	prenylcysteine oxidase 1 like	258.5	278.1	268.30
PWWP2B	PWWP domain containing 2B	266.0	270.5	268.25
SCUBE2	signal peptide, CUB domain, EGF-like 2	250.0	286.4	268.23
RSPRY1	ring finger and SPRY domain containing 1	259.4	276.8	268.06
MMP23B	matrix metallopeptidase 23B	261.0	275.0	268.00
SLC5A9	solute carrier family 5 (sodium/sugar cotransporter), member 9	260.0	275.8	267.94
ABCB11	ATP-binding cassette, sub-family B (MDR/TAP), member 11	250.0	285.7	267.83
LOC104914588	uncharacterized LOC104914588	272.0	263.3	267.66
IL8	interleukin 8	256.8	278.2	267.51

PHYH	phytanoyl-CoA 2-hydroxylase	255.1	279.8	267.45
XRCC2	X-ray repair complementing defective repair in Chinese hamster cells 2	252.4	281.9	267.15
LOC104915423	ribosomal protein S6 kinase delta-1-like	242.5	291.8	267.13
DOLK	dolichol kinase	236.5	297.4	266.96
SPATA5L1	spermatogenesis associated 5-like 1	256.7	277.1	266.94
CCDC127	coiled-coil domain containing 127	244.1	289.7	266.86
TMEM243	transmembrane protein 243, mitochondrial	261.8	271.9	266.85
SOAT1	sterol O-acyltransferase 1	248.2	285.0	266.64
LOC100546181	sorting nexin-9-like	261.8	271.0	266.39
LOC100549531	ankyrin repeat and KH domain-containing protein 1	246.5	286.2	266.35
LOC104911104	oxysterols receptor LXR-alpha-like	297.4	235.2	266.29
LOC104917613	platelet-derived growth factor D-like	262.8	269.8	266.26
MAP2K3	mitogen-activated protein kinase kinase 3	262.7	269.7	266.19
CHST2	carbohydrate (N-acetylglucosamine-6-O) sulfotransferase 2	280.3	251.7	266.02
LANCL2	LanC lantibiotic synthetase component C-like 2 (bacterial)	313.3	218.6	265.97
GLCCI1	glucocorticoid induced transcript 1	264.4	267.5	265.94
LRRC73	leucine rich repeat containing 73	268.5	263.3	265.89
LOC100544577	arginine-glutamic acid dipeptide repeats protein-like	241.7	289.8	265.72
SLN	sarcolipin	277.0	254.1	265.54
ANGPT2	angiopoietin 2	242.5	288.5	265.49
LOC104909521	ninein-like protein	248.4	282.4	265.38
LOC104917361	nanos homolog 1-like	269.5	260.9	265.19
MAFA	v-maf avian musculoaponeurotic fibrosarcoma oncogene homolog A	273.7	256.5	265.09
HDAC4	histone deacetylase 4	267.6	262.0	264.83
GSTZ1	glutathione S-transferase zeta 1	271.1	258.5	264.79
POP1	processing of precursor 1, ribonuclease P/MRP subunit (S. cerevisiae)	255.0	274.1	264.57
CASP3	caspase 3, apoptosis-related cysteine peptidase	254.2	274.9	264.55
LOC100545689	cytoplasmic dynein 1 light intermediate chain 2-like	237.2	291.7	264.48
KIAA1467	KIAA1467 ortholog	266.0	262.8	264.40
LOC104913068	DNA replication factor Cdt1-like	290.5	238.3	264.38
VCPKMT	valosin containing protein lysine (K) methyltransferase	272.7	255.7	264.17
BBS2	Bardet-Biedl syndrome 2	261.0	267.3	264.15
RPP38	ribonuclease P/MRP 38kDa subunit	264.2	264.0	264.11
GJC2	gap junction protein, gamma 2, 47kDa	269.4	258.8	264.09
LOC104911750	obg-like ATPase 1	269.4	258.3	263.85
KIF11	kinesin family member 11	294.7	232.2	263.45
RAVER2	ribonucleoprotein, PTB-binding 2	259.2	267.6	263.43
LOC100542915	integrator complex subunit 3-like	284.7	242.1	263.42
NAAA	N-acylethanolamine acid amidase	249.1	277.6	263.36
NAV3	neuron navigator 3	262.8	263.8	263.26
ACVR2A	activin A receptor, type IIA	261.9	264.6	263.24
SLC26A11	solute carrier family 26 (anion exchanger), member 11	267.8	258.6	263.21
LOC100543914	tyrosine-protein kinase Fyn	243.3	282.8	263.06
ABHD17C	abhydrolase domain containing 17C	241.7	284.2	262.95
XRRA1	X-ray radiation resistance associated 1	261.9	263.9	262.90
GAL3ST4	galactose-3-O-sulfotransferase 4	268.8	257.0	262.88
RAD51	RAD51 recombinase	278.5	246.9	262.70
FBXL13	F-box and leucine-rich repeat protein 13	275.2	250.0	262.57
C17H12orf49	chromosome 17 open reading frame, human C12orf49	280.5	244.6	262.54
LOC100541464	synaptotagmin-like protein 2	263.5	261.6	262.53
LOC104909304	rab GTPase-activating protein 1-like	262.6	262.4	262.46
ITSN1	intersectin 1 (SH3 domain protein)	248.4	276.5	262.45
ITGB3	integrin, beta 3 (platelet glycoprotein IIIa, antigen CD61)	297.3	227.6	262.45
EARS2	glutamyl-tRNA synthetase 2, mitochondrial	260.2	264.7	262.42
LOC100541500	serine/threonine-protein kinase Sgk1	233.1	291.7	262.39

DBT	dihydrolipoamide branched chain transacylase E2	265.1	259.4	262.26
TRAF4	TNF receptor-associated factor 4	267.0	257.5	262.22
HAUS8	HAUS augmin-like complex, subunit 8	256.6	267.7	262.14
COMP	cartilage oligomeric matrix protein	288.6	235.6	262.11
SLC35A1	solute carrier family 35 (CMP-sialic acid transporter), member A1	261.8	262.3	262.05
C9HXorf57	chromosome 9 open reading frame, human CXorf57	255.1	268.9	261.99
RBM43	RNA binding motif protein 43	266.9	257.0	261.92
LOC104913690	uncharacterized LOC104913690	274.4	249.4	261.91
LOC100546708	cholesterol 24-hydroxylase	282.9	240.8	261.86
TMEM9	transmembrane protein 9	268.5	254.9	261.69
LOC104910682	tyrosine-protein phosphatase non-receptor type 13-like	271.0	252.3	261.68
LOC100544203	cadherin-4	247.6	275.5	261.57
EPM2A	epilepsy, progressive myoclonus type 2A, Lafora disease (laforin)	254.1	269.0	261.55
CDADC1	cytidine and dCMP deaminase domain containing 1	254.2	268.8	261.50
NUAK2	NUAK family, SNF1-like kinase, 2	266.1	256.4	261.26
LOC100539278	FYVE, RhoGEF and PH domain-containing protein 4	255.0	267.4	261.20
LOC100544321	ephrin type-B receptor 5-like	249.2	273.2	261.19
BTBD2	BTB (POZ) domain containing 2	313.4	208.5	260.96
ECE1	endothelin converting enzyme 1	279.7	241.9	260.76
PARP6	poly (ADP-ribose) polymerase family, member 6	294.0	227.4	260.69
CDK9	cyclin-dependent kinase 9	271.1	249.9	260.51
RASGEF1C	RasGEF domain family, member 1C	242.4	278.3	260.37
LOC100539565	talin-2	250.9	269.7	260.31
CAMK1	calcium/calmodulin-dependent protein kinase I	291.4	229.2	260.28
KLHL25	kelch-like family member 25	262.6	258.0	260.27
LOC100547156	glycogen phosphorylase, brain form	266.8	253.6	260.19
TMED8	transmembrane emp24 protein transport domain containing 8	251.7	268.6	260.15
CDK6	cyclin-dependent kinase 6	255.8	264.3	260.09
PTER	phosphotriesterase related	270.1	250.0	260.08
SMCR8	Smith-Magenis syndrome chromosome region, candidate 8	281.3	238.8	260.05
TNFAIP8L1	tumor necrosis factor, alpha-induced protein 8-like 1	251.7	268.2	259.94
IRF8	interferon regulatory factor 8	264.4	255.4	259.90
LOC104911665	uncharacterized LOC104911665	223.8	296.0	259.88
LOC104911899	uncharacterized LOC104911899	242.4	277.2	259.81
PODN	podocan	239.9	279.6	259.79
LOC100544738	ribosome-releasing factor 2, mitochondrial-like	248.3	270.7	259.47
METTL22	methyltransferase like 22	268.5	250.4	259.44
FAIM	Fas apoptotic inhibitory molecule	236.5	282.4	259.44
PDXDC1	pyridoxal-dependent decarboxylase domain containing 1	242.5	276.4	259.43
DOPEY1	dopey family member 1	253.3	265.3	259.33
B3GALT2	UDP-Gal:betaGlcNAc beta 1,3-galactosyltransferase, polypeptide 2	251.8	266.7	259.21
LOC104916751	LIX1-like protein	293.0	225.3	259.14
DEF8	differentially expressed in FDCP 8 homolog (mouse)	292.3	225.9	259.06
KCTD10	potassium channel tetramerization domain containing 10	272.0	245.7	258.84
ZC3H10	zinc finger CCCH-type containing 10	288.8	228.2	258.55
PEX2	peroxisomal biogenesis factor 2	263.5	253.1	258.31
FAM171B	family with sequence similarity 171, member B	266.8	249.6	258.19
PIGW	phosphatidylinositol glycan anchor biosynthesis, class W	266.9	249.3	258.09
LOC104916188	ataxin-7-like protein 3	266.1	249.9	257.98
FANCE	Fanconi anemia, complementation group E	301.5	214.4	257.93
RMI1	RecQ mediated genome instability 1	255.0	260.8	257.88
LOC100546369	argininosuccinate lyase	256.0	259.7	257.84
WDR6	WD repeat domain 6	267.8	247.9	257.81
NPAT	nuclear protein, ataxia-telangiectasia locus	253.4	261.2	257.28
PIGP	phosphatidylinositol glycan anchor biosynthesis, class P	246.4	267.9	257.17
TMEM140	transmembrane protein 140	249.2	265.0	257.10
TMEM141	transmembrane protein 141	251.6	262.4	257.03
LOC100545319	eyes absent homolog 1	277.8	236.1	256.95

ANKIB1	ankyrin repeat and IBR domain containing 1	234.9	278.6	256.78
FAM64A	family with sequence similarity 64, member A	287.8	225.7	256.77
CEP162	centrosomal protein 162kDa	255.8	257.6	256.74
LOC100550740	putative fatty acid-binding protein 5-like protein 3	293.8	219.5	256.68
FAM118B	family with sequence similarity 118, member B	244.1	268.6	256.37
LOC100543330	myosin heavy chain, skeletal muscle-like	280.4	232.2	256.34
LOC104917432	integral membrane protein GPR180-like	230.5	281.9	256.21
LOC100544196	uncharacterized LOC100544196	259.2	253.2	256.18
ANAPC5	anaphase promoting complex subunit 5	260.1	252.2	256.15
ZNF76	zinc finger protein 76	267.8	244.3	256.05
CEP85	centrosomal protein 85kDa	283.8	227.8	255.79
LOC100540713	uncharacterized LOC100540713	260.8	250.5	255.69
C1H22orf23	chromosome 1 open reading frame, human C22orf23	262.8	247.9	255.35
ARVCF	armadillo repeat gene deleted in velocardiofacial syndrome	250.9	259.8	255.31
LOC104914598	mediator of RNA polymerase II transcription subunit 24-like	293.9	216.4	255.14
LOC100540806	Krueppel-like factor 13	234.8	275.2	255.00
ITSN2	intersectin 2	256.0	253.6	254.79
PIK3CA	phosphatidylinositol-4,5-bisphosphate 3-kinase, catalytic subunit alpha	245.0	264.2	254.62
ZNF518B	zinc finger protein 518B	257.5	250.7	254.10
FAM76A	family with sequence similarity 76, member A	254.3	253.9	254.09
HIPK1	homeodomain interacting protein kinase 1	213.8	294.2	253.97
DBNDD1	dysbindin (dystrobrevin binding protein 1) domain containing 1	253.4	253.3	253.37
HIP1R	huntingtin interacting protein 1 related	255.1	251.6	253.35
LOC104915924	uncharacterized LOC104915924	269.5	237.1	253.29
LRRC49	leucine rich repeat containing 49	229.7	276.7	253.20
UTP23	UTP23, small subunit (SSU) processome component, homolog (yeast)	250.0	256.2	253.08
ZNF341	zinc finger protein 341	248.3	257.8	253.07
SPAG5	sperm associated antigen 5	255.8	250.3	253.01
DNAJC15	DnaJ (Hsp40) homolog, subfamily C, member 15	231.4	274.1	252.74
TMEM161A	transmembrane protein 161A	267.8	237.7	252.71
LOC100549393	maternal embryonic leucine zipper kinase	263.3	242.1	252.70
GTPBP3	GTP binding protein 3 (mitochondrial)	267.8	237.6	252.70
LOC104912958	uncharacterized LOC104912958	262.6	242.6	252.63
MTERF2	mitochondrial transcription termination factor 2	238.1	267.1	252.62
TMEM109	transmembrane protein 109	263.5	241.7	252.61
MDM1	Mdm1 nuclear protein homolog (mouse)	244.1	260.8	252.45
DDX6	DEAD (Asp-Glu-Ala-Asp) box helicase 6	237.3	267.5	252.44
LOC104916035	death domain-associated protein 6-like	266.1	238.7	252.42
RASAL2	RAS protein activator like 2	257.7	247.0	252.33
LIFR	leukemia inhibitory factor receptor alpha	241.6	263.1	252.32
LOC104916228	uncharacterized LOC104916228	290.5	214.0	252.27
LOC100539364	methylcrotonoyl-CoA carboxylase beta chain, mitochondrial-like	242.3	262.2	252.26
DFFA	DNA fragmentation factor, 45kDa, alpha polypeptide	241.5	262.8	252.17
SKA3	spindle and kinetochore associated complex subunit 3	264.3	239.9	252.09
LOC104916022	uncharacterized LOC104916022	265.1	239.0	252.08
PRKD1	protein kinase D1	249.1	255.0	252.07
PRKRIR	protein-kinase, interferon-inducible double stranded RNA dependent inhibitor, repressor of (P58 repressor)	240.7	263.1	251.89
CPEB2	cytoplasmic polyadenylation element binding protein 2	250.0	252.9	251.47
LOC104915782	histone-lysine N-methyltransferase 2D-like	252.6	249.9	251.25
PHIP	pleckstrin homology domain interacting protein	257.6	244.7	251.14
LOC100547218	uncharacterized LOC100547218	264.4	237.8	251.10
LOC104909405	uncharacterized LOC104909405	247.5	254.7	251.07
LOC100541118	zinc finger ZZ-type and EF-hand domain-containing protein 1	278.0	224.0	251.01
LOC100550296	T-cell immunomodulatory protein	248.3	253.6	250.95
TGFB2	transforming growth factor, beta 2	250.8	251.0	250.91

GEN1	GEN1 Holliday junction 5' flap endonuclease	247.4	254.4	250.90
CAMK2G	calcium/calmodulin-dependent protein kinase II gamma	255.2	246.1	250.66
JMJD7	jumonji domain containing 7	243.2	258.0	250.59
P4HA3	prolyl 4-hydroxylase, alpha polypeptide III	252.5	248.5	250.49
XRCC4	X-ray repair complementing defective repair in Chinese hamster cells 4	250.7	250.3	250.47
ADIPOQ	adiponectin, C1Q and collagen domain containing	247.5	253.2	250.38
C8G	complement component 8, gamma polypeptide	250.1	250.6	250.33
CCDC97	coiled-coil domain containing 97	295.7	204.7	250.16
LOC104913034	condensin complex subunit 1-like	268.5	231.7	250.13
PPAP2A	phosphatidic acid phosphatase type 2A	238.2	261.8	249.97
LOC104914711	uncharacterized LOC104914711	248.3	251.5	249.94
IQCC	IQ motif containing C	259.3	240.6	249.93
LOC100541794	uncharacterized LOC100541794	250.7	249.1	249.93
ALDH6A1	aldehyde dehydrogenase 6 family, member A1	255.1	244.7	249.92
PARP8	poly (ADP-ribose) polymerase family, member 8	242.4	257.0	249.71
ZNF865	zinc finger protein 865	285.6	213.4	249.52
LARP1	La ribonucleoprotein domain family, member 1	249.2	249.6	249.44
ERMP1	endoplasmic reticulum metallopeptidase 1	260.0	238.9	249.43
OSBPL10	oxysterol binding protein-like 10	273.6	224.9	249.27
ATAD2	ATPase family, AAA domain containing 2	256.7	241.5	249.12
LOC104914045	nuclear receptor coactivator 3-like	220.4	277.6	249.01
LOC104914185	vacuolar protein sorting-associated protein 13D-like	212.9	284.8	248.82
TTLL1	tubulin tyrosine ligase-like family, member 1	266.8	230.4	248.61
TBX4	T-box 4	242.4	254.6	248.46
TOR2A	torsin family 2, member A	245.7	251.2	248.43
LOC104909382	protocadherin Fat 3-like	260.9	235.9	248.40
LOC104909362	G1/S-specific cyclin-D2	244.1	252.6	248.37
LOC104914455	uncharacterized LOC104914455	271.1	225.4	248.28
LOC100547709	endoplasmic reticulum-Golgi intermediate compartment protein 2	237.3	259.0	248.13
ARNTL	aryl hydrocarbon receptor nuclear translocator-like	228.8	267.3	248.07
DPH2	DPH2 homolog (S. cerevisiae)	267.0	228.6	247.81
C3H5orf22	chromosome 3 open reading frame, human C5orf22	241.5	254.1	247.78
LOC104914713	splicing factor, proline- and glutamine-rich-like	278.6	216.8	247.70
SOX4	SRY (sex determining region Y)-box 4	285.4	209.8	247.62
C2H2orf43	chromosome 2 open reading frame, human C2orf43	257.6	237.1	247.38
LOC100540676	interleukin-1 receptor type 1-like	215.4	279.4	247.37
MXD1	MAX dimerization protein 1	245.9	248.5	247.17
TMEM130	transmembrane protein 130	248.3	245.8	247.07
SENP2	SUMO1/sentrin/SMT3 specific peptidase 2	256.7	237.3	246.98
STK16	serine/threonine kinase 16	261.9	231.6	246.79
LOC100540389	DNA annealing helicase and endonuclease ZRANB3-like	226.3	267.2	246.72
ALG11	ALG11, alpha-1,2-mannosyltransferase	241.6	251.4	246.47
CEP83	centrosomal protein 83kDa	253.3	238.8	246.07
DUSP26	dual specificity phosphatase 26 (putative)	258.4	233.7	246.04
PRKG1	protein kinase, cGMP-dependent, type I	256.8	235.2	246.02
CEP19	centrosomal protein 19kDa	248.3	243.5	245.91
EXOC8	exocyst complex component 8	254.2	237.6	245.87
LOC104909514	lysocardiolipin acyltransferase 1-like	215.3	276.4	245.84
RPAIN	RPA interacting protein	243.2	248.4	245.80
TICRR	TOPBP1-interacting checkpoint and replication regulator	261.0	230.5	245.74
NAGPA	N-acetylglucosamine-1-phosphodiester alpha-N-acetylglucosaminidase	249.2	242.1	245.68
LONRF3	LON peptidase N-terminal domain and ring finger 3	247.4	243.3	245.40
SAMHD1	SAM domain and HD domain 1	235.7	254.7	245.17
PKP2	plakophilin 2	262.4	227.7	245.07
PHF19	PHD finger protein 19	277.9	212.2	245.06
CHRAC1	chromatin accessibility complex 1	250.8	239.1	244.98

DCDC2B	doublecortin domain containing 2B	235.7	254.2	244.92
RNF217	ring finger protein 217	219.6	269.9	244.76
RNASEL	ribonuclease L (2',5'-oligoisoadenylate synthetase-dependent)	237.3	252.1	244.71
LOC100542822	fibulin-1	218.8	270.3	244.57
WIF1	WNT inhibitory factor 1	213.7	275.3	244.50
LOC104909595	uncharacterized LOC104909595	255.9	232.8	244.32
LOC100541195	high affinity cAMP-specific 3',5'-cyclic phosphodiesterase 7A	249.9	237.8	243.88
LOC100541969	uncharacterized LOC100541969	238.1	249.5	243.84
STXBP5	syntaxin binding protein 5 (tomosyn)	220.4	267.1	243.74
LOC104915693	dynactin subunit 1-like	294.9	192.4	243.69
VEGFC	vascular endothelial growth factor C	231.4	255.9	243.65
L3HYPDH	L-3-hydroxyproline dehydratase (trans-)	262.7	224.5	243.57
POLR3K	polymerase (RNA) III (DNA directed) polypeptide K, 12.3 kDa	268.5	218.6	243.55
CENPQ	centromere protein Q	253.3	233.7	243.49
ARL5B	ADP-ribosylation factor-like 5B	231.3	255.4	243.35
TRMT5	tRNA methyltransferase 5	251.6	235.0	243.32
FZR1	fizzy/cell division cycle 20 related 1 (Drosophila)	253.5	232.8	243.16
RHBG	Rh family, B glycoprotein	269.4	216.8	243.09
PECR	peroxisomal trans-2-enoyl-CoA reductase	244.0	242.1	243.06
LOC100551187	WD repeat-containing protein 36-like	250.9	234.9	242.88
LOC104917222	spindle and kinetochore-associated protein 1-like	253.3	232.4	242.87
GPR161	G protein-coupled receptor 161	236.5	249.1	242.78
BLM	Bloom syndrome, RecQ helicase-like	255.9	229.6	242.76
OPCML	opioid binding protein/cell adhesion molecule-like	236.7	248.7	242.66
GABPB1	GA binding protein transcription factor, beta subunit 1	221.3	263.9	242.62
PRMT7	protein arginine methyltransferase 7	228.0	257.2	242.60
LOC104911910	uncharacterized LOC104911910	233.0	252.1	242.57
CNNM4	cyclin and CBS domain divalent metal cation transport mediator 4	245.9	239.2	242.50
NDUFAF7	NADH dehydrogenase (ubiquinone) complex I, assembly factor 7	247.4	237.5	242.44
PHOSPHO2	phosphatase, orphan 2	233.1	251.8	242.44
FASTKD3	FAST kinase domains 3	259.2	225.6	242.40
XRCC3	X-ray repair complementing defective repair in Chinese hamster cells 3	237.3	247.3	242.26
ZBTB26	zinc finger and BTB domain containing 26	229.8	254.7	242.25
GRIP2	glutamate receptor interacting protein 2	229.8	254.6	242.23
LOC100545629	putative Polycomb group protein ASXL3	229.7	254.4	242.07
USP40	ubiquitin specific peptidase 40	239.8	244.3	242.06
LOC104913907	uncharacterized LOC104913907	228.1	255.9	242.04
CASC5	cancer susceptibility candidate 5	249.9	233.7	241.79
SLC25A42	solute carrier family 25, member 42	228.9	254.5	241.72
NRBP2	nuclear receptor binding protein 2	282.0	201.1	241.58
FGD3	FYVE, RhoGEF and PH domain containing 3	244.1	239.0	241.54
DDX55	DEAD (Asp-Glu-Ala-Asp) box polypeptide 55	256.7	226.1	241.40
NPY	neuropeptide Y	195.4	287.1	241.29
SUMF2	sulfatase modifying factor 2	240.7	241.6	241.15
MYO19	myosin XIX	235.7	246.3	241.01
LOC100547196	probable global transcription activator SNF2L2	244.9	237.1	241.00
CDK10	cyclin-dependent kinase 10	243.2	238.6	240.91
C10H1orf21	chromosome 10 open reading frame, human C1orf21	227.1	254.7	240.89
LOC104912327	transmembrane protein 121	225.5	255.9	240.69
VAT1L	vesicle amine transport 1-like	226.3	254.8	240.53
SLC4A3	solute carrier family 4 (anion exchanger), member 3	268.7	212.3	240.49
FAM134B	family with sequence similarity 134, member B	263.4	217.6	240.47
IGSF9	immunoglobulin superfamily, member 9	264.5	216.4	240.40
DHX8	DEAH (Asp-Glu-Ala-His) box polypeptide 8	224.7	255.7	240.20
SLC7A6OS	solute carrier family 7, member 6 opposite strand	242.4	237.9	240.14
RUFY2	RUN and FYVE domain containing 2	243.2	236.0	239.60
LOC104910217	leucine-rich repeat-containing protein 16A-like	247.4	231.6	239.50

ACOX3	acyl-CoA oxidase 3, pristanoyl	220.5	258.5	239.50
HOXA9	homeobox A9	211.1	267.8	239.43
SPP1	secreted phosphoprotein 1	267.5	211.4	239.42
LOC104916182	TSC22 domain family protein 1-like	256.9	222.0	239.42
SLC35D1	solute carrier family 35 (UDP-GlcA/UDP-GalNAc transporter), member D1	241.4	237.3	239.36
MTFR2	mitochondrial fission regulator 2	245.6	232.1	238.88
ANKRD27	ankyrin repeat domain 27 (VPS9 domain)	196.0	281.4	238.70
MACF1	microtubule-actin crosslinking factor 1	279.9	197.4	238.69
LOC100538587	lipid phosphate phosphatase-related protein type 5	231.4	245.1	238.25
STK26	serine/threonine protein kinase 26	213.7	262.8	238.21
PPP1R16B	protein phosphatase 1, regulatory subunit 16B	194.3	282.0	238.12
CALHM2	calcium homeostasis modulator 2	222.9	253.2	238.10
LOC104915877	dual specificity tyrosine-phosphorylation-regulated kinase 1B-like	229.0	247.0	238.02
LOC104913418	E3 ubiquitin-protein ligase RNF216-like	244.2	231.7	237.96
LOC100545241	nuclear factor erythroid 2-related factor 1 pseudogene	240.8	234.9	237.86
MAGI3	membrane associated guanylate kinase, WW and PDZ domain containing 3	240.7	234.6	237.62
NEURL1	neuralized E3 ubiquitin protein ligase 1	232.4	242.4	237.41
RGMB	repulsive guidance molecule family member b	218.8	255.9	237.36
BRCA1	breast cancer 1, early onset	260.1	214.3	237.17
ERCC6L	excision repair cross-complementation group 6-like	244.0	230.1	237.07
LOC104910908	transforming acidic coiled-coil-containing protein 3-like	246.5	227.7	237.07
LOC104912287	uncharacterized LOC104912287	236.4	237.6	237.03
TTC25	tetratricopeptide repeat domain 25	246.6	227.2	236.92
WDR35	WD repeat domain 35	241.6	232.2	236.92
GNPTAB	N-acetylglucosamine-1-phosphate transferase, alpha and beta subunits	240.7	232.7	236.70
MAFB	v-maf avian musculoaponeurotic fibrosarcoma oncogene homolog B	240.7	232.7	236.69
PANK1	pantothenate kinase 1	236.4	236.9	236.66
WWC2	WW and C2 domain containing 2	234.0	239.0	236.48
GANC	glucosidase, alpha; neutral C	227.3	245.7	236.46
TMEM79	transmembrane protein 79	258.4	214.3	236.34
ECHDC3	enoyl CoA hydratase domain containing 3	250.7	221.9	236.30
ZMAT5	zinc finger, matrin-type 5	245.7	226.9	236.29
LOC104911425	uncharacterized LOC104911425	212.0	260.4	236.22
OTUD3	OTU deubiquitinase 3	232.2	239.8	236.02
POMK	protein-O-mannose kinase	244.9	226.9	235.90
LOC104911143	RNA-binding protein Nova-1	259.2	212.1	235.65
LOC104910178	protein cordon-bleu-like	208.6	262.5	235.58
PYROXD1	pyridine nucleotide-disulphide oxidoreductase domain 1	232.3	238.8	235.55
GARNL3	GTPase activating Rap/RanGAP domain-like 3	217.0	254.0	235.50
RRH	retinal pigment epithelium-derived rhodopsin homolog	227.9	243.0	235.49
LOC104916090	extended synaptotagmin-1-like	271.2	199.6	235.41
WARS2	tryptophanyl tRNA synthetase 2, mitochondrial	209.6	261.2	235.39
GCLC	glutamate-cysteine ligase, catalytic subunit	217.9	252.5	235.20
PAOX	polyamine oxidase (exo-N4-amino)	230.6	239.6	235.11
CENPT	centromere protein T	241.5	228.3	234.91
CNGA4	cyclic nucleotide gated channel alpha 4	261.9	207.8	234.84
EPHX4	epoxide hydrolase 4	223.8	245.5	234.69
TBC1D22B	TBC1 domain family, member 22B	234.1	234.6	234.36
PLK3	polo-like kinase 3	249.0	219.6	234.29
GCAT	glycine C-acetyltransferase	287.1	181.4	234.26
LOC100543188	serine/threonine-protein phosphatase 2B catalytic subunit gamma isoform-like	225.7	242.6	234.14
HMBOX1	homeobox containing 1	218.7	249.3	233.99
TMEM192	transmembrane protein 192	241.5	226.3	233.86

SEC14L2	SEC14-like 2 (S. cerevisiae)	255.0	212.7	233.85
SULT4A1	sulfotransferase family 4A, member 1	216.3	251.3	233.79
FBXL14	F-box and leucine-rich repeat protein 14	233.2	234.3	233.74
PANK4	pantothenate kinase 4	211.1	256.3	233.70
MIOS	missing oocyte, meiosis regulator, homolog (Drosophila)	239.0	227.7	233.34
CDC37L1	cell division cycle 37-like 1	226.3	239.8	233.02
ATF7IP	activating transcription factor 7 interacting protein	228.9	237.2	233.00
IPP	intracisternal A particle-promoted polypeptide	231.5	234.4	232.93
AP4S1	adaptor-related protein complex 4, sigma 1 subunit	243.9	221.9	232.90
LOC104912358	ral guanine nucleotide dissociation stimulator-like 1	214.6	250.9	232.72
DTX3L	deltex 3 like, E3 ubiquitin ligase	217.0	248.0	232.51
AKAP10	A kinase (PRKA) anchor protein 10	219.6	245.0	232.29
MIIP	migration and invasion inhibitory protein	234.0	230.2	232.06
SMG5	SMG5 nonsense mediated mRNA decay factor	282.3	181.9	232.06
PURG	purine-rich element binding protein G	239.8	224.2	231.99
LOC104915932	melanoma-associated antigen G1-like	244.9	219.1	231.98
RPUSD2	RNA pseudouridylate synthase domain containing 2	228.8	234.9	231.84
ACTR5	ARP5 actin-related protein 5 homolog (yeast)	246.6	216.8	231.71
CDYL2	chromodomain protein, Y-like 2	223.8	238.9	231.34
LOC104912839	uncharacterized LOC104912839	234.7	227.8	231.22
DCUN1D3	DCN1, defective in cullin neddylation 1, domain containing 3	216.2	246.2	231.21
RPAP2	RNA polymerase II associated protein 2	233.9	228.5	231.16
CCDC153	coiled-coil domain containing 153	228.1	233.8	230.97
UBR1	ubiquitin protein ligase E3 component n-recognin 1	228.1	233.6	230.83
LOC104911804	protein disulfide-isomerase A5-like	213.7	247.7	230.74
LOC104909740	plasma alpha-L-fucosidase-like	225.6	235.9	230.73
ZNF770	zinc finger protein 770	211.1	250.3	230.69
TBC1D12	TBC1 domain family, member 12	222.9	238.3	230.62
FAM92A1	family with sequence similarity 92, member A1	221.3	239.8	230.56
DSCC1	DNA replication and sister chromatid cohesion 1	243.1	217.9	230.49
AASDHPPT	aminoadipate-semialdehyde dehydrogenase-phosphopantetheinyl transferase	216.1	244.6	230.39
LOC104914946	BEN domain-containing protein 2-like	228.8	232.0	230.37
LOC100544843	transducin-like enhancer protein 4	235.6	225.1	230.36
LOC100540378	magnesium transporter NIPA2-like	224.6	235.9	230.27
UNC93B1	unc-93 homolog B1 (C. elegans)	233.1	227.4	230.27
TNRC6B	trinucleotide repeat containing 6B	215.5	244.9	230.18
GMPR	guanosine monophosphate reductase	229.8	230.1	229.97
TPMT	thiopurine S-methyltransferase	218.7	241.1	229.90
TBC1D19	TBC1 domain family, member 19	231.3	228.5	229.90
TLL2	tolloid-like 2	236.6	223.2	229.89
FAM120B	family with sequence similarity 120B	248.1	211.4	229.74
PARP11	poly (ADP-ribose) polymerase family, member 11	222.1	237.4	229.73
ZMYM5	zinc finger, MYM-type 5	221.3	237.7	229.50
TGIF2	TGFB-induced factor homeobox 2	250.0	208.9	229.47
LOC104910037	ribosome biogenesis protein bop1-like	246.7	212.2	229.43
BAZ1B	bromodomain adjacent to zinc finger domain, 1B	243.4	215.0	229.23
TRIM37	tripartite motif containing 37	230.6	227.4	229.01
LOC100543611	protein cordon-bleu-like	212.8	244.8	228.80
LRP5	low density lipoprotein receptor-related protein 5	244.2	213.2	228.66
CMAS	cytidine monophosphate N-acetylneuraminic acid synthetase	238.0	219.3	228.66
ADAMTS6	ADAM metallopeptidase with thrombospondin type 1 motif, 6	211.2	245.8	228.53
UBE2W	ubiquitin-conjugating enzyme E2W (putative)	213.6	243.4	228.49
RPRD1A	regulation of nuclear pre-mRNA domain containing 1A	201.8	255.1	228.46
TRMT12	tRNA methyltransferase 12 homolog (S. cerevisiae)	224.6	231.9	228.25
HSD11B2	hydroxysteroid (11-beta) dehydrogenase 2	233.2	223.1	228.15
PPP1R26	protein phosphatase 1, regulatory subunit 26	223.0	233.2	228.10
GAS8	growth arrest-specific 8	244.8	211.2	228.00

FSD1	fibronectin type III and SPRY domain containing 1	242.4	213.5	227.96
ADORA2A	adenosine A2a receptor	237.3	218.5	227.94
SEC22A	SEC22 vesicle trafficking protein homolog A (S. cerevisiae)	248.3	207.5	227.89
LOC104910232	zinc finger protein 407-like	248.3	207.4	227.85
KDM1B	lysine (K)-specific demethylase 1B	212.0	243.5	227.77
INSR	insulin receptor	237.4	218.1	227.74
LOC104915180	inositol hexakisphosphate and diphosphoinositol-pentakisphosphate kinase 2-like	218.8	236.4	227.56
TYSND1	trypsin domain containing 1	228.9	225.9	227.43
LOC104913553	uncharacterized LOC104913553	214.5	240.2	227.34
LOC104916337	uncharacterized LOC104916337	242.4	212.2	227.28
JPH2	junctophilin 2	237.5	216.6	227.10
PLA1A	phospholipase A1 member A	216.2	237.9	227.09
IFNAR1	interferon (alpha, beta and omega) receptor 1	207.9	246.1	226.99
LOC104916259	sortilin-like	239.8	213.9	226.88
ALPK2	alpha-kinase 2	227.2	226.3	226.76
FARS2	phenylalanyl-tRNA synthetase 2, mitochondrial	223.7	229.5	226.61
TSEN2	TSEN2 tRNA splicing endonuclease subunit	240.7	212.5	226.57
RNFT2	ring finger protein, transmembrane 2	217.9	234.9	226.42
LOC104909494	uncharacterized LOC104909494	200.2	252.7	226.41
LRRC8B	leucine rich repeat containing 8 family, member B	227.9	224.8	226.36
CDK5R1	cyclin-dependent kinase 5, regulatory subunit 1 (p35)	239.1	213.6	226.35
NPAS2	neuronal PAS domain protein 2	228.1	224.6	226.33
MPP4	membrane protein, palmitoylated 4 (MAGUK p55 subfamily member 4)	216.2	236.4	226.32
LOC100549940	long-chain-fatty-acid--CoA ligase 3-like	230.7	221.7	226.16
ADAMTS20	ADAM metallopeptidase with thrombospondin type 1 motif, 20	223.8	228.3	226.02
TTC13	tetratricopeptide repeat domain 13	217.9	234.1	226.00
EDC3	enhancer of mRNA decapping 3	218.8	233.1	225.95
MND1	meiotic nuclear divisions 1 homolog (S. cerevisiae)	229.6	221.7	225.64
CCDC34	coiled-coil domain containing 34	210.2	240.6	225.39
SWT1	SWT1 RNA endoribonuclease homolog (S. cerevisiae)	229.7	221.0	225.38
LOC104916865	hydroxysteroid dehydrogenase-like protein 2	224.7	225.8	225.24
DEPTOR	DEP domain containing MTOR-interacting protein	192.5	257.9	225.19
STRADB	STE20-related kinase adaptor beta	223.8	226.6	225.18
APITD1	apoptosis-inducing, TAF9-like domain 1	227.1	223.2	225.17
LOC100546747	guanine nucleotide-binding protein G(olf) subunit alpha	220.5	229.5	224.99
STXBP3	syntaxin binding protein 3	216.2	233.6	224.92
LOC104916301	uncharacterized LOC104916301	245.8	203.7	224.72
KANSL1	KAT8 regulatory NSL complex subunit 1	238.2	211.2	224.71
DIRAS1	DIRAS family, GTP-binding RAS-like 1	249.3	199.9	224.60
MPDZ	multiple PDZ domain protein	211.1	238.0	224.54
RAB5A	RAB5A, member RAS oncogene family	216.2	232.8	224.52
IMPACT	impact RWD domain protein	215.3	233.6	224.48
LOC100543391	WD40 repeat-containing protein SMU1	209.5	239.1	224.32
LOC104911452	dnaJ homolog subfamily C member 1-like	206.9	241.5	224.18
LOC100550735	centrosomal protein of 85 kDa-like	203.5	244.8	224.15
SEMA3D	sema domain, immunoglobulin domain (Ig), short basic domain, secreted, (semaphorin) 3D	202.8	245.5	224.13
TMEM39B	transmembrane protein 39B	240.7	207.4	224.05
GPKOW	G patch domain and KOW motifs	241.5	206.5	224.02
LOC104916463	uncharacterized LOC104916463	257.8	190.0	223.86
SPATA13	spermatogenesis associated 13	219.7	228.1	223.86
DFNB59	deafness, autosomal recessive 59	219.6	227.9	223.75
C8H10orf54	chromosome 8 open reading frame, human C10orf54	230.6	216.7	223.63
CHDH	choline dehydrogenase	201.0	246.0	223.51
DNAJC6	DnaJ (Hsp40) homolog, subfamily C, member 6	213.7	233.2	223.50
LOC104916215	KAT8 regulatory NSL complex subunit 2-like	239.9	206.9	223.39

GCH1	GTP cyclohydrolase 1	222.1	224.2	223.17
LOC104909288	uncharacterized LOC104909288	215.3	231.0	223.16
MTMR10	myotubularin related protein 10	223.1	222.9	223.04
LOC104913914	RNA-binding protein Musashi homolog 2-like	217.0	229.0	223.03
IMMP1L	IMP1 inner mitochondrial membrane peptidase-like (S. cerevisiae)	237.2	208.6	222.93
LOC100547303	EVI5-like protein	240.0	205.8	222.91
LOC104910865	probable tRNA (uracil-O(2)-)-methyltransferase	218.7	227.0	222.86
NGFR	nerve growth factor receptor	216.3	229.4	222.85
FBXO8	F-box protein 8	216.2	229.4	222.82
LOC104914527	ankyrin repeat and SAM domain-containing protein 1A-like	212.0	233.6	222.81
LOC100542094	exportin-2	218.8	226.5	222.63
CDC25A	cell division cycle 25A	247.4	197.5	222.45
LOC104912087	WD repeat-containing protein 11-like	208.6	236.0	222.29
GIMD1	GIMAP family P-loop NTPase domain containing 1	209.4	235.1	222.24
NRDE2	NRDE-2, necessary for RNA interference, domain containing	205.2	238.9	222.08
LOC100544745	epidermal growth factor receptor kinase substrate 8-like	170.7	273.5	222.07
MSRA	methionine sulfoxide reductase A	239.7	204.2	221.96
UBN1	ubinuclein 1	206.1	237.7	221.90
PLS1	plastin 1	213.7	230.0	221.85
LOC104915960	acyl-coenzyme A thioesterase THEM4-like	248.2	195.4	221.83
LOC100548334	N(4)-(beta-N-acetylglucosaminyl)-L-asparaginase-like	209.4	234.1	221.78
NCAPG	non-SMC condensin I complex, subunit G	223.7	219.6	221.67
LOC104917160	transcription intermediary factor 1-beta-like	240.0	203.1	221.56
LOC104916768	uncharacterized LOC104916768	242.4	200.7	221.53
KATNAL2	katanin p60 subunit A-like 2	223.7	219.2	221.48
FBXO34	F-box protein 34	255.8	186.7	221.26
LOC104910552	extracellular matrix protein FRAS1-like	244.8	197.6	221.22
GATSL3	GATS protein-like 3	244.1	198.0	221.05
LOC104916486	dehydrogenase/reductase SDR family member 4-like	248.3	193.8	221.04
LOC100549111	uncharacterized LOC100549111	227.1	215.0	221.04
C13H16orf87	chromosome 13 open reading frame, human C16orf87	222.1	219.8	220.95
EML1	echinoderm microtubule associated protein like 1	192.7	248.9	220.80
LOC104916374	evolutionarily conserved signaling intermediate in Toll pathway, mitochondrial-like	245.8	195.7	220.77
LOC104917114	uncharacterized LOC104917114	231.5	209.6	220.51
FADD	Fas (TNFRSF6)-associated via death domain	217.8	223.2	220.49
C2H6orf120	chromosome 2 open reading frame, human C6orf120	201.9	238.9	220.41
CMTM4	CKLF-like MARVEL transmembrane domain containing 4	198.4	242.4	220.40
C2CD2	C2 calcium-dependent domain containing 2	206.1	234.5	220.29
SNX10	sorting nexin 10	215.3	225.2	220.26
LOC104912991	uncharacterized LOC104912991	212.0	228.4	220.20
LOC100543305	mitotic checkpoint serine/threonine-protein kinase BUB1 beta-like	247.4	192.7	220.04
FHIT	fragile histidine triad	211.1	229.0	220.02
IFT43	intraflagellar transport 43	213.7	225.9	219.83
KIN	Kin17 DNA and RNA binding protein	202.6	237.0	219.82
PARP4	poly (ADP-ribose) polymerase family, member 4	201.8	237.8	219.80
LOC104910131	uncharacterized LOC104910131	210.2	229.0	219.64
TTI1	TELO2 interacting protein 1	206.0	232.9	219.48
LOC100545707	uncharacterized LOC100545707	235.7	203.3	219.46
CBFA2T2	core-binding factor, runt domain, alpha subunit 2; translocated to, 2	230.6	208.3	219.45
VPS13A	vacuolar protein sorting 13 homolog A (S. cerevisiae)	208.7	230.2	219.42
LOC100546568	semaphorin-3D-like	229.7	208.8	219.22
AGPAT4	1-acylglycerol-3-phosphate O-acyltransferase 4	202.7	235.4	219.05
LOC104913950	uncharacterized LOC104913950	215.4	222.6	218.99
LOC104913523	uncharacterized LOC104913523	184.3	253.5	218.90

RALGPS1	Ral GEF with PH domain and SH3 binding motif 1	210.3	227.5	218.86
CBLB	Cbl proto-oncogene B, E3 ubiquitin protein ligase	224.7	212.9	218.80
LOC104913498	protein sidekick-1-like	207.8	229.6	218.74
ELL	elongation factor RNA polymerase II	218.8	218.6	218.71
TCTN1	tectonic family member 1	225.4	212.0	218.70
ERMARD	ER membrane-associated RNA degradation	192.6	244.7	218.65
EFCAB7	EF-hand calcium binding domain 7	220.3	216.6	218.45
LOC104910842	DNA excision repair protein ERCC-6-like 2	220.4	216.5	218.45
CZH5orf51	chromosome Z open reading frame, human C5orf51	218.6	218.2	218.42
MAPK14	mitogen-activated protein kinase 14	212.0	224.8	218.41
ZBTB6	zinc finger and BTB domain containing 6	231.3	205.3	218.31
CD36	CD36 molecule (thrombospondin receptor)	202.8	233.8	218.30
CCDC88A	coiled-coil domain containing 88A	236.6	199.9	218.29
TRIM32	tripartite motif containing 32	229.7	206.8	218.29
FRA10AC1	fragile site, folic acid type, rare, fra(10)(q23.3) or fra(10)(q24.2) candidate 1	219.4	217.0	218.23
MIF4GD	MIF4G domain containing	208.7	227.6	218.18
NACC2	NACC family member 2, BEN and BTB (POZ) domain containing	225.5	210.8	218.15
GPR158	G protein-coupled receptor 158	217.1	219.2	218.12
CRAT	carnitine O-acetyltransferase	225.5	210.7	218.11
LOC104914182	natriuretic peptides A	207.8	228.3	218.03
NTHL1	nth endonuclease III-like 1 (E. coli)	224.7	211.2	217.94
CHIC1	cysteine-rich hydrophobic domain 1	206.0	229.8	217.88
BBIP1	BBSome interacting protein 1	207.7	228.0	217.84
LOC104910558	uncharacterized LOC104910558	239.0	196.5	217.74
R3HCC1L	R3H domain and coiled-coil containing 1-like	229.7	205.2	217.45
LOC104914649	putative nuclease HARBI1	250.8	184.1	217.44
LOC104915278	ubiquitin-like-conjugating enzyme ATG10	226.3	208.5	217.40
COQ10A	coenzyme Q10 homolog A (S. cerevisiae)	213.7	220.9	217.26
SLC9A7	solute carrier family 9, subfamily A (NHE7, cation proton antiporter 7), member 7	206.1	228.3	217.22
RBM41	RNA binding motif protein 41	212.0	222.2	217.07
LOC100544136	myc box-dependent-interacting protein 1	202.0	232.1	217.01
RASSF1	Ras association (RalGDS/AF-6) domain family member 1	256.8	177.2	216.99
ORAOV1	oral cancer overexpressed 1	225.4	208.0	216.72
LOC104911581	cholinephosphotransferase 1-like	224.6	208.8	216.68
DNAH17	dynein, axonemal, heavy chain 17	212.8	220.6	216.67
B4GALT3	UDP-Gal:betaGlcNAc beta 1,4- galactosyltransferase, polypeptide 3	240.9	192.2	216.57
E2F8	E2F transcription factor 8	248.2	184.9	216.54
IFI30	interferon, gamma-inducible protein 30	202.0	231.0	216.51
LOC104912447	transcription factor BTF3 homolog 4	253.4	179.5	216.42
KCNAB1	potassium voltage-gated channel, shaker-related subfamily, beta member 1	203.6	229.2	216.41
LIG4	ligase IV, DNA, ATP-dependent	217.8	214.9	216.38
LOC104910222	myelin basic protein-like	199.4	232.8	216.13
LYST	lysosomal trafficking regulator	199.4	232.8	216.09
LOC104912584	uncharacterized LOC104912584	202.7	229.3	215.96
MIS18BP1	MIS18 binding protein 1	211.2	220.6	215.87
UAP1L1	UDP-N-acetylglucosamine pyrophosphorylase 1 like 1	199.4	232.2	215.83
ASB15	ankyrin repeat and SOCS box containing 15	212.7	218.9	215.81
FMN1	formin 1	206.0	225.5	215.75
BRIP1	BRCA1 interacting protein C-terminal helicase 1	223.8	207.6	215.71
DNAJC17	DnaJ (Hsp40) homolog, subfamily C, member 17	215.2	215.8	215.49
LOC104917197	peptidyl-prolyl cis-trans isomerase CWC27 homolog	206.1	224.9	215.49
SLC41A2	solute carrier family 41 (magnesium transporter), member 2	213.6	217.2	215.40
LOC100543078	aprataxin-like	206.1	224.4	215.24
DDHD1	DDHD domain containing 1	205.3	225.2	215.22

RTEL1	regulator of telomere elongation helicase 1	197.0	233.1	215.02
MAST3	microtubule associated serine/threonine kinase 3	228.1	201.9	215.01
RGS20	regulator of G-protein signaling 20	218.7	211.2	214.95
C7H2orf76	chromosome 7 open reading frame, human C2orf76	219.4	210.4	214.88
LOC104912486	structure-specific endonuclease subunit SLX1-like	221.2	208.5	214.83
NFKBIZ	nuclear factor of kappa light polypeptide gene enhancer in B-cells inhibitor, zeta	215.3	214.0	214.69
STAT2	signal transducer and activator of transcription 2, 113kDa	235.7	193.0	214.35
MRS2	MRS2 magnesium transporter	212.0	216.3	214.13
MNX1	motor neuron and pancreas homeobox 1	179.2	248.7	213.96
LOC104909237	multidrug resistance protein 3-like	221.9	205.9	213.91
ATF7	activating transcription factor 7	225.6	202.1	213.85
RBM33	RNA binding motif protein 33	210.3	217.4	213.83
GABRB1	gamma-aminobutyric acid (GABA) A receptor, beta 1	202.7	224.4	213.53
GPR162	G protein-coupled receptor 162	229.0	198.1	213.53
LOC100551000	myotubularin-related protein 7-like	195.9	231.1	213.53
LOC104916108	chromosome unknown open reading frame, human C6orf136	194.2	232.7	213.45
STK11IP	serine/threonine kinase 11 interacting protein	218.8	207.5	213.14
LOC104912343	aspartate--tRNA ligase, mitochondrial-like	215.4	210.8	213.07
NLRX1	NLR family member X1	232.3	193.4	212.83
SPAG1	sperm associated antigen 1	201.0	224.4	212.69
STBD1	starch binding domain 1	227.3	198.1	212.69
PPT2	palmitoyl-protein thioesterase 2	232.3	193.0	212.69
RIC1	RAB6A GEF complex partner 1	195.1	230.2	212.68
LOC104913209	SLIT-ROBO Rho GTPase-activating protein 3	218.8	206.4	212.59
LOC104913369	ubiquitin carboxyl-terminal hydrolase 7-like	200.2	224.7	212.47
NXPE3	neurexophilin and PC-esterase domain family, member 3	214.4	210.5	212.46
C4H4orf48	chromosome 4 open reading frame, human C4orf48	169.1	255.7	212.40
TIAM2	T-cell lymphoma invasion and metastasis 2	196.8	227.5	212.18
MCM10	minichromosome maintenance complex component 10	224.6	199.8	212.17
N4BP3	NEDD4 binding protein 3	251.7	172.6	212.14
CZH5orf34	chromosome Z open reading frame, human C5orf34	210.3	213.8	212.05
PODXL2	podocalyxin-like 2	229.7	193.9	211.82
TTC7A	tetratricopeptide repeat domain 7A	217.2	206.4	211.81
WDSUB1	WD repeat, sterile alpha motif and U-box domain containing 1	216.2	207.2	211.75
LOC104913510	uncharacterized LOC104913510	211.3	211.6	211.42
NHLRC2	NHL repeat containing 2	185.8	236.8	211.30
PPEF1	protein phosphatase, EF-hand calcium binding domain 1	216.3	206.3	211.29
CHTF8	CTF8, chromosome transmission fidelity factor 8 homolog (S. cerevisiae)	214.4	208.1	211.28
SLA2	Src-like-adaptor 2	207.8	214.7	211.23
PEA15	phosphoprotein enriched in astrocytes 15	237.4	184.5	210.95
TELO2	telomere maintenance 2	233.8	188.0	210.90
PLEKHG4	pleckstrin homology domain containing, family G (with RhoGef domain) member 4	204.4	217.2	210.79
OPRD1	opioid receptor, delta 1	192.7	228.7	210.71
DERA	deoxyribose-phosphate aldolase (putative)	191.7	229.5	210.56
NUDT19	nudix (nucleoside diphosphate linked moiety X)-type motif 19	208.6	212.2	210.40
ABLIM1	actin binding LIM protein 1	196.2	224.6	210.40
SDC1	syndecan 1	187.6	233.0	210.32
LOC104910076	little elongation complex subunit 1-like	202.6	217.5	210.09
FAM175A	family with sequence similarity 175, member A	207.7	212.5	210.06
CGN	cingulin	225.5	194.5	210.02
RMDN1	regulator of microtubule dynamics 1	211.1	208.2	209.66
LOC100548742	zinc finger homeobox protein 4-like	190.9	228.2	209.55
ATXN7	ataxin 7	212.9	206.2	209.54
ERI1	exoribonuclease 1	194.2	224.5	209.35
LOC104915800	UDP-galactose translocator-like	242.5	176.2	209.33

GHDC	GH3 domain containing	228.0	190.5	209.23
STOML1	stomatin (EPB72)-like 1	211.1	207.2	209.15
RAD51AP1	RAD51 associated protein 1	227.0	191.3	209.13
MFF	mitochondrial fission factor	215.3	202.9	209.09
LOC100546305	E3 ubiquitin-protein ligase HECTD3-like	199.3	218.8	209.05
NUDT13	nudix (nucleoside diphosphate linked moiety X)-type motif 13	190.1	228.0	209.04
LOC104909219	two pore calcium channel protein 1-like	202.7	215.0	208.82
PCTP	phosphatidylcholine transfer protein	211.0	206.5	208.73
LIPT1	lipoyltransferase 1	217.0	200.3	208.68
LOC100539075	transcription factor 4-like	218.8	198.5	208.67
BATF	basic leucine zipper transcription factor, ATF-like	217.9	199.4	208.65
LOC100539460	solute carrier family 22 member 15-like	214.5	202.5	208.52
AIFM2	apoptosis-inducing factor, mitochondrion-associated, 2	193.4	223.6	208.48
LOC100540888	V-type proton ATPase catalytic subunit A-like	222.9	193.8	208.36
CZH9orf41	chromosome Z open reading frame, human C9orf41	218.7	197.9	208.27
RRNAD1	ribosomal RNA adenine dimethylase domain containing 1	219.6	196.7	208.16
ZBTB38	zinc finger and BTB domain containing 38	206.1	210.2	208.14
SPATA7	spermatogenesis associated 7	192.6	223.6	208.11
HBEGF	heparin-binding EGF-like growth factor	217.8	198.3	208.04
LOC104914277	complement C2-like	200.3	215.8	208.03
CENPN	centromere protein N	233.9	182.1	208.00
TMEM194A	transmembrane protein 194A	205.2	210.4	207.80
MREG	melanoregulin	191.8	223.8	207.79
WNT4	wingless-type MMTV integration site family, member 4	187.6	228.0	207.78
LOC104909254	arf-GAP with dual PH domain-containing protein 1-like	207.1	208.4	207.75
LOC100539104	unconventional myosin-Va	195.2	219.8	207.49
TOP3A	topoisomerase (DNA) III alpha	206.9	207.9	207.40
IFT81	intraflagellar transport 81	203.5	211.2	207.35
LOC104915214	uncharacterized LOC104915214	210.3	204.1	207.19
PLCE1	phospholipase C, epsilon 1	184.2	230.1	207.18
GCLM	glutamate-cysteine ligase, modifier subunit	190.0	223.3	206.65
PIK3C3	phosphatidylinositol 3-kinase, catalytic subunit type 3	220.4	192.6	206.48
USP3	ubiquitin specific peptidase 3	191.8	221.1	206.42
TNS4	tensin 4	221.3	191.5	206.40
DIRC2	disrupted in renal carcinoma 2	206.9	205.3	206.11
DHRS3	dehydrogenase/reductase (SDR family) member 3	222.9	189.2	206.06
FOXK2	forkhead box K2	209.5	202.4	205.96
ALKBH8	alkB, alkylation repair homolog 8 (E. coli)	208.6	203.3	205.96
C16H8orf33	chromosome 16 open reading frame, human C8orf33	213.7	197.6	205.64
LOC100538719	exportin-1-like	190.0	221.2	205.62
TRMT10A	tRNA methyltransferase 10 homolog A (S. cerevisiae)	194.3	216.6	205.45
EXD1	exonuclease 3'-5' domain containing 1	198.5	212.3	205.39
LOC104911358	serine/threonine-protein kinase greatwall-like	204.3	206.4	205.34
LOC100546578	zinc finger protein castor homolog 1-like	199.5	211.0	205.25
PLXND1	plexin D1	191.8	218.3	205.08
PLEKHM1	pleckstrin homology domain containing, family M (with RUN domain) member 1	248.4	161.7	205.07
LOC100540394	putative gonadotropin-releasing hormone II receptor	212.8	197.3	205.05
FAM46C	family with sequence similarity 46, member C	195.9	214.2	205.05
ATAD5	ATPase family, AAA domain containing 5	203.5	206.5	205.01
NHS	Nance-Horan syndrome (congenital cataracts and dental anomalies)	179.1	230.8	204.93
LOC104912550	uncharacterized LOC104912550	216.2	193.7	204.92
CDT1	chromatin licensing and DNA replication factor 1	236.5	173.1	204.84
MAP2K6	mitogen-activated protein kinase kinase 6	189.2	220.4	204.83
USP46	ubiquitin specific peptidase 46	201.8	207.5	204.64
LOC104917422	propionyl-CoA carboxylase alpha chain, mitochondrial-like	221.2	187.6	204.41
RIF1	replication timing regulatory factor 1	226.4	182.3	204.32

TAF5	TAF5 RNA polymerase II, TATA box binding protein (TBP)-associated factor, 100kDa	201.0	207.4	204.21
IRF1	interferon regulatory factor 1	161.4	247.0	204.18
LOC104911276	uncharacterized LOC104911276	206.9	201.0	203.95
LOC104915502	nucleotide exchange factor SIL1-like	195.1	212.8	203.93
TMEM5	transmembrane protein 5	190.8	217.1	203.91
LOC100550649	transcription factor Sp3	197.6	209.8	203.72
PDPK1	3-phosphoinositide dependent protein kinase 1	204.4	203.1	203.72
LOC100540047	chromodomain-helicase-DNA-binding protein 1-like	197.5	209.8	203.66
TSHZ2	teashirt zinc finger homeobox 2	206.2	201.0	203.57
LOC100547773	transmembrane protein 121-like	193.4	213.3	203.35
APPL2	adaptor protein, phosphotyrosine interaction, PH domain and leucine zipper containing 2	178.3	228.3	203.29
RFX5	regulatory factor X, 5 (influences HLA class II expression)	223.0	183.5	203.28
LOC104909493	neuronal PAS domain-containing protein 4-like	218.8	187.8	203.26
RNF213	ring finger protein 213	207.9	198.4	203.14
LOC723980	avidin	187.6	218.3	202.96
ABI3BP	ABI family, member 3 (NESH) binding protein	231.3	174.6	202.93
LOC104910147	uncharacterized LOC104910147	199.3	206.5	202.88
LOC100547383	uncharacterized LOC100547383	223.8	181.9	202.84
LOC104917527	von Willebrand factor A domain-containing protein 8-like	194.3	211.2	202.74
LOC100550445	rho-related BTB domain-containing protein 1-like	206.8	198.6	202.72
ZBTB37	zinc finger and BTB domain containing 37	205.4	199.9	202.62
LOC104911939	uncharacterized LOC104911939	185.8	219.4	202.60
LOC100546974	caspase recruitment domain-containing protein 8-like	201.8	203.3	202.59
SLC37A1	solute carrier family 37 (glucose-6-phosphate transporter), member 1	213.7	191.5	202.59
CLIP4	CAP-GLY domain containing linker protein family, member 4	195.1	209.8	202.44
PPARA	peroxisome proliferator-activated receptor alpha	195.1	209.6	202.35
LSM14A	LSM14A, SCD6 homolog A (S. cerevisiae)	193.4	211.1	202.28
LOC100545069	disintegrin and metalloproteinase domain-containing protein 10-like	183.3	221.2	202.25
CCDC66	coiled-coil domain containing 66	191.0	213.4	202.20
NOL6	nucleolar protein 6 (RNA-associated)	201.1	203.2	202.18
FASTKD5	FAST kinase domains 5	201.8	202.4	202.13
CRIM1	cysteine rich transmembrane BMP regulator 1 (chordin-like)	184.1	219.8	201.97
LRP11	low density lipoprotein receptor-related protein 11	209.5	194.2	201.86
LOC100548692	threonylcarbamoyladenosine tRNA methylthiotransferase-like	176.5	227.2	201.84
CHRD	chordin	214.6	189.0	201.78
LOC104915863	bromodomain-containing protein 4-like	212.9	190.6	201.76
TBC1D24	TBC1 domain family, member 24	194.3	209.2	201.73
LOC104911167	fibrinogen-like protein 1	190.9	212.4	201.66
FSD2	fibronectin type III and SPRY domain containing 2	201.1	202.1	201.60
IRAK1BP1	interleukin-1 receptor-associated kinase 1 binding protein 1	181.5	221.5	201.54
LOC104911651	nucleoporin Nup37-like	198.4	204.5	201.42
KLHL34	kelch-like family member 34	173.2	229.5	201.38
FEM1C	fem-1 homolog c (C. elegans)	198.4	204.0	201.21
SEMA4C	sema domain, immunoglobulin domain (Ig), transmembrane domain (TM) and short cytoplasmic domain, (semaphorin) 4C	235.7	166.5	201.09
FANCF	Fanconi anemia, complementation group F	217.9	184.3	201.08
DEPDC5	DEP domain containing 5	186.8	215.2	201.00
CRTC1	CREB regulated transcription coactivator 1	208.5	193.2	200.85
OGFOD2	2-oxoglutarate and iron-dependent oxygenase domain containing 2	205.3	196.4	200.85
LOC104916019	protein SCO2 homolog, mitochondrial	216.2	185.2	200.74
LOC100541781	histone H2A	213.6	187.4	200.50
SRPK1	SRSF protein kinase 1	186.7	214.3	200.50
RXRA	retinoid X receptor, alpha	199.4	201.5	200.46

PPM1J	protein phosphatase, Mg2+/Mn2+ dependent, 1J	226.4	174.5	200.44
LOC104915124	protein patched homolog 1-like	195.1	205.8	200.42
ST7	suppression of tumorigenicity 7	200.1	200.3	200.23
LOC104910260	uncharacterized LOC104910260	204.4	196.0	200.22
ZNF142	zinc finger protein 142	216.3	184.1	200.21
MAT1A	methionine adenosyltransferase I, alpha	230.3	169.9	200.12
LOC100546408	glycerol-3-phosphate dehydrogenase, mitochondrial	208.7	191.4	200.03
LOC104913010	G1/S-specific cyclin-E1-like	189.9	210.1	199.99
LOC100551267	glycogen debranching enzyme	185.9	213.6	199.74
LOC104916088	uncharacterized LOC104916088	201.8	197.6	199.74
PPCDC	phosphopantothenoylcysteine decarboxylase	214.4	184.7	199.58
LOC104914262	nucleolar pre-ribosomal-associated protein 1-like	211.1	187.8	199.45
PAK3	p21 protein (Cdc42/Rac)-activated kinase 3	207.0	191.9	199.44
CWF19L2	CWF19-like 2, cell cycle control (S. pombe)	198.4	200.4	199.39
TCEANC2	transcription elongation factor A (SII) N-terminal and central domain containing 2	182.4	216.0	199.24
LOC100547495	nuclear envelope pore membrane protein POM 121-like	212.1	186.1	199.08
SNED1	sushi, nidogen and EGF-like domains 1	200.1	197.9	198.99
LOC100549000	transcription factor E2F3-like	197.6	200.3	198.95
CLMN	calmin (calponin-like, transmembrane)	222.0	175.9	198.92
LOC104913932	uncharacterized LOC104913932	179.1	218.6	198.86
CENPF	centromere protein F, 350/400kDa	214.4	182.9	198.67
LOC100540107	transcription factor SPT20 homolog	198.5	198.5	198.51
LOC104913600	EMI domain-containing protein 1-like	177.3	219.7	198.50
PRKAB1	protein kinase, AMP-activated, beta 1 non-catalytic subunit	201.8	195.1	198.45
OVCA2	ovarian tumor suppressor candidate 2	204.4	192.2	198.30
ALDH1L2	aldehyde dehydrogenase 1 family, member L2	217.8	178.6	198.19
USB1	U6 snRNA biogenesis 1	200.2	196.1	198.12
LOC104915227	zinc finger protein 462-like	206.9	189.1	197.98
WDHD1	WD repeat and HMG-box DNA binding protein 1	193.4	202.5	197.93
LOC104915743	dynamin-2	229.0	166.8	197.91
BMPR1B	bone morphogenetic protein receptor, type IB	175.7	219.9	197.82
DHDH	dihydrodiol dehydrogenase (dimeric)	196.8	198.7	197.78
LOC100542534	inactive phospholipase C-like protein 2	185.0	210.5	197.73
LOC100539898	lysine-specific demethylase 6A	199.4	196.0	197.70
SRSF4	serine/arginine-rich splicing factor 4	191.6	203.6	197.61
FADS2	fatty acid desaturase 2	225.4	169.8	197.59
GPRIN1	G protein regulated inducer of neurite outgrowth 1	148.8	246.3	197.58
C7H2orf69	chromosome 7 open reading frame, human C2orf69	218.7	176.4	197.57
EXD2	exonuclease 3'-5' domain containing 2	192.6	202.3	197.42
TNFRSF9	tumor necrosis factor receptor superfamily, member 9	175.6	219.1	197.34
PLAC9	placenta-specific 9	188.4	206.2	197.32
DHX57	DEAH (Asp-Glu-Ala-Asp/His) box polypeptide 57	204.4	189.7	197.02
GALT	galactose-1-phosphate uridylyltransferase	208.6	185.4	197.01
ZBTB45	zinc finger and BTB domain containing 45	212.7	181.1	196.90
LOC104913353	uncharacterized LOC104913353	199.3	194.1	196.72
SEMA6A	sema domain, transmembrane domain (TM), and cytoplasmic domain, (semaphorin) 6A	189.4	203.9	196.67
TXLNB	taxilin beta	186.7	206.2	196.44
LOC104912332	myomegalin	229.0	163.8	196.44
MVB12B	multivesicular body subunit 12B	205.4	187.5	196.42
GYLTL1B	glycosyltransferase-like 1B	193.5	199.3	196.38
MOGS	mannosyl-oligosaccharide glucosidase	206.1	186.6	196.35
CASP2	caspase 2, apoptosis-related cysteine peptidase	199.3	193.3	196.32
HYI	hydroxypyruvate isomerase (putative)	206.8	185.8	196.31
ACRV1	acrosomal vesicle protein 1	223.0	169.5	196.25
CCP110	centriolar coiled coil protein 110kDa	182.4	210.1	196.24
JARID2	jumonji, AT rich interactive domain 2	185.9	206.6	196.24

MLLT3	myeloid/lymphoid or mixed-lineage leukemia (trithorax homolog, Drosophila); translocated to, 3	183.3	209.2	196.22
UBAC1	UBA domain containing 1	199.3	192.9	196.13
POLR3D	polymerase (RNA) III (DNA directed) polypeptide D, 44kDa	216.2	175.9	196.02
C28H1orf74	chromosome 28 open reading frame, human C1orf74	201.0	190.7	195.84
NIPAL3	NIPA-like domain containing 3	168.9	222.3	195.64
THNSL2	threonine synthase-like 2 (S. cerevisiae)	188.4	202.6	195.47
SIL1	SIL1 nucleotide exchange factor	204.4	186.3	195.35
C7H2orf88	chromosome 7 open reading frame, human C2orf88	178.4	212.2	195.25
RTCB	RNA 2',3'-cyclic phosphate and 5'-OH ligase	203.7	186.6	195.11
CEP95	centrosomal protein 95kDa	215.3	174.9	195.09
CTPS2	CTP synthase 2	192.5	197.5	194.99
LOC104916686	transcription activator BRG1-like	218.0	171.9	194.97
PQLC2	PQ loop repeat containing 2	202.0	187.8	194.89
SLK	STE20-like kinase	171.5	218.2	194.84
RNF207	ring finger protein 207	201.0	188.5	194.74
LRR1	leucine rich repeat protein 1	204.3	184.8	194.59
METTL17	methyltransferase like 17	222.9	166.2	194.54
LOXL2	lysyl oxidase-like 2	199.4	189.4	194.42
CDKL2	cyclin-dependent kinase-like 2 (CDC2-related kinase)	174.9	213.9	194.37
LOC104916717	zinc finger MIZ domain-containing protein 2-like	212.0	176.7	194.34
LOC104909631	baculoviral IAP repeat-containing protein 6-like	172.3	215.5	193.93
APOOL	apolipoprotein O-like	191.7	196.1	193.89
HELB	helicase (DNA) B	196.8	190.9	193.86
ZFYVE26	zinc finger, FYVE domain containing 26	199.3	188.3	193.82
LOC104916159	ribosome biogenesis protein BOP1-like	199.4	188.2	193.78
LOC104916309	neuroligin-2	213.0	174.4	193.72
LRRC39	leucine rich repeat containing 39	190.9	196.1	193.49
ARHGEF3	Rho guanine nucleotide exchange factor (GEF) 3	179.1	207.9	193.47
SEC63	SEC63 homolog (S. cerevisiae)	181.6	205.3	193.47
LMLN	leishmanolysin-like (metallopeptidase M8 family)	201.9	184.7	193.30
SNX13	sorting nexin 13	180.8	205.7	193.28
LOC104909839	tubulin epsilon chain-like	192.5	193.8	193.16
ORAI1	ORAI calcium release-activated calcium modulator 1	198.4	187.9	193.16
TMEM120B	transmembrane protein 120B	200.9	185.4	193.14
LOC104914344	uncharacterized LOC104914344	170.6	215.3	192.96
LOC100546995	uncharacterized LOC100546995	176.5	209.3	192.92
LOC100538468	epidermal retinol dehydrogenase 2-like	178.2	207.6	192.91
SLC12A7	solute carrier family 12 (potassium/chloride transporter), member 7	199.2	186.4	192.81
QKI	QKI, KH domain containing, RNA binding	174.8	210.8	192.80
RBMS2	RNA binding motif, single stranded interacting protein 2	217.0	168.2	192.61
STARD13	StAR-related lipid transfer (START) domain containing 13	198.4	186.8	192.60
GAMT	guanidinoacetate N-methyltransferase	206.8	178.3	192.52
LOC104914753	ectopic P granules protein 5 homolog	153.7	231.1	192.39
POLE4	polymerase (DNA-directed), epsilon 4, accessory subunit	185.8	198.9	192.36
GRHPR	glyoxylate reductase/hydroxypyruvate reductase	198.4	186.3	192.33
RIMBP2	RIMS binding protein 2	180.6	203.9	192.27
LOC100542006	heterogeneous nuclear ribonucleoprotein L-like	193.5	190.4	191.93
DLL1	delta-like 1 (Drosophila)	200.2	183.7	191.92
KIAA1211	KIAA1211 ortholog	194.2	189.3	191.78
LOC100551292	arginine and glutamate-rich protein 1	174.8	208.7	191.76
CREM	cAMP responsive element modulator	185.0	198.5	191.73
WDR59	WD repeat domain 59	199.4	183.9	191.68
CNST	consortin, connexin sorting protein	185.0	198.4	191.67
TMEM129	transmembrane protein 129, E3 ubiquitin protein ligase	195.9	187.4	191.66
LOC100538358	paraspeckle component 1-like	193.4	189.8	191.57
SLC45A4	solute carrier family 45, member 4	199.4	183.6	191.49

PIP5K1A	phosphatidylinositol-4-phosphate 5-kinase, type I, alpha	198.6	184.3	191.49
LOC104913779	uncharacterized LOC104913779	206.1	176.8	191.44
ZMIZ2	zinc finger, MIZ-type containing 2	223.9	158.7	191.31
NEK8	NIMA-related kinase 8	206.1	176.1	191.11
PIGL	phosphatidylinositol glycan anchor biosynthesis, class L	189.2	192.9	191.07
LOC104912573	cullin-3-B-like	186.6	195.4	191.00
SSTR2	somatostatin receptor 2	197.7	184.2	190.94
LOC104912224	uncharacterized LOC104912224	182.5	199.3	190.91
TGM2	transglutaminase 2	165.7	216.1	190.87
LOC104917219	myosin-7-like	178.4	203.2	190.82
TRAF3IP1	TNF receptor-associated factor 3 interacting protein 1	198.5	183.0	190.80
FAM151B	family with sequence similarity 151, member B	194.2	187.2	190.72
CSPG5	chondroitin sulfate proteoglycan 5 (neuroglycan C)	217.9	163.3	190.59
LOC100550986	microtubule-associated protein 4-like	207.9	173.2	190.58
LOC104916268	uncharacterized LOC104916268	197.7	183.4	190.57
FANCD2	Fanconi anemia, complementation group D2	199.4	181.8	190.56
THNSL1	threonine synthase-like 1 (S. cerevisiae)	189.1	191.9	190.52
PTBP3	polypyrimidine tract binding protein 3	176.5	204.5	190.51
CCDC170	coiled-coil domain containing 170	181.5	199.3	190.39
CEP41	centrosomal protein 41kDa	200.1	180.6	190.34
FSD1L	fibronectin type III and SPRY domain containing 1-like	184.1	196.3	190.17
LCORL	ligand dependent nuclear receptor corepressor-like	187.4	192.7	190.08
SPRED2	sprouty-related, EVH1 domain containing 2	189.2	190.9	190.07
NPY2R	neuropeptide Y receptor Y2	176.6	203.5	190.04
TTC26	tetratricopeptide repeat domain 26	173.0	207.0	190.00
RNF166	ring finger protein 166	201.1	178.7	189.92
IL1RAP	interleukin 1 receptor accessory protein	163.1	216.8	189.91
LOC104914456	uncharacterized LOC104914456	182.5	197.1	189.81
CGNL1	cingulin-like 1	196.8	182.8	189.79
LOC104909350	double-stranded RNA-binding protein Staufen homolog 2-like	184.1	195.3	189.74
HIF1A	hypoxia inducible factor 1, alpha subunit (basic helix-loop-helix transcription factor)	169.0	210.1	189.56
TBC1D32	TBC1 domain family, member 32	179.8	199.3	189.56
LOC104915434	ranBP-type and C3HC4-type zinc finger-containing protein 1-like	201.0	178.1	189.54
LOC104909525	ribosome-binding protein 1-like	171.5	207.1	189.28
LOC104916859	uncharacterized LOC104916859	175.7	202.9	189.28
LOC104914589	arf-GAP with GTPase, ANK repeat and PH domain-containing protein 3-like	224.7	153.8	189.26
LOC104913412	retinoic acid-induced protein 1-like	201.9	176.4	189.13
LOC100551128	cryptochrome-1-like	177.4	200.7	189.06
LOC104911394	kinesin-1 heavy chain	174.8	203.3	189.02
PVRL1	poliovirus receptor-related 1 (herpesvirus entry mediator C)	188.4	189.6	189.00
CCDC18	coiled-coil domain containing 18	196.6	181.3	188.95
LOC100549299	protein FAM46A pseudogene	184.1	193.4	188.76
S100Z	S100 calcium binding protein Z	168.1	209.4	188.74
LOC100547373	opsin-VA-like	171.5	205.8	188.64
ENTPD5	ectonucleoside triphosphate diphosphohydrolase 5	201.0	176.0	188.53
LOC100550908	uncharacterized LOC100550908	182.3	194.6	188.47
TAL2	T-cell acute lymphocytic leukemia 2	259.7	117.1	188.40
CARF	calcium responsive transcription factor	176.4	200.3	188.39
ADAMTS1	ADAM metallopeptidase with thrombospondin type 1 motif, 1	210.1	166.5	188.33
GOLGA7B	golgin A7 family, member B	199.5	177.2	188.32
DNAAF1	dynein, axonemal, assembly factor 1	179.1	197.5	188.32
LOC104916034	F-box only protein 46-like	190.0	186.4	188.21
P2RX5	purinergic receptor P2X, ligand-gated ion channel, 5	174.0	202.3	188.15
ELL2	elongation factor, RNA polymerase II, 2	162.2	213.9	188.02
YJEFN3	YjeF N-terminal domain containing 3	192.5	182.7	187.62
FAM105A	family with sequence similarity 105, member A	200.0	175.1	187.59

SLC24A3	solute carrier family 24 (sodium/potassium/calcium exchanger), member 3	197.6	177.3	187.43
ADAMTSL3	ADAMTS-like 3	185.0	189.7	187.38
LOC104913512	uncharacterized LOC104913512	156.5	218.3	187.36
ARMC10	armadillo repeat containing 10	189.1	185.3	187.20
SCP2D1	SCP2 sterol-binding domain containing 1	210.1	164.2	187.17
PIGK	phosphatidylinositol glycan anchor biosynthesis, class K	161.3	212.7	187.01
DUS2	dihydrouridine synthase 2	186.7	187.1	186.88
TMEM175	transmembrane protein 175	186.6	187.1	186.85
HTR1D	5-hydroxytryptamine (serotonin) receptor 1D, G protein-coupled	179.0	194.4	186.74
WNT10A	wingless-type MMTV integration site family, member 10A	198.6	174.7	186.67
VRK1	vaccinia related kinase 1	179.9	193.4	186.65
TNRC6C	trinucleotide repeat containing 6C	194.3	178.5	186.40
LOC104911305	zinc finger protein 577-like	188.4	184.3	186.35
LOC104917111	chromodomain-helicase-DNA-binding protein 8-like	220.6	152.0	186.30
WDR73	WD repeat domain 73	218.7	153.7	186.22
LOC100543905	histone H2B 1/2/3/4/6	181.6	190.7	186.16
MSH3	mutS homolog 3	189.1	183.2	186.15
RDH16	retinol dehydrogenase 16 (all-trans)	175.0	197.1	186.05
SNTB2	syntrophin, beta 2 (dystrophin-associated protein A1, 59kDa, basic component 2)	196.0	176.1	186.03
METRN	meteorin, glial cell differentiation regulator	216.9	155.0	185.94
FLRT2	fibronectin leucine rich transmembrane protein 2	191.7	179.9	185.82
LOC104915572	pumilio domain-containing protein KIAA0020-like	196.7	174.9	185.79
SH3BP2	SH3-domain binding protein 2	158.1	213.0	185.52
TSPAN5	tetraspanin 5	180.7	190.2	185.44
JAKMIP2	janus kinase and microtubule interacting protein 2	183.3	187.5	185.41
LOC104911842	uncharacterized LOC104911842	177.3	193.4	185.39
SLC24A1	solute carrier family 24 (sodium/potassium/calcium exchanger), member 1	187.4	183.3	185.38
ANGPTL1	angiopoietin-like 1	171.4	199.2	185.33
LOC100547805	E3 ubiquitin-protein ligase TRIM39-like	197.7	172.9	185.30
OGFRL1	opioid growth factor receptor-like 1	172.3	197.8	185.02
MT2A	uncharacterized MT2A	195.0	174.9	184.94
TMPRSS13	transmembrane protease, serine 13	200.1	169.8	184.94
MAU2	MAU2 sister chromatid cohesion factor	179.9	189.9	184.91
FAN1	FANCD2/FANCI-associated nuclease 1	182.5	187.2	184.85
IGFN1	immunoglobulin-like and fibronectin type III domain containing 1	179.1	190.5	184.79
ATP13A2	ATPase type 13A2	192.6	176.8	184.69
SIK2	salt-inducible kinase 2	186.7	182.5	184.59
PIBF1	progesterone immunomodulatory binding factor 1	190.8	178.2	184.49
CDC40	cell division cycle 40	173.1	195.5	184.30
SBSPON	somatomedin B and thrombospondin, type 1 domain containing	188.3	180.3	184.29
LGR4	leucine-rich repeat containing G protein-coupled receptor 4	165.5	203.1	184.28
LOC104916749	zinc finger protein 664-like	194.3	174.0	184.15
CEP89	centrosomal protein 89kDa	172.3	195.7	183.98
LRRC14	leucine rich repeat containing 14	179.1	188.6	183.88
PRDM5	PR domain containing 5	176.5	191.2	183.86
LOC104911120	uncharacterized LOC104911120	176.5	190.8	183.68
TYW3	tRNA-yW synthesizing protein 3 homolog (S. cerevisiae)	180.7	186.6	183.64
STON2	stonin 2	169.8	197.4	183.61
PROM1	prominin 1	193.4	173.5	183.45
LOC100546777	methylcrotonoyl-CoA carboxylase beta chain, mitochondrial-like	192.5	174.3	183.41
SOCS4	suppressor of cytokine signaling 4	191.8	174.9	183.33
LOC100550605	tumor suppressor p53-binding protein 1	181.7	184.6	183.14
IDUA	iduronidase, alpha-L-	168.1	198.0	183.06
MKRN2OS	MKRN2 opposite strand	186.6	179.3	182.97
LRRC71	leucine rich repeat containing 71	184.1	181.7	182.91

LOC100550348	leucine-rich repeat-containing protein 41	188.3	177.5	182.88
LLGL2	lethal giant larvae homolog 2 (Drosophila)	180.8	184.9	182.83
DCLRE1C	DNA cross-link repair 1C	190.0	175.5	182.78
ZDHHC7	zinc finger, DHHC-type containing 7	190.9	174.7	182.77
FAM214B	family with sequence similarity 214, member B	172.3	193.2	182.75
ZRANB2	zinc finger, RAN-binding domain containing 2	179.9	185.4	182.68
LOC104912076	uncharacterized LOC104912076	149.6	215.6	182.58
LOC104914006	receptor-type tyrosine-protein phosphatase T-like	175.8	189.3	182.54
LOC104910469	uncharacterized LOC104910469	190.8	174.2	182.52
LOC104916149	homeobox protein SIX2-like	185.8	179.0	182.38
BOD1L1	biorientation of chromosomes in cell division 1-like 1	169.9	194.6	182.21
FAM169B	family with sequence similarity 169, member B	185.8	178.6	182.18
GRK6	G protein-coupled receptor kinase 6	181.6	182.7	182.10
LOC100549110	aldehyde dehydrogenase family 3 member B1-like	190.1	173.7	181.92
ITGB5	integrin, beta 5	176.7	186.8	181.74
DRD5	dopamine receptor D5	175.7	187.5	181.62
LOC100544818	disks large homolog 3	177.4	185.8	181.59
LOC104914828	A disintegrin and metalloproteinase with thrombospondin motifs 12-like	177.4	185.8	181.57
HYAL1	hyaluronoglucosaminidase 1	180.0	183.0	181.51
GDF1	growth differentiation factor 1	217.0	146.0	181.50
MID1	midline 1	195.1	167.8	181.46
LOC104910710	histone H2A-IV	177.4	185.3	181.35
MSI1	musashi RNA-binding protein 1	174.0	187.9	180.92
POLE	polymerase (DNA directed), epsilon, catalytic subunit	205.3	156.4	180.83
LOC100544524	signal peptide, CUB and EGF-like domain-containing protein 3	178.9	182.6	180.75
TMEM150C	transmembrane protein 150C	162.2	199.1	180.64
NAPB	N-ethylmaleimide-sensitive factor attachment protein, beta	161.3	199.9	180.64
HINFP	histone H4 transcription factor	179.0	182.1	180.56
ASRGL1	asparaginase like 1	186.7	174.3	180.53
NHSL2	NHS-like 2	191.8	169.2	180.48
LOC104913625	zinc finger protein CKR1 pseudogene	189.1	171.6	180.34
AXIN2	axin 2	170.7	189.7	180.19
LOC100547611	T-box transcription factor TBX3-like	174.9	185.3	180.09
IQCK	IQ motif containing K	179.0	181.1	180.04
EXOG	endo/exonuclease (5'-3'), endonuclease G-like	185.8	174.2	179.98
LOC104912797	uncharacterized LOC104912797	174.0	185.8	179.89
C19H16orf93	chromosome 19 open reading frame, human C16orf93	182.4	177.1	179.76
LOC100542828	interleukin-1 receptor type 1-like	172.2	187.2	179.72
GRHL3	grainyhead-like 3 (Drosophila)	177.4	181.9	179.63
LOC104915349	uncharacterized LOC104915349	187.4	171.3	179.35
DCX	doublecortin	146.2	212.4	179.30
CENPP	centromere protein P	195.8	162.7	179.27
LOC104912824	uncharacterized LOC104912824	163.9	194.4	179.17
NEU3	sialidase 3 (membrane sialidase)	175.6	182.7	179.15
BACE2	beta-site APP-cleaving enzyme 2	181.6	176.5	179.06
GPATCH3	G patch domain containing 3	169.7	188.4	179.06
GFOD2	glucose-fructose oxidoreductase domain containing 2	185.0	173.1	179.05
AGBL1	ATP/GTP binding protein-like 1	195.0	162.6	178.79
LOC104917089	uncharacterized LOC104917089	172.3	185.0	178.69
LOC100545225	fatty acyl-CoA hydrolase precursor, medium chain-like	162.1	195.2	178.64
ANKRD13B	ankyrin repeat domain 13B	215.4	141.6	178.49
CSF1	colony stimulating factor 1 (macrophage)	201.1	155.7	178.40
LOC104909440	cytochrome c oxidase assembly factor 4 homolog, mitochondrial	178.2	178.3	178.27
LOC104913300	uncharacterized LOC104913300	188.3	168.2	178.25
LOC104913777	coiled-coil domain-containing protein 57-like	169.8	186.6	178.21
BRD3	bromodomain containing 3	176.5	179.8	178.14
CHEK1	checkpoint kinase 1	196.0	160.2	178.06

LRRC61	leucine rich repeat containing 61	194.3	161.8	178.04
ADRA2A	adrenoceptor alpha 2A	179.9	176.1	177.98
LOC104915797	amyloid beta A4 precursor protein-binding family B member 1-like	206.2	149.7	177.93
LOC100548407	uncharacterized LOC100548407	178.2	177.6	177.89
SUOX	sulfite oxidase	191.7	164.0	177.89
LOC104911168	uncharacterized LOC104911168	179.8	175.8	177.82
CEP55	centrosomal protein 55kDa	188.3	167.0	177.63
NFYA	nuclear transcription factor Y, alpha	175.7	179.2	177.44
TTC30B	tetratricopeptide repeat domain 30B	186.6	168.2	177.36
C19H9orf142	chromosome 19 open reading frame, human C9orf142	184.1	170.6	177.32
LOC104916068	DNA-directed RNA polymerase II subunit RPB1-like	192.7	161.8	177.27
ZUFSP	zinc finger with UFM1-specific peptidase domain	166.3	188.2	177.24
HAUS3	HAUS augmin-like complex, subunit 3	192.5	161.8	177.17
TPGS1	tubulin polyglutamylase complex subunit 1	185.7	167.6	176.64
SCAMP1	secretory carrier membrane protein 1	148.6	204.6	176.60
CDCP1	CUB domain containing protein 1	168.1	185.0	176.52
LOC104911715	retinol dehydrogenase 7-like	156.3	196.4	176.38
NAB2	NGFI-A binding protein 2 (EGR1 binding protein 2)	194.2	158.3	176.28
LOC100549411	tomoregulin-1-like	186.6	165.9	176.24
LOC100549735	hepatoma-derived growth factor-related protein 3	176.5	175.5	176.02
LOC104913072	large neutral amino acids transporter small subunit 1-like	175.6	176.3	175.96
HHEX	hematopoietically expressed homeobox	177.3	174.5	175.94
KLHL30	kelch-like family member 30	199.3	152.0	175.64
LOC100549522	DNA excision repair protein ERCC-6	187.6	163.6	175.59
POC1A	POC1 centriolar protein A	182.5	168.7	175.57
MAP9	microtubule-associated protein 9	177.3	173.8	175.56
OSGIN1	oxidative stress induced growth inhibitor 1	174.0	177.0	175.50
LOC104910780	sec1 family domain-containing protein 2-like	172.3	178.5	175.38
LOC104910841	biorientation of chromosomes in cell division protein 1-like 1	169.7	180.8	175.29
LOC104913812	uncharacterized LOC104913812	165.6	184.8	175.20
PUS7L	pseudouridylate synthase 7 homolog (S. cerevisiae)-like	173.0	177.0	175.02
RNF165	ring finger protein 165	146.2	203.6	174.92
NEO1	neogenin 1	154.6	194.8	174.71
DDX11	DEAD/H (Asp-Glu-Ala-Asp/His) box helicase 11	190.1	159.3	174.67
SPATA2	spermatogenesis associated 2	159.6	189.7	174.63
CEP70	centrosomal protein 70kDa	189.1	160.0	174.58
CBLN1	cerebellin 1 precursor	184.8	164.3	174.54
SLC25A44	solute carrier family 25, member 44	176.6	172.3	174.42
FAM110D	family with sequence similarity 110, member D	171.5	177.1	174.30
ACYP1	acylphosphatase 1, erythrocyte (common) type	189.2	159.3	174.23
GCNT7	glucosaminyl (N-acetyl) transferase family member 7	162.2	186.0	174.09
PPAPDC1B	phosphatidic acid phosphatase type 2 domain containing 1B	194.3	153.7	174.00
LOC104914402	uncharacterized LOC104914402	153.7	194.1	173.92
ZNF507	zinc finger protein 507	179.0	168.6	173.80
ERO1LB	ERO1-like beta (S. cerevisiae)	180.6	166.9	173.78
GJA9	gap junction protein, alpha 9, 59kDa	147.8	199.6	173.72
KLHL21	kelch-like family member 21	178.2	169.1	173.66
TTLL6	tubulin tyrosine ligase-like family, member 6	177.4	169.6	173.50
TAF4	TAF4 RNA polymerase II, TATA box binding protein (TBP)-associated factor, 135kDa	155.4	191.6	173.50
CTIF	CBP80/20-dependent translation initiation factor	196.9	150.0	173.45
LOC104911082	ubiquitin-conjugating enzyme E2 L3-like	174.8	171.7	173.29
LOC104916377	ras association domain-containing protein 1-like	213.7	132.9	173.27
SH3GL3	SH3-domain GRB2-like 3	168.0	178.5	173.26
C16H16orf52	chromosome 16 open reading frame, human C16orf52	175.6	170.9	173.26
DCLRE1B	DNA cross-link repair 1B	176.6	169.9	173.26
LOC100540665	focadhesin-like	174.0	172.5	173.23
FGD6	FYVE, RhoGEF and PH domain containing 6	176.5	169.6	173.07

TP63	tumor protein p63	161.3	184.7	172.97
METTL18	methyltransferase like 18	172.2	173.7	172.96
SOCS6	suppressor of cytokine signaling 6	164.7	181.3	172.96
LOC104915260	arrestin domain-containing protein 3-like	144.4	201.3	172.84
LOC100550331	inversin	152.1	193.4	172.71
MTRF1L	mitochondrial translational release factor 1-like	175.6	169.6	172.62
PSEN1	presenilin 1	158.8	186.4	172.60
AMPD2	adenosine monophosphate deaminase 2	167.3	177.9	172.60
PRIMPOL	primase and polymerase (DNA-directed)	192.5	152.7	172.58
LOC100542863	transcription factor AP-4-like	174.8	170.3	172.54
B3GNT2	UDP-GlcNAc:betaGal beta-1,3-N-acetylglucosaminyltransferase 2	174.0	171.0	172.46
LOC104916967	integrin alpha-3-like	196.1	148.5	172.32
LOC104916508	probable ATP-dependent RNA helicase DDX23	197.7	146.8	172.24
LOC104909310	membrane-bound transcription factor site-2 protease-like	169.7	174.8	172.22
LOC104914348	zonadhesin-like	166.4	177.9	172.15
LOC104912637	2-phosphoxylose phosphatase 1-like	171.4	172.8	172.10
RASGRP1	RAS guanyl releasing protein 1 (calcium and DAG-regulated)	171.4	172.7	172.03
SLC19A3	solute carrier family 19 (thiamine transporter), member 3	181.5	162.5	172.02
PIGN	phosphatidylinositol glycan anchor biosynthesis, class N	174.0	169.8	171.90
LOC104916254	TATA box-binding protein-associated factor RNA polymerase I subunit C-like	180.7	162.8	171.75
PRRC2B	proline-rich coiled-coil 2B	158.9	184.4	171.64
LOC104911679	probable hydrolase PNKD	183.3	159.7	171.51
LOC100549646	protein AF-17-like	179.1	163.6	171.37
PWWP2A	PWWP domain containing 2A	182.4	160.2	171.28
C8H10orf11	chromosome 8 open reading frame, human C10orf11	173.9	168.6	171.28
LOC100539892	small conductance calcium-activated potassium channel protein 2-like	185.1	157.3	171.18
DBF4B	DBF4 zinc finger B	174.0	168.4	171.17
LOC104909340	core histone macro-H2A.1	175.7	166.6	171.13
LOC104913084	protein CBFA2T3	178.3	163.9	171.11
PIKFYVE	phosphoinositide kinase, FYVE finger containing	165.6	175.7	170.67
POLR3A	polymerase (RNA) III (DNA directed) polypeptide A, 155kDa	163.0	177.9	170.45
DNAJC27	DnaJ (Hsp40) homolog, subfamily C, member 27	177.3	163.6	170.44
SFXN2	sideroflexin 2	174.0	166.9	170.43
SPON1	spondin 1, extracellular matrix protein	125.3	215.4	170.35
PTCHD1	patched domain containing 1	156.3	184.3	170.30
HEG1	heart development protein with EGF-like domains 1	161.2	179.1	170.18
NNT	nicotinamide nucleotide transhydrogenase	168.1	172.0	170.03
LOC104910075	checkpoint protein HUS1-like	172.1	167.9	170.02
LOC100539183	periostin	173.2	166.8	170.00
IFT140	intraflagellar transport 140	175.6	164.3	169.96
KIAA1715	KIAA1715 ortholog	163.0	176.8	169.93
SLC1A2	solute carrier family 1 (glial high affinity glutamate transporter), member 2	153.8	186.0	169.88
LOC104916836	pyruvate dehydrogenase (acetyl-transferring) kinase isozyme 2, mitochondrial-like	169.8	169.5	169.65
TNFRSF11B	tumor necrosis factor receptor superfamily, member 11b	184.0	155.3	169.62
CEP290	centrosomal protein 290kDa	193.4	145.8	169.59
SIMC1	SUMO-interacting motifs containing 1	147.0	192.2	169.57
LOC104916382	zinc finger protein ZFPM1-like	181.7	157.3	169.49
LRRC23	leucine rich repeat containing 23	163.8	175.2	169.48
MANEA	mannosidase, endo-alpha	160.4	178.2	169.30
LOC104916317	uncharacterized LOC104916317	196.8	141.8	169.28
ZNF148	zinc finger protein 148	180.8	157.4	169.09
DENND4A	DENN/MADD domain containing 4A	168.1	170.1	169.08
BRF2	BRF2, RNA polymerase III transcription initiation factor 50 kDa subunit	161.4	176.7	169.01

SNX16	sorting nexin 16	166.3	171.6	168.96
LOC104912497	zinc finger protein GLIS3-like	176.6	161.2	168.92
TCEANC	transcription elongation factor A (SII) N-terminal and central domain containing	168.0	169.8	168.90
LOC104914783	uncharacterized LOC104914783	173.2	164.6	168.87
UBE2T	ubiquitin-conjugating enzyme E2T (putative)	171.3	166.4	168.85
LOC100543679	ecotropic viral integration site 5 protein homolog	172.3	165.2	168.78
COQ2	coenzyme Q2 4-hydroxybenzoate polyprenyltransferase	179.9	157.5	168.68
LOC104910607	WD repeat and FYVE domain-containing protein 3-like	170.6	166.7	168.67
MYCBPAP	MYCBP associated protein	188.3	149.0	168.64
LOC104910451	interferon-induced guanylate-binding protein 1-like	163.8	172.9	168.38
RCL1	RNA terminal phosphate cyclase-like 1	164.6	172.1	168.35
LRRC58	leucine rich repeat containing 58	159.7	176.9	168.31
SLC25A19	solute carrier family 25 (mitochondrial thiamine pyrophosphate carrier), member 19	182.4	154.2	168.29
COL15A1	collagen, type XV, alpha 1	168.9	167.6	168.27
LOC100544461	TOM1-like protein 2	183.3	153.0	168.16
LOC100543009	CMP-N-acetylneuraminate-beta-1,4-galactoside alpha-2,3-sialyltransferase	167.3	168.6	167.96
CDYL	chromodomain protein, Y-like	155.4	180.4	167.90
LOC104914470	lymphoid-restricted membrane protein-like	165.6	170.2	167.90
OPA1	optic atrophy 1 (autosomal dominant)	158.8	177.0	167.87
ABCB10	ATP-binding cassette, sub-family B (MDR/TAP), member 10	176.5	159.2	167.84
SNRNP48	small nuclear ribonucleoprotein 48kDa (U11/U12)	191.6	144.0	167.82
HLCS	holocarboxylase synthetase (biotin-(proprionyl-CoA-carboxylase (ATP-hydrolysing)) ligase)	143.6	191.8	167.72
CCDC173	coiled-coil domain containing 173	157.9	177.4	167.66
SORBS2	sorbin and SH3 domain containing 2	179.8	155.5	167.64
MTR	5-methyltetrahydrofolate-homocysteine methyltransferase	156.2	179.1	167.62
SOCS7	suppressor of cytokine signaling 7	179.1	156.1	167.61
LOC104910621	polycystin-2-like	160.4	174.6	167.50
BEND7	BEN domain containing 7	165.5	169.4	167.47
TRIQK	triple QxxK/R motif containing	143.6	191.2	167.40
AMIGO2	adhesion molecule with Ig-like domain 2	160.4	174.3	167.35
GSG1	germ cell associated 1	184.1	150.5	167.34
PDSS1	prenyl (decaprenyl) diphosphate synthase, subunit 1	159.6	174.7	167.13
SNX18	sorting nexin 18	162.1	171.9	167.05
SEMA4D	sema domain, immunoglobulin domain (Ig), transmembrane domain (TM) and short cytoplasmic domain, (semaphorin) 4D	170.6	163.2	166.89
PLK4	polo-like kinase 4	173.1	160.4	166.73
LOC104916707	leucine-rich repeat extensin-like protein 5	173.1	160.3	166.73
LOC100545581	signal transducing adapter molecule 1	153.7	179.7	166.72
ZNF414	zinc finger protein 414	182.4	150.9	166.66
MBOAT1	membrane bound O-acyltransferase domain containing 1	166.3	167.0	166.62
ARHGEF37	Rho guanine nucleotide exchange factor (GEF) 37	177.3	155.7	166.54
ACSL1	acyl-CoA synthetase long-chain family member 1	176.5	156.2	166.33
ACSS1	acyl-CoA synthetase short-chain family member 1	169.7	162.8	166.25
PCGF1	polycomb group ring finger 1	173.1	158.9	166.04
RALGAPA2	Ral GTPase activating protein, alpha subunit 2 (catalytic)	158.0	174.0	165.99
LOC104917309	putative protein TPRXL	155.6	176.4	165.99
LOC100543400	matrix metalloproteinase-27-like	135.2	196.7	165.96
GSG2	germ cell associated 2 (haspin)	174.0	157.8	165.91
RAPGEF6	Rap guanine nucleotide exchange factor (GEF) 6	163.9	167.9	165.91
LOC104916890	E3 ubiquitin-protein ligase synoviolin-like	180.8	150.9	165.83
ERICH1	glutamate-rich 1	163.9	167.5	165.71
METTL25	methyltransferase like 25	178.1	152.9	165.50
CAMSAP2	calmodulin regulated spectrin-associated protein family, member 2	134.4	196.5	165.44

LOC104912761	protein PML-like	174.8	155.9	165.37
TIMP4	TIMP metallopeptidase inhibitor 4	155.4	175.3	165.33
LOC100542602	uncharacterized LOC100542602	172.3	158.2	165.27
PRKAR2A	protein kinase, cAMP-dependent, regulatory, type II, alpha	173.2	157.3	165.23
KCTD18	potassium channel tetramerization domain containing 18	171.4	158.0	164.68
BOK	BCL2-related ovarian killer	177.4	151.9	164.66
SLC35A3	solute carrier family 35 (UDP-N-acetylglucosamine (UDP-GlcNAc) transporter), member A3	173.1	156.1	164.58
LOC100546358	SLAIN motif-containing protein-like	170.5	158.7	164.57
RDH10	retinol dehydrogenase 10 (all-trans)	158.8	170.3	164.52
GPAM	glycerol-3-phosphate acyltransferase, mitochondrial	154.5	174.4	164.47
LOC104911422	GTP-binding protein 10-like	161.3	167.6	164.43
CCDC40	coiled-coil domain containing 40	168.9	159.9	164.42
SYTL4	synaptotagmin-like 4	155.5	172.9	164.23
LECT1	leukocyte cell derived chemotaxin 1	174.0	154.0	163.99
KIAA0408	KIAA0408 ortholog	165.6	162.3	163.94
LOC104912270	uncharacterized LOC104912270	168.0	159.8	163.93
LOC104909225	kalirin-like	165.7	162.0	163.86
KNTC1	kinetochore associated 1	172.3	155.3	163.81
FBLN5	fibulin 5	172.3	155.2	163.76
CNTD1	cyclin N-terminal domain containing 1	179.9	147.6	163.75
LOC100546326	snRNA-activating protein complex subunit 3-like	179.8	147.6	163.70
SLC43A2	solute carrier family 43 (amino acid system L transporter), member 2	148.6	178.7	163.67
FUCA2	fucosidase, alpha-L- 2, plasma	148.7	178.6	163.64
ATG2B	autophagy related 2B	142.8	184.5	163.64
MIS18A	MIS18 kinetochore protein A	171.3	155.9	163.64
LOC104915486	monocarboxylate transporter 8-like	151.1	176.1	163.64
LOC104910138	uncharacterized LOC104910138	145.2	182.0	163.62
LOC100541931	protein kinase C delta type-like	168.9	158.3	163.60
LOC104913791	unconventional myosin-XVB-like	213.4	113.7	163.57
S1PR3	sphingosine-1-phosphate receptor 3	169.8	157.3	163.53
LOC104909671	interferon-induced, double-stranded RNA-activated protein kinase-like	163.8	163.2	163.53
ENTPD4	ectonucleoside triphosphate diphosphohydrolase 4	157.9	169.1	163.53
HJURP	Holliday junction recognition protein	175.6	151.5	163.51
FAM72A	family with sequence similarity 72, member A	167.1	159.9	163.51
LOC104913168	SRSF protein kinase 3-like	164.7	162.3	163.51
LOC104913665	uncharacterized LOC104913665	152.1	174.9	163.50
LOC104912668	uncharacterized LOC104912668	164.6	161.9	163.27
FAM180A	family with sequence similarity 180, member A	143.7	182.8	163.25
FAM132A	family with sequence similarity 132, member A	139.4	186.9	163.18
LOC104915319	intraflagellar transport protein 74 homolog	162.1	164.1	163.09
LCA5	Leber congenital amaurosis 5	191.6	134.5	163.00
PIGO	phosphatidylinositol glycan anchor biosynthesis, class O	186.7	139.3	163.00
ADAMTS9	ADAM metallopeptidase with thrombospondin type 1 motif, 9	170.6	155.2	162.90
LOC100540140	trinucleotide repeat-containing gene 18 protein-like	176.7	149.1	162.90
STK40	serine/threonine kinase 40	174.9	150.8	162.85
LOC104911765	lymphocyte antigen 75-like	145.3	180.4	162.83
PLLP	plasmolipin	162.2	163.1	162.62
LOC100540017	poly(A) RNA polymerase, mitochondrial-like	169.7	155.3	162.54
MOCS3	molybdenum cofactor synthesis 3	173.1	152.0	162.54
SEPT2	septin 2	166.3	158.5	162.44
WDR34	WD repeat domain 34	178.3	146.5	162.40
MPHOSPH9	M-phase phosphoprotein 9	163.9	160.8	162.36
LOC100546441	NEDD4-binding protein 2-like 2	149.5	174.7	162.09
LOC104911339	sodium channel protein type 5 subunit alpha-like	147.0	177.0	162.03
SFMBT1	Scm-like with four mbt domains 1	174.8	148.8	161.79

BRF1	BRF1, RNA polymerase III transcription initiation factor 90 kDa subunit	167.2	156.3	161.76
LOC104914945	uncharacterized LOC104914945	149.5	174.0	161.74
LOC100548115	pumilio domain-containing protein KIAA0020-like	152.9	170.5	161.70
GBGT1	globoside alpha-1,3-N-acetylgalactosaminyltransferase 1	164.7	158.5	161.61
KPNA3	karyopherin alpha 3 (importin alpha 4)	141.1	182.1	161.59
ANKDD1A	ankyrin repeat and death domain containing 1A	154.6	168.5	161.54
PIP5K1C	phosphatidylinositol-4-phosphate 5-kinase, type I, gamma	168.1	154.7	161.41
LOC104911355	uncharacterized LOC104911355	151.2	171.6	161.40
LOC104916294	transcriptional activator protein Pur-alpha	179.1	143.7	161.37
C4H4orf33	chromosome 4 open reading frame, human C4orf33	156.3	166.3	161.28
WDR7	WD repeat domain 7	151.3	171.2	161.22
LOC104916335	uncharacterized LOC104916335	181.6	140.2	160.94
LOC104911278	actin-related protein 10-like	172.4	149.5	160.91
FBXL12	F-box and leucine-rich repeat protein 12	164.7	156.9	160.81
LOC104912081	dedicator of cytokinesis protein 1-like	151.1	170.4	160.79
E2F2	E2F transcription factor 2	190.7	130.7	160.69
FAM53A	family with sequence similarity 53, member A	174.8	146.3	160.52
CLUAP1	clusterin associated protein 1	168.8	151.9	160.36
LOC100540379	attractin-like	160.5	160.1	160.29
LMO2	LIM domain only 2 (rhombotin-like 1)	178.1	142.4	160.25
LOC100546546	ethylmalonyl-CoA decarboxylase	147.8	172.7	160.24
LOC104914600	voltage-dependent L-type calcium channel subunit beta-1-like	181.7	138.7	160.19
FZD7	frizzled class receptor 7	179.9	140.3	160.12
CDKN2C	cyclin-dependent kinase inhibitor 2C (p18, inhibits CDK4)	172.3	147.6	159.97
MAPK15	mitogen-activated protein kinase 15	157.1	162.4	159.77
GRAMD1C	GRAM domain containing 1C	171.4	147.9	159.64
CPPED1	calcineurin-like phosphoesterase domain containing 1	160.4	158.8	159.57
LOC100538780	ras GTPase-activating protein-binding protein 2-like	168.9	150.2	159.50
P4HTM	prolyl 4-hydroxylase, transmembrane (endoplasmic reticulum)	164.8	154.1	159.45
LOC104915406	uncharacterized LOC104915406	168.2	150.6	159.39
LOC104916107	uncharacterized LOC104916107	166.3	152.3	159.30
IGF2	insulin-like growth factor 2	159.7	158.5	159.11
GTPBP6	GTP binding protein 6 (putative)	152.0	166.0	158.98
SCARF2	scavenger receptor class F, member 2	174.0	143.9	158.93
KLF4	Kruppel-like factor 4 (gut)	158.8	158.8	158.81
LOC104915548	E3 ubiquitin-protein ligase MIB2-like	165.7	151.9	158.76
DNAJC5B	DnaJ (Hsp40) homolog, subfamily C, member 5 beta	172.2	145.2	158.70
LOC104914014	uncharacterized LOC104914014	179.9	137.4	158.65
LOC104913069	zinc finger CCCH domain-containing protein 18-like	151.3	166.0	158.62
LOC104916958	class E basic helix-loop-helix protein 40-like	193.4	123.7	158.58
LOC104913634	carboxy-terminal kinesin 2-like	185.8	130.7	158.27
LOC104915233	uncharacterized LOC104915233	145.3	171.1	158.20
LOC100547091	HMG box-containing protein 1-like	143.6	172.7	158.13
FAM210B	family with sequence similarity 210, member B	150.4	165.7	158.03
STK36	serine/threonine kinase 36	174.1	141.8	157.96
MAZ	MYC-associated zinc finger protein (purine-binding transcription factor)	179.1	136.8	157.96
SEMA3C	sema domain, immunoglobulin domain (Ig), short basic domain, secreted, (semaphorin) 3C	150.2	165.6	157.91
ELK3	ELK3, ETS-domain protein (SRF accessory protein 2)	161.4	154.0	157.69
RSBN1	round spermatid basic protein 1	156.3	159.0	157.64
CEP44	centrosomal protein 44kDa	170.4	144.6	157.50
CLN5	ceroid-lipofuscinosis, neuronal 5	148.6	166.4	157.48
LOC100540667	tudor and KH domain-containing protein-like	168.9	146.0	157.47
LOC104912190	uncharacterized LOC104912190	143.5	171.3	157.45
LOC104912314	zinc finger and BTB domain-containing protein 41-like	149.6	165.2	157.43
CHRNB2	cholinergic receptor, nicotinic, beta 2 (neuronal)	152.1	162.7	157.41

RNPC3	RNA-binding region (RNP1, RRM) containing 3	157.8	157.0	157.41
FIBIN	fin bud initiation factor homolog (zebrafish)	166.4	148.4	157.35
BBX	bobby sox homolog (Drosophila)	154.5	160.1	157.29
DUS1L	dihydrouridine synthase 1-like (S. cerevisiae)	163.1	151.1	157.11
RASD1	RAS, dexamethasone-induced 1	175.6	138.4	157.01
LOC100542638	BCL-6 corepressor-like protein 1	143.7	170.3	156.99
DHX33	DEAH (Asp-Glu-Ala-His) box polypeptide 33	171.4	142.3	156.84
LOC104910675	uncharacterized LOC104910675	109.9	203.7	156.82
D2HGDH	D-2-hydroxyglutarate dehydrogenase	169.7	143.9	156.80
LOC104910870	protein Dok-7-like	170.6	143.0	156.76
LOC100551087	ETS translocation variant 5	158.0	155.5	156.71
SLC9A3R2	solute carrier family 9, subfamily A (NHE3, cation proton antiporter 3), member 3 regulator 2	159.6	153.7	156.68
LOC104914811	gamma-secretase subunit APH-1A-like	179.1	134.2	156.67
OBFC1	oligonucleotide/oligosaccharide-binding fold containing 1	178.3	134.8	156.54
CEP135	centrosomal protein 135kDa	171.4	141.6	156.52
SLC27A1	solute carrier family 27 (fatty acid transporter), member 1	152.0	161.0	156.51
RHOU	ras homolog family member U	162.1	150.7	156.38
LYRM5	LYR motif containing 5	152.8	159.8	156.32
LOC104916927	procollagen galactosyltransferase 1-like	161.4	151.1	156.25
TERF1	telomeric repeat binding factor (NIMA-interacting) 1	156.2	156.2	156.21
LOC104909204	UPF0687 protein C20orf27-like	176.5	135.7	156.09
LOXHD1	lipoxygenase homology domains 1	140.2	171.9	156.07
URGCP	upregulator of cell proliferation	144.5	167.3	155.93
ELMO3	engulfment and cell motility 3	147.0	164.8	155.93
C4H4orf47	chromosome 4 open reading frame, human C4orf47	160.5	151.2	155.85
LACE1	lactation elevated 1	144.4	166.8	155.57
CCNJ	cyclin J	153.7	157.3	155.45
CD82	CD82 molecule	152.0	158.9	155.45
POF1B	premature ovarian failure, 1B	149.5	161.2	155.37
TCEA3	transcription elongation factor A (SII), 3	177.3	133.2	155.28
SPIDR	scaffolding protein involved in DNA repair	171.5	138.9	155.18
LOC100540575	uncharacterized LOC100540575	141.0	169.2	155.08
LOC100549359	ellis-van Creveld syndrome protein	150.3	159.8	155.05
FABP3	fatty acid binding protein 3, muscle and heart	185.7	124.4	155.05
DAGLA	diacylglycerol lipase, alpha	159.6	150.5	155.04
ID2	inhibitor of DNA binding 2, dominant negative helix-loop-helix protein	158.8	151.2	154.97
SMS	spermine synthase	138.5	171.4	154.95
LOC104909337	complement C4-like	162.9	146.8	154.88
MASP2	mannan-binding lectin serine peptidase 2	150.4	158.9	154.64
PTPRU	protein tyrosine phosphatase, receptor type, U	124.4	184.8	154.61
ZGRF1	zinc finger, GRF-type containing 1	163.9	145.2	154.55
RFWD3	ring finger and WD repeat domain 3	172.3	136.8	154.53
MEGF9	multiple EGF-like-domains 9	152.8	155.8	154.33
KLHL26	kelch-like family member 26	153.7	154.9	154.28
LOC100540839	NEDD4-like E3 ubiquitin-protein ligase WWP1	135.9	172.5	154.22
LOC104912103	sestrin-3-like	160.5	147.9	154.20
CHEK2	checkpoint kinase 2	138.5	169.8	154.15
LOC100542170	selenoprotein O-like	158.0	150.1	154.04
LOC104916993	filamin-C-like	192.7	115.4	154.04
UBXN2B	UBX domain protein 2B	119.9	188.0	153.97
LYN	LYN proto-oncogene, Src family tyrosine kinase	168.8	138.9	153.88
LOC100547338	proton-coupled amino acid transporter 1	142.8	164.9	153.85
ACAP3	ArfGAP with coiled-coil, ankyrin repeat and PH domains 3	135.2	172.4	153.82
SLC30A1	solute carrier family 30 (zinc transporter), member 1	150.3	157.2	153.76
LOC104910397	nuclear transcription factor Y subunit gamma-like	142.8	164.5	153.62
GIN1	gypsy retrotransposon integrase 1	138.5	168.7	153.60

LOC100549557	exosome complex exonuclease RRP44-like	141.8	165.2	153.50
FAM69A	family with sequence similarity 69, member A	149.5	157.4	153.48
NSUN6	NOP2/Sun domain family, member 6	163.8	142.9	153.37
LOC100542219	estradiol 17-beta-dehydrogenase 11-like	135.1	171.5	153.31
SLC30A7	solute carrier family 30 (zinc transporter), member 7	155.4	150.9	153.15
LOC100544327	general transcription factor IIF subunit 2-like	147.0	159.2	153.09
LOC104917565	periostin-like	153.7	152.3	153.02
LOC104916513	epsilon-sarcoglycan-like	163.9	142.0	152.93
GTSE1	G-2 and S-phase expressed 1	160.4	145.2	152.81
SLC36A4	solute carrier family 36 (proton/amino acid symporter), member 4	140.1	165.4	152.75
LOC104910456	uncharacterized LOC104910456	147.8	157.4	152.59
NOS2	nitric oxide synthase 2, inducible	136.8	168.3	152.53
LOC104916906	uncharacterized LOC104916906	169.8	135.0	152.39
RNF4	ring finger protein 4	157.0	147.6	152.32
OGG1	8-oxoguanine DNA glycosylase	160.5	144.1	152.26
LOC104909695	uncharacterized LOC104909695	144.4	160.1	152.21
SALL1	spalt-like transcription factor 1	176.4	127.8	152.11
NT5DC3	5'-nucleotidase domain containing 3	126.8	177.4	152.10
LOC104917584	neurobeachin-like	147.9	156.3	152.08
DNALI1	dynein, axonemal, light intermediate chain 1	146.0	158.1	152.06
KLHL8	kelch-like family member 8	150.3	153.4	151.88
PNPO	pyridoxamine 5'-phosphate oxidase	163.1	140.7	151.87
TNFRSF6B	tumor necrosis factor receptor superfamily, member 6b, decoy	139.5	164.2	151.86
ANKRD40	ankyrin repeat domain 40	158.0	145.8	151.86
HYLS1	hydrolethalus syndrome 1	163.0	140.3	151.65
DMTF1	cyclin D binding myb-like transcription factor 1	156.3	146.9	151.58
CHIC2	cysteine-rich hydrophobic domain 2	149.4	153.7	151.55
SYT15	synaptotagmin XV	165.5	137.4	151.46
KIAA1210	KIAA1210 ortholog	130.9	171.9	151.42
MIB2	mindbomb E3 ubiquitin protein ligase 2	158.0	144.6	151.34
LOC104913863	zinc finger ZZ-type and EF-hand domain-containing protein 1-like	136.9	165.8	151.33
HUS1	HUS1 checkpoint homolog (S. pombe)	159.6	143.0	151.27
LOC104909224	heat shock protein 105 kDa-like	157.1	145.4	151.26
LOC100548799	aldehyde dehydrogenase family 3 member B1-like	150.4	152.1	151.23
PPP1R3D	protein phosphatase 1, regulatory subunit 3D	136.8	165.6	151.20
LOC104912253	ecotropic viral integration site 5 protein homolog	130.9	171.5	151.20
LOC104911807	tyrosine-protein phosphatase non-receptor type 4-like	145.2	157.1	151.16
TECRL	trans-2,3-enoyl-CoA reductase-like	302.3	0.0	151.15
LOC104914707	uncharacterized LOC104914707	152.9	149.4	151.14
PM20D1	peptidase M20 domain containing 1	142.9	159.3	151.10
LOC104909638	uncharacterized LOC104909638	161.3	140.9	151.07
LOC104916823	maestro heat-like repeat-containing protein family member 1	165.6	136.4	150.96
LOC100541106	tyrosine-protein kinase RYK	145.2	156.6	150.89
STAP1	signal transducing adaptor family member 1	144.4	157.2	150.79
HEYL	hes-related family bHLH transcription factor with YRPW motif-like	170.7	130.9	150.76
ARL11	ADP-ribosylation factor-like 11	162.1	139.3	150.69
B3GALTL	beta 1,3-galactosyltransferase-like	125.9	175.5	150.68
M1AP	meiosis 1 associated protein	160.5	140.7	150.59
LOC104912616	basic salivary proline-rich protein 1-like	162.1	139.0	150.54
TRIM65	tripartite motif containing 65	163.0	138.0	150.48
FAM161A	family with sequence similarity 161, member A	141.9	159.0	150.42
WDR19	WD repeat domain 19	136.8	164.0	150.40
LXN	latexin	151.2	149.6	150.37
DPH1	diphthamide biosynthesis 1	154.7	146.1	150.37
LOC104915351	transcription factor TFIIIB component B'' homolog	163.0	137.4	150.25
KAT6A	K(lysine) acetyltransferase 6A	150.4	149.9	150.17
ZNF438	zinc finger protein 438	143.5	156.7	150.14
CRYAA	crystallin, alpha A	117.5	182.7	150.10

BTD	biotinidase	166.4	133.8	150.08
LETM2	leucine zipper-EF-hand containing transmembrane protein 2	149.5	150.2	149.86
PARP9	poly (ADP-ribose) polymerase family, member 9	134.3	165.4	149.86
C13H19orf40	chromosome 13 open reading frame, human C19orf40	176.4	123.1	149.75
PDS5B	PDS5 cohesin associated factor B	139.4	160.0	149.71
RBKS	ribokinase	149.5	149.6	149.57
CTNS	cystinosin, lysosomal cystine transporter	154.4	144.5	149.47
LOC104912722	anoctamin-2-like	137.6	161.1	149.34
MANSC1	MANSC domain containing 1	142.8	155.6	149.22
LOC104910882	dysferlin-like	158.2	140.0	149.11
LOC104912999	uncharacterized LOC104912999	134.3	163.9	149.09
LOC104909448	caseinolytic peptidase B protein homolog	161.4	136.7	149.01
ZBTB24	zinc finger and BTB domain containing 24	130.9	167.0	148.98
RNF170	ring finger protein 170	145.3	152.6	148.96
CELF2	CUGBP, Elav-like family member 2	146.1	151.7	148.93
LOC104915241	uncharacterized LOC104915241	146.9	150.9	148.90
DLGAP3	discs, large (Drosophila) homolog-associated protein 3	147.9	149.7	148.81
LOC104912108	semaphorin-3A-like	138.4	159.2	148.80
GTSF1	gametocyte specific factor 1	161.4	135.6	148.48
CEP120	centrosomal protein 120kDa	138.5	158.4	148.46
ARSB	arylsulfatase B	132.6	164.2	148.40
WWC3	WWC family member 3	152.0	144.7	148.38
A4GALT	alpha 1,4-galactosyltransferase	130.0	166.0	148.02
LOC104911023	uncharacterized LOC104911023	154.6	141.4	148.00
STK24	serine/threonine kinase 24	134.2	161.7	147.96
WRB	tryptophan rich basic protein	145.2	150.5	147.88
MYCBP2	MYC binding protein 2, E3 ubiquitin protein ligase	145.3	150.4	147.85
LOC104916957	phosphatidylinositol 3,4,5-trisphosphate 5-phosphatase 2-like	166.5	129.1	147.76
NKD1	naked cuticle homolog 1 (Drosophila)	120.0	175.2	147.61
FAM150B	family with sequence similarity 150, member B	152.0	142.8	147.39
LOC104917568	uncharacterized LOC104917568	144.4	150.2	147.33
C2H1orf131	chromosome 2 open reading frame, human C1orf131	137.6	157.0	147.30
SOCS3	suppressor of cytokine signaling 3	156.3	138.0	147.11
SPRY1	sprouty homolog 1, antagonist of FGF signaling (Drosophila)	144.5	149.5	146.96
AP5M1	adaptor-related protein complex 5, mu 1 subunit	157.9	135.9	146.91
NR4A1	nuclear receptor subfamily 4, group A, member 1	170.7	123.1	146.89
HSD17B4	hydroxysteroid (17-beta) dehydrogenase 4	158.7	135.1	146.87
LOC100549281	7,8-dihydro-8-oxoguanine triphosphatase-like	159.6	134.1	146.85
WDR66	WD repeat domain 66	127.5	166.2	146.83
LOC100542442	histone-lysine N-methyltransferase SETMAR	144.4	148.7	146.55
GRIN2A	glutamate receptor, ionotropic, N-methyl D-aspartate 2A	154.6	138.4	146.50
POMGNT2	protein O-linked mannose N-acetylglucosaminyltransferase 2 (beta 1,4-)	139.4	153.6	146.48
PXDN	peroxidasin homolog (Drosophila)	128.4	164.5	146.44
LOC104911129	estradiol 17-beta-dehydrogenase 12-like	138.5	154.1	146.28
FBXO2	F-box protein 2	156.2	136.3	146.27
ITGB3BP	integrin beta 3 binding protein (beta3-endonexin)	153.6	138.7	146.18
LOC104909230	SPATS2-like protein	131.8	160.5	146.15
LOC104910273	interleukin-15 receptor subunit alpha-like	145.3	146.9	146.10
RAB28	RAB28, member RAS oncogene family	144.4	147.8	146.10
CBX4	chromobox homolog 4	142.7	149.3	146.02
TRUB1	TruB pseudouridine (psi) synthase family member 1	130.9	161.1	145.98
TIMELESS	timeless circadian clock	168.1	123.9	145.97
LOC104917388	uncharacterized LOC104917388	156.3	135.6	145.96
ZNF462	zinc finger protein 462	141.1	150.8	145.95
LOC100544508	transmembrane protein 2-like	112.5	179.3	145.86
PLXDC2	plexin domain containing 2	142.7	149.0	145.85
CMIP	c-Maf inducing protein	162.1	129.6	145.83

CNTF	ciliary neurotrophic factor	141.9	149.3	145.60
LOC104916378	alpha-sarcoglycan-like	152.9	138.1	145.51
LOC104914900	uncharacterized LOC104914900	146.0	144.8	145.40
LOC100538693	protein C19orf12 homolog pseudogene	134.3	156.3	145.30
LOC100551134	ARF GTPase-activating protein GIT1-like	159.7	130.8	145.24
LOC100542221	attractin-like	157.9	132.5	145.21
LOC100542691	mitogen-activated protein kinase kinase kinase 3-like	158.7	131.7	145.20
GLMN	glomulin, FKBP associated protein	146.9	143.5	145.19
LOC100548247	protein CXorf40A-like	148.6	141.5	145.08
VAT1	vesicle amine transport 1	150.2	139.9	145.06
RAB20	RAB20, member RAS oncogene family	146.9	143.1	144.98
LOC104912802	transmembrane protein C15orf27-like	156.3	133.6	144.94
LOC100548965	cytochrome P450 2J3-like	146.9	143.0	144.94
LOC100539929	uncharacterized LOC100539929	139.3	150.5	144.93
LOC104915185	peptidyl-glycine alpha-amidating monooxygenase-like	140.2	149.3	144.77
LOC100549172	phosphatidylinositol 4,5-bisphosphate 3-kinase catalytic subunit beta isoform-like	150.4	139.0	144.68
TFAP4	transcription factor AP-4 (activating enhancer binding protein 4)	161.3	127.9	144.58
FAM213A	family with sequence similarity 213, member A	154.5	134.5	144.49
KIF25	kinesin family member 25	154.5	134.5	144.48
PUSL1	pseudouridylate synthase-like 1	121.7	167.2	144.44
CRLF3	cytokine receptor-like factor 3	153.7	135.0	144.36
TDG	thymine-DNA glycosylase	130.1	158.6	144.33
LOC104914891	superkiller viralicidic activity 2-like 2	140.1	148.3	144.21
LOC104917206	uncharacterized LOC104917206	156.3	132.0	144.15
LOC104912335	dynamin-3-like	149.4	138.6	144.02
LOC104915558	vacuolar protein sorting-associated protein 11 homolog	152.9	135.1	144.01
LOC100540040	interferon regulatory factor 4-like	152.9	134.8	143.82
MYOM2	myomesin 2	162.0	125.4	143.70
LOC100545985	WD repeat and FYVE domain-containing protein 3	136.9	150.5	143.69
LOC100546750	uncharacterized LOC100546750	134.4	152.9	143.63
KIAA0226	KIAA0226 ortholog	140.3	146.9	143.61
LOC104914363	uncharacterized LOC104914363	135.1	152.0	143.55
B3GNT5	UDP-GlcNAc:betaGal beta-1,3-N-acetylglucosaminyltransferase 5	131.7	155.2	143.46
LOC104913253	E3 ubiquitin-protein ligase TRIM17-like	155.4	131.2	143.32
LOC104916931	uncharacterized LOC104916931	148.6	137.9	143.27
SMARCAL1	SWI/SNF related, matrix associated, actin dependent regulator of chromatin, subfamily a-like 1	141.0	145.0	143.01
LSM10	LSM10, U7 small nuclear RNA associated	150.3	135.6	142.99
SLC25A25	solute carrier family 25 (mitochondrial carrier; phosphate carrier), member 25	130.9	155.0	142.95
IMPG2	interphotoreceptor matrix proteoglycan 2	142.0	143.6	142.81
ASIC4	acid-sensing (proton-gated) ion channel family member 4	148.6	136.9	142.76
C1H7orf60	chromosome 1 open reading frame, human C7orf60	150.3	135.0	142.63
LOC104914770	uncharacterized LOC104914770	132.6	152.3	142.43
ABHD6	abhydrolase domain containing 6	159.6	125.2	142.38
PLAA	phospholipase A2-activating protein	131.8	152.9	142.31
LOC104916042	max dimerization protein 3-like	159.6	124.8	142.19
NUPL2	nucleoporin like 2	141.0	143.2	142.12
LOC104917600	paraspeckle component 1	121.7	162.4	142.03
BRCA2	breast cancer 2, early onset	125.8	158.1	141.97
SLC8B1	solute carrier family 8 (sodium/lithium/calcium exchanger), member B1	154.5	129.2	141.86
RBP1	retinol binding protein 1, cellular	142.7	141.0	141.83
RUFY1	RUN and FYVE domain containing 1	132.6	150.7	141.65
LOC100549678	adrenodoxin-like	127.6	155.6	141.60
SHQ1	SHQ1, H/ACA ribonucleoprotein assembly factor	142.8	140.4	141.58
TPBG	trophoblast glycoprotein	140.2	142.6	141.40

LOC104910864	carboxypeptidase Z	124.2	158.5	141.31
LOC104914944	zinc transporter 5-like	140.2	142.1	141.19
LOC104911107	myosin-binding protein C, cardiac-type-like	118.3	163.8	141.10
MKNK1	MAP kinase interacting serine/threonine kinase 1	146.9	135.3	141.09
LOC104916356	zinc finger protein GLIS2-like	159.7	122.4	141.07
LOC104914687	minor histocompatibility protein HA-1-like	122.5	159.6	141.05
ACER2	alkaline ceramidase 2	142.7	139.2	140.97
ACSL5	acyl-CoA synthetase long-chain family member 5	152.9	128.9	140.90
LOC100547826	reticulon-4-interacting protein 1, mitochondrial-like	157.0	124.7	140.88
LPCAT1	lysophosphatidylcholine acyltransferase 1	141.8	139.8	140.83
LOC104914341	uncharacterized LOC104914341	141.0	140.6	140.76
LOC104912048	PDZ domain-containing protein 8-like	132.6	148.6	140.63
TRIM9	tripartite motif containing 9	153.7	127.3	140.53
BCL7A	B-cell CLL/lymphoma 7A	139.3	141.6	140.47
LOC104916448	uncharacterized LOC104916448	155.4	125.4	140.40
IFIH1	interferon induced with helicase C domain 1	141.9	138.8	140.36
MFSD8	major facilitator superfamily domain containing 8	125.0	155.5	140.26
KCTD7	potassium channel tetramerization domain containing 7	152.0	128.4	140.22
CLIC3	chloride intracellular channel 3	141.9	138.5	140.19
RAI1	retinoic acid induced 1	164.8	115.5	140.16
LOC104916940	diacylglycerol kinase alpha-like	138.6	141.7	140.11
LOC104915702	uncharacterized LOC104915702	114.1	166.2	140.11
CST7	cystatin F (leukocystatin)	141.9	138.4	140.11
LOC104910280	receptor-type tyrosine-protein phosphatase mu-like	157.0	123.2	140.10
FRS2	fibroblast growth factor receptor substrate 2	136.8	143.1	139.98
LOC104913736	caseinolytic peptidase B protein homolog	157.9	122.0	139.97
LOC104909791	uncharacterized LOC104909791	125.8	154.1	139.94
LOC100540453	melanotransferrin-like	134.3	145.5	139.90
TCTEX1D2	Tctex1 domain containing 2	149.4	130.3	139.86
LOC104912888	uncharacterized LOC104912888	140.2	139.5	139.84
DUSP15	dual specificity phosphatase 15	142.7	136.8	139.78
PLCL1	phospholipase C-like 1	128.4	151.0	139.68
LOC100540050	histone H2B 1/2/3/4/6	138.6	140.4	139.49
LOC104913631	class I histocompatibility antigen, F10 alpha chain-like	145.3	133.7	139.47
RASGRF1	Ras protein-specific guanine nucleotide-releasing factor 1	144.6	134.3	139.46
B3GALT4	UDP-Gal:betaGlcNAc beta 1,3-galactosyltransferase, polypeptide 4	155.5	123.5	139.46
SESN3	sestrin 3	147.0	131.8	139.37
CIPC	CLOCK-interacting pacemaker	149.5	129.0	139.24
LOC104909949	DNA primase large subunit-like	139.3	139.1	139.24
LOC104914301	uncharacterized LOC104914301	116.7	161.7	139.19
CHST10	carbohydrate sulfotransferase 10	136.0	142.2	139.09
LOC100549452	kelch-like protein 42	147.7	130.3	139.02
DENND6A	DENN/MADD domain containing 6A	132.5	145.4	138.95
CCDC30	coiled-coil domain containing 30	151.2	126.4	138.81
LOC104913620	antigen peptide transporter 2-like	157.1	120.5	138.80
LOC104911796	band 4.1-like protein 5	145.3	132.3	138.80
MTX3	metaxin 3	141.0	136.4	138.67
ST14	suppression of tumorigenicity 14 (colon carcinoma)	166.4	110.9	138.65
LOC104912284	uncharacterized LOC104912284	143.6	133.5	138.55
RWDD3	RWD domain containing 3	125.9	151.2	138.54
LOC100549525	lipoma-preferred partner homolog	125.8	151.0	138.41
MFAP2	microfibrillar-associated protein 2	146.1	130.7	138.37
C3H18orf42	chromosome 3 open reading frame, human C18orf42	118.2	158.5	138.33
PIAS2	protein inhibitor of activated STAT, 2	121.7	155.0	138.32
LOC104912741	uncharacterized LOC104912741	120.8	155.7	138.25
LOC104914807	talin-1-like	157.2	119.2	138.24
LOC100549446	inactive phospholipase C-like protein 2	165.5	110.8	138.16
OXR1	oxidation resistance 1	151.2	125.1	138.13

SENP8	SUMO/sentrin specific peptidase family member 8	137.7	138.6	138.13
EAF2	ELL associated factor 2	135.2	141.0	138.09
PEX26	peroxisomal biogenesis factor 26	148.6	127.4	137.99
LPIN2	lipin 2	135.1	140.2	137.67
RAD54B	RAD54 homolog B (S. cerevisiae)	126.7	148.6	137.63
RHOQ	ras homolog family member Q	144.4	130.5	137.48
FAM195A	family with sequence similarity 195, member A	144.4	130.5	137.43
SLC25A22	solute carrier family 25 (mitochondrial carrier: glutamate), member 22	153.8	121.1	137.42
MIS12	MIS12 kinetochore complex component	128.3	146.5	137.41
EOGT	EGF domain-specific O-linked N-acetylglucosamine (GlcNAc) transferase	140.2	134.3	137.24
GALNT16	polypeptide N-acetylgalactosaminyltransferase 16	135.2	139.3	137.23
MNS1	meiosis-specific nuclear structural 1	135.1	139.3	137.23
LRBA	LPS-responsive vesicle trafficking, beach and anchor containing	147.0	127.4	137.18
SAMD4B	sterile alpha motif domain containing 4B	143.7	130.7	137.17
LOC104917537	nucleoporin p58/p45	129.3	144.5	136.89
AP3B2	adaptor-related protein complex 3, beta 2 subunit	131.8	141.6	136.73
GATAD2B	GATA zinc finger domain containing 2B	137.7	135.7	136.73
LOC104917520	TSC22 domain family protein 3 pseudogene	135.2	138.3	136.72
LOC100548480	filamin-A-interacting protein 1	120.8	152.5	136.66
C11H3orf58	chromosome 11 open reading frame, human C3orf58	124.2	148.9	136.57
LOC104915981	E3 ubiquitin-protein ligase HERC2-like	140.3	132.5	136.42
AATF	apoptosis antagonizing transcription factor	133.4	139.4	136.42
LOC104914083	tissue factor pathway inhibitor 2-like	129.3	143.4	136.36
SLC7A11	solute carrier family 7 (anionic amino acid transporter light chain, xc- system), member 11	142.6	129.9	136.26
LOC100540906	formin-binding protein 1-like	130.0	142.5	136.26
LOC104914619	uncharacterized LOC104914619	152.9	119.6	136.23
PARD6G	par-6 family cell polarity regulator gamma	125.8	146.6	136.23
SLC35E4	solute carrier family 35, member E4	156.2	116.2	136.17
SHMT1	serine hydroxymethyltransferase 1 (soluble)	136.8	135.4	136.12
MSANTD3	Myb/SANT-like DNA-binding domain containing 3	130.8	141.2	136.03
REM1	RAS (RAD and GEM)-like GTP-binding 1	163.1	108.9	136.00
DAZAP2	DAZ associated protein 2	117.5	154.5	136.00
SYT17	synaptotagmin XVII	135.1	136.4	135.79
TNPO1	transportin 1	139.3	132.3	135.79
UPP2	uridine phosphorylase 2	134.3	136.9	135.58
LOC100550690	kinesin-like protein KIF20A	132.7	138.0	135.35
LOC104917358	zinc finger protein 22-like	135.2	135.4	135.31
F13A1	coagulation factor XIII, A1 polypeptide	131.8	138.6	135.22
LOC100546146	uncharacterized LOC100546146	150.3	120.1	135.20
LOC100543285	zinc finger protein 239-like	157.8	112.5	135.18
LOC104915787	oxysterol-binding protein-related protein 7-like	157.1	113.2	135.16
LOC104915931	neural Wiskott-Aldrich syndrome protein-like	141.0	129.2	135.10
LOC104917129	uncharacterized LOC104917129	143.5	126.7	135.09
ANKRD66	ankyrin repeat domain 66	131.7	138.4	135.09
LOC104916953	lysine-specific demethylase 5C-like	151.3	118.6	134.93
LRRK1	leucine-rich repeat kinase 1	136.0	133.9	134.93
TULP3	tubby like protein 3	130.9	138.9	134.89
LOC104912440	single-stranded DNA-binding protein 3-like	145.2	124.5	134.87
RGS5	regulator of G-protein signaling 5	124.2	145.6	134.87
LOC104915707	uncharacterized LOC104915707	132.6	137.0	134.83
THOC6	THO complex 6 homolog (Drosophila)	135.1	134.2	134.69
LOC104909312	ectonucleotide pyrophosphatase/phosphodiesterase family member 1-like	118.3	151.1	134.68
CDKN3	cyclin-dependent kinase inhibitor 3	142.7	126.5	134.61
WASF2	WAS protein family, member 2	151.3	117.9	134.59

PSKH1	protein serine kinase H1	125.1	144.1	134.57
LOC104909270	neurolysin, mitochondrial-like	133.4	135.5	134.47
GINS1	GINS complex subunit 1 (Psf1 homolog)	149.5	119.5	134.46
LOC100539663	calcium-binding mitochondrial carrier protein Aralar1-like	141.8	127.1	134.46
SLC52A3	solute carrier family 52 (riboflavin transporter), member 3	150.3	118.6	134.44
LOC100550081	calcium uptake protein 3, mitochondrial-like	134.3	134.5	134.37
LOC100547566	poly(rC)-binding protein 3-like	120.0	148.7	134.32
CENPK	centromere protein K	143.5	125.1	134.29
AASDH	aminoadipate-semialdehyde dehydrogenase	128.4	140.0	134.19
KLF12	Kruppel-like factor 12	162.9	105.4	134.16
PRDM11	PR domain containing 11	149.5	118.8	134.15
RASSF5	Ras association (RalGDS/AF-6) domain family member 5	124.2	144.0	134.10
ADCK2	aarF domain containing kinase 2	138.5	129.7	134.08
ADAMTS17	ADAM metallopeptidase with thrombospondin type 1 motif, 17	132.7	135.5	134.07
LRRCC1	leucine rich repeat and coiled-coil centrosomal protein 1	141.0	127.1	134.05
LOC104914959	uncharacterized LOC104914959	125.0	143.0	134.02
AAK1	AP2 associated kinase 1	131.8	136.1	133.99
LMOD3	leiomodin 3 (fetal)	136.8	131.1	133.98
SEC22C	SEC22 vesicle trafficking protein homolog C (S. cerevisiae)	134.2	133.7	133.94
LOC104911838	von Willebrand factor D and EGF domain-containing protein-like	133.4	134.5	133.94
FHDC1	FH2 domain containing 1	131.7	136.0	133.88
TMEM209	transmembrane protein 209	122.5	145.2	133.84
SEMA3F	sema domain, immunoglobulin domain (Ig), short basic domain, secreted, (semaphorin) 3F	131.8	135.6	133.72
ZNF518A	zinc finger protein 518A	124.9	142.4	133.68
C1H7orf49	chromosome 1 open reading frame, human C7orf49	126.7	140.6	133.64
GRAMD2	GRAM domain containing 2	142.7	124.5	133.63
LINGO1	leucine rich repeat and Ig domain containing 1	149.5	117.7	133.60
LOC104916896	uncharacterized LOC104916896	139.3	127.8	133.58
TIGD5	tigger transposable element derived 5	129.2	137.8	133.51
LOC100545615	zinc finger protein 70-like	147.8	119.1	133.48
LOC104916732	transducin-like enhancer protein 2	146.1	120.8	133.45
TET1	tet methylcytosine dioxygenase 1	125.0	141.8	133.41
LOC100541237	propionyl-CoA carboxylase alpha chain, mitochondrial-like	129.2	137.4	133.35
LOC100546931	class I histocompatibility antigen, F10 alpha chain-like	131.0	135.6	133.29
LRP8	low density lipoprotein receptor-related protein 8, apolipoprotein e receptor	131.7	134.8	133.28
CCDC27	coiled-coil domain containing 27	130.9	135.5	133.22
LOC104917056	phosphatidylinositol 3,4,5-trisphosphate 5-phosphatase 2-like	163.2	103.2	133.18
RAB7B	RAB7B, member RAS oncogene family	135.1	131.1	133.12
TECR	trans-2,3-enoyl-CoA reductase	129.2	137.0	133.11
LOC100540176	probable E3 ubiquitin-protein ligase MID2	119.2	146.9	133.07
CECR1	cat eye syndrome chromosome region, candidate 1	125.0	141.0	133.02
PLEKHG1	pleckstrin homology domain containing, family G (with RhoGef domain) member 1	134.4	131.6	132.97
PRKCE	protein kinase C, epsilon	138.5	127.3	132.90
LOC104915330	uncharacterized LOC104915330	129.3	136.5	132.89
FANCG	Fanconi anemia, complementation group G	132.6	133.1	132.85
TRAF5	TNF receptor-associated factor 5	146.0	119.6	132.81
LOC104914131	uncharacterized LOC104914131	141.9	123.7	132.78
LOC104910826	epididymis-specific alpha-mannosidase-like	124.2	141.2	132.71
LOC104909317	polypeptide N-acetylgalactosaminyltransferase 13-like	142.7	122.3	132.49
FGGY	FGGY carbohydrate kinase domain containing	140.2	124.3	132.28
OASL	2'-5'-oligoadenylate synthetase-like	126.7	137.8	132.24
ALG8	ALG8, alpha-1,3-glucosyltransferase	141.1	123.0	132.04
VDR	vitamin D (1,25- dihydroxyvitamin D3) receptor	136.0	128.0	131.98
SEPT10	septin 10	130.9	132.9	131.91
LOC104917533	uncharacterized LOC104917533	120.8	142.9	131.88

NUDT8	nudix (nucleoside diphosphate linked moiety X)-type motif 8	118.2	145.4	131.83
LOC100540872	hydroxysteroid 11-beta-dehydrogenase 1-like protein	136.8	126.8	131.80
SAP30L	SAP30-like	122.5	141.0	131.76
IFT27	intraflagellar transport 27	131.8	131.5	131.64
ZNF488	zinc finger protein 488	156.9	106.3	131.62
FDXACB1	ferredoxin-fold anticodon binding domain containing 1	127.5	135.6	131.56
ASAP2	ArfGAP with SH3 domain, ankyrin repeat and PH domain 2	130.1	132.9	131.48
LOC104917226	chromosome unknown open reading frame, human C19orf26	152.1	110.7	131.41
LOC104909867	uncharacterized LOC104909867	127.5	135.3	131.40
LOC104913142	protein BTG1-like	136.8	125.9	131.35
OMA1	OMA1 zinc metallopeptidase	141.9	120.8	131.35
RNF24	ring finger protein 24	137.7	125.0	131.33
LOC104913218	uncharacterized LOC104913218	128.4	134.1	131.24
TMEM18	transmembrane protein 18	117.3	145.2	131.24
LOC100539605	tumor necrosis factor receptor superfamily member 6-like	120.0	142.3	131.17
PARD3	par-3 family cell polarity regulator	132.6	129.7	131.13
LOC104909279	pancreatic alpha-amylase-like	120.8	141.3	131.02
LOC104915898	amyloid beta A4 precursor protein-binding family B member 1-like	141.9	119.8	130.87
LOC100549601	zinc finger protein 668-like	146.1	115.6	130.86
VWA5B2	von Willebrand factor A domain containing 5B2	147.1	114.6	130.85
LOC100548280	band 4.1-like protein 3	123.4	138.3	130.84
LOC104914699	transcription factor 4-like	131.8	129.5	130.63
BCOR	BCL6 corepressor	124.1	137.0	130.57
LOC104913256	uncharacterized LOC104913256	132.6	128.2	130.39
LRCH2	leucine-rich repeats and calponin homology (CH) domain containing 2	130.1	130.0	130.02
LOC104911095	uncharacterized LOC104911095	135.2	124.8	129.99
ING2	inhibitor of growth family, member 2	114.0	145.9	129.92
CCDC82	coiled-coil domain containing 82	146.1	113.5	129.81
ARHGAP20	Rho GTPase activating protein 20	113.2	146.2	129.68
CDC42EP2	CDC42 effector protein (Rho GTPase binding) 2	120.0	139.2	129.64
NT5DC1	5'-nucleotidase domain containing 1	142.7	116.5	129.60
BARD1	BRCA1 associated RING domain 1	141.9	117.3	129.57
GP1BB	glycoprotein Ib (platelet), beta polypeptide	115.7	143.3	129.51
LOC100548047	SNF-related serine/threonine-protein kinase-like	136.0	122.8	129.37
LRRC10B	leucine rich repeat containing 10B	139.4	119.3	129.37
RCAN1	regulator of calcineurin 1	126.7	132.0	129.36
FOXK1	forkhead box K1	126.7	132.0	129.34
CNNM2	cyclin and CBS domain divalent metal cation transport mediator 2	143.6	115.0	129.31
BBS9	Bardet-Biedl syndrome 9	124.1	134.3	129.22
SHC2	SHC (Src homology 2 domain containing) transforming protein 2	134.3	123.7	129.03
KAT6B	K(lysine) acetyltransferase 6B	118.3	139.6	128.92
LOC104913970	uncharacterized LOC104913970	125.8	132.0	128.91
RAP1GAP2	RAP1 GTPase activating protein 2	134.3	123.5	128.90
TMEM63A	transmembrane protein 63A	128.4	129.3	128.83
LOC104917495	uncharacterized LOC104917495	131.8	125.9	128.82
LOC100548468	ETS translocation variant 3-like	152.1	105.4	128.76
LOC104917112	chromodomain-helicase-DNA-binding protein 8-like	133.4	124.0	128.74
LOC104916524	putative uncharacterized zinc finger protein 814	139.4	118.0	128.70
UHRF2	ubiquitin-like with PHD and ring finger domains 2, E3 ubiquitin protein ligase	129.2	128.1	128.66
LOC104915542	uncharacterized LOC104915542	125.0	132.2	128.59
ARHGAP35	Rho GTPase activating protein 35	150.5	106.1	128.32
LOC104916672	histone H1.10	130.9	125.2	128.07
DZIP1L	DAZ interacting zinc finger protein 1-like	134.3	121.6	127.96
FIGNL2	fidgetin-like 2	122.5	133.3	127.93
TMEM220	transmembrane protein 220	124.2	131.6	127.88
WBSCR16	Williams-Beuren syndrome chromosome region 16	133.4	122.2	127.82

PAQR3	progestin and adipoQ receptor family member III	130.9	124.7	127.78
LOC100547436	protoheme IX farnesyltransferase, mitochondrial	142.7	112.6	127.66
LOC104915857	bud site selection protein 27-like	142.8	112.5	127.64
ARHGAP29	Rho GTPase activating protein 29	118.3	137.0	127.62
ZNF236	zinc finger protein 236	120.8	134.3	127.54
PTPN21	protein tyrosine phosphatase, non-receptor type 21	123.4	131.6	127.49
LOC104914242	uncharacterized LOC104914242	128.4	126.5	127.46
SESN2	sestrin 2	157.9	96.8	127.35
LOC104916716	arf-GAP with Rho-GAP domain, ANK repeat and PH domain-containing protein 1-like	140.3	114.4	127.35
XRCC1	X-ray repair complementing defective repair in Chinese hamster cells 1	134.3	120.2	127.27
LIN9	lin-9 DREAM MuvB core complex component	120.7	133.8	127.27
DYNLL1	dynein, light chain, LC8-type 1	125.9	128.5	127.21
OSR2	odd-skipped related transciption factor 2	141.0	113.4	127.20
NCAPG2	non-SMC condensin II complex, subunit G2	136.8	117.4	127.12
LOC104912151	ribosomal biogenesis protein LAS1L-like	129.2	124.8	127.01
PURA	purine-rich element binding protein A	131.0	123.0	126.97
TP53I3	tumor protein p53 inducible protein 3	139.2	114.4	126.80
LOC104916769	5'-AMP-activated protein kinase subunit gamma-1-like	153.8	99.7	126.74
WDR60	WD repeat domain 60	129.2	123.9	126.59
ARID3B	AT rich interactive domain 3B (BRIGHT-like)	142.0	111.0	126.49
SNCG	synuclein, gamma (breast cancer-specific protein 1)	114.8	138.1	126.47
LOC104917018	protein Wiz-like	134.4	118.4	126.38
MLH1	mutL homolog 1	120.7	132.0	126.34
DIXDC1	DIX domain containing 1	118.3	134.3	126.32
DZIP1	DAZ interacting zinc finger protein 1	133.4	118.9	126.17
GJA5	gap junction protein, alpha 5, 40kDa	114.1	137.9	126.02
COG3	component of oligomeric golgi complex 3	119.1	132.8	125.93
LOC100550035	integral membrane protein GPR155-like	125.0	126.8	125.92
GALNS	galactosamine (N-acetyl)-6-sulfatase	113.2	138.6	125.86
ARHGAP28	Rho GTPase activating protein 28	107.4	144.3	125.84
LOC104912962	uncharacterized LOC104912962	118.2	133.4	125.81
POU2F1	POU class 2 homeobox 1	117.4	134.1	125.73
LOC104910673	electrogenic sodium bicarbonate cotransporter 1-like	118.2	133.2	125.70
ID4	inhibitor of DNA binding 4, dominant negative helix-loop-helix protein	125.1	126.3	125.68
GMEB1	glucocorticoid modulatory element binding protein 1	141.1	110.2	125.66
SHCBP1	SHC SH2-domain binding protein 1	119.9	131.4	125.64
DHX58	DEXH (Asp-Glu-X-His) box polypeptide 58	123.4	127.8	125.59
FRY	furry homolog (Drosophila)	117.4	133.7	125.56
TF	transferrin	123.3	127.5	125.44
LOC100541650	microtubule-associated tumor suppressor candidate 2	122.5	128.3	125.44
CHD1L	chromodomain helicase DNA binding protein 1-like	136.8	113.8	125.30
LPAR2	lysophosphatidic acid receptor 2	134.4	116.1	125.25
CXCL12	chemokine (C-X-C motif) ligand 12	119.1	131.3	125.20
LOC104915171	uncharacterized LOC104915171	123.3	126.5	124.89
EPGN	epithelial mitogen	118.3	131.5	124.88
LOC100538525	desumoylating isopeptidase 2-like	124.2	125.5	124.84
LOC104917250	serine/threonine-protein phosphatase 5-like	122.5	127.1	124.81
LOC104913572	unconventional myosin-XVIIIb-like	145.4	104.0	124.68
LOC104914479	uncharacterized LOC104914479	133.4	115.6	124.50
LOC100541177	thrombospondin-4	140.2	108.5	124.35
FAM188B	family with sequence similarity 188, member B	125.9	122.6	124.22
MAPKBP1	mitogen-activated protein kinase binding protein 1	118.3	130.1	124.18
LOC100543474	glutathione S-transferase omega-1-like	147.7	100.7	124.18
PDZD7	PDZ domain containing 7	110.7	137.5	124.10
SOCS1	suppressor of cytokine signaling 1	115.7	132.3	124.02

LOC100539357	tubulin polyglutamylase TTLL4	127.5	120.2	123.87
ALKBH2	alkB, alkylation repair homolog 2 (E. coli)	113.2	134.5	123.84
C5H15orf52	chromosome 5 open reading frame, human C15orf52	104.7	142.9	123.82
NOL4L	nucleolar protein 4-like	119.1	128.2	123.67
APBA2	amyloid beta (A4) precursor protein-binding, family A, member 2	114.0	133.1	123.51
SETBP1	SET binding protein 1	105.7	141.3	123.48
GGCT	gamma-glutamylcyclotransferase	121.6	125.2	123.41
STX3	syntaxin 3	147.9	98.9	123.36
SNX24	sorting nexin 24	114.8	131.6	123.19
PMS1	PMS1 postmeiotic segregation increased 1 (S. cerevisiae)	123.3	122.7	122.99
LOC104910021	uncharacterized LOC104910021	131.0	114.6	122.82
NUP210	nucleoporin 210kDa	140.9	104.6	122.75
LOC100548654	14 kDa phosphohistidine phosphatase-like	124.9	120.5	122.72
LOC100549869	p53 apoptosis effector related to PMP-22-like	117.4	128.0	122.71
LOC100548902	cytoskeleton-associated protein 5	125.1	120.3	122.67
MTF1	metal-regulatory transcription factor 1	114.1	131.1	122.60
PKNOX1	PBX/knotted 1 homeobox 1	117.5	127.5	122.51
LOC104910625	uncharacterized LOC104910625	141.0	103.9	122.45
LOC104911158	uncharacterized LOC104911158	122.4	122.4	122.40
LOC104916721	mitogen-activated protein kinase 7-like	128.5	116.3	122.39
RWDD2A	RWD domain containing 2A	118.3	126.3	122.31
LOC100541896	histone H2B 8-like	116.5	128.1	122.30
LOC104915080	uncharacterized LOC104915080	122.4	122.1	122.27
RICTOR	RPTOR independent companion of MTOR, complex 2	127.6	116.8	122.21
ARL13B	ADP-ribosylation factor-like 13B	115.7	128.7	122.16
TET3	tet methylcytosine dioxygenase 3	136.8	107.5	122.14
TMEM128	transmembrane protein 128	116.5	127.7	122.14
LOC100550377	putative N-acetylated-alpha-linked acidic dipeptidase	140.1	104.1	122.09
DHX35	DEAH (Asp-Glu-Ala-His) box polypeptide 35	116.6	127.6	122.08
SLC3A1	solute carrier family 3 (amino acid transporter heavy chain), member 1	108.9	135.2	122.07
HAS2	hyaluronan synthase 2	113.1	130.9	122.03
LOC104917161	transcription intermediary factor 1-beta-like	144.5	99.4	121.95
LOC104916180	cell division control protein 6 homolog	138.5	105.1	121.84
ARC	activity-regulated cytoskeleton-associated protein	115.7	127.8	121.74
CEP152	centrosomal protein 152kDa	123.3	120.1	121.71
LOC100545095	1,4-alpha-glucan-branching enzyme-like	111.5	131.9	121.66
PRRG1	proline rich Gla (G-carboxyglutamic acid) 1	99.7	143.6	121.66
ZDHHC23	zinc finger, DHHC-type containing 23	132.6	110.7	121.64
GSTK1	glutathione S-transferase kappa 1	122.4	120.8	121.63
PSMC3IP	PSMC3 interacting protein	119.0	123.8	121.42
GOSR1	golgi SNAP receptor complex member 1	107.3	135.3	121.31
LOC100545397	autophagy-related protein 9A-like	133.5	109.1	121.30
PREX1	phosphatidylinositol-3,4,5-trisphosphate-dependent Rac exchange factor 1	110.8	131.5	121.15
NRSN2	neurensin 2	135.9	106.2	121.06
FAM173B	family with sequence similarity 173, member B	111.5	130.6	121.04
TMCO4	transmembrane and coiled-coil domains 4	143.5	98.5	121.02
LOC104916962	synaptogyrin-2-like	130.9	111.0	120.97
LOC100544767	uncharacterized LOC100544767	108.9	132.7	120.83
LOC104915092	uncharacterized LOC104915092	125.0	116.5	120.74
LOC104911263	uncharacterized LOC104911263	123.3	118.0	120.67
WDYHV1	WDYHV motif containing 1	120.0	121.3	120.66
LOC104909563	serine/arginine-rich splicing factor 7-like	122.5	118.8	120.65
LOC100546355	prominin-1-A-like	116.6	124.7	120.64
LOC100539483	uncharacterized LOC100539483	111.5	129.6	120.58
PPL	periplakin	130.1	110.9	120.51
PKD2	polycystic kidney disease 2 (autosomal dominant)	125.0	116.0	120.48

LOC100551355	CD2-associated protein-like	114.0	127.0	120.47
UVSSA	UV-stimulated scaffold protein A	115.7	125.2	120.47
ADAMTS13	ADAM metallopeptidase with thrombospondin type 1 motif, 13	108.2	132.8	120.47
PHLPP1	PH domain and leucine rich repeat protein phosphatase 1	135.2	105.7	120.43
LOC100550056	probable glutamate receptor	104.8	135.9	120.30
LOC104914631	synaptonemal complex protein SC65-like	104.8	135.8	120.29
AMER1	APC membrane recruitment protein 1	120.7	119.8	120.27
IFT88	intraflagellar transport 88	136.0	104.5	120.26
LOC100544340	heme-binding protein 2-like	107.3	133.1	120.20
LOC104909676	ribokinase-like	109.0	131.2	120.08
LOC104912609	serine/threonine-protein phosphatase 2A regulatory subunit B'' subunit alpha-like	107.3	132.8	120.02
SLBP	stem-loop binding protein	124.9	115.1	119.98
LOC100546243	ethanolaminephosphotransferase 1-like	120.0	119.9	119.94
PSAT1	phosphoserine aminotransferase 1	145.9	94.0	119.94
LOC104909798	A-kinase anchor protein 7 isoforms alpha and beta-like	102.2	137.6	119.90
LOC100550180	uncharacterized LOC100550180	119.9	119.9	119.89
RPRD1B	regulation of nuclear pre-mRNA domain containing 1B	135.9	103.8	119.87
NRAS	neuroblastoma RAS viral (v-ras) oncogene homolog	130.9	108.7	119.81
CSRNP2	cysteine-serine-rich nuclear protein 2	125.9	113.4	119.68
ARRDC4	arrestin domain containing 4	115.7	123.6	119.64
LOC100549911	methyl-CpG-binding domain protein 2-like	114.8	124.3	119.55
KIAA0895L	KIAA0895-like ortholog	125.8	113.2	119.54
LOC100546484	arylsulfatase D-like	120.8	118.3	119.54
LOC104914032	uncharacterized LOC104914032	108.2	130.8	119.49
LOC100543128	histone H3-like	115.6	123.3	119.43
MTHFS	5,10-methenyltetrahydrofolate synthetase (5-formyltetrahydrofolate cyclo-ligase)	106.4	132.4	119.38
VSTM2L	V-set and transmembrane domain containing 2 like	136.8	101.8	119.31
GGPS1	geranylgeranyl diphosphate synthase 1	105.6	133.0	119.30
AKAP11	A kinase (PRKA) anchor protein 11	127.5	111.0	119.21
CHST12	carbohydrate (chondroitin 4) sulfotransferase 12	116.5	121.8	119.16
THRA	thyroid hormone receptor, alpha	135.2	103.1	119.16
MEPCE	methylphosphate capping enzyme	147.1	91.2	119.13
LOC104911453	uncharacterized LOC104911453	101.4	136.5	118.93
LOC100539048	dual specificity protein phosphatase 13 isoform A-like	127.4	110.4	118.93
OSBPL6	oxysterol binding protein-like 6	114.8	122.9	118.87
OSCP1	organic solute carrier partner 1	132.6	105.0	118.79
MTCP1	mature T-cell proliferation 1	117.4	120.1	118.78
OVCH2	ovochymase 2	118.2	119.0	118.62
ZBTB21	zinc finger and BTB domain containing 21	114.0	123.2	118.58
LOC100539290	little elongation complex subunit 1-like	115.7	121.4	118.56
TPPP	tubulin polymerization promoting protein	119.1	118.0	118.55
RAB26	RAB26, member RAS oncogene family	130.8	106.3	118.52
METTL11B	methyltransferase like 11B	108.2	128.8	118.47
LOC104910843	DNA excision repair protein ERCC-6-like 2	114.1	122.8	118.46
CZH5orf63	chromosome Z open reading frame, human C5orf63	115.6	121.3	118.45
WDR90	WD repeat domain 90	135.2	101.6	118.41
B3GNT9	UDP-GlcNAc:betaGal beta-1,3-N-acetylglucosaminyltransferase 9	129.3	107.5	118.41
ANPEP	alanyl (membrane) aminopeptidase	128.4	108.4	118.40
LOC104913453	uncharacterized LOC104913453	110.6	125.8	118.21
LOC104913419	uncharacterized LOC104913419	121.7	114.6	118.15
CMTM3	CKLF-like MARVEL transmembrane domain containing 3	115.7	120.5	118.07
PUS7	pseudouridylate synthase 7 (putative)	115.7	120.4	118.04
RCSD1	RCSD domain containing 1	114.0	122.0	118.00
DNAH3	dynein, axonemal, heavy chain 3	120.7	115.1	117.91
LOC100551043	ubiquitin carboxyl-terminal hydrolase 45-like	111.5	124.3	117.87
INPP1	inositol polyphosphate-1-phosphatase	142.8	92.9	117.86

PKD1	polycystic kidney disease 1 (autosomal dominant)	122.5	113.1	117.81
PEX7	peroxisomal biogenesis factor 7	109.8	125.6	117.67
LOC100541881	ketimine reductase mu-crystallin-like	145.1	90.0	117.60
ZMYND12	zinc finger, MYND-type containing 12	106.4	128.5	117.44
DGKQ	diacylglycerol kinase, theta 110kDa	116.6	118.2	117.41
LOC100541592	X-linked retinitis pigmentosa GTPase regulator-like	118.2	116.6	117.38
SOX13	SRY (sex determining region Y)-box 13	131.8	102.9	117.37
IRF7	interferon regulatory factor 7	114.9	119.8	117.33
RASSF8	Ras association (RalGDS/AF-6) domain family (N-terminal) member 8	116.7	117.9	117.27
WNT5B	wingless-type MMTV integration site family, member 5B	130.1	104.4	117.27
LOC104913588	RING finger protein 10-like	120.0	114.5	117.24
ARL16	ADP-ribosylation factor-like 16	128.4	106.1	117.23
ETNK1	ethanolamine kinase 1	121.6	112.7	117.17
LOC100538605	transmembrane protein 8B-like	114.1	120.2	117.13
LOC100548295	pancreatic alpha-amylase	114.9	119.4	117.13
PINX1	PIN2/TERF1 interacting, telomerase inhibitor 1	114.9	119.4	117.13
LOC104917594	probable ATP-dependent RNA helicase DDX10	117.4	116.6	117.00
FAM217B	family with sequence similarity 217, member B	106.4	127.5	116.91
SLC22A23	solute carrier family 22, member 23	134.1	99.6	116.86
B3GALNT2	beta-1,3-N-acetylgalactosaminyltransferase 2	107.2	126.5	116.85
C10H1orf112	chromosome 10 open reading frame, human C1orf112	125.0	108.7	116.85
C9H8orf48	chromosome 9 open reading frame, human C8orf48	106.4	127.2	116.77
LOC104913520	uncharacterized LOC104913520	118.2	115.3	116.76
LRFN5	leucine rich repeat and fibronectin type III domain containing 5	115.7	117.8	116.74
MEF2B	myocyte enhancer factor 2B	130.8	102.6	116.73
LOC104910786	suppressor of cytokine signaling 1-like	105.6	127.7	116.69
ICOSLG	inducible T-cell co-stimulator ligand	125.8	107.5	116.68
ADCK1	aarF domain containing kinase 1	119.9	113.4	116.67
TRANK1	tetratricopeptide repeat and ankyrin repeat containing 1	108.1	125.2	116.65
CRYGN	crystallin, gamma N	119.1	114.1	116.60
CELF6	CUGBP, Elav-like family member 6	106.5	126.7	116.57
LRRC8C	leucine rich repeat containing 8 family, member C	98.0	134.8	116.41
KRT80	keratin 80	113.3	119.5	116.39
LOC104912065	BRCA2 and CDKN1A-interacting protein-like	124.9	107.8	116.35
TSPAN8	tetraspanin 8	103.0	129.7	116.35
LOC104910268	tyrosine-protein phosphatase non-receptor type 2-like	117.4	115.2	116.31
BACH2	BTB and CNC homology 1, basic leucine zipper transcription factor 2	108.1	124.5	116.30
LOC104911909	uncharacterized LOC104911909	120.0	112.6	116.28
KIF3C	kinesin family member 3C	116.6	116.0	116.26
FAM189A1	family with sequence similarity 189, member A1	139.2	93.2	116.24
LOC104914210	uncharacterized LOC104914210	107.3	125.0	116.15
MPP5	membrane protein, palmitoylated 5 (MAGUK p55 subfamily member 5)	118.2	114.0	116.12
ATP13A3	ATPase type 13A3	101.4	130.9	116.11
RC3H2	ring finger and CCCH-type domains 2	111.5	120.5	116.03
FBXO5	F-box protein 5	123.3	108.8	116.03
LARP4	La ribonucleoprotein domain family, member 4	123.4	108.6	116.00
PYGO2	pygopus family PHD finger 2	125.8	106.0	115.91
LOC100546012	protein CBFA2T3-like	116.6	115.2	115.89
SENP1	SUMO1/sentrin specific peptidase 1	119.2	112.6	115.87
LOC100551442	tenascin-X-like	136.8	95.0	115.87
LOC100539100	guanylate cyclase soluble subunit beta-2-like	112.3	119.3	115.80
CITED4	Cbp/p300-interacting transactivator, with Glu/Asp-rich carboxy-terminal domain, 4	114.9	116.7	115.79
LOC104914124	calsyntenin-1-like	114.8	116.4	115.64
ARG2	arginase 2	108.1	123.1	115.59

LOC100539493	epoxide hydrolase 1-like	119.9	111.1	115.49
NUDT6	nudix (nucleoside diphosphate linked moiety X)-type motif 6	114.8	116.1	115.46
PDE8B	phosphodiesterase 8B	93.8	136.8	115.30
SPEF1	sperm flagellar 1	136.0	94.6	115.26
RNLS	renalase, FAD-dependent amine oxidase	124.9	105.5	115.24
LOC104909332	deleted in malignant brain tumors 1 protein-like	108.1	122.3	115.22
GGCX	gamma-glutamyl carboxylase	127.6	102.8	115.19
CZH5orf30	chromosome Z open reading frame, human C5orf30	119.0	111.2	115.11
ANKRD52	ankyrin repeat domain 52	104.8	125.4	115.09
NR3C2	nuclear receptor subfamily 3, group C, member 2	99.7	130.0	114.83
HIPK3	homeodomain interacting protein kinase 3	98.0	131.6	114.78
SYCP2	synaptonemal complex protein 2	113.2	116.3	114.75
LOC104912120	serine/threonine-protein kinase WNK4-like	112.3	117.2	114.75
IL17RD	interleukin 17 receptor D	91.3	138.2	114.73
KDM8	lysine (K)-specific demethylase 8	112.3	117.2	114.73
LOC100542421	rotatin	108.1	121.1	114.59
LOC104916260	sestrin-2-like	138.4	90.7	114.56
DOK1	docking protein 1, 62kDa (downstream of tyrosine kinase 1)	111.5	117.6	114.56
LOC104915748	integrator complex subunit 3-like	119.9	109.0	114.45
MIPOL1	mirror-image polydactyly 1	124.1	104.7	114.43
LAMA1	laminin, alpha 1	113.2	115.6	114.40
AAED1	AhpC/TSA antioxidant enzyme domain containing 1	125.9	102.9	114.38
LOC104914734	E3 ubiquitin-protein ligase NEDD4-like	104.7	124.0	114.36
HTATIP2	HIV-1 Tat interactive protein 2, 30kDa	122.5	106.1	114.28
PGAP1	post-GPI attachment to proteins 1	114.9	113.6	114.25
CENPC	centromere protein C	123.2	105.1	114.16
LOC104911486	F-box only protein 7-like	97.1	131.1	114.08
GATA2	GATA binding protein 2	108.1	119.6	113.87
PIAS4	protein inhibitor of activated STAT, 4	114.8	112.7	113.74
APOPT1	apoptogenic 1, mitochondrial	114.0	113.3	113.67
RILP	Rab interacting lysosomal protein	117.4	109.8	113.61
LOC100550389	general transcription factor IIE subunit 1-like	104.8	122.3	113.53
LOC104917342	cyclin-dependent kinase 2-like	135.2	91.8	113.52
TLR3	toll-like receptor 3	112.3	114.5	113.41
LOC104913862	uncharacterized LOC104913862	122.5	104.3	113.37
LOC104911397	cullin-2-like	119.1	107.6	113.35
LOC100550661	derlin-2-like	106.4	120.1	113.28
MYO7B	myosin VIIB	108.1	118.4	113.22
BBS7	Bardet-Biedl syndrome 7	113.9	112.5	113.22
LOC104912256	retinal-specific ATP-binding cassette transporter-like	119.0	107.5	113.21
METTL3	methyltransferase like 3	116.6	109.9	113.21
SLC22A3	solute carrier family 22 (organic cation transporter), member 3	108.1	118.2	113.16
PTK2	protein tyrosine kinase 2	110.6	115.4	113.04
LOC100549648	class II histocompatibility antigen, M alpha chain-like	121.6	104.4	113.02
COLGALT2	collagen beta(1-O)galactosyltransferase 2	114.9	111.2	113.01
LOC104916866	transcription activator BRG1-like	124.2	101.7	112.93
LOC100549118	monocarboxylate transporter 8-like	113.1	112.7	112.91
ANGPTL5	angiopoietin-like 5	95.4	130.1	112.75
LOC104916163	N-acetylserotonin O-methyltransferase-like protein	98.0	127.5	112.72
HPDL	4-hydroxyphenylpyruvate dioxygenase-like	110.7	114.4	112.53
CXCR4	chemokine (C-X-C motif) receptor 4	109.8	115.2	112.52
KIAA1407	KIAA1407 ortholog	111.5	113.4	112.47
FNDC4	fibronectin type III domain containing 4	111.5	113.3	112.39
LOC100544425	zinc finger protein 383-like	109.8	114.8	112.34
RFX2	regulatory factor X, 2 (influences HLA class II expression)	119.1	105.6	112.34
CHAD	chondroadherin	129.2	95.5	112.34
ABCC2	ATP-binding cassette, sub-family C (CFTR/MRP), member 2	117.5	107.1	112.30

LOC104912822	S phase cyclin A-associated protein in the endoplasmic reticulum-like	101.4	123.2	112.29
LOC104916946	putative tRNA (cytidine(32)/guanosine(34)-2'-O)-methyltransferase	134.3	90.0	112.14
DLG1	discs, large homolog 1 (Drosophila)	106.4	117.4	111.95
LOC104917099	histone-lysine N-methyltransferase 2D-like	127.6	96.2	111.93
LOC104913281	uncharacterized LOC104913281	108.2	115.5	111.85
LOC100540769	protein kinase C-binding protein 1	97.2	126.4	111.77
VCPIP1	valosin containing protein (p97)/p47 complex interacting protein 1	105.6	117.6	111.62
LCAT	lecithin-cholesterol acyltransferase	121.6	101.6	111.61
LOC104916782	titin-like	109.0	114.2	111.58
LOC104916874	uncharacterized LOC104916874	113.1	110.0	111.57
LOC100551282	dephospho-CoA kinase domain-containing protein-like	110.6	112.3	111.47
BBS10	Bardet-Biedl syndrome 10	112.4	110.5	111.46
LOC104916405	ATP-binding cassette sub-family F member 3-like	141.2	81.5	111.35
LOC100539942	excitatory amino acid transporter 5-like	112.3	110.2	111.28
DTL	denticleless E3 ubiquitin protein ligase homolog (Drosophila)	116.5	106.0	111.25
LOC100540985	laminin subunit alpha-4-like	121.6	100.7	111.17
CEP97	centrosomal protein 97kDa	107.2	115.1	111.17
UBN2	ubinuclein 2	107.3	115.0	111.15
CRABP1	cellular retinoic acid binding protein 1	99.7	122.6	111.12
LOC104913537	uncharacterized LOC104913537	108.1	114.1	111.12
LOC100544491	glutathione S-transferase C-terminal domain-containing protein	111.4	110.5	110.97
TSPAN2	tetraspanin 2	108.1	113.8	110.97
TMOD4	tropomodulin 4 (muscle)	127.5	94.3	110.93
TSTD3	thiosulfate sulfurtransferase (rhodanese)-like domain containing 3	114.0	107.8	110.88
PPP1R1B	protein phosphatase 1, regulatory (inhibitor) subunit 1B	114.8	106.8	110.83
LOC104910719	uncharacterized LOC104910719	109.8	111.8	110.81
TMTC4	transmembrane and tetratricopeptide repeat containing 4	116.5	104.9	110.73
ARHGAP18	Rho GTPase activating protein 18	104.8	116.6	110.71
HDAC11	histone deacetylase 11	103.0	118.2	110.63
LOC100538883	oxidation resistance protein 1	114.0	107.2	110.58
LOXL4	lysyl oxidase-like 4	124.2	96.7	110.45
CDK18	cyclin-dependent kinase 18	114.0	106.8	110.43
LOC104913556	gamma-glutamyltransferase 5-like	95.5	125.4	110.43
TAPBPL	TAP binding protein-like	118.3	102.5	110.40
MOK	MOK protein kinase	109.8	110.8	110.31
NICN1	nicolin 1	119.8	100.7	110.26
PGF	placental growth factor	103.2	117.2	110.21
LOC100546124	COBW domain-containing protein 2-like	103.8	116.6	110.20
PXK	PX domain containing serine/threonine kinase	110.6	109.7	110.15
LOC100544088	uncharacterized LOC100544088	119.9	100.3	110.13
NSUN7	NOP2/Sun domain family, member 7	105.6	114.6	110.12
LOC100539591	probable ATP-dependent RNA helicase DDX10	117.4	102.7	110.06
CCDC84	coiled-coil domain containing 84	108.9	111.0	109.96
ALPK1	alpha-kinase 1	105.6	114.3	109.94
SAMD8	sterile alpha motif domain containing 8	94.6	125.1	109.84
LOC104915497	protein C19orf12 pseudogene	89.6	130.1	109.82
LOC104911430	integrin alpha-8-like	158.6	60.9	109.76
LOC104909482	uncharacterized LOC104909482	102.2	117.3	109.74
LOC104913414	ubiquitin carboxyl-terminal hydrolase 22-A-like	104.8	114.7	109.72
LOC104916455	arylsulfatase E-like	96.3	123.0	109.63
HYAL3	hyaluronoglucosaminidase 3	115.7	103.3	109.52
OXTR	oxytocin receptor	101.4	117.5	109.45
FANCI	Fanconi anemia, complementation group I	125.8	93.1	109.44
IDNK	idnK, gluconokinase homolog (E. coli)	104.7	114.2	109.44
VGLL4	vestigial-like family member 4	103.1	115.6	109.32

PGS1	phosphatidylglycerophosphate synthase 1	106.4	112.2	109.31
TMEM100	transmembrane protein 100	104.8	113.0	108.94
LOC104913496	fascin pseudogene	129.3	88.4	108.85
LOC104915299	sushi domain-containing protein 1-like	118.2	99.3	108.77
LOC100543750	poly [ADP-ribose] polymerase 12-like	115.7	101.9	108.77
PTHLH	parathyroid hormone-like hormone	115.7	101.8	108.74
LOC104914590	uncharacterized LOC104914590	111.5	106.0	108.74
CCDC149	coiled-coil domain containing 149	97.1	120.3	108.70
PGR	progesterone receptor	88.7	128.6	108.66
NRSN1	neurensin 1	101.4	115.8	108.63
UBE2Z	ubiquitin-conjugating enzyme E2Z	107.3	109.7	108.52
BCAT1	branched chain amino-acid transaminase 1, cytosolic	112.3	104.7	108.52
NOXO1	NADPH oxidase organizer 1	103.1	113.8	108.41
LOC104916502	CLK4-associating serine/arginine rich protein-like	119.2	97.6	108.40
SGMS2	sphingomyelin synthase 2	114.9	101.9	108.40
TCF3	transcription factor 3	110.7	106.1	108.38
LOC104917058	interleukin enhancer-binding factor 3-like	127.6	89.1	108.33
LOC104916527	zinc finger and BTB domain-containing protein 9-like	125.0	91.6	108.31
DUSP7	dual specificity phosphatase 7	102.2	114.4	108.30
INTU	inturned planar cell polarity protein	102.2	114.3	108.22
LOC104911695	unconventional myosin-X-like	102.2	114.2	108.20
B4GALNT4	beta-1,4-N-acetyl-galactosaminyl transferase 4	130.9	85.4	108.18
TACSTD2	tumor-associated calcium signal transducer 2	92.1	124.2	108.15
LOC100549707	GRAM domain-containing protein 4	88.7	127.6	108.12
LOC100547403	NACHT, LRR and PYD domains-containing protein 1-like	103.9	112.3	108.09
OSR1	odd-skipped related transciption factor 1	113.2	102.9	108.04
LOC104916384	ankyrin repeat domain-containing protein 34C-like	108.1	107.9	108.00
ADHFE1	alcohol dehydrogenase, iron containing, 1	113.2	102.7	107.94
SARM1	sterile alpha and TIR motif containing 1	101.4	114.4	107.90
LAMA3	laminin, alpha 3	93.0	122.8	107.85
LOC104911548	C-C chemokine receptor type 8-like	101.4	114.2	107.79
VANGL1	VANGL planar cell polarity protein 1	114.9	100.7	107.79
LOC100550341	corepressor interacting with RBPJ 1	109.0	106.6	107.77
BORA	bora, aurora kinase A activator	109.8	105.7	107.75
MDFI	MyoD family inhibitor	117.4	98.0	107.73
LOC104915781	all-trans-retinol 13,14-reductase-like	109.9	105.5	107.70
LOC100543979	PHD finger protein 14	109.7	105.7	107.70
LOC104913958	protein AATF-like	117.4	98.0	107.69
LOC104912918	uncharacterized protein KIAA0355-like	95.5	119.8	107.66
ANKRD34C	ankyrin repeat domain 34C	125.9	89.4	107.63
LOC104910827	serine/threonine-protein phosphatase 2A 55 kDa regulatory subunit B gamma isoform-like	96.3	118.9	107.61
ZNF395	zinc finger protein 395	109.9	105.3	107.60
LOC104913653	cysteine-rich tail protein 1-like	109.8	105.1	107.46
LOC104910282	tyrosine-protein kinase Yes-like	102.2	112.2	107.22
PRICKLE4	prickle homolog 4 (Drosophila)	115.7	98.6	107.16
LOC104912871	uncharacterized LOC104912871	100.6	113.7	107.15
LOC104910195	uncharacterized LOC104910195	99.6	114.4	107.04
LOC104913701	pleckstrin homology-like domain family B member 2	104.8	109.1	106.94
KBTBD11	kelch repeat and BTB (POZ) domain containing 11	109.7	104.0	106.86
LRP3	low density lipoprotein receptor-related protein 3	109.0	104.7	106.83
LOC104911538	uncharacterized LOC104911538	89.5	124.0	106.78
CCDC14	coiled-coil domain containing 14	103.9	109.5	106.68
FANCL	Fanconi anemia, complementation group L	114.8	98.5	106.67
TEX11	testis expressed 11	103.9	109.5	106.67
LOC100545963	ubiquitin conjugation factor E4 B	98.9	114.3	106.57
GNA13	guanine nucleotide binding protein (G protein), alpha 13	104.7	108.4	106.56
KLHL36	kelch-like family member 36	104.8	108.1	106.42

LOC100547042	probable global transcription activator SNF2L2	113.2	99.6	106.40
LOC104912150	stAR-related lipid transfer protein 8-like	109.8	102.9	106.35
TRIP13	thyroid hormone receptor interactor 13	123.2	89.4	106.34
LOC104914708	uncharacterized LOC104914708	104.0	108.6	106.29
DAAM1	dishevelled associated activator of morphogenesis 1	109.8	102.7	106.22
LOC100541211	leucine-rich repeat-containing protein 40-like	101.3	111.1	106.21
LOC100546474	general transcription factor IIH subunit 2	101.4	111.0	106.19
NUF2	NUF2, NDC80 kinetochore complex component	127.5	84.9	106.18
TSHZ3	teashirt zinc finger homeobox 3	108.9	103.3	106.11
PIH1D2	PIH1 domain containing 2	98.8	113.4	106.10
SLCO5A1	solute carrier organic anion transporter family, member 5A1	132.5	79.4	105.97
LOC104917310	putative protein TPRXL	107.3	104.5	105.89
YIPF4	Yip1 domain family, member 4	99.6	112.1	105.86
LOC104914426	uncharacterized LOC104914426	109.7	102.0	105.85
LOC104916023	zinc finger protein 853-like	112.3	99.4	105.83
SH3BGRL2	SH3 domain binding glutamate-rich protein like 2	88.7	122.7	105.73
PTDSS2	phosphatidylserine synthase 2	119.2	92.2	105.68
PIGA	phosphatidylinositol glycan anchor biosynthesis, class A	88.6	122.6	105.62
FSTL3	follistatin-like 3 (secreted glycoprotein)	93.0	118.2	105.59
GNB4	guanine nucleotide binding protein (G protein), beta polypeptide 4	95.5	115.5	105.50
GRAMD1B	GRAM domain containing 1B	97.1	113.9	105.50
LOC104913521	uncharacterized LOC104913521	98.0	113.0	105.48
MAPRE2	microtubule-associated protein, RP/EB family, member 2	92.1	118.5	105.31
CCDC77	coiled-coil domain containing 77	111.5	99.0	105.23
LOC104910213	polypeptide N-acetylgalactosaminyltransferase 12-like	92.1	118.4	105.23
LOC100544081	NACHT, LRR and PYD domains-containing protein 3-like	119.1	91.3	105.22
PDP2	pyruvate dehyrogenase phosphatase catalytic subunit 2	103.0	107.3	105.19
RNF150	ring finger protein 150	107.2	103.1	105.19
SLC8A3	solute carrier family 8 (sodium/calcium exchanger), member 3	112.4	97.8	105.10
LOC104911470	RUN domain-containing protein 3B-like	93.7	116.2	104.95
DNASE2B	deoxyribonuclease II beta	95.4	114.5	104.94
LOC100544253	E3 ubiquitin/ISG15 ligase TRIM25-like	108.9	100.9	104.90
KIF21A	kinesin family member 21A	96.3	113.5	104.90
CD247	CD247 molecule	119.1	90.7	104.88
LOC104914977	ribosome-releasing factor 2, mitochondrial-like	107.3	102.5	104.86
RAB39B	RAB39B, member RAS oncogene family	106.4	103.1	104.79
LOC104915359	S-methyl-5'-thioadenosine phosphorylase-like	104.7	104.7	104.72
LOC104915664	tubulin alpha chain-like	116.6	92.8	104.71
BAIAP2L1	BAI1-associated protein 2-like 1	96.3	113.0	104.67
LOC104909962	dystonin-like	98.0	111.0	104.51
SFT2D2	SFT2 domain containing 2	108.9	100.1	104.49
KCNA5	potassium voltage-gated channel, shaker-related subfamily, member 5	100.4	108.4	104.40
PIGQ	phosphatidylinositol glycan anchor biosynthesis, class Q	107.2	101.3	104.28
ENO2	enolase 2 (gamma, neuronal)	92.1	116.3	104.18
MFHAS1	malignant fibrous histiocytoma amplified sequence 1	115.7	92.5	104.13
LOC104911971	kinesin-like protein KIF20B	109.7	98.3	103.98
LDHD	lactate dehydrogenase D	102.2	105.3	103.77
CD99L2	CD99 molecule-like 2	104.8	102.7	103.75
SYTL2	synaptotagmin-like 2	100.5	107.0	103.74
LOC104909330	centrosomal protein of 192 kDa-like	109.8	97.6	103.71
LOC104909613	uncharacterized LOC104909613	98.8	108.7	103.71
LOC104912785	little elongation complex subunit 2-like	97.9	109.5	103.69
NDUFAF4	NADH dehydrogenase (ubiquinone) complex I, assembly factor 4	112.2	95.2	103.69
FAM168A	family with sequence similarity 168, member A	103.9	103.4	103.67
LOC104910574	E3 ISG15--protein ligase HERC5-like	117.4	89.9	103.64
SYNE3	spectrin repeat containing, nuclear envelope family member 3	102.2	105.0	103.62

LOC104916237	uncharacterized LOC104916237	101.4	105.8	103.59
MPLKIP	M-phase specific PLK1 interacting protein	95.4	111.2	103.30
LOC100544756	DENN domain-containing protein 5A	94.6	111.9	103.27
LOC104912917	SUMO-activating enzyme subunit 2-like	101.3	105.1	103.20
RPA3	replication protein A3, 14kDa	96.2	110.1	103.19
S1PR4	sphingosine-1-phosphate receptor 4	113.2	93.2	103.18
L3MBTL1	l(3)mbt-like 1 (Drosophila)	93.8	112.6	103.16
MST1R	macrophage stimulating 1 receptor (c-met-related tyrosine kinase)	119.1	87.3	103.15
LOC104913797	uncharacterized LOC104913797	94.7	111.5	103.13
LOC100551250	mitogen-activated protein kinase kinase kinase 5	110.7	95.6	103.12
SLC6A6	solute carrier family 6 (neurotransmitter transporter), member 6	91.3	114.8	103.09
ELP2	elongator acetyltransferase complex subunit 2	111.4	94.7	103.06
LOC104909666	adenylosuccinate synthetase isozyme 2-like	106.3	99.6	102.94
LIMK1	LIM domain kinase 1	114.1	91.8	102.93
PLEKHH3	pleckstrin homology domain containing, family H (with MyTH4 domain) member 3	127.6	78.2	102.90
LOC104914570	uncharacterized LOC104914570	94.7	111.0	102.83
LOC104912474	E3 ubiquitin-protein ligase RNF220-like	97.9	107.7	102.82
LOC100545547	WD and tetratricopeptide repeats protein 1	116.6	89.0	102.80
LSMEM2	leucine-rich single-pass membrane protein 2	111.4	94.1	102.78
SCN8A	sodium channel, voltage gated, type VIII, alpha subunit	82.0	123.5	102.76
PLAT	plasminogen activator, tissue	91.2	114.3	102.72
LOC100541378	1,25-dihydroxyvitamin D(3) 24-hydroxylase, mitochondrial	92.9	112.5	102.68
LOC100541993	glutamate receptor ionotropic, NMDA 3B-like	98.9	106.4	102.65
LOC100540894	lymphocyte antigen 6E	100.5	104.7	102.57
LOC104911331	uncharacterized LOC104911331	92.1	112.9	102.48
LOC104912526	uncharacterized LOC104912526	103.9	101.0	102.42
ERG	v-ets avian erythroblastosis virus E26 oncogene homolog	99.7	105.0	102.32
PLAG1	pleiomorphic adenoma gene 1	82.8	121.9	102.32
LOC104912835	CDC42 small effector protein 2-B-like	107.2	97.3	102.26
LOC100548019	beta-1,4-galactosyltransferase 3-like	112.3	92.0	102.16
MST1	macrophage stimulating 1 (hepatocyte growth factor-like)	103.1	101.2	102.16
LOC104913824	ankyrin repeat domain-containing protein 40-like	88.7	115.5	102.12
WDR18	WD repeat domain 18	88.7	115.5	102.10
FGF2	fibroblast growth factor 2 (basic)	105.4	98.6	102.00
KCNK1	potassium channel, subfamily K, member 1	107.1	96.8	101.99
LOC104911721	E3 ubiquitin-protein ligase UBR3-like	97.2	106.7	101.93
LOC104911590	uncharacterized LOC104911590	87.9	115.7	101.79
LOC104916547	dual specificity protein phosphatase 1 pseudogene	108.1	95.5	101.78
LOC104913011	phosphorylase b kinase regulatory subunit beta-like	108.9	94.6	101.77
GLI2	GLI family zinc finger 2	99.7	103.7	101.71
PHC3	polyhomeotic homolog 3 (Drosophila)	86.1	117.3	101.71
LOC100540082	xaa-Pro dipeptidase	107.2	96.2	101.70
SIRT4	sirtuin 4	92.9	110.5	101.66
IGHMBP2	immunoglobulin mu binding protein 2	93.8	109.5	101.66
CILP	cartilage intermediate layer protein, nucleotide pyrophosphohydrolase	98.8	104.4	101.60
LOC100549810	high affinity cGMP-specific 3',5'-cyclic phosphodiesterase 9A	103.8	99.4	101.60
LOC104916869	tripartite motif-containing protein 2-like	117.4	85.7	101.54
ALG14	ALG14, UDP-N-acetylglucosaminyltransferase subunit	108.0	94.9	101.47
FIGNL1	fidgetin-like 1	108.0	94.8	101.42
KDELC1	KDEL (Lys-Asp-Glu-Leu) containing 1	98.8	103.9	101.36
LOC100542198	UPF0505 protein C16orf62-like	103.0	99.5	101.28
LOC104912853	rap1 GTPase-activating protein 1-like	92.0	110.5	101.27
GALNT7	polypeptide N-acetylgalactosaminyltransferase 7	102.2	100.2	101.23
SLC31A2	solute carrier family 31 (copper transporter), member 2	98.0	104.5	101.21
LOC100545421	tetratricopeptide repeat protein 17	89.5	112.6	101.06

LOC104910322	putative Polycomb group protein ASXL3	98.8	103.2	101.00
BCL9L	B-cell CLL/lymphoma 9-like	104.0	97.6	100.77
CAMK2B	calcium/calmodulin-dependent protein kinase II beta	100.5	100.9	100.72
SUPT7L	suppressor of Ty 7 (S. cerevisiae)-like	99.7	101.6	100.66
LMBR1	limb development membrane protein 1	92.9	108.4	100.65
MFSD12	major facilitator superfamily domain containing 12	114.9	86.4	100.65
LOC104914634	kelch-like protein 24	115.7	85.5	100.58
LOC104912912	cytoplasmic dynein 1 light intermediate chain 2-like	106.5	94.7	100.57
LOC104917251	uncharacterized LOC104917251	98.7	102.4	100.57
TAF6	TAF6 RNA polymerase II, TATA box binding protein (TBP)-associated factor, 80kDa	120.0	80.9	100.43
ZBTB5	zinc finger and BTB domain containing 5	93.7	107.0	100.36
LOC104910753	LON peptidase N-terminal domain and RING finger protein 1-like	92.0	108.7	100.35
LOC100547702	myotubularin-related protein 9-like	95.5	105.2	100.34
LOC100541374	beta-adrenergic receptor kinase 2	104.7	96.0	100.33
SENP7	SUMO1/sentrin specific peptidase 7	97.1	103.3	100.22
LOC104911306	uncharacterized LOC104911306	103.0	97.3	100.16
LOC104916563	uncharacterized LOC104916563	97.2	103.1	100.15
LOC100543381	solute carrier family 2, facilitated glucose transporter member 11-like	97.1	102.9	100.00
LOC104911953	uncharacterized LOC104911953	102.2	97.7	99.94
LOC104916198	nuclear RNA export factor 1-like	87.1	112.6	99.83
WDR37	WD repeat domain 37	93.8	105.9	99.81
LOC104912983	uncharacterized oxidoreductase C663.06c-like	101.3	98.2	99.75
LOC104911515	uncharacterized LOC104911515	100.4	99.0	99.74
LOC104912375	laforin, isoform 9-like	97.2	102.3	99.74
ORC5	origin recognition complex, subunit 5	99.7	99.4	99.55
LGALSL	lectin, galactoside-binding-like	97.1	101.9	99.53
EPB41	erythrocyte membrane protein band 4.1	95.5	103.3	99.41
LOC104915526	myosin heavy chain, skeletal muscle-like	108.1	90.6	99.40
RPS6KB1	ribosomal protein S6 kinase, 70kDa, polypeptide 1	108.9	89.7	99.31
RPH3AL	rabphilin 3A-like (without C2 domains)	93.0	105.6	99.27
GPR75	G protein-coupled receptor 75	100.5	97.9	99.19
UHRF1BP1	UHRF1 binding protein 1	93.0	105.4	99.18
LOC104910494	bromodomain adjacent to zinc finger domain protein 2A-like	120.0	78.3	99.16
MAD2L1BP	MAD2L1 binding protein	98.8	99.3	99.05
OTOR	otoraplin	95.5	102.6	99.04
LOC100538679	cytochrome c oxidase assembly protein COX16 homolog, mitochondrial	107.2	90.8	99.03
LOC104911003	protein-methionine sulfoxide oxidase MICAL2-like	93.0	104.6	98.80
LOC100546538	MICOS complex subunit MIC19-like	94.6	102.8	98.68
LOC104913957	acetyl-CoA carboxylase-like	114.9	82.2	98.59
APOLD1	apolipoprotein L domain containing 1	95.4	101.7	98.58
AQP3	aquaporin 3 (Gill blood group)	92.9	104.3	98.57
MYLK3	myosin light chain kinase 3	99.7	97.4	98.54
LOC104914112	uncharacterized LOC104914112	98.8	98.3	98.53
LOC104916863	plectin-like	125.1	71.9	98.50
DTNB	dystrobrevin, beta	96.3	100.4	98.36
SRC	SRC proto-oncogene, non-receptor tyrosine kinase	104.8	91.8	98.30
STK10	serine/threonine kinase 10	98.0	98.5	98.24
LOC104913816	uncharacterized LOC104913816	98.9	97.5	98.19
FEZ1	fasciculation and elongation protein zeta 1 (zygin I)	110.6	85.8	98.18
NAMPT	nicotinamide phosphoribosyltransferase	101.4	94.8	98.10
LOC100543114	uncharacterized LOC100543114	95.4	100.8	98.09
LOC104909463	uncharacterized LOC104909463	102.2	93.8	98.00
CAMK4	calcium/calmodulin-dependent protein kinase IV	87.9	108.1	97.99
SHB	Src homology 2 domain containing adaptor protein B	95.5	100.4	97.97
CENPU	centromere protein U	113.1	82.7	97.91

LOC104915140	uncharacterized LOC104915140	96.3	99.4	97.88
LOC100548521	breast cancer type 1 susceptibility protein homolog	90.4	105.3	97.82
AJAP1	adherens junctions associated protein 1	91.3	104.1	97.70
RBBP7	retinoblastoma binding protein 7	111.4	84.0	97.70
MLLT1	myeloid/lymphoid or mixed-lineage leukemia (trithorax homolog, Drosophila); translocated to, 1	98.8	96.6	97.69
NPNT	nephronectin	85.3	110.0	97.68
ZDHHC15	zinc finger, DHHC-type containing 15	103.8	91.4	97.65
LOC100542484	serine/threonine-protein kinase ATR-like	108.1	87.2	97.62
DHRS11	dehydrogenase/reductase (SDR family) member 11	104.7	90.3	97.54
LOC100550623	mitogen-activated protein kinase kinase kinase 1	90.4	104.5	97.45
LOC104912561	uncharacterized LOC104912561	98.0	96.9	97.45
SH2D4A	SH2 domain containing 4A	97.1	97.8	97.45
QTRT1	queuine tRNA-ribosyltransferase 1	108.1	86.4	97.25
LYPLAL1	lysophospholipase-like 1	101.3	93.1	97.17
LOC104915841	chromosome unknown open reading frame, human C7orf43	109.8	84.4	97.13
LOC100550217	HLA class II histocompatibility antigen, DM beta chain-like	92.9	101.3	97.12
LOC104913359	zinc finger protein 501-like	93.8	100.3	97.06
NSUN3	NOP2/Sun domain family, member 3	95.5	98.5	97.01
LOC104911703	uncharacterized LOC104911703	102.2	91.8	96.96
BNC2	basonuclin 2	106.4	87.4	96.90
DOC2B	double C2-like domains, beta	95.5	98.2	96.85
SMIM5	small integral membrane protein 5	94.6	99.0	96.81
VWA8	von Willebrand factor A domain containing 8	97.9	95.6	96.78
LOC104909873	uncharacterized LOC104909873	91.2	102.3	96.78
LOC104911837	von Willebrand factor D and EGF domain-containing protein-like	103.0	90.4	96.72
RNF44	ring finger protein 44	109.0	84.4	96.71
LOC100547676	rho-related GTP-binding protein RhoG-like	94.6	98.8	96.69
AASS	aminoadipate-semialdehyde synthase	87.8	105.4	96.60
MUTYH	mutY homolog	105.6	87.5	96.56
LOC104910957	1-phosphatidylinositol 4,5-bisphosphate phosphodiesterase beta-2-like	97.1	96.0	96.55
CASC1	cancer susceptibility candidate 1	110.6	82.5	96.54
PARP15	poly (ADP-ribose) polymerase family, member 15	95.5	97.3	96.40
ELP4	elongator acetyltransferase complex subunit 4	88.6	104.1	96.39
AHI1	Abelson helper integration site 1	82.8	109.9	96.33
LOC104915161	uncharacterized LOC104915161	93.8	98.9	96.32
LOC100545726	interferon regulatory factor 2-like	89.6	103.0	96.31
LOC100540920	breast carcinoma-amplified sequence 3	106.4	85.9	96.15
ZNF800	zinc finger protein 800	101.3	90.9	96.07
STXBP5L	syntaxin binding protein 5-like	91.2	100.8	96.02
ATMIN	ATM interactor	90.3	101.7	96.00
LOC104909307	E3 ubiquitin-protein ligase TRIM33-like	98.8	93.2	95.98
TMEM63C	transmembrane protein 63C	105.6	86.2	95.93
PTPN1	protein tyrosine phosphatase, non-receptor type 1	87.8	104.0	95.90
PANX3	pannexin 3	92.1	99.6	95.87
LOC104915545	heterogeneous nuclear ribonucleoprotein U-like protein 1	109.0	82.7	95.81
SERINC5	serine incorporator 5	93.8	97.7	95.78
LRRC7	leucine rich repeat containing 7	100.5	91.0	95.77
LOC104914998	KN motif and ankyrin repeat domain-containing protein 1-like	88.6	102.8	95.74
LOC100541738	histone H4	93.0	98.5	95.73
SPATS2L	spermatogenesis associated, serine-rich 2-like	83.6	107.8	95.72
LOC104915912	uncharacterized LOC104915912	103.0	88.3	95.69
GAS2L1	growth arrest-specific 2 like 1	125.0	66.2	95.63
PRR14L	proline rich 14-like	94.6	96.4	95.49
CITED2	Cbp/p300-interacting transactivator, with Glu/Asp-rich carboxy-terminal domain, 2	95.4	95.4	95.41
DDAH1	dimethylarginine dimethylaminohydrolase 1	95.4	95.2	95.33

LOC104911028	nucleobindin-2-like	91.2	99.5	95.33
SRPX	sushi-repeat containing protein, X-linked	96.2	94.4	95.30
LOC104916112	inositol-3-phosphate synthase 1-A-like	84.5	105.9	95.18
LOC104914392	myotubularin-related protein 11-like	103.9	86.4	95.16
LOC104916521	E3 ubiquitin-protein ligase TRIM39-like	109.0	81.3	95.15
PEX12	peroxisomal biogenesis factor 12	103.0	87.2	95.12
LOC100551403	DNA polymerase delta subunit 3-like	103.9	86.3	95.06
LOC104915152	acyl-coenzyme A thioesterase 9, mitochondrial-like	95.4	94.6	95.04
LOC100539830	dysferlin-like	103.1	86.9	95.02
IYD	iodotyrosine deiodinase	109.7	80.3	95.00
LOC104911850	nck-associated protein 5-like	86.2	103.7	94.97
LOC100540025	dimethylaniline monooxygenase [N-oxide-forming] 5-like	106.4	83.3	94.83
LOC104917184	uncharacterized LOC104917184	94.7	94.7	94.72
CEP57L1	centrosomal protein 57kDa-like 1	92.0	97.4	94.70
MIEN1	migration and invasion enhancer 1	101.4	88.0	94.68
PLEKHG5	pleckstrin homology domain containing, family G (with RhoGef domain) member 5	109.8	79.1	94.45
CRISPLD2	cysteine-rich secretory protein LCCL domain containing 2	81.1	107.8	94.45
LURAP1	leucine rich adaptor protein 1	94.6	94.2	94.42
LOC104916146	extensin-like	114.0	74.6	94.34
NBN	nibrin	102.2	86.4	94.29
KIAA0930	KIAA0930 ortholog	103.8	84.7	94.24
LOC100547721	ankyrin repeat and SAM domain-containing protein 6-like	87.8	100.5	94.20
LOC100542457	putative homeodomain transcription factor 2	83.6	104.7	94.13
LOC104913413	phosphatidylethanolamine N-methyltransferase-like	93.0	95.3	94.12
LOC104911776	serine/threonine-protein kinase tousled-like 1	91.2	96.7	93.97
FANCB	Fanconi anemia, complementation group B	90.4	97.6	93.97
LOC104910425	ER membrane protein complex subunit 2-like	98.8	89.1	93.94
LOC100546696	BRCA1-A complex subunit BRE	102.2	85.4	93.81
TRAPPC13	trafficking protein particle complex 13	92.0	95.6	93.80
LOC104910939	palmitoyltransferase ZDHHC13-like	99.7	87.9	93.80
LOC104916157	mothers against decapentaplegic homolog 4	105.6	81.9	93.79
LOC104916287	proto-oncogene c-Fos-like	78.6	108.6	93.60
LOC104913956	tapasin-related protein-like	102.2	84.9	93.56
LOC104913967	adenine phosphoribosyltransferase 1-like	108.1	78.9	93.48
MYOC	myocilin, trabecular meshwork inducible glucocorticoid response	97.2	89.7	93.45
SKI	SKI proto-oncogene	91.2	95.7	93.44
IL11RA	interleukin 11 receptor, alpha	96.3	90.5	93.41
IGF2BP3	insulin-like growth factor 2 mRNA binding protein 3	102.1	84.7	93.40
LOC104914114	taste receptor type 1 member 1	91.3	95.4	93.33
PI4K2B	phosphatidylinositol 4-kinase type 2 beta	94.6	92.0	93.31
LOC104916964	uncharacterized LOC104916964	110.7	75.9	93.29
ATG5	autophagy related 5	97.9	88.7	93.29
FCHSD1	FCH and double SH3 domains 1	114.0	72.5	93.26
TBCC	tubulin folding cofactor C	97.1	89.3	93.22
DNAJC28	DnaJ (Hsp40) homolog, subfamily C, member 28	92.8	93.4	93.12
MEGF10	multiple EGF-like-domains 10	97.2	89.1	93.12
INSL5	insulin-like 5	90.3	95.9	93.10
VKORC1L1	vitamin K epoxide reductase complex, subunit 1-like 1	92.0	94.1	93.08
SAMD13	sterile alpha motif domain containing 13	93.7	92.4	93.07
TEX9	testis expressed 9	94.6	91.3	92.94
VPS9D1	VPS9 domain containing 1	103.9	81.7	92.80
LOC100545373	dedicator of cytokinesis protein 1-like	87.0	98.5	92.76
GPR123	G protein-coupled receptor 123	99.6	85.7	92.65
IGDCC4	immunoglobulin superfamily, DCC subclass, member 4	83.7	101.6	92.64
FCHSD2	FCH and double SH3 domains 2	87.9	97.0	92.45
LOC100544369	mothers against decapentaplegic homolog 2-like	89.5	95.3	92.42
ALDH5A1	aldehyde dehydrogenase 5 family, member A1	88.7	96.0	92.34

APLF	aprataxin and PNKP like factor	88.7	95.9	92.27
CCDC171	coiled-coil domain containing 171	87.0	97.4	92.21
LRRC52	leucine rich repeat containing 52	104.7	79.6	92.14
NMNAT1	nicotinamide nucleotide adenylyltransferase 1	97.1	87.2	92.14
AGO3	argonaute RISC catalytic component 3	93.8	90.4	92.12
MFSD7	major facilitator superfamily domain containing 7	88.7	95.3	91.98
LOC104912460	DNA repair and recombination protein RAD54-like	88.7	95.2	91.97
LOC104910818	uncharacterized LOC104910818	99.6	84.3	91.96
KIAA0040	KIAA0040 ortholog	97.1	86.7	91.94
SLC16A5	solute carrier family 16 (monocarboxylate transporter), member 5	96.3	87.5	91.92
LOC104911707	cordon-bleu protein-like 1	92.0	91.6	91.85
GAB2	GRB2-associated binding protein 2	90.4	93.2	91.81
PRICKLE2	prickle homolog 2 (Drosophila)	101.4	82.0	91.69
LOC104910848	limbin-like	106.4	76.9	91.65
TSHZ1	teashirt zinc finger homeobox 1	87.8	95.5	91.64
LOC100549832	rho GTPase-activating protein 20-like	88.7	94.6	91.62
LOC104912471	translation initiation factor eIF-2B subunit gamma-like	92.9	90.2	91.55
LOC104916083	EGF-containing fibulin-like extracellular matrix protein 2	101.4	81.6	91.50
LOC104913014	adenylate cyclase type 7-like	91.2	91.8	91.47
ZFAND5	zinc finger, AN1-type domain 5	77.7	105.1	91.43
LOC104916709	uncharacterized LOC104916709	106.5	76.3	91.42
CEMIP	cell migration inducing protein, hyaluronan binding	70.3	112.5	91.39
USP2	ubiquitin specific peptidase 2	106.4	76.3	91.38
LOC104910248	myomesin-1-like	89.5	93.2	91.37
LOC104913628	uncharacterized LOC104913628	81.9	100.5	91.23
LOC104914272	pumilio homolog 1-like	96.3	86.1	91.23
LOC104910276	methyltransferase-like protein 4	93.8	88.7	91.22
ANKLE1	ankyrin repeat and LEM domain containing 1	92.1	90.2	91.13
DMGDH	dimethylglycine dehydrogenase	94.6	87.7	91.12
LOC104917180	uncharacterized LOC104917180	87.8	94.4	91.09
NMNAT3	nicotinamide nucleotide adenylyltransferase 3	97.1	85.1	91.08
LOC104914802	motile sperm domain-containing protein 2-like	76.8	105.3	91.08
ASB18	ankyrin repeat and SOCS box containing 18	104.7	77.3	90.99
LOC104909917	sentrin-specific protease 6-like	84.5	97.5	90.97
LOC104909963	dystonin-like	83.7	98.2	90.91
TCP11L1	t-complex 11, testis-specific-like 1	87.0	94.6	90.81
STMN4	stathmin-like 4	102.2	79.4	90.79
PRRT1	proline-rich transmembrane protein 1	98.0	83.4	90.71
ATP1B4	ATPase, Na+/K+ transporting, beta 4 polypeptide	87.0	94.4	90.70
ASTE1	asteroid homolog 1 (Drosophila)	91.2	90.2	90.69
LOC104909947	dystonin-like	83.8	97.5	90.62
E2F7	E2F transcription factor 7	96.2	84.9	90.57
DYNC2LI1	dynein, cytoplasmic 2, light intermediate chain 1	98.8	82.3	90.51
PER2	period circadian clock 2	74.4	106.6	90.50
PMAIP1	phorbol-12-myristate-13-acetate-induced protein 1	94.6	86.3	90.46
LOC104909672	baculoviral IAP repeat-containing protein 6-like	91.3	89.5	90.40
LOC104914056	ADP-ribosylation factor GTPase-activating protein 1-like	92.9	87.9	90.39
ZNF710	zinc finger protein 710	96.4	84.4	90.38
CNEP1R1	CTD nuclear envelope phosphatase 1 regulatory subunit 1	76.9	103.9	90.36
LOC104910097	uncharacterized LOC104910097	75.2	105.5	90.35
LOC104912361	cell division cycle 7-related protein kinase-like	97.9	82.7	90.33
ZNF467	zinc finger protein 467	98.0	82.6	90.30
LOC104909799	eyes absent homolog 4-like	77.7	102.6	90.14
LOC100551422	ferric-chelate reductase 1	89.5	90.8	90.12
DYX1C1	dyslexia susceptibility 1 candidate 1	87.0	92.9	89.95
LOC104913769	pleckstrin homology-like domain family B member 2	87.9	91.9	89.87
LOC104910987	AMP deaminase 3-like	86.2	93.5	89.85
IL17RA	interleukin 17 receptor A	83.7	95.6	89.63

LOC104914556	uncharacterized LOC104914556	101.4	77.8	89.61
LOC104910406	uncharacterized LOC104910406	96.2	82.7	89.49
LOC104915511	UPF0505 protein C16orf62 homolog	86.2	92.6	89.40
LOC104917546	uncharacterized LOC104917546	90.4	88.3	89.35
LOC100539384	antimicrobial peptide NK-lysin-like	92.8	85.7	89.28
SNAP29	synaptosomal-associated protein, 29kDa	85.3	93.0	89.17
HESX1	HESX homeobox 1	103.0	75.2	89.12
LOC104914300	protein dopey-2-like	83.6	94.5	89.05
DRC7	dynein regulatory complex subunit 7	88.7	89.3	89.02
LOC104914665	uncharacterized LOC104914665	109.8	68.2	89.00
PKN3	protein kinase N3	71.8	106.2	88.97
LOC100542349	cyclin-G-associated kinase-like	88.7	89.2	88.96
RAB40C	RAB40C, member RAS oncogene family	96.3	81.5	88.91
NCMAP	noncompact myelin associated protein	95.4	82.3	88.88
SIX2	SIX homeobox 2	87.0	90.6	88.83
CCDC142	coiled-coil domain containing 142	87.9	89.6	88.71
HACL1	2-hydroxyacyl-CoA lyase 1	87.8	89.6	88.69
NR2E1	nuclear receptor subfamily 2, group E, member 1	86.1	91.0	88.57
PDK4	pyruvate dehydrogenase kinase, isozyme 4	85.2	91.9	88.56
HOXA3	homeobox A3	98.0	79.1	88.54
WDR83	WD repeat domain 83	103.0	74.0	88.53
LOC104909565	uncharacterized LOC104909565	87.0	90.1	88.53
CEP164	centrosomal protein 164kDa	86.2	90.9	88.52
LOC104916966	homeobox protein GBX-2	92.0	85.0	88.49
BHLHE41	basic helix-loop-helix family, member e41	83.7	93.1	88.41
LOC104917316	homeobox protein SIX2	101.4	75.4	88.40
LOC100543050	polypeptide N-acetylgalactosaminyltransferase 18	86.1	90.4	88.29
WIPF1	WAS/WASL interacting protein family, member 1	93.7	82.7	88.22
KPTN	kaptin (actin binding protein)	97.1	79.3	88.22
CASP7	caspase 7, apoptosis-related cysteine peptidase	95.4	80.9	88.16
PIDD1	p53-induced death domain protein 1	92.1	84.3	88.16
LOC104909478	putative tRNA pseudouridine synthase Pus10	97.1	79.1	88.09
CHST13	carbohydrate (chondroitin 4) sulfotransferase 13	92.0	84.1	88.06
LOC104911138	uncharacterized LOC104911138	79.5	96.6	88.04
LOC104912073	uncharacterized LOC104912073	85.3	90.6	87.98
LOC100540261	F-box only protein 7-like	79.4	96.6	87.96
LOC104913119	ubiquitin-like modifier-activating enzyme 7	87.8	88.1	87.95
OPN3	opsin 3	105.4	70.5	87.94
LOC100546110	glutamate receptor 1-like	77.7	98.1	87.91
LOC104909637	uncharacterized LOC104909637	83.6	92.0	87.83
ITPR2	inositol 1,4,5-trisphosphate receptor, type 2	79.5	96.2	87.81
LOC104913920	uncharacterized LOC104913920	106.4	69.0	87.69
LOC104915207	uncharacterized LOC104915207	91.2	84.1	87.67
ZNF384	zinc finger protein 384	98.0	77.3	87.62
SLC25A30	solute carrier family 25, member 30	83.5	91.6	87.58
LOC104912855	uncharacterized LOC104912855	81.1	94.0	87.56
LOC104910357	uncharacterized LOC104910357	78.6	96.5	87.55
GK	glycerol kinase	96.3	78.6	87.45
ACP5	acid phosphatase 5, tartrate resistant	94.6	80.0	87.30
LOC104917212	very long-chain acyl-CoA synthetase-like	86.1	88.4	87.25
LOC104911242	uncharacterized LOC104911242	93.8	80.7	87.22
LOC104913064	acyl-CoA synthetase family member 3, mitochondrial-like	104.7	69.6	87.18
NEURL2	neuralized E3 ubiquitin protein ligase 2	97.1	77.1	87.10
LOC104911101	activating molecule in BECN1-regulated autophagy protein 1-like	91.3	82.8	87.02
LOC104917339	ADM-like	94.6	79.3	86.97
LOC104912892	uncharacterized LOC104912892	85.3	88.6	86.95
KIAA0556	KIAA0556 ortholog	78.5	95.1	86.81
TXNRD1	thioredoxin reductase 1	83.6	89.9	86.78

MILR1	mast cell immunoglobulin-like receptor 1	88.7	84.9	86.77
LOC100544702	glyoxylate reductase/hydroxypyruvate reductase-like	100.5	72.9	86.68
LOC104916842	carboxy-terminal kinesin 2-like	97.9	75.2	86.57
HNF1A	HNF1 homeobox A	87.8	85.0	86.38
BCDIN3D	BCDIN3 domain containing	90.4	82.4	86.38
VGLL1	vestigial-like family member 1	82.0	90.6	86.30
TRPT1	tRNA phosphotransferase 1	90.4	82.2	86.30
LOC100547437	probable low-specificity L-threonine aldolase 2	78.6	94.0	86.28
CHRM5	cholinergic receptor, muscarinic 5	77.7	94.8	86.23
SCRN1	secernin 1	84.4	87.9	86.14
SLC45A3	solute carrier family 45, member 3	80.3	91.7	86.00
LOC104916303	mRNA cap guanine-N7 methyltransferase-like	101.4	70.5	85.99
TMEM17	transmembrane protein 17	73.5	98.4	85.95
LOC104916678	cyclin-dependent kinase 12-like	80.3	91.5	85.87
RPE65	retinal pigment epithelium-specific protein 65kDa	94.5	77.1	85.83
LOC100539738	histone H1.03-like	81.2	90.3	85.74
LOC100549160	cytochrome P450 4V2	95.4	76.1	85.74
LOC100545820	host cell factor 2-like	76.0	95.4	85.70
ARNT2	aryl-hydrocarbon receptor nuclear translocator 2	78.6	92.8	85.69
LOC104912401	uncharacterized LOC104912401	75.2	96.2	85.66
CAPN6	calpain 6	69.3	101.7	85.50
LOC104914902	uncharacterized LOC104914902	82.8	88.1	85.44
LOC100547979	junctophilin-1	86.1	84.5	85.33
LOC100539640	selenoprotein O	83.6	86.9	85.22
ELMO1	engulfment and cell motility 1	91.2	79.1	85.15
EFCAB1	EF-hand calcium binding domain 1	81.9	88.2	85.05
LOC104917237	SH3 and multiple ankyrin repeat domains protein 3-like	92.0	77.8	84.89
ZNF653	zinc finger protein 653	89.5	80.1	84.84
LOC104910859	G protein-coupled receptor kinase 4-like	69.2	100.4	84.81
LOC104913884	WD repeat-containing protein 81-like	98.0	71.4	84.71
LOC104916175	aquaporin-4-like	74.3	94.8	84.55
CDH6	cadherin 6, type 2, K-cadherin (fetal kidney)	124.7	44.3	84.52
LOC100541742	Na(+)/H(+) exchange regulatory cofactor NHE-RF3-like	71.8	97.1	84.44
KLHL3	kelch-like family member 3	85.3	83.5	84.40
LOC104917434	UDP-glucose:glycoprotein glucosyltransferase 2-like	87.0	81.7	84.35
LOC104915499	uncharacterized LOC104915499	90.3	78.2	84.25
NTM	neurotrimin	93.8	74.7	84.24
CREB1	cAMP responsive element binding protein 1	81.1	87.4	84.24
LOC100549340	uncharacterized LOC100549340	110.6	57.8	84.22
ABCC9	ATP-binding cassette, sub-family C (CFTR/MRP), member 9	88.7	79.7	84.19
LOC104916346	AT-hook DNA-binding motif-containing protein 1-like	93.0	75.4	84.16
LOC104912140	protein Shroom4-like	115.0	53.4	84.15
LOC104917073	hyphally regulated cell wall protein 3-like	93.9	74.4	84.15
PAQR5	progestin and adipoQ receptor family member V	88.7	79.6	84.15
LOC100540432	estradiol 17-beta-dehydrogenase 11-like	86.1	81.9	84.02
LOC104917103	circumsporozoite protein-like	88.7	79.3	84.02
CIB2	calcium and integrin binding family member 2	83.6	84.3	83.95
LOC100545705	dmX-like protein 1	81.9	86.0	83.94
LOC104916371	cytoplasmic FMR1-interacting protein 1-like	85.3	82.5	83.92
LOC104912473	uncharacterized protein C1orf228 homolog	76.9	90.8	83.86
LOC100547482	myotubularin	72.6	95.0	83.82
CAMKV	CaM kinase-like vesicle-associated	83.7	83.9	83.80
LOC100548070	histone-lysine N-methyltransferase SUV39H1-like	90.4	77.2	83.80
PCMTD2	protein-L-isoaspartate (D-aspartate) O-methyltransferase domain containing 2	77.7	89.9	83.79
LOC100551294	transmembrane protein 135	74.3	93.1	83.73
IGSF11	immunoglobulin superfamily, member 11	77.7	89.5	83.63
LOC100548792	collagen alpha-1(XII) chain-like	84.6	82.7	83.61

GALNT9	polypeptide N-acetylgalactosaminyltransferase 9	91.2	76.0	83.60
GJD2	gap junction protein, delta 2, 36kDa	70.9	96.2	83.59
LOC104915181	inositol hexakisphosphate and diphosphoinositol-pentakisphosphate kinase 2-like	72.7	94.4	83.55
RPUSD1	RNA pseudouridylate synthase domain containing 1	98.8	68.3	83.55
LOC100541693	integrin-alpha FG-GAP repeat-containing protein 2	84.5	82.4	83.45
PTPRK	protein tyrosine phosphatase, receptor type, K	87.8	78.9	83.37
CLSPN	claspin	92.8	73.9	83.37
PDZD3	PDZ domain containing 3	84.5	81.9	83.18
EME1	essential meiotic structure-specific endonuclease 1	92.9	73.5	83.17
LOC104912217	X-linked interleukin-1 receptor accessory protein-like 2	80.3	86.0	83.14
LOC104914761	nucleolar protein 6-like	91.3	75.0	83.11
LOC104916939	thyroid hormone receptor alpha-like	93.8	72.4	83.11
KIAA0513	KIAA0513 ortholog	81.9	84.3	83.09
CNRIP1	cannabinoid receptor interacting protein 1	85.2	80.9	83.05
LOC104909309	kinesin-like protein KIF27	82.8	83.1	82.95
LOC104917248	serine/arginine-rich splicing factor 6-like	88.8	77.1	82.95
POU6F1	POU class 6 homeobox 1	61.7	104.1	82.89
LOC104912858	uncharacterized LOC104912858	90.4	75.4	82.86
PITPNM3	PITPNM family member 3	81.9	83.8	82.85
ZNF451	zinc finger protein 451	80.3	85.4	82.81
MAP10	microtubule-associated protein 10	72.6	92.9	82.78
LOC104917027	uncharacterized LOC104917027	95.5	70.0	82.71
LOC104914990	pentatricopeptide repeat-containing protein 2, mitochondrial-like	79.4	85.9	82.65
RWDD2B	RWD domain containing 2B	77.6	87.6	82.65
LOC104911108	myosin-binding protein C, cardiac-type-like	77.7	87.3	82.52
REL	v-rel avian reticuloendotheliosis viral oncogene homolog	71.0	94.0	82.50
GNG2	guanine nucleotide binding protein (G protein), gamma 2	90.4	74.5	82.42
LOC100548974	uncharacterized LOC100548974	76.9	87.9	82.40
KIAA0895	KIAA0895 ortholog	95.4	69.4	82.40
PCBP3	poly(rC) binding protein 3	76.9	87.9	82.38
RAB43	RAB43, member RAS oncogene family	76.9	87.9	82.37
LOC100547518	phosphorylase b kinase regulatory subunit alpha, liver isoform-like	92.9	71.8	82.30
C10H1orf226	chromosome 10 open reading frame, human C1orf226	70.1	94.4	82.29
LOC104914563	KAT8 regulatory NSL complex subunit 1-like	82.8	81.6	82.22
NRIP1	nuclear receptor interacting protein 1	82.8	81.6	82.17
MATN2	matrilin 2	81.0	83.3	82.16
LOC104916339	small conductance calcium-activated potassium channel protein 1-like	94.7	69.6	82.13
LOC104916959	WD repeat-containing protein 55-like	97.1	67.1	82.11
PEX11A	peroxisomal biogenesis factor 11 alpha	78.5	85.7	82.11
PRKCH	protein kinase C, eta	76.9	87.3	82.10
KCNMB2	potassium large conductance calcium-activated channel, subfamily M, beta member 2	73.5	90.7	82.10
LOC104912346	uncharacterized LOC104912346	91.2	73.0	82.09
LOC104914814	rho guanine nucleotide exchange factor 39-like	99.6	64.5	82.06
LRAT	lecithin retinol acyltransferase (phosphatidylcholine--retinol O-acyltransferase)	70.9	93.0	81.95
LOC100543124	purpurin	82.8	81.0	81.90
KIAA1522	KIAA1522 ortholog	87.9	75.8	81.82
PARD6B	par-6 family cell polarity regulator beta	87.8	75.8	81.79
TVP23A	trans-golgi network vesicle protein 23 homolog A (S. cerevisiae)	69.2	94.3	81.77
LOC100548954	aldehyde dehydrogenase family 3 member B1	88.7	74.7	81.69
HPCAL4	hippocalcin like 4	81.1	82.2	81.66
GPD1	glycerol-3-phosphate dehydrogenase 1 (soluble)	87.8	75.5	81.66
LOC100542179	putative glycerol kinase 5	67.6	95.7	81.65
UBE3D	ubiquitin protein ligase E3D	87.0	76.3	81.65

BDNF	brain-derived neurotrophic factor	76.9	86.4	81.64
LOC104916369	ATP-binding cassette sub-family F member 1-like	99.7	63.6	81.63
LOC104909370	5-hydroxytryptamine receptor 7-like	87.0	76.1	81.55
INTS5	integrator complex subunit 5	106.4	56.7	81.54
LOC104910185	uncharacterized LOC104910185	76.0	86.9	81.46
FUT4	fucosyltransferase 4 (alpha (1,3) fucosyltransferase, myeloid-specific)	75.9	86.8	81.38
USP30	ubiquitin specific peptidase 30	81.9	80.8	81.37
MOV10	Mov10 RISC complex RNA helicase	98.0	64.6	81.27
EPT1	ethanolaminephosphotransferase 1 (CDP-ethanolamine-specific)	70.9	91.5	81.22
C28H6orf132	chromosome 28 open reading frame, human C6orf132	87.0	75.4	81.20
LOC100540051	thiamine transporter 1	82.7	79.7	81.19
ATP7A	ATPase, Cu++ transporting, alpha polypeptide	70.1	92.3	81.19
LOC100550673	protein FAM214B-like	92.1	70.1	81.10
AMIGO1	adhesion molecule with Ig-like domain 1	83.6	78.6	81.10
LOC100303681	somatotropin	92.0	70.1	81.06
CCR7	chemokine (C-C motif) receptor 7	77.7	84.2	80.98
CAPS2	calcyphosine 2	70.1	91.7	80.91
LOC104915326	aprataxin-like	89.5	72.1	80.83
LOC100548716	chitobiosyldiphosphodolichol beta-mannosyltransferase-like	80.2	81.5	80.83
KCTD21	potassium channel tetramerization domain containing 21	82.8	78.9	80.83
LOC104914536	inositol 1,4,5-trisphosphate receptor type 3-like	82.0	79.7	80.82
SZT2	seizure threshold 2 homolog (mouse)	87.1	74.5	80.78
CPEB3	cytoplasmic polyadenylation element binding protein 3	54.1	107.4	80.73
SLC38A9	solute carrier family 38, member 9	76.9	84.5	80.70
LOC104917410	uncharacterized LOC104917410	85.3	76.0	80.64
LOC104912942	T-cell immunomodulatory protein-like	76.0	85.2	80.61
SLC9A3R1	solute carrier family 9, subfamily A (NHE3, cation proton antiporter 3), member 3 regulator 1	87.8	73.3	80.57
LOC100545886	uncharacterized LOC100545886	83.6	77.2	80.41
CYYR1	cysteine/tyrosine-rich 1	88.6	72.2	80.40
LOC100540607	E3 ubiquitin-protein ligase RNF216	79.5	81.3	80.38
GEMIN5	gem (nuclear organelle) associated protein 5	87.9	72.7	80.28
LOC104916314	histone-lysine N-methyltransferase SUV39H1-like	85.3	75.2	80.24
LOC100542004	CWF19-like protein 2	69.3	91.2	80.23
R3HDML	R3H domain containing-like	78.6	81.7	80.18
SOX7	SRY (sex determining region Y)-box 7	73.5	86.7	80.09
LOC104911171	fibrous sheath-interacting protein 1-like	77.7	82.5	80.08
LOC100546123	G protein-activated inward rectifier potassium channel 1-like	87.9	72.3	80.07
LOC104914292	uncharacterized LOC104914292	81.9	78.2	80.06
ANK3	ankyrin 3, node of Ranvier (ankyrin G)	73.6	86.4	79.97
LOC104911843	cat eye syndrome critical region protein 2-like	82.8	77.1	79.94
PTPRC	protein tyrosine phosphatase, receptor type, C	75.1	84.7	79.93
INPP5A	inositol polyphosphate-5-phosphatase, 40kDa	73.5	86.3	79.91
FER	fer (fps/fes related) tyrosine kinase	60.8	99.0	79.91
SEPSECS	Sep (O-phosphoserine) tRNA:Sec (selenocysteine) tRNA synthase	76.0	83.6	79.80
LOC104909483	outer dense fiber protein 3-like	76.0	83.5	79.77
LOC104912316	pancreatic alpha-amylase-like	76.9	82.3	79.63
LOC100539085	serine/threonine-protein kinase PDIK1L-like	86.1	73.0	79.54
LOC104916785	homeobox protein GHOX-7	82.8	76.3	79.53
LOC104916759	phosphatidylinositol 3,4,5-trisphosphate 5-phosphatase 2-like	86.2	72.8	79.52
TUBB1	tubulin, beta 1 class VI	83.6	75.3	79.47
LHPP	phospholysine phosphohistidine inorganic pyrophosphate phosphatase	78.5	80.4	79.45
NOS1AP	nitric oxide synthase 1 (neuronal) adaptor protein	89.6	69.3	79.43
KCNMB1	potassium large conductance calcium-activated channel, subfamily M, beta member 1	75.1	83.7	79.40
KIF18A	kinesin family member 18A	91.2	67.4	79.33

LOC104915256	arrestin domain-containing protein 3-like	58.3	100.3	79.32
EBF3	early B-cell factor 3	76.8	81.7	79.24
LOC104917612	cytoplasmic dynein 2 heavy chain 1-like	65.9	92.5	79.22
LOC100547387	transmembrane protein 180-like	70.1	88.2	79.16
CRAMP1L	Crm, cramped-like (Drosophila)	78.5	79.8	79.16
LOC104911413	coiled-coil domain-containing protein 132-like	76.0	82.2	79.08
ZMAT3	zinc finger, matrin-type 3	79.4	78.7	79.08
PLEK	pleckstrin	83.5	74.6	79.07
IL17RE	interleukin 17 receptor E	92.1	65.8	78.98
ATXN1	ataxin 1	77.7	80.2	78.95
TRAK2	trafficking protein, kinesin binding 2	83.7	74.1	78.87
LOC104913166	uncharacterized LOC104913166	89.5	68.2	78.85
LOC100547363	double-stranded RNA-binding protein Staufen homolog 2-like	82.0	75.7	78.82
LOC104916390	signal-induced proliferation-associated 1-like protein 3	94.8	62.7	78.71
LOC104910352	uncharacterized LOC104910352	91.2	66.2	78.68
EXO1	exonuclease 1	80.2	77.1	78.67
SETD9	SET domain containing 9	79.4	77.8	78.59
THSD1	thrombospondin, type I, domain containing 1	78.5	78.6	78.57
LOC100541035	lysocardiolipin acyltransferase 1-like	78.5	78.6	78.55
LOC100544170	uncharacterized LOC100544170	79.4	77.7	78.54
LOC104916867	flocculation protein FLO11-like	87.9	69.2	78.54
OPA3	optic atrophy 3 (autosomal recessive, with chorea and spastic paraplegia)	87.8	69.2	78.53
LOC100548582	5-hydroxytryptamine receptor 2A-like	72.7	84.4	78.52
RSPH3	radial spoke 3 homolog (Chlamydomonas)	79.3	77.6	78.48
NEK11	NIMA-related kinase 11	70.1	86.8	78.48
TMEM86A	transmembrane protein 86A	75.2	81.7	78.47
ANK2	ankyrin 2, neuronal	81.0	75.8	78.37
FBXO40	F-box protein 40	82.7	74.0	78.36
LOC104914830	A disintegrin and metalloproteinase with thrombospondin motifs 12-like	91.2	65.3	78.26
DNAJB13	DnaJ (Hsp40) homolog, subfamily B, member 13	85.3	71.2	78.23
LOC104912415	SH3-containing GRB2-like protein 3-interacting protein 1	70.9	85.5	78.22
LOC100541880	BTB/POZ domain-containing protein KCTD5-like	81.0	75.4	78.19
DCP1B	decapping mRNA 1B	80.2	76.1	78.16
ASB12	ankyrin repeat and SOCS box containing 12	76.1	80.2	78.12
MITD1	MIT, microtubule interacting and transport, domain containing 1	87.0	69.1	78.04
LOC104917341	glutamate dehydrogenase 1, mitochondrial pseudogene	75.2	80.8	78.00
SCFD2	sec1 family domain containing 2	76.0	79.9	77.98
MYOT	myotilin	66.7	89.0	77.87
NDST3	N-deacetylase/N-sulfotransferase (heparan glucosaminyl) 3	81.1	74.6	77.86
LOC100546034	tetraspanin-18-like	80.3	75.4	77.84
SWAP70	SWAP switching B-cell complex 70kDa subunit	85.3	70.3	77.79
ITIH2	inter-alpha-trypsin inhibitor heavy chain 2	87.9	67.6	77.75
LOC104915770	DNA-directed RNA polymerase II subunit RPB1-like	82.8	72.6	77.73
LIPG	lipase, endothelial	98.7	56.7	77.68
FBXL15	F-box and leucine-rich repeat protein 15	75.2	80.1	77.65
ARHGAP26	Rho GTPase activating protein 26	72.6	82.4	77.50
LOC104914722	uncharacterized LOC104914722	87.1	67.6	77.35
LOC104909793	laminin subunit alpha-2-like	81.1	73.6	77.34
LOC104913336	uncharacterized LOC104913336	67.6	87.1	77.30
MYOZ3	myozenin 3	75.2	79.3	77.26
LOC104915554	uncharacterized LOC104915554	72.6	81.5	77.07
LOC100541519	metal-response element-binding transcription factor 2-like	74.3	79.8	77.04
C12H15orf61	chromosome 12 open reading frame, human C15orf61	81.9	72.0	76.96
EVPL	envoplakin	76.0	77.8	76.93
LOC104915134	uncharacterized LOC104915134	76.8	76.9	76.88

SEMA6D	sema domain, transmembrane domain (TM), and cytoplasmic domain, (semaphorin) 6D	78.6	75.2	76.88
LOC104914090	neurofilament heavy polypeptide-like	94.6	59.1	76.87
LOC104916961	zinc finger protein 621-like	81.1	72.6	76.85
VEGFA	vascular endothelial growth factor A	66.7	87.0	76.84
NADSYN1	NAD synthetase 1	87.8	65.8	76.82
LOC104915430	double zinc ribbon and ankyrin repeat-containing protein 1-like	72.7	81.0	76.82
BAMBI	BMP and activin membrane-bound inhibitor	71.0	82.6	76.80
LOC100545360	serine/threonine-protein kinase MRCK alpha-like	76.1	77.4	76.74
ROPN1L	rhophilin associated tail protein 1-like	71.8	81.4	76.61
DNA2	DNA replication helicase/nuclease 2	78.5	74.7	76.59
DNM1	dynamin 1	76.9	76.3	76.58
ENG	endoglin	76.1	76.9	76.50
LOC104912391	rab GTPase-activating protein 1-like	74.3	78.6	76.49
LOC104912876	uncharacterized LOC104912876	93.7	59.3	76.47
LOC104910851	histone-lysine N-methyltransferase NSD2-like	76.8	76.1	76.46
LIM2	lens intrinsic membrane protein 2, 19kDa	94.6	58.2	76.41
PLCL2	phospholipase C-like 2	62.5	90.0	76.25
LOC104916451	uncharacterized LOC104916451	82.7	69.8	76.23
LOC100549758	pleckstrin homology domain-containing family A member 5-like	71.0	81.4	76.22
LOC104916941	TERF1-interacting nuclear factor 2-like	80.2	72.1	76.14
LOC104917149	structural maintenance of chromosomes protein 5-like	76.9	75.4	76.11
RNMT	RNA (guanine-7-) methyltransferase	67.5	84.7	76.10
TSPEAR	thrombospondin-type laminin G domain and EAR repeats	74.3	77.7	76.02
YPEL4	yippee-like 4 (Drosophila)	71.8	80.2	75.99
LOC104915809	uncharacterized LOC104915809	81.1	70.8	75.99
ZBTB4	zinc finger and BTB domain containing 4	95.5	56.4	75.96
LOC104912509	uncharacterized LOC104912509	81.9	70.0	75.96
C1H12orf75	chromosome 1 open reading frame, human C12orf75	60.8	91.1	75.95
LOC104909928	uncharacterized LOC104909928	62.5	89.2	75.88
LSM11	LSM11, U7 small nuclear RNA associated	67.6	84.2	75.88
LOC104914800	TATA box-binding protein-associated factor, RNA polymerase I, subunit C-like	71.0	80.7	75.83
LOC104912652	cohesin subunit SA-1-like	71.0	80.5	75.76
FAM167B	family with sequence similarity 167, member B	92.9	58.6	75.74
LOC104917009	mitogen-activated protein kinase kinase kinase 12-like	82.8	68.6	75.72
KLHL31	kelch-like family member 31	75.2	76.1	75.65
LOC104912884	chloride channel protein 1-like	71.7	79.5	75.62
ACRC	acidic repeat containing	79.4	71.7	75.57
LOC100545035	C-C motif chemokine 4-like	67.5	83.6	75.55
LOC104915759	josephin-2-like	79.4	71.6	75.50
DCBLD1	discoidin, CUB and LCCL domain containing 1	70.1	80.8	75.48
LOC104914890	ATP-dependent RNA helicase DHX29-like	71.0	79.9	75.43
ACSBG1	acyl-CoA synthetase bubblegum family member 1	76.8	73.9	75.38
LOC104910316	DNA-dependent protein kinase catalytic subunit-like	77.7	73.0	75.36
HK3	hexokinase 3 (white cell)	66.7	83.9	75.31
LOC104909268	leucine-rich repeat-containing protein 41-like	83.6	67.0	75.31
GLRX	glutaredoxin (thioltransferase)	59.2	91.2	75.16
LOC100541121	G protein-activated inward rectifier potassium channel 4-like	66.8	83.5	75.15
NXN	nucleoredoxin	69.3	80.9	75.12
C12H15orf39	chromosome 12 open reading frame, human C15orf39	71.0	79.2	75.07
ORAI2	ORAI calcium release-activated calcium modulator 2	78.5	71.6	75.05
LOC104910644	ectonucleotide pyrophosphatase/phosphodiesterase family member 6-like	97.0	53.0	75.01
FUT8	fucosyltransferase 8 (alpha (1,6) fucosyltransferase)	76.8	73.2	75.00
DRC1	dynein regulatory complex subunit 1	78.5	71.4	74.97
CCK	cholecystokinin	74.3	75.6	74.94
ANKRD42	ankyrin repeat domain 42	76.9	73.0	74.92

SMAD6	SMAD family member 6	75.1	74.4	74.74
PATZ1	POZ (BTB) and AT hook containing zinc finger 1	76.9	72.6	74.71
DSG2	desmoglein 2	73.5	75.9	74.69
LOC100538821	FRAS1-related extracellular matrix protein 2-like	65.1	84.3	74.68
EFHD2	EF-hand domain family, member D2	77.7	71.6	74.63
LOC100551377	histamine H3 receptor-like	70.9	78.3	74.61
RERGL	RERG/RAS-like	75.1	74.1	74.59
LOC104909859	absent in melanoma 1 protein-like	81.0	68.1	74.58
LOC100539332	nucleoside diphosphate kinase 6-like	66.7	82.3	74.49
LOC100539745	probable ATP-dependent RNA helicase DDX10	78.6	70.2	74.41
LOC104909395	serine/threonine-protein kinase PAK 1	75.1	73.2	74.18
IL6R	interleukin 6 receptor	79.4	68.9	74.16
MYH11	myosin, heavy chain 11, smooth muscle	62.5	85.6	74.06
TNFAIP8	tumor necrosis factor, alpha-induced protein 8	75.1	72.9	74.02
LOC104911537	DENN domain-containing protein 2A-like	86.1	61.8	73.98
C17H12orf43	chromosome 17 open reading frame, human C12orf43	79.4	68.4	73.91
DFFB	DNA fragmentation factor, 40kDa, beta polypeptide (caspase-activated DNase)	71.8	75.9	73.86
LOC104917062	filamin-C-like	91.2	56.4	73.84
LOC104914841	uncharacterized LOC104914841	67.5	80.1	73.83
TEX30	testis expressed 30	73.4	74.0	73.71
OSBPL7	oxysterol binding protein-like 7	84.5	62.9	73.71
LOC100548583	sacsin	71.0	76.4	73.70
ABL2	ABL proto-oncogene 2, non-receptor tyrosine kinase	66.0	81.4	73.67
LOC100548603	uncharacterized LOC100548603	66.7	80.6	73.66
LOC104909218	integrator complex subunit 10	70.1	77.2	73.64
LOC104917203	E3 SUMO-protein ligase PIAS2 pseudogene	73.5	73.6	73.58
KIAA1328	KIAA1328 ortholog	74.3	72.7	73.51
LOC104915264	uncharacterized LOC104915264	66.8	80.2	73.49
BMP6	bone morphogenetic protein 6	71.0	76.0	73.49
LOC100543818	lethal(3)malignant brain tumor-like protein 3	69.3	77.6	73.46
LOC104911022	potassium voltage-gated channel subfamily C member 1 pseudogene	74.3	72.5	73.38
LOC104911762	striated muscle preferentially expressed protein kinase-like	86.3	60.5	73.36
KANK1	KN motif and ankyrin repeat domains 1	65.9	80.6	73.28
DOCK2	dedicator of cytokinesis 2	81.9	64.5	73.19
MAATS1	MYCBP-associated, testis expressed 1	67.5	78.7	73.14
LOC104911111	CUGBP Elav-like family member 1	67.6	78.4	73.02
LOC104909457	uncharacterized LOC104909457	76.9	69.1	73.00
LOC100550431	grainyhead-like protein 1 homolog	87.8	58.1	72.94
LOC104913115	RNA-binding protein 6-like	77.7	68.1	72.91
CAMKMT	calmodulin-lysine N-methyltransferase	82.0	63.6	72.76
HES4	hes family bHLH transcription factor 4	74.3	71.2	72.75
LOC104915538	uncharacterized LOC104915538	73.5	72.0	72.75
LOC104913288	uncharacterized LOC104913288	71.0	74.5	72.72
LOC104913925	protein phosphatase 1D-like	69.3	76.0	72.63
LOC100540610	myelin-oligodendrocyte glycoprotein-like	82.0	63.3	72.63
LOC104911519	uncharacterized LOC104911519	65.0	80.2	72.63
TCF24	transcription factor 24	75.1	70.2	72.62
XG	Xg blood group	66.8	78.5	72.62
ESF1	ESF1, nucleolar pre-rRNA processing protein, homolog (S. cerevisiae)	79.4	65.9	72.62
LOC104911277	actin-related protein 10-like	82.0	63.3	72.61
KLHL10	kelch-like family member 10	67.6	77.5	72.55
SYNGR3	synaptogyrin 3	71.8	73.2	72.52
SP4	Sp4 transcription factor	65.0	79.9	72.48
SLC10A4	solute carrier family 10, member 4	61.7	83.2	72.46
LOC104915633	splicing factor 3A subunit 2-like	70.9	73.9	72.39

HYDIN	HYDIN, axonemal central pair apparatus protein	74.4	70.4	72.38
CCDC113	coiled-coil domain containing 113	69.3	75.4	72.37
NRXN3	neurexin 3	74.4	70.3	72.32
ADAMTS4	ADAM metallopeptidase with thrombospondin type 1 motif, 4	86.1	58.5	72.30
GPR160	G protein-coupled receptor 160	65.8	78.7	72.29
LOC104911610	uncharacterized LOC104911610	65.1	79.3	72.18
LOC104912157	uncharacterized LOC104912157	71.7	72.4	72.05
LOC100545443	dynein heavy chain 9, axonemal	73.5	70.5	72.02
RARRES1	retinoic acid receptor responder (tazarotene induced) 1	76.8	67.0	71.92
CNNM1	cyclin and CBS domain divalent metal cation transport mediator 1	78.6	65.1	71.85
CLMP	CXADR-like membrane protein	73.5	70.0	71.73
LOC104909746	synaptojanin-2-like	65.8	77.6	71.73
RET	ret proto-oncogene	97.0	46.3	71.66
THAP11	THAP domain containing 11	72.7	70.6	71.64
LOC104913893	S-adenosyl-L-methionine-dependent tRNA 4-demethylwyosine synthase-like	69.3	74.0	71.63
TJAP1	tight junction associated protein 1 (peripheral)	70.1	73.1	71.62
NETO2	neuropilin (NRP) and tolloid (TLL)-like 2	59.2	84.0	71.60
MBLAC1	metallo-beta-lactamase domain containing 1	86.2	57.0	71.60
LOC104910861	uncharacterized LOC104910861	81.0	62.1	71.54
LOC104912350	angiopoietin-related protein 1 pseudogene	65.9	76.9	71.41
SDR42E1	short chain dehydrogenase/reductase family 42E, member 1	72.6	69.9	71.28
SPECC1L	sperm antigen with calponin homology and coiled-coil domains 1-like	72.6	69.8	71.22
ENPP4	ectonucleotide pyrophosphatase/phosphodiesterase 4 (putative)	70.1	72.3	71.19
EPHX1	epoxide hydrolase 1, microsomal (xenobiotic)	70.1	72.3	71.18
RIPK2	receptor-interacting serine-threonine kinase 2	74.2	68.1	71.16
DOK5	docking protein 5	67.6	74.7	71.16
LOC104912581	phosphatidylinositol 4,5-bisphosphate 3-kinase catalytic subunit beta isoform-like	70.9	71.2	71.05
LOC100547765	palmitoyltransferase ZDHHC3-like	75.2	66.8	71.01
LOC104914376	AT-rich interactive domain-containing protein 1A-like	71.7	70.3	71.01
LOC104914729	asparagine--tRNA ligase, cytoplasmic-like	65.9	76.1	71.00
EPHB2	EPH receptor B2	66.7	75.3	71.00
FGF12	fibroblast growth factor 12	75.2	66.7	70.95
LOC104917135	gem-associated protein 8-like	69.3	72.6	70.94
PLEKHH2	pleckstrin homology domain containing, family H (with MyTH4 domain) member 2	70.1	71.8	70.93
RIC3	RIC3 acetylcholine receptor chaperone	68.4	73.4	70.91
MMACHC	methylmalonic aciduria (cobalamin deficiency) cblC type, with homocystinuria	70.1	71.7	70.89
LOC100542821	histone H2A.J	72.6	69.0	70.84
PARK2	parkin RBR E3 ubiquitin protein ligase	65.0	76.7	70.84
LOC104916811	uncharacterized LOC104916811	70.1	71.6	70.83
RBPJ	recombination signal binding protein for immunoglobulin kappa J region	67.6	74.0	70.77
LOC104914903	uncharacterized LOC104914903	68.4	73.2	70.76
USP12	ubiquitin specific peptidase 12	66.8	74.7	70.72
P2RX3	purinergic receptor P2X, ligand-gated ion channel, 3	85.3	56.1	70.70
DHTKD1	dehydrogenase E1 and transketolase domain containing 1	71.8	69.3	70.55
LOC104917205	reticulon-3-like	75.2	65.9	70.54
LOC104909729	vacuolar protein 8-like	74.3	66.8	70.54
GYPC	glycophorin C (Gerbich blood group)	70.0	70.9	70.48
FBXO4	F-box protein 4	78.5	62.4	70.48
LOC104913670	uncharacterized LOC104913670	55.8	85.1	70.46
AMOTL1	angiomotin like 1	76.0	64.8	70.42
LOC104917314	14-3-3 protein gamma-B	63.4	77.4	70.40
TRIM7	tripartite motif containing 7	91.2	49.5	70.33

E2F5	E2F transcription factor 5, p130-binding	66.7	73.9	70.30
GLYCTK	glycerate kinase	76.0	64.6	70.30
GRP	gastrin-releasing peptide	65.0	75.4	70.22
LOC104916195	heparan-sulfate 6-O-sulfotransferase 1-like	71.8	68.6	70.19
C15H5orf24	chromosome 15 open reading frame, human C5orf24	66.8	73.6	70.18
PIGH	phosphatidylinositol glycan anchor biosynthesis, class H	72.7	67.6	70.15
CREG2	cellular repressor of E1A-stimulated genes 2	69.2	71.0	70.09
LOC104909989	kinase D-interacting substrate of 220 kDa-like	70.1	70.0	70.08
LOC100541791	protein TANC2	70.2	69.8	69.97
INO80D	INO80 complex subunit D	65.9	74.0	69.93
LOC104909520	ninein-like protein	74.4	65.3	69.85
LOC100544245	nebulin	43.1	96.5	69.82
SMPDL3A	sphingomyelin phosphodiesterase, acid-like 3A	76.9	62.4	69.66
CPSF7	cleavage and polyadenylation specific factor 7, 59kDa	82.8	56.5	69.65
ACSS3	acyl-CoA synthetase short-chain family member 3	65.9	73.2	69.56
INPP4B	inositol polyphosphate-4-phosphatase, type II, 105kDa	76.8	62.3	69.55
LOC104913525	uncharacterized LOC104913525	69.3	69.4	69.36
LOC104916459	mucin-5AC-like	82.0	56.7	69.35
LOC104910286	uncharacterized LOC104910286	72.6	66.0	69.32
LOC100547244	leucine-rich repeat and fibronectin type III domain-containing protein 1-like protein	75.2	63.4	69.29
LOC100538841	inactive dipeptidyl peptidase 10	53.3	85.3	69.26
LOC104912617	LIM and senescent cell antigen-like-containing domain protein 2	75.2	63.3	69.25
RBM44	RNA binding motif protein 44	77.7	60.8	69.23
RNF212B	ring finger protein 212B	71.8	66.6	69.23
LOC104913345	uncharacterized LOC104913345	74.3	63.8	69.06
DIO3	deiodinase, iodothyronine, type III	62.6	75.5	69.03
LOC104913048	uncharacterized LOC104913048	62.5	75.4	68.99
LOC100542468	dystonin	60.0	77.9	68.94
LOC104911110	uncharacterized LOC104911110	55.8	81.9	68.86
DSTYK	dual serine/threonine and tyrosine protein kinase	74.4	63.3	68.85
LOC104916338	protein Smaug homolog 2-like	79.4	58.2	68.79
LOC104915801	uncharacterized LOC104915801	72.7	64.8	68.77
STIL	SCL/TAL1 interrupting locus	73.5	64.0	68.77
LOC104916948	sortilin-like	69.3	68.2	68.75
LOC100538666	centromere protein J-like	78.5	58.9	68.74
MOB3C	MOB kinase activator 3C	72.6	64.8	68.69
LOC104915556	uncharacterized LOC104915556	64.2	73.2	68.68
LOC104914760	nucleolar protein 6-like	72.6	64.6	68.58
LOC104909964	dystonin-like	65.9	71.1	68.51
LOC104913742	myosin-6-like	72.7	64.2	68.44
LOC104911466	1-aminocyclopropane-1-carboxylate synthase-like protein 1	56.6	80.2	68.41
LOC100547242	interferon-induced guanylate-binding protein 1-like	77.7	59.1	68.40
SLC35F2	solute carrier family 35, member F2	76.8	59.9	68.39
LOC104917305	myelin-oligodendrocyte glycoprotein-like	66.7	70.0	68.36
TXNL4B	thioredoxin-like 4B	68.4	68.2	68.32
LOC104911778	mitogen-activated protein kinase kinase kinase MLT-like	64.2	72.4	68.30
LOC104913979	ubiquinol-cytochrome-c reductase complex assembly factor 1-like	69.2	67.3	68.27
KCNJ2	potassium inwardly-rectifying channel, subfamily J, member 2	71.0	65.5	68.24
OCSTAMP	osteoclast stimulatory transmembrane protein	81.1	55.4	68.22
LOC104915976	putative pre-mRNA-splicing factor ATP-dependent RNA helicase DHX16	75.2	61.3	68.22
WDR25	WD repeat domain 25	76.0	60.4	68.21
LOC104909504	uncharacterized LOC104909504	73.5	62.9	68.16
LOC104912175	uncharacterized LOC104912175	72.7	63.6	68.12
LOC104912994	nuclear pore complex protein Nup93-like	70.9	65.3	68.10
LOC104911498	DNA-binding protein SATB1	60.0	76.2	68.10
EXOC3L1	exocyst complex component 3-like 1	75.1	61.1	68.09

LOC100543145	adenomatous polyposis coli protein-like	55.8	80.3	68.05
XYLB	xylulokinase homolog (H. influenzae)	66.7	69.3	67.99
LOC100550622	microtubule-actin cross-linking factor 1, isoforms 1/2/3/5	65.1	70.8	67.95
LOC104914701	metallophosphoesterase domain-containing protein 1-like	67.6	68.3	67.93
SERINC2	serine incorporator 2	66.7	69.1	67.93
LOC100542733	pantetheinase-like	66.7	69.1	67.92
ORC2	origin recognition complex, subunit 2	74.4	61.4	67.86
LOC104912468	uncharacterized LOC104912468	66.7	69.0	67.84
GPR137C	G protein-coupled receptor 137C	65.9	69.8	67.82
PIGM	phosphatidylinositol glycan anchor biosynthesis, class M	70.1	65.4	67.77
LOC104911340	uncharacterized LOC104911340	70.1	65.4	67.76
NIM1K	NIM1 serine/threonine protein kinase	69.2	66.3	67.74
LOC104914121	kinesin-like protein KIF1B	64.2	71.2	67.73
LOC104909432	uncharacterized LOC104909432	77.7	57.7	67.70
LOC100542364	DNA polymerase zeta catalytic subunit	60.8	74.5	67.67
CCR4	chemokine (C-C motif) receptor 4	59.1	76.2	67.66
LOC104910399	OTU domain-containing protein 6B-like	63.3	72.0	67.65
FAM20A	family with sequence similarity 20, member A	76.0	59.1	67.56
WNK2	WNK lysine deficient protein kinase 2	54.9	80.1	67.49
SPTLC2	serine palmitoyltransferase, long chain base subunit 2	60.9	74.1	67.47
LOC104915487	semaphorin-4C-like	77.7	57.1	67.43
LOC104912422	inaD-like protein	59.1	75.6	67.39
LOC104915439	uncharacterized LOC104915439	81.9	52.8	67.34
IKZF4	IKAROS family zinc finger 4 (Eos)	65.9	68.7	67.33
TMCC3	transmembrane and coiled-coil domain family 3	102.9	31.6	67.22
LOC104916029	CDK5 regulatory subunit-associated protein 3-like	74.3	60.0	67.15
ENHO	energy homeostasis associated	73.5	60.8	67.12
TBC1D22A	TBC1 domain family, member 22A	66.7	67.5	67.11
SLC12A5	solute carrier family 12 (potassium/chloride transporter), member 5	72.7	61.1	66.93
MEX3A	mex-3 RNA binding family member A	71.0	62.9	66.91
LOC104914829	A disintegrin and metalloproteinase with thrombospondin motifs 12-like	65.1	68.7	66.90
ABCG5	ATP-binding cassette, sub-family G (WHITE), member 5	65.9	67.6	66.76
LOC100546006	F-box only protein 6-like	65.9	67.6	66.75
LOC100545944	LON peptidase N-terminal domain and RING finger protein 3	60.8	72.7	66.75
LOC104911938	uncharacterized LOC104911938	70.1	63.2	66.62
LOC104910769	uncharacterized LOC104910769	70.1	63.1	66.60
AADAC	arylacetamide deacetylase	65.9	67.3	66.60
LOC104912128	uncharacterized LOC104912128	62.5	70.4	66.49
SVOPL	SVOP-like	55.8	77.1	66.43
CAMLG	calcium modulating ligand	65.0	67.8	66.41
LOC100542164	DNA primase large subunit-like	66.0	66.8	66.36
FAR2	fatty acyl CoA reductase 2	60.0	72.7	66.36
PLEKHA2	pleckstrin homology domain containing, family A (phosphoinositide binding specific) member 2	76.0	56.7	66.36
LOC100549755	UPF0687 protein C20orf27 homolog	75.2	57.5	66.33
TMEM246	transmembrane protein 246	69.2	63.2	66.20
LOC100547828	methyltransferase-like protein 4	65.1	67.3	66.18
LOC104917090	mediator of RNA polymerase II transcription subunit 25-like	71.8	60.5	66.17
ZNF423	zinc finger protein 423	75.2	57.1	66.13
GALNT18	polypeptide N-acetylgalactosaminyltransferase 18	64.2	68.0	66.12
EVA1A	eva-1 homolog A (C. elegans)	50.7	81.5	66.12
USP18	ubiquitin specific peptidase 18	69.2	62.9	66.05
USP42	ubiquitin specific peptidase 42	63.3	68.5	65.93
FLRT3	fibronectin leucine rich transmembrane protein 3	75.2	56.6	65.89
BTBD7	BTB (POZ) domain containing 7	73.5	58.2	65.88
LOC100543530	glypican-5-like	49.8	81.9	65.86

FAM63B	family with sequence similarity 63, member B	65.9	65.8	65.83
DCP1A	decapping mRNA 1A	58.3	73.4	65.83
CD38	CD38 molecule	66.8	64.9	65.82
PALM2	paralemmin 2	64.2	67.4	65.81
LOC104910657	TBC domain-containing protein kinase-like protein	60.8	70.8	65.79
SERPINI1	serpin peptidase inhibitor, clade I (neuroserpin), member 1	60.8	70.7	65.77
SORCS1	sortilin-related VPS10 domain containing receptor 1	58.3	73.2	65.76
LOC104913644	kelch-like protein 9	68.4	63.1	65.75
TPCN2	two pore segment channel 2	59.1	72.3	65.71
CACNA1E	calcium channel, voltage-dependent, R type, alpha 1E subunit	68.4	63.0	65.70
LOC104916790	single-strand selective monofunctional uracil DNA glycosylase-like	79.4	52.0	65.69
LOC100546494	mitochondrial inner membrane protein COX18	67.6	63.7	65.61
USP24	ubiquitin specific peptidase 24	63.4	67.8	65.61
OTUD7A	OTU deubiquitinase 7A	66.7	64.5	65.61
XKR4	XK, Kell blood group complex subunit-related family, member 4	65.0	66.1	65.57
GALC	galactosylceramidase	64.2	66.9	65.56
LOC104914093	uncharacterized LOC104914093	81.9	49.2	65.54
LOC104913060	NEDD4-like E3 ubiquitin-protein ligase WWP2	57.4	73.5	65.45
LOC104910734	uncharacterized LOC104910734	69.2	61.5	65.37
LOC104914968	DNA polymerase kappa-like	64.2	66.5	65.31
CCDC92	coiled-coil domain containing 92	70.1	60.5	65.31
LOC104913621	antigen peptide transporter 1-like	73.5	57.1	65.30
IGFBP5	insulin-like growth factor binding protein 5	61.7	68.7	65.21
LOC100539441	uncharacterized LOC100539441	65.0	65.3	65.18
LOC104913639	integrator complex subunit 3-like	76.1	54.2	65.14
IL13RA2	interleukin 13 receptor, alpha 2	57.5	72.7	65.11
LOC104914049	uncharacterized LOC104914049	62.5	67.6	65.09
SERINC4	serine incorporator 4	59.1	71.0	65.09
LOC104916266	noggin-2-like	68.4	61.5	64.96
LOC100550074	DNA helicase MCM9	63.3	66.6	64.93
ADAMTS7	ADAM metallopeptidase with thrombospondin type 1 motif, 7	61.7	68.2	64.92
DCAF12	DDB1 and CUL4 associated factor 12	60.8	68.9	64.84
LOC104917548	uncharacterized LOC104917548	57.4	72.2	64.81
PTPRE	protein tyrosine phosphatase, receptor type, E	68.4	61.2	64.80
LOC104913452	zinc finger CCCH domain-containing protein 7A-like	68.4	61.1	64.76
LOC104912315	uncharacterized LOC104912315	63.4	66.1	64.74
ADAP2	ArfGAP with dual PH domains 2	65.8	63.5	64.65
LOC104910663	uncharacterized LOC104910663	58.3	70.7	64.49
PPARGC1A	peroxisome proliferator-activated receptor gamma, coactivator 1 alpha	56.7	72.2	64.43
LOC104914856	5'-AMP-activated protein kinase catalytic subunit alpha-1	65.9	62.9	64.41
LOC104916950	protein FAM110A-like	65.9	62.7	64.33
MSTN	myostatin	49.0	79.6	64.31
LOC104909269	ribosome-releasing factor 2, mitochondrial-like	64.2	64.4	64.28
LOC100545947	UDP-glucose:glycoprotein glucosyltransferase 1	62.5	66.0	64.27
LOC104913439	SUN domain-containing protein 1-like	73.5	55.0	64.24
LOC104916093	uncharacterized LOC104916093	80.3	48.1	64.20
FNDC1	fibronectin type III domain containing 1	48.2	80.1	64.17
SLX4IP	SLX4 interacting protein	66.7	61.6	64.14
LOC104917345	ryanodine receptor 1-like	73.6	54.7	64.14
NHLH1	nescient helix loop helix 1	76.8	51.4	64.13
SMIM18	small integral membrane protein 18	70.1	58.1	64.07
LOC100550524	mucosal pentraxin-like	84.5	43.6	64.06
LOC104910410	uncharacterized LOC104910410	54.1	74.0	64.04
CLCN2	chloride channel, voltage-sensitive 2	68.4	59.7	64.03
DNAJC24	DnaJ (Hsp40) homolog, subfamily C, member 24	62.5	65.5	64.00
ZBTB16	zinc finger and BTB domain containing 16	67.6	60.4	63.98

LOC104910301	uncharacterized LOC104910301	50.7	77.2	63.95
FAM161B	family with sequence similarity 161, member B	61.6	66.2	63.91
LOC104914750	ectopic P granules protein 5 homolog	56.6	71.1	63.85
C16H16orf59	chromosome 16 open reading frame, human C16orf59	66.7	61.0	63.84
ARHGAP39	Rho GTPase activating protein 39	82.9	44.8	63.83
LOC104910382	uncharacterized LOC104910382	70.1	57.5	63.80
ATE1	arginyltransferase 1	66.7	60.8	63.78
LOC104915951	homeobox protein Hox-A10-like	63.4	64.2	63.77
MAP3K13	mitogen-activated protein kinase kinase kinase 13	59.2	68.2	63.68
PLAU	plasminogen activator, urokinase	54.9	72.4	63.67
LOC100550956	uncharacterized LOC100550956	54.9	72.4	63.63
PCDH1	protocadherin 1	63.3	63.9	63.58
LOC104916298	zinc finger protein 36, C3H1 type-like 2	59.1	67.9	63.51
LOC104909948	dystonin-like	56.7	70.3	63.48
CDH22	cadherin 22, type 2	81.9	45.0	63.45
FN3K	fructosamine 3 kinase	54.0	72.8	63.44
LOC104917315	spore wall protein 2-like	67.6	59.3	63.42
LOC104917082	sericin-1-like	74.4	52.3	63.35
LOC104913329	transcription elongation regulator 1-like	60.8	65.7	63.26
LOC100542702	uncharacterized protein KIAA0355-like	67.6	58.9	63.25
LOC100550977	ceramide synthase 4-like	62.5	63.9	63.23
LOC104913379	uncharacterized LOC104913379	61.7	64.7	63.23
KCNH2	potassium voltage-gated channel, subfamily H (eag-related), member 2	67.6	58.8	63.21
LOC104910974	uncharacterized LOC104910974	70.9	55.5	63.20
NOX1	NADPH oxidase 1	63.4	62.9	63.13
LOC100540213	uncharacterized protein KIAA1841-like	62.5	63.7	63.08
LOC104910054	CD2-associated protein-like	60.8	65.3	63.08
LOC104911408	CAS1 domain-containing protein 1-like	49.8	76.2	63.01
IMMP2L	IMP2 inner mitochondrial membrane peptidase-like (S. cerevisiae)	66.7	59.2	62.94
SSBP2	single-stranded DNA binding protein 2	64.1	61.7	62.90
SLCO4A1	solute carrier organic anion transporter family, member 4A1	61.6	64.1	62.87
LOC100551119	protein sidekick-1	66.8	59.0	62.87
LOC104912760	uncharacterized LOC104912760	66.7	59.0	62.84
SNX22	sorting nexin 22	57.4	68.2	62.81
RAB3IP	RAB3A interacting protein	63.3	62.3	62.80
MYLK4	myosin light chain kinase family, member 4	58.3	67.2	62.77
TRAF6	TNF receptor-associated factor 6, E3 ubiquitin protein ligase	55.7	69.8	62.76
CYFIP2	cytoplasmic FMR1 interacting protein 2	65.8	59.6	62.70
LOC100550818	potassium channel subfamily K member 9-like	57.5	67.9	62.69
LOC104912649	vacuolar protein sorting-associated protein 8 homolog	71.0	54.3	62.63
ALS2CL	ALS2 C-terminal like	61.6	63.6	62.61
SYN2	synapsin II	62.5	62.7	62.60
LOC104911002	dickkopf-related protein 3-like	60.8	64.3	62.52
LOC104915967	transmembrane protein 198-like	56.6	68.4	62.52
LOC100550173	zinc finger X-chromosomal protein-like	51.5	73.5	62.49
GPT2	glutamic pyruvate transaminase (alanine aminotransferase) 2	63.3	61.5	62.42
INPP5J	inositol polyphosphate-5-phosphatase J	71.8	52.9	62.40
LOC100550289	ankyrin-3-like	59.1	65.6	62.36
LOC100543282	histone H2B 1/2/3/4/6	58.3	66.3	62.30
RNF157	ring finger protein 157	70.1	54.5	62.28
KIDINS220	kinase D-interacting substrate, 220kDa	55.0	69.6	62.28
LIN54	lin-54 DREAM MuvB core complex component	69.2	55.2	62.23
LOC100539654	nudC domain-containing protein 1-like	52.4	71.9	62.14
CACFD1	calcium channel flower domain containing 1	54.9	69.2	62.09
LOC104917118	proline-rich protein 4-like	65.9	58.1	62.05
LOC104914244	calsenilin-like	63.3	60.7	62.04

LOC104915443	NF-X1-type zinc finger protein NFXL1-like	59.1	64.9	62.01
AGK	acylglycerol kinase	68.4	55.6	62.00
LOC100539335	condensin complex subunit 1-like	71.0	53.0	61.98
LOC100540561	CMP-N-acetylneuraminate-beta-galactosamide-alpha-2,3-sialyltransferase 2-like	60.8	63.1	61.96
LOC104912093	uncharacterized LOC104912093	63.4	60.5	61.94
DEPDC1B	DEP domain containing 1B	72.6	51.2	61.88
LOC104915182	uncharacterized LOC104915182	65.9	57.9	61.88
LOC100542357	trifunctional purine biosynthetic protein adenosine-3-like	71.8	51.9	61.86
LOC104911615	uncharacterized LOC104911615	65.9	57.8	61.85
LOC104914235	uncharacterized LOC104914235	55.7	67.9	61.82
SCNN1A	sodium channel, non-voltage-gated 1 alpha subunit	51.6	72.1	61.82
LOC104912328	uncharacterized LOC104912328	76.0	47.4	61.72
LOC104912005	uncharacterized LOC104912005	49.0	74.4	61.70
LOC104914215	uncharacterized protein FLJ45252-like	65.1	58.2	61.64
RAB39A	RAB39A, member RAS oncogene family	59.1	64.1	61.62
TMEM98	transmembrane protein 98	66.7	56.5	61.62
GSTA1.1	glutathione S-transferase alpha class A1.1	62.5	60.7	61.60
LOC100541400	Y+L amino acid transporter 2-like	65.8	57.3	61.58
NCOA2	nuclear receptor coactivator 2	63.4	59.7	61.53
LOC100544543	glyoxylate reductase/hydroxypyruvate reductase-like	64.2	58.8	61.50
AGTR1	angiotensin II receptor, type 1	56.6	66.2	61.39
LOC104913804	uncharacterized LOC104913804	58.2	64.5	61.37
LOC104912547	uncharacterized LOC104912547	68.4	54.3	61.33
LOC104916516	uncharacterized LOC104916516	61.7	60.9	61.27
LPAR4	lysophosphatidic acid receptor 4	51.5	70.9	61.23
LOC104914255	uncharacterized LOC104914255	54.1	68.4	61.23
LOC104912184	uncharacterized LOC104912184	55.8	66.6	61.23
LOC104912430	E3 ubiquitin-protein ligase RNF170-like	62.5	59.9	61.22
LOC104913726	myosin-7-like	71.0	51.4	61.19
FHL2	four and a half LIM domains 2	53.2	69.2	61.18
LOC104909329	histone-lysine N-methyltransferase SMYD3-like	62.5	59.8	61.16
LOC104916862	transcription factor E3-like	68.4	53.8	61.12
UNK	unkempt family zinc finger	63.4	58.8	61.10
H6PD	hexose-6-phosphate dehydrogenase (glucose 1-dehydrogenase)	58.2	63.9	61.07
LOC100547297	MAP kinase-interacting serine/threonine-protein kinase 2-like	65.9	55.8	60.88
LRRC14B	leucine rich repeat containing 14B	63.3	58.4	60.86
WASF1	WAS protein family, member 1	54.9	66.8	60.83
MAP3K8	mitogen-activated protein kinase kinase kinase 8	54.0	67.6	60.82
PDE7B	phosphodiesterase 7B	55.8	65.8	60.80
ENGASE	endo-beta-N-acetylglucosaminidase	67.6	54.0	60.79
LOC104916989	DNA-directed RNA polymerase II subunit RPB1-like	57.5	64.0	60.75
ASB13	ankyrin repeat and SOCS box containing 13	65.0	56.3	60.68
LOC104909272	WASH complex subunit 7-like	61.7	59.7	60.68
LOC104916726	chromosome unknown open reading frame, human C8orf82	67.6	53.7	60.65
OSBP2	oxysterol binding protein 2	56.6	64.5	60.58
LOC104910266	uncharacterized LOC104910266	59.2	61.9	60.53
B3GNTL1	UDP-GlcNAc:betaGal beta-1,3-N-acetylglucosaminyltransferase-like 1	54.0	67.0	60.52
FBXL2	F-box and leucine-rich repeat protein 2	63.3	57.7	60.49
INHA	inhibin, alpha	60.8	60.2	60.49
LOC104916914	zinc finger protein ubi-d4-like	51.6	69.4	60.49
LOC104910472	protein NDRG1-like	55.8	65.2	60.46
B3GAT2	beta-1,3-glucuronyltransferase 2	51.5	69.4	60.44
LOC104912507	microtubule-associated serine/threonine-protein kinase 2-like	65.9	54.8	60.31
LOC104916465	nucleoside diphosphate kinase 6-like	59.1	61.5	60.29
LOC104913610	uncharacterized LOC104913610	69.3	51.3	60.27
LOC104915621	tetratricopeptide repeat protein 38-like	60.0	60.3	60.15

LOC104916758	homeobox protein Hox-A4-like	57.4	62.8	60.11
LOC104912500	ras-related protein Rab-3A-like	56.6	63.5	60.05
RELT	RELT tumor necrosis factor receptor	64.2	55.9	60.04
COL16A1	collagen, type XVI, alpha 1	82.0	38.1	60.04
NEK3	NIMA-related kinase 3	58.3	61.7	60.03
LOC104913318	uncharacterized LOC104913318	59.9	60.0	59.98
MYCN	v-myc avian myelocytomatosis viral oncogene neuroblastoma derived homolog	55.7	64.2	59.94
PCSK9	proprotein convertase subtilisin/kexin type 9	67.6	52.2	59.90
NPL	N-acetylneuraminate pyruvate lyase (dihydrodipicolinate synthase)	59.9	59.7	59.80
LOC104915350	methylcrotonoyl-CoA carboxylase beta chain, mitochondrial-like	56.6	62.9	59.75
LOC100540591	CAS1 domain-containing protein 1	59.1	60.3	59.68
PAQR8	progestin and adipoQ receptor family member VIII	59.1	60.2	59.66
RAB3A	RAB3A, member RAS oncogene family	60.8	58.5	59.64
ACBD7	acyl-CoA binding domain containing 7	51.5	67.7	59.61
MEF2C	myocyte enhancer factor 2C	54.1	65.1	59.60
ACAD10	acyl-CoA dehydrogenase family, member 10	71.8	47.3	59.53
CABIN1	calcineurin binding protein 1	64.2	54.7	59.46
ZNF654	zinc finger protein 654	51.5	67.4	59.46
LOC104914920	kinesin-like protein KIF2A	62.5	56.4	59.45
LOC100551044	nucleolar protein 10	52.3	66.5	59.42
LOC104912544	uncharacterized LOC104912544	54.1	64.7	59.40
LOC104913962	clathrin heavy chain 1-like	69.3	49.5	59.39
LOC104914995	phosphoglucomutase-like protein 5	68.4	50.4	59.39
ARHGAP1	Rho GTPase activating protein 1	62.5	56.2	59.37
LOC104915493	fatty acyl-CoA hydrolase precursor, medium chain-like	54.9	63.7	59.30
LOC104914821	PDZ domain-containing protein 2-like	60.8	57.7	59.27
CPNE2	copine II	50.7	67.8	59.22
C23H1orf86	chromosome 23 open reading frame, human C1orf86	70.1	48.4	59.21
LOC104913784	putative sodium-coupled neutral amino acid transporter 10	64.2	54.1	59.17
LOC100541905	tubulin epsilon chain	61.7	56.7	59.16
RNF2	ring finger protein 2	55.8	62.4	59.08
LOC100547584	Krueppel-like factor 5	67.6	50.6	59.06
TSPAN13	tetraspanin 13	67.5	50.6	59.06
GPATCH2	G patch domain containing 2	50.7	67.4	59.04
LOC104911256	ras GTPase-activating-like protein IQGAP3	69.3	48.8	59.03
LOC104909677	BRCA1-A complex subunit BRE-like	55.8	62.3	59.03
RELL1	RELT-like 1	56.7	61.3	58.96
PTPLAD2	protein tyrosine phosphatase-like A domain containing 2	55.7	62.2	58.94
DAO	D-amino-acid oxidase	54.9	62.9	58.94
ELFN1	extracellular leucine-rich repeat and fibronectin type III domain containing 1	63.4	54.5	58.94
JMY	junction mediating and regulatory protein, p53 cofactor	70.9	46.9	58.92
STOX2	storkhead box 2	50.7	67.1	58.91
LOC104911250	uncharacterized LOC104911250	48.1	69.7	58.91
COL4A5	collagen, type IV, alpha 5	64.9	52.9	58.91
LOC104912859	uncharacterized LOC104912859	53.2	64.5	58.88
XPNPEP2	X-prolyl aminopeptidase (aminopeptidase P) 2, membrane-bound	62.5	55.1	58.81
EGLN3	egl-9 family hypoxia-inducible factor 3	60.8	56.7	58.76
CPNE8	copine VIII	63.3	54.2	58.76
LINS	lines homolog (Drosophila)	67.6	49.8	58.70
TAF4B	TAF4b RNA polymerase II, TATA box binding protein (TBP)-associated factor, 105kDa	67.6	49.7	58.64
DOK7	docking protein 7	59.1	58.1	58.57
PROX1	prospero homeobox 1	49.0	68.1	58.53
LOC104912647	ephrin type-B receptor 1-like	60.1	56.9	58.47
NCF2	neutrophil cytosolic factor 2	63.4	53.5	58.44

ZDHHC2	zinc finger, DHHC-type containing 2	54.9	61.9	58.42
LOC104914108	uncharacterized LOC104914108	58.3	58.5	58.36
LOC100538454	potassium voltage-gated channel subfamily V member 2-like	57.5	59.2	58.34
LOC104917603	uncharacterized LOC104917603	56.6	59.9	58.23
SLC41A1	solute carrier family 41 (magnesium transporter), member 1	56.6	59.7	58.17
LOC104914577	pleckstrin homology domain-containing family M member 1-like	66.7	49.6	58.16
LOC100547893	aspartate--tRNA ligase, mitochondrial	65.0	51.3	58.16
LOC104913942	uncharacterized LOC104913942	65.0	51.2	58.12
HHLA2	HERV-H LTR-associating 2	51.5	64.6	58.09
LOC100543118	interferon-induced guanylate-binding protein 1-like	74.3	41.9	58.08
LOC104915229	uncharacterized LOC104915229	52.4	63.7	58.03
HYKK	hydroxylysine kinase	60.8	55.1	57.98
LOC104909927	uncharacterized LOC104909927	50.7	65.3	57.97
CDC14B	cell division cycle 14B	59.1	56.8	57.96
LOC104913752	uncharacterized LOC104913752	66.7	49.2	57.93
LH-BETA	luteinizing hormone beta subunit	59.1	56.7	57.91
LOC104914813	uncharacterized LOC104914813	58.3	57.5	57.91
LOC104909364	zinc finger protein 131-like	58.3	57.5	57.86
SLC25A37	solute carrier family 25 (mitochondrial iron transporter), member 37	67.6	48.2	57.86
LOC104917200	uncharacterized LOC104917200	44.8	70.9	57.82
LOC100540242	lysine-specific demethylase 3B	62.5	53.2	57.82
IFITM10	interferon induced transmembrane protein 10	56.6	59.0	57.80
LOC100542976	ataxin-10-like	59.9	55.4	57.66
BTBD19	BTB (POZ) domain containing 19	60.0	55.3	57.61
TMEM173	transmembrane protein 173	49.9	65.3	57.56
MB21D1	Mab-21 domain containing 1	57.4	57.5	57.48
FMNL2	formin-like 2	45.7	69.2	57.43
LOC104911372	uncharacterized LOC104911372	53.2	61.6	57.39
LOC104914744	dymeclin-like	54.0	60.6	57.33
LOC104913340	cyclin-F-like	56.6	58.0	57.28
LOC104909528	methylglutaconyl-CoA hydratase, mitochondrial-like	62.5	52.0	57.26
MFI2	antigen p97 (melanoma associated) identified by monoclonal antibodies 133.2 and 96.5	44.7	69.8	57.25
PASK	PAS domain containing serine/threonine kinase	48.2	66.2	57.19
LOC100550886	cystathionine beta-synthase-like	54.9	59.3	57.14
LOC100549197	retinoblastoma-associated protein	59.1	55.1	57.13
LOC100550324	MAP7 domain-containing protein 2	59.1	55.1	57.12
SKIDA1	SKI/DACH domain containing 1	54.1	60.1	57.08
VTI1A	vesicle transport through interaction with t-SNAREs 1A	53.2	60.9	57.04
LOC104911926	uncharacterized protein C10orf107 homolog	55.0	59.1	57.02
LOC104912138	caprin-2-like	61.6	52.3	56.99
NDC80	NDC80 kinetochore complex component	48.1	65.9	56.99
DGKB	diacylglycerol kinase, beta 90kDa	41.4	72.5	56.97
LOC100541460	WAS/WASL-interacting protein family member 3-like	45.7	68.2	56.95
SFXN5	sideroflexin 5	47.3	66.4	56.83
RNF169	ring finger protein 169	57.5	56.1	56.82
LOC100541582	tubulin alpha-1A chain pseudogene	48.2	65.4	56.80
APOA5	apolipoprotein A-V	57.5	56.0	56.76
TAGLN3	transgelin 3	54.9	58.4	56.64
ADAMTSL1	ADAMTS-like 1	59.9	53.1	56.50
OSGIN2	oxidative stress induced growth inhibitor family member 2	60.8	52.2	56.48
PIWIL1	piwi-like RNA-mediated gene silencing 1	46.5	66.4	56.45
LOC104915975	diacylglycerol kinase alpha-like	64.3	48.6	56.43
LOC104914853	5'-AMP-activated protein kinase catalytic subunit alpha-1-like	54.1	58.6	56.34
LOC104917577	vascular endothelial growth factor receptor 1-like	54.0	58.6	56.31
LOC104913085	piezo-type mechanosensitive ion channel component 1-like	71.8	40.7	56.28
PRR33	proline rich 33	47.3	65.2	56.27

LOC104913635	intercellular adhesion molecule 5-like	63.4	49.2	56.26
PNPLA4	patatin-like phospholipase domain containing 4	55.7	56.7	56.19
CMPK2	cytidine monophosphate (UMP-CMP) kinase 2, mitochondrial	54.9	57.5	56.19
PDE4C	phosphodiesterase 4C, cAMP-specific	54.0	58.3	56.18
PRMT8	protein arginine methyltransferase 8	53.2	59.1	56.16
LOC104911233	endoribonuclease Dicer-like	54.0	58.3	56.16
LOC100548169	zinc finger protein 586-like	51.5	60.8	56.14
LOC100543395	gamma-secretase-activating protein	57.4	54.8	56.10
SGSM1	small G protein signaling modulator 1	49.8	62.3	56.07
GLT1D1	glycosyltransferase 1 domain containing 1	49.0	63.1	56.02
LOC104913564	uncharacterized LOC104913564	54.0	57.9	55.99
SCNN1G	sodium channel, non-voltage-gated 1, gamma subunit	55.8	56.1	55.94
UCHL1	ubiquitin carboxyl-terminal esterase L1 (ubiquitin thiolesterase)	58.2	53.6	55.93
PITPNC1	phosphatidylinositol transfer protein, cytoplasmic 1	49.0	62.8	55.89
TMEM254	transmembrane protein 254	54.9	56.7	55.82
LOC104914875	molybdopterin synthase sulfur carrier subunit-like	53.2	58.4	55.77
LOC104917433	dnaJ homolog subfamily C member 3-like	59.2	52.3	55.71
MCF2L2	MCF.2 cell line derived transforming sequence-like 2	51.5	59.8	55.68
LOC100545925	GDNF-inducible zinc finger protein 1-like	43.1	68.2	55.68
USH2A	Usher syndrome 2A (autosomal recessive, mild)	49.0	62.3	55.66
LOC100550418	receptor-type tyrosine-protein phosphatase delta-like	49.0	62.3	55.64
QRFPR	pyroglutamylated RFamide peptide receptor	50.7	60.5	55.62
MPEG1	macrophage expressed 1	58.3	52.9	55.57
LOC100547972	nucleoporin p58/p45-like	56.6	54.4	55.54
TMEM144	transmembrane protein 144	56.6	54.4	55.49
DUSP4	dual specificity phosphatase 4	57.4	53.6	55.48
PAG1	phosphoprotein membrane anchor with glycosphingolipid microdomains 1	61.6	49.4	55.48
AKAP5	A kinase (PRKA) anchor protein 5	53.2	57.7	55.48
TMEM41B	transmembrane protein 41B	47.3	63.5	55.40
LOC104915498	solute carrier family 12 member 2-like	54.0	56.8	55.40
LOC104910368	uncharacterized LOC104910368	47.3	63.5	55.39
LOC104911321	uncharacterized LOC104911321	54.9	55.8	55.35
LOC104911406	neurabin-1-like	51.6	58.9	55.23
XYLT1	xylosyltransferase I	54.1	56.3	55.22
LOC100546071	receptor tyrosine-protein kinase erbB-3-like	69.2	41.2	55.22
LOC100539161	serine/threonine-protein phosphatase 2A 55 kDa regulatory subunit B beta isoform	49.9	60.6	55.22
LOC104910474	PHD finger protein 20-like protein 1	49.8	60.6	55.20
LOC100546634	arylsulfatase D-like	56.6	53.7	55.18
LOC104915950	early growth response protein 3-like	67.6	42.8	55.17
TRAF3	TNF receptor-associated factor 3	49.8	60.5	55.15
LOC104909881	cyclin-C-like	60.8	49.4	55.12
LOC100545452	protein phosphatase 1 regulatory subunit 12B	59.1	51.0	55.07
ZBTB14	zinc finger and BTB domain containing 14	52.3	57.7	55.05
LOC104914743	dymeclin-like	54.0	56.0	55.03
LOC104910980	importin-7-like	48.2	61.9	55.01
LOC100542230	E3 ubiquitin-protein ligase UBR3-like	47.4	62.7	55.00
LOC100540851	raftlin-like	55.7	54.3	55.00
LOC104916081	heterogeneous nuclear ribonucleoprotein U-like protein 1	61.7	48.3	54.99
ESAM	endothelial cell adhesion molecule	63.3	46.7	54.99
CORO7	coronin 7	56.6	53.2	54.90
GPR21	G protein-coupled receptor 21	54.0	55.7	54.86
LOC104915336	uncharacterized LOC104915336	54.9	54.5	54.71
C16H16orf45	chromosome 16 open reading frame, human C16orf45	47.3	62.0	54.65
LOC100539204	sterol 26-hydroxylase, mitochondrial	56.6	52.7	54.64
LOC100550697	carboxypeptidase Z-like	54.1	55.2	54.64
LOC104917158	serine/threonine-protein kinase MARK2-like	63.4	45.8	54.60

LRFN1	leucine rich repeat and fibronectin type III domain containing 1	64.2	45.0	54.60
SLC4A8	solute carrier family 4, sodium bicarbonate cotransporter, member 8	61.6	47.6	54.60
LOC104915313	round spermatid basic protein 1-like protein	56.5	52.6	54.55
LOC104916481	frizzled-1-like	56.6	52.4	54.53
LOC104910250	uncharacterized LOC104910250	45.6	63.4	54.51
LOC104910855	uncharacterized LOC104910855	39.7	69.2	54.47
SCML2	sex comb on midleg-like 2 (Drosophila)	49.0	59.8	54.42
LOC104916851	ryanodine receptor 1-like	61.7	47.1	54.42
RAB33A	RAB33A, member RAS oncogene family	51.5	57.3	54.41
LOC104916932	NAD-dependent malic enzyme, mitochondrial-like	54.1	54.7	54.41
DCAKD	dephospho-CoA kinase domain containing	57.5	51.3	54.39
GSC	goosecoid homeobox	58.2	50.5	54.38
TROVE2	TROVE domain family, member 2	52.4	56.3	54.34
CENPI	centromere protein I	54.1	54.5	54.30
ASCL3	achaete-scute family bHLH transcription factor 3	51.6	56.9	54.24
LOC100545470	laminin subunit alpha-2-like	53.2	55.2	54.23
LOC104911021	protein SAAL1-like	66.7	41.7	54.20
CFD	complement factor D (adipsin)	51.5	56.8	54.18
LOC104912605	centrosomal protein of 63 kDa-like	54.1	54.2	54.16
LOC104916393	ephrin-A4-like	60.0	48.2	54.11
ACVR1B	activin A receptor, type IB	52.4	55.8	54.08
LOC104912446	terminal uridylyltransferase 4-like	49.9	58.2	54.03
LOC104912520	rho GTPase-activating protein 32-like	65.0	43.0	54.01
LOC104913432	7,8-dihydro-8-oxoguanine triphosphatase-like	65.9	42.0	53.91
LDB2	LIM domain binding 2	54.0	53.8	53.89
HCK	HCK proto-oncogene, Src family tyrosine kinase	60.9	46.8	53.84
CCND2	cyclin D2	53.2	54.4	53.83
SLIT2	slit homolog 2 (Drosophila)	59.9	47.5	53.68
CRYBG3	beta-gamma crystallin domain containing 3	60.0	47.2	53.62
LOC104916500	single-strand selective monofunctional uracil-DNA glycosylase pseudogene	67.5	39.6	53.58
ZDHHC1	zinc finger, DHHC-type containing 1	54.1	52.9	53.46
C23H1orf174	chromosome 23 open reading frame, human C1orf174	45.6	61.2	53.41
LOC104909594	uncharacterized LOC104909594	47.3	59.5	53.40
LOC104911401	uncharacterized LOC104911401	49.8	57.0	53.39
LOC104910198	uncharacterized LOC104910198	65.8	40.6	53.24
LOC104909543	serine palmitoyltransferase 3-like	48.2	58.2	53.17
LOC104913696	uncharacterized LOC104913696	50.7	55.5	53.12
ACOX2	acyl-CoA oxidase 2, branched chain	54.9	51.3	53.10
LOC104914804	RAB6A-GEF complex partner protein 2-like	55.7	50.4	53.08
LOC100543777	sodium channel protein type 5 subunit alpha-like	49.0	57.1	53.05
PTPN22	protein tyrosine phosphatase, non-receptor type 22 (lymphoid)	50.6	55.4	53.03
LOC104915567	scaffold attachment factor B1-like	42.2	63.8	53.01
LOC104912770	uncharacterized LOC104912770	49.9	56.0	52.92
LOC104912555	E3 ubiquitin-protein ligase TRIP12-like	47.3	58.5	52.92
LOC104913890	general transcription factor II-I-like	64.2	41.6	52.87
LOC104915987	zinc finger protein OZF-like	60.0	45.7	52.86
LOC104909449	caseinolytic peptidase B protein homolog	66.8	38.9	52.84
LOC104909796	lethal(3)malignant brain tumor-like protein 3	51.5	54.1	52.81
LOC104909351	lipid phosphate phosphatase-related protein type 4	55.8	49.8	52.80
TBC1D30	TBC1 domain family, member 30	49.0	56.5	52.75
FRZB	frizzled-related protein	52.4	53.1	52.73
LOC100540641	neutral ceramidase	51.5	53.9	52.69
LOC104912274	uncharacterized LOC104912274	45.6	59.7	52.68
LOC100542745	von Willebrand factor D and EGF domain-containing protein-like	54.1	51.3	52.68
SPDYA	speedy/RINGO cell cycle regulator family member A	49.8	55.5	52.68
BTBD9	BTB (POZ) domain containing 9	45.6	59.5	52.54

LOC104911862	uncharacterized LOC104911862	47.3	57.8	52.54
LOC104915440	uncharacterized LOC104915440	49.0	56.1	52.52
LOC104917392	zinc finger protein 572-like	49.0	55.9	52.47
LOC104916774	potassium voltage-gated channel subfamily C member 1 pseudogene	56.6	48.2	52.42
LOC100541518	deleted in malignant brain tumors 1 protein-like	45.6	59.2	52.41
STK35	serine/threonine kinase 35	49.0	55.7	52.35
GPR68	G protein-coupled receptor 68	37.2	67.4	52.31
LOC104912000	cilia- and flagella-associated protein 43-like	52.4	52.2	52.28
PATL2	protein associated with topoisomerase II homolog 2 (yeast)	51.5	52.8	52.14
LOC100544119	PCNA-interacting partner	48.1	56.1	52.13
TET2	tet methylcytosine dioxygenase 2	51.5	52.6	52.07
LOC104916868	uncharacterized LOC104916868	60.8	43.3	52.06
RAB19	RAB19, member RAS oncogene family	54.9	49.1	52.00
LOC104914243	uncharacterized LOC104914243	53.2	50.7	51.94
FUT10	fucosyltransferase 10 (alpha (1,3) fucosyltransferase)	53.2	50.7	51.94
ADAT1	adenosine deaminase, tRNA-specific 1	52.4	51.4	51.89
LOC104914728	maestro heat-like repeat-containing protein family member 2B	50.7	53.1	51.87
LOC100538554	protein FAM49A-like	53.2	50.5	51.86
LOC104915798	histone-lysine N-methyltransferase 2D-like	65.1	38.5	51.84
LOC100550234	uncharacterized LOC100550234	52.3	51.3	51.82
FZD3	frizzled class receptor 3	49.8	53.7	51.74
LOC104912651	cohesin subunit SA-1-like	50.7	52.8	51.73
LOC104909335	putative homeodomain transcription factor 2	49.8	53.6	51.72
PYGO1	pygopus family PHD finger 1	49.0	54.4	51.69
LOC104911371	uncharacterized LOC104911371	43.9	59.3	51.61
LOC104910999	vitamin D 25-hydroxylase-like	43.9	59.2	51.55
LOC100545760	membrane primary amine oxidase-like	52.4	50.6	51.51
LOC104911683	non-homologous end-joining factor 1-like	50.6	52.3	51.47
PDE6C	phosphodiesterase 6C, cGMP-specific, cone, alpha prime	54.8	48.0	51.44
ADA	adenosine deaminase	44.8	58.1	51.44
LOC104915561	uncharacterized LOC104915561	62.5	40.4	51.44
LOC104915317	intraflagellar transport protein 74 homolog	49.0	53.9	51.42
LOC100540743	tyrosine-protein phosphatase non-receptor type 13	52.4	50.4	51.40
SLC26A1	solute carrier family 26 (anion exchanger), member 1	49.8	52.9	51.37
NRF1	nuclear respiratory factor 1	51.5	51.2	51.34
LOC104917598	poly [ADP-ribose] polymerase 4-like	59.1	43.6	51.33
TYW1	tRNA-yW synthesizing protein 1 homolog (S. cerevisiae)	56.6	46.0	51.32
LOC104913727	myosin-8-like	53.3	49.3	51.27
LOC104910371	eyes absent homolog 1-like	53.2	49.3	51.27
LOC104916505	DNA-directed RNA polymerase III subunit RPC7-like	58.3	44.2	51.27
KLHL17	kelch-like family member 17	53.2	49.2	51.21
LOC100548322	cytochrome P450 2D17	54.9	47.5	51.19
LOC100544344	serine protease 23-like	56.6	45.8	51.19
PROZ	protein Z, vitamin K-dependent plasma glycoprotein	34.6	67.6	51.14
LAT2	linker for activation of T cells family, member 2	52.4	49.9	51.13
POLQ	polymerase (DNA directed), theta	52.3	49.9	51.11
LOC104909356	poly [ADP-ribose] polymerase 8-like	54.1	48.2	51.11
BIRC6	baculoviral IAP repeat containing 6	43.9	58.2	51.08
SUGCT	succinyl-CoA:glutarate-CoA transferase	42.2	59.7	50.95
LOC100550721	perilipin-3-like	58.3	43.6	50.94
LOC104914866	growth hormone receptor-like	60.8	41.1	50.94
EVA1B	eva-1 homolog B (C. elegans)	54.9	46.9	50.90
HSF4	heat shock transcription factor 4	58.3	43.5	50.90
SERTAD2	SERTA domain containing 2	47.3	54.4	50.88
LOC100545318	ras-related protein Rab-10-like	54.9	46.8	50.83
CREBRF	CREB3 regulatory factor	37.2	64.5	50.82
OTULIN	OTU deubiquitinase with linear linkage specificity	54.0	47.6	50.82

LOC104912059	dedicator of cytokinesis protein 1-like	48.1	53.5	50.80
KIAA0753	KIAA0753 ortholog	45.6	55.9	50.76
SLC23A2	solute carrier family 23 (ascorbic acid transporter), member 2	52.3	49.2	50.75
JAG1	jagged 1	34.6	66.7	50.68
PPP4R4	protein phosphatase 4, regulatory subunit 4	39.7	61.6	50.65
LOC100541494	protein bicaudal D homolog 1	57.4	43.8	50.62
LOC104912027	transcription factor 7-like 2	45.6	55.6	50.60
AKNAD1	AKNA domain containing 1	52.3	48.7	50.50
LOC104912067	erythroid differentiation-related factor 1-like	53.2	47.8	50.49
LOC104911816	glycerol-3-phosphate dehydrogenase, mitochondrial-like	56.6	44.3	50.44
LOC104916602	DDB1- and CUL4-associated factor 12-like	56.6	44.2	50.40
LOC100542320	cellular nucleic acid-binding protein pseudogene	41.4	59.3	50.36
LOC104912227	AF4/FMR2 family member 2-like	42.2	58.4	50.31
LOC104914647	GRAM domain-containing protein 2-like	51.6	49.0	50.28
ARHGEF10	Rho guanine nucleotide exchange factor (GEF) 10	45.6	54.9	50.27
RND2	Rho family GTPase 2	49.0	51.5	50.23
ANKS1B	ankyrin repeat and sterile alpha motif domain containing 1B	53.1	47.2	50.19
COL19A1	collagen, type XIX, alpha 1	61.6	38.8	50.19
SATB2	SATB homeobox 2	47.3	52.9	50.13
LOC104911730	cytoplasmic dynein 1 intermediate chain 2-like	38.0	62.2	50.08
COLEC10	collectin sub-family member 10 (C-type lectin)	54.8	45.3	50.08
UNC80	unc-80 homolog (C. elegans)	55.8	44.2	50.02
SHROOM1	shroom family member 1	52.4	47.6	49.98
CDHR2	cadherin-related family member 2	49.9	50.1	49.98
DCK	deoxycytidine kinase	54.9	45.0	49.96
RASGEF1A	RasGEF domain family, member 1A	45.6	54.3	49.92
LOC104917440	UDP-glucose:glycoprotein glucosyltransferase 2-like	51.5	48.3	49.90
LOC100551401	SH3 domain-containing kinase-binding protein 1	54.0	45.6	49.81
LOC100548109	multidrug and toxin extrusion protein 2-like	59.9	39.7	49.80
IRS1	insulin receptor substrate 1	50.7	48.9	49.78
LOC104916222	uncharacterized protein YBL113C-like	56.6	42.9	49.75
ERCC8	excision repair cross-complementation group 8	60.8	38.6	49.73
LOC104913214	protocadherin beta-15-like	54.9	44.4	49.67
CERK	ceramide kinase	46.4	52.9	49.65
LOC104917553	NEDD4-binding protein 2-like 2	37.2	62.1	49.61
NQO2	NAD(P)H dehydrogenase, quinone 2	53.1	46.1	49.61
HS3ST3B1	heparan sulfate (glucosamine) 3-O-sulfotransferase 3B1	54.9	44.3	49.60
SPSB4	splA/ryanodine receptor domain and SOCS box containing 4	54.9	44.3	49.60
LOC100541737	leucine-rich repeats and immunoglobulin-like domains protein 3	53.2	46.0	49.60
LRRC46	leucine rich repeat containing 46	50.7	48.5	49.60
LOC104912086	protein-methionine sulfoxide oxidase MICAL3-like	53.2	45.9	49.58
MPL	MPL proto-oncogene, thrombopoietin receptor	53.3	45.7	49.50
LOC104913283	protein FAM13B-like	43.9	54.9	49.44
LOC104915069	transmembrane protein 2-like	44.7	54.1	49.43
LOC104912180	uncharacterized protein CXorf21 homolog	47.3	51.5	49.37
TLCD2	TLC domain containing 2	58.3	40.5	49.36
GABRR2	gamma-aminobutyric acid (GABA) A receptor, rho 2	49.8	48.8	49.34
STKLD1	serine/threonine kinase-like domain containing 1	57.4	41.1	49.25
LOC104916884	natural resistance-associated macrophage protein 2-like	58.3	40.2	49.24
MAPK8IP2	mitogen-activated protein kinase 8 interacting protein 2	53.2	45.2	49.22
LOC100544490	rho guanine nucleotide exchange factor 10	40.6	57.8	49.20
LOC100550448	dimethylaniline monooxygenase [N-oxide-forming] 3-like	50.7	47.7	49.19
KIF5C	kinesin family member 5C	43.9	54.3	49.13
LOC104911997	diacylglycerol kinase iota-like	42.2	55.9	49.07
LOC104909876	activating signal cointegrator 1 complex subunit 3-like	50.6	47.5	49.06
LRRN4	leucine rich repeat neuronal 4	48.2	49.7	48.93
USP53	ubiquitin specific peptidase 53	45.6	52.2	48.93
IL22RA1	interleukin 22 receptor, alpha 1	43.9	53.9	48.92

SYBU	syntabulin (syntaxin-interacting)	44.0	53.9	48.91
HDHD1	haloacid dehalogenase-like hydrolase domain containing 1	45.6	52.2	48.88
LITAF	lipopolysaccharide-induced TNF factor	38.1	59.7	48.86
HDX	highly divergent homeobox	52.3	45.3	48.83
RGS22	regulator of G-protein signaling 22	54.0	43.6	48.82
SPO11	SPO11 meiotic protein covalently bound to DSB	63.3	34.2	48.77
GRHL2	grainyhead-like 2 (Drosophila)	48.1	49.2	48.67
DZANK1	double zinc ribbon and ankyrin repeat domains 1	41.4	55.9	48.63
BPHL	biphenyl hydrolase-like (serine hydrolase)	46.5	50.8	48.63
HDAC10	histone deacetylase 10	43.1	54.2	48.61
LOC104917051	EVI5-like protein	61.7	35.5	48.60
SHISA5	shisa family member 5	48.1	49.0	48.59
ELOVL7	ELOVL fatty acid elongase 7	41.4	55.7	48.58
LOC104917435	UDP-glucose:glycoprotein glucosyltransferase 2-like	46.4	50.6	48.51
SLC1A7	solute carrier family 1 (glutamate transporter), member 7	36.3	60.7	48.51
LOC100540503	zinc transporter ZIP11-like	48.1	48.9	48.51
TADA2B	transcriptional adaptor 2B	48.1	48.8	48.48
RASD2	RASD family, member 2	44.8	52.1	48.44
LOC100547664	leucine-rich repeat and calponin homology domain-containing protein 1	36.3	60.5	48.40
CDKL1	cyclin-dependent kinase-like 1 (CDC2-related kinase)	43.9	52.8	48.38
ASS1	argininosuccinate synthase 1	52.3	44.4	48.37
CREG1	cellular repressor of E1A-stimulated genes 1	44.7	52.0	48.37
LOC104914302	testis development-related protein-like	52.4	44.4	48.36
LOC104916031	zinc finger protein 865-like	56.6	40.1	48.36
ZNF469	zinc finger protein 469	50.7	45.9	48.30
LOC104909786	uncharacterized LOC104909786	49.0	47.4	48.23
LOC100543837	NADH-cytochrome b5 reductase-like	38.8	57.5	48.19
KCNC1	potassium voltage-gated channel, Shaw-related subfamily, member 1	47.3	49.1	48.18
LOC100541961	pantetheinase-like	54.9	41.4	48.14
CCDC39	coiled-coil domain containing 39	43.1	52.9	48.00
LOC104910694	bifunctional 3'-phosphoadenosine 5'-phosphosulfate synthase 1-like	43.1	52.9	47.97
RAB32	RAB32, member RAS oncogene family	47.3	48.7	47.97
LBX1	ladybird homeobox 1	60.0	35.9	47.93
VSIG1	V-set and immunoglobulin domain containing 1	41.4	54.4	47.92
NKIRAS1	NFKB inhibitor interacting Ras-like 1	49.8	46.0	47.89
WNT6	wingless-type MMTV integration site family, member 6	49.0	46.7	47.85
ZNF827	zinc finger protein 827	53.2	42.5	47.84
PPP1R9A	protein phosphatase 1, regulatory subunit 9A	46.4	49.1	47.78
ASMTL	acetylserotonin O-methyltransferase-like	49.0	46.5	47.73
ZDHHC18	zinc finger, DHHC-type containing 18	44.8	50.6	47.69
LOC104913909	uncharacterized LOC104913909	49.0	46.2	47.59
LOC100545545	protein FAM83D-like	49.8	45.2	47.51
LOC104912781	uncharacterized LOC104912781	48.2	46.8	47.49
LOC104912459	uncharacterized LOC104912459	43.1	51.9	47.48
PTN	pleiotrophin	71.7	23.3	47.47
GUCY1A3	guanylate cyclase 1, soluble, alpha 3	50.7	44.2	47.45
C2H6orf165	chromosome 2 open reading frame, human C6orf165	38.9	56.0	47.44
REEP3	receptor accessory protein 3	43.1	51.8	47.42
LOC104914423	serum amyloid P-component-like	48.2	46.6	47.41
RANBP17	RAN binding protein 17	48.1	46.7	47.40
LOC104915681	nuclear pore complex protein Nup88-like	44.8	49.8	47.29
LOC100538632	threo-3-hydroxyaspartate ammonia-lyase-like	50.7	43.8	47.21
LOC104915760	platelet glycoprotein VI-like	41.4	52.9	47.18
HDC	histidine decarboxylase	39.7	54.6	47.14

SLC28A2	solute carrier family 28 (concentrative nucleoside transporter), member 2	52.4	41.9	47.12
METAP1D	methionyl aminopeptidase type 1D (mitochondrial)	40.5	53.7	47.08
DAB2	Dab, mitogen-responsive phosphoprotein, homolog 2 (Drosophila)	43.1	51.1	47.08
WSCD2	WSC domain containing 2	49.0	45.2	47.08
LOC104916340	gastrula zinc finger protein XlCGF49.1-like	55.7	38.4	47.07
GPR156	G protein-coupled receptor 156	49.8	44.3	47.06
MAD1L1	MAD1 mitotic arrest deficient-like 1 (yeast)	43.9	50.0	46.99
LOC104913930	mediator of RNA polymerase II transcription subunit 13-like	49.9	44.0	46.94
LOC100541042	25-hydroxycholesterol 7-alpha-hydroxylase	45.6	48.3	46.93
LOC104911030	phosphatidylinositol 4-phosphate 3-kinase C2 domain-containing subunit alpha-like	36.4	57.3	46.81
LOC100545524	myotubularin-related protein 13-like	54.0	39.5	46.79
PREPL	prolyl endopeptidase-like	39.7	53.9	46.78
LOC100541274	uncharacterized LOC100541274	49.9	43.6	46.72
MPZL1	myelin protein zero-like 1	49.9	43.6	46.71
LOC100541052	serine/threonine-protein kinase tousled-like 1	50.7	42.7	46.70
PIK3AP1	phosphoinositide-3-kinase adaptor protein 1	41.4	52.0	46.69
TPH2	tryptophan hydroxylase 2	48.2	45.2	46.68
SLC46A2	solute carrier family 46, member 2	41.4	51.9	46.68
FRMD1	FERM domain containing 1	47.3	45.9	46.62
EXOC1	exocyst complex component 1	49.0	44.2	46.57
FBXO48	F-box protein 48	42.2	50.5	46.34
LOC104915143	uncharacterized LOC104915143	43.9	48.7	46.28
LOC104916837	ribonucleoprotein PTB-binding 1-like	59.2	33.4	46.28
PECAM1	platelet/endothelial cell adhesion molecule 1	47.3	45.1	46.21
LOC104914857	5'-AMP-activated protein kinase catalytic subunit alpha-1-like	43.9	48.3	46.08
ANKRD9	ankyrin repeat domain 9	52.4	39.8	46.08
LOC100543214	probable ATP-dependent DNA helicase HFM1	46.4	45.7	46.08
LOC104909452	uncharacterized LOC104909452	42.2	49.8	46.02
LOC104917492	uncharacterized LOC104917492	49.9	42.2	46.01
LOC104915868	pyruvate carboxylase, mitochondrial-like	37.2	54.8	46.01
FRAS1	Fraser extracellular matrix complex subunit 1	43.1	48.9	46.00
CHGB	chromogranin B (secretogranin 1)	42.2	49.8	45.99
LOC104913819	nucleoside diphosphate kinase	43.9	48.0	45.97
CX3CL1	chemokine (C-X3-C motif) ligand 1	58.3	33.6	45.92
LOC100539298	inner centromere protein-like	38.0	53.7	45.89
LOC104912228	mastermind-like domain-containing protein 1	40.6	51.1	45.85
SEMA5A	sema domain, seven thrombospondin repeats (type 1 and type 1-like), transmembrane domain (TM) and short cytoplasmic domain, (semaphorin) 5A	47.2	44.4	45.83
SPATA1	spermatogenesis associated 1	43.9	47.7	45.81
LOC104915786	uncharacterized LOC104915786	48.1	43.4	45.80
TINAGL1	tubulointerstitial nephritis antigen-like 1	48.2	43.4	45.78
EFCAB11	EF-hand calcium binding domain 11	35.5	56.0	45.75
ZNF385D	zinc finger protein 385D	32.1	59.3	45.74
RASSF2	Ras association (RalGDS/AF-6) domain family member 2	45.6	45.8	45.72
ASPG	asparaginase	42.2	49.2	45.72
LOC104915524	uncharacterized LOC104915524	41.4	50.0	45.70
LOC104917210	semaphorin-4C-like	54.1	37.3	45.67
LOC104912384	uncharacterized LOC104912384	51.5	39.7	45.61
IGF2BP1	insulin-like growth factor 2 mRNA binding protein 1	60.0	31.1	45.56
LHFPL2	lipoma HMGIC fusion partner-like 2	49.8	41.2	45.53
LOC100547885	UDP-glucuronosyltransferase 1-1-like	39.7	51.4	45.52
LOC104911636	UPF0606 protein KIAA1549-like	49.9	41.0	45.44
LYRM9	LYR motif containing 9	38.8	52.0	45.41
MYRF	myelin regulatory factor	54.0	36.7	45.36

N4BP2	NEDD4 binding protein 2	44.8	45.9	45.34
FAM26E	family with sequence similarity 26, member E	44.8	45.9	45.34
LOC104915251	multiple C2 and transmembrane domain-containing protein 1-like	45.6	45.0	45.30
SLC37A2	solute carrier family 37 (glucose-6-phosphate transporter), member 2	44.0	46.6	45.29
ASAP3	ArfGAP with SH3 domain, ankyrin repeat and PH domain 3	54.0	36.5	45.25
MECOM	MDS1 and EVI1 complex locus	42.3	48.2	45.23
WDPCP	WD repeat containing planar cell polarity effector	40.5	49.9	45.21
PDK3	pyruvate dehydrogenase kinase, isozyme 3	50.7	39.5	45.12
LOC100549070	non-canonical poly(A) RNA polymerase PAPD5-like	45.6	44.6	45.10
LOC104913609	T-box transcription factor TBX3-like	40.5	49.6	45.09
TMEM136	transmembrane protein 136	47.3	42.8	45.08
GPR62	G protein-coupled receptor 62	44.0	46.2	45.07
FGF10	fibroblast growth factor 10	38.0	52.1	45.06
GAREM	GRB2 associated, regulator of MAPK1	39.7	50.4	45.06
F2	coagulation factor II (thrombin)	43.9	46.2	45.05
PTGES	prostaglandin E synthase	57.4	32.7	45.04
STRN	striatin, calmodulin binding protein	47.3	42.8	45.03
LOC104909261	interleukin-12 receptor subunit beta-2-like	46.5	43.5	45.01
KIAA0141	KIAA0141 ortholog	46.5	43.4	44.96
LOC104917402	uncharacterized LOC104917402	34.6	55.2	44.92
LOC104911293	uncharacterized LOC104911293	44.7	45.0	44.88
LOC104910573	probable E3 ubiquitin-protein ligase HERC4	45.6	44.1	44.87
LOC104916975	uncharacterized LOC104916975	54.1	35.6	44.85
LOC104913751	uncharacterized LOC104913751	41.4	48.3	44.84
HAL	histidine ammonia-lyase	41.4	48.3	44.84
LOC104909732	mitogen-activated protein kinase kinase kinase 5-like	43.9	45.6	44.76
PANX2	pannexin 2	42.2	47.3	44.75
LOC104916086	diacylglycerol kinase alpha-like	52.4	37.1	44.75
LOC100549863	solute carrier family 25 member 33-like	51.5	38.0	44.73
AMDHD1	amidohydrolase domain containing 1	37.2	52.2	44.70
LOC104917417	basic immunoglobulin-like variable motif-containing protein	42.2	47.2	44.70
METTL15	methyltransferase like 15	45.6	43.7	44.65
LOC104910568	uncharacterized LOC104910568	44.8	44.4	44.58
LOC100544832	acetyl-CoA carboxylase	48.2	40.9	44.55
LOC104913984	myosin-7B-like	45.7	43.4	44.55
LOC104910450	metastasis suppressor protein 1-like	48.2	40.9	44.53
FIG4	FIG4 phosphoinositide 5-phosphatase	46.5	42.5	44.50
CRYBA1	crystallin, beta A1	43.9	45.0	44.49
PCGF2	polycomb group ring finger 2	43.1	45.9	44.48
LOC104913344	uncharacterized LOC104913344	54.1	34.8	44.45
LOC104913994	uncharacterized LOC104913994	45.6	43.2	44.43
LOC104909731	mitogen-activated protein kinase kinase kinase 5-like	42.2	46.6	44.43
RUNX1	runt-related transcription factor 1	44.8	44.0	44.40
BBS12	Bardet-Biedl syndrome 12	38.8	50.0	44.38
LOC104917441	uncharacterized LOC104917441	41.4	47.4	44.37
RHPN1	rhophilin, Rho GTPase binding protein 1	38.9	49.9	44.37
LOC104914530	uncharacterized LOC104914530	39.7	49.0	44.36
LOC104915163	colorectal mutant cancer protein-like	34.6	54.0	44.30
LOC104917120	neuroblast differentiation-associated protein AHNAK-like	49.1	39.5	44.29
HCN2	hyperpolarization activated cyclic nucleotide-gated potassium channel 2	48.1	40.4	44.27
LOC104913358	uncharacterized LOC104913358	43.9	44.5	44.23
CENPW	centromere protein W	46.4	42.0	44.22
LOC104912988	CCR4-NOT transcription complex subunit 1-like	50.7	37.7	44.20
SLC2A4RG	SLC2A4 regulator	43.0	45.2	44.14
LOC100543595	histone H4	38.0	50.1	44.02
GAN	gigaxonin	41.4	46.6	44.01

DEPDC7	DEP domain containing 7	43.1	45.0	44.01
LOC100549762	hydroxyacid oxidase 1-like	47.3	40.6	43.92
LOC104911414	coiled-coil domain-containing protein 132-like	41.4	46.4	43.88
LOC104916870	glycogen phosphorylase, muscle form-like	47.3	40.4	43.86
WISP2	WNT1 inducible signaling pathway protein 2	44.8	42.8	43.84
ENTPD3	ectonucleoside triphosphate diphosphohydrolase 3	44.8	42.7	43.76
MEST	mesoderm specific transcript	43.1	44.4	43.75
LOC104912134	uncharacterized LOC104912134	51.5	36.0	43.74
LOC100545893	ras-like protein family member 11A-like	43.1	44.3	43.72
GAB3	GRB2-associated binding protein 3	40.6	46.8	43.70
SLC9A2	solute carrier family 9, subfamily A (NHE2, cation proton antiporter 2), member 2	44.7	42.7	43.69
ODF2L	outer dense fiber of sperm tails 2-like	43.9	43.5	43.69
LOC104916581	7-dehydrocholesterol reductase-like	40.6	46.8	43.68
LOC104914425	cell adhesion molecule 3-like	49.0	38.3	43.66
FTSJ2	FtsJ RNA methyltransferase homolog 2 (E. coli)	40.5	46.8	43.66
LOC100540511	leucyl-cystinyl aminopeptidase-like	34.6	52.7	43.65
APOD	apolipoprotein D	40.6	46.7	43.64
SLC2A12	solute carrier family 2 (facilitated glucose transporter), member 12	41.4	45.8	43.62
LOC104915585	transmembrane channel-like protein 1	38.9	48.4	43.61
LOC100546590	heat shock protein 105 kDa	45.6	41.6	43.61
LOC104916453	zinc finger and BTB domain-containing protein 22-like	39.7	47.5	43.60
LOC104914751	ectopic P granules protein 5 homolog	36.3	50.8	43.59
LOC104913311	lysine-specific demethylase 3B-like	41.4	45.7	43.56
RAB38	RAB38, member RAS oncogene family	36.3	50.8	43.56
LOC104915071	transmembrane protein 2-like	40.5	46.6	43.54
P2RY1	purinergic receptor P2Y, G-protein coupled, 1	50.7	36.3	43.51
LOC104913743	myosin-10-like	47.3	39.7	43.50
LOC100542038	vacuolar protein sorting-associated protein 13D	36.4	50.6	43.45
TIFA	TRAF-interacting protein with forkhead-associated domain	45.6	41.2	43.43
LOC104910799	toll-like receptor 6	43.1	43.7	43.42
LOC104915352	transcription factor TFIIIB component B'' homolog	44.8	42.0	43.38
POLR3C	polymerase (RNA) III (DNA directed) polypeptide C (62kD)	44.8	42.0	43.38
CFI	complement factor I	46.5	40.3	43.36
ABTB2	ankyrin repeat and BTB (POZ) domain containing 2	37.2	49.5	43.34
PLCXD2	phosphatidylinositol-specific phospholipase C, X domain containing 2	45.6	41.1	43.32
LOC104917142	protein FAM71B-like	40.5	46.0	43.25
PDCL2	phosducin-like 2	42.2	44.2	43.24
NALCN	sodium leak channel, non-selective	38.0	48.3	43.18
LOC104914552	uncharacterized LOC104914552	49.8	36.5	43.17
LOC104916207	kallikrein-8-like	47.3	39.1	43.16
LOC104909844	uncharacterized LOC104909844	47.3	38.9	43.10
LOC104916677	uncharacterized LOC104916677	49.0	37.2	43.10
LOC100545905	RNA-binding protein Musashi homolog 2-like	45.6	40.6	43.09
LOC104912064	disintegrin and metalloproteinase domain-containing protein 12-like	37.2	49.0	43.07
SV2B	synaptic vesicle glycoprotein 2B	32.2	53.9	43.03
LOC104915980	zinc finger protein 574-like	57.5	28.5	43.02
ATP2C2	ATPase, Ca++ transporting, type 2C, member 2	38.0	48.0	43.02
CA5A	carbonic anhydrase VA, mitochondrial	38.9	47.2	43.01
LOC104914656	uncharacterized LOC104914656	33.8	52.2	43.00
LOC100548015	zinc finger protein ubi-d4-like	47.3	38.6	42.95
LOC100542236	rhophilin-2-like	41.4	44.5	42.94
FOXP2	forkhead box P2	42.3	43.6	42.93
LOC104916221	uncharacterized LOC104916221	39.7	46.1	42.91
INSRR	insulin receptor-related receptor	52.3	33.5	42.90
ABHD3	abhydrolase domain containing 3	32.1	53.5	42.82

LOC104914466	protein phosphatase 1 regulatory subunit 12B-like	53.2	32.4	42.81
XKR7	XK, Kell blood group complex subunit-related family, member 7	51.6	34.0	42.80
LOC104916984	rho GTPase-activating protein 39-like	51.6	33.9	42.76
HSF5	heat shock transcription factor family member 5	33.8	51.6	42.68
MMP7	matrix metallopeptidase 7 (matrilysin, uterine)	43.1	42.3	42.67
LOC104917620	uncharacterized LOC104917620	45.6	39.7	42.66
PGBD5	piggyBac transposable element derived 5	45.6	39.7	42.62
ATP8A1	ATPase, aminophospholipid transporter (APLT), class I, type 8A, member 1	43.9	41.2	42.59
LOC104911964	uncharacterized LOC104911964	34.6	50.5	42.58
TNFSF4	tumor necrosis factor (ligand) superfamily, member 4	42.2	42.9	42.58
LOC104910281	microtubule cross-linking factor 1-like	38.0	47.1	42.57
NRTN	neurturin	33.0	52.1	42.53
LOC100546766	protocadherin alpha-C2-like	40.6	44.4	42.51
ROBO1	roundabout, axon guidance receptor, homolog 1 (Drosophila)	34.7	50.2	42.46
FRMPD4	FERM and PDZ domain containing 4	34.6	50.3	42.46
LOC100540339	little elongation complex subunit 2	42.2	42.5	42.38
HHIPL2	HHIP-like 2	37.2	47.5	42.36
LOC104909795	rho GTPase-activating protein 18-like	38.0	46.6	42.34
LOC104916395	transcription factor COE3-like	36.3	48.4	42.34
LOC100544731	C-C motif chemokine 4 homolog	43.1	41.6	42.32
LOC104911403	islet cell autoantigen 1-like	43.9	40.6	42.22
LOC104911795	leucine-zipper-like transcriptional regulator 1	43.1	41.4	42.21
GEM	GTP binding protein overexpressed in skeletal muscle	33.8	50.6	42.17
LOC100544459	SLIT-ROBO Rho GTPase-activating protein 3	54.9	29.4	42.17
EPB41L2	erythrocyte membrane protein band 4.1-like 2	49.0	35.3	42.13
ELFN2	extracellular leucine-rich repeat and fibronectin type III domain containing 2	42.2	42.0	42.12
LOC104916538	class I histocompatibility antigen, F10 alpha chain pseudogene	43.9	40.3	42.09
DIO2	deiodinase, iodothyronine, type II	40.5	43.6	42.07
MAPK9	mitogen-activated protein kinase 9	41.4	42.7	42.06
COL4A6	collagen, type IV, alpha 6	41.3	42.8	42.06
GDPD5	glycerophosphodiester phosphodiesterase domain containing 5	35.5	48.6	42.03
CAPNS2	calpain, small subunit 2	40.6	43.5	42.03
HOMER2	homer homolog 2 (Drosophila)	51.5	32.5	42.01
BMPER	BMP binding endothelial regulator	27.9	56.1	41.98
RSPH9	radial spoke head 9 homolog (Chlamydomonas)	35.5	48.5	41.98
ZDHHC4	zinc finger, DHHC-type containing 4	43.9	40.0	41.94
HHATL	hedgehog acyltransferase-like	48.1	35.7	41.92
LOC100544547	extracellular sulfatase Sulf-1-like	36.3	47.5	41.92
LOC100545338	uncharacterized LOC100545338	41.4	42.3	41.89
LOC104915678	uncharacterized LOC104915678	45.6	38.1	41.85
IPCEF1	interaction protein for cytohesin exchange factors 1	30.4	53.1	41.74
LOC104916535	egl nine homolog 1-like	41.4	41.9	41.65
LOC104911737	mitogen-activated protein kinase kinase kinase MLT-like	42.2	41.1	41.64
LOC100544592	serologically defined colon cancer antigen 8 homolog	48.9	34.3	41.60
LOC104914263	CAAX prenyl protease 1 homolog	40.5	42.6	41.60
C7H2orf66	chromosome 7 open reading frame, human C2orf66	40.5	42.6	41.58
ASB7	ankyrin repeat and SOCS box containing 7	39.7	43.4	41.53
LOC104917587	transcriptional coactivator YAP1-like	41.4	41.7	41.53
PYROXD2	pyridine nucleotide-disulphide oxidoreductase domain 2	53.2	29.7	41.46
LOC100541773	protein BANP-like	43.1	39.7	41.40
LOC104910336	DNA repair-scaffolding protein-like	51.5	31.2	41.35
LOC100548761	arylamine N-acetyltransferase, pineal gland isozyme NAT-10	42.2	40.5	41.33
LOC104917153	small conductance calcium-activated potassium channel protein 3	39.7	42.9	41.29
LOC100539427	chromodomain-helicase-DNA-binding protein 1-like	30.4	52.1	41.27
TMEM204	transmembrane protein 204	38.0	44.5	41.26
LOC104909294	hypermethylated in cancer 1 protein	42.2	40.3	41.25

ASB14	ankyrin repeat and SOCS box containing 14	42.2	40.2	41.22
LOC100544020	TBC domain-containing protein kinase-like protein	49.0	33.4	41.20
LOC104911091	MAX gene-associated protein-like	39.7	42.6	41.15
LOC104911389	uncharacterized LOC104911389	47.3	35.0	41.13
LOC104915412	uncharacterized LOC104915412	38.0	44.2	41.10
LOC104912006	uncharacterized LOC104912006	37.1	45.0	41.10
KLF15	Kruppel-like factor 15	35.5	46.7	41.08
OVOL2	ovo-like zinc finger 2	43.9	38.3	41.07
LOC104912793	uncharacterized LOC104912793	38.0	44.1	41.07
LOC104913063	Fanconi anemia group A protein-like	32.9	49.1	41.01
LOC100542574	HEAT repeat-containing protein 5B	33.8	48.2	40.99
LOC104912465	microtubule-associated serine/threonine-protein kinase 2-like	43.1	38.9	40.99
NRCAM	neuronal cell adhesion molecule	43.1	38.9	40.96
LOC104911292	uncharacterized LOC104911292	43.0	38.9	40.96
STS	steroid sulfatase (microsomal), isozyme S	40.5	41.4	40.95
TMEM119	transmembrane protein 119	41.4	40.5	40.93
LOC100538692	inositol hexakisphosphate and diphosphoinositol-pentakisphosphate kinase 1-like	43.1	38.8	40.93
LOC100541119	tyrosine-protein phosphatase non-receptor type substrate 1-like	39.7	42.1	40.89
AAR2	AAR2 splicing factor homolog (S. cerevisiae)	39.7	42.0	40.84
LOC104916925	uncharacterized LOC104916925	41.4	40.2	40.80
ZBTB42	zinc finger and BTB domain containing 42	41.4	40.0	40.70
RHBDL3	rhomboid, veinlet-like 3 (Drosophila)	43.1	38.2	40.66
CLEC16A	C-type lectin domain family 16, member A	39.7	41.4	40.56
LOC104916668	retinol dehydrogenase 7-like	38.9	42.2	40.56
PLXNA2	plexin A2	33.8	47.3	40.54
LOC104911812	bromodomain adjacent to zinc finger domain protein 2B-like	35.5	45.6	40.54
LOC104913906	uncharacterized LOC104913906	38.0	43.0	40.53
WT1	Wilms tumor 1	39.7	41.4	40.52
PRUNE2	prune homolog 2 (Drosophila)	37.2	43.8	40.51
MB	myoglobin	33.8	47.2	40.50
ADRA1D	adrenoceptor alpha 1D	46.5	34.5	40.49
LOC104909467	translation initiation factor IF-2, mitochondrial-like	41.4	39.5	40.47
LOC104912499	origin recognition complex subunit 1-like	43.9	36.8	40.34
HELZ2	helicase with zinc finger 2, transcriptional coactivator	36.3	44.3	40.33
LOC104910493	bromodomain adjacent to zinc finger domain protein 2A-like	57.5	23.2	40.33
LOC104914812	uncharacterized LOC104914812	43.9	36.7	40.30
LOC104914359	uncharacterized LOC104914359	38.0	42.6	40.30
KCNRG	potassium channel regulator	38.0	42.6	40.30
CALCRL	calcitonin receptor-like	41.4	39.2	40.28
LOC104913312	putative E3 ubiquitin-protein ligase SH3RF2	38.9	41.7	40.27
LOC104913486	leucine carboxyl methyltransferase 1-like	43.9	36.6	40.26
LOC100544294	dedicator of cytokinesis protein 1-like	37.2	43.4	40.26
PLAGL1	pleiomorphic adenoma gene-like 1	44.8	35.7	40.25
PHYKPL	5-phosphohydroxy-L-lysine phospho-lyase	33.8	46.6	40.21
RMDN2	regulator of microtubule dynamics 2	42.2	38.2	40.20
CPAMD8	C3 and PZP-like, alpha-2-macroglobulin domain containing 8	33.8	46.3	40.07
LOC100548437	latrophilin-3	38.0	42.1	40.06
LOC104916026	protein ADP-ribosylarginine hydrolase-like	49.0	31.0	39.99
LOC104916058	uncharacterized LOC104916058	48.2	31.8	39.97
LOC104915425	protein LTV1 homolog	38.8	41.0	39.90
LOC104913513	rab-like protein 2A	34.7	45.1	39.88
LOC104909892	uncharacterized LOC104909892	45.6	34.1	39.88
LOC100549541	alkaline phosphatase, tissue-nonspecific isozyme-like	37.2	42.6	39.87
LOC104910875	actin filament-associated protein 1-like	36.3	43.4	39.86
LOC104912355	rab GTPase-activating protein 1-like	35.5	44.2	39.85
LOC104914919	importin-11-like	45.6	34.1	39.83
LOC104916066	uncharacterized LOC104916066	33.8	45.9	39.82

LOC104915277	uncharacterized LOC104915277	36.3	43.3	39.82
LOC100547415	MAX gene-associated protein	29.6	50.0	39.78
DNAH12	dynein, axonemal, heavy chain 12	40.5	39.0	39.75
EGFL6	EGF-like-domain, multiple 6	30.5	49.0	39.74
CRB2	crumbs family member 2	35.5	44.0	39.74
LOC104917139	germin-like protein subfamily 2 member 2	36.3	43.1	39.71
NECAB2	N-terminal EF-hand calcium binding protein 2	38.0	41.4	39.70
CPNE9	copine family member IX	38.8	40.6	39.70
LOC104912586	calcium-binding protein 39-like	43.1	36.3	39.69
LOC104911205	actin-fragmin kinase-like	35.5	43.9	39.68
ACACB	acetyl-CoA carboxylase beta	39.7	39.7	39.68
LOC104910355	uncharacterized LOC104910355	43.9	35.5	39.67
NFAM1	NFAT activating protein with ITAM motif 1	38.0	41.3	39.67
LOC104911114	uncharacterized LOC104911114	40.6	38.7	39.65
LOC104912195	BTB/POZ domain-containing protein KCTD12-like	45.6	33.5	39.59
ABCG2	ATP-binding cassette, sub-family G (WHITE), member 2 (Junior blood group)	38.0	41.2	39.57
LOC100540178	dimethylaniline monooxygenase [N-oxide-forming] 5-like	45.6	33.5	39.57
ICK	intestinal cell (MAK-like) kinase	49.8	29.3	39.57
LOC100545658	calsyntenin-1-like	38.8	40.3	39.55
LOC104910387	copine-3-like	46.4	32.7	39.55
NAT6	N-acetyltransferase 6 (GCN5-related)	53.2	25.8	39.53
PIF1	PIF1 5'-to-3' DNA helicase	42.2	36.8	39.49
KIAA2022	KIAA2022 ortholog	37.1	41.8	39.48
ROR1	receptor tyrosine kinase-like orphan receptor 1	45.6	33.3	39.47
LOC104916350	visinin-like	41.4	37.4	39.40
DYDC1	DPY30 domain containing 1	43.9	34.9	39.40
LOC104909587	uncharacterized LOC104909587	41.3	37.5	39.39
LOC104910706	histone H2A-IV-like	43.1	35.7	39.39
ST6GALNAC1	ST6 (alpha-N-acetyl-neuraminyl-2,3-beta-galactosyl-1,3)-N-acetylgalactosaminide alpha-2,6-sialyltransferase 1	40.6	38.1	39.33
FBXL17	F-box and leucine-rich repeat protein 17	41.4	37.2	39.29
CRYBB1	crystallin, beta B1	29.6	49.0	39.29
GNAO1	guanine nucleotide binding protein (G protein), alpha activating activity polypeptide O	40.5	38.0	39.28
LOC104915832	protocadherin gamma-B2-like	49.0	29.5	39.25
TRIM14	tripartite motif containing 14	34.6	43.8	39.22
TUBB3	tubulin, beta 3 class III	36.3	42.1	39.19
HRH1	histamine receptor H1	37.1	41.2	39.19
ADAMTS8	ADAM metallopeptidase with thrombospondin type 1 motif, 8	35.5	42.9	39.19
LOC104913130	condensin complex subunit 1-like	47.3	31.0	39.17
LOC104912533	uncharacterized LOC104912533	43.1	35.1	39.10
LOC104911454	uncharacterized LOC104911454	43.0	35.1	39.09
LOC104917477	progesterone-induced-blocking factor 1-like	43.1	35.1	39.06
SLC26A2	solute carrier family 26 (anion exchanger), member 2	36.3	41.8	39.06
LOC104909357	UPF0606 protein KIAA1549-like	38.1	40.1	39.05
PRRG4	proline rich Gla (G-carboxyglutamic acid) 4 (transmembrane)	32.1	46.0	39.03
CHRM4	cholinergic receptor, muscarinic 4	40.6	37.5	39.02
LOC104911951	DENN domain-containing protein 5B-like	43.1	34.9	38.97
SUSD4	sushi domain containing 4	32.1	45.8	38.95
LOC104912452	FAS-associated factor 1-like	34.7	43.2	38.95
LOC104913759	ephrin type-A receptor 3-like	37.2	40.7	38.93
LOC100550412	connective tissue growth factor-like	38.0	39.8	38.91
SPI1	Spi-1 proto-oncogene	30.4	47.4	38.91
LOC104909923	uncharacterized LOC104909923	39.7	38.1	38.90
LOC100539703	LON peptidase N-terminal domain and RING finger protein 1-like	41.4	36.4	38.90
ZNF574	zinc finger protein 574	48.2	29.6	38.87
LOC104912943	phosphorylase b kinase regulatory subunit beta-like	38.0	39.7	38.85

LOC104910344	uncharacterized LOC104910344	35.5	42.1	38.81
NCKAP5	NCK-associated protein 5	28.7	48.8	38.76
UPK1B	uroplakin 1B	54.0	23.5	38.74
LOC100541411	parafibromin-like	34.6	42.8	38.72
LOC104912923	uncharacterized LOC104912923	34.6	42.8	38.70
LAMC3	laminin, gamma 3	34.7	42.6	38.65
LOC104917352	spectrin beta chain, non-erythrocytic 2-like	39.7	37.5	38.60
LOC104909866	leucine-rich repeats and immunoglobulin-like domains protein 3	29.6	47.6	38.58
LOC104913760	uncharacterized protein C16orf45 homolog	38.9	38.3	38.58
AMMECR1	Alport syndrome, mental retardation, midface hypoplasia and elliptocytosis chromosomal region gene 1	32.1	45.0	38.54
SLC35G1	solute carrier family 35, member G1	30.4	46.6	38.52
WEE2	WEE1 homolog 2 (S. pombe)	37.2	39.9	38.51
SSNA1	Sjogren syndrome nuclear autoantigen 1	41.4	35.6	38.50
LOC104912648	centrosomal protein of 63 kDa-like	39.7	37.3	38.49
LOC100545512	tubulin alpha-2 chain	38.8	38.1	38.48
SAMD15	sterile alpha motif domain containing 15	41.4	35.6	38.48
ARSG	arylsulfatase G	40.5	36.4	38.47
LOC104915365	uncharacterized LOC104915365	29.5	47.3	38.43
APEX1	APEX nuclease (multifunctional DNA repair enzyme) 1	29.6	47.2	38.39
ZFHX3	zinc finger homeobox 3	43.1	33.6	38.36
VWA5B1	von Willebrand factor A domain containing 5B1	32.9	43.7	38.34
LOC104912551	ras GTPase-activating protein 2-like	27.9	48.8	38.32
WNT8B	wingless-type MMTV integration site family, member 8B	35.5	41.1	38.30
MITF	microphthalmia-associated transcription factor	34.6	41.9	38.28
LOC104910409	uncharacterized LOC104910409	43.1	33.5	38.27
PDE6G	phosphodiesterase 6G, cGMP-specific, rod, gamma	43.1	33.5	38.27
LOC104913676	uncharacterized LOC104913676	35.5	41.0	38.24
LOC100538933	probable ATP-dependent RNA helicase DDX60	34.6	41.8	38.20
LOC100540309	rho guanine nucleotide exchange factor 39-like	42.2	34.1	38.17
LOC100541057	retinol dehydrogenase 8-like	39.7	36.6	38.15
LOC104916608	UPF0392 protein F13G3.3-like	40.5	35.7	38.14
LOC100542633	acyl-coenzyme A thioesterase 1-like	39.7	36.6	38.13
SFR1	SWI5-dependent recombination repair 1	37.2	39.1	38.11
LOC100545437	D-beta-hydroxybutyrate dehydrogenase, mitochondrial-like	35.5	40.7	38.10
IQCH	IQ motif containing H	45.6	30.5	38.05
TEAD1	TEA domain family member 1 (SV40 transcriptional enhancer factor)	32.9	43.1	38.00
STK32C	serine/threonine kinase 32C	33.0	42.8	37.89
GCK	glucokinase (hexokinase 4)	41.3	34.4	37.86
SIDT1	SID1 transmembrane family, member 1	30.4	45.2	37.84
SOX9	SRY (sex determining region Y)-box 9	42.2	33.4	37.82
LOC104912590	fibulin-1-like	44.0	31.6	37.79
POLN	polymerase (DNA directed) nu	31.2	44.3	37.78
LOC104912911	uncharacterized LOC104912911	40.5	34.9	37.71
FERMT1	fermitin family member 1	38.8	36.6	37.71
GPR22	G protein-coupled receptor 22	39.7	35.7	37.70
SH2B3	SH2B adaptor protein 3	38.9	36.5	37.70
OXSM	3-oxoacyl-ACP synthase, mitochondrial	40.5	34.9	37.69
LOC100546989	fructose-1,6-bisphosphatase 1-like	38.8	36.5	37.66
HAPLN3	hyaluronan and proteoglycan link protein 3	38.0	37.3	37.65
LOC104909849	monocarboxylate transporter 10-like	39.7	35.6	37.65
LOC104914772	dynein intermediate chain 1, axonemal-like	45.6	29.6	37.59
LOC104916153	methyltransferase-like protein 16	38.8	36.3	37.58
LOC104909491	exportin-1-like	38.0	37.1	37.57
ARHGAP44	Rho GTPase activating protein 44	33.0	42.1	37.55
EDARADD	EDAR-associated death domain	43.1	32.0	37.54
PPEF2	protein phosphatase, EF-hand calcium binding domain 2	33.8	41.2	37.53

FNDC7	fibronectin type III domain containing 7	35.5	39.5	37.51
LOC104911017	uncharacterized LOC104911017	36.3	38.7	37.50
LOC104916871	zinc finger protein 541-like	43.9	31.0	37.48
LOC100550569	receptor-type tyrosine-protein phosphatase delta-like	31.3	43.7	37.48
PEBP4	phosphatidylethanolamine-binding protein 4	39.7	35.1	37.40
LOC100541254	PCTP-like protein	32.1	42.7	37.40
FRRS1L	ferric-chelate reductase 1-like	42.2	32.5	37.34
UBAP1L	ubiquitin associated protein 1-like	38.9	35.8	37.34
LOC104914624	beclin-1-like	38.9	35.8	37.32
B4GALT1	UDP-Gal:betaGlcNAc beta 1,4- galactosyltransferase, polypeptide 1	40.5	34.1	37.32
LOC104910351	centrosome and spindle pole associated protein 1-like	28.7	45.8	37.29
SAMD12	sterile alpha motif domain containing 12	25.4	49.2	37.27
LOC100545932	dystrobrevin alpha	38.9	35.5	37.21
CLYBL	citrate lyase beta like	36.3	38.1	37.21
SLC46A1	solute carrier family 46 (folate transporter), member 1	33.8	40.5	37.16
KCNC2	potassium voltage-gated channel, Shaw-related subfamily, member 2	31.3	43.0	37.16
P2RX7	purinergic receptor P2X, ligand-gated ion channel, 7	33.0	41.3	37.15
HKDC1	hexokinase domain containing 1	43.9	30.3	37.13
LOC104915427	zinc finger protein 345-like	34.7	39.6	37.12
LOC104914769	ubiquitin-associated protein 2-like	27.0	47.2	37.10
GPR20	G protein-coupled receptor 20	33.8	40.4	37.10
FAXDC2	fatty acid hydroxylase domain containing 2	41.4	32.7	37.03
KIAA1755	KIAA1755 ortholog	38.9	35.1	37.01
LOC104914484	uncharacterized LOC104914484	36.3	37.7	36.99
CIT	citron rho-interacting serine/threonine kinase	40.6	33.4	36.97
LAPTM4B	lysosomal protein transmembrane 4 beta	40.5	33.4	36.97
LOC104915905	arf-GAP with GTPase, ANK repeat and PH domain-containing protein 3-like	38.9	35.0	36.96
TMED6	transmembrane emp24 protein transport domain containing 6	38.9	34.9	36.92
PLB1	phospholipase B1	31.2	42.5	36.88
LOC104912495	histone H2A deubiquitinase MYSM1-like	39.7	34.0	36.88
LOC104911694	unconventional myosin-X-like	38.0	35.7	36.87
SETD4	SET domain containing 4	38.8	34.9	36.85
ESRRG	estrogen-related receptor gamma	29.5	44.1	36.84
LOC104917462	conserved oligomeric Golgi complex subunit 5-like	34.6	39.0	36.83
TJP1	tight junction protein 1	37.2	36.5	36.81
SEPP1	selenoprotein P, plasma, 1	26.2	47.4	36.81
LOC100545386	transmembrane channel-like protein 7	36.3	37.1	36.74
SUSD3	sushi domain containing 3	43.1	30.4	36.73
SLC22A15	solute carrier family 22, member 15	33.8	39.7	36.72
TACR2	tachykinin receptor 2	34.6	38.8	36.71
LOC100550505	rho guanine nucleotide exchange factor 40-like	43.1	30.2	36.66
VTCN1	V-set domain containing T cell activation inhibitor 1	30.4	42.9	36.65
LOC100540688	G protein-coupled receptor kinase 4	31.3	42.0	36.64
GAS2L3	growth arrest-specific 2 like 3	30.4	42.8	36.60
LOC100540396	hepatocyte nuclear factor 4-beta-like	28.7	44.4	36.57
LOC100545125	ras-related and estrogen-regulated growth inhibitor-like protein	31.3	41.9	36.55
WISP3	WNT1 inducible signaling pathway protein 3	38.0	35.0	36.50
ARAP2	ArfGAP with RhoGAP domain, ankyrin repeat and PH domain 2	45.6	27.4	36.48
LOC104910823	TBC1 domain family member 14-like	35.5	37.3	36.41
LHFPL3	lipoma HMGIC fusion partner-like 3	34.7	38.1	36.39
LOC104914201	alkaline phosphatase, tissue-nonspecific isozyme-like	33.0	39.8	36.39
CPNE4	copine IV	35.4	37.3	36.39
LOC100542020	roquin-1-like	28.7	44.0	36.38
LOC104915269	ras GTPase-activating protein 1-like	35.5	37.3	36.37
LOC104916770	serine/arginine-rich splicing factor 6 pseudogene	37.2	35.5	36.36

LOC104916781	titin-like	28.7	43.9	36.34
COL9A3	collagen, type IX, alpha 3	33.8	38.9	36.33
CTNNAL1	catenin (cadherin-associated protein), alpha-like 1	42.2	30.4	36.33
MGMT	O-6-methylguanine-DNA methyltransferase	37.1	35.5	36.32
SLC25A21	solute carrier family 25 (mitochondrial oxoadipate carrier), member 21	34.6	38.0	36.29
LOC104917370	zinc finger protein 282-like	40.6	32.0	36.29
LOC104917403	uncharacterized LOC104917403	34.6	37.9	36.27
LOC104911954	uncharacterized LOC104911954	42.2	30.3	36.27
LOC104914091	bactericidal permeability-increasing protein-like	38.9	33.7	36.26
LOC104915170	uncharacterized LOC104915170	39.7	32.8	36.25
B9D1	B9 protein domain 1	26.2	46.3	36.25
F5	coagulation factor V (proaccelerin, labile factor)	27.9	44.6	36.23
IL6	interleukin 6	32.1	40.4	36.22
LOC100540939	teneurin-4	28.7	43.7	36.22
KDM7A	lysine (K)-specific demethylase 7A	33.8	38.6	36.17
LOC100548849	transmembrane protease serine 11E-like	27.9	44.4	36.14
SMTNL1	smoothelin-like 1	38.0	34.1	36.09
DGKH	diacylglycerol kinase, eta	34.6	37.5	36.07
PPARGC1B	peroxisome proliferator-activated receptor gamma, coactivator 1 beta	38.9	33.3	36.06
LOC104909937	rho guanine nucleotide exchange factor 10-like	35.5	36.6	36.03
LOC104911443	glycosyltransferase-like protein LARGE1	36.3	35.7	35.99
LOC100546176	arylsulfatase D-like	43.9	28.0	35.98
CCDC103	coiled-coil domain containing 103	42.2	29.7	35.96
LOC104910200	dynein heavy chain 8, axonemal-like	34.6	37.3	35.95
LOC104911019	Hermansky-Pudlak syndrome 5 protein-like	36.3	35.5	35.90
UNC5B	unc-5 homolog B (C. elegans)	33.8	37.9	35.87
COL8A1	collagen, type VIII, alpha 1	28.7	43.0	35.84
LOC104909438	prolyl 4-hydroxylase subunit alpha-3-like	46.5	25.0	35.75
AK8	adenylate kinase 8	32.1	39.4	35.75
RYR1	ryanodine receptor 1 (skeletal)	43.1	28.4	35.74
LOC100548128	1-phosphatidylinositol 4,5-bisphosphate phosphodiesterase beta-1-like	31.2	40.2	35.73
LOC104912353	ras/Rap GTPase-activating protein SynGAP-like	39.7	31.7	35.70
APBA1	amyloid beta (A4) precursor protein-binding, family A, member 1	37.2	34.2	35.69
LOC104910445	uncharacterized LOC104910445	36.3	35.1	35.69
LOC100543404	muscular LMNA-interacting protein-like	32.9	38.4	35.67
LOC104917094	noelin-2-like	38.9	32.4	35.64
CAPN13	calpain 13	34.6	36.5	35.59
LOC104911232	endoribonuclease Dicer-like	26.2	45.0	35.58
LOC104913987	uncharacterized LOC104913987	42.2	28.7	35.44
LOC104911135	uncharacterized LOC104911135	31.2	39.6	35.43
LOC104913349	rab11 family-interacting protein 3-like	32.9	37.8	35.38
CCDC157	coiled-coil domain containing 157	28.7	42.0	35.36
LOC104917574	phospholipid-transporting ATPase IB-like	36.3	34.3	35.33
LOC100549351	protocadherin Fat 3-like	38.0	32.6	35.33
RAP2B	RAP2B, member of RAS oncogene family	38.8	31.7	35.30
ZC2HC1A	zinc finger, C2HC-type containing 1A	29.6	41.0	35.29
LOC100539157	G1/S-specific cyclin-E1-like	28.7	41.9	35.28
LOC100550208	serine/threonine-protein kinase 4-like	36.3	34.3	35.28
LOC100539498	leucine-rich repeat-containing protein 16A	36.3	34.2	35.24
FAM102A	family with sequence similarity 102, member A	43.1	27.4	35.22
LOC104912957	chromodomain-helicase-DNA-binding protein 9-like	30.4	40.0	35.21
LOC104914061	protein kinase C-binding protein 1-like	36.3	34.1	35.20
LOC100546512	serine/threonine-protein kinase Nek7-like	42.2	28.2	35.19
LOC100541200	NADH-cytochrome b5 reductase 2	32.1	38.3	35.19
ABCA13	ATP-binding cassette, sub-family A (ABC1), member 13	44.7	25.6	35.18

MTBP	MDM2 binding protein	32.1	38.2	35.15
LOC104916655	uncharacterized LOC104916655	31.3	38.8	35.04
RGS4	regulator of G-protein signaling 4	38.0	32.0	35.03
LOC104912945	lon protease homolog 2, peroxisomal-like	32.9	37.1	35.02
LOC100547920	cat eye syndrome critical region protein 2-like	40.6	29.4	35.00
SPAM1	sperm adhesion molecule 1 (PH-20 hyaluronidase, zona pellucida binding)	29.6	40.3	34.91
LOC100544436	L-threonine 3-dehydrogenase, mitochondrial	22.8	47.0	34.91
LOC104913284	protein FAM13B-like	37.2	32.6	34.89
SLC39A9	solute carrier family 39, member 9	28.7	40.9	34.82
LOC100538976	complement C4-like	26.2	43.3	34.76
LOC104915985	uncharacterized LOC104915985	33.8	35.7	34.73
SNN	stannin	32.9	36.5	34.72
RHBDD1	rhomboid domain containing 1	25.3	44.1	34.71
LOC100545410	rho GTPase-activating protein 42	34.7	34.7	34.70
RBMS3	RNA binding motif, single stranded interacting protein 3	31.2	38.1	34.67
SMOC1	SPARC related modular calcium binding 1	32.1	37.2	34.62
ARHGAP22	Rho GTPase activating protein 22	39.7	29.5	34.59
AGTRAP	angiotensin II receptor-associated protein	28.7	40.5	34.58
LOC100548307	cytohesin-3	27.0	42.0	34.53
CDCA2	cell division cycle associated 2	39.7	29.4	34.53
LOC100540465	myosin-IIIb-like	37.2	31.8	34.50
LOC104914207	polyhomeotic-like protein 2	37.2	31.7	34.47
LOC104916039	N6-adenosine-methyltransferase 70 kDa subunit-like	34.7	34.3	34.46
TMEM106C	transmembrane protein 106C	33.8	35.1	34.45
LOC100541529	interaptin-like	38.1	30.8	34.44
KCNN2	potassium intermediate/small conductance calcium-activated channel, subfamily N, member 2	32.1	36.7	34.42
LOC104915019	uncharacterized LOC104915019	34.6	34.2	34.42
LOC100540262	synapse-associated protein 1-like	43.9	24.9	34.41
NME5	NME/NM23 family member 5	35.5	33.3	34.41
BTK	Bruton agammaglobulinemia tyrosine kinase	43.1	25.7	34.37
LOC100543316	cilia- and flagella-associated protein 43-like	29.6	39.2	34.37
LOC104916357	vegetative cell wall protein gp1-like	31.3	37.5	34.36
LOC104915799	uncharacterized LOC104915799	31.3	37.3	34.32
LOC104914200	uncharacterized LOC104914200	32.1	36.5	34.30
FAM185A	family with sequence similarity 185, member A	39.7	28.9	34.28
CLSTN3	calsyntenin 3	32.1	36.4	34.26
LOC104915721	hepatic lectin-like	25.3	43.2	34.25
LOC104917186	beta-arrestin-1-like	33.0	35.5	34.24
LOC104913039	uncharacterized LOC104913039	39.7	28.6	34.16
ARL6	ADP-ribosylation factor-like 6	27.8	40.5	34.14
LOC104914972	collagen type IV alpha-3-binding protein-like	28.7	39.5	34.10
PBX1	pre-B-cell leukemia homeobox 1	32.9	35.3	34.10
TCTE3	t-complex-associated-testis-expressed 3	36.3	31.9	34.10
NFXL1	nuclear transcription factor, X-box binding-like 1	39.7	28.5	34.08
LIF	leukemia inhibitory factor	38.0	30.1	34.05
APCDD1L	adenomatosis polyposis coli down-regulated 1-like	33.0	35.1	34.03
LOC100539124	solute carrier family 23 member 1-like	33.8	34.3	34.02
PGM5	phosphoglucomutase 5	33.8	34.2	33.98
LOC104909281	meckelin-like	35.5	32.5	33.96
LOC104911645	uncharacterized LOC104911645	40.6	27.3	33.94
METTL20	methyltransferase like 20	40.6	27.2	33.91
LOC104917199	uncharacterized LOC104917199	31.3	36.5	33.89
VAV3	vav 3 guanine nucleotide exchange factor	33.8	34.0	33.89
SIX4	SIX homeobox 4	34.6	33.1	33.89
ULK4	unc-51 like kinase 4	39.7	28.0	33.86
LOC104917472	progesterone-induced-blocking factor 1-like	31.2	36.5	33.85

SLC16A9	solute carrier family 16, member 9	38.8	28.9	33.85
LOC104914742	uncharacterized LOC104914742	38.8	28.8	33.81
LOC104915541	uncharacterized LOC104915541	27.9	39.7	33.81
LOC104911458	PHD finger protein 14-like	38.8	28.8	33.78
LOC104915198	E3 ubiquitin-protein ligase RNF38-like	27.0	40.5	33.78
FAM184B	family with sequence similarity 184, member B	37.2	30.4	33.77
LOC104911010	uncharacterized LOC104911010	27.0	40.5	33.77
LOC104910320	dystrobrevin alpha-like	29.6	37.9	33.75
CLRN1	clarin 1	35.5	31.9	33.70
LOC104911907	uncharacterized LOC104911907	35.4	31.9	33.69
ENTPD2	ectonucleoside triphosphate diphosphohydrolase 2	34.6	32.7	33.69
WNT5A	wingless-type MMTV integration site family, member 5A	35.5	31.8	33.66
TRAPPC9	trafficking protein particle complex 9	30.4	36.9	33.65
ZBTB11	zinc finger and BTB domain containing 11	33.8	33.5	33.65
NTNG2	netrin G2	35.5	31.8	33.65
LOC100550121	uncharacterized LOC100550121	30.4	36.9	33.65
LOC104913026	enhancer of mRNA-decapping protein 4-like	39.7	27.6	33.65
LOC104911952	uncharacterized LOC104911952	24.5	42.8	33.64
SLC4A11	solute carrier family 4, sodium borate transporter, member 11	41.4	25.9	33.62
MYB	v-myb avian myeloblastosis viral oncogene homolog	34.6	32.6	33.59
LOC104914283	stromal membrane-associated protein 2-like	31.3	35.9	33.58
LOC100543675	glycosyltransferase-like domain-containing protein 1	33.8	33.4	33.58
ARHGEF16	Rho guanine nucleotide exchange factor (GEF) 16	40.5	26.6	33.55
IL18BP	interleukin 18 binding protein	28.7	38.4	33.54
LOC100542902	neurotrypsin-like	32.1	35.0	33.53
ZNF185	zinc finger protein 185 (LIM domain)	39.7	27.4	33.52
ATP6V1C2	ATPase, H+ transporting, lysosomal 42kDa, V1 subunit C2	29.6	37.4	33.51
LOC104915739	uncharacterized LOC104915739	33.8	33.3	33.51
B3GALNT1	beta-1,3-N-acetylgalactosaminyltransferase 1 (globoside blood group)	32.1	34.9	33.49
LOC104911395	partitioning defective 3 homolog	34.6	32.3	33.48
CENPO	centromere protein O	32.1	34.9	33.48
LOC100549513	wolframin-like	30.4	36.5	33.47
LOC104915358	uncharacterized LOC104915358	26.2	40.6	33.42
SLC18B1	solute carrier family 18, subfamily B, member 1	29.5	37.2	33.38
RAB31	RAB31, member RAS oncogene family	36.3	30.4	33.35
DICER1	dicer 1, ribonuclease type III	31.2	35.4	33.33
CELF5	CUGBP, Elav-like family member 5	36.3	30.3	33.33
ZC2HC1C	zinc finger, C2HC-type containing 1C	27.9	38.8	33.32
LOC104912299	uncharacterized LOC104912299	32.1	34.5	33.31
SCAPER	S-phase cyclin A-associated protein in the ER	36.3	30.3	33.30
AGO2	argonaute RISC catalytic component 2	32.1	34.4	33.27
IGSF5	immunoglobulin superfamily, member 5	35.5	31.0	33.23
GGACT	gamma-glutamylamine cyclotransferase	36.3	30.1	33.21
LOC104914171	eukaryotic translation initiation factor 4 gamma 3-like	34.6	31.7	33.17
ADAMTS5	ADAM metallopeptidase with thrombospondin type 1 motif, 5	29.6	36.7	33.14
LOC104913446	uncharacterized LOC104913446	33.0	33.3	33.13
DYNLRB2	dynein, light chain, roadblock-type 2	32.9	33.3	33.13
LOC104909515	son of sevenless homolog 1-like	32.9	33.3	33.12
LOC104917428	uncharacterized LOC104917428	32.9	33.3	33.09
LOC104910814	uncharacterized LOC104910814	27.0	39.1	33.06
CHST1	carbohydrate (keratan sulfate Gal-6) sulfotransferase 1	39.7	26.4	33.05
LOC104910459	zinc finger CCCH domain-containing protein 3-like	30.4	35.7	33.04
GPR17	G protein-coupled receptor 17	31.3	34.8	33.02
LOC104911241	uncharacterized LOC104911241	27.1	38.9	33.01
LOC104909840	laminin subunit alpha-4-like	35.5	30.5	32.99
LOC100541356	cytoplasmic dynein 1 intermediate chain 1-like	34.7	31.3	32.96
LOC100548578	transcription intermediary factor 1-alpha	26.2	39.6	32.91

LOC104914048	protein kinase C-binding protein 1-like	32.9	32.8	32.86
ALDH1A3	aldehyde dehydrogenase 1 family, member A3	24.5	41.2	32.85
LOC104909635	latent-transforming growth factor beta-binding protein 1-like	28.7	37.0	32.84
SLC25A47	solute carrier family 25, member 47	34.6	31.1	32.84
LOC104916267	ADP-ribosylation factor-like protein 2	33.8	31.8	32.81
DENND2A	DENN/MADD domain containing 2A	33.0	32.6	32.79
LOC104912359	uncharacterized LOC104912359	32.1	33.5	32.78
CHST15	carbohydrate (N-acetylgalactosamine 4-sulfate 6-O) sulfotransferase 15	22.0	43.5	32.73
PKDCC	protein kinase domain containing, cytoplasmic	41.4	24.1	32.73
LOC100539447	carbonic anhydrase 13-like	31.3	34.2	32.72
PFN4	profilin family, member 4	32.1	33.3	32.72
KIF17	kinesin family member 17	32.1	33.3	32.71
MALL	mal, T-cell differentiation protein-like	38.9	26.6	32.71
LOC104913851	uncharacterized LOC104913851	36.4	28.9	32.63
NCKAP1L	NCK-associated protein 1-like	38.0	27.2	32.60
LOC100542556	protein-glutamine gamma-glutamyltransferase 6-like	33.8	31.4	32.59
ISCA1	iron-sulfur cluster assembly 1	32.1	33.1	32.59
LOC104915142	serine palmitoyltransferase 1-like	27.0	38.1	32.59
LOC104909484	centrosomal protein kizuna-like	36.3	28.8	32.57
LOC104915392	uncharacterized LOC104915392	28.7	36.3	32.50
LOC104916095	retinol dehydrogenase 12-like	36.3	28.6	32.47
LOC104909548	uncharacterized LOC104909548	22.0	42.9	32.47
LOC104912719	anoctamin-2-like	24.5	40.4	32.46
GRK7	G protein-coupled receptor kinase 7	35.5	29.4	32.45
TMEM82	transmembrane protein 82	33.8	31.0	32.42
PTGER2	prostaglandin E receptor 2 (subtype EP2), 53kDa	33.8	31.0	32.41
LOC104913683	uncharacterized LOC104913683	31.3	33.5	32.40
LOC104909856	uncharacterized LOC104909856	23.6	41.2	32.40
CCDC57	coiled-coil domain containing 57	32.9	31.8	32.39
TRAF1	TNF receptor-associated factor 1	35.5	29.3	32.38
TECTA	tectorin alpha	33.8	30.9	32.37
LOC104913700	uncharacterized LOC104913700	43.1	21.6	32.35
LOC100545311	two pore calcium channel protein 2-like	39.7	25.0	32.33
FAM168B	family with sequence similarity 168, member B	27.0	37.6	32.30
FOXN2	forkhead box N2	31.2	33.3	32.29
GPATCH1	G patch domain containing 1	30.4	34.2	32.29
LOC104917075	zinc finger protein 501-like	34.6	30.0	32.28
LOC100538756	protein kinase C zeta type	37.1	27.4	32.28
AGPAT5	1-acylglycerol-3-phosphate O-acyltransferase 5	28.7	35.9	32.27
LOC104909707	uncharacterized LOC104909707	32.1	32.4	32.27
LOC104909383	protocadherin Fat 3-like	37.2	27.3	32.23
AMPD1	adenosine monophosphate deaminase 1	35.5	28.9	32.20
LOC100548121	poly [ADP-ribose] polymerase 12-like	37.1	27.3	32.20
LOC104913527	uncharacterized LOC104913527	36.3	28.0	32.16
LOC104915909	meteorin-like	37.1	27.1	32.12
LOC100544458	epidermal differentiation-specific protein-like	37.2	27.0	32.08
LOC104914428	uncharacterized LOC104914428	33.8	30.3	32.02
ARFGEF1	ADP-ribosylation factor guanine nucleotide-exchange factor 1 (brefeldin A-inhibited)	27.9	36.2	32.02
ENKUR	enkurin, TRPC channel interacting protein	33.0	31.1	32.02
LOC104915089	uncharacterized LOC104915089	25.3	38.7	32.00
LOC100542699	protein FAM73A-like	31.3	32.7	31.99
LOC100543123	transient receptor potential cation channel subfamily M member 6-like	33.8	30.2	31.98
LOC104911370	uncharacterized LOC104911370	29.5	34.4	31.96
LOC100546025	probable palmitoyltransferase ZDHHC14	32.9	30.9	31.94
LOC100545423	diphthine--ammonia ligase-like	31.3	32.6	31.92

LOC104911950	uncharacterized LOC104911950	27.9	35.9	31.92
APPL1	adaptor protein, phosphotyrosine interaction, PH domain and leucine zipper containing 1	29.5	34.2	31.88
FCHO1	FCH domain only 1	28.7	35.0	31.84
LOC100540753	WD repeat-containing protein 11-like	27.9	35.8	31.84
TGDS	TDP-glucose 4,6-dehydratase	29.5	34.1	31.82
MGAT4A	mannosyl (alpha-1,3-)-glycoprotein beta-1,4-N-acetylglucosaminyltransferase, isozyme A	35.5	28.2	31.81
LOC100539082	myelin and lymphocyte protein-like	35.5	28.1	31.79
LOC100545816	microtubule-associated protein 1B-like	29.5	34.0	31.78
LOC104917211	protein patched homolog 1-like	34.6	28.9	31.77
ESR1	estrogen receptor 1	29.6	33.9	31.72
EVA1C	eva-1 homolog C (C. elegans)	32.9	30.5	31.71
TRIM25	tripartite motif containing 25	36.4	27.0	31.69
LOC100539971	coiled-coil domain-containing protein 132-like	25.3	38.0	31.66
LOC104916880	neuroblast differentiation-associated protein AHNAK-like	39.7	23.6	31.65
LOC104913269	uncharacterized LOC104913269	34.6	28.7	31.63
LOC104909399	probable asparagine--tRNA ligase, mitochondrial	32.9	30.3	31.63
LOC104913538	uncharacterized LOC104913538	34.6	28.6	31.62
GFER	growth factor, augmenter of liver regeneration	34.6	28.5	31.58
RHO	rhodopsin	30.4	32.7	31.57
FABP1	fatty acid binding protein 1, liver	30.4	32.7	31.55
LOC104912720	caspase recruitment domain-containing protein 8-like	30.4	32.7	31.55
ATP2B2	ATPase, Ca++ transporting, plasma membrane 2	29.5	33.5	31.50
MAR1	membrane-associated ring finger (C3HC4) 1, E3 ubiquitin protein ligase	31.2	31.7	31.49
LOC104916783	class II histocompatibility antigen, B-L beta chain-like	28.7	34.2	31.45
KPNA5	karyopherin alpha 5 (importin alpha 6)	33.8	29.1	31.43
LOC104914663	transducin-like enhancer protein 2	39.7	23.2	31.42
LOC100551049	uncharacterized LOC100551049	27.0	35.7	31.36
WRNIP1	Werner helicase interacting protein 1	35.5	27.2	31.34
LOC100546134	serine/threonine-protein kinase D3-like	33.8	28.7	31.24
LOC100551004	uncharacterized LOC100551004	32.9	29.5	31.20
LOC104917049	uncharacterized LOC104917049	30.4	31.9	31.18
SFMBT2	Scm-like with four mbt domains 2	31.3	31.0	31.16
LOC100548127	pannexin-1-like	32.1	30.2	31.15
LOC104916408	adenylate cyclase type 6-like	42.3	20.0	31.13
OXCT1	3-oxoacid CoA transferase 1	31.2	31.0	31.12
LOC104912443	uncharacterized LOC104912443	28.7	33.5	31.09
LOC100548900	otogelin-like	37.1	25.0	31.05
PLCD1	phospholipase C, delta 1	27.9	34.2	31.02
LOC104910917	DNA-directed RNA polymerase I subunit RPA1-like	27.9	34.1	30.99
LOC104913827	uncharacterized LOC104913827	33.8	28.2	30.99
CLEC19A	C-type lectin domain family 19, member A	34.6	27.3	30.94
LOC104910134	uncharacterized LOC104910134	32.1	29.7	30.92
ZNF407	zinc finger protein 407	27.9	33.9	30.91
CCKBR	cholecystokinin B receptor	33.8	28.0	30.90
LOC104916072	protein NDRG2-like	33.8	28.0	30.88
LOC100542552	myosin heavy chain, skeletal muscle-like	40.6	21.2	30.88
LOC104916534	pre-mRNA cleavage complex 2 protein Pcf11 pseudogene	32.9	28.8	30.86
LOC104909406	uncharacterized LOC104909406	32.1	29.6	30.84
LOC104912273	uncharacterized LOC104912273	32.1	29.6	30.83
LOC104914057	cadherin-4-like	33.8	27.8	30.83
LOC104917188	uncharacterized LOC104917188	30.4	31.2	30.81
MAK	male germ cell-associated kinase	31.3	30.3	30.79
LOC104916400	forkhead box protein D1-like	31.3	30.3	30.77
NUP210L	nucleoporin 210kDa-like	29.6	31.9	30.77
C1R	complement component 1, r subcomponent	28.7	32.8	30.75

LOC100541987	myelin-oligodendrocyte glycoprotein-like	31.2	30.3	30.75
LOC104912491	dedicator of cytokinesis protein 7-like	24.5	37.0	30.75
LOC104909620	spastin-like	31.3	30.2	30.72
C1S	complement component 1, s subcomponent	32.1	29.4	30.72
DDIAS	DNA damage-induced apoptosis suppressor	29.6	31.8	30.70
LOC104915398	histone H2A-IV-like	20.3	41.1	30.66
LOC104911012	transient receptor potential cation channel subfamily M member 5-like	30.4	30.9	30.66
LOC104910388	NEDD4-like E3 ubiquitin-protein ligase WWP1	34.6	26.6	30.62
CSGALNACT1	chondroitin sulfate N-acetylgalactosaminyltransferase 1	26.2	35.0	30.62
LOC104912791	probable E3 ubiquitin-protein ligase HERC1	27.1	34.1	30.60
LOC100547485	E3 ubiquitin-protein ligase NEDD4	27.8	33.3	30.59
LOC104910318	uncharacterized LOC104910318	37.2	24.0	30.59
TMEM55A	transmembrane protein 55A	27.0	34.1	30.56
CARTPT	CART prepropeptide	27.0	34.1	30.55
LOC104910584	uncharacterized LOC104910584	27.9	33.2	30.54
LOC104909376	centrosomal protein KIAA1731 homolog	35.5	25.6	30.54
SUPT3H	suppressor of Ty 3 homolog (S. cerevisiae)	33.8	27.3	30.53
HS3ST2	heparan sulfate (glucosamine) 3-O-sulfotransferase 2	34.6	26.5	30.52
LOC104910738	protein FAM149A-like	34.6	26.4	30.52
LOC104913612	F-box/WD repeat-containing protein 8-like	34.6	26.3	30.48
RSAD1	radical S-adenosyl methionine domain containing 1	32.9	28.0	30.47
DHX34	DEAH (Asp-Glu-Ala-His) box polypeptide 34	43.9	17.0	30.46
GDF15	growth differentiation factor 15	33.0	27.9	30.42
LOC104909445	C2 domain-containing protein 3-like	32.9	27.9	30.41
LOC104917431	integral membrane protein GPR180-like	31.3	29.5	30.38
PIGG	phosphatidylinositol glycan anchor biosynthesis, class G	21.1	39.7	30.38
KCNJ11	potassium inwardly-rectifying channel, subfamily J, member 11	38.8	21.9	30.38
KCNJ8	potassium inwardly-rectifying channel, subfamily J, member 8	32.1	28.7	30.36
LOC104914926	uncharacterized LOC104914926	28.7	32.0	30.36
ANKRD31	ankyrin repeat domain 31	30.4	30.3	30.36
ZFYVE9	zinc finger, FYVE domain containing 9	33.8	26.9	30.35
LOC104915401	histone H1-like	31.3	29.4	30.35
CRYBA4	crystallin, beta A4	28.7	31.9	30.31
ARHGEF33	Rho guanine nucleotide exchange factor (GEF) 33	29.5	31.0	30.28
NATD1	N-acetyltransferase domain containing 1	31.2	29.3	30.27
BAIAP2	BAI1-associated protein 2	27.0	33.5	30.25
BOLL	boule-like RNA-binding protein	34.6	25.8	30.22
ITGB2	integrin, beta 2 (complement component 3 receptor 3 and 4 subunit)	25.4	35.1	30.22
SNAP91	synaptosomal-associated protein, 91kDa	25.3	35.1	30.22
ABLIM2	actin binding LIM protein family, member 2	27.9	32.6	30.22
COL26A1	collagen, type XXVI, alpha 1	26.2	34.2	30.19
IFNLR1	interferon, lambda receptor 1	25.4	35.0	30.19
LOC100551071	glucocorticoid receptor-like	27.9	32.4	30.16
LOC104914980	rho guanine nucleotide exchange factor 28-like	34.6	25.7	30.14
KIT	v-kit Hardy-Zuckerman 4 feline sarcoma viral oncogene homolog	24.5	35.7	30.13
LOC104911505	uncharacterized LOC104911505	32.1	28.1	30.12
SPRY3	sprouty homolog 3 (Drosophila)	33.8	26.4	30.11
SLC16A6	solute carrier family 16, member 6	32.1	28.1	30.10
LOC104909336	netrin-G1-like	30.4	29.7	30.06
DEPDC1	DEP domain containing 1	32.1	28.0	30.01
LOC100541707	serine/threonine-protein kinase greatwall	23.6	36.3	29.99
LOC104916285	E3 ubiquitin-protein ligase TRIM39 pseudogene	30.4	29.5	29.99
MAF	v-maf avian musculoaponeurotic fibrosarcoma oncogene homolog	31.3	28.7	29.97
LOC104912669	uncharacterized LOC104912669	29.6	30.3	29.96
LOC104909519	atlastin-2-like	31.2	28.7	29.95
HLF	hepatic leukemia factor	29.6	30.3	29.94

FAXC	failed axon connections homolog (Drosophila)	27.8	32.0	29.94
PLEKHA6	pleckstrin homology domain containing, family A member 6	29.6	30.3	29.92
LOC104910608	WD repeat and FYVE domain-containing protein 3	18.6	41.2	29.91
LOC104909673	tetratricopeptide repeat protein 27-like	28.7	31.1	29.91
CGREF1	cell growth regulator with EF-hand domain 1	22.8	37.0	29.91
CCSAP	centriole, cilia and spindle-associated protein	35.5	24.2	29.82
LOC104914491	uncharacterized LOC104914491	26.2	33.4	29.81
ABCB9	ATP-binding cassette, sub-family B (MDR/TAP), member 9	27.8	31.7	29.79
LOC100539139	transcription factor E2F5-like	30.4	29.1	29.77
LOC104910214	zinc finger protein 516-like	26.2	33.3	29.76
STYXL1	serine/threonine/tyrosine interacting-like 1	26.2	33.3	29.75
CORO2B	coronin, actin binding protein, 2B	32.9	26.6	29.74
LOC104912990	estradiol 17-beta-dehydrogenase 2-like	22.8	36.6	29.72
LOC104912998	uncharacterized LOC104912998	29.6	29.8	29.70
PPP1R3A	protein phosphatase 1, regulatory subunit 3A	29.6	29.8	29.69
LOC104916734	uncharacterized LOC104916734	31.2	28.1	29.68
LOC104914177	uncharacterized LOC104914177	31.2	28.1	29.64
PALMD	palmdelphin	32.1	27.2	29.64
LOC104913651	uncharacterized LOC104913651	30.4	28.8	29.59
PIGZ	phosphatidylinositol glycan anchor biosynthesis, class Z	29.6	29.5	29.55
MATN3	matrilin 3	27.9	31.2	29.55
LOC100545666	uncharacterized LOC100545666	25.3	33.7	29.52
LOC104917409	uncharacterized LOC104917409	30.4	28.6	29.49
SPSB1	splA/ryanodine receptor domain and SOCS box containing 1	29.6	29.4	29.48
LOC104913692	uncharacterized LOC104913692	25.3	33.6	29.48
LOC100549732	dual specificity protein phosphatase CDC14A-like	29.6	29.4	29.45
LOC100546382	lamin-B1-like	27.0	31.9	29.45
PRKAG3	protein kinase, AMP-activated, gamma 3 non-catalytic subunit	32.9	26.0	29.45
IPPK	inositol 1,3,4,5,6-pentakisphosphate 2-kinase	18.6	40.3	29.43
LOC104914347	uncharacterized LOC104914347	26.2	32.7	29.41
PLEKHN1	pleckstrin homology domain containing, family N member 1	33.8	25.0	29.40
LOC104910963	palmitoyltransferase ZDHHC13-like	33.8	25.0	29.38
LOC104913254	uncharacterized LOC104913254	23.7	35.1	29.36
LRRC1	leucine rich repeat containing 1	30.4	28.3	29.34
CNDP1	carnosine dipeptidase 1 (metallopeptidase M20 family)	32.1	26.6	29.33
LOC104913674	uncharacterized LOC104913674	33.0	25.6	29.29
LOC100550164	chondroitin sulfate synthase 3	32.1	26.5	29.29
LOC100541852	alkylated DNA repair protein alkB homolog 8-like	32.1	26.4	29.24
ETS1	v-ets avian erythroblastosis virus E26 oncogene homolog 1	30.4	28.1	29.24
LOC104912075	fibroblast growth factor receptor 2-like	31.2	27.2	29.24
LOC100548520	gametocyte-specific factor 1-like	32.1	26.3	29.18
LOC104915894	transcription factor COE1-like	29.6	28.8	29.18
S1PR1	sphingosine-1-phosphate receptor 1	28.7	29.5	29.12
LOC104911630	transcription intermediary factor 1-alpha-like	29.5	28.7	29.11
CHRNA5	cholinergic receptor, nicotinic, alpha 5 (neuronal)	27.9	30.3	29.10
C5H11orf94	chromosome 5 open reading frame, human C11orf94	26.2	32.0	29.09
LOC104910604	uncharacterized LOC104910604	27.0	31.1	29.09
LOC104909850	tyrosine-protein kinase Fyn-like	26.2	31.9	29.05
C5H11orf16	chromosome 5 open reading frame, human C11orf16	27.9	30.2	29.02
LOC100546870	5-hydroxyisourate hydrolase-like	32.1	25.9	29.01
HSBP1L1	heat shock factor binding protein 1-like 1	32.1	25.9	28.99
ARHGAP25	Rho GTPase activating protein 25	23.7	34.3	28.96
PRKX	protein kinase, X-linked	24.5	33.3	28.92
LOC104917038	E3 ubiquitin-protein ligase HUWE1-like	29.6	28.1	28.84
LOC104910984	myotubularin-related protein 13-like	31.2	26.4	28.83
LOC104911418	A-kinase anchor protein 9-like	32.1	25.6	28.81
S1PR2	sphingosine-1-phosphate receptor 2	31.3	26.2	28.77
DNAH5	dynein, axonemal, heavy chain 5	22.8	34.7	28.75

LOC100539535	alpha-2-macroglobulin-like protein 1	28.7	28.8	28.75
LOC100550814	uncharacterized LOC100550814	30.5	27.0	28.73
LOC100544288	von Willebrand factor D and EGF domain-containing protein-like	37.9	19.5	28.71
SRRM4	serine/arginine repetitive matrix 4	29.6	27.8	28.70
LOC104913316	glucocorticoid receptor-like	30.4	27.0	28.69
THPO	thrombopoietin	36.3	21.0	28.69
ZAR1L	zygote arrest 1-like	27.0	30.3	28.68
AZIN2	antizyme inhibitor 2	35.5	21.8	28.67
LOC104914859	uncharacterized LOC104914859	24.5	32.7	28.60
LOC104910467	focal adhesion kinase 1-like	27.9	29.3	28.59
NANP	N-acetylneuraminic acid phosphatase	25.4	31.8	28.57
IL20RA	interleukin 20 receptor, alpha	22.8	34.3	28.56
LOC104913502	mitotic spindle assembly checkpoint protein MAD1-like	33.0	24.2	28.56
FILIP1L	filamin A interacting protein 1-like	30.4	26.7	28.54
LOC104914104	cerebral cavernous malformations 2 protein-like	22.0	35.1	28.53
LOC104910327	E3 ubiquitin-protein ligase RNF138-like	22.8	34.2	28.51
CD93	CD93 molecule	22.0	35.0	28.50
ANKRD6	ankyrin repeat domain 6	30.4	26.6	28.50
LOC104911374	uncharacterized LOC104911374	21.1	35.9	28.48
LOC104913377	uncharacterized LOC104913377	31.2	25.7	28.46
PCDH12	protocadherin 12	23.7	33.3	28.46
EDAR	ectodysplasin A receptor	32.1	24.8	28.44
CADPS2	Ca++-dependent secretion activator 2	32.0	24.8	28.43
LOC100538975	lysozyme g-like	29.5	27.3	28.43
LOC104915148	chondroitin sulfate synthase 3-like	32.1	24.8	28.41
IL16	interleukin 16	21.1	35.7	28.40
LOC104910566	uncharacterized LOC104910566	27.9	28.9	28.38
LOC100540238	probable C-mannosyltransferase DPY19L3	22.0	34.8	28.37
LOC100542975	histone H3-like	27.1	29.7	28.37
LOC104913101	MAP kinase-activated protein kinase 3-like	27.9	28.8	28.36
FGL1	fibrinogen-like 1	27.9	28.8	28.33
LOC100546656	serine-rich coiled-coil domain-containing protein 2-like	21.1	35.5	28.31
LOC100547151	endonuclease domain-containing 1 protein-like	23.7	32.9	28.26
LRRC43	leucine rich repeat containing 43	27.0	29.4	28.24
N4BP2L1	NEDD4 binding protein 2-like 1	26.2	30.3	28.24
SOST	sclerostin	27.0	29.4	28.24
LRP6	low density lipoprotein receptor-related protein 6	26.2	30.3	28.23
SNTB1	syntrophin, beta 1 (dystrophin-associated protein A1, 59kDa, basic component 1)	32.9	23.5	28.23
LOC104913105	uncharacterized LOC104913105	27.9	28.6	28.21
PTGFR	prostaglandin F receptor (FP)	27.0	29.4	28.21
CHGA	chromogranin A (parathyroid secretory protein 1)	25.4	31.0	28.20
LOC100544062	uncharacterized LOC100544062	36.3	20.1	28.19
ERBB4	v-erb-b2 avian erythroblastic leukemia viral oncogene homolog 4	25.3	31.0	28.18
LOC104916936	microtubule-actin cross-linking factor 1-like	33.8	22.6	28.18
LOC100541513	caspase-8-like	25.4	31.0	28.18
LOC104913350	nucleolar protein 58-like	22.8	33.5	28.16
FAT4	FAT atypical cadherin 4	25.3	31.0	28.16
LOC100540910	protein NLRC5-like	31.3	25.0	28.15
LOC104912771	uncharacterized LOC104912771	23.7	32.6	28.13
LOC100538808	Fc receptor-like protein 4	31.2	25.0	28.09
LOC100538862	WAP four-disulfide core domain protein 2-like	22.0	34.1	28.05
NAALADL2	N-acetylated alpha-linked acidic dipeptidase-like 2	24.5	31.6	28.04
LOC104914002	uncharacterized LOC104914002	30.4	25.7	28.04
LOC104914044	extracellular sulfatase Sulf-2-like	27.9	28.2	28.03
EPHB6	EPH receptor B6	32.9	23.1	28.03
LOC100540209	amine oxidase [flavin-containing] B-like	32.1	24.0	28.03
LOC100539684	fatty acid-binding protein, liver	31.3	24.8	28.02

LOC104912657	uncharacterized LOC104912657	28.7	27.4	28.02
LOC100549996	cyclin-I-like	18.6	37.4	28.00
LOC100549766	E3 ubiquitin-protein ligase NRDP1-like	17.7	38.2	27.95
CCDC79	coiled-coil domain containing 79	27.0	28.8	27.92
LOC104910172	uncharacterized LOC104910172	24.5	31.3	27.91
LOC104913695	uncharacterized LOC104913695	25.3	30.5	27.91
NPAS3	neuronal PAS domain protein 3	25.3	30.5	27.91
HOXA2	homeobox A2	27.9	27.8	27.86
FGF22	fibroblast growth factor 22	33.8	21.9	27.86
LAD1	ladinin 1	26.2	29.5	27.85
LOC104909490	uncharacterized LOC104909490	28.7	27.0	27.83
ANKRD33	ankyrin repeat domain 33	23.7	32.0	27.83
LOC104910204	inversin-like	26.2	29.5	27.83
LOC104916919	uncharacterized LOC104916919	33.8	21.8	27.80
FIGN	fidgetin	26.2	29.4	27.80
LOC100548226	protein-methionine sulfoxide oxidase MICAL3	34.6	21.0	27.80
LOC104913071	protein BANP-like	26.2	29.4	27.77
LOC104913035	uncharacterized LOC104913035	33.8	21.7	27.76
LOC104913535	uncharacterized LOC104913535	32.9	22.5	27.73
LOC100549361	uncharacterized LOC100549361	22.0	33.5	27.73
STYK1	serine/threonine/tyrosine kinase 1	30.4	25.0	27.72
CRYM	crystallin, mu	31.2	24.2	27.69
LOC104911987	uncharacterized LOC104911987	28.7	26.6	27.63
LOC104912374	protein crumbs homolog 1-like	31.2	24.0	27.63
LOC104916943	uncharacterized LOC104916943	27.9	27.4	27.63
LOC104913880	peripheral-type benzodiazepine receptor-associated protein 1-like	19.4	35.8	27.62
SLC30A2	solute carrier family 30 (zinc transporter), member 2	29.6	25.7	27.62
SCN2B	sodium channel, voltage-gated, type II, beta subunit	38.9	16.4	27.61
GDNF	glial cell derived neurotrophic factor	27.0	28.2	27.61
IKZF2	IKAROS family zinc finger 2 (Helios)	27.9	27.4	27.61
LOC104913735	uncharacterized LOC104913735	26.2	29.0	27.58
LOC104913894	S-adenosyl-L-methionine-dependent tRNA 4-demethylwyosine synthase-like	27.9	27.3	27.57
LOC104911240	uncharacterized LOC104911240	31.2	23.9	27.57
LOC104914986	uncharacterized LOC104914986	28.7	26.4	27.51
RIMS1	regulating synaptic membrane exocytosis 1	26.2	28.8	27.50
ST8SIA5	ST8 alpha-N-acetyl-neuraminide alpha-2,8-sialyltransferase 5	24.5	30.4	27.45
ZBTB7C	zinc finger and BTB domain containing 7C	27.0	27.8	27.44
LOC104912451	uncharacterized LOC104912451	27.9	27.0	27.42
LOC104916791	uncharacterized LOC104916791	35.5	19.3	27.42
LOC104914690	polypyrimidine tract-binding protein 1-like	27.9	27.0	27.41
LOC104911174	ryanodine receptor 3-like	26.2	28.6	27.39
LOC100538504	gasdermin-A2-like	35.4	19.3	27.39
DMP1	dentin matrix acidic phosphoprotein 1	30.4	24.3	27.35
LOC104911600	WASH complex subunit CCDC53-like	23.7	31.0	27.35
LOC104917247	extensin-like	32.1	22.5	27.33
LOC104911789	uncharacterized LOC104911789	22.8	31.8	27.33
CENPA	centromere protein A	30.4	24.2	27.31
G6PC	glucose-6-phosphatase, catalytic subunit	30.4	24.2	27.27
SLC22A5	solute carrier family 22 (organic cation/carnitine transporter), member 5	24.5	30.0	27.27
NKX6-3	NK6 homeobox 3	30.4	24.1	27.26
LOC104913569	T-box-containing protein TBX6L-like	29.5	25.0	27.25
METTL7A	methyltransferase like 7A	27.9	26.5	27.20
LOC104916401	E3 ubiquitin-protein ligase RNF103 pseudogene	26.2	28.2	27.20
MGAT5	mannosyl (alpha-1,6-)-glycoprotein beta-1,6-N-acetyl-glucosaminyltransferase	21.1	33.2	27.18

TMOD2	tropomodulin 2 (neuronal)	26.2	28.1	27.15
SPINK2	serine peptidase inhibitor, Kazal type 2 (acrosin-trypsin inhibitor)	27.9	26.4	27.15
LOC104914574	periodic tryptophan protein 2 homolog	25.3	28.9	27.15
LOC100549030	transmembrane protein 136-like	24.5	29.7	27.14
HORMAD2	HORMA domain containing 2	28.7	25.6	27.13
LOC104912549	uncharacterized LOC104912549	25.3	28.9	27.13
LOC100543707	N-alpha-acetyltransferase 16, NatA auxiliary subunit-like	16.9	37.3	27.12
LOC104915156	probable ATP-dependent RNA helicase YTHDC2	20.3	33.9	27.09
UGCG	UDP-glucose ceramide glucosyltransferase	25.4	28.8	27.07
LUM	lumican	24.5	29.6	27.06
LOC104912389	uncharacterized LOC104912389	25.4	28.7	27.03
GALNT5	polypeptide N-acetylgalactosaminyltransferase 5	23.7	30.3	27.00
LOC104915858	leucine-rich repeat-containing protein 51-like	24.5	29.5	27.00
LOC100540368	serine-protein kinase ATM	24.5	29.5	26.98
SOCS2	suppressor of cytokine signaling 2	33.8	20.2	26.96
IBSP	integrin-binding sialoprotein	24.5	29.4	26.95
MAP3K9	mitogen-activated protein kinase kinase kinase 9	32.1	21.8	26.94
ATP12A	ATPase, H+/K+ transporting, nongastric, alpha polypeptide	22.0	31.9	26.92
LOC104912866	uncharacterized LOC104912866	31.2	22.6	26.91
LOC104915037	centlein-like	32.9	20.9	26.90
C1H2orf40	chromosome 1 open reading frame, human C2orf40	27.9	25.8	26.86
LOC104914148	uncharacterized LOC104914148	32.9	20.8	26.86
TRPS1	trichorhinophalangeal syndrome I	27.0	26.6	26.80
TTC12	tetratricopeptide repeat domain 12	26.2	27.4	26.79
LOC104912127	lysine-specific demethylase 5A-like	31.3	22.3	26.78
RGL1	ral guanine nucleotide dissociation stimulator-like 1	27.1	26.5	26.77
TRHDE	thyrotropin-releasing hormone degrading enzyme	28.7	24.8	26.77
LOC104916402	SWI/SNF complex subunit SMARCC2-like	28.7	24.8	26.76
EGFL7	EGF-like-domain, multiple 7	30.4	23.1	26.76
LOC104915244	uncharacterized LOC104915244	30.4	23.1	26.75
VSNL1	visinin-like 1	27.0	26.5	26.75
SLC25A34	solute carrier family 25, member 34	25.3	28.1	26.71
SLC16A4	solute carrier family 16, member 4	25.4	28.0	26.69
MAVS	mitochondrial antiviral signaling protein	25.4	28.0	26.66
LOC104912632	WD repeat and FYVE domain-containing protein 1-like	23.7	29.6	26.62
LOC104911356	uncharacterized LOC104911356	25.3	27.9	26.62
LOC100541754	lateral signaling target protein 2 homolog	23.7	29.5	26.58
LOC104913623	myelin-oligodendrocyte glycoprotein-like	23.7	29.4	26.54
ASIC1	acid-sensing (proton-gated) ion channel 1	30.4	22.6	26.50
ELK4	ELK4, ETS-domain protein (SRF accessory protein 1)	31.2	21.8	26.50
DHRS12	dehydrogenase/reductase (SDR family) member 12	29.5	23.4	26.49
LOC104916421	zinc finger and BTB domain-containing protein 12-like	30.4	22.4	26.42
LOC104909682	uncharacterized LOC104909682	28.7	24.1	26.42
TASP1	taspase, threonine aspartase, 1	28.7	24.1	26.41
HOMER1	homer homolog 1 (Drosophila)	29.6	23.2	26.40
LOC104915303	structural maintenance of chromosomes protein 2-like	28.7	24.0	26.38
LOC104913549	protein SFI1 homolog	27.9	24.8	26.34
LOC104916101	uncharacterized LOC104916101	29.6	23.1	26.33
LOC104911751	integral membrane protein GPR155-like	26.2	26.4	26.32
GNG4	guanine nucleotide binding protein (G protein), gamma 4	25.3	27.3	26.30
TRDN	triadin	27.0	25.6	26.29
LOC104911785	uncharacterized LOC104911785	23.7	28.9	26.27
LOC104913049	uncharacterized LOC104913049	25.3	27.2	26.26
LOC104913098	E3 ubiquitin-protein ligase RNF123-like	26.2	26.4	26.26
FOXN3	forkhead box N3	26.2	26.3	26.24
LOC104912992	E3 ubiquitin-protein ligase RFWD3-like	22.8	29.6	26.23
LOC100544165	rap1 GTPase-activating protein 1	25.3	27.0	26.18
SPATA18	spermatogenesis associated 18	24.5	27.9	26.18

VWA2	von Willebrand factor A domain containing 2	32.1	20.2	26.15
STRIP2	striatin interacting protein 2	21.1	31.1	26.10
LOC104911504	serine/threonine-protein kinase B-raf-like	22.0	30.2	26.09
GABRR1	gamma-aminobutyric acid (GABA) A receptor, rho 1	28.7	23.4	26.07
LOC104916280	glioma tumor suppressor candidate region gene 1 protein-like	30.4	21.7	26.07
PLA2G15	phospholipase A2, group XV	21.1	31.0	26.06
LOC104917408	cadherin EGF LAG seven-pass G-type receptor 1-like	20.3	31.8	26.05
MAP3K6	mitogen-activated protein kinase kinase kinase 6	28.7	23.4	26.04
FAM131B	family with sequence similarity 131, member B	27.9	24.2	26.03
GLYATL3	glycine-N-acyltransferase-like 3	27.9	24.2	26.03
NTN1	netrin 1	25.4	26.6	25.98
LOC100548228	connector enhancer of kinase suppressor of ras 2	27.8	24.1	25.98
IRS2	insulin receptor substrate 2	27.8	24.1	25.98
LOC104913462	WAP, Kazal, immunoglobulin, Kunitz and NTR domain-containing protein 1-like	36.3	15.6	25.95
LOC104911409	probable acyl-CoA dehydrogenase 6	27.9	24.0	25.94
IL18R1	interleukin 18 receptor 1	24.5	27.4	25.94
LOC104912650	serine/threonine-protein phosphatase 2A regulatory subunit B'' subunit delta-like	25.4	26.5	25.93
OTOA	otoancorin	26.2	25.6	25.93
CTTNBP2	cortactin binding protein 2	27.0	24.8	25.91
ELMSAN1	ELM2 and Myb/SANT-like domain containing 1	25.4	26.4	25.87
STX17	syntaxin 17	22.8	28.9	25.86
CCNE2	cyclin E2	33.0	18.8	25.86
LOC104912886	uncharacterized LOC104912886	24.5	27.2	25.86
LOC104911410	uncharacterized LOC104911410	24.5	27.2	25.82
ADAMTSL5	ADAMTS-like 5	25.3	26.3	25.82
HSPB3	heat shock 27kDa protein 3	24.5	27.1	25.80
LOC100538867	putative protocadherin beta-18	22.0	29.6	25.80
LOC104910284	guanine nucleotide-binding protein G(olf) subunit alpha-like	22.8	28.8	25.77
LOC104916200	runt-related transcription factor 1-like	22.8	28.8	25.77
SLC17A9	solute carrier family 17 (vesicular nucleotide transporter), member 9	22.0	29.5	25.74
VGLL2	vestigial-like family member 2	20.3	31.1	25.71
LOC100544156	arylacetamide deacetylase-like 4	20.3	31.1	25.70
LOC104910201	ankyrin repeat and SAM domain-containing protein 6-like	29.5	21.8	25.68
STON1	stonin 1	30.4	20.9	25.68
LOC104915208	DNA polymerase alpha catalytic subunit-like	28.7	22.6	25.67
LOC104910525	uncharacterized LOC104910525	28.7	22.6	25.66
ITGB8	integrin, beta 8	30.4	20.9	25.63
LOC104911702	dipeptidyl peptidase 4-like	25.3	25.8	25.57
APOC3	apolipoprotein C-III	22.8	28.2	25.51
LOC104912851	uncharacterized LOC104912851	25.3	25.7	25.51
LOC100550127	receptor-type tyrosine-protein phosphatase mu	26.2	24.8	25.50
LOC100540273	nudC domain-containing protein 1-like	26.2	24.8	25.49
PRRX2	paired related homeobox 2	24.5	26.5	25.49
LOC100542634	probable G-protein coupled receptor 158	25.3	25.6	25.48
LOC104917426	tripeptidyl-peptidase 2-like	27.9	23.0	25.46
ARID3A	AT rich interactive domain 3A (BRIGHT-like)	22.8	28.1	25.44
B3GALT5	UDP-Gal:betaGlcNAc beta 1,3-galactosyltransferase, polypeptide 5	24.5	26.3	25.43
LOC104911392	6-methylsalicylic acid synthase-like	32.1	18.7	25.42
LOC104912979	uncharacterized oxidoreductase YKL071W-like	24.5	26.3	25.42
C16H16orf96	chromosome 16 open reading frame, human C16orf96	31.3	19.6	25.41
LOC100548608	arylamine N-acetyltransferase, pineal gland isozyme NAT-3	22.0	28.8	25.37
CATIP	ciliogenesis associated TTC17 interacting protein	22.8	27.9	25.37
LOC104911547	uncharacterized LOC104911547	24.5	26.2	25.36
LRRC70	leucine rich repeat containing 70	22.8	27.9	25.35
TWIST2	twist family bHLH transcription factor 2	22.8	27.9	25.33

LOC104910616	uncharacterized LOC104910616	30.4	20.2	25.32
LRRC72	leucine rich repeat containing 72	19.4	31.2	25.32
NEIL3	nei endonuclease VIII-like 3 (E. coli)	22.8	27.8	25.31
LOC104909797	uncharacterized LOC104909797	20.3	30.3	25.31
RFK	riboflavin kinase	22.0	28.6	25.28
LOC100544007	AP-4 complex subunit epsilon-1-like	28.7	21.8	25.26
PTGR1	prostaglandin reductase 1	28.7	21.8	25.26
LOC104917456	uncharacterized LOC104917456	28.7	21.8	25.24
LOC104914028	collagen alpha-1(XX) chain-like	24.5	25.9	25.22
LOC104912696	uncharacterized LOC104912696	25.4	25.0	25.20
LOC104917505	uncharacterized LOC104917505	16.9	33.5	25.18
LOC104912368	collagen alpha-1(XI) chain-like	18.6	31.7	25.17
CSRNP3	cysteine-serine-rich nuclear protein 3	26.1	24.1	25.13
GALNT10	polypeptide N-acetylgalactosaminyltransferase 10	27.9	22.4	25.13
ANKS1A	ankyrin repeat and sterile alpha motif domain containing 1A	27.9	22.4	25.12
LOC104911826	uncharacterized LOC104911826	25.3	24.9	25.12
LOC104909883	activating signal cointegrator 1 complex subunit 3-like	25.3	24.9	25.11
LOC104909564	son of sevenless homolog 1-like	26.2	24.0	25.11
MPND	MPN domain containing	25.3	24.9	25.11
PCDH20	protocadherin 20	25.3	24.9	25.11
LOC104915221	uncharacterized LOC104915221	25.3	24.8	25.06
LOC100538963	potassium/sodium hyperpolarization-activated cyclic nucleotide-gated channel 4-like	32.9	17.1	25.04
LOC100541562	putative P2Y purinoceptor 10	22.0	28.1	25.03
TSPAN15	tetraspanin 15	22.0	28.1	25.02
LOC100548805	myosin X-like	22.0	28.1	25.01
LOC100549353	activating signal cointegrator 1 complex subunit 3	24.5	25.5	25.01
LOC100543599	nucleolar pre-ribosomal-associated protein 1	24.5	25.5	25.00
LOC104915118	aminopeptidase O-like	22.0	28.0	24.99
ZBTB34	zinc finger and BTB domain containing 34	31.3	18.7	24.98
VWA3A	von Willebrand factor A domain containing 3A	28.7	21.2	24.97
LOC100543441	histone H2B 5	19.5	30.4	24.94
APLN	apelin	21.1	28.8	24.94
LOC104911808	tyrosine-protein phosphatase non-receptor type 4-like	22.8	27.0	24.93
AQP11	aquaporin 11	21.9	27.9	24.92
STRBP	spermatid perinuclear RNA binding protein	30.4	19.4	24.88
LOC104914976	putative ankyrin repeat domain-containing protein 31	23.6	26.1	24.87
LOC104909693	uncharacterized LOC104909693	21.2	28.6	24.87
PLEKHA5	pleckstrin homology domain containing, family A member 5	22.0	27.8	24.87
LOC100549851	eukaryotic translation initiation factor 4 gamma 3-like	28.7	21.0	24.87
LOC104916558	patched domain-containing protein 3-like	39.6	10.1	24.85
LOC104913016	uncharacterized LOC104913016	30.4	19.3	24.85
LOC104912078	aldose reductase-related protein 1-like	28.7	21.0	24.84
MCF2L	MCF.2 cell line derived transforming sequence-like	21.1	28.6	24.82
LURAP1L	leucine rich adaptor protein 1-like	28.7	21.0	24.82
CCDC148	coiled-coil domain containing 148	27.0	22.6	24.81
LOC104914128	uncharacterized LOC104914128	20.3	29.3	24.80
ATXN7L1	ataxin 7-like 1	16.9	32.7	24.80
FAM19A3	family with sequence similarity 19 (chemokine (C-C motif)-like), member A3	16.9	32.7	24.79
LOC104913803	centrosomal protein of 112 kDa-like	25.3	24.2	24.76
TNIP2	TNFAIP3 interacting protein 2	25.3	24.2	24.74
LOC104916651	ATP-dependent DNA helicase Q4-like	27.9	21.6	24.73
LOC100547451	DNA excision repair protein ERCC-6-like 2	25.3	24.1	24.73
KIAA2018	KIAA2018 ortholog	25.4	24.1	24.72
LOC104913360	uncharacterized LOC104913360	25.3	24.1	24.71
LOC100539130	lysozyme g	22.8	26.6	24.71
TCF7	transcription factor 7 (T-cell specific, HMG-box)	24.5	24.8	24.67

LOC100541835	uncharacterized LOC100541835	25.4	24.0	24.66
ARHGAP6	Rho GTPase activating protein 6	22.0	27.4	24.65
LOC104910950	ras-related protein Rab-15-like	20.3	29.0	24.63
PRKDC	protein kinase, DNA-activated, catalytic polypeptide	33.7	15.5	24.62
ST3GAL1	ST3 beta-galactoside alpha-2,3-sialyltransferase 1	22.0	27.3	24.62
LOC104910737	uncharacterized LOC104910737	20.3	28.8	24.53
LOC104912909	protein BEAN1-like	29.5	19.5	24.52
POLD3	polymerase (DNA-directed), delta 3, accessory subunit	21.1	27.9	24.49
LOC104915397	histone H1.11R	20.3	28.6	24.47
EPB41L4B	erythrocyte membrane protein band 4.1 like 4B	16.0	32.7	24.38
LOC100542367	BTB/POZ domain-containing protein KCTD1-like	26.2	22.6	24.36
LENG8	leukocyte receptor cluster (LRC) member 8	28.7	20.0	24.36
LOC104910705	uncharacterized LOC104910705	22.8	25.9	24.33
NRROS	negative regulator of reactive oxygen species	25.4	23.3	24.32
LOC104913449	radial spoke head 10 homolog B-like	25.4	23.3	24.32
LOC104912246	uncharacterized LOC104912246	22.8	25.8	24.30
BCHE	butyrylcholinesterase	27.0	21.5	24.28
LOC100547274	protein FAM13C-like	22.8	25.8	24.27
LOC104915788	protein phosphatase 1 regulatory subunit 37-like	25.4	23.2	24.26
LOC104909895	uncharacterized LOC104909895	20.3	28.2	24.25
PLXDC1	plexin domain containing 1	23.7	24.8	24.23
FAM126B	family with sequence similarity 126, member B	22.0	26.4	24.20
GALNT3	polypeptide N-acetylgalactosaminyltransferase 3	28.7	19.7	24.20
LOC100550268	serine/threonine-protein kinase B-raf-like	24.5	23.9	24.18
LOC104916118	uncharacterized LOC104916118	29.6	18.8	24.17
LOC104914697	transcription factor E2-alpha-like	31.3	17.1	24.15
LOC104911902	catenin alpha-3-like	23.6	24.6	24.14
LOC104914806	talin-1-like	23.7	24.6	24.14
EDNRA	endothelin receptor type A	22.0	26.3	24.13
RPL3L	ribosomal protein L3-like	28.7	19.4	24.07
LOC104909736	uncharacterized LOC104909736	26.2	21.9	24.06
LOC104916952	gap junction delta-2 protein-like	27.9	20.2	24.05
LOC104912514	protein FAM73A-like	27.9	20.2	24.04
LOC104909559	serine/threonine-protein kinase MRCK alpha-like	16.9	31.1	24.00
ACVR2B	activin A receptor, type IIB	24.5	23.5	23.99
NEDD4	neural precursor cell expressed, developmentally down-regulated 4, E3 ubiquitin protein ligase	26.2	21.8	23.98
LOC104914454	importin-9-like	27.9	20.1	23.98
KLHL23	kelch-like family member 23	25.3	22.6	23.95
LOC104915076	sarcoplasmic reticulum histidine-rich calcium-binding protein-like	27.0	20.8	23.94
NOXA1	NADPH oxidase activator 1	24.5	23.4	23.93
VTN	vitronectin	22.0	25.8	23.90
PTGDR	prostaglandin D2 receptor (DP)	25.3	22.4	23.89
LOC104916893	uncharacterized LOC104916893	22.8	25.0	23.88
UPB1	ureidopropionase, beta	25.3	22.4	23.87
SH2D5	SH2 domain containing 5	22.8	24.9	23.86
SULT6B1	sulfotransferase family, cytosolic, 6B, member 1	22.8	24.9	23.86
LOC104914782	protein unc-13 homolog B-like	22.8	24.9	23.85
LOC104913045	WW domain-containing oxidoreductase-like	22.0	25.7	23.84
ESR2	estrogen receptor 2 (ER beta)	22.8	24.8	23.81
RGS11	regulator of G-protein signaling 11	22.0	25.6	23.80
PTCD2	pentatricopeptide repeat domain 2	18.6	29.0	23.80
LOC100545133	receptor-type tyrosine-protein phosphatase T	23.7	23.9	23.80
LOC104910668	protein transport protein Sec24B-like	22.8	24.8	23.79
LOC104909256	N-alpha-acetyltransferase 16, NatA auxiliary subunit-like	24.5	23.0	23.78
LOC104915216	protein Hook homolog 3-like	22.0	25.6	23.76
LOC104912015	ATP-dependent DNA helicase Q1-like	27.9	19.6	23.73
C1H11orf70	chromosome 1 open reading frame, human C11orf70	19.4	28.0	23.73

LOC100549374	zinc finger protein 423-like	29.6	17.9	23.71
LOC104915661	fibronectin-like	28.7	18.7	23.70
BAIAP2L2	BAI1-associated protein 2-like 2	25.3	21.9	23.62
TMPPE	transmembrane protein with metallophosphoesterase domain	19.4	27.8	23.61
LOC104912978	enhancer of mRNA-decapping protein 4-like	27.9	19.3	23.60
LOC104913778	ATP-dependent DNA helicase Q5-like	27.0	20.1	23.58
LOC100549016	coagulation factor IX-like	26.2	20.9	23.55
EML5	echinoderm microtubule associated protein like 5	24.5	22.6	23.53
LOC104914326	brain-specific angiogenesis inhibitor 2-like	23.7	23.4	23.52
PELI2	pellino E3 ubiquitin protein ligase family member 2	24.5	22.5	23.50
LOC104911159	uncharacterized LOC104911159	24.5	22.5	23.49
LOC104911916	ankyrin-3-like	25.3	21.6	23.49
LOC104913838	uncharacterized LOC104913838	22.8	24.2	23.48
CDK14	cyclin-dependent kinase 14	32.9	14.0	23.46
CLRN2	clarin 2	20.3	26.6	23.45
LOC104916418	U8 snoRNA-decapping enzyme-like	32.1	14.8	23.44
LOC104910594	uncharacterized LOC104910594	22.0	24.9	23.42
LOC104917463	uncharacterized LOC104917463	22.8	24.0	23.41
SLC9A5	solute carrier family 9, subfamily A (NHE5, cation proton antiporter 5), member 5	22.8	24.0	23.39
TBXA2R	thromboxane A2 receptor	30.4	16.4	23.38
FZD8	frizzled class receptor 8	28.7	18.0	23.34
TOM1L1	target of myb1 (chicken)-like 1	21.2	25.4	23.29
LOC104911086	cytosolic phospholipase A2 beta-like	28.7	17.9	23.29
LOC104915544	uncharacterized LOC104915544	16.0	30.5	23.27
LOC104912257	retinal-specific ATP-binding cassette transporter-like	17.7	28.8	23.26
LOC104915838	solute carrier family 2, facilitated glucose transporter member 4-like	26.2	20.3	23.24
LOC100540589	fatty acyl-CoA reductase 1	21.1	25.4	23.23
LOC104910872	probable tRNA (uracil-O(2)-)-methyltransferase	16.0	30.4	23.23
LOC104916853	death-associated protein kinase 1-like	28.7	17.7	23.22
LOC104914241	phosphatidylinositol 4,5-bisphosphate 3-kinase catalytic subunit gamma isoform-like	18.6	27.8	23.22
LOC104916474	uncharacterized LOC104916474	26.2	20.2	23.21
LOC100543589	60S ribosomal protein L17	27.9	18.5	23.21
LOC104912377	parafibromin-like	27.0	19.4	23.19
LOC104912417	tyrosine-protein kinase JAK1-like	18.6	27.8	23.19
LOC100543947	uncharacterized LOC100543947	24.5	21.9	23.18
RAC2	ras-related C3 botulinum toxin substrate 2 (rho family, small GTP binding protein Rac2)	24.5	21.8	23.17
LEPR	leptin receptor	26.2	20.1	23.17
LOC104915977	uncharacterized LOC104915977	27.0	19.3	23.15
LOC100550836	glutamate carboxypeptidase 2-like	27.0	19.2	23.13
LOC104916472	adenylate cyclase type 6-like	25.4	20.9	23.13
GLUL	glutamate-ammonia ligase	25.3	20.9	23.10
LOC104909433	uncharacterized LOC104909433	22.8	23.4	23.09
LOC104911798	uncharacterized LOC104911798	23.7	22.5	23.08
LOC104910299	uncharacterized LOC104910299	13.5	32.5	23.03
LOC104913872	putative polypeptide N-acetylgalactosaminyltransferase-like protein 3	20.3	25.8	23.02
GABRD	gamma-aminobutyric acid (GABA) A receptor, delta	22.8	23.2	23.01
LOC104909776	rho GTPase-activating protein 18-like	21.1	24.9	23.00
NACC1	nucleus accumbens associated 1, BEN and BTB (POZ) domain containing	22.0	24.0	23.00
LOC104910553	uncharacterized LOC104910553	21.9	24.0	22.97
DCTD	dCMP deaminase	21.1	24.8	22.96
LOC100548663	disintegrin and metalloproteinase domain-containing protein 21-like	18.6	27.2	22.91

GPR182	G protein-coupled receptor 182	17.8	28.0	22.86
CYHR1	cysteine/histidine-rich 1	26.2	19.5	22.85
DMC1	DNA meiotic recombinase 1	16.9	28.8	22.84
LOC104910954	mitotic checkpoint serine/threonine-protein kinase BUB1 beta-like	16.1	29.6	22.83
LOC104913926	breast carcinoma-amplified sequence 3-like	16.0	29.6	22.83
LOC100549485	myosin-7-like	17.7	27.9	22.82
RFFL	ring finger and FYVE-like domain containing E3 ubiquitin protein ligase	18.6	27.1	22.82
LOC104911918	ankyrin-3-like	28.7	16.9	22.82
SOGA3	SOGA family member 3	27.0	18.6	22.81
LOC104912308	uncharacterized LOC104912308	24.5	21.1	22.81
LOC104915078	uncharacterized LOC104915078	27.0	18.6	22.78
LOC104910302	uncharacterized LOC104910302	24.5	21.0	22.77
LOC104913601	uncharacterized LOC104913601	24.5	21.0	22.73
LOC104912247	uncharacterized LOC104912247	26.2	19.3	22.73
BEND4	BEN domain containing 4	27.0	18.5	22.72
ASPHD2	aspartate beta-hydroxylase domain containing 2	29.6	15.8	22.67
LOC104911513	serum response factor homolog A-like	23.6	21.7	22.66
NCF1	neutrophil cytosolic factor 1	32.0	13.2	22.63
LOC104912900	ras GTPase-activating-like protein IQGAP1	21.1	24.1	22.61
CHRNB4	cholinergic receptor, nicotinic, beta 4 (neuronal)	24.5	20.7	22.60
SYNDIG1L	synapse differentiation inducing 1-like	19.4	25.8	22.60
GPR124	G protein-coupled receptor 124	22.0	23.2	22.60
LOC104911747	uncharacterized LOC104911747	17.7	27.5	22.60
LOC104917487	SRSF protein kinase 2-like	22.0	23.2	22.58
LOC100542589	annexin A8-like	20.3	24.9	22.57
LOC104917183	uncharacterized LOC104917183	20.3	24.9	22.57
LSAMP	limbic system-associated membrane protein	19.4	25.6	22.54
LOC104915867	calcium/calmodulin-dependent protein kinase type II subunit beta-like	18.6	26.5	22.53
TMEM67	transmembrane protein 67	19.4	25.5	22.47
LOC104910654	uncharacterized LOC104910654	25.4	19.6	22.46
PDE6B	phosphodiesterase 6B, cGMP-specific, rod, beta	26.2	18.7	22.45
LOC104911161	formin	19.4	25.4	22.44
LOC100549479	ephrin type-A receptor 4-like	25.4	19.5	22.43
LOC104909284	protein fantom-like	26.2	18.7	22.43
ITIH5	inter-alpha-trypsin inhibitor heavy chain family, member 5	25.4	19.4	22.38
LOC104910966	ras-related protein Rab-15-like	23.7	21.1	22.37
LOC104912941	uncharacterized LOC104912941	24.5	20.2	22.36
SLC35D2	solute carrier family 35 (UDP-GlcNAc/UDP-glucose transporter), member D2	19.4	25.2	22.33
LOC104911845	DNA annealing helicase and endonuclease ZRANB3-like	24.5	20.2	22.33
FAM181A	family with sequence similarity 181, member A	22.8	21.8	22.32
LOC104910777	uncharacterized LOC104910777	23.7	21.0	22.31
LOC104916372	constitutive coactivator of PPAR-gamma-like protein 1	25.4	19.2	22.30
LOC100544896	myosin-binding protein C, slow-type-like	20.3	24.3	22.30
LOC104916394	CCAAT/enhancer-binding protein alpha-like	24.5	20.1	22.30
HPCA	hippocalcin	21.2	23.4	22.29
ZNF618	zinc finger protein 618	22.0	22.6	22.26
LOC104916165	uncharacterized LOC104916165	32.1	12.5	22.26
LOC104915054	focadhesin-like	23.7	20.8	22.23
RBP4	retinol binding protein 4, plasma	21.1	23.3	22.22
LOC104911468	cyclin-dependent kinase 14-like	22.8	21.6	22.21
MUC	mucin protein	20.3	24.1	22.21
LOC104910328	uncharacterized LOC104910328	20.3	24.1	22.19
LOC104914064	protein FAM65C-like	22.8	21.5	22.18
LOC100538944	dynamin-3-like	19.4	24.9	22.15

LOC104915899	sodium/potassium-transporting ATPase subunit alpha-2	31.2	13.1	22.15
F9	coagulation factor IX	20.3	24.0	22.14
LOC100551326	kazal-type serine protease inhibitor domain-containing protein 1-like	27.9	16.3	22.10
FAM83H	family with sequence similarity 83, member H	18.6	25.6	22.08
LOC104909821	protein FAM184A-like	25.3	18.8	22.06
ZC4H2	zinc finger, C4H2 domain containing	24.5	19.6	22.03
TTBK2	tau tubulin kinase 2	18.6	25.4	22.02
LOC104911691	rac GTPase-activating protein 1-like	24.5	19.5	22.00
MAPT	microtubule-associated protein tau	25.4	18.6	21.96
PPFIA2	protein tyrosine phosphatase, receptor type, f polypeptide (PTPRF), interacting protein (liprin), alpha 2	24.5	19.4	21.95
ERICH6	glutamate-rich 6	24.5	19.4	21.95
PI16	peptidase inhibitor 16	22.8	21.1	21.94
PREX2	phosphatidylinositol-3,4,5-trisphosphate-dependent Rac exchange factor 2	13.5	30.3	21.92
DLGAP2	discs, large (Drosophila) homolog-associated protein 2	22.0	21.8	21.88
LOC100546675	bactericidal permeability-increasing protein-like	18.6	25.2	21.87
UNC5C	unc-5 homolog C (C. elegans)	20.3	23.4	21.85
PDE2A	phosphodiesterase 2A, cGMP-stimulated	19.5	24.2	21.81
LOC100551127	transcription factor HES-5-like	20.3	23.4	21.81
LOC100544139	gamma-aminobutyric acid receptor subunit pi-like	20.3	23.3	21.80
ETV7	ets variant 7	19.4	24.2	21.78
LOC104913581	uncharacterized LOC104913581	19.5	24.0	21.75
NFIA	nuclear factor I/A	15.2	28.2	21.70
APOA4	apolipoprotein A-IV	16.0	27.3	21.66
LOC104912158	uncharacterized LOC104912158	19.4	23.9	21.64
CREB5	cAMP responsive element binding protein 5	24.5	18.7	21.62
LOC104916965	uncharacterized LOC104916965	25.3	17.9	21.61
LOC104910620	uncharacterized LOC104910620	24.5	18.6	21.59
LOC104913264	uncharacterized LOC104913264	23.6	19.5	21.56
LOC104917458	uncharacterized LOC104917458	22.8	20.3	21.56
LOC104914660	signal-transducing adaptor protein 2-like	30.4	12.6	21.50
LOC104910946	uncharacterized LOC104910946	13.5	29.5	21.50
LOC104911801	poly [ADP-ribose] polymerase 14-like	22.0	21.0	21.46
LOC100546398	DNA repair-scaffolding protein-like	21.9	21.0	21.46
ACR	acrosin	19.4	23.4	21.44
PCYT1B	phosphate cytidylyltransferase 1, choline, beta	22.8	20.0	21.43
LOC104915004	uncharacterized LOC104915004	20.3	22.6	21.43
BEST1	bestrophin 1	21.1	21.7	21.42
LOC104915187	uncharacterized LOC104915187	19.4	23.4	21.42
LOC104909869	protein MMS22-like	21.1	21.7	21.41
LOC104910768	WD repeat-containing protein 36-like	27.9	14.8	21.34
PML	promyelocytic leukemia	20.3	22.4	21.34
LOC104915444	uncharacterized LOC104915444	27.8	14.8	21.33
TMEFF1	transmembrane protein with EGF-like and two follistatin-like domains 1	18.6	24.1	21.32
LOC104912255	uncharacterized LOC104912255	27.9	14.8	21.31
LOC104910653	glutathione S-transferase C-terminal domain-containing protein-like	17.7	24.9	21.31
LOC104913617	uncharacterized LOC104913617	26.2	16.4	21.30
LOC104914186	uncharacterized LOC104914186	17.8	24.8	21.28
GFAP	glial fibrillary acidic protein	26.2	16.4	21.27
RBP2	retinol binding protein 2, cellular	26.2	16.3	21.27
LOC104912121	BCL-6 corepressor-like protein 1	26.2	16.3	21.25
LOC104912815	unconventional myosin-Ie-like	19.4	23.1	21.25
LOC100539729	group IIE secretory phospholipase A2-like	27.0	15.5	21.23
LOC104911759	non-homologous end-joining factor 1-like	24.5	17.9	21.21

ERI2	ERI1 exoribonuclease family member 2	20.3	22.2	21.21
LOC104917373	histone-lysine N-methyltransferase 2B-like	26.2	16.3	21.21
SERPINC1	serpin peptidase inhibitor, clade C (antithrombin), member 1	22.8	19.6	21.20
LOC100548822	schlafen family member 13-like	24.5	17.9	21.19
LOC104914493	uncharacterized LOC104914493	21.9	20.4	21.19
LRRC4C	leucine rich repeat containing 4C	16.1	26.3	21.18
LOC104910655	uncharacterized LOC104910655	16.0	26.3	21.18
LOC104912382	dynamin-3-like	22.0	20.4	21.17
CENPL	centromere protein L	22.8	19.5	21.16
MYLK	myosin light chain kinase	15.2	27.1	21.14
LOC104916850	uncharacterized LOC104916850	33.8	8.5	21.14
USP31	ubiquitin specific peptidase 31	23.7	18.6	21.14
LOC104914842	nipped-B-like protein	22.8	19.4	21.13
LOC100543147	cytochrome P450 2U1	19.4	22.8	21.10
LOC100549696	peroxidasin homolog	20.3	21.9	21.09
LOC100546988	SET-binding protein-like	22.8	19.3	21.08
LOC104911271	uncharacterized LOC104911271	21.9	20.2	21.07
FAM109A	family with sequence similarity 109, member A	18.6	23.6	21.07
PLCXD1	phosphatidylinositol-specific phospholipase C, X domain containing 1	22.8	19.3	21.07
TCFL5	transcription factor-like 5 (basic helix-loop-helix)	20.3	21.8	21.04
SEMA3B	sema domain, immunoglobulin domain (Ig), short basic domain, secreted, (semaphorin) 3B	20.3	21.7	21.00
PPP1R3C	protein phosphatase 1, regulatory subunit 3C	17.7	24.2	20.98
CCDC73	coiled-coil domain containing 73	16.9	25.0	20.96
FST	follistatin	17.7	24.1	20.93
LOC100548653	ligand of Numb protein X 2-like	16.0	25.8	20.93
TMEM59L	transmembrane protein 59-like	25.3	16.5	20.91
MGST2	microsomal glutathione S-transferase 2	27.8	14.0	20.91
SPATA17	spermatogenesis associated 17	19.4	22.4	20.90
LOC104916154	extensin-like	27.9	13.8	20.84
FRMPD2	FERM and PDZ domain containing 2	25.3	16.3	20.83
LOC104917621	exportin-4-like	25.4	16.3	20.83
LOC100543167	transcription initiation factor TFIID subunit 4-like	23.7	18.0	20.81
TMEM125	transmembrane protein 125	26.2	15.4	20.79
LOC104912442	uncharacterized LOC104912442	26.1	15.4	20.78
LOC104915106	patched domain-containing protein 1-like	22.0	19.6	20.77
LOC104911166	uncharacterized LOC104911166	25.4	16.2	20.77
LOC104916693	AT-hook-containing transcription factor-like	22.8	18.6	20.74
LOC104913402	mesothelin-like	23.7	17.8	20.73
TTC16	tetratricopeptide repeat domain 16	22.0	19.5	20.73
LOC104915706	aryl hydrocarbon receptor nuclear translocator-like protein 2	13.5	27.9	20.72
CELF1	CUGBP, Elav-like family member 1	16.9	24.5	20.71
GLP2R	glucagon-like peptide 2 receptor	21.1	20.2	20.66
KIF21B	kinesin family member 21B	15.2	26.1	20.66
LOC100542427	myotubularin-related protein 13-like	22.0	19.3	20.65
LOC104911561	uncharacterized LOC104911561	21.1	20.2	20.64
AMIGO3	adhesion molecule with Ig-like domain 3	23.7	17.6	20.63
HOXA11	homeobox A11	21.1	20.1	20.62
LOC100542428	mucin-5B	17.8	23.5	20.62
LOC104909777	uncharacterized LOC104909777	18.6	22.6	20.59
LOC104913339	protein Jade-2-like	20.3	20.9	20.59
MTMR8	myotubularin related protein 8	21.1	20.0	20.56
CAMK1D	calcium/calmodulin-dependent protein kinase ID	18.6	22.5	20.56
FGF23	fibroblast growth factor 23	17.7	23.4	20.55
C11H2orf82	chromosome 11 open reading frame, human C2orf82	18.6	22.5	20.54
SLC46A3	solute carrier family 46, member 3	17.7	23.3	20.53

LOC104911029	phosphatidylinositol 4-phosphate 3-kinase C2 domain-containing subunit alpha-like	17.8	23.2	20.50
LOC104912658	uncharacterized LOC104912658	14.4	26.6	20.49
CUBN	cubilin (intrinsic factor-cobalamin receptor)	16.9	24.0	20.48
LOC100545163	alcohol dehydrogenase 1	25.3	15.6	20.47
LOC104913075	dual specificity protein phosphatase 22-A-like	15.2	25.7	20.45
ACMSD	aminocarboxymuconate semialdehyde decarboxylase	24.5	16.4	20.43
SSX2IP	synovial sarcoma, X breakpoint 2 interacting protein	24.5	16.4	20.43
LOC104914568	serine/threonine-protein kinase tousled-like 2	25.4	15.5	20.42
RAP1GAP	RAP1 GTPase activating protein	22.0	18.8	20.40
OGN	osteoglycin	15.2	25.6	20.37
LOC104912924	uncharacterized LOC104912924	22.0	18.8	20.36
LOC104910129	ovalbumin-related protein X	21.1	19.6	20.35
LOC104917337	neuralized-like protein 2	23.6	17.1	20.35
LOC100546764	hypoxanthine-guanine phosphoribosyltransferase-like	23.6	17.0	20.32
THSD7A	thrombospondin, type I, domain containing 7A	20.3	20.3	20.29
LOC104916821	uncharacterized LOC104916821	21.1	19.4	20.28
HRH3	histamine receptor H3	21.2	19.4	20.28
LOC104911407	neurabin-1 pseudogene	22.8	17.7	20.28
LOC104913597	translational activator GCN1-like	19.4	21.1	20.27
LOC104913919	acetyl-CoA carboxylase-like	22.8	17.7	20.27
LOC104916074	transcription factor COE1-like	22.0	18.6	20.27
LOC104910809	uncharacterized LOC104910809	19.4	21.1	20.26
LOC104910761	uncharacterized LOC104910761	11.0	29.5	20.26
LOC100551463	MAP kinase-activating death domain protein-like	12.7	27.8	20.25
LOC104914788	protein unc-13 homolog B	22.0	18.5	20.23
SOWAHB	sosondowah ankyrin repeat domain family member B	19.4	21.0	20.23
LOC100547145	aminopeptidase O-like	20.3	20.2	20.23
LOC104913384	centriolar coiled-coil protein of 110 kDa-like	21.1	19.3	20.21
RASEF	RAS and EF-hand domain containing	27.0	13.4	20.20
DUOX2	dual oxidase 2	19.4	21.0	20.19
ABCA12	ATP-binding cassette, sub-family A (ABC1), member 12	18.6	21.8	20.18
LOC104915275	versican core protein-like	18.6	21.8	20.17
LOC100549632	paired box protein Pax-3	26.2	14.1	20.15
LOC100547680	actin-related protein 10	18.6	21.7	20.13
LOC100541297	ras-related and estrogen-regulated growth inhibitor-like	16.1	24.2	20.13
NRARP	NOTCH-regulated ankyrin repeat protein	16.9	23.4	20.12
TOX	thymocyte selection-associated high mobility group box	16.1	24.2	20.11
LOC100550530	N-acetylated-alpha-linked acidic dipeptidase 2	16.9	23.4	20.11
NXPH2	neurexophilin 2	25.4	14.8	20.08
MEI1	meiosis inhibitor 1	16.1	24.1	20.08
GUCA1B	guanylate cyclase activator 1B (retina)	26.2	13.9	20.06
LOC104911144	uncharacterized LOC104911144	17.8	22.4	20.06
CRYGS	crystallin, gamma S	17.7	22.4	20.04
C5H14orf79	chromosome 5 open reading frame, human C14orf79	22.8	17.2	20.03
LRRC4	leucine rich repeat containing 4	24.5	15.5	20.00
SEC16B	SEC16 homolog B (S. cerevisiae)	22.0	18.0	20.00
LOC100538510	heat shock factor 2-binding protein-like	15.2	24.8	19.99
RBP5	retinol binding protein 5, cellular	21.9	18.0	19.95
LOC104917040	36.4 kDa proline-rich protein-like	22.0	17.9	19.94
LOC104911940	uncharacterized LOC104911940	19.4	20.4	19.93
LOC104909986	uncharacterized LOC104909986	21.1	18.7	19.90
LOC100303695	serum paraoxonase/arylesterase 2	16.1	23.7	19.88
CERS3	ceramide synthase 3	21.1	18.6	19.87
KCNK13	potassium channel, subfamily K, member 13	18.6	21.1	19.85
PRDM8	PR domain containing 8	21.9	17.7	19.84
HTR6	5-hydroxytryptamine (serotonin) receptor 6, G protein-coupled	19.4	20.2	19.84
GREB1L	growth regulation by estrogen in breast cancer-like	18.6	21.0	19.82

LOC104916515	uncharacterized LOC104916515	20.2	19.4	19.81
CEP128	centrosomal protein 128kDa	17.7	21.9	19.80
LOC104911958	beta-microseminoprotein-like	18.6	21.0	19.77
LOC100548027	gamma-aminobutyric acid type B receptor subunit 2	18.6	21.0	19.77
CCDC181	coiled-coil domain containing 181	18.6	20.9	19.76
LOC104916972	RNA-binding protein 4 pseudogene	16.1	23.4	19.75
MASP1	mannan-binding lectin serine peptidase 1 (C4/C2 activating component of Ra-reactive factor)	18.6	20.8	19.72
GDPGP1	GDP-D-glucose phosphorylase 1	27.0	12.4	19.72
LOC104914119	heterogeneous nuclear ribonucleoprotein U-like protein 1	27.0	12.4	19.71
MYZAP	myocardial zonula adherens protein	27.0	12.3	19.68
SYCP1	synaptonemal complex protein 1	24.5	14.9	19.68
LOC104913366	4-aminobutyrate aminotransferase, mitochondrial-like	18.6	20.7	19.67
LOC104914411	uncharacterized LOC104914411	16.9	22.4	19.65
LOC104912309	uncharacterized LOC104912309	14.3	25.0	19.65
LOC104909554	tau-tubulin kinase 1-like	24.5	14.7	19.63
LOC104917540	uncharacterized LOC104917540	23.6	15.6	19.62
POLR3G	polymerase (RNA) III (DNA directed) polypeptide G (32kD)	15.2	24.0	19.62
STK38	serine/threonine kinase 38	22.8	16.4	19.61
LOC104910889	uncharacterized LOC104910889	23.6	15.6	19.61
LOC104913458	uncharacterized LOC104913458	22.8	16.4	19.59
LOC104914987	microtubule-associated protein 1B-like	22.0	17.2	19.56
LOC100542299	minor histocompatibility protein HA-1-like	15.2	23.9	19.56
C5H11orf74	chromosome 5 open reading frame, human C11orf74	22.0	17.1	19.53
PMFBP1	polyamine modulated factor 1 binding protein 1	20.2	18.8	19.53
LOC104912372	calmodulin-regulated spectrin-associated protein 2-like	14.4	24.7	19.52
BAAT	bile acid CoA:amino acid N-acyltransferase	21.1	17.9	19.51
ST8SIA6	ST8 alpha-N-acetyl-neuraminide alpha-2,8-sialyltransferase 6	20.3	18.7	19.50
LOC104914312	zinc finger MYM-type protein 4-like	22.8	16.2	19.48
CRHR2	corticotropin releasing hormone receptor 2	21.9	17.0	19.47
LOC100549855	serine palmitoyltransferase 1-like	19.4	19.4	19.44
LOC104911396	uncharacterized LOC104911396	22.0	16.9	19.43
LOC104912001	transcription factor SOX-5-like	20.3	18.5	19.40
CDR2L	cerebellar degeneration-related protein 2-like	16.9	21.9	19.39
SMOC2	SPARC related modular calcium binding 2	18.6	20.2	19.39
LOC104913088	zinc finger CCHC domain-containing protein 2-like	18.6	20.2	19.38
LOC104915070	transmembrane protein 2-like	19.4	19.3	19.36
LOC104916787	neutral and basic amino acid transport protein rBAT-like	19.5	19.2	19.35
SYT14	synaptotagmin XIV	16.9	21.8	19.35
KIAA1033	KIAA1033 ortholog	21.1	17.5	19.33
LOC104915995	uncharacterized LOC104915995	24.5	14.1	19.33
LOC104911047	uncharacterized LOC104911047	16.1	22.5	19.29
CCDC71L	coiled-coil domain containing 71-like	16.0	22.5	19.29
LOC104914280	uncharacterized LOC104914280	25.4	13.2	19.27
LOC104909904	uncharacterized LOC104909904	15.2	23.3	19.25
LOC100539391	ankyrin repeat domain-containing protein 42-like	24.5	14.0	19.25
CPSF1	cleavage and polyadenylation specific factor 1, 160kDa	25.4	13.1	19.24
LOC100541957	DENN domain-containing protein 5B-like	16.1	22.4	19.23
LOC104917020	6-phosphofructo-2-kinase/fructose-2,6-bisphosphatase-like	22.8	15.7	19.22
LOC104914710	X-linked retinitis pigmentosa GTPase regulator-like	15.2	23.2	19.20
UROC1	urocanate hydratase 1	22.8	15.6	19.18
LOC104909782	mediator of RNA polymerase II transcription subunit 23-like	21.1	17.1	19.13
LOC104916275	cytoplasmic tRNA 2-thiolation protein 1-like	22.8	15.4	19.12
LOC104911948	uncharacterized LOC104911948	20.3	17.9	19.07
LOC104916396	gap junction delta-2 protein-like	21.1	17.0	19.06
LOC104913842	myosin-7-like	22.0	16.1	19.06
PLCG2	phospholipase C, gamma 2 (phosphatidylinositol-specific)	18.6	19.5	19.06
CDO1	cysteine dioxygenase type 1	17.7	20.4	19.05

LOC104914218	dedicator of cytokinesis protein 5-like	17.7	20.4	19.05
LOC104913442	uncharacterized LOC104913442	11.0	27.1	19.04
CCDC42B	coiled-coil domain containing 42B	20.3	17.8	19.03
LOC104913693	putative UDP-GlcNAc:betaGal beta-1,3-N-acetylglucosaminyltransferase LOC100288842	18.6	19.4	19.01
LECT2	leukocyte cell-derived chemotaxin 2	25.3	12.7	18.99
GPAT2	glycerol-3-phosphate acyltransferase 2, mitochondrial	16.9	21.0	18.98
SMAD7	SMAD family member 7	16.9	21.0	18.98
C5H11orf49	chromosome 5 open reading frame, human C11orf49	16.9	21.0	18.95
LOC104912337	protein PRRC2C-like	18.6	19.3	18.94
LOC104911823	uncharacterized LOC104911823	16.0	21.8	18.94
LOC104916894	uncharacterized LOC104916894	16.9	21.0	18.94
LOC104911697	lymphocyte antigen 75-like	17.7	20.1	18.94
PIPOX	pipecolic acid oxidase	18.6	19.3	18.93
PRLH	prolactin releasing hormone	16.1	21.8	18.93
LOC104910909	dysferlin-like	14.4	23.4	18.87
LOC104912612	PERQ amino acid-rich with GYF domain-containing protein 2-like	18.6	19.1	18.85
LOC104916358	arf-GAP with coiled-coil, ANK repeat and PH domain-containing protein 1-like	26.1	11.5	18.84
LOC104913365	uncharacterized LOC104913365	26.1	11.5	18.84
LOC104916786	fibrinogen-like protein 1	16.9	20.8	18.83
LOC104916861	carnitine O-acetyltransferase-like	24.5	13.1	18.82
PARD6A	par-6 family cell polarity regulator alpha	22.0	15.7	18.81
LOC100550137	glycerol-3-phosphate dehydrogenase [NAD(+)], cytoplasmic-like	22.8	14.8	18.81
LOC100539746	protocadherin Fat 4-like	14.4	23.2	18.80
CNTN1	contactin 1	14.4	23.2	18.78
LOC100539821	2-hydroxyacylsphingosine 1-beta-galactosyltransferase-like	19.4	18.0	18.73
LOC104912167	uncharacterized LOC104912167	21.9	15.5	18.70
BDKRB1	bradykinin receptor B1	22.0	15.4	18.69
LOC100543020	myosin-7-like	21.1	16.3	18.68
UGGT2	UDP-glucose glycoprotein glucosyltransferase 2	20.3	17.1	18.68
MCMDC2	minichromosome maintenance domain containing 2	11.8	25.5	18.67
LHFPL4	lipoma HMGIC fusion partner-like 4	21.1	16.2	18.66
ASPA	aspartoacylase	19.4	17.9	18.65
LOC104913624	zinc finger protein RFP-like	18.6	18.7	18.65
LOC104912280	spindle assembly abnormal protein 6 homolog	17.7	19.6	18.64
LOC104913878	very large A-kinase anchor protein-like	17.7	19.5	18.63
LOC100548696	radial spoke head protein 6 homolog A-like	18.6	18.6	18.60
LOC100541789	transcription factor HIVEP3-like	18.6	18.6	18.59
RHCG	Rh family, C glycoprotein	16.1	21.0	18.55
MPP6	membrane protein, palmitoylated 6 (MAGUK p55 subfamily member 6)	17.7	19.4	18.55
LOC104909378	hephaestin-like protein 1	16.1	21.0	18.55
LOC104912101	uncharacterized LOC104912101	16.9	20.2	18.54
LOC100541803	vascular endothelial growth factor receptor 1	17.7	19.3	18.53
LOC100541423	centrosomal protein of 112 kDa	16.0	21.0	18.52
LOC104911650	N-acetylglucosamine-1-phosphotransferase subunits alpha/beta-like	18.6	18.5	18.51
PLXNC1	plexin C1	15.2	21.8	18.51
SLC35F3	solute carrier family 35, member F3	16.9	20.1	18.51
LOC104916079	solute carrier family 22 member 6-like	16.0	21.0	18.50
TP73	tumor protein p73	15.2	21.8	18.48
LOC104915164	adenomatous polyposis coli protein-like	16.1	20.9	18.47
LOC100541117	regulator of G-protein signaling 9	15.2	21.7	18.46
LOC100541270	PR domain zinc finger protein 2-like	16.0	20.8	18.44
LOC104914394	OTU domain-containing protein 7B-like	13.5	23.4	18.44
LOC100549945	uncharacterized LOC100549945	22.0	14.9	18.43
SLC7A4	solute carrier family 7, member 4	16.9	19.9	18.41

LOC104916425	potassium voltage-gated channel subfamily C member 3-like	24.5	12.3	18.41
LOC104913565	uncharacterized LOC104913565	14.4	22.4	18.39
LOC104909735	brefeldin A-inhibited guanine nucleotide-exchange protein 3-like	10.1	26.6	18.37
MYO1F	myosin IF	22.8	13.9	18.36
LOC104915846	arf-GAP with Rho-GAP domain, ANK repeat and PH domain-containing protein 1-like	20.3	16.4	18.35
LOC104912985	uncharacterized LOC104912985	22.8	13.9	18.35
LOC104912664	mucin-4-like	22.8	13.9	18.34
LOC104916848	glutamate receptor ionotropic, kainate 5-like	18.6	18.1	18.33
ESM1	endothelial cell-specific molecule 1	21.1	15.5	18.31
MTNR1A	melatonin receptor 1A	19.4	17.2	18.31
CDK15	cyclin-dependent kinase 15	18.6	18.0	18.29
SPTSSB	serine palmitoyltransferase, small subunit B	19.4	17.1	18.28
LOC104913290	uncharacterized LOC104913290	19.4	17.1	18.25
USP43	ubiquitin specific peptidase 43	21.1	15.3	18.24
LOC104909252	myosin-3-like	17.7	18.7	18.23
LOC104911887	ELKS/Rab6-interacting/CAST family member 1-like	17.8	18.7	18.22
CNTN3	contactin 3 (plasmacytoma associated)	15.2	21.2	18.20
IL13RA1	interleukin 13 receptor, alpha 1	18.6	17.8	18.20
LOC100542756	neuron-specific protein family member 2	17.7	18.6	18.16
SMPD3	sphingomyelin phosphodiesterase 3, neutral membrane (neutral sphingomyelinase II)	15.2	21.0	18.13
LOC100546489	tubby-related protein 4-like	16.1	20.2	18.12
LOC104910636	N(4)-(beta-N-acetylglucosaminyl)-L-asparaginase-like	16.1	20.2	18.11
TTC33	tetratricopeptide repeat domain 33	14.4	21.8	18.10
CEP192	centrosomal protein 192kDa	25.4	10.8	18.07
LOC100542248	protein-glutamine gamma-glutamyltransferase E-like	17.7	18.4	18.06
LOC104911351	phosphoribosyltransferase domain-containing protein 1-like	23.6	12.5	18.05
NCOA3	nuclear receptor coactivator 3	16.1	20.0	18.03
DHX32	DEAH (Asp-Glu-Ala-His) box polypeptide 32	22.7	13.2	17.98
PROCA1	protein interacting with cyclin A1	19.4	16.5	17.95
RAD54L	RAD54-like (S. cerevisiae)	22.8	13.1	17.95
MPP2	membrane protein, palmitoylated 2 (MAGUK p55 subfamily member 2)	21.1	14.8	17.94
FAM124B	family with sequence similarity 124B	20.3	15.6	17.94
GUCA1C	guanylate cyclase activator 1C	12.7	23.2	17.94
SERTM1	serine-rich and transmembrane domain containing 1	21.1	14.7	17.93
LGI3	leucine-rich repeat LGI family, member 3	22.8	13.0	17.92
GREM1	gremlin 1, DAN family BMP antagonist	12.7	23.2	17.91
CLCF1	cardiotrophin-like cytokine factor 1	19.5	16.3	17.89
TLR5	toll-like receptor 5	20.3	15.5	17.89
LOC104915457	uncharacterized LOC104915457	19.4	16.3	17.88
NTSR1	neurotensin receptor 1 (high affinity)	17.8	18.0	17.88
LOC104914197	uncharacterized LOC104914197	20.3	15.5	17.87
SYT1	synaptotagmin I	18.6	17.2	17.86
CCDC110	coiled-coil domain containing 110	19.4	16.3	17.86
MMD2	monocyte to macrophage differentiation-associated 2	18.6	17.1	17.86
LOC100546571	arf-GAP with dual PH domain-containing protein 1-like	19.4	16.3	17.85
LOC100548964	dual specificity testis-specific protein kinase 2	18.6	17.1	17.85
SLC29A3	solute carrier family 29 (equilibrative nucleoside transporter), member 3	20.3	15.4	17.84
CHD9	chromodomain helicase DNA binding protein 9	18.6	17.1	17.83
TCTEX1D1	Tctex1 domain containing 1	18.6	17.1	17.83
ASB9	ankyrin repeat and SOCS box containing 9	17.8	17.9	17.83
LOC104911781	N-chimaerin-like	15.2	20.4	17.82
LOC104911099	uncharacterized LOC104911099	17.7	17.9	17.81
LOC104911073	uncharacterized LOC104911073	17.8	17.8	17.79
LOC104917329	protein FAM53B-like	16.1	19.5	17.78

SPAG6	sperm associated antigen 6	17.7	17.8	17.78
LOC104909636	uncharacterized LOC104909636	16.9	18.6	17.76
IRF5	interferon regulatory factor 5	15.2	20.3	17.75
LOC104909471	uncharacterized LOC104909471	14.4	21.1	17.75
LOC104917101	keratin-associated protein 16-1	25.4	10.1	17.74
LOC104910782	uncharacterized LOC104910782	16.9	18.6	17.74
LOC104910350	centrosome and spindle pole-associated protein 1-like	16.9	18.6	17.73
SLC7A10	solute carrier family 7 (neutral amino acid transporter light chain, asc system), member 10	16.0	19.4	17.73
FAM228B	family with sequence similarity 228, member B	23.6	11.8	17.70
CCDC89	coiled-coil domain containing 89	12.7	22.7	17.69
LOC100549062	kalirin-like	14.4	21.0	17.68
LOC104915253	uncharacterized protein KIAA0825-like	14.4	21.0	17.68
LOC104915519	myelin-oligodendrocyte glycoprotein-like	22.8	12.5	17.64
LOC104912061	uncharacterized LOC104912061	22.8	12.5	17.64
LOC104910133	uncharacterized LOC104910133	24.4	10.8	17.63
LOC104911116	tetraspanin-18-like	14.4	20.8	17.61
POLA1	polymerase (DNA directed), alpha 1, catalytic subunit	24.5	10.7	17.60
LOC104910609	uncharacterized LOC104910609	15.2	20.0	17.59
DYNC2H1	dynein, cytoplasmic 2, heavy chain 1	21.1	14.0	17.57
LOC104913756	uncharacterized LOC104913756	19.4	15.7	17.56
IL1R2	interleukin 1 receptor, type II	12.7	22.4	17.55
ESRRB	estrogen-related receptor beta	18.6	16.5	17.54
DIO1	deiodinase, iodothyronine, type I	19.4	15.6	17.53
LOC100550702	solute carrier family 2, facilitated glucose transporter member 5-like	19.4	15.6	17.50
LOC104916702	uncharacterized LOC104916702	19.4	15.6	17.49
PMEL	premelanosome protein	19.4	15.5	17.49
LOC104915255	uncharacterized LOC104915255	19.4	15.5	17.48
AIM1	absent in melanoma 1	18.6	16.4	17.48
SUSD1	sushi domain containing 1	19.4	15.5	17.46
LOC104909751	AT-rich interactive domain-containing protein 1B-like	17.7	17.1	17.44
SGOL2	shugoshin-like 2 (S. pombe)	19.4	15.5	17.44
LOC104909346	gamma-secretase-activating protein-like	13.5	21.4	17.43
CMTM8	CKLF-like MARVEL transmembrane domain containing 8	17.7	17.1	17.41
MCPH1	microcephalin 1	16.0	18.8	17.40
HOOK1	hook microtubule-tethering protein 1	7.6	27.2	17.40
LOC104910850	DNA excision repair protein ERCC-6-like 2	15.2	19.6	17.39
CNTNAP1	contactin associated protein 1	16.9	17.9	17.38
LOC104909851	protein SCAF11-like	17.7	16.9	17.34
LOC104914932	protein LAP2-like	16.9	17.8	17.33
IQCG	IQ motif containing G	14.4	20.3	17.32
LOC104909410	uncharacterized LOC104909410	16.9	17.7	17.31
LOC104909601	uncharacterized LOC104909601	15.2	19.4	17.30
LOC100541825	disintegrin and metalloproteinase domain-containing protein 9-like	12.7	21.9	17.28
LOC104917512	ETS-related transcription factor Elf-1-like	16.1	18.5	17.27
LOC104910571	sodium channel and clathrin linker 1-like	15.2	19.3	17.26
LYPD1	LY6/PLAUR domain containing 1	13.5	21.0	17.23
LOC104914063	uncharacterized LOC104914063	13.5	20.9	17.21
KRT23	keratin 23 (histone deacetylase inducible)	19.4	14.9	17.18
LOC104914602	protein AF-17-like	19.4	14.9	17.18
RDH5	retinol dehydrogenase 5 (11-cis/9-cis)	21.1	13.3	17.18
LOC100546521	delta-1 crystallin	11.8	22.4	17.13
IGSF10	immunoglobulin superfamily, member 10	16.9	17.3	17.11
LOC104912896	inositol hexakisphosphate and diphosphoinositol-pentakisphosphate kinase 1-like	17.7	16.5	17.11
FBXO39	F-box protein 39	18.6	15.6	17.10

LOC100541044	lymphocyte antigen 6E-like	18.6	15.6	17.07
LOC104909456	uncharacterized LOC104909456	16.9	17.2	17.03
GPR126	G protein-coupled receptor 126	17.7	16.3	17.01
AQP9	aquaporin 9	18.6	15.4	17.00
LOC104909805	triadin-like	16.9	17.1	17.00
LOC104911846	DNA annealing helicase and endonuclease ZRANB3-like	16.0	17.9	16.98
PLCB2	phospholipase C, beta 2	15.2	18.7	16.96
TC2N	tandem C2 domains, nuclear	17.7	16.2	16.96
GNAQ	guanine nucleotide binding protein (G protein), q polypeptide	16.9	17.0	16.95
LOC104909292	EF-hand domain-containing family member C2-like	17.8	16.1	16.95
PIH1D3	PIH1 domain containing 3	13.5	20.4	16.94
LOC104909898	uncharacterized LOC104909898	15.2	18.6	16.92
LOC100544448	cytochrome P450 2C9-like	14.4	19.4	16.91
LOC104914818	PDZ domain-containing protein 2-like	13.5	20.3	16.90
SGPP2	sphingosine-1-phosphate phosphatase 2	13.5	20.2	16.89
C3H5orf49	chromosome 3 open reading frame, human C5orf49	16.0	17.7	16.88
LOC104916647	histone H2A-like	14.4	19.4	16.87
LOC104912501	tetratricopeptide repeat protein 39A-like	16.0	17.7	16.87
IQCE	IQ motif containing E	11.8	21.9	16.86
LOC100546503	integrin alpha-8	23.6	10.0	16.82
RERG	RAS-like, estrogen-regulated, growth inhibitor	22.0	11.7	16.81
LOC104909271	1,4-alpha-glucan-branching enzyme-like	12.7	21.0	16.81
LOC104910956	mitotic checkpoint serine/threonine-protein kinase BUB1 beta-like	22.0	11.6	16.80
LOC104911586	uncharacterized LOC104911586	10.1	23.4	16.79
LOC100544740	cGMP-dependent protein kinase 2-like	21.1	12.5	16.79
LOC100303670	hepatocyte growth factor activator	21.1	12.4	16.77
C1QTNF1	C1q and tumor necrosis factor related protein 1	19.4	14.1	16.77
LOC104911693	anion exchange protein 3-like	11.8	21.7	16.76
LOC104913229	ankyrin repeat and KH domain-containing protein 1-like	22.0	11.5	16.75
COL4A4	collagen, type IV, alpha 4	20.3	13.2	16.74
EFCAB12	EF-hand calcium binding domain 12	9.3	24.1	16.71
LOC104915235	uncharacterized LOC104915235	18.6	14.8	16.70
MMP17	matrix metallopeptidase 17 (membrane-inserted)	17.7	15.6	16.68
LOC104913598	RNA-binding protein Musashi homolog 1-like	18.6	14.7	16.67
SLC24A4	solute carrier family 24 (sodium/potassium/calcium exchanger), member 4	18.6	14.8	16.67
LOC104915189	chromodomain-helicase-DNA-binding protein 1-like	18.6	14.7	16.66
GABRP	gamma-aminobutyric acid (GABA) A receptor, pi	16.9	16.4	16.66
LOC104913124	acylamino-acid-releasing enzyme-like	15.2	18.1	16.65
LOC104910060	fibrocystin-like	16.1	17.2	16.62
GRAP2	GRB2-related adaptor protein 2	17.7	15.5	16.61
LOC104910565	uncharacterized LOC104910565	16.1	17.1	16.59
LOC104917179	zinc finger protein 501-like	16.9	16.3	16.59
LOC104909853	uncharacterized LOC104909853	16.0	17.1	16.58
LOC100545341	parvalbumin, thymic	13.5	19.6	16.58
LOC104915515	myelin-oligodendrocyte glycoprotein-like	17.7	15.4	16.58
FLI1	Fli-1 proto-oncogene, ETS transcription factor	16.0	17.1	16.56
LOC104910139	uncharacterized LOC104910139	16.9	16.3	16.56
LOC104911611	uncharacterized LOC104911611	13.5	19.6	16.54
NAPEPLD	N-acyl phosphatidylethanolamine phospholipase D	11.8	21.2	16.51
LOC104909675	potassium channel subfamily K member 17-like	15.2	17.8	16.50
TMEM74	transmembrane protein 74	14.4	18.6	16.48
CCDC91	coiled-coil domain containing 91	14.3	18.6	16.48
LOC104914159	zinc finger protein castor homolog 1-like	15.2	17.7	16.46
LOC104910783	WD repeat-containing protein 36-like	13.5	19.4	16.45
LOC104912543	uncharacterized LOC104912543	11.8	21.0	16.43
LOC104915050	uncharacterized LOC104915050	16.0	16.8	16.43

LOC104915340	dmX-like protein 1	11.8	20.9	16.38
CHRDL2	chordin-like 2	18.6	14.2	16.38
RNF32	ring finger protein 32	19.4	13.3	16.36
GNA14	guanine nucleotide binding protein (G protein), alpha 14	21.1	11.6	16.36
LOC104916152	solute carrier family 2, facilitated glucose transporter member 1-like	20.3	12.4	16.35
LOC104913574	uncharacterized LOC104913574	16.9	15.8	16.35
LOC100548266	arg8-vasotocin receptor-like	19.4	13.3	16.34
LOC104916460	interleukin-36 receptor antagonist protein-like	18.6	14.1	16.34
A2M	alpha-2-macroglobulin	18.6	14.1	16.34
LOC100540439	C-X-C chemokine receptor type 1-like	17.8	14.9	16.33
SCARA5	scavenger receptor class A, member 5	21.1	11.5	16.32
LOC104912823	S phase cyclin A-associated protein in the endoplasmic reticulum-like	21.1	11.5	16.31
PPFIBP2	PTPRF interacting protein, binding protein 2 (liprin beta 2)	17.8	14.9	16.31
LOC104914801	uncharacterized LOC104914801	18.6	14.1	16.30
LOC104909861	uncharacterized LOC104909861	16.9	15.7	16.28
LOC104915116	uncharacterized LOC104915116	16.0	16.5	16.27
LOC104909988	kinase D-interacting substrate of 220 kDa-like	18.6	13.9	16.23
MEGF11	multiple EGF-like-domains 11	15.2	17.2	16.21
LOC104915946	ryanodine receptor 2-like	17.8	14.7	16.21
CCDC176	coiled-coil domain containing 176	17.7	14.7	16.20
LOC104910876	VPS10 domain-containing receptor SorCS2-like	7.6	24.8	16.19
PROK2	prokineticin 2	14.3	18.0	16.18
LOC104910708	uncharacterized LOC104910708	15.2	17.1	16.18
LOC100542775	alpha-2-macroglobulin-like protein 1	15.2	17.1	16.16
FECH	ferrochelatase	16.1	16.3	16.16
LOC104917328	ras-related protein Rab-33B	16.1	16.3	16.16
SPARCL1	SPARC-like 1 (hevin)	14.4	17.9	16.15
LOC104912946	uncharacterized LOC104912946	22.0	10.3	16.15
LOC104909420	transmembrane protein 135-like	13.5	18.7	16.12
LOC104910181	uncharacterized LOC104910181	12.7	19.6	16.12
LOC104915053	focadhesin-like	15.2	17.0	16.10
LOC104915342	transcription factor TFIIIB component B'' homolog	12.7	19.4	16.04
LOC104915184	inositol hexakisphosphate and diphosphoinositol-pentakisphosphate kinase 2-like	13.5	18.5	16.03
TMEM229B	transmembrane protein 229B	12.7	19.4	16.02
LOC104914377	uncharacterized LOC104914377	20.2	11.8	16.01
KITLG	KIT ligand	21.1	10.9	16.01
RPGRIP1L	RPGRIP1-like	11.8	20.1	15.96
LOC104910481	trafficking protein particle complex subunit 9-like	18.6	13.3	15.94
LOC104917442	uncharacterized LOC104917442	16.9	15.0	15.93
LOC100548130	PR domain zinc finger protein 1	20.3	11.5	15.92
LOC104910561	uncharacterized LOC104910561	11.0	20.8	15.92
LOC104917059	histone-lysine N-methyltransferase SETD1A-like	19.4	12.4	15.91
SLC11A1	solute carrier family 11 (proton-coupled divalent metal ion transporter), member 1	18.6	13.2	15.90
LOC104911352	probable G-protein coupled receptor 158	16.9	14.9	15.90
LOC104916536	uncharacterized LOC104916536	20.3	11.5	15.90
LOC104914253	uncharacterized LOC104914253	20.3	11.5	15.89
MLPH	melanophilin	18.6	13.2	15.89
LOC104912976	uncharacterized LOC104912976	17.7	14.0	15.88
ADORA2B	adenosine A2b receptor	17.7	14.0	15.85
LOC104909286	anthrax toxin receptor 1-like	8.5	23.2	15.85
LRRC56	leucine rich repeat containing 56	18.6	13.1	15.84
LOC104916576	uncharacterized LOC104916576	16.1	15.6	15.84
LOC104910099	uncharacterized LOC104910099	14.4	17.3	15.82
LOC104917365	polycomb group RING finger protein 6-like	16.9	14.7	15.81

LOC104910868	huntingtin-like	16.1	15.5	15.80
LOC104916462	CCAAT/enhancer-binding protein alpha-like	16.9	14.7	15.80
ADRA2B	adrenoceptor alpha 2B	18.6	13.0	15.78
LOC104910221	uncharacterized LOC104910221	14.3	17.2	15.78
LOC100538484	dedicator of cytokinesis protein 10	15.2	16.3	15.77
LOC100543496	keratin, type I cytoskeletal 14	15.2	16.3	15.75
LOC104912806	unconventional myosin-Vc-like	15.2	16.3	15.73
MTHFD2L	methylenetetrahydrofolate dehydrogenase (NADP+ dependent) 2-like	14.4	17.1	15.73
LOC104911589	uncharacterized LOC104911589	12.7	18.7	15.70
LOC104912932	neural-cadherin-like	13.5	17.9	15.70
LOC104910258	receptor-type tyrosine-protein phosphatase mu-like	14.3	17.1	15.70
SEMA6C	sema domain, transmembrane domain (TM), and cytoplasmic domain, (semaphorin) 6C	14.3	17.1	15.70
LOC100540105	synapsin-3-like	11.8	19.5	15.68
LOC100546652	protein FAM171A1-like	14.4	16.9	15.65
DPP6	dipeptidyl-peptidase 6	20.3	11.0	15.64
LOC104913234	uncharacterized LOC104913234	11.0	20.3	15.64
NMRK1	nicotinamide riboside kinase 1	13.5	17.7	15.61
LOC104912700	transmembrane and TPR repeat-containing protein 2-like	11.0	20.2	15.61
LNPEP	leucyl/cystinyl aminopeptidase	13.5	17.7	15.60
KIAA1324	KIAA1324 ortholog	22.0	9.2	15.57
KCNE4	potassium voltage-gated channel, Isk-related family, member 4	10.1	21.0	15.57
CLCN4	chloride channel, voltage-sensitive 4	21.1	10.0	15.53
LOC104910307	uncharacterized LOC104910307	17.7	13.3	15.52
LOC104911871	uncharacterized LOC104911871	17.7	13.3	15.51
PHYHIPL	phytanoyl-CoA 2-hydroxylase interacting protein-like	9.3	21.7	15.51
LOC100540803	delta and Notch-like epidermal growth factor-related receptor	16.9	14.1	15.48
LMOD1	leiomodin 1 (smooth muscle)	17.7	13.2	15.48
DOPEY2	dopey family member 2	17.7	13.2	15.47
SEPT4	septin 4	18.5	12.4	15.46
LOC100541434	histone H2B 1/2/3/4/6	15.2	15.7	15.45
LOC104914666	MAP kinase-interacting serine/threonine-protein kinase 2-like	18.6	12.3	15.44
LOC104917468	uncharacterized LOC104917468	15.2	15.7	15.43
LOC104916908	mucin-2-like	15.2	15.7	15.43
LOC104910503	uncharacterized LOC104910503	17.7	13.1	15.42
LOC100548557	protein FAM188B2-like	15.2	15.6	15.41
C19H9orf9	chromosome 19 open reading frame, human C9orf9	16.0	14.8	15.41
TCHH	trichohyalin	13.5	17.2	15.38
LOC104915044	sperm-associated antigen 4 protein-like	14.4	16.3	15.35
LOC104916741	sterile alpha motif domain-containing protein 14-like	15.2	15.5	15.34
SLC29A4	solute carrier family 29 (equilibrative nucleoside transporter), member 4	14.3	16.3	15.33
FOXE3	forkhead box E3	14.4	16.3	15.33
LOC100538416	ensconsin-like	14.4	16.2	15.29
LOC104915166	colorectal mutant cancer protein-like	10.1	20.4	15.29
LOC100547251	acetylserotonin O-methyltransferase	11.0	19.6	15.28
SLC25A48	solute carrier family 25, member 48	12.7	17.9	15.28
ADAMTS15	ADAM metallopeptidase with thrombospondin type 1 motif, 15	11.8	18.7	15.28
LOC104909739	uncharacterized LOC104909739	13.5	17.0	15.27
ST6GAL1	ST6 beta-galactosamide alpha-2,6-sialyltranferase 1	13.5	17.0	15.26
MFSD4	major facilitator superfamily domain containing 4	11.8	18.7	15.26
TTLL2	tubulin tyrosine ligase-like family, member 2	8.5	22.0	15.23
LOC104912009	uncharacterized LOC104912009	14.4	16.1	15.22
TTPA	tocopherol (alpha) transfer protein	19.4	11.0	15.22
LOC100550910	relaxin receptor 1-like	21.1	9.3	15.22
LOC100540472	ELKS/Rab6-interacting/CAST family member 1	12.7	17.7	15.21
LOC104912132	transmembrane and TPR repeat-containing protein 1-like	11.0	19.4	15.17

LOC104912159	plakophilin-2-like	18.6	11.7	15.16
LOC104912769	transcription factor 12-like	12.7	17.6	15.15
ZFAT	zinc finger and AT hook domain containing	18.6	11.7	15.15
LOC100547382	WW domain-containing oxidoreductase-like	11.0	19.3	15.13
LOC104913330	putative E3 ubiquitin-protein ligase SH3RF2	8.5	21.8	15.13
LGI1	leucine-rich, glioma inactivated 1	19.4	10.8	15.11
LOC100539718	C2 calcium-dependent domain-containing protein 4C-like	16.9	13.3	15.11
MLANA	melan-A	16.9	13.3	15.11
FAM154A	family with sequence similarity 154, member A	16.9	13.3	15.09
CHRNA2	cholinergic receptor, nicotinic, alpha 2 (neuronal)	9.3	20.9	15.09
ZMYND10	zinc finger, MYND-type containing 10	16.0	14.1	15.08
CFAP44	cilia and flagella associated protein 44	16.1	14.1	15.08
CPS1	carbamoyl-phosphate synthase 1, mitochondrial	17.7	12.4	15.05
CA14	carbonic anhydrase XIV	15.2	14.9	15.04
LOC104909708	uncharacterized LOC104909708	15.2	14.9	15.04
PLAC8	placenta-specific 8	16.9	13.2	15.04
SLC5A1	solute carrier family 5 (sodium/glucose cotransporter), member 1	16.9	13.2	15.03
LOC104911225	uncharacterized LOC104911225	16.1	14.0	15.01
HTR2C	5-hydroxytryptamine (serotonin) receptor 2C, G protein-coupled	14.4	15.7	15.01
LOC104913357	CREB-binding protein-like	16.0	14.0	15.01
LOC100549639	uncharacterized LOC100549639	15.2	14.8	15.01
LOC104913106	protein kinase C delta type-like	12.7	17.3	14.99
C23H1orf158	chromosome 23 open reading frame, human C1orf158	15.2	14.8	14.99
LOC104914752	ectopic P granules protein 5 homolog	15.2	14.8	14.99
CNGA3	cyclic nucleotide gated channel alpha 3	6.8	23.2	14.98
RASL10B	RAS-like, family 10, member B	15.2	14.7	14.97
LOC100544056	dymeclin-like	14.4	15.6	14.96
GPR37L1	G protein-coupled receptor 37 like 1	13.5	16.4	14.96
LOC104915289	uncharacterized LOC104915289	16.0	13.8	14.93
LOC104910646	uncharacterized LOC104910646	13.5	16.3	14.92
KIF27	kinesin family member 27	11.8	18.0	14.90
LOC104914325	uncharacterized LOC104914325	12.7	17.1	14.90
LOC104909339	flocculation protein FLO11-like	15.2	14.6	14.89
LOC104912083	dedicator of cytokinesis protein 1-like	13.5	16.3	14.88
FKBP6	FK506 binding protein 6, 36kDa	13.5	16.2	14.87
SIGLEC15	sialic acid binding Ig-like lectin 15	13.5	16.2	14.87
CCDC13	coiled-coil domain containing 13	12.7	17.0	14.84
LOC104909314	DENN domain-containing protein 1A-like	11.8	17.8	14.82
LOC100544614	protein BEAN1-like	19.4	10.1	14.78
DMXL2	Dmx-like 2	10.1	19.4	14.75
LOC104910702	uncharacterized LOC104910702	18.5	10.9	14.74
LOC104909724	utrophin-like	8.5	21.0	14.73
VWCE	von Willebrand factor C and EGF domains	17.8	11.7	14.73
LOC100547620	cAMP and cAMP-inhibited cGMP 3',5'-cyclic phosphodiesterase 10A-like	18.6	10.8	14.69
VIL1	villin 1	16.9	12.5	14.69
LOC104914184	vacuolar protein sorting-associated protein 13D-like	8.5	20.9	14.69
LOC104910709	uncharacterized LOC104910709	18.6	10.8	14.69
LMBR1L	limb development membrane protein 1-like	16.9	12.5	14.67
LOC104915495	fatty acyl-CoA hydrolase precursor, medium chain-like	15.2	14.1	14.65
LOC100548963	wiskott-Aldrich syndrome protein family member 3-like	17.7	11.5	14.64
LOC104917080	uncharacterized LOC104917080	8.5	20.8	14.64
LOC104912689	uncharacterized LOC104912689	15.2	14.0	14.61
LOC104915791	piwi-like protein 2	16.0	13.2	14.61
LOC100543035	potassium voltage-gated channel subfamily D member 2	16.9	12.3	14.61
LOC104914786	Fanconi anemia group G protein homolog	16.9	12.3	14.60
LPAR1	lysophosphatidic acid receptor 1	15.2	13.9	14.59
LOC104911404	uncharacterized LOC104911404	15.2	14.0	14.59

LOC100550580	proto-oncogene tyrosine-protein kinase ROS	13.5	15.7	14.58
IGDCC3	immunoglobulin superfamily, DCC subclass, member 3	14.4	14.8	14.58
LOC104916274	potassium voltage-gated channel subfamily A member 7-like	15.2	13.9	14.58
LOC100541783	hypermethylated in cancer 1 protein-like	16.1	13.1	14.58
LOC104913223	ankyrin repeat and KH domain-containing protein 1-like	16.0	13.1	14.57
CAPN14	calpain 14	15.2	13.9	14.57
LOC104909938	M-protein, striated muscle-like	15.2	13.9	14.57
EFCAB5	EF-hand calcium binding domain 5	15.2	13.9	14.57
LOC104911463	neurabin-1-like	14.3	14.7	14.54
LOC104910852	uncharacterized LOC104910852	15.2	13.9	14.53
LOC104913298	LON peptidase N-terminal domain and RING finger protein 1-like	12.7	16.4	14.53
LOC104915506	protocadherin gamma-A5-like	14.4	14.7	14.51
LOC104910118	uncharacterized LOC104910118	12.7	16.3	14.49
LOC104910639	teneurin-3-like	11.8	17.1	14.49
LOC100541903	putative tRNA pseudouridine synthase Pus10	12.7	16.3	14.49
DSEL	dermatan sulfate epimerase-like	14.4	14.6	14.49
NR6A1	nuclear receptor subfamily 6, group A, member 1	11.0	17.9	14.46
FRMD7	FERM domain containing 7	12.7	16.3	14.45
OPN4	opsin 4	10.1	18.7	14.44
LOC104910023	uncharacterized LOC104910023	18.6	10.3	14.43
CCDC177	coiled-coil domain containing 177	20.3	8.6	14.43
ADCY8	adenylate cyclase 8 (brain)	20.2	8.6	14.43
FGFRL1	fibroblast growth factor receptor-like 1	9.3	19.6	14.43
PROSER2	proline and serine rich 2	11.0	17.9	14.41
LOC104914298	uncharacterized LOC104914298	20.3	8.5	14.40
ERICH6B	glutamate-rich 6B	7.6	21.2	14.38
LOC104916299	sprT-like domain-containing protein Spartan	11.8	16.9	14.38
LOC100547719	unconventional myosin-VI-like	18.6	10.1	14.35
LOC104910345	uncharacterized LOC104910345	5.9	22.7	14.32
HAP1	huntingtin-associated protein 1	10.1	18.5	14.32
LOC104911906	uncharacterized LOC104911906	15.2	13.4	14.31
PKP3	plakophilin 3	16.0	12.5	14.27
LOC100545943	transcription factor 7-like 2	16.1	12.5	14.26
CILP2	cartilage intermediate layer protein 2	16.1	12.5	14.26
LOC104914314	claspin-like	16.0	12.5	14.25
LOC104910680	WD repeat and FYVE domain-containing protein 3-like	16.9	11.6	14.24
LOC104914639	lamin-B2 pseudogene	16.1	12.4	14.24
LOC104909819	uncharacterized LOC104909819	14.4	14.1	14.23
LOC100541450	leucine zipper putative tumor suppressor 3	16.1	12.4	14.23
LOC104913837	uncharacterized LOC104913837	16.9	11.5	14.22
LOC104910771	atrial natriuretic peptide-converting enzyme-like	16.0	12.4	14.20
LOC104914510	uncharacterized LOC104914510	15.2	13.2	14.19
LOC104917146	uncharacterized LOC104917146	16.0	12.3	14.19
LOC104910537	uncharacterized LOC104910537	14.4	14.0	14.19
LOC104917145	uncharacterized LOC104917145	16.1	12.3	14.18
CRMP1	collapsin response mediator protein 1	13.5	14.9	14.18
MCTP2	multiple C2 domains, transmembrane 2	15.2	13.1	14.17
GPNMB	glycoprotein (transmembrane) nmb	16.0	12.3	14.17
LOC104914270	synaptojanin-1-like	12.7	15.7	14.16
LOC104916771	uncharacterized LOC104916771	14.4	13.9	14.15
LOC104913643	uncharacterized LOC104913643	13.5	14.8	14.15
LOC104915265	uncharacterized LOC104915265	12.7	15.6	14.14
MKI67	marker of proliferation Ki-67	11.8	16.4	14.12
LOC104912364	dihydropyrimidine dehydrogenase [NADP(+)]-like	11.8	16.4	14.10
CHST9	carbohydrate (N-acetylgalactosamine 4-0) sulfotransferase 9	12.7	15.5	14.09
PPP1R3B	protein phosphatase 1, regulatory subunit 3B	13.5	14.7	14.09
LOC104912783	transient receptor potential cation channel subfamily M member 1-like	11.0	17.2	14.08

LIX1	Lix1 homolog (chicken)	11.8	16.3	14.08
LOC100540978	uncharacterized LOC100540978	11.8	16.3	14.08
LOC104915783	uncharacterized LOC104915783	11.8	16.3	14.08
LOC104911253	inverted formin-2-like	11.0	17.1	14.06
LOC104917405	GRAM domain-containing protein 4-like	11.8	16.3	14.06
LOC104915020	receptor-type tyrosine-protein phosphatase delta-like	12.7	15.5	14.06
LOC104913921	uncharacterized LOC104913921	11.8	16.3	14.06
FAM84B	family with sequence similarity 84, member B	12.7	15.4	14.06
MDH1B	malate dehydrogenase 1B, NAD (soluble)	10.1	18.0	14.05
LOC104911521	serine/threonine-protein phosphatase 6 regulatory ankyrin repeat subunit A-like	10.1	18.0	14.05
C10H1orf111	chromosome 10 open reading frame, human C1orf111	11.8	16.2	14.02
DPYSL4	dihydropyrimidinase-like 4	11.0	17.0	14.01
LOC104914036	phosphatase and actin regulator 3-like	13.5	14.5	14.00
LOC104916517	GRAM domain-containing protein 3-like	10.1	17.9	13.99
LOC104910869	regulator of G-protein signaling 12-like	9.3	18.7	13.99
LOC100541160	junctophilin-3-like	18.6	9.4	13.98
LOC104915979	uncharacterized LOC104915979	16.9	11.0	13.94
LOC100545624	laminin subunit alpha-2-like	10.1	17.7	13.91
LOC104911164	ryanodine receptor 3-like	14.4	13.4	13.89
LOC104909683	uncharacterized LOC104909683	16.0	11.7	13.89
LOC104914582	GTPase IMAP family member 1-like	16.9	10.9	13.89
CERS1	ceramide synthase 1	17.7	10.0	13.87
PTCRA	pre T-cell antigen receptor alpha	16.0	11.7	13.87
LOC100538588	cytochrome P450 4B1-like	16.9	10.9	13.87
LOC100546836	fructose-1,6-bisphosphatase isozyme 2	16.9	10.8	13.83
ADAMTS3	ADAM metallopeptidase with thrombospondin type 1 motif, 3	13.5	14.1	13.83
LOC104909941	uncharacterized LOC104909941	15.2	12.5	13.82
LOC104915411	DNA polymerase alpha catalytic subunit-like	14.4	13.3	13.81
LOC104915000	uncharacterized LOC104915000	15.2	12.4	13.79
PTPN6	protein tyrosine phosphatase, non-receptor type 6	14.4	13.2	13.79
LOC100548691	L-gulonolactone oxidase-like	13.5	14.1	13.78
LOC104913795	uncharacterized LOC104913795	14.3	13.2	13.78
LRRC19	leucine rich repeat containing 19	14.3	13.2	13.78
CRABP2	cellular retinoic acid binding protein 2	11.8	15.7	13.78
LOC104912967	uncharacterized LOC104912967	14.4	13.2	13.78
PPIL6	peptidylprolyl isomerase (cyclophilin)-like 6	12.7	14.9	13.76
TRPM2	transient receptor potential cation channel, subfamily M, member 2	14.3	13.1	13.74
LOC100542110	caprin-2	14.4	13.1	13.74
SCARF1	scavenger receptor class F, member 1	13.5	13.9	13.73
KRTCAP3	keratinocyte associated protein 3	12.7	14.8	13.73
KIAA1549L	KIAA1549-like ortholog	13.5	13.9	13.73
LOC104910533	uncharacterized LOC104910533	14.3	13.1	13.72
FGF16	fibroblast growth factor 16	12.7	14.7	13.71
NFATC2	nuclear factor of activated T-cells, cytoplasmic, calcineurin-dependent 2	11.8	15.5	13.68
LOC104910652	rho guanine nucleotide exchange factor 38-like	12.7	14.7	13.68
LOC100540751	dual specificity protein phosphatase 13 isoform B-like	12.7	14.7	13.68
LOC104915057	structural maintenance of chromosomes protein 5-like	13.5	13.8	13.67
LOC104913712	fibril-forming collagen alpha chain-like	11.0	16.3	13.65
GPR52	G protein-coupled receptor 52	11.0	16.3	13.65
LOC104910729	uncharacterized LOC104910729	9.3	18.0	13.65
CBX7	chromobox homolog 7	11.0	16.3	13.64
LOC104909200	interferon-induced guanylate-binding protein 1-like	18.6	8.7	13.63
XKR5	XK, Kell blood group complex subunit-related family, member 5	18.6	8.6	13.61
LIME1	Lck interacting transmembrane adaptor 1	17.7	9.5	13.60
C23H1orf159	chromosome 23 open reading frame, human C1orf159	11.0	16.2	13.60

LOC104911844	uncharacterized LOC104911844	9.3	17.9	13.59
ESRP2	epithelial splicing regulatory protein 2	10.1	17.0	13.57
SLC16A14	solute carrier family 16, member 14	10.1	17.0	13.57
LOC100547159	uncharacterized LOC100547159	17.7	9.4	13.57
LOC104912699	mucin-4-like	18.6	8.5	13.54
LOC104909502	uncharacterized LOC104909502	17.7	9.3	13.53
LOC100546791	synaptotagmin-like protein 3	5.9	21.1	13.52
MME	membrane metallo-endopeptidase	17.7	9.3	13.52
LOC104913025	copine-2-like	13.5	13.5	13.50
THEMIS2	thymocyte selection associated family member 2	15.2	11.7	13.49
LOC104914156	1-phosphatidylinositol 4,5-bisphosphate phosphodiesterase eta-2-like	16.1	10.9	13.48
COL28A1	collagen, type XXVIII, alpha 1	16.0	10.9	13.47
LOC104915068	uncharacterized LOC104915068	9.3	17.7	13.47
ANKRD24	ankyrin repeat domain 24	16.0	10.9	13.46
LOC100547427	BTB/POZ domain-containing protein KCTD16-like	14.4	12.5	13.45
PTCHD2	patched domain containing 2	14.4	12.5	13.43
LOC100548929	uncharacterized LOC100548929	13.5	13.3	13.42
RASSF7	Ras association (RalGDS/AF-6) domain family (N-terminal) member 7	14.4	12.4	13.38
LOC100539639	acrosin-like	12.7	14.1	13.37
CLCN1	chloride channel, voltage-sensitive 1	11.0	15.7	13.35
LOC104913933	uncharacterized LOC104913933	12.7	14.0	13.34
PNLDC1	poly(A)-specific ribonuclease (PARN)-like domain containing 1	12.7	14.0	13.34
ABCC8	ATP-binding cassette, sub-family C (CFTR/MRP), member 8	13.5	13.1	13.33
NPTX1	neuronal pentraxin I	11.8	14.8	13.32
LOC104916211	uncharacterized LOC104916211	12.7	13.9	13.32
FASLG	Fas ligand (TNF superfamily, member 6)	11.0	15.7	13.31
LOC104909855	uncharacterized LOC104909855	10.1	16.5	13.31
LOC100548376	transitional endoplasmic reticulum ATPase	13.5	13.1	13.30
TEN1	TEN1 CST complex subunit	11.0	15.6	13.29
B3GAT1	beta-1,3-glucuronyltransferase 1	11.8	14.7	13.28
GAS6	growth arrest-specific 6	12.7	13.9	13.28
LOC100548610	PH and SEC7 domain-containing protein 2-like	11.0	15.6	13.27
LOC100548925	DEP domain-containing mTOR-interacting protein-like	11.8	14.7	13.26
RAB11FIP5	RAB11 family interacting protein 5 (class I)	10.2	16.3	13.25
B3GALT1	UDP-Gal:betaGlcNAc beta 1,3-galactosyltransferase, polypeptide 1	19.4	7.0	13.22
LOC100547793	melatonin receptor type 1C-like	10.1	16.3	13.22
SYCN	syncollin	10.1	16.3	13.21
DCP2	decapping mRNA 2	10.2	16.3	13.20
SS18L1	synovial sarcoma translocation gene on chromosome 18-like 1	9.3	17.1	13.17
LOC100546734	transmembrane protein 45B-like	7.6	18.7	13.16
GABRA1	gamma-aminobutyric acid (GABA) A receptor, alpha 1	8.5	17.9	13.15
LOC104912367	uncharacterized oxidoreductase ZK1290.5-like	17.7	8.5	13.12
DENND2D	DENN/MADD domain containing 2D	15.2	11.0	13.12
LOC104911813	protein TANC1-like	15.2	10.9	13.08
HPSE	heparanase	16.0	10.1	13.08
LOC104916363	protein-lysine methyltransferase METTL21B-like	16.1	10.1	13.06
THBS4	thrombospondin 4	16.0	10.1	13.05
LOC100539825	spermatogenesis-associated protein 6-like	15.2	10.9	13.03
SLC2A5	solute carrier family 2 (facilitated glucose/fructose transporter), member 5	16.0	10.0	13.03
LOC100540703	intestinal-type alkaline phosphatase-like	14.4	11.7	13.03
ACKR4	atypical chemokine receptor 4	14.4	11.6	12.99
GPR135	G protein-coupled receptor 135	13.5	12.5	12.98
LOC100546910	semaphorin-5B	15.2	10.8	12.98
RAB11FIP4	RAB11 family interacting protein 4 (class II)	12.7	13.3	12.96
LOC100542776	cell cycle control protein 50C-like	12.7	13.2	12.95

AIRE	autoimmune regulator	11.8	14.0	12.91
LOC104916835	sterile alpha motif domain-containing protein 14-like	12.7	13.1	12.91
TEKT2	tektin 2 (testicular)	12.7	13.1	12.90
LOC104912656	lipoma-preferred partner homolog	9.3	16.5	12.90
NPTX2	neuronal pentraxin II	10.1	15.7	12.89
LOC100546324	ubiquitin carboxyl-terminal hydrolase 34-like	11.0	14.8	12.89
LOC104912505	spermatogenesis-associated protein 6-like	10.1	15.6	12.88
LOC104910982	uncharacterized LOC104910982	11.8	13.9	12.88
LOC104909501	uncharacterized LOC104909501	21.1	4.7	12.87
LOC104910665	uncharacterized LOC104910665	9.3	16.4	12.86
LOC104915316	uncharacterized LOC104915316	10.1	15.6	12.86
APCDD1	adenomatosis polyposis coli down-regulated 1	11.0	14.7	12.86
LOC104912557	ataxin-10-like	9.3	16.3	12.81
FABP7	fatty acid binding protein 7, brain	16.0	9.5	12.75
HIVEP3	human immunodeficiency virus type I enhancer binding protein 3	16.9	8.6	12.75
TMEM213	transmembrane protein 213	17.7	7.8	12.74
LOC104917067	pyruvate carboxylase, mitochondrial-like	16.1	9.4	12.72
LOC104913802	centrosomal protein of 112 kDa-like	16.9	8.5	12.70
SH3YL1	SH3 and SYLF domain containing 1	15.2	10.2	12.70
CLIC5	chloride intracellular channel 5	16.9	8.5	12.69
HMGCS2	3-hydroxy-3-methylglutaryl-CoA synthase 2 (mitochondrial)	15.2	10.2	12.69
STEAP4	STEAP family member 4	14.4	11.0	12.69
CDS1	CDP-diacylglycerol synthase (phosphatidate cytidylyltransferase) 1	15.2	10.1	12.66
LOC100541480	tyrosine-protein phosphatase non-receptor type 11-like	15.2	10.1	12.65
IRG1	immunoresponsive 1 homolog (mouse)	15.2	10.1	12.64
CD79A	CD79a molecule, immunoglobulin-associated alpha	13.5	11.7	12.63
MADCAM1	mucosal vascular addressin cell adhesion molecule 1	13.5	11.7	12.63
LOC100549764	solute carrier family 22 member 2-like	15.2	10.1	12.61
LOC104917061	IQ motif and SEC7 domain-containing protein 2-like	14.4	10.8	12.60
SYT12	synaptotagmin XII	11.8	13.3	12.59
LOC104912904	ras and EF-hand domain-containing protein-like	13.5	11.7	12.59
LOC100545617	collagen alpha-1(VII) chain-like	13.5	11.7	12.58
RBM15	RNA binding motif protein 15	13.5	11.7	12.58
EPHA1	EPH receptor A1	13.5	11.6	12.57
LOC104913289	uncharacterized LOC104913289	11.8	13.3	12.56
EPB42	erythrocyte membrane protein band 4.2	12.7	12.4	12.55
LOC104911915	ankyrin-3-like	12.7	12.4	12.54
SLC39A8	solute carrier family 39 (zinc transporter), member 8	14.4	10.7	12.54
LOC104911105	MAP kinase-activating death domain protein-like	13.5	11.5	12.53
LOC104915779	uncharacterized LOC104915779	12.7	12.3	12.52
PTPRR	protein tyrosine phosphatase, receptor type, R	11.0	14.0	12.50
LOC104910277	structural maintenance of chromosomes flexible hinge domain-containing protein 1-like	12.7	12.3	12.49
LEF1	lymphoid enhancer-binding factor 1	10.1	14.8	12.47
FANCA	Fanconi anemia, complementation group A	11.8	13.1	12.47
LOC104911075	uncharacterized LOC104911075	9.3	15.7	12.47
LOC100549134	smoothelin-like protein 2	8.4	16.5	12.47
LOC104910337	dystrobrevin alpha-like	11.0	13.9	12.46
CLDN10	claudin 10	10.1	14.8	12.45
LOC104912485	kelch-like protein 42 pseudogene	9.3	15.6	12.45
LOC104910641	uncharacterized LOC104910641	10.1	14.7	12.44
LOC104915366	uncharacterized LOC104915366	10.1	14.7	12.44
LOC104910046	uncharacterized LOC104910046	19.4	5.5	12.43
LOC104912568	uncharacterized LOC104912568	11.0	13.9	12.43
SYTL5	synaptotagmin-like 5	10.1	14.7	12.42
C2H6orf163	chromosome 2 open reading frame, human C6orf163	10.1	14.7	12.42
LOC104913117	uncharacterized LOC104913117	19.4	5.4	12.41

LOC104911638	UPF0606 protein KIAA1549-like	10.2	14.6	12.39
SPACA1	sperm acrosome associated 1	17.7	6.9	12.32
LOC104911344	5'-AMP-activated protein kinase subunit gamma-2-like	16.0	8.6	12.32
LOC104913891	general transcription factor II-I-like	15.2	9.4	12.31
LOC104915141	tyrosine-protein kinase transmembrane receptor ROR2-like	15.2	9.4	12.30
LOC104912142	interleukin-22 receptor subunit alpha-1-like	15.2	9.3	12.28
IGFALS	insulin-like growth factor binding protein, acid labile subunit	14.4	10.2	12.27
LOC104915729	E3 ubiquitin-protein ligase HUWE1-like	14.4	10.1	12.27
YBEY	ybeY metallopeptidase (putative)	14.4	10.1	12.25
LOC104916134	zinc finger protein 586-like	12.7	11.8	12.25
LOC104910257	receptor-type tyrosine-protein phosphatase mu-like	14.3	10.1	12.25
PDE10A	phosphodiesterase 10A	15.2	9.3	12.24
LOC100539847	zinc finger MYM-type protein 3-like	11.8	12.7	12.24
LOC104909711	uncharacterized LOC104909711	15.2	9.3	12.23
CPNE7	copine VII	13.5	10.9	12.23
SPRY4	sprouty homolog 4 (Drosophila)	15.2	9.2	12.22
CA6	carbonic anhydrase VI	14.4	10.1	12.21
LOC104914767	dynein intermediate chain 1, axonemal-like	14.4	10.1	12.21
MAPK10	mitogen-activated protein kinase 10	13.5	10.9	12.21
UPF3A	UPF3 regulator of nonsense transcripts homolog A (yeast)	12.7	11.7	12.21
LOC104914060	extracellular sulfatase Sulf-2-like	14.4	10.0	12.20
FRMPD1	FERM and PDZ domain containing 1	12.7	11.7	12.20
LOC104911949	uncharacterized LOC104911949	14.4	10.0	12.19
DNMT3B	DNA (cytosine-5-)-methyltransferase 3 beta	13.5	10.9	12.18
LOC100549167	mannose-binding protein A-like	11.8	12.5	12.18
LOC104912764	unconventional myosin-Va-like	13.5	10.8	12.17
LOC104911637	uncharacterized LOC104911637	13.5	10.8	12.17
LOC104917595	maestro heat-like repeat-containing protein family member 2B	11.8	12.5	12.17
PRF1	perforin 1 (pore forming protein)	12.7	11.7	12.17
LOC104916277	ras/Rap GTPase-activating protein SynGAP-like	13.5	10.8	12.15
LOC104916857	homeobox protein OTX1-like	12.7	11.6	12.14
SEMA3G	sema domain, immunoglobulin domain (Ig), short basic domain, secreted, (semaphorin) 3G	11.0	13.3	12.14
COL8A2	collagen, type VIII, alpha 2	12.7	11.6	12.14
LOC104915703	zinc finger protein 501-like	13.5	10.7	12.13
MANEAL	mannosidase, endo-alpha-like	11.8	12.5	12.13
LOC104915830	chromosome unknown open reading frame, human C1orf106	11.8	12.4	12.12
LOC104913161	uncharacterized LOC104913161	11.0	13.3	12.12
SLC44A5	solute carrier family 44, member 5	11.0	13.2	12.10
LOC104909506	uncharacterized LOC104909506	11.0	13.2	12.10
LOC100548071	cytosolic purine 5'-nucleotidase-like	12.7	11.5	12.09
LOC100540132	adenylate kinase isoenzyme 5	10.1	14.1	12.09
C3H8orf37	chromosome 3 open reading frame, human C8orf37	11.0	13.2	12.08
COL24A1	collagen, type XXIV, alpha 1	21.1	3.1	12.07
LOC104913314	uncharacterized LOC104913314	9.3	14.8	12.06
LOC104913964	EF-hand calcium-binding domain-containing protein 5-like	11.0	13.1	12.04
STK39	serine threonine kinase 39	11.8	12.3	12.04
EPHA7	EPH receptor A7	18.5	5.5	12.03
EREG	epiregulin	16.9	7.1	12.01
LOC100543639	uncharacterized LOC100543639	8.4	15.6	12.01
PABPC1L	poly(A) binding protein, cytoplasmic 1-like	10.1	13.9	12.00
GALR2	galanin receptor 2	6.8	17.2	11.99
LOC104916998	ryanodine receptor 1-like	18.6	5.4	11.98
LOC104916885	ras GTPase-activating-like protein IQGAP3	16.9	7.0	11.96
DNAH10	dynein, axonemal, heavy chain 10	6.8	17.1	11.95
LOC104913351	uncharacterized LOC104913351	15.2	8.6	11.91
GIPC3	GIPC PDZ domain containing family, member 3	16.1	7.7	11.90
LOC104916109	zinc transporter ZIP5-like	14.4	9.4	11.90

ECE2	endothelin converting enzyme 2	15.2	8.6	11.89
LOC100551023	phospholipase A2 crotoxin basic chain-like	14.3	9.4	11.88
LOC104914803	transmembrane protein 8A-like	15.2	8.5	11.87
LOC100542432	histamine N-methyltransferase-like	14.3	9.4	11.85
LOC104913927	breast carcinoma-amplified sequence 3-like	13.5	10.2	11.85
LOC104916229	transcription factor 4-like	15.2	8.5	11.84
LOC104910819	uncharacterized LOC104910819	14.3	9.3	11.82
LOC100542876	keratin, type I cytoskeletal 9-like	12.7	11.0	11.82
LOC104910247	uncharacterized LOC104910247	13.5	10.1	11.82
BIRC7	baculoviral IAP repeat containing 7	14.3	9.3	11.82
CCDC112	coiled-coil domain containing 112	13.5	10.1	11.81
LOC100545285	dynein heavy chain 9, axonemal-like	13.5	10.1	11.81
LOC104914473	uncharacterized LOC104914473	12.7	10.9	11.81
LOC104915199	uncharacterized LOC104915199	12.7	10.9	11.81
LOC104909513	uncharacterized LOC104909513	11.8	11.7	11.79
LOC104911204	uncharacterized LOC104911204	11.8	11.7	11.78
RNF144B	ring finger protein 144B	12.7	10.9	11.78
LOC100551228	filamin-interacting protein FAM101A-like	11.8	11.7	11.78
LOC104917473	uncharacterized LOC104917473	11.8	11.7	11.77
LOC104914463	uncharacterized LOC104914463	12.7	10.9	11.77
LOC104915666	uncharacterized LOC104915666	11.8	11.7	11.76
MOCOS	molybdenum cofactor sulfurase	12.7	10.8	11.75
LOC104909847	uncharacterized LOC104909847	12.7	10.8	11.74
LOC104912115	uncharacterized LOC104912115	10.1	13.3	11.74
C30H19orf45	chromosome 30 open reading frame, human C19orf45	11.8	11.7	11.74
PPP1R42	protein phosphatase 1, regulatory subunit 42	10.1	13.3	11.72
LOC104909387	uncharacterized LOC104909387	12.7	10.7	11.71
ARTN	artemin	11.8	11.6	11.70
LOC100543177	kinesin-like protein KIF1A	12.7	10.7	11.70
LOC104915725	mitochondrial ribosome-associated GTPase 1-like	9.3	14.1	11.70
CHMP4C	charged multivesicular body protein 4C	11.0	12.4	11.69
GPC3	glypican 3	9.3	14.1	11.69
SLC30A10	solute carrier family 30, member 10	11.8	11.5	11.68
LOC104910385	extracellular matrix protein FRAS1-like	10.1	13.2	11.67
LOC104915318	intraflagellar transport protein 74 homolog	10.1	13.2	11.67
LOC104910098	uncharacterized LOC104910098	7.6	15.7	11.63
LOC104913493	voltage-dependent T-type calcium channel subunit alpha-1H-like	9.3	13.9	11.61
LOC104913633	inhibitor of growth protein 3 pseudogene	8.4	14.8	11.61
LOC100544944	transmembrane protein 222-like	16.9	6.3	11.61
LOC104910873	uncharacterized LOC104910873	10.2	13.1	11.61
LRG1	leucine-rich alpha-2-glycoprotein 1	7.6	15.6	11.60
LOC100545266	uncharacterized LOC100545266	9.3	13.9	11.59
LOC104909409	uncharacterized LOC104909409	8.5	14.7	11.59
LOC104911644	uncharacterized LOC104911644	6.8	16.4	11.58
LOC104913749	uncharacterized LOC104913749	9.3	13.9	11.58
LOC100542618	uncharacterized LOC100542618	9.3	13.9	11.57
LOC104912905	creatine kinase U-type, mitochondrial-like	16.9	6.2	11.57
LOC104909479	uncharacterized protein KIAA1841-like	8.5	14.7	11.56
LOC104911354	amyloid beta A4 precursor protein-binding family B member 1-interacting protein-like	16.0	7.0	11.54
LOC104917605	serine/threonine-protein kinase LATS2-like	7.6	15.5	11.53
LOC104916930	uncharacterized LOC104916930	15.2	7.8	11.51
PPP2R2B	protein phosphatase 2, regulatory subunit B, beta	5.9	17.1	11.50
WSCD1	WSC domain containing 1	14.4	8.6	11.50
KIAA1324L	KIAA1324-like ortholog	14.3	8.6	11.49
LOC100546426	fibrinogen-like protein A	15.2	7.7	11.48
STAB1	stabilin 1	14.4	8.6	11.48
LOC104916353	uncharacterized LOC104916353	11.8	11.1	11.44

LOC104910251	disks large-associated protein 1-like	12.7	10.2	11.44
LOC100548991	aldose reductase-like	12.7	10.2	11.44
BCL2L13	BCL2-like 13 (apoptosis facilitator)	13.5	9.3	11.43
PCDH19	protocadherin 19	15.2	7.6	11.43
LOC104915093	vacuolar protein sorting-associated protein 13A-like	5.9	16.9	11.42
LOC104909668	histone-lysine N-methyltransferase SMYD3-like	12.7	10.2	11.42
SHISA6	shisa family member 6	12.7	10.2	11.42
MGLL	monoglyceride lipase	5.1	17.8	11.41
RSPO3	R-spondin 3	11.0	11.8	11.41
LOC104913426	uncharacterized LOC104913426	12.7	10.1	11.41
LIPH	lipase, member H	11.8	11.0	11.41
IL17C	interleukin 17C	12.7	10.1	11.40
LOC104916468	AT-hook-containing transcription factor-like	12.7	10.1	11.39
LTK	leukocyte receptor tyrosine kinase	11.8	10.9	11.38
LOC104914393	uncharacterized LOC104914393	11.8	10.9	11.37
LOC100548836	transmembrane O-methyltransferase	12.7	10.1	11.37
OMD	osteomodulin	11.8	10.9	11.37
LOC104909749	uncharacterized LOC104909749	11.8	10.9	11.36
PLCH1	phospholipase C, eta 1	10.1	12.6	11.36
LOC100539753	carbonic anhydrase 2	11.0	11.7	11.35
GGT1	gamma-glutamyltransferase 1	10.1	12.6	11.35
LOC104913431	sorting nexin-8-like	11.8	10.9	11.35
SPATA4	spermatogenesis associated 4	11.8	10.9	11.35
LOC104910042	uncharacterized LOC104910042	11.0	11.7	11.35
LOC104913946	active breakpoint cluster region-related protein-like	12.7	10.0	11.33
LOC104913199	uncharacterized LOC104913199	8.5	14.2	11.33
LOC104913629	uncharacterized LOC104913629	11.8	10.8	11.32
LOC104910986	uncharacterized LOC104910986	11.0	11.7	11.32
LOC104913913	uncharacterized LOC104913913	11.0	11.7	11.32
RHBDF2	rhomboid 5 homolog 2 (Drosophila)	11.0	11.6	11.30
BNC1	basonuclin 1	10.1	12.5	11.29
LOC104914232	uncharacterized LOC104914232	10.1	12.5	11.29
LOC104909978	phosphatidate phosphatase LPIN1-like	11.8	10.7	11.28
GOLPH3L	golgi phosphoprotein 3-like	10.1	12.4	11.27
LOC104916290	uncharacterized LOC104916290	11.0	11.5	11.27
LOC104909998	kinase D-interacting substrate of 220 kDa-like	8.4	14.1	11.27
RD3	retinal degeneration 3	9.3	13.2	11.25
RGCC	regulator of cell cycle	10.1	12.3	11.23
LOC104913309	uncharacterized LOC104913309	9.3	13.2	11.23
FBXO47	F-box protein 47	8.5	14.0	11.22
LOC104909371	centrosomal protein KIAA1731-like	10.1	12.3	11.22
TRPC3	transient receptor potential cation channel, subfamily C, member 3	9.3	13.1	11.21
LOC104917326	adenylyltransferase and sulfurtransferase MOCS3-like	7.6	14.8	11.20
RARB	retinoic acid receptor, beta	7.6	14.7	11.17
LOC104914368	uncharacterized LOC104914368	15.2	7.1	11.16
SHROOM2	shroom family member 2	15.2	7.1	11.16
RSPH10B	radial spoke head 10 homolog B (Chlamydomonas)	8.4	13.9	11.15
LEFTY1	left-right determination factor 1	6.7	15.5	11.12
SOSTDC1	sclerostin domain containing 1	7.6	14.6	11.10
LOC104916947	uncharacterized LOC104916947	16.0	6.2	11.10
LOC104914622	uncharacterized LOC104914622	14.4	7.8	11.09
CHST11	carbohydrate (chondroitin 4) sulfotransferase 11	14.4	7.8	11.07
LOC100538897	dihydropyrimidine dehydrogenase [NADP(+)]-like	13.5	8.6	11.07
LOC104912044	NHL repeat-containing protein 2-like	13.5	8.6	11.05
LOC104915494	fatty acyl-CoA hydrolase precursor, medium chain-like	11.8	10.2	11.03
LOC104909993	uncharacterized LOC104909993	12.7	9.4	11.03
LOC104915555	uncharacterized LOC104915555	12.7	9.4	11.02

LOC100543835	transforming growth factor beta receptor type 3-like	5.9	16.1	11.02
LOC104914835	NAD kinase 2, mitochondrial-like	12.7	9.3	11.00
LOC104915357	adenylate cyclase type 10-like	13.5	8.5	11.00
LOC104913110	uncharacterized LOC104913110	13.5	8.5	10.99
LOC100538956	multidrug and toxin extrusion protein 1-like	11.0	11.0	10.99
NXPH3	neurexophilin 3	11.8	10.1	10.99
LOC104912131	uncharacterized LOC104912131	13.5	8.5	10.98
LOC104911898	serine-rich coiled-coil domain-containing protein 2-like	11.0	11.0	10.98
MFAP5	microfibrillar associated protein 5	11.8	10.1	10.98
LOC100544113	keratin, type II cytoskeletal 4-like	12.7	9.3	10.97
TSNAXIP1	translin-associated factor X interacting protein 1	10.1	11.8	10.96
TNNI3K	TNNI3 interacting kinase	13.5	8.4	10.95
LOC104916365	uncharacterized LOC104916365	10.1	11.8	10.95
IL17REL	interleukin 17 receptor E-like	5.1	16.8	10.94
LOC104913291	uncharacterized LOC104913291	11.0	10.9	10.94
CABP2	calcium binding protein 2	9.3	12.5	10.92
FAIM2	Fas apoptotic inhibitory molecule 2	11.0	10.8	10.91
LOC104915130	leucine-rich repeat-containing protein 2-like	10.1	11.7	10.91
TRPV1	transient receptor potential cation channel, subfamily V, member 1	8.4	13.4	10.91
SLC15A2	solute carrier family 15 (oligopeptide transporter), member 2	9.3	12.5	10.90
LOC104915072	guanine deaminase-like	8.4	13.3	10.89
LOC104912109	voltage-dependent calcium channel subunit alpha-2/delta-4-like	8.4	13.3	10.87
ARL10	ADP-ribosylation factor-like 10	8.5	13.3	10.86
DKK3	dickkopf WNT signaling pathway inhibitor 3	10.1	11.6	10.85
LOC104912779	protein FAM81A-like	8.4	13.3	10.85
LOC100540409	mucosa-associated lymphoid tissue lymphoma translocation protein 1-like	16.9	4.8	10.83
LOC104909290	E3 ubiquitin-protein ligase TRIM33-like	11.0	10.7	10.83
KLHDC8B	kelch domain containing 8B	8.5	13.2	10.82
TEC	tec protein tyrosine kinase	9.3	12.3	10.82
CD7	CD7 molecule	5.9	15.7	10.78
LOC104909508	uncharacterized LOC104909508	5.9	15.7	10.78
LOC104915416	kinesin-like protein KIF3C	7.6	13.9	10.75
FADS6	fatty acid desaturase 6	6.8	14.7	10.73
LOC104916680	notchless protein homolog 1-like	6.8	14.7	10.73
LOC104913548	uncharacterized LOC104913548	14.3	7.1	10.73
LOC100544435	A-kinase anchor protein 7 isoform gamma-like	14.3	7.0	10.69
EPS8L2	EPS8-like 2	5.9	15.5	10.68
BAI2	brain-specific angiogenesis inhibitor 2	13.5	7.8	10.68
LOC104917366	feline leukemia virus subgroup C receptor-related protein 1-like	14.4	7.0	10.67
FAM83C	family with sequence similarity 83, member C	6.8	14.6	10.67
TMEM35	transmembrane protein 35	13.5	7.8	10.67
LOC104917418	DNA repair protein complementing XP-G cells-like	14.4	6.9	10.65
S100A16	S100 calcium binding protein A16	13.5	7.7	10.63
LOC104915764	myosin light chain kinase, smooth muscle-like	14.3	6.9	10.63
BDKRB2	bradykinin receptor B2	13.5	7.7	10.62
AMN1	antagonist of mitotic exit network 1 homolog (S. cerevisiae)	11.8	9.4	10.62
LOC104910234	F-box only protein 15-like	13.5	7.7	10.62
LOC104909863	sine oculis-binding protein homolog	12.7	8.5	10.61
LOC100303709	multidrug resistance protein 1	11.0	10.2	10.60
LOC100545656	fibrinogen-like protein 1	11.8	9.4	10.60
LOC104914378	von Willebrand factor A domain-containing protein 5A-like	11.8	9.3	10.59
KCNK5	potassium channel, subfamily K, member 5	10.1	11.0	10.58
LOC104911296	uncharacterized LOC104911296	11.0	10.2	10.58
LOC104910497	uncharacterized LOC104910497	11.8	9.3	10.57
LOC104912252	uncharacterized LOC104912252	10.1	11.0	10.56
LOC104914861	F-box only protein 4-like	10.1	11.0	10.56

LOC104914178	uncharacterized LOC104914178	11.0	10.1	10.56
LOC104913632	class II histocompatibility antigen, B-L beta chain-like	11.8	9.3	10.55
GPR87	G protein-coupled receptor 87	11.8	9.3	10.55
LOC104913230	uncharacterized LOC104913230	11.0	10.1	10.55
LOC104910530	uncharacterized LOC104910530	11.8	9.3	10.55
LOC100542849	TBC1 domain family member 5	11.0	10.1	10.54
STOX1	storkhead box 1	11.8	9.3	10.54
LOC100545776	vesicular inhibitory amino acid transporter-like	9.3	11.8	10.53
LOC104910921	uncharacterized LOC104910921	10.1	10.9	10.53
CIB3	calcium and integrin binding family member 3	11.0	10.1	10.52
FOXS1	forkhead box S1	11.0	10.1	10.52
LOC104914733	maestro heat-like repeat-containing protein family member 1	10.1	10.9	10.52
GRXCR2	glutaredoxin, cysteine rich 2	9.3	11.7	10.52
LOC100548288	transcription factor PU.1-like	11.0	10.1	10.51
GPR18	G protein-coupled receptor 18	8.4	12.6	10.51
LOC104912349	uncharacterized LOC104912349	11.0	10.0	10.51
C12H15orf26	chromosome 12 open reading frame, human C15orf26	10.1	10.9	10.50
LOC104909752	uncharacterized LOC104909752	11.0	10.0	10.50
LOC100543196	interferon-induced, double-stranded RNA-activated protein kinase-like	11.0	10.0	10.50
CNTNAP2	contactin associated protein-like 2	8.4	12.5	10.49
LOC104913562	uncharacterized LOC104913562	11.0	10.0	10.48
LOC100545207	uncharacterized LOC100545207	9.3	11.7	10.48
FAM117B	family with sequence similarity 117, member B	10.1	10.8	10.48
LOC104915658	uncharacterized LOC104915658	10.1	10.8	10.48
UNC79	unc-79 homolog (C. elegans)	10.1	10.8	10.48
LOC104915736	gem-associated protein 8 pseudogene	9.3	11.7	10.48
LOC100548957	RUN domain-containing protein 3B-like	9.3	11.7	10.47
LOC100541941	somatomedin-B and thrombospondin type-1 domain-containing protein-like	7.6	13.3	10.47
MGARP	mitochondria-localized glutamic acid-rich protein	9.3	11.6	10.46
LOC100539006	hemoglobin subunit alpha-A	7.6	13.3	10.45
LOC104909935	rho guanine nucleotide exchange factor 10-like	9.3	11.6	10.45
LOC104911520	serine/threonine-protein phosphatase 6 regulatory ankyrin repeat subunit A-like	9.3	11.6	10.45
GATM	glycine amidinotransferase (L-arginine:glycine amidinotransferase)	9.3	11.6	10.45
LOC104914917	zinc finger SWIM domain-containing protein 6-like	9.3	11.6	10.44
LOC100550849	cationic amino acid transporter 2-like	8.5	12.4	10.42
SYT16	synaptotagmin XVI	8.5	12.4	10.41
STAR	steroidogenic acute regulatory protein	8.4	12.3	10.39
LOC104911252	uncharacterized LOC104911252	7.6	13.2	10.39
LOC104913809	uncharacterized LOC104913809	6.8	14.0	10.37
GSTA3	glutathione S-transferase	5.9	14.8	10.36
LOC104914007	receptor-type tyrosine-protein phosphatase T-like	6.8	13.9	10.36
LOC100550893	taste receptor type 2 member 9-like	6.8	13.9	10.35
LOC100542437	uncharacterized LOC100542437	6.8	13.9	10.35
PRPH	peripherin	6.7	13.9	10.32
LOC100547524	vitamin D 25-hydroxylase	13.5	7.1	10.31
C2CD3	C2 calcium-dependent domain containing 3	7.6	13.0	10.31
RHOF	ras homolog family member F (in filopodia)	5.1	15.5	10.30
LOC104916532	zinc finger CCCH domain-containing protein 4-like	15.2	5.4	10.30
GPR83	G protein-coupled receptor 83	14.3	6.2	10.26
TRMT112	tRNA methyltransferase 11-2 homolog (S. cerevisiae)	13.5	7.0	10.26
LOC104915844	E3 ubiquitin-protein ligase HUWE1-like	12.7	7.8	10.26
CABYR	calcium binding tyrosine-(Y)-phosphorylation regulated	11.8	8.7	10.25
CLCA2	chloride channel accessory 2	11.8	8.7	10.24
PRR22	proline rich 22	12.7	7.8	10.23

LOC100540834	putative dimethylaniline monooxygenase [N-oxide-forming] 6	11.8	8.6	10.23
LOC104912820	long-chain-fatty-acid--CoA ligase ACSBG1-like	11.8	8.6	10.22
RND1	Rho family GTPase 1	12.7	7.8	10.22
LOC104911381	uncharacterized LOC104911381	11.8	8.6	10.21
CPN1	carboxypeptidase N, polypeptide 1	10.1	10.3	10.20
LOC104911662	uncharacterized LOC104911662	11.8	8.6	10.20
CFAP99	cilia and flagella associated protein 99	12.6	7.7	10.20
LOC100542617	neuronal pentraxin receptor-like	12.7	7.7	10.19
LOC104909629	uncharacterized LOC104909629	10.1	10.2	10.18
LOC100543242	serine/threonine-protein kinase PAK 1-like	11.8	8.5	10.16
LOC104912371	netrin-G1-like	11.8	8.5	10.16
LOC104912259	uncharacterized LOC104912259	11.0	9.3	10.15
RUNX2	runt-related transcription factor 2	11.0	9.3	10.15
ABCA4	ATP-binding cassette, sub-family A (ABC1), member 4	10.1	10.1	10.14
ZBTB20	zinc finger and BTB domain containing 20	11.0	9.3	10.14
LOC104912135	uncharacterized LOC104912135	7.6	12.6	10.10
LOC104910470	zinc finger protein ZFAT-like	9.3	10.9	10.09
LOC104917000	G-protein coupled receptor 98-like	10.1	10.1	10.09
LOC104910576	CCR4-NOT transcription complex subunit 6-like	11.0	9.2	10.09
LOC100544441	glycerol-3-phosphate acyltransferase 3-like	9.3	10.9	10.08
LOC100546792	sine oculis-binding protein homolog	8.5	11.7	10.08
LOC100545914	ATP synthase subunit alpha, mitochondrial-like	10.1	10.0	10.08
C7H21orf58	chromosome 7 open reading frame, human C21orf58	8.4	11.7	10.05
LOC104910229	uncharacterized LOC104910229	8.4	11.7	10.05
LOC104916167	butyrophilin subfamily 1 member A1-like	17.7	2.4	10.05
CLDN25	claudin 25	7.6	12.4	10.02
PEX5L	peroxisomal biogenesis factor 5-like	7.6	12.4	10.01
C19H9orf172	chromosome 19 open reading frame, human C9orf172	7.6	12.4	9.99
LOC100543156	solute carrier family 22 member 13-like	7.6	12.3	9.97
LOC104911357	uncharacterized LOC104911357	7.6	12.3	9.95
MEGF6	multiple EGF-like-domains 6	6.8	13.1	9.93
LOC100538538	creatine kinase U-type, mitochondrial	14.3	5.5	9.93
LOC104915828	piwi-like protein 2	15.2	4.6	9.93
GPR171	G protein-coupled receptor 171	5.9	13.9	9.90
KBTBD12	kelch repeat and BTB (POZ) domain containing 12	13.5	6.3	9.90
HORMAD1	HORMA domain containing 1	5.9	13.9	9.88
LOC104913973	outer dense fiber protein 3-like	11.8	7.9	9.87
LAMB3	laminin, beta 3	14.4	5.3	9.84
LOC104909264	multidrug resistance-associated protein 4-like	12.7	7.0	9.83
TLE2	transducin-like enhancer of split 2	12.7	7.0	9.83
NPS	neuropeptide S	11.8	7.8	9.80
ADRB2	adrenoceptor beta 2, surface	11.0	8.6	9.80
LOC100542243	dynein light chain LC6, flagellar outer arm-like	11.8	7.7	9.79
PKHD1	polycystic kidney and hepatic disease 1 (autosomal recessive)	11.0	8.6	9.78
LOC100542871	taste receptor type 1 member 3	11.8	7.7	9.77
C2H1orf101	chromosome 2 open reading frame, human C1orf101	10.1	9.4	9.77
IKZF3	IKAROS family zinc finger 3 (Aiolos)	9.3	10.2	9.76
LOC104916158	uncharacterized LOC104916158	9.3	10.2	9.76
LOC104910922	uncharacterized LOC104910922	10.1	9.4	9.75
KBTBD3	kelch repeat and BTB (POZ) domain containing 3	10.2	9.3	9.75
LOC104916305	sporozoite surface protein 2-like	11.0	8.5	9.73
LOC104909599	uncharacterized LOC104909599	11.0	8.5	9.73
LOC104916578	uncharacterized LOC104916578	10.2	9.3	9.73
LOC104911459	host cell factor 2-like	11.0	8.5	9.73
LRRC31	leucine rich repeat containing 31	9.3	10.1	9.72
FAM178B	family with sequence similarity 178, member B	9.3	10.1	9.71
LOC104913409	chemokine-like receptor 1	10.1	9.3	9.71
TMEM132D	transmembrane protein 132D	9.3	10.1	9.70

LOC104912169	uncharacterized LOC104912169	9.3	10.1	9.70
LOC104913055	uncharacterized LOC104913055	9.3	10.1	9.70
LOC104909656	uncharacterized LOC104909656	10.1	9.3	9.69
LOC104912455	uncharacterized LOC104912455	8.4	10.9	9.69
LOC100549795	msx2-interacting protein-like	10.1	9.2	9.69
NR0B2	nuclear receptor subfamily 0, group B, member 2	5.9	13.5	9.68
LOC100548199	transmembrane protein C15orf27-like	8.5	10.9	9.68
LOC104909748	uncharacterized LOC104909748	8.5	10.9	9.68
LOC104914166	arginine-glutamic acid dipeptide repeats protein-like	8.5	10.9	9.68
ZNF385C	zinc finger protein 385C	8.5	10.9	9.68
CCDC36	coiled-coil domain containing 36	10.1	9.2	9.67
LOC100546153	dipeptidase 2-like	6.8	12.6	9.66
LOC104917498	uncharacterized LOC104917498	10.1	9.2	9.66
RPS6KA2	ribosomal protein S6 kinase, 90kDa, polypeptide 2	8.4	10.9	9.65
LOC104913861	uncharacterized LOC104913861	5.9	13.4	9.65
LOC104911334	uncharacterized LOC104911334	7.6	11.7	9.63
LOC104913331	putative E3 ubiquitin-protein ligase SH3RF2	7.6	11.7	9.63
CA12	carbonic anhydrase XII	7.6	11.6	9.61
MMRN1	multimerin 1	5.9	13.3	9.61
LOC104917057	tetratricopeptide repeat protein 39B-like	5.9	13.3	9.60
LOC104909816	uncharacterized LOC104909816	5.9	13.3	9.58
KMO	kynurenine 3-monooxygenase (kynurenine 3-hydroxylase)	6.8	12.4	9.57
LOC104910473	thyroglobulin-like	5.9	13.2	9.54
LOC104913178	uncharacterized LOC104913178	13.5	5.4	9.43
LOC104911070	uncharacterized LOC104911070	10.2	8.7	9.41
CABLES2	Cdk5 and Abl enzyme substrate 2	11.0	7.8	9.41
LOC100542387	tumor necrosis factor receptor type 1-associated DEATH domain protein-like	11.8	6.9	9.39
LOC100541366	origin recognition complex subunit 1	11.8	6.9	9.39
SLC4A10	solute carrier family 4, sodium bicarbonate transporter, member 10	11.0	7.8	9.39
FGD5	FYVE, RhoGEF and PH domain containing 5	10.1	8.6	9.37
LOC104913849	proton myo-inositol cotransporter-like	10.1	8.6	9.36
PAK7	p21 protein (Cdc42/Rac)-activated kinase 7	10.1	8.6	9.36
TMEM114	transmembrane protein 114	10.1	8.6	9.36
WDR31	WD repeat domain 31	10.1	8.6	9.36
LOC100539289	phosphatidate phosphatase LPIN1	10.1	8.6	9.35
NOX3	NADPH oxidase 3	9.3	9.4	9.34
LOC104910064	uncharacterized LOC104910064	10.1	8.5	9.33
LOC100545648	tubulin alpha-3 chain	11.0	7.7	9.33
LOC100548067	cryptic protein-like	11.0	7.7	9.31
LHX6	LIM homeobox 6	9.3	9.3	9.31
LOC104916661	proprotein convertase subtilisin/kexin type 5-like	9.3	9.3	9.31
ADAM22	ADAM metallopeptidase domain 22	10.1	8.5	9.31
BTBD8	BTB (POZ) domain containing 8	8.5	10.1	9.30
LOC104915110	uncharacterized LOC104915110	8.5	10.1	9.30
TRIM67	tripartite motif containing 67	7.6	11.0	9.30
LOC104913732	uncharacterized LOC104913732	9.3	9.3	9.30
VIP	vasoactive intestinal peptide	10.1	8.5	9.30
LOC104909602	uncharacterized LOC104909602	8.4	10.1	9.29
LOC100549268	aldehyde oxidase 2-like	9.3	9.3	9.28
LOC100550655	vitamin D3 hydroxylase-associated protein-like	9.3	9.3	9.28
RASL12	RAS-like, family 12	9.3	9.3	9.28
LOC104911456	uncharacterized LOC104911456	8.5	10.1	9.28
NRG2	neuregulin 2	8.5	10.1	9.28
SORL1	sortilin-related receptor, L(DLR class) A repeats containing	8.4	10.1	9.27
LOC104909687	uncharacterized LOC104909687	8.4	10.1	9.27
LOC100539065	ganglioside-induced differentiation-associated protein 1-like 1	9.3	9.3	9.27

LOC104911560	uncharacterized LOC104911560	8.4	10.1	9.27
LOC100539445	tensin-3	9.3	9.2	9.26
GYG2	glycogenin 2	8.4	10.1	9.25
VAMP1	vesicle-associated membrane protein 1 (synaptobrevin 1)	5.1	13.4	9.24
LOC104910488	protein EFR3 homolog A-like	9.3	9.2	9.23
LOC104917532	uncharacterized LOC104917532	6.8	11.7	9.23
LOC104911740	uncharacterized LOC104911740	7.6	10.9	9.23
PVRL4	poliovirus receptor-related 4	6.8	11.7	9.23
FAM19A1	family with sequence similarity 19 (chemokine (C-C motif)-like), member A1	8.5	10.0	9.22
LOC100547109	transmembrane protein 151B-like	7.6	10.8	9.21
SUCNR1	succinate receptor 1	6.8	11.7	9.21
LOC104911579	uncharacterized LOC104911579	7.6	10.8	9.20
LOC100541541	voltage-dependent calcium channel subunit alpha-2/delta-1-like	8.4	9.9	9.19
KCNH5	potassium voltage-gated channel, subfamily H (eag-related), member 5	5.1	13.3	9.19
LOC104913051	uncharacterized LOC104913051	6.8	11.6	9.19
C2H1orf115	chromosome 2 open reading frame, human C1orf115	7.6	10.7	9.17
MAB21L3	mab-21-like 3 (C. elegans)	6.8	11.5	9.15
LOC104912916	fatty acyl-CoA hydrolase precursor, medium chain-like	13.5	4.8	9.14
LOC100546312	protein Jade-2-like	5.1	13.1	9.11
LOC104911661	striated muscle-specific serine/threonine-protein kinase-like	4.2	13.9	9.08
LOC104916385	carbohydrate sulfotransferase 7-like	12.7	5.5	9.07
LOC100546440	ecto-NOX disulfide-thiol exchanger 1-like	4.2	13.9	9.07
UNC5D	unc-5 homolog D (C. elegans)	5.9	12.2	9.07
LOC100546493	teneurin-3-like	5.1	13.1	9.06
C12H15orf48	chromosome 12 open reading frame, human C15orf48	10.2	7.9	9.05
LOC100548568	mitochondrial peptide methionine sulfoxide reductase-like	12.7	5.4	9.02
EGFLAM	EGF-like, fibronectin type III and laminin G domains	11.0	7.1	9.02
LOC100539648	mitogen-activated protein kinase kinase kinase MLK4	11.0	7.1	9.02
LOC100545337	sulfotransferase family cytosolic 2B member 1-like	11.8	6.2	9.01
RAPGEFL1	Rap guanine nucleotide exchange factor (GEF)-like 1	12.7	5.4	9.00
RASAL1	RAS protein activator like 1 (GAP1 like)	9.3	8.7	9.00
LOC104915462	low-density lipoprotein receptor-related protein 1-like	11.0	7.0	8.99
VSIG8	V-set and immunoglobulin domain containing 8	11.0	7.0	8.99
F3	coagulation factor III (thromboplastin, tissue factor)	11.0	7.0	8.98
LOC100551033	deleted in malignant brain tumors 1 protein-like	11.0	7.0	8.98
LOC104913937	double C2-like domain-containing protein beta	11.0	7.0	8.98
LOC104914652	platelet glycoprotein Ib alpha chain-like	11.0	6.9	8.97
TMEM40	transmembrane protein 40	10.1	7.8	8.96
LOC104912080	uncharacterized LOC104912080	9.3	8.6	8.95
LOC104916248	uncharacterized LOC104916248	11.0	6.9	8.95
TMEM108	transmembrane protein 108	8.5	9.4	8.94
LOC104909389	uncharacterized LOC104909389	11.0	6.9	8.94
HOXD8	homeobox D8	8.5	9.4	8.94
FOXL2	forkhead box L2	9.3	8.6	8.94
FAM159A	family with sequence similarity 159, member A	10.1	7.7	8.94
LOC104915986	ankyrin repeat and SOCS box protein 16-like	8.4	9.4	8.93
LOC104914467	uncharacterized LOC104914467	10.1	7.7	8.93
MEOX1	mesenchyme homeobox 1	8.4	9.4	8.92
CAMK1G	calcium/calmodulin-dependent protein kinase IG	10.1	7.7	8.92
KRT222	keratin 222	10.1	7.7	8.92
DHX9	DEAH (Asp-Glu-Ala-His) box helicase 9	8.4	9.4	8.91
LOC104910749	uncharacterized LOC104910749	8.4	9.4	8.91
LOC104912431	uncharacterized LOC104912431	8.4	9.4	8.91
FZD5	frizzled class receptor 5	9.3	8.5	8.90
LOC104911420	uncharacterized LOC104911420	9.3	8.5	8.90
LOC100547714	membrane-bound transcription factor site-2 protease-like	8.4	9.3	8.89

TRIM71	tripartite motif containing 71, E3 ubiquitin protein ligase	9.3	8.5	8.89
LOC104910283	piezo-type mechanosensitive ion channel component 2-like	8.5	9.3	8.88
LOC104913814	immunoglobulin superfamily member 3-like	9.3	8.5	8.88
LOC104912792	probable E3 ubiquitin-protein ligase HERC1	7.6	10.1	8.88
LOC104913786	regulatory-associated protein of mTOR-like	9.3	8.5	8.88
LOC100550777	bone morphogenetic protein 8A-like	8.4	9.3	8.87
LOC100545878	alpha-fetoprotein	7.6	10.1	8.87
LOC104913136	importin subunit alpha-5-like	6.8	11.0	8.87
PTPN7	protein tyrosine phosphatase, non-receptor type 7	8.5	9.3	8.86
LOC104913764	uncharacterized LOC104913764	8.4	9.3	8.85
LOC104909997	grainyhead-like protein 1 homolog	9.3	8.4	8.84
AMZ1	archaelysin family metallopeptidase 1	7.6	10.1	8.84
LOC100541145	vasotocin-neurophysin VT	7.6	10.1	8.84
LOC100540211	phosphatidylinositol 3,4,5-trisphosphate 3-phosphatase TPTE2-like	6.8	10.9	8.83
LOC104911150	diphthine--ammonia ligase-like	6.8	10.9	8.83
LOC104916646	histone H2B 1/2/3/4/6	6.8	10.9	8.81
STAC	SH3 and cysteine rich domain	6.8	10.9	8.81
MLN	motilin	7.6	10.0	8.81
KCNE2	potassium voltage-gated channel, Isk-related family, member 2	6.7	10.9	8.80
GCHFR	GTP cyclohydrolase I feedback regulator	5.1	12.5	8.80
EFCC1	EF-hand and coiled-coil domain containing 1	5.1	12.5	8.79
LOC104914961	uncharacterized LOC104914961	5.9	11.7	8.78
DGKI	diacylglycerol kinase, iota	5.9	11.6	8.77
FBN2	fibrillin 2	5.1	12.5	8.77
NMNAT2	nicotinamide nucleotide adenylyltransferase 2	5.1	12.4	8.74
ENO4	enolase family member 4	14.4	3.1	8.73
LYVE1	lymphatic vessel endothelial hyaluronan receptor 1	6.8	10.7	8.73
LOC104909319	myotubularin-related protein 13-like	5.9	11.5	8.72
LOC104913592	uncharacterized LOC104913592	13.5	3.9	8.69
LOC104911284	uncharacterized LOC104911284	11.8	5.5	8.68
BSN	bassoon presynaptic cytomatrix protein	11.8	5.5	8.67
LOC104916498	glucosylceramidase-like	5.1	12.3	8.66
LGALS2	lectin, galactoside-binding, soluble, 2	4.2	13.1	8.66
LOC104913943	nuclear receptor corepressor 1-like	12.7	4.6	8.65
LOC104913664	uncharacterized LOC104913664	11.8	5.5	8.65
LOC100546965	uncharacterized protein KIAA1614-like	11.0	6.3	8.62
LOC104909646	uncharacterized LOC104909646	10.1	7.1	8.61
LOC104911569	uncharacterized LOC104911569	11.8	5.4	8.60
LOC104915045	uncharacterized LOC104915045	11.0	6.2	8.60
LOC104909558	protein jagged-1b-like	10.1	7.0	8.59
SYT13	synaptotagmin XIII	10.1	7.0	8.58
CAPN9	calpain 9	9.3	7.9	8.58
LOC100546815	2-phosphoxylose phosphatase 1	9.3	7.9	8.58
DLX6	distal-less homeobox 6	10.1	7.0	8.57
LOC104912052	nebulin-related-anchoring protein-like	11.0	6.2	8.57
LOC104917340	uncharacterized protein MGC39606-like	9.3	7.8	8.57
LOC104910373	zinc finger homeobox protein 4-like	10.1	7.0	8.57
LETMD1	LETM1 domain containing 1	9.3	7.8	8.56
LOC100545464	semaphorin-3A	9.3	7.8	8.56
GRIN2C	glutamate receptor, ionotropic, N-methyl D-aspartate 2C	7.6	9.5	8.55
PPP1R10	protein phosphatase 1, regulatory subunit 10	9.3	7.8	8.55
LHX2	LIM homeobox 2	9.3	7.8	8.55
SLC44A3	solute carrier family 44, member 3	11.0	6.1	8.54
MAB21L1	mab-21-like 1 (C. elegans)	7.6	9.5	8.54
LOC104913785	regulatory-associated protein of mTOR-like	11.0	6.1	8.54
LOC100545461	antigen-presenting glycoprotein CD1d-like	9.3	7.8	8.53
LOC104911591	uncharacterized LOC104911591	9.3	7.8	8.53

LOC104912476	uncharacterized LOC104912476	8.4	8.6	8.53
LOC104915123	coiled-coil domain-containing protein 146-like	8.4	8.6	8.53
LOC100550995	epidermal retinol dehydrogenase 2-like	9.3	7.7	8.52
LOC104914256	uncharacterized LOC104914256	9.3	7.7	8.52
LOC104910744	uncharacterized LOC104910744	8.4	8.6	8.51
LOC104914916	gamma-taxilin-like	8.5	8.5	8.50
WNK4	WNK lysine deficient protein kinase 4	7.6	9.4	8.50
LOC104917499	uncharacterized LOC104917499	7.6	9.4	8.49
LOC104909652	uncharacterized LOC104909652	7.6	9.4	8.49
LOC104912602	aquaporin-12-like	7.6	9.4	8.49
LOC104912964	uncharacterized LOC104912964	7.6	9.3	8.48
LOC104910049	uncharacterized LOC104910049	8.5	8.5	8.48
LOC100542989	zinc finger protein Helios-like	9.3	7.7	8.48
CACNG2	calcium channel, voltage-dependent, gamma subunit 2	7.6	9.3	8.47
LOC104915154	uncharacterized LOC104915154	8.5	8.5	8.46
LOC100539852	urotensin-2 receptor-like	8.5	8.5	8.46
TMTC2	transmembrane and tetratricopeptide repeat containing 2	7.6	9.3	8.46
LOC104909779	uncharacterized LOC104909779	9.3	7.6	8.46
EPB41L4A	erythrocyte membrane protein band 4.1 like 4A	7.6	9.3	8.46
LOC104909301	keratin, type I cytoskeletal 13-like	8.4	8.5	8.46
LOC100551010	uricase-like	9.3	7.6	8.45
LOC104910190	uncharacterized LOC104910190	6.8	10.1	8.45
ALB	albumin	7.6	9.3	8.44
LOC100539788	neurolysin, mitochondrial-like	8.5	8.4	8.44
LOC100550023	p53 apoptosis effector related to PMP-22-like	6.8	10.1	8.44
LOC104910617	tyrosine-protein phosphatase non-receptor type 13-like	7.6	9.3	8.44
LOC104909209	anosmin-1-like	7.6	9.3	8.43
LOC104911013	uncharacterized LOC104911013	5.9	10.9	8.43
LOC100544354	uncharacterized LOC100544354	7.6	9.2	8.42
PFKFB2	6-phosphofructo-2-kinase/fructose-2,6-biphosphatase 2	6.8	10.1	8.41
LOC100539736	nuclear receptor subfamily 4 group A member 2-like	6.7	10.1	8.40
LOC104912060	protein FAM196A-like	7.6	9.2	8.40
LOC104910659	uncharacterized LOC104910659	5.9	10.9	8.40
LOC100548828	complement receptor type 1-like	6.8	10.0	8.39
MRVI1	murine retrovirus integration site 1 homolog	5.9	10.9	8.39
MUC3A	mucin 3A, cell surface associated	16.0	0.8	8.38
ETNPPL	ethanolamine-phosphate phospho-lyase	5.9	10.8	8.38
RTKN2	rhotekin 2	5.1	11.7	8.37
LOC100542220	N-acetylaspartate synthetase-like	5.9	10.8	8.37
NYX	nyctalopin	5.1	11.6	8.34
LOC104912954	ubiquitin carboxyl-terminal hydrolase CYLD-like	5.1	11.6	8.34
DKK2	dickkopf WNT signaling pathway inhibitor 2	5.1	11.6	8.34
LOC100541147	actin, alpha skeletal muscle B-like	3.4	13.3	8.32
LOC104912516	uncharacterized LOC104912516	5.9	10.7	8.31
LOC104914193	uncharacterized LOC104914193	13.5	3.1	8.31
LOC104915708	uncharacterized LOC104915708	12.7	3.8	8.26
LOC104912995	uncharacterized LOC104912995	12.6	3.9	8.26
ATP2A3	ATPase, Ca++ transporting, ubiquitous	11.8	4.7	8.26
LOC100546372	BPI fold-containing family B member 4-like	11.8	4.7	8.25
LOC104912528	uncharacterized LOC104912528	11.0	5.5	8.23
PPP1R27	protein phosphatase 1, regulatory subunit 27	11.8	4.6	8.23
LOC104910529	uncharacterized LOC104910529	11.8	4.6	8.22
PTGS2	prostaglandin-endoperoxide synthase 2 (prostaglandin G/H synthase and cyclooxygenase)	11.8	4.6	8.22
LOC104915035	centlein-like	11.8	4.6	8.22
LOC104913856	uroplakin-3b-like protein	10.1	6.3	8.20
LOC104915052	uncharacterized LOC104915052	10.1	6.3	8.20
KIF19	kinesin family member 19	9.3	7.1	8.18

CNKSR1	connector enhancer of kinase suppressor of Ras 1	10.1	6.2	8.18
LOC104909298	mitogen-activated protein kinase kinase kinase 15-like	9.3	7.1	8.18
ADARB2	adenosine deaminase, RNA-specific, B2 (non-functional)	7.6	8.7	8.17
LOC104911701	secretory phospholipase A2 receptor-like	9.3	7.0	8.15
LOC104909994	uncharacterized LOC104909994	10.1	6.2	8.15
LOC104917539	uncharacterized LOC104917539	8.5	7.8	8.14
PLSCR5	phospholipid scramblase family, member 5	8.5	7.8	8.14
LOC104912233	uncharacterized LOC104912233	8.5	7.8	8.13
LOC104911172	RNA-binding protein Nova-1-like	9.3	6.9	8.12
FAM110C	family with sequence similarity 110, member C	7.6	8.6	8.11
LOC100539008	glutamine synthetase	5.9	10.3	8.10
LOC104910879	uncharacterized LOC104910879	7.6	8.6	8.10
KLHL38	kelch-like family member 38	8.5	7.7	8.10
LOC104914684	protein ENL-like	8.5	7.7	8.10
LOC104911149	uncharacterized LOC104911149	6.8	9.4	8.09
TAGAP	T-cell activation RhoGTPase activating protein	7.6	8.6	8.09
LRRC74A	leucine rich repeat containing 74A	6.8	9.4	8.09
CERS6	ceramide synthase 6	7.6	8.6	8.09
LOC104911658	uncharacterized LOC104911658	7.6	8.6	8.09
PPP1R1C	protein phosphatase 1, regulatory (inhibitor) subunit 1C	7.6	8.6	8.09
KIAA1377	KIAA1377 ortholog	5.9	10.3	8.09
LOC100543003	DNA-binding protein SATB1-like	7.6	8.5	8.08
SCNN1B	sodium channel, non-voltage-gated 1, beta subunit	6.8	9.4	8.07
LOC104912841	uncharacterized LOC104912841	7.6	8.5	8.07
LOC104916183	N-acetylserotonin O-methyltransferase-like protein	8.4	7.7	8.07
LOC104910431	regulating synaptic membrane exocytosis protein 2-like	9.3	6.9	8.07
RHNO1	RAD9-HUS1-RAD1 interacting nuclear orphan 1	6.8	9.4	8.07
LOC104913575	uncharacterized LOC104913575	6.8	9.4	8.07
UPP1	uridine phosphorylase 1	6.8	9.4	8.07
GRIP1	glutamate receptor interacting protein 1	5.9	10.2	8.07
LOC104910896	uncharacterized LOC104910896	5.9	10.2	8.06
LOC100543080	folate receptor alpha-like	7.6	8.5	8.06
LOC104912156	uncharacterized LOC104912156	7.6	8.5	8.05
LOC100547088	class II histocompatibility antigen, B-L beta chain-like	6.8	9.3	8.05
UNC119	unc-119 homolog (C. elegans)	6.8	9.3	8.05
LOC100542157	G-protein coupled receptor 183-like	5.9	10.2	8.04
LOC104910940	membrane-spanning 4-domains subfamily A member 15-like	7.6	8.5	8.04
FAM179A	family with sequence similarity 179, member A	5.9	10.1	8.03
LOC104917582	neurobeachin-like	6.8	9.3	8.03
PPM1H	protein phosphatase, Mg2+/Mn2+ dependent, 1H	6.7	9.3	8.03
LOC100550739	band 4.1-like protein 3	7.6	8.4	8.02
CAPN8	calpain 8	5.1	10.9	8.01
LOC104917513	ETS-related transcription factor Elf-1-like	6.8	9.2	8.00
NOTCH2NL	notch 2 N-terminal like	5.9	10.1	7.99
LOC100541041	zinc finger protein 407	6.8	9.2	7.97
LOC104912449	oxysterol-binding protein-related protein 9-like	6.7	9.2	7.96
LOC104911865	voltage-dependent L-type calcium channel subunit alpha-1C-like	5.1	10.8	7.94
LOC104914309	uncharacterized LOC104914309	4.2	11.6	7.92
LOC104915167	uncharacterized LOC104915167	3.4	12.3	7.86
LOC104915047	uncharacterized LOC104915047	11.8	3.9	7.86
LOC104917132	MARVEL domain-containing protein 3-like	11.0	4.7	7.84
ALOX5	arachidonate 5-lipoxygenase	11.0	4.7	7.84
MAMDC4	MAM domain containing 4	11.0	4.7	7.83
LOC100546659	caltractin-like	9.3	6.3	7.82
SERGEF	secretion regulating guanine nucleotide exchange factor	9.3	6.3	7.82
TRPV3	transient receptor potential cation channel, subfamily V, member 3	11.0	4.6	7.80
LOC104913278	uncharacterized LOC104913278	10.1	5.5	7.80

LOC104909625	cysteine-rich motor neuron 1 protein-like	10.1	5.4	7.79
IL10RA	interleukin 10 receptor, alpha	9.3	6.3	7.78
LOC104911754	epidermal growth factor receptor kinase substrate 8-like	8.5	7.1	7.78
ST8SIA1	ST8 alpha-N-acetyl-neuraminide alpha-2,8-sialyltransferase 1	8.4	7.1	7.78
FAM84A	family with sequence similarity 84, member A	9.3	6.3	7.78
DRAM1	DNA-damage regulated autophagy modulator 1	9.3	6.2	7.76
LOC104909728	ensconsin-like	9.3	6.2	7.76
LOC104913536	uncharacterized LOC104913536	8.5	7.1	7.76
LOC104912593	ETS translocation variant 5-like	9.3	6.2	7.76
PLP1	proteolipid protein 1	9.3	6.2	7.76
TRIM66	tripartite motif containing 66	7.6	7.9	7.76
LOC100544274	voltage-dependent calcium channel subunit alpha-2/delta-4	8.5	7.1	7.76
LOC100550755	probable G-protein coupled receptor 83	10.1	5.4	7.76
LOC104917525	diacylglycerol kinase eta-like	8.4	7.1	7.76
C19H9orf173	chromosome 19 open reading frame, human C9orf173	9.3	6.2	7.75
IL1RL1	interleukin 1 receptor-like 1	8.5	7.0	7.74
LOC104912324	uncharacterized LOC104912324	8.5	7.0	7.74
LOC104910341	uncharacterized LOC104910341	8.4	7.0	7.74
LOC104917133	uncharacterized LOC104917133	8.4	7.0	7.74
NCF4	neutrophil cytosolic factor 4, 40kDa	9.3	6.2	7.74
C7	complement component 7	10.1	5.4	7.74
LOC104909829	centrosomal protein of 85 kDa-like pseudogene	6.7	8.7	7.72
LOC104917384	PHD finger protein 7-like	9.3	6.2	7.72
ENPP2	ectonucleotide pyrophosphatase/phosphodiesterase 2	8.5	7.0	7.72
LOC104915119	protein patched homolog 1-like	8.5	7.0	7.72
SLC12A3	solute carrier family 12 (sodium/chloride transporter), member 3	8.5	7.0	7.72
LOC104912323	uncharacterized LOC104912323	7.6	7.8	7.72
LOC104914013	receptor-type tyrosine-protein phosphatase T-like	7.6	7.8	7.72
LOC100543059	cell division cycle 7-related protein kinase	8.4	7.0	7.72
LOC100550050	uncharacterized LOC100550050	8.4	7.0	7.72
LOC104910611	uncharacterized LOC104910611	8.4	7.0	7.72
SLC5A3	solute carrier family 5 (sodium/myo-inositol cotransporter), member 3	6.8	8.7	7.72
LRGUK	leucine-rich repeats and guanylate kinase domain containing	8.4	7.0	7.71
LOC104912628	serine/threonine-protein kinase ATR-like	6.8	8.7	7.71
AKAP14	A kinase (PRKA) anchor protein 14	6.7	8.7	7.71
LOC104909285	exocyst complex component 6B-like	7.6	7.8	7.70
LOC104911996	uncharacterized LOC104911996	7.6	7.8	7.70
C5H15orf62	chromosome 5 open reading frame, human C15orf62	7.6	7.8	7.69
LOC104911692	uncharacterized LOC104911692	7.6	7.8	7.69
THBS2	thrombospondin 2	7.6	7.8	7.69
FAM149A	family with sequence similarity 149, member A	7.6	7.8	7.69
LOC104910463	secreted Ly-6/uPAR-related protein 1-like	6.7	8.6	7.68
LOC104912245	probable ATP-dependent DNA helicase HFM1	7.6	7.7	7.68
LOC100539575	anthrax toxin receptor 1-like	5.9	9.4	7.67
COL14A1	collagen, type XIV, alpha 1	6.7	8.6	7.66
CLDN11	claudin 11	6.8	8.5	7.66
LOC104914906	zinc finger SWIM domain-containing protein 6-like	7.6	7.7	7.66
LOC104913373	protein CLEC16A-like	6.8	8.5	7.66
FAH	fumarylacetoacetate hydrolase (fumarylacetoacetase)	5.9	9.4	7.65
LOC104914271	cAMP-dependent protein kinase inhibitor beta-like	6.8	8.5	7.65
LOC104911635	uncharacterized LOC104911635	5.9	9.4	7.65
LOC104913443	uncharacterized LOC104913443	5.9	9.4	7.65
TMIGD1	transmembrane and immunoglobulin domain containing 1	7.6	7.7	7.65
S100B	S100 calcium binding protein B	5.9	9.4	7.65
LOC104911071	uncharacterized LOC104911071	5.9	9.4	7.65
LOC104910866	N-acetylaspartate synthetase-like	6.7	8.5	7.65
LOC104915091	vacuolar protein sorting-associated protein 13A-like	6.7	8.5	7.65

LOC104914942	sex comb on midleg-like protein 1	6.8	8.5	7.64
B3GNT7	UDP-GlcNAc:betaGal beta-1,3-N-acetylglucosaminyltransferase 7	6.8	8.5	7.63
LOC104916466	protein asteroid homolog 1-like	7.6	7.7	7.63
LOC104917580	sister chromatid cohesion protein PDS5 homolog B-like	5.1	10.2	7.62
LOC104909884	activating signal cointegrator 1 complex subunit 3-like	7.6	7.6	7.62
LOC104909669	kinesin-like protein KIF28P	6.8	8.5	7.61
LOC104917538	voltage-dependent calcium channel subunit alpha-2/delta-1-like	4.2	11.0	7.61
LOC100549626	rap1 GTPase-GDP dissociation stimulator 1-like	5.9	9.3	7.60
RAD21L1	RAD21-like 1 (S. pombe)	5.9	9.3	7.60
LRIT3	leucine-rich repeat, immunoglobulin-like and transmembrane domains 3	6.8	8.4	7.59
MATK	megakaryocyte-associated tyrosine kinase	5.9	9.3	7.59
PRLR	prolactin receptor	5.9	9.3	7.59
LOC104915522	uncharacterized LOC104915522	5.1	10.1	7.59
LOC104912572	uncharacterized LOC104912572	4.2	10.9	7.58
LOC104915039	centlein-like	5.1	10.1	7.58
TDRD9	tudor domain containing 9	5.1	10.1	7.57
LOC104917618	serine-protein kinase ATM-like	5.9	9.2	7.57
LOC104911092	MAX gene-associated protein-like	5.1	10.1	7.57
LOC104909581	uncharacterized LOC104909581	5.1	10.0	7.55
LOC104909291	myosin heavy chain, skeletal muscle, adult-like	3.4	11.7	7.54
RPH3A	rabphilin 3A	4.2	10.8	7.51
BMP3	bone morphogenetic protein 3	5.1	9.9	7.50
LOC104909718	uncharacterized LOC104909718	3.4	11.6	7.48
LOC104915122	protein patched homolog 1-like	3.4	11.6	7.47
RNF182	ring finger protein 182	1.7	13.2	7.46
LOC100538559	C-type lectin domain family 2 member B	11.0	3.9	7.45
LOC104911559	endogenous retrovirus group FC1 Env polyprotein	11.0	3.9	7.45
MCM9	minichromosome maintenance complex component 9	2.5	12.3	7.44
NXNL2	nucleoredoxin-like 2	11.0	3.9	7.43
LOC100541319	dynein light chain 2, cytoplasmic-like	9.3	5.5	7.43
LOC104909277	T-cell receptor-associated transmembrane adapter 1	10.1	4.7	7.43
CCL24	chemokine (C-C motif) ligand 24	11.0	3.8	7.41
LOC104912807	unconventional myosin-Va-like	11.0	3.8	7.41
LOC100544231	coiled-coil domain-containing protein 178-like	9.3	5.5	7.40
LOC104913375	uncharacterized LOC104913375	10.1	4.7	7.40
PRPS2	phosphoribosyl pyrophosphate synthetase 2	9.3	5.5	7.39
LOC104911148	intron-binding protein aquarius-like	8.5	6.3	7.38
HVCN1	hydrogen voltage-gated channel 1	9.3	5.5	7.38
CCNO	cyclin O	7.6	7.1	7.38
LOC100544367	alpha-(1,3)-fucosyltransferase 6-like	7.6	7.1	7.37
LOC100540905	fibroblast growth factor receptor 2	8.4	6.3	7.37
LOC104911439	signal transducing adapter molecule 1	9.3	5.4	7.36
LOC104916518	riboflavin kinase-like	9.3	5.4	7.36
NKAIN1	Na+/K+ transporting ATPase interacting 1	9.3	5.4	7.36
LOC100546639	transmembrane protein 181	10.1	4.6	7.36
LOC100548666	GTP-binding protein Rhes-like	7.6	7.1	7.36
LOC104911502	TBC1 domain family member 5-like	8.5	6.3	7.36
GDAP1	ganglioside induced differentiation associated protein 1	8.4	6.3	7.35
LOC104917500	uncharacterized LOC104917500	8.5	6.2	7.34
HAND2	heart and neural crest derivatives expressed 2	8.5	6.2	7.34
SLC18A1	solute carrier family 18 (vesicular monoamine transporter), member 1	8.5	6.2	7.34
LOC100548001	monocyte differentiation antigen CD14-like	7.6	7.1	7.34
LOC104912212	uncharacterized LOC104912212	9.3	5.4	7.34
LOC100546220	gastricsin-like	7.6	7.1	7.34
ITGA9	integrin, alpha 9	7.6	7.1	7.33
LOC104909818	uncharacterized LOC104909818	8.5	6.2	7.33

LOC104909477	uncharacterized LOC104909477	9.3	5.4	7.32
LOC104910189	uncharacterized LOC104910189	7.6	7.0	7.32
LOC100545599	ATP-binding cassette sub-family A member 9	7.6	7.0	7.32
LOC104915362	E3 ubiquitin-protein ligase TRIM36-like	7.6	7.0	7.32
LOC104910026	uncharacterized LOC104910026	8.4	6.2	7.31
LOC104914965	uncharacterized LOC104914965	8.4	6.2	7.31
LOC104911830	uncharacterized LOC104911830	6.8	7.9	7.31
PLEKHD1	pleckstrin homology domain containing, family D (with coiled-coil domains) member 1	7.6	7.0	7.30
AICDA	activation-induced cytidine deaminase	7.6	7.0	7.30
LOC104914495	uncharacterized LOC104914495	8.4	6.2	7.30
LOC104910681	tyrosine-protein phosphatase non-receptor type 13-like	9.3	5.3	7.30
LOC104912481	uncharacterized LOC104912481	6.8	7.8	7.30
LOC104913440	uncharacterized LOC104913440	7.6	7.0	7.30
ANKMY1	ankyrin repeat and MYND domain containing 1	7.6	7.0	7.29
LHFPL5	lipoma HMGIC fusion partner-like 5	6.8	7.8	7.29
PRR16	proline rich 16	7.6	7.0	7.29
LOC104916889	hemojuvelin pseudogene	5.1	9.5	7.28
LOC104912879	uncharacterized LOC104912879	6.8	7.8	7.28
LOC104912913	uncharacterized LOC104912913	7.6	6.9	7.27
LOC104909924	uncharacterized LOC104909924	5.9	8.6	7.27
LOC104917558	neurobeachin-like	5.9	8.6	7.27
LOC104909649	uncharacterized LOC104909649	7.6	6.9	7.27
LOC104911713	uncharacterized LOC104911713	7.6	6.9	7.27
FGF5	fibroblast growth factor 5	6.8	7.8	7.27
CSAD	cysteine sulfinic acid decarboxylase	7.6	6.9	7.26
LOC104909560	neurexin-1-like	6.8	7.7	7.26
DSP	desmoplakin	5.9	8.6	7.24
LOC100547693	secreted frizzled-related protein 5-like	4.2	10.3	7.24
MELK	maternal embryonic leucine zipper kinase	7.6	6.9	7.24
LOC100538434	cytochrome P450 4B1-like	5.9	8.5	7.23
LOC100549708	cytoglobin-1-like	5.9	8.5	7.23
LOC100551216	interferon-induced protein with tetratricopeptide repeats 5-like	5.1	9.4	7.23
LOC104915676	uncharacterized LOC104915676	5.9	8.5	7.22
OLFM1	olfactomedin 1	6.7	7.7	7.22
LOC100550253	ankyrin repeat and fibronectin type-III domain-containing protein 1-like	5.1	9.4	7.22
LOC104914227	uncharacterized LOC104914227	4.2	10.2	7.22
LOC104910731	uncharacterized LOC104910731	5.9	8.5	7.21
LOC104909622	uncharacterized LOC104909622	5.1	9.3	7.21
LOC104915356	aminopeptidase Q-like	5.1	9.3	7.21
LOC104915202	DNA polymerase alpha catalytic subunit-like	6.7	7.7	7.20
LOC104910932	uncharacterized LOC104910932	4.2	10.2	7.20
LOC100545676	neural-cadherin-like	5.9	8.5	7.20
LOC104916271	osteocalcin-like	5.9	8.5	7.20
LOC100549275	nuclear factor 1 A-type-like	5.1	9.3	7.19
CHADL	chondroadherin-like	5.1	9.3	7.19
LOC104917608	rho GTPase-activating protein 42-like	5.1	9.3	7.18
RAMP2	receptor (G protein-coupled) activity modifying protein 2	5.9	8.4	7.18
LOC100550971	P2Y purinoceptor 1-like	4.2	10.1	7.17
BCO1	beta-carotene oxygenase 1	5.1	9.3	7.16
DNAH1	dynein, axonemal, heavy chain 1	5.1	9.2	7.15
LOC104909966	zinc finger protein 707-like	4.2	10.1	7.14
BEST3	bestrophin 3	12.7	1.6	7.12
DACH1	dachshund family transcription factor 1	11.0	3.2	7.08
LOC104915765	uncharacterized LOC104915765	11.0	3.2	7.07
LOC104909767	uncharacterized LOC104909767	11.0	3.2	7.07
SCX	scleraxis basic helix-loop-helix transcription factor	11.0	3.1	7.05

HIC2	hypermethylated in cancer 2	4.2	9.9	7.05
LOC104916351	zinc finger CCCH domain-containing protein 4-like	10.1	3.9	7.01
LOC104910183	uncharacterized LOC104910183	9.3	4.7	7.01
MDGA1	MAM domain containing glycosylphosphatidylinositol anchor 1	9.3	4.7	7.00
CAB39L	calcium binding protein 39-like	10.1	3.9	7.00
LOC104913122	E3 ubiquitin-protein ligase RNF123-like	9.3	4.7	7.00
ABHD8	abhydrolase domain containing 8	10.1	3.8	6.98
LOC104909960	transcription elongation factor B polypeptide 3-like	8.4	5.5	6.98
LOC104915569	integrin alpha-1-like	10.1	3.8	6.97
LOC104910813	zinc finger protein 501-like	9.3	4.6	6.97
LOC104910112	uncharacterized LOC104910112	9.3	4.6	6.96
LOC104912966	uncharacterized LOC104912966	9.3	4.6	6.96
LOC100545453	metabotropic glutamate receptor 4-like	8.5	5.5	6.96
LOC104914889	ATP-dependent RNA helicase DHX29-like	8.5	5.5	6.96
LOC100538873	phosphorylase b kinase regulatory subunit alpha, liver isoform	9.3	4.6	6.96
ARL14EPL	ADP-ribosylation factor-like 14 effector protein-like	8.5	5.5	6.96
ADAM11	ADAM metallopeptidase domain 11	9.3	4.6	6.96
LOC104913107	uncharacterized LOC104913107	7.6	6.3	6.96
LOC104914698	AT-rich interactive domain-containing protein 3A-like	9.3	4.6	6.96
LOC104911763	rac GTPase-activating protein 1-like	8.4	5.5	6.96
LOC104915018	lysine-specific demethylase 4C-like	8.4	5.5	6.96
LOC100538825	protein MMS22-like	8.4	5.5	6.95
CD55	CD55 molecule, decay accelerating factor for complement (Cromer blood group)	9.3	4.6	6.94
LOC100540249	fer-1-like protein 4	8.5	5.4	6.94
LOC104909404	synaptotagmin-like protein 2	7.6	6.3	6.94
LOC104911927	ankyrin repeat and protein kinase domain-containing protein 1-like	7.6	6.3	6.94
LOC104909959	angiopoietin-2-like	8.4	5.4	6.94
LOC104911080	synaptotagmin-5-like	7.6	6.3	6.94
LOC104914164	arginine-glutamic acid dipeptide repeats protein-like	7.6	6.3	6.94
LOC104913257	uncharacterized LOC104913257	7.6	6.3	6.93
LOC100538957	signal-regulatory protein delta-like	8.5	5.4	6.92
LOC104910825	uncharacterized LOC104910825	8.5	5.4	6.92
LOC104916379	insulin gene enhancer protein ISL-2-like	8.4	5.4	6.92
LOC104916032	growth/differentiation factor 6-like	7.6	6.2	6.92
LRRC4B	leucine rich repeat containing 4B	7.6	6.2	6.91
MOGAT1	monoacylglycerol O-acyltransferase 1	7.6	6.2	6.91
LOC100543744	proprotein convertase subtilisin/kexin type 5-like	8.5	5.4	6.90
BSPRY	B-box and SPRY domain containing	7.6	6.2	6.90
SHC4	SHC (Src homology 2 domain containing) family, member 4	8.5	5.4	6.90
LOC104912553	uncharacterized LOC104912553	7.6	6.2	6.90
FGF7	fibroblast growth factor 7	7.6	6.2	6.90
PCDH15	protocadherin-related 15	7.6	6.2	6.90
LOC104914688	minor histocompatibility protein HA-1-like	6.8	7.0	6.89
CRISPLD1	cysteine-rich secretory protein LCCL domain containing 1	8.4	5.4	6.89
ATP6AP1L	ATPase, H+ transporting, lysosomal accessory protein 1-like	8.4	5.4	6.89
LOC104916080	receptor tyrosine-protein kinase erbB-3-like	6.8	7.0	6.89
MEIOB	meiosis specific with OB domains	6.8	7.0	6.89
SBK3	SH3 domain binding kinase family, member 3	5.9	7.9	6.89
LOC100539800	neurobeachin-like	7.6	6.2	6.88
CALR3	calreticulin 3	6.8	7.0	6.87
PACRGL	PARK2 co-regulated-like	6.8	7.0	6.87
LOC100545215	band 4.1-like protein 5	5.9	7.8	6.87
CSF1R	colony stimulating factor 1 receptor	7.6	6.1	6.86
LOC100550809	vitamin D3 hydroxylase-associated protein	5.9	7.8	6.85
LOC104909956	CUB and sushi domain-containing protein 1-like	5.9	7.8	6.85
LOC104911704	interferon-induced helicase C domain-containing protein 1-like	5.9	7.8	6.85

LOC104910752	uncharacterized LOC104910752	6.8	6.9	6.85
GFRA3	GDNF family receptor alpha 3	7.6	6.1	6.85
GNGT2	guanine nucleotide binding protein (G protein), gamma transducing activity polypeptide 2	5.9	7.8	6.85
LOC104909827	proto-oncogene tyrosine-protein kinase ROS-like	5.9	7.8	6.85
LOC104914979	rho guanine nucleotide exchange factor 28-like	5.9	7.8	6.85
LOC104910032	nuclear GTPase SLIP-GC-like	6.8	6.9	6.85
GHRL	ghrelin/obestatin prepropeptide	5.9	7.8	6.85
TMEM88B	transmembrane protein 88B	5.9	7.7	6.83
LOC104911832	glycosyltransferase-like domain-containing protein 1	6.8	6.9	6.83
LOC104913112	uncharacterized LOC104913112	5.1	8.6	6.83
LOC104914360	uncharacterized LOC104914360	5.9	7.7	6.83
LOC104909891	uncharacterized LOC104909891	5.1	8.6	6.83
LOC104909539	protein jagged-1-like	5.9	7.7	6.82
LOC100539773	zinc finger protein 536	5.9	7.7	6.81
AANAT	aralkylamine N-acetyltransferase	5.9	7.7	6.81
KCNMB4	potassium large conductance calcium-activated channel, subfamily M, beta member 4	5.1	8.5	6.81
RADIL	Ras association and DIL domains	5.1	8.5	6.81
PIANP	PILR alpha associated neural protein	5.9	7.7	6.81
LOC104913036	uncharacterized LOC104913036	5.1	8.5	6.80
LOC104917479	protein aurora borealis-like	5.9	7.7	6.80
LOC104909441	uncharacterized LOC104909441	5.1	8.5	6.79
LOC104917347	heparan-sulfate 6-O-sulfotransferase 2-like	5.1	8.5	6.79
LOC104914565	uncharacterized LOC104914565	5.1	8.5	6.79
LOC104909397	probable dolichyl pyrophosphate Glc1Man9GlcNAc2 alpha-1,3-glucosyltransferase	4.2	9.3	6.78
GPR64	G protein-coupled receptor 64	5.1	8.5	6.78
LOC104915327	ribosomal protein S6 kinase alpha-3-like	4.2	9.3	6.76
WASF3	WAS protein family, member 3	4.2	9.3	6.76
LOC104914896	interleukin-31 receptor subunit alpha-like	5.9	7.6	6.74
LOC104909263	deleted in malignant brain tumors 1 protein-like	3.4	10.1	6.74
CIDEC	cell death-inducing DFFA-like effector c	2.5	10.9	6.74
LOC104911270	uncharacterized LOC104911270	3.4	10.1	6.72
LOC104910785	protein furry homolog-like	3.4	10.0	6.71
GPR63	G protein-coupled receptor 63	3.4	10.0	6.70
LOC104910992	uncharacterized LOC104910992	4.2	9.1	6.68
LOC104915923	uncharacterized LOC104915923	10.1	3.2	6.66
ANGPT4	angiopoietin 4	9.3	4.0	6.63
CYSLTR1	cysteinyl leukotriene receptor 1	10.1	3.1	6.63
SLC38A3	solute carrier family 38, member 3	10.2	3.1	6.61
LOC100548708	epithelial chloride channel protein-like	9.3	4.0	6.61
LOC100550020	dachshund homolog 1	9.3	3.9	6.61
LOC104909557	hydroxyacid oxidase 1-like	10.1	3.1	6.60
LOC104911829	uncharacterized LOC104911829	9.3	3.9	6.60
GPR114	G protein-coupled receptor 114	7.6	5.6	6.59
LOC100546322	hemopexin-like	7.6	5.5	6.58
LOC104909246	neurolysin, mitochondrial-like	7.6	5.5	6.58
CFAP45	cilia and flagella associated protein 45	7.6	5.5	6.58
CCDC83	coiled-coil domain containing 83	9.3	3.9	6.57
LOC104917509	epithelial-stromal interaction protein 1-like	8.4	4.7	6.56
LOC100543786	heat shock protein beta-7-like	7.6	5.5	6.56
PLEK2	pleckstrin 2	7.6	5.5	6.55
LOC100543179	phosphatase and actin regulator 3	9.3	3.8	6.55
LOC104916980	neurogenic locus notch homolog protein 4-like	9.3	3.8	6.55
LOC104916915	ETS translocation variant 3-like protein	8.5	4.6	6.54
LOC104913096	PHD finger protein 7-like	7.6	5.5	6.54
LOC104912956	chromodomain-helicase-DNA-binding protein 9 pseudogene	7.6	5.5	6.54

ASIP	agouti signaling protein	6.8	6.3	6.53
LOC104914265	uncharacterized LOC104914265	7.6	5.4	6.52
LOC100540365	amine oxidase [flavin-containing] A-like	8.5	4.6	6.52
LOC104909460	uncharacterized LOC104909460	6.8	6.3	6.52
LYPD6	LY6/PLAUR domain containing 6	8.4	4.6	6.52
LOC100546808	cordon-bleu protein-like 1	8.5	4.6	6.50
LOC104917235	uncharacterized LOC104917235	7.6	5.4	6.50
LOC104916283	uncharacterized LOC104916283	6.8	6.2	6.50
SPIRE2	spire-type actin nucleation factor 2	6.8	6.2	6.49
LOC104910767	uncharacterized LOC104910767	6.7	6.2	6.49
LOC100538662	protein spire homolog 1-like	5.1	7.9	6.49
LOC104917611	cytoplasmic dynein 2 heavy chain 1-like	5.1	7.9	6.49
LOC104915790	maestro heat-like repeat-containing protein family member 2B	7.6	5.4	6.48
LOC104910677	tyrosine-protein kinase SgK223	5.1	7.9	6.48
LOC104910393	matrix metalloproteinase-16-like	5.9	7.1	6.48
LOC100551231	urotensin-2 receptor-like	6.8	6.2	6.48
KCNQ5	potassium voltage-gated channel, KQT-like subfamily, member 5	6.8	6.2	6.48
LOC104911571	thromboxane-A synthase-like	5.9	7.0	6.48
LOC104912152	protein FAM162B-like	6.8	6.2	6.48
ARHGAP8	Rho GTPase activating protein 8	6.8	6.2	6.47
LOC104911957	uncharacterized LOC104911957	6.8	6.2	6.47
LOC104913528	uncharacterized LOC104913528	6.8	6.2	6.47
PDE11A	phosphodiesterase 11A	5.9	7.0	6.46
SBK2	SH3 domain binding kinase family, member 2	6.8	6.2	6.46
RTN1	reticulon 1	5.9	7.0	6.45
FILIP1	filamin A interacting protein 1	6.8	6.2	6.45
LOC104911385	rho GTPase-activating protein 21-like	5.9	7.0	6.45
LOC104914994	uncharacterized LOC104914994	5.9	7.0	6.45
KAZN	kazrin, periplakin interacting protein	5.1	7.8	6.45
LOC104917493	uncharacterized LOC104917493	5.9	7.0	6.45
LOC104910985	uncharacterized LOC104910985	5.1	7.8	6.45
FTCD	formimidoyltransferase cyclodeaminase	5.9	7.0	6.45
PCNXL2	pecanex-like 2 (Drosophila)	5.9	7.0	6.45
LOC100547963	erythroblast NAD(P)(+)--arginine ADP-ribosyltransferase-like	5.9	7.0	6.44
LOC104917155	alpha-mannosidase 2-like	5.9	6.9	6.43
LOC104912922	uncharacterized LOC104912922	5.1	7.8	6.43
LOC104910353	uncharacterized LOC104910353	5.9	6.9	6.43
LOC100541990	probable phospholipid-transporting ATPase IIA	5.9	6.9	6.43
NLGN1	neuroligin 1	5.9	6.9	6.43
LCOR	ligand dependent nuclear receptor corepressor	5.1	7.8	6.42
LOC104915361	E3 ubiquitin-protein ligase TRIM36-like	5.1	7.8	6.42
LOC100550711	hexosaminidase D-like	5.1	7.7	6.41
HTR3A	5-hydroxytryptamine (serotonin) receptor 3A, ionotropic	5.1	7.7	6.41
LOC104913144	uncharacterized LOC104913144	5.9	6.9	6.41
MLNR	motilin receptor	4.2	8.6	6.41
SST	somatostatin	5.1	7.7	6.41
LOC104911806	CLIP-associating protein 1-like	4.2	8.6	6.41
LOC104910534	uncharacterized LOC104910534	4.2	8.6	6.40
LOC104915686	histone-lysine N-methyltransferase 2D-like	5.9	6.9	6.40
LOC104912304	uncharacterized LOC104912304	5.1	7.7	6.39
LOC104916490	NAD-dependent malic enzyme, mitochondrial-like	5.1	7.7	6.39
TDRD12	tudor domain containing 12	4.2	8.5	6.39
SLC26A3	solute carrier family 26 (anion exchanger), member 3	5.1	7.7	6.38
LOC104914230	uncharacterized LOC104914230	5.1	7.7	6.37
ISM1	isthmin 1, angiogenesis inhibitor	3.4	9.3	6.36
PARP14	poly (ADP-ribose) polymerase family, member 14	4.2	8.5	6.36
LOC104916324	uncharacterized LOC104916324	3.4	9.3	6.36
LOC104913723	growth arrest-specific protein 7-like	5.1	7.6	6.35

STMN3	stathmin-like 3	3.4	9.3	6.34
LOC104917317	bromodomain adjacent to zinc finger domain protein 2A pseudogene	2.5	10.1	6.34
REC8	REC8 meiotic recombination protein	2.5	10.1	6.34
PAWR	PRKC, apoptosis, WT1, regulator	3.4	9.3	6.34
GHSR	growth hormone secretagogue receptor	2.5	10.1	6.34
LOC104913460	sorting nexin-29-like	2.5	10.1	6.32
LOC100544297	ras-related protein Rab-3B-like	1.7	10.9	6.31
LOC104910298	uncharacterized LOC104910298	2.5	10.1	6.30
LOC104916656	uncharacterized LOC104916656	10.1	2.4	6.25
MEOX2	mesenchyme homeobox 2	10.1	2.4	6.23
LOC104916618	adenylate cyclase type 10-like	10.1	2.3	6.23
HTR1B	5-hydroxytryptamine (serotonin) receptor 1B, G protein-coupled	8.4	4.0	6.22
LOC104910426	uncharacterized LOC104910426	9.3	3.2	6.21
CFAP74	cilia and flagella associated protein 74	9.3	3.1	6.21
CREB3L3	cAMP responsive element binding protein 3-like 3	8.4	4.0	6.19
LOC104912365	dihydropyrimidine dehydrogenase [NADP(+)]-like	8.5	3.9	6.18
LOC104914779	uncharacterized LOC104914779	8.5	3.9	6.18
LOC100540290	uncharacterized LOC100540290	9.3	3.1	6.18
LOC104912125	lysine-specific demethylase 5A-like	7.6	4.8	6.18
LOC104912768	uncharacterized LOC104912768	8.4	3.9	6.17
LOC104909838	sodium-coupled neutral amino acid transporter 4-like	7.6	4.7	6.16
LOC104913627	uncharacterized LOC104913627	7.6	4.7	6.16
LOC104915794	uncharacterized LOC104915794	8.4	3.9	6.16
LOC104915594	kinesin-like protein KIF20B	6.8	5.5	6.15
LOC104916270	POU domain, class 3, transcription factor 3	7.6	4.7	6.15
ANO9	anoctamin 9	7.6	4.7	6.14
GRM8	glutamate receptor, metabotropic 8	7.6	4.7	6.14
LOC104909944	uncharacterized LOC104909944	7.6	4.7	6.14
ATP8B1	ATPase, aminophospholipid transporter, class I, type 8B, member 1	7.6	4.6	6.12
LOC104915036	endophilin-A1	7.6	4.6	6.12
LOC104914496	Krueppel-like factor 15	6.8	5.5	6.12
LOC104916013	fibronectin type III domain-containing protein 9-like	5.9	6.3	6.11
LOC104911318	uncharacterized LOC104911318	7.6	4.6	6.11
CAMKK1	calcium/calmodulin-dependent protein kinase kinase 1, alpha	5.9	6.3	6.11
LOC104913763	heparan sulfate glucosamine 3-O-sulfotransferase 3A1-like	7.6	4.6	6.11
LOC104910427	oxidation resistance protein 1-like	6.7	5.5	6.11
LOC104910548	uncharacterized LOC104910548	7.6	4.6	6.10
LOC104910448	uncharacterized LOC104910448	5.1	7.1	6.10
LOC100545042	rho guanine nucleotide exchange factor 28-like	7.6	4.6	6.10
AQP8	aquaporin 8	5.9	6.3	6.09
ADTRP	androgen-dependent TFPI-regulating protein	7.6	4.6	6.09
LRRN1	leucine rich repeat neuronal 1	6.8	5.4	6.09
LOC100543508	ankyrin repeat domain-containing protein 9-like	5.1	7.1	6.09
LOC104909846	coiled-coil domain-containing protein 162-like	5.9	6.3	6.09
NOS3	nitric oxide synthase 3 (endothelial cell)	5.9	6.3	6.09
LOC104915017	lysine-specific demethylase 4C-like	4.2	7.9	6.09
LRRC34	leucine rich repeat containing 34	5.9	6.3	6.09
GUCY2C	guanylate cyclase 2C (heat stable enterotoxin receptor)	5.1	7.1	6.08
ST8SIA2	ST8 alpha-N-acetyl-neuraminide alpha-2,8-sialyltransferase 2	6.8	5.4	6.08
LPAR6	lysophosphatidic acid receptor 6	6.8	5.4	6.08
LOC104912348	uncharacterized LOC104912348	6.8	5.4	6.08
LOC104913232	proteinase-activated receptor 3-like	5.9	6.2	6.07
TNXB	tenascin XB	5.9	6.2	6.07
LOC100544871	polypeptide N-acetylgalactosaminyltransferase 13	6.8	5.4	6.07
LOC104913718	protein CIP2A homolog	5.9	6.2	6.07
LYPD6B	LY6/PLAUR domain containing 6B	5.9	6.2	6.07

LOC104912738	glucose-dependent insulinotropic receptor-like	5.1	7.1	6.07
LOC104917547	uncharacterized LOC104917547	5.9	6.2	6.07
LOC100549668	serine/threonine-protein phosphatase 2A 55 kDa regulatory subunit B gamma isoform	5.1	7.1	6.06
LOC104911720	E3 ubiquitin-protein ligase UBR3-like	6.8	5.4	6.06
MX1	MX dynamin-like GTPase 1	5.9	6.2	6.05
LOC104917171	very long-chain acyl-CoA synthetase-like	5.9	6.2	6.05
LOC104911914	rho-related BTB domain-containing protein 1-like	4.2	7.9	6.05
LOC104912322	uncharacterized LOC104912322	5.1	7.0	6.05
LOC104913659	uncharacterized LOC104913659	5.1	7.0	6.05
LOC104916834	uncharacterized LOC104916834	4.2	7.9	6.04
LOC100540386	D-serine dehydratase-like	5.9	6.2	6.03
LOC104911009	uncharacterized LOC104911009	5.1	7.0	6.03
NEK5	NIMA-related kinase 5	5.1	7.0	6.03
SLC38A8	solute carrier family 38, member 8	5.1	7.0	6.03
COL20A1	collagen, type XX, alpha 1	4.2	7.8	6.03
LOC104916607	polyadenylate-binding protein 4	5.1	7.0	6.03
LOC100547835	pleckstrin homology domain-containing family A member 7	5.9	6.1	6.02
LOC100546647	photoreceptor outer segment membrane glycoprotein 2	5.9	6.1	6.01
LOC104913447	uncharacterized LOC104913447	5.1	6.9	6.01
LOC104911774	low-density lipoprotein receptor-related protein 2-like	5.1	6.9	6.01
LOC104912261	uncharacterized LOC104912261	5.1	6.9	6.01
LOC104916737	class II histocompatibility antigen, B-L beta chain-like	5.1	6.9	6.01
LOC104911313	uncharacterized LOC104911313	3.4	8.6	6.00
FOXC1	forkhead box C1	2.5	9.5	6.00
DOK3	docking protein 3	3.4	8.6	6.00
LOC104912525	probable cation-transporting ATPase 13A4	5.1	6.9	5.99
LOC104915059	uncharacterized LOC104915059	4.2	7.7	5.99
PET117	PET117 homolog (S. cerevisiae)	4.2	7.7	5.99
PABPN1L	poly(A) binding protein, nuclear 1-like (cytoplasmic)	3.4	8.6	5.98
ADAMTS19	ADAM metallopeptidase with thrombospondin type 1 motif, 19	5.1	6.9	5.98
LOC104910224	leucine-rich repeat-containing protein 16A-like	4.2	7.7	5.97
LOC104910466	focal adhesion kinase 1-like	4.2	7.7	5.97
LOC100546837	solute carrier family 12 member 2-like	3.4	8.5	5.96
CTCFL	CCCTC-binding factor (zinc finger protein)-like	4.2	7.7	5.96
LOC104909900	uncharacterized LOC104909900	4.2	7.7	5.96
LOC100551492	nuclear factor 1 B-type	2.5	9.4	5.96
LOC104911558	uncharacterized LOC104911558	3.4	8.5	5.96
GRIN3A	glutamate receptor, ionotropic, N-methyl-D-aspartate 3A	4.2	7.7	5.95
LOC104910867	huntingtin-like	3.4	8.5	5.94
LOC104913860	uncharacterized LOC104913860	2.5	9.3	5.94
CACNA2D3	calcium channel, voltage-dependent, alpha 2/delta subunit 3	2.5	9.3	5.92
LOC104914165	arginine-glutamic acid dipeptide repeats protein-like	2.5	9.3	5.92
LOC104913196	uncharacterized LOC104913196	2.5	9.3	5.92
LOC104912260	ATP-binding cassette sub-family D member 3-like	3.4	8.4	5.90
TMC3	transmembrane channel-like 3	1.7	10.1	5.90
TNR	tenascin R	1.7	10.1	5.88
CLUL1	clusterin-like 1 (retinal)	10.1	1.6	5.86
LOC104915610	cytochrome P450 2C4-like	9.3	2.4	5.85
LOC104911677	uncharacterized LOC104911677	10.1	1.6	5.85
DGKK	diacylglycerol kinase, kappa	10.1	1.5	5.83
LOC104912413	interleukin-23 receptor-like	9.3	2.4	5.83
OPRM1	opioid receptor, mu 1	9.3	2.4	5.82
TMEM61	transmembrane protein 61	9.3	2.3	5.81
LOC104910961	uncharacterized LOC104910961	9.3	2.4	5.81
LOC104912929	rhophilin-2-like	8.5	3.1	5.78
RAB40B	RAB40B, member RAS oncogene family	7.6	4.0	5.78
HOXB2	homeobox B2	8.4	3.1	5.78

LOC100539329	potassium voltage-gated channel subfamily KQT member 4-like	8.4	3.1	5.78
LOC104913491	uncharacterized LOC104913491	8.4	3.1	5.78
COL2A1	collagen, type II, alpha 1	7.6	4.0	5.78
LOC104913417	E3 ubiquitin-protein ligase SMURF1-like	8.4	3.1	5.77
FSCN2	fascin actin-bundling protein 2, retinal	7.6	3.9	5.77
LOC104909879	protein MMS22-like	7.6	3.9	5.76
LOC104914157	uncharacterized LOC104914157	7.6	3.9	5.76
LOC104913123	maestro heat-like repeat-containing protein family member 7	6.8	4.8	5.76
LOC104914557	Ig-like V-type domain-containing protein FAM187A	7.6	3.9	5.76
GABRA4	gamma-aminobutyric acid (GABA) A receptor, alpha 4	8.4	3.1	5.75
LOC104911451	protein AF-10-like	7.6	3.9	5.74
LOC104910374	uncharacterized LOC104910374	7.6	3.9	5.74
LOC104910947	sn1-specific diacylglycerol lipase alpha-like	6.8	4.7	5.74
LOC104912794	TBC1 domain family member 2B-like	6.8	4.7	5.74
ETNK2	ethanolamine kinase 2	6.8	4.7	5.73
LOC104917494	uncharacterized LOC104917494	6.8	4.7	5.73
ANKRD61	ankyrin repeat domain 61	5.9	5.5	5.73
ARR3	arrestin 3, retinal (X-arrestin)	6.8	4.7	5.72
LOC104910765	uncharacterized LOC104910765	6.8	4.7	5.72
LOC104915002	transcription factor RFX3-like	6.8	4.7	5.72
LOC100549687	ADP-ribosylation factor-like protein 3	5.9	5.5	5.71
S100A13	S100 calcium binding protein A13	5.9	5.5	5.70
EPCAM	epithelial cell adhesion molecule	6.8	4.6	5.70
LOC104911076	uncharacterized LOC104911076	5.9	5.5	5.69
BCAS1	breast carcinoma amplified sequence 1	6.8	4.6	5.69
LOC104915344	MARVEL domain-containing protein 2-like	5.9	5.5	5.69
RBPMS2	RNA binding protein with multiple splicing 2	5.9	5.5	5.69
LOC100543839	thrombospondin type-1 domain-containing protein 4	6.8	4.6	5.69
LOC104909926	uncharacterized LOC104909926	5.1	6.3	5.69
C10H1orf228	chromosome 10 open reading frame, human C1orf228	5.9	5.5	5.69
LOC100542571	zinc finger protein OZF-like	5.1	6.3	5.69
LOC104910840	uncharacterized LOC104910840	5.1	6.3	5.69
LOC104910995	tubulin alpha chain-like	5.9	5.5	5.68
LOC104914870	uncharacterized LOC104914870	5.9	5.4	5.68
LOC104910091	uncharacterized LOC104910091	5.9	5.4	5.67
BEGAIN	brain-enriched guanylate kinase-associated	5.1	6.3	5.67
LOC104910642	uncharacterized LOC104910642	5.1	6.3	5.67
LOC104911700	secretory phospholipase A2 receptor-like	5.1	6.3	5.67
LOC104914873	integrin alpha-2-like	5.9	5.4	5.67
TSPO2	translocator protein 2	5.1	6.3	5.67
NECAB1	N-terminal EF-hand calcium binding protein 1	5.1	6.3	5.66
LOC104915902	uncharacterized LOC104915902	4.2	7.1	5.66
LOC104913005	putative inactive carboxylesterase 4	6.8	4.6	5.66
LOC100546896	serine palmitoyltransferase 3	5.1	6.2	5.65
LOC100546217	protein TENP	5.9	5.4	5.65
LOC104912458	MAP kinase-interacting serine/threonine-protein kinase 1-like	6.7	4.6	5.65
LOC104916223	glucosylceramidase-like	5.1	6.2	5.65
EGR4	early growth response 4	5.1	6.2	5.63
LOC104915024	FRAS1-related extracellular matrix protein 1-like	5.1	6.2	5.63
LOC104915546	kinesin-like protein KIF1B	5.9	5.4	5.63
TENM2	teneurin transmembrane protein 2	5.1	6.2	5.63
LOC104917425	ATP-binding cassette sub-family D member 2-like	5.9	5.4	5.63
LOC100549247	ATP-binding cassette sub-family D member 2	4.2	7.0	5.63
LOC100550328	brefeldin A-inhibited guanine nucleotide-exchange protein 3-like	5.9	5.4	5.63
LOC104916327	semaphorin-6C-like	3.4	7.9	5.62
SLC13A2	solute carrier family 13 (sodium-dependent dicarboxylate transporter), member 2	4.2	7.0	5.62
LOC104915300	uncharacterized LOC104915300	4.2	7.0	5.62

LOC104914515	SLIT-ROBO Rho GTPase-activating protein 2B-like	4.2	7.0	5.61
RASL11A	RAS-like, family 11, member A	4.2	7.0	5.61
PTCH2	patched 2	3.4	7.8	5.60
MYBPC1	myosin binding protein C, slow type	4.2	7.0	5.60
MSX2	msh homeobox 2	5.1	6.2	5.60
LOC100548556	beta-galactoside alpha-2,6-sialyltransferase 1-like	4.2	7.0	5.60
LOC104911497	uncharacterized LOC104911497	3.4	7.8	5.60
PAQR9	progestin and adipoQ receptor family member IX	3.4	7.8	5.60
LOC104917363	extended synaptotagmin-2-A-like	5.1	6.1	5.60
MYO1A	myosin IA	4.2	6.9	5.59
SCG5	secretogranin V (7B2 protein)	3.4	7.8	5.58
SLC13A4	solute carrier family 13 (sodium/sulfate symporter), member 4	3.4	7.8	5.58
LOC104909604	uncharacterized LOC104909604	4.2	6.9	5.58
LOC104909461	uncharacterized LOC104909461	2.5	8.6	5.58
LOC104914145	taste receptor type 1 member 3-like	1.7	9.5	5.58
LOC104913217	ankyrin repeat and KH domain-containing protein 1-like	5.1	6.1	5.57
LOC104912116	serine/threonine-protein kinase WNK3-like	4.2	6.9	5.57
LOC100542440	pancreatic secretory granule membrane major glycoprotein GP2-like	3.4	7.7	5.57
LOC104912773	uncharacterized LOC104912773	4.2	6.9	5.57
CYTH4	cytohesin 4	2.5	8.6	5.56
LOC104915347	uncharacterized LOC104915347	3.4	7.7	5.56
LOC104912181	uncharacterized LOC104912181	0.8	10.3	5.55
C23H1orf167	chromosome 23 open reading frame, human C1orf167	4.2	6.9	5.55
LOC104914487	V-set domain-containing T-cell activation inhibitor 1-like	2.5	8.5	5.54
KCNJ12	potassium inwardly-rectifying channel, subfamily J, member 12	3.4	7.7	5.53
LOC104915490	dmX-like protein 2	3.4	7.7	5.52
TP53TG5	TP53 target 5	3.4	7.7	5.52
LOC104911173	ryanodine receptor 3-like	11.0	0.0	5.48
LOC104915513	histone deacetylase 7-like	10.9	0.0	5.47
MORN2	MORN repeat containing 2	2.5	8.3	5.44
LOC104911267	uncharacterized LOC104911267	9.3	1.6	5.43
LOC100542202	histone H2B 7	9.3	1.6	5.42
GATA6	GATA binding protein 6	8.4	2.4	5.41
LOC104909345	myosin heavy chain, skeletal muscle, adult-like	8.5	2.3	5.39
LOC104916997	uncharacterized LOC104916997	7.6	3.2	5.39
HNF4A	hepatocyte nuclear factor 4, alpha	7.6	3.2	5.38
CSF3	colony stimulating factor 3 (granulocyte)	7.6	3.2	5.38
LOC104909289	limbin-like	7.6	3.2	5.38
LOC104914238	uncharacterized LOC104914238	7.6	3.2	5.38
PTK2B	protein tyrosine kinase 2 beta	7.6	3.2	5.38
PDE6H	phosphodiesterase 6H, cGMP-specific, cone, gamma	8.4	2.3	5.38
LOC100544815	scavenger receptor cysteine-rich type 1 protein M130-like	7.6	3.2	5.37
ROBO2	roundabout, axon guidance receptor, homolog 2 (Drosophila)	8.4	2.3	5.37
LOC100551070	leukotriene C4 synthase-like	8.5	2.3	5.36
LOC104916654	uncharacterized LOC104916654	7.6	3.1	5.36
LOC104911508	uncharacterized protein C11orf16 homolog	6.8	4.0	5.36
LOC104911580	uncharacterized LOC104911580	7.6	3.1	5.36
FMOD	fibromodulin	6.8	4.0	5.35
LOC104912041	pleckstrin homology domain-containing family A member 5-like	6.8	4.0	5.35
LOC104913568	beta-adrenergic receptor kinase 2-like	7.6	3.1	5.34
LOC104913729	myosin heavy chain, skeletal muscle-like	6.8	3.9	5.34
LOC104910762	microtubule-associated tumor suppressor 1 homolog	6.8	3.9	5.34
LOC104917557	neurobeachin-like	6.8	3.9	5.34
LOC104914053	signal-regulatory protein beta-1 isoform 3-like	5.9	4.8	5.33
LOC104917289	histone H1-like	5.9	4.8	5.33
LCP1	lymphocyte cytosolic protein 1 (L-plastin)	6.8	3.9	5.33
LOC104910059	fibrocystin-like	7.6	3.1	5.33

SLC16A12	solute carrier family 16, member 12	5.9	4.8	5.33
LOC104912885	uncharacterized LOC104912885	5.1	5.6	5.33
LOC104917021	sialidase-3-like	6.8	3.9	5.32
ALCAM	activated leukocyte cell adhesion molecule	7.6	3.0	5.32
LOC104916291	sortilin-like	6.8	3.9	5.32
LOC104911606	uncharacterized LOC104911606	6.8	3.9	5.31
CUX2	cut-like homeobox 2	5.9	4.7	5.31
LOC100546746	interleukin-17A-like	5.9	4.7	5.31
LOC104911942	uncharacterized LOC104911942	5.9	4.7	5.31
LOC104912842	uncharacterized LOC104912842	5.9	4.7	5.31
LOC104913422	thiamine transporter 1-like	5.9	4.7	5.31
ITK	IL2-inducible T-cell kinase	5.9	4.7	5.31
NINJ2	ninjurin 2	5.9	4.7	5.31
LOC104910679	death-associated protein kinase 1-like	5.1	5.5	5.31
LOC104914202	rap1 GTPase-activating protein 1-like	6.8	3.8	5.30
CCSER1	coiled-coil serine-rich protein 1	5.9	4.7	5.30
LOC104915672	notchless protein homolog 1-like	5.9	4.7	5.30
MAB21L2	mab-21-like 2 (C. elegans)	5.9	4.7	5.30
LOC100547703	semaphorin-4D-like	5.9	4.7	5.29
NMUR1	neuromedin U receptor 1	5.9	4.7	5.29
LOC100542048	histone H4	5.9	4.7	5.29
LOC104911146	uncharacterized LOC104911146	6.7	3.8	5.29
LOC104911633	V-type proton ATPase 116 kDa subunit a isoform 4-like	6.7	3.8	5.29
LOC104911602	PCNA-interacting partner-like	5.1	5.5	5.29
DNAI2	dynein, axonemal, intermediate chain 2	5.1	5.5	5.28
LOC104911378	uncharacterized LOC104911378	5.1	5.5	5.28
ASTN2	astrotactin 2	5.9	4.6	5.27
LOC104913425	caspase recruitment domain-containing protein 11-like	5.1	5.5	5.27
LOC104913865	uncharacterized LOC104913865	5.1	5.5	5.27
C19H9orf91	chromosome 19 open reading frame, human C9orf91	5.1	5.5	5.27
LOC104912338	uncharacterized LOC104912338	5.1	5.5	5.27
LOC104914781	uncharacterized LOC104914781	4.2	6.3	5.26
LOC104913945	uncharacterized LOC104913945	5.1	5.5	5.26
LOC100541485	mast cell protease 1A-like	4.2	6.3	5.26
SPON2	spondin 2, extracellular matrix protein	4.2	6.3	5.26
ERC2	ELKS/RAB6-interacting/CAST family member 2	5.9	4.6	5.25
CKMT2	creatine kinase, mitochondrial 2 (sarcomeric)	5.1	5.4	5.25
LRRN3	leucine rich repeat neuronal 3	5.9	4.6	5.25
LOC104914367	NF-kappa-B inhibitor zeta-like	5.1	5.4	5.25
LOC104909965	plectin-like	3.4	7.1	5.24
SEC14L5	SEC14-like 5 (S. cerevisiae)	5.1	5.4	5.23
AR	androgen receptor	4.2	6.2	5.23
HOXB3	homeobox B3	5.1	5.4	5.23
FAM183A	family with sequence similarity 183, member A	4.2	6.2	5.23
LOC100550948	interferon alpha-inducible protein 27-like protein 2B	4.2	6.2	5.23
LOC104913489	testis-expressed sequence 2 protein-like	4.2	6.2	5.23
CD180	CD180 molecule	5.1	5.4	5.22
SLC12A8	solute carrier family 12, member 8	3.4	7.1	5.22
LOC100542744	cytochrome P450 27C1	4.2	6.2	5.22
LOC104912279	uncharacterized LOC104912279	5.1	5.4	5.21
LOC104914284	CUB and sushi domain-containing protein 2-like	4.2	6.2	5.21
LOC104917545	uncharacterized LOC104917545	4.2	6.2	5.21
LOC104910678	tankyrase-1-like	4.2	6.2	5.21
TGFA	transforming growth factor, alpha	4.2	6.2	5.20
LOC100544450	disintegrin and metalloproteinase domain-containing protein 12-like	3.4	7.0	5.20
LOC104917001	uncharacterized LOC104917001	5.1	5.3	5.19
LOC104911943	uncharacterized LOC104911943	4.2	6.2	5.19

LOC104916388	DNA polymerase subunit gamma-1-like	4.2	6.2	5.19
LOC104915656	maestro heat-like repeat-containing protein family member 2B	2.5	7.8	5.18
LOC100548173	galactosylgalactosylxylosylprotein 3-beta-glucuronosyltransferase 1-like	4.2	6.2	5.18
LOC104913529	uncharacterized LOC104913529	4.2	6.2	5.18
LOC104910725	uncharacterized LOC104910725	3.4	7.0	5.18
LOC100545842	galanin receptor type 1-like	3.4	6.9	5.17
LOC104913678	uncharacterized LOC104913678	4.2	6.1	5.17
LOC100547944	riboflavin-binding protein	3.4	6.9	5.17
LOC104917323	kinesin-like protein KIF13A	3.4	6.9	5.17
LOC104913822	uncharacterized LOC104913822	4.2	6.1	5.17
LOC104915155	probable ATP-dependent RNA helicase YTHDC2	2.5	7.8	5.16
LOC100550133	galectin-related protein A-like	3.4	6.9	5.16
LOC104910511	uncharacterized LOC104910511	3.4	6.9	5.16
LOC104911285	uncharacterized LOC104911285	3.4	6.9	5.16
LOC104914880	uncharacterized LOC104914880	3.4	6.9	5.16
LOC104910041	24-hydroxycholesterol 7-alpha-hydroxylase	2.5	7.8	5.16
LOC104912666	uncharacterized LOC104912666	2.5	7.8	5.16
LOC104915518	uncharacterized LOC104915518	3.4	6.9	5.15
LOC100538380	protein Wnt-11b-like	3.4	6.9	5.15
LOC104911163	ryanodine receptor 3-like	1.7	8.6	5.14
STRA8	stimulated by retinoic acid 8	3.4	6.9	5.12
LOC104909745	transmembrane protein 181-like	1.7	8.5	5.12
SLC6A17	solute carrier family 6 (neutral amino acid transporter), member 17	8.4	1.6	5.02
C24H8orf4	chromosome 24 open reading frame, human C8orf4	9.3	0.8	5.01
LOC100549719	dynein heavy chain 5, axonemal-like	7.6	2.4	5.00
LOC104917596	uncharacterized LOC104917596	7.6	2.4	4.99
LOC104909388	protein EMSY-like	7.6	2.4	4.98
CNR2	cannabinoid receptor 2 (macrophage)	7.6	2.3	4.96
LOC104911488	leucine-rich repeat-containing protein 16B-like	7.6	2.3	4.96
LOC100542562	keratin, type I cytoskeletal 16-like	6.8	3.2	4.96
LOC104909306	tenascin-N-like	6.8	3.2	4.95
LOC100544338	ryanodine receptor 3-like	7.6	2.3	4.95
IGSF21	immunoglobin superfamily, member 21	6.7	3.2	4.95
LOC100545586	microsomal triglyceride transfer protein large subunit-like	6.7	3.2	4.95
LOC104909320	von Willebrand factor A domain-containing protein 5B1-like	6.8	3.1	4.94
LOC100538446	1-phosphatidylinositol 4,5-bisphosphate phosphodiesterase eta-2-like	5.9	4.0	4.94
LOC104917105	heat shock factor protein 1-like	5.9	4.0	4.94
LOC104912098	uncharacterized LOC104912098	6.8	3.1	4.94
GAL	galanin/GMAP prepropeptide	6.8	3.1	4.93
KCNE1	potassium voltage-gated channel, Isk-related family, member 1	5.9	4.0	4.93
GIF	gastric intrinsic factor (vitamin B synthesis)	6.7	3.1	4.93
SAMD7	sterile alpha motif domain containing 7	5.9	3.9	4.92
LOC100550280	low-density lipoprotein receptor-related protein 2-like	5.9	3.9	4.92
LOC104912510	uncharacterized LOC104912510	6.8	3.1	4.91
LOC104915153	probable ATP-dependent RNA helicase YTHDC2	6.8	3.1	4.91
AGRP	agouti related neuropeptide	5.1	4.8	4.91
HMGCLL1	3-hydroxymethyl-3-methylglutaryl-CoA lyase-like 1	5.9	3.9	4.91
LOC104915938	proto-oncogene Mas-like	5.9	3.9	4.91
DBH	dopamine beta-hydroxylase (dopamine beta-monooxygenase)	6.7	3.1	4.91
LOC100549337	uncharacterized LOC100549337	5.1	4.8	4.91
PRRT3	proline-rich transmembrane protein 3	5.9	3.9	4.91
LOC104916815	mitogen-activated protein kinase 7-like	6.8	3.0	4.90
DACH2	dachshund family transcription factor 2	5.9	3.9	4.90
LOC104910339	myosin-6-like	6.8	3.0	4.90
LINGO2	leucine rich repeat and Ig domain containing 2	5.9	3.9	4.89

LOC100541844	dedicator of cytokinesis protein 2-like	5.9	3.9	4.89
LOC104912836	transient receptor potential cation channel subfamily M member 7-like	5.9	3.9	4.89
AKR1D1	aldo-keto reductase family 1, member D1	5.1	4.7	4.89
RBM46	RNA binding motif protein 46	5.9	3.9	4.89
DHRS13	dehydrogenase/reductase (SDR family) member 13	4.2	5.5	4.89
CCDC68	coiled-coil domain containing 68	5.1	4.7	4.89
LOC104916251	folate receptor beta-like	5.9	3.9	4.89
LOC104914643	thaicobrin-like	3.4	6.4	4.88
IGSF9B	immunoglobulin superfamily, member 9B	5.9	3.8	4.88
LOC104909372	centrosomal protein KIAA1731-like	5.9	3.8	4.88
LOC104916122	putative solute carrier family 22 member 31	5.9	3.8	4.87
LOC100545203	neurexin-1a-like	4.2	5.5	4.87
LOC104911546	uncharacterized LOC104911546	5.1	4.7	4.87
LOC104913745	protein shisa-6 homolog	5.1	4.7	4.87
LOC104914153	protein kinase C zeta type-like	5.1	4.7	4.87
LOC104913162	uncharacterized LOC104913162	4.2	5.5	4.87
LOC104912082	protein FAM196A-like	5.1	4.7	4.86
OMG	oligodendrocyte myelin glycoprotein	4.2	5.5	4.85
LOC104909848	cyclin-dependent kinase 19-like	5.1	4.6	4.85
PACRG	PARK2 co-regulated	5.1	4.6	4.85
TOX3	TOX high mobility group box family member 3	5.1	4.6	4.85
LOC104909589	uncharacterized LOC104909589	5.9	3.8	4.85
FAM180B	family with sequence similarity 180, member B	4.2	5.5	4.85
LOC104909328	collagen alpha-1(XXI) chain-like	4.2	5.5	4.85
LOC104913271	bromodomain-containing protein 8-like	5.9	3.8	4.84
LOC104909690	uncharacterized LOC104909690	3.4	6.3	4.84
TPD52L1	tumor protein D52-like 1	3.4	6.3	4.84
LOC104913169	uncharacterized LOC104913169	4.2	5.5	4.84
TMC5	transmembrane channel-like 5	3.4	6.3	4.84
CD84	CD84 molecule	4.2	5.4	4.83
LOC100538782	calcium uptake protein 3, mitochondrial-like	5.1	4.6	4.83
LOC104912118	probable E3 ubiquitin-protein ligase MID2	3.4	6.3	4.83
LOC104913386	uncharacterized LOC104913386	4.2	5.4	4.83
LOC104915354	transcription factor TFIIIB component B'' homolog	4.2	5.4	4.83
MYRIP	myosin VIIA and Rab interacting protein	4.2	5.4	4.83
SERPINB10	serpin peptidase inhibitor, clade B (ovalbumin), member 10	4.2	5.4	4.83
LOC104909614	uncharacterized LOC104909614	3.4	6.3	4.83
LOC100546575	angiopoietin-related protein 1-like	4.2	5.4	4.83
LOC104910024	uncharacterized LOC104910024	4.2	5.4	4.83
LOC104911185	uncharacterized LOC104911185	5.1	4.6	4.82
LOC104917344	carbohydrate sulfotransferase 7-like	4.2	5.4	4.82
LOC100539388	transcription factor SOX-5	5.1	4.6	4.81
LOC104914789	uncharacterized LOC104914789	4.2	5.4	4.81
TRIL	TLR4 interactor with leucine-rich repeats	4.2	5.4	4.81
LOC104909792	receptor-type tyrosine-protein phosphatase kappa-like	3.4	6.2	4.81
LOC104910907	uncharacterized LOC104910907	3.4	6.2	4.81
LOC104914223	uncharacterized LOC104914223	4.2	5.4	4.81
LOC104913766	serine/threonine-protein kinase LMTK1-like	3.4	6.2	4.81
LOC104915021	uncharacterized LOC104915021	4.2	5.4	4.81
LOC100539435	EF-hand domain-containing family member C2	3.4	6.2	4.80
TMEM51	transmembrane protein 51	3.4	6.2	4.80
LOC100546347	RNA-binding protein Nova-1-like	3.4	6.2	4.80
LOC104910993	uncharacterized LOC104910993	3.4	6.2	4.80
CADPS	Ca++-dependent secretion activator	3.4	6.2	4.79
OLFM3	olfactomedin 3	3.4	6.2	4.79
LOC104911483	uncharacterized LOC104911483	2.5	7.0	4.78
FBXL4	F-box and leucine-rich repeat protein 4	2.5	7.0	4.78

LOC100544938	cytochrome P450 26B1	2.5	7.0	4.78
ARHGEF9	Cdc42 guanine nucleotide exchange factor (GEF) 9	3.4	6.2	4.78
LOC100539804	eyes absent homolog 4-like	2.5	7.0	4.78
LOC100548588	cysteine-rich venom protein-like	2.5	7.0	4.76
LOC104913397	Na(+)/H(+) exchange regulatory cofactor NHE-RF2-like	2.5	7.0	4.76
LOC104910775	protein furry homolog-like	3.4	6.2	4.76
LOC104916132	uncharacterized LOC104916132	3.4	6.2	4.76
KIF26B	kinesin family member 26B	3.4	6.1	4.75
CALN1	calneuron 1	2.5	6.9	4.74
LDLRAD3	low density lipoprotein receptor class A domain containing 3	2.5	6.9	4.74
LOC104911501	uncharacterized LOC104911501	1.7	7.7	4.70
ELMOD1	ELMO/CED-12 domain containing 1	0.8	8.5	4.69
LOC104915104	uncharacterized LOC104915104	8.4	0.8	4.61
LOC100549617	coagulation factor XI	8.4	0.8	4.60
CTXN3	cortexin 3	7.6	1.6	4.60
CDC20B	cell division cycle 20B	6.8	2.4	4.58
LOC100540054	lysozyme g-like	7.6	1.6	4.58
LOC104913543	uncharacterized LOC104913543	7.6	1.6	4.57
LOC104909919	collagen alpha-1(XII) chain-like	7.6	1.5	4.57
LOC100546839	probable carboxypeptidase X1	6.8	2.4	4.56
LOC104910577	extracellular matrix protein FRAS1-like	6.8	2.4	4.56
RBFOX3	RNA binding protein, fox-1 homolog (C. elegans) 3	6.8	2.4	4.56
LOC104912219	AF4/FMR2 family member 2-like	5.9	3.2	4.55
HCRT	hypocretin (orexin) neuropeptide precursor	6.8	2.4	4.55
LOC104911326	cathelicidin-3	5.9	3.2	4.54
LOC104914208	ephrin type-B receptor 2-like	6.8	2.3	4.54
LOC100545183	glutamate receptor 1	6.8	2.3	4.54
LOC104910633	uncharacterized LOC104910633	6.8	2.3	4.54
PDCD1	programmed cell death 1	5.1	4.0	4.53
EDN1	endothelin 1	5.9	3.2	4.53
LOC104915162	colorectal mutant cancer protein-like	5.9	3.2	4.53
EBF2	early B-cell factor 2	6.8	2.3	4.53
LOC104909455	vinculin-like	5.1	4.0	4.53
LOC104911738	mitogen-activated protein kinase kinase kinase MLT-like	5.1	4.0	4.53
HHIP	hedgehog interacting protein	6.7	2.3	4.53
LOC100548269	arf-GAP with dual PH domain-containing protein 1-like	6.7	2.3	4.53
LOC104917065	tetra-peptide repeat homeobox protein 1-like	6.7	2.3	4.53
LOC100540763	urotensin-2 receptor-like	5.9	3.2	4.53
LRRTM3	leucine rich repeat transmembrane neuronal 3	5.9	3.2	4.53
FFAR4	free fatty acid receptor 4	5.1	4.0	4.53
LOC104914549	von Willebrand factor A domain-containing protein 5A-like	5.9	3.1	4.52
NKAIN3	Na+/K+ transporting ATPase interacting 3	5.9	3.1	4.52
LOC104911229	adenylate kinase 7-like	6.8	2.3	4.52
LOC104911152	uncharacterized LOC104911152	5.9	3.1	4.51
LOC104912748	uncharacterized LOC104912748	5.9	3.1	4.51
CHST4	carbohydrate (N-acetylglucosamine 6-O) sulfotransferase 4	6.8	2.3	4.51
LOC104910321	uncharacterized LOC104910321	6.7	2.3	4.51
LOC104915547	uncharacterized LOC104915547	5.1	4.0	4.51
LOC104911499	uncharacterized LOC104911499	5.9	3.1	4.51
LOC100550263	lysine-specific demethylase 4C-like	5.9	3.1	4.50
LOC104910256	receptor-type tyrosine-protein phosphatase mu-like	5.9	3.1	4.50
LOC104916990	uncharacterized aarF domain-containing protein kinase 5-like	5.1	3.9	4.50
LOC104917338	spectrin beta chain, non-erythrocytic 1-like	5.9	3.1	4.50
ACKR3	atypical chemokine receptor 3	5.1	3.9	4.50
DLK1	delta-like 1 homolog (Drosophila)	5.9	3.1	4.49
LOC104909641	uncharacterized LOC104909641	5.9	3.1	4.49
LOC104912687	uncharacterized LOC104912687	5.9	3.1	4.49

BRINP2	bone morphogenetic protein/retinoic acid inducible neural-specific 2	5.1	3.9	4.49
LOC104917025	heterogeneous nuclear ribonucleoprotein 87F-like	5.1	3.9	4.49
RNF224	ring finger protein 224	5.1	3.9	4.49
LOC104915016	lysine-specific demethylase 4C-like	5.9	3.1	4.49
LOC104914254	uncharacterized LOC104914254	5.9	3.0	4.47
LOC104916637	spectrin beta chain, non-erythrocytic 4-like	5.1	3.9	4.47
LOC104917254	uncharacterized LOC104917254	5.1	3.9	4.47
LOC100542196	guanine deaminase-like	5.9	3.0	4.47
LOC104912728	uncharacterized LOC104912728	5.9	3.0	4.47
MIOX	myo-inositol oxygenase	5.9	3.0	4.47
FAM162B	family with sequence similarity 162, member B	5.1	3.9	4.47
LOC104911911	AT-rich interactive domain-containing protein 5B-like	5.1	3.9	4.47
TCP11	t-complex 11, testis-specific	5.1	3.9	4.47
SNX20	sorting nexin 20	5.9	3.0	4.47
LOC104913554	breakpoint cluster region protein-like	4.2	4.7	4.47
LOC104916443	zinc finger protein neuro-d4-like	4.2	4.7	4.47
PLSCR1	phospholipid scramblase 1	4.2	4.7	4.47
LOC100544485	stromelysin-1-like	5.1	3.9	4.47
LOC104917306	putative ADCY10-like protein	4.2	4.7	4.47
MACC1	metastasis associated in colon cancer 1	5.1	3.9	4.47
SH3TC2	SH3 domain and tetratricopeptide repeats 2	4.2	4.7	4.46
TNN	tenascin N	4.2	4.7	4.46
LOC104914775	uncharacterized LOC104914775	3.4	5.5	4.46
LOC104910002	uncharacterized LOC104910002	5.1	3.8	4.46
LOC104909476	uncharacterized LOC104909476	5.1	3.8	4.45
C1H12orf50	chromosome 1 open reading frame, human C12orf50	4.2	4.7	4.45
LOC100540871	keratin, type I cytoskeletal 19-like	4.2	4.7	4.45
TUSC3	tumor suppressor candidate 3	4.2	4.7	4.45
USP50	ubiquitin specific peptidase 50	5.1	3.8	4.45
LOC104913924	ubiquitin carboxyl-terminal hydrolase 32-like	3.4	5.5	4.45
LOC104914567	uncharacterized LOC104914567	3.4	5.5	4.45
PRSS55	protease, serine, 55	3.4	5.5	4.45
DLGAP1	discs, large (Drosophila) homolog-associated protein 1	4.2	4.7	4.45
LOC100538725	carboxymethylenebutenolidase homolog	4.2	4.7	4.45
LOC104910476	brain-specific angiogenesis inhibitor 1-like	4.2	4.7	4.45
MAP3K19	mitogen-activated protein kinase kinase kinase 19	4.2	4.7	4.45
NEU2	sialidase 2 (cytosolic sialidase)	4.2	4.7	4.45
LOC104913108	dedicator of cytokinesis protein 3-like	3.4	5.5	4.44
C20H17orf67	chromosome 20 open reading frame, human C17orf67	4.2	4.7	4.44
ELOVL3	ELOVL fatty acid elongase 3	4.2	4.7	4.44
LOC104915074	guanine deaminase-like	5.1	3.8	4.44
SH2D4B	SH2 domain containing 4B	5.1	3.8	4.44
LOC104910465	uncharacterized LOC104910465	4.2	4.6	4.43
LOC104913263	serine protease inhibitor Kazal-type 6-like	3.4	5.5	4.43
LOC104917163	maestro heat-like repeat-containing protein family member 2B	3.4	5.5	4.43
ABCG4	ATP-binding cassette, sub-family G (WHITE), member 4	4.2	4.6	4.43
MUC2	mucin 2, oligomeric mucus/gel-forming	4.2	4.6	4.43
NEIL2	nei endonuclease VIII-like 2 (E. coli)	4.2	4.6	4.43
LOC100544433	neurexin-1-beta-like	4.2	4.6	4.43
CHRFAM7A	CHRNA7 (cholinergic receptor, nicotinic, alpha 7, exons 5-10) and FAM7A (family with sequence similarity 7A, exons A-E) fusion	3.4	5.5	4.42
LOC100542515	toll-like receptor 2	3.4	5.5	4.42
EFEMP1	EGF containing fibulin-like extracellular matrix protein 1	4.2	4.6	4.42
GPR37	G protein-coupled receptor 37 (endothelin receptor type B-like)	4.2	4.6	4.42
LOC104911728	uncharacterized LOC104911728	4.2	4.6	4.42
LOC100546802	potassium channel subfamily K member 16-like	4.2	4.6	4.42
LOC104913469	periplakin-like	4.2	4.6	4.41

LOC104917232	alpha-mannosidase 2-like	3.4	5.4	4.41
LOC104915234	lamin-B1-like	4.2	4.6	4.41
LOC104914973	uncharacterized LOC104914973	3.4	5.4	4.41
PFKFB3	6-phosphofructo-2-kinase/fructose-2,6-biphosphatase 3	3.4	5.4	4.40
LOC104914317	type II inositol 1,4,5-trisphosphate 5-phosphatase-like	3.4	5.4	4.40
LOC104911215	uncharacterized LOC104911215	2.5	6.3	4.40
LOC104914930	ralBP1-associated Eps domain-containing protein 2-like	2.5	6.3	4.40
MLXIPL	MLX interacting protein-like	3.4	5.4	4.40
LOC104915407	interleukin-12 receptor subunit beta-1-like	3.4	5.4	4.38
SLC30A8	solute carrier family 30 (zinc transporter), member 8	3.4	5.4	4.38
TRIM50	tripartite motif containing 50	3.4	5.4	4.38
TTC39B	tetratricopeptide repeat domain 39B	4.2	4.6	4.38
LOC104909743	uncharacterized LOC104909743	2.5	6.2	4.38
CD79B	CD79b molecule, immunoglobulin-associated beta	3.4	5.4	4.38
LOC104911243	uncharacterized LOC104911243	3.4	5.4	4.38
LOC104912191	uncharacterized LOC104912191	1.7	7.1	4.38
LOC100549590	merlin-like	3.4	5.4	4.38
LOC100538375	homeobox protein Hox-A1-like	1.7	7.1	4.38
CNTN4	contactin 4	2.5	6.2	4.37
LOC100538773	FRAS1-related extracellular matrix protein 2-like	2.5	6.2	4.37
LOC104911235	uncharacterized LOC104911235	3.4	5.4	4.36
LOC104914149	uncharacterized LOC104914149	3.4	5.4	4.36
LOC104909815	proto-oncogene tyrosine-protein kinase ROS-like	2.5	6.2	4.36
COLQ	collagen-like tail subunit (single strand of homotrimer) of asymmetric acetylcholinesterase	2.5	6.2	4.36
LOC104913154	uncharacterized LOC104913154	1.7	7.0	4.36
LOC104914398	nucleoporin-like protein 2	3.4	5.3	4.35
LOC104913287	uncharacterized LOC104913287	2.5	6.2	4.35
FAM155B	family with sequence similarity 155, member B	3.4	5.3	4.34
GNG13	guanine nucleotide binding protein (G protein), gamma 13	2.5	6.2	4.34
LOC104913796	uncharacterized LOC104913796	0.8	7.8	4.33
LOC104911878	uncharacterized LOC104911878	1.7	6.9	4.32
RS1	retinoschisin 1	7.6	0.8	4.19
LOC104909713	uncharacterized LOC104909713	7.6	0.8	4.18
LRRTM2	leucine rich repeat transmembrane neuronal 2	6.8	1.6	4.17
LOC100538901	uncharacterized oxidoreductase C663.09c-like	6.8	1.6	4.16
LOC104914342	uncharacterized LOC104914342	5.9	2.4	4.16
PAX9	paired box 9	6.8	1.6	4.16
LOC104909955	disks large-associated protein 2-like	5.9	2.4	4.16
LOC104917014	E3 ubiquitin-protein ligase HUWE1-like	5.9	2.4	4.16
RNF223	ring finger protein 223	5.9	2.4	4.15
FAM131A	family with sequence similarity 131, member A	6.7	1.6	4.15
LOC104909413	serine/threonine-protein kinase PAK 1-like	6.8	1.5	4.14
LOC104917346	uncharacterized LOC104917346	5.9	2.4	4.13
NR5A1	nuclear receptor subfamily 5, group A, member 1	5.9	2.4	4.13
LOC104915195	uncharacterized LOC104915195	5.9	2.4	4.13
LOC104913622	HLA class II histocompatibility antigen, DM beta chain-like	5.1	3.2	4.13
REC114	REC114 meiotic recombination protein	5.1	3.2	4.13
FXYD2	FXYD domain containing ion transport regulator 2	5.9	2.4	4.13
TPPP2	tubulin polymerization-promoting protein family member 2	5.9	2.4	4.13
LOC104916103	phytanoyl-CoA hydroxylase-interacting protein	5.9	2.3	4.12
LOC100540301	protocadherin gamma-B1-like	5.1	3.2	4.11
LOC100549029	ras-related protein Rab-39B-like	5.1	3.2	4.11
LOC104910743	toll-like receptor 2	5.1	3.2	4.11
WAS	Wiskott-Aldrich syndrome	5.1	3.2	4.11
HSPA12A	heat shock 70kDa protein 12A	5.9	2.3	4.11
LOC100549920	protein FAM184A-like	5.9	2.3	4.11
SLC15A1	solute carrier family 15 (oligopeptide transporter), member 1	5.1	3.2	4.10

KLHL32	kelch-like family member 32	5.9	2.3	4.10
LOC104913675	uncharacterized LOC104913675	5.9	2.3	4.10
HEPHL1	hephaestin-like 1	5.1	3.1	4.09
LOC104909544	uncharacterized LOC104909544	5.1	3.1	4.09
LOC104912739	uncharacterized LOC104912739	4.2	4.0	4.09
HPD	4-hydroxyphenylpyruvate dioxygenase	5.1	3.1	4.09
LOC104910671	uncharacterized LOC104910671	5.1	3.1	4.09
LOC104910457	uncharacterized LOC104910457	5.1	3.1	4.09
LOC100548914	adenylate cyclase type 7	5.9	2.3	4.09
KCNIP4	Kv channel interacting protein 4	5.1	3.1	4.09
LOC104911179	uncharacterized LOC104911179	4.2	4.0	4.08
ATP10D	ATPase, class V, type 10D	4.2	3.9	4.07
LOC100545463	histone H4-like	4.2	3.9	4.07
LOC104909574	uncharacterized LOC104909574	5.1	3.1	4.07
LOC104911613	G2/M phase-specific E3 ubiquitin-protein ligase-like	5.1	3.1	4.07
LOC100544389	extracellular sulfatase Sulf-1-like	4.2	3.9	4.07
LOC104914150	uncharacterized LOC104914150	5.1	3.1	4.07
ATP7B	ATPase, Cu++ transporting, beta polypeptide	4.2	3.9	4.07
LOC104913746	uncharacterized LOC104913746	3.4	4.8	4.07
LOC104914773	dynein intermediate chain 1, axonemal-like	3.4	4.8	4.07
GPR61	G protein-coupled receptor 61	4.2	3.9	4.07
LOC100550192	PDZ domain-containing protein 8	4.2	3.9	4.07
LOC100551131	receptor-type tyrosine-protein phosphatase alpha-like	4.2	3.9	4.07
LOC104914453	neuron navigator 1-like	4.2	3.9	4.07
SLC4A5	solute carrier family 4 (sodium bicarbonate cotransporter), member 5	4.2	3.9	4.07
TEX33	testis expressed 33	4.2	3.9	4.07
FBXO16	F-box protein 16	4.2	3.9	4.06
LOC104912126	type-1 angiotensin II receptor-like	4.2	3.9	4.06
LOC104911601	uncharacterized LOC104911601	5.1	3.0	4.06
SOX2	SRY (sex determining region Y)-box 2	4.2	3.9	4.05
LOC104915400	PR domain zinc finger protein 15-like	5.1	3.0	4.05
ENPP3	ectonucleotide pyrophosphatase/phosphodiesterase 3	4.2	3.9	4.05
COL6A6	collagen, type VI, alpha 6	4.2	3.9	4.05
LOC104911221	uncharacterized LOC104911221	4.2	3.9	4.05
LOC104909532	1-phosphatidylinositol 4,5-bisphosphate phosphodiesterase beta-1-like	3.4	4.7	4.05
RIN3	Ras and Rab interactor 3	3.4	4.7	4.05
UMODL1	uromodulin-like 1	3.4	4.7	4.05
ADPRHL1	ADP-ribosylhydrolase like 1	4.2	3.9	4.05
GCNT4	glucosaminyl (N-acetyl) transferase 4, core 2	4.2	3.9	4.05
LOC104910291	uncharacterized LOC104910291	4.2	3.9	4.05
TMEM132C	transmembrane protein 132C	4.2	3.9	4.05
ANKDD1B	ankyrin repeat and death domain containing 1B	3.4	4.7	4.04
PTGFRN	prostaglandin F2 receptor inhibitor	3.4	4.7	4.04
TDRD6	tudor domain containing 6	3.4	4.7	4.04
LOC104914585	oocyte zinc finger protein XlCOF6-like	4.2	3.9	4.04
LOC100546066	protein SCARECROW 1-like	3.4	4.7	4.04
LOC104914004	uncharacterized LOC104914004	4.2	3.8	4.03
FUT7	fucosyltransferase 7 (alpha (1,3) fucosyltransferase)	3.4	4.7	4.03
LOC104915287	uncharacterized protein KIAA1958-like	3.4	4.7	4.03
C21H17orf97	chromosome 21 open reading frame, human C17orf97	4.2	3.8	4.03
LOC104911160	uncharacterized LOC104911160	4.2	3.8	4.03
LOC104912360	ATP synthase subunit alpha, mitochondrial-like	4.2	3.8	4.03
LOC104914127	uncharacterized LOC104914127	2.5	5.5	4.03
LOC104910647	uncharacterized LOC104910647	3.4	4.7	4.03
MCTP1	multiple C2 domains, transmembrane 1	2.5	5.5	4.02
LOC104916084	putative solute carrier family 22 member 31	3.4	4.7	4.02

EMCN	endomucin	2.5	5.5	4.02
LOC104912402	uncharacterized LOC104912402	3.4	4.6	4.01
CBX2	chromobox homolog 2	3.4	4.6	4.01
DLL4	delta-like 4 (Drosophila)	2.5	5.5	4.01
LOC100540009	uncharacterized LOC100540009	3.4	4.6	4.01
LOC100550812	caspase recruitment domain-containing protein 11	3.4	4.6	4.01
LOC104909248	limbin-like	3.4	4.6	4.01
LOC100541607	acetyl-coenzyme A synthetase 2-like, mitochondrial	2.5	5.5	4.00
LOC104917554	uncharacterized LOC104917554	2.5	5.5	4.00
LOC100541471	sorting nexin-29-like	3.4	4.6	4.00
LOC104910346	uncharacterized LOC104910346	1.7	6.3	4.00
BCL2A1	BCL2-related protein A1	2.5	5.5	4.00
TNMD	tenomodulin	2.5	5.5	4.00
LOC104911428	synapsin-3-like	3.4	4.6	3.99
REEP6	receptor accessory protein 6	3.4	4.6	3.99
LOC104911937	uncharacterized LOC104911937	3.4	4.6	3.99
EMILIN3	elastin microfibril interfacer 3	2.5	5.4	3.98
GALNT15	polypeptide N-acetylgalactosaminyltransferase 15	2.5	5.4	3.98
LOC104914005	uncharacterized LOC104914005	1.7	6.3	3.98
BMP2	bone morphogenetic protein 2	2.5	5.4	3.98
LOC104912930	putative ATP-dependent RNA helicase TDRD12	1.7	6.3	3.98
LOC104914286	CUB and sushi domain-containing protein 2-like	1.7	6.3	3.98
LOC104916540	zinc transporter 1-like	2.5	5.4	3.97
LOC104912921	uncharacterized LOC104912921	3.4	4.6	3.97
GRM7	glutamate receptor, metabotropic 7	2.5	5.4	3.96
LOC104909634	uncharacterized LOC104909634	2.5	5.4	3.96
LOC104910113	uncharacterized LOC104910113	2.5	5.4	3.96
LOC104910269	uncharacterized LOC104910269	1.7	6.2	3.96
PLXNA4	plexin A4	1.7	6.2	3.96
ZBBX	zinc finger, B-box domain containing	1.7	6.2	3.96
CFAP57	cilia and flagella associated protein 57	2.5	5.4	3.96
LOC100539909	matrix metalloproteinase-16	1.7	6.2	3.96
LOC100539697	integrin beta-like protein 1	2.5	5.4	3.95
LOC104917561	uncharacterized LOC104917561	2.5	5.4	3.94
LOC104909906	uncharacterized LOC104909906	1.7	6.2	3.94
LOC100543904	putative cation exchanger C521.04c	2.5	5.4	3.94
LOC104910401	uncharacterized LOC104910401	1.7	6.2	3.94
LOC104913519	uncharacterized LOC104913519	1.7	6.2	3.94
LOC104912222	uncharacterized LOC104912222	2.5	5.3	3.92
LOC100543920	opsin-5-like	1.7	6.2	3.92
ANKK1	ankyrin repeat and kinase domain containing 1	0.8	7.0	3.92
LOC100541465	cytochrome P450 2J2-like	0.8	7.0	3.92
LOC104912852	uncharacterized LOC104912852	0.8	7.0	3.92
LOC104914947	uncharacterized LOC104914947	1.7	6.1	3.90
LOC100546225	cytosolic carboxypeptidase 1	1.7	6.1	3.90
AGMAT	agmatine ureohydrolase (agmatinase)	0.8	6.9	3.88
LOC104915158	uncharacterized LOC104915158	7.6	0.0	3.79
LOC104912915	fatty acyl-CoA hydrolase precursor, medium chain-like	6.7	0.8	3.77
ASPDH	aspartate dehydrogenase domain containing	5.9	1.6	3.76
LOC104916621	maestro heat-like repeat-containing protein family member 1	5.9	1.6	3.76
SHC3	SHC (Src homology 2 domain containing) transforming protein 3	5.9	1.6	3.76
RALYL	RALY RNA binding protein-like	6.7	0.8	3.75
LOC104917072	zinc finger protein 502-like	6.7	0.8	3.75
LOC104914144	uncharacterized LOC104914144	5.9	1.6	3.74
LOC104911297	uncharacterized LOC104911297	5.9	1.6	3.74
TFAP2C	transcription factor AP-2 gamma (activating enhancer binding protein 2 gamma)	5.9	1.6	3.74
DPT	dermatopontin	5.9	1.6	3.74

LOC100547424	dipeptidyl peptidase 4-like	5.9	1.6	3.74
MEP1A	meprin A, alpha (PABA peptide hydrolase)	5.1	2.4	3.74
ANKRD55	ankyrin repeat domain 55	5.9	1.6	3.74
LOC104909663	WD repeat-containing protein 64-like	5.9	1.6	3.74
LOC104912112	tyrosine-protein kinase BTK-like	5.9	1.6	3.73
MFAP3L	microfibrillar-associated protein 3-like	5.1	2.4	3.73
FAM163A	family with sequence similarity 163, member A	5.9	1.6	3.73
RHOH	ras homolog family member H	5.9	1.6	3.73
LOC104910496	amphiphysin-like	5.1	2.4	3.72
CAPSL	calcyphosine-like	5.9	1.5	3.72
ATOH7	atonal homolog 7 (Drosophila)	5.1	2.4	3.71
PADI2	peptidyl arginine deiminase, type II	5.1	2.4	3.71
LOC100550045	toll-like receptor 13	5.9	1.5	3.71
ERP27	endoplasmic reticulum protein 27	5.1	2.4	3.71
SUSD5	sushi domain containing 5	5.1	2.4	3.71
RGS7	regulator of G-protein signaling 7	5.1	2.4	3.71
LOC100540595	dual specificity protein phosphatase 13 isoform B-like	4.2	3.2	3.70
GRTP1	growth hormone regulated TBC protein 1	5.1	2.3	3.69
GABRA3	gamma-aminobutyric acid (GABA) A receptor, alpha 3	4.2	3.2	3.69
LOC104914523	metabotropic glutamate receptor 4-like	5.1	2.3	3.69
LOC104916038	C5a anaphylatoxin chemotactic receptor 1-like	5.1	2.3	3.69
TMEM132B	transmembrane protein 132B	5.1	2.3	3.69
LOC104911217	uncharacterized LOC104911217	4.2	3.2	3.69
LOC104912269	uncharacterized LOC104912269	4.2	3.2	3.69
TCTE1	t-complex-associated-testis-expressed 1	5.1	2.3	3.69
LOC100548996	dynein heavy chain 8, axonemal	3.4	4.0	3.69
LOC104910379	junctophilin-1-like	5.1	2.3	3.69
LOC104916225	uncharacterized protein PB18E9.04c-like	5.1	2.3	3.69
SLC26A6	solute carrier family 26 (anion exchanger), member 6	5.1	2.3	3.69
FLT3	fms-related tyrosine kinase 3	3.4	4.0	3.68
LOC100546913	selenoprotein Pb-like	4.2	3.1	3.67
LOC104912615	vitamin K-dependent protein C-like	5.1	2.3	3.67
LOC104916067	mucin-21-like	5.1	2.3	3.67
GNAZ	guanine nucleotide binding protein (G protein), alpha z polypeptide	4.2	3.1	3.67
LOC100538406	killer cell lectin-like receptor subfamily B member 1B allele A	4.2	3.1	3.67
LOC100542877	tyrosine-protein kinase ZAP-70	4.2	3.1	3.67
LOC104911436	uncharacterized LOC104911436	4.2	3.1	3.67
LOC104916060	uncharacterized LOC104916060	4.2	3.1	3.67
LOC104917474	uncharacterized LOC104917474	4.2	3.1	3.67
TULP1	tubby like protein 1	4.2	3.1	3.67
LOC104910579	extracellular matrix protein FRAS1-like	3.4	4.0	3.67
LOC104912305	uncharacterized LOC104912305	3.4	4.0	3.67
LOC104917074	uncharacterized LOC104917074	4.2	3.1	3.67
RORA	RAR-related orphan receptor A	4.2	3.1	3.67
WDR17	WD repeat domain 17	5.1	2.3	3.67
GPR82	G protein-coupled receptor 82	3.4	4.0	3.67
LOC104910542	uncharacterized LOC104910542	4.2	3.1	3.66
LOC100541429	perilipin-3-like	4.2	3.1	3.66
LOC104912077	G protein-coupled receptor kinase 5-like	3.4	4.0	3.66
TESC	tescalcin	3.4	4.0	3.66
LOC104917414	uncharacterized LOC104917414	4.2	3.1	3.65
LOC100546313	voltage-dependent T-type calcium channel subunit alpha-1H-like	3.4	3.9	3.65
LOC104916934	probable N-acetyltransferase CML3	3.4	3.9	3.65
LOC100539486	protocadherin gamma-A2-like	4.2	3.1	3.65
LOC100543185	keratin, type I cytoskeletal 13-like	4.2	3.1	3.65
LOC104910123	uncharacterized LOC104910123	4.2	3.1	3.65
LOC104916003	adenylate cyclase type 10-like	4.2	3.1	3.65

LOC100541174	vasoactive intestinal polypeptide receptor 1-like	3.4	3.9	3.65
LOC104910126	uncharacterized LOC104910126	3.4	3.9	3.65
LOC104911155	uncharacterized LOC104911155	3.4	3.9	3.65
LOC104911766	uncharacterized LOC104911766	3.4	3.9	3.65
LOC104912398	uncharacterized LOC104912398	3.4	3.9	3.65
LOC104915947	tetratricopeptide repeat protein 36-like	3.4	3.9	3.65
NPFFR1	neuropeptide FF receptor 1	3.4	3.9	3.65
SLC5A8	solute carrier family 5 (sodium/monocarboxylate cotransporter), member 8	4.2	3.1	3.65
LOC100548077	disheveled-associated activator of morphogenesis 2	3.4	3.9	3.65
LOC104909394	uncharacterized LOC104909394	3.4	3.9	3.65
LOC104913325	uncharacterized LOC104913325	3.4	3.9	3.65
LOC104909385	erythroblast NAD(P)(+)--arginine ADP-ribosyltransferase pseudogene	2.5	4.8	3.64
ACVRL1	activin A receptor type II-like 1	3.4	3.9	3.64
CRHBP	corticotropin releasing hormone binding protein	3.4	3.9	3.64
LOC104917521	ecto-NOX disulfide-thiol exchanger 1-like	3.4	3.9	3.64
NTNG1	netrin G1	3.4	3.9	3.64
LOC104913341	uncharacterized LOC104913341	1.7	5.6	3.64
LOC100548529	tubulin alpha-4 chain	2.5	4.8	3.64
SLC45A2	solute carrier family 45, member 2	4.2	3.0	3.63
B4GALT6	UDP-Gal:betaGlcNAc beta 1,4- galactosyltransferase, polypeptide 6	3.4	3.9	3.63
LOC104910541	uncharacterized LOC104910541	3.4	3.9	3.63
LOC104911264	uncharacterized LOC104911264	3.4	3.9	3.63
DENND1B	DENN/MADD domain containing 1B	4.2	3.0	3.63
LOC104914008	ganglioside-induced differentiation-associated protein 1-like 1	4.2	3.0	3.63
LOC104914847	uncharacterized LOC104914847	2.5	4.7	3.63
ABI3	ABI family, member 3	4.2	3.0	3.63
LOC104912045	phosphatidylinositol 4-phosphate 3-kinase C2 domain-containing subunit gamma-like	4.2	3.0	3.63
LOC100538412	mitogen-activated protein kinase kinase kinase 15-like	2.5	4.7	3.62
LOC100543893	TOX high mobility group box family member 2-like	2.5	4.7	3.62
ARAP3	ArfGAP with RhoGAP domain, ankyrin repeat and PH domain 3	3.4	3.9	3.62
LOC100551192	group 10 secretory phospholipase A2-like	3.4	3.9	3.62
BAI3	brain-specific angiogenesis inhibitor 3	2.5	4.7	3.62
TSPAN32	tetraspanin 32	2.5	4.7	3.62
LOC104910563	SH2 domain-containing protein 4A-like	3.4	3.9	3.62
LOC104916161	uncharacterized LOC104916161	3.4	3.9	3.62
RIMS4	regulating synaptic membrane exocytosis 4	3.4	3.9	3.62
LOC104914499	neurofascin-like	2.5	4.7	3.62
LOC100548275	hephaestin-like protein 1	3.4	3.8	3.61
NELL1	NEL-like 1 (chicken)	3.4	3.8	3.61
IL10	interleukin 10	3.4	3.8	3.61
LOC100540115	myelin basic protein-like	2.5	4.7	3.61
LOC100545313	sulfotransferase 6B1-like	2.5	4.7	3.61
LOC104916792	uncharacterized LOC104916792	3.4	3.8	3.61
LOC104917467	uncharacterized LOC104917467	3.4	3.8	3.61
ADRA1B	adrenoceptor alpha 1B	2.5	4.7	3.60
LOC104911592	uncharacterized LOC104911592	2.5	4.7	3.60
TBPL2	TATA box binding protein like 2	2.5	4.7	3.60
XKR6	XK, Kell blood group complex subunit-related family, member 6	2.5	4.7	3.60
LOC104913531	uncharacterized LOC104913531	3.4	3.8	3.60
LOC104914531	uncharacterized LOC104914531	3.4	3.8	3.60
LOC100538433	lipid phosphate phosphatase-related protein type 4-like	2.5	4.7	3.60
LOC104912865	uncharacterized LOC104912865	2.5	4.7	3.60
LOC104917583	neurobeachin-like	2.5	4.7	3.60
KIAA0226L	KIAA0226-like ortholog	1.7	5.5	3.60

LOC100542486	cytochrome P450 1A4	1.7	5.5	3.60
LOC104910820	uncharacterized LOC104910820	1.7	5.5	3.60
LOC104910928	uncharacterized LOC104910928	0.8	6.3	3.60
LOC100550636	C2 domain-containing protein 3	3.4	3.8	3.59
CDHR4	cadherin-related family member 4	2.5	4.6	3.59
KCNJ3	potassium inwardly-rectifying channel, subfamily J, member 3	3.4	3.8	3.59
LOC104911382	uncharacterized LOC104911382	2.5	4.6	3.58
LOC104910218	GLIPR1-like protein 1	1.7	5.5	3.58
ATP6V0A4	ATPase, H+ transporting, lysosomal V0 subunit a4	2.5	4.6	3.58
LOC100546335	olfactory receptor 51E2-like	2.5	4.6	3.58
LOC104915209	cyclin-G-associated kinase-like	2.5	4.6	3.58
LOC104916424	microtubule-actin cross-linking factor 1-like	2.5	4.6	3.58
LOC104911049	uncharacterized LOC104911049	2.5	4.6	3.58
MTL5	metallothionein-like 5, testis-specific (tesmin)	1.7	5.5	3.58
RAB44	RAB44, member RAS oncogene family	1.7	5.5	3.58
LOC104913551	uncharacterized LOC104913551	0.0	7.1	3.57
LOC104917421	uncharacterized LOC104917421	2.5	4.6	3.56
LOC104910615	uncharacterized LOC104910615	1.7	5.4	3.56
LOC104913579	leucine-rich repeat-containing protein 74B-like	1.7	5.4	3.56
AOAH	acyloxyacyl hydrolase (neutrophil)	2.5	4.6	3.56
DRD4	dopamine receptor D4	0.8	6.3	3.56
BMP15	bone morphogenetic protein 15	1.7	5.4	3.54
LOC104915183	uncharacterized LOC104915183	0.8	6.2	3.54
LOC104915121	protein patched homolog 1-like	0.8	6.2	3.52
LOC100542547	T-cell immunoglobulin and mucin domain-containing protein 4-like	6.8	0.0	3.39
LOC104910208	dynein heavy chain 5, axonemal-like	6.8	0.0	3.38
WDFY4	WDFY family member 4	5.9	0.8	3.36
NLGN4X	neuroligin 4, X-linked	5.9	0.8	3.36
LOC104915006	transcription factor RFX3	5.1	1.6	3.34
LOC104911969	kinesin-like protein KIF20B	5.1	1.6	3.33
LOC104910906	uncharacterized LOC104910906	5.9	0.8	3.33
RBM11	RNA binding motif protein 11	5.9	0.8	3.33
LOC104911646	uncharacterized LOC104911646	5.1	1.6	3.33
CA7	carbonic anhydrase VII	5.1	1.6	3.32
RYR2	ryanodine receptor 2 (cardiac)	5.1	1.6	3.32
LOC104910888	uncharacterized LOC104910888	5.1	1.6	3.31
LOC104912936	uncharacterized LOC104912936	4.2	2.4	3.31
FAM154B	family with sequence similarity 154, member B	5.1	1.6	3.31
LOC100545752	ATP-binding cassette sub-family A member 10	5.1	1.6	3.31
LOC104911921	protein FAM13A-like	5.1	1.6	3.31
FMO4	flavin containing monooxygenase 4	4.2	2.4	3.31
ITGB6	integrin, beta 6	4.2	2.4	3.31
LOC104911207	uncharacterized LOC104911207	4.2	2.4	3.31
RXFP3	relaxin/insulin-like family peptide receptor 3	5.1	1.6	3.31
LOC104909699	uncharacterized LOC104909699	4.2	2.4	3.31
KLHL14	kelch-like family member 14	5.1	1.6	3.30
ATP8B3	ATPase, aminophospholipid transporter, class I, type 8B, member 3	5.1	1.5	3.30
MTTP	microsomal triglyceride transfer protein	4.2	2.4	3.30
LOC104909628	opsin-5-like	5.1	1.5	3.29
TRPA1	transient receptor potential cation channel, subfamily A, member 1	5.1	1.5	3.29
LOC104914541	probable E3 ubiquitin-protein ligase makorin-1	5.1	1.5	3.29
LOC104910532	uncharacterized LOC104910532	4.2	2.4	3.29
LOC104910656	uncharacterized LOC104910656	4.2	2.4	3.29
LOC100545731	acyl-coenzyme A thioesterase 5-like	3.4	3.2	3.29
CLDN20	claudin 20	4.2	2.4	3.29

ILDR2	immunoglobulin-like domain containing receptor 2	4.2	2.4	3.29
LOC104912221	uncharacterized LOC104912221	4.2	2.4	3.29
LOC104913472	glucagon receptor-like	4.2	2.4	3.29
LOC104916380	frizzled-8-like	4.2	2.4	3.29
LOC104914158	uncharacterized LOC104914158	5.1	1.5	3.29
LOC104914079	uncharacterized LOC104914079	3.4	3.2	3.29
ENTPD1	ectonucleoside triphosphate diphosphohydrolase 1	4.2	2.4	3.29
LOC104912295	uncharacterized LOC104912295	3.4	3.2	3.28
CDH17	cadherin 17, LI cadherin (liver-intestine)	4.2	2.3	3.28
AGBL3	ATP/GTP binding protein-like 3	4.2	2.3	3.27
LOC104910853	uncharacterized protein PB18E9.04c-like	4.2	2.3	3.27
CCR8	chemokine (C-C motif) receptor 8	4.2	2.3	3.27
DPEP1	dipeptidase 1 (renal)	4.2	2.3	3.27
GPRC5C	G protein-coupled receptor, class C, group 5, member C	4.2	2.3	3.27
LOC100544693	histone H3.3-like	4.2	2.3	3.27
LOC100545729	prolactin-releasing peptide receptor-like	4.2	2.3	3.27
LOC104910774	uncharacterized LOC104910774	4.2	2.3	3.27
LOC104911020	serum amyloid A protein-like	4.2	2.3	3.27
LOC104915845	uncharacterized LOC104915845	4.2	2.3	3.27
SPNS3	spinster homolog 3 (Drosophila)	4.2	2.3	3.27
LOC100540841	electrogenic sodium bicarbonate cotransporter 1-like	3.4	3.2	3.27
LOC100540856	intestinal-type alkaline phosphatase-like	3.4	3.2	3.27
LOC104909318	elastin-like	3.4	3.2	3.27
LOC104913127	HAUS augmin-like complex subunit 3	3.4	3.2	3.27
LOC104913387	uncharacterized LOC104913387	3.4	3.2	3.27
P2RY14	purinergic receptor P2Y, G-protein coupled, 14	3.4	3.2	3.27
CCDC96	coiled-coil domain containing 96	4.2	2.3	3.27
LOC100539248	prostatic acid phosphatase-like	4.2	2.3	3.27
LOC104912977	uncharacterized LOC104912977	4.2	2.3	3.27
LOC104914606	uncharacterized LOC104914606	4.2	2.3	3.27
LOC104914240	disintegrin and metalloproteinase domain-containing protein 32-like	2.5	4.0	3.27
LOC104910180	uncharacterized LOC104910180	3.4	3.2	3.27
TRAPPC3L	trafficking protein particle complex 3-like	3.4	3.2	3.27
GREM2	gremlin 2, DAN family BMP antagonist	4.2	2.3	3.27
HAVCR2	hepatitis A virus cellular receptor 2	4.2	2.3	3.27
LOC104916410	uncharacterized LOC104916410	4.2	2.3	3.27
MC1R	melanocortin 1 receptor (alpha melanocyte stimulating hormone receptor)	4.2	2.3	3.27
PAMR1	peptidase domain containing associated with muscle regeneration 1	4.2	2.3	3.27
MC5R	melanocortin 5 receptor	2.5	4.0	3.26
LOC104915489	lipoma-preferred partner homolog	3.4	3.2	3.26
PIK3R5	phosphoinositide-3-kinase, regulatory subunit 5	3.4	3.2	3.26
CTNND2	catenin (cadherin-associated protein), delta 2	4.2	2.3	3.26
LOC104909952	protein eyes shut homolog	3.4	3.2	3.26
IRF6	interferon regulatory factor 6	4.2	2.3	3.25
LOC104915355	aminopeptidase Q-like	4.2	2.3	3.25
LOC104911341	uncharacterized LOC104911341	3.4	3.1	3.25
LOC104912968	uncharacterized LOC104912968	2.5	4.0	3.25
C3	complement component 3	3.4	3.1	3.25
LOC100543622	sodium channel protein type 5 subunit alpha-like	3.4	3.1	3.25
LOC100547769	interleukin-15 receptor subunit alpha-like	3.4	3.1	3.25
TACR1	tachykinin receptor 1	3.4	3.1	3.25
LOC104910759	myotubularin-related protein 7-like	4.2	2.3	3.25
LOC104910650	uncharacterized LOC104910650	2.5	4.0	3.24
LOC100541180	G-protein coupled receptor 183-like	3.4	3.1	3.24
LOC100545122	G-protein coupled receptor 35-like	3.4	3.1	3.24

LOC104910241	rotatin-like	3.4	3.1	3.24
LOC104912165	uncharacterized LOC104912165	3.4	3.1	3.24
PLEKHS1	pleckstrin homology domain containing, family S member 1	3.4	3.1	3.24
TDO2	tryptophan 2,3-dioxygenase	3.4	3.1	3.24
LOC104915428	maestro heat-like repeat-containing protein family member 2B	2.5	4.0	3.24
SLC25A40	solute carrier family 25, member 40	2.5	4.0	3.24
PKP1	plakophilin 1	3.4	3.1	3.24
ACSM3	acyl-CoA synthetase medium-chain family member 3	3.4	3.1	3.23
HPX	hemopexin	3.4	3.1	3.23
LOC100548707	collagen alpha-1(XI) chain-like	3.4	3.1	3.23
LOC104911338	GTPase IMAP family member 8-like	3.4	3.1	3.23
LOC104916970	microtubule-associated serine/threonine-protein kinase 1-like	3.4	3.1	3.23
LOC104912607	uncharacterized LOC104912607	3.4	3.1	3.23
LOC104913722	growth arrest-specific protein 7-like	3.4	3.1	3.23
LOC104912145	protein bicaudal D homolog 1-like	3.4	3.1	3.23
LOC100547876	polypeptide N-acetylgalactosaminyltransferase 12-like	2.5	3.9	3.22
LOC100550763	junctophilin-3	2.5	3.9	3.22
TMEM171	transmembrane protein 171	2.5	3.9	3.22
LOC104914738	uncharacterized LOC104914738	3.4	3.1	3.22
SPOCK3	sparc/osteonectin, cwcv and kazal-like domains proteoglycan (testican) 3	3.4	3.1	3.22
UPK3B	uroplakin 3B	3.4	3.1	3.22
LOC104909465	uncharacterized LOC104909465	1.7	4.8	3.22
LOC100550975	adenosine receptor A3-like	3.4	3.1	3.22
LOC104916100	uncharacterized LOC104916100	2.5	3.9	3.22
GABRR3	gamma-aminobutyric acid (GABA) A receptor, rho 3	0.8	5.6	3.22
LOC100539637	ATP-dependent RNA helicase DDX25-like	2.5	3.9	3.21
SLC7A14	solute carrier family 7, member 14	2.5	3.9	3.21
LOC104909215	coagulation factor VII-like	3.4	3.0	3.21
LOC104917496	uncharacterized LOC104917496	2.5	3.9	3.20
LOC104916244	protein MANBAL-like	3.4	3.0	3.20
IL23A	interleukin 23, alpha subunit p19	1.7	4.7	3.20
LOC104911819	formin-like protein 2	1.7	4.7	3.20
LOC104913190	uncharacterized LOC104913190	1.7	4.7	3.20
LOC104917415	sodium leak channel non-selective protein-like	1.7	4.7	3.20
LOC100542853	transcription factor SOX-3-like	2.5	3.9	3.20
LOC100549671	fatty acid desaturase 1-like	1.7	4.7	3.20
LOC104910047	uncharacterized LOC104910047	1.7	4.7	3.20
FMR1NB	fragile X mental retardation 1 neighbor	2.5	3.9	3.20
FIBCD1	fibrinogen C domain containing 1	1.7	4.7	3.20
LOC104910756	uncharacterized LOC104910756	0.8	5.5	3.20
LOC104915640	uncharacterized protein KIAA1755 homolog	2.5	3.8	3.19
LOC104916420	adenylate cyclase type 10-like	2.5	3.8	3.19
LOC104917420	uncharacterized LOC104917420	2.5	3.8	3.19
ARRDC5	arrestin domain containing 5	1.7	4.7	3.18
LOC100547461	metalloendopeptidase homolog PEX-like	1.7	4.7	3.18
LOC100538447	liprin-alpha-4-like	2.5	3.8	3.18
LOC104911165	uncharacterized LOC104911165	2.5	3.8	3.18
RNF43	ring finger protein 43	2.5	3.8	3.18
GIMAP8	GTPase, IMAP family member 8	1.7	4.7	3.18
LOC100538506	non-specific lipid-transfer protein pseudogene	1.7	4.7	3.18
LOC104909817	uncharacterized LOC104909817	1.7	4.7	3.18
LOC104911154	uncharacterized LOC104911154	1.7	4.7	3.18
LOC104913445	uncharacterized LOC104913445	1.7	4.7	3.18
LOC104915382	uncharacterized LOC104915382	1.7	4.7	3.18
LRRC48	leucine rich repeat containing 48	1.7	4.7	3.18
RCAN2	regulator of calcineurin 2	1.7	4.7	3.18

TRPM8	transient receptor potential cation channel, subfamily M, member 8	1.7	4.7	3.18
SLC6A1	solute carrier family 6 (neurotransmitter transporter), member 1	2.5	3.8	3.18
LOC100545050	putative protein FAM172B	0.8	5.5	3.18
LOC104914691	uncharacterized protein KIAA1958-like	0.8	5.5	3.18
TMPRSS5	transmembrane protease, serine 5	0.8	5.5	3.18
CD34	CD34 molecule	1.7	4.7	3.18
NOV	nephroblastoma overexpressed	1.7	4.7	3.18
VIPR2	vasoactive intestinal peptide receptor 2	1.7	4.7	3.18
LOC104910271	uncharacterized LOC104910271	1.7	4.6	3.16
LOC104912271	uncharacterized LOC104912271	1.7	4.6	3.16
BRICD5	BRICHOS domain containing 5	1.7	4.6	3.16
C4H4orf17	chromosome 4 open reading frame, human C4orf17	1.7	4.6	3.16
DLX5	distal-less homeobox 5	1.7	4.6	3.16
LOC100549159	transmembrane protease serine 11E-like	1.7	4.6	3.16
LOC104910479	DENN domain-containing protein 3-like	1.7	4.6	3.16
LOC104916543	voltage-dependent calcium channel subunit alpha-2/delta-1-like	1.7	4.6	3.16
LOC104912039	uncharacterized LOC104912039	1.7	4.6	3.14
LOC100544371	uncharacterized LOC100544371	1.7	4.6	3.14
LOC104912566	uncharacterized LOC104912566	0.8	5.4	3.14
LOC104916901	A-kinase anchor protein 9-like	1.7	4.6	3.12
LOC104909653	disheveled-associated activator of morphogenesis 2-like	0.8	5.4	3.12
LOC104915025	MAP7 domain-containing protein 2-like	0.8	5.4	3.12
LOC104917286	uncharacterized LOC104917286	0.8	5.4	3.12
DRP2	dystrophin related protein 2	0.8	5.4	3.12
TMEM247	transmembrane protein 247	0.8	5.4	3.12
LOC104917424	integrin beta-like protein 1	0.8	5.3	3.08
LOC104915067	membrane-bound transcription factor site-2 protease-like	0.8	5.3	3.08
SLC14A2	solute carrier family 14 (urea transporter), member 2	0.0	6.2	3.08
LOC104916272	uncharacterized LOC104916272	5.9	0.0	2.95
LOC104911655	uncharacterized LOC104911655	5.9	0.0	2.95
LOC104915744	voltage-dependent P/Q-type calcium channel subunit alpha-1A-like	5.1	0.8	2.94
LOC104914065	pendrin-like	5.1	0.8	2.93
LOC104909870	protein MMS22-like	5.1	0.8	2.93
LOC100539126	cell surface glycoprotein CD200 receptor 1-A-like	5.1	0.8	2.92
LOC104909459	proteasome activator complex subunit 4-like	5.1	0.8	2.92
ARMC3	armadillo repeat containing 3	5.1	0.8	2.91
LOC100539633	excitatory amino acid transporter 4-like	5.1	0.8	2.91
GCGR	glucagon receptor	4.2	1.6	2.91
LOC104911053	uncharacterized LOC104911053	4.2	1.6	2.91
LOC104917007	uncharacterized LOC104917007	4.2	1.6	2.90
LOC100549986	alpha N-terminal protein methyltransferase 1B-like	4.2	1.6	2.89
LOC104914642	platelet glycoprotein Ib alpha chain-like	4.2	1.6	2.89
LOC104914645	maestro heat-like repeat-containing protein family member 2B	4.2	1.6	2.89
LOC104916572	uncharacterized LOC104916572	4.2	1.6	2.89
LOC104909260	uncharacterized LOC104909260	3.4	2.4	2.89
BATF3	basic leucine zipper transcription factor, ATF-like 3	4.2	1.6	2.89
LOC104911853	uncharacterized LOC104911853	4.2	1.6	2.89
LOC100545149	histone H3.3-like	3.4	2.4	2.89
SPAG17	sperm associated antigen 17	3.4	2.4	2.89
FHL5	four and a half LIM domains 5	4.2	1.6	2.89
LOC104909359	periphilin-1-like	4.2	1.6	2.89
LOC104910701	uncharacterized LOC104910701	4.2	1.6	2.89
SEBOX	SEBOX homeobox	4.2	1.6	2.89
LOC104913054	uncharacterized LOC104913054	3.4	2.4	2.89
LOC104913948	uncharacterized LOC104913948	3.4	2.4	2.89
LOC104916784	class II histocompatibility antigen, B-L beta chain-like	3.4	2.4	2.89

TNFRSF13B	tumor necrosis factor receptor superfamily, member 13B	3.4	2.4	2.89
GFPT2	glutamine-fructose-6-phosphate transaminase 2	4.2	1.6	2.89
LOC104912591	uncharacterized LOC104912591	4.2	1.6	2.89
TMEM196	transmembrane protein 196	4.2	1.6	2.89
ADIRF	adipogenesis regulatory factor	4.2	1.6	2.88
LOC100548817	disintegrin and metalloproteinase domain-containing protein 20-like	4.2	1.6	2.88
LOC104917357	plexin-D1-like	4.2	1.5	2.88
COLEC11	collectin sub-family member 11	4.2	1.5	2.87
LOC104911365	uncharacterized LOC104911365	4.2	1.5	2.87
LOC104914194	uncharacterized LOC104914194	4.2	1.5	2.87
CCL20	chemokine (C-C motif) ligand 20	4.2	1.5	2.87
LOC100550424	uncharacterized LOC100550424	4.2	1.5	2.87
LOC104911626	uncharacterized LOC104911626	4.2	1.5	2.87
LOC104915520	uncharacterized LOC104915520	4.2	1.5	2.87
FGF4	fibroblast growth factor 4	3.4	2.4	2.87
LOC100539895	sodium- and chloride-dependent GABA transporter 2	3.4	2.4	2.87
LOC104911384	sickle tail protein homolog	3.4	2.4	2.87
LOC104911955	cytochrome P450 2H2	3.4	2.4	2.87
LOC104913847	apoptosis-inducing factor 3-like	3.4	2.4	2.87
LOC104917113	adenylate cyclase type 10-like	3.4	2.4	2.87
LOC100542834	ras-like GTP-binding protein rhoA	2.5	3.2	2.87
LOC100551338	cathepsin L1-like	2.5	3.2	2.87
ADAD1	adenosine deaminase domain containing 1 (testis-specific)	3.4	2.4	2.87
LOC100544999	histone H1-like	3.4	2.4	2.87
LOC104913687	uncharacterized LOC104913687	3.4	2.4	2.87
RBP7	retinol binding protein 7, cellular	3.4	2.4	2.87
SLC22A7	solute carrier family 22 (organic anion transporter), member 7	3.4	2.4	2.87
USHBP1	Usher syndrome 1C binding protein 1	3.4	2.4	2.87
LOC104915712	uncharacterized LOC104915712	4.2	1.5	2.87
REG4	regenerating islet-derived family, member 4	4.2	1.5	2.87
ANXA13	annexin A13	2.5	3.2	2.86
LOC104915224	uncharacterized LOC104915224	2.5	3.2	2.86
LOC100540274	40S ribosomal protein S6 pseudogene	3.4	2.4	2.86
PKDREJ	polycystin (PKD) family receptor for egg jelly	3.4	2.4	2.86
LOC104913686	uncharacterized LOC104913686	2.5	3.2	2.86
C1HXorf36	chromosome 1 open reading frame, human CXorf36	3.4	2.3	2.85
LOC104913514	uncharacterized LOC104913514	3.4	2.3	2.85
CLRN3	clarin 3	3.4	2.3	2.85
LOC100540450	rho GTPase-activating protein 7-like	3.4	2.3	2.85
LOC100545105	CUB and sushi domain-containing protein 1-like	3.4	2.3	2.85
LOC104910315	chloride channel protein C-like	3.4	2.3	2.85
LOC104912774	uncharacterized LOC104912774	3.4	2.3	2.85
LOC104912847	uncharacterized LOC104912847	3.4	2.3	2.85
LOC104913731	myosin heavy chain, skeletal muscle, adult-like	3.4	2.3	2.85
LOC104917287	putative ADCY10-like protein	3.4	2.3	2.85
LOC100544848	histone H2A-IV-like	3.4	2.3	2.85
LOC104910699	uncharacterized LOC104910699	3.4	2.3	2.85
LOC104915762	uncharacterized LOC104915762	3.4	2.3	2.85
STAT4	signal transducer and activator of transcription 4	3.4	2.3	2.85
LOC104910314	uncharacterized LOC104910314	3.4	2.3	2.84
BCO2	beta-carotene oxygenase 2	2.5	3.2	2.84
C3H8orf22	chromosome 3 open reading frame, human C8orf22	2.5	3.2	2.84
HAPLN2	hyaluronan and proteoglycan link protein 2	2.5	3.2	2.84
RAB3C	RAB3C, member RAS oncogene family	2.5	3.2	2.84
LOC104909766	uncharacterized LOC104909766	3.4	2.3	2.84
LOC104910199	dynein heavy chain 5, axonemal-like	3.4	2.3	2.84
FSTL5	follistatin-like 5	1.7	4.0	2.84

LOC100550460	RNA binding protein fox-1 homolog 1-like	2.5	3.2	2.84
LOC104914497	uncharacterized LOC104914497	2.5	3.2	2.84
FGG	fibrinogen gamma chain	1.7	4.0	2.84
G6PC2	glucose-6-phosphatase, catalytic, 2	3.4	2.3	2.83
LOC104913558	uncharacterized LOC104913558	3.4	2.3	2.83
IGSF6	immunoglobulin superfamily, member 6	2.5	3.1	2.83
LOC104913433	uncharacterized LOC104913433	2.5	3.1	2.83
LOC104916506	uncharacterized LOC104916506	2.5	3.1	2.83
LOC104917507	ecto-NOX disulfide-thiol exchanger 1-like	2.5	3.1	2.83
ALDH8A1	aldehyde dehydrogenase 8 family, member A1	3.4	2.3	2.83
LOC104910394	carbonic anhydrase 2-like	3.4	2.3	2.83
LOC104910910	exocyst complex component 6B-like	3.4	2.3	2.83
LOC104911851	uncharacterized LOC104911851	3.4	2.3	2.83
LOC104912873	uncharacterized LOC104912873	3.4	2.3	2.83
LY86	lymphocyte antigen 86	3.4	2.3	2.83
PLD4	phospholipase D family, member 4	3.4	2.3	2.83
ARL13A	ADP-ribosylation factor-like 13A	2.5	3.1	2.83
LOC100542046	calmodulin, striated muscle	2.5	3.1	2.83
LOC100544544	ovalbumin-related protein Y	2.5	3.1	2.83
LOC104910206	dynein heavy chain 5, axonemal-like	2.5	3.1	2.83
LOC104910745	uncharacterized LOC104910745	2.5	3.1	2.83
LOC104913389	uncharacterized LOC104913389	2.5	3.1	2.83
MEIS2	Meis homeobox 2	2.5	3.1	2.83
LOC100545856	dmX-like protein 1	1.7	4.0	2.82
KRT20	keratin 20	2.5	3.1	2.82
LOC104913501	uncharacterized LOC104913501	2.5	3.1	2.82
LOC104917300	outer dense fiber protein 3-like	2.5	3.1	2.82
LOC100546360	calsyntenin-2	3.4	2.3	2.82
LOC104911685	uncharacterized LOC104911685	3.4	2.3	2.82
LOC104909537	uncharacterized LOC104909537	1.7	4.0	2.82
LOC104913755	ephrin type-A receptor 3	1.7	4.0	2.82
LOC104915895	uncharacterized LOC104915895	1.7	4.0	2.82
IL5RA	interleukin 5 receptor, alpha	2.5	3.1	2.82
LOC100543691	dual specificity testis-specific protein kinase 1-like	2.5	3.1	2.82
LOC104912321	uncharacterized LOC104912321	2.5	3.1	2.82
LOC104912582	uncharacterized LOC104912582	2.5	3.1	2.82
LOC104917411	uncharacterized LOC104917411	2.5	3.1	2.82
MYO3A	myosin IIIA	2.5	3.1	2.82
LOC100544043	myosin-8-like	0.8	4.8	2.82
AGT	angiotensinogen (serpin peptidase inhibitor, clade A, member 8)	2.5	3.1	2.81
ERICH3	glutamate-rich 3	2.5	3.1	2.81
LOC104916024	uncharacterized LOC104916024	2.5	3.1	2.81
C6H9orf152	chromosome 6 open reading frame, human C9orf152	2.5	3.1	2.81
LOC104911330	uncharacterized LOC104911330	2.5	3.1	2.81
LOC104913198	uncharacterized LOC104913198	2.5	3.1	2.81
LOC104913390	multidrug resistance-associated protein 1-like	2.5	3.1	2.81
LOC104915231	uncharacterized LOC104915231	2.5	3.1	2.81
TMEM178B	transmembrane protein 178B	2.5	3.1	2.81
ANKRD60	ankyrin repeat domain 60	1.7	3.9	2.80
EVC	Ellis van Creveld syndrome	1.7	3.9	2.80
CCDC3	coiled-coil domain containing 3	2.5	3.1	2.80
LOC100547425	protein FAM13C-like	2.5	3.1	2.80
LOC104910895	zinc finger matrin-type protein 1-like	2.5	3.1	2.80
LOC104915083	transient receptor potential cation channel subfamily M member 6-like	2.5	3.1	2.80
LOC104916203	mediator of RNA polymerase II transcription subunit 25-like	2.5	3.1	2.80
OTOL1	otolin 1	2.5	3.1	2.80
LOC100545120	ES1 protein homolog, mitochondrial-like	1.7	3.9	2.80

LOC104911169	uncharacterized LOC104911169	1.7	3.9	2.80
LOC104911775	glutamate decarboxylase 1-like	1.7	3.9	2.80
LOC104913864	uncharacterized LOC104913864	1.7	3.9	2.80
LOC104914233	uncharacterized LOC104914233	1.7	3.9	2.80
LOC104915878	uncharacterized LOC104915878	1.7	3.9	2.80
ZP2	zona pellucida glycoprotein 2 (sperm receptor)	2.5	3.1	2.80
AQP10	aquaporin 10	1.7	3.9	2.80
LOC104909903	tyrosine-protein kinase SYK	1.7	3.9	2.80
LOC104911818	uncharacterized LOC104911818	2.5	3.0	2.79
LOC104912242	uncharacterized LOC104912242	2.5	3.0	2.79
LOC104914142	uncharacterized LOC104914142	2.5	3.0	2.79
LOC104911162	formin-1-like	1.7	3.9	2.78
LOC104914410	uncharacterized LOC104914410	1.7	3.9	2.78
LOC100544277	follicle-stimulating hormone receptor-like	2.5	3.0	2.78
LOC104916114	interleukin-5 receptor subunit alpha-like	2.5	3.0	2.78
LOC100543342	cell adhesion molecule 3-like	1.7	3.9	2.78
LOC100546529	muellerian-inhibiting factor-like	1.7	3.9	2.78
LOC104911824	uncharacterized LOC104911824	1.7	3.9	2.78
LOC104911990	uncharacterized LOC104911990	1.7	3.9	2.78
LOC104909664	uncharacterized LOC104909664	0.8	4.7	2.78
CH25H	cholesterol 25-hydroxylase	1.7	3.9	2.78
RHOG	ras homolog family member G	1.7	3.9	2.78
GPR139	G protein-coupled receptor 139	1.7	3.8	2.76
LOC104916219	mucin-6-like	1.7	3.8	2.76
SPHKAP	SPHK1 interactor, AKAP domain containing	1.7	3.8	2.76
C5H14orf37	chromosome 5 open reading frame, human C14orf37	1.7	3.8	2.76
LOC104910643	uncharacterized LOC104910643	1.7	3.8	2.76
LOC104911462	cytoplasmic dynein 1 intermediate chain 1-like	1.7	3.8	2.76
LOC104916736	uncharacterized LOC104916736	1.7	3.8	2.76
P2RX2	purinergic receptor P2X, ligand-gated ion channel, 2	1.7	3.8	2.76
LOC104910815	uncharacterized LOC104910815	0.8	4.7	2.76
PIK3R1	phosphoinositide-3-kinase, regulatory subunit 1 (alpha)	1.7	3.8	2.76
LOC100540766	E3 ubiquitin-protein ligase TRIM39-like	0.8	4.7	2.76
LOC104909756	ensconsin-like	0.8	4.7	2.76
LOC104916650	histone H3	0.0	5.5	2.76
LOC104915647	adenylate cyclase type 10-like	1.7	3.8	2.74
CPA2	carboxypeptidase A2 (pancreatic)	0.8	4.6	2.74
LOC104913499	protein sidekick-1-like	1.7	3.8	2.74
LOC104914911	zinc finger SWIM domain-containing protein 6-like	1.7	3.8	2.74
UTS2	urotensin 2	0.8	4.6	2.74
LOC104916897	coiled-coil domain-containing protein 81-like	0.0	5.5	2.74
LOC104910119	uncharacterized LOC104910119	0.8	4.6	2.72
LOC104912418	uncharacterized LOC104912418	0.8	4.6	2.72
LOC104913953	smoothelin-like protein 2	0.8	4.6	2.72
IRF4	interferon regulatory factor 4	0.0	5.4	2.72
LOC104913282	uncharacterized LOC104913282	0.0	5.4	2.68
LOC100549312	UDP-glucuronosyltransferase 2C1-like	5.1	0.0	2.53
LOC100546187	interleukin-8-like	4.2	0.8	2.51
LOC104913317	uncharacterized LOC104913317	4.2	0.8	2.51
PKD1L2	polycystic kidney disease 1-like 2	4.2	0.8	2.51
ANKS4B	ankyrin repeat and sterile alpha motif domain containing 4B	4.2	0.8	2.51
LOC104912202	uncharacterized LOC104912202	4.2	0.8	2.51
LOC104916051	uncharacterized LOC104916051	4.2	0.8	2.51
GGT5	gamma-glutamyltransferase 5	4.2	0.8	2.49
LOC104911811	uncharacterized LOC104911811	4.2	0.8	2.49
TIE1	tyrosine kinase with immunoglobulin-like and EGF-like domains 1	4.2	0.8	2.49
TCAP	titin-cap	3.4	1.6	2.49
LOC104911435	uncharacterized LOC104911435	4.2	0.8	2.49

NKPD1	NTPase, KAP family P-loop domain containing 1	4.2	0.8	2.49
KLHDC7A	kelch domain containing 7A	3.4	1.6	2.49
LOC100546979	solute carrier family 17 member 9-like	3.4	1.6	2.49
ANGPTL7	angiopoietin-like 7	4.2	0.8	2.49
CNTN2	contactin 2 (axonal)	4.2	0.8	2.49
GPER1	G protein-coupled estrogen receptor 1	4.2	0.8	2.49
KCNB2	potassium voltage-gated channel, Shab-related subfamily, member 2	4.2	0.8	2.49
LOC104913424	uncharacterized LOC104913424	4.2	0.8	2.49
LOC104915239	solute carrier family 12 member 2-like	4.2	0.8	2.49
LOC104916293	adenylate cyclase type 10-like	4.2	0.8	2.49
COPRS	coordinator of PRMT5, differentiation stimulator	3.4	1.6	2.49
FABP2	fatty acid binding protein 2, intestinal	3.4	1.6	2.49
LOC104910114	uncharacterized LOC104910114	3.4	1.6	2.49
LOC104916164	uncharacterized LOC104916164	3.4	1.6	2.49
IQCD	IQ motif containing D	4.2	0.8	2.49
LOC104913165	uncharacterized LOC104913165	4.2	0.8	2.49
LOC104913662	uncharacterized LOC104913662	4.2	0.8	2.48
LOC104911945	uncharacterized LOC104911945	3.4	1.6	2.48
LCK	LCK proto-oncogene, Src family tyrosine kinase	3.4	1.6	2.47
LOC104910877	keratin, type II cytoskeletal 3-like	3.4	1.6	2.47
RGS19	regulator of G-protein signaling 19	3.4	1.6	2.47
ASB10	ankyrin repeat and SOCS box containing 10	3.4	1.6	2.47
CLDN2	claudin 2	3.4	1.6	2.47
LOC100547627	sulfotransferase family cytosolic 1B member 1	3.4	1.6	2.47
LOC104910760	cationic amino acid transporter 2-like	3.4	1.6	2.47
LOC104910930	uncharacterized LOC104910930	3.4	1.6	2.47
LOC104911061	uncharacterized LOC104911061	3.4	1.6	2.47
LOC104914881	uncharacterized LOC104914881	3.4	1.6	2.47
LOC104915240	solute carrier family 12 member 2-like	3.4	1.6	2.47
PCDHAC1	protocadherin alpha subfamily C, 1	3.4	1.6	2.47
SLC6A5	solute carrier family 6 (neurotransmitter transporter), member 5	3.4	1.6	2.47
AQP1	aquaporin 1 (Colton blood group)	2.5	2.4	2.47
LOC104909488	uncharacterized LOC104909488	2.5	2.4	2.47
GLDN	gliomedin	3.4	1.6	2.47
LOC100544298	claudin-19-like	3.4	1.6	2.47
LOC104914883	granzyme K-like	3.4	1.6	2.47
LOC100538614	liprin-alpha-2-like	2.5	2.4	2.46
LOC100546859	extracellular tyrosine-protein kinase PKDCC-like	2.5	2.4	2.46
LOC104915833	GTPase IMAP family member 1-like	2.5	2.4	2.46
CCDC183	coiled-coil domain containing 183	3.4	1.5	2.45
LOC104910423	syntabulin-like	3.4	1.5	2.45
LOC100544929	extracellular fatty acid-binding protein	3.4	1.5	2.45
LOC104913321	uncharacterized LOC104913321	3.4	1.5	2.45
SELPLG	selectin P ligand	3.4	1.5	2.45
ASB11	ankyrin repeat and SOCS box containing 11, E3 ubiquitin protein ligase	2.5	2.4	2.45
LOC104913793	glutamine-rich protein 2-like	2.5	2.4	2.45
PLVAP	plasmalemma vesicle associated protein	2.5	2.4	2.45
ELAVL4	ELAV like neuron-specific RNA binding protein 4	3.4	1.5	2.45
LOC100548260	disintegrin and metalloproteinase domain-containing protein 18-like	3.4	1.5	2.45
LOC104911103	uncharacterized LOC104911103	3.4	1.5	2.45
LOC104911182	uncharacterized LOC104911182	3.4	1.5	2.45
LOC104914012	receptor-type tyrosine-protein phosphatase T-like	3.4	1.5	2.45
TRIM29	tripartite motif containing 29	3.4	1.5	2.45
DHH	desert hedgehog	2.5	2.4	2.45
IFITM5	interferon induced transmembrane protein 5	2.5	2.4	2.45

LOC100542763	chymotrypsin-like elastase family member 2A	2.5	2.4	2.45
LOC104910724	uncharacterized LOC104910724	2.5	2.4	2.45
LOC104910794	uncharacterized LOC104910794	2.5	2.4	2.45
LOC104910885	uncharacterized LOC104910885	2.5	2.4	2.45
LOC104911848	uncharacterized LOC104911848	2.5	2.4	2.45
LOC104914077	uncharacterized LOC104914077	2.5	2.4	2.45
MORN3	MORN repeat containing 3	2.5	2.4	2.45
TMEM71	transmembrane protein 71	2.5	2.4	2.45
FES	FES proto-oncogene, tyrosine kinase	3.4	1.5	2.45
LOC104913301	uncharacterized LOC104913301	3.4	1.5	2.45
CACNA1G	calcium channel, voltage-dependent, T type, alpha 1G subunit	1.7	3.2	2.44
LOC100538764	ras-related protein Rab-11A-like	2.5	2.4	2.44
LOC104910403	uncharacterized LOC104910403	2.5	2.4	2.44
LOC104911933	uncharacterized LOC104911933	2.5	2.4	2.44
LOC104912030	shootin-1-like	2.5	2.4	2.44
LOC104917297	uncharacterized LOC104917297	2.5	2.4	2.44
ZP1	zona pellucida glycoprotein 1 (sperm receptor)	2.5	2.4	2.44
LOC104916496	zinc finger protein 665-like	3.4	1.5	2.44
LOC104910156	cadherin-12-like	1.7	3.2	2.44
LOC104912110	probable G-protein coupled receptor 34	1.7	3.2	2.44
LOC104917590	uncharacterized LOC104917590	1.7	3.2	2.44
LOC104912920	uncharacterized LOC104912920	2.5	2.4	2.44
BTBD17	BTB (POZ) domain containing 17	2.5	2.3	2.43
SCRT2	scratch family zinc finger 2	2.5	2.3	2.43
ACSL6	acyl-CoA synthetase long-chain family member 6	2.5	2.3	2.43
EPS8L3	EPS8-like 3	2.5	2.3	2.43
KDF1	keratinocyte differentiation factor 1	2.5	2.3	2.43
LOC104910219	secretagogin	2.5	2.3	2.43
LOC104912508	dual specificity testis-specific protein kinase 2-like	2.5	2.3	2.43
LOC104915727	adenylyl cyclase-associated protein 1-like	2.5	2.3	2.43
RSAD2	radical S-adenosyl methionine domain containing 2	2.5	2.3	2.43
ZC3H12D	zinc finger CCCH-type containing 12D	2.5	2.3	2.43
LOC100541187	neurobeachin-like	1.7	3.2	2.42
LOC100544235	exocyst complex component 6B-like	1.7	3.2	2.42
LOC100550375	E3 SUMO-protein ligase RanBP2-like	1.7	3.2	2.42
LOC104910407	uncharacterized LOC104910407	1.7	3.2	2.42
LOC104911015	receptor-type tyrosine-protein phosphatase eta-like	1.7	3.2	2.42
LOC104911629	uncharacterized LOC104911629	1.7	3.2	2.42
MARCH11	membrane-associated ring finger (C3HC4) 11	1.7	3.2	2.42
ARMC2	armadillo repeat containing 2	2.5	2.3	2.42
C1H11orf97	chromosome 1 open reading frame, human C11orf97	2.5	2.3	2.42
EXOC3L4	exocyst complex component 3-like 4	2.5	2.3	2.42
LOC100539826	SH3-containing GRB2-like protein 3-interacting protein 1	2.5	2.3	2.42
LOC100542130	cytochrome P450 26C1	2.5	2.3	2.42
LOC100543887	leukotriene C4 synthase-like	2.5	2.3	2.42
LOC104909852	forkhead box protein O3-like	2.5	2.3	2.42
LOC104913091	uncharacterized LOC104913091	2.5	2.3	2.42
NGF	nerve growth factor (beta polypeptide)	2.5	2.3	2.42
LOC100547592	ectonucleoside triphosphate diphosphohydrolase 8-like	1.7	3.2	2.42
LOC104911711	sodium channel protein type 2 subunit alpha-like	1.7	3.2	2.42
LOC104912752	mucin-5B-like	1.7	3.2	2.42
LOC100546239	UPF0762 protein C6orf58 homolog	2.5	2.3	2.42
LOC100547089	C-type lectin domain family 18 member A-like	2.5	2.3	2.42
LOC104910040	24-hydroxycholesterol 7-alpha-hydroxylase-like	0.8	4.0	2.42
LOC104910160	uncharacterized LOC104910160	0.8	4.0	2.42
LOC104911716	uncharacterized LOC104911716	1.7	3.2	2.42
MGAT4D	MGAT4 family, member D	1.7	3.2	2.42
LOC104911912	uncharacterized LOC104911912	0.8	4.0	2.42

LOC104909836	uncharacterized LOC104909836	2.5	2.3	2.41
LOC104915485	collagen alpha-5(IV) chain-like	2.5	2.3	2.41
LOC104915813	histone-lysine N-methyltransferase 2D-like	2.5	2.3	2.41
RTN4RL1	reticulon 4 receptor-like 1	2.5	2.3	2.41
LOC104911006	dual specificity protein phosphatase 8-like	2.5	2.3	2.41
LOC104913021	epithelial splicing regulatory protein 2-like	2.5	2.3	2.41
LOC104913885	scavenger receptor cysteine-rich domain-containing group B protein-like	2.5	2.3	2.41
LOC104916795	maestro heat-like repeat-containing protein family member 2B	2.5	2.3	2.41
TDRD1	tudor domain containing 1	2.5	2.3	2.41
CLVS1	clavesin 1	1.7	3.1	2.40
ZNF536	zinc finger protein 536	1.7	3.1	2.40
BANK1	B-cell scaffold protein with ankyrin repeats 1	2.5	2.3	2.40
C8H10orf90	chromosome 8 open reading frame, human C10orf90	2.5	2.3	2.40
LOC100540180	thiamine transporter 2-like	2.5	2.3	2.40
LOC104916171	E3 ubiquitin-protein ligase TRIM39-like	2.5	2.3	2.40
LOC104917335	ras-related protein Rap-2a-like	2.5	2.3	2.40
C28H1orf116	chromosome 28 open reading frame, human C1orf116	1.7	3.1	2.40
DPF3	D4, zinc and double PHD fingers, family 3	1.7	3.1	2.40
FBXO43	F-box protein 43	1.7	3.1	2.40
LOC100540289	interleukin-12 receptor subunit beta-2-like	1.7	3.1	2.40
LOC100541078	lipid phosphate phosphatase-related protein type 3	1.7	3.1	2.40
LOC100547473	transmembrane emp24 domain-containing protein 11-like	1.7	3.1	2.40
LOC104912833	protein FAM227B-like	1.7	3.1	2.40
NT5E	5'-nucleotidase, ecto (CD73)	1.7	3.1	2.40
POU2AF1	POU class 2 associating factor 1	1.7	3.1	2.40
LOC104909910	uncharacterized LOC104909910	0.8	4.0	2.40
LOC104917465	uncharacterized LOC104917465	0.8	4.0	2.40
SAMD3	sterile alpha motif domain containing 3	0.8	4.0	2.40
WDR78	WD repeat domain 78	0.8	4.0	2.40
LOC100545877	ectonucleotide pyrophosphatase/phosphodiesterase family member 6-like	1.7	3.1	2.40
LOC104910444	uncharacterized LOC104910444	1.7	3.1	2.40
LOC104913134	uncharacterized LOC104913134	1.7	3.1	2.40
LOC104909763	uncharacterized LOC104909763	0.0	4.8	2.40
ADD2	adducin 2 (beta)	1.7	3.1	2.38
CD274	CD274 molecule	1.7	3.1	2.38
EIF4E1B	eukaryotic translation initiation factor 4E family member 1B	1.7	3.1	2.38
GADL1	glutamate decarboxylase-like 1	1.7	3.1	2.38
LOC100540410	uncharacterized LOC100540410	1.7	3.1	2.38
LOC104911506	uncharacterized LOC104911506	1.7	3.1	2.38
SPAG8	sperm associated antigen 8	1.7	3.1	2.38
SSPO	SCO-spondin	1.7	3.1	2.38
ATP13A5	ATPase type 13A5	1.7	3.1	2.38
JAK3	Janus kinase 3	1.7	3.1	2.38
LOC104915616	zinc finger CCCH domain-containing protein 11A-like	1.7	3.1	2.38
TNFSF13B	tumor necrosis factor (ligand) superfamily, member 13b	1.7	3.1	2.38
CFH	complement factor H	0.8	3.9	2.38
LOC100544920	leukotriene B4 receptor 1-like	0.8	3.9	2.38
LOC104917455	uncharacterized LOC104917455	0.8	3.9	2.38
TMEM200C	transmembrane protein 200C	0.8	3.9	2.38
SRGN	serglycin	1.7	3.1	2.38
LOC100542678	b(0,+)-type amino acid transporter 1-like	0.8	3.9	2.38
LOC100548534	uncharacterized LOC100548534	0.8	3.9	2.38
LOC104914796	killer cell lectin-like receptor subfamily F member 1	0.8	3.9	2.38
SERPIND1	serpin peptidase inhibitor, clade D (heparin cofactor), member 1	0.8	3.9	2.38
LOC104913109	dedicator of cytokinesis protein 3-like	0.0	4.8	2.38
LOC100538582	homeobox protein DLX-3-like	1.7	3.0	2.36

LOC104913184	PHD finger protein 7-like	1.7	3.0	2.36
LOC104917475	uncharacterized LOC104917475	1.7	3.0	2.36
KCNS2	potassium voltage-gated channel, delayed-rectifier, subfamily S, member 2	0.8	3.9	2.36
MPP7	membrane protein, palmitoylated 7 (MAGUK p55 subfamily member 7)	0.8	3.9	2.36
LOC104913544	uncharacterized LOC104913544	0.8	3.9	2.36
LOC104914285	CUB and sushi domain-containing protein 2-like	0.8	3.9	2.36
RNF152	ring finger protein 152	0.8	3.9	2.36
BCL11A	B-cell CLL/lymphoma 11A (zinc finger protein)	0.8	3.8	2.34
KCNA4	potassium voltage-gated channel, shaker-related subfamily, member 4	0.8	3.8	2.34
KCNJ4	potassium inwardly-rectifying channel, subfamily J, member 4	0.8	3.8	2.34
KPNA7	karyopherin alpha 7 (importin alpha 8)	0.8	3.8	2.34
LOC104910728	uncharacterized LOC104910728	0.8	3.8	2.34
TRPM1	transient receptor potential cation channel, subfamily M, member 1	0.8	3.8	2.34
LOC104909774	receptor-type tyrosine-protein phosphatase kappa-like	0.8	3.8	2.34
LOC104910834	cytokine-dependent hematopoietic cell linker-like	0.8	3.8	2.34
TEX12	testis expressed 12	0.8	3.8	2.34
LOC104911803	kalirin-like	0.0	4.7	2.34
LOC104914214	uncharacterized LOC104914214	0.8	3.8	2.32
LOC104913394	uncharacterized LOC104913394	0.0	4.6	2.32
CYP3A80	cytochrome P450 3A80	0.0	4.6	2.30
LOC104912816	thrombospondin type-1 domain-containing protein 4-like	0.0	4.6	2.28
LOC104912319	uncharacterized LOC104912319	4.2	0.0	2.12
LOC100548679	uncharacterized protein KIAA0825-like	4.2	0.0	2.11
LOC100549208	limbin-like	4.2	0.0	2.11
LOC104912524	uncharacterized LOC104912524	4.2	0.0	2.11
LOC104915657	maestro heat-like repeat-containing protein family member 1	4.2	0.0	2.11
ZP4	zona pellucida glycoprotein 4	4.2	0.0	2.11
LOC100549193	pseudouridine-metabolizing bifunctional protein C1861.05-like	4.2	0.0	2.11
LOC104910197	uncharacterized LOC104910197	4.2	0.0	2.11
LOC104911476	uncharacterized LOC104911476	4.2	0.0	2.11
LOC100543549	coiled-coil domain-containing protein 146	4.2	0.0	2.11
LOC100546753	transcription factor SOX-6	4.2	0.0	2.11
LOC104911052	uncharacterized LOC104911052	4.2	0.0	2.11
OLIG3	oligodendrocyte transcription factor 3	4.2	0.0	2.11
SGCZ	sarcoglycan, zeta	4.2	0.0	2.11
CACNB2	calcium channel, voltage-dependent, beta 2 subunit	3.4	0.8	2.09
LOC104911854	nuclear receptor subfamily 4 group A member 2-like	3.4	0.8	2.09
LOC104915614	interferon-like	3.4	0.8	2.09
LOC100546113	T-box-containing protein TBX6L	3.4	0.8	2.09
LOC104914397	synaptic vesicle glycoprotein 2A-like	3.4	0.8	2.09
LOC104916827	cadherin EGF LAG seven-pass G-type receptor 2-like	3.4	0.8	2.09
VILL	villin-like	3.4	0.8	2.09
DMRTB1	DMRT-like family B with proline-rich C-terminal, 1	3.4	0.8	2.09
LOC104910793	uncharacterized LOC104910793	3.4	0.8	2.09
LOC104917530	uncharacterized LOC104917530	3.4	0.8	2.09
LOC100544046	scavenger receptor cysteine-rich domain-containing group B protein-like	3.4	0.8	2.08
ICOS	inducible T-cell co-stimulator	3.4	0.8	2.07
LOC104910989	uncharacterized LOC104910989	3.4	0.8	2.07
LOC104914395	synaptic vesicle glycoprotein 2A-like	3.4	0.8	2.07
RANBP1	RAN binding protein 1	3.4	0.8	2.07
C10H1orf146	chromosome 10 open reading frame, human C1orf146	3.4	0.8	2.07
GPR133	G protein-coupled receptor 133	3.4	0.8	2.07
LOC104909430	NAD(P)(+)--arginine ADP-ribosyltransferase 2-like	3.4	0.8	2.07

SI	sucrase-isomaltase (alpha-glucosidase)	3.4	0.8	2.07
LOC104910658	uncharacterized LOC104910658	2.5	1.6	2.07
LOC104913430	uncharacterized LOC104913430	2.5	1.6	2.07
LOC104917549	uncharacterized LOC104917549	2.5	1.6	2.07
SLC24A2	solute carrier family 24 (sodium/potassium/calcium exchanger), member 2	2.5	1.6	2.07
CCDC85C	coiled-coil domain containing 85C	3.4	0.8	2.07
LOC100541314	serine protease inhibitor Kazal-type 5-like	3.4	0.8	2.07
LOC104909555	tau-tubulin kinase 1-like	3.4	0.8	2.07
LOC104910564	uncharacterized LOC104910564	3.4	0.8	2.07
LOC104911956	uncharacterized LOC104911956	3.4	0.8	2.07
LOC104912154	uncharacterized LOC104912154	3.4	0.8	2.07
LOC104913737	uncharacterized LOC104913737	3.4	0.8	2.07
LOC104915697	uncharacterized LOC104915697	3.4	0.8	2.07
LOC104916629	protocadherin alpha-5-like	3.4	0.8	2.07
LOC100540528	histamine H3 receptor-like	2.5	1.6	2.07
RGS6	regulator of G-protein signaling 6	2.5	1.6	2.07
EFCAB2	EF-hand calcium binding domain 2	3.4	0.8	2.07
LOC104914581	uncharacterized LOC104914581	3.4	0.8	2.07
LOC104915034	centlein-like	3.4	0.8	2.07
LOC100547689	zonadhesin-like	2.5	1.6	2.06
LOC104910104	synaptonemal complex protein 2-like	2.5	1.6	2.06
LOC104912290	uncharacterized LOC104912290	2.5	1.6	2.06
LOC104913473	glucagon receptor-like	2.5	1.6	2.06
LRRN2	leucine rich repeat neuronal 2	3.4	0.8	2.06
LOC100541658	F-box only protein 15-like	2.5	1.6	2.06
GPR119	G protein-coupled receptor 119	2.5	1.6	2.05
LOC100550363	alpha-1,3-mannosyl-glycoprotein 4-beta-N-acetylglucosaminyltransferase C	2.5	1.6	2.05
LOC104914618	uncharacterized LOC104914618	2.5	1.6	2.05
LOC104915314	E3 ubiquitin-protein ligase Topors-like	2.5	1.6	2.05
UNC13D	unc-13 homolog D (C. elegans)	2.5	1.6	2.05
ACOT12	acyl-CoA thioesterase 12	2.5	1.6	2.05
AMH	anti-Mullerian hormone	2.5	1.6	2.05
CLDN1	claudin 1	2.5	1.6	2.05
LOC100539361	guanine nucleotide exchange factor VAV3	2.5	1.6	2.05
LOC100550576	zinc finger protein Rlf-like	2.5	1.6	2.05
LOC104910619	uncharacterized LOC104910619	2.5	1.6	2.05
LOC104911254	uncharacterized LOC104911254	2.5	1.6	2.05
LOC104911652	uncharacterized LOC104911652	2.5	1.6	2.05
LOC104911999	diacylglycerol kinase iota-like	2.5	1.6	2.05
LOC104912106	voltage-dependent calcium channel subunit alpha-2/delta-4-like	2.5	1.6	2.05
LOC104912302	uncharacterized LOC104912302	2.5	1.6	2.05
LOC104912545	serine/threonine-protein kinase ATR-like	2.5	1.6	2.05
LOC104913517	integrin alpha-D-like	2.5	1.6	2.05
LOC104913918	uncharacterized LOC104913918	2.5	1.6	2.05
LOC104914731	phospholipid-transporting ATPase IC-like	2.5	1.6	2.05
LOC104912688	uncharacterized LOC104912688	1.7	2.4	2.04
LOC104909857	uncharacterized LOC104909857	1.7	2.4	2.04
MYO1G	myosin IG	1.7	2.4	2.04
PI15	peptidase inhibitor 15	1.7	2.4	2.04
DLEC1	deleted in lung and esophageal cancer 1	2.5	1.6	2.04
LOC104912198	uncharacterized LOC104912198	2.5	1.6	2.04
LOC104913073	ovostatin-like	2.5	1.6	2.04
LOC104915022	uncharacterized LOC104915022	2.5	1.6	2.04
PTPRN2	protein tyrosine phosphatase, receptor type, N polypeptide 2	2.5	1.6	2.04
TSPAN19	tetraspanin 19	2.5	1.6	2.04
LOC104909588	uncharacterized LOC104909588	1.7	2.4	2.04

NIPAL2	NIPA-like domain containing 2	1.7	2.4	2.04
CASR	calcium-sensing receptor	2.5	1.5	2.03
LOC100543615	keratin, type II cytoskeletal 5-like	2.5	1.5	2.03
LOC100548078	uncharacterized LOC100548078	2.5	1.5	2.03
LOC104912002	uncharacterized LOC104912002	2.5	1.5	2.03
LOC104914038	uncharacterized LOC104914038	2.5	1.5	2.03
LOC104915051	focadhesin-like	2.5	1.5	2.03
ATRNL1	attractin-like 1	2.5	1.5	2.03
LOC100546506	sodium channel protein type 2 subunit alpha	2.5	1.5	2.03
LOC104912354	uncharacterized LOC104912354	2.5	1.5	2.03
RP1L1	retinitis pigmentosa 1-like 1	2.5	1.5	2.03
FAM81A	family with sequence similarity 81, member A	1.7	2.4	2.02
GRIN2B	glutamate receptor, ionotropic, N-methyl D-aspartate 2B	1.7	2.4	2.02
LOC100550511	alpha-N-acetylgalactosaminide alpha-2,6-sialyltransferase 2-like	1.7	2.4	2.02
LOC104911248	exocyst complex component 3-like protein	1.7	2.4	2.02
LOC104912989	uncharacterized LOC104912989	1.7	2.4	2.02
LOC104913435	uncharacterized LOC104913435	1.7	2.4	2.02
LOC104914335	alpha-actinin-2-like	1.7	2.4	2.02
PDZRN4	PDZ domain containing ring finger 4	1.7	2.4	2.02
POMC	proopiomelanocortin	1.7	2.4	2.02
RAPGEF3	Rap guanine nucleotide exchange factor (GEF) 3	1.7	2.4	2.02
SMIM24	small integral membrane protein 24	1.7	2.4	2.02
LOC104917526	membrane-associated guanylate kinase, WW and PDZ domain-containing protein 2-like	2.5	1.5	2.02
PRKAR2B	protein kinase, cAMP-dependent, regulatory, type II, beta	2.5	1.5	2.02
FGFBP2	fibroblast growth factor binding protein 2	1.7	2.4	2.02
FOXI1	forkhead box I1	1.7	2.4	2.02
GPR115	G protein-coupled receptor 115	1.7	2.4	2.02
GPR85	G protein-coupled receptor 85	1.7	2.4	2.02
LAPTM5	lysosomal protein transmembrane 5	1.7	2.4	2.02
LOC100550869	putative polypeptide N-acetylgalactosaminyltransferase-like protein 3	1.7	2.4	2.02
LOC104910596	uncharacterized LOC104910596	1.7	2.4	2.02
LOC104912200	heat shock transcription factor, X-linked-like	1.7	2.4	2.02
LOC104915073	uncharacterized LOC104915073	1.7	2.4	2.02
LOC104917037	uncharacterized LOC104917037	1.7	2.4	2.02
NFKBID	nuclear factor of kappa light polypeptide gene enhancer in B-cells inhibitor, delta	1.7	2.4	2.02
ZMAT4	zinc finger, matrin-type 4	1.7	2.4	2.02
CDH11	cadherin 11, type 2, OB-cadherin (osteoblast)	2.5	1.5	2.02
CDHR3	cadherin-related family member 3	2.5	1.5	2.02
LOC104911432	uncharacterized LOC104911432	2.5	1.5	2.02
LOC104914876	integrin alpha-1-like	2.5	1.5	2.02
LOC100543865	alkaline ceramidase 3-like	0.8	3.2	2.02
LOC104914110	uncharacterized LOC104914110	0.8	3.2	2.02
AP1S3	adaptor-related protein complex 1, sigma 3 subunit	1.7	2.4	2.02
ARID3C	AT rich interactive domain 3C (BRIGHT-like)	1.7	2.4	2.02
C25H1orf216	chromosome 25 open reading frame, human C1orf216	1.7	2.4	2.02
CCDC78	coiled-coil domain containing 78	1.7	2.4	2.02
LOC104910526	uncharacterized LOC104910526	1.7	2.4	2.02
RANBP3L	RAN binding protein 3-like	1.7	2.4	2.02
SLC16A8	solute carrier family 16 (monocarboxylate transporter), member 8	1.7	2.4	2.02
VSX1	visual system homeobox 1	1.7	2.4	2.02
CUZD1	CUB and zona pellucida-like domains 1	1.7	2.3	2.01
LOC100543562	uncharacterized LOC100543562	1.7	2.3	2.01
LOC104909822	protein FAM184A-like	1.7	2.3	2.01
LOC104910022	fibrinogen-like protein 1	1.7	2.3	2.01
LOC104911175	uncharacterized LOC104911175	1.7	2.3	2.01

LOC104915023	nuclear factor 1 B-type	1.7	2.3	2.01
LOC104915650	signal-regulatory protein beta-1-like	1.7	2.3	2.01
LOC104917319	forkhead box protein D3-like	1.7	2.3	2.01
PTAFR	platelet-activating factor receptor	1.7	2.3	2.01
SOX10	SRY (sex determining region Y)-box 10	1.7	2.3	2.01
CFAP61	cilia and flagella associated protein 61	1.7	2.3	2.00
CFAP69	cilia and flagella associated protein 69	1.7	2.3	2.00
COCH	cochlin	1.7	2.3	2.00
GRIK1	glutamate receptor, ionotropic, kainate 1	1.7	2.3	2.00
LOC100544286	solute carrier family 2, facilitated glucose transporter member 9-like	1.7	2.3	2.00
LOC104909527	uncharacterized LOC104909527	1.7	2.3	2.00
LOC104911123	MAX gene-associated protein-like	1.7	2.3	2.00
LOC104913252	uncharacterized LOC104913252	1.7	2.3	2.00
LOC104913589	zona pellucida sperm-binding protein 3-like	1.7	2.3	2.00
TMPRSS9	transmembrane protease, serine 9	1.7	2.3	2.00
LOC100538868	hematopoietic lineage cell-specific protein-like	0.8	3.2	2.00
LOC104910990	uncharacterized LOC104910990	0.8	3.2	2.00
LOC104911223	uncharacterized LOC104911223	0.8	3.2	2.00
LOC104916816	outer dense fiber protein 3-like	0.8	3.2	2.00
RNF17	ring finger protein 17	0.8	3.2	2.00
LOC100303701	uncharacterized LOC100303701	1.7	2.3	2.00
LOC100548146	vitellogenin-2	1.7	2.3	2.00
LOC104909647	uncharacterized LOC104909647	1.7	2.3	2.00
LOC104911048	uncharacterized LOC104911048	1.7	2.3	2.00
LOC104912423	inaD-like protein	1.7	2.3	2.00
LOC104914237	uncharacterized LOC104914237	1.7	2.3	2.00
LOC104914507	apoptosis facilitator Bcl-2-like protein 14	1.7	2.3	2.00
NEFH	neurofilament, heavy polypeptide	1.7	2.3	2.00
RHOV	ras homolog family member V	1.7	2.3	2.00
LOC104909824	amine sulfotransferase-like	0.8	3.2	2.00
LOC104916780	leucine-rich repeat-containing G-protein coupled receptor 4 pseudogene	0.8	3.2	2.00
PITX1	paired-like homeodomain 1	0.8	3.2	2.00
LOC100543069	atrial natriuretic peptide receptor 1-like	1.7	2.3	1.99
LOC104909654	uncharacterized LOC104909654	1.7	2.3	1.99
LOC104911641	dynein heavy chain 7, axonemal-like	1.7	2.3	1.99
LOC104915367	adenylate cyclase type 10-like	1.7	2.3	1.99
SV2C	synaptic vesicle glycoprotein 2C	1.7	2.3	1.99
WISP1	WNT1 inducible signaling pathway protein 1	1.7	2.3	1.99
LOC104912801	uncharacterized LOC104912801	1.7	2.3	1.98
ABCG1	ATP-binding cassette, sub-family G (WHITE), member 1	0.8	3.1	1.98
CPLX2	complexin 2	0.8	3.1	1.98
FGFR3	fibroblast growth factor receptor 3	0.8	3.1	1.98
LOC100550885	ephrin type-A receptor 3-like	0.8	3.1	1.98
LOC104910216	uncharacterized LOC104910216	0.8	3.1	1.98
LOC104910942	membrane-spanning 4-domains subfamily A member 12-like	0.8	3.1	1.98
LOC104912662	uncharacterized LOC104912662	0.8	3.1	1.98
LOC104914948	microtubule-associated serine/threonine-protein kinase 4-like	0.8	3.1	1.98
LOC104915500	protocadherin beta-15-like	0.8	3.1	1.98
LOC104915991	uncharacterized LOC104915991	0.8	3.1	1.98
PRPH2	peripherin 2 (retinal degeneration, slow)	0.8	3.1	1.98
CXADR	coxsackie virus and adenovirus receptor	1.7	2.3	1.98
KIF12	kinesin family member 12	1.7	2.3	1.98
LOC100541644	erythroblast NAD(P)(+)--arginine ADP-ribosyltransferase-like	1.7	2.3	1.98
LOC104916983	uncharacterized LOC104916983	1.7	2.3	1.98
LOC104917446	uncharacterized LOC104917446	1.7	2.3	1.98
GPC5	glypican 5	0.8	3.1	1.98

LOC104910874	actin-binding LIM protein 2-like	0.8	3.1	1.98
LOC104912092	uncharacterized LOC104912092	0.8	3.1	1.98
LOC104912211	testis-specific serine/threonine-protein kinase 6-like	0.8	3.1	1.98
LOC104912434	uncharacterized LOC104912434	0.8	3.1	1.98
NEK10	NIMA-related kinase 10	0.8	3.1	1.98
AGMO	alkylglycerol monooxygenase	0.0	4.0	1.98
LOC104909754	uncharacterized LOC104909754	0.0	4.0	1.98
LOC100540973	guanine nucleotide-binding protein G(t) subunit alpha-2	0.8	3.1	1.96
LOC104909832	adenylate kinase 9-like	0.8	3.1	1.96
LOC104910902	uncharacterized LOC104910902	0.8	3.1	1.96
LOC104911087	spectrin beta chain, non-erythrocytic 4-like	0.8	3.1	1.96
LOC104913265	uncharacterized LOC104913265	0.8	3.1	1.96
LOC104913492	voltage-dependent T-type calcium channel subunit alpha-1H-like	0.8	3.1	1.96
SLC6A4	solute carrier family 6 (neurotransmitter transporter), member 4	0.8	3.1	1.96
IL21R	interleukin 21 receptor	0.8	3.1	1.96
LOC104911627	uncharacterized LOC104911627	0.8	3.1	1.96
LOC104911963	FYVE, RhoGEF and PH domain-containing protein 4-like	0.8	3.1	1.96
GDF9	growth differentiation factor 9	0.0	3.9	1.96
LOC104909893	uncharacterized LOC104909893	0.0	3.9	1.96
LOC104911817	uncharacterized LOC104911817	0.0	3.9	1.96
LOC104914732	uncharacterized LOC104914732	0.8	3.0	1.94
LOC104914791	protein unc-13 homolog B-like	0.8	3.0	1.94
LOC104912004	uncharacterized LOC104912004	0.8	3.0	1.94
LOC104917454	uncharacterized LOC104917454	0.8	3.0	1.94
LOC100542931	alpha-2-macroglobulin-like protein 1	0.0	3.9	1.94
LOC100546662	vitamin K-dependent protein C-like	0.0	3.9	1.94
LOC104910535	uncharacterized LOC104910535	0.0	3.9	1.94
LOC104916030	uncharacterized LOC104916030	0.0	3.9	1.94
LOC100541159	neural-cadherin-like	0.0	3.8	1.90
LOC100547665	phospholipid-transporting ATPase IB-like	0.0	3.8	1.90
LOC104916981	all-trans-retinol 13,14-reductase-like	3.4	0.0	1.69
LOC100550586	tyrosine-protein kinase SgK223-like	3.4	0.0	1.69
LOC104910205	dynein heavy chain 5, axonemal-like	3.4	0.0	1.69
LOC104915668	uncharacterized LOC104915668	3.4	0.0	1.69
LOC104917187	uncharacterized LOC104917187	3.4	0.0	1.69
YIPF7	Yip1 domain family, member 7	3.4	0.0	1.69
IRX4	iroquois homeobox 4	3.4	0.0	1.69
LOC100539603	neurophysin 1-like	3.4	0.0	1.69
LOC100540188	acyl-coenzyme A synthetase ACSM3, mitochondrial-like	3.4	0.0	1.69
LOC100542279	thrombospondin type-1 domain-containing protein 7B-like	3.4	0.0	1.69
LOC104916232	tripartite motif-containing protein 7-like	3.4	0.0	1.69
ST6GALNAC2	ST6 (alpha-N-acetyl-neuraminyl-2,3-beta-galactosyl-1,3)-N-acetylgalactosaminide alpha-2,6-sialyltransferase 2	3.4	0.0	1.69
FABP6	fatty acid binding protein 6, ileal	3.4	0.0	1.69
LOC104912574	uncharacterized LOC104912574	3.4	0.0	1.69
LOC100538449	myosin light chain kinase, smooth muscle-like	3.4	0.0	1.68
TBX18	T-box 18	3.4	0.0	1.68
FOXN1	forkhead box N1	2.5	0.8	1.67
LOC100546179	olfactory receptor 51G2-like	2.5	0.8	1.67
LOC104912635	ephrin type-A receptor 4-like	2.5	0.8	1.67
LOC104915553	collagen alpha-1(XVI) chain-like	2.5	0.8	1.67
LOC104915958	uncharacterized LOC104915958	2.5	0.8	1.67
AK5	adenylate kinase 5	2.5	0.8	1.67
LOC100545013	keratin, type II cytoskeletal 75-like	2.5	0.8	1.67
LOC104909830	protein FAM184A-like	2.5	0.8	1.67
LOC104912681	transmembrane and TPR repeat-containing protein 2 pseudogene	2.5	0.8	1.67
LOC104912948	uncharacterized LOC104912948	2.5	0.8	1.67
LOC104913604	uncharacterized LOC104913604	2.5	0.8	1.67

LOC104914628	uncharacterized LOC104914628	2.5	0.8	1.67
LOC104914981	rho guanine nucleotide exchange factor 28-like	2.5	0.8	1.67
LOC104915660	G-protein coupled receptor 98-like	2.5	0.8	1.67
TMEM215	transmembrane protein 215	2.5	0.8	1.67
DNASE1L3	deoxyribonuclease I-like 3	2.5	0.8	1.66
GDF5	growth differentiation factor 5	2.5	0.8	1.66
KCNJ16	potassium inwardly-rectifying channel, subfamily J, member 16	2.5	0.8	1.66
LOC100549440	neuritin-like	2.5	0.8	1.66
LOC104910243	rotatin-like	2.5	0.8	1.66
LOC104911676	uncharacterized LOC104911676	2.5	0.8	1.66
LOC104912008	transcription factor SOX-5-like	2.5	0.8	1.66
LOC104912811	protein unc-13 homolog C-like	2.5	0.8	1.66
SPTBN5	spectrin, beta, non-erythrocytic 5	2.5	0.8	1.66
LOC104910741	uncharacterized LOC104910741	2.5	0.8	1.66
LOC104911984	uncharacterized LOC104911984	2.5	0.8	1.66
LOC104912799	nuclear receptor-interacting protein 2-like	2.5	0.8	1.66
LOC104915689	protocadherin alpha-13-like	2.5	0.8	1.66
TMSB4X	thymosin beta 4, X-linked	2.5	0.8	1.66
CYTIP	cytohesin 1 interacting protein	2.5	0.8	1.65
LOC104911469	uncharacterized protein C7orf63 homolog	2.5	0.8	1.65
LOC104911934	steroidogenic acute regulatory protein, mitochondrial-like	2.5	0.8	1.65
LOC104913767	brain-specific angiogenesis inhibitor 1-associated protein 2-like	2.5	0.8	1.65
VWA3B	von Willebrand factor A domain containing 3B	2.5	0.8	1.65
XK	X-linked Kx blood group	2.5	0.8	1.65
ANKRD34B	ankyrin repeat domain 34B	2.5	0.8	1.65
DCLK3	doublecortin-like kinase 3	2.5	0.8	1.65
LOC104911324	uncharacterized LOC104911324	2.5	0.8	1.65
LOC104911472	armadillo repeat-containing protein 3-like	2.5	0.8	1.65
LOC104913427	uncharacterized LOC104913427	2.5	0.8	1.65
LOC104914826	atrial natriuretic peptide receptor 3-like	2.5	0.8	1.65
LRRD1	leucine-rich repeats and death domain containing 1	2.5	0.8	1.65
TMC2	transmembrane channel-like 2	2.5	0.8	1.65
INPP5D	inositol polyphosphate-5-phosphatase, 145kDa	1.7	1.6	1.65
LOC104909764	uncharacterized LOC104909764	1.7	1.6	1.65
LOC104913524	uncharacterized LOC104913524	1.7	1.6	1.65
LOC104913782	glutamine-rich protein 2-like	1.7	1.6	1.65
LOC104914239	disintegrin and metalloproteinase domain-containing protein 32-like	1.7	1.6	1.65
LOC104915627	protein MIS12 homolog	1.7	1.6	1.65
LOC104915808	synaptic vesicle glycoprotein 2C-like	1.7	1.6	1.65
LOC104916803	V-type proton ATPase subunit G 1-like	1.7	1.6	1.65
RFESD	Rieske (Fe-S) domain containing	1.7	1.6	1.65
KL	klotho	2.5	0.8	1.64
LOC100546061	BPI fold-containing family B member 3-like	2.5	0.8	1.64
LOC104909500	uncharacterized LOC104909500	2.5	0.8	1.64
LOC104909979	phosphatidate phosphatase LPIN1-like	2.5	0.8	1.64
LOC104913980	uncharacterized LOC104913980	2.5	0.8	1.64
LOC104915597	sperm-associated antigen 4 protein-like	2.5	0.8	1.64
LOC104916214	mamu class II histocompatibility antigen, DR alpha chain-like	2.5	0.8	1.64
LOC104916387	uncharacterized LOC104916387	2.5	0.8	1.64
LOC104916822	proline-rich protein 2-like	2.5	0.8	1.64
RGS12	regulator of G-protein signaling 12	2.5	0.8	1.64
TMEM132E	transmembrane protein 132E	2.5	0.8	1.64
FAM3B	family with sequence similarity 3, member B	1.7	1.6	1.64
LIPC	lipase, hepatic	1.7	1.6	1.64
LOC100539021	T-cell surface glycoprotein CD1b-3-like	1.7	1.6	1.64
LOC100541770	cytochrome P450 2J2-like	1.7	1.6	1.64
LOC104913277	uncharacterized LOC104913277	1.7	1.6	1.64

LOC104914046	uncharacterized LOC104914046	1.7	1.6	1.64
LOC104915320	uncharacterized LOC104915320	1.7	1.6	1.64
LOC104915552	uncharacterized LOC104915552	1.7	1.6	1.64
PVALB	parvalbumin	1.7	1.6	1.64
T	T, brachyury homolog (mouse)	1.7	1.6	1.64
ADCYAP1R1	adenylate cyclase activating polypeptide 1 (pituitary) receptor type I	2.5	0.8	1.64
LOC100539767	catenin alpha-3	2.5	0.8	1.64
LOC104910058	fibrocystin-like	2.5	0.8	1.64
LOC104910391	leucine-rich repeat and coiled-coil domain-containing protein 1-like	2.5	0.8	1.64
LOC104910766	myotubularin-related protein 7-like	2.5	0.8	1.64
LOC104911415	uncharacterized LOC104911415	2.5	0.8	1.64
LOC104911790	uncharacterized LOC104911790	2.5	0.8	1.64
MATN1	matrilin 1, cartilage matrix protein	2.5	0.8	1.64
LOC100544098	sulfate transporter	1.7	1.6	1.64
LOC100549720	nebulette-like	1.7	1.6	1.64
LOC100550430	cytochrome P450 4F22	1.7	1.6	1.64
LOC104911316	uncharacterized LOC104911316	1.7	1.6	1.64
LOC104911424	uncharacterized LOC104911424	1.7	1.6	1.64
LOC104916806	adenylate cyclase type 10-like	1.7	1.6	1.64
SYT6	synaptotagmin VI	1.7	1.6	1.64
TLDC2	TBC/LysM-associated domain containing 2	1.7	1.6	1.64
CCNA1	cyclin A1	1.7	1.6	1.63
CORO2A	coronin, actin binding protein, 2A	1.7	1.6	1.63
LOC100546330	multidrug resistance-associated protein 1-like	1.7	1.6	1.63
LOC100546946	gamma-2-syntrophin-like	1.7	1.6	1.63
LOC104909804	uncharacterized LOC104909804	1.7	1.6	1.63
LOC104911880	uncharacterized LOC104911880	1.7	1.6	1.63
LOC104911993	uncharacterized LOC104911993	1.7	1.6	1.63
LOC104913215	protocadherin alpha-5-like	1.7	1.6	1.63
LOC104913279	uncharacterized LOC104913279	1.7	1.6	1.63
LOC104913724	putative germ cell-specific gene 1-like protein 2	1.7	1.6	1.63
LOC104915687	fibrous sheath CABYR-binding protein-like	1.7	1.6	1.63
LOC104916055	G2/M phase-specific E3 ubiquitin-protein ligase-like	1.7	1.6	1.63
SLITRK4	SLIT and NTRK-like family, member 4	1.7	1.6	1.63
TAT	tyrosine aminotransferase	1.7	1.6	1.63
TMPRSS7	transmembrane protease, serine 7	1.7	1.6	1.63
C6H7orf31	chromosome 6 open reading frame, human C7orf31	1.7	1.6	1.62
EDNRB	endothelin receptor type B	1.7	1.6	1.62
GLI1	GLI family zinc finger 1	1.7	1.6	1.62
GPRIN2	G protein regulated inducer of neurite outgrowth 2	1.7	1.6	1.62
INHBB	inhibin, beta B	1.7	1.6	1.62
LOC100539236	AF4/FMR2 family member 3-like	1.7	1.6	1.62
LOC100543038	sodium- and chloride-dependent GABA transporter 1-like	1.7	1.6	1.62
LOC100550016	heat shock 70 kDa protein 12A-like	1.7	1.6	1.62
LOC104909468	uncharacterized LOC104909468	1.7	1.6	1.62
LOC104909972	uncharacterized LOC104909972	1.7	1.6	1.62
LOC104910264	piezo-type mechanosensitive ion channel component 2-like	1.7	1.6	1.62
LOC104910860	hepatocyte growth factor activator-like	1.7	1.6	1.62
LOC104911500	uncharacterized LOC104911500	1.7	1.6	1.62
LOC104912388	astrotactin-1-like	1.7	1.6	1.62
LOC104912504	uncharacterized LOC104912504	1.7	1.6	1.62
LOC104913367	lipopolysaccharide-induced tumor necrosis factor-alpha factor homolog	1.7	1.6	1.62
LOC104913734	uncharacterized LOC104913734	1.7	1.6	1.62
LOC104914219	uncharacterized LOC104914219	1.7	1.6	1.62
NCAN	neurocan	1.7	1.6	1.62

SHANK3	SH3 and multiple ankyrin repeat domains 3	1.7	1.6	1.62
SNAPC3	small nuclear RNA activating complex, polypeptide 3, 50kDa	1.7	1.6	1.62
WNT2B	wingless-type MMTV integration site family, member 2B	1.7	1.6	1.62
C16H7orf62	chromosome 16 open reading frame, human C7orf62	0.8	2.4	1.62
HGD	homogentisate 1,2-dioxygenase	0.8	2.4	1.62
LOC100549962	transmembrane protease serine 7-like	0.8	2.4	1.62
LOC104912042	uncharacterized LOC104912042	0.8	2.4	1.62
LOC104912808	WD repeat-containing protein 72-like	0.8	2.4	1.62
LOC104914027	potassium voltage-gated channel subfamily KQT member 2-like	0.8	2.4	1.62
NDNF	neuron-derived neurotrophic factor	0.8	2.4	1.62
CDH18	cadherin 18, type 2	1.7	1.6	1.62
GLDC	glycine dehydrogenase (decarboxylating)	1.7	1.6	1.62
LOC100544777	POU domain, class 3, transcription factor 3-like	1.7	1.6	1.62
LOC104911369	uncharacterized LOC104911369	1.7	1.6	1.62
LOC104913074	uncharacterized LOC104913074	1.7	1.6	1.62
LOC104914490	interleukin-20-like	1.7	1.6	1.62
LOC104917607	uncharacterized LOC104917607	1.7	1.6	1.62
PDE1A	phosphodiesterase 1A, calmodulin-dependent	1.7	1.6	1.62
SDCBP2	syndecan binding protein (syntenin) 2	1.7	1.6	1.62
WDR27	WD repeat domain 27	1.7	1.6	1.62
LOC100544311	arylacetamide deacetylase-like 3	0.8	2.4	1.62
LOC104914544	uncharacterized LOC104914544	0.8	2.4	1.62
GCNT3	glucosaminyl (N-acetyl) transferase 3, mucin type	1.7	1.5	1.61
LOC100543828	adseverin-like	1.7	1.5	1.61
LOC100545419	atrial natriuretic peptide-converting enzyme-like	1.7	1.5	1.61
LOC104910364	uncharacterized LOC104910364	1.7	1.5	1.61
LOC104911187	uncharacterized LOC104911187	1.7	1.5	1.61
LOC104911743	uncharacterized LOC104911743	1.7	1.5	1.61
LOC104915011	uncharacterized LOC104915011	1.7	1.5	1.61
LOC104915496	uncharacterized LOC104915496	1.7	1.5	1.61
LOC104915709	myosin-7-like	1.7	1.5	1.61
LOC104916606	polycystic kidney disease and receptor for egg jelly-related protein-like	1.7	1.5	1.61
MYL2	myosin, light chain 2, regulatory, cardiac, slow	1.7	1.5	1.61
P2RX6	purinergic receptor P2X, ligand-gated ion channel, 6	1.7	1.5	1.61
ABCC3	ATP-binding cassette, sub-family C (CFTR/MRP), member 3	1.7	1.5	1.60
CLIC6	chloride intracellular channel 6	1.7	1.5	1.60
LOC100545481	adenylate kinase 7	1.7	1.5	1.60
LOC100548731	cystine/glutamate transporter-like	1.7	1.5	1.60
LOC104909390	alkaline ceramidase 3-like	1.7	1.5	1.60
LOC104912032	PHD finger protein 7-like	1.7	1.5	1.60
LOC104912262	uncharacterized LOC104912262	1.7	1.5	1.60
LOC104915418	uncharacterized LOC104915418	1.7	1.5	1.60
LOC104916711	integrin alpha-IIb-like	1.7	1.5	1.60
LOC104917017	uncharacterized LOC104917017	1.7	1.5	1.60
LOC104917383	interferon-like	1.7	1.5	1.60
SLCO2A1	solute carrier organic anion transporter family, member 2A1	1.7	1.5	1.60
C2H2orf71	chromosome 2 open reading frame, human C2orf71	0.8	2.4	1.60
LNX1	ligand of numb-protein X 1, E3 ubiquitin protein ligase	0.8	2.4	1.60
LOC100549093	proton myo-inositol cotransporter-like	0.8	2.4	1.60
LOC100550618	CMRF35-like molecule 8	0.8	2.4	1.60
LOC104909681	uncharacterized LOC104909681	0.8	2.4	1.60
LOC104909783	uncharacterized LOC104909783	0.8	2.4	1.60
LOC104911093	uncharacterized LOC104911093	0.8	2.4	1.60
LOC104911249	uncharacterized LOC104911249	0.8	2.4	1.60
LOC104911562	uncharacterized LOC104911562	0.8	2.4	1.60
LOC104911908	uncharacterized LOC104911908	0.8	2.4	1.60
LOC104912313	uncharacterized LOC104912313	0.8	2.4	1.60

LOC104912534	uncharacterized LOC104912534	0.8	2.4	1.60
LOC104913191	uncharacterized LOC104913191	0.8	2.4	1.60
LOC104914343	uncharacterized LOC104914343	0.8	2.4	1.60
LOC104916295	glycine-rich cell wall structural protein 1.0-like	0.8	2.4	1.60
LOC104917130	uncharacterized LOC104917130	0.8	2.4	1.60
MYO15A	myosin XVA	0.8	2.4	1.60
SERPINB5	serpin peptidase inhibitor, clade B (ovalbumin), member 5	0.8	2.4	1.60
SLC34A2	solute carrier family 34 (type II sodium/phosphate cotransporter), member 2	0.8	2.4	1.60
TAL1	T-cell acute lymphocytic leukemia 1	0.8	2.4	1.60
ASCL4	achaete-scute family bHLH transcription factor 4	1.7	1.5	1.60
B3GNT3	UDP-GlcNAc:betaGal beta-1,3-N-acetylglucosaminyltransferase 3	1.7	1.5	1.60
COL9A2	collagen, type IX, alpha 2	1.7	1.5	1.60
CSMD2	CUB and Sushi multiple domains 2	1.7	1.5	1.60
HPGDS	hematopoietic prostaglandin D synthase	1.7	1.5	1.60
LOC104909572	uncharacterized LOC104909572	1.7	1.5	1.60
LOC104910384	uncharacterized LOC104910384	1.7	1.5	1.60
LOC104911522	cyclic AMP-responsive element-binding protein 5-like	1.7	1.5	1.60
LOC104913808	uncharacterized LOC104913808	1.7	1.5	1.60
VRTN	vertebrae development associated	1.7	1.5	1.60
C14H3orf67	chromosome 14 open reading frame, human C3orf67	0.8	2.4	1.60
GPR116	G protein-coupled receptor 116	0.8	2.4	1.60
KIAA1456	KIAA1456 ortholog	0.8	2.4	1.60
LOC100543851	guanylin-like	0.8	2.4	1.60
LOC100545500	fibrinogen-like protein 1	0.8	2.4	1.60
LOC104910726	uncharacterized LOC104910726	0.8	2.4	1.60
LOC104911147	uncharacterized LOC104911147	0.8	2.4	1.60
LOC104912182	uncharacterized LOC104912182	0.8	2.4	1.60
LOC104914371	uncharacterized LOC104914371	0.8	2.4	1.60
LOC104915270	uncharacterized LOC104915270	0.8	2.4	1.60
LOC104915637	adenylate cyclase type 10-like	0.8	2.4	1.60
LOC104916414	runt-related transcription factor 2 pseudogene	0.8	2.4	1.60
DDX43	DEAD (Asp-Glu-Ala-Asp) box polypeptide 43	0.0	3.2	1.60
LOC104911593	uncharacterized LOC104911593	0.0	3.2	1.60
LOC104917581	neurobeachin-like	0.0	3.2	1.60
CGA	glycoprotein hormones, alpha polypeptide	0.8	2.3	1.58
DOCK5	dedicator of cytokinesis 5	0.8	2.3	1.58
GJA8	gap junction protein, alpha 8, 50kDa	0.8	2.3	1.58
KCNJ6	potassium inwardly-rectifying channel, subfamily J, member 6	0.8	2.3	1.58
LOC100540974	sodium-coupled neutral amino acid transporter 4-like	0.8	2.3	1.58
LOC104910560	uncharacterized LOC104910560	0.8	2.3	1.58
LOC104911362	uncharacterized LOC104911362	0.8	2.3	1.58
LOC104911891	ELKS/Rab6-interacting/CAST family member 1-like	0.8	2.3	1.58
LOC104912268	uncharacterized LOC104912268	0.8	2.3	1.58
LOC104912394	uncharacterized LOC104912394	0.8	2.3	1.58
LOC104916121	potassium voltage-gated channel subfamily A member 7-like	0.8	2.3	1.58
LOC104916624	uncharacterized LOC104916624	0.8	2.3	1.58
MAP2	microtubule-associated protein 2	0.8	2.3	1.58
PCSK4	proprotein convertase subtilisin/kexin type 4	0.8	2.3	1.58
TM4SF19	transmembrane 4 L six family member 19	0.8	2.3	1.58
FAM83A	family with sequence similarity 83, member A	0.8	2.3	1.58
KCNH1	potassium voltage-gated channel, subfamily H (eag-related), member 1	0.8	2.3	1.58
LOC100549545	granzyme A-like	0.8	2.3	1.58
LOC104909814	proto-oncogene tyrosine-protein kinase ROS-like	0.8	2.3	1.58
LOC104910539	uncharacterized LOC104910539	0.8	2.3	1.58
LOC104910605	uncharacterized LOC104910605	0.8	2.3	1.58
LOC104912310	receptor-type tyrosine-protein phosphatase C-like	0.8	2.3	1.58

LOC104912795	uncharacterized LOC104912795	0.8	2.3	1.58
LOC104916343	homeobox protein SIX3 pseudogene	0.8	2.3	1.58
CD80	CD80 molecule	0.0	3.2	1.58
GFRA2	GDNF family receptor alpha 2	0.0	3.2	1.58
LOC104910128	uncharacterized LOC104910128	0.0	3.2	1.58
LOC104914538	metabotropic glutamate receptor 4-like	0.0	3.2	1.58
LOC104915972	AP-1 complex subunit mu-2-like	0.0	3.2	1.58
CPED1	cadherin-like and PC-esterase domain containing 1	0.8	2.3	1.56
DTWD2	DTW domain containing 2	0.8	2.3	1.56
LOC100539521	uncharacterized LOC100539521	0.8	2.3	1.56
LOC104910130	uncharacterized LOC104910130	0.8	2.3	1.56
LOC104912529	uncharacterized LOC104912529	0.8	2.3	1.56
CLPS	colipase, pancreatic	0.8	2.3	1.56
GPR146	G protein-coupled receptor 146	0.8	2.3	1.56
LOC100550754	lysosomal acid lipase/cholesteryl ester hydrolase-like	0.8	2.3	1.56
LOC104910010	uncharacterized LOC104910010	0.8	2.3	1.56
LOC104912185	uncharacterized LOC104912185	0.8	2.3	1.56
LOC104914031	uncharacterized LOC104914031	0.8	2.3	1.56
PPP1R36	protein phosphatase 1, regulatory subunit 36	0.8	2.3	1.56
SMCO3	single-pass membrane protein with coiled-coil domains 3	0.8	2.3	1.56
STOML3	stomatin (EPB72)-like 3	0.8	2.3	1.56
LOC100545469	sulfotransferase 6B1-like	0.0	3.1	1.56
LOC104911050	uncharacterized LOC104911050	0.0	3.1	1.56
LOC104912598	uncharacterized LOC104912598	0.0	3.1	1.56
LOC104913192	uncharacterized LOC104913192	0.0	3.1	1.56
LOC104915298	uncharacterized LOC104915298	0.0	3.1	1.56
LOC104917033	rho guanine nucleotide exchange factor 9-like	0.0	3.1	1.56
NTRK1	neurotrophic tyrosine kinase, receptor, type 1	0.0	3.1	1.56
PROX2	prospero homeobox 2	0.0	3.1	1.56
LOC100541074	ephrin type-A receptor 7-like	0.0	3.1	1.54
LOC104916036	zinc finger and BTB domain-containing protein 7B-like	0.0	3.1	1.54
SCGN	secretagogin, EF-hand calcium binding protein	0.0	3.1	1.54
SSTR5	somatostatin receptor 5	0.0	3.1	1.54
FGL2	fibrinogen-like 2	0.0	3.0	1.52
PTH1R	parathyroid hormone 1 receptor	0.0	3.0	1.52
HNF1B	HNF1 homeobox B	2.5	0.0	1.27
LOC104909561	follicle-stimulating hormone receptor	2.5	0.0	1.27
LOC104910555	uncharacterized LOC104910555	2.5	0.0	1.27
LOC104912675	uncharacterized LOC104912675	2.5	0.0	1.27
LOC104913747	uncharacterized LOC104913747	2.5	0.0	1.27
LOC104915983	uncharacterized LOC104915983	2.5	0.0	1.27
LOC104916767	uncharacterized LOC104916767	2.5	0.0	1.27
LOC104917302	forkhead box protein D3	2.5	0.0	1.27
RLBP1	retinaldehyde binding protein 1	2.5	0.0	1.27
KNG1	kininogen 1	2.5	0.0	1.27
LOC104910342	lipoxygenase homology domain-containing protein 1-like	2.5	0.0	1.27
LOC104912040	uncharacterized LOC104912040	2.5	0.0	1.27
LOC104916748	uncharacterized LOC104916748	2.5	0.0	1.27
LOC104917307	glutamic acid-rich protein-like	2.5	0.0	1.27
RAB25	RAB25, member RAS oncogene family	2.5	0.0	1.27
RBM47	RNA binding motif protein 47	2.5	0.0	1.27
SLC39A12	solute carrier family 39 (zinc transporter), member 12	2.5	0.0	1.27
SLCO2B1	solute carrier organic anion transporter family, member 2B1	2.5	0.0	1.27
GJA4	gap junction protein, alpha 4, 37kDa	2.5	0.0	1.27
LOC100542972	histone H2A type 2-B-like	2.5	0.0	1.27
LOC100546724	cytochrome P450 2K4-like	2.5	0.0	1.27
LOC104914357	uncharacterized LOC104914357	2.5	0.0	1.27
LOC104914849	uncharacterized LOC104914849	2.5	0.0	1.27

LOC104916483	beta-citrylglutamate synthase B-like	2.5	0.0	1.27
SPP2	secreted phosphoprotein 2, 24kDa	2.5	0.0	1.27
SYT2	synaptotagmin II	2.5	0.0	1.27
CCDC138	coiled-coil domain containing 138	2.5	0.0	1.26
CLGN	calmegin	2.5	0.0	1.26
LOC100543590	uncharacterized LOC100543590	2.5	0.0	1.26
LOC104912634	paired box protein Pax-3-like	2.5	0.0	1.26
LOC104915885	uncharacterized LOC104915885	2.5	0.0	1.26
LOC104916375	gonadotropin-releasing hormone II receptor-like	2.5	0.0	1.26
PDE1C	phosphodiesterase 1C, calmodulin-dependent 70kDa	2.5	0.0	1.26
RAB17	RAB17, member RAS oncogene family	2.5	0.0	1.26
DGAT2	diacylglycerol O-acyltransferase 2	1.7	0.8	1.25
LOC100543754	cytochrome b-245 heavy chain	1.7	0.8	1.25
LOC104909400	teneurin-4-like	1.7	0.8	1.25
LOC104909538	uncharacterized LOC104909538	1.7	0.8	1.25
LOC104909758	brefeldin A-inhibited guanine nucleotide-exchange protein 3-like	1.7	0.8	1.25
LOC104910020	uncharacterized LOC104910020	1.7	0.8	1.25
LOC104911885	uncharacterized LOC104911885	1.7	0.8	1.25
LOC104912425	uncharacterized LOC104912425	1.7	0.8	1.25
LOC104912488	interleukin-23 receptor-like	1.7	0.8	1.25
LOC104912788	uncharacterized LOC104912788	1.7	0.8	1.25
LOC104913905	uncharacterized LOC104913905	1.7	0.8	1.25
LOC104914405	T-lymphocyte surface antigen Ly-9-like	1.7	0.8	1.25
AGXT2	alanine--glyoxylate aminotransferase 2	1.7	0.8	1.24
CABP4	calcium binding protein 4	1.7	0.8	1.24
CD164L2	CD164 sialomucin-like 2	1.7	0.8	1.24
IL15	interleukin 15	1.7	0.8	1.24
LOC100542224	alpha-1-antitrypsin-like	1.7	0.8	1.24
LOC100543889	myosin-8-like	1.7	0.8	1.24
LOC100543921	carboxypeptidase A6	1.7	0.8	1.24
LOC100545300	granzyme M-like	1.7	0.8	1.24
LOC100548429	high affinity choline transporter 1-like	1.7	0.8	1.24
LOC104909398	GRB2-associated-binding protein 2-like	1.7	0.8	1.24
LOC104909734	sulfotransferase family cytosolic 2B member 1-like	1.7	0.8	1.24
LOC104910904	uncharacterized LOC104910904	1.7	0.8	1.24
LOC104910996	uncharacterized LOC104910996	1.7	0.8	1.24
LOC104911894	glutamate receptor ionotropic, delta-1-like	1.7	0.8	1.24
LOC104912692	uncharacterized LOC104912692	1.7	0.8	1.24
LOC104912959	uncharacterized LOC104912959	1.7	0.8	1.24
LOC104913177	uncharacterized LOC104913177	1.7	0.8	1.24
LOC104913315	uncharacterized LOC104913315	1.7	0.8	1.24
LOC104913482	uncharacterized LOC104913482	1.7	0.8	1.24
LOC104913515	uncharacterized LOC104913515	1.7	0.8	1.24
LOC104917011	uncharacterized LOC104917011	1.7	0.8	1.24
TRPC5	transient receptor potential cation channel, subfamily C, member 5	1.7	0.8	1.24
DRAXIN	dorsal inhibitory axon guidance protein	1.7	0.8	1.24
EOMES	eomesodermin	1.7	0.8	1.24
GABRA2	gamma-aminobutyric acid (GABA) A receptor, alpha 2	1.7	0.8	1.24
IL18RAP	interleukin 18 receptor accessory protein	1.7	0.8	1.24
KBTBD13	kelch repeat and BTB (POZ) domain containing 13	1.7	0.8	1.24
KCNB1	potassium voltage-gated channel, Shab-related subfamily, member 1	1.7	0.8	1.24
LOC100539381	avidin-related protein 1-like	1.7	0.8	1.24
LOC100541858	protein CBFA2T1	1.7	0.8	1.24
LOC104909311	mucin-2-like	1.7	0.8	1.24
LOC104910009	uncharacterized LOC104910009	1.7	0.8	1.24
LOC104910740	uncharacterized LOC104910740	1.7	0.8	1.24

LOC104911096	uncharacterized LOC104911096	1.7	0.8	1.24
LOC104913463	WAP, Kazal, immunoglobulin, Kunitz and NTR domain-containing protein 1-like	1.7	0.8	1.24
POU3F1	POU class 3 homeobox 1	1.7	0.8	1.24
SPAG16	sperm associated antigen 16	1.7	0.8	1.24
LOC100540007	uncharacterized LOC100540007	1.7	0.8	1.23
LOC100542160	ubiquitin carboxyl-terminal hydrolase 35	1.7	0.8	1.23
LOC100543299	desmocollin-2	1.7	0.8	1.23
LOC104910359	phosphatidylinositol 3,4,5-trisphosphate-dependent Rac exchanger 2 protein-like	1.7	0.8	1.23
LOC104910610	uncharacterized LOC104910610	1.7	0.8	1.23
LOC104911510	uncharacterized LOC104911510	1.7	0.8	1.23
LOC104911659	uncharacterized LOC104911659	1.7	0.8	1.23
LOC104912539	uncharacterized LOC104912539	1.7	0.8	1.23
LOC104913007	putative ATP-dependent RNA helicase TDRD12	1.7	0.8	1.23
LOC104913702	uncharacterized LOC104913702	1.7	0.8	1.23
LOC104913904	uncharacterized LOC104913904	1.7	0.8	1.23
LOC104914351	uncharacterized LOC104914351	1.7	0.8	1.23
LOC104914579	protein TANC2-like	1.7	0.8	1.23
LOC104916683	uncharacterized LOC104916683	1.7	0.8	1.23
LOC104916832	uncharacterized protein DDB_G0271670-like	1.7	0.8	1.23
LOC104916891	interleukin-36 receptor antagonist protein-like	1.7	0.8	1.23
LOC104916960	larval cuticle protein F1-like	1.7	0.8	1.23
LOC104917003	zonadhesin-like	1.7	0.8	1.23
LRRIQ4	leucine-rich repeats and IQ motif containing 4	1.7	0.8	1.23
PKD2L1	polycystic kidney disease 2-like 1	1.7	0.8	1.23
RELL2	RELT-like 2	1.7	0.8	1.23
TMEM156	transmembrane protein 156	1.7	0.8	1.23
AIFM3	apoptosis-inducing factor, mitochondrion-associated, 3	1.7	0.8	1.22
BRINP1	bone morphogenetic protein/retinoic acid inducible neural-specific 1	1.7	0.8	1.22
CNGB1	cyclic nucleotide gated channel beta 1	1.7	0.8	1.22
ERMN	ermin, ERM-like protein	1.7	0.8	1.22
LOC100538811	FRAS1-related extracellular matrix protein 1-like	1.7	0.8	1.22
LOC100539660	sterile alpha motif domain-containing protein 9-like	1.7	0.8	1.22
LOC100540008	protein-lysine methyltransferase METTL21E-like	1.7	0.8	1.22
LOC104909547	uncharacterized LOC104909547	1.7	0.8	1.22
LOC104911081	uncharacterized LOC104911081	1.7	0.8	1.22
LOC104911925	uncharacterized protein C10orf107-like	1.7	0.8	1.22
LOC104911941	uncharacterized LOC104911941	1.7	0.8	1.22
LOC104912028	uncharacterized LOC104912028	1.7	0.8	1.22
LOC104913222	PH and SEC7 domain-containing protein 2-like	1.7	0.8	1.22
LOC104914983	rho guanine nucleotide exchange factor 28-like	1.7	0.8	1.22
LOC104915179	uncharacterized LOC104915179	1.7	0.8	1.22
LOC104916658	maestro heat-like repeat-containing protein family member 2B	1.7	0.8	1.22
LOC104916682	uncharacterized LOC104916682	1.7	0.8	1.22
NME9	NME/NM23 family member 9	1.7	0.8	1.22
NTF3	neurotrophin 3	1.7	0.8	1.22
PDCD1LG2	programmed cell death 1 ligand 2	1.7	0.8	1.22
PRSS56	protease, serine, 56	1.7	0.8	1.22
PSCA	prostate stem cell antigen	1.7	0.8	1.22
TEKT5	tektin 5	1.7	0.8	1.22
TEX36	testis expressed 36	1.7	0.8	1.22
TMEM37	transmembrane protein 37	1.7	0.8	1.22
C5	complement component 5	0.8	1.6	1.22
CALHM3	calcium homeostasis modulator 3	0.8	1.6	1.22
CCDC65	coiled-coil domain containing 65	0.8	1.6	1.22
CDRT1	CMT1A duplicated region transcript 1	0.8	1.6	1.22

EPHA4	EPH receptor A4	0.8	1.6	1.22
LOC100539032	N-acetyllactosaminide beta-1,6-N-acetylglucosaminyl-transferase, isoform C-like	0.8	1.6	1.22
LOC100540306	bile acid receptor-like	0.8	1.6	1.22
LOC100543637	parvalbumin beta-like	0.8	1.6	1.22
LOC104909408	uncharacterized LOC104909408	0.8	1.6	1.22
LOC104909789	uncharacterized LOC104909789	0.8	1.6	1.22
LOC104911224	uncharacterized LOC104911224	0.8	1.6	1.22
LOC104911946	uncharacterized LOC104911946	0.8	1.6	1.22
LOC104912250	bromodomain testis-specific protein-like	0.8	1.6	1.22
LOC104913041	uncharacterized LOC104913041	0.8	1.6	1.22
LOC104913459	sorting nexin-29-like	0.8	1.6	1.22
LOC104913478	protein disulfide-isomerase A2-like	0.8	1.6	1.22
LOC104914427	uncharacterized LOC104914427	0.8	1.6	1.22
LOC104914659	uncharacterized LOC104914659	0.8	1.6	1.22
LOC104917181	maestro heat-like repeat-containing protein family member 2B	0.8	1.6	1.22
NKX6-2	NK6 homeobox 2	0.8	1.6	1.22
SLC6A15	solute carrier family 6 (neutral amino acid transporter), member 15	0.8	1.6	1.22
TPBGL	trophoblast glycoprotein-like	0.8	1.6	1.22
VIT	vitrin	0.8	1.6	1.22
ABCB5	ATP-binding cassette, sub-family B (MDR/TAP), member 5	1.7	0.8	1.22
DRD3	dopamine receptor D3	1.7	0.8	1.22
FAM43B	family with sequence similarity 43, member B	1.7	0.8	1.22
KCNV2	potassium channel, subfamily V, member 2	1.7	0.8	1.22
LOC100541069	E3 ubiquitin-protein ligase TRIM39-like	1.7	0.8	1.22
LOC100549076	glutamate receptor ionotropic, NMDA 2A-like	1.7	0.8	1.22
LOC104909228	tyrosine-protein phosphatase non-receptor type substrate 1-like	1.7	0.8	1.22
LOC104909392	glycerophosphodiester phosphodiesterase domain-containing protein 4-like	1.7	0.8	1.22
LOC104909719	uncharacterized LOC104909719	1.7	0.8	1.22
LOC104909727	uncharacterized LOC104909727	1.7	0.8	1.22
LOC104910674	electrogenic sodium bicarbonate cotransporter 1-like	1.7	0.8	1.22
LOC104911132	uncharacterized LOC104911132	1.7	0.8	1.22
LOC104911529	uncharacterized LOC104911529	1.7	0.8	1.22
LOC104914703	uncharacterized LOC104914703	1.7	0.8	1.22
LOC104917127	maestro heat-like repeat-containing protein family member 2B	1.7	0.8	1.22
PLEKHB1	pleckstrin homology domain containing, family B (evectins) member 1	1.7	0.8	1.22
CALB1	calbindin 1, 28kDa	0.8	1.6	1.22
CIITA	class II, major histocompatibility complex, transactivator	0.8	1.6	1.22
DUOXA2	dual oxidase maturation factor 2	0.8	1.6	1.22
FA2H	fatty acid 2-hydroxylase	0.8	1.6	1.22
LOC100549814	metabotropic glutamate receptor 5-like	0.8	1.6	1.22
LOC104910808	uncharacterized LOC104910808	0.8	1.6	1.22
LOC104911368	uncharacterized LOC104911368	0.8	1.6	1.22
LOC104911377	uncharacterized LOC104911377	0.8	1.6	1.22
LOC104911478	uncharacterized LOC104911478	0.8	1.6	1.22
LOC104916609	uncharacterized LOC104916609	0.8	1.6	1.22
NRIP3	nuclear receptor interacting protein 3	0.8	1.6	1.22
VSTM5	V-set and transmembrane domain containing 5	0.8	1.6	1.22
C5H14orf180	chromosome 5 open reading frame, human C14orf180	0.8	1.6	1.20
CHRNA4	cholinergic receptor, nicotinic, alpha 4 (neuronal)	0.8	1.6	1.20
F8	coagulation factor VIII, procoagulant component	0.8	1.6	1.20
GPR97	G protein-coupled receptor 97	0.8	1.6	1.20
HPSE2	heparanase 2 (inactive)	0.8	1.6	1.20
IL20RB	interleukin 20 receptor beta	0.8	1.6	1.20
LINGO3	leucine rich repeat and Ig domain containing 3	0.8	1.6	1.20

LOC100540438	uncharacterized LOC100540438	0.8	1.6	1.20
LOC100544424	band 3 anion transport protein-like	0.8	1.6	1.20
LOC100545870	olfactory receptor 52I2-like	0.8	1.6	1.20
LOC100549614	transmembrane protein 68-like	0.8	1.6	1.20
LOC104909691	uncharacterized LOC104909691	0.8	1.6	1.20
LOC104909742	uncharacterized LOC104909742	0.8	1.6	1.20
LOC104910159	uncharacterized LOC104910159	0.8	1.6	1.20
LOC104910220	uncharacterized LOC104910220	0.8	1.6	1.20
LOC104910449	uncharacterized LOC104910449	0.8	1.6	1.20
LOC104910559	uncharacterized LOC104910559	0.8	1.6	1.20
LOC104910690	uncharacterized LOC104910690	0.8	1.6	1.20
LOC104910796	uncharacterized LOC104910796	0.8	1.6	1.20
LOC104911380	uncharacterized LOC104911380	0.8	1.6	1.20
LOC104911554	uncharacterized LOC104911554	0.8	1.6	1.20
LOC104912532	carboxypeptidase N subunit 2-like	0.8	1.6	1.20
LOC104913533	uncharacterized LOC104913533	0.8	1.6	1.20
LOC104914718	uncharacterized LOC104914718	0.8	1.6	1.20
LOC104914931	A disintegrin and metalloproteinase with thrombospondin motifs 6-like	0.8	1.6	1.20
LOC104915740	putative N-acetyltransferase 8B	0.8	1.6	1.20
LOC104915769	maestro heat-like repeat-containing protein family member 2B	0.8	1.6	1.20
LOC104916454	integrin alpha-10-like	0.8	1.6	1.20
REP15	RAB15 effector protein	0.8	1.6	1.20
SLC35D3	solute carrier family 35, member D3	0.8	1.6	1.20
TMPRSS4	transmembrane protease, serine 4	0.8	1.6	1.20
APOB	apolipoprotein B	0.8	1.6	1.20
FZD10	frizzled class receptor 10	0.8	1.6	1.20
GLRA1	glycine receptor, alpha 1	0.8	1.6	1.20
LNP1	leukemia NUP98 fusion partner 1	0.8	1.6	1.20
LOC100538965	centlein-like	0.8	1.6	1.20
LOC100539643	C-type natriuretic peptide 2-like	0.8	1.6	1.20
LOC100551190	glioma pathogenesis-related protein 1-like	0.8	1.6	1.20
LOC104910116	uncharacterized LOC104910116	0.8	1.6	1.20
LOC104910338	coiled-coil domain-containing protein 178-like	0.8	1.6	1.20
LOC104910593	tyrosine-protein kinase SgK223-like	0.8	1.6	1.20
LOC104911274	cingulin-like	0.8	1.6	1.20
LOC104911426	uncharacterized LOC104911426	0.8	1.6	1.20
LOC104911516	uncharacterized LOC104911516	0.8	1.6	1.20
LOC104912074	fibroblast growth factor receptor 2-like	0.8	1.6	1.20
LOC104912576	uncharacterized LOC104912576	0.8	1.6	1.20
LOC104912877	uncharacterized LOC104912877	0.8	1.6	1.20
LOC104913704	uncharacterized LOC104913704	0.8	1.6	1.20
LOC104916485	class I histocompatibility antigen, F10 alpha chain-like	0.8	1.6	1.20
LOC104916973	transcription factor Sp5	0.8	1.6	1.20
LRFN2	leucine rich repeat and fibronectin type III domain containing 2	0.8	1.6	1.20
LRRC3C	leucine rich repeat containing 3C	0.8	1.6	1.20
SLC17A8	solute carrier family 17 (vesicular glutamate transporter), member 8	0.8	1.6	1.20
TRH	thyrotropin-releasing hormone	0.8	1.6	1.20
DNASE1L2	deoxyribonuclease I-like 2	0.0	2.4	1.20
LAMP3	lysosomal-associated membrane protein 3	0.0	2.4	1.20
LOC100543241	interstitial collagenase-like	0.0	2.4	1.20
LOC100546809	protein NDNF-like	0.0	2.4	1.20
LOC104909825	uncharacterized LOC104909825	0.0	2.4	1.20
LOC104909954	protein eyes shut homolog	0.0	2.4	1.20
LOC104913850	uncharacterized LOC104913850	0.0	2.4	1.20
LOC104917201	chromosome unknown open reading frame, human C9orf24	0.0	2.4	1.20
OTOGL	otogelin-like	0.0	2.4	1.20

PAPPA	pregnancy-associated plasma protein A, pappalysin 1	0.0	2.4	1.20
TBX19	T-box 19	0.0	2.4	1.20
C26H11orf63	chromosome 26 open reading frame, human C11orf63	0.8	1.5	1.18
CAPN5	calpain 5	0.8	1.5	1.18
CDH1	cadherin 1, type 1, E-cadherin (epithelial)	0.8	1.5	1.18
LOC100539600	carbonic anhydrase 3-like	0.8	1.5	1.18
LOC100550599	probable G-protein coupled receptor 83	0.8	1.5	1.18
LOC104909576	uncharacterized LOC104909576	0.8	1.5	1.18
LOC104909579	uncharacterized LOC104909579	0.8	1.5	1.18
LOC104911246	uncharacterized LOC104911246	0.8	1.5	1.18
LOC104911625	uncharacterized LOC104911625	0.8	1.5	1.18
LOC104912096	uncharacterized LOC104912096	0.8	1.5	1.18
LOC104913992	uncharacterized LOC104913992	0.8	1.5	1.18
LOC104915579	maestro heat-like repeat-containing protein family member 2B	0.8	1.5	1.18
LOC104915683	putative protein TPRXL	0.8	1.5	1.18
LOC104915918	uncharacterized LOC104915918	0.8	1.5	1.18
NOXRED1	NADP-dependent oxidoreductase domain containing 1	0.8	1.5	1.18
SAMD10	sterile alpha motif domain containing 10	0.8	1.5	1.18
CACNA1D	calcium channel, voltage-dependent, L type, alpha 1D subunit	0.8	1.5	1.18
CECR6	cat eye syndrome chromosome region, candidate 6	0.8	1.5	1.18
GMIP	GEM interacting protein	0.8	1.5	1.18
LOC100543749	osteocalcin-like	0.8	1.5	1.18
LOC100545478	keratin, type II cytoskeletal cochleal-like	0.8	1.5	1.18
LOC100550358	serine/threonine-protein kinase LMTK1-like	0.8	1.5	1.18
LOC104909618	kinesin-like protein KIF28P	0.8	1.5	1.18
LOC104909686	uncharacterized LOC104909686	0.8	1.5	1.18
LOC104909987	membrane-bound O-acyltransferase domain-containing protein 2	0.8	1.5	1.18
LOC104910976	uncharacterized LOC104910976	0.8	1.5	1.18
LOC104911727	myosin-IIIb-like	0.8	1.5	1.18
LOC104912220	uncharacterized LOC104912220	0.8	1.5	1.18
LOC104912395	uncharacterized LOC104912395	0.8	1.5	1.18
LOC104912564	uncharacterized LOC104912564	0.8	1.5	1.18
LOC104913241	uncharacterized LOC104913241	0.8	1.5	1.18
LOC104913406	voltage-dependent T-type calcium channel subunit alpha-1H-like	0.8	1.5	1.18
LOC104917291	uncharacterized LOC104917291	0.8	1.5	1.18
MANSC4	MANSC domain containing 4	0.8	1.5	1.18
MMP13	matrix metallopeptidase 13 (collagenase 3)	0.8	1.5	1.18
MOS	v-mos Moloney murine sarcoma viral oncogene homolog	0.8	1.5	1.18
MSANTD1	Myb/SANT-like DNA-binding domain containing 1	0.8	1.5	1.18
PCDH8	protocadherin 8	0.8	1.5	1.18
BRSK2	BR serine/threonine kinase 2	0.0	2.4	1.18
DBX2	developing brain homeobox 2	0.0	2.4	1.18
FGF18	fibroblast growth factor 18	0.0	2.4	1.18
FOXJ1	forkhead box J1	0.0	2.4	1.18
IL22RA2	interleukin 22 receptor, alpha 2	0.0	2.4	1.18
KIAA1211L	KIAA1211-like ortholog	0.0	2.4	1.18
LOC100542906	bromodomain testis-specific protein-like	0.0	2.4	1.18
LOC100544134	thrombospondin type-1 domain-containing protein 7A	0.0	2.4	1.18
LOC100545091	reelin	0.0	2.4	1.18
LOC104909650	kinesin-like protein KIF6	0.0	2.4	1.18
LOC104910460	uncharacterized LOC104910460	0.0	2.4	1.18
LOC104910929	uncharacterized LOC104910929	0.0	2.4	1.18
LOC104913275	uncharacterized LOC104913275	0.0	2.4	1.18
LOC104913673	uncharacterized LOC104913673	0.0	2.4	1.18
LOC104914390	uromodulin-like 1	0.0	2.4	1.18
NLGN3	neuroligin 3	0.0	2.4	1.18
NTS	neurotensin	0.0	2.4	1.18
CHRNB3	cholinergic receptor, nicotinic, beta 3 (neuronal)	0.0	2.3	1.16

GPR112	G protein-coupled receptor 112	0.0	2.3	1.16
LOC100542090	myosin-3-like	0.0	2.3	1.16
LOC104910811	uncharacterized LOC104910811	0.0	2.3	1.16
LOC104911628	uncharacterized LOC104911628	0.0	2.3	1.16
LOC104912679	uncharacterized LOC104912679	0.0	2.3	1.16
LOC104912812	uncharacterized LOC104912812	0.0	2.3	1.16
LOC104913363	uncharacterized LOC104913363	0.0	2.3	1.16
LOC104913555	gamma-glutamyltranspeptidase 1-like	0.0	2.3	1.16
LOC104914502	neurofascin-like	0.0	2.3	1.16
LOC104916909	cytochrome P450 2C31-like	0.0	2.3	1.16
S100A12	S100 calcium binding protein A12	0.0	2.3	1.16
TDRP	testis development related protein	0.0	2.3	1.16
TFAP2E	transcription factor AP-2 epsilon (activating enhancer binding protein 2 epsilon)	0.0	2.3	1.16
C1H3orf52	chromosome 1 open reading frame, human C3orf52	0.0	2.3	1.14
LOC100543573	zona pellucida sperm-binding protein 3	0.0	2.3	1.14
LOC100545353	erythroblast NAD(P)(+)--arginine ADP-ribosyltransferase-like	0.0	2.3	1.14
LOC104909931	uncharacterized LOC104909931	0.0	2.3	1.14
LOC104910215	uncharacterized LOC104910215	0.0	2.3	1.14
LOC104912301	uncharacterized LOC104912301	0.0	2.3	1.14
LOC104914898	mitogen-activated protein kinase kinase kinase 1-like	0.0	2.3	1.14
LRP2	low density lipoprotein receptor-related protein 2	0.0	2.3	1.14
PROKR1	prokineticin receptor 1	0.0	2.3	1.14
RGS17	regulator of G-protein signaling 17	0.0	2.3	1.14
SAG	S-antigen; retina and pineal gland (arrestin)	0.0	2.3	1.14
CDX1	caudal type homeobox 1	1.7	0.0	0.85
DEGS2	delta(4)-desaturase, sphingolipid 2	1.7	0.0	0.85
FNDC9	fibronectin type III domain containing 9	1.7	0.0	0.85
IMPG1	interphotoreceptor matrix proteoglycan 1	1.7	0.0	0.85
LOC100544825	chymotrypsinogen A-like	1.7	0.0	0.85
LOC100547576	UDP-glucuronosyltransferase 1-1-like	1.7	0.0	0.85
LOC100547722	claudin-22-like	1.7	0.0	0.85
LOC104909932	uncharacterized LOC104909932	1.7	0.0	0.85
LOC104911202	uncharacterized LOC104911202	1.7	0.0	0.85
LOC104911431	uncharacterized LOC104911431	1.7	0.0	0.85
LOC104911542	uncharacterized LOC104911542	1.7	0.0	0.85
LOC104911900	uncharacterized LOC104911900	1.7	0.0	0.85
LOC104912094	uncharacterized LOC104912094	1.7	0.0	0.85
LOC104912155	uncharacterized LOC104912155	1.7	0.0	0.85
LOC104912183	rho GTPase-activating protein 6-like	1.7	0.0	0.85
LOC104912570	uncharacterized LOC104912570	1.7	0.0	0.85
LOC104915109	uncharacterized LOC104915109	1.7	0.0	0.85
LOC104915504	MARVEL domain-containing protein 2-like	1.7	0.0	0.85
LOC104915648	probable JmjC domain-containing histone demethylation protein 2C	1.7	0.0	0.85
LOC104915734	zinc finger protein 532-like	1.7	0.0	0.85
LOC104916569	myosin-8	1.7	0.0	0.85
LOC104917086	myoferlin-like	1.7	0.0	0.85
LRRC30	leucine rich repeat containing 30	1.7	0.0	0.85
LYZ	lysozyme	1.7	0.0	0.85
MYRFL	myelin regulatory factor-like	1.7	0.0	0.85
PTPRJ	protein tyrosine phosphatase, receptor type, J	1.7	0.0	0.85
RAG2	recombination activating gene 2	1.7	0.0	0.85
RNF222	ring finger protein 222	1.7	0.0	0.85
VWC2L	von Willebrand factor C domain containing protein 2-like	1.7	0.0	0.85
WDR93	WD repeat domain 93	1.7	0.0	0.85
ARX	aristaless related homeobox	1.7	0.0	0.84
CALB2	calbindin 2	1.7	0.0	0.84

CALCA	calcitonin-related polypeptide alpha	1.7	0.0	0.84
CD28	CD28 molecule	1.7	0.0	0.84
CHRNA6	cholinergic receptor, nicotinic, alpha 6 (neuronal)	1.7	0.0	0.84
FRMPD3	FERM and PDZ domain containing 3	1.7	0.0	0.84
GPLD1	glycosylphosphatidylinositol specific phospholipase D1	1.7	0.0	0.84
LOC100538829	carbonic anhydrase 3-like	1.7	0.0	0.84
LOC100539646	uncharacterized LOC100539646	1.7	0.0	0.84
LOC100542183	gonadotropin-releasing hormone II receptor-like	1.7	0.0	0.84
LOC100542866	myosin heavy chain, skeletal muscle, adult-like	1.7	0.0	0.84
LOC100547334	protein unc-13 homolog C-like	1.7	0.0	0.84
LOC100548732	uncharacterized LOC100548732	1.7	0.0	0.84
LOC100548843	solute carrier family 22 member 2-like	1.7	0.0	0.84
LOC104909305	protein fantom-like	1.7	0.0	0.84
LOC104909567	uncharacterized LOC104909567	1.7	0.0	0.84
LOC104909933	rho GTPase-activating protein 32-like	1.7	0.0	0.84
LOC104910270	uncharacterized LOC104910270	1.7	0.0	0.84
LOC104910464	uncharacterized LOC104910464	1.7	0.0	0.84
LOC104911176	uncharacterized LOC104911176	1.7	0.0	0.84
LOC104911745	uncharacterized LOC104911745	1.7	0.0	0.84
LOC104911768	sodium-driven chloride bicarbonate exchanger-like	1.7	0.0	0.84
LOC104911805	semaphorin-5B-like	1.7	0.0	0.84
LOC104912012	uncharacterized LOC104912012	1.7	0.0	0.84
LOC104912429	protein Hook homolog 1-like	1.7	0.0	0.84
LOC104912732	uncharacterized LOC104912732	1.7	0.0	0.84
LOC104913207	uncharacterized LOC104913207	1.7	0.0	0.84
LOC104913457	uncharacterized LOC104913457	1.7	0.0	0.84
LOC104913748	uncharacterized LOC104913748	1.7	0.0	0.84
LOC104914134	uncharacterized LOC104914134	1.7	0.0	0.84
LOC104914992	dedicator of cytokinesis protein 8-like	1.7	0.0	0.84
LOC104915469	uncharacterized LOC104915469	1.7	0.0	0.84
LOC104916243	uncharacterized protein C19orf60 homolog	1.7	0.0	0.84
LOC104916588	fibronectin-like	1.7	0.0	0.84
LOC104916649	histone H4	1.7	0.0	0.84
LOC104916744	uncharacterized LOC104916744	1.7	0.0	0.84
LOC104917249	annexin A4-like	1.7	0.0	0.84
LOC104917393	ATP-dependent RNA helicase DDX25-like	1.7	0.0	0.84
MFNG	MFNG O-fucosylpeptide 3-beta-N-acetylglucosaminyltransferase	1.7	0.0	0.84
MFSD2B	major facilitator superfamily domain containing 2B	1.7	0.0	0.84
NTRK2	neurotrophic tyrosine kinase, receptor, type 2	1.7	0.0	0.84
PODXL	podocalyxin-like	1.7	0.0	0.84
RGSL1	regulator of G-protein signaling like 1	1.7	0.0	0.84
SLC30A3	solute carrier family 30 (zinc transporter), member 3	1.7	0.0	0.84
UNC93A	unc-93 homolog A (C. elegans)	1.7	0.0	0.84
VSX2	visual system homeobox 2	1.7	0.0	0.84
XCR1	chemokine (C motif) receptor 1	1.7	0.0	0.84
ALX3	ALX homeobox 3	1.7	0.0	0.84
DCST1	DC-STAMP domain containing 1	1.7	0.0	0.84
EXPH5	exophilin 5	1.7	0.0	0.84
FAM221A	family with sequence similarity 221, member A	1.7	0.0	0.84
KCNA1	potassium voltage-gated channel, shaker-related subfamily, member 1 (episodic ataxia with myokymia)	1.7	0.0	0.84
KCNK10	potassium channel, subfamily K, member 10	1.7	0.0	0.84
KCNK2	potassium channel, subfamily K, member 2	1.7	0.0	0.84
LIMD2	LIM domain containing 2	1.7	0.0	0.84
LOC100540103	secreted frizzled-related protein 2-like	1.7	0.0	0.84
LOC100544780	arylacetamide deacetylase-like 4	1.7	0.0	0.84
LOC100546899	piezo-type mechanosensitive ion channel component 2-like	1.7	0.0	0.84

LOC100551363	disintegrin and metalloproteinase domain-containing protein 21-like	1.7	0.0	0.84
LOC104909842	uncharacterized LOC104909842	1.7	0.0	0.84
LOC104910107	N-acetyllactosaminide beta-1,6-N-acetylglucosaminyl-transferase, isoform C pseudogene	1.7	0.0	0.84
LOC104910366	uncharacterized LOC104910366	1.7	0.0	0.84
LOC104910575	interleukin-8-like	1.7	0.0	0.84
LOC104910742	uncharacterized LOC104910742	1.7	0.0	0.84
LOC104911068	uncharacterized LOC104911068	1.7	0.0	0.84
LOC104911831	uncharacterized LOC104911831	1.7	0.0	0.84
LOC104912565	uncharacterized LOC104912565	1.7	0.0	0.84
LOC104912950	uncharacterized LOC104912950	1.7	0.0	0.84
LOC104913129	uncharacterized LOC104913129	1.7	0.0	0.84
LOC104913219	uncharacterized LOC104913219	1.7	0.0	0.84
LOC104914514	plexin-A2-like	1.7	0.0	0.84
LOC104914629	zinc finger protein 385A-like	1.7	0.0	0.84
LOC104915077	uncharacterized LOC104915077	1.7	0.0	0.84
LOC104915196	uncharacterized LOC104915196	1.7	0.0	0.84
LOC104915420	uncharacterized LOC104915420	1.7	0.0	0.84
LOC104916028	annexin A4-like	1.7	0.0	0.84
LOC104916830	uncharacterized LOC104916830	1.7	0.0	0.84
MSS51	MSS51 mitochondrial translational activator	1.7	0.0	0.84
NWD2	NACHT and WD repeat domain containing 2	1.7	0.0	0.84
PRL	prolactin	1.7	0.0	0.84
RGS21	regulator of G-protein signaling 21	1.7	0.0	0.84
A1CF	APOBEC1 complementation factor	0.8	0.8	0.82
CD2	CD2 molecule	0.8	0.8	0.82
CELF4	CUGBP, Elav-like family member 4	0.8	0.8	0.82
CLDN5	claudin 5	0.8	0.8	0.82
DRD1	dopamine receptor D1	0.8	0.8	0.82
DUPD1	dual specificity phosphatase and pro isomerase domain containing 1	0.8	0.8	0.82
FGF14	fibroblast growth factor 14	0.8	0.8	0.82
GRB7	growth factor receptor-bound protein 7	0.8	0.8	0.82
HCN4	hyperpolarization activated cyclic nucleotide-gated potassium channel 4	0.8	0.8	0.82
HTR5A	5-hydroxytryptamine (serotonin) receptor 5A, G protein-coupled	0.8	0.8	0.82
IL7R	interleukin 7 receptor	0.8	0.8	0.82
LOC100541131	lymphotactin-like	0.8	0.8	0.82
LOC100541750	V-set and transmembrane domain-containing protein 1-like	0.8	0.8	0.82
LOC100544821	carboxypeptidase B-like	0.8	0.8	0.82
LOC100545010	meprin A subunit beta	0.8	0.8	0.82
LOC100549666	uncharacterized LOC100549666	0.8	0.8	0.82
LOC104909266	uncharacterized LOC104909266	0.8	0.8	0.82
LOC104909386	uncharacterized LOC104909386	0.8	0.8	0.82
LOC104909738	uncharacterized LOC104909738	0.8	0.8	0.82
LOC104909784	uncharacterized LOC104909784	0.8	0.8	0.82
LOC104909828	proto-oncogene tyrosine-protein kinase ROS-like	0.8	0.8	0.82
LOC104909922	unconventional myosin-VI-like	0.8	0.8	0.82
LOC104910164	uncharacterized LOC104910164	0.8	0.8	0.82
LOC104910531	uncharacterized LOC104910531	0.8	0.8	0.82
LOC104910691	teneurin-3-like	0.8	0.8	0.82
LOC104910807	uncharacterized LOC104910807	0.8	0.8	0.82
LOC104910887	kyphoscoliosis peptidase-like	0.8	0.8	0.82
LOC104911045	uncharacterized LOC104911045	0.8	0.8	0.82
LOC104911578	uncharacterized LOC104911578	0.8	0.8	0.82
LOC104911594	uncharacterized LOC104911594	0.8	0.8	0.82
LOC104911640	uncharacterized LOC104911640	0.8	0.8	0.82

LOC104911839	uncharacterized LOC104911839	0.8	0.8	0.82
LOC104911870	uncharacterized LOC104911870	0.8	0.8	0.82
LOC104911881	uncharacterized LOC104911881	0.8	0.8	0.82
LOC104911983	homeobox protein VENTX-like	0.8	0.8	0.82
LOC104912099	uncharacterized LOC104912099	0.8	0.8	0.82
LOC104912174	uncharacterized LOC104912174	0.8	0.8	0.82
LOC104912339	uncharacterized LOC104912339	0.8	0.8	0.82
LOC104912540	uncharacterized LOC104912540	0.8	0.8	0.82
LOC104912830	uncharacterized LOC104912830	0.8	0.8	0.82
LOC104912869	uncharacterized LOC104912869	0.8	0.8	0.82
LOC104913111	uncharacterized LOC104913111	0.8	0.8	0.82
LOC104913605	uncharacterized LOC104913605	0.8	0.8	0.82
LOC104913844	uncharacterized LOC104913844	0.8	0.8	0.82
LOC104914095	probable phospholipid-transporting ATPase IIA	0.8	0.8	0.82
LOC104914327	potassium voltage-gated channel subfamily KQT member 4-like	0.8	0.8	0.82
LOC104914716	uncharacterized LOC104914716	0.8	0.8	0.82
LOC104915659	E3 SUMO-protein ligase RanBP2-like	0.8	0.8	0.82
LOC104916549	uncharacterized LOC104916549	0.8	0.8	0.82
LOC104917280	synaptobrevin homolog YKT6-like	0.8	0.8	0.82
LOC104917506	uncharacterized LOC104917506	0.8	0.8	0.82
PALM	paralemmin	0.8	0.8	0.82
PRR29	proline rich 29	0.8	0.8	0.82
SASH3	SAM and SH3 domain containing 3	0.8	0.8	0.82
SBK1	SH3 domain binding kinase 1	0.8	0.8	0.82
SERPINA10	serpin peptidase inhibitor, clade A (alpha-1 antiproteinase, antitrypsin), member 10	0.8	0.8	0.82
SH3TC1	SH3 domain and tetratricopeptide repeats 1	0.8	0.8	0.82
SYCE3	synaptonemal complex central element protein 3	0.8	0.8	0.82
TAC1	tachykinin, precursor 1	0.8	0.8	0.82
TMEM221	transmembrane protein 221	0.8	0.8	0.82
TSGA10	testis specific, 10	0.8	0.8	0.82
BFSP1	beaded filament structural protein 1, filensin	0.8	0.8	0.82
C13H16orf74	chromosome 13 open reading frame, human C16orf74	0.8	0.8	0.82
CRYBB3	crystallin, beta B3	0.8	0.8	0.82
EFCAB3	EF-hand calcium binding domain 3	0.8	0.8	0.82
F11R	F11 receptor	0.8	0.8	0.82
FBXL16	F-box and leucine-rich repeat protein 16	0.8	0.8	0.82
GNB3	guanine nucleotide binding protein (G protein), beta polypeptide 3	0.8	0.8	0.82
HAS3	hyaluronan synthase 3	0.8	0.8	0.82
KCNA6	potassium voltage-gated channel, shaker-related subfamily, member 6	0.8	0.8	0.82
LOC100538926	AF4/FMR2 family member 3 pseudogene	0.8	0.8	0.82
LOC100540406	arf-GAP with SH3 domain, ANK repeat and PH domain-containing protein 2-like	0.8	0.8	0.82
LOC100547762	aquaporin-7-like	0.8	0.8	0.82
LOC100549896	vacuolar protein sorting-associated protein 29-like	0.8	0.8	0.82
LOC100550455	monocarboxylate transporter 2-like	0.8	0.8	0.82
LOC104909297	macrophage mannose receptor 1-like	0.8	0.8	0.82
LOC104909531	uncharacterized LOC104909531	0.8	0.8	0.82
LOC104910111	uncharacterized LOC104910111	0.8	0.8	0.82
LOC104910242	rotatin-like	0.8	0.8	0.82
LOC104910446	uncharacterized LOC104910446	0.8	0.8	0.82
LOC104910550	uncharacterized LOC104910550	0.8	0.8	0.82
LOC104911319	uncharacterized LOC104911319	0.8	0.8	0.82
LOC104911473	uncharacterized LOC104911473	0.8	0.8	0.82
LOC104911710	uncharacterized LOC104911710	0.8	0.8	0.82
LOC104911896	protein FAM13C-like	0.8	0.8	0.82

LOC104912037	uncharacterized LOC104912037	0.8	0.8	0.82
LOC104912236	uncharacterized LOC104912236	0.8	0.8	0.82
LOC104912462	uncharacterized LOC104912462	0.8	0.8	0.82
LOC104912619	uncharacterized LOC104912619	0.8	0.8	0.82
LOC104912861	uncharacterized LOC104912861	0.8	0.8	0.82
LOC104913093	uncharacterized LOC104913093	0.8	0.8	0.82
LOC104913100	uncharacterized LOC104913100	0.8	0.8	0.82
LOC104913516	integrin alpha-D-like	0.8	0.8	0.82
LOC104914067	probable phospholipid-transporting ATPase IIA	0.8	0.8	0.82
LOC104914431	tyrosine-protein kinase receptor TYRO3-like	0.8	0.8	0.82
LOC104914658	activated RNA polymerase II transcriptional coactivator p15-like	0.8	0.8	0.82
LOC104914662	SH2 domain-containing adapter protein D-like	0.8	0.8	0.82
LOC104915249	multiple C2 and transmembrane domain-containing protein 1-like	0.8	0.8	0.82
LOC104915514	rac GTPase-activating protein 1-like	0.8	0.8	0.82
LOC104916245	uncharacterized LOC104916245	0.8	0.8	0.82
LOC104917004	IgGFc-binding protein-like	0.8	0.8	0.82
LOC104917556	uncharacterized LOC104917556	0.8	0.8	0.82
LOC104917606	uncharacterized LOC104917606	0.8	0.8	0.82
MLKL	mixed lineage kinase domain-like	0.8	0.8	0.82
PLEKHG7	pleckstrin homology domain containing, family G (with RhoGef domain) member 7	0.8	0.8	0.82
SLC34A1	solute carrier family 34 (type II sodium/phosphate cotransporter), member 1	0.8	0.8	0.82
SLC6A2	solute carrier family 6 (neurotransmitter transporter, noradrenalin), member 2	0.8	0.8	0.82
STRA6	stimulated by retinoic acid 6	0.8	0.8	0.82
UNC13A	unc-13 homolog A (C. elegans)	0.8	0.8	0.82
XKR9	XK, Kell blood group complex subunit-related family, member 9	0.8	0.8	0.82
ALDOB	aldolase B, fructose-bisphosphate	0.8	0.8	0.80
ANO4	anoctamin 4	0.8	0.8	0.80
APC2	adenomatosis polyposis coli 2	0.8	0.8	0.80
C1H21orf62	chromosome 1 open reading frame, human C21orf62	0.8	0.8	0.80
CNGB3	cyclic nucleotide gated channel beta 3	0.8	0.8	0.80
DCHS2	dachsous cadherin-related 2	0.8	0.8	0.80
DEF6	differentially expressed in FDCP 6 homolog (mouse)	0.8	0.8	0.80
DOCK3	dedicator of cytokinesis 3	0.8	0.8	0.80
ESRP1	epithelial splicing regulatory protein 1	0.8	0.8	0.80
GABRB2	gamma-aminobutyric acid (GABA) A receptor, beta 2	0.8	0.8	0.80
GHRH	growth hormone releasing hormone	0.8	0.8	0.80
GPR128	G protein-coupled receptor 128	0.8	0.8	0.80
GRIA2	glutamate receptor, ionotropic, AMPA 2	0.8	0.8	0.80
IL2RG	interleukin 2 receptor, gamma	0.8	0.8	0.80
LOC100541567	multiple epidermal growth factor-like domains protein 6	0.8	0.8	0.80
LOC100541906	uncharacterized LOC100541906	0.8	0.8	0.80
LOC100544290	contactin-associated protein-like 5	0.8	0.8	0.80
LOC100544917	rho guanine nucleotide exchange factor 4	0.8	0.8	0.80
LOC100545280	zona pellucida sperm-binding protein 3-like	0.8	0.8	0.80
LOC100546803	7-alpha-hydroxycholest-4-en-3-one 12-alpha-hydroxylase	0.8	0.8	0.80
LOC100547726	cytosolic phospholipase A2 beta-like	0.8	0.8	0.80
LOC100548218	nuclear receptor ROR-beta-like	0.8	0.8	0.80
LOC100549460	cadherin-12	0.8	0.8	0.80
LOC104910105	synaptonemal complex protein 2-like	0.8	0.8	0.80
LOC104910557	uncharacterized LOC104910557	0.8	0.8	0.80
LOC104911514	uncharacterized LOC104911514	0.8	0.8	0.80
LOC104911616	uncharacterized LOC104911616	0.8	0.8	0.80
LOC104911976	uncharacterized LOC104911976	0.8	0.8	0.80
LOC104912007	uncharacterized LOC104912007	0.8	0.8	0.80
LOC104913160	uncharacterized LOC104913160	0.8	0.8	0.80

LOC104913691	uncharacterized LOC104913691	0.8	0.8	0.80
LOC104913789	cell surface glycoprotein CD200 receptor 1-B-like	0.8	0.8	0.80
LOC104913798	uncharacterized LOC104913798	0.8	0.8	0.80
LOC104913895	laminin subunit beta-4-like	0.8	0.8	0.80
LOC104914042	E3 ubiquitin-protein ligase RNF8-B-like	0.8	0.8	0.80
LOC104914250	uncharacterized LOC104914250	0.8	0.8	0.80
LOC104914266	oxysterol-binding protein 1-like	0.8	0.8	0.80
LOC104914458	uncharacterized LOC104914458	0.8	0.8	0.80
LOC104914498	heat shock factor 2-binding protein-like	0.8	0.8	0.80
LOC104915232	maestro heat-like repeat-containing protein family member 7	0.8	0.8	0.80
LOC104915505	occludin-like	0.8	0.8	0.80
LOC104915509	protocadherin gamma-A11-like	0.8	0.8	0.80
LOC104916991	uncharacterized LOC104916991	0.8	0.8	0.80
LOC104917022	uncharacterized LOC104917022	0.8	0.8	0.80
LOC104917100	receptor-type tyrosine-protein phosphatase kappa-like	0.8	0.8	0.80
LOC104917394	neurofilament medium polypeptide-like	0.8	0.8	0.80
LOC104917430	uncharacterized LOC104917430	0.8	0.8	0.80
LOC104917457	uncharacterized LOC104917457	0.8	0.8	0.80
LOC104917459	uncharacterized LOC104917459	0.8	0.8	0.80
LOC104917616	guanylate cyclase soluble subunit alpha-2-like	0.8	0.8	0.80
MRAP2	melanocortin 2 receptor accessory protein 2	0.8	0.8	0.80
MYO7A	myosin VIIA	0.8	0.8	0.80
P2RY13	purinergic receptor P2Y, G-protein coupled, 13	0.8	0.8	0.80
PRKAA2	protein kinase, AMP-activated, alpha 2 catalytic subunit	0.8	0.8	0.80
RAB36	RAB36, member RAS oncogene family	0.8	0.8	0.80
RGS16	regulator of G-protein signaling 16	0.8	0.8	0.80
SH3BP1	SH3-domain binding protein 1	0.8	0.8	0.80
SLC51A	solute carrier family 51, alpha subunit	0.8	0.8	0.80
SPOCK1	sparc/osteonectin, cwcv and kazal-like domains proteoglycan (testican) 1	0.8	0.8	0.80
UTS2B	urotensin 2B	0.8	0.8	0.80
ACKR2	atypical chemokine receptor 2	0.8	0.8	0.80
AGBL4	ATP/GTP binding protein-like 4	0.8	0.8	0.80
C1H3orf30	chromosome 1 open reading frame, human C3orf30	0.8	0.8	0.80
C1HXorf22	chromosome 1 open reading frame, human CXorf22	0.8	0.8	0.80
CLDN3	claudin 3	0.8	0.8	0.80
CX3CR1	chemokine (C-X3-C motif) receptor 1	0.8	0.8	0.80
DMRTA2	DMRT-like family A2	0.8	0.8	0.80
DYTN	dystrotelin	0.8	0.8	0.80
ELF3	E74-like factor 3 (ets domain transcription factor, epithelial-specific)	0.8	0.8	0.80
FAM107A	family with sequence similarity 107, member A	0.8	0.8	0.80
FAM169A	family with sequence similarity 169, member A	0.8	0.8	0.80
GAL3ST2	galactose-3-O-sulfotransferase 2	0.8	0.8	0.80
GALR1	galanin receptor 1	0.8	0.8	0.80
GC	group-specific component (vitamin D binding protein)	0.8	0.8	0.80
GCNT1	glucosaminyl (N-acetyl) transferase 1, core 2	0.8	0.8	0.80
KCNE3	potassium voltage-gated channel, Isk-related family, member 3	0.8	0.8	0.80
KEL	Kell blood group, metallo-endopeptidase	0.8	0.8	0.80
LGR6	leucine-rich repeat containing G protein-coupled receptor 6	0.8	0.8	0.80
LIN28B	lin-28 homolog B (C. elegans)	0.8	0.8	0.80
LOC100538712	fibrillin-2-like	0.8	0.8	0.80
LOC100540787	neural Wiskott-Aldrich syndrome protein pseudogene	0.8	0.8	0.80
LOC100542874	uncharacterized LOC100542874	0.8	0.8	0.80
LOC100543064	tubulin monoglycylase TTLL3	0.8	0.8	0.80
LOC100543537	ficolin-2-like	0.8	0.8	0.80
LOC104909419	PHD finger protein 7-like	0.8	0.8	0.80
LOC104909905	uncharacterized LOC104909905	0.8	0.8	0.80

LOC104910011	uncharacterized LOC104910011	0.8	0.8	0.80
LOC104910356	uncharacterized LOC104910356	0.8	0.8	0.80
LOC104910618	uncharacterized LOC104910618	0.8	0.8	0.80
LOC104911228	alpha-1-antitrypsin-like	0.8	0.8	0.80
LOC104911639	uncharacterized LOC104911639	0.8	0.8	0.80
LOC104911698	uncharacterized LOC104911698	0.8	0.8	0.80
LOC104911977	synaptotagmin-10-like	0.8	0.8	0.80
LOC104912055	phosphatidylinositol 4-phosphate 3-kinase C2 domain-containing subunit gamma-like	0.8	0.8	0.80
LOC104912133	uncharacterized LOC104912133	0.8	0.8	0.80
LOC104912173	uncharacterized LOC104912173	0.8	0.8	0.80
LOC104912210	G-protein coupled receptor 12-like	0.8	0.8	0.80
LOC104912515	vitellogenin-1-like	0.8	0.8	0.80
LOC104912702	uncharacterized LOC104912702	0.8	0.8	0.80
LOC104912800	uncharacterized LOC104912800	0.8	0.8	0.80
LOC104912825	uncharacterized LOC104912825	0.8	0.8	0.80
LOC104912827	uncharacterized LOC104912827	0.8	0.8	0.80
LOC104912965	uncharacterized LOC104912965	0.8	0.8	0.80
LOC104913095	uncharacterized LOC104913095	0.8	0.8	0.80
LOC104913149	uncharacterized LOC104913149	0.8	0.8	0.80
LOC104913211	histone H1oo	0.8	0.8	0.80
LOC104913368	uncharacterized LOC104913368	0.8	0.8	0.80
LOC104913703	uncharacterized LOC104913703	0.8	0.8	0.80
LOC104913740	uncharacterized LOC104913740	0.8	0.8	0.80
LOC104913917	uncharacterized LOC104913917	0.8	0.8	0.80
LOC104914482	complement component receptor 1-like protein	0.8	0.8	0.80
LOC104914674	tyrosine-protein kinase ZAP-70-like	0.8	0.8	0.80
LOC104915698	uncharacterized LOC104915698	0.8	0.8	0.80
LOC104915789	long-chain fatty acid transport protein 6-like	0.8	0.8	0.80
LOC104916191	uncharacterized LOC104916191	0.8	0.8	0.80
LOC104917541	voltage-dependent calcium channel subunit alpha-2/delta-1-like	0.8	0.8	0.80
MYO3B	myosin IIIB	0.8	0.8	0.80
NTRK3	neurotrophic tyrosine kinase, receptor, type 3	0.8	0.8	0.80
P2RX1	purinergic receptor P2X, ligand-gated ion channel, 1	0.8	0.8	0.80
RGS1	regulator of G-protein signaling 1	0.8	0.8	0.80
RHBDL1	rhomboid, veinlet-like 1 (Drosophila)	0.8	0.8	0.80
SALL3	spalt-like transcription factor 3	0.8	0.8	0.80
SLC17A6	solute carrier family 17 (vesicular glutamate transporter), member 6	0.8	0.8	0.80
SLC35F1	solute carrier family 35, member F1	0.8	0.8	0.80
SLCO3A1	solute carrier organic anion transporter family, member 3A1	0.8	0.8	0.80
TCTEX1D4	Tctex1 domain containing 4	0.8	0.8	0.80
GCM1	glial cells missing homolog 1 (Drosophila)	0.0	1.6	0.80
KIAA1644	KIAA1644 ortholog	0.0	1.6	0.80
LOC100539056	inter-alpha-trypsin inhibitor heavy chain H3	0.0	1.6	0.80
LOC100541175	keratin, type I cytoskeletal 13-like	0.0	1.6	0.80
LOC100546438	tyrosine 3-monooxygenase-like	0.0	1.6	0.80
LOC100546728	angiopoietin-related protein 1-like	0.0	1.6	0.80
LOC100549178	torsin-1A-like	0.0	1.6	0.80
LOC100549516	fatty acid desaturase 1-like	0.0	1.6	0.80
LOC100549991	arylamine N-acetyltransferase, liver isozyme	0.0	1.6	0.80
LOC100550029	olfactory receptor 1052-like	0.0	1.6	0.80
LOC100550042	inaD-like protein	0.0	1.6	0.80
LOC104909412	unconventional myosin-VIIa	0.0	1.6	0.80
LOC104909462	uncharacterized LOC104909462	0.0	1.6	0.80
LOC104909696	uncharacterized LOC104909696	0.0	1.6	0.80
LOC104909778	uncharacterized LOC104909778	0.0	1.6	0.80
LOC104909995	phosphatidate phosphatase LPIN3-like	0.0	1.6	0.80

LOC104910746	alcohol dehydrogenase 1-like	0.0	1.6	0.80
LOC104911097	uncharacterized LOC104911097	0.0	1.6	0.80
LOC104911100	uncharacterized LOC104911100	0.0	1.6	0.80
LOC104911491	uncharacterized LOC104911491	0.0	1.6	0.80
LOC104911733	uncharacterized LOC104911733	0.0	1.6	0.80
LOC104911901	uncharacterized LOC104911901	0.0	1.6	0.80
LOC104912034	uncharacterized LOC104912034	0.0	1.6	0.80
LOC104912249	uncharacterized LOC104912249	0.0	1.6	0.80
LOC104912435	uncharacterized LOC104912435	0.0	1.6	0.80
LOC104912527	uncharacterized LOC104912527	0.0	1.6	0.80
LOC104912672	uncharacterized LOC104912672	0.0	1.6	0.80
LOC104913571	tetratricopeptide repeat protein 28-like	0.0	1.6	0.80
LOC104914141	uncharacterized LOC104914141	0.0	1.6	0.80
LOC104915288	uncharacterized protein KIAA1958-like	0.0	1.6	0.80
LOC104915421	potassium channel subfamily K member 2	0.0	1.6	0.80
LOC104916278	mothers against decapentaplegic homolog 6-like	0.0	1.6	0.80
LOC104916426	uncharacterized LOC104916426	0.0	1.6	0.80
LOC104916779	uncharacterized LOC104916779	0.0	1.6	0.80
PKIB	protein kinase (cAMP-dependent, catalytic) inhibitor beta	0.0	1.6	0.80
SLC6A11	solute carrier family 6 (neurotransmitter transporter), member 11	0.0	1.6	0.80
TLR7	toll-like receptor 7	0.0	1.6	0.80
ANKFN1	ankyrin-repeat and fibronectin type III domain containing 1	0.0	1.6	0.78
ANXA10	annexin A10	0.0	1.6	0.78
C8H10orf128	chromosome 8 open reading frame, human C10orf128	0.0	1.6	0.78
CCDC67	coiled-coil domain containing 67	0.0	1.6	0.78
CDK3	cyclin-dependent kinase 3	0.0	1.6	0.78
DMRT1	doublesex and mab-3 related transcription factor 1	0.0	1.6	0.78
EDN2	endothelin 2	0.0	1.6	0.78
EFHB	EF-hand domain family, member B	0.0	1.6	0.78
EPAS1	endothelial PAS domain protein 1	0.0	1.6	0.78
EVI2A	ecotropic viral integration site 2A	0.0	1.6	0.78
FGF20	fibroblast growth factor 20	0.0	1.6	0.78
GRIK3	glutamate receptor, ionotropic, kainate 3	0.0	1.6	0.78
HOXA13	homeobox A13	0.0	1.6	0.78
IL2RB	interleukin 2 receptor, beta	0.0	1.6	0.78
IRX1	iroquois homeobox 1	0.0	1.6	0.78
ITGA11	integrin, alpha 11	0.0	1.6	0.78
KCTD17	potassium channel tetramerization domain containing 17	0.0	1.6	0.78
LMX1B	LIM homeobox transcription factor 1, beta	0.0	1.6	0.78
LOC100541564	potassium channel subfamily T member 2	0.0	1.6	0.78
LOC100541927	cytochrome P450 2J2-like	0.0	1.6	0.78
LOC100544530	pro-neuregulin-1, membrane-bound isoform-like	0.0	1.6	0.78
LOC100545471	WD repeat-containing protein C2orf44 homolog	0.0	1.6	0.78
LOC100546469	ectonucleoside triphosphate diphosphohydrolase 8	0.0	1.6	0.78
LOC100548257	myeloperoxidase-like	0.0	1.6	0.78
LOC104909526	uncharacterized LOC104909526	0.0	1.6	0.78
LOC104909535	uncharacterized LOC104909535	0.0	1.6	0.78
LOC104909542	uncharacterized LOC104909542	0.0	1.6	0.78
LOC104909639	uncharacterized LOC104909639	0.0	1.6	0.78
LOC104909991	uncharacterized LOC104909991	0.0	1.6	0.78
LOC104910092	uncharacterized LOC104910092	0.0	1.6	0.78
LOC104910211	gamma-aminobutyric acid type B receptor subunit 2-like	0.0	1.6	0.78
LOC104910365	uncharacterized LOC104910365	0.0	1.6	0.78
LOC104910404	protein CBFA2T1-like	0.0	1.6	0.78
LOC104910498	uncharacterized LOC104910498	0.0	1.6	0.78
LOC104910632	uncharacterized LOC104910632	0.0	1.6	0.78
LOC104910651	uncharacterized LOC104910651	0.0	1.6	0.78
LOC104910692	teneurin-3-like	0.0	1.6	0.78

LOC104911043	ATP-binding cassette sub-family C member 8-like	0.0	1.6	0.78
LOC104911170	uncharacterized LOC104911170	0.0	1.6	0.78
LOC104911329	uncharacterized LOC104911329	0.0	1.6	0.78
LOC104911449	nebulette-like	0.0	1.6	0.78
LOC104911494	dynein heavy chain 11, axonemal-like	0.0	1.6	0.78
LOC104911734	uncharacterized LOC104911734	0.0	1.6	0.78
LOC104911897	uncharacterized LOC104911897	0.0	1.6	0.78
LOC104912278	uncharacterized LOC104912278	0.0	1.6	0.78
LOC104912621	rho guanine nucleotide exchange factor 4-like	0.0	1.6	0.78
LOC104912863	uncharacterized LOC104912863	0.0	1.6	0.78
LOC104912938	uncharacterized LOC104912938	0.0	1.6	0.78
LOC104913002	cadherin-8-like	0.0	1.6	0.78
LOC104913023	uncharacterized LOC104913023	0.0	1.6	0.78
LOC104913560	solute carrier family 2, facilitated glucose transporter member 11-like	0.0	1.6	0.78
LOC104913783	uncharacterized LOC104913783	0.0	1.6	0.78
LOC104914167	agrin-like	0.0	1.6	0.78
LOC104914373	uncharacterized LOC104914373	0.0	1.6	0.78
LOC104914894	interleukin-6 receptor subunit beta-like	0.0	1.6	0.78
LOC104914941	uncharacterized LOC104914941	0.0	1.6	0.78
LOC104914952	E3 ubiquitin-protein ligase Topors-like	0.0	1.6	0.78
LOC104915219	cyclin-A1-like	0.0	1.6	0.78
LOC104915695	uncharacterized LOC104915695	0.0	1.6	0.78
LOC104916209	kallikrein-14-like	0.0	1.6	0.78
LOC104916615	protein MANBAL-like	0.0	1.6	0.78
LOC104916648	histone H2B-like	0.0	1.6	0.78
LOC104917279	fibrinogen-like protein A	0.0	1.6	0.78
LOC104917395	uncharacterized LOC104917395	0.0	1.6	0.78
LOC104917543	uncharacterized LOC104917543	0.0	1.6	0.78
LUZP2	leucine zipper protein 2	0.0	1.6	0.78
MC4R	melanocortin 4 receptor	0.0	1.6	0.78
NME4	NME/NM23 nucleoside diphosphate kinase 4	0.0	1.6	0.78
NOX4	NADPH oxidase 4	0.0	1.6	0.78
NR2E3	nuclear receptor subfamily 2, group E, member 3	0.0	1.6	0.78
P2RY8	purinergic receptor P2Y, G-protein coupled, 8	0.0	1.6	0.78
PCDH7	protocadherin 7	0.0	1.6	0.78
PCDH9	protocadherin 9	0.0	1.6	0.78
PLIN1	perilipin 1	0.0	1.6	0.78
RFX4	regulatory factor X, 4 (influences HLA class II expression)	0.0	1.6	0.78
TMEM240	transmembrane protein 240	0.0	1.6	0.78
TNFRSF18	tumor necrosis factor receptor superfamily, member 18	0.0	1.6	0.78
TNFRSF25	tumor necrosis factor receptor superfamily, member 25	0.0	1.6	0.78
TNIP3	TNFAIP3 interacting protein 3	0.0	1.6	0.78
TRPC6	transient receptor potential cation channel, subfamily C, member 6	0.0	1.6	0.78
ADCY3	adenylate cyclase 3	0.0	1.5	0.76
ASIC2	acid-sensing (proton-gated) ion channel 2	0.0	1.5	0.76
BCL2L15	BCL2-like 15	0.0	1.5	0.76
C12H15orf59	chromosome 12 open reading frame, human C15orf59	0.0	1.5	0.76
C17H11orf85	chromosome 17 open reading frame, human C11orf85	0.0	1.5	0.76
CCR9	chemokine (C-C motif) receptor 9	0.0	1.5	0.76
CD163	CD163 molecule	0.0	1.5	0.76
CD8B	CD8b molecule	0.0	1.5	0.76
CZH9orf84	chromosome Z open reading frame, human C9orf84	0.0	1.5	0.76
DLX1	distal-less homeobox 1	0.0	1.5	0.76
FLT4	fms-related tyrosine kinase 4	0.0	1.5	0.76
GPR149	G protein-coupled receptor 149	0.0	1.5	0.76
ITGB4	integrin, beta 4	0.0	1.5	0.76

KCNIP2	Kv channel interacting protein 2	0.0	1.5	0.76
KCNJ15	potassium inwardly-rectifying channel, subfamily J, member 15	0.0	1.5	0.76
LOC100538436	proto-oncogene Mas-like	0.0	1.5	0.76
LOC100541890	transient receptor potential cation channel subfamily M member 3-like	0.0	1.5	0.76
LOC100543510	uncharacterized LOC100543510	0.0	1.5	0.76
LOC100545420	cytochrome P450 1B1 pseudogene	0.0	1.5	0.76
LOC100547434	leucine-rich repeat-containing protein 74B-like	0.0	1.5	0.76
LOC100551109	rap guanine nucleotide exchange factor 4	0.0	1.5	0.76
LOC104909379	pannexin-1-like	0.0	1.5	0.76
LOC104909772	uncharacterized LOC104909772	0.0	1.5	0.76
LOC104910381	uncharacterized LOC104910381	0.0	1.5	0.76
LOC104910540	uncharacterized LOC104910540	0.0	1.5	0.76
LOC104910587	electrogenic sodium bicarbonate cotransporter 1-like	0.0	1.5	0.76
LOC104911112	uncharacterized LOC104911112	0.0	1.5	0.76
LOC104911474	uncharacterized LOC104911474	0.0	1.5	0.76
LOC104911810	contactin-associated protein-like 5	0.0	1.5	0.76
LOC104912404	uncharacterized LOC104912404	0.0	1.5	0.76
LOC104912601	uncharacterized LOC104912601	0.0	1.5	0.76
LOC104912631	dedicator of cytokinesis protein 9-like	0.0	1.5	0.76
LOC104912857	uncharacterized LOC104912857	0.0	1.5	0.76
LOC104912906	ephrin type-A receptor 1-like	0.0	1.5	0.76
LOC104912935	uncharacterized LOC104912935	0.0	1.5	0.76
LOC104913231	uncharacterized LOC104913231	0.0	1.5	0.76
LOC104913255	uncharacterized LOC104913255	0.0	1.5	0.76
LOC104913294	uncharacterized LOC104913294	0.0	1.5	0.76
LOC104913922	lysophosphatidic acid receptor 1-B-like	0.0	1.5	0.76
LOC104913974	immunoglobulin superfamily member 1-like	0.0	1.5	0.76
LOC104913999	uncharacterized LOC104913999	0.0	1.5	0.76
LOC104914073	uncharacterized LOC104914073	0.0	1.5	0.76
LOC104914681	uncharacterized LOC104914681	0.0	1.5	0.76
LOC104914838	ran-binding protein 3-like	0.0	1.5	0.76
LOC104914929	A disintegrin and metalloproteinase with thrombospondin motifs 6-like	0.0	1.5	0.76
LOC104915205	uncharacterized LOC104915205	0.0	1.5	0.76
LOC104915415	olfactory receptor 14C36-like	0.0	1.5	0.76
LOC104915461	uncharacterized LOC104915461	0.0	1.5	0.76
LOC104915620	adenylate cyclase type 10-like	0.0	1.5	0.76
LOC104916662	uncharacterized LOC104916662	0.0	1.5	0.76
LPAR5	lysophosphatidic acid receptor 5	0.0	1.5	0.76
MIP	major intrinsic protein of lens fiber	0.0	1.5	0.76
OC90	otoconin 90	0.0	1.5	0.76
PACSIN1	protein kinase C and casein kinase substrate in neurons 1	0.0	1.5	0.76
S100A14	S100 calcium binding protein A14	0.0	1.5	0.76
SIGIRR	single immunoglobulin and toll-interleukin 1 receptor (TIR) domain	0.0	1.5	0.76
UCN3	urocortin 3	0.0	1.5	0.76
USH1G	Usher syndrome 1G (autosomal recessive)	0.0	1.5	0.76
WDR88	WD repeat domain 88	0.0	1.5	0.76
WNT2	wingless-type MMTV integration site family member 2	0.0	1.5	0.76
ADRA2C	adrenoceptor alpha 2C	0.8	0.0	0.42
AMER2	APC membrane recruitment protein 2	0.8	0.0	0.42
AMN	amnion associated transmembrane protein	0.8	0.0	0.42
ANKRD33B	ankyrin repeat domain 33B	0.8	0.0	0.42
APOBEC4	apolipoprotein B mRNA editing enzyme, catalytic polypeptide-like 4 (putative)	0.8	0.0	0.42
AVPR1B	arginine vasopressin receptor 1B	0.8	0.0	0.42
C10H1orf210	chromosome 10 open reading frame, human C1orf210	0.8	0.0	0.42

CCDC37	coiled-coil domain containing 37	0.8	0.0	0.42
CDH5	cadherin 5, type 2 (vascular endothelium)	0.8	0.0	0.42
CLDN18	claudin 18	0.8	0.0	0.42
CNTN6	contactin 6	0.8	0.0	0.42
DGKG	diacylglycerol kinase, gamma 90kDa	0.8	0.0	0.42
DNAJB8	DnaJ (Hsp40) homolog, subfamily B, member 8	0.8	0.0	0.42
DND1	DND microRNA-mediated repression inhibitor 1	0.8	0.0	0.42
EMID1	EMI domain containing 1	0.8	0.0	0.42
ENPEP	glutamyl aminopeptidase (aminopeptidase A)	0.8	0.0	0.42
F2RL3	coagulation factor II (thrombin) receptor-like 3	0.8	0.0	0.42
FAM166A	family with sequence similarity 166, member A	0.8	0.0	0.42
FTO	fat mass and obesity associated	0.8	0.0	0.42
GABRA6	gamma-aminobutyric acid (GABA) A receptor, alpha 6	0.8	0.0	0.42
GALR3	galanin receptor 3	0.8	0.0	0.42
GAS2L2	growth arrest-specific 2 like 2	0.8	0.0	0.42
GDF11	growth differentiation factor 11	0.8	0.0	0.42
GFRA4	GDNF family receptor alpha 4	0.8	0.0	0.42
GJB2	gap junction protein, beta 2, 26kDa	0.8	0.0	0.42
GPHB5	glycoprotein hormone beta 5	0.8	0.0	0.42
GPR143	G protein-coupled receptor 143	0.8	0.0	0.42
GRM2	glutamate receptor, metabotropic 2	0.8	0.0	0.42
IL12A	interleukin 12A	0.8	0.0	0.42
IL17B	interleukin 17B	0.8	0.0	0.42
JAKMIP3	Janus kinase and microtubule interacting protein 3	0.8	0.0	0.42
KCNG4	potassium voltage-gated channel, subfamily G, member 4	0.8	0.0	0.42
KERA	keratocan	0.8	0.0	0.42
KSR2	kinase suppressor of ras 2	0.8	0.0	0.42
LMO3	LIM domain only 3 (rhombotin-like 2)	0.8	0.0	0.42
LOC100538374	cathelicidin-2	0.8	0.0	0.42
LOC100538528	cathelicidin-B1-like	0.8	0.0	0.42
LOC100539194	collagen alpha-1(XXII) chain-like	0.8	0.0	0.42
LOC100539644	ceramide synthase 4-like	0.8	0.0	0.42
LOC100539757	olfactory receptor 1052-like	0.8	0.0	0.42
LOC100540083	forkhead box protein C2-like	0.8	0.0	0.42
LOC100540418	BPI fold-containing family C protein-like	0.8	0.0	0.42
LOC100540502	forkhead box protein J1-like	0.8	0.0	0.42
LOC100540904	cytochrome P450 26A1	0.8	0.0	0.42
LOC100541197	lymphocyte antigen 6E-like	0.8	0.0	0.42
LOC100541261	transient receptor potential cation channel subfamily M member 1-like	0.8	0.0	0.42
LOC100541369	interferon-induced guanylate-binding protein 1-like	0.8	0.0	0.42
LOC100541632	potassium voltage-gated channel subfamily B member 1	0.8	0.0	0.42
LOC100541995	inhibin beta E chain-like	0.8	0.0	0.42
LOC100542495	3 beta-hydroxysteroid dehydrogenase type 7-like	0.8	0.0	0.42
LOC100543277	transient receptor potential cation channel subfamily M member 6-like	0.8	0.0	0.42
LOC100543476	testis-specific serine/threonine-protein kinase 6-like	0.8	0.0	0.42
LOC100544385	serpin B10-like	0.8	0.0	0.42
LOC100544478	trypsin II-P29-like	0.8	0.0	0.42
LOC100544625	arylacetamide deacetylase-like 4	0.8	0.0	0.42
LOC100545365	toll-like receptor 2 type-1	0.8	0.0	0.42
LOC100545742	homeobox protein CDX-4-like	0.8	0.0	0.42
LOC100547030	cytochrome P450 2W1-like	0.8	0.0	0.42
LOC100547361	interleukin-17F-like	0.8	0.0	0.42
LOC100547603	acidic mammalian chitinase-like	0.8	0.0	0.42
LOC100547801	cationic amino acid transporter 2-like	0.8	0.0	0.42
LOC100548108	1-acyl-sn-glycerol-3-phosphate acyltransferase alpha-like	0.8	0.0	0.42
LOC100549042	regucalcin-like	0.8	0.0	0.42

LOC100549370	E-selectin-like	0.8	0.0	0.42
LOC100549507	single-minded homolog 1	0.8	0.0	0.42
LOC100550010	protocadherin beta-15-like	0.8	0.0	0.42
LOC100550136	interferon lambda-3	0.8	0.0	0.42
LOC100550333	thyroglobulin-like	0.8	0.0	0.42
LOC100550517	corticosteroid 11-beta-dehydrogenase isozyme 1-like	0.8	0.0	0.42
LOC100551424	rho-related GTP-binding protein RhoF-like	0.8	0.0	0.42
LOC104909300	RANBP2-like and GRIP domain-containing protein 1	0.8	0.0	0.42
LOC104909324	mitogen-activated protein kinase kinase kinase 15-like	0.8	0.0	0.42
LOC104909352	phosphatidylinositol 3-kinase regulatory subunit alpha-like	0.8	0.0	0.42
LOC104909374	uncharacterized LOC104909374	0.8	0.0	0.42
LOC104909381	melatonin receptor type 1B	0.8	0.0	0.42
LOC104909415	teneurin-4-like	0.8	0.0	0.42
LOC104909505	CD63 antigen-like	0.8	0.0	0.42
LOC104909545	uncharacterized LOC104909545	0.8	0.0	0.42
LOC104909553	carbohydrate sulfotransferase 8-like	0.8	0.0	0.42
LOC104909562	TFIIA-alpha and beta-like factor	0.8	0.0	0.42
LOC104909575	uncharacterized LOC104909575	0.8	0.0	0.42
LOC104909684	uncharacterized LOC104909684	0.8	0.0	0.42
LOC104909712	uncharacterized LOC104909712	0.8	0.0	0.42
LOC104909721	uncharacterized LOC104909721	0.8	0.0	0.42
LOC104909771	UPF0762 protein C6orf58 homolog	0.8	0.0	0.42
LOC104909800	maestro heat-like repeat-containing protein family member 2B	0.8	0.0	0.42
LOC104909868	kelch-like protein 32	0.8	0.0	0.42
LOC104909971	myelin transcription factor 1-like protein	0.8	0.0	0.42
LOC104910074	uncharacterized LOC104910074	0.8	0.0	0.42
LOC104910094	uncharacterized LOC104910094	0.8	0.0	0.42
LOC104910360	carboxypeptidase A6-like	0.8	0.0	0.42
LOC104910583	uncharacterized LOC104910583	0.8	0.0	0.42
LOC104910599	uncharacterized LOC104910599	0.8	0.0	0.42
LOC104910600	uncharacterized LOC104910600	0.8	0.0	0.42
LOC104910630	uncharacterized LOC104910630	0.8	0.0	0.42
LOC104910649	neuromedin-K receptor-like	0.8	0.0	0.42
LOC104910698	uncharacterized LOC104910698	0.8	0.0	0.42
LOC104910747	uncharacterized LOC104910747	0.8	0.0	0.42
LOC104910770	probable phospholipid-transporting ATPase VD	0.8	0.0	0.42
LOC104910812	NACHT, LRR and PYD domains-containing protein 1-like	0.8	0.0	0.42
LOC104910903	uncharacterized LOC104910903	0.8	0.0	0.42
LOC104911074	uncharacterized LOC104911074	0.8	0.0	0.42
LOC104911126	uncharacterized LOC104911126	0.8	0.0	0.42
LOC104911230	uncharacterized LOC104911230	0.8	0.0	0.42
LOC104911257	uncharacterized LOC104911257	0.8	0.0	0.42
LOC104911269	histone H3.3	0.8	0.0	0.42
LOC104911419	uncharacterized LOC104911419	0.8	0.0	0.42
LOC104911445	MAM and LDL-receptor class A domain-containing protein 1-like	0.8	0.0	0.42
LOC104911457	adseverin-like	0.8	0.0	0.42
LOC104911536	uncharacterized LOC104911536	0.8	0.0	0.42
LOC104911574	uncharacterized LOC104911574	0.8	0.0	0.42
LOC104911709	sodium channel protein type 2 subunit alpha-like	0.8	0.0	0.42
LOC104911771	potassium voltage-gated channel subfamily H member 7-like	0.8	0.0	0.42
LOC104911886	uncharacterized LOC104911886	0.8	0.0	0.42
LOC104912137	disks large homolog 3-like	0.8	0.0	0.42
LOC104912176	connector enhancer of kinase suppressor of ras 2-like	0.8	0.0	0.42
LOC104912189	uncharacterized LOC104912189	0.8	0.0	0.42
LOC104912293	uncharacterized LOC104912293	0.8	0.0	0.42
LOC104912366	uncharacterized LOC104912366	0.8	0.0	0.42
LOC104912397	uncharacterized LOC104912397	0.8	0.0	0.42
LOC104912426	uncharacterized LOC104912426	0.8	0.0	0.42

LOC104912560	uncharacterized LOC104912560	0.8	0.0	0.42
LOC104912680	uncharacterized LOC104912680	0.8	0.0	0.42
LOC104912694	uncharacterized LOC104912694	0.8	0.0	0.42
LOC104912709	uncharacterized LOC104912709	0.8	0.0	0.42
LOC104912712	uncharacterized LOC104912712	0.8	0.0	0.42
LOC104912829	uncharacterized LOC104912829	0.8	0.0	0.42
LOC104912910	uncharacterized LOC104912910	0.8	0.0	0.42
LOC104912914	uncharacterized LOC104912914	0.8	0.0	0.42
LOC104912955	uncharacterized LOC104912955	0.8	0.0	0.42
LOC104913193	uncharacterized LOC104913193	0.8	0.0	0.42
LOC104913266	uncharacterized LOC104913266	0.8	0.0	0.42
LOC104913637	uncharacterized LOC104913637	0.8	0.0	0.42
LOC104913684	uncharacterized LOC104913684	0.8	0.0	0.42
LOC104913799	uncharacterized LOC104913799	0.8	0.0	0.42
LOC104913846	uncharacterized LOC104913846	0.8	0.0	0.42
LOC104914133	uncharacterized LOC104914133	0.8	0.0	0.42
LOC104914355	complement receptor type 1-like	0.8	0.0	0.42
LOC104914384	laminin subunit gamma-2-like	0.8	0.0	0.42
LOC104914420	keratin-associated protein 10-4-like	0.8	0.0	0.42
LOC104914459	uncharacterized LOC104914459	0.8	0.0	0.42
LOC104914464	tetraspanin-18-like	0.8	0.0	0.42
LOC104914480	uncharacterized LOC104914480	0.8	0.0	0.42
LOC104914571	acid-sensing ion channel 2-like	0.8	0.0	0.42
LOC104914969	ankyrin repeat and death domain-containing protein 1B-like	0.8	0.0	0.42
LOC104914982	rho guanine nucleotide exchange factor 28-like	0.8	0.0	0.42
LOC104915177	uncharacterized LOC104915177	0.8	0.0	0.42
LOC104915393	polycystic kidney disease and receptor for egg jelly-related protein-like	0.8	0.0	0.42
LOC104915409	epiplakin-like	0.8	0.0	0.42
LOC104915507	protocadherin alpha-13-like	0.8	0.0	0.42
LOC104915593	uncharacterized LOC104915593	0.8	0.0	0.42
LOC104915669	probable ATP-dependent RNA helicase DDX4	0.8	0.0	0.42
LOC104915677	protocadherin alpha-5-like	0.8	0.0	0.42
LOC104915738	uncharacterized LOC104915738	0.8	0.0	0.42
LOC104915843	uncharacterized LOC104915843	0.8	0.0	0.42
LOC104915874	uncharacterized LOC104915874	0.8	0.0	0.42
LOC104916352	ephrin type-B receptor 4-like	0.8	0.0	0.42
LOC104916417	uncharacterized LOC104916417	0.8	0.0	0.42
LOC104916458	dachshund homolog 1-like	0.8	0.0	0.42
LOC104916491	glutamate receptor ionotropic, NMDA 3A-like	0.8	0.0	0.42
LOC104916529	uncharacterized LOC104916529	0.8	0.0	0.42
LOC104916622	maestro heat-like repeat-containing protein family member 7	0.8	0.0	0.42
LOC104916626	pyruvate dehydrogenase (acetyl-transferring) kinase isozyme 1, mitochondrial-like	0.8	0.0	0.42
LOC104916627	3-keto-steroid reductase-like	0.8	0.0	0.42
LOC104916633	estradiol 17-beta-dehydrogenase 2-like	0.8	0.0	0.42
LOC104916665	histone H3-like	0.8	0.0	0.42
LOC104916666	histone H3-like	0.8	0.0	0.42
LOC104916681	spectrin beta chain, non-erythrocytic 4-like	0.8	0.0	0.42
LOC104916904	maestro heat-like repeat-containing protein family member 2B	0.8	0.0	0.42
LOC104916917	tetratricopeptide repeat protein 24-like	0.8	0.0	0.42
LOC104916923	uncharacterized LOC104916923	0.8	0.0	0.42
LOC104916969	uncharacterized LOC104916969	0.8	0.0	0.42
LOC104917008	sialoadhesin-like	0.8	0.0	0.42
LOC104917016	dematin-like	0.8	0.0	0.42
LOC104917048	rho GTPase-activating protein 27-like	0.8	0.0	0.42
LOC104917052	protein patched homolog 1-like	0.8	0.0	0.42
LOC104917076	uncharacterized LOC104917076	0.8	0.0	0.42

LOC104917119	uncharacterized LOC104917119	0.8	0.0	0.42
LOC104917122	IgGFc-binding protein-like	0.8	0.0	0.42
LOC104917176	uncharacterized LOC104917176	0.8	0.0	0.42
LOC104917423	sodium leak channel non-selective protein-like	0.8	0.0	0.42
LOC104917490	uncharacterized LOC104917490	0.8	0.0	0.42
LOC104917573	uncharacterized LOC104917573	0.8	0.0	0.42
LOC104917610	uncharacterized LOC104917610	0.8	0.0	0.42
LRRC55	leucine rich repeat containing 55	0.8	0.0	0.42
LRRC6	leucine rich repeat containing 6	0.8	0.0	0.42
LRTM2	leucine-rich repeats and transmembrane domains 2	0.8	0.0	0.42
MGAT5B	mannosyl (alpha-1,6-)-glycoprotein beta-1,6-N-acetyl-glucosaminyltransferase, isozyme B	0.8	0.0	0.42
MISP	mitotic spindle positioning	0.8	0.0	0.42
MMEL1	membrane metallo-endopeptidase-like 1	0.8	0.0	0.42
NETO1	neuropilin (NRP) and tolloid (TLL)-like 1	0.8	0.0	0.42
NGEF	neuronal guanine nucleotide exchange factor	0.8	0.0	0.42
NOS1	nitric oxide synthase 1 (neuronal)	0.8	0.0	0.42
NR0B1	nuclear receptor subfamily 0, group B, member 1	0.8	0.0	0.42
NR1H4	nuclear receptor subfamily 1, group H, member 4	0.8	0.0	0.42
PAX5	paired box 5	0.8	0.0	0.42
PEAR1	platelet endothelial aggregation receptor 1	0.8	0.0	0.42
PFN3	profilin 3	0.8	0.0	0.42
PHACTR1	phosphatase and actin regulator 1	0.8	0.0	0.42
PNP	purine nucleoside phosphorylase	0.8	0.0	0.42
PNPLA1	patatin-like phospholipase domain containing 1	0.8	0.0	0.42
POU3F2	POU class 3 homeobox 2	0.8	0.0	0.42
REPS2	RALBP1 associated Eps domain containing 2	0.8	0.0	0.42
RIMS3	regulating synaptic membrane exocytosis 3	0.8	0.0	0.42
SFRP1	secreted frizzled-related protein 1	0.8	0.0	0.42
SHANK2	SH3 and multiple ankyrin repeat domains 2	0.8	0.0	0.42
SLC18A2	solute carrier family 18 (vesicular monoamine transporter), member 2	0.8	0.0	0.42
SLC38A11	solute carrier family 38, member 11	0.8	0.0	0.42
SLC6A20	solute carrier family 6 (proline IMINO transporter), member 20	0.8	0.0	0.42
SLC7A9	solute carrier family 7 (amino acid transporter light chain, bo,+ system), member 9	0.8	0.0	0.42
SNAP25	synaptosomal-associated protein, 25kDa	0.8	0.0	0.42
SOWAHA	sosondowah ankyrin repeat domain family member A	0.8	0.0	0.42
SOX14	SRY (sex determining region Y)-box 14	0.8	0.0	0.42
SRD5A2	steroid-5-alpha-reductase, alpha polypeptide 2 (3-oxo-5 alpha-steroid delta 4-dehydrogenase alpha 2)	0.8	0.0	0.42
SRMS	src-related kinase lacking C-terminal regulatory tyrosine and N-terminal myristylation sites	0.8	0.0	0.42
SYPL1	synaptophysin-like 1	0.8	0.0	0.42
TBX2	T-box 2	0.8	0.0	0.42
TH	tyrosine hydroxylase	0.8	0.0	0.42
TLX1	T-cell leukemia homeobox 1	0.8	0.0	0.42
TMEM139	transmembrane protein 139	0.8	0.0	0.42
TMEM27	transmembrane protein 27	0.8	0.0	0.42
TPL	pancreatic lipase	0.8	0.0	0.42
VWF	von Willebrand factor	0.8	0.0	0.42
ZNF804A	zinc finger protein 804A	0.8	0.0	0.42
ADGB	androglobin	0.8	0.0	0.42
ALDH1A1	aldehyde dehydrogenase 1 family, member A1	0.8	0.0	0.42
ANGPT1	angiopoietin 1	0.8	0.0	0.42
ATCAY	ataxia, cerebellar, Cayman type	0.8	0.0	0.42
BLNK	B-cell linker	0.8	0.0	0.42
C1QTNF5	C1q and tumor necrosis factor related protein 5	0.8	0.0	0.42

C26H11orf1	chromosome 26 open reading frame, human C11orf1	0.8	0.0	0.42
C3AR1	complement component 3a receptor 1	0.8	0.0	0.42
C5H14orf132	chromosome 5 open reading frame, human C14orf132	0.8	0.0	0.42
CARD10	caspase recruitment domain family, member 10	0.8	0.0	0.42
CPO	carboxypeptidase O	0.8	0.0	0.42
CTRL	chymotrypsin-like	0.8	0.0	0.42
DRGX	dorsal root ganglia homeobox	0.8	0.0	0.42
DSCAM	Down syndrome cell adhesion molecule	0.8	0.0	0.42
DTX1	deltex 1, E3 ubiquitin ligase	0.8	0.0	0.42
EMB	embigin	0.8	0.0	0.42
FAM19A4	family with sequence similarity 19 (chemokine (C-C motif)-like), member A4	0.8	0.0	0.42
FER1L6	fer-1-like family member 6	0.8	0.0	0.42
FGF8	fibroblast growth factor 8 (androgen-induced)	0.8	0.0	0.42
FMNL1	formin-like 1	0.8	0.0	0.42
GABRG1	gamma-aminobutyric acid (GABA) A receptor, gamma 1	0.8	0.0	0.42
GABRG3	gamma-aminobutyric acid (GABA) A receptor, gamma 3	0.8	0.0	0.42
GAP43	growth associated protein 43	0.8	0.0	0.42
GBX1	gastrulation brain homeobox 1	0.8	0.0	0.42
GLRA4	glycine receptor, alpha 4	0.8	0.0	0.42
GPR132	G protein-coupled receptor 132	0.8	0.0	0.42
GPR19	G protein-coupled receptor 19	0.8	0.0	0.42
GPR65	G protein-coupled receptor 65	0.8	0.0	0.42
HOXD4	homeobox D4	0.8	0.0	0.42
HS3ST1	heparan sulfate (glucosamine) 3-O-sulfotransferase 1	0.8	0.0	0.42
IGLL1	immunoglobulin lambda-like polypeptide 1	0.8	0.0	0.42
INHBC	inhibin, beta C	0.8	0.0	0.42
KCNA2	potassium voltage-gated channel, shaker-related subfamily, member 2	0.8	0.0	0.42
KCNK9	potassium channel, subfamily K, member 9	0.8	0.0	0.42
KHK	ketohexokinase (fructokinase)	0.8	0.0	0.42
KIAA1024	KIAA1024 ortholog	0.8	0.0	0.42
KRT18	keratin 18	0.8	0.0	0.42
LOC100539227	1-acyl-sn-glycerol-3-phosphate acyltransferase alpha-like	0.8	0.0	0.42
LOC100540476	tau-tubulin kinase 1-like	0.8	0.0	0.42
LOC100540586	brain-specific angiogenesis inhibitor 1	0.8	0.0	0.42
LOC100540701	sodium/hydrogen exchanger 2-like	0.8	0.0	0.42
LOC100540786	fibrinogen-like protein 1	0.8	0.0	0.42
LOC100541086	poly [ADP-ribose] polymerase 11-like	0.8	0.0	0.42
LOC100541557	amyloid beta A4 precursor protein-binding family B member 1-interacting protein	0.8	0.0	0.42
LOC100541871	coagulation factor XIII B chain-like	0.8	0.0	0.42
LOC100542159	guanylate cyclase soluble subunit alpha-2	0.8	0.0	0.42
LOC100543157	uncharacterized LOC100543157	0.8	0.0	0.42
LOC100544022	PGAP2-interacting protein-like	0.8	0.0	0.42
LOC100544636	uncharacterized LOC100544636	0.8	0.0	0.42
LOC100544802	adenylate kinase 9	0.8	0.0	0.42
LOC100545106	rho guanine nucleotide exchange factor 38-like	0.8	0.0	0.42
LOC100545251	sulfotransferase 1C1-like	0.8	0.0	0.42
LOC100546434	dedicator of cytokinesis protein 8-like	0.8	0.0	0.42
LOC100546811	uncharacterized LOC100546811	0.8	0.0	0.42
LOC100546895	transmembrane 4 L6 family member 1-like	0.8	0.0	0.42
LOC100547860	fibulin-7-like	0.8	0.0	0.42
LOC100548920	tektin-5	0.8	0.0	0.42
LOC100548930	GTP-binding protein Di-Ras2-like	0.8	0.0	0.42
LOC100550174	kelch-like protein 1	0.8	0.0	0.42
LOC100550270	uncharacterized LOC100550270	0.8	0.0	0.42
LOC100550830	twist-related protein 2-like	0.8	0.0	0.42

LOC100550877	uncharacterized LOC100550877	0.8	0.0	0.42
LOC104909316	low-density lipoprotein receptor-related protein 1B-like	0.8	0.0	0.42
LOC104909422	metabotropic glutamate receptor 5-like	0.8	0.0	0.42
LOC104909512	uncharacterized LOC104909512	0.8	0.0	0.42
LOC104909529	uncharacterized LOC104909529	0.8	0.0	0.42
LOC104909533	estradiol 17-beta-dehydrogenase 2-like	0.8	0.0	0.42
LOC104909534	potassium voltage-gated channel subfamily D member 2	0.8	0.0	0.42
LOC104909592	uncharacterized LOC104909592	0.8	0.0	0.42
LOC104909679	uncharacterized LOC104909679	0.8	0.0	0.42
LOC104909874	single-minded homolog 1-like	0.8	0.0	0.42
LOC104909940	uncharacterized LOC104909940	0.8	0.0	0.42
LOC104909951	uncharacterized LOC104909951	0.8	0.0	0.42
LOC104909974	uncharacterized LOC104909974	0.8	0.0	0.42
LOC104910005	uncharacterized LOC104910005	0.8	0.0	0.42
LOC104910073	uncharacterized LOC104910073	0.8	0.0	0.42
LOC104910125	uncharacterized LOC104910125	0.8	0.0	0.42
LOC104910166	uncharacterized LOC104910166	0.8	0.0	0.42
LOC104910207	dynein heavy chain 8, axonemal-like	0.8	0.0	0.42
LOC104910285	cadherin-7	0.8	0.0	0.42
LOC104910289	uncharacterized LOC104910289	0.8	0.0	0.42
LOC104910377	uncharacterized LOC104910377	0.8	0.0	0.42
LOC104910522	uncharacterized LOC104910522	0.8	0.0	0.42
LOC104910578	extracellular matrix protein FRAS1-like	0.8	0.0	0.42
LOC104910662	uncharacterized LOC104910662	0.8	0.0	0.42
LOC104910683	cGMP-dependent protein kinase 2-like	0.8	0.0	0.42
LOC104910697	uncharacterized LOC104910697	0.8	0.0	0.42
LOC104910703	uncharacterized LOC104910703	0.8	0.0	0.42
LOC104910704	uncharacterized LOC104910704	0.8	0.0	0.42
LOC104910722	uncharacterized LOC104910722	0.8	0.0	0.42
LOC104910801	uncharacterized LOC104910801	0.8	0.0	0.42
LOC104910839	uncharacterized LOC104910839	0.8	0.0	0.42
LOC104910965	uncharacterized LOC104910965	0.8	0.0	0.42
LOC104910973	uncharacterized LOC104910973	0.8	0.0	0.42
LOC104911153	uncharacterized LOC104911153	0.8	0.0	0.42
LOC104911275	uncharacterized LOC104911275	0.8	0.0	0.42
LOC104911350	uncharacterized LOC104911350	0.8	0.0	0.42
LOC104911379	uncharacterized LOC104911379	0.8	0.0	0.42
LOC104911388	uncharacterized LOC104911388	0.8	0.0	0.42
LOC104911391	lovastatin diketide synthase LovF-like	0.8	0.0	0.42
LOC104911448	uncharacterized LOC104911448	0.8	0.0	0.42
LOC104911490	uncharacterized LOC104911490	0.8	0.0	0.42
LOC104911530	uncharacterized LOC104911530	0.8	0.0	0.42
LOC104911532	uncharacterized LOC104911532	0.8	0.0	0.42
LOC104911744	uncharacterized LOC104911744	0.8	0.0	0.42
LOC104911757	desert hedgehog protein-like	0.8	0.0	0.42
LOC104911786	uncharacterized LOC104911786	0.8	0.0	0.42
LOC104911793	uncharacterized LOC104911793	0.8	0.0	0.42
LOC104911820	uncharacterized LOC104911820	0.8	0.0	0.42
LOC104911905	uncharacterized LOC104911905	0.8	0.0	0.42
LOC104911928	DNA replication ATP-dependent helicase/nuclease DNA2-like	0.8	0.0	0.42
LOC104911972	uncharacterized LOC104911972	0.8	0.0	0.42
LOC104912051	phosphatidylinositol 4-phosphate 3-kinase C2 domain-containing subunit gamma-like	0.8	0.0	0.42
LOC104912194	uncharacterized LOC104912194	0.8	0.0	0.42
LOC104912196	BTB/POZ domain-containing protein KCTD16-like	0.8	0.0	0.42
LOC104912267	uncharacterized LOC104912267	0.8	0.0	0.42
LOC104912294	uncharacterized LOC104912294	0.8	0.0	0.42
LOC104912298	uncharacterized LOC104912298	0.8	0.0	0.42

LOC104912307	uncharacterized LOC104912307	0.8	0.0	0.42
LOC104912453	uncharacterized LOC104912453	0.8	0.0	0.42
LOC104912457	uncharacterized LOC104912457	0.8	0.0	0.42
LOC104912563	uncharacterized LOC104912563	0.8	0.0	0.42
LOC104912577	uncharacterized LOC104912577	0.8	0.0	0.42
LOC104912686	AF4/FMR2 family member 3-like	0.8	0.0	0.42
LOC104912872	uncharacterized LOC104912872	0.8	0.0	0.42
LOC104912949	uncharacterized LOC104912949	0.8	0.0	0.42
LOC104912953	adenylate cyclase type 7-like	0.8	0.0	0.42
LOC104913089	uncharacterized LOC104913089	0.8	0.0	0.42
LOC104913159	uncharacterized LOC104913159	0.8	0.0	0.42
LOC104913201	uncharacterized LOC104913201	0.8	0.0	0.42
LOC104913346	uncharacterized LOC104913346	0.8	0.0	0.42
LOC104913381	putative short-chain dehydrogenase/reductase family 42E member 2	0.8	0.0	0.42
LOC104913392	interleukin-9 receptor-like	0.8	0.0	0.42
LOC104913488	caskin-1-like	0.8	0.0	0.42
LOC104913526	uncharacterized LOC104913526	0.8	0.0	0.42
LOC104913681	OX-2 membrane glycoprotein-like	0.8	0.0	0.42
LOC104913706	uncharacterized LOC104913706	0.8	0.0	0.42
LOC104913952	endogenous retrovirus group K member 25 Pol protein-like	0.8	0.0	0.42
LOC104913990	uncharacterized LOC104913990	0.8	0.0	0.42
LOC104914152	gamma-aminobutyric acid receptor subunit delta	0.8	0.0	0.42
LOC104914211	phospholipase A2, membrane associated-like	0.8	0.0	0.42
LOC104914488	uncharacterized LOC104914488	0.8	0.0	0.42
LOC104914500	uncharacterized LOC104914500	0.8	0.0	0.42
LOC104914580	GTPase IMAP family member 7-like	0.8	0.0	0.42
LOC104914878	integrin alpha-1-like	0.8	0.0	0.42
LOC104915014	uncharacterized LOC104915014	0.8	0.0	0.42
LOC104915046	interferon kappa-like	0.8	0.0	0.42
LOC104915100	FERM domain-containing protein 3-like	0.8	0.0	0.42
LOC104915331	connector enhancer of kinase suppressor of ras 2-like	0.8	0.0	0.42
LOC104915360	uncharacterized LOC104915360	0.8	0.0	0.42
LOC104915402	uncharacterized LOC104915402	0.8	0.0	0.42
LOC104915404	epiplakin-like	0.8	0.0	0.42
LOC104915638	putative uncharacterized protein ENSP00000383309	0.8	0.0	0.42
LOC104915643	uncharacterized LOC104915643	0.8	0.0	0.42
LOC104915673	membrane-associated guanylate kinase, WW and PDZ domain-containing protein 2-like	0.8	0.0	0.42
LOC104915771	alpha-aminoadipic semialdehyde dehydrogenase-like	0.8	0.0	0.42
LOC104915884	uncharacterized LOC104915884	0.8	0.0	0.42
LOC104916069	neuronal PAS domain-containing protein 4-like	0.8	0.0	0.42
LOC104916142	uncharacterized LOC104916142	0.8	0.0	0.42
LOC104916282	1,25-dihydroxyvitamin D(3) 24-hydroxylase, mitochondrial-like	0.8	0.0	0.42
LOC104916312	forkhead box protein C2-like	0.8	0.0	0.42
LOC104916360	protocadherin-10-like	0.8	0.0	0.42
LOC104916557	PHD finger protein 7-like	0.8	0.0	0.42
LOC104916674	outer dense fiber protein 3-like	0.8	0.0	0.42
LOC104916738	E3 ubiquitin-protein ligase TRIM36-like	0.8	0.0	0.42
LOC104916809	uncharacterized LOC104916809	0.8	0.0	0.42
LOC104916985	uncharacterized LOC104916985	0.8	0.0	0.42
LOC104917115	protein piccolo-like	0.8	0.0	0.42
LOC104917213	leukocyte immunoglobulin-like receptor subfamily A member 6	0.8	0.0	0.42
LOC104917522	epithelial-stromal interaction protein 1-like	0.8	0.0	0.42
LOC104917563	uncharacterized LOC104917563	0.8	0.0	0.42
LOC104917592	uncharacterized LOC104917592	0.8	0.0	0.42
LRRIQ1	leucine-rich repeats and IQ motif containing 1	0.8	0.0	0.42
MARCO	macrophage receptor with collagenous structure	0.8	0.0	0.42

MCOLN3	mucolipin 3	0.8	0.0	0.42
MKX	mohawk homeobox	0.8	0.0	0.42
MMRN2	multimerin 2	0.8	0.0	0.42
MOB3B	MOB kinase activator 3B	0.8	0.0	0.42
MUC6	mucin 6, oligomeric mucus/gel-forming	0.8	0.0	0.42
NLRC3	NLR family, CARD domain containing 3	0.8	0.0	0.42
NPBWR1	neuropeptides B/W receptor 1	0.8	0.0	0.42
OLFM4	olfactomedin 4	0.8	0.0	0.42
OSTN	osteocrin	0.8	0.0	0.42
OTOS	otospiralin	0.8	0.0	0.42
P2RY12	purinergic receptor P2Y, G-protein coupled, 12	0.8	0.0	0.42
PAK6	p21 protein (Cdc42/Rac)-activated kinase 6	0.8	0.0	0.42
PAPOLG	poly(A) polymerase gamma	0.8	0.0	0.42
PLXNB1	plexin B1	0.8	0.0	0.42
PMCH	pro-melanin-concentrating hormone	0.8	0.0	0.42
POU6F2	POU class 6 homeobox 2	0.8	0.0	0.42
RAB37	RAB37, member RAS oncogene family	0.8	0.0	0.42
RUFY4	RUN and FYVE domain containing 4	0.8	0.0	0.42
SAMSN1	SAM domain, SH3 domain and nuclear localization signals 1	0.8	0.0	0.42
SHH	sonic hedgehog	0.8	0.0	0.42
SHISA9	shisa family member 9	0.8	0.0	0.42
SLC13A1	solute carrier family 13 (sodium/sulfate symporter), member 1	0.8	0.0	0.42
SLC4A9	solute carrier family 4, sodium bicarbonate cotransporter, member 9	0.8	0.0	0.42
TDRD5	tudor domain containing 5	0.8	0.0	0.42
TEKT3	tektin 3	0.8	0.0	0.42
WFDC3	WAP four-disulfide core domain 3	0.8	0.0	0.42
WNT9A	wingless-type MMTV integration site family, member 9A	0.8	0.0	0.42
XDH	xanthine dehydrogenase	0.8	0.0	0.42
AHSG	alpha-2-HS-glycoprotein	0.0	0.8	0.40
ANKRD22	ankyrin repeat domain 22	0.0	0.8	0.40
C12H15orf65	chromosome 12 open reading frame, human C15orf65	0.0	0.8	0.40
C30H19orf71	chromosome 30 open reading frame, human C19orf71	0.0	0.8	0.40
CBLN4	cerebellin 4 precursor	0.0	0.8	0.40
CCL19	chemokine (C-C motif) ligand 19	0.0	0.8	0.40
CCR5	chemokine (C-C motif) receptor 5	0.0	0.8	0.40
CD9	CD9 molecule	0.0	0.8	0.40
CDCP2	CUB domain containing protein 2	0.0	0.8	0.40
CFAP58	cilia and flagella associated protein 58	0.0	0.8	0.40
CHRNA10	cholinergic receptor, nicotinic, alpha 10 (neuronal)	0.0	0.8	0.40
CHRNA9	cholinergic receptor, nicotinic, alpha 9 (neuronal)	0.0	0.8	0.40
COLCA2	colorectal cancer associated 2	0.0	0.8	0.40
CXCL14	chemokine (C-X-C motif) ligand 14	0.0	0.8	0.40
DAW1	dynein assembly factor with WDR repeat domains 1	0.0	0.8	0.40
DNTT	DNA nucleotidylexotransferase	0.0	0.8	0.40
ECSCR	endothelial cell surface expressed chemotaxis and apoptosis regulator	0.0	0.8	0.40
ELOVL4	ELOVL fatty acid elongase 4	0.0	0.8	0.40
ESPN	espin	0.0	0.8	0.40
ESPNL	espin-like	0.0	0.8	0.40
FGFBP1	fibroblast growth factor binding protein 1	0.0	0.8	0.40
GJB5	gap junction protein, beta 5, 31.1kDa	0.0	0.8	0.40
GLRA2	glycine receptor, alpha 2	0.0	0.8	0.40
GLRB	glycine receptor, beta	0.0	0.8	0.40
GPA33	glycoprotein A33 (transmembrane)	0.0	0.8	0.40
GPR6	G protein-coupled receptor 6	0.0	0.8	0.40
GSG1L	GSG1-like	0.0	0.8	0.40
IQSEC3	IQ motif and Sec7 domain 3	0.0	0.8	0.40

KAL1	Kallmann syndrome 1 sequence	0.0	0.8	0.40
KCNC4	potassium voltage-gated channel, Shaw-related subfamily, member 4	0.0	0.8	0.40
KCNF1	potassium voltage-gated channel, subfamily F, member 1	0.0	0.8	0.40
KCNG3	potassium voltage-gated channel, subfamily G, member 3	0.0	0.8	0.40
KCTD4	potassium channel tetramerization domain containing 4	0.0	0.8	0.40
KRT8	keratin 8	0.0	0.8	0.40
LOC100538407	guanylyl cyclase-activating protein 1-like	0.0	0.8	0.40
LOC100539108	galanin receptor type 2-like	0.0	0.8	0.40
LOC100539174	cornulin-like	0.0	0.8	0.40
LOC100539666	G protein-coupled receptor kinase 5-like	0.0	0.8	0.40
LOC100539913	leucine zipper protein 2-like	0.0	0.8	0.40
LOC100540170	astacin-like metalloendopeptidase	0.0	0.8	0.40
LOC100540203	histone H2B 1/2/3/4/6-like	0.0	0.8	0.40
LOC100540643	diacylglycerol O-acyltransferase 2-like	0.0	0.8	0.40
LOC100540823	retinoic acid-induced protein 3-like	0.0	0.8	0.40
LOC100541395	thyroid hormone-inducible hepatic protein-like	0.0	0.8	0.40
LOC100543544	excitatory amino acid transporter 1	0.0	0.8	0.40
LOC100543646	parathyroid hormone/parathyroid hormone-related peptide receptor-like	0.0	0.8	0.40
LOC100543930	uncharacterized LOC100543930	0.0	0.8	0.40
LOC100544866	alpha-1-antiproteinase-like	0.0	0.8	0.40
LOC100545228	follistatin-related protein 4-like	0.0	0.8	0.40
LOC100545609	mucin-16-like	0.0	0.8	0.40
LOC100545931	leukocyte elastase inhibitor-like	0.0	0.8	0.40
LOC100546606	probable cation-transporting ATPase 13A4	0.0	0.8	0.40
LOC100546609	A disintegrin and metalloproteinase with thrombospondin motifs 18	0.0	0.8	0.40
LOC100548577	noggin-1-like	0.0	0.8	0.40
LOC100548837	aldose reductase-like	0.0	0.8	0.40
LOC100550169	putative maltase-glucoamylase-like protein FLJ16351	0.0	0.8	0.40
LOC100550364	corticosteroid 11-beta-dehydrogenase isozyme 1-like	0.0	0.8	0.40
LOC100551284	receptor-type tyrosine-protein phosphatase F-like	0.0	0.8	0.40
LOC104909229	nuclear receptor subfamily 5 group A member 2-like	0.0	0.8	0.40
LOC104909259	protein decapentaplegic-like	0.0	0.8	0.40
LOC104909380	uncharacterized LOC104909380	0.0	0.8	0.40
LOC104909541	uncharacterized LOC104909541	0.0	0.8	0.40
LOC104909593	uncharacterized LOC104909593	0.0	0.8	0.40
LOC104909662	WD repeat-containing protein 64-like	0.0	0.8	0.40
LOC104909715	uncharacterized LOC104909715	0.0	0.8	0.40
LOC104909864	PR domain zinc finger protein 1-like	0.0	0.8	0.40
LOC104909958	CUB and sushi domain-containing protein 1-like	0.0	0.8	0.40
LOC104909992	uncharacterized LOC104909992	0.0	0.8	0.40
LOC104910089	uncharacterized LOC104910089	0.0	0.8	0.40
LOC104910244	uncharacterized LOC104910244	0.0	0.8	0.40
LOC104910347	uncharacterized LOC104910347	0.0	0.8	0.40
LOC104910370	transient receptor potential cation channel subfamily A member 1-like	0.0	0.8	0.40
LOC104910375	uncharacterized LOC104910375	0.0	0.8	0.40
LOC104910416	uncharacterized LOC104910416	0.0	0.8	0.40
LOC104910536	uncharacterized LOC104910536	0.0	0.8	0.40
LOC104910685	uncharacterized LOC104910685	0.0	0.8	0.40
LOC104910720	uncharacterized LOC104910720	0.0	0.8	0.40
LOC104910776	PGAP2-interacting protein-like	0.0	0.8	0.40
LOC104910803	uncharacterized LOC104910803	0.0	0.8	0.40
LOC104910806	uncharacterized LOC104910806	0.0	0.8	0.40
LOC104910914	catenin alpha-2-like	0.0	0.8	0.40
LOC104910927	uncharacterized LOC104910927	0.0	0.8	0.40

LOC104911127	embryonic protein UVS.2-like	0.0	0.8	0.40
LOC104911210	uncharacterized LOC104911210	0.0	0.8	0.40
LOC104911332	uncharacterized LOC104911332	0.0	0.8	0.40
LOC104911364	uncharacterized LOC104911364	0.0	0.8	0.40
LOC104911411	calcitonin receptor-like	0.0	0.8	0.40
LOC104911450	nebulette-like	0.0	0.8	0.40
LOC104911487	uncharacterized LOC104911487	0.0	0.8	0.40
LOC104911549	uncharacterized LOC104911549	0.0	0.8	0.40
LOC104911834	kynureninase-like	0.0	0.8	0.40
LOC104911852	uncharacterized LOC104911852	0.0	0.8	0.40
LOC104911872	uncharacterized LOC104911872	0.0	0.8	0.40
LOC104911879	proline-rich acidic protein 1-like	0.0	0.8	0.40
LOC104912079	uncharacterized LOC104912079	0.0	0.8	0.40
LOC104912095	uncharacterized LOC104912095	0.0	0.8	0.40
LOC104912166	uncharacterized LOC104912166	0.0	0.8	0.40
LOC104912201	uncharacterized LOC104912201	0.0	0.8	0.40
LOC104912229	uncharacterized LOC104912229	0.0	0.8	0.40
LOC104912286	uncharacterized LOC104912286	0.0	0.8	0.40
LOC104912464	phosphatidylinositol 3-kinase regulatory subunit alpha-like	0.0	0.8	0.40
LOC104912538	uncharacterized LOC104912538	0.0	0.8	0.40
LOC104912599	uncharacterized LOC104912599	0.0	0.8	0.40
LOC104912674	uncharacterized LOC104912674	0.0	0.8	0.40
LOC104912838	uncharacterized LOC104912838	0.0	0.8	0.40
LOC104912874	uncharacterized LOC104912874	0.0	0.8	0.40
LOC104912882	uncharacterized LOC104912882	0.0	0.8	0.40
LOC104912934	uncharacterized LOC104912934	0.0	0.8	0.40
LOC104913024	protein NLRC5-like	0.0	0.8	0.40
LOC104913158	urocortin-3-like	0.0	0.8	0.40
LOC104913206	uncharacterized LOC104913206	0.0	0.8	0.40
LOC104913410	uncharacterized LOC104913410	0.0	0.8	0.40
LOC104913428	uncharacterized LOC104913428	0.0	0.8	0.40
LOC104913578	uncharacterized LOC104913578	0.0	0.8	0.40
LOC104913593	uncharacterized LOC104913593	0.0	0.8	0.40
LOC104913688	uncharacterized LOC104913688	0.0	0.8	0.40
LOC104913820	uncharacterized LOC104913820	0.0	0.8	0.40
LOC104913934	uncharacterized LOC104913934	0.0	0.8	0.40
LOC104913997	uncharacterized LOC104913997	0.0	0.8	0.40
LOC104914003	uncharacterized LOC104914003	0.0	0.8	0.40
LOC104914088	BPI fold-containing family B member 4-like	0.0	0.8	0.40
LOC104914102	BPI fold-containing family B member 3-like	0.0	0.8	0.40
LOC104914179	uncharacterized LOC104914179	0.0	0.8	0.40
LOC104914191	basic phospholipase A2 Cdr-13-like	0.0	0.8	0.40
LOC104914236	nucleoplasmin ATPase-like	0.0	0.8	0.40
LOC104914350	uncharacterized LOC104914350	0.0	0.8	0.40
LOC104914483	complement decay-accelerating factor-like	0.0	0.8	0.40
LOC104914501	neurofascin-like	0.0	0.8	0.40
LOC104914543	uncharacterized LOC104914543	0.0	0.8	0.40
LOC104914583	GTPase IMAP family member 1-like	0.0	0.8	0.40
LOC104914651	uncharacterized LOC104914651	0.0	0.8	0.40
LOC104914657	granzyme G-like	0.0	0.8	0.40
LOC104915248	uncharacterized LOC104915248	0.0	0.8	0.40
LOC104915395	histone H4-like	0.0	0.8	0.40
LOC104915451	uncharacterized LOC104915451	0.0	0.8	0.40
LOC104915458	inositol 1,4,5-trisphosphate receptor-interacting protein-like 1	0.0	0.8	0.40
LOC104915466	uncharacterized LOC104915466	0.0	0.8	0.40
LOC104915470	nebulin-like	0.0	0.8	0.40
LOC104915477	UDP-glucuronosyltransferase 1-6 pseudogene	0.0	0.8	0.40
LOC104915521	uncharacterized LOC104915521	0.0	0.8	0.40

LOC104915580	uncharacterized LOC104915580	0.0	0.8	0.40
LOC104915629	protein-glutamine gamma-glutamyltransferase 2-like	0.0	0.8	0.40
LOC104915641	niban-like protein 1	0.0	0.8	0.40
LOC104915714	uncharacterized membrane protein YFL067W-like	0.0	0.8	0.40
LOC104915726	uncharacterized LOC104915726	0.0	0.8	0.40
LOC104915778	uncharacterized LOC104915778	0.0	0.8	0.40
LOC104915815	protocadherin alpha-13-like	0.0	0.8	0.40
LOC104915818	uncharacterized LOC104915818	0.0	0.8	0.40
LOC104916311	copine-8-like	0.0	0.8	0.40
LOC104916612	feather keratin 1-like	0.0	0.8	0.40
LOC104916797	kinesin-like protein KIF20B	0.0	0.8	0.40
LOC104916938	putative protein TPRXL	0.0	0.8	0.40
LOC104917039	cohesin subunit SA-3-like	0.0	0.8	0.40
LOC104917054	uncharacterized LOC104917054	0.0	0.8	0.40
LOC104917177	zinc finger protein 501-like	0.0	0.8	0.40
LOC104917332	uncharacterized LOC104917332	0.0	0.8	0.40
LOC104917404	uncharacterized LOC104917404	0.0	0.8	0.40
LOC104917464	uncharacterized LOC104917464	0.0	0.8	0.40
LOC104917551	uncharacterized LOC104917551	0.0	0.8	0.40
LOC104917569	uncharacterized LOC104917569	0.0	0.8	0.40
LOC104917593	uncharacterized LOC104917593	0.0	0.8	0.40
LTB4R	leukotriene B4 receptor	0.0	0.8	0.40
MEIG1	meiosis/spermiogenesis associated 1	0.0	0.8	0.40
MZB1	marginal zone B and B1 cell-specific protein	0.0	0.8	0.40
ONECUT3	one cut homeobox 3	0.0	0.8	0.40
OTOP1	otopetrin 1	0.0	0.8	0.40
PAICS	phosphoribosylaminoimidazole carboxylase, phosphoribosylaminoimidazole succinocarboxamide synthetase	0.0	0.8	0.40
POU1F1	POU class 1 homeobox 1	0.0	0.8	0.40
POU2F3	POU class 2 homeobox 3	0.0	0.8	0.40
POU4F3	POU class 4 homeobox 3	0.0	0.8	0.40
PROK1	prokineticin 1	0.0	0.8	0.40
PRR15L	proline rich 15-like	0.0	0.8	0.40
SCARA3	scavenger receptor class A, member 3	0.0	0.8	0.40
SEMA3E	sema domain, immunoglobulin domain (Ig), short basic domain, secreted, (semaphorin) 3E	0.0	0.8	0.40
SFN	stratifin	0.0	0.8	0.40
SLC16A7	solute carrier family 16 (monocarboxylate transporter), member 7	0.0	0.8	0.40
SLC26A7	solute carrier family 26 (anion exchanger), member 7	0.0	0.8	0.40
SLC35F4	solute carrier family 35, member F4	0.0	0.8	0.40
STMN2	stathmin 2	0.0	0.8	0.40
STX11	syntaxin 11	0.0	0.8	0.40
SVOP	SV2 related protein homolog (rat)	0.0	0.8	0.40
TECTB	tectorin beta	0.0	0.8	0.40
TERT	telomerase reverse transcriptase	0.0	0.8	0.40
TMEM255B	transmembrane protein 255B	0.0	0.8	0.40
TNFSF8	tumor necrosis factor (ligand) superfamily, member 8	0.0	0.8	0.40
TRIM2	tripartite motif containing 2	0.0	0.8	0.40
UPK3A	uroplakin 3A	0.0	0.8	0.40
VMO1	vitelline membrane outer layer 1 homolog (chicken)	0.0	0.8	0.40
ZDHHC22	zinc finger, DHHC-type containing 22	0.0	0.8	0.40
ZNF831	zinc finger protein 831	0.0	0.8	0.40
ABCB4	ATP-binding cassette, sub-family B (MDR/TAP), member 4	0.0	0.8	0.38
ACE2	angiotensin I converting enzyme 2	0.0	0.8	0.38
ADAM19	ADAM metallopeptidase domain 19	0.0	0.8	0.38
AGBL2	ATP/GTP binding protein-like 2	0.0	0.8	0.38
AGR3	anterior gradient 3	0.0	0.8	0.38
APOH	apolipoprotein H (beta-2-glycoprotein I)	0.0	0.8	0.38

ARL14	ADP-ribosylation factor-like 14	0.0	0.8	0.38
ASB4	ankyrin repeat and SOCS box containing 4	0.0	0.8	0.38
AVPR1A	arginine vasopressin receptor 1A	0.0	0.8	0.38
C1QTNF6	C1q and tumor necrosis factor related protein 6	0.0	0.8	0.38
C3H7orf57	chromosome 3 open reading frame, human C7orf57	0.0	0.8	0.38
CCDC81	coiled-coil domain containing 81	0.0	0.8	0.38
CHRM3	cholinergic receptor, muscarinic 3	0.0	0.8	0.38
CLDN23	claudin 23	0.0	0.8	0.38
CR1	complement component (3b/4b) receptor 1 (Knops blood group)	0.0	0.8	0.38
CSMD1	CUB and Sushi multiple domains 1	0.0	0.8	0.38
DDC	dopa decarboxylase (aromatic L-amino acid decarboxylase)	0.0	0.8	0.38
DEXI	Dexi homolog (mouse)	0.0	0.8	0.38
DRD2	dopamine receptor D2	0.0	0.8	0.38
EN1	engrailed homeobox 1	0.0	0.8	0.38
EPHA8	EPH receptor A8	0.0	0.8	0.38
FAM124A	family with sequence similarity 124A	0.0	0.8	0.38
FAM81B	family with sequence similarity 81, member B	0.0	0.8	0.38
FBXO41	F-box protein 41	0.0	0.8	0.38
FGA	fibrinogen alpha chain	0.0	0.8	0.38
FYB	FYN binding protein	0.0	0.8	0.38
GALNT6	polypeptide N-acetylgalactosaminyltransferase 6	0.0	0.8	0.38
GDF10	growth differentiation factor 10	0.0	0.8	0.38
GDF2	growth differentiation factor 2	0.0	0.8	0.38
GKN2	gastrokine 2	0.0	0.8	0.38
GPR12	G protein-coupled receptor 12	0.0	0.8	0.38
GSC2	goosecoid homeobox 2	0.0	0.8	0.38
HEMGN	hemogen	0.0	0.8	0.38
HOXD10	homeobox D10	0.0	0.8	0.38
KCNA10	potassium voltage-gated channel, shaker-related subfamily, member 10	0.0	0.8	0.38
KCNJ10	potassium inwardly-rectifying channel, subfamily J, member 10	0.0	0.8	0.38
KCNQ1	potassium voltage-gated channel, KQT-like subfamily, member 1	0.0	0.8	0.38
KCNS1	potassium voltage-gated channel, delayed-rectifier, subfamily S, member 1	0.0	0.8	0.38
KRBA1	KRAB-A domain containing 1	0.0	0.8	0.38
LOC100538367	octopamine receptor-like	0.0	0.8	0.38
LOC100538453	ephrin-A2-like	0.0	0.8	0.38
LOC100538523	tetraspanin-15-like	0.0	0.8	0.38
LOC100539035	cholesterol 7-alpha-monooxygenase	0.0	0.8	0.38
LOC100539461	putative P2Y purinoceptor 10	0.0	0.8	0.38
LOC100539699	NAD(P)(+)--arginine ADP-ribosyltransferase 2-like	0.0	0.8	0.38
LOC100539818	uncharacterized LOC100539818	0.0	0.8	0.38
LOC100540172	C-C chemokine receptor type 5-like	0.0	0.8	0.38
LOC100540222	aryl-hydrocarbon-interacting protein-like 1	0.0	0.8	0.38
LOC100540415	LIM homeobox transcription factor 1-alpha-like	0.0	0.8	0.38
LOC100541067	sodium-dependent serotonin transporter-like	0.0	0.8	0.38
LOC100541206	heparan sulfate glucosamine 3-O-sulfotransferase 1-like	0.0	0.8	0.38
LOC100541413	delta and Notch-like epidermal growth factor-related receptor	0.0	0.8	0.38
LOC100541447	glutamate receptor ionotropic, delta-2-like	0.0	0.8	0.38
LOC100541755	catenin alpha-2	0.0	0.8	0.38
LOC100541793	complexin-3-like	0.0	0.8	0.38
LOC100541860	vascular endothelial growth factor receptor 2	0.0	0.8	0.38
LOC100541947	cytochrome b561	0.0	0.8	0.38
LOC100542192	uncharacterized LOC100542192	0.0	0.8	0.38
LOC100542400	protein-glutamine gamma-glutamyltransferase 6-like	0.0	0.8	0.38
LOC100542403	uncharacterized LOC100542403	0.0	0.8	0.38
LOC100543986	transmembrane protein 56-B-like	0.0	0.8	0.38
LOC100544684	la-related protein 6-like	0.0	0.8	0.38

LOC100544973	G-protein coupled receptor 35-like	0.0	0.8	0.38
LOC100545427	macrophage mannose receptor 1-like	0.0	0.8	0.38
LOC100545430	testis-specific serine/threonine-protein kinase 6-like	0.0	0.8	0.38
LOC100545863	ovostatin-like	0.0	0.8	0.38
LOC100546507	glutamate receptor ionotropic, delta-1	0.0	0.8	0.38
LOC100546620	gap junction beta-3 protein-like	0.0	0.8	0.38
LOC100546874	cytochrome P450 2W1-like	0.0	0.8	0.38
LOC100546884	transmembrane protein 45B-like	0.0	0.8	0.38
LOC100546941	olfactory receptor 51A4-like	0.0	0.8	0.38
LOC100547818	OX-2 membrane glycoprotein-like	0.0	0.8	0.38
LOC100548022	olfactory receptor 52B2-like	0.0	0.8	0.38
LOC100548043	vascular endothelial growth factor receptor kdr-like	0.0	0.8	0.38
LOC100548106	syntaxin-binding protein 4-like	0.0	0.8	0.38
LOC100548422	histone H5	0.0	0.8	0.38
LOC100548643	polypeptide N-acetylgalactosaminyltransferase-like 6	0.0	0.8	0.38
LOC100548793	membrane-bound O-acyltransferase domain-containing protein 2-like	0.0	0.8	0.38
LOC100548941	organic solute transporter subunit alpha-like	0.0	0.8	0.38
LOC100549054	janus kinase and microtubule-interacting protein 3-like	0.0	0.8	0.38
LOC100549453	coagulation factor X-like	0.0	0.8	0.38
LOC100549494	antigen peptide transporter 2-like	0.0	0.8	0.38
LOC100549505	melatonin receptor type 1B-like	0.0	0.8	0.38
LOC100549706	semaphorin-3A-like	0.0	0.8	0.38
LOC100550436	T-cell surface glycoprotein CD8 alpha chain-like	0.0	0.8	0.38
LOC100550526	poly(U)-specific endoribonuclease-like	0.0	0.8	0.38
LOC104909239	synaptotagmin-9-like	0.0	0.8	0.38
LOC104909474	uncharacterized LOC104909474	0.0	0.8	0.38
LOC104909841	uncharacterized LOC104909841	0.0	0.8	0.38
LOC104909911	maestro heat-like repeat-containing protein family member 2B	0.0	0.8	0.38
LOC104910003	uncharacterized LOC104910003	0.0	0.8	0.38
LOC104910063	uncharacterized LOC104910063	0.0	0.8	0.38
LOC104910067	protein eva-1 homolog C-like	0.0	0.8	0.38
LOC104910102	uncharacterized LOC104910102	0.0	0.8	0.38
LOC104910152	uncharacterized LOC104910152	0.0	0.8	0.38
LOC104910194	uncharacterized LOC104910194	0.0	0.8	0.38
LOC104910292	uncharacterized LOC104910292	0.0	0.8	0.38
LOC104910333	chloride channel protein B-like	0.0	0.8	0.38
LOC104910402	uncharacterized LOC104910402	0.0	0.8	0.38
LOC104910580	uncharacterized LOC104910580	0.0	0.8	0.38
LOC104910640	uncharacterized LOC104910640	0.0	0.8	0.38
LOC104910991	serologically defined colon cancer antigen 3 homolog	0.0	0.8	0.38
LOC104911037	solute carrier family 22 member 18-like	0.0	0.8	0.38
LOC104911044	ATP-binding cassette sub-family C member 8-like	0.0	0.8	0.38
LOC104911046	transcription factor SOX-6-like	0.0	0.8	0.38
LOC104911128	astacin-like metalloendopeptidase	0.0	0.8	0.38
LOC104911130	uncharacterized LOC104911130	0.0	0.8	0.38
LOC104911145	uncharacterized LOC104911145	0.0	0.8	0.38
LOC104911213	uncharacterized LOC104911213	0.0	0.8	0.38
LOC104911219	uncharacterized LOC104911219	0.0	0.8	0.38
LOC104911322	uncharacterized LOC104911322	0.0	0.8	0.38
LOC104911353	uncharacterized LOC104911353	0.0	0.8	0.38
LOC104911543	uncharacterized LOC104911543	0.0	0.8	0.38
LOC104911619	uncharacterized LOC104911619	0.0	0.8	0.38
LOC104911642	baculoviral IAP repeat-containing protein 5.1-like	0.0	0.8	0.38
LOC104911670	uncharacterized LOC104911670	0.0	0.8	0.38
LOC104911682	uncharacterized LOC104911682	0.0	0.8	0.38
LOC104911722	uncharacterized LOC104911722	0.0	0.8	0.38
LOC104911791	uncharacterized LOC104911791	0.0	0.8	0.38

LOC104911809	contactin-associated protein-like 5	0.0	0.8	0.38
LOC104911858	thrombospondin type-1 domain-containing protein 7B-like	0.0	0.8	0.38
LOC104912129	uncharacterized LOC104912129	0.0	0.8	0.38
LOC104912177	uncharacterized LOC104912177	0.0	0.8	0.38
LOC104912342	uncharacterized LOC104912342	0.0	0.8	0.38
LOC104912369	collagen alpha-1(XI) chain-like	0.0	0.8	0.38
LOC104912554	uncharacterized LOC104912554	0.0	0.8	0.38
LOC104912558	uncharacterized LOC104912558	0.0	0.8	0.38
LOC104912567	uncharacterized LOC104912567	0.0	0.8	0.38
LOC104912718	serine palmitoyltransferase small subunit B-like	0.0	0.8	0.38
LOC104912721	uncharacterized LOC104912721	0.0	0.8	0.38
LOC104912726	uncharacterized LOC104912726	0.0	0.8	0.38
LOC104912821	ovostatin homolog	0.0	0.8	0.38
LOC104912826	uncharacterized LOC104912826	0.0	0.8	0.38
LOC104912848	uncharacterized LOC104912848	0.0	0.8	0.38
LOC104912925	uncharacterized LOC104912925	0.0	0.8	0.38
LOC104913059	uncharacterized LOC104913059	0.0	0.8	0.38
LOC104913090	uncharacterized LOC104913090	0.0	0.8	0.38
LOC104913097	serine/arginine repetitive matrix protein 1-like	0.0	0.8	0.38
LOC104913116	protein bassoon-like	0.0	0.8	0.38
LOC104913148	ovostatin-like	0.0	0.8	0.38
LOC104913197	uncharacterized LOC104913197	0.0	0.8	0.38
LOC104913205	uncharacterized LOC104913205	0.0	0.8	0.38
LOC104913268	uncharacterized LOC104913268	0.0	0.8	0.38
LOC104913293	calcium/calmodulin-dependent protein kinase type II subunit alpha-like	0.0	0.8	0.38
LOC104913297	uncharacterized LOC104913297	0.0	0.8	0.38
LOC104913308	glutamate receptor 1-like	0.0	0.8	0.38
LOC104913705	uncharacterized LOC104913705	0.0	0.8	0.38
LOC104913806	uncharacterized LOC104913806	0.0	0.8	0.38
LOC104913810	uncharacterized LOC104913810	0.0	0.8	0.38
LOC104914162	uncharacterized LOC104914162	0.0	0.8	0.38
LOC104914168	serine/arginine repetitive matrix protein 2-like	0.0	0.8	0.38
LOC104914287	uncharacterized LOC104914287	0.0	0.8	0.38
LOC104914311	P2Y purinoceptor 4-like	0.0	0.8	0.38
LOC104914345	uncharacterized LOC104914345	0.0	0.8	0.38
LOC104914553	uncharacterized LOC104914553	0.0	0.8	0.38
LOC104914641	uncharacterized LOC104914641	0.0	0.8	0.38
LOC104914678	uncharacterized LOC104914678	0.0	0.8	0.38
LOC104914705	uncharacterized LOC104914705	0.0	0.8	0.38
LOC104914822	PDZ domain-containing protein 2-like	0.0	0.8	0.38
LOC104914840	sperm flagellar protein 2-like	0.0	0.8	0.38
LOC104915005	zinc finger protein GLIS3-like	0.0	0.8	0.38
LOC104915027	FRAS1-related extracellular matrix protein 1-like	0.0	0.8	0.38
LOC104915028	uncharacterized LOC104915028	0.0	0.8	0.38
LOC104915102	uncharacterized LOC104915102	0.0	0.8	0.38
LOC104915120	protein patched homolog 1-like	0.0	0.8	0.38
LOC104915335	uncharacterized LOC104915335	0.0	0.8	0.38
LOC104915422	uncharacterized LOC104915422	0.0	0.8	0.38
LOC104915491	uncharacterized LOC104915491	0.0	0.8	0.38
LOC104915517	uncharacterized LOC104915517	0.0	0.8	0.38
LOC104915523	tripartite motif-containing protein 10-like	0.0	0.8	0.38
LOC104915568	uncharacterized LOC104915568	0.0	0.8	0.38
LOC104915619	adenylate cyclase type 10-like	0.0	0.8	0.38
LOC104915626	uncharacterized LOC104915626	0.0	0.8	0.38
LOC104915751	uncharacterized LOC104915751	0.0	0.8	0.38
LOC104915919	SUN domain-containing protein 3-like	0.0	0.8	0.38
LOC104915935	class II histocompatibility antigen, B-L beta chain-like	0.0	0.8	0.38

LOC104916027	myb-related transcription factor, partner of profilin-like	0.0	0.8	0.38
LOC104916082	sodium/potassium-transporting ATPase subunit alpha-3-like	0.0	0.8	0.38
LOC104916166	E3 ubiquitin-protein ligase TRIM39-like	0.0	0.8	0.38
LOC104916172	E3 ubiquitin-protein ligase TRIM39-like	0.0	0.8	0.38
LOC104916218	uncharacterized LOC104916218	0.0	0.8	0.38
LOC104916265	AP-1 complex subunit mu-2-like	0.0	0.8	0.38
LOC104916349	uncharacterized LOC104916349	0.0	0.8	0.38
LOC104916403	UPF0500 protein C1orf216-like	0.0	0.8	0.38
LOC104916470	potassium channel subfamily K member 1-like	0.0	0.8	0.38
LOC104916482	phenylethanolamine N-methyltransferase-like	0.0	0.8	0.38
LOC104916512	adenylate cyclase type 10-like	0.0	0.8	0.38
LOC104916533	IgGFc-binding protein-like	0.0	0.8	0.38
LOC104916545	uncharacterized LOC104916545	0.0	0.8	0.38
LOC104916573	zinc finger CCCH domain-containing protein 11A-like	0.0	0.8	0.38
LOC104916595	erythroblast NAD(P)(+)--arginine ADP-ribosyltransferase-like	0.0	0.8	0.38
LOC104916645	transmembrane protein FLJ37396-like	0.0	0.8	0.38
LOC104916763	G2/M phase-specific E3 ubiquitin-protein ligase-like	0.0	0.8	0.38
LOC104916852	uncharacterized LOC104916852	0.0	0.8	0.38
LOC104917042	uncharacterized LOC104917042	0.0	0.8	0.38
LOC104917174	maestro heat-like repeat-containing protein family member 1	0.0	0.8	0.38
LOC104917351	uncharacterized LOC104917351	0.0	0.8	0.38
LOC104917436	UDP-glucose:glycoprotein glucosyltransferase 2-like	0.0	0.8	0.38
LOC104917482	RNA-directed DNA polymerase from mobile element jockey-like	0.0	0.8	0.38
LOC104917489	uncharacterized LOC104917489	0.0	0.8	0.38
LRRC26	leucine rich repeat containing 26	0.0	0.8	0.38
MATN4	matrilin 4	0.0	0.8	0.38
MCHR1	melanin-concentrating hormone receptor 1	0.0	0.8	0.38
NKAIN2	Na+/K+ transporting ATPase interacting 2	0.0	0.8	0.38
NMB	neuromedin B	0.0	0.8	0.38
NODAL	nodal growth differentiation factor	0.0	0.8	0.38
NXNL1	nucleoredoxin-like 1	0.0	0.8	0.38
OXGR1	oxoglutarate (alpha-ketoglutarate) receptor 1	0.0	0.8	0.38
P2RY2	purinergic receptor P2Y, G-protein coupled, 2	0.0	0.8	0.38
PAX2	paired box 2	0.0	0.8	0.38
PAX6	paired box 6	0.0	0.8	0.38
PLA2G12B	phospholipase A2, group XIIB	0.0	0.8	0.38
PTPRB	protein tyrosine phosphatase, receptor type, B	0.0	0.8	0.38
QRFP	pyroglutamylated RFamide peptide	0.0	0.8	0.38
RAG1	recombination activating gene 1	0.0	0.8	0.38
RBP3	retinol binding protein 3, interstitial	0.0	0.8	0.38
RGR	retinal G protein coupled receptor	0.0	0.8	0.38
ROBO4	roundabout, axon guidance receptor, homolog 4 (Drosophila)	0.0	0.8	0.38
RUNDC3A	RUN domain containing 3A	0.0	0.8	0.38
SCNN1D	sodium channel, non-voltage-gated 1, delta subunit	0.0	0.8	0.38
SCUBE1	signal peptide, CUB domain, EGF-like 1	0.0	0.8	0.38
SLC12A1	solute carrier family 12 (sodium/potassium/chloride transporter), member 1	0.0	0.8	0.38
SLC2A2	solute carrier family 2 (facilitated glucose transporter), member 2	0.0	0.8	0.38
SLC5A5	solute carrier family 5 (sodium/iodide cotransporter), member 5	0.0	0.8	0.38
SLC6A19	solute carrier family 6 (neutral amino acid transporter), member 19	0.0	0.8	0.38
SLITRK6	SLIT and NTRK-like family, member 6	0.0	0.8	0.38
SSC5D	scavenger receptor cysteine rich family, 5 domains	0.0	0.8	0.38
STAB2	stabilin 2	0.0	0.8	0.38
SYT8	synaptotagmin VIII	0.0	0.8	0.38
TCERG1L	transcription elongation regulator 1-like	0.0	0.8	0.38
TLX2	T-cell leukemia homeobox 2	0.0	0.8	0.38
TMEM154	transmembrane protein 154	0.0	0.8	0.38

TMEM174	transmembrane protein 174	0.0	0.8	0.38
TMEM244	transmembrane protein 244	0.0	0.8	0.38
TMEM26	transmembrane protein 26	0.0	0.8	0.38
TNFRSF1B	tumor necrosis factor receptor superfamily, member 1B	0.0	0.8	0.38
TNFRSF8	tumor necrosis factor receptor superfamily, member 8	0.0	0.8	0.38
TNFSF15	tumor necrosis factor (ligand) superfamily, member 15	0.0	0.8	0.38
TSNARE1	t-SNARE domain containing 1	0.0	0.8	0.38
TTC29	tetratricopeptide repeat domain 29	0.0	0.8	0.38
TTC36	tetratricopeptide repeat domain 36	0.0	0.8	0.38
TXK	TXK tyrosine kinase	0.0	0.8	0.38
ZNF366	zinc finger protein 366	0.0	0.8	0.38
