# Supplementary material for: Response of Turkey Muscle Satellite Cells to Thermal Challenge. II. Transcriptome Effects in Differentiating Cells
Source: Front Physiol. 2017 Nov 30;8:948. doi: 10.3389/fphys.2017.00948 (PMC5714890; doi:10.3389/fphys.2017.00948)
Supplement: Supplementary file 9 [file Table3.PDF]

Table S3. Summary of pairwise differential gene expression (DESeq) analysis of *p. major* satellite cell transcriptomes. Comparisons highlighted in blue have significant FDR p-values (< 0.05) and |Log_2FC| > 2.0. Comparisons highlighted in brown have significant FDR p-values (< 0.05) but with |Log_2FC| < 2.0 and > 1.0.

	Cold				Hot				CNTL		Cold		Hot	
	33R vs 38R		33F vs 38F		43R vs 38R		43F vs 38F		38F vs 38R		33F vs 33R		43F vs 43R	
Feature ID	FDR p-value correction	Log_2FC	FDR p-value correction	Log_2FC	FDR p-value correction	Log_2FC	FDR p-value correction	Log_2FC	FDR p-value correction	Log_2FC	FDR p-value correction	Log_2FC	FDR p-value correction	Log_2FC
A1CF	0.6881	1.4503	0.9148	1.2162	0.9361	1.2064	0.6172	1.7722	1.0000	-0.1033	1.0000	-0.3316	1.0000	0.4629
A2M	0.7263	-0.4263	1.0000	0.0383	1.0000	0.1503	0.2404	0.7352	1.0000	-0.4143	1.0000	0.0605	1.0000	0.1762
A4GALT	0.0187	0.6474	0.0826	0.3579	0.2007	0.3906	0.1685	0.2729	1.0000	0.3377	1.0000	0.0615	0.5215	0.2282
A4GNT	1.0000	0.0000	1.0000	0.0000	1.0000	0.0000	1.0000	0.0000	1.0000	0.0000	1.0000	0.0000	1.0000	0.0000
AAAS	1.0000	0.0005	0.6167	0.0807	0.0000	-1.1151	0.0000	-0.7790	1.0000	-0.3534	0.1202	-0.2610	1.0000	-0.0116
AACS	0.0000	0.8934	0.0000	0.9099	0.0000	0.7591	0.0000	0.4103	1.0000	-0.1134	0.9155	-0.0851	0.0000	-0.4566
AADAC	0.8479	0.2105	0.4612	0.3023	0.2479	0.4684	1.0000	0.0103	1.0000	0.0196	1.0000	0.1241	0.3113	-0.4294
AADAT	0.9434	0.0809	0.6072	-0.0992	0.1746	0.2900	0.0549	0.2481	1.0000	-0.0207	0.5044	-0.1885	1.0000	-0.0573
AAED1	0.0019	0.7884	0.0000	0.9648	0.5638	-0.2668	0.5800	0.2050	1.0000	-0.3019	1.0000	-0.1100	0.9144	0.1783
AAGAB	0.1707	0.3498	0.0004	0.4847	0.0004	0.7374	0.0000	0.6679	1.0000	-0.1026	1.0000	0.0450	0.6130	-0.1668
AAK1	1.0000	0.0122	0.3513	0.2981	0.0001	1.1297	0.0000	1.2373	1.0000	0.0352	0.6181	0.3344	0.8110	0.1488
AAMDC	0.9810	-0.1059	0.0007	-0.4866	0.7624	0.1682	0.8920	0.0704	1.0000	0.0305	0.2563	-0.3370	1.0000	-0.0606
AAMP	0.0005	-0.5277	0.0000	-0.3947	0.0000	-0.6756	0.0001	-0.3229	1.0000	-0.0973	1.0000	0.0478	0.0334	0.2609
AANAT	0.2959	-2.0245	0.5298	-1.1388	0.1846	-2.7129	0.3395	-1.2622	1.0000	0.3679	1.0000	1.2787	0.8937	1.8362
AAR2	0.5611	0.3828	0.4429	0.3299	0.0604	0.8482	0.4370	0.3840	1.0000	0.0670	1.0000	0.0258	0.6944	-0.3915
AARS	1.0000	0.0382	0.0383	0.1633	1.0000	-0.0220	0.4144	-0.0768	1.0000	-0.2493	0.6519	-0.1116	0.0006	-0.2987
AARS2	0.9899	-0.0733	0.0984	0.2283	0.0012	-0.6669	0.1344	-0.1944	1.0000	-0.2752	1.0000	0.0387	0.4002	0.2035
AARSD1	0.1421	-0.2666	0.0000	-0.3760	0.0000	-0.9757	0.0000	-0.8793	1.0000	0.0251	0.9908	-0.0720	0.6338	0.1267
AASDH	0.0009	0.8270	0.0003	0.6500	0.2062	0.4214	0.0087	0.4579	1.0000	0.1110	1.0000	-0.0536	0.9032	0.1512
AASDHPPT	0.6272	-0.2033	0.0671	-0.3247	0.0191	-0.6022	0.0011	-0.5358	1.0000	0.1634	1.0000	0.0556	0.5701	0.2358
AASS	0.3149	0.3863	0.5390	0.2003	0.3013	-0.4112	0.1602	-0.3906	1.0000	0.2490	1.0000	0.0775	0.7040	0.2759
AATF	1.0000	0.0353	0.3609	-0.2624	0.2718	-0.3746	0.0624	-0.4202	1.0000	0.0481	0.8785	-0.2375	1.0000	0.0078
ABAT	0.4509	-0.1920	0.0144	-0.2086	0.1413	-0.2878	0.5194	0.0736	1.0000	0.2229	0.2700	0.2188	0.0000	0.5898
ABCA1	0.0000	-0.9727	0.0000	-0.9154	0.0000	-1.1276	0.0017	-0.2669	1.0000	0.2499	0.1381	0.3197	0.0000	1.1133
ABCA12	0.2068	0.8149	0.4549	0.4664	0.4809	-0.7432	0.2686	-0.7045	1.0000	0.2124	1.0000	-0.1235	1.0000	0.2574
ABCA13	1.0000	-0.0492	0.4440	0.4169	0.1052	-0.8782	1.0000	-0.1292	1.0000	-0.8140	0.8785	-0.3361	1.0000	-0.0568
ABCA2	0.0034	-0.7781	0.0000	-0.7149	0.0000	1.0846	0.0000	1.3128	1.0000	-0.2329	0.9154	-0.1576	1.0000	0.0001
ABCA3	0.0000	0.9914	0.0000	1.1270	0.0078	0.4501	0.0000	0.7569	1.0000	-0.3440	0.2514	-0.1952	1.0000	-0.0312
ABCA4	0.7252	-0.7597	0.2409	-1.0468	0.4261	0.7284	1.0000	0.2098	1.0000	-0.0156	1.0000	-0.2918	0.8486	-0.5290
ABCA5	0.8059	-0.1455	0.8546	-0.0633	0.0748	0.4412	0.0000	0.6824	1.0000	0.2148	0.1086	0.3108	0.0003	0.4603
ABCA7	0.0227	-0.4633	0.0000	-0.6079	0.0000	-1.9307	0.0000	-1.8548	1.0000	-0.1599	0.0906	-0.2921	1.0000	-0.0803
ABCB10	0.0014	0.7473	0.0000	0.7765	1.0000	0.0253	1.0000	0.0264	1.0000	-0.1643	0.9375	-0.1248	0.8438	-0.1608
ABCB11	0.3089	-0.3035	0.0005	-0.5530	0.0005	-0.8267	0.0001	-0.5748	1.0000	0.1779	1.0000	-0.0592	0.0834	0.4341
ABCB4	1.0000	0.0000	1.0000	-2.3959	1.0000	2.2428	1.0000	-2.3175	1.0000	2.2674	1.0000	0.0000	1.0000	-2.2887
ABCB5	1.0000	-1.1585	0.9062	1.2235	1.0000	-0.1754	1.0000	0.0076	1.0000	-0.9638	1.0000	1.4325	1.0000	-0.7804
ABCB6	0.0003	0.7883	0.0000	0.7547	0.0163	-0.6110	0.0008	-0.4106	1.0000	0.0909	1.0000	0.0694	0.1766	0.2980
ABCB7	0.0015	0.6518	0.0004	0.3998	0.0000	0.9381	0.0000	0.4828	1.0000	0.2389	1.0000	-0.0005	0.2798	-0.2111
ABCB9	0.5568	0.5570	0.9952	0.1605	0.8986	0.2791	0.5398	0.3496	1.0000	0.1707	1.0000	-0.2133	1.0000	0.2563
ABCC1	0.0000	1.0564	0.0000	0.9581	1.0000	0.0271	0.1645	0.1611	1.0000	0.1078	1.0000	0.0223	0.0900	0.2475
ABCC10	0.0668	0.4098	0.0021	0.5516	0.0012	-0.7032	0.0024	-0.6301	1.0000	-0.1407	1.0000	0.0117	1.0000	-0.0614
ABCC2	0.0451	-0.7097	0.0113	-0.6145	0.1298	-0.5493	1.0000	-0.0559	1.0000	-0.1438	1.0000	-0.0341	0.4057	0.3566
ABCC3	1.0000	-1.1562	1.0000	-0.9973	1.0000	-0.1811	1.0000	0.5370	1.0000	-0.1210	1.0000	0.0515	1.0000	0.6042
ABCC5	0.0146	0.4398	0.0125	0.2376	0.1912	0.2798	0.0385	0.2017	1.0000	0.0498	0.5063	-0.1398	1.0000	-0.0240
ABCC6	0.0150	0.5960	0.0157	0.3251	0.0240	-0.6036	0.0007	-0.4285	1.0000	0.0375	0.4527	-0.2207	0.4588	0.2181
ABCC8	0.8582	-0.4423	0.0340	-1.8908	1.0000	0.0451	0.7977	0.3751	1.0000	-0.0468	0.3998	-1.4818	1.0000	0.2896
ABCC9	0.0000	-4.2165	0.0000	-4.4683	0.6758	0.2350	0.3654	0.3011	1.0000	-0.1654	1.0000	-0.4053	1.0000	-0.0911
ABCD4	0.8830	-0.0972	0.0013	-0.4052	0.0568	-0.4004	0.0178	-0.3114	1.0000	0.1641	0.8553	-0.1326	0.2212	0.2577
ABCE1	1.0000	-0.0284	1.0000	-0.0112	0.1459	-0.2579	0.0004	-0.2929	1.0000	-0.0242	1.0000	0.0055	1.0000	-0.0537
ABCF2	0.8885	0.0715	0.0056	0.1871	0.0000	-0.9760	0.0000	-0.8867	1.0000	-0.0598	0.8785	0.0683	1.0000	0.0350
ABCF3	0.1096	-0.2816	0.2839	-0.1091	0.0000	-0.7548	0.0000	-0.4440	1.0000	-0.2245	1.0000	-0.0398	0.7894	0.0916
ABCG1	1.0000	-0.2881	1.0000	-0.5563	1.0000	-2.4055	0.9786	-0.9107	1.0000	1.6505	1.0000	1.4306	1.0000	3.2066
ABCG2	0.0001	1.5427	0.4374	0.3563	0.0849	0.7900	0.0232	-0.9066	1.0000	0.1004	0.0008	-1.0746	0.0000	-1.5918
ABCG4	0.0122	2.0759	0.1748	1.2024	1.0000	0.2578	1.0000	-0.5490	1.0000	0.1233	0.6701	-0.7375	1.0000	-0.6831
ABCG5	1.0000	0.0716	0.9214	0.1302	0.2899	-0.4624	0.3159	-0.3464	1.0000	0.0243	1.0000	0.0969	1.0000	0.1462
ABCG8	1.0000	0.0000	1.0000	0.0000	1.0000	0.0000	1.0000	0.0000	1.0000	0.0000	1.0000	0.0000	1.0000	0.0000
ABHD1	1.0000	0.0279	0.0510	0.2962	0.0072	-0.7418	0.0924	-0.2626	1.0000	-0.0711	0.4723	0.2088	0.1952	0.4139
ABHD10	0.1955	0.2998	0.0028	0.3732	0.0021	-0.6305	0.0000	-0.6138	1.0000	-0.1005	1.0000	-0.0135	1.0000	-0.0780
ABHD11	0.0006	-0.8998	0.0000	-1.0838	0.2315	-0.4014	0.0007	-0.4744	1.0000	0.2464	1.0000	0.0738	0.8434	0.1809
ABHD12	1.0000	-0.0192	0.1032	0.2413	0.7158	0.1318	0.0077	0.3550	1.0000	0.0439	0.0419	0.3164	0.0792	0.2718
ABHD12B	1.0000	0.0507	0.0066	0.3420	1.0000	0.0406	0.4000	0.1450	1.0000	-0.2389	1.0000	0.0634	0.8973	-0.1286
ABHD13	0.7539	0.1234	0.1046	0.2289	0.7906	-0.1163	0.1086	-0.2081	1.0000	-0.0055	0.9307	0.1125	0.9489	-0.0925
ABHD17A	0.0000	-0.9038	0.0000	-0.8176	0.1626	-0.2620	0.6134	-0.0707	1.0000	-0.1464	1.0000	-0.0481	1.0000	0.0506
ABHD17B	0.0599	-0.4553	0.0000	-0.5202	0.0902	-0.4140	0.0000	-0.7488	1.0000	0.2539	0.4588	0.2028	1.0000	-0.0751
ABHD17C	0.6760	-0.2020	0.1959	-0.2252	0.0624	0.4893	0.0044	0.3841	1.0000	0.2218	0.6497	0.2112	0.8297	0.1213
ABHD2	0.0000	1.4691	0.0000	1.2376	0.2713	0.2403	0.5357	-0.0928	1.0000	0.1625	1.0000	-0.0573	0.2944	-0.1662
ABHD3	0.1462	-0.9601	0.0009	-1.1528	0.1825	-0.8568	1.0000	-0.0779	1.0000	0.7226	0.7552	0.5439	0.0003	1.5088
ABHD5	0.2410	-0.3116	0.0101	-0.3386	0.0367	0.4510	0.0051	0.2919	1.0000	0.0144	1.0000	0.0005	0.6667	-0.1396
ABHD6	0.0000	0.9955	0.0000	1.3846	0.0000	-1.1617	0.0005	-0.7517	1.0000	-0.3647	1.0000	0.0362	1.0000	0.0513
ABHD8	0.6673	0.6249	0.0842	1.5393	0.2531	-1.1673	1.0000	-0.6844	1.0000	-1.3530	1.0000	-0.4307	1.0000	-0.8669
ABI1	0.0613	0.3655	0.0000	0.5000	0.0000	0.9907	0.0000	0.9404	1.0000	0.0258	0.4102	0.1736	1.0000	-0.0183
ABI2	0.0110	-0.4040	0.0000	-0.4862	0.9979	-0.0550	0.1908	-0.1271	1.0000	0.1016	1.0000	0.0320	1.0000	0.0349
ABI3	1.0000	0.1207	0.1841	1.4701	0.7692	-1.4082	0.9957	-0.9063	1.0000	-0.4413	0.8875	0.9295	1.0000	0.0717
ABI3BP	0.0000	-1.3869	0.0000	-1.0279	0.5871	0.2233	0.0002	0.5820	1.0000	-0.4222	1.0000	-0.0517	1.0000	-0.0610
ABL1	1.0000	-0.0018	0.4419	0.0791	0.0072	0.4533	0.0000	0.3742	1.0000	0.0144	0.6210	0.1078	0.9303	-0.0598
ABL2	0.9925	-0.1913	0.5001	-0.2639	0.0001	1.5090	0.0000	1.2937	1.0000	0.2937	1.0000	0.2333	1.0000	0.0837
ABLIM1	1.0000	0.0341	0.0002	0.6395	1.0000	-0.1219	0.3193	0.2291	1.0000	0.1863	0.0000	0.8060	0.0218	0.5399
ABLIM2	0.0015	-2.0541	0.0040	-1.3898	0.0015	1.3532	0.0000	1.4180	1.0000	0.2110	0.7818	0.8922	0.7468	0.2863
ABLIM3	0.0000	-4.4737	0.0000	-4.3419	0.0001	-0.6930	0.0000	-0.5023	1.0000	-0.0341	1.0000	0.1085	0.3338	0.1613
ABR	0.0415	-0.3750	0.0000	-0.4690	0.0897	-0.3195	0.0085	-0.2434	1.0000	0.0575	1.0000	-0.0241	0.4605	0.1385
ABRA	0.0018	-0.5186	0.0000	-1.0174	0.5071	-0.1585	0.9053	-0.0437	1.0000	0.0908	0.0020	-0.3952	0.1399	0.2111
ABRACL	0.0062	-0.6398	0.0002	-0.5294	1.0000	0.0237	1.0000	0.0459	1.0000	0.0396	0.8607	0.1617	1.0000	0.0669
ABTB1	1.0000	-0.0315	0.0123	-0.2313	0.1864	0.2367	0.0292	0.1895	1.0000	0.1566	1.0000	-0.0306	0.4752	0.1148
ABTB2	0.0328	0.8866	0.0528	0.6023	0.0000	1.4632	0.0658	0.5614	1.0000	0.4013	1.0000	0.1306	0.1640	-0.4924
ACAA1	0.0003	0.6460	0.0000	0.4937	0.8456	-0.1066	0.0002	-0.4945	1.0000	-0.0237	0.4152	-0.1645	0.0243	-0.4059
ACAA2	0.0000	-0.6363	0.0000	-0.6205	0.0000	-1.2229	0.0000	-1.3405	1.0000	-0.0147	1.0000	0.0134	0.3494	-0.1268

ACACB	0.0000	1.5535	0.0060	0.8559	0.0000	-2.2679	0.0000	-3.2799	1.0000	-0.0148	0.0238	-0.6998	0.6819	-1.0214
ACAD10	0.0049	0.8748	0.0000	1.1870	0.0107	-1.0175	0.4475	-0.3670	1.0000	-0.6121	0.7323	-0.2866	1.0000	0.0451
ACAD11	0.8407	0.1106	0.7713	-0.0753	0.8011	0.1183	0.0522	0.2464	1.0000	0.1081	1.0000	-0.0662	0.1854	0.2409
ACAD8	0.3775	-0.2210	0.2673	-0.1676	0.0025	-0.6328	0.0002	-0.4672	1.0000	-0.1962	0.8549	-0.1303	1.0000	-0.0247
ACAD9	0.8733	0.0745	0.3104	-0.0941	0.0001	-0.5721	0.0000	-0.5793	1.0000	0.0191	0.3460	-0.1372	1.0000	0.0174
ACADL	0.4874	-0.1969	0.0244	-0.1751	0.0540	-0.4050	0.0000	-0.6022	1.0000	-0.1268	0.7487	-0.0925	0.0007	-0.3178
ACADM	0.0000	-0.9840	0.0000	-1.1046	0.0035	-0.4951	0.0000	-0.6429	1.0000	0.1013	1.0000	-0.0066	1.0000	-0.0409
ACADS	0.0000	-0.9524	0.0000	-0.9475	0.0564	-0.3409	0.0007	-0.3232	1.0000	-0.0103	1.0000	0.0065	1.0000	0.0123
ACADSB	0.0482	0.3480	0.0101	0.2366	1.0000	0.0430	0.3210	-0.1186	1.0000	0.0405	1.0000	-0.0582	0.6600	-0.1155
ACAN	1.0000	0.0000	1.0000	0.0000	1.0000	0.0000	1.0000	0.0000	1.0000	0.0000	1.0000	0.0000	1.0000	0.0000
ACAP2	0.0067	0.4045	0.0000	0.3242	0.2263	-0.2198	0.1597	-0.1156	1.0000	0.0751	1.0000	0.0071	0.1479	0.1845
ACAP3	0.0001	-1.3772	0.0000	-1.1243	0.0244	0.6899	0.0072	0.4756	1.0000	0.3388	0.1585	0.6019	0.8965	0.1257
ACAT1	0.7155	0.1274	0.0023	0.2828	0.2356	-0.2618	0.0003	-0.3772	1.0000	-0.1557	1.0000	0.0126	0.0917	-0.2645
ACAT2	0.0000	0.7833	0.0000	0.8592	1.0000	0.0358	0.0005	-0.3439	1.0000	-0.1182	1.0000	-0.0303	0.0000	-0.4920
ACBD3	0.0009	-0.5229	0.0000	-0.5451	0.2104	-0.2346	0.1544	-0.1381	1.0000	0.0288	1.0000	0.0194	0.4901	0.1308
ACBD5	0.8478	-0.1011	0.7338	0.0749	0.8033	0.1066	0.4546	0.1125	1.0000	-0.1001	1.0000	0.0885	0.8776	-0.0881
ACBD6	1.0000	-0.0243	0.5207	-0.1089	0.0000	-0.8669	0.0000	-0.7768	1.0000	0.0738	1.0000	0.0015	0.6738	0.1697
ACBD7	0.0006	1.1920	0.1323	0.4317	0.2609	-0.5727	0.0019	-0.9404	1.0000	0.3783	0.5907	-0.3685	1.0000	0.0166
ACCS	1.0000	-0.0649	0.0062	-0.3956	1.0000	-0.0676	1.0000	0.0266	1.0000	0.0027	0.1687	-0.3160	0.9248	0.1013
ACE	0.0000	-3.9125	0.0000	-3.6297	1.0000	-0.0611	0.7519	-0.0964	1.0000	-0.1139	1.0000	0.1753	0.7435	-0.1462
ACE2	0.8226	3.0199	0.4135	1.9103	1.0000	2.2472	1.0000	-2.3176	1.0000	2.2674	1.0000	1.2774	1.0000	-2.2909
ACER1	1.0000	0.0000	1.0000	0.0000	1.0000	0.0000	1.0000	0.0000	1.0000	0.0000	1.0000	0.0000	1.0000	0.0000
ACER2	0.2519	0.3677	0.0013	0.5533	0.6151	-0.2253	1.0000	0.0236	1.0000	-0.0501	0.9397	0.1471	0.7287	0.2054
ACKR2	1.0000	-0.2892	0.9062	1.2235	1.0000	-0.1597	1.0000	0.8538	1.0000	-0.1089	1.0000	1.4314	1.0000	0.9149
ACKR3	0.4781	1.0255	0.5340	0.9221	1.0000	-0.4550	1.0000	0.2585	1.0000	-0.3755	1.0000	-0.4709	1.0000	0.3321
ACKR4	0.0036	-2.7531	0.0698	-1.4625	0.6794	-0.5786	0.0478	-1.8358	1.0000	-0.3095	1.0000	1.0010	0.2944	-1.5622
ACLY	0.0000	0.6379	0.0000	0.6061	0.0000	0.7898	0.0000	0.5241	1.0000	0.0073	1.0000	-0.0123	0.0046	-0.2531
ACMSD	0.0010	-2.8320	0.0008	-2.8833	0.0217	-1.7120	0.0351	-1.5444	1.0000	-0.5916	1.0000	-0.6350	1.0000	-0.4203
ACO1	0.2478	0.2333	0.0030	0.2730	0.1334	0.2783	0.1976	0.1361	1.0000	-0.0374	1.0000	0.0150	0.2322	-0.1737
ACO2	0.0000	-1.5458	0.0000	-1.3464	0.0409	0.3256	0.0001	0.2643	1.0000	-0.0502	0.3216	0.1617	0.5215	-0.1062
ACOT11	0.0000	-1.0596	0.0000	-1.3311	0.2590	-0.2275	0.0000	-0.3217	1.0000	-0.0517	0.0167	-0.3105	0.3569	-0.1410
ACOT12	1.0000	0.0468	0.9762	0.7526	1.0000	0.5042	0.7524	1.2353	1.0000	-0.6504	1.0000	0.0600	1.0000	0.0793
ACOT13	0.0296	-0.5611	0.0000	-0.5988	0.1837	-0.3970	0.0000	-0.4883	1.0000	-0.0348	1.0000	-0.0615	0.9206	-0.1196
ACOT7	0.4096	0.2275	0.0000	0.5918	0.4255	-0.2296	0.8317	0.0770	1.0000	-0.2155	0.6450	0.1608	0.9887	0.0962
ACOT8	0.0000	-0.8395	0.0000	-1.1044	0.0000	-1.2444	0.0000	-1.1936	1.0000	-0.1334	0.0173	-0.3868	1.0000	-0.0767
ACOT9	1.0000	0.0350	0.0158	-0.1949	1.0000	0.0472	0.2231	-0.1164	1.0000	0.1029	0.5859	-0.1147	0.9940	-0.0553
ACOX1	0.0088	0.4214	0.0002	0.2974	0.0430	-0.3537	0.0028	-0.2527	1.0000	-0.0415	0.3521	-0.1534	1.0000	0.0639
ACOX2	0.3182	-0.5030	0.2728	0.3734	0.0066	0.9387	0.5621	0.2661	1.0000	-0.1110	0.0599	0.7773	0.0167	-0.7799
ACOX3	0.2928	0.3350	0.0153	0.3503	1.0000	0.0425	1.0000	-0.0011	1.0000	0.2164	0.4839	0.2448	0.6707	0.1768
ACP1	0.0366	-0.4528	0.0000	-0.5535	0.0143	-0.5113	0.0000	-0.6738	1.0000	0.0222	1.0000	-0.0665	0.5443	-0.1348
ACP2	0.3888	0.2091	0.0013	0.3126	0.4987	0.1737	0.2139	0.1535	1.0000	0.0341	0.5881	0.1505	1.0000	0.0191
ACP5	0.0572	0.5895	0.0069	0.6081	0.0000	-1.5752	0.0000	-2.0241	1.0000	-0.2545	0.8125	-0.2231	0.3841	-0.6963
ACP6	0.6468	-0.1577	0.0002	-0.5386	0.0000	-0.8489	0.0000	-0.9456	1.0000	0.1284	0.3509	-0.2410	1.0000	0.0367
ACR	0.2340	-0.9673	0.0383	-1.1682	0.2745	0.7337	1.0000	0.1436	1.0000	0.2515	1.0000	0.0610	0.9139	-0.3328
ACRBP	1.0000	0.0000	1.0000	0.0000	1.0000	0.0000	1.0000	0.0000	1.0000	0.0000	1.0000	0.0000	1.0000	0.0000
ACRC	0.0135	0.8498	0.0000	1.1696	0.3427	0.3981	0.9573	-0.1070	1.0000	-0.1608	1.0000	0.1706	0.0492	-0.6602
ACRV1	0.0000	-1.9240	0.0000	-1.5368	0.9040	-0.1103	0.0000	0.6780	1.0000	-0.4086	1.0000	-0.0074	0.0560	0.3855
ACSBG1	1.0000	-0.0395	0.2237	-0.3756	0.0039	-1.0338	0.0175	-0.6773	1.0000	-0.0705	0.6036	-0.3951	0.8260	0.2908
ACSBG2	0.0000	0.9408	0.0000	1.1712	0.0000	0.9607	0.0000	1.0340	1.0000	-0.2873	1.0000	-0.0436	0.1208	-0.2077
ACSF2	0.8249	0.0988	1.0000	0.0349	0.0007	-0.6094	0.2493	-0.1434	1.0000	0.0347	1.0000	-0.0168	0.0001	0.5060
ACSL1	0.0001	0.9020	0.0000	1.2109	0.0035	0.6807	0.0004	0.5844	1.0000	-0.1904	0.9420	0.1307	0.2748	-0.2816
ACSL3	0.2295	-0.3084	0.2169	-0.1833	0.0022	-0.6401	0.0001	-0.4794	1.0000	0.0666	0.6574	0.2044	0.3897	0.2334
ACSL4	0.7494	-0.1214	0.0000	-0.5422	0.0021	0.5616	0.0320	0.2297	1.0000	0.2813	0.8004	-0.1265	1.0000	-0.0452
ACSL5	0.7574	-0.2104	0.4574	-0.2154	0.0609	-0.6167	0.0007	-0.7362	1.0000	-0.2586	0.8785	-0.2499	0.5105	-0.3699
ACSL6	1.0000	-0.8620	1.0000	-0.1673	1.0000	0.5002	1.0000	0.0069	1.0000	-0.1232	1.0000	0.5871	1.0000	-0.6149
ACSM3	0.5086	1.1783	0.6518	0.7875	0.7002	-1.9426	0.2455	-4.0833	1.0000	-0.1268	1.0000	-0.5085	1.0000	-2.2887
ACSS1	0.0181	0.6252	0.0000	0.7964	0.6721	0.1865	0.0354	0.3766	1.0000	-0.0755	1.0000	0.1061	0.9667	0.1194
ACSS2	0.6457	-0.1222	0.1949	-0.1255	0.0000	-0.7621	0.0000	-0.6044	1.0000	-0.0880	0.9061	-0.0788	0.9147	0.0755
ACSS3	0.0305	-1.0083	0.0000	-1.3042	0.2610	-0.4847	1.0000	-0.0882	1.0000	0.1375	1.0000	-0.1456	0.2476	0.5385
ACTA1	0.0000	-3.2046	0.0000	-3.3473	0.0000	-2.8612	0.0000	-3.1927	1.0000	-0.0710	0.3459	-0.2007	0.0033	-0.3960
ACTA2	0.0043	0.6053	0.0000	0.9118	0.0199	0.5186	0.2037	-0.1236	1.0000	0.1115	0.0000	0.4301	0.0000	-0.5262
ACTB	0.5388	0.1314	0.0936	0.1421	0.0000	-0.6395	0.0000	-0.6514	1.0000	-0.0016	1.0000	0.0215	1.0000	-0.0080
ACTC1	0.0000	-3.8406	0.0000	-4.1141	0.0004	-0.5141	0.0000	-0.6397	1.0000	0.0815	0.8165	-0.1809	1.0000	-0.0390
ACTL6A	0.0000	0.9956	0.0000	1.1499	0.0007	0.6399	0.0170	0.3276	1.0000	0.0870	0.1558	0.2546	0.2396	-0.2190
ACTL9	1.0000	0.0000	1.0000	0.0000	1.0000	0.0000	1.0000	0.0000	1.0000	0.0000	1.0000	0.0000	1.0000	0.0000
ACTN1	0.0126	0.3847	0.0000	0.5446	1.0000	0.0077	0.7093	0.0452	1.0000	0.0487	0.0095	0.2210	0.6118	0.0914
ACTN2	0.0000	-1.4588	0.0000	-1.8580	1.0000	0.0041	0.0002	-0.3210	1.0000	0.1482	0.0677	-0.2391	0.2041	-0.1718
ACTR1A	0.0000	-0.6657	0.0000	-0.6114	0.0405	-0.3197	0.0000	-0.3016	1.0000	-0.0019	0.9860	0.0646	1.0000	0.0216
ACTR2	0.0026	0.5201	0.0000	0.7753	0.9594	0.0676	0.3940	-0.0984	1.0000	-0.0854	0.2473	0.1820	0.0404	-0.2457
ACTR3	0.1825	0.2328	0.0000	0.3819	1.0000	0.0337	0.7210	-0.0465	1.0000	0.0371	0.0692	0.1987	1.0000	-0.0375
ACTR3B	0.0473	0.3444	0.0000	0.3305	0.0000	-0.7536	0.0000	-0.7715	1.0000	-0.0021	1.0000	-0.0036	1.0000	-0.0145
ACTR5	1.0000	-0.0707	0.1998	0.2816	0.0311	-0.5268	0.0119	-0.4542	1.0000	-0.2004	0.9264	0.1637	0.9927	-0.1242
ACTR6	0.0024	-0.5184	0.0000	-0.4749	0.0015	-0.5434	0.0000	-0.6134	1.0000	-0.0026	1.0000	0.0531	0.9382	-0.0672
ACTR8	0.6353	-0.1428	0.3093	-0.1320	0.4671	-0.1836	0.6310	-0.0814	1.0000	0.1208	0.6655	0.1445	0.1851	0.2278
ACTRT2	1.0000	0.0000	1.0000	0.0000	1.0000	0.0000	1.0000	0.0000	1.0000	0.0000	1.0000	0.0000	1.0000	0.0000
ACVR1	0.5054	0.1472	0.1687	0.1308	0.0950	0.2792	0.0000	0.3902	1.0000	0.0767	0.9442	0.0726	0.0958	0.1929
ACVR1B	1.0000	0.0384	0.4658	0.2933	0.0000	1.4729	0.0000	1.4422	1.0000	0.0798	0.7181	0.3481	1.0000	0.0581
ACVR1C	1.0000	2.1903	1.0000	0.0000	1.0000	2.2427	1.0000	2.3258	1.0000	0.0000	1.0000	-2.2993	1.0000	0.0660
ACVR2A	0.0000	0.8456	0.0000	0.8169	0.0000	1.0257	0.0000	0.8279	1.0000	0.0020	1.0000	-0.0131	0.4169	-0.1891
ACVR2B	0.8412	0.2988	0.3139	-0.6904	1.0000	-0.1527	0.2520	-0.7233	1.0000	-0.0786	0.1608	-1.0600	0.6642	-0.6483
ACVRL1	1.0000	-0.7208	0.7189	-1.3897	0.5405	0.9322	1.0000	0.4744	1.0000	0.1799	1.0000	-0.4781	1.0000	-0.2726
ACY1	0.8374	0.0989	0.4877	0.1178	0.0001	-0.6951	0.0004	-0.4489	1.0000	-0.1279	0.9599	-0.0978	0.7927	0.1232
ACYP1	0.0078	-0.7323	0.3328	-0.2436	0.0010	-0.8484	0.9065	-0.0994	1.0000	-0.2615	0.8219	0.2412	0.1140	0.4929
ACYP2	0.0025	-0.6319	0.0000	-0.9275	0.0000	-1.0636	0.0000	-1.1539	1.0000	0.0796	0.3106	-0.2029	1.0000	-0.0042
ADA	0.0298	-1.0404	0.0138	-0.9086	0.0484	0.7527	0.0066	0.7080	1.0000	0.3634	0.7644	0.5090	0.5621	0.3250
ADAD1	1.0000	-1.2498	1.0000	-0.1658	0.6597	-1.9401	1.0000	0.3938	1.0000	-0.5096	1.0000	0.5888	0.8937	1.8362
ADAL	0.0000	-0.8480	0.0000	-1.2510	0.0923	-0.3633	0.0000	-0.5609	1.0000	0.2063	0.7971	-0.1842	1.0000	0.0124
ADAM11	0.2392	-1.4491	1.0000	0.0400	0.2130	-1.5960	1.0000	0.2212	1.0000	-0.9781	1.0000	0.5242	1.0000	0.8467
ADAM17	0.0465	0.3313	0.0000	0.3346	0.0826	0.3017	0.0017	0.2543	1.0000	0.0756	0.8156	0.0912	1.0000	0.0331
ADAM19	1.0000	0.0000	1.0000	-0.1449	0.8033	3.0892	1.0000	0.0076	1.0000	2.2674	1.0000	2.3454	1.0000	-0.7804
ADAM22	1.0000	0.0510	0.3340	0.8066	0.0780	1.1999	0.0334	1.4784	1.0000	-0.2562	1.0000	0.5141	1.0000	0.0297
ADAM23	0.0119	0.4099	1.0000	-0.0244	0.0001	0.6300	0.0000	0.4877	1.0000	0.0714	0.0009	-0.3498	0.9760	-0.0651
ADAM33	0.0000	-1.6800	0.0000	-1.7366	0.0789	-0.3730	0.0002	-0.3146	1.0000	-0.0308	1.0000	-0.0754	1.0000	0.0323

ADAM8	1.0000	-0.0215	0.0201	0.2033	0.1569	0.3132	0.0000	0.3724	1.0000	0.0574	0.0202	0.2945	0.5531	0.1214
ADAM9	0.4578	0.1833	0.7763	0.0566	0.0002	0.5978	0.0000	0.4796	1.0000	-0.0154	0.7355	-0.1303	0.4891	-0.1285
ADAMTS1	0.8583	0.1619	0.0083	-0.5043	0.0305	0.6201	1.0000	0.0074	1.0000	-0.3528	0.0000	-1.0080	0.0000	-0.9604
ADAMTS10	0.0000	-2.6279	0.0000	-2.7105	0.0135	-0.5202	0.0000	0.4127	1.0000	0.0105	1.0000	-0.0583	0.0000	0.9498
ADAMTS13	0.0002	1.0040	0.1244	0.3275	0.2564	0.4027	0.2949	0.2411	1.0000	0.2835	0.1801	-0.3792	0.9904	0.1265
ADAMTS14	0.0000	-1.0179	0.0000	-0.8790	0.0000	0.9897	0.0000	1.1880	1.0000	-0.0112	0.5902	0.1397	0.0584	0.1920
ADAMTS15	0.0712	1.1386	0.2994	0.5852	0.1439	-1.3874	0.0007	-2.5055	1.0000	0.6380	1.0000	0.0962	1.0000	-0.4776
ADAMTS17	0.0018	0.8399	0.0000	0.7451	0.0023	0.7987	0.0001	0.6618	1.0000	0.0178	1.0000	-0.0646	0.9607	-0.1150
ADAMTS19	1.0000	0.4988	1.0000	-0.3345	0.0206	2.0221	0.8777	0.5422	1.0000	0.4224	1.0000	-0.3876	0.3897	-1.0533
ADAMTS2	0.0002	-0.9025	0.0000	-0.6100	0.0000	1.4366	0.0000	1.4595	1.0000	0.2249	0.0000	0.5293	0.0076	0.2523
ADAMTS20	0.9969	0.0885	1.0000	0.0044	0.0023	0.6715	0.0000	0.5768	1.0000	0.0142	1.0000	-0.0571	1.0000	-0.0744
ADAMTS3	0.0002	1.8711	0.0005	1.5186	0.0005	1.8856	0.2426	0.7349	1.0000	0.0439	0.9094	-0.3020	0.0235	-1.1110
ADAMTS4	0.1666	-0.5741	0.1437	-0.5020	1.0000	-0.0117	0.0115	0.6591	1.0000	-0.5735	0.4699	-0.4898	1.0000	0.1040
ADAMTS5	0.6906	0.3622	0.8361	0.1949	0.0000	2.0318	0.0000	1.6156	1.0000	0.2939	1.0000	0.1373	1.0000	-0.1217
ADAMTS6	0.5640	0.2474	0.1776	0.2710	0.0550	0.4990	0.0000	0.6691	1.0000	0.2065	0.6450	0.2443	0.0223	0.3837
ADAMTS7	0.0020	-1.3382	0.0000	-1.5423	0.1117	0.6153	0.0000	1.4107	1.0000	0.1331	1.0000	-0.0580	0.0000	0.9342
ADAMTS8	0.3042	-0.7005	0.0207	-0.9593	0.5586	0.3836	0.8072	0.2025	1.0000	0.2579	1.0000	0.0111	1.0000	0.0811
ADAMTS9	0.0000	-1.7490	0.0002	-0.9933	0.0005	-0.9362	1.0000	0.0670	1.0000	-0.1496	0.3331	0.6175	0.0003	0.8614
ADAMTSL1	0.1894	0.6835	1.0000	-0.0604	0.0420	0.9267	0.2878	0.3772	1.0000	-0.1931	0.0026	-0.9271	0.0085	-0.7422
ADAMTSL2	1.0000	0.0026	0.7819	-0.0422	0.0332	-0.5388	0.0000	-0.5443	1.0000	0.1650	0.6804	0.1316	0.2206	0.1637
ADAMTSL3	0.0251	0.5824	0.0004	0.7585	0.0000	1.2321	0.0000	1.0288	1.0000	0.0239	0.7436	0.2138	0.5114	-0.1729
ADAMTSL5	0.0014	-2.4180	0.0000	-4.0969	0.7194	-0.4065	0.1557	0.6173	1.0000	0.0427	0.7608	-1.6308	0.0374	1.0741
ADAP2	0.9646	0.1676	0.1291	0.4723	0.3575	-0.4392	1.0000	0.0274	1.0000	-0.0665	0.8875	0.2523	0.5071	0.4102
ADAR	0.4389	-0.1959	0.7871	-0.0621	1.0000	0.0502	0.0184	0.2442	1.0000	-0.0325	0.8185	0.1130	0.3536	0.1663
ADARB1	0.0886	-0.4535	0.0028	-0.4630	0.0002	0.7456	0.0000	0.7842	1.0000	0.1339	1.0000	0.1384	0.4483	0.1783
ADARB2	0.0148	-5.3982	0.0090	-5.6048	0.0190	-5.3113	0.6742	-0.8537	1.0000	0.1692	1.0000	0.0000	0.2129	4.6838
ADAT1	0.9018	0.1881	0.0518	0.5664	0.9763	-0.1786	1.0000	-0.1066	1.0000	-0.0411	0.6946	0.3479	1.0000	0.0344
ADCK1	0.6353	0.2269	0.0437	0.4165	1.0000	-0.1249	0.9077	-0.1054	1.0000	-0.0944	1.0000	0.1080	1.0000	-0.0670
ADCK2	0.0000	1.0187	0.0000	0.8357	0.0019	-0.8718	0.0050	-0.6691	1.0000	-0.1108	0.3181	-0.2832	1.0000	0.0951
ADCK3	0.0108	-0.4004	0.0000	-0.6847	0.0993	0.2950	0.0152	0.2291	1.0000	0.0458	0.1164	-0.2257	1.0000	-0.0142
ADCY2	0.0000	-1.3164	0.0000	-1.9665	1.0000	0.0040	0.9163	-0.0428	1.0000	0.1843	0.0770	-0.4533	0.4840	0.1430
ADCY3	1.0000	2.1902	1.0000	-0.9973	0.0110	5.4000	0.5168	1.4855	1.0000	3.1056	1.0000	0.0489	0.8620	-0.7694
ADCY5	0.3916	0.2316	0.7808	0.0739	0.2135	0.2778	0.8651	-0.0581	1.0000	0.2065	1.0000	0.0615	0.7369	-0.1241
ADCY8	0.0002	-6.7963	0.0022	-5.5849	0.6756	0.5284	0.0134	1.4341	1.0000	-1.2375	1.0000	0.0000	0.9409	-0.3312
ADCYAP1	0.7446	-0.1034	0.2935	0.0960	0.1144	-0.2683	0.0038	-0.2222	1.0000	0.0210	0.0789	0.2326	0.8749	0.0726
ADCYAP1R1	0.2739	1.6759	0.1790	2.3721	0.4809	1.3147	0.9164	1.3891	1.0000	-1.4982	0.9052	-0.8097	0.5671	-1.4301
ADD1	1.0000	-0.0363	1.0000	-0.0056	0.0845	0.2969	0.0003	0.2683	1.0000	0.0137	1.0000	0.0569	1.0000	-0.0093
ADD2	0.7938	1.1369	1.0000	-0.1698	0.2725	1.6862	0.9786	-0.9103	1.0000	0.7940	1.0000	-0.4972	0.4791	-1.7986
ADD3	0.5530	0.2040	0.4991	0.1445	0.1293	0.3759	0.0048	0.3994	1.0000	0.1034	1.0000	0.0580	0.7271	0.1350
ADGB	1.0000	-2.4788	1.0000	2.2506	1.0000	-2.4061	1.0000	0.0000	1.0000	-2.3771	1.0000	2.3454	1.0000	0.0000
ADHFE1	0.8157	-0.1741	1.0000	0.0046	1.0000	-0.0285	0.6471	-0.1663	1.0000	-0.1540	1.0000	0.0367	0.5422	-0.2863
ADI1	0.4795	0.2168	0.0006	0.4448	1.0000	0.0310	1.0000	0.0429	1.0000	-0.2011	1.0000	0.0407	0.5311	-0.1832
ADIPOQ	0.2254	0.3319	0.0000	-0.6562	0.0560	-0.5558	0.0002	-0.7113	1.0000	0.0196	0.0000	-0.9567	1.0000	-0.1315
ADIPOR1	0.5054	-0.1522	0.0016	-0.2778	0.3178	-0.2059	0.0001	-0.3296	1.0000	0.0747	1.0000	-0.0387	1.0000	-0.0435
ADIPOR2	0.0005	0.5040	0.0000	0.5105	0.8296	0.0816	1.0000	-0.0006	1.0000	0.0441	0.9538	0.0629	1.0000	-0.0331
ADIRF	0.8760	-0.6513	0.2268	1.7061	0.5166	-2.2541	0.7701	-3.1666	1.0000	-1.3480	1.0000	1.0157	1.0000	-2.2887
ADK	0.0005	-0.5176	0.0000	-0.3987	0.0001	-0.6184	0.0000	-0.7110	1.0000	0.0272	0.2123	0.1583	0.9932	-0.0602
ADNP	0.0361	-0.3308	0.0000	-0.4032	0.2596	0.2089	0.1100	0.1509	1.0000	0.0739	1.0000	0.0139	1.0000	0.0210
ADNP2	0.4759	-0.2090	0.0061	-0.3479	0.0001	0.7382	0.0000	0.5258	1.0000	0.1893	1.0000	0.0637	1.0000	-0.0173
ADORA2A	0.0000	0.9594	0.0000	1.0035	0.0000	1.2683	0.0000	1.1173	1.0000	-0.1328	1.0000	-0.0756	0.1128	-0.2794
ADORA2B	0.0747	-1.5583	0.0433	-1.5078	0.0011	1.5075	0.0385	1.0368	1.0000	-0.3517	1.0000	-0.2896	0.0936	-0.8148
ADPGK	0.4938	0.1810	0.0001	0.4267	0.0018	0.5583	0.0000	0.6268	1.0000	-0.0581	0.3004	0.1991	1.0000	0.0145
ADPRH	0.0010	0.4883	0.0000	0.4250	0.2885	0.2033	0.0225	0.1892	1.0000	0.0303	1.0000	-0.0208	1.0000	0.0214
ADPRHL1	0.1857	-4.5768	0.4021	-2.2287	0.7660	-1.4098	1.0000	0.0073	1.0000	-0.1273	1.0000	2.3480	0.9835	1.2979
ADPRHL2	0.4766	0.1686	0.6291	0.0701	0.0030	0.5023	0.0000	0.6613	1.0000	0.0334	1.0000	-0.0523	0.1398	0.1989
ADPRM	0.0888	-0.3451	0.0098	-0.2878	0.0000	-0.9196	0.0000	-0.9153	1.0000	-0.0863	1.0000	-0.0171	1.0000	-0.0779
ADRA1A	1.0000	0.0000	1.0000	2.2535	0.8011	3.0840	0.4370	3.7037	1.0000	0.0000	1.0000	2.3480	1.0000	0.6041
ADRA1B	1.0000	-0.3294	1.0000	0.2241	1.0000	-0.7104	0.0709	-4.6403	1.0000	0.8189	0.8605	1.3970	1.0000	-3.1370
ADRA1D	0.6294	-0.4059	0.3356	-0.5551	1.0000	0.1307	0.6452	-0.4207	1.0000	-0.4449	0.7147	-0.5829	0.0902	-0.9874
ADRA2A	0.0000	-1.2392	0.0589	-0.3732	0.0000	-1.9747	0.0231	-0.4205	1.0000	-0.0454	0.0013	0.8317	0.0000	1.5148
ADRA2B	0.6723	0.4242	1.0000	0.0628	0.7705	-0.4834	0.3904	0.7773	1.0000	-0.5137	0.3849	-0.8592	0.4346	0.7590
ADRA2C	0.9545	1.0652	1.0000	0.0000	1.0000	0.6823	1.0000	2.3257	1.0000	-2.3757	0.8607	-3.6719	1.0000	-0.7804
ADRB1	1.0000	0.0000	1.0000	2.2535	1.0000	0.0000	1.0000	0.0000	1.0000	0.0000	1.0000	2.3480	1.0000	0.0000
ADRB2	0.0297	-2.3727	0.0390	-2.4926	0.0521	1.2483	1.0000	0.2425	1.0000	-0.3640	1.0000	-0.4752	0.0435	-1.3702
ADRB3	1.0000	0.0000	1.0000	0.0000	1.0000	0.0000	1.0000	0.0000	1.0000	0.0000	1.0000	0.0000	1.0000	0.0000
ADRBK1	0.5519	-0.1550	0.7959	-0.0522	0.0033	-0.5166	0.0386	-0.1948	1.0000	-0.2152	0.8265	-0.1005	0.6736	0.1118
ADSL	1.0000	-0.0421	0.0628	-0.1897	0.0033	-0.4823	0.0000	-0.3965	1.0000	-0.0101	0.5013	-0.1457	0.8908	0.0813
ADSSL1	1.0000	-0.0505	0.0121	0.2121	0.0614	-0.3286	0.0000	-0.3571	1.0000	0.1432	0.0001	0.4183	0.6233	0.1196
ADTRP	0.6166	-0.9169	0.5228	-1.6394	0.5879	-1.0103	0.2608	-2.3113	1.0000	-0.6993	0.9082	-1.4179	0.6865	-1.9992
AEBP1	0.0000	-1.2167	0.0000	-1.0137	1.0000	0.0501	0.0000	0.5412	1.0000	-0.0745	0.5921	0.1404	0.0145	0.4214
AEBP2	0.2182	0.3329	0.0040	0.4035	0.1638	0.3507	0.2642	0.1876	1.0000	0.0641	0.7187	0.1484	0.9356	-0.0916
AEN	0.0122	-0.5298	0.0010	-0.3925	0.6017	-0.1691	0.5106	0.1065	1.0000	-0.0918	1.0000	0.0579	0.3721	0.1890
AFAP1	0.0000	-0.8956	0.0000	-0.7628	0.0000	1.2660	0.0000	1.2459	1.0000	0.1123	0.0238	0.2578	0.7006	0.0974
AFAP1L1	0.0001	-0.6672	0.0000	-0.8225	0.0145	-0.4288	0.0008	-0.3388	1.0000	-0.1064	0.0219	-0.2494	1.0000	-0.0111
AFAP1L2	0.0000	-0.8414	0.0000	-0.7062	0.1341	0.2745	0.0002	0.3079	1.0000	-0.0391	0.6553	0.1084	1.0000	-0.0010
AFF1	0.0021	0.4687	0.0000	0.4532	0.0000	0.6143	0.0000	0.5491	1.0000	-0.1326	0.3768	-0.1357	0.0757	-0.1927
AFF4	0.0007	0.7896	0.0000	0.8900	0.0000	1.7562	0.0000	1.4482	1.0000	0.2033	0.1566	0.3170	0.8556	-0.0992
AFG3L2	0.4681	0.1611	1.0000	-0.0131	0.8007	-0.0960	0.0564	-0.1660	1.0000	0.0812	0.8619	-0.0807	1.0000	0.0163
AFMID	0.0007	-0.7388	0.0000	-0.6281	1.0000	-0.0042	1.0000	0.0132	1.0000	-0.2506	1.0000	-0.1275	0.3178	-0.2275
AFTPH	1.0000	0.0171	0.2853	0.1653	0.0509	0.3979	0.0840	0.2136	1.0000	0.1007	0.2366	0.2623	0.9814	-0.0773
AGAP1	0.9043	0.0840	1.0000	-0.0259	0.0000	0.7632	0.0000	0.9224	1.0000	-0.0513	0.6178	-0.1486	0.6185	0.1132
AGAP3	0.0030	-0.5257	0.0345	-0.2500	0.0039	-0.5722	1.0000	-0.0247	1.0000	-0.2969	1.0000	-0.0080	0.2070	0.2579
AGBL1	0.0000	-3.2038	0.0000	-3.1216	0.0015	-0.9454	0.0000	-1.8879	1.0000	-0.2784	1.0000	-0.1835	0.0000	-1.2159
AGBL2	1.0000	2.1902	1.0000	0.6956	1.0000	2.2427	1.0000	-2.3176	1.0000	2.2674	1.0000	0.8994	1.0000	-2.2888
AGBL3	1.0000	0.4722	0.2891	1.3372	1.0000	-0.4948	1.0000	0.3938	1.0000	-0.8165	1.0000	0.0585	1.0000	0.0763
AGBL4	1.0000	0.5414	1.0000	-2.3959	1.0000	-2.4061	1.0000	0.8545	1.0000	-0.1089	1.0000	-3.1429	1.0000	3.2066
AGBL5	0.4006	-0.3244	0.4575	-0.1513	0.0105	-0.7745	0.0453	-0.3140	1.0000	-0.0532	0.9658	0.1330	0.0755	0.4139
AGFG1	0.1533	-0.2748	0.0121	-0.2282	1.0000	-0.0440	0.3054	-0.1213	1.0000	0.0608	0.7086	0.1198	1.0000	-0.0114
AGGF1	0.8371	0.1027	0.4833	-0.1166	0.9318	-0.0843	1.0000	-0.0117	1.0000	-0.1170	0.0421	-0.3233	1.0000	-0.0387
AGK	0.0270	0.7228	0.0000	1.1682	1.0000	-0.1174	1.0000	0.0330	1.0000	-0.3093	1.0000	0.1511	1.0000	-0.1532
AGMAT	1.0000	-2.4788	0.3205	-1.6790	0.1166	2.5445	0.7001	-0.8087	1.0000	2.7626	0.8785	3.7233	1.0000	-0.5745
AGMO	1.0000	2.1902	0.1476	-4.4851	0.5077	3.6020	1.0000	-0.6864	1.0000	4.3367	1.0000	-2.2993	1.0000	0.0762
AGO1	0.1807	0.3312	0.0050	0.5152	0.0000	1.7140	0.0000	1.5787	1.0000	0.0124	0.4548	0.2098	0.7036	-0.1171

AGO2	1.0000	-0.0195	1.0000	-0.0313	0.0195	1.3223	0.0000	1.2007	1.0000	0.0836	1.0000	0.0838	1.0000	-0.0390
AGO3	0.1575	0.5570	0.0008	0.6995	0.0000	1.1850	0.0000	1.0355	1.0000	-0.0656	1.0000	0.0862	0.6347	-0.2136
AGO4	0.0749	-0.3379	0.0002	-0.4411	0.0113	0.4454	0.0000	0.4492	1.0000	0.0059	1.0000	-0.0844	1.0000	0.0151
AGPAT2	0.1870	-0.2904	0.0647	-0.2545	0.0000	-0.9935	0.0000	-0.7429	1.0000	-0.2722	0.3425	-0.2246	1.0000	-0.0165
AGPAT3	0.2036	0.3208	0.0122	0.3434	0.3982	0.2422	1.0000	-0.0276	1.0000	0.0053	1.0000	0.0388	0.3793	-0.2608
AGPAT4	0.0000	1.2171	0.0000	0.9834	1.0000	0.0142	0.0921	-0.2865	1.0000	0.2017	1.0000	-0.0187	1.0000	-0.0912
AGPAT5	0.0012	1.4768	0.0000	1.3022	0.0000	1.6808	0.0000	1.2719	1.0000	0.3018	1.0000	0.1364	1.0000	-0.1023
AGPAT6	0.8833	0.0772	0.5851	0.0721	0.0792	-0.3055	0.0019	-0.2684	1.0000	-0.0030	1.0000	0.0039	1.0000	0.0391
AGPAT9	0.5320	0.1967	1.0000	-0.0048	0.1460	-0.3709	0.5768	-0.1172	1.0000	-0.0526	0.3609	-0.2423	0.5469	0.2076
AGPS	0.4315	-0.1824	0.2429	-0.1306	1.0000	-0.0125	1.0000	-0.0137	1.0000	-0.0280	1.0000	0.0368	1.0000	-0.0234
AGR2	1.0000	0.0000	1.0000	0.0000	1.0000	0.0000	1.0000	0.0000	1.0000	0.0000	1.0000	0.0000	1.0000	0.0000
AGR3	1.0000	2.1847	0.9148	1.2198	1.0000	0.0000	1.0000	-2.3176	1.0000	2.2674	1.0000	1.4294	1.0000	0.0000
AGRN	0.0000	-1.6785	0.0000	-1.3454	0.2213	0.2782	0.0000	0.9474	1.0000	-0.0640	0.3056	0.2821	0.0000	0.6113
AGRP	0.7153	0.7376	1.0000	-0.1785	1.0000	0.2084	1.0000	-0.2458	1.0000	-0.1211	0.7635	-1.0281	1.0000	-0.5740
AGT	0.6733	0.9995	0.6518	0.7876	1.0000	-0.1865	1.0000	0.5636	1.0000	0.2613	1.0000	0.0621	1.0000	1.0199
AGTPBP1	0.5384	3.5543	0.1463	4.3063	0.2829	3.9942	1.0000	0.0000	1.0000	0.0000	1.0000	0.7437	0.5105	-4.0491
AGTR1	1.0000	0.0590	0.5713	0.2299	0.0805	0.6795	0.9425	-0.1169	1.0000	0.2106	0.6058	0.3927	0.1785	-0.5797
AGTR2	1.0000	0.0000	1.0000	2.2535	0.8011	3.0840	1.0000	0.0000	1.0000	0.0000	1.0000	2.3480	1.0000	-3.1344
AGTRAP	0.0420	0.9506	1.0000	0.0714	1.0000	-0.0392	0.0533	-0.7813	1.0000	0.4776	0.7385	-0.3910	1.0000	-0.2585
AGXT	1.0000	0.0000	1.0000	0.0000	1.0000	0.0000	1.0000	0.0000	1.0000	0.0000	1.0000	0.0000	1.0000	0.0000
AGXT2	0.6066	1.3438	0.0007	3.7050	1.0000	-1.0191	1.0000	-2.3200	1.0000	-0.9587	0.4155	1.3929	1.0000	-2.2888
AHCTF1	0.1411	0.2958	0.0002	0.3542	0.0492	0.3766	0.0016	0.2952	1.0000	0.0332	0.8269	0.1042	1.0000	-0.0430
AHCY	0.0000	-0.9742	0.0000	-1.2158	0.0000	-0.7103	0.0000	-0.7974	1.0000	0.0140	0.1283	-0.2157	0.7770	-0.0677
AHCYL1	1.0000	-0.0256	0.5283	-0.1214	0.9551	0.0852	0.0880	0.2390	1.0000	0.0038	1.0000	-0.0815	0.5628	0.1622
AHCYL2	0.0000	-1.1384	0.0000	-0.9510	0.2574	0.2426	0.7146	0.0560	1.0000	0.0907	0.0224	0.2904	0.7263	-0.0911
AHI1	0.0024	0.8775	0.2256	0.2996	0.1524	0.4695	0.3393	-0.2620	1.0000	0.3933	0.9188	-0.1738	0.4059	-0.3336
AHNAK2	0.0000	1.5028	0.0000	1.1368	0.0000	1.4518	0.0000	1.0153	1.0000	0.0507	0.0214	-0.3023	0.0004	-0.3802
AHR	0.1380	0.3990	0.0003	0.6166	0.0000	1.0248	0.0000	1.3108	1.0000	0.1706	0.0319	0.4011	0.0015	0.4624
AHRR	0.5722	-0.1565	0.5491	-0.0704	0.8011	-0.1071	0.0281	0.1766	1.0000	0.1483	0.0153	0.2470	0.0000	0.4370
AHSA1	0.0000	-0.9566	0.0000	-0.9429	0.0261	-0.3559	0.0708	-0.1563	1.0000	0.0460	0.9687	0.0721	0.0218	0.2510
AHSA2	0.7712	0.1109	1.0000	0.0381	0.0524	0.3668	0.0000	0.4044	1.0000	0.0205	1.0000	-0.0396	1.0000	0.0634
AHSG	1.0000	2.1902	1.0000	-2.3986	1.0000	0.0000	1.0000	0.0057	1.0000	2.2732	1.0000	-2.2993	1.0000	2.3555
AICDA	0.5008	0.7806	0.1223	1.1519	0.0768	-3.0668	0.1383	-2.0325	1.0000	-0.1290	1.0000	0.2580	1.0000	0.9168
AIDA	0.0004	-0.5736	0.0000	-0.5819	0.2574	-0.2265	0.0000	-0.3682	1.0000	0.0441	1.0000	0.0477	0.8002	-0.0921
AIF1L	0.0000	-1.5039	0.0000	-1.1120	0.6298	-0.1210	0.0000	-0.5012	1.0000	-0.0189	0.0002	0.3853	0.0000	-0.3935
AIFM1	0.6135	-0.1277	0.3233	-0.1008	0.0006	-0.5309	0.0000	-0.4639	1.0000	-0.0187	1.0000	0.0204	1.0000	0.0538
AIFM2	0.0443	0.5050	0.0134	0.4876	0.0053	-0.7269	0.0001	-0.8934	1.0000	0.1941	0.6519	0.1889	1.0000	0.0339
AIFM3	0.2959	1.7028	0.9062	1.2235	1.0000	0.3438	1.0000	0.8538	1.0000	-0.9614	0.6957	-1.4508	1.0000	-0.4550
AIG1	0.5623	0.1715	0.7064	-0.0824	0.0025	-0.5999	0.0000	-0.8344	1.0000	0.0070	0.3018	-0.2338	0.3963	-0.2214
AIM1	0.0000	2.1988	0.0000	1.9399	0.0000	2.0869	0.2178	0.6958	1.0000	-0.1960	0.7475	-0.4413	0.0001	-1.5859
AIM1L	1.0000	0.0000	1.0000	2.2507	0.8033	3.0892	1.0000	2.3257	1.0000	0.0000	1.0000	2.3455	1.0000	-0.7804
AIMP1	0.0040	-0.5367	0.0000	-0.5839	0.2467	-0.2491	0.0000	-0.3871	1.0000	0.0683	1.0000	0.0333	1.0000	-0.0640
AIMP2	0.8069	-0.0944	0.6000	-0.0762	0.0007	-0.5534	0.0000	-0.5201	1.0000	-0.0261	1.0000	0.0042	1.0000	0.0128
AIP	0.9124	-0.0838	0.5486	0.1138	0.0114	-0.4995	0.0092	-0.3437	1.0000	-0.0738	0.7323	0.1350	0.9982	0.0876
AIRE	0.5077	0.7406	0.2646	0.6854	1.0000	-0.0983	1.0000	-0.0705	1.0000	0.2265	1.0000	0.1882	1.0000	0.2601
AJAP1	0.0015	-1.8154	0.0598	-0.4780	0.5243	0.4431	0.0000	1.2845	1.0000	0.1801	0.0000	1.5363	0.0000	1.0309
AK1	0.0000	-0.7553	0.0000	-0.8617	0.0000	-1.0432	0.0000	-0.5521	1.0000	0.1407	1.0000	0.0464	0.0000	0.6381
AK2	0.6195	-0.1423	0.0584	0.1831	0.0009	-0.5891	0.0230	-0.2164	1.0000	-0.1797	0.4092	0.1583	0.2021	0.1976
AK3	0.2394	0.2919	0.0731	0.3289	0.0259	-0.5058	0.0004	-0.6647	1.0000	0.0831	0.8372	0.1309	1.0000	-0.0706
AK4	1.0000	0.0035	1.0000	-0.0082	0.3692	0.2163	0.0000	0.4791	1.0000	0.0445	1.0000	0.0451	0.0239	0.3136
AK5	1.0000	0.0574	0.0113	3.2442	1.0000	-0.7104	0.9096	1.3845	1.0000	-1.4912	0.4254	1.6976	1.0000	0.6034
AK6	0.0245	-0.4507	0.0037	-0.3031	0.0010	-0.6310	0.0000	-0.7406	1.0000	0.0294	0.3972	0.1895	0.9851	-0.0749
AK8	0.1168	-0.9620	0.0000	-2.1274	0.9279	0.2442	0.6303	-0.3037	1.0000	0.2835	0.6105	-0.8692	1.0000	-0.2583
AKAP1	0.8255	-0.0881	0.1070	0.1448	0.0547	0.3372	0.0256	0.1853	1.0000	-0.1019	0.3972	0.1437	0.0352	-0.2486
AKAP10	0.7746	0.1470	0.1131	0.2570	0.0000	1.2014	0.0000	1.1632	1.0000	0.1438	0.3555	0.2669	0.7643	0.1108
AKAP11	0.0020	0.8699	0.0000	1.3165	0.0000	1.2469	0.0000	1.4685	1.0000	-0.2152	0.4663	0.2449	1.0000	0.0124
AKAP12	0.7082	-0.1053	0.0000	-0.7237	0.0000	1.1587	0.0000	0.6058	1.0000	0.0240	0.0000	-0.5819	0.0000	-0.5237
AKAP13	0.0006	-0.9965	0.0000	-0.9330	0.0000	1.9406	0.0000	1.6292	1.0000	0.0352	1.0000	0.1119	0.1829	-0.2714
AKAP14	0.2233	-2.2131	0.4801	-0.8193	0.7662	-0.6007	1.0000	0.0063	1.0000	0.3234	0.7896	1.7409	0.8600	0.9288
AKAP17A	0.5908	-0.1441	0.0000	-0.4623	1.0000	0.0168	0.0122	-0.2371	1.0000	0.1858	0.7293	-0.1195	1.0000	-0.0628
AKAP5	0.0024	-1.3768	0.0011	-1.1011	0.7002	-0.2942	0.3950	-0.3430	1.0000	0.1019	0.9217	0.3896	1.0000	0.0568
AKAP6	0.0000	-1.2773	0.0000	-1.5438	0.0191	0.4366	0.8728	-0.0497	1.0000	0.1866	1.0000	-0.0672	0.0511	-0.2950
AKAP8	0.0060	-0.4574	0.0000	-0.4388	0.0022	-0.5243	0.0374	-0.1862	1.0000	-0.2030	0.3603	-0.1721	0.4901	0.1405
AKAP8L	0.7530	-0.1139	0.9979	-0.0312	0.0739	-0.3431	0.0021	-0.2741	1.0000	0.0183	0.7290	0.1133	0.7900	0.0919
AKAP9	0.7482	-0.1234	0.0361	-0.2169	0.0002	0.6981	0.0000	0.7500	1.0000	0.0736	1.0000	-0.0070	0.5531	0.1303
AKIP1	0.0803	-0.4305	0.0542	-0.2994	0.0810	-0.4255	0.7781	-0.0848	1.0000	-0.1022	1.0000	0.0414	0.4055	0.2441
AKIRIN2	0.0037	-0.4833	0.0000	-0.6623	0.0005	-0.5548	0.0000	-0.5682	1.0000	0.0767	0.9314	-0.0897	0.9304	0.0694
AKNAD1	0.8905	-0.2281	0.4927	-0.4359	0.0029	-1.4473	0.0001	-1.8540	1.0000	-0.1235	0.9508	-0.3217	0.7898	-0.5255
AKR1A1	0.0653	0.3291	0.0000	0.2900	0.0000	-0.6897	0.0000	-0.7048	1.0000	-0.1221	0.1961	-0.1487	0.3356	-0.1315
AKR1D1	0.0673	-4.8289	0.5029	-1.6425	0.2894	-2.4999	1.0000	-0.2449	1.0000	-0.1248	1.0000	3.1976	0.6865	2.1417
AKT1	0.6190	0.1290	0.2275	-0.1164	0.1024	0.2845	0.9260	0.0343	1.0000	0.1626	0.9808	-0.0706	0.8020	-0.0828
AKT3	0.0678	-0.3484	0.0000	-0.4689	0.0038	0.4864	0.0000	0.5238	1.0000	0.0426	1.0000	-0.0655	0.8274	0.0852
AKTIP	0.7150	-0.1272	0.0059	-0.2646	0.0008	-0.6286	0.0000	-0.9022	1.0000	0.1254	1.0000	0.0006	0.6254	-0.1413
ALAD	1.0000	0.0512	0.3267	0.1702	0.7568	0.1379	0.2502	0.2020	1.0000	-0.3523	0.5322	-0.2211	0.1730	-0.2832
ALAS1	0.0080	-0.3983	0.0013	-0.2454	0.5373	0.1394	0.0222	-0.1684	1.0000	0.1495	0.0003	0.3144	0.2066	-0.1532
ALB	1.0000	0.3745	1.0000	0.3159	1.0000	-0.2020	1.0000	0.1205	1.0000	0.2712	1.0000	0.2347	1.0000	0.5981
ALCAM	0.1399	-1.8538	1.0000	-0.5518	0.7889	-0.7560	0.4715	1.1301	1.0000	-1.2533	1.0000	0.0569	1.0000	0.6427
ALDH18A1	0.4901	0.1679	0.0000	0.2956	0.0000	0.8220	0.0000	0.5471	1.0000	-0.0738	0.9591	0.0662	0.0000	-0.3430
ALDH1A1	1.0000	-0.2931	1.0000	2.2535	1.0000	-2.4061	1.0000	0.0000	1.0000	-2.3771	1.0000	0.0515	1.0000	0.0000
ALDH1A2	0.0000	-1.1999	0.0000	-1.4930	0.0363	-0.5167	0.5021	-0.1683	1.0000	0.2601	1.0000	-0.0191	0.0007	0.6157
ALDH1A3	0.0717	-1.6021	0.0000	-1.9830	0.4365	-0.8030	0.0001	1.0312	1.0000	0.7341	1.0000	0.3749	0.0000	2.5725
ALDH1L2	0.0533	-0.5910	0.0000	-0.9911	1.0000	-0.0585	0.0000	-0.8684	1.0000	-0.3019	0.0171	-0.6898	0.0000	-1.1052
ALDH2	0.2032	-0.2234	0.0166	-0.2132	0.1271	-0.2571	0.3408	-0.0983	1.0000	-0.0596	1.0000	-0.0373	0.5915	0.1046
ALDH3A2	0.0070	0.4459	0.0000	0.3588	0.3605	0.1922	0.2044	0.1274	1.0000	-0.1558	0.0299	-0.2303	0.0703	-0.2146
ALDH4A1	0.0376	-0.3493	0.0002	-0.3159	1.0000	-0.0424	0.5269	-0.0743	1.0000	-0.0727	1.0000	-0.0269	0.7124	-0.0992
ALDH5A1	0.0000	1.3113	0.0000	1.2219	0.0000	1.4460	0.0371	0.5053	1.0000	0.1020	1.0000	0.0266	0.0000	-0.8320
ALDH6A1	0.0407	-0.5343	0.0001	-0.6767	0.0001	0.8694	0.0000	0.7781	1.0000	-0.0729	0.8219	-0.2023	0.6625	-0.1593
ALDH7A1	0.3123	0.2071	0.0492	0.1548	0.9859	-0.0606	0.4202	-0.0800	1.0000	-0.0443	0.7896	-0.0842	0.9835	-0.0576
ALDH8A1	1.0000	-0.7263	1.0000	-0.1665	1.0000	-1.1037	0.4639	-3.6898	1.0000	-0.5150	1.0000	0.0554	1.0000	-3.1317
ALDH9A1	0.1004	-0.2843	0.0045	-0.2420	0.6723	-0.1213	0.0844	-0.1497	1.0000	-0.0042	1.0000	0.0503	1.0000	-0.0268
ALDOB	1.0000	0.5464	1.0000	0.6956	1.0000	-2.4055	1.0000	0.0076	1.0000	-0.1078	1.0000	0.0531	1.0000	2.3555
ALDOC	0.9742	0.0555	0.0000	0.2975	0.0087	-0.4116	0.2835	-0.0912	1.0000	-0.2523	1.0000	0.0022	0.8937	0.0733
ALG10	0.0004	0.6806	0.0000	0.6771	0.2186	0.2871	0.8152	-0.0639	1.0000	0.0950	0.8855	0.1028	0.1506	-0.2518

ALG11	0.0003	0.7627	0.0000	0.7212	0.4831	-0.2276	0.3804	-0.1907	1.0000	0.0440	1.0000	0.0158	1.0000	0.0868
ALG12	0.4685	0.1767	0.0060	0.2490	0.0000	0.8501	0.0000	0.6381	1.0000	0.0164	0.7972	0.1013	0.1366	-0.1899
ALG13	0.8225	-0.1195	0.4999	-0.1420	0.0000	-0.9041	0.0000	-0.7586	1.0000	0.0512	1.0000	0.0410	0.6386	0.2016
ALG14	0.3270	0.4188	0.5478	0.2004	0.1064	-0.6716	0.0513	-0.4962	1.0000	-0.2033	0.2686	-0.4111	1.0000	-0.0193
ALG2	0.6171	0.1793	0.4137	0.1513	0.0268	-0.5163	0.0007	-0.5094	1.0000	0.1585	0.8341	0.1418	0.7512	0.1709
ALG3	0.1613	-0.2988	0.0020	-0.3772	0.0084	-0.5078	0.0001	-0.4550	1.0000	-0.0740	0.7264	-0.1410	1.0000	-0.0165
ALG5	0.0000	1.0732	0.0000	0.9591	0.6130	-0.1857	0.0157	-0.3609	1.0000	-0.0077	0.9388	-0.1096	0.6714	-0.1767
ALG6	0.0103	0.4691	0.0047	0.2790	0.3323	-0.2364	0.0551	-0.2151	1.0000	0.1117	1.0000	-0.0666	0.6915	0.1385
ALG8	0.0596	0.5885	0.0000	0.8580	0.0000	1.1856	0.0000	0.9598	1.0000	-0.2108	1.0000	0.0695	0.0218	-0.4307
ALG9	0.2589	0.2209	0.0880	0.1903	0.1137	-0.2912	0.0326	-0.2319	1.0000	-0.0174	1.0000	-0.0362	1.0000	0.0468
ALK	0.0000	-1.2005	0.0000	-0.7663	0.0000	-0.8932	0.0000	-1.0621	1.0000	-0.1243	0.2884	0.3224	0.1815	-0.2888
ALKBH1	0.5170	-0.2068	0.7062	-0.0938	0.0037	-0.6379	0.0003	-0.4485	1.0000	0.0297	0.8385	0.1554	0.4096	0.2246
ALKBH2	0.6004	0.2446	0.2745	-0.2823	0.9262	0.1485	0.1386	-0.3393	1.0000	0.2352	0.6924	-0.2785	0.7602	-0.2456
ALKBH3	0.0661	0.3908	0.8078	0.0666	0.0990	-0.3593	0.1783	-0.2044	1.0000	0.1320	0.5897	-0.1796	0.1798	0.2932
ALKBH4	0.6733	-0.1609	0.0061	-0.3772	0.3332	-0.2528	0.2348	-0.1851	1.0000	-0.0004	0.5898	-0.2032	1.0000	0.0738
ALKBH5	0.8048	-0.1103	0.4486	-0.1221	0.0480	0.3753	0.0019	0.3651	1.0000	0.0745	1.0000	0.0745	1.0000	0.0682
ALKBH8	1.0000	0.0732	1.0000	-0.0399	0.2013	-0.3658	0.0051	-0.4813	1.0000	-0.0515	0.8915	-0.1525	0.8496	-0.1616
ALMS1	0.2496	0.2605	0.0011	0.4181	0.0025	0.5524	0.0000	0.6800	1.0000	0.0226	0.3388	0.1935	0.4440	0.1563
ALOX5	0.2760	-1.2231	1.0000	-0.1730	0.3221	0.8435	0.0049	1.9633	1.0000	-1.2117	1.0000	-0.1537	1.0000	-0.0880
ALOX5AP	1.0000	0.0000	1.0000	2.2535	1.0000	0.0000	1.0000	0.0000	1.0000	0.0000	1.0000	2.3480	1.0000	0.0000
ALPK1	0.0001	1.0430	0.0000	0.7320	0.2494	-0.4136	0.0415	-0.4775	1.0000	0.1005	0.7617	-0.1970	1.0000	0.0408
ALPK2	0.3154	-0.3123	0.0165	-0.4000	0.8620	0.1212	0.0001	-0.6534	1.0000	-0.0195	1.0000	-0.0955	0.0000	-0.7900
ALPK3	1.0000	0.0076	1.0000	0.0178	0.1544	0.3088	0.0000	0.6278	1.0000	-0.1866	0.3247	-0.1632	0.4523	0.1374
ALS2	0.7269	-0.1267	0.0094	-0.3733	0.5595	-0.1534	0.0210	-0.2266	1.0000	0.1869	1.0000	-0.0478	0.6819	0.1187
ALS2CL	0.0000	1.6260	0.0000	1.7943	0.0174	0.7671	0.2561	0.3642	1.0000	0.0315	0.7051	0.2132	0.3839	-0.3636
ALX1	1.0000	0.0000	1.0000	0.0000	1.0000	0.0000	1.0000	0.0000	1.0000	0.0000	1.0000	0.0000	1.0000	0.0000
ALX3	0.8249	-3.3439	1.0000	0.0000	1.0000	-0.1833	1.0000	2.3242	1.0000	-3.2319	1.0000	0.0000	1.0000	-0.7782
ALX4	0.0000	0.6618	0.0000	0.4935	0.0001	0.6341	0.0000	0.7367	1.0000	0.0805	0.9090	-0.0753	0.1404	0.1885
ALYREF	0.1851	-0.2544	0.3144	-0.1256	0.0000	-0.8924	0.0000	-0.8834	1.0000	-0.0733	1.0000	0.0675	0.9838	-0.0589
AMACR	0.8360	0.1163	0.2841	0.1468	0.0053	-0.6267	0.0000	-0.6918	1.0000	-0.1036	1.0000	-0.0598	0.5724	-0.1626
AMBP	1.0000	2.1902	1.0000	0.0000	1.0000	0.0000	1.0000	0.0000	1.0000	0.0000	1.0000	-2.2992	1.0000	0.0000
AMD1	0.3572	0.1848	0.0000	0.3364	1.0000	0.0023	1.0000	0.0205	1.0000	-0.0588	0.6036	0.1051	1.0000	-0.0354
AMDHD1	0.0039	1.2033	0.1080	0.4922	0.0213	-1.1916	0.0053	-1.0085	1.0000	0.4742	0.9798	-0.2236	0.5128	0.6619
AMDHD2	0.1790	-0.2821	0.3880	-0.1169	0.0021	-0.5763	0.0011	-0.3350	1.0000	-0.0409	0.6790	0.1372	0.2926	0.2068
AMER1	0.6482	0.2237	0.0111	0.4946	0.0000	0.9918	0.0003	0.6343	1.0000	-0.0262	0.6211	0.2568	0.0898	-0.3776
AMER2	1.0000	-2.4776	1.0000	0.0000	1.0000	-2.4055	1.0000	2.3242	1.0000	-2.3757	1.0000	0.0000	1.0000	2.3543
AMER3	1.0000	0.0000	1.0000	0.0000	1.0000	0.0000	1.0000	0.0000	1.0000	0.0000	1.0000	0.0000	1.0000	0.0000
AMFR	0.0162	0.3889	0.0000	0.4506	0.3017	-0.2146	0.6271	-0.0668	1.0000	-0.0177	1.0000	0.0562	0.5436	0.1360
AMH	1.0000	-0.8591	1.0000	-0.1598	1.0000	-0.1865	0.7701	-3.1666	1.0000	-0.6504	1.0000	0.0559	0.7287	-3.6634
AMICA1	1.0000	2.1848	0.7674	3.0957	1.0000	0.0000	1.0000	2.3242	1.0000	0.0000	1.0000	0.9036	1.0000	2.3543
AMIGO1	0.3953	0.3573	1.0000	0.0079	0.4428	0.3219	0.0082	0.5960	1.0000	-0.1033	0.3609	-0.4401	0.9029	0.1778
AMIGO2	0.0000	-2.9244	0.0000	-3.6491	0.1470	0.4057	0.0274	0.3693	1.0000	0.1044	0.7635	-0.6086	1.0000	0.0745
AMIGO3	0.5086	0.4972	0.2495	0.8039	0.5806	0.4894	0.0028	1.3381	1.0000	-0.4258	1.0000	-0.1134	0.6654	0.4410
AMMECR1	0.0002	1.5859	0.0000	1.2966	0.0000	2.2186	0.0000	1.8467	1.0000	0.4728	1.0000	0.1958	1.0000	0.1058
AMMECR1L	0.1134	-0.2907	0.0000	-0.4743	0.0139	-0.4404	0.0013	-0.2884	1.0000	0.0806	0.9073	-0.0901	0.1287	0.2372
AMN	1.0000	-2.4776	0.4431	3.6192	1.0000	-0.1591	1.0000	2.3257	1.0000	-2.3757	0.8607	3.7287	1.0000	0.0649
AMN1	0.9867	0.2875	0.1362	1.0256	0.3942	0.7363	0.0171	1.3577	1.0000	-0.3454	1.0000	0.4040	1.0000	0.2781
AMOT	0.0001	0.6418	0.0000	0.6604	0.5719	0.1495	0.3183	-0.1193	1.0000	0.0600	0.8785	0.0906	0.1717	-0.2038
AMOTL1	0.7721	-0.2232	0.0958	0.5406	0.0000	1.4272	0.0000	1.5469	1.0000	-0.2402	0.1724	0.5392	1.0000	-0.1088
AMOTL2	0.0004	-0.6427	0.0000	-0.4146	0.8388	0.0997	0.0000	0.3173	1.0000	0.0451	0.0084	0.2854	0.0083	0.2677
AMPD1	0.0000	-4.5715	0.0000	-5.0612	0.2635	0.5568	0.0254	0.8152	1.0000	-0.3094	1.0000	-0.7956	1.0000	-0.0471
AMPD2	0.9489	0.1268	1.0000	-0.0601	0.0059	0.7927	0.0000	0.9123	1.0000	0.0767	1.0000	-0.0972	0.5781	0.2040
AMPD3	0.0000	-0.8940	0.0000	-1.1820	0.0000	1.0806	0.0000	0.6373	1.0000	0.2396	1.0000	-0.0361	0.1952	-0.1993
AMPH	1.0000	0.0000	1.0000	0.0000	1.0000	2.2472	1.0000	0.0000	1.0000	0.0000	1.0000	0.0000	1.0000	-2.2909
AMT	0.1374	-0.3852	0.0447	-0.3052	0.0007	-0.8112	0.0001	-0.5524	1.0000	-0.0323	1.0000	0.0590	0.6292	0.2327
AMZ1	0.1463	-2.3768	0.0006	-5.8188	0.4037	-1.3142	0.1985	-1.3282	1.0000	0.3875	1.0000	-3.1429	1.0000	0.3817
ANAPC1	0.1949	0.2436	0.0119	0.2553	1.0000	0.0394	0.4757	-0.0924	1.0000	0.0114	1.0000	0.0354	0.5972	-0.1160
ANAPC10	0.2712	-0.3149	0.3969	-0.1665	0.0452	-0.4742	0.0051	-0.4224	1.0000	-0.0961	1.0000	0.0632	1.0000	-0.0391
ANAPC13	0.0613	-0.4781	0.2489	-0.2363	0.0000	-0.9255	0.0000	-0.7205	1.0000	-0.0965	0.9744	0.1584	1.0000	0.1151
ANAPC15	0.0000	-0.7358	0.0000	-0.8391	0.0000	-1.0085	0.0000	-0.7984	1.0000	-0.1308	0.3015	-0.2218	0.9809	0.0851
ANAPC16	0.0000	-0.6483	0.0000	-0.6847	0.0000	-0.8190	0.0000	-0.5642	1.0000	-0.0493	1.0000	-0.0734	0.1557	0.2114
ANAPC2	0.2891	0.2743	0.0003	0.4628	1.0000	-0.0337	0.3413	0.1690	1.0000	-0.2401	1.0000	-0.0407	1.0000	-0.0318
ANAPC4	0.8336	0.0935	0.9645	0.0353	0.8033	-0.0978	0.0001	-0.3604	1.0000	0.0581	1.0000	0.0124	0.1769	-0.1983
ANAPC5	0.0000	0.9407	0.0000	1.2463	0.0585	0.4543	0.4934	0.1603	1.0000	-0.0591	0.2413	0.2567	0.0876	-0.3481
ANAPC7	0.0434	0.4095	0.0060	0.3177	0.8783	0.0963	0.2920	-0.1493	1.0000	0.1049	1.0000	0.0244	0.6749	-0.1354
ANGEL1	0.7381	-0.1110	0.4534	-0.0895	0.0000	0.6612	0.0000	0.6998	1.0000	0.1261	0.2872	0.1603	0.1325	0.1702
ANGEL2	0.0000	-1.4159	0.0000	-1.6046	0.0000	-1.1923	0.0000	-0.9921	1.0000	0.0631	0.8175	-0.1130	0.0169	0.2683
ANGPT1	1.0000	-2.4788	0.7666	3.0922	1.0000	-0.1630	1.0000	2.3257	1.0000	-2.3771	1.0000	3.1976	1.0000	0.0660
ANGPT2	0.0016	-0.8186	0.0000	-0.8327	0.0000	-1.0635	0.1285	-0.2377	1.0000	0.2378	0.6728	0.2367	0.0000	1.0670
ANGPT4	0.0794	-2.6562	1.0000	-0.1749	0.6927	-0.8251	1.0000	-0.6895	1.0000	-1.2217	1.0000	1.2775	0.8734	-1.0836
ANGPTL1	0.0021	-0.8602	0.0000	-1.0512	0.0000	-1.5389	0.0000	-1.2493	1.0000	0.2020	1.0000	0.0220	0.2386	0.4985
ANGPTL2	1.0000	-0.0035	0.2059	-0.1612	0.0000	1.9742	0.0000	1.7055	1.0000	0.2801	0.8565	0.1349	1.0000	0.0175
ANGPTL3	0.8226	3.0199	1.0000	0.0000	1.0000	0.0000	1.0000	0.0000	1.0000	0.0000	1.0000	-3.1429	1.0000	0.0000
ANGPTL4	0.0000	0.9339	0.0000	0.7969	0.9832	-0.0614	0.0002	0.3474	1.0000	0.0698	1.0000	-0.0547	0.0000	0.4843
ANGPTL5	0.0000	-2.1728	0.0000	-2.4686	0.1470	-0.7126	0.0111	-0.5964	1.0000	0.4308	1.0000	0.1480	0.5000	0.5476
ANGPTL7	1.0000	0.1206	1.0000	-0.1429	0.7660	-1.4098	0.9096	1.3854	1.0000	-2.1881	0.5011	-2.4835	1.0000	0.6050
ANK1	0.0000	-3.2639	0.0000	-3.8093	0.0022	-0.6692	0.0248	-0.2791	1.0000	0.1047	0.7487	-0.4274	0.0021	0.4979
ANK2	0.0003	1.2277	0.0004	0.8843	0.6800	-0.3143	0.0284	-0.7416	1.0000	-0.1143	0.1090	-0.4397	0.2981	-0.5338
ANK3	0.5198	0.3934	1.0000	-0.0482	0.0000	2.0232	0.0000	1.4205	1.0000	0.2216	0.9521	-0.2063	0.1825	-0.3745
ANKDD1A	0.0000	-1.6258	0.0000	-1.2952	0.6553	-0.2032	0.0069	-0.5242	1.0000	0.1111	0.4575	0.4560	0.7602	-0.2047
ANKDD1B	1.0000	-1.2473	0.2550	-2.4832	1.0000	-0.5781	0.7732	-0.9367	1.0000	0.4324	1.0000	-0.8002	1.0000	0.0768
ANKEF1	1.0000	0.0000	1.0000	0.0000	1.0000	2.2472	1.0000	0.0000	1.0000	0.0000	1.0000	0.0000	1.0000	-2.2909
ANKFN1	0.5086	3.5431	1.0000	-0.1625	1.0000	0.0000	0.7701	-3.1666	1.0000	3.1126	1.0000	-0.4752	1.0000	0.0000
ANKFY1	1.0000	-0.0394	0.1744	0.1383	0.0020	0.5052	0.0000	0.5754	1.0000	-0.1278	1.0000	0.0623	1.0000	-0.0523
ANKH	0.6111	-0.1600	0.4253	-0.1222	0.1074	0.3312	0.4133	0.1247	1.0000	-0.0274	1.0000	0.0226	0.1370	-0.2295
ANKIB1	0.0016	0.8343	0.0000	0.9754	0.0000	1.6194	0.0000	1.4499	1.0000	0.2344	0.0120	0.3889	0.9649	0.0714
ANKK1	1.0000	0.5464	0.0121	-5.3003	0.1954	2.5403	0.6831	-0.8102	1.0000	2.7696	1.0000	-3.1469	1.0000	-0.5732
ANKLE1	0.2060	0.5111	0.0058	0.5824	0.2816	0.3710	0.8573	-0.1229	1.0000	-0.0432	1.0000	0.0403	0.0882	-0.5311
ANKLE2	0.0576	0.3594	0.0013	0.3430	0.0000	1.0722	0.0000	0.8477	1.0000	0.1763	0.4136	0.1730	1.0000	-0.0427
ANKMY1	0.0972	1.3508	0.0483	1.3268	0.5881	-0.7621	1.0000	0.1553	1.0000	-0.1302	1.0000	-0.1433	0.9862	0.7925
ANKMY2	1.0000	-0.0319	0.0256	-0.1881	0.0060	-0.4636	0.0000	-0.6535	1.0000	0.1867	1.0000	0.0432	1.0000	0.0019
ANKRA2	0.4131	0.1846	0.4567	0.0939	0.9933	0.0612	1.0000	0.0158	1.0000	0.0544	1.0000	-0.0241	1.0000	0.0137
ANKRD1	0.0000	-1.9421	0.0000	-2.1037	0.0000	-0.7079	0.0000	-0.8280	1.0000	0.0987	1.0000	-0.0508	1.0000	-0.0161

ANKRD10	0.0000	-0.7057	0.0000	-0.9067	0.2017	0.2710	0.0496	0.1872	1.0000	0.0613	0.6519	-0.1266	1.0000	-0.0169
ANKRD11	1.0000	0.0176	0.0115	0.2535	0.0000	0.9360	0.0000	0.9556	1.0000	-0.0011	0.0271	0.2478	1.0000	0.0237
ANKRD12	1.0000	0.0023	0.2378	-0.2076	0.8829	-0.1041	1.0000	-0.0347	1.0000	0.2281	1.0000	0.0324	0.1071	0.3038
ANKRD13A	0.6428	0.1165	0.2229	0.1065	0.5252	-0.1394	0.0376	-0.1623	1.0000	0.0425	1.0000	0.0448	1.0000	0.0249
ANKRD13B	0.0000	-1.2876	0.0000	-1.0292	0.6906	-0.1850	0.0523	0.3575	1.0000	-0.6182	0.6187	-0.3469	1.0000	-0.0686
ANKRD13C	0.0112	-0.4167	0.0000	-0.3868	0.0022	0.4817	0.0000	0.5011	1.0000	-0.0183	1.0000	0.0242	1.0000	0.0064
ANKRD16	0.1134	-0.3816	0.0469	-0.3029	0.5851	-0.1877	0.0700	-0.2707	1.0000	-0.0198	1.0000	0.0717	1.0000	-0.0956
ANKRD17	0.9257	-0.0697	0.0303	-0.2082	0.0076	0.4304	0.0001	0.3247	1.0000	0.0902	1.0000	-0.0356	1.0000	-0.0104
ANKRD2	0.0000	-3.5174	0.0000	-3.4556	0.0000	-1.3351	0.0000	-1.0656	1.0000	-0.1037	1.0000	-0.0307	0.5736	0.1713
ANKRD22	1.0000	2.1902	0.9062	1.2214	1.0000	0.0000	1.0000	-2.3200	1.0000	2.2733	1.0000	1.4306	1.0000	0.0000
ANKRD24	0.0711	1.0541	0.0001	1.7760	0.0042	-2.7591	0.2318	-1.1818	1.0000	-0.5653	1.0000	0.1693	1.0000	1.0199
ANKRD27	0.0414	0.5275	1.0000	-0.0511	0.4166	0.2548	0.0007	-0.4972	1.0000	0.5082	1.0000	-0.0566	0.4216	-0.2374
ANKRD28	0.9037	0.1027	0.2459	-0.1652	0.3384	0.2582	0.4861	-0.1107	1.0000	0.3238	1.0000	0.0683	1.0000	-0.0412
ANKRD29	0.0000	1.0124	0.0000	1.1918	0.0025	0.6331	0.0003	0.4574	1.0000	-0.0092	0.3978	0.1818	0.5010	-0.1815
ANKRD31	0.0439	0.9060	0.0896	0.6777	0.7693	-0.2836	0.0733	0.6728	1.0000	-0.0177	1.0000	-0.2331	0.0425	0.9432
ANKRD32	0.0017	0.6051	0.0000	0.6959	0.1622	-0.3302	0.1405	-0.2562	1.0000	-0.0366	1.0000	0.0662	1.0000	0.0424
ANKRD33	0.0000	-3.9922	0.0000	-3.4554	0.5638	-0.6055	0.3617	-0.4889	1.0000	0.4188	1.0000	0.9743	0.6819	0.5410
ANKRD33B	0.9650	1.0748	1.0000	2.2506	1.0000	-2.4055	1.0000	2.3242	1.0000	-2.3757	1.0000	-1.3320	1.0000	2.3543
ANKRD34B	0.5600	1.3115	0.6501	1.6027	1.0000	0.2017	0.2439	2.3286	1.0000	-1.4938	0.7765	-1.2119	1.0000	0.6332
ANKRD34C	0.7202	0.2080	0.0000	0.9197	0.0002	0.9201	0.0000	1.5482	1.0000	-0.5057	0.8175	0.2172	0.8939	0.1265
ANKRD40	0.7791	0.1697	0.8824	0.0988	0.0169	-0.6699	0.1322	-0.3413	1.0000	-0.1299	0.8840	-0.1898	0.8277	0.2028
ANKRD42	1.0000	0.0617	0.8027	0.1464	0.0001	-1.5726	0.0001	-1.1843	1.0000	-0.0870	1.0000	0.0120	0.9680	0.3074
ANKRD44	0.0000	1.5027	0.0000	1.2197	0.0000	1.0722	0.0000	0.6217	1.0000	0.1493	0.7561	-0.1197	0.0533	-0.2947
ANKRD46	0.2685	-0.2215	0.0058	-0.2813	0.9144	-0.0748	0.3894	-0.1040	1.0000	-0.0564	0.8433	-0.1033	0.8724	-0.0800
ANKRD49	0.2611	-0.3278	0.0007	-0.4322	0.5041	-0.2233	0.0000	-0.6911	1.0000	0.2135	0.9428	0.1215	0.3100	-0.2482
ANKRD50	0.0000	-0.7526	0.0000	-0.5897	0.0000	0.8291	0.0000	0.8620	1.0000	0.0326	0.5907	0.2086	0.9721	0.0709
ANKRD52	0.0076	0.8935	0.0000	0.9144	0.0000	1.5493	0.0000	1.1876	1.0000	0.2474	0.6565	0.2822	0.9591	-0.1067
ANKRD54	0.5322	0.1655	0.2019	0.1653	0.4669	0.1875	0.0125	0.2725	1.0000	-0.1334	0.7603	-0.1221	1.0000	-0.0427
ANKRD55	0.9241	-0.5614	1.0000	0.3667	0.8692	-0.9639	1.0000	0.5371	1.0000	-1.8108	1.0000	-0.8861	1.0000	-0.3110
ANKRD6	0.9867	-0.2079	1.0000	0.0158	0.8297	0.3233	0.0368	0.7978	1.0000	-0.2083	1.0000	0.0249	0.8939	0.2637
ANKRD60	1.0000	-0.3217	0.8634	0.4770	0.0859	2.2539	0.8620	0.6591	1.0000	1.1024	0.6123	1.9312	1.0000	-0.4903
ANKRD61	0.0387	1.8291	0.0381	1.5636	0.9860	0.5561	0.6138	0.7627	1.0000	-0.1208	1.0000	-0.3829	1.0000	0.0819
ANKRD66	1.0000	-0.0122	0.8192	-0.1167	0.0303	-0.6741	0.0377	-0.4446	1.0000	0.0571	1.0000	-0.0349	0.6385	0.2944
ANKRD9	0.2068	-0.6132	0.3286	-0.4587	0.0664	-0.8614	1.0000	-0.0799	1.0000	-0.4101	1.0000	-0.2443	0.8165	0.3776
ANKS1A	1.0000	0.0170	0.7674	0.2831	0.0009	1.2887	0.0000	1.7702	1.0000	-0.3218	1.0000	-0.0418	1.0000	0.1692
ANKS1B	0.0992	0.7534	0.0000	1.0565	0.0040	1.1346	0.0033	0.8165	1.0000	-0.1864	1.0000	0.1333	0.1389	-0.4966
ANKS3	0.5349	-0.1886	0.2501	-0.1666	1.0000	-0.0574	0.8423	-0.0616	1.0000	-0.0980	1.0000	-0.0635	0.9060	-0.0968
ANKS4B	0.8178	0.7447	1.0000	-0.1473	0.0160	2.0124	0.6172	1.7722	1.0000	-2.1858	0.1322	-3.1129	0.0072	-2.4339
ANKZF1	0.5674	-0.1866	0.5439	-0.1314	0.0423	-0.4513	0.0441	-0.3230	1.0000	-0.1055	1.0000	-0.0385	1.0000	0.0273
ANLN	0.0000	1.3937	0.0000	1.8633	0.0000	0.8020	0.0275	0.2321	1.0000	-0.2729	0.1542	0.2088	0.0000	-0.8373
ANO1	0.0000	-3.2041	0.0000	-3.4090	0.0260	0.5727	0.0011	-0.5297	1.0000	-0.3509	0.6050	-0.5434	0.0000	-1.4467
ANO10	0.0524	0.4010	0.0005	0.4074	0.2609	0.2639	1.0000	0.0380	1.0000	0.0980	0.8269	0.1156	0.7287	-0.1240
ANO3	0.0011	-0.9312	0.0000	-1.3980	0.1064	0.4605	0.0000	0.8826	1.0000	0.3832	1.0000	-0.0726	0.0000	0.8069
ANO4	0.9650	1.0594	1.0000	0.6956	1.0000	-0.1624	1.0000	0.0062	1.0000	-0.1078	1.0000	-0.4715	1.0000	0.0652
ANO5	0.0000	1.1825	0.0000	1.2766	0.0000	1.8924	0.0000	1.4946	1.0000	0.2953	0.0052	0.4027	0.8364	-0.0970
ANO8	0.0000	-1.1407	0.0000	-1.0475	0.0324	0.4453	0.0000	0.6612	1.0000	-0.2233	0.9979	-0.1173	1.0000	-0.0011
ANO9	0.0701	-5.4052	0.0776	-4.7349	0.4243	-1.7086	0.2466	-2.3158	1.0000	-0.6929	1.0000	0.0000	1.0000	-1.3081
ANP32A	0.7763	0.1101	1.0000	0.0279	0.9615	-0.0697	1.0000	0.0136	1.0000	0.0884	1.0000	0.0186	0.3356	0.1762
ANP32E	0.0003	0.5186	0.0000	0.4917	1.0000	0.0362	0.7744	-0.0428	1.0000	0.0272	1.0000	0.0124	1.0000	-0.0466
ANPEP	0.9222	0.1316	0.0880	0.3974	0.5800	-0.2392	0.8544	-0.1122	1.0000	-0.2584	1.0000	0.0191	1.0000	-0.1279
ANTXRL	0.6551	-0.1692	1.0000	-0.0350	0.0013	0.6434	0.0000	1.0515	1.0000	-0.3349	0.6450	-0.1885	0.9660	0.0784
ANXA10	0.3148	3.9252	1.0000	-0.1625	0.8033	3.0790	1.0000	0.0059	1.0000	3.1126	1.0000	-0.8610	1.0000	0.0729
ANXA11	1.0000	-0.0278	0.0342	-0.2002	0.3763	0.1834	0.0041	0.2561	1.0000	0.1437	1.0000	-0.0161	0.0548	0.2224
ANXA13	1.0000	0.3418	0.6547	-1.9366	0.9361	-1.5509	0.9875	-0.9155	1.0000	0.2728	0.8723	-2.0057	1.0000	0.9164
ANXA2	0.0046	-0.4105	0.0000	-0.3275	0.4449	0.1604	0.7685	0.0449	1.0000	0.0437	0.2687	0.1392	0.9149	-0.0663
ANXA4	0.0046	-0.4413	0.0000	-0.5205	1.0000	-0.0253	0.8502	-0.0405	1.0000	0.1312	1.0000	0.0644	0.5701	0.1216
ANXA5	0.0000	-0.6783	0.0000	-0.7519	0.0070	0.4408	0.0067	0.1726	1.0000	0.0605	1.0000	-0.0005	0.0282	-0.2020
ANXA6	0.0045	-0.5027	0.0000	-0.4835	0.4049	-0.1954	0.0024	-0.2036	1.0000	-0.0126	1.0000	0.0194	1.0000	-0.0161
ANXA7	0.5362	0.1492	0.4391	-0.0807	0.0000	-0.8208	0.0000	-0.8705	1.0000	0.0391	0.1387	-0.1785	1.0000	-0.0057
AOAH	0.0001	3.2506	0.0033	1.9507	0.2417	1.5980	0.1233	1.3860	1.0000	0.8125	0.9277	-0.4665	0.9835	0.6103
AOX1	0.0000	0.6966	0.0000	0.7736	0.6691	-0.1392	0.6172	-0.0882	1.0000	-0.0740	1.0000	0.0152	1.0000	-0.0171
AP1B1	1.0000	-0.0105	0.3389	0.1114	0.2953	0.2165	0.0000	0.3553	1.0000	-0.1831	1.0000	-0.0495	1.0000	-0.0394
AP1G1	0.0074	0.5291	0.0000	0.6765	0.0000	0.8751	0.0000	0.9644	1.0000	-0.1108	1.0000	0.0495	1.0000	-0.0161
AP1M1	1.0000	-0.0080	0.2885	0.0955	0.5231	-0.1563	0.0054	-0.2072	1.0000	0.0203	0.3566	0.1363	1.0000	-0.0252
AP1S2	0.0000	1.1212	0.0000	1.0858	0.0000	0.9323	0.0000	0.7812	1.0000	-0.0570	1.0000	-0.0787	0.2861	-0.2002
AP1S3	1.0000	0.1993	0.4431	-3.7876	1.0000	-1.0206	0.4370	-3.6973	1.0000	0.4112	0.8607	-3.6719	1.0000	-2.2888
AP2A2	1.0000	-0.0215	0.0312	0.1686	0.0950	0.2878	0.0000	0.3076	1.0000	-0.0932	0.6331	0.1095	0.8378	-0.0677
AP2B1	0.1879	-0.2326	0.7819	-0.0454	0.0041	0.4465	0.0000	0.5214	1.0000	-0.1532	1.0000	0.0462	0.8752	-0.0729
AP2M1	0.0316	0.3418	0.0000	0.4533	0.0019	0.4660	0.0000	0.4450	1.0000	-0.1059	1.0000	0.0182	0.4484	-0.1213
AP3B1	0.5173	0.1543	0.1904	0.1250	0.1280	0.2778	0.3855	0.0939	1.0000	0.0629	1.0000	0.0461	0.5856	-0.1157
AP3B2	0.7412	-0.2219	0.4244	-0.2132	1.0000	0.0160	0.0589	0.3802	1.0000	0.0916	1.0000	0.1116	0.0918	0.4611
AP3D1	0.0960	-0.2845	0.0006	-0.2713	0.1779	0.2392	0.0000	0.3002	1.0000	-0.0083	1.0000	0.0172	0.9692	0.0579
AP3M1	0.0000	-0.7786	0.0000	-0.7377	0.5503	-0.1317	0.0089	-0.1771	1.0000	0.0888	0.5561	0.1415	0.9896	0.0487
AP3M2	0.0000	0.7143	0.0000	0.6662	0.0285	-0.4348	0.0000	-0.4729	1.0000	0.0574	1.0000	0.0220	1.0000	0.0243
AP3S1	0.0077	0.5451	0.0000	0.4906	0.5444	0.1868	1.0000	-0.0107	1.0000	0.1649	0.8547	0.1226	1.0000	-0.0270
AP4B1	1.0000	-0.0611	1.0000	0.0048	0.3017	-0.2359	0.0648	-0.2365	1.0000	0.0577	0.7800	0.1362	1.0000	0.0637
AP4S1	0.2465	-0.4230	0.0738	-0.3144	0.0045	-0.8611	0.0000	-1.3257	1.0000	-0.1538	1.0000	-0.0322	0.0198	-0.6131
AP5M1	0.5086	-0.2613	0.2143	0.2971	0.1371	-0.4455	0.0499	-0.4598	1.0000	-0.2321	0.3818	0.3361	0.7140	-0.2440
AP5S1	0.0043	-0.6060	0.0000	-0.5887	0.0000	-1.0945	0.0000	-0.5821	1.0000	-0.1736	0.8895	-0.1433	0.3766	0.3468
AP5Z1	1.0000	-0.0273	0.9876	-0.0429	0.0031	-0.5959	0.0019	-0.3826	1.0000	-0.1114	0.9050	-0.1151	0.9469	0.1085
APAF1	0.4078	0.2156	0.8432	-0.0680	0.6362	-0.1614	0.0004	-0.4206	1.0000	0.1915	1.0000	-0.0786	1.0000	-0.0624
APBA1	1.0000	-0.1814	0.1370	0.5468	0.0028	1.0929	0.0004	1.1263	1.0000	-0.1321	0.3957	0.6069	1.0000	-0.0925
APBA2	0.0018	-1.3192	0.0000	-1.2743	0.0000	2.0409	0.0000	1.1353	1.0000	0.2074	1.0000	0.2645	0.0000	-0.6930
APBA3	0.0000	-1.0336	0.0000	-0.8454	0.0058	-0.4837	0.9868	0.0365	1.0000	-0.2322	1.0000	-0.0321	0.0224	0.2929
APBB2	0.0001	-0.7385	0.0000	-0.6523	1.0000	0.0453	0.0151	-0.2556	1.0000	0.1896	0.2746	0.2884	0.8749	-0.1068
APC	0.0000	-1.0290	0.0000	-1.1131	0.0263	0.3897	0.0167	0.1978	1.0000	0.1569	0.9514	0.0853	1.0000	-0.0302
APC2	1.0000	-0.2920	1.0000	-0.1429	1.0000	0.6783	0.9164	1.3892	1.0000	-0.1078	1.0000	0.0515	1.0000	0.6054
APCDD1	0.3668	-1.0386	0.1462	-1.0870	1.0000	-0.1967	0.0581	0.9602	1.0000	0.4071	1.0000	0.3731	0.0141	1.5716
APCDD1L	0.0001	1.4898	0.0029	0.9226	0.0015	1.2120	0.3733	-0.4838	1.0000	0.0762	0.3109	-0.4789	0.0000	-1.6147
APEH	0.0505	-0.3636	0.0241	-0.2357	0.0064	-0.4776	0.0004	-0.3553	1.0000	-0.1746	1.0000	-0.0351	1.0000	-0.0468
APEX1	0.3194	-0.6764	0.1546	-0.6833	0.0000	2.2253	0.0000	1.5651	1.0000	0.6609	0.6096	0.6672	1.0000	0.0098
APH1A	1.0000	0.0295	0.7997	-0.0555	0.0602	-0.3760	0.0015	-0.3201	1.0000	-0.0402	0.7681	-0.1129	1.0000	0.0221
API5	0.4649	0.1609	0.0003	0.2777	0.0213	0.3827	0.0000	0.3191	1.0000	0.0118	0.3932	0.1411	1.0000	-0.0465

APIP	1.0000	-0.0503	0.5915	-0.1053	0.7860	-0.1289	0.0170	-0.3056	1.0000	0.1228	1.0000	0.0799	1.0000	-0.0478
APITD1	0.0009	0.7351	0.0000	0.8979	0.0291	-0.5759	0.0000	-0.9666	1.0000	-0.0401	0.8607	0.1346	0.1563	-0.4239
APLF	0.0241	0.6771	0.2391	0.3098	1.0000	0.1186	0.3141	-0.2913	1.0000	0.0969	0.6568	-0.2586	0.5510	-0.3082
APLN	0.0702	-1.4076	0.0000	-2.5392	0.0344	-1.5004	0.0823	-0.8717	1.0000	0.4286	1.0000	-0.6949	0.3841	1.0630
APLNR	0.8249	3.0255	1.0000	0.0000	1.0000	0.0000	1.0000	0.0000	1.0000	0.0000	1.0000	-3.1469	1.0000	0.0000
APLP2	0.1870	0.2324	0.0002	0.2484	0.0007	0.5082	0.0000	0.5687	1.0000	0.1203	0.2066	0.1489	0.0509	0.1858
APMAP	1.0000	0.0202	0.3971	0.1208	0.1120	0.3410	0.0000	0.4739	1.0000	0.0553	0.4801	0.1673	0.2809	0.1933
APOA1	0.0000	-3.1105	0.0000	-3.1125	0.0000	-4.1998	0.0000	-2.4038	1.0000	0.1647	0.8785	0.1757	0.0000	1.9687
APOA4	0.1256	1.0700	0.5968	0.3324	0.5939	-0.6328	0.0357	-1.1112	1.0000	0.7424	1.0000	0.0177	1.0000	0.2681
APOA5	0.3425	-0.5882	0.1713	-0.4912	0.3425	-0.5562	0.0055	-0.9476	1.0000	-0.0472	1.0000	0.0617	0.6594	-0.4338
APOB	1.0000	-2.4788	0.7666	-3.2534	0.4424	1.8933	0.3378	1.6944	1.0000	0.7357	1.0000	0.0000	1.0000	0.5434
APOBEC2	0.0000	-1.5768	0.0000	-2.0697	0.1795	-0.2458	0.0000	-0.3846	1.0000	0.0497	0.0006	-0.4307	0.7521	-0.0834
APOBEC4	1.0000	-2.4776	1.0000	0.0000	1.0000	-2.4055	1.0000	0.0000	1.0000	-2.3757	1.0000	0.0000	1.0000	0.0000
APOC3	0.3540	-0.7864	0.5498	-0.4414	0.0029	-2.5657	0.0011	-1.8174	1.0000	0.2880	0.6943	0.6426	0.8140	1.0425
APOD	0.0003	-2.8961	0.0000	-2.2598	1.0000	0.0522	0.0441	0.6062	1.0000	0.1924	0.9130	0.8468	0.0474	0.7530
APOH	1.0000	0.0000	1.0000	-2.3959	1.0000	0.0000	1.0000	0.8538	1.0000	2.2674	1.0000	0.0000	1.0000	3.2048
APOLD1	0.0001	-1.4835	0.0000	-1.5945	1.0000	-0.1045	0.6048	-0.1922	1.0000	0.0783	1.0000	-0.0214	1.0000	-0.0040
APOO	0.0003	-0.6231	0.0000	-0.7119	0.0569	-0.3618	0.0000	-0.4452	1.0000	0.0009	1.0000	-0.0756	0.9579	-0.0766
APOOL	0.0075	0.6214	0.0000	0.7884	0.0042	0.6365	0.0000	0.8929	1.0000	0.0188	0.5836	0.2005	0.1778	0.2825
APOPT1	0.0000	-1.4122	0.0001	-0.9579	0.6101	-0.2332	1.0000	-0.0219	1.0000	-0.0220	0.5831	0.4468	0.8261	0.1962
APP	0.3329	-0.1866	0.0003	-0.2391	0.0000	0.8433	0.0000	0.8917	1.0000	0.1712	0.4400	0.1313	0.0115	0.2250
APPBP2	1.0000	-0.0435	0.2032	-0.1307	1.0000	0.0181	0.0140	-0.2160	1.0000	0.0952	1.0000	0.0204	0.5137	-0.1341
APPL1	0.0000	1.8255	0.0000	1.7547	0.0000	2.3943	0.0000	1.8825	1.0000	0.1946	1.0000	0.1341	0.5554	-0.3133
APPL2	0.0000	1.1563	0.0000	1.0820	0.0000	1.5853	0.0000	1.0112	1.0000	0.3441	0.1273	0.2830	0.3691	-0.2253
APRT	0.0001	-0.8575	0.0000	-0.8811	0.0509	-0.4394	1.0000	-0.0176	1.0000	-0.2921	0.2884	-0.3037	0.7231	0.1365
AQP1	1.0000	-0.8561	0.4469	1.3289	0.0155	2.2966	0.0019	2.6455	1.0000	-0.1163	0.5035	2.0915	1.0000	0.2302
AQP10	0.0338	2.6811	0.2857	1.1600	1.0000	-0.1833	1.0000	-0.6852	1.0000	1.1010	1.0000	-0.4021	1.0000	0.6046
AQP11	0.8249	0.3569	0.9311	-0.2197	1.0000	-0.1984	0.1053	-0.8323	1.0000	0.3297	1.0000	-0.2334	1.0000	-0.2943
AQP2	1.0000	0.0000	1.0000	0.0000	1.0000	0.0000	1.0000	0.0000	1.0000	0.0000	1.0000	0.0000	1.0000	0.0000
AQP3	0.0000	-2.3301	0.0000	-3.4153	1.0000	-0.0931	0.1716	-0.3841	1.0000	0.1513	0.5202	-0.9222	1.0000	-0.1349
AQP4	1.0000	0.0000	1.0000	0.0000	1.0000	0.0000	1.0000	2.3242	1.0000	0.0000	1.0000	0.0000	1.0000	2.3543
AQP8	0.3070	-2.0239	0.0953	-2.8843	0.1916	-2.7146	0.0891	-2.7166	1.0000	0.0609	1.0000	-0.7966	1.0000	0.0652
AQP9	0.0353	1.1445	0.0000	1.7348	1.0000	-0.0752	0.5282	-0.6030	1.0000	-0.2715	0.8219	0.3327	0.5358	-0.7918
AQR	0.0530	0.5072	0.0000	0.6105	0.0000	1.1178	0.0000	0.9295	1.0000	-0.0411	1.0000	0.0760	0.3797	-0.2231
AR	0.5544	-2.3893	0.8617	-0.8266	1.0000	-0.1879	1.0000	0.3198	1.0000	0.5270	0.8269	2.1245	0.8155	1.0426
ARAP2	0.0000	1.5419	0.0000	1.4773	0.2848	0.5351	0.9203	-0.2153	1.0000	-0.7511	0.0019	-0.8073	0.0000	-1.4973
ARAP3	1.0000	-1.2537	0.4021	-2.2294	1.0000	-0.1896	0.1318	-4.3887	1.0000	0.1779	1.0000	-0.7943	0.5093	-4.0498
ARC	0.0000	-4.4166	0.0000	-5.8346	0.6045	-0.3234	0.2665	0.2870	1.0000	0.1277	0.8512	-1.2831	0.0744	0.7423
ARCN1	0.6220	-0.1182	1.0000	-0.0148	0.1507	-0.2415	0.8069	-0.0381	1.0000	-0.0293	0.7864	0.0863	0.0863	0.1793
AREG	1.0000	2.1902	1.0000	0.0000	1.0000	0.0000	1.0000	0.0000	1.0000	0.0000	1.0000	-2.2992	1.0000	0.0000
AREL1	0.2601	-0.2333	0.2337	-0.1602	0.0000	0.7873	0.0000	0.8609	1.0000	-0.1018	1.0000	-0.0160	1.0000	-0.0226
ARF1	0.0165	0.4038	0.0000	0.4093	0.0027	0.4918	0.0000	0.4538	1.0000	0.0579	0.8423	0.0760	1.0000	0.0256
ARF4	0.2082	-0.2216	0.0000	-0.3069	0.2785	-0.1991	0.0149	-0.1690	1.0000	0.1409	0.9744	0.0678	0.1268	0.1761
ARF6	0.2791	-0.2207	0.5641	-0.0823	0.9361	0.0717	0.4086	0.1036	1.0000	-0.0297	0.6920	0.1208	1.0000	0.0077
ARFGAP2	0.1218	-0.2983	0.6413	-0.0668	0.0036	-0.5031	0.0025	-0.2817	1.0000	-0.1243	0.6752	0.1192	0.7495	0.1021
ARFGAP3	0.6642	0.1285	0.3307	0.1245	0.4680	0.1749	0.2556	0.1365	1.0000	0.1449	0.4309	0.1537	0.6855	0.1120
ARFGEF1	0.0001	1.5483	0.0000	1.4338	0.0000	2.5227	0.0000	1.7079	1.0000	0.3640	0.8156	0.2666	0.1576	-0.4402
ARFGEF2	0.7655	-0.1078	0.0458	-0.2021	0.0727	0.3311	0.0102	0.2355	1.0000	0.0632	1.0000	-0.0188	1.0000	-0.0280
ARFIP1	0.5920	0.1356	0.3231	0.1030	0.6976	-0.1149	0.0233	-0.2003	1.0000	0.1078	0.8607	0.0880	1.0000	0.0284
ARFIP2	0.1481	-0.2765	0.7704	-0.0663	0.0001	-0.6379	0.6142	-0.0813	1.0000	-0.2905	1.0000	-0.0682	0.0864	0.2717
ARFRP1	0.5378	-0.1847	0.0001	-0.4868	0.0140	-0.4919	0.0000	-0.6186	1.0000	0.2097	1.0000	-0.0801	1.0000	0.0866
ARG2	0.8226	-0.1739	0.0066	-0.6024	1.0000	0.0190	0.5231	-0.1884	1.0000	0.1718	0.8328	-0.2453	1.0000	-0.0303
ARGLU1	0.4318	-0.1825	0.0000	-0.4247	0.5185	-0.1589	0.1000	-0.1673	1.0000	0.1411	0.9218	-0.0884	0.5119	0.1383
ARHGAP1	0.0000	1.8595	0.0000	2.1192	0.0000	1.8234	0.0000	1.4136	1.0000	-0.1629	1.0000	0.1159	0.0042	-0.5636
ARHGAP10	0.1550	0.2710	0.8641	-0.0423	0.0213	-0.4110	0.0000	-0.8567	1.0000	0.2188	0.8915	-0.0821	0.1259	-0.2211
ARHGAP11A	0.0000	1.1300	0.0000	1.3686	0.1820	0.3804	0.4635	-0.1519	1.0000	-0.1260	0.7323	0.1250	0.0001	-0.6532
ARHGAP12	0.0000	1.0886	0.0000	0.8482	0.0000	1.0719	0.0000	0.8659	1.0000	0.2229	1.0000	-0.0044	1.0000	0.0214
ARHGAP17	0.0065	-0.4036	0.0000	-0.4250	0.1323	-0.2510	0.0011	-0.2422	1.0000	0.0506	1.0000	0.0416	0.8932	0.0648
ARHGAP18	0.0717	-0.6721	0.4583	-0.2284	0.0000	1.2284	0.0014	0.6036	1.0000	0.1424	0.1058	0.5985	0.0343	-0.4789
ARHGAP19	0.0000	1.4978	0.0000	1.7330	0.0030	0.5434	0.4147	0.1472	1.0000	-0.1939	1.0000	0.0532	0.0000	-0.5849
ARHGAP20	0.0001	-1.2764	0.0000	-1.2257	0.3196	-0.3728	1.0000	-0.0077	1.0000	0.3551	0.5790	0.4182	0.0022	0.7250
ARHGAP21	0.0570	-0.3303	0.0000	-0.4392	0.0094	0.4326	0.0000	0.3590	1.0000	0.1045	1.0000	0.0082	1.0000	0.0355
ARHGAP22	0.3089	0.5235	0.1655	0.5917	0.9143	0.2025	0.0025	1.1261	1.0000	-0.4383	0.8145	-0.3572	0.5599	0.4911
ARHGAP23	0.0003	-1.1300	0.0008	-0.6323	0.2882	0.3736	0.0000	0.9021	1.0000	-0.5835	1.0000	-0.0727	1.0000	-0.0496
ARHGAP24	0.0048	-0.4576	0.0000	-0.6889	0.6616	-0.1235	0.2868	-0.1106	1.0000	0.2293	1.0000	0.0108	0.0399	0.2472
ARHGAP25	0.0001	-3.0843	0.0000	-3.2518	0.0031	-1.9739	0.0009	-1.5299	1.0000	0.5167	1.0000	0.3643	0.6913	0.9654
ARHGAP26	0.0376	0.6782	0.2446	0.3190	0.5851	0.2816	0.3246	0.2902	1.0000	0.1673	0.9872	-0.1782	0.9555	0.1838
ARHGAP27	0.8249	3.0255	1.0000	0.0000	1.0000	0.0000	1.0000	0.0000	1.0000	0.0000	1.0000	-3.1469	1.0000	0.0000
ARHGAP28	0.0000	-1.8523	0.0000	-1.6902	0.0000	1.5807	0.0000	1.1452	1.0000	0.4151	0.4450	0.5896	1.0000	-0.0152
ARHGAP29	0.0249	0.6927	0.0325	0.5080	0.0001	1.0060	0.0031	0.6218	1.0000	0.1993	1.0000	0.0282	0.7274	-0.1794
ARHGAP31	0.0000	-1.2643	0.0000	-1.5934	0.0010	0.6410	0.7563	-0.0566	1.0000	-0.1808	0.0017	-0.4977	0.0000	-0.8732
ARHGAP32	0.0172	-0.7120	0.0003	-0.4843	0.0012	0.7991	0.0000	0.9656	1.0000	0.2255	0.0224	0.4673	0.0012	0.3971
ARHGAP35	0.6231	-0.3216	0.0693	0.4222	0.0000	1.6887	0.0000	2.0600	1.0000	-0.5120	0.7356	0.2463	0.7291	-0.1332
ARHGAP39	0.0238	-1.1100	0.6858	-0.2810	0.0338	0.9472	0.0000	1.6626	1.0000	-0.8914	1.0000	-0.0443	1.0000	-0.1684
ARHGAP40	0.0000	-3.6453	0.0000	-4.4849	0.0129	0.4366	1.0000	-0.0229	1.0000	0.1683	0.0987	-0.6592	0.0279	-0.2863
ARHGAP44	0.0000	-3.9380	0.0000	-3.2959	1.0000	0.1967	0.6457	-0.2862	1.0000	0.3397	1.0000	1.0010	1.0000	-0.1344
ARHGAP5	0.0002	0.6308	0.0000	0.5831	0.0000	0.7808	0.0000	0.4761	1.0000	0.1098	1.0000	0.0752	0.3429	-0.1896
ARHGAP6	0.0829	-1.2405	0.0497	-1.0532	0.1079	0.8221	1.0000	0.0872	1.0000	0.2962	1.0000	0.4951	0.6667	-0.4343
ARHGAP8	1.0000	-0.3552	0.0288	1.4926	0.9346	-0.5946	0.9343	0.4550	1.0000	-0.1299	0.0828	1.7313	0.8501	0.9269
ARHGDIA	0.2197	-0.2129	0.1028	-0.1236	0.0416	-0.3181	0.0073	-0.1779	1.0000	-0.1068	1.0000	-0.0049	1.0000	0.0389
ARHGDIB	1.0000	-0.0238	0.1253	-0.1311	0.0000	-0.9448	0.0000	-0.7413	1.0000	-0.1269	0.0421	-0.2218	0.8790	0.0822
ARHGDIG	0.2584	0.2902	0.0009	0.4554	0.0037	-0.6359	0.5918	-0.1207	1.0000	-0.1858	1.0000	-0.0083	0.1261	0.3344
ARHGEF10	0.2028	0.6444	0.0323	0.6899	0.0000	2.0143	0.0000	1.9410	1.0000	0.2563	0.8194	0.3157	0.7299	0.1893
ARHGEF10L	0.1793	-0.3673	0.0000	-0.6232	0.7438	-0.1534	0.5071	-0.1288	1.0000	0.0654	0.6245	-0.1775	0.9033	0.0969
ARHGEF11	1.0000	0.0690	0.0281	0.2367	0.9400	-0.0857	0.0101	0.2361	1.0000	-0.1851	1.0000	-0.0048	0.4386	0.1414
ARHGEF12	0.1816	0.2997	0.0014	0.3067	0.0000	0.8463	0.0000	0.7839	1.0000	0.1237	0.5035	0.1436	0.9222	0.0662
ARHGEF16	0.2537	-0.7006	0.0001	-2.2348	1.0000	0.1020	0.0096	0.9366	1.0000	-0.6239	0.0028	-2.1517	1.0000	0.2172
ARHGEF17	0.0794	-0.4211	0.2273	-0.1407	0.2602	0.2941	0.0000	0.5937	1.0000	-0.2618	1.0000	0.0314	1.0000	0.0435
ARHGEF18	1.0000	0.0514	0.0296	0.2185	0.0021	0.5057	0.0000	0.3312	1.0000	0.1210	0.0113	0.3013	1.0000	-0.0481
ARHGEF26	0.0036	-0.5699	0.0000	-0.7545	0.9020	0.0837	0.9546	-0.0430	1.0000	0.0159	0.7904	-0.1561	0.7687	-0.1064
ARHGEF3	0.8117	0.1524	0.7356	-0.1124	0.0055	0.6371	0.0000	0.7651	1.0000	0.2010	1.0000	-0.0517	0.0885	0.3340
ARHGEF33	0.0425	-1.2331	0.0548	-0.9100	0.2102	-0.7984	0.0277	-1.0557	1.0000	0.0567	1.0000	0.3941	1.0000	-0.1921
ARHGEF37	0.0733	-0.5153	0.0672	-0.3818	0.6419	-0.1962	0.0239	-0.4364	1.0000	-0.2020	1.0000	-0.0569	0.1112	-0.4386

ARHGEF5	1.0000	0.0000	1.0000	0.0000	1.0000	0.0000	1.0000	0.0000	1.0000	0.0000	1.0000	0.0000	1.0000	0.0000
ARHGEF6	0.9698	-0.0594	0.4271	-0.1059	0.0504	0.3158	0.0154	0.2535	1.0000	-0.1359	0.3020	-0.1700	0.1591	-0.1927
ARHGEF7	0.0006	-0.6476	0.0000	-0.6906	1.0000	-0.0594	0.1373	-0.1563	1.0000	0.0177	1.0000	-0.0123	0.9835	-0.0736
ARHGEF9	0.6135	1.0553	1.0000	-0.3621	0.5405	0.9322	0.4937	0.7959	1.0000	0.8282	1.0000	-0.5746	0.9362	0.7023
ARID1B	1.0000	-0.0408	0.8541	0.0566	0.0000	0.8030	0.0000	0.9346	1.0000	-0.0146	0.9320	0.0958	0.6071	0.1223
ARID2	0.8284	-0.1084	0.0159	-0.2981	0.0262	0.4447	0.0569	0.2193	1.0000	0.2389	1.0000	0.0626	1.0000	0.0187
ARID3A	0.4790	-0.6556	0.0018	-1.6310	1.0000	0.1695	0.9411	0.1936	1.0000	0.2820	0.8720	-0.6831	0.9702	0.3082
ARID3B	1.0000	-0.1230	0.6673	0.1587	0.3656	0.3767	0.0000	0.8097	1.0000	-0.3654	1.0000	-0.0714	1.0000	0.0753
ARID3C	0.8249	-3.3439	0.4431	-3.7876	1.0000	0.3467	1.0000	-0.5248	1.0000	0.4112	1.0000	0.0000	1.0000	-0.4570
ARID4A	0.1516	0.3413	0.0000	0.5279	0.0269	0.4656	0.0000	0.5423	1.0000	0.0084	0.4333	0.2067	0.9382	0.0890
ARID4B	1.0000	0.0272	0.0610	-0.2431	0.7276	0.1305	0.6725	-0.0884	1.0000	0.1812	1.0000	-0.0756	1.0000	-0.0324
ARID5A	1.0000	0.0617	0.0132	0.2825	0.0032	-0.6130	0.0002	-0.4153	1.0000	-0.1695	1.0000	0.0632	1.0000	0.0335
ARID5B	1.0000	-0.0027	0.2484	0.1555	0.0126	0.4536	0.0000	0.5608	1.0000	-0.0445	0.7781	0.1267	1.0000	0.0680
ARIH1	0.6787	-0.1200	0.0634	-0.1737	0.0156	0.4000	0.0000	0.3360	1.0000	0.0508	1.0000	0.0099	1.0000	-0.0081
ARIH2	0.3149	-0.2014	0.0001	-0.3411	0.1752	-0.2521	0.0478	-0.1812	1.0000	0.0863	1.0000	-0.0406	0.2841	0.1628
ARL1	1.0000	0.0152	0.0447	-0.1779	0.0000	-0.6696	0.0000	-0.7868	1.0000	0.1602	1.0000	-0.0207	1.0000	0.0489
ARL10	0.8824	-0.6659	1.0000	0.1236	0.8772	-0.6852	0.4463	-0.7434	1.0000	0.6253	0.3249	1.4288	1.0000	0.5783
ARL11	0.4017	-0.3406	1.0000	-0.0293	0.0001	-1.2352	0.0000	-1.3858	1.0000	-0.2344	1.0000	0.0895	0.5788	-0.3825
ARL13A	0.5086	-3.8790	0.2495	-4.1757	0.9361	-1.5509	0.6142	-1.7592	1.0000	0.2637	1.0000	0.0000	1.0000	0.0641
ARL13B	0.2818	0.3989	0.0652	0.3990	0.0032	0.7951	0.0005	0.6443	1.0000	0.1387	0.9773	0.1524	1.0000	-0.0049
ARL14	1.0000	0.0000	0.9062	1.2225	1.0000	2.2428	1.0000	-2.3176	1.0000	2.2675	0.8607	3.7287	1.0000	-2.2888
ARL14EP	0.9177	-0.0883	0.9062	0.0527	0.6189	0.1541	0.0282	0.2651	1.0000	-0.0674	1.0000	0.0864	1.0000	0.0505
ARL14EPL	1.0000	-0.0970	1.0000	0.4573	0.0800	-2.3751	0.1480	-2.5298	1.0000	-0.6270	1.0000	-0.0629	1.0000	-0.7795
ARL16	0.7150	0.2010	0.0614	0.4668	0.0003	-1.0543	0.8626	-0.1160	1.0000	-0.2900	1.0000	-0.0122	0.0442	0.6524
ARL2BP	0.5221	-0.1928	0.0010	-0.4135	0.0010	0.6045	0.0000	0.4788	1.0000	0.0734	0.8605	-0.1349	1.0000	-0.0474
ARL3	0.0361	-0.5537	0.0689	-0.3163	0.0552	0.4268	0.0000	0.5866	1.0000	-0.1598	1.0000	0.0905	1.0000	0.0068
ARL4A	0.0875	0.4593	0.0010	0.4448	0.6791	0.1931	0.8794	0.0701	1.0000	-0.0605	1.0000	-0.0625	0.6583	-0.1790
ARL4C	0.0000	-1.6769	0.0000	-1.4380	1.0000	0.0109	0.0000	0.3610	1.0000	0.1034	0.0027	0.3550	0.0000	0.4583
ARL5A	0.0795	-0.3504	0.0001	-0.5083	1.0000	-0.0150	1.0000	0.0271	1.0000	0.1421	1.0000	-0.0025	0.2354	0.1909
ARL5B	0.5889	0.2081	0.5793	-0.1412	0.9110	0.1106	1.0000	-0.0268	1.0000	0.1273	0.6469	-0.2091	1.0000	-0.0023
ARL6	0.2050	0.8532	0.0155	0.7450	0.0589	1.0828	0.0759	0.5902	1.0000	0.5163	0.5286	0.4224	1.0000	0.0277
ARL6IP1	0.0026	0.4471	0.0000	0.3973	0.8377	-0.0795	0.0001	-0.2847	1.0000	-0.0013	1.0000	-0.0385	0.0578	-0.2012
ARL6IP4	0.2831	0.2389	0.8299	0.0570	0.3323	-0.2331	0.1820	-0.1745	1.0000	-0.0304	0.3103	-0.1991	1.0000	0.0346
ARL6IP5	0.4160	0.1691	0.8528	0.0433	0.0232	0.3641	0.0410	0.2011	1.0000	0.0916	1.0000	-0.0216	0.9541	-0.0662
ARL8A	0.0000	-1.1441	0.0000	-0.8974	0.9468	0.0702	0.0520	0.1987	1.0000	-0.0883	0.6334	0.1709	1.0000	0.0455
ARL8B	0.0046	-0.4364	0.0000	-0.3458	0.9361	0.0635	0.4617	-0.0761	1.0000	0.0357	0.4158	0.1387	0.6395	-0.0986
ARL9	0.0075	-0.4401	0.0000	-0.7295	0.0000	-0.9003	0.0000	-1.0350	1.0000	0.2915	1.0000	0.0147	0.4058	0.1626
ARMC1	0.4848	-0.2059	0.2893	-0.1432	0.0729	0.4050	0.0430	0.2291	1.0000	0.0293	0.8759	0.1048	0.6438	-0.1408
ARMC10	0.9916	0.0970	1.0000	-0.0051	0.4956	-0.2460	0.7241	-0.1253	1.0000	-0.0451	1.0000	-0.1364	1.0000	0.0814
ARMC2	1.0000	0.5960	1.0000	0.2177	0.3373	1.4677	0.1325	1.7976	1.0000	-0.1237	1.0000	-0.4961	1.0000	0.2121
ARMC3	1.0000	0.0469	0.0945	2.5620	1.0000	-0.7524	0.9096	1.3854	1.0000	-2.4397	1.0000	0.0607	1.0000	-0.3077
ARMC4	1.0000	2.1848	1.0000	0.0000	1.0000	0.0000	1.0000	0.0000	1.0000	0.0000	1.0000	-2.2956	1.0000	0.0000
ARMC6	0.0549	-0.3349	0.0204	-0.2433	0.0131	-0.4082	0.0005	-0.3344	1.0000	0.0602	0.4316	0.1637	0.4564	0.1391
ARMC7	0.0000	-1.3815	0.0000	-1.4911	0.0000	-1.6647	0.0000	-1.3077	1.0000	-0.0080	0.9348	-0.1046	0.0109	0.3548
ARMC8	0.1278	-0.3073	0.0017	-0.3073	0.1815	0.2685	0.0001	0.3509	1.0000	0.0733	0.9410	0.0862	0.2853	0.1619
ARMC9	0.0254	0.4718	0.0000	0.5380	0.0413	0.4308	0.0000	0.6502	1.0000	-0.1925	0.8759	-0.1130	1.0000	0.0337
ARNT	0.0535	-0.4116	0.0074	-0.2455	0.4423	-0.2099	0.1524	0.1320	1.0000	-0.1922	1.0000	-0.0138	0.3035	0.1547
ARNT2	0.0196	0.8678	0.0000	1.0144	0.0000	1.6802	0.0000	0.9626	1.0000	0.2274	0.1862	0.3851	0.0097	-0.4883
ARNTL	0.0004	0.7524	0.0033	0.4251	1.0000	0.0278	0.1239	-0.2903	1.0000	0.2094	1.0000	-0.1047	1.0000	-0.1034
ARNTL2	0.7378	-0.1132	0.0002	-0.3070	0.0030	-0.4867	0.0000	-0.5711	1.0000	0.1862	1.0000	0.0049	0.6865	0.1069
ARPC1A	0.4762	0.1757	0.5802	0.0678	0.0396	-0.3839	0.0000	-0.4728	1.0000	-0.1350	0.0130	-0.2305	0.0498	-0.2180
ARPC1B	1.0000	0.0090	0.0216	0.2158	0.3809	0.2014	0.0000	0.4246	1.0000	-0.2297	1.0000	-0.0110	1.0000	-0.0008
ARPC2	1.0000	-0.0195	0.4640	0.0736	0.1928	-0.2396	0.0603	-0.1401	1.0000	-0.0014	0.5902	0.1040	0.5646	0.1038
ARPC3	0.4369	-0.1912	0.0012	-0.2935	0.0233	-0.4304	0.0000	-0.4391	1.0000	0.0187	0.9227	-0.0714	1.0000	0.0157
ARPC4	0.0000	-0.7087	0.0000	-0.5917	0.0000	-0.9445	0.0000	-0.8297	1.0000	-0.0656	1.0000	0.0637	1.0000	0.0550
ARPC5	0.0062	-0.4314	0.0000	-0.4785	0.0037	-0.4627	0.0000	-0.4540	1.0000	0.0807	1.0000	0.0457	0.6682	0.0952
ARPC5L	0.0031	-0.5421	0.0000	-0.6057	0.0000	-0.9385	0.0000	-0.7110	1.0000	-0.0523	0.9755	-0.1038	0.4081	0.1805
ARPP19	0.0140	-0.4032	0.0000	-0.5669	0.9014	-0.0741	0.2895	-0.0894	1.0000	0.0559	0.7510	-0.0955	1.0000	0.0462
ARPP21	0.0000	-1.1100	0.0000	-1.4438	0.0025	-0.5379	0.0000	-0.5527	1.0000	0.1131	0.5816	-0.2075	0.8556	0.1041
ARR3	0.3917	-1.2996	1.0000	0.0386	1.0000	-0.3609	1.0000	-0.5493	1.0000	-0.5273	1.0000	0.8270	1.0000	-0.7042
ARRDC1	0.0000	-1.9293	0.0000	-1.8297	0.1954	0.3175	0.5013	-0.1363	1.0000	-0.1367	1.0000	-0.0245	0.0001	-0.5846
ARRDC2	0.0000	-1.0641	0.0000	-1.0043	1.0000	0.0362	0.0673	0.1843	1.0000	0.1730	0.4816	0.2465	0.0053	0.3268
ARRDC4	0.0000	1.0475	0.0001	0.7258	0.0206	0.6294	0.2786	0.2489	1.0000	0.0808	0.6218	-0.2287	0.3442	-0.2943
ARRDC5	1.0000	0.8880	1.0000	0.0407	0.8033	-3.2622	0.4937	-1.4671	1.0000	1.3521	1.0000	0.5261	1.0000	3.2066
ARSA	0.0797	-0.4232	0.4127	-0.1074	0.6802	0.1592	0.0000	0.4493	1.0000	-0.0655	0.0970	0.2622	0.1074	0.2303
ARSB	0.0000	1.2767	0.0000	1.1470	1.0000	0.0570	0.3235	-0.2217	1.0000	0.2938	0.7694	0.1757	1.0000	0.0192
ARSG	0.1036	-0.8581	0.1056	-0.7306	0.1746	0.6186	0.0251	0.7358	1.0000	-0.1660	1.0000	-0.0256	1.0000	-0.0420
ARSI	0.0000	1.0768	0.0000	1.2182	0.1031	0.3826	0.0000	0.6476	1.0000	0.0091	0.4754	0.1615	0.1746	0.2791
ARSJ	0.1011	-0.2893	0.0229	-0.1980	0.0300	0.3590	0.0000	0.3871	1.0000	0.1383	0.0532	0.2425	0.1723	0.1720
ARSK	0.0000	1.0378	0.0000	1.2521	0.0033	-0.5484	0.0001	-0.4129	1.0000	-0.1032	0.6388	0.1231	1.0000	0.0381
ART4	1.0000	0.0000	1.0000	0.0000	1.0000	0.0000	1.0000	0.0000	1.0000	0.0000	1.0000	0.0000	1.0000	0.0000
ARTN	0.8230	-0.5679	0.3143	-0.8979	0.7547	-0.5397	0.3992	-0.8787	1.0000	-0.0353	1.0000	-0.3508	1.0000	-0.3683
ARV1	0.9919	0.0798	0.3178	-0.1555	0.1863	0.3059	0.0940	0.2209	1.0000	0.1427	1.0000	-0.0798	1.0000	0.0632
ARVCF	0.0013	0.6876	0.0000	0.7965	0.0000	1.4513	0.0000	1.2812	1.0000	0.0366	0.8840	0.1583	0.6855	-0.1291
ARX	1.0000	-0.3227	1.0000	0.0000	0.8011	-3.2629	1.0000	0.0000	1.0000	-3.2304	1.0000	-3.1429	1.0000	0.0000
AS3MT	0.0000	-1.0146	0.0000	-1.0887	0.2882	-0.2843	0.5020	-0.1398	1.0000	-0.1346	0.8553	-0.1963	1.0000	0.0143
ASAH1	1.0000	0.0246	1.0000	-0.0269	0.3914	0.1804	0.4801	0.0900	1.0000	0.2399	0.1811	0.2003	0.3016	0.1545
ASAP1	0.4624	0.2409	1.0000	0.0352	0.1521	0.3831	0.0002	0.4809	1.0000	0.1815	1.0000	-0.0114	0.2017	0.2832
ASAP2	0.3416	-0.3659	0.4960	-0.2737	0.0000	1.4137	0.0000	1.0790	1.0000	0.0177	1.0000	0.1208	0.2526	-0.3108
ASAP3	0.0015	-1.3557	0.0002	-1.6327	0.0002	1.1926	0.0000	1.9309	1.0000	-0.5774	0.4769	-0.8421	0.9222	0.1695
ASB1	0.6410	-0.1381	0.7956	-0.0562	0.7429	0.1138	0.9662	0.0375	1.0000	0.0305	0.7092	0.1248	1.0000	-0.0398
ASB10	0.3089	-4.2696	1.0000	-0.1625	0.2785	-4.1857	1.0000	-0.8416	1.0000	-1.0389	1.0000	3.1945	1.0000	2.3555
ASB11	0.5391	-3.8767	0.4431	-3.7876	1.0000	0.1972	0.5997	1.1668	1.0000	-0.1188	1.0000	0.0000	1.0000	0.8521
ASB12	0.2915	-0.6113	0.0056	-0.7574	0.7678	0.2443	1.0000	0.0893	1.0000	0.0636	1.0000	-0.0698	1.0000	-0.0889
ASB13	1.0000	-0.0627	1.0000	0.0817	0.9586	0.1577	0.2745	0.4560	1.0000	-0.2177	1.0000	-0.0618	1.0000	0.0845
ASB14	0.0078	1.0812	0.0000	1.1817	0.0000	1.3186	0.0000	1.3582	1.0000	-0.0807	1.0000	0.0332	1.0000	-0.0336
ASB15	0.0000	-3.6254	0.0000	-4.1285	0.2892	-0.3266	0.0000	-0.9292	1.0000	0.0255	0.9270	-0.4638	0.0147	-0.5698
ASB18	0.0000	-5.6068	0.0000	-5.6364	0.0230	-0.7424	0.3118	0.3199	1.0000	-0.4538	1.0000	-0.4724	0.1042	0.6133
ASB2	0.0000	-1.3486	0.0000	-1.5489	0.0000	-0.9103	0.0000	-1.4307	1.0000	0.2032	1.0000	0.0153	0.3374	-0.3137
ASB3	0.1877	-0.3703	0.3720	-0.1734	0.7872	-0.1421	0.0113	-0.4013	1.0000	0.0576	0.3779	0.2668	0.5343	-0.1982
ASB4	1.0000	0.0000	1.0000	-0.1449	1.0000	2.2472	1.0000	-2.3176	1.0000	2.2675	1.0000	2.3455	1.0000	-2.2909
ASB5	0.0000	-1.3996	0.0000	-1.8821	0.0621	-0.3665	0.0000	-0.5439	1.0000	0.0107	0.0000	-0.4593	0.2327	-0.1609
ASB6	0.0444	-0.3873	0.0000	-0.6307	1.0000	-0.0244	0.6507	0.0751	1.0000	0.0415	0.4828	-0.1897	0.4778	0.1453

ASB7	0.1893	0.6175	0.3509	0.3857	0.0031	1.1520	0.0000	1.0921	1.0000	0.1161	1.0000	-0.1020	1.0000	0.0608
ASB8	0.2078	-0.2669	1.0000	-0.0228	0.0007	-0.6461	0.0000	-0.5120	1.0000	-0.1161	0.6642	0.1394	1.0000	0.0238
ASB9	1.0000	-0.1088	0.0122	1.0685	0.9777	0.2655	0.0000	1.5805	1.0000	0.0017	0.0275	1.1907	0.0028	1.3222
ASCC1	0.0016	-0.4927	0.0000	-0.4302	0.0000	-0.9054	0.0000	-0.7430	1.0000	-0.1145	1.0000	-0.0401	1.0000	0.0534
ASCC2	0.0033	-0.5093	0.0000	-0.3900	0.2708	-0.2286	1.0000	0.0028	1.0000	0.0098	0.4420	0.1416	0.0136	0.2459
ASCL1	1.0000	0.0000	1.0000	0.0000	1.0000	0.0000	1.0000	0.0000	1.0000	0.0000	1.0000	0.0000	1.0000	0.0000
ASCL3	0.4395	0.4665	1.0000	-0.0675	1.0000	0.0790	0.2991	-0.3992	1.0000	0.1306	0.6375	-0.3903	0.7345	-0.3447
ASCL4	1.0000	-1.1585	1.0000	0.3746	1.0000	-0.1793	1.0000	0.5377	1.0000	-0.1245	1.0000	1.4371	1.0000	0.6041
ASH1L	0.1443	0.2719	0.0001	0.3526	0.0000	0.9745	0.0000	1.0552	1.0000	-0.1217	1.0000	-0.0280	1.0000	-0.0358
ASH2L	0.8271	-0.0880	0.3057	-0.1148	0.6836	-0.1190	0.6364	-0.0665	1.0000	-0.0319	1.0000	-0.0464	1.0000	0.0263
ASIC1	0.0047	-1.7050	0.0211	-1.3206	0.7092	0.3628	0.0003	1.2710	1.0000	-0.4402	1.0000	-0.0433	0.5490	0.4756
ASIC2	1.0000	0.0000	0.7674	-3.2498	1.0000	0.0000	1.0000	0.5377	1.0000	3.1056	1.0000	0.0000	0.7287	3.7384
ASIC4	0.0000	-4.0930	0.0000	-3.9128	0.2193	0.3564	0.8364	0.1022	1.0000	-0.1328	1.0000	0.0607	0.1295	-0.3812
ASIC5	1.0000	0.0000	1.0000	0.0000	1.0000	0.0000	1.0000	0.0000	1.0000	0.0000	1.0000	0.0000	1.0000	0.0000
ASIP	0.0902	-3.0436	0.0266	-5.1393	1.0000	0.2608	0.5003	0.7947	1.0000	-0.1247	1.0000	-2.2992	1.0000	0.4170
ASMTL	0.0410	0.7960	0.0000	1.4934	1.0000	0.0517	0.2544	0.4895	1.0000	-0.0871	0.0369	0.6236	0.7368	0.3571
ASNA1	0.7079	-0.1272	0.2237	-0.1624	0.1058	-0.3272	0.0159	-0.2767	1.0000	-0.0713	0.9979	-0.0946	1.0000	-0.0149
ASNS	1.0000	0.0396	0.5801	0.0703	0.4837	-0.1961	0.0000	-0.5543	1.0000	-0.0948	1.0000	-0.0519	0.0000	-0.4473
ASNSD1	0.9949	-0.0724	1.0000	-0.0127	0.2586	0.2745	0.0219	0.2805	1.0000	-0.0384	1.0000	0.0339	1.0000	-0.0283
ASPA	1.0000	-0.2446	1.0000	-0.0048	0.0249	-1.6935	0.0303	-1.4870	1.0000	-0.1321	1.0000	0.1179	1.0000	0.0802
ASPDH	1.0000	-0.3525	1.0000	-0.1616	0.6370	-1.3481	1.0000	-0.8429	1.0000	-1.8071	0.7704	-1.6281	1.0000	-1.3081
ASPG	0.0317	0.8698	0.0000	1.0472	1.0000	0.0392	0.3716	-0.3840	1.0000	0.2034	0.4595	0.3894	1.0000	-0.2159
ASPH	0.0000	0.6583	0.0000	0.6538	0.0008	0.5142	0.0065	0.2638	1.0000	-0.0061	1.0000	0.0015	0.0283	-0.2516
ASPHD2	0.8712	-0.2790	1.0000	0.2409	0.0822	-1.0712	0.4974	0.5342	1.0000	-0.9229	1.0000	-0.3916	0.4979	0.6838
ASPM	0.0000	0.9649	0.0000	1.3886	0.0000	1.1533	0.0000	0.7277	1.0000	-0.2110	0.1383	0.2234	0.0000	-0.6328
ASPN	0.0045	0.4741	0.0000	0.4530	0.0292	0.3749	0.0000	1.1749	1.0000	0.2114	0.0995	0.2027	0.0000	1.0163
ASPSCR1	0.4390	-0.1786	0.0000	-0.3861	0.2556	-0.2310	0.0168	-0.2216	1.0000	0.0600	0.5720	-0.1347	0.8988	0.0753
ASRGL1	0.7650	0.1687	0.0388	0.3650	0.0000	-1.3908	0.0001	-0.7329	1.0000	-0.1117	1.0000	0.0973	0.1162	0.5484
ASS1	0.8484	-0.2770	0.1505	-0.5855	0.6895	0.3318	1.0000	0.0567	1.0000	-0.2534	0.4857	-0.5532	0.2638	-0.5257
ASTE1	0.7172	-0.2459	0.3982	-0.2702	0.7267	-0.2274	1.0000	0.0194	1.0000	-0.0309	1.0000	-0.0414	0.8395	0.2224
ASTN2	0.2959	-2.0245	0.8978	0.3923	1.0000	0.4434	0.4347	0.9793	1.0000	-0.3431	0.4765	2.0979	1.0000	0.2014
ASUN	0.8589	-0.0780	0.5303	-0.0680	0.4869	0.1508	0.2592	0.0977	1.0000	-0.0381	1.0000	-0.0156	0.6608	-0.0856
ASXL1	0.4225	0.2103	0.0000	0.6460	0.3952	0.2138	0.0000	0.5152	1.0000	-0.3830	1.0000	0.0640	0.9862	-0.0776
ASXL2	0.1884	0.2930	0.0003	0.3949	0.0030	0.5497	0.0000	0.5450	1.0000	-0.0151	0.9169	0.0987	1.0000	-0.0155
ASXL3	0.8207	-0.1046	0.0076	-0.3275	0.3212	0.2194	0.0013	0.3665	1.0000	0.1454	1.0000	-0.0638	0.0189	0.2984
ASZ1	1.0000	0.0000	1.0000	0.0000	1.0000	2.2428	1.0000	0.0000	1.0000	0.0000	1.0000	0.0000	1.0000	-2.2888
ATAD1	0.0003	0.5837	0.0000	0.7397	0.1072	-0.3002	0.0047	-0.2389	1.0000	-0.0917	0.8732	0.0766	1.0000	-0.0247
ATAD2	0.0000	1.0727	0.0000	1.4319	0.0000	0.9422	0.0000	0.7826	1.0000	-0.1029	0.1086	0.2670	0.1564	-0.2592
ATAD2B	0.3366	0.2594	0.6488	0.0960	0.0004	0.7176	0.0071	0.3437	1.0000	0.2204	1.0000	0.0710	0.6730	-0.1480
ATAD3A	1.0000	-0.0369	0.9967	0.0347	0.0000	-0.6970	0.0000	-0.6151	1.0000	-0.1404	1.0000	-0.0568	1.0000	-0.0538
ATAD5	0.0000	1.0972	0.0000	1.0483	0.0008	0.7959	0.0005	0.5111	1.0000	0.0067	1.0000	-0.0281	0.3178	-0.2727
ATCAY	1.0000	-2.4788	0.7666	3.0922	1.0000	-0.1630	1.0000	0.0000	1.0000	-2.3771	1.0000	3.1976	1.0000	-2.2888
ATE1	0.0005	1.1585	0.0000	1.4812	0.0000	1.5118	0.0000	1.5272	1.0000	-0.1482	1.0000	0.1849	1.0000	-0.1302
ATF1	0.0308	0.4759	0.0004	0.5320	0.0000	0.8944	0.0000	0.6854	1.0000	0.0863	0.7106	0.1555	0.6687	-0.1166
ATF2	0.6009	-0.1429	0.0019	-0.3135	1.0000	0.0468	0.7063	0.0667	1.0000	0.0784	0.9927	-0.0790	0.6865	0.1042
ATF3	1.0000	0.0503	0.5814	0.0975	0.0008	0.6542	0.0000	0.5816	1.0000	0.0367	0.9998	0.0977	1.0000	-0.0299
ATF4	0.0000	-0.9207	0.0000	-0.9204	0.0239	-0.3421	0.0000	-0.2939	1.0000	0.0503	1.0000	0.0625	0.4560	0.1037
ATF6	0.0483	-0.3432	0.2528	-0.1323	1.0000	0.0142	0.1962	0.1318	1.0000	-0.1490	1.0000	0.0741	1.0000	-0.0263
ATF7	0.1610	-0.4856	0.9062	-0.0971	0.0443	0.5686	0.0000	0.7356	1.0000	-0.1704	0.6888	0.2324	1.0000	0.0034
ATF7IP	0.1662	0.3552	0.0013	0.4748	0.0000	0.9196	0.0000	0.6402	1.0000	0.0370	0.7549	0.1693	0.2518	-0.2360
ATG12	1.0000	-0.0420	0.9638	0.0412	0.0000	-0.8156	0.0000	-0.6007	1.0000	-0.0554	1.0000	0.0401	0.5776	0.1648
ATG13	0.3486	0.1796	0.9169	0.0317	0.7758	-0.0936	0.0657	-0.1452	1.0000	-0.0043	0.3555	-0.1396	1.0000	-0.0502
ATG14	0.0248	-0.4581	0.0000	-0.5361	1.0000	0.0260	1.0000	-0.0081	1.0000	-0.0191	1.0000	-0.0848	1.0000	-0.0480
ATG16L1	0.0001	-0.6198	0.0000	-0.5824	0.2622	-0.2257	0.0160	-0.2246	1.0000	0.0028	1.0000	0.0528	1.0000	0.0101
ATG2B	0.0019	0.8255	0.0001	0.6367	0.0000	1.7137	0.0000	1.1230	1.0000	0.3570	0.7491	0.1815	0.4103	-0.2278
ATG3	1.0000	0.0274	1.0000	-0.0176	0.0510	-0.3605	0.0000	-0.5205	1.0000	-0.0088	1.0000	-0.0413	0.3790	-0.1627
ATG4A	0.0259	-0.3917	0.0000	-0.4635	0.0944	-0.2993	0.0000	-0.3902	1.0000	0.0605	1.0000	0.0012	1.0000	-0.0257
ATG4B	1.0000	0.0464	0.0188	0.2750	0.0002	0.6624	0.0000	0.7131	1.0000	-0.1268	0.8501	0.1154	0.9610	-0.0699
ATG4C	0.1120	0.3255	0.3442	0.1401	0.6415	0.1461	0.7516	-0.0738	1.0000	0.0634	0.8840	-0.1098	0.5732	-0.1523
ATG5	0.0288	0.7503	0.0000	0.9906	0.0003	1.0507	0.0000	1.0367	1.0000	-0.1591	1.0000	0.0922	0.7554	-0.1681
ATG7	0.5832	0.1787	1.0000	-0.0124	1.0000	-0.0725	0.1735	-0.2021	1.0000	0.1479	1.0000	-0.0308	1.0000	0.0249
ATG9A	0.0144	0.5308	0.0000	0.8102	0.5754	0.1735	0.0000	0.4052	1.0000	-0.2812	1.0000	0.0107	1.0000	-0.0435
ATHL1	0.0264	0.5015	0.0000	0.6771	0.0001	-0.8613	0.0255	-0.3982	1.0000	-0.2640	1.0000	-0.0782	0.6422	0.2020
ATIC	0.0000	-0.6077	0.0000	-0.4820	0.0000	-0.7232	0.0000	-0.6852	1.0000	-0.1429	1.0000	-0.0049	0.6456	-0.0997
ATL1	1.0000	0.0597	0.5807	0.0968	0.0025	0.5370	0.0000	0.5718	1.0000	0.0268	1.0000	0.0775	1.0000	0.0683
ATL2	1.0000	0.0053	0.2542	-0.1404	0.4507	-0.1795	0.0946	-0.1783	1.0000	-0.0202	0.4743	-0.1542	1.0000	-0.0152
ATMIN	0.0453	0.6210	0.0092	0.5527	0.1520	0.5025	0.1793	0.3335	1.0000	0.1558	1.0000	0.1012	1.0000	-0.0078
ATOH7	1.0000	-0.3423	0.7823	0.7692	1.0000	-0.4402	1.0000	0.3933	1.0000	-1.0667	1.0000	0.0511	1.0000	-0.2297
ATOH8	0.6571	0.1318	0.0910	-0.1780	0.0000	0.8122	0.0000	0.6850	1.0000	0.1295	0.3074	-0.1673	1.0000	0.0090
ATOX1	0.0440	-0.3514	0.0000	-0.4508	0.0000	-1.2156	0.0000	-1.1865	1.0000	-0.0714	0.4857	-0.1586	1.0000	-0.0366
ATP10A	0.0296	0.4240	0.0001	0.4461	0.0000	1.0625	0.0000	0.7174	1.0000	0.1143	0.6123	0.1485	0.1028	-0.2262
ATP10B	1.0000	0.0000	1.0000	0.0000	1.0000	0.0000	1.0000	0.0000	1.0000	0.0000	1.0000	0.0000	1.0000	0.0000
ATP10D	0.5669	1.1758	0.1518	1.3612	0.0293	1.8223	0.5115	0.9723	1.0000	-0.1212	1.0000	0.0643	0.4561	-0.9720
ATP11A	0.0000	-1.1749	0.0000	-1.5290	0.2395	-0.2246	0.0000	-0.6573	1.0000	0.2102	0.7745	-0.1315	0.0925	-0.2175
ATP11B	0.0018	0.4966	0.0000	0.5097	0.0000	0.8975	0.0000	0.7494	1.0000	-0.0200	1.0000	0.0056	0.2791	-0.1630
ATP11C	0.3239	0.1962	0.0001	0.3162	0.0584	0.3307	0.0000	0.3763	1.0000	0.0599	0.1327	0.1922	0.6334	0.1099
ATP12A	0.0002	1.5837	0.0000	1.2895	0.0349	-1.4205	0.0418	-0.9497	1.0000	0.5202	0.9217	0.2374	0.4968	0.9971
ATP13A1	0.4998	-0.1925	1.0000	0.0070	1.0000	0.0620	0.1143	0.1608	1.0000	-0.1234	1.0000	0.0884	1.0000	-0.0197
ATP13A2	1.0000	-0.0428	1.0000	-0.0253	0.1831	-0.4146	0.2661	-0.2413	1.0000	-0.1370	1.0000	-0.1086	1.0000	0.0427
ATP13A3	0.0000	1.4442	0.0000	1.3280	0.0000	2.5882	0.0000	1.8554	1.0000	0.3552	0.7433	0.2533	0.0819	-0.3700
ATP13A5	1.0000	-0.3195	0.9762	-1.0825	1.0000	-1.0171	1.0000	-0.3772	1.0000	0.7940	1.0000	0.0532	1.0000	1.4497
ATP1A1	0.3546	-0.1811	0.0000	-0.3041	0.2731	0.1958	0.0115	-0.1889	1.0000	0.0112	0.7510	-0.0989	0.0000	-0.3683
ATP1B1	0.0000	1.3552	0.0000	0.7885	0.0000	1.1313	0.0106	0.2910	1.0000	-0.1642	0.0000	-0.7192	0.0000	-0.9993
ATP1B3	0.0057	0.4046	0.0000	0.5047	0.2213	0.2134	0.0862	0.1335	1.0000	0.0840	0.0319	0.1965	1.0000	0.0093
ATP1B4	0.0026	0.8572	0.1129	0.4041	0.5476	0.2729	0.7394	-0.1735	1.0000	0.1025	0.4057	-0.3389	0.5237	-0.3389
ATP2A2	0.0000	-0.7130	0.0000	-0.8313	0.2286	0.2118	0.7294	-0.0448	1.0000	0.0698	1.0000	-0.0360	0.0767	-0.1815
ATP2A3	0.0083	-3.8318	0.4991	-1.6416	0.0026	1.6196	0.0000	4.1088	1.0000	-1.3163	1.0000	0.8994	0.0002	1.1754
ATP2B1	0.0000	-0.7593	0.0000	-0.4406	1.0000	0.0510	0.1136	-0.1318	1.0000	0.1144	0.0000	0.4454	1.0000	-0.0635
ATP2B2	0.0064	1.2366	0.0014	0.9729	0.0416	-1.3219	0.2135	-0.6077	1.0000	0.1629	1.0000	-0.0874	0.3540	0.8792
ATP2B4	0.0000	0.9814	0.0000	1.1118	0.0000	1.5944	0.0000	1.5061	1.0000	-0.0471	0.9773	0.0964	0.6132	-0.1300
ATP2C1	0.3148	0.2040	0.0019	0.2473	0.0000	0.6996	0.0000	0.4869	1.0000	0.0887	0.3631	0.1442	0.5418	-0.1191
ATP2C2	0.0579	0.8473	0.0069	0.7590	0.2283	-0.6876	0.0060	-1.0321	1.0000	0.3242	0.9072	0.2528	1.0000	-0.0117
ATP4B	1.0000	2.1848	1.0000	0.0000	1.0000	0.0000	1.0000	0.0000	1.0000	0.0000	1.0000	-2.2956	1.0000	0.0000

ATP5A1	1.0000	0.0394	0.1658	0.1344	0.2361	-0.2106	0.0002	-0.2939	1.0000	-0.1016	1.0000	0.0063	0.0990	-0.1794
ATP5B	0.0008	-0.4896	0.0000	-0.4756	0.0000	-0.7835	0.0000	-0.5490	1.0000	-0.1863	0.2788	-0.1594	1.0000	0.0536
ATP5C1	0.0734	-0.3135	0.0000	-0.3234	0.0079	-0.4345	0.0000	-0.5520	1.0000	-0.0578	1.0000	-0.0553	0.1278	-0.1697
ATP5D	0.0000	-0.8092	0.0000	-0.8129	0.0000	-0.9860	0.0000	-0.8771	1.0000	-0.0909	0.9031	-0.0822	1.0000	0.0239
ATP5E	0.0000	-1.0850	0.0000	-1.0426	0.0000	-1.1066	0.0000	-1.0273	1.0000	-0.1180	1.0000	-0.0633	1.0000	-0.0320
ATP5F1	0.0256	-0.3569	0.0000	-0.3376	0.0000	-0.7451	0.0000	-0.7935	1.0000	-0.0583	1.0000	-0.0268	0.6435	-0.1011
ATP5G1	0.0038	-0.5120	0.0000	-0.4597	0.0000	-0.9303	0.0000	-0.9662	1.0000	-0.1401	0.9441	-0.0755	0.2792	-0.1697
ATP5G3	0.0079	-0.4501	0.0000	-0.5129	0.0000	-0.9341	0.0000	-1.0054	1.0000	0.0031	1.0000	-0.0473	0.9960	-0.0622
ATP5H	0.0000	-1.1106	0.0000	-1.3356	0.0000	-1.3345	0.0000	-1.2302	1.0000	0.0875	0.4462	-0.1250	0.0793	0.1971
ATP5I	0.0000	-0.6268	0.0000	-0.5242	0.0000	-1.4879	0.0000	-1.3270	1.0000	-0.1970	0.8759	-0.0819	1.0000	-0.0303
ATP5J	0.0085	-0.4366	0.0000	-0.4792	0.0000	-0.9425	0.0000	-0.8650	1.0000	-0.1015	0.3922	-0.1318	1.0000	-0.0180
ATP5J2	0.0012	-0.4897	0.0000	-0.4823	0.0000	-0.8736	0.0000	-0.7831	1.0000	-0.0543	1.0000	-0.0348	1.0000	0.0419
ATP5O	0.7836	0.0954	1.0000	0.0262	0.0000	-0.8389	0.0000	-0.8509	1.0000	-0.1166	0.2736	-0.1735	0.6652	-0.1230
ATP5S	0.0006	0.6347	0.0000	0.6127	0.0000	-1.5239	0.0000	-1.5704	1.0000	-0.0930	0.8875	-0.1020	0.9702	-0.1345
ATP6AP1	0.0000	0.9162	0.0000	0.8621	0.2569	0.2068	0.3376	-0.1147	1.0000	0.0812	1.0000	0.0398	0.0133	-0.2350
ATP6AP1L	0.8246	0.4736	0.1658	1.1592	0.6601	-0.6945	1.0000	0.3665	1.0000	-0.6402	1.0000	0.0619	1.0000	0.4312
ATP6AP2	0.8860	0.0801	1.0000	0.0004	1.0000	-0.0065	0.0009	-0.2955	1.0000	0.1422	0.9727	0.0746	0.4640	-0.1412
ATP6V0A1	0.0063	0.4574	0.0005	0.2554	0.0230	-0.3969	0.0196	-0.1772	1.0000	0.0102	0.1705	-0.1796	0.0233	0.2350
ATP6V0A2	0.0011	0.5670	0.0000	0.5840	0.0292	0.3989	0.0026	0.2968	1.0000	0.0894	0.7760	0.1183	1.0000	-0.0078
ATP6V0A4	0.2060	1.7932	0.5045	-1.6415	1.0000	0.5040	1.0000	0.4074	1.0000	0.8165	0.1201	-2.6123	1.0000	0.7290
ATP6V0B	0.1330	-0.2837	0.0000	-0.4522	0.0008	-0.5835	0.0000	-0.6056	1.0000	0.0630	0.9285	-0.0933	1.0000	0.0467
ATP6V0C	0.1092	-0.2940	0.0000	-0.3800	0.3989	-0.1838	0.0004	-0.2402	1.0000	0.0383	1.0000	-0.0354	1.0000	-0.0123
ATP6V0D1	0.0000	-0.6684	0.0000	-0.6980	0.0005	-0.5840	0.0000	-0.4886	1.0000	0.0307	1.0000	0.0137	0.5773	0.1321
ATP6V0E1	0.0000	-1.2814	0.0000	-1.3006	0.0000	-0.8176	0.0000	-0.7483	1.0000	0.0578	1.0000	0.0510	0.2893	0.1327
ATP6V1A	0.8536	-0.0814	0.3053	-0.1060	0.1625	0.2530	0.5797	0.0690	1.0000	0.0399	1.0000	0.0277	0.3933	-0.1391
ATP6V1B2	0.6491	0.1159	0.1741	0.1214	0.1046	-0.2733	0.0000	-0.3841	1.0000	0.0813	0.7013	0.0991	1.0000	-0.0245
ATP6V1C1	0.0002	-0.6408	0.0000	-0.6411	0.1600	-0.2837	0.0000	-0.4758	1.0000	0.0232	1.0000	0.0357	0.3406	-0.1624
ATP6V1C2	0.8905	-0.3551	0.1612	-0.6337	1.0000	0.1657	0.1761	0.5075	1.0000	0.3267	1.0000	0.0604	0.1814	0.6734
ATP6V1D	0.0047	-0.4487	0.0000	-0.4906	0.0000	-1.0481	0.0000	-1.2311	1.0000	0.0266	1.0000	-0.0029	0.3117	-0.1509
ATP6V1E1	0.1025	-0.3281	0.0001	-0.3609	0.9331	-0.0780	0.0028	-0.2551	1.0000	0.0925	1.0000	0.0725	0.8556	-0.0788
ATP6V1G1	0.0259	-0.3628	0.0000	-0.3934	0.0000	-0.8127	0.0000	-0.8839	1.0000	0.0209	1.0000	0.0027	1.0000	-0.0443
ATP6V1G3	1.0000	0.0000	1.0000	2.2507	1.0000	0.0000	1.0000	0.0000	1.0000	0.0000	1.0000	2.3455	1.0000	0.0000
ATP6V1H	0.3240	-0.2210	0.1881	-0.1388	1.0000	-0.0064	0.0110	-0.2355	1.0000	0.0961	0.2581	0.1904	0.5550	-0.1277
ATP7A	0.0000	1.8502	0.0000	1.5635	0.0000	2.5295	0.0000	1.7713	1.0000	0.3829	1.0000	0.1096	0.1301	-0.3693
ATP7B	1.0000	-0.3444	1.0000	0.2913	0.4733	1.0227	1.0000	0.0070	1.0000	-0.1251	1.0000	0.5242	0.5722	-1.1413
ATP8A1	0.0000	1.9121	0.0000	2.2053	0.0000	1.4600	0.0000	1.5313	1.0000	-0.1035	0.8265	0.2002	1.0000	-0.0281
ATP8B1	0.5857	0.7057	0.0089	1.8951	0.6062	-0.7614	1.0000	-0.5490	1.0000	-0.6946	0.9457	0.5082	1.0000	-0.4788
ATP8B3	0.1350	-4.8236	1.0000	-0.9972	1.0000	-0.1952	0.0074	2.7678	1.0000	-1.6009	1.0000	2.3480	0.3998	1.3659
ATP9B	0.8249	0.1193	0.0002	0.4637	1.0000	-0.0522	0.5248	-0.1262	1.0000	-0.0870	0.2678	0.2697	0.6865	-0.1547
ATPAF1	0.4889	0.2285	1.0000	-0.0238	0.0001	-0.9105	0.0000	-0.9398	1.0000	-0.0235	0.3223	-0.2622	1.0000	-0.0457
ATPAF2	0.0010	-0.6288	0.0011	-0.4411	0.0000	-1.1800	0.0000	-0.8839	1.0000	-0.3403	0.8617	-0.1403	1.0000	-0.0385
ATPIF1	0.0020	-0.4884	0.0000	-0.3471	0.0000	-1.2775	0.0000	-1.0023	1.0000	-0.2075	1.0000	-0.0542	1.0000	0.0734
ATRAID	0.0000	-0.9344	0.0000	-0.7802	0.0000	-1.0362	0.0000	-0.5013	1.0000	-0.2976	0.7401	-0.1316	0.3793	0.2436
ATRIP	0.0015	0.5290	0.0000	0.6219	0.0065	-0.5016	0.0002	-0.4053	1.0000	-0.1479	1.0000	-0.0424	1.0000	-0.0460
ATRNL1	1.0000	0.6085	0.7315	1.0551	0.0784	1.9398	0.0005	3.1771	1.0000	-0.6533	1.0000	-0.1989	0.8254	0.5873
ATRX	0.0000	0.9573	0.0000	0.9187	0.0000	1.4921	0.0000	1.1642	1.0000	0.1658	0.8607	0.1391	0.6442	-0.1581
ATXN1	0.0450	0.6424	0.0025	0.6768	0.0000	1.9907	0.0000	1.7276	1.0000	0.0309	1.0000	0.0767	0.4111	-0.2290
ATXN1L	0.4423	0.2063	0.0241	0.2900	0.2362	0.2638	0.0000	0.4908	1.0000	-0.0971	1.0000	-0.0007	0.5306	0.1355
ATXN2	0.4327	-0.1898	0.0489	-0.2193	0.0018	0.5296	0.0000	0.5370	1.0000	0.0218	1.0000	0.0040	1.0000	0.0341
ATXN3	1.0000	0.0631	0.1392	0.2420	0.0901	0.3491	0.0080	0.3817	1.0000	-0.0222	0.4998	0.1701	1.0000	0.0155
ATXN7	0.0145	0.5735	0.0031	0.4675	0.8288	0.1323	0.0890	0.2843	1.0000	-0.0592	0.8354	-0.1526	1.0000	0.0967
ATXN7L1	0.0319	1.1380	0.7909	-0.2149	0.0000	1.9596	0.0001	1.1651	1.0000	0.9303	0.8240	-0.4110	1.0000	0.1429
AUH	0.0006	-0.7757	0.0000	-0.8488	0.4130	-0.2461	0.0064	-0.4113	1.0000	0.1050	1.0000	0.0449	1.0000	-0.0549
AUP1	0.3331	-0.2063	0.4108	-0.1188	0.2912	-0.2299	0.7469	-0.0678	1.0000	-0.0655	1.0000	0.0336	0.8304	0.1021
AURKA	0.0000	0.8566	0.0000	1.4568	0.0736	0.3774	0.6351	0.1131	1.0000	-0.3550	0.3199	0.2571	0.0000	-0.6141
AURKAIP1	0.0002	-0.6234	0.0001	-0.4669	0.0000	-0.8093	0.0000	-0.5953	1.0000	-0.1793	1.0000	-0.0105	1.0000	0.0399
AUTS2	0.9650	-0.0713	0.5294	-0.0983	0.0000	0.7761	0.0000	0.7040	1.0000	0.0239	1.0000	0.0097	1.0000	-0.0424
AVEN	0.0404	-0.4400	0.0006	-0.4142	0.0000	-1.0769	0.0000	-0.9323	1.0000	-0.0359	1.0000	0.0023	0.9562	0.1123
AVL9	0.0008	0.6746	0.0002	0.4479	0.6677	-0.1688	0.4197	-0.1395	1.0000	0.1707	1.0000	-0.0435	0.4791	0.2042
AVPR1A	1.0000	2.1902	1.0000	-2.3959	1.0000	2.2428	1.0000	-2.3176	1.0000	2.2674	1.0000	-2.2992	1.0000	-2.2888
AVPR1B	1.0000	-0.2881	1.0000	2.2507	1.0000	-2.4055	1.0000	0.0000	1.0000	-2.3757	1.0000	0.0470	1.0000	0.0000
AXIN1	0.0000	-0.7620	0.0000	-0.7163	0.2312	-0.2553	0.4995	-0.0988	1.0000	0.0323	1.0000	0.0910	0.2906	0.1940
AXIN2	0.1354	-0.5363	0.0018	-0.6397	1.0000	0.0228	0.0045	0.4766	1.0000	0.1403	1.0000	0.0466	0.0008	0.5951
AZI2	0.4829	-0.1977	0.0804	-0.2108	1.0000	0.0557	1.0000	-0.0129	1.0000	0.0594	1.0000	0.0589	1.0000	-0.0037
AZIN1	0.8249	-0.0930	0.4341	-0.0828	0.3599	0.1928	0.1319	0.1268	1.0000	-0.0676	1.0000	-0.0450	0.3515	-0.1280
AZIN2	1.0000	-0.1415	0.7158	0.3336	0.0029	-1.7686	0.2026	-0.7908	1.0000	-0.7093	1.0000	-0.2241	1.0000	0.2699
B2M	0.1107	-0.2664	0.0146	-0.2093	1.0000	-0.0090	0.0000	0.3474	1.0000	-0.0841	1.0000	-0.0146	0.0057	0.2780
B3GALNT1	0.6683	0.3389	0.1600	0.5361	0.0013	1.1909	0.3254	0.4438	1.0000	0.1084	0.8607	0.3183	0.1389	-0.6313
B3GALNT2	0.0250	0.8203	0.0296	0.4354	0.0731	0.6534	0.0554	0.3801	1.0000	0.2215	0.9979	-0.1509	1.0000	-0.0431
B3GALT1	0.0268	1.1279	0.0177	1.6591	1.0000	-0.0813	0.7955	0.5259	1.0000	-1.4594	0.1322	-0.9149	0.4627	-0.8496
B3GALT2	0.0000	-2.0698	0.0000	-2.0721	0.0344	-0.5589	0.0000	-1.0269	1.0000	0.0700	1.0000	0.0808	0.1641	-0.3931
B3GALT4	0.6103	-0.2393	0.5409	-0.1897	0.1400	-0.4672	1.0000	-0.0389	1.0000	-0.3439	0.6427	-0.2799	1.0000	0.0898
B3GALT5	0.1947	-1.2019	0.0005	-1.9173	0.0451	1.1415	0.0004	1.1817	1.0000	0.0943	0.9589	-0.6046	1.0000	0.1395
B3GALT6	0.0000	0.8746	0.0000	0.8812	1.0000	0.0390	0.1008	-0.2638	1.0000	0.2271	0.2363	0.2457	1.0000	-0.0720
B3GALTL	0.0121	0.6983	0.4791	0.1644	0.0007	0.8715	0.0047	0.4644	1.0000	0.4660	1.0000	-0.0542	1.0000	0.0642
B3GAT1	1.0000	0.0760	0.0546	-1.3996	0.2209	-1.1704	1.0000	-0.0689	1.0000	0.3016	0.4348	-1.1625	0.2840	1.4115
B3GAT2	1.0000	-0.1617	0.0082	-0.7971	0.6901	-0.2992	1.0000	-0.0590	1.0000	0.4126	1.0000	-0.2101	0.1174	0.6559
B3GNT1	0.4047	0.2039	0.0000	0.5559	0.0191	-0.4778	0.1457	0.1867	1.0000	-0.6029	0.0986	-0.2393	1.0000	0.0675
B3GNT2	1.0000	0.0334	0.1482	0.2769	0.5077	-0.2373	0.9261	-0.0858	1.0000	-0.0391	0.6888	0.2178	1.0000	0.1179
B3GNT3	1.0000	-1.1585	0.7674	-3.2498	0.8033	-3.2636	0.7710	-3.1631	1.0000	-0.1245	1.0000	-2.2957	1.0000	0.0000
B3GNT4	0.2143	-0.3593	0.0000	-0.6445	0.1783	-0.3581	0.0000	-0.8833	1.0000	0.2399	1.0000	-0.0319	0.4356	-0.2813
B3GNT5	1.0000	0.0314	0.2040	0.2834	0.0005	0.8329	0.0007	0.6093	1.0000	0.2223	0.0668	0.4884	1.0000	0.0045
B3GNT7	1.0000	0.3387	0.6058	-0.8091	1.0000	0.2542	1.0000	0.0074	1.0000	0.3167	0.9101	-0.8151	1.0000	0.0783
B3GNT9	0.4300	-0.3332	0.7067	0.1522	1.0000	-0.0126	0.9648	0.0889	1.0000	-0.2781	0.8605	0.2187	0.9103	-0.1715
B3GNTL1	0.3639	-0.4999	0.0004	-1.0809	0.8043	0.2238	0.3051	-0.3518	1.0000	0.2935	1.0000	-0.2749	0.7779	-0.2779
B4GALNT3	0.0602	0.4245	0.0001	0.5118	0.0577	0.4175	0.6023	-0.1213	1.0000	-0.1634	1.0000	-0.0646	0.0000	-0.6975
B4GALNT4	0.2828	-0.3905	0.4590	0.2380	0.0063	0.7403	0.0000	0.9664	1.0000	-0.6272	1.0000	0.0143	0.1112	-0.3931
B4GALT1	0.1274	0.6627	0.0000	1.2979	0.0002	1.2788	0.0000	1.3376	1.0000	-0.2594	0.5459	0.3890	0.8873	-0.1919
B4GALT2	0.0021	-0.4756	0.0000	-0.3382	0.0001	-0.6193	0.0000	-0.4147	1.0000	-0.1160	1.0000	0.0337	0.7035	0.0937
B4GALT3	0.0643	-0.6664	0.6224	-0.1451	0.7347	0.2077	0.0000	0.7169	1.0000	-0.3353	0.8555	0.1993	0.6389	0.1797
B4GALT4	0.0001	0.8471	0.0000	0.6263	0.5288	0.2151	0.8875	0.0729	1.0000	0.0072	0.4723	-0.2024	0.8608	-0.1291
B4GALT5	0.0113	-0.4623	0.0000	-0.3364	0.8176	-0.0989	0.7837	0.0445	1.0000	0.0911	0.0750	0.2299	0.0179	0.2395
B4GALT6	1.0000	0.4279	0.7215	-1.3897	0.0268	2.0654	1.0000	-0.6847	1.0000	0.1796	0.7635	-1.6323	0.0131	-2.5639

B4GALT7	0.0217	-0.4046	0.0004	-0.3982	0.0000	-1.1458	0.0000	-1.0537	1.0000	0.0594	1.0000	0.0777	0.6274	0.1570
B9D1	0.0005	1.4408	0.1484	0.5622	0.0058	1.1604	0.8180	0.2296	1.0000	0.8065	1.0000	-0.0587	1.0000	-0.1158
BAAT	0.0578	-1.4018	0.9449	-0.3088	0.9660	0.2413	0.3507	0.5661	1.0000	-0.2504	0.7488	0.8558	1.0000	0.0803
BABAM1	0.0002	-0.5823	0.0000	-0.6020	0.0028	-0.5042	0.0050	-0.3063	1.0000	-0.0582	1.0000	-0.0660	0.6118	0.1453
BACE1	0.0000	-0.7786	0.0000	-0.7221	1.0000	-0.0472	0.0857	0.1291	1.0000	0.0535	0.4713	0.1226	0.0118	0.2345
BACE2	0.8764	0.1223	0.0629	0.3329	0.0494	0.4822	0.0050	0.5140	1.0000	-0.0545	0.8553	0.1686	1.0000	-0.0171
BACH1	0.0019	-0.5426	0.0000	-0.5557	0.0581	0.3567	0.0020	0.3366	1.0000	0.1582	0.4618	0.1578	0.4260	0.1436
BACH2	0.4392	0.3148	0.7509	0.1640	0.0000	1.9067	0.0000	1.4832	1.0000	0.1903	1.0000	0.0538	0.4060	-0.2259
BAG1	1.0000	-0.0126	0.0603	-0.2464	0.0008	-0.7663	0.0000	-0.8239	1.0000	0.1032	0.8875	-0.1186	1.0000	0.0504
BAG2	0.0000	-0.7048	0.0000	-0.6687	0.0000	-0.6728	0.0000	-0.4372	1.0000	-0.0153	1.0000	0.0330	0.0283	0.2257
BAG3	0.0000	-0.6364	0.0000	-0.7134	1.0000	0.0259	0.0201	0.1685	1.0000	0.0990	1.0000	0.0345	0.0071	0.2471
BAG4	0.0215	-0.4113	0.0008	-0.3137	0.0101	-0.4493	0.0002	-0.3366	1.0000	0.1000	0.2482	0.2097	0.1467	0.2184
BAG5	0.2542	-0.2563	0.0000	-0.4321	0.5139	0.1693	0.5717	0.0846	1.0000	0.2883	0.8269	0.1253	0.1970	0.2084
BAHCC1	0.0083	-0.5594	0.0001	-0.3682	0.6654	0.1539	0.0000	0.6934	1.0000	-0.1677	1.0000	0.0365	0.0003	0.3775
BAHD1	0.3891	0.1886	0.0053	0.2668	0.4707	0.1669	0.0822	0.1802	1.0000	0.0135	0.7635	0.1048	1.0000	0.0330
BAI2	1.0000	-0.2720	0.3862	0.8036	0.0003	-6.1241	0.0305	-3.0293	1.0000	-0.7915	1.0000	0.2923	1.0000	2.3554
BAI3	1.0000	-0.8627	1.0000	-0.1756	1.0000	0.2014	1.0000	-0.5497	1.0000	0.8205	0.9094	1.5312	1.0000	0.0761
BAIAP2	0.0062	-1.8690	0.0009	-1.5919	1.0000	-0.1102	0.8726	-0.2087	1.0000	0.2924	1.0000	0.5859	1.0000	0.1999
BAIAP2L1	0.0249	0.6612	0.0634	0.3986	0.3855	-0.4241	0.0558	-0.4577	1.0000	0.2163	1.0000	-0.0350	1.0000	0.1898
BAIAP2L2	0.1512	-0.9405	0.8939	-0.2325	0.7153	-0.4083	0.6555	-0.4043	1.0000	-0.2272	0.8785	0.4907	1.0000	-0.2229
BAK1	0.0000	-1.1931	0.0003	-0.5188	0.1539	0.3410	0.0000	0.5165	1.0000	-0.3501	0.1849	0.3374	0.4317	-0.1676
BAMBI	0.8060	-0.2296	0.3757	-0.2939	0.0237	-0.9197	0.0768	-0.4907	1.0000	0.2053	1.0000	0.1527	0.1712	0.6359
BANK1	1.0000	0.3554	0.3053	1.3411	0.9361	-1.5532	0.4639	-3.6898	1.0000	-0.1283	1.0000	0.8759	1.0000	-2.2888
BAP1	0.2766	-0.2163	0.0848	-0.1663	0.0664	-0.3268	0.5913	-0.0699	1.0000	-0.1794	0.6439	-0.1172	0.8225	0.0827
BARD1	0.0000	1.4477	0.0000	1.9497	0.0000	1.0854	0.0042	0.5623	1.0000	-0.2881	0.3704	0.2265	0.0000	-0.8059
BARHL1	1.0000	0.0000	1.0000	0.0000	1.0000	0.0000	1.0000	0.0000	1.0000	0.0000	1.0000	0.0000	1.0000	0.0000
BARHL2	1.0000	0.0000	1.0000	0.0000	1.0000	0.0000	1.0000	0.0000	1.0000	0.0000	1.0000	0.0000	1.0000	0.0000
BARX2	0.0000	-4.2099	0.0000	-3.7868	0.0000	-0.9410	0.0000	-0.7248	1.0000	0.2932	0.2083	0.7307	0.0003	0.5149
BASP1	0.9336	0.0657	0.0008	0.2484	0.3295	0.1824	0.3372	0.0915	1.0000	0.0016	0.1202	0.1969	0.7215	-0.0839
BATF	0.6145	-0.1981	1.0000	-0.0173	0.0000	-1.6251	0.0000	-1.2905	1.0000	-0.1422	1.0000	0.0508	0.9076	0.1965
BATF3	0.6590	0.8738	0.2310	1.8698	1.0000	-0.4970	0.3400	1.6957	1.0000	-1.3443	1.0000	-0.3466	1.0000	0.8494
BAZ1A	0.1905	0.2576	0.0000	0.3636	0.0620	0.3328	0.4025	0.0981	1.0000	-0.0366	0.9234	0.0818	0.0209	-0.2662
BAZ1B	0.1099	0.5299	0.0000	0.9621	0.0000	1.5333	0.0000	1.5190	1.0000	-0.1899	0.5523	0.2561	0.4054	-0.1988
BAZ2B	0.0515	0.3577	0.6119	0.0845	0.5283	0.1646	0.5317	0.0907	1.0000	0.0944	0.4311	-0.1655	1.0000	0.0260
BBIP1	0.0493	-0.5841	0.0000	-0.8700	0.3560	0.3065	0.1180	0.3042	1.0000	0.1187	1.0000	-0.1527	0.9783	0.1252
BBOX1	0.1560	0.2925	0.0000	0.5315	0.0001	-0.7618	0.0000	-0.5523	1.0000	-0.1109	0.6229	0.1400	0.9791	0.1021
BBS10	0.6336	0.2541	0.0910	0.3765	0.7681	-0.2098	0.7210	0.1635	1.0000	-0.0362	1.0000	0.0978	0.4840	0.3420
BBS12	0.0901	0.8373	1.0000	-0.0325	0.5745	0.4366	0.4179	0.3278	1.0000	0.3433	0.3504	-0.5157	0.9347	0.2341
BBS2	0.3755	-0.2632	0.2042	-0.2376	0.9090	-0.1051	0.3837	0.1755	1.0000	0.0202	1.0000	0.0575	0.1864	0.3057
BBS4	0.3900	0.2598	0.3356	0.1422	1.0000	0.0311	0.0326	0.2514	1.0000	0.0888	1.0000	-0.0166	0.0349	0.3146
BBS5	0.0012	0.6386	0.0013	0.4161	0.2507	0.2766	1.0000	0.0068	1.0000	0.0959	0.9212	-0.1129	0.5458	-0.1681
BBS7	0.0026	0.9071	0.0035	0.6415	0.8011	0.1837	0.8211	-0.1328	1.0000	-0.0360	0.5171	-0.2925	0.3976	-0.3468
BBS9	0.0000	1.2108	0.0000	1.0169	0.5099	0.2570	1.0000	0.0404	1.0000	0.0994	1.0000	-0.0806	1.0000	-0.1102
BBX	0.0026	0.7305	0.0001	0.7969	0.0000	1.6218	0.0000	1.5021	1.0000	0.0364	1.0000	0.1159	1.0000	-0.0779
BCAN	0.0000	-4.0613	0.0000	-3.8387	0.0000	-2.3763	0.0000	-2.0945	1.0000	0.0031	1.0000	0.2381	0.2252	0.2903
BCAP29	0.0000	1.0828	0.0000	0.9046	0.0000	0.7305	0.0000	0.4735	1.0000	0.1575	1.0000	-0.0080	0.7287	-0.0938
BCAR3	1.0000	-0.0140	0.9880	0.0417	0.1287	-0.3233	0.0094	-0.3091	1.0000	0.0380	0.8669	0.1051	1.0000	0.0577
BCAS1	1.0000	0.0966	1.0000	0.0393	0.7491	-0.5983	1.0000	0.2214	1.0000	-0.5300	1.0000	-0.5765	1.0000	0.2934
BCAS2	0.0006	-0.6002	0.0000	-0.7186	0.0000	-0.8257	0.0000	-0.6504	1.0000	-0.0189	0.7596	-0.1254	0.5215	0.1619
BCAT1	0.0000	1.4079	0.0000	1.7774	0.0000	1.9050	0.0000	1.8627	1.0000	-0.1143	0.5245	0.2682	0.8937	-0.1509
BCDIN3D	0.4173	-0.3604	0.6317	0.1899	0.9291	-0.1454	0.9046	-0.1184	1.0000	-0.1466	0.4413	0.4175	1.0000	-0.1128
BCHE	0.2829	-0.7673	0.2606	-0.8850	0.0000	1.9921	0.0000	2.4425	1.0000	-0.3318	1.0000	-0.4346	1.0000	0.1233
BCKDHB	0.5634	0.2003	1.0000	0.0146	0.0083	-0.6753	0.0000	-0.8196	1.0000	0.0515	0.9350	-0.1221	1.0000	-0.0898
BCL10	0.3550	-0.3177	0.0563	-0.3016	0.9497	0.1006	0.9444	0.0565	1.0000	-0.0638	1.0000	-0.0343	0.9591	-0.1007
BCL11A	0.6881	1.7543	0.7304	-1.3883	0.0039	3.5202	1.0000	0.4723	1.0000	1.9501	1.0000	-1.1674	0.4538	-1.0856
BCL11B	1.0000	0.0000	1.0000	0.0000	1.0000	0.0000	1.0000	0.0000	1.0000	0.0000	1.0000	0.0000	1.0000	0.0000
BCL2	0.0000	-0.6919	0.0000	-0.8375	0.0000	-0.6782	0.0000	-0.4730	1.0000	0.2026	1.0000	0.0696	0.0000	0.4136
BCL2A1	0.3256	1.5706	1.0000	0.1753	1.0000	0.4998	1.0000	-0.4586	1.0000	1.0331	1.0000	-0.3460	1.0000	0.0784
BCL2L1	0.0972	-0.3432	0.4642	0.0963	0.0001	-0.7456	0.0000	-0.4609	1.0000	-0.0457	0.0000	0.4054	0.1541	0.2442
BCL2L13	0.6692	0.4851	0.1531	1.0238	0.0045	1.4817	0.0182	1.4063	1.0000	-0.5374	1.0000	0.0112	0.5002	-0.6104
BCL2L14	0.5086	3.5456	1.0000	0.0000	1.0000	0.0000	1.0000	0.0000	1.0000	0.0000	0.8607	-3.6732	1.0000	0.0000
BCL2L15	0.8226	3.0199	1.0000	-0.9997	1.0000	2.2428	0.7710	-3.1631	1.0000	3.1056	1.0000	-0.7966	1.0000	-2.2888
BCL6	0.0000	-1.0240	0.0000	-1.0785	0.0000	1.1711	0.0000	1.1766	1.0000	0.1930	0.6843	0.1522	0.0596	0.2037
BCL7A	0.0453	-0.5997	1.0000	-0.0549	0.0000	1.7743	0.0000	1.3404	1.0000	0.0095	0.0476	0.5683	0.0283	-0.4173
BCL7B	0.0857	-0.3214	0.6560	-0.0821	0.0296	-0.4231	0.2374	-0.1416	1.0000	-0.2636	1.0000	-0.0120	1.0000	0.0243
BCL9	0.0293	-0.5415	0.0010	-0.5065	0.0000	0.8876	0.0000	1.1562	1.0000	-0.0459	1.0000	0.0027	0.1717	0.2278
BCL9L	0.0751	0.6786	0.0000	1.2281	0.0000	2.1341	0.0000	2.0646	1.0000	-0.1014	0.0933	0.4637	0.6747	-0.1650
BCLAF1	0.0115	0.3989	0.0000	0.3901	0.2383	-0.2249	0.0001	-0.2974	1.0000	-0.0133	1.0000	-0.0097	0.8133	-0.0801
BCO1	0.6877	-0.8995	1.0000	-0.0633	0.6866	0.8912	0.0830	-1.9064	1.0000	0.8373	0.4139	1.6993	0.1717	-1.9507
BCO2	1.0000	-0.8568	1.0000	-0.1688	1.0000	0.5040	1.0000	0.0059	1.0000	0.2656	1.0000	0.9758	1.0000	-0.2284
BCOR	0.0000	1.4681	0.0000	0.9026	0.0000	1.0485	0.0000	0.7321	1.0000	0.1268	0.0503	-0.4261	0.6361	-0.1846
BCR	0.0018	-0.6020	0.0000	-0.8871	0.0228	0.4264	0.0046	0.2817	1.0000	0.2399	1.0000	-0.0331	0.7924	0.0998
BDH1	0.0000	-1.7450	0.0000	-1.5725	0.0951	0.2826	0.0525	-0.1612	1.0000	0.1074	0.0254	0.2928	0.0007	-0.3315
BDH2	0.9037	0.0930	0.6636	-0.0895	0.4781	-0.1895	1.0000	0.0030	1.0000	0.2468	1.0000	0.0774	0.0006	0.4450
BDKRB1	0.1475	0.7897	0.0000	1.9933	0.1616	-0.9823	1.0000	0.2080	1.0000	-0.5100	0.1306	0.7090	0.6707	0.6886
BDKRB2	0.3846	-1.0233	0.8463	0.4861	0.2119	-1.1751	1.0000	-0.3059	1.0000	-0.7976	0.9738	0.7241	1.0000	0.0786
BDNF	0.0112	-1.1090	0.0000	-1.1146	0.0156	-1.0339	0.0705	-0.4819	1.0000	0.1556	1.0000	0.1631	0.0923	0.7104
BEGAIN	0.0673	-4.8289	0.0231	-5.1361	0.0942	-4.7437	0.0892	-2.7162	1.0000	0.2750	1.0000	0.0000	1.0000	2.3554
BEND3	1.0000	-0.0422	1.0000	0.0389	1.0000	0.0577	0.3548	0.1626	1.0000	0.1045	0.6058	0.1972	0.4132	0.2148
BEND4	0.0527	1.0254	0.0884	0.8447	1.0000	-0.0770	0.4782	0.5095	1.0000	-0.5576	0.1753	-0.7159	1.0000	0.0402
BEND5	0.0000	-1.0078	0.0000	-1.0814	0.0001	-0.8732	0.0000	-0.7518	1.0000	-0.0479	1.0000	-0.1096	1.0000	0.0774
BEND6	0.0652	0.4168	1.0000	-0.0258	0.0450	-0.4790	0.0007	-0.4941	1.0000	0.3308	1.0000	-0.0990	0.2199	0.3207
BEND7	0.1564	0.4737	0.7868	0.1006	0.5959	0.2081	0.0873	0.3001	1.0000	0.0193	0.4857	-0.3413	0.9852	0.1187
BEST1	1.0000	0.0350	0.6890	-0.3421	0.1483	-1.0244	0.3634	-0.6217	1.0000	0.0295	1.0000	-0.3359	1.0000	0.4384
BEST3	0.0030	-3.1020	1.0000	-0.1612	0.9653	0.2781	0.4962	1.4805	1.0000	-2.8839	1.0000	0.0563	0.1014	-1.6857
BEST4	1.0000	0.0000	1.0000	0.0000	1.0000	2.2428	1.0000	2.3242	1.0000	0.0000	1.0000	0.0000	1.0000	0.0652
BET1	0.0000	-1.0205	0.0000	-1.0400	1.0000	-0.0324	0.0443	-0.2503	1.0000	-0.0217	1.0000	-0.0280	0.1193	-0.2326
BET1L	0.0156	0.4067	0.0000	0.4469	0.0732	-0.3307	0.6667	-0.0683	1.0000	-0.0978	1.0000	-0.0454	0.3202	0.1698
BFAR	0.0001	0.7260	0.0000	0.5842	0.0002	0.7037	0.0040	0.4302	1.0000	0.1283	1.0000	-0.0002	0.5236	-0.1385
BFSP1	1.0000	0.5370	1.0000	-0.1452	1.0000	0.6740	1.0000	-2.3200	1.0000	-0.1045	1.0000	-0.7919	1.0000	-3.1317
BFSP2	0.8226	3.0199	1.0000	0.0000	1.0000	0.0000	1.0000	2.3257	1.0000	0.0000	1.0000	-3.1429	1.0000	2.3554
BHLHA15	0.0001	-0.9817	0.0000	-0.8322	0.0006	-0.7812	0.0000	-0.9486	1.0000	0.0701	0.8607	0.2305	1.0000	-0.0940
BHLHA9	1.0000	2.1902	1.0000	0.0000	1.0000	0.0000	1.0000	0.0000	1.0000	0.0000	1.0000	-2.2992	1.0000	0.0000

BHLHE23	1.0000	0.0000	0.7666	3.0922	0.8011	3.0840	1.0000	2.3242	1.0000	0.0000	1.0000	3.1976	1.0000	-0.7795
BHLHE40	0.0009	-0.5149	0.0000	-0.4909	0.7953	-0.0934	0.1416	-0.1260	1.0000	0.1523	0.2482	0.1887	0.4438	0.1250
BHLHE41	0.8226	-0.2685	1.0000	0.0912	0.1174	-0.7795	0.0169	-0.6689	1.0000	0.1443	0.3049	0.5208	0.8894	0.2625
BICC1	0.0557	0.3505	0.0056	0.2995	0.0000	0.6862	0.0000	0.6056	1.0000	0.0667	1.0000	0.0283	1.0000	-0.0087
BICD2	1.0000	-0.0054	0.6846	0.0625	0.0000	0.9082	0.0000	0.7157	1.0000	0.2337	0.0007	0.3142	1.0000	0.0459
BID	1.0000	0.0168	0.4954	0.1006	0.2484	0.2667	0.3314	0.1237	1.0000	-0.0767	1.0000	0.0190	0.1789	-0.2133
BIN2	0.5086	3.5431	1.0000	2.2506	1.0000	0.0000	1.0000	0.0000	1.0000	0.0000	1.0000	-1.3254	1.0000	0.0000
BIRC2	0.0653	0.3227	0.0000	0.4042	0.8114	-0.0920	0.0202	0.2204	1.0000	-0.0691	1.0000	0.0248	0.0509	0.2485
BIRC6	0.0000	1.6532	0.0000	1.1264	0.0000	2.5187	0.0000	2.0595	1.0000	0.3930	1.0000	-0.1205	1.0000	-0.0623
BIRC7	0.8899	-0.4434	0.3254	-1.1463	1.0000	-0.0436	1.0000	0.0093	1.0000	-0.6283	0.4336	-1.3193	0.8965	-0.5703
BLCAP	0.3892	-0.1762	0.0055	-0.2115	0.0000	-0.8805	0.0000	-0.6684	1.0000	-0.0314	1.0000	-0.0547	0.1635	0.1862
BLK	1.0000	0.0000	1.0000	0.0000	1.0000	0.0000	1.0000	0.0000	1.0000	0.0000	1.0000	0.0000	1.0000	0.0000
BLM	0.0000	0.9070	0.0000	1.0819	0.0672	0.4321	1.0000	0.0313	1.0000	-0.1705	1.0000	0.0163	0.0007	-0.5676
BLMH	1.0000	-0.0085	0.0727	0.1510	0.0608	-0.3151	0.0000	-0.3737	1.0000	-0.0851	0.7814	0.0867	0.3532	-0.1383
BLNK	1.0000	-2.4788	1.0000	0.0000	1.0000	-2.4061	1.0000	0.0000	1.0000	-2.3771	1.0000	0.0000	1.0000	0.0000
BLOC1S1	0.0000	-1.0364	0.0000	-0.7015	0.0000	-1.0099	0.0000	-0.6890	1.0000	-0.1608	0.8269	0.1866	0.6687	0.1653
BLOC1S2	0.8833	0.0924	1.0000	-0.0029	0.0001	-0.6800	0.0000	-0.8838	1.0000	-0.0280	0.8785	-0.1108	0.1766	-0.2255
BLOC1S4	0.0000	-0.7095	0.0000	-0.8205	0.0000	-0.7209	0.0000	-0.6751	1.0000	-0.0707	0.4723	-0.1692	1.0000	-0.0190
BLOC1S5	0.6207	-0.1420	0.0013	-0.2508	0.6969	0.1238	0.3210	-0.0954	1.0000	-0.1217	0.0468	-0.2179	0.0002	-0.3350
BLOC1S6	0.7821	-0.1429	0.4929	-0.1488	0.2524	-0.3016	0.0159	-0.3886	1.0000	0.0117	1.0000	0.0200	1.0000	-0.0693
BLVRA	0.0000	-2.4929	0.0000	-2.4477	0.0000	-1.5004	0.0000	-1.8154	1.0000	-0.0468	1.0000	0.0109	0.2400	-0.3550
BLZF1	0.0449	-0.4008	0.0003	-0.4180	0.8571	0.0953	0.2321	0.1604	1.0000	-0.1276	0.7971	-0.1315	1.0000	-0.0569
BMF	0.0135	0.4181	1.0000	-0.0125	1.0000	0.0090	0.0010	0.2721	1.0000	0.2156	0.2727	-0.2026	0.0000	0.4833
BMP10	1.0000	0.0000	1.0000	0.0000	1.0000	0.0000	1.0000	0.0000	1.0000	0.0000	1.0000	0.0000	1.0000	0.0000
BMP15	1.0000	0.2007	1.0000	-0.3890	0.7628	1.0365	0.0401	-4.8533	1.0000	1.5621	1.0000	0.9997	0.3369	-4.3541
BMP2	1.0000	0.3554	1.0000	-0.6395	0.0577	2.0349	1.0000	-0.2070	1.0000	1.0314	1.0000	0.0565	0.4068	-1.2071
BMP2K	0.0000	-1.1080	0.0000	-0.9129	1.0000	0.0402	0.3885	-0.1022	1.0000	0.0821	0.0139	0.2900	1.0000	-0.0555
BMP3	0.0065	2.0586	0.0000	2.7607	0.0101	2.0274	0.5092	0.7002	1.0000	0.9380	0.0000	1.6690	1.0000	-0.3785
BMP4	0.0000	-1.0125	0.0000	-1.5114	0.0003	0.7787	0.0000	0.6506	1.0000	-0.2983	0.0001	-0.7846	0.0096	-0.4200
BMP5	1.0000	0.0000	1.0000	0.0000	1.0000	0.0000	1.0000	0.0000	1.0000	0.0000	1.0000	0.0000	1.0000	0.0000
BMP6	0.2891	0.4533	0.8973	-0.1227	0.5474	-0.3411	0.9993	0.0957	1.0000	0.0872	0.2718	-0.4749	0.2265	0.5324
BMP7	0.0000	-1.9096	0.0000	-1.4307	0.6343	-0.2112	0.0000	0.4470	1.0000	0.3793	0.0000	0.8721	0.0000	1.0423
BMPER	0.3810	0.6195	0.0000	1.0900	1.0000	-0.2406	0.0087	-0.8729	1.0000	0.9902	0.0000	1.4775	0.8793	0.3641
BMPR1A	0.4185	0.1910	1.0000	-0.0138	0.0080	0.4582	0.0000	0.4453	1.0000	0.1064	0.9927	-0.0855	0.7383	0.0989
BMPR1B	0.0000	-1.9714	0.0000	-1.6930	0.0000	-1.4386	0.0000	-0.8981	1.0000	0.3106	0.2035	0.6021	0.0026	0.8567
BMPR2	0.3650	0.1880	0.4012	0.0989	0.0089	0.4524	0.0000	0.4578	1.0000	0.0563	1.0000	-0.0203	0.9979	0.0665
BMS1	0.0459	0.3557	0.0000	0.4009	0.6450	0.1329	0.0127	0.2461	1.0000	-0.0934	1.0000	-0.0360	1.0000	0.0246
BNC1	0.4884	0.7422	1.0000	0.2081	0.7002	0.5273	0.4762	0.5852	1.0000	0.2774	1.0000	-0.2442	0.9886	0.3408
BNC2	0.3451	-0.4259	0.6925	-0.1982	0.1018	0.4868	0.0000	0.8554	1.0000	-0.2992	1.0000	-0.0607	1.0000	0.0733
BNIP1	0.1434	-0.3527	0.0085	-0.3708	0.1954	-0.3124	0.3960	-0.1453	1.0000	0.0001	1.0000	-0.0059	0.6054	0.1719
BNIP2	0.8311	-0.1029	0.5902	-0.0880	0.2011	0.2785	0.1303	0.1731	1.0000	0.1214	0.4899	0.1497	1.0000	0.0218
BNIP3	1.0000	0.0549	0.4882	0.0971	0.0262	-0.4808	0.0002	-0.3889	1.0000	-0.0580	1.0000	-0.0030	1.0000	0.0398
BNIP3L	0.4693	0.2123	0.0743	0.2326	0.0000	1.0051	0.0000	0.8920	1.0000	0.0959	0.8534	0.1288	1.0000	-0.0107
BOC	0.0269	0.4667	0.1885	0.1828	0.8982	-0.0950	0.4012	-0.1263	1.0000	0.0493	0.3394	-0.2229	1.0000	0.0229
BOD1	0.3433	-0.2346	0.0035	-0.3079	0.0007	-0.6500	0.0000	-0.8441	1.0000	0.0958	1.0000	0.0347	0.8482	-0.0934
BOD1L1	0.0004	0.9878	0.0000	0.9031	0.0000	1.9809	0.0000	1.4647	1.0000	0.1842	1.0000	0.1132	0.2264	-0.3256
BOK	0.0000	-2.1857	0.0000	-2.4592	0.1957	-0.3819	0.0556	-0.3908	1.0000	-0.2373	0.6983	-0.4979	0.6340	-0.2413
BOLA3	0.0000	-0.9078	0.0000	-0.6062	0.0000	-1.1150	0.0000	-0.6924	1.0000	-0.2811	1.0000	0.0331	0.7015	0.1483
BOLL	1.0000	0.0324	0.6526	-0.3666	0.0711	-0.9678	0.7183	-0.3350	1.0000	-0.4395	0.2530	-0.8295	1.0000	0.1951
BORA	0.0152	0.6842	0.0000	0.9007	0.1172	0.4804	0.6261	0.1768	1.0000	-0.0685	0.8932	0.1605	0.2507	-0.3676
BPGM	0.0859	0.3307	0.0000	0.4051	0.1018	0.3175	0.0414	0.1962	1.0000	-0.0967	1.0000	-0.0098	0.1113	-0.2129
BPHL	0.5282	0.3781	1.0000	0.0956	0.0616	-0.8496	0.0289	-0.8025	1.0000	0.1119	1.0000	-0.1592	1.0000	0.1609
BPIFB6	1.0000	0.0000	1.0000	0.0000	1.0000	0.0000	1.0000	0.0000	1.0000	0.0000	1.0000	0.0000	1.0000	0.0000
BPNT1	0.7959	0.1441	0.5282	-0.1311	0.9673	0.0906	0.3627	-0.1638	1.0000	0.0547	0.5784	-0.2081	0.5328	-0.1939
BPTF	1.0000	0.0368	0.9431	-0.0518	0.0000	1.2657	0.0000	1.0866	1.0000	0.1396	1.0000	0.0648	1.0000	-0.0336
BRAP	0.0001	0.6165	0.0000	0.3789	0.7056	0.1166	0.7710	-0.0542	1.0000	0.1225	0.7323	-0.1024	1.0000	-0.0433
BRAT1	0.1150	-0.3686	0.0000	-0.4913	0.6216	-0.1668	1.0000	0.0053	1.0000	0.1497	1.0000	0.0401	0.0540	0.3256
BRCA1	1.0000	0.0761	0.1635	0.2498	0.8960	0.1074	0.6533	-0.1264	1.0000	-0.2946	1.0000	-0.1086	0.0081	-0.5229
BRCA2	0.0000	1.2739	0.0000	0.9339	0.0322	0.5727	0.6594	-0.1351	1.0000	0.3140	1.0000	-0.0145	0.1235	-0.3894
BRCC3	0.3295	-0.2286	0.2073	-0.1605	0.0003	-0.6577	0.0000	-0.7796	1.0000	0.1291	0.3233	0.2086	1.0000	0.0119
BRD1	0.4597	0.1798	1.0000	0.0177	0.0200	0.4243	0.0000	0.4276	1.0000	0.1622	1.0000	0.0132	0.3415	0.1709
BRD3	1.0000	0.0776	1.0000	0.0628	0.0000	0.9084	0.0000	0.7823	1.0000	0.0120	1.0000	0.0091	0.9728	-0.1098
BRD4	0.9059	-0.0980	0.7674	0.0880	0.0000	1.0017	0.0000	0.9695	1.0000	-0.1655	1.0000	0.0331	0.3895	-0.1914
BRD7	0.0923	-0.2893	0.0000	-0.3567	0.0312	-0.3558	0.0000	-0.3207	1.0000	0.1029	1.0000	0.0480	0.3291	0.1434
BRD8	0.6813	-0.1200	0.0354	-0.1986	1.0000	-0.0562	0.1502	-0.1512	1.0000	-0.0409	0.7636	-0.1070	0.5410	-0.1305
BRD9	1.0000	0.0607	0.0180	-0.3173	0.0016	-0.6596	0.0000	-0.8097	1.0000	0.0236	0.0401	-0.3427	0.8825	-0.1217
BRF1	0.2100	0.3639	0.0156	0.4286	0.1224	0.4124	0.0001	0.6221	1.0000	-0.1115	1.0000	-0.0344	1.0000	0.1040
BRF2	0.1441	0.4332	0.1407	0.2765	0.0107	-0.7511	0.0011	-0.6181	1.0000	0.1174	1.0000	-0.0263	0.7219	0.2574
BRI3	0.0000	-0.7620	0.0000	-0.9447	0.0000	-0.6993	0.0000	-0.5934	1.0000	0.0431	0.8269	-0.1271	0.5105	0.1544
BRI3BP	0.0000	0.7104	0.0000	0.7849	0.0242	0.3742	0.0000	0.3823	1.0000	-0.0652	1.0000	0.0215	1.0000	-0.0523
BRICD5	0.8226	-3.3425	0.2541	-2.4812	1.0000	-0.1789	0.4992	-1.4670	1.0000	1.3496	1.0000	2.3455	1.0000	0.0717
BRINP1	0.0058	3.0241	0.0118	3.2442	1.0000	-0.1811	0.9164	1.3892	1.0000	-0.9614	0.7865	-0.7493	1.0000	0.6087
BRINP2	0.0648	-4.8294	1.0000	-0.4768	0.8151	-1.1321	1.0000	-0.6852	1.0000	-0.3775	0.6427	4.1168	1.0000	0.0749
BRINP3	1.0000	0.0000	1.0000	0.0000	1.0000	0.0000	1.0000	0.0000	1.0000	0.0000	1.0000	0.0000	1.0000	0.0000
BRIP1	0.4925	0.2279	0.0023	0.4626	0.0647	0.4310	0.0683	0.3121	1.0000	-0.1223	0.9441	0.1253	0.4062	-0.2359
BRIX1	0.5144	-0.1797	0.0070	-0.2288	0.0009	-0.6265	0.0000	-0.6126	1.0000	-0.0042	1.0000	-0.0408	1.0000	0.0155
BRMS1L	0.2182	0.2751	0.9716	0.0430	0.4784	-0.1909	0.0110	-0.2943	1.0000	0.1016	0.8341	-0.1189	1.0000	0.0026
BROX	1.0000	0.0167	0.2196	-0.1587	1.0000	-0.0263	0.0097	-0.2899	1.0000	0.1024	1.0000	-0.0600	0.5814	-0.1563
BRPF1	0.0927	-0.3940	0.0164	-0.3293	0.8615	-0.1101	0.8840	0.0614	1.0000	-0.3002	0.4357	-0.2234	0.8094	-0.1234
BRPF3	0.2710	-0.2349	0.0001	-0.3579	0.0013	-0.5677	0.0001	-0.3871	1.0000	0.1278	1.0000	0.0178	0.0467	0.3130
BRS3	1.0000	0.0000	1.0000	0.0000	1.0000	0.0000	1.0000	0.0000	1.0000	0.0000	1.0000	0.0000	1.0000	0.0000
BRSK2	1.0000	2.1902	0.9062	-1.5359	0.8011	3.0840	1.0000	-0.5236	1.0000	3.6422	1.0000	0.0470	1.0000	0.0728
BRWD1	1.0000	0.0369	1.0000	-0.0047	1.0000	0.0505	0.7305	0.0719	1.0000	0.1226	0.9942	0.0945	0.5003	0.1492
BRWD3	0.7308	0.1664	0.0890	-0.2795	0.0000	1.1472	0.0000	0.6204	1.0000	0.3453	1.0000	-0.0872	0.7504	-0.1764
BSDC1	0.7196	-0.1536	0.1284	-0.2379	1.0000	0.0498	0.0881	0.2825	1.0000	-0.1307	0.6842	-0.2020	0.9675	0.1066
BSG	0.0079	0.4060	0.0000	0.5936	0.0019	0.4823	0.0000	0.6451	1.0000	-0.0295	0.2013	0.1707	0.4805	0.1385
BSN	0.2584	-1.3186	0.4066	0.9855	0.3031	0.8432	0.0000	2.4042	1.0000	-1.0988	0.6064	1.2202	0.7408	0.4650
BSND	1.0000	0.0000	1.0000	0.0000	1.0000	0.0000	1.0000	0.0000	1.0000	0.0000	1.0000	0.0000	1.0000	0.0000
BSPRY	1.0000	-0.0729	0.6413	-1.1287	0.6055	-1.0111	0.7710	0.5777	1.0000	-0.2945	0.6442	-1.3437	0.5528	1.3011
BST1	0.1052	0.3591	0.0154	0.3136	0.1835	-0.3265	0.0000	-0.6162	1.0000	-0.0064	1.0000	-0.0402	0.2041	-0.2907
BSX	1.0000	0.0000	1.0000	0.0000	1.0000	0.0000	1.0000	0.0000	1.0000	0.0000	1.0000	0.0000	1.0000	0.0000
BTAF1	0.5264	-0.1798	0.0271	-0.2154	0.2825	0.2567	1.0000	-0.0235	1.0000	0.1178	0.8840	0.0950	0.4437	-0.1585
BTBD1	0.0229	-0.3742	0.0000	-0.5521	0.0197	-0.3834	0.0000	-0.3664	1.0000	0.0911	0.9278	-0.0745	0.5546	0.1135

BTBD10	0.4050	0.2149	0.0419	0.2484	0.0512	0.3920	0.0830	0.2110	1.0000	0.1353	0.4488	0.1820	1.0000	-0.0394
BTBD11	0.0000	-1.2929	0.0000	-1.4047	0.0000	-1.6017	0.0000	-2.1199	1.0000	0.2266	1.0000	0.1258	0.4853	-0.2874
BTBD17	0.9671	-1.6927	0.4431	-3.7865	0.5042	-3.7955	0.4370	-3.6968	1.0000	-0.1227	1.0000	-2.2957	1.0000	0.0000
BTBD19	0.0551	-0.8079	0.0508	-0.6841	0.2998	0.4160	0.0009	0.8210	1.0000	-0.1321	1.0000	0.0047	0.6649	0.2781
BTBD2	0.3969	-0.2537	0.0552	0.3511	1.0000	-0.0608	0.0016	0.4609	1.0000	-0.6006	1.0000	0.0162	1.0000	-0.0725
BTBD3	1.0000	-0.0089	1.0000	0.0052	0.0063	0.5077	0.0000	0.6690	1.0000	-0.1081	0.8793	-0.0814	1.0000	0.0588
BTBD6	0.0001	-0.8570	0.0000	-0.9157	0.0019	-0.6937	0.0000	-0.8383	1.0000	-0.3457	0.1246	-0.3926	0.0139	-0.4867
BTBD7	0.0447	0.8232	0.0000	1.4823	0.0000	1.8486	0.0000	1.9916	1.0000	-0.3461	0.5779	0.3250	0.7043	-0.1988
BTBD8	0.3137	1.1415	1.0000	0.1169	0.6879	0.6330	0.2612	0.8184	1.0000	0.2435	0.7851	-0.7686	0.8768	0.4300
BTBD9	0.0001	1.3884	0.0028	0.8420	0.0000	2.1520	0.0000	1.3760	1.0000	0.3696	1.0000	-0.1628	0.3891	-0.3988
BTC	0.4274	0.1870	0.0641	0.2069	0.0000	-1.7695	0.0000	-1.2035	1.0000	-0.2415	0.2344	-0.2092	0.0981	0.3303
BTD	0.6380	0.2077	0.0000	0.7193	0.6746	0.1923	0.0013	0.5740	1.0000	-0.3283	0.7682	0.1952	1.0000	0.0599
BTF3	0.7118	0.1142	1.0000	0.0218	0.0133	-0.4135	0.0000	-0.5635	1.0000	-0.0018	0.8395	-0.0820	0.2171	-0.1462
BTF3L4	0.0006	-0.5488	0.0000	-0.6186	0.0027	-0.4912	0.0000	-0.6829	1.0000	0.0598	1.0000	0.0025	0.4143	-0.1260
BTG1	0.0006	0.5800	0.0000	0.4152	0.5745	-0.1471	0.0000	-0.2721	1.0000	0.0132	0.3296	-0.1395	0.4815	-0.1060
BTG2	0.3451	-0.1881	0.1434	-0.1426	0.2216	0.2235	0.0001	0.2941	1.0000	-0.0622	1.0000	-0.0043	1.0000	0.0137
BTG4	1.0000	0.0000	1.0000	0.0000	1.0000	0.0000	1.0000	2.3242	1.0000	0.0000	1.0000	0.0000	1.0000	2.3543
BTK	1.0000	-0.0159	0.6303	0.3267	0.0299	-1.0582	0.6032	-0.3895	1.0000	-0.7563	0.8269	-0.4033	1.0000	-0.0815
BTRC	0.0413	-0.4816	0.0042	-0.3899	0.0000	0.9591	0.0000	0.9060	1.0000	-0.1023	1.0000	0.0016	0.5692	-0.1507
BUB1	0.0000	1.2364	0.0000	1.6338	0.0008	0.6007	0.0095	0.2965	1.0000	0.0296	0.0000	0.4383	0.0543	-0.2698
BUB3	0.0292	0.4620	0.0000	0.6784	0.9120	0.0901	0.3026	0.1058	1.0000	-0.1245	0.7745	0.1045	0.6506	-0.1027
BUD13	0.4021	-0.2143	0.0034	-0.3767	0.7021	-0.1407	0.1294	-0.2004	1.0000	0.0571	1.0000	-0.0930	1.0000	0.0031
BUD31	0.0017	-0.4753	0.0000	-0.5192	0.0000	-0.6809	0.0000	-0.5578	1.0000	-0.0140	1.0000	-0.0458	0.5810	0.1145
BVES	0.0000	-1.1628	0.0000	-0.9684	0.0000	-0.7709	0.0000	-0.6031	1.0000	0.2149	0.0025	0.4221	0.0006	0.3873
BYSL	0.0000	-0.8634	0.0000	-0.8927	0.0000	-0.7440	0.0000	-0.5528	1.0000	-0.0468	1.0000	-0.0646	0.5348	0.1497
BZRAP1	0.7930	-0.1280	0.0045	-0.4486	0.0000	0.7887	0.0000	0.9134	1.0000	0.1557	0.8607	-0.1515	0.0480	0.2856
BZW1	0.0081	-0.4152	0.0000	-0.3524	0.0842	-0.2961	0.0003	-0.2356	1.0000	-0.0261	1.0000	0.0491	1.0000	0.0399
BZW2	0.0066	-0.4956	0.0000	-0.4879	0.0000	-0.7660	0.0000	-0.8518	1.0000	-0.0456	1.0000	-0.0254	0.3760	-0.1257
C10H14orf80	1.0000	-0.0422	0.1628	0.2296	0.0001	-0.8688	0.0000	-0.7755	1.0000	-0.2887	1.0000	-0.0040	0.7263	-0.1905
C10H1orf111	1.0000	-0.2556	0.1366	-1.0944	0.8381	0.3794	0.0121	1.1633	1.0000	0.4421	1.0000	-0.3803	0.0266	1.2344
C10H1orf112	0.0000	1.0510	0.0000	1.1804	0.1282	0.4547	0.2647	0.2704	1.0000	-0.2147	1.0000	-0.0721	0.1568	-0.3912
C10H1orf123	0.3747	0.2120	0.9691	-0.0424	0.0000	-0.9112	0.0000	-0.7256	1.0000	-0.0009	0.1915	-0.2437	0.5697	0.1909
C10H1orf146	1.0000	-0.7259	0.2741	2.1685	0.6647	-1.9417	0.6142	1.7722	1.0000	-1.8819	1.0000	1.0051	0.8937	1.8364
C10H1orf168	1.0000	0.0000	1.0000	2.2507	1.0000	0.0000	1.0000	0.0000	1.0000	0.0000	1.0000	2.3455	1.0000	0.0000
C10H1orf177	0.8249	3.0140	1.0000	2.2507	1.0000	0.0000	1.0000	0.0000	1.0000	0.0000	1.0000	-0.7943	1.0000	0.0000
C10H1orf21	0.9925	-0.0948	0.2264	-0.2215	0.1783	0.3611	1.0000	-0.0148	1.0000	0.1494	1.0000	0.0347	0.4805	-0.2208
C10H1orf210	1.0000	-2.4776	1.0000	0.0000	1.0000	-2.4055	1.0000	0.0000	1.0000	-2.3757	1.0000	0.0000	1.0000	0.0000
C10H1orf226	0.0350	-0.9561	0.0004	-0.9495	0.4973	-0.3635	0.6895	-0.1708	1.0000	0.4149	0.6847	0.4303	0.0934	0.6112
C10H1orf228	1.0000	-0.0016	0.9383	0.5813	0.9777	0.5595	0.9476	0.4995	1.0000	-0.1277	1.0000	0.4667	1.0000	-0.1802
C10H1orf27	0.5523	0.1545	0.4330	0.1037	0.0178	-0.4361	0.0000	-0.5472	1.0000	0.0913	1.0000	0.0525	1.0000	-0.0152
C10H1orf52	1.0000	-0.0612	0.0007	-0.4922	0.1323	-0.4043	0.0000	-0.5806	1.0000	0.0927	0.4857	-0.3252	1.0000	-0.0792
C11H18orf32	0.0165	-0.4064	0.0000	-0.4099	0.0233	-0.3878	0.0000	-0.4147	1.0000	0.1645	0.3922	0.1735	0.4509	0.1426
C11H21orf2	0.1782	-0.2628	0.0187	-0.2261	0.0000	-0.6854	0.0000	-0.5872	1.0000	-0.0880	1.0000	-0.0388	1.0000	0.0169
C11H2orf54	1.0000	0.0000	1.0000	0.0000	1.0000	2.2472	1.0000	0.0000	1.0000	0.0000	1.0000	0.0000	1.0000	-2.2909
C11H2orf82	0.1483	-1.2222	0.1041	-0.9408	0.2480	-1.0621	0.0104	-1.5053	1.0000	0.2601	1.0000	0.5545	1.0000	-0.1774
C11H3orf33	0.0001	-0.7148	0.0000	-0.8589	0.0006	-0.6241	0.0744	-0.2219	1.0000	0.0216	0.9589	-0.1107	0.0030	0.4288
C11H3orf58	0.8805	0.1645	0.0120	0.4743	0.0079	0.7789	0.0002	0.6327	1.0000	0.2499	0.0105	0.5730	0.9815	0.1109
C11H3orf70	0.3051	0.2547	0.0113	0.3164	0.1072	0.3450	0.0001	0.4649	1.0000	0.0081	1.0000	0.0827	0.6861	0.1335
C12H15orf26	0.5685	-0.9250	0.2719	-0.9651	1.0000	-0.0798	0.6658	-0.6166	1.0000	0.0874	1.0000	0.0602	1.0000	-0.4433
C12H15orf39	0.5649	-0.3115	0.0755	-0.5124	1.0000	0.1067	0.7701	-0.1565	1.0000	0.1455	1.0000	-0.0421	1.0000	-0.1113
C12H15orf40	0.3214	-0.2300	0.0016	-0.3111	0.0000	-0.9966	0.0000	-0.7460	1.0000	-0.0471	0.7886	-0.1154	0.3089	0.2087
C12H15orf43	1.0000	0.0000	1.0000	0.0000	1.0000	2.2472	1.0000	2.3257	1.0000	0.0000	1.0000	0.0000	1.0000	0.0649
C12H15orf48	0.9844	0.3710	0.0125	1.5586	1.0000	0.2969	0.0467	1.4150	1.0000	-0.3627	0.4754	0.8191	0.4787	0.7411
C12H15orf59	1.0000	2.1902	1.0000	-0.1594	0.8011	3.0840	1.0000	0.0074	1.0000	3.1056	1.0000	0.8994	1.0000	0.0723
C12H15orf61	0.0156	0.7885	0.0123	0.6105	0.0690	-0.7122	0.0745	-0.5953	1.0000	-0.1990	0.3515	-0.3619	1.0000	-0.0743
C12H15orf65	1.0000	2.1847	1.0000	-2.3986	1.0000	2.2428	1.0000	-2.3200	1.0000	2.2732	1.0000	-2.2957	1.0000	-2.2888
C13H16orf70	0.2797	-0.2211	0.0000	-0.6789	0.4449	-0.1729	0.0004	-0.3159	1.0000	0.2215	0.1720	-0.2233	0.8486	0.0839
C13H16orf74	0.9545	1.0672	1.0000	-0.1452	1.0000	-0.1597	1.0000	-2.3200	1.0000	-0.1045	1.0000	-1.3257	1.0000	-2.2909
C13H16orf87	0.4680	0.2440	0.2383	0.2209	0.0487	-0.6451	0.0008	-0.5852	1.0000	-0.0301	1.0000	-0.0409	1.0000	0.0364
C13H19orf40	1.0000	-0.0858	0.0000	0.8486	1.0000	0.0335	0.0944	0.3765	1.0000	-0.5354	0.1239	0.4091	0.7759	-0.1873
C14H3orf14	0.6016	-0.1914	1.0000	-0.0197	0.4809	0.2094	0.0309	0.2760	1.0000	-0.0978	1.0000	0.0852	1.0000	-0.0260
C14H3orf18	0.0032	0.6024	0.0000	0.8792	0.2069	0.3016	0.0000	0.4825	1.0000	-0.0809	0.3760	0.2088	0.7303	0.1062
C14H3orf67	0.0903	2.7156	0.0393	2.0555	1.0000	0.6780	0.9096	-1.3722	1.0000	1.2654	1.0000	0.6313	1.0000	-0.7792
C14H9orf89	0.0597	0.3054	0.0000	0.3460	1.0000	-0.0378	0.0215	0.1981	1.0000	0.0392	0.7505	0.0922	0.0079	0.2802
C15H5orf15	0.2770	0.2061	0.0037	0.2126	0.9127	0.0711	0.0027	0.2306	1.0000	0.0467	0.9301	0.0656	0.0606	0.2116
C15H5orf24	0.0001	1.2375	0.0000	1.4012	0.0000	2.1059	0.0000	1.7029	1.0000	0.1299	0.4520	0.3082	0.3839	-0.2669
C15H5orf45	0.4083	0.2868	0.4004	0.1500	1.0000	0.0311	0.0025	-0.3957	1.0000	-0.2519	0.0130	-0.3780	0.0000	-0.6724
C15H5orf58	1.0000	0.0000	1.0000	0.0000	1.0000	0.0000	1.0000	0.0000	1.0000	0.0000	1.0000	0.0000	1.0000	0.0000
C16H16orf45	0.7421	0.2730	0.5751	-0.2742	1.0000	0.0521	1.0000	0.0973	1.0000	0.3771	1.0000	-0.1561	0.5498	0.4277
C16H16orf52	0.7205	-0.1886	0.0554	-0.3768	0.3544	0.2917	0.0025	0.5034	1.0000	-0.0542	0.7370	-0.2296	0.7334	0.1637
C16H16orf59	0.0446	0.7540	0.1444	0.4607	0.6177	-0.3125	1.0000	-0.0924	1.0000	-0.1464	0.3206	-0.4317	1.0000	0.0791
C16H16orf72	1.0000	0.0081	0.7223	-0.0483	0.2292	-0.2270	0.0000	-0.2857	1.0000	0.0392	1.0000	-0.0047	1.0000	-0.0138
C16H16orf96	0.1765	0.6552	0.0352	0.9229	0.0023	-1.8016	0.8445	-0.3130	1.0000	-0.6894	0.6780	-0.4112	0.7040	0.8038
C16H7orf26	0.4401	-0.2334	0.2184	-0.2317	1.0000	-0.0663	0.0864	-0.2833	1.0000	0.0813	1.0000	0.0947	0.8914	-0.1306
C16H7orf50	1.0000	-0.0061	0.1519	0.2344	0.1055	-0.4422	0.0000	-0.7014	1.0000	-0.1990	1.0000	0.0533	0.0515	-0.4511
C16H7orf62	1.0000	0.5381	1.0000	-0.1716	1.0000	-0.1624	1.0000	-0.5258	1.0000	1.2739	1.0000	0.5871	1.0000	0.9186
C16H8orf33	0.0386	0.4894	0.0001	0.5678	1.0000	-0.0002	0.8927	0.0791	1.0000	-0.1269	1.0000	-0.0362	1.0000	-0.0428
C17H11orf85	1.0000	0.0000	0.7674	-3.2498	0.0938	4.5491	1.0000	-0.8404	1.0000	3.1056	1.0000	0.0000	0.5105	-2.2502
C17H12orf43	1.0000	-0.1213	1.0000	0.0752	0.1170	-0.6030	0.2928	-0.3625	1.0000	-0.2278	1.0000	-0.0181	1.0000	0.0193
C17H12orf49	0.0029	0.6334	0.0000	0.9458	1.0000	-0.0581	0.9759	0.0578	1.0000	-0.2099	0.9028	0.1158	1.0000	-0.0866
C17H12orf65	0.0361	-0.4086	0.0018	-0.3662	0.0719	-0.3518	0.0005	-0.4147	1.0000	-0.0184	1.0000	0.0363	1.0000	-0.0769
C19H16orf93	0.5212	-0.2555	0.0237	-0.4923	0.0111	-0.6787	0.0015	-0.6310	1.0000	-0.0579	0.6091	-0.2840	1.0000	-0.0057
C19H9orf114	0.0088	-0.6444	0.0002	-0.5655	0.0049	-0.6765	0.0536	-0.3142	1.0000	-0.1801	1.0000	-0.0900	0.7485	0.1890
C19H9orf142	0.0034	-0.8586	0.0000	-0.9120	0.0216	-0.6370	0.8056	-0.0954	1.0000	-0.1253	1.0000	-0.1684	0.1224	0.4217
C19H9orf16	1.0000	0.0478	0.0741	0.1957	0.0003	-0.6307	0.0000	-0.4277	1.0000	-0.1806	1.0000	-0.0197	1.0000	0.0281
C19H9orf171	1.0000	0.0000	1.0000	0.0000	1.0000	0.0000	1.0000	0.0000	1.0000	0.0000	1.0000	0.0000	1.0000	0.0000
C19H9orf172	1.0000	0.2730	0.9214	0.2742	1.0000	-0.1976	1.0000	0.2567	1.0000	0.6812	0.8799	0.7003	0.4112	1.1425
C19H9orf173	1.0000	-0.1290	0.2552	1.0452	0.1310	-1.9822	1.0000	-0.3926	1.0000	-0.5776	1.0000	0.6025	1.0000	1.0209
C19H9orf69	0.0429	-0.4753	0.0017	-0.4771	0.0652	-0.4156	0.2355	-0.2037	1.0000	0.0542	1.0000	0.0649	0.2139	0.2716
C19H9orf78	0.6491	-0.1543	0.0076	-0.3327	1.0000	0.0360	0.3115	0.1338	1.0000	0.0538	0.9726	-0.1115	0.5015	0.1568
C19H9orf9	1.0000	0.0861	1.0000	-0.1789	0.0023	1.5208	0.0007	1.4201	1.0000	-0.1310	1.0000	-0.3855	0.9625	-0.2241
C19H9orf91	0.6568	-0.9013	0.3422	-1.8566	0.2857	-2.5000	0.1480	-2.5296	1.0000	0.0873	1.0000	-0.8613	1.0000	0.0660
C1D	1.0000	-0.0549	0.1483	-0.1562	0.0021	-0.7034	0.0000	-0.8227	1.0000	0.0187	1.0000	-0.0701	0.8768	-0.0956

C1GALT1	0.2366	0.2653	0.1084	0.1706	0.0420	0.4005	0.0016	0.2909	1.0000	0.1017	1.0000	0.0192	1.0000	-0.0031
C1GALT1C1	0.1235	0.2821	0.0000	0.3924	1.0000	0.0297	0.3416	-0.1089	1.0000	-0.0030	0.6300	0.1191	0.4752	-0.1362
C1H11orf30	0.4621	-0.2175	0.0197	-0.3293	1.0000	0.0586	0.9801	0.0495	1.0000	0.0156	1.0000	-0.0836	1.0000	0.0125
C1H11orf54	0.0034	0.5809	0.0082	0.3370	0.1656	-0.3687	0.0202	-0.3230	1.0000	0.1538	1.0000	-0.0769	0.5813	0.2078
C1H11orf70	0.0110	1.2577	0.1216	0.6456	0.8566	0.3160	0.9151	-0.2068	1.0000	0.5098	1.0000	-0.0886	1.0000	-0.0087
C1H11orf73	0.4882	-0.1764	0.1267	-0.1623	0.0026	-0.5561	0.0000	-0.7099	1.0000	-0.0946	1.0000	-0.0679	0.1552	-0.2415
C1H11orf87	0.0075	0.4365	0.0213	0.2079	0.0071	-0.4447	0.0000	0.4114	1.0000	0.0100	0.1355	-0.2065	0.0000	0.8715
C1H11orf97	0.9545	-1.6907	1.0000	0.5219	0.4809	-3.7970	1.0000	0.3938	1.0000	-0.1237	0.8269	2.1235	0.5105	4.1259
C1H12orf29	0.4476	0.2096	0.0085	0.3160	0.3885	-0.2227	0.0000	-0.4959	1.0000	-0.0513	1.0000	0.0665	0.0798	-0.3190
C1H12orf4	0.0645	0.4024	0.0001	0.4745	0.3148	-0.2633	0.0007	-0.4208	1.0000	-0.1147	1.0000	-0.0304	0.1876	-0.2664
C1H12orf40	1.0000	2.1902	0.7666	3.0922	1.0000	0.0000	1.0000	0.0000	1.0000	0.0000	1.0000	0.8994	1.0000	0.0000
C1H12orf45	0.0013	-0.5997	0.0000	-0.6137	0.0000	-0.9010	0.0000	-0.9503	1.0000	0.0105	1.0000	0.0093	1.0000	-0.0313
C1H12orf5	1.0000	-0.0357	0.0043	-0.3125	0.0001	-0.7962	0.0000	-0.9851	1.0000	0.1102	0.6454	-0.1533	1.0000	-0.0733
C1H12orf50	0.5340	1.0871	0.0180	1.7798	1.0000	-0.8813	0.7754	-0.9357	1.0000	0.1250	0.7147	0.8291	1.0000	0.0765
C1H12orf57	0.0000	-1.2301	0.0000	-1.0791	0.0000	-1.1627	0.0000	-0.8610	1.0000	-0.0139	0.5247	0.1498	0.0262	0.2940
C1H12orf66	0.0000	-0.7295	0.0000	-0.8077	0.0000	-0.8192	0.0000	-0.8222	1.0000	0.1218	1.0000	0.0559	0.7148	0.1235
C1H12orf73	0.0000	-0.9395	0.0000	-0.9624	0.0199	-0.3786	0.0000	-0.3334	1.0000	0.1007	0.9808	0.0904	0.3213	0.1515
C1H12orf75	0.2963	0.4455	0.7391	-0.1686	0.0049	0.8746	0.3165	0.2855	1.0000	0.5669	1.0000	-0.0339	1.0000	-0.0174
C1H21orf140	1.0000	0.0000	1.0000	0.0000	1.0000	0.0000	1.0000	0.0000	1.0000	0.0000	1.0000	0.0000	1.0000	0.0000
C1H21orf33	1.0000	-0.0107	0.8717	0.0482	0.0235	-0.5748	0.0000	-0.5218	1.0000	-0.2694	0.3105	-0.1978	0.2184	-0.2102
C1H21orf59	1.0000	-0.0148	0.8109	-0.0626	0.3815	-0.2082	0.0225	-0.2627	1.0000	-0.0828	0.8269	-0.1185	0.6831	-0.1324
C1H21orf62	1.0000	-0.2920	0.2526	2.1629	1.0000	-2.4056	1.0000	-2.3176	1.0000	-0.1078	0.6450	2.3766	1.0000	0.0000
C1H21orf91	0.4753	-0.2232	0.2620	-0.1977	1.0000	-0.0268	0.0208	0.3215	1.0000	-0.1285	1.0000	-0.0889	0.3380	0.2275
C1H22orf23	0.0022	0.6916	0.0001	0.5157	0.0018	-0.8003	0.0017	-0.5422	1.0000	-0.0961	0.2413	-0.2584	0.8908	0.1676
C1H2orf40	0.9338	-0.2762	0.5841	-0.4195	0.0927	0.7954	0.3234	0.5139	1.0000	-0.1271	1.0000	-0.2593	0.6843	-0.4063
C1H2orf49	0.0001	-0.6494	0.0000	-0.5114	0.2083	-0.2484	0.0309	-0.2117	1.0000	-0.0099	0.7228	0.1407	1.0000	0.0322
C1H3orf17	0.0000	-1.0176	0.0000	-0.8226	0.0002	-0.6804	0.0000	-0.5423	1.0000	0.0396	0.4102	0.2471	0.4551	0.1819
C1H3orf30	1.0000	-2.4788	0.9062	1.2235	1.0000	0.6780	1.0000	-2.3176	1.0000	-0.1089	0.8607	3.7297	1.0000	-3.1344
C1H3orf38	0.2043	0.3377	0.0020	0.4117	0.0416	-0.5197	0.0009	-0.4808	1.0000	0.0058	1.0000	0.0903	1.0000	0.0476
C1H3orf52	1.0000	0.0000	0.4637	-3.7802	0.5076	3.6021	1.0000	-0.5176	1.0000	3.6298	1.0000	0.0000	1.0000	-0.4518
C1H7orf49	0.8161	0.1670	1.0000	0.0057	0.0309	-0.6584	0.0039	-0.7115	1.0000	0.1363	1.0000	-0.0127	1.0000	0.0885
C1H7orf55	1.0000	0.0652	0.0153	0.3095	0.0000	-1.2310	0.0000	-1.4758	1.0000	-0.1980	1.0000	0.0589	0.1909	-0.4379
C1H7orf60	0.4333	0.2934	0.0047	0.5600	0.0000	1.0941	0.0000	1.2330	1.0000	-0.1694	1.0000	0.1110	1.0000	-0.0238
C1HXorf22	1.0000	0.5453	1.0000	-2.3959	1.0000	0.6780	1.0000	-2.3176	1.0000	-0.1089	1.0000	-3.1469	1.0000	-3.1344
C1HXorf23	0.0728	0.3377	0.1351	0.1727	0.8011	-0.1071	0.0145	-0.2652	1.0000	0.0339	0.7510	-0.1195	0.7320	-0.1204
C1HXorf30	1.0000	2.1848	0.4431	3.6192	0.8011	3.0840	1.0000	0.0000	1.0000	0.0000	1.0000	1.4317	1.0000	-3.1344
C1HXorf36	1.0000	0.6151	0.2891	1.3372	0.3228	-4.1836	0.4370	-3.6968	1.0000	-0.5105	1.0000	0.2240	1.0000	0.0000
C1QA	1.0000	0.0000	1.0000	0.0000	1.0000	0.0000	1.0000	0.0000	1.0000	0.0000	1.0000	0.0000	1.0000	0.0000
C1QB	1.0000	0.0000	1.0000	0.0000	1.0000	0.0000	1.0000	0.0000	1.0000	0.0000	1.0000	0.0000	1.0000	0.0000
C1QBP	0.0053	-0.4201	0.0000	-0.4072	0.0000	-0.8972	0.0000	-0.7692	1.0000	-0.0899	0.9169	-0.0646	1.0000	0.0437
C1QC	1.0000	0.0000	1.0000	0.0000	1.0000	0.0000	1.0000	0.0000	1.0000	0.0000	1.0000	0.0000	1.0000	0.0000
C1QL1	0.0000	-2.1332	0.0000	-2.4648	0.0000	-2.9418	0.0000	-2.1619	1.0000	-0.1627	0.0976	-0.4819	0.0036	0.6228
C1QL4	1.0000	2.1848	1.0000	0.0000	1.0000	2.2472	1.0000	0.0000	1.0000	0.0000	1.0000	-2.2957	1.0000	-2.2909
C1QTNF1	0.6921	0.4306	0.3902	0.5949	0.9520	-0.3166	1.0000	-0.2523	1.0000	-0.4766	1.0000	-0.3038	0.9852	-0.4049
C1QTNF2	1.0000	0.0000	0.7674	3.0957	1.0000	0.0000	0.7710	3.1753	1.0000	0.0000	1.0000	3.2003	1.0000	3.2081
C1QTNF3	0.0000	-0.9263	0.0014	-0.3384	0.0000	2.1293	0.0000	1.9425	1.0000	0.0715	0.0000	0.6726	0.5423	-0.1096
C1QTNF4	0.0000	1.1065	0.0000	1.1675	0.0000	-1.1073	0.0000	-1.0700	1.0000	0.0871	0.3134	0.1605	0.6233	0.1303
C1QTNF5	1.0000	-0.2931	1.0000	0.0000	1.0000	-2.4061	1.0000	0.0000	1.0000	-2.3771	1.0000	-2.2957	1.0000	0.0000
C1QTNF6	0.8249	3.0141	1.0000	0.6988	0.4809	3.6101	1.0000	0.0062	1.0000	2.2674	1.0000	0.0598	1.0000	-1.3084
C1QTNF7	1.0000	0.0000	1.0000	2.2534	1.0000	0.0000	1.0000	0.0000	1.0000	0.0000	1.0000	2.3480	1.0000	0.0000
C1QTNF8	1.0000	0.0000	1.0000	0.0000	1.0000	0.0000	0.7701	3.1732	1.0000	0.0000	1.0000	0.0000	1.0000	3.2066
C1R	0.1169	-0.9803	0.2065	-0.6151	0.0015	-1.9393	0.0001	-1.9021	1.0000	0.1733	0.8607	0.5514	1.0000	0.2141
C1S	1.0000	-0.0847	1.0000	0.0025	0.0017	-1.8431	0.0394	-1.0581	1.0000	-0.1369	1.0000	-0.0354	0.8937	0.6555
C20H17orf58	0.0000	-2.1099	0.0000	-1.7429	0.3428	-0.2519	0.0000	0.4643	1.0000	0.2491	0.0061	0.6287	0.0000	0.9707
C20H17orf62	1.0000	-0.0249	1.0000	-0.0082	0.0000	-0.8390	0.0000	-1.0630	1.0000	0.2204	0.2107	0.2504	1.0000	0.0018
C20H17orf67	0.6664	0.8698	1.0000	0.2256	0.4564	-2.2491	0.0709	-4.6403	1.0000	0.1241	1.0000	-0.5099	1.0000	-2.2888
C20H17orf70	0.1470	-0.2764	0.0000	-0.5063	0.6901	0.1218	0.0018	0.2867	1.0000	-0.1793	0.0008	-0.3969	1.0000	-0.0086
C20H17orf75	0.0013	0.5614	0.2938	0.1276	0.7668	0.1155	1.0000	-0.0124	1.0000	-0.0334	0.0000	-0.4540	0.3721	-0.1554
C20H17orf80	0.2958	0.2699	0.8033	0.0744	0.0544	-0.4681	0.3218	-0.1720	1.0000	-0.0342	0.4709	-0.2168	0.3574	0.2689
C20H17orf89	0.0000	-0.8041	0.1274	-0.2247	0.0000	-1.7013	0.0000	-1.0567	1.0000	-0.3452	0.3335	0.2479	0.3411	0.3064
C21H17orf64	1.0000	0.0000	1.0000	0.0000	1.0000	0.0000	1.0000	0.0000	1.0000	0.0000	1.0000	0.0000	1.0000	0.0000
C21H17orf85	0.3293	-0.2026	0.0005	-0.3099	0.0252	-0.3797	0.0000	-0.5273	1.0000	0.0300	1.0000	-0.0643	0.6636	-0.1123
C21H17orf97	1.0000	0.1168	0.8681	0.4802	0.7390	0.7713	1.0000	0.4748	1.0000	-0.1298	1.0000	0.2467	1.0000	-0.4203
C22H20orf195	1.0000	0.0000	1.0000	2.2506	1.0000	0.0000	1.0000	0.0000	1.0000	0.0000	1.0000	2.3455	1.0000	0.0000
C22H20orf24	0.1292	-0.2813	0.0253	-0.2051	0.0215	-0.3856	0.0000	-0.4406	1.0000	-0.0080	0.9094	0.0804	1.0000	-0.0580
C23H1orf158	1.0000	0.1642	0.2112	0.7308	0.9468	-0.3632	0.2663	-0.9020	1.0000	-0.0541	0.8062	0.5249	0.8534	-0.5864
C23H1orf159	0.3727	0.7826	1.0000	0.1256	0.6209	0.5480	0.1637	-1.0435	1.0000	0.5468	1.0000	-0.0957	0.3788	-1.0380
C23H1orf167	0.6692	0.8758	0.7307	-0.9818	1.0000	0.0724	0.0817	-2.8681	1.0000	0.6786	0.7069	-1.1645	0.5920	-2.2634
C23H1orf174	0.0067	0.9655	0.0027	0.7522	0.2579	0.5010	1.0000	-0.0616	1.0000	0.4107	0.9146	0.2118	1.0000	-0.1465
C23H1orf50	0.2763	-0.3280	0.0407	-0.3186	0.0023	-0.7003	0.0000	-0.8212	1.0000	-0.1859	0.9091	-0.1633	0.3446	-0.3003
C23H1orf86	0.1062	0.5909	0.0275	0.6681	0.2114	-0.5319	0.3101	-0.4220	1.0000	-0.5506	0.2530	-0.4621	0.5572	-0.4375
C24H2orf42	0.1070	-0.4164	0.0145	-0.4309	0.0031	-0.6673	0.0000	-0.5865	1.0000	0.0644	1.0000	0.0625	0.8550	0.1498
C24H8orf4	0.1562	-2.6578	1.0000	-2.3958	1.0000	-0.2002	0.9096	1.3854	1.0000	-3.2976	1.0000	-3.1469	0.3257	-1.7139
C25H1orf216	1.0000	0.8825	0.9062	-1.5349	0.5567	1.2856	0.5798	1.1649	1.0000	0.4112	0.8354	-2.0132	1.0000	0.2935
C26H11orf1	1.0000	-2.4788	1.0000	0.0000	0.6966	1.5793	0.1321	4.3960	1.0000	-2.3771	1.0000	0.0000	1.0000	0.3841
C26H11orf52	0.0000	-1.2808	0.0000	-1.6287	0.0088	-0.4057	0.0000	-0.4563	1.0000	0.0464	0.0373	-0.2892	1.0000	0.0010
C26H11orf57	0.1033	-0.3334	0.0004	-0.4069	0.0040	-0.5412	0.0539	-0.2288	1.0000	-0.0726	0.7956	-0.1337	0.1809	0.2441
C26H11orf63	1.0000	0.5420	0.9787	0.7551	0.1945	2.3625	1.0000	0.5427	1.0000	0.7318	1.0000	0.9748	0.8736	-1.0814
C26H11orf88	1.0000	0.0000	1.0000	0.0000	1.0000	0.0000	1.0000	0.0000	1.0000	0.0000	1.0000	0.0000	1.0000	0.0000
C28H1orf116	0.0100	2.8975	0.6359	0.7838	0.7628	1.0365	0.6142	-1.7590	1.0000	0.7963	0.2911	-1.3043	0.6865	-1.9990
C28H1orf186	1.0000	0.0000	1.0000	0.0000	1.0000	0.0000	1.0000	0.0000	1.0000	0.0000	1.0000	0.0000	1.0000	0.0000
C28H1orf74	0.5416	-0.2366	1.0000	-0.0042	0.0001	-1.0066	0.0000	-0.6617	1.0000	-0.0906	0.9220	0.1535	0.6226	0.2600
C28H6orf106	1.0000	-0.0279	0.9074	-0.0454	0.8468	-0.0950	0.5800	0.0845	1.0000	-0.0952	0.8719	-0.1007	0.8495	0.0900
C28H6orf132	0.0014	-1.1391	0.0154	-0.7116	0.0005	-1.3830	0.0001	-1.1612	1.0000	-0.2193	1.0000	0.2212	1.0000	0.0111
C28H6orf89	1.0000	0.0596	0.1627	0.1960	0.0218	0.4533	0.0000	0.5028	1.0000	-0.0307	0.8785	0.1182	1.0000	0.0242
C29H17orf104	1.0000	0.0000	1.0000	0.0000	1.0000	0.0000	0.4370	3.7037	1.0000	0.0000	1.0000	0.0000	0.7287	3.7384
C2CD2	0.0000	1.1087	0.0000	1.0607	0.0000	1.2306	0.0000	0.8598	1.0000	0.1722	0.7973	0.1389	0.6999	-0.1927
C2CD2L	0.8219	0.1254	0.9450	-0.0574	1.0000	0.0019	0.9880	0.0513	1.0000	0.0634	1.0000	-0.1081	0.8184	0.1159
C2CD3	0.0025	1.9919	0.0202	1.1838	0.0000	2.6203	0.0091	1.3027	1.0000	0.7569	1.0000	-0.0317	0.3791	-0.5460
C2CD4C	1.0000	0.0000	1.0000	0.0000	1.0000	0.0000	1.0000	2.3257	1.0000	0.0000	1.0000	0.0000	1.0000	2.3554
C2CD5	0.3325	0.2181	1.0000	-0.0290	0.5711	0.1579	0.5856	-0.0796	1.0000	0.1834	1.0000	-0.0513	1.0000	-0.0493
C2H1orf101	0.1876	1.0417	0.2517	0.9165	1.0000	0.0235	0.9538	-0.3983	1.0000	-0.1262	1.0000	-0.2396	0.9352	-0.5444
C2H1orf115	0.1749	1.2605	0.3392	0.7591	0.4153	-1.3099	0.0981	-1.7311	1.0000	0.4861	1.0000	0.0060	1.0000	0.0744

C2H1orf131	1.0000	0.0723	0.6352	-0.1637	0.4809	-0.2628	0.3376	-0.2451	1.0000	0.1736	1.0000	-0.0502	0.7944	0.1958
C2H1orf198	0.0527	0.3388	0.0175	0.1877	0.1013	0.2923	0.0339	0.1721	1.0000	-0.0584	0.1130	-0.1971	0.1509	-0.1727
C2H2orf43	0.6711	0.1774	0.0025	0.4849	0.0977	0.3997	0.0006	0.4689	1.0000	-0.1329	0.7563	0.1869	1.0000	-0.0588
C2H2orf50	0.8249	3.0255	1.0000	2.2507	1.0000	0.0000	1.0000	0.0000	1.0000	0.0000	1.0000	-0.8002	1.0000	0.0000
C2H2orf70	1.0000	0.0000	1.0000	0.0000	1.0000	0.0000	1.0000	0.0000	1.0000	0.0000	1.0000	0.0000	1.0000	0.0000
C2H2orf71	1.0000	0.5464	1.0000	-0.1708	1.0000	-2.4056	0.4370	-3.6973	1.0000	1.2661	1.0000	0.5792	1.0000	0.0000
C2H6orf120	0.9522	0.1129	0.9435	-0.0598	0.1790	0.3886	0.0257	0.3265	1.0000	0.2298	1.0000	0.0698	0.6285	0.1716
C2H6orf163	0.0146	-3.6168	0.0024	-2.7385	1.0000	-0.0832	0.1217	-1.2141	1.0000	0.5192	1.0000	1.4317	0.9094	-0.6042
C2H6orf165	0.0293	-1.4804	0.0000	-1.9458	0.0716	-1.1858	1.0000	0.0705	1.0000	0.5139	1.0000	0.0605	0.0002	1.7782
C2H6orf203	0.5086	-0.1785	0.5172	-0.0959	0.0000	-0.8780	0.0000	-0.6474	1.0000	-0.1263	1.0000	-0.0321	0.8523	0.1081
C2H6orf211	1.0000	-0.0310	0.0723	0.2343	0.0585	0.4602	0.4609	0.1220	1.0000	0.1157	0.0100	0.3937	0.5492	-0.2178
C2H6orf57	0.0000	-1.2175	0.0000	-1.3675	0.0000	-0.7445	0.0000	-0.5124	1.0000	0.1762	1.0000	0.0387	0.0008	0.4136
C2HXorf21	1.0000	0.0000	1.0000	0.0000	1.0000	0.0000	1.0000	0.0000	1.0000	0.0000	1.0000	0.0000	1.0000	0.0000
C3	0.6877	-2.0809	0.9762	-1.0826	0.2785	-4.1857	0.2426	-4.0840	1.0000	-0.1247	1.0000	0.9006	1.0000	0.0000
C30H19orf10	0.0009	-0.5032	0.0000	-0.4681	0.0079	-0.4197	0.0364	-0.1696	1.0000	0.0090	1.0000	0.0566	0.0112	0.2648
C30H19orf35	1.0000	0.0000	1.0000	0.0000	1.0000	2.2428	1.0000	2.3257	1.0000	0.0000	1.0000	0.0000	1.0000	0.0660
C30H19orf44	0.0062	-0.5669	0.0000	-0.5149	0.0032	-0.6148	0.0001	-0.4620	1.0000	0.1566	0.3725	0.2209	0.0549	0.3147
C30H19orf45	0.7466	0.5312	0.1099	0.9466	0.8908	-0.4167	0.2604	-1.0654	1.0000	-0.0332	1.0000	0.3989	0.8844	-0.6773
C30H19orf60	0.0000	-0.8375	0.0000	-0.6831	0.0432	-0.4322	0.3069	-0.1368	1.0000	-0.1752	1.0000	-0.0086	0.8431	0.1267
C30H19orf70	0.0000	-0.8001	0.0000	-0.7808	0.0000	-1.0694	0.0000	-1.0250	1.0000	-0.2029	0.5089	-0.1712	0.6602	-0.1518
C30H19orf71	0.3574	3.9146	1.0000	-2.3986	1.0000	2.2472	1.0000	-2.3200	1.0000	2.2733	0.6983	-4.0460	1.0000	-2.2909
C3AR1	1.0000	-2.4788	1.0000	0.0000	1.0000	-2.4061	1.0000	0.0000	1.0000	-2.3771	1.0000	0.0000	1.0000	0.0000
C3H18orf21	1.0000	0.0703	0.1813	0.1751	0.0000	-1.2179	0.0000	-1.0497	1.0000	-0.1435	1.0000	-0.0270	1.0000	0.0312
C3H18orf42	0.2945	-0.4121	0.0000	-0.9324	1.0000	-0.0811	0.7667	-0.1172	1.0000	0.4075	1.0000	-0.0981	0.2331	0.3793
C3H18orf63	1.0000	0.0000	1.0000	0.0000	1.0000	0.0000	1.0000	0.0000	1.0000	0.0000	1.0000	0.0000	1.0000	0.0000
C3H18orf8	1.0000	0.0012	0.9762	-0.0439	0.7377	-0.1272	0.1127	-0.2104	1.0000	0.1000	1.0000	0.0671	1.0000	0.0205
C3H5orf22	0.0566	0.4693	0.0151	0.3862	1.0000	-0.0840	0.0042	-0.4440	1.0000	0.0583	1.0000	-0.0119	0.3101	-0.2969
C3H5orf49	0.0325	-1.9776	0.0002	-3.5336	0.7518	0.4061	0.2717	0.6946	1.0000	0.1328	0.9082	-1.4164	0.8204	0.4305
C3H6orf52	0.7225	0.1435	0.0364	0.2092	0.5446	-0.1877	0.4278	-0.1079	1.0000	-0.0256	1.0000	0.0520	1.0000	0.0592
C3H6orf62	0.5957	0.1292	0.8268	0.0357	0.0095	-0.4078	0.0000	-0.5490	1.0000	0.0624	1.0000	-0.0188	0.7695	-0.0732
C3H7orf57	1.0000	2.1902	1.0000	-0.1429	1.0000	0.0000	1.0000	-2.3176	1.0000	2.2674	1.0000	0.0489	1.0000	0.0000
C3H7orf72	1.0000	0.0000	1.0000	0.0000	1.0000	0.0000	1.0000	0.0000	1.0000	0.0000	1.0000	0.0000	1.0000	0.0000
C3H8orf22	0.7163	1.1642	1.0000	0.3823	0.4809	-3.7970	1.0000	0.5626	1.0000	0.2656	1.0000	-0.5064	0.2118	4.6832
C3H8orf37	0.0301	1.3800	0.1766	0.8088	0.0033	1.6345	0.0007	1.4918	1.0000	0.2494	1.0000	-0.3092	1.0000	0.1118
C3H8orf46	1.0000	0.0000	1.0000	0.0000	1.0000	0.0000	1.0000	0.0000	1.0000	0.0000	1.0000	0.0000	1.0000	0.0000
C3H8orf76	0.0067	0.4349	0.0069	0.2517	0.0000	-0.6921	0.0000	-0.7125	1.0000	-0.0480	0.0809	-0.2191	1.0000	-0.0631
C4H20orf194	0.0769	0.2888	0.8356	0.0604	1.0000	-0.0061	0.3808	-0.1026	1.0000	-0.0491	0.1145	-0.2652	0.5275	-0.1403
C4H4orf17	0.0822	2.2109	0.5045	-1.6415	0.2673	1.6861	0.7723	-0.9362	1.0000	1.3496	0.1732	-2.4960	0.7040	-1.2668
C4H4orf19	1.0000	0.0000	1.0000	0.0000	1.0000	0.0000	1.0000	0.0000	1.0000	0.0000	1.0000	0.0000	1.0000	0.0000
C4H4orf27	0.0611	0.3918	0.0000	0.4627	0.0233	-0.5011	0.0000	-0.5226	1.0000	-0.2412	0.5327	-0.1583	0.2489	-0.2555
C4H4orf29	0.8355	-0.0984	0.0467	-0.2258	1.0000	-0.0544	0.7260	-0.0691	1.0000	0.0292	1.0000	-0.0855	1.0000	0.0205
C4H4orf33	0.0378	0.5599	0.0004	0.6557	0.0002	0.8584	0.0000	0.8084	1.0000	0.0765	0.8407	0.1852	1.0000	0.0319
C4H4orf46	0.5189	-0.2131	0.7465	0.1060	0.2798	-0.3321	0.7982	0.0817	1.0000	-0.1942	0.9671	0.1367	0.5940	0.2261
C4H4orf47	0.0000	1.1657	0.0000	1.0887	0.0021	-0.8365	0.0020	-0.6261	1.0000	-0.1003	0.7665	-0.1657	1.0000	0.1156
C4H4orf48	0.0666	-0.7351	0.0000	-0.8057	0.0009	-1.2581	0.0000	-1.3992	1.0000	0.5859	0.0778	0.5295	0.2532	0.4497
C4H4orf50	1.0000	0.0000	1.0000	0.0000	1.0000	0.0000	1.0000	0.0000	1.0000	0.0000	1.0000	0.0000	1.0000	0.0000
C5	0.6881	1.7520	1.0000	-1.0027	1.0000	0.6783	1.0000	-0.8443	1.0000	0.7416	0.8269	-2.0158	1.0000	-0.7795
C5H11orf16	0.0622	0.8915	0.0011	1.0688	0.3275	0.5832	0.9674	0.1814	1.0000	0.1037	0.8528	0.2960	0.9094	-0.2906
C5H11orf24	1.0000	-0.0495	0.0082	0.2586	0.0206	-0.4356	0.0746	-0.1801	1.0000	0.0051	0.0068	0.3255	0.0500	0.2659
C5H11orf31	0.0062	-0.4689	0.7545	0.0727	0.0000	-0.8843	0.0001	-0.4714	1.0000	-0.4189	0.6210	0.1342	1.0000	-0.0002
C5H11orf49	0.3191	0.7396	0.0607	0.8140	0.1819	0.8642	0.2360	0.6056	1.0000	0.2976	0.8785	0.3872	1.0000	0.0458
C5H11orf58	0.5387	-0.1526	0.0105	-0.1919	0.0000	-1.0372	0.0000	-1.0005	1.0000	-0.1129	0.3561	-0.1399	0.8839	-0.0707
C5H11orf74	0.4498	-0.6657	0.4850	0.5280	0.0598	-1.2983	0.2435	-0.8537	1.0000	-0.3697	0.4316	0.8367	1.0000	0.0797
C5H11orf94	0.8873	0.2375	0.6358	-0.3276	0.0244	-1.4341	0.0002	-1.8673	1.0000	0.2711	1.0000	-0.2821	1.0000	-0.1567
C5H14orf105	1.0000	0.0000	1.0000	2.2507	1.0000	0.0000	1.0000	0.0000	1.0000	0.0000	1.0000	2.3455	1.0000	0.0000
C5H14orf132	0.4743	2.0027	0.4696	3.6125	1.0000	-2.4061	1.0000	0.0000	1.0000	-2.3771	1.0000	-0.8870	1.0000	0.0000
C5H14orf159	0.9786	-0.0879	0.1906	-0.2359	0.0000	-1.0280	0.0000	-1.8939	1.0000	-0.2213	0.0985	-0.3551	0.0000	-1.0815
C5H14orf166	0.2068	-0.2551	0.0009	-0.2855	0.0000	-0.8078	0.0000	-0.7351	1.0000	-0.0666	0.8939	-0.0848	1.0000	0.0121
C5H14orf169	0.8799	-0.1118	0.6221	-0.1114	0.0233	-0.5473	0.8332	-0.0747	1.0000	-0.1476	0.8968	-0.1351	0.1453	0.3308
C5H14orf180	1.0000	-2.4776	0.7666	-3.2534	0.1954	2.5403	1.0000	0.0066	1.0000	0.7365	1.0000	0.0000	0.4877	-1.7975
C5H14orf2	0.0000	-0.7443	0.0000	-0.6676	0.0000	-0.9500	0.0000	-0.8842	1.0000	-0.1856	0.8028	-0.0966	0.7000	-0.1142
C5H14orf37	0.2953	1.7012	0.5418	0.9240	0.0328	2.4749	0.8972	0.6680	1.0000	1.0964	1.0000	0.3412	0.8110	-0.7077
C5H14orf39	1.0000	0.0000	1.0000	2.2507	1.0000	0.0000	1.0000	2.3242	1.0000	0.0000	1.0000	2.3455	1.0000	2.3543
C5H14orf79	0.2382	-0.8598	0.8243	-0.3906	0.6647	-0.4260	1.0000	0.0066	1.0000	-0.4189	1.0000	0.0604	1.0000	0.0169
C5H15orf52	0.0003	0.9347	0.1537	0.2964	0.0009	-1.0628	0.0002	-0.8146	1.0000	0.4342	0.8025	-0.1906	0.1017	0.6885
C5H15orf57	1.0000	0.0445	1.0000	0.0302	0.3904	-0.2468	0.2027	-0.2077	1.0000	-0.0005	1.0000	-0.0033	1.0000	0.0436
C5H15orf62	0.9743	-0.5206	0.3978	-1.1409	1.0000	0.2091	0.9147	-0.4924	1.0000	0.0192	1.0000	-0.5920	0.8833	-0.6778
C6	1.0000	0.0000	1.0000	0.0000	1.0000	0.0000	1.0000	0.0000	1.0000	0.0000	1.0000	0.0000	1.0000	0.0000
C6H1orf35	1.0000	-0.0057	0.0996	0.2043	0.0000	-0.8620	0.0000	-0.6626	1.0000	-0.2940	1.0000	-0.0721	0.9835	-0.0894
C6H7orf25	0.8226	-0.1288	0.1631	-0.2279	1.0000	-0.0034	0.3533	0.1627	1.0000	-0.0531	0.8759	-0.1404	0.8620	0.1167
C6H7orf31	1.0000	0.8767	1.0000	-1.0008	0.8011	-3.2629	1.0000	-0.8419	1.0000	-0.1179	0.8723	-2.0004	1.0000	2.3543
C6H9orf152	0.5086	-3.8790	0.9779	-1.0833	0.9361	-1.5509	0.2455	-4.0833	1.0000	0.2615	1.0000	3.1945	1.0000	-2.2909
C7	1.0000	0.2175	0.9536	0.3380	1.0000	-0.3194	1.0000	-0.4564	1.0000	-0.8958	0.8059	-0.7701	0.6819	-1.0214
C7H21orf58	0.0937	-2.0025	0.0002	-6.0226	0.1721	-1.8509	0.0203	-2.2231	1.0000	0.4429	0.8607	-3.6719	1.0000	0.0768
C7H2orf47	0.3092	0.2278	0.0191	0.2270	0.0000	-1.0940	0.0000	-1.3058	1.0000	-0.1034	0.8723	-0.0917	0.0835	-0.3099
C7H2orf66	0.3663	0.4764	0.4201	0.3597	1.0000	0.0450	0.3218	-0.4481	1.0000	0.0604	1.0000	-0.0422	0.6707	-0.4265
C7H2orf69	0.3507	-0.2909	1.0000	-0.0038	0.3963	-0.2716	0.2668	0.2145	1.0000	-0.3239	1.0000	-0.0239	0.7821	0.1671
C7H2orf76	0.8478	-0.1697	0.9762	0.0639	0.0952	-0.6185	0.0000	-0.8302	1.0000	-0.0788	0.8656	0.1656	0.6200	-0.2823
C7H2orf88	0.0952	-0.6436	0.0224	-0.4428	0.0000	-2.3590	0.0000	-2.0165	1.0000	0.2400	0.1517	0.4528	0.4976	0.5848
C8A	1.0000	0.0000	1.0000	0.0000	1.0000	0.0000	1.0000	0.0000	1.0000	0.0000	1.0000	0.0000	1.0000	0.0000
C8B	1.0000	0.0000	1.0000	0.0000	1.0000	0.0000	1.0000	0.0000	1.0000	0.0000	1.0000	0.0000	1.0000	0.0000
C8G	0.2813	0.3372	0.9952	0.0544	0.0000	-1.2764	0.0000	-1.0458	1.0000	-0.0099	0.4097	-0.2809	0.7495	0.2273
C8H10orf11	0.0119	0.6319	0.0000	0.6683	0.1801	-0.4054	0.0001	-0.7305	1.0000	-0.0596	1.0000	-0.0102	0.2779	-0.3794
C8H10orf12	0.0172	-0.4116	0.0000	-0.6372	0.0040	-0.5197	0.0000	0.5061	1.0000	-0.0709	0.0136	-0.2840	0.0000	0.9606
C8H10orf128	1.0000	0.0000	0.7666	-3.2534	1.0000	0.0000	1.0000	-0.8416	1.0000	3.1126	1.0000	0.0000	1.0000	2.3554
C8H10orf2	0.0002	-0.6317	0.0020	-0.2910	0.0000	-0.7634	0.0000	-0.5012	1.0000	-0.2005	0.4973	0.1526	1.0000	0.0680
C8H10orf32	1.0000	0.0501	0.0179	-0.3746	0.0182	0.5127	0.5256	-0.1494	1.0000	0.4161	1.0000	0.0055	0.3476	-0.2397
C8H10orf54	0.7860	0.1462	0.1193	0.2767	0.0001	0.8172	0.0000	0.7739	1.0000	-0.1038	1.0000	0.0387	0.7644	-0.1420
C8H10orf71	0.0000	-2.2566	0.0000	-2.5306	0.0000	1.7856	0.0000	1.4303	1.0000	0.1550	1.0000	-0.1062	0.2572	-0.1946
C8H10orf76	0.0040	-0.4796	0.0161	-0.2454	0.2450	-0.2349	0.9691	0.0358	1.0000	-0.1545	0.8852	0.0925	0.6246	0.1228
C8H10orf88	0.0015	-0.5698	0.0000	-0.5276	0.0607	-0.3623	0.0001	-0.4182	1.0000	-0.1064	1.0000	-0.0518	0.4983	-0.1567
C8H10orf90	0.5086	-3.8796	0.4636	-3.7802	1.0000	0.1975	1.0000	0.7034	1.0000	-0.1283	1.0000	0.0000	1.0000	0.3834
C9H8orf48	0.4318	0.3368	1.0000	0.0025	0.7769	0.1873	1.0000	-0.0568	1.0000	0.2406	1.0000	-0.0826	1.0000	0.0004

C9HXorf56	0.0010	-0.5438	0.0000	-0.4871	0.8991	-0.0777	0.3102	0.1171	1.0000	-0.0521	1.0000	0.0165	0.4176	0.1477
C9HXorf57	0.0003	0.7959	0.0000	0.7607	0.0000	1.5141	0.0000	1.0769	1.0000	0.0627	1.0000	0.0407	0.0038	-0.3679
C9HXorf65	1.0000	0.0000	1.0000	0.0000	1.0000	0.0000	1.0000	2.3257	1.0000	0.0000	1.0000	0.0000	1.0000	2.3554
CA10	1.0000	2.1902	1.0000	0.0000	0.8033	3.0892	1.0000	2.3242	1.0000	0.0000	1.0000	-2.2992	1.0000	-0.7815
CA12	1.0000	-0.0788	1.0000	-0.0851	0.0000	2.5791	0.0075	1.3344	1.0000	0.5903	1.0000	0.5972	0.2481	-0.6457
CA14	1.0000	-0.2828	0.4238	-0.7198	0.0068	-2.2978	0.0034	-2.5605	1.0000	-0.0507	1.0000	-0.4783	1.0000	-0.3106
CA4	1.0000	0.0000	1.0000	0.0000	1.0000	0.0000	1.0000	0.0000	1.0000	0.0000	1.0000	0.0000	1.0000	0.0000
CA5A	0.0002	1.5171	0.0000	1.3303	0.0000	2.4265	0.0000	2.1133	1.0000	0.2678	1.0000	0.0965	1.0000	-0.0376
CA6	1.0000	-0.2817	0.6648	-0.5475	0.0000	2.3892	0.0059	1.4590	1.0000	-0.5123	0.8870	-0.7691	0.0002	-1.4354
CA7	1.0000	0.2200	0.9762	0.7526	1.0000	-0.7510	1.0000	-0.8416	1.0000	-1.5917	0.8841	-1.0630	0.8939	-1.6940
CA8	0.0000	-1.1124	0.0000	-1.1489	0.0621	-0.3314	0.0056	-0.2322	1.0000	-0.0137	1.0000	-0.0376	0.8047	0.0906
CAAP1	1.0000	-0.0079	0.8463	0.0574	0.0000	-1.2147	0.0000	-1.2432	1.0000	-0.1642	0.9847	-0.0873	0.6500	-0.1864
CAB39L	0.5690	-0.9257	0.7186	-1.3890	1.0000	-0.3192	0.2772	1.2295	1.0000	-1.3516	0.6123	-1.8167	1.0000	0.2020
CABIN1	0.3115	0.4445	0.0086	0.8623	0.0000	1.9254	0.0000	1.8870	1.0000	-0.2401	0.9749	0.1944	0.3456	-0.2689
CABLES1	0.0431	-0.4120	0.0225	-0.3079	0.7006	-0.1351	0.2802	0.1579	1.0000	-0.0483	1.0000	0.0690	0.1322	0.2507
CABLES2	1.0000	0.0252	0.1074	1.1227	0.1521	1.0352	0.0325	1.3641	1.0000	-0.4979	0.8555	0.6107	1.0000	-0.1686
CABP1	1.0000	2.1847	1.0000	2.2534	1.0000	0.0000	1.0000	2.3242	1.0000	0.0000	1.0000	0.0515	1.0000	2.3543
CABP2	1.0000	0.1724	0.8079	0.3357	0.4770	0.7196	0.7146	-0.5240	1.0000	0.4036	0.9832	0.5776	0.5697	-0.8376
CABP4	1.0000	0.2029	1.0000	-0.1452	0.8011	-3.2629	1.0000	0.8541	1.0000	-0.9587	1.0000	-1.3257	1.0000	3.2066
CABP7	1.0000	0.0000	1.0000	0.0000	1.0000	0.0000	1.0000	2.3242	1.0000	0.0000	1.0000	0.0000	1.0000	2.3543
CABYR	0.4029	0.7732	0.4233	0.7370	1.0000	-0.0078	0.3004	0.8541	1.0000	-0.4649	0.8964	-0.4951	1.0000	0.3975
CACFD1	1.0000	-0.0821	1.0000	0.0440	0.0000	2.5408	0.0000	2.2090	1.0000	0.3216	0.4626	0.4589	1.0000	-0.0063
CACHD1	1.0000	-0.0132	0.9450	0.0475	0.1139	0.3253	0.0000	0.4505	1.0000	0.0925	0.5560	0.1666	0.1481	0.2237
CACNA1B	1.0000	0.0000	0.7666	3.0922	1.0000	0.0000	0.7701	3.1732	1.0000	0.0000	1.0000	3.1976	1.0000	3.2066
CACNA1D	0.0888	2.8600	0.0382	2.3885	0.4424	1.8933	0.9960	0.9228	1.0000	0.7304	1.0000	0.2895	1.0000	-0.2288
CACNA1E	0.2175	0.4839	1.0000	0.0018	0.0083	0.8209	0.4383	0.2816	1.0000	-0.1324	0.1164	-0.6029	0.0193	-0.6678
CACNA1G	1.0000	-1.1514	0.6543	-1.9314	1.0000	-0.1821	1.0000	-0.3834	1.0000	0.8117	1.0000	0.0489	1.0000	0.6050
CACNA1I	1.0000	0.0000	1.0000	0.0000	1.0000	0.0000	1.0000	0.0000	1.0000	0.0000	1.0000	0.0000	1.0000	0.0000
CACNA1S	0.0000	-3.6385	0.0000	-3.9110	0.0409	0.4159	0.0000	0.4198	1.0000	-0.0126	0.6957	-0.2735	1.0000	-0.0040
CACNA2D2	0.0000	0.9944	0.0000	1.2294	0.3113	-0.2587	0.2037	-0.1840	1.0000	-0.1997	1.0000	0.0483	0.8275	-0.1190
CACNA2D3	1.0000	0.0491	0.0568	-2.0866	1.0000	-0.7128	0.0099	-3.2859	1.0000	1.7871	1.0000	-0.3300	1.0000	-0.7792
CACNB2	1.0000	0.2134	0.6215	1.6060	1.0000	-0.1920	1.0000	0.8541	1.0000	-1.8737	1.0000	-0.4985	1.0000	-0.8428
CACNB4	0.3574	3.9146	0.7674	3.0888	0.2780	3.9963	0.4370	3.7040	1.0000	0.0000	1.0000	-0.8566	1.0000	-0.3112
CACNG1	0.0000	-4.2425	0.0000	-5.0169	0.0539	-0.3228	0.0000	-0.5305	1.0000	-0.1159	0.0101	-0.8781	0.0047	-0.3188
CACNG2	0.4300	-1.1674	0.3242	-1.1467	1.0000	0.4244	0.2765	-1.2152	1.0000	0.2758	1.0000	0.3106	0.3534	-1.3610
CACNG3	0.0001	-0.6659	0.0000	-0.6035	0.3403	-0.2250	0.0002	0.3537	1.0000	0.1197	0.2765	0.1943	0.0000	0.7037
CACNG4	0.0000	-3.9796	0.0000	-3.6145	0.0000	0.8029	0.0000	0.7485	1.0000	-0.2195	1.0000	0.1586	0.0530	-0.2670
CACNG5	0.6665	0.1941	0.6884	0.1141	0.0574	-0.5381	0.0024	-0.5463	1.0000	0.1492	1.0000	0.0808	0.8872	0.1449
CACTIN	0.5255	-0.1599	0.1546	-0.1462	0.0070	-0.4852	0.0002	-0.3294	1.0000	0.0064	1.0000	0.0322	0.3458	0.1675
CACUL1	0.6155	-0.1567	0.0484	-0.2306	0.7811	0.1154	0.1148	0.1903	1.0000	-0.0647	0.7914	-0.1259	1.0000	0.0153
CACYBP	1.0000	-0.0114	0.3110	-0.1405	0.1635	0.2956	0.9932	-0.0423	1.0000	0.0903	1.0000	-0.0266	0.1727	-0.2424
CAD	1.0000	-0.0022	0.2662	0.1541	1.0000	0.0078	0.0030	0.3516	1.0000	-0.2979	0.7607	-0.1289	1.0000	0.0527
CADM1	0.0000	-3.2509	0.0000	-4.1314	0.0032	0.6420	0.8295	0.0632	1.0000	-0.0703	0.0241	-0.9390	0.0000	-0.6439
CADPS	1.0000	-0.3433	0.4235	-1.5160	0.8736	0.5762	0.8653	-0.6450	1.0000	0.8291	1.0000	-0.3301	1.0000	-0.3874
CADPS2	1.0000	0.0568	0.2461	-0.6522	0.1792	0.8988	0.2867	0.5281	1.0000	-0.3858	0.0824	-1.0805	0.0910	-0.7488
CALB1	0.3168	2.2149	1.0000	-0.1616	1.0000	0.6740	1.0000	-0.8443	1.0000	0.7402	0.7635	-1.6300	1.0000	-0.7782
CALB2	1.0000	-1.1562	1.0000	0.0000	0.8011	-3.2629	1.0000	0.0000	1.0000	-3.2304	1.0000	-2.2957	1.0000	0.0000
CALCA	1.0000	-1.1562	1.0000	0.0000	0.5476	1.2880	0.0245	5.2243	1.0000	-3.2304	1.0000	-2.2957	1.0000	0.6484
CALCOCO2	0.0350	0.3979	0.0000	0.4268	0.9361	0.0778	1.0000	0.0225	1.0000	-0.0286	1.0000	0.0126	0.8937	-0.0788
CALCRL	0.5514	-0.4178	1.0000	-0.0105	1.0000	0.0641	0.4537	0.4516	1.0000	-0.0897	0.9569	0.3345	0.7894	0.3061
CALD1	0.7819	-0.1070	0.1175	0.2114	0.0000	1.6554	0.0000	1.6913	1.0000	0.0943	0.0000	0.4249	0.4308	0.1352
CALHM1	1.0000	0.0000	1.0000	0.0000	1.0000	0.0000	1.0000	2.3242	1.0000	0.0000	1.0000	0.0000	1.0000	2.3543
CALHM2	1.0000	-0.0469	0.8084	0.0937	0.0000	0.9088	0.0001	0.5844	1.0000	0.1689	0.3216	0.3219	0.7435	-0.1500
CALHM3	1.0000	0.5464	1.0000	-0.1616	1.0000	-0.1624	1.0000	0.0031	1.0000	0.7416	1.0000	0.0540	1.0000	0.9164
CALM1	1.0000	-0.0227	0.1716	-0.1093	0.2014	-0.2470	0.0000	-0.2864	1.0000	0.0646	1.0000	-0.0096	1.0000	0.0307
CALM2	1.0000	-0.0433	1.0000	0.0049	0.1973	-0.2381	0.0000	-0.3160	1.0000	0.0421	0.6012	0.1028	1.0000	-0.0300
CALML4	0.0000	-0.8880	0.0000	-0.8250	0.0000	-0.7508	0.0000	-0.6123	1.0000	-0.0029	1.0000	0.0725	0.5213	0.1414
CALN1	1.0000	-0.8591	0.8949	-0.7399	0.4809	-3.7967	0.0114	-5.2052	1.0000	1.3811	0.9052	1.5308	1.0000	0.0000
CALR	0.0059	-0.4759	0.0000	-0.3735	0.2453	-0.2353	0.8589	0.0423	1.0000	-0.0523	1.0000	0.0633	0.0288	0.2303
CALR3	1.0000	-0.3552	0.9131	-0.5268	1.0000	0.2523	1.0000	0.2876	1.0000	0.0357	1.0000	-0.1248	1.0000	0.0768
CAMK1	0.0001	-0.9316	0.0000	-0.9562	0.3325	-0.2685	0.2180	0.2128	1.0000	-0.3599	0.4115	-0.3716	0.8804	0.1283
CAMK1D	0.0608	-1.4734	0.1045	-0.9405	0.1519	0.9100	1.0000	0.1484	1.0000	0.2631	0.8690	0.8115	0.6944	-0.4967
CAMK1G	0.2036	0.9917	0.0006	1.8673	0.4005	-1.1620	1.0000	0.1419	1.0000	-0.3894	0.7730	0.4983	0.8602	0.9247
CAMK2A	0.0000	-2.7455	0.0000	-2.8713	1.0000	0.0598	0.5321	-0.0602	1.0000	0.1754	1.0000	0.0619	0.9029	0.0598
CAMK2B	0.0033	-1.0479	0.0000	-1.1997	0.5750	-0.2760	0.7710	0.1352	1.0000	-0.0067	1.0000	-0.1463	0.2393	0.4104
CAMK2D	0.0000	-0.8111	0.0000	-0.9022	0.0001	0.6561	0.0000	0.5556	1.0000	0.3784	0.0073	0.2997	0.0022	0.2827
CAMK2G	1.0000	-0.0733	1.0000	0.0553	0.0025	0.7781	0.0000	0.7478	1.0000	-0.0637	1.0000	0.0799	0.9760	-0.0888
CAMK4	1.0000	-0.0263	0.0949	0.3880	0.1107	-0.7490	1.0000	-0.0338	1.0000	0.2868	0.0120	0.7149	0.0004	1.0067
CAMKK1	0.8460	0.6206	0.4353	0.8860	0.4586	0.9674	0.1209	1.2291	1.0000	0.0626	1.0000	0.3368	1.0000	0.3248
CAMKK2	0.8575	0.1258	0.9002	-0.0757	0.0000	1.4541	0.0000	1.4960	1.0000	0.0384	0.9058	-0.1503	0.9162	0.0862
CAMKMT	1.0000	0.0195	0.5471	0.2427	1.0000	0.0313	0.9573	-0.1181	1.0000	-0.3762	1.0000	-0.1372	0.1999	-0.5200
CAMKV	0.0000	-3.2597	0.0000	-3.4451	0.0589	-0.8217	0.8915	-0.1169	1.0000	-0.0066	1.0000	-0.1769	0.0715	0.7076
CAMLG	0.0235	0.9065	0.0000	0.9348	0.0000	1.2840	0.0000	1.2620	1.0000	0.0442	1.0000	0.0808	1.0000	0.0265
CAMSAP1	0.1513	0.3196	0.1553	0.2474	0.0019	0.5648	0.0071	0.4010	1.0000	-0.0216	1.0000	-0.0807	0.3501	-0.1791
CAMSAP2	0.0043	0.9504	0.0000	0.8114	0.0000	2.1881	0.0000	1.5066	1.0000	0.5361	0.0745	0.4109	0.7937	-0.1391
CAMTA1	0.6881	0.1397	1.0000	-0.0042	0.7367	-0.1266	0.0216	-0.2185	1.0000	0.1696	1.0000	0.0382	0.8620	0.0829
CAND1	0.1605	0.2403	0.0000	0.3258	0.3035	0.1989	0.1958	0.1203	1.0000	-0.0271	0.9273	0.0710	0.7180	-0.1005
CANT1	1.0000	-0.0316	0.0421	-0.2555	0.3729	-0.2600	0.7367	0.0728	1.0000	-0.0300	0.2244	-0.2412	0.1521	0.3100
CANX	0.0029	0.4693	0.0000	0.5538	0.0000	1.3178	0.0000	0.8400	1.0000	0.1590	0.0272	0.2563	0.0003	-0.3137
CAP1	0.0076	0.3989	0.0000	0.2908	0.0169	-0.3737	0.0000	-0.5745	1.0000	0.0474	1.0000	-0.0484	0.2282	-0.1481
CAP2	0.0000	-1.0155	0.0000	-0.9814	0.0000	-0.6842	0.0000	-0.5248	1.0000	0.1358	0.2520	0.1825	0.0019	0.2998
CAPG	0.0000	-0.9557	0.0000	-0.8396	0.5794	-0.1952	1.0000	-0.0167	1.0000	0.0070	0.6915	0.1349	0.5475	0.1910
CAPN10	0.4488	0.2302	0.8559	-0.0689	0.5067	-0.2097	0.0002	-0.5127	1.0000	0.0640	0.5088	-0.2230	0.4180	-0.2335
CAPN11	0.3313	-0.2060	0.0253	-0.1507	0.2793	-0.2213	0.0135	-0.1568	1.0000	-0.0735	1.0000	-0.0055	1.0000	-0.0042
CAPN13	0.0095	-1.4621	0.0013	-1.4744	0.1972	0.6076	0.0776	0.6206	1.0000	0.0640	1.0000	0.0639	1.0000	0.0818
CAPN14	1.0000	0.1662	0.5585	0.5003	1.0000	0.0207	0.2461	-0.9726	1.0000	-0.1328	1.0000	0.2150	0.3458	-1.1201
CAPN15	0.0950	-0.4612	0.1120	-0.2980	0.0058	0.6715	0.0000	1.0894	1.0000	-0.3562	0.4748	-0.1800	0.9427	0.0663
CAPN2	0.5902	0.1256	0.1854	0.1203	0.5895	-0.1263	0.2004	-0.1099	1.0000	-0.0011	1.0000	0.0058	1.0000	0.0206
CAPN3	0.0000	-3.7304	0.0000	-4.2151	0.0000	-1.1344	0.0000	-1.0915	1.0000	-0.0200	0.3178	-0.4921	1.0000	0.0279
CAPN5	1.0000	-2.4776	1.0000	-0.1594	1.0000	-2.4056	0.7710	-3.1631	1.0000	0.7317	1.0000	3.1976	1.0000	0.0000
CAPN6	0.0000	-2.6970	0.0000	-1.9573	0.0000	-3.2026	0.0000	-2.6187	1.0000	0.5417	0.0886	1.2971	0.3839	1.1314
CAPN7	0.9637	0.0868	0.2424	-0.2134	0.2850	0.2752	0.5985	0.1187	1.0000	0.2158	1.0000	-0.0711	1.0000	0.0653
CAPN8	0.8399	0.6138	0.0096	1.3189	1.0000	-0.7492	0.0738	-1.7384	1.0000	1.0623	0.0051	1.7791	1.0000	0.0775

CAPN9	0.0803	1.2874	0.8552	0.4826	0.0137	1.6118	0.9213	-0.4931	1.0000	-0.2601	0.2637	-1.0561	0.0006	-2.3607
CAPNS2	0.3467	0.5116	0.2299	0.4367	0.7984	0.2847	0.4915	-0.3388	1.0000	0.0889	1.0000	0.0281	0.4497	-0.5259
CAPRIN1	0.4121	-0.1689	0.0023	-0.2084	0.3916	0.1720	0.3319	0.0805	1.0000	0.0140	1.0000	-0.0130	0.7708	-0.0720
CAPS2	1.0000	-0.0521	1.0000	0.0348	0.8011	0.2128	0.0780	-0.4648	1.0000	0.3731	0.2987	0.4729	0.6888	-0.3012
CAPSL	0.6754	0.7995	0.0049	2.7691	0.3905	-1.8727	1.0000	0.5384	1.0000	-1.8129	1.0000	0.1526	1.0000	0.6038
CAPZA1	0.1055	-0.2952	0.0001	-0.2926	0.0696	-0.3190	0.0000	-0.4687	1.0000	0.0267	1.0000	0.0417	0.4513	-0.1172
CAPZA2	0.3472	0.2190	0.0006	0.3209	0.0013	0.5509	0.0017	0.2822	1.0000	0.0590	0.3709	0.1730	0.1235	-0.2042
CAPZA3	1.0000	0.0000	1.0000	2.2534	1.0000	0.0000	1.0000	0.0000	1.0000	0.0000	1.0000	2.3480	1.0000	0.0000
CAPZB	0.0132	-0.3816	0.0000	-0.5290	0.0000	-0.6134	0.0000	-0.6663	1.0000	0.0728	0.9348	-0.0622	1.0000	0.0255
CARD10	1.0000	-0.2892	1.0000	0.0000	1.0000	0.6780	1.0000	2.3257	1.0000	-2.3771	1.0000	-2.2992	1.0000	-0.7792
CARF	0.0026	0.7334	0.0149	0.4179	1.0000	0.0919	1.0000	0.0022	1.0000	0.1677	0.9344	-0.1337	1.0000	0.0857
CARHSP1	0.8069	-0.0994	0.7542	-0.0636	0.0010	-0.5544	0.0000	-0.4376	1.0000	0.0386	0.9234	0.0862	0.4380	0.1605
CARKD	1.0000	0.0422	0.7674	0.0649	0.0046	-0.5487	0.0000	-0.5765	1.0000	-0.0774	1.0000	-0.0415	0.9749	-0.0989
CARNS1	0.0000	-2.1946	0.0000	-2.3794	0.0002	-0.8110	0.0000	-0.4962	1.0000	-0.0635	0.5828	-0.2361	0.1021	0.2563
CARS	0.0341	-0.3825	0.0001	-0.2909	0.8581	0.0860	0.8597	-0.0375	1.0000	-0.0577	1.0000	0.0463	0.1306	-0.1756
CARS2	0.3472	-0.2114	0.0000	-0.4727	0.0000	-1.0775	0.0000	-1.0195	1.0000	0.1760	1.0000	-0.0731	0.2812	0.2397
CARTPT	0.0493	-1.1799	0.0404	-1.2901	0.0003	-2.3506	0.0002	-1.8439	1.0000	0.3213	1.0000	0.2234	0.8854	0.8362
CASC1	0.0076	-0.9306	0.1601	-0.4157	0.2619	-0.4003	0.2497	-0.3607	1.0000	-0.4376	1.0000	0.0914	0.4877	-0.3922
CASC3	0.1169	-0.3083	0.7726	-0.0609	0.9519	0.0692	0.0076	0.2525	1.0000	-0.2715	1.0000	-0.0123	0.8768	-0.0830
CASC4	0.8709	0.0740	0.7017	0.0516	1.0000	-0.0234	0.4192	0.0768	1.0000	0.0625	1.0000	0.0524	0.1398	0.1680
CASC5	0.0000	1.4737	0.0000	1.8857	0.0000	1.2111	0.0027	0.4774	1.0000	-0.1121	0.0539	0.3130	0.0000	-0.8390
CASK	0.4553	0.1810	0.7384	-0.0667	0.0056	0.4810	0.0001	0.4084	1.0000	0.1777	1.0000	-0.0568	0.7549	0.1103
CASKIN2	0.0000	-1.8486	0.0000	-1.6834	0.0039	0.7240	0.0001	0.5374	1.0000	-0.0612	1.0000	0.1173	0.0756	-0.2435
CASP10	0.5034	0.2035	0.2417	0.1964	0.3893	0.2293	0.4616	-0.1405	1.0000	0.0692	1.0000	0.0753	0.1175	-0.2947
CASP2	0.0006	0.7607	0.0000	0.8514	0.5802	0.2042	1.0000	-0.0178	1.0000	-0.0588	1.0000	0.0436	0.3754	-0.2755
CASP3	0.9671	-0.0913	1.0000	-0.0489	0.0002	0.7613	0.0001	0.5934	1.0000	0.0990	0.8875	0.1551	1.0000	-0.0629
CASP6	0.1369	-0.3720	0.0171	-0.3448	1.0000	-0.0628	0.8808	-0.0672	1.0000	0.1619	0.6256	0.2000	0.6575	0.1625
CASP7	0.0482	0.6684	0.0009	0.7586	0.7340	0.2104	0.0259	0.5521	1.0000	-0.2525	1.0000	-0.1513	1.0000	0.0958
CASP8AP2	0.0403	0.4634	0.0399	0.2898	0.0025	0.6149	0.0014	0.4308	1.0000	0.0941	1.0000	-0.0656	1.0000	-0.0844
CASP9	0.0000	-0.9447	0.0000	-1.0188	0.0099	-0.5263	0.1126	-0.2203	1.0000	-0.2105	0.3768	-0.2724	0.9440	0.1011
CASQ2	0.0000	-2.1860	0.0000	-2.4907	0.5230	0.1417	0.0010	-0.2643	1.0000	0.0053	0.0280	-0.2872	0.0000	-0.3959
CASR	0.5391	-3.8767	1.0000	-0.9997	1.0000	-0.1928	1.0000	0.0074	1.0000	-0.6546	1.0000	2.3455	1.0000	-0.4550
CAST	0.3086	0.1865	0.5871	0.0579	0.1618	-0.2379	0.0000	-0.3329	1.0000	0.0697	1.0000	-0.0466	1.0000	-0.0197
CAT	0.0035	0.6166	0.7671	0.0797	0.7723	0.1423	0.1365	0.2069	1.0000	0.2385	0.1330	-0.2847	0.0850	0.3106
CATIP	1.0000	0.1624	1.0000	0.1074	0.2956	0.6553	0.4539	0.4195	1.0000	0.2791	1.0000	0.2353	1.0000	0.0500
CAV1	0.0000	0.5854	0.0000	0.6958	0.0007	0.4953	0.0007	0.2215	1.0000	-0.0097	0.4825	0.1130	0.0008	-0.2781
CAV2	0.9742	0.0612	0.7652	-0.0546	0.4076	0.1783	0.2505	0.1157	1.0000	0.1049	1.0000	0.0014	1.0000	0.0474
CAV3	0.0000	-3.0893	0.0000	-3.1080	0.0000	-1.3993	0.0000	-1.3140	1.0000	0.0537	1.0000	0.0477	0.4454	0.1438
CBFA2T2	0.4200	0.2579	0.0156	0.4358	0.8651	-0.1254	0.1615	0.2451	1.0000	-0.1598	1.0000	0.0303	0.5202	0.2154
CBFB	0.0000	-0.7501	0.0000	-0.5014	1.0000	-0.0172	0.0935	-0.1741	1.0000	-0.0801	0.4762	0.1809	0.0975	-0.2324
CBL	0.3672	-0.1899	0.0000	-0.3963	0.6642	-0.1228	0.0090	-0.2071	1.0000	0.0907	0.8328	-0.1032	1.0000	0.0113
CBLB	0.6675	-0.1846	0.9512	0.0721	0.0002	0.7742	0.0000	0.6328	1.0000	-0.0909	0.8764	0.1786	0.3660	-0.2262
CBLL1	0.4470	-0.2023	0.5151	-0.1039	0.5699	-0.1644	1.0000	0.0334	1.0000	-0.0607	1.0000	0.0511	0.6247	0.1431
CBLN1	0.0011	-1.1747	0.0000	-1.6706	1.0000	0.1109	0.0000	0.7973	1.0000	-0.1878	0.0906	-0.6731	0.0075	0.5017
CBLN2	1.0000	0.0000	0.7666	3.0922	1.0000	0.0000	1.0000	0.0000	1.0000	0.0000	1.0000	3.1976	1.0000	0.0000
CBLN4	1.0000	0.0000	1.0000	-0.1473	1.0000	0.0000	1.0000	0.0057	1.0000	2.2733	1.0000	2.3455	1.0000	2.3554
CBR1	0.0017	0.5378	0.0000	0.5903	0.0779	-0.3703	0.0001	-0.4381	1.0000	0.2419	0.0075	0.3062	0.5760	0.1781
CBR4	0.4265	0.2687	0.3717	0.1920	0.0000	-1.1303	0.0000	-0.9970	1.0000	-0.0759	0.9289	-0.1410	1.0000	0.0642
CBX1	0.4367	-0.2085	0.8177	-0.0640	0.0000	0.9062	0.0000	0.8882	1.0000	0.0250	0.5963	0.1824	1.0000	0.0119
CBX2	0.6923	-2.0798	0.7726	-1.1137	0.0012	2.6385	0.0047	1.9635	1.0000	0.4290	1.0000	1.4306	1.0000	-0.2387
CBX3	0.9712	-0.0613	0.0256	-0.1681	1.0000	-0.0533	0.1801	-0.1068	1.0000	0.0393	1.0000	-0.0551	1.0000	-0.0089
CBX4	1.0000	-0.0084	0.3185	-0.2560	0.3718	-0.3229	0.9520	-0.0775	1.0000	0.0508	0.9263	-0.1839	0.4513	0.3035
CBX7	0.3383	0.8757	0.0263	-1.5416	0.7433	0.4796	0.3187	-0.7866	1.0000	0.5500	0.0298	-1.8576	0.7135	-0.7121
CBY1	0.3913	0.2164	0.0000	0.5150	0.4809	-0.2195	0.0000	-0.4988	1.0000	-0.2074	0.8171	0.1030	0.0081	-0.4809
CC2D1B	0.3200	0.2075	0.1877	0.1486	0.0997	-0.3039	0.1557	-0.1594	1.0000	0.0596	1.0000	0.0134	0.1438	0.2106
CC2D2A	0.1265	-0.3910	0.0902	-0.2876	0.7319	-0.1463	0.8055	-0.0812	1.0000	-0.2176	1.0000	-0.1029	0.7580	-0.1492
CCAR1	1.0000	0.0279	0.3282	-0.1005	0.1468	-0.2696	0.0000	-0.3599	1.0000	0.0414	0.9844	-0.0748	1.0000	-0.0440
CCBL1	0.1153	0.3670	0.0254	0.3306	0.1031	-0.3852	0.0013	-0.4874	1.0000	-0.0581	1.0000	-0.0827	0.7901	-0.1564
CCBL2	0.0000	0.9498	0.0000	0.8450	0.5806	-0.2085	0.0053	-0.5271	1.0000	-0.1347	0.3659	-0.2252	0.0455	-0.4455
CCDC101	0.9420	-0.0736	0.1196	-0.1568	0.0000	-1.1668	0.0000	-0.8041	1.0000	-0.0631	0.5611	-0.1342	0.1216	0.3058
CCDC102A	0.1623	-0.3191	0.2531	-0.1862	0.0000	-0.8244	0.0541	-0.2626	1.0000	-0.1517	1.0000	-0.0071	0.0119	0.4151
CCDC102B	1.0000	0.0000	1.0000	0.0000	1.0000	0.0000	1.0000	0.0000	1.0000	0.0000	1.0000	0.0000	1.0000	0.0000
CCDC103	0.0307	-1.0950	1.0000	-0.1011	0.0262	-1.0837	0.0942	-0.8414	1.0000	-0.5229	0.9297	0.4859	1.0000	-0.2780
CCDC107	0.4779	0.1853	0.1033	0.1876	0.9361	0.0793	1.0000	0.0075	1.0000	0.1244	0.6630	0.1393	1.0000	0.0572
CCDC108	1.0000	0.0000	1.0000	0.0000	1.0000	0.0000	1.0000	0.0000	1.0000	0.0000	1.0000	0.0000	1.0000	0.0000
CCDC109B	0.0611	-0.4382	0.0000	-0.6409	1.0000	-0.0589	0.0125	-0.3242	1.0000	0.2249	1.0000	0.0343	1.0000	-0.0355
CCDC110	0.1980	0.7832	0.0386	1.0028	0.0432	1.0207	0.1438	0.7784	1.0000	-0.2618	1.0000	-0.0281	0.5774	-0.4997
CCDC112	0.4950	0.6319	0.1446	0.9635	0.0735	-1.8225	0.3152	-1.0768	1.0000	-0.4249	1.0000	-0.0808	1.0000	0.3284
CCDC113	0.0264	0.8439	0.0724	0.4566	0.2150	-0.5781	0.2193	-0.3814	1.0000	0.1104	0.7851	-0.2657	0.7466	0.3127
CCDC117	0.9980	-0.0660	0.4006	-0.1124	1.0000	0.0452	0.4697	0.0990	1.0000	0.1033	1.0000	0.0690	0.4484	0.1613
CCDC12	0.0113	-0.4541	0.0006	-0.4644	0.0083	-0.5379	0.0000	-0.5911	1.0000	0.0066	1.0000	0.0085	1.0000	-0.0407
CCDC122	1.0000	0.0000	1.0000	0.0000	1.0000	0.0000	1.0000	0.0000	1.0000	0.0000	1.0000	0.0000	1.0000	0.0000
CCDC124	0.0000	-0.7262	0.0000	-0.8020	0.1071	-0.2969	0.1209	-0.1359	1.0000	-0.0051	1.0000	-0.0685	0.2693	0.1611
CCDC125	0.0004	0.6837	0.0000	0.6626	0.0143	-0.5734	0.0000	-0.8579	1.0000	-0.0911	0.9808	-0.0991	0.1811	-0.3711
CCDC126	0.4123	0.2632	0.7035	0.0895	1.0000	0.0654	0.0488	-0.2654	1.0000	0.0899	1.0000	-0.0710	0.3394	-0.2365
CCDC127	0.0000	0.9328	0.0000	0.7137	0.1541	0.3541	1.0000	0.0395	1.0000	0.2327	1.0000	0.0276	1.0000	-0.0757
CCDC129	1.0000	0.0000	1.0000	0.0000	1.0000	0.0000	1.0000	0.0000	1.0000	0.0000	1.0000	0.0000	1.0000	0.0000
CCDC13	0.0000	2.3497	0.0029	1.3034	0.7562	0.4701	0.0522	-1.4239	1.0000	0.4112	0.3456	-0.6207	0.1169	-1.4766
CCDC134	0.3926	0.1996	0.0099	0.3064	0.0000	-0.9840	0.0000	-0.5730	1.0000	-0.3225	0.3504	-0.2040	0.9522	0.0935
CCDC137	0.8455	-0.1174	0.4841	-0.1425	0.0000	-1.1745	0.0000	-0.8125	1.0000	-0.0582	1.0000	-0.0716	0.3434	0.3090
CCDC138	1.0000	0.0476	1.0000	0.0000	0.6975	1.1391	1.0000	0.0000	1.0000	-3.7658	0.6427	-4.0589	0.1033	-4.9980
CCDC14	0.0148	0.6880	0.0003	0.6909	1.0000	0.1023	0.5256	-0.2115	1.0000	0.0623	1.0000	0.0787	0.7138	-0.2460
CCDC141	0.0000	-2.1481	0.0000	-2.3352	0.0000	0.7135	0.0408	0.2426	1.0000	0.1576	1.0000	-0.0173	0.0088	-0.3080
CCDC142	0.1835	-0.5376	0.3151	-0.3249	0.1418	-0.5426	0.0116	-0.6468	1.0000	0.0138	0.9689	0.2389	1.0000	-0.0861
CCDC148	0.5016	-0.6004	0.0486	-1.1190	1.0000	-0.0353	0.3579	0.5033	1.0000	-0.2712	0.5810	-0.7776	1.0000	0.2685
CCDC149	0.0000	1.1136	0.0000	0.9675	0.0001	0.9735	0.0000	0.8208	1.0000	0.2945	0.9085	0.1614	0.8586	0.1479
CCDC15	0.0125	0.4712	0.0000	0.5620	0.0066	-0.5438	0.0000	-0.6751	1.0000	-0.2145	0.8785	-0.1121	0.0847	-0.3399
CCDC153	0.0000	-3.1393	0.0000	-3.0843	0.0882	-0.5062	0.4145	-0.1748	1.0000	0.0234	1.0000	0.0901	0.1671	0.3623
CCDC157	1.0000	-0.2370	0.0008	-1.3419	1.0000	0.1373	0.7701	-0.2230	1.0000	0.5312	0.7598	-0.5604	1.0000	0.1770
CCDC166	1.0000	0.0000	1.0000	2.2507	1.0000	0.0000	1.0000	0.0000	1.0000	0.0000	1.0000	2.3455	1.0000	0.0000
CCDC167	1.0000	0.0506	1.0000	0.0020	0.0335	-0.4080	0.0000	-0.6585	1.0000	-0.0384	1.0000	-0.0753	0.0491	-0.2837
CCDC169	0.0000	-3.3514	0.0000	-3.5497	0.0000	-0.8604	0.0000	-1.1624	1.0000	0.1630	1.0000	-0.0226	0.5661	-0.1341
CCDC17	1.0000	0.0000	1.0000	2.2535	1.0000	2.2472	1.0000	0.0000	1.0000	0.0000	1.0000	2.3480	1.0000	-2.2909

CCDC170	0.0743	0.5175	0.6929	-0.1348	0.0629	0.4848	0.2587	0.2324	1.0000	0.1191	0.0338	-0.5185	0.8622	-0.1250
CCDC171	0.7406	0.2319	0.5948	0.2120	0.0000	1.0843	0.0072	0.6482	1.0000	0.1507	1.0000	0.1441	0.4180	-0.2761
CCDC172	1.0000	0.0000	1.0000	0.0000	1.0000	0.0000	1.0000	0.0000	1.0000	0.0000	1.0000	0.0000	1.0000	0.0000
CCDC173	0.0538	-0.6094	0.0012	-0.5875	0.0664	-0.5355	0.0015	-0.5673	1.0000	0.1532	1.0000	0.1861	1.0000	0.1280
CCDC174	0.9930	0.0723	0.0393	0.2711	0.9533	0.0794	0.6142	0.1000	1.0000	-0.0608	0.7228	0.1512	1.0000	-0.0343
CCDC176	0.0671	1.1150	0.0464	0.9549	0.1216	-1.4000	0.0718	-1.4010	1.0000	-0.2808	0.7744	-0.4263	1.0000	-0.2726
CCDC177	0.1343	0.8523	0.0685	1.1991	0.0798	0.9055	0.0006	1.8108	1.0000	-1.2378	0.1905	-0.8822	0.8939	-0.3301
CCDC18	0.0925	0.5174	0.6204	0.1343	0.5719	-0.2571	1.0000	0.0012	1.0000	-0.1347	0.0066	-0.5058	0.9633	0.1310
CCDC181	0.0295	1.1881	0.0766	0.7877	0.7107	0.4967	1.0000	-0.1025	1.0000	0.1599	1.0000	-0.2287	0.8776	-0.4378
CCDC183	1.0000	-0.3436	1.0000	-0.1557	0.2028	1.5618	0.0874	2.4438	1.0000	-1.0431	1.0000	-0.8584	1.0000	-0.1577
CCDC186	0.1149	0.3859	0.0016	0.4358	0.1085	0.3845	0.0001	0.5147	1.0000	0.0399	0.9945	0.1032	0.5149	0.1762
CCDC25	0.0967	-0.3510	0.0000	-0.3623	0.0000	-0.7910	0.0000	-0.7455	1.0000	-0.0896	0.9158	-0.0884	1.0000	-0.0380
CCDC27	0.6011	0.2407	0.8757	0.0905	0.0036	-0.9619	0.0000	-0.9274	1.0000	0.0357	1.0000	-0.1015	1.0000	0.0750
CCDC28A	0.0004	-0.6565	0.0000	-0.5294	0.0024	-0.5981	0.0004	-0.5363	1.0000	-0.1215	1.0000	0.0186	1.0000	-0.0538
CCDC28B	0.2875	-0.2721	0.1723	-0.2274	0.0000	-1.5491	0.0000	-0.8555	1.0000	-0.3836	0.1446	-0.3265	0.4394	0.3174
CCDC3	0.5086	-3.8796	0.2535	-4.1743	1.0000	0.5023	1.0000	0.3124	1.0000	0.2609	1.0000	0.0000	1.0000	0.0777
CCDC30	0.1415	-0.5097	0.0340	-0.5151	0.7811	0.1644	0.0001	0.6872	1.0000	-0.2702	0.8607	-0.2606	0.4359	0.2584
CCDC33	1.0000	0.0000	1.0000	0.0000	1.0000	0.0000	1.0000	0.0000	1.0000	0.0000	1.0000	0.0000	1.0000	0.0000
CCDC34	0.2158	0.3476	0.5263	0.1417	0.7950	-0.1447	0.0000	-0.6343	1.0000	0.1788	1.0000	-0.0152	0.2902	-0.3058
CCDC36	0.0048	-5.8059	0.0679	-2.0823	0.5804	-0.7723	0.1748	-1.5178	1.0000	-0.1389	0.8607	3.7297	0.9145	-0.8781
CCDC37	1.0000	-2.4776	1.0000	2.2507	1.0000	-2.4056	1.0000	0.0000	1.0000	-2.3757	1.0000	2.3455	1.0000	0.0000
CCDC39	0.1103	0.6939	0.0027	0.7740	0.8407	0.2494	1.0000	0.1093	1.0000	0.2820	0.5459	0.3763	1.0000	0.1434
CCDC40	0.1688	-0.4268	0.7674	-0.1180	1.0000	-0.0429	0.9164	0.0767	1.0000	-0.0922	0.7771	0.2311	1.0000	0.0349
CCDC42	1.0000	0.0000	1.0000	0.0000	1.0000	0.0000	1.0000	2.3242	1.0000	0.0000	1.0000	0.0000	1.0000	2.3543
CCDC42B	0.0283	1.1109	0.0848	0.8724	0.7335	-0.4567	1.0000	-0.1919	1.0000	-0.1955	0.7488	-0.4196	1.0000	0.0785
CCDC43	0.0000	-0.8431	0.0000	-0.9473	0.0000	-0.8203	0.0000	-0.6751	1.0000	0.0807	1.0000	-0.0110	0.0664	0.2312
CCDC47	0.0792	0.3069	0.0003	0.3377	1.0000	0.0304	0.0099	-0.2621	1.0000	0.0464	0.8607	0.0894	0.0590	-0.2414
CCDC50	0.0000	1.3851	0.0000	1.4862	0.0000	1.7963	0.0000	1.8817	1.0000	-0.1498	1.0000	-0.0343	0.9693	-0.0585
CCDC51	0.5973	-0.1615	0.2783	-0.1541	0.0319	-0.4325	0.0005	-0.3978	1.0000	0.0178	1.0000	0.0374	1.0000	0.0578
CCDC57	0.0124	-1.3892	0.0268	-1.0717	0.8589	-0.2366	0.7413	0.2633	1.0000	-0.0612	1.0000	0.2682	0.6830	0.4453
CCDC58	0.0425	-0.4967	0.0040	-0.4194	0.2593	-0.3094	0.0007	-0.4423	1.0000	-0.1152	1.0000	-0.0252	0.3493	-0.2413
CCDC59	0.5442	-0.1896	0.0976	-0.2212	0.0234	-0.4951	0.0000	-0.6419	1.0000	-0.0229	1.0000	-0.0419	0.6584	-0.1631
CCDC6	0.8475	0.0869	0.3731	0.0928	0.7448	-0.1102	0.3879	-0.0932	1.0000	0.0433	1.0000	0.0620	0.9519	0.0658
CCDC60	1.0000	0.0000	1.0000	0.0000	1.0000	0.0000	1.0000	0.0000	1.0000	0.0000	1.0000	0.0000	1.0000	0.0000
CCDC61	1.0000	-0.0377	0.3591	-0.1604	0.0000	-0.9379	0.0000	-0.8107	1.0000	-0.1545	0.2184	-0.2656	1.0000	-0.0218
CCDC63	1.0000	0.0000	1.0000	0.0000	1.0000	2.2472	1.0000	0.0000	1.0000	0.0000	1.0000	0.0000	1.0000	-2.2909
CCDC64	0.0002	-0.7525	0.0000	-0.6268	0.0000	-0.9503	0.0000	-0.8198	1.0000	-0.0350	1.0000	0.1036	0.9767	0.1018
CCDC65	0.6881	1.7520	1.0000	-1.0027	0.4426	1.8953	0.9801	0.9240	1.0000	0.7416	0.8269	-2.0158	1.0000	-0.2278
CCDC66	0.9714	0.1063	0.9544	0.0651	0.9007	-0.1282	0.1998	-0.2605	1.0000	0.1484	1.0000	0.1221	1.0000	0.0211
CCDC67	1.0000	2.1902	1.0000	-1.0008	1.0000	0.0000	1.0000	0.0059	1.0000	3.1126	1.0000	0.0489	1.0000	3.2048
CCDC68	0.6568	-0.9013	0.8984	0.5359	0.2857	-2.5000	0.0731	-4.6410	1.0000	-0.1255	0.7702	1.3278	1.0000	-2.2888
CCDC69	0.0000	-5.3480	0.0000	-4.0150	0.0000	-3.7001	0.0000	-2.1379	1.0000	0.3940	0.0006	1.7456	0.0000	1.9575
CCDC71	0.9731	-0.0687	0.0676	-0.1860	1.0000	0.0569	0.5557	0.0829	1.0000	0.0830	1.0000	-0.0214	0.6701	0.1149
CCDC71L	0.3495	0.7093	1.0000	0.1296	0.2542	0.8239	1.0000	0.0550	1.0000	0.4716	1.0000	-0.0928	1.0000	-0.2941
CCDC73	0.3791	0.7080	1.0000	-0.0530	0.7660	0.4252	0.1725	0.6473	1.0000	0.5430	1.0000	-0.2047	0.2439	0.7678
CCDC77	0.1190	0.5016	0.0241	0.4868	1.0000	-0.0433	0.0338	-0.5372	1.0000	-0.1863	0.8871	-0.1909	0.0335	-0.6746
CCDC78	1.0000	0.1923	0.4431	-3.7876	1.0000	-0.1793	0.9096	-1.3726	1.0000	0.4112	0.8785	-3.6646	1.0000	-0.7795
CCDC79	1.0000	-0.1127	1.0000	-0.1428	0.0308	1.0704	0.0044	0.9632	1.0000	0.0763	1.0000	0.0592	1.0000	-0.0300
CCDC80	0.1272	0.3096	0.0467	0.1830	0.0000	0.6019	0.0000	0.9077	1.0000	0.1290	1.0000	0.0156	0.0000	0.4402
CCDC81	1.0000	0.0000	1.0000	-2.3958	1.0000	0.0000	1.0000	0.0062	1.0000	2.2674	1.0000	0.0000	1.0000	2.3543
CCDC82	1.0000	0.0248	0.0400	0.4285	0.3191	-0.3539	1.0000	-0.0020	1.0000	-0.3775	1.0000	0.0375	1.0000	-0.0216
CCDC83	1.0000	-0.4906	1.0000	-0.1708	0.7112	-0.6472	1.0000	0.0073	1.0000	-1.2316	0.9277	-0.9051	1.0000	-0.5735
CCDC84	1.0000	-0.0881	0.2709	-0.3331	0.0170	-0.7505	0.4698	-0.2377	1.0000	0.0108	1.0000	-0.2230	0.1340	0.5273
CCDC85A	1.0000	2.1902	1.0000	2.2507	1.0000	0.0000	1.0000	0.0000	1.0000	0.0000	1.0000	0.0470	1.0000	0.0000
CCDC85C	0.6877	-2.0832	0.1015	2.5606	0.2909	1.3356	0.0100	3.2998	1.0000	-1.8822	0.3757	2.7756	1.0000	0.0795
CCDC86	0.0002	-0.5754	0.0000	-0.4822	0.0000	-1.1688	0.0000	-1.1655	1.0000	-0.1191	1.0000	-0.0137	0.8237	-0.1096
CCDC88A	0.0387	0.6339	0.0000	0.9331	0.0000	1.5208	0.0000	1.7389	1.0000	-0.2539	1.0000	0.0592	1.0000	-0.0289
CCDC88C	0.0000	-2.0016	0.0000	-1.8957	1.0000	0.0349	0.6210	-0.0647	1.0000	0.2508	0.0006	0.3690	0.3477	0.1555
CCDC89	0.0394	1.2991	0.2819	0.6015	1.0000	-0.1053	1.0000	0.0065	1.0000	0.8128	1.0000	0.1245	0.3312	0.9283
CCDC90B	0.0895	-0.3033	0.0019	-0.2866	0.7500	-0.1114	0.0494	-0.1776	1.0000	0.0357	1.0000	0.0647	1.0000	-0.0260
CCDC91	0.2263	-1.2501	0.2417	-0.8467	0.0008	1.6526	0.6895	0.3741	1.0000	0.3582	0.9519	0.7800	0.0723	-0.9132
CCDC92	0.8092	-0.2665	0.4787	-0.2959	0.9577	-0.1459	0.0118	0.6520	1.0000	-0.2234	1.0000	-0.2410	0.1053	0.5836
CCDC93	0.7303	0.1109	0.3737	0.1043	0.4741	-0.1660	0.1430	-0.1496	1.0000	-0.0366	1.0000	-0.0310	1.0000	-0.0151
CCDC94	0.0026	-0.5338	0.0007	-0.3856	0.0000	-0.8615	0.0000	-0.4802	1.0000	-0.2334	1.0000	-0.0733	0.6362	0.1535
CCDC96	1.0000	-0.3424	0.5798	0.9870	1.0000	-0.8771	1.0000	0.0069	1.0000	-0.8174	1.0000	0.5236	1.0000	0.0732
CCDC97	1.0000	-0.0723	0.1435	0.2855	1.0000	0.0426	0.0000	0.6374	1.0000	-0.5429	0.7554	-0.1716	1.0000	0.0608
CCK	0.0260	-1.0433	0.0000	-1.4555	0.4435	-0.4350	0.0018	-0.8895	1.0000	0.0083	0.8808	-0.3954	0.5240	-0.4445
CCKAR	1.0000	2.1902	1.0000	0.0000	1.0000	0.0000	1.0000	0.0000	1.0000	0.0000	1.0000	-2.2992	1.0000	0.0000
CCKBR	0.0001	-2.6422	0.0000	-2.5001	0.0122	-1.3417	0.0083	-1.3397	1.0000	-0.2832	1.0000	-0.1287	1.0000	-0.2767
CCL1	1.0000	0.0000	1.0000	0.0000	1.0000	0.0000	1.0000	0.0000	1.0000	0.0000	1.0000	0.0000	1.0000	0.0000
CCL13	1.0000	0.0000	1.0000	0.0000	1.0000	0.0000	1.0000	0.0000	1.0000	0.0000	1.0000	0.0000	1.0000	0.0000
CCL17	1.0000	0.0000	1.0000	0.0000	1.0000	0.0000	1.0000	0.0000	1.0000	0.0000	1.0000	0.0000	1.0000	0.0000
CCL19	1.0000	0.0000	1.0000	-2.3986	1.0000	0.0000	1.0000	-2.3200	1.0000	2.2733	1.0000	0.0000	1.0000	0.0000
CCL20	1.0000	-0.6461	0.9762	0.7528	0.9164	0.4632	1.0000	0.0074	1.0000	-1.3466	1.0000	0.0569	0.4910	-1.8008
CCL21	1.0000	2.1847	0.2920	4.0165	1.0000	0.0000	1.0000	2.3242	1.0000	0.0000	1.0000	1.8280	1.0000	2.3543
CCL24	0.0107	1.5087	0.0000	3.0551	0.0551	-2.2196	1.0000	-0.6807	1.0000	-1.4659	1.0000	0.0906	1.0000	0.0765
CCL26	1.0000	2.1847	0.2542	4.0037	1.0000	0.0000	1.0000	0.0000	1.0000	0.0000	1.0000	1.8188	1.0000	0.0000
CCL5	1.0000	0.0000	1.0000	0.0000	1.0000	0.0000	1.0000	0.0000	1.0000	0.0000	1.0000	0.0000	1.0000	0.0000
CCM2	0.6834	0.1256	0.0002	0.3338	0.0042	0.4938	0.0000	0.4140	1.0000	-0.0457	0.2993	0.1747	0.6270	-0.1198
CCM2L	0.0008	-0.7448	0.0070	-0.4429	0.7482	-0.1436	0.0000	0.5797	1.0000	-0.3688	1.0000	-0.0545	0.0586	0.3597
CCNA1	1.0000	-1.1514	1.0000	0.3667	1.0000	-0.1742	0.7701	-3.1666	1.0000	-0.1170	1.0000	1.4314	1.0000	-3.1370
CCNA2	0.0000	0.8734	0.0000	1.1547	0.7313	0.1356	0.0244	-0.2345	1.0000	-0.1258	0.4005	0.1673	0.0000	-0.4899
CCNB2	0.0003	0.5856	0.0000	0.7416	0.9743	-0.0593	0.0000	-0.4150	1.0000	0.0072	0.3932	0.1758	0.0002	-0.3433
CCNB3	0.0019	0.6051	0.0000	1.0243	0.5476	-0.1604	0.0000	-0.5811	1.0000	-0.2139	0.4754	0.2178	0.0000	-0.6297
CCND1	0.0001	-0.7008	0.0000	-0.9623	0.5106	0.1750	0.5772	-0.0856	1.0000	0.1395	0.9755	-0.1095	0.7096	-0.1163
CCND2	0.0005	1.1702	0.0000	1.1886	0.0196	0.8589	0.0289	0.6042	1.0000	0.0194	1.0000	0.0495	0.8254	-0.2326
CCND3	0.0000	1.0238	0.0000	1.1448	0.4809	-0.1937	0.2490	0.1665	1.0000	-0.2192	0.9757	-0.0869	0.6600	0.1465
CCNDBP1	0.1372	-0.2708	0.0000	-0.4070	0.0001	-0.6157	0.0000	-0.7842	1.0000	-0.0003	0.6091	-0.1242	0.3515	-0.1630
CCNE2	0.2889	0.6806	0.0002	1.4128	0.3939	0.5287	0.0844	0.8398	1.0000	-0.8225	1.0000	-0.0831	0.5103	-0.5112
CCNF	0.0003	0.7034	0.0000	0.8315	0.0248	0.4776	0.1122	0.2197	1.0000	-0.3399	0.3223	-0.1993	0.0000	-0.5915
CCNG1	1.0000	0.0229	0.4025	-0.0907	0.8434	0.0784	0.0994	-0.1463	1.0000	0.0631	1.0000	-0.0379	0.1668	-0.1563
CCNG2	0.0137	0.4021	0.0086	0.2341	0.0000	0.6878	0.0000	0.4409	1.0000	0.0711	0.8785	-0.0841	0.1917	-0.1702
CCNH	0.4520	0.1661	0.1864	0.1211	0.0624	-0.3248	0.0008	-0.2681	1.0000	0.1201	0.8269	0.0875	0.1774	0.1820

CCNI	0.0000	-0.7669	0.0000	-1.0192	1.0000	-0.0040	0.0176	0.1595	1.0000	0.0826	0.2227	-0.1572	0.0046	0.2514
CCNJ	0.1177	-0.5044	0.0009	-0.6496	0.5942	0.2391	0.0274	0.3767	1.0000	0.0176	1.0000	-0.1156	0.8682	0.1581
CCNJL	1.0000	2.1902	1.0000	0.0000	1.0000	0.0000	1.0000	0.0000	1.0000	0.0000	1.0000	-2.2992	1.0000	0.0000
CCNK	0.4445	-0.1842	0.0038	-0.2701	0.3939	0.1855	0.8321	0.0481	1.0000	0.1622	0.9773	0.0894	1.0000	0.0305
CCNL1	0.6608	0.1246	0.0016	-0.2353	0.0000	-1.0815	0.0000	-0.8306	1.0000	0.0544	0.0468	-0.2938	0.0015	0.3105
CCNL2	0.0000	-0.8344	0.0000	-1.1359	0.0005	-0.5261	0.0000	-0.4735	1.0000	0.1813	0.7896	-0.1078	0.0137	0.2390
CCNO	0.6463	0.6912	1.0000	0.1002	1.0000	0.0890	1.0000	0.1488	1.0000	-0.1155	0.9476	-0.7031	1.0000	-0.0558
CCNT1	0.3321	-0.3079	0.5987	-0.1088	0.5541	-0.2263	0.7339	-0.0880	1.0000	0.0108	0.4052	0.2221	0.7006	0.1539
CCNT2	0.0042	-0.5659	0.0000	-0.6828	0.0151	0.4704	0.0003	0.4374	1.0000	0.1259	1.0000	0.0227	0.8560	0.0983
CCNYL1	1.0000	-0.0031	0.0139	0.1838	0.6545	-0.1160	1.0000	0.0135	1.0000	-0.0660	0.3739	0.1331	0.8776	0.0689
CCP110	0.0411	0.5247	0.0489	0.3356	0.0000	0.9624	0.0000	0.8154	1.0000	0.1890	1.0000	0.0130	1.0000	0.0475
CCPG1	0.0259	0.3744	0.0000	0.4979	0.0000	0.8748	0.0000	0.6906	1.0000	-0.0648	0.9399	0.0713	0.0164	-0.2435
CCR10	1.0000	0.0000	1.0000	0.0000	1.0000	2.2472	0.7701	3.1732	1.0000	0.0000	1.0000	0.0000	1.0000	0.9165
CCR4	0.0031	0.9563	0.0093	0.6044	1.0000	0.0435	0.2857	-0.3938	1.0000	0.3513	1.0000	0.0120	1.0000	-0.0806
CCR5	1.0000	0.0000	1.0000	-2.3986	0.8033	3.0892	1.0000	-2.3200	1.0000	2.2734	1.0000	0.0000	1.0000	-3.1370
CCR6	1.0000	0.0000	1.0000	0.0000	1.0000	0.0000	1.0000	0.0000	1.0000	0.0000	1.0000	0.0000	1.0000	0.0000
CCR7	0.1700	0.5382	1.0000	0.0627	0.4809	0.3429	0.0000	1.1286	1.0000	0.1022	0.4706	-0.3635	0.0005	0.8926
CCR8	0.3181	-2.3898	0.4431	-3.7865	0.4426	-2.2459	1.0000	-0.5229	1.0000	-0.8169	1.0000	-2.2957	1.0000	0.9169
CCR9	1.0000	2.1902	0.9762	0.7528	1.0000	2.2472	0.7710	-3.1631	1.0000	3.1056	1.0000	1.8187	1.0000	-2.2909
CCRN4L	0.0003	-0.8221	0.0000	-0.9094	1.0000	-0.0659	0.8164	0.0688	1.0000	0.0694	1.0000	-0.0062	0.3117	0.2087
CCSAP	0.8970	0.2223	0.0015	1.2009	0.0464	0.8263	0.0007	1.1761	1.0000	-0.5656	0.7070	0.4246	0.9853	-0.2136
CCSER1	1.0000	0.4078	0.7708	-1.1144	0.0002	2.4156	0.0390	1.9777	1.0000	-0.3399	0.3468	-1.8568	0.5057	-0.7816
CCT2	0.0021	-0.4921	0.0000	-0.4204	0.3634	-0.1881	0.0124	-0.1935	1.0000	-0.0150	0.9146	0.0691	1.0000	-0.0148
CCT3	0.1551	-0.2438	0.8523	0.0423	0.0011	-0.5195	0.0089	-0.2084	1.0000	-0.3529	1.0000	-0.0543	1.0000	-0.0365
CCT4	0.2752	-0.2086	0.1114	-0.1346	0.0339	-0.3393	0.0000	-0.3082	1.0000	-0.0802	1.0000	0.0064	1.0000	-0.0437
CCT5	0.1845	-0.2292	0.1360	-0.1171	0.7806	-0.0920	0.3371	-0.0814	1.0000	-0.0838	1.0000	0.0407	0.8264	-0.0677
CCT6A	0.0086	-0.4459	0.0000	-0.3041	0.1854	-0.2540	0.0015	-0.2170	1.0000	-0.0394	0.5289	0.1149	1.0000	0.0033
CCT7	0.2423	-0.2291	0.0142	-0.1848	0.1164	-0.2716	0.0101	-0.1846	1.0000	-0.1296	1.0000	-0.0726	1.0000	-0.0369
CCT8	0.4306	-0.1791	0.1302	-0.1251	0.8862	0.0775	1.0000	0.0236	1.0000	-0.0452	1.0000	0.0214	0.6067	-0.0936
CCZ1	0.0167	0.3965	0.0949	0.1557	0.7903	-0.0999	0.0071	-0.2344	1.0000	0.0663	0.2736	-0.1623	0.9811	-0.0631
CD109	0.0000	1.5464	0.0000	1.6220	1.0000	0.0372	0.3034	-0.1365	1.0000	0.0178	0.6830	0.1055	0.5521	-0.1513
CD151	0.6303	-0.1189	0.0002	-0.2912	0.2816	-0.1957	1.0000	0.0195	1.0000	0.0249	0.4538	-0.1348	0.0063	0.2454
CD163	1.0000	2.1902	1.0000	0.3654	1.0000	0.0000	0.7210	1.2296	1.0000	3.1056	1.0000	1.4259	0.3369	4.4316
CD164	0.4470	0.1535	0.0135	0.1813	0.6851	-0.1081	0.6078	-0.0583	1.0000	0.0467	0.7050	0.0869	0.5244	0.1018
CD164L2	1.0000	-0.3195	1.0000	-0.1452	0.8011	-3.2629	1.0000	0.0057	1.0000	-0.9587	1.0000	-0.7977	1.0000	2.3554
CD180	0.6033	0.8398	0.0776	1.4442	0.8305	-1.1352	1.0000	0.0067	1.0000	0.0813	0.8269	0.7036	0.8768	1.2328
CD2	1.0000	-2.4776	1.0000	0.6944	1.0000	-2.4056	1.0000	-2.3200	1.0000	-0.1033	1.0000	3.1976	1.0000	0.0000
CD200R1	1.0000	2.1847	1.0000	0.0000	1.0000	2.2428	1.0000	0.0000	1.0000	0.0000	1.0000	-2.2957	1.0000	-2.2888
CD24	0.0001	-0.9079	0.0001	-0.5429	0.1468	0.3259	0.0046	0.3634	1.0000	0.1513	0.0436	0.5303	0.4082	0.1941
CD247	0.2490	-0.4324	0.5021	-0.2477	0.0889	-0.5316	1.0000	0.0019	1.0000	-0.4086	0.9421	-0.2120	1.0000	0.1300
CD274	0.4241	1.5381	0.6403	0.7839	1.0000	-1.0194	0.9801	-0.9105	1.0000	0.7940	1.0000	0.0592	1.0000	0.9164
CD276	0.0782	-0.3090	0.0500	-0.1986	0.0010	0.5214	0.0000	0.4739	1.0000	-0.0840	1.0000	0.0385	0.4936	-0.1263
CD28	1.0000	-1.1562	1.0000	0.0000	0.8011	-3.2629	1.0000	0.0000	1.0000	-3.2304	1.0000	-2.2957	1.0000	0.0000
CD320	0.0072	-0.7148	0.0000	-0.5610	0.0125	-0.6607	0.0000	-0.7877	1.0000	0.0129	0.7635	0.1783	1.0000	-0.1098
CD34	1.0000	-0.3236	0.4991	-1.6416	0.8033	-3.2636	0.7713	-0.9365	1.0000	1.3509	1.0000	0.0547	0.7287	3.7384
CD36	0.0000	2.3004	0.0000	2.4788	0.0000	-2.6056	0.0000	-2.1310	1.0000	0.1924	0.0017	0.3818	0.1916	0.6729
CD38	0.8587	-0.2068	1.0000	-0.0478	0.0000	-1.7938	0.0036	-0.9729	1.0000	-0.0548	1.0000	0.1142	0.2750	0.7678
CD3E	0.3092	4.2299	0.0412	4.7731	1.0000	2.2472	1.0000	2.3242	1.0000	0.0000	1.0000	0.5247	1.0000	0.0641
CD4	1.0000	0.0000	1.0000	0.0000	1.0000	0.0000	1.0000	0.0000	1.0000	0.0000	1.0000	0.0000	1.0000	0.0000
CD40	0.0000	1.4983	0.0000	1.5829	0.9357	-0.1012	0.5707	0.1425	1.0000	-0.5474	0.0005	-0.4499	0.2727	-0.2967
CD40LG	1.0000	0.0000	1.0000	0.0000	1.0000	0.0000	1.0000	0.0000	1.0000	0.0000	1.0000	0.0000	1.0000	0.0000
CD44	0.0000	-0.7795	0.0000	-0.2713	0.0000	0.7210	0.0000	0.6112	1.0000	0.0449	0.0000	0.5655	0.8705	-0.0595
CD46	0.3792	0.2283	0.0000	0.4704	0.0000	1.3354	0.0000	1.1744	1.0000	0.0314	0.0925	0.2858	0.5208	-0.1234
CD47	0.0000	-0.6945	0.0000	-0.7758	0.0012	0.5078	0.0004	0.2743	1.0000	0.2325	0.3185	0.1636	1.0000	0.0040
CD5	0.8249	3.0140	0.4431	3.6192	1.0000	0.0000	1.0000	0.0000	1.0000	0.0000	1.0000	0.5871	1.0000	0.0000
CD55	0.0010	1.9589	0.0004	2.3185	0.5798	0.6529	0.5149	-1.4651	1.0000	-0.9799	0.5536	-0.6113	0.0037	-3.0952
CD59	0.5876	0.1279	1.0000	0.0162	0.0001	0.5615	0.0000	0.5566	1.0000	0.0739	1.0000	-0.0255	0.7917	0.0743
CD6	0.5086	3.5431	1.0000	0.0000	1.0000	0.0000	1.0000	0.0000	1.0000	0.0000	0.8607	-3.6719	1.0000	0.0000
CD63	0.1330	-0.2807	0.0124	-0.2114	0.0000	-0.6990	0.0000	-0.4240	1.0000	-0.0391	1.0000	0.0432	0.1015	0.2411
CD7	0.1380	-2.8557	0.0004	-3.3415	1.0000	-0.1948	0.1392	-1.1239	1.0000	1.3555	1.0000	0.8994	1.0000	0.4303
CD74	1.0000	-0.0360	0.1742	-0.2373	0.3004	-0.3000	0.0060	-0.4037	1.0000	-0.1973	0.0296	-0.3876	0.1209	-0.2959
CD79A	0.4783	-0.7616	0.2806	0.7609	0.1796	0.8757	0.0039	1.4070	1.0000	-0.2191	0.1686	1.3168	0.9593	0.3137
CD79B	1.0000	-0.7247	0.0433	-4.9468	1.0000	-1.1048	0.8272	-0.7630	1.0000	0.6404	0.8607	-3.6732	1.0000	0.9922
CD80	0.8226	3.0199	0.6224	-1.9244	0.2829	3.9942	0.6172	-1.7602	1.0000	4.0300	1.0000	-0.7956	0.8939	-1.6944
CD81	0.7533	0.1009	0.0639	0.1750	0.8828	0.0742	0.0413	0.1841	1.0000	-0.0806	1.0000	0.0056	1.0000	0.0345
CD82	0.0001	0.9544	0.0121	0.4453	0.0061	0.6874	0.7280	-0.1186	1.0000	0.0486	0.0216	-0.4490	0.0000	-0.7540
CD83	1.0000	0.0000	1.0000	0.0000	1.0000	0.0000	1.0000	0.0000	1.0000	0.0000	1.0000	0.0000	1.0000	0.0000
CD84	1.0000	0.4658	1.0000	0.0100	1.0000	0.0572	1.0000	0.3578	1.0000	0.3394	1.0000	-0.1054	1.0000	0.6436
CD86	1.0000	0.0000	1.0000	0.0000	1.0000	0.0000	1.0000	0.0000	1.0000	0.0000	1.0000	0.0000	1.0000	0.0000
CD8B	1.0000	2.1848	1.0000	-0.9997	1.0000	2.2428	0.7710	-3.1632	1.0000	3.1056	1.0000	0.0495	1.0000	-2.2888
CD9	1.0000	0.0000	1.0000	-2.3986	1.0000	0.0000	1.0000	-2.3200	1.0000	2.2735	1.0000	0.0000	1.0000	0.0000
CD93	0.2118	0.7413	0.9754	-0.1487	0.2593	-0.8852	0.0006	-1.5619	1.0000	0.6548	1.0000	-0.2216	1.0000	-0.0140
CD96	1.0000	0.0000	1.0000	2.2535	0.8033	3.0790	1.0000	2.3241	1.0000	0.0000	1.0000	2.3480	1.0000	-0.7782
CD99	0.8566	0.0770	0.0000	0.4229	0.0332	-0.3607	0.0000	-0.5536	1.0000	0.1832	0.0000	0.5414	1.0000	-0.0050
CD99L2	0.0009	1.0089	0.0000	1.4525	0.0000	1.5822	0.0000	1.6601	1.0000	-0.0399	0.1033	0.4176	1.0000	0.0454
CDADC1	0.1676	-0.3901	0.0265	-0.3554	0.0078	0.5684	0.0000	0.8119	1.0000	0.0660	1.0000	0.1113	0.0504	0.3141
CDAN1	0.4230	0.2023	1.0000	0.0259	0.8926	0.0902	0.1153	0.1806	1.0000	0.1914	1.0000	0.0280	0.0369	0.2870
CDC123	0.0000	0.9615	0.0000	0.5504	0.9880	-0.0722	0.0014	-0.3472	1.0000	0.1874	0.1936	-0.2106	0.9753	-0.0811
CDC14B	0.0001	1.2248	0.0000	1.3118	0.7663	0.2209	0.0225	0.6103	1.0000	-0.0719	1.0000	0.0267	0.5694	0.3211
CDC16	0.6365	0.1698	0.1257	0.1954	0.5494	-0.1922	0.0002	-0.4277	1.0000	-0.0098	1.0000	0.0285	0.1828	-0.2387
CDC20	0.0830	-0.3642	0.1302	-0.2026	0.0000	-0.7709	0.0000	-0.6737	1.0000	0.0585	0.3972	0.2327	0.3671	0.1610
CDC20B	0.0230	1.6928	0.0008	2.6649	0.7513	-0.8421	0.9162	-1.3768	1.0000	-1.4652	0.9442	-0.4950	0.6944	-2.0012
CDC23	0.0015	0.5303	0.0000	0.4664	0.0110	-0.4673	0.0000	-0.5703	1.0000	-0.0059	1.0000	-0.0581	0.8328	-0.1040
CDC25A	0.1252	0.3898	0.0000	0.7796	1.0000	0.0125	0.8040	0.0909	1.0000	-0.3395	1.0000	0.0635	0.6185	-0.2533
CDC25B	0.0006	-0.5777	0.0000	-0.5853	0.0000	-0.7731	0.0000	-0.5820	1.0000	-0.2857	0.0021	-0.2810	0.8804	-0.0885
CDC26	0.0799	-0.4206	0.0724	-0.3085	0.0000	-1.5106	0.0000	-1.5912	1.0000	-0.1174	1.0000	0.0070	0.9158	-0.1922
CDC27	0.0032	0.5439	0.0002	0.5002	0.0000	0.8467	0.0000	0.6045	1.0000	0.1094	1.0000	0.0790	0.6747	-0.1273
CDC34	1.0000	-0.0538	0.0226	-0.3017	1.0000	0.0460	0.5827	0.1253	1.0000	0.0852	0.7971	-0.1504	0.6795	0.1697
CDC37	0.0001	-0.6565	0.0000	-0.4692	0.0000	-0.8215	0.0000	-0.5463	1.0000	-0.2373	1.0000	-0.0382	1.0000	0.0442
CDC37L1	0.0773	-0.4903	0.0000	-0.7069	1.0000	-0.0766	0.0481	-0.3291	1.0000	0.0681	1.0000	-0.1358	0.7233	-0.1788
CDC40	0.0005	0.9212	0.0000	0.9304	0.0000	1.1532	0.0000	0.8728	1.0000	0.1608	0.8212	0.1835	0.8637	-0.1125
CDC42	0.1301	-0.2476	0.0163	-0.1848	1.0000	-0.0351	1.0000	-0.0200	1.0000	-0.0344	1.0000	0.0408	1.0000	-0.0139
CDC42BPB	0.1310	0.2554	0.0000	0.3004	0.0039	0.4465	0.0000	0.2900	1.0000	0.0764	0.3339	0.1339	0.8125	-0.0751

CDC42EP1	1.0000	-0.0447	0.5924	0.0843	0.0027	0.5136	0.0000	0.4983	1.0000	-0.2230	1.0000	-0.0825	0.1305	-0.2330
CDC42EP2	0.0010	-1.3350	0.0000	-1.1548	0.1712	0.5372	0.0436	0.3747	1.0000	0.2034	0.6156	0.3977	1.0000	0.0480
CDC42EP3	0.1117	-0.2958	0.0002	-0.3421	1.0000	0.0201	0.6554	0.0681	1.0000	-0.0282	1.0000	-0.0622	1.0000	0.0254
CDC42EP4	0.0000	-0.7441	0.0000	-0.4340	0.0002	-0.6795	0.0000	-0.4777	1.0000	-0.1089	0.2494	0.2140	0.8973	0.0996
CDC42SE1	0.0002	-0.5404	0.0000	-0.4987	0.0108	-0.4201	0.0107	-0.2219	1.0000	-0.1272	0.9514	-0.0732	0.9296	0.0767
CDC42SE2	0.1841	0.3032	0.0813	0.2142	0.0048	-0.5892	0.0000	-0.6912	1.0000	0.0936	1.0000	0.0164	1.0000	-0.0035
CDC45	0.0000	0.9478	0.0000	1.2117	0.0146	0.4999	0.5353	-0.1206	1.0000	-0.1048	0.4324	0.1710	0.0000	-0.7194
CDC6	0.3510	-0.2111	0.2153	-0.1624	0.0281	-0.4360	0.0049	-0.3229	1.0000	-0.2812	0.2488	-0.2203	0.6247	-0.1625
CDCA2	0.0007	1.2264	0.0000	2.0491	0.0000	1.4881	0.0001	1.2309	1.0000	-0.4411	0.3224	0.3960	0.0157	-0.6895
CDCA3	0.0120	0.5745	0.0000	0.8766	1.0000	-0.0473	0.0033	-0.3775	1.0000	-0.2183	0.8820	0.0953	0.0008	-0.5422
CDCA4	0.1413	0.2646	0.0036	0.2291	0.2962	-0.2065	0.0006	-0.2688	1.0000	0.0281	1.0000	0.0050	1.0000	-0.0286
CDCA7	0.0000	0.8393	0.0000	0.9605	0.2294	0.3063	0.3093	0.1584	1.0000	-0.1741	1.0000	-0.0419	0.0366	-0.3163
CDCA7L	0.0970	0.3117	0.0408	0.1738	0.0015	-0.5449	0.0000	-0.5566	1.0000	0.1023	1.0000	-0.0233	0.7423	0.0959
CDCA8	1.0000	0.0162	0.2101	0.1437	1.0000	0.0088	1.0000	-0.0235	1.0000	-0.4257	0.0146	-0.2866	0.0020	-0.4521
CDCP1	0.0358	0.5405	0.0000	0.6615	0.2972	0.3112	0.0335	0.3516	1.0000	0.1244	0.3858	0.2590	0.7050	0.1704
CDCP2	1.0000	2.1903	1.0000	-2.3986	1.0000	0.0000	1.0000	-2.3200	1.0000	2.2735	1.0000	-2.2992	1.0000	0.0000
CDH1	1.0000	0.5420	0.7674	-3.2498	0.9361	1.2064	0.1081	2.1928	1.0000	0.7317	1.0000	-3.1429	0.4602	1.7312
CDH11	0.8461	0.8153	0.9762	0.7528	1.0000	-0.7189	0.7710	-3.1632	1.0000	-0.6592	1.0000	-0.7122	1.0000	-3.1317
CDH13	0.0020	0.5679	0.0000	0.6917	0.0000	1.3059	0.0000	1.1926	1.0000	0.1832	0.0023	0.3198	0.8439	0.0746
CDH17	0.2220	1.4477	0.4200	1.3366	1.0000	-0.8757	0.9096	-1.3717	1.0000	-0.8156	0.6497	-0.9208	1.0000	-1.3086
CDH18	1.0000	-1.1585	0.7666	-3.2534	1.0000	-0.1754	1.0000	0.0066	1.0000	-0.1187	1.0000	-2.2957	1.0000	0.0716
CDH2	0.0001	-0.5397	0.0000	-0.7633	0.0000	0.7494	0.0000	0.6846	1.0000	0.0919	0.6066	-0.1193	1.0000	0.0322
CDH20	1.0000	0.0000	1.0000	0.0000	1.0000	0.0000	1.0000	0.0000	1.0000	0.0000	1.0000	0.0000	1.0000	0.0000
CDH22	0.0000	2.9293	0.0000	2.7048	0.6334	-0.3067	1.0000	0.0802	1.0000	-0.8752	0.0000	-1.0865	0.3532	-0.4789
CDH23	0.9763	0.0844	0.0041	0.3087	0.6477	0.1743	0.2443	0.1426	1.0000	-0.1850	1.0000	0.0510	0.2089	-0.2138
CDH5	1.0000	-2.4776	1.0000	0.0000	1.0000	-0.1591	1.0000	0.0000	1.0000	-2.3757	1.0000	0.0000	1.0000	-2.2909
CDH6	0.0150	1.3836	0.0042	0.9551	1.0000	0.1830	0.8799	0.1740	1.0000	-1.5142	0.0000	-1.9292	0.0000	-1.5148
CDHR1	1.0000	0.0000	1.0000	0.0000	1.0000	0.0000	1.0000	0.0000	1.0000	0.0000	1.0000	0.0000	1.0000	0.0000
CDHR2	0.6334	-0.4054	0.0156	-0.9336	0.0001	-2.2528	0.0007	-1.3422	1.0000	-0.0064	0.6519	-0.5232	0.4112	0.9058
CDHR3	0.0383	2.2895	0.1250	2.0289	0.5035	-3.7981	1.0000	0.5377	1.0000	-0.6592	0.6300	-0.9182	0.7287	3.7384
CDHR4	1.0000	0.3545	1.0000	0.0406	0.3579	1.4625	1.0000	0.2204	1.0000	0.8171	1.0000	0.5246	1.0000	-0.4205
CDHR5	1.0000	0.0000	1.0000	0.0000	1.0000	0.0000	1.0000	0.0000	1.0000	0.0000	1.0000	0.0000	1.0000	0.0000
CDIP1	0.9349	-0.0666	1.0000	0.0051	0.0000	-0.8028	0.0000	-0.4971	1.0000	-0.2508	0.3216	-0.1667	1.0000	0.0606
CDK1	0.0013	0.7508	0.0000	1.1188	0.8825	0.1061	0.1084	-0.1895	1.0000	-0.2930	1.0000	0.0874	0.0000	-0.5825
CDK10	0.0825	-0.4567	0.0000	-0.7735	0.2745	-0.3062	0.1685	-0.2619	1.0000	-0.0424	0.3254	-0.3481	1.0000	0.0053
CDK12	0.5717	0.1901	0.0004	0.4116	0.0009	0.6725	0.0000	0.7253	1.0000	-0.1688	1.0000	0.0648	0.7495	-0.1111
CDK13	0.0064	-0.4410	0.0000	-0.5124	0.1845	0.2473	0.0047	0.2277	1.0000	0.0965	1.0000	0.0375	0.8127	0.0816
CDK14	0.0969	-0.9439	0.1811	0.7691	0.0494	0.8129	0.0001	1.6315	1.0000	-1.2367	0.8607	0.4868	0.5410	-0.4133
CDK15	0.0381	-1.7876	0.0726	-1.2273	0.2366	-1.0538	0.0912	-1.1762	1.0000	-0.0650	1.0000	0.5072	1.0000	-0.1822
CDK17	0.8450	-0.0873	0.1860	-0.1568	0.5195	0.1629	0.2760	0.1312	1.0000	0.0338	1.0000	-0.0228	1.0000	0.0075
CDK18	0.0000	-2.2188	0.0000	-1.7508	0.0000	1.2997	0.0000	1.0529	1.0000	-0.1085	0.9217	0.3726	0.1339	-0.3526
CDK19	0.0005	0.6086	0.0010	0.3652	0.0003	0.6278	0.0000	0.5182	1.0000	0.0580	0.4711	-0.1722	1.0000	-0.0453
CDK2	1.0000	0.0564	0.0010	0.4134	0.1312	-0.2855	0.0009	-0.4520	1.0000	-0.2639	0.7721	0.1049	0.0005	-0.4245
CDK2AP1	1.0000	0.0040	0.0157	-0.2101	0.4863	0.1555	0.0000	0.3626	1.0000	0.1064	0.7881	-0.0954	0.0006	0.3189
CDK3	0.3090	3.9281	0.0939	2.1531	1.0000	0.0000	1.0000	-0.8416	1.0000	3.1126	0.6381	1.4636	1.0000	2.3554
CDK5	0.0001	-0.7601	0.0000	-0.5943	0.2750	-0.2599	0.3882	0.1353	1.0000	-0.1826	1.0000	-0.0046	0.3167	0.2178
CDK5R1	0.0000	-1.9900	0.0000	-2.2776	0.0027	-0.8009	0.0817	-0.4084	1.0000	-0.1750	0.6001	-0.4489	0.6506	0.2220
CDK5R2	1.0000	0.0000	1.0000	0.0000	1.0000	0.0000	1.0000	0.0000	1.0000	0.0000	1.0000	0.0000	1.0000	0.0000
CDK5RAP1	0.3092	-0.2474	0.1696	-0.2228	0.2550	-0.2759	0.0057	-0.4042	1.0000	-0.2961	0.2687	-0.2582	0.0253	-0.4169
CDK5RAP2	0.0001	0.7624	0.0000	0.9128	0.0646	0.4038	0.0477	0.2821	1.0000	0.0029	0.5832	0.1669	0.8597	-0.1132
CDK5RAP3	0.8785	0.0831	0.1706	0.1483	0.0023	-0.5728	0.0000	-0.4692	1.0000	-0.1031	1.0000	-0.0258	1.0000	0.0066
CDK6	1.0000	0.0573	0.1410	0.2505	0.0319	0.4929	0.2562	0.2045	1.0000	0.0317	0.4411	0.2356	0.2409	-0.2535
CDK8	0.0010	-0.6122	0.0000	-0.5087	0.0000	-0.9627	0.0000	-0.5295	1.0000	0.0515	0.6501	0.1673	0.0019	0.4891
CDK9	0.8435	0.1294	0.0959	0.2691	0.5813	0.1894	0.0024	0.4187	1.0000	-0.1317	1.0000	0.0189	0.9716	0.1038
CDKAL1	0.0886	-0.4596	0.0016	-0.4558	0.0149	-0.6127	0.0000	-0.6354	1.0000	-0.0989	1.0000	-0.0824	0.9777	-0.1161
CDKL1	0.0000	1.3939	0.0033	0.7735	0.9055	0.2194	1.0000	0.0894	1.0000	0.2525	0.4632	-0.3541	1.0000	0.1248
CDKL2	1.0000	-0.0381	1.0000	-0.0456	0.0000	-1.1874	0.0000	-1.1866	1.0000	0.2771	0.4236	0.2827	0.7139	0.2858
CDKL5	0.5806	-0.1982	0.0297	-0.3230	0.1618	0.3391	1.0000	0.0032	1.0000	0.1469	1.0000	0.0348	0.5358	-0.1855
CDKN1A	0.9871	0.0692	0.2541	0.1250	0.0201	0.4635	0.0001	0.3387	1.0000	0.0333	0.8405	0.1009	0.8932	-0.0865
CDKN1B	0.0000	-1.3360	0.0000	-1.4025	0.0000	-0.9557	0.0000	-0.9719	1.0000	0.0623	1.0000	0.0076	0.9816	0.0515
CDKN2AIP	1.0000	-0.0050	0.9224	-0.0365	1.0000	-0.0270	0.5559	-0.0772	1.0000	-0.0684	0.8553	-0.0874	0.6401	-0.1124
CDKN2C	1.0000	-0.0032	0.2003	0.3318	0.2955	0.3068	0.0000	0.7140	1.0000	-0.2361	1.0000	0.1110	0.6819	0.1776
CDKN3	0.0002	0.9052	0.0000	0.8683	0.0000	1.0843	0.0100	0.4853	1.0000	-0.1874	0.6607	-0.2115	0.0000	-0.7788
CDO1	1.0000	-0.0565	0.7822	-0.3546	0.0953	0.9561	0.0025	1.3453	1.0000	0.1779	1.0000	-0.1101	0.4901	0.5707
CDON	0.0000	0.7254	0.0000	0.7947	0.2866	0.2239	0.0000	0.3853	1.0000	-0.2780	0.1434	-0.1962	0.7287	-0.1104
CDPF1	0.1137	-0.3191	0.0145	-0.2764	0.2324	-0.2535	0.0001	-0.4422	1.0000	0.0589	0.8860	0.1148	0.7450	-0.1242
CDR2	0.0783	-0.3238	0.0123	-0.2461	0.7954	-0.1007	1.0000	-0.0145	1.0000	-0.0044	0.9311	0.0852	0.8212	0.0869
CDR2L	0.7145	0.4415	0.5859	-0.4013	0.2002	0.8323	0.1711	0.6545	1.0000	0.3517	0.8733	-0.4797	1.0000	0.1804
CDRT1	1.0000	-2.4776	1.0000	-1.0051	1.0000	-2.4056	0.7710	-3.1693	1.0000	0.7416	1.0000	2.3455	1.0000	0.0000
CDS1	0.0031	1.5478	0.0000	2.2869	0.1882	-1.1791	1.0000	-0.2288	1.0000	-0.5918	1.0000	0.1606	1.0000	0.3621
CDS2	0.0265	0.3789	0.0001	0.3703	0.0052	0.4622	0.0000	0.4644	1.0000	0.0004	1.0000	0.0037	1.0000	0.0075
CDT1	0.2128	0.3373	0.0039	0.5440	1.0000	-0.0668	1.0000	-0.0532	1.0000	-0.4634	0.5211	-0.2459	0.0338	-0.4459
CDV3	0.0499	-0.3465	0.2743	-0.1133	0.0453	0.3431	0.0000	0.3281	1.0000	-0.0919	0.3313	0.1540	0.5837	-0.1010
CDX1	1.0000	-1.1514	1.0000	0.0000	0.8033	-3.2622	1.0000	2.3257	1.0000	-3.2289	1.0000	-2.2992	1.0000	2.3554
CDYL	0.0461	0.5280	0.0785	0.3096	0.0426	0.5131	0.0158	0.3979	1.0000	0.2010	1.0000	-0.0041	1.0000	0.0914
CDYL2	0.0869	0.4471	0.0611	0.2970	0.0512	0.4677	0.4547	0.1459	1.0000	0.0794	1.0000	-0.0594	0.3574	-0.2363
CEBPB	0.0000	-0.7621	0.0000	-0.4698	0.4426	0.1799	0.0000	0.6004	1.0000	-0.1236	0.4754	0.1817	0.0219	0.3033
CEBPG	0.0000	-0.7138	0.0000	-0.5082	0.0659	-0.3225	0.0008	-0.2502	1.0000	0.0211	0.1631	0.2391	0.6285	0.0991
CEBPZ	0.4363	0.1723	0.3680	0.1008	0.0002	0.5736	0.0000	0.4326	1.0000	0.0698	1.0000	0.0109	0.9317	-0.0655
CECR1	0.0631	0.5520	0.0000	0.9962	0.0555	-0.6252	0.0001	-0.7771	1.0000	0.1598	0.0048	0.6157	1.0000	0.0135
CECR5	1.0000	0.0516	0.1048	-0.2172	1.0000	-0.0212	0.0625	-0.2319	1.0000	0.1786	1.0000	-0.0770	1.0000	-0.0264
CECR6	1.0000	-2.4788	0.9784	0.7520	0.1166	2.5445	1.0000	0.0106	1.0000	0.7304	0.6450	4.1157	0.4805	-1.7987
CEL	1.0000	0.0000	1.0000	0.0000	1.0000	0.0000	1.0000	0.0000	1.0000	0.0000	1.0000	0.0000	1.0000	0.0000
CELA1	1.0000	0.0000	1.0000	0.0000	1.0000	0.0000	1.0000	0.0000	1.0000	0.0000	1.0000	0.0000	1.0000	0.0000
CELF1	0.0234	1.3208	0.3090	0.6334	0.0164	1.2210	0.3260	0.6010	1.0000	0.5237	1.0000	-0.1486	1.0000	-0.0877
CELF2	0.0358	0.6104	0.0000	0.7768	0.0000	1.8521	0.0000	1.6503	1.0000	0.0403	0.7070	0.2180	0.6865	-0.1577
CELF4	1.0000	-2.4776	1.0000	-2.3986	1.0000	-2.4056	1.0000	-2.3199	1.0000	-0.1034	1.0000	0.0000	1.0000	0.0000
CELF5	0.6806	-0.4877	1.0000	-0.1087	0.4112	-0.5760	0.0215	0.8058	1.0000	-0.2712	1.0000	0.1238	0.0078	1.1181
CELF6	1.0000	0.0524	0.7786	-0.1419	0.3949	0.3309	0.5609	0.1675	1.0000	0.2381	1.0000	0.0565	1.0000	0.0814
CELSR1	1.0000	-0.0048	0.0012	-0.2507	0.6104	0.1292	1.0000	-0.0149	1.0000	0.1303	0.7284	-0.1031	1.0000	-0.0088
CELSR3	0.0002	-0.8123	0.0002	-0.5415	0.9102	0.0956	0.0343	0.2917	1.0000	-0.1141	0.8785	0.1696	0.9886	0.0879
CEMIP	0.0072	-2.4521	0.0000	-1.5204	0.0504	1.3379	0.0000	1.5449	1.0000	0.6742	0.0062	1.6333	0.0000	0.8905
CEND1	1.0000	0.0000	1.0000	2.2507	0.4809	3.6101	1.0000	2.3241	1.0000	0.0000	1.0000	2.3455	1.0000	-1.3084

CENPA	0.1992	-0.8858	0.3734	0.4746	1.0000	0.0561	1.0000	0.0961	1.0000	-0.3436	0.1099	1.0257	0.9691	-0.2992
CENPC	0.0003	1.0331	0.0000	1.5542	0.0038	0.8025	0.1858	0.3409	1.0000	-0.2460	0.4791	0.2865	0.0012	-0.7021
CENPE	0.0000	1.5009	0.0000	1.7875	0.0000	1.1564	0.0000	0.5809	1.0000	-0.2215	0.9636	0.0776	0.0000	-0.7915
CENPF	0.0000	1.3874	0.0000	1.6747	0.0000	1.2082	0.0000	0.7547	1.0000	-0.2453	1.0000	0.0534	0.0000	-0.6938
CENPH	0.0002	0.7427	0.0000	0.8447	0.0964	0.4021	1.0000	-0.0198	1.0000	-0.1020	1.0000	0.0111	0.0002	-0.5181
CENPI	0.0000	1.8748	0.0000	2.2157	0.0000	1.7239	0.0000	1.3728	1.0000	-0.0015	0.2197	0.3500	0.2961	-0.3493
CENPK	0.0000	1.3525	0.0000	1.7355	0.0016	0.8892	0.0705	0.3626	1.0000	-0.2144	0.5879	0.1815	0.0000	-0.7349
CENPL	0.0001	1.6135	0.0000	2.4182	0.0002	1.4960	0.0607	0.8489	1.0000	-0.2397	0.1387	0.5755	0.0247	-0.8827
CENPM	0.0018	0.5697	0.0000	0.8685	1.0000	0.0057	0.0130	-0.2610	1.0000	-0.0723	0.0536	0.2386	0.0100	-0.3330
CENPN	0.0000	1.0548	0.0000	1.4684	0.4119	0.2450	0.8177	-0.0949	1.0000	-0.3758	1.0000	0.0495	0.0000	-0.7095
CENPO	0.0174	1.0654	0.0011	0.9973	0.0000	2.0410	0.0421	0.7407	1.0000	0.1077	1.0000	0.0522	0.0000	-1.1847
CENPP	0.0090	0.7086	0.0000	0.9325	0.8011	-0.1786	0.4334	-0.1841	1.0000	-0.2838	1.0000	-0.0486	0.4865	-0.2817
CENPQ	0.0011	0.6946	0.0000	0.7467	0.6164	0.1835	1.0000	-0.0369	1.0000	-0.1317	1.0000	-0.0682	0.0904	-0.3478
CENPT	0.0000	0.9416	0.0000	1.2593	1.0000	0.0636	1.0000	0.0129	1.0000	-0.0956	0.3460	0.2348	0.8554	-0.1382
CENPU	0.0002	0.9802	0.0000	1.5966	0.4786	0.2845	0.6446	-0.1981	1.0000	-0.4680	0.8785	0.1582	0.0001	-0.9476
CENPW	0.1098	0.6610	0.0000	1.4869	0.0198	0.8679	0.0154	0.7917	1.0000	-0.1589	0.0197	0.6797	0.9390	-0.2307
CEP104	0.0067	0.5627	0.0000	0.6151	0.0000	0.8816	0.0000	0.8675	1.0000	-0.1420	1.0000	-0.0786	0.5135	-0.1511
CEP120	0.0219	0.6195	0.0000	0.6924	0.0000	1.6105	0.0000	1.2535	1.0000	0.1802	0.4417	0.2670	0.6516	-0.1717
CEP128	0.0000	1.9895	0.0001	1.3436	0.0197	1.3134	1.0000	-0.0461	1.0000	0.2815	0.6888	-0.3529	0.0846	-1.0736
CEP131	1.0000	-0.0522	0.6571	-0.0696	0.0010	-0.6762	0.0000	-0.4276	1.0000	-0.1416	0.5245	-0.1466	0.8215	0.1128
CEP135	0.0000	0.9771	0.0000	1.4542	0.2833	0.3221	1.0000	0.0228	1.0000	-0.2901	0.5168	0.1986	0.0035	-0.5844
CEP152	0.0611	0.5343	0.0477	0.4276	0.0501	0.5539	0.0304	0.4716	1.0000	-0.0529	0.9773	-0.1486	0.9936	-0.1300
CEP162	0.4751	0.2345	1.0000	-0.0117	0.0416	0.4754	0.0075	0.4206	1.0000	-0.0048	0.4790	-0.2364	1.0000	-0.0530
CEP164	0.3675	0.3632	0.6049	0.1952	0.0120	0.8048	0.0007	0.6752	1.0000	0.0639	1.0000	-0.0917	1.0000	-0.0619
CEP170	0.0003	0.6659	0.0000	0.6500	0.0000	1.6826	0.0000	1.3749	1.0000	0.2059	0.3185	0.2034	0.8024	-0.0956
CEP170B	0.7811	0.1027	1.0000	-0.0002	0.5852	-0.1463	0.7025	-0.0655	1.0000	0.1350	1.0000	0.0449	0.0780	0.2207
CEP19	0.9282	-0.1036	0.5637	-0.1435	0.0013	-0.7687	0.3578	-0.1742	1.0000	-0.0423	1.0000	-0.0699	0.0071	0.5587
CEP192	0.0182	1.1632	0.0000	2.5908	0.0000	2.0612	0.0000	2.3888	1.0000	-1.2202	1.0000	0.2192	0.0020	-0.8865
CEP250	0.4161	0.2359	0.3710	0.1468	0.1963	0.3132	0.0134	0.3095	1.0000	0.0495	1.0000	-0.0272	1.0000	0.0494
CEP290	0.0938	0.4411	0.0000	0.8208	0.7098	0.1832	0.0460	0.3891	1.0000	-0.4225	1.0000	-0.0306	0.6583	-0.2145
CEP350	1.0000	-0.0869	0.5040	0.1462	0.0000	1.2111	0.0000	1.0955	1.0000	-0.0328	0.3648	0.2130	0.7237	-0.1432
CEP41	0.4562	-0.2636	0.9880	-0.0668	0.0022	-0.7932	0.0266	-0.4074	1.0000	-0.1633	1.0000	0.0457	0.6865	0.2285
CEP44	0.0211	0.8497	0.0000	1.0841	1.0000	-0.1241	0.6508	-0.1479	1.0000	-0.2563	1.0000	-0.0100	0.5135	-0.2707
CEP55	0.0000	1.0126	0.0000	1.3147	0.0663	0.4571	1.0000	0.0258	1.0000	-0.1891	0.9123	0.1233	0.0005	-0.6163
CEP57	0.4422	-0.2361	0.0345	-0.2930	0.8974	-0.1031	0.0031	-0.3810	1.0000	0.1193	1.0000	0.0745	0.6818	-0.1537
CEP57L1	1.0000	0.0677	1.0000	0.0623	0.0140	-0.8361	0.0840	-0.4493	1.0000	0.0657	1.0000	0.0719	0.3658	0.4584
CEP68	0.0040	-0.6106	0.0001	-0.5214	0.0055	-0.5871	0.0662	-0.2494	1.0000	-0.1629	1.0000	-0.0612	0.5802	0.1806
CEP70	0.0004	0.8001	0.0000	0.9676	0.2905	-0.3285	0.0568	-0.4160	1.0000	-0.2568	1.0000	-0.0788	0.2972	-0.3403
CEP76	0.2626	0.2735	0.4453	0.1281	0.0004	-0.7562	0.0000	-0.7549	1.0000	0.1131	1.0000	-0.0195	0.9408	0.1199
CEP78	0.3017	0.2410	0.8817	-0.0558	0.0068	-0.5829	0.0000	-1.0243	1.0000	0.1721	0.8607	-0.1113	0.3782	-0.2647
CEP83	0.0008	0.7102	0.0014	0.4484	0.2140	-0.3447	0.0015	-0.5122	1.0000	-0.0999	0.0558	-0.3488	0.4472	-0.2627
CEP85	0.0637	0.4230	0.0000	0.8107	0.1355	-0.3693	0.5206	-0.1487	1.0000	-0.3305	1.0000	0.0703	1.0000	-0.1045
CEP89	1.0000	0.0145	0.9732	-0.0644	0.2068	0.3656	0.0482	0.3686	1.0000	0.1696	1.0000	0.1044	0.7613	0.1778
CEP95	0.0000	1.2782	0.0000	1.4994	0.2511	-0.3604	0.2246	-0.2786	1.0000	-0.3158	1.0000	-0.0848	0.6298	-0.2322
CEP97	0.2656	0.4005	0.1526	0.3227	0.8104	0.1647	0.8477	0.1190	1.0000	0.0871	1.0000	0.0211	1.0000	0.0457
CEPT1	1.0000	0.0242	0.0973	-0.1801	1.0000	-0.0604	0.6941	-0.0687	1.0000	0.0291	0.4531	-0.1626	1.0000	0.0271
CER1	1.0000	0.0000	1.0000	0.0000	1.0000	2.2428	1.0000	2.3257	1.0000	0.0000	1.0000	0.0000	1.0000	0.0660
CERCAM	0.0566	-0.4353	0.0000	-0.4846	0.7191	-0.1382	0.0000	0.6894	1.0000	0.0662	1.0000	0.0293	0.0000	0.8983
CERK	0.0037	1.0393	0.0030	0.8349	0.0000	1.7368	0.0000	1.3698	1.0000	0.1718	1.0000	-0.0204	0.8297	-0.1909
CERKL	1.0000	2.1847	1.0000	2.2535	1.0000	0.0000	1.0000	0.0000	1.0000	0.0000	1.0000	0.0515	1.0000	0.0000
CERS1	0.1511	0.8803	0.0046	1.4979	0.0000	2.1596	0.0000	2.5510	1.0000	-0.8167	1.0000	-0.1871	0.3828	-0.4185
CERS2	0.4523	0.2322	0.0000	0.7011	0.0002	0.7594	0.0000	1.1323	1.0000	-0.2542	0.3181	0.2271	0.6703	0.1255
CERS3	1.0000	-0.0982	1.0000	-0.2400	0.9282	-0.2596	1.0000	-0.0534	1.0000	-0.1909	1.0000	-0.3195	1.0000	0.0199
CERS5	0.0081	0.4250	0.0000	0.4797	0.4539	-0.1724	1.0000	-0.0039	1.0000	-0.0706	1.0000	-0.0039	0.7287	0.1031
CERS6	0.6768	0.6219	0.6048	-0.6238	0.0145	1.6469	0.7220	0.5387	1.0000	0.1534	0.5279	-1.0838	0.3051	-0.9514
CETN2	0.0020	0.5759	0.0000	0.4760	0.3962	-0.2178	0.0000	-0.3857	1.0000	-0.0119	0.8607	-0.0994	0.3369	-0.1737
CETN3	0.8836	0.1040	0.3810	0.1090	0.0006	-0.7388	0.0000	-0.6624	1.0000	-0.1063	0.9047	-0.0892	1.0000	-0.0239
CETP	1.0000	0.0000	1.0000	0.0000	0.8033	3.0790	1.0000	2.3241	1.0000	0.0000	1.0000	0.0000	1.0000	-0.7782
CFAP20	0.8094	-0.0973	1.0000	-0.0148	0.0004	-0.6116	0.0001	-0.4871	1.0000	-0.1206	1.0000	-0.0264	1.0000	0.0096
CFAP221	1.0000	0.0000	1.0000	0.0000	1.0000	0.0000	1.0000	0.0000	1.0000	0.0000	1.0000	0.0000	1.0000	0.0000
CFAP36	0.0012	-0.5064	0.0000	-0.6789	0.0014	-0.4946	0.0000	-0.4275	1.0000	0.1059	1.0000	-0.0543	0.1700	0.1779
CFAP44	1.0000	-0.1505	1.0000	0.0371	1.0000	0.1955	1.0000	-0.0766	1.0000	-0.2029	1.0000	-0.0061	0.8920	-0.4713
CFAP45	0.1400	-2.3773	0.3882	-1.8677	0.2388	-1.6989	1.0000	0.0052	1.0000	-0.4735	1.0000	0.0533	0.8620	1.2343
CFAP52	1.0000	0.0000	1.0000	0.0000	1.0000	0.0000	1.0000	0.0000	1.0000	0.0000	1.0000	0.0000	1.0000	0.0000
CFAP54	0.0006	0.6778	0.0000	0.6445	0.3526	0.2419	0.9619	-0.0361	1.0000	-0.0340	1.0000	-0.0551	0.0072	-0.3069
CFAP57	1.0000	0.0486	1.0000	-0.3865	0.4702	1.1552	1.0000	0.3588	1.0000	1.0291	1.0000	0.6155	1.0000	0.2431
CFAP58	0.3699	4.2134	0.1545	2.3755	0.4809	3.6122	1.0000	-2.3199	1.0000	2.2735	1.0000	0.5281	0.7287	-3.6637
CFAP61	1.0000	0.8875	1.0000	0.5243	1.0000	0.3474	0.9096	-1.3721	1.0000	0.4094	1.0000	0.0604	1.0000	-1.3084
CFAP69	1.0000	0.8894	0.4312	1.1755	1.0000	-0.1811	1.0000	0.6991	1.0000	0.4094	1.0000	0.7123	0.9835	1.2979
CFAP74	1.0000	0.2619	1.0000	0.3847	1.0000	0.2403	1.0000	-0.3802	1.0000	-1.5310	0.3500	-1.4048	0.0823	-2.1525
CFAP97	1.0000	-0.0313	0.8956	-0.0586	1.0000	0.0220	0.3760	0.1334	1.0000	-0.1589	0.5842	-0.1740	1.0000	-0.0431
CFAP99	1.0000	0.2441	0.0794	1.1783	0.5070	0.7512	0.0303	1.3642	1.0000	-0.7082	1.0000	0.2364	1.0000	-0.0794
CFD	0.6987	0.2781	0.1304	-0.5370	0.0000	-5.7958	0.0000	-3.1429	1.0000	0.1270	0.1774	-0.6753	0.2614	2.7964
CFDP1	0.0002	-0.6566	0.0000	-0.5560	0.0085	-0.4799	0.6637	-0.0928	1.0000	-0.1852	1.0000	-0.0729	0.2409	0.2068
CFH	1.0000	-2.4776	1.0000	-0.4768	1.0000	-2.4056	0.1321	-4.3889	1.0000	1.9558	0.6427	4.1168	1.0000	0.0000
CFI	0.4940	0.4155	0.0178	0.7773	0.2395	-0.5865	0.2482	-0.5189	1.0000	-0.2159	1.0000	0.1619	1.0000	-0.1404
CFL2	0.0197	-0.3556	0.0000	-0.4774	0.0033	-0.4392	0.0000	-0.6317	1.0000	0.0459	0.9155	-0.0634	0.2241	-0.1413
CFLAR	0.3603	-0.2161	0.4253	-0.1537	0.5858	0.1513	0.0000	0.5700	1.0000	-0.1487	1.0000	-0.0728	0.0331	0.2765
CFTR	1.0000	0.0000	1.0000	0.0000	1.0000	0.0000	1.0000	0.0000	1.0000	0.0000	1.0000	0.0000	1.0000	0.0000
CGA	0.3168	2.2155	1.0000	0.5219	1.0000	-0.1624	0.4370	-3.6968	1.0000	1.2635	1.0000	-0.4066	1.0000	-2.2888
CGGBP1	0.0132	0.4455	0.0007	0.3369	0.3166	0.2365	0.0110	0.2570	1.0000	0.0948	1.0000	-0.0014	0.7287	0.1196
CGN	0.3918	-0.2788	1.0000	-0.0231	0.0000	-1.6109	0.0000	-1.5307	1.0000	-0.2268	1.0000	0.0411	1.0000	-0.1427
CGNL1	0.0606	0.4784	0.0087	0.5080	0.0215	0.5387	0.0000	0.5898	1.0000	-0.1200	1.0000	-0.0776	1.0000	-0.0634
CGREF1	1.0000	-0.0346	0.0296	-1.2851	0.0623	-1.2401	0.0153	-1.8585	1.0000	0.6762	0.8250	-0.5599	1.0000	0.0741
CGRRF1	0.3062	0.2706	0.4743	0.1191	0.8700	-0.1100	0.1245	-0.2079	1.0000	0.2520	0.9220	0.1114	0.6280	0.1591
CH25H	0.2602	1.8591	1.0000	0.0792	0.8033	-3.2636	1.0000	-0.6849	1.0000	1.0986	1.0000	-0.6583	0.7287	3.7384
CHAC1	0.7721	-0.1317	0.0022	0.2887	0.0001	-0.8430	0.0000	-0.5143	1.0000	-0.2533	0.3972	0.1803	1.0000	0.0825
CHAD	0.0000	1.8108	0.0000	1.9763	0.3227	0.3663	0.1268	0.4359	1.0000	-0.4521	0.2366	-0.2782	0.3100	-0.3770
CHADL	1.0000	0.3664	1.0000	0.2311	0.9425	0.3767	0.2803	-1.2153	1.0000	0.8411	0.9755	0.7246	0.9735	-0.7435
CHAF1A	0.0016	0.6518	0.0000	0.8688	1.0000	-0.0457	0.0308	-0.2702	1.0000	-0.0747	0.6218	0.1536	0.0741	-0.2937
CHAF1B	0.8366	0.1216	0.0680	0.2453	0.9260	0.0969	0.3234	-0.1541	1.0000	0.2326	0.0452	0.3704	1.0000	-0.0123
CHAMP1	1.0000	0.0365	0.0391	0.2158	1.0000	-0.0237	1.0000	-0.0032	1.0000	-0.0791	0.7990	0.1134	1.0000	-0.0530
CHAT	1.0000	0.0000	1.0000	0.0000	1.0000	0.0000	1.0000	0.0000	1.0000	0.0000	1.0000	0.0000	1.0000	0.0000

CHCHD1	0.3175	-0.2638	0.0000	-0.4417	0.0001	-0.8057	0.0000	-0.9133	1.0000	0.0316	0.7364	-0.1331	1.0000	-0.0700
CHCHD2	0.0005	-0.5480	0.0000	-0.5004	0.0000	-1.1177	0.0000	-0.8848	1.0000	-0.0902	1.0000	-0.0303	0.2715	0.1486
CHCHD3	1.0000	0.0575	0.6860	0.0640	0.0221	-0.4162	0.0000	-0.5773	1.0000	0.0313	1.0000	0.0499	0.6395	-0.1236
CHCHD4	1.0000	-0.0564	1.0000	-0.0086	0.0002	-0.6706	0.0000	-0.8262	1.0000	-0.1016	1.0000	-0.0410	0.2441	-0.2512
CHCHD6	0.0249	-0.4078	0.0019	-0.2962	0.0000	-0.7630	0.0000	-0.6484	1.0000	-0.0783	1.0000	0.0461	1.0000	0.0414
CHCHD7	0.0000	-0.8985	0.0000	-1.0556	0.0000	-1.2711	0.0000	-1.2659	1.0000	0.1233	1.0000	-0.0214	0.5289	0.1340
CHD1L	0.0000	1.0422	0.0000	1.5422	0.3442	-0.3593	0.1357	-0.3747	1.0000	-0.2809	0.4603	0.2308	0.5886	-0.2909
CHD2	0.0056	-0.4745	0.0000	-0.6217	0.1109	0.3061	1.0000	0.0294	1.0000	0.0846	1.0000	-0.0496	0.1765	-0.1874
CHD4	0.7620	-0.0983	0.6850	-0.0541	1.0000	-0.0191	0.0153	0.1787	1.0000	-0.0642	1.0000	-0.0075	0.3233	0.1386
CHD5	0.1793	0.3229	0.0936	0.3160	0.3745	0.2382	0.0008	0.5365	1.0000	-0.2900	0.2236	-0.2831	1.0000	0.0141
CHD7	0.0005	-0.6453	0.0000	-0.9296	0.8106	0.1177	1.0000	0.0072	1.0000	0.1870	1.0000	-0.0845	1.0000	0.0807
CHD9	0.0170	1.3750	0.0003	1.4571	0.0000	2.4733	0.0000	2.5893	1.0000	-0.1314	1.0000	-0.0383	1.0000	-0.0133
CHDC2	1.0000	0.0000	1.0000	0.0000	1.0000	0.0000	1.0000	0.0000	1.0000	0.0000	1.0000	0.0000	1.0000	0.0000
CHDH	0.3434	0.2899	0.2491	0.2188	0.0000	-1.1777	0.0000	-1.1787	1.0000	0.2777	0.5505	0.2208	0.5880	0.2817
CHEK1	0.0000	1.2217	0.0000	1.4566	0.0513	-0.5249	0.0022	-0.5909	1.0000	-0.3041	1.0000	-0.0572	0.3163	-0.3637
CHEK2	0.4102	0.3029	0.4160	0.1891	0.3616	-0.3198	0.0187	-0.4387	1.0000	0.2787	0.8620	0.1774	0.9287	0.1644
CHERP	0.9937	0.0808	0.0022	0.3109	0.0481	0.4614	0.0000	0.6294	1.0000	0.0716	0.0213	0.3138	0.1547	0.2453
CHFR	0.0792	0.3595	0.1971	0.1772	0.0117	0.4759	0.8866	0.0608	1.0000	0.1862	1.0000	0.0171	0.2096	-0.2228
CHGA	1.0000	0.0914	0.4752	-0.4562	0.1661	-1.0870	0.0000	-2.2790	1.0000	0.2797	1.0000	-0.2526	0.6108	-0.9027
CHGB	0.0003	1.3580	0.0005	0.9039	0.0006	1.1386	1.0000	0.0948	1.0000	0.2219	0.9504	-0.2209	0.0070	-0.8170
CHIC1	0.6881	0.1859	0.9958	0.0549	0.3988	0.2814	0.5923	0.1257	1.0000	0.1420	1.0000	0.0249	1.0000	-0.0081
CHIC2	0.3953	-0.3547	0.6580	-0.1680	0.9260	0.1285	0.5633	0.1601	1.0000	0.0248	0.8875	0.2250	1.0000	0.0625
CHID1	1.0000	-0.0168	0.2671	-0.1651	0.0000	-0.7511	0.0000	-0.8694	1.0000	0.1485	1.0000	0.0124	1.0000	0.0358
CHKA	0.0000	-0.9508	0.0000	-1.0935	0.0000	-0.7949	0.0000	-0.9718	1.0000	0.1364	1.0000	0.0059	1.0000	-0.0355
CHL1	0.0000	-2.8439	0.0000	-3.2464	0.2443	-0.3814	0.3816	0.1426	1.0000	-0.1019	0.5622	-0.4923	0.0131	0.4269
CHM	0.1377	0.4395	0.0363	0.3429	0.0004	0.7635	0.0006	0.5504	1.0000	0.1576	1.0000	0.0743	1.0000	-0.0507
CHMP1A	0.9034	-0.0761	0.3098	-0.1093	0.0007	-0.5395	0.0000	-0.3857	1.0000	-0.1876	0.1950	-0.2087	1.0000	-0.0284
CHMP1B	0.0200	-0.4027	0.0000	-0.3457	0.2694	-0.2223	0.0000	-0.3155	1.0000	0.0272	0.8016	0.0967	0.9503	-0.0606
CHMP2A	0.4318	-0.2449	1.0000	-0.0058	0.0110	0.5364	0.0000	0.6847	1.0000	-0.1903	1.0000	0.0621	1.0000	-0.0343
CHMP2B	0.0062	0.5675	0.0000	0.5396	0.3596	-0.2534	0.0000	-0.4595	1.0000	-0.0018	1.0000	-0.0175	0.2972	-0.2032
CHMP3	1.0000	0.0551	0.5480	-0.0803	0.3740	-0.2079	0.0147	-0.2255	1.0000	0.0115	0.6861	-0.1121	1.0000	-0.0004
CHMP4B	0.0975	-0.3110	0.0000	-0.4327	0.0005	-0.5807	0.0000	-0.5601	1.0000	0.0037	0.6816	-0.1056	1.0000	0.0304
CHMP4C	0.0012	1.8248	0.0054	1.2979	0.7445	-0.5660	0.0631	-1.6228	1.0000	0.1620	0.8820	-0.3520	0.8120	-0.8874
CHMP5	0.6612	-0.1383	0.6926	-0.0652	0.3526	-0.2141	0.0099	-0.2231	1.0000	-0.0249	1.0000	0.0606	1.0000	-0.0278
CHMP6	0.9996	0.0589	0.3752	0.0959	0.0085	-0.4625	0.3887	-0.0916	1.0000	0.0116	1.0000	0.0607	0.0001	0.3877
CHMP7	0.2816	-0.2089	0.0354	-0.2050	0.0442	-0.3369	0.2329	-0.1140	1.0000	-0.1202	0.8372	-0.1036	0.6231	0.1087
CHN2	0.0432	0.4362	0.0776	0.1575	0.3599	0.2382	0.4762	-0.0822	1.0000	-0.0540	0.0005	-0.3198	0.0001	-0.3682
CHODL	0.8226	3.0199	1.0000	0.0000	1.0000	2.2428	1.0000	0.0000	1.0000	0.0000	1.0000	-3.1429	1.0000	-2.2887
CHORDC1	0.9436	-0.0748	1.0000	-0.0200	0.2598	0.2430	0.0569	0.1985	1.0000	-0.0354	1.0000	0.0319	0.9631	-0.0751
CHP1	0.7008	-0.1146	0.3872	-0.0927	1.0000	0.0550	0.0650	0.1728	1.0000	0.1285	0.2687	0.1631	0.0410	0.2516
CHPF	0.0012	-0.6380	0.0000	-0.4421	0.4334	0.2220	0.0000	0.4248	1.0000	0.0335	0.1315	0.2411	0.1727	0.2413
CHPF2	0.9963	-0.0555	1.0000	-0.0084	0.0010	-0.5215	0.0000	-0.3866	1.0000	0.0959	0.2339	0.1553	0.0266	0.2359
CHRAC1	0.3631	0.2630	0.2385	0.2713	0.9176	0.1029	0.5454	-0.1744	1.0000	-0.0836	1.0000	-0.0624	0.1063	-0.3562
CHRD	0.4875	0.2503	0.0000	0.7060	0.3962	0.2829	0.0000	0.7977	1.0000	-0.1959	0.3978	0.2717	0.2138	0.3261
CHRDL1	0.2650	-0.2853	0.0000	-0.5925	0.0291	0.4414	0.0000	0.8149	1.0000	-0.1778	0.0027	-0.4721	0.2098	0.2026
CHRDL2	0.8819	0.3060	0.9148	-0.3502	0.0031	-2.2793	0.0873	-1.5425	1.0000	-0.4103	0.2444	-1.0572	1.0000	0.3308
CHRFAM7A	0.7625	0.9225	1.0000	0.4524	1.0000	0.1136	0.3342	-1.6813	1.0000	0.6450	1.0000	0.1909	0.9835	-1.1475
CHRM2	1.0000	0.0000	1.0000	0.0000	1.0000	0.0000	1.0000	0.0000	1.0000	0.0000	1.0000	0.0000	1.0000	0.0000
CHRM3	0.8226	3.0199	1.0000	-2.3959	0.1614	4.2993	0.9167	1.3833	1.0000	2.2674	1.0000	-3.1429	1.0000	-0.6160
CHRM4	0.0000	-2.8967	0.0004	-1.5060	0.0097	-1.6733	0.1032	-0.7148	1.0000	-0.1268	0.4461	1.2816	0.4460	0.8410
CHRM5	0.0000	1.5847	0.0000	0.8240	0.0069	-1.0166	0.0001	-1.0377	1.0000	0.2723	0.0333	-0.4744	1.0000	0.2578
CHRNA1	0.0000	-1.0081	0.0000	-1.1564	0.8369	-0.0833	0.0000	-0.4242	1.0000	0.1324	1.0000	-0.0039	0.0394	-0.2035
CHRNA10	1.0000	0.0000	1.0000	-2.3986	1.0000	0.0000	1.0000	-2.3199	1.0000	2.2736	1.0000	0.0000	1.0000	0.0000
CHRNA2	0.0000	2.6887	0.3729	0.5507	0.0000	2.7951	0.8621	-0.2836	1.0000	1.1421	0.0362	-0.9814	0.0000	-1.9262
CHRNA3	0.0779	-0.4256	0.0000	-0.8487	0.0000	-1.0025	0.0000	-1.1658	1.0000	0.3386	1.0000	-0.0710	0.7051	0.1800
CHRNA4	1.0000	-2.4776	0.7666	-3.2534	1.0000	0.6783	0.7701	-3.1666	1.0000	0.7365	1.0000	0.0000	1.0000	-3.1344
CHRNA5	0.0155	1.1208	0.4430	0.4130	0.3772	0.5552	0.7338	0.2745	1.0000	0.1072	0.3460	-0.5907	1.0000	-0.1702
CHRNA6	0.8226	-3.3425	1.0000	0.0000	0.8011	-3.2629	1.0000	0.0000	1.0000	-3.2304	1.0000	0.0000	1.0000	0.0000
CHRNA9	1.0000	0.0000	1.0000	-2.3986	0.8011	3.0840	1.0000	-2.3199	1.0000	2.2736	1.0000	0.0000	1.0000	-3.1344
CHRNB2	0.0038	-1.0662	0.0000	-1.1307	0.1130	-0.6127	1.0000	-0.0489	1.0000	0.0859	1.0000	0.0354	0.0049	0.6545
CHRNB3	1.0000	0.0000	0.4431	-3.7865	1.0000	2.2428	0.4370	-3.6968	1.0000	3.6396	1.0000	0.0000	1.0000	-2.2888
CHRNB4	0.2457	0.7060	0.2181	0.7388	0.8928	-0.3007	1.0000	-0.0387	1.0000	-0.2479	1.0000	-0.1968	1.0000	0.0240
CHRND	0.0000	-1.2190	0.0000	-1.1142	0.4913	-0.1528	0.0011	-0.3083	1.0000	-0.0594	1.0000	0.0576	0.1026	-0.2093
CHRNG	0.0000	-1.1294	0.0000	-1.3281	0.0000	-1.4010	0.0000	-1.4706	1.0000	-0.0547	0.0324	-0.2409	0.6205	-0.1187
CHST1	0.0000	-3.0567	0.0000	-3.5713	0.0000	1.4397	0.0000	1.7216	1.0000	-0.5974	0.9957	-1.1063	0.5740	-0.3097
CHST10	0.0000	1.2029	0.0000	1.1118	0.1332	0.4212	0.1586	0.2860	1.0000	0.0509	1.0000	-0.0280	1.0000	-0.0785
CHST11	1.0000	0.1878	0.0181	1.4737	0.0412	1.1900	0.0088	1.5676	1.0000	-0.8807	0.9491	0.4143	0.6424	-0.5002
CHST12	0.1109	0.4812	0.0001	0.6897	0.0675	0.5254	0.0035	0.5521	1.0000	0.0501	0.4632	0.2724	1.0000	0.0856
CHST13	0.0005	-1.2472	0.0000	-2.2245	0.0124	0.7198	0.0000	1.4662	1.0000	-0.1454	0.0521	-1.1121	0.0067	0.6050
CHST14	0.1847	0.2608	0.0632	0.1678	0.1721	-0.2665	0.2412	-0.1164	1.0000	0.1138	1.0000	0.0331	0.0155	0.2690
CHST15	0.3095	-0.8800	0.0002	-1.5181	0.0530	0.9741	0.0477	0.6422	1.0000	0.9672	1.0000	0.3447	0.1485	0.6408
CHST2	1.0000	0.0058	1.0000	0.0431	0.0226	-0.5413	0.0170	-0.3833	1.0000	-0.1701	0.9773	-0.1199	1.0000	-0.0048
CHST3	0.0195	-0.4057	0.0250	-0.2309	0.0332	0.3794	0.0000	0.3849	1.0000	-0.0824	0.8496	0.1047	0.9701	-0.0714
CHST4	0.9536	-0.5386	0.9148	-1.5313	0.4695	-1.5338	1.0000	0.3951	1.0000	-1.4736	0.4857	-2.4818	1.0000	0.4623
CHST8	1.0000	2.1903	1.0000	0.0000	1.0000	0.0000	1.0000	0.0000	1.0000	0.0000	1.0000	-2.2992	1.0000	0.0000
CHST9	1.0000	-0.0227	0.5417	-0.5877	0.7662	0.4706	0.0293	1.0336	1.0000	0.2766	1.0000	-0.2755	0.2085	0.8464
CHSY1	0.0336	-0.3973	0.0011	-0.3067	0.4575	0.1780	0.0689	0.1746	1.0000	0.1223	0.1932	0.2256	0.5560	0.1239
CHTF18	0.4875	0.1978	0.0000	0.5223	1.0000	0.0300	0.0034	0.3822	1.0000	-0.2728	1.0000	0.0628	1.0000	0.0849
CHTF8	0.0016	0.7402	0.0000	0.7965	1.0000	-0.0510	0.0000	-0.8455	1.0000	-0.0591	1.0000	0.0080	0.0087	-0.8496
CHTOP	0.0528	-0.3103	0.0002	-0.2949	0.0000	-0.6776	0.0000	-0.4600	1.0000	-0.1278	0.7310	-0.1002	0.7287	0.0953
CHUK	0.0384	-0.3359	0.0000	-0.4519	0.0000	-0.6814	0.0000	-0.5170	1.0000	0.0058	0.7702	-0.0979	0.1937	0.1755
CHURC1	0.0000	-0.9082	0.0000	-0.8630	0.0000	-1.0285	0.0000	-0.6401	1.0000	-0.1305	1.0000	-0.0731	0.0342	0.2637
CIAO1	0.0416	-0.3954	0.0034	-0.3418	0.0000	-0.8462	0.0000	-0.7121	1.0000	-0.1410	1.0000	-0.0750	1.0000	0.0003
CIAPIN1	0.0078	-0.4171	0.0000	-0.3821	0.0066	-0.4287	0.0000	-0.3335	1.0000	-0.0400	1.0000	0.0073	0.9836	0.0603
CIART	0.0000	-2.1647	0.0000	-2.1665	0.0002	-1.0500	0.0276	-0.3253	1.0000	0.2206	1.0000	0.2331	0.0000	0.9500
CIB1	0.0122	-0.4179	0.0000	-0.3828	0.0014	-0.5220	0.0003	-0.2980	1.0000	-0.0724	1.0000	-0.0252	0.3073	0.1567
CIB2	0.0832	0.5833	0.0967	0.4375	0.0059	-1.0176	0.0179	-0.6804	1.0000	-0.0042	1.0000	-0.1397	0.7783	0.3383
CIB3	0.7815	-0.4699	0.4301	-0.8652	0.4205	-0.8796	0.4641	-0.8626	1.0000	-0.1316	1.0000	-0.5162	1.0000	-0.1082
CIDEA	0.0086	-0.4479	0.0000	-0.7583	0.0131	-0.4561	0.0000	-0.6703	1.0000	0.2499	1.0000	-0.0476	1.0000	0.0402
CIDEC	0.8366	0.8162	0.0234	-2.3056	0.0000	3.3666	0.0311	1.1855	1.0000	2.0080	0.9744	-1.0996	1.0000	-0.1660
CIITA	0.9545	1.0672	0.9947	0.7546	1.0000	0.6780	0.9786	0.9238	1.0000	0.7402	1.0000	0.4472	1.0000	0.9919
CILP	1.0000	0.0910	0.0036	-0.7084	0.0000	1.2974	0.0000	0.9060	1.0000	0.0643	0.0319	-0.7228	0.1777	-0.3229
CILP2	0.2692	0.7795	0.3561	0.6698	0.0019	1.4939	0.0121	1.5651	1.0000	-0.3745	0.8915	-0.4708	1.0000	-0.2954

CINP	0.1783	-0.3551	0.0362	-0.3239	0.0322	-0.5135	0.0000	-0.6588	1.0000	-0.0684	1.0000	-0.0249	0.6435	-0.2078
CIPC	0.0524	0.5289	0.0001	0.6714	0.0081	0.6673	0.0000	0.7760	1.0000	-0.2263	1.0000	-0.0714	0.9934	-0.1130
CIRBP	0.0534	-0.3328	0.0000	-0.3789	0.0000	-0.8460	0.0000	-0.7370	1.0000	-0.0397	0.8171	-0.0734	0.7104	0.0751
CIRH1A	1.0000	0.0273	0.0004	0.3583	0.0000	-0.9916	0.0000	-0.5596	1.0000	-0.2610	0.9846	0.0814	0.4840	0.1766
CISD1	0.0076	-0.4753	0.0000	-0.3889	1.0000	-0.0586	0.5844	0.0633	1.0000	-0.0635	1.0000	0.0355	0.9016	0.0642
CISD2	0.0237	0.3980	0.0052	0.3122	0.5710	-0.1596	0.4755	-0.1095	1.0000	0.0658	1.0000	-0.0083	0.7112	0.1214
CISD3	0.0002	-0.6914	0.0016	-0.3853	0.0000	-1.3177	0.0000	-0.7552	1.0000	-0.1492	0.7364	0.1698	0.1182	0.4204
CISH	0.1384	-0.2644	0.5962	-0.0602	0.0000	-0.7536	0.0000	-0.5427	1.0000	-0.0391	0.0976	0.1775	0.1414	0.1767
CIT	0.0000	1.8243	0.0000	2.7786	0.0000	2.4340	0.0000	1.9415	1.0000	-0.2906	0.0021	0.6751	0.0001	-0.7787
CITED2	0.0039	0.8001	0.0033	0.6108	0.4685	0.2938	0.4197	0.2637	1.0000	-0.0134	0.8553	-0.1878	1.0000	-0.0363
CITED4	0.0673	-0.6054	0.0087	-0.6027	0.0124	-0.7645	0.0252	-0.5642	1.0000	0.0089	1.0000	0.0232	0.9631	0.2137
CIZ1	0.1211	0.2756	0.0031	0.2688	0.0012	-0.5557	0.0000	-0.3710	1.0000	-0.0943	0.8676	-0.0886	0.8486	0.0969
CKAP2	0.0000	0.7954	0.0000	1.1878	0.0108	0.4803	1.0000	0.0096	1.0000	-0.3042	0.8875	0.1008	0.0000	-0.7695
CKAP2L	0.0000	0.7359	0.0000	1.0950	0.0651	0.3822	0.2800	0.1704	1.0000	-0.1308	0.1671	0.2398	0.0445	-0.3371
CKAP4	0.0009	0.5220	0.0000	0.6630	0.0000	1.3038	0.0000	1.2671	1.0000	0.1152	0.0134	0.2689	0.8026	0.0838
CKB	0.0000	-1.9474	0.0000	-1.9921	0.0000	-1.1115	0.0000	-1.2773	1.0000	0.2662	0.0257	0.2336	0.5617	0.1051
CKLF	0.0008	-0.5109	0.0000	-0.3877	0.0152	-0.4057	0.0045	-0.2360	1.0000	-0.0126	0.5510	0.1228	0.3237	0.1625
CKMT2	0.2092	-2.6409	0.0410	-4.9481	0.2858	-2.4984	0.3356	-1.6813	1.0000	0.0858	1.0000	-2.2992	1.0000	0.9149
CKS1B	0.5560	-0.1521	0.0611	0.2232	0.0005	-0.6360	0.0000	-0.5336	1.0000	-0.3904	1.0000	-0.0032	0.1421	-0.2821
CKS2	0.0220	0.6029	0.0000	0.7118	1.0000	-0.0928	0.0133	-0.4084	1.0000	-0.3094	0.7147	-0.1896	0.0003	-0.6195
CLASP1	0.1561	0.3230	0.0041	0.4109	0.0000	0.8986	0.0000	0.8387	1.0000	-0.0126	1.0000	0.0884	1.0000	-0.0670
CLASP2	0.0835	0.3029	0.3959	0.1053	0.0620	0.3252	0.0268	0.2144	1.0000	0.2297	1.0000	0.0452	0.5159	0.1243
CLCA2	0.7973	0.4647	1.0000	0.3513	1.0000	0.2331	0.5912	0.6224	1.0000	-0.4668	0.9002	-0.5737	1.0000	-0.0777
CLCC1	0.0892	0.3681	0.0009	0.4564	1.0000	0.0120	1.0000	-0.0223	1.0000	-0.1616	1.0000	-0.0627	0.4627	-0.1914
CLCF1	0.4459	-0.7851	0.0254	-1.5424	0.2134	-1.1184	1.0000	0.0737	1.0000	-0.2590	0.6178	-1.0043	0.4340	0.9408
CLCN1	0.0030	1.7215	0.0040	1.2912	0.0001	1.9907	0.0626	0.9591	1.0000	0.4880	1.0000	0.0630	0.4482	-0.5405
CLCN2	0.0000	-3.8422	0.0000	-2.6172	1.0000	0.0888	1.0000	-0.0488	1.0000	-0.2090	0.8278	1.0293	0.6110	-0.3382
CLCN3	0.5412	0.1519	0.4093	0.0894	0.8768	0.0842	0.0480	0.1612	1.0000	0.1251	0.9301	0.0750	0.0834	0.2067
CLCN4	0.9545	0.2770	0.2559	0.8107	0.0833	1.0667	0.0114	1.3783	1.0000	-1.0723	0.8553	-0.5198	0.2582	-0.7526
CLCN5	0.1009	0.3254	0.1309	0.2021	0.0000	-0.8709	0.0000	-0.6641	1.0000	0.0478	1.0000	-0.0632	0.3096	0.2606
CLCN6	0.0015	0.6636	0.0000	0.6869	0.0228	0.5057	0.0001	0.4972	1.0000	-0.0585	1.0000	-0.0219	1.0000	-0.0600
CLCN7	0.5414	0.1605	0.0442	0.1973	0.4411	-0.1863	0.4488	-0.0966	1.0000	-0.1071	1.0000	-0.0585	1.0000	-0.0122
CLDN1	0.5086	-3.8790	0.7666	-3.2534	0.4809	-3.7967	1.0000	0.0066	1.0000	-0.6504	1.0000	0.0000	1.0000	3.2066
CLDN10	0.0022	1.7354	1.0000	0.0297	0.3900	-0.9548	0.1204	-1.2146	1.0000	0.5211	0.0626	-1.1715	1.0000	0.2674
CLDN11	0.1320	-3.0378	0.0904	-1.9625	0.6435	0.7790	0.1697	0.9912	1.0000	0.3194	1.0000	1.4336	0.7523	0.5339
CLDN12	0.0014	0.6602	0.0022	0.4034	0.0095	0.5671	0.0008	0.4104	1.0000	0.0036	0.3057	-0.2405	0.6865	-0.1487
CLDN14	1.0000	0.0000	1.0000	2.2535	1.0000	0.0000	1.0000	0.0000	1.0000	0.0000	1.0000	2.3480	1.0000	0.0000
CLDN16	1.0000	0.0000	1.0000	0.0000	1.0000	0.0000	1.0000	0.0000	1.0000	0.0000	1.0000	0.0000	1.0000	0.0000
CLDN18	1.0000	-2.4776	1.0000	0.0000	1.0000	-2.4056	1.0000	0.0000	1.0000	-2.3757	1.0000	0.0000	1.0000	0.0000
CLDN19	1.0000	0.0000	1.0000	0.0000	1.0000	0.0000	1.0000	0.0000	1.0000	0.0000	1.0000	0.0000	1.0000	0.0000
CLDN2	0.6877	-2.0809	1.0000	-0.1612	0.2785	-4.1857	1.0000	-0.8416	1.0000	-1.0389	1.0000	0.8994	1.0000	2.3554
CLDN20	1.0000	0.1184	0.2899	1.3372	0.7660	-1.4098	1.0000	-0.5239	1.0000	-0.8154	1.0000	0.4110	1.0000	0.0730
CLDN23	1.0000	0.0000	1.0000	-2.3959	1.0000	2.2427	1.0000	-2.3176	1.0000	2.2674	1.0000	0.0000	1.0000	-2.2888
CLDN25	0.3027	-1.4644	0.1528	-1.1537	0.0504	1.5941	0.2528	0.7534	1.0000	0.6830	1.0000	1.0164	1.0000	-0.1577
CLDN3	1.0000	-0.2931	1.0000	-2.3959	1.0000	-0.1597	1.0000	-2.3176	1.0000	-0.1089	1.0000	-2.2957	1.0000	-2.2909
CLDN5	0.9650	1.0594	1.0000	0.6959	0.9361	1.2045	0.6172	1.7722	1.0000	-0.1034	1.0000	-0.4672	1.0000	0.4634
CLDND1	0.0240	-0.3719	0.0000	-0.3631	0.6146	0.1259	0.1892	0.1249	1.0000	0.0818	0.7069	0.1028	0.7529	0.0861
CLEC16A	0.1905	0.6214	0.0050	0.8408	0.0108	1.0286	0.2200	0.4665	1.0000	0.0439	0.8717	0.2748	0.2570	-0.5185
CLEC19A	0.0363	-1.2501	0.5796	-0.3998	0.2067	-0.7936	0.0303	-1.1101	1.0000	-0.3588	0.9188	0.5047	0.6285	-0.6676
CLEC3A	1.0000	2.1903	1.0000	2.2535	1.0000	0.0000	1.0000	2.3257	1.0000	0.0000	1.0000	0.0489	1.0000	2.3554
CLEC3B	0.0000	-1.1959	0.0000	-1.5004	0.0000	-1.0684	0.0035	-0.4781	1.0000	0.3603	1.0000	0.0683	0.0000	0.9531
CLGN	0.9671	-1.6906	1.0000	2.2535	1.0000	-0.7136	0.7710	3.1753	1.0000	-3.7658	1.0000	0.0489	1.0000	0.0727
CLIC2	1.0000	0.0512	0.3993	0.0787	0.0000	-0.7218	0.0000	-0.5157	1.0000	-0.0164	1.0000	0.0235	0.0956	0.1952
CLIC3	0.9712	0.1111	0.3594	-0.3045	0.0044	-0.8092	0.0000	-1.1589	1.0000	-0.0500	0.1774	-0.4541	0.4850	-0.3952
CLIC4	0.0010	-0.4847	0.0000	-0.3721	0.1117	-0.2594	0.0049	-0.1885	1.0000	-0.0276	0.7563	0.0971	0.9746	0.0485
CLIC5	0.0000	-6.5317	0.3091	-1.2756	0.9743	-0.3501	1.0000	-0.1271	1.0000	-0.9817	0.5389	4.4097	0.6491	-0.7527
CLIC6	1.0000	-1.1562	1.0000	-0.9972	1.0000	-1.0191	1.0000	0.0074	1.0000	-0.1210	1.0000	0.0515	1.0000	0.9168
CLINT1	1.0000	-0.0139	0.9952	-0.0272	0.0080	0.4549	0.0215	0.1888	1.0000	0.0527	1.0000	0.0520	0.0728	-0.2076
CLIP1	0.5897	-0.1361	0.0181	-0.2264	0.9495	0.0650	0.5057	-0.0847	1.0000	0.0423	1.0000	-0.0349	0.6687	-0.1015
CLIP2	0.6846	-0.1401	0.0766	0.1934	0.0002	0.6531	0.0000	1.0548	1.0000	-0.1226	0.2426	0.2245	0.0099	0.2843
CLIP4	0.1235	0.4079	0.0056	0.4250	0.7016	-0.1765	0.2127	0.2385	1.0000	0.0909	0.9838	0.1216	0.0130	0.5117
CLK1	0.0122	-0.4256	0.0000	-0.5416	0.0141	-0.4181	0.0442	-0.1863	1.0000	0.0081	0.9375	-0.0955	0.0676	0.2443
CLK2	0.2113	-0.2815	0.0139	-0.3308	0.1749	-0.2961	0.0510	-0.2650	1.0000	0.0088	1.0000	-0.0288	1.0000	0.0444
CLK3	0.1585	-0.2695	0.0021	-0.3063	0.1397	-0.2818	0.2501	-0.1263	1.0000	-0.0496	1.0000	-0.0739	0.6608	0.1117
CLK4	0.0000	-0.6695	0.0000	-0.8128	0.0839	-0.3170	0.4333	-0.0992	1.0000	0.0328	0.9375	-0.0978	0.0540	0.2565
CLMN	0.0001	0.9499	0.0000	0.7145	0.0725	0.5091	0.1613	0.2682	1.0000	-0.3527	0.0001	-0.5761	0.0006	-0.5882
CLMP	0.0000	1.7247	0.0000	2.1308	0.0000	1.4340	0.0000	1.8439	1.0000	-0.0835	0.1745	0.3343	0.1519	0.3307
CLN5	0.0002	0.8961	0.0000	0.7283	0.0154	0.6061	0.3121	0.2359	1.0000	0.1466	1.0000	-0.0100	0.5075	-0.2197
CLN6	1.0000	-0.0420	0.6740	0.0996	0.0065	-0.5531	0.2060	-0.2006	1.0000	-0.2049	1.0000	-0.0521	0.6286	0.1524
CLN8	0.6824	0.1371	0.0041	0.2913	0.0870	0.3426	0.0019	0.3043	1.0000	-0.0485	0.8269	0.1177	0.9603	-0.0824
CLNS1A	0.5769	0.1450	0.0470	0.1911	0.0631	-0.3405	0.0016	-0.2896	1.0000	0.0013	1.0000	0.0601	1.0000	0.0578
CLOCK	1.0000	-0.0453	0.0341	-0.2983	0.0331	0.4380	0.0004	0.4511	1.0000	0.0909	0.8247	-0.1486	0.8553	0.1091
CLP1	0.4494	-0.2234	0.9069	-0.0597	0.0136	-0.5418	0.1403	-0.2859	1.0000	-0.0790	1.0000	0.0968	0.7640	0.1821
CLPP	1.0000	0.0000	1.0000	0.0000	1.0000	0.0000	1.0000	0.0000	1.0000	0.0000	1.0000	0.0000	1.0000	0.0000
CLPS	1.0000	-0.2892	1.0000	0.2173	1.0000	-0.1630	0.4638	-3.6898	1.0000	1.2548	1.0000	1.8106	1.0000	-2.2888
CLPTM1	0.1758	0.4423	0.0000	0.8426	0.0071	0.6646	0.0000	0.8439	1.0000	-0.3391	1.0000	0.0752	0.5438	-0.1525
CLPTM1L	0.8985	0.0795	0.2384	0.1574	1.0000	0.0232	0.5621	0.0935	1.0000	-0.0095	0.9979	0.0804	0.9885	0.0655
CLPX	0.2793	0.2193	0.8617	0.0383	0.4335	-0.1792	0.0000	-0.3606	1.0000	0.1254	1.0000	-0.0432	1.0000	-0.0509
CLRN1	0.4334	-0.5420	0.0129	-1.1367	0.7664	-0.3073	0.3242	0.4555	1.0000	-0.1652	0.5846	-0.7486	0.3569	0.6014
CLRN2	0.8858	-0.3517	0.1784	-0.8038	0.1507	-1.0669	0.0330	-1.1616	1.0000	0.3708	1.0000	-0.0679	1.0000	0.2816
CLRN3	1.0000	0.6109	0.7819	0.7709	1.0000	-0.5733	0.4370	-3.6968	1.0000	-0.5112	1.0000	-0.3427	0.7287	-3.6637
CLSPN	0.0109	0.8458	0.0000	1.3496	0.0033	0.9221	0.0063	0.6260	1.0000	-0.3452	0.8810	0.1703	0.0034	-0.6351
CLSTN3	0.2702	0.5942	0.2946	0.4348	1.0000	0.0111	0.6541	-0.2971	1.0000	0.1707	1.0000	0.0243	1.0000	-0.1300
CLTA	0.2134	0.2786	0.0003	0.3562	0.0059	-0.5502	0.0000	-0.6332	1.0000	-0.0213	1.0000	0.0677	0.8686	-0.0983
CLTB	0.0000	-0.6678	0.0000	-0.5860	0.0001	-0.6039	0.0000	-0.4043	1.0000	-0.0723	1.0000	0.0218	0.4447	0.1324
CLTCL1	0.8905	-0.0778	0.8716	-0.0385	0.6822	0.1165	0.0000	-0.3759	1.0000	-0.1128	1.0000	-0.0611	0.0000	-0.5997
CLU	0.4033	-0.2595	0.2970	-0.1196	0.0008	-0.7818	0.5022	-0.0794	1.0000	0.0868	0.1188	0.2400	0.0000	0.7936
CLUAP1	0.1184	0.4666	0.0023	0.5331	0.9383	0.1157	1.0000	0.0215	1.0000	-0.1687	1.0000	-0.0909	0.4850	-0.2572
CLUH	1.0000	0.0150	0.0325	0.1967	0.5333	0.1507	0.6666	-0.0700	1.0000	-0.0086	0.2100	0.1856	0.1141	-0.2238
CLUL1	0.7859	0.5607	0.0010	3.1274	0.0792	-2.1067	0.7710	-3.1693	1.0000	-2.5672	1.0000	-0.0020	0.7287	-3.6634
CLVS1	1.0000	0.5864	0.2445	1.3532	0.2725	1.6862	1.0000	-0.3802	1.0000	0.7970	0.5164	1.5864	0.7040	-1.2668
CLVS2	1.0000	2.1847	1.0000	0.0000	0.5080	3.6209	0.7710	3.1708	1.0000	0.0000	1.0000	-2.2957	1.0000	-0.4617
CLYBL	0.4785	-0.5048	0.0618	-0.7911	1.0000	0.1292	0.2185	-0.5589	1.0000	0.0545	1.0000	-0.2214	0.3693	-0.6259

CMAS	0.6443	0.2016	0.0044	0.4208	0.3159	0.3089	0.0933	0.2735	1.0000	-0.1347	1.0000	0.0966	0.6795	-0.1640
CMC1	0.0450	-0.4548	0.0000	-0.5133	0.3637	-0.2408	0.0685	-0.2399	1.0000	0.0363	1.0000	-0.0097	1.0000	0.0425
CMC2	1.0000	-0.0535	0.4270	0.1626	0.2088	-0.3975	0.0499	-0.3126	1.0000	-0.1698	1.0000	0.0594	1.0000	-0.0783
CMC4	0.0658	-0.4296	0.0000	-0.6159	0.0012	-0.6835	0.0002	-0.3950	1.0000	0.0958	1.0000	-0.0784	0.0423	0.3911
CMIP	0.1550	0.4327	0.0003	0.7047	0.2099	0.3806	0.0023	0.5890	1.0000	-0.3393	1.0000	-0.0574	0.9443	-0.1258
CMKLR1	0.0000	-2.5178	0.0000	-2.0354	0.0011	0.5432	0.2039	0.1698	1.0000	-0.2474	0.5622	0.2479	0.0000	-0.6156
CMPK1	0.0188	0.4959	0.0000	0.5808	0.0000	0.8724	0.0000	0.6930	1.0000	0.0813	0.5245	0.1805	0.8742	-0.0910
CMPK2	1.0000	-0.0101	1.0000	-0.0140	0.0000	1.3711	0.0000	1.4458	1.0000	0.0533	1.0000	0.0625	1.0000	0.1362
CMSS1	1.0000	-0.0446	0.3757	0.1640	1.0000	-0.0472	0.0405	0.2920	1.0000	-0.1125	1.0000	0.1070	0.4031	0.2341
CMTM3	0.0004	0.9346	0.0000	1.1493	0.0001	0.9994	0.0004	0.6260	1.0000	0.0435	0.3603	0.2717	0.1854	-0.3229
CMTM4	0.0000	1.2729	0.0000	1.2749	0.0000	1.6326	0.0000	1.2562	1.0000	0.2735	0.2872	0.2885	1.0000	-0.0972
CMTM7	0.0000	0.8959	0.0000	0.5235	0.7680	-0.1012	0.2606	0.1151	1.0000	0.1384	0.0291	-0.2217	0.0004	0.3599
CMTM8	0.1947	-1.0386	0.0086	-1.7945	1.0000	0.1612	1.0000	0.1315	1.0000	-0.0663	0.8820	-0.8102	1.0000	-0.0884
CMTR1	0.3130	0.2266	0.0009	0.3216	0.5365	0.1687	0.0001	0.3783	1.0000	-0.1047	1.0000	0.0027	0.7287	0.1095
CMTR2	0.5805	0.1962	0.4524	0.1376	0.1834	0.3374	0.1494	0.2163	1.0000	0.0968	1.0000	0.0503	1.0000	-0.0195
CMYA5	0.0000	-2.8497	0.0000	-3.1993	0.5634	-0.1650	0.0079	-0.3587	1.0000	0.1781	1.0000	-0.1608	1.0000	-0.0100
CNBP	0.2141	0.2516	0.0083	0.1768	0.0000	-0.9534	0.0000	-0.8953	1.0000	-0.0110	0.8212	-0.0735	0.9641	0.0529
CNDP1	0.0077	1.1173	0.0000	1.4128	0.0000	-3.7503	0.0008	-1.8819	1.0000	-0.2888	1.0000	0.0189	0.5565	1.5862
CNDP2	1.0000	0.0175	0.9727	0.0424	0.0130	-0.4421	0.0000	-0.5163	1.0000	-0.1209	0.9436	-0.0838	0.2936	-0.1898
CNEP1R1	0.0111	0.7737	0.7890	0.1354	0.0074	0.7873	0.4632	0.2364	1.0000	0.4180	0.8607	-0.2087	1.0000	-0.1306
CNGA1	1.0000	0.0000	1.0000	0.0000	1.0000	0.0000	1.0000	0.0000	1.0000	0.0000	1.0000	0.0000	1.0000	0.0000
CNGA2	0.0250	-0.5474	0.0000	-0.6323	0.0000	-1.2379	0.0000	-0.9601	1.0000	0.0809	1.0000	0.0094	0.1163	0.3653
CNGA3	0.3843	-1.3025	0.7150	-0.3285	0.1282	-2.9014	0.3945	-0.5710	1.0000	1.7408	0.0031	2.7404	0.0008	4.0904
CNGA4	0.0000	-3.4190	0.0000	-3.5846	0.0000	-1.1820	0.0000	-1.0750	1.0000	-0.3464	0.8306	-0.5003	0.7226	-0.2356
CNGB1	1.0000	-0.3257	1.0000	0.6988	1.0000	0.7317	0.6172	1.7729	1.0000	-0.9614	1.0000	0.0598	1.0000	0.0775
CNGB3	0.6936	1.4536	1.0000	-2.3959	1.0000	0.6783	0.2460	2.3284	1.0000	-0.1078	0.6450	-4.0607	0.7908	1.5489
CNIH1	0.4871	-0.1519	0.9907	-0.0265	0.0145	-0.4016	0.0002	-0.2950	1.0000	-0.0910	1.0000	0.0467	1.0000	0.0214
CNIH3	1.0000	2.1847	0.7666	3.0922	1.0000	0.0000	1.0000	2.3257	1.0000	0.0000	1.0000	0.9006	1.0000	2.3554
CNIH4	0.1510	0.3169	0.0542	0.2289	0.0755	-0.3884	0.0000	-0.5143	1.0000	0.0907	1.0000	0.0143	1.0000	-0.0303
CNKSR1	0.8905	-0.3549	1.0000	-0.0108	0.7026	-0.5992	0.6385	0.6917	1.0000	-0.6991	1.0000	-0.3451	1.0000	0.5980
CNKSR3	0.0000	0.7472	0.0000	0.5824	0.0428	0.3503	0.0155	0.1913	1.0000	0.0362	0.5525	-0.1160	0.4907	-0.1172
CNN2	0.0000	-1.4619	0.0000	-1.4546	0.0000	-0.7164	0.0000	-0.4985	1.0000	-0.0668	1.0000	-0.0471	0.3209	0.1566
CNN3	0.1468	0.2675	0.0002	0.2846	0.0341	0.3595	0.0000	0.4960	1.0000	-0.0039	1.0000	0.0252	0.2216	0.1383
CNNM1	1.0000	0.0228	0.2302	-0.4199	0.6074	-0.3444	0.0957	0.4886	1.0000	-0.2809	0.0856	-0.7069	0.1750	0.5592
CNNM2	1.0000	0.0892	0.0138	0.4859	0.0000	1.2099	0.0000	1.3581	1.0000	-0.3325	1.0000	0.0762	0.6155	-0.1795
CNNM4	0.4664	0.2495	0.0000	0.5747	0.0262	0.5384	0.0000	0.7853	1.0000	-0.0522	0.2131	0.2862	0.4247	0.2023
CNOT1	0.3039	0.1956	0.0001	0.2857	0.0221	0.3605	0.0000	0.3016	1.0000	-0.0600	1.0000	0.0427	0.4742	-0.1137
CNOT10	0.9367	0.0749	1.0000	0.0230	0.1686	-0.2874	0.0002	-0.3617	1.0000	0.0922	1.0000	0.0527	1.0000	0.0243
CNOT11	0.3753	-0.2059	1.0000	-0.0306	0.4274	-0.1867	0.1212	-0.1753	1.0000	-0.0882	0.9523	0.1002	0.9863	-0.0702
CNOT2	0.0257	-0.4209	0.0121	-0.2536	0.1766	-0.2858	0.6334	-0.0749	1.0000	-0.1005	1.0000	0.0797	0.6982	0.1153
CNOT4	0.0243	-0.4889	0.0001	-0.5315	0.4576	0.2017	0.9361	0.0567	1.0000	0.0752	1.0000	0.0445	1.0000	-0.0648
CNOT6	0.5734	-0.1448	0.0094	-0.2472	0.0083	0.4619	0.0531	0.1811	1.0000	0.1986	0.7749	0.1090	0.9515	-0.0771
CNOT7	0.4741	-0.2227	0.7503	-0.0841	0.0228	0.4687	0.0005	0.3952	1.0000	-0.0381	1.0000	0.1132	0.8493	-0.1059
CNOT8	1.0000	-0.0122	1.0000	0.0134	0.0570	-0.3448	0.0007	-0.3091	1.0000	-0.0104	1.0000	0.0277	1.0000	0.0310
CNP	0.4157	-0.1650	0.0000	-0.3973	0.3639	-0.1752	0.0001	-0.2842	1.0000	-0.1378	0.0000	-0.3579	0.0109	-0.2413
CNPPD1	0.0005	0.5221	0.0000	0.4422	0.0000	-0.9210	0.0000	-0.8385	1.0000	-0.0202	0.8269	-0.0877	0.9814	0.0678
CNPY3	0.0000	-1.0382	0.0000	-0.7996	0.0007	-0.5947	0.3613	-0.1004	1.0000	-0.0595	0.4032	0.1912	0.0001	0.4407
CNR1	0.0000	1.1861	0.0000	0.9491	0.0014	0.6174	0.0012	0.3880	1.0000	-0.0416	0.0698	-0.2656	0.1489	-0.2658
CNR2	0.2144	1.0963	0.0360	2.0580	0.6063	-1.0166	1.0000	0.7001	1.0000	-1.6354	0.7456	-0.6681	1.0000	0.0854
CNRIP1	0.0000	-1.8433	0.0000	-2.4079	0.1584	0.5284	0.0860	0.4250	1.0000	-0.0925	0.7334	-0.6438	0.8425	-0.1911
CNST	0.4104	-0.2876	0.0058	-0.4818	0.9361	0.1085	0.1117	0.2654	1.0000	0.0867	1.0000	-0.0958	0.4359	0.2474
CNTD1	0.0000	-1.6750	0.0000	-1.0452	1.0000	-0.0390	0.0056	0.4884	1.0000	-0.2983	0.8944	0.3463	0.5019	0.2361
CNTF	0.0142	-0.7214	0.0000	-0.9969	0.1855	-0.4139	0.0000	-0.8214	1.0000	0.0593	0.9839	-0.2039	0.4329	-0.3431
CNTN1	0.0096	-2.3708	0.0269	-1.2626	0.0675	1.0571	1.0000	0.1450	1.0000	0.6754	0.3249	1.8051	1.0000	-0.2296
CNTN2	0.5780	-1.5569	1.0000	-0.1429	0.7660	-1.4058	0.3945	2.0770	1.0000	-2.1881	1.0000	-0.7956	0.9835	1.2966
CNTN3	0.7643	-0.5218	0.8190	-0.2987	0.2480	-1.0283	0.1245	-1.0387	1.0000	0.4541	0.8607	0.6915	1.0000	0.4484
CNTN4	0.5391	-3.8767	0.2310	-2.0430	0.2459	1.6000	0.1345	1.2322	1.0000	1.2174	1.0000	3.1945	0.6029	0.8566
CNTN5	1.0000	0.0000	1.0000	0.0000	1.0000	0.0000	1.0000	0.0000	1.0000	0.0000	1.0000	0.0000	1.0000	0.0000
CNTN6	1.0000	-2.4776	1.0000	0.0000	1.0000	-2.4056	1.0000	0.0000	1.0000	-2.3757	1.0000	0.0000	1.0000	0.0000
CNTNAP1	0.5980	0.5240	0.1303	0.7810	0.0443	1.1145	0.0078	1.1198	1.0000	0.0678	0.9773	0.3404	1.0000	0.0806
CNTNAP2	0.6374	0.7566	0.8033	0.3372	0.0325	1.4534	0.0068	1.3153	1.0000	0.5379	1.0000	0.1269	0.8133	0.4008
CNTRL	0.0001	0.6257	0.0000	0.5660	1.0000	0.0057	0.0221	-0.2454	1.0000	-0.0545	0.8219	-0.1018	0.0245	-0.3006
COASY	0.0000	-1.0752	0.0000	-0.9792	0.0000	-1.2808	0.0000	-0.7979	1.0000	-0.1265	1.0000	-0.0181	0.1169	0.3622
COCH	0.7972	0.8927	1.0000	-0.1673	0.0920	2.2507	0.4057	1.3511	1.0000	0.4094	1.0000	-0.6369	1.0000	-0.4892
COG1	0.2024	0.2567	0.3476	0.1191	0.4758	-0.2009	0.4556	-0.1021	1.0000	0.0146	0.7933	-0.1111	0.8546	0.1198
COG2	0.8033	0.1147	0.1157	0.1996	0.2649	-0.2618	0.2626	-0.1598	1.0000	0.0421	0.7224	0.1397	0.6579	0.1492
COG3	0.4271	0.3085	0.1946	0.2915	0.0000	1.4532	0.0000	0.8607	1.0000	0.1435	1.0000	0.1392	0.0475	-0.4440
COG4	0.6365	0.1242	0.0036	0.2335	0.0000	-0.6891	0.0000	-0.5428	1.0000	-0.0647	1.0000	0.0569	0.8111	0.0871
COG5	0.3089	0.2982	0.0405	0.3083	0.0000	0.8781	0.0000	0.7026	1.0000	-0.0350	1.0000	-0.0113	0.3431	-0.2057
COG6	0.0666	-0.3909	0.0425	-0.2590	0.1031	0.3399	0.2193	0.1588	1.0000	-0.0103	0.8163	0.1351	0.3383	-0.1858
COG7	0.8941	-0.0820	0.6841	0.0772	0.1496	-0.2798	0.2305	-0.1499	1.0000	-0.0495	0.7475	0.1221	0.8686	0.0868
COG8	0.0061	-0.5741	0.0007	-0.4510	0.0230	-0.5043	0.0475	-0.2690	1.0000	-0.1762	1.0000	-0.0417	1.0000	0.0649
COIL	0.4324	-0.2149	1.0000	-0.0179	0.3697	-0.2331	0.9084	-0.0557	1.0000	-0.0449	0.6980	0.1643	0.7345	0.1383
COL10A1	1.0000	0.0000	1.0000	0.0000	1.0000	2.2427	1.0000	0.0000	1.0000	0.0000	1.0000	0.0000	1.0000	-2.2888
COL13A1	0.0000	-4.4834	0.0000	-3.6067	0.0128	-0.7743	0.0000	-0.7522	1.0000	0.2538	0.0130	1.1407	0.1558	0.2786
COL14A1	1.0000	-0.0356	1.0000	-0.0544	0.5292	0.8643	1.0000	0.2433	1.0000	0.3179	1.0000	0.3163	1.0000	-0.3002
COL15A1	0.0181	-0.6678	0.0102	-0.5165	0.0000	1.2164	0.0000	0.9559	1.0000	-0.0245	1.0000	0.1398	0.2316	-0.2794
COL16A1	0.0000	-4.3162	0.0000	-2.9411	0.1031	-0.6694	0.0139	0.7648	1.0000	-1.1127	1.0000	0.2778	0.6869	0.3262
COL17A1	0.0002	-0.6160	0.0000	-0.5839	0.0000	-1.1132	0.0000	-0.7743	1.0000	0.0599	0.8999	0.1042	0.0002	0.4044
COL18A1	0.0000	-0.9242	0.0000	-0.6682	0.0005	0.7714	0.0000	1.2220	1.0000	0.0232	0.0081	0.2918	0.0000	0.4781
COL19A1	1.0000	0.0262	0.5628	-0.3313	0.4188	0.4140	0.0823	0.6452	1.0000	-0.6808	0.0138	-1.0264	0.3821	-0.4413
COL1A2	0.0000	-1.0139	0.0000	-1.0681	0.0701	0.3280	0.0000	0.6484	1.0000	0.2329	0.0957	0.1912	0.0000	0.5580
COL20A1	0.6015	-1.5590	0.0038	-5.4498	0.0357	1.8865	0.2046	0.9893	1.0000	0.8403	1.0000	-3.1382	1.0000	-0.0553
COL23A1	0.0000	-1.0464	0.0000	-1.0981	0.1384	0.2848	0.0025	0.2798	1.0000	-0.0599	1.0000	-0.0997	1.0000	-0.0593
COL24A1	0.4664	0.7073	0.8234	0.5981	0.0163	1.4248	0.3371	1.2778	1.0000	-2.7097	0.0000	-2.8150	0.0000	-2.8508
COL25A1	1.0000	0.0000	1.0000	0.0000	1.0000	0.0000	1.0000	2.3241	1.0000	0.0000	1.0000	0.0000	1.0000	2.3543
COL26A1	0.4056	0.5430	0.4770	0.3588	0.7411	0.3342	0.4570	-0.4047	1.0000	0.3701	1.0000	0.2018	0.8333	-0.3635
COL27A1	0.7425	-0.2123	0.1211	0.2697	0.1798	-0.4960	0.9287	0.0707	1.0000	-0.0179	0.0136	0.4802	0.0029	0.5529
COL28A1	0.8746	-0.4334	0.3913	0.6457	0.5990	-0.6340	0.4927	-0.7813	1.0000	-0.5641	1.0000	0.5277	0.7939	-0.7084
COL2A1	1.0000	-0.2087	1.0000	-0.1749	0.2248	1.0658	0.0442	1.7226	1.0000	-0.9420	0.9301	-0.9047	1.0000	-0.2863
COL3A1	0.0000	-2.9028	0.0000	-2.3822	0.0000	1.6595	0.0000	1.9231	1.0000	0.3961	0.0000	0.9293	0.0000	0.6637
COL4A1	0.0000	-1.6059	0.0000	-1.2239	0.0000	1.2173	0.0000	1.6851	1.0000	0.3655	0.0000	0.7600	0.0000	0.8374
COL4A2	0.0001	-1.2675	0.0000	-0.7431	0.0001	1.2879	0.0000	1.8648	1.0000	0.2560	0.0000	0.7932	0.0000	0.8366

COL4A3	1.0000	0.0029	0.8588	0.0735	0.8787	-0.1299	0.1572	-0.2460	1.0000	-0.0977	1.0000	-0.0148	0.7683	-0.2068
COL4A3BP	0.3843	-0.2511	0.7848	-0.0744	1.0000	0.0205	1.0000	0.0159	1.0000	0.0150	0.5392	0.2040	1.0000	0.0162
COL4A4	1.0000	-0.2465	0.0012	1.4743	1.0000	-0.0854	0.2644	0.7726	1.0000	-0.6226	0.0523	1.1097	1.0000	0.2396
COL4A5	0.0771	-1.0446	0.1572	-0.5377	1.0000	-0.0784	0.0000	1.9630	1.0000	-0.3157	1.0000	0.2043	0.0000	1.7329
COL4A6	0.0012	-1.7916	0.0000	-2.0365	0.9431	-0.2291	0.0000	1.1876	1.0000	0.0320	1.0000	-0.1992	0.0000	1.4561
COL5A1	0.0065	-0.5183	0.0000	-0.3642	0.0000	1.2516	0.0000	1.2190	1.0000	0.3395	0.0000	0.5059	0.0001	0.3115
COL5A2	0.3159	-0.1841	0.0185	-0.1629	0.0000	0.8513	0.0000	0.8808	1.0000	0.0968	0.3460	0.1304	0.2833	0.1315
COL6A1	0.0000	-1.0872	0.0000	-0.7220	0.0039	0.6192	0.0000	0.8048	1.0000	0.1852	0.0000	0.5626	0.0000	0.3750
COL6A2	0.0000	-0.8692	0.0000	-0.3752	0.0272	0.5083	0.0000	0.6809	1.0000	0.1729	0.0000	0.6794	0.0000	0.3498
COL6A3	0.0000	-2.1716	0.0000	-1.5714	1.0000	0.0246	0.0000	0.5379	1.0000	0.3153	0.0000	0.9278	0.0000	0.8321
COL6A6	1.0000	0.3037	1.0000	-0.4754	0.4625	1.0317	1.0000	0.2591	1.0000	-0.1269	1.0000	-0.8977	0.7287	-0.8929
COL7A1	0.0000	-1.4285	0.0000	-0.8011	0.3212	-0.3909	0.0000	0.6176	1.0000	0.0188	0.0000	0.6590	0.0000	1.0309
COL8A1	0.0006	-2.2505	0.0000	-3.3232	0.0510	0.8758	1.0000	0.1580	1.0000	0.5613	1.0000	-0.4990	1.0000	-0.1527
COL8A2	0.7038	-0.5592	0.1345	-1.2501	0.0000	4.2025	0.0000	4.1181	1.0000	-0.1333	0.8910	-0.8125	0.6887	-0.2085
COL9A1	0.8249	3.0140	1.0000	0.0000	0.2779	3.9963	0.1317	4.3958	1.0000	0.0000	1.0000	-3.1382	1.0000	0.3817
COL9A2	1.0000	-0.3189	1.0000	-0.1608	0.0859	2.2532	0.4962	1.4812	1.0000	-0.1245	1.0000	0.0496	0.7283	-0.8912
COL9A3	0.4347	0.4790	0.9119	-0.1804	1.0000	0.1741	0.9959	0.1440	1.0000	0.1879	0.6630	-0.4608	1.0000	0.1617
COLCA2	0.1096	4.6934	0.4670	1.9211	1.0000	0.0000	1.0000	-2.3199	1.0000	2.2736	1.0000	-0.4023	1.0000	0.0000
COLEC10	0.0144	1.0315	0.0000	1.3651	0.0057	-1.4614	0.0239	-0.8918	1.0000	-0.2938	1.0000	0.0465	1.0000	0.2787
COLEC11	0.8470	-0.6501	1.0000	-0.1557	1.0000	-0.1921	0.9786	0.9248	1.0000	-1.3469	1.0000	-0.8584	1.0000	-0.2278
COLEC12	0.0034	0.4309	0.0000	0.3161	1.0000	0.0388	0.5092	-0.0669	1.0000	0.0873	1.0000	-0.0151	1.0000	-0.0133
COLGALT2	0.0077	-0.8332	0.1862	-0.4330	0.0000	1.4307	0.0000	1.2857	1.0000	-0.0628	0.6691	0.3510	0.4837	-0.2054
COLQ	0.9545	-1.6907	1.0000	-0.5749	0.1230	1.8319	0.6321	0.6914	1.0000	1.2167	0.6450	2.3757	1.0000	0.0815
COMMD1	0.7211	-0.1617	0.0673	-0.2422	0.0003	-0.9243	0.0000	-0.8830	1.0000	-0.0694	0.8022	-0.1369	1.0000	-0.0197
COMMD2	0.0014	-0.6035	0.0000	-0.6344	0.0940	-0.3379	0.0000	-0.4086	1.0000	-0.0596	1.0000	-0.0779	0.5886	-0.1247
COMMD4	0.0000	-0.9087	0.0000	-0.7637	0.0000	-0.9639	0.0000	-0.6775	1.0000	-0.1599	1.0000	-0.0031	0.6548	0.1322
COMMD6	0.1670	0.2728	0.0005	0.2948	1.0000	0.0110	0.0248	-0.2039	1.0000	0.0149	1.0000	0.0493	0.1846	-0.1949
COMMD7	0.1521	-0.2947	0.0000	-0.4757	0.0025	-0.5339	0.0000	-0.4823	1.0000	-0.1103	0.0590	-0.2793	1.0000	-0.0532
COMMD8	0.9545	-0.0906	0.8771	-0.0588	0.8971	-0.1008	0.1706	-0.1914	1.0000	-0.0405	1.0000	0.0044	0.8050	-0.1244
COMMD9	0.2138	0.2757	0.0452	0.2379	0.0059	-0.5492	0.0000	-0.5279	1.0000	-0.0250	1.0000	-0.0503	1.0000	0.0020
COMP	0.0000	1.0327	0.0002	0.5708	0.9950	-0.1014	0.0075	-0.4786	1.0000	-0.3101	0.0000	-0.7607	0.0003	-0.6814
COMT	0.6208	-0.1227	0.0000	-0.4482	0.3867	0.1720	1.0000	-0.0182	1.0000	0.1430	0.2541	-0.1700	1.0000	-0.0421
COMTD1	0.2704	0.2626	0.4075	0.0922	0.0100	-0.5170	0.0000	-0.5712	1.0000	-0.1113	0.0101	-0.2691	0.3511	-0.1595
COPA	0.0536	0.3442	0.0000	0.5221	0.4261	0.1797	0.0002	0.2640	1.0000	-0.0249	0.1785	0.1653	0.8632	0.0642
COPB1	0.0301	0.3454	0.0000	0.4396	0.3259	0.1842	0.2075	0.1046	1.0000	-0.0023	0.6615	0.1046	0.7486	-0.0766
COPB2	0.9681	0.0577	0.5190	0.0685	0.3617	0.1734	1.0000	0.0027	1.0000	0.1147	0.4092	0.1380	0.9791	-0.0507
COPE	1.0000	-0.0120	0.2244	0.1003	0.0001	-0.5950	0.0000	-0.5637	1.0000	-0.0112	0.4814	0.1134	1.0000	0.0259
COPG1	0.8513	-0.0815	0.8830	0.0353	1.0000	-0.0545	0.2999	0.0901	1.0000	0.0159	0.3609	0.1451	0.2120	0.1657
COPG2	0.3744	0.1840	0.0005	0.2947	1.0000	0.0307	0.2330	-0.1190	1.0000	-0.0175	0.7202	0.1053	0.2715	-0.1619
COPRS	0.6877	-2.0809	1.0000	-0.1630	0.7033	0.7691	0.1448	2.0465	1.0000	-1.0363	1.0000	0.8994	1.0000	0.2432
COPS2	0.4852	0.1681	1.0000	0.0027	0.6383	-0.1308	0.0063	-0.2262	1.0000	0.0071	0.4103	-0.1460	0.8047	-0.0830
COPS3	0.0877	-0.2881	0.0605	-0.1784	0.0014	-0.4922	0.0000	-0.4586	1.0000	-0.1091	1.0000	0.0129	0.9032	-0.0703
COPS4	1.0000	-0.0481	0.8952	-0.0380	0.0358	-0.3843	0.0000	-0.4486	1.0000	-0.0554	1.0000	-0.0331	0.5610	-0.1141
COPS5	1.0000	-0.0474	1.0000	-0.0098	0.0331	-0.4423	0.0000	-0.4775	1.0000	-0.0020	1.0000	0.0472	1.0000	-0.0318
COPS7A	0.5390	-0.1408	0.0715	-0.1602	0.0001	-0.5869	0.0000	-0.4557	1.0000	-0.0111	1.0000	-0.0179	0.5091	0.1259
COPS7B	0.3767	0.2045	0.0008	0.3914	0.0534	-0.3876	0.4257	-0.1342	1.0000	-0.3401	0.6194	-0.1416	1.0000	-0.0814
COPS8	0.0136	-0.4642	0.0000	-0.5245	0.0012	-0.5865	0.0000	-0.6240	1.0000	-0.0344	0.8406	-0.0825	0.8937	-0.0659
COQ10A	0.1694	-0.3909	0.0014	-0.5480	0.8011	-0.1476	0.7701	-0.0981	1.0000	0.0334	1.0000	-0.1106	1.0000	0.0896
COQ10B	0.0179	-0.4490	0.0000	-0.8429	0.0000	-0.7474	0.0000	-0.9516	1.0000	0.1447	0.3504	-0.2363	1.0000	-0.0543
COQ2	0.0882	-0.4997	0.6429	-0.1561	0.0267	-0.5877	0.7719	-0.1226	1.0000	-0.2067	1.0000	0.1479	0.6093	0.2626
COQ3	0.4680	-0.1789	0.2286	-0.1458	0.0000	-1.0661	0.0000	-0.9538	1.0000	-0.0895	1.0000	-0.0437	1.0000	0.0291
COQ4	0.3855	-0.2075	0.0787	-0.2299	0.0000	-0.8740	0.0000	-0.6547	1.0000	-0.1162	0.8172	-0.1270	0.8937	0.1070
COQ5	0.9907	-0.0659	1.0000	-0.0316	0.0031	-0.5479	0.0000	-0.6768	1.0000	-0.1019	1.0000	-0.0559	0.1442	-0.2255
COQ6	0.0432	-0.3930	0.8419	-0.0601	0.0000	-0.9343	0.0000	-0.9100	1.0000	-0.0823	0.1527	0.2635	1.0000	-0.0530
COQ7	1.0000	0.0300	1.0000	0.0021	0.0411	-0.4561	0.0001	-0.4814	1.0000	-0.1781	0.5821	-0.1939	0.5805	-0.1968
COQ9	0.0000	-0.8642	0.0000	-0.7425	0.0000	-1.0121	0.0000	-0.7096	1.0000	-0.0960	1.0000	0.0374	0.2241	0.2120
CORO1B	0.9938	-0.0564	0.0121	0.2255	0.3666	-0.2089	0.3403	0.0990	1.0000	-0.2216	0.9712	0.0724	0.9116	0.0916
CORO1C	0.7233	-0.1017	0.4172	0.0884	0.8099	-0.0844	0.6899	-0.0524	1.0000	-0.0621	0.4071	0.1403	1.0000	-0.0248
CORO2A	0.7902	1.1393	0.5155	1.3035	1.0000	-0.1785	0.5092	1.4828	1.0000	-0.1170	1.0000	0.0574	0.8003	1.5506
CORO2B	0.2455	0.7682	0.1415	0.6335	0.1245	-1.2227	0.0071	-1.4778	1.0000	-0.3266	0.5536	-0.4555	0.8020	-0.5743
CORO6	0.0000	-1.2190	0.0000	-1.3607	0.0000	-1.2632	0.0000	-1.2866	1.0000	-0.0220	0.8171	-0.1516	1.0000	-0.0408
CORO7	0.0147	0.9129	0.1072	0.6019	0.0237	0.7660	0.0026	0.9145	1.0000	-0.0993	0.5345	-0.3979	1.0000	0.0587
CORT	0.1877	4.4789	0.2495	4.0050	1.0000	2.2427	0.7701	3.1732	1.0000	0.0000	1.0000	-0.4972	1.0000	0.9168
COTL1	0.0127	0.3711	0.0000	0.2811	0.3522	-0.1828	0.1605	-0.1027	1.0000	0.0889	1.0000	0.0113	0.1711	0.1744
COX3	1.0000	0.0000	1.0000	0.0000	1.0000	0.0000	1.0000	0.0000	1.0000	0.0000	1.0000	0.0000	1.0000	0.0000
CP	0.0134	-0.5362	0.0000	-1.0302	0.0108	-0.5318	0.0000	-0.6222	1.0000	0.3055	0.6637	-0.1752	0.2144	0.2198
CPA2	0.4949	1.7587	0.7782	-1.1113	1.0000	0.6783	0.4962	-1.4667	1.0000	2.2035	1.0000	-0.6351	1.0000	0.0723
CPAMD8	0.0282	-1.2813	0.0391	-0.8686	0.0000	1.5081	0.3178	0.4083	1.0000	0.4432	0.3181	0.8712	0.0574	-0.6472
CPB2	1.0000	0.0000	1.0000	0.0000	1.0000	0.0000	1.0000	0.0000	1.0000	0.0000	1.0000	0.0000	1.0000	0.0000
CPD	0.0009	0.8139	0.0000	1.0374	0.0000	1.5375	0.0000	1.7672	1.0000	-0.0525	0.6489	0.1846	0.1397	0.1829
CPE	0.0000	0.7255	0.0000	0.5433	0.0095	0.4730	0.0094	0.2746	1.0000	0.2110	1.0000	0.0412	1.0000	0.0179
CPEB1	0.0000	-1.3107	0.0000	-1.2927	0.0000	-1.1288	0.0000	-1.0963	1.0000	0.1966	0.6141	0.2279	0.3500	0.2339
CPEB2	0.1580	0.3640	0.0002	0.5458	0.0000	1.1449	0.0003	0.5953	1.0000	0.0034	0.5245	0.1990	0.0022	-0.5407
CPEB3	0.0234	0.9506	1.0000	0.0123	0.0000	1.5764	0.0004	0.6593	1.0000	0.9746	1.0000	0.0467	1.0000	0.0615
CPEB4	0.0000	-0.9447	0.0000	-1.0786	0.0091	0.5184	0.7326	0.0654	1.0000	0.0848	1.0000	-0.0366	0.0455	-0.3636
CPED1	0.1418	2.5637	0.5864	0.9876	0.0218	3.2328	1.0000	0.0084	1.0000	1.2565	1.0000	-0.2912	0.1809	-1.9525
CPLX2	0.6957	1.7486	0.6180	-1.9239	0.4424	1.8936	1.0000	0.0070	1.0000	1.6505	0.8354	-2.0147	1.0000	-0.2278
CPLX3	1.0000	0.0000	1.0000	0.0000	1.0000	0.0000	1.0000	0.0000	1.0000	0.0000	1.0000	0.0000	1.0000	0.0000
CPLX4	1.0000	0.0000	1.0000	0.0000	1.0000	0.0000	1.0000	2.3241	1.0000	0.0000	1.0000	0.0000	1.0000	2.3543
CPM	0.3201	-0.2057	0.2459	-0.1037	0.0121	0.4270	0.0157	0.1792	1.0000	0.1415	0.0061	0.2558	0.5430	-0.1014
CPN1	1.0000	0.1390	0.2802	-1.0528	0.7035	-0.6019	0.9814	-0.3663	1.0000	-0.0069	0.4752	-1.1911	1.0000	0.2274
CPNE1	0.0254	0.3571	0.3630	0.0979	1.0000	-0.0316	0.6785	-0.0593	1.0000	0.1039	0.3709	-0.1433	0.7977	0.0810
CPNE2	0.0023	1.0124	0.6413	0.2076	1.0000	-0.1506	1.0000	-0.0954	1.0000	0.4032	0.3998	-0.3901	0.4082	0.4648
CPNE4	1.0000	0.0453	0.0594	-0.8072	0.6959	-0.3893	0.8117	-0.2193	1.0000	0.0577	0.3097	-0.7855	1.0000	0.2290
CPNE7	0.1098	-1.5171	0.0536	-1.9200	0.4185	-0.8664	1.0000	-0.2110	1.0000	-0.3181	1.0000	-0.7117	1.0000	0.3392
CPNE8	0.0057	0.9932	0.0000	1.5882	0.0000	1.5368	0.0000	1.6508	1.0000	-0.2375	0.3074	0.3750	0.9934	-0.1146
CPNE9	0.1715	-0.7913	0.0128	-1.0177	0.0124	-1.2553	0.0219	-0.9321	1.0000	0.0454	1.0000	-0.1681	0.9248	0.3731
CPO	1.0000	-2.4788	1.0000	2.2507	1.0000	-2.4061	1.0000	0.0000	1.0000	-2.3771	1.0000	2.3455	1.0000	0.0000
CPOX	1.0000	-0.0129	1.0000	-0.0294	0.0001	-0.7257	0.0000	-0.5129	1.0000	-0.1149	0.8507	-0.1188	0.9057	0.1022
CPPED1	0.0972	-0.5588	0.0091	-0.6512	0.0063	0.7178	0.0003	0.7357	1.0000	-0.0314	1.0000	-0.1132	1.0000	-0.0099
CPQ	0.4768	0.1652	0.0043	0.2712	0.0003	-0.5936	0.0000	-0.4622	1.0000	0.0315	0.4632	0.1493	0.3507	0.1683
CPS1	1.0000	0.1937	0.0918	-1.3409	0.2949	0.7529	0.5744	-0.6578	1.0000	-0.5219	0.0132	-2.0472	0.0017	-1.9280
CPSF1	0.3772	0.6765	0.0181	1.1475	0.0043	1.4954	0.0000	2.4293	1.0000	-0.9458	0.7740	-0.4617	1.0000	0.0023

CPSF2	1.0000	0.0208	0.7666	-0.0566	0.4216	0.1968	0.6781	-0.0659	1.0000	0.1148	1.0000	0.0498	0.5105	-0.1431
CPSF3	0.4743	-0.1565	0.6080	-0.0679	0.1661	-0.2483	0.0150	-0.2093	1.0000	-0.0990	1.0000	0.0018	1.0000	-0.0551
CPSF3L	0.0674	-0.3204	0.0127	-0.2354	0.1023	-0.3155	0.0013	-0.2900	1.0000	0.0312	0.6450	0.1286	1.0000	0.0626
CPSF4	1.0000	-0.0021	0.7968	0.0608	0.6637	-0.1304	0.0249	-0.2574	1.0000	-0.0127	1.0000	0.0624	0.5978	-0.1344
CPSF6	0.0119	0.4125	0.0000	0.4386	0.0982	-0.2965	0.0039	-0.2570	1.0000	-0.0529	1.0000	-0.0147	1.0000	-0.0081
CPSF7	1.0000	0.0863	0.0091	0.7055	0.1207	0.5060	0.0005	0.8610	1.0000	-0.5596	1.0000	0.0732	0.8137	-0.1962
CPT1A	0.4842	0.1522	1.0000	0.0228	1.0000	-0.0245	0.5772	-0.0776	1.0000	0.1614	1.0000	0.0447	0.6362	0.1134
CPT2	0.4304	0.2032	0.0190	0.2712	0.4616	-0.1965	0.0150	-0.2892	1.0000	-0.2273	0.6102	-0.1474	0.0430	-0.3153
CPTP	1.0000	0.0112	0.0477	0.3472	0.7615	0.1435	0.0327	0.3025	1.0000	-0.0964	0.5111	0.2520	1.0000	0.0679
CPXM2	0.1851	4.4812	1.0000	2.2535	0.0691	4.7549	0.2426	4.0910	1.0000	0.0000	0.6450	-2.2675	1.0000	-0.6904
CR1	0.8226	3.0199	1.0000	-2.3958	1.0000	0.0000	1.0000	-2.3176	1.0000	2.2674	1.0000	-3.1430	1.0000	0.0000
CRABP1	0.0000	-1.5952	0.0000	-1.5458	1.0000	-0.0215	0.0000	0.7423	1.0000	0.2851	0.9380	0.3486	0.0000	1.0592
CRABP2	0.9366	-0.3530	0.0513	-1.8740	1.0000	-0.0081	1.0000	0.0037	1.0000	0.3838	0.6975	-1.1284	1.0000	0.3973
CRACR2B	0.3100	0.3081	0.1808	0.2424	0.0268	0.5418	0.0000	1.0053	1.0000	-0.4351	0.0026	-0.4869	1.0000	0.0353
CRADD	1.0000	-0.0259	0.7523	0.0930	0.0000	-1.0951	0.0000	-1.1600	1.0000	-0.2431	1.0000	-0.1127	0.4112	-0.3006
CRAMP1L	0.1847	0.5023	0.6319	0.2190	0.0000	1.3944	0.0000	1.2506	1.0000	0.0102	0.8607	-0.2598	1.0000	-0.1277
CRAT	0.1149	0.3995	0.0001	0.6172	0.0379	0.4911	0.0000	0.6796	1.0000	-0.1116	1.0000	0.1190	1.0000	0.0833
CRB1	0.5487	0.1747	0.6202	-0.0976	0.7944	-0.1169	0.0001	0.4222	1.0000	-0.1830	0.0011	-0.4420	0.0074	0.3624
CRB2	0.0802	-1.0996	0.1883	-0.6428	0.3553	-0.6362	0.1780	-0.6829	1.0000	0.2986	0.4048	0.7702	1.0000	0.2561
CRBN	0.2634	0.2680	0.3033	0.1529	0.6254	0.1663	0.6099	-0.0977	1.0000	0.1836	1.0000	0.0814	1.0000	-0.0758
CRCP	0.6423	0.1359	0.3535	0.1017	0.0000	-0.8811	0.0000	-0.9334	1.0000	-0.0391	1.0000	-0.0606	0.9428	-0.0844
CREB1	0.0003	1.0257	0.0001	0.8771	0.0000	1.9665	0.0000	1.6216	1.0000	0.0955	1.0000	-0.0384	0.5093	-0.2416
CREB3	0.2580	-0.2028	1.0000	-0.0145	0.0611	-0.3011	0.0107	-0.2095	1.0000	-0.0655	0.3834	0.1351	1.0000	0.0314
CREB3L1	0.4581	-0.2183	0.1748	-0.1445	0.0500	0.4332	0.0000	0.7152	1.0000	0.1161	0.2494	0.2022	0.0000	0.4027
CREB3L2	0.0000	0.7736	0.0000	0.9584	0.0000	1.5128	0.0000	1.7395	1.0000	0.0099	0.2085	0.2078	0.0489	0.2420
CREB3L3	0.5531	0.8250	0.2516	1.3628	0.0424	-3.2137	0.7302	-1.2190	1.0000	-1.0896	0.8840	-0.5475	1.0000	0.9149
CREB5	1.0000	0.1689	0.1701	0.7192	0.0072	1.1839	0.0000	1.8870	1.0000	-0.3999	1.0000	0.1604	0.7221	0.3077
CREBL2	0.0013	0.5540	0.0000	0.4030	0.0600	-0.3642	0.0002	-0.3332	1.0000	0.0671	1.0000	-0.0712	0.7387	0.1038
CREBRF	0.0909	0.7583	1.0000	-0.0325	0.0004	1.2653	0.0536	0.5140	1.0000	0.7788	1.0000	0.0006	1.0000	0.0332
CREG1	0.0000	2.2260	0.0000	2.3957	0.0206	0.9421	1.0000	0.0074	1.0000	0.2019	0.0844	0.3859	0.0993	-0.7291
CREG2	0.2823	0.4562	0.3651	0.2973	0.3768	0.3909	1.0000	0.0963	1.0000	0.0188	1.0000	-0.1295	0.7853	-0.2723
CRELD1	0.0000	-3.0344	0.0000	-3.0566	0.0164	-0.5555	0.0002	0.4625	1.0000	-0.3820	0.7086	-0.3910	0.0001	0.6430
CRELD2	0.0037	0.5038	0.0000	0.3940	0.1015	0.3170	0.3026	0.1232	1.0000	0.1553	1.0000	0.0575	1.0000	-0.0330
CREM	0.5354	0.2281	0.0197	0.3708	0.7085	0.1686	0.0149	0.3742	1.0000	0.0875	0.4742	0.2422	0.2103	0.2980
CRH	0.0013	-0.7799	0.0000	-0.8687	0.0000	-1.5215	0.0000	-0.8868	1.0000	0.1445	1.0000	0.0685	0.0011	0.7868
CRHBP	1.0000	-0.7313	0.7218	-1.3908	0.2835	-4.1860	0.7210	-1.2154	1.0000	0.1799	1.0000	-0.4724	1.0000	3.2081
CRHR1	1.0000	0.0000	1.0000	0.0000	1.0000	0.0000	1.0000	0.0000	1.0000	0.0000	1.0000	0.0000	1.0000	0.0000
CRHR2	1.0000	-0.1521	0.0397	-1.4438	1.0000	0.0503	0.9694	0.2479	1.0000	-0.3748	0.0561	-1.6535	1.0000	-0.1699
CRIM1	0.0324	0.5622	0.0071	0.4126	0.0000	1.8262	0.0000	1.5840	1.0000	0.2419	1.0000	0.1047	1.0000	0.0050
CRIP1	0.0000	-2.5922	0.0000	-2.4130	0.0000	-1.9290	0.0000	-1.1688	1.0000	-0.1686	1.0000	0.0238	0.0000	0.5981
CRIPT	0.1062	-0.3670	0.0000	-0.4861	0.1290	0.3263	0.0412	0.1987	1.0000	0.0154	0.9810	-0.0916	0.6865	-0.1062
CRISPLD1	0.1010	1.3750	0.0017	2.1030	0.0267	1.4974	0.4700	0.8786	1.0000	-0.6364	1.0000	0.1040	0.1169	-1.2466
CRISPLD2	0.0540	-0.7351	0.0054	-0.6965	0.0006	0.9420	0.0000	1.3324	1.0000	0.3973	0.4785	0.4506	0.0000	0.7957
CRK	0.2385	-0.2275	0.0087	-0.2091	0.1157	0.2786	0.2203	0.1097	1.0000	0.0382	0.9442	0.0693	0.3839	-0.1249
CRKL	0.1261	-0.2795	0.0041	-0.2357	1.0000	0.0525	0.2050	-0.1155	1.0000	-0.0490	1.0000	0.0071	0.0552	-0.2116
CRLF1	0.0219	-0.7092	0.0000	-0.6569	0.0000	1.4829	0.0000	1.8027	1.0000	-0.8070	0.0000	-0.7427	0.0015	-0.4797
CRLF2	1.0000	0.0000	1.0000	0.0000	1.0000	2.2427	1.0000	0.0000	1.0000	0.0000	1.0000	0.0000	1.0000	-2.2888
CRLF3	0.2096	0.4105	0.0001	0.7155	0.0000	1.5088	0.0000	1.5208	1.0000	-0.1999	1.0000	0.1179	0.5306	-0.1820
CRLS1	0.4570	-0.2226	0.0001	-0.3864	0.3559	-0.2509	0.0090	-0.2579	1.0000	0.0705	1.0000	-0.0808	0.9887	0.0688
CRMP1	0.2833	-1.1701	0.1477	-1.0879	1.0000	-0.0298	1.0000	0.1497	1.0000	0.1151	1.0000	0.2083	1.0000	0.2996
CRNKL1	0.1463	0.2764	0.0226	0.2172	1.0000	-0.0267	0.2875	-0.1215	1.0000	0.0655	1.0000	0.0187	1.0000	-0.0238
CROCC	1.0000	-0.0029	0.0263	0.3101	0.0021	-0.6832	0.4989	-0.1273	1.0000	-0.3057	1.0000	0.0196	0.3051	0.2563
CROT	0.0000	0.7157	0.0000	0.6182	0.0395	0.3707	0.6667	0.0723	1.0000	0.2028	0.7101	0.1178	0.8297	-0.0898
CRTAC1	0.0000	-1.6098	0.0000	-1.4844	0.0022	0.5620	0.0000	0.6075	1.0000	-0.3214	0.8269	-0.1843	0.0461	-0.2706
CRTAM	1.0000	0.0000	1.0000	0.0000	1.0000	0.0000	1.0000	0.0000	1.0000	0.0000	1.0000	0.0000	1.0000	0.0000
CRTAP	0.4763	0.1553	1.0000	-0.0055	0.0940	-0.2981	0.0008	-0.2895	1.0000	0.2173	0.9436	0.0689	0.1149	0.2317
CRTC1	0.2138	0.3775	0.0327	0.3591	0.0180	0.5730	0.0000	0.5995	1.0000	-0.1262	0.9773	-0.1341	0.9919	-0.0955
CRY1	1.0000	0.0271	0.0123	-0.2183	0.0624	-0.3273	0.0000	-0.5006	1.0000	0.0482	0.1975	-0.1844	0.5598	-0.1192
CRY2	0.0000	-0.9273	0.0000	-0.8199	0.0000	1.2644	0.0000	1.6201	1.0000	0.0866	0.4584	0.2073	0.0000	0.4477
CRYAA	0.0000	-2.0730	0.0000	-2.5581	0.1250	-0.8163	0.0000	-0.9137	1.0000	0.6265	1.0000	0.1590	0.2054	0.5382
CRYAB	0.0000	-4.5459	0.0000	-5.5103	0.0085	0.4020	0.0000	0.5642	1.0000	0.0370	0.0006	-0.9149	0.1824	0.2044
CRYBA1	0.3317	-0.5365	0.0251	-0.8652	0.1298	-0.7699	0.0261	-0.8456	1.0000	0.0241	1.0000	-0.2918	1.0000	-0.0473
CRYBA2	1.0000	0.0000	1.0000	0.0000	1.0000	0.0000	1.0000	0.0000	1.0000	0.0000	1.0000	0.0000	1.0000	0.0000
CRYBA4	1.0000	0.0693	1.0000	0.0465	0.0004	-2.6548	0.0000	-2.3149	1.0000	0.1356	1.0000	0.1237	1.0000	0.4791
CRYBB1	0.2036	0.7125	0.7507	0.1981	0.1105	-0.9976	0.0000	-1.7089	1.0000	0.7124	1.0000	0.2095	1.0000	0.0069
CRYBB2	1.0000	0.0000	1.0000	0.0000	1.0000	0.0000	1.0000	0.0000	1.0000	0.0000	1.0000	0.0000	1.0000	0.0000
CRYBB3	1.0000	-0.2892	1.0000	-2.3986	1.0000	0.6780	1.0000	0.0045	1.0000	-0.1045	1.0000	-2.2992	1.0000	-0.7795
CRYBG3	0.0164	0.8261	0.0001	1.0154	0.0002	1.1389	0.0000	1.6292	1.0000	-0.3532	1.0000	-0.1491	0.9639	0.1445
CRYGN	0.6775	-0.2338	0.2573	-0.3834	0.0254	-0.6957	0.0012	-0.7636	1.0000	-0.0744	1.0000	-0.2122	1.0000	-0.1360
CRYGS	0.8872	0.3814	1.0000	0.0922	0.5513	-0.6767	0.0660	-1.1322	1.0000	0.3209	1.0000	0.0525	1.0000	-0.1232
CRYM	0.2606	0.6131	0.0910	0.6931	0.0004	1.3515	0.0322	0.8546	1.0000	-0.3854	0.9413	-0.2942	0.0102	-0.8773
CRYZ	0.7712	0.1132	0.0476	0.1963	0.0472	-0.3785	0.0739	-0.1853	1.0000	-0.0456	1.0000	0.0506	0.4262	0.1540
CRYZL1	0.5308	0.1774	0.6107	0.1019	0.0031	-0.5818	0.0000	-0.6480	1.0000	-0.0071	1.0000	-0.0703	1.0000	-0.0686
CS	0.0000	-0.5936	0.0000	-0.4007	0.0029	-0.4495	0.0002	-0.2614	1.0000	-0.1844	1.0000	0.0209	1.0000	0.0092
CSAD	1.0000	-0.3433	0.3086	-1.6781	0.1387	-2.2259	0.1561	-2.0305	1.0000	-0.1339	0.7260	-1.4559	1.0000	0.0717
CSDC2	1.0000	2.1847	1.0000	0.0000	1.0000	2.2427	1.0000	0.0000	1.0000	0.0000	1.0000	-2.2957	1.0000	-2.2888
CSDE1	0.3128	-0.1897	0.0005	-0.2342	1.0000	0.0400	0.7626	-0.0419	1.0000	0.0678	1.0000	0.0356	1.0000	-0.0086
CSF1	0.8152	-0.1898	1.0000	0.0285	0.3409	0.3324	0.0000	0.8662	1.0000	-0.3803	1.0000	-0.1488	0.6865	0.1621
CSF1R	0.0719	-3.2060	0.4543	-1.5095	0.2649	-1.7002	0.4409	-1.3304	1.0000	-0.3022	1.0000	1.4306	1.0000	0.0752
CSF3	0.1528	1.2572	0.0097	2.2121	0.0176	-5.3108	0.6172	-1.7596	1.0000	-1.2469	1.0000	-0.2855	1.0000	2.3554
CSF3R	0.8249	3.0140	1.0000	0.0000	1.0000	0.0000	0.7710	3.1708	1.0000	0.0000	1.0000	-3.1382	1.0000	3.2048
CSGALNACT1	0.2841	0.6580	1.0000	0.1073	0.1548	0.7909	0.0042	0.8869	1.0000	0.4045	1.0000	-0.1341	0.3394	0.5015
CSGALNACT2	0.0017	-0.5100	0.0002	-0.3816	0.0201	-0.3957	0.0537	-0.1976	1.0000	0.0432	0.3894	0.1847	0.0487	0.2473
CSK	0.3510	0.2198	0.0214	0.2918	0.0002	0.6361	0.0000	0.7499	1.0000	-0.0076	1.0000	0.0765	0.6942	0.1114
CSMD1	1.0000	2.1847	1.0000	0.6987	1.0000	0.0000	1.0000	-2.3177	1.0000	2.2674	1.0000	0.9035	1.0000	0.0000
CSMD2	0.8249	-3.3439	0.7674	-3.2498	0.2696	1.6874	1.0000	0.0106	1.0000	-0.1245	1.0000	0.0000	0.4805	-1.7987
CSNK1A1	0.4345	0.1650	0.0804	0.1324	0.0672	0.3062	0.1960	0.1018	1.0000	0.0590	1.0000	0.0387	0.2705	-0.1399
CSNK1D	1.0000	0.0004	0.1628	-0.1372	0.3287	0.2010	0.0234	0.1925	1.0000	0.0733	1.0000	-0.0514	0.8781	0.0704
CSNK1E	0.1253	-0.3369	0.0984	-0.2219	0.0000	1.2215	0.0000	0.7856	1.0000	0.1661	0.0995	0.2939	0.0262	-0.2632
CSNK1G1	1.0000	-0.0020	1.0000	0.0305	0.8829	-0.0826	0.4528	0.0946	1.0000	-0.0358	1.0000	0.0093	0.4394	0.1464
CSNK1G2	0.1064	-0.3762	0.9062	0.0557	0.7513	0.1329	0.0000	0.7564	1.0000	-0.3602	1.0000	0.0838	0.0813	0.2697
CSNK2A1	0.8516	-0.0854	0.3887	-0.0960	1.0000	0.0034	0.3589	-0.0983	1.0000	0.0915	0.8175	0.0937	1.0000	-0.0048
CSNK2A2	0.1511	0.2559	0.0000	0.4225	0.0781	0.3019	0.0000	0.3893	1.0000	-0.0987	0.8764	0.0801	1.0000	-0.0060

CSPG4	0.0082	-0.6133	1.0000	-0.0226	0.0000	1.3155	0.0000	1.1204	1.0000	-0.3066	0.2024	0.2961	0.0005	-0.4954
CSPG5	0.0522	0.4766	0.0000	0.8863	0.0015	-0.7757	1.0000	-0.0594	1.0000	-0.4308	1.0000	-0.0101	0.4325	0.2896
CSPP1	1.0000	-0.0437	1.0000	-0.0373	0.1324	-0.3362	0.0362	-0.2676	1.0000	-0.1380	0.8666	-0.1181	1.0000	-0.0642
CSRNP1	0.6481	0.1629	0.0006	0.4334	0.0000	1.0286	0.0000	0.8349	1.0000	0.1081	0.0188	0.3911	0.9206	-0.0800
CSRNP2	0.0958	-0.6171	0.8854	-0.1125	0.9776	0.1234	0.0096	0.5261	1.0000	-0.1618	0.5445	0.3547	0.6362	0.2486
CSRNP3	0.0196	1.2255	0.0006	1.1802	0.0569	1.0427	0.0152	0.9299	1.0000	-0.1344	1.0000	-0.1695	0.9515	-0.2441
CSRP1	0.0000	-0.8080	0.0000	-0.8230	0.0000	-1.0732	0.0000	-1.0460	1.0000	-0.0560	1.0000	-0.0588	1.0000	-0.0234
CSRP2	0.0000	1.3322	0.0000	1.4180	0.0000	-0.8505	0.0000	-0.3442	1.0000	0.2412	0.0003	0.3391	0.0000	0.7529
CSRP2BP	0.5919	-0.1600	0.9266	-0.0486	0.5699	0.1605	0.0023	0.3362	1.0000	-0.1942	1.0000	-0.0706	1.0000	-0.0138
CSRP3	0.0000	-2.2941	0.0000	-2.4025	0.0000	-1.6719	0.0000	-1.7581	1.0000	0.1044	1.0000	0.0082	1.0000	0.0232
CST7	0.0310	0.5791	0.0000	0.8215	0.2634	0.3642	0.5017	0.1754	1.0000	-0.0505	0.6770	0.2049	0.6294	-0.2317
CSTA	0.0000	-1.1744	0.0000	-1.2100	0.2955	-0.2036	0.0000	-0.4518	1.0000	0.1106	1.0000	0.0877	0.4669	-0.1327
CSTB	0.0000	-0.7747	0.0000	-0.8792	1.0000	-0.0152	0.0004	-0.3107	1.0000	0.1766	0.9957	0.0850	0.6362	-0.1136
CSTF1	0.2250	-0.2558	0.0155	-0.2846	1.0000	0.0328	0.6018	0.0833	1.0000	-0.0415	1.0000	-0.0579	1.0000	0.0145
CSTF2	0.4695	-0.1600	0.5482	-0.0837	0.0000	-0.9136	0.0000	-0.6868	1.0000	-0.1078	1.0000	-0.0195	0.7485	0.1246
CSTF3	0.4970	0.1579	0.6215	-0.0722	0.1609	-0.2641	0.0000	-0.5204	1.0000	0.0876	0.5881	-0.1301	0.3476	-0.1631
CTAGE5	0.0093	0.5020	0.0000	0.7741	0.7688	0.1201	1.0000	-0.0118	1.0000	-0.0581	0.2652	0.2258	0.4095	-0.1854
CTBP1	0.0005	-0.5316	0.0000	-0.6033	0.8981	-0.0721	0.2426	-0.0952	1.0000	0.0961	1.0000	0.0367	0.7600	0.0784
CTBP2	0.6423	0.1318	0.9022	0.0418	0.0376	0.3752	0.0001	0.3418	1.0000	-0.0951	0.3460	-0.1726	0.6247	-0.1226
CTBS	0.0317	0.3945	0.0002	0.3478	0.5417	-0.1583	1.0000	0.0264	1.0000	0.1551	0.7475	0.1205	0.0049	0.3453
CTC1	0.1744	-0.2938	0.0288	-0.2532	0.5854	0.1513	0.6179	-0.0846	1.0000	-0.0399	1.0000	0.0139	0.0639	-0.2695
CTCF	0.0029	0.5105	0.0000	0.5357	0.0000	0.7757	0.0000	0.6511	1.0000	-0.0083	1.0000	0.0296	0.5366	-0.1269
CTCFL	0.6572	0.8705	0.9366	-0.3232	0.7628	0.7646	1.0000	0.2662	1.0000	0.8338	1.0000	-0.3446	1.0000	0.3407
CTDP1	0.1308	0.3376	0.4437	0.1183	1.0000	0.0087	1.0000	0.0219	1.0000	0.1246	1.0000	-0.0814	0.6029	0.1448
CTDSP1	1.0000	-0.0597	0.7287	0.1034	0.1152	0.3639	0.0000	0.8537	1.0000	-0.2588	1.0000	-0.0818	0.2205	0.2391
CTDSPL	0.0198	0.3813	0.1982	0.1296	0.9361	0.0691	0.0407	0.1838	1.0000	0.0553	0.1963	-0.1837	0.2043	0.1757
CTDSPL2	0.2182	0.2377	0.0088	0.2507	0.1819	0.2601	0.1196	0.1630	1.0000	-0.0284	1.0000	-0.0025	0.6265	-0.1203
CTH	0.0000	-0.8033	0.0000	-0.7949	0.0527	-0.3623	0.0000	-0.5051	1.0000	-0.0318	1.0000	-0.0109	0.2505	-0.1692
CTHRC1	0.3628	0.1846	0.0000	0.5103	1.0000	0.0454	1.0000	-0.0102	1.0000	-0.0228	0.0179	0.3151	0.9536	-0.0725
CTIF	1.0000	0.0303	0.0096	0.6353	0.0000	1.3602	0.0000	1.4802	1.0000	-0.4034	0.7595	0.2161	0.0935	-0.2757
CTLA4	1.0000	0.0000	1.0000	0.0000	1.0000	0.0000	1.0000	0.0000	1.0000	0.0000	1.0000	0.0000	1.0000	0.0000
CTNNA1	0.0013	0.5530	0.0000	0.7013	0.0000	0.9001	0.0000	0.7835	1.0000	0.0016	0.5390	0.1628	0.4438	-0.1098
CTNNAL1	0.9094	-0.2468	0.9087	0.2055	0.3939	0.4367	0.0055	0.9266	1.0000	-0.4864	1.0000	-0.0213	1.0000	0.0057
CTNNB1	0.8947	0.0700	0.1779	0.1023	0.7042	-0.1058	0.0000	-0.2730	1.0000	0.0523	0.5853	0.0969	0.4171	-0.1094
CTNNBIP1	0.0145	-0.4447	0.0000	-0.4150	0.0000	-0.7571	0.0000	-0.6791	1.0000	-0.0071	1.0000	0.0347	0.9185	0.0761
CTNNBL1	0.0283	-0.3419	0.0000	-0.3819	0.0015	-0.4732	0.0000	-0.3241	1.0000	-0.0032	1.0000	-0.0309	0.2718	0.1512
CTNND1	0.0309	-0.3543	0.0000	-0.3402	0.3866	-0.1799	0.0000	-0.2925	1.0000	0.0288	1.0000	0.0554	0.7239	-0.0789
CTNND2	0.8760	-0.6513	0.4431	-3.7865	0.7693	0.7731	0.9096	-1.3717	1.0000	-0.8223	0.6427	-4.0589	0.1105	-2.9638
CTNS	0.9680	-0.1245	0.9431	0.0949	0.6597	0.2391	0.0288	0.4742	1.0000	-0.1135	1.0000	0.1178	0.9835	0.1237
CTPS1	0.4664	-0.1650	0.2635	0.1330	0.0009	-0.5631	0.0018	-0.3109	1.0000	-0.2620	1.0000	0.0477	1.0000	-0.0044
CTPS2	0.0000	1.0876	0.0000	1.1679	1.0000	-0.0762	0.9211	-0.0779	1.0000	0.0209	0.9587	0.1117	1.0000	0.0224
CTRC	1.0000	0.0000	1.0000	2.2507	1.0000	0.0000	1.0000	0.0000	1.0000	0.0000	1.0000	2.3455	1.0000	0.0000
CTRL	1.0000	-2.4788	1.0000	2.2507	1.0000	-2.4061	1.0000	2.3257	1.0000	-2.3771	1.0000	2.3455	1.0000	2.3554
CTSA	0.0000	-1.3038	0.0000	-1.1021	0.0001	-0.6375	0.0000	-0.4303	1.0000	-0.0418	0.1614	0.1725	0.1777	0.1705
CTSB	0.0144	0.4666	0.0000	0.3937	0.0008	0.6046	0.0000	0.4768	1.0000	0.0921	1.0000	0.0315	1.0000	-0.0306
CTSC	0.1867	0.2406	0.0016	0.2409	0.7242	-0.1068	0.1683	-0.1239	1.0000	0.1570	0.2687	0.1697	0.3282	0.1453
CTSD	0.0407	-0.3297	0.0000	-0.3279	0.8104	-0.0868	0.0000	-0.4244	1.0000	0.0807	0.6156	0.0951	0.0014	-0.2519
CTSE	1.0000	0.0000	1.0000	0.0000	1.0000	0.0000	1.0000	0.0000	1.0000	0.0000	1.0000	0.0000	1.0000	0.0000
CTSH	0.0003	-0.6171	0.0000	-0.4799	0.0000	-0.7406	0.2597	0.1098	1.0000	0.0457	0.1485	0.1951	0.0000	0.9009
CTSK	0.0000	-3.0660	0.0000	-3.3348	0.0000	-1.4779	0.0000	-0.9299	1.0000	0.1226	1.0000	-0.1342	0.0000	0.6760
CTSL	0.0000	0.9072	0.0000	0.7361	0.4809	0.1569	0.0003	-0.2576	1.0000	0.2543	0.6980	0.0956	0.2056	-0.1551
CTSO	0.4694	0.1918	1.0000	-0.0133	0.0020	0.5587	0.0000	0.5881	1.0000	0.0205	0.5444	-0.1727	1.0000	0.0547
CTSS	0.4202	0.2327	0.0022	0.4575	0.9361	0.1297	1.0000	0.0478	1.0000	-0.2457	1.0000	-0.0104	0.6072	-0.3216
CTSZ	0.7448	-0.0990	0.0002	-0.2843	0.0381	-0.3439	0.0000	-0.3350	1.0000	0.1688	1.0000	-0.0041	0.1864	0.1833
CTTN	0.6932	0.1083	0.1920	0.1055	0.5041	0.1439	0.1872	0.1045	1.0000	0.0893	0.6450	0.0990	0.9411	0.0553
CTTNBP2	0.0015	1.2883	0.3943	0.4856	0.0007	1.3966	0.0256	0.8606	1.0000	-0.1343	0.0360	-0.9232	0.1235	-0.6609
CTTNBP2NL	1.0000	0.0207	1.0000	-0.0363	0.1271	0.3332	0.0001	0.4103	1.0000	0.0861	1.0000	0.0415	0.4805	0.1672
CTU2	0.3150	-0.2666	1.0000	0.0001	0.0059	-0.6693	0.0016	-0.4879	1.0000	-0.2764	1.0000	0.0024	1.0000	-0.0897
CTXN1	1.0000	2.1847	1.0000	0.0000	1.0000	0.0000	1.0000	2.3257	1.0000	0.0000	1.0000	-2.2957	1.0000	2.3554
CTXN2	0.1101	4.6954	0.2920	3.9943	1.0000	0.0000	1.0000	0.0000	1.0000	0.0000	1.0000	-0.7149	1.0000	0.0000
CTXN3	0.6215	-0.9150	0.1484	2.0180	0.4042	-1.3149	1.0000	0.5318	1.0000	-2.1613	1.0000	0.7727	1.0000	-0.3121
CUBN	1.0000	-0.2271	0.3664	-0.5456	1.0000	0.0023	0.5796	-0.4189	1.0000	0.4937	1.0000	0.1863	1.0000	0.0804
CUEDC1	1.0000	0.0563	0.2876	0.1246	0.1035	0.2881	0.0000	0.5219	1.0000	-0.1719	0.8759	-0.0906	0.9165	0.0678
CUEDC2	0.7426	0.1146	0.2723	0.1213	0.2408	-0.2420	0.0018	-0.2963	1.0000	-0.1416	0.6605	-0.1226	0.2264	-0.1904
CUL1	0.0909	-0.2889	0.0073	-0.2315	0.9341	-0.0680	0.1629	-0.1258	1.0000	0.0465	0.6496	0.1161	1.0000	-0.0063
CUL3	0.8343	-0.0830	0.0632	-0.1571	0.0298	-0.3588	0.0000	-0.4293	1.0000	0.0191	1.0000	-0.0427	1.0000	-0.0462
CUL4A	0.3617	0.2040	0.0314	0.2646	0.0006	-0.6246	0.0000	-0.5665	1.0000	0.2243	0.0280	0.2977	0.0792	0.2893
CUL4B	0.0666	0.3189	0.0006	0.3089	0.0001	0.6334	0.0000	0.5098	1.0000	0.0584	1.0000	0.0609	0.9834	-0.0605
CUL5	0.0040	0.5309	0.0498	0.3056	0.0000	0.9841	0.0000	0.5945	1.0000	0.1904	1.0000	-0.0216	0.3290	-0.1937
CUTA	0.6231	-0.1191	0.0000	-0.4495	0.0000	-0.8118	0.0000	-0.7502	1.0000	0.0822	0.0154	-0.2358	0.2735	0.1492
CUTC	0.3790	-0.2092	0.0001	-0.4131	0.0000	-0.7854	0.0000	-0.7323	1.0000	0.1172	1.0000	-0.0740	0.5476	0.1770
CUX1	0.0023	-0.5384	0.0000	-0.6657	0.6560	0.1304	0.4927	0.0962	1.0000	-0.1929	0.0766	-0.3069	0.1236	-0.2217
CUX2	0.2958	-1.5010	0.5029	-1.6425	0.9739	0.5601	1.0000	0.4076	1.0000	-0.3398	1.0000	-0.4744	1.0000	-0.4905
CUZD1	0.8249	-3.3411	0.4431	-3.7865	1.0000	0.3498	1.0000	0.3941	1.0000	0.4097	1.0000	0.0000	1.0000	0.4627
CWC15	0.0566	-0.3249	0.0000	-0.3863	0.2129	-0.2342	0.0598	-0.1553	1.0000	0.0009	1.0000	-0.0481	0.7574	0.0856
CWC22	0.1542	0.2783	1.0000	0.0311	0.7600	-0.1136	0.2634	-0.1402	1.0000	0.0973	0.5657	-0.1384	0.9592	0.0745
CWC25	0.5963	-0.1756	0.3550	-0.1630	0.4426	-0.2106	0.1170	-0.2385	1.0000	-0.0122	1.0000	0.0143	1.0000	-0.0336
CWC27	0.7380	-0.1463	0.5390	-0.0983	0.0002	-0.8192	0.0000	-0.4931	1.0000	-0.1819	0.7881	-0.1215	0.6069	0.1505
CWF19L1	0.3295	0.2104	0.0000	0.4069	0.0000	-0.7316	0.0000	-0.5391	1.0000	-0.2284	1.0000	-0.0194	1.0000	-0.0296
CWF19L2	0.8226	0.1581	0.7250	-0.1216	0.0438	-0.5932	0.0004	-0.6672	1.0000	-0.0018	0.4823	-0.2696	1.0000	-0.0711
CX3CL1	0.6340	0.3167	0.0066	0.9087	0.0001	-1.6576	0.0115	-1.2411	1.0000	-0.8093	1.0000	-0.2076	1.0000	-0.3891
CX3CR1	1.0000	-2.4788	1.0000	-2.3958	1.0000	-2.4061	1.0000	-2.3177	1.0000	-0.1089	1.0000	0.0000	1.0000	0.0000
CXADR	1.0000	0.8866	1.0000	-0.1559	0.7662	1.0359	1.0000	-0.5176	1.0000	0.3999	1.0000	-0.6319	0.9835	-1.1470
CXCL12	0.2202	0.4023	1.0000	0.0690	0.0000	2.8093	0.0000	1.2562	1.0000	0.1274	0.8606	-0.1934	0.0000	-1.4193
CXCL14	1.0000	2.1847	1.0000	-2.3986	1.0000	0.0000	1.0000	-2.3199	1.0000	2.2736	1.0000	-2.2957	1.0000	0.0000
CXCR4	0.8191	-0.1850	0.0001	-0.9302	0.0550	-0.6795	0.9973	-0.0735	1.0000	0.0558	0.0505	-0.6777	0.0352	0.6678
CXCR5	1.0000	0.0272	0.8476	0.0541	0.6098	0.1424	0.0255	0.2272	1.0000	-0.1510	0.8553	-0.1116	1.0000	-0.0605
CXXC5	0.0000	-0.7404	0.0000	-0.7857	1.0000	0.0583	0.9096	-0.0372	1.0000	0.0678	1.0000	0.0352	1.0000	-0.0221
CYC	0.0000	-0.7806	0.0000	-0.8154	0.0004	-0.5985	0.0000	-0.6993	1.0000	-0.0497	0.8999	-0.0721	0.2901	-0.1445
CYFIP2	0.0044	-1.3617	0.0000	-1.4837	0.9987	0.1572	1.0000	-0.1067	1.0000	-0.1591	1.0000	-0.2676	0.4402	-0.4119
CYGB	0.0000	-2.4776	0.0000	-2.7395	0.0006	-1.2194	0.0000	0.8024	1.0000	0.3800	1.0000	0.1337	0.0000	2.4091
CYHR1	0.0032	1.2870	0.0000	1.6699	0.0001	1.5067	0.0000	1.7626	1.0000	-0.4374	1.0000	-0.0446	0.9868	-0.1800
CYLD	0.0016	-0.5729	0.0000	-0.6520	0.6063	-0.1491	1.0000	0.0152	1.0000	0.0654	1.0000	-0.0010	0.1564	0.2347

CYP1A5	0.8226	3.0199	1.0000	0.0000	0.8011	3.0840	1.0000	2.3241	1.0000	0.0000	1.0000	-3.1430	1.0000	-0.7795
CYP3A37	1.0000	2.1903	1.0000	2.2535	1.0000	0.0000	1.0000	0.0000	1.0000	0.0000	1.0000	0.0489	1.0000	0.0000
CYP3A80	0.2012	4.2368	0.5159	-1.6382	1.0000	2.2472	0.0805	-4.6366	1.0000	4.5742	1.0000	-1.1683	1.0000	-2.2909
CYR61	0.0028	0.4651	0.0000	0.9688	0.0135	0.4252	0.0000	0.7885	1.0000	-0.1243	0.0000	0.3916	0.0662	0.2445
CYSLTR1	0.7257	-0.7583	0.2504	1.3520	1.0000	0.1194	0.6312	0.9640	1.0000	-1.6542	1.0000	0.4640	0.6853	-0.8071
CYSLTR2	1.0000	0.0000	1.0000	0.0000	1.0000	0.0000	1.0000	0.0000	1.0000	0.0000	1.0000	0.0000	1.0000	0.0000
CYTH1	0.0251	-0.4430	0.0539	-0.2420	0.0012	-0.5877	0.9986	-0.0369	1.0000	-0.2495	1.0000	-0.0352	0.0430	0.3070
CYTH4	1.0000	-0.3358	0.0912	-1.9632	0.6300	0.9670	0.0173	-3.1634	1.0000	1.6666	1.0000	0.0603	0.3615	-2.4641
CYTIP	1.0000	0.0467	1.0000	0.6940	1.0000	-0.7120	0.9096	1.3850	1.0000	-1.4938	1.0000	-0.8610	1.0000	0.6041
CYYR1	1.0000	-0.1045	0.2791	-0.3612	0.2006	0.5124	0.1723	0.3998	1.0000	-0.3113	0.2252	-0.5557	0.2665	-0.4142
CZH18orf25	0.0387	0.5018	0.0064	0.3467	0.0192	0.5516	0.2905	0.1649	1.0000	0.4176	0.1502	0.2754	1.0000	0.0357
CZH5orf28	0.2249	0.3169	0.0000	0.6378	0.0129	0.5376	0.0000	0.4975	1.0000	-0.1298	0.4785	0.2031	0.5376	-0.1650
CZH5orf30	1.0000	-0.0998	0.1630	-0.4771	0.0444	0.6260	0.0045	0.5337	1.0000	-0.1140	0.5182	-0.4795	0.6865	-0.2018
CZH5orf34	0.0751	0.4443	0.0034	0.4402	0.2507	-0.3384	0.2479	-0.2192	1.0000	0.0097	1.0000	0.0182	0.9554	0.1359
CZH5orf42	0.0210	0.4580	0.4601	0.1240	0.0701	0.3730	0.9096	0.0569	1.0000	-0.1030	0.0014	-0.4239	0.0037	-0.4129
CZH5orf51	0.0348	0.5693	0.0016	0.4931	0.0018	-0.8790	0.0004	-0.6138	1.0000	-0.0193	1.0000	-0.0846	0.6004	0.2498
CZH5orf63	0.4743	0.3340	0.2638	0.2614	0.0001	-1.4180	0.0000	-2.1398	1.0000	0.0522	1.0000	-0.0089	0.3395	-0.6643
CZH9orf41	0.6725	-0.1908	1.0000	0.0533	0.5016	0.2437	0.6698	0.1211	1.0000	-0.1598	1.0000	0.0958	0.3995	-0.2798
CZH9orf64	0.1920	-0.2739	0.0262	-0.2395	0.0174	-0.4413	0.0000	-0.5727	1.0000	0.0619	0.8627	0.1087	1.0000	-0.0641
CZH9orf72	0.1423	0.2835	0.1836	0.1624	0.0224	0.4156	1.0000	0.0341	1.0000	0.0721	1.0000	-0.0355	0.0236	-0.3040
CZH9orf84	1.0000	0.0000	1.0000	-0.9997	1.0000	0.0000	0.7710	-3.1631	1.0000	3.1056	1.0000	2.3455	1.0000	0.0000
D2HGDH	0.0056	0.7390	0.0000	0.8370	0.7160	-0.1934	0.1889	-0.2944	1.0000	-0.2533	0.9146	-0.1440	0.2944	-0.3477
DAAM1	0.0035	0.8423	0.0004	0.6860	0.1743	0.4253	0.0000	0.7763	1.0000	-0.1111	0.6450	-0.2563	0.5399	0.2448
DAB1	1.0000	0.0000	1.0000	0.0000	1.0000	0.0000	1.0000	0.0000	1.0000	0.0000	1.0000	0.0000	1.0000	0.0000
DAB2	0.0577	0.7806	0.0035	0.8440	0.1413	0.6149	0.0107	0.8424	1.0000	0.2340	0.7374	0.3103	0.3713	0.4683
DAB2IP	1.0000	-0.0615	0.3094	0.1470	0.0002	0.8611	0.0000	1.0299	1.0000	0.0137	0.1104	0.2356	0.1398	0.1886
DACH1	0.3161	0.8310	0.0922	1.6637	1.0000	-0.0899	0.2700	1.4110	1.0000	-1.7637	0.4455	-0.9276	1.0000	-0.2617
DACH2	0.2056	1.1973	1.0000	0.2924	0.9987	0.5690	1.0000	0.2591	1.0000	-0.5930	0.1950	-1.4911	0.7446	-0.8945
DACT1	0.0000	-0.9090	0.0000	-0.5879	0.0000	0.7644	0.0000	0.5261	1.0000	-0.2568	1.0000	0.0775	0.0000	-0.4888
DACT2	0.8226	3.0199	1.0000	2.2534	1.0000	2.2472	1.0000	2.3241	1.0000	0.0000	1.0000	-0.7956	1.0000	0.0641
DAD1	0.0152	-0.3699	0.0001	-0.3774	0.0000	-0.9277	0.0000	-0.7179	1.0000	0.0193	1.0000	0.0240	0.0540	0.2345
DAG1	0.1607	0.3255	0.0000	0.4875	0.0000	0.9865	0.0000	0.8852	1.0000	0.0032	0.1648	0.1778	0.7387	-0.0935
DAGLA	0.7012	-0.1949	0.6299	-0.1461	1.0000	0.0027	1.0000	0.0660	1.0000	-0.1001	1.0000	-0.0391	1.0000	-0.0335
DAGLB	0.7778	0.1203	1.0000	-0.0101	0.1621	0.3100	0.0001	0.4065	1.0000	0.0194	0.9606	-0.0978	0.7363	0.1207
DAK	0.0005	-0.6912	0.0000	-0.7404	0.0384	-0.4225	0.9040	-0.0622	1.0000	-0.2126	0.3554	-0.2506	0.6181	0.1525
DALRD3	0.0000	-0.8745	0.0015	-0.4791	0.0032	-0.5737	0.0794	-0.2499	1.0000	-0.2476	0.8581	0.1594	1.0000	0.0809
DAO	0.3333	-0.5743	0.0025	-0.9614	0.2426	-0.7177	0.0140	-0.7748	1.0000	0.1847	1.0000	-0.1893	1.0000	0.1374
DAP	0.1577	0.2546	0.0351	0.1506	0.0214	-0.3669	0.0000	-0.2777	1.0000	0.1106	1.0000	0.0187	0.0337	0.2053
DAP3	1.0000	0.0388	0.9245	0.0398	0.0000	-0.8362	0.0000	-0.7432	1.0000	-0.1807	0.2235	-0.1675	0.9091	-0.0820
DAPK1	0.7721	-0.1093	0.0009	-0.3560	0.0243	-0.4333	0.7118	-0.0713	1.0000	0.0996	0.6991	-0.1339	0.0003	0.4664
DAPK2	0.0000	-2.1564	0.0000	-2.4520	0.0000	-0.6517	0.0000	-0.5721	1.0000	0.1400	0.7141	-0.1432	0.1406	0.2245
DAPK3	0.2317	0.2606	0.0000	0.8094	0.0000	0.7301	0.0000	1.2262	1.0000	-0.6126	1.0000	-0.0504	0.6508	-0.1101
DAPP1	1.0000	-0.0405	0.1635	-0.2029	0.0000	-0.7967	0.0000	-0.9159	1.0000	-0.0712	0.4452	-0.2215	0.5773	-0.1856
DARS	0.0545	0.3146	0.0000	0.3311	0.0000	-0.6636	0.0000	-0.6963	1.0000	0.0094	1.0000	0.0382	1.0000	-0.0178
DAW1	1.0000	0.0000	1.0000	-0.1473	1.0000	0.0000	1.0000	-2.3199	1.0000	2.2736	1.0000	2.3456	1.0000	0.0000
DAZAP1	1.0000	-0.0122	0.6368	-0.0592	0.0002	-0.5755	0.0000	-0.5954	1.0000	0.0560	1.0000	0.0213	1.0000	0.0414
DAZAP2	0.9015	-0.1869	1.0000	0.0472	0.2186	0.4789	0.0128	0.4525	1.0000	0.3839	0.0333	0.6352	0.1747	0.3623
DAZL	1.0000	0.0000	1.0000	0.0000	1.0000	0.0000	1.0000	2.3257	1.0000	0.0000	1.0000	0.0000	1.0000	2.3554
DBF4	0.4307	0.2246	0.0106	0.3082	0.9236	-0.0958	0.0056	-0.3294	1.0000	-0.0626	1.0000	0.0334	0.0923	-0.2893
DBF4B	0.7019	0.1783	0.6882	0.1530	1.0000	-0.0601	0.0311	-0.4266	1.0000	-0.0621	1.0000	-0.0758	0.1235	-0.4239
DBH	0.2232	-2.2131	0.9762	-1.0825	0.2806	-2.0622	0.2349	1.4116	1.0000	-1.0874	1.0000	0.0547	0.1717	2.3977
DBI	1.0000	0.0078	0.5968	0.0653	0.0000	-0.9857	0.0000	-1.0237	1.0000	-0.1421	0.8756	-0.0724	0.2017	-0.1740
DBN1	0.0642	-0.3153	0.0713	-0.1475	0.0007	-0.5460	0.0000	-0.3626	1.0000	-0.2521	0.8619	-0.0719	0.9091	-0.0637
DBNDD1	0.0002	-0.9122	0.0000	-1.2688	0.0609	-0.4732	0.0020	-0.5186	1.0000	-0.0137	0.4709	-0.3575	1.0000	-0.0525
DBNDD2	0.0001	-0.5654	0.0000	-0.7349	0.0000	-1.4057	0.0000	-1.2899	1.0000	0.0340	0.5501	-0.1232	0.5926	0.1560
DBNL	0.0000	-0.7403	0.0000	-0.5469	0.0071	-0.4566	0.9547	0.0347	1.0000	-0.3868	0.4206	-0.1814	0.7200	0.1104
DBR1	0.0203	0.4204	0.0001	0.3897	1.0000	-0.0304	0.7603	-0.0629	1.0000	-0.0075	1.0000	-0.0263	1.0000	-0.0343
DBT	0.6203	-0.2032	1.0000	-0.0038	0.0998	-0.4541	0.0031	-0.4624	1.0000	-0.0469	0.8785	0.1658	1.0000	-0.0496
DBX1	0.0000	-0.6778	0.0000	-1.1792	0.0450	-0.3577	0.0000	0.8057	1.0000	-0.0265	0.0000	-0.5154	0.0000	1.1428
DBX2	0.5086	3.5431	0.4160	1.1721	0.8033	3.0790	1.0000	-0.5247	1.0000	3.6422	0.8372	1.4025	1.0000	0.0729
DCAF10	0.1858	0.2470	0.1173	0.1540	0.8020	0.0953	0.2791	0.1151	1.0000	0.0358	1.0000	-0.0444	0.9919	0.0611
DCAF12	0.3024	0.4532	0.0284	0.6039	0.0000	1.4016	0.0046	0.8244	1.0000	0.1667	0.6072	0.3313	0.3691	-0.4041
DCAF13	0.4823	0.1603	0.0146	0.1923	0.9173	-0.0700	0.0029	-0.2241	1.0000	0.0359	0.8999	0.0803	0.5105	-0.1126
DCAF17	0.0450	-0.3881	0.0000	-0.4394	0.0011	-0.6073	0.0001	-0.3885	1.0000	0.0395	1.0000	0.0003	0.1339	0.2629
DCAF4	0.9427	0.0753	0.1257	-0.1997	0.0095	-0.4855	0.0000	-0.4963	1.0000	0.1013	0.4754	-0.1620	0.9196	0.0951
DCAF5	0.6300	0.1495	0.0260	0.2575	0.1536	-0.3139	0.0650	-0.2264	1.0000	-0.0711	1.0000	0.0492	1.0000	0.0217
DCAF7	0.2751	0.2169	0.2635	0.1257	0.5559	0.1622	0.4418	0.0938	1.0000	0.0605	1.0000	-0.0180	1.0000	-0.0030
DCAF8	0.4367	-0.2683	1.0000	-0.0011	0.0183	0.5445	0.0000	0.5490	1.0000	-0.1052	0.7962	0.1744	0.8333	-0.0937
DCAKD	0.1434	-0.7701	0.0016	-1.0910	0.1046	-0.7981	0.0914	-0.6080	1.0000	-0.1742	0.7833	-0.4812	1.0000	0.0172
DCBLD1	0.3036	0.4425	0.3293	0.2892	1.0000	0.0579	1.0000	-0.0900	1.0000	0.1923	1.0000	0.0523	1.0000	0.0517
DCBLD2	0.0166	0.4189	0.0015	0.2913	0.0180	0.4199	0.0000	0.4431	1.0000	0.0025	0.7168	-0.1126	1.0000	0.0310
DCDC1	1.0000	0.0000	1.0000	0.0000	1.0000	0.0000	1.0000	0.0000	1.0000	0.0000	1.0000	0.0000	1.0000	0.0000
DCDC2	0.8249	3.0140	1.0000	2.2535	0.8011	3.0840	1.0000	0.0000	1.0000	0.0000	1.0000	-0.7919	1.0000	-3.1344
DCDC2B	0.5543	-0.2143	0.0009	-0.5648	0.3452	-0.2810	0.5465	-0.1389	1.0000	0.0950	0.5902	-0.2438	0.4438	0.2409
DCHS1	0.8606	-0.1088	0.6893	0.0865	0.0000	1.3740	0.0000	1.5038	1.0000	-0.0479	0.6565	0.1605	0.9052	0.0882
DCHS2	1.0000	-2.4776	1.0000	-2.3958	1.0000	-2.4056	0.9096	1.3854	1.0000	-0.1078	1.0000	0.0000	0.7287	3.7387
DCK	0.0254	0.8643	0.0000	1.5863	0.0000	1.3694	0.0000	1.2016	1.0000	-0.2979	0.2691	0.4353	0.1312	-0.4609
DCLK1	0.0000	-1.8596	0.0000	-1.5521	0.0000	0.8554	0.0008	0.4120	1.0000	0.1564	0.1186	0.4773	0.0708	-0.2813
DCLK2	1.0000	-0.0063	0.3241	0.1060	0.0002	-0.6504	0.0000	-0.7238	1.0000	0.0529	0.2459	0.1777	1.0000	-0.0156
DCLK3	1.0000	-0.8620	1.0000	-0.1429	1.0000	0.1978	0.6172	1.7729	1.0000	-1.4938	1.0000	-0.7919	1.0000	0.0775
DCLRE1A	0.0011	0.7363	0.0000	0.6133	1.0000	-0.0568	0.1821	-0.1907	1.0000	0.0388	1.0000	-0.0708	0.9853	-0.0891
DCLRE1B	0.7453	0.1963	0.0414	0.3715	0.0365	0.5411	0.8484	0.0967	1.0000	-0.0684	1.0000	0.1171	0.0118	-0.5080
DCLRE1C	0.1836	0.3668	0.1950	0.2518	0.4697	-0.2461	1.0000	-0.0429	1.0000	-0.1280	0.5742	-0.2293	1.0000	0.0817
DCN	0.0000	-2.2650	0.0000	-2.1872	0.6794	-0.1623	0.0000	0.7255	1.0000	0.5836	0.0003	0.6746	0.0000	1.4757
DCP1A	0.0061	0.9157	0.0004	0.8945	0.0000	1.6879	0.0000	1.1875	1.0000	0.3195	0.5465	0.3133	0.8254	-0.1732
DCP1B	0.9545	0.1430	0.1440	0.4025	0.7716	0.2096	0.0438	0.5125	1.0000	-0.0894	1.0000	0.1833	0.8554	0.2216
DCP2	0.2535	0.9967	0.0884	0.8127	0.0771	1.3202	1.0000	0.0740	1.0000	0.6624	0.7581	0.4960	0.6802	-0.5813
DCPS	0.7697	0.1356	0.1876	0.2200	0.0423	-0.4710	0.0002	-0.5961	1.0000	-0.0861	1.0000	0.0093	0.6004	-0.2077
DCST1	0.8249	-3.3439	1.0000	0.0000	0.8033	-3.2636	1.0000	0.0000	1.0000	-3.2319	1.0000	0.0000	1.0000	0.0000
DCSTAMP	1.0000	0.0000	1.0000	0.0000	1.0000	0.0000	1.0000	0.0000	1.0000	0.0000	1.0000	0.0000	1.0000	0.0000
DCT	1.0000	0.0000	1.0000	0.0000	1.0000	0.0000	1.0000	0.0000	1.0000	0.0000	1.0000	0.0000	1.0000	0.0000
DCTD	0.0004	1.5591	0.0000	1.6940	0.0575	0.9297	0.8972	-0.2353	1.0000	0.2197	0.6438	0.3703	0.0750	-0.9389
DCTN3	0.0857	-0.2901	0.0016	-0.2610	0.0065	-0.4289	0.0000	-0.3986	1.0000	-0.0952	1.0000	-0.0540	0.9862	-0.0593

DCTN4	0.3832	0.1815	0.0011	0.2504	0.0223	-0.3695	0.0002	-0.2814	1.0000	-0.0957	1.0000	-0.0143	1.0000	-0.0023
DCTN5	0.1082	0.3220	0.0041	0.2971	0.0135	-0.4876	0.0000	-0.5983	1.0000	-0.1026	0.7561	-0.1161	0.3569	-0.2071
DCTN6	0.0485	-0.3475	0.6771	-0.0783	0.0001	-0.6679	0.0005	-0.3566	1.0000	-0.2398	1.0000	0.0412	0.9955	0.0772
DCUN1D1	1.0000	-0.0079	0.9486	-0.0479	0.0561	0.3734	0.0015	0.3641	1.0000	-0.0091	1.0000	-0.0358	1.0000	-0.0128
DCUN1D2	0.0204	-0.4475	0.0000	-0.5164	0.0002	-0.6838	0.0000	-0.8509	1.0000	0.1388	1.0000	0.0834	1.0000	-0.0229
DCUN1D3	0.6653	0.1891	0.7025	0.1043	0.5079	0.2235	1.0000	-0.0418	1.0000	0.1736	1.0000	0.1014	1.0000	-0.0841
DCUN1D4	0.8337	0.1031	0.8190	0.0604	0.0132	-0.5006	0.0000	-0.4727	1.0000	-0.0735	0.8820	-0.1031	1.0000	-0.0401
DCUN1D5	0.0003	-0.6536	0.0000	-0.7670	0.0745	-0.3608	0.0000	-0.4916	1.0000	-0.0502	0.5890	-0.1506	0.3778	-0.1760
DCX	0.0000	-2.1990	0.0000	-1.9867	0.0000	1.8150	0.0000	1.0733	1.0000	0.5267	0.1811	0.7563	0.6016	-0.2091
DCXR	0.3010	-0.2327	0.0000	-0.4504	0.0000	-1.2048	0.0000	-0.8814	1.0000	0.1436	1.0000	-0.0613	0.0050	0.4739
DDA1	1.0000	-0.0182	1.0000	0.0106	0.9353	0.0823	0.3603	-0.1439	1.0000	0.0916	0.8119	0.1335	0.7284	-0.1291
DDAH1	0.0000	1.3365	0.0000	1.9724	0.0000	2.3639	0.0000	2.0374	1.0000	-0.0155	0.0003	0.6351	0.0675	-0.3337
DDB1	0.0030	-0.4497	0.0000	-0.4326	0.0237	-0.3607	0.0000	-0.3120	1.0000	0.0088	1.0000	0.0384	0.8620	0.0626
DDB2	0.0504	-0.3862	0.0000	-0.6201	0.0000	-0.8868	0.0000	-0.7603	1.0000	0.2583	1.0000	0.0362	0.0032	0.3903
DDC	1.0000	0.0000	1.0000	-2.3958	1.0000	2.2427	1.0000	0.0062	1.0000	2.2674	1.0000	0.0000	1.0000	0.0652
DDHD1	0.1303	-0.4350	0.0001	-0.6994	0.0023	0.6826	0.0000	0.6736	1.0000	0.1203	1.0000	-0.1309	0.8496	0.1170
DDHD2	0.0001	-0.8447	0.0000	-0.9503	0.0338	-0.4838	0.2268	-0.1501	1.0000	0.0899	1.0000	-0.0036	0.0012	0.4278
DDIAS	0.4962	0.5191	0.4013	0.4133	0.3313	0.5483	0.2326	0.5053	1.0000	0.0939	1.0000	-0.0012	1.0000	0.0564
DDO	0.6235	0.1297	0.0041	0.2789	0.0000	-0.6788	0.0000	-0.8361	1.0000	-0.0850	0.9304	0.0761	0.1074	-0.2379
DDOST	0.1669	0.2436	0.0013	0.3122	1.0000	-0.0351	1.0000	0.0189	1.0000	-0.0115	1.0000	0.0698	1.0000	0.0480
DDR2	0.7266	-0.1133	0.0052	0.2420	1.0000	0.0485	0.0002	0.3093	1.0000	-0.3176	1.0000	0.0503	1.0000	-0.0511
DDRGK1	0.1308	-0.3340	0.0000	-0.5198	1.0000	0.0171	0.2681	-0.1461	1.0000	-0.0076	0.6427	-0.1816	0.4355	-0.1657
DDX1	0.2782	0.2064	0.0025	0.2216	0.8712	0.0749	0.6070	-0.0594	1.0000	0.0053	1.0000	0.0329	0.4082	-0.1236
DDX11	0.1978	0.3745	0.0025	0.5108	0.6386	0.2011	0.8091	0.1048	1.0000	-0.2676	1.0000	-0.1180	0.2259	-0.3592
DDX17	0.1699	0.2891	0.0041	0.3161	0.0000	1.0208	0.0000	0.9989	1.0000	0.0836	0.7549	0.1235	0.9475	0.0668
DDX18	0.5471	0.1477	0.0006	0.3115	0.1227	-0.2860	0.5931	-0.0769	1.0000	-0.0378	0.4839	0.1380	0.2582	0.1760
DDX20	0.0176	0.4477	0.0005	0.3692	0.9468	0.0790	1.0000	-0.0100	1.0000	0.0789	1.0000	0.0123	1.0000	-0.0053
DDX23	0.0000	-1.0489	0.0000	-0.3981	0.7950	-0.1059	0.0000	0.3690	1.0000	-0.4027	0.0539	0.2602	0.9059	0.0778
DDX24	1.0000	-0.0418	1.0000	-0.0293	0.8011	-0.1019	0.0186	-0.2167	1.0000	0.1266	0.4413	0.1512	1.0000	0.0170
DDX28	0.0057	-0.6563	0.0118	-0.3988	0.2176	-0.3194	0.1769	-0.2302	1.0000	-0.0713	0.7962	0.1990	1.0000	0.0227
DDX31	0.1345	0.3463	0.0002	0.4840	1.0000	-0.0180	1.0000	-0.0301	1.0000	-0.1767	1.0000	-0.0284	0.5572	-0.1832
DDX3X	0.5442	-0.1399	0.0001	-0.2888	0.0000	0.7044	0.0000	0.6081	1.0000	0.1160	1.0000	-0.0206	1.0000	0.0251
DDX4	1.0000	0.0000	1.0000	0.0000	1.0000	0.0000	1.0000	0.0000	1.0000	0.0000	1.0000	0.0000	1.0000	0.0000
DDX41	0.7255	0.1187	0.0217	0.2084	0.0162	-0.4460	0.0342	-0.1969	1.0000	-0.1222	1.0000	-0.0205	0.6029	0.1326
DDX42	0.1154	-0.2898	0.0000	-0.4241	0.1209	0.2792	0.0176	0.2267	1.0000	0.0558	1.0000	-0.0653	1.0000	0.0090
DDX43	1.0000	0.0000	0.2920	-4.1891	1.0000	0.0000	0.2782	-4.0944	1.0000	4.0426	1.0000	0.0000	1.0000	0.0000
DDX46	0.2380	0.2405	0.1330	0.1541	0.2372	0.2354	0.1059	0.1581	1.0000	0.0244	1.0000	-0.0493	1.0000	-0.0477
DDX47	0.4571	0.2043	0.0007	0.3850	1.0000	-0.0331	0.8399	0.0640	1.0000	-0.1776	1.0000	0.0144	1.0000	-0.0755
DDX49	0.4958	-0.1673	0.0875	0.2109	0.0002	-0.6428	0.0033	-0.3592	1.0000	-0.1997	0.2563	0.1902	0.9122	0.0886
DDX5	0.0000	0.7163	0.0000	0.4225	0.0001	-0.5818	0.0000	-0.5508	1.0000	0.0528	0.0043	-0.2285	0.5843	0.0892
DDX51	1.0000	0.0458	0.8331	0.0566	0.1222	-0.3241	0.0089	-0.2842	1.0000	-0.0331	1.0000	-0.0098	1.0000	0.0127
DDX52	0.3733	0.2288	0.0289	0.2554	0.9950	-0.0671	0.4280	-0.1172	1.0000	-0.0624	1.0000	-0.0244	0.8175	-0.1073
DDX54	0.0161	-0.4217	0.0081	-0.2803	0.0188	-0.4446	0.0203	-0.2440	1.0000	-0.1894	1.0000	-0.0365	1.0000	0.0166
DDX55	0.3203	-0.3142	0.8744	-0.0833	0.2182	-0.3407	0.3232	-0.2310	1.0000	-0.1978	1.0000	0.0469	1.0000	-0.0823
DDX59	0.0000	-0.8267	0.0000	-0.6724	0.0776	-0.3674	0.0000	-0.8695	1.0000	0.0300	0.5970	0.1968	0.0024	-0.4675
DDX6	0.0000	1.0536	0.0000	0.8908	0.0000	1.5556	0.0000	1.2442	1.0000	0.1589	1.0000	0.0080	0.5508	-0.1481
DEAF1	0.0966	-0.4154	0.0001	-0.5555	0.5591	-0.2011	0.2198	-0.2025	1.0000	0.0888	1.0000	-0.0385	1.0000	0.0924
DECR1	0.9614	0.1223	0.0173	0.2510	1.0000	0.0691	0.0522	-0.2126	1.0000	-0.4413	0.0271	-0.2999	0.0000	-0.7165
DECR2	0.4622	0.1731	0.6846	-0.1042	0.0201	-0.4437	0.0605	-0.2531	1.0000	-0.0265	0.1446	-0.2921	0.5584	0.1697
DEDD	0.6290	-0.1518	1.0000	-0.0021	1.0000	-0.0277	0.1607	0.1664	1.0000	-0.2128	1.0000	-0.0515	1.0000	-0.0135
DEF6	0.6957	1.7486	0.9062	1.2235	1.0000	-2.4056	1.0000	0.0062	1.0000	-0.1078	1.0000	-0.6318	1.0000	2.3543
DEF8	0.0012	-0.7588	0.0001	-0.6535	0.0392	-0.5274	0.1975	-0.2418	1.0000	-0.3853	0.5831	-0.2686	1.0000	-0.0935
DEGS1	0.0000	0.7218	0.0000	0.6541	0.0000	0.8946	0.0000	0.5646	1.0000	0.1176	1.0000	0.0630	0.1206	-0.2072
DEGS2	1.0000	-1.1514	1.0000	0.0000	1.0000	-0.1742	1.0000	0.0000	1.0000	-3.2289	1.0000	-2.2992	1.0000	-3.1370
DEK	0.0000	1.2020	0.0000	1.3580	0.0000	1.3423	0.0000	0.9969	1.0000	-0.0891	0.9048	0.0797	0.0000	-0.4277
DENND1A	1.0000	0.0103	0.5225	-0.0874	1.0000	-0.0241	1.0000	-0.0187	1.0000	0.0311	1.0000	-0.0538	1.0000	0.0420
DENND1B	0.6557	0.8719	0.6486	0.9505	0.9164	0.4629	0.2455	1.4122	1.0000	-0.4373	1.0000	-0.3454	1.0000	0.5247
DENND2A	0.1216	0.8017	0.0004	1.0821	0.4702	0.4413	0.1735	0.5436	1.0000	-0.0259	0.9420	0.2664	1.0000	0.0815
DENND2C	0.1047	-0.3982	0.0000	-0.3808	0.7813	-0.1311	0.4791	-0.0834	1.0000	0.0941	0.7609	0.1232	0.3941	0.1456
DENND2D	0.5819	0.5858	0.0890	1.0427	0.0078	1.3711	0.0000	2.1105	1.0000	-0.4811	1.0000	-0.0186	0.8937	0.2564
DENND3	0.2927	0.2865	0.7738	0.0805	1.0000	0.0120	0.8718	0.0658	1.0000	0.1009	1.0000	-0.0914	0.6615	0.1620
DENND4A	0.0175	0.5898	0.0000	0.6737	0.0000	0.9450	0.0000	0.9508	1.0000	0.0037	1.0000	0.1014	1.0000	0.0145
DENND4C	0.0313	0.4485	0.0201	0.3658	0.0008	0.6881	0.0000	0.5564	1.0000	0.0688	1.0000	-0.0007	1.0000	-0.0574
DENND5A	1.0000	0.0171	0.0531	-0.1923	1.0000	0.0526	0.5214	-0.0831	1.0000	0.0904	0.7635	-0.1061	1.0000	-0.0398
DENND6A	0.0135	0.6881	0.0002	0.6238	0.0000	1.0365	0.0000	0.8947	1.0000	0.1174	1.0000	0.0657	1.0000	-0.0187
DENND6B	0.1318	-0.3617	0.0005	-0.4630	0.7713	-0.1311	1.0000	0.0197	1.0000	-0.0093	1.0000	-0.0976	0.6842	0.1472
DENR	0.9135	0.0844	0.2880	0.1192	0.0000	-1.0347	0.0000	-0.9163	1.0000	-0.1502	0.7806	-0.1036	1.0000	-0.0262
DEPDC1	0.0001	1.6594	0.0000	1.7745	0.0000	1.5763	0.0033	1.0029	1.0000	-0.2111	1.0000	-0.0831	0.0117	-0.7792
DEPDC1B	0.0050	0.8921	0.0000	1.6301	0.0004	1.0014	0.0040	0.7822	1.0000	-0.5161	0.7323	0.2348	0.0031	-0.7270
DEPDC5	0.5397	0.2769	0.9947	0.0698	0.0330	0.5977	0.4399	0.1971	1.0000	0.1928	1.0000	-0.0010	0.5339	-0.2023
DEPDC7	0.0009	1.1777	0.0000	1.3330	0.1105	0.6471	0.0799	0.5738	1.0000	0.0495	0.8691	0.2180	1.0000	-0.0165
DEPTOR	0.8824	0.1274	0.0000	-1.1698	0.0020	0.7036	0.0000	0.7036	1.0000	0.4062	0.0000	-0.8769	0.0635	0.4118
DERA	0.0729	0.4641	0.0196	0.3551	0.0250	-0.6038	0.0000	-0.6728	1.0000	0.2443	0.8475	0.1479	0.8395	0.1794
DERL1	0.0423	0.4181	0.0001	0.3860	0.0003	0.6359	0.0000	0.6103	1.0000	-0.0521	1.0000	-0.0721	0.9515	-0.0725
DERL2	0.4395	0.1846	0.2649	0.1462	0.0114	-0.4618	0.0016	-0.4114	1.0000	-0.0652	0.9052	-0.0919	1.0000	-0.0097
desmin	0.0000	-1.7721	0.0000	-1.7233	0.0000	-1.3538	0.0000	-0.8455	1.0000	-0.2226	0.1628	-0.1613	0.0027	0.2904
DESI1	0.5953	0.1636	1.0000	-0.0120	0.0000	0.7739	0.0000	0.7787	1.0000	0.0079	0.8607	-0.1549	1.0000	0.0184
DESI2	0.2923	-0.2221	0.1326	-0.1449	0.9468	0.0679	0.6508	0.0656	1.0000	-0.0012	0.9234	0.0888	1.0000	0.0018
DET1	0.0070	0.5653	0.0038	0.4516	0.0000	0.9814	0.0000	0.6772	1.0000	0.1914	1.0000	0.0910	0.8052	-0.1065
DEXI	1.0000	2.1848	0.9148	1.2198	1.0000	2.2472	1.0000	0.0062	1.0000	2.2674	1.0000	1.4294	1.0000	0.0641
DFFA	1.0000	-0.0735	0.9084	-0.0726	0.0411	-0.5033	0.0696	-0.2955	1.0000	0.1076	1.0000	0.1207	0.2394	0.3206
DFFB	0.3070	0.4867	0.6622	0.1943	0.3596	0.3653	0.8455	0.1354	1.0000	0.0688	1.0000	-0.2101	1.0000	-0.1540
DFNA5	0.6231	0.1775	0.0004	0.4268	0.0000	-0.9401	0.0000	-0.6028	1.0000	-0.1984	1.0000	0.0648	0.8672	0.1452
DFNB31	1.0000	0.0000	1.0000	0.0000	0.8033	3.0790	0.7710	3.1753	1.0000	0.0000	1.0000	0.0000	1.0000	0.0753
DFNB59	0.1033	0.4062	0.3118	-0.2007	1.0000	-0.0090	0.8188	-0.0842	1.0000	0.0394	0.0020	-0.5548	1.0000	-0.0301
DGAT2	1.0000	0.8880	0.0701	2.8679	1.0000	0.7314	0.0532	2.8933	1.0000	-0.9563	0.9437	1.0219	0.7631	1.1998
DGCR14	0.9259	-0.0820	0.0017	-0.3866	0.3578	-0.2144	0.0013	-0.3782	1.0000	0.0497	0.2355	-0.2428	0.8022	-0.1090
DGCR2	1.0000	-0.0190	0.0412	-0.1850	1.0000	0.0086	0.0973	0.1470	1.0000	-0.0469	0.1427	-0.2005	0.6865	0.0973
DGCR6L	0.0146	-0.4103	0.0000	-0.4380	0.0595	-0.3314	0.0058	-0.2659	1.0000	-0.0856	0.8372	-0.1010	1.0000	-0.0147
DGCR8	0.0184	0.4846	0.0000	0.6055	0.0000	1.2311	0.0000	1.1527	1.0000	-0.0882	1.0000	0.0462	0.4271	-0.1603
DGKB	0.4445	-0.5455	0.0761	-0.5299	0.4809	-0.4926	0.0000	-1.2416	1.0000	0.7927	0.1175	0.8209	1.0000	0.0450
DGKD	0.0013	-0.5166	0.0000	-0.7484	0.9716	-0.0644	0.0396	-0.2452	1.0000	0.0970	0.6857	-0.1221	0.9017	-0.0791
DGKE	0.0012	0.5322	0.0000	0.6186	0.0167	0.4248	0.0016	0.3016	1.0000	0.0217	0.6649	0.1200	0.7931	-0.0974

DGKG	1.0000	-2.4776	1.0000	0.0000	1.0000	-0.1591	0.4370	3.7037	1.0000	-2.3757	1.0000	0.0000	1.0000	1.4483
DGKH	0.0057	-1.6141	0.0001	-1.6654	0.1786	-0.8019	0.0432	-0.8714	1.0000	0.0979	1.0000	0.0592	1.0000	0.0286
DGKI	1.0000	-0.1680	0.0345	-2.0172	1.0000	-0.0117	1.0000	-0.1952	1.0000	0.9423	1.0000	-0.8964	0.9122	0.7638
DGKK	0.1830	-1.5751	1.0000	-0.1608	0.6971	-0.6008	0.7296	1.2292	1.0000	-2.5702	1.0000	-1.1645	0.9562	-0.7396
DGKQ	1.0000	-0.0781	0.0889	0.4121	0.4940	0.2730	0.0650	0.3666	1.0000	0.0072	0.1596	0.5100	1.0000	0.1047
DGKZ	0.0014	-0.6863	0.0000	-0.7358	0.0001	-0.8251	0.0000	-0.3860	1.0000	0.0434	1.0000	0.0067	0.0000	0.4869
DGUOK	0.1621	-0.3315	0.0006	-0.4572	0.0000	-1.0679	0.0000	-0.5665	1.0000	-0.1648	0.2457	-0.2785	0.2784	0.3438
DHCR24	0.0000	0.9134	0.0000	0.9772	0.1386	0.2956	0.8680	-0.0551	1.0000	-0.0705	1.0000	0.0050	0.0003	-0.4158
DHDDS	0.8670	-0.0842	0.7878	-0.0526	0.1638	-0.2677	0.7552	-0.0562	1.0000	-0.0900	1.0000	-0.0459	0.5686	0.1260
DHDH	1.0000	-0.0425	1.0000	0.0228	0.0000	-1.2404	0.0001	-0.7857	1.0000	0.0003	1.0000	0.0781	0.2797	0.4607
DHFR	0.2433	-0.3333	0.1167	-0.2011	0.3999	0.2401	0.0733	0.2076	1.0000	-0.0131	0.8661	0.1311	1.0000	-0.0389
DHH	0.5086	-3.8790	1.0000	-0.6969	1.0000	-0.7104	0.9096	-1.3722	1.0000	-0.1210	1.0000	3.1945	1.0000	-0.7804
DHODH	0.1823	0.2551	0.0014	0.3461	0.3240	-0.2127	0.1657	-0.1686	1.0000	-0.1491	1.0000	-0.0465	0.8090	-0.0996
DHRS11	0.2101	-0.5275	1.0000	-0.0850	0.1540	-0.5121	0.3872	-0.2652	1.0000	-0.2272	0.9846	0.2292	1.0000	0.0229
DHRS12	0.3357	0.5700	0.6128	0.3353	0.0023	1.2023	0.0000	1.4511	1.0000	-0.3507	0.4436	-0.5753	1.0000	-0.0988
DHRS13	1.0000	-0.0945	1.0000	0.3207	1.0000	0.4494	0.8146	0.6404	1.0000	0.3497	1.0000	0.7732	1.0000	0.5410
DHRS3	0.0648	-0.5400	0.0000	-0.9810	0.0254	-0.6027	0.0000	1.2581	1.0000	-0.2513	0.0206	-0.6792	0.0000	1.6169
DHRS7	0.0241	-0.3802	0.0000	-0.4329	0.0001	-0.6362	0.0000	-0.4635	1.0000	0.0738	1.0000	0.0330	0.1398	0.2518
DHRS7B	0.6750	-0.1449	1.0000	0.0141	1.0000	-0.0301	1.0000	0.0364	1.0000	-0.0117	0.6300	0.1594	1.0000	0.0605
DHRS7C	0.0000	-2.9832	0.0000	-3.5652	0.0008	-0.5793	0.0000	-0.7253	1.0000	0.0191	0.1631	-0.5496	0.9656	-0.1219
DHRS9	0.2028	0.2975	0.4786	-0.1159	0.0000	-0.9513	0.0000	-0.6549	1.0000	0.0642	0.0766	-0.3373	0.0570	0.3667
DHTKD1	0.0564	-0.7500	0.1061	-0.5065	0.0000	1.3810	0.0000	1.3093	1.0000	-0.0653	1.0000	0.1889	0.9382	-0.1323
DHX15	0.8226	-0.0922	0.3472	-0.0973	0.0027	0.4737	0.0005	0.2702	1.0000	0.0966	0.6644	0.1041	0.6110	-0.1013
DHX30	0.3428	-0.2071	0.0842	-0.1774	0.0646	0.3383	0.0000	0.5002	1.0000	-0.0456	1.0000	-0.0033	0.5598	0.1221
DHX32	0.4055	-1.1073	0.0368	-1.6431	0.6009	0.6974	1.0000	0.0074	1.0000	-0.7993	0.4699	-1.3231	0.0070	-1.4879
DHX33	0.0114	0.6324	0.0000	0.9469	0.5605	0.2192	0.2542	0.2462	1.0000	-0.2833	1.0000	0.0440	0.4627	-0.2510
DHX34	1.0000	-0.1324	0.0008	1.3756	0.1629	0.6392	0.0000	2.5231	1.0000	-1.3666	1.0000	0.1544	0.1558	0.5275
DHX35	0.0000	1.1465	0.0000	1.1174	1.0000	0.0791	1.0000	-0.0087	1.0000	0.1165	1.0000	0.0989	1.0000	0.0332
DHX36	0.9438	0.0666	0.0020	0.2616	0.2895	-0.2087	0.1186	-0.1491	1.0000	-0.1856	1.0000	0.0219	0.5656	-0.1209
DHX37	0.1355	0.2754	0.0000	0.3503	0.1219	-0.2874	0.0003	-0.3637	1.0000	0.0251	0.6954	0.1121	1.0000	-0.0463
DHX38	1.0000	0.0416	0.0041	0.2303	0.3323	-0.1902	0.4934	-0.0783	1.0000	-0.1493	1.0000	0.0518	1.0000	-0.0322
DHX40	0.1830	0.2946	0.6477	0.0898	0.0882	-0.3655	0.0003	-0.4290	1.0000	-0.0178	0.3228	-0.2106	1.0000	-0.0759
DHX57	0.0513	0.5261	0.0000	0.8025	0.0011	0.7086	0.0000	0.7026	1.0000	-0.1228	0.8788	0.1638	0.8165	-0.1257
DHX58	0.1271	-0.5655	0.0032	-0.6696	0.4866	-0.3188	1.0000	-0.0107	1.0000	0.0382	1.0000	-0.0559	0.4248	0.3526
DHX8	0.0259	0.5413	0.0066	0.4565	0.0000	1.0051	0.0000	0.7400	1.0000	0.1733	1.0000	0.1021	0.9707	-0.0851
DHX9	1.0000	0.0136	0.6396	-0.5821	1.0000	0.1731	0.7290	0.5009	1.0000	0.1291	1.0000	-0.4567	1.0000	0.4606
DIABLO	1.0000	-0.0359	0.5613	-0.0852	0.1098	-0.3031	0.0001	-0.3727	1.0000	-0.2239	0.0845	-0.2604	0.0264	-0.2880
DIAPH2	1.0000	0.0637	0.3073	-0.0994	0.0502	0.3743	0.0056	0.2129	1.0000	0.0741	0.8840	-0.0763	0.7643	-0.0815
DIAPH3	0.0000	0.9071	0.0000	1.2209	0.0000	1.0662	0.0003	0.4319	1.0000	-0.2888	1.0000	0.0368	0.0000	-0.9178
DICER1	0.0000	1.6374	0.0000	1.3598	0.0000	2.1239	0.0000	1.6723	1.0000	0.1700	1.0000	-0.0908	0.6213	-0.2720
DIDO1	1.0000	0.0415	0.5558	0.0993	0.0002	0.6222	0.0000	0.4008	1.0000	0.0547	0.7475	0.1257	0.3640	-0.1610
DIEXF	0.6832	-0.1464	1.0000	-0.0065	1.0000	-0.0637	0.2299	-0.1447	1.0000	0.0323	0.4627	0.1843	1.0000	-0.0431
DIMT1	0.6962	0.1475	0.0021	0.4382	0.3447	0.2432	0.1922	0.2008	1.0000	-0.0087	0.2049	0.2955	1.0000	-0.0458
DIO1	1.0000	0.1953	0.1369	0.8115	0.0992	-1.3735	0.2940	-0.8413	1.0000	-0.3269	1.0000	0.2967	1.0000	0.2120
DIO2	1.0000	-0.1436	0.4867	-0.3742	0.1207	-0.7842	0.9993	-0.1273	1.0000	0.0897	1.0000	-0.1303	0.2400	0.7495
DIO3	0.0648	-1.1374	0.1517	-0.4418	0.8011	-0.3375	1.0000	0.0365	1.0000	0.2615	0.0459	0.9691	0.1010	0.6417
DIP2A	0.0000	0.9482	0.0000	0.7318	0.0000	1.1264	0.0000	0.6636	1.0000	0.3637	0.6848	0.1614	0.9287	-0.0934
DIP2B	0.9434	0.0660	0.8890	-0.0362	0.0000	0.8876	0.0000	0.5153	1.0000	0.1425	1.0000	0.0529	0.0190	-0.2244
DIP2C	0.0000	-1.3087	0.0000	-1.5980	1.0000	0.0157	0.0633	-0.1686	1.0000	0.0990	0.4447	-0.1775	0.8216	-0.0800
DIRAS1	0.0000	-2.7511	0.0000	-2.3733	0.0959	-0.4794	0.4322	0.1614	1.0000	-0.3299	1.0000	0.0618	0.2053	0.3158
DIRC2	0.5927	0.2016	0.0664	0.3525	0.0095	0.5799	0.0113	0.4405	1.0000	-0.0259	0.9468	0.1370	0.6865	-0.1613
DIS3	0.8941	0.1021	0.2526	-0.1871	0.3516	-0.2589	0.0073	-0.3579	1.0000	0.1888	1.0000	-0.0887	0.9671	0.0948
DIS3L	0.0093	0.4683	0.0237	0.2558	0.9496	-0.0778	0.0848	-0.2042	1.0000	0.0687	0.6450	-0.1322	1.0000	-0.0545
DIS3L2	0.3174	0.2224	0.0021	0.3585	0.0003	-0.6650	0.0140	-0.3127	1.0000	-0.1543	1.0000	-0.0067	0.4145	0.2035
DISC1	0.7746	-0.1112	0.0581	-0.2864	0.9183	-0.0826	1.0000	0.0373	1.0000	0.0493	0.9356	-0.1129	0.4272	0.1742
DISP1	1.0000	0.0218	0.9039	0.0438	0.0055	-0.4690	0.4490	0.0792	1.0000	0.0599	0.9234	0.0944	0.0000	0.6129
DISP2	1.0000	0.0000	1.0000	0.0000	1.0000	0.0000	1.0000	0.0000	1.0000	0.0000	1.0000	0.0000	1.0000	0.0000
DIXDC1	0.1527	0.5011	1.0000	-0.0329	0.0000	-1.4653	0.0000	-1.1015	1.0000	0.1713	0.5143	-0.3495	0.2802	0.5431
DKC1	0.3738	0.1929	0.0263	0.1851	0.0907	-0.2996	0.0000	-0.3496	1.0000	-0.0824	0.9375	-0.0778	0.5093	-0.1267
DKK2	0.1257	1.7004	0.0711	1.0287	0.0037	2.2117	0.9961	-0.3088	1.0000	1.1528	0.8820	0.4891	0.0541	-1.3602
DKK3	0.0472	-2.2594	0.7280	-0.4907	0.0000	3.2037	0.0000	2.5426	1.0000	0.1813	0.3571	1.9738	0.2862	-0.4700
DLAT	0.0343	0.4010	0.0000	0.7010	0.0206	0.4401	0.0002	0.4051	1.0000	-0.1329	0.3656	0.1807	0.4594	-0.1625
DLD	0.2625	0.2289	0.3833	0.0906	0.5990	-0.1379	0.0130	-0.1946	1.0000	0.0478	0.9587	-0.0780	1.0000	-0.0034
DLEC1	1.0000	0.0513	0.7249	1.0539	1.0000	-0.7189	1.0000	0.0066	1.0000	-0.6522	1.0000	0.3600	1.0000	0.0730
DLEU7	1.0000	0.0000	1.0000	0.0000	1.0000	0.0000	1.0000	0.0000	1.0000	0.0000	1.0000	0.0000	1.0000	0.0000
DLG1	0.0418	0.6761	0.0029	0.7101	0.0000	2.3720	0.0000	2.1022	1.0000	0.1295	0.9254	0.1770	0.7749	-0.1332
DLG2	0.0000	-2.3307	0.0000	-3.0357	0.3222	0.2055	0.0062	-0.2522	1.0000	-0.0574	0.0000	-0.7505	0.0000	-0.5099
DLG5	0.2326	0.2729	0.5899	-0.1379	0.0000	1.0037	0.0002	0.4671	1.0000	0.1546	0.4252	-0.2429	0.0034	-0.3761
DLGAP1	1.0000	0.1206	1.0000	-0.4260	1.0000	-0.1909	1.0000	-0.2439	1.0000	0.1246	1.0000	-0.4100	1.0000	0.0777
DLGAP2	1.0000	0.1029	1.0000	0.0574	0.1929	0.7410	0.4837	0.4440	1.0000	-0.0249	1.0000	-0.0586	0.8941	-0.3156
DLGAP3	0.0000	-2.5156	0.0000	-1.6187	0.0000	-2.1221	0.0000	-1.1714	1.0000	0.0067	0.0714	0.9202	0.0079	0.9635
DLGAP4	0.0000	-1.0590	0.0000	-1.0713	1.0000	-0.0508	1.0000	-0.0019	1.0000	-0.0666	1.0000	-0.0668	1.0000	-0.0123
DLGAP5	0.0000	1.0102	0.0000	1.4842	0.0003	0.6295	0.0263	0.3145	1.0000	-0.2680	0.3998	0.2173	0.0000	-0.5781
DLK1	1.0000	-0.1680	1.0000	0.1329	0.3903	-1.8765	0.6172	-1.7581	1.0000	-0.8985	1.0000	-0.5920	1.0000	-0.7769
DLK2	0.0000	-4.2450	0.0000	-4.3357	0.4770	0.2652	0.0000	0.5757	1.0000	0.0726	1.0000	-0.0030	0.0144	0.3910
DLL1	0.0000	1.2600	0.0000	1.0208	0.9341	-0.1023	0.5086	-0.2000	1.0000	-0.1372	0.0258	-0.3625	0.6201	-0.2283
DLL4	0.5391	-3.8767	0.3422	-1.8566	1.0000	0.2002	1.0000	0.3589	1.0000	1.0348	1.0000	3.1976	0.7629	1.2003
DLST	0.2263	-0.2404	1.0000	-0.0298	0.0398	0.3431	0.0107	0.2730	1.0000	-0.1212	0.8786	0.1017	0.1613	-0.1853
DLX1	1.0000	0.0000	0.7674	-3.2498	1.0000	2.2427	0.7710	-3.1631	1.0000	3.1056	1.0000	0.0000	1.0000	-2.2888
DLX5	1.0000	-1.1537	1.0000	-0.1734	1.0000	0.7355	1.0000	0.4129	1.0000	1.3496	0.6450	2.3757	0.9410	1.0365
DLX6	0.1147	-1.8740	0.6849	-0.9912	0.2833	-1.4169	0.8938	-0.5584	1.0000	-0.5340	1.0000	0.3634	1.0000	0.3294
DMAP1	1.0000	0.0595	0.0874	0.2305	1.0000	0.0661	0.2377	0.1750	1.0000	-0.1042	1.0000	0.0786	1.0000	0.0099
DMBX1	1.0000	0.0000	1.0000	0.0000	1.0000	0.0000	1.0000	0.0000	1.0000	0.0000	1.0000	0.0000	1.0000	0.0000
DMC1	0.0205	1.1884	0.1758	0.5609	0.1035	1.0303	0.7599	-0.3484	1.0000	0.7478	1.0000	0.1330	0.7009	-0.6292
DMD	0.0000	-0.6456	0.0000	-0.8986	0.0928	0.2874	0.0888	0.1240	1.0000	0.1345	0.7020	-0.1062	1.0000	-0.0236
DMGDH	1.0000	-0.0857	1.0000	0.0111	0.0000	1.6965	0.0000	1.2676	1.0000	-0.1225	1.0000	-0.0130	0.0051	-0.5456
DMP1	0.0000	2.9745	0.0000	3.1215	1.0000	0.0581	0.3334	-0.6206	1.0000	-0.3403	0.9348	-0.1821	0.1279	-1.0156
DMRT1	1.0000	2.1903	0.7666	-3.2534	0.0535	4.7653	0.7246	1.2302	1.0000	3.1126	1.0000	-2.2992	1.0000	-0.3874
DMRT2	0.0000	-3.7478	0.0000	-3.5288	0.0000	-1.0908	0.3070	-0.1786	1.0000	0.3544	0.5859	0.5852	0.0000	1.2690
DMRT3	1.0000	0.0000	1.0000	0.0000	1.0000	0.0000	1.0000	0.0000	1.0000	0.0000	1.0000	0.0000	1.0000	0.0000
DMRTA2	0.9650	1.0733	1.0000	-2.3958	0.4584	1.8985	0.3945	2.0770	1.0000	-0.1089	0.8764	-3.6794	1.0000	0.0763
DMRTB1	0.3150	-4.2704	1.0000	-2.3986	0.6647	-1.9424	1.0000	0.0057	1.0000	-1.8802	1.0000	0.0000	1.0000	0.0660
DMTF1	0.2184	0.3670	0.2164	0.2822	0.0131	0.6019	0.0095	0.4665	1.0000	-0.1023	0.9049	-0.1743	0.4818	-0.2322

DMXL2	0.4882	0.7418	1.0000	-0.0121	0.0000	2.7729	0.0013	1.2385	1.0000	0.9109	1.0000	0.1707	0.1528	-0.6179
DNA2	0.0000	1.6962	0.0000	1.7309	0.0062	0.8997	0.6428	0.2038	1.0000	-0.0882	1.0000	-0.0418	0.0011	-0.7790
DNAAF1	0.0200	0.6176	0.0000	0.6213	0.0211	-0.6404	0.4004	-0.1801	1.0000	0.1282	0.9334	0.1457	0.0082	0.5952
DNAAF5	0.2916	0.2561	1.0000	0.0339	0.0528	0.3955	0.1055	0.2096	1.0000	0.1088	0.8915	-0.1011	1.0000	-0.0715
DNAH1	1.0000	-0.3500	0.7001	-0.7342	1.0000	-0.7493	0.1955	-1.5131	1.0000	0.8367	1.0000	0.4673	1.0000	0.0801
DNAH10	0.0038	1.9670	0.4586	0.5232	1.0000	0.3718	0.0019	-2.3810	1.0000	1.3022	1.0000	-0.1269	0.3976	-1.4478
DNAH12	0.0222	0.8921	0.5641	0.2821	1.0000	-0.1704	0.1102	-0.6784	1.0000	-0.0722	0.1304	-0.6719	0.4633	-0.5759
DNAH14	1.0000	0.0000	1.0000	0.0000	1.0000	0.0000	1.0000	0.0000	1.0000	0.0000	1.0000	0.0000	1.0000	0.0000
DNAH17	0.0913	-0.4837	0.0004	-0.6123	0.6742	-0.1923	0.9460	-0.0665	1.0000	0.0362	1.0000	-0.0815	0.7783	0.1681
DNAH3	0.0022	0.8028	0.0042	0.5784	0.0081	-0.8560	0.0000	-0.9521	1.0000	-0.0840	0.4508	-0.2954	1.0000	-0.1767
DNAH5	0.2288	-0.8606	0.0006	-1.8392	0.8211	0.3234	0.1959	-0.6674	1.0000	0.5916	1.0000	-0.3732	0.8686	-0.3926
DNAI2	0.4507	-1.8112	0.1616	-2.6990	0.3106	-2.4982	0.3402	-1.6822	1.0000	0.0895	1.0000	-0.7966	1.0000	0.9164
DNAJA1	0.0000	-1.5801	0.0000	-2.0460	0.0000	0.9796	0.0000	1.0552	1.0000	-0.0181	0.0015	-0.4712	0.8846	0.0629
DNAJA2	0.0054	-0.4338	0.0000	-0.6179	0.0004	-0.5398	0.0000	-0.6908	1.0000	0.0948	0.8913	-0.0768	1.0000	-0.0508
DNAJA3	0.2934	-0.2191	0.4527	-0.0938	1.0000	-0.0563	0.8228	-0.0497	1.0000	0.0181	0.4187	0.1566	1.0000	0.0300
DNAJA4	0.0260	-0.4527	0.0000	-0.5464	0.3148	0.2293	0.0296	0.2424	1.0000	0.0647	1.0000	-0.0161	0.9270	0.0840
DNAJB1	0.0020	-0.5808	0.0000	-0.5401	0.1888	0.2803	0.0004	0.3341	1.0000	-0.0185	1.0000	0.0342	1.0000	0.0399
DNAJB11	0.3297	-0.1920	1.0000	-0.0168	0.0028	-0.4744	0.0159	-0.2055	1.0000	-0.0451	0.4101	0.1424	0.0472	0.2289
DNAJB12	0.0827	-0.3072	0.0109	-0.2026	0.3946	-0.1843	0.3656	0.0889	1.0000	0.0432	0.2929	0.1605	0.0009	0.3212
DNAJB13	0.3313	-0.4381	0.0088	-0.8575	0.0161	-0.8566	0.0001	-1.3689	1.0000	-0.2775	0.1446	-0.6873	0.1268	-0.7856
DNAJB14	0.0189	-0.3748	0.0000	-0.4036	1.0000	-0.0501	0.1013	-0.1405	1.0000	0.0197	1.0000	0.0033	0.9017	-0.0654
DNAJB2	0.0000	-0.7049	0.0000	-0.3680	0.0000	-0.6990	0.0000	-0.3185	1.0000	-0.3211	1.0000	0.0280	0.9689	0.0649
DNAJB4	0.1245	-0.2792	0.0000	-0.3397	0.2392	0.2257	0.0001	0.3118	1.0000	0.1558	0.7275	0.1081	0.0211	0.2475
DNAJB5	0.0000	-2.2126	0.0000	-2.0204	0.0178	0.4690	0.0001	0.4354	1.0000	0.0572	0.4857	0.2619	1.0000	0.0280
DNAJB6	0.2743	0.1957	0.1181	0.1312	0.4276	0.1595	0.4684	-0.0751	1.0000	0.0623	1.0000	0.0102	0.1361	-0.1672
DNAJB8	0.9650	1.0594	0.2495	4.0050	1.0000	-2.4056	0.4370	3.7037	1.0000	-2.3757	1.0000	0.4467	0.7287	3.7384
DNAJB9	1.0000	-0.0002	0.0041	-0.2932	0.0132	0.4464	0.9956	0.0344	1.0000	0.3107	1.0000	0.0303	0.8110	-0.0955
DNAJC1	0.0605	-0.3404	0.0000	-0.4321	0.0000	-0.8249	0.0000	-0.6380	1.0000	0.0438	1.0000	-0.0356	0.1094	0.2357
DNAJC10	0.4522	0.1562	0.0580	0.1572	0.1436	0.2538	0.0211	0.1792	1.0000	0.0439	1.0000	0.0572	1.0000	-0.0259
DNAJC11	1.0000	-0.0190	0.6048	-0.0773	0.2028	-0.2437	0.0003	-0.3371	1.0000	-0.0517	0.8395	-0.0979	0.4964	-0.1407
DNAJC12	0.0001	0.8526	0.0488	0.2923	0.0031	-0.7781	0.0000	-0.7656	1.0000	0.3916	0.6427	-0.1581	0.0880	0.4072
DNAJC13	0.0303	-0.3557	0.0000	-0.4638	0.2881	0.2123	0.3100	0.1056	1.0000	-0.0094	0.7635	-0.1053	0.6789	-0.1115
DNAJC14	0.0279	-0.3911	0.0000	-0.4582	0.0495	-0.3871	0.4373	-0.1089	1.0000	-0.1462	0.3053	-0.2015	0.6894	0.1375
DNAJC15	0.0962	-0.4431	0.0000	-0.7024	0.1404	-0.3866	0.0000	-0.7655	1.0000	0.2296	1.0000	-0.0173	0.8941	-0.1449
DNAJC16	0.3892	0.2233	0.0891	0.2182	0.5952	-0.1708	0.1234	-0.2039	1.0000	0.0159	1.0000	0.0242	1.0000	-0.0122
DNAJC17	0.0008	0.8415	0.0000	0.7301	0.0192	-0.6977	0.0001	-0.6546	1.0000	-0.0134	0.9658	-0.1137	1.0000	0.0334
DNAJC18	0.5486	-0.1437	0.0064	-0.2454	0.0001	-0.6134	0.0000	-0.5990	1.0000	0.0005	0.8496	-0.0890	1.0000	0.0202
DNAJC19	0.3884	0.2464	0.2530	0.1799	0.0228	-0.5526	0.0000	-0.7420	1.0000	-0.0104	1.0000	-0.0650	0.6276	-0.1933
DNAJC2	0.3630	-0.1986	0.2674	-0.1155	0.0000	-0.7877	0.0000	-0.7476	1.0000	-0.0339	1.0000	0.0616	1.0000	0.0116
DNAJC21	0.0544	0.4537	0.0005	0.4289	1.0000	0.0182	1.0000	-0.0086	1.0000	-0.0104	1.0000	-0.0234	1.0000	-0.0327
DNAJC22	1.0000	0.0000	1.0000	0.0000	1.0000	0.0000	1.0000	2.3257	1.0000	0.0000	1.0000	0.0000	1.0000	2.3554
DNAJC24	0.4594	0.3703	1.0000	0.0972	0.3729	-0.4554	0.2320	-0.4067	1.0000	0.0516	0.9681	-0.2096	1.0000	0.1041
DNAJC25	1.0000	-0.0699	0.4847	-0.1587	0.3040	0.2595	0.3593	0.1796	1.0000	0.0900	1.0000	0.0125	1.0000	0.0132
DNAJC27	0.3329	-0.3315	0.3601	-0.2247	0.0655	-0.5288	0.0970	-0.3657	1.0000	-0.1318	1.0000	-0.0140	1.0000	0.0375
DNAJC28	0.0789	-0.6986	0.0250	-0.5789	1.0000	0.0914	0.2347	-0.3288	1.0000	-0.0079	1.0000	0.1253	0.2997	-0.4239
DNAJC3	0.0000	0.9016	0.0000	0.7321	0.0008	0.5252	0.0000	0.4225	1.0000	0.1544	1.0000	-0.0030	1.0000	0.0563
DNAJC5	0.1992	0.2658	0.0000	0.5100	0.5386	0.1585	0.0103	0.2604	1.0000	-0.0536	0.2323	0.2023	1.0000	0.0528
DNAJC5B	0.0461	-0.5836	0.4126	-0.2328	0.0010	-0.9159	0.0114	-0.5381	1.0000	-0.2611	1.0000	0.1031	1.0000	0.1250
DNAJC6	0.0034	0.7359	0.0015	0.4773	0.9510	-0.1034	0.0003	-0.5624	1.0000	0.1132	0.9957	-0.1319	0.2085	-0.3416
DNAJC7	0.0000	-0.9513	0.0000	-1.0485	0.0000	-0.8564	0.0000	-0.9083	1.0000	0.1023	1.0000	0.0175	0.9875	0.0556
DNAJC8	0.0039	-0.4651	0.0000	-0.4328	0.0010	-0.5194	0.0000	-0.4613	1.0000	-0.0566	1.0000	-0.0120	1.0000	0.0069
DNAJC9	0.1250	0.3241	0.0000	0.5034	0.6415	-0.1548	0.1812	-0.1859	1.0000	-0.1719	1.0000	0.0194	0.3930	-0.1973
DNAL1	0.8226	-0.1394	0.1136	-0.2253	0.0055	0.6014	0.0000	0.5366	1.0000	-0.0004	1.0000	-0.0734	1.0000	-0.0603
DNAL4	1.0000	-0.0531	0.7199	-0.0713	0.1163	-0.3585	0.0180	-0.2632	1.0000	-0.1015	0.8676	-0.1069	1.0000	0.0007
DNALI1	0.5349	-0.3173	0.0000	-0.9512	0.6579	-0.2615	0.0183	-0.4583	1.0000	0.0973	0.1298	-0.5261	1.0000	-0.0943
DNASE1L2	0.8249	3.0255	1.0000	-0.7038	1.0000	0.0000	1.0000	-0.5262	1.0000	3.6521	1.0000	0.0496	1.0000	3.2066
DNASE1L3	1.0000	0.6016	0.1551	2.3761	0.9361	-1.5512	1.0000	-2.3199	1.0000	-1.4918	1.0000	0.2735	1.0000	-2.2909
DNASE2B	0.0000	-1.8894	0.0000	-2.0188	0.7557	0.2119	0.0954	-0.4821	1.0000	0.2478	1.0000	0.1326	0.2400	-0.4402
DND1	1.0000	-2.4776	1.0000	0.0000	1.0000	-2.4056	1.0000	0.0000	1.0000	-2.3757	1.0000	0.0000	1.0000	0.0000
DNM1	0.1813	0.6426	0.0292	0.7027	1.0000	-0.1368	0.7701	-0.1648	1.0000	-0.0241	1.0000	0.0508	1.0000	-0.0469
DNM1L	0.2239	-0.2279	0.0000	-0.3536	0.0763	0.2939	0.2412	0.1090	1.0000	0.1173	1.0000	0.0043	0.9356	-0.0623
DNMBP	0.7769	0.1349	0.0239	0.2712	0.5719	-0.1877	0.0242	0.2821	1.0000	-0.1286	1.0000	0.0203	0.0243	0.3472
DNMT1	0.8800	-0.0936	0.0294	0.2347	0.0437	0.3972	0.0000	0.5305	1.0000	-0.1989	0.5962	0.1415	1.0000	-0.0605
DNMT3A	0.0000	-0.9824	0.0000	-0.7820	1.0000	-0.0004	0.0956	0.1917	1.0000	-0.1220	1.0000	0.0908	0.9777	0.0752
DNMT3B	0.0014	-4.0261	0.0021	-3.6725	0.0355	-1.8257	0.9825	-0.3333	1.0000	-0.3212	1.0000	0.0495	0.6855	1.1787
DNTT	1.0000	2.1903	1.0000	-2.3986	1.0000	0.0000	1.0000	-2.3199	1.0000	2.2736	1.0000	-2.2992	1.0000	0.0000
DNTTIP1	0.2806	-0.2083	0.0041	-0.2857	0.0000	-1.0278	0.0000	-0.8415	1.0000	-0.1758	0.0849	-0.2411	1.0000	0.0163
DNTTIP2	1.0000	0.0255	0.2885	0.1026	0.3300	-0.2004	0.0066	-0.2184	1.0000	-0.0189	0.9572	0.0705	1.0000	-0.0320
DOC2B	0.0021	-1.0904	0.0000	-1.6303	0.4561	0.3130	0.1584	0.3668	1.0000	0.0272	0.5668	-0.5006	1.0000	0.0862
DOCK11	0.6505	0.1711	0.4295	0.1564	0.0028	0.6063	0.0000	0.8462	1.0000	-0.1120	0.9987	-0.1132	0.6643	0.1338
DOCK2	1.0000	-0.0795	1.0000	-0.0031	0.3986	0.3940	1.0000	0.0589	1.0000	-0.3594	0.8797	-0.2694	0.0224	-0.6894
DOCK3	1.0000	0.5381	1.0000	-2.3958	0.0280	3.1142	0.0006	3.7931	1.0000	-0.1078	1.0000	-3.1382	0.9919	0.5734
DOCK4	0.0461	-0.5013	0.0000	-1.0661	0.0694	0.4581	0.7835	0.0893	1.0000	0.4031	0.9942	-0.1483	1.0000	0.0389
DOCK5	0.0890	2.7125	0.0392	2.0564	0.1846	2.3580	0.0125	2.3291	1.0000	1.2635	1.0000	0.6328	0.4393	1.2438
DOCK8	1.0000	0.0000	1.0000	2.2507	1.0000	2.2472	1.0000	2.3241	1.0000	0.0000	1.0000	2.3456	1.0000	0.0641
DOCK9	0.2164	0.2640	0.0021	0.3311	0.0003	0.6286	0.0162	0.2649	1.0000	0.1174	0.2780	0.1982	0.0729	-0.2403
DOHH	0.0000	-0.9069	0.0000	-0.7151	0.0000	-0.9316	0.0000	-0.6130	1.0000	-0.1407	1.0000	0.0633	0.3995	0.1838
DOK1	0.8525	0.1565	0.7917	0.1190	0.6821	-0.2905	0.4904	0.1949	1.0000	0.0626	1.0000	0.0377	0.2481	0.5558
DOK2	0.0000	-1.6052	0.0000	-1.0677	1.0000	-0.0175	0.0910	0.2410	1.0000	0.1754	0.0067	0.7267	0.0063	0.4401
DOK3	0.6801	1.0626	0.4431	-1.0254	1.0000	0.1151	0.2388	-1.3978	1.0000	1.2797	0.9773	-0.7924	1.0000	-0.2280
DOK4	0.0000	-2.3612	0.0000	-1.7172	0.0000	-1.0855	0.0068	-0.4076	1.0000	-0.3006	0.6020	0.3554	0.1132	0.3820
DOK5	0.0000	-4.0375	0.0000	-3.5583	0.5017	-0.4243	1.0000	-0.0370	1.0000	0.1316	1.0000	0.6250	0.4317	0.5283
DOK7	0.0004	1.1739	0.0000	1.1570	0.0000	1.5947	0.0000	1.4980	1.0000	-0.0392	1.0000	-0.0415	0.9619	-0.1274
DOLK	0.1950	0.3610	0.2385	0.1961	0.7986	-0.1453	0.1485	-0.2305	1.0000	0.3169	0.7609	0.1644	0.3972	0.2360
DOLPP1	0.7842	-0.1208	0.0435	-0.2625	0.2316	-0.2731	0.0000	-0.5439	1.0000	0.0700	1.0000	-0.0596	0.4147	-0.1964
DONSON	0.9287	0.1023	0.0362	0.3136	0.0034	-0.6989	0.0000	-0.8281	1.0000	-0.1578	1.0000	0.0639	0.3357	-0.2840
DOPEY1	0.3514	0.2719	0.0050	0.4370	0.5907	0.1885	0.0830	0.2924	1.0000	0.0516	0.3973	0.2278	0.6741	0.1595
DOPEY2	0.1754	1.0285	0.0003	1.6690	0.0000	1.7839	0.0000	2.0155	1.0000	-0.4324	1.0000	0.2165	1.0000	-0.1975
DOT1L	0.4618	-0.2392	0.7512	-0.0736	0.0098	0.5697	0.0000	0.4242	1.0000	0.0867	0.1761	0.2657	1.0000	-0.0546
DPAGT1	0.5348	-0.1561	0.0549	-0.1913	0.5942	-0.1420	0.3396	-0.1091	1.0000	0.0138	1.0000	-0.0092	1.0000	0.0517
DPCD	0.0006	-0.6356	0.0033	-0.3531	0.0000	-1.0860	0.0000	-0.6379	1.0000	0.1090	0.0166	0.4040	0.0001	0.5634
DPEP1	0.8359	-0.6482	0.9062	-1.5348	0.4426	-2.2477	0.9096	-1.3717	1.0000	-0.8169	1.0000	-1.7124	1.0000	0.0660
DPF3	0.7849	1.1386	0.3475	1.2297	0.5505	1.2863	0.6142	-1.7592	1.0000	0.7963	0.9640	0.9069	0.5105	-2.2502

DPH1	1.0000	0.1066	0.0555	0.3866	0.6687	0.2318	0.0000	0.7235	1.0000	-0.0933	0.8269	0.2003	0.0659	0.4037
DPH2	0.7442	-0.1720	0.2958	0.2021	0.0078	-0.7138	0.0839	-0.2969	1.0000	-0.2358	0.8875	0.1514	0.7133	0.1874
DPH3	0.0006	-0.7709	0.0000	-0.9879	0.9168	-0.0963	0.0070	-0.3151	1.0000	0.1576	1.0000	-0.0476	1.0000	-0.0551
DPH5	0.0016	0.6461	0.0000	0.6477	0.0022	-0.6703	0.0000	-0.6579	1.0000	0.1398	0.7284	0.1532	0.8252	0.1576
DPH7	0.0100	0.5135	0.0001	0.5218	0.0918	0.3672	0.0000	0.7223	1.0000	-0.0836	1.0000	-0.0623	0.0828	0.2784
DPM2	0.0070	-0.4614	0.0001	-0.3990	0.0000	-0.7806	0.0000	-0.6636	1.0000	-0.0849	1.0000	-0.0105	1.0000	0.0384
DPM3	0.5252	-0.1716	0.7432	-0.0815	0.0000	-1.2652	0.0000	-1.2323	1.0000	-0.2041	0.9792	-0.1012	0.8353	-0.1637
DPP6	0.0005	-2.8661	0.0648	-1.9239	0.7209	-0.3930	0.2529	-1.1839	1.0000	-0.8932	1.0000	0.0619	0.1082	-1.6841
DPP7	1.0000	-0.0357	1.0000	-0.0097	0.0009	-0.6387	0.0002	-0.3747	1.0000	0.0511	0.9082	0.0886	0.0172	0.3201
DPP8	1.0000	0.0088	1.0000	-0.0145	0.6929	0.1208	0.3884	-0.1076	1.0000	0.0476	1.0000	0.0364	0.2798	-0.1758
DPP9	0.0000	0.7719	0.0000	0.6975	1.0000	-0.0051	0.9963	-0.0382	1.0000	-0.0037	1.0000	-0.0653	1.0000	-0.0315
DPT	0.9263	-0.5627	0.7249	1.0539	0.6371	-1.3481	1.0000	-0.8419	1.0000	-1.8102	1.0000	-0.1938	1.0000	-1.3084
DPY19L1	0.9258	0.0730	0.8232	0.0450	0.0207	0.3932	0.0000	0.3627	1.0000	0.0205	1.0000	0.0049	1.0000	-0.0044
DPY19L4	0.6131	0.1487	0.0121	0.2847	0.3013	0.2313	0.0001	0.3982	1.0000	-0.0640	0.9853	0.0849	0.7504	0.1084
DPY30	0.0177	-0.4826	0.0000	-0.5252	0.1126	-0.3481	0.0003	-0.3975	1.0000	0.0991	1.0000	0.0693	1.0000	0.0563
DPYS	1.0000	0.0000	1.0000	0.0000	1.0000	0.0000	1.0000	0.0000	1.0000	0.0000	1.0000	0.0000	1.0000	0.0000
DPYSL4	0.3671	-1.4304	0.5881	-0.5451	1.0000	0.0040	0.2654	0.6744	1.0000	0.6151	0.3460	1.5244	0.0541	1.2915
DPYSL5	0.0000	-2.6937	0.0000	-2.8487	0.0010	-0.6532	0.0000	-0.5078	1.0000	-0.1337	0.5746	-0.2759	1.0000	0.0175
DQX1	1.0000	-0.0214	1.0000	0.0517	1.0000	-0.0340	1.0000	0.0109	1.0000	-0.2076	0.9592	-0.1240	0.7557	-0.1580
DR1	1.0000	0.0474	0.7782	0.0599	0.6471	0.1380	0.3714	0.1142	1.0000	0.0927	0.7688	0.1176	0.9479	0.0740
DRAM1	0.4695	0.8086	0.2101	1.1200	0.3567	-1.0463	0.8642	-0.6453	1.0000	-0.5759	1.0000	-0.2554	1.0000	-0.1728
DRAM2	0.9125	-0.0900	0.1812	-0.1770	0.4630	0.1918	1.0000	0.0245	1.0000	0.0555	1.0000	-0.0189	0.8259	-0.1061
DRAXIN	0.0053	3.0252	0.0946	2.5612	0.2738	1.8694	0.9096	1.3845	1.0000	-0.9587	0.2377	-1.4338	0.6247	-1.4423
DRC1	0.8580	0.1778	1.0000	-0.0322	1.0000	0.0548	1.0000	-0.0738	1.0000	-0.1500	0.6439	-0.3457	0.7603	-0.2720
DRC7	0.0000	-4.2105	0.0000	-3.9809	0.0000	-2.2591	0.0000	-2.0725	1.0000	-0.0022	1.0000	0.2378	1.0000	0.1906
DRD1	0.9545	1.0679	0.0209	3.1296	1.0000	0.6746	0.9096	1.3849	1.0000	-0.1033	0.3531	1.9712	1.0000	0.6050
DRD2	1.0000	0.0000	1.0000	-2.3959	1.0000	0.0000	1.0000	-2.3177	1.0000	2.2674	1.0000	0.0000	1.0000	0.0000
DRD3	0.8109	1.1304	0.4029	1.9126	1.0000	0.3405	0.6142	1.7722	1.0000	-0.9637	1.0000	-0.1898	1.0000	0.4639
DRD4	0.6936	1.4527	0.2330	-2.0449	1.0000	-2.4061	0.2202	-1.8680	1.0000	2.6071	1.0000	-0.8645	1.0000	3.2066
DRD5	0.0117	-0.7659	0.0000	-0.9415	0.5870	0.2290	0.1723	0.2762	1.0000	0.0809	1.0000	-0.0817	0.9237	0.1332
DRG1	1.0000	0.0003	0.8521	0.0456	0.0028	-0.4815	0.0000	-0.5838	1.0000	-0.0921	1.0000	-0.0347	0.1806	-0.1892
DRG2	0.0951	-0.2848	0.0001	-0.3639	0.0000	-0.7722	0.0000	-0.6964	1.0000	-0.0475	0.6628	-0.1143	1.0000	0.0333
DRGX	1.0000	-2.4788	1.0000	0.0000	1.0000	-0.1597	1.0000	0.0000	1.0000	-2.3771	1.0000	0.0000	1.0000	-2.2909
DROSHA	0.1824	0.3193	0.4447	0.1351	0.2443	0.2861	0.5298	-0.1225	1.0000	0.1896	1.0000	0.0172	0.3774	-0.2155
DRP2	0.0023	3.6191	0.0113	1.7331	0.3084	2.1386	1.0000	0.1957	1.0000	2.4153	0.8607	0.5624	1.0000	0.4827
DSCAM	1.0000	-2.4788	1.0000	0.0000	1.0000	-2.4061	1.0000	0.0000	1.0000	-2.3771	1.0000	0.0000	1.0000	0.0000
DSCAML1	0.3089	3.9281	0.7666	3.0922	0.8011	3.0840	0.7701	3.1732	1.0000	0.0000	1.0000	-0.8613	1.0000	0.0723
DSCC1	0.0000	1.0421	0.0000	1.3638	0.9005	-0.1336	0.0209	-0.4155	1.0000	-0.1748	0.6297	0.1584	0.0539	-0.4508
DSCR3	0.1409	-0.3322	0.0051	-0.3151	0.0683	-0.3852	0.0001	-0.3987	1.0000	-0.1209	1.0000	-0.0920	0.6448	-0.1288
DSE	0.9566	-0.0850	0.8260	-0.0748	0.0766	0.3758	0.0038	0.3661	1.0000	0.0589	1.0000	0.0825	1.0000	0.0556
DSEL	0.5993	0.5896	0.2362	0.7345	0.5996	0.5131	1.0000	0.0822	1.0000	0.0206	1.0000	0.1827	1.0000	-0.4003
DSG2	0.0000	2.1108	0.0000	2.4175	0.4260	0.3529	1.0000	-0.0051	1.0000	0.0348	0.2488	0.3559	0.5842	-0.3171
DSN1	1.0000	-0.0027	0.7887	0.0779	0.0030	-0.5727	0.0000	-0.5561	1.0000	-0.0920	1.0000	0.0003	1.0000	-0.0695
DSP	0.9303	-0.5620	1.0000	-0.0551	0.2242	1.2129	0.3711	0.7821	1.0000	0.5054	0.8607	1.0295	1.0000	0.0800
DSTN	0.0051	0.4354	0.0000	0.4252	0.0009	-0.5107	0.0000	-0.5929	1.0000	0.0206	1.0000	0.0230	0.8904	-0.0558
DSTYK	0.3082	0.4683	0.0022	0.8127	0.0001	1.2634	0.0000	1.2368	1.0000	-0.2406	1.0000	0.1180	0.5864	-0.2605
DTD1	0.5902	0.1676	0.3789	-0.1232	0.0038	-0.6005	0.0000	-0.9455	1.0000	0.1480	0.7268	-0.1302	0.4840	-0.1907
DTD2	1.0000	0.0170	0.7945	-0.0877	0.0001	-1.4592	0.0000	-1.7535	1.0000	-0.3179	0.0271	-0.4089	0.0816	-0.6003
DTHD1	1.0000	0.0000	1.0000	0.0000	1.0000	0.0000	1.0000	0.0000	1.0000	0.0000	1.0000	0.0000	1.0000	0.0000
DTL	0.1056	0.4883	0.0010	0.6493	1.0000	0.0717	0.0090	-0.6199	1.0000	-0.1522	1.0000	0.0206	0.0015	-0.8406
DTNB	1.0000	0.0996	1.0000	0.0266	0.7662	0.1849	0.7966	0.1354	1.0000	0.0464	1.0000	-0.0141	1.0000	0.0018
DTNBP1	0.0054	-0.5376	0.0007	-0.3807	0.1057	-0.3351	0.0967	-0.1964	1.0000	-0.0994	1.0000	0.0698	1.0000	0.0444
DTWD1	0.0001	0.7615	0.0004	0.4700	0.2838	-0.2901	0.0116	-0.3592	1.0000	0.1762	0.9753	-0.1027	0.9376	0.1123
DTWD2	0.7367	1.7384	1.0000	-0.6912	0.6966	1.5803	1.0000	0.3951	1.0000	1.2565	1.0000	-1.1565	1.0000	0.0784
DTX1	1.0000	-2.4788	1.0000	0.0000	1.0000	-2.4061	1.0000	0.0000	1.0000	-2.3771	1.0000	0.0000	1.0000	0.0000
DTX2	0.0001	-0.5891	0.0000	-0.4018	0.0001	-0.6163	0.0000	-0.3683	1.0000	-0.1181	0.9213	0.0816	0.4006	0.1353
DTX3L	0.0494	0.5321	0.2125	0.2285	0.0150	0.6577	0.0792	0.2889	1.0000	0.1772	1.0000	-0.1158	0.7609	-0.1892
DTX4	0.3194	0.2324	0.0000	0.4237	0.0528	0.3866	0.0000	0.6898	1.0000	-0.0740	0.6435	0.1299	0.0900	0.2352
DTYMK	1.0000	0.0048	0.5642	0.0856	0.0074	-0.5162	0.0000	-0.4085	1.0000	-0.1602	1.0000	-0.0678	1.0000	-0.0462
DUOX2	0.8402	-0.3627	0.9876	-0.1809	0.3095	-0.8095	0.0118	-1.5513	1.0000	0.0966	1.0000	0.2899	0.8260	-0.6409
DUOXA1	1.0000	0.0000	1.0000	0.0000	1.0000	0.0000	1.0000	0.0000	1.0000	0.0000	1.0000	0.0000	1.0000	0.0000
DUOXA2	1.0000	-2.4788	1.0000	-1.0027	1.0000	-2.4061	0.7710	-3.1692	1.0000	0.7402	1.0000	2.3480	1.0000	0.0000
DUPD1	1.0000	-2.4776	1.0000	-2.3986	1.0000	-2.4056	1.0000	0.0045	1.0000	-0.1034	1.0000	0.0000	1.0000	2.3543
DUS1L	0.0766	0.5249	0.0000	0.6851	0.5729	-0.2661	0.7697	-0.1181	1.0000	-0.1219	1.0000	0.0494	1.0000	0.0312
DUS2	1.0000	0.0839	0.7113	0.1204	0.1682	-0.4033	0.0007	-0.6142	1.0000	-0.0110	1.0000	0.0372	0.6942	-0.2179
DUS3L	1.0000	0.0677	0.9966	0.0529	0.2394	-0.3155	0.1441	-0.2371	1.0000	-0.0984	1.0000	-0.1004	1.0000	-0.0129
DUS4L	0.8226	0.1112	0.0390	-0.2503	0.1736	-0.2987	0.0229	-0.2614	1.0000	0.1564	0.4743	-0.1925	0.3660	0.1990
DUSP1	0.0000	-2.8838	0.0000	-3.4009	0.2288	-0.2342	0.1576	-0.1692	1.0000	0.1054	0.2788	-0.3990	0.3376	0.1750
DUSP10	1.0000	-0.0022	0.4563	-0.1192	0.0001	0.6793	0.0000	0.5278	1.0000	0.1466	1.0000	0.0429	1.0000	0.0012
DUSP11	0.0876	-0.3570	0.6243	-0.0947	0.0001	-0.7249	0.0000	-0.4963	1.0000	-0.1498	0.8572	0.1239	1.0000	0.0840
DUSP12	1.0000	0.0560	0.0907	-0.2198	0.0000	-0.8404	0.0000	-0.9449	1.0000	0.1499	0.9038	-0.1142	1.0000	0.0495
DUSP14	0.0000	-2.5452	0.0000	-2.3837	0.0000	-0.9577	0.0230	-0.1667	1.0000	0.2508	0.0090	0.4255	0.0000	1.0466
DUSP15	0.0000	-1.4949	0.0000	-1.5078	0.0247	-0.6449	0.0000	-1.6149	1.0000	-0.0748	1.0000	-0.0754	0.0007	-1.0382
DUSP16	0.5246	0.1791	1.0000	-0.0254	0.0012	-0.6395	0.0000	-0.6725	1.0000	0.1382	1.0000	-0.0544	0.8858	0.1104
DUSP18	1.0000	0.0000	1.0000	0.0000	1.0000	0.0000	1.0000	0.0000	1.0000	0.0000	1.0000	0.0000	1.0000	0.0000
DUSP19	0.2490	0.2657	0.2555	0.1680	0.5800	-0.1704	0.0081	-0.3306	1.0000	-0.0206	0.9111	-0.1060	0.5351	-0.1755
DUSP22	0.0000	1.1347	0.0000	1.1286	0.0023	0.6516	0.1179	0.3543	1.0000	0.2410	0.0778	0.2477	1.0000	-0.0518
DUSP23	0.0000	-1.2856	0.0000	-1.4355	0.0000	-0.9056	0.0028	-0.4690	1.0000	-0.3331	0.1615	-0.4695	1.0000	0.1105
DUSP26	0.0058	0.6136	0.0067	0.4062	1.0000	0.0738	1.0000	-0.0466	1.0000	-0.1601	0.0489	-0.3564	0.4385	-0.2740
DUSP27	0.0000	-1.0131	0.0000	-1.3271	1.0000	0.0233	0.0002	-0.4392	1.0000	0.1594	0.8468	-0.1413	0.1209	-0.2981
DUSP28	0.0000	-1.2430	0.0000	-1.2012	0.0000	-1.3066	0.0000	-1.0447	1.0000	-0.2037	0.9309	-0.1491	1.0000	0.0644
DUSP3	0.0000	-1.3764	0.0000	-1.4560	0.0311	-0.4115	0.0000	-0.5140	1.0000	0.1485	1.0000	0.0823	1.0000	0.0519
DUSP4	0.0171	-1.0202	0.7936	-0.1999	0.5957	-0.3502	0.4741	-0.3168	1.0000	-0.1134	0.2560	0.7205	1.0000	-0.0706
DUSP5	0.0000	-1.1818	0.0000	-0.6167	0.0000	0.9248	0.0000	0.8795	1.0000	-0.0210	0.0416	0.5568	1.0000	-0.0615
DUSP6	0.0000	-2.1594	0.0000	-1.7553	0.3016	-0.2728	0.0205	-0.3313	1.0000	0.1071	0.0460	0.5243	1.0000	0.0514
DUSP7	0.6877	0.2514	0.0011	0.6127	0.0000	1.1309	0.0000	1.0637	1.0000	0.1483	0.0985	0.5205	1.0000	0.0859
DUSP8	0.0111	-0.4440	0.0048	-0.3042	0.7772	0.1045	0.0000	0.4369	1.0000	-0.0881	1.0000	0.0644	0.0757	0.2498
DUT	0.1027	0.3184	0.0000	0.5637	0.0978	-0.3279	0.0000	-0.4422	1.0000	-0.1658	0.8702	0.0914	0.0283	-0.2745
DVL1	0.9157	0.0773	0.3171	-0.1099	0.0046	0.4674	0.0072	0.2307	1.0000	-0.0293	0.2497	-0.2037	0.0146	-0.2604
DVL3	0.3563	0.2440	0.0120	0.3069	0.0879	0.3722	0.0000	0.4948	1.0000	-0.1052	1.0000	-0.0293	1.0000	0.0238
DYDC1	0.2075	0.7270	0.0004	1.0634	0.0000	-3.2624	0.0000	-2.0002	1.0000	-0.3452	1.0000	0.0065	0.8535	0.9272
DYNC1H1	0.8521	0.0777	0.0152	0.1649	0.0001	0.5755	0.0000	0.4746	1.0000	-0.0255	0.8219	0.0742	0.3382	-0.1209
DYNC1I2	0.0072	-0.4095	0.0000	-0.5393	0.0038	-0.4400	0.0000	-0.4259	1.0000	0.0801	1.0000	-0.0371	0.6416	0.0995

DYNC1LI1	0.1564	-0.2709	0.0026	-0.2735	0.0948	-0.3076	0.0028	-0.2496	1.0000	0.0490	1.0000	0.0588	0.6232	0.1131
DYNC2H1	0.9176	0.3110	0.0004	1.5195	0.0001	1.7656	0.0000	2.0510	1.0000	-0.5991	0.4412	0.6200	0.7784	-0.3127
DYNC2LI1	0.0038	0.8673	0.0002	0.8062	0.1790	0.4753	0.0871	0.4185	1.0000	-0.2786	0.3834	-0.3261	0.3764	-0.3265
DYNLL1	0.0193	-0.8728	0.1336	-0.3483	0.0019	-1.0906	0.0105	-0.5631	1.0000	0.0172	0.1948	0.5558	0.1925	0.5517
DYNLL2	0.1082	0.3053	0.0000	0.4243	0.0007	-0.5779	0.0000	-0.5828	1.0000	-0.1254	1.0000	0.0057	0.5147	-0.1250
DYNLRB1	0.0438	-0.3332	0.0000	-0.4149	0.0005	-0.5389	0.0000	-0.5488	1.0000	-0.0793	0.3216	-0.1488	0.7613	-0.0839
DYNLRB2	0.5416	-0.5124	0.1757	-0.6964	0.5274	-0.4864	0.1643	-0.6587	1.0000	0.0046	1.0000	-0.1656	1.0000	-0.1620
DYNLT1	0.2691	-0.2604	0.0939	-0.2126	0.9990	0.0692	0.4390	-0.1234	1.0000	0.0288	1.0000	0.0894	0.6087	-0.1580
DYNLT3	0.9394	0.0798	0.5751	0.0851	0.5149	-0.1816	0.0046	-0.2836	1.0000	0.0314	1.0000	0.0490	1.0000	-0.0659
DYRK1A	0.0000	-0.6478	0.0000	-0.8252	0.0085	-0.4342	0.0000	-0.5806	1.0000	0.1034	1.0000	-0.0611	1.0000	-0.0378
DYRK2	0.0000	-0.9821	0.0000	-1.2699	0.2602	-0.2264	0.0000	-0.4041	1.0000	0.2045	1.0000	-0.0703	1.0000	0.0320
DYRK3	0.0000	-1.6940	0.0000	-1.7853	0.0016	-0.4899	0.0000	-0.7148	1.0000	0.0988	1.0000	0.0192	0.5419	-0.1213
DYTN	0.9545	1.0672	1.0000	-0.1429	1.0000	-2.4061	1.0000	0.0062	1.0000	-0.1089	1.0000	-1.3257	1.0000	2.3543
DYX1C1	0.0029	0.8568	0.0075	0.6398	0.1638	-0.5314	0.0415	-0.5920	1.0000	0.0820	1.0000	-0.1196	1.0000	0.0279
DZANK1	0.4664	0.4278	0.0564	0.5426	0.6006	-0.3894	1.0000	-0.0130	1.0000	0.4190	0.2426	0.5465	0.0833	0.8006
DZIP1	0.0063	0.7074	0.0027	0.5731	1.0000	-0.0492	0.2089	0.2885	1.0000	-0.1801	0.3604	-0.3014	0.8975	0.1617
DZIP1L	0.7549	-0.1901	0.4316	-0.2304	1.0000	-0.0784	0.6588	0.1629	1.0000	-0.1575	0.9773	-0.1857	1.0000	0.0883
E2F1	0.0000	0.7548	0.0000	1.0832	0.4815	0.1633	0.0647	0.1973	1.0000	-0.3521	1.0000	-0.0121	0.0064	-0.3130
E2F2	0.2645	0.4030	0.0132	0.5650	0.1820	0.4661	0.2965	0.3221	1.0000	-0.5631	0.0985	-0.3919	0.0022	-0.7007
E2F4	0.0051	-0.5135	0.0000	-0.5846	0.0030	-0.5463	0.0000	-0.5745	1.0000	0.2094	0.6141	0.1509	0.3546	0.1854
E2F5	0.0576	0.6611	0.8515	0.1303	0.3740	0.4072	1.0000	0.0373	1.0000	0.1329	0.4563	-0.3860	0.8899	-0.2293
E2F6	0.3961	0.2419	0.3833	0.1518	0.0576	-0.4499	0.0011	-0.4653	1.0000	0.0479	1.0000	-0.0288	1.0000	0.0372
E2F7	0.0000	1.1219	0.0000	1.3152	0.0024	0.8629	0.0043	0.6219	1.0000	-0.1965	1.0000	0.0076	0.1298	-0.4309
E2F8	0.0000	1.4946	0.0000	1.8678	0.0496	0.4867	0.5443	0.1562	1.0000	-0.4410	1.0000	-0.0579	0.0000	-0.7678
E4F1	0.0775	0.3405	0.0562	0.2206	0.9780	-0.0698	1.0000	-0.0346	1.0000	0.0232	0.9741	-0.0836	1.0000	0.0638
EAF1	0.1794	0.2703	0.0145	0.2231	1.0000	-0.0466	0.0318	-0.2107	1.0000	0.0765	1.0000	0.0419	0.8832	-0.0819
EAF2	0.1065	-0.5609	0.1348	-0.3412	0.3048	0.3473	0.0002	0.6301	1.0000	0.0475	0.7354	0.2804	0.1887	0.3342
EAPP	0.2815	-0.2519	0.4260	-0.1091	0.0006	-0.6267	0.0000	-0.6338	1.0000	0.0087	0.6197	0.1641	1.0000	0.0058
EARS2	0.2124	0.3329	0.5866	0.1224	0.0007	-0.8274	0.0000	-0.7143	1.0000	0.0112	0.6450	-0.1873	1.0000	0.1306
EBAG9	0.0001	-0.6460	0.0000	-0.4659	0.0000	-0.7415	0.0000	-0.5308	1.0000	-0.0752	0.8269	0.1177	0.5874	0.1416
EBF1	0.0000	0.8499	0.0000	0.8638	0.6280	-0.1549	0.0546	-0.2661	1.0000	0.0686	0.8793	0.0945	1.0000	-0.0375
EBF2	0.2074	-1.6889	1.0000	-0.1666	0.4692	-1.5355	0.0324	2.1396	1.0000	-1.4708	1.0000	0.0576	0.1169	2.2101
EBF3	0.4509	0.3581	1.0000	0.0699	0.5778	-0.3456	0.0029	-0.8691	1.0000	0.0722	0.9634	-0.2025	0.6218	-0.4429
EBNA1BP2	0.0011	-0.5230	0.0000	-0.4687	0.0000	-0.6520	0.0000	-0.6462	1.0000	-0.0002	1.0000	0.0665	1.0000	0.0108
ECD	0.0328	0.3915	0.0001	0.3447	0.1783	-0.2692	0.0019	-0.3206	1.0000	0.0363	1.0000	0.0020	1.0000	-0.0100
ECE1	0.0478	0.5132	0.0000	0.6114	0.0001	0.8063	0.0000	1.1423	1.0000	-0.2217	1.0000	-0.1118	0.7311	0.1198
ECE2	1.0000	0.0483	0.1174	1.0439	0.7091	-0.5526	0.2332	0.9253	1.0000	-0.8277	1.0000	0.1766	0.7472	0.6564
ECEL1	1.0000	2.1903	1.0000	0.0000	1.0000	0.0000	1.0000	2.3241	1.0000	0.0000	1.0000	-2.2992	1.0000	2.3543
ECH1	0.2454	-0.2460	0.2393	-0.1496	0.0000	-0.9580	0.0000	-0.5501	1.0000	-0.2367	0.7325	-0.1287	0.6118	0.1771
ECHDC2	0.2702	0.2417	0.7162	0.0545	0.0000	-1.1352	0.0000	-1.3389	1.0000	-0.0185	0.1188	-0.1931	0.2130	-0.2155
ECHDC3	0.2672	-0.3746	0.0720	-0.3136	0.0028	-0.7923	0.0004	-0.5853	1.0000	-0.1924	1.0000	-0.1183	1.0000	0.0204
ECHS1	0.0015	-0.5293	0.0001	-0.4441	0.0000	-0.8729	0.0000	-0.6869	1.0000	-0.1574	1.0000	-0.0599	1.0000	0.0357
ECI1	0.8820	-0.0869	1.0000	0.0210	0.0003	-0.6443	0.0002	-0.3806	1.0000	-0.2519	0.6629	-0.1324	1.0000	0.0175
ECI2	0.0071	0.4850	0.0001	0.3513	0.0273	-0.4271	0.0000	-0.6604	1.0000	0.0644	1.0000	-0.0569	0.4876	-0.1633
ECM1	0.0000	-0.9248	0.0000	-0.8470	0.3271	0.2745	0.0037	0.2548	1.0000	0.1016	0.0708	0.1923	1.0000	0.0868
ECM2	0.2720	-0.2308	0.0002	-0.2650	0.5273	-0.1600	1.0000	-0.0008	1.0000	0.2446	0.0267	0.2228	0.0000	0.4087
ECSCR	0.8226	3.0199	1.0000	-0.1452	1.0000	0.0000	1.0000	0.8541	1.0000	2.2736	1.0000	-0.7956	1.0000	3.2066
ECT2	0.0002	0.7238	0.0000	0.9877	0.0005	0.6647	0.0001	0.4494	1.0000	-0.0609	0.3615	0.2154	0.1172	-0.2692
EDA	1.0000	-0.0437	0.6921	-0.0786	0.7674	-0.1240	0.0585	-0.2253	1.0000	0.1796	0.6442	0.1570	0.9980	0.0838
EDA2R	0.0000	-0.7181	0.0048	-0.2975	0.0010	0.5185	0.0000	0.7562	1.0000	-0.1201	0.0586	0.3134	0.5617	0.1232
EDAR	0.7499	-0.4128	0.6323	-0.3695	0.1363	-0.8593	0.3426	-0.5945	1.0000	-0.3813	1.0000	-0.3255	1.0000	-0.1104
EDARADD	0.1423	0.6331	0.0002	1.1715	0.2106	-0.6969	0.5982	-0.3484	1.0000	-0.4430	1.0000	0.1054	1.0000	-0.0892
EDC3	0.8249	0.1361	0.6846	0.1195	0.0497	0.4613	0.1285	0.2689	1.0000	0.0776	1.0000	0.0726	0.9134	-0.1109
EDEM1	0.2906	-0.2176	0.0326	-0.1718	1.0000	0.0400	0.0007	0.2423	1.0000	0.0695	0.4997	0.1278	0.0024	0.2766
EDEM2	0.8489	-0.1155	0.3913	-0.1480	0.0750	-0.3885	0.5038	-0.1150	1.0000	-0.0364	1.0000	-0.0561	0.2750	0.2426
EDEM3	0.0594	0.3244	0.0000	0.3694	0.5762	0.1397	0.0184	0.1947	1.0000	-0.1228	1.0000	-0.0652	0.9418	-0.0622
EDF1	0.0003	-0.5272	0.0000	-0.5831	0.0025	-0.4587	0.0000	-0.6004	1.0000	0.0145	1.0000	-0.0290	0.4078	-0.1216
EDIL3	1.0000	0.0000	1.0000	0.0000	1.0000	2.2472	1.0000	2.3257	1.0000	0.0000	1.0000	0.0000	1.0000	0.0649
EDN1	0.4958	0.8964	0.0784	1.6682	0.0352	1.6636	0.0053	2.2588	1.0000	-0.8948	1.0000	-0.1172	1.0000	-0.2959
EDN2	1.0000	2.1903	1.0000	-1.0017	0.0318	4.9524	0.2272	1.8802	1.0000	3.1126	1.0000	0.0470	1.0000	0.0774
EDNRA	0.0414	0.9969	0.0087	0.9382	0.3579	0.5852	0.5480	0.3773	1.0000	0.2488	1.0000	0.2048	1.0000	0.0484
EDNRB	1.0000	-0.3227	0.7666	-3.2534	0.0328	2.4749	0.1387	2.0465	1.0000	-0.1179	1.0000	-3.1430	0.9333	-0.5439
EEA1	0.1390	0.2632	0.0031	0.2737	0.4134	0.1795	0.0008	0.2763	1.0000	-0.0798	1.0000	-0.0566	1.0000	0.0221
EED	0.0161	0.5368	0.0000	0.6009	1.0000	-0.0172	0.5044	-0.1448	1.0000	-0.0232	1.0000	0.0535	0.8183	-0.1454
EEF1A1	0.3492	0.2009	0.0013	0.2116	0.0011	-0.5427	0.0000	-0.4294	1.0000	-0.1082	0.7168	-0.0852	1.0000	0.0109
EEF1A2	0.0000	-5.5792	0.0000	-6.3705	0.0000	-0.7718	0.0000	-0.9539	1.0000	-0.3177	0.0026	-1.0985	0.0000	-0.4945
EEF1B2	0.0496	0.3269	0.0370	0.1577	0.0000	-0.7524	0.0000	-0.7022	1.0000	-0.0981	0.0025	-0.2546	1.0000	-0.0422
EEF1D	0.0004	-0.5389	0.0000	-0.6454	0.0000	-0.8121	0.0000	-0.7207	1.0000	-0.1108	0.0165	-0.2049	1.0000	-0.0137
EEF1E1	0.1985	-0.3002	0.0067	-0.2696	0.0038	-0.5610	0.0000	-0.5083	1.0000	-0.0770	1.0000	-0.0334	1.0000	-0.0187
EEF2	0.0013	0.4720	0.0000	0.4817	0.9676	0.0577	0.0016	0.1879	1.0000	-0.1414	0.4675	-0.1190	1.0000	-0.0061
EEF2K	0.0007	0.6226	0.0004	0.3977	1.0000	0.0018	1.0000	-0.0204	1.0000	0.0883	0.7771	-0.1228	1.0000	0.0717
EEF2KMT	0.4834	-0.1967	0.0000	-0.6761	0.0002	-0.7164	0.0000	-0.8853	1.0000	0.1383	0.1370	-0.3287	1.0000	-0.0236
EEFSEC	0.9650	-0.0872	0.6846	0.0940	0.6424	-0.1670	0.9424	-0.0536	1.0000	-0.0651	0.8785	0.1275	1.0000	0.0526
EEPD1	0.4437	0.2220	0.0301	-0.2824	0.9468	-0.0885	0.0531	-0.2957	1.0000	0.0985	0.0104	-0.3938	0.9852	-0.1030
EFCAB1	0.0065	0.8038	0.0004	0.7444	0.5364	-0.2948	0.0001	-1.1124	1.0000	0.0902	1.0000	0.0415	0.0952	-0.7237
EFCAB11	0.8413	-0.2943	0.6829	-0.2418	0.1895	-0.7311	0.5957	-0.2551	1.0000	0.6421	0.2482	0.7075	0.0186	1.1229
EFCAB12	0.3638	0.9161	0.9057	-0.2271	0.0540	1.3221	0.4347	-0.5513	1.0000	1.3441	1.0000	0.2126	0.7288	-0.5240
EFCAB14	1.0000	0.0314	0.9994	-0.0273	0.0769	0.2971	0.0036	0.2264	1.0000	-0.0620	0.6550	-0.1084	0.4210	-0.1277
EFCAB2	1.0000	-0.3395	1.0000	0.6956	0.2835	-4.1860	1.0000	-2.3177	1.0000	-1.8834	1.0000	-0.8625	1.0000	0.0000
EFCAB3	1.0000	-0.2931	1.0000	-2.3986	1.0000	-2.4061	1.0000	-2.3199	1.0000	-0.1045	1.0000	-2.2957	1.0000	0.0000
EFCAB5	0.3618	0.7095	1.0000	-0.2598	0.1087	-1.3411	0.9238	0.2936	1.0000	-0.1331	0.1811	-1.0891	0.1079	1.5107
EFCAB7	0.0002	0.8449	0.0000	0.8984	0.1167	-0.4486	0.0062	-0.4784	1.0000	-0.0405	1.0000	0.0273	1.0000	-0.0634
EFCC1	0.2384	-2.6422	0.0234	-1.8070	0.8450	-1.1378	0.0638	-1.6232	1.0000	1.2525	0.8354	2.1233	1.0000	0.7689
EFEMP1	0.0000	3.0641	0.0000	2.5435	0.5862	0.9044	1.0000	-0.5500	1.0000	0.1217	0.8860	-0.3863	0.5105	-1.3270
EFHB	1.0000	2.1903	1.0000	0.3667	1.0000	2.2472	1.0000	0.0066	1.0000	3.1126	1.0000	1.4314	1.0000	0.9164
EFHC1	0.0000	-0.8921	0.0000	-0.9525	0.0000	-0.6864	0.0000	-0.5355	1.0000	0.0773	1.0000	0.0293	0.0703	0.2331
EFHD1	0.4464	-0.1618	0.0554	-0.1444	1.0000	0.0291	0.0005	-0.2276	1.0000	0.0277	1.0000	0.0572	0.0151	-0.2236
EFHD2	0.0927	0.5643	0.0051	0.8108	0.0097	0.9125	0.0000	1.0892	1.0000	-0.1323	1.0000	0.1272	1.0000	0.0519
EFNA5	1.0000	0.0000	1.0000	0.0000	1.0000	2.2428	1.0000	2.3241	1.0000	0.0000	1.0000	0.0000	1.0000	0.0652
EFNB2	0.0063	-0.6896	0.0031	-0.4418	0.0000	1.4661	0.0000	1.5547	1.0000	0.0526	0.2993	0.3136	0.5774	0.1459
EFR3A	0.4910	0.1967	0.2425	0.1487	1.0000	0.0245	0.9080	-0.0566	1.0000	-0.0575	0.9176	-0.0926	0.7202	-0.1324
EFR3B	0.0000	-0.6146	0.0000	-0.7222	0.0035	-0.4591	0.0000	-0.4904	1.0000	-0.0060	0.8341	-0.1009	1.0000	-0.0320
EFTUD1	1.0000	0.0104	0.0397	-0.2190	1.0000	-0.0253	0.8429	0.0502	1.0000	0.0421	0.3562	-0.1743	0.6087	0.1245

EFTUD2	0.3594	0.1966	0.0000	0.3593	0.6654	-0.1183	1.0000	0.0092	1.0000	-0.2235	1.0000	-0.0483	0.7644	-0.0905
EGF	0.0000	-6.2578	0.0000	-5.9125	0.4189	-0.2355	0.0000	-0.6815	1.0000	0.1999	1.0000	0.5593	0.3369	-0.2418
EGFL6	0.2005	-1.0569	0.0000	-1.8228	1.0000	0.0604	0.5604	0.3266	1.0000	0.6752	1.0000	-0.0753	0.0880	0.9467
EGFL7	0.0408	-1.1992	0.9222	-0.2254	0.5930	-0.4620	0.9065	-0.2507	1.0000	-0.3989	0.8847	0.5905	1.0000	-0.1799
EGFLAM	1.0000	-0.2499	0.3630	0.8788	0.4639	0.6775	0.3367	0.9065	1.0000	-0.6458	1.0000	0.4938	1.0000	-0.4119
EGFR	0.2643	-0.2963	0.0687	-0.2406	0.0013	0.6960	0.0000	0.5734	1.0000	0.3323	0.0009	0.4012	0.1829	0.2142
EGLN1	0.3891	-0.2630	0.0025	-0.4145	0.0536	0.4252	0.6709	0.0950	1.0000	0.1267	1.0000	-0.0116	0.4346	-0.1989
EGLN3	0.0041	-1.2043	0.1045	-0.5611	0.0000	1.3170	0.0000	1.4218	1.0000	-0.1132	0.5800	0.5438	1.0000	-0.0040
EGR1	0.0000	-2.2353	0.0000	-1.9639	0.0884	0.3419	0.0000	0.8992	1.0000	-0.0964	0.7635	0.1869	0.0000	0.4668
EGR4	0.0801	-4.8275	0.0943	-2.8827	0.5460	0.9944	0.0003	2.0609	1.0000	0.2718	1.0000	2.3456	0.0349	1.3451
EHBP1	0.3917	0.1956	0.7877	0.0544	0.0566	0.3533	0.0031	0.2668	1.0000	-0.0900	0.0917	-0.2190	0.2117	-0.1720
EHD4	0.0528	-0.3919	0.0013	-0.3829	1.0000	0.0643	0.0085	-0.3166	1.0000	0.1003	0.8619	0.1228	0.0942	-0.2751
EHF	1.0000	0.0000	1.0000	0.0000	1.0000	0.0000	1.0000	0.0000	1.0000	0.0000	1.0000	0.0000	1.0000	0.0000
EHHADH	0.0002	0.7289	0.0000	0.7227	0.2088	-0.3243	0.0011	-0.5529	1.0000	-0.0171	1.0000	-0.0117	0.5612	-0.2407
EHMT1	0.7972	-0.1078	1.0000	-0.0195	0.0252	0.4078	0.0097	0.2491	1.0000	0.1031	0.2781	0.2044	1.0000	-0.0497
EI24	0.0000	-0.6129	0.0000	-0.5704	0.1954	-0.2423	0.4987	0.0700	1.0000	-0.0906	1.0000	-0.0357	0.0733	0.2271
EIF1	0.0000	-0.8577	0.0000	-0.8559	0.0000	-0.8922	0.0000	-0.6781	1.0000	-0.0290	1.0000	-0.0149	0.1235	0.1908
EIF1AX	1.0000	-0.0495	0.0221	-0.1738	0.0051	-0.4606	0.0000	-0.5469	1.0000	0.0837	1.0000	-0.0281	1.0000	0.0030
EIF1B	0.0000	-0.7010	0.0000	-0.5441	0.5503	-0.1499	0.4481	-0.0836	1.0000	-0.0970	1.0000	0.0728	1.0000	-0.0248
EIF2A	0.0698	-0.3495	0.0011	-0.3056	1.0000	-0.0253	0.3632	-0.1200	1.0000	-0.0204	1.0000	0.0364	0.8058	-0.1084
EIF2AK1	0.4139	-0.1971	0.0000	-0.4852	0.0811	-0.3442	0.0067	-0.2791	1.0000	0.1083	0.4773	-0.1679	0.3507	0.1775
EIF2AK3	0.0000	0.9277	0.0000	1.2231	0.0000	0.8660	0.0000	1.0028	1.0000	0.0265	0.0269	0.3336	0.4182	0.1683
EIF2AK4	0.6878	-0.1152	0.2960	-0.1025	0.3846	-0.1807	0.0001	-0.3009	1.0000	0.0734	0.7407	0.0986	1.0000	-0.0413
EIF2B1	1.0000	0.0633	1.0000	0.0245	0.0620	-0.3846	0.0000	-0.4900	1.0000	-0.0003	1.0000	-0.0261	0.8425	-0.0995
EIF2B2	0.1250	-0.2769	0.0001	-0.3397	0.0055	-0.4781	0.0000	-0.4168	1.0000	0.0493	1.0000	-0.0015	0.6912	0.1162
EIF2B3	0.5022	-0.1768	0.1046	-0.1893	0.0000	-0.8190	0.0000	-0.8603	1.0000	-0.0476	1.0000	-0.0484	0.9610	-0.0831
EIF2B4	0.0456	-0.3561	0.0439	-0.2215	0.0002	-0.6148	0.0004	-0.3636	1.0000	-0.1873	1.0000	-0.0409	1.0000	0.0690
EIF2B5	0.4083	-0.1824	0.3287	-0.1100	0.6939	-0.1230	0.0676	-0.1736	1.0000	-0.0449	1.0000	0.0398	0.8526	-0.0912
EIF2D	0.0054	-0.5004	0.0000	-0.6879	0.0097	-0.4581	0.0000	-0.4704	1.0000	-0.0216	0.3468	-0.1962	1.0000	-0.0284
EIF2S1	0.0285	-0.3616	0.0003	-0.2874	0.0017	-0.4941	0.0000	-0.4534	1.0000	-0.0629	1.0000	0.0236	1.0000	-0.0168
EIF2S2	0.0228	-0.4043	0.0001	-0.2779	0.0007	-0.5726	0.0000	-0.6368	1.0000	-0.0517	0.7660	0.0871	0.5105	-0.1104
EIF2S3	0.0345	-0.4032	0.0000	-0.4786	0.8682	-0.0907	0.0092	-0.1774	1.0000	-0.0375	0.6365	-0.1004	0.3841	-0.1182
EIF3A	0.9919	-0.0536	0.5207	-0.0681	0.2907	0.1951	0.0543	0.1387	1.0000	-0.0434	1.0000	-0.0455	0.5760	-0.0943
EIF3B	0.8249	0.0828	0.9056	0.0308	0.1023	0.2709	0.2243	0.0978	1.0000	0.0598	1.0000	0.0203	0.5007	-0.1077
EIF3D	0.2634	0.2218	0.0319	0.1767	0.0001	-0.6355	0.0000	-0.6208	1.0000	-0.0965	0.3713	-0.1292	0.8357	-0.0760
EIF3E	0.1876	0.2391	0.0021	0.2040	0.0017	-0.4871	0.0000	-0.4842	1.0000	-0.0079	1.0000	-0.0305	1.0000	0.0007
EIF3F	1.0000	-0.0072	0.0151	-0.1824	0.0001	-0.6170	0.0000	-0.5054	1.0000	-0.0827	0.0078	-0.2456	1.0000	0.0347
EIF3H	0.9854	-0.0626	0.0074	-0.1999	0.0002	-0.6292	0.0000	-0.6573	1.0000	-0.0335	0.2026	-0.1585	0.9835	-0.0556
EIF3I	0.0525	-0.3022	0.0000	-0.4119	0.0000	-0.6272	0.0000	-0.6646	1.0000	-0.0308	0.3445	-0.1282	0.9200	-0.0626
EIF3J	0.0165	-0.3902	0.0000	-0.4196	0.0359	-0.3554	0.0001	-0.3711	1.0000	0.0485	1.0000	0.0313	1.0000	0.0381
EIF3K	0.1258	-0.2610	0.0000	-0.3427	0.0000	-1.2466	0.0000	-0.9757	1.0000	-0.0923	0.2735	-0.1617	0.2305	0.1845
EIF3L	0.4783	-0.1490	0.0016	-0.2233	0.0042	-0.4312	0.0000	-0.4486	1.0000	-0.0910	0.1871	-0.1530	0.5343	-0.1030
EIF3M	0.0004	0.5442	0.0000	0.4784	0.0001	-0.6272	0.0000	-0.6786	1.0000	-0.0475	0.5640	-0.1009	0.7100	-0.0929
EIF4A2	0.0015	-0.5041	0.0000	-0.5919	0.0551	0.3305	0.0049	0.1793	1.0000	0.0732	1.0000	-0.0023	0.7420	-0.0722
EIF4A3	0.0333	-0.3316	0.0075	-0.2207	0.0019	-0.4607	0.0002	-0.2978	1.0000	-0.1074	1.0000	0.0158	0.9420	0.0605
EIF4B	0.2936	-0.1993	0.0036	-0.2702	0.0000	-1.1426	0.0000	-0.9139	1.0000	-0.1285	0.1621	-0.1874	0.8519	0.1060
EIF4E	0.0210	-0.4139	0.0001	-0.2917	0.0857	-0.3228	0.0009	-0.2460	1.0000	-0.1076	1.0000	0.0270	1.0000	-0.0250
EIF4E1B	1.0000	0.8845	1.0000	-0.1724	0.8033	-3.2622	0.2455	-4.0833	1.0000	0.7940	1.0000	-0.2469	1.0000	0.0000
EIF4E2	0.0000	-0.8373	0.0000	-0.8415	0.0000	-1.0384	0.0000	-0.8345	1.0000	-0.0613	1.0000	-0.0533	0.4114	0.1478
EIF4E3	0.0001	-0.8181	0.0000	-0.8658	0.5307	-0.2063	0.0475	-0.2216	1.0000	0.2302	0.6563	0.1956	0.3741	0.2219
EIF4EBP1	0.0000	-0.6692	0.0000	-0.7141	0.0000	-0.8523	0.0000	-0.8527	1.0000	-0.0267	1.0000	-0.0594	1.0000	-0.0213
EIF4EBP3	0.1577	-0.3570	0.0014	-0.6000	0.0000	-1.1265	0.0000	-0.9648	1.0000	0.0315	0.7638	-0.1999	0.7287	0.1987
EIF4ENIF1	0.4581	0.1910	1.0000	0.0233	0.4210	0.1979	0.0330	0.2278	1.0000	0.1045	1.0000	-0.0508	0.5469	0.1404
EIF4G1	0.2782	-0.2621	1.0000	-0.0054	1.0000	-0.0025	0.0112	0.2029	1.0000	-0.2390	1.0000	0.0303	1.0000	-0.0290
EIF4G2	0.2715	-0.1936	0.0002	-0.2389	0.0138	0.3687	0.0002	0.2220	1.0000	0.0498	1.0000	0.0170	0.5483	-0.0915
EIF4G3	0.7781	-0.1212	0.9054	0.0480	0.0000	1.0512	0.0000	0.9445	1.0000	-0.0821	0.9140	0.0994	0.1929	-0.1835
EIF4H	1.0000	-0.0224	0.2341	0.1242	0.1335	0.2516	0.0016	0.2702	1.0000	0.0082	0.1523	0.1673	1.0000	0.0321
EIF5	0.0947	-0.2776	0.0000	-0.3769	0.1096	-0.2725	0.0014	-0.2528	1.0000	0.0127	0.8405	-0.0741	1.0000	0.0379
EIF5A2	0.9586	0.0658	0.2701	0.0886	0.0022	-0.5053	0.0000	-0.5196	1.0000	-0.1714	0.3162	-0.1362	0.0793	-0.1800
EIF5B	1.0000	-0.0460	0.1309	-0.1430	0.0016	-0.5130	0.0000	-0.6994	1.0000	-0.0277	0.6728	-0.1122	0.0377	-0.2086
EIF6	0.0005	-0.5406	0.0000	-0.4156	0.0705	-0.3084	0.0002	-0.3395	1.0000	-0.0416	0.8269	0.0955	0.9300	-0.0679
ELAC2	0.0872	-0.3107	0.0000	-0.4077	0.0397	-0.3606	0.0000	-0.3816	1.0000	-0.0170	0.8496	-0.1014	1.0000	-0.0327
ELAVL1	0.0004	-0.5573	0.0000	-0.5873	1.0000	0.0436	0.5843	-0.0610	1.0000	0.0650	1.0000	0.0475	1.0000	-0.0341
ELAVL2	1.0000	0.0000	1.0000	0.0000	1.0000	0.0000	1.0000	0.0000	1.0000	0.0000	1.0000	0.0000	1.0000	0.0000
ELAVL4	1.0000	-0.3415	0.7674	-3.2498	0.6597	-1.9401	1.0000	0.5377	1.0000	-1.0414	0.6427	-4.0589	1.0000	1.4483
ELF1	1.0000	-0.0578	0.9982	-0.0391	0.1817	-0.2792	0.0946	-0.2120	1.0000	0.0537	0.9606	0.0860	0.6246	0.1278
ELF2	0.8585	-0.0833	0.4301	-0.0948	0.8786	-0.0803	0.4104	-0.0971	1.0000	0.0590	1.0000	0.0602	1.0000	0.0476
ELF3	0.9545	1.0644	1.0000	0.6988	1.0000	-0.1630	1.0000	0.8545	1.0000	-0.1089	1.0000	-0.4731	1.0000	0.9168
ELF5	1.0000	0.0000	1.0000	0.0000	1.0000	0.0000	1.0000	0.0000	1.0000	0.0000	1.0000	0.0000	1.0000	0.0000
ELFN1	0.0771	-0.9751	0.6180	-0.2647	0.0279	-1.1320	0.0133	0.6711	1.0000	-0.2287	0.6450	0.4916	0.0000	1.5836
ELFN2	0.0000	-2.9612	0.0000	-3.0817	0.1543	0.6131	0.0001	1.0565	1.0000	-0.0211	1.0000	-0.1287	0.3095	0.4281
ELK3	1.0000	0.0386	0.0266	0.3889	0.0066	0.6690	0.0000	1.0158	1.0000	-0.0800	0.4452	0.2834	0.3196	0.2720
ELK4	0.1577	0.7226	0.0007	1.2133	0.0000	1.6225	0.0000	1.8490	1.0000	-0.5319	1.0000	-0.0312	0.6362	-0.3023
ELL	0.2706	0.3224	0.0017	0.4583	0.0184	0.5626	0.0000	0.6278	1.0000	-0.0143	0.9084	0.1332	1.0000	0.0572
ELL2	0.1855	0.3777	0.2891	0.2026	0.9406	0.1067	1.0000	-0.0396	1.0000	0.3849	0.6054	0.2223	0.4990	0.2433
ELMO1	0.0000	1.3223	0.0000	1.8249	0.0315	-0.7443	0.6809	-0.2041	1.0000	-0.2177	0.2580	0.2988	0.6741	0.3299
ELMO2	0.4509	-0.1565	0.6790	-0.0532	0.0029	-0.4523	0.0001	-0.2595	1.0000	-0.1477	1.0000	-0.0322	1.0000	0.0507
ELMO3	0.5780	0.2476	0.4635	-0.1931	0.3204	0.3680	0.6982	-0.1349	1.0000	0.1524	0.6061	-0.2760	0.3520	-0.3427
ELMOD1	0.0002	4.3259	0.0681	1.1995	0.0742	2.7101	0.7552	-0.6266	1.0000	3.0514	1.0000	-0.0393	1.0000	-0.2729
ELMOD2	0.0001	0.6795	0.0000	0.5814	0.3837	0.2093	1.0000	0.0129	1.0000	0.0309	1.0000	-0.0551	0.3544	-0.1602
ELMOD3	0.0057	-0.5411	0.0000	-0.7303	0.0191	-0.4551	0.0762	-0.2203	1.0000	-0.0593	0.4618	-0.2368	0.4180	0.1801
ELMSAN1	0.0142	1.1482	0.0001	1.2792	0.0000	2.8096	0.0000	2.5785	1.0000	0.0470	1.0000	0.1897	0.8274	-0.1801
ELOF1	0.0000	-1.0452	0.0000	-0.6700	0.0000	-1.0980	0.0000	-0.8213	1.0000	-0.2930	0.9755	0.0950	1.0000	-0.0100
ELOVL1	0.0004	0.8348	0.0000	1.3387	0.0354	0.4918	0.0000	0.6093	1.0000	-0.1871	0.0950	0.3287	1.0000	-0.0643
ELOVL2	1.0000	0.0000	1.0000	2.2507	0.8011	3.0840	1.0000	2.3257	1.0000	0.0000	1.0000	2.3456	1.0000	-0.7792
ELOVL3	1.0000	-0.3452	1.0000	-0.7281	0.4564	-2.2491	0.0709	-4.6403	1.0000	0.1241	1.0000	-0.2464	1.0000	-2.2888
ELOVL4	1.0000	0.0000	1.0000	-2.3986	1.0000	0.0000	1.0000	-2.3199	1.0000	2.2737	1.0000	0.0000	1.0000	0.0000
ELOVL5	0.1505	0.3008	0.0000	0.3833	0.0011	0.5860	0.0000	0.4185	1.0000	-0.1124	1.0000	-0.0176	0.0080	-0.2744
ELOVL6	0.8583	0.0838	0.0253	0.2697	0.0006	0.5482	0.0000	0.5221	1.0000	-0.1321	1.0000	0.0665	0.3095	-0.1532
ELOVL7	0.0000	-2.6209	0.0000	-2.8573	0.7917	0.2865	0.1711	-0.5136	1.0000	0.4171	1.0000	0.1960	0.7393	-0.3780
ELP2	0.7000	0.2128	0.1491	0.3570	0.0003	0.9312	0.0000	0.9528	1.0000	-0.2491	1.0000	-0.0916	0.5433	-0.2210
ELP3	0.9650	-0.0683	0.7124	0.0847	0.0000	-0.7241	0.0000	-0.5370	1.0000	-0.1745	1.0000	-0.0099	1.0000	0.0170
ELP4	0.0015	0.9426	0.0006	0.6532	0.9929	0.1281	0.2116	-0.3427	1.0000	0.2166	1.0000	-0.0597	0.7477	-0.2477

ELP6	0.2713	0.2390	1.0000	-0.0032	0.0000	-0.7592	0.0000	-0.7135	1.0000	0.1033	0.6878	-0.1275	0.5838	0.1537
ELTD1	0.0472	5.0373	0.4695	3.6125	0.8033	3.0892	1.0000	0.0000	1.0000	0.0000	0.7323	-1.4486	1.0000	-3.1370
EMB	0.2138	2.4036	0.0348	4.9502	1.0000	0.6817	1.0000	2.3257	1.0000	-2.3771	1.0000	0.0538	1.0000	-0.7804
EMC1	0.0003	0.6134	0.0000	0.7290	0.5616	0.1480	0.0546	0.1766	1.0000	0.0454	0.3116	0.1735	0.8133	0.0786
EMC2	0.9941	-0.0759	0.0038	-0.3066	0.0116	-0.5372	0.0000	-0.7822	1.0000	-0.0231	0.2317	-0.2409	0.1425	-0.2630
EMC3	0.0013	0.4904	0.0000	0.3652	0.0000	-0.6956	0.0000	-0.5542	1.0000	0.0101	0.6861	-0.1031	0.3891	0.1564
EMC4	0.0302	-0.3596	0.0226	-0.2106	0.0000	-1.1177	0.0000	-0.7258	1.0000	-0.2840	0.6427	-0.1226	0.7898	0.1142
EMC6	0.0000	-0.7106	0.0000	-0.6668	0.0000	-0.8418	0.0000	-0.6041	1.0000	-0.1014	1.0000	-0.0451	0.5057	0.1419
EMC7	1.0000	-0.0238	0.8783	-0.0478	0.5307	-0.1636	0.0281	-0.2383	1.0000	0.0419	1.0000	0.0301	1.0000	-0.0267
EMC8	0.0711	-0.3730	0.0012	-0.3494	0.0417	-0.4076	0.0000	-0.4390	1.0000	-0.1462	0.8793	-0.1102	0.4104	-0.1730
EMCN	0.0001	3.2473	0.1124	1.4448	0.8151	0.7539	0.5798	-1.1513	1.0000	1.0356	0.6015	-0.7554	1.0000	-0.8668
EME1	0.0002	1.0208	0.0000	1.2262	0.4967	0.2839	1.0000	-0.0119	1.0000	-0.3547	1.0000	-0.1408	0.0321	-0.6460
EME2	0.0030	-0.7011	0.0000	-0.8295	0.0024	-0.6912	0.0001	-0.5508	1.0000	0.0400	1.0000	-0.0775	0.7256	0.1860
EMG1	0.7974	-0.1190	0.1752	0.2365	0.0029	-0.6190	0.0031	-0.4765	1.0000	-0.2017	0.6045	0.1653	1.0000	-0.0537
EMID1	1.0000	-2.4776	1.0000	0.0000	1.0000	-2.4056	1.0000	2.3257	1.0000	-2.3757	1.0000	0.0000	1.0000	2.3554
EMILIN1	0.2542	-0.2425	0.0012	-0.2909	0.0000	-0.8520	0.0014	-0.3241	1.0000	-0.2078	0.0652	-0.2436	0.1398	0.3271
EMILIN2	1.0000	0.0087	0.0008	0.2626	0.0286	0.4005	0.0000	0.5256	1.0000	0.0453	0.0007	0.3113	0.1389	0.1750
EMILIN3	0.9545	-1.6900	0.1541	-2.6957	0.0003	2.8772	0.0328	1.5588	1.0000	1.0319	1.0000	0.0489	1.0000	-0.2787
EML1	0.1657	0.4347	0.0001	0.5427	0.3029	0.3442	0.0275	0.3181	1.0000	0.3576	0.0050	0.4782	0.0964	0.3351
EML4	0.7172	-0.1160	0.6027	-0.0721	0.0001	0.6116	1.0000	-0.0285	1.0000	0.0112	0.9927	0.0677	0.0000	-0.6228
EML5	0.7788	0.3812	0.0235	0.9085	0.0013	1.3903	0.0000	2.0941	1.0000	-0.1317	0.8269	0.4024	0.0876	0.5754
EML6	0.0000	-1.0328	0.0000	-1.4393	0.0000	-0.8845	0.0000	-0.8975	1.0000	0.0057	0.0001	-0.3885	1.0000	-0.0013
EMP1	0.0000	-1.1867	0.0000	-1.0861	0.2593	0.2006	0.1541	0.1118	1.0000	0.1684	0.0133	0.2811	0.6529	0.0848
EMP2	1.0000	-0.0048	1.0000	-0.0370	0.0786	0.3298	0.0000	0.4460	1.0000	0.0117	1.0000	-0.0089	0.5304	0.1325
EMX1	1.0000	0.0000	1.0000	0.0000	1.0000	0.0000	1.0000	0.0000	1.0000	0.0000	1.0000	0.0000	1.0000	0.0000
EMX2	0.0972	-0.3592	0.6477	-0.0966	0.3043	0.2379	0.0057	0.3403	1.0000	-0.2022	1.0000	0.0729	0.9041	-0.0932
EN1	1.0000	2.1903	1.0000	-2.3959	1.0000	2.2428	1.0000	0.8538	1.0000	2.2674	1.0000	-2.2992	1.0000	0.9164
EN2	1.0000	0.0000	1.0000	2.2507	1.0000	0.0000	1.0000	0.0000	1.0000	0.0000	1.0000	2.3456	1.0000	0.0000
ENAH	0.3092	-0.2055	0.0000	-0.2803	1.0000	0.0327	0.5125	-0.0658	1.0000	0.0474	1.0000	-0.0150	1.0000	-0.0455
ENC1	0.0000	-1.5047	0.0000	-1.5480	0.0041	0.4880	0.0000	0.4499	1.0000	-0.0937	0.9618	-0.1239	0.5487	-0.1259
ENDOD1	0.4391	0.2041	0.0243	0.2694	0.0020	0.5533	0.0227	0.2641	1.0000	-0.1802	0.9356	-0.1036	0.0001	-0.4640
ENDOG	0.0002	-0.7336	0.0000	-0.5746	0.0084	-0.5683	0.0000	-0.4999	1.0000	-0.2456	1.0000	-0.0743	0.6022	-0.1711
ENDOU	1.0000	0.0000	1.0000	0.0000	1.0000	0.0000	1.0000	0.0000	1.0000	0.0000	1.0000	0.0000	1.0000	0.0000
ENDOV	0.4347	-0.2045	0.0012	-0.4078	0.0000	-0.8281	0.0000	-0.6738	1.0000	-0.1130	0.0850	-0.3040	1.0000	0.0455
ENG	0.8235	-0.2502	0.9958	0.0841	0.5645	0.3662	0.0000	0.9464	1.0000	0.0061	0.6424	0.3549	0.0177	0.5942
ENGASE	0.0936	0.5919	0.0005	0.9430	0.0132	-0.9930	0.0644	-0.7090	1.0000	-0.3395	1.0000	0.0222	1.0000	-0.0507
ENHO	0.0000	1.6346	0.0000	2.1131	0.4440	-0.3900	0.8077	-0.1686	1.0000	-0.2884	0.7110	0.2014	1.0000	-0.0598
ENKD1	0.9899	-0.0856	0.3034	-0.1905	0.0097	-0.6160	0.0065	-0.4339	1.0000	-0.0805	0.7714	-0.1742	1.0000	0.1070
ENKUR	0.0771	0.9010	0.0014	1.0170	0.0567	-1.2983	0.0000	-2.2791	1.0000	-0.0949	1.0000	0.0325	0.4003	-1.0672
ENO1	0.0003	0.5234	0.0000	0.4720	0.6918	0.1136	0.0056	-0.1781	1.0000	-0.0008	1.0000	-0.0395	0.0049	-0.2873
ENO2	0.0383	0.6447	1.0000	-0.0158	0.0000	1.7829	0.0000	1.2843	1.0000	0.3238	0.4709	-0.3217	0.6758	-0.1683
ENO4	0.3050	0.8561	0.0000	3.0677	0.0102	-2.6009	0.9786	-0.9107	1.0000	-2.1489	1.0000	0.0643	1.0000	-0.4568
ENOPH1	0.3603	-0.1786	0.1771	-0.1190	0.0010	-0.4946	0.0000	-0.5373	1.0000	0.0184	0.8219	0.0902	1.0000	-0.0191
ENOSF1	1.0000	0.0000	1.0000	0.0000	1.0000	0.0000	1.0000	0.0000	1.0000	0.0000	1.0000	0.0000	1.0000	0.0000
ENOX2	0.0002	-0.5830	0.0000	-0.4254	0.4922	0.1543	0.1203	0.1397	1.0000	0.1043	0.0252	0.2747	0.6886	0.0951
ENPEP	1.0000	-0.2920	1.0000	2.2534	1.0000	-2.4056	0.7710	3.1708	1.0000	-2.3757	1.0000	0.0515	1.0000	3.2048
ENPP2	0.1007	-2.0044	0.1463	-2.2081	0.4639	-0.9118	0.0001	2.0552	1.0000	-0.2775	1.0000	-0.4715	0.0000	2.6934
ENPP3	0.6015	-1.5518	1.0000	0.0768	0.9143	0.4613	0.2773	1.2295	1.0000	-0.1264	0.9234	1.5250	1.0000	0.6489
ENPP4	0.0180	0.7795	0.1300	0.4072	0.0139	0.7520	0.7701	0.1685	1.0000	0.0304	0.5998	-0.3285	0.0595	-0.5478
ENPP7	1.0000	0.0000	1.0000	0.0000	0.8011	3.0840	0.7701	3.1732	1.0000	0.0000	1.0000	0.0000	1.0000	0.0723
ENSA	0.0075	-0.4377	0.0000	-0.4033	0.0179	-0.3900	0.0752	-0.1676	1.0000	-0.1182	1.0000	-0.0708	0.6386	0.1096
ENTHD2	0.3146	-0.2830	0.4364	-0.1445	0.0068	-0.6315	0.9147	0.0547	1.0000	-0.2820	0.8785	-0.1325	0.0076	0.4091
ENTPD1	0.3366	-2.3921	1.0000	-0.1674	0.4564	-2.2491	1.0000	0.3899	1.0000	-0.8158	1.0000	1.4325	0.9136	1.8339
ENTPD2	1.0000	0.1100	0.3159	0.5164	0.0792	-0.9621	0.0818	-0.8568	1.0000	-0.0956	0.9944	0.3232	1.0000	0.0129
ENTPD3	0.0121	1.0018	0.0000	1.0788	0.0057	-1.3946	0.0016	-1.2412	1.0000	-0.0788	1.0000	0.0096	1.0000	0.0804
ENTPD4	0.0006	0.8030	0.0004	0.5677	0.0002	0.8354	0.0018	0.5156	1.0000	0.0840	0.8875	-0.1403	0.4147	-0.2322
ENTPD5	0.0280	0.5560	0.0000	1.0224	0.0038	0.6894	0.0000	0.6375	1.0000	-0.2054	0.3092	0.2717	0.3741	-0.2516
ENTPD6	0.1065	0.3310	0.0000	0.7005	1.0000	0.0465	1.0000	0.0243	1.0000	-0.1499	0.1335	0.2327	0.4990	-0.1656
ENTPD7	0.3595	-0.2586	0.0862	-0.3150	0.3990	-0.2477	0.9870	-0.0508	1.0000	-0.0448	1.0000	-0.0894	0.7106	0.1576
ENY2	1.0000	0.0275	1.0000	-0.0255	0.0048	-0.6370	0.0000	-0.7477	1.0000	-0.0063	1.0000	-0.0471	0.9796	-0.1103
EOGT	0.0004	0.8700	0.0000	1.2066	0.5741	-0.2378	0.3946	-0.2786	1.0000	-0.0753	0.3315	0.2743	1.0000	-0.1105
EOMES	0.8249	-3.3439	1.0000	-2.3986	0.8033	-3.2636	1.0000	-2.3199	1.0000	-0.9587	1.0000	0.0000	1.0000	0.0000
EP300	1.0000	0.0043	0.0280	0.2763	0.0000	1.0635	0.0000	1.1625	1.0000	-0.0307	0.0625	0.2547	0.8830	0.0742
EP400	0.8611	-0.0898	0.5635	-0.0932	0.0199	0.4019	0.0000	0.4353	1.0000	0.0297	1.0000	0.0388	0.9562	0.0678
EPAS1	0.1096	4.6934	0.9754	0.7517	0.0177	5.1160	1.0000	0.5390	1.0000	3.1126	1.0000	-0.7116	0.5628	-1.4317
EPB41	0.0000	1.5443	0.0000	1.3161	0.0005	1.0981	0.0000	0.8697	1.0000	0.1022	1.0000	-0.1136	0.9914	-0.1225
EPB41L1	0.0001	0.7726	0.0000	0.7429	0.0000	1.0426	0.0000	0.9604	1.0000	0.0323	1.0000	0.0156	1.0000	-0.0442
EPB41L2	0.0000	1.8590	0.0000	2.1065	0.0000	1.9653	0.0000	1.9958	1.0000	-0.4818	0.8332	-0.2165	0.1142	-0.4414
EPB41L4A	0.6157	-0.9156	0.1238	-1.6992	1.0000	0.0824	1.0000	0.1205	1.0000	0.2751	1.0000	-0.4956	1.0000	0.3165
EPB41L4B	0.0456	-1.7650	0.0000	-3.4888	0.6344	0.4534	0.0359	0.7979	1.0000	1.0033	1.0000	-0.7096	0.0027	1.3532
EPB42	0.0026	1.6248	0.0585	1.1906	0.4210	0.7322	0.1642	0.8561	1.0000	-0.0394	0.8601	-0.4592	1.0000	0.0867
EPC1	1.0000	0.0274	1.0000	-0.0390	0.7692	-0.1135	0.8327	-0.0631	1.0000	-0.0133	1.0000	-0.0664	1.0000	0.0429
EPC2	0.3991	0.2239	0.5310	0.1157	0.0860	0.3787	0.0270	0.2760	1.0000	0.1019	1.0000	0.0068	1.0000	0.0060
EPCAM	0.0313	1.5679	0.0178	1.7798	0.1186	-2.9014	0.2460	-2.3148	1.0000	-0.5297	1.0000	-0.3057	1.0000	0.0660
EPDR1	0.8352	0.1037	0.1041	-0.1504	0.2352	0.2677	0.0003	0.2766	1.0000	0.3176	0.9987	0.0760	0.0004	0.3308
EPGN	1.0000	-0.0332	0.4306	-0.2074	1.0000	0.0067	1.0000	-0.0625	1.0000	0.1392	1.0000	-0.0230	1.0000	0.0734
EPHA1	0.0599	1.2788	0.0002	1.6527	0.0054	-3.0410	0.0216	-2.2232	1.0000	-0.2237	1.0000	0.1666	1.0000	0.6029
EPHA2	1.0000	-0.0060	0.0000	0.5282	0.0000	0.8349	0.0000	0.8985	1.0000	-0.1262	0.0004	0.4201	1.0000	-0.0569
EPHA4	0.4949	1.7587	0.3896	1.5233	0.0915	2.8490	0.5147	1.4765	1.0000	0.7416	1.0000	0.5255	1.0000	-0.6329
EPHA5	0.2113	-0.2784	0.2568	-0.1535	0.9153	0.0806	0.0003	0.3695	1.0000	-0.0842	1.0000	0.0525	0.1874	0.2092
EPHA7	0.4944	0.7391	0.6403	0.7980	0.0928	1.1535	0.3561	0.9815	1.0000	-1.7451	0.0187	-1.6879	0.0003	-1.9233
EPHA8	0.8226	3.0199	1.0000	-0.1429	1.0000	0.0000	1.0000	-2.3177	1.0000	2.2673	1.0000	-0.7956	1.0000	0.0000
EPHB1	0.2445	-0.4717	0.0006	-0.3947	0.8589	0.1647	0.0000	0.5481	1.0000	0.2179	0.0945	0.3075	0.0000	0.6060
EPHB2	1.0000	-0.1229	1.0000	-0.0529	0.0000	1.2998	0.0000	1.1137	1.0000	0.1608	0.9606	0.2457	1.0000	-0.0185
EPHB3	0.0424	-0.5896	0.0014	-0.4270	0.2358	0.3503	0.0000	0.4874	1.0000	-0.0360	0.9009	0.1405	0.8032	0.1067
EPHB6	0.0678	0.8214	0.0000	1.6605	0.0047	1.0633	0.0000	2.1692	1.0000	-0.5135	0.7202	0.3406	0.0474	0.6023
EPHX1	0.0299	0.7105	0.0017	0.7133	0.0218	0.7171	0.0007	0.7525	1.0000	0.0303	1.0000	0.0456	1.0000	0.0720
EPHX2	0.0000	1.2967	0.0000	1.2170	0.0959	-0.3511	0.1057	-0.1627	1.0000	-0.3377	0.0132	-0.4043	0.6824	-0.1432
EPHX4	0.0729	0.4355	0.0001	0.5375	1.0000	0.0151	0.5022	-0.1481	1.0000	0.1194	0.3771	0.2325	1.0000	-0.0405
EPM2A	0.0022	-0.8838	0.0000	-0.8570	0.2565	-0.3451	0.0183	-0.4337	1.0000	0.0661	1.0000	0.1053	1.0000	-0.0164
EPN2	0.0097	-0.4109	0.0000	-0.4065	0.0004	0.5405	0.0000	0.4787	1.0000	0.0617	0.9217	0.0788	1.0000	0.0054
EPN3	0.1096	0.4184	0.0000	0.6658	0.3973	-0.2939	0.0318	-0.3089	1.0000	-0.3695	1.0000	-0.1100	0.1895	-0.3780
EPRS	1.0000	-0.0093	1.0000	0.0237	0.2020	0.2230	0.3966	0.0798	1.0000	0.0175	0.9449	0.0630	0.3840	-0.1202

EPS15	1.0000	0.0330	1.0000	-0.0180	0.0000	1.0964	0.0000	1.1549	1.0000	0.0623	1.0000	0.0244	0.5488	0.1261
EPS15L1	0.0765	0.3747	0.0080	0.3116	0.0238	0.4308	0.0104	0.2888	1.0000	0.1155	1.0000	0.0651	1.0000	-0.0210
EPS8L2	0.0070	1.8864	0.0883	0.8470	0.0769	1.5424	0.6416	0.4360	1.0000	1.3489	1.0000	0.3279	1.0000	0.2492
EPS8L3	1.0000	-0.3395	0.9062	-1.5348	0.9361	-1.5509	0.9096	-1.3721	1.0000	-0.1232	1.0000	-1.3217	1.0000	0.0641
EPT1	0.0000	1.4320	0.0000	1.4298	0.0000	1.8894	0.0000	1.3008	1.0000	0.3535	0.1856	0.3657	0.5014	-0.2291
EPYC	1.0000	0.0000	1.0000	0.0000	1.0000	0.0000	1.0000	0.0000	1.0000	0.0000	1.0000	0.0000	1.0000	0.0000
ERAL1	0.1877	-0.2586	0.0020	-0.3007	0.2592	-0.2299	1.0000	-0.0311	1.0000	-0.0042	1.0000	-0.0342	0.1943	0.1998
ERAP1	0.0325	0.3616	0.0003	0.2740	0.0000	0.6108	0.0000	0.6579	1.0000	0.0938	1.0000	0.0188	0.3113	0.1462
ERBB2	0.2083	-0.2333	0.0013	-0.2903	0.0138	-0.4173	0.3618	-0.1060	1.0000	-0.2347	0.0132	-0.2797	0.8988	0.0822
ERBB4	0.0003	-2.6291	0.0000	-2.1517	0.0033	1.3215	1.0000	0.0784	1.0000	0.2785	1.0000	0.7720	0.0336	-0.9596
ERC2	0.9529	-0.5533	1.0000	0.2243	1.0000	-0.1936	0.2612	-2.3127	1.0000	-0.3462	1.0000	0.4280	0.3619	-2.4646
ERCC3	1.0000	-0.0205	0.2458	-0.1318	0.1657	0.2755	0.6502	0.0695	1.0000	0.0298	1.0000	-0.0687	0.2958	-0.1706
ERCC4	1.0000	0.0704	0.0068	0.3857	0.0156	0.5125	0.0091	0.3696	1.0000	-0.0344	0.2970	0.2937	0.5388	-0.1714
ERCC5	0.7058	0.1349	0.7891	-0.0700	0.0000	-0.8056	0.0000	-0.6803	1.0000	-0.1116	0.0593	-0.3044	1.0000	0.0179
ERCC6L	0.0000	1.0860	0.0000	1.4639	0.0001	0.8316	0.1411	0.2603	1.0000	-0.1001	0.0844	0.2878	0.0000	-0.6663
ERCC8	0.2419	0.5297	0.0000	1.4024	0.9093	0.1926	0.0094	0.8739	1.0000	-0.6611	1.0000	0.2227	1.0000	0.0251
EREG	1.0000	-0.1495	0.2775	1.0123	1.0000	-0.1248	0.0138	1.5655	1.0000	-1.2493	1.0000	-0.0776	0.8364	0.4391
ERG	0.0001	1.0407	0.0000	1.1328	0.0255	0.6216	0.6172	0.1786	1.0000	0.0609	0.8621	0.1647	0.2215	-0.3770
ERGIC1	1.0000	-0.0160	0.7909	0.0468	0.0000	0.6822	0.0000	0.5599	1.0000	0.0792	0.2762	0.1545	1.0000	-0.0376
ERGIC3	0.5574	0.1533	0.0196	0.2331	0.2405	0.2472	0.0030	0.2813	1.0000	-0.0496	1.0000	0.0418	1.0000	-0.0100
ERH	0.1848	-0.2708	0.0001	-0.2981	0.0000	-0.6892	0.0000	-0.7363	1.0000	-0.0616	0.8762	-0.0766	0.6054	-0.1030
ERI1	0.0119	0.6616	0.0000	0.7395	0.3267	0.3083	0.6572	0.1138	1.0000	0.1938	0.3216	0.2861	1.0000	0.0076
ERI2	0.0027	1.4954	0.0020	1.5173	0.0019	1.3531	0.6899	0.4301	1.0000	0.1082	1.0000	0.1337	0.0792	-0.8157
ERI3	0.5118	-0.2108	1.0000	-0.0199	0.0255	0.4694	0.0000	0.5464	1.0000	-0.0658	0.8676	0.1381	1.0000	0.0172
ERICH1	0.7294	0.1898	0.6049	0.1412	0.6901	-0.1946	0.6142	0.1470	1.0000	0.0182	1.0000	-0.0199	0.1779	0.3626
ERICH3	1.0000	0.5846	1.0000	-0.1686	1.0000	0.5052	0.2353	1.4118	1.0000	0.2613	1.0000	-0.4826	0.6819	1.1780
ERICH6	0.4166	0.5310	0.6518	0.3832	0.0379	-1.3404	0.1576	-0.9228	1.0000	-0.3450	0.7466	-0.4804	1.0000	0.0795
ERICH6B	1.0000	-0.0626	0.0050	-1.7391	1.0000	0.0853	0.2087	-0.8314	1.0000	1.4331	1.0000	-0.2277	1.0000	0.5191
ERLEC1	0.8632	-0.0837	0.4552	0.0766	0.0005	-0.5738	0.0000	-0.4881	1.0000	0.1433	0.0004	0.3160	0.0297	0.2342
ERLIN1	0.5364	0.1898	0.0723	0.2521	0.0181	0.4663	0.0002	0.4459	1.0000	-0.0717	1.0000	0.0027	0.9429	-0.0864
ERLIN2	0.7605	0.0987	0.0150	0.1797	0.9320	-0.0670	0.5833	-0.0576	1.0000	-0.0178	0.8377	0.0757	1.0000	-0.0030
ERMARD	0.0000	0.9073	0.0001	0.5285	0.0001	-1.0020	0.0000	-0.9771	1.0000	0.3317	1.0000	-0.0336	0.3510	0.3613
ERMN	1.0000	-1.1537	1.0000	0.6956	1.0000	0.7355	1.0000	-2.3177	1.0000	-0.9614	1.0000	0.8994	0.5105	-4.0506
ERMP1	0.0000	1.1046	0.0000	1.2046	0.0143	0.5833	0.0110	0.3750	1.0000	-0.1385	1.0000	-0.0267	0.0560	-0.3411
ERN1	0.0000	0.7938	0.0000	0.7111	0.3834	0.2033	0.1382	0.1749	1.0000	0.0980	1.0000	0.0284	0.9593	0.0751
ERN2	0.1035	-0.3638	0.0120	-0.3184	0.3193	0.2425	0.0006	0.3699	1.0000	-0.1986	0.8175	-0.1408	1.0000	-0.0658
ERO1L	0.0000	0.8943	0.0000	0.9721	0.6741	-0.1333	0.0166	-0.2672	1.0000	0.1587	0.0178	0.2485	1.0000	0.0297
ERO1LB	0.0000	1.1789	0.0000	1.4589	0.0000	1.0759	0.0000	1.0173	1.0000	-0.1299	0.7487	0.1631	0.5162	-0.1819
ERP27	0.2092	-2.6409	0.4431	-3.7876	0.5476	-1.6600	1.0000	-0.5239	1.0000	-1.0673	1.0000	-2.2992	1.0000	0.0723
ERP29	0.0000	-0.6500	0.0000	-0.6154	0.0189	0.3797	0.0000	0.4408	1.0000	0.1385	0.2236	0.1851	0.0597	0.2045
ERP44	0.9545	0.0622	0.1204	0.1706	0.4082	0.1708	0.0910	0.1761	1.0000	0.0984	0.0480	0.2189	0.5346	0.1085
ERRFI1	0.2888	0.2440	1.0000	0.0388	0.8901	-0.0913	0.0509	-0.2467	1.0000	0.1477	1.0000	-0.0440	1.0000	-0.0017
ESAM	0.0000	1.9580	0.0000	2.1164	0.0120	-1.3387	0.0000	-2.2909	1.0000	-0.4553	0.5330	-0.2870	0.0599	-1.4029
ESCO1	0.6929	0.1577	0.4411	0.1224	0.7438	-0.1422	0.0879	-0.2376	1.0000	0.1170	1.0000	0.0951	1.0000	0.0279
ESCO2	0.2405	0.2925	0.0000	0.5216	0.9381	-0.0847	0.9156	-0.0611	1.0000	-0.1905	1.0000	0.0510	0.6771	-0.1603
ESD	1.0000	-0.0487	0.0823	-0.1456	0.0327	-0.3502	0.0120	-0.2077	1.0000	-0.0518	0.3734	-0.1365	0.6801	0.0961
ESF1	0.0328	0.7000	0.0000	1.5124	0.0075	0.8297	0.0000	1.1142	1.0000	-0.2809	0.0300	0.5447	1.0000	0.0093
ESM1	0.0000	3.2681	0.0000	3.0434	0.0454	1.0859	1.0000	-0.0671	1.0000	-0.4514	0.0039	-0.6644	0.0048	-1.5986
ESPL1	0.0003	0.7147	0.0000	1.2288	0.0000	0.8269	0.0000	0.7501	1.0000	-0.4240	0.9301	0.1003	0.0008	-0.4963
ESPN	0.8249	3.0255	1.0000	-0.1473	0.8011	3.0840	1.0000	-2.3199	1.0000	2.2737	1.0000	-0.8002	1.0000	-3.1344
ESPNL	1.0000	0.0000	1.0000	-0.1473	1.0000	0.0000	1.0000	0.0045	1.0000	2.2737	1.0000	2.3456	1.0000	2.3543
ESR1	0.6468	0.3901	0.4230	-0.5873	0.0000	2.1940	0.0000	1.6763	1.0000	0.1861	0.3015	-0.7749	0.5502	-0.3223
ESR2	0.8758	-0.3590	0.1035	-0.9223	1.0000	0.0870	0.6984	-0.3449	1.0000	0.1098	1.0000	-0.4400	1.0000	-0.3160
ESRP1	1.0000	0.5464	1.0000	-2.3959	1.0000	-2.4056	1.0000	0.8568	1.0000	-0.1078	1.0000	-3.1469	1.0000	3.2081
ESRP2	0.0000	3.3117	0.0000	2.3246	0.2523	-1.4161	0.0751	-1.2580	1.0000	0.7272	0.8607	-0.2453	0.9538	0.8953
ESRRB	0.4804	0.5648	1.0000	0.2311	0.3308	0.6880	0.9273	-0.2949	1.0000	-0.1914	0.8328	-0.5172	0.0804	-1.1756
ESRRG	1.0000	0.1193	0.0436	-0.7980	0.0012	1.2748	0.1185	0.5313	1.0000	0.5629	0.9892	-0.3406	1.0000	-0.1740
ESYT2	0.0000	0.8889	0.0000	0.9833	0.0813	0.2952	0.0011	0.2429	1.0000	0.1223	0.0120	0.2292	0.7915	0.0752
ESYT3	1.0000	0.0000	0.2920	3.9943	1.0000	0.0000	0.4624	3.6988	1.0000	0.0000	0.6951	4.1075	0.7455	3.7355
ETAA1	1.0000	0.0166	0.4723	0.1400	0.7761	-0.1284	1.0000	0.0287	1.0000	-0.0950	1.0000	0.0404	1.0000	0.0679
ETF1	0.0000	-0.9882	0.0000	-1.0012	0.0001	-0.5585	0.0000	-0.6242	1.0000	0.0939	0.8740	0.0930	1.0000	0.0334
ETFA	0.0385	0.3459	0.0000	0.4055	0.0056	-0.4425	0.0000	-0.5005	1.0000	-0.1528	0.9234	-0.0806	0.0942	-0.2054
ETFDH	0.0131	0.4661	0.0000	0.6508	0.0001	0.7088	0.0026	0.3333	1.0000	0.0284	0.2541	0.2252	0.0257	-0.3427
ETNK1	0.0450	0.5601	0.0005	0.6436	0.0036	0.8444	0.0001	0.6907	1.0000	-0.1230	1.0000	-0.0263	0.5723	-0.2732
ETNK2	0.9516	-0.5382	1.0000	-0.7289	1.0000	0.2498	0.1651	1.2938	1.0000	-0.5267	1.0000	-0.7110	1.0000	0.5190
ETNPPL	0.4695	-2.0070	0.1040	-1.6152	0.2777	1.2133	0.0320	1.1862	1.0000	0.8438	1.0000	1.2775	0.3096	0.8251
ETS1	0.0099	1.2221	0.0239	0.9412	0.0033	1.1598	0.0124	0.8992	1.0000	-0.1303	0.8354	-0.3993	0.5624	-0.3884
ETS2	1.0000	0.0465	0.1949	0.1971	0.0181	0.4951	0.0008	0.4378	1.0000	0.2366	0.0399	0.3989	0.5024	0.1848
ETV1	0.0388	-0.5275	0.2189	0.2068	0.0004	0.7314	0.0000	0.8093	1.0000	-0.0771	0.0002	0.6706	1.0000	0.0056
ETV4	0.0000	-0.9542	0.0002	-0.6061	0.0000	0.8333	0.0000	1.0623	1.0000	-0.0745	0.5611	0.2855	0.5531	0.1602
ETV6	0.0000	-1.0695	0.0000	-0.8727	0.1657	-0.2637	1.0000	0.0230	1.0000	-0.0310	0.5245	0.1784	0.0311	0.2613
ETV7	0.0000	2.1597	0.0000	1.6640	0.0033	-2.3420	0.0005	-2.1007	1.0000	0.2952	1.0000	-0.1914	1.0000	0.5433
EVA1A	0.1762	-0.7363	0.0011	-0.9158	0.0006	-1.6335	0.0007	-0.9475	1.0000	0.6717	0.6666	0.5070	0.0063	1.3666
EVA1B	0.1468	-0.7422	0.1964	-0.5318	0.1147	0.6374	0.0667	0.5884	1.0000	-0.2424	1.0000	-0.0232	0.6927	-0.2869
EVA1C	0.3619	0.5413	0.7671	0.2574	0.0308	0.8846	0.0001	1.1913	1.0000	-0.1288	0.7375	-0.4011	1.0000	0.1810
EVC	0.0053	3.0262	0.0001	2.5704	0.0009	3.1187	0.0078	2.0240	1.0000	1.1024	0.5903	0.6620	1.0000	0.0135
EVI2A	1.0000	0.0000	1.0000	-1.0017	1.0000	0.0000	0.7701	-3.1666	1.0000	3.1126	1.0000	2.3456	1.0000	0.0000
EVI2B	1.0000	0.0000	0.4696	3.6125	1.0000	0.0000	1.0000	0.0000	1.0000	0.0000	0.8785	3.7233	1.0000	0.0000
EVL	0.4972	0.1630	0.2528	0.1272	0.1945	0.2572	0.0000	0.4696	1.0000	0.0371	1.0000	0.0137	0.0419	0.2558
EVPL	0.0000	1.3656	0.0009	0.7419	0.0016	-1.1996	0.0000	-1.3101	1.0000	0.0202	0.0985	-0.5922	1.0000	-0.0876
EVX1	1.0000	0.0000	1.0000	0.0000	1.0000	0.0000	1.0000	2.3257	1.0000	0.0000	1.0000	0.0000	1.0000	2.3554
EVX2	1.0000	0.0000	1.0000	0.0000	1.0000	0.0000	1.0000	0.0000	1.0000	0.0000	1.0000	0.0000	1.0000	0.0000
EWSR1	0.9381	-0.0678	1.0000	0.0077	0.0002	-0.5866	0.0002	-0.3271	1.0000	-0.1085	1.0000	-0.0209	0.3521	0.1559
EXD1	0.0012	0.7320	0.0000	0.7962	0.0000	-1.2187	0.0000	-1.3823	1.0000	0.0831	0.8156	0.1593	1.0000	-0.0753
EXD2	0.0341	0.5217	0.1433	0.2630	1.0000	-0.0677	0.6176	0.1338	1.0000	0.0569	0.6864	-0.1892	0.4266	0.2638
EXD3	0.6881	0.1539	0.7595	0.0853	0.0619	-0.4462	0.0001	-0.6161	1.0000	-0.1347	0.5431	-0.1913	0.3207	-0.2994
EXO1	0.0000	1.7089	0.0000	2.1629	0.0359	0.7062	0.9816	0.1051	1.0000	-0.0725	0.1255	0.3911	0.0175	-0.6716
EXOC1	0.0000	1.5102	0.0000	1.8275	0.0000	1.9538	0.0000	2.0330	1.0000	-0.1607	0.9944	0.1693	1.0000	-0.0735
EXOC2	0.2056	0.3086	0.0207	0.2241	0.9359	-0.0925	0.0187	-0.2331	1.0000	0.0935	1.0000	0.0223	1.0000	-0.0418
EXOC3	0.3370	0.2077	0.0602	0.1756	0.0017	-0.5352	0.0000	-0.6016	1.0000	0.0143	1.0000	-0.0056	1.0000	-0.0459
EXOC3L1	0.8525	-0.2305	0.3426	-0.4026	0.4237	-0.4080	0.1746	-0.5237	1.0000	-0.3171	0.6450	-0.4782	0.5430	-0.4290
EXOC3L4	1.0000	0.5997	1.0000	0.5210	1.0000	-0.1902	1.0000	0.0069	1.0000	-0.1237	1.0000	-0.1929	1.0000	0.0762
EXOC4	0.6623	-0.1337	0.2315	-0.1557	0.1658	0.2627	0.0074	0.2798	1.0000	-0.1073	0.8278	-0.1176	0.8306	-0.0858

EXOC5	0.2673	-0.2304	0.4291	-0.0813	0.9455	-0.0668	0.1021	-0.1338	1.0000	0.0389	0.1761	0.2005	1.0000	-0.0226
EXOC6	0.0006	-0.5789	0.0000	-0.8896	1.0000	0.0236	0.1055	-0.1789	1.0000	0.1168	0.4868	-0.1812	0.9757	-0.0807
EXOC7	0.9509	-0.0657	0.1994	0.1387	1.0000	0.0483	0.5629	0.0769	1.0000	-0.0871	0.6050	0.1295	1.0000	-0.0532
EXOC8	1.0000	0.0016	0.4077	0.1695	0.4944	0.2206	0.0057	0.4058	1.0000	-0.1118	1.0000	0.0687	1.0000	0.0790
EXOG	1.0000	0.0815	0.1838	0.2762	0.1479	-0.4123	0.0221	-0.4242	1.0000	-0.1073	1.0000	0.0994	1.0000	-0.1135
EXOSC1	0.3614	-0.1943	0.0202	-0.2818	0.0000	-1.1545	0.0000	-0.9043	1.0000	-0.1177	0.3313	-0.1932	0.6042	0.1379
EXOSC10	0.8905	0.0787	0.0584	0.2078	0.7763	-0.1106	0.1616	-0.1520	1.0000	-0.0135	0.6696	0.1279	1.0000	-0.0492
EXOSC2	1.0000	-0.0246	0.5974	0.0746	0.0000	-0.7077	0.0000	-0.7817	1.0000	-0.0335	0.9821	0.0784	0.8237	-0.1024
EXOSC3	0.3504	-0.2932	0.1422	-0.2955	0.0138	-0.6168	0.0428	-0.3802	1.0000	-0.2123	0.6861	-0.2035	1.0000	0.0288
EXOSC4	0.6619	-0.1770	0.3012	0.1971	0.0002	-1.1338	0.0033	-0.4634	1.0000	-0.3648	1.0000	0.0214	0.6600	0.3125
EXOSC7	1.0000	-0.0167	1.0000	-0.0346	0.0004	-0.6803	0.0000	-0.7063	1.0000	0.0760	1.0000	0.0695	1.0000	0.0540
EXOSC8	0.8597	-0.1073	1.0000	0.0358	0.0002	-0.7395	0.0000	-0.6484	1.0000	-0.1099	1.0000	0.0449	1.0000	-0.0132
EXOSC9	0.6109	0.1551	0.0060	0.2950	0.2357	-0.2719	0.7606	-0.0681	1.0000	-0.1731	1.0000	-0.0217	1.0000	0.0350
EXPH5	0.2087	1.8491	0.4437	3.6202	0.0275	2.5773	0.0001	6.1954	1.0000	-3.2319	0.5657	-1.5986	1.0000	0.3387
EXT1	0.2026	0.2756	0.0000	0.4949	0.0002	0.6499	0.0000	0.7342	1.0000	0.0029	0.1381	0.2335	0.8275	0.0918
EXT2	0.0000	1.2828	0.0000	1.4628	0.0000	1.4932	0.0000	1.2881	1.0000	0.1742	0.0176	0.3663	1.0000	-0.0263
EXTL1	0.0775	-0.3422	0.0006	-0.4079	0.1169	-0.3259	0.2065	-0.2485	1.0000	-0.4451	0.0002	-0.4987	0.1762	-0.3622
EXTL2	0.0000	0.9968	0.0000	1.0550	0.0010	-0.7984	0.0000	-0.8349	1.0000	0.0309	0.8741	0.1025	1.0000	-0.0012
EXTL3	0.0023	-0.5435	0.0006	-0.2460	1.0000	0.0267	0.3290	0.0930	1.0000	-0.0749	0.0155	0.2350	1.0000	-0.0039
EYA2	0.0000	-4.1711	0.0000	-4.4514	0.0000	-1.6325	0.0000	-1.8101	1.0000	0.2951	1.0000	0.0267	0.6054	0.1223
EYA3	0.2892	-0.2264	0.2974	-0.1078	0.6130	-0.1441	0.5974	-0.0686	1.0000	-0.0418	0.8553	0.0892	1.0000	0.0382
EZH1	0.7643	-0.1152	0.0001	-0.4148	0.4910	-0.1750	0.1122	0.1739	1.0000	0.0157	0.0845	-0.2713	0.0011	0.3703
EZH2	0.0002	0.5957	0.0000	0.5132	0.8765	0.0855	0.0008	-0.3233	1.0000	0.0308	1.0000	-0.0398	0.0011	-0.3725
EZR	0.5705	-0.1302	0.1651	-0.1211	1.0000	0.0291	0.1638	-0.1195	1.0000	0.1087	0.4117	0.1303	1.0000	-0.0345
F10	0.0051	-0.5249	0.4074	0.1066	0.2018	-0.2796	0.0000	0.4975	1.0000	0.0280	0.0000	0.6710	0.0000	0.8110
F11R	0.6881	1.4496	0.9148	1.2162	1.0000	-2.4061	1.0000	-2.3199	1.0000	-0.1045	1.0000	-0.3316	1.0000	0.0000
F13A1	0.2285	-0.5110	0.5003	0.1819	0.8484	-0.1743	0.0032	-0.5971	1.0000	0.0605	0.0027	0.7630	0.3596	-0.3599
F2	0.9901	0.1656	0.5159	0.3076	0.0233	-1.0876	0.2011	-0.5531	1.0000	0.0547	1.0000	0.2069	0.5105	0.5916
F2R	0.8441	-0.0917	0.6102	-0.0750	0.0000	0.9741	0.0000	0.9140	1.0000	0.0858	0.7289	0.1149	1.0000	0.0319
F2RL1	1.0000	0.0237	1.0000	0.0177	0.0000	1.2540	0.0000	1.2093	1.0000	0.0380	1.0000	0.0440	1.0000	-0.0017
F2RL2	0.0001	-0.9135	0.0000	-0.8998	0.0090	0.5547	0.0002	0.4656	1.0000	0.1157	1.0000	0.1422	1.0000	0.0311
F2RL3	1.0000	-2.4776	1.0000	0.0000	1.0000	-2.4055	1.0000	0.0000	1.0000	-2.3757	1.0000	0.0000	1.0000	0.0000
F3	0.3673	-1.0389	0.9774	0.4490	0.6048	-0.8733	0.5343	0.7296	1.0000	-0.6487	0.9277	0.8522	0.7255	0.9638
F5	1.0000	0.0846	0.9277	-0.1806	0.4082	0.5080	0.0801	0.5805	1.0000	0.6567	0.8720	0.4028	0.0673	0.7326
F7	1.0000	0.0000	1.0000	2.2507	1.0000	0.0000	1.0000	0.0000	1.0000	0.0000	1.0000	2.3456	1.0000	0.0000
F8	0.4801	2.0058	0.5693	1.2942	1.0000	-0.1591	0.7701	-3.1666	1.0000	0.7365	1.0000	0.0489	1.0000	-2.2909
F9	0.0001	-3.7695	0.0000	-4.8051	0.1564	-1.0685	0.4013	-0.5486	1.0000	0.2322	1.0000	-0.7966	0.6280	0.7611
FA2H	0.0289	3.0887	0.5895	1.2843	0.1846	2.3577	1.0000	-0.8443	1.0000	0.7402	0.8260	-1.0370	0.3615	-2.4643
FAAH	0.2345	-0.2536	0.0000	-0.3890	0.0007	-0.5942	0.0000	-0.6130	1.0000	-0.1004	0.1971	-0.2230	0.7148	-0.1135
FAAH2	0.1866	-0.3542	1.0000	-0.0422	0.0005	-0.7983	0.0000	-0.6629	1.0000	-0.1573	0.7875	0.1670	1.0000	-0.0174
FABP1	0.0007	-2.0449	0.0000	-2.7206	0.8601	0.2926	0.0040	0.9517	1.0000	0.0896	1.0000	-0.5742	0.1313	0.7518
FABP2	0.3089	-4.2696	1.0000	-0.1630	0.6597	-1.9401	1.0000	-0.8429	1.0000	-1.0363	1.0000	3.1976	1.0000	0.0649
FABP3	0.0000	-5.2844	0.0000	-4.8577	0.0000	-3.0201	0.0000	-3.9593	1.0000	-0.5935	1.0000	-0.1543	0.0409	-1.5283
FABP6	1.0000	-0.3395	0.4437	3.6202	1.0000	-1.0994	1.0000	0.0000	1.0000	-4.1522	1.0000	-0.3305	1.0000	-3.1370
FABP7	1.0000	0.1443	0.6497	0.5439	1.0000	-0.1955	0.9681	-0.4019	1.0000	-0.7773	1.0000	-0.3686	0.4438	-0.9797
FADD	0.0138	0.6219	0.0000	0.8318	0.0000	1.3190	0.0000	1.1146	1.0000	0.0193	0.3684	0.2411	0.4993	-0.1799
FADS1	0.0000	0.9059	0.0000	1.1141	0.2857	0.2148	0.0553	0.1831	1.0000	-0.1316	0.7953	0.0889	0.3458	-0.1576
FADS2	0.0062	0.6515	0.0000	1.5119	0.0000	2.2928	0.0000	1.9393	1.0000	-0.4245	0.0060	0.4467	0.0000	-0.7728
FADS6	0.4368	0.8675	1.0000	-0.2522	0.7276	0.6908	1.0000	-0.1508	1.0000	1.0901	1.0000	-0.0145	1.0000	0.2570
FAF1	0.4221	0.2405	0.0964	0.2900	0.0157	0.6013	0.0001	0.5642	1.0000	-0.0004	1.0000	0.0635	1.0000	-0.0321
FAF2	1.0000	-0.0101	0.3760	-0.1104	0.2689	-0.2291	0.0139	-0.2423	1.0000	0.0846	1.0000	-0.0040	0.9424	0.0758
FAH	0.5318	-1.1144	0.0577	-2.0877	0.6822	-1.3531	0.0100	-3.2866	1.0000	0.6316	1.0000	-0.3300	1.0000	-1.3057
FAHD1	0.1497	-0.3852	0.3049	-0.2051	0.0029	-0.6866	0.0088	-0.4764	1.0000	-0.1255	1.0000	0.0669	1.0000	0.0902
FAHD2A	0.0013	-0.5532	0.0000	-0.5021	0.0000	-1.3786	0.0000	-1.0764	1.0000	-0.1617	0.9413	-0.0987	0.6707	0.1468
FAIM	0.0309	0.5084	0.4395	0.1450	0.8658	-0.1299	0.1659	-0.2247	1.0000	0.2418	1.0000	-0.1098	0.8758	0.1542
FAIM2	0.2827	-1.4403	0.1041	-1.6147	1.0000	0.0963	1.0000	-0.2079	1.0000	-0.0270	1.0000	-0.1898	1.0000	-0.3261
FAM101B	0.0000	-0.8440	0.0000	-0.9445	0.0081	-0.4024	0.0000	-0.2884	1.0000	0.0873	1.0000	-0.0011	0.0288	0.2066
FAM102A	0.0446	0.8361	0.0534	0.7947	0.0607	0.7371	0.0000	1.4842	1.0000	-0.6682	0.1286	-0.6987	1.0000	0.0808
FAM102B	0.8338	-0.1142	0.3236	-0.1717	0.6038	-0.1756	0.0296	-0.3198	1.0000	0.0969	1.0000	0.0528	1.0000	-0.0421
FAM103A1	0.9196	-0.0896	0.9457	-0.0394	0.1059	-0.3426	0.0000	-0.4763	1.0000	-0.0785	1.0000	-0.0149	0.1700	-0.2056
FAM104A	0.1013	-0.3619	0.0438	-0.2860	0.6260	-0.1557	0.3257	-0.1550	1.0000	-0.0268	1.0000	0.0617	1.0000	-0.0214
FAM105A	0.0028	0.7770	0.0000	0.7602	0.6014	0.2197	0.2138	0.2322	1.0000	-0.2083	0.5860	-0.2138	0.6415	-0.1916
FAM107A	1.0000	-2.4788	1.0000	-0.1449	1.0000	-0.1630	0.9167	1.3833	1.0000	-0.1089	1.0000	2.3456	1.0000	1.4474
FAM107B	0.0191	0.5009	0.4833	0.1264	1.0000	0.0776	0.0001	-0.5304	1.0000	0.3023	1.0000	-0.0604	0.1773	-0.3001
FAM109A	0.6231	-0.5677	0.1119	-0.9937	0.8878	-0.2664	1.0000	-0.1454	1.0000	0.3185	1.0000	-0.0973	0.8487	0.4408
FAM109B	0.0017	-0.4907	0.0000	-0.6121	0.8629	-0.0817	0.9792	0.0271	1.0000	0.1704	1.0000	0.0616	0.0026	0.2844
FAM110B	0.7575	-0.1369	1.0000	0.0057	0.1137	-0.3673	0.4008	-0.1545	1.0000	-0.0212	0.8863	0.1344	0.5692	0.1976
FAM110C	0.1160	1.3056	0.7380	0.5196	0.9564	-0.5439	0.3888	-1.0931	1.0000	0.1557	0.7952	-0.6197	1.0000	-0.3888
FAM110D	0.2279	-0.4014	0.1463	-0.3166	0.0015	0.7249	0.0003	0.5917	1.0000	0.0326	1.0000	0.1273	0.9853	-0.0976
FAM114A1	0.0511	-0.3757	0.0155	-0.2795	0.2468	0.2444	0.0003	0.3613	1.0000	0.2066	0.0470	0.3162	0.0068	0.3289
FAM114A2	0.4487	0.1709	0.0508	0.1945	0.0090	-0.4439	0.0029	-0.2858	1.0000	-0.0876	1.0000	-0.0519	0.9362	0.0754
FAM117A	0.0249	0.3827	0.0000	0.4805	0.0201	-0.4086	0.5917	0.0792	1.0000	-0.2472	0.5273	-0.1368	0.0712	0.2470
FAM117B	0.2930	1.0269	0.0399	1.4065	0.0054	1.7101	0.0001	1.7371	1.0000	0.0857	1.0000	0.4795	1.0000	0.1154
FAM118B	1.0000	-0.0264	1.0000	-0.0250	1.0000	-0.0465	1.0000	0.0134	1.0000	0.1246	0.9094	0.1392	0.6110	0.1924
FAM120A	0.0029	0.4827	0.0002	0.3002	0.0000	1.1953	0.0000	0.9823	1.0000	0.2492	0.8626	0.0793	1.0000	0.0412
FAM120B	0.3933	0.3099	0.0002	0.5737	1.0000	-0.0795	1.0000	0.0189	1.0000	-0.2485	1.0000	0.0251	0.9053	-0.1466
FAM122B	0.6695	0.1501	0.0020	0.3926	0.0000	1.0244	0.0000	0.9679	1.0000	-0.0432	0.3633	0.2122	0.8155	-0.0935
FAM124A	1.0000	0.0000	1.0000	-2.3959	1.0000	0.0000	1.0000	-2.3177	1.0000	2.2673	1.0000	0.0000	1.0000	0.0000
FAM124B	0.6952	0.3731	0.7666	0.3522	1.0000	0.1181	0.7783	-0.4035	1.0000	-0.3901	1.0000	-0.3993	0.3248	-0.9082
FAM126A	0.0000	0.8938	0.0000	1.0058	0.0074	0.4817	0.0003	0.3389	1.0000	-0.0303	0.7289	0.0948	0.2301	-0.1668
FAM126B	0.4837	0.5475	0.1128	0.6357	0.0019	1.3717	0.0038	1.0226	1.0000	0.2523	0.9008	0.3532	1.0000	-0.0942
FAM129A	0.0000	-1.1426	0.0000	-1.1194	0.0402	-0.5985	0.0000	-1.1982	1.0000	0.2241	0.7010	0.2595	0.2606	-0.3685
FAM129B	0.6329	-0.1380	0.0020	0.2244	0.0721	-0.3458	0.6770	-0.0575	1.0000	-0.0942	0.0007	0.2807	0.1213	0.1991
FAM131A	0.3935	-1.3021	0.5021	1.3057	1.0000	0.1131	0.1387	2.0465	1.0000	-1.9990	1.0000	0.6132	1.0000	-0.0678
FAM131B	0.6177	0.3998	0.1212	0.6682	0.0000	1.5138	0.0000	1.8388	1.0000	-0.2194	1.0000	0.0612	1.0000	0.1078
FAM132A	0.1566	0.4723	1.0000	0.0295	0.0233	0.6652	0.9238	-0.0771	1.0000	0.4107	1.0000	-0.0179	0.2594	-0.3227
FAM133B	0.1501	-0.2596	0.0044	-0.2419	0.0001	-0.6164	0.0000	-0.6119	1.0000	-0.0160	1.0000	0.0140	1.0000	-0.0062
FAM134A	1.0000	0.0378	1.0000	-0.0006	0.0214	-0.4053	0.0151	-0.2146	1.0000	-0.0826	0.6442	-0.1088	0.5999	0.1131
FAM134B	0.0000	-1.7016	0.0000	-1.5004	0.6140	-0.2041	0.0122	0.4197	1.0000	-0.2915	1.0000	-0.0759	0.1715	0.3397
FAM134C	0.9006	-0.0831	0.9581	-0.0408	0.3922	-0.1979	0.3326	-0.1340	1.0000	0.0017	1.0000	0.0555	1.0000	0.0709
FAM135A	0.5690	0.1416	0.0328	0.1726	0.0000	0.8418	0.0000	0.5186	1.0000	-0.0507	1.0000	-0.0073	0.0000	-0.3686
FAM136A	0.0766	0.3710	0.0000	0.5054	0.0000	-1.7032	0.0000	-1.5277	1.0000	-0.3157	0.6287	-0.1695	0.9554	-0.1351
FAM13A	0.1481	-0.2571	0.0000	-0.4652	0.0000	0.8946	0.0000	0.8002	1.0000	0.0724	0.6336	-0.1229	1.0000	-0.0165

FAM149A	0.0273	1.6138	0.9863	0.3971	0.2214	1.0700	0.2840	0.9827	1.0000	0.0185	0.2073	-1.1876	1.0000	-0.0614
FAM149B1	0.0003	0.5630	0.0000	0.4378	1.0000	0.0018	0.3716	-0.1160	1.0000	0.0919	1.0000	-0.0214	1.0000	-0.0209
FAM150B	0.0038	-0.8322	0.0000	-1.3594	0.0432	-0.6426	0.0068	-0.5713	1.0000	-0.1041	0.1308	-0.6183	1.0000	-0.0234
FAM151B	0.0128	0.6732	0.0000	0.8009	0.0001	0.8469	0.0000	0.9982	1.0000	-0.0675	1.0000	0.0727	0.9733	0.0903
FAM154A	0.1392	0.8904	0.2495	0.7223	0.5978	0.5190	0.2869	0.7172	1.0000	-0.3602	0.7436	-0.5194	1.0000	-0.1568
FAM154B	0.8826	-0.5976	0.9762	0.7508	1.0000	-0.7493	1.0000	0.5390	1.0000	-1.5965	1.0000	-0.2478	1.0000	-0.3089
FAM155A	1.0000	0.0000	1.0000	0.0000	1.0000	2.2428	1.0000	2.3241	1.0000	0.0000	1.0000	0.0000	1.0000	0.0652
FAM155B	1.0000	-0.0351	1.0000	-0.3598	0.0522	1.8267	1.0000	0.3702	1.0000	0.6229	1.0000	0.3103	0.6170	-0.8190
FAM159A	0.1876	-1.5728	0.9402	-0.3257	0.0124	-3.4715	0.5124	-0.9578	1.0000	-0.3890	1.0000	0.8734	0.6867	2.1416
FAM160A1	0.0345	-0.5173	0.0364	-0.3470	0.0071	-0.6147	0.0001	-0.6237	1.0000	0.0241	0.6986	0.2081	1.0000	0.0216
FAM160A2	0.0000	-1.6915	0.0000	-1.4695	0.8341	-0.1074	0.0116	0.3168	1.0000	-0.2825	1.0000	-0.0470	0.4196	0.1479
FAM160B1	0.5972	0.1462	0.0885	0.1937	0.9950	-0.0637	1.0000	0.0325	1.0000	0.0430	0.8666	0.1032	0.5293	0.1443
FAM161A	0.4004	0.2952	0.8438	0.0998	1.0000	0.0947	0.7302	-0.1185	1.0000	0.1490	1.0000	-0.0344	1.0000	-0.0590
FAM161B	1.0000	0.0774	0.9017	-0.1477	0.5847	0.3055	0.9043	0.1365	1.0000	0.0865	1.0000	-0.1256	1.0000	-0.0793
FAM162A	0.8396	0.0942	1.0000	-0.0192	0.0000	-0.7159	0.0000	-0.9120	1.0000	0.0722	1.0000	-0.0292	0.7924	-0.1189
FAM162B	0.6606	-0.8994	1.0000	-0.8629	1.0000	-0.4446	1.0000	-0.6849	1.0000	-0.3791	1.0000	-0.3347	1.0000	-0.6156
FAM163A	0.0427	-5.0455	1.0000	-1.0017	1.0000	-0.4101	1.0000	-0.8419	1.0000	-1.8119	1.0000	2.3456	0.5105	-2.2502
FAM163B	0.0000	-1.5414	0.0000	-1.7985	0.0000	-1.3912	0.0000	-1.6516	1.0000	-0.1151	0.2832	-0.3598	0.1854	-0.3684
FAM166A	1.0000	0.5381	0.4431	3.6192	1.0000	-0.1591	1.0000	0.0000	1.0000	-2.3757	1.0000	0.5871	1.0000	-2.2909
FAM167A	1.0000	0.0000	1.0000	0.0000	1.0000	0.0000	1.0000	0.0000	1.0000	0.0000	1.0000	0.0000	1.0000	0.0000
FAM167B	0.0023	-1.0541	0.0047	-0.9483	0.0001	-1.3353	0.0624	-0.6350	1.0000	-0.6802	0.4525	-0.5638	1.0000	0.0226
FAM168A	1.0000	0.0864	1.0000	-0.0164	0.0159	0.6764	0.0000	0.8463	1.0000	-0.0196	1.0000	-0.1101	0.8425	0.1560
FAM168B	0.5633	0.4653	1.0000	0.1131	0.0000	1.8018	0.0013	0.9720	1.0000	0.4549	1.0000	0.1115	0.4265	-0.3743
FAM169A	1.0000	-2.4788	1.0000	-2.3959	0.9361	1.2042	0.9096	1.3854	1.0000	-0.1089	1.0000	0.0000	1.0000	0.0752
FAM169B	0.0936	-0.4921	0.0008	-0.6547	0.0092	-0.6971	0.0001	-0.7871	1.0000	-0.0723	0.8269	-0.2232	0.9562	-0.1586
FAM171B	0.0000	1.0404	0.0000	1.0249	1.0000	0.0071	0.3138	0.1767	1.0000	-0.1119	0.8646	-0.1140	1.0000	0.0623
FAM172A	0.0148	0.4763	0.0001	0.4482	0.0919	0.3516	0.1359	0.1919	1.0000	-0.0797	0.9744	-0.0953	0.1821	-0.2342
FAM173A	0.2791	-0.3146	0.0647	-0.2868	0.0003	-0.8379	0.0000	-0.7423	1.0000	-0.0193	1.0000	0.0198	1.0000	0.0809
FAM173B	0.8510	0.1540	1.0000	0.0695	0.1945	-0.4524	0.7317	-0.1542	1.0000	0.2139	1.0000	0.1421	0.1319	0.5174
FAM175A	0.0000	0.9950	0.0000	0.9221	0.7764	-0.1663	0.3640	-0.1903	1.0000	0.0167	1.0000	-0.0446	1.0000	-0.0013
FAM175B	0.0218	-0.4260	0.0000	-0.5390	0.2660	-0.2454	0.0000	-0.3919	1.0000	0.0694	1.0000	-0.0307	0.9940	-0.0723
FAM177A1	0.0023	0.6791	0.0000	0.5246	0.2453	-0.3235	0.0000	-0.6460	1.0000	-0.1099	0.0275	-0.2516	0.0007	-0.4250
FAM178A	0.2099	-0.3061	1.0000	0.0137	1.0000	0.0671	0.0039	0.3111	1.0000	-0.1605	0.5273	0.1712	0.8749	0.0883
FAM178B	0.4147	0.8606	1.0000	0.0240	0.4910	-0.8288	0.0516	-2.0205	1.0000	0.1069	0.7531	-0.7212	0.8620	-1.0809
FAM179A	1.0000	-0.3470	0.6623	-0.5480	1.0000	-0.0002	1.0000	-0.2290	1.0000	0.7435	1.0000	0.5590	1.0000	0.5254
FAM179B	0.0008	-0.6207	0.0000	-0.6072	1.0000	0.0479	0.0107	0.3009	1.0000	-0.0825	1.0000	-0.0554	0.3627	0.1771
FAM180A	0.9650	-0.1464	1.0000	0.0550	1.0000	0.0985	0.0000	-0.8659	1.0000	0.3364	0.0252	0.5510	0.0760	-0.6202
FAM180B	0.8069	0.7475	0.6547	0.8005	1.0000	-0.8813	0.3342	-1.6813	1.0000	0.3396	1.0000	0.4053	1.0000	-0.4568
FAM181A	0.0000	-3.9412	0.0000	-3.2945	0.0940	-1.1358	0.0265	-1.3213	1.0000	-0.0783	1.0000	0.5858	1.0000	-0.2608
FAM183A	1.0000	-0.3444	1.0000	-0.5750	0.7628	-1.4076	0.0872	-2.7160	1.0000	0.5250	1.0000	0.3105	1.0000	-0.7795
FAM184B	0.4123	0.4635	0.2914	0.4850	0.3536	-0.5287	0.0966	-0.8758	1.0000	-0.3040	0.9314	-0.2698	0.6286	-0.6461
FAM185A	0.0022	1.2057	0.0033	1.0315	0.0492	0.8732	0.0043	1.0028	1.0000	-0.4734	0.0953	-0.6385	0.6233	-0.3382
FAM188A	0.1383	0.3086	0.0000	0.4402	0.0064	-0.5316	0.0000	-0.4914	1.0000	-0.0340	0.8047	0.1097	1.0000	0.0116
FAM188B	0.0682	0.5461	0.0000	0.9120	1.0000	0.0006	0.2461	0.2741	1.0000	-0.0505	0.2289	0.3292	0.6531	0.2317
FAM189A1	0.0266	0.7543	0.0000	0.8820	0.4364	0.3346	0.0013	0.6632	1.0000	-0.5949	0.1059	-0.4553	0.5463	-0.2575
FAM189A2	0.3706	0.2052	0.0001	0.3860	0.5321	-0.1697	1.0000	0.0108	1.0000	0.0246	0.1532	0.2182	0.2769	0.2099
FAM192A	0.7308	-0.1288	0.2813	-0.1159	0.0001	-0.7324	0.0000	-0.6160	1.0000	-0.1713	0.4709	-0.1461	1.0000	-0.0490
FAM193A	0.4306	0.2232	0.2913	0.1685	0.0000	0.9593	0.0000	0.7154	1.0000	0.0892	1.0000	0.0475	0.5095	-0.1482
FAM193B	0.0002	-0.7030	0.0000	-0.5825	0.0828	-0.3636	1.0000	-0.0029	1.0000	-0.1203	1.0000	0.0121	0.1277	0.2452
FAM195A	0.0011	-0.9701	0.0000	-1.3986	0.0050	0.6834	0.0000	0.7851	1.0000	-0.1603	0.2571	-0.5748	1.0000	-0.0520
FAM198A	1.0000	0.0000	1.0000	0.0000	1.0000	0.0000	1.0000	2.3241	1.0000	0.0000	1.0000	0.0000	1.0000	2.3543
FAM198B	0.0001	-0.6694	0.0000	-0.4526	0.0015	-0.5440	0.1513	-0.1298	1.0000	0.1474	0.0027	0.3767	0.0000	0.5657
FAM199X	0.5954	0.1364	0.2817	0.1141	0.4106	0.1832	0.5984	0.0707	1.0000	0.0445	1.0000	0.0347	1.0000	-0.0632
FAM19A1	0.8226	0.4806	1.0000	0.0263	0.0323	-3.2126	0.0015	-5.7190	1.0000	0.2344	1.0000	-0.2015	1.0000	-2.2909
FAM19A2	1.0000	0.0000	1.0000	0.0000	1.0000	2.2428	1.0000	0.0000	1.0000	0.0000	1.0000	0.0000	1.0000	-2.2888
FAM19A3	0.1196	-1.4832	0.0127	-1.2387	0.1768	-1.1787	0.0057	-1.2852	1.0000	0.9305	0.4489	1.1935	0.7450	0.8300
FAM19A4	1.0000	-2.4788	1.0000	0.0000	1.0000	0.6817	1.0000	0.0000	1.0000	-2.3771	1.0000	0.0000	1.0000	-3.1370
FAM204A	1.0000	0.0421	1.0000	0.0143	0.0000	-1.2552	0.0000	-0.9558	1.0000	-0.1244	0.7635	-0.1400	0.7291	0.1826
FAM206A	0.0334	-0.4865	0.2803	-0.1945	0.0001	-0.8630	0.0005	-0.5087	1.0000	-0.2232	1.0000	0.0820	0.8732	0.1380
FAM207A	1.0000	0.0281	0.7091	-0.0652	0.0000	-1.0463	0.0000	-0.8310	1.0000	-0.0193	0.7682	-0.1004	0.1786	0.2023
FAM208A	0.2012	0.2446	0.7674	0.0542	1.0000	-0.0101	0.2412	-0.1208	1.0000	0.1213	1.0000	-0.0567	1.0000	0.0157
FAM208B	0.0563	0.4906	0.0000	0.6718	0.0000	1.0232	0.0000	0.9334	1.0000	-0.1633	1.0000	0.0310	0.1191	-0.2468
FAM20A	0.0001	-1.6027	0.0000	-1.5146	0.5182	0.3163	0.0107	0.6526	1.0000	-0.3763	1.0000	-0.2774	1.0000	-0.0349
FAM20B	0.7546	0.1320	0.8552	0.0626	0.6369	0.1564	0.0002	0.3921	1.0000	0.0585	1.0000	0.0008	0.0525	0.2985
FAM20C	0.6876	0.1190	0.2740	-0.1225	1.0000	-0.0409	0.6452	0.0676	1.0000	0.1480	0.9428	-0.0812	0.0278	0.2620
FAM210A	0.3299	0.2375	0.7269	0.0771	0.0494	0.3920	0.0389	0.2373	1.0000	-0.0127	0.6066	-0.1603	0.4563	-0.1613
FAM210B	0.0087	0.6909	0.0000	0.9671	0.0000	1.0935	0.0000	1.1662	1.0000	0.1267	0.0587	0.4150	0.4139	0.2042
FAM212B	0.0080	-0.4856	0.0000	-0.6205	0.0004	-0.6550	0.0002	-0.4125	1.0000	-0.1119	0.3216	-0.2335	0.7287	0.1375
FAM213A	0.5274	0.2455	0.0006	0.6209	0.0000	-1.6248	0.0116	-0.5311	1.0000	-0.2148	0.8715	0.1739	0.0033	0.8858
FAM214A	0.7988	-0.1054	0.0349	-0.1972	0.0038	0.4814	0.0001	0.3289	1.0000	0.1932	0.8243	0.1137	1.0000	0.0452
FAM214B	0.1542	-0.4501	0.0003	-0.6430	0.2679	-0.3772	0.2001	-0.2538	1.0000	0.1510	1.0000	-0.0304	0.5057	0.2804
FAM217B	0.1070	0.5287	0.9379	0.0949	0.0000	1.6286	0.0000	1.1330	1.0000	0.2451	0.9773	-0.1757	0.3897	-0.2456
FAM219A	0.0125	0.4322	0.0124	0.2019	0.6730	0.1304	1.0000	0.0152	1.0000	0.1506	0.8874	-0.0674	1.0000	0.0401
FAM219B	0.0145	-0.5489	0.0000	-0.7476	0.0008	-0.7187	0.0000	-0.5944	1.0000	0.1129	1.0000	-0.0734	0.3818	0.2418
FAM21C	0.6235	0.1304	0.2278	0.1136	1.0000	0.0429	0.9770	-0.0297	1.0000	0.0655	1.0000	0.0611	1.0000	-0.0016
FAM221A	1.0000	-0.3288	0.7674	3.0957	0.8033	-3.2636	1.0000	0.0000	1.0000	-3.2319	1.0000	0.0598	1.0000	0.0000
FAM222A	1.0000	0.0000	1.0000	0.0000	1.0000	0.0000	1.0000	0.0000	1.0000	0.0000	1.0000	0.0000	1.0000	0.0000
FAM222B	0.3191	-0.2947	0.0262	-0.3175	1.0000	0.0007	0.3847	0.1468	1.0000	-0.0290	1.0000	-0.0386	0.8519	0.1232
FAM228B	0.4348	0.5518	0.6082	0.5445	0.8011	0.3153	0.9040	0.3424	1.0000	-1.0172	0.1290	-1.0159	0.1834	-0.9855
FAM26D	1.0000	2.1903	1.0000	0.0000	1.0000	0.0000	1.0000	0.0000	1.0000	0.0000	1.0000	-2.2992	1.0000	0.0000
FAM26E	0.1580	-0.7317	0.6169	-0.2559	1.0000	-0.1080	0.5769	-0.2891	1.0000	0.0220	0.6450	0.5090	1.0000	-0.1576
FAM26F	1.0000	0.0000	1.0000	2.2507	1.0000	2.2428	0.2461	4.0904	1.0000	0.0000	1.0000	2.3456	0.8939	1.8363
FAM32A	0.0013	-0.5014	0.0000	-0.4891	0.0000	-0.7855	0.0000	-0.6850	1.0000	-0.0716	1.0000	-0.0471	1.0000	0.0349
FAM35A	0.0415	-0.4836	0.0000	-0.6721	0.6897	0.1558	0.2679	-0.1788	1.0000	0.0179	0.8175	-0.1581	0.0720	-0.3118
FAM3B	1.0000	0.2029	1.0000	-1.0027	0.7694	1.0409	1.0000	0.5367	1.0000	-0.1146	1.0000	-1.3257	1.0000	-0.6166
FAM3C	0.0105	0.4322	0.0005	0.3319	1.0000	-0.0377	0.8426	-0.0519	1.0000	0.0445	1.0000	-0.0432	1.0000	0.0363
FAM3D	1.0000	2.1847	1.0000	2.2507	1.0000	0.0000	1.0000	0.0000	1.0000	0.0000	1.0000	0.0495	1.0000	0.0000
FAM43A	0.1230	-0.5863	0.0013	-0.4715	0.0034	0.9404	0.0000	1.5496	1.0000	-0.1120	1.0000	0.0171	0.0004	0.5043
FAM43B	1.0000	-1.1585	0.9062	1.2225	0.5528	1.2878	0.6142	1.7722	1.0000	-0.9638	1.0000	1.4317	1.0000	-0.4791
FAM45A	1.0000	0.0729	0.8894	-0.0545	0.7358	-0.1437	0.2404	-0.1556	1.0000	0.0391	1.0000	-0.0753	1.0000	0.0345
FAM46A	0.0024	-0.7735	0.0038	-0.4200	0.0000	1.2235	0.0000	1.3434	1.0000	0.2719	0.0001	0.6386	0.0003	0.3974
FAM46C	0.0097	0.6093	1.0000	0.0449	0.0002	0.8631	0.0000	1.1574	1.0000	0.1135	0.0271	-0.4386	0.0188	0.4105
FAM46D	0.8249	3.0256	0.4431	3.6192	0.0938	4.5491	0.0005	5.8367	1.0000	0.0000	1.0000	0.5843	0.5010	1.2686

FAM49A	0.0000	-0.8530	0.0000	-0.8863	1.0000	-0.0186	0.0867	-0.1613	1.0000	0.0765	1.0000	0.0557	0.9847	-0.0606
FAM49B	0.0923	-0.3469	0.0011	-0.2945	0.1040	-0.3352	0.0001	-0.3350	1.0000	-0.0540	1.0000	0.0108	1.0000	-0.0478
FAM53A	0.3308	0.3086	0.0872	0.3346	0.7641	0.1589	0.8120	0.1038	1.0000	-0.2717	0.6061	-0.2336	0.2363	-0.3221
FAM57A	0.6650	0.1374	0.0229	0.1937	0.3798	-0.2127	0.0361	-0.1629	1.0000	0.0363	0.6450	0.1050	0.7148	0.0909
FAM60A	0.8158	-0.1282	0.7523	0.0868	0.0150	0.4894	0.0000	0.4598	1.0000	0.2673	0.0061	0.4948	0.1399	0.2432
FAM63A	0.0169	-0.4722	0.0000	-0.4496	0.0794	-0.3722	1.0000	0.0296	1.0000	-0.2573	0.2090	-0.2227	0.5095	0.1504
FAM63B	0.0151	0.8006	0.0001	0.9401	0.0000	1.3618	0.0000	1.2118	1.0000	-0.0137	1.0000	0.1411	0.8548	-0.1553
FAM64A	0.0000	1.0445	0.0000	1.3988	0.0000	0.8911	0.0428	0.3067	1.0000	-0.3671	1.0000	-0.0013	0.0000	-0.9454
FAM65A	1.0000	-0.0009	0.4027	0.1007	0.8322	0.0933	0.2202	0.1288	1.0000	-0.0312	0.8932	0.0831	1.0000	0.0103
FAM65B	0.0000	-2.2019	0.0000	-2.4984	0.9660	-0.0596	0.0000	-0.3910	1.0000	0.0947	0.6426	-0.1903	0.0482	-0.2315
FAM69A	0.2050	-0.4122	0.0020	-0.6296	0.1107	0.4357	0.0072	0.4813	1.0000	0.0602	1.0000	-0.1457	1.0000	0.1106
FAM69B	0.3694	-0.2214	0.0067	-0.3234	0.0036	-0.5599	0.0202	-0.2672	1.0000	0.0060	1.0000	-0.0833	0.0696	0.3039
FAM69C	1.0000	2.1848	1.0000	2.2507	1.0000	0.0000	1.0000	0.0000	1.0000	0.0000	1.0000	0.0495	1.0000	0.0000
FAM71D	1.0000	0.0000	1.0000	0.0000	1.0000	0.0000	1.0000	0.0000	1.0000	0.0000	1.0000	0.0000	1.0000	0.0000
FAM72A	0.0000	1.0509	0.0000	1.3425	0.0001	0.9336	0.0000	0.7653	1.0000	-0.0807	0.4307	0.2208	0.3369	-0.2464
FAM73B	0.0340	0.5105	1.0000	-0.0177	0.0592	0.4609	0.0320	0.2910	1.0000	0.1925	0.1277	-0.3221	1.0000	0.0272
FAM76A	0.3148	0.2938	0.0052	0.4224	0.0485	0.4788	0.0000	0.5750	1.0000	-0.0149	0.9169	0.1273	1.0000	0.0864
FAM76B	0.6264	0.1763	0.8037	0.0841	1.0000	0.0688	1.0000	-0.0139	1.0000	0.1546	1.0000	0.0759	1.0000	0.0799
FAM78A	0.0000	-2.0792	0.0000	-2.4048	0.0005	0.6473	0.0059	0.3640	1.0000	-0.0804	0.5496	-0.3944	0.0124	-0.3592
FAM78B	0.0000	-0.8630	0.0000	-0.9159	0.0010	-0.6602	0.0001	-0.4322	1.0000	0.0493	1.0000	0.0088	0.1549	0.2805
FAM81A	1.0000	-0.3164	1.0000	-0.6943	1.0000	0.3475	0.9096	-1.3726	1.0000	0.4129	1.0000	0.0540	1.0000	-1.3084
FAM81B	0.3092	4.2299	0.6215	1.6074	1.0000	0.0000	1.0000	0.0062	1.0000	2.2673	1.0000	-0.2469	1.0000	2.3543
FAM83A	0.9545	1.0644	1.0000	0.2184	0.9468	1.2118	0.4370	-3.6968	1.0000	1.2628	1.0000	0.4448	0.7455	-3.6684
FAM83B	1.0000	0.0000	1.0000	0.0000	1.0000	0.0000	1.0000	0.0000	1.0000	0.0000	1.0000	0.0000	1.0000	0.0000
FAM83C	0.6607	0.7067	0.7162	-0.5087	1.0000	0.1181	0.1097	-1.3943	1.0000	1.0817	1.0000	-0.1165	1.0000	-0.4208
FAM83F	1.0000	0.0000	1.0000	2.2507	1.0000	0.0000	1.0000	0.0000	1.0000	0.0000	1.0000	2.3456	1.0000	0.0000
FAM83G	1.0000	0.0000	1.0000	0.0000	1.0000	0.0000	1.0000	0.0000	1.0000	0.0000	1.0000	0.0000	1.0000	0.0000
FAM83H	1.0000	-0.1679	0.6395	-0.3639	0.0038	-2.2812	0.0146	-1.3194	1.0000	0.4458	1.0000	0.2678	0.4552	1.4156
FAM84A	0.1423	-1.7551	0.2859	1.0471	0.1890	1.0282	0.0836	1.3026	1.0000	-0.5754	0.0925	2.2434	1.0000	-0.2952
FAM84B	1.0000	0.2537	0.9620	0.2515	0.0080	1.5801	0.0001	1.5576	1.0000	0.2729	1.0000	0.2902	0.9420	0.2624
FAM8A1	0.7017	-0.1193	0.0032	-0.3000	0.0262	-0.3831	0.0001	-0.3732	1.0000	0.1116	1.0000	-0.0569	0.5518	0.1256
FAM91A1	1.0000	0.0388	0.6224	0.0851	0.1688	0.3064	0.0194	0.2522	1.0000	-0.0171	1.0000	0.0428	1.0000	-0.0653
FAM92A1	0.2038	0.3434	0.8070	0.0865	0.0601	0.4494	0.6007	0.1291	1.0000	0.1020	0.8906	-0.1424	0.5215	-0.2144
FAM96A	0.6044	-0.2212	0.4972	-0.1321	0.1352	-0.4440	0.0006	-0.4844	1.0000	0.0431	0.8875	0.1424	1.0000	0.0094
FAM96B	1.0000	-0.0475	0.0973	-0.1589	0.1584	-0.2813	0.0006	-0.2878	1.0000	0.0255	0.9609	-0.0737	1.0000	0.0250
FAM98A	0.8746	-0.0758	0.0000	-0.3306	0.4048	-0.1732	0.0002	-0.2752	1.0000	0.1552	0.8269	-0.0870	0.9904	0.0584
FAM98B	0.0226	0.4878	0.0000	0.6292	0.9408	0.0966	0.8726	0.0757	1.0000	-0.1195	1.0000	0.0333	0.8831	-0.1366
FAN1	1.0000	-0.0145	1.0000	-0.0371	0.6552	0.2117	0.4215	0.1821	1.0000	0.0246	1.0000	0.0171	1.0000	0.0023
FANCA	0.0001	2.0757	0.0000	1.9264	0.0100	1.5353	0.4130	0.6138	1.0000	0.1400	1.0000	0.0049	0.2874	-0.7690
FANCB	0.0207	0.6939	0.0167	0.5300	0.0000	1.0831	0.7050	0.1695	1.0000	0.0947	1.0000	-0.0592	0.0000	-0.8160
FANCC	0.3437	-0.2829	0.0018	-0.3693	0.0026	-0.7470	0.0000	-0.8763	1.0000	0.0675	1.0000	-0.0055	1.0000	-0.0564
FANCD2	0.0005	0.8402	0.0000	1.1052	0.0000	1.1244	0.0000	0.6488	1.0000	-0.1470	0.9592	0.1282	0.0000	-0.6187
FANCE	0.0385	0.4609	0.0000	0.8725	0.0041	-0.6717	0.0042	-0.5126	1.0000	-0.5066	1.0000	-0.0842	0.2698	-0.3421
FANCF	0.0000	1.3328	0.0000	1.4277	0.0126	-0.7288	0.0011	-0.6787	1.0000	-0.2566	0.7475	-0.1519	0.8939	-0.2013
FANCG	0.8407	0.1531	0.9645	0.0797	0.1544	0.4250	0.8848	0.0977	1.0000	-0.0090	1.0000	-0.0715	0.3257	-0.3300
FANCI	0.0000	1.2235	0.0000	1.9331	0.0000	1.2948	0.0000	0.9955	1.0000	-0.4477	0.4530	0.2752	0.0000	-0.7385
FANCL	0.1018	0.5013	0.0000	0.9993	0.8807	0.1436	0.3023	-0.3288	1.0000	-0.2376	0.5813	0.2703	0.0075	-0.7076
FANCM	0.0002	-0.6762	0.0000	-0.6987	0.0476	-0.3795	1.0000	0.0280	1.0000	-0.0434	1.0000	-0.0538	0.0003	0.3685
FAR2	0.0003	1.1381	0.0000	1.2132	0.0000	1.6457	0.0000	0.9462	1.0000	0.2662	0.3027	0.3579	0.1771	-0.4269
FARP1	1.0000	0.0642	0.5387	0.1128	0.0009	0.6146	0.0000	0.5587	1.0000	0.0587	0.8879	0.1201	1.0000	0.0100
FARP2	0.4597	0.2413	0.0490	0.2779	0.0004	0.7261	0.0000	1.3422	1.0000	0.0398	1.0000	0.0882	0.0000	0.6606
FARS2	0.0014	0.7254	0.0206	0.3633	0.6691	-0.1879	0.1860	-0.2804	1.0000	0.0210	0.1151	-0.3287	1.0000	-0.0668
FARSA	0.0205	-0.4119	0.0096	-0.3025	0.0015	-0.5924	0.0000	-0.5097	1.0000	-0.1012	1.0000	0.0198	1.0000	-0.0130
FARSB	0.5509	0.1403	0.7997	0.0630	0.0000	-0.8645	0.0000	-0.8583	1.0000	-0.2082	0.0081	-0.2733	0.3635	-0.1961
FAS	0.0000	-1.0086	0.0000	-1.1815	0.0000	-0.9573	0.0000	-1.0773	1.0000	0.2417	1.0000	0.0812	0.7552	0.1269
FASLG	0.3341	0.8364	1.0000	0.0187	0.2050	0.9446	1.0000	0.1427	1.0000	0.4838	1.0000	-0.3213	1.0000	-0.3143
FASN	0.0204	0.5005	0.0000	0.6377	0.0000	1.3926	0.0000	1.5202	1.0000	0.0523	0.2735	0.2032	0.3168	0.1855
FASTK	0.0057	-0.6218	0.4376	-0.1515	0.2171	0.3216	0.0000	0.7715	1.0000	-0.4543	1.0000	0.0287	1.0000	0.0033
FASTKD1	0.2619	-0.2751	0.1234	-0.2247	0.7944	-0.1299	1.0000	-0.0470	1.0000	-0.0912	1.0000	-0.0289	1.0000	-0.0041
FASTKD2	0.4552	0.2328	0.0045	0.3677	0.6471	-0.1772	0.6808	-0.1010	1.0000	-0.1256	1.0000	0.0204	1.0000	-0.0441
FASTKD3	1.0000	0.0217	0.0039	0.4229	0.6353	0.1738	0.2432	0.2023	1.0000	-0.2154	0.6044	0.1983	0.5887	-0.1808
FASTKD5	0.0152	-0.6666	0.0000	-0.7119	0.6308	-0.1939	0.2805	-0.2204	1.0000	-0.0104	1.0000	-0.0449	1.0000	-0.0314
FAT1	0.4833	-0.1483	0.0785	-0.1577	0.0000	1.0228	0.0000	0.6718	1.0000	0.1313	0.3506	0.1344	0.0728	-0.2146
FAT2	1.0000	0.0000	1.0000	2.2507	1.0000	2.2472	1.0000	0.0000	1.0000	0.0000	1.0000	2.3456	1.0000	-2.2909
FAT4	0.0000	2.0251	0.0000	1.7849	0.1130	0.7688	0.0399	0.7481	1.0000	0.2773	1.0000	0.0514	0.8751	0.2635
FAU	0.3394	-0.1969	0.0002	-0.3153	0.0000	-1.1945	0.0000	-1.0084	1.0000	-0.1178	0.0353	-0.2239	0.8988	0.0743
FAXC	0.0043	1.2945	0.0000	1.5048	0.0000	1.7525	0.0000	1.6756	1.0000	0.1811	0.6450	0.4018	1.0000	0.1047
FAXDC2	0.0602	-0.9713	0.5778	-0.3249	0.0232	-1.1593	0.6005	-0.3383	1.0000	-0.3529	1.0000	0.3058	0.7455	0.4759
FBF1	0.9303	-0.0863	0.5740	-0.0977	0.2257	-0.2882	0.4084	-0.1292	1.0000	-0.1561	0.6050	-0.1558	1.0000	0.0079
FBLIM1	0.6678	-0.1695	0.7701	-0.0859	0.5932	-0.1846	0.9096	-0.0699	1.0000	-0.0537	1.0000	0.0426	1.0000	0.0678
FBLN2	0.0000	-1.5727	0.0000	-0.7091	0.0264	0.7624	0.0000	1.2053	1.0000	0.5107	0.0000	1.3871	0.0000	0.9565
FBLN5	0.1731	-0.4288	0.9404	0.0810	0.0147	0.5952	0.0000	0.8408	1.0000	-0.1650	0.3104	0.3552	1.0000	0.0827
FBLN7	0.0000	-1.6784	0.0000	-1.6187	0.0000	-1.2691	0.0051	-0.3855	1.0000	0.0943	0.9107	0.1681	0.0000	0.9835
FBN1	0.0033	0.5911	0.0000	0.4543	0.0000	1.0445	0.0000	1.0240	1.0000	0.2552	0.6966	0.1308	0.1047	0.2408
FBN2	0.0802	-4.8275	0.0001	-6.1146	1.0000	-0.1847	1.0000	0.2523	1.0000	1.2508	1.0000	0.0000	0.1123	1.7018
FBN3	1.0000	0.0000	1.0000	0.0000	1.0000	2.2472	1.0000	2.3241	1.0000	0.0000	1.0000	0.0000	1.0000	0.0641
FBRSL1	0.0073	-0.5517	0.0000	-0.6999	1.0000	0.0314	0.5855	0.1082	1.0000	-0.0135	0.8122	-0.1492	1.0000	0.0678
FBXL12	0.5206	-0.2724	0.0276	-0.5020	0.4169	-0.2975	1.0000	-0.0023	1.0000	-0.0841	0.6450	-0.3034	0.7040	0.2157
FBXL13	0.2629	0.3365	0.1126	0.2560	0.5073	-0.2439	0.1248	-0.2589	1.0000	-0.1551	0.4279	-0.2238	0.7437	-0.1660
FBXL14	0.2281	-0.3753	0.1662	-0.2565	0.7653	0.1518	0.0818	0.2982	1.0000	-0.0059	1.0000	0.1239	0.8175	0.1458
FBXL15	0.1456	0.5518	0.4271	0.2503	0.0001	1.1023	0.0000	0.9192	1.0000	0.0786	0.9091	-0.2111	1.0000	-0.0999
FBXL16	1.0000	-2.4788	1.0000	-2.3986	1.0000	-0.1597	1.0000	-2.3199	1.0000	-0.1045	1.0000	0.0000	1.0000	-2.2909
FBXL17	0.0365	0.8841	0.0005	1.0262	0.0010	1.1220	0.0000	1.1874	1.0000	-0.1644	1.0000	-0.0100	1.0000	-0.0923
FBXL18	0.7616	0.1525	0.2824	0.1981	0.5478	0.2182	0.0099	0.3392	1.0000	-0.0320	1.0000	0.0255	1.0000	0.0951
FBXL2	0.9324	-0.1698	0.4789	-0.2824	0.7505	0.2398	0.6134	0.2403	1.0000	-0.1508	0.9920	-0.2537	1.0000	-0.1467
FBXL20	0.9409	0.0698	0.5471	-0.0855	0.9985	0.0588	0.1241	0.1671	1.0000	-0.0702	0.1383	-0.2135	1.0000	0.0427
FBXL21	0.0030	0.5364	0.0027	0.3292	0.0157	-0.4898	0.0002	-0.4166	1.0000	0.0819	0.8187	-0.1129	0.6077	0.1615
FBXL22	0.0000	-1.0646	0.0000	-1.0799	0.0007	-0.5244	0.0000	-0.5282	1.0000	0.0813	0.9655	0.0786	0.8024	0.0823
FBXL3	0.0012	0.5916	0.0428	0.2334	0.3005	0.2453	0.5710	-0.0929	1.0000	0.2336	0.8219	-0.1119	0.8475	-0.0992
FBXL4	0.1274	1.9763	0.0672	1.2709	0.0000	3.4695	0.0000	2.4190	1.0000	1.3845	0.7531	0.6967	0.8942	0.3370
FBXL5	0.9545	0.0712	1.0000	0.0145	0.3373	-0.2086	0.0019	-0.3077	1.0000	0.0500	1.0000	0.0055	1.0000	-0.0438
FBXL7	0.0001	-0.7335	0.0000	-0.6557	0.0111	-0.5023	0.0000	-0.5782	1.0000	0.0340	0.9188	0.1249	1.0000	-0.0371
FBXL8	0.0122	0.5652	0.0002	0.5558	0.0824	-0.4342	0.0177	-0.4220	1.0000	-0.0192	1.0000	-0.0170	1.0000	-0.0014

FBXO11	0.6466	0.1294	0.6221	0.0716	0.6105	0.1477	0.8534	-0.0462	1.0000	-0.0028	1.0000	-0.0482	0.3378	-0.1921
FBXO16	1.0000	0.4663	0.6859	0.6426	0.5916	0.9026	0.5189	0.9716	1.0000	-0.1259	1.0000	0.0603	1.0000	-0.0549
FBXO18	0.0605	-0.3984	0.0000	-0.4879	0.0007	0.6028	0.0000	0.5610	1.0000	-0.0325	0.9755	-0.1101	0.9936	-0.0699
FBXO2	0.0137	0.6412	0.0059	0.5070	0.0019	-0.9693	0.0001	-0.8280	1.0000	-0.2115	0.3057	-0.3345	1.0000	-0.0637
FBXO21	0.7695	-0.1222	0.1105	-0.2340	0.0148	-0.4911	0.0000	-0.5347	1.0000	0.0506	1.0000	-0.0494	1.0000	0.0116
FBXO22	0.8249	-0.0957	0.9125	0.0436	0.7346	0.1139	0.0755	0.1842	1.0000	0.0303	0.3566	0.1822	0.6932	0.1066
FBXO25	0.0079	0.6106	0.0001	0.4532	1.0000	-0.0404	0.0438	-0.2697	1.0000	0.2237	1.0000	0.0797	1.0000	0.0010
FBXO28	0.0010	-0.6074	0.0000	-0.5748	0.0357	-0.4066	0.0001	-0.4180	1.0000	-0.0232	1.0000	0.0225	1.0000	-0.0284
FBXO3	0.0303	-0.4189	0.0000	-0.4087	1.0000	0.0396	0.7710	0.0591	1.0000	0.0008	1.0000	0.0236	1.0000	0.0263
FBXO30	0.0249	-0.3809	0.0000	-0.4042	0.0118	0.4151	0.0243	0.1895	1.0000	0.1910	0.3235	0.1806	1.0000	-0.0294
FBXO31	0.0414	-0.3466	0.0000	-0.5756	1.0000	-0.0234	0.8118	0.0510	1.0000	-0.0995	0.0095	-0.3164	1.0000	-0.0199
FBXO32	0.0000	1.1536	0.0000	1.2827	0.0000	2.0510	0.0000	1.6668	1.0000	0.0587	0.3097	0.2012	0.0046	-0.3192
FBXO33	0.6675	0.1452	0.0443	0.2265	0.3138	0.2311	1.0000	0.0157	1.0000	0.0460	0.6846	0.1393	0.4333	-0.1640
FBXO34	0.2326	-0.3455	0.4767	0.1758	1.0000	0.0386	0.0019	0.5247	1.0000	-0.4694	1.0000	0.0641	1.0000	0.0222
FBXO38	1.0000	0.0205	0.1580	0.1799	0.6859	0.1339	0.8938	0.0511	1.0000	-0.0326	0.7225	0.1390	0.7678	-0.1101
FBXO39	0.4895	0.5668	0.0079	1.2159	1.0000	0.1430	1.0000	0.0068	1.0000	-0.2644	0.8641	0.3959	1.0000	-0.3995
FBXO4	0.0880	0.5929	0.0001	0.9380	0.4230	0.3394	0.0033	0.7501	1.0000	-0.3467	1.0000	0.0086	1.0000	0.0697
FBXO40	1.0000	-0.0605	1.0000	-0.0629	0.7742	-0.2156	0.2830	-0.3529	1.0000	-0.1772	1.0000	-0.1680	0.7024	-0.3091
FBXO41	1.0000	0.0000	1.0000	-2.3959	1.0000	2.2472	1.0000	-2.3177	1.0000	2.2673	1.0000	0.0000	1.0000	-2.2909
FBXO42	0.0095	-0.4779	0.0880	-0.2599	0.2139	-0.2635	0.5992	-0.1135	1.0000	-0.0894	0.7291	0.1402	1.0000	0.0658
FBXO43	0.0938	2.1061	1.0000	0.3835	0.5524	1.2899	0.9786	-0.9103	1.0000	0.7963	0.8607	-0.9117	0.7921	-1.3990
FBXO45	0.0030	0.4757	0.0027	0.2632	1.0000	-0.0408	0.9362	-0.0384	1.0000	0.0817	0.6429	-0.1183	0.8720	0.0903
FBXO47	1.0000	0.1183	0.7666	-0.4378	0.8771	-0.5145	0.0126	-2.0949	1.0000	0.7032	1.0000	0.1587	0.9181	-0.8772
FBXO48	0.1656	0.6445	0.7614	0.1882	0.9519	-0.1779	0.1361	-0.5523	1.0000	0.2428	1.0000	-0.2020	1.0000	-0.1275
FBXO5	0.0110	0.6887	0.0000	1.1223	0.0152	0.6500	0.0256	0.4560	1.0000	-0.1946	0.4885	0.2521	0.1645	-0.3834
FBXO8	1.0000	-0.0401	0.9402	-0.0736	0.9660	0.0880	0.4785	0.1635	1.0000	0.0710	1.0000	0.0488	0.7772	0.1494
FBXO9	0.2877	-0.2033	0.0004	-0.2665	0.2208	-0.2191	0.6695	-0.0541	1.0000	-0.0130	1.0000	-0.0638	0.2054	0.1574
FBXW11	0.6754	-0.1173	0.5519	-0.0704	1.0000	-0.0550	0.2913	-0.1015	1.0000	-0.0286	1.0000	0.0308	0.9036	-0.0700
FBXW2	0.8691	0.0951	0.1792	-0.1651	0.3648	0.2158	0.4477	0.1063	1.0000	0.1648	1.0000	-0.0817	1.0000	0.0613
FBXW4	0.3437	-0.2075	0.0016	-0.3361	0.0982	-0.3357	0.0658	-0.2055	1.0000	-0.1287	0.1371	-0.2451	1.0000	0.0073
FBXW5	0.0306	-0.3550	0.0000	-0.3550	0.0171	-0.3968	0.0000	-0.4090	1.0000	0.0153	1.0000	0.0277	1.0000	0.0086
FBXW7	0.7498	-0.1529	0.4497	-0.1415	0.6275	-0.1806	0.0442	-0.3085	1.0000	0.1751	0.6450	0.1987	1.0000	0.0521
FBXW8	0.9088	-0.0928	1.0000	-0.0050	0.0305	-0.4961	0.6260	-0.1090	1.0000	-0.2360	0.8601	-0.1352	0.7307	0.1587
FCF1	0.0771	-0.3408	0.0078	-0.2457	0.0000	-0.7663	0.0000	-0.8427	1.0000	0.0176	0.6607	0.1252	1.0000	-0.0532
FCHO1	1.0000	0.1911	0.5872	-0.3131	1.0000	0.1963	0.7649	0.2423	1.0000	0.2690	1.0000	-0.2227	0.8648	0.3175
FCHO2	0.9722	0.0706	1.0000	-0.0255	1.0000	-0.0152	0.4801	-0.1052	1.0000	0.1296	1.0000	0.0464	1.0000	0.0454
FCHSD1	0.3075	0.3687	0.0024	0.7218	0.5213	-0.2968	0.5798	0.2224	1.0000	-0.6668	0.5389	-0.3020	1.0000	-0.1419
FCHSD2	1.0000	-0.1024	0.9383	-0.1009	0.1591	0.4741	0.0040	0.6207	1.0000	0.1307	1.0000	0.1448	0.5065	0.2840
FDFT1	0.4192	0.1603	0.5366	0.0691	0.3291	0.1853	0.0819	0.1403	1.0000	-0.1021	0.0786	-0.1808	0.2871	-0.1416
FDPS	1.0000	-0.0467	0.2728	0.1340	0.5844	0.1379	0.0449	0.2047	1.0000	-0.2904	0.7634	-0.0975	0.1028	-0.2180
FDX1	0.5079	0.1958	0.0516	0.2392	0.0271	-0.4810	0.1507	-0.1793	1.0000	-0.1431	1.0000	-0.0868	0.5215	0.1650
FDXACB1	0.3557	0.3218	1.0000	-0.0472	0.6315	-0.2198	0.6514	-0.1514	1.0000	0.0734	0.5273	-0.2846	1.0000	0.1464
FDXR	0.5382	0.1561	0.9907	0.0373	0.0002	-0.6466	0.0216	-0.2559	1.0000	-0.0415	0.5611	-0.1471	0.0089	0.3554
FECH	0.0000	2.3968	0.0000	2.6515	0.0054	1.4440	0.0041	1.2488	1.0000	0.0098	0.7407	0.2776	1.0000	-0.1806
FEM1A	0.5560	0.1522	0.0053	0.3617	0.0000	1.1626	0.0000	1.0683	1.0000	-0.0312	0.3313	0.1913	0.5215	-0.1197
FEM1B	0.6145	-0.1372	1.0000	0.0012	0.0196	0.3961	0.0000	0.4230	1.0000	-0.0561	0.8607	0.0947	1.0000	-0.0237
FEM1C	0.9730	0.0999	0.2772	0.2408	0.0000	1.2611	0.0000	0.9790	1.0000	0.0245	0.8785	0.1778	0.3635	-0.2541
FEN1	0.0000	0.7586	0.0000	0.9866	0.2436	0.2423	1.0000	-0.0239	1.0000	-0.1587	0.9052	0.0811	0.0009	-0.4189
FER	0.0083	0.9363	0.9289	0.1079	0.0000	1.4354	0.0483	0.5556	1.0000	0.6889	1.0000	-0.1260	0.8936	-0.1846
FER1L6	0.3176	2.2169	0.4431	3.6192	1.0000	-0.1630	1.0000	2.3257	1.0000	-2.3771	0.9744	-1.1001	1.0000	0.0660
FERD3L	1.0000	0.0000	1.0000	0.0000	1.0000	0.0000	1.0000	0.0000	1.0000	0.0000	1.0000	0.0000	1.0000	0.0000
FERMT1	0.0000	1.4470	0.0004	1.0285	0.0243	-1.1879	0.0249	-0.9551	1.0000	-0.1015	0.1623	-0.5086	1.0000	0.1394
FERMT2	0.0000	-0.7140	0.0000	-0.7383	0.0956	-0.2713	0.0000	-0.3492	1.0000	0.1233	0.6015	0.1110	0.9562	0.0505
FES	0.3150	-4.2704	0.7674	-3.2498	1.0000	-1.1020	1.0000	-0.8388	1.0000	-1.0429	1.0000	0.0000	1.0000	-0.7793
FETUB	0.8249	3.0141	1.0000	0.0000	1.0000	2.2472	1.0000	0.0000	1.0000	0.0000	1.0000	-3.1382	1.0000	-2.2909
FEV	1.0000	0.0000	1.0000	0.0000	1.0000	0.0000	1.0000	0.0000	1.0000	0.0000	1.0000	0.0000	1.0000	0.0000
FEZ1	0.0140	-0.8254	0.0003	-0.9837	0.0573	-0.7189	0.0875	-0.4872	1.0000	-0.3826	0.3504	-0.5297	1.0000	-0.1437
FEZF1	1.0000	0.0000	1.0000	0.0000	1.0000	2.2472	1.0000	0.0000	1.0000	0.0000	1.0000	0.0000	1.0000	-2.2909
FEZF2	1.0000	0.0000	1.0000	0.0000	1.0000	0.0000	1.0000	0.0000	1.0000	0.0000	1.0000	0.0000	1.0000	0.0000
FFAR4	0.6640	-1.2846	1.0000	-0.8704	1.0000	-0.4433	1.0000	-0.6907	1.0000	-0.3692	1.0000	0.0567	1.0000	-0.6157
FGA	1.0000	0.0000	1.0000	-2.3959	1.0000	2.2428	1.0000	0.0076	1.0000	2.2673	1.0000	0.0000	1.0000	0.0659
FGB	1.0000	0.0000	1.0000	0.0000	1.0000	0.0000	1.0000	0.0000	1.0000	0.0000	1.0000	0.0000	1.0000	0.0000
FGD3	0.0631	0.4485	0.0000	0.6563	0.0023	0.6545	0.0026	0.4660	1.0000	-0.0438	0.6342	0.1781	0.3266	-0.2255
FGD5	0.8505	0.4367	1.0000	0.1621	0.8590	-0.4526	1.0000	-0.2754	1.0000	-0.2507	0.9423	-0.5161	1.0000	-0.0689
FGD6	1.0000	-0.0689	0.6703	0.1403	0.5800	0.2082	0.2686	-0.2371	1.0000	-0.0715	1.0000	0.1503	0.0210	-0.5128
FGF10	0.1009	0.7259	1.0000	-0.0781	0.0277	0.8594	0.0144	0.6673	1.0000	0.4394	0.7323	-0.3527	0.7461	0.2537
FGF12	0.0000	-2.8159	0.0000	-2.4192	0.0558	-0.7233	0.2567	0.3924	1.0000	-0.1838	1.0000	0.2276	0.0156	0.9373
FGF13	0.0000	-4.5035	0.0000	-4.6049	0.0139	-0.5090	0.0000	-0.4869	1.0000	0.2474	1.0000	0.1599	0.0531	0.2741
FGF14	0.6881	1.4503	1.0000	-0.1452	0.9361	1.2045	0.6142	1.7719	1.0000	-0.1034	1.0000	-1.7117	1.0000	0.4629
FGF16	0.0028	1.6237	0.0006	1.4679	0.7850	-0.5114	1.0000	-0.0695	1.0000	0.2046	1.0000	0.0621	0.8911	0.6545
FGF18	0.8226	3.0199	0.9062	-1.5349	1.0000	2.2472	0.4370	-3.6973	1.0000	3.6422	1.0000	-0.7956	1.0000	-2.2909
FGF2	0.0113	0.9812	0.0004	0.7118	0.3550	-0.5334	0.0684	-0.5042	1.0000	-0.1169	0.1971	-0.3702	1.0000	-0.0760
FGF20	1.0000	2.1848	1.0000	-1.0008	1.0000	0.0000	0.7701	-3.1666	1.0000	3.1126	1.0000	0.0515	1.0000	0.0000
FGF22	0.0562	-1.0892	0.1474	-0.8932	0.6741	0.3326	0.0001	1.4373	1.0000	-0.6386	0.9944	-0.4317	0.4348	0.4687
FGF23	0.6568	0.4499	0.8980	-0.2287	0.7752	-0.5034	1.0000	0.0076	1.0000	0.3789	1.0000	-0.2878	0.2908	0.8928
FGF3	1.0000	0.0000	1.0000	0.0000	1.0000	0.0000	1.0000	0.0000	1.0000	0.0000	1.0000	0.0000	1.0000	0.0000
FGF4	0.6923	-2.0829	0.4431	-3.7876	1.0000	-0.5786	0.7718	0.9503	1.0000	-0.5088	1.0000	-2.2957	1.0000	1.0208
FGF5	0.0902	-3.0436	0.7614	-0.4906	1.0000	-0.1967	0.7426	-0.7091	1.0000	0.1840	0.3755	2.7740	1.0000	-0.3238
FGF6	0.8226	3.0199	1.0000	0.0000	1.0000	2.2472	1.0000	0.0000	1.0000	0.0000	1.0000	-3.1430	1.0000	-2.2909
FGF7	0.0133	-5.3972	0.0227	-5.1345	0.0731	-3.0652	0.2196	-1.8673	1.0000	-0.2950	1.0000	0.0000	1.0000	0.9164
FGF8	1.0000	-2.4788	1.0000	2.2507	1.0000	-2.4061	0.7710	3.1708	1.0000	-2.3771	1.0000	2.3456	1.0000	3.2048
FGFBP1	0.8249	3.0140	1.0000	-0.1473	1.0000	0.0000	1.0000	-2.3199	1.0000	2.2737	1.0000	-0.7943	1.0000	0.0000
FGFBP2	1.0000	-0.3227	1.0000	0.5197	1.0000	-1.0171	1.0000	0.6986	1.0000	0.4115	1.0000	1.2774	0.6865	2.1414
FGFR1	0.0634	-0.3238	0.5029	-0.0868	0.0068	0.4379	0.0000	0.9870	1.0000	-0.2128	1.0000	0.0368	0.0003	0.3417
FGFR1OP	0.0000	-1.5982	0.0000	-1.6951	0.0088	-0.4698	0.0000	-0.3801	1.0000	0.1296	1.0000	0.0456	0.1877	0.2241
FGFR1OP2	0.2611	0.2446	0.0117	0.2403	0.8485	-0.0964	0.0640	-0.1924	1.0000	0.1009	0.7543	0.1092	1.0000	0.0114
FGFR3	0.0570	2.8490	0.3475	1.2297	0.1908	2.3558	0.9786	-0.9111	1.0000	1.6505	1.0000	0.0592	0.6308	-1.6122
FGFR4	0.0142	0.3726	0.0000	0.3461	0.0918	-0.2794	0.0000	0.2994	1.0000	-0.1805	0.0660	-0.1945	0.0000	0.4037
FGFRL1	0.5634	0.7552	0.9723	-0.1807	0.0001	2.1000	0.7819	0.3145	1.0000	1.0391	1.0000	0.1172	0.2093	-0.7419
FGG	0.8249	-3.3439	0.7642	-1.4014	1.0000	-1.0206	0.1711	-4.4026	1.0000	1.1162	1.0000	3.1976	1.0000	-2.2888
FGGY	0.4532	0.2787	0.0168	0.5109	0.0000	-1.1833	0.0000	-1.2665	1.0000	-0.1861	1.0000	0.0590	0.8295	-0.2648
FGL1	0.2913	-0.7540	0.0407	-0.9901	0.0701	0.8349	0.0000	1.3626	1.0000	0.0321	1.0000	-0.1909	0.1906	0.5637
FGL2	0.1876	4.2322	0.1771	1.4677	0.5080	3.6209	0.9877	-0.9060	1.0000	4.0114	0.5831	1.3947	1.0000	-0.4587

FH	0.1883	-0.2398	0.0005	-0.2974	0.1996	-0.2373	0.0038	-0.2401	1.0000	0.0263	1.0000	-0.0192	1.0000	0.0290
FHAD1	1.0000	0.0000	1.0000	0.0000	1.0000	2.2472	0.7710	3.1708	1.0000	0.0000	1.0000	0.0000	1.0000	0.9149
FHDC1	0.0002	0.9469	0.0000	0.8184	0.4784	0.2707	1.0000	0.0078	1.0000	0.0321	1.0000	-0.0844	0.6865	-0.2267
FHIT	0.2684	-0.3536	0.0273	-0.3761	0.0222	-0.6119	0.0008	-0.7047	1.0000	0.1016	1.0000	0.0901	1.0000	0.0141
FHL1	0.0031	-0.4696	0.0000	-0.8704	0.0015	-0.4661	0.0000	-0.8399	1.0000	0.0541	0.0081	-0.3349	0.0001	-0.3143
FHL2	0.0034	-1.2895	0.0000	-1.8400	0.0000	-1.9169	0.0000	-1.7585	1.0000	0.3634	1.0000	-0.1746	0.7481	0.5268
FHL3	0.0001	0.5816	0.0000	0.5425	0.3507	0.1803	0.0282	0.1559	1.0000	-0.0279	1.0000	-0.0544	1.0000	-0.0467
FHL5	1.0000	-0.3449	0.5029	1.3071	0.7390	0.7713	0.5080	1.4790	1.0000	-1.3447	1.0000	0.3105	0.9885	-0.6358
FHOD1	0.0000	-1.0483	0.0000	-1.0623	0.0000	-0.9367	0.0000	-0.5374	1.0000	-0.2422	0.0686	-0.2437	0.3584	0.1625
FHOD3	0.0076	-0.4196	0.0000	-0.4394	0.0000	0.8002	0.0032	0.3093	1.0000	0.1331	0.5245	0.1259	0.0072	-0.3528
FIBCD1	0.8249	-3.3439	0.5066	-1.6418	1.0000	-0.1793	0.4996	-1.4673	1.0000	1.3534	1.0000	3.2003	1.0000	0.0727
FIBIN	0.0008	-0.9478	0.0012	-0.6788	0.2071	-0.4654	0.0978	0.3535	1.0000	-0.1791	1.0000	0.1042	0.0560	0.6463
FICD	0.9629	-0.0865	0.4915	0.1264	1.0000	-0.0182	0.9096	0.0687	1.0000	-0.0964	0.8904	0.1290	1.0000	-0.0040
FIG4	0.0362	0.8506	0.0000	1.4778	0.0000	1.5685	0.0000	1.8719	1.0000	-0.1364	0.1703	0.5050	0.9934	0.1725
FIGF	1.0000	0.0000	1.0000	0.0000	1.0000	0.0000	1.0000	0.0000	1.0000	0.0000	1.0000	0.0000	1.0000	0.0000
FIGN	0.0484	1.0200	0.0349	0.7784	0.0000	1.7853	0.0000	1.5739	1.0000	0.1566	1.0000	-0.0685	1.0000	-0.0498
FIGNL1	0.0058	0.8279	0.0001	0.7964	0.8763	0.1606	0.3498	0.2568	1.0000	-0.2042	0.7635	-0.2234	1.0000	-0.0984
FIGNL2	0.0002	-1.2356	0.0000	-1.1570	0.0133	0.6885	0.0244	0.4642	1.0000	0.1102	1.0000	0.2020	1.0000	-0.1076
FILIP1	0.3770	-1.6842	0.4272	-1.5128	0.0240	1.7597	0.0699	1.3721	1.0000	-0.1320	1.0000	0.0544	0.8598	-0.5174
FILIP1L	0.5855	-0.4889	1.0000	0.0485	1.0000	0.2177	0.0148	1.0214	1.0000	-0.2063	1.0000	0.3375	0.3629	0.6015
FIP1L1	0.6923	0.1384	0.4636	-0.1195	1.0000	-0.0069	0.3028	-0.1542	1.0000	0.1629	1.0000	-0.0817	1.0000	0.0209
FITM2	0.0029	0.4766	0.0000	0.5233	0.0468	-0.3569	0.0000	-0.5054	1.0000	0.1989	0.0107	0.2579	1.0000	0.0548
FKBP10	0.7619	-0.1566	1.0000	0.0052	0.3942	0.2740	0.0000	0.7029	1.0000	0.0505	0.0986	0.2245	0.0000	0.4839
FKBP14	0.0417	-0.4049	0.0000	-0.5313	0.1511	0.2986	0.0002	0.3107	1.0000	0.1159	1.0000	0.0016	0.4698	0.1334
FKBP15	0.7601	0.1101	0.8498	-0.0605	0.6103	-0.1418	0.4483	-0.1038	1.0000	-0.0147	0.5445	-0.1725	1.0000	0.0298
FKBP1A	0.0000	-0.7810	0.0000	-0.8706	0.4809	-0.1501	0.3940	-0.0957	1.0000	0.0230	1.0000	-0.0541	0.7916	0.0829
FKBP1B	0.0000	-1.5282	0.0000	-1.7482	0.3057	-0.2091	0.2051	-0.1370	1.0000	0.1254	1.0000	-0.0816	0.1671	0.2027
FKBP3	0.0000	-1.2665	0.0000	-1.2830	0.0000	-0.9875	0.0000	-0.7955	1.0000	0.0259	1.0000	0.0217	0.0792	0.2236
FKBP4	0.1480	0.2787	0.2500	0.1229	0.0010	0.5468	0.0000	0.3644	1.0000	-0.0590	0.1315	-0.2025	0.0371	-0.2358
FKBP5	0.0000	1.2813	0.0000	1.3683	0.0000	0.8756	0.0000	0.9654	1.0000	-0.4521	0.0010	-0.3531	0.0005	-0.3564
FKBP6	0.9036	0.3335	0.3076	0.5921	1.0000	-0.2944	0.5454	-0.5676	1.0000	0.2525	0.7591	0.5267	1.0000	-0.0155
FKBP7	0.1169	0.2709	0.3195	0.1053	0.4274	-0.1675	0.6265	0.0638	1.0000	0.0850	0.9963	-0.0683	0.0011	0.3214
FKBP8	0.0053	-0.4346	0.0051	-0.2441	0.0001	-0.6499	0.0001	-0.3233	1.0000	-0.2077	1.0000	-0.0051	0.6546	0.1245
FKBP9	0.0001	0.5650	0.0000	0.5650	1.0000	-0.0545	0.1131	-0.1418	1.0000	-0.0145	1.0000	-0.0022	0.6584	-0.0967
FKRP	0.0000	-0.9162	0.0000	-0.9535	0.0000	-0.8887	0.0000	-0.6888	1.0000	-0.1241	0.7902	-0.1491	1.0000	0.0817
FKTN	0.0215	0.4031	1.0000	-0.0180	0.0318	-0.3870	0.0000	-0.5856	1.0000	-0.0024	0.0000	-0.4107	0.1569	-0.1950
FLAD1	0.5741	-0.1900	0.1493	-0.2710	0.0421	-0.4726	0.3469	-0.2072	1.0000	0.0345	1.0000	-0.0354	0.1907	0.3040
FLCN	0.0892	0.3153	0.0001	0.4074	0.8599	0.0890	0.6591	0.0803	1.0000	-0.0663	1.0000	0.0386	0.9492	-0.0689
FLI1	1.0000	-0.0897	0.6157	0.4442	0.0022	1.4927	0.0013	1.2932	1.0000	0.0764	0.6646	0.6225	1.0000	-0.1175
FLII	0.0632	-0.3174	0.1342	-0.1466	0.1315	-0.2565	1.0000	-0.0223	1.0000	-0.2739	0.9050	-0.0907	1.0000	-0.0346
FLNB	0.0000	0.7103	0.0000	0.8665	0.0022	0.4495	0.0000	0.5659	1.0000	0.0348	0.0387	0.2035	0.2882	0.1563
FLOT2	0.4647	-0.1872	0.0793	-0.2003	0.0115	-0.4622	0.0000	-0.6350	1.0000	-0.0458	1.0000	-0.0473	0.1862	-0.2135
FLRT2	0.0544	-0.5371	0.0044	-0.5422	0.0000	2.0787	0.0000	1.5760	1.0000	-0.1058	1.0000	-0.0989	0.0001	-0.6053
FLRT3	0.9996	0.1341	0.0154	0.6611	0.6077	0.3068	0.0000	1.3096	1.0000	-0.4196	1.0000	0.1196	0.0888	0.5926
FLT3	0.4916	1.1802	0.7056	0.7867	0.3180	1.3276	0.0318	1.8038	1.0000	0.1909	1.0000	-0.1976	0.8939	0.6568
FLT4	1.0000	0.0000	1.0000	-0.1608	1.0000	2.2472	1.0000	0.5384	1.0000	3.1056	1.0000	3.1945	1.0000	1.4485
FLVCR1	1.0000	-0.0193	1.0000	0.0001	0.5708	-0.1969	0.7452	-0.0900	1.0000	0.0514	1.0000	0.0841	0.7896	0.1637
FLVCR2	0.0275	-0.3702	0.0014	-0.3009	0.0000	-0.8251	0.0000	-0.6922	1.0000	-0.1254	1.0000	-0.0440	1.0000	0.0132
FMN1	1.0000	-0.0432	0.4009	-0.2133	0.2027	0.3641	0.3943	0.1922	1.0000	0.1148	1.0000	-0.0402	1.0000	-0.0508
FMNL1	0.9650	1.0733	1.0000	2.2534	1.0000	0.6740	0.4370	3.7040	1.0000	-2.3771	1.0000	-1.3292	1.0000	0.6050
FMNL2	0.5407	0.4366	0.9813	0.0986	0.0014	1.2979	0.0000	1.0616	1.0000	0.5883	0.8946	0.2625	0.2784	0.3556
FMO4	1.0000	-0.3418	1.0000	-0.6986	1.0000	-0.8796	0.9162	-1.3749	1.0000	-0.8115	1.0000	-1.1683	1.0000	-1.3086
FMOD	0.3649	-1.3036	0.1771	1.4537	0.9346	-0.3817	1.0000	-0.6859	1.0000	-0.7765	0.1830	1.9942	0.8620	-1.0805
FMR1	0.2097	0.2780	0.1095	0.2617	0.0045	0.5178	0.0027	0.3939	1.0000	0.0681	1.0000	0.0651	1.0000	-0.0496
FMR1NB	1.0000	-0.3384	0.6845	0.7908	1.0000	-0.1880	0.1318	-4.3887	1.0000	0.5651	0.5658	1.7158	0.7287	-3.6634
FN1	0.1298	0.5130	0.0000	1.0621	0.0000	1.4311	0.0000	1.6338	1.0000	0.2805	0.0000	0.8420	0.0000	0.4870
FN3K	0.0892	0.6471	1.0000	-0.0205	0.8921	-0.1825	0.5753	-0.2602	1.0000	0.4166	0.9052	-0.2360	0.7243	0.3440
FN3KRP	1.0000	0.0339	0.4700	-0.0924	0.5996	0.1513	1.0000	-0.0002	1.0000	0.1187	1.0000	0.0053	1.0000	-0.0275
FNBP1	0.0064	0.4720	0.0000	0.4154	0.0000	0.9364	0.0000	1.0509	1.0000	0.0176	1.0000	-0.0264	0.4453	0.1372
FNBP4	0.0002	-0.6174	0.0000	-0.6910	0.0000	-0.8993	0.0000	-0.5541	1.0000	-0.0586	0.5382	-0.1198	0.0076	0.2918
FNDC1	0.0000	-4.4849	0.0000	-4.4744	0.7100	0.3399	0.0034	0.6513	1.0000	0.7216	1.0000	0.7503	0.0001	1.0361
FNDC3A	0.0141	0.4366	0.0003	0.3457	0.8011	0.1086	0.0161	0.2500	1.0000	-0.0102	0.9052	-0.0889	0.5935	0.1356
FNDC3B	0.0336	0.3899	0.0000	0.6529	0.0000	1.4390	0.0000	1.3101	1.0000	0.2834	0.0000	0.5593	0.3931	0.1595
FNDC4	1.0000	0.0194	1.0000	-0.0323	0.0179	-0.7708	0.8515	-0.1196	1.0000	0.0081	1.0000	-0.0332	0.0360	0.6603
FNDC5	0.0000	-1.1292	0.0000	-1.1752	0.0033	-0.5392	0.0000	-0.3703	1.0000	-0.0211	1.0000	-0.0548	0.3406	0.1526
FNDC7	0.0122	0.9939	0.0876	0.5718	1.0000	-0.1547	1.0000	0.1162	1.0000	0.1444	0.8856	-0.2648	0.7779	0.4264
FNDC9	0.8249	-3.3411	1.0000	0.0000	0.8033	-3.2622	1.0000	0.0000	1.0000	-3.2289	1.0000	0.0000	1.0000	0.0000
FNIP1	0.4780	0.2700	0.1898	0.2604	0.0000	1.6783	0.0000	1.5179	1.0000	0.1758	0.5247	0.1797	1.0000	0.0211
FNIP2	0.0892	-0.3809	0.0000	-0.5487	0.0531	0.3891	0.6364	0.0800	1.0000	0.2558	1.0000	0.1007	1.0000	-0.0479
FNTA	0.9227	0.0831	0.5248	0.0971	0.9291	-0.0828	0.1729	-0.1759	1.0000	-0.1720	0.5232	-0.1465	0.0878	-0.2592
FOPNL	0.0263	0.4368	0.0191	0.2631	0.0120	-0.5151	0.0000	-0.7218	1.0000	-0.0621	0.1665	-0.2232	0.1862	-0.2626
FOSL2	0.0000	0.6378	0.0000	0.7528	0.0000	0.9327	0.0000	0.9107	1.0000	0.0687	0.1162	0.1964	0.9790	0.0521
FOXA1	1.0000	0.0000	1.0000	0.0000	1.0000	0.0000	1.0000	0.0000	1.0000	0.0000	1.0000	0.0000	1.0000	0.0000
FOXA2	1.0000	2.1848	1.0000	0.0000	1.0000	0.0000	1.0000	0.0000	1.0000	0.0000	1.0000	-2.2957	1.0000	0.0000
FOXB1	1.0000	0.0000	1.0000	0.0000	1.0000	0.0000	1.0000	0.0000	1.0000	0.0000	1.0000	0.0000	1.0000	0.0000
FOXC1	0.0017	2.8217	0.3867	0.7450	0.0002	2.9793	0.0676	1.1599	1.0000	1.7966	1.0000	-0.2687	1.0000	-0.0218
FOXE3	1.0000	0.2503	0.7981	-0.3984	0.1975	0.8295	0.5498	0.4685	1.0000	0.1691	0.9301	-0.4651	1.0000	-0.1878
FOXF1	0.8226	3.0199	0.7666	3.0922	1.0000	0.0000	1.0000	2.3257	1.0000	0.0000	1.0000	0.0547	1.0000	2.3554
FOXF2	1.0000	0.0000	1.0000	0.0000	1.0000	0.0000	1.0000	0.0000	1.0000	0.0000	1.0000	0.0000	1.0000	0.0000
FOXG1	1.0000	0.0000	1.0000	0.0000	1.0000	2.2472	1.0000	0.0000	1.0000	0.0000	1.0000	0.0000	1.0000	-2.2909
FOXI1	1.0000	0.8875	1.0000	-0.6969	1.0000	0.7335	1.0000	0.0077	1.0000	0.4115	1.0000	-1.1662	1.0000	-0.3104
FOXI2	1.0000	0.0000	1.0000	0.0000	1.0000	0.0000	1.0000	0.0000	1.0000	0.0000	1.0000	0.0000	1.0000	0.0000
FOXI3	1.0000	0.0000	1.0000	0.0000	0.8033	3.0892	1.0000	0.0000	1.0000	0.0000	1.0000	0.0000	1.0000	-3.1370
FOXJ1	1.0000	0.0000	1.0000	-0.6953	1.0000	0.0000	0.9096	-1.3722	1.0000	3.6422	1.0000	3.1976	1.0000	2.3554
FOXJ2	0.8586	0.0808	0.5841	-0.0706	0.2824	-0.2083	0.0123	-0.2180	1.0000	0.0338	0.7475	-0.1049	1.0000	0.0295
FOXK1	0.7781	0.1762	0.7928	0.1176	0.3893	0.2964	0.0065	0.4933	1.0000	0.0459	1.0000	0.0009	0.4897	0.2487
FOXK2	0.3466	0.2952	0.2818	0.2229	0.0000	1.0304	0.0000	0.9714	1.0000	-0.0621	0.9941	-0.1199	0.8482	-0.1136
FOXL1	1.0000	0.0000	1.0000	0.0000	1.0000	2.2472	1.0000	0.0000	1.0000	0.0000	1.0000	0.0000	1.0000	-2.2909
FOXL2	1.0000	0.1737	0.0700	1.1505	0.2132	0.9703	1.0000	-0.2756	1.0000	-0.1287	0.5349	0.8598	0.1311	-1.3699
FOXM1	0.0000	0.8278	0.0000	0.9997	0.0000	0.9629	0.0046	0.3376	1.0000	-0.0821	0.8590	0.1022	0.0000	-0.7012
FOXN1	0.5391	-3.8768	1.0000	-2.3986	0.9468	-1.5535	1.0000	-2.3199	1.0000	-1.4875	1.0000	0.0000	1.0000	-2.2888
FOXN2	0.3183	0.5511	0.0007	1.0325	0.0000	1.5120	0.0000	1.2061	1.0000	0.0814	0.2600	0.5752	0.8718	-0.2189
FOXN3	0.0343	1.0007	0.2660	0.5114	0.0019	1.3414	0.0005	1.1825	1.0000	-0.0032	0.5473	-0.4773	1.0000	-0.1564

FOXN4	0.5086	3.5456	1.0000	2.2534	1.0000	0.0000	1.0000	0.0000	1.0000	0.0000	1.0000	-1.3257	1.0000	0.0000
FOXO1	0.6905	0.1269	0.4259	0.1038	0.0316	0.3808	0.0000	0.4712	1.0000	0.0346	1.0000	0.0235	0.5195	0.1305
FOXO3	0.1150	0.3413	0.4954	0.1115	0.0110	0.4998	0.0022	0.3306	1.0000	0.0924	0.8064	-0.1256	1.0000	-0.0707
FOXO4	0.9115	0.0917	0.0244	0.2586	0.0000	0.8163	0.0000	1.1166	1.0000	-0.1335	1.0000	0.0464	0.1917	0.1723
FOXO6	0.0000	-0.9949	0.0000	-0.7937	0.0000	1.3905	0.0000	1.9046	1.0000	-0.2501	1.0000	-0.0357	0.1231	0.2718
FOXP1	1.0000	0.0296	0.2707	-0.1631	1.0000	0.0494	0.0258	0.2685	1.0000	0.1473	1.0000	-0.0331	0.0169	0.3698
FOXP2	0.0021	1.1952	0.0054	0.8019	0.0000	1.9876	0.0000	1.6370	1.0000	0.0322	0.6488	-0.3510	0.3976	-0.3148
FOXP4	0.1126	-0.3230	0.4971	0.0906	0.1394	0.3089	0.0000	0.4978	1.0000	-0.2174	0.1948	0.2087	1.0000	-0.0231
FOXRED1	0.0000	-0.8689	0.0000	-0.8261	0.0788	-0.3948	0.4614	-0.1373	1.0000	-0.2195	0.9238	-0.1640	1.0000	0.0436
FOXRED2	0.6007	0.1467	0.8285	0.0507	0.0567	-0.3722	0.0004	-0.3390	1.0000	0.1315	1.0000	0.0479	0.4021	0.1703
FOXS1	1.0000	-0.1526	0.3682	0.6939	1.0000	-0.1977	1.0000	0.2103	1.0000	-0.1316	0.8175	0.7297	1.0000	0.2827
FPGS	0.5086	0.1675	0.0007	0.3738	0.0000	-0.8366	0.0172	-0.2915	1.0000	-0.3947	0.3920	-0.1768	0.6795	0.1560
FPGT	1.0000	0.0370	0.5490	-0.1141	0.0816	-0.3979	0.0195	-0.2934	1.0000	0.0035	0.8759	-0.1343	0.9091	0.1125
FRA10AC1	0.5634	0.2873	0.6646	-0.1219	0.0040	0.7555	0.0011	0.4645	1.0000	-0.0331	0.2781	-0.4312	0.0855	-0.3175
FRAS1	0.0019	-1.7720	0.0000	-2.5477	0.0000	2.2964	0.0000	1.1373	1.0000	0.1706	0.9345	-0.5937	0.0000	-0.9838
FRG1	0.1882	0.2767	0.0679	0.2204	0.4331	-0.1923	0.0914	-0.2202	1.0000	0.0189	1.0000	-0.0247	1.0000	-0.0033
FRK	1.0000	0.0000	1.0000	2.2507	1.0000	0.0000	1.0000	0.0000	1.0000	0.0000	1.0000	2.3456	1.0000	0.0000
FRMD1	0.5570	0.3657	0.1369	-0.5690	0.9210	-0.1953	0.2797	-0.4469	1.0000	-0.0562	0.0196	-0.9800	0.8741	-0.3005
FRMD3	0.0000	-2.2423	0.0000	-2.6865	0.0000	-0.9435	0.0000	-1.2221	1.0000	0.1480	0.5666	-0.2861	0.7032	-0.1256
FRMD4A	0.0577	-0.3907	0.0000	-0.5084	0.1564	0.2745	0.0006	0.2955	1.0000	0.1358	1.0000	0.0310	0.3063	0.1617
FRMD4B	0.0000	-1.2522	0.0000	-1.3745	0.0077	-0.4512	0.0069	-0.2122	1.0000	0.1900	1.0000	0.0795	0.0000	0.4342
FRMD5	0.1105	-0.3538	0.0001	-0.5716	0.0024	0.5542	0.0000	0.5992	1.0000	-0.3172	0.0033	-0.5234	0.0609	-0.2673
FRMD6	0.0036	-0.5235	0.0000	-0.4635	0.1322	0.2901	0.0000	0.7150	1.0000	-0.1681	1.0000	-0.0956	0.0296	0.2621
FRMD7	1.0000	-0.2684	1.0000	-0.1192	0.9090	0.3538	0.9575	0.2575	1.0000	0.3429	1.0000	0.5058	1.0000	0.2613
FRMPD1	0.7603	-0.6883	0.7193	-0.4933	0.3425	0.7912	0.0000	2.1479	1.0000	-0.1265	1.0000	0.0800	0.0028	1.2332
FRMPD2	0.9871	-0.2244	0.8772	0.2787	0.0153	1.0745	0.0057	1.2212	1.0000	-0.6410	1.0000	-0.1260	0.4812	-0.4902
FRMPD3	0.8226	-3.3425	1.0000	0.0000	1.0000	-0.1765	0.4623	3.6988	1.0000	-3.2304	1.0000	0.0000	1.0000	0.5998
FRMPD4	0.0549	0.8305	1.0000	-0.0925	0.0180	-1.2262	0.0009	-1.3042	1.0000	0.5247	0.6450	-0.3834	0.8237	0.4545
FRRS1L	0.2564	-0.6442	0.2185	-0.6605	0.9361	-0.1968	1.0000	-0.0262	1.0000	-0.3868	1.0000	-0.3882	1.0000	-0.2069
FRS2	0.0006	0.8528	0.0000	0.9315	0.0000	1.5553	0.0000	1.3054	1.0000	0.0520	0.8875	0.1446	0.5010	-0.1910
FRS3	0.4757	-0.1940	0.0543	-0.2329	1.0000	-0.0147	0.1398	-0.1790	1.0000	0.0375	1.0000	0.0106	0.7256	-0.1215
FRY	0.9545	0.1397	0.8913	-0.1261	0.0000	-1.8553	0.0011	-0.9265	1.0000	0.1741	1.0000	-0.0774	0.0012	1.1093
FRZB	0.3239	-0.5064	0.0002	-1.3556	0.0594	0.6767	0.0018	0.8363	1.0000	0.0036	0.2314	-0.8343	1.0000	0.1671
FSBP	0.0970	-0.4294	0.0000	-0.8584	0.0821	0.4024	1.0000	-0.0151	1.0000	0.2943	1.0000	-0.1225	0.8239	-0.1180
FSCN2	0.0684	-5.3897	0.1392	-4.4820	0.5729	-1.3100	1.0000	0.2595	1.0000	-0.9345	1.0000	0.0000	1.0000	0.6349
FSD1	0.0000	-1.1745	0.0000	-0.9846	0.8407	-0.1278	1.0000	0.0389	1.0000	-0.1969	1.0000	0.0057	1.0000	-0.0264
FSD1L	0.7596	-0.1793	0.0718	-0.3820	0.7628	-0.1628	0.2554	-0.2461	1.0000	0.0778	1.0000	-0.1111	1.0000	0.0016
FSD2	0.0000	-1.3221	0.0000	-1.7889	0.0113	-0.6774	0.0007	-0.5711	1.0000	-0.0052	0.4632	-0.4580	1.0000	0.1063
FSHB	1.0000	0.0000	1.0000	0.0000	1.0000	0.0000	1.0000	0.0000	1.0000	0.0000	1.0000	0.0000	1.0000	0.0000
FST	0.0018	1.5532	0.0001	1.3010	0.0000	3.0097	0.0000	2.1818	1.0000	0.4257	1.0000	0.1839	0.2590	-0.3960
FSTL1	0.0022	0.4600	0.0000	0.6792	0.0001	0.5811	0.0000	0.5473	1.0000	0.0320	0.0018	0.2637	1.0000	0.0033
FSTL3	1.0000	-0.1071	1.0000	-0.0712	0.0124	0.8682	0.0000	1.8219	1.0000	0.3347	0.4026	0.3846	0.0000	1.2968
FSTL5	1.0000	0.2029	0.7641	-1.4013	0.8011	-3.2629	0.7524	-1.2240	1.0000	1.1146	1.0000	-0.4752	1.0000	3.2066
FTCD	0.8509	0.6197	0.6876	0.6357	0.5823	0.8787	0.6831	-0.8102	1.0000	0.2216	1.0000	0.2504	0.2750	-1.4611
FTH1	1.0000	0.0392	0.1568	-0.1427	0.0000	-0.9705	0.0000	-0.9711	1.0000	-0.1444	0.0001	-0.3136	0.3862	-0.1387
FTO	0.7251	1.4394	0.0255	4.9567	0.0526	2.9863	0.4370	3.7040	1.0000	-2.3757	1.0000	1.0169	0.3391	-1.7122
FTSJ2	0.7532	0.2994	0.7874	0.1962	0.5990	-0.3932	0.0815	-0.6883	1.0000	0.1896	1.0000	0.0985	1.0000	-0.1017
FTSJ3	0.3350	-0.1960	0.7674	0.0638	0.0184	-0.4045	0.9018	-0.0436	1.0000	-0.2096	1.0000	0.0625	0.4172	0.1567
FUBP1	0.0031	0.5201	0.0000	0.5563	0.0052	0.4600	0.0001	0.3690	1.0000	0.0391	0.9592	0.0884	1.0000	-0.0458
FUBP3	0.0002	0.5921	0.0000	0.4380	0.5255	0.1596	0.9689	-0.0395	1.0000	0.0818	1.0000	-0.0595	0.6929	-0.1120
FUCA1	1.0000	0.0300	0.9454	0.0445	0.0003	-0.7065	0.0346	-0.2439	1.0000	0.0910	0.7582	0.1172	0.0000	0.5591
FUCA2	0.2057	0.4426	1.0000	0.0468	0.0000	1.0167	0.0000	0.6982	1.0000	0.2504	1.0000	-0.1327	1.0000	-0.0638
FUK	0.3582	-0.2677	0.3450	0.1909	0.1977	-0.3314	0.0127	0.4009	1.0000	-0.4881	1.0000	-0.0179	0.4328	0.2498
FUNDC1	0.2453	0.2375	0.0165	0.2139	0.0004	-0.6081	0.0000	-0.5319	1.0000	-0.0119	1.0000	-0.0231	0.9809	0.0698
FUNDC2	0.0010	-0.4743	0.0000	-0.4889	0.1130	-0.2613	0.0127	-0.1825	1.0000	-0.0076	1.0000	-0.0097	0.7558	0.0762
FUT10	0.7050	0.3028	1.0000	-0.0957	0.6846	0.2942	0.0150	0.6973	1.0000	-0.0861	0.4709	-0.4731	0.6008	0.3201
FUT11	0.0868	0.2957	0.0354	0.1949	0.0144	0.4071	0.0000	0.3960	1.0000	0.0111	0.9183	-0.0775	1.0000	0.0056
FUT4	0.0801	0.6811	0.4200	0.2508	0.4396	0.3949	1.0000	0.0595	1.0000	0.1756	0.7800	-0.2391	1.0000	-0.1486
FUT7	0.6923	-2.0829	1.0000	-0.7281	1.0000	0.1138	0.7748	-0.9371	1.0000	0.4310	1.0000	1.8209	1.0000	-0.6160
FUT8	0.0000	1.2731	0.0000	1.5287	0.0872	0.5685	0.0407	0.5067	1.0000	-0.0855	0.8607	0.1812	1.0000	-0.1408
FUT9	1.0000	0.0000	1.0000	0.0000	1.0000	2.2428	1.0000	2.3257	1.0000	0.0000	1.0000	0.0000	1.0000	0.0659
FXN	0.1929	0.2524	0.0005	0.3070	0.0227	0.3866	0.2078	0.1335	1.0000	0.1194	0.2872	0.1865	0.5237	-0.1288
FXR1	0.5514	-0.1359	0.0166	-0.2047	0.1356	0.2637	0.4102	0.0830	1.0000	0.0827	1.0000	0.0266	0.7363	-0.0926
FXYD2	0.0446	1.6601	0.5796	0.9858	1.0000	-0.6652	1.0000	-0.5247	1.0000	-1.2828	0.0195	-1.9542	0.9863	-1.1468
FXYD6	0.5079	0.1437	0.8313	0.0374	0.0000	-0.9372	0.0000	-0.5608	1.0000	-0.1586	0.0026	-0.2526	0.0694	0.2235
FYB	1.0000	2.1903	1.0000	-2.3959	1.0000	0.0000	1.0000	0.0062	1.0000	2.2673	1.0000	-2.2992	1.0000	2.3543
FYCO1	0.0010	0.5673	0.0000	0.6575	0.0000	1.2558	0.0000	1.1747	1.0000	-0.0013	0.8607	0.1022	0.8927	-0.0767
FYTTD1	0.6152	0.1310	0.4663	0.0818	0.4415	-0.1693	0.0259	-0.1937	1.0000	0.0653	1.0000	0.0285	1.0000	0.0465
FZD1	1.0000	0.0497	0.5151	0.1359	0.0000	1.4655	0.0000	1.5235	1.0000	-0.0221	1.0000	0.0772	1.0000	0.0425
FZD10	0.2122	2.3996	0.9754	0.7517	0.0787	2.7123	1.0000	0.0059	1.0000	0.7357	1.0000	-0.8977	0.3585	-1.9655
FZD3	0.9719	0.1637	1.0000	0.1023	0.0033	1.0035	0.0005	0.8622	1.0000	0.0921	1.0000	0.0439	1.0000	-0.0418
FZD4	1.0000	0.0370	0.4298	0.1220	0.0000	0.6944	0.0000	0.9966	1.0000	-0.0571	1.0000	0.0410	0.0439	0.2511
FZD5	0.9455	0.4172	0.2622	0.8704	0.0516	-2.5097	0.3866	-1.0919	1.0000	-0.1314	1.0000	0.3344	0.9835	1.2974
FZD6	0.7844	0.0992	1.0000	0.0123	0.0013	0.5366	0.0059	0.2251	1.0000	0.0809	1.0000	0.0066	0.1072	-0.2253
FZD7	1.0000	-0.0943	0.2735	0.2569	0.0022	0.7064	0.0001	0.6687	1.0000	-0.3706	1.0000	-0.0063	0.0492	-0.3999
FZD8	0.0150	1.0350	0.0101	1.0936	1.0000	0.0400	0.5792	0.4337	1.0000	-0.6886	0.2538	-0.6204	1.0000	-0.2882
FZD9	0.0000	-0.9471	0.0005	-0.3785	0.4242	-0.2390	0.0000	0.6406	1.0000	-0.5286	1.0000	0.0522	0.0006	0.3567
FZR1	0.1278	0.4609	0.0000	0.7499	0.0016	0.7111	0.0000	0.7706	1.0000	-0.1345	0.8690	0.1676	1.0000	-0.0678
G0S2	0.0468	0.3339	0.0000	0.7680	0.1529	-0.2989	0.0000	-0.4719	1.0000	0.1017	0.0000	0.5478	1.0000	-0.0657
G2E3	0.0000	0.9990	0.0000	1.1626	0.0000	1.2635	0.0000	0.7498	1.0000	0.0903	0.1086	0.2671	0.0009	-0.4169
G3BP1	0.0110	-0.4481	0.0000	-0.3494	0.3860	0.1945	0.0080	0.1952	1.0000	-0.0957	1.0000	0.0154	0.6616	-0.0892
G6PC	0.4903	0.5177	0.9078	-0.2259	0.0984	-0.9695	0.2417	-0.6918	1.0000	-0.3456	0.1383	-1.0763	1.0000	-0.0625
G6PC2	0.3638	1.4092	0.0236	2.1500	0.8736	0.5762	0.4105	1.3517	1.0000	-0.5152	1.0000	0.2451	1.0000	0.2657
G6PC3	0.1855	-0.3835	0.9119	0.0665	0.1130	0.3970	0.0000	0.7858	1.0000	-0.3084	0.9681	0.1526	1.0000	0.0866
GAA	0.7675	-0.1307	0.7735	-0.0775	0.0770	-0.4615	0.9264	-0.0554	1.0000	-0.0428	1.0000	0.0215	0.1140	0.3686
GAB1	0.0438	-0.4107	0.0000	-0.4361	0.7266	-0.1283	0.1377	-0.1666	1.0000	0.1876	0.5381	0.1755	0.4724	0.1547
GAB2	0.0000	1.7029	0.0000	2.1600	0.0000	1.2386	0.0000	1.1650	1.0000	0.0319	0.0009	0.5015	1.0000	-0.0344
GAB3	0.3428	-0.5859	1.0000	-0.0884	1.0000	0.1193	0.1689	0.4728	1.0000	0.1922	0.2884	0.7008	0.2531	0.5474
GABARAPL1	0.1503	0.2542	0.0110	0.2073	0.3501	-0.1895	0.0324	-0.1728	1.0000	-0.0125	1.0000	-0.0470	1.0000	0.0094
GABARAPL2	1.0000	-0.0401	0.0010	-0.2713	0.0006	-0.6147	0.0000	-0.5756	1.0000	0.0418	0.3109	-0.1773	0.7868	0.0865
GABPA	0.0183	0.3859	0.0000	0.3580	1.0000	-0.0342	0.7017	-0.0568	1.0000	-0.0163	1.0000	-0.0317	1.0000	-0.0337
GABPB1	0.0385	0.4945	0.1963	0.2242	0.0023	0.7151	0.0552	0.2926	1.0000	0.2406	1.0000	-0.0164	0.6511	-0.1767
GABPB2	0.2526	-0.2920	0.8991	-0.0674	0.5264	-0.2008	0.7913	-0.0879	1.0000	-0.1103	0.8932	0.1273	1.0000	0.0103

GABRA1	0.0097	1.6854	0.0000	2.1453	0.0495	1.4140	0.0041	1.1758	1.0000	1.0507	0.0000	1.5247	0.1568	0.8217
GABRA2	0.4017	1.7176	1.0000	0.6912	0.7756	1.0325	0.2442	2.3283	1.0000	-0.9587	0.5934	-2.0021	1.0000	0.3300
GABRA3	0.5927	-1.5556	1.0000	-0.5554	0.7692	-1.4072	0.1617	1.5342	1.0000	-0.4263	1.0000	0.5888	0.1169	2.5208
GABRA4	1.0000	-0.3458	0.6520	0.7876	0.5849	0.7138	0.0728	1.7527	1.0000	-1.4028	1.0000	-0.2566	1.0000	-0.3545
GABRA5	1.0000	0.0000	1.0000	0.0000	1.0000	0.0000	1.0000	0.0000	1.0000	0.0000	1.0000	0.0000	1.0000	0.0000
GABRA6	1.0000	-2.4776	1.0000	0.0000	1.0000	-0.1624	1.0000	0.0000	1.0000	-2.3757	1.0000	0.0000	1.0000	-2.2888
GABRB1	0.5074	-0.2555	0.0000	-0.9759	0.0026	-0.7884	0.0001	-0.6712	1.0000	0.1317	0.0381	-0.5761	0.6093	0.2517
GABRB2	1.0000	-2.4776	1.0000	-0.1429	0.9361	1.2045	1.0000	0.8545	1.0000	-0.1078	1.0000	2.3480	1.0000	-0.4568
GABRB3	1.0000	0.0000	1.0000	0.0000	1.0000	0.0000	1.0000	2.3257	1.0000	0.0000	1.0000	0.0000	1.0000	2.3554
GABRD	0.0741	0.9005	0.0629	0.7660	0.0988	-1.1311	0.0629	-1.0775	1.0000	0.0161	1.0000	-0.1031	1.0000	0.0790
GABRE	1.0000	0.0000	1.0000	0.0000	1.0000	0.0000	1.0000	0.0000	1.0000	0.0000	1.0000	0.0000	1.0000	0.0000
GABRG1	0.3247	2.2123	1.0000	0.0000	1.0000	-0.1597	1.0000	0.0000	1.0000	-2.3771	0.2181	-4.8268	1.0000	-2.2908
GABRG2	0.5086	3.5431	1.0000	0.0000	1.0000	2.2472	1.0000	2.3257	1.0000	0.0000	0.8607	-3.6719	1.0000	0.0649
GABRG3	1.0000	-2.4788	1.0000	0.0000	1.0000	-0.1597	1.0000	0.0000	1.0000	-2.3771	1.0000	0.0000	1.0000	-2.2908
GABRP	0.0029	1.4533	0.0598	0.9413	0.6797	0.4749	1.0000	0.0070	1.0000	-0.0588	0.4754	-0.5616	0.7869	-0.5221
GABRR1	0.5046	-0.5361	0.4290	-0.4993	1.0000	0.1081	0.1339	-0.8908	1.0000	-0.3089	1.0000	-0.2598	0.0427	-1.3031
GABRR2	0.0807	-0.8140	1.0000	0.0314	0.0071	-1.1717	0.0295	-0.7950	1.0000	-0.0403	0.1311	0.8199	0.9274	0.3422
GABRR3	0.0157	3.1846	1.0000	-0.0010	1.0000	-0.1597	0.4274	-1.6986	1.0000	2.4430	0.9098	-0.7274	1.0000	0.9164
GAD2	1.0000	0.0000	1.0000	0.0000	1.0000	0.0000	1.0000	0.0000	1.0000	0.0000	1.0000	0.0000	1.0000	0.0000
GADD45A	0.0045	-0.4133	0.0000	-0.4050	0.0000	-1.0676	0.0000	-1.0735	1.0000	-0.0089	1.0000	0.0118	1.0000	-0.0093
GADD45G	0.0000	-1.1149	0.0000	-1.4575	0.0085	-0.4498	0.0000	-0.9044	1.0000	0.0850	0.3560	-0.2449	0.0062	-0.3647
GADD45GIP1	0.0001	-0.8600	0.0007	-0.4807	0.0055	-0.6746	0.0095	-0.3501	1.0000	-0.4956	1.0000	-0.1033	0.8175	-0.1636
GADL1	0.0329	2.6792	1.0000	0.3842	0.3910	1.5011	1.0000	0.3121	1.0000	0.7940	0.2781	-1.4861	1.0000	-0.3877
GAL	1.0000	-0.0384	0.4952	0.9518	1.0000	0.2472	0.8222	0.7777	1.0000	-1.0840	1.0000	-0.0872	1.0000	-0.5533
GAL3ST1	1.0000	2.1848	1.0000	0.0000	1.0000	2.2472	1.0000	0.0000	1.0000	0.0000	1.0000	-2.2957	1.0000	-2.2908
GAL3ST2	0.4950	1.7576	1.0000	-2.3959	1.0000	0.6780	1.0000	-2.3177	1.0000	-0.1089	0.4711	-4.3669	1.0000	-3.1344
GAL3ST4	0.0002	-1.1758	0.0000	-0.7874	0.0154	0.6894	0.0000	1.3370	1.0000	-0.0754	0.4548	0.3253	0.0001	0.5804
GALC	1.0000	0.1005	0.3855	0.2972	0.0000	1.9103	0.0000	1.5269	1.0000	0.0462	0.8946	0.2540	0.1469	-0.3320
GALE	0.0429	-0.4370	0.5179	-0.0975	0.2477	-0.2804	0.3767	0.1163	1.0000	-0.2397	0.8840	0.1114	0.4805	0.1628
GALK1	0.8549	-0.1066	0.5659	-0.1146	0.0000	-1.8329	0.0000	-1.7846	1.0000	-0.0886	1.0000	-0.0842	1.0000	-0.0356
GALK2	0.0619	0.3698	0.0078	0.2712	0.1666	-0.3137	0.0000	-0.5972	1.0000	0.0143	1.0000	-0.0713	0.1524	-0.2645
GALM	0.0447	-0.4103	0.0000	-0.6060	0.1472	-0.3099	0.0003	-0.3505	1.0000	0.2512	1.0000	0.0686	0.1970	0.2160
GALNS	0.0058	0.7364	0.0047	0.5073	0.0000	1.3659	0.0000	0.9360	1.0000	0.2771	1.0000	0.0608	0.7704	-0.1471
GALNT1	0.0284	0.4008	0.0000	0.4950	0.0000	1.0895	0.0000	0.8455	1.0000	0.0635	0.3321	0.1703	0.2777	-0.1754
GALNT10	0.0000	1.8644	0.0000	2.2277	0.0000	3.0093	0.0000	2.7763	1.0000	-0.3217	1.0000	0.0558	0.0099	-0.5466
GALNT11	1.0000	0.0459	1.0000	-0.0224	1.0000	-0.0204	1.0000	0.0240	1.0000	-0.1683	0.4684	-0.2253	0.7880	-0.1193
GALNT14	0.8566	-0.0905	0.0000	-0.4798	0.4126	-0.1913	0.0000	-0.5266	1.0000	-0.0628	0.0008	-0.4387	0.0028	-0.3919
GALNT15	0.7162	1.1646	1.0000	0.1753	1.0000	-0.1865	1.0000	-0.4588	1.0000	1.0319	1.0000	0.0594	1.0000	0.7678
GALNT16	0.0036	0.7744	0.0000	0.6957	1.0000	0.0915	1.0000	0.0627	1.0000	0.0303	1.0000	-0.0362	1.0000	0.0059
GALNT18	0.1953	-0.5865	0.1070	-0.5588	0.5678	0.3229	0.8090	-0.1717	1.0000	0.0682	1.0000	0.1057	0.4282	-0.4218
GALNT2	0.0047	0.4903	0.0000	0.5098	0.0000	1.0723	0.0000	0.9548	1.0000	0.1560	0.2648	0.1876	1.0000	0.0436
GALNT3	0.9207	-0.3172	0.6516	-0.5112	1.0000	0.1333	0.0052	-2.5836	1.0000	-0.5598	0.5657	-0.7411	0.0000	-3.2684
GALNT4	0.1071	0.3382	0.0000	0.4774	1.0000	-0.0096	0.7097	0.0734	1.0000	0.0522	0.2689	0.2039	0.5814	0.1397
GALNT5	0.0084	-1.8169	0.0010	-1.6408	1.0000	0.2072	0.1806	0.5520	1.0000	0.3436	1.0000	0.5351	0.2797	0.6985
GALNT6	1.0000	0.0000	1.0000	-2.3959	1.0000	0.0000	1.0000	-2.3177	1.0000	2.2673	1.0000	0.0000	1.0000	0.0000
GALNT7	0.0003	1.0306	0.0000	1.1101	0.0010	0.8880	0.0073	0.5670	1.0000	-0.0408	1.0000	0.0507	0.2396	-0.3571
GALNT9	0.0006	-1.2816	0.0000	-2.3213	0.6297	-0.2716	0.0375	0.5011	1.0000	-0.2761	0.0187	-1.3025	0.1317	0.5031
GALR1	1.0000	-2.4788	1.0000	-2.3959	1.0000	-2.4061	0.1532	2.5440	1.0000	-0.1089	1.0000	0.0000	0.1360	4.8986
GALR2	0.7565	0.6228	0.7064	-0.4659	1.0000	0.3718	0.1179	-1.1118	1.0000	1.3048	1.0000	0.2285	1.0000	-0.1772
GALR3	1.0000	0.5465	1.0000	0.0000	1.0000	-2.4055	0.7701	3.1732	1.0000	-2.3757	1.0000	-3.1469	1.0000	3.2066
GALT	1.0000	0.0767	0.4176	0.1917	0.0025	-0.7797	0.0359	-0.4153	1.0000	-0.1831	1.0000	-0.0551	0.8216	0.1895
GAMT	0.4346	0.2984	0.0022	0.4862	0.0000	-1.3931	0.0000	-1.3373	1.0000	-0.2311	1.0000	-0.0311	1.0000	-0.1681
GAN	0.3744	0.4638	0.4289	0.3204	1.0000	0.0607	0.8214	0.1874	1.0000	0.1584	1.0000	0.0280	0.8560	0.2878
GANC	0.0104	0.6220	0.0293	0.3497	0.0855	0.4423	0.0243	0.3636	1.0000	0.0992	0.7510	-0.1627	1.0000	0.0243
GAP43	1.0000	-2.4788	1.0000	0.0000	1.0000	-0.1630	1.0000	2.3241	1.0000	-2.3771	1.0000	0.0000	1.0000	0.0652
GAPDH	0.4525	-0.1546	0.0157	-0.1660	0.0379	-0.3496	0.0000	-0.2652	1.0000	-0.1156	0.4069	-0.1144	1.0000	-0.0260
GAPVD1	1.0000	0.0112	0.3523	-0.0975	1.0000	-0.0060	1.0000	-0.0111	1.0000	0.1735	0.9598	0.0772	0.2085	0.1728
GAR1	0.0389	0.3856	0.0023	0.3169	0.0000	-1.0322	0.0000	-1.0598	1.0000	-0.0775	0.5714	-0.1342	0.9447	-0.1011
GAREM	0.0544	0.8447	0.0000	1.3239	0.0000	1.9959	0.0000	2.0994	1.0000	0.3315	0.0067	0.8234	0.0994	0.4397
GAREML	1.0000	0.0000	1.0000	0.0000	1.0000	0.0000	1.0000	2.3241	1.0000	0.0000	1.0000	0.0000	1.0000	2.3543
GARNL3	0.0001	0.8409	0.0016	0.4538	0.0039	0.6711	0.0006	0.4678	1.0000	0.2116	0.6968	-0.1643	1.0000	0.0111
GARS	0.2359	-0.2142	0.8363	-0.0373	1.0000	0.0427	0.0380	-0.1510	1.0000	-0.1352	1.0000	0.0543	0.0001	-0.3235
GART	0.0303	0.3732	0.0286	0.2345	0.0000	-1.0027	0.0000	-0.8972	1.0000	0.0104	0.8161	-0.1158	0.7287	0.1209
GAS1	0.0000	0.9529	0.0000	1.0791	0.0906	0.3801	0.0000	0.7364	1.0000	-0.1432	1.0000	-0.0039	0.3229	0.2194
GAS2	0.1793	0.3799	0.1742	0.2328	0.2752	-0.3112	0.0130	-0.4017	1.0000	0.1371	1.0000	0.0035	1.0000	0.0515
GAS2L1	0.7744	-0.2082	0.0067	0.6554	0.4459	0.2985	0.0000	0.9801	1.0000	-0.9286	1.0000	-0.0550	0.6247	-0.2420
GAS2L2	1.0000	-0.2881	1.0000	0.0000	1.0000	0.6783	1.0000	2.3257	1.0000	-2.3757	1.0000	-2.2992	1.0000	-0.7792
GAS2L3	0.0001	1.5368	0.0000	1.5292	0.0000	2.5589	0.0000	1.2418	1.0000	0.4765	0.3181	0.4816	0.0005	-0.8345
GAS6	1.0000	0.2481	0.3295	0.6424	0.0001	1.9402	0.0009	1.4401	1.0000	0.1231	0.7933	0.5326	0.6795	-0.3682
GAS8	0.6531	-0.1960	0.0950	-0.3096	1.0000	-0.0575	0.3871	0.1772	1.0000	-0.2292	0.3398	-0.3303	1.0000	0.0111
GATA2	0.0011	0.9090	0.0029	0.5628	0.0000	1.1734	0.1210	0.3328	1.0000	0.1326	0.7976	-0.2012	0.0012	-0.7003
GATA3	1.0000	0.0000	1.0000	0.0000	1.0000	0.0000	1.0000	2.3257	1.0000	0.0000	1.0000	0.0000	1.0000	2.3554
GATA5	1.0000	0.0000	1.0000	0.0000	1.0000	0.0000	1.0000	0.0000	1.0000	0.0000	1.0000	0.0000	1.0000	0.0000
GATA6	1.0000	0.1176	1.0000	-0.1698	1.0000	-0.0587	0.9162	-1.3768	1.0000	-1.7763	0.1904	-2.0738	0.0732	-3.0989
GATAD1	0.6655	0.1649	0.8167	0.0737	0.0190	-0.5513	0.0286	-0.3281	1.0000	0.1144	1.0000	0.0358	0.1854	0.3442
GATAD2A	0.0035	0.5729	0.0000	0.6078	0.0205	0.4745	0.0042	0.3430	1.0000	-0.0252	1.0000	0.0210	0.6062	-0.1529
GATAD2B	0.7198	-0.2297	0.2091	-0.3131	0.0001	1.0059	0.0001	0.6766	1.0000	-0.0336	1.0000	-0.1078	0.0797	-0.3597
GATB	1.0000	0.0616	1.0000	0.0363	1.0000	-0.0006	0.8251	0.0750	1.0000	-0.0598	1.0000	-0.0714	1.0000	0.0231
GATM	0.4664	-1.2103	0.1344	-1.2501	0.2797	1.0172	1.0000	0.0994	1.0000	0.3042	1.0000	0.2736	0.8027	-0.6097
GATSL2	0.0000	-0.6726	0.0000	-0.7996	0.0165	-0.4148	0.0000	-0.5050	1.0000	-0.0642	0.4213	-0.1788	0.4853	-0.1500
GATSL3	0.0048	-0.7335	0.0900	-0.3343	0.9577	-0.0908	0.0010	0.5267	1.0000	-0.3150	1.0000	0.0966	0.1947	0.3085
GBA2	0.1968	-0.2403	0.0716	-0.1709	0.0000	-0.8492	0.0000	-0.5240	1.0000	-0.1743	0.8607	-0.0927	0.5046	0.1570
GBAS	0.0305	-0.3735	0.0000	-0.5121	0.0477	-0.3486	0.0000	-0.4246	1.0000	0.0075	0.6050	-0.1185	0.9835	-0.0626
GBF1	0.7381	-0.1224	0.3495	0.0848	0.6112	0.1505	0.0000	0.3923	1.0000	0.0434	0.0038	0.2631	0.0013	0.2901
GBGT1	0.9867	0.1012	0.0463	0.3612	0.0832	0.4576	0.0024	0.5383	1.0000	-0.0683	0.7293	0.2047	1.0000	0.0180
GBX1	1.0000	-2.4788	1.0000	0.0000	1.0000	-2.4061	1.0000	2.3257	1.0000	-2.3771	1.0000	0.0000	1.0000	2.3554
GC	1.0000	-2.4788	1.0000	-2.3959	1.0000	-2.4061	1.0000	-2.3178	1.0000	-0.1089	1.0000	0.0000	1.0000	0.0000
GCAT	0.4046	0.2472	0.0004	0.5427	0.6539	-0.1772	0.9283	-0.0817	1.0000	-0.6768	0.0778	-0.3701	0.0046	-0.5750
GCC1	0.0857	-0.3688	0.7439	-0.0833	0.1347	-0.3250	0.1976	-0.1847	1.0000	-0.1031	0.5253	0.1953	1.0000	0.0434
GCC2	0.0023	0.5674	0.0000	0.5993	1.0000	0.0316	1.0000	-0.0253	1.0000	0.0092	1.0000	0.0532	1.0000	-0.0429
GCFC2	1.0000	-0.0044	0.0008	-0.3018	0.0001	-0.6094	0.0000	-0.6942	1.0000	0.1477	0.6110	-0.1371	0.9579	0.0684
GCG	0.0000	-1.3887	0.0000	-1.7325	0.0000	-1.4710	0.0000	-1.6688	1.0000	0.2685	1.0000	-0.0620	0.9919	0.0765
GCGR	0.0323	1.8558	0.1555	2.0154	1.0000	0.0643	0.9801	0.9225	1.0000	-1.3418	0.3049	-1.1829	1.0000	-0.4822

GCH1	0.1768	-0.3796	0.1853	-0.2479	0.0706	-0.5320	0.0000	-0.9329	1.0000	-0.0008	0.9618	0.1428	0.3229	-0.3986
GCHFR	1.0000	0.2150	0.4720	-0.7120	0.8150	0.7715	1.0000	0.1736	1.0000	1.2525	1.0000	0.3403	0.8937	0.6553
GCK	1.0000	-0.0948	0.3248	-0.5098	0.0219	-1.2211	0.0312	-0.9850	1.0000	-0.2844	0.3581	-0.6887	1.0000	-0.0437
GCLC	0.1441	0.3977	0.0198	0.4254	0.0000	1.0666	0.0000	0.7120	1.0000	0.1987	0.4461	0.2395	0.6314	-0.1493
GCLM	0.0001	0.8718	0.0000	0.7880	0.0000	0.9110	0.0000	0.6196	1.0000	0.2182	0.7681	0.1465	1.0000	-0.0685
GCM1	0.8249	3.0141	0.7674	-3.2570	1.0000	0.0000	0.7710	-3.1692	1.0000	3.1191	1.0000	-3.1382	1.0000	0.0000
GCM2	1.0000	2.1902	1.0000	2.2534	0.8033	3.0892	1.0000	2.3241	1.0000	0.0000	1.0000	0.0489	1.0000	-0.7815
GCN1L1	0.0361	-0.3514	0.0126	-0.2071	0.1178	0.2762	0.0750	0.1483	1.0000	-0.0403	0.6008	0.1167	0.2352	-0.1628
GCNT1	0.9545	1.0672	1.0000	-0.1449	1.0000	0.6740	1.0000	0.0076	1.0000	-0.1089	1.0000	-1.3266	1.0000	-0.7769
GCNT3	0.8249	-3.3411	0.7674	-3.2498	1.0000	-1.0194	1.0000	0.0072	1.0000	-0.1218	1.0000	0.0000	1.0000	0.9164
GCNT4	1.0000	-0.3466	1.0000	-0.1726	1.0000	0.4572	0.2773	1.2295	1.0000	-0.1273	1.0000	0.0586	1.0000	0.6497
GCNT7	0.0000	1.4411	0.0000	0.7925	0.8033	0.1499	1.0000	0.0456	1.0000	0.1846	0.0065	-0.4491	1.0000	0.0872
GCSH	1.0000	0.0723	0.0560	0.2827	0.0003	-0.8832	0.0000	-1.0366	1.0000	-0.2196	1.0000	0.0020	0.1918	-0.3679
GDAP1	0.3234	-1.3162	0.4195	-1.5160	1.0000	0.0553	1.0000	0.1702	1.0000	-0.4408	1.0000	-0.6335	1.0000	-0.3246
GDAP2	0.2527	-0.2569	0.0237	-0.2636	0.7224	0.1251	0.2904	-0.1326	1.0000	0.0499	1.0000	0.0561	0.2298	-0.2028
GDE1	0.0000	0.8777	0.0000	0.9353	0.4843	0.2014	0.1667	0.1807	1.0000	0.0530	0.7085	0.1229	1.0000	0.0372
GDF1	1.0000	0.0200	1.0000	0.0193	0.0551	0.4808	0.0000	0.8103	1.0000	-0.5858	0.0071	-0.5725	0.3407	-0.2483
GDF10	1.0000	0.0000	1.0000	-0.1429	1.0000	0.0000	1.0000	-2.3177	1.0000	2.2673	1.0000	2.3479	1.0000	0.0000
GDF11	1.0000	-2.4776	1.0000	0.0000	0.0787	2.7127	0.0059	5.3610	1.0000	-2.3757	1.0000	0.0000	1.0000	0.2263
GDF15	1.0000	0.0555	0.0012	1.1394	0.1404	0.7249	0.0000	1.5594	1.0000	-0.2487	0.0725	0.8506	0.1241	0.5969
GDF2	1.0000	0.0000	1.0000	-2.3959	1.0000	0.0000	1.0000	-2.3177	1.0000	2.2673	1.0000	0.0000	1.0000	0.0000
GDF5	0.5086	-3.8796	1.0000	0.6944	0.9361	-1.5532	1.0000	0.0045	1.0000	-1.4918	1.0000	3.1976	1.0000	0.0652
GDF7	1.0000	0.0000	1.0000	0.0000	1.0000	2.2428	1.0000	0.0000	1.0000	0.0000	1.0000	0.0000	1.0000	-2.2888
GDF9	1.0000	2.1902	0.1392	-4.4820	0.8011	3.0840	1.0000	-0.3002	1.0000	4.3319	1.0000	-2.2992	1.0000	0.9893
GDI2	0.0692	-0.2909	0.0000	-0.3557	1.0000	0.0221	0.8423	-0.0383	1.0000	0.0407	1.0000	-0.0117	1.0000	-0.0142
GDNF	0.1131	-1.0348	0.0882	-0.8857	0.5322	0.4119	0.6303	0.3588	1.0000	0.0416	1.0000	0.1999	1.0000	-0.0085
GDPD1	0.0114	-0.4911	0.0000	-0.4932	0.7352	-0.1239	0.1681	-0.1684	1.0000	0.0176	1.0000	0.0282	1.0000	-0.0226
GDPD2	1.0000	0.0000	1.0000	0.0000	1.0000	2.2427	1.0000	0.0000	1.0000	0.0000	1.0000	0.0000	1.0000	-2.2888
GDPD5	0.1038	-1.0430	0.4140	-0.4087	0.2131	-0.7813	1.0000	-0.0463	1.0000	0.4364	0.0633	1.0816	0.0174	1.1725
GDPGP1	0.2322	0.6544	0.0427	1.0568	0.0908	-1.0987	1.0000	-0.1819	1.0000	-1.1207	0.2884	-0.7080	1.0000	-0.1966
GEM	1.0000	-0.1021	0.3661	-0.3695	0.0000	1.4744	0.2313	0.4102	1.0000	0.5651	0.9957	0.3090	0.2041	-0.4921
GEMIN2	0.8249	-0.1077	0.1931	-0.1856	0.6334	-0.1444	0.1102	-0.2043	1.0000	0.0544	1.0000	-0.0120	1.0000	-0.0011
GEMIN4	0.5294	-0.2010	0.4307	0.1504	1.0000	-0.0243	0.3800	0.1559	1.0000	-0.2142	0.8269	0.1482	1.0000	-0.0298
GEMIN5	0.0000	1.3059	0.0000	1.9557	0.0000	2.2411	0.0000	2.0262	1.0000	-0.2835	0.2328	0.3811	0.0018	-0.4902
GEMIN6	0.0054	-0.5432	0.7767	-0.0866	0.1945	-0.3075	0.1006	-0.2449	1.0000	-0.1241	0.0824	0.3442	1.0000	-0.0559
GEN1	0.0672	0.4491	0.0876	0.2718	1.0000	0.0655	0.6364	-0.1169	1.0000	0.0250	0.8607	-0.1401	0.7638	-0.1527
GET4	1.0000	-0.0374	1.0000	0.0011	0.1290	-0.2723	0.0001	-0.3274	1.0000	0.0340	0.8808	0.0848	1.0000	-0.0159
GFAP	1.0000	-0.1840	0.9605	0.2315	0.3701	0.5125	0.0692	0.8923	1.0000	-0.6873	1.0000	-0.2593	0.8830	-0.3040
GFER	0.2456	-0.7162	1.0000	-0.0267	0.5143	0.4538	0.1055	0.7818	1.0000	-0.2861	0.8793	0.4173	1.0000	0.0464
GFI1	1.0000	0.0000	1.0000	0.0000	1.0000	0.0000	1.0000	0.0000	1.0000	0.0000	1.0000	0.0000	1.0000	0.0000
GFI1B	1.0000	0.0000	1.0000	0.0000	1.0000	0.0000	1.0000	0.0000	1.0000	0.0000	1.0000	0.0000	1.0000	0.0000
GFM1	0.2264	-0.2552	0.0219	-0.2574	1.0000	0.0200	0.2959	-0.1353	1.0000	-0.0429	1.0000	-0.0331	0.2607	-0.1931
GFOD1	1.0000	0.0659	0.7915	0.0746	0.0001	0.7340	0.0000	0.7408	1.0000	0.0801	1.0000	0.1018	0.8939	0.0923
GFOD2	0.8574	0.1300	0.0732	0.3388	0.9596	0.1008	0.0437	0.3658	1.0000	-0.1095	1.0000	0.1109	0.7638	0.1588
GFPT1	0.0423	0.4043	0.0000	0.6353	0.0000	1.0932	0.0000	1.1033	1.0000	-0.2039	1.0000	0.0390	0.1893	-0.1896
GFPT2	1.0000	0.4742	0.0933	2.1526	0.4564	-2.2460	0.7701	-3.1666	1.0000	-1.3455	1.0000	0.3396	1.0000	-2.2908
GFRA2	1.0000	0.0000	0.6224	-1.9244	0.8011	3.0840	1.0000	0.5605	1.0000	4.0300	1.0000	2.3479	0.7978	1.5478
GFRA3	0.2026	1.1979	0.0049	2.0116	0.0110	1.6892	0.3203	0.9891	1.0000	-0.3033	0.8607	0.5207	0.2429	-0.9945
GFRA4	1.0000	-2.4776	1.0000	0.0000	1.0000	-2.4055	1.0000	0.0000	1.0000	-2.3757	1.0000	0.0000	1.0000	0.0000
GFRAL	1.0000	0.0000	1.0000	0.0000	1.0000	0.0000	1.0000	0.0000	1.0000	0.0000	1.0000	0.0000	1.0000	0.0000
GGA1	0.5086	-0.1591	0.0075	-0.2469	0.8151	0.0934	0.4025	0.0978	1.0000	0.0030	1.0000	-0.0723	1.0000	0.0128
GGA2	0.6933	-0.1650	0.8895	-0.0680	0.6280	-0.1755	0.4969	0.1332	1.0000	-0.1202	1.0000	-0.0118	0.5291	0.1935
GGA3	0.9505	-0.0709	0.0002	-0.3625	0.7086	-0.1192	1.0000	-0.0057	1.0000	-0.0184	0.0300	-0.2972	0.8116	0.1008
GGACT	0.9427	0.2170	0.0516	0.7422	0.2915	-0.6221	0.2038	-0.6852	1.0000	-0.2773	1.0000	0.2659	1.0000	-0.3314
GGCT	0.3218	0.3519	0.0001	0.6862	0.8035	-0.1690	0.2186	-0.3003	1.0000	0.0282	0.2117	0.3760	1.0000	-0.0963
GGCX	0.5928	-0.2702	1.0000	0.0590	0.0370	0.6232	0.0000	1.2513	1.0000	-0.3237	1.0000	0.0164	0.2061	0.3096
GGH	0.1464	-0.2668	0.0000	-0.5708	0.0390	0.3407	0.0000	0.3055	1.0000	0.1933	0.8808	-0.0981	0.2028	0.1634
GGNBP2	1.0000	-0.0578	1.0000	-0.0239	0.3749	-0.2073	0.0204	-0.2345	1.0000	0.0688	0.7591	0.1152	1.0000	0.0463
GGPS1	1.0000	0.0953	1.0000	-0.0001	0.0000	1.2588	0.0000	0.8751	1.0000	0.3191	0.8171	0.2352	1.0000	-0.0605
GGT1	0.8990	-0.3575	0.8884	-0.3722	1.0000	-0.3223	0.8641	-0.4019	1.0000	0.2823	1.0000	0.2782	1.0000	0.2029
GGT5	1.0000	-0.0945	0.1018	2.8637	1.0000	-0.8743	0.3949	2.0773	1.0000	-2.1873	1.0000	0.7652	1.0000	0.7673
GGT7	0.0007	0.6844	0.0000	0.5840	0.0000	-1.6874	0.0000	-1.3105	1.0000	0.1973	0.8607	0.1103	0.0496	0.5792
GHDC	0.0811	0.4236	0.0022	0.4789	0.0104	-0.6528	0.7507	-0.1147	1.0000	-0.2738	0.5859	-0.2058	0.4901	0.2719
GHITM	0.3748	0.1831	0.0395	0.1521	0.0222	-0.3655	0.0000	-0.4087	1.0000	-0.0193	1.0000	-0.0377	0.9211	-0.0570
GHRH	1.0000	-0.2881	1.0000	0.6940	1.0000	-0.1624	1.0000	-2.3178	1.0000	-0.1078	1.0000	0.8962	1.0000	-2.2888
GHRHR	1.0000	0.0000	1.0000	0.0000	1.0000	2.2428	1.0000	0.0000	1.0000	0.0000	1.0000	0.0000	1.0000	-2.2888
GHRL	0.7448	0.6246	1.0000	-0.0421	0.4009	-1.8726	0.5115	-0.9580	1.0000	0.3704	1.0000	-0.2815	0.9835	1.2969
GHSR	0.8848	0.9987	1.0000	-0.1782	1.0000	0.5040	0.6898	-0.6716	1.0000	1.9020	1.0000	0.7438	1.0000	0.7385
GID4	0.2243	-0.2701	0.0534	-0.2299	0.0988	-0.3296	0.0285	-0.2425	1.0000	-0.0659	1.0000	-0.0141	1.0000	0.0257
GID8	0.3117	-0.2184	0.0262	-0.2262	0.2356	0.2414	0.1424	0.1518	1.0000	0.0323	1.0000	0.0374	1.0000	-0.0520
GIF	1.0000	-0.0490	0.0337	1.8463	0.0356	-5.1463	1.0000	-0.3791	1.0000	-1.0847	0.8785	0.8134	0.7453	3.7416
GIMAP8	0.2036	1.8496	0.5021	-1.6423	1.0000	0.7335	0.4937	-1.4671	1.0000	1.3515	0.3579	-2.1309	1.0000	-0.8431
GIMD1	0.0000	-1.7435	0.0000	-2.1313	0.2221	-0.3495	0.1392	-0.2538	1.0000	0.1515	1.0000	-0.2237	0.4453	0.2514
GIN1	0.6982	-0.2064	0.0126	-0.4898	0.5616	-0.2354	0.0037	-0.5538	1.0000	0.2703	1.0000	-0.0007	1.0000	-0.0420
GINM1	0.3351	0.2062	0.1352	0.1582	0.8793	0.0826	0.0044	0.2922	1.0000	0.0815	1.0000	0.0454	0.0226	0.2961
GINS1	0.0472	0.5406	0.0001	0.7631	0.0367	-0.6311	0.0009	-0.8429	1.0000	-0.3389	1.0000	-0.1054	0.1568	-0.5451
GINS2	1.0000	-0.0012	0.3882	0.1806	0.1548	-0.3322	0.0060	-0.4761	1.0000	-0.2734	1.0000	-0.0808	0.0263	-0.4117
GINS3	0.0257	-0.3989	0.0000	-0.4246	0.0352	-0.3717	0.0292	-0.2085	1.0000	-0.0338	1.0000	-0.0472	0.5105	0.1352
GINS4	1.0000	0.0067	0.1235	0.1895	1.0000	0.0213	0.9164	0.0527	1.0000	-0.1825	1.0000	0.0121	0.5783	-0.1460
GIP	1.0000	0.0000	1.0000	0.0000	1.0000	0.0000	1.0000	0.0000	1.0000	0.0000	1.0000	0.0000	1.0000	0.0000
GIPC1	0.0207	-0.6993	0.0230	-0.3714	0.7352	-0.1825	0.0179	0.3615	1.0000	-0.3948	1.0000	-0.0521	0.7485	0.1556
GIPC2	1.0000	0.0310	0.5915	0.0966	0.3497	-0.2247	0.0003	-0.4435	1.0000	0.1291	0.3393	0.2063	1.0000	-0.0855
GIPC3	0.9465	0.3384	0.7580	-0.4895	0.0462	-1.6055	0.3340	-1.2628	1.0000	-1.0401	0.0237	-1.8605	1.0000	-0.6929
GIT1	0.0003	-0.7615	0.0000	-0.6507	0.0009	-0.7553	0.1311	-0.2260	1.0000	-0.3016	0.6901	-0.1785	0.4493	0.2330
GIT2	0.0009	0.6981	0.0000	0.7799	0.0000	1.3480	0.0000	1.0365	1.0000	0.1489	0.2357	0.2436	0.4982	-0.1575
GJA1	0.0000	-0.7886	0.0000	-0.4431	0.0003	0.6043	0.0000	0.8917	1.0000	0.1345	0.0000	0.4918	0.0000	0.4259
GJA10	1.0000	0.0000	1.0000	0.0000	1.0000	0.0000	0.7701	3.1732	1.0000	0.0000	1.0000	0.0000	1.0000	3.2066
GJA3	1.0000	0.0000	1.0000	0.0000	1.0000	0.0000	1.0000	0.0000	1.0000	0.0000	1.0000	0.0000	1.0000	0.0000
GJA4	0.5086	-3.8796	1.0000	0.0000	0.4809	-3.7970	1.0000	0.0000	1.0000	-3.7635	1.0000	0.0000	1.0000	0.0000
GJA5	0.0000	-5.0411	0.0000	-4.7906	0.5908	-0.3254	0.0026	-0.6644	1.0000	0.2625	1.0000	0.5262	1.0000	-0.0754
GJA8	1.0000	0.5420	0.4431	-3.7865	1.0000	-2.4055	1.0000	0.0060	1.0000	1.2635	1.0000	-3.1430	0.7455	3.7355
GJA9	0.0000	-2.1072	0.0000	-1.9334	0.0133	-0.7069	0.0010	-0.7022	1.0000	0.4189	0.3972	0.6046	0.3378	0.4285
GJB1	1.0000	0.0000	1.0000	0.0000	1.0000	0.0000	1.0000	2.3241	1.0000	0.0000	1.0000	0.0000	1.0000	2.3543

GJB2	1.0000	-2.4776	1.0000	0.0000	1.0000	-2.4055	1.0000	0.0000	1.0000	-2.3757	1.0000	0.0000	1.0000	0.0000
GJB5	1.0000	0.0000	1.0000	-0.1473	1.0000	0.0000	1.0000	-2.3200	1.0000	2.2737	1.0000	2.3456	1.0000	0.0000
GJC1	0.0053	-0.4898	0.0000	-0.5091	0.0005	-0.6023	0.0000	-0.3623	1.0000	0.1002	0.8122	0.0935	0.0007	0.3453
GJC2	0.0000	0.8882	0.0000	1.2185	0.0000	0.9153	0.0000	0.6198	1.0000	-0.0727	0.2111	0.2686	0.0164	-0.3634
GJD2	0.0000	-5.0427	0.0000	-4.0902	0.5154	-0.3291	1.0000	0.0083	1.0000	0.4260	0.8605	1.3970	0.0134	0.7681
GJD4	0.0000	-3.1572	0.0000	-3.8626	0.0000	1.0364	0.0000	0.6559	1.0000	0.2909	0.6036	-0.4029	0.9058	-0.0852
GK	0.0857	0.5393	0.0000	1.0950	0.0212	-0.7471	0.0025	-0.8658	1.0000	-0.3063	0.6123	0.2621	0.5682	-0.4198
GKAP1	0.0001	0.9353	0.0000	0.6359	1.0000	0.0736	0.1720	-0.2310	1.0000	0.1550	0.8269	-0.1326	0.8306	-0.1427
GKN2	1.0000	2.1902	1.0000	0.6940	1.0000	0.0000	1.0000	-2.3178	1.0000	2.2673	1.0000	0.8962	1.0000	0.0000
GLA	0.3747	-0.2369	0.0000	-0.5319	0.0095	-0.5541	0.0000	-0.7061	1.0000	0.2393	1.0000	-0.0440	0.9368	0.0927
GLB1	0.1061	0.2947	0.0279	0.1979	0.4889	0.1645	0.9581	-0.0338	1.0000	0.1043	1.0000	0.0198	0.7915	-0.0887
GLB1L	0.5818	0.1796	0.7338	0.0698	0.0000	-1.2338	0.0000	-0.7109	1.0000	0.0863	1.0000	-0.0120	0.0001	0.6133
GLCCI1	0.4097	0.2454	0.1464	0.2289	0.0000	0.8158	0.0000	0.8240	1.0000	0.0034	1.0000	-0.0002	1.0000	0.0166
GLCE	0.4866	0.1794	0.0404	0.2344	0.0056	0.4934	0.0000	0.8051	1.0000	0.1087	0.4307	0.1761	0.0001	0.4256
GLDC	1.0000	-1.1585	0.7186	1.0553	0.7662	1.0359	1.0000	-0.8416	1.0000	-0.1187	0.8269	2.1245	0.6865	-1.9990
GLDN	1.0000	-1.2504	0.5029	1.3071	0.3977	1.2106	1.0000	0.0066	1.0000	-1.0395	0.9082	1.5316	0.1717	-2.2460
GLE1	0.7954	0.1308	0.9297	0.0525	0.4841	0.2066	0.9852	0.0460	1.0000	0.1137	1.0000	0.0484	1.0000	-0.0417
GLG1	0.0000	1.2306	0.0000	1.5466	0.0000	1.6780	0.0000	1.5432	1.0000	-0.1075	0.6331	0.2230	0.0548	-0.2371
GLI1	0.8226	-3.3425	0.7666	-3.2534	1.0000	-1.0171	0.4937	1.4808	1.0000	-0.1179	1.0000	0.0000	0.5105	2.3934
GLI2	0.9073	0.1699	0.0156	0.5553	0.0000	1.7360	0.0000	1.9967	1.0000	0.0457	0.1951	0.4450	0.1186	0.3130
GLI3	0.0485	-0.3680	0.0701	-0.2027	0.6134	0.1383	0.0321	0.2164	1.0000	0.0343	0.2671	0.2120	0.6106	0.1172
GLIPR2	0.8427	0.1103	0.7488	-0.0521	1.0000	-0.0587	0.0000	-0.4122	1.0000	-0.1494	0.0019	-0.2991	0.0000	-0.4967
GLIS1	0.0909	-0.3593	0.0000	-0.4295	0.0428	0.3862	0.0028	0.3007	1.0000	0.1890	0.8219	0.1314	0.7059	0.1081
GLMN	0.3676	0.3166	0.0115	0.4820	0.9333	-0.1279	0.9732	0.0822	1.0000	-0.0502	1.0000	0.1264	0.8812	0.1645
GLO1	0.1394	0.2925	0.1577	0.1144	0.9716	0.0666	0.2259	-0.1034	1.0000	-0.0478	0.0233	-0.2134	0.0399	-0.2121
GLOD4	0.0468	-0.3176	0.0000	-0.4000	0.0003	-0.5449	0.0000	-0.3354	1.0000	0.0693	1.0000	-0.0007	0.0064	0.2843
GLOD5	1.0000	0.0000	1.0000	0.0000	1.0000	0.0000	1.0000	0.0000	1.0000	0.0000	1.0000	0.0000	1.0000	0.0000
GLP1R	0.7094	0.1265	0.0362	0.2388	0.0000	-1.1391	0.0000	-1.0453	1.0000	0.0038	0.7248	0.1283	1.0000	0.1037
GLP2R	1.0000	-0.0995	0.4231	0.5370	0.7270	0.3614	0.7048	0.3487	1.0000	-0.0760	0.6953	0.5735	1.0000	-0.0829
GLRA1	1.0000	-2.4788	0.7666	-3.2534	1.0000	-2.4061	1.0000	-0.8419	1.0000	0.7357	1.0000	0.0000	1.0000	2.3543
GLRA2	1.0000	0.0000	1.0000	-2.3986	1.0000	0.0000	1.0000	-2.3200	1.0000	2.2737	1.0000	0.0000	1.0000	0.0000
GLRA4	1.0000	-2.4788	0.4437	3.6202	1.0000	0.6740	1.0000	0.0000	1.0000	-2.3771	0.8607	3.7297	1.0000	-3.1317
GLRB	1.0000	2.1902	1.0000	0.6912	0.2779	3.9963	1.0000	0.8518	1.0000	2.2736	1.0000	0.8962	1.0000	-0.8434
GLRX	0.0293	-0.9961	0.0032	-0.7826	0.0602	-0.8930	0.0000	-1.7274	1.0000	0.6093	0.0950	0.8345	1.0000	-0.2187
GLRX2	0.0084	-0.5075	0.0000	-0.6621	0.0107	-0.4924	0.0000	-0.5264	1.0000	-0.0086	0.5480	-0.1502	1.0000	-0.0365
GLRX3	0.0786	-0.3774	0.0003	-0.3275	0.0821	-0.3701	0.0000	-0.5377	1.0000	-0.0994	1.0000	-0.0372	0.0150	-0.2613
GLRX5	0.8333	-0.1072	0.0412	-0.1892	0.1406	-0.3266	0.0000	-0.4897	1.0000	-0.0308	0.7962	-0.1007	0.1895	-0.1882
GLS	0.0215	0.5523	0.0000	0.5881	0.0000	1.3364	0.0000	1.3048	1.0000	0.1079	0.8156	0.1565	0.8890	0.0824
GLT1D1	1.0000	0.1179	1.0000	-0.0305	0.6746	-0.3015	0.0123	-0.7747	1.0000	0.3488	1.0000	0.2122	1.0000	-0.1186
GLT8D1	0.1260	-0.2782	0.0028	-0.2981	1.0000	0.0060	0.0275	0.2015	1.0000	0.0076	1.0000	-0.0001	0.1010	0.2082
GLT8D2	0.0003	-0.6223	0.0000	-0.5834	0.0000	-1.0882	0.0000	-0.9247	1.0000	0.0697	0.8240	0.1213	0.1142	0.2377
GLTP	0.7187	0.1574	0.2953	0.1519	0.0000	-1.1815	0.0000	-1.3157	1.0000	-0.2639	0.1055	-0.2580	0.0630	-0.3913
GLTSCR1L	0.0027	0.5476	0.1259	0.2510	0.0006	-0.6926	0.0000	-0.7895	1.0000	0.0894	0.4635	-0.1950	1.0000	-0.0004
GLUD1	0.0000	0.6160	0.0000	0.4518	0.6067	-0.1262	0.0000	-0.3662	1.0000	0.0890	0.9247	-0.0627	0.2476	-0.1454
GLUL	0.0001	1.6034	0.0059	1.0585	0.1881	-0.9305	1.0000	-0.1602	1.0000	-0.2864	0.0328	-0.8177	0.7908	0.4909
GLYATL3	0.0391	-1.2243	0.3594	-0.5461	0.0005	-2.2072	0.0664	-1.0283	1.0000	-0.2196	1.0000	0.4708	0.6865	0.9650
GLYCTK	0.0150	-0.9416	0.0020	-0.9669	1.0000	-0.1426	0.4102	0.2897	1.0000	-0.2484	1.0000	-0.2599	1.0000	0.1914
GLYR1	0.7101	-0.1037	0.0668	-0.1458	0.0929	0.2827	0.4160	0.0756	1.0000	0.0038	1.0000	-0.0259	0.1008	-0.1981
GM2A	0.0047	-1.1505	0.0000	-0.9077	0.0000	-1.7155	0.0000	-1.1136	1.0000	0.5242	0.0000	0.7800	0.0000	1.1306
GMCL1	0.2687	-0.2683	0.1509	-0.2152	0.0914	-0.3622	1.0000	0.0027	1.0000	-0.2673	0.5013	-0.2023	0.9277	0.1019
GMDS	1.0000	-0.0519	1.0000	0.0243	0.0077	-0.4459	0.0003	-0.3917	1.0000	-0.1000	1.0000	-0.0113	1.0000	-0.0403
GMEB1	0.5850	-0.2855	1.0000	0.0622	0.7334	0.2101	0.0076	0.5505	1.0000	-0.3674	1.0000	-0.0056	1.0000	-0.0204
GMEB2	1.0000	-0.0060	0.3295	0.1453	0.0000	-0.8256	0.0002	-0.4334	1.0000	-0.2527	1.0000	-0.0893	0.7036	0.1443
GMFB	0.2477	0.2229	0.1195	0.1323	0.1096	0.2839	1.0000	-0.0089	1.0000	0.0183	0.9960	-0.0598	0.0034	-0.2688
GMIP	1.0000	-2.4788	0.7674	-3.2498	1.0000	-2.4061	0.7710	-3.1632	1.0000	0.7304	1.0000	0.0000	1.0000	0.0000
GMNC	1.0000	0.0000	1.0000	0.0000	1.0000	0.0000	1.0000	0.0000	1.0000	0.0000	1.0000	0.0000	1.0000	0.0000
GMNN	0.1118	0.3961	0.0445	0.2927	0.6654	-0.1740	0.0014	-0.4656	1.0000	0.0104	1.0000	-0.0818	0.1561	-0.2769
GMPPA	0.0015	-0.6016	0.0000	-0.5620	0.1189	-0.3349	0.6874	-0.0649	1.0000	-0.0620	1.0000	-0.0109	0.1692	0.2132
GMPPB	0.0030	-0.4892	0.0000	-0.5460	0.0000	-0.9138	0.0000	-0.5893	1.0000	0.0116	1.0000	-0.0329	0.0093	0.3420
GMPR	0.0289	-0.6592	0.0016	-0.5330	0.0096	0.6687	0.3627	0.1770	1.0000	-0.0105	1.0000	0.1271	0.0160	-0.4985
GMPS	0.2704	0.2152	0.0008	0.2940	0.8700	0.0807	0.1235	0.1543	1.0000	0.0106	0.7877	0.1020	0.7915	0.0897
GNA11	0.0184	-0.4184	0.0000	-0.4536	0.0000	-0.7289	0.0000	-0.7310	1.0000	0.0059	1.0000	-0.0170	1.0000	0.0093
GNA12	0.0002	-0.5359	0.0000	-0.5713	0.1993	0.2308	0.0008	0.2356	1.0000	0.1190	0.7800	0.0960	0.4523	0.1288
GNA13	0.0072	0.7618	0.0000	0.8553	0.0000	1.6199	0.0000	1.2799	1.0000	0.0371	0.9673	0.1448	0.4068	-0.2960
GNA14	0.0305	-1.8033	0.7212	-0.4921	1.0000	-0.0416	1.0000	0.2668	1.0000	-0.8622	1.0000	0.4653	0.8234	-0.5490
GNAI1	0.9180	0.0900	1.0000	-0.0195	0.6777	0.1453	0.6921	0.0957	1.0000	0.0330	1.0000	-0.0630	1.0000	-0.0101
GNAI2	0.3025	0.2152	0.0000	0.3775	1.0000	-0.0104	0.0008	0.2818	1.0000	-0.1486	1.0000	0.0257	0.5346	0.1489
GNAO1	1.0000	0.0065	1.0000	-0.0126	0.1362	0.6374	0.0120	0.7988	1.0000	-0.1041	1.0000	-0.1098	1.0000	0.0636
GNAQ	0.0012	1.5512	0.0001	1.5000	0.0000	1.9386	0.0000	1.6242	1.0000	0.0018	1.0000	-0.0358	0.7588	-0.3061
GNAS	1.0000	-0.0180	0.2855	-0.1449	0.0180	-0.4658	0.0000	-0.5314	1.0000	0.0859	1.0000	-0.0290	1.0000	0.0237
GNAT1	1.0000	0.0000	1.0000	2.2534	1.0000	0.0000	1.0000	0.0000	1.0000	0.0000	1.0000	2.3479	1.0000	0.0000
GNAT3	1.0000	0.0000	1.0000	0.0000	1.0000	0.0000	1.0000	0.0000	1.0000	0.0000	1.0000	0.0000	1.0000	0.0000
GNAZ	0.6571	0.8709	0.4787	0.9496	0.5829	0.9036	0.0080	2.1863	1.0000	-0.4301	1.0000	-0.3439	0.6751	0.8550
GNB1	0.4925	0.1562	0.0194	0.1687	0.0009	0.5272	0.0000	0.3614	1.0000	0.0779	0.5963	0.1027	0.7455	-0.0823
GNB1L	0.2036	-0.3257	0.0003	-0.5358	0.6990	-0.1501	0.0000	-0.5731	1.0000	-0.1078	0.2253	-0.3047	0.0005	-0.5235
GNB2L1	0.4922	-0.1486	0.0354	-0.1757	0.0000	-0.7272	0.0000	-0.5796	1.0000	-0.1384	0.2168	-0.1531	1.0000	0.0148
GNB3	0.0373	2.9719	0.0933	2.5619	1.0000	0.6740	1.0000	0.8541	1.0000	-0.1045	1.0000	-0.5110	1.0000	0.0730
GNB4	0.0000	1.3014	0.0000	1.3033	0.0000	2.5491	0.0000	2.0298	1.0000	0.2613	0.3757	0.2773	0.3499	-0.2515
GNB5	0.7054	-0.1337	0.0006	-0.4036	1.0000	0.0550	0.4486	0.1195	1.0000	0.0011	0.1692	-0.2568	1.0000	0.0700
GNE	0.0835	0.3248	0.0058	0.2935	0.0119	0.4422	0.0000	0.4493	1.0000	-0.0048	1.0000	-0.0233	1.0000	0.0085
GNG11	0.0000	-1.8451	0.0000	-1.8957	0.0000	-1.3186	0.0000	-1.4042	1.0000	-0.2181	0.4823	-0.2561	0.1659	-0.2963
GNG12	0.5640	0.1516	0.9254	0.0373	0.1222	0.2922	0.0000	0.4039	1.0000	-0.0639	0.3257	-0.1661	1.0000	0.0534
GNG13	1.0000	-0.3302	0.1009	-2.8810	0.9361	-1.5512	0.4160	-1.3358	1.0000	1.2138	1.0000	-1.3320	1.0000	1.4483
GNG2	0.0000	-1.7042	0.0000	-1.4742	0.0000	1.1264	0.0000	1.1578	1.0000	-0.2909	1.0000	-0.0472	0.5215	-0.2536
GNG4	0.0611	0.9360	0.0660	0.7298	0.2061	-0.9241	0.1314	-0.7926	1.0000	0.0893	1.0000	-0.1040	1.0000	0.2287
GNG5	0.0028	-0.5024	0.0000	-0.4195	0.0024	-0.5242	0.0000	-0.4511	1.0000	-0.0217	0.9576	0.0736	1.0000	0.0575
GNGT2	0.0098	1.8443	0.7161	0.5738	1.0000	-0.0066	0.1917	-1.6497	1.0000	0.3712	0.4295	-0.8862	0.7121	-1.2671
GNL2	0.5582	0.1442	0.0228	0.2172	0.6160	-0.1359	0.3479	-0.1144	1.0000	-0.0266	1.0000	0.0584	1.0000	0.0003
GNL3	0.5086	0.1478	0.1619	0.1253	0.0106	-0.4124	0.0000	-0.3804	1.0000	-0.0325	1.0000	-0.0426	1.0000	0.0045
GNMT	0.0000	-1.0058	0.0000	-0.7776	0.0000	-1.4143	0.0000	-1.1342	1.0000	-0.0691	0.3061	0.1714	0.0440	0.2162
GNPAT	0.0697	-0.3065	0.0000	-0.4005	0.0024	-0.4856	0.0000	-0.5570	1.0000	0.1149	1.0000	0.0334	1.0000	0.0481
GNPDA2	0.0002	0.6788	0.0025	0.3535	0.5487	-0.1788	0.0116	-0.3041	1.0000	0.0585	0.1315	-0.2553	1.0000	-0.0627
GNPNAT1	0.0568	-0.3336	0.0055	-0.2595	0.0823	-0.3107	0.0041	-0.2597	1.0000	0.0273	0.7284	0.1135	0.8402	0.0833

GNPTAB	0.0000	1.1581	0.0000	1.0357	0.0000	1.6111	0.0000	1.2257	1.0000	-0.0629	0.7510	-0.1729	0.0003	-0.4441
GNPTG	1.0000	0.0004	1.0000	-0.0243	0.0052	-0.5294	0.0000	-0.4479	1.0000	-0.1139	0.6668	-0.1264	1.0000	-0.0265
GNS	0.5240	-0.1375	0.0060	-0.1956	0.0016	0.4771	0.0001	0.2558	1.0000	0.1188	0.8785	0.0733	0.6621	-0.0974
GOLGA1	0.5997	0.1429	0.8144	-0.0547	0.2360	0.2489	0.5284	0.0887	1.0000	0.1770	1.0000	-0.0080	1.0000	0.0211
GOLGA2	0.8069	-0.1008	0.0327	-0.1638	0.0072	-0.4691	0.0000	-0.3317	1.0000	0.0747	1.0000	0.0243	0.0351	0.2171
GOLGA3	0.0233	-0.3917	0.0000	-0.4177	0.1975	0.2502	0.0418	0.1659	1.0000	0.1413	0.5890	0.1281	0.9435	0.0619
GOLGA4	0.1448	0.2862	1.0000	0.0296	0.0361	0.3856	0.1052	0.1809	1.0000	0.1901	1.0000	-0.0532	1.0000	-0.0092
GOLGA5	0.8358	0.1031	1.0000	-0.0115	1.0000	0.0575	0.3973	0.1248	1.0000	-0.0298	0.7173	-0.1312	1.0000	0.0443
GOLGA7	0.6096	0.1419	0.0654	0.1660	0.5166	-0.1603	0.2022	-0.1215	1.0000	-0.0975	1.0000	-0.0613	1.0000	-0.0535
GOLGA7B	0.0000	-2.9833	0.0000	-1.9821	0.0148	-0.8560	0.0007	-0.7111	1.0000	-0.1816	0.1811	0.8331	1.0000	-0.0312
GOLGB1	0.1288	0.4209	0.0002	0.5113	0.0000	1.6266	0.0000	1.5681	1.0000	0.1105	0.5245	0.2134	1.0000	0.0574
GOLIM4	0.0131	0.4535	0.0002	0.4051	0.0000	1.4032	0.0000	1.5019	1.0000	0.1858	0.5452	0.1508	0.0072	0.2900
GOLM1	1.0000	-0.0174	0.3060	-0.1324	0.5319	0.1677	0.2197	-0.1481	1.0000	0.1247	1.0000	0.0217	0.3258	-0.1871
GOLPH3	1.0000	-0.0214	0.0009	-0.2816	0.9210	0.0737	0.3068	-0.1059	1.0000	0.1444	0.7505	-0.1031	1.0000	-0.0295
GOLPH3L	0.0456	-2.2630	0.0029	-3.0220	0.0792	-2.1067	0.0001	-6.0186	1.0000	0.2762	1.0000	-0.4715	0.7287	-3.6634
GOLT1A	1.0000	0.0000	1.0000	0.0000	1.0000	2.2472	1.0000	0.0000	1.0000	0.0000	1.0000	0.0000	1.0000	-2.2908
GOLT1B	0.2773	0.2546	0.0254	0.2032	1.0000	0.0127	0.4628	0.0871	1.0000	0.0653	1.0000	0.0265	0.4977	0.1450
GON4L	0.9390	0.0690	0.0799	-0.1758	0.1819	0.2498	0.9905	0.0299	1.0000	-0.0544	0.0136	-0.2865	0.0102	-0.2690
GOPC	0.2901	0.2218	0.0703	0.1692	1.0000	-0.0079	0.6577	-0.0641	1.0000	0.0441	1.0000	0.0043	1.0000	-0.0065
GORAB	0.0000	-0.7653	0.0000	-0.7926	0.0334	-0.3823	0.0001	-0.3708	1.0000	0.1275	0.9819	0.1132	0.5134	0.1442
GORASP1	0.5982	-0.1591	0.7865	-0.0606	0.2848	0.2366	0.0008	0.3344	1.0000	-0.0922	1.0000	0.0186	1.0000	0.0104
GORASP2	0.0015	-0.4688	0.0000	-0.4668	1.0000	0.0039	0.8476	-0.0379	1.0000	0.0580	0.9234	0.0724	1.0000	0.0217
GOSR1	0.0003	0.9567	0.0006	0.7588	0.0000	1.3260	0.0000	0.9390	1.0000	0.3221	0.9773	0.1376	1.0000	-0.0560
GOSR2	0.1975	-0.2628	0.1290	-0.1642	0.8033	-0.1001	1.0000	-0.0032	1.0000	0.0112	0.7608	0.1231	0.6717	0.1131
GOT1	0.1024	0.2896	0.0333	0.2059	0.0002	-0.6032	0.0000	-0.7122	1.0000	-0.0830	0.4216	-0.1542	0.2892	-0.1858
GOT2	0.0004	-0.5123	0.0008	-0.2556	0.0000	-0.8573	0.0000	-0.7234	1.0000	-0.1493	0.4839	0.1198	1.0000	-0.0101
GP1BB	0.6956	0.2229	0.7666	-0.1197	0.9260	-0.1281	0.0705	-0.3766	1.0000	0.2940	1.0000	-0.0352	1.0000	0.0503
GP5	0.2180	0.2286	0.0675	0.1531	0.0806	0.3029	0.1371	0.1277	1.0000	0.1279	0.9764	0.0649	1.0000	-0.0420
GP9	1.0000	0.0000	1.0000	0.0000	1.0000	0.0000	1.0000	0.0000	1.0000	0.0000	1.0000	0.0000	1.0000	0.0000
GPA33	1.0000	0.0000	1.0000	-0.1472	1.0000	0.0000	1.0000	-2.3200	1.0000	2.2736	1.0000	2.3456	1.0000	0.0000
GPALPP1	1.0000	-0.0095	0.0005	-0.4214	0.0376	-0.4547	0.0000	-0.7861	1.0000	0.1366	0.2046	-0.2625	0.5598	-0.1896
GPAM	0.9680	0.1165	1.0000	0.0155	0.7784	0.1601	1.0000	-0.0127	1.0000	0.1593	1.0000	0.0689	1.0000	-0.0081
GPAT2	0.3149	-0.9681	0.0025	-1.9026	0.0367	-2.1331	0.5206	-0.4943	1.0000	0.2996	1.0000	-0.6243	0.0834	1.9513
GPATCH1	0.0141	1.0732	0.0011	0.9706	0.0001	1.4895	0.0041	0.9380	1.0000	0.1547	1.0000	0.0634	0.4901	-0.3924
GPATCH11	1.0000	0.0075	0.2429	-0.1898	0.1105	-0.4102	0.0000	-0.5593	1.0000	-0.0005	0.6424	-0.1851	0.7718	-0.1452
GPATCH2	0.0621	0.7462	0.0804	0.4857	0.0000	1.7373	0.0000	1.3171	1.0000	0.3982	1.0000	0.1507	1.0000	-0.0153
GPATCH2L	0.3356	0.2195	1.0000	-0.0044	0.0019	0.5752	0.0005	0.4218	1.0000	0.1078	0.8841	-0.1025	1.0000	-0.0401
GPATCH3	0.7845	-0.1709	0.0216	-0.4272	0.0449	-0.5839	0.0001	-0.7013	1.0000	0.1351	1.0000	-0.1100	1.0000	0.0236
GPATCH8	0.1045	-0.4751	0.6016	0.1231	0.9809	0.0975	0.0000	1.0054	1.0000	-0.5305	1.0000	0.0813	0.0046	0.3849
GPBP1	1.0000	-0.0117	0.0165	-0.2555	0.7701	-0.1227	0.9284	-0.0424	1.0000	0.1783	1.0000	-0.0532	0.0559	0.2647
GPC1	0.0868	0.3845	0.0000	0.6232	0.0001	0.7909	0.0000	0.4994	1.0000	0.0783	0.0001	0.3294	0.0303	-0.2088
GPC3	1.0000	-0.4901	1.0000	-0.1773	0.4908	-1.0430	1.0000	0.0872	1.0000	0.5705	0.8269	0.8976	0.1723	1.7087
GPC4	0.7251	-0.0999	0.1044	0.1411	0.9260	0.0656	0.0121	-0.2019	1.0000	0.0944	0.0000	0.3478	0.1522	-0.1678
GPC5	0.9545	1.0644	0.6180	-1.9232	0.9361	1.2061	1.0000	0.0067	1.0000	1.6499	1.0000	-1.3245	1.0000	0.4622
GPCPD1	0.0009	-0.5207	0.0000	-0.5404	0.0363	-0.3471	0.0201	-0.2049	1.0000	0.1962	0.2289	0.1892	0.0002	0.3438
GPD1	0.0309	-0.8485	0.0013	-0.9209	0.6303	-0.2695	0.0852	0.4514	1.0000	-0.2328	0.9348	-0.2923	0.1737	0.4950
GPD1L	0.0006	-0.5546	0.0000	-0.6921	0.1585	-0.2729	0.0000	-0.5906	1.0000	0.0735	1.0000	-0.0518	0.0525	-0.2389
GPER1	0.8360	-0.6486	1.0000	-0.1429	1.0000	0.0575	0.4038	2.0762	1.0000	-2.1881	1.0000	-1.7117	1.0000	-0.1738
GPHB5	1.0000	-0.2881	1.0000	0.0000	1.0000	-2.4055	1.0000	0.0000	1.0000	-2.3757	1.0000	-2.2992	1.0000	0.0000
GPHN	0.7134	0.1540	0.4310	0.1402	0.2954	0.2639	1.0000	0.0113	1.0000	-0.0497	1.0000	-0.0510	0.0729	-0.2962
GPI	0.0000	0.9303	0.0000	0.9264	0.0029	0.4405	0.0000	0.3407	1.0000	-0.0021	1.0000	0.0067	0.5531	-0.0964
GPKOW	0.0124	-0.6157	0.3369	-0.2076	0.0000	-1.0543	0.0002	-0.6716	1.0000	-0.2396	0.8626	0.1820	1.0000	0.1502
GPLD1	1.0000	0.2007	1.0000	0.0000	1.0000	-1.0191	1.0000	0.0000	1.0000	-3.2304	0.8607	-3.6719	1.0000	-2.2888
GPM6B	0.0677	0.3067	0.0000	0.3503	0.0000	0.8617	0.0000	0.6597	1.0000	0.1040	0.2629	0.1598	0.6500	-0.0931
GPN1	1.0000	-0.0529	0.8530	0.0486	0.0002	-0.6738	0.0000	-0.4637	1.0000	-0.2567	0.5331	-0.1428	1.0000	-0.0416
GPN2	0.6027	-0.1727	0.0045	-0.3621	0.0297	-0.4746	0.0001	-0.4888	1.0000	0.0241	0.7098	-0.1536	1.0000	0.0151
GPN3	1.0000	0.0491	0.3628	-0.1237	0.0006	-0.6140	0.0000	-0.8224	1.0000	0.1699	1.0000	0.0092	1.0000	-0.0333
GPNMB	0.5696	-0.6980	0.1752	-1.1517	1.0000	0.1290	1.0000	-0.1802	1.0000	-0.3832	0.8314	-0.8252	0.6669	-0.6855
GPR1	0.1152	-0.2612	0.0006	-0.2521	0.0324	0.3352	0.0000	0.3097	1.0000	0.0588	0.8607	0.0803	1.0000	0.0387
GPR107	0.6295	0.1303	0.0339	0.1797	0.1621	0.2583	0.2786	0.1032	1.0000	0.0498	0.6427	0.1116	0.6362	-0.0998
GPR112	1.0000	0.0000	1.0000	-0.6928	1.0000	2.2427	0.4370	-3.6968	1.0000	3.6396	1.0000	3.2003	1.0000	-2.2888
GPR114	0.8207	-0.7086	1.0000	-0.1903	0.1189	-2.2270	1.0000	-0.4700	1.0000	-0.4666	1.0000	0.0620	0.9852	1.2981
GPR115	1.0000	-1.1562	0.9062	-1.5359	1.0000	0.7355	0.7718	0.9503	1.0000	0.4115	1.0000	0.0495	1.0000	0.6332
GPR116	1.0000	-0.2892	0.9062	-1.5349	1.0000	0.6780	1.0000	0.0068	1.0000	1.2654	1.0000	0.0489	1.0000	0.6043
GPR119	0.6881	0.9962	0.2268	1.7061	0.8524	0.7638	0.9875	0.9278	1.0000	-0.6490	1.0000	0.0612	1.0000	-0.4807
GPR12	1.0000	0.0000	1.0000	-2.3959	1.0000	0.0000	1.0000	0.0062	1.0000	2.2673	1.0000	0.0000	1.0000	2.3543
GPR123	0.0000	1.5583	0.0000	1.6630	0.1460	-0.5288	0.8523	-0.1313	1.0000	-0.2331	1.0000	-0.1161	1.0000	0.1691
GPR124	0.1749	0.7880	0.2288	0.5806	0.0001	1.5484	0.0000	1.6768	1.0000	0.0706	1.0000	-0.1235	0.9463	0.2056
GPR125	0.0482	-0.3635	0.0000	-0.4257	0.0002	0.6047	0.0032	0.2585	1.0000	0.1523	0.8620	0.1023	0.1568	-0.1884
GPR126	0.2546	-0.9330	0.7981	-0.3984	0.0000	2.1337	0.0000	2.4474	1.0000	-0.1328	1.0000	0.4166	0.9553	0.1861
GPR128	1.0000	-2.4776	1.0000	-2.3959	0.4426	1.8953	0.0890	2.7296	1.0000	-0.1078	1.0000	0.0000	1.0000	0.7303
GPR132	1.0000	-0.2931	1.0000	2.2534	0.9361	1.2061	1.0000	2.3241	1.0000	-2.3771	1.0000	0.0515	1.0000	-1.3089
GPR133	1.0000	-0.0391	0.9062	1.2235	1.0000	-0.5753	1.0000	-2.3178	1.0000	-1.8819	1.0000	-0.6335	0.7287	-3.6634
GPR135	1.0000	-0.2793	0.7024	0.3980	0.1912	-1.1747	0.6817	0.4610	1.0000	-0.1306	0.9234	0.5549	0.1124	1.5115
GPR137B	0.8252	-0.1243	0.2662	-0.1657	0.0246	-0.5307	0.0135	-0.3120	1.0000	-0.0346	1.0000	-0.0625	0.5339	0.1907
GPR137C	0.0011	1.0523	0.0152	0.6024	0.5836	0.3034	0.4181	0.2706	1.0000	0.0703	0.4548	-0.3667	1.0000	0.0429
GPR139	0.8249	-3.3411	0.4125	-2.2277	1.0000	0.7338	0.7238	-1.2144	1.0000	1.0963	1.0000	2.3456	1.0000	-0.8431
GPR141	1.0000	0.0000	1.0000	0.0000	1.0000	0.0000	1.0000	0.0000	1.0000	0.0000	1.0000	0.0000	1.0000	0.0000
GPR142	1.0000	0.0000	1.0000	0.0000	1.0000	2.2472	1.0000	2.3257	1.0000	0.0000	1.0000	0.0000	1.0000	0.0649
GPR143	1.0000	0.5420	0.4695	3.6125	1.0000	-0.1591	1.0000	0.0000	1.0000	-2.3757	1.0000	0.5836	1.0000	-2.2907
GPR144	0.0191	0.3932	0.0051	0.2902	0.5222	-0.1549	0.4608	-0.1067	1.0000	-0.0635	0.2872	-0.1541	1.0000	-0.0107
GPR146	0.2242	2.3963	1.0000	0.2204	1.0000	0.6780	0.4637	-3.6898	1.0000	1.2548	1.0000	-0.8964	1.0000	-3.1344
GPR148	1.0000	0.0000	1.0000	0.0000	1.0000	0.0000	1.0000	0.0000	1.0000	0.0000	1.0000	0.0000	1.0000	0.0000
GPR149	1.0000	0.0000	0.7674	-3.2498	0.8011	3.0840	1.0000	-0.8404	1.0000	3.1056	1.0000	0.0000	1.0000	-0.7795
GPR15	1.0000	0.0000	1.0000	0.0000	1.0000	0.0000	1.0000	0.0000	1.0000	0.0000	1.0000	0.0000	1.0000	0.0000
GPR156	0.2626	-0.6001	0.5271	-0.3645	0.1702	-0.6527	0.1895	0.4759	1.0000	-0.1824	1.0000	0.0661	0.0250	0.9526
GPR157	0.0000	-0.9729	0.0000	-0.9561	0.8666	-0.1163	0.2968	0.1718	1.0000	-0.1784	0.9944	-0.1477	0.8812	0.1143
GPR158	0.5153	0.2268	0.3315	-0.2095	0.0016	0.6858	1.0000	0.0649	1.0000	-0.0006	0.0849	-0.4249	0.0001	-0.6181
GPR160	1.0000	0.0230	1.0000	0.0538	0.7446	-0.2580	0.0701	-0.5202	1.0000	0.2403	0.8343	0.2825	1.0000	-0.0178
GPR161	0.0171	0.5662	0.0000	0.6849	0.0334	0.4899	0.0027	0.4585	1.0000	0.0613	0.6555	0.1944	1.0000	0.0364
GPR162	0.0023	0.7109	0.0000	0.9101	0.7660	-0.1668	0.9786	0.0739	1.0000	-0.2216	1.0000	-0.0107	1.0000	0.0234
GPR17	0.0008	-2.0818	0.0020	-1.4877	0.0083	-1.5832	0.0000	-3.0948	1.0000	0.1443	0.9012	0.7554	0.3691	-1.3614
GPR171	0.5086	-1.4999	0.5098	-0.6384	1.0000	-0.6580	0.0055	-2.4811	1.0000	1.1973	0.2620	2.0860	1.0000	-0.6159

GPR174	1.0000	0.0000	1.0000	0.0000	1.0000	0.0000	1.0000	2.3241	1.0000	0.0000	1.0000	0.0000	1.0000	2.3543
GPR176	0.0077	-0.5386	0.0000	-0.6398	0.1117	-0.3486	0.0000	-0.4336	1.0000	0.2821	0.3474	0.1941	0.2053	0.2028
GPR18	1.0000	0.1200	0.1681	-1.1574	0.6264	-0.6957	0.1310	-1.3742	1.0000	0.5398	0.9086	-0.7281	1.0000	-0.1359
GPR182	0.6866	-0.6490	0.7652	-0.3033	0.1202	-1.9455	0.1422	-0.7627	1.0000	0.6409	0.3241	1.0078	0.0552	1.8442
GPR19	0.0093	3.4706	0.0006	5.8456	1.0000	-2.4061	1.0000	0.0000	1.0000	-2.3771	1.0000	-0.1198	1.0000	0.0000
GPR20	0.0009	-1.9612	0.0002	-1.5474	0.6170	0.3581	0.1978	0.4749	1.0000	0.2441	0.9273	0.6720	0.6298	0.3668
GPR21	0.6990	0.3044	0.7640	0.1896	0.7773	-0.2693	0.0001	-1.5069	1.0000	0.0290	1.0000	-0.0709	0.0096	-1.1982
GPR22	0.0160	-1.3263	0.0010	-1.4442	1.0000	0.1018	1.0000	0.1271	1.0000	-0.1647	1.0000	-0.2715	1.0000	-0.1326
GPR26	1.0000	0.0000	1.0000	0.0000	1.0000	0.0000	1.0000	0.0000	1.0000	0.0000	1.0000	0.0000	1.0000	0.0000
GPR27	0.0000	-0.9088	0.0000	-0.7362	0.0012	0.6680	0.0000	0.4923	1.0000	-0.1855	1.0000	0.0006	0.0029	-0.3537
GPR34	1.0000	0.0000	1.0000	0.0000	0.8033	3.0790	1.0000	2.3257	1.0000	0.0000	1.0000	0.0000	1.0000	-0.7769
GPR37	0.0147	2.1814	0.0001	2.3989	0.0053	2.3367	0.0373	1.6303	1.0000	0.1217	1.0000	0.3580	0.7722	-0.5738
GPR37L1	0.0634	-1.7327	0.0025	-2.1941	0.8609	0.3783	0.9167	-0.2937	1.0000	0.2582	1.0000	-0.1923	1.0000	-0.4107
GPR39	1.0000	2.1848	1.0000	0.0000	1.0000	0.0000	1.0000	0.0000	1.0000	0.0000	1.0000	-2.2957	1.0000	0.0000
GPR52	1.0000	0.1873	1.0000	0.1251	0.1906	-1.5312	0.1532	-1.0444	1.0000	0.5502	1.0000	0.5045	0.8120	1.0440
GPR55	1.0000	0.0000	1.0000	2.2507	1.0000	0.0000	1.0000	0.0000	1.0000	0.0000	1.0000	2.3456	1.0000	0.0000
GPR56	0.0000	-0.8976	0.0000	-0.9541	0.0000	-0.8498	0.0000	-0.7225	1.0000	0.0063	1.0000	-0.0378	0.3378	0.1385
GPR6	1.0000	2.1902	1.0000	-2.3986	1.0000	0.0000	1.0000	0.0045	1.0000	2.2736	1.0000	-2.2992	1.0000	2.3543
GPR61	0.5259	0.9842	0.8644	0.4781	0.7660	-1.4058	0.3949	-2.0643	1.0000	-0.1255	1.0000	-0.6229	1.0000	-0.7815
GPR62	0.4333	-0.6301	0.0003	-1.4717	0.3240	-0.6873	0.0681	-0.7433	1.0000	0.0596	0.4515	-0.7737	1.0000	0.0018
GPR63	0.0000	3.3501	0.0011	1.5935	0.0000	3.1526	0.0001	1.8419	1.0000	1.5090	1.0000	-0.2275	1.0000	0.2082
GPR64	0.0154	1.9661	0.0000	2.0640	0.0803	1.6370	0.9389	-0.4403	1.0000	0.7126	0.2158	0.8296	0.1498	-1.3528
GPR65	1.0000	-2.4788	1.0000	0.0000	1.0000	-2.4061	1.0000	0.0000	1.0000	-2.3771	1.0000	0.0000	1.0000	0.0000
GPR68	0.0000	-2.9661	0.0000	-3.0065	0.9351	-0.2370	0.0073	-1.1333	1.0000	0.8425	1.0000	0.8166	1.0000	-0.0538
GPR75	0.0000	1.1285	0.0000	1.0215	0.6681	0.2297	0.0389	0.4554	1.0000	-0.0513	0.9437	-0.1454	0.9124	0.1819
GPR78	1.0000	0.0000	1.0000	0.0000	1.0000	0.0000	1.0000	0.0000	1.0000	0.0000	1.0000	0.0000	1.0000	0.0000
GPR82	1.0000	-0.7294	1.0000	-0.1739	0.3982	1.2094	0.3918	1.1054	1.0000	0.1831	1.0000	0.7522	1.0000	0.0797
GPR83	0.8578	0.3503	0.7936	0.6120	1.0000	0.1679	0.9343	0.4550	1.0000	-1.1988	0.4711	-0.9263	0.4898	-0.9039
GPR85	0.5779	1.3527	1.0000	-0.1674	0.3860	1.5009	0.9096	-1.3726	1.0000	0.4115	0.9744	-1.0996	0.3615	-2.4643
GPR87	0.6889	-0.5743	0.9743	-0.2981	0.2445	-1.1690	0.2857	-1.2148	1.0000	-0.3504	1.0000	-0.0614	1.0000	-0.3886
GPR89B	0.0036	0.6519	0.0000	0.5351	0.5047	-0.2287	0.0000	-0.6480	1.0000	0.2407	0.7714	0.1376	0.7291	-0.1712
GPR97	1.0000	-0.2920	0.7666	-3.2534	1.0000	0.6746	0.7701	-3.1666	1.0000	0.7365	1.0000	-2.2957	1.0000	-3.1317
GPRC5B	1.0000	0.0321	0.1250	-0.3406	0.0000	1.5685	0.0000	1.1026	1.0000	0.0599	0.3398	-0.2992	0.0012	-0.4000
GPRC5C	0.3181	-2.3875	0.9062	-1.5339	1.0000	-0.1883	0.9096	-1.3717	1.0000	-0.8169	1.0000	0.0489	0.6944	-2.0004
GPRC6A	1.0000	0.0000	1.0000	0.0000	1.0000	0.0000	1.0000	0.0000	1.0000	0.0000	1.0000	0.0000	1.0000	0.0000
GPRIN1	0.0635	-0.8744	0.0000	-0.6933	0.4008	-0.4643	0.0000	-0.6561	1.0000	0.7176	0.0001	0.9125	0.0397	0.5335
GPRIN2	0.2054	1.8480	0.5029	1.3071	1.0000	0.3474	1.0000	-0.8416	1.0000	-0.1179	1.0000	-0.6531	1.0000	-1.3081
GPRIN3	0.0608	-0.4899	0.0000	-1.0445	0.1017	0.4051	0.0001	0.3978	1.0000	-0.1757	0.0000	-0.7175	0.3196	-0.1767
GPS1	0.0002	-0.5946	0.0000	-0.5240	0.8964	0.0772	0.5080	0.0837	1.0000	-0.0500	1.0000	0.0329	1.0000	-0.0383
GPSM1	0.0000	1.4434	0.0000	1.1303	0.0105	0.5114	0.0000	0.5800	1.0000	-0.0083	0.0094	-0.3084	1.0000	0.0663
GPSM2	0.1944	0.3167	0.0002	0.3744	0.0756	0.3869	0.2787	0.1371	1.0000	-0.0583	1.0000	0.0111	0.0176	-0.3028
GPT2	0.2087	-0.6118	0.9479	-0.1285	0.0000	1.7703	0.0000	1.7068	1.0000	-0.0578	0.6541	0.4383	1.0000	-0.1142
GPX1	0.0021	-0.4451	0.0002	-0.2997	0.0000	-1.0989	0.0000	-0.6648	1.0000	-0.1697	1.0000	-0.0119	0.1780	0.2700
GPX2	1.0000	2.1848	1.0000	0.0000	1.0000	0.0000	1.0000	0.0000	1.0000	0.0000	1.0000	-2.2957	1.0000	0.0000
GPX3	0.0000	-0.9126	0.0000	-0.9998	0.0000	-2.1967	0.0000	-1.2317	1.0000	0.0403	1.0000	-0.0347	0.0000	1.0106
GPX4	0.0000	-0.9429	0.0000	-1.1413	0.0000	-1.3393	0.0000	-1.0810	1.0000	-0.2579	0.0000	-0.4439	1.0000	0.0065
GPX7	0.2118	-0.3337	0.0023	-0.3641	0.0018	-0.7199	0.0021	-0.3426	1.0000	0.4027	0.0205	0.3846	0.0000	0.7832
GPX8	0.0390	0.4240	1.0000	0.0331	0.9417	-0.0809	0.0109	-0.2707	1.0000	0.1879	0.3117	-0.1916	1.0000	0.0032
GRAMD1B	0.3663	-0.3742	0.0753	-0.4408	0.0000	1.0535	0.0000	0.9031	1.0000	0.2136	1.0000	0.1587	1.0000	0.0660
GRAMD1C	0.0390	0.5507	0.0002	0.7616	0.1658	-0.4314	0.0287	-0.5843	1.0000	-0.2287	1.0000	-0.0076	0.3009	-0.3760
GRAMD2	0.8028	0.1626	0.0389	0.4220	0.3581	-0.3178	1.0000	-0.0573	1.0000	-0.2109	1.0000	0.0614	1.0000	0.0536
GRAP	1.0000	0.0000	1.0000	0.0000	1.0000	2.2428	1.0000	0.0000	1.0000	0.0000	1.0000	0.0000	1.0000	-2.2889
GRAP2	0.9196	-0.2927	1.0000	0.0824	0.0023	-2.5181	0.0001	-4.0109	1.0000	-0.2034	1.0000	0.1857	0.8939	-1.6944
GRB10	0.0000	-1.2449	0.0000	-1.1029	1.0000	0.0214	0.3602	-0.0945	1.0000	0.0190	0.2566	0.1735	0.7557	-0.0913
GRB2	1.0000	0.0002	0.0917	-0.1324	0.6446	0.1150	0.7979	0.0397	1.0000	0.0812	1.0000	-0.0391	1.0000	0.0111
GRB7	1.0000	-2.4776	1.0000	-2.3986	1.0000	-2.4056	1.0000	-2.3200	1.0000	-0.1034	1.0000	0.0000	1.0000	0.0000
GREB1L	0.1520	-1.3310	0.0362	-1.2194	1.0000	-0.0790	0.9660	-0.2219	1.0000	0.1630	1.0000	0.2912	1.0000	0.0208
GREM1	0.9859	0.3114	1.0000	0.0835	0.9535	0.2790	0.0000	1.4332	1.0000	0.8516	0.6014	0.6410	0.0000	2.0150
GREM2	1.0000	0.4663	1.0000	-0.6953	0.0667	1.7512	0.5741	1.1646	1.0000	-0.8186	0.4739	-1.9826	0.2099	-1.4024
GRHL2	0.0000	-3.8017	0.0000	-2.6715	0.3465	-0.4789	0.0247	-0.8377	1.0000	0.0149	0.8265	1.1602	0.8623	-0.3417
GRHL3	0.0000	-1.5033	0.0000	-1.7439	0.1439	0.3929	0.3390	0.1987	1.0000	0.0228	1.0000	-0.2043	0.7476	-0.1656
GRHPR	0.0008	0.8058	0.0045	0.4635	0.0060	-0.8150	0.0001	-0.9681	1.0000	-0.1070	0.0203	-0.4374	0.8804	-0.2554
GRIA2	1.0000	-2.4776	1.0000	-2.3959	1.0000	-0.1591	1.0000	-2.3178	1.0000	-0.1078	1.0000	0.0000	1.0000	-2.2907
GRIA3	0.0000	-1.4165	0.0000	-1.5671	0.0007	-0.7086	0.0000	-1.1567	1.0000	0.1226	1.0000	-0.0151	0.1528	-0.3224
GRID2IP	1.0000	0.0000	1.0000	0.0000	1.0000	0.0000	1.0000	0.0000	1.0000	0.0000	1.0000	0.0000	1.0000	0.0000
GRIK1	1.0000	0.2029	1.0000	-0.1688	1.0000	0.7335	1.0000	0.0072	1.0000	0.4094	1.0000	0.0533	1.0000	-0.3112
GRIK3	0.8249	3.0256	1.0000	-1.0008	0.5080	3.6209	1.0000	0.5354	1.0000	3.1126	1.0000	-0.7977	1.0000	0.0690
GRIK4	1.0000	0.0000	1.0000	0.0000	1.0000	2.2428	1.0000	0.0000	1.0000	0.0000	1.0000	0.0000	1.0000	-2.2889
GRIN1	1.0000	0.0000	1.0000	0.0000	1.0000	0.0000	1.0000	2.3257	1.0000	0.0000	1.0000	0.0000	1.0000	2.3554
GRIN2A	0.0000	-2.4710	0.0000	-1.8988	0.0000	1.1149	0.0000	1.3540	1.0000	-0.1725	1.0000	0.4039	1.0000	0.0719
GRIN2B	1.0000	-0.3217	1.0000	-0.6953	1.0000	0.3498	1.0000	0.0065	1.0000	0.4129	1.0000	0.0547	1.0000	0.0749
GRIN2C	0.4916	0.7797	1.0000	0.1290	0.6263	0.7018	0.5295	0.6546	1.0000	0.2875	1.0000	-0.3518	1.0000	0.2383
GRIN3A	0.8226	0.7451	0.7439	-0.8853	0.5908	0.9035	0.9780	0.3795	1.0000	0.8304	0.9736	-0.7863	1.0000	0.3163
GRIP1	0.3224	1.0529	0.9941	-0.2918	0.9739	0.5599	0.3326	-1.0792	1.0000	0.7454	0.9110	-0.5899	0.7257	-0.8919
GRIP2	0.0410	-0.6200	0.0000	-1.1184	0.9468	-0.1157	0.9355	-0.0684	1.0000	0.1357	0.3768	-0.3482	0.6738	0.1877
GRK6	0.1448	0.4060	0.0893	0.3041	0.4570	-0.2742	1.0000	0.0078	1.0000	-0.0057	1.0000	-0.0954	0.4508	0.2803
GRK7	1.0000	-0.1407	1.0000	-0.0029	0.1903	-0.7310	0.0220	-1.1402	1.0000	-0.2779	1.0000	-0.1268	0.6484	-0.6810
GRM1	1.0000	0.0000	1.0000	0.0000	1.0000	0.0000	1.0000	0.0000	1.0000	0.0000	1.0000	0.0000	1.0000	0.0000
GRM2	1.0000	-2.4776	1.0000	0.0000	1.0000	0.6746	1.0000	0.0000	1.0000	-2.3757	1.0000	0.0000	1.0000	-3.1317
GRM3	1.0000	0.0000	1.0000	0.0000	1.0000	0.0000	1.0000	0.0000	1.0000	0.0000	1.0000	0.0000	1.0000	0.0000
GRM7	0.9545	-1.6928	0.3512	-1.8556	0.1862	1.7171	0.9162	0.5070	1.0000	1.0297	1.0000	0.8992	1.0000	-0.1763
GRM8	0.4302	-1.1654	1.0000	-0.4251	1.0000	0.0792	0.4400	0.9765	1.0000	-0.6935	1.0000	0.0580	1.0000	0.2046
GRP	0.0009	-1.4494	0.0000	-1.7279	0.0091	-1.1703	0.2575	-0.3631	1.0000	0.1989	1.0000	-0.0650	0.0335	1.0138
GRPEL1	0.0469	-0.3917	0.0066	-0.2386	0.0000	-0.8282	0.0000	-0.8085	1.0000	-0.1212	1.0000	0.0445	0.7809	-0.0948
GRPEL2	0.3820	-0.1847	0.5594	-0.0796	0.2280	0.2283	0.3853	0.0959	1.0000	0.0341	0.4735	0.1517	0.7300	-0.0929
GRPR	1.0000	0.0000	1.0000	0.0000	1.0000	2.2472	1.0000	0.0000	1.0000	0.0000	1.0000	0.0000	1.0000	-2.2907
GRSF1	0.6546	-0.1324	0.0103	-0.2467	0.1793	-0.2765	0.0000	-0.4214	1.0000	0.0461	1.0000	-0.0555	0.8775	-0.0921
GRTP1	0.8905	-0.6012	0.9062	-1.5348	1.0000	-0.7474	0.5745	1.1648	1.0000	-1.0688	0.8354	-2.0147	1.0000	0.8481
GRXCR1	1.0000	0.0000	1.0000	0.0000	1.0000	0.0000	1.0000	0.0000	1.0000	0.0000	1.0000	0.0000	1.0000	0.0000
GRXCR2	0.0046	-5.6805	0.0013	-3.7721	0.2170	1.0218	1.0000	0.2662	1.0000	0.3112	1.0000	2.3456	0.9907	-0.4430
GSC	1.0000	-0.0271	0.0113	0.7013	0.3000	-0.4996	1.0000	-0.1076	1.0000	-0.2198	0.2691	0.5230	1.0000	0.1795
GSC2	1.0000	0.0000	1.0000	-2.3959	1.0000	0.0000	1.0000	-2.3178	1.0000	2.2673	1.0000	0.0000	1.0000	0.0000
GSE1	0.0000	-0.7849	0.0000	-0.6859	0.0852	0.3611	0.0000	0.4986	1.0000	-0.0347	1.0000	0.0777	0.8120	0.1088

GSG1	0.0000	-2.2440	0.0000	-1.7973	1.0000	-0.0142	0.8426	0.1020	1.0000	-0.3031	1.0000	0.1567	0.7455	-0.1795
GSG1L	1.0000	0.0000	1.0000	-2.3986	0.2233	4.3199	1.0000	-2.3200	1.0000	2.2736	1.0000	0.0000	0.4036	-4.3683
GSG2	0.0202	0.5873	0.0000	0.9638	0.0405	0.5124	0.5589	0.1789	1.0000	-0.1549	0.4436	0.2319	0.0109	-0.4860
GSK3B	0.0010	-0.5263	0.0000	-0.4726	0.0893	0.2957	0.0082	0.2449	1.0000	0.0439	0.8581	0.1101	1.0000	-0.0020
GSKIP	1.0000	-0.0679	0.9642	-0.0467	0.5170	0.1808	0.0046	0.3680	1.0000	-0.0984	1.0000	-0.0639	0.9323	0.0949
GSN	0.1228	-0.2498	0.0007	-0.2172	0.0000	-0.7014	0.0000	-0.3825	1.0000	0.0642	0.4575	0.1092	0.0000	0.3884
GSPT1	1.0000	0.0349	1.0000	-0.0090	0.5895	-0.1338	0.0020	-0.2691	1.0000	0.0879	1.0000	0.0560	1.0000	-0.0423
GSR	0.0549	0.3627	0.0768	0.1903	0.0000	-0.8100	0.0000	-0.8731	1.0000	-0.1331	0.0177	-0.2938	0.3252	-0.1906
GSS	0.0000	-1.6470	0.0000	-1.9528	0.0000	-1.3033	0.0000	-0.7416	1.0000	0.1435	0.5603	-0.1498	0.0000	0.7104
GSTA1.1	0.0001	-1.8606	0.0000	-1.6006	0.0015	-1.3600	0.0000	-1.8732	1.0000	-0.0570	1.0000	0.2157	0.7106	-0.5651
GSTA1.3	1.0000	0.0068	0.4592	0.0820	0.0208	-0.4557	0.0000	-0.7038	1.0000	-0.1093	1.0000	-0.0217	0.0022	-0.3524
GSTA2	1.0000	-0.0424	0.0690	-0.2084	0.6097	0.1345	1.0000	0.0054	1.0000	0.1885	1.0000	0.0345	1.0000	0.0642
GSTA3	1.0000	-0.1635	0.0542	-1.4004	0.4810	0.9663	0.6248	-0.5315	1.0000	1.2793	1.0000	0.0585	1.0000	-0.2139
GSTA4	0.9545	0.0935	0.7888	-0.0710	0.2680	-0.3039	0.0530	-0.2577	1.0000	0.0338	0.8875	-0.1183	1.0000	0.0851
GSTK1	0.0105	-0.8009	0.0078	-0.6235	0.1544	0.4299	0.0013	0.5939	1.0000	-0.0348	1.0000	0.1539	0.9382	0.1321
GSTZ1	0.2587	-0.3148	0.0114	-0.4024	0.4589	-0.2237	0.4490	-0.1715	1.0000	-0.0830	0.8841	-0.1588	1.0000	-0.0259
GSX1	1.0000	0.0000	1.0000	0.0000	1.0000	2.2428	1.0000	0.0000	1.0000	0.0000	1.0000	0.0000	1.0000	-2.2889
GSX2	1.0000	0.0000	1.0000	0.0000	1.0000	0.0000	1.0000	0.0000	1.0000	0.0000	1.0000	0.0000	1.0000	0.0000
GTF2A1	0.0001	0.5975	0.0000	0.3789	0.4327	0.1849	0.4019	-0.0951	1.0000	0.1967	1.0000	-0.0091	0.9583	-0.0784
GTF2A2	0.0005	-0.5875	0.0000	-0.6242	0.0000	-0.6970	0.0000	-0.8457	1.0000	-0.0297	1.0000	-0.0536	0.2892	-0.1725
GTF2B	0.3753	-0.1857	0.0002	-0.3061	0.0100	-0.4254	0.0000	-0.5642	1.0000	0.0950	1.0000	-0.0131	1.0000	-0.0382
GTF2E1	0.5568	0.1471	0.0226	0.2152	0.3014	-0.2127	0.0015	-0.2990	1.0000	0.0337	0.6931	0.1144	1.0000	-0.0469
GTF2E2	0.6061	-0.1474	0.0221	-0.2427	0.0025	-0.5634	0.0000	-0.6621	1.0000	-0.0099	0.9348	-0.0923	0.8565	-0.1039
GTF2F2	0.0000	-0.7446	0.0000	-0.7774	0.0208	-0.4167	0.0000	-0.4228	1.0000	0.0035	1.0000	-0.0172	1.0000	0.0029
GTF2H1	0.0673	-0.3394	0.0000	-0.4179	0.0180	-0.4041	0.0000	-0.4758	1.0000	0.0445	1.0000	-0.0215	1.0000	-0.0222
GTF2H3	0.1653	0.3082	0.0030	0.3656	0.4330	-0.2126	0.0158	-0.3012	1.0000	-0.1095	1.0000	-0.0405	0.4420	-0.1925
GTF2H5	0.0040	-0.5545	0.0000	-0.5623	0.0041	-0.5710	0.0112	-0.2727	1.0000	-0.0818	1.0000	-0.0777	0.2488	0.2202
GTF3C1	0.2538	-0.2233	0.0180	-0.2035	0.1862	-0.2494	0.0036	-0.2472	1.0000	0.0255	1.0000	0.0576	1.0000	0.0327
GTF3C2	0.0446	-0.3663	0.0503	-0.2556	0.0035	-0.5547	0.2799	-0.1455	1.0000	-0.2926	0.5548	-0.1704	0.8182	0.1221
GTF3C3	0.0823	0.3204	0.0484	0.2240	0.1055	-0.3288	0.0807	-0.2038	1.0000	-0.0298	0.7635	-0.1144	0.8749	0.0988
GTF3C4	0.2691	0.2797	0.0002	0.4611	0.1076	0.3572	0.0040	0.3627	1.0000	-0.0096	0.5317	0.1839	1.0000	0.0006
GTF3C5	1.0000	0.0463	0.8884	-0.0673	0.0376	-0.4975	0.0103	-0.4019	1.0000	0.0728	1.0000	-0.0286	0.7325	0.1741
GTF3C6	0.8487	-0.1244	1.0000	-0.0182	0.0000	-1.1775	0.0000	-0.9074	1.0000	-0.0928	1.0000	0.0240	0.8163	0.1829
GTPBP1	1.0000	0.0193	0.2634	0.1164	0.3733	-0.1912	1.0000	0.0276	1.0000	-0.1685	1.0000	-0.0593	1.0000	0.0557
GTPBP2	0.8505	-0.0842	0.5693	-0.0774	0.0372	-0.3588	0.3583	-0.1033	1.0000	-0.0725	1.0000	-0.0530	0.1886	0.1885
GTPBP3	0.0270	-0.5499	0.0099	-0.4292	0.0000	-1.7700	0.0000	-1.2329	1.0000	-0.1860	1.0000	-0.0530	0.6294	0.3579
GTPBP4	0.3834	0.1789	0.0081	0.2328	0.0029	-0.4696	0.0002	-0.3136	1.0000	-0.1731	0.6450	-0.1069	1.0000	-0.0120
GTPBP6	0.4027	-0.2990	0.0024	-0.5849	1.0000	0.0502	0.8523	-0.0920	1.0000	0.1115	1.0000	-0.1623	1.0000	-0.0252
GTPBP8	0.1264	-0.3045	0.0000	-0.5315	1.0000	0.0468	1.0000	0.0218	1.0000	0.1715	1.0000	-0.0425	0.4370	0.1523
GTSE1	0.0000	1.1575	0.0000	1.7448	0.0467	0.5196	1.0000	0.0075	1.0000	-0.1580	0.0134	0.4419	0.0014	-0.6649
GTSF1	0.4392	0.2902	1.0000	0.0611	0.0819	-0.5359	1.0000	0.0315	1.0000	-0.2643	0.0776	-0.4831	0.4482	0.3088
GUCA1A	1.0000	0.0000	1.0000	0.0000	1.0000	0.0000	1.0000	0.0000	1.0000	0.0000	1.0000	0.0000	1.0000	0.0000
GUCA1B	0.0000	-3.2276	0.0000	-6.2812	0.0000	2.9903	0.0000	3.3158	1.0000	-0.9088	0.6450	-4.0607	0.0187	-0.5791
GUCA1C	0.0078	-2.5769	0.0000	-7.0093	1.0000	0.1366	0.3230	0.5240	1.0000	0.8535	0.8607	-3.6719	0.0254	1.2488
GUCD1	0.2050	0.3061	0.0352	0.2725	1.0000	0.0371	0.7710	0.0751	1.0000	-0.1100	0.8354	-0.1304	1.0000	-0.0675
GUCY1A3	0.2500	0.5238	0.9676	0.1364	0.2236	0.5051	0.5742	0.2824	1.0000	-0.2077	0.2339	-0.5814	0.4227	-0.4236
GUCY1B3	0.1877	0.3209	0.0003	0.4244	0.9343	0.0893	0.2763	0.1648	1.0000	-0.1248	1.0000	-0.0082	1.0000	-0.0440
GUCY2C	0.8856	-0.5980	1.0000	0.3377	0.8251	-1.1370	0.0622	-2.8846	1.0000	0.4434	0.5866	1.3941	1.0000	-1.3039
GUCY2F	1.0000	0.0000	1.0000	0.0000	1.0000	0.0000	1.0000	0.0000	1.0000	0.0000	1.0000	0.0000	1.0000	0.0000
GUF1	0.8028	-0.1189	1.0000	-0.0435	0.8964	-0.0941	0.1318	-0.2162	1.0000	-0.0773	1.0000	0.0103	0.4854	-0.1945
GUK1	0.0521	0.3971	0.2840	0.1728	0.8709	0.0984	0.2503	0.1675	1.0000	-0.0890	0.1015	-0.3002	1.0000	-0.0133
GULP1	0.5679	0.1656	0.7666	0.0562	0.0143	0.4701	0.0457	0.1881	1.0000	0.2913	0.2214	0.1943	1.0000	0.0139
GUSB	0.0019	0.4717	0.0000	0.5085	0.0000	-0.7283	0.0000	-0.7379	1.0000	0.0699	0.5657	0.1189	0.9835	0.0657
GXYLT1	0.1468	0.3434	0.1617	0.1797	0.0029	0.6020	0.1306	0.1817	1.0000	0.0107	0.6519	-0.1405	0.0006	-0.4030
GXYLT2	0.0000	-0.5979	0.0000	-0.5111	0.4222	-0.1602	0.0008	0.2174	1.0000	0.1069	0.0763	0.2058	0.0000	0.4897
GYG1	0.4587	0.1713	0.5985	-0.0713	0.1945	0.2529	0.5873	0.0697	1.0000	0.1234	0.7253	-0.1068	1.0000	-0.0545
GYG2	0.9893	-0.5022	1.0000	-0.0731	0.6284	-0.6969	0.4654	-0.8618	1.0000	0.2389	1.0000	0.6830	1.0000	0.0801
GYLTL1B	0.1858	-0.4133	0.6504	-0.1262	0.4867	0.2459	0.0125	0.3955	1.0000	0.0298	0.3332	0.3288	0.6259	0.1842
GYPC	0.0278	0.8177	0.0012	0.8029	0.0000	1.3380	0.0165	0.6076	1.0000	0.0002	1.0000	-0.0042	0.0017	-0.7264
GYS2	1.0000	0.0000	1.0000	0.0000	1.0000	0.0000	1.0000	0.0000	1.0000	0.0000	1.0000	0.0000	1.0000	0.0000
GZF1	0.1366	0.3038	0.0026	0.3410	0.0495	0.3715	0.0012	0.3489	1.0000	-0.0884	1.0000	-0.0392	0.7362	-0.1065
H3F3A	0.0019	-0.5074	0.0000	-0.8134	0.0160	-0.3984	0.0000	-0.3836	1.0000	0.1330	0.3561	-0.1610	0.2358	0.1535
H3F3B	1.0000	-0.0481	1.0000	-0.0054	0.0000	-0.6896	0.0000	-0.4727	1.0000	-0.0132	1.0000	0.0419	0.0324	0.2092
H6PD	0.0002	1.2618	0.0000	1.1463	0.0000	1.4888	0.0000	1.0410	1.0000	0.1165	1.0000	0.0117	0.4555	-0.3278
HAAO	1.0000	0.0000	1.0000	2.2534	1.0000	2.2428	1.0000	0.0000	1.0000	0.0000	1.0000	2.3479	1.0000	-2.2888
HABP4	0.3240	0.2698	0.6527	0.0978	0.8896	0.1013	1.0000	-0.0457	1.0000	-0.0096	0.6957	-0.1702	0.6800	-0.1522
HACE1	0.0005	0.5719	0.0000	0.5620	0.2962	0.2217	1.0000	-0.0073	1.0000	0.0727	0.9504	0.0754	0.4097	-0.1510
HACL1	1.0000	0.0875	0.9282	0.1120	0.4210	0.3500	1.0000	0.0193	1.0000	0.0130	1.0000	0.0502	0.5598	-0.3154
HADH	0.7252	-0.1264	0.0048	-0.2407	0.0019	-0.5649	0.0000	-0.6473	1.0000	0.0237	0.9127	-0.0782	1.0000	-0.0533
HADHA	0.4439	0.1545	0.0023	0.2321	0.0002	-0.5523	0.0000	-0.5447	1.0000	-0.1161	1.0000	-0.0261	0.6384	-0.1030
HADHB	0.5299	-0.1416	0.0008	-0.2351	0.0000	-1.0041	0.0000	-0.9483	1.0000	0.0358	1.0000	-0.0451	0.6202	0.0972
HAGH	1.0000	-0.0376	0.6100	-0.0715	1.0000	-0.0206	0.0461	0.1766	1.0000	-0.0396	1.0000	-0.0613	0.1748	0.1630
HAL	0.0771	0.7494	0.2220	0.4311	1.0000	-0.1415	0.0444	-0.7336	1.0000	0.2065	1.0000	-0.0991	0.7457	-0.3798
HAND1	1.0000	0.0000	1.0000	0.0000	1.0000	0.0000	1.0000	0.0000	1.0000	0.0000	1.0000	0.0000	1.0000	0.0000
HAND2	0.0960	-2.5257	0.6368	-1.1302	0.4513	-1.1597	1.0000	-0.1789	1.0000	-0.4417	1.0000	0.9738	1.0000	0.5445
HAO2	1.0000	0.0000	1.0000	0.0000	1.0000	2.2472	1.0000	2.3241	1.0000	0.0000	1.0000	0.0000	1.0000	0.0641
HAP1	0.0253	1.4312	0.2735	0.6567	1.0000	0.2119	0.1171	-1.0972	1.0000	0.8482	1.0000	0.0934	0.9809	-0.4510
HAPLN1	0.3159	3.9314	0.2920	4.0165	0.8011	3.0840	1.0000	2.3257	1.0000	0.0000	1.0000	0.0593	1.0000	-0.7793
HAPLN2	1.0000	-0.3341	0.6224	-1.9258	1.0000	0.2014	0.6172	-1.7596	1.0000	0.2656	1.0000	-1.3266	0.8939	-1.6953
HAPLN3	0.5673	0.3777	0.3114	0.4026	0.9640	-0.1984	0.8037	-0.2187	1.0000	-0.0399	1.0000	0.0001	1.0000	-0.0533
HAPLN4	1.0000	0.0000	1.0000	0.0000	1.0000	0.0000	1.0000	0.0000	1.0000	0.0000	1.0000	0.0000	1.0000	0.0000
HARBI1	1.0000	-0.0450	0.1196	0.2650	0.0000	-1.0494	0.0000	-0.7483	1.0000	-0.2534	1.0000	0.0691	1.0000	0.0555
HARS	0.0416	-0.3397	0.0001	-0.3560	0.2317	-0.2258	0.0083	-0.2185	1.0000	-0.0552	1.0000	-0.0589	1.0000	-0.0419
HAS2	0.9412	-0.1341	0.9822	0.0778	0.3621	0.3381	0.0003	0.7037	1.0000	0.1942	0.3027	0.4182	0.0179	0.5645
HAS3	0.0392	2.9665	0.1546	2.3755	1.0000	-2.4061	1.0000	-2.3200	1.0000	-0.1045	0.9986	-0.6959	1.0000	0.0000
HAT1	0.0000	0.9104	0.0000	0.8231	1.0000	-0.0558	0.0023	-0.3169	1.0000	0.0713	1.0000	-0.0043	0.3278	-0.1845
HAUS1	0.0007	0.8828	0.0000	1.3822	0.7517	0.1687	0.3593	-0.2190	1.0000	-0.3579	0.7309	0.1526	0.0000	-0.7385
HAUS2	0.6012	0.1958	0.0442	0.2492	0.4831	-0.2359	0.0875	-0.2285	1.0000	-0.0558	1.0000	0.0113	1.0000	-0.0407
HAUS3	0.0511	0.5244	0.0000	0.9368	0.0000	1.0108	0.0000	1.0102	1.0000	-0.2655	0.8496	0.1582	0.1880	-0.2611
HAUS6	0.0000	0.7991	0.0000	1.0152	0.0566	0.4059	0.6265	0.0793	1.0000	-0.1271	0.7531	0.1012	0.0000	-0.4483
HAUS8	0.0001	0.8746	0.0000	0.8048	0.1818	0.3887	1.0000	0.0362	1.0000	0.0443	1.0000	-0.0137	0.1672	-0.3011
HAVCR2	0.8108	0.7480	0.1333	1.7417	0.7692	-1.4082	1.0000	0.0060	1.0000	-0.8186	1.0000	0.1829	1.0000	0.6029
HBEGF	0.1891	-0.4248	0.0883	-0.3308	0.0273	-0.5898	0.4812	-0.1895	1.0000	-0.1512	1.0000	-0.0459	0.5842	0.2553

HBS1L	0.5611	-0.1489	0.1518	-0.1418	0.2225	-0.2445	0.0000	-0.3393	1.0000	0.0553	0.9928	0.0749	1.0000	-0.0345
HCK	0.0036	-1.6112	0.0333	-0.8354	0.0001	-2.1838	0.0001	-1.4964	1.0000	-0.3880	0.9314	0.4006	1.0000	0.3003
HCN1	1.0000	0.0000	1.0000	0.0000	1.0000	0.0000	1.0000	0.0000	1.0000	0.0000	1.0000	0.0000	1.0000	0.0000
HCN2	1.0000	-0.1284	1.0000	-0.0487	0.0122	0.8975	0.0000	1.5407	1.0000	-0.2647	1.0000	-0.1718	0.3427	0.3863
HCN4	1.0000	-0.2920	1.0000	0.6944	1.0000	-0.1591	1.0000	0.8541	1.0000	-0.1034	1.0000	0.9006	1.0000	0.9164
HCRT	0.9538	-0.5370	1.0000	-0.6953	0.5332	-1.1512	0.5798	1.1649	1.0000	-1.4689	0.7610	-1.6314	1.0000	0.8494
HDAC1	0.0000	0.6495	0.0000	0.5681	0.0001	0.6213	0.0000	0.6443	1.0000	0.1389	0.9318	0.0697	0.1964	0.1667
HDAC10	0.0000	1.3597	0.0000	1.0289	0.0002	1.2223	0.0020	0.8875	1.0000	0.3179	1.0000	0.0016	1.0000	-0.0082
HDAC11	0.4997	0.2840	0.1396	-0.3978	0.6683	-0.2243	0.0332	-0.4841	1.0000	0.1839	0.2349	-0.4854	1.0000	-0.0701
HDAC2	0.7992	-0.0941	0.0015	-0.2213	0.3809	-0.1792	0.0011	-0.2228	1.0000	0.0463	0.8929	-0.0685	1.0000	0.0083
HDAC3	1.0000	0.0081	1.0000	-0.0266	0.1093	-0.3225	0.0212	-0.2489	1.0000	0.0401	1.0000	0.0169	0.7307	0.1190
HDAC4	1.0000	0.0677	0.5137	-0.1388	0.9357	0.1014	0.5591	0.1301	1.0000	-0.0462	0.4716	-0.2392	1.0000	-0.0111
HDAC7	0.0049	-0.5047	0.0017	-0.2472	1.0000	-0.0505	0.0007	0.2374	1.0000	-0.0619	0.0789	0.2079	0.0361	0.2311
HDAC8	0.3909	-0.1830	0.2361	-0.1267	0.0020	-0.5047	0.0000	-0.3743	1.0000	-0.0571	1.0000	0.0114	0.8937	0.0780
HDAC9	0.1807	-0.3181	0.0002	-0.4750	0.1056	0.3392	1.0000	0.0057	1.0000	0.1145	1.0000	-0.0294	0.3011	-0.2130
HDC	0.4946	-0.4841	0.0322	-0.7541	0.7834	0.2407	0.8633	0.1625	1.0000	0.4420	1.0000	0.1850	0.6242	0.3660
HDDC2	0.0147	-0.4056	0.0000	-0.4176	0.0000	-1.1266	0.0000	-0.9707	1.0000	0.0202	1.0000	0.0206	0.3178	0.1812
HDGF	0.0006	-0.5186	0.0000	-0.5435	0.0000	-0.8111	0.0000	-0.6708	1.0000	-0.1206	0.4528	-0.1331	1.0000	0.0255
HDHD1	0.1539	0.6442	0.2257	0.4095	0.0010	1.1048	0.0040	0.7590	1.0000	0.1783	1.0000	-0.0460	0.9826	-0.1639
HDHD2	0.8259	0.1151	0.3165	-0.1609	1.0000	0.0302	1.0000	-0.0342	1.0000	0.0688	0.5088	-0.1941	1.0000	0.0106
HDHD3	0.0735	-0.3037	0.0003	-0.2851	0.0000	-1.3058	0.0000	-1.0756	1.0000	-0.1119	0.9043	-0.0809	0.7248	0.1245
HDLBP	0.0140	-0.3966	0.0015	-0.2157	0.7347	0.1089	0.0068	0.1782	1.0000	-0.0947	0.5607	0.0987	1.0000	-0.0202
HDX	1.0000	0.0327	0.6898	0.2431	0.0001	1.1965	0.0000	1.2223	1.0000	-0.2251	1.0000	-0.0066	0.8381	-0.1978
HEATR1	0.2113	0.2346	0.0000	0.4530	1.0000	0.0065	0.8943	0.0433	1.0000	-0.1984	1.0000	0.0321	0.3549	-0.1571
HEATR5A	0.4523	0.1890	0.5405	0.0986	0.0000	0.7301	0.0000	0.8441	1.0000	0.2553	0.3106	0.1777	0.0003	0.3745
HEATR6	0.7278	0.1261	0.5026	0.1134	0.8586	-0.0983	0.5859	-0.1029	1.0000	0.0110	1.0000	0.0102	1.0000	0.0112
HEBP1	1.0000	-0.0042	0.3058	0.1800	0.0023	0.5852	0.0000	0.6202	1.0000	0.0748	0.3558	0.2729	0.7601	0.1166
HECA	0.5899	0.1488	0.0948	0.1481	0.4578	0.1782	0.3696	0.0923	1.0000	-0.0405	1.0000	-0.0290	0.5475	-0.1214
HECTD1	1.0000	-0.0086	0.1608	-0.1214	0.0884	0.2854	0.6740	0.0561	1.0000	0.1559	1.0000	0.0555	0.9194	-0.0684
HECTD2	0.2156	-0.2832	0.0000	-0.7401	1.0000	0.0531	1.0000	-0.0117	1.0000	0.1253	0.2557	-0.3188	1.0000	0.0659
HECTD4	1.0000	0.0176	0.0076	-0.2614	0.0001	0.5901	0.0000	0.4764	1.0000	-0.0048	0.0438	-0.2712	0.6403	-0.1132
HECW1	0.0002	0.6144	0.0000	0.7054	0.0164	0.4189	0.0000	0.4124	1.0000	0.0134	0.6957	0.1163	1.0000	0.0114
HECW2	0.0021	0.5228	0.0000	0.6315	0.0031	0.5011	0.0000	0.5939	1.0000	-0.1167	1.0000	0.0047	1.0000	-0.0180
HEG1	0.8992	0.1363	1.0000	-0.0193	0.0000	1.2426	0.0000	0.9227	1.0000	0.1355	1.0000	-0.0078	0.5408	-0.1807
HELB	0.7021	-0.1774	0.7503	0.1132	0.8953	-0.1194	0.0867	-0.3414	1.0000	-0.0569	0.5907	0.2467	0.4513	-0.2736
HELLS	0.1966	0.2437	0.0088	0.2216	1.0000	-0.0219	0.0016	-0.2646	1.0000	-0.0224	1.0000	-0.0324	0.0179	-0.2596
HELQ	1.0000	0.0597	0.5981	-0.1074	0.3261	-0.2578	1.0000	0.0103	1.0000	0.0732	1.0000	-0.0820	0.0442	0.3473
HELZ	1.0000	-0.0235	0.4747	0.1180	0.0001	0.7999	0.0000	0.6617	1.0000	0.0919	0.2252	0.2455	1.0000	-0.0430
HELZ2	0.5114	0.4217	0.4130	0.3424	0.0000	1.8142	0.0000	1.8566	1.0000	0.2733	1.0000	0.2083	0.3574	0.3181
HEMGN	1.0000	0.0000	1.0000	-2.3959	1.0000	2.2427	1.0000	0.0062	1.0000	2.2673	1.0000	0.0000	1.0000	0.0652
HEMK1	0.0128	0.4301	0.0011	0.4111	0.0000	-0.8736	0.0000	-0.6533	1.0000	-0.0493	1.0000	-0.0571	0.5335	0.1763
HENMT1	1.0000	-0.0146	1.0000	0.0426	0.0000	-1.1350	0.0000	-1.2380	1.0000	-0.0341	1.0000	0.0368	1.0000	-0.1306
HEP21	1.0000	0.0000	1.0000	0.0000	1.0000	0.0000	1.0000	0.0000	1.0000	0.0000	1.0000	0.0000	1.0000	0.0000
HEPACAM	1.0000	0.0000	1.0000	0.0000	1.0000	0.0000	1.0000	0.0000	1.0000	0.0000	1.0000	0.0000	1.0000	0.0000
HEPACAM2	1.0000	0.0000	1.0000	0.0000	1.0000	0.0000	1.0000	0.0000	1.0000	0.0000	1.0000	0.0000	1.0000	0.0000
HEPH	0.3178	-0.2403	0.0010	-0.3143	0.0127	-0.4925	0.0327	-0.2169	1.0000	0.1650	0.8797	0.1033	0.0002	0.4465
HEPHL1	0.6971	0.7304	0.0553	1.7616	0.2898	-2.4979	0.2426	-4.0840	1.0000	-0.6819	1.0000	0.3568	1.0000	-2.2907
HERC3	0.1580	-0.2952	0.0000	-0.7601	0.8291	-0.0980	0.0112	-0.2780	1.0000	0.2902	0.6681	-0.1622	0.7079	0.1151
HERC4	0.6520	0.1442	1.0000	-0.0111	0.0000	0.8790	0.0000	0.7123	1.0000	0.0474	0.9106	-0.0952	0.6543	-0.1135
HERPUD1	0.0001	-0.7701	0.0000	-0.4769	0.7788	-0.1177	0.7474	0.0666	1.0000	-0.0534	0.2354	0.2525	0.6089	0.1375
HERPUD2	0.0798	-0.3082	0.0000	-0.4082	0.0121	-0.4125	0.0000	-0.3909	1.0000	0.0613	1.0000	-0.0263	0.7339	0.0881
HES1	0.0000	-0.9869	0.0000	-0.8011	0.0000	-0.8841	0.0000	-0.7575	1.0000	-0.1245	1.0000	0.0735	1.0000	0.0087
HES4	0.0550	-0.7561	0.0002	-1.2376	0.8023	0.2028	1.0000	0.0718	1.0000	-0.0740	0.5603	-0.5422	0.9885	-0.1944
HES6	0.0004	0.5834	0.0000	0.4705	0.0858	-0.3409	0.0527	-0.2078	1.0000	-0.1632	0.0645	-0.2636	1.0000	-0.0230
HESX1	0.0001	1.0265	0.0000	1.1503	0.5510	-0.2904	0.4334	-0.3038	1.0000	-0.4659	0.3204	-0.3285	0.3431	-0.4739
HEXA	0.5846	-0.1347	0.8474	-0.0411	0.2202	-0.2364	0.5098	0.0817	1.0000	-0.0206	0.8607	0.0853	0.0143	0.3031
HEXB	0.0000	0.7811	0.0000	0.7425	0.0020	-0.6393	0.0001	-0.4798	1.0000	0.2091	0.2838	0.1817	0.0078	0.3733
HEXDC	0.3052	-0.2536	0.0000	-0.5224	0.0000	-0.8988	0.0000	-1.0130	1.0000	0.2731	1.0000	0.0175	0.7248	0.1659
HEY1	0.0685	0.3386	0.0000	0.3561	0.0604	-0.3518	0.0000	-0.4357	1.0000	0.0817	0.7186	0.1114	1.0000	0.0023
HEY2	0.0033	-0.7719	0.0000	-0.6731	0.0119	-0.6685	0.0000	-0.8858	1.0000	0.3111	0.0371	0.4239	1.0000	0.0995
HEYL	0.0000	-1.8431	0.0000	-0.9275	1.0000	0.0618	0.0000	1.2166	1.0000	-0.3957	0.3998	0.5336	0.0000	0.7641
HGD	1.0000	-2.4776	0.4643	-3.7945	1.0000	-2.4056	0.4547	-3.7024	1.0000	1.2739	1.0000	0.0000	1.0000	0.0000
HGF	1.0000	2.1848	0.4695	3.6125	1.0000	2.2472	1.0000	0.0000	1.0000	0.0000	1.0000	1.4294	1.0000	-2.2907
HGH1	0.0171	-0.5160	0.0001	-0.6826	0.0000	-1.3836	0.0000	-1.4219	1.0000	-0.1502	0.4603	-0.3045	0.7097	-0.1830
HGS	0.3145	-0.2048	0.2032	-0.1230	0.7534	-0.1132	1.0000	0.0199	1.0000	-0.1194	1.0000	-0.0252	1.0000	0.0192
HGSNAT	0.0000	0.7814	0.0000	0.8268	0.0009	-0.7217	0.0000	-0.5850	1.0000	0.1799	0.0477	0.2373	0.0609	0.3225
HHAT	0.0000	-0.8924	0.0000	-0.7632	0.0742	-0.3741	0.0016	-0.3384	1.0000	0.0737	0.6072	0.2166	0.7750	0.1146
HHATL	0.0333	-1.0659	0.0002	-1.7787	0.0250	0.8510	0.0000	1.6786	1.0000	-0.4411	0.2446	-1.1413	0.3476	0.3956
HHEX	0.1896	0.3727	0.0001	0.6129	0.0862	-0.5179	0.6666	-0.1285	1.0000	-0.0380	0.5959	0.2135	0.3458	0.3575
HHIP	0.9567	-0.5374	0.5793	0.9864	1.0000	-0.0278	1.0000	0.0072	1.0000	-1.4719	1.0000	0.0589	0.5628	-1.4327
HHIPL1	0.0004	-0.9057	0.0104	-0.2806	0.0064	0.6318	0.0000	1.0447	1.0000	0.1622	0.0000	0.8018	0.0000	0.5803
HHIPL2	1.0000	-0.0679	1.0000	0.0577	0.5632	-0.4482	1.0000	-0.0655	1.0000	0.3402	0.6237	0.4759	0.2054	0.7257
HHLA1	1.0000	0.0000	1.0000	0.0000	1.0000	0.0000	1.0000	0.0000	1.0000	0.0000	1.0000	0.0000	1.0000	0.0000
HHLA2	0.3062	0.4858	1.0000	0.0289	0.0000	1.4858	0.0000	0.9516	1.0000	0.3120	1.0000	-0.1334	0.6865	-0.2186
HIAT1	0.0384	0.3533	0.0000	0.4247	0.1306	0.2764	0.0009	0.2903	1.0000	-0.0015	0.9107	0.0823	1.0000	0.0178
HIBADH	0.5780	-0.1529	0.0000	-0.4293	0.0000	-0.9016	0.0000	-1.1505	1.0000	0.0485	0.1652	-0.2153	0.3236	-0.1955
HIBCH	0.0000	-0.6406	0.0000	-0.6655	0.4957	-0.1523	0.0000	-0.3609	1.0000	0.0181	1.0000	0.0058	0.1541	-0.1848
HIC2	0.0011	2.4344	0.4438	0.7737	0.0000	2.8800	0.0283	1.5859	1.0000	1.1776	0.9458	-0.4606	1.0000	-0.0932
HID1	0.4293	0.2262	0.1675	0.1985	0.0000	-1.3832	0.0000	-0.8166	1.0000	0.0787	1.0000	0.0637	0.0004	0.6494
HIF1A	0.0000	1.3010	0.0000	1.2210	0.0000	1.7224	0.0000	1.5151	1.0000	0.3022	0.6728	0.2367	0.9498	0.1009
HIF1AN	0.0047	-0.4533	0.0000	-0.4097	0.0337	-0.3580	0.0000	-0.3677	1.0000	0.0491	0.7806	0.1051	1.0000	0.0444
HIGD1A	0.0004	0.7710	0.0000	0.5965	0.0698	-0.4406	0.0000	-0.4664	1.0000	-0.1725	0.0001	-0.3349	0.2052	-0.1923
HIGD2A	0.0000	-0.9038	0.0000	-0.6673	0.0000	-1.4504	0.0000	-0.9731	1.0000	-0.1882	1.0000	0.0609	0.0494	0.2953
HINFP	0.0075	0.6498	0.0267	0.3775	0.8784	-0.1318	0.2439	-0.2411	1.0000	0.0096	0.4236	-0.2504	1.0000	-0.0931
HINT1	0.0023	-0.5227	0.0000	-0.5103	0.0000	-0.9670	0.0000	-0.8396	1.0000	-0.1719	0.3240	-0.1472	1.0000	-0.0384
HINT3	0.2624	-0.2680	0.3353	-0.1424	0.7965	-0.1193	0.0528	-0.2531	1.0000	-0.0159	0.8607	0.1214	0.7271	-0.1456
HIP1	0.0460	0.4674	0.0000	0.9340	0.4652	0.2227	0.0000	0.6227	1.0000	-0.4030	1.0000	0.0765	1.0000	0.0032
HIP1R	0.1577	0.3552	0.0403	0.3333	0.0874	-0.4284	0.2027	-0.2477	1.0000	-0.0332	1.0000	-0.0417	0.8178	0.1534
HIPK1	0.2822	0.3474	0.0780	0.3638	0.0000	2.2395	0.0000	1.7079	1.0000	0.4476	0.0098	0.4779	0.9779	-0.0779
HIPK2	0.0158	-0.4410	0.0001	-0.3913	1.0000	-0.0565	0.1272	0.1691	1.0000	0.1389	0.1932	0.2009	0.0003	0.3693
HIPK3	0.0027	0.9021	0.0006	0.7149	0.0000	2.0523	0.0000	1.4444	1.0000	0.4119	0.7323	0.2383	0.5150	-0.1886
HIVEP1	0.6203	-0.1745	0.0279	-0.3003	0.0000	0.8555	0.0000	0.8212	1.0000	0.1622	1.0000	0.0499	0.5965	0.1333
HIVEP2	1.0000	0.0148	0.1597	0.1591	0.1973	-0.2652	0.0261	-0.2298	1.0000	-0.1138	1.0000	0.0432	1.0000	-0.0738

HIVEP3	0.5077	-0.7673	1.0000	-0.1773	0.0014	1.7231	0.0000	2.8239	1.0000	-0.9755	1.0000	-0.3777	1.0000	0.1233
HJURP	0.0000	1.1645	0.0000	1.5982	0.0010	0.8475	0.0111	0.4389	1.0000	-0.2294	0.5716	0.2169	0.0001	-0.6308
HK1	0.2749	0.2814	0.0000	0.3547	0.0045	0.5591	0.0000	0.5091	1.0000	0.0858	0.4563	0.1720	1.0000	0.0403
HK2	0.8025	-0.1076	0.5665	-0.0799	0.0004	0.6482	0.0000	0.8528	1.0000	-0.0677	1.0000	-0.0271	0.5362	0.1426
HK3	0.1752	-0.5781	0.0092	-0.7250	1.0000	0.0193	1.0000	0.0591	1.0000	0.3164	1.0000	0.1823	0.5042	0.3625
HKDC1	0.0041	1.0591	0.0000	1.5720	0.3751	0.4665	0.1274	0.6015	1.0000	-0.5447	1.0000	-0.0215	0.6130	-0.4072
HLCS	0.0455	0.5447	0.2830	0.2111	0.0653	0.4927	1.0000	-0.0039	1.0000	0.4042	1.0000	0.0841	1.0000	-0.0858
HLF	0.0510	0.8983	0.0007	1.1123	1.0000	0.1258	0.8754	0.2126	1.0000	0.0236	0.9442	0.2512	1.0000	0.1137
HM13	0.0181	0.3691	0.0003	0.4395	0.0001	-0.5844	0.0000	-0.5285	1.0000	0.0197	0.6614	0.1029	0.7715	0.0813
HMBOX1	0.3961	-0.2757	0.0001	-0.6548	0.1050	-0.4237	0.0000	-0.9062	1.0000	0.1741	0.8505	-0.1913	0.3950	-0.3023
HMBS	0.5407	-0.1492	0.6121	-0.0963	0.4037	-0.1847	0.8806	-0.0542	1.0000	-0.0744	1.0000	-0.0097	1.0000	0.0614
HMCES	0.0144	-0.4591	0.0494	-0.2370	0.0014	-0.6312	0.0956	-0.2038	1.0000	-0.0442	0.4048	0.1892	0.0183	0.3889
HMCN1	0.1611	0.3785	0.0000	0.4536	1.0000	-0.0700	0.0007	0.2851	1.0000	0.1223	0.0713	0.2095	0.0000	0.4815
HMG20A	0.9458	0.0754	0.5418	0.0820	0.0354	-0.3949	0.0000	-0.5172	1.0000	-0.0262	1.0000	-0.0066	0.4965	-0.1428
HMG20B	1.0000	0.0321	0.0246	0.2229	0.0229	-0.4188	0.0030	-0.2861	1.0000	-0.1588	1.0000	0.0437	1.0000	-0.0206
HMGB1	0.0000	0.7412	0.0000	0.6943	1.0000	-0.0522	0.0705	-0.1673	1.0000	-0.0978	0.3762	-0.1324	0.1051	-0.2071
HMGB2	0.0000	0.7355	0.0000	1.0221	0.0035	-0.5377	0.0000	-0.5548	1.0000	-0.2505	1.0000	0.0478	0.0632	-0.2618
HMGB3	0.4371	0.1901	0.0000	0.3219	0.9608	-0.0698	0.0198	-0.1908	1.0000	-0.0689	0.8755	0.0751	0.1310	-0.1844
HMGCL	0.0524	-0.3802	0.0004	-0.4528	0.0052	-0.5165	0.0001	-0.4531	1.0000	-0.0814	0.8158	-0.1419	1.0000	-0.0128
HMGCLL1	0.2960	-2.0249	1.0000	-0.8626	0.3865	-1.8748	0.7210	-1.2163	1.0000	-0.5921	1.0000	0.5860	1.0000	0.0717
HMGCR	0.0000	0.8218	0.0000	0.8612	0.0000	0.8668	0.0000	0.4916	1.0000	-0.1087	1.0000	-0.0570	0.0000	-0.4781
HMGCS1	0.0000	1.7017	0.0000	1.8984	0.0000	1.2178	0.0000	0.8218	1.0000	-0.1367	1.0000	0.0734	0.0000	-0.5263
HMGCS2	0.8601	-0.4408	1.0000	0.1132	0.0163	-1.9933	0.3201	-1.0774	1.0000	-0.5905	1.0000	-0.0262	1.0000	0.3294
HMGN1	0.0930	0.3870	0.0000	0.5283	0.1830	-0.3212	0.0000	-0.3823	1.0000	-0.1813	1.0000	-0.0277	0.0243	-0.2361
HMGN2	0.7187	-0.1117	0.3338	-0.1055	0.0191	-0.3919	0.0000	-0.4447	1.0000	-0.0254	1.0000	-0.0070	0.8274	-0.0724
HMGN3	1.0000	-0.0234	0.0011	-0.2325	0.0097	-0.5702	0.0000	-0.6165	1.0000	-0.0771	0.0069	-0.2739	0.4727	-0.1172
HMGXB3	0.8941	0.0941	0.6413	0.0805	0.0357	0.4426	0.0000	0.5717	1.0000	-0.0551	1.0000	-0.0556	0.9904	0.0786
HMGXB4	0.2412	-0.2369	0.0194	-0.2350	0.0052	-0.4813	0.0000	-0.4820	1.0000	0.0239	1.0000	0.0380	1.0000	0.0287
HMMR	0.0000	1.0923	0.0000	1.2750	0.0121	0.5636	1.0000	-0.0037	1.0000	-0.1425	1.0000	0.0509	0.0000	-0.7041
HMOX1	0.8249	-0.0911	0.0662	0.2105	0.0060	-0.4462	0.0000	-0.9450	1.0000	-0.0876	0.1277	0.2260	0.0000	-0.5809
HMOX2	0.0581	0.3458	0.1042	0.1772	0.0001	-0.6629	0.0001	-0.3745	1.0000	-0.2070	0.0006	-0.3636	0.8180	0.0866
HMX1	1.0000	0.0000	1.0000	0.0000	1.0000	0.0000	1.0000	0.0000	1.0000	0.0000	1.0000	0.0000	1.0000	0.0000
HMX2	1.0000	0.0000	1.0000	0.0000	1.0000	0.0000	1.0000	0.0000	1.0000	0.0000	1.0000	0.0000	1.0000	0.0000
HMX3	1.0000	0.0000	1.0000	2.2507	1.0000	2.2472	1.0000	0.0000	1.0000	0.0000	1.0000	2.3456	1.0000	-2.2907
HN1	0.0000	-1.6704	0.0000	-1.8023	0.0000	-1.1805	0.0000	-1.2099	1.0000	0.1816	1.0000	0.0616	0.1465	0.1572
HN1L	0.4039	-0.1675	0.9592	0.0303	0.1678	-0.2393	0.0780	-0.1426	1.0000	0.0618	0.0061	0.2721	0.1854	0.1636
HNF1A	0.4761	-0.4559	0.3713	-0.2931	0.2722	-0.5246	0.0217	-0.6311	1.0000	-0.0640	1.0000	0.1101	1.0000	-0.1651
HNF1B	0.5391	-3.8767	1.0000	0.0000	0.5042	-3.7955	1.0000	0.0000	1.0000	-3.7606	1.0000	0.0000	1.0000	0.0000
HNF4A	1.0000	-0.0699	0.6561	0.9435	0.6002	-0.7638	0.2388	1.4108	1.0000	-1.2460	1.0000	-0.2246	0.8581	0.9274
HNF4G	1.0000	0.0000	1.0000	0.0000	1.0000	0.0000	1.0000	0.0000	1.0000	0.0000	1.0000	0.0000	1.0000	0.0000
HNRNPA0	0.1039	0.3152	0.0000	0.8641	0.6617	0.1414	0.0000	0.5787	1.0000	-0.3776	0.3849	0.1829	1.0000	0.0652
HNRNPA2B1	0.0353	-0.3218	0.0000	-0.3168	0.0055	-0.4112	0.0000	-0.3495	1.0000	-0.0169	1.0000	0.0006	0.9723	0.0502
HNRNPA3	0.1940	-0.2334	1.0000	-0.0217	1.0000	-0.0503	0.0573	-0.1435	1.0000	-0.1055	0.4145	0.1186	0.0989	-0.1932
HNRNPAB	0.2658	-0.2110	0.0250	-0.1917	0.2357	-0.2205	0.0000	-0.3217	1.0000	0.0176	1.0000	0.0492	0.7533	-0.0781
HNRNPD	1.0000	-0.0121	0.8160	-0.0461	0.0000	-0.9470	0.0000	-0.8869	1.0000	-0.0335	1.0000	-0.0554	1.0000	0.0321
HNRNPDL	0.0001	-0.6345	0.0000	-0.5665	0.8033	-0.0942	1.0000	0.0156	1.0000	-0.0264	1.0000	0.0538	0.6395	0.0890
HNRNPH1	0.7225	0.1019	0.0659	0.1292	1.0000	0.0145	0.6665	0.0493	1.0000	-0.0297	1.0000	0.0097	1.0000	0.0106
HNRNPH3	0.4169	0.1596	0.3565	0.0859	0.1774	-0.2294	0.0000	-0.2916	1.0000	0.0501	1.0000	-0.0113	1.0000	-0.0069
HNRNPK	0.0215	0.4028	0.0000	0.5187	0.0200	0.3994	0.0204	0.2068	1.0000	0.0116	0.4455	0.1402	0.1491	-0.1751
HNRNPL	0.5100	-0.1705	0.1050	-0.1633	0.9167	-0.0883	0.7710	-0.0534	1.0000	-0.1192	0.8269	-0.1003	0.9904	-0.0793
HNRNPM	1.0000	0.0016	0.8289	0.0373	1.0000	-0.0456	0.4085	-0.0802	1.0000	-0.0780	1.0000	-0.0298	0.5378	-0.1071
HNRNPR	0.0024	-0.4987	0.0000	-0.5434	0.0087	-0.4192	0.0000	-0.4499	1.0000	0.0263	1.0000	-0.0061	1.0000	0.0008
HNRNPU	0.2396	0.2086	0.0066	0.1944	1.0000	0.0231	1.0000	0.0091	1.0000	0.0134	1.0000	0.0115	1.0000	0.0048
HOGA1	0.0000	-4.2268	0.0000	-4.4389	0.0000	-1.0268	0.0074	-0.3759	1.0000	-0.2118	0.9522	-0.4126	0.0124	0.4430
HOMER1	0.4978	0.4619	0.0310	0.9051	0.0048	1.1223	0.0002	1.2834	1.0000	-0.3549	1.0000	0.1023	1.0000	-0.1876
HOMER2	1.0000	0.0633	0.9762	-0.1437	0.1692	-0.7671	0.7001	-0.2952	1.0000	-0.6734	0.0827	-0.8658	1.0000	-0.1897
HOMER3	0.0003	-0.5660	0.0000	-0.6077	0.0558	-0.3292	0.0000	-0.3045	1.0000	0.0341	1.0000	0.0048	0.9323	0.0635
HOOK1	0.0000	2.4833	0.0003	1.1910	0.0000	2.5016	1.0000	0.0873	1.0000	1.7990	0.3698	0.5221	0.3583	-0.6110
HOOK3	0.4260	-0.1708	0.0034	-0.2288	0.0003	-0.5402	0.0000	-0.3266	1.0000	-0.0280	1.0000	-0.0737	0.0966	0.1911
HOPX	0.0000	-2.4145	0.0000	-2.8505	0.3264	-0.1862	0.0682	-0.1637	1.0000	0.0211	0.0000	-0.4022	1.0000	0.0490
HORMAD1	0.0264	1.7457	0.3992	0.5987	0.0449	1.6066	0.2717	-0.9711	1.0000	1.1956	1.0000	0.0640	0.1368	-1.3706
HORMAD2	0.7460	-0.3982	0.9156	-0.2231	0.7437	-0.3787	0.2730	-0.6380	1.0000	-0.1775	1.0000	0.0136	0.9338	-0.4291
HOXA10	1.0000	0.0479	0.9880	0.0484	0.7156	-0.1460	0.0631	-0.2783	1.0000	0.0212	1.0000	0.0333	0.9059	-0.1059
HOXA11	0.6788	-0.4777	0.8176	0.2861	0.0079	1.2510	0.0008	1.2538	1.0000	-0.0784	0.5264	0.7005	1.0000	-0.0707
HOXA13	1.0000	2.1848	1.0000	0.3667	0.2779	3.9963	1.0000	0.5373	1.0000	3.1126	1.0000	1.4325	1.0000	-0.3112
HOXA2	0.0296	-1.4773	0.0300	-1.1680	0.1319	-1.0586	0.7460	-0.3010	1.0000	-0.0106	1.0000	0.3166	0.5105	0.7597
HOXA3	1.0000	-0.0687	0.5813	-0.2374	0.9306	-0.1537	0.0477	0.4944	1.0000	-0.3201	0.3016	-0.4739	0.4520	0.3337
HOXA9	0.6554	-0.1980	0.0000	-0.9988	0.0104	-0.6949	0.0000	-0.8171	1.0000	0.3275	0.0808	-0.4611	0.7437	0.2075
HOXB1	1.0000	0.0000	1.0000	0.0000	1.0000	0.0000	1.0000	0.0000	1.0000	0.0000	1.0000	0.0000	1.0000	0.0000
HOXB13	1.0000	0.0000	1.0000	0.0000	1.0000	0.0000	1.0000	0.0000	1.0000	0.0000	1.0000	0.0000	1.0000	0.0000
HOXB2	0.4804	-1.0648	0.4787	0.9496	0.6231	-0.6954	0.0033	2.3286	1.0000	-1.3971	1.0000	0.6259	0.1245	1.6304
HOXB3	0.6926	0.7328	1.0000	0.1782	1.0000	0.0181	0.9162	0.5072	1.0000	0.0840	1.0000	-0.4580	1.0000	0.5790
HOXB5	1.0000	0.0000	1.0000	0.0000	1.0000	0.0000	1.0000	0.0000	1.0000	0.0000	1.0000	0.0000	1.0000	0.0000
HOXB8	1.0000	0.0000	1.0000	0.0000	1.0000	0.0000	1.0000	0.0000	1.0000	0.0000	1.0000	0.0000	1.0000	0.0000
HOXB9	1.0000	0.0000	1.0000	0.0000	1.0000	0.0000	1.0000	0.0000	1.0000	0.0000	1.0000	0.0000	1.0000	0.0000
HOXC10	1.0000	0.0000	1.0000	0.0000	1.0000	0.0000	1.0000	0.0000	1.0000	0.0000	1.0000	0.0000	1.0000	0.0000
HOXC11	1.0000	0.0000	1.0000	0.0000	1.0000	0.0000	1.0000	0.0000	1.0000	0.0000	1.0000	0.0000	1.0000	0.0000
HOXC12	1.0000	0.0000	1.0000	0.0000	1.0000	0.0000	1.0000	0.0000	1.0000	0.0000	1.0000	0.0000	1.0000	0.0000
HOXC5	1.0000	0.0000	1.0000	0.0000	1.0000	0.0000	1.0000	0.0000	1.0000	0.0000	1.0000	0.0000	1.0000	0.0000
HOXC6	1.0000	0.0000	1.0000	0.0000	1.0000	0.0000	1.0000	0.0000	1.0000	0.0000	1.0000	0.0000	1.0000	0.0000
HOXC8	1.0000	0.0000	1.0000	0.0000	1.0000	0.0000	1.0000	0.0000	1.0000	0.0000	1.0000	0.0000	1.0000	0.0000
HOXD10	1.0000	2.1902	1.0000	0.6956	1.0000	2.2427	1.0000	0.0076	1.0000	2.2673	1.0000	0.8994	1.0000	0.0659
HOXD11	1.0000	0.0000	1.0000	0.0000	1.0000	0.0000	1.0000	0.0000	1.0000	0.0000	1.0000	0.0000	1.0000	0.0000
HOXD12	1.0000	0.0000	1.0000	0.0000	1.0000	0.0000	1.0000	0.0000	1.0000	0.0000	1.0000	0.0000	1.0000	0.0000
HOXD4	1.0000	-2.4788	1.0000	0.0000	1.0000	-2.4061	1.0000	0.0000	1.0000	-2.3771	1.0000	0.0000	1.0000	0.0000
HOXD8	0.5966	0.6260	0.1045	1.0763	0.6617	-0.7003	1.0000	0.2251	1.0000	0.1326	0.7352	0.5919	0.6705	1.0572
HP1BP3	0.8558	-0.0814	0.0313	-0.1560	0.0342	-0.3509	0.0000	-0.3697	1.0000	-0.0172	0.8175	-0.0793	1.0000	-0.0302
HPCA	1.0000	-0.1014	0.4678	-0.5579	0.0197	-2.4511	0.0145	-1.4207	1.0000	0.1371	1.0000	-0.3116	0.6865	1.1767
HPCAL1	0.0000	-0.6884	0.0000	-0.6507	0.0000	-0.9926	0.0000	-0.9376	1.0000	0.0853	0.6308	0.1359	0.5346	0.1462
HPCAL4	0.0000	-1.6532	0.0000	-1.6157	1.0000	-0.1109	0.9096	0.1139	1.0000	0.0080	1.0000	0.0604	0.8003	0.2410
HPD	0.2092	-2.6429	0.2495	-4.1757	1.0000	-0.7493	0.2426	-4.0840	1.0000	-0.6824	1.0000	-2.2957	0.5105	-4.0491
HPDL	0.3830	0.3258	0.0223	0.4581	0.0466	-0.7338	0.0000	-0.9810	1.0000	0.0342	0.9094	0.1794	1.0000	-0.2074
HPGD	1.0000	0.0000	1.0000	0.0000	1.0000	0.0000	1.0000	0.0000	1.0000	0.0000	1.0000	0.0000	1.0000	0.0000

HPGDS	0.0033	3.0905	0.3454	1.5205	1.0000	-0.1793	0.7710	-3.1632	1.0000	-0.1245	0.0861	-1.6826	1.0000	-3.1344
HPRT1	0.0000	-0.9680	0.0000	-0.8646	0.0338	-0.4996	0.0000	-0.6610	1.0000	-0.1656	1.0000	-0.0493	0.0083	-0.3208
HPS1	0.0004	-0.5887	0.0000	-0.4669	0.0001	-0.6523	0.0000	-0.4848	1.0000	-0.1945	1.0000	-0.0612	1.0000	-0.0218
HPS3	0.3495	-0.2887	0.0000	-0.6717	1.0000	0.0533	0.1298	-0.2410	1.0000	0.2325	1.0000	-0.1379	1.0000	-0.0552
HPS4	0.1048	0.3631	0.0000	0.5386	1.0000	-0.0443	0.9868	0.0490	1.0000	-0.1028	1.0000	0.0849	1.0000	-0.0054
HPS5	1.0000	-0.0302	0.9082	-0.0513	0.4614	0.1897	0.0015	-0.3607	1.0000	-0.0059	1.0000	-0.0138	0.0000	-0.5501
HPS6	0.0024	-0.5065	0.0054	-0.2812	0.9669	-0.0677	0.0024	0.2796	1.0000	-0.1397	0.9008	0.0984	0.1890	0.2140
HPSE	1.0000	0.0807	0.0111	1.3499	0.0135	1.2702	0.5181	0.6187	1.0000	-0.6696	0.6954	0.6073	0.0118	-1.3155
HPSE2	1.0000	-2.4776	1.0000	-1.0017	1.0000	-2.4056	1.0000	-0.8416	1.0000	0.7365	1.0000	2.3456	1.0000	2.3554
HPX	1.0000	0.4157	1.0000	-0.1698	1.0000	0.3662	1.0000	-0.3794	1.0000	-0.1268	1.0000	-0.7078	1.0000	-0.8668
HRAS	0.0000	-1.4523	0.0000	-1.2768	0.0000	-0.7272	0.0000	-0.3744	1.0000	-0.1016	1.0000	0.0861	0.0691	0.2568
HRASLS	0.2132	-0.2711	0.0017	-0.3773	1.0000	-0.0604	1.0000	0.0051	1.0000	-0.0230	0.8732	-0.1174	1.0000	0.0474
HRG	1.0000	2.1848	1.0000	0.0000	1.0000	0.0000	1.0000	0.0000	1.0000	0.0000	1.0000	-2.2956	1.0000	0.0000
HRH1	0.4727	0.4392	0.8851	0.1771	0.0018	1.1430	0.0000	1.4016	1.0000	0.1346	1.0000	-0.1167	0.4194	0.3994
HRH2	1.0000	2.1848	1.0000	0.0000	1.0000	0.0000	1.0000	0.0000	1.0000	0.0000	1.0000	-2.2956	1.0000	0.0000
HRH3	0.8757	-0.4212	1.0000	-0.0682	0.0859	-1.4987	0.1576	-0.9228	1.0000	-0.1298	1.0000	0.2333	1.0000	0.4501
HRSP12	0.8303	-0.1297	0.0041	-0.4024	0.2069	-0.3147	0.0011	-0.4781	1.0000	0.0600	0.7623	-0.2013	1.0000	-0.0985
HS1BP3	0.9154	0.0778	0.8397	-0.0460	1.0000	0.0131	0.0547	-0.1804	1.0000	-0.0229	0.5288	-0.1339	0.1496	-0.2112
HS2ST1	0.1565	0.3058	0.0000	0.4260	0.7244	0.1257	1.0000	0.0002	1.0000	0.0054	0.6072	0.1376	0.5722	-0.1151
HS3ST1	1.0000	0.5453	1.0000	2.2534	1.0000	-2.4061	1.0000	0.0000	1.0000	-2.3771	1.0000	-0.7977	1.0000	0.0000
HS3ST2	0.0001	-3.3293	0.0001	-2.2317	0.0565	1.0259	0.5643	0.3781	1.0000	-0.4029	1.0000	0.7109	0.0100	-1.0413
HS3ST3B1	1.0000	0.0922	1.0000	-0.0839	0.6900	-0.2720	0.8515	-0.1805	1.0000	-0.3217	0.5235	-0.4849	1.0000	-0.2265
HS3ST5	0.0000	-1.7811	0.0000	-1.4879	0.8967	-0.1318	0.0468	0.2756	1.0000	0.2562	0.0860	0.5602	0.0001	0.6643
HS3ST6	1.0000	0.0000	1.0000	0.0000	1.0000	0.0000	1.0000	0.0000	1.0000	0.0000	1.0000	0.0000	1.0000	0.0000
HS6ST1	0.0016	-0.5273	0.0000	-0.4540	0.1292	0.2905	0.0000	0.5904	1.0000	0.0498	0.6218	0.1353	0.0012	0.3551
HS6ST2	0.0325	-0.5145	0.0000	-0.5560	0.0244	-0.5377	0.0000	-0.4989	1.0000	0.2970	0.1324	0.2688	0.0210	0.3419
HS6ST3	0.0000	-2.2120	0.0000	-2.9635	0.0029	0.7350	0.2490	0.2325	1.0000	0.2989	0.5963	-0.4383	0.5457	-0.1991
HSBP1	0.0058	-0.4239	0.0000	-0.3421	0.0000	-0.6705	0.0000	-0.5707	1.0000	0.0435	0.3468	0.1377	0.2337	0.1489
HSBP1L1	1.0000	-0.1831	0.4230	-0.5250	0.1427	-0.8557	0.6284	-0.3915	1.0000	-0.3285	0.6033	-0.6599	1.0000	0.1386
HSCB	0.0041	-0.5465	0.0000	-0.7122	0.0006	-0.6500	0.0002	-0.4395	1.0000	-0.1398	0.1682	-0.2932	1.0000	0.0747
HSD11B2	0.0000	-3.0328	0.0000	-3.5262	0.0000	-1.1359	0.0000	-0.9627	1.0000	-0.0761	0.7428	-0.5593	1.0000	0.1012
HSD17B10	0.6865	-0.1308	0.7568	-0.0707	0.0019	-0.5659	0.0000	-0.5078	1.0000	-0.2646	0.3834	-0.1932	0.3529	-0.2012
HSD17B12	0.0000	-1.0061	0.0000	-0.9248	0.0023	-0.4666	0.0000	-0.4662	1.0000	0.0037	0.8770	0.0972	1.0000	0.0095
HSD17B2	1.0000	0.0000	1.0000	0.0000	1.0000	0.0000	1.0000	0.0000	1.0000	0.0000	1.0000	0.0000	1.0000	0.0000
HSD17B4	0.0000	1.3206	0.0000	1.5848	0.0000	1.3767	0.0000	1.4346	1.0000	-0.2486	1.0000	0.0299	0.4840	-0.1825
HSD17B7	0.0000	0.7472	0.0000	0.9113	0.0074	-0.5112	0.0023	-0.3352	1.0000	-0.2910	0.7416	-0.1152	0.8606	-0.1091
HSDL1	0.1343	0.2985	0.0000	0.4692	0.1124	-0.3152	0.0002	-0.3356	1.0000	0.2547	0.0000	0.4381	0.1015	0.2390
HSF2	0.0000	-0.6765	0.0000	-0.7678	0.0014	-0.5074	0.0000	-0.4596	1.0000	0.0532	1.0000	-0.0257	0.5213	0.1065
HSF4	1.0000	0.0543	0.5084	0.3136	1.0000	0.0976	0.1052	0.5380	1.0000	-0.4322	1.0000	-0.1591	1.0000	0.0121
HSF5	0.5079	0.4433	0.1772	-0.5268	0.0184	-1.2642	0.0000	-1.6346	1.0000	0.5913	0.8256	-0.3674	1.0000	0.2230
HSP90AA1	0.0000	-1.0928	0.0000	-0.9383	0.0000	1.1237	0.0000	1.2130	1.0000	-0.0626	0.7971	0.1046	1.0000	0.0322
HSP90AB1	0.6459	0.1194	0.4156	0.0768	0.0000	-1.0849	0.0000	-1.1257	1.0000	-0.1360	0.1962	-0.1660	0.1594	-0.1715
HSP90B1	0.0022	-0.4765	0.0000	-0.3146	0.0000	0.8951	0.0000	0.8868	1.0000	0.0405	0.1112	0.2152	1.0000	0.0373
HSPA12A	0.9899	0.4039	0.0083	2.3977	0.0708	1.4843	0.0102	2.4137	1.0000	-1.2839	0.9271	0.7149	1.0000	-0.3512
HSPA13	0.0000	0.8405	0.0000	0.8945	1.0000	0.0044	0.6796	0.1050	1.0000	-0.0714	1.0000	-0.0034	1.0000	0.0351
HSPA14	0.4919	0.1716	0.2570	0.1314	0.0000	-0.7320	0.0000	-0.8517	1.0000	0.0890	1.0000	0.0610	1.0000	-0.0260
HSPA2	0.8048	0.0923	0.0077	0.2317	1.0000	-0.0183	0.0000	0.2995	1.0000	-0.0282	0.6072	0.1234	0.0019	0.2954
HSPA4	0.0009	-0.5683	0.0000	-0.5449	0.6654	0.1273	0.3548	0.0863	1.0000	-0.0908	1.0000	-0.0550	0.3764	-0.1262
HSPA4L	0.9899	0.0596	0.0547	0.1713	0.0001	0.6114	0.0000	0.4994	1.0000	0.0192	0.3979	0.1430	0.7749	-0.0877
HSPA5	0.6835	0.1127	0.0005	0.2935	0.0251	0.3422	0.0264	0.1971	1.0000	0.0517	0.0237	0.2454	0.6228	-0.0879
HSPA8	0.3151	-0.1891	0.8999	-0.0305	0.1624	-0.2332	0.0098	-0.1827	1.0000	-0.0620	0.5904	0.1096	1.0000	-0.0061
HSPA9	0.5043	0.1482	0.0000	0.3043	0.0367	-0.3383	0.0000	-0.3192	1.0000	-0.0844	0.7635	0.0840	0.8973	-0.0596
HSPB1	0.0000	-1.4364	0.0000	-1.5305	0.0010	-0.5412	0.0000	-0.5032	1.0000	0.0105	0.9519	-0.0712	1.0000	0.0541
HSPB11	0.0398	0.4758	1.0000	0.0453	0.0010	-0.7151	0.0000	-0.9881	1.0000	0.2211	0.7020	-0.1975	1.0000	-0.0472
HSPB2	0.0000	-4.1198	0.0000	-4.5158	0.0009	-0.5023	0.0001	-0.3934	1.0000	0.0044	0.3106	-0.3804	0.6865	0.1182
HSPB3	0.0409	-1.3958	0.0000	-2.6701	0.0236	-1.4551	0.1356	-0.7917	1.0000	0.1347	0.6439	-1.1282	0.6029	0.8053
HSPB7	0.0049	-0.5306	0.0000	-0.4538	0.0000	-2.5013	0.0000	-2.6014	1.0000	-0.0699	1.0000	0.0188	0.8330	-0.1639
HSPB8	0.0000	-2.8000	0.0000	-2.9770	0.0000	-1.4253	0.0000	-1.3791	1.0000	-0.0113	0.6664	-0.1767	1.0000	0.0406
HSPBAP1	1.0000	-0.0565	0.6560	-0.1071	0.0056	-0.6639	0.0001	-0.5911	1.0000	0.0812	1.0000	0.0428	0.8254	0.1602
HSPBP1	0.0305	-0.3741	1.0000	-0.0232	0.0000	-0.7528	0.0930	-0.1764	1.0000	-0.3368	1.0000	0.0259	0.0798	0.2451
HSPD1	0.0049	-0.4271	0.0000	-0.3087	0.0343	0.3404	0.0000	0.2733	1.0000	-0.0259	0.6141	0.1047	0.6800	-0.0877
HSPE1	0.0103	-0.4402	0.0000	-0.4789	0.0012	-0.5262	0.0000	-0.4440	1.0000	-0.1180	0.4205	-0.1444	1.0000	-0.0302
HSPG2	1.0000	-0.0134	0.0001	0.6821	0.0000	1.8245	0.0000	2.7396	1.0000	-0.5773	0.9442	0.1312	0.0000	0.3409
HTATIP2	0.7382	0.1921	0.1839	0.3457	0.0073	-0.7945	0.2818	-0.2996	1.0000	-0.2219	1.0000	-0.0571	0.6865	0.2767
HTATSF1	0.4768	0.1876	0.0768	0.1780	0.7891	0.1145	0.9156	-0.0446	1.0000	0.0574	1.0000	0.0607	0.8278	-0.0963
HTR1A	1.0000	0.0000	1.0000	0.0000	1.0000	0.0000	1.0000	0.0000	1.0000	0.0000	1.0000	0.0000	1.0000	0.0000
HTR1B	1.0000	-0.1003	0.5899	1.0428	1.0000	0.2796	0.5287	0.9704	1.0000	-1.0862	1.0000	0.0609	1.0000	-0.3961
HTR1D	0.0652	0.4711	0.0025	0.4679	1.0000	-0.0102	0.2810	0.2014	1.0000	0.1047	1.0000	0.1142	0.1883	0.3219
HTR1E	1.0000	2.1848	1.0000	0.0000	1.0000	0.0000	1.0000	0.0000	1.0000	0.0000	1.0000	-2.2956	1.0000	0.0000
HTR1F	1.0000	0.0000	1.0000	0.0000	1.0000	0.0000	1.0000	0.0000	1.0000	0.0000	1.0000	0.0000	1.0000	0.0000
HTR2B	0.0341	0.4789	0.0000	0.7404	1.0000	-0.0475	0.0217	0.3348	1.0000	-0.1936	1.0000	0.0803	0.5150	0.1943
HTR2C	0.4213	-0.9702	0.4422	-0.6877	0.0620	-1.6594	0.0507	-1.4755	1.0000	0.1055	1.0000	0.4001	1.0000	0.2923
HTR3A	1.0000	-0.3478	0.3948	-1.1398	0.3428	1.1863	0.0018	1.7878	1.0000	0.5844	1.0000	-0.1930	0.1209	1.1940
HTR4	0.0002	-0.7878	0.8032	-0.0736	0.0000	-0.8964	0.0014	-0.4673	1.0000	-0.1723	0.0007	0.5538	0.3897	0.2618
HTR5A	1.0000	-2.4776	1.0000	-2.3986	1.0000	-0.1624	1.0000	0.0057	1.0000	-0.1034	1.0000	0.0000	1.0000	0.0659
HTR6	0.3006	-0.8720	0.0075	-1.6834	0.8974	-0.2621	0.1310	0.7628	1.0000	0.0443	0.8607	-0.7552	0.0901	1.0746
HTR7	0.0000	0.9330	0.0000	0.7165	0.1746	0.3792	0.9024	0.0641	1.0000	0.2400	1.0000	0.0361	1.0000	-0.0705
HTRA1	0.0028	-0.4426	0.0000	-0.3264	0.0000	-0.8790	0.0022	-0.2325	1.0000	0.1358	0.0046	0.2649	0.0000	0.7878
HTRA2	0.2072	-0.2326	0.5880	-0.0887	0.0000	-0.8261	0.0000	-0.4876	1.0000	-0.3001	0.5761	-0.1442	1.0000	0.0441
HTRA3	0.6008	-0.1977	0.8029	-0.0985	0.1679	0.3486	0.3905	0.1831	1.0000	0.3132	0.0679	0.4240	0.7263	0.1520
HUNK	1.0000	0.0000	1.0000	0.0000	1.0000	0.0000	1.0000	0.0000	1.0000	0.0000	1.0000	0.0000	1.0000	0.0000
HUS1	0.0000	0.9624	0.0000	1.3228	0.0375	-0.6292	0.0245	-0.5219	1.0000	-0.1746	0.5877	0.1959	1.0000	-0.0615
HVCN1	0.1420	-2.1336	0.8315	-0.9418	1.0000	-0.1961	1.0000	-0.4586	1.0000	-0.7621	1.0000	0.4441	0.6819	-1.0212
HYAL1	0.0000	-1.6039	0.0000	-1.2270	0.3599	-0.3557	0.8364	-0.1091	1.0000	0.0126	0.5607	0.4023	0.5336	0.2659
HYAL2	0.0000	-0.8826	0.0000	-0.7328	0.0046	-0.6041	0.0052	-0.4008	1.0000	-0.0574	1.0000	0.1039	0.7798	0.1513
HYAL3	0.0785	-0.6132	0.0050	-0.6942	1.0000	0.0860	0.0105	0.5146	1.0000	-0.1760	0.9052	-0.2435	0.5518	0.2595
HYDIN	1.0000	-0.0740	1.0000	0.0155	0.5627	-0.3910	1.0000	0.1035	1.0000	-0.0892	1.0000	0.0189	0.4143	0.4135
HYI	0.2949	0.3412	0.2525	0.2258	0.0000	-1.2145	0.0000	-0.8582	1.0000	-0.1706	0.3807	-0.2736	0.8939	0.1945
HYKK	0.3920	0.3948	0.5293	0.2685	0.0321	-0.8740	0.0150	-0.8161	1.0000	-0.1536	0.8607	-0.2677	1.0000	-0.0884
HYLS1	0.1562	0.4558	0.0000	0.9542	0.3134	-0.3269	0.0172	-0.5078	1.0000	-0.2311	0.5250	0.2774	0.2266	-0.4084
HYOU1	0.0489	0.3529	0.0000	0.4327	0.0186	0.3790	0.0000	0.5154	1.0000	-0.0019	0.9158	0.0905	0.2882	0.1396
HYPK	0.0000	-0.9675	0.0000	-0.8900	0.0000	-1.1788	0.0000	-0.9669	1.0000	-0.0303	1.0000	0.0594	0.3591	0.1873
IAH1	0.0069	-0.6055	0.0000	-0.9121	0.0815	-0.4102	0.0006	-0.4438	1.0000	-0.3026	0.0004	-0.5972	0.0834	-0.3301

IAPP	1.0000	0.0000	1.0000	0.0000	1.0000	0.0000	1.0000	0.0000	1.0000	0.0000	1.0000	0.0000	1.0000	0.0000
IARS	0.1754	-0.2616	1.0000	-0.0178	0.0061	0.4327	0.0072	0.2002	1.0000	-0.1615	0.8885	0.0950	0.0000	-0.3883
IARS2	0.0000	0.6534	0.0000	0.6925	0.9743	0.0637	0.8597	0.0457	1.0000	-0.0931	1.0000	-0.0418	0.6822	-0.1058
IBA57	0.1847	-0.3349	0.0032	-0.3681	0.0439	0.4325	0.0677	0.2238	1.0000	0.2778	0.3001	0.2568	1.0000	0.0738
IBSP	0.6957	0.3644	0.6226	-0.3787	0.4265	-0.5922	0.0009	-1.7568	1.0000	0.2519	0.8219	-0.4763	0.4907	-0.9043
IBTK	0.0034	-0.4523	0.0000	-0.4314	1.0000	0.0518	1.0000	0.0227	1.0000	0.0938	0.5732	0.1275	0.9878	0.0698
ICA1	0.4438	0.2396	1.0000	-0.0684	0.0011	-0.8106	0.0000	-0.9019	1.0000	0.0512	0.4712	-0.2433	1.0000	-0.0372
ICK	0.6669	0.3034	0.0000	1.3613	0.0163	0.8314	0.0000	1.7314	1.0000	-0.7695	0.8143	0.3023	1.0000	0.1396
ICOS	1.0000	-0.3341	1.0000	0.6987	1.0000	-0.1836	1.0000	-2.3178	1.0000	-1.8818	1.0000	-0.8617	0.5105	-4.0506
ICOSLG	0.0254	0.6118	0.0000	1.2084	0.0000	0.9992	0.0000	0.8160	1.0000	-0.2416	0.1255	0.3665	0.0574	-0.4193
ICT1	0.0705	-0.4282	0.0022	-0.3280	0.0000	-1.2041	0.0000	-1.0446	1.0000	-0.1512	1.0000	-0.0387	1.0000	0.0154
ID1	0.0000	-0.8398	0.0000	-0.5469	0.0000	-1.0485	0.0000	-0.8606	1.0000	-0.1991	0.8138	0.1064	1.0000	-0.0049
ID2	1.0000	-0.0709	1.0000	0.0195	0.0053	0.6767	0.0009	0.5512	1.0000	-0.0857	1.0000	0.0168	0.5649	-0.2056
ID3	0.0829	0.3360	0.1249	0.1844	0.0000	1.0026	0.0000	1.0030	1.0000	-0.0764	0.2252	-0.2158	0.9584	-0.0698
ID4	1.0000	-0.0295	0.0016	0.6546	0.0003	1.1267	0.0000	1.1427	1.0000	0.0030	0.0033	0.7018	1.0000	0.0284
IDE	0.7023	0.1147	1.0000	-0.0059	0.4000	0.1862	0.5061	-0.0797	1.0000	0.1076	1.0000	-0.0004	0.3521	-0.1528
IDH1	0.8249	-0.0852	0.2828	-0.0886	0.0000	-0.7276	0.0000	-0.8986	1.0000	0.0281	1.0000	0.0372	0.3169	-0.1372
IDH2	1.0000	-0.0386	1.0000	0.0221	0.0005	-0.5309	0.0014	-0.2402	1.0000	-0.2458	0.1830	-0.1727	1.0000	0.0506
IDH3A	0.1876	-0.2344	0.0000	-0.3685	0.0000	-0.6753	0.0000	-0.8234	1.0000	0.0735	1.0000	-0.0482	0.9031	-0.0694
IDH3B	0.0000	-0.8102	0.0000	-0.6303	0.0000	-0.6734	0.0000	-0.4448	1.0000	-0.2542	1.0000	-0.0619	1.0000	-0.0199
IDNK	0.5832	-0.2838	0.0229	-0.5943	0.0262	-0.7769	0.0104	-0.6061	1.0000	0.1101	1.0000	-0.1874	0.7729	0.2887
IDO2	1.0000	0.0000	1.0000	0.0000	1.0000	0.0000	1.0000	0.0000	1.0000	0.0000	1.0000	0.0000	1.0000	0.0000
IDS	0.0001	0.7209	0.0000	0.5605	0.0015	-0.6867	0.0000	-0.8446	1.0000	0.1904	1.0000	0.0421	1.0000	0.0373
IDUA	0.0922	0.4603	0.0001	0.5991	0.5120	0.2372	1.0000	-0.0546	1.0000	0.2231	0.0771	0.3757	1.0000	-0.0633
IER3IP1	0.0002	-0.6175	0.0000	-0.6012	0.0001	-0.6550	0.0000	-0.5888	1.0000	0.0258	1.0000	0.0544	0.8254	0.0969
IER5	0.0008	-0.5871	0.0000	-0.5442	0.0002	-0.6406	0.2462	-0.1529	1.0000	-0.2185	0.6004	-0.1634	0.0364	0.2757
IFFO1	0.0000	-0.6786	0.0000	-0.9190	0.0000	-0.8180	0.0000	-0.7228	1.0000	-0.0714	0.0773	-0.2998	1.0000	0.0293
IFFO2	0.0038	-0.5565	0.0000	-0.6123	0.0003	-0.6948	0.0000	-1.0190	1.0000	0.0875	1.0000	0.0443	0.4039	-0.2311
IFI30	1.0000	-0.0538	0.5422	-0.1697	0.0001	-1.3906	0.0000	-1.4366	1.0000	0.1825	1.0000	0.0805	1.0000	0.1464
IFI35	0.5451	0.1516	1.0000	0.0254	0.1496	-0.2815	0.1398	-0.1809	1.0000	-0.0782	0.2314	-0.1926	1.0000	0.0274
IFIH1	0.0758	0.4907	0.0000	0.7201	0.2384	0.3530	0.0019	0.5584	1.0000	-0.0444	0.7201	0.1983	0.7821	0.1672
IFITM10	1.0000	0.0133	0.5585	-0.2765	0.0325	-0.9716	0.0648	-0.6234	1.0000	0.0475	1.0000	-0.2294	0.8120	0.4027
IFITM5	0.8425	0.8187	0.5798	0.9863	0.6305	0.9686	1.0000	0.0046	1.0000	-0.1210	1.0000	0.0584	0.8664	-1.0818
IFNAR1	0.4026	0.3027	0.0555	0.3314	0.0273	0.5975	0.0022	0.4242	1.0000	0.2315	0.4139	0.2722	1.0000	0.0633
IFNAR2	0.0000	0.7680	0.0000	0.6527	0.0498	-0.4014	0.0010	-0.4042	1.0000	0.0186	0.9357	-0.0855	1.0000	0.0199
IFNG	1.0000	0.0000	1.0000	0.0000	1.0000	0.0000	1.0000	0.0000	1.0000	0.0000	1.0000	0.0000	1.0000	0.0000
IFNGR1	0.4442	-0.1775	0.7003	-0.0732	1.0000	0.0199	1.0000	-0.0261	1.0000	-0.0403	1.0000	0.0762	0.9362	-0.0825
IFNGR2	0.1248	0.2640	0.0597	0.1686	0.0370	-0.3447	0.0000	-0.4683	1.0000	0.0640	1.0000	-0.0190	1.0000	-0.0542
IFNLR1	0.1195	0.8155	0.2226	-0.5826	0.0356	0.9535	0.0662	0.6420	1.0000	0.4504	0.0831	-0.9349	1.0000	0.1440
IFRD1	1.0000	-0.0551	0.7802	-0.0480	0.0003	-0.6504	0.0000	-0.6480	1.0000	0.0090	1.0000	0.0284	1.0000	0.0169
IFT122	1.0000	0.0258	0.0919	-0.2484	0.0423	0.4800	0.0031	0.3397	1.0000	-0.3301	0.0000	-0.5913	0.0001	-0.4644
IFT140	0.4966	0.2678	1.0000	0.0393	0.8388	0.1441	0.0825	0.3189	1.0000	-0.1115	0.3346	-0.3294	1.0000	0.0662
IFT172	0.1824	0.3011	1.0000	0.0353	0.1205	0.3361	0.0040	0.3607	1.0000	-0.2519	0.0002	-0.5051	0.2820	-0.2211
IFT20	1.0000	0.0084	0.3983	0.1042	0.0507	-0.4009	0.4147	-0.1028	1.0000	-0.2198	0.7488	-0.1113	0.9610	0.0851
IFT22	1.0000	-0.0022	0.6884	-0.1011	0.5444	0.1827	0.3591	0.1543	1.0000	-0.0472	0.8269	-0.1332	1.0000	-0.0695
IFT27	1.0000	-0.0448	1.0000	-0.0274	0.0094	0.6634	0.0000	0.6935	1.0000	-0.0165	1.0000	0.0121	1.0000	0.0190
IFT43	0.7877	0.1529	0.1597	-0.2647	1.0000	-0.0857	0.5287	-0.1709	1.0000	0.0666	0.2357	-0.3399	1.0000	-0.0137
IFT46	0.6290	-0.1627	0.0000	-0.5103	0.4935	-0.1936	0.3016	-0.1558	1.0000	0.0925	0.3598	-0.2424	0.7872	0.1360
IFT52	0.0894	0.3033	0.0016	0.2666	1.0000	-0.0523	0.6358	-0.0707	1.0000	0.0353	1.0000	0.0108	1.0000	0.0225
IFT57	1.0000	-0.0578	0.0728	-0.2421	0.6714	-0.1440	0.0203	-0.2795	1.0000	0.0628	0.9371	-0.1094	1.0000	-0.0685
IFT80	0.7220	-0.1594	0.6670	-0.0968	0.7640	0.1407	0.7290	0.0827	1.0000	-0.0350	1.0000	0.0395	0.9356	-0.0880
IFT81	1.0000	-0.0934	0.3374	-0.2352	0.0237	0.5611	0.0022	0.4886	1.0000	0.0381	1.0000	-0.0917	1.0000	-0.0313
IFT88	0.7097	0.2028	0.0023	0.6567	0.0003	0.8680	0.0000	1.0681	1.0000	-0.3909	1.0000	0.0785	0.6460	-0.1841
IGBP1	0.6357	-0.1289	0.3800	-0.1014	0.0000	-0.7849	0.0000	-0.6366	1.0000	-0.0695	1.0000	-0.0292	0.9868	0.0857
IGDCC3	0.1250	-1.8037	0.7802	-0.4213	0.1686	0.9521	0.1943	0.7520	1.0000	0.0301	0.4816	1.4384	1.0000	-0.1654
IGDCC4	0.0325	-0.8759	0.0002	-0.9802	1.0000	0.0988	0.0002	0.9469	1.0000	0.2688	1.0000	0.1805	0.0001	1.1223
IGF1R	0.0297	-0.4758	0.0000	-0.6883	0.1873	-0.2949	0.0000	-0.5624	1.0000	0.0748	1.0000	-0.1259	0.4819	-0.1893
IGF2	0.0000	-1.9878	0.0000	-1.4761	0.1985	0.3983	0.0003	0.6362	1.0000	-0.0226	0.5345	0.5023	0.5786	0.2213
IGF2BP1	1.0000	-0.0122	0.4632	0.3788	0.3390	-0.4684	1.0000	0.0429	1.0000	-0.9550	0.5758	-0.5507	0.6280	-0.4404
IGF2BP2	0.0000	-0.7755	0.0000	-0.8293	1.0000	0.0608	0.0006	0.3537	1.0000	-0.1843	0.3972	-0.2252	0.7096	0.1141
IGF2BP3	0.0248	0.6967	0.0001	0.8365	0.0094	0.7804	0.0520	0.4482	1.0000	-0.2856	1.0000	-0.1332	0.0149	-0.6129
IGF2R	0.5865	-0.1384	0.6904	-0.0675	0.0018	0.4958	0.0002	0.3443	1.0000	0.0670	0.4520	0.1508	0.8630	-0.0791
IGFALS	0.0033	-2.7551	0.0382	-2.2009	1.0000	-0.1975	0.8903	0.3886	1.0000	-0.5087	1.0000	0.0576	1.0000	0.0811
IGFBP1	1.0000	0.0000	1.0000	0.0000	1.0000	2.2428	1.0000	0.0000	1.0000	0.0000	1.0000	0.0000	1.0000	-2.2888
IGFBP2	0.0000	1.8821	0.0000	2.1200	0.0000	1.2819	0.0000	1.0953	1.0000	-0.3787	0.7556	-0.1289	0.0001	-0.5594
IGFBP3	0.4010	0.2217	0.0464	0.2400	0.6819	-0.1331	0.3886	-0.1316	1.0000	0.0301	1.0000	0.0600	1.0000	0.0356
IGFBP4	0.0113	0.4596	0.0000	0.6861	0.0001	0.8147	0.0000	1.7205	1.0000	-0.3353	0.8899	-0.0965	0.0003	0.5767
IGFBP5	0.9338	0.1783	0.4182	0.2978	0.2694	-0.5546	0.0000	-1.4017	1.0000	0.1422	0.8607	0.2732	0.2302	-0.7025
IGFBP7	0.5415	-0.1471	0.1165	-0.1384	0.6972	-0.1168	1.0000	0.0126	1.0000	0.0927	0.7561	0.1137	0.0819	0.2277
IGFN1	0.0000	-2.3831	0.0000	-2.6830	0.0000	0.9492	0.0000	1.0538	1.0000	0.0758	1.0000	-0.2102	0.6433	0.1850
IGHMBP2	0.1557	0.5050	0.0038	0.5731	0.0159	0.7432	0.1000	0.4022	1.0000	0.2114	0.5392	0.2916	1.0000	-0.1226
IGJ	0.0000	-1.5857	0.0000	-1.5586	0.2215	0.2196	0.0626	-0.1571	1.0000	0.0546	0.8808	0.0941	0.0025	-0.3170
IGLL1	1.0000	-2.4788	1.0000	0.0000	0.9468	1.1983	1.0000	0.0000	1.0000	-2.3771	1.0000	0.0000	0.7477	-3.6583
IGSF10	0.3979	0.6648	0.9969	0.2584	0.0286	1.1398	0.0101	1.1512	1.0000	0.0140	0.9979	-0.3855	1.0000	0.0235
IGSF11	0.0000	-4.7881	0.0000	-5.3227	0.3794	-0.4026	0.0000	-1.4751	1.0000	0.1902	1.0000	-0.3361	0.0308	-0.8788
IGSF21	0.3679	0.9386	0.0395	1.8475	0.7657	-0.8420	1.0000	0.0058	1.0000	-1.0820	1.0000	-0.1690	1.0000	-0.2297
IGSF3	0.0000	1.5526	0.0000	1.7006	0.0000	2.4844	0.0000	2.3980	1.0000	-0.1657	1.0000	-0.0042	0.0160	-0.2455
IGSF5	0.0638	-0.9929	0.0341	-1.0350	0.0948	-0.8877	0.0833	-0.8475	1.0000	-0.2044	1.0000	-0.2333	1.0000	-0.1581
IGSF6	1.0000	0.3477	1.0000	0.1330	0.3656	1.4722	0.9786	-0.9111	1.0000	0.2648	1.0000	0.0597	0.2640	-2.1145
IGSF9	0.0001	-1.0232	0.0012	-0.5453	0.0959	0.4502	0.0000	0.7491	1.0000	-0.3018	0.8875	0.1874	1.0000	0.0034
IGSF9B	1.0000	0.0037	1.0000	-0.1716	0.0043	2.0015	0.0000	2.7131	1.0000	-0.5955	1.0000	-0.7595	1.0000	0.1232
IK	0.3766	-0.1853	0.9477	-0.0309	0.0006	-0.5359	0.0000	-0.4394	1.0000	-0.1941	1.0000	-0.0273	0.7395	-0.0920
IKBIP	0.9630	-0.0895	0.0610	0.2366	0.9629	-0.0923	0.7190	0.0801	1.0000	0.2815	0.0000	0.6208	0.0059	0.4571
IKBKAP	0.0010	-0.5184	0.0082	-0.2316	0.1990	0.2353	0.0995	0.1498	1.0000	-0.0342	0.0287	0.2647	0.5550	-0.1143
IKBKB	0.0504	0.3567	0.0035	0.3220	1.0000	0.0293	0.1254	-0.2004	1.0000	0.1153	0.8664	0.0938	0.7805	-0.1089
IKBKE	0.0000	-2.1265	0.0000	-1.5473	0.0989	-0.4141	0.0000	-0.6202	1.0000	0.0496	0.0237	0.6439	0.7973	-0.1508
IKZF1	1.0000	0.0000	1.0000	0.0000	1.0000	0.0000	1.0000	0.0000	1.0000	0.0000	1.0000	0.0000	1.0000	0.0000
IKZF2	0.5141	0.4500	0.2545	0.5447	1.0000	-0.1582	0.7328	-0.3123	1.0000	-0.0449	1.0000	0.0615	1.0000	-0.1965
IKZF3	0.1719	-1.7573	0.0179	-2.7299	0.0045	-5.5929	0.2095	-1.3304	1.0000	0.1132	1.0000	-0.8494	0.3369	4.4316
IKZF4	0.4920	-0.4413	0.8969	-0.1710	0.1255	0.6019	0.0833	0.6202	1.0000	0.0501	0.8512	0.3320	1.0000	0.0768
IKZF5	0.9472	-0.0761	0.4228	-0.1036	0.0000	0.7843	0.0000	0.5717	1.0000	0.1363	0.7401	0.1217	0.9409	-0.0712
IL10	0.3148	-4.2689	1.0000	-0.8617	0.6970	0.9273	1.0000	0.4723	1.0000	0.1761	0.8785	3.7233	1.0000	-0.2729
IL10RA	1.0000	0.1682	1.0000	0.3936	0.3445	-1.0441	0.7864	0.5786	1.0000	-0.5742	1.0000	-0.3423	0.7425	1.0527

IL10RB	0.0000	0.9300	0.0000	0.8412	0.0040	-0.7717	0.0000	-0.8683	1.0000	0.1284	1.0000	0.0518	1.0000	0.0393
IL11RA	0.4419	-0.3740	0.1696	-0.4126	1.0000	0.0400	0.1393	0.3775	1.0000	-0.1028	1.0000	-0.1304	0.9934	0.2416
IL12A	1.0000	-2.4776	1.0000	0.0000	1.0000	0.6746	1.0000	0.0000	1.0000	-2.3757	1.0000	0.0000	1.0000	-3.1317
IL12B	1.0000	0.0000	1.0000	0.0000	1.0000	0.0000	1.0000	0.0000	1.0000	0.0000	1.0000	0.0000	1.0000	0.0000
IL13RA1	0.2644	0.7308	1.0000	-0.0596	0.8788	-0.4067	0.8377	0.3351	1.0000	-0.0696	0.3396	-0.8450	0.6087	0.6776
IL13RA2	0.0005	-2.1739	0.0227	-0.7454	1.0000	-0.1260	0.0006	0.8667	1.0000	0.3283	0.0005	1.7738	0.0000	1.3274
IL15	0.8226	-3.3425	1.0000	-0.1452	1.0000	-0.1789	1.0000	0.8547	1.0000	-0.9587	1.0000	2.3479	1.0000	0.0727
IL16	0.0051	1.2921	0.0029	0.8920	1.0000	0.0154	0.0233	-0.9847	1.0000	0.7395	0.7064	0.3551	1.0000	-0.2519
IL17B	1.0000	-2.4776	1.0000	2.2507	1.0000	-2.4056	1.0000	0.0000	1.0000	-2.3757	1.0000	2.3456	1.0000	0.0000
IL17C	0.9650	-0.3508	0.1480	-1.5119	0.1669	-1.2697	0.0516	-2.0206	1.0000	-0.3327	0.4482	-1.4877	0.8616	-1.0807
IL17RA	0.0000	1.5180	0.0000	1.6145	0.0000	1.4639	0.0000	1.2448	1.0000	0.1813	0.3424	0.2905	1.0000	-0.0325
IL17RC	0.1315	-0.3426	0.5522	0.0920	0.0001	-0.7442	1.0000	-0.0261	1.0000	-0.3712	1.0000	0.0758	0.0066	0.3530
IL17RD	0.2720	0.4828	0.3784	0.2132	0.0000	1.1729	0.0000	0.9655	1.0000	0.5858	0.6427	0.3309	0.0638	0.3855
IL17RE	1.0000	0.0639	0.0562	0.5897	0.0450	-0.8004	0.5792	-0.3054	1.0000	-0.4964	1.0000	0.0396	1.0000	-0.0005
IL17REL	0.6628	-1.2857	0.0128	-2.9159	0.7803	0.6513	0.1245	0.9427	1.0000	1.6830	1.0000	0.0603	0.0003	1.9894
IL18	0.0248	-0.4630	0.0030	-0.3080	1.0000	0.0264	0.2783	-0.1363	1.0000	-0.2283	1.0000	-0.0606	0.0011	-0.3857
IL18BP	0.0005	1.5133	0.0000	1.3246	0.0007	-2.0868	0.0000	-2.4087	1.0000	0.3975	0.9967	0.2168	1.0000	0.0791
IL18R1	0.9859	-0.2201	0.1037	-0.8453	0.2752	0.6418	0.3227	0.5483	1.0000	0.1409	0.9888	-0.4743	1.0000	0.0527
IL18RAP	0.4304	1.5391	0.2526	2.1622	1.0000	-1.0167	1.0000	-2.3200	1.0000	-0.9587	1.0000	-0.3419	1.0000	-2.2908
IL1R2	0.3793	-1.0743	0.0005	-2.1885	0.3178	0.7869	0.9801	-0.2042	1.0000	0.8030	1.0000	-0.2933	1.0000	-0.1798
IL1RAP	0.2796	0.4285	0.5814	0.1346	0.0000	1.0615	0.0000	1.0221	1.0000	0.3977	1.0000	0.1175	0.0392	0.3642
IL1RAPL1	1.0000	0.0000	1.0000	0.0000	1.0000	0.0000	1.0000	0.0000	1.0000	0.0000	1.0000	0.0000	1.0000	0.0000
IL1RAPL2	0.9545	-0.0802	0.4383	-0.1374	0.9173	-0.0920	0.0658	-0.2584	1.0000	0.0062	1.0000	-0.0373	0.6242	-0.1546
IL1RL1	1.0000	0.2164	1.0000	-0.3410	1.0000	-0.3225	0.0055	1.6682	1.0000	-0.2761	0.8269	-0.8251	0.0323	1.7371
IL1RN	1.0000	0.0000	1.0000	0.0000	1.0000	2.2472	1.0000	0.0000	1.0000	0.0000	1.0000	0.0000	1.0000	-2.2907
IL20RA	0.0628	-1.3935	0.0388	-0.9360	0.3502	-0.7027	0.0047	-1.2706	1.0000	0.5705	0.5052	1.0417	1.0000	0.0039
IL20RB	0.6881	1.7543	0.3421	1.5203	0.9468	1.2126	1.0000	0.5373	1.0000	0.7365	1.0000	0.5246	1.0000	0.0738
IL21	1.0000	0.0000	1.0000	0.0000	1.0000	0.0000	1.0000	2.3257	1.0000	0.0000	1.0000	0.0000	1.0000	2.3554
IL21R	0.9545	1.0644	0.2535	-4.1743	0.2892	2.1431	0.6172	-1.7586	1.0000	1.6470	0.8607	-3.6719	0.5105	-2.2502
IL22	1.0000	0.0000	1.0000	0.0000	1.0000	0.0000	1.0000	0.0000	1.0000	0.0000	1.0000	0.0000	1.0000	0.0000
IL22RA1	0.0001	-2.0415	0.0000	-2.9368	0.4513	-0.4386	0.0001	-1.3934	1.0000	0.2781	0.9689	-0.6055	0.3971	-0.6730
IL22RA2	0.8249	3.0141	1.0000	0.2167	0.8033	3.0790	1.0000	0.0065	1.0000	3.6423	1.0000	0.9743	1.0000	0.6046
IL23A	0.8108	1.1326	0.5029	-1.6425	1.0000	-0.1821	1.0000	0.0070	1.0000	1.3548	0.9185	-1.4135	0.7916	1.5501
IL26	1.0000	0.0000	1.0000	0.0000	1.0000	0.0000	1.0000	0.0000	1.0000	0.0000	1.0000	0.0000	1.0000	0.0000
IL2RB	1.0000	2.1849	0.7666	-3.2534	1.0000	0.0000	1.0000	-0.8416	1.0000	3.1126	1.0000	-2.2956	1.0000	2.3554
IL2RG	1.0000	0.5464	0.6215	1.6074	0.3910	2.1681	0.6142	1.7721	1.0000	-0.1078	1.0000	0.9709	1.0000	-0.4863
IL31RA	0.0049	5.5592	0.2920	3.9943	0.0949	4.5529	0.7701	3.1732	1.0000	0.0000	0.4324	-1.5817	0.7908	-1.3992
IL34	0.0000	-0.9244	0.0000	-0.7387	0.0000	-1.1696	0.0000	-0.9084	1.0000	-0.3789	0.7760	-0.1795	0.9847	-0.1104
IL4I1	1.0000	0.0000	1.0000	0.0000	1.0000	0.0000	1.0000	0.0000	1.0000	0.0000	1.0000	0.0000	1.0000	0.0000
IL4R	0.2876	-0.2259	0.3290	-0.1277	0.8337	0.0923	0.0001	0.3453	1.0000	-0.1222	1.0000	-0.0117	0.4773	0.1363
IL5RA	1.0000	0.0435	1.0000	0.1346	0.0564	2.0391	0.6327	0.9639	1.0000	0.2623	1.0000	0.3662	0.6865	-0.8074
IL6	0.3907	0.5129	0.0094	0.8281	0.0167	-1.5221	0.0718	-0.7340	1.0000	0.3152	0.1921	0.6432	0.1708	1.1142
IL6R	0.4361	-0.3805	0.1417	-0.5043	0.0000	-1.9901	0.1590	-0.5351	1.0000	-0.2148	0.8269	-0.3258	0.0124	1.2468
IL7R	0.7251	1.4394	0.9062	1.2223	1.0000	-2.4056	1.0000	-2.3200	1.0000	-0.1034	1.0000	-0.3235	1.0000	0.0000
IL8	0.0081	0.5985	0.0000	0.8887	0.0117	-0.6626	0.0033	-0.4563	1.0000	0.1022	0.0081	0.4038	0.3128	0.3144
ILDR1	1.0000	2.1902	0.7674	3.0888	1.0000	2.2428	1.0000	0.0000	1.0000	0.0000	1.0000	0.8962	1.0000	-2.2888
ILDR2	1.0000	0.1248	0.2019	1.6179	0.1471	1.5958	0.0006	2.7825	1.0000	-0.8154	1.0000	0.6890	0.9809	0.3684
ILF2	0.0008	-0.4843	0.0000	-0.5510	0.0000	-0.6324	0.0000	-0.5993	1.0000	0.0128	1.0000	-0.0417	1.0000	0.0513
ILF3	0.6221	0.1617	0.0000	0.5464	0.1811	0.3074	0.0000	0.5272	1.0000	-0.2951	0.8269	0.1015	0.9760	-0.0700
ILK	0.0059	-0.4103	0.0000	-0.3779	0.0001	-0.5881	0.0000	-0.3676	1.0000	-0.1053	1.0000	-0.0606	0.5416	0.1205
ILKAP	0.0382	-0.3490	0.0000	-0.4060	0.0000	-0.7982	0.0000	-0.5959	1.0000	-0.0736	0.6726	-0.1187	0.5419	0.1343
IMMP1L	0.8403	-0.1352	0.7309	0.1157	0.1320	-0.4270	0.0468	-0.3892	1.0000	-0.2004	1.0000	0.0651	0.8937	-0.1572
IMMP2L	0.0719	-0.8781	0.0358	-0.7249	0.0408	-0.9726	0.0024	-0.9879	1.0000	-0.1880	1.0000	-0.0206	1.0000	-0.1949
IMMT	0.0024	-0.4700	0.0020	-0.3422	0.0195	-0.3802	0.0001	-0.4008	1.0000	0.0267	0.3764	0.1666	1.0000	0.0114
IMP3	0.0000	-0.8125	0.0000	-0.4201	0.0000	-1.0680	0.0000	-0.4425	1.0000	-0.2833	0.6277	0.1219	0.0283	0.3487
IMP4	0.0014	-0.4841	0.0033	-0.2879	0.0005	-0.5860	0.0000	-0.3374	1.0000	-0.1594	1.0000	0.0492	0.8620	0.0952
IMPA1	0.0000	0.9021	0.0000	0.7418	0.0877	-0.3355	0.0000	-0.5612	1.0000	0.1129	1.0000	-0.0348	0.8133	-0.1080
IMPA2	1.0000	0.0239	0.0388	-0.1917	0.0000	-0.7126	0.0000	-0.6392	1.0000	0.0511	0.5080	-0.1518	0.5537	0.1301
IMPACT	1.0000	-0.0390	0.0068	-0.4499	0.5253	-0.2426	0.0262	-0.3834	1.0000	0.1025	0.4632	-0.2973	1.0000	-0.0325
IMPAD1	0.4202	0.1840	0.9365	0.0436	0.0029	-0.5248	0.0000	-0.4712	1.0000	-0.0316	0.4321	-0.1602	1.0000	0.0259
IMPDH2	0.0000	-0.9224	0.0000	-0.7539	0.0000	-0.6808	0.0000	-0.5722	1.0000	-0.1782	1.0000	0.0027	0.8924	-0.0642
IMPG1	0.8249	-3.3411	0.7674	3.0957	1.0000	-0.1742	1.0000	0.0000	1.0000	-3.2289	1.0000	3.2003	1.0000	-3.1370
IMPG2	0.0069	-0.9442	0.0000	-1.3686	0.3291	-0.4404	0.1445	-0.3333	1.0000	0.0056	0.5382	-0.4048	1.0000	0.1154
INA	0.0000	-2.6029	0.0000	-2.1860	0.0705	-0.4770	0.0001	-0.5707	1.0000	-0.0559	0.7603	0.3743	0.8705	-0.1428
INCENP	0.0000	1.0471	0.0000	1.4353	0.0338	0.4222	1.0000	0.0288	1.0000	-0.2357	0.7215	0.1640	0.0000	-0.6240
INF2	0.3212	-0.2772	0.3596	0.1356	0.0000	1.0268	0.0000	0.7932	1.0000	0.2598	0.0000	0.6866	1.0000	0.0329
ING1	1.0000	-0.0552	0.0296	-0.2730	0.0134	-0.5449	0.0000	-0.5784	1.0000	0.1528	1.0000	-0.0520	0.7939	0.1254
ING2	0.2592	0.4218	0.9225	0.0940	1.0000	-0.0588	0.0006	-0.7191	1.0000	0.3393	1.0000	0.0231	0.4790	-0.3170
ING3	1.0000	0.0420	0.3773	-0.1395	0.5077	-0.2096	0.1681	-0.1885	1.0000	0.0809	1.0000	-0.0889	0.8937	0.1080
ING4	0.0641	-0.3414	0.0968	-0.2002	0.7564	-0.1108	0.4077	-0.1048	1.0000	-0.0421	0.8723	0.1115	1.0000	-0.0308
ING5	0.0000	0.8681	0.0000	0.7460	0.0019	0.5814	0.0000	0.5855	1.0000	0.0976	1.0000	-0.0124	0.7908	0.1066
INHA	0.0000	-5.3464	0.0000	-6.1154	0.0000	1.2051	0.0000	1.1805	1.0000	-0.0320	1.0000	-0.7977	1.0000	-0.0531
INHBA	0.7408	-0.1823	0.5727	0.1257	0.0001	-1.2644	0.0012	-0.4944	1.0000	0.2402	0.0004	0.5585	0.0000	1.0168
INHBB	1.0000	-0.3195	1.0000	-1.0008	1.0000	0.7355	1.0000	0.0066	1.0000	-0.1179	1.0000	-0.7977	1.0000	-0.8435
INHBC	1.0000	-2.4788	1.0000	0.0000	1.0000	-2.4061	1.0000	0.0000	1.0000	-2.3771	1.0000	0.0000	1.0000	0.0000
INIP	1.0000	-0.0597	0.8892	-0.0452	0.0674	-0.3907	0.0000	-0.4713	1.0000	-0.0962	1.0000	-0.0699	0.4031	-0.1710
INO80	0.6374	0.1400	0.7635	0.0637	0.3684	0.2004	0.0011	0.3043	1.0000	0.0909	1.0000	0.0276	0.1665	0.2002
INO80B	0.0000	-0.8512	0.0014	-0.4304	0.0000	-0.9659	0.0000	-0.7524	1.0000	-0.2523	0.7577	0.1810	1.0000	-0.0308
INO80C	0.0789	-0.4430	0.0000	-0.5655	1.0000	-0.0197	0.9873	-0.0476	1.0000	0.2140	1.0000	0.1051	0.5116	0.1904
INO80D	0.8691	-0.2085	0.1950	-0.4099	0.0977	0.6369	0.0759	0.4592	1.0000	0.1528	1.0000	-0.0379	1.0000	-0.0226
INPP1	0.0138	-0.8169	0.3093	-0.3294	0.0874	-0.5940	0.0031	0.6576	1.0000	-0.6294	1.0000	-0.1265	0.0256	0.6269
INPP4A	0.0674	0.3556	0.7263	-0.0729	0.5723	0.1635	0.0000	0.5527	1.0000	0.2695	0.5245	-0.1458	0.0000	0.6635
INPP4B	0.0169	-1.1922	0.0000	-1.7397	1.0000	-0.0557	0.0008	-1.0623	1.0000	-0.3186	0.3051	-0.8538	0.0001	-1.3229
INPP5A	0.0872	0.5875	0.0034	0.6468	0.0002	1.0714	0.0023	0.6381	1.0000	0.2182	0.6533	0.2910	0.7248	-0.2115
INPP5B	0.0040	0.4879	0.1066	0.1803	1.0000	0.0546	0.4146	-0.1132	1.0000	0.2340	1.0000	-0.0618	0.9880	0.0709
INPP5D	0.8249	-3.3411	0.7674	-3.2570	1.0000	0.7314	1.0000	0.0061	1.0000	-0.1113	1.0000	0.0000	1.0000	-0.8419
INPP5E	1.0000	0.0441	0.4632	0.1100	0.1588	0.2654	0.6455	0.0829	1.0000	0.0352	0.7407	0.1127	0.4325	-0.1429
INPP5F	0.8021	0.1009	0.9831	-0.0379	0.0253	0.3905	0.0108	0.2580	1.0000	0.0882	1.0000	-0.0374	1.0000	-0.0388
INPP5J	0.0002	-1.7182	0.0009	-1.1341	0.0159	-1.0795	1.0000	-0.1000	1.0000	-0.4502	1.0000	0.1451	0.5079	0.5347
INPP5K	0.0000	0.7503	0.0000	0.7077	0.8011	0.1144	1.0000	0.0360	1.0000	0.0170	1.0000	-0.0132	1.0000	-0.0575
INS	1.0000	0.0000	1.0000	0.0000	1.0000	0.0000	1.0000	0.0000	1.0000	0.0000	1.0000	0.0000	1.0000	0.0000
INSC	1.0000	0.0000	1.0000	0.0000	1.0000	0.0000	1.0000	0.0000	1.0000	0.0000	1.0000	0.0000	1.0000	0.0000
INSIG1	0.0000	0.7527	0.0000	0.6903	0.0000	0.8095	0.0000	0.5656	1.0000	-0.0767	0.3997	-0.1269	0.0007	-0.3151

INSIG2	0.6932	0.1293	0.9742	-0.0386	0.0018	0.5388	0.0000	0.4051	1.0000	0.1961	1.0000	0.0407	0.9809	0.0666
INSL5	0.6467	0.2526	1.0000	-0.0261	0.0000	-1.6128	0.0000	-1.2318	1.0000	0.0693	0.9238	-0.1977	0.6595	0.4560
INSR	0.0010	0.7226	0.0000	0.9800	0.0000	1.3748	0.0000	1.1857	1.0000	-0.1356	0.8314	0.1328	0.0487	-0.3216
INSRR	0.4687	0.3985	0.3651	0.4124	0.0000	-1.9716	0.0737	-0.8280	1.0000	-0.6577	0.1932	-0.6310	0.8088	0.4901
INTS1	0.1504	0.2626	0.0552	0.1968	0.0758	0.3123	0.0004	0.3207	1.0000	-0.0373	0.8607	-0.0911	1.0000	-0.0238
INTS10	0.9366	0.0920	0.5683	0.1102	0.0007	-0.7517	0.0000	-0.7002	1.0000	0.0285	1.0000	0.0593	1.0000	0.0856
INTS12	0.0304	0.4968	0.0442	0.2924	0.4841	-0.2240	0.0010	-0.5034	1.0000	0.2873	1.0000	0.0965	1.0000	0.0149
INTS2	0.8952	0.1123	0.1089	0.1437	1.0000	0.0524	1.0000	-0.0085	1.0000	-0.2585	0.0596	-0.2145	0.0012	-0.3130
INTS4	0.7974	0.1095	0.3171	0.1342	0.1881	0.2688	0.0722	0.2011	1.0000	0.0626	0.8899	0.1000	1.0000	0.0015
INTS5	0.9545	0.1386	0.0000	1.1663	0.0019	0.8698	0.0000	1.5824	1.0000	-0.9193	1.0000	0.1204	0.7350	-0.1989
INTS6	0.0000	0.7685	0.0000	0.4191	0.7139	-0.1284	0.0414	-0.2140	1.0000	0.2878	1.0000	-0.0494	0.2199	0.2063
INTS7	0.8249	0.1074	0.0155	0.3015	0.8082	-0.1122	0.0039	-0.3471	1.0000	0.0338	0.2501	0.2404	0.4598	-0.1964
INTS8	0.8848	0.0853	0.9797	-0.0342	1.0000	-0.0370	0.5875	-0.0781	1.0000	0.0268	0.9773	-0.0799	1.0000	-0.0081
INTS9	1.0000	-0.0592	0.0433	-0.2310	0.5638	-0.1551	0.0777	-0.1820	1.0000	0.0498	0.8732	-0.1095	1.0000	0.0273
INTU	0.0659	0.5772	0.4669	0.2047	0.0214	0.6475	0.0018	0.6998	1.0000	0.1466	0.8232	-0.2121	0.7991	0.2043
IP6K1	0.0019	0.5362	0.0000	0.5591	0.5667	-0.1643	0.0011	0.3413	1.0000	-0.1315	0.8607	-0.0957	0.0013	0.3809
IP6K2	0.0056	0.4660	0.0003	0.3350	0.2329	0.2408	1.0000	0.0308	1.0000	0.1869	1.0000	0.0680	1.0000	-0.0181
IP6K3	0.0000	-3.6331	0.0000	-3.7592	0.0034	-0.4633	0.0000	-0.7556	1.0000	-0.0524	0.9773	-0.1661	0.0017	-0.3397
IPCEF1	0.0001	1.4839	0.3833	0.3425	0.7840	-0.3636	0.0000	-2.0636	1.0000	0.7838	0.6439	-0.3462	0.3837	-0.9101
IPMK	0.6985	0.1437	0.2952	0.1574	0.4472	0.2088	0.0239	0.3134	1.0000	0.0387	1.0000	0.0656	0.6865	0.1486
IPO13	0.7885	0.1234	0.7198	-0.0827	0.9598	0.0814	1.0000	0.0267	1.0000	-0.1014	0.0668	-0.2946	0.6417	-0.1488
IPO5	0.0256	0.3722	0.0000	0.3826	1.0000	-0.0146	0.2556	-0.1046	1.0000	-0.1658	0.3106	-0.1432	0.0118	-0.2502
IPO8	0.8271	0.0962	0.9371	0.0428	0.1774	0.2819	1.0000	0.0187	1.0000	0.0464	1.0000	0.0058	0.2351	-0.2117
IPP	0.8226	0.1487	0.3062	0.2113	0.0676	0.4761	0.0011	0.4525	1.0000	0.0050	1.0000	0.0792	1.0000	-0.0159
IPPK	0.0001	1.7052	0.0025	0.9836	0.0000	2.3428	0.0002	1.0456	1.0000	1.0966	0.6497	0.3896	0.9437	-0.1955
IQCA1	1.0000	2.1902	1.0000	2.2534	0.8033	3.0892	1.0000	2.3257	1.0000	0.0000	1.0000	0.0489	1.0000	-0.7804
IQCB1	0.6411	0.1604	0.7338	-0.0708	0.0000	-1.0120	0.0000	-0.7919	1.0000	0.1842	1.0000	-0.0340	0.0169	0.4095
IQCC	0.4399	-0.2457	0.0002	-0.5890	0.0256	-0.5330	0.0072	-0.4315	1.0000	-0.1223	0.0515	-0.4536	1.0000	-0.0166
IQCD	1.0000	-0.0985	1.0000	0.6940	1.0000	-0.4958	0.6172	1.7719	1.0000	-2.1898	0.9094	-1.4169	1.0000	0.0763
IQCE	0.0003	1.9021	0.0113	1.0038	0.0010	1.7267	0.0344	0.8930	1.0000	0.8598	1.0000	-0.0268	1.0000	0.0271
IQCG	1.0000	-0.1254	0.1135	-0.9634	0.1008	0.9494	1.0000	-0.0482	1.0000	0.4759	1.0000	-0.3496	0.7040	-0.5176
IQCH	1.0000	0.1178	0.0624	0.6985	0.5935	-0.3426	0.4071	0.4191	1.0000	-0.5959	1.0000	-0.0038	1.0000	0.1668
IQCJ	1.0000	0.0000	1.0000	0.0000	1.0000	0.0000	1.0000	0.0000	1.0000	0.0000	1.0000	0.0000	1.0000	0.0000
IQCK	0.6353	0.2211	0.7666	-0.1085	0.0000	-1.4188	0.0000	-1.7650	1.0000	0.0016	0.4452	-0.3142	0.6196	-0.3379
IQGAP1	0.0000	0.8169	0.0000	0.9023	0.0000	0.7423	0.0000	0.5648	1.0000	0.0474	0.2620	0.1453	0.3549	-0.1246
IQGAP2	0.0131	-0.4579	0.0001	-0.4222	0.0149	0.4800	0.0000	0.9576	1.0000	0.3038	0.0061	0.3530	0.0000	0.7864
IQSEC1	0.7015	-0.1185	0.0001	-0.3667	0.9058	-0.0776	0.8018	0.0492	1.0000	0.1150	0.7355	-0.1205	0.0404	0.2472
IQSEC3	0.5378	3.5350	0.4660	1.8951	0.8011	3.0840	1.0000	-2.3200	1.0000	2.2737	1.0000	0.7481	1.0000	-3.1344
IQUB	0.0000	-1.3244	0.0000	-1.4381	0.0037	-0.7770	0.0000	-1.0958	1.0000	-0.2775	0.3730	-0.3788	0.0141	-0.5904
IRAK1BP1	0.3050	-0.3423	0.0001	-0.6829	0.0025	-0.8068	0.0000	-1.2749	1.0000	0.2722	1.0000	-0.0551	0.8876	-0.1885
IRAK2	0.0022	0.4870	0.0000	0.4961	1.0000	-0.0270	0.1635	-0.1427	1.0000	0.0816	0.7364	0.1030	1.0000	-0.0296
IRAK4	0.0007	0.6045	0.0053	0.3060	0.1151	-0.3376	0.0000	-0.5248	1.0000	0.1317	0.5515	-0.1537	1.0000	-0.0493
IREB2	0.2537	-0.2268	0.4744	-0.0916	0.1893	0.2457	0.2114	0.1233	1.0000	0.0313	0.3382	0.1787	0.7968	-0.0861
IRF1	0.8249	-0.1659	0.0000	-0.8622	0.2558	0.4085	1.0000	0.0197	1.0000	0.6009	1.0000	-0.0843	0.6600	0.2180
IRF2	0.8035	-0.1152	0.2970	-0.1514	0.2857	-0.2489	0.1023	-0.2125	1.0000	-0.1027	0.7701	-0.1254	1.0000	-0.0595
IRF2BP2	0.0000	-0.7482	0.0000	-0.8446	0.0000	-1.0983	0.0000	-0.7710	1.0000	0.0279	1.0000	-0.0564	0.0015	0.3612
IRF2BPL	0.4670	0.1716	0.0068	0.2496	0.7662	0.1061	0.2025	0.1328	1.0000	-0.0418	1.0000	0.0489	1.0000	-0.0093
IRF4	0.5384	3.5543	0.5793	-1.3282	1.0000	2.2428	0.3356	-1.6813	1.0000	4.7948	1.0000	0.0544	1.0000	0.9164
IRF5	0.8885	-0.4395	0.2307	-0.7871	1.0000	-0.1972	0.7350	-0.3672	1.0000	0.3967	1.0000	0.0596	1.0000	0.2302
IRF6	1.0000	-0.3485	0.9148	-1.5280	1.0000	-0.1922	0.7772	0.9510	1.0000	-0.8200	0.8354	-2.0132	1.0000	0.3292
IRF7	1.0000	0.0232	1.0000	0.0673	0.3536	-0.3683	1.0000	0.0169	1.0000	0.0469	1.0000	0.1024	0.1697	0.4378
IRF8	0.1170	0.3823	0.0000	0.7918	1.0000	-0.0401	0.8780	0.0751	1.0000	-0.0636	0.0344	0.3572	1.0000	0.0573
IRG1	0.0519	-1.9035	0.1491	-1.2609	0.2297	-1.1849	0.7921	-0.5112	1.0000	-0.5947	1.0000	0.0584	1.0000	0.0803
IRS1	0.2601	0.5119	0.0002	0.9585	0.0001	1.2846	0.0004	0.9229	1.0000	-0.0636	0.4531	0.3978	0.2427	-0.4167
IRS2	0.0425	-1.5926	0.0268	-1.2145	0.6017	-0.5502	0.7167	-0.3564	1.0000	-0.2232	1.0000	0.1613	1.0000	-0.0247
IRS4	0.0000	0.8280	0.0000	0.7379	0.0002	0.5872	0.0000	0.4894	1.0000	0.0997	1.0000	0.0222	1.0000	0.0071
IRX1	1.0000	0.0000	1.0000	-1.0017	1.0000	0.0000	0.7701	-3.1666	1.0000	3.1126	1.0000	2.3456	1.0000	0.0000
IRX4	1.0000	0.2135	0.4694	3.6125	0.2785	-4.1857	1.0000	2.3257	1.0000	-4.1514	1.0000	-0.8889	1.0000	2.3554
ISCA1	1.0000	0.1915	1.0000	-0.1694	1.0000	-0.0877	0.2653	-0.8067	1.0000	0.0341	0.9921	-0.3150	0.4920	-0.6854
ISCA2	0.0164	-0.4098	0.0000	-0.6057	0.0001	-0.6771	0.0000	-0.5191	1.0000	0.1650	1.0000	-0.0179	0.0146	0.3279
ISCU	0.2847	0.2283	0.0033	0.2525	0.0000	-0.6963	0.0000	-0.6106	1.0000	-0.1388	0.7290	-0.1023	1.0000	-0.0473
ISG20L2	0.2432	-0.3115	0.7371	-0.0978	0.1116	-0.3942	0.2536	-0.2107	1.0000	-0.1639	1.0000	0.0621	1.0000	0.0255
ISL1	1.0000	0.0000	1.0000	0.0000	1.0000	2.2472	1.0000	0.0000	1.0000	0.0000	1.0000	0.0000	1.0000	-2.2907
ISM1	0.6229	1.0597	0.0065	-3.4546	0.8954	0.5880	0.9578	-0.3988	1.0000	1.4006	0.1253	-3.1172	1.0000	0.4233
ISM2	0.0001	-1.1738	0.0000	-1.1250	0.3332	0.3274	0.0001	0.5412	1.0000	0.1582	0.8046	0.2223	0.0086	0.3790
ISPD	0.0244	0.5175	0.0001	0.5033	0.1007	-0.4205	0.0087	-0.4250	1.0000	0.0484	1.0000	0.0467	1.0000	0.0498
IST1	0.1931	-0.2274	0.0094	-0.2160	0.0056	-0.4223	0.0010	-0.2716	1.0000	-0.0885	0.9619	-0.0648	0.8841	0.0674
ISX	0.8249	3.0141	1.0000	0.0000	1.0000	0.0000	1.0000	0.0000	1.0000	0.0000	1.0000	-3.1383	1.0000	0.0000
ISY1	0.9650	0.0630	1.0000	-0.0006	0.0220	-0.3863	0.0000	-0.4836	1.0000	0.0251	1.0000	-0.0261	0.9691	-0.0666
ITCH	1.0000	-0.0120	0.5185	-0.1033	0.1362	0.3025	0.7253	0.0827	1.0000	0.1235	1.0000	0.0446	0.9342	-0.0914
ITFG2	0.8256	-0.1055	0.0312	-0.2371	0.3466	-0.2336	0.0081	-0.2990	1.0000	0.0537	1.0000	-0.0650	1.0000	-0.0050
ITFG3	0.1036	-0.3010	1.0000	-0.0214	0.0165	0.3975	0.0000	0.4658	1.0000	0.0438	0.0027	0.3354	0.4981	0.1168
ITGA11	1.0000	0.0000	0.7666	-3.2534	1.0000	2.2472	0.7546	1.2230	1.0000	3.1126	1.0000	0.0000	0.7287	2.1333
ITGA4	0.0113	0.5199	0.0001	0.4223	1.0000	-0.0421	0.0035	0.3202	1.0000	0.2692	0.3275	0.1852	0.0000	0.6375
ITGA6	0.0457	0.3068	0.0000	0.4958	0.0000	0.8206	0.0000	0.5508	1.0000	0.0772	0.0003	0.2788	0.0444	-0.1872
ITGA7	0.0050	-0.7517	0.0000	-0.7994	0.0000	-1.5520	0.0000	-2.0741	1.0000	-0.1148	1.0000	-0.1511	0.0494	-0.6333
ITGA9	0.4123	0.8413	0.2547	1.0161	0.0000	3.2207	0.0000	2.5853	1.0000	-0.1267	1.0000	0.0591	0.0552	-0.7629
ITGAV	0.0000	1.6489	0.0000	1.6409	0.0000	2.5074	0.0000	1.8506	1.0000	0.2832	0.2184	0.2889	0.0167	-0.3685
ITGB1	0.1918	0.2261	0.0000	0.2940	0.0000	0.8270	0.0000	0.7440	1.0000	0.0647	0.2545	0.1452	1.0000	-0.0129
ITGB1BP1	0.4104	-0.2129	0.0232	-0.3161	0.0013	-0.6644	0.0000	-0.5431	1.0000	-0.1297	0.3468	-0.2208	1.0000	-0.0026
ITGB1BP2	0.0000	-1.2762	0.0000	-1.5509	0.0000	-1.7828	0.0000	-1.6050	1.0000	-0.0549	0.1246	-0.3176	0.8042	0.1287
ITGB2	0.0002	-4.0909	0.0000	-2.8197	0.6533	-0.6356	0.0055	-1.2259	1.0000	0.4556	0.7635	1.7464	1.0000	-0.1290
ITGB3	0.0000	-6.7250	0.0000	-6.2831	0.0000	-1.3660	0.0000	-1.0969	1.0000	-0.3987	1.0000	0.0586	1.0000	-0.1241
ITGB3BP	0.0010	0.8352	0.0000	1.0309	0.7475	0.1798	0.0791	-0.3813	1.0000	-0.1636	1.0000	0.0434	0.0009	-0.7198
ITGB4	1.0000	0.0000	0.7674	-3.2498	1.0000	2.2472	0.7710	-3.1632	1.0000	3.1056	1.0000	0.0000	1.0000	-2.2907
ITGB5	0.8060	0.2040	0.0021	0.5311	0.0036	0.9056	0.0000	1.4945	1.0000	0.0702	0.0512	0.4122	0.0000	0.6663
ITGB6	0.1856	-4.5762	0.4643	-3.7945	0.0018	2.3770	0.0129	2.4163	1.0000	-0.8115	1.0000	0.0000	0.5139	-0.7754
ITGB8	0.1360	-1.0055	0.0033	-1.8981	0.0000	2.2767	0.0000	2.7012	1.0000	-0.5478	0.2620	-1.4303	1.0000	-0.1163
ITIH2	0.0254	0.6768	0.0000	1.7054	0.0470	-0.7091	0.9117	-0.1294	1.0000	-0.3891	0.0017	0.6517	1.0000	0.1961
ITIH5	0.0015	1.6805	0.0014	1.2742	0.2899	-1.1009	0.9593	-0.2416	1.0000	-0.3917	0.0387	-0.7834	0.9557	0.4676
ITK	0.0271	1.7022	0.0000	2.8021	0.1921	-2.7131	0.7731	-0.9370	1.0000	-0.3403	0.3445	0.7669	1.0000	1.4483
ITM2A	0.1931	-0.2312	0.0000	-0.3235	0.1210	0.2604	0.0000	0.3283	1.0000	0.1779	0.7456	0.0979	0.0125	0.2509
ITM2B	0.0000	0.8468	0.0000	0.9919	0.1312	0.3527	0.0000	0.5775	1.0000	0.1472	0.0585	0.3046	0.0032	0.3762

ITM2C	0.7355	-0.1485	0.6850	-0.0994	0.8079	-0.1198	0.9287	0.0667	1.0000	0.0114	1.0000	0.0727	0.5656	0.2026
ITPA	0.0115	-0.5835	0.0042	-0.3471	0.0000	-1.0752	0.0000	-0.8371	1.0000	-0.2264	1.0000	0.0222	1.0000	0.0187
ITPK1	0.0000	-2.0706	0.0000	-2.2167	0.0000	-1.2170	0.0000	-1.2552	1.0000	0.0030	0.8625	-0.1309	1.0000	-0.0308
ITPKA	0.8083	-0.0934	0.7569	-0.0556	0.0100	0.4149	0.0014	0.2753	1.0000	0.0088	1.0000	0.0594	0.4972	-0.1254
ITPKB	0.0001	-0.7815	0.0000	-0.8590	1.0000	-0.0703	0.5740	0.1044	1.0000	-0.0755	0.8607	-0.1418	0.9035	0.1045
ITPR1	0.0001	0.6398	0.0000	0.7705	0.0000	0.6701	0.0000	0.5466	1.0000	-0.0271	0.7635	0.1164	0.4393	-0.1455
ITPR2	0.0288	0.8307	0.0001	0.8307	0.0620	0.7223	0.1725	0.3652	1.0000	0.2639	0.5611	0.2725	1.0000	-0.0913
ITPR3	0.0000	-2.0490	0.0000	-2.1948	0.9357	0.0712	0.0000	-0.5359	1.0000	0.0780	1.0000	-0.0556	0.0000	-0.5242
ITPRIP	0.0390	0.4668	0.0341	0.4603	0.0000	1.1333	0.0000	1.0509	1.0000	0.0474	1.0000	0.0540	1.0000	-0.0271
ITSN1	0.0048	0.6790	0.0000	0.7071	0.0000	1.4609	0.0000	1.4474	1.0000	0.1419	0.7582	0.1834	0.7287	0.1345
ITSN2	0.0030	0.6742	0.0000	0.7169	0.0002	0.7799	0.0000	0.8071	1.0000	-0.0265	1.0000	0.0279	1.0000	0.0049
IVD	0.0000	-1.2387	0.0000	-1.2074	0.9649	-0.0742	0.5067	0.1255	1.0000	-0.0345	1.0000	0.0093	0.4901	0.1690
IVNS1ABP	1.0000	0.0242	0.7560	0.0498	1.0000	0.0208	0.7039	0.0542	1.0000	-0.0245	1.0000	0.0137	1.0000	0.0144
IWS1	1.0000	-0.0206	0.6755	-0.0595	0.1554	-0.2541	0.0007	-0.2741	1.0000	0.0029	1.0000	-0.0237	1.0000	-0.0120
IYD	0.0000	-1.5433	0.0000	-1.8604	0.2434	0.3982	0.0592	0.4592	1.0000	-0.4670	0.3713	-0.7730	0.1707	-0.4009
JADE1	0.0000	1.1456	0.0000	1.0817	0.5503	0.1769	0.3394	0.1578	1.0000	-0.0273	0.9773	-0.0781	1.0000	-0.0406
JADE3	0.5745	-0.1453	1.0000	-0.0017	0.2432	0.2313	0.0090	0.4843	1.0000	0.0087	0.3874	0.1648	1.0000	0.0248
JAG1	0.0002	1.4593	0.0004	0.8092	0.0000	2.0526	0.0000	1.4180	1.0000	0.9297	0.7173	0.2919	0.4097	0.2999
JAG2	0.1575	0.2501	0.0000	0.3435	0.3812	0.1794	0.0128	-0.2034	1.0000	0.0872	0.0998	0.1932	0.0039	-0.2902
JAGN1	0.2789	-0.2373	0.6418	-0.0905	0.0000	-0.8710	0.0000	-0.4783	1.0000	-0.0712	1.0000	0.0875	0.1553	0.3279
JAK2	0.4106	0.2629	0.2732	0.1928	0.0120	0.6037	0.0000	0.7033	1.0000	-0.0438	1.0000	-0.0999	1.0000	0.0612
JAK3	0.4227	1.5394	1.0000	-0.1640	1.0000	0.3474	1.0000	0.0071	1.0000	0.7940	1.0000	-0.8974	1.0000	0.4627
JAKMIP2	0.4414	-0.2951	0.0153	-0.5258	0.0000	1.4774	0.0000	1.4383	1.0000	0.0200	0.8688	-0.1959	1.0000	-0.0129
JAKMIP3	1.0000	-0.2881	1.0000	2.2506	1.0000	-2.4056	1.0000	2.3257	1.0000	-2.3757	1.0000	0.0470	1.0000	2.3554
JAM2	0.0606	0.4320	0.0412	0.2910	0.3604	0.2539	0.0083	0.3625	1.0000	0.0102	0.8759	-0.1190	0.7853	0.1226
JAM3	0.2659	-0.1998	0.0078	-0.1814	0.7499	0.0981	0.0350	-0.1449	1.0000	-0.0082	1.0000	0.0224	0.0080	-0.2458
JARID2	0.0000	1.9475	0.0000	1.7244	0.0030	0.6951	0.0060	0.5333	1.0000	0.1398	1.0000	-0.0680	1.0000	-0.0161
JDP2	0.0040	-0.6043	0.0025	-0.4338	0.4166	0.2123	0.0640	0.2585	1.0000	-0.0665	0.9920	0.1182	1.0000	-0.0125
JKAMP	0.2460	0.3088	0.0002	0.4532	0.9204	0.0991	1.0000	0.0356	1.0000	-0.1927	1.0000	-0.0372	0.2266	-0.2513
JMJD1C	0.9087	-0.0783	0.2908	-0.1399	0.0000	0.8162	0.0000	0.8541	1.0000	0.0001	1.0000	-0.0485	1.0000	0.0436
JMJD4	1.0000	-0.0488	0.4487	-0.1036	0.5177	0.1575	0.3940	0.1074	1.0000	0.0064	1.0000	-0.0351	1.0000	-0.0379
JMJD6	0.0003	-0.6908	0.0000	-0.6059	0.2582	0.2497	0.7748	0.0589	1.0000	-0.0245	1.0000	0.0729	0.1574	-0.2100
JMJD7	0.8338	-0.1317	0.1627	0.2417	0.0000	-1.1393	0.0000	-0.9972	1.0000	0.0700	0.0166	0.4551	0.7541	0.2175
JMJD8	0.0353	-0.4375	0.0000	-0.5379	0.0041	-0.6963	0.0123	-0.3035	1.0000	0.0142	1.0000	-0.0748	0.0902	0.4126
JMY	0.2123	0.4893	0.0001	1.0758	0.0414	0.6839	0.0000	1.0623	1.0000	-0.6124	1.0000	-0.0163	0.7548	-0.2299
JOSD1	0.1104	0.3063	0.0305	0.2180	0.6556	0.1359	0.0640	0.1914	1.0000	-0.0252	0.8269	-0.1008	1.0000	0.0365
JPH2	0.0000	-2.2369	0.0000	-1.9786	0.0417	0.7267	0.0031	0.6031	1.0000	-0.1432	1.0000	0.1321	0.4925	-0.2620
JUN	0.3874	-0.1813	0.2785	-0.1127	0.1559	0.2617	0.0000	0.4515	1.0000	0.0010	0.9002	0.0821	0.1773	0.1966
JUND	0.0000	-0.7383	0.0000	-0.8131	0.0000	-1.2378	0.0000	-0.8525	1.0000	-0.1336	0.1705	-0.1962	0.1916	0.2576
JUP	0.6415	0.1169	0.2809	0.0959	0.1238	0.2572	0.0000	0.2743	1.0000	-0.0103	1.0000	-0.0188	1.0000	0.0123
KAL1	1.0000	2.1849	0.6182	1.6074	0.0080	5.5129	0.0010	3.7068	1.0000	2.2736	1.0000	1.8196	1.0000	0.4887
KANK1	0.1678	0.6654	0.0628	0.4587	0.0002	1.2871	0.0000	1.0139	1.0000	0.2793	1.0000	0.0861	1.0000	0.0109
KANK3	0.0026	-0.5161	0.0000	-0.3694	0.1203	-0.3044	0.1878	-0.1076	1.0000	-0.0613	0.7320	0.0977	0.3446	0.1407
KANK4	0.0000	-1.5961	0.0000	-1.4146	0.5750	-0.2024	0.0000	0.6045	1.0000	0.1961	0.0827	0.3920	0.0000	1.0101
KANSL1	0.5358	0.2305	0.0111	0.4212	0.1158	0.4319	0.0000	0.7997	1.0000	-0.1863	1.0000	0.0173	0.5773	0.1897
KANSL1L	0.2164	-0.2664	0.0013	-0.3381	0.5881	0.1526	0.0458	0.2081	1.0000	0.1166	1.0000	0.0579	0.3153	0.1772
KANSL3	0.7355	-0.1115	0.8737	-0.0430	0.0024	-0.5175	0.0002	-0.3311	1.0000	-0.1320	1.0000	-0.0514	1.0000	0.0597
KARS	0.0652	0.3060	0.0000	0.3497	0.0070	-0.4251	0.0000	-0.3893	1.0000	-0.0777	1.0000	-0.0217	1.0000	-0.0362
KAT2A	0.3130	-0.2721	1.0000	-0.0277	1.0000	0.0463	0.0051	0.3636	1.0000	-0.3305	1.0000	-0.0736	1.0000	-0.0065
KAT2B	0.0013	0.5851	0.0000	0.4838	0.0028	0.5508	0.0000	0.6153	1.0000	0.1626	1.0000	0.0735	0.1368	0.2320
KAT6A	0.0001	1.0377	0.0000	1.0588	0.0000	2.0385	0.0000	1.9267	1.0000	-0.0164	1.0000	0.0199	0.7117	-0.1201
KAT6B	0.0105	0.7029	0.0097	0.5500	0.0152	0.6880	0.0066	0.5052	1.0000	0.2263	1.0000	0.0876	1.0000	0.0491
KAT7	0.5286	0.1480	0.1616	0.1456	0.9994	-0.0589	0.0142	-0.2413	1.0000	0.1057	0.6375	0.1161	0.9659	-0.0705
KATNA1	0.0391	0.4344	0.0001	0.5892	0.0130	0.4961	0.5618	0.1366	1.0000	-0.0945	1.0000	0.0715	0.0009	-0.4499
KATNAL1	0.0027	0.4917	0.0897	0.1733	1.0000	0.0475	0.1711	0.1477	1.0000	0.1473	0.3939	-0.1589	0.0693	0.2520
KATNAL2	0.6001	-0.2171	0.0353	-0.3663	0.0144	-0.6424	0.0000	-0.6800	1.0000	-0.0448	0.8553	-0.1804	1.0000	-0.0754
KATNB1	1.0000	0.0172	0.6510	0.0991	0.4048	-0.2440	0.4313	0.1335	1.0000	-0.0244	1.0000	0.0686	0.0302	0.3580
KATNBL1	0.6131	-0.1647	0.7984	-0.0622	0.9466	0.0787	0.9194	0.0438	1.0000	0.0221	0.8204	0.1366	1.0000	-0.0085
KAZALD1	0.0000	-2.6803	0.0000	-2.0277	0.0000	0.8922	0.0000	0.7605	1.0000	-0.1878	0.0233	0.4772	0.0050	-0.3148
KAZN	0.7001	0.8343	0.2507	1.1075	0.0057	2.0783	0.1566	1.0592	1.0000	0.5876	0.7913	0.8795	0.9362	-0.4286
KBTBD11	0.1190	-0.5765	0.0330	-0.5433	0.6179	-0.2605	0.0071	0.5410	1.0000	-0.0927	1.0000	-0.0450	0.0025	0.7148
KBTBD12	0.0006	-6.2100	0.0952	-2.8836	0.0117	-3.0359	1.0000	-0.3936	1.0000	-1.1027	1.0000	2.3480	0.7908	1.5487
KBTBD13	0.8249	-3.3439	1.0000	-0.1452	0.3911	1.5005	1.0000	0.0057	1.0000	-0.9587	1.0000	2.3480	0.3615	-2.4641
KBTBD2	0.0087	-0.4364	0.0000	-0.4860	0.0390	0.3499	0.0001	0.3515	1.0000	0.0438	1.0000	0.0072	1.0000	0.0510
KBTBD3	0.7150	0.6274	0.2189	0.8646	0.9468	0.3791	0.4082	0.7333	1.0000	-0.1271	1.0000	0.1195	1.0000	0.2310
KBTBD4	0.1010	-0.3195	0.0904	-0.1913	0.0014	-0.5561	0.0000	-0.4329	1.0000	-0.0466	0.9437	0.0931	0.9689	0.0816
KBTBD8	0.4552	-0.2308	0.6860	-0.0999	0.1850	0.3225	0.6241	0.1049	1.0000	-0.0299	0.9956	0.1143	0.2654	-0.2427
KCMF1	1.0000	0.0000	1.0000	0.0000	1.0000	0.0000	1.0000	0.0000	1.0000	0.0000	1.0000	0.0000	1.0000	0.0000
KCNA1	0.8249	-3.3439	1.0000	2.2506	1.0000	-1.0167	0.7710	3.1753	1.0000	-3.2319	1.0000	2.3456	1.0000	0.9169
KCNA10	1.0000	2.1902	1.0000	-2.3959	1.0000	0.0000	1.0000	-2.3178	1.0000	2.2673	1.0000	-2.2992	1.0000	0.0000
KCNA2	1.0000	-2.4788	1.0000	2.2506	1.0000	-0.1597	0.7701	3.1732	1.0000	-2.3771	1.0000	2.3456	1.0000	0.9164
KCNA4	0.6881	1.7543	1.0000	-0.8596	0.4424	1.8936	0.6886	0.8258	1.0000	1.9501	1.0000	-0.6343	0.9565	0.8961
KCNA5	0.0096	0.8062	0.0011	0.6397	0.0001	1.1294	0.0038	0.5797	1.0000	0.0922	1.0000	-0.0629	0.0601	-0.4516
KCNA6	1.0000	0.5370	1.0000	-2.3986	0.9361	1.2061	0.6172	1.7712	1.0000	-0.1045	1.0000	-3.1383	1.0000	0.4623
KCNAB1	0.0000	-1.2745	0.0000	-1.3366	0.0763	0.4489	0.0000	0.6384	1.0000	0.1572	1.0000	0.1063	0.0730	0.3490
KCNAB2	0.0005	-0.7636	0.0000	-0.7974	0.0052	-0.5781	0.0002	-0.4269	1.0000	0.0021	1.0000	-0.0180	0.6608	0.1600
KCNB1	1.0000	-1.1585	0.6182	1.6074	0.7663	1.0380	1.0000	0.0045	1.0000	-0.9587	1.0000	1.8196	0.6865	-1.9994
KCNB2	1.0000	-0.0974	1.0000	0.6956	1.0000	-0.1886	0.1523	2.5422	1.0000	-2.1881	0.9075	-1.4162	1.0000	0.5416
KCNC1	0.1019	-0.8405	0.0067	-1.0631	0.1889	-0.6501	0.0145	-0.8776	1.0000	0.0375	1.0000	-0.1713	1.0000	-0.1866
KCNC2	0.0201	1.0360	0.0309	0.6909	0.0030	1.1712	0.0027	0.8795	1.0000	0.4435	1.0000	0.1058	1.0000	0.1535
KCNC4	1.0000	0.0000	1.0000	-2.3986	1.0000	0.0000	1.0000	-2.3201	1.0000	2.2736	1.0000	0.0000	1.0000	0.0000
KCNE1	0.1381	-2.8562	1.0000	-0.8686	1.0000	-0.6594	0.7246	-1.2170	1.0000	-0.5899	1.0000	1.4259	0.9835	-1.1475
KCNE2	1.0000	0.3200	0.1750	-1.3643	0.9398	-0.5973	0.0326	-2.1253	1.0000	0.6569	0.7798	-1.0184	1.0000	-0.8666
KCNE3	0.6881	1.7537	0.9062	1.2235	1.0000	-2.4061	1.0000	-2.3178	1.0000	-0.1089	1.0000	-0.6343	1.0000	0.0000
KCNE4	0.8943	-0.3580	0.0001	-2.5525	0.0106	-3.4728	0.0002	-2.6737	1.0000	1.0227	0.7085	-1.1624	0.8939	1.8363
KCNF1	1.0000	0.0000	1.0000	-2.3986	1.0000	2.2428	1.0000	0.0057	1.0000	2.2736	1.0000	0.0000	1.0000	0.0659
KCNG1	1.0000	2.1902	1.0000	0.0000	1.0000	2.2428	1.0000	0.0000	1.0000	0.0000	1.0000	-2.2992	1.0000	-2.2888
KCNG2	1.0000	0.0000	0.7674	3.0957	1.0000	0.0000	1.0000	0.0000	1.0000	0.0000	1.0000	3.2003	1.0000	0.0000
KCNG3	1.0000	2.1902	1.0000	-2.3986	1.0000	0.0000	1.0000	-2.3201	1.0000	2.2736	1.0000	-2.2991	1.0000	0.0000
KCNG4	0.9545	1.0679	0.7666	3.0922	1.0000	-2.4056	1.0000	0.0000	1.0000	-2.3757	1.0000	-0.4752	1.0000	0.0000
KCNH1	1.0000	0.5370	0.7852	0.7768	0.6654	1.5927	1.0000	-0.5236	1.0000	1.2628	0.9257	1.5374	1.0000	-0.8435
KCNH2	0.0002	-1.6771	0.0007	-1.4621	0.2745	-0.5412	0.4257	-0.3922	1.0000	-0.2141	1.0000	0.0145	1.0000	-0.0629
KCNH4	1.0000	2.1902	1.0000	0.0000	0.4809	3.6122	1.0000	0.0000	1.0000	0.0000	1.0000	-2.2991	0.7287	-3.6637

KCNH5	0.5582	-1.7937	0.2603	-0.9300	1.0000	-0.4439	0.1409	-1.2439	1.0000	1.3417	0.3609	2.2437	1.0000	0.5437
KCNH6	0.0000	-1.1001	0.0000	-1.3279	1.0000	0.0076	0.0009	-0.3594	1.0000	-0.0221	0.6878	-0.2378	0.0039	-0.3864
KCNH8	1.0000	0.0000	1.0000	0.0000	1.0000	0.0000	1.0000	0.0000	1.0000	0.0000	1.0000	0.0000	1.0000	0.0000
KCNIP1	0.0824	-0.4895	0.0005	-0.5066	0.0000	-1.7841	0.0000	-2.8189	1.0000	-0.3552	0.0126	-0.3610	0.0000	-1.3850
KCNIP2	0.5086	3.5431	0.3461	1.5213	0.8033	3.0790	1.0000	0.0106	1.0000	3.1056	0.9744	1.2162	1.0000	0.0753
KCNIP4	0.9794	0.4966	0.4787	0.9496	0.9378	0.5168	1.0000	0.0070	1.0000	-0.6837	1.0000	-0.2220	0.6274	-1.1919
KCNJ1	1.0000	0.0000	1.0000	0.0000	1.0000	0.0000	1.0000	0.0000	1.0000	0.0000	1.0000	0.0000	1.0000	0.0000
KCNJ10	1.0000	0.0000	1.0000	-2.3959	1.0000	2.2472	1.0000	-2.3178	1.0000	2.2674	1.0000	0.0000	1.0000	-2.2907
KCNJ11	0.0000	-3.7954	0.0048	-1.7957	0.0639	-0.9658	0.8717	-0.2743	1.0000	-0.8399	0.9217	1.1740	1.0000	-0.1471
KCNJ12	0.9382	0.7752	1.0000	-0.3220	0.0340	2.2787	1.0000	0.2726	1.0000	1.1348	1.0000	0.0599	0.6346	-0.8534
KCNJ13	0.8249	3.0256	1.0000	0.0000	1.0000	0.0000	1.0000	0.0000	1.0000	0.0000	1.0000	-3.1469	1.0000	0.0000
KCNJ15	1.0000	0.0000	1.0000	-0.9997	0.2840	3.9988	0.9801	0.9262	1.0000	3.1056	1.0000	2.3456	1.0000	0.0763
KCNJ16	0.9545	-1.6935	1.0000	-2.3986	1.0000	-0.1868	1.0000	0.8541	1.0000	-1.4918	1.0000	-2.2956	1.0000	-0.4568
KCNJ2	0.7674	-0.2664	0.5461	0.2452	0.0000	2.0015	0.0000	1.8829	1.0000	-0.1290	0.6209	0.3913	0.3698	-0.2460
KCNJ3	0.3089	-4.2696	0.1802	-4.4668	0.0260	1.9826	0.2087	1.3447	1.0000	0.1692	1.0000	0.0000	1.0000	-0.4580
KCNJ4	1.0000	-0.2881	0.4124	-2.2261	0.3084	2.1392	0.4035	-2.0617	1.0000	1.9501	1.0000	0.0489	0.5236	-2.2473
KCNJ5	0.0000	-5.3435	0.0000	-5.6351	1.0000	0.0670	0.1312	-2.2117	1.0000	0.2426	1.0000	-0.0344	1.0000	-0.0298
KCNJ6	1.0000	-2.4776	0.4431	-3.7865	1.0000	-2.4056	0.9096	-1.3717	1.0000	1.2635	1.0000	0.0000	1.0000	2.3554
KCNJ8	0.7172	-0.4372	0.0024	-1.5643	0.0000	2.0627	0.0000	1.7103	1.0000	-0.1764	0.1071	-1.2874	0.0822	-0.5165
KCNK1	0.0003	1.2574	0.0000	0.9850	0.0071	0.9325	1.0000	0.0628	1.0000	-0.1648	0.1125	-0.4296	0.0000	-1.0307
KCNK10	1.0000	-0.3288	1.0000	0.0000	1.0000	0.7306	0.4370	3.7037	1.0000	-3.2319	1.0000	-3.1383	1.0000	-0.3110
KCNK12	1.0000	2.1849	1.0000	0.0000	1.0000	2.2427	1.0000	0.0000	1.0000	0.0000	1.0000	-2.2955	1.0000	-2.2889
KCNK13	0.6236	-0.6387	0.1376	-0.9243	0.5679	0.5819	1.0000	0.0054	1.0000	0.1669	1.0000	-0.1059	0.8652	-0.4091
KCNK16	1.0000	0.0000	1.0000	0.0000	1.0000	0.0000	1.0000	0.0000	1.0000	0.0000	1.0000	0.0000	1.0000	0.0000
KCNK18	1.0000	0.0000	1.0000	0.0000	1.0000	0.0000	1.0000	0.0000	1.0000	0.0000	1.0000	0.0000	1.0000	0.0000
KCNK2	0.8249	-3.3439	1.0000	2.2534	1.0000	-1.0167	1.0000	2.3241	1.0000	-3.2319	1.0000	2.3480	1.0000	0.0641
KCNK3	1.0000	0.0000	1.0000	0.0000	1.0000	0.0000	1.0000	0.0000	1.0000	0.0000	1.0000	0.0000	1.0000	0.0000
KCNK5	0.0070	1.7281	0.0058	1.4183	0.0003	2.3454	0.0000	1.8811	1.0000	0.0954	1.0000	-0.2091	0.9057	-0.3738
KCNK9	1.0000	-2.4788	1.0000	0.0000	1.0000	-0.1630	1.0000	0.0000	1.0000	-2.3771	1.0000	0.0000	1.0000	-2.2888
KCNMA1	1.0000	-0.0149	0.2542	-0.1779	0.0000	1.5754	0.0000	1.4475	1.0000	0.3580	0.5213	0.2081	0.0593	0.2361
KCNMB1	0.8784	-0.1827	0.1192	-0.4814	0.6581	0.2512	1.0000	-0.0080	1.0000	0.1373	1.0000	-0.1519	1.0000	-0.1201
KCNMB2	0.0443	0.6777	0.0172	0.5422	0.8730	0.1759	0.6341	-0.2029	1.0000	0.2910	0.9681	0.1702	1.0000	-0.0805
KCNMB4	0.8905	-0.6012	0.1711	-1.5777	0.5560	-1.6619	0.0532	-2.3149	1.0000	0.7194	1.0000	-0.2435	1.0000	0.0730
KCNN2	1.0000	0.1208	1.0000	-0.0361	1.0000	0.1361	0.4355	-0.4168	1.0000	0.1775	1.0000	0.0327	0.7908	-0.3726
KCNQ1	1.0000	0.0000	1.0000	-2.3959	1.0000	0.0000	1.0000	0.0062	1.0000	2.2674	1.0000	0.0000	1.0000	2.3543
KCNQ2	1.0000	0.0000	1.0000	0.0000	1.0000	0.0000	1.0000	0.0000	1.0000	0.0000	1.0000	0.0000	1.0000	0.0000
KCNQ3	1.0000	0.0000	1.0000	2.2506	1.0000	0.0000	1.0000	0.0000	1.0000	0.0000	1.0000	2.3456	1.0000	0.0000
KCNQ5	1.0000	0.3262	0.0599	1.3794	0.1590	1.2363	0.0587	1.3724	1.0000	-0.1294	0.5437	0.9353	1.0000	0.0137
KCNRG	0.9427	-0.2118	0.2201	-0.5322	0.9655	-0.2017	0.3705	-0.4133	1.0000	0.1522	1.0000	-0.1546	1.0000	-0.0524
KCNS1	1.0000	0.0000	1.0000	-0.1429	1.0000	0.0000	1.0000	0.0076	1.0000	2.2674	1.0000	2.3480	1.0000	2.3554
KCNS2	1.0000	-0.2881	0.1388	-4.4811	0.6601	1.5906	0.3949	-2.0636	1.0000	1.9536	1.0000	-2.2991	0.8937	-1.6947
KCNS3	1.0000	0.0000	1.0000	0.0000	1.0000	0.0000	1.0000	0.0000	1.0000	0.0000	1.0000	0.0000	1.0000	0.0000
KCNT1	0.0000	-0.9098	0.0000	-1.0301	0.0663	-0.3831	0.0000	-0.3524	1.0000	-0.1002	0.1990	-0.2080	0.9950	-0.0652
KCNU1	1.0000	2.1902	1.0000	0.0000	0.8033	3.0892	0.7710	3.1753	1.0000	0.0000	1.0000	-2.2991	1.0000	0.0717
KCNV1	1.0000	0.0000	1.0000	2.2534	1.0000	0.0000	0.7710	3.1708	1.0000	0.0000	1.0000	2.3480	1.0000	3.2048
KCNV2	1.0000	-0.3236	1.0000	-2.3959	1.0000	-1.0167	1.0000	0.8545	1.0000	-0.9637	1.0000	-3.1430	1.0000	0.9164
KCTD10	0.0001	0.8339	0.0000	1.4722	0.0000	1.5391	0.0000	1.5740	1.0000	-0.1597	0.0004	0.4923	0.6742	-0.1182
KCTD14	1.0000	0.0000	1.0000	0.0000	1.0000	0.0000	1.0000	0.0000	1.0000	0.0000	1.0000	0.0000	1.0000	0.0000
KCTD15	0.0423	0.4229	0.6467	0.1020	1.0000	0.0459	0.6482	0.0990	1.0000	0.0895	0.3768	-0.2174	0.6093	0.1480
KCTD16	1.0000	2.1849	1.0000	2.2534	0.0919	4.5509	0.4370	3.7041	1.0000	0.0000	1.0000	0.0515	1.0000	-0.8668
KCTD17	1.0000	2.1849	1.0000	-0.1598	0.0535	4.7653	0.0154	2.6722	1.0000	3.1126	1.0000	0.9035	0.6700	1.0552
KCTD18	0.1853	0.3927	0.0166	0.4151	0.0086	-0.7718	0.0041	-0.5487	1.0000	-0.1331	1.0000	-0.0983	1.0000	0.0932
KCTD19	1.0000	2.1902	0.2542	4.0037	0.5080	3.6209	0.2831	4.0829	1.0000	0.0000	1.0000	1.8175	1.0000	0.4541
KCTD2	0.8591	-0.0754	0.8740	0.0357	0.1459	-0.2460	0.0010	-0.2323	1.0000	-0.0257	0.7183	0.0978	1.0000	-0.0066
KCTD20	0.6557	0.1154	0.2000	0.1159	0.3024	-0.1978	0.0076	-0.2103	1.0000	-0.0281	1.0000	-0.0154	1.0000	-0.0352
KCTD21	0.5086	-0.3426	0.6824	-0.1840	0.0001	1.0270	0.0000	1.0613	1.0000	-0.0849	1.0000	0.0859	1.0000	-0.0486
KCTD3	0.2197	-0.2247	0.0000	-0.4191	0.1483	-0.2562	0.0031	-0.2281	1.0000	0.0694	0.6345	-0.1125	0.6226	0.1027
KCTD4	1.0000	2.1902	1.0000	-2.3986	1.0000	0.0000	1.0000	-2.3201	1.0000	2.2736	1.0000	-2.2991	1.0000	0.0000
KCTD6	0.4306	-0.2179	0.0416	-0.2908	0.0038	-0.5876	0.0008	-0.4522	1.0000	0.0827	1.0000	0.0208	0.3734	0.2232
KCTD7	0.4073	-0.3132	0.4438	0.1998	1.0000	0.0926	0.1141	0.3335	1.0000	-0.2573	0.6450	0.2687	1.0000	-0.0127
KCTD8	1.0000	2.1849	1.0000	0.0000	1.0000	0.0000	1.0000	0.0000	1.0000	0.0000	1.0000	-2.2955	1.0000	0.0000
KCTD9	0.0150	0.4198	0.0002	0.3872	0.0602	-0.3639	0.0726	-0.2118	1.0000	0.0242	1.0000	0.0036	0.3821	0.1799
KDELC1	0.0055	0.8269	0.0000	0.9183	0.0000	1.6661	0.0000	1.4818	1.0000	0.0595	0.9383	0.1639	0.9886	-0.1195
KDELC2	0.0013	0.5249	0.0000	0.7465	0.1954	0.2532	0.3137	0.1348	1.0000	-0.1449	0.8607	0.0883	0.0435	-0.2582
KDELR2	0.0026	-0.4374	0.0000	-0.3447	1.0000	-0.0181	0.6734	0.0553	1.0000	0.0375	0.2712	0.1427	0.4196	0.1162
KDELR3	0.0600	0.3977	0.0593	0.2344	0.0000	0.8327	0.0000	0.7524	1.0000	0.0966	1.0000	-0.0552	1.0000	0.0211
KDF1	0.5619	1.3148	1.0000	0.5243	1.0000	0.5042	1.0000	0.0072	1.0000	-0.1232	0.9306	-0.9059	1.0000	-0.6157
KDM1A	0.4328	-0.1710	0.4822	-0.0760	0.0562	-0.3317	0.0003	-0.2808	1.0000	-0.0297	0.8915	0.0777	1.0000	0.0258
KDM1B	1.0000	0.0792	0.3346	-0.2161	0.1614	0.3639	0.0958	0.2960	1.0000	0.1858	1.0000	-0.0985	0.8995	0.1203
KDM2A	0.9013	-0.0896	1.0000	-0.0154	1.0000	-0.0247	0.6618	0.0794	1.0000	-0.0346	1.0000	0.0527	0.9317	0.0759
KDM2B	0.6754	-0.1347	1.0000	-0.0255	0.1328	0.2934	0.0004	0.3632	1.0000	-0.0432	1.0000	0.0784	1.0000	0.0318
KDM3A	1.0000	-0.0500	0.0598	-0.1683	0.0002	0.5634	0.0000	0.3425	1.0000	0.1068	1.0000	0.0009	0.6241	-0.1091
KDM4A	0.2854	0.2216	0.3110	0.1220	0.8033	0.0980	0.2722	0.1245	1.0000	0.0457	1.0000	-0.0419	0.8939	0.0767
KDM4B	0.0380	0.3844	0.0031	0.2846	0.1174	0.3041	0.0114	0.2487	1.0000	0.1689	0.9676	0.0814	0.6386	0.1188
KDM5B	0.0481	-0.3703	0.0000	-0.4261	1.0000	0.0290	0.0002	0.2993	1.0000	0.0591	1.0000	0.0163	0.0006	0.3344
KDM7A	1.0000	-0.0419	0.4651	-0.4273	0.0000	2.0074	0.0000	1.5409	1.0000	0.1799	1.0000	-0.1899	0.7200	-0.2779
KDM8	0.8941	-0.1744	0.0212	-0.6075	0.1635	-0.5176	0.0902	-0.4417	1.0000	0.0447	0.6375	-0.3770	1.0000	0.1220
KDSR	0.0450	0.3851	0.0000	0.4775	0.8991	0.0954	1.0000	-0.0058	1.0000	0.1621	0.0510	0.2672	1.0000	0.0653
KEAP1	0.0000	-0.9724	0.0001	-0.5096	1.0000	0.0604	0.0000	0.6365	1.0000	-0.2782	0.6465	0.1981	0.0553	0.3044
KEL	1.0000	-2.4788	1.0000	-2.3959	1.0000	0.6817	1.0000	0.8545	1.0000	-0.1089	1.0000	0.0000	1.0000	0.0716
KERA	1.0000	-2.4776	1.0000	0.0000	1.0000	-2.4056	0.4623	3.6988	1.0000	-2.3757	1.0000	0.0000	0.7455	3.7355
KHDRBS1	1.0000	0.0435	0.1353	0.2100	0.0019	0.6086	0.0000	0.5322	1.0000	0.0023	0.6102	0.1808	1.0000	-0.0694
KHDRBS3	0.8252	0.1075	0.9213	-0.0363	0.0019	-0.6053	0.0000	-0.3576	1.0000	-0.0198	0.3778	-0.1514	0.0471	0.2335
KHK	0.6927	1.4466	0.7674	3.0957	1.0000	0.6816	1.0000	0.0000	1.0000	-2.3771	1.0000	-0.8584	1.0000	-3.1370
KIAA0040	1.0000	-0.0680	0.8824	0.1314	0.6128	-0.2736	0.2219	-0.3950	1.0000	-0.1786	1.0000	0.0313	0.7561	-0.2950
KIAA0100	0.9611	-0.0714	0.4180	0.1107	0.5842	-0.1569	0.0017	0.2939	1.0000	-0.2836	0.9238	-0.0897	0.3434	0.1725
KIAA0141	0.0746	0.7466	0.0014	0.8921	1.0000	0.1292	0.1525	0.5019	1.0000	-0.1074	1.0000	0.0506	0.8400	0.2737
KIAA0195	0.0000	-0.6790	0.0000	-0.7795	0.4744	-0.1596	0.0007	-0.3178	1.0000	0.0808	1.0000	-0.0073	0.8937	-0.0725
KIAA0196	0.6371	-0.1335	0.9214	-0.0428	0.5967	0.1402	1.0000	0.0095	1.0000	0.0366	0.5718	0.1406	0.8331	-0.0878
KIAA0226	0.8428	0.2000	0.0620	0.4034	1.0000	0.0251	0.0226	0.3994	1.0000	0.0560	0.6328	0.2722	0.0570	0.4353
KIAA0226L	0.1362	2.2197	0.9699	0.4609	0.2647	1.6876	1.0000	0.0067	1.0000	1.5688	1.0000	-0.1730	1.0000	-0.1077
KIAA0232	0.0354	0.4404	0.0056	0.4858	0.0000	1.4636	0.0000	1.1640	1.0000	-0.0097	1.0000	0.0489	0.0893	-0.3036
KIAA0319	0.0731	0.4584	0.4890	0.1157	0.0048	-0.6854	0.0000	-0.9317	1.0000	0.0685	0.2745	-0.2617	0.6942	-0.1726

KIAA0319L	0.1532	0.2607	0.0100	0.2248	0.5929	0.1374	0.2459	-0.1230	1.0000	0.0987	0.9318	0.0751	0.3530	-0.1565
KIAA0368	0.5975	0.1317	0.5529	0.0928	0.3106	0.2035	0.3964	0.1250	1.0000	-0.0269	1.0000	-0.0530	0.7701	-0.1005
KIAA0391	0.0088	-0.6199	0.0070	-0.3946	0.2648	0.2892	0.0136	0.3135	1.0000	-0.0960	0.9270	0.1407	1.0000	-0.0676
KIAA0408	0.0318	-0.6180	0.0553	-0.4212	0.7029	0.1813	0.1104	0.3307	1.0000	-0.0415	1.0000	0.1685	1.0000	0.1129
KIAA0430	0.0037	0.4768	0.0040	0.2952	0.0004	0.5952	0.0002	0.3738	1.0000	0.1309	1.0000	-0.0377	0.8877	-0.0851
KIAA0513	0.0024	-1.1728	0.0000	-1.5738	1.0000	-0.0597	0.0522	0.4703	1.0000	0.0249	0.9311	-0.3635	0.0638	0.5583
KIAA0556	0.0009	0.9816	0.0061	0.6676	0.0000	1.1957	0.0018	0.7673	1.0000	0.2619	1.0000	-0.0354	0.9237	-0.1593
KIAA0586	0.1149	-0.6128	0.4770	0.1382	0.9604	0.1263	0.0004	0.4389	1.0000	0.2986	0.0000	1.0649	0.0000	0.6166
KIAA0753	0.0003	1.2618	0.0000	1.1855	0.3322	0.4808	0.3642	0.3285	1.0000	0.2793	0.8316	0.2164	1.0000	0.1310
KIAA0895	0.0033	0.8473	0.0000	1.0128	0.0453	0.6111	0.0004	0.7962	1.0000	-0.4747	0.4515	-0.2986	0.4772	-0.2872
KIAA0895L	0.0324	-0.7182	0.0009	-0.8390	0.1881	-0.4449	0.2702	0.3102	1.0000	-0.1674	0.8803	-0.2760	0.0536	0.5905
KIAA0907	0.0372	0.5669	0.0000	0.6351	0.0000	0.8743	0.0000	0.8308	1.0000	0.0469	1.0000	0.1278	1.0000	0.0088
KIAA0922	1.0000	-0.0147	0.4749	-0.1023	0.3305	-0.2191	0.9399	-0.0422	1.0000	0.0482	1.0000	-0.0275	0.1363	0.2290
KIAA0930	0.3425	0.3839	0.0000	0.9273	0.0118	0.7430	0.0007	0.6976	1.0000	-0.3095	0.6869	0.2464	0.2058	-0.3478
KIAA1024	1.0000	-2.4788	1.0000	0.0000	1.0000	0.6780	1.0000	0.0000	1.0000	-2.3771	1.0000	0.0000	1.0000	-3.1344
KIAA1024L	1.0000	0.0000	1.0000	0.0000	1.0000	0.0000	1.0000	0.0000	1.0000	0.0000	1.0000	0.0000	1.0000	0.0000
KIAA1033	0.2122	0.8636	0.0004	1.7900	0.0000	2.8344	0.0000	2.6565	1.0000	-0.2730	0.2344	0.6714	0.1385	-0.4305
KIAA1045	1.0000	0.0000	1.0000	0.0000	1.0000	2.2472	1.0000	0.0000	1.0000	0.0000	1.0000	0.0000	1.0000	-2.2907
KIAA1107	0.0861	-0.3685	0.0001	-0.4595	1.0000	0.0591	0.4217	-0.1201	1.0000	0.2862	0.4687	0.2076	0.8523	0.1101
KIAA1109	0.1920	0.2644	0.0274	0.2290	0.0571	0.3458	0.0153	0.2327	1.0000	0.0719	1.0000	0.0496	1.0000	-0.0356
KIAA1143	0.0000	0.7065	0.0000	0.5111	0.0000	-0.7289	0.0000	-0.9278	1.0000	0.2328	1.0000	0.0500	1.0000	0.0386
KIAA1147	0.0093	0.5335	1.0000	-0.0098	0.1338	-0.3711	0.0053	-0.3511	1.0000	0.2003	0.0419	-0.3304	0.4008	0.2238
KIAA1161	0.1082	0.2949	0.0019	0.2844	0.0263	-0.3963	0.0000	-0.4270	1.0000	0.0794	0.9035	0.0808	1.0000	0.0533
KIAA1191	0.5383	-0.2357	0.0313	-0.2556	0.3227	-0.3193	0.0015	-0.3644	1.0000	0.3057	0.1012	0.2986	0.1753	0.2663
KIAA1210	0.3583	-0.3539	0.0001	-0.7488	0.0000	1.4165	0.0000	1.1847	1.0000	0.3790	1.0000	-0.0039	0.6819	0.1511
KIAA1211	1.0000	0.0134	0.9785	-0.0665	0.1158	-0.4376	1.0000	-0.0064	1.0000	-0.0508	1.0000	-0.1162	0.1798	0.3871
KIAA1211L	0.8226	3.0199	1.0000	-0.6969	1.0000	2.2427	0.4370	-3.6973	1.0000	3.6423	1.0000	0.0533	1.0000	-2.2888
KIAA1217	0.0002	-0.7543	0.0000	-0.9571	0.0000	1.0845	0.0000	0.6733	1.0000	0.1635	1.0000	-0.0269	0.0552	-0.2420
KIAA1279	0.1010	0.2851	0.0186	0.2041	0.1310	-0.2731	0.0000	-0.3870	1.0000	0.2159	0.3921	0.1476	0.6654	0.1075
KIAA1324	0.6011	-0.5362	0.5654	0.6223	0.8231	-0.3140	0.0247	1.3597	1.0000	-1.2366	1.0000	-0.0672	0.8013	0.4460
KIAA1324L	0.4139	-0.8558	1.0000	-0.0550	0.0174	1.3120	0.0001	1.9501	1.0000	-0.7450	1.0000	0.0636	1.0000	-0.1057
KIAA1328	0.0007	0.9964	0.0000	1.2920	0.0000	1.2551	0.0000	1.2342	1.0000	-0.0433	0.5610	0.2683	1.0000	-0.0559
KIAA1377	1.0000	0.1621	1.0000	-0.0770	1.0000	0.3032	1.0000	0.0048	1.0000	0.7497	1.0000	0.5283	1.0000	0.4514
KIAA1407	1.0000	0.0861	0.7674	-0.1406	0.0003	0.9074	0.0015	0.6606	1.0000	0.0119	0.9129	-0.2011	0.6097	-0.2293
KIAA1429	0.4106	0.1869	0.4655	-0.0956	0.5834	0.1396	0.6221	-0.0694	1.0000	0.2085	1.0000	-0.0617	1.0000	0.0048
KIAA1456	0.9650	1.0733	1.0000	-0.6953	1.0000	0.6780	0.9096	-1.3726	1.0000	1.2654	1.0000	-0.4781	1.0000	-0.7795
KIAA1462	0.0002	0.7064	0.0000	0.8405	0.8196	0.1106	1.0000	-0.0174	1.0000	0.1195	0.0153	0.2654	1.0000	-0.0048
KIAA1467	1.0000	-0.0744	0.6506	-0.1151	0.8467	0.1193	0.8607	-0.0756	1.0000	-0.0320	1.0000	-0.0601	0.4376	-0.2221
KIAA1468	0.1666	0.2915	0.1436	0.1626	1.0000	0.0331	0.0385	-0.2212	1.0000	0.1231	1.0000	0.0068	0.6564	-0.1247
KIAA1522	1.0000	0.1083	0.6742	0.2173	0.1313	0.5443	0.0891	0.4959	1.0000	-0.2239	1.0000	-0.0990	0.6149	-0.2637
KIAA1524	0.0000	1.2150	0.0000	1.5204	0.0001	0.8026	0.1723	0.2113	1.0000	-0.1724	0.6107	0.1436	0.0000	-0.7586
KIAA1549L	0.9009	0.3353	1.0000	-0.0285	1.0000	-0.0339	0.7529	-0.4544	1.0000	0.0350	1.0000	-0.3144	1.0000	-0.3811
KIAA1551	0.0650	0.4075	0.1998	0.1961	0.0214	0.4750	0.0011	0.4003	1.0000	0.0405	0.6890	-0.1582	1.0000	-0.0275
KIAA1644	0.5086	3.5456	1.0000	-1.0027	1.0000	0.0000	0.7710	-3.1692	1.0000	3.1191	1.0000	-1.3257	1.0000	0.0000
KIAA1671	0.0000	-0.9659	0.0000	-1.1418	0.0000	1.0799	0.0000	0.8611	1.0000	0.1928	1.0000	0.0294	1.0000	-0.0203
KIAA1715	0.0297	0.5645	0.0000	0.6698	0.0002	0.8816	0.0012	0.5418	1.0000	0.1044	0.5246	0.2242	0.4393	-0.2270
KIAA1755	0.8210	-0.3367	0.7616	-0.2479	0.1768	-0.9003	0.0879	0.6211	1.0000	-0.1581	1.0000	-0.0630	0.0015	1.3728
KIAA1919	0.0036	0.5296	0.0000	0.6070	0.6927	0.1382	1.0000	-0.0355	1.0000	0.0078	0.9192	0.0971	0.5075	-0.1615
KIAA2013	0.0030	0.4403	0.0000	0.3776	1.0000	-0.0381	0.0868	-0.1435	1.0000	0.0915	1.0000	0.0410	1.0000	-0.0088
KIAA2018	0.1054	0.8392	0.0053	1.0084	0.0000	2.2008	0.0000	2.2065	1.0000	-0.0846	1.0000	0.0993	1.0000	-0.0755
KIAA2022	0.9108	0.2212	1.0000	-0.1288	0.0263	0.9308	0.1254	0.5371	1.0000	0.1574	1.0000	-0.1779	0.8939	-0.2303
KIAA2026	0.5308	-0.1889	0.0015	-0.4196	1.0000	-0.0021	0.8855	0.0565	1.0000	0.1062	0.9862	-0.1113	0.4643	0.1699
KIDINS220	0.0017	1.5970	0.0000	1.5810	0.0000	3.2928	0.0000	2.4814	1.0000	0.3306	0.9052	0.3309	0.0088	-0.4727
KIF11	0.0000	1.3559	0.0000	2.0846	0.0000	0.8030	0.0001	0.5688	1.0000	-0.3589	0.0414	0.3811	0.0000	-0.5892
KIF12	0.7955	1.1406	1.0000	-0.1633	1.0000	-0.1793	1.0000	0.0081	1.0000	0.3999	1.0000	-0.8861	1.0000	0.6029
KIF13A	0.1176	-0.2679	0.0026	-0.2349	0.2807	0.2009	0.6628	0.0564	1.0000	0.0479	0.7812	0.0934	0.7068	-0.0915
KIF13B	0.0000	-1.9184	0.0000	-1.8609	0.0002	0.5801	0.0000	0.4124	1.0000	-0.1175	1.0000	-0.0473	0.0039	-0.2796
KIF14	0.0000	1.0588	0.0000	1.5699	0.4320	0.2328	1.0000	-0.0137	1.0000	-0.2342	0.0333	0.2885	0.0005	-0.4760
KIF15	0.0000	1.4824	0.0000	1.9109	0.0000	1.4445	0.0000	0.7897	1.0000	-0.2214	0.1744	0.2183	0.0000	-0.8710
KIF16B	1.0000	0.0524	0.2697	0.1723	1.0000	-0.0462	0.7797	-0.0810	1.0000	-0.1019	1.0000	0.0310	0.6616	-0.1312
KIF17	0.0015	-2.2494	0.0002	-1.8825	1.0000	0.1639	0.2490	0.5124	1.0000	0.0431	1.0000	0.4283	0.6425	0.3974
KIF18A	0.0109	0.7499	0.0000	1.3420	0.1378	0.4802	0.1228	0.4223	1.0000	-0.4464	0.9572	0.1588	0.1074	-0.4957
KIF18B	0.8636	0.1019	0.0028	0.3878	1.0000	-0.0132	0.5769	0.1091	1.0000	-0.2837	1.0000	0.0140	0.6001	-0.1560
KIF19	0.0294	-3.4900	0.0594	-3.0507	0.5430	-0.8351	1.0000	-0.3456	1.0000	-0.4070	1.0000	0.0470	1.0000	0.0800
KIF1B	1.0000	0.0302	1.0000	0.0031	0.0000	1.9034	0.0000	1.4099	1.0000	0.1019	1.0000	0.0875	0.0091	-0.3872
KIF20A	0.0000	1.3379	0.0000	1.8032	0.0000	1.3971	0.0000	0.9274	1.0000	-0.2781	0.5980	0.1992	0.0000	-0.7437
KIF21A	0.0000	1.5842	0.0000	1.4764	0.0222	0.6825	0.8206	0.1203	1.0000	0.2235	0.9339	0.1276	0.4058	-0.3348
KIF21B	1.0000	0.0420	0.0415	-1.3350	0.0040	1.4719	0.0003	1.2943	1.0000	0.7632	0.9254	-0.6022	0.1887	0.5972
KIF23	0.0000	1.0269	0.0000	1.0979	0.0000	1.1549	0.0000	0.6432	1.0000	-0.0775	1.0000	0.0048	0.0000	-0.5848
KIF24	0.0000	-1.5847	0.0000	-1.5386	0.0062	-0.4254	0.0000	-0.8084	1.0000	0.1469	0.1761	0.2056	0.0343	-0.2310
KIF25	0.0034	0.7276	0.0000	0.7634	0.0000	-1.7193	0.0000	-1.7780	1.0000	-0.2152	0.8042	-0.1662	0.9382	-0.2700
KIF26A	0.0000	-6.4855	0.0000	-5.5894	1.0000	-0.0478	0.0000	0.6306	1.0000	0.5245	0.4439	1.4394	0.0000	1.2057
KIF26B	1.0000	0.2187	0.9461	0.3962	1.0000	0.1187	1.0000	-0.3903	1.0000	0.8210	0.8609	1.0280	1.0000	0.3278
KIF27	0.2568	0.9095	1.0000	0.0998	0.0002	2.1129	0.0000	1.5392	1.0000	0.5792	1.0000	-0.2185	1.0000	0.0030
KIF2B	1.0000	0.0000	1.0000	0.0000	1.0000	0.0000	1.0000	0.0000	1.0000	0.0000	1.0000	0.0000	1.0000	0.0000
KIF2C	0.0000	0.8574	0.0000	1.2024	0.0312	0.4018	1.0000	0.0368	1.0000	-0.1793	0.3309	0.1769	0.0000	-0.5397
KIF3A	0.0462	0.3742	0.0000	0.3796	0.9084	0.0839	0.8525	-0.0515	1.0000	0.0695	0.8845	0.0869	1.0000	-0.0613
KIF3B	0.9278	-0.0861	1.0000	-0.0358	0.3008	0.2380	0.0163	0.2773	1.0000	-0.0333	1.0000	0.0296	1.0000	0.0111
KIF3C	1.0000	-0.0137	0.3436	-0.2609	0.0151	0.6510	0.1680	0.3114	1.0000	-0.0215	0.7644	-0.2573	0.1925	-0.3552
KIF4A	0.0000	1.0531	0.0000	1.5982	0.0467	0.3681	0.6353	0.0971	1.0000	-0.3628	0.3056	0.1937	0.0000	-0.6289
KIF5C	0.0000	1.5943	0.0000	1.2959	0.0016	1.2078	0.0008	0.8420	1.0000	0.2935	1.0000	0.0079	1.0000	-0.0687
KIF7	0.7631	0.1106	0.0000	0.3874	0.0826	0.3246	0.0000	0.5218	1.0000	-0.2242	1.0000	0.0645	1.0000	-0.0213
KIF9	0.0000	-1.2088	0.0000	-1.0277	0.0199	-0.5100	0.0028	-0.4167	1.0000	-0.1092	1.0000	0.0837	1.0000	-0.0105
KIFAP3	0.0000	-0.8146	0.0000	-0.6814	0.1224	0.2650	0.0000	0.4647	1.0000	0.1193	0.0154	0.2648	0.0004	0.3240
KIFC3	0.3961	0.1815	1.0000	0.0201	0.0141	-0.4104	0.0009	-0.2567	1.0000	-0.3315	0.0000	-0.4803	0.1766	-0.1717
KIN	1.0000	0.0445	0.5233	-0.1538	0.0561	0.4662	0.5120	0.1537	1.0000	0.2109	1.0000	0.0260	1.0000	-0.0951
KIRREL	0.1024	0.2944	0.0000	0.3507	0.1115	0.3126	0.0000	0.5876	1.0000	-0.0769	1.0000	-0.0083	0.2021	0.2033
KIRREL3	0.8249	3.0141	1.0000	0.0000	0.8011	3.0840	0.7710	3.1708	1.0000	0.0000	1.0000	-3.1383	1.0000	0.0717
KIT	0.1861	-1.1203	0.0000	-2.8502	0.0024	-2.4270	0.0014	-1.4151	1.0000	0.5301	0.4823	-1.1891	0.2478	1.5454
KITLG	0.1992	-0.9935	1.0000	0.1785	0.0272	-1.4981	0.8163	-0.4679	1.0000	-0.9555	1.0000	0.2280	1.0000	0.0795
KL	1.0000	-0.3341	1.0000	0.6987	1.0000	0.2013	1.0000	0.8538	1.0000	-1.4945	1.0000	-0.4744	1.0000	-0.8446
KLB	0.0000	-2.0079	0.0000	-2.1142	0.0000	1.0599	0.0000	1.0494	1.0000	-0.0939	1.0000	-0.1886	0.8725	-0.1001
KLC1	0.0003	0.6610	0.0000	0.6059	0.0000	1.1390	0.0000	1.0097	1.0000	0.0579	1.0000	0.0160	1.0000	-0.0660

KLC4	0.0002	-0.5274	0.0000	-0.8114	0.0589	-0.3039	0.0000	-0.4724	1.0000	-0.2169	0.0000	-0.4885	0.0000	-0.3799
KLF10	0.0035	0.5758	0.0000	1.1077	0.7262	0.1222	0.0003	0.3934	1.0000	-0.3687	0.6066	0.1759	0.8908	-0.0913
KLF11	0.7417	0.1134	0.2478	0.1426	1.0000	-0.0196	0.1055	0.1799	1.0000	0.0032	1.0000	0.0446	0.1404	0.2071
KLF12	0.1524	0.4525	1.0000	0.0045	0.0398	0.5937	0.0000	0.9502	1.0000	-0.6446	0.0000	-1.0812	0.3154	-0.2870
KLF15	0.0000	-2.7123	0.0000	-3.6971	0.0039	1.1003	0.0485	0.6068	1.0000	0.3803	1.0000	-0.5928	1.0000	-0.1094
KLF2	0.0000	-1.3349	0.0000	-1.2397	0.1989	-0.2685	1.0000	0.0110	1.0000	-0.0790	1.0000	0.0292	0.1535	0.2072
KLF3	0.5700	-0.1650	0.0094	-0.3131	0.2954	0.2311	0.1887	0.1619	1.0000	0.0158	0.8863	-0.1199	1.0000	-0.0478
KLF4	0.8773	-0.1450	0.7577	-0.1193	0.9262	0.1167	0.9324	-0.0806	1.0000	-0.0137	1.0000	0.0253	0.6746	-0.2053
KLF5	0.0000	-1.1988	0.0000	-1.3988	0.0117	-0.4283	0.0000	-0.6327	1.0000	0.1541	1.0000	-0.0336	1.0000	-0.0447
KLF6	0.0069	-0.5207	0.0000	-0.3403	0.1819	0.2892	0.0008	0.2700	1.0000	0.0069	0.1315	0.1999	1.0000	-0.0064
KLF7	0.0000	0.9185	0.0000	0.8926	0.0000	1.0049	0.0000	0.8635	1.0000	0.1880	0.3820	0.1749	1.0000	0.0525
KLF8	0.0000	-0.8697	0.0000	-0.5796	0.0000	-0.8475	0.0000	-0.5623	1.0000	-0.0934	0.4307	0.2099	0.5105	0.1987
KLHDC1	0.4979	-0.1735	0.0078	-0.2961	0.0505	-0.3735	0.0224	-0.2497	1.0000	-0.0015	0.8723	-0.1121	0.6739	0.1271
KLHDC10	0.0295	-0.4251	0.0002	-0.4506	0.3144	-0.2332	0.1183	-0.1980	1.0000	-0.0019	1.0000	-0.0154	1.0000	0.0385
KLHDC2	0.0215	-0.3667	0.0000	-0.3344	0.0041	-0.4425	0.0000	-0.3441	1.0000	-0.1624	0.5978	-0.1175	0.9773	-0.0584
KLHDC4	0.9639	0.0706	0.9784	0.0385	0.2088	-0.2652	0.6628	-0.0760	1.0000	-0.2267	0.0562	-0.2470	1.0000	-0.0324
KLHDC7A	0.0001	2.8953	0.0000	3.8006	0.2833	-4.1854	0.7710	-3.1692	1.0000	-1.0347	1.0000	-0.1301	1.0000	0.0000
KLHDC8A	0.0000	-2.1619	0.0000	-2.1155	0.5109	-0.1975	0.0505	-0.2724	1.0000	-0.2203	1.0000	-0.1623	0.1309	-0.2898
KLHDC8B	1.0000	-0.0968	1.0000	-0.0969	0.8551	-0.5064	0.0438	-1.7090	1.0000	0.6209	1.0000	0.6360	1.0000	-0.5745
KLHL10	1.0000	-0.0271	0.1185	-0.6205	0.4882	-0.3671	1.0000	-0.0572	1.0000	0.1823	0.6968	-0.4013	0.3276	0.4976
KLHL11	0.0000	0.8684	0.0000	0.7896	0.4809	0.1879	0.0010	0.3215	1.0000	0.0688	1.0000	0.0022	0.2786	0.2068
KLHL12	0.0923	-0.3913	0.0011	-0.3995	0.0781	-0.4215	0.8283	-0.0585	1.0000	-0.0762	1.0000	-0.0730	0.1326	0.2923
KLHL13	0.0001	0.6728	0.0000	0.4948	0.1597	-0.3230	0.0000	-0.6936	1.0000	0.1976	1.0000	0.0330	0.6110	-0.1682
KLHL14	1.0000	0.1996	0.7189	1.0558	0.6654	-1.6733	0.9786	0.9240	1.0000	-1.6010	1.0000	-0.7546	1.0000	0.9922
KLHL15	0.2187	0.2823	0.0000	0.4637	0.3659	0.2280	0.2836	0.1389	1.0000	-0.1394	1.0000	0.0552	0.1608	-0.2228
KLHL17	1.0000	0.0545	1.0000	0.1064	1.0000	0.0509	0.1488	0.4877	1.0000	-0.1284	1.0000	-0.0650	0.6865	0.3114
KLHL18	0.0423	-0.4199	0.5749	-0.0976	0.2845	-0.2556	0.0465	0.2218	1.0000	-0.1502	0.4639	0.1847	0.0133	0.3329
KLHL2	0.3401	0.2014	0.0001	0.3507	0.5683	-0.1471	0.1392	-0.1615	1.0000	-0.0990	1.0000	0.0628	0.7219	-0.1080
KLHL20	0.0639	0.3938	0.0000	0.4912	0.1692	0.3169	0.0011	0.3490	1.0000	-0.0702	1.0000	0.0395	1.0000	-0.0317
KLHL21	0.5929	0.2223	0.0328	0.3924	0.0000	1.0548	0.0000	0.9989	1.0000	-0.0887	1.0000	0.0964	0.7705	-0.1387
KLHL22	0.3963	0.2363	0.8753	0.0753	0.0271	0.4761	0.0004	0.4376	1.0000	0.1279	1.0000	-0.0198	0.9304	0.0947
KLHL23	1.0000	0.1224	0.5243	0.4167	0.3441	-0.7161	0.7701	-0.3238	1.0000	-0.1799	1.0000	0.1270	1.0000	0.2142
KLHL24	0.1338	-0.2610	0.0000	-0.3842	0.6757	0.1148	0.2543	0.1051	1.0000	-0.0730	0.2160	-0.1837	0.7996	-0.0771
KLHL25	0.1838	0.3530	0.2191	0.2260	0.1972	0.3345	0.8614	-0.0752	1.0000	-0.0413	0.8269	-0.1561	0.0110	-0.4462
KLHL26	0.4360	-0.2985	0.4160	-0.1963	0.1577	0.3980	0.0000	0.6831	1.0000	-0.0038	1.0000	0.1113	0.2515	0.2859
KLHL28	0.0112	-0.5078	0.0095	-0.3100	0.4822	0.1830	0.0052	0.3261	1.0000	-0.0812	0.8607	0.1291	1.0000	0.0662
KLHL29	0.0000	-0.9039	0.0000	-1.0611	0.0715	-0.4014	0.0129	-0.3053	1.0000	0.0930	1.0000	-0.0506	0.4646	0.1938
KLHL3	0.5420	-0.3219	0.8584	-0.1494	0.7583	0.2040	1.0000	-0.0694	1.0000	-0.0436	1.0000	0.1429	0.5758	-0.3105
KLHL30	0.0000	-1.5712	0.0000	-1.5963	0.5285	-0.2692	0.1585	-0.3054	1.0000	-0.4059	0.5676	-0.4196	0.2556	-0.4360
KLHL31	0.0000	-2.2979	0.0000	-3.7516	0.0000	2.0701	0.0000	1.2845	1.0000	0.0053	0.1932	-1.4363	0.0058	-0.7753
KLHL32	0.0558	-5.0419	0.4636	-3.7802	0.2180	-2.7100	1.0000	-0.5213	1.0000	-1.2878	1.0000	0.0000	1.0000	0.9164
KLHL33	0.0000	-1.1548	0.0000	-0.9305	0.0000	-1.0134	0.0000	-0.8175	1.0000	-0.0737	0.8607	0.1626	0.8321	0.1277
KLHL34	0.0000	-3.8474	0.0000	-3.6719	0.0000	-1.7694	0.0000	-1.6865	1.0000	0.3937	0.8625	0.5842	0.3434	0.4799
KLHL35	1.0000	0.0000	1.0000	0.0000	1.0000	0.0000	1.0000	0.0000	1.0000	0.0000	1.0000	0.0000	1.0000	0.0000
KLHL36	0.0013	1.1118	0.0000	1.3914	0.0000	2.1069	0.0000	2.1644	1.0000	0.0329	0.5672	0.3278	0.9671	0.0985
KLHL38	0.1951	-1.6192	0.2042	-1.8297	0.0193	1.5450	0.4395	0.7585	1.0000	-0.1302	1.0000	-0.3301	0.3154	-0.9096
KLHL4	0.5086	3.5431	1.0000	0.0000	1.0000	2.2427	1.0000	2.3241	1.0000	0.0000	0.8607	-3.6719	1.0000	0.0652
KLHL40	0.0000	-1.6945	0.0000	-2.0517	0.0002	-0.6043	0.0000	-0.5736	1.0000	0.0149	0.0338	-0.3301	1.0000	0.0502
KLHL41	0.3958	-0.2043	0.0297	-0.4398	0.0168	0.4320	0.5798	0.1647	1.0000	0.0429	0.4553	-0.1806	0.1907	-0.2203
KLHL5	0.0001	0.6704	0.0000	0.6070	0.9534	-0.0736	0.5748	-0.0935	1.0000	0.0817	1.0000	0.0309	1.0000	0.0669
KLHL6	0.8226	3.0199	1.0000	0.0000	1.0000	0.0000	0.7701	3.1732	1.0000	0.0000	1.0000	-3.1429	1.0000	3.2066
KLHL7	0.7873	-0.1224	1.0000	-0.0073	0.2605	0.2568	0.5119	0.1069	1.0000	-0.0172	0.9055	0.1108	0.4669	-0.1608
KLHL8	0.8905	0.1315	1.0000	-0.0220	0.8709	-0.1304	0.0073	-0.5264	1.0000	0.0149	1.0000	-0.1273	0.2489	-0.3768
KMO	0.3673	-1.6866	0.0001	-6.1134	0.2641	-2.0615	0.1181	-1.3708	1.0000	0.8461	0.8607	-3.6732	0.7894	1.5491
KMT2A	0.9899	0.0937	1.0000	-0.0409	0.0000	0.9813	0.0000	1.1245	1.0000	0.0155	1.0000	-0.1044	0.4602	0.1649
KMT2C	0.3010	-0.2201	0.0000	-0.4005	0.0000	0.8088	0.0000	0.8630	1.0000	0.1245	1.0000	-0.0428	0.2667	0.1839
KMT2E	0.0499	-0.4146	0.0000	-0.7566	0.0000	0.9254	0.0000	0.8052	1.0000	0.1864	0.6225	-0.1421	0.9420	0.0712
KNDC1	0.1876	4.2322	1.0000	0.0000	1.0000	0.0000	1.0000	0.0000	1.0000	0.0000	0.4563	-4.3639	1.0000	0.0000
KNG1	0.8362	0.8144	0.2920	3.9943	0.9361	-1.5529	1.0000	0.0000	1.0000	-3.7629	1.0000	-0.7134	1.0000	-2.2888
KNOP1	0.0002	-0.5744	0.0000	-0.8262	0.0022	-0.5000	0.0000	-0.6020	1.0000	0.0790	0.3626	-0.1604	1.0000	-0.0180
KNSTRN	0.0029	0.6979	0.0000	0.9563	0.3234	-0.3056	0.1116	-0.2720	1.0000	-0.3489	1.0000	-0.0803	0.1236	-0.3100
KNTC1	0.0000	1.4369	0.0000	1.7551	0.0000	1.5518	0.0000	0.8749	1.0000	-0.1627	0.6424	0.1684	0.0000	-0.8331
KPNA2	0.0000	0.7688	0.0000	1.3098	0.5110	0.1657	0.0465	-0.2120	1.0000	-0.2110	0.0252	0.3430	0.0000	-0.5831
KPNA3	0.0139	0.6634	0.0239	0.4430	0.0000	1.5539	0.0000	1.1042	1.0000	0.3551	0.9569	0.1481	0.9875	-0.0879
KPNA4	0.0653	-0.3125	0.0000	-0.3181	0.0285	-0.3630	0.0000	-0.4192	1.0000	-0.0371	1.0000	-0.0303	0.7229	-0.0879
KPNA5	0.0009	1.4217	0.0000	1.9280	0.0001	1.4901	0.0001	1.2979	1.0000	-0.2338	0.8641	0.2775	0.3431	-0.4303
KPNA6	0.0000	-0.7720	0.0000	-0.7550	0.5184	-0.1459	0.2391	-0.1107	1.0000	0.0053	1.0000	0.0348	1.0000	0.0456
KPNA7	1.0000	-2.4776	0.1456	-4.4788	1.0000	-2.4056	0.4037	-2.0626	1.0000	1.9501	1.0000	0.0000	1.0000	2.3543
KPTN	1.0000	0.0267	1.0000	-0.0332	1.0000	0.0518	0.4413	0.2536	1.0000	-0.3052	0.5898	-0.3537	1.0000	-0.0976
KRAS	1.0000	0.0079	0.5199	-0.0725	0.8893	0.0789	1.0000	0.0150	1.0000	0.0800	1.0000	0.0122	1.0000	0.0214
KRBA1	0.3159	3.9314	0.6215	1.6074	1.0000	2.2472	1.0000	0.0076	1.0000	2.2674	1.0000	0.0557	1.0000	0.0649
KREMEN1	0.3865	0.1704	0.3866	0.0806	0.0267	0.3495	0.0001	0.2505	1.0000	0.0406	1.0000	-0.0369	0.9412	-0.0534
KRIT1	1.0000	-0.0228	0.8164	-0.0486	0.0000	0.6608	0.0000	0.5471	1.0000	-0.0117	1.0000	-0.0249	0.5200	-0.1203
KRR1	0.1539	-0.2897	0.0001	-0.4094	0.7209	0.1198	0.7773	0.0602	1.0000	0.0659	1.0000	-0.0419	1.0000	0.0114
KRT18	1.0000	-2.4788	1.0000	0.0000	1.0000	-2.4061	1.0000	0.0000	1.0000	-2.3771	1.0000	0.0000	1.0000	0.0000
KRT20	0.7247	1.1679	0.9762	-1.0844	0.9361	-1.5532	0.6142	-1.7592	1.0000	0.2632	0.4839	-1.9851	1.0000	0.0652
KRT222	0.8476	0.5016	0.9366	-0.3232	1.0000	0.1221	0.5594	0.6729	1.0000	-0.3902	0.3263	-1.2050	1.0000	0.1707
KRT23	0.0341	1.1068	0.0000	2.2418	0.0422	1.0529	0.0000	1.8112	1.0000	-0.3968	0.0633	0.7453	0.7170	0.3634
KRT8	0.5378	3.5350	1.0000	-0.1472	0.8011	3.0840	1.0000	0.8541	1.0000	2.2736	1.0000	-1.3217	1.0000	0.0723
KRT80	0.3158	0.4294	0.0968	0.3855	0.0103	-1.0313	0.0000	-1.1869	1.0000	0.0665	1.0000	0.0311	1.0000	-0.0844
KRTCAP3	0.2621	0.8520	0.0755	0.8854	0.9174	-0.4018	0.4891	-0.6448	1.0000	0.2059	1.0000	0.2528	1.0000	-0.0333
KSR1	0.0609	0.3491	0.0313	0.2744	0.0035	0.5151	0.0596	0.2403	1.0000	0.2220	0.4251	0.1606	1.0000	-0.0475
KSR2	1.0000	-2.4776	1.0000	0.0000	1.0000	-2.4056	1.0000	0.0000	1.0000	-2.3757	1.0000	0.0000	1.0000	0.0000
KTN1	0.3596	0.1828	0.0467	0.1647	0.5881	0.1322	0.4380	0.0880	1.0000	0.0289	1.0000	0.0232	1.0000	-0.0101
KXD1	0.0789	-0.3307	0.0307	-0.2558	0.0000	-0.9562	0.0000	-0.8577	1.0000	-0.0765	1.0000	0.0103	1.0000	0.0274
KY	1.0000	0.0000	1.0000	0.0000	1.0000	0.0000	1.0000	0.0000	1.0000	0.0000	1.0000	0.0000	1.0000	0.0000
L2HGDH	0.0004	0.6891	0.0125	0.3600	0.0409	-0.4733	0.0046	-0.4471	1.0000	0.0155	0.0826	-0.3006	1.0000	0.0467
L3HYPDH	0.7885	0.1392	0.3590	0.2284	0.0000	-1.5122	0.0000	-0.9527	1.0000	-0.2408	1.0000	-0.1399	0.4805	0.3239
L3MBTL1	0.1534	0.4837	0.8390	0.1142	0.5757	0.2504	0.9643	0.0850	1.0000	0.2504	1.0000	-0.1064	1.0000	0.0907
L3MBTL2	1.0000	0.0384	0.0579	0.2290	0.8601	0.0936	0.1497	0.1857	1.0000	-0.0923	0.8785	0.1105	1.0000	0.0055
LACC1	1.0000	0.0000	0.7674	3.0888	1.0000	0.0000	1.0000	2.3241	1.0000	0.0000	1.0000	3.1946	1.0000	2.3543
LACE1	0.4252	-0.3159	0.1232	-0.3480	0.6723	0.1985	0.2724	-0.2772	1.0000	0.1932	0.9944	0.1739	0.5073	-0.2769
LACTB	0.1906	-0.3010	0.0118	-0.3128	0.7787	-0.1191	0.3906	-0.1260	1.0000	0.0073	1.0000	0.0086	1.0000	0.0070

LACTB2	0.1966	0.2965	0.0003	0.3987	0.2325	0.2744	1.0000	-0.0138	1.0000	-0.0247	0.9847	0.0905	0.0637	-0.3075
LACTBL1	1.0000	0.0000	1.0000	2.2506	0.4809	3.6101	1.0000	0.0000	1.0000	0.0000	1.0000	2.3456	0.7287	-3.6634
LAD1	0.0088	-2.4558	0.0000	-3.0408	0.3187	-0.9010	1.0000	0.0446	1.0000	0.1604	1.0000	-0.4072	0.0783	1.1084
LAG3	1.0000	0.0000	1.0000	0.0000	1.0000	0.0000	1.0000	0.0000	1.0000	0.0000	1.0000	0.0000	1.0000	0.0000
LAMA1	0.0012	0.8530	0.0000	1.3538	0.0005	0.8660	0.0000	0.8451	1.0000	0.0178	0.0039	0.5316	1.0000	0.0032
LAMA3	0.1079	0.5579	0.0034	0.6001	0.0116	0.8304	0.0000	0.7635	1.0000	0.3886	0.1735	0.4445	0.3273	0.3303
LAMA4	0.1929	0.2498	0.0335	0.1974	0.0236	-0.3993	0.0231	0.2434	1.0000	0.0818	1.0000	0.0420	0.0000	0.7295
LAMA5	0.3820	0.1987	0.0000	0.3406	0.0000	0.8573	0.0000	1.0869	1.0000	0.0569	0.0178	0.2113	0.0001	0.2913
LAMB1	0.0001	0.6914	0.0000	0.3959	0.0000	0.7667	0.0000	1.4380	1.0000	0.1434	0.5273	-0.1401	0.0000	0.8198
LAMB2	0.0001	-0.6641	0.0000	-0.4735	0.0016	-0.5708	1.0000	0.0119	1.0000	-0.1522	1.0000	0.0509	0.0000	0.4354
LAMB3	0.5803	0.5963	0.0000	2.6998	1.0000	-0.2770	1.0000	0.2036	1.0000	-1.3908	0.3686	0.7360	0.6029	-0.9004
LAMC1	0.0109	0.4512	0.0000	0.5468	0.0000	0.9926	0.0000	1.3975	1.0000	0.0443	0.2781	0.1526	0.0000	0.4542
LAMC2	0.0000	1.3733	0.0000	1.4283	0.0000	-1.4803	0.0000	-0.9876	1.0000	-0.0826	1.0000	-0.0158	0.0167	0.4148
LAMC3	0.8721	0.2553	1.0000	0.1013	0.0429	-1.1569	0.0210	-0.9092	1.0000	0.2881	1.0000	0.1477	0.6830	0.5420
LAMP1	1.0000	-0.0295	1.0000	-0.0055	0.8033	0.0853	0.0709	0.1372	1.0000	0.1356	0.0915	0.1720	0.0508	0.1928
LAMP2	0.0002	0.7966	0.0000	1.1453	0.0000	1.6681	0.0000	1.4154	1.0000	0.0718	0.0060	0.4336	0.4317	-0.1750
LAMP3	0.3159	3.9314	1.0000	-0.6986	0.8033	3.0790	0.9162	-1.3767	1.0000	3.6521	1.0000	-0.8625	1.0000	-0.7782
LAMP5	0.0004	0.6318	0.0000	0.9947	0.0000	1.1829	0.0000	1.2931	1.0000	-0.0905	0.0369	0.2847	1.0000	0.0253
LAMTOR1	0.0000	-0.8887	0.0000	-0.7390	0.0270	-0.4043	0.1562	-0.1630	1.0000	-0.1760	1.0000	-0.0144	1.0000	0.0709
LAMTOR2	0.0000	-0.7048	0.0000	-0.5839	0.0000	-1.0981	0.0000	-0.8624	1.0000	-0.1514	1.0000	-0.0183	0.9853	0.0906
LAMTOR3	0.0002	-0.5728	0.0000	-0.7092	0.0061	-0.4555	0.0000	-0.4167	1.0000	0.0654	1.0000	-0.0587	0.6687	0.1088
LAMTOR4	0.0000	-0.8395	0.0000	-1.0252	0.0000	-1.0333	0.0000	-0.8588	1.0000	0.0335	0.8626	-0.1392	0.5764	0.2153
LAMTOR5	0.0000	-0.7481	0.0000	-0.7111	0.0000	-0.8078	0.0000	-0.7613	1.0000	-0.0489	1.0000	-0.0001	1.0000	0.0036
LANCL1	0.3832	0.2138	0.0000	0.4732	0.9598	-0.0719	0.5456	-0.0996	1.0000	-0.1036	0.4754	0.1680	0.6583	-0.1267
LANCL2	0.0000	0.8054	0.0000	1.2398	0.0017	0.6175	0.0000	0.9927	1.0000	-0.5324	1.0000	-0.0841	0.6584	-0.1505
LANCL3	1.0000	0.0000	1.0000	2.2534	1.0000	0.0000	1.0000	0.0000	1.0000	0.0000	1.0000	2.3480	1.0000	0.0000
LAP3	0.7710	0.1004	0.2164	0.1403	0.0000	-0.7920	0.0000	-0.8816	1.0000	0.0449	0.7745	0.0967	1.0000	-0.0398
LAPTM4A	0.8180	-0.0920	1.0000	-0.0240	0.0070	0.4243	0.0000	0.4503	1.0000	0.0457	0.5720	0.1257	0.7961	0.0764
LAPTM4B	0.8293	0.2587	0.0043	0.8968	0.0019	-1.5260	0.3262	-0.5086	1.0000	-0.2907	0.7089	0.3595	0.4472	0.7321
LAPTM5	0.1353	2.2183	0.0902	1.8555	0.8011	-3.2629	1.0000	-0.5239	1.0000	0.4115	1.0000	0.0612	1.0000	3.2066
LARP1	0.0358	0.5682	0.0000	0.9154	0.0000	1.7533	0.0000	1.5820	1.0000	-0.0101	0.2404	0.3505	0.3789	-0.1749
LARP1B	0.0195	0.5008	0.0020	0.3744	0.8033	0.1260	1.0000	0.0219	1.0000	0.0969	1.0000	-0.0168	1.0000	-0.0014
LARP4	0.0003	1.0417	0.0000	1.6060	0.0000	1.9056	0.0000	1.5807	1.0000	-0.1945	0.0978	0.3835	0.0017	-0.5140
LARP4B	0.0001	-0.5961	0.0000	-0.7390	1.0000	-0.0355	0.0390	-0.1939	1.0000	0.0337	0.8860	-0.0966	0.5275	-0.1192
LARP6	0.1374	0.2836	0.0515	0.2013	0.0224	0.3983	0.0000	0.4636	1.0000	-0.0328	0.7953	-0.1023	1.0000	0.0392
LARP7	0.8757	-0.0927	0.5911	-0.0883	0.6375	-0.1483	0.0160	-0.2616	1.0000	0.1192	0.6999	0.1365	1.0000	0.0124
LARS	1.0000	-0.0398	0.2568	0.1143	0.6373	-0.1440	0.0167	-0.2046	1.0000	-0.0728	0.8158	0.0934	0.4807	-0.1278
LARS2	0.4310	0.2015	0.2154	0.1607	0.3853	-0.2161	0.0053	-0.3210	1.0000	0.1250	0.9429	0.0963	1.0000	0.0254
LASP1	0.0000	0.7396	0.0000	0.8822	0.5412	0.1435	0.0000	0.3110	1.0000	-0.1102	1.0000	0.0451	0.9835	0.0626
LAT2	0.0061	-1.3057	0.2720	-0.4251	0.5760	-0.3468	0.9524	-0.1347	1.0000	-0.0849	0.2545	0.8096	1.0000	0.1311
LATS1	0.0002	0.7450	0.0000	0.7730	0.0000	1.1030	0.0000	0.9473	1.0000	0.0058	1.0000	0.0475	0.5737	-0.1436
LATS2	1.0000	0.0004	1.0000	-0.0069	0.0000	1.6032	0.0000	1.2291	1.0000	0.2608	0.3594	0.2672	0.7908	-0.1071
LBH	0.0000	1.7928	0.0000	2.2565	0.0000	1.1029	0.0000	1.5206	1.0000	-0.1879	0.1029	0.2892	0.0186	0.2354
LBR	0.0000	0.9981	0.0000	1.1255	0.0876	0.3346	0.0956	0.1720	1.0000	-0.0940	1.0000	0.0458	0.0698	-0.2512
LBX1	0.2540	-0.5324	0.3442	0.4010	1.0000	0.1047	0.0015	0.9554	1.0000	-0.7536	1.0000	0.1913	1.0000	0.0986
LCA5	0.5680	0.2719	0.0153	0.4617	0.6280	0.2471	0.1779	0.2802	1.0000	-0.5279	0.2646	-0.3245	0.0145	-0.4871
LCA5L	0.2948	0.3030	0.6044	0.1025	0.0876	-0.4550	0.0000	-0.5706	1.0000	-0.0267	0.3997	-0.2133	0.8120	-0.1343
LCAT	0.0001	0.9814	0.0487	0.4719	0.0000	-2.0048	0.0000	-1.4887	1.0000	-0.2745	0.0001	-0.7729	1.0000	0.2459
LCK	1.0000	-0.3393	0.7666	-3.2534	0.7001	-1.9425	0.0607	2.3306	1.0000	-1.0362	0.6427	-4.0589	0.0798	3.2447
LCMT1	1.0000	-0.0536	0.9202	0.0513	0.0009	-0.6695	0.0009	-0.4171	1.0000	-0.1714	1.0000	-0.0536	1.0000	0.0876
LCMT2	0.0006	-0.5898	0.0000	-0.7204	0.0032	-0.5231	0.0000	-0.4261	1.0000	-0.0395	0.6424	-0.1579	1.0000	0.0618
LCOR	0.0351	2.1583	0.0003	1.9297	0.0000	2.8346	0.0000	2.5590	1.0000	0.5851	1.0000	0.3639	0.8939	0.3140
LCORL	0.1441	0.4041	0.0044	0.4495	0.0015	0.7145	0.0002	0.5494	1.0000	0.0251	1.0000	0.0838	0.7978	-0.1334
LCP1	0.1613	1.2042	0.1727	1.3665	0.2719	-2.0598	1.0000	0.0068	1.0000	-0.7790	0.8607	-0.6089	0.9835	1.2966
LCP2	1.0000	0.0000	1.0000	0.0000	1.0000	0.0000	1.0000	0.0000	1.0000	0.0000	1.0000	0.0000	1.0000	0.0000
LCT	0.0002	-0.7750	0.0000	-0.5844	0.0001	-0.7728	0.0000	-1.1093	1.0000	0.0859	0.2873	0.2904	0.3998	-0.2449
LDB1	0.5690	-0.2135	0.1288	0.2618	0.0029	0.6572	0.0000	1.0804	1.0000	-0.2751	0.5166	0.2143	0.5786	0.1550
LDB2	0.0751	0.7525	0.0000	1.1984	1.0000	-0.0300	0.0201	-0.8129	1.0000	-0.0241	0.2628	0.4331	0.0928	-0.8010
LDB3	0.0000	-6.2139	0.0000	-6.0606	0.0639	-0.3909	0.0000	-0.4070	1.0000	0.1543	1.0000	0.3209	0.6701	0.1426
LDHB	0.4670	0.1693	0.0010	0.2572	0.0018	-0.5287	0.0000	-0.5372	1.0000	-0.1424	1.0000	-0.0420	0.3229	-0.1449
LDHC	0.3190	0.2037	1.0000	0.0189	0.0000	1.3546	0.0000	0.9988	1.0000	0.3341	0.3097	0.1620	1.0000	-0.0162
LDHD	0.0264	-0.8348	0.0704	-0.5648	1.0000	0.0240	0.0778	0.4191	1.0000	0.0296	0.9107	0.3120	0.2276	0.4296
LDLRAD1	1.0000	0.0420	0.6005	0.1083	0.0063	-0.5924	0.0048	-0.3872	1.0000	-0.1225	1.0000	-0.0440	1.0000	0.0882
LDLRAD3	0.0010	2.9578	0.0941	1.2126	0.0001	3.0254	0.0368	1.3978	1.0000	1.3811	1.0000	-0.3484	1.0000	-0.2386
LDLRAD4	0.0001	-0.6577	0.0000	-0.7284	0.6903	0.1227	1.0000	0.0111	1.0000	0.0854	1.0000	0.0266	1.0000	-0.0214
LDLRAP1	0.0791	0.3430	0.3011	0.1261	0.8442	-0.0952	0.0875	-0.1769	1.0000	-0.0019	0.2339	-0.2067	0.8937	-0.0780
LECT1	0.1016	-0.5059	0.0093	-0.5738	0.0000	1.5597	0.0000	0.9882	1.0000	-0.1898	0.8799	-0.2462	0.0000	-0.7553
LECT2	0.0070	1.2575	0.0000	1.8229	0.0198	-1.5050	0.2722	-1.1684	1.0000	-1.0178	0.5737	-0.4505	0.8878	-0.6770
LEF1	0.0027	-5.8038	0.0001	-4.1075	0.0030	1.6843	0.0000	1.9574	1.0000	0.5225	1.0000	2.3456	0.1007	0.7993
LEFTY1	0.0309	1.6964	1.0000	0.0815	1.0000	0.3724	0.5111	-0.6043	1.0000	1.1624	1.0000	-0.4369	1.0000	0.1931
LEKR1	0.0049	-0.7079	0.1059	-0.3468	0.0015	0.6733	0.0000	1.0295	1.0000	-0.0921	0.4515	0.2819	0.0864	0.2717
LEMD2	1.0000	-0.0132	1.0000	-0.0190	0.2936	0.2143	0.0352	0.2010	1.0000	0.0602	1.0000	0.0667	1.0000	0.0514
LEMD3	0.0000	0.8727	0.0000	0.9519	0.0003	0.6705	0.0000	0.5312	1.0000	0.0499	0.6450	0.1425	0.9515	-0.0831
LENG8	0.3308	0.6309	0.1799	0.7027	0.6176	0.4110	0.0002	1.5542	1.0000	-0.5252	0.7531	-0.4376	0.2050	0.6273
LEO1	0.3378	-0.2166	0.0170	-0.2746	0.2405	0.2426	0.1009	0.1778	1.0000	0.0810	1.0000	0.0351	1.0000	0.0205
LEPR	0.6061	0.4388	0.0903	0.7854	1.0000	-0.1061	0.3709	0.5499	1.0000	-0.3846	1.0000	-0.0221	1.0000	0.2800
LEPREL2	0.6418	0.1529	0.9614	0.0376	0.0642	-0.4044	0.0040	-0.2901	1.0000	0.0659	1.0000	-0.0374	0.3793	0.1863
LEPROT	0.1510	0.2713	0.0275	0.1746	0.0000	0.6815	0.0000	0.6737	1.0000	0.1022	1.0000	0.0179	0.5659	0.1001
LEPROTL1	1.0000	-0.0402	0.8678	-0.0420	1.0000	0.0195	0.7711	0.0519	1.0000	0.0450	1.0000	0.0555	0.8328	0.0834
LETM1	0.0064	0.5688	0.0035	0.3603	0.0000	0.8800	0.0010	0.4162	1.0000	0.1109	1.0000	-0.0848	0.0761	-0.3474
LETM2	0.2990	0.3294	0.1880	0.2700	0.5474	-0.2538	0.0408	-0.4256	1.0000	-0.0071	1.0000	-0.0543	0.9210	-0.1757
LETMD1	0.7336	0.5619	0.1244	1.1186	0.0025	1.7747	0.0011	1.7888	1.0000	-0.2621	1.0000	0.3068	1.0000	-0.2468
LFNG	0.0289	-0.4677	0.2689	-0.1326	0.0001	0.7149	0.0000	0.7011	1.0000	-0.2396	0.8841	0.1070	0.0814	-0.2472
LGALS1	0.0000	-0.9269	0.0000	-0.7794	0.0000	-0.8278	0.0000	-0.7037	1.0000	0.0054	0.2570	0.1658	0.3854	0.1348
LGALS2	1.0000	-0.3449	0.2759	-0.9267	1.0000	-0.4936	0.0520	-1.7074	1.0000	1.5818	0.9238	1.0238	1.0000	0.3802
LGALS3	0.0896	-0.3283	0.0000	-0.5744	0.0103	-0.4874	0.0000	-0.4769	1.0000	0.1860	1.0000	-0.0477	0.1672	0.2017
LGALS8	1.0000	-0.0102	0.4200	-0.0840	1.0000	0.0203	1.0000	0.0221	1.0000	0.2102	0.3377	0.1488	0.0487	0.2172
LGALSL	0.0125	0.7290	0.0003	0.7250	0.3108	-0.4534	0.2133	-0.3248	1.0000	0.0553	1.0000	0.0641	1.0000	0.1865
LGI1	0.3365	0.6309	0.0594	1.0875	1.0000	-0.2010	1.0000	0.1059	1.0000	-0.8405	0.9655	-0.3714	0.8493	-0.5279
LGI2	1.0000	-0.0205	0.7696	0.0851	0.9540	0.0902	0.2818	0.1902	1.0000	0.1542	0.3057	0.2723	0.3081	0.2613
LGI3	0.0298	-1.5101	0.1124	-1.6294	0.0438	-1.3523	0.7593	0.4424	1.0000	-0.8003	0.8605	-0.9113	0.3644	1.0043
LGMN	0.3454	0.2003	0.0000	0.3799	0.0599	0.3251	0.0000	0.5372	1.0000	0.1580	0.0003	0.3498	0.0000	0.3752
LGR4	0.0041	0.6946	0.0004	0.5181	0.6946	-0.1918	0.0288	-0.3762	1.0000	0.2798	0.9773	0.1161	1.0000	0.1021

LGR5	0.5814	0.1887	0.0286	0.2075	0.1323	0.3623	0.0000	-0.6205	1.0000	0.2053	0.0925	0.2365	0.0000	-0.7730
LGR6	1.0000	-2.4788	1.0000	-2.3959	1.0000	-0.1630	1.0000	-2.3178	1.0000	-0.1089	1.0000	0.0000	1.0000	-2.2888
LGSN	1.0000	0.0000	1.0000	0.0000	1.0000	0.0000	1.0000	0.0000	1.0000	0.0000	1.0000	0.0000	1.0000	0.0000
LH-BETA	0.0001	-1.8359	0.0000	-1.9651	0.0009	-1.4681	0.0010	-1.0892	1.0000	-0.0738	1.0000	-0.1889	0.9930	0.3092
LHCGR	1.0000	0.0000	1.0000	0.0000	1.0000	0.0000	1.0000	0.0000	1.0000	0.0000	1.0000	0.0000	1.0000	0.0000
LHFP	0.0000	1.3841	0.0000	1.2218	0.0028	0.6492	0.9612	-0.0504	1.0000	-0.2590	0.0003	-0.4084	0.0000	-0.9505
LHFPL2	0.0444	0.7736	0.0000	1.1951	0.0000	1.6137	0.0000	1.3302	1.0000	-0.2860	1.0000	0.1474	0.0795	-0.5668
LHFPL3	0.0018	-1.8917	0.0053	-1.1441	0.0001	1.3910	0.0000	1.7880	1.0000	0.1252	0.6300	0.8860	0.0624	0.5267
LHFPL4	1.0000	0.0755	0.2605	0.6309	0.8743	0.2818	0.1318	0.8173	1.0000	-0.3839	1.0000	0.1837	1.0000	0.1595
LHFPL5	0.0401	1.5167	0.0424	1.3341	0.1512	1.2355	0.1142	1.1254	1.0000	0.1855	1.0000	0.0130	1.0000	0.0803
LHPP	0.4395	-0.3790	0.6224	-0.1967	1.0000	-0.0547	0.4824	-0.2529	1.0000	0.0171	1.0000	0.2099	1.0000	-0.1780
LHX1	1.0000	0.0000	1.0000	2.2534	1.0000	0.0000	1.0000	0.0000	1.0000	0.0000	1.0000	2.3480	1.0000	0.0000
LHX2	0.0055	1.8278	0.4814	0.7315	1.0000	0.2444	1.0000	-0.1403	1.0000	-0.2637	0.0265	-1.3514	0.8350	-0.6417
LHX3	1.0000	0.0000	1.0000	0.0000	1.0000	0.0000	1.0000	0.0000	1.0000	0.0000	1.0000	0.0000	1.0000	0.0000
LHX4	0.5086	3.5431	1.0000	0.0000	1.0000	0.0000	1.0000	0.0000	1.0000	0.0000	0.8607	-3.6719	1.0000	0.0000
LHX5	1.0000	2.1849	1.0000	2.2506	1.0000	0.0000	1.0000	0.0000	1.0000	0.0000	1.0000	0.0495	1.0000	0.0000
LHX6	1.0000	0.2623	1.0000	0.0403	0.2198	-1.5960	0.1602	-1.5201	1.0000	-0.0072	1.0000	-0.2159	1.0000	0.0761
LHX8	1.0000	2.1902	1.0000	2.2506	1.0000	2.2427	1.0000	0.0000	1.0000	0.0000	1.0000	0.0470	1.0000	-2.2888
LHX9	1.0000	2.1902	1.0000	0.0000	1.0000	0.0000	1.0000	0.0000	1.0000	0.0000	1.0000	-2.2991	1.0000	0.0000
LIAS	1.0000	-0.0435	0.0633	-0.2048	0.1049	-0.3211	0.0000	-0.4044	1.0000	-0.0040	0.6497	-0.1527	0.8817	-0.0808
LIF	0.0002	-2.1290	0.5654	-0.6126	0.0002	1.2881	0.0000	1.3772	1.0000	-0.3434	0.6210	1.1775	0.7870	-0.2468
LIFR	0.0000	1.0285	0.0000	0.8732	0.6305	0.1770	0.3118	0.1864	1.0000	0.1090	1.0000	-0.0335	0.8937	0.1228
LIG1	0.0035	-0.5358	0.0143	-0.3219	0.1814	-0.2785	1.0000	-0.0073	1.0000	-0.3504	0.8553	-0.1251	1.0000	-0.0737
LIG3	0.5598	-0.1402	1.0000	-0.0245	0.2956	-0.2048	0.5361	-0.0792	1.0000	-0.1067	1.0000	0.0212	1.0000	0.0237
LIG4	0.6044	-0.2208	0.6504	-0.1451	0.0154	0.5526	0.0001	0.5982	1.0000	-0.0340	1.0000	0.0551	1.0000	0.0193
LIM2	0.0000	-2.1139	0.0000	-1.8742	0.2125	-0.4667	0.4942	0.3858	1.0000	-0.7106	0.9289	-0.4578	1.0000	0.1466
LIMA1	0.8721	0.0730	0.0713	0.1397	0.8011	-0.0895	0.0978	-0.1237	1.0000	0.0159	0.7173	0.0949	1.0000	-0.0132
LIMCH1	0.0000	-4.9767	0.0000	-5.2660	0.0000	1.3870	0.0000	0.7889	1.0000	0.0184	1.0000	-0.2575	0.0001	-0.5758
LIMD1	0.0000	-0.5995	0.0000	-0.5581	0.7500	-0.1003	0.5089	0.0652	1.0000	0.0434	0.6983	0.0971	0.0266	0.2141
LIMD2	1.0000	-0.3236	0.4693	3.6125	1.0000	-1.0206	1.0000	0.0000	1.0000	-3.2319	1.0000	0.5836	1.0000	-2.2888
LIME1	0.0433	-1.7197	0.8127	-0.4376	0.3227	-0.8787	1.0000	0.1157	1.0000	-0.9191	1.0000	0.3730	1.0000	0.0787
LIMK1	0.1039	-0.6128	0.4519	-0.2551	0.1755	-0.4833	1.0000	0.0435	1.0000	-0.3253	1.0000	0.0468	0.8822	0.2056
LIMK2	1.0000	0.0603	1.0000	0.0137	0.0872	0.3754	0.0014	0.3696	1.0000	0.1829	0.8390	0.1497	0.4216	0.1831
LIMS1	0.1928	0.2682	0.0001	0.3743	1.0000	-0.0379	0.8285	-0.0547	1.0000	-0.0543	1.0000	0.0651	0.9987	-0.0650
LIN28A	1.0000	0.0000	1.0000	0.0000	1.0000	2.2427	1.0000	0.0000	1.0000	0.0000	1.0000	0.0000	1.0000	-2.2888
LIN28B	1.0000	-0.2892	1.0000	-2.3959	1.0000	-0.1630	1.0000	0.8568	1.0000	-0.1089	1.0000	-2.2991	1.0000	0.9186
LIN52	0.8351	-0.1172	1.0000	0.0167	0.5284	0.1959	0.7328	0.1006	1.0000	0.0029	0.8764	0.1497	1.0000	-0.0871
LIN54	0.0001	1.3130	0.0000	1.5545	0.0001	1.0959	0.0000	1.1588	1.0000	-0.3399	1.0000	-0.0871	0.5138	-0.2722
LIN7A	1.0000	-0.0180	0.8827	0.0693	1.0000	0.0236	0.1589	0.2150	1.0000	0.0696	0.8175	0.1712	0.2150	0.2682
LIN7C	0.3128	0.2509	0.0768	0.1920	0.0018	0.6083	0.0000	0.4861	1.0000	-0.1006	0.5831	-0.1473	0.1775	-0.2176
LIN9	0.0081	0.7387	0.0000	0.7930	0.0400	0.5796	0.5946	0.1580	1.0000	0.1327	0.7013	0.1999	0.3816	-0.2838
LINGO1	0.0775	-0.5260	0.0698	-0.4431	0.0098	0.6893	0.0003	0.6621	1.0000	-0.3593	0.7667	-0.2650	0.1806	-0.3811
LINGO2	0.6011	0.8110	0.6852	0.7903	1.0000	-0.1964	1.0000	-0.6849	1.0000	-0.5934	0.9595	-0.6050	0.8648	-1.0802
LINGO3	1.0000	-2.4776	0.7666	-3.2534	1.0000	0.6746	1.0000	0.0072	1.0000	0.7365	1.0000	0.0000	1.0000	0.0753
LINS	1.0000	-0.0266	0.7934	-0.2061	1.0000	-0.0315	0.5377	-0.2910	1.0000	-0.4526	0.2488	-0.6199	0.0820	-0.7075
LIPA	0.0000	0.9173	0.0000	0.8705	0.0094	0.3965	0.8726	0.0391	1.0000	0.1829	0.2302	0.1484	0.1476	-0.1694
LIPC	1.0000	0.8846	1.0000	0.3638	1.0000	-0.1789	0.7710	-3.1692	1.0000	-0.1146	1.0000	-0.6328	1.0000	-3.1344
LIPG	0.1426	-0.7404	1.0000	0.0042	0.8924	0.2001	0.1288	0.4761	1.0000	-0.8171	1.0000	-0.0565	0.0972	-0.5340
LIPH	0.6748	-0.6965	0.0094	-2.8345	0.4697	0.6836	0.0287	1.2299	1.0000	-0.1260	0.2657	-2.2644	0.8276	0.4224
LIPI	1.0000	0.0000	1.0000	0.0000	1.0000	0.0000	1.0000	0.0000	1.0000	0.0000	1.0000	0.0000	1.0000	0.0000
LIPT1	0.8226	-0.1388	0.4746	-0.1708	0.0619	-0.4790	0.6412	-0.1344	1.0000	-0.1305	0.9421	-0.1506	0.6099	0.2180
LITAF	0.9681	-0.2383	0.0819	-0.6103	0.5431	0.4733	0.3206	-0.3741	1.0000	0.6376	1.0000	0.2797	1.0000	-0.2045
LIX1	0.0179	1.3975	0.0000	1.6452	0.0065	1.5548	0.2733	0.6537	1.0000	0.4459	0.2236	0.7048	0.7526	-0.4525
LLGL1	0.7164	0.1174	0.0117	0.2530	0.4330	-0.1882	0.1171	0.1783	1.0000	-0.2594	0.7547	-0.1114	0.7603	0.1129
LLGL2	0.0369	0.5431	0.0003	0.5888	0.8033	-0.1552	0.3763	-0.2167	1.0000	0.0187	1.0000	0.0754	1.0000	-0.0401
LLPH	0.0000	-1.0833	0.0000	-1.0539	0.0000	-1.0341	0.0000	-0.8648	1.0000	-0.0120	1.0000	0.0300	0.5135	0.1635
LMAN1	0.0017	0.4674	0.0000	0.5749	0.5828	-0.1311	0.2979	-0.1213	1.0000	0.0598	0.1169	0.1795	0.8613	0.0745
LMAN2	1.0000	0.0337	0.6161	0.0692	0.0597	-0.3257	0.3436	-0.0999	1.0000	0.0321	0.9306	0.0798	0.0251	0.2636
LMAN2L	0.3365	-0.2278	0.0274	-0.2044	0.0085	-0.4999	0.0000	-0.3970	1.0000	-0.0985	1.0000	-0.0633	1.0000	0.0089
LMBR1	0.0001	1.0462	0.0000	1.0111	0.0000	1.0582	0.0000	0.8363	1.0000	0.2068	0.8269	0.1817	1.0000	-0.0119
LMBR1L	1.0000	-0.2206	0.7966	0.3378	0.9343	0.3329	1.0000	0.0940	1.0000	-0.4489	1.0000	0.1230	0.6595	-0.6815
LMBRD1	1.0000	0.0382	0.2471	-0.1542	0.0123	0.4598	1.0000	-0.0073	1.0000	0.0884	1.0000	-0.0927	0.0029	-0.3732
LMBRD2	0.1231	0.3236	0.0021	0.3532	0.0120	0.4914	0.0081	0.3158	1.0000	0.0099	1.0000	0.0522	0.5773	-0.1613
LMCD1	0.0000	-0.5812	0.0000	-0.4183	0.0000	-1.0878	0.0000	-0.8923	1.0000	0.1502	0.0001	0.3252	0.0000	0.3508
LMF1	0.7526	0.1413	0.0316	0.3148	0.7920	0.1304	0.6747	0.1130	1.0000	-0.1082	1.0000	0.0760	0.8481	-0.1206
LMF2	0.8250	0.1228	0.0000	0.4789	1.0000	0.0467	0.0000	0.4013	1.0000	-0.2223	0.4729	0.1465	0.4068	0.1368
LMLN	0.0033	0.7172	0.0000	0.6496	0.9571	0.0954	1.0000	0.0406	1.0000	-0.1410	0.7428	-0.1954	0.7287	-0.1906
LMNB2	0.8078	0.1126	0.0000	0.4542	0.3258	0.2314	0.0127	0.2203	1.0000	-0.2622	0.7166	0.0919	0.0024	-0.2673
LMO2	0.0003	1.0223	0.0000	0.8831	0.0426	-0.7079	0.0000	-0.9073	1.0000	-0.3393	0.0133	-0.4654	0.1277	-0.5358
LMO3	1.0000	-2.4776	0.7674	3.0888	1.0000	-2.4056	1.0000	2.3241	1.0000	-2.3757	1.0000	3.1946	1.0000	2.3543
LMO4	0.0011	0.6368	0.0000	0.5687	0.2182	0.2859	0.0042	0.2455	1.0000	-0.2123	0.0111	-0.2681	0.0251	-0.2468
LMO7	0.0000	-1.0052	0.0000	-1.2291	0.0160	0.3675	0.8663	-0.0335	1.0000	0.0322	0.1592	-0.1793	0.0000	-0.3636
LMOD1	1.0000	0.0480	0.3688	0.6320	0.6079	0.4916	0.0074	1.2652	1.0000	-0.4319	1.0000	0.1628	0.8937	0.3485
LMOD2	0.0000	-4.4551	0.0000	-4.6652	0.0013	-0.4801	0.0001	-0.4216	1.0000	0.0717	1.0000	-0.1275	0.5628	0.1352
LMOD3	0.0000	-5.0502	0.0000	-5.4867	0.3362	-0.3388	1.0000	0.0307	1.0000	-0.0761	1.0000	-0.5279	0.5042	0.2979
LMTK2	0.0623	0.3392	0.0627	0.2133	0.0001	0.7054	0.0001	0.3928	1.0000	0.2411	0.6604	0.1285	1.0000	-0.0661
LMX1A	1.0000	0.0000	1.0000	0.0000	1.0000	2.2427	1.0000	0.0000	1.0000	0.0000	1.0000	0.0000	1.0000	-2.2888
LMX1B	0.8249	3.0141	0.9754	0.7517	0.8011	3.0840	1.0000	-0.8419	1.0000	3.1126	1.0000	0.9750	1.0000	-0.7795
LNP1	1.0000	0.5414	0.4991	1.3064	1.0000	-0.1630	0.7701	-3.1666	1.0000	0.7357	0.9052	1.5308	1.0000	-2.2888
LNPEP	0.0000	2.5892	0.0000	2.3189	0.0000	3.1096	0.0000	2.2669	1.0000	0.3781	1.0000	0.1249	0.1535	-0.4538
LNX1	1.0000	-0.2881	1.0000	-0.1682	1.0000	-2.4056	0.9096	-1.3726	1.0000	1.2661	1.0000	1.4306	1.0000	2.3543
LNX2	0.1655	-0.3540	0.0021	-0.4357	0.0037	0.5741	0.0000	0.6950	1.0000	-0.1246	0.7010	-0.1924	1.0000	0.0018
LOC100126556	0.8226	3.0199	1.0000	2.2534	1.0000	0.0000	1.0000	0.0000	1.0000	0.0000	1.0000	-0.7956	1.0000	0.0000
LOC100303663	0.0000	-3.0699	0.0000	-3.1196	0.0000	-1.1854	0.0000	-1.2999	1.0000	-0.2954	0.8269	-0.3324	0.1534	-0.4043
LOC100303669	1.0000	0.0000	1.0000	0.0000	1.0000	0.0000	1.0000	0.0000	1.0000	0.0000	1.0000	0.0000	1.0000	0.0000
LOC100303670	1.0000	0.1032	0.7045	0.4564	0.0011	-2.4602	0.1919	-1.1569	1.0000	-0.7670	1.0000	-0.4036	1.0000	0.5436
LOC100303673	0.0000	-2.1678	0.0000	-2.3045	0.0000	-1.1113	0.0000	-1.0607	1.0000	0.0732	1.0000	-0.0518	0.3168	0.1291
LOC100303679	1.0000	0.0000	1.0000	0.0000	1.0000	0.0000	1.0000	0.0000	1.0000	0.0000	1.0000	0.0000	1.0000	0.0000
LOC100303681	0.0639	-0.8207	0.0056	-0.8386	0.0001	-1.5399	0.0000	-1.3092	1.0000	-0.4081	0.7428	-0.4138	1.0000	-0.1739
LOC100303683	1.0000	0.0000	1.0000	0.0000	1.0000	0.0000	1.0000	0.0000	1.0000	0.0000	1.0000	0.0000	1.0000	0.0000
LOC100303687	1.0000	0.0000	1.0000	0.0000	1.0000	0.0000	1.0000	0.0000	1.0000	0.0000	1.0000	0.0000	1.0000	0.0000
LOC100303688	1.0000	0.0000	1.0000	0.0000	1.0000	0.0000	1.0000	2.3257	1.0000	0.0000	1.0000	0.0000	1.0000	2.3554
LOC100303695	1.0000	0.1396	0.6989	-0.4580	1.0000	-0.0571	0.9132	-0.3253	1.0000	0.5404	1.0000	-0.0476	1.0000	0.2717

LOC100303701	0.2102	1.8473	0.5865	0.9883	0.1278	2.1369	0.4077	1.3509	1.0000	0.4081	1.0000	-0.4384	1.0000	-0.3694
LOC100303705	1.0000	2.1902	1.0000	0.0000	1.0000	0.0000	1.0000	0.0000	1.0000	0.0000	1.0000	-2.2991	1.0000	0.0000
LOC100303707	1.0000	0.0000	1.0000	2.2534	1.0000	0.0000	1.0000	2.3241	1.0000	0.0000	1.0000	2.3479	1.0000	2.3543
LOC100303709	0.0673	-1.9887	0.0052	-3.5706	0.0000	2.1010	0.3418	0.7541	1.0000	-0.1249	1.0000	-1.7117	0.0010	-1.4715
LOC100538357	0.0048	-0.4913	0.0000	-0.6163	0.0017	-0.5454	0.0000	-0.5046	1.0000	0.0701	1.0000	-0.0429	0.5636	0.1167
LOC100538358	0.2979	-0.3323	1.0000	-0.0510	0.6547	-0.1979	0.2332	-0.2499	1.0000	-0.0417	0.6022	0.2521	1.0000	-0.0864
LOC100538367	1.0000	0.0000	1.0000	-2.3959	1.0000	2.2472	0.4057	2.0793	1.0000	2.2674	1.0000	0.0000	0.6944	2.1422
LOC100538371	0.5539	-0.1749	0.0338	-0.2384	0.7110	-0.1299	0.2808	-0.1525	1.0000	0.1446	1.0000	0.0935	0.7380	0.1268
LOC100538374	1.0000	-2.4776	1.0000	0.0000	1.0000	-2.4056	1.0000	0.0000	1.0000	-2.3757	1.0000	0.0000	1.0000	0.0000
LOC100538375	1.0000	-0.3195	0.0979	-2.2099	0.3860	1.5009	1.0000	0.0068	1.0000	1.9193	1.0000	0.0532	1.0000	0.4305
LOC100538380	0.6923	-2.0798	0.0153	-5.2960	1.0000	-1.1039	0.6922	-0.8085	1.0000	0.9898	1.0000	-2.2991	0.9835	1.2976
LOC100538403	1.0000	0.0000	1.0000	0.0000	1.0000	0.0000	1.0000	0.0000	1.0000	0.0000	1.0000	0.0000	1.0000	0.0000
LOC100538406	1.0000	0.3082	0.0774	1.6687	0.1614	-4.4916	0.9786	-0.9107	1.0000	-0.4301	0.8286	0.9448	1.0000	3.2066
LOC100538407	1.0000	0.0000	1.0000	-0.1472	1.0000	0.0000	1.0000	-2.3201	1.0000	2.2736	1.0000	2.3456	1.0000	0.0000
LOC100538412	1.0000	0.6084	0.2550	-2.4822	1.0000	0.5002	1.0000	0.0068	1.0000	0.8210	0.6703	-2.2721	1.0000	0.3300
LOC100538416	0.0033	1.5620	0.0000	1.6069	1.0000	0.0393	0.7596	0.3676	1.0000	0.1664	1.0000	0.2280	0.8475	0.5079
LOC100538424	1.0000	0.0000	1.0000	0.0000	1.0000	0.0000	1.0000	0.0000	1.0000	0.0000	1.0000	0.0000	1.0000	0.0000
LOC100538433	0.0992	2.0582	0.3631	1.1102	0.4671	1.3190	1.0000	0.4077	1.0000	0.8185	1.0000	-0.1165	1.0000	-0.0862
LOC100538434	0.7224	-0.8138	0.2860	-1.2745	0.0043	1.9997	0.0364	1.2838	1.0000	0.5051	1.0000	0.0586	1.0000	-0.2062
LOC100538436	1.0000	0.0000	0.7674	-3.2498	1.0000	0.0000	0.7710	-3.1632	1.0000	3.1056	1.0000	0.0000	1.0000	0.0000
LOC100538440	0.0001	-0.6307	0.0000	-0.6963	0.0414	-0.3571	0.6555	-0.0710	1.0000	0.0826	1.0000	0.0289	0.0001	0.3734
LOC100538445	0.3092	4.2299	0.7674	3.0957	0.8011	3.0840	1.0000	2.3257	1.0000	0.0000	1.0000	-1.1643	1.0000	-0.7793
LOC100538446	0.4101	0.9774	0.0713	1.6992	0.2933	1.1336	0.2815	1.2287	1.0000	-0.5879	1.0000	0.1393	0.9934	-0.4948
LOC100538447	1.0000	0.3524	1.0000	0.2921	0.0539	2.0367	1.0000	0.4731	1.0000	0.5635	1.0000	0.5233	0.5419	-0.9934
LOC100538449	0.6353	1.0542	1.0000	2.2506	1.0000	0.1151	1.0000	0.0000	1.0000	-4.1552	0.1185	-3.1153	0.3373	-4.3543
LOC100538453	0.8249	3.0255	1.0000	-2.3959	0.2840	3.9988	1.0000	-2.3178	1.0000	2.2674	1.0000	-3.1468	0.5105	-4.0506
LOC100538454	0.0000	-3.2432	0.0000	-2.0225	0.0008	-1.8774	0.0000	-2.0557	1.0000	0.0317	0.3809	1.2668	1.0000	-0.1399
LOC100538455	0.8848	-0.1081	0.2593	0.2477	0.5994	0.1779	0.1888	0.2456	1.0000	-0.5435	0.8385	-0.1765	0.0077	-0.4707
LOC100538468	1.0000	-0.0395	0.3608	-0.1965	1.0000	0.0237	0.5038	-0.1578	1.0000	0.2060	1.0000	0.0619	1.0000	0.0291
LOC100538484	0.0000	2.0239	0.0002	1.7108	0.0000	2.6053	0.0000	2.0778	1.0000	0.0884	1.0000	-0.2105	0.4325	-0.4370
LOC100538487	0.0010	0.6533	0.0000	0.6987	0.0024	0.6198	0.0000	0.6949	1.0000	0.0670	0.8377	0.1249	0.6321	0.1466
LOC100538504	1.0000	0.1019	0.5004	0.4569	0.9714	-0.2038	0.6172	0.4061	1.0000	-0.8794	0.6450	-0.5095	1.0000	-0.2632
LOC100538505	1.0000	0.0000	1.0000	0.0000	1.0000	0.0000	1.0000	0.0000	1.0000	0.0000	1.0000	0.0000	1.0000	0.0000
LOC100538506	1.0000	-0.3227	0.5021	-1.6423	1.0000	0.7355	1.0000	-0.5493	1.0000	1.3515	1.0000	0.0533	1.0000	0.0761
LOC100538510	0.0260	1.2383	0.0014	1.1516	0.0914	1.0138	0.9953	-0.1834	1.0000	0.6871	0.3571	0.6154	0.6687	-0.5008
LOC100538523	1.0000	0.0000	1.0000	-2.3959	0.8033	3.0892	1.0000	0.0076	1.0000	2.2674	1.0000	0.0000	1.0000	-0.7804
LOC100538525	0.1059	0.4849	0.2416	0.2750	0.1318	-0.4823	0.0000	-1.0931	1.0000	0.0010	0.8371	-0.1984	0.0769	-0.6068
LOC100538528	1.0000	-2.4776	1.0000	0.0000	1.0000	-2.4056	1.0000	0.0000	1.0000	-2.3757	1.0000	0.0000	1.0000	0.0000
LOC100538538	0.0543	-1.8166	1.0000	0.1799	0.6968	-0.5766	1.0000	-0.2076	1.0000	-1.3778	1.0000	0.6295	0.6110	-1.0059
LOC100538539	0.0036	-0.4410	0.0002	-0.3526	0.0001	-0.5835	0.0000	-0.5342	1.0000	-0.0904	1.0000	0.0101	1.0000	-0.0356
LOC100538554	0.0000	-2.4060	0.0000	-2.5919	0.0038	-1.3625	0.0068	-1.0099	1.0000	-0.0876	1.0000	-0.2582	1.0000	0.2700
LOC100538555	0.0000	-0.9307	0.0000	-0.8507	0.2411	-0.3055	0.0156	-0.3322	1.0000	0.1609	0.5963	0.2539	0.8150	0.1389
LOC100538558	0.0000	-1.1652	0.0000	-1.1310	0.0001	-0.7699	0.0000	-0.6646	1.0000	-0.0698	1.0000	-0.0231	1.0000	0.0410
LOC100538559	0.8636	0.3925	0.0006	2.3503	0.4371	-1.0629	1.0000	-0.3002	1.0000	-1.4624	0.9072	0.5036	1.0000	-0.6969
LOC100538560	0.7651	0.0979	0.0527	-0.1895	0.7003	0.1113	0.0437	0.1769	1.0000	-0.0252	0.0045	-0.3003	1.0000	0.0456
LOC100538570	1.0000	0.0000	1.0000	0.0000	1.0000	0.0000	1.0000	0.0000	1.0000	0.0000	1.0000	0.0000	1.0000	0.0000
LOC100538580	0.0014	0.5286	0.0000	0.5747	0.0002	0.6175	0.0000	0.4731	1.0000	-0.1486	0.6987	-0.0904	0.0035	-0.2873
LOC100538582	1.0000	-0.3217	1.0000	-0.5518	1.0000	-1.0194	0.2831	-4.0731	1.0000	0.7852	1.0000	0.5860	1.0000	-2.2888
LOC100538586	1.0000	0.0222	1.0000	0.0317	0.0050	0.4864	0.0070	0.2826	1.0000	0.0135	1.0000	0.0351	0.2843	-0.1860
LOC100538587	0.2575	-0.3416	0.0001	-0.6344	0.1264	0.4315	0.9069	0.0769	1.0000	0.0683	0.7434	-0.2119	0.4766	-0.2829
LOC100538588	0.0004	-4.3430	0.0021	-3.6721	0.3189	-0.9220	0.6682	-0.6149	1.0000	-0.6398	1.0000	0.0489	1.0000	-0.3255
LOC100538591	1.0000	0.0000	1.0000	0.0000	1.0000	0.0000	1.0000	0.0000	1.0000	0.0000	1.0000	0.0000	1.0000	0.0000
LOC100538595	0.0000	0.6792	0.0000	0.4279	0.0189	-0.3724	0.0000	-0.7130	1.0000	0.0743	0.1889	-0.1649	0.0156	-0.2613
LOC100538605	0.0003	-1.2237	0.0001	-0.9953	0.1929	-0.4678	0.2146	-0.3486	1.0000	0.0637	0.9318	0.3065	0.9515	0.1904
LOC100538613	1.0000	0.0000	1.0000	0.0000	1.0000	0.0000	1.0000	0.0000	1.0000	0.0000	1.0000	0.0000	1.0000	0.0000
LOC100538614	1.0000	-0.3364	0.9149	-1.5421	1.0000	0.1994	1.0000	0.0043	1.0000	-0.1172	1.0000	-1.3254	1.0000	-0.3113
LOC100538629	0.1196	-0.2875	0.0043	-0.2684	0.5460	0.1512	1.0000	0.0087	1.0000	0.0210	1.0000	0.0523	0.5421	-0.1161
LOC100538632	0.7859	0.2488	0.6998	0.2545	0.1039	-0.7489	0.1911	-0.5500	1.0000	-0.2281	1.0000	-0.2125	1.0000	-0.0274
LOC100538652	1.0000	0.0000	1.0000	0.0000	1.0000	0.0000	1.0000	0.0000	1.0000	0.0000	1.0000	0.0000	1.0000	0.0000
LOC100538662	1.0000	0.3613	0.9574	-0.3277	0.5513	-1.6581	0.3654	-1.2673	1.0000	0.5934	1.0000	-0.0867	1.0000	0.9915
LOC100538666	0.0006	0.9870	0.0000	1.3943	0.3515	0.3841	0.5182	0.2674	1.0000	-0.4251	1.0000	-0.0033	0.1425	-0.5368
LOC100538679	0.6904	-0.2201	1.0000	0.0147	0.0285	-0.7410	0.0005	-0.9290	1.0000	-0.2529	1.0000	-0.0037	0.5162	-0.4329
LOC100538692	0.1256	0.7109	0.0094	0.8030	0.0000	1.7278	0.0000	1.9664	1.0000	-0.1624	1.0000	-0.0578	1.0000	0.0857
LOC100538693	0.3648	0.3469	1.0000	0.0132	0.2310	0.3700	1.0000	-0.0589	1.0000	0.2050	1.0000	-0.1171	0.6260	-0.2175
LOC100538694	0.4310	-0.1655	0.0059	-0.2561	0.0000	-0.7617	0.0000	-0.7611	1.0000	-0.0334	0.6944	-0.1116	1.0000	-0.0270
LOC100538708	0.1947	0.2499	0.0144	0.2364	0.0021	0.4996	0.0000	0.4754	1.0000	-0.0099	1.0000	-0.0105	1.0000	-0.0284
LOC100538712	1.0000	-2.4788	1.0000	-2.3959	1.0000	-2.4061	1.0000	-2.3178	1.0000	-0.1089	1.0000	0.0000	1.0000	0.0000
LOC100538719	0.0000	1.0765	0.0000	1.1788	0.0000	2.1488	0.0000	1.5655	1.0000	0.2046	0.3260	0.3208	0.0024	-0.3717
LOC100538725	0.5836	-1.5593	0.7753	-1.1128	1.0000	0.0474	0.0842	1.4713	1.0000	0.1246	1.0000	0.5934	0.2735	1.5454
LOC100538736	1.0000	2.1849	1.0000	0.0000	1.0000	2.2472	1.0000	0.0000	1.0000	0.0000	1.0000	-2.2955	1.0000	-2.2907
LOC100538756	0.0014	1.2601	0.0000	1.4387	0.9333	-0.2359	0.3257	-0.5947	1.0000	-0.4555	0.8732	-0.2688	0.2593	-0.8141
LOC100538764	1.0000	-0.8627	0.4431	-3.7875	1.0000	0.4924	0.5789	1.1637	1.0000	-0.1216	1.0000	-3.1383	1.0000	0.5459
LOC100538773	0.6757	1.0044	1.0000	0.2708	0.0001	3.1169	1.0000	0.3204	1.0000	1.2174	1.0000	0.5059	0.0258	-1.5735
LOC100538779	0.2674	0.2941	0.2306	0.2033	0.1137	0.3707	0.4861	0.1397	1.0000	0.0179	1.0000	-0.0613	0.3309	-0.2090
LOC100538780	0.0001	0.9422	0.0000	1.2796	0.0027	0.7056	0.0000	0.8460	1.0000	-0.1839	0.7635	0.1677	1.0000	-0.0349
LOC100538782	0.8360	0.6136	0.0444	1.5202	0.0024	2.2180	0.0818	1.7212	1.0000	-0.1315	0.8480	0.7872	0.7287	-0.6331
LOC100538789	1.0000	2.1902	1.0000	0.0000	1.0000	0.0000	1.0000	0.0000	1.0000	0.0000	1.0000	-2.2991	1.0000	0.0000
LOC100538796	0.0001	-0.5907	0.0000	-0.6385	0.8033	0.0930	0.0768	0.1473	1.0000	0.0717	1.0000	0.0365	0.4351	0.1309
LOC100538805	0.0005	-0.5222	0.0000	-0.4798	0.0002	-0.5771	0.0000	-0.4800	1.0000	-0.0360	1.0000	0.0185	0.9441	0.0666
LOC100538808	0.1143	-1.0405	0.8190	-0.2716	0.6526	0.3934	0.3900	0.4636	1.0000	-0.3397	0.9994	0.4415	0.9693	-0.2652
LOC100538811	0.1384	2.2168	0.1594	2.3753	0.3860	1.5009	0.0000	4.2841	1.0000	-0.9614	0.8856	-0.8100	0.0505	1.8209
LOC100538813	0.3073	-0.2234	0.0405	-0.2202	0.5080	0.1612	0.0010	0.3084	1.0000	-0.0506	1.0000	-0.0345	0.7287	0.1019
LOC100538821	0.7902	0.2165	0.1615	-0.5365	0.2889	0.4520	0.1550	0.3795	1.0000	0.3590	0.7565	-0.3804	0.5843	0.2902
LOC100538825	0.0705	1.3822	0.0001	2.2586	0.0106	1.6236	0.0004	2.1608	1.0000	-0.6281	1.0000	0.2576	1.0000	-0.0875
LOC100538829	1.0000	-1.1562	0.2920	4.0165	1.0000	-1.0191	0.2426	4.0910	1.0000	-3.2304	1.0000	1.8280	0.8937	1.8364
LOC100538839	0.4266	0.1935	0.0002	0.4161	0.0001	0.6672	0.0000	0.5743	1.0000	-0.1183	0.7635	0.1174	0.1712	-0.2048
LOC100538841	0.1600	-0.8061	0.0030	-0.8719	0.0016	1.1697	0.0000	1.8725	1.0000	0.6669	0.4313	0.6188	0.0000	1.3787
LOC100538845	0.0990	0.3795	0.0781	0.2393	0.9874	-0.0778	0.5792	-0.1086	1.0000	0.1589	1.0000	0.0319	0.7908	0.1351
LOC100538849	1.0000	-0.0254	0.7674	-0.0629	0.0020	-0.5467	0.0000	-0.4844	1.0000	0.0408	1.0000	0.0155	0.7287	0.1075
LOC100538852	1.0000	-0.0051	0.0766	-0.3165	0.3122	-0.2835	0.0001	-0.5998	1.0000	0.0037	0.2773	-0.2952	0.1872	-0.3052
LOC100538860	0.2017	-0.2942	0.0296	0.2957	0.0659	0.3627	0.0000	0.5197	1.0000	-0.3586	0.2687	0.2440	0.2633	-0.1954
LOC100538862	1.0000	-0.0648	0.6562	-0.2811	0.1170	-1.0785	0.9647	-0.1644	1.0000	0.6202	0.8793	0.4168	0.0159	1.5426
LOC100538867	0.2324	0.7444	1.0000	0.1542	0.0638	1.0141	0.0004	1.1817	1.0000	0.4133	1.0000	-0.1631	0.3395	0.5835

LOC100538868	0.6927	1.4475	0.9762	-1.0846	0.9468	1.2126	1.0000	-0.3805	1.0000	1.6538	1.0000	-0.8603	1.0000	0.0738
LOC100538873	0.0001	2.1315	0.0000	3.5053	0.1772	1.0814	0.0399	1.6299	1.0000	-0.9777	0.6337	0.4052	1.0000	-0.4228
LOC100538883	0.0178	0.8252	0.0001	0.7209	0.0000	1.5836	0.0000	1.5776	1.0000	-0.1030	0.9920	-0.1944	1.0000	-0.1037
LOC100538884	1.0000	0.0000	1.0000	0.0000	1.0000	0.0000	1.0000	0.0000	1.0000	0.0000	1.0000	0.0000	1.0000	0.0000
LOC100538887	1.0000	0.0000	1.0000	0.0000	1.0000	0.0000	1.0000	0.0000	1.0000	0.0000	1.0000	0.0000	1.0000	0.0000
LOC100538893	0.0000	-1.0018	0.0000	-0.9665	0.0000	-1.7883	0.0000	-1.5234	1.0000	-0.0817	1.0000	-0.0339	0.2175	0.1885
LOC100538897	0.0000	3.0942	0.0000	3.5884	0.7702	0.4369	0.1339	1.0536	1.0000	-0.6587	1.0000	-0.1551	1.0000	-0.0389
LOC100538901	0.3843	-1.3025	0.3421	1.5203	1.0000	-0.0324	0.9786	0.9243	1.0000	-1.9965	1.0000	0.8294	0.7609	-1.0436
LOC100538902	0.0060	-0.6183	0.0000	-0.7574	0.0000	-1.5876	0.0000	-1.0824	1.0000	-0.1487	0.0082	-0.2753	0.0101	0.3634
LOC100538920	0.0021	-0.4547	0.0000	-0.4437	0.0000	-0.8252	0.0000	-0.8862	1.0000	-0.0308	1.0000	-0.0076	0.8234	-0.0861
LOC100538921	0.7743	0.0918	0.0001	0.2841	0.9176	-0.0691	0.0013	0.2263	1.0000	-0.3160	0.5245	-0.1112	1.0000	-0.0151
LOC100538926	1.0000	-2.4788	1.0000	-0.1452	1.0000	-2.4061	1.0000	-2.3201	1.0000	-0.1045	1.0000	2.3479	1.0000	0.0000
LOC100538928	1.0000	0.0000	1.0000	0.0000	1.0000	2.2427	1.0000	2.3241	1.0000	0.0000	1.0000	0.0000	1.0000	0.0652
LOC100538933	0.8249	0.2959	0.7468	-0.2618	0.2149	0.6300	0.4221	0.3613	1.0000	0.2574	1.0000	-0.2867	1.0000	-0.0003
LOC100538944	0.0216	1.1498	0.0131	0.9235	0.0159	1.2369	0.1403	0.6476	1.0000	0.3401	1.0000	0.1264	1.0000	-0.2464
LOC100538956	0.5408	-0.8696	0.4201	-0.8050	1.0000	-0.1930	0.5021	-0.7818	1.0000	-0.0186	1.0000	0.0574	0.9122	-0.6046
LOC100538957	1.0000	0.0141	0.2772	0.9889	0.0864	-2.3747	1.0000	-0.2060	1.0000	-0.6302	1.0000	0.3560	0.7908	1.5494
LOC100538959	0.0005	-0.5130	0.0000	-0.6563	1.0000	0.0213	0.0143	-0.2033	1.0000	0.1014	1.0000	-0.0295	0.5038	-0.1178
LOC100538963	1.0000	0.0529	0.0995	0.8124	1.0000	-0.1813	0.1951	0.7108	1.0000	-0.9496	1.0000	-0.1785	1.0000	-0.0536
LOC100538965	1.0000	0.5370	0.3415	1.5198	0.1954	2.5399	1.0000	0.0066	1.0000	0.7357	0.7635	1.7457	0.4877	-1.7976
LOC100538966	0.0646	0.3792	0.0004	0.3059	0.0000	0.7889	0.0000	0.5618	1.0000	0.0027	1.0000	-0.0577	0.0831	-0.2180
LOC100538971	0.0000	-1.0437	0.0000	-1.2532	0.0000	-1.2867	0.0000	-1.5932	1.0000	0.0293	0.2582	-0.1678	0.0067	-0.2715
LOC100538974	0.6544	-0.1575	0.2915	0.1442	0.6903	-0.1484	0.0151	-0.2842	1.0000	-0.0346	0.0743	0.2786	0.4901	-0.1654
LOC100538975	0.9546	-0.2033	1.0000	-0.1823	0.1174	-0.9290	0.1779	-0.7230	1.0000	-0.1305	1.0000	-0.0973	1.0000	0.0803
LOC100538976	0.7157	-0.4497	0.0004	-1.5126	0.0577	-1.2336	0.2508	-0.5090	1.0000	0.7121	1.0000	-0.3349	0.0137	1.4449
LOC100538984	1.0000	0.0000	1.0000	0.0000	1.0000	0.0000	1.0000	0.0000	1.0000	0.0000	1.0000	0.0000	1.0000	0.0000
LOC100538986	1.0000	0.0000	1.0000	0.0000	1.0000	0.0000	1.0000	0.0000	1.0000	0.0000	1.0000	0.0000	1.0000	0.0000
LOC100539001	0.2639	-0.4019	0.0000	-0.7119	0.2681	-0.3826	0.0033	-0.3809	1.0000	0.2924	1.0000	-0.0036	0.1787	0.2990
LOC100539002	0.0000	-0.7852	0.0000	-0.7848	0.0411	0.3602	0.0001	0.3764	1.0000	0.0579	1.0000	0.0717	0.8663	0.0798
LOC100539006	0.1121	1.3509	0.4786	0.5775	0.0880	-3.0633	0.0006	-3.7804	1.0000	0.7733	1.0000	0.0113	1.0000	0.0649
LOC100539008	0.8454	0.6216	0.2501	0.8568	1.0000	-0.6579	0.0980	-2.0322	1.0000	0.7547	0.4103	0.9965	1.0000	-0.6157
LOC100539010	0.1644	-0.3542	0.0193	-0.3282	0.0000	-1.1421	0.0000	-0.8371	1.0000	0.0110	1.0000	0.0497	0.3290	0.3203
LOC100539020	1.0000	0.0000	1.0000	0.0000	1.0000	0.0000	1.0000	0.0000	1.0000	0.0000	1.0000	0.0000	1.0000	0.0000
LOC100539021	1.0000	-1.1537	0.7674	-3.2570	0.8011	-3.2629	1.0000	-0.8443	1.0000	-0.1146	1.0000	-2.2991	1.0000	2.3543
LOC100539027	1.0000	-0.0469	0.0412	-0.2155	0.4547	0.1790	0.2111	0.1523	1.0000	0.1593	1.0000	0.0038	0.6307	0.1376
LOC100539032	0.4782	2.0011	0.3572	1.5204	1.0000	-0.1624	1.0000	0.5362	1.0000	0.7416	1.0000	0.2753	1.0000	1.4486
LOC100539035	0.8226	3.0199	1.0000	-0.1429	0.8033	3.0789	1.0000	0.0076	1.0000	2.2673	1.0000	-0.7956	1.0000	-0.7769
LOC100539037	1.0000	0.0000	1.0000	0.0000	1.0000	0.0000	1.0000	0.0000	1.0000	0.0000	1.0000	0.0000	1.0000	0.0000
LOC100539040	0.5086	3.5431	1.0000	2.2534	1.0000	0.0000	1.0000	0.0000	1.0000	0.0000	1.0000	-1.3246	1.0000	0.0000
LOC100539041	1.0000	0.0000	1.0000	0.0000	1.0000	0.0000	1.0000	0.0000	1.0000	0.0000	1.0000	0.0000	1.0000	0.0000
LOC100539048	0.0000	-3.9864	0.0000	-4.2864	0.0015	-1.0626	0.0000	-1.2089	1.0000	-0.2231	1.0000	-0.5115	0.6585	-0.3633
LOC100539056	1.0000	0.0000	1.0000	-0.1630	1.0000	0.0000	0.7710	-3.1692	1.0000	3.1191	1.0000	3.1976	1.0000	0.0000
LOC100539065	0.6770	0.6844	0.1746	0.9212	1.0000	-0.0728	0.7701	0.4997	1.0000	-0.0117	1.0000	0.2370	1.0000	0.5696
LOC100539073	1.0000	0.0000	1.0000	0.0000	1.0000	0.0000	1.0000	0.0000	1.0000	0.0000	1.0000	0.0000	1.0000	0.0000
LOC100539075	0.2746	0.3501	0.0075	0.4633	1.0000	0.0501	0.0026	0.5274	1.0000	-0.1525	1.0000	-0.0263	0.2263	0.3300
LOC100539082	0.0151	-1.3543	0.1390	-0.7605	0.0086	-1.3326	0.0762	-0.9063	1.0000	-0.3505	1.0000	0.2532	1.0000	0.0800
LOC100539085	0.0003	1.0893	0.0000	1.5138	0.0001	1.1542	0.0065	0.6413	1.0000	-0.2533	0.8388	0.1844	0.0003	-0.7576
LOC100539092	0.8249	3.0141	1.0000	0.0000	1.0000	2.2472	1.0000	0.0000	1.0000	0.0000	1.0000	-3.1383	1.0000	-2.2907
LOC100539100	0.0000	2.0628	0.0000	1.7085	0.0002	-1.2093	0.0000	-1.0814	1.0000	0.0728	0.1981	-0.2659	1.0000	0.2073
LOC100539104	0.0049	0.7597	0.0000	0.8846	0.0000	1.4782	0.0000	1.1007	1.0000	0.1592	0.2872	0.2969	0.3799	-0.2136
LOC100539108	1.0000	2.1902	1.0000	-2.3986	1.0000	0.0000	1.0000	0.0057	1.0000	2.2736	1.0000	-2.2991	1.0000	2.3554
LOC100539112	0.0101	-0.4356	0.0000	-0.4727	0.5243	-0.1567	0.0000	0.3192	1.0000	-0.0401	0.9773	-0.0650	0.0000	0.4405
LOC100539124	0.1881	-0.7711	0.0053	-1.2379	0.3739	-0.5634	0.2669	-0.5412	1.0000	0.0053	1.0000	-0.4490	1.0000	0.0336
LOC100539126	0.6866	-0.8964	0.6224	1.6101	0.3093	-2.5001	1.0000	0.0062	1.0000	-2.4391	1.0000	0.0576	1.0000	0.0652
LOC100539130	0.0001	1.6172	0.0640	0.7689	0.0011	-2.3212	0.0033	-1.7381	1.0000	0.2024	0.1655	-0.6355	0.9955	0.7905
LOC100539136	1.0000	2.1902	0.7674	3.0888	1.0000	0.0000	1.0000	2.3241	1.0000	0.0000	1.0000	0.8962	1.0000	2.3542
LOC100539139	0.4525	0.4667	0.1042	0.8093	0.0102	1.0166	0.0704	0.8098	1.0000	-0.0802	1.0000	0.2708	0.7708	-0.2875
LOC100539142	0.0254	0.4225	0.5171	0.1226	1.0000	0.0157	0.0376	0.2421	1.0000	0.0537	0.2806	-0.2346	0.2172	0.2858
LOC100539152	0.9299	-0.0696	0.0572	0.1713	0.0029	0.4792	0.0000	0.7132	1.0000	-0.0489	0.1129	0.2044	0.1339	0.1902
LOC100539157	0.0075	1.1007	0.0547	0.6175	0.0327	0.9594	0.0897	0.6672	1.0000	0.5289	1.0000	0.0610	0.9809	0.2424
LOC100539161	0.3372	-0.5675	1.0000	0.0413	0.4174	0.4276	0.2909	0.3504	1.0000	0.2686	0.0318	0.8927	0.9662	0.2002
LOC100539169	1.0000	-0.0160	0.0045	-0.2572	0.3217	0.2170	0.0003	0.2986	1.0000	0.1887	1.0000	-0.0399	0.0112	0.2750
LOC100539172	1.0000	0.0206	0.6850	0.0898	0.1876	0.3019	0.0002	0.4204	1.0000	-0.2124	0.8232	-0.1303	0.9264	-0.0875
LOC100539174	1.0000	0.0000	1.0000	-2.3986	1.0000	2.2427	1.0000	-2.3201	1.0000	2.2736	1.0000	0.0000	1.0000	-2.2888
LOC100539176	1.0000	0.0000	1.0000	0.0000	1.0000	0.0000	1.0000	0.0000	1.0000	0.0000	1.0000	0.0000	1.0000	0.0000
LOC100539183	0.0000	-1.2062	0.1533	0.2857	0.0000	2.7470	0.0000	2.8092	1.0000	-0.0664	0.0000	1.4401	1.0000	0.0020
LOC100539194	1.0000	0.5420	1.0000	2.2534	1.0000	-0.1624	1.0000	0.0000	1.0000	-2.3757	1.0000	-0.7956	1.0000	-2.2888
LOC100539204	0.0179	-1.1425	0.0001	-1.4509	0.9098	-0.1963	0.9355	0.1488	1.0000	-0.1139	1.0000	-0.4100	0.9352	0.2402
LOC100539216	0.0003	0.6338	0.0000	0.4402	0.0037	0.5536	0.0000	0.6827	1.0000	-0.0473	0.0550	-0.2281	0.8768	0.0873
LOC100539227	0.4950	1.7576	0.4431	3.6192	1.0000	-2.4061	1.0000	0.0000	1.0000	-2.3771	1.0000	-0.6369	1.0000	0.0000
LOC100539228	1.0000	0.0000	1.0000	0.0000	1.0000	0.0000	1.0000	0.0000	1.0000	0.0000	1.0000	0.0000	1.0000	0.0000
LOC100539236	0.1877	1.9952	0.0244	2.4949	0.8011	1.0501	1.0000	0.5373	1.0000	-0.1179	1.0000	0.3977	1.0000	-0.6204
LOC100539239	1.0000	0.0000	1.0000	0.0000	1.0000	2.2427	1.0000	2.3241	1.0000	0.0000	1.0000	0.0000	1.0000	0.0652
LOC100539246	0.1512	-0.2693	0.0132	-0.2637	0.1009	-0.2997	0.2123	-0.1402	1.0000	-0.1071	0.9305	-0.0889	1.0000	0.0588
LOC100539247	1.0000	0.0292	0.4126	0.1305	0.0015	-0.7018	0.0000	-0.5466	1.0000	-0.1458	1.0000	-0.0322	1.0000	0.0160
LOC100539248	0.0244	1.9708	0.0360	2.0580	1.0000	-0.4936	1.0000	0.0092	1.0000	-0.8174	0.6478	-0.7232	1.0000	-0.3113
LOC100539273	1.0000	0.0000	1.0000	0.0000	1.0000	0.0000	1.0000	0.0000	1.0000	0.0000	1.0000	0.0000	1.0000	0.0000
LOC100539278	0.0000	-1.2506	0.0000	-1.2556	0.4074	0.2473	0.9238	-0.0681	1.0000	0.0536	1.0000	0.0621	0.3867	-0.2566
LOC100539280	0.0150	-0.4121	0.0000	-0.5412	0.0000	-0.9375	0.0000	-0.9159	1.0000	0.0083	0.8064	-0.1088	1.0000	0.0349
LOC100539289	0.7098	0.6193	0.0685	1.1990	0.0005	1.8660	0.0226	1.3867	1.0000	-0.2524	1.0000	0.3338	0.2625	-0.7277
LOC100539290	0.0000	1.1574	0.0000	1.2631	0.0000	1.5620	0.0000	1.1799	1.0000	0.0554	0.7635	0.1723	0.0970	-0.3227
LOC100539298	0.0069	1.0638	0.0001	0.9554	0.0000	1.4590	0.0294	0.6115	1.0000	0.4837	0.4575	0.3886	0.4263	-0.3616
LOC100539302	0.0000	-2.1999	0.0000	-1.7963	0.0000	-0.9805	0.0083	-0.2062	1.0000	-0.1776	0.3555	0.2388	0.0000	0.6024
LOC100539311	0.7808	-0.1321	0.0616	-0.2382	0.0009	-0.7196	0.0000	-0.7011	1.0000	0.0764	1.0000	-0.0173	0.9809	0.1012
LOC100539329	0.8249	-0.6648	0.4744	1.0969	1.0000	0.3798	0.1100	1.6473	1.0000	-1.3975	1.0000	0.3728	1.0000	-0.1249
LOC100539332	1.0000	-0.0255	0.0469	-0.5677	0.3729	-0.4157	0.0013	-0.8834	1.0000	0.2881	0.9908	-0.2416	1.0000	-0.1724
LOC100539335	0.0000	1.7990	0.0000	2.3285	0.0000	1.2907	0.0000	1.0969	1.0000	-0.4343	1.0000	0.1057	0.0105	-0.6223
LOC100539357	0.0488	0.5527	0.0091	0.5257	0.0000	0.9770	0.0000	0.8557	1.0000	-0.0978	1.0000	-0.1107	0.5105	-0.2120
LOC100539361	1.0000	0.0491	1.0000	-1.0017	0.0784	1.9398	1.0000	0.0066	1.0000	-0.6504	1.0000	-1.7124	0.0524	-2.5867
LOC100539364	0.3880	0.2644	0.0675	0.2769	0.0005	-0.8601	0.0000	-0.6743	1.0000	0.0983	0.9428	0.1239	0.3984	0.2883
LOC100539365	0.0080	-0.6004	0.0000	-0.6611	0.0006	-0.7680	0.0000	-0.7510	1.0000	0.0474	1.0000	-0.0009	1.0000	0.0707
LOC100539368	1.0000	0.0000	1.0000	0.0000	1.0000	0.0000	1.0000	0.0000	1.0000	0.0000	1.0000	0.0000	1.0000	0.0000
LOC100539376	0.6992	-0.1162	0.0003	-0.3488	1.0000	-0.0221	1.0000	-0.0211	1.0000	0.2866	1.0000	0.0668	0.0105	0.2930

LOC100539381	1.0000	-1.1537	1.0000	-2.3986	0.8033	-3.2636	1.0000	-2.3201	1.0000	-0.9587	1.0000	-2.2991	1.0000	0.0000
LOC100539382	0.0000	-1.6078	0.0000	-1.7285	0.0026	-0.5117	0.0000	-0.5615	1.0000	0.0501	1.0000	-0.0580	1.0000	0.0050
LOC100539384	0.0210	-0.9167	0.0060	-0.8151	0.0312	-0.8296	0.0364	-0.5631	1.0000	-0.1320	1.0000	-0.0197	1.0000	0.1404
LOC100539388	1.0000	0.3564	0.1130	1.3766	0.5560	-1.6619	1.0000	-0.5357	1.0000	-0.1387	0.8156	0.8971	1.0000	0.9931
LOC100539391	1.0000	-0.1826	0.0606	0.9634	0.9468	-0.2495	0.0892	0.9158	1.0000	-0.8118	1.0000	0.3450	0.9094	0.3588
LOC100539397	1.0000	0.0000	1.0000	0.0000	1.0000	0.0000	1.0000	2.3241	1.0000	0.0000	1.0000	0.0000	1.0000	2.3542
LOC100539427	0.1428	0.8164	0.0803	0.5192	0.0000	2.2234	0.0000	1.0774	1.0000	0.7607	0.4558	0.4736	0.3550	-0.3833
LOC100539435	1.0000	-0.3436	0.6395	-1.1296	0.8672	0.5795	1.0000	0.0050	1.0000	0.8304	1.0000	0.0600	1.0000	0.2621
LOC100539441	0.0000	-2.0288	0.0000	-2.3866	0.5764	-0.3178	0.7427	0.1846	1.0000	-0.0072	1.0000	-0.3518	0.2498	0.5001
LOC100539445	0.0012	2.2693	0.0000	2.3214	0.0000	2.7905	0.0000	3.0258	1.0000	-0.0116	1.0000	0.0561	0.8138	0.2336
LOC100539447	0.5457	-0.4787	0.0008	-1.5393	0.6150	0.3722	0.5604	0.3285	1.0000	0.1161	0.3892	-0.9321	1.0000	0.0804
LOC100539460	1.0000	0.0319	1.0000	0.0307	0.0261	-0.5616	0.3548	-0.2132	1.0000	-0.0975	1.0000	-0.0869	0.5528	0.2552
LOC100539461	1.0000	0.0000	1.0000	-2.3959	1.0000	0.0000	1.0000	-2.3178	1.0000	2.2674	1.0000	0.0000	1.0000	0.0000
LOC100539476	1.0000	0.0000	1.0000	0.0000	1.0000	0.0000	1.0000	0.0000	1.0000	0.0000	1.0000	0.0000	1.0000	0.0000
LOC100539477	0.4654	-0.1751	0.2250	-0.1468	0.0011	-0.5593	0.0000	-0.4252	1.0000	-0.1311	0.9519	-0.0897	1.0000	0.0086
LOC100539483	0.0012	-1.1962	0.0000	-1.3646	0.2378	-0.4855	0.0120	-0.5871	1.0000	0.2038	1.0000	0.0456	1.0000	0.1067
LOC100539484	0.0131	-0.4326	0.0103	-0.2040	0.4809	-0.1621	0.0129	-0.1927	1.0000	-0.0091	0.1187	0.2317	1.0000	-0.0347
LOC100539486	0.6590	0.8738	0.6391	0.9489	0.0443	1.9455	0.0000	3.2506	1.0000	-0.4321	1.0000	-0.3460	0.2088	0.8736
LOC100539487	0.1253	-0.3397	0.0000	-0.5361	0.0000	-1.2867	0.0000	-0.9380	1.0000	-0.1550	0.0833	-0.3391	0.5944	0.1988
LOC100539489	1.0000	2.1902	1.0000	0.0000	1.0000	0.0000	1.0000	0.0000	1.0000	0.0000	1.0000	-2.2991	1.0000	0.0000
LOC100539493	0.0215	0.6369	0.0001	0.8493	1.0000	0.0437	0.9296	0.1110	1.0000	-0.1265	1.0000	0.0962	1.0000	-0.0546
LOC100539497	1.0000	2.1849	1.0000	0.0000	1.0000	0.0000	1.0000	0.0000	1.0000	0.0000	1.0000	-2.2955	1.0000	0.0000
LOC100539498	0.0439	0.9409	0.0002	1.0829	0.0131	0.9843	0.0821	0.6332	1.0000	-0.1009	1.0000	0.0512	0.3975	-0.4446
LOC100539521	1.0000	-2.4776	0.4636	-3.7802	1.0000	-2.4056	0.4635	-3.6899	1.0000	1.2565	1.0000	0.0000	1.0000	0.0000
LOC100539530	0.3031	-0.2448	0.0000	-0.5283	0.8043	-0.1052	0.4569	-0.0796	1.0000	0.1744	0.9075	-0.0966	0.0816	0.2047
LOC100539535	0.3268	-0.7905	0.0792	-0.8592	0.0057	-1.8008	0.0060	-1.3789	1.0000	-0.0102	1.0000	-0.0618	1.0000	0.4199
LOC100539550	0.8399	0.1279	0.0034	0.4390	0.0000	1.2705	0.0000	1.2049	1.0000	0.1430	0.0328	0.4660	0.9412	0.0827
LOC100539553	0.2624	0.3335	0.9534	0.0903	0.0000	2.2537	0.0000	1.7812	1.0000	0.2135	1.0000	-0.0169	0.1895	-0.2544
LOC100539565	0.2644	0.3143	0.2555	0.2415	0.0000	1.4464	0.0000	1.0976	1.0000	0.0913	1.0000	0.0324	0.4096	-0.2514
LOC100539575	0.9917	0.5147	0.0327	1.2609	0.0000	3.8700	0.0000	3.5242	1.0000	0.6311	0.0891	1.3886	0.6042	0.2898
LOC100539580	1.0000	0.0000	1.0000	0.0000	1.0000	0.0000	1.0000	0.0000	1.0000	0.0000	1.0000	0.0000	1.0000	0.0000
LOC100539591	1.0000	0.1006	0.0191	0.4870	0.1610	-0.4641	0.1162	-0.3922	1.0000	-0.2068	0.8847	0.1909	1.0000	-0.1312
LOC100539600	1.0000	-0.2881	1.0000	-0.1594	1.0000	-2.4056	0.7710	-3.1632	1.0000	0.7317	1.0000	0.8994	1.0000	0.0000
LOC100539601	0.4875	-0.1683	0.0005	-0.3197	0.1544	0.2673	0.0159	0.2183	1.0000	0.1509	1.0000	0.0122	0.6637	0.1074
LOC100539603	1.0000	-0.3436	0.7674	3.0888	1.0000	0.1177	0.2426	4.0910	1.0000	-4.1514	1.0000	-0.8610	1.0000	-0.2289
LOC100539604	1.0000	0.0000	1.0000	0.0000	1.0000	0.0000	1.0000	0.0000	1.0000	0.0000	1.0000	0.0000	1.0000	0.0000
LOC100539605	0.7717	-0.2386	0.0436	0.4517	0.0634	-0.7217	1.0000	0.0071	1.0000	0.2344	0.0007	0.9389	0.0001	0.9660
LOC100539619	0.3247	0.2680	0.1628	0.2990	0.0005	0.6950	0.0003	0.6432	1.0000	0.1122	0.7597	0.1574	1.0000	0.0664
LOC100539623	0.2246	-0.2355	0.0000	-0.4505	0.0296	-0.3844	0.0000	-0.3703	1.0000	-0.0615	0.0054	-0.2642	1.0000	-0.0416
LOC100539630	0.4891	0.1881	0.8192	0.0496	0.0000	0.7326	0.0000	0.5899	1.0000	0.0576	1.0000	-0.0685	0.8499	-0.0803
LOC100539632	0.0000	-1.9378	0.0000	-2.3145	0.0000	-0.7867	0.0000	-0.7949	1.0000	0.1286	0.4871	-0.2355	0.6362	0.1253
LOC100539633	0.2092	-2.6409	0.9062	1.2225	0.0919	-4.7440	1.0000	0.0062	1.0000	-2.4397	1.0000	1.4306	1.0000	2.3542
LOC100539637	1.0000	0.0545	0.6906	0.7895	0.6394	0.9672	1.0000	0.4730	1.0000	0.5674	0.7749	1.3264	1.0000	0.0788
LOC100539639	0.8249	0.4309	0.8250	0.3443	0.9174	-0.4018	1.0000	0.1573	1.0000	0.1311	1.0000	0.0581	0.7180	0.6925
LOC100539640	0.0002	1.0463	0.0000	1.1284	0.0001	1.0653	0.0000	0.9479	1.0000	0.0416	1.0000	0.1386	1.0000	-0.0697
LOC100539641	0.5645	0.1483	0.3975	0.1124	0.0180	-0.4176	0.0000	-0.4697	1.0000	0.0947	1.0000	0.0709	1.0000	0.0476
LOC100539643	1.0000	0.5414	0.7268	1.0575	1.0000	-0.1597	0.7701	-3.1666	1.0000	0.7357	1.0000	1.2809	1.0000	-2.2907
LOC100539644	1.0000	-2.4776	1.0000	0.0000	1.0000	-0.1624	1.0000	0.0000	1.0000	-2.3757	1.0000	0.0000	1.0000	-2.2888
LOC100539646	0.0739	2.2242	0.0299	5.1397	1.0000	-1.0191	0.4370	3.7037	1.0000	-3.2304	1.0000	-0.4577	1.0000	1.4486
LOC100539648	1.0000	0.0258	0.9725	0.4458	1.0000	0.2584	0.6843	-0.8104	1.0000	-0.6458	1.0000	-0.2156	0.1688	-1.7152
LOC100539649	1.0000	0.0000	1.0000	0.0000	1.0000	0.0000	1.0000	0.0000	1.0000	0.0000	1.0000	0.0000	1.0000	0.0000
LOC100539654	0.0042	0.9551	0.0000	1.0474	0.0000	1.4825	0.0001	0.8873	1.0000	0.4442	0.0633	0.5520	0.9662	-0.1434
LOC100539660	1.0000	0.2029	1.0000	0.6956	1.0000	-0.1765	0.0517	2.8944	1.0000	-0.9614	1.0000	-0.4752	0.3521	2.1149
LOC100539663	0.0000	1.2433	0.0000	1.5169	0.3045	0.3329	0.1160	0.3520	1.0000	-0.1727	0.9606	0.1151	0.8936	-0.1440
LOC100539666	0.3089	3.9281	0.4154	1.9132	0.0562	4.7614	1.0000	0.8518	1.0000	2.2736	1.0000	0.3644	0.6308	-1.6122
LOC100539678	1.0000	0.0000	1.0000	0.0000	1.0000	0.0000	1.0000	0.0000	1.0000	0.0000	1.0000	0.0000	1.0000	0.0000
LOC100539680	1.0000	0.0000	1.0000	2.2534	1.0000	0.0000	1.0000	0.0000	1.0000	0.0000	1.0000	2.3479	1.0000	0.0000
LOC100539684	0.0059	-2.5105	0.0026	-1.6968	1.0000	-0.0508	0.1878	0.6202	1.0000	-0.3419	1.0000	0.4912	0.8908	0.3372
LOC100539689	1.0000	0.0692	0.0000	0.3320	0.0012	0.6099	0.0000	0.6551	1.0000	-0.1958	0.9606	0.0799	0.2324	-0.1458
LOC100539697	1.0000	-0.8577	0.2329	1.0795	1.0000	-0.7120	0.1071	1.3397	1.0000	1.0237	0.0319	2.9982	0.0088	3.0957
LOC100539699	1.0000	0.0000	1.0000	-2.3959	0.8033	3.0892	1.0000	-2.3178	1.0000	2.2674	1.0000	0.0000	1.0000	-3.1370
LOC100539703	0.9671	0.2083	0.4555	0.3510	1.0000	-0.0918	1.0000	0.0376	1.0000	-0.1947	1.0000	-0.0392	1.0000	-0.0607
LOC100539718	0.6400	0.5703	1.0000	-0.0207	0.0567	-1.6779	0.4427	-0.7441	1.0000	-0.3565	0.4092	-0.9345	1.0000	0.5786
LOC100539719	0.4820	0.2239	1.0000	-0.0253	0.4568	0.2245	0.7723	0.0940	1.0000	0.1792	1.0000	-0.0557	1.0000	0.0546
LOC100539727	0.0000	1.0812	0.0000	0.7606	1.0000	0.0590	0.5146	-0.0841	1.0000	-0.1664	0.0000	-0.4747	0.0073	-0.3037
LOC100539729	1.0000	-0.0782	1.0000	-0.1784	0.1310	-1.1084	1.0000	-0.2233	1.0000	-0.8083	0.3203	-0.8945	1.0000	0.0814
LOC100539736	0.9567	-0.5374	0.5032	-0.6952	1.0000	0.2520	0.6384	0.5457	1.0000	0.5510	1.0000	0.4106	0.6865	0.8532
LOC100539738	0.4007	-0.5034	0.0889	-0.4693	0.0069	-1.3161	0.0004	-0.9170	1.0000	0.1441	1.0000	0.1881	0.3971	0.5498
LOC100539745	0.0001	1.1991	0.0000	1.6781	0.0003	1.0364	0.0000	1.0349	1.0000	-0.1718	0.4480	0.3225	0.8664	-0.1667
LOC100539746	0.4585	0.6720	0.9318	0.1978	0.2134	0.9027	0.2392	-0.7206	1.0000	0.6766	1.0000	0.2170	0.3186	-0.9417
LOC100539753	0.9729	0.3899	0.9107	0.2981	0.0000	2.6034	0.0020	1.5718	1.0000	0.0742	1.0000	-0.0065	0.0211	-0.9509
LOC100539757	1.0000	-2.4776	1.0000	0.0000	1.0000	-2.4056	1.0000	0.0000	1.0000	-2.3757	1.0000	0.0000	1.0000	0.0000
LOC100539767	1.0000	0.3491	1.0000	-2.3959	0.0055	2.5133	0.0031	3.5176	1.0000	-1.4982	0.4563	-4.3630	0.9237	-0.4959
LOC100539772	0.0005	-0.6292	0.0000	-0.4303	0.0001	-0.7187	0.0002	-0.3628	1.0000	-0.2411	1.0000	-0.0298	0.6942	0.1204
LOC100539773	1.0000	0.3987	1.0000	0.0817	0.0012	2.1663	0.0000	2.3572	1.0000	0.3680	1.0000	0.0636	0.4602	0.5682
LOC100539788	0.8249	-0.6671	0.8773	0.4440	0.1673	1.1548	0.3283	0.8566	1.0000	-0.0023	0.6236	1.1255	1.0000	-0.2932
LOC100539791	1.0000	0.0000	1.0000	0.0000	1.0000	0.0000	1.0000	0.0000	1.0000	0.0000	1.0000	0.0000	1.0000	0.0000
LOC100539800	0.0542	1.4463	0.0112	1.7019	0.1994	1.2008	0.0140	1.6217	1.0000	-0.2979	1.0000	-0.0292	1.0000	0.1377
LOC100539804	0.9672	-1.6972	0.0563	-3.0485	0.1973	1.8182	0.5225	0.7284	1.0000	1.3837	1.0000	0.0515	1.0000	0.2868
LOC100539811	0.6091	-0.1360	0.0290	-0.2031	0.0070	-0.4421	0.0000	-0.3494	1.0000	0.0378	1.0000	-0.0170	0.4874	0.1353
LOC100539812	0.0000	1.2620	0.0000	1.2805	0.0000	1.6313	0.0000	1.3820	1.0000	0.0047	1.0000	0.0370	0.0528	-0.2388
LOC100539818	0.1150	4.6907	0.6224	1.6101	1.0000	2.2427	0.9096	1.3854	1.0000	2.2674	1.0000	-0.7096	1.0000	1.4489
LOC100539819	0.1659	-0.2662	0.0000	-0.3583	0.0000	-0.9805	0.0318	0.1665	1.0000	0.1695	0.9216	0.0897	0.0000	1.3207
LOC100539820	0.0000	-1.0063	0.0000	-0.8193	0.8712	-0.1106	0.4946	-0.1405	1.0000	-0.0452	1.0000	0.1539	1.0000	-0.0703
LOC100539821	0.0673	1.0333	0.2536	0.6460	0.1626	0.8258	0.0334	0.9669	1.0000	-0.1268	0.6300	-0.5073	1.0000	0.0175
LOC100539825	0.0814	1.0417	0.0005	1.6386	0.9880	0.2677	0.1733	0.8890	1.0000	-0.4882	1.0000	0.1199	1.0000	0.1373
LOC100539826	0.5086	-3.8796	1.0000	-0.6944	0.0354	2.1290	0.1233	1.7985	1.0000	-0.1237	1.0000	3.1976	0.9794	-0.4506
LOC100539830	0.0002	-1.6729	0.0014	-1.4870	0.0010	1.0949	0.0000	1.5534	1.0000	-0.2572	1.0000	-0.0604	0.5348	0.2064
LOC100539836	0.1381	-0.3701	0.0002	-0.5230	0.6861	0.1543	0.4370	-0.1372	1.0000	0.1745	1.0000	0.0351	0.8155	-0.1117
LOC100539842	1.0000	0.0000	1.0000	0.0000	1.0000	0.0000	1.0000	0.0000	1.0000	0.0000	1.0000	0.0000	1.0000	0.0000
LOC100539846	1.0000	2.1849	0.4437	3.6202	1.0000	0.0000	1.0000	0.0000	1.0000	0.0000	1.0000	1.4325	1.0000	0.0000
LOC100539847	0.6734	-0.6939	0.8363	-0.4963	1.0000	-0.2900	0.7516	-0.6626	1.0000	0.0723	1.0000	0.2909	1.0000	-0.2977
LOC100539849	0.0002	-0.7001	0.0000	-0.4502	0.9260	0.0837	0.0003	0.3391	1.0000	-0.2952	1.0000	-0.0334	1.0000	-0.0345

LOC100539852	0.3351	-1.3150	0.1433	-1.9616	0.0898	-2.3721	0.2463	-1.3950	1.0000	0.0011	1.0000	-0.6341	1.0000	0.9917
LOC100539860	0.1152	0.3707	0.0000	0.6306	0.6452	-0.1637	0.0003	-0.3028	1.0000	-0.1925	0.8788	0.0796	0.0019	-0.3252
LOC100539868	0.0001	-0.9011	0.0000	-0.7199	0.0005	0.7500	0.0021	0.4278	1.0000	0.2354	0.0765	0.4296	1.0000	-0.0814
LOC100539883	0.1835	-0.3543	0.3761	-0.1440	0.5540	0.2012	0.0000	0.4512	1.0000	0.0694	0.1054	0.2922	0.0257	0.3240
LOC100539892	0.0012	-1.0127	0.0000	-1.2565	1.0000	-0.0828	0.0434	0.4149	1.0000	-0.2456	0.3346	-0.4739	0.4865	0.2612
LOC100539893	1.0000	0.0000	1.0000	0.0000	1.0000	0.0000	1.0000	0.0000	1.0000	0.0000	1.0000	0.0000	1.0000	0.0000
LOC100539895	1.0000	0.6129	0.7735	0.7731	1.0000	-1.0986	1.0000	0.0068	1.0000	-0.5088	1.0000	-0.3414	1.0000	0.6038
LOC100539898	0.0264	0.5723	0.0013	0.5666	0.0000	1.1357	0.0000	1.2178	1.0000	-0.0369	1.0000	-0.0280	1.0000	0.0509
LOC100539904	1.0000	0.0000	1.0000	0.0000	1.0000	0.0000	1.0000	0.0000	1.0000	0.0000	1.0000	0.0000	1.0000	0.0000
LOC100539909	0.4430	1.5313	0.2032	1.1166	0.0004	3.3048	0.4995	0.7962	1.0000	1.7509	0.3807	1.3556	0.6213	-0.7466
LOC100539913	1.0000	0.0000	1.0000	-2.3986	1.0000	0.0000	1.0000	-2.3201	1.0000	2.2736	1.0000	0.0000	1.0000	0.0000
LOC100539914	0.0001	-0.7977	0.0027	-0.3232	0.0000	-1.2518	0.0000	-1.0022	1.0000	-0.1992	0.0713	0.2873	1.0000	0.0572
LOC100539922	1.0000	2.1902	1.0000	0.0000	1.0000	0.0000	1.0000	0.0000	1.0000	0.0000	1.0000	-2.2991	1.0000	0.0000
LOC100539929	0.6923	-0.2068	1.0000	-0.0466	0.3718	0.3053	0.0916	0.3449	1.0000	0.0959	0.6410	0.2669	0.9420	0.1408
LOC100539934	0.6612	0.1690	0.8846	0.0565	0.0000	1.3709	0.0000	1.1266	1.0000	0.1787	1.0000	0.0789	1.0000	-0.0590
LOC100539940	0.6096	-0.1342	0.0014	-0.2498	0.3273	-0.1972	0.0114	-0.1995	1.0000	0.0680	1.0000	-0.0351	0.8549	0.0713
LOC100539942	0.6465	-0.2468	0.2681	-0.3170	0.3130	-0.3754	0.8364	0.1153	1.0000	-0.0401	1.0000	-0.0965	0.1705	0.4557
LOC100539943	0.0000	1.5185	0.0000	1.6753	0.0000	1.9308	0.0000	1.4893	1.0000	0.1816	0.0242	0.3519	0.0445	-0.2535
LOC100539971	0.0622	0.9313	0.1151	0.5802	0.0000	1.6137	0.0206	0.8146	1.0000	0.5689	1.0000	0.2307	0.9122	-0.2229
LOC100539974	0.0069	-0.3999	0.3768	-0.1080	0.0000	-0.8806	0.0000	-0.6918	1.0000	-0.2188	0.9052	0.0857	1.0000	-0.0245
LOC100539980	0.1096	4.6934	0.1384	4.3086	0.5076	3.6021	1.0000	2.3241	1.0000	0.0000	1.0000	-0.4066	1.0000	-1.3057
LOC100539992	1.0000	-0.0006	0.9639	-0.0382	0.0154	-0.5695	0.0000	-0.6025	1.0000	-0.0816	0.8278	-0.1079	0.7552	-0.1093
LOC100540000	0.0000	-1.7860	0.0000	-1.6838	0.0000	-1.2730	0.0000	-1.3763	1.0000	-0.0406	1.0000	0.0737	0.3669	-0.1383
LOC100540001	1.0000	0.0000	1.0000	0.0000	1.0000	0.0000	1.0000	0.0000	1.0000	0.0000	1.0000	0.0000	1.0000	0.0000
LOC100540007	0.8249	-3.3411	1.0000	-0.1449	0.8033	-3.2622	1.0000	-2.3178	1.0000	-0.9614	1.0000	2.3456	1.0000	0.0000
LOC100540008	0.8226	-3.3425	1.0000	-2.3959	0.7628	1.0365	0.0304	3.0427	1.0000	-0.9614	1.0000	0.0000	0.8120	1.0440
LOC100540009	0.2335	1.4987	1.0000	-0.4239	1.0000	-0.5754	1.0000	0.4092	1.0000	0.4287	0.3998	-1.4810	0.7145	1.4225
LOC100540017	0.2158	0.3637	0.0158	0.5202	0.0023	0.7068	0.0000	0.8000	1.0000	-0.1417	1.0000	0.0278	1.0000	-0.0432
LOC100540025	0.0013	0.8610	0.0014	0.6898	0.0124	-0.8031	0.0443	-0.5425	1.0000	-0.3677	0.0356	-0.5274	1.0000	-0.1028
LOC100540035	1.0000	0.0000	1.0000	0.0000	1.0000	0.0000	1.0000	0.0000	1.0000	0.0000	1.0000	0.0000	1.0000	0.0000
LOC100540040	0.1880	0.4138	0.3485	0.2564	0.0079	-0.7352	0.0034	-0.6063	1.0000	-0.1961	0.4546	-0.3419	1.0000	-0.0636
LOC100540041	0.0014	0.5362	0.0000	0.7401	0.0003	0.5924	0.0000	0.5866	1.0000	-0.1532	1.0000	0.0637	0.3691	-0.1532
LOC100540047	0.4289	-0.3489	0.0714	-0.3704	0.6381	0.2257	0.5047	-0.1701	1.0000	0.0700	1.0000	0.0607	0.2049	-0.3220
LOC100540049	0.0000	-3.6890	0.0000	-4.1088	0.0003	-1.0680	0.0000	-0.8178	1.0000	0.3397	1.0000	-0.0689	0.0053	0.5948
LOC100540050	1.0000	0.0324	1.0000	0.0049	0.0001	-1.4051	0.0001	-0.7975	1.0000	0.0071	1.0000	-0.0086	0.0946	0.6164
LOC100540051	0.0000	1.2724	0.0001	0.8501	0.5020	0.3358	1.0000	-0.0661	1.0000	-0.0719	0.0418	-0.4838	0.2964	-0.4733
LOC100540054	0.3843	0.8449	0.0039	2.8528	0.2389	-1.6991	1.0000	0.0059	1.0000	-2.1632	1.0000	-0.1578	1.0000	-0.4576
LOC100540056	0.0727	-0.3763	0.0009	-0.3752	0.1407	0.2959	0.1141	0.2021	1.0000	0.1163	0.8607	0.1301	1.0000	0.0274
LOC100540064	1.0000	2.1849	1.0000	0.0000	1.0000	0.0000	1.0000	0.0000	1.0000	0.0000	1.0000	-2.2955	1.0000	0.0000
LOC100540081	0.0052	-0.4848	0.0060	-0.2715	0.0000	-1.8440	0.0000	-0.9649	1.0000	-0.1207	0.8535	0.1049	0.0000	0.7640
LOC100540082	0.0000	1.3243	0.0000	1.4302	0.0809	0.5477	0.0401	0.4539	1.0000	-0.1710	1.0000	-0.0518	0.5381	-0.2583
LOC100540083	0.9650	1.0594	1.0000	0.0000	1.0000	0.6746	1.0000	0.0000	1.0000	-2.3757	0.8785	-3.6646	1.0000	-3.1317
LOC100540086	0.8050	-0.1225	0.0011	0.4314	0.1159	0.3354	0.1023	0.2273	1.0000	-0.2669	0.1629	0.2987	0.0106	-0.3702
LOC100540096	0.7134	-0.1208	0.0062	-0.2577	0.0635	-0.3473	0.0054	-0.2712	1.0000	0.0232	0.8841	-0.1014	0.7999	0.1049
LOC100540100	1.0000	0.0000	1.0000	0.0000	1.0000	0.0000	1.0000	0.0000	1.0000	0.0000	1.0000	0.0000	1.0000	0.0000
LOC100540103	1.0000	-1.1537	1.0000	0.0000	1.0000	-0.1833	1.0000	0.0000	1.0000	-3.2319	1.0000	-2.2991	1.0000	-3.1317
LOC100540105	0.5930	0.6384	0.8573	-0.2386	0.6171	-0.6748	0.0712	-1.1593	1.0000	0.6973	1.0000	-0.1646	1.0000	0.2147
LOC100540107	0.0149	0.6012	0.0000	0.9010	0.0000	1.0495	0.0000	1.0088	1.0000	-0.0134	0.1835	0.2977	1.0000	-0.0504
LOC100540115	0.2484	1.5802	0.3631	1.1102	0.4766	1.1542	0.8938	0.5727	1.0000	0.8198	1.0000	0.3723	1.0000	0.2439
LOC100540124	0.0006	-0.8359	0.0000	-0.9787	0.0000	-1.1236	0.0000	-1.1959	1.0000	0.4484	0.3106	0.3201	0.0878	0.3802
LOC100540132	0.0738	1.2469	1.0000	-0.1795	1.0000	0.0262	0.0556	-1.5390	1.0000	0.4457	0.2806	-0.9692	0.5155	-1.1127
LOC100540135	0.6540	-0.1347	0.8304	0.0482	0.3097	-0.2254	1.0000	-0.0172	1.0000	-0.1708	1.0000	0.0242	1.0000	0.0426
LOC100540140	0.2314	0.5103	0.0025	0.7726	0.0000	1.4225	0.0000	1.6816	1.0000	-0.2548	1.0000	0.0217	1.0000	0.0114
LOC100540154	0.0000	-3.0610	0.0000	-2.1340	0.0028	-1.1781	0.0028	-0.3913	1.0000	0.4908	0.0000	1.4312	0.0000	1.2825
LOC100540167	1.0000	0.0000	1.0000	0.0000	1.0000	0.0000	1.0000	0.0000	1.0000	0.0000	1.0000	0.0000	1.0000	0.0000
LOC100540170	1.0000	2.1849	1.0000	-2.3986	1.0000	0.0000	1.0000	0.0045	1.0000	2.2736	1.0000	-2.2955	1.0000	2.3542
LOC100540172	0.8226	3.0199	1.0000	0.6987	1.0000	2.2472	1.0000	-2.3178	1.0000	2.2674	1.0000	0.0559	1.0000	-2.2907
LOC100540176	0.0687	-0.9291	0.0000	-0.8352	0.6103	-0.3612	0.0109	0.4455	1.0000	0.2924	0.5389	0.3979	0.0000	1.1033
LOC100540178	0.0990	0.6851	0.2847	0.4564	0.1973	-0.6289	0.9696	-0.1692	1.0000	-0.4574	0.1232	-0.6760	1.0000	0.0077
LOC100540180	0.5086	-3.8796	0.9148	-1.5313	0.4809	-3.7970	0.4635	-3.6899	1.0000	-0.1283	1.0000	2.3456	1.0000	0.0000
LOC100540188	1.0000	0.4278	1.0000	0.0000	0.6597	-1.9401	0.2831	4.0829	1.0000	-4.1514	0.2235	-4.8314	0.9124	1.8308
LOC100540196	0.0208	0.4945	0.0003	0.4889	1.0000	-0.0519	0.3771	-0.1752	1.0000	0.0371	1.0000	0.0419	1.0000	-0.0823
LOC100540203	1.0000	2.1849	1.0000	-2.3986	1.0000	0.0000	1.0000	-2.3201	1.0000	2.2736	1.0000	-2.2955	1.0000	0.0000
LOC100540209	0.0000	1.5211	0.0000	2.2918	0.0000	1.8002	0.0000	1.8056	1.0000	-0.4269	0.4563	0.3574	0.3518	-0.4170
LOC100540211	0.1381	1.3211	0.0952	0.9989	1.0000	-0.0246	1.0000	0.1969	1.0000	0.6597	1.0000	0.3542	0.7263	0.8914
LOC100540213	0.0000	1.7381	0.0000	1.8208	0.2955	0.4212	0.8411	0.1575	1.0000	0.0138	1.0000	0.1090	0.7967	-0.2434
LOC100540222	1.0000	2.1849	1.0000	-2.3959	1.0000	0.0000	1.0000	-2.3178	1.0000	2.2674	1.0000	-2.2955	1.0000	0.0000
LOC100540231	0.0000	-2.0963	0.0000	-2.3296	0.0072	-0.4200	0.0000	-0.5983	1.0000	0.0017	0.2142	-0.2189	0.3349	-0.1711
LOC100540238	0.0000	1.6959	0.0001	1.1694	0.0000	2.1921	0.0000	1.6617	1.0000	0.6490	1.0000	0.1384	1.0000	0.1253
LOC100540242	0.2303	0.4930	0.0002	1.1061	0.0000	1.5851	0.0000	1.5394	1.0000	-0.2448	0.3984	0.3860	0.3793	-0.2810
LOC100540249	0.6578	-0.8520	0.8230	-0.9436	1.0000	-0.1989	0.7645	0.6404	1.0000	-0.6286	1.0000	-0.7121	1.0000	0.2139
LOC100540256	1.0000	0.0000	1.0000	0.0000	1.0000	0.0000	1.0000	0.0000	1.0000	0.0000	1.0000	0.0000	1.0000	0.0000
LOC100540261	0.0000	1.2415	0.0000	1.3669	0.0000	1.1790	0.0002	0.7319	1.0000	0.2667	0.1105	0.4028	0.7830	-0.1753
LOC100540262	0.4029	0.4434	0.0000	1.4738	0.8033	0.2528	0.0000	1.4270	1.0000	-0.8272	1.0000	0.2144	0.6511	0.3554
LOC100540273	0.0030	1.3017	0.0000	2.1863	0.0000	2.0052	0.0000	1.9532	1.0000	-0.0880	0.0077	0.8082	1.0000	-0.1341
LOC100540274	1.0000	-0.3421	1.0000	0.2211	1.0000	-0.5759	1.0000	0.3938	1.0000	-0.5100	1.0000	0.0611	1.0000	0.4629
LOC100540275	1.0000	0.0000	1.0000	2.2534	1.0000	2.2427	1.0000	0.0000	1.0000	0.0000	1.0000	2.3479	1.0000	-2.2888
LOC100540278	1.0000	0.0000	1.0000	0.0000	1.0000	0.0000	0.7710	3.1708	1.0000	0.0000	1.0000	0.0000	1.0000	3.2048
LOC100540282	1.0000	0.0000	0.7674	3.0957	1.0000	0.0000	1.0000	0.0000	1.0000	0.0000	1.0000	3.2002	1.0000	0.0000
LOC100540289	0.8226	-3.3425	0.6180	-1.9232	0.7694	1.0409	0.1601	1.5344	1.0000	0.7963	1.0000	2.3479	0.5572	1.3003
LOC100540290	0.5671	0.7461	0.0785	1.6695	0.7333	0.5860	0.4820	1.1283	1.0000	-1.5335	0.8915	-0.6058	0.5177	-0.9881
LOC100540301	1.0000	-0.1338	0.3469	1.2296	1.0000	0.0181	0.0488	1.8498	1.0000	-0.6796	1.0000	0.6932	0.5421	1.1522
LOC100540302	0.5036	0.2016	0.6224	0.1020	0.0879	-0.4049	0.7783	-0.0789	1.0000	0.0594	1.0000	-0.0277	0.0280	0.3914
LOC100540306	1.0000	0.5420	0.9763	0.7505	1.0000	0.6783	0.7710	-3.1692	1.0000	0.7416	1.0000	0.9738	1.0000	-3.1344
LOC100540309	0.0000	1.4120	0.0000	1.6494	0.3326	0.4736	1.0000	-0.1288	1.0000	-0.3180	1.0000	-0.0683	0.0231	-0.9141
LOC100540318	0.7356	0.1349	0.9520	0.0455	0.0207	0.4756	0.0000	0.5651	1.0000	0.0168	1.0000	-0.0603	0.8431	0.1129
LOC100540331	0.0000	-1.5277	0.0000	-1.8980	0.8114	-0.1156	0.3751	0.1421	1.0000	-0.3323	0.0051	-0.6903	1.0000	-0.0684
LOC100540334	0.4559	0.1816	0.4245	-0.0825	1.0000	0.0147	0.0030	0.2234	1.0000	-0.0613	0.0007	-0.3128	0.2212	0.1526
LOC100540339	0.0409	0.7984	0.0353	0.7354	0.1625	0.6092	0.0089	0.7946	1.0000	0.0000	1.0000	-0.0479	0.9371	0.1922
LOC100540340	0.8699	0.1141	0.0983	0.2077	0.1459	0.3598	0.0000	0.6276	1.0000	-0.2066	0.9752	-0.1015	1.0000	0.0664
LOC100540358	0.5318	-0.1422	0.0122	-0.2172	0.1366	-0.2595	0.0186	-0.1913	1.0000	0.0156	1.0000	-0.0467	0.7221	0.0893
LOC100540364	1.0000	0.0000	1.0000	0.0000	1.0000	0.0000	1.0000	0.0000	1.0000	0.0000	1.0000	0.0000	1.0000	0.0000
LOC100540365	0.5242	-1.0726	0.7940	-1.1058	0.8033	-0.5099	0.3362	1.0915	1.0000	-0.8455	1.0000	-0.8586	0.9145	0.7625

LOC100540368	0.0006	1.4955	0.0001	1.2382	0.0000	2.9789	0.0000	2.4036	1.0000	0.2538	1.0000	0.0082	0.4688	-0.3166
LOC100540378	0.7053	-0.1734	0.0132	-0.4183	0.0038	-0.7144	0.0085	-0.4222	1.0000	0.0559	0.8688	-0.1762	0.2302	0.3542
LOC100540379	0.0000	1.3405	0.0000	1.5300	0.0000	1.8012	0.0000	1.6920	1.0000	-0.0166	0.6630	0.1873	0.7387	-0.1185
LOC100540386	0.3070	-2.0239	0.1009	-2.8810	0.1918	-2.7122	0.4160	-1.3358	1.0000	0.0547	1.0000	-0.7966	1.0000	1.4483
LOC100540389	0.6582	0.1870	0.6493	0.1168	0.7657	0.1548	0.0005	-0.5475	1.0000	0.2242	0.8401	0.1680	0.0183	-0.4710
LOC100540394	0.2392	-0.3722	1.0000	0.0554	0.1927	0.3517	0.0016	0.4745	1.0000	-0.1230	0.4131	0.3170	1.0000	0.0076
LOC100540396	0.0024	-1.9601	0.0000	-1.9966	0.0041	-1.8030	0.0000	-1.9013	1.0000	0.6112	1.0000	0.5901	1.0000	0.5184
LOC100540406	0.9545	1.0672	1.0000	0.6944	1.0000	-0.1630	1.0000	0.0045	1.0000	-0.1045	1.0000	-0.4752	1.0000	0.0652
LOC100540409	0.5911	-0.6769	1.0000	0.3893	0.6373	-0.6086	0.2396	1.1957	1.0000	-1.8192	0.8607	-0.7507	1.0000	-0.0173
LOC100540410	0.8085	0.8951	1.0000	0.3842	0.7663	1.0386	0.3641	1.2758	1.0000	0.7940	1.0000	0.3079	0.8275	1.0409
LOC100540413	1.0000	0.0000	1.0000	0.0000	1.0000	0.0000	1.0000	0.0000	1.0000	0.0000	1.0000	0.0000	1.0000	0.0000
LOC100540415	1.0000	0.0000	1.0000	-2.3959	1.0000	0.0000	1.0000	0.0076	1.0000	2.2674	1.0000	0.0000	1.0000	2.3554
LOC100540418	1.0000	-2.4776	1.0000	0.0000	1.0000	-2.4056	1.0000	2.3241	1.0000	-2.3757	1.0000	0.0000	1.0000	2.3542
LOC100540428	0.0002	0.8260	0.0065	0.3557	0.0005	0.7940	0.0489	0.2622	1.0000	0.4517	1.0000	-0.0054	1.0000	-0.0755
LOC100540432	0.0472	-0.7484	0.0140	-0.8040	0.5297	-0.3029	1.0000	0.0196	1.0000	-0.0884	1.0000	-0.1321	0.7943	0.2382
LOC100540438	1.0000	0.5464	1.0000	-1.0017	1.0000	0.6746	0.7701	-3.1666	1.0000	0.7365	1.0000	-0.8001	1.0000	-3.1317
LOC100540439	0.8395	-0.3586	0.9127	-0.3393	1.0000	-0.3352	1.0000	0.0043	1.0000	-0.2667	1.0000	-0.2401	1.0000	0.0772
LOC100540450	1.0000	0.6109	0.2099	1.4877	0.6647	-1.9394	1.0000	0.0092	1.0000	-0.5112	1.0000	0.3742	1.0000	1.4497
LOC100540451	0.0003	-0.7209	0.0001	-0.3937	0.0000	0.8814	0.0000	1.0914	1.0000	-0.0860	0.1715	0.2540	0.4553	0.1288
LOC100540453	0.0565	0.5626	0.0001	0.6600	0.0056	0.6967	0.3175	0.2215	1.0000	0.1014	0.7531	0.2103	0.1414	-0.3701
LOC100540457	0.4828	-0.1673	0.0023	-0.2822	0.0030	0.4968	0.0000	0.3566	1.0000	0.1420	1.0000	0.0400	1.0000	0.0071
LOC100540462	0.6551	-0.1319	0.0000	-0.3501	0.3213	-0.2104	0.0001	-0.3168	1.0000	0.2329	1.0000	0.0274	0.5046	0.1324
LOC100540465	0.0428	0.8602	0.0000	1.4786	0.0065	1.0341	0.0083	0.8747	1.0000	-0.2340	0.4709	0.3970	0.4752	-0.3888
LOC100540472	0.0650	1.2052	0.0403	0.9627	0.0000	2.0988	0.0000	1.6835	1.0000	0.4722	1.0000	0.2438	1.0000	0.0638
LOC100540476	1.0000	0.5414	0.0530	4.7669	0.1166	2.5445	0.0406	4.8626	1.0000	-2.3771	0.7896	1.7409	1.0000	-0.1073
LOC100540496	0.2537	-0.3222	0.0323	-0.3713	1.0000	-0.0830	0.3441	0.1883	1.0000	-0.0919	1.0000	-0.1288	0.7087	0.1849
LOC100540502	1.0000	-2.4776	1.0000	0.0000	1.0000	-2.4056	1.0000	0.0000	1.0000	-2.3757	1.0000	0.0000	1.0000	0.0000
LOC100540503	0.0000	1.4978	0.0000	1.8317	0.1316	0.5795	0.0590	0.5842	1.0000	0.0098	0.3460	0.3561	1.0000	0.0214
LOC100540511	0.0000	1.5940	0.0000	1.2678	0.0000	2.0143	0.0000	1.0269	1.0000	0.5905	0.6906	0.2785	0.5651	-0.3904
LOC100540515	0.8249	3.0141	1.0000	0.0000	1.0000	0.0000	1.0000	0.0000	1.0000	0.0000	1.0000	-3.1383	1.0000	0.0000
LOC100540527	1.0000	0.0000	1.0000	0.0000	1.0000	0.0000	1.0000	0.0000	1.0000	0.0000	1.0000	0.0000	1.0000	0.0000
LOC100540528	0.0346	2.2908	0.0037	2.8468	1.0000	-0.7128	0.7710	-3.1693	1.0000	-0.6472	1.0000	-0.0896	1.0000	-3.1344
LOC100540532	0.0722	0.3566	0.6390	0.0879	0.0010	0.5952	0.0000	0.4496	1.0000	0.1865	1.0000	-0.0693	1.0000	0.0457
LOC100540561	1.0000	0.0849	1.0000	-0.0609	0.5743	-0.3250	1.0000	0.0239	1.0000	0.0390	1.0000	-0.0937	0.5603	0.3922
LOC100540564	0.5690	-0.1603	0.0000	-0.3854	0.0503	-0.3791	0.0003	-0.3621	1.0000	0.0262	0.3460	-0.1860	1.0000	0.0483
LOC100540568	1.0000	0.0000	1.0000	0.0000	1.0000	0.0000	1.0000	0.0000	1.0000	0.0000	1.0000	0.0000	1.0000	0.0000
LOC100540575	0.0031	0.8120	0.0000	0.6879	0.0001	1.0286	0.0000	0.7323	1.0000	0.2469	0.9048	0.1365	1.0000	-0.0430
LOC100540586	1.0000	-2.4788	1.0000	2.2506	0.1945	2.3621	0.4370	3.7037	1.0000	-2.3771	1.0000	2.3456	0.8664	-1.0820
LOC100540589	0.0010	1.5480	0.0094	1.0948	0.0000	2.2718	0.0000	2.0663	1.0000	0.2522	1.0000	-0.1837	1.0000	0.0610
LOC100540591	0.0000	2.1339	0.0000	1.9896	0.0694	0.7047	0.0174	0.6558	1.0000	0.0098	0.9844	-0.1269	1.0000	-0.0400
LOC100540595	1.0000	-0.3476	0.9968	-1.0984	1.0000	-0.1910	0.9946	-0.9209	1.0000	-0.4224	1.0000	-1.1662	0.9835	-1.1482
LOC100540607	0.3572	0.5331	0.0109	0.6224	0.0655	0.8228	0.0002	0.8552	1.0000	0.0234	1.0000	0.1274	1.0000	0.0649
LOC100540610	0.0571	-0.8459	0.9107	-0.1468	1.0000	0.0713	0.0000	1.3782	1.0000	-0.3855	0.8627	0.3259	0.0000	0.9234
LOC100540612	0.0727	-0.3258	0.0000	-0.6465	0.0007	0.5565	0.0102	0.2306	1.0000	0.1634	0.5971	-0.1446	0.3582	-0.1573
LOC100540620	0.0628	0.3155	0.0003	0.3018	0.0020	-0.5065	0.0000	-0.8926	1.0000	0.2372	0.0437	0.2358	0.4901	-0.1432
LOC100540622	0.0107	-0.8210	0.0000	-0.6135	0.5990	0.2668	0.0000	0.6433	1.0000	0.3221	0.0001	0.5409	0.0001	0.7032
LOC100540623	0.1242	0.3344	0.8422	-0.0652	0.0009	-0.6853	0.0000	-0.8477	1.0000	0.0487	0.0250	-0.3376	0.9886	-0.1077
LOC100540635	0.3961	-0.2124	0.1065	-0.2124	0.0829	-0.3500	0.0101	-0.3049	1.0000	-0.0797	1.0000	-0.0661	1.0000	-0.0282
LOC100540641	0.0001	-1.8857	0.0000	-1.6365	0.0215	-1.1626	0.0018	-1.0958	1.0000	0.0476	1.0000	0.3094	1.0000	0.1212
LOC100540643	1.0000	0.0000	1.0000	-2.3986	1.0000	0.0000	1.0000	0.8541	1.0000	2.2736	1.0000	0.0000	1.0000	3.2066
LOC100540665	0.2416	0.3496	0.0029	0.4840	0.0000	0.9230	0.0006	0.5522	1.0000	-0.0260	1.0000	0.1210	0.0484	-0.3918
LOC100540667	0.0055	0.7577	0.0305	0.3991	1.0000	-0.0121	0.1401	-0.3575	1.0000	-0.2232	0.0277	-0.5687	0.0323	-0.5626
LOC100540668	0.0002	0.7836	0.0000	0.8150	0.0000	2.1854	0.0000	1.8040	1.0000	0.1088	0.8269	0.1535	0.0301	-0.2662
LOC100540670	1.0000	0.0000	1.0000	0.0000	1.0000	0.0000	1.0000	0.0000	1.0000	0.0000	1.0000	0.0000	1.0000	0.0000
LOC100540676	0.0000	1.0081	0.0016	0.4352	0.0624	0.4525	0.3903	0.1590	1.0000	0.3611	0.5245	-0.1991	1.0000	0.0713
LOC100540688	0.0001	1.4890	0.0420	0.7153	0.4695	0.4764	0.4411	0.3387	1.0000	0.4111	0.7181	-0.3493	0.9055	0.2825
LOC100540701	1.0000	-2.4788	0.7674	3.0957	1.0000	-2.4061	0.7710	3.1753	1.0000	-2.3771	1.0000	3.2002	1.0000	3.2081
LOC100540703	0.3855	-0.8490	1.0000	-0.1791	0.0491	-1.6617	0.6966	-0.5674	1.0000	-0.3077	1.0000	0.3752	0.9981	0.7914
LOC100540713	0.0083	0.6267	0.0001	0.5380	1.0000	0.0713	0.5350	-0.1472	1.0000	-0.0743	0.7777	-0.1513	0.3100	-0.2864
LOC100540723	1.0000	0.0000	1.0000	0.0000	1.0000	0.0000	0.7710	3.1753	1.0000	0.0000	1.0000	0.0000	1.0000	3.2081
LOC100540739	0.8226	0.0941	0.9245	-0.0351	0.0000	-0.7314	0.0000	-0.8500	1.0000	0.1333	1.0000	0.0167	1.0000	0.0211
LOC100540742	1.0000	0.0000	1.0000	0.0000	1.0000	0.0000	1.0000	0.0000	1.0000	0.0000	1.0000	0.0000	1.0000	0.0000
LOC100540743	0.0069	1.0033	0.0000	1.1235	0.0000	2.0995	0.0000	1.9587	1.0000	-0.0659	1.0000	0.0686	0.6422	-0.2014
LOC100540751	0.0031	-3.9330	0.0000	-6.3578	1.0000	-0.1975	0.1212	-1.2146	1.0000	0.2012	1.0000	-2.2991	0.6865	-0.8074
LOC100540753	0.0010	1.3254	0.0000	1.3647	0.0000	1.9033	0.0000	1.2585	1.0000	0.3445	0.5558	0.3954	0.6506	-0.2964
LOC100540763	1.0000	0.1487	0.6545	0.7848	0.8898	-0.9564	0.6355	0.9639	1.0000	-0.8953	1.0000	-0.2529	0.9194	1.0334
LOC100540766	0.0890	2.7120	1.0000	-0.4247	0.4428	1.8950	0.2441	-2.3153	1.0000	2.2052	0.9238	-0.9052	0.6865	-1.9992
LOC100540769	0.2287	0.4332	0.9286	-0.0939	0.0000	1.8101	0.0000	1.0040	1.0000	0.3668	1.0000	-0.1469	0.0184	-0.4332
LOC100540781	1.0000	0.0000	1.0000	0.0000	1.0000	0.0000	1.0000	0.0000	1.0000	0.0000	1.0000	0.0000	1.0000	0.0000
LOC100540786	1.0000	0.5370	0.7674	3.0888	1.0000	0.6740	0.7710	3.1753	1.0000	-2.3771	1.0000	0.0554	1.0000	0.0753
LOC100540787	0.3635	2.2263	1.0000	0.6956	1.0000	-0.1630	0.2467	2.3292	1.0000	-0.1089	0.7955	-1.6375	0.5109	2.3941
LOC100540799	1.0000	0.0000	0.4437	3.6202	1.0000	2.2472	1.0000	2.3241	1.0000	0.0000	0.8607	3.7297	1.0000	0.0641
LOC100540803	0.0032	-2.6017	0.0096	-2.2781	0.0001	1.7504	0.0000	1.9734	1.0000	-0.2789	1.0000	0.0572	1.0000	-0.0530
LOC100540806	0.0077	-0.6784	0.0000	-0.8497	1.0000	-0.0394	0.2710	-0.2046	1.0000	0.2144	1.0000	0.0554	1.0000	0.0533
LOC100540816	0.0000	-0.6331	0.0000	-0.6130	0.0000	-1.1854	0.0000	-0.9196	1.0000	-0.1240	0.8820	-0.0912	0.2826	0.1470
LOC100540821	0.5780	-0.1272	0.7604	-0.0437	0.0000	-0.5957	0.0000	-0.4688	1.0000	-0.1076	1.0000	-0.0118	1.0000	0.0248
LOC100540823	1.0000	0.0000	1.0000	-2.3986	1.0000	0.0000	1.0000	-2.3201	1.0000	2.2736	1.0000	0.0000	1.0000	0.0000
LOC100540824	1.0000	0.0000	1.0000	0.0000	1.0000	0.0000	1.0000	0.0000	1.0000	0.0000	1.0000	0.0000	1.0000	0.0000
LOC100540834	0.6873	-0.6917	0.6089	-0.8107	0.6018	-0.6708	1.0000	0.1295	1.0000	-0.4683	1.0000	-0.5765	1.0000	0.3360
LOC100540839	0.0404	0.5795	0.7272	0.1169	0.2908	0.3428	1.0000	-0.0569	1.0000	0.3283	1.0000	-0.1216	1.0000	-0.0656
LOC100540841	1.0000	-0.0391	1.0000	0.1327	0.0361	1.9078	0.0496	1.8505	1.0000	-0.1215	1.0000	0.0591	1.0000	-0.1785
LOC100540846	0.0000	0.8983	0.0000	0.8362	0.4707	0.1744	0.4278	-0.0976	1.0000	-0.1002	0.3652	-0.1503	0.0003	-0.3670
LOC100540850	1.0000	0.0000	1.0000	0.0000	1.0000	0.0000	1.0000	0.0000	1.0000	0.0000	1.0000	0.0000	1.0000	0.0000
LOC100540851	0.0000	1.3547	0.0000	1.8817	0.0000	2.3495	0.0000	1.9564	1.0000	-0.0508	0.0452	0.4893	0.0527	-0.4381
LOC100540856	1.0000	-0.7191	0.6224	-1.9258	1.0000	-1.1014	1.0000	-0.3809	1.0000	-0.1215	1.0000	-1.3320	1.0000	0.6041
LOC100540871	0.5834	-1.5587	0.5022	-1.6409	0.7422	0.7730	1.0000	0.0069	1.0000	0.1251	1.0000	0.0598	0.9863	-0.6357
LOC100540872	1.0000	0.0106	0.4789	0.2439	0.9361	0.1199	0.2312	0.3285	1.0000	-0.1228	1.0000	0.1232	1.0000	0.0944
LOC100540873	0.0223	0.4883	0.0000	0.5571	0.9001	-0.0974	0.4330	-0.1313	1.0000	-0.0870	1.0000	-0.0055	0.8164	-0.1138
LOC100540876	1.0000	0.0000	1.0000	0.0000	1.0000	0.0000	1.0000	0.0000	1.0000	0.0000	1.0000	0.0000	1.0000	0.0000
LOC100540882	1.0000	0.0000	1.0000	0.0000	1.0000	2.2426	1.0000	0.0000	1.0000	0.0000	1.0000	0.0000	1.0000	-2.2888
LOC100540888	1.0000	-0.0221	1.0000	0.0567	0.0040	-0.8123	0.0000	-0.7627	1.0000	-0.2169	1.0000	-0.1254	1.0000	-0.1617
LOC100540893	1.0000	0.0000	1.0000	0.0000	1.0000	0.0000	1.0000	0.0000	1.0000	0.0000	1.0000	0.0000	1.0000	0.0000
LOC100540894	0.0000	-2.2882	0.0000	-1.9644	0.6647	-0.2587	0.0000	1.3039	1.0000	0.0452	1.0000	0.3799	0.0000	1.6166

LOC100540904	1.0000	-2.4776	1.0000	2.2506	1.0000	-2.4056	0.7710	3.1753	1.0000	-2.3757	1.0000	2.3456	1.0000	3.2081
LOC100540905	1.0000	-0.3506	1.0000	-0.1810	0.8984	0.4688	0.2995	0.9848	1.0000	-0.4383	1.0000	-0.2578	1.0000	0.0799
LOC100540906	0.0003	1.1011	0.0000	1.1031	0.0000	2.0544	0.0000	1.9071	1.0000	0.1176	1.0000	0.1337	1.0000	-0.0219
LOC100540910	0.0006	1.3329	0.0000	1.4260	0.1137	-0.9391	0.1562	-0.8156	1.0000	-0.3361	0.9306	-0.2319	1.0000	-0.2077
LOC100540915	0.1508	0.3126	0.5707	0.1000	0.0054	0.5155	0.0010	0.3740	1.0000	0.1062	1.0000	-0.0935	1.0000	-0.0302
LOC100540918	1.0000	0.0000	1.0000	0.0000	1.0000	0.0000	1.0000	0.0000	1.0000	0.0000	1.0000	0.0000	1.0000	0.0000
LOC100540920	0.6011	0.2543	0.5814	0.2371	0.0299	0.6257	0.0234	0.5362	1.0000	-0.3245	0.6659	-0.3303	0.1325	-0.4121
LOC100540921	0.0289	-0.5054	0.1565	-0.2274	0.2854	0.2717	0.0004	-0.5089	1.0000	0.1426	0.0056	0.4323	0.0000	-0.6350
LOC100540933	1.0000	0.0000	1.0000	0.0000	1.0000	0.0000	1.0000	0.0000	1.0000	0.0000	1.0000	0.0000	1.0000	0.0000
LOC100540939	0.7175	-0.4003	0.0009	-1.3369	0.2297	-0.7603	0.0002	-1.5405	1.0000	0.5865	1.0000	-0.3376	1.0000	-0.1915
LOC100540942	0.9070	-0.0741	1.0000	-0.0234	0.0000	-2.4263	0.0000	-1.7077	1.0000	-0.2858	0.0531	-0.2229	0.0045	0.4379
LOC100540945	1.0000	0.0000	1.0000	0.0000	1.0000	0.0000	1.0000	0.0000	1.0000	0.0000	1.0000	0.0000	1.0000	0.0000
LOC100540961	0.3159	3.9314	1.0000	2.2506	0.0002	6.0933	0.0816	4.6508	1.0000	0.0000	1.0000	-1.7140	0.1845	-1.4659
LOC100540962	0.2753	0.2461	0.0769	0.2105	0.0812	-0.3558	0.0033	-0.3331	1.0000	-0.0151	1.0000	-0.0374	1.0000	0.0140
LOC100540965	1.0000	-0.0116	0.3837	-0.1589	0.0228	-0.4912	0.1426	-0.2280	1.0000	0.0177	0.9238	-0.1181	0.2152	0.2864
LOC100540970	1.0000	0.0000	1.0000	0.0000	1.0000	0.0000	1.0000	0.0000	1.0000	0.0000	1.0000	0.0000	1.0000	0.0000
LOC100540973	0.4976	1.9967	0.6403	0.7839	0.0526	2.9863	0.8238	0.7780	1.0000	1.6478	1.0000	0.4612	1.0000	-0.5533
LOC100540974	0.5454	1.7708	1.0000	-0.6928	1.0000	-0.1591	0.9096	-1.3717	1.0000	1.2635	1.0000	-1.1747	1.0000	0.0649
LOC100540978	0.0011	1.7677	0.0000	1.6812	0.0315	1.3936	0.1810	0.7380	1.0000	0.4459	0.8187	0.3721	1.0000	-0.2073
LOC100540985	0.7744	0.1874	0.1027	0.3894	0.6968	0.2046	0.0000	0.9709	1.0000	-0.2869	1.0000	-0.0745	0.0297	0.4811
LOC100540989	0.9448	-0.0758	0.9324	0.0416	0.0080	0.4550	0.0001	0.3520	1.0000	-0.0799	1.0000	0.0506	0.2241	-0.1770
LOC100541022	0.0000	-3.0659	0.0000	-3.4224	0.2067	-0.2641	0.0000	-0.6310	1.0000	0.1192	0.7608	-0.2252	0.0704	-0.2424
LOC100541035	0.0000	1.2061	0.0000	1.3290	0.0000	1.1148	0.0000	0.8551	1.0000	-0.0141	1.0000	0.1202	0.4333	-0.2686
LOC100541041	0.5678	-1.0043	1.0000	0.2325	0.1597	1.2309	0.6843	0.5847	1.0000	0.4303	0.3263	1.6891	1.0000	-0.2056
LOC100541042	0.0003	1.2346	0.0000	1.2362	0.8785	0.2102	1.0000	0.0073	1.0000	0.0663	1.0000	0.0793	1.0000	-0.1331
LOC100541044	0.2082	-1.0008	1.0000	0.1395	0.0096	-2.0313	1.0000	0.0060	1.0000	-0.2672	0.5550	0.8865	0.1233	1.7749
LOC100541052	0.3090	0.5283	0.0002	0.9997	0.0000	1.5012	0.0000	1.8208	1.0000	-0.2581	1.0000	0.2239	1.0000	0.0665
LOC100541057	0.8249	0.2518	0.0122	0.8005	1.0000	-0.1674	0.1260	0.5585	1.0000	-0.1314	0.5396	0.4296	0.2798	0.6009
LOC100541067	1.0000	0.0000	1.0000	0.6987	1.0000	0.0000	1.0000	-2.3178	1.0000	2.2674	1.0000	3.2002	1.0000	0.0000
LOC100541069	1.0000	-1.1585	1.0000	-2.3959	1.0000	-0.1834	1.0000	0.0076	1.0000	-0.9638	1.0000	-2.2955	1.0000	-0.7769
LOC100541072	1.0000	0.0000	1.0000	0.0000	1.0000	0.0000	1.0000	0.0000	1.0000	0.0000	1.0000	0.0000	1.0000	0.0000
LOC100541074	1.0000	2.1902	1.0000	-0.5546	1.0000	2.2426	0.9801	-0.9105	1.0000	4.0238	1.0000	1.4314	1.0000	0.9164
LOC100541078	0.4183	1.5375	1.0000	-0.5563	0.7629	1.0380	0.8219	0.7775	1.0000	0.7963	0.8375	-1.2860	1.0000	0.5433
LOC100541081	0.3765	-0.1791	0.0803	-0.1614	0.0000	-1.2298	0.0000	-0.9802	1.0000	-0.1969	0.3181	-0.1662	0.9264	0.0579
LOC100541083	1.0000	0.0000	1.0000	0.0000	1.0000	0.0000	1.0000	0.0000	1.0000	0.0000	1.0000	0.0000	1.0000	0.0000
LOC100541086	1.0000	-0.2892	1.0000	0.0000	1.0000	-2.4061	1.0000	0.0000	1.0000	-2.3771	1.0000	-2.2991	1.0000	0.0000
LOC100541106	0.0022	0.7900	0.0000	0.9059	0.0000	1.4611	0.0000	1.1988	1.0000	0.0934	0.5234	0.2243	0.5781	-0.1602
LOC100541107	0.0000	-1.0596	0.0000	-0.9761	0.0000	-0.9247	0.0000	-1.2675	1.0000	-0.0933	1.0000	0.0025	0.0003	-0.4307
LOC100541117	0.6835	0.6172	0.6707	0.3318	0.0001	1.9228	0.0138	0.9754	1.0000	0.4976	1.0000	0.2242	0.5071	-0.4403
LOC100541118	0.0011	0.7626	0.0000	1.0660	0.0000	1.3928	0.0000	1.4948	1.0000	-0.3230	1.0000	-0.0058	0.3237	-0.2149
LOC100541119	1.0000	-0.1335	1.0000	0.0635	1.0000	-0.1137	0.4432	0.3384	1.0000	0.0682	1.0000	0.2783	0.3494	0.5232
LOC100541121	0.0000	-3.1652	0.0000	-3.0935	0.7115	-0.3300	0.9408	-0.1050	1.0000	0.3111	1.0000	0.3987	0.1916	0.5391
LOC100541131	1.0000	-0.2920	1.0000	-2.3986	1.0000	-2.4056	1.0000	0.0057	1.0000	-0.1034	1.0000	-2.2955	1.0000	2.3554
LOC100541134	0.0280	0.3464	0.0008	0.2738	0.0000	0.6701	0.0000	0.5598	1.0000	0.0081	1.0000	-0.0519	0.6777	-0.0967
LOC100541135	1.0000	0.0000	1.0000	0.0000	1.0000	0.0000	1.0000	0.0000	1.0000	0.0000	1.0000	0.0000	1.0000	0.0000
LOC100541138	1.0000	0.0000	1.0000	0.0000	1.0000	0.0000	1.0000	0.0000	1.0000	0.0000	1.0000	0.0000	1.0000	0.0000
LOC100541145	1.0000	-0.3531	0.1468	-1.5104	0.4089	-1.3121	0.0522	-2.0202	1.0000	0.3875	1.0000	-0.7579	1.0000	-0.3119
LOC100541147	0.0254	2.0988	0.7526	-0.4546	0.6927	0.7664	0.4397	-0.7440	1.0000	1.8942	0.8723	-0.6449	1.0000	0.3916
LOC100541155	0.0000	-3.6824	0.0000	-3.1522	0.0000	0.8911	0.0000	1.3865	1.0000	0.0435	0.0976	0.5852	0.0000	0.5444
LOC100541159	0.8226	3.0199	1.0000	-0.8548	0.0195	5.2592	1.0000	-0.6653	1.0000	4.3117	1.0000	0.5860	0.4492	-1.5774
LOC100541160	0.0569	-1.6234	0.4706	-0.9334	0.4604	-0.7467	0.2577	0.8688	1.0000	-0.9906	1.0000	-0.2914	0.6733	0.6275
LOC100541166	1.0000	2.1849	1.0000	2.2506	1.0000	2.2472	1.0000	0.0000	1.0000	0.0000	1.0000	0.0495	1.0000	-2.2907
LOC100541174	1.0000	0.2101	0.4025	-2.2302	1.0000	-0.1868	0.3952	-2.0640	1.0000	0.1810	0.6450	-2.2673	0.8939	-1.6953
LOC100541175	1.0000	0.0000	0.7674	-3.2570	1.0000	0.0000	0.7710	-3.1693	1.0000	3.1191	1.0000	0.0000	1.0000	0.0000
LOC100541177	0.0000	1.1617	0.0000	1.0838	0.2461	-0.3785	0.0074	-0.6692	1.0000	-0.3849	0.0149	-0.4520	0.0269	-0.6732
LOC100541180	1.0000	-0.7247	0.9754	-1.0835	0.8700	0.5794	0.9786	-0.9107	1.0000	-0.1253	1.0000	-0.4752	0.6246	-1.6128
LOC100541187	0.0453	2.3259	0.0153	2.1453	0.2725	1.6862	0.2364	1.4114	1.0000	0.8007	0.9498	0.6355	1.0000	0.5271
LOC100541195	1.0000	-0.0219	0.0635	0.3089	0.0002	0.7694	0.0000	0.7840	1.0000	-0.0870	0.3662	0.2552	1.0000	-0.0692
LOC100541197	1.0000	-2.4776	1.0000	0.0000	1.0000	-2.4056	1.0000	2.3241	1.0000	-2.3757	1.0000	0.0000	1.0000	2.3542
LOC100541200	0.0791	-1.0181	0.0033	-1.3317	0.3570	0.5029	0.9965	0.1460	1.0000	0.2358	1.0000	-0.0659	1.0000	-0.1177
LOC100541203	1.0000	0.0000	1.0000	0.0000	1.0000	0.0000	1.0000	0.0000	1.0000	0.0000	1.0000	0.0000	1.0000	0.0000
LOC100541205	1.0000	0.0000	1.0000	0.0000	1.0000	0.0000	1.0000	0.0000	1.0000	0.0000	1.0000	0.0000	1.0000	0.0000
LOC100541206	1.0000	0.0000	1.0000	-2.3959	1.0000	0.0000	1.0000	-2.3178	1.0000	2.2674	1.0000	0.0000	1.0000	0.0000
LOC100541207	0.0000	-0.9707	0.0000	-1.2587	0.0000	-0.8399	0.0000	-0.7659	1.0000	0.0847	0.2017	-0.1913	0.1906	0.1641
LOC100541211	0.0003	0.9555	0.0000	0.7626	0.0001	0.9614	0.0000	0.9804	1.0000	0.1185	1.0000	-0.0619	0.8718	0.1435
LOC100541213	1.0000	-0.0593	0.7594	-0.0939	0.0533	-0.5472	0.0897	-0.2676	1.0000	-0.0839	1.0000	-0.1063	0.7307	0.2038
LOC100541214	0.0000	1.1525	0.0000	1.4225	0.0086	-0.4614	0.0003	-0.3614	1.0000	0.0365	0.0021	0.3191	0.5646	0.1427
LOC100541229	0.1672	-0.2349	0.0014	-0.2280	0.0012	0.4801	0.0000	0.5724	1.0000	0.0782	0.6671	0.0975	0.0793	0.1758
LOC100541237	0.1303	0.4604	0.3683	0.2209	0.3880	0.3053	0.1178	-0.3614	1.0000	0.0759	0.9944	-0.1506	0.0219	-0.5835
LOC100541254	0.0004	1.3490	0.0015	0.9042	0.3212	-0.6349	0.0264	-0.8614	1.0000	0.3966	1.0000	-0.0348	1.0000	0.1787
LOC100541257	1.0000	0.0000	1.0000	0.0000	1.0000	0.0000	1.0000	0.0000	1.0000	0.0000	1.0000	0.0000	1.0000	0.0000
LOC100541261	1.0000	-2.4776	1.0000	0.0000	1.0000	-2.4056	1.0000	0.0000	1.0000	-2.3757	1.0000	0.0000	1.0000	0.0000
LOC100541270	0.6128	0.5464	1.0000	0.1946	0.1707	0.8658	1.0000	0.0101	1.0000	0.3640	1.0000	0.0249	0.7231	-0.4820
LOC100541274	1.0000	0.1602	0.2030	0.4532	0.6976	0.2903	0.0001	1.0297	1.0000	-0.2057	1.0000	0.0977	0.1306	0.5379
LOC100541286	1.0000	0.0000	1.0000	0.0000	1.0000	0.0000	1.0000	0.0000	1.0000	0.0000	1.0000	0.0000	1.0000	0.0000
LOC100541290	0.0943	-0.3792	0.0065	-0.3431	0.0688	0.3673	1.0000	-0.0226	1.0000	-0.0766	1.0000	-0.0269	0.0000	-0.4596
LOC100541291	1.0000	2.1902	1.0000	0.0000	1.0000	0.0000	1.0000	2.3241	1.0000	0.0000	1.0000	-2.2991	1.0000	2.3542
LOC100541293	1.0000	0.0000	1.0000	0.0000	1.0000	0.0000	1.0000	0.0000	1.0000	0.0000	1.0000	0.0000	1.0000	0.0000
LOC100541297	0.7820	0.4901	0.4400	-0.4879	0.5083	-0.7321	0.1412	-0.8499	1.0000	0.5705	1.0000	-0.3944	1.0000	0.4599
LOC100541314	0.3089	-4.2696	1.0000	-2.3959	0.2785	-4.1857	1.0000	-2.3178	1.0000	-1.8822	1.0000	0.0000	1.0000	0.0000
LOC100541319	0.2958	-1.4454	1.0000	0.1744	0.0314	-3.3447	1.0000	-0.2095	1.0000	-0.7484	1.0000	0.8784	0.5105	2.3934
LOC100541326	1.0000	0.0000	1.0000	0.0000	1.0000	0.0000	1.0000	0.0000	1.0000	0.0000	1.0000	0.0000	1.0000	0.0000
LOC100541332	1.0000	2.1849	1.0000	0.0000	1.0000	0.0000	1.0000	0.0000	1.0000	0.0000	1.0000	-2.2954	1.0000	0.0000
LOC100541340	1.0000	0.0000	1.0000	0.0000	1.0000	0.0000	1.0000	0.0000	1.0000	0.0000	1.0000	0.0000	1.0000	0.0000
LOC100541343	0.0000	0.7895	0.0000	0.5869	0.0000	1.0248	0.0000	1.1113	1.0000	0.4047	0.2897	0.2150	0.0000	0.4964
LOC100541356	0.0321	0.9822	0.0250	0.7773	1.0000	0.0259	0.6715	-0.3564	1.0000	-0.1617	0.7510	-0.3555	0.5904	-0.5423
LOC100541366	0.0007	2.0497	0.0000	3.0433	0.0609	1.2901	0.0266	1.4560	1.0000	-0.7546	1.0000	0.2495	0.6482	-0.5878
LOC100541369	1.0000	-0.2881	1.0000	0.0000	1.0000	0.6783	0.7710	3.1708	1.0000	-2.3757	1.0000	-2.2992	1.0000	0.0717
LOC100541374	0.7346	0.2188	0.7492	0.1614	1.0000	0.0272	0.0362	0.5073	1.0000	-0.1387	1.0000	-0.1810	0.4472	0.3470
LOC100541378	0.7421	-0.2246	0.0000	-1.0736	0.0146	-1.0616	0.0000	-1.2138	1.0000	0.2616	0.2401	-0.5739	1.0000	0.1192
LOC100541387	1.0000	0.0000	1.0000	0.0000	1.0000	0.0000	1.0000	0.0000	1.0000	0.0000	1.0000	0.0000	1.0000	0.0000
LOC100541391	0.0118	-0.4873	0.0000	-0.5186	0.3095	0.2303	0.8408	0.0560	1.0000	-0.0646	1.0000	-0.0837	0.1687	-0.2325
LOC100541395	1.0000	2.1849	1.0000	-0.1452	1.0000	0.0000	1.0000	-2.3201	1.0000	2.2736	1.0000	0.0514	1.0000	0.0000

LOC100541400	0.0000	1.4737	0.0000	1.2734	0.0000	1.5112	0.0341	0.6533	1.0000	-0.2142	0.1886	-0.3978	0.0000	-1.0623
LOC100541411	0.1016	0.8231	0.0000	1.1094	0.0000	1.9359	0.0000	1.1996	1.0000	0.2897	0.2142	0.5847	0.2489	-0.4445
LOC100541413	1.0000	2.1849	1.0000	-2.3959	0.0001	6.1696	0.0004	3.9481	1.0000	2.2674	1.0000	-2.2954	1.0000	0.0792
LOC100541423	0.0000	1.9156	0.0000	1.6684	0.0000	1.8593	0.0000	1.5103	1.0000	0.3694	1.0000	0.1353	1.0000	0.0258
LOC100541429	0.0364	1.9716	0.0069	2.1486	0.8140	-1.4015	0.6142	-1.7590	1.0000	-0.4331	1.0000	-0.2442	1.0000	-0.7804
LOC100541434	0.7833	-0.5203	0.9673	0.2484	0.1835	-1.1775	0.4175	-0.7205	1.0000	0.0255	0.6256	0.8064	1.0000	0.4843
LOC100541445	1.0000	0.0000	1.0000	0.0000	1.0000	0.0000	1.0000	0.0000	1.0000	0.0000	1.0000	0.0000	1.0000	0.0000
LOC100541447	1.0000	0.0000	1.0000	-2.3959	1.0000	0.0000	1.0000	-2.3178	1.0000	2.2674	1.0000	0.0000	1.0000	0.0000
LOC100541450	0.8335	-0.5092	1.0000	0.0618	0.0038	1.5414	0.0017	1.4530	1.0000	-0.3762	1.0000	0.2100	0.5374	-0.4571
LOC100541451	1.0000	2.1849	1.0000	0.0000	1.0000	0.0000	1.0000	0.0000	1.0000	0.0000	1.0000	-2.2954	1.0000	0.0000
LOC100541460	0.8383	-0.2805	0.0000	-1.7241	0.0001	1.4910	0.0011	0.7466	1.0000	0.5685	0.2000	-0.8601	0.8815	-0.1697
LOC100541463	0.0000	-2.2487	0.0000	-1.8779	1.0000	-0.0386	0.0000	0.3447	1.0000	0.0530	0.0037	0.4362	0.0000	0.4407
LOC100541464	0.5062	0.2172	0.2064	0.2182	0.8033	-0.1310	0.1604	0.2306	1.0000	-0.0247	1.0000	-0.0094	0.0860	0.3428
LOC100541465	1.0000	-2.4776	0.0121	-5.3003	1.0000	-2.4056	0.0109	-5.2058	1.0000	2.7696	1.0000	0.0000	1.0000	0.0000
LOC100541468	0.0001	0.6668	0.0000	0.9370	0.0460	0.3770	0.0019	0.2724	1.0000	-0.3121	1.0000	-0.0297	0.0000	-0.4116
LOC100541469	1.0000	2.1848	1.0000	0.0000	1.0000	0.0000	1.0000	0.0000	1.0000	0.0000	1.0000	-2.2954	1.0000	0.0000
LOC100541471	0.8249	0.9059	0.5663	0.7952	0.4071	1.2138	0.5724	0.8547	1.0000	0.4278	1.0000	0.3228	1.0000	0.0773
LOC100541480	0.3652	0.7066	0.0275	1.2322	0.0633	-1.5274	1.0000	-0.2293	1.0000	-0.5925	1.0000	-0.0564	1.0000	0.7115
LOC100541485	0.0746	1.7891	0.3442	0.9674	0.4564	-2.2460	0.0961	-2.7180	1.0000	0.5293	1.0000	-0.2862	1.0000	0.0649
LOC100541489	1.0000	0.0000	1.0000	0.0000	1.0000	0.0000	1.0000	0.0000	1.0000	0.0000	1.0000	0.0000	1.0000	0.0000
LOC100541494	0.1780	-0.6649	0.9684	-0.1569	1.0000	-0.1438	0.9664	-0.1567	1.0000	-0.4066	1.0000	0.1122	0.6635	-0.4187
LOC100541498	0.1578	0.3417	0.0000	0.5599	0.7935	-0.1330	1.0000	-0.0460	1.0000	-0.0865	0.7800	0.1428	1.0000	0.0049
LOC100541500	0.0010	0.7529	0.1612	0.2170	0.0009	0.7011	0.0157	0.3274	1.0000	0.3091	0.5944	-0.2147	1.0000	-0.0592
LOC100541513	0.0000	1.7812	0.0240	0.7797	0.9020	0.2892	0.2361	-0.6089	1.0000	0.2785	0.0477	-0.7072	0.5379	-0.6083
LOC100541518	0.0000	-2.5030	0.0000	-2.8169	1.0000	-0.1463	0.0542	0.5397	1.0000	0.3614	1.0000	0.0612	0.0008	1.0523
LOC100541519	0.1942	0.5521	0.1298	0.4099	0.0000	1.1701	0.0010	0.7301	1.0000	0.0892	1.0000	-0.0395	0.2417	-0.3421
LOC100541523	0.0710	-0.3137	0.0000	-0.5208	0.0000	-0.7731	0.0000	-0.7127	1.0000	0.0584	0.6050	-0.1358	0.6531	0.1236
LOC100541529	0.7256	-0.3907	1.0000	-0.0674	0.4851	0.4722	0.0006	1.1419	1.0000	-0.3081	1.0000	0.0295	0.6077	0.3700
LOC100541538	1.0000	0.0000	1.0000	0.0000	1.0000	0.0000	1.0000	0.0000	1.0000	0.0000	1.0000	0.0000	1.0000	0.0000
LOC100541541	1.0000	-0.3557	0.2095	-1.4990	0.0000	2.8529	0.0001	1.8745	1.0000	0.2285	0.9238	-0.9052	0.1079	-0.7376
LOC100541557	1.0000	0.5370	0.4431	3.6192	1.0000	-0.1597	1.0000	2.3257	1.0000	-2.3771	1.0000	0.5871	1.0000	0.0649
LOC100541562	0.3561	-0.8060	0.3158	-0.5952	0.9547	0.2763	0.9552	0.1944	1.0000	0.3381	0.8885	0.5598	1.0000	0.2656
LOC100541564	0.8226	3.0199	1.0000	-1.0017	1.0000	0.0000	1.0000	-0.8416	1.0000	3.1126	1.0000	-0.7966	1.0000	2.3554
LOC100541567	1.0000	-2.4776	1.0000	-2.3959	1.0000	-2.4056	1.0000	-2.3178	1.0000	-0.1078	1.0000	0.0000	1.0000	0.0000
LOC100541582	0.0036	1.0300	0.0158	0.6052	0.0768	-0.8188	0.0010	-1.0236	1.0000	0.4272	1.0000	0.0136	1.0000	0.2272
LOC100541592	0.0089	-0.9228	0.0012	-0.7365	0.0108	0.7334	0.0323	0.4288	1.0000	-0.0354	1.0000	0.1633	0.2214	-0.3338
LOC100541596	0.2626	-0.4031	0.6732	0.0954	0.3187	-0.3745	0.0121	0.2952	1.0000	0.1356	0.0001	0.6479	0.0000	0.8123
LOC100541598	1.0000	0.0000	1.0000	0.0000	1.0000	0.0000	1.0000	0.0000	1.0000	0.0000	1.0000	0.0000	1.0000	0.0000
LOC100541600	1.0000	0.0000	1.0000	0.0000	1.0000	0.0000	1.0000	0.0000	1.0000	0.0000	1.0000	0.0000	1.0000	0.0000
LOC100541603	1.0000	0.0000	1.0000	0.0000	1.0000	0.0000	1.0000	0.0000	1.0000	0.0000	1.0000	0.0000	1.0000	0.0000
LOC100541604	1.0000	0.0000	1.0000	0.0000	1.0000	0.0000	1.0000	0.0000	1.0000	0.0000	1.0000	0.0000	1.0000	0.0000
LOC100541605	0.0739	0.3488	0.1656	0.1774	1.0000	0.0539	1.0000	0.0304	1.0000	-0.0565	0.2818	-0.2156	1.0000	-0.0762
LOC100541607	1.0000	-0.3358	1.0000	0.0102	0.4809	-3.7967	0.5746	-1.1508	1.0000	1.0336	0.8385	1.4020	0.7287	3.7384
LOC100541608	0.3747	0.2059	0.0005	0.2517	1.0000	-0.0348	0.0689	-0.1432	1.0000	-0.1035	1.0000	-0.0454	0.0434	-0.2063
LOC100541609	0.0830	-0.3161	0.0000	-0.4755	0.6806	-0.1228	0.0014	-0.2743	1.0000	0.0464	0.8372	-0.1003	0.7271	-0.0999
LOC100541617	1.0000	2.1902	1.0000	0.0000	1.0000	2.2426	1.0000	0.0000	1.0000	0.0000	1.0000	-2.2991	1.0000	-2.2888
LOC100541624	0.1784	0.2504	0.0000	0.4717	0.0049	-0.4762	0.0008	-0.3023	1.0000	-0.2987	1.0000	-0.0650	0.6770	-0.1185
LOC100541632	1.0000	-2.4776	1.0000	0.0000	1.0000	-0.1624	0.7710	3.1708	1.0000	-2.3757	1.0000	0.0000	1.0000	0.9164
LOC100541641	0.1077	-0.3227	0.0000	-0.4760	0.0084	-0.4826	0.0000	-0.5563	1.0000	-0.1228	0.0205	-0.2640	0.0703	-0.1907
LOC100541644	0.8249	-3.3439	0.4636	-3.7802	1.0000	0.3467	1.0000	-0.5214	1.0000	0.3999	1.0000	0.0000	1.0000	-0.4568
LOC100541646	0.5095	-0.1574	0.0002	-0.3285	0.0881	-0.3066	0.0000	-0.4559	1.0000	0.1006	1.0000	-0.0584	1.0000	-0.0433
LOC100541650	0.8218	0.1962	0.0001	0.6752	0.0557	0.6138	0.1151	0.3982	1.0000	0.0552	0.0234	0.5449	0.9460	-0.1553
LOC100541658	0.8461	0.8153	0.2306	1.7052	0.9468	-1.5556	1.0000	0.5319	1.0000	-0.6465	1.0000	0.2457	1.0000	1.4474
LOC100541666	1.0000	-0.0271	1.0000	0.0021	0.8945	0.1022	0.4560	-0.1362	1.0000	-0.1048	1.0000	-0.0640	0.0741	-0.3369
LOC100541679	0.0121	-0.8146	0.0000	-0.8904	0.0007	-1.0523	0.0002	-0.4936	1.0000	0.4349	0.1367	0.3721	0.0000	0.9954
LOC100541687	1.0000	0.0000	1.0000	0.0000	1.0000	0.0000	1.0000	0.0000	1.0000	0.0000	1.0000	0.0000	1.0000	0.0000
LOC100541689	1.0000	0.0000	1.0000	0.0000	1.0000	0.0000	1.0000	0.0000	1.0000	0.0000	1.0000	0.0000	1.0000	0.0000
LOC100541693	0.0318	0.7395	0.0000	0.8652	0.0053	0.8741	0.0000	1.0395	1.0000	-0.0481	1.0000	0.0897	0.9893	0.1220
LOC100541694	1.0000	0.0000	1.0000	0.0000	1.0000	0.0000	1.0000	0.0000	1.0000	0.0000	1.0000	0.0000	1.0000	0.0000
LOC100541702	0.8226	3.0199	1.0000	0.0000	1.0000	0.0000	1.0000	0.0000	1.0000	0.0000	1.0000	-3.1429	1.0000	0.0000
LOC100541707	0.0000	2.3067	0.0000	2.0913	0.0000	2.6633	0.0000	1.2823	1.0000	0.6054	0.3791	0.4042	0.0030	-0.7685
LOC100541714	0.7147	0.1399	0.6047	0.1120	0.0000	-1.1018	0.0000	-0.5864	1.0000	-0.1541	0.7010	-0.1697	0.0769	0.3680
LOC100541737	0.8249	0.2288	0.1925	0.4415	0.0000	1.2939	0.0000	2.0388	1.0000	-0.2245	1.0000	-0.0007	0.0162	0.5239
LOC100541738	0.1108	-0.7292	0.0000	-1.2574	0.0010	-1.3881	0.0043	-0.7483	1.0000	0.0726	0.5907	-0.4400	0.1225	0.7220
LOC100541742	1.0000	-0.0449	0.0677	-0.4731	0.7182	0.2231	0.4040	-0.2699	1.0000	0.4207	1.0000	0.0059	1.0000	-0.0648
LOC100541746	0.3491	-0.2341	0.3465	-0.1610	0.0000	-1.1241	0.0000	-0.6509	1.0000	0.0145	1.0000	0.1001	0.0109	0.4935
LOC100541750	1.0000	-2.4776	1.0000	-2.3986	1.0000	-0.1591	1.0000	-2.3200	1.0000	-0.1034	1.0000	0.0000	1.0000	-2.2907
LOC100541754	0.0001	1.6881	0.0000	1.5181	0.0000	1.9348	0.0000	1.2615	1.0000	0.3049	1.0000	0.1463	0.5023	-0.3639
LOC100541755	1.0000	0.0000	1.0000	-0.1449	1.0000	2.2427	1.0000	0.0062	1.0000	2.2674	1.0000	2.3456	1.0000	0.0652
LOC100541756	1.0000	0.0000	1.0000	0.0000	1.0000	0.0000	1.0000	0.0000	1.0000	0.0000	1.0000	0.0000	1.0000	0.0000
LOC100541770	0.8226	-3.3425	1.0000	-1.0051	0.8011	-3.2629	1.0000	-0.8429	1.0000	-0.1146	1.0000	2.3456	1.0000	2.3554
LOC100541773	1.0000	0.0553	1.0000	0.0781	0.3384	0.4672	0.2550	0.4416	1.0000	-0.1309	1.0000	-0.0950	1.0000	-0.1503
LOC100541776	0.8483	0.0943	0.6883	0.0733	0.0000	1.0426	0.0000	0.9008	1.0000	0.1093	0.8607	0.1010	1.0000	-0.0267
LOC100541781	0.5578	0.2205	0.0071	0.4288	0.0000	-1.1754	0.0000	-0.8380	1.0000	-0.2039	1.0000	0.0177	1.0000	0.1374
LOC100541783	0.0274	-1.9769	0.0025	-3.1052	0.0000	2.3803	0.0000	2.6845	1.0000	-0.2933	0.9075	-1.4162	1.0000	0.0182
LOC100541789	0.0004	-3.1233	0.0583	-1.2875	0.6154	0.4635	0.0538	0.8752	1.0000	-0.0089	0.5004	1.8456	0.7692	0.4090
LOC100541791	0.0410	-1.1661	0.2017	-0.4422	0.0000	1.7285	0.0000	1.8843	1.0000	-0.0174	0.2808	0.7265	0.7908	0.1446
LOC100541793	1.0000	0.0000	1.0000	-2.3959	1.0000	0.0000	1.0000	0.0075	1.0000	2.2675	1.0000	0.0000	1.0000	2.3554
LOC100541794	0.0544	-0.5112	0.0110	-0.4697	0.0000	1.1003	0.0000	0.6793	1.0000	-0.0248	1.0000	0.0299	0.0076	-0.4397
LOC100541803	1.0000	0.0529	0.9657	-0.2968	0.0106	1.2544	0.0019	1.2137	1.0000	0.1141	1.0000	-0.2205	1.0000	0.0809
LOC100541825	1.0000	0.0508	1.0000	0.0105	0.1948	-1.4754	0.1495	-0.8827	1.0000	0.7611	0.5245	0.7327	0.4180	1.3633
LOC100541835	0.0186	1.0831	0.3968	0.5039	0.0316	-1.3894	1.0000	0.1410	1.0000	-0.0881	0.3216	-0.6524	0.0211	1.4506
LOC100541844	0.9961	0.4077	1.0000	-0.1721	0.0230	1.7679	0.0002	2.4850	1.0000	-0.5934	0.7069	-1.1645	1.0000	0.1296
LOC100541845	1.0000	0.0000	1.0000	0.0000	1.0000	0.0000	1.0000	0.0000	1.0000	0.0000	1.0000	0.0000	1.0000	0.0000
LOC100541846	0.1264	0.3223	0.1538	0.1917	0.0000	0.9424	0.0000	0.7183	1.0000	0.1392	1.0000	0.0218	0.8887	-0.0797
LOC100541852	0.1270	0.7262	0.0034	1.0314	0.5554	0.4040	0.1732	0.6184	1.0000	-0.2923	1.0000	0.0261	1.0000	-0.0731
LOC100541858	1.0000	0.2026	1.0000	-0.1452	1.0000	0.7306	0.6142	1.7719	1.0000	-0.9587	1.0000	-1.3257	1.0000	0.0769
LOC100541860	1.0000	0.0000	1.0000	-2.3959	1.0000	0.0000	1.0000	-2.3178	1.0000	2.2674	1.0000	0.0000	1.0000	0.0000
LOC100541862	0.6878	-0.1347	0.0587	-0.2055	0.3391	-0.2259	0.1010	-0.1756	1.0000	-0.1145	0.4115	-0.1730	1.0000	-0.0597
LOC100541865	1.0000	0.0000	1.0000	0.0000	1.0000	0.0000	1.0000	2.3257	1.0000	0.0000	1.0000	0.0000	1.0000	2.3554
LOC100541871	1.0000	-2.4788	0.7674	3.0888	1.0000	-0.1597	1.0000	0.0000	1.0000	-2.3771	1.0000	3.1946	1.0000	-2.2907
LOC100541880	0.0003	1.1116	0.0000	1.2158	0.0000	1.4960	0.0000	1.3711	1.0000	-0.1199	1.0000	-0.0038	0.7140	-0.2404
LOC100541881	0.1877	0.4826	0.1121	0.3946	0.0055	0.8238	0.0028	0.6167	1.0000	-0.7047	0.0002	-0.7792	0.0000	-0.9047

LOC100541890	0.8226	3.0199	0.7674	-3.2498	1.0000	0.0000	0.7710	-3.1632	1.0000	3.1056	1.0000	-3.1429	1.0000	0.0000
LOC100541896	0.8645	0.1492	1.0000	-0.0741	0.0001	-1.2421	0.0287	-0.5182	1.0000	0.1202	1.0000	-0.0926	0.0075	0.8465
LOC100541903	0.8967	0.3713	0.1540	0.7764	0.0012	1.6556	0.0324	0.9988	1.0000	0.3473	0.4014	0.7673	0.9049	-0.3057
LOC100541905	0.0000	1.3517	0.0000	1.4445	0.2691	0.4411	1.0000	0.0463	1.0000	-0.1338	1.0000	-0.0275	0.1842	-0.5221
LOC100541906	1.0000	0.5464	0.9148	1.2289	0.9361	1.2064	1.0000	-2.3177	1.0000	-0.1078	1.0000	0.5866	0.7287	-3.6637
LOC100541913	1.0000	0.0000	1.0000	0.0000	1.0000	0.0000	1.0000	0.0000	1.0000	0.0000	1.0000	0.0000	1.0000	0.0000
LOC100541927	1.0000	0.0000	0.7666	-3.2534	0.8033	3.0790	0.9877	0.9208	1.0000	3.1126	1.0000	0.0000	1.0000	0.9911
LOC100541930	0.0002	-0.6756	0.0000	-0.5536	0.0055	-0.5089	0.0030	-0.3195	1.0000	-0.1866	1.0000	-0.0525	1.0000	0.0086
LOC100541931	0.0835	0.4732	0.0222	0.4334	0.0120	-0.6938	0.0227	-0.6431	1.0000	-0.1088	0.9688	-0.1372	1.0000	-0.0529
LOC100541941	0.4064	0.8399	0.2754	0.8330	0.6289	0.7008	1.0000	0.0066	1.0000	0.7735	0.7073	0.7862	1.0000	0.0805
LOC100541947	1.0000	0.0000	1.0000	-2.3959	1.0000	0.0000	1.0000	-2.3177	1.0000	2.2674	1.0000	0.0000	1.0000	0.0000
LOC100541954	1.0000	0.0000	1.0000	0.0000	1.0000	0.0000	1.0000	2.3257	1.0000	0.0000	1.0000	0.0000	1.0000	2.3554
LOC100541957	0.5475	-0.6947	0.0326	-1.3184	0.0068	1.3490	0.2723	0.5731	1.0000	0.4682	1.0000	-0.1416	0.8526	-0.2984
LOC100541961	0.3539	0.4437	0.0003	1.1463	0.0000	-2.0378	0.0000	-2.1135	1.0000	-0.4223	0.8723	0.2924	0.9913	-0.4952
LOC100541966	0.0456	0.4167	0.0201	0.2999	0.1155	-0.3700	0.0002	-0.4834	1.0000	0.0459	1.0000	-0.0586	1.0000	-0.0617
LOC100541969	0.0002	-0.8863	0.0001	-0.7051	0.0210	0.5464	0.0014	0.4722	1.0000	0.0524	0.7303	0.2458	1.0000	-0.0192
LOC100541987	0.7844	-0.3274	0.0263	-1.0653	0.1178	0.7381	0.2964	0.4747	1.0000	-0.0587	0.4857	-0.7860	0.8252	-0.3131
LOC100541990	0.2875	1.1214	0.0357	1.3812	0.0012	2.1614	0.0005	2.0895	1.0000	0.2205	0.8840	0.4914	1.0000	0.1550
LOC100541993	0.0011	-1.5485	0.0000	-1.4417	0.1337	0.6497	0.0000	0.8736	1.0000	0.0954	1.0000	0.2191	0.3838	0.3279
LOC100541995	1.0000	-2.4776	1.0000	0.0000	1.0000	-2.4056	1.0000	0.0000	1.0000	-2.3757	1.0000	0.0000	1.0000	0.0000
LOC100541996	1.0000	0.0000	1.0000	0.0000	1.0000	0.0000	1.0000	0.0000	1.0000	0.0000	1.0000	0.0000	1.0000	0.0000
LOC100542004	0.0090	0.8588	0.0711	0.4300	0.0114	0.7625	1.0000	0.0558	1.0000	0.3811	1.0000	-0.0377	0.4732	-0.3210
LOC100542006	0.7070	0.1952	0.0231	0.4717	0.0047	0.7032	0.0000	0.7992	1.0000	-0.0355	0.5458	0.2534	1.0000	0.0663
LOC100542016	1.0000	0.0000	1.0000	0.0000	1.0000	0.0000	1.0000	0.0000	1.0000	0.0000	1.0000	0.0000	1.0000	0.0000
LOC100542017	0.0002	0.6317	0.0000	0.8239	0.7064	0.1217	1.0000	0.0071	1.0000	0.0523	0.1047	0.2563	1.0000	-0.0572
LOC100542020	0.0004	1.3991	0.1183	0.5565	0.0000	2.3785	0.0000	1.4382	1.0000	0.6031	0.9819	-0.2248	0.4165	-0.3296
LOC100542032	0.0460	-0.3373	0.0000	-0.3082	0.0000	-0.7730	0.0000	-0.7940	1.0000	-0.2286	0.0867	-0.1871	0.0122	-0.2440
LOC100542038	0.0283	1.0714	0.0169	0.9427	0.0000	2.1573	0.0000	1.7360	1.0000	0.4627	0.9238	0.3479	1.0000	0.0436
LOC100542043	1.0000	0.0000	1.0000	0.0000	1.0000	0.0000	1.0000	0.0000	1.0000	0.0000	1.0000	0.0000	1.0000	0.0000
LOC100542046	1.0000	0.3530	1.0000	0.3847	0.6305	0.9686	0.3397	1.2766	1.0000	0.2637	1.0000	0.3105	1.0000	0.5776
LOC100542048	1.0000	0.1489	0.5649	0.7947	1.0000	-0.6614	0.8940	0.5728	1.0000	-0.3419	1.0000	0.3163	0.9562	0.8963
LOC100542049	1.0000	0.0000	1.0000	0.0000	1.0000	0.0000	1.0000	0.0000	1.0000	0.0000	1.0000	0.0000	1.0000	0.0000
LOC100542067	1.0000	0.0000	1.0000	0.0000	0.8011	3.0840	1.0000	2.3257	1.0000	0.0000	1.0000	0.0000	1.0000	-0.7793
LOC100542070	1.0000	0.0000	1.0000	0.0000	1.0000	0.0000	1.0000	0.0000	1.0000	0.0000	1.0000	0.0000	1.0000	0.0000
LOC100542090	0.8249	3.0255	0.4431	-3.7865	0.0318	4.9524	0.7799	0.9494	1.0000	3.6396	1.0000	-3.1468	1.0000	-0.3239
LOC100542094	0.0000	1.2877	0.0000	1.5050	0.0000	1.4817	0.0000	1.2307	1.0000	0.0363	0.6165	0.2661	0.2199	-0.2099
LOC100542101	0.3089	3.9281	1.0000	2.2507	1.0000	2.2427	1.0000	0.0000	1.0000	0.0000	1.0000	-1.7124	1.0000	-2.2888
LOC100542106	1.0000	0.0000	1.0000	0.0000	1.0000	0.0000	1.0000	0.0000	1.0000	0.0000	1.0000	0.0000	1.0000	0.0000
LOC100542110	0.4327	0.7051	0.0041	1.3348	0.0987	1.0226	0.3441	0.6680	1.0000	-0.1352	0.7554	0.5065	0.7138	-0.4818
LOC100542113	0.0000	1.6344	0.0000	1.7582	0.0000	2.3293	0.0000	2.2718	1.0000	0.3815	0.0001	0.5191	0.0005	0.3296
LOC100542122	0.0011	0.5415	0.0000	0.5593	0.0175	-0.4248	0.0001	-0.5038	1.0000	-0.0242	1.0000	0.0061	0.8275	-0.0971
LOC100542130	1.0000	-0.8597	1.0000	-0.6953	0.8184	0.7558	1.0000	0.6991	1.0000	-0.1237	1.0000	0.0533	1.0000	-0.1741
LOC100542155	1.0000	0.0000	1.0000	2.2534	1.0000	0.0000	1.0000	2.3257	1.0000	0.0000	1.0000	2.3479	1.0000	2.3554
LOC100542157	1.0000	0.3978	0.1493	-1.5116	0.4170	1.0588	0.6317	-0.6767	1.0000	0.7429	0.7010	-1.1613	0.4957	-0.9860
LOC100542159	1.0000	-0.2931	1.0000	0.0000	1.0000	0.6740	0.7701	3.1732	1.0000	-2.3771	1.0000	-2.2954	1.0000	0.0730
LOC100542160	1.0000	-0.3217	0.6224	1.6101	1.0000	0.3474	0.4562	2.0902	1.0000	-0.9614	1.0000	0.9748	1.0000	0.7732
LOC100542161	0.1248	-0.2987	0.0000	-0.3841	0.0001	-0.6844	0.0000	-0.6524	1.0000	-0.0212	0.7561	-0.0944	1.0000	0.0164
LOC100542164	0.0010	1.4154	0.0000	1.9591	0.0008	1.2994	0.0000	1.3822	1.0000	0.0089	0.0474	0.5646	1.0000	0.0963
LOC100542170	0.0869	0.5314	0.0011	0.5560	0.1244	-0.5176	0.9923	-0.0689	1.0000	-0.0861	1.0000	-0.0481	0.2554	0.3679
LOC100542173	0.6217	-0.1345	0.5813	0.0794	0.0000	-0.7731	0.0000	-0.6730	1.0000	-0.1819	1.0000	0.0443	0.9001	-0.0761
LOC100542179	0.0055	0.8764	0.0190	0.5129	0.2738	0.4503	0.6336	0.1839	1.0000	0.4875	1.0000	0.1365	0.7718	0.2244
LOC100542183	0.8226	-3.3425	1.0000	0.0000	1.0000	0.3474	1.0000	0.0000	1.0000	-3.2304	1.0000	0.0000	0.7287	-3.6634
LOC100542184	0.7382	-0.1568	1.0000	0.0085	0.0513	-0.4734	0.0034	-0.4286	1.0000	0.0154	0.7141	0.1925	1.0000	0.0652
LOC100542185	1.0000	0.0000	1.0000	0.0000	0.8011	3.0840	1.0000	2.3257	1.0000	0.0000	1.0000	0.0000	1.0000	-0.7793
LOC100542192	0.8226	3.0199	0.6180	1.6082	0.4809	3.6101	0.6172	1.7729	1.0000	2.2674	1.0000	0.9738	1.0000	0.4634
LOC100542196	1.0000	-0.1685	0.9862	-1.0774	1.0000	0.4364	0.4792	1.1300	1.0000	-0.9026	0.6242	-1.8150	1.0000	-0.2029
LOC100542198	0.0005	1.0430	0.0000	1.0212	0.0000	1.2666	0.0000	1.1042	1.0000	-0.0637	1.0000	-0.0740	0.6093	-0.2222
LOC100542202	0.8598	-0.6310	0.1467	1.8706	0.3377	-1.2929	0.5092	1.4828	1.0000	-2.4455	1.0000	0.0567	1.0000	0.3300
LOC100542208	0.0000	-1.0454	0.0000	-1.1265	0.0002	0.6113	0.0174	0.2291	1.0000	0.0297	1.0000	-0.0382	0.0008	-0.3468
LOC100542210	0.0023	-0.4359	0.0000	-0.4776	0.2227	-0.2106	0.0000	-0.2930	1.0000	0.1183	0.6861	0.0890	1.0000	0.0412
LOC100542217	0.0000	-2.7186	0.0000	-3.5122	0.0000	-1.1596	0.0000	-1.2102	1.0000	-0.0454	0.1691	-0.8269	1.0000	-0.0906
LOC100542219	0.6100	0.2508	1.0000	-0.0395	0.2778	-0.3625	0.0020	-0.5949	1.0000	0.3284	1.0000	0.0501	1.0000	0.1005
LOC100542220	0.1381	-2.8562	0.1041	-1.6147	0.8229	0.6748	1.0000	-0.0977	1.0000	0.8430	0.8269	2.1242	1.0000	0.0787
LOC100542221	0.0494	0.5127	0.0000	0.8461	1.0000	-0.0697	0.4196	0.2279	1.0000	-0.2679	1.0000	0.0773	1.0000	0.0337
LOC100542222	1.0000	0.0000	1.0000	0.0000	1.0000	0.0000	1.0000	0.0000	1.0000	0.0000	1.0000	0.0000	1.0000	0.0000
LOC100542224	1.0000	0.2007	1.0000	-0.1472	0.8011	-3.2629	1.0000	-2.3200	1.0000	-0.9587	1.0000	-1.3254	1.0000	0.0000
LOC100542230	0.0211	1.0543	0.0002	0.9224	0.0000	2.1746	0.0000	1.6432	1.0000	0.3939	0.6761	0.2769	0.9322	-0.1299
LOC100542236	0.0004	1.2906	0.0028	0.8651	0.0009	1.1338	0.0204	0.7110	1.0000	0.0876	0.6497	-0.3292	0.5275	-0.3323
LOC100542241	0.4526	0.1964	0.3566	0.1332	0.0000	0.8233	0.0000	0.8891	1.0000	0.0479	1.0000	-0.0021	0.5228	0.1191
LOC100542243	0.6840	-0.6923	0.2573	0.9385	0.0844	-1.6400	0.9205	-0.4930	1.0000	-0.6063	0.6036	1.0379	1.0000	0.5464
LOC100542248	0.1447	0.9714	1.0000	0.1494	0.2201	0.7951	1.0000	-0.0381	1.0000	0.0436	0.4247	-0.7587	0.3431	-0.7815
LOC100542256	1.0000	0.0000	1.0000	0.0000	1.0000	0.0000	1.0000	0.0000	1.0000	0.0000	1.0000	0.0000	1.0000	0.0000
LOC100542258	0.0000	-0.8350	0.0000	-0.8393	0.0000	-0.9706	0.0000	-0.7031	1.0000	-0.0429	1.0000	-0.0350	0.0383	0.2299
LOC100542269	1.0000	0.0000	1.0000	0.0000	1.0000	0.0000	1.0000	0.0000	1.0000	0.0000	1.0000	0.0000	1.0000	0.0000
LOC100542272	0.7214	0.1183	0.0098	0.2446	0.0257	0.3930	0.0000	0.4641	1.0000	-0.1203	1.0000	0.0179	1.0000	-0.0433
LOC100542279	1.0000	-0.7263	1.0000	2.2534	0.6597	-1.9418	0.4622	3.6989	1.0000	-4.1514	1.0000	-1.3246	1.0000	1.4474
LOC100542292	0.0001	-0.9584	0.0000	-0.8838	0.0001	-0.9330	0.0008	-0.5378	1.0000	0.0532	1.0000	0.1419	0.0399	0.4556
LOC100542299	0.0358	-2.8220	0.0000	-3.9570	1.0000	-0.3628	0.3690	-0.6137	1.0000	0.6370	1.0000	-0.4772	1.0000	0.3978
LOC100542304	1.0000	0.0000	1.0000	0.0000	1.0000	0.0000	1.0000	0.0000	1.0000	0.0000	1.0000	0.0000	1.0000	0.0000
LOC100542306	1.0000	2.1848	1.0000	0.0000	1.0000	2.2472	1.0000	0.0000	1.0000	0.0000	1.0000	-2.2954	1.0000	-2.2907
LOC100542307	0.0000	1.3555	0.0000	1.0139	0.1243	0.3277	0.7988	0.0608	1.0000	0.2589	1.0000	-0.0705	1.0000	-0.0035
LOC100542320	0.0060	1.0285	0.2868	0.3732	1.0000	-0.1406	0.0156	-0.8113	1.0000	0.5014	1.0000	-0.1428	1.0000	-0.1643
LOC100542349	0.0000	1.3776	0.0000	1.5335	0.0000	1.5552	0.0000	1.4319	1.0000	-0.0034	0.8756	0.1655	1.0000	-0.1218
LOC100542357	1.0000	0.0097	1.0000	-0.0761	0.0002	-1.4722	0.0002	-1.3483	1.0000	-0.4770	0.2759	-0.5487	0.9852	-0.3450
LOC100542364	0.0000	1.6414	0.0000	1.1206	0.0000	2.3140	0.0000	2.1342	1.0000	0.2798	0.7247	-0.2283	1.0000	0.1049
LOC100542367	0.1022	-1.1343	0.7923	-0.2803	0.9357	0.2371	0.2157	0.6044	1.0000	-0.2279	0.8219	0.6402	1.0000	0.1465
LOC100542387	0.2172	0.9516	0.0016	1.8334	0.0266	1.3344	0.0007	1.9814	1.0000	-0.7545	1.0000	0.1408	1.0000	-0.1038
LOC100542388	1.0000	-0.0582	0.6739	-0.0961	1.0000	-0.0035	0.0745	-0.2712	1.0000	0.1142	1.0000	0.0892	0.7445	-0.1477
LOC100542393	0.0011	0.7440	0.0000	0.9256	0.0000	1.5015	0.0000	1.2623	1.0000	0.0147	0.2872	0.2097	0.0287	-0.2193
LOC100542400	0.5086	3.5456	0.9062	1.2235	0.1614	4.2993	1.0000	0.8568	1.0000	2.2674	1.0000	0.0567	0.9835	-1.1470
LOC100542403	1.0000	2.1902	1.0000	-0.1429	1.0000	2.2472	1.0000	-2.3177	1.0000	2.2674	1.0000	0.0489	1.0000	-2.2907
LOC100542409	1.0000	0.0000	1.0000	0.0000	1.0000	0.0000	1.0000	0.0000	1.0000	0.0000	1.0000	0.0000	1.0000	0.0000
LOC100542421	0.0000	1.4829	0.0000	1.2146	0.4984	0.2825	0.0907	-0.4578	1.0000	0.1495	1.0000	-0.1051	0.0671	-0.5841

LOC100542427	0.0002	1.5837	0.0000	2.2807	0.0000	2.6368	0.0000	2.2228	1.0000	-0.1902	0.3399	0.5201	0.0505	-0.5992
LOC100542428	0.0196	1.2149	0.0004	1.3268	1.0000	-0.2698	0.2422	-0.7225	1.0000	0.3836	0.5314	0.5064	1.0000	-0.0691
LOC100542432	0.6662	-0.7470	0.0011	-5.7072	0.0000	3.1030	0.0000	2.9662	1.0000	-0.6249	0.0120	-5.6973	0.0030	-0.7569
LOC100542433	1.0000	0.0000	1.0000	0.0000	1.0000	0.0000	1.0000	0.0000	1.0000	0.0000	1.0000	0.0000	1.0000	0.0000
LOC100542437	0.7620	-0.7506	0.0445	-1.5085	1.0000	0.2511	0.0569	-1.5385	1.0000	1.0136	1.0000	0.2725	0.8501	-0.7693
LOC100542440	1.0000	0.2172	0.2491	-1.4439	1.0000	0.3633	0.0884	-2.1801	1.0000	1.1424	1.0000	-0.4997	0.7915	-1.3979
LOC100542442	0.0030	0.7320	0.0002	0.6515	0.4075	-0.2957	1.0000	0.0081	1.0000	0.0275	1.0000	-0.0405	0.3496	0.3369
LOC100542445	0.0802	0.3201	0.0006	0.3153	1.0000	0.0264	1.0000	0.0282	1.0000	-0.0502	1.0000	-0.0422	1.0000	-0.0425
LOC100542446	0.0000	-0.8610	0.0000	-0.8359	0.0620	-0.3098	0.0003	-0.2600	1.0000	-0.0763	1.0000	-0.0388	1.0000	-0.0209
LOC100542457	0.0207	0.7309	0.0000	0.8203	0.0000	1.4368	0.0000	1.0692	1.0000	0.3099	0.1761	0.4126	1.0000	-0.0507
LOC100542468	0.0022	1.0125	0.2765	0.3589	0.1136	0.5664	1.0000	0.0058	1.0000	0.3620	0.8008	-0.2792	0.9242	-0.1943
LOC100542473	1.0000	0.0000	1.0000	0.0000	1.0000	0.0000	1.0000	0.0000	1.0000	0.0000	1.0000	0.0000	1.0000	0.0000
LOC100542475	1.0000	0.0000	1.0000	0.0000	1.0000	0.0000	1.0000	0.0000	1.0000	0.0000	1.0000	0.0000	1.0000	0.0000
LOC100542484	0.0000	1.8539	0.0000	2.3032	0.0000	1.3327	0.0000	1.1289	1.0000	-0.3250	0.8278	0.1356	0.0067	-0.5251
LOC100542486	0.4323	1.5330	0.5872	-1.3300	1.0000	0.7335	0.3441	-1.6833	1.0000	1.5688	0.8512	-1.2831	1.0000	-0.8434
LOC100542495	1.0000	-0.2920	1.0000	0.0000	1.0000	-2.4056	1.0000	0.0000	1.0000	-2.3757	1.0000	-2.2954	1.0000	0.0000
LOC100542503	1.0000	0.0000	1.0000	2.2507	1.0000	2.2427	1.0000	0.0000	1.0000	0.0000	1.0000	2.3456	1.0000	-2.2888
LOC100542515	1.0000	-0.0377	0.6483	0.7958	1.0000	0.3608	1.0000	0.0071	1.0000	0.6450	0.4944	1.4972	1.0000	0.2939
LOC100542519	0.0223	-0.3823	0.7922	-0.0512	0.6199	0.1305	0.0000	0.5555	1.0000	0.0398	0.0001	0.3827	0.0000	0.4697
LOC100542520	0.0708	-0.4114	0.0019	-0.4991	1.0000	-0.0333	0.8130	0.0798	1.0000	-0.0215	1.0000	-0.0979	0.9460	0.0963
LOC100542525	1.0000	0.0000	1.0000	0.0000	1.0000	0.0000	1.0000	0.0000	1.0000	0.0000	1.0000	0.0000	1.0000	0.0000
LOC100542534	0.0361	-0.6026	0.0000	-0.9999	0.1314	-0.4276	0.0231	-0.3942	1.0000	0.1728	0.9618	-0.2112	0.6686	0.2132
LOC100542537	1.0000	0.0210	0.7629	-0.0495	0.0000	-0.7875	0.0000	-0.8707	1.0000	-0.0609	0.4765	-0.1191	0.4839	-0.1380
LOC100542547	0.1383	1.3178	0.0168	5.1296	0.5168	-1.5304	0.0729	4.6479	1.0000	-5.1068	0.1402	-1.4439	1.0000	1.0201
LOC100542552	0.0000	-7.7807	0.0000	-6.8644	0.1610	-0.9762	0.9046	0.2494	1.0000	-0.9455	1.0000	0.0000	1.0000	0.2728
LOC100542556	0.6579	0.3872	0.1075	0.6584	0.4646	-0.5213	1.0000	0.0047	1.0000	-0.1235	1.0000	0.1587	0.8133	0.4013
LOC100542562	0.0227	-5.2317	0.6224	-1.9244	0.0304	-5.1455	0.6172	-1.7602	1.0000	-1.0818	1.0000	2.3480	1.0000	2.3542
LOC100542571	0.8856	-0.5980	1.0000	-0.1870	0.8183	-1.1322	1.0000	-0.1851	1.0000	0.2768	1.0000	0.7055	0.8768	1.2314
LOC100542574	0.0044	1.1994	0.0011	0.8621	0.0000	2.6924	0.0000	1.9867	1.0000	0.4967	1.0000	0.1702	0.7509	-0.2048
LOC100542589	0.7720	0.3373	0.0225	-1.2607	0.0499	-1.4390	0.0063	-1.5119	1.0000	0.2792	0.0950	-1.3071	1.0000	0.2135
LOC100542602	0.8060	-0.1675	0.8212	-0.1058	0.8201	-0.1508	1.0000	-0.0653	1.0000	-0.1371	1.0000	-0.0628	1.0000	-0.0469
LOC100542612	0.0043	-0.5788	0.0001	-0.3792	0.2691	0.2656	0.0000	0.7174	1.0000	-0.3072	0.9146	-0.0951	0.4180	0.1506
LOC100542617	0.0339	1.3001	0.0080	1.7893	0.0000	2.0838	0.0000	2.8252	1.0000	-0.7060	1.0000	-0.2037	1.0000	0.0419
LOC100542618	0.4640	0.8083	1.0000	0.0425	0.0000	2.2028	0.0008	1.4694	1.0000	0.5619	1.0000	-0.1879	1.0000	-0.1584
LOC100542621	1.0000	0.0000	1.0000	0.0000	1.0000	0.0000	1.0000	0.0000	1.0000	0.0000	1.0000	0.0000	1.0000	0.0000
LOC100542624	0.4664	-0.1762	0.0495	-0.1972	0.0855	-0.3262	0.0001	-0.3558	1.0000	0.1008	0.8875	0.0921	0.9423	0.0756
LOC100542626	1.0000	0.0000	1.0000	0.0000	0.0919	4.5509	1.0000	0.0000	1.0000	0.0000	1.0000	0.0000	0.2088	-4.6054
LOC100542630	1.0000	0.0000	1.0000	2.2534	1.0000	0.0000	1.0000	0.0000	1.0000	0.0000	1.0000	2.3480	1.0000	0.0000
LOC100542633	0.3081	-0.5925	1.0000	-0.0932	1.0000	-0.1439	1.0000	-0.0875	1.0000	-0.1319	0.9036	0.3797	1.0000	-0.0727
LOC100542634	0.0000	1.6959	0.0018	1.0729	1.0000	-0.0579	0.0045	-1.5561	1.0000	0.0032	0.1687	-0.6086	0.0455	-1.4871
LOC100542638	0.1748	-0.6487	0.0003	-0.6893	0.2882	0.3922	0.0000	0.6837	1.0000	0.2338	1.0000	0.2046	0.0040	0.5318
LOC100542660	0.8226	3.0199	1.0000	0.0000	1.0000	0.0000	1.0000	0.0000	1.0000	0.0000	1.0000	-3.1429	1.0000	0.0000
LOC100542665	0.0640	-0.3460	0.0487	-0.1873	0.8785	-0.0938	0.4197	0.0926	1.0000	-0.0596	0.7475	0.1119	0.6819	0.1329
LOC100542676	0.0000	1.0190	0.0000	1.0112	0.0453	-0.3847	0.0000	-0.6479	1.0000	0.0965	0.7704	0.1010	0.5217	-0.1620
LOC100542678	1.0000	-0.2892	1.0000	0.2918	0.5084	1.8778	0.7190	-1.2157	1.0000	1.9552	0.4857	2.5903	1.0000	-1.1412
LOC100542684	1.0000	0.0000	1.0000	0.0000	1.0000	0.0000	1.0000	0.0000	1.0000	0.0000	1.0000	0.0000	1.0000	0.0000
LOC100542686	0.7505	-0.1138	0.4251	-0.0950	0.0201	-0.4163	0.0022	-0.2734	1.0000	-0.0688	1.0000	-0.0381	0.8725	0.0793
LOC100542689	1.0000	0.0000	1.0000	0.0000	1.0000	0.0000	1.0000	0.0000	1.0000	0.0000	1.0000	0.0000	1.0000	0.0000
LOC100542691	1.0000	0.0190	0.2911	0.2442	0.0000	0.9400	0.0000	0.8534	1.0000	-0.2850	1.0000	-0.0479	0.0535	-0.3673
LOC100542699	1.0000	-0.0404	0.8317	0.2062	0.0004	1.3873	0.0000	1.3368	1.0000	0.0515	1.0000	0.3133	1.0000	0.0028
LOC100542702	0.0074	0.8917	0.0000	1.3372	0.0000	1.6642	0.0000	2.0878	1.0000	-0.2084	0.6832	0.2518	0.5764	0.2205
LOC100542710	0.0115	0.4460	0.0000	0.4666	0.0007	0.5785	0.0000	0.6443	1.0000	-0.0320	1.0000	0.0012	1.0000	0.0391
LOC100542725	0.0000	-0.7738	0.0000	-0.8628	0.0001	-0.7062	0.0000	-0.5685	1.0000	-0.0959	0.7464	-0.1728	1.0000	0.0478
LOC100542733	0.1725	0.5200	0.1748	0.3994	1.0000	-0.0954	0.3755	-0.3176	1.0000	0.0378	1.0000	-0.0703	1.0000	-0.1784
LOC100542744	1.0000	0.4641	0.5327	0.8005	0.1527	1.5840	0.9351	0.4544	1.0000	0.5241	0.8553	0.8735	0.9390	-0.6097
LOC100542745	1.0000	0.1308	0.7647	0.1838	0.0000	1.4729	0.0000	2.0505	1.0000	-0.0879	1.0000	-0.0210	0.0393	0.4939
LOC100542752	0.2584	-0.2228	0.0699	-0.1941	0.0000	0.9348	0.0000	0.7347	1.0000	-0.0016	1.0000	0.0399	0.1011	-0.1958
LOC100542756	0.0139	-2.1218	0.0007	-2.6853	0.2981	0.6869	0.6851	0.3737	1.0000	0.0569	1.0000	-0.4934	1.0000	-0.2484
LOC100542757	0.0001	-0.6452	0.0000	-0.5651	0.2795	-0.2036	0.0759	-0.1493	1.0000	-0.0299	1.0000	0.0621	1.0000	0.0295
LOC100542763	0.8939	0.7981	0.0183	2.2315	0.4809	-3.7967	1.0000	0.6976	1.0000	-0.1210	0.6009	1.3142	0.3446	4.4306
LOC100542770	1.0000	0.0000	1.0000	0.0000	1.0000	0.0000	1.0000	0.0000	1.0000	0.0000	1.0000	0.0000	1.0000	0.0000
LOC100542772	0.1876	4.2322	1.0000	2.2507	0.0742	4.7762	0.4370	3.7041	1.0000	0.0000	0.8269	-2.0174	0.8804	-1.0842
LOC100542775	0.0000	2.4068	0.0000	2.2519	0.0401	-1.9904	0.0019	-2.3810	1.0000	0.1547	1.0000	0.0125	1.0000	-0.2287
LOC100542776	1.0000	0.1905	0.8907	-0.3549	0.9128	-0.3982	0.2905	0.7205	1.0000	0.0474	0.9773	-0.4845	0.1936	1.1730
LOC100542779	1.0000	0.0000	1.0000	0.0000	1.0000	0.0000	1.0000	0.0000	1.0000	0.0000	1.0000	0.0000	1.0000	0.0000
LOC100542789	1.0000	0.0000	1.0000	0.0000	1.0000	0.0000	1.0000	2.3257	1.0000	0.0000	1.0000	0.0000	1.0000	2.3555
LOC100542796	1.0000	0.0000	1.0000	0.0000	1.0000	0.0000	0.1396	4.3976	1.0000	0.0000	1.0000	0.0000	0.3459	4.4328
LOC100542800	0.0000	-0.6198	0.0000	-0.4822	0.0005	-0.5088	0.0000	-0.4196	1.0000	0.0009	0.4346	0.1510	0.6785	0.0952
LOC100542816	0.0000	1.4747	0.0000	1.6407	0.0000	1.1497	0.0000	0.7631	1.0000	0.0707	0.4709	0.2510	0.0017	-0.3102
LOC100542821	0.0000	1.4510	0.0000	1.4649	0.5959	-0.3027	0.1671	-0.4440	1.0000	-0.0851	1.0000	-0.0576	0.9662	-0.2185
LOC100542822	0.0000	0.9759	0.0000	1.2670	0.0153	0.6247	0.0000	1.3339	1.0000	0.2923	0.0000	0.5960	0.0000	1.0091
LOC100542828	0.0000	1.0408	0.0184	0.3898	0.2155	-0.3899	0.0000	-0.8149	1.0000	0.1051	0.0010	-0.5321	0.4594	-0.3135
LOC100542830	0.0091	-0.4613	0.0000	-0.7430	0.0001	-0.6649	0.0000	-0.8965	1.0000	0.1407	0.6367	-0.1285	0.8901	-0.0850
LOC100542834	1.0000	-0.8632	0.8738	0.5802	0.9468	-1.5484	1.0000	0.0045	1.0000	0.2811	0.8009	1.7431	0.8939	1.8365
LOC100542849	0.0007	1.8686	0.0000	2.0514	0.0015	1.7477	0.0000	2.1680	1.0000	-0.1307	1.0000	0.0621	0.8475	0.2916
LOC100542853	0.0092	2.6592	0.1007	1.4544	0.2376	1.5996	0.8618	0.6593	1.0000	0.5665	0.8607	-0.6280	1.0000	-0.3682
LOC100542860	0.0000	-0.9809	0.0000	-0.6396	0.0000	-1.0204	0.0000	-0.6065	1.0000	-0.1863	0.7558	0.1668	0.6531	0.2338
LOC100542863	0.9996	-0.0924	1.0000	0.0045	0.9823	0.0957	0.0382	0.3463	1.0000	-0.0520	1.0000	0.0570	0.6018	0.2054
LOC100542866	0.8226	-3.3425	1.0000	0.0000	0.7679	1.0340	1.0000	2.3257	1.0000	-3.2304	1.0000	0.0000	0.6942	-1.9975
LOC100542871	0.8249	-0.4643	1.0000	-0.0403	0.0032	1.7231	0.0000	2.5627	1.0000	-0.6076	1.0000	-0.1729	0.9814	0.2344
LOC100542873	0.2100	0.2614	1.0000	0.0100	1.0000	-0.0252	0.0017	-0.3641	1.0000	0.1504	0.9568	-0.0877	0.3585	-0.1827
LOC100542874	1.0000	-0.2931	1.0000	-0.1449	0.4428	1.8950	0.1476	2.5427	1.0000	-0.1089	1.0000	0.0495	1.0000	0.5433
LOC100542876	0.0022	-3.9324	0.0004	-5.9270	0.0089	-2.9509	0.1425	-1.4345	1.0000	-0.2254	1.0000	-2.2991	0.9835	1.2976
LOC100542877	0.3181	-2.3875	0.2495	-4.1757	1.0000	0.0481	0.9786	-0.9107	1.0000	-0.4301	1.0000	-2.2991	0.8318	-1.3891
LOC100542888	1.0000	0.0000	1.0000	0.0000	0.8033	3.0892	1.0000	0.0000	1.0000	0.0000	1.0000	0.0000	1.0000	-3.1370
LOC100542889	1.0000	2.1848	0.2542	4.0037	1.0000	2.2427	1.0000	2.3257	1.0000	0.0000	1.0000	1.8188	1.0000	0.0659
LOC100542901	1.0000	0.0000	1.0000	0.0000	1.0000	0.0000	1.0000	0.0000	1.0000	0.0000	1.0000	0.0000	1.0000	0.0000
LOC100542902	0.9346	0.2195	0.9896	0.1337	0.6014	0.3811	0.6895	0.2692	1.0000	0.1097	1.0000	0.0362	1.0000	0.0034
LOC100542906	1.0000	0.0000	1.0000	-0.6943	1.0000	2.2472	0.4370	-3.6973	1.0000	3.6423	1.0000	3.2002	1.0000	-2.2906
LOC100542915	1.0000	0.0150	0.1502	0.2455	0.5761	0.1999	0.0000	0.5620	1.0000	-0.2464	1.0000	-0.0033	0.8519	0.1214
LOC100542919	1.0000	0.0000	1.0000	2.2507	1.0000	0.0000	1.0000	0.0000	1.0000	0.0000	1.0000	2.3456	1.0000	0.0000
LOC100542926	1.0000	0.0000	1.0000	0.0000	1.0000	0.0000	1.0000	0.0000	1.0000	0.0000	1.0000	0.0000	1.0000	0.0000
LOC100542931	1.0000	0.0000	0.1388	-4.4811	0.2781	3.9963	0.7186	-1.2155	1.0000	4.3298	1.0000	0.0000	1.0000	-0.8431

LOC100542970	0.0010	0.5009	0.0000	0.6018	0.5585	-0.1318	0.0000	-0.3937	1.0000	0.0499	0.3025	0.1635	0.0271	-0.2065
LOC100542972	0.5086	-3.8796	1.0000	0.0000	0.9361	-1.5512	1.0000	0.0000	1.0000	-3.7635	1.0000	0.0000	1.0000	-2.2907
LOC100542975	0.1492	-1.1745	0.2525	-0.6176	0.9601	-0.2960	0.6575	-0.3314	1.0000	0.1209	0.7211	0.6910	1.0000	0.0820
LOC100542976	0.0086	0.9156	0.0000	0.9881	0.7139	-0.2820	0.1655	-0.4969	1.0000	-0.1306	1.0000	-0.0488	0.7363	-0.3403
LOC100542989	0.3999	0.8663	1.0000	-0.3188	0.0563	-2.5081	0.0389	-3.0234	1.0000	-0.2710	0.1322	-1.4445	1.0000	-0.7804
LOC100542991	1.0000	-0.0260	1.0000	0.0137	0.0000	-0.7350	0.0000	-0.5565	1.0000	-0.1373	0.7474	-0.0852	1.0000	0.0467
LOC100543002	1.0000	0.0000	1.0000	0.0000	0.1621	4.3010	1.0000	2.3241	1.0000	0.0000	1.0000	0.0000	0.6865	-1.9994
LOC100543003	0.1694	1.2505	0.3342	0.8047	1.0000	0.4245	1.0000	0.1302	1.0000	0.1531	1.0000	-0.2826	1.0000	-0.1378
LOC100543005	0.0000	-1.2843	0.0000	-1.7310	0.0000	-1.0848	0.0000	-1.2175	1.0000	0.1022	0.0125	-0.3321	1.0000	-0.0246
LOC100543007	1.0000	0.0000	1.0000	0.0000	1.0000	0.0000	1.0000	0.0000	1.0000	0.0000	1.0000	0.0000	1.0000	0.0000
LOC100543009	0.8482	-0.1561	0.0724	-0.4297	0.0001	0.9243	0.0006	0.5624	1.0000	-0.0009	0.8496	-0.2623	0.0782	-0.3570
LOC100543013	0.0088	-0.4626	0.0000	-0.4430	0.1179	-0.3055	0.4902	-0.0971	1.0000	-0.1590	0.7774	-0.1269	1.0000	0.0558
LOC100543018	1.0000	-0.0640	0.0434	0.3113	0.0010	-0.6983	0.0068	-0.4114	1.0000	-0.4950	0.9591	-0.1090	0.6001	-0.2028
LOC100543020	0.0000	-3.8281	0.0002	-3.4090	0.0074	1.1924	0.0045	1.2207	1.0000	-0.3824	1.0000	0.0496	0.7865	-0.3468
LOC100543023	0.0891	-0.4020	0.2376	-0.1849	1.0000	-0.0217	0.0004	0.4125	1.0000	-0.1727	1.0000	0.0561	0.1280	0.2667
LOC100543030	1.0000	0.0000	1.0000	0.0000	1.0000	2.2472	1.0000	0.0000	1.0000	0.0000	1.0000	0.0000	1.0000	-2.2907
LOC100543035	0.7701	0.4339	0.2072	0.8133	0.3348	-0.9237	1.0000	-0.2882	1.0000	-0.4553	1.0000	-0.0634	1.0000	0.1890
LOC100543038	0.8226	-3.3425	0.7666	-3.2534	0.8011	-3.2629	0.7701	-3.1666	1.0000	-0.1179	1.0000	0.0000	1.0000	0.0000
LOC100543050	0.4179	-0.3763	0.1834	-0.4133	0.6972	0.2244	1.0000	-0.0318	1.0000	0.0545	1.0000	0.0297	0.8814	-0.1972
LOC100543055	1.0000	0.0000	1.0000	0.0000	1.0000	2.2427	0.7701	3.1732	1.0000	0.0000	1.0000	0.0000	1.0000	0.9168
LOC100543059	0.0001	2.2204	0.0000	2.6398	0.0051	1.7014	0.0353	1.3977	1.0000	-0.2778	1.0000	0.1526	0.6093	-0.5769
LOC100543064	1.0000	-0.2931	1.0000	-0.1449	1.0000	-2.4061	0.2467	2.3292	1.0000	-0.1089	1.0000	0.0495	0.2129	4.6838
LOC100543069	1.0000	-1.1514	0.4636	-3.7802	0.7753	1.0335	1.0000	0.3943	1.0000	0.4032	1.0000	-2.2992	1.0000	-0.2277
LOC100543078	0.0039	-0.7595	0.0000	-0.9516	0.2856	-0.3341	0.0099	-0.4447	1.0000	0.1093	1.0000	-0.0688	1.0000	0.0035
LOC100543080	0.1456	-1.8534	0.4413	-0.8100	1.0000	-0.0507	1.0000	0.0077	1.0000	0.1515	0.9773	1.2150	1.0000	0.2147
LOC100543103	0.0000	-1.1213	0.0000	-1.0654	0.4652	-0.2120	1.0000	-0.0041	1.0000	0.0034	1.0000	0.0728	0.4081	0.2176
LOC100543106	1.0000	-0.0323	0.0131	-0.2367	0.9053	-0.0756	0.1278	-0.1609	1.0000	-0.0396	0.1024	-0.2319	0.5880	-0.1196
LOC100543114	0.0001	1.1014	0.0000	0.8520	0.9050	-0.1535	0.4116	-0.2715	1.0000	0.0654	0.9227	-0.1706	1.0000	-0.0468
LOC100543118	0.3153	0.4207	0.0000	1.3377	0.0003	0.9891	0.0000	2.1053	1.0000	-0.8367	1.0000	0.0927	0.3904	0.2870
LOC100543119	1.0000	0.0000	1.0000	0.0000	1.0000	0.0000	1.0000	0.0000	1.0000	0.0000	1.0000	0.0000	1.0000	0.0000
LOC100543123	0.2961	-0.6837	0.0127	-1.2813	0.0001	-2.3190	0.0020	-1.5584	1.0000	-0.1719	0.6861	-0.7573	1.0000	0.5976
LOC100543124	0.0021	0.9754	0.0000	1.0373	0.7911	-0.2391	0.0450	-0.5629	1.0000	-0.0443	1.0000	0.0297	0.6205	-0.3619
LOC100543128	0.0000	1.4875	0.0000	1.1691	0.0000	-2.7555	0.0000	-2.4799	1.0000	0.0743	0.5290	-0.2337	1.0000	0.3592
LOC100543133	0.0000	-1.0908	0.0000	-1.2241	1.0000	-0.0343	0.0320	0.2452	1.0000	0.0103	1.0000	-0.1105	0.0501	0.2942
LOC100543145	0.8357	-0.2153	0.0767	-0.5295	0.0000	1.8780	0.0000	1.1057	1.0000	0.5114	1.0000	0.2090	0.6218	-0.2604
LOC100543147	0.0135	1.2754	0.0001	1.4410	0.0000	2.1462	0.0000	1.5998	1.0000	0.2092	0.6723	0.3815	0.5378	-0.3403
LOC100543156	0.4778	0.7778	0.3485	-0.8397	0.4153	-1.3099	0.0387	-1.9267	1.0000	0.6791	0.6096	-0.9228	1.0000	0.0641
LOC100543157	1.0000	-2.4788	1.0000	0.0000	1.0000	-2.4061	1.0000	0.0000	1.0000	-2.3771	1.0000	0.0000	1.0000	0.0000
LOC100543159	1.0000	0.0540	0.6645	0.0616	0.0912	-0.3027	0.0003	-0.3056	1.0000	-0.0059	1.0000	0.0139	1.0000	-0.0031
LOC100543166	0.8998	0.0832	0.0030	0.3255	0.0079	-0.5519	0.0009	-0.3801	1.0000	-0.2171	1.0000	0.0366	1.0000	-0.0394
LOC100543167	0.8905	-0.3225	0.4380	0.5342	0.6713	0.4079	0.0253	1.0928	1.0000	-0.4103	1.0000	0.4554	0.9886	0.2791
LOC100543177	1.0000	0.3194	0.5080	0.8472	0.0527	-2.0356	0.0940	-1.7331	1.0000	-0.2355	1.0000	0.2864	1.0000	0.0761
LOC100543179	0.1625	-2.1534	1.0000	-0.8596	0.0001	2.2474	0.0020	2.2150	1.0000	-1.2410	1.0000	0.0604	0.0066	-1.2567
LOC100543185	0.1856	-4.5762	0.2535	-4.1743	0.7660	-1.4056	0.9801	-0.9105	1.0000	-0.4321	1.0000	0.0000	1.0000	0.0696
LOC100543188	0.6152	-0.2651	0.1564	-0.2627	0.0025	0.8810	0.0000	0.7640	1.0000	0.0938	1.0000	0.1094	1.0000	-0.0196
LOC100543189	1.0000	2.1848	1.0000	2.2507	1.0000	0.0000	1.0000	0.0000	1.0000	0.0000	1.0000	0.0495	1.0000	0.0000
LOC100543196	0.0062	1.5656	0.0001	1.8550	0.0004	1.8359	0.0038	1.5365	1.0000	-0.1344	1.0000	0.1709	0.6687	-0.4249
LOC100543199	1.0000	0.0000	1.0000	0.0000	1.0000	0.0000	1.0000	0.0000	1.0000	0.0000	1.0000	0.0000	1.0000	0.0000
LOC100543202	1.0000	0.0000	1.0000	0.0000	1.0000	0.0000	1.0000	0.0000	1.0000	0.0000	1.0000	0.0000	1.0000	0.0000
LOC100543206	0.0001	0.7385	0.0000	0.8277	0.0134	0.5013	0.0000	0.5700	1.0000	-0.0971	1.0000	0.0050	1.0000	-0.0229
LOC100543214	0.1502	-0.7474	0.2112	-0.5357	0.8967	-0.1991	0.0002	-1.5414	1.0000	-0.0350	1.0000	0.1918	0.0095	-1.3699
LOC100543226	0.4869	0.1609	0.7502	0.0582	0.2018	-0.2479	1.0000	-0.0094	1.0000	0.0638	1.0000	-0.0260	0.0140	0.3082
LOC100543235	0.0001	-0.7648	0.0000	-1.2788	0.0380	0.4053	0.0000	0.6067	1.0000	-0.1214	0.0010	-0.6225	0.9150	0.0866
LOC100543241	1.0000	2.1848	0.4643	-3.7945	0.4809	3.6122	1.0000	0.3926	1.0000	3.6521	1.0000	-2.2955	1.0000	0.4627
LOC100543242	0.8137	0.4626	0.8572	0.4388	0.8385	0.3787	0.9888	0.3479	1.0000	-0.4739	1.0000	-0.4878	0.9281	-0.4975
LOC100543251	0.0000	0.8862	0.0000	0.8064	0.1231	-0.3266	0.0000	-0.6369	1.0000	0.0494	1.0000	-0.0173	0.1668	-0.2542
LOC100543260	1.0000	0.0000	1.0000	0.0000	1.0000	0.0000	1.0000	0.0000	1.0000	0.0000	1.0000	0.0000	1.0000	0.0000
LOC100543264	0.1071	-0.2962	0.0029	-0.3034	0.0000	-0.7361	0.0000	-0.4317	1.0000	-0.0764	1.0000	-0.0714	0.1268	0.2335
LOC100543274	1.0000	0.0000	1.0000	0.0000	1.0000	2.2472	1.0000	2.3257	1.0000	0.0000	1.0000	0.0000	1.0000	0.0649
LOC100543277	1.0000	-2.4776	0.7674	3.0957	1.0000	-2.4056	1.0000	0.0000	1.0000	-2.3757	1.0000	3.2002	1.0000	0.0000
LOC100543282	0.2319	0.4925	1.0000	0.0517	0.7276	0.2475	1.0000	0.0569	1.0000	0.1702	0.8607	-0.2598	1.0000	-0.0148
LOC100543283	1.0000	0.0000	1.0000	0.0000	1.0000	2.2427	1.0000	0.0000	1.0000	0.0000	1.0000	0.0000	1.0000	-2.2888
LOC100543285	0.4707	0.3010	0.1514	0.3344	0.4810	0.2867	1.0000	-0.0219	1.0000	-0.5036	0.0869	-0.4570	0.0001	-0.8026
LOC100543297	0.9798	-0.0812	0.0127	0.2907	1.0000	0.0093	1.0000	-0.0136	1.0000	-0.0452	0.0146	0.3392	0.9747	-0.0627
LOC100543299	0.8249	-3.3411	1.0000	0.6956	1.0000	-1.0156	1.0000	0.8545	1.0000	-0.9614	1.0000	3.1976	1.0000	0.9164
LOC100543301	0.0000	0.9332	0.0000	1.1227	0.0000	1.1286	0.0000	1.0136	1.0000	0.0182	0.4839	0.2204	0.8620	-0.0912
LOC100543303	1.0000	0.0286	0.0768	-0.1992	0.4300	-0.1989	0.0000	-0.5305	1.0000	0.1691	1.0000	-0.0458	0.5939	-0.1576
LOC100543305	0.0000	1.4717	0.0000	1.9301	0.0000	0.8806	0.0008	0.5939	1.0000	-0.3760	1.0000	0.0927	0.0000	-0.6591
LOC100543307	1.0000	0.0000	1.0000	0.0000	1.0000	0.0000	1.0000	0.0000	1.0000	0.0000	1.0000	0.0000	1.0000	0.0000
LOC100543316	0.1436	0.7125	0.0809	0.6287	0.0055	1.0910	0.2106	0.5073	1.0000	0.3859	0.8269	0.3109	0.9853	-0.1953
LOC100543320	0.0000	0.7205	0.0000	0.8211	0.0002	-0.7162	0.0000	-0.6224	1.0000	-0.1766	1.0000	-0.0634	1.0000	-0.0759
LOC100543329	1.0000	0.0000	1.0000	0.0000	1.0000	0.0000	1.0000	0.0000	1.0000	0.0000	1.0000	0.0000	1.0000	0.0000
LOC100543330	0.0000	-8.3810	0.0000	-10.3165	0.9276	-0.0998	0.1316	-0.3485	1.0000	-0.2854	1.0000	-2.2955	0.0031	-0.5299
LOC100543336	0.0000	-0.8888	0.0000	-1.0847	0.0000	-1.3486	0.0000	-0.9527	1.0000	-0.0781	0.4411	-0.2615	0.1205	0.3237
LOC100543342	0.7877	1.1367	0.3011	1.1630	1.0000	0.3490	1.0000	0.4728	1.0000	1.0995	0.7635	1.1468	0.8620	1.2340
LOC100543343	0.0001	-0.5853	0.0000	-0.5366	0.0000	-1.0580	0.0000	-0.8991	1.0000	-0.1093	1.0000	-0.0485	1.0000	0.0552
LOC100543349	1.0000	-0.0207	0.0516	-0.2014	0.0628	0.3743	0.3834	0.1043	1.0000	0.2299	1.0000	0.0621	1.0000	-0.0348
LOC100543356	1.0000	0.0000	1.0000	0.0000	1.0000	0.0000	1.0000	2.3257	1.0000	0.0000	1.0000	0.0000	1.0000	2.3555
LOC100543359	1.0000	0.0000	1.0000	0.0000	1.0000	0.0000	1.0000	0.0000	1.0000	0.0000	1.0000	0.0000	1.0000	0.0000
LOC100543367	1.0000	0.0000	1.0000	0.0000	1.0000	0.0000	1.0000	0.0000	1.0000	0.0000	1.0000	0.0000	1.0000	0.0000
LOC100543379	0.3135	-0.3277	0.0077	-0.4344	0.0683	-0.5155	0.8206	-0.0859	1.0000	0.1637	1.0000	0.0691	0.0024	0.5988
LOC100543381	0.6733	-0.2501	0.0216	-0.5675	0.6971	-0.2364	0.5722	-0.1917	1.0000	0.0678	0.9240	-0.2371	1.0000	0.1181
LOC100543391	0.0014	0.8071	0.0000	0.9101	0.0000	1.3523	0.0000	1.1132	1.0000	0.1776	0.2753	0.2939	1.0000	-0.0556
LOC100543395	0.0000	1.4929	0.0000	1.3635	1.0000	0.0283	1.0000	0.0744	1.0000	-0.0791	0.8607	-0.1924	1.0000	-0.0280
LOC100543400	0.0000	-9.5139	0.0000	-4.8053	0.2149	-0.5221	0.2761	-0.2264	1.0000	0.5289	0.0520	5.3880	0.0001	0.8290
LOC100543401	1.0000	0.0000	1.0000	0.0000	1.0000	0.0000	1.0000	0.0000	1.0000	0.0000	1.0000	0.0000	1.0000	0.0000
LOC100543404	0.0000	-2.6069	0.0000	-3.7174	0.7976	-0.2764	0.2422	-0.6508	1.0000	0.2016	1.0000	-0.8987	1.0000	-0.1701
LOC100543408	1.0000	2.1902	1.0000	0.0000	1.0000	0.0000	0.7710	3.1753	1.0000	0.0000	1.0000	-2.2992	1.0000	3.2081
LOC100543412	0.0255	-0.3623	0.0022	-0.2666	0.0002	-0.5776	0.0000	-0.3505	1.0000	-0.1746	1.0000	-0.0667	1.0000	0.0579
LOC100543417	1.0000	2.1902	1.0000	0.0000	1.0000	0.0000	1.0000	0.0000	1.0000	0.0000	1.0000	-2.2992	1.0000	0.0000
LOC100543419	0.0009	-0.8324	0.0402	-0.3435	1.0000	-0.0380	0.0000	0.6495	1.0000	0.0430	0.0105	0.5450	0.0000	0.7393
LOC100543433	1.0000	0.0000	1.0000	0.0000	1.0000	0.0000	1.0000	0.0000	1.0000	0.0000	1.0000	0.0000	1.0000	0.0000
LOC100543439	1.0000	0.0000	1.0000	0.0000	1.0000	0.0000	1.0000	0.0000	1.0000	0.0000	1.0000	0.0000	1.0000	0.0000

LOC100543441	0.7760	-0.4825	0.0357	-0.9989	0.1645	-1.3759	0.0001	-2.0783	1.0000	0.6307	1.0000	0.1272	1.0000	-0.0690
LOC100543444	1.0000	0.0000	1.0000	0.0000	1.0000	0.0000	1.0000	0.0000	1.0000	0.0000	1.0000	0.0000	1.0000	0.0000
LOC100543458	1.0000	0.0000	0.2552	4.0066	1.0000	0.0000	1.0000	0.0000	1.0000	0.0000	0.6450	4.1180	1.0000	0.0000
LOC100543464	0.0000	-3.0734	0.0000	-2.7258	0.0331	0.4178	0.0014	0.2503	1.0000	0.1555	0.0183	0.5157	1.0000	-0.0073
LOC100543469	1.0000	2.1901	1.0000	0.0000	1.0000	0.0000	1.0000	0.0000	1.0000	0.0000	1.0000	-2.2992	1.0000	0.0000
LOC100543473	1.0000	0.0000	1.0000	0.0000	1.0000	2.2472	1.0000	2.3241	1.0000	0.0000	1.0000	0.0000	1.0000	0.0641
LOC100543474	0.0000	1.5871	0.0000	1.6661	0.2875	-0.4212	0.0011	-0.8545	1.0000	-0.5700	0.0033	-0.4819	0.0005	-0.9976
LOC100543476	0.6881	1.7520	0.7666	3.0922	1.0000	-2.4056	1.0000	0.0000	1.0000	-2.3757	1.0000	-1.1654	1.0000	0.0000
LOC100543487	1.0000	0.0000	1.0000	0.0000	1.0000	0.0000	1.0000	0.0000	1.0000	0.0000	1.0000	0.0000	1.0000	0.0000
LOC100543496	0.0359	-1.8999	0.0002	-3.4096	1.0000	-0.1192	1.0000	0.1985	1.0000	0.0879	0.9094	-1.4169	0.8939	0.4134
LOC100543498	1.0000	0.0000	1.0000	0.0000	1.0000	0.0000	1.0000	0.0000	1.0000	0.0000	1.0000	0.0000	1.0000	0.0000
LOC100543500	0.0372	-0.5096	0.0007	-0.5061	0.0008	-0.7643	0.0128	-0.3402	1.0000	-0.0573	1.0000	-0.0405	0.1098	0.3739
LOC100543508	0.6606	-0.9008	0.3732	-1.2997	1.0000	-0.7492	0.0622	-2.8846	1.0000	0.4447	1.0000	0.0579	0.8939	-1.6940
LOC100543510	1.0000	0.0000	0.7674	-3.2498	1.0000	0.0000	0.7710	-3.1632	1.0000	3.1056	1.0000	0.0000	1.0000	0.0000
LOC100543523	1.0000	0.0000	1.0000	0.0000	1.0000	0.0000	1.0000	0.0000	1.0000	0.0000	1.0000	0.0000	1.0000	0.0000
LOC100543527	0.8374	0.0899	0.0494	0.1640	0.1155	-0.2972	0.0000	-0.4601	1.0000	-0.0249	1.0000	0.0618	0.2021	-0.1824
LOC100543530	0.0041	0.9800	0.3239	0.2925	0.0000	1.7310	0.2794	0.3144	1.0000	0.6990	1.0000	0.0224	0.0160	-0.7156
LOC100543537	0.6927	1.4466	0.4156	1.9155	1.0000	-2.4061	1.0000	-2.3177	1.0000	-0.1089	1.0000	0.3662	1.0000	0.0000
LOC100543540	0.5086	-0.1615	0.0052	-0.3004	0.0860	0.3102	0.0730	0.1899	1.0000	0.1644	1.0000	0.0374	1.0000	0.0490
LOC100543544	0.8249	3.0141	1.0000	-2.3986	1.0000	0.0000	1.0000	0.8518	1.0000	2.2736	1.0000	-3.1383	1.0000	3.2048
LOC100543546	0.0461	-0.4172	0.0154	-0.3409	0.1966	-0.3036	0.7434	0.0842	1.0000	-0.2650	0.6497	-0.1770	0.8133	0.1284
LOC100543548	1.0000	0.0000	1.0000	0.0000	1.0000	0.0000	1.0000	0.0000	1.0000	0.0000	1.0000	0.0000	1.0000	0.0000
LOC100543549	0.8475	-1.0318	0.0774	4.5589	0.1707	-4.4923	1.0000	2.3257	1.0000	-4.4584	1.0000	1.0003	1.0000	2.3554
LOC100543552	0.0000	0.9925	0.0000	1.2060	0.0374	-0.3911	0.1313	-0.1672	1.0000	-0.0363	0.1581	0.1893	0.3056	0.1934
LOC100543556	0.0000	-4.4014	0.0000	-4.2932	0.0019	-0.7587	0.0000	-0.6925	1.0000	0.1513	1.0000	0.2736	0.3845	0.2209
LOC100543562	0.0971	2.1066	0.2954	1.3388	1.0000	0.7314	1.0000	0.3946	1.0000	0.4097	1.0000	-0.3446	1.0000	0.0775
LOC100543567	0.1881	0.2615	0.0285	0.2236	0.0000	-1.0142	0.0000	-0.9575	1.0000	0.0511	1.0000	0.0255	0.8694	0.1133
LOC100543573	1.0000	2.1848	0.4636	-3.7802	1.0000	0.0000	0.4634	-3.6899	1.0000	3.6298	1.0000	-2.2955	1.0000	0.0000
LOC100543582	1.0000	0.0000	1.0000	0.0000	1.0000	0.0000	1.0000	0.0000	1.0000	0.0000	1.0000	0.0000	1.0000	0.0000
LOC100543589	0.0047	-1.7972	0.4883	-0.6594	0.0784	-1.2238	0.2198	-0.8630	1.0000	-0.5903	1.0000	0.5591	1.0000	-0.2180
LOC100543590	0.9672	-1.6972	0.1463	4.3063	1.0000	-0.1848	0.0816	4.6507	1.0000	-3.7658	0.8354	2.1233	1.0000	1.0209
LOC100543595	1.0000	-0.1848	0.0296	-0.8995	0.6587	-0.3691	0.0026	-1.1818	1.0000	0.3780	1.0000	-0.3249	0.7924	-0.4307
LOC100543599	0.0030	1.3942	0.0000	1.8430	0.0000	2.4909	0.0000	2.3471	1.0000	0.0491	0.3982	0.5103	1.0000	-0.0876
LOC100543611	0.0000	-2.5295	0.0000	-2.7438	0.0319	-0.5582	0.0000	-0.8692	1.0000	0.1871	1.0000	-0.0163	1.0000	-0.1208
LOC100543612	0.8249	3.0141	1.0000	0.0000	0.2829	3.9942	1.0000	2.3257	1.0000	0.0000	1.0000	-3.1383	0.8939	-1.6940
LOC100543615	1.0000	0.0467	1.0000	0.3671	1.0000	-0.7120	1.0000	-0.8388	1.0000	-0.6546	1.0000	-0.3291	1.0000	-0.7793
LOC100543617	0.6865	-0.1116	0.1476	-0.1523	0.3789	-0.1783	0.0075	-0.2728	1.0000	0.0104	1.0000	-0.0180	0.8848	-0.0786
LOC100543622	0.6877	-2.0809	1.0000	-0.1706	0.6597	-1.9401	0.6142	-1.7592	1.0000	-0.1247	1.0000	1.8187	1.0000	0.0641
LOC100543634	1.0000	0.0000	1.0000	0.0000	1.0000	0.0000	1.0000	0.0000	1.0000	0.0000	1.0000	0.0000	1.0000	0.0000
LOC100543636	1.0000	0.0000	1.0000	0.0000	1.0000	0.0000	1.0000	0.0000	1.0000	0.0000	1.0000	0.0000	1.0000	0.0000
LOC100543637	1.0000	-2.4776	0.7674	-3.2570	1.0000	-0.1624	0.7710	-3.1693	1.0000	0.7416	1.0000	0.0000	1.0000	-2.2888
LOC100543639	0.0358	-3.3575	0.0013	-2.8129	0.1875	1.0440	0.0833	-1.2884	1.0000	0.8524	1.0000	1.4306	0.1072	-1.4759
LOC100543646	1.0000	0.0000	1.0000	-2.3986	1.0000	0.0000	1.0000	-2.3200	1.0000	2.2736	1.0000	0.0000	1.0000	0.0000
LOC100543661	0.4342	-0.2044	0.0222	-0.3355	0.0011	0.5732	0.0117	0.3060	1.0000	0.3093	0.5692	0.1903	1.0000	0.0476
LOC100543673	1.0000	0.0000	1.0000	0.0000	1.0000	0.0000	1.0000	0.0000	1.0000	0.0000	1.0000	0.0000	1.0000	0.0000
LOC100543675	1.0000	0.0980	0.6479	-0.2836	0.5056	-0.4342	0.8657	-0.2079	1.0000	-0.0299	0.8607	-0.3971	1.0000	0.2020
LOC100543679	0.0566	0.4991	0.0000	0.7039	0.0000	1.3861	0.0000	1.3280	1.0000	-0.0731	0.8840	0.1453	0.8709	-0.1260
LOC100543691	1.0000	0.5920	1.0000	0.1320	1.0000	0.1989	1.0000	-0.3791	1.0000	0.2623	1.0000	-0.1913	1.0000	-0.3104
LOC100543701	1.0000	0.0000	1.0000	0.0000	1.0000	0.0000	1.0000	0.0000	1.0000	0.0000	1.0000	0.0000	1.0000	0.0000
LOC100543707	0.0395	1.1355	1.0000	0.1406	0.0000	1.9503	0.0658	0.6296	1.0000	1.1212	1.0000	0.1379	1.0000	-0.1968
LOC100543736	0.8649	-0.1115	1.0000	0.0125	0.0000	-1.1414	0.0000	-0.8555	1.0000	-0.0015	0.9107	0.1345	0.3129	0.2879
LOC100543737	1.0000	0.0000	1.0000	0.0000	1.0000	0.0000	1.0000	0.0000	1.0000	0.0000	1.0000	0.0000	1.0000	0.0000
LOC100543743	0.5659	-0.1290	0.0000	-0.3004	0.0000	-0.8758	0.0000	-0.6068	1.0000	-0.0735	0.0139	-0.2323	0.0526	0.2008
LOC100543744	0.0652	1.4733	0.0000	2.5878	0.0232	1.5908	0.0011	2.1672	1.0000	-0.6337	0.7394	0.5007	1.0000	-0.0488
LOC100543747	1.0000	0.0000	1.0000	0.0000	1.0000	0.0000	1.0000	0.0000	1.0000	0.0000	1.0000	0.0000	1.0000	0.0000
LOC100543749	1.0000	-2.4788	1.0000	-0.9972	1.0000	-2.4061	1.0000	-0.8404	1.0000	0.7304	1.0000	2.3480	1.0000	2.3542
LOC100543750	0.0182	0.6740	0.0000	0.8223	0.0000	1.0741	0.0000	1.1820	1.0000	-0.1979	1.0000	-0.0363	1.0000	-0.0842
LOC100543754	0.8249	-3.3411	1.0000	-2.3986	1.0000	-1.0194	1.0000	0.0057	1.0000	-0.9563	1.0000	0.0000	1.0000	0.0659
LOC100543777	1.0000	-0.0262	1.0000	0.0015	0.0034	1.0908	0.0846	0.5290	1.0000	0.2039	1.0000	0.2438	0.5518	-0.3581
LOC100543785	0.0263	-0.3587	0.0000	-0.4083	0.9361	0.0659	0.0154	0.1692	1.0000	0.1414	0.7800	0.1040	0.0199	0.2500
LOC100543786	0.0565	-3.2087	0.1616	-2.6990	0.0176	-5.3108	0.0408	-4.8559	1.0000	-0.4770	1.0000	0.0470	1.0000	0.0000
LOC100543796	0.0000	0.6959	0.0000	1.1092	0.2112	0.2483	0.0001	0.3995	0.8479	-0.5872	0.5392	-0.1614	0.0001	-0.4301
LOC100543818	1.0000	-0.0195	0.7444	-0.1801	0.0000	1.2898	0.0000	0.8932	1.0000	0.1521	1.0000	0.0050	0.5779	-0.2389
LOC100543828	1.0000	0.5864	1.0000	0.3671	0.7662	1.0366	0.0539	2.3290	1.0000	-0.1218	1.0000	-0.3300	0.6818	1.1781
LOC100543834	0.0933	0.3362	0.1701	0.1821	0.0001	0.6572	0.0000	0.6167	1.0000	0.0303	0.8627	-0.1116	1.0000	-0.0050
LOC100543835	0.0033	2.1096	0.0718	0.9143	0.0000	3.1372	0.0000	1.6499	1.0000	1.4113	1.0000	0.2337	1.0000	-0.0631
LOC100543837	0.0053	1.0290	0.0572	0.5265	0.8368	-0.3207	0.2053	-0.4452	1.0000	0.5503	1.0000	0.0608	0.7492	0.4359
LOC100543839	0.0397	1.6048	0.0102	1.8963	0.0000	3.5112	0.0000	3.7769	1.0000	-0.5306	1.0000	-0.2306	0.7661	-0.2621
LOC100543851	1.0000	0.5370	0.4431	-3.7875	1.0000	-2.4061	1.0000	-0.5236	1.0000	1.2654	1.0000	-3.1383	1.0000	3.2081
LOC100543857	1.0000	0.0000	1.0000	0.0000	1.0000	0.0000	1.0000	0.0000	1.0000	0.0000	1.0000	0.0000	1.0000	0.0000
LOC100543858	1.0000	0.0000	1.0000	0.0000	1.0000	0.0000	1.0000	0.0000	1.0000	0.0000	1.0000	0.0000	1.0000	0.0000
LOC100543865	0.0065	3.4597	0.0508	1.9264	0.0003	3.9087	0.1166	1.6457	1.0000	1.6647	1.0000	0.1432	0.8105	-0.6019
LOC100543880	1.0000	0.0000	1.0000	0.0000	1.0000	0.0000	1.0000	0.0000	1.0000	0.0000	1.0000	0.0000	1.0000	0.0000
LOC100543887	1.0000	0.3554	1.0000	-0.1673	0.2603	1.6058	1.0000	0.6991	1.0000	-0.1237	1.0000	-0.6369	0.6942	-1.0229
LOC100543889	0.8226	-3.3425	1.0000	-2.3986	0.8011	-3.2629	1.0000	-2.3200	1.0000	-0.9587	1.0000	0.0000	1.0000	0.0000
LOC100543893	0.5086	-3.8790	0.5029	-1.6425	0.8151	0.7542	1.0000	-0.5497	1.0000	0.8210	1.0000	3.1976	1.0000	-0.4791
LOC100543903	1.0000	0.0000	1.0000	0.0000	1.0000	0.0000	1.0000	0.0000	1.0000	0.0000	1.0000	0.0000	1.0000	0.0000
LOC100543904	0.2406	1.6814	0.3908	0.8968	0.9361	-1.5532	0.5961	-1.1475	1.0000	1.0240	1.0000	0.2624	1.0000	1.4486
LOC100543905	0.2832	-0.3484	0.0190	-0.4247	0.0000	-1.3619	0.0000	-1.0330	1.0000	0.0567	1.0000	-0.0073	0.3721	0.3904
LOC100543914	0.0000	0.9773	0.0000	0.8468	0.0995	0.4255	0.0025	0.4356	1.0000	0.2042	1.0000	0.0878	0.4143	0.2193
LOC100543920	1.0000	0.5881	0.1008	-2.8797	1.0000	-1.0171	0.2313	-1.8664	1.0000	1.7468	1.0000	-1.7130	1.0000	0.9149
LOC100543921	0.8226	-3.3425	1.0000	-0.1452	1.0000	0.3474	0.9162	1.3863	1.0000	-0.9587	1.0000	2.3480	1.0000	0.0765
LOC100543930	1.0000	0.0000	0.9148	1.2162	0.3268	4.0111	1.0000	0.0045	1.0000	2.2736	0.8785	3.7234	0.9124	-1.7022
LOC100543947	0.5196	0.4766	1.0000	0.0547	0.0935	-1.1415	0.0165	-1.4558	1.0000	-0.1799	0.5791	-0.5910	0.9641	-0.4921
LOC100543953	1.0000	0.0000	1.0000	0.0000	1.0000	0.0000	1.0000	0.0000	1.0000	0.0000	1.0000	0.0000	1.0000	0.0000
LOC100543957	0.0156	0.4959	0.1356	0.2210	0.0576	0.3991	0.1189	0.2285	1.0000	0.0561	0.3768	-0.2046	0.8155	-0.1075
LOC100543967	1.0000	0.0000	1.0000	0.0000	1.0000	0.0000	1.0000	0.0000	1.0000	0.0000	1.0000	0.0000	1.0000	0.0000
LOC100543969	1.0000	-0.0502	0.0001	0.3161	0.0120	0.4094	0.0000	0.9562	1.0000	0.1253	0.0000	0.5040	0.0000	0.6773
LOC100543979	0.3386	0.3793	0.0022	0.5888	0.0251	0.7391	0.0000	0.9128	1.0000	-0.0702	0.9815	0.1519	1.0000	0.1073
LOC100543986	0.3159	3.9314	1.0000	0.6940	0.0349	4.9458	0.9096	1.3854	1.0000	2.2674	1.0000	-0.8645	0.7121	-1.2654
LOC100544007	0.0270	0.9542	0.0000	1.3954	0.0000	1.5590	0.0000	1.6176	1.0000	-0.4089	1.0000	0.0438	0.5526	-0.3479
LOC100544020	0.4884	0.3753	0.0008	1.0032	0.0002	1.1845	0.0003	1.0845	1.0000	-0.5623	1.0000	0.0769	0.0238	-0.6573
LOC100544022	1.0000	-0.2931	1.0000	0.0000	1.0000	-2.4061	1.0000	0.0000	1.0000	-2.3771	1.0000	-2.2955	1.0000	0.0000

LOC100544043	1.0000	-2.4776	0.1126	-4.7517	0.1167	2.7088	0.3140	-2.3336	1.0000	2.2290	1.0000	0.0000	0.1658	-2.8155
LOC100544046	1.0000	-1.2606	1.0000	-0.1452	1.0000	-0.5736	0.9096	1.3845	1.0000	-1.8802	1.0000	-0.7919	1.0000	0.0749
LOC100544056	0.1981	0.9291	0.0201	1.1017	0.0000	2.0424	0.0000	2.1795	1.0000	0.1006	1.0000	0.2833	0.8746	0.2463
LOC100544060	1.0000	0.0000	1.0000	0.0000	1.0000	0.0000	1.0000	0.0000	1.0000	0.0000	1.0000	0.0000	1.0000	0.0000
LOC100544062	0.3020	0.5303	0.0154	0.9961	0.0246	-1.1575	0.1867	-0.8723	1.0000	-0.8564	0.7510	-0.3763	0.8274	-0.5629
LOC100544066	0.3155	-0.2580	0.0000	-0.4216	0.0145	-0.5178	0.0000	-0.4062	1.0000	0.0457	0.8870	-0.1048	0.4509	0.1619
LOC100544071	1.0000	0.0000	1.0000	0.0000	1.0000	0.0000	1.0000	0.0000	1.0000	0.0000	1.0000	0.0000	1.0000	0.0000
LOC100544072	0.0526	-0.4577	0.0000	-0.5344	0.0495	0.4254	0.0004	0.4095	1.0000	0.1843	0.9640	0.1205	0.4552	0.1749
LOC100544079	1.0000	0.0000	1.0000	0.0000	1.0000	0.0000	1.0000	0.0000	1.0000	0.0000	1.0000	0.0000	1.0000	0.0000
LOC100544081	0.1947	0.4266	0.0000	1.0290	0.1869	-0.5125	0.1041	-0.4908	1.0000	-0.3972	0.7552	0.2151	0.6186	-0.3712
LOC100544088	0.0001	0.9450	0.0000	1.2033	0.0482	0.5899	0.6822	0.1656	1.0000	-0.2709	1.0000	-0.0005	0.0063	-0.6885
LOC100544098	0.8087	0.8930	0.9784	0.7485	1.0000	-1.0206	0.4992	1.4798	1.0000	-0.1139	1.0000	-0.2500	0.5105	2.3935
LOC100544105	0.0000	1.1443	0.0000	1.2439	0.0000	1.6177	0.0000	1.1283	1.0000	0.1480	0.1771	0.2611	0.0032	-0.3356
LOC100544107	0.8249	3.0256	1.0000	2.2507	1.0000	0.0000	1.0000	2.3242	1.0000	0.0000	1.0000	-0.8001	1.0000	2.3542
LOC100544113	0.0484	-1.8923	0.3360	-0.9326	0.0086	-2.9494	0.2818	-1.2144	1.0000	-0.4489	1.0000	0.5251	0.9835	1.2974
LOC100544119	0.5157	0.3733	0.0010	0.8468	1.0000	0.0523	0.0506	-0.6678	1.0000	0.2048	0.0433	0.6904	0.4139	-0.5114
LOC100544123	0.4587	-0.2737	0.0003	-0.4637	0.0540	-0.5189	0.3627	-0.1513	1.0000	0.4753	0.4236	0.2986	0.0000	0.8460
LOC100544124	0.1394	-0.3088	0.0002	-0.3825	0.0001	-0.7321	0.0000	-0.8844	1.0000	0.0266	1.0000	-0.0344	0.7916	-0.1198
LOC100544125	0.0079	5.4486	0.0001	6.1038	0.2840	3.9988	0.0203	5.0480	1.0000	0.0000	1.0000	0.6359	0.9118	1.0342
LOC100544134	0.0164	5.4352	0.1487	1.7376	0.0001	6.2375	0.0324	2.1395	1.0000	3.6423	1.0000	0.0639	1.0000	-0.4250
LOC100544136	0.8573	0.1493	0.1405	0.3435	0.0000	2.0580	0.0000	1.6403	1.0000	0.1881	0.0770	0.3958	0.2856	-0.2233
LOC100544139	0.2484	-0.9337	0.2689	-0.6886	0.0000	-6.7027	0.0008	-2.0536	1.0000	0.1875	1.0000	0.4474	0.1307	4.8977
LOC100544143	0.0095	-0.4190	0.0000	-0.5916	0.0000	-0.6833	0.0000	-0.5361	1.0000	-0.0248	0.2678	-0.1850	0.5565	0.1272
LOC100544156	0.1473	0.8543	0.5760	0.3513	1.0000	0.1230	0.4215	-0.4531	1.0000	0.5992	1.0000	0.1094	1.0000	0.0313
LOC100544159	1.0000	0.0000	1.0000	0.0000	1.0000	0.0000	1.0000	0.0000	1.0000	0.0000	1.0000	0.0000	1.0000	0.0000
LOC100544162	1.0000	0.0000	1.0000	0.0000	1.0000	0.0000	1.0000	0.0000	1.0000	0.0000	1.0000	0.0000	1.0000	0.0000
LOC100544165	0.2926	0.6648	0.0215	0.8584	0.2816	0.5896	1.0000	0.0883	1.0000	0.0834	0.9957	0.2953	0.6915	-0.4079
LOC100544169	1.0000	0.0000	1.0000	0.0000	1.0000	0.0000	1.0000	0.0000	1.0000	0.0000	1.0000	0.0000	1.0000	0.0000
LOC100544170	0.0000	-1.6993	0.0000	-2.1690	0.0000	-1.8869	0.0001	-1.1387	1.0000	-0.0446	0.8875	-0.5009	0.3610	0.7111
LOC100544185	1.0000	0.0000	1.0000	0.0000	1.0000	0.0000	1.0000	0.0000	1.0000	0.0000	1.0000	0.0000	1.0000	0.0000
LOC100544196	1.0000	0.0789	0.2310	0.2160	0.8448	0.1344	0.0530	0.2920	1.0000	-0.0498	1.0000	0.0994	1.0000	0.1144
LOC100544198	0.0000	-7.4799	0.0000	-11.2182	0.0000	1.1495	0.0000	0.7926	1.0000	-0.2223	0.6983	-4.0460	0.0000	-0.5738
LOC100544199	0.0501	-0.4123	0.0000	-0.5098	0.7181	0.1309	0.4929	-0.1092	1.0000	0.1406	1.0000	0.0554	0.8937	-0.0943
LOC100544203	0.8413	-0.1524	0.7666	-0.0907	0.4671	-0.2925	0.0000	-0.7385	1.0000	0.1426	0.5937	0.2155	0.3515	-0.2970
LOC100544221	1.0000	0.0238	0.7893	0.0611	0.0414	-0.3928	0.0000	-0.5268	1.0000	0.1355	0.4639	0.1857	1.0000	0.0070
LOC100544224	1.0000	0.0000	1.0000	0.0000	1.0000	0.0000	1.0000	0.0000	1.0000	0.0000	1.0000	0.0000	1.0000	0.0000
LOC100544231	1.0000	-0.2315	0.5872	-1.3300	0.1184	-1.9832	1.0000	0.3582	1.0000	-0.7591	0.3440	-1.8556	0.5565	1.5864
LOC100544232	1.0000	0.0000	1.0000	0.0000	1.0000	2.2427	1.0000	0.0000	1.0000	0.0000	1.0000	0.0000	1.0000	-2.2888
LOC100544235	0.5833	1.3514	0.2468	1.3526	0.0001	3.4608	0.0484	1.9448	1.0000	0.8006	0.9942	0.8168	0.6546	-0.7157
LOC100544245	0.3003	0.6944	0.0000	-2.4249	0.0000	1.9910	1.0000	0.0756	1.0000	1.1475	0.0014	-1.9618	0.0064	-0.7669
LOC100544252	0.3557	0.2244	0.1860	0.1485	0.0000	0.8205	0.0000	0.8381	1.0000	0.1483	0.9238	0.0856	0.2232	0.1720
LOC100544253	0.9465	-0.1328	1.0000	-0.0521	1.0000	0.0736	0.0865	0.4586	1.0000	-0.1241	1.0000	-0.0284	0.7059	0.2673
LOC100544274	0.0370	-3.3570	0.0132	-5.3023	0.0288	-3.2151	1.0000	0.2891	1.0000	-0.2743	1.0000	-2.2992	0.0703	3.2423
LOC100544277	0.5086	-3.8796	0.2920	-4.1632	1.0000	-0.1850	0.9959	-0.9001	1.0000	0.2544	1.0000	0.0000	1.0000	-0.4568
LOC100544286	0.8226	-3.3425	0.4431	-3.7865	0.8011	-3.2629	0.4370	-3.6968	1.0000	0.4094	1.0000	0.0000	1.0000	0.0000
LOC100544288	0.1657	-1.0946	0.2526	-0.8154	1.0000	-0.0789	1.0000	0.0623	1.0000	-0.9760	0.6719	-0.6857	0.1925	-0.8282
LOC100544290	1.0000	-2.4776	1.0000	-2.3959	1.0000	-0.1591	1.0000	-2.3177	1.0000	-0.1078	1.0000	0.0000	1.0000	-2.2906
LOC100544291	0.8035	-0.1906	0.7655	-0.0891	0.5235	-0.2933	1.0000	-0.0343	1.0000	0.1053	0.5070	0.2201	0.0609	0.3715
LOC100544294	0.0090	1.2073	0.0000	1.2562	0.0000	2.3451	0.0000	2.0227	1.0000	0.2104	0.9155	0.2721	1.0000	-0.1058
LOC100544297	0.0696	2.2212	0.2736	-0.9659	1.0000	0.3467	0.0344	-2.1254	1.0000	2.5403	1.0000	-0.6245	1.0000	0.0765
LOC100544298	1.0000	0.2089	0.0955	2.1519	0.6647	-1.9401	1.0000	-0.8416	1.0000	-1.0395	0.9659	0.9075	1.0000	0.0649
LOC100544306	0.4098	-0.1769	0.2818	-0.1109	0.7214	0.1101	0.0067	0.2249	1.0000	-0.1076	1.0000	-0.0291	1.0000	0.0124
LOC100544311	1.0000	-2.4788	0.4642	-3.7944	1.0000	-2.4061	0.4547	-3.7024	1.0000	1.2721	1.0000	0.0000	1.0000	0.0000
LOC100544321	0.9650	0.0926	0.9084	-0.0642	0.0100	-0.6221	1.0000	-0.0184	1.0000	0.1188	1.0000	-0.0253	0.0000	0.7281
LOC100544322	1.0000	0.0000	1.0000	0.0000	1.0000	0.0000	1.0000	0.0000	1.0000	0.0000	1.0000	0.0000	1.0000	0.0000
LOC100544327	0.1139	0.4507	0.0236	0.4018	0.9268	-0.1331	0.0242	-0.4519	1.0000	0.1021	1.0000	0.0664	0.8131	-0.2125
LOC100544335	0.0293	0.4996	0.6632	0.1079	0.0000	1.0850	0.0000	0.8497	1.0000	0.3585	1.0000	-0.0193	0.6911	0.1286
LOC100544338	0.9055	0.4570	0.0055	2.3933	0.6235	0.7702	0.0048	2.4968	1.0000	-1.6379	1.0000	0.3049	1.0000	0.0847
LOC100544340	0.9246	0.1594	0.1347	-0.3405	0.4032	0.3664	0.5800	0.1592	1.0000	0.2984	0.9741	-0.1884	1.0000	0.0962
LOC100544344	0.0087	-1.1027	0.0012	-1.2482	0.0626	-0.8044	0.5090	-0.3189	1.0000	-0.3164	0.9118	-0.4494	1.0000	0.1754
LOC100544354	0.0147	-5.3966	0.0028	-5.7016	0.0028	1.9543	0.0095	1.4484	1.0000	0.2709	1.0000	0.0000	1.0000	-0.2290
LOC100544355	1.0000	-0.0459	0.4842	-0.1112	0.0000	-0.9753	0.0000	-0.9243	1.0000	0.0150	1.0000	-0.0384	1.0000	0.0709
LOC100544367	0.0566	-3.2110	0.0699	-3.0678	0.0458	-3.0671	0.2119	-2.0429	1.0000	-0.1185	1.0000	0.0495	1.0000	0.9186
LOC100544369	0.4106	0.3648	0.0097	0.6647	0.0000	1.5550	0.0000	1.3985	1.0000	0.0778	0.4447	0.3901	1.0000	-0.0708
LOC100544371	0.0139	2.8931	0.1013	1.3694	0.0000	3.7401	0.0022	2.1310	1.0000	1.3429	1.0000	-0.1580	1.0000	-0.2509
LOC100544372	0.1710	-0.3273	0.0147	-0.2921	0.0000	-0.8817	0.0509	-0.2418	1.0000	-0.1001	1.0000	-0.0530	0.0002	0.5448
LOC100544385	1.0000	0.5464	0.2495	4.0050	1.0000	-0.1591	1.0000	0.0000	1.0000	-2.3757	1.0000	0.9726	1.0000	-2.2906
LOC100544389	0.9604	0.6124	0.1261	1.5398	0.9250	0.4648	1.0000	0.0070	1.0000	-0.1241	0.8759	0.8118	1.0000	-0.5752
LOC100544391	1.0000	0.0000	1.0000	0.0000	1.0000	0.0000	1.0000	0.0000	1.0000	0.0000	1.0000	0.0000	1.0000	0.0000
LOC100544408	0.5972	-0.1594	0.4027	-0.1344	0.0741	-0.3688	0.0320	-0.3029	1.0000	-0.1557	0.8799	-0.1185	1.0000	-0.0841
LOC100544421	0.1436	0.4210	0.0000	0.7130	0.0000	0.8694	0.0000	1.1775	1.0000	-0.4170	1.0000	-0.1110	0.8725	-0.1009
LOC100544424	1.0000	-2.4776	0.7666	-3.2534	1.0000	-2.4056	1.0000	-0.8416	1.0000	0.7365	1.0000	0.0000	1.0000	2.3554
LOC100544425	0.1253	0.5192	0.0034	0.6007	0.0000	1.2232	0.0000	1.1595	1.0000	0.0525	1.0000	0.1467	1.0000	-0.0059
LOC100544433	0.6646	0.8687	0.3694	1.0094	0.0114	2.0712	0.0000	2.6210	1.0000	0.1229	1.0000	0.2770	0.4066	0.6797
LOC100544434	0.2686	0.3305	0.0000	0.5872	0.0207	-0.6026	0.0000	-0.4278	1.0000	-0.2373	1.0000	0.0318	1.0000	-0.0561
LOC100544435	0.6894	0.5415	0.0002	2.0490	0.2866	0.7493	0.1133	1.3444	1.0000	-1.0289	0.8016	0.4873	0.9553	-0.4326
LOC100544436	0.0972	0.9281	1.0000	0.1185	0.9361	-0.2535	0.0531	-0.8124	1.0000	1.0204	1.0000	0.2232	0.7455	0.4641
LOC100544441	0.0615	1.3315	0.7704	0.3978	0.7515	-0.6576	0.9950	-0.3309	1.0000	0.2105	0.5676	-0.7102	1.0000	0.5586
LOC100544443	0.0000	1.0280	0.0000	1.0755	0.0000	1.7041	0.0000	1.2758	1.0000	0.1155	0.5211	0.1765	0.0237	-0.3072
LOC100544448	0.3159	-0.9720	0.0346	-1.3448	1.0000	-0.0447	0.0056	1.1105	1.0000	0.4198	1.0000	0.0602	0.0011	1.5759
LOC100544450	0.6926	-2.0812	0.0122	-5.3009	0.0000	4.0910	0.0000	3.1269	1.0000	0.9956	1.0000	-2.2992	1.0000	0.0345
LOC100544458	1.0000	-0.0116	0.6840	0.3055	0.4426	-0.5612	1.0000	-0.1644	1.0000	-0.4655	1.0000	-0.1318	1.0000	-0.0600
LOC100544459	0.0803	0.6656	0.0000	1.5551	0.0013	1.0283	0.0000	2.3887	1.0000	-0.9047	1.0000	-0.0029	0.0963	0.4608
LOC100544461	1.0000	-0.0611	0.5213	0.1683	0.0000	1.0152	0.0000	0.8896	1.0000	-0.2731	1.0000	-0.0309	0.0253	-0.3933
LOC100544468	1.0000	2.1848	1.0000	0.0000	1.0000	0.0000	1.0000	2.3242	1.0000	0.0000	1.0000	-2.2955	1.0000	2.3542
LOC100544471	0.6400	0.1318	0.1087	0.1736	0.0005	0.5709	0.0000	0.6063	1.0000	-0.0293	1.0000	0.0256	1.0000	0.0118
LOC100544478	1.0000	-2.4776	1.0000	2.2507	1.0000	-2.4056	1.0000	0.0000	1.0000	-2.3757	1.0000	2.3456	1.0000	0.0000
LOC100544485	0.6750	-1.2899	0.1388	-4.4811	0.0943	-4.7443	0.1318	-4.3887	1.0000	-0.3796	0.8785	-3.6646	1.0000	0.0000
LOC100544490	0.8703	0.2404	0.0273	-0.7471	0.0184	1.0258	0.0001	0.9239	1.0000	0.4964	0.6156	-0.4823	0.3395	0.3916
LOC100544491	0.2167	0.4119	0.0029	0.5880	0.3769	-0.3539	0.9096	0.1051	1.0000	-0.0277	0.9569	0.1608	0.2135	0.4345
LOC100544502	0.0013	-0.5914	0.0000	-0.5041	1.0000	-0.0178	0.3427	0.0966	1.0000	-0.1075	1.0000	-0.0080	1.0000	0.0131
LOC100544508	0.0000	-3.4808	0.0000	-2.3531	1.0000	-0.1260	0.3653	0.2009	1.0000	0.6628	0.0015	1.8085	0.0000	1.0020
LOC100544511	0.1511	-0.2760	0.0001	-0.3723	0.0000	-0.8514	0.0000	-0.6803	1.0000	0.0727	1.0000	-0.0117	0.1089	0.2494

LOC100544513	0.3827	-0.2070	0.2786	-0.1436	0.0447	-0.3965	0.0116	-0.2545	1.0000	0.0150	1.0000	0.0910	0.4901	0.1634
LOC100544524	0.0207	-0.7355	0.0000	-1.1826	0.3128	-0.3661	0.0000	-0.8410	1.0000	0.0119	0.3309	-0.4248	0.1130	-0.4582
LOC100544528	1.0000	0.0000	1.0000	0.0000	0.8011	3.0840	1.0000	0.0000	1.0000	0.0000	1.0000	0.0000	1.0000	-3.1344
LOC100544530	1.0000	0.0000	0.7666	-3.2534	1.0000	0.0000	1.0000	-0.8419	1.0000	3.1126	1.0000	0.0000	1.0000	2.3542
LOC100544531	1.0000	0.0000	1.0000	0.0000	1.0000	0.0000	1.0000	0.0000	1.0000	0.0000	1.0000	0.0000	1.0000	0.0000
LOC100544543	1.0000	-0.1379	1.0000	0.1247	0.6218	0.2787	1.0000	0.0766	1.0000	-0.1431	1.0000	0.1285	0.5515	-0.3421
LOC100544544	1.0000	0.0573	0.9762	-1.0844	0.9361	-1.5529	0.9786	-0.9107	1.0000	0.2637	1.0000	-0.8736	1.0000	0.9168
LOC100544547	1.0000	0.0147	0.3415	0.3809	0.1948	-0.7693	0.0229	-0.8315	1.0000	0.3710	0.1665	0.7495	1.0000	0.3103
LOC100544550	1.0000	0.0000	1.0000	0.0000	1.0000	0.0000	1.0000	0.0000	1.0000	0.0000	1.0000	0.0000	1.0000	0.0000
LOC100544552	1.0000	0.0000	1.0000	0.0000	1.0000	0.0000	1.0000	0.0000	1.0000	0.0000	1.0000	0.0000	1.0000	0.0000
LOC100544555	1.0000	0.0000	1.0000	0.0000	1.0000	0.0000	1.0000	0.0000	1.0000	0.0000	1.0000	0.0000	1.0000	0.0000
LOC100544563	1.0000	0.0000	1.0000	0.0000	1.0000	0.0000	1.0000	0.0000	1.0000	0.0000	1.0000	0.0000	1.0000	0.0000
LOC100544568	0.3143	0.3504	0.0003	0.5479	0.0000	1.7177	0.0000	1.6500	1.0000	0.0438	0.0943	0.2544	1.0000	-0.0185
LOC100544577	0.9900	-0.0986	0.0109	-0.3921	0.0000	1.5195	0.0000	1.1338	1.0000	0.2497	1.0000	-0.0316	0.6072	-0.1302
LOC100544580	0.0001	-0.6890	0.0000	-0.6686	0.3117	0.2574	0.0000	0.4910	1.0000	0.1298	0.2530	0.1627	0.0077	0.3682
LOC100544583	0.1882	0.2200	0.0062	0.2128	0.9227	0.0685	0.0278	0.1541	1.0000	-0.0679	0.9553	-0.0628	1.0000	0.0232
LOC100544584	1.0000	0.0000	1.0000	2.2507	1.0000	0.0000	1.0000	2.3242	1.0000	0.0000	1.0000	2.3456	1.0000	2.3542
LOC100544585	0.8249	0.1403	0.3062	-0.1764	0.9127	-0.1180	0.7668	-0.0890	1.0000	0.2275	1.0000	-0.0747	0.3200	0.2609
LOC100544587	0.6853	-0.1220	0.0005	-0.3644	1.0000	-0.0418	0.0697	-0.2038	1.0000	0.2322	1.0000	0.0028	0.9054	0.0756
LOC100544588	1.0000	0.0000	1.0000	0.0000	1.0000	0.0000	1.0000	2.3257	1.0000	0.0000	1.0000	0.0000	1.0000	2.3554
LOC100544592	0.1483	0.6784	0.0000	1.4625	0.2137	0.5967	0.0034	0.9194	1.0000	-0.5299	0.8175	0.2638	0.9810	-0.2011
LOC100544598	1.0000	0.0000	1.0000	0.0000	1.0000	0.0000	1.0000	0.0000	1.0000	0.0000	1.0000	0.0000	1.0000	0.0000
LOC100544614	0.4509	0.5702	0.5510	0.5678	0.1083	-1.2448	1.0000	0.0078	1.0000	-0.9417	0.2386	-0.9332	1.0000	0.3163
LOC100544619	0.1644	0.2491	0.0000	0.4286	0.0007	0.5136	0.0000	0.4934	1.0000	-0.0831	0.6450	0.1088	0.6268	-0.0979
LOC100544620	0.2034	-0.2352	0.0003	-0.2363	0.0048	-0.4502	0.0000	-0.4977	1.0000	0.0136	1.0000	0.0248	1.0000	-0.0282
LOC100544625	1.0000	-2.4776	0.4431	3.6192	1.0000	-2.4056	1.0000	0.0000	1.0000	-2.3758	0.8607	3.7287	1.0000	0.0000
LOC100544628	1.0000	0.0000	1.0000	0.0000	1.0000	0.0000	1.0000	0.0000	1.0000	0.0000	1.0000	0.0000	1.0000	0.0000
LOC100544636	1.0000	-2.4788	1.0000	2.2507	1.0000	-2.4061	1.0000	0.0000	1.0000	-2.3771	1.0000	2.3456	1.0000	0.0000
LOC100544640	1.0000	0.0000	0.7674	3.0957	1.0000	2.2427	1.0000	0.0000	1.0000	0.0000	1.0000	3.2002	1.0000	-2.2888
LOC100544642	1.0000	0.0000	1.0000	0.0000	1.0000	0.0000	1.0000	0.0000	1.0000	0.0000	1.0000	0.0000	1.0000	0.0000
LOC100544649	0.0551	0.3794	0.0019	0.3450	0.1102	-0.3424	0.0583	-0.2360	1.0000	-0.0633	0.9689	-0.0849	1.0000	0.0499
LOC100544657	0.3089	3.9281	1.0000	2.2507	0.8011	3.0840	1.0000	2.3242	1.0000	0.0000	1.0000	-1.7124	1.0000	-0.7795
LOC100544679	0.5706	0.1491	0.2212	0.1483	0.1506	-0.2795	0.0003	-0.3523	1.0000	0.0862	0.8689	0.0978	1.0000	0.0184
LOC100544684	1.0000	2.1848	1.0000	0.6956	1.0000	0.0000	1.0000	-2.3176	1.0000	2.2674	1.0000	0.9006	1.0000	0.0000
LOC100544690	0.5378	3.5350	1.0000	0.0000	1.0000	0.0000	1.0000	0.0000	1.0000	0.0000	0.8785	-3.6646	1.0000	0.0000
LOC100544693	1.0000	-0.3444	0.4431	-3.7865	0.4426	-2.2477	1.0000	0.0072	1.0000	-0.8169	0.4563	-4.3639	1.0000	1.4489
LOC100544702	0.3048	-0.4271	0.7666	0.1596	0.0198	-0.8587	0.9237	-0.1370	1.0000	-0.4750	1.0000	0.1245	0.9276	0.2545
LOC100544708	0.9609	0.0595	0.1043	0.1431	0.1421	-0.2499	0.0000	-0.3333	1.0000	0.1012	0.1099	0.1970	1.0000	0.0227
LOC100544709	1.0000	0.0000	1.0000	0.0000	1.0000	0.0000	1.0000	0.0000	1.0000	0.0000	1.0000	0.0000	1.0000	0.0000
LOC100544713	1.0000	0.0000	1.0000	0.0000	1.0000	0.0000	1.0000	0.0000	1.0000	0.0000	1.0000	0.0000	1.0000	0.0000
LOC100544724	0.0000	-1.1606	0.0000	-1.1697	0.4878	0.1881	0.0000	0.7299	1.0000	-0.4513	0.0000	-0.4481	0.6891	0.0954
LOC100544731	0.1894	0.6115	0.0022	0.9429	0.0040	-1.4056	1.0000	-0.1374	1.0000	-0.0689	0.8847	0.2728	0.0432	1.2017
LOC100544738	0.2972	0.2938	0.4876	-0.1601	0.0008	-0.7778	0.0000	-0.9971	1.0000	0.1096	0.1727	-0.3319	1.0000	-0.1054
LOC100544740	0.4193	-0.6802	0.7325	-0.4725	0.4265	-0.6679	0.8536	-0.4002	1.0000	-0.7664	1.0000	-0.5495	0.9919	-0.4948
LOC100544745	0.0027	-0.9122	0.0000	-1.1850	0.0004	-1.0232	0.0000	-1.9886	1.0000	0.6670	0.4089	0.4074	0.6777	-0.2917
LOC100544750	1.0000	0.0000	1.0000	0.0000	1.0000	0.0000	1.0000	0.0000	1.0000	0.0000	1.0000	0.0000	1.0000	0.0000
LOC100544756	0.0000	1.4596	0.0000	1.3832	0.0000	2.1668	0.0000	1.4857	1.0000	0.2295	0.8741	0.1672	0.0063	-0.4452
LOC100544767	0.0055	0.7911	0.0393	0.4437	0.0000	1.1602	0.0003	0.6760	1.0000	0.2707	1.0000	-0.0640	0.5842	-0.2070
LOC100544777	1.0000	0.5849	0.9754	0.7517	1.0000	-0.1833	1.0000	0.0073	1.0000	-0.1187	1.0000	0.0579	1.0000	0.0753
LOC100544780	1.0000	-0.3288	1.0000	2.2507	1.0000	-1.0206	1.0000	2.3257	1.0000	-3.2319	1.0000	-0.7943	1.0000	0.0659
LOC100544787	1.0000	0.0000	1.0000	0.0000	1.0000	0.0000	1.0000	0.0000	1.0000	0.0000	1.0000	0.0000	1.0000	0.0000
LOC100544792	1.0000	0.0000	1.0000	0.0000	1.0000	0.0000	1.0000	0.0000	1.0000	0.0000	1.0000	0.0000	1.0000	0.0000
LOC100544802	1.0000	-2.4788	0.7666	3.0922	1.0000	-2.4061	0.7710	3.1708	1.0000	-2.3771	1.0000	3.1976	1.0000	3.2048
LOC100544805	1.0000	0.0000	1.0000	0.0000	1.0000	0.0000	1.0000	0.0000	1.0000	0.0000	1.0000	0.0000	1.0000	0.0000
LOC100544806	0.0000	-2.6852	0.0000	-2.5407	0.0000	-1.1654	0.0000	-1.2126	1.0000	-0.0324	0.8269	0.1251	0.8699	-0.0743
LOC100544815	0.8249	0.5387	0.6409	0.7823	0.6508	-1.0145	0.6172	-1.7602	1.0000	-1.2471	0.6201	-1.0037	0.6865	-1.9991
LOC100544818	1.0000	0.0482	0.7153	0.1125	0.1189	-0.4402	0.0010	-0.6154	1.0000	0.0534	1.0000	0.1310	1.0000	-0.1166
LOC100544821	1.0000	-0.2881	0.6505	1.6133	1.0000	-0.1624	1.0000	0.0057	1.0000	-0.1034	1.0000	1.8233	1.0000	0.0659
LOC100544825	0.8249	-3.3411	1.0000	2.2507	0.8033	-3.2622	1.0000	0.0000	1.0000	-3.2289	1.0000	2.3456	1.0000	0.0000
LOC100544832	0.0511	1.0486	0.0000	1.5554	0.0000	2.3814	0.0000	2.7915	1.0000	-0.2430	0.7623	0.2809	0.6687	0.1771
LOC100544843	0.0000	0.9643	0.0000	0.9743	0.1435	0.3727	0.0000	0.6163	1.0000	-0.0799	1.0000	-0.0564	0.6135	0.1696
LOC100544848	1.0000	-0.7294	1.0000	-0.6944	0.6597	-1.9401	0.4370	-3.6968	1.0000	-0.5115	1.0000	-0.4715	1.0000	-2.2906
LOC100544862	0.5086	3.5456	1.0000	0.0000	1.0000	0.0000	1.0000	0.0000	1.0000	0.0000	0.8607	-3.6732	1.0000	0.0000
LOC100544866	0.5086	3.5456	0.1791	2.3688	1.0000	0.0000	1.0000	0.0057	1.0000	2.2736	0.9937	1.2106	1.0000	2.3554
LOC100544870	0.5086	3.5456	0.4671	3.6266	1.0000	0.0000	1.0000	0.0000	1.0000	0.0000	1.0000	0.0582	1.0000	0.0000
LOC100544871	1.0000	0.4333	0.2747	0.9884	1.0000	-0.0346	0.7701	0.6438	1.0000	-0.3171	1.0000	0.2504	1.0000	0.3661
LOC100544877	0.1233	-0.3932	0.0101	-0.3320	0.0034	-0.6795	0.0000	-0.5395	1.0000	0.0143	1.0000	0.0878	0.7263	0.1584
LOC100544881	1.0000	2.1848	0.1384	4.3086	1.0000	2.2427	0.7710	3.1709	1.0000	0.0000	0.8269	2.1245	1.0000	0.9164
LOC100544896	0.0382	-1.4640	0.0001	-2.5017	0.5098	-0.6063	1.0000	-0.1908	1.0000	0.2404	0.9731	-0.7875	0.7170	0.6600
LOC100544897	1.0000	0.0000	1.0000	0.0000	1.0000	0.0000	1.0000	0.0000	1.0000	0.0000	1.0000	0.0000	1.0000	0.0000
LOC100544899	0.0042	-0.4313	0.0000	-0.3436	0.0003	0.5284	0.0000	0.4427	1.0000	-0.0321	0.9052	0.0680	0.3986	-0.1122
LOC100544913	1.0000	0.0224	0.1395	-0.2174	0.7272	0.1340	1.0000	-0.0005	1.0000	-0.1304	0.0654	-0.3576	0.1306	-0.2601
LOC100544917	1.0000	-0.2921	1.0000	-2.3959	1.0000	-2.4056	0.9096	1.3850	1.0000	-0.1078	1.0000	-2.2955	0.7287	3.7384
LOC100544920	0.0059	3.5370	0.6859	0.6426	0.0280	3.1142	0.2776	1.2293	1.0000	1.9558	0.7785	-0.9160	1.0000	0.0794
LOC100544929	1.0000	-1.2458	1.0000	0.3654	0.6925	0.9291	0.0119	2.6702	1.0000	-1.0414	1.0000	0.5792	0.9331	0.7031
LOC100544938	0.0365	2.2962	0.0002	2.0129	0.9361	-1.5509	0.4211	0.8205	1.0000	1.3845	0.1330	1.1208	0.0068	3.7735
LOC100544939	1.0000	0.0000	1.0000	0.0000	1.0000	0.0000	1.0000	0.0000	1.0000	0.0000	1.0000	0.0000	1.0000	0.0000
LOC100544943	1.0000	0.0000	1.0000	0.0000	1.0000	0.0000	1.0000	0.0000	1.0000	0.0000	1.0000	0.0000	1.0000	0.0000
LOC100544944	0.8226	-0.5094	1.0000	-0.0131	1.0000	-0.1975	0.6342	0.6899	1.0000	-1.4182	0.6507	-0.9194	0.9017	-0.5320
LOC100544946	1.0000	0.0000	1.0000	0.0000	1.0000	2.2427	1.0000	0.0000	1.0000	0.0000	1.0000	0.0000	1.0000	-2.2888
LOC100544965	1.0000	0.0000	1.0000	0.0000	1.0000	0.0000	1.0000	0.0000	1.0000	0.0000	1.0000	0.0000	1.0000	0.0000
LOC100544972	0.0000	-1.4784	0.0000	-1.2585	0.0001	-0.8122	0.0043	-0.3887	1.0000	-0.2585	1.0000	-0.0273	0.6831	0.1702
LOC100544973	0.8226	3.0199	1.0000	-2.3959	1.0000	0.0000	1.0000	-2.3176	1.0000	2.2674	1.0000	-3.1429	1.0000	0.0000
LOC100544984	1.0000	0.0000	1.0000	0.0000	1.0000	0.0000	1.0000	0.0000	1.0000	0.0000	1.0000	0.0000	1.0000	0.0000
LOC100544999	0.3765	1.2898	0.7726	0.7717	1.0000	0.1152	1.0000	-0.5247	1.0000	-0.5096	0.7488	-1.0240	0.9835	-1.1482
LOC100545010	1.0000	-2.4776	1.0000	-2.3986	1.0000	-0.1591	1.0000	0.0045	1.0000	-0.1034	1.0000	0.0000	1.0000	0.0641
LOC100545013	0.1425	1.9714	0.6499	1.5975	1.0000	0.1996	1.0000	-2.3201	1.0000	-1.4912	0.2189	-1.8744	0.5093	-4.0498
LOC100545035	0.1124	0.6131	0.0024	0.7090	0.0024	-1.2310	0.0000	-1.4463	1.0000	0.2894	0.3543	0.3945	1.0000	0.0792
LOC100545042	0.0054	1.9573	0.0007	2.2353	0.0002	2.2106	0.0000	2.5835	1.0000	-0.6974	0.9421	-0.4103	0.9407	-0.3202
LOC100545050	0.0088	3.3775	1.0000	-0.1753	0.2151	2.3502	1.0000	-0.4591	1.0000	2.4237	0.5830	-1.1039	1.0000	-0.3849
LOC100545053	0.0360	0.4275	0.0331	0.2692	0.7080	-0.1516	1.0000	-0.0258	1.0000	-0.0880	0.1594	-0.2329	1.0000	0.0425
LOC100545069	0.0000	1.4726	0.0000	1.4392	0.0000	2.2869	0.0000	1.9016	1.0000	0.2576	0.4969	0.2384	0.7420	-0.1213
LOC100545074	0.0523	-0.3275	0.0000	-0.3989	0.4199	-0.1701	0.1011	-0.1581	1.0000	-0.0062	1.0000	-0.0652	1.0000	0.0107

LOC100545080	0.0000	0.7519	0.0001	-0.5053	0.0000	-2.0273	0.0000	-1.3702	1.0000	0.1584	0.0000	-1.0859	0.0004	0.8236
LOC100545091	0.2537	4.2501	1.0000	-0.6953	0.1621	4.3010	1.0000	0.3938	1.0000	3.6423	1.0000	-1.1756	1.0000	-0.2280
LOC100545094	1.0000	0.0000	1.0000	0.0000	1.0000	0.0000	1.0000	0.0000	1.0000	0.0000	1.0000	0.0000	1.0000	0.0000
LOC100545095	0.0000	1.4282	0.0000	1.4715	0.0283	0.6319	0.0132	0.4809	1.0000	0.2282	0.2349	0.2849	1.0000	0.0827
LOC100545101	0.0000	-0.8984	0.0000	-0.8795	0.0000	-0.8295	0.0001	-0.3713	1.0000	-0.1558	0.9072	-0.1234	0.0284	0.3076
LOC100545105	0.9398	0.7696	0.5843	0.9857	0.0247	1.9864	0.0204	2.2376	1.0000	-0.5112	1.0000	-0.2903	1.0000	-0.2560
LOC100545106	1.0000	-0.2892	0.4431	3.6191	0.9361	1.2061	1.0000	2.3242	1.0000	-2.3771	1.0000	1.4306	1.0000	-1.3089
LOC100545114	1.0000	0.0000	1.0000	0.0000	1.0000	0.0000	1.0000	0.0000	1.0000	0.0000	1.0000	0.0000	1.0000	0.0000
LOC100545120	0.8226	-3.3425	0.7218	-1.3890	1.0000	0.7317	1.0000	-0.2977	1.0000	1.1014	1.0000	3.2002	1.0000	0.0775
LOC100545121	0.8536	-0.1157	0.8812	0.0664	0.0051	0.5719	0.0000	0.5855	1.0000	-0.0562	0.8856	0.1380	1.0000	-0.0357
LOC100545122	1.0000	0.2132	1.0000	0.1346	0.2836	-4.1861	0.6142	-1.7590	1.0000	-0.1253	1.0000	-0.1926	1.0000	2.3554
LOC100545125	0.0228	0.9803	1.0000	-0.1002	0.6218	-0.4070	0.0010	-1.3440	1.0000	0.4080	0.2355	-0.6580	0.7792	-0.5215
LOC100545133	0.1124	-1.2500	0.3988	-0.6016	0.0002	1.6907	0.0025	1.1140	1.0000	0.0089	0.8875	0.6712	0.2557	-0.5617
LOC100545134	0.0569	-0.3146	0.0008	-0.3262	0.0000	-1.0189	0.0000	-0.8648	1.0000	-0.0617	1.0000	-0.0611	0.7180	0.0973
LOC100545141	0.1512	0.2630	0.0001	0.3415	0.0000	-0.7717	0.0000	-0.6941	1.0000	-0.1309	1.0000	-0.0402	1.0000	-0.0476
LOC100545142	1.0000	0.0000	1.0000	0.0000	1.0000	0.0000	1.0000	0.0000	1.0000	0.0000	1.0000	0.0000	1.0000	0.0000
LOC100545149	0.2891	1.3971	0.0440	2.1525	1.0000	-0.5753	0.4547	-3.7024	1.0000	-0.5040	1.0000	0.2534	0.7287	-3.6634
LOC100545163	0.7765	0.3146	0.7893	-0.4117	0.1002	-1.0955	0.0121	-1.9405	1.0000	-0.7096	0.0194	-1.4252	0.1950	-1.5513
LOC100545164	0.0049	0.8388	0.0000	1.4526	0.0000	-1.6409	0.0000	-1.6219	1.0000	0.1021	0.0000	0.7278	1.0000	0.1241
LOC100545183	0.0501	1.4711	0.0000	3.4204	1.0000	-0.3804	1.0000	0.7001	1.0000	-1.4703	0.7984	0.4847	1.0000	-0.3874
LOC100545184	1.0000	0.0000	1.0000	0.0000	1.0000	0.0000	1.0000	0.0000	1.0000	0.0000	1.0000	0.0000	1.0000	0.0000
LOC100545191	0.8249	3.0142	1.0000	0.0000	0.8033	3.0790	1.0000	2.3242	1.0000	0.0000	1.0000	-3.1383	1.0000	-0.7782
LOC100545203	0.1188	1.5939	0.0249	1.6229	0.0000	3.0027	0.0000	3.8525	1.0000	0.3435	1.0000	0.3807	0.0002	1.1924
LOC100545207	0.5807	0.6968	0.5798	0.4905	0.7965	0.4998	0.9960	-0.3082	1.0000	0.3090	1.0000	0.1192	0.9947	-0.4944
LOC100545208	0.0161	-0.4435	0.0008	-0.3825	0.0189	0.4330	0.0000	0.4402	1.0000	0.0925	0.3764	0.1666	0.6595	0.1046
LOC100545215	0.1639	1.3784	0.0007	1.8662	0.0001	2.4719	0.0031	1.7495	1.0000	0.3721	0.3505	0.8687	0.8877	-0.3484
LOC100545217	1.0000	0.0021	0.2833	-0.1286	0.0001	-0.8276	0.0000	-0.7490	1.0000	-0.1762	0.0054	-0.2952	0.8164	-0.0918
LOC100545218	0.3280	-0.2627	1.0000	0.0035	0.0086	0.5180	0.0000	0.5143	1.0000	-0.0446	0.4333	0.2351	1.0000	-0.0426
LOC100545219	1.0000	-0.0301	0.2660	0.1510	0.0009	-0.6407	0.0027	-0.3482	1.0000	-0.2048	1.0000	-0.0121	0.9885	0.0910
LOC100545225	0.5693	0.2639	0.2137	-0.2455	0.0000	-2.2497	0.0000	-2.4656	1.0000	0.2517	0.6615	-0.2438	1.0000	0.0455
LOC100545228	1.0000	0.0000	1.0000	-2.3986	1.0000	0.0000	1.0000	0.0057	1.0000	2.2736	1.0000	0.0000	1.0000	2.3555
LOC100545232	1.0000	0.0000	1.0000	0.0000	1.0000	0.0000	1.0000	0.0000	1.0000	0.0000	1.0000	0.0000	1.0000	0.0000
LOC100545236	1.0000	0.0000	1.0000	0.0000	1.0000	0.0000	1.0000	0.0000	1.0000	0.0000	1.0000	0.0000	1.0000	0.0000
LOC100545241	0.0686	0.5697	0.0006	0.5387	0.0000	1.3991	0.0000	1.2515	1.0000	-0.0476	1.0000	-0.0647	0.3290	-0.1887
LOC100545242	1.0000	0.0000	1.0000	0.0000	1.0000	0.0000	1.0000	0.0000	1.0000	0.0000	1.0000	0.0000	1.0000	0.0000
LOC100545251	1.0000	-2.4788	1.0000	0.0000	1.0000	-2.4061	1.0000	0.0000	1.0000	-2.3771	1.0000	0.0000	1.0000	0.0000
LOC100545254	1.0000	0.0000	1.0000	0.0000	1.0000	0.0000	1.0000	0.0000	1.0000	0.0000	1.0000	0.0000	1.0000	0.0000
LOC100545259	0.1103	-0.4499	0.0000	-0.6600	0.2123	-0.3594	0.4415	-0.1554	1.0000	0.0882	1.0000	-0.1087	0.2400	0.2975
LOC100545266	0.0748	1.2912	0.1409	0.8119	1.0000	0.1467	0.7587	-0.4539	1.0000	0.5642	1.0000	0.1012	1.0000	-0.0266
LOC100545269	1.0000	0.0000	1.0000	0.0000	1.0000	0.0000	1.0000	0.0000	1.0000	0.0000	1.0000	0.0000	1.0000	0.0000
LOC100545276	1.0000	0.0000	1.0000	0.0000	1.0000	0.0000	1.0000	0.0000	1.0000	0.0000	1.0000	0.0000	1.0000	0.0000
LOC100545280	1.0000	-2.4776	1.0000	-2.3959	1.0000	0.6823	1.0000	0.8545	1.0000	-0.1078	1.0000	0.0000	1.0000	0.0716
LOC100545285	1.0000	-0.1907	0.5412	0.5695	1.0000	0.0450	1.0000	-0.1044	1.0000	-0.4249	1.0000	0.3470	0.8965	-0.5703
LOC100545300	0.8226	-3.3425	1.0000	-0.1472	1.0000	-1.0191	1.0000	-2.3201	1.0000	-0.9587	1.0000	2.3456	1.0000	-2.2888
LOC100545311	0.0886	0.7502	0.0002	1.3317	0.4910	0.4134	0.0000	1.3749	1.0000	-0.6809	1.0000	-0.0881	0.7908	0.2849
LOC100545312	1.0000	0.0180	0.0228	0.1860	0.6171	-0.1232	0.3287	-0.0935	1.0000	-0.1229	1.0000	0.0576	0.7135	-0.0878
LOC100545313	0.5391	-3.8767	0.0754	-4.7338	0.9468	-1.5535	0.0709	-4.6403	1.0000	0.8198	1.0000	0.0000	1.0000	-2.2888
LOC100545318	0.0543	0.7500	0.0013	0.8742	0.0292	-1.0067	0.0546	-0.7264	1.0000	-0.2462	1.0000	-0.1118	1.0000	0.0400
LOC100545319	0.2471	-0.3205	0.2391	-0.2329	0.0023	0.6384	0.0000	0.6518	1.0000	-0.2491	0.8973	-0.1484	0.2959	-0.2299
LOC100545322	0.0000	1.4323	0.0000	0.7912	0.4424	-0.3364	0.0000	-1.7705	1.0000	-0.2824	0.0000	-0.9103	0.0000	-1.7079
LOC100545337	0.0701	-1.7917	0.4176	-1.5147	0.0818	-1.9367	1.0000	0.3204	1.0000	-0.9187	1.0000	-0.6335	0.6295	1.3473
LOC100545338	0.7972	0.3424	0.0005	1.1284	0.4143	0.5546	0.0007	1.0996	1.0000	0.0231	0.0107	0.8259	0.0998	0.5760
LOC100545341	0.0145	-2.2840	0.0000	-4.5045	1.0000	-0.1100	0.2423	0.6422	1.0000	0.5124	1.0000	-1.7117	0.0332	1.2663
LOC100545343	0.0021	0.6221	0.0000	0.9068	0.0000	1.4729	0.0000	1.3399	1.0000	-0.1485	0.7057	0.1493	0.0851	-0.2755
LOC100545344	0.0000	-4.9753	0.0000	-4.7162	0.0000	-2.9877	0.0000	-2.1688	1.0000	-0.3251	1.0000	-0.0539	0.0607	0.4993
LOC100545353	1.0000	0.0000	0.4635	-3.7803	1.0000	0.0000	0.9167	-1.3669	1.0000	3.6298	1.0000	0.0000	1.0000	2.3555
LOC100545360	0.0069	0.8962	0.0000	1.1446	0.0000	2.4016	0.0000	2.1662	1.0000	0.0154	0.6717	0.2778	0.5555	-0.2130
LOC100545362	1.0000	2.1849	0.7666	3.0922	1.0000	0.0000	1.0000	0.0000	1.0000	0.0000	1.0000	0.9006	1.0000	0.0000
LOC100545365	0.6881	1.7520	0.7674	3.0888	1.0000	0.6746	1.0000	0.0000	1.0000	-2.3758	1.0000	-1.1662	1.0000	-3.1317
LOC100545373	0.0225	0.7093	0.0039	0.6109	0.0000	1.3235	0.0000	1.3394	1.0000	0.1667	1.0000	0.0816	0.6106	0.1890
LOC100545386	0.0010	1.2604	0.0000	1.1731	0.0000	1.7666	0.0001	1.1474	1.0000	0.0224	1.0000	-0.0507	0.0366	-0.5898
LOC100545389	1.0000	2.1901	1.0000	2.2507	1.0000	0.0000	1.0000	0.0000	1.0000	0.0000	1.0000	0.0470	1.0000	0.0000
LOC100545392	1.0000	2.1849	1.0000	0.0000	1.0000	0.0000	1.0000	2.3257	1.0000	0.0000	1.0000	-2.2955	1.0000	2.3554
LOC100545397	0.0657	-0.7099	0.3504	-0.2663	0.0333	-0.7980	1.0000	-0.0150	1.0000	-0.3030	1.0000	0.1516	0.2186	0.4854
LOC100545399	1.0000	2.1849	1.0000	0.0000	1.0000	2.2427	1.0000	0.0000	1.0000	0.0000	1.0000	-2.2955	1.0000	-2.2888
LOC100545410	0.0272	1.0011	0.0000	1.3118	0.0000	2.3547	0.0000	2.2515	1.0000	-0.0040	0.7487	0.3218	1.0000	-0.0995
LOC100545415	0.2786	0.2407	0.0073	0.3126	0.2182	-0.2681	0.0034	-0.3651	1.0000	0.0139	0.9303	0.0988	1.0000	-0.0771
LOC100545419	0.2081	1.8502	0.7332	1.0605	1.0000	-1.0156	0.7710	-3.1632	1.0000	-0.1218	0.9277	-0.9051	1.0000	-2.2906
LOC100545420	0.3089	3.9281	0.2913	1.7214	0.8033	3.0790	0.7710	-3.1632	1.0000	3.1056	1.0000	1.0212	1.0000	-3.1317
LOC100545421	0.0006	1.0703	0.0000	0.9803	0.0000	1.8345	0.0000	1.3965	1.0000	0.3169	0.8079	0.2403	0.9001	-0.1156
LOC100545422	0.0510	-0.3662	0.0000	-0.4477	1.0000	-0.0607	0.1569	-0.1495	1.0000	-0.0202	0.9500	-0.0886	0.7287	-0.1035
LOC100545423	0.1719	0.6535	0.0044	0.9157	0.2913	0.5609	0.8375	0.2309	1.0000	0.0484	0.7934	0.3242	0.9802	-0.2766
LOC100545427	0.8249	3.0256	1.0000	0.6956	1.0000	0.0000	1.0000	0.8545	1.0000	2.2674	1.0000	0.0532	1.0000	3.2066
LOC100545429	1.0000	0.0000	1.0000	0.0000	1.0000	2.2472	1.0000	2.3242	1.0000	0.0000	1.0000	0.0000	1.0000	0.0641
LOC100545430	1.0000	0.0000	1.0000	-2.3959	1.0000	0.0000	1.0000	-2.3176	1.0000	2.2675	1.0000	0.0000	1.0000	0.0000
LOC100545434	0.0000	-0.7883	0.0000	-0.8216	0.0000	-0.7262	0.0000	-0.7726	1.0000	0.0151	1.0000	-0.0058	1.0000	-0.0258
LOC100545437	0.0014	-1.8259	0.0115	-1.1222	1.0000	-0.0447	1.0000	-0.0814	1.0000	0.1801	0.5244	0.8973	1.0000	0.1428
LOC100545443	0.0751	-0.8005	0.0862	-0.7328	0.0047	-1.2134	0.0065	-1.0253	1.0000	-0.0727	1.0000	0.0099	1.0000	0.1153
LOC100545452	0.6927	0.2857	0.0017	0.8724	0.0002	1.1330	0.0004	0.9400	1.0000	-0.2225	0.5071	0.3785	0.2184	-0.4077
LOC100545453	1.0000	0.1125	0.0081	1.7855	0.0000	2.8578	0.0000	3.1522	1.0000	-0.6263	0.3157	1.0530	0.7194	-0.3284
LOC100545461	1.0000	-0.3598	0.5661	-0.8896	0.2188	-1.5995	0.9149	-0.4923	1.0000	-0.2642	0.9744	-0.7868	1.0000	0.8486
LOC100545463	1.0000	-0.6388	1.0000	-0.4776	0.8011	-1.4055	0.7190	-1.2157	1.0000	-0.1212	1.0000	0.0557	1.0000	0.0723
LOC100545464	0.0803	1.3610	0.0047	1.6419	0.0009	1.8737	0.0000	2.2119	1.0000	-0.2621	1.0000	0.0255	1.0000	0.0804
LOC100545469	1.0000	2.1849	0.6180	-1.9239	1.0000	0.0000	0.2426	-4.0840	1.0000	4.0267	1.0000	0.0495	1.0000	0.0000
LOC100545470	0.0001	1.4733	0.0000	1.5083	0.0000	1.9115	0.0000	1.8481	1.0000	0.0401	1.0000	0.0869	1.0000	-0.0177
LOC100545471	1.0000	0.0000	0.7666	-3.2534	1.0000	2.2427	1.0000	-0.8419	1.0000	3.1126	1.0000	0.0000	1.0000	0.0652
LOC100545477	1.0000	0.0409	0.0266	0.2044	0.0000	-0.6677	0.0000	-0.7581	1.0000	-0.1099	1.0000	0.0659	0.0668	-0.1946
LOC100545478	0.6881	1.7514	1.0000	-0.9997	1.0000	-2.4061	0.7710	-3.1633	1.0000	0.7304	0.8269	-2.0165	1.0000	0.0000
LOC100545481	0.7849	1.1386	1.0000	0.3671	1.0000	-1.0191	0.9786	0.9248	1.0000	-0.1210	1.0000	-0.8858	0.8937	1.8364
LOC100545485	1.0000	2.1849	1.0000	0.0000	0.2829	3.9942	1.0000	0.0000	1.0000	0.0000	1.0000	-2.2955	0.5105	-4.0491
LOC100545500	1.0000	0.5414	0.9062	-1.5359	1.0000	-2.4061	0.9096	-1.3722	1.0000	1.2654	1.0000	-0.7966	1.0000	2.3554
LOC100545512	0.5095	0.4139	0.6391	0.2947	0.2998	-0.6305	0.1664	-0.6034	1.0000	-0.0416	1.0000	-0.1491	1.0000	-0.0069
LOC100545524	0.0019	1.1199	0.0000	1.7570	0.0000	1.6380	0.0000	2.0442	1.0000	-0.4608	0.9750	0.1885	1.0000	-0.0489

LOC100545545	0.2463	0.5300	0.0813	0.5587	0.4045	-0.4396	1.0000	0.0557	1.0000	-0.1557	1.0000	-0.1170	0.7170	0.3430
LOC100545547	0.0088	0.9470	0.0000	1.3265	0.0000	1.1164	0.0000	1.4185	1.0000	-0.3997	1.0000	-0.0045	1.0000	-0.0908
LOC100545558	1.0000	0.0000	1.0000	0.0000	1.0000	0.0000	1.0000	0.0000	1.0000	0.0000	1.0000	0.0000	1.0000	0.0000
LOC100545577	1.0000	0.0127	0.3266	-0.1284	1.0000	-0.0084	0.2094	-0.1506	1.0000	-0.0340	0.4548	-0.1631	0.3574	-0.1721
LOC100545581	0.0011	0.7964	0.0000	0.6615	0.0000	1.6377	0.0000	1.2014	1.0000	0.2114	1.0000	0.0888	0.4643	-0.2214
LOC100545583	0.8249	3.0142	1.0000	0.0000	1.0000	0.0000	1.0000	0.0000	1.0000	0.0000	1.0000	-3.1383	1.0000	0.0000
LOC100545586	1.0000	0.2160	0.4777	1.0963	0.8692	0.5953	1.0000	0.0059	1.0000	-1.0820	1.0000	-0.1967	0.2268	-1.6689
LOC100545589	1.0000	0.0000	1.0000	0.0000	1.0000	0.0000	1.0000	0.0000	1.0000	0.0000	1.0000	0.0000	1.0000	0.0000
LOC100545599	0.2948	-1.4677	0.0562	-3.0491	0.6213	-1.0052	0.1343	1.1375	1.0000	-0.1265	1.0000	-1.7124	0.0547	2.0256
LOC100545600	1.0000	2.1849	1.0000	0.0000	1.0000	0.0000	1.0000	0.0000	1.0000	0.0000	1.0000	-2.2955	1.0000	0.0000
LOC100545607	1.0000	0.0000	1.0000	0.0000	1.0000	0.0000	1.0000	0.0000	1.0000	0.0000	1.0000	0.0000	1.0000	0.0000
LOC100545609	1.0000	0.0000	1.0000	-0.1452	1.0000	2.2427	1.0000	0.0045	1.0000	2.2736	1.0000	2.3480	1.0000	0.0652
LOC100545615	0.2205	-0.4327	1.0000	-0.0108	0.3319	0.3146	0.0019	0.5696	1.0000	-0.3246	1.0000	0.1092	1.0000	-0.0643
LOC100545617	0.7333	-0.5472	0.7129	-0.4942	0.9346	-0.3877	0.6837	-0.5657	1.0000	-0.2237	1.0000	-0.1582	1.0000	-0.3956
LOC100545624	0.0000	2.4366	0.0250	1.0174	0.0000	2.7286	0.0000	1.5835	1.0000	0.7833	0.3147	-0.6171	0.6215	-0.3481
LOC100545629	0.2382	0.3182	0.4577	0.1873	0.0000	-1.1184	0.0000	-0.7357	1.0000	0.1327	1.0000	0.0131	0.0402	0.5185
LOC100545648	1.0000	0.1088	1.0000	0.2998	0.9475	0.3414	0.3660	0.8405	1.0000	-0.5063	1.0000	-0.2973	1.0000	0.0030
LOC100545656	0.8861	0.4134	0.4310	0.6823	1.0000	0.1632	0.4921	0.6589	1.0000	-0.3468	1.0000	-0.0652	1.0000	0.1553
LOC100545658	0.0308	0.9959	0.0000	1.3336	0.0000	1.7859	0.0000	1.9746	1.0000	0.0402	0.6367	0.3903	0.6096	0.2373
LOC100545666	0.9650	0.2152	1.0000	-0.1168	0.3562	0.5314	1.0000	0.1562	1.0000	0.3897	1.0000	0.0664	1.0000	0.0205
LOC100545668	0.3435	0.2160	0.5341	0.0682	0.0010	-0.5747	0.0000	-0.6378	1.0000	-0.0671	0.1405	-0.2026	0.5482	-0.1247
LOC100545675	0.9925	-0.0705	0.6702	-0.0824	0.0236	-0.4800	0.0001	-0.4450	1.0000	-0.0897	0.9420	-0.0898	1.0000	-0.0511
LOC100545676	1.0000	-0.1602	0.7453	0.5206	0.9331	-0.9590	0.5688	-0.8396	1.0000	0.4999	0.5253	1.2048	1.0000	0.6337
LOC100545683	1.0000	2.1849	1.0000	0.0000	1.0000	0.0000	1.0000	0.0000	1.0000	0.0000	1.0000	-2.2955	1.0000	0.0000
LOC100545689	0.0000	1.1225	0.0000	0.8579	0.0000	1.2569	0.0000	0.6783	1.0000	0.2825	1.0000	0.0312	0.1252	-0.2899
LOC100545705	0.0033	0.8484	0.0073	0.6601	0.0592	0.5874	0.1337	0.4032	1.0000	0.0570	1.0000	-0.1168	1.0000	-0.1207
LOC100545707	0.1471	-0.4039	0.6424	-0.1613	1.0000	0.0201	0.0000	0.6437	1.0000	-0.2270	1.0000	0.0277	0.0805	0.4026
LOC100545720	0.8249	3.0142	1.0000	0.0000	1.0000	0.0000	1.0000	0.0000	1.0000	0.0000	1.0000	-3.1383	1.0000	0.0000
LOC100545726	0.0097	0.8747	0.0018	0.6538	0.0000	1.3179	0.0000	0.9995	1.0000	0.1908	1.0000	-0.0151	0.9814	-0.1209
LOC100545729	1.0000	-0.3462	0.4431	-3.7865	0.7628	-1.4076	1.0000	-0.5240	1.0000	-0.8169	0.4563	-4.3630	1.0000	0.0718
LOC100545731	1.0000	-1.2491	0.2920	-4.1891	1.0000	-0.5733	1.0000	-0.3848	1.0000	-0.1148	1.0000	-3.1429	1.0000	0.0749
LOC100545735	1.0000	0.0548	0.0000	0.3817	0.0050	-0.5442	0.3937	-0.1029	1.0000	-0.1896	0.3986	0.1494	0.0700	0.2570
LOC100545741	0.0000	-0.9991	0.0000	-1.2576	0.0014	-0.4781	0.0000	-0.6038	1.0000	-0.0359	0.0224	-0.2819	0.2426	-0.1562
LOC100545742	1.0000	-0.2881	1.0000	0.0000	1.0000	-2.4056	1.0000	0.0000	1.0000	-2.3757	1.0000	-2.2992	1.0000	0.0000
LOC100545745	0.0019	-0.4694	0.0000	-0.5238	0.0000	-0.9281	0.0000	-0.9475	1.0000	0.0207	1.0000	-0.0215	1.0000	0.0072
LOC100545752	0.4195	-1.8096	1.0000	-1.0017	0.2857	-2.5000	0.7190	1.2294	1.0000	-1.5965	1.0000	-0.7966	0.6865	2.1419
LOC100545757	1.0000	0.0091	0.9503	0.0265	0.0000	-1.3764	0.0000	-0.2785	1.0000	-0.0670	1.0000	-0.0372	0.0000	1.0359
LOC100545760	0.1738	0.6310	0.5486	0.2732	0.9876	-0.1751	1.0000	0.1137	1.0000	-0.0623	0.6424	-0.4084	0.9356	0.2318
LOC100545769	0.8249	3.0142	0.7666	3.0922	0.2829	3.9942	0.1317	4.3958	1.0000	0.0000	1.0000	0.0563	1.0000	0.3821
LOC100545776	0.1396	-1.7532	0.1363	-1.2512	0.4972	-0.8303	0.0003	-5.9292	1.0000	0.3126	1.0000	0.8294	0.1303	-4.8192
LOC100545780	1.0000	0.0000	1.0000	2.2507	1.0000	2.2472	1.0000	0.0000	1.0000	0.0000	1.0000	2.3456	1.0000	-2.2906
LOC100545797	0.4555	-0.2134	0.0197	-0.3039	0.0000	-1.2497	0.0000	-1.3912	1.0000	0.0534	1.0000	-0.0245	1.0000	-0.0835
LOC100545800	1.0000	-0.0205	0.6342	-0.0916	0.5047	-0.1848	0.1249	0.2117	1.0000	0.0848	1.0000	0.0271	0.0012	0.4863
LOC100545806	0.3573	3.9147	0.7674	3.0957	1.0000	2.2472	1.0000	2.3242	1.0000	0.0000	1.0000	-0.8494	1.0000	0.0641
LOC100545814	0.0274	-0.4978	0.0001	-0.4514	0.8901	-0.0975	0.6424	-0.0870	1.0000	0.0061	1.0000	0.0664	1.0000	0.0231
LOC100545816	1.0000	0.0333	0.5207	-0.3887	0.0046	1.2137	0.0130	0.8313	1.0000	0.1889	1.0000	-0.2148	1.0000	-0.1857
LOC100545817	0.0081	0.4721	0.0000	0.6495	0.0005	0.6114	0.0000	0.9068	1.0000	-0.1489	1.0000	0.0409	0.4622	0.1521
LOC100545820	0.0960	0.5697	0.1758	0.3362	0.0000	1.6242	0.0000	1.1025	1.0000	0.3141	1.0000	0.0946	0.6174	-0.2013
LOC100545821	1.0000	0.0705	1.0000	-0.0160	0.0274	-0.4793	0.0010	-0.3826	1.0000	-0.0034	1.0000	-0.0774	0.9460	0.1001
LOC100545842	0.0078	2.3849	0.0130	1.5784	0.3227	-4.1836	0.6874	-0.8087	1.0000	0.9930	1.0000	0.2057	0.3459	4.4328
LOC100545856	0.0117	2.8964	0.0355	1.8985	0.0001	3.5063	0.0648	1.6369	1.0000	1.1071	1.0000	0.1257	0.5649	-0.7660
LOC100545863	1.0000	0.0000	1.0000	-2.3959	1.0000	0.0000	1.0000	-2.3176	1.0000	2.2675	1.0000	0.0000	1.0000	0.0000
LOC100545870	1.0000	0.5381	1.0000	-1.0017	1.0000	-0.1624	0.7701	-3.1666	1.0000	0.7365	1.0000	-0.7943	1.0000	-2.2888
LOC100545877	0.0365	2.5226	0.8221	0.5980	0.0000	4.1678	0.0030	2.3291	1.0000	0.7956	0.6421	-1.1104	0.0942	-1.0401
LOC100545878	0.9248	0.4667	0.6504	0.5023	0.1215	-2.2251	0.6347	-0.6760	1.0000	0.3899	1.0000	0.4424	0.4959	1.9504
LOC100545885	1.0000	0.0000	1.0000	2.2507	1.0000	0.0000	1.0000	0.0000	1.0000	0.0000	1.0000	2.3456	1.0000	0.0000
LOC100545886	0.0018	0.9505	0.0000	1.0326	0.6974	-0.2307	0.0012	-0.9190	1.0000	-0.1301	1.0000	-0.0382	0.0309	-0.8151
LOC100545893	0.7306	0.3395	0.3550	-0.4273	0.9860	-0.2148	0.6353	-0.2693	1.0000	0.0298	0.1645	-0.7255	1.0000	-0.0126
LOC100545895	0.0004	-0.9484	0.0000	-1.0555	0.0000	-1.3827	0.0000	-1.6184	1.0000	0.2323	1.0000	0.1382	1.0000	-0.0003
LOC100545905	1.0000	0.0517	0.6054	0.2874	1.0000	0.1070	1.0000	0.0880	1.0000	-0.1842	1.0000	0.0626	1.0000	-0.2006
LOC100545914	0.0685	1.2463	0.4153	0.6996	0.3449	0.8441	0.7657	0.4694	1.0000	-0.0208	0.8501	-0.5564	0.9838	-0.3883
LOC100545924	0.0526	0.3103	0.0000	0.3908	0.0100	0.4098	0.0095	0.1983	1.0000	0.1365	0.0200	0.2295	0.9086	-0.0700
LOC100545925	0.0186	0.9395	0.0414	0.5383	0.0521	-1.0599	0.0012	-0.9873	1.0000	0.6500	0.7902	0.2632	0.3004	0.7287
LOC100545931	0.0389	5.0454	0.6215	1.6060	0.2781	3.9963	0.6142	1.7719	1.0000	2.2736	0.8840	-1.0640	1.0000	0.0765
LOC100545932	0.0042	-1.6999	0.0000	-2.4970	0.0000	1.7031	0.0000	1.5346	1.0000	-0.1367	0.6497	-0.9203	0.5394	-0.2974
LOC100545943	0.0959	1.0786	0.0052	1.2978	0.0027	1.5794	0.0000	2.1190	1.0000	-0.3728	1.0000	-0.1452	1.0000	0.1666
LOC100545944	0.9776	0.1504	0.2660	-0.4270	0.3939	0.3696	1.0000	-0.0508	1.0000	0.2450	0.8194	-0.3190	1.0000	-0.1677
LOC100545946	0.0901	-0.3268	0.0001	-0.3852	1.0000	0.0352	0.2592	-0.1454	1.0000	0.0355	1.0000	-0.0107	0.5935	-0.1402
LOC100545947	0.0000	1.9916	0.0000	2.4104	0.0000	3.2617	0.0000	2.6952	1.0000	0.0660	0.3897	0.4981	0.0003	-0.4955
LOC100545950	0.0026	0.4428	0.0001	0.4350	0.0000	1.5412	0.0000	1.5084	1.0000	0.1003	0.6178	0.1047	0.8844	0.0726
LOC100545957	0.1144	0.3244	0.1421	0.1785	1.0000	0.0251	0.9429	-0.0432	1.0000	0.1171	1.0000	-0.0166	1.0000	0.0544
LOC100545963	0.1245	0.5627	0.0003	0.6728	0.0000	1.8291	0.0000	1.4514	1.0000	0.1965	0.4916	0.3177	0.6203	-0.1766
LOC100545973	0.5086	0.2212	0.6777	-0.0863	0.0430	-0.5192	0.0000	-0.8693	1.0000	-0.0317	0.0421	-0.3268	0.0548	-0.3739
LOC100545977	0.7410	-0.1471	0.2943	-0.1405	0.0000	0.8521	0.0000	0.6928	1.0000	0.1520	0.5128	0.1712	1.0000	-0.0021
LOC100545985	0.0010	0.9001	0.0000	0.7698	0.0000	2.1164	0.0000	1.8706	1.0000	0.1247	1.0000	0.0075	0.8530	-0.1153
LOC100546006	0.0000	1.5330	0.0000	1.1289	0.1818	0.5291	1.0000	-0.0259	1.0000	0.0239	0.2110	-0.3678	0.1998	-0.5277
LOC100546012	0.0002	-1.4273	0.0009	-0.7456	0.8448	0.1821	0.0000	1.0541	1.0000	-0.0301	0.1817	0.6615	0.0001	0.8502
LOC100546025	1.0000	0.0177	0.1103	0.6246	0.0212	0.9195	0.0235	0.8127	1.0000	-0.1000	0.5412	0.5193	0.9835	-0.1980
LOC100546034	0.0000	1.6035	0.0000	2.2398	0.0000	2.2099	0.0000	1.7666	1.0000	-0.1023	0.0338	0.5464	0.0007	-0.5414
LOC100546043	0.8249	3.0142	1.0000	0.0000	1.0000	0.0000	1.0000	0.0000	1.0000	0.0000	1.0000	-3.1382	1.0000	0.0000
LOC100546061	1.0000	-0.3341	1.0000	0.6987	1.0000	-0.7107	1.0000	0.8538	1.0000	-1.4945	1.0000	-0.4744	1.0000	0.0695
LOC100546064	1.0000	2.1849	1.0000	0.0000	1.0000	0.0000	1.0000	0.0000	1.0000	0.0000	1.0000	-2.2955	1.0000	0.0000
LOC100546066	1.0000	0.4209	1.0000	-0.4260	1.0000	-1.1048	0.7732	-0.9367	1.0000	0.4321	1.0000	-0.4059	1.0000	0.6050
LOC100546071	0.0000	-2.6109	0.0000	-4.7355	0.8033	0.1975	0.2638	0.4276	1.0000	-0.7602	0.0326	-2.8837	0.2091	-0.5231
LOC100546086	0.6017	-0.2243	0.1910	-0.1357	0.0862	-0.4915	0.0000	-0.5713	1.0000	0.6427	0.0000	0.7449	0.0000	0.5672
LOC100546087	0.0000	-2.9870	0.0000	-3.2807	0.0023	-0.4937	0.0000	-0.4884	1.0000	0.0866	0.8377	-0.1964	0.7492	0.0966
LOC100546100	1.0000	-0.0596	0.1714	-0.1553	0.0224	-0.4342	0.0000	-0.4477	1.0000	0.0142	1.0000	-0.0689	1.0000	0.0071
LOC100546110	0.0005	1.0217	0.0000	0.9941	0.0024	-1.0769	0.0201	-0.5760	1.0000	0.3212	0.4233	0.3066	0.0337	0.8271
LOC100546113	0.0993	2.0847	0.0071	3.3478	0.5081	1.0966	0.0515	2.8939	1.0000	-1.8788	0.9819	-0.6339	1.0000	-0.0769
LOC100546114	0.0000	-1.0155	0.0000	-1.0358	0.0000	-0.8986	0.0000	-0.6737	1.0000	-0.0298	1.0000	-0.0376	0.0894	0.2003
LOC100546118	1.0000	0.0000	1.0000	0.0000	1.0000	0.0000	1.0000	0.0000	1.0000	0.0000	1.0000	0.0000	1.0000	0.0000
LOC100546123	0.0000	-1.7328	0.0000	-2.1236	0.0065	-0.9897	0.0003	-1.0356	1.0000	-0.2933	0.5758	-0.6718	0.8066	-0.3325
LOC100546124	0.0042	0.8493	0.0000	0.7451	0.3432	0.3529	1.0000	-0.0714	1.0000	0.1510	1.0000	0.0590	0.5565	-0.2680

LOC100546134	0.0022	1.3691	0.0000	1.7342	0.0000	2.3102	0.0000	2.3829	1.0000	-0.2452	1.0000	0.1330	0.9001	-0.1681
LOC100546136	1.0000	0.0000	1.0000	0.0000	1.0000	0.0000	1.0000	0.0000	1.0000	0.0000	1.0000	0.0000	1.0000	0.0000
LOC100546141	0.0000	-1.2261	0.0000	-1.2110	0.7006	-0.1177	0.5542	0.0835	1.0000	0.0664	1.0000	0.0938	0.0200	0.2723
LOC100546146	0.0431	-0.6206	0.6000	-0.1906	1.0000	-0.0196	0.0169	0.4738	1.0000	-0.3391	1.0000	0.1016	0.8429	0.1577
LOC100546153	0.2320	1.0772	1.0000	0.1362	0.5295	-1.1494	0.0368	-1.9302	1.0000	0.8536	1.0000	-0.0759	1.0000	0.0765
LOC100546173	0.0000	-1.6868	0.0000	-1.8752	0.0000	-0.8278	0.0000	-0.9085	1.0000	0.0724	0.9520	-0.1031	1.0000	-0.0028
LOC100546176	0.0002	-1.8746	0.1907	-0.7034	0.0458	0.7404	0.0003	1.1539	1.0000	-0.6588	1.0000	0.5243	0.8333	-0.2402
LOC100546177	1.0000	0.0000	1.0000	0.0000	1.0000	0.0000	1.0000	0.0000	1.0000	0.0000	1.0000	0.0000	1.0000	0.0000
LOC100546179	1.0000	-0.3354	1.0000	-0.1472	0.5041	-3.7956	1.0000	0.8541	1.0000	-1.4875	1.0000	-1.3254	1.0000	3.2066
LOC100546181	0.4566	0.2321	0.0872	0.3597	0.0000	1.2326	0.0000	1.0320	1.0000	0.0355	0.7299	0.1774	0.5822	-0.1590
LOC100546187	0.2944	1.3593	0.1546	2.3755	1.0000	-0.1921	0.9096	1.3849	1.0000	-2.1835	0.5029	-1.1880	1.0000	-0.6154
LOC100546195	0.4166	-0.1965	0.4279	-0.1401	0.5259	0.1716	0.0340	0.2705	1.0000	-0.1031	1.0000	-0.0342	1.0000	0.0029
LOC100546203	0.0852	-0.4630	0.0298	-0.3754	0.0315	-0.5445	0.0001	-0.6415	1.0000	-0.0225	1.0000	0.0759	0.9980	-0.1148
LOC100546217	0.0387	-5.0448	0.0433	-4.9468	0.0216	1.7666	0.0000	3.3039	1.0000	-0.1308	1.0000	0.0000	0.0005	1.4145
LOC100546220	0.8026	-0.7036	0.2061	-1.6829	1.0000	0.2064	0.9162	0.5008	1.0000	-0.1262	0.9741	-1.0994	1.0000	0.1828
LOC100546225	0.0949	2.1046	0.4769	-1.5108	1.0000	0.7355	0.2510	-1.8613	1.0000	1.7404	0.3524	-1.8561	1.0000	-0.8436
LOC100546239	1.0000	0.3443	0.7725	0.7725	0.9468	-1.5556	0.4370	-3.6968	1.0000	-0.1257	1.0000	0.3111	1.0000	-2.2888
LOC100546242	1.0000	0.0000	1.0000	0.0000	1.0000	0.0000	1.0000	2.3257	1.0000	0.0000	1.0000	0.0000	1.0000	2.3554
LOC100546243	1.0000	-0.0839	0.8870	0.1046	0.0069	-0.8413	0.0041	-0.6289	1.0000	-0.0140	0.9589	0.1871	0.8939	0.2025
LOC100546254	0.1449	-0.3031	0.9169	-0.0416	0.0001	-0.7376	0.0000	-0.7107	1.0000	-0.1815	0.9282	0.0920	0.6972	-0.1483
LOC100546258	1.0000	-0.0121	0.5740	-0.1270	0.3524	0.2549	0.8520	0.0777	1.0000	0.0702	1.0000	-0.0322	0.9810	-0.1020
LOC100546272	0.1630	-0.3087	0.0000	-0.4782	1.0000	0.0322	1.0000	0.0194	1.0000	0.0824	1.0000	-0.0747	0.9368	0.0739
LOC100546305	0.1664	0.3701	0.4217	0.1681	0.1282	-0.4187	1.0000	-0.0176	1.0000	0.1198	1.0000	-0.0696	0.0223	0.5257
LOC100546312	0.0538	1.8113	0.1466	1.0005	0.0003	2.4518	0.0007	1.6046	1.0000	1.3328	0.9773	0.5369	0.6516	0.4926
LOC100546313	0.3642	-4.2658	0.1392	-4.4820	1.0000	-0.5613	0.3952	-2.0640	1.0000	0.1832	1.0000	0.0000	1.0000	-1.3110
LOC100546322	0.3010	-1.4705	0.9244	-0.6632	0.6213	-1.0052	0.2795	1.0790	1.0000	-0.4683	1.0000	0.3602	0.2747	1.6169
LOC100546323	0.2647	-0.2289	0.0419	-0.1806	0.1803	0.2598	0.0000	0.5093	1.0000	-0.1102	1.0000	-0.0491	0.3885	0.1449
LOC100546324	0.0259	1.5977	0.0289	1.0847	0.0000	2.8836	0.0000	2.1387	1.0000	0.4121	1.0000	-0.0876	0.8475	-0.3312
LOC100546326	1.0000	-0.0526	0.9645	0.0711	0.0018	-0.8683	0.0027	-0.6064	1.0000	-0.3000	0.9413	-0.1625	1.0000	-0.0306
LOC100546330	0.6235	1.3434	0.3415	1.5198	1.0000	0.3475	0.9786	0.9243	1.0000	-0.1170	1.0000	0.0620	1.0000	0.4629
LOC100546335	0.5086	-3.8796	0.0768	-4.7329	0.4809	-3.7970	0.4962	-1.4667	1.0000	0.8165	1.0000	0.0000	1.0000	3.2066
LOC100546341	0.3372	0.2545	0.0023	0.4090	0.0583	0.4052	0.0001	0.4875	1.0000	-0.1646	1.0000	0.0030	1.0000	-0.0758
LOC100546345	1.0000	2.1849	1.0000	0.0000	1.0000	0.0000	1.0000	0.0000	1.0000	0.0000	1.0000	-2.2955	1.0000	0.0000
LOC100546347	0.0252	2.1598	0.0098	1.6484	0.0117	2.2561	1.0000	0.1712	1.0000	0.8300	1.0000	0.3324	0.2235	-1.2502
LOC100546349	1.0000	0.0000	1.0000	0.0000	1.0000	0.0000	1.0000	2.3257	1.0000	0.0000	1.0000	0.0000	1.0000	2.3554
LOC100546350	0.0718	-0.3309	0.0000	-0.4092	0.0000	-1.0281	0.0000	-0.7568	1.0000	-0.0688	0.5816	-0.1347	0.1375	0.2072
LOC100546353	0.5810	-0.1829	0.0000	-0.5236	0.0035	-0.6227	0.0004	0.3056	1.0000	0.2066	0.7487	-0.1216	0.0000	1.1398
LOC100546354	1.0000	0.0000	1.0000	2.2534	1.0000	0.0000	1.0000	0.0000	1.0000	0.0000	1.0000	2.3480	1.0000	0.0000
LOC100546355	1.0000	-0.0881	0.1941	-0.3326	0.2287	-0.4250	0.1327	-0.3486	1.0000	0.0832	1.0000	-0.1502	1.0000	0.1639
LOC100546358	0.2913	0.3762	0.0224	0.3988	0.3361	0.3298	0.1344	0.2837	1.0000	-0.1204	1.0000	-0.0839	0.7827	-0.1598
LOC100546360	0.9650	0.7826	0.1366	1.7431	1.0000	-0.1903	0.5792	1.1652	1.0000	-0.5259	1.0000	0.4630	1.0000	0.8479
LOC100546369	0.0001	-0.9968	0.0000	-0.7700	0.0193	-0.6154	0.0000	-0.8903	1.0000	0.0081	0.7035	0.2478	0.5382	-0.2606
LOC100546371	1.0000	0.0000	1.0000	0.0000	1.0000	0.0000	1.0000	0.0000	1.0000	0.0000	1.0000	0.0000	1.0000	0.0000
LOC100546372	0.0055	-3.8342	0.2517	-2.4814	0.6283	-0.6667	0.0086	1.9015	1.0000	-1.3178	1.0000	0.0489	0.2841	1.2571
LOC100546378	0.0000	-0.6188	0.0000	-0.6400	0.0007	-0.5496	0.0000	-0.3554	1.0000	-0.1287	0.4242	-0.1376	0.9837	0.0710
LOC100546381	0.4688	0.1627	0.5057	-0.0753	0.0000	-1.2879	0.0000	-1.1335	1.0000	-0.0240	0.0585	-0.2495	0.5436	0.1355
LOC100546382	0.1145	0.8009	0.0003	1.1461	0.0236	1.0045	0.1740	0.5536	1.0000	0.2231	0.3052	0.5804	0.9950	-0.2216
LOC100546398	0.0057	1.2666	0.0000	1.5797	0.0111	1.1355	0.1431	0.7062	1.0000	-0.0790	0.9592	0.2460	0.4627	-0.5021
LOC100546401	0.2430	0.2628	0.9225	0.0527	0.7747	0.1181	0.5933	0.1006	1.0000	0.1027	0.9671	-0.0959	0.9333	0.0893
LOC100546403	0.7718	-0.1259	0.0118	-0.3246	0.0001	-0.7625	0.0003	-0.4426	1.0000	0.0070	0.5720	-0.1795	0.0703	0.3325
LOC100546408	0.0095	0.6893	0.0000	0.9887	0.0093	0.6073	0.0000	0.8746	1.0000	-0.1369	0.8064	0.1763	0.7485	0.1372
LOC100546418	0.2112	-0.4290	0.0067	-0.4152	0.7091	-0.2052	0.1869	0.2050	1.0000	-0.0970	1.0000	-0.0683	0.1658	0.3217
LOC100546426	1.0000	-0.1404	0.3687	0.8690	0.7403	-0.5519	0.4412	0.7582	1.0000	-0.9624	1.0000	0.0584	1.0000	0.3550
LOC100546433	0.0000	-1.0070	0.0000	-1.4925	0.0001	-0.7406	0.0000	-1.1262	1.0000	0.2742	0.6963	-0.1983	0.9111	-0.1066
LOC100546434	1.0000	-2.4788	1.0000	0.0000	1.0000	-0.1597	1.0000	0.0000	1.0000	-2.3771	1.0000	0.0000	1.0000	-2.2906
LOC100546435	0.2028	-0.4622	0.8224	0.0623	0.0000	1.7184	0.0000	2.1214	1.0000	-0.1602	0.0001	0.3766	0.0040	0.2465
LOC100546438	1.0000	0.0000	0.7674	-3.2570	0.8011	3.0840	0.7710	-3.1693	1.0000	3.1191	1.0000	0.0000	1.0000	-3.1344
LOC100546440	0.8249	-1.5528	0.0013	-3.1878	0.2810	1.2453	1.0000	0.0169	1.0000	1.6651	1.0000	0.0547	1.0000	0.4421
LOC100546441	0.4768	-0.2943	0.1404	-0.3137	0.8519	-0.1394	0.2263	-0.3167	1.0000	0.2111	0.8762	0.2041	1.0000	0.0387
LOC100546443	0.9545	-0.0771	0.0021	-0.3136	0.0020	0.5926	0.0040	0.2871	1.0000	0.1644	1.0000	-0.0586	0.6531	-0.1355
LOC100546458	1.0000	0.0382	0.4287	0.1065	0.6026	0.1455	0.0006	0.3276	1.0000	-0.1347	1.0000	-0.0541	1.0000	0.0528
LOC100546462	1.0000	2.1849	1.0000	2.2507	1.0000	0.0000	1.0000	0.0000	1.0000	0.0000	1.0000	0.0495	1.0000	0.0000
LOC100546469	0.5378	3.5350	0.7666	-3.2534	1.0000	2.2472	0.4937	1.4808	1.0000	3.1126	0.8785	-3.6647	0.5105	2.3934
LOC100546474	0.0008	1.0295	0.0000	1.2123	0.0000	1.4532	0.0000	1.1501	1.0000	0.1186	0.3555	0.3141	0.7004	-0.1795
LOC100546477	1.0000	0.0000	1.0000	0.0000	1.0000	0.0000	1.0000	0.0000	1.0000	0.0000	1.0000	0.0000	1.0000	0.0000
LOC100546484	0.0000	-1.4288	0.0001	-0.8728	0.0035	-0.8850	0.8799	0.1080	1.0000	-0.0439	0.3861	0.5259	0.0001	0.9529
LOC100546489	1.0000	0.0830	0.6421	-0.4835	0.0003	1.7102	0.0000	1.7057	1.0000	0.3159	1.0000	-0.2402	0.7303	0.3181
LOC100546493	0.0551	1.9500	0.0035	1.3655	0.0000	3.2662	0.0000	2.2797	1.0000	1.3269	0.5389	0.7586	0.6979	0.3500
LOC100546494	1.0000	-0.0431	0.6228	-0.2340	0.4703	0.3328	0.3187	0.3354	1.0000	-0.0983	0.8950	-0.2769	1.0000	-0.0901
LOC100546503	0.1362	0.8422	0.0405	1.1914	0.0000	2.9799	0.0000	3.3300	1.0000	-1.2295	0.1460	-0.8679	0.0010	-0.8723
LOC100546506	1.0000	-0.3294	1.0000	-0.9997	0.9361	-1.5529	0.7710	-3.1633	1.0000	-0.6533	1.0000	-1.3320	1.0000	-2.2888
LOC100546507	1.0000	0.0000	1.0000	-0.1449	1.0000	2.2427	1.0000	-2.3176	1.0000	2.2675	1.0000	2.3456	1.0000	-2.2888
LOC100546512	0.0000	1.3652	0.0000	2.1404	0.0166	0.8779	0.0000	1.5171	1.0000	-0.5999	0.9146	0.1855	1.0000	0.0406
LOC100546519	1.0000	0.0000	1.0000	0.0000	1.0000	0.0000	1.0000	0.0000	1.0000	0.0000	1.0000	0.0000	1.0000	0.0000
LOC100546521	0.9278	-0.3575	0.2069	-0.7821	1.0000	-0.1997	0.0852	-1.0284	1.0000	0.9034	1.0000	0.4944	1.0000	0.0814
LOC100546529	1.0000	0.8894	0.4021	-2.2287	0.8011	-3.2629	0.3945	-2.0639	1.0000	1.0995	0.8269	-2.0167	1.0000	2.3542
LOC100546534	0.0039	-0.4748	0.0002	-0.3718	0.8825	0.0796	0.0736	0.1981	1.0000	-0.0449	0.9514	0.0703	0.8535	0.0785
LOC100546535	1.0000	0.0000	1.0000	0.0000	1.0000	0.0000	1.0000	0.0000	1.0000	0.0000	1.0000	0.0000	1.0000	0.0000
LOC100546538	0.0416	0.6337	0.0007	0.6615	0.3005	0.3710	0.9005	0.1120	1.0000	0.1045	1.0000	0.1434	0.9852	-0.1515
LOC100546539	0.3161	0.1898	0.0000	0.3762	0.0000	-0.6566	0.0000	-0.3838	1.0000	0.0627	0.0037	0.2613	0.0001	0.3412
LOC100546546	0.8641	0.1411	0.3613	-0.2507	0.3217	0.3126	0.2761	0.2190	1.0000	0.2099	1.0000	-0.1694	0.9767	0.1217
LOC100546547	0.0549	-0.4485	0.0020	-0.5259	0.0000	-0.8922	0.0079	-0.4109	1.0000	-0.1773	0.5465	-0.2431	0.2015	0.3089
LOC100546553	0.0016	0.5468	0.0000	0.6339	0.1068	-0.3213	0.0000	-0.5353	1.0000	-0.0719	1.0000	0.0274	0.0208	-0.2796
LOC100546568	0.0021	0.6678	0.1724	0.2760	0.5385	-0.2172	0.1135	-0.2985	1.0000	-0.1528	0.0044	-0.5320	0.5550	-0.2294
LOC100546571	0.0341	-1.5387	0.1818	-0.9726	0.7393	0.4362	0.9502	0.2563	1.0000	-0.2630	1.0000	0.3173	0.8425	-0.4396
LOC100546575	0.8095	0.7518	1.0000	0.0078	1.0000	-0.4955	0.3356	-1.6813	1.0000	0.3377	1.0000	-0.3938	1.0000	-0.8434
LOC100546578	0.2254	0.4585	0.0874	0.3169	0.0000	1.2461	0.0000	1.0154	1.0000	0.0707	1.0000	-0.0556	0.7771	-0.1553
LOC100546590	0.7652	-0.2816	0.9505	0.1561	0.0000	1.8277	0.0000	2.2451	1.0000	-0.1419	0.9442	0.3130	0.4757	0.2851
LOC100546606	1.0000	0.0000	0.2555	2.1629	1.0000	0.0000	1.0000	-2.3200	1.0000	2.2736	0.3216	4.6747	1.0000	0.0000
LOC100546609	1.0000	0.0000	1.0000	-2.3986	1.0000	0.0000	1.0000	-2.3200	1.0000	2.2736	1.0000	0.0000	1.0000	0.0000
LOC100546611	1.0000	0.0000	1.0000	0.0000	1.0000	0.0000	1.0000	2.3242	1.0000	0.0000	1.0000	0.0000	1.0000	2.3542
LOC100546620	1.0000	0.0000	1.0000	-2.3959	1.0000	0.0000	1.0000	-2.3176	1.0000	2.2675	1.0000	0.0000	1.0000	0.0000
LOC100546621	0.6644	0.1482	1.0000	-0.0101	0.0001	-0.7918	0.0000	-1.0034	1.0000	0.1055	1.0000	-0.0398	1.0000	-0.1009

LOC100546622	0.6019	-0.1384	0.0739	-0.1563	0.0708	-0.3162	0.0000	-0.3642	1.0000	-0.0327	1.0000	-0.0384	0.8744	-0.0753
LOC100546634	1.0000	0.0281	0.1135	0.4770	0.0003	1.2391	0.0000	1.9005	1.0000	-0.0883	0.6450	0.3711	0.0243	0.5742
LOC100546637	1.0000	0.0000	1.0000	0.0000	1.0000	0.0000	1.0000	0.0000	1.0000	0.0000	1.0000	0.0000	1.0000	0.0000
LOC100546639	0.0045	1.6478	0.0000	3.2038	0.0000	2.3192	0.0000	3.1520	1.0000	-1.1030	0.7457	0.4645	0.9196	-0.2647
LOC100546640	1.0000	0.0039	0.4379	-0.0920	0.0244	-0.3768	0.0001	-0.3384	1.0000	0.0557	1.0000	-0.0280	0.6688	0.0997
LOC100546647	1.0000	-0.0048	1.0000	0.1398	0.3684	1.0554	0.0222	1.5640	1.0000	0.0500	1.0000	0.2096	0.6865	0.5716
LOC100546652	0.0070	1.4907	0.1304	0.7855	0.0030	1.7332	0.0001	1.6081	1.0000	0.2294	0.7015	-0.4619	1.0000	0.1110
LOC100546653	1.0000	0.0502	0.0000	-0.6348	0.0000	-1.0828	0.0000	-0.8212	1.0000	0.2741	0.1284	-0.3977	0.0225	0.5417
LOC100546655	1.0000	0.0000	1.0000	0.0000	0.5080	3.6208	1.0000	2.3242	1.0000	0.0000	1.0000	0.0000	1.0000	-1.3126
LOC100546656	0.0862	0.9393	0.2666	0.5044	0.0000	2.3976	0.0000	1.3135	1.0000	0.7348	0.8899	0.3157	0.4845	-0.3412
LOC100546659	1.0000	0.3490	0.9752	0.5038	0.3371	-1.2945	1.0000	0.0036	1.0000	-0.5692	1.0000	-0.4051	1.0000	0.7304
LOC100546662	1.0000	2.1849	0.4021	-2.2287	0.0175	5.1145	1.0000	-0.6847	1.0000	4.3298	1.0000	0.0514	0.5565	-1.4317
LOC100546675	0.9265	-0.2975	0.1225	-1.0056	0.5491	-0.6536	0.1578	-0.9000	1.0000	0.4131	1.0000	-0.2864	1.0000	0.1672
LOC100546688	0.0000	-1.0808	0.0000	-0.9220	0.0462	-0.5260	1.0000	-0.0014	1.0000	-0.0616	1.0000	0.1094	0.0025	0.4682
LOC100546696	0.2903	0.4512	0.0000	1.0952	0.0010	0.8549	0.0000	0.9459	1.0000	-0.2711	0.4857	0.3848	0.7554	-0.1743
LOC100546708	1.0000	0.0252	1.0000	0.0109	0.0153	-0.5645	0.0949	-0.2908	1.0000	-0.2463	0.4245	-0.2468	1.0000	0.0346
LOC100546724	0.5086	-3.8796	1.0000	2.2507	1.0000	-0.1868	0.1393	4.3943	1.0000	-3.7635	1.0000	2.3456	1.0000	0.7678
LOC100546728	0.8226	3.0199	0.7674	-3.2570	1.0000	0.0000	0.7710	-3.1693	1.0000	3.1191	1.0000	-3.1429	1.0000	0.0000
LOC100546734	0.0148	-5.3982	0.0000	-4.4412	0.4085	-1.3169	0.0358	-1.3833	1.0000	1.2614	1.0000	2.3456	0.7602	1.2003
LOC100546746	1.0000	0.2841	1.0000	0.0386	0.1845	-2.7146	0.4996	-1.4683	1.0000	-0.3398	1.0000	-0.5755	1.0000	0.9164
LOC100546747	0.0676	0.4632	0.0006	0.5419	0.0478	0.4797	0.0000	0.6176	1.0000	0.0450	0.8811	0.1387	0.5077	0.1887
LOC100546750	0.0025	0.8335	0.0000	0.6768	0.0000	1.2124	0.0000	1.0102	1.0000	0.1747	1.0000	0.0327	1.0000	-0.0199
LOC100546753	0.1225	-4.5779	1.0000	2.2507	0.3489	1.1387	0.1317	4.3958	1.0000	-4.4584	1.0000	2.3456	0.4483	-1.2567
LOC100546764	1.0000	-0.0860	1.0000	0.1157	0.0694	0.8953	1.0000	0.0709	1.0000	-0.4793	1.0000	-0.2657	0.0078	-1.2952
LOC100546766	0.8161	-0.3507	0.1833	-0.5524	0.6370	-0.3920	0.7210	-0.2393	1.0000	0.1182	1.0000	-0.0686	1.0000	0.2733
LOC100546777	0.6430	0.2093	0.0439	0.3461	0.0000	-1.3036	0.0000	-1.1397	1.0000	-0.1583	1.0000	-0.0086	1.0000	0.0115
LOC100546791	1.0000	-0.0010	0.3946	-0.6098	1.0000	-0.0068	0.0344	-1.4086	1.0000	1.7837	0.4732	1.1902	1.0000	0.3849
LOC100546792	0.0289	1.5409	0.3045	0.7566	0.1017	1.2623	0.7710	0.4164	1.0000	0.4451	1.0000	-0.3272	1.0000	-0.3992
LOC100546802	1.0000	0.3047	1.0000	0.0406	0.8011	-1.4096	0.2460	-2.3148	1.0000	0.1184	1.0000	-0.1273	1.0000	-0.7793
LOC100546803	1.0000	-2.4776	1.0000	-2.3959	1.0000	-0.1591	1.0000	0.8545	1.0000	-0.1078	1.0000	0.0000	1.0000	0.9164
LOC100546808	0.0538	1.4233	0.0209	1.7255	0.0000	3.0122	0.0000	3.0985	1.0000	-0.8506	0.8785	-0.5374	0.0877	-0.7558
LOC100546809	0.8249	3.0142	1.0000	-0.1698	1.0000	0.0000	0.4547	-3.7024	1.0000	3.6521	1.0000	0.5881	1.0000	0.0000
LOC100546811	1.0000	-0.2931	1.0000	0.0000	1.0000	-2.4061	1.0000	0.0000	1.0000	-2.3771	1.0000	-2.2956	1.0000	0.0000
LOC100546813	1.0000	2.1901	1.0000	0.0000	1.0000	0.0000	1.0000	0.0000	1.0000	0.0000	1.0000	-2.2993	1.0000	0.0000
LOC100546815	0.6353	0.6265	0.0041	1.6837	0.0040	1.6747	0.0000	2.1805	1.0000	-0.2601	0.4065	0.8064	1.0000	0.2461
LOC100546824	0.0000	-0.9323	0.0000	-0.9932	1.0000	0.0417	1.0000	-0.0151	1.0000	0.0752	1.0000	0.0267	1.0000	0.0237
LOC100546836	0.0934	-1.4856	0.2961	-1.1471	1.0000	-0.2758	0.8236	-0.4668	1.0000	-0.6420	1.0000	-0.2914	0.6583	-0.8278
LOC100546837	0.0018	2.9135	0.0076	1.5517	0.0000	3.7631	0.0003	1.8468	1.0000	1.2771	1.0000	-0.0671	0.2843	-0.6356
LOC100546839	0.1905	1.1400	0.7726	0.7717	0.9394	-0.5960	0.0017	2.6466	1.0000	-1.4669	0.0661	-1.8356	0.0985	1.7757
LOC100546840	1.0000	0.0000	1.0000	0.0000	1.0000	0.0000	1.0000	0.0000	1.0000	0.0000	1.0000	0.0000	1.0000	0.0000
LOC100546851	0.0005	-0.8319	0.0000	-0.7519	0.0524	-0.5017	0.9490	0.0507	1.0000	-0.1463	1.0000	-0.0537	0.0237	0.4134
LOC100546859	0.5086	-3.8796	0.9149	-1.5421	0.2602	1.6058	0.2836	1.5152	1.0000	-0.1172	1.0000	2.3456	1.0000	-0.2049
LOC100546870	0.7155	0.3207	0.0655	0.8124	0.0001	-2.4114	0.0000	-2.9674	1.0000	-0.3269	1.0000	0.1743	0.9122	-0.8792
LOC100546874	1.0000	0.0000	1.0000	-0.1449	1.0000	2.2472	1.0000	0.0062	1.0000	2.2675	1.0000	2.3456	1.0000	0.0641
LOC100546884	1.0000	2.1849	1.0000	-2.3959	1.0000	0.0000	1.0000	-2.3176	1.0000	2.2675	1.0000	-2.2955	1.0000	0.0000
LOC100546887	0.1737	-0.4104	0.0000	-0.7931	0.0006	0.7689	0.0000	0.6109	1.0000	0.1082	0.5330	-0.2604	1.0000	-0.0445
LOC100546891	0.0001	0.7669	0.0001	0.5200	0.7553	0.1435	0.8430	0.0724	1.0000	0.0113	0.3448	-0.2232	1.0000	-0.0531
LOC100546893	0.0529	-0.4630	1.0000	-0.0372	0.0000	0.9403	0.0000	1.1085	1.0000	-0.1114	0.0716	0.3271	1.0000	0.0632
LOC100546894	1.0000	0.0000	0.2542	4.0037	1.0000	2.2471	1.0000	0.0000	1.0000	0.0000	0.6450	4.1157	1.0000	-2.2906
LOC100546895	1.0000	0.5414	0.7674	3.0888	1.0000	-0.1630	1.0000	2.3242	1.0000	-2.3771	1.0000	0.0533	1.0000	0.0652
LOC100546896	0.2380	1.2703	0.9566	0.5054	0.0551	1.7038	0.0847	1.3024	1.0000	0.2739	1.0000	-0.4793	1.0000	-0.1205
LOC100546899	0.8249	-3.3439	1.0000	0.0000	1.0000	-0.1793	1.0000	2.3257	1.0000	-3.2319	1.0000	0.0000	1.0000	-0.7793
LOC100546910	0.0013	1.9318	0.0000	2.4174	0.0000	2.2880	0.0004	1.7058	1.0000	-0.4957	1.0000	0.0028	0.0017	-1.0651
LOC100546913	1.0000	0.3058	0.6504	0.9458	1.0000	-0.8812	0.6142	-1.7592	1.0000	-0.4273	1.0000	0.2229	1.0000	-1.3084
LOC100546924	1.0000	0.0013	0.5405	0.0979	0.5089	-0.1743	0.8893	0.0479	1.0000	-0.0223	1.0000	0.0868	0.2339	0.2041
LOC100546929	0.0000	1.5218	0.0000	1.1645	0.0049	0.5279	0.0229	0.2108	1.0000	-0.1287	0.0000	-0.4735	0.0000	-0.4404
LOC100546931	0.1988	-0.4677	0.2802	-0.2780	0.7615	0.1822	0.0000	0.7383	1.0000	0.0374	0.8607	0.2385	0.0019	0.5974
LOC100546932	1.0000	0.0000	1.0000	0.0000	1.0000	0.0000	1.0000	0.0000	1.0000	0.0000	1.0000	0.0000	1.0000	0.0000
LOC100546933	0.0230	0.5139	0.1178	0.2249	1.0000	-0.0673	0.0000	-0.6488	1.0000	0.3610	1.0000	0.0848	0.4993	-0.2154
LOC100546941	1.0000	0.0000	1.0000	-2.3959	1.0000	0.0000	1.0000	-2.3176	1.0000	2.2675	1.0000	0.0000	1.0000	0.0000
LOC100546946	0.8249	-3.3411	0.7666	-3.2534	1.0000	-1.0156	1.0000	-0.8419	1.0000	-0.1170	1.0000	0.0000	1.0000	0.0640
LOC100546961	1.0000	0.0000	1.0000	0.0000	1.0000	0.0000	1.0000	2.3242	1.0000	0.0000	1.0000	0.0000	1.0000	2.3542
LOC100546964	1.0000	0.0000	1.0000	2.2507	1.0000	2.2471	1.0000	0.0000	1.0000	0.0000	1.0000	2.3456	1.0000	-2.2906
LOC100546965	1.0000	-0.1519	1.0000	0.1348	1.0000	-0.1984	1.0000	-0.3936	1.0000	-0.8119	1.0000	-0.5153	0.6069	-1.0051
LOC100546971	0.0010	-0.8207	0.0000	-0.6873	0.8948	-0.1083	0.1752	-0.2184	1.0000	-0.0887	1.0000	0.0572	0.4279	-0.1925
LOC100546974	1.0000	0.0734	0.6226	0.1295	0.9124	0.1073	0.8417	0.1090	1.0000	-0.0040	1.0000	0.0644	1.0000	0.0030
LOC100546979	0.1660	1.6702	0.0137	2.5905	1.0000	0.3655	0.7210	1.2285	1.0000	-1.0347	1.0000	-0.1168	1.0000	-0.1739
LOC100546986	0.0001	-0.6688	0.0000	-0.4436	0.0109	-0.4665	1.0000	0.0174	1.0000	-0.1977	1.0000	0.0400	0.0063	0.2914
LOC100546988	0.4339	-0.7804	0.0232	-1.4776	1.0000	0.1353	0.3973	0.5309	1.0000	-0.2441	0.6218	-0.9252	1.0000	0.1621
LOC100546989	0.1369	0.6928	0.8299	-0.2097	0.0065	-1.3901	0.3517	-0.4567	1.0000	-0.1032	0.0160	-0.9939	0.2740	0.8383
LOC100546992	0.0001	-0.6446	0.0000	-0.8171	0.6150	-0.1376	0.0157	-0.1917	1.0000	-0.0046	0.3382	-0.1649	0.9950	-0.0536
LOC100546995	0.3248	0.3060	0.6055	-0.1407	0.0003	-0.9426	0.0000	-1.1081	1.0000	0.2315	0.7757	-0.2027	1.0000	0.0704
LOC100547028	1.0000	0.0000	1.0000	0.0000	1.0000	0.0000	1.0000	0.0000	1.0000	0.0000	1.0000	0.0000	1.0000	0.0000
LOC100547030	1.0000	-2.4776	1.0000	2.2534	1.0000	-2.4056	1.0000	2.3242	1.0000	-2.3758	1.0000	2.3480	1.0000	2.3542
LOC100547042	0.0129	0.9298	0.0000	1.2370	0.0000	2.3561	0.0000	2.2149	1.0000	-0.1960	1.0000	0.1264	0.1206	-0.3285
LOC100547060	1.0000	2.1901	1.0000	0.0000	1.0000	0.0000	1.0000	0.0000	1.0000	0.0000	1.0000	-2.2992	1.0000	0.0000
LOC100547061	0.0000	-1.2819	0.0000	-1.5359	0.9203	0.0728	1.0000	0.0129	1.0000	0.0890	0.6218	-0.1528	1.0000	0.0337
LOC100547088	1.0000	0.0866	1.0000	-0.1779	0.1163	-2.8999	0.7837	-0.5614	1.0000	0.4413	1.0000	0.1861	0.2994	2.7982
LOC100547089	1.0000	-0.3275	1.0000	0.2177	1.0000	0.1849	0.7725	0.9503	1.0000	-0.1257	1.0000	0.4396	1.0000	0.6350
LOC100547091	0.0012	0.8033	0.0071	0.5213	0.0000	1.4607	0.0000	1.3406	1.0000	0.2525	1.0000	-0.0142	0.7477	0.1390
LOC100547096	1.0000	0.0000	1.0000	0.0000	1.0000	0.0000	1.0000	0.0000	1.0000	0.0000	1.0000	0.0000	1.0000	0.0000
LOC100547097	0.1564	-0.4177	0.1302	-0.1986	0.2903	-0.3380	0.0527	-0.2341	1.0000	0.2721	0.0004	0.5018	0.0221	0.3814
LOC100547098	0.0795	0.3053	0.0054	0.2461	0.0000	-0.8684	0.0000	-0.8498	1.0000	-0.0401	0.8851	-0.0870	1.0000	-0.0163
LOC100547103	1.0000	0.0000	1.0000	0.0000	1.0000	0.0000	1.0000	0.0000	1.0000	0.0000	1.0000	0.0000	1.0000	0.0000
LOC100547109	0.4379	-1.1680	1.0000	-0.1780	0.1932	1.2023	0.0777	1.0453	1.0000	0.4904	0.4766	1.4995	1.0000	0.3479
LOC100547124	0.0038	-0.4806	0.0000	-0.5812	0.7749	-0.1018	0.0908	-0.1461	1.0000	0.0697	1.0000	-0.0184	1.0000	0.0299
LOC100547129	1.0000	0.0000	1.0000	2.2507	1.0000	0.0000	1.0000	0.0000	1.0000	0.0000	1.0000	2.3456	1.0000	0.0000
LOC100547145	0.0073	1.3717	0.0100	1.0644	0.0022	1.3551	0.0001	1.3860	1.0000	-0.0184	0.9234	-0.3125	1.0000	0.0189
LOC100547151	0.0581	0.9331	0.5599	0.3509	0.1551	-0.9938	0.2253	-0.6281	1.0000	0.4538	1.0000	-0.1172	0.4809	0.8242
LOC100547156	0.0001	0.8199	0.0000	0.8937	0.0939	0.4186	0.0611	0.3146	1.0000	-0.0890	1.0000	-0.0044	0.5403	-0.1878
LOC100547159	0.6759	0.4467	0.9782	-0.3044	1.0000	0.2645	0.1701	-1.5219	1.0000	-0.9225	0.0238	-1.6663	0.0009	-2.7059
LOC100547196	0.0018	0.7166	0.0000	0.8957	0.0000	1.8746	0.0000	1.7805	1.0000	-0.0607	0.9072	0.1318	0.6684	-0.1481
LOC100547197	1.0000	0.0000	1.0000	0.0000	0.2232	4.3199	0.4622	3.6989	1.0000	0.0000	1.0000	0.0000	1.0000	-0.6299

LOC100547210	1.0000	0.0000	1.0000	0.0000	1.0000	0.0000	1.0000	0.0000	1.0000	0.0000	1.0000	0.0000	1.0000	0.0000
LOC100547218	1.0000	0.0448	0.5840	0.1277	0.2715	-0.2974	0.8499	0.0849	1.0000	-0.1662	1.0000	-0.0699	0.5257	0.2213
LOC100547219	0.0000	0.8028	0.0000	0.7920	0.2328	0.2502	0.0287	0.2181	1.0000	0.1407	0.6988	0.1424	0.7248	0.1143
LOC100547230	0.6382	-0.1347	0.1331	-0.1939	0.0000	-1.0657	0.0000	-0.7722	1.0000	-0.1887	0.2325	-0.2356	0.8838	0.1103
LOC100547239	0.0000	-1.3111	0.0000	-0.9715	0.0588	0.3778	0.0000	0.5655	1.0000	0.0200	0.0238	0.3721	0.0855	0.2124
LOC100547241	0.7914	0.1349	0.0695	0.2784	0.5358	0.1976	0.0029	0.3872	1.0000	-0.0892	1.0000	0.0673	0.8315	0.1085
LOC100547242	0.5614	-0.3163	1.0000	0.0303	0.0005	-1.3251	0.4361	-0.3085	1.0000	-0.4072	1.0000	-0.0483	0.2851	0.6162
LOC100547244	0.0012	1.0106	0.0000	1.5079	1.0000	-0.0295	0.1234	-0.5514	1.0000	-0.2604	0.6450	0.2441	0.0361	-0.7791
LOC100547251	1.0000	0.2536	1.0000	0.0314	0.8629	0.4788	0.7296	-0.3849	1.0000	0.8057	0.7370	0.5921	1.0000	-0.0612
LOC100547252	1.0000	0.0000	1.0000	0.0000	0.2840	3.9988	0.2426	4.0910	1.0000	0.0000	1.0000	0.0000	1.0000	0.0761
LOC100547253	1.0000	0.0000	1.0000	0.0000	0.4809	3.6122	1.0000	0.0000	1.0000	0.0000	1.0000	0.0000	0.7287	-3.6637
LOC100547273	0.0000	-0.7524	0.0000	-0.7460	0.6249	-0.1484	0.0000	-0.4502	1.0000	0.3176	0.0012	0.3364	1.0000	0.0203
LOC100547274	0.0026	1.4377	0.0000	1.4106	0.5049	-0.5603	0.5071	-0.4482	1.0000	0.1564	1.0000	0.1372	1.0000	0.2714
LOC100547276	1.0000	0.0000	1.0000	0.0000	1.0000	0.0000	1.0000	0.0000	1.0000	0.0000	1.0000	0.0000	1.0000	0.0000
LOC100547278	1.0000	0.0000	1.0000	0.0000	1.0000	0.0000	1.0000	0.0000	1.0000	0.0000	1.0000	0.0000	1.0000	0.0000
LOC100547295	1.0000	0.0000	1.0000	0.0000	1.0000	0.0000	1.0000	0.0000	1.0000	0.0000	1.0000	0.0000	1.0000	0.0000
LOC100547297	0.4489	-0.4732	1.0000	-0.1018	0.1301	0.6514	0.0000	1.0545	1.0000	-0.2485	1.0000	0.1388	0.9479	0.1608
LOC100547303	0.9545	0.1183	0.8990	-0.0808	0.3617	0.3252	0.0751	0.3360	1.0000	-0.2327	0.0521	-0.4182	0.5656	-0.2152
LOC100547331	0.0000	0.7551	0.0000	0.7874	0.0010	0.5381	0.0000	0.4260	1.0000	-0.0146	1.0000	0.0306	0.5531	-0.1207
LOC100547334	1.0000	-0.3257	1.0000	2.2507	1.0000	-1.0191	0.4370	3.7041	1.0000	-3.2304	1.0000	-0.7943	1.0000	1.4489
LOC100547338	1.0000	0.1072	0.7666	-0.1313	0.6333	0.2205	0.9367	-0.0768	1.0000	0.1950	1.0000	-0.0311	1.0000	-0.0962
LOC100547348	0.7522	0.1463	0.0064	0.3991	0.0000	0.9293	0.0000	1.0343	1.0000	-0.1547	1.0000	0.1113	1.0000	-0.0441
LOC100547350	0.0000	-1.3112	0.0000	-1.3864	0.0000	-0.9587	0.0000	-0.8581	1.0000	0.0570	1.0000	-0.0060	0.5311	0.1628
LOC100547354	0.4959	-0.1696	0.0000	-0.3273	0.1652	-0.2734	0.0000	-0.3620	1.0000	-0.0107	0.3268	-0.1559	0.7229	-0.0935
LOC100547356	1.0000	0.0000	1.0000	0.0000	1.0000	0.0000	1.0000	0.0000	1.0000	0.0000	1.0000	0.0000	1.0000	0.0000
LOC100547361	1.0000	-0.2921	1.0000	0.0000	1.0000	-2.4056	1.0000	0.0000	1.0000	-2.3757	1.0000	-2.2955	1.0000	0.0000
LOC100547363	0.0002	1.1148	0.0000	1.2390	0.0000	1.4567	0.0000	1.6620	1.0000	-0.1253	1.0000	0.0139	1.0000	0.0902
LOC100547373	0.1014	-0.4944	0.0000	-0.8992	1.0000	0.0142	0.9442	-0.0745	1.0000	0.2499	1.0000	-0.1413	0.8210	0.1664
LOC100547382	0.0003	1.9404	0.0006	1.3184	0.0100	1.5048	0.1967	0.6820	1.0000	0.7943	1.0000	0.1888	1.0000	-0.0213
LOC100547383	0.0001	-1.0505	0.0001	-0.6931	0.0007	-0.8230	0.0131	-0.4529	1.0000	-0.3129	1.0000	0.0582	1.0000	0.0636
LOC100547387	0.0719	0.6812	0.0636	0.4871	0.5205	0.3075	1.0000	-0.0227	1.0000	0.3187	1.0000	0.1380	1.0000	-0.0060
LOC100547397	0.7187	0.1259	0.0758	0.1813	0.7356	0.1229	0.0000	0.4925	1.0000	-0.1500	0.9689	-0.0826	0.1285	0.2251
LOC100547402	0.1413	0.3222	0.0251	0.2708	1.0000	-0.0403	0.0005	-0.4346	1.0000	-0.0096	1.0000	-0.0492	0.0218	-0.3983
LOC100547403	0.2093	0.4379	0.4835	0.2200	0.0033	-0.9915	0.1340	-0.4380	1.0000	0.0995	1.0000	-0.1031	0.0755	0.6586
LOC100547404	1.0000	0.0000	1.0000	0.0000	1.0000	0.0000	1.0000	0.0000	1.0000	0.0000	1.0000	0.0000	1.0000	0.0000
LOC100547415	0.0267	0.9650	0.8783	0.1574	0.0036	1.2131	0.1445	0.4810	1.0000	0.7378	1.0000	-0.0581	1.0000	0.0057
LOC100547424	0.9263	-0.5627	0.3415	1.5198	1.0000	0.3064	0.0532	2.3288	1.0000	-1.8102	1.0000	0.2731	1.0000	0.2133
LOC100547425	0.0210	2.5980	1.0000	0.3842	0.8276	0.7489	1.0000	0.0074	1.0000	0.2609	0.1017	-1.9460	1.0000	-0.4776
LOC100547427	0.0227	-2.0683	0.1536	-1.1564	1.0000	-0.2862	1.0000	0.0065	1.0000	-0.2138	1.0000	0.7115	1.0000	0.0804
LOC100547434	1.0000	2.1849	1.0000	-0.9997	0.5080	3.6209	0.7710	-3.1633	1.0000	3.1056	1.0000	0.0495	0.7455	-3.6684
LOC100547435	1.0000	0.0518	0.2189	-0.2104	0.9361	-0.1011	0.0040	-0.4172	1.0000	0.1186	0.9649	-0.1309	0.6583	-0.1913
LOC100547436	0.3770	0.3030	0.0040	0.5578	0.7827	0.1621	0.1706	0.3117	1.0000	-0.3549	1.0000	-0.0862	0.7453	-0.1972
LOC100547437	0.5269	0.3147	0.0454	0.4462	0.0243	-0.8299	0.0479	-0.5180	1.0000	0.2454	0.3248	0.3893	0.2409	0.5636
LOC100547441	0.0458	-0.3822	0.0208	-0.2600	0.4279	0.1886	0.3632	0.1304	1.0000	-0.0557	1.0000	0.0794	0.7915	-0.1087
LOC100547442	0.5690	-0.1508	0.0347	0.2864	0.0001	-0.6980	0.0000	-0.8155	1.0000	-0.1947	0.1892	0.2543	0.0757	-0.3068
LOC100547447	1.0000	0.0000	1.0000	0.0000	1.0000	0.0000	1.0000	0.0000	1.0000	0.0000	1.0000	0.0000	1.0000	0.0000
LOC100547451	0.7619	0.3408	0.0758	0.7649	0.5096	0.4444	0.9238	0.2206	1.0000	-0.0847	0.9217	0.3512	0.9337	-0.3030
LOC100547453	0.0300	-0.3810	0.0009	-0.2648	0.0000	-0.7528	0.0001	-0.2894	1.0000	-0.4068	0.0844	-0.2775	1.0000	0.0626
LOC100547459	1.0000	0.0000	1.0000	0.0000	1.0000	2.2471	1.0000	2.3242	1.0000	0.0000	1.0000	0.0000	1.0000	0.0640
LOC100547461	1.0000	0.5833	1.0000	-0.4251	1.0000	0.7467	0.7212	0.7208	1.0000	1.3521	1.0000	0.3636	0.6367	1.3461
LOC100547471	0.0392	0.3381	1.0000	0.0221	0.0023	0.4786	0.0033	0.2860	1.0000	0.1323	0.2439	-0.1711	1.0000	-0.0552
LOC100547473	0.8226	-3.3425	0.2495	-4.1757	0.8011	-3.2629	0.2426	-4.0840	1.0000	0.7963	1.0000	0.0000	1.0000	0.0000
LOC100547482	0.0247	0.7249	0.0163	0.6673	0.0000	1.6100	0.0000	1.1654	1.0000	0.3739	0.4723	0.3317	1.0000	-0.0631
LOC100547485	0.1520	0.9155	0.0006	1.0924	0.0001	1.7276	0.0000	1.3316	1.0000	0.2425	0.6912	0.4320	1.0000	-0.1483
LOC100547495	0.0733	0.5235	0.0000	0.7991	0.0000	1.0134	0.0000	1.2213	1.0000	-0.2002	1.0000	0.0896	1.0000	0.0153
LOC100547499	1.0000	0.0000	1.0000	0.0000	1.0000	0.0000	0.7701	3.1732	1.0000	0.0000	1.0000	0.0000	1.0000	3.2066
LOC100547506	1.0000	0.0000	1.0000	0.0000	1.0000	0.0000	1.0000	0.0000	1.0000	0.0000	1.0000	0.0000	1.0000	0.0000
LOC100547507	0.4260	-0.2748	0.0017	-0.4184	0.0000	1.3146	0.0000	1.1174	1.0000	0.5228	0.0162	0.3922	0.0016	0.3315
LOC100547518	0.0000	1.3766	0.0000	1.6581	0.0273	-0.8465	0.0012	-0.9586	1.0000	-0.3882	1.0000	-0.0959	0.4779	-0.4939
LOC100547524	0.8226	0.3890	0.1486	1.1465	0.8033	0.3857	0.0029	1.7630	1.0000	-0.9365	1.0000	-0.1700	0.8133	0.4447
LOC100547530	1.0000	0.0068	0.4422	0.0810	0.0223	-0.3667	0.0257	-0.1876	1.0000	-0.0222	0.9979	0.0642	0.2529	0.1618
LOC100547559	0.0000	-1.5318	0.0000	-1.6381	1.0000	-0.0497	1.0000	-0.0286	1.0000	0.1432	1.0000	0.0500	0.5972	0.1680
LOC100547566	0.0000	-2.0719	0.0000	-2.1389	0.1846	-0.4660	0.0000	0.6954	1.0000	0.2960	1.0000	0.2411	0.0000	1.4625
LOC100547576	1.0000	-1.1561	1.0000	0.0000	0.8033	-3.2622	1.0000	0.0000	1.0000	-3.2289	1.0000	-2.2955	1.0000	0.0000
LOC100547584	0.0000	-4.0363	0.0000	-4.1165	1.0000	0.0908	1.0000	-0.0603	1.0000	-0.4307	1.0000	-0.5029	0.1774	-0.5770
LOC100547592	0.4183	1.5375	1.0000	-0.5567	1.0000	-1.0171	0.9786	-0.9114	1.0000	0.7988	0.8375	-1.2856	1.0000	0.9164
LOC100547598	0.0000	-1.1378	0.0000	-1.1379	0.0000	-1.5307	0.0000	-1.2753	1.0000	-0.0764	1.0000	-0.0639	0.3322	0.1842
LOC100547599	0.3755	-0.1876	0.6115	-0.0764	0.2548	-0.2236	0.6172	-0.0802	1.0000	-0.0834	1.0000	0.0398	1.0000	0.0645
LOC100547603	1.0000	-2.4776	1.0000	0.0000	1.0000	-2.4056	1.0000	2.3242	1.0000	-2.3758	1.0000	0.0000	1.0000	2.3542
LOC100547610	0.4960	-0.2142	0.0748	0.2714	0.0000	-1.3780	0.0000	-0.8875	1.0000	-0.3276	0.7150	0.1709	0.8651	0.1709
LOC100547611	0.0678	-0.5446	0.0014	-0.6432	0.5284	0.2279	0.5158	0.1689	1.0000	0.0710	1.0000	-0.0129	1.0000	0.0183
LOC100547618	0.0573	-0.3373	0.0014	-0.3427	1.0000	-0.0417	0.6527	0.0679	1.0000	-0.1368	0.7597	-0.1299	1.0000	-0.0209
LOC100547620	0.0123	1.5129	0.0000	2.3316	0.0027	1.4344	0.0044	1.4320	1.0000	-0.7783	1.0000	0.0503	0.1154	-0.7747
LOC100547627	1.0000	-0.7211	0.5021	1.3057	0.6597	-1.9418	1.0000	-0.8419	1.0000	-1.0389	1.0000	0.9982	1.0000	0.0652
LOC100547642	1.0000	0.0000	1.0000	0.0000	1.0000	0.0000	1.0000	0.0000	1.0000	0.0000	1.0000	0.0000	1.0000	0.0000
LOC100547653	1.0000	0.0000	1.0000	0.0000	1.0000	2.2427	1.0000	0.0000	1.0000	0.0000	1.0000	0.0000	1.0000	-2.2888
LOC100547657	1.0000	2.1901	1.0000	2.2507	0.0058	5.5185	0.0038	5.4938	1.0000	0.0000	1.0000	0.0471	1.0000	-0.0437
LOC100547664	0.0185	0.9729	0.4628	-0.2945	0.0002	1.5284	0.0685	0.5157	1.0000	0.7206	0.3550	-0.5337	0.7583	-0.2875
LOC100547665	1.0000	0.0000	0.7585	-1.3791	1.0000	0.0000	1.0000	-0.6794	1.0000	4.3117	1.0000	3.1976	0.7287	3.7384
LOC100547676	0.0001	1.1214	0.0000	1.2511	0.0000	1.7348	0.0000	1.2557	1.0000	0.0490	0.7635	0.1904	0.0896	-0.4265
LOC100547680	0.7308	-0.4228	0.2873	0.5626	0.3492	-0.8430	1.0000	0.1084	1.0000	0.2109	0.0633	1.2129	0.1232	1.1688
LOC100547687	0.4270	-0.2022	0.0147	-0.2756	0.8524	-0.0977	0.7710	-0.0636	1.0000	0.1764	0.8470	0.1164	0.1756	0.2165
LOC100547689	1.0000	0.0486	0.3458	1.5188	1.0000	0.1923	0.7710	-3.1693	1.0000	-0.6479	1.0000	0.8290	0.5469	-4.0409
LOC100547693	1.0000	0.1184	0.6962	-0.5525	1.0000	-0.8815	0.6639	-0.6796	1.0000	1.2162	1.0000	0.5590	0.7054	1.4214
LOC100547702	0.0067	-0.9320	0.0000	-1.4220	0.0036	-1.0589	0.0159	-0.5767	1.0000	0.1259	0.8269	-0.3524	0.1712	0.6151
LOC100547703	0.8760	0.6136	1.0000	0.2251	1.0000	-0.0042	0.0143	1.8374	1.0000	-0.3415	0.9388	-0.7242	0.1731	1.5070
LOC100547709	0.2817	0.2945	0.4876	0.1459	0.0000	1.1067	0.0000	0.7761	1.0000	0.1114	1.0000	-0.0241	0.3312	-0.2143
LOC100547714	1.0000	0.1156	1.0000	-0.0657	0.4950	0.7917	0.2763	-1.2153	1.0000	0.1277	1.0000	-0.0436	0.0721	-1.8733
LOC100547716	1.0000	2.1849	1.0000	0.0000	1.0000	0.0000	1.0000	0.0000	1.0000	0.0000	1.0000	-2.2955	1.0000	0.0000
LOC100547719	0.1134	0.8983	0.0001	1.8267	0.0000	1.7383	0.0001	1.8106	1.0000	-0.8783	1.0000	0.0608	0.0432	-0.8016
LOC100547721	0.0001	1.0796	0.0000	0.9656	0.0280	0.6572	0.0000	0.8285	1.0000	0.1798	1.0000	0.0769	0.2580	0.3542
LOC100547722	1.0000	-1.1561	1.0000	2.2507	1.0000	-1.0156	1.0000	2.3257	1.0000	-3.2289	1.0000	0.0495	1.0000	0.0649
LOC100547726	0.9650	1.0748	1.0000	0.6940	0.6654	1.5931	1.0000	0.8568	1.0000	-0.1078	1.0000	-0.4823	1.0000	-0.8435

LOC100547732	0.0662	-0.3024	0.0000	-0.4538	0.0012	-0.5031	0.0000	-0.5911	1.0000	0.0879	1.0000	-0.0513	1.0000	0.0049
LOC100547734	0.1871	0.2890	0.0000	0.4641	1.0000	0.0235	0.3012	-0.1370	1.0000	-0.0058	0.4725	0.1816	0.4988	-0.1616
LOC100547760	1.0000	-0.0595	0.0351	-0.2199	0.9361	-0.0737	0.0007	-0.3198	1.0000	-0.0142	0.5382	-0.1615	0.0406	-0.2540
LOC100547762	0.9650	1.0580	1.0000	-0.1472	1.0000	-2.4061	1.0000	0.0057	1.0000	-0.1045	1.0000	-1.3218	1.0000	2.3554
LOC100547765	1.0000	-0.1106	1.0000	-0.0800	0.4809	0.3256	0.2069	0.3887	1.0000	-0.1820	1.0000	-0.1391	1.0000	-0.1116
LOC100547767	0.0113	0.4654	0.0000	0.6273	0.1947	-0.2878	0.1298	0.1894	1.0000	-0.3964	0.1668	-0.2217	0.9248	0.0877
LOC100547769	1.0000	-1.2473	0.6180	-1.9239	0.6597	-1.9401	1.0000	0.5635	1.0000	-0.1247	1.0000	-0.8001	0.5105	2.3934
LOC100547773	0.6374	0.2006	0.4704	-0.1901	0.6361	-0.2207	0.6768	0.1459	1.0000	0.1268	0.5594	-0.2538	0.0574	0.4988
LOC100547793	1.0000	-0.1361	0.0787	-1.2297	0.1051	1.1533	0.0077	1.1602	1.0000	0.6638	1.0000	-0.4155	0.3378	0.6770
LOC100547794	0.0000	-0.8641	0.0000	-0.8633	0.2372	-0.3321	0.0000	-0.6236	1.0000	-0.2796	0.3257	-0.2665	0.0047	-0.5649
LOC100547801	1.0000	-2.4776	1.0000	0.0000	1.0000	-2.4056	1.0000	0.0000	1.0000	-2.3758	1.0000	0.0000	1.0000	0.0000
LOC100547805	0.0000	1.4454	0.0000	1.3928	0.0044	-0.7380	0.0135	-0.4825	1.0000	-0.2065	0.2425	-0.2479	1.0000	0.0542
LOC100547818	1.0000	2.1901	1.0000	-0.1449	0.2829	3.9942	1.0000	0.0075	1.0000	2.2675	1.0000	0.0471	0.8939	-1.6940
LOC100547821	1.0000	0.0000	1.0000	0.0000	1.0000	0.0000	1.0000	0.0000	1.0000	0.0000	1.0000	0.0000	1.0000	0.0000
LOC100547826	0.1729	0.4258	0.0000	0.9899	0.0075	-0.7877	0.0016	-0.7583	1.0000	-0.3481	0.5784	0.2265	0.6491	-0.3151
LOC100547828	0.0635	-0.8723	0.0802	-0.6106	0.0129	0.8435	0.0000	0.9949	1.0000	0.0347	0.9773	0.3056	0.7909	0.1860
LOC100547835	1.0000	0.0028	0.9477	0.3984	1.0000	-0.6589	1.0000	-0.1765	1.0000	0.0484	1.0000	0.4654	1.0000	0.5434
LOC100547843	0.8249	0.1069	0.7628	0.0708	0.0052	-0.5306	0.0000	-0.5797	1.0000	0.1388	0.8854	0.1151	0.9429	0.0945
LOC100547855	1.0000	0.0000	1.0000	0.0000	1.0000	0.0000	1.0000	0.0000	1.0000	0.0000	1.0000	0.0000	1.0000	0.0000
LOC100547860	0.6927	1.4466	1.0000	2.2534	1.0000	0.6780	1.0000	0.0000	1.0000	-2.3771	1.0000	-1.7102	1.0000	-3.1344
LOC100547871	1.0000	0.0000	1.0000	0.0000	1.0000	0.0000	1.0000	0.0000	1.0000	0.0000	1.0000	0.0000	1.0000	0.0000
LOC100547876	0.0017	2.8227	0.0000	2.9304	0.0028	2.5824	0.0019	2.2148	1.0000	0.5689	0.4452	0.6901	1.0000	0.2048
LOC100547877	0.0010	-0.8062	0.0000	-0.8170	0.0048	-0.6644	0.0000	-0.6335	1.0000	0.1100	1.0000	0.1122	0.8804	0.1463
LOC100547883	0.0000	0.9928	0.0000	0.9261	1.0000	0.0090	0.1019	0.1403	1.0000	-0.2148	0.0036	-0.2693	0.8866	-0.0778
LOC100547885	0.5512	0.3842	0.5478	0.2492	0.2022	-0.7072	0.9535	-0.1296	1.0000	0.3552	0.9815	0.2327	0.0552	0.9384
LOC100547893	0.0199	0.7842	0.0000	1.0011	0.2623	0.4577	0.0022	0.8182	1.0000	-0.3546	1.0000	-0.1258	1.0000	0.0096
LOC100547897	0.0000	-1.0075	0.0000	-0.9625	1.0000	0.0406	0.0003	0.3745	1.0000	-0.0384	1.0000	0.0180	0.0415	0.3008
LOC100547911	0.0000	-3.4439	0.0000	-3.7130	0.0000	-1.8240	0.0000	-1.2086	1.0000	-0.0492	0.1016	-0.3055	0.0001	0.5715
LOC100547916	0.9813	0.1103	0.0000	0.5273	0.0002	0.9571	0.0000	1.3572	1.0000	-0.3136	0.9443	0.1168	0.7287	0.0922
LOC100547917	0.0082	-0.4665	0.0061	-0.2183	0.0030	-0.5263	0.4441	-0.0811	1.0000	-0.4733	0.0869	-0.2128	1.0000	-0.0227
LOC100547920	0.9545	0.1786	0.0001	1.1811	0.0009	1.2501	0.0000	1.4951	1.0000	-0.4689	0.3117	0.5463	0.8550	-0.2195
LOC100547923	0.5598	-0.1814	0.0897	-0.2397	1.0000	0.0028	0.0006	0.4030	1.0000	-0.1755	0.4065	-0.2224	0.2057	0.2290
LOC100547927	0.0030	-0.5825	0.0015	-0.4266	1.0000	-0.0746	0.6532	0.0930	1.0000	-0.2145	1.0000	-0.0459	1.0000	-0.0396
LOC100547929	1.0000	0.0000	1.0000	0.0000	1.0000	0.0000	1.0000	0.0000	1.0000	0.0000	1.0000	0.0000	1.0000	0.0000
LOC100547940	0.0000	-1.8181	0.0000	-2.5360	1.0000	-0.0300	0.0702	-0.1703	1.0000	0.0605	0.0000	-0.6451	0.7777	-0.0755
LOC100547944	0.3148	-4.2688	0.0579	-3.0476	0.2833	-4.1854	0.0114	-5.2052	1.0000	0.9931	1.0000	2.3456	1.0000	0.0000
LOC100547963	0.1065	-5.0513	0.0121	-5.3003	1.0000	-0.6581	1.0000	-0.3443	1.0000	0.2178	1.0000	0.0000	1.0000	0.5433
LOC100547971	0.0004	-0.7302	0.0052	-0.4021	0.0035	-0.6283	0.0075	-0.3837	1.0000	-0.1351	0.5465	0.2047	0.9139	0.1152
LOC100547972	0.1743	0.6652	0.0000	1.1400	0.0000	1.4828	0.0000	0.9840	1.0000	-0.0673	0.3682	0.4186	0.0253	-0.5635
LOC100547979	0.0000	-4.1750	0.0000	-3.9039	0.0000	2.1980	0.0000	1.7225	1.0000	-0.0402	1.0000	0.2458	0.0083	-0.5094
LOC100548001	0.8137	-0.7052	1.0000	0.3404	0.1032	1.3079	0.0002	2.0167	1.0000	-0.1254	0.8840	0.9305	0.5403	0.5855
LOC100548015	0.8360	0.2421	0.0297	0.7314	0.0001	1.1969	0.0000	1.3386	1.0000	-0.3007	1.0000	0.2048	0.9966	-0.1502
LOC100548019	1.0000	-0.0565	0.5556	0.2012	0.7763	0.1850	0.0055	0.6059	1.0000	-0.3030	1.0000	-0.0360	1.0000	0.1216
LOC100548022	1.0000	0.0000	1.0000	-2.3959	1.0000	0.0000	1.0000	-2.3176	1.0000	2.2675	1.0000	0.0000	1.0000	0.0000
LOC100548025	0.0093	0.6078	0.0002	0.5429	0.2500	0.3214	0.3543	0.1782	1.0000	-0.0063	1.0000	-0.0564	0.7187	-0.1423
LOC100548026	1.0000	0.0000	1.0000	0.0000	1.0000	0.0000	1.0000	0.0000	1.0000	0.0000	1.0000	0.0000	1.0000	0.0000
LOC100548027	0.0198	1.1909	0.6558	0.3471	1.0000	0.2441	0.0420	0.8614	1.0000	0.1599	0.3223	-0.6715	0.1986	0.7839
LOC100548043	0.3092	4.2299	0.6500	1.6027	1.0000	2.2427	1.0000	-2.3176	1.0000	2.2675	1.0000	-0.2486	1.0000	-2.2888
LOC100548047	0.5425	-0.2578	0.0899	-0.4302	0.9327	0.1333	0.5611	0.1712	1.0000	-0.1607	0.6068	-0.3201	1.0000	-0.1148
LOC100548053	0.0000	-1.1203	0.0000	-1.0090	0.0000	-0.8917	0.0000	-0.9816	1.0000	-0.2086	1.0000	-0.0850	0.0949	-0.2930
LOC100548064	0.0000	0.7580	0.0000	0.5362	0.0920	-0.3883	1.0000	-0.0485	1.0000	-0.2701	0.0000	-0.4790	1.0000	0.0769
LOC100548067	0.9859	0.3223	1.0000	0.0820	0.6626	0.6089	1.0000	-0.1385	1.0000	-0.5115	0.7457	-0.7344	0.2191	-1.2491
LOC100548069	0.0000	-1.2009	0.0000	-1.1440	0.0089	-0.4500	0.3791	0.1093	1.0000	-0.3180	0.2553	-0.2480	0.0756	0.2478
LOC100548070	0.7482	-0.2224	0.7794	-0.1796	0.0468	-0.7645	0.0206	-0.6669	1.0000	-0.2419	1.0000	-0.1878	1.0000	-0.1389
LOC100548071	1.0000	-0.1015	1.0000	-0.1742	0.2666	-1.0822	1.0000	-0.3031	1.0000	-0.1389	1.0000	-0.1974	1.0000	0.6498
LOC100548072	0.0000	1.2461	0.0000	1.1579	0.0000	-0.7886	0.0000	-0.7873	1.0000	0.0102	0.9309	-0.0657	1.0000	0.0171
LOC100548077	0.0005	2.7032	0.0090	2.0307	0.0000	3.2178	0.0008	2.3291	1.0000	0.1803	0.8760	-0.4802	0.3445	-0.7049
LOC100548078	0.9671	-1.6863	0.1496	1.8720	0.8235	0.7547	1.0000	-0.8404	1.0000	-0.6546	0.2589	2.9420	0.5105	-2.2505
LOC100548083	1.0000	0.0000	1.0000	0.0000	0.8011	3.0840	1.0000	0.0000	1.0000	0.0000	1.0000	0.0000	1.0000	-3.1344
LOC100548086	0.0286	0.4516	0.6250	0.1029	0.8539	0.1077	1.0000	0.0035	1.0000	0.1351	0.4426	-0.2001	1.0000	0.0374
LOC100548097	0.0000	-0.8510	0.0000	-0.9642	1.0000	-0.0427	0.9830	0.0436	1.0000	0.1559	1.0000	0.0553	0.1753	0.2472
LOC100548098	0.0000	-0.6932	0.0000	-0.6589	0.0000	-0.9363	0.0000	-0.8028	1.0000	-0.1467	0.7247	-0.1001	1.0000	-0.0074
LOC100548105	1.0000	0.0441	0.1348	-0.1541	0.7377	-0.1253	0.5569	0.0747	1.0000	-0.1762	0.0015	-0.3617	1.0000	0.0307
LOC100548106	1.0000	0.0000	0.9062	1.2235	1.0000	2.2471	1.0000	-2.3176	1.0000	2.2676	0.8607	3.7297	1.0000	-2.2907
LOC100548108	1.0000	-2.4776	1.0000	0.0000	1.0000	-2.4056	0.7710	3.1753	1.0000	-2.3758	1.0000	0.0000	1.0000	3.2081
LOC100548109	0.5751	0.3173	0.0016	0.9118	1.0000	-0.1035	0.0287	0.6899	1.0000	-0.6086	1.0000	-0.0023	1.0000	0.1904
LOC100548115	0.0032	0.8505	0.0000	0.7519	0.0000	0.9979	0.0076	0.4714	1.0000	0.1444	1.0000	0.0593	0.0502	-0.3752
LOC100548116	1.0000	0.0000	1.0000	0.0000	1.0000	2.2427	1.0000	0.0000	1.0000	0.0000	1.0000	0.0000	1.0000	-2.2888
LOC100548118	1.0000	0.0000	1.0000	0.0000	1.0000	0.0000	1.0000	0.0000	1.0000	0.0000	1.0000	0.0000	1.0000	0.0000
LOC100548121	0.7197	-0.3616	0.7503	-0.3087	1.0000	0.1162	0.7996	-0.3192	1.0000	-0.4602	0.9443	-0.3957	0.2373	-0.8892
LOC100548127	0.0684	0.8278	0.0002	1.1902	0.0001	1.4039	0.0000	1.6475	1.0000	-0.0974	0.8875	0.2781	1.0000	0.1539
LOC100548128	0.1626	0.6885	1.0000	0.0032	0.0000	1.8948	0.0000	1.5783	1.0000	0.3510	0.8785	-0.3203	1.0000	0.0430
LOC100548130	1.0000	0.0986	0.0031	1.4889	0.0370	1.0895	0.0000	1.9421	1.0000	-0.8049	0.5977	0.5999	1.0000	0.0568
LOC100548137	0.8226	-0.0975	0.1180	-0.1677	1.0000	-0.0122	1.0000	-0.0301	1.0000	-0.0086	1.0000	-0.0665	1.0000	-0.0217
LOC100548146	0.7938	1.1355	1.0000	0.5219	0.8033	-3.2636	1.0000	-0.5240	1.0000	0.4081	1.0000	-0.1923	1.0000	3.2049
LOC100548147	1.0000	0.0646	0.2292	0.2113	0.0000	-0.9495	0.0000	-0.7468	1.0000	-0.1610	1.0000	-0.0023	1.0000	0.0470
LOC100548148	1.0000	0.0000	1.0000	0.0000	1.0000	0.0000	1.0000	0.0000	1.0000	0.0000	1.0000	0.0000	1.0000	0.0000
LOC100548153	1.0000	0.0000	1.0000	0.0000	1.0000	0.0000	1.0000	0.0000	1.0000	0.0000	1.0000	0.0000	1.0000	0.0000
LOC100548169	0.4925	-0.4390	0.0736	-0.5827	1.0000	0.0751	0.4260	-0.3242	1.0000	0.2223	1.0000	0.0883	1.0000	-0.1735
LOC100548173	0.3366	-2.3921	0.1008	-2.8797	1.0000	0.2691	0.2979	0.9862	1.0000	0.5188	1.0000	0.0514	0.4461	1.2445
LOC100548193	0.0283	0.4784	0.0074	0.4193	0.0000	1.2111	0.0000	0.8829	1.0000	0.3010	0.1932	0.2555	1.0000	-0.0216
LOC100548196	1.0000	0.0000	1.0000	0.0000	1.0000	0.0000	1.0000	0.0000	1.0000	0.0000	1.0000	0.0000	1.0000	0.0000
LOC100548199	0.8573	-0.6731	0.1041	-1.3662	0.8954	0.4683	1.0000	0.2824	1.0000	0.3466	1.0000	-0.3376	1.0000	0.1656
LOC100548214	0.0001	-0.7302	0.0000	-0.9799	0.1031	-0.3544	0.0000	-0.3873	1.0000	0.0223	0.1533	-0.2151	1.0000	-0.0058
LOC100548218	1.0000	-0.2921	1.0000	-0.1449	0.9361	1.2064	1.0000	0.0075	1.0000	-0.1078	1.0000	0.0495	1.0000	-1.3086
LOC100548226	0.9734	-0.1956	0.0904	0.7598	0.0273	0.8940	0.0001	1.3736	1.0000	-0.7304	1.0000	0.2376	0.8939	-0.2472
LOC100548228	0.0000	-4.2306	0.0000	-4.8066	1.0000	0.1406	0.1810	-0.7687	1.0000	-0.2223	1.0000	-0.7943	0.0893	-1.1293
LOC100548247	0.5565	0.2404	0.0636	0.3559	0.0369	-0.5826	0.0060	-0.5915	1.0000	-0.0848	1.0000	0.0443	1.0000	-0.0877
LOC100548251	0.5934	-0.1349	0.5793	-0.0775	0.0371	-0.3533	0.0000	-0.3311	1.0000	-0.1129	1.0000	-0.0434	0.7827	-0.0851
LOC100548257	0.8249	3.0255	0.7666	-3.2534	1.0000	0.0000	0.7701	-3.1666	1.0000	3.1126	1.0000	-3.1468	1.0000	0.0000
LOC100548260	0.6877	-2.0832	0.9762	0.7528	1.0000	-0.5781	0.9801	0.9262	1.0000	-1.0414	1.0000	1.8196	1.0000	0.4653
LOC100548266	0.0297	-1.5378	0.0314	-1.6413	0.0115	-1.8794	0.7420	-0.4857	1.0000	-0.5607	1.0000	-0.6542	0.8997	0.8395
LOC100548269	0.1021	-3.0439	1.0000	0.5225	0.7629	-0.8500	1.0000	0.0060	1.0000	-1.4719	0.8269	2.1242	1.0000	-0.6151

LOC100548270	0.1772	-0.2819	0.0080	-0.2683	0.0209	-0.4415	0.0031	-0.2938	1.0000	0.0900	0.7913	0.1161	0.1235	0.2436
LOC100548272	0.9451	-0.0651	0.0598	0.1524	0.2682	0.2072	0.0012	0.2359	1.0000	-0.0221	0.0702	0.2078	1.0000	0.0117
LOC100548274	0.0778	0.3490	0.0092	0.2930	0.5405	-0.1790	0.0000	-0.4705	1.0000	0.0700	1.0000	0.0270	0.3540	-0.2145
LOC100548275	1.0000	0.4279	1.0000	0.2849	1.0000	0.1171	0.7296	-1.2146	1.0000	0.1751	1.0000	0.0494	0.9835	-1.1482
LOC100548279	1.0000	0.0000	1.0000	0.0000	1.0000	0.0000	1.0000	0.0000	1.0000	0.0000	1.0000	0.0000	1.0000	0.0000
LOC100548280	0.0001	-1.4637	0.0000	-2.1904	0.0000	-1.5058	0.1452	-0.3475	1.0000	0.1530	0.5379	-0.5625	0.0000	1.3156
LOC100548286	0.5615	0.1354	0.0086	0.1909	0.0064	-0.4288	0.0000	-0.3610	1.0000	0.1093	0.0833	0.1772	0.0842	0.1821
LOC100548287	0.0000	-1.3147	0.0000	-1.0848	0.2683	-0.2743	0.3015	-0.0977	1.0000	0.0376	0.0182	0.2802	0.0409	0.2188
LOC100548288	0.2864	-1.2208	0.0387	-2.2002	0.0273	-2.7494	0.0200	-2.5508	1.0000	-0.1333	0.9877	-1.1043	1.0000	0.0730
LOC100548295	0.9791	0.1124	0.6833	0.1523	1.0000	0.0573	1.0000	0.0066	1.0000	0.0410	1.0000	0.0916	1.0000	-0.0040
LOC100548298	1.0000	0.0000	1.0000	0.0000	1.0000	0.0000	1.0000	0.0000	1.0000	0.0000	1.0000	0.0000	1.0000	0.0000
LOC100548306	0.0000	-0.6418	0.0000	-0.6013	0.0000	-1.4328	0.0000	-1.1552	1.0000	-0.1468	0.8372	-0.0943	0.5628	0.1364
LOC100548307	0.0114	1.4349	0.0008	1.0297	0.0000	2.5655	0.0000	1.4576	1.0000	0.6189	1.0000	0.2294	0.1137	-0.4867
LOC100548320	1.0000	0.0000	1.0000	0.0000	1.0000	0.0000	1.0000	0.0000	1.0000	0.0000	1.0000	0.0000	1.0000	0.0000
LOC100548321	0.5086	3.5456	1.0000	2.2507	1.0000	2.2471	0.4370	3.7041	1.0000	0.0000	1.0000	-1.3266	1.0000	1.4485
LOC100548322	0.2376	-0.6241	0.1198	-0.5862	0.4669	-0.4602	1.0000	-0.0405	1.0000	-0.2233	1.0000	-0.1721	1.0000	0.2052
LOC100548327	1.0000	0.0000	1.0000	0.0000	1.0000	0.0000	1.0000	0.0000	1.0000	0.0000	1.0000	0.0000	1.0000	0.0000
LOC100548330	0.0006	-0.5962	0.0000	-0.6015	0.0000	-1.1169	0.0000	-1.0597	1.0000	-0.1073	0.7581	-0.1005	1.0000	-0.0445
LOC100548331	0.6599	-0.1129	0.0002	-0.2336	0.1954	-0.2215	0.0000	-0.3193	1.0000	0.0232	0.7635	-0.0854	0.7609	-0.0691
LOC100548332	0.0074	-0.5981	0.0001	-0.5872	0.0267	-0.5028	0.0004	-0.5214	1.0000	0.0007	1.0000	0.0234	1.0000	-0.0130
LOC100548334	0.1906	-0.3789	0.0002	-0.6004	0.1212	0.3801	0.0008	0.4936	1.0000	0.1463	1.0000	-0.0620	0.2779	0.2655
LOC100548337	0.0002	-0.5991	0.0000	-0.5347	0.0000	-1.0746	0.0000	-0.7793	1.0000	-0.1400	1.0000	-0.0634	0.4742	0.1610
LOC100548350	1.0000	0.0609	0.0019	-0.3976	0.0043	0.5457	0.0063	0.3508	1.0000	0.3292	0.9217	-0.1164	0.6747	0.1408
LOC100548360	0.0033	-0.7045	0.0000	-0.5765	0.0720	0.4377	0.3619	0.1321	1.0000	0.1335	0.1948	0.2747	0.4243	-0.1673
LOC100548372	1.0000	0.0000	1.0000	0.0000	1.0000	0.0000	1.0000	0.0000	1.0000	0.0000	1.0000	0.0000	1.0000	0.0000
LOC100548375	1.0000	0.0000	1.0000	0.0000	1.0000	0.0000	1.0000	0.0000	1.0000	0.0000	1.0000	0.0000	1.0000	0.0000
LOC100548376	0.8340	0.3901	0.9426	0.2561	0.7881	0.4353	1.0000	0.0899	1.0000	-0.0520	1.0000	-0.1686	1.0000	-0.3878
LOC100548381	0.0127	-0.4721	0.0002	-0.5005	0.8736	0.0972	0.0002	0.3685	1.0000	-0.1194	0.8839	-0.1358	0.5656	0.1575
LOC100548382	1.0000	0.0000	1.0000	0.0000	1.0000	0.0000	1.0000	0.0000	1.0000	0.0000	1.0000	0.0000	1.0000	0.0000
LOC100548407	0.0004	0.8225	0.0000	0.8701	0.0007	0.7548	0.0347	0.3488	1.0000	-0.0195	1.0000	0.0389	0.0266	-0.4203
LOC100548422	0.5086	3.5456	1.0000	0.6987	0.5076	3.6021	1.0000	0.8545	1.0000	2.2676	1.0000	-0.4744	1.0000	-0.4550
LOC100548429	1.0000	0.5832	0.2547	2.1612	1.0000	0.7335	0.4037	2.0750	1.0000	-0.9587	1.0000	0.6151	1.0000	0.3813
LOC100548433	1.0000	0.0000	1.0000	0.0000	1.0000	0.0000	1.0000	0.0000	1.0000	0.0000	1.0000	0.0000	1.0000	0.0000
LOC100548437	0.0000	1.9930	0.0000	1.8076	0.0000	2.1794	0.0000	1.7228	1.0000	0.1319	1.0000	-0.0436	0.3000	-0.3201
LOC100548454	0.0081	0.5890	0.0128	0.3564	0.1642	0.3514	0.0027	0.3863	1.0000	0.0300	0.4569	-0.1893	1.0000	0.0719
LOC100548465	1.0000	-0.0558	0.7292	-0.0602	1.0000	0.0480	0.6393	-0.0735	1.0000	-0.0187	1.0000	-0.0102	0.5335	-0.1348
LOC100548468	0.2934	0.3811	0.0000	0.9058	0.0001	0.9702	0.0000	1.4739	1.0000	-0.5389	1.0000	-0.0008	1.0000	-0.0267
LOC100548480	0.0000	-1.3902	0.0000	-2.5813	0.0000	1.2719	0.0015	0.6337	1.0000	0.3233	0.1244	-0.8546	0.3489	-0.3094
LOC100548498	0.0446	-0.3413	0.0000	-0.4018	0.8136	0.0947	0.5833	0.0714	1.0000	-0.0640	0.6465	-0.1121	0.8939	-0.0815
LOC100548520	0.0176	-1.3501	0.1092	-0.8672	0.9318	-0.1995	0.0055	1.0037	1.0000	-0.2953	1.0000	0.2020	0.0391	0.9165
LOC100548521	0.0001	1.0375	0.0000	0.9477	0.0000	2.0995	0.0000	1.0041	1.0000	0.2070	1.0000	0.1317	0.0000	-0.8802
LOC100548527	1.0000	0.0000	1.0000	0.0000	1.0000	0.0000	1.0000	0.0000	1.0000	0.0000	1.0000	0.0000	1.0000	0.0000
LOC100548529	0.2582	1.6787	0.9513	0.5392	0.8235	0.7536	1.0000	-0.2488	1.0000	0.8260	1.0000	-0.3058	1.0000	-0.1743
LOC100548534	1.0000	-2.4788	0.4024	-2.2295	1.0000	0.6780	0.7190	-1.2157	1.0000	1.9552	1.0000	2.3480	1.0000	0.0723
LOC100548539	1.0000	0.0000	1.0000	0.0000	1.0000	0.0000	1.0000	0.0000	1.0000	0.0000	1.0000	0.0000	1.0000	0.0000
LOC100548555	0.4296	0.1763	0.0001	0.3900	1.0000	-0.0110	0.5059	0.0964	1.0000	-0.1379	0.8791	0.0874	1.0000	-0.0259
LOC100548556	0.0018	2.3928	0.8176	0.5436	1.0000	0.2734	0.8949	-0.5582	1.0000	0.6880	0.1783	-1.1481	1.0000	-0.1358
LOC100548557	0.0359	-1.8999	0.0094	-1.8735	1.0000	-0.1238	0.4509	0.5377	1.0000	0.0212	1.0000	0.0592	0.5014	0.6845
LOC100548568	0.8513	0.4144	0.0016	2.0204	0.0280	-2.4238	1.0000	-0.4581	1.0000	-1.2059	0.9755	0.4064	1.0000	0.7682
LOC100548577	1.0000	0.0000	1.0000	-2.3986	1.0000	0.0000	1.0000	-2.3200	1.0000	2.2736	1.0000	0.0000	1.0000	0.0000
LOC100548578	0.0732	0.9059	0.0726	0.6035	0.0027	1.2800	0.0056	0.8239	1.0000	0.5816	0.8619	0.2917	1.0000	0.1302
LOC100548579	0.0000	-1.5877	0.0000	-1.6369	0.0000	1.3917	0.0000	0.7133	1.0000	0.0216	1.0000	-0.0149	0.0000	-0.6522
LOC100548582	1.0000	0.0012	0.0063	0.6537	0.0000	1.2435	0.0000	0.8618	1.0000	0.2040	0.0021	0.8695	0.8436	-0.1712
LOC100548583	0.2093	0.5080	0.1993	0.3631	0.0011	1.0572	0.0045	0.6658	1.0000	0.0925	1.0000	-0.0425	0.5474	-0.2979
LOC100548588	1.0000	-0.8632	1.0000	-0.3405	1.0000	-0.7120	0.6798	-0.8099	1.0000	1.3834	0.6178	1.9334	0.9835	1.2974
LOC100548591	1.0000	0.0609	0.0096	0.2405	0.8087	-0.1076	0.1554	-0.1713	1.0000	-0.0834	0.7561	0.1083	0.5656	-0.1415
LOC100548603	0.0022	0.9790	0.0000	0.8937	0.0000	1.7245	0.0000	1.1875	1.0000	0.2590	0.8841	0.1871	0.3972	-0.2704
LOC100548608	0.9280	0.2946	1.0000	0.0642	0.1887	-0.9770	0.1484	-0.7361	1.0000	0.3734	1.0000	0.1555	0.7692	0.6234
LOC100548610	0.0279	-2.3724	0.0096	-1.8736	1.0000	0.1012	0.0123	-1.9401	1.0000	0.4815	1.0000	0.9980	0.2252	-1.5533
LOC100548634	1.0000	0.0000	1.0000	0.0000	0.4809	3.6101	1.0000	0.0000	1.0000	0.0000	1.0000	0.0000	0.7287	-3.6634
LOC100548643	1.0000	0.0000	1.0000	-2.3959	1.0000	0.0000	1.0000	-2.3176	1.0000	2.2676	1.0000	0.0000	1.0000	0.0000
LOC100548653	0.9961	0.3019	0.4776	-0.4688	0.3053	0.7977	0.9096	-0.2290	1.0000	0.6608	1.0000	-0.0963	0.9515	-0.3594
LOC100548654	0.0000	1.1362	0.0000	0.8285	0.0027	-0.9982	0.0029	-0.6676	1.0000	-0.0679	0.1286	-0.3628	0.7673	0.2700
LOC100548663	0.3386	0.6670	0.6622	-0.3546	0.7074	-0.5588	0.0481	-1.0233	1.0000	0.5332	0.8547	-0.4749	1.0000	0.0785
LOC100548666	0.1624	-1.8529	0.5613	-0.9993	0.0879	-3.0633	0.4815	-1.1174	1.0000	-0.1192	1.0000	0.7487	0.8937	1.8362
LOC100548675	1.0000	0.0000	1.0000	0.0000	1.0000	0.0000	1.0000	0.0000	1.0000	0.0000	1.0000	0.0000	1.0000	0.0000
LOC100548678	0.3698	-0.3805	0.0567	-0.3894	0.0001	1.1186	0.0000	0.8824	1.0000	0.1732	0.8006	0.1785	1.0000	-0.0576
LOC100548679	1.0000	-0.0932	0.0121	5.1239	0.0443	1.9455	0.0001	6.0261	1.0000	-4.4568	1.0000	0.6242	0.9675	-0.4344
LOC100548686	0.5609	-0.2294	0.1902	0.1503	0.0727	-0.4949	0.0078	0.2843	1.0000	-0.1357	0.1856	0.2570	0.0000	0.6486
LOC100548691	0.0000	4.1930	0.0000	3.3800	0.8291	0.3907	1.0000	0.0070	1.0000	0.0382	0.0001	-0.7651	1.0000	-0.3377
LOC100548692	0.0098	0.6277	0.0073	0.4246	0.0008	0.7479	0.0140	0.3564	1.0000	0.3494	0.8459	0.1583	1.0000	-0.0373
LOC100548693	1.0000	0.0000	1.0000	0.0000	1.0000	0.0000	1.0000	0.0000	1.0000	0.0000	1.0000	0.0000	1.0000	0.0000
LOC100548696	1.0000	-0.2300	0.9539	-0.3035	1.0000	0.2405	0.0685	0.8423	1.0000	-0.0082	1.0000	-0.0664	0.4923	0.5974
LOC100548705	0.4546	0.2188	0.0158	0.3046	0.0097	0.5119	0.0000	0.6559	1.0000	-0.0087	1.0000	0.0890	0.6333	0.1400
LOC100548706	0.0633	0.3766	0.0001	0.4276	0.0090	0.4931	0.0000	0.4482	1.0000	0.0747	0.6334	0.1388	1.0000	0.0358
LOC100548707	1.0000	-0.0384	1.0000	0.1329	0.0000	3.3413	0.0310	1.9421	1.0000	-0.1268	1.0000	0.0579	0.0048	-1.5196
LOC100548708	0.5100	0.8089	0.2558	1.3597	1.0000	-0.2050	0.4057	-2.0667	1.0000	-1.2240	0.7406	-0.6682	0.0775	-3.0959
LOC100548709	0.0028	-0.4518	0.0000	-0.4219	0.2688	0.2004	0.5630	0.0648	1.0000	0.0143	1.0000	0.0562	0.3998	-0.1159
LOC100548710	0.0012	0.5508	0.0000	0.5338	0.1947	0.2905	0.9520	0.0441	1.0000	0.0918	0.9101	0.0880	0.6616	-0.1500
LOC100548711	0.0038	-0.5156	0.0002	-0.3058	0.0621	-0.3572	0.0005	-0.2727	1.0000	-0.1170	0.7879	0.1056	1.0000	-0.0261
LOC100548716	0.0076	0.8372	0.2783	0.3138	0.0020	0.9082	0.4102	0.2587	1.0000	0.0081	0.1105	-0.5022	0.0122	-0.6330
LOC100548731	0.7972	0.8927	1.0000	0.3671	1.0000	0.3474	0.7302	1.2324	1.0000	-0.1210	1.0000	-0.6369	1.0000	0.7692
LOC100548732	0.8226	-3.3425	0.7666	3.0922	1.0000	-1.0171	1.0000	0.0000	1.0000	-3.2304	1.0000	3.1976	1.0000	-2.2907
LOC100548734	0.0385	-0.4767	0.0000	-0.7938	0.0000	-1.5658	0.0000	-1.4625	1.0000	0.0394	0.4945	-0.2651	0.8425	0.1475
LOC100548741	0.0001	-0.7255	0.0000	-0.9025	0.0040	-0.4842	0.0000	-0.8603	1.0000	-0.0009	0.6983	-0.1664	0.0000	-0.3714
LOC100548742	1.0000	0.0238	0.3828	-0.1935	0.0000	1.2104	0.0000	1.1226	1.0000	0.2443	1.0000	0.0413	0.6456	0.1617
LOC100548747	1.0000	0.0000	1.0000	0.0000	1.0000	0.0000	1.0000	0.0000	1.0000	0.0000	1.0000	0.0000	1.0000	0.0000
LOC100548761	0.0261	-1.4103	0.0000	-2.0556	1.0000	0.1266	1.0000	0.0354	1.0000	-0.0775	0.7563	-0.7119	1.0000	-0.1653
LOC100548773	0.2057	0.2451	0.0001	0.2792	0.2024	0.2442	0.0149	0.1835	1.0000	-0.0382	1.0000	0.0081	0.6130	-0.0934
LOC100548778	0.0105	-0.5536	0.7906	-0.0813	0.1437	-0.4236	0.9260	0.0540	1.0000	-0.0558	0.0247	0.4288	0.1264	0.4284
LOC100548787	0.5496	-0.1879	0.0031	-0.3649	0.0151	0.4885	0.1195	0.1952	1.0000	-0.0690	0.3346	-0.2328	0.0050	-0.3565
LOC100548792	0.0000	2.0611	0.0000	2.7199	0.0000	3.3332	0.0000	2.6519	1.0000	-0.0415	0.1105	0.6370	0.0000	-0.7130
LOC100548793	1.0000	0.0000	1.0000	-2.3959	0.2829	3.9942	1.0000	-2.3176	1.0000	2.2676	1.0000	0.0000	0.5105	-4.0491

LOC100548799	0.2955	-0.3925	0.4470	-0.2148	0.3631	-0.3673	0.0111	-0.5549	1.0000	0.0030	0.9482	0.1928	0.9427	-0.1789
LOC100548805	0.0916	0.8853	0.0000	1.3000	0.0014	1.3723	0.0550	0.7406	1.0000	0.3353	0.0657	0.7620	0.8204	-0.2939
LOC100548808	0.5299	0.1567	0.3174	0.1266	0.6948	0.1219	0.3516	-0.1184	1.0000	-0.0144	1.0000	-0.0315	0.0438	-0.2485
LOC100548817	0.8760	-0.6513	1.0000	0.3692	1.0000	0.0619	1.0000	0.0059	1.0000	-1.3480	1.0000	-0.3278	0.7921	-1.3999
LOC100548822	0.0115	1.3341	0.0005	1.4511	1.0000	-0.1027	0.4547	-0.6014	1.0000	-0.4636	0.8619	-0.3369	0.2525	-0.9555
LOC100548827	0.0000	0.6824	0.0000	0.8929	0.1542	-0.2823	0.0000	-0.5118	1.0000	-0.1588	1.0000	0.0641	0.0007	-0.3824
LOC100548828	0.7559	0.6250	1.0000	0.1152	1.0000	-0.0296	0.8102	-0.5109	1.0000	0.5505	1.0000	0.0564	1.0000	0.0777
LOC100548829	1.0000	0.0000	1.0000	0.0000	1.0000	0.0000	1.0000	0.0000	1.0000	0.0000	1.0000	0.0000	1.0000	0.0000
LOC100548833	1.0000	0.0000	1.0000	0.0000	1.0000	0.0000	1.0000	0.0000	1.0000	0.0000	1.0000	0.0000	1.0000	0.0000
LOC100548836	1.0000	0.2546	0.1862	0.9141	0.1803	-1.4784	0.9737	-0.3631	1.0000	-0.3344	1.0000	0.3406	0.9947	0.7894
LOC100548837	1.0000	2.1849	1.0000	0.6959	1.0000	0.0000	1.0000	-2.3200	1.0000	2.2736	1.0000	0.9035	1.0000	0.0000
LOC100548843	1.0000	-1.1537	1.0000	2.2534	0.8011	-3.2629	1.0000	2.3242	1.0000	-3.2304	1.0000	0.0489	1.0000	2.3542
LOC100548849	0.0909	0.9689	0.4125	0.3419	0.8822	0.2790	1.0000	-0.0444	1.0000	0.6553	1.0000	0.0427	0.8631	0.3366
LOC100548859	0.5463	0.1535	0.6078	-0.0866	0.2533	-0.2428	0.0001	-0.3756	1.0000	0.1116	0.8377	-0.1159	1.0000	-0.0168
LOC100548864	1.0000	2.1901	1.0000	0.0000	0.8011	3.0840	0.7701	3.1732	1.0000	0.0000	1.0000	-2.2993	1.0000	0.0723
LOC100548894	0.3902	-0.1982	0.0000	-0.4091	0.1414	-0.2804	0.0000	-0.5692	1.0000	0.1354	1.0000	-0.0642	0.5583	-0.1480
LOC100548900	0.3772	-0.5753	0.9160	-0.2276	0.2543	-0.7056	0.6734	0.3270	1.0000	-0.5878	1.0000	-0.2305	0.7388	0.4469
LOC100548902	0.0000	1.6408	0.0000	2.0000	0.0000	2.4311	0.0000	1.9812	1.0000	-0.0677	0.5830	0.3057	0.0003	-0.5118
LOC100548914	1.0000	-0.3568	0.2103	1.6226	0.3635	1.2676	0.0210	2.2378	1.0000	-1.2936	1.0000	0.6959	1.0000	-0.3227
LOC100548920	0.9650	1.0580	1.0000	2.2534	1.0000	-2.4061	1.0000	2.3242	1.0000	-2.3771	1.0000	-1.3189	1.0000	2.3542
LOC100548925	0.0066	1.5190	0.2056	0.7328	0.2206	-1.1700	0.1371	-1.2116	1.0000	0.3009	0.8172	-0.4701	1.0000	0.2670
LOC100548929	0.4900	0.6297	1.0000	0.1829	0.4265	0.6483	0.0042	1.3330	1.0000	-0.0408	0.9188	-0.4760	0.4081	0.6452
LOC100548930	1.0000	-2.4788	1.0000	0.0000	1.0000	-2.4061	1.0000	0.0000	1.0000	-2.3771	1.0000	0.0000	1.0000	0.0000
LOC100548938	0.1199	-0.3341	0.0084	-0.2946	0.8114	-0.1137	1.0000	0.0008	1.0000	0.0457	0.9908	0.0984	0.5628	0.1670
LOC100548941	1.0000	2.1902	1.0000	0.6956	1.0000	2.2427	1.0000	-2.3176	1.0000	2.2676	1.0000	0.8994	1.0000	-2.2888
LOC100548946	0.0000	0.7292	0.0000	0.9701	0.0000	1.0420	0.0000	0.8480	1.0000	0.0466	0.0167	0.3001	0.3352	-0.1427
LOC100548949	0.0013	-0.6516	0.0000	-0.7313	0.0001	0.6943	0.0076	0.2833	1.0000	0.2789	0.5629	0.2119	0.6347	-0.1281
LOC100548954	0.5588	0.2796	0.2274	0.3749	0.0364	0.6244	0.0059	0.6490	1.0000	-0.2612	1.0000	-0.1539	0.6944	-0.2307
LOC100548957	1.0000	0.2625	0.5801	0.4902	0.0836	1.3115	0.4541	0.6157	1.0000	0.3083	0.9944	0.5511	0.9950	-0.3873
LOC100548962	1.0000	0.0000	1.0000	0.0000	1.0000	0.0000	1.0000	0.0000	1.0000	0.0000	1.0000	0.0000	1.0000	0.0000
LOC100548963	0.0022	-2.6699	0.0519	-2.0086	0.1189	-1.2515	0.0259	-2.2205	1.0000	-0.6139	1.0000	0.0593	0.4393	-1.5791
LOC100548964	0.1988	0.8408	0.0136	1.1078	1.0000	0.2451	0.7587	0.3519	1.0000	-0.1307	1.0000	0.1471	1.0000	-0.0181
LOC100548965	0.0247	-0.8107	0.0001	-0.8326	0.1012	-0.5444	0.0000	-1.0546	1.0000	-0.0545	1.0000	-0.0620	0.1028	-0.5573
LOC100548974	0.0156	0.7829	0.0078	0.5753	0.0000	-2.0549	0.0000	-1.1088	1.0000	0.1801	1.0000	-0.0138	0.0268	1.1301
LOC100548984	1.0000	0.0000	1.0000	0.0000	1.0000	0.0000	1.0000	0.0000	1.0000	0.0000	1.0000	0.0000	1.0000	0.0000
LOC100548989	0.0000	-2.3555	0.0000	-2.2767	0.0000	-0.8571	0.0000	-0.5202	1.0000	0.0087	1.0000	0.0990	0.0006	0.3505
LOC100548991	0.4816	0.6277	0.2085	0.8612	0.0942	1.0467	0.3458	0.7536	1.0000	-0.3293	1.0000	-0.0879	0.5968	-0.6229
LOC100548996	1.0000	-0.0481	1.0000	-0.8704	0.2833	-4.1854	0.7524	-1.2239	1.0000	0.1935	1.0000	-0.6248	1.0000	3.2066
LOC100549000	0.1119	0.4295	0.2628	0.2946	0.1327	-0.4614	0.0007	-0.8189	1.0000	0.0044	0.9892	-0.1193	0.3618	-0.3504
LOC100549006	1.0000	0.0000	1.0000	0.0000	1.0000	0.0000	1.0000	0.0000	1.0000	0.0000	1.0000	0.0000	1.0000	0.0000
LOC100549016	0.0056	-1.9631	0.0147	-1.5839	0.9127	-0.2965	0.2903	0.5709	1.0000	-0.3315	1.0000	0.0627	0.5877	0.5420
LOC100549021	0.0003	-0.7145	0.0000	-0.7076	0.1892	-0.2851	0.8102	0.0676	1.0000	-0.1177	1.0000	-0.0972	0.1998	0.2416
LOC100549028	0.0000	-0.6574	0.0000	-0.7196	0.0013	-0.5240	0.0055	-0.2590	1.0000	-0.2020	0.0953	-0.2518	0.9835	0.0684
LOC100549029	0.8318	0.7284	0.4777	1.0963	0.8177	-1.1335	0.6172	-1.7602	1.0000	-0.6796	1.0000	-0.3078	1.0000	-1.3084
LOC100549030	0.0541	-1.4963	0.0801	-0.9628	1.0000	-0.1490	0.2820	-0.5931	1.0000	0.2638	0.7760	0.8089	1.0000	-0.1801
LOC100549042	1.0000	-2.4776	1.0000	0.0000	1.0000	-2.4056	0.4370	3.7041	1.0000	-2.3758	1.0000	0.0000	0.7287	3.7387
LOC100549046	1.0000	0.0000	1.0000	0.0000	1.0000	0.0000	1.0000	0.0000	1.0000	0.0000	1.0000	0.0000	1.0000	0.0000
LOC100549047	1.0000	0.0000	1.0000	0.0000	1.0000	0.0000	1.0000	0.0000	1.0000	0.0000	1.0000	0.0000	1.0000	0.0000
LOC100549054	1.0000	0.0000	1.0000	-2.3959	1.0000	2.2471	1.0000	-2.3176	1.0000	2.2676	1.0000	0.0000	1.0000	-2.2907
LOC100549055	0.5506	-0.2564	0.0000	0.3335	1.0000	-0.0567	0.0000	0.8820	1.0000	0.5995	0.0000	1.2015	0.0000	1.5421
LOC100549062	0.1553	0.9658	0.0067	1.0572	0.0000	2.8756	0.0000	2.4125	1.0000	0.5289	0.3799	0.6325	1.0000	0.0714
LOC100549070	0.6126	0.3670	0.2644	0.4248	0.0451	0.7288	0.0205	0.7383	1.0000	-0.0500	1.0000	0.0157	1.0000	-0.0365
LOC100549073	0.0000	-1.0655	0.0000	-0.8826	0.0000	-1.2249	0.0000	-0.8561	1.0000	-0.2356	1.0000	-0.0409	0.6830	0.1390
LOC100549076	1.0000	-1.1537	1.0000	-0.1449	0.0038	2.9742	0.1523	2.5422	1.0000	-0.9637	1.0000	0.0471	0.2394	-1.4005
LOC100549082	0.0000	-1.9734	0.0000	-2.1628	0.0000	-1.6540	0.0000	-1.1201	1.0000	0.1383	1.0000	-0.0394	0.0004	0.6765
LOC100549086	0.0010	0.5852	0.0018	0.4273	0.0000	-1.6579	0.0000	-1.6345	1.0000	-0.0488	0.4491	-0.1951	1.0000	-0.0204
LOC100549091	1.0000	0.0000	1.0000	0.0000	1.0000	0.0000	1.0000	0.0000	1.0000	0.0000	1.0000	0.0000	1.0000	0.0000
LOC100549093	0.0653	2.8427	0.2913	1.4842	0.0000	4.5106	0.0000	3.0748	1.0000	1.2661	1.0000	-0.0729	1.0000	-0.1628
LOC100549101	0.0122	0.3978	0.0043	0.2251	1.0000	-0.0500	0.6831	0.0591	1.0000	0.1005	1.0000	-0.0598	0.0716	0.2151
LOC100549107	0.0353	-0.4382	0.0000	-0.4217	0.0012	-0.6465	0.0000	-0.8384	1.0000	-0.1826	0.1871	-0.1537	0.0003	-0.3682
LOC100549110	0.0303	-0.6454	0.3326	-0.2207	0.0012	-1.0393	0.0001	-0.7057	1.0000	-0.1426	0.5517	0.2929	0.9338	0.1976
LOC100549111	0.8601	0.1191	0.2752	0.2094	0.0000	-1.0539	0.0000	-0.8710	1.0000	-0.0948	1.0000	0.0070	1.0000	0.0925
LOC100549117	1.0000	0.0000	1.0000	0.0000	1.0000	0.0000	1.0000	0.0000	1.0000	0.0000	1.0000	0.0000	1.0000	0.0000
LOC100549118	0.0062	0.7457	0.0022	0.5813	0.4261	0.2996	0.4607	0.2023	1.0000	-0.0204	0.8732	-0.1711	1.0000	-0.1128
LOC100549121	1.0000	2.1901	1.0000	0.0000	1.0000	0.0000	1.0000	0.0000	1.0000	0.0000	1.0000	-2.2993	1.0000	0.0000
LOC100549134	0.0939	-2.0008	0.0455	-1.5525	1.0000	0.2775	1.0000	-0.1401	1.0000	0.9273	0.8665	1.3952	1.0000	0.5133
LOC100549138	0.0660	0.3133	0.0001	0.3211	0.1939	-0.2433	0.4942	-0.0831	1.0000	-0.1114	0.8183	-0.0917	1.0000	0.0541
LOC100549140	1.0000	0.0000	1.0000	0.0000	1.0000	0.0000	1.0000	0.0000	1.0000	0.0000	1.0000	0.0000	1.0000	0.0000
LOC100549141	0.0000	-0.8641	0.0000	-0.7416	0.0000	-1.3491	0.0176	-0.3600	1.0000	-0.2318	1.0000	-0.0962	0.0007	0.7650
LOC100549146	0.0212	-0.3886	0.0000	-0.5420	0.0000	-1.0681	0.0000	-0.7108	1.0000	-0.1345	0.0404	-0.2757	0.4202	0.2291
LOC100549159	1.0000	0.2026	0.2541	-2.4804	0.7662	1.0359	0.4992	-1.4670	1.0000	1.3485	1.0000	-1.3257	0.9835	-1.1477
LOC100549160	0.2915	-0.4712	0.0009	-1.0121	0.0061	0.8222	0.0006	0.7677	1.0000	-0.3406	0.0405	-0.8693	0.1702	-0.3881
LOC100549167	0.0029	1.6703	0.0103	1.2321	0.0046	-3.6920	0.0012	-3.6952	1.0000	0.0616	0.8732	-0.3678	1.0000	0.0652
LOC100549172	0.0237	0.6395	0.0000	1.2060	0.0000	1.3100	0.0000	1.5666	1.0000	-0.1257	0.0162	0.4554	0.7363	0.1367
LOC100549177	0.3576	0.2454	0.1493	0.2217	0.0948	0.3670	1.0000	0.0267	1.0000	-0.0149	1.0000	-0.0272	0.0291	-0.3513
LOC100549178	0.5086	3.5431	0.7231	1.0549	0.0304	4.9505	1.0000	-0.8429	1.0000	3.1191	1.0000	0.7503	0.2476	-2.6505
LOC100549188	1.0000	2.1849	1.0000	0.0000	1.0000	0.0000	1.0000	2.3257	1.0000	0.0000	1.0000	-2.2955	1.0000	2.3554
LOC100549193	1.0000	-0.0937	0.7674	3.0956	1.0000	-0.8815	0.4370	3.7041	1.0000	-4.4573	0.9101	-1.4174	1.0000	0.0752
LOC100549197	0.0025	1.0095	0.0000	1.5609	0.0000	2.0542	0.0000	1.5197	1.0000	-0.1132	0.1428	0.4498	0.0015	-0.6432
LOC100549201	1.0000	0.0000	1.0000	0.0000	1.0000	0.0000	1.0000	0.0000	1.0000	0.0000	1.0000	0.0000	1.0000	0.0000
LOC100549202	1.0000	0.0000	1.0000	0.0000	1.0000	2.2427	1.0000	0.0000	1.0000	0.0000	1.0000	0.0000	1.0000	-2.2888
LOC100549208	0.1824	1.7732	0.0000	6.5709	0.0184	2.0094	0.2426	4.0910	1.0000	-4.4568	1.0000	0.1960	0.0079	-2.4329
LOC100549217	0.0016	-0.6293	0.0000	-1.0066	0.0000	-0.9082	0.0000	-0.7013	1.0000	0.0973	0.3681	-0.2684	0.1618	0.3101
LOC100549224	0.3019	0.3016	0.1201	0.2405	0.0084	0.5389	0.0007	0.4312	1.0000	-0.0021	1.0000	-0.0507	0.8751	-0.1048
LOC100549245	0.0472	5.0373	1.0000	0.0000	1.0000	2.2427	1.0000	2.3257	1.0000	0.0000	0.1007	-5.1752	1.0000	0.0660
LOC100549247	0.1237	1.5997	0.0375	1.4314	0.0000	2.9710	0.0009	1.8948	1.0000	0.6914	0.9652	0.5387	0.8028	-0.3830
LOC100549259	1.0000	0.0000	1.0000	0.0000	1.0000	2.2426	1.0000	2.3257	1.0000	0.0000	1.0000	0.0000	1.0000	0.0660
LOC100549268	0.8489	-0.6364	0.9762	-0.3005	0.0171	1.4888	0.0013	1.6505	1.0000	-0.0092	1.0000	0.3412	1.0000	0.1587
LOC100549271	1.0000	0.0000	1.0000	0.0000	1.0000	0.0000	1.0000	0.0000	1.0000	0.0000	1.0000	0.0000	1.0000	0.0000
LOC100549275	0.0585	1.9508	0.6332	0.5466	0.1183	1.4968	1.0000	0.2256	1.0000	0.8414	0.9618	-0.5518	1.0000	-0.4259
LOC100549281	1.0000	-0.0135	0.1283	0.3219	0.0035	-0.8075	0.0004	-0.7885	1.0000	-0.2664	1.0000	0.0798	0.7661	-0.2427
LOC100549283	1.0000	0.0000	1.0000	0.0000	1.0000	0.0000	1.0000	0.0000	1.0000	0.0000	1.0000	0.0000	1.0000	0.0000
LOC100549289	0.0014	-0.5962	0.0000	-0.8388	0.1188	-0.3178	0.2421	-0.1630	1.0000	-0.2043	0.0109	-0.4346	1.0000	-0.0447

LOC100549299	0.8549	-0.1367	0.7666	0.1031	0.4053	0.2655	0.3235	0.2118	1.0000	0.0570	0.3324	0.3103	1.0000	0.0103
LOC100549312	0.6671	-1.2886	1.0000	2.2534	0.2858	-2.4984	0.4370	3.7041	1.0000	-4.7091	1.0000	-1.3189	1.0000	1.4485
LOC100549326	0.0303	-0.4421	0.0000	-0.4049	0.0000	-1.1652	0.0000	-1.1609	1.0000	0.0459	1.0000	0.0963	1.0000	0.0548
LOC100549331	0.0000	-5.4667	0.0000	-6.4119	0.0000	1.2746	0.0000	1.3905	1.0000	0.1987	0.2039	-0.7341	0.0037	0.3183
LOC100549337	1.0000	0.2050	1.0000	0.2188	0.4345	1.0882	0.0838	1.4711	1.0000	-0.1224	1.0000	-0.1006	1.0000	0.2585
LOC100549340	0.6227	0.2750	0.0103	0.7127	0.0668	0.6245	0.0002	0.9452	1.0000	-0.9511	0.3217	-0.5039	0.0773	-0.6249
LOC100549350	1.0000	0.0000	0.7674	3.0888	1.0000	0.0000	1.0000	0.0000	1.0000	0.0000	1.0000	3.1946	1.0000	0.0000
LOC100549351	0.0383	0.8702	0.3749	0.4235	0.0000	1.8617	0.0000	1.9317	1.0000	-0.2310	0.1381	-0.6652	0.9594	-0.1569
LOC100549353	0.0000	2.1266	0.0000	2.3791	0.0000	2.6993	0.0000	2.2762	1.0000	0.0500	0.8607	0.3158	0.3816	-0.3678
LOC100549354	0.2502	-0.2340	0.4690	-0.0950	0.7731	0.1032	0.0146	0.2239	1.0000	-0.0021	0.5244	0.1493	0.5423	0.1241
LOC100549359	0.0021	0.8028	0.0000	0.7408	0.6411	0.2190	0.6307	0.1523	1.0000	0.0726	1.0000	0.0213	1.0000	0.0112
LOC100549361	1.0000	0.2345	1.0000	-0.0514	1.0000	0.1441	0.7781	-0.2481	1.0000	0.5894	1.0000	0.3194	1.0000	0.2033
LOC100549369	0.5542	-0.1487	0.0134	-0.2185	0.8994	-0.0782	0.0060	-0.2161	1.0000	0.1765	0.6439	0.1190	1.0000	0.0441
LOC100549370	0.3247	2.2130	0.0076	5.5188	1.0000	-2.4056	1.0000	0.0000	1.0000	-2.3758	1.0000	0.8135	1.0000	0.0000
LOC100549374	1.0000	-0.1717	0.1304	0.7810	0.2472	-0.7415	0.1583	0.7258	1.0000	-0.7328	1.0000	0.2310	0.3434	0.7388
LOC100549389	0.0366	-0.3555	0.0002	-0.3300	0.4886	-0.1575	0.4421	-0.0904	1.0000	-0.0673	1.0000	-0.0298	1.0000	0.0047
LOC100549393	0.0001	0.9212	0.0000	1.1970	1.0000	0.0660	0.0644	-0.3648	1.0000	-0.1383	0.7487	0.1468	0.0023	-0.5635
LOC100549411	0.0000	-1.1858	0.0000	-1.3797	0.0000	0.9475	0.0126	0.4519	1.0000	-0.1845	0.6448	-0.3650	0.0000	-0.6726
LOC100549421	1.0000	0.0143	0.0601	0.2924	1.0000	0.0578	0.0033	0.4282	1.0000	-0.1080	0.6102	0.1815	0.2724	0.2678
LOC100549425	1.0000	0.0164	0.4567	0.0831	0.0505	0.3254	0.0000	0.3827	1.0000	0.0389	0.6054	0.1182	0.6106	0.1017
LOC100549427	1.0000	0.0000	1.0000	0.0000	1.0000	0.0000	1.0000	0.0000	1.0000	0.0000	1.0000	0.0000	1.0000	0.0000
LOC100549440	1.0000	0.3476	0.0599	2.8723	1.0000	-0.7130	1.0000	0.8518	1.0000	-1.4918	0.9343	1.0246	1.0000	0.0718
LOC100549445	1.0000	0.0000	1.0000	0.0000	1.0000	0.0000	1.0000	0.0000	1.0000	0.0000	1.0000	0.0000	1.0000	0.0000
LOC100549446	0.0000	-1.1928	0.0000	-1.0095	1.0000	-0.0915	0.0396	0.4407	1.0000	-0.5939	0.5942	-0.3986	1.0000	-0.0566
LOC100549451	0.8249	3.0255	1.0000	0.0000	1.0000	0.0000	1.0000	0.0000	1.0000	0.0000	1.0000	-3.1468	1.0000	0.0000
LOC100549452	0.7197	0.1918	0.4007	0.2558	0.8211	-0.1533	0.6037	-0.1677	1.0000	-0.1975	1.0000	-0.1220	0.7287	-0.2085
LOC100549453	1.0000	2.1902	1.0000	-0.1449	1.0000	2.2471	1.0000	-2.3176	1.0000	2.2676	1.0000	0.0471	1.0000	-2.2907
LOC100549460	0.7251	1.4394	1.0000	-0.1449	1.0000	0.6783	1.0000	-2.3176	1.0000	-0.1078	1.0000	-1.7053	1.0000	-3.1344
LOC100549461	1.0000	0.0000	1.0000	0.0000	1.0000	0.0000	1.0000	0.0000	1.0000	0.0000	1.0000	0.0000	1.0000	0.0000
LOC100549463	0.1331	0.3717	0.0000	0.6802	0.1142	-0.4474	0.0345	0.3290	1.0000	-0.1724	0.7636	0.1469	0.0023	0.6095
LOC100549470	0.0191	0.4930	0.0000	0.5604	0.0719	0.3874	0.1460	0.1469	1.0000	-0.1334	1.0000	-0.0540	0.0000	-0.3685
LOC100549479	0.8226	0.3189	0.0985	0.7842	0.7742	0.3543	0.1070	0.7824	1.0000	-0.3889	1.0000	0.0900	1.0000	0.0449
LOC100549485	0.0006	-3.0567	0.0000	-3.2668	0.0685	1.0165	0.7094	0.2956	1.0000	0.6369	1.0000	0.4448	1.0000	-0.0745
LOC100549494	0.5378	3.5350	1.0000	-0.1449	0.4809	3.6101	1.0000	0.0062	1.0000	2.2676	1.0000	-1.3218	1.0000	-1.3084
LOC100549505	1.0000	2.1901	1.0000	0.6956	1.0000	2.2471	1.0000	0.0062	1.0000	2.2676	1.0000	0.8994	1.0000	0.0640
LOC100549507	1.0000	0.5464	1.0000	2.2534	1.0000	-0.1591	0.7701	3.1732	1.0000	-2.3758	1.0000	-0.7977	1.0000	0.9164
LOC100549511	1.0000	0.0000	1.0000	0.0000	1.0000	0.0000	1.0000	0.0000	1.0000	0.0000	1.0000	0.0000	1.0000	0.0000
LOC100549513	0.0001	1.7188	0.0000	1.7268	0.0000	2.2541	0.0000	1.7964	1.0000	0.2502	0.8565	0.2693	0.7879	-0.2040
LOC100549516	0.1174	4.6986	1.0000	-0.1630	1.0000	0.0000	0.7710	-3.1693	1.0000	3.1191	0.7681	-1.6329	1.0000	0.0000
LOC100549522	0.0462	0.5988	0.0000	0.8194	0.0005	0.9729	0.0000	0.9309	1.0000	-0.2088	1.0000	0.0256	0.4509	-0.2453
LOC100549525	0.4695	-0.3368	0.0006	-0.6935	0.0000	1.3835	0.0000	0.9217	1.0000	0.2480	1.0000	-0.0965	0.4483	-0.2082
LOC100549531	0.8226	-0.1358	0.1119	-0.2568	0.1439	0.3689	0.2831	0.1854	1.0000	0.1996	1.0000	0.0913	1.0000	0.0216
LOC100549535	0.0041	-0.5047	0.0000	-0.7083	0.7056	-0.1191	0.0140	-0.2196	1.0000	0.1260	1.0000	-0.0652	1.0000	0.0307
LOC100549541	0.0513	-1.0589	0.4809	-0.3734	0.0000	1.6610	0.0000	1.7537	1.0000	0.1848	0.2017	0.8858	0.5215	0.2885
LOC100549545	0.6881	1.7514	0.9062	-1.5339	1.0000	-0.1597	1.0000	0.3938	1.0000	1.2628	0.8269	-2.0158	0.8939	1.8357
LOC100549552	0.0000	0.8356	0.0000	0.8576	0.0417	-0.4005	0.0705	-0.2197	1.0000	0.0179	1.0000	0.0513	0.3232	0.2040
LOC100549557	0.0002	0.9370	0.0000	0.7398	0.0002	0.9002	0.0002	0.6373	1.0000	0.2034	1.0000	0.0168	1.0000	-0.0550
LOC100549568	1.0000	0.0000	1.0000	0.0000	1.0000	0.0000	1.0000	0.0000	1.0000	0.0000	1.0000	0.0000	1.0000	0.0000
LOC100549590	0.3089	1.4942	1.0000	0.1770	0.7004	-1.9462	0.8250	-0.7628	1.0000	0.6374	0.9341	-0.6612	0.8937	1.8364
LOC100549601	0.4026	-0.3127	0.9689	0.0909	1.0000	0.0796	0.5580	0.1967	1.0000	-0.3526	1.0000	0.0617	0.6247	-0.2320
LOC100549614	1.0000	-2.4776	1.0000	0.3667	1.0000	0.6746	1.0000	0.0059	1.0000	0.7365	0.8607	3.7297	1.0000	0.0729
LOC100549616	1.0000	0.0000	1.0000	0.0000	1.0000	0.0000	1.0000	0.0000	1.0000	0.0000	1.0000	0.0000	1.0000	0.0000
LOC100549617	0.8249	-0.6674	0.2555	2.1641	0.1875	1.0440	0.0532	2.8939	1.0000	-3.1543	1.0000	-0.3400	0.1673	-1.3108
LOC100549626	0.3135	-2.0285	0.2067	-1.3973	1.0000	-0.4132	1.0000	-0.2501	1.0000	0.6258	1.0000	1.2798	0.9940	0.7930
LOC100549632	0.7124	-0.4118	0.0120	1.2493	0.5084	-0.5598	0.2038	0.7809	1.0000	-0.9035	0.3485	0.7647	0.8257	0.4427
LOC100549639	1.0000	0.2183	1.0000	-0.0397	0.0022	-3.2088	0.3719	-0.7677	1.0000	-0.0533	1.0000	-0.2996	0.1720	2.3979
LOC100549646	0.0102	-0.7634	0.0000	-1.0547	0.4809	-0.2570	0.2949	0.2309	1.0000	-0.1424	0.4761	-0.4205	0.1817	0.3517
LOC100549648	0.0000	1.5184	0.0000	1.7054	0.0353	-0.6537	0.0710	-0.4436	1.0000	-0.2344	1.0000	-0.0361	1.0000	-0.0191
LOC100549666	1.0000	-2.4776	1.0000	-0.1452	1.0000	-2.4056	1.0000	-2.3200	1.0000	-0.1034	1.0000	2.3480	1.0000	0.0000
LOC100549668	0.4252	-1.8101	1.0000	0.1021	0.5378	0.9949	0.8982	-0.5593	1.0000	0.4400	0.2853	2.3773	0.5016	-1.1108
LOC100549671	0.0000	4.7782	0.0000	3.4295	1.0000	-1.0191	0.2469	-2.3163	1.0000	1.3536	1.0000	0.0228	1.0000	0.0652
LOC100549678	0.0000	-1.4475	0.0000	-1.6319	0.1803	-0.4715	0.0934	-0.3511	1.0000	0.2736	1.0000	0.1014	0.2614	0.3992
LOC100549687	1.0000	-0.1608	1.0000	-0.1767	1.0000	-0.6594	0.5798	-1.1513	1.0000	-0.1253	1.0000	-0.1293	1.0000	-0.6154
LOC100549689	1.0000	0.0000	1.0000	0.0000	1.0000	0.0000	1.0000	0.0000	1.0000	0.0000	1.0000	0.0000	1.0000	0.0000
LOC100549696	0.0511	-1.7420	0.0099	-1.5085	0.0796	-1.5772	0.0000	1.4947	1.0000	0.0951	1.0000	0.3414	0.0000	3.1775
LOC100549706	0.0080	5.4471	0.6215	1.6074	0.0004	6.0113	0.0000	4.2845	1.0000	2.2676	0.5211	-1.4682	0.6913	0.5717
LOC100549707	0.0027	0.8458	0.0059	0.5098	0.1002	0.5100	0.8552	0.1008	1.0000	0.5094	0.8607	0.1858	1.0000	0.1058
LOC100549708	1.0000	-0.3504	0.4339	-0.8100	1.0000	-0.6665	0.9344	-0.4400	1.0000	0.5051	1.0000	0.0594	1.0000	0.7337
LOC100549719	0.1503	-2.3791	1.0000	0.5202	0.5868	-1.0113	1.0000	0.6974	1.0000	-1.6298	1.0000	1.2807	1.0000	0.0775
LOC100549720	0.0448	2.4187	0.0070	2.7638	1.0000	-1.0167	0.7710	-3.1693	1.0000	-0.1139	1.0000	0.2373	1.0000	-2.2907
LOC100549722	1.0000	0.0000	1.0000	0.0000	1.0000	2.2426	0.2461	4.0903	1.0000	0.0000	1.0000	0.0000	0.8939	1.8363
LOC100549728	0.6161	-0.1533	0.0000	-0.4209	0.0590	-0.3575	0.0001	-0.3679	1.0000	0.0555	0.3939	-0.2002	1.0000	0.0506
LOC100549732	0.1474	0.7305	0.0145	0.9910	0.0178	0.9846	0.0620	0.7598	1.0000	-0.0192	1.0000	0.2556	0.9273	-0.2378
LOC100549733	0.0646	-0.3313	0.0050	-0.2899	0.0641	0.3303	0.0692	0.1932	1.0000	-0.0742	1.0000	-0.0199	0.1022	-0.2059
LOC100549735	0.0001	0.8803	0.0000	1.0195	0.0001	0.8590	0.0000	0.9418	1.0000	-0.0219	0.8875	0.1305	1.0000	0.0676
LOC100549737	0.0004	0.6684	0.0000	0.5944	0.5474	-0.1896	0.0331	-0.2958	1.0000	0.1091	1.0000	0.0472	1.0000	0.0085
LOC100549755	0.2658	0.4299	0.2243	0.3913	1.0000	0.1253	0.0175	0.6295	1.0000	-0.3987	0.4348	-0.4237	1.0000	0.1146
LOC100549758	1.0000	0.0917	0.7666	-0.1788	0.0675	0.8103	0.0044	0.6498	1.0000	0.1876	1.0000	-0.0686	1.0000	0.0309
LOC100549762	0.2199	0.5864	0.0004	1.0944	1.0000	-0.0300	1.0000	-0.0791	1.0000	-0.2373	0.8875	0.2830	0.8937	-0.2813
LOC100549764	0.8784	-0.5265	0.4534	0.6339	0.9361	-0.4581	0.3201	-1.0762	1.0000	-0.6025	0.9002	0.5775	0.3986	-1.2079
LOC100549766	0.0002	1.7231	0.0079	0.8161	0.0019	-2.9034	0.0001	-1.6840	1.0000	1.0819	1.0000	0.1860	0.0861	2.3092
LOC100549788	1.0000	2.1902	1.0000	2.2507	1.0000	2.2426	1.0000	0.0000	1.0000	0.0000	1.0000	0.0471	1.0000	-2.2888
LOC100549795	0.2705	0.9458	0.0058	1.5014	0.0000	2.8071	0.0000	2.6075	1.0000	-0.1350	0.8688	0.4365	0.8331	-0.3279
LOC100549801	0.0000	-1.2330	0.0000	-1.2378	0.0002	-0.5760	0.0000	-0.5444	1.0000	-0.0025	1.0000	0.0052	1.0000	0.0346
LOC100549803	0.9234	-0.0838	0.5182	0.1044	0.7536	-0.1286	0.3356	0.1325	1.0000	-0.2705	1.0000	-0.0706	1.0000	-0.0034
LOC100549810	0.0000	1.5574	0.0000	1.4904	0.0000	2.1689	0.0000	2.0672	1.0000	-0.0781	1.0000	-0.1321	0.5342	-0.1716
LOC100549814	1.0000	-2.4788	0.7674	-3.2570	1.0000	-0.1597	0.7710	-3.1693	1.0000	0.7402	1.0000	0.0000	1.0000	-2.2907
LOC100549822	1.0000	0.0184	0.5168	0.0837	1.0000	0.0278	0.6127	-0.0741	1.0000	0.1863	0.0074	0.2638	0.7290	0.0892
LOC100549823	0.1667	0.3451	0.0000	0.6923	0.0001	0.7441	0.0000	0.7493	1.0000	-0.0567	0.1215	0.3021	1.0000	-0.0467
LOC100549824	0.7070	0.1232	0.2938	0.1244	0.0419	-0.3788	0.0000	-0.4201	1.0000	0.0983	0.7833	0.1118	1.0000	0.0622
LOC100549826	0.9684	-0.0595	0.0696	-0.1360	0.0010	-0.5042	0.0000	-0.4884	1.0000	0.0189	1.0000	-0.0454	1.0000	0.0401
LOC100549829	0.0000	1.1944	0.0000	1.1889	0.0000	-1.2616	0.0000	-0.6899	1.0000	-0.0699	1.0000	-0.0639	0.0097	0.5079
LOC100549832	0.2571	0.4199	0.2557	0.3070	0.0000	1.1420	0.0000	1.3364	1.0000	0.0798	1.0000	-0.0196	0.3644	0.2801

LOC100549837	0.0174	0.4383	0.0250	0.2544	0.2292	-0.2665	0.1556	-0.1616	1.0000	0.0552	0.8154	-0.1160	0.4382	0.1655
LOC100549851	0.7131	0.3778	0.0066	1.0346	0.0000	2.1437	0.0000	2.6075	1.0000	-0.4605	1.0000	0.2063	1.0000	0.0058
LOC100549854	0.4216	-0.2001	0.5704	-0.0759	0.0066	-0.4936	0.0001	-0.3272	1.0000	-0.0376	0.8889	0.0989	0.4996	0.1347
LOC100549855	0.0001	1.8761	0.0000	2.1976	0.0000	2.2486	0.0000	2.1718	1.0000	-0.0122	0.8162	0.3190	1.0000	-0.0861
LOC100549857	1.0000	0.0000	1.0000	0.0000	1.0000	0.0000	1.0000	2.3242	1.0000	0.0000	1.0000	0.0000	1.0000	2.3542
LOC100549863	1.0000	0.0292	0.8536	-0.2069	0.4571	-0.4281	1.0000	0.0081	1.0000	-0.4506	0.2470	-0.6726	1.0000	-0.0049
LOC100549869	0.0624	-0.6499	0.0215	-0.5837	0.3027	-0.3774	0.3999	-0.2326	1.0000	0.1102	1.0000	0.1872	0.6442	0.2573
LOC100549877	1.0000	0.0000	1.0000	0.0000	1.0000	0.0000	1.0000	0.0000	1.0000	0.0000	1.0000	0.0000	1.0000	0.0000
LOC100549885	1.0000	0.0000	1.0000	2.2534	0.0349	4.9458	0.4547	3.7078	1.0000	0.0000	1.0000	2.3480	0.7202	-1.2637
LOC100549893	0.0337	-0.3409	0.0001	-0.2856	0.0001	-0.5695	0.0000	-0.5875	1.0000	-0.0364	1.0000	0.0313	1.0000	-0.0494
LOC100549894	0.0635	0.3227	0.0001	0.3371	0.0005	0.5598	0.0000	0.4391	1.0000	0.0451	0.9415	0.0723	0.8791	-0.0703
LOC100549896	1.0000	0.5370	1.0000	0.6959	1.0000	-0.1597	1.0000	0.8518	1.0000	-0.1045	1.0000	0.0598	1.0000	0.9149
LOC100549911	0.1878	-0.4704	0.0444	-0.5872	1.0000	-0.0392	0.0846	-0.4865	1.0000	0.0976	1.0000	-0.0076	0.4702	-0.3456
LOC100549918	0.3414	0.3587	0.0000	0.7389	0.0000	1.0813	0.0000	1.3280	1.0000	-0.2244	0.7641	0.1691	1.0000	0.0288
LOC100549920	0.0067	1.9271	0.0013	2.6016	0.4651	0.9659	0.2792	1.5161	1.0000	-1.2839	0.7202	-0.6039	0.7179	-0.7328
LOC100549940	0.0020	0.7507	0.0000	1.0593	0.0002	0.8391	0.0000	0.8863	1.0000	-0.0698	0.3280	0.2503	1.0000	-0.0182
LOC100549945	0.5463	0.4895	0.0671	0.9536	0.6547	-0.5014	0.4996	-0.6461	1.0000	-0.5740	1.0000	-0.1005	0.6076	-0.7154
LOC100549955	0.0000	-0.8754	0.0000	-0.6238	0.0000	-0.9937	0.0002	-0.3616	1.0000	-0.4087	0.7247	-0.1449	0.1846	0.2296
LOC100549962	1.0000	-2.4776	0.4642	-3.7944	1.0000	-2.4056	1.0000	0.6947	1.0000	1.2739	1.0000	0.0000	0.3446	4.4306
LOC100549969	0.0062	0.4748	0.0000	0.3908	0.0001	-0.7242	0.0000	-0.5743	1.0000	0.0671	1.0000	-0.0049	0.2726	0.2216
LOC100549986	0.1974	-4.5752	0.7666	-3.2534	0.9250	0.4648	0.0534	2.3289	1.0000	-1.3434	1.0000	0.0000	1.0000	0.5257
LOC100549990	0.1788	-0.2949	0.0000	-0.3569	1.0000	0.0571	0.4367	-0.0856	1.0000	-0.0129	1.0000	-0.0621	0.3052	-0.1498
LOC100549991	1.0000	0.0000	0.7674	-3.2570	1.0000	0.0000	0.7710	-3.1693	1.0000	3.1191	1.0000	0.0000	1.0000	0.0000
LOC100549995	1.0000	0.0000	1.0000	0.0000	1.0000	0.0000	1.0000	0.0000	1.0000	0.0000	1.0000	0.0000	1.0000	0.0000
LOC100549996	1.0000	-0.0138	0.0179	-1.0058	0.8859	0.3298	0.0966	-0.7149	1.0000	0.9870	1.0000	0.0075	1.0000	-0.0573
LOC100550004	1.0000	0.0000	1.0000	0.0000	1.0000	0.0000	1.0000	0.0000	1.0000	0.0000	1.0000	0.0000	1.0000	0.0000
LOC100550010	1.0000	-0.2881	1.0000	0.0000	0.9361	1.2064	1.0000	0.0000	1.0000	-2.3758	1.0000	-2.2992	0.7287	-3.6637
LOC100550016	1.0000	0.1965	1.0000	0.3635	1.0000	-1.0191	0.7701	-3.1666	1.0000	-0.1179	1.0000	0.0581	1.0000	-2.2889
LOC100550020	0.9628	0.4243	1.0000	0.4717	0.3446	0.8523	1.0000	0.2585	1.0000	-1.2250	0.4868	-1.1689	0.0369	-1.8167
LOC100550023	0.1319	-3.0378	0.0006	-5.8196	1.0000	0.1215	0.4594	-0.8626	1.0000	0.5553	1.0000	-2.2992	1.0000	-0.4209
LOC100550029	1.0000	2.1849	0.7231	1.0549	0.8033	3.0892	0.7710	-3.1693	1.0000	3.1191	0.8269	2.1252	1.0000	-3.1370
LOC100550035	0.0000	1.5422	0.0000	1.2152	0.0000	1.0366	0.0348	0.3997	1.0000	0.0070	0.3598	-0.3070	0.0010	-0.6263
LOC100550042	1.0000	0.0000	0.7674	-3.2570	0.4809	3.6101	1.0000	-0.8442	1.0000	3.1191	1.0000	0.0000	1.0000	-1.3084
LOC100550044	1.0000	0.0133	0.6224	-0.1084	1.0000	0.0395	0.0100	-0.3661	1.0000	0.0896	1.0000	-0.0187	0.1211	-0.3102
LOC100550045	0.4996	-1.4996	1.0000	-0.9973	0.4586	0.9672	1.0000	0.0106	1.0000	-1.8135	1.0000	-1.3257	0.0222	-2.7758
LOC100550050	0.6157	0.6977	1.0000	-0.1768	0.0280	-3.2137	0.1383	-2.0325	1.0000	-0.2778	0.4012	-1.1413	1.0000	0.9164
LOC100550056	1.0000	-0.1143	0.0033	-0.6304	0.4046	-0.3335	0.0443	-0.4419	1.0000	0.3619	1.0000	-0.1393	0.6819	0.2606
LOC100550062	0.7782	0.1056	0.0016	0.2268	0.0033	0.4837	0.0166	0.2133	1.0000	0.1631	0.0387	0.2962	0.7901	-0.1017
LOC100550063	0.2090	0.3903	0.0000	0.8738	0.0000	0.9486	0.0000	1.1806	1.0000	-0.4698	1.0000	0.0276	0.1514	-0.2299
LOC100550074	0.0000	1.4034	0.0000	1.3716	1.0000	-0.0913	0.1387	-0.5462	1.0000	0.0537	1.0000	0.0305	0.5528	-0.3989
LOC100550081	0.3034	0.3444	0.3458	0.2175	0.0002	0.8837	0.0806	0.3595	1.0000	-0.0121	1.0000	-0.1267	0.0130	-0.5297
LOC100550096	1.0000	0.0000	1.0000	0.0000	1.0000	0.0000	1.0000	0.0000	1.0000	0.0000	1.0000	0.0000	1.0000	0.0000
LOC100550104	1.0000	0.0000	1.0000	0.0000	1.0000	0.0000	1.0000	0.0000	1.0000	0.0000	1.0000	0.0000	1.0000	0.0000
LOC100550119	0.0205	0.7102	0.0003	0.5746	0.0000	1.7129	0.0000	1.6378	1.0000	0.1674	1.0000	0.0454	0.9645	0.0975
LOC100550121	0.7615	0.3250	1.0000	0.1342	0.0001	1.4287	0.0213	0.8873	1.0000	0.2663	1.0000	0.0866	0.7472	-0.2654
LOC100550127	0.0000	1.7267	0.0003	1.2236	0.0030	1.3836	0.0006	1.2285	1.0000	-0.0889	0.1886	-0.5801	0.9668	-0.2410
LOC100550128	0.0000	1.0830	0.0000	1.6258	0.0619	-0.4027	0.0000	-1.8569	1.0000	0.1601	0.0000	0.7142	0.0000	-1.2867
LOC100550133	1.0000	-0.0395	1.0000	-0.3408	1.0000	-1.1017	0.6829	-0.8095	1.0000	0.9928	1.0000	0.7111	0.9835	1.2974
LOC100550136	1.0000	-2.4776	1.0000	0.0000	1.0000	-2.4056	1.0000	0.0000	1.0000	-2.3757	1.0000	0.0000	1.0000	0.0000
LOC100550137	0.0771	0.9016	0.0000	1.9152	0.4957	-0.5630	0.1841	-1.0505	1.0000	-0.6317	0.6983	0.3944	0.3841	-1.1177
LOC100550153	0.1271	-0.2786	0.3075	-0.1099	0.0000	-0.9329	0.0000	-0.7265	1.0000	-0.1184	1.0000	0.0626	0.8686	0.0942
LOC100550164	0.5662	-0.4417	0.7409	-0.3125	0.0002	1.3750	0.2539	0.5314	1.0000	-0.2905	1.0000	-0.1513	0.0006	-1.1313
LOC100550169	0.0908	4.8672	0.1791	2.3688	0.8011	3.0840	1.0000	-2.3200	1.0000	2.2735	1.0000	-0.1238	1.0000	-3.1344
LOC100550170	0.0006	-0.5303	0.0000	-0.4255	0.0340	-0.3490	0.0001	-0.3141	1.0000	0.0752	0.1968	0.1926	0.5608	0.1154
LOC100550171	1.0000	0.0000	1.0000	0.0000	1.0000	0.0000	1.0000	0.0000	1.0000	0.0000	1.0000	0.0000	1.0000	0.0000
LOC100550173	0.0015	1.0670	0.0222	0.5909	0.0000	1.5766	0.0003	0.8924	1.0000	0.4982	1.0000	0.0364	0.8306	-0.1794
LOC100550174	0.9545	1.0672	0.2920	4.0165	1.0000	0.6816	0.7701	3.1732	1.0000	-2.3771	1.0000	0.4471	1.0000	0.0716
LOC100550180	0.9890	-0.1052	0.6450	0.1639	0.0000	1.0542	0.0000	1.0759	1.0000	-0.0153	0.7635	0.2656	1.0000	0.0105
LOC100550182	0.8653	-0.0885	0.0230	-0.2292	0.0836	-0.3378	0.0000	-0.4628	1.0000	0.1413	1.0000	0.0131	1.0000	0.0207
LOC100550192	0.0036	2.2632	0.0001	2.6483	0.0000	3.5860	0.0000	3.2661	1.0000	-0.1255	1.0000	0.2687	0.5443	-0.4426
LOC100550202	0.2175	0.2751	0.5814	0.1000	0.8073	-0.1103	0.0525	-0.2446	1.0000	0.0337	0.7854	-0.1294	0.9371	-0.0960
LOC100550208	0.0000	1.7734	0.0000	1.9968	0.0001	1.3441	0.0000	1.4370	1.0000	-0.0987	1.0000	0.1357	1.0000	-0.0014
LOC100550216	0.0000	1.3494	0.0000	1.9416	0.0000	2.8544	0.0000	2.2966	1.0000	0.0824	0.0000	0.6884	0.0000	-0.4699
LOC100550217	0.0024	-1.0547	0.0000	-1.5268	0.7030	-0.2256	0.4199	-0.2476	1.0000	0.1100	0.8475	-0.3508	1.0000	0.0930
LOC100550226	1.0000	0.0000	1.0000	0.0000	1.0000	0.0000	1.0000	2.3257	1.0000	0.0000	1.0000	0.0000	1.0000	2.3554
LOC100550229	0.0046	0.5348	0.0000	0.4486	0.6922	0.1282	0.6770	-0.0756	1.0000	0.0027	1.0000	-0.0713	0.2562	-0.1957
LOC100550231	0.0683	0.3516	0.0000	0.5679	0.0178	0.4357	0.3178	0.1407	1.0000	0.0323	0.0663	0.2604	0.0704	-0.2586
LOC100550234	1.0000	0.0229	1.0000	0.0051	0.0000	1.6856	0.0000	1.8481	1.0000	-0.0437	1.0000	-0.0483	0.9353	0.1247
LOC100550249	1.0000	0.0000	1.0000	0.0000	1.0000	0.0000	1.0000	0.0000	1.0000	0.0000	1.0000	0.0000	1.0000	0.0000
LOC100550253	0.0616	1.6576	0.4385	0.6830	0.0460	1.6966	0.7710	-0.5636	1.0000	0.8436	1.0000	-0.1196	0.1479	-1.4146
LOC100550262	0.7184	0.1115	0.0004	0.3159	0.2515	-0.2256	0.2855	-0.1193	1.0000	-0.1578	1.0000	0.0585	1.0000	-0.0461
LOC100550263	1.0000	0.1478	0.0536	1.7595	0.3948	1.1264	0.2443	1.4104	1.0000	-0.9014	0.9652	0.7236	0.8856	-0.6175
LOC100550264	1.0000	0.0000	1.0000	0.0000	1.0000	0.0000	1.0000	0.0000	1.0000	0.0000	1.0000	0.0000	1.0000	0.0000
LOC100550265	0.3148	3.9252	1.0000	0.0000	1.0000	0.0000	0.7710	3.1709	1.0000	0.0000	0.6450	-4.0575	1.0000	3.2049
LOC100550268	0.0834	0.8623	0.0318	0.8844	0.1875	0.7160	0.0006	1.2383	1.0000	-0.0456	1.0000	-0.0045	0.3812	0.4881
LOC100550270	1.0000	-2.4788	1.0000	2.2507	1.0000	-0.1630	1.0000	0.0000	1.0000	-2.3771	1.0000	2.3456	1.0000	-2.2889
LOC100550272	0.0827	-0.3239	0.0000	-0.5953	0.0111	-0.4227	0.0059	-0.2223	1.0000	0.0909	0.4725	-0.1680	0.0043	0.2965
LOC100550279	0.0000	1.6270	0.0000	1.9277	0.0000	-1.3391	0.0000	-2.6944	1.0000	0.0923	0.0007	0.4060	0.0000	-1.2573
LOC100550280	0.5384	-1.5000	0.7218	-1.3908	0.8943	-0.9573	1.0000	0.0068	1.0000	-0.5892	1.0000	-0.4752	1.0000	0.3812
LOC100550282	0.0000	0.8527	0.0000	0.7848	0.0000	2.2541	0.0000	1.8895	1.0000	0.2712	0.1381	0.2162	0.7693	-0.0873
LOC100550289	0.0000	-2.2994	0.0000	-2.7447	0.7674	-0.2428	0.0016	-0.9892	1.0000	0.1339	1.0000	-0.2988	0.2644	-0.6106
LOC100550291	0.9691	-0.0785	0.4677	-0.0939	0.4733	-0.2024	0.3989	0.1018	1.0000	0.1834	0.3961	0.1814	0.0000	0.4920
LOC100550296	1.0000	-0.0626	0.4347	0.1744	0.0000	0.9606	0.0000	1.0054	1.0000	0.0161	0.3768	0.2646	1.0000	0.0631
LOC100550313	0.0001	0.5921	0.0000	0.7639	0.1686	0.2664	0.0011	0.2868	1.0000	-0.1514	1.0000	0.0333	0.5723	-0.1255
LOC100550314	0.0457	0.3852	0.0000	0.4747	0.4495	-0.1927	0.0000	-0.4217	1.0000	0.0842	0.3380	0.1859	0.5740	-0.1404
LOC100550316	1.0000	0.0000	1.0000	0.0000	1.0000	0.0000	1.0000	0.0000	1.0000	0.0000	1.0000	0.0000	1.0000	0.0000
LOC100550324	0.0000	-2.0253	0.0000	-2.4030	0.0108	-1.0796	0.0002	-1.3256	1.0000	-0.1130	1.0000	-0.4779	0.9515	-0.3536
LOC100550328	0.6230	0.8135	0.7666	0.5820	0.0000	2.6242	0.0002	2.2050	1.0000	-0.1364	1.0000	-0.3490	0.4860	-0.5436
LOC100550331	1.0000	0.0088	0.3422	-0.2162	0.0932	0.4787	0.0558	0.3195	1.0000	0.3335	1.0000	0.1216	0.6802	0.1792
LOC100550333	1.0000	0.5381	1.0000	2.2507	0.1846	2.3580	0.2426	4.0910	1.0000	-2.3757	1.0000	-0.7943	1.0000	-0.6929
LOC100550341	0.8450	0.1565	0.6224	0.1732	0.8033	0.1632	1.0000	-0.0245	1.0000	-0.0454	1.0000	-0.0164	0.6944	-0.2276
LOC100550348	0.7924	-0.1535	0.9062	-0.0932	0.0589	-0.5167	0.0026	-0.6931	1.0000	-0.1010	1.0000	-0.0301	0.5470	-0.2729
LOC100550358	1.0000	-0.2892	1.0000	-0.1608	0.0729	2.8572	0.0609	2.3282	1.0000	0.7304	1.0000	0.8962	1.0000	0.2128

LOC100550363	1.0000	-0.3354	1.0000	0.3667	0.3916	1.3283	0.4962	1.4811	1.0000	-0.6490	1.0000	0.0576	1.0000	-0.4889
LOC100550364	1.0000	2.1849	0.9062	1.2223	0.8011	3.0840	1.0000	0.0057	1.0000	2.2735	1.0000	1.4325	1.0000	-0.7793
LOC100550375	1.0000	-0.3163	0.6394	0.7831	1.0000	-1.0156	1.0000	0.3113	1.0000	0.8006	0.6141	1.9309	0.6865	2.1414
LOC100550376	0.0596	-0.5468	0.0000	-0.6882	0.2899	-0.3239	0.0835	-0.2751	1.0000	-0.0403	0.9052	-0.1712	1.0000	0.0133
LOC100550377	0.2156	0.4365	0.9513	-0.0974	0.2960	0.3746	1.0000	-0.0455	1.0000	-0.4444	0.0000	-0.9660	0.0003	-0.8557
LOC100550389	0.0615	0.5729	0.0119	0.5206	0.0358	-0.7114	0.0006	-0.7629	1.0000	0.2094	0.9436	0.1695	1.0000	0.1614
LOC100550402	1.0000	0.0000	1.0000	0.0000	1.0000	0.0000	1.0000	0.0000	1.0000	0.0000	1.0000	0.0000	1.0000	0.0000
LOC100550412	0.0000	1.9058	0.0000	1.3711	0.7553	-0.3312	0.0266	-0.9043	1.0000	0.0515	0.1675	-0.4743	0.6504	-0.5177
LOC100550418	0.5412	0.3816	0.5571	0.2605	0.0343	0.8973	0.0009	0.7925	1.0000	0.3317	1.0000	0.2237	0.8344	0.2285
LOC100550420	0.0013	-0.8372	0.0014	-0.5839	0.5879	-0.2221	0.0000	0.8310	1.0000	-0.3898	1.0000	-0.1239	0.0000	0.6673
LOC100550421	0.3840	0.1938	0.0000	0.4375	0.0000	1.2589	0.0000	1.0233	1.0000	-0.0244	0.0995	0.2308	0.0591	-0.2551
LOC100550423	0.4078	-0.2020	0.1826	-0.1811	0.0000	-1.3381	0.0000	-0.9851	1.0000	-0.1279	0.9718	-0.0952	0.3890	0.2305
LOC100550424	0.0864	1.6624	0.0000	3.4793	0.7660	-1.4056	0.7710	-3.1633	1.0000	-1.3466	0.9626	0.4718	1.0000	-3.1369
LOC100550425	1.0000	0.0000	1.0000	0.0000	1.0000	0.0000	1.0000	0.0000	1.0000	0.0000	1.0000	0.0000	1.0000	0.0000
LOC100550430	0.8249	-3.3439	1.0000	-1.0027	1.0000	0.7332	1.0000	-0.8429	1.0000	-0.1140	1.0000	2.3480	0.8937	-1.6947
LOC100550431	0.0042	-1.1279	0.0003	-1.3157	0.0493	0.6489	0.6400	-0.2425	1.0000	-0.6081	0.3018	-0.7818	0.0000	-1.4921
LOC100550436	1.0000	0.0000	1.0000	-2.3959	1.0000	2.2426	1.0000	0.0062	1.0000	2.2677	1.0000	0.0000	1.0000	0.0652
LOC100550445	0.4726	0.2805	0.0662	0.3331	0.3374	-0.3493	0.0000	-1.1284	1.0000	-0.0747	1.0000	-0.0100	0.0010	-0.8514
LOC100550448	0.1263	-0.7805	0.1881	-0.5593	0.1794	-0.6473	0.0729	-0.8311	1.0000	-0.1043	1.0000	0.1260	1.0000	-0.2836
LOC100550455	0.4801	2.0051	0.0560	2.7264	0.9361	1.2061	0.9096	1.3849	1.0000	-0.1045	1.0000	0.6242	1.0000	0.0750
LOC100550460	0.9671	-1.6906	0.9784	-1.0837	1.0000	0.1849	0.9801	-0.9127	1.0000	0.2660	1.0000	0.9006	1.0000	-0.8395
LOC100550466	0.1080	-0.3462	0.0012	-0.3263	0.4524	-0.1966	0.0000	-0.4223	1.0000	0.0146	1.0000	0.0476	0.2215	-0.2057
LOC100550472	1.0000	0.0000	1.0000	0.0000	1.0000	0.0000	1.0000	0.0000	1.0000	0.0000	1.0000	0.0000	1.0000	0.0000
LOC100550475	1.0000	0.0000	1.0000	0.0000	1.0000	0.0000	1.0000	0.0000	1.0000	0.0000	1.0000	0.0000	1.0000	0.0000
LOC100550490	0.0000	-2.9988	0.0000	-2.8679	0.0000	-1.5133	0.0000	-0.8633	1.0000	0.2115	0.5756	0.3523	0.0000	0.8659
LOC100550494	0.0000	1.1383	0.0000	0.8785	1.0000	0.0351	0.1034	-0.2317	1.0000	-0.0706	0.0257	-0.3179	0.0606	-0.3319
LOC100550496	0.4935	-0.1918	1.0000	-0.0217	0.2231	-0.2921	0.0015	-0.3800	1.0000	-0.0483	0.8395	0.1341	0.8360	-0.1293
LOC100550503	0.3181	0.2647	0.0000	0.5575	0.0000	1.3268	0.0000	1.1571	1.0000	-0.0160	0.0516	0.2899	0.2475	-0.1799
LOC100550505	0.0490	0.9617	0.0000	1.6785	0.0000	2.5341	0.0000	2.7805	1.0000	-0.5186	1.0000	0.2120	0.4163	-0.2674
LOC100550511	1.0000	-1.1514	1.0000	-0.6969	0.7780	1.0427	1.0000	-0.5239	1.0000	0.4129	1.0000	0.8962	0.9852	-1.1488
LOC100550516	0.0000	-0.6467	0.0000	-0.9307	1.0000	0.0496	0.0074	-0.2201	1.0000	0.1678	0.8306	-0.1038	0.6456	-0.0971
LOC100550517	1.0000	-2.4776	1.0000	0.0000	1.0000	-2.4056	1.0000	0.0000	1.0000	-2.3758	1.0000	0.0000	1.0000	0.0000
LOC100550520	0.0001	0.6539	0.0000	0.8145	0.0000	0.7886	0.0000	0.6059	1.0000	-0.0332	0.6982	0.1406	0.0308	-0.2105
LOC100550523	1.0000	0.0000	1.0000	0.0000	1.0000	0.0000	1.0000	0.0000	1.0000	0.0000	1.0000	0.0000	1.0000	0.0000
LOC100550524	1.0000	-0.0803	0.0221	0.6944	0.3530	-0.4549	0.0000	1.2944	1.0000	-0.9635	1.0000	-0.1796	0.0070	0.7869
LOC100550526	1.0000	0.0000	1.0000	0.6987	1.0000	0.0000	1.0000	0.0075	1.0000	2.2676	1.0000	3.2002	1.0000	2.3554
LOC100550530	1.0000	0.2144	0.0359	-1.1671	0.2920	0.8537	1.0000	0.0540	1.0000	0.4456	0.4350	-0.9265	0.8949	-0.3534
LOC100550532	0.0110	0.6004	0.0000	0.6681	1.0000	0.0659	0.0002	-0.5107	1.0000	-0.0995	1.0000	-0.0206	0.0000	-0.6697
LOC100550551	1.0000	0.0000	1.0000	2.2507	1.0000	0.0000	1.0000	0.0000	1.0000	0.0000	1.0000	2.3456	1.0000	0.0000
LOC100550557	1.0000	0.0000	1.0000	0.0000	0.4809	3.6122	0.0402	4.8608	1.0000	0.0000	1.0000	0.0000	0.8652	1.2335
LOC100550565	1.0000	0.0000	1.0000	0.0000	1.0000	0.0000	1.0000	0.0000	1.0000	0.0000	1.0000	0.0000	1.0000	0.0000
LOC100550569	0.1929	0.6741	0.1943	0.5123	0.0000	1.9034	0.0000	1.1848	1.0000	0.4659	0.8840	0.3165	0.6837	-0.2510
LOC100550576	0.2623	1.7818	0.0223	2.4930	0.0113	2.3715	0.0010	3.1048	1.0000	-0.6504	1.0000	0.0620	1.0000	0.0820
LOC100550580	0.0002	-6.2121	0.0000	-6.4338	0.1201	-1.3610	0.0878	-1.2892	1.0000	0.1898	1.0000	0.0000	1.0000	0.2650
LOC100550583	1.0000	0.0000	1.0000	0.0000	1.0000	0.0000	1.0000	0.0000	1.0000	0.0000	1.0000	0.0000	1.0000	0.0000
LOC100550586	1.0000	-1.2491	1.0000	2.2534	1.0000	0.3637	0.0016	5.6182	1.0000	-4.1507	1.0000	-0.7956	0.7260	1.0498
LOC100550587	1.0000	0.0000	1.0000	0.0000	1.0000	0.0000	1.0000	0.0000	1.0000	0.0000	1.0000	0.0000	1.0000	0.0000
LOC100550588	0.0000	1.3023	0.0000	1.3588	0.6995	0.1380	0.2038	-0.1575	1.0000	-0.0706	1.0000	-0.0026	0.0014	-0.3615
LOC100550589	0.1349	0.3820	0.0003	0.5068	0.0195	0.5331	0.0059	0.3923	1.0000	0.0272	0.7284	0.1645	0.9177	-0.1067
LOC100550598	1.0000	0.0000	1.0000	0.0000	1.0000	0.0000	1.0000	0.0000	1.0000	0.0000	1.0000	0.0000	1.0000	0.0000
LOC100550599	1.0000	0.5381	0.5241	1.3050	0.6971	1.6025	0.2228	1.8823	1.0000	0.7317	0.9238	1.5299	0.9194	1.0334
LOC100550605	0.1131	0.5855	0.0001	0.6255	0.0000	1.7579	0.0000	1.6067	1.0000	0.0114	1.0000	0.0656	0.6855	-0.1337
LOC100550606	0.0014	0.6074	0.0000	0.8044	0.0000	0.7374	0.0000	0.6330	1.0000	-0.0623	0.6968	0.1478	0.4627	-0.1610
LOC100550610	1.0000	0.0000	1.0000	2.2507	1.0000	0.0000	1.0000	2.3257	1.0000	0.0000	1.0000	2.3456	1.0000	2.3554
LOC100550618	1.0000	-2.4776	0.4431	-3.7875	0.6971	1.6025	0.9096	-1.3722	1.0000	1.2661	1.0000	0.0000	0.9122	-1.6997
LOC100550620	1.0000	0.0000	1.0000	0.0000	1.0000	0.0000	1.0000	0.0000	1.0000	0.0000	1.0000	0.0000	1.0000	0.0000
LOC100550621	0.9788	-0.0665	0.4750	0.0936	0.0001	0.6323	0.0000	0.5611	1.0000	-0.0099	0.4220	0.1625	0.8818	-0.0751
LOC100550622	1.0000	-0.1163	0.9372	-0.1212	0.0000	2.4971	0.0000	2.2174	1.0000	0.1105	1.0000	0.1190	1.0000	-0.1663
LOC100550623	0.2003	0.4822	0.0916	0.4020	0.0000	1.1412	0.0000	0.9086	1.0000	0.1970	1.0000	0.1302	1.0000	-0.0282
LOC100550625	1.0000	0.0000	1.0000	0.0000	1.0000	0.0000	1.0000	0.0000	1.0000	0.0000	1.0000	0.0000	1.0000	0.0000
LOC100550626	1.0000	0.0000	1.0000	0.0000	1.0000	0.0000	1.0000	0.0000	1.0000	0.0000	1.0000	0.0000	1.0000	0.0000
LOC100550636	0.3818	1.2901	1.0000	0.4805	0.0196	2.0549	0.3974	1.1088	1.0000	0.1686	1.0000	-0.6237	0.6809	-0.7668
LOC100550642	0.8520	-0.1304	0.0004	-0.4334	0.0202	0.5659	0.0000	0.6908	1.0000	0.3631	1.0000	0.0725	0.0000	0.4923
LOC100550646	1.0000	0.0000	1.0000	0.0000	1.0000	0.0000	1.0000	0.0000	1.0000	0.0000	1.0000	0.0000	1.0000	0.0000
LOC100550649	0.2977	0.3088	0.0039	0.5358	0.0000	1.3555	0.0000	1.3259	1.0000	0.0726	0.1705	0.3132	1.0000	0.0485
LOC100550655	1.0000	-0.3583	0.2128	-1.1460	1.0000	-0.3361	0.4093	0.7324	1.0000	-0.0092	0.9744	-0.7868	0.4948	1.0630
LOC100550661	0.2875	0.3797	1.0000	0.0395	0.0017	-1.0237	0.0021	-0.7573	1.0000	0.1619	1.0000	-0.1655	0.4296	0.4368
LOC100550667	0.3323	-0.2138	0.1306	-0.1703	0.1975	-0.2437	0.0430	-0.2241	1.0000	0.0666	0.8240	0.1221	0.8081	0.0913
LOC100550673	0.8619	-0.2020	0.2008	0.3825	0.1197	0.5616	0.0000	1.0049	1.0000	-0.4051	1.0000	0.1899	1.0000	0.0415
LOC100550679	1.0000	0.0131	1.0000	0.0190	0.0356	-0.3260	0.0000	-0.4029	1.0000	0.1576	0.1427	0.1756	0.6247	0.0858
LOC100550690	0.8627	-0.1614	0.0929	-0.3935	0.7083	-0.2169	0.9244	0.0983	1.0000	0.0444	0.9908	-0.1755	0.3431	0.3642
LOC100550693	1.0000	0.0000	1.0000	0.0000	1.0000	0.0000	1.0000	0.0000	1.0000	0.0000	1.0000	0.0000	1.0000	0.0000
LOC100550697	1.0000	0.1119	1.0000	0.0847	0.0006	1.1672	0.0000	1.4786	1.0000	0.0173	1.0000	0.0022	0.4317	0.3360
LOC100550702	0.6551	-0.4892	0.3259	-0.7909	0.4108	-0.7121	0.6402	-0.4997	1.0000	-0.3294	0.8605	-0.6209	1.0000	-0.1103
LOC100550711	1.0000	-0.3440	1.0000	-0.0452	0.7953	0.6570	0.2033	0.9898	1.0000	0.5847	0.9953	0.9006	0.4936	0.9274
LOC100550717	1.0000	0.0539	0.0102	-0.2817	0.0009	-0.5944	0.0000	-0.7253	1.0000	-0.1295	0.0004	-0.4524	0.2553	-0.2561
LOC100550719	1.0000	0.0000	1.0000	0.0000	1.0000	0.0000	1.0000	0.0000	1.0000	0.0000	1.0000	0.0000	1.0000	0.0000
LOC100550721	0.5508	-0.3401	0.9371	-0.1301	0.0000	-2.8217	0.0030	-1.1484	1.0000	-0.4314	1.0000	-0.2091	0.1692	1.2465
LOC100550735	1.0000	-0.0258	1.0000	-0.0121	0.7438	0.1750	0.4440	0.1590	1.0000	0.2513	0.4371	0.2793	0.5114	0.2395
LOC100550736	0.2064	0.2845	0.2265	0.1791	0.9361	-0.0912	0.2797	-0.1496	1.0000	0.0399	1.0000	-0.0525	1.0000	-0.0140
LOC100550739	0.4762	-1.1597	1.0000	-0.1761	1.0000	0.4293	0.0000	2.0475	1.0000	0.1449	0.8607	1.1491	0.0006	1.7784
LOC100550740	0.0565	0.4913	0.0000	0.7692	0.1290	0.3746	0.0074	-0.5351	1.0000	-0.4356	0.9128	-0.1461	0.0000	-1.3394
LOC100550742	1.0000	0.0000	1.0000	0.0000	1.0000	2.2471	1.0000	0.0000	1.0000	0.0000	1.0000	0.0000	1.0000	-2.2907
LOC100550744	0.3175	0.2797	0.6280	0.1140	0.0456	-0.4953	0.0000	-0.6036	1.0000	0.2195	1.0000	0.0661	0.9439	0.1161
LOC100550749	0.1138	-0.2967	0.0000	-0.4007	0.0719	-0.3385	0.0017	-0.2879	1.0000	0.0617	1.0000	-0.0295	0.7235	0.1168
LOC100550754	0.5565	1.9825	0.2078	1.4876	0.9468	1.1984	0.4634	-3.6899	1.0000	1.2548	1.0000	0.7801	0.7477	-3.6583
LOC100550755	0.1938	-1.5771	1.0000	-0.1736	0.2627	-1.1664	0.3529	0.9825	1.0000	-0.8894	1.0000	0.5262	0.4974	1.2680
LOC100550763	1.0000	-0.3358	1.0000	-0.4768	0.0042	2.5166	0.2309	1.3455	1.0000	0.5689	1.0000	0.4448	0.9352	-0.6000
LOC100550767	0.0010	-0.5435	0.0000	-0.6272	1.0000	0.0377	0.5235	-0.0834	1.0000	0.0591	1.0000	-0.0115	1.0000	-0.0564
LOC100550777	1.0000	0.1233	1.0000	-0.1775	1.0000	-0.0690	0.1872	0.9319	1.0000	0.1264	1.0000	-0.1597	0.2033	1.1288
LOC100550780	1.0000	0.0000	1.0000	0.0000	1.0000	0.0000	1.0000	0.0000	1.0000	0.0000	1.0000	0.0000	1.0000	0.0000
LOC100550794	0.0016	-0.5053	0.0000	-0.5299	0.0000	-0.6704	0.0000	-0.4436	1.0000	0.1192	0.8437	0.1067	0.0011	0.3509
LOC100550801	1.0000	-0.0524	0.1081	-0.1675	0.0070	-0.5154	0.0000	-0.4873	1.0000	0.0036	0.8607	-0.0989	1.0000	0.0379

LOC100550809	0.7224	-0.8138	1.0000	0.0796	1.0000	0.5755	0.0309	1.3637	1.0000	0.3717	0.6953	1.2817	0.2232	1.1758
LOC100550812	0.7643	0.9210	0.5855	0.7934	1.0000	-0.5738	0.7723	-0.9362	1.0000	0.4287	1.0000	0.3160	1.0000	0.0749
LOC100550814	0.1644	1.0217	0.0001	1.2711	0.1330	1.0725	0.0006	1.1784	1.0000	-0.1775	1.0000	0.0920	1.0000	-0.0577
LOC100550818	0.6531	-0.3211	0.0013	-1.0392	0.0851	-0.9152	1.0000	0.0722	1.0000	0.2270	0.6637	-0.4810	0.0064	1.2233
LOC100550820	1.0000	0.0000	1.0000	0.0000	1.0000	0.0000	1.0000	0.0000	1.0000	0.0000	1.0000	0.0000	1.0000	0.0000
LOC100550826	0.6723	-0.1524	0.8588	0.0588	0.9098	0.0919	0.0003	0.3855	1.0000	0.0789	0.0528	0.3014	0.0041	0.3775
LOC100550829	0.0000	-0.6910	0.0000	-0.7711	0.0000	-0.9084	0.0000	-0.9084	1.0000	-0.0481	0.8607	-0.1158	1.0000	-0.0416
LOC100550830	1.0000	-0.2931	0.4692	3.6126	1.0000	-0.1630	1.0000	0.0000	1.0000	-2.3771	1.0000	1.4294	1.0000	-2.2889
LOC100550832	0.1747	-0.2305	0.0000	-0.3062	0.4252	-0.1617	0.4284	-0.0726	1.0000	0.0610	1.0000	-0.0023	0.2133	0.1551
LOC100550836	0.0004	1.4590	0.0028	1.3364	0.0000	1.6578	0.0033	1.3173	1.0000	-0.4926	0.3074	-0.5999	0.0271	-0.8279
LOC100550847	1.0000	0.0000	1.0000	0.0000	1.0000	0.0000	1.0000	0.0000	1.0000	0.0000	1.0000	0.0000	1.0000	0.0000
LOC100550849	0.1708	1.1992	0.0911	0.9383	0.0011	1.9463	0.0550	1.0404	1.0000	0.5330	1.0000	0.2838	0.8373	-0.3645
LOC100550859	0.7631	-0.0992	0.3712	-0.0972	0.0000	-0.7415	0.0000	-0.5213	1.0000	-0.0876	0.9320	-0.0733	0.4894	0.1380
LOC100550869	1.0000	0.2029	0.9062	-1.5349	0.5505	1.2863	1.0000	0.3933	1.0000	0.4115	1.0000	-1.3257	1.0000	-0.4790
LOC100550877	1.0000	-2.4788	1.0000	0.0000	1.0000	-2.4061	1.0000	0.0000	1.0000	-2.3771	1.0000	0.0000	1.0000	0.0000
LOC100550884	1.0000	0.0000	1.0000	0.0000	1.0000	0.0000	1.0000	0.0000	1.0000	0.0000	1.0000	0.0000	1.0000	0.0000
LOC100550885	0.9650	1.0594	0.2282	1.4569	0.0000	4.2641	0.0000	3.2507	1.0000	1.6505	0.3886	2.0819	0.4393	0.6439
LOC100550886	0.3288	-0.5703	0.0000	-1.8940	0.0127	-1.2572	0.0000	-2.0563	1.0000	0.0981	0.0502	-1.2139	0.6062	-0.6961
LOC100550893	0.0513	1.5193	0.9446	0.2336	0.5168	-1.5304	0.3503	-0.8247	1.0000	1.0143	1.0000	-0.2564	0.4332	1.7339
LOC100550906	0.2775	0.2268	0.0000	0.4755	0.0000	-0.9187	0.0000	-0.5653	1.0000	-0.2422	1.0000	0.0188	0.7261	0.1172
LOC100550908	0.0730	-0.5470	0.0000	-0.8673	0.0000	-1.1485	0.0000	-1.0742	1.0000	0.0777	0.9022	-0.2298	1.0000	0.1567
LOC100550909	1.0000	2.1902	1.0000	0.0000	1.0000	0.0000	1.0000	0.0000	1.0000	0.0000	1.0000	-2.2992	1.0000	0.0000
LOC100550910	0.3089	0.6887	0.2752	0.8067	0.2225	-0.9258	0.5098	0.6608	1.0000	-1.1750	0.1094	-1.0466	1.0000	0.4171
LOC100550934	1.0000	0.0000	1.0000	0.0000	1.0000	0.0000	1.0000	0.0000	1.0000	0.0000	1.0000	0.0000	1.0000	0.0000
LOC100550942	1.0000	0.0000	1.0000	0.0000	1.0000	0.0000	1.0000	0.0000	1.0000	0.0000	1.0000	0.0000	1.0000	0.0000
LOC100550948	1.0000	0.3037	0.2290	-2.0437	0.5830	0.9036	1.0000	-0.1795	1.0000	0.5250	0.6141	-1.8180	1.0000	-0.5547
LOC100550949	1.0000	0.0000	1.0000	0.0000	1.0000	0.0000	1.0000	0.0000	1.0000	0.0000	1.0000	0.0000	1.0000	0.0000
LOC100550954	0.0580	-0.3503	0.0000	-0.4472	1.0000	-0.0284	0.0001	0.3389	1.0000	-0.1091	0.1545	-0.1940	0.1013	0.2634
LOC100550956	0.0674	0.6814	0.0143	0.6165	0.0000	1.5416	0.0001	0.8417	1.0000	0.3832	0.6123	0.3315	0.3460	-0.3114
LOC100550971	0.8467	-1.0326	0.2402	-1.0464	0.2629	1.2481	1.0000	0.0077	1.0000	1.2076	0.9741	1.2157	1.0000	-0.0252
LOC100550975	1.0000	-1.2453	0.8234	0.5981	0.3227	-4.1878	0.9801	-0.9105	1.0000	-0.1316	0.7635	1.7439	1.0000	3.2049
LOC100550977	0.6881	-0.2850	1.0000	0.0729	0.1090	0.6307	0.0156	0.6460	1.0000	0.0189	0.6450	0.3881	1.0000	0.0365
LOC100550978	0.0000	-2.4115	0.0000	-2.5400	0.3211	-0.2514	0.0181	-0.2044	1.0000	-0.0422	0.7952	-0.1584	1.0000	0.0103
LOC100550986	0.0636	0.7200	0.0000	1.1935	0.0000	1.6766	0.0000	1.8222	1.0000	-0.2733	0.8632	0.2132	0.7287	-0.1233
LOC100550995	1.0000	-0.3551	0.7150	0.5731	0.3482	-1.0461	1.0000	-0.3062	1.0000	-0.2648	0.9816	0.6768	1.0000	0.4797
LOC100551000	1.0000	0.0080	0.0976	-0.3018	0.0401	-0.5352	0.0000	-0.8481	1.0000	0.2238	1.0000	-0.0736	1.0000	-0.0840
LOC100551004	0.0011	1.2435	0.0000	1.7147	0.1311	0.6824	0.1347	0.6140	1.0000	-0.1712	0.6727	0.3147	0.9950	-0.2316
LOC100551007	1.0000	0.0000	1.0000	0.0000	1.0000	0.0000	1.0000	0.0000	1.0000	0.0000	1.0000	0.0000	1.0000	0.0000
LOC100551010	0.4025	-1.2031	0.0669	-3.1835	0.8011	0.5002	0.7701	-0.6994	1.0000	-0.2800	0.6450	-2.2673	0.1780	-1.4671
LOC100551022	0.2817	0.1942	0.0001	0.2785	0.1546	0.2385	0.0003	0.2393	1.0000	0.0183	0.5587	0.1148	1.0000	0.0245
LOC100551023	0.0201	1.3430	0.0330	1.2606	0.0158	-2.5998	0.1701	-1.5219	1.0000	-0.6213	0.3783	-0.6970	1.0000	0.4623
LOC100551027	0.7688	-0.0993	0.7170	0.0623	0.9350	-0.0703	0.9338	-0.0371	1.0000	-0.1536	1.0000	0.0201	0.6336	-0.1150
LOC100551033	0.2164	-1.4348	0.9125	-0.3410	0.0309	1.2918	0.0004	1.9771	1.0000	-0.6487	1.0000	0.4592	1.0000	0.0411
LOC100551038	0.0705	-0.4284	0.0010	-0.3767	0.0060	0.5571	0.0209	0.2417	1.0000	-0.1355	1.0000	-0.0714	0.0001	-0.4460
LOC100551040	0.0000	0.7027	0.0000	0.8048	0.0000	-0.9166	0.0000	-0.6922	1.0000	-0.1216	1.0000	-0.0074	0.7687	0.1081
LOC100551042	1.0000	0.0000	1.0000	0.0000	1.0000	0.0000	1.0000	0.0000	1.0000	0.0000	1.0000	0.0000	1.0000	0.0000
LOC100551043	0.0001	1.0084	0.0000	0.9222	0.4280	0.2988	0.2788	0.2485	1.0000	0.1428	1.0000	0.0698	1.0000	0.0979
LOC100551044	0.1017	0.7686	0.0037	0.8525	0.0000	1.4295	0.0057	0.7114	1.0000	0.3310	0.6317	0.4287	0.2835	-0.3796
LOC100551049	0.4670	-0.5531	0.8186	-0.2115	0.0691	-1.1837	0.0707	-0.8099	1.0000	0.3869	0.3814	0.7415	0.5692	0.7682
LOC100551066	0.9545	0.1321	0.0000	0.6645	0.0098	0.7601	0.0000	1.2087	1.0000	-0.3115	0.4588	0.2328	0.5416	0.1423
LOC100551070	0.0960	-2.5257	1.0000	-0.1652	0.1689	-1.8488	1.0000	0.6997	1.0000	-1.7857	1.0000	0.5860	1.0000	0.7682
LOC100551071	0.0021	1.3591	0.0033	0.9786	0.0000	1.7773	0.0000	1.5562	1.0000	0.2082	1.0000	-0.1535	1.0000	-0.0063
LOC100551072	1.0000	0.0000	1.0000	0.0000	1.0000	0.0000	1.0000	0.0000	1.0000	0.0000	1.0000	0.0000	1.0000	0.0000
LOC100551074	1.0000	-0.0195	0.2195	-0.1712	0.0001	0.6888	0.0000	0.7101	1.0000	0.1521	1.0000	0.0128	0.2599	0.1797
LOC100551087	0.0000	-1.3329	0.0016	-0.7290	0.0244	0.5713	0.0000	0.8187	1.0000	-0.0362	0.3263	0.5787	0.4856	0.2183
LOC100551088	0.0073	-0.4059	0.0011	-0.4263	0.0408	-0.4071	0.1645	-0.2046	1.0000	0.0058	1.0000	-0.0018	0.4180	0.2138
LOC100551109	1.0000	0.0000	1.0000	-0.9997	1.0000	0.0000	1.0000	-0.8388	1.0000	3.1056	1.0000	2.3456	1.0000	2.3554
LOC100551117	0.0000	-0.8162	0.0000	-0.6851	0.0051	-0.5558	1.0000	0.0044	1.0000	-0.2750	0.7475	-0.1315	0.0625	0.2913
LOC100551119	0.0037	1.0855	0.0000	1.1525	0.0000	1.3990	0.0000	1.4780	1.0000	-0.1891	1.0000	-0.1099	1.0000	-0.1040
LOC100551121	1.0000	0.0000	1.0000	0.0000	1.0000	0.0000	1.0000	0.0000	1.0000	0.0000	1.0000	0.0000	1.0000	0.0000
LOC100551127	0.5378	0.5021	1.0000	-0.0875	0.5075	-0.6100	0.0155	-1.4208	1.0000	0.1875	0.9688	-0.3926	0.7850	-0.6192
LOC100551128	1.0000	0.0687	0.3610	-0.1974	0.0002	-1.0433	0.0000	-1.7521	1.0000	0.1651	1.0000	-0.0893	0.1541	-0.5389
LOC100551131	0.5836	-1.5593	1.0000	-0.4768	0.9164	0.4629	0.7210	-1.2163	1.0000	-0.1255	1.0000	0.9750	0.4972	-1.8022
LOC100551134	0.7841	-0.1913	0.6584	0.1467	0.3213	0.3467	0.0001	0.6948	1.0000	-0.2999	1.0000	0.0488	1.0000	0.0530
LOC100551145	0.8230	-0.0891	0.1680	-0.1334	0.0000	0.8306	0.0000	0.7293	1.0000	0.1514	0.6367	0.1195	1.0000	0.0548
LOC100551155	0.0000	-0.9356	0.0000	-1.2251	0.0001	-0.8257	0.0000	-0.7439	1.0000	0.0918	0.8607	-0.1850	0.7171	0.1767
LOC100551159	1.0000	0.0000	1.0000	0.0000	1.0000	0.0000	1.0000	0.0000	1.0000	0.0000	1.0000	0.0000	1.0000	0.0000
LOC100551160	0.0009	-0.5441	0.0000	-0.4826	0.2465	0.2466	0.0000	0.4401	1.0000	-0.0867	1.0000	-0.0131	0.7171	0.1120
LOC100551166	1.0000	0.0000	1.0000	0.0000	1.0000	0.0000	1.0000	0.0000	1.0000	0.0000	1.0000	0.0000	1.0000	0.0000
LOC100551187	0.0341	0.5564	0.0000	0.7351	0.0002	0.7863	0.0010	0.4517	1.0000	-0.1084	1.0000	0.0824	0.0057	-0.4388
LOC100551190	0.0373	2.9719	0.2761	1.8615	0.9361	1.2061	0.3123	1.8951	1.0000	0.7357	1.0000	-0.3513	0.8232	1.4312
LOC100551192	1.0000	-0.0351	1.0000	-0.1734	1.0000	-0.5739	0.8618	0.6593	1.0000	0.1779	1.0000	0.0542	0.7035	1.4207
LOC100551205	1.0000	0.0000	1.0000	0.0000	1.0000	0.0000	1.0000	0.0000	1.0000	0.0000	1.0000	0.0000	1.0000	0.0000
LOC100551216	1.0000	0.0596	1.0000	0.3292	0.3218	1.3691	0.0000	2.4852	1.0000	0.8439	0.8242	1.1266	0.0000	1.9798
LOC100551225	0.0741	0.4462	0.0000	0.7386	0.2734	-0.3184	0.0001	-0.4439	1.0000	-0.2656	1.0000	0.0378	0.0075	-0.3849
LOC100551228	0.8125	-0.4624	1.0000	-0.0015	0.9468	0.3076	0.4570	0.6152	1.0000	-0.0308	1.0000	0.4411	1.0000	0.2796
LOC100551229	0.0009	-0.6010	1.0000	0.0180	0.0000	0.8661	0.0000	0.9424	1.0000	-0.3198	0.0006	0.3119	0.2220	-0.2383
LOC100551231	0.1320	-3.0439	0.0943	-2.8827	1.0000	0.1255	0.4942	0.7958	1.0000	-0.1290	1.0000	0.0495	1.0000	0.5537
LOC100551250	0.0000	1.1639	0.0000	1.3658	0.0134	0.6885	0.0000	0.8399	1.0000	-0.2237	1.0000	-0.0098	1.0000	-0.0672
LOC100551262	0.4743	0.2157	0.2913	0.2553	0.0000	1.5222	0.0000	1.3465	1.0000	0.2191	0.1715	0.2721	1.0000	0.0485
LOC100551267	0.6103	0.2312	1.0000	0.0439	0.0000	1.6630	0.0000	1.0375	1.0000	0.1866	1.0000	0.0111	0.0589	-0.4343
LOC100551269	0.4115	0.1627	0.0036	0.2118	0.0042	0.4316	0.0000	0.3975	1.0000	-0.0040	0.9957	0.0576	1.0000	-0.0327
LOC100551278	0.8751	-0.0891	1.0000	0.0112	0.2836	0.2297	0.7710	0.0625	1.0000	-0.0651	1.0000	0.0481	0.1170	-0.2262
LOC100551279	1.0000	2.1849	1.0000	0.0000	0.8033	3.0789	0.7710	3.1753	1.0000	0.0000	1.0000	-2.2955	1.0000	0.0752
LOC100551282	0.0000	-1.8352	0.0000	-2.0611	0.0000	-1.6104	0.0000	-1.1629	1.0000	0.0085	1.0000	-0.2046	0.6362	0.4635
LOC100551284	0.5384	3.5543	0.4029	1.9116	0.2829	3.9942	0.2442	2.3283	1.0000	2.2735	1.0000	0.7480	1.0000	0.6341
LOC100551287	1.0000	0.0000	1.0000	0.0000	1.0000	0.0000	1.0000	0.0000	1.0000	0.0000	1.0000	0.0000	1.0000	0.0000
LOC100551292	0.0587	0.4993	0.0045	0.4722	0.0000	1.1170	0.0000	0.8686	1.0000	0.2413	0.5412	0.2275	1.0000	-0.0007
LOC100551294	0.0000	1.5272	0.0000	1.0662	0.0000	1.2919	0.0007	0.6920	1.0000	0.3120	0.9741	-0.1358	0.3928	-0.2809
LOC100551296	1.0000	0.0044	0.0034	-0.2041	0.0000	-0.6872	0.0000	-0.4585	1.0000	0.1103	0.7184	-0.0858	0.0004	0.3445
LOC100551326	0.0000	1.7657	0.0000	2.3806	0.2223	-0.7764	0.1036	-1.1919	1.0000	-0.7771	1.0000	-0.1512	0.2358	-1.1868
LOC100551338	1.0000	0.0467	0.6547	-1.9365	0.3450	1.4650	0.3232	1.4118	1.0000	0.2811	1.0000	-1.7113	1.0000	0.2173
LOC100551343	1.0000	0.0000	1.0000	0.0000	1.0000	0.0000	1.0000	0.0000	1.0000	0.0000	1.0000	0.0000	1.0000	0.0000

LOC100551355	0.0001	1.0115	0.0000	0.9078	0.0000	1.5655	0.0000	1.1870	1.0000	0.1414	1.0000	0.0513	0.3437	-0.2286
LOC100551358	1.0000	2.1849	1.0000	0.0000	1.0000	0.0000	1.0000	0.0000	1.0000	0.0000	1.0000	-2.2955	1.0000	0.0000
LOC100551363	1.0000	0.5849	0.0066	5.4059	0.8033	-3.2636	1.0000	0.0000	1.0000	-3.2319	0.6375	1.4633	1.0000	0.0000
LOC100551372	0.0000	-0.7662	0.0002	-0.3306	0.0007	0.6254	0.0000	0.9386	1.0000	-0.1466	0.0194	0.3019	0.3139	0.1720
LOC100551374	0.4207	0.2250	0.0098	0.3224	0.0000	-1.0741	0.0000	-0.8310	1.0000	-0.1187	1.0000	-0.0096	0.9139	0.1301
LOC100551377	0.2667	-0.4876	0.0033	-0.8790	0.0727	0.6275	0.0009	0.7335	1.0000	0.1290	1.0000	-0.2490	0.6855	0.2445
LOC100551379	0.2432	0.3243	0.0217	0.3355	0.0386	-0.5375	0.0001	-0.5883	1.0000	-0.2507	0.3867	-0.2270	0.3005	-0.2954
LOC100551380	1.0000	-0.0557	0.2089	-0.2119	0.0604	-0.5120	0.0000	-0.6738	1.0000	-0.0804	0.5071	-0.2242	0.5422	-0.2362
LOC100551381	1.0000	0.0000	1.0000	0.0000	1.0000	0.0000	1.0000	0.0000	1.0000	0.0000	1.0000	0.0000	1.0000	0.0000
LOC100551394	1.0000	0.0000	1.0000	0.0000	1.0000	0.0000	1.0000	0.0000	1.0000	0.0000	1.0000	0.0000	1.0000	0.0000
LOC100551399	0.4238	-0.1963	0.3894	-0.1286	0.1378	0.2927	0.0981	0.1958	1.0000	0.1694	0.1129	0.2506	0.8937	0.0781
LOC100551401	0.0047	1.0393	0.0001	1.0437	0.2507	0.5087	0.0248	0.6789	1.0000	-0.2569	0.8601	-0.2373	1.0000	-0.0777
LOC100551403	0.1825	0.4409	0.0017	0.6671	1.0000	-0.0299	1.0000	0.0960	1.0000	-0.2814	1.0000	-0.0426	1.0000	-0.1469
LOC100551412	1.0000	-0.0490	1.0000	-0.0160	0.0021	-0.5839	0.0042	-0.3430	1.0000	-0.0356	1.0000	0.0090	0.3839	0.2110
LOC100551413	1.0000	0.0000	1.0000	0.0000	1.0000	0.0000	1.0000	0.0000	1.0000	0.0000	1.0000	0.0000	1.0000	0.0000
LOC100551422	0.0105	0.7845	0.0138	0.5764	0.7421	-0.2253	0.0065	-0.8770	1.0000	0.0057	0.9052	-0.1878	0.1980	-0.6392
LOC100551424	1.0000	-2.4776	1.0000	2.2507	1.0000	-2.4056	1.0000	0.0000	1.0000	-2.3757	1.0000	2.3455	1.0000	0.0000
LOC100551434	0.0074	-0.4408	0.0025	-0.3325	0.0366	-0.3829	0.0101	-0.2899	1.0000	-0.0820	1.0000	0.0384	1.0000	0.0166
LOC100551438	1.0000	2.1849	1.0000	0.0000	1.0000	0.0000	1.0000	0.0000	1.0000	0.0000	1.0000	-2.2956	1.0000	0.0000
LOC100551442	0.0012	-0.9594	0.0020	-0.7707	0.0000	-2.0410	0.0000	-1.1129	1.0000	-0.5409	0.7247	-0.3399	0.7485	0.3934
LOC100551461	1.0000	0.0417	0.0156	0.2866	0.6796	-0.1413	0.3044	-0.1432	1.0000	0.0163	0.1270	0.2726	1.0000	0.0184
LOC100551463	0.0000	3.1753	0.0000	1.8030	0.0000	3.7489	0.0000	1.6655	1.0000	1.1134	0.8269	-0.2438	0.0001	-0.9629
LOC100551492	0.4306	1.4463	0.2158	-1.1489	1.0000	0.1978	1.0000	-0.2500	1.0000	1.7901	0.9702	-0.7894	0.6298	1.3488
LOC104909199	0.0845	-0.4211	0.0000	-0.4993	0.8011	0.1307	0.0000	0.4835	1.0000	0.1270	0.9940	0.0611	0.0000	0.4842
LOC104909200	1.0000	0.0333	0.6786	0.5856	1.0000	0.2429	0.0362	1.3318	1.0000	-1.1070	0.8808	-0.5436	1.0000	-0.0184
LOC104909202	1.0000	0.0000	1.0000	0.0000	0.8033	3.0892	1.0000	0.0000	1.0000	0.0000	1.0000	0.0000	1.0000	-3.1369
LOC104909203	1.0000	0.0000	1.0000	0.0000	1.0000	0.0000	1.0000	0.0000	1.0000	0.0000	1.0000	0.0000	1.0000	0.0000
LOC104909204	0.2744	-0.3495	0.7182	0.1385	0.0042	-0.7492	0.0246	-0.5101	1.0000	-0.3922	1.0000	0.1093	1.0000	-0.1469
LOC104909205	0.0000	0.9317	0.0000	0.9753	0.0000	0.8047	0.0000	0.9463	1.0000	-0.0781	1.0000	-0.0224	1.0000	0.0689
LOC104909207	1.0000	0.0000	1.0000	0.0000	1.0000	0.0000	1.0000	0.0000	1.0000	0.0000	1.0000	0.0000	1.0000	0.0000
LOC104909208	1.0000	0.0000	1.0000	0.0000	1.0000	0.0000	1.0000	0.0000	1.0000	0.0000	1.0000	0.0000	1.0000	0.0000
LOC104909209	0.4164	0.8386	0.9718	-0.2990	0.0001	2.2138	0.0012	1.6869	1.0000	0.2720	0.7345	-0.8546	1.0000	-0.2472
LOC104909210	0.0477	-0.3755	0.0001	-0.4593	0.0165	-0.4399	0.0000	-0.5200	1.0000	0.1097	1.0000	0.0383	1.0000	0.0344
LOC104909211	1.0000	0.0000	1.0000	0.0000	1.0000	0.0000	0.7701	3.1732	1.0000	0.0000	1.0000	0.0000	1.0000	3.2066
LOC104909212	1.0000	0.0000	1.0000	0.0000	1.0000	0.0000	1.0000	0.0000	1.0000	0.0000	1.0000	0.0000	1.0000	0.0000
LOC104909213	0.0577	0.4249	0.0000	0.5697	0.1602	-0.3356	0.7849	0.0856	1.0000	-0.3360	0.6497	-0.1797	1.0000	0.0905
LOC104909215	0.6877	-2.0832	0.9947	-1.0788	0.0521	1.8258	0.2034	1.5427	1.0000	-0.1316	1.0000	0.8992	1.0000	-0.4119
LOC104909216	1.0000	0.0000	1.0000	0.0000	1.0000	0.0000	1.0000	0.0000	1.0000	0.0000	1.0000	0.0000	1.0000	0.0000
LOC104909218	0.0048	0.9410	0.0000	1.1470	0.0005	0.9829	0.0000	0.8761	1.0000	0.1231	0.4488	0.3387	1.0000	0.0195
LOC104909219	1.0000	0.0439	1.0000	0.0013	0.0044	-0.7143	0.0008	-0.6210	1.0000	0.0710	1.0000	0.0425	0.8720	0.1713
LOC104909220	1.0000	0.0000	1.0000	0.0000	1.0000	0.0000	1.0000	0.0000	1.0000	0.0000	1.0000	0.0000	1.0000	0.0000
LOC104909221	1.0000	0.0000	1.0000	0.0000	1.0000	0.0000	1.0000	0.0000	1.0000	0.0000	1.0000	0.0000	1.0000	0.0000
LOC104909224	0.0048	-0.8114	0.0002	-0.7383	0.0003	0.8266	0.0000	1.0031	1.0000	-0.1251	1.0000	-0.0386	1.0000	0.0577
LOC104909225	0.0224	-0.8856	0.0075	-0.5341	1.0000	-0.0281	0.0000	0.6986	1.0000	-0.0419	0.6050	0.3211	0.0001	0.6863
LOC104909228	1.0000	0.5849	1.0000	-0.1449	1.0000	0.7332	1.0000	0.0075	1.0000	-0.9637	1.0000	-1.7124	0.8937	-1.6947
LOC104909229	1.0000	2.1902	1.0000	-2.3986	0.8033	3.0789	1.0000	-2.3200	1.0000	2.2735	1.0000	-2.2993	1.0000	-3.1317
LOC104909230	1.0000	-0.0476	0.2354	-0.2952	0.3166	0.3495	0.0089	0.4708	1.0000	0.2716	1.0000	0.0383	0.0946	0.3983
LOC104909231	0.0001	-0.7136	0.0000	-0.7626	0.0000	-1.0863	0.0000	-0.9433	1.0000	-0.1419	0.6584	-0.1789	1.0000	0.0069
LOC104909233	0.2817	0.2374	0.2620	0.1390	1.0000	-0.0602	0.8484	-0.0537	1.0000	-0.0575	0.6291	-0.1442	1.0000	-0.0465
LOC104909235	1.0000	0.0000	1.0000	0.0000	1.0000	2.2471	1.0000	2.3242	1.0000	0.0000	1.0000	0.0000	1.0000	0.0640
LOC104909236	0.5738	-0.1423	0.0001	-0.3435	0.0032	-0.4884	0.0000	-0.4468	1.0000	0.0060	0.3109	-0.1823	1.0000	0.0528
LOC104909237	1.0000	0.0953	0.0049	-0.4887	0.0067	-1.0172	0.0000	-1.1329	1.0000	-0.1268	0.0002	-0.6968	0.7952	-0.2375
LOC104909238	1.0000	0.0000	1.0000	0.0000	1.0000	2.2471	1.0000	0.0000	1.0000	0.0000	1.0000	0.0000	1.0000	-2.2908
LOC104909239	1.0000	0.0000	1.0000	-2.3960	1.0000	0.0000	1.0000	-2.3176	1.0000	2.2677	1.0000	0.0000	1.0000	0.0000
LOC104909240	1.0000	0.0000	1.0000	0.0000	1.0000	0.0000	1.0000	0.0000	1.0000	0.0000	1.0000	0.0000	1.0000	0.0000
LOC104909241	1.0000	0.0000	1.0000	0.0000	1.0000	0.0000	1.0000	0.0000	1.0000	0.0000	1.0000	0.0000	1.0000	0.0000
LOC104909245	0.3063	0.3088	0.0000	0.6143	0.0000	1.0758	0.0000	1.0802	1.0000	-0.0600	0.4324	0.2580	1.0000	-0.0500
LOC104909246	0.8947	0.4573	0.6918	0.7979	0.4276	0.8602	0.1616	1.2571	1.0000	-0.4717	1.0000	-0.1264	1.0000	-0.0774
LOC104909248	0.0058	2.3826	0.0007	2.2348	0.0534	1.9172	0.0043	2.0238	1.0000	0.4287	1.0000	0.2959	0.8560	0.5469
LOC104909250	0.0000	-0.9334	0.0000	-0.7536	0.0025	-0.6225	0.0000	-0.7293	1.0000	0.0107	0.6218	0.2023	1.0000	-0.0903
LOC104909252	0.0000	-6.5999	0.0000	-6.6931	0.0396	1.0715	0.0031	1.1945	1.0000	0.0608	1.0000	0.0000	1.0000	0.1884
LOC104909254	0.0000	-2.1392	0.0000	-1.7857	0.0020	-0.9734	0.0000	-0.9286	1.0000	-0.0015	0.7953	0.3655	1.0000	0.0491
LOC104909256	1.0000	0.2026	0.4511	0.4998	0.0037	1.2543	0.0447	1.0421	1.0000	-0.0939	1.0000	0.2196	0.8316	-0.3043
LOC104909257	0.3619	-0.1971	0.3304	-0.1254	0.0104	-0.4377	0.8415	-0.0522	1.0000	-0.2250	0.5912	-0.1414	0.3757	0.1655
LOC104909259	1.0000	0.0000	1.0000	-2.3986	1.0000	2.2471	1.0000	-2.3200	1.0000	2.2735	1.0000	0.0000	1.0000	-2.2908
LOC104909260	0.3572	1.4874	1.0000	0.5202	1.0000	-0.5830	1.0000	0.0052	1.0000	-0.4952	0.4124	-1.4746	1.0000	0.0768
LOC104909261	0.2320	-0.7161	0.4960	0.3141	1.0000	-0.0295	0.0367	0.6566	1.0000	-0.1050	0.0473	0.9360	0.2051	0.5843
LOC104909263	0.6923	-2.0798	0.0372	-2.2007	1.0000	-0.1891	0.4622	-0.8629	1.0000	1.5130	1.0000	1.4259	1.0000	0.8475
LOC104909264	0.0000	2.2170	0.0000	3.4077	0.0000	2.2069	0.0000	2.3288	1.0000	-0.8514	0.7635	0.3481	0.1761	-0.7285
LOC104909265	1.0000	2.1849	1.0000	0.0000	1.0000	0.0000	1.0000	0.0000	1.0000	0.0000	1.0000	-2.2955	1.0000	0.0000
LOC104909266	1.0000	-0.2881	1.0000	-2.3986	0.4424	1.8936	0.0011	3.7064	1.0000	-0.1034	1.0000	-2.2993	0.2011	1.7085
LOC104909267	1.0000	0.0392	0.1998	0.1766	0.0598	-0.3910	0.2176	-0.1755	1.0000	-0.1111	1.0000	0.0378	0.8727	0.1100
LOC104909268	0.1930	0.5192	0.0000	1.1975	0.4113	0.3476	0.0121	0.6131	1.0000	-0.3331	0.3768	0.3554	1.0000	-0.0645
LOC104909269	0.1732	0.5994	0.1033	0.4658	1.0000	0.0883	1.0000	0.0767	1.0000	-0.0083	1.0000	-0.1295	1.0000	-0.0138
LOC104909270	0.0011	-0.9777	0.0010	-0.7302	0.1594	-0.4493	0.3312	-0.2357	1.0000	0.0073	0.8811	0.2670	0.7287	0.2255
LOC104909271	0.0001	2.1472	0.0000	1.8866	0.0215	1.3360	1.0000	0.1096	1.0000	0.7038	0.5330	0.4534	0.6109	-0.5164
LOC104909272	0.2261	0.4989	0.0138	0.6486	0.0000	1.6645	0.0000	1.4185	1.0000	-0.0583	1.0000	0.1045	0.3431	-0.2982
LOC104909274	1.0000	0.0000	1.0000	0.0000	1.0000	0.0000	1.0000	0.0000	1.0000	0.0000	1.0000	0.0000	1.0000	0.0000
LOC104909277	0.4399	0.8004	0.0026	2.0498	1.0000	0.1197	1.0000	0.4076	1.0000	-1.0965	1.0000	0.1623	0.6865	-0.8069
LOC104909278	1.0000	2.1902	1.0000	0.0000	0.8033	3.0892	1.0000	2.3257	1.0000	0.0000	1.0000	-2.2992	1.0000	-0.7804
LOC104909279	0.0003	0.9022	0.0004	0.6721	0.0000	1.2680	0.0001	0.7061	1.0000	0.2122	1.0000	-0.0051	0.1299	-0.3429
LOC104909281	0.4275	0.4710	0.0709	0.6722	1.0000	0.0950	0.3583	0.4486	1.0000	-0.1370	1.0000	0.0796	1.0000	0.2225
LOC104909282	1.0000	2.1849	1.0000	0.0000	1.0000	0.0000	1.0000	0.0000	1.0000	0.0000	1.0000	-2.2955	1.0000	0.0000
LOC104909283	0.0252	-0.4312	0.0000	-0.8590	1.0000	0.0604	0.1659	-0.1512	1.0000	0.0977	0.0258	-0.3174	0.7140	-0.1078
LOC104909284	0.8227	-0.3693	0.8919	0.2761	1.0000	-0.0245	1.0000	0.0656	1.0000	-0.4978	1.0000	0.1566	0.8973	-0.4012
LOC104909285	0.2206	-2.3800	0.9836	0.3944	0.0492	1.6390	0.0428	1.3078	1.0000	0.0218	0.0705	2.8178	1.0000	-0.3134
LOC104909286	0.6011	0.6229	1.0000	-0.0003	0.0000	3.2026	0.0000	1.6780	1.0000	1.4285	0.4133	0.8199	1.0000	-0.0882
LOC104909287	1.0000	0.0000	1.0000	0.0000	1.0000	0.0000	1.0000	0.0000	1.0000	0.0000	1.0000	0.0000	1.0000	0.0000
LOC104909288	1.0000	-0.0712	0.0975	-0.3216	0.6963	-0.1838	0.0469	-0.3302	1.0000	0.0866	1.0000	-0.1511	1.0000	-0.0563
LOC104909289	0.0000	2.5886	0.0000	3.5237	0.0006	2.0585	0.0001	2.7401	1.0000	-1.2469	0.8915	-0.3064	0.5423	-0.5657
LOC104909290	0.1133	1.1309	0.2192	1.0675	0.0036	1.7450	0.0022	1.6466	1.0000	-0.0441	1.0000	-0.0986	1.0000	-0.1280
LOC104909291	0.6877	-2.0832	0.0002	-6.0231	0.2888	1.3329	0.9938	-0.3088	1.0000	1.7166	1.0000	-2.2956	1.0000	0.0799
LOC104909292	0.0017	-3.5748	0.0482	-1.5327	0.0979	-1.3969	0.8215	-0.3740	1.0000	-0.1397	0.6181	1.9314	0.7148	0.8928

LOC104909293	0.0005	-0.5691	0.0000	-0.8088	0.5638	-0.1398	0.0001	-0.2847	1.0000	0.1063	0.8219	-0.1213	1.0000	-0.0334
LOC104909294	0.0000	-2.2812	0.0000	-3.4893	0.0000	1.8756	0.0000	2.2335	1.0000	-0.0789	0.5819	-1.2762	0.4006	0.2892
LOC104909297	1.0000	-2.4788	1.0000	-2.3986	1.0000	-0.1630	1.0000	-2.3200	1.0000	-0.1045	1.0000	0.0000	1.0000	-2.2889
LOC104909298	1.0000	-0.3548	0.7325	-0.5297	0.6783	-0.6463	0.6843	-0.8104	1.0000	-0.4084	1.0000	-0.5746	1.0000	-0.5726
LOC104909299	1.0000	0.0000	1.0000	0.0000	1.0000	0.0000	1.0000	0.0000	1.0000	0.0000	1.0000	0.0000	1.0000	0.0000
LOC104909300	1.0000	-2.4776	1.0000	0.0000	1.0000	-0.1591	1.0000	2.3242	1.0000	-2.3757	1.0000	0.0000	1.0000	0.0640
LOC104909301	0.0358	-3.3593	0.0044	-5.5802	0.0280	-3.2137	1.0000	-0.4195	1.0000	0.0009	1.0000	-2.2956	0.3840	2.8048
LOC104909304	0.9784	0.0905	0.1126	0.2481	0.0006	0.7371	0.0000	0.5818	1.0000	-0.0169	0.8238	0.1530	0.5282	-0.1678
LOC104909305	0.1430	1.9811	0.0076	5.5188	0.1507	2.1245	0.7710	3.1753	1.0000	-3.2304	1.0000	0.1804	0.2138	-2.2382
LOC104909306	0.2151	-1.6916	0.6224	-1.9258	1.0000	0.1245	1.0000	0.5633	1.0000	-1.0821	1.0000	-1.3218	1.0000	-0.6380
LOC104909307	0.2872	0.3797	0.0003	0.7187	0.0082	0.7856	0.0044	0.5994	1.0000	-0.0980	0.6269	0.2543	0.5528	-0.2800
LOC104909308	1.0000	0.0000	1.0000	0.0000	1.0000	0.0000	1.0000	0.0000	1.0000	0.0000	1.0000	0.0000	1.0000	0.0000
LOC104909309	0.9545	0.1318	1.0000	0.0547	0.0043	0.8234	0.0001	0.7880	1.0000	-0.0070	1.0000	-0.0704	1.0000	-0.0381
LOC104909310	0.0118	0.6820	0.0004	0.5900	0.6103	-0.2366	0.1082	-0.3656	1.0000	0.0261	1.0000	-0.0563	1.0000	-0.0982
LOC104909311	0.8249	-3.3439	1.0000	-0.1472	1.0000	-1.0206	1.0000	0.0057	1.0000	-0.9587	1.0000	2.3455	1.0000	0.0660
LOC104909312	0.0172	0.6696	0.2184	0.2634	0.0032	-1.1276	0.0000	-0.9471	1.0000	0.3392	1.0000	-0.0543	0.3627	0.5275
LOC104909314	0.0885	1.1050	0.3394	0.5762	0.0007	1.7779	0.0206	1.0303	1.0000	0.5732	1.0000	0.0601	1.0000	-0.1698
LOC104909316	1.0000	-2.4788	1.0000	0.0000	1.0000	-2.4061	1.0000	0.0000	1.0000	-2.3771	1.0000	0.0000	1.0000	0.0000
LOC104909317	1.0000	0.0085	0.1789	0.3120	0.0014	-0.9720	0.0018	-0.6861	1.0000	-0.2382	1.0000	0.0764	1.0000	0.0521
LOC104909318	0.3148	-4.2689	0.6224	-1.9258	0.5329	1.0793	0.0000	3.1767	1.0000	-0.1215	1.0000	2.3455	0.0015	1.9794
LOC104909319	0.0001	2.4905	0.0002	1.7339	0.0000	3.1778	0.0000	1.8387	1.0000	0.9359	1.0000	0.1961	0.6232	-0.3912
LOC104909320	1.0000	0.3349	0.0221	1.9277	1.0000	-0.0307	1.0000	0.5622	1.0000	-1.0832	1.0000	0.5207	1.0000	-0.4876
LOC104909321	0.0389	5.0454	1.0000	2.2507	1.0000	0.0000	0.7710	3.1753	1.0000	0.0000	0.2592	-2.8337	1.0000	3.2081
LOC104909322	1.0000	0.0000	1.0000	0.0000	0.0121	5.3919	0.0114	5.2126	1.0000	0.0000	1.0000	0.0000	1.0000	-0.2022
LOC104909324	1.0000	-2.4776	0.0781	4.5608	0.9361	1.2064	0.2786	4.0987	1.0000	-2.3757	0.3216	4.6747	1.0000	0.4642
LOC104909325	1.0000	0.0000	1.0000	0.0000	0.8011	3.0840	1.0000	0.0000	1.0000	0.0000	1.0000	0.0000	1.0000	-3.1344
LOC104909326	0.0415	-0.4673	0.0000	-0.7863	0.0000	0.8343	0.0000	1.2457	1.0000	-0.3155	0.0004	-0.6218	0.8495	0.1020
LOC104909327	1.0000	0.0000	1.0000	0.0000	1.0000	0.0000	1.0000	0.0000	1.0000	0.0000	1.0000	0.0000	1.0000	0.0000
LOC104909328	0.8374	-1.0335	0.3422	-1.8566	0.1953	1.3402	1.0000	-0.2069	1.0000	0.3392	1.0000	-0.4744	0.4073	-1.2063
LOC104909329	0.1506	-0.6369	0.8309	-0.1629	0.0000	-1.6485	0.0419	-0.6706	1.0000	-0.0760	0.7486	0.4117	0.1408	0.9074
LOC104909330	0.0340	0.6171	0.0012	0.7155	0.1096	0.4757	1.0000	0.0284	1.0000	-0.1849	1.0000	-0.0766	0.0083	-0.6294
LOC104909332	0.0000	-1.8947	0.0000	-2.1828	0.0516	-0.6454	0.0000	-1.2841	1.0000	0.1638	1.0000	-0.1113	0.3625	-0.4712
LOC104909334	1.0000	0.0000	1.0000	0.0000	1.0000	0.0000	1.0000	0.0000	1.0000	0.0000	1.0000	0.0000	1.0000	0.0000
LOC104909335	0.2235	0.5905	0.0019	0.7958	0.0000	1.5928	0.0000	1.4956	1.0000	0.0917	0.7904	0.3078	1.0000	-0.0020
LOC104909336	0.2530	0.6932	0.8736	-0.2206	1.0000	-0.1663	0.9366	-0.1963	1.0000	-0.0508	0.0520	-0.9547	1.0000	-0.0826
LOC104909337	1.0000	-0.0544	0.4874	-0.2015	0.4361	-0.2954	0.2062	0.2712	1.0000	-0.1656	0.5516	-0.2991	0.1299	0.4071
LOC104909339	0.0070	-2.4508	0.0156	-2.3439	0.1563	0.9476	0.0316	1.2198	1.0000	-0.0643	1.0000	0.0569	1.0000	0.2164
LOC104909340	0.0935	0.5116	0.0000	0.7090	0.0037	0.6707	0.0000	0.8311	1.0000	-0.0907	1.0000	0.1178	1.0000	0.0740
LOC104909342	1.0000	0.0000	1.0000	0.0000	1.0000	0.0000	1.0000	0.0000	1.0000	0.0000	1.0000	0.0000	1.0000	0.0000
LOC104909344	1.0000	0.0000	1.0000	0.0000	1.0000	0.0000	1.0000	0.0000	1.0000	0.0000	1.0000	0.0000	1.0000	0.0000
LOC104909345	0.0562	-5.5379	0.4431	-3.7865	0.2350	1.2152	0.0002	2.9669	1.0000	-1.7802	1.0000	0.0000	1.0000	-0.0284
LOC104909346	0.0000	2.2179	0.0030	1.5437	0.7906	0.4331	0.7167	-0.5948	1.0000	0.6393	1.0000	-0.0138	1.0000	-0.3866
LOC104909348	1.0000	0.0000	1.0000	0.0000	1.0000	0.0000	1.0000	0.0000	1.0000	0.0000	1.0000	0.0000	1.0000	0.0000
LOC104909349	1.0000	0.0000	1.0000	0.0000	1.0000	0.0000	1.0000	0.0000	1.0000	0.0000	1.0000	0.0000	1.0000	0.0000
LOC104909350	0.0143	0.6228	0.0000	0.6378	0.0000	0.9331	0.0000	0.7801	1.0000	0.0712	1.0000	0.0974	1.0000	-0.0783
LOC104909351	0.2627	0.5601	0.0000	1.0753	0.0871	0.7318	0.0000	1.0724	1.0000	-0.1738	0.5658	0.3538	0.9527	0.1711
LOC104909352	1.0000	-0.2881	1.0000	0.0000	1.0000	0.6783	1.0000	0.0000	1.0000	-2.3757	1.0000	-2.2992	1.0000	-3.1344
LOC104909353	1.0000	0.0000	1.0000	0.0000	1.0000	0.0000	1.0000	0.0000	1.0000	0.0000	1.0000	0.0000	1.0000	0.0000
LOC104909356	0.6882	-0.3134	0.2578	-0.4602	1.0000	-0.0167	0.1866	0.4605	1.0000	-0.1784	0.9318	-0.3104	0.7777	0.3016
LOC104909357	0.0096	1.1636	0.0001	1.2975	0.0000	2.1799	0.0000	2.0800	1.0000	0.0662	0.9377	0.2178	1.0000	-0.0238
LOC104909358	1.0000	0.0000	1.0000	0.0000	1.0000	0.0000	1.0000	0.0000	1.0000	0.0000	1.0000	0.0000	1.0000	0.0000
LOC104909359	1.0000	0.1184	1.0000	0.3635	1.0000	-0.4955	1.0000	0.0066	1.0000	-1.3447	0.9773	-1.1014	1.0000	-0.8431
LOC104909362	0.0000	0.9369	0.0000	0.9134	0.0006	0.7244	0.0017	0.4812	1.0000	0.0353	1.0000	0.0227	0.4598	-0.2046
LOC104909364	0.0005	1.0880	0.0001	0.9196	1.0000	0.0116	0.3896	-0.3428	1.0000	-0.0335	0.9238	-0.1884	0.6154	-0.3817
LOC104909368	1.0000	2.1902	1.0000	0.0000	1.0000	0.0000	1.0000	0.0000	1.0000	0.0000	1.0000	-2.2992	1.0000	0.0000
LOC104909369	0.0000	-1.0527	0.0000	-1.1704	0.0000	-0.8703	0.0000	-0.7286	1.0000	-0.0546	0.4861	-0.1596	0.7640	0.0930
LOC104909370	0.6963	0.2725	0.8651	0.1339	0.0005	0.9178	0.0003	0.7836	1.0000	-0.2053	0.8156	-0.3323	0.2779	-0.3335
LOC104909371	0.9869	0.4287	0.3712	0.6718	0.0464	1.2829	1.0000	0.2532	1.0000	0.2711	0.8607	0.5271	0.4823	-0.7505
LOC104909372	0.0791	1.5050	0.0004	2.4431	0.0000	2.6466	0.0020	2.2150	1.0000	-0.5955	1.0000	0.3567	0.0936	-1.0245
LOC104909373	1.0000	-0.0035	1.0000	0.0118	0.1399	-0.3583	0.0412	-0.2797	1.0000	-0.1176	1.0000	-0.0898	1.0000	-0.0332
LOC104909374	0.9545	1.0679	1.0000	0.0000	0.9361	1.2064	0.7701	3.1732	1.0000	-2.3757	0.8607	-3.6732	1.0000	-0.4570
LOC104909376	0.0450	0.8397	0.0000	1.7382	0.0000	1.6939	0.0000	1.6562	1.0000	-0.4788	0.5382	0.4313	0.0991	-0.5113
LOC104909378	0.7928	0.4291	0.4410	0.4529	0.0503	-1.6040	0.0005	-2.3686	1.0000	0.3719	0.8841	0.4074	1.0000	-0.3874
LOC104909379	1.0000	2.1902	1.0000	0.3654	0.4809	3.6101	1.0000	0.5384	1.0000	3.1056	1.0000	1.4259	1.0000	0.0752
LOC104909380	1.0000	0.0000	1.0000	-2.3986	1.0000	0.0000	1.0000	-2.3200	1.0000	2.2735	1.0000	0.0000	1.0000	0.0000
LOC104909381	1.0000	-0.2881	1.0000	0.0000	1.0000	-2.4056	0.7701	3.1732	1.0000	-2.3757	1.0000	-2.2992	1.0000	3.2066
LOC104909382	0.0764	0.4738	0.5008	-0.1917	0.0008	0.7135	0.0002	0.5464	1.0000	-0.1600	0.0007	-0.8137	0.0904	-0.3212
LOC104909383	0.2106	-0.7344	0.0310	-1.2085	0.0015	1.1885	0.0000	1.4259	1.0000	-0.4584	0.5587	-0.9187	0.9124	-0.2197
LOC104909384	1.0000	2.1902	1.0000	0.0000	1.0000	0.0000	1.0000	0.0000	1.0000	0.0000	1.0000	-2.2992	1.0000	0.0000
LOC104909385	0.5086	-3.8790	0.0881	-4.7397	0.9361	-1.5529	0.5166	-1.4694	1.0000	0.8255	1.0000	0.0000	1.0000	0.9186
LOC104909386	1.0000	-0.2920	1.0000	-0.1472	1.0000	-0.1624	0.6429	1.7753	1.0000	-0.1034	1.0000	0.0495	0.9124	1.8420
LOC104909387	1.0000	0.0568	0.7685	-0.4999	0.6601	0.5299	0.2489	0.8340	1.0000	-0.2349	0.7966	-0.7878	1.0000	0.0799
LOC104909388	0.5212	0.7658	0.0292	2.3009	0.4221	0.8598	0.0102	2.4128	1.0000	-1.6341	1.0000	-0.0893	1.0000	-0.0790
LOC104909389	0.0089	-3.7308	0.0655	-3.0450	0.0824	1.2642	1.0000	0.2906	1.0000	-0.6525	1.0000	0.0495	0.0344	-1.6161
LOC104909390	1.0000	0.8894	0.3280	1.8593	0.0039	2.9053	0.0350	2.4516	1.0000	-0.1210	1.0000	0.8545	0.8939	-0.5717
LOC104909392	0.8249	-3.3439	1.0000	-2.3959	1.0000	-1.0206	1.0000	-2.3176	1.0000	-0.9638	1.0000	0.0000	1.0000	-2.2889
LOC104909393	1.0000	2.1849	1.0000	0.0000	1.0000	0.0000	1.0000	0.0000	1.0000	0.0000	1.0000	-2.2956	1.0000	0.0000
LOC104909394	1.0000	0.4239	0.7407	0.7792	0.0016	2.5893	0.5142	0.9725	1.0000	0.1803	1.0000	0.5530	0.0648	-1.4329
LOC104909395	1.0000	-0.1193	0.4374	-0.2766	1.0000	0.0347	0.0170	-0.6870	1.0000	-0.0534	1.0000	-0.2017	0.0329	-0.7717
LOC104909396	1.0000	2.1849	1.0000	0.0000	1.0000	2.2471	1.0000	2.3242	1.0000	0.0000	1.0000	-2.2956	1.0000	0.0640
LOC104909397	0.0028	2.4640	0.0000	2.1864	0.0000	3.6438	0.0000	2.2818	1.0000	1.0943	0.2939	0.8273	0.8937	-0.2630
LOC104909398	0.4209	1.5357	0.9148	1.2162	0.7628	1.0365	0.6172	1.7712	1.0000	-0.9587	0.8496	-1.2861	1.0000	-0.2280
LOC104909399	0.0933	0.7736	0.0061	0.9223	0.3272	0.5104	0.8749	0.2127	1.0000	-0.1322	1.0000	0.0287	0.5963	-0.4247
LOC104909400	0.8249	-3.3411	1.0000	-2.3986	0.8033	-3.2622	1.0000	-2.3200	1.0000	-0.9563	1.0000	0.0000	1.0000	0.0000
LOC104909401	1.0000	0.0000	1.0000	0.0000	1.0000	0.0000	1.0000	0.0000	1.0000	0.0000	1.0000	0.0000	1.0000	0.0000
LOC104909404	0.1456	-1.8534	0.9020	-0.5785	1.0000	-0.1927	1.0000	-0.1792	1.0000	-0.2913	1.0000	0.9997	1.0000	-0.2730
LOC104909405	0.0864	0.4128	0.7534	0.0971	0.7161	-0.1602	0.4370	-0.1677	1.0000	0.0272	0.2626	-0.2770	1.0000	0.0250
LOC104909406	1.0000	0.1447	0.6086	0.3239	0.0005	1.2616	0.0000	1.5475	1.0000	-0.1312	1.0000	0.0608	1.0000	0.1605
LOC104909408	0.6936	1.4536	0.7226	1.0540	1.0000	-2.4056	1.0000	0.0058	1.0000	0.7416	1.0000	0.3618	1.0000	3.2066
LOC104909409	0.6919	0.5544	1.0000	-0.1789	1.0000	0.1744	0.1210	-1.2152	1.0000	0.7780	1.0000	0.0599	0.9124	-0.6051
LOC104909410	0.6994	-0.5105	0.0322	-1.5050	0.0000	3.5567	0.0000	2.3490	1.0000	0.0623	0.6546	-0.9198	0.0001	-1.1393
LOC104909411	0.5378	0.1846	0.4826	-0.1214	0.0004	0.6604	0.0000	0.6126	1.0000	0.1599	0.7394	-0.1330	0.6246	0.1187
LOC104909412	1.0000	0.0000	0.7674	-3.2570	1.0000	0.0000	0.7710	-3.1693	1.0000	3.1191	1.0000	0.0000	1.0000	0.0000

LOC104909413	0.7736	-0.7545	0.1736	1.8784	0.7352	0.6868	0.2198	1.8817	1.0000	-1.9997	1.0000	0.6330	0.6888	-0.8059
LOC104909414	1.0000	0.0000	1.0000	0.0000	1.0000	0.0000	1.0000	0.0000	1.0000	0.0000	1.0000	0.0000	1.0000	0.0000
LOC104909415	1.0000	-2.4776	1.0000	2.2507	1.0000	-0.1624	1.0000	0.0000	1.0000	-2.3758	1.0000	2.3455	1.0000	-2.2889
LOC104909416	1.0000	0.0000	1.0000	0.0000	1.0000	0.0000	1.0000	0.0000	1.0000	0.0000	1.0000	0.0000	1.0000	0.0000
LOC104909417	1.0000	0.0000	1.0000	0.0000	1.0000	0.0000	1.0000	0.0000	1.0000	0.0000	1.0000	0.0000	1.0000	0.0000
LOC104909419	1.0000	-2.4788	1.0000	0.6956	1.0000	0.6740	1.0000	0.0075	1.0000	-0.1089	1.0000	3.1976	1.0000	-0.7769
LOC104909420	0.0252	1.3484	0.0062	1.1086	0.0036	1.5134	0.0196	0.9996	1.0000	0.4480	1.0000	0.2175	1.0000	-0.0616
LOC104909421	1.0000	0.0000	1.0000	0.0000	1.0000	2.2471	1.0000	2.3242	1.0000	0.0000	1.0000	0.0000	1.0000	0.0640
LOC104909422	1.0000	-2.4788	1.0000	0.0000	1.0000	-2.4061	1.0000	0.0000	1.0000	-2.3771	1.0000	0.0000	1.0000	0.0000
LOC104909425	1.0000	0.0000	1.0000	0.0000	1.0000	0.0000	1.0000	0.0000	1.0000	0.0000	1.0000	0.0000	1.0000	0.0000
LOC104909426	1.0000	0.0000	1.0000	0.0000	1.0000	0.0000	1.0000	0.0000	1.0000	0.0000	1.0000	0.0000	1.0000	0.0000
LOC104909427	1.0000	0.0000	1.0000	0.0000	1.0000	0.0000	1.0000	0.0000	1.0000	0.0000	1.0000	0.0000	1.0000	0.0000
LOC104909429	1.0000	0.0000	1.0000	0.0000	1.0000	0.0000	1.0000	0.0000	1.0000	0.0000	1.0000	0.0000	1.0000	0.0000
LOC104909430	0.6923	-2.0829	1.0000	-2.3959	0.2833	-4.1854	1.0000	-2.3176	1.0000	-1.8819	1.0000	-2.2956	1.0000	0.0000
LOC104909432	0.0154	-0.9315	0.0273	-0.7458	0.9441	-0.1445	0.8164	0.1718	1.0000	-0.4438	1.0000	-0.2475	1.0000	-0.1261
LOC104909433	1.0000	0.0134	1.0000	0.0404	0.2979	0.6256	1.0000	0.0078	1.0000	0.0205	1.0000	0.0604	0.4598	-0.5925
LOC104909434	1.0000	0.0000	1.0000	0.0000	1.0000	0.0000	1.0000	0.0000	1.0000	0.0000	1.0000	0.0000	1.0000	0.0000
LOC104909436	1.0000	0.0000	1.0000	0.0000	1.0000	0.0000	1.0000	0.0000	1.0000	0.0000	1.0000	0.0000	1.0000	0.0000
LOC104909438	0.0357	0.9522	0.0000	1.6055	1.0000	0.0870	0.3415	0.4952	1.0000	-0.9050	1.0000	-0.2425	0.4842	-0.4924
LOC104909440	0.4622	0.2633	0.1924	0.2609	0.0000	-1.6135	0.0000	-1.3538	1.0000	-0.0135	1.0000	-0.0031	0.8686	0.2521
LOC104909441	1.0000	0.3595	1.0000	0.1632	0.7917	0.6512	0.8364	0.4466	1.0000	0.7180	1.0000	0.5359	1.0000	0.5192
LOC104909445	0.4833	0.4575	0.1518	0.6857	0.2606	0.5552	0.0040	1.0025	1.0000	-0.2491	1.0000	-0.0074	0.9990	0.2055
LOC104909448	0.0308	-0.7046	0.0007	-0.7312	1.0000	-0.0430	0.4517	0.1923	1.0000	-0.2518	0.8607	-0.2660	1.0000	-0.0097
LOC104909449	0.2096	-0.5957	0.6786	-0.2967	0.1723	-0.6176	0.4348	0.3625	1.0000	-0.7877	0.6968	-0.4768	1.0000	0.1980
LOC104909451	0.1167	0.3341	0.0027	0.3801	0.0124	-0.5319	0.0094	-0.3532	1.0000	-0.0519	1.0000	0.0056	0.8191	0.1302
LOC104909452	0.1820	0.6194	1.0000	0.0471	0.5107	0.4026	0.7550	-0.2109	1.0000	0.2220	0.7635	-0.3382	0.6474	-0.3851
LOC104909453	0.6155	0.1767	0.4431	0.1435	0.3698	-0.2576	0.3602	-0.1632	1.0000	-0.0540	1.0000	-0.0740	1.0000	0.0459
LOC104909454	1.0000	0.0000	1.0000	0.0000	1.0000	0.0000	1.0000	0.0000	1.0000	0.0000	1.0000	0.0000	1.0000	0.0000
LOC104909455	0.6754	-1.2803	0.8870	0.4740	0.8177	-1.1335	1.0000	0.0031	1.0000	-0.3674	0.8496	1.4013	1.0000	0.7684
LOC104909456	0.6380	0.4811	0.0084	1.1592	1.0000	0.1789	0.6787	-0.4464	1.0000	0.0062	0.3725	0.6962	0.7027	-0.6132
LOC104909457	0.0572	0.6348	0.0003	0.8246	0.0001	1.0715	0.0000	1.1400	1.0000	-0.1652	1.0000	0.0372	1.0000	-0.0913
LOC104909458	0.2482	-0.3113	0.4919	-0.1327	0.0012	-0.7649	0.0000	-0.6675	1.0000	0.1380	0.1631	0.3291	0.5364	0.2421
LOC104909459	0.3119	1.1911	0.0001	3.9925	0.0051	2.1238	0.0000	4.2241	1.0000	-2.4391	1.0000	0.3479	1.0000	-0.3455
LOC104909460	0.2578	-1.6868	0.0953	-2.8836	0.5160	-1.5348	0.8636	-0.6451	1.0000	-0.1229	1.0000	-1.3246	1.0000	0.7684
LOC104909461	0.1988	1.6954	0.4431	-1.0242	0.6552	0.9728	0.0567	-2.3163	1.0000	1.6700	0.7962	-1.0294	0.6338	-1.6147
LOC104909462	1.0000	2.1849	1.0000	0.3653	1.0000	2.2471	1.0000	-0.8442	1.0000	3.1191	1.0000	1.4325	1.0000	0.0640
LOC104909463	0.0003	-1.2116	0.0000	-1.3066	0.0082	-0.8495	0.0092	-0.7303	1.0000	-0.1391	1.0000	-0.2226	1.0000	-0.0159
LOC104909464	0.0380	-0.3786	0.0010	-0.5416	0.0000	1.5272	0.0000	0.9131	1.0000	0.2012	1.0000	0.0500	0.0003	-0.4083
LOC104909465	0.6235	1.3434	1.0000	-0.1830	0.5528	1.2884	0.9053	0.5720	1.0000	1.3612	1.0000	-0.1528	1.0000	0.6446
LOC104909466	0.5384	3.5543	1.0000	0.0000	0.8011	3.0840	1.0000	0.0000	1.0000	0.0000	0.8764	-3.6794	1.0000	-3.1344
LOC104909467	0.0010	1.3067	0.0000	1.1993	0.0059	0.9869	0.0000	1.2740	1.0000	-0.0766	1.0000	-0.1710	0.8494	0.2165
LOC104909468	1.0000	0.8875	0.7249	1.0539	0.3962	1.5048	1.0000	-0.8419	1.0000	-0.1179	1.0000	0.0579	0.3704	-2.4658
LOC104909469	1.0000	0.0000	1.0000	0.0000	1.0000	0.0000	1.0000	0.0000	1.0000	0.0000	1.0000	0.0000	1.0000	0.0000
LOC104909470	1.0000	0.0000	1.0000	0.0000	1.0000	0.0000	1.0000	0.0000	1.0000	0.0000	1.0000	0.0000	1.0000	0.0000
LOC104909471	0.7821	0.4006	1.0000	-0.0300	0.6065	-0.6965	1.0000	-0.1601	1.0000	0.5339	1.0000	0.1110	0.3019	1.0734
LOC104909472	0.8249	3.0142	1.0000	0.0000	1.0000	0.0000	1.0000	0.0000	1.0000	0.0000	1.0000	-3.1382	1.0000	0.0000
LOC104909473	1.0000	0.0000	1.0000	0.0000	1.0000	2.2426	1.0000	0.0000	1.0000	0.0000	1.0000	0.0000	1.0000	-2.2889
LOC104909474	1.0000	0.0000	1.0000	-2.3959	1.0000	0.0000	1.0000	-2.3176	1.0000	2.2677	1.0000	0.0000	1.0000	0.0000
LOC104909475	1.0000	0.0000	1.0000	0.0000	1.0000	0.0000	1.0000	0.0000	1.0000	0.0000	1.0000	0.0000	1.0000	0.0000
LOC104909476	1.0000	-0.3483	0.1456	-4.4788	0.8011	0.5265	1.0000	0.2591	1.0000	-0.3812	0.3176	-4.6147	1.0000	-0.6396
LOC104909477	1.0000	0.2607	0.0006	2.1470	0.0171	1.4888	0.0010	2.0684	1.0000	-0.7676	0.1840	1.1332	1.0000	-0.1805
LOC104909478	0.3271	-0.4148	0.7097	-0.1935	0.7581	-0.2159	0.5680	-0.2370	1.0000	-0.3092	1.0000	-0.0741	0.5998	-0.3238
LOC104909479	0.0000	2.6565	0.0000	1.8069	0.0009	1.9731	0.0371	1.0357	1.0000	0.7748	1.0000	-0.0604	1.0000	-0.1555
LOC104909481	0.1590	-0.2724	0.0001	-0.2925	0.2468	0.2284	0.4424	0.0823	1.0000	-0.0171	1.0000	-0.0246	0.2249	-0.1573
LOC104909482	0.5130	0.2883	0.7554	0.1416	0.6094	-0.2536	0.1264	-0.4111	1.0000	0.1846	1.0000	0.0515	1.0000	0.0314
LOC104909483	0.8228	-0.2090	0.8498	0.1320	0.8033	-0.2561	0.1042	0.4142	1.0000	0.1208	0.3216	0.4728	0.0203	0.7985
LOC104909484	0.0431	0.8520	0.0072	0.9602	0.7006	-0.3734	1.0000	0.0819	1.0000	-0.3467	1.0000	-0.2263	1.0000	0.1146
LOC104909488	1.0000	0.6043	0.0039	2.4643	0.6305	0.9686	0.7950	0.9502	1.0000	-0.1163	0.1679	1.7545	1.0000	-0.1353
LOC104909489	1.0000	2.1850	1.0000	2.2533	1.0000	0.0000	1.0000	0.0000	1.0000	0.0000	1.0000	0.0514	1.0000	0.0000
LOC104909490	0.0097	1.2447	0.0544	0.7776	0.1147	-1.4479	0.0045	-1.6359	1.0000	-0.1029	0.3279	-0.5500	1.0000	-0.2706
LOC104909491	0.0004	1.3074	0.0000	1.5611	0.0000	1.9882	0.0000	1.4407	1.0000	-0.0431	0.8785	0.2240	0.0692	-0.5849
LOC104909493	0.0000	-1.3678	0.0000	-1.1264	0.0000	-1.1508	0.0001	-0.7746	1.0000	-0.2345	1.0000	0.0188	1.0000	0.1464
LOC104909494	0.4083	-0.2792	0.0146	-0.4697	0.0012	-0.8246	0.0000	-0.9968	1.0000	0.3220	0.9755	0.1451	0.9524	0.1549
LOC104909495	1.0000	0.0000	1.0000	0.0000	1.0000	0.0000	1.0000	0.0000	1.0000	0.0000	1.0000	0.0000	1.0000	0.0000
LOC104909497	1.0000	0.0000	1.0000	0.0000	1.0000	0.0000	0.7701	3.1732	1.0000	0.0000	1.0000	0.0000	1.0000	3.2066
LOC104909499	1.0000	0.0000	1.0000	0.0000	1.0000	0.0000	1.0000	0.0000	1.0000	0.0000	1.0000	0.0000	1.0000	0.0000
LOC104909500	1.0000	-0.3341	1.0000	0.6987	0.9361	-1.5512	1.0000	-2.3176	1.0000	-1.4945	1.0000	-0.4744	1.0000	-2.2908
LOC104909501	0.4903	0.6832	1.0000	0.0398	0.5634	-0.7587	1.0000	-0.5491	1.0000	-2.1486	0.0000	-2.7885	0.0847	-1.9412
LOC104909502	0.0105	1.3815	0.8698	0.3969	0.0902	1.0155	0.4257	-0.9638	1.0000	-0.9268	0.0000	-1.9029	0.0000	-2.9000
LOC104909504	0.1423	0.5152	0.1905	0.3859	0.0481	-0.7795	0.0238	-0.7140	1.0000	-0.2372	0.5089	-0.3528	1.0000	-0.1627
LOC104909505	1.0000	-0.2882	1.0000	0.0000	1.0000	-2.4056	1.0000	0.0000	1.0000	-2.3758	1.0000	-2.2992	1.0000	0.0000
LOC104909506	0.0068	1.5675	0.0001	1.6955	0.0212	-2.7463	0.0025	-2.9317	1.0000	0.2498	0.8349	0.3924	1.0000	0.0718
LOC104909507	1.0000	0.0000	1.0000	0.0000	1.0000	0.0000	1.0000	0.0000	1.0000	0.0000	1.0000	0.0000	1.0000	0.0000
LOC104909508	0.7153	-0.8125	0.0042	-2.4286	1.0000	-0.4060	0.0019	-2.6333	1.0000	1.3555	1.0000	-0.2470	1.0000	-0.8675
LOC104909510	1.0000	0.0000	1.0000	0.0000	1.0000	0.0000	1.0000	0.0000	1.0000	0.0000	1.0000	0.0000	1.0000	0.0000
LOC104909512	1.0000	-2.4788	1.0000	0.0000	1.0000	0.6780	1.0000	0.0000	1.0000	-2.3771	1.0000	0.0000	1.0000	-3.1344
LOC104909513	1.0000	-0.2599	0.5668	-0.6173	1.0000	-0.0066	1.0000	0.0989	1.0000	-0.0294	1.0000	-0.3777	1.0000	0.0801
LOC104909514	0.0000	1.3326	0.0000	1.1350	0.0000	0.9838	0.0000	0.7447	1.0000	0.3448	0.5915	0.1605	0.8013	0.1131
LOC104909515	0.0546	0.8387	0.0001	1.1582	0.0003	1.3850	0.0000	1.4087	1.0000	0.0051	0.7202	0.3382	1.0000	0.0334
LOC104909517	0.4197	-0.2542	0.1039	-0.2517	0.0006	-0.8009	0.0000	-0.5978	1.0000	-0.1586	0.8759	-0.1422	1.0000	0.0513
LOC104909518	0.0032	-0.5618	0.0000	-0.4763	0.3061	-0.2375	1.0000	-0.0325	1.0000	0.0450	0.7247	0.1426	0.0885	0.2553
LOC104909519	0.0166	1.0760	0.0000	1.3493	0.0013	1.2660	0.0000	1.4069	1.0000	-0.1348	1.0000	0.1508	1.0000	0.0110
LOC104909520	0.9246	0.1821	0.0019	0.7284	1.0000	-0.1200	0.8411	0.1540	1.0000	-0.1978	0.4857	0.3629	1.0000	0.0812
LOC104909521	0.6758	-0.1901	0.0354	-0.3310	0.0000	-1.2378	0.0000	-1.0277	1.0000	0.1724	1.0000	0.0444	0.2162	0.3882
LOC104909522	1.0000	0.0000	1.0000	0.0000	1.0000	2.2426	1.0000	0.0000	1.0000	0.0000	1.0000	0.0000	1.0000	-2.2889
LOC104909523	1.0000	0.0000	1.0000	0.0000	1.0000	0.0000	1.0000	0.0000	1.0000	0.0000	1.0000	0.0000	1.0000	0.0000
LOC104909525	0.0137	0.7061	0.0000	0.7714	0.0000	2.0115	0.0000	1.8160	1.0000	0.2590	0.3107	0.3379	1.0000	0.0712
LOC104909526	0.0016	5.7633	0.3790	1.6993	0.0195	5.2592	0.2197	1.8812	1.0000	3.1126	0.8385	-0.8283	1.0000	-0.2335
LOC104909527	1.0000	-0.3227	0.4431	-3.7865	0.8011	-3.2629	0.9096	-1.3721	1.0000	0.4094	1.0000	-3.1429	1.0000	2.3542
LOC104909528	0.2072	-0.5906	0.9756	-0.1375	1.0000	-0.0210	1.0000	-0.0140	1.0000	-0.2764	1.0000	0.1914	0.8877	-0.2650
LOC104909529	1.0000	-2.4788	1.0000	0.0000	1.0000	-0.1630	1.0000	0.0000	1.0000	-2.3771	1.0000	0.0000	1.0000	-2.2889
LOC104909530	1.0000	0.0000	1.0000	0.0000	1.0000	0.0000	1.0000	0.0000	1.0000	0.0000	1.0000	0.0000	1.0000	0.0000
LOC104909531	1.0000	0.5414	1.0000	-2.3986	1.0000	-2.4061	1.0000	-2.3200	1.0000	-0.1045	1.0000	-3.1429	1.0000	0.0000

LOC104909532	1.0000	0.4262	0.7356	0.6692	0.2195	1.4499	0.4332	0.9770	1.0000	0.4333	1.0000	0.6914	1.0000	-0.0349
LOC104909533	1.0000	-0.2931	0.7666	3.0922	1.0000	-2.4061	1.0000	0.0000	1.0000	-2.3771	1.0000	0.9006	1.0000	0.0000
LOC104909534	0.9545	1.0644	0.0781	4.5608	1.0000	-0.1597	0.7710	3.1709	1.0000	-2.3771	1.0000	1.0025	1.0000	0.9149
LOC104909535	1.0000	0.0000	1.0000	-0.1612	1.0000	0.0000	0.7701	-3.1666	1.0000	3.1126	1.0000	3.1976	1.0000	0.0000
LOC104909536	1.0000	0.0000	1.0000	0.0000	1.0000	0.0000	1.0000	0.0000	1.0000	0.0000	1.0000	0.0000	1.0000	0.0000
LOC104909537	0.0668	2.4099	0.5440	0.9198	0.0352	2.4721	1.0000	0.2585	1.0000	1.1048	1.0000	-0.3741	0.5105	-1.1080
LOC104909538	1.0000	0.2116	0.1546	2.3755	1.0000	-0.1821	0.6172	1.7712	1.0000	-0.9563	0.9773	1.2136	1.0000	0.9922
LOC104909539	0.8647	0.5176	1.0000	0.2981	0.1429	1.3565	0.1590	1.0602	1.0000	0.3678	1.0000	0.1648	1.0000	0.0804
LOC104909540	0.8226	3.0199	1.0000	0.0000	1.0000	0.0000	1.0000	0.0000	1.0000	0.0000	1.0000	-3.1429	1.0000	0.0000
LOC104909541	1.0000	2.1850	0.9062	1.2214	1.0000	0.0000	1.0000	-2.3200	1.0000	2.2735	1.0000	1.4317	1.0000	0.0000
LOC104909542	1.0000	2.1850	0.7666	-3.2534	0.0919	4.5509	0.0534	2.3289	1.0000	3.1126	1.0000	-2.2956	0.8501	0.9269
LOC104909543	1.0000	0.0549	0.0683	-0.6258	1.0000	-0.0150	0.8299	0.1705	1.0000	0.2606	0.6954	-0.4051	0.4566	0.4503
LOC104909544	0.4624	1.0264	0.0515	1.7604	0.0606	1.6342	1.0000	0.5632	1.0000	-0.6819	1.0000	0.0605	0.0726	-1.7523
LOC104909545	1.0000	-0.2921	1.0000	0.0000	0.9361	1.2045	1.0000	0.0000	1.0000	-2.3758	1.0000	-2.2955	0.7287	-3.6634
LOC104909546	1.0000	0.0000	1.0000	0.0000	1.0000	0.0000	1.0000	0.0000	1.0000	0.0000	1.0000	0.0000	1.0000	0.0000
LOC104909547	0.8226	-3.3425	0.2544	2.1622	0.8011	-3.2629	1.0000	0.0062	1.0000	-0.9614	0.3206	4.6730	1.0000	2.3543
LOC104909548	0.1335	-1.5745	0.1181	-0.6379	0.0013	-4.5729	1.0000	0.1316	1.0000	0.9519	0.0042	1.9003	0.0000	5.6638
LOC104909549	1.0000	0.0000	1.0000	0.0000	1.0000	0.0000	1.0000	0.0000	1.0000	0.0000	1.0000	0.0000	1.0000	0.0000
LOC104909550	1.0000	0.0000	1.0000	0.0000	1.0000	0.0000	1.0000	0.0000	1.0000	0.0000	1.0000	0.0000	1.0000	0.0000
LOC104909551	1.0000	2.1902	1.0000	0.0000	1.0000	0.0000	1.0000	0.0000	1.0000	0.0000	1.0000	-2.2992	1.0000	0.0000
LOC104909552	1.0000	0.0000	1.0000	0.0000	1.0000	0.0000	1.0000	0.0000	1.0000	0.0000	1.0000	0.0000	1.0000	0.0000
LOC104909553	1.0000	-0.2920	1.0000	2.2533	0.4584	1.8990	1.0000	2.3242	1.0000	-2.3758	1.0000	0.0514	0.6944	-2.0012
LOC104909554	1.0000	0.1021	0.0001	1.5590	0.0013	1.4629	0.0000	1.9935	1.0000	-0.7345	0.2459	0.7356	0.9676	-0.1950
LOC104909555	0.6877	-2.0809	1.0000	0.6940	1.0000	-0.5738	0.9167	1.3833	1.0000	-1.8822	1.0000	0.8962	1.0000	0.0735
LOC104909557	1.0000	-0.2417	0.0775	1.7692	0.6943	-0.6022	0.1088	1.6478	1.0000	-1.6562	1.0000	0.3579	1.0000	0.5979
LOC104909558	1.0000	-0.0398	1.0000	-0.0291	0.9270	0.3780	0.3201	0.9062	1.0000	-0.5329	1.0000	-0.5131	1.0000	-0.0012
LOC104909559	0.0650	1.0356	0.4411	0.4017	0.0002	1.6720	0.0286	0.7691	1.0000	0.8596	1.0000	0.2393	1.0000	-0.0399
LOC104909560	0.0102	1.8581	0.0000	2.1984	0.0000	2.4631	0.0000	3.2502	1.0000	0.1836	0.6123	0.5379	0.0164	0.9757
LOC104909561	0.5391	-3.8767	1.0000	0.0000	0.5041	-3.7956	1.0000	0.0000	1.0000	-3.7606	1.0000	0.0000	1.0000	0.0000
LOC104909562	1.0000	-2.4776	1.0000	2.2534	1.0000	-2.4056	1.0000	0.0000	1.0000	-2.3758	1.0000	2.3480	1.0000	0.0000
LOC104909563	0.8937	-0.1563	0.5222	0.2098	0.5556	0.2507	0.0170	0.4638	1.0000	-0.0568	0.7069	0.3227	0.8749	0.1640
LOC104909564	0.1427	0.7900	0.0002	1.2165	0.0000	1.8360	0.0000	1.9875	1.0000	-0.1334	0.8820	0.3084	1.0000	0.0242
LOC104909565	0.0000	-1.5523	0.0000	-2.3266	0.4056	-0.3627	0.0506	-0.5833	1.0000	0.0369	0.4791	-0.7248	1.0000	-0.1777
LOC104909566	1.0000	0.0000	1.0000	0.0000	1.0000	0.0000	1.0000	0.0000	1.0000	0.0000	1.0000	0.0000	1.0000	0.0000
LOC104909567	1.0000	-1.1537	1.0000	0.0000	1.0000	-1.0172	1.0000	0.0000	1.0000	-3.2304	1.0000	-2.2992	1.0000	-2.2907
LOC104909569	1.0000	0.0000	1.0000	0.0000	1.0000	0.0000	1.0000	0.0000	1.0000	0.0000	1.0000	0.0000	1.0000	0.0000
LOC104909570	1.0000	0.0000	1.0000	0.0000	1.0000	0.0000	1.0000	0.0000	1.0000	0.0000	1.0000	0.0000	1.0000	0.0000
LOC104909571	1.0000	0.0000	1.0000	0.0000	0.8033	3.0892	1.0000	0.0000	1.0000	0.0000	1.0000	0.0000	1.0000	-3.1369
LOC104909572	1.0000	0.1993	1.0000	-0.9973	0.0826	2.2584	0.2198	1.8817	1.0000	-0.1245	1.0000	-1.3246	1.0000	-0.4914
LOC104909573	1.0000	0.0000	1.0000	2.2508	1.0000	0.0000	0.7710	3.1753	1.0000	0.0000	1.0000	2.3455	1.0000	3.2081
LOC104909574	1.0000	0.3648	1.0000	0.1329	0.5513	-1.6597	1.0000	0.3124	1.0000	-0.6840	0.9301	-0.9079	0.9835	1.2974
LOC104909575	1.0000	-2.4776	1.0000	0.0000	1.0000	-2.4056	1.0000	2.3242	1.0000	-2.3758	1.0000	0.0000	1.0000	2.3543
LOC104909576	1.0000	-2.4776	0.7674	-3.2498	1.0000	-2.4056	0.7710	-3.1633	1.0000	0.7318	1.0000	0.0000	1.0000	0.0000
LOC104909579	0.2097	2.4021	1.0000	-0.1594	0.6647	1.5885	1.0000	0.5384	1.0000	0.7318	0.6102	-1.8174	1.0000	-0.3106
LOC104909580	1.0000	-0.0829	0.0284	-0.2880	0.0079	-0.6357	0.0000	-0.9432	1.0000	0.1542	1.0000	-0.0399	0.7894	-0.1472
LOC104909581	0.0158	1.9752	0.3737	0.6945	1.0000	-0.7474	0.2111	-1.3260	1.0000	0.9519	1.0000	-0.3083	1.0000	0.3825
LOC104909582	1.0000	0.0000	1.0000	0.0000	1.0000	0.0000	1.0000	0.0000	1.0000	0.0000	1.0000	0.0000	1.0000	0.0000
LOC104909583	1.0000	0.0000	1.0000	0.0000	1.0000	0.0000	1.0000	0.0000	1.0000	0.0000	1.0000	0.0000	1.0000	0.0000
LOC104909584	1.0000	0.0000	1.0000	2.2534	1.0000	0.0000	1.0000	0.0000	1.0000	0.0000	1.0000	2.3480	1.0000	0.0000
LOC104909585	1.0000	0.0000	1.0000	0.0000	1.0000	0.0000	1.0000	0.0000	1.0000	0.0000	1.0000	0.0000	1.0000	0.0000
LOC104909586	1.0000	0.0000	1.0000	0.0000	1.0000	0.0000	1.0000	0.0000	1.0000	0.0000	1.0000	0.0000	1.0000	0.0000
LOC104909587	0.8679	-0.2559	0.0927	-0.9662	0.3248	-0.6916	0.4037	0.4157	1.0000	-0.1612	0.4483	-0.8586	0.1188	0.9468
LOC104909588	1.0000	-0.3236	0.9148	-1.5387	0.8033	-3.2636	1.0000	-0.5257	1.0000	0.4178	1.0000	-0.7956	1.0000	3.2080
LOC104909589	0.5086	-1.4999	1.0000	-0.4643	0.3949	-1.8751	0.4562	-2.0485	1.0000	-0.6040	1.0000	0.4447	1.0000	-0.7793
LOC104909590	1.0000	0.0000	1.0000	0.0000	1.0000	0.0000	1.0000	0.0000	1.0000	0.0000	1.0000	0.0000	1.0000	0.0000
LOC104909591	0.0002	0.8385	0.0000	1.0612	0.0000	1.8430	0.0000	1.5565	1.0000	0.0208	0.4999	0.2569	0.0248	-0.2612
LOC104909592	1.0000	-2.4788	1.0000	0.0000	1.0000	-0.1630	1.0000	0.0000	1.0000	-2.3771	1.0000	0.0000	1.0000	-2.2889
LOC104909593	0.8226	3.0199	1.0000	0.6912	0.8033	3.0789	1.0000	-2.3200	1.0000	2.2735	1.0000	0.0533	1.0000	-3.1316
LOC104909594	0.5925	0.3692	1.0000	0.1002	1.0000	0.0580	0.6734	-0.3523	1.0000	0.3185	1.0000	0.0628	1.0000	-0.0861
LOC104909595	0.0000	1.2051	0.0000	1.0640	0.0194	-0.5945	0.0000	-0.6539	1.0000	-0.1511	0.1058	-0.2807	0.7256	-0.2039
LOC104909596	1.0000	0.0000	1.0000	0.0000	1.0000	0.0000	1.0000	0.0000	1.0000	0.0000	1.0000	0.0000	1.0000	0.0000
LOC104909597	0.8249	3.0142	0.4431	3.6192	1.0000	2.2426	0.7710	3.1709	1.0000	0.0000	1.0000	0.5871	1.0000	0.9164
LOC104909599	0.6330	0.6963	0.1745	0.9879	0.1429	-1.8292	0.1385	-1.7832	1.0000	-0.3698	1.0000	-0.0594	1.0000	-0.3137
LOC104909601	0.0606	-1.6869	0.1314	-1.0037	0.9856	0.2684	0.2274	-0.8173	1.0000	0.3359	0.7842	1.0351	0.6777	-0.7444
LOC104909602	0.2365	-2.0020	0.0006	-5.8201	0.4887	-0.9122	0.0516	-2.0206	1.0000	0.2404	0.8607	-3.6732	1.0000	-0.8666
LOC104909604	0.5780	-1.5569	0.0579	-3.0476	1.0000	0.2755	0.6868	-0.8099	1.0000	0.6869	1.0000	-0.7966	1.0000	-0.3899
LOC104909608	1.0000	0.0000	1.0000	0.0000	1.0000	0.0000	1.0000	0.0000	1.0000	0.0000	1.0000	0.0000	1.0000	0.0000
LOC104909609	1.0000	0.0000	1.0000	0.0000	1.0000	0.0000	1.0000	0.0000	1.0000	0.0000	1.0000	0.0000	1.0000	0.0000
LOC104909610	1.0000	0.0000	1.0000	0.0000	1.0000	0.0000	1.0000	0.0000	1.0000	0.0000	1.0000	0.0000	1.0000	0.0000
LOC104909611	1.0000	0.0000	1.0000	0.0000	1.0000	2.2426	1.0000	0.0000	1.0000	0.0000	1.0000	0.0000	1.0000	-2.2889
LOC104909612	0.7746	0.1393	0.1485	-0.2097	0.7625	-0.1500	0.0248	-0.2771	1.0000	0.2630	1.0000	-0.0738	0.7770	0.1417
LOC104909613	0.0009	0.9318	0.0000	0.9586	0.4700	-0.3400	0.0024	-0.8089	1.0000	0.1203	0.9050	0.1578	0.5809	-0.3431
LOC104909614	0.8069	0.9326	0.5366	0.8008	1.0000	0.3655	0.6353	-0.9506	1.0000	0.8328	0.9773	0.7228	1.0000	-0.4794
LOC104909616	1.0000	0.0000	1.0000	0.0000	1.0000	0.0000	1.0000	0.0000	1.0000	0.0000	1.0000	0.0000	1.0000	0.0000
LOC104909618	1.0000	-2.4788	0.7674	-3.2498	0.9361	1.2061	1.0000	0.0106	1.0000	0.7304	1.0000	0.0000	1.0000	-0.4568
LOC104909620	0.2044	0.6512	0.1028	0.6397	0.0000	1.9658	0.0000	1.7706	1.0000	-0.0593	1.0000	-0.0576	0.6917	-0.2473
LOC104909621	1.0000	0.0409	0.7699	0.0639	1.0000	0.0638	0.2168	0.1595	1.0000	0.0738	0.8528	0.1093	0.4279	0.1746
LOC104909622	0.1358	1.4134	0.4289	0.6826	0.1535	1.4245	0.7722	-0.5636	1.0000	0.8429	1.0000	0.1269	0.4052	-1.1424
LOC104909623	1.0000	0.0000	1.0000	0.0000	1.0000	0.0000	1.0000	0.0000	1.0000	0.0000	1.0000	0.0000	1.0000	0.0000
LOC104909624	1.0000	0.0000	1.0000	0.0000	1.0000	0.0000	1.0000	2.3242	1.0000	0.0000	1.0000	0.0000	1.0000	2.3543
LOC104909625	0.0532	1.3221	0.0000	2.3669	0.0000	2.8890	0.0000	3.7826	1.0000	-0.8851	1.0000	0.1688	1.0000	0.0110
LOC104909626	1.0000	-0.0204	1.0000	-0.0391	0.4337	0.2277	0.6770	0.0940	1.0000	0.1841	0.7345	0.1780	1.0000	0.0547
LOC104909627	1.0000	2.1902	1.0000	2.2534	1.0000	2.2471	1.0000	0.0000	1.0000	0.0000	1.0000	0.0489	1.0000	-2.2908
LOC104909628	0.4302	-1.8115	1.0000	-0.9997	0.5560	-1.6619	0.9786	0.9248	1.0000	-1.5985	1.0000	-0.7943	1.0000	0.9923
LOC104909629	1.0000	0.0532	0.3092	0.8053	0.0372	1.3239	0.1120	1.0475	1.0000	-0.0119	0.7581	0.7514	1.0000	-0.2885
LOC104909630	1.0000	0.0000	1.0000	0.0000	1.0000	0.0000	1.0000	0.0000	1.0000	0.0000	1.0000	0.0000	1.0000	0.0000
LOC104909631	0.0004	0.8280	0.0003	0.7802	0.0000	1.5510	0.0000	1.0164	1.0000	0.3088	0.2183	0.2743	0.2980	-0.2208
LOC104909632	0.9545	-0.0735	0.1000	-0.1643	0.0518	-0.3492	0.0008	-0.3066	1.0000	0.0281	1.0000	-0.0506	0.9157	0.0763
LOC104909633	0.2948	-0.2998	0.0145	-0.3107	0.0000	-1.2316	0.0000	-1.3065	1.0000	-0.0647	1.0000	-0.0629	0.9116	-0.1327
LOC104909634	0.5619	1.3148	0.5846	-1.3270	0.4712	1.3161	1.0000	0.0075	1.0000	1.0297	0.5698	-1.5992	1.0000	-0.2723
LOC104909635	0.0000	2.8753	0.0000	3.0391	0.0000	4.0770	0.0000	3.7164	1.0000	0.3524	0.3199	0.5360	1.0000	0.0047
LOC104909636	0.0183	-2.0499	0.0038	-1.9194	0.2200	-1.0465	0.0199	-1.5480	1.0000	0.1277	1.0000	0.2725	1.0000	-0.3679
LOC104909637	0.0013	1.0223	0.0000	1.2509	0.0000	1.4819	0.0000	1.2249	1.0000	0.1258	0.3402	0.3686	0.9441	-0.1248

LOC104909638	0.7505	-0.2309	1.0000	0.0329	0.0001	-1.0745	0.0000	-0.9847	1.0000	-0.2100	1.0000	0.0657	1.0000	-0.1152
LOC104909639	1.0000	2.1849	1.0000	-1.0017	1.0000	2.2426	1.0000	0.5390	1.0000	3.1126	1.0000	0.0495	1.0000	1.4520
LOC104909640	1.0000	0.0000	1.0000	0.0000	0.5076	3.6021	1.0000	0.0000	1.0000	0.0000	1.0000	0.0000	0.7477	-3.6583
LOC104909641	1.0000	-0.1632	1.0000	0.3848	1.0000	0.4406	0.1621	1.5345	1.0000	-0.8989	1.0000	-0.3419	1.0000	0.2015
LOC104909642	0.0697	-0.3374	0.0000	-0.4850	0.0001	-0.6392	0.0000	-0.4269	1.0000	-0.0213	0.3768	-0.1566	0.0821	0.1960
LOC104909644	0.0379	-0.4181	0.0007	-0.2769	0.0348	-0.4256	0.0144	-0.1692	1.0000	-0.3483	0.1424	-0.1945	0.6385	-0.0873
LOC104909646	0.5807	-0.9212	1.0000	-0.0294	0.5414	-0.7659	1.0000	-0.1587	1.0000	-0.5309	1.0000	0.3724	1.0000	0.0786
LOC104909647	0.8109	1.1304	1.0000	-0.1688	0.2687	1.8513	0.4057	1.3511	1.0000	0.4081	1.0000	-0.8837	1.0000	-0.0860
LOC104909648	0.0011	-0.5568	0.0000	-0.7276	0.0924	0.3210	0.1216	0.1487	1.0000	0.0912	1.0000	-0.0666	0.9420	-0.0758
LOC104909649	0.4379	-1.1680	0.0579	-3.0467	1.0000	-0.0407	0.0114	-5.2052	1.0000	-0.1321	0.8269	-2.0158	0.0400	-5.3251
LOC104909650	0.3089	3.9281	0.5798	0.9863	1.0000	2.2426	0.4370	-3.6973	1.0000	3.6423	1.0000	0.8294	1.0000	-2.2889
LOC104909651	1.0000	0.0000	1.0000	0.0000	1.0000	0.0000	1.0000	0.0000	1.0000	0.0000	1.0000	0.0000	1.0000	0.0000
LOC104909652	0.0031	1.9034	1.0000	0.1361	0.1189	-2.2270	0.0100	-3.2866	1.0000	0.2772	0.0233	-1.4784	1.0000	-0.7795
LOC104909653	0.0000	4.5771	0.0090	1.8344	0.0000	5.1942	0.0047	1.8636	1.0000	2.4160	1.0000	-0.2918	0.2212	-0.9068
LOC104909654	0.8367	0.9078	1.0000	-0.1559	1.0000	-0.1785	0.9167	-1.3669	1.0000	0.4032	1.0000	-0.6419	1.0000	-0.7793
LOC104909655	1.0000	0.0000	1.0000	0.0000	1.0000	2.2426	1.0000	0.0000	1.0000	0.0000	1.0000	0.0000	1.0000	-2.2889
LOC104909656	1.0000	0.2987	1.0000	-0.1770	1.0000	-0.3223	0.7728	-0.5621	1.0000	-0.1339	0.9158	-0.5939	1.0000	-0.3674
LOC104909657	0.0005	-0.6017	0.0000	-0.5814	1.0000	-0.0218	0.0669	-0.1768	1.0000	0.0955	0.8172	0.1287	1.0000	-0.0534
LOC104909658	1.0000	0.0000	1.0000	0.0000	1.0000	0.0000	1.0000	0.0000	1.0000	0.0000	1.0000	0.0000	1.0000	0.0000
LOC104909659	1.0000	2.1902	1.0000	0.0000	1.0000	0.0000	1.0000	0.0000	1.0000	0.0000	1.0000	-2.2993	1.0000	0.0000
LOC104909661	1.0000	0.0000	1.0000	2.2534	1.0000	2.2426	1.0000	0.0000	1.0000	0.0000	1.0000	2.3480	1.0000	-2.2889
LOC104909662	1.0000	0.0000	1.0000	-0.1452	1.0000	0.0000	1.0000	-2.3200	1.0000	2.2735	1.0000	2.3480	1.0000	0.0000
LOC104909663	1.0000	0.3987	1.0000	-0.1612	1.0000	-0.1934	1.0000	0.0066	1.0000	-1.8108	0.2158	-2.3834	0.6246	-1.6128
LOC104909664	0.0566	2.8474	1.0000	-0.4260	1.0000	0.6783	0.7732	-0.9367	1.0000	2.2082	0.8219	-1.0402	1.0000	0.6043
LOC104909665	1.0000	0.0000	1.0000	2.2534	1.0000	0.0000	1.0000	0.0000	1.0000	0.0000	1.0000	2.3480	1.0000	0.0000
LOC104909666	0.0000	1.3522	0.0000	1.5313	0.0019	0.9216	0.0000	0.8997	1.0000	-0.1120	1.0000	0.0787	0.9527	-0.1279
LOC104909667	1.0000	0.0000	1.0000	0.0000	1.0000	2.2471	1.0000	0.0000	1.0000	0.0000	1.0000	0.0000	1.0000	-2.2909
LOC104909668	0.8401	-0.4540	1.0000	0.2833	1.0000	-0.2962	0.6327	-0.6770	1.0000	-0.3314	1.0000	0.4173	0.7915	-0.7094
LOC104909669	0.9409	0.4501	0.5121	0.6704	1.0000	-0.0280	1.0000	0.0081	1.0000	0.3143	1.0000	0.5549	1.0000	0.3609
LOC104909670	0.5928	-0.1869	0.3702	-0.1674	0.3624	0.2455	0.0079	0.3535	1.0000	-0.0661	1.0000	-0.0342	1.0000	0.0462
LOC104909671	0.5229	0.2412	0.0956	0.3097	0.0655	-0.5579	0.4353	-0.1904	1.0000	-0.0196	1.0000	0.0619	0.3469	0.3562
LOC104909672	0.0023	1.1506	0.0000	1.3295	0.0000	2.2431	0.0000	2.0069	1.0000	-0.0384	0.9183	0.1549	0.2530	-0.2661
LOC104909673	0.0802	0.8289	0.0009	1.0610	0.0004	1.3328	0.0369	0.7476	1.0000	0.1009	0.7577	0.3447	0.3311	-0.4803
LOC104909674	1.0000	0.0000	1.0000	0.0000	1.0000	0.0000	1.0000	0.0000	1.0000	0.0000	1.0000	0.0000	1.0000	0.0000
LOC104909675	0.6788	-0.6187	0.2992	-0.7861	0.4645	-0.8858	0.0931	-1.1744	1.0000	0.2134	1.0000	0.0608	1.0000	-0.0673
LOC104909676	0.0950	0.5921	0.0128	0.5065	1.0000	-0.0498	0.8502	-0.1194	1.0000	0.2535	0.9633	0.1797	0.8703	0.1876
LOC104909677	0.7250	0.2629	0.0469	0.5290	0.5274	0.3499	1.0000	-0.0296	1.0000	0.1456	0.3858	0.4228	0.8939	-0.2316
LOC104909678	1.0000	0.0000	1.0000	0.0000	1.0000	0.0000	1.0000	0.0000	1.0000	0.0000	1.0000	0.0000	1.0000	0.0000
LOC104909679	1.0000	-2.4788	1.0000	0.0000	1.0000	-2.4061	1.0000	0.0000	1.0000	-2.3771	1.0000	0.0000	1.0000	0.0000
LOC104909680	1.0000	0.0000	1.0000	0.0000	1.0000	0.0000	1.0000	0.0000	1.0000	0.0000	1.0000	0.0000	1.0000	0.0000
LOC104909681	0.6936	1.4536	0.5849	0.9846	1.0000	-2.4056	0.9096	-1.3726	1.0000	1.2661	1.0000	0.8270	1.0000	2.3543
LOC104909682	0.3319	0.5881	0.1973	0.5896	0.0375	-1.2751	0.6934	-0.3593	1.0000	-0.2635	1.0000	-0.2512	0.6072	0.6570
LOC104909683	0.2180	0.8421	0.0209	1.1844	0.2379	0.8337	0.0362	1.1317	1.0000	-0.4651	1.0000	-0.1122	1.0000	-0.1604
LOC104909684	0.6957	1.7486	0.7674	3.0888	0.6971	1.6025	0.4370	3.7041	1.0000	-2.3757	1.0000	-1.1645	1.0000	-0.3138
LOC104909685	1.0000	0.0000	1.0000	0.0000	1.0000	0.0000	1.0000	0.0000	1.0000	0.0000	1.0000	0.0000	1.0000	0.0000
LOC104909686	0.9545	1.0672	0.7674	-3.2499	0.1908	2.3554	0.2228	1.8823	1.0000	0.7304	0.8607	-3.6732	1.0000	0.2661
LOC104909687	0.1957	-1.6201	0.0134	-2.7274	0.0803	-2.3755	0.3172	-1.0766	1.0000	0.2399	1.0000	-0.8603	0.7908	1.5494
LOC104909688	0.4606	0.2140	0.0932	0.2411	0.0000	-1.1377	0.0000	-0.9190	1.0000	-0.1169	1.0000	-0.0768	1.0000	0.1075
LOC104909690	1.0000	-0.0419	0.4949	-1.1319	0.8677	0.7633	1.0000	-0.3952	1.0000	0.8350	1.0000	-0.2435	1.0000	-0.3220
LOC104909691	0.0000	4.4066	0.0006	3.0693	0.9361	1.2045	0.2178	1.8814	1.0000	0.7365	0.7702	-0.5843	0.7027	1.4211
LOC104909693	0.8218	0.4289	1.0000	0.0716	1.0000	0.0136	0.0121	0.9433	1.0000	0.4257	1.0000	0.0924	0.0012	1.3642
LOC104909695	1.0000	0.0481	0.2235	-0.2734	0.0007	-0.9873	0.0000	-1.0040	1.0000	0.1335	0.9486	-0.1737	1.0000	0.1240
LOC104909696	0.8249	3.0142	0.7674	-3.2570	1.0000	2.2471	1.0000	-0.8429	1.0000	3.1191	1.0000	-3.1382	1.0000	0.0649
LOC104909699	0.2072	1.4425	0.3071	1.3368	0.4564	-2.2491	0.9162	-1.3767	1.0000	-0.8113	0.6567	-0.9183	1.0000	0.0652
LOC104909700	1.0000	0.0000	1.0000	0.0000	1.0000	0.0000	1.0000	0.0000	1.0000	0.0000	1.0000	0.0000	1.0000	0.0000
LOC104909701	1.0000	0.0000	1.0000	0.0000	1.0000	0.0000	1.0000	0.0000	1.0000	0.0000	1.0000	0.0000	1.0000	0.0000
LOC104909702	1.0000	0.0000	1.0000	0.0000	1.0000	2.2426	0.7710	3.1709	1.0000	0.0000	1.0000	0.0000	1.0000	0.9164
LOC104909705	1.0000	0.0000	1.0000	2.2534	1.0000	0.0000	1.0000	0.0000	1.0000	0.0000	1.0000	2.3480	1.0000	0.0000
LOC104909707	0.0000	-3.9028	0.0007	-1.8440	1.0000	0.0089	0.6350	0.3157	1.0000	0.0062	0.2849	2.0877	0.8967	0.3209
LOC104909708	0.0124	-3.3558	0.0006	-3.2679	0.0347	-2.6875	0.0009	-3.0904	1.0000	-0.0461	1.0000	0.0547	1.0000	-0.4550
LOC104909710	1.0000	0.0000	0.4431	3.6192	1.0000	0.0000	1.0000	0.0000	1.0000	0.0000	0.8607	3.7288	1.0000	0.0000
LOC104909711	0.0012	1.5837	0.0000	2.1426	0.0092	1.3472	0.0134	1.4468	1.0000	-0.7079	1.0000	-0.1368	0.4210	-0.6009
LOC104909712	0.7367	1.7385	1.0000	2.2508	1.0000	-2.4056	1.0000	0.0000	1.0000	-2.3757	0.8723	-2.0057	1.0000	0.0000
LOC104909713	0.2778	1.0923	0.0004	3.7821	0.0458	-3.0654	0.9167	1.3833	1.0000	-3.0063	1.0000	-0.3314	1.0000	1.4453
LOC104909714	1.0000	0.0000	1.0000	0.0000	1.0000	0.0000	1.0000	0.0000	1.0000	0.0000	1.0000	0.0000	1.0000	0.0000
LOC104909715	0.8226	3.0199	1.0000	-2.3986	0.1053	4.5444	1.0000	0.0045	1.0000	2.2735	1.0000	-3.1429	0.5236	-2.2483
LOC104909716	1.0000	0.0000	1.0000	2.2534	1.0000	0.0000	1.0000	0.0000	1.0000	0.0000	1.0000	2.3480	1.0000	0.0000
LOC104909717	0.0864	-0.3513	0.1869	-0.1880	0.7663	-0.1236	0.6384	0.0895	1.0000	-0.3230	0.7367	-0.1482	0.8932	-0.1044
LOC104909718	1.0000	-0.3393	0.0002	-6.0208	0.2088	1.4467	1.0000	-0.3068	1.0000	1.7121	0.6450	-4.0608	1.0000	-0.0328
LOC104909719	1.0000	-0.3236	1.0000	0.6956	1.0000	-1.0167	1.0000	-2.3176	1.0000	-0.9638	1.0000	0.0547	1.0000	-2.2908
LOC104909721	0.6927	1.4475	1.0000	2.2534	1.0000	-2.4056	1.0000	0.0000	1.0000	-2.3757	1.0000	-1.7102	1.0000	0.0000
LOC104909723	1.0000	0.0000	1.0000	0.0000	1.0000	2.2426	1.0000	2.3242	1.0000	0.0000	1.0000	0.0000	1.0000	0.0652
LOC104909724	0.0019	1.9317	0.1593	0.6741	0.0000	3.2894	0.0000	1.7350	1.0000	1.2804	1.0000	0.0346	0.7326	-0.2706
LOC104909725	0.0000	-0.8022	0.0000	-1.0716	0.0000	-1.3803	0.0000	-0.6246	1.0000	-0.0803	0.0101	-0.3372	0.0000	0.6795
LOC104909726	0.0190	-0.6066	0.0000	-0.5955	0.1738	-0.3828	0.9397	0.0548	1.0000	-0.0791	1.0000	-0.0562	0.0433	0.3627
LOC104909727	1.0000	-0.3236	0.6215	1.6074	1.0000	-1.0206	1.0000	-2.3177	1.0000	-0.9638	1.0000	0.9730	1.0000	-2.2889
LOC104909728	1.0000	-0.0037	0.1489	1.1866	0.1184	-1.9832	0.4060	-1.3369	1.0000	-0.5759	0.9853	0.6296	1.0000	0.0752
LOC104909729	0.0844	0.5954	0.0005	0.7925	0.0000	-1.5663	0.0003	-1.1284	1.0000	-0.1675	1.0000	0.0439	1.0000	0.2767
LOC104909730	0.0001	0.7725	0.0000	0.8498	0.7499	-0.1470	1.0000	-0.0397	1.0000	-0.0438	1.0000	0.0468	1.0000	0.0717
LOC104909731	0.0046	1.0443	0.0000	1.0786	1.0000	0.0612	0.5873	0.2697	1.0000	0.1286	1.0000	0.1756	0.6830	0.3432
LOC104909732	0.0000	1.8083	0.0000	1.8676	0.0000	1.5579	0.0000	1.2918	1.0000	0.0437	1.0000	0.1171	0.8066	-0.2154
LOC104909734	0.8226	-3.3425	1.0000	-0.1452	0.8011	-3.2629	1.0000	-2.3200	1.0000	-0.9587	1.0000	2.3480	1.0000	0.0000
LOC104909735	0.3793	0.8509	1.0000	-0.0605	0.0000	3.1195	0.0000	1.3053	1.0000	1.3581	0.8521	0.4592	0.2617	-0.4558
LOC104909736	1.0000	-0.0324	0.2516	0.5912	0.1972	-0.8959	0.4509	-0.5474	1.0000	-0.2729	0.9273	0.3597	1.0000	0.0795
LOC104909737	1.0000	0.0000	1.0000	0.0000	1.0000	0.0000	1.0000	0.0000	1.0000	0.0000	1.0000	0.0000	1.0000	0.0000
LOC104909738	1.0000	0.5464	0.9062	1.2214	1.0000	-2.4056	1.0000	-2.3200	1.0000	-0.1034	1.0000	0.5843	1.0000	0.0000
LOC104909739	1.0000	0.2458	0.4423	-0.7143	0.5233	-0.7281	0.0007	-2.7673	1.0000	0.3198	1.0000	-0.6230	0.3256	-1.7141
LOC104909740	0.9585	-0.1047	0.0245	-0.3743	0.6712	-0.1883	1.0000	0.0508	1.0000	0.0515	0.7275	-0.2058	0.3209	0.2959
LOC104909742	0.4801	2.0058	0.9762	0.7508	0.9361	1.2045	0.7701	-3.1666	1.0000	0.7365	1.0000	-0.4997	0.7287	-3.6634
LOC104909743	1.0000	0.0491	1.0000	-0.0162	0.4809	-3.7967	0.0872	-2.7160	1.0000	1.2188	0.9217	1.1740	1.0000	2.3543
LOC104909744	1.0000	2.1849	1.0000	2.2508	0.4809	3.6101	1.0000	2.3242	1.0000	0.0000	1.0000	0.0495	1.0000	-1.3084
LOC104909745	0.0000	4.0134	0.0001	1.9774	0.0000	3.9962	0.8535	0.4484	1.0000	2.1981	1.0000	0.1820	0.1010	-1.3428
LOC104909746	0.9846	-0.1602	1.0000	-0.0694	0.0004	1.0993	0.0001	0.8056	1.0000	0.2219	0.7598	0.3250	1.0000	-0.0664

LOC104909747	0.7735	-0.1325	0.3143	-0.2068	0.0207	0.4670	0.0119	0.3783	1.0000	0.0397	1.0000	-0.0209	1.0000	-0.0426
LOC104909748	0.1041	-2.5251	1.0000	-0.0805	1.0000	0.3790	0.6565	-0.6161	1.0000	0.3466	0.0621	2.8183	0.8237	-0.6423
LOC104909749	1.0000	0.1456	0.7865	0.4000	0.2243	-1.3832	0.4927	-0.7813	1.0000	-0.1304	1.0000	0.1354	1.0000	0.4786
LOC104909750	1.0000	0.0000	1.0000	0.0000	1.0000	2.2426	1.0000	0.0000	1.0000	0.0000	1.0000	0.0000	1.0000	-2.2889
LOC104909751	0.2925	0.7871	0.0809	0.8752	0.0000	2.1732	0.0000	2.1488	1.0000	-0.0637	1.0000	0.0331	1.0000	-0.0851
LOC104909752	0.1176	1.0941	0.0674	1.1055	0.1920	-1.5279	0.1163	-1.6324	1.0000	-0.1337	1.0000	-0.1077	1.0000	-0.2297
LOC104909753	1.0000	0.0000	1.0000	2.2508	1.0000	0.0000	1.0000	2.3256	1.0000	0.0000	1.0000	2.3455	1.0000	2.3554
LOC104909754	0.8226	3.0199	0.4147	-2.2315	1.0000	0.0000	0.7246	-1.2170	1.0000	4.3367	1.0000	-0.7956	1.0000	3.2066
LOC104909756	0.0009	3.7629	0.0379	1.6557	0.4424	1.8933	1.0000	0.0062	1.0000	2.2052	1.0000	0.1276	1.0000	0.3286
LOC104909758	0.5833	1.3514	0.6215	1.6060	0.0000	3.6020	0.0010	3.7072	1.0000	-0.9563	1.0000	-0.7121	0.4305	-0.8611
LOC104909759	0.8249	3.0255	1.0000	0.0000	1.0000	0.0000	1.0000	2.3242	1.0000	0.0000	1.0000	-3.1468	1.0000	2.3543
LOC104909760	0.0100	0.4766	0.0005	0.3526	0.6927	0.1378	0.4025	-0.1172	1.0000	0.2873	0.3862	0.1761	1.0000	0.0371
LOC104909761	0.0476	-0.4834	0.0001	-0.6307	0.0011	0.6952	0.0000	0.5152	1.0000	0.1927	1.0000	0.0578	1.0000	0.0198
LOC104909763	0.1096	4.6934	0.1126	-4.7517	1.0000	2.2426	1.0000	-0.5581	1.0000	4.6059	0.2100	-4.8283	0.8937	1.8364
LOC104909764	0.8249	-3.3411	1.0000	-0.1668	0.8033	-3.2622	0.7710	-3.1693	1.0000	-0.1113	1.0000	3.1946	1.0000	0.0000
LOC104909765	0.5086	3.5431	1.0000	2.2508	1.0000	0.0000	1.0000	0.0000	1.0000	0.0000	1.0000	-1.3254	1.0000	0.0000
LOC104909766	1.0000	-0.7401	1.0000	-0.6954	0.7004	-1.9462	1.0000	-0.5236	1.0000	-0.5152	1.0000	-0.4724	1.0000	0.9168
LOC104909767	0.3676	-1.0390	0.6395	0.7830	0.1053	-1.8340	0.6172	-1.7602	1.0000	-1.7664	1.0000	0.0602	0.8937	-1.6949
LOC104909768	0.0054	-0.5986	0.0002	-0.3261	0.0000	-0.9692	0.0000	-0.9095	1.0000	0.0424	0.0112	0.3278	0.7743	0.1074
LOC104909771	1.0000	0.5464	0.2552	4.0066	1.0000	0.6823	1.0000	0.0000	1.0000	-2.3757	1.0000	0.9734	1.0000	-3.1369
LOC104909772	1.0000	0.0000	0.7674	-3.2498	1.0000	0.0000	0.7710	-3.1633	1.0000	3.1056	1.0000	0.0000	1.0000	0.0000
LOC104909773	1.0000	0.0000	1.0000	0.0000	1.0000	0.0000	1.0000	0.0000	1.0000	0.0000	1.0000	0.0000	1.0000	0.0000
LOC104909774	0.3168	2.2149	0.8772	0.4830	0.0173	3.2264	0.5555	0.9786	1.0000	1.9491	1.0000	0.2479	1.0000	-0.2891
LOC104909775	0.0000	-1.4933	0.0000	-1.5059	0.0304	-0.3647	0.0000	-0.6924	1.0000	0.1748	0.4779	0.1750	0.4066	-0.1475
LOC104909776	1.0000	-0.2637	0.6304	-0.3714	0.0006	1.5339	0.0001	1.3224	1.0000	0.2217	1.0000	0.1236	1.0000	0.0123
LOC104909777	0.4171	0.5995	1.0000	-0.1288	1.0000	0.0933	0.6560	-0.3874	1.0000	0.2644	0.9048	-0.4523	1.0000	-0.2119
LOC104909778	1.0000	0.0000	0.7674	-3.2570	1.0000	0.0000	1.0000	-0.8429	1.0000	3.1191	1.0000	0.0000	1.0000	2.3554
LOC104909779	0.0000	2.2230	0.0000	2.6396	0.0001	2.1202	0.0000	2.3613	1.0000	-0.2750	1.0000	0.1634	1.0000	-0.0232
LOC104909781	1.0000	0.0000	1.0000	0.0000	1.0000	0.0000	1.0000	2.3242	1.0000	0.0000	1.0000	0.0000	1.0000	2.3543
LOC104909782	0.0571	0.9855	0.0006	1.3719	0.0000	1.6855	0.0003	1.4205	1.0000	-0.3130	1.0000	0.0840	0.1819	-0.5733
LOC104909783	1.0000	-0.2882	0.2017	1.4853	1.0000	-0.1625	1.0000	-0.5239	1.0000	1.2661	0.1785	3.0905	1.0000	0.9168
LOC104909784	1.0000	-0.2921	1.0000	-2.3986	0.9468	1.2126	0.9096	1.3849	1.0000	-0.1034	1.0000	-2.2955	1.0000	0.0738
LOC104909786	0.0018	-1.6846	0.0057	-1.1472	0.0160	0.9276	0.0000	1.8989	1.0000	-0.0582	0.9094	0.4949	0.0000	0.9177
LOC104909787	1.0000	0.0000	1.0000	0.0000	0.8011	3.0840	1.0000	2.3256	1.0000	0.0000	1.0000	0.0000	1.0000	-0.7793
LOC104909788	1.0000	0.0000	1.0000	0.0000	1.0000	2.2471	0.0710	4.6474	1.0000	0.0000	1.0000	0.0000	0.5105	2.3934
LOC104909789	0.6957	1.7486	0.0957	2.1524	1.0000	-2.4055	1.0000	0.0058	1.0000	0.7416	0.8265	1.1602	1.0000	3.2066
LOC104909791	0.7975	-0.1752	0.0299	-0.4517	0.0000	-1.3516	0.0000	-1.0656	1.0000	0.2770	1.0000	0.0123	0.1842	0.5679
LOC104909792	0.0746	1.9055	0.0781	1.3174	0.0004	2.8193	0.0019	1.8821	1.0000	0.8319	1.0000	0.2598	1.0000	-0.0988
LOC104909793	0.0025	0.8876	0.0000	1.1073	0.0012	0.9040	0.0001	0.8545	1.0000	-0.1512	1.0000	0.0820	0.7784	-0.1939
LOC104909794	1.0000	-0.0497	0.0489	-0.2310	0.0000	-1.3071	0.0000	-0.8819	1.0000	-0.2398	0.0041	-0.4091	0.4150	0.1914
LOC104909795	0.8583	-0.2717	0.0236	-0.8355	0.0003	1.3134	0.1455	0.4919	1.0000	0.2816	1.0000	-0.2715	0.1551	-0.5354
LOC104909796	0.8671	0.2085	1.0000	0.0157	0.0000	1.7409	0.0000	1.3381	1.0000	0.0596	1.0000	-0.1201	0.3929	-0.3364
LOC104909797	0.1174	-1.2236	0.0020	-1.5427	0.8033	-0.3250	0.1678	-0.6872	1.0000	0.5630	1.0000	0.2602	1.0000	0.2052
LOC104909798	0.1402	0.4775	0.1084	0.3228	1.0000	-0.0027	0.1367	-0.3302	1.0000	0.4146	0.5520	0.2722	1.0000	0.0919
LOC104909799	0.0000	-3.5267	0.0000	-3.2043	0.4954	0.2982	0.3386	0.2483	1.0000	0.3863	0.9652	0.7236	0.3733	0.3434
LOC104909800	1.0000	-2.4776	1.0000	0.0000	1.0000	-2.4056	1.0000	0.0000	1.0000	-2.3757	1.0000	0.0000	1.0000	0.0000
LOC104909802	1.0000	0.0000	1.0000	0.0000	1.0000	0.0000	1.0000	0.0000	1.0000	0.0000	1.0000	0.0000	1.0000	0.0000
LOC104909803	1.0000	0.0000	1.0000	0.0000	1.0000	0.0000	1.0000	0.0000	1.0000	0.0000	1.0000	0.0000	1.0000	0.0000
LOC104909804	0.8249	-3.3411	0.7666	-3.2534	1.0000	-1.0194	1.0000	0.5390	1.0000	-0.1170	1.0000	0.0000	1.0000	1.4520
LOC104909805	0.0001	-6.5285	0.0000	-6.5680	0.4344	-0.8080	0.5511	-0.5375	1.0000	0.0053	1.0000	0.0000	1.0000	0.2826
LOC104909807	0.0035	-0.5712	0.0000	-0.8190	1.0000	0.0003	0.0000	0.4449	1.0000	0.1528	1.0000	-0.0816	0.0000	0.6024
LOC104909808	0.0000	-2.8439	0.0000	-2.6450	0.0000	-1.3693	0.0000	-1.5110	1.0000	-0.0149	1.0000	0.1963	0.7288	-0.1517
LOC104909809	1.0000	0.0000	1.0000	0.0000	1.0000	0.0000	1.0000	0.0000	1.0000	0.0000	1.0000	0.0000	1.0000	0.0000
LOC104909810	1.0000	0.0000	1.0000	0.0000	1.0000	0.0000	1.0000	0.0000	1.0000	0.0000	1.0000	0.0000	1.0000	0.0000
LOC104909811	1.0000	0.0000	1.0000	0.0000	1.0000	0.0000	1.0000	0.0000	1.0000	0.0000	1.0000	0.0000	1.0000	0.0000
LOC104909812	1.0000	2.1849	1.0000	0.0000	1.0000	2.2471	1.0000	2.3257	1.0000	0.0000	1.0000	-2.2956	1.0000	0.0649
LOC104909813	1.0000	0.0000	1.0000	2.2534	1.0000	0.0000	1.0000	0.0000	1.0000	0.0000	1.0000	2.3480	1.0000	0.0000
LOC104909814	1.0000	-2.4788	0.4431	-3.7865	1.0000	-0.1597	0.9096	-1.3717	1.0000	1.2628	1.0000	0.0000	1.0000	0.0649
LOC104909815	0.5086	-3.8790	0.0227	-5.1345	0.4809	-3.7967	0.0207	-5.0405	1.0000	1.2172	1.0000	0.0000	1.0000	0.0000
LOC104909816	0.7154	-0.8130	0.1730	-1.0775	0.2925	1.1382	0.7964	0.3741	1.0000	1.1219	1.0000	0.8752	1.0000	0.3650
LOC104909817	0.8226	-3.3425	0.0754	-4.7338	1.0000	0.3474	1.0000	-0.5496	1.0000	1.3515	1.0000	0.0000	1.0000	0.4627
LOC104909818	0.1245	-2.5238	0.8628	-0.8260	0.0417	-3.2109	0.4136	-1.3371	1.0000	-0.4426	1.0000	1.2787	1.0000	1.4453
LOC104909819	0.0046	-3.2756	0.0003	-4.0312	0.0276	-2.2144	0.0053	-2.4833	1.0000	-0.0453	1.0000	-0.7977	1.0000	-0.3113
LOC104909821	0.0005	1.5262	0.0084	1.1526	1.0000	-0.1535	0.3880	-0.6629	1.0000	-0.4471	0.0545	-0.8123	0.3252	-0.9546
LOC104909822	0.0018	3.2616	0.0157	2.2342	0.2687	1.8519	1.0000	-0.5236	1.0000	0.4098	0.7953	-0.6020	0.3496	-1.9637
LOC104909823	0.1389	-0.2704	0.0047	-0.2403	0.1511	-0.2577	0.0054	-0.2233	1.0000	0.0043	1.0000	0.0469	1.0000	0.0440
LOC104909824	0.9650	1.0580	1.0000	-0.5554	0.4012	2.3337	0.6172	-1.7602	1.0000	1.6529	1.0000	0.0673	0.4941	-2.4480
LOC104909825	0.5086	3.5456	1.0000	-0.1794	1.0000	2.2471	0.9162	-1.3749	1.0000	3.6521	1.0000	0.0533	1.0000	0.0649
LOC104909826	0.1415	0.3096	0.2235	0.1359	1.0000	0.0097	0.1683	-0.1531	1.0000	0.0377	0.6954	-0.1228	0.6575	-0.1195
LOC104909827	0.1380	-2.8557	0.0066	-5.4489	0.3864	-1.8746	0.9162	-0.4920	1.0000	0.3712	1.0000	-2.2993	0.6295	1.7641
LOC104909828	1.0000	-2.4776	1.0000	-2.3986	1.0000	-0.1591	1.0000	0.8541	1.0000	-0.1034	1.0000	0.0000	1.0000	0.9164
LOC104909829	1.0000	0.2160	0.5317	0.6664	0.4998	0.8667	0.0807	1.1721	1.0000	0.3281	0.8341	0.7857	0.6080	0.6302
LOC104909830	0.0005	2.9640	0.0067	3.3498	0.2798	1.5911	0.6142	1.7719	1.0000	-1.4912	0.2444	-1.1174	0.5331	-1.3220
LOC104909831	1.0000	0.0000	1.0000	0.0000	1.0000	0.0000	1.0000	0.0000	1.0000	0.0000	1.0000	0.0000	1.0000	0.0000
LOC104909832	0.6881	1.4503	0.9762	-1.0825	1.0000	-0.1591	0.6172	-1.7581	1.0000	1.6478	1.0000	-0.8613	1.0000	0.0649
LOC104909834	0.4291	-0.2011	0.0090	-0.3050	0.1171	-0.3213	0.0000	-0.4804	1.0000	0.0260	1.0000	-0.0657	0.6586	-0.1286
LOC104909835	1.0000	0.0000	1.0000	0.0000	1.0000	0.0000	1.0000	2.3256	1.0000	0.0000	1.0000	0.0000	1.0000	2.3554
LOC104909836	0.0909	2.2334	0.1378	1.7425	0.8270	0.7523	0.7772	0.9510	1.0000	-0.1299	0.9498	-0.5990	1.0000	0.0782
LOC104909837	0.1060	0.3153	0.1358	0.1671	0.2004	-0.2685	1.0000	0.0132	1.0000	-0.0105	0.6036	-0.1456	0.0491	0.2764
LOC104909838	0.2791	-1.4664	0.7802	-1.1137	0.7845	-0.7579	1.0000	0.0070	1.0000	-0.6910	1.0000	-0.3294	1.0000	0.0779
LOC104909839	0.0005	0.7935	0.0001	0.6403	0.0435	-0.5641	0.0000	-0.8961	1.0000	-0.0056	0.8655	-0.1466	0.4081	-0.3311
LOC104909840	0.0018	1.2033	0.0002	1.1849	0.0002	1.3299	0.0000	1.7491	1.0000	-0.2345	0.9094	-0.2444	0.9032	0.1860
LOC104909841	0.8249	3.0142	1.0000	-2.3960	1.0000	2.2426	1.0000	-2.3177	1.0000	2.2677	1.0000	-3.1382	1.0000	-2.2889
LOC104909842	0.8249	-3.3439	1.0000	0.0000	0.8033	-3.2636	0.7710	3.1753	1.0000	-3.2319	1.0000	0.0000	1.0000	3.2081
LOC104909843	1.0000	0.0000	1.0000	0.0000	1.0000	0.0000	1.0000	0.0000	1.0000	0.0000	1.0000	0.0000	1.0000	0.0000
LOC104909844	0.0011	1.1963	0.0000	1.4718	0.5076	-0.3938	0.9047	-0.1759	1.0000	-0.2951	1.0000	-0.0070	1.0000	-0.0729
LOC104909845	1.0000	0.0000	1.0000	0.0000	1.0000	2.2426	1.0000	0.0000	1.0000	0.0000	1.0000	0.0000	1.0000	-2.2889
LOC104909846	0.9242	-0.5619	1.0000	-0.3622	0.0393	1.6042	0.0602	1.4339	1.0000	0.0596	1.0000	0.2729	1.0000	-0.1044
LOC104909847	1.0000	0.2445	0.3643	0.7541	0.0012	1.6294	0.0284	1.2293	1.0000	-0.2307	1.0000	0.2903	0.4097	-0.6254
LOC104909848	0.2885	1.1920	0.0170	1.8354	0.0006	2.3392	0.0033	2.0222	1.0000	-0.1288	1.0000	0.5274	0.8275	-0.4407
LOC104909849	0.0000	2.1392	0.0000	2.6442	0.0626	0.7480	0.2007	0.5285	1.0000	-0.1664	0.2746	0.3519	0.5880	-0.3796
LOC104909850	0.0008	1.4673	0.0000	1.5317	0.0000	1.6293	0.0000	1.6101	1.0000	0.2701	0.6342	0.3445	0.7246	0.2536
LOC104909851	0.0775	0.9943	0.2073	0.7462	0.1782	0.8553	0.0271	1.0947	1.0000	-0.0712	1.0000	-0.3023	1.0000	0.1756

LOC104909852	0.9545	-1.6907	1.0000	0.5219	1.0000	0.2013	1.0000	0.7001	1.0000	-0.1237	0.8269	2.1235	1.0000	0.3818
LOC104909853	1.0000	0.1380	0.2785	-0.8223	1.0000	-0.0567	0.7940	-0.3600	1.0000	0.0775	0.5727	-0.8737	1.0000	-0.2206
LOC104909855	1.0000	-0.1349	0.2747	-0.9724	0.9232	0.3764	0.7633	0.3644	1.0000	0.6695	1.0000	-0.1558	0.5460	0.6587
LOC104909856	0.9650	0.2559	0.1325	-0.6206	0.8726	0.2786	1.0000	-0.0192	1.0000	0.7800	1.0000	-0.0812	0.6099	0.4929
LOC104909857	1.0000	0.2007	1.0000	0.2165	1.0000	-0.1811	1.0000	-0.5262	1.0000	0.4165	1.0000	0.4459	1.0000	0.0730
LOC104909858	1.0000	-0.0281	1.0000	-0.0192	0.0000	-1.5327	0.0000	-1.6447	1.0000	-0.1763	0.7864	-0.1555	0.6277	-0.2817
LOC104909859	0.0011	0.9445	0.0000	1.0782	0.5373	-0.3115	0.3785	-0.3985	1.0000	-0.2680	1.0000	-0.1255	0.6924	-0.3495
LOC104909861	0.1562	-1.3372	0.0042	-2.1250	0.1102	-1.3292	0.9096	-0.3106	1.0000	-0.1268	0.9234	-0.9062	0.7179	0.8933
LOC104909862	1.0000	0.0000	1.0000	0.0000	1.0000	0.0000	1.0000	0.0000	1.0000	0.0000	1.0000	0.0000	1.0000	0.0000
LOC104909863	0.1176	1.0725	0.2567	0.8688	0.0082	1.4305	0.0000	2.0153	1.0000	-0.5689	0.5254	-0.7641	1.0000	0.0189
LOC104909864	0.8226	3.0199	1.0000	-0.1452	0.4809	3.6101	0.9162	1.3863	1.0000	2.2735	1.0000	-0.7956	1.0000	0.0765
LOC104909865	1.0000	0.0000	1.0000	0.0000	1.0000	0.0000	1.0000	0.0000	1.0000	0.0000	1.0000	0.0000	1.0000	0.0000
LOC104909866	0.3017	0.6747	0.3296	-0.4365	0.0000	1.7502	0.0001	1.0280	1.0000	0.6702	0.7434	-0.4310	1.0000	-0.0510
LOC104909867	0.0019	-0.9403	0.0034	-0.6234	0.2509	-0.3942	0.0183	-0.5040	1.0000	0.0712	0.5052	0.4013	1.0000	-0.0328
LOC104909868	1.0000	-2.4776	1.0000	2.2507	1.0000	0.6783	0.7710	3.1753	1.0000	-2.3757	1.0000	2.3455	1.0000	0.0727
LOC104909869	0.2542	0.7453	0.0059	1.0501	0.0938	0.8765	0.9946	0.1988	1.0000	0.0279	0.8856	0.3485	0.3273	-0.6426
LOC104909870	0.3762	1.3203	0.0001	3.9248	0.1296	1.5615	0.0106	3.2984	1.0000	-2.4384	1.0000	0.1369	0.7113	-0.7137
LOC104909871	1.0000	0.0000	1.0000	0.0000	1.0000	0.0000	1.0000	0.0000	1.0000	0.0000	1.0000	0.0000	1.0000	0.0000
LOC104909872	1.0000	2.1902	1.0000	0.0000	1.0000	0.0000	1.0000	2.3256	1.0000	0.0000	1.0000	-2.2993	1.0000	2.3555
LOC104909873	1.0000	0.0940	0.1215	0.3744	0.5600	0.2685	0.0381	0.4860	1.0000	0.1530	0.3730	0.4457	0.3290	0.3760
LOC104909874	1.0000	0.5414	1.0000	0.0000	0.9361	1.2042	0.4370	3.7041	1.0000	-2.3771	1.0000	-3.1429	1.0000	0.0752
LOC104909875	0.5061	0.1940	0.6128	0.1047	0.0053	-0.6205	0.0000	-0.6380	1.0000	0.0692	1.0000	-0.0079	1.0000	0.0583
LOC104909876	0.0016	1.1401	0.0000	1.1885	0.0152	0.8657	0.0000	1.1287	1.0000	-0.1091	1.0000	-0.0504	0.9458	0.1590
LOC104909877	1.0000	0.0000	1.0000	0.0000	1.0000	0.0000	1.0000	0.0000	1.0000	0.0000	1.0000	0.0000	1.0000	0.0000
LOC104909878	1.0000	0.0000	1.0000	0.0000	1.0000	0.0000	1.0000	0.0000	1.0000	0.0000	1.0000	0.0000	1.0000	0.0000
LOC104909879	0.1066	1.3522	0.0202	1.9065	0.0054	1.8145	0.1532	1.4492	1.0000	-0.9418	1.0000	-0.3822	0.0716	-1.3059
LOC104909881	0.0125	0.8944	0.0000	1.2694	0.0003	1.0750	0.0000	1.1257	1.0000	-0.3080	1.0000	0.0814	0.6435	-0.2495
LOC104909883	0.0109	1.1642	0.0000	1.3817	0.0006	1.3902	0.0000	1.3576	1.0000	-0.0394	1.0000	0.1889	1.0000	-0.0684
LOC104909884	0.0415	1.7513	0.0004	1.9963	0.0000	2.8218	0.0000	2.2160	1.0000	0.0077	1.0000	0.2688	0.3338	-0.5874
LOC104909885	1.0000	0.0000	1.0000	0.0000	1.0000	0.0000	1.0000	0.0000	1.0000	0.0000	1.0000	0.0000	1.0000	0.0000
LOC104909886	1.0000	0.0000	1.0000	0.0000	1.0000	0.0000	1.0000	0.0000	1.0000	0.0000	1.0000	0.0000	1.0000	0.0000
LOC104909887	1.0000	0.0000	1.0000	0.0000	1.0000	0.0000	1.0000	0.0000	1.0000	0.0000	1.0000	0.0000	1.0000	0.0000
LOC104909888	1.0000	0.0000	1.0000	0.0000	1.0000	0.0000	1.0000	0.0000	1.0000	0.0000	1.0000	0.0000	1.0000	0.0000
LOC104909889	1.0000	0.0000	1.0000	0.0000	1.0000	0.0000	1.0000	0.0000	1.0000	0.0000	1.0000	0.0000	1.0000	0.0000
LOC104909890	1.0000	0.0000	1.0000	0.0000	1.0000	0.0000	1.0000	0.0000	1.0000	0.0000	1.0000	0.0000	1.0000	0.0000
LOC104909891	0.4238	-1.8118	0.0368	-2.4916	0.8151	-1.1321	0.0534	-2.3151	1.0000	0.7204	1.0000	0.0563	1.0000	-0.4570
LOC104909892	0.0000	-5.7650	0.0000	-3.9404	0.0101	0.9431	0.0796	0.6327	1.0000	-0.4272	1.0000	1.4306	0.0478	-0.7339
LOC104909893	0.3089	3.9281	0.4024	-2.2295	0.1614	4.2993	0.3952	-2.0640	1.0000	4.3319	1.0000	-1.7117	0.6865	-1.9990
LOC104909895	1.0000	-0.1416	0.9612	-0.1848	0.0026	1.3138	0.1928	0.5865	1.0000	0.4544	0.9052	0.4211	0.9122	-0.2715
LOC104909896	0.7308	-0.1149	0.7627	-0.0590	0.0775	-0.3301	0.0000	-0.4232	1.0000	0.0159	0.9441	0.0842	1.0000	-0.0716
LOC104909897	1.0000	0.0000	1.0000	0.0000	1.0000	0.0000	1.0000	0.0000	1.0000	0.0000	1.0000	0.0000	1.0000	0.0000
LOC104909898	1.0000	-0.2096	0.6296	-0.5100	1.0000	-0.0496	0.0020	-2.2000	1.0000	0.2778	1.0000	-0.0097	0.0548	-1.8680
LOC104909900	0.6645	0.8687	0.3687	0.8826	0.3439	1.1433	0.7400	0.5786	1.0000	0.8338	0.7293	0.8627	1.0000	0.2771
LOC104909901	1.0000	0.0000	1.0000	0.0000	1.0000	0.0000	1.0000	0.0000	1.0000	0.0000	1.0000	0.0000	1.0000	0.0000
LOC104909903	1.0000	0.5815	1.0000	-0.1727	1.0000	-1.0206	0.3949	-2.0643	1.0000	1.1010	1.0000	0.3643	1.0000	0.0652
LOC104909904	0.0482	-1.8970	0.0044	-1.6054	1.0000	0.1541	0.8176	0.2687	1.0000	0.5974	0.9640	0.9069	0.3764	0.7210
LOC104909905	1.0000	-0.2931	1.0000	-0.1449	1.0000	-2.4061	1.0000	-2.3177	1.0000	-0.1089	1.0000	0.0495	1.0000	0.0000
LOC104909906	1.0000	-0.3262	0.4174	-1.5152	1.0000	-1.0194	1.0000	-0.1791	1.0000	1.7500	1.0000	0.5871	0.3618	2.6079
LOC104909907	1.0000	0.0000	1.0000	0.0000	1.0000	0.0000	1.0000	0.0000	1.0000	0.0000	1.0000	0.0000	1.0000	0.0000
LOC104909908	1.0000	0.0000	1.0000	0.0000	1.0000	0.0000	1.0000	0.0000	1.0000	0.0000	1.0000	0.0000	1.0000	0.0000
LOC104909910	1.0000	-0.2920	1.0000	-0.8635	1.0000	0.6823	0.1399	-4.3913	1.0000	1.9604	1.0000	1.4325	1.0000	-3.1369
LOC104909911	1.0000	2.1902	1.0000	-0.1429	1.0000	2.2471	1.0000	-2.3177	1.0000	2.2677	1.0000	0.0489	1.0000	-2.2909
LOC104909912	1.0000	0.0000	1.0000	0.0000	1.0000	0.0000	1.0000	0.0000	1.0000	0.0000	1.0000	0.0000	1.0000	0.0000
LOC104909913	1.0000	0.0000	1.0000	0.0000	1.0000	0.0000	1.0000	0.0000	1.0000	0.0000	1.0000	0.0000	1.0000	0.0000
LOC104909914	1.0000	0.0000	1.0000	0.0000	1.0000	0.0000	1.0000	0.0000	1.0000	0.0000	1.0000	0.0000	1.0000	0.0000
LOC104909916	0.0000	-2.2336	0.0000	-2.8314	0.0166	-0.5942	0.0000	-0.8993	1.0000	0.0212	0.5058	-0.5688	0.3640	-0.2788
LOC104909917	0.0000	1.2301	0.0000	1.0637	0.0000	1.7151	0.0000	1.2157	1.0000	0.1941	1.0000	0.0404	0.2542	-0.2981
LOC104909918	0.8158	0.1302	0.0589	-0.2231	1.0000	-0.0566	0.0000	-0.4992	1.0000	0.0431	0.0238	-0.2964	0.0012	-0.3930
LOC104909919	0.3234	1.1335	0.0000	4.0935	0.0000	2.4529	0.0000	4.2756	1.0000	-2.1648	0.5250	0.7891	0.8448	-0.3444
LOC104909920	0.0000	-1.2903	0.0000	-1.1243	0.0000	-1.3718	0.0000	-1.2693	1.0000	-0.2059	1.0000	-0.0275	0.9984	-0.0977
LOC104909922	0.0023	3.6196	0.0000	4.2271	0.0000	5.1392	0.0000	4.4527	1.0000	-0.1034	0.9388	0.5073	0.3162	-0.7976
LOC104909923	0.0615	0.8920	0.0004	1.0213	0.2124	-0.7100	0.0100	-1.0754	1.0000	-0.0734	1.0000	0.0707	0.8852	-0.4347
LOC104909924	1.0000	0.5133	1.0000	0.2616	1.0000	0.2980	0.7625	-0.6279	1.0000	0.5087	1.0000	0.2674	1.0000	-0.4187
LOC104909925	0.8249	3.0255	1.0000	0.0000	1.0000	2.2471	1.0000	0.0000	1.0000	0.0000	1.0000	-3.1468	1.0000	-2.2908
LOC104909926	0.4717	1.0290	0.5543	0.8005	0.0919	-4.7440	0.6419	-0.9514	1.0000	0.2770	1.0000	0.0601	0.5093	4.1263
LOC104909927	0.8437	0.2199	0.0900	-0.5506	0.5487	0.3484	0.3792	0.3008	1.0000	0.3501	0.6578	-0.4080	0.6172	0.3077
LOC104909928	0.0091	0.9057	0.7701	-0.1750	0.8287	0.1919	0.4593	-0.2909	1.0000	0.4980	0.1015	-0.5709	1.0000	0.0194
LOC104909930	1.0000	2.1902	1.0000	0.0000	1.0000	0.0000	1.0000	0.0000	1.0000	0.0000	1.0000	-2.2992	1.0000	0.0000
LOC104909931	0.2081	4.4899	0.4635	-3.7803	0.0578	4.7682	1.0000	-0.5218	1.0000	3.6299	0.3448	-4.6208	0.6338	-1.6147
LOC104909932	1.0000	-0.3163	1.0000	2.2534	1.0000	-0.1742	0.7710	3.1709	1.0000	-3.2289	1.0000	-0.7977	1.0000	0.0695
LOC104909933	1.0000	0.2007	0.0128	5.1230	1.0000	-1.0172	0.7710	3.1753	1.0000	-3.2304	0.6991	1.5669	1.0000	0.9169
LOC104909935	0.0746	1.4343	0.7868	0.3662	0.0000	2.2884	0.0000	2.3100	1.0000	0.3070	0.5859	-0.7514	0.7260	0.3324
LOC104909937	1.0000	0.0762	1.0000	0.1211	0.0004	1.2596	0.0000	1.4924	1.0000	0.0302	1.0000	0.0865	0.6541	0.2674
LOC104909938	0.0357	-1.9022	0.0002	-4.0293	0.0025	1.4755	1.0000	-0.0740	1.0000	-0.1328	0.6655	-2.2644	0.0005	-1.6757
LOC104909939	0.0000	-4.0478	0.0000	-4.8707	0.0000	-1.8619	0.0000	-3.4455	1.0000	-0.4110	0.1771	-1.2233	0.0000	-1.9897
LOC104909940	1.0000	-0.2931	1.0000	0.0000	1.0000	0.6780	1.0000	2.3242	1.0000	-2.3771	1.0000	-2.2956	1.0000	-0.7795
LOC104909941	0.6873	0.5414	0.0350	1.1935	1.0000	0.1589	1.0000	-0.1822	1.0000	-0.2994	1.0000	0.3630	0.6865	-0.6296
LOC104909942	0.8226	0.0998	0.0454	0.2149	1.0000	0.0406	0.8476	-0.0528	1.0000	-0.0511	1.0000	0.0764	0.5425	-0.1392
LOC104909943	0.1721	0.3015	0.1187	0.1992	0.1337	-0.3236	0.1243	-0.2001	1.0000	0.1052	1.0000	0.0162	0.2539	0.2341
LOC104909944	0.5780	0.6972	0.2799	1.2033	0.2468	-1.6967	1.0000	-0.5493	1.0000	-0.6935	1.0000	-0.1797	1.0000	0.4616
LOC104909945	1.0000	0.0000	1.0000	0.0000	1.0000	0.0000	1.0000	0.0000	1.0000	0.0000	1.0000	0.0000	1.0000	0.0000
LOC104909946	1.0000	2.1902	1.0000	0.0000	0.4809	3.6101	1.0000	2.3242	1.0000	0.0000	1.0000	-2.2992	1.0000	-1.3084
LOC104909947	0.5174	0.5521	0.0882	0.4371	0.0000	2.6551	0.0000	2.3869	1.0000	0.2122	1.0000	0.1147	1.0000	-0.0477
LOC104909948	1.0000	-0.0801	0.0936	-0.5238	0.0097	1.4521	0.0000	1.1631	1.0000	0.3039	1.0000	-0.1292	1.0000	0.0085
LOC104909949	0.0010	0.7974	0.0000	1.1671	0.0396	0.5305	0.0002	0.6159	1.0000	-0.0162	0.1585	0.3660	1.0000	0.0756
LOC104909950	1.0000	0.0000	1.0000	0.0000	1.0000	0.0000	1.0000	0.0000	1.0000	0.0000	1.0000	0.0000	1.0000	0.0000
LOC104909951	1.0000	-2.4788	1.0000	0.0000	1.0000	-2.4061	1.0000	0.0000	1.0000	-2.3771	1.0000	0.0000	1.0000	0.0000
LOC104909952	0.7340	-2.0895	0.2545	-4.1774	1.0000	0.3613	0.9786	-0.9114	1.0000	-0.1223	1.0000	-2.2956	0.7908	-1.3984
LOC104909953	1.0000	0.0000	1.0000	0.0000	1.0000	0.0000	1.0000	0.0000	1.0000	0.0000	1.0000	0.0000	1.0000	0.0000
LOC104909954	1.0000	0.0000	0.4642	-3.7944	1.0000	0.0000	0.9162	-1.3749	1.0000	3.6521	1.0000	0.0000	1.0000	2.3554
LOC104909955	0.4518	0.9716	0.1394	1.7412	0.1318	1.3538	0.0358	2.1379	1.0000	-1.2768	1.0000	-0.5103	0.9237	-0.4964
LOC104909956	0.0426	1.6071	0.0578	1.2838	0.1310	1.3560	1.0000	0.2644	1.0000	0.3717	1.0000	0.0607	0.6919	-0.7149
LOC104909958	0.3159	3.9314	1.0000	-0.1472	0.1614	4.2993	0.9096	1.3849	1.0000	2.2735	1.0000	-1.7140	1.0000	-0.6154

LOC104909959	0.8226	0.5540	0.0002	2.2216	1.0000	0.3798	0.0003	2.1604	1.0000	-0.6289	0.2128	1.0507	0.1382	1.1595
LOC104909960	0.5204	0.7618	0.2362	1.1546	0.2910	-1.4631	0.3402	-1.6822	1.0000	-0.6259	1.0000	-0.2249	1.0000	-0.8431
LOC104909961	0.0000	-0.7208	0.0000	-0.8255	1.0000	0.0183	0.0596	0.1460	1.0000	0.1484	1.0000	0.0558	0.0029	0.2811
LOC104909962	1.0000	0.0628	0.9862	-0.0955	0.0000	1.8052	0.0000	1.7199	1.0000	0.1676	1.0000	0.0222	1.0000	0.0872
LOC104909963	0.5086	0.3445	0.4272	0.3379	0.0000	2.1845	0.0000	2.1247	1.0000	0.2193	0.9932	0.2267	0.6915	0.1673
LOC104909964	0.7050	0.2929	0.3175	0.3429	0.0000	2.5830	0.0000	2.4738	1.0000	0.0987	1.0000	0.1637	1.0000	-0.0015
LOC104909965	0.0455	1.9728	0.4725	0.7992	1.0000	-0.5754	1.0000	-0.3526	1.0000	1.0017	1.0000	-0.1599	0.8768	1.2328
LOC104909966	0.9522	0.6154	0.3649	-0.8601	1.0000	-0.1829	0.6351	-0.6763	1.0000	1.2053	1.0000	-0.2538	1.0000	0.7234
LOC104909967	1.0000	0.0000	1.0000	0.0000	1.0000	0.0000	1.0000	0.0000	1.0000	0.0000	1.0000	0.0000	1.0000	0.0000
LOC104909969	1.0000	0.0000	1.0000	0.0000	1.0000	0.0000	1.0000	0.0000	1.0000	0.0000	1.0000	0.0000	1.0000	0.0000
LOC104909971	1.0000	-0.2882	1.0000	0.0000	1.0000	-2.4056	1.0000	0.0000	1.0000	-2.3757	1.0000	-2.2993	1.0000	0.0000
LOC104909972	0.0256	2.7609	0.0001	3.3176	0.0019	3.0461	0.0024	2.9455	1.0000	-0.1179	1.0000	0.4501	1.0000	-0.2195
LOC104909974	1.0000	-2.4788	1.0000	0.0000	1.0000	-2.4061	1.0000	0.0000	1.0000	-2.3771	1.0000	0.0000	1.0000	0.0000
LOC104909975	0.8360	-0.0943	0.0335	-0.2546	0.0299	0.3886	0.0007	0.3541	1.0000	-0.0166	0.4185	-0.1636	1.0000	-0.0443
LOC104909976	1.0000	0.0000	0.4437	3.6202	0.8033	3.0789	0.7701	3.1732	1.0000	0.0000	0.8607	3.7297	1.0000	0.0730
LOC104909977	0.6394	-0.1876	0.4800	-0.1403	0.4371	0.2251	1.0000	-0.0228	1.0000	-0.0421	1.0000	0.0182	0.1884	-0.2855
LOC104909978	0.6946	0.5225	0.2297	0.8628	0.0040	1.5579	0.0656	1.0950	1.0000	-0.1382	1.0000	0.2179	0.4555	-0.5920
LOC104909979	0.5601	1.3110	0.0560	2.7270	0.0003	2.9256	0.1477	2.5429	1.0000	-1.4945	1.0000	-0.0879	0.0162	-1.8822
LOC104909980	0.2006	-0.3559	0.0225	-0.3932	0.3067	0.2713	0.7687	0.0859	1.0000	0.0720	1.0000	0.0475	0.9237	-0.1080
LOC104909981	1.0000	0.0000	1.0000	0.0000	1.0000	0.0000	1.0000	2.3256	1.0000	0.0000	1.0000	0.0000	1.0000	2.3554
LOC104909982	0.5851	-0.1769	0.0318	-0.2763	0.9419	-0.0822	0.2141	-0.1776	1.0000	0.0575	1.0000	-0.0293	1.0000	-0.0330
LOC104909983	1.0000	2.1849	1.0000	2.2507	1.0000	2.2426	1.0000	0.0000	1.0000	0.0000	1.0000	0.0495	1.0000	-2.2889
LOC104909984	0.0009	0.5662	0.0000	0.7581	0.0000	0.9928	0.0000	0.9997	1.0000	-0.0208	0.3358	0.1841	1.0000	-0.0084
LOC104909986	1.0000	-0.0986	0.6128	-0.5122	0.6375	-0.5137	0.3779	-0.6623	1.0000	-0.1894	0.8149	-0.5912	1.0000	-0.3319
LOC104909987	0.9545	1.0644	0.9943	0.7489	0.1166	2.5445	0.9786	0.9248	1.0000	0.7304	1.0000	0.4416	0.9099	-0.8793
LOC104909988	0.1047	0.9739	0.0011	1.4195	0.0000	2.4841	0.0000	2.4513	1.0000	-0.4221	1.0000	0.0373	0.2362	-0.4470
LOC104909989	0.1073	0.6401	0.0000	1.0324	0.0000	2.1437	0.0000	2.0646	1.0000	-0.0147	0.3566	0.3881	1.0000	-0.0901
LOC104909990	0.0000	-0.6807	0.0000	-0.7328	1.0000	-0.0531	0.8114	-0.0445	1.0000	-0.0049	1.0000	-0.0442	1.0000	0.0091
LOC104909991	0.8226	3.0199	1.0000	-1.0017	1.0000	0.0000	0.7701	-3.1666	1.0000	3.1126	1.0000	-0.7966	1.0000	0.0000
LOC104909992	1.0000	2.1849	1.0000	-2.3986	1.0000	0.0000	1.0000	0.0045	1.0000	2.2735	1.0000	-2.2956	1.0000	2.3543
LOC104909993	0.2194	0.8874	0.0376	1.2591	0.0017	1.6022	0.0000	1.9553	1.0000	-0.4446	1.0000	-0.0634	1.0000	-0.0897
LOC104909994	0.2534	0.9455	0.2611	1.0464	0.0123	-3.4726	1.0000	-0.3927	1.0000	-0.7024	0.8162	-0.5904	0.5105	2.3935
LOC104909995	0.0657	4.8781	0.2507	1.8645	0.0033	5.6323	0.5147	1.4765	1.0000	3.1191	1.0000	0.2238	0.6135	-1.0057
LOC104909996	1.0000	0.0000	1.0000	0.0000	1.0000	0.0000	1.0000	0.0000	1.0000	0.0000	1.0000	0.0000	1.0000	0.0000
LOC104909997	1.0000	0.1784	0.4888	-1.2581	0.0290	1.4086	0.8794	0.4514	1.0000	-0.1425	0.3160	-1.5718	0.1299	-1.0873
LOC104909998	0.0000	2.3918	0.0003	1.5427	0.0000	3.7551	0.0000	2.6148	1.0000	0.7047	1.0000	-0.1368	0.2275	-0.4350
LOC104910000	1.0000	0.0000	1.0000	0.0000	1.0000	0.0000	1.0000	0.0000	1.0000	0.0000	1.0000	0.0000	1.0000	0.0000
LOC104910001	1.0000	0.0000	1.0000	0.0000	1.0000	0.0000	1.0000	0.0000	1.0000	0.0000	1.0000	0.0000	1.0000	0.0000
LOC104910002	1.0000	0.3619	1.0000	-0.4757	0.8461	-1.1252	1.0000	0.4731	1.0000	-0.3815	0.7636	-1.2111	0.8719	1.2320
LOC104910003	0.5384	3.5543	1.0000	0.6956	0.4809	3.6122	0.9096	1.3854	1.0000	2.2677	1.0000	-0.4781	1.0000	0.0750
LOC104910004	1.0000	0.0000	1.0000	0.0000	1.0000	2.2426	1.0000	0.0000	1.0000	0.0000	1.0000	0.0000	1.0000	-2.2889
LOC104910005	1.0000	-2.4788	1.0000	0.0000	0.9361	1.2042	1.0000	0.0000	1.0000	-2.3771	1.0000	0.0000	0.7287	-3.6634
LOC104910007	1.0000	0.0000	1.0000	0.0000	1.0000	0.0000	1.0000	0.0000	1.0000	0.0000	1.0000	0.0000	1.0000	0.0000
LOC104910008	1.0000	0.0000	1.0000	0.0000	1.0000	0.0000	1.0000	0.0000	1.0000	0.0000	1.0000	0.0000	1.0000	0.0000
LOC104910009	0.0001	3.5427	0.0000	4.2297	0.2725	1.6855	0.4037	2.0749	1.0000	-0.9587	1.0000	-0.2799	1.0000	-0.5740
LOC104910010	1.0000	-0.2931	0.9148	-1.5313	1.0000	-2.4061	0.9167	-1.3690	1.0000	1.2548	1.0000	0.0495	1.0000	2.3543
LOC104910011	0.1110	2.7046	0.1621	2.3788	1.0000	0.6780	1.0000	-2.3177	1.0000	-0.1089	1.0000	-0.4333	1.0000	-3.1344
LOC104910012	1.0000	0.0000	1.0000	0.0000	1.0000	0.0000	1.0000	0.0000	1.0000	0.0000	1.0000	0.0000	1.0000	0.0000
LOC104910013	1.0000	0.0000	1.0000	0.0000	1.0000	0.0000	1.0000	0.0000	1.0000	0.0000	1.0000	0.0000	1.0000	0.0000
LOC104910014	1.0000	0.0000	1.0000	0.0000	1.0000	0.0000	1.0000	0.0000	1.0000	0.0000	1.0000	0.0000	1.0000	0.0000
LOC104910015	1.0000	0.0000	1.0000	0.0000	1.0000	0.0000	1.0000	0.0000	1.0000	0.0000	1.0000	0.0000	1.0000	0.0000
LOC104910019	0.5271	-0.1670	0.0024	-0.2678	0.0000	-1.9545	0.0000	-1.7129	1.0000	0.0126	1.0000	-0.0755	0.0427	0.2597
LOC104910020	1.0000	-0.3262	1.0000	0.6944	0.8033	-3.2622	1.0000	-2.3200	1.0000	-0.9563	1.0000	0.0563	1.0000	0.0000
LOC104910021	0.0163	0.7542	0.0000	1.0969	0.0000	1.3942	0.0000	1.6862	1.0000	-0.2027	0.9420	0.1540	1.0000	0.0991
LOC104910022	0.8249	-3.3412	0.4431	-3.7865	1.0000	-0.1821	0.4370	-3.6968	1.0000	0.4098	1.0000	0.0000	1.0000	-3.1316
LOC104910023	0.0002	-3.6450	0.0077	-3.5777	0.0488	-1.6228	1.0000	-0.1096	1.0000	-0.8678	1.0000	-0.7966	1.0000	0.6482
LOC104910024	1.0000	-0.3449	0.8204	0.5689	0.7390	0.7713	1.0000	0.0072	1.0000	0.3377	0.8269	1.2656	1.0000	-0.4208
LOC104910026	0.5210	0.8257	0.5340	0.8009	0.0803	-2.3755	0.0881	-2.7157	1.0000	-0.4439	0.9773	-0.4567	1.0000	-0.7795
LOC104910027	0.0204	-0.5874	0.0005	-0.4727	0.0126	-0.5873	0.0121	-0.3526	1.0000	0.0470	0.8875	0.1745	0.3380	0.2877
LOC104910030	1.0000	0.0000	1.0000	0.0000	1.0000	0.0000	1.0000	2.3256	1.0000	0.0000	1.0000	0.0000	1.0000	2.3555
LOC104910032	1.0000	0.3288	0.5796	0.7245	0.9276	0.6087	1.0000	0.2899	1.0000	0.0335	1.0000	0.4426	1.0000	-0.2718
LOC104910034	1.0000	0.0000	1.0000	0.0000	1.0000	0.0000	1.0000	0.0000	1.0000	0.0000	1.0000	0.0000	1.0000	0.0000
LOC104910037	1.0000	0.0738	0.0697	0.2995	0.1537	0.3577	0.0004	0.5191	1.0000	-0.2300	1.0000	0.0077	1.0000	-0.0628
LOC104910038	0.4270	0.1851	0.6602	-0.0791	0.4789	0.1694	0.1055	0.1711	1.0000	0.0514	0.3222	-0.2007	1.0000	0.0580
LOC104910040	0.9545	1.0652	1.0000	0.0722	0.0202	3.2228	1.0000	0.0028	1.0000	1.9737	1.0000	1.0017	0.4643	-1.2545
LOC104910041	0.0076	2.5531	0.8359	0.4872	0.1684	1.7246	0.4381	0.7582	1.0000	1.5316	0.8923	-0.5169	0.9919	0.5733
LOC104910042	0.5143	-0.8708	0.1258	-1.4630	0.2014	-1.5304	0.0935	-1.5314	1.0000	0.0745	1.0000	-0.5060	1.0000	0.0775
LOC104910043	0.0000	-0.8589	0.0000	-0.9981	0.0000	-0.8416	0.0000	-0.7926	1.0000	0.0757	1.0000	-0.0505	0.6830	0.1305
LOC104910044	0.0337	0.4508	0.0022	0.3906	0.1083	0.3496	0.0007	0.4050	1.0000	0.0397	1.0000	-0.0081	0.8817	0.0997
LOC104910045	1.0000	0.0000	1.0000	0.0000	1.0000	0.0000	1.0000	0.0000	1.0000	0.0000	1.0000	0.0000	1.0000	0.0000
LOC104910046	0.0003	1.7181	0.0708	1.4389	0.0758	-1.5324	0.8240	-0.7634	1.0000	-1.8118	0.0000	-2.0827	0.7633	-1.0431
LOC104910047	1.0000	0.5856	0.7802	-1.1137	1.0000	-0.1789	0.0731	-4.6410	1.0000	1.3536	1.0000	-0.3278	1.0000	-3.1344
LOC104910049	0.4958	-1.0639	1.0000	0.1673	0.0322	-3.2126	0.0177	-3.1630	1.0000	0.0034	0.6549	1.2514	1.0000	0.0640
LOC104910050	1.0000	0.0000	1.0000	0.0000	1.0000	0.0000	1.0000	0.0000	1.0000	0.0000	1.0000	0.0000	1.0000	0.0000
LOC104910051	1.0000	0.0000	1.0000	0.0000	1.0000	0.0000	1.0000	0.0000	1.0000	0.0000	1.0000	0.0000	1.0000	0.0000
LOC104910052	1.0000	0.0000	1.0000	0.0000	1.0000	0.0000	1.0000	0.0000	1.0000	0.0000	1.0000	0.0000	1.0000	0.0000
LOC104910053	1.0000	0.0000	1.0000	0.0000	1.0000	0.0000	1.0000	0.0000	1.0000	0.0000	1.0000	0.0000	1.0000	0.0000
LOC104910054	0.0015	1.1377	0.0000	1.0610	0.0000	1.6740	0.0000	1.4958	1.0000	0.0897	1.0000	0.0242	1.0000	-0.0842
LOC104910057	1.0000	0.0000	1.0000	0.0000	1.0000	0.0000	1.0000	0.0000	1.0000	0.0000	1.0000	0.0000	1.0000	0.0000
LOC104910058	0.0000	3.5938	0.4135	1.9103	0.3428	1.4659	1.0000	0.0075	1.0000	-1.4982	0.0000	-3.1943	0.1085	-2.9634
LOC104910059	0.0037	2.0001	0.0358	1.8478	0.9660	-0.5460	0.9786	-0.9103	1.0000	-1.2536	0.0478	-1.3951	0.6246	-1.6128
LOC104910060	0.0008	1.6277	1.0000	-0.1796	0.2978	-0.9708	0.0002	-3.2994	1.0000	0.0809	0.0001	-1.7162	0.1743	-2.2466
LOC104910061	1.0000	0.0000	1.0000	0.0000	1.0000	0.0000	1.0000	0.0000	1.0000	0.0000	1.0000	0.0000	1.0000	0.0000
LOC104910062	1.0000	0.0000	1.0000	0.0000	1.0000	0.0000	1.0000	0.0000	1.0000	0.0000	1.0000	0.0000	1.0000	0.0000
LOC104910063	1.0000	0.0000	1.0000	-2.3960	1.0000	0.0000	1.0000	0.0075	1.0000	2.2677	1.0000	0.0000	1.0000	2.3555
LOC104910064	0.0034	-5.8049	0.0019	-5.5832	0.0911	-2.1027	0.7590	-0.6262	1.0000	-0.2540	1.0000	0.0000	0.8728	1.2338
LOC104910065	1.0000	0.0000	1.0000	0.0000	1.0000	0.0000	1.0000	0.0000	1.0000	0.0000	1.0000	0.0000	1.0000	0.0000
LOC104910066	1.0000	0.0000	1.0000	0.0000	1.0000	0.0000	1.0000	0.0000	1.0000	0.0000	1.0000	0.0000	1.0000	0.0000
LOC104910067	1.0000	0.0000	0.9148	1.2198	1.0000	0.0000	1.0000	-2.3177	1.0000	2.2677	0.8785	3.7234	1.0000	0.0000
LOC104910068	1.0000	0.0000	1.0000	0.0000	1.0000	0.0000	1.0000	0.0000	1.0000	0.0000	1.0000	0.0000	1.0000	0.0000
LOC104910069	1.0000	2.1849	1.0000	0.0000	0.8011	3.0840	1.0000	2.3256	1.0000	0.0000	1.0000	-2.2956	1.0000	-0.7793
LOC104910070	1.0000	0.0000	1.0000	0.0000	1.0000	0.0000	1.0000	0.0000	1.0000	0.0000	1.0000	0.0000	1.0000	0.0000
LOC104910071	1.0000	2.1849	1.0000	2.2534	1.0000	2.2426	1.0000	0.0000	1.0000	0.0000	1.0000	0.0514	1.0000	-2.2889

LOC104910072	1.0000	0.0000	1.0000	0.0000	1.0000	0.0000	1.0000	0.0000	1.0000	0.0000	1.0000	0.0000	1.0000	0.0000
LOC104910073	1.0000	-2.4788	1.0000	0.0000	1.0000	-2.4061	0.7701	3.1732	1.0000	-2.3771	1.0000	0.0000	1.0000	3.2066
LOC104910074	0.9650	1.0594	1.0000	0.0000	1.0000	0.6746	0.0729	4.6479	1.0000	-2.3757	0.8785	-3.6647	0.7916	1.5501
LOC104910075	0.3935	0.3727	0.0000	0.6898	0.0055	-1.0257	0.0000	-1.0453	1.0000	-0.0542	0.3580	0.2771	1.0000	-0.0650
LOC104910076	0.8490	-0.1389	0.0882	-0.3244	0.0071	-0.7380	0.0000	-0.7422	1.0000	0.0865	1.0000	-0.0867	1.0000	0.0884
LOC104910077	1.0000	0.0000	1.0000	0.0000	1.0000	0.0000	1.0000	0.0000	1.0000	0.0000	1.0000	0.0000	1.0000	0.0000
LOC104910078	0.0002	0.7792	0.0000	0.5279	0.1002	0.3964	0.0303	0.2982	1.0000	0.1881	1.0000	-0.0516	0.9757	0.0941
LOC104910081	1.0000	2.1849	0.4437	3.6202	0.8011	3.0840	1.0000	0.0000	1.0000	0.0000	1.0000	1.4325	1.0000	-3.1344
LOC104910083	1.0000	2.1902	1.0000	0.0000	1.0000	0.0000	1.0000	2.3256	1.0000	0.0000	1.0000	-2.2993	1.0000	2.3555
LOC104910085	1.0000	2.1902	1.0000	0.0000	1.0000	0.0000	1.0000	2.3242	1.0000	0.0000	1.0000	-2.2992	1.0000	2.3543
LOC104910088	1.0000	0.0000	1.0000	0.0000	1.0000	0.0000	1.0000	0.0000	1.0000	0.0000	1.0000	0.0000	1.0000	0.0000
LOC104910089	0.5378	3.5350	1.0000	-2.3986	1.0000	0.0000	1.0000	-2.3200	1.0000	2.2735	0.8785	-3.6647	1.0000	0.0000
LOC104910090	1.0000	2.1902	1.0000	0.0000	1.0000	0.0000	1.0000	0.0000	1.0000	0.0000	1.0000	-2.2992	1.0000	0.0000
LOC104910091	0.7149	-0.8142	1.0000	-0.3878	1.0000	-0.6592	0.9147	0.5069	1.0000	-0.1285	1.0000	0.3112	0.8110	1.0438
LOC104910092	1.0000	0.0000	0.7666	-3.2534	1.0000	0.0000	1.0000	-0.8416	1.0000	3.1126	1.0000	0.0000	1.0000	2.3555
LOC104910094	1.0000	-2.4776	1.0000	0.0000	1.0000	-0.1625	1.0000	0.0000	1.0000	-2.3757	1.0000	0.0000	1.0000	-2.2889
LOC104910095	1.0000	0.0000	1.0000	0.0000	1.0000	0.0000	1.0000	0.0000	1.0000	0.0000	1.0000	0.0000	1.0000	0.0000
LOC104910097	0.5280	-0.4513	0.0001	-1.0347	0.0916	-0.8977	1.0000	-0.0343	1.0000	0.4763	1.0000	-0.0882	0.0000	1.3417
LOC104910098	1.0000	-0.3520	0.4377	-0.6870	0.3379	0.9325	1.0000	-0.1421	1.0000	1.0035	1.0000	0.6830	1.0000	-0.0677
LOC104910099	0.2879	-0.9748	0.1791	-1.0421	1.0000	0.1011	1.0000	-0.0070	1.0000	0.2423	1.0000	0.1854	1.0000	0.1441
LOC104910100	1.0000	0.0000	1.0000	0.0000	1.0000	0.0000	1.0000	0.0000	1.0000	0.0000	1.0000	0.0000	1.0000	0.0000
LOC104910102	1.0000	2.1849	1.0000	-2.3960	1.0000	2.2426	1.0000	0.0075	1.0000	2.2676	1.0000	-2.2956	1.0000	0.0661
LOC104910104	0.2452	1.6798	0.9787	0.7514	0.9361	-1.5532	1.0000	0.5367	1.0000	-0.6479	0.4187	-1.5799	1.0000	1.4489
LOC104910105	0.4743	2.0033	1.0000	0.6940	0.9468	1.2126	1.0000	-2.3177	1.0000	-0.1078	0.9082	-1.4179	0.7455	-3.6684
LOC104910106	0.0013	-0.6164	0.0000	-0.6048	0.2125	-0.2756	0.0000	-0.5734	1.0000	-0.1350	0.9101	-0.1110	0.0011	-0.4271
LOC104910107	0.2034	1.9953	0.0081	5.4031	0.8033	-3.2636	1.0000	0.0000	1.0000	-3.2319	1.0000	0.0482	1.0000	0.0000
LOC104910108	1.0000	0.0000	1.0000	0.0000	1.0000	0.0000	1.0000	0.0000	1.0000	0.0000	1.0000	0.0000	1.0000	0.0000
LOC104910109	1.0000	0.0000	1.0000	0.0000	1.0000	0.0000	1.0000	0.0000	1.0000	0.0000	1.0000	0.0000	1.0000	0.0000
LOC104910110	1.0000	0.0000	1.0000	0.0000	1.0000	0.0000	1.0000	0.0000	1.0000	0.0000	1.0000	0.0000	1.0000	0.0000
LOC104910111	1.0000	-2.4788	1.0000	-2.3986	1.0000	-2.4061	1.0000	0.0045	1.0000	-0.1045	1.0000	0.0000	1.0000	2.3543
LOC104910112	0.3946	-1.2029	0.7726	-1.1137	0.6904	0.5749	0.0012	2.1317	1.0000	-0.9775	1.0000	-0.8831	0.6498	0.5823
LOC104910113	1.0000	0.3483	0.3472	-1.8549	1.0000	0.5071	0.3374	-1.6805	1.0000	1.0297	1.0000	-1.1637	0.9852	-1.1488
LOC104910114	1.0000	-0.3393	1.0000	-0.1616	1.0000	-1.0997	1.0000	0.5367	1.0000	-1.0363	1.0000	-0.8617	1.0000	0.6038
LOC104910115	1.0000	0.0000	1.0000	0.0000	1.0000	0.0000	1.0000	0.0000	1.0000	0.0000	1.0000	0.0000	1.0000	0.0000
LOC104910116	1.0000	-0.2892	1.0000	-0.1612	1.0000	0.6780	0.7701	-3.1666	1.0000	0.7357	1.0000	0.8994	1.0000	-3.1344
LOC104910117	0.8226	3.0199	1.0000	0.0000	1.0000	2.2425	1.0000	2.3242	1.0000	0.0000	1.0000	-3.1429	1.0000	0.0652
LOC104910118	0.2620	0.8495	0.4211	0.5093	0.1777	-1.2685	0.0590	-1.3576	1.0000	0.3468	1.0000	0.0196	1.0000	0.2651
LOC104910119	1.0000	0.5381	0.5159	-1.6382	0.4424	1.8936	0.7798	-0.9341	1.0000	2.1988	1.0000	0.0563	1.0000	-0.6154
LOC104910121	0.0000	-0.9140	0.0000	-0.9021	0.0000	-0.7490	0.0000	-0.7861	1.0000	0.0275	1.0000	0.0518	1.0000	-0.0042
LOC104910123	1.0000	-0.3444	0.4737	1.0970	1.0000	-0.1907	1.0000	0.0116	1.0000	-0.4321	0.9234	1.0243	1.0000	-0.2269
LOC104910124	1.0000	0.0000	1.0000	0.0000	1.0000	0.0000	1.0000	0.0000	1.0000	0.0000	1.0000	0.0000	1.0000	0.0000
LOC104910125	1.0000	-0.2892	0.7666	3.0922	1.0000	-2.4061	1.0000	0.0000	1.0000	-2.3771	1.0000	0.8994	1.0000	0.0000
LOC104910126	1.0000	0.2121	0.1281	1.5410	0.6647	-1.9394	0.7210	-1.2163	1.0000	0.1810	0.3519	1.5252	1.0000	0.9149
LOC104910128	0.3159	3.9314	1.0000	-0.5567	1.0000	2.2471	0.2461	-4.0849	1.0000	4.0300	1.0000	-0.3305	1.0000	-2.2908
LOC104910129	0.4811	0.5443	0.0821	0.8627	1.0000	0.0622	0.3914	0.5281	1.0000	-0.1272	1.0000	0.2034	0.9248	0.3415
LOC104910130	1.0000	-0.2882	0.9148	-1.5280	1.0000	0.6783	0.9167	-1.3669	1.0000	1.2565	1.0000	0.0489	1.0000	-0.7793
LOC104910131	0.7510	-0.1669	0.0729	-0.4053	0.0004	0.7736	0.0010	0.6249	1.0000	0.1079	1.0000	-0.1176	1.0000	-0.0359
LOC104910132	1.0000	0.0000	1.0000	0.0000	1.0000	0.0000	1.0000	0.0000	1.0000	0.0000	1.0000	0.0000	1.0000	0.0000
LOC104910133	0.0893	1.1773	1.0000	-0.1799	0.7804	0.4672	0.9825	-0.3334	1.0000	-1.1809	0.0000	-2.5211	0.0007	-1.9624
LOC104910134	0.0064	1.1233	0.1627	0.6082	0.2399	-0.6887	0.6734	0.3096	1.0000	-0.1272	0.1945	-0.6330	0.1521	0.8741
LOC104910135	1.0000	0.0000	1.0000	0.0000	1.0000	0.0000	1.0000	0.0000	1.0000	0.0000	1.0000	0.0000	1.0000	0.0000
LOC104910136	1.0000	0.0000	1.0000	0.0000	1.0000	0.0000	1.0000	0.0000	1.0000	0.0000	1.0000	0.0000	1.0000	0.0000
LOC104910137	1.0000	0.0000	1.0000	0.0000	1.0000	0.0000	1.0000	0.0000	1.0000	0.0000	1.0000	0.0000	1.0000	0.0000
LOC104910138	0.7070	-0.1974	0.0004	-0.6411	1.0000	-0.0438	1.0000	-0.0232	1.0000	0.3104	1.0000	-0.1208	0.2493	0.3361
LOC104910139	0.2071	0.8873	1.0000	0.1229	1.0000	-0.2021	0.4687	0.5170	1.0000	-0.0664	0.3651	-0.8179	0.5150	0.6596
LOC104910145	0.0000	-1.5601	0.0000	-1.6267	0.0000	-1.2765	0.0000	-1.1927	1.0000	-0.0032	1.0000	-0.0573	0.7383	0.0862
LOC104910147	1.0000	0.0291	0.1128	-0.3100	0.8311	0.1367	0.4921	0.1621	1.0000	0.0366	0.4034	-0.2892	1.0000	0.0675
LOC104910148	1.0000	0.0000	1.0000	0.0000	1.0000	0.0000	1.0000	0.0000	1.0000	0.0000	1.0000	0.0000	1.0000	0.0000
LOC104910149	1.0000	0.0000	1.0000	0.0000	1.0000	0.0000	1.0000	0.0000	1.0000	0.0000	1.0000	0.0000	1.0000	0.0000
LOC104910150	1.0000	0.0000	1.0000	2.2534	1.0000	0.0000	1.0000	0.0000	1.0000	0.0000	1.0000	2.3480	1.0000	0.0000
LOC104910151	1.0000	0.0000	1.0000	0.0000	1.0000	0.0000	1.0000	0.0000	1.0000	0.0000	1.0000	0.0000	1.0000	0.0000
LOC104910152	1.0000	2.1902	1.0000	-0.1429	1.0000	2.2425	1.0000	-2.3177	1.0000	2.2676	1.0000	0.0489	1.0000	-2.2889
LOC104910153	1.0000	0.0000	1.0000	0.0000	1.0000	0.0000	1.0000	0.0000	1.0000	0.0000	1.0000	0.0000	1.0000	0.0000
LOC104910154	1.0000	0.0000	1.0000	0.0000	1.0000	2.2425	1.0000	0.0000	1.0000	0.0000	1.0000	0.0000	1.0000	-2.2889
LOC104910155	1.0000	0.0000	1.0000	0.0000	1.0000	0.0000	1.0000	0.0000	1.0000	0.0000	1.0000	0.0000	1.0000	0.0000
LOC104910156	1.0000	0.1965	1.0000	0.1281	1.0000	-0.1789	0.9946	-0.9209	1.0000	0.8060	1.0000	0.7511	1.0000	0.0718
LOC104910157	1.0000	0.0000	1.0000	0.0000	1.0000	0.0000	1.0000	0.0000	1.0000	0.0000	1.0000	0.0000	1.0000	0.0000
LOC104910158	1.0000	0.0000	1.0000	0.0000	1.0000	0.0000	1.0000	0.0000	1.0000	0.0000	1.0000	0.0000	1.0000	0.0000
LOC104910159	1.0000	0.5464	0.7666	-3.2534	1.0000	-0.1591	0.7701	-3.1666	1.0000	0.7365	1.0000	-3.1468	1.0000	-2.2908
LOC104910160	0.9545	1.0679	0.7641	-1.4013	1.0000	0.6783	0.4562	-2.0801	1.0000	1.9737	1.0000	-0.4752	1.0000	-0.7795
LOC104910161	1.0000	0.0000	1.0000	0.0000	1.0000	0.0000	1.0000	0.0000	1.0000	0.0000	1.0000	0.0000	1.0000	0.0000
LOC104910162	1.0000	0.0000	1.0000	0.0000	1.0000	0.0000	1.0000	2.3242	1.0000	0.0000	1.0000	0.0000	1.0000	2.3543
LOC104910163	1.0000	0.0000	1.0000	0.0000	1.0000	0.0000	1.0000	0.0000	1.0000	0.0000	1.0000	0.0000	1.0000	0.0000
LOC104910164	1.0000	-2.4776	1.0000	-2.3986	1.0000	-2.4055	1.0000	-2.3200	1.0000	-0.1034	1.0000	0.0000	1.0000	0.0000
LOC104910166	1.0000	0.5414	1.0000	2.2507	1.0000	-2.4061	1.0000	0.0000	1.0000	-2.3771	1.0000	-0.7966	1.0000	0.0000
LOC104910167	1.0000	0.0000	1.0000	0.0000	1.0000	0.0000	1.0000	0.0000	1.0000	0.0000	1.0000	0.0000	1.0000	0.0000
LOC104910168	1.0000	0.0000	1.0000	0.0000	1.0000	0.0000	1.0000	0.0000	1.0000	0.0000	1.0000	0.0000	1.0000	0.0000
LOC104910169	1.0000	0.0000	1.0000	0.0000	1.0000	0.0000	1.0000	0.0000	1.0000	0.0000	1.0000	0.0000	1.0000	0.0000
LOC104910170	1.0000	0.0000	1.0000	0.0000	1.0000	0.0000	1.0000	0.0000	1.0000	0.0000	1.0000	0.0000	1.0000	0.0000
LOC104910171	1.0000	0.0000	1.0000	0.0000	1.0000	2.2471	1.0000	0.0000	1.0000	0.0000	1.0000	0.0000	1.0000	-2.2908
LOC104910172	0.3056	-0.8163	0.1740	-0.7417	0.3040	-0.8034	0.1951	-0.6666	1.0000	0.3352	1.0000	0.4225	0.8047	0.4722
LOC104910173	1.0000	0.0000	1.0000	0.0000	1.0000	0.0000	1.0000	0.0000	1.0000	0.0000	1.0000	0.0000	1.0000	0.0000
LOC104910174	1.0000	0.0000	1.0000	0.0000	1.0000	0.0000	1.0000	0.0000	1.0000	0.0000	1.0000	0.0000	1.0000	0.0000
LOC104910175	0.8226	3.0199	1.0000	0.0000	1.0000	0.0000	1.0000	2.3256	1.0000	0.0000	1.0000	-3.1429	1.0000	2.3554
LOC104910176	1.0000	2.1902	1.0000	0.0000	1.0000	0.0000	1.0000	0.0000	1.0000	0.0000	1.0000	-2.2992	1.0000	0.0000
LOC104910177	1.0000	2.1849	1.0000	0.0000	1.0000	0.0000	1.0000	0.0000	1.0000	0.0000	1.0000	-2.2956	1.0000	0.0000
LOC104910178	0.0000	-1.9858	0.0000	-2.5069	0.1333	0.3837	1.0000	0.0159	1.0000	0.3176	1.0000	-0.1919	1.0000	-0.0458
LOC104910179	0.0193	-0.4103	0.0000	-0.7355	0.1797	0.2546	0.0002	0.3254	1.0000	0.1642	0.6100	-0.1478	0.0535	0.2405
LOC104910180	0.9265	0.7776	1.0000	0.5761	1.0000	-0.1951	1.0000	0.3120	1.0000	-0.1226	1.0000	-0.3055	1.0000	0.3870
LOC104910181	0.4366	-0.9300	0.2916	-0.7290	1.0000	-0.1962	0.0472	-1.2941	1.0000	0.5997	0.8785	0.8135	0.9614	-0.4939
LOC104910182	1.0000	0.0000	1.0000	2.2534	1.0000	0.0000	1.0000	0.0000	1.0000	0.0000	1.0000	2.3480	1.0000	0.0000
LOC104910183	0.3011	-1.4565	0.2550	-2.4832	0.0222	-3.3467	0.2469	-2.3163	1.0000	-0.9727	0.8723	-2.0057	1.0000	0.0640
LOC104910184	1.0000	0.0000	1.0000	0.0000	1.0000	0.0000	1.0000	0.0000	1.0000	0.0000	1.0000	0.0000	1.0000	0.0000

LOC104910185	0.5661	-0.3253	0.0022	-0.8357	0.0007	-1.3356	0.0661	-0.5314	1.0000	0.1801	0.8372	-0.3154	0.0259	0.9879
LOC104910186	1.0000	0.0000	1.0000	0.0000	1.0000	0.0000	1.0000	0.0000	1.0000	0.0000	1.0000	0.0000	1.0000	0.0000
LOC104910187	1.0000	0.0000	1.0000	0.0000	1.0000	0.0000	1.0000	0.0000	1.0000	0.0000	1.0000	0.0000	1.0000	0.0000
LOC104910188	1.0000	0.0000	1.0000	0.0000	1.0000	0.0000	1.0000	0.0000	1.0000	0.0000	1.0000	0.0000	1.0000	0.0000
LOC104910189	0.5255	-1.1698	1.0000	-0.1773	0.0351	-5.3084	0.0519	-2.8813	1.0000	-0.1245	1.0000	0.8767	1.0000	2.3543
LOC104910190	0.1108	1.3132	1.0000	0.2818	0.2719	-2.0598	0.0197	-2.5512	1.0000	0.5547	1.0000	-0.4645	1.0000	0.0716
LOC104910191	1.0000	0.0000	1.0000	0.0000	1.0000	0.0000	1.0000	0.0000	1.0000	0.0000	1.0000	0.0000	1.0000	0.0000
LOC104910193	1.0000	0.0000	1.0000	0.0000	1.0000	0.0000	1.0000	0.0000	1.0000	0.0000	1.0000	0.0000	1.0000	0.0000
LOC104910194	0.8249	3.0255	0.4135	1.9103	0.0320	5.1126	0.2439	2.3286	1.0000	2.2676	1.0000	1.2747	1.0000	-0.4865
LOC104910195	0.0000	-3.0551	0.0000	-2.2850	1.0000	0.0720	0.0000	0.9252	1.0000	0.1844	0.3284	0.9684	0.0000	1.0455
LOC104910196	0.0010	-0.6155	0.0000	-0.6899	0.0054	-0.5452	0.0001	-0.5002	1.0000	0.0531	1.0000	-0.0088	0.9139	0.1039
LOC104910197	0.3182	-2.3881	0.0566	4.7816	1.0000	-0.1925	0.1393	4.3943	1.0000	-4.4573	0.5366	2.5945	1.0000	0.0771
LOC104910198	0.0169	-1.0009	1.0000	-0.1055	0.0201	0.8019	0.0000	1.4313	1.0000	-0.7122	1.0000	0.1952	1.0000	-0.0824
LOC104910199	1.0000	-0.3390	0.9062	-1.5348	1.0000	-0.1862	1.0000	0.3946	1.0000	-0.5152	1.0000	-1.7140	1.0000	0.0763
LOC104910200	0.0337	-1.2486	0.0255	-1.0005	0.0163	-1.3749	0.0001	-1.7527	1.0000	0.0923	1.0000	0.3556	1.0000	-0.2798
LOC104910201	0.4306	0.5469	0.0044	1.1877	0.8768	-0.3325	0.2110	0.6211	1.0000	-0.4511	1.0000	0.2022	0.6338	0.5043
LOC104910202	1.0000	0.0000	1.0000	0.0000	1.0000	0.0000	1.0000	0.0000	1.0000	0.0000	1.0000	0.0000	1.0000	0.0000
LOC104910204	0.0087	1.1806	0.0006	1.0901	0.0000	1.7417	0.0004	1.1295	1.0000	0.1581	1.0000	0.0794	0.2705	-0.4489
LOC104910205	1.0000	-0.7234	1.0000	0.0000	1.0000	0.1113	1.0000	2.3256	1.0000	-4.1507	0.8607	-3.6732	0.6942	-1.9975
LOC104910206	0.9545	-1.6928	1.0000	-0.1706	1.0000	0.5026	1.0000	0.0070	1.0000	0.2637	1.0000	1.8196	1.0000	-0.2278
LOC104910207	1.0000	-0.2931	1.0000	2.2507	1.0000	-0.1630	1.0000	2.3242	1.0000	-2.3771	1.0000	0.0495	1.0000	0.0652
LOC104910208	0.1011	-3.0444	0.1463	4.3063	0.4809	-1.5325	1.0000	2.3256	1.0000	-5.1088	0.8354	2.1233	1.0000	-1.3086
LOC104910211	0.2081	4.4899	0.3421	1.5203	0.1702	4.2960	0.0865	2.1946	1.0000	3.1126	1.0000	0.2714	0.8125	1.0442
LOC104910212	0.0232	5.1905	0.0069	5.2727	0.0312	4.9489	0.2460	4.0916	1.0000	0.0000	1.0000	0.0610	0.9124	-0.8785
LOC104910213	0.2847	0.4029	0.9960	0.0793	0.3009	-0.4115	0.0086	-0.6386	1.0000	0.3478	1.0000	0.0373	1.0000	0.1266
LOC104910214	0.0023	1.3573	0.0000	1.4863	0.0000	1.9567	0.0000	2.0432	1.0000	0.3351	0.3460	0.4758	0.1563	0.4293
LOC104910215	1.0000	2.1902	0.4635	-3.7803	0.8033	3.0789	0.4634	-3.6900	1.0000	3.6299	1.0000	-2.2992	1.0000	-3.1317
LOC104910216	0.2243	2.3971	0.0826	1.6674	0.1203	2.7069	0.4667	1.1295	1.0000	1.6505	0.8372	0.9466	1.0000	0.0803
LOC104910217	0.3843	0.2657	0.1456	0.2478	0.5678	-0.2045	0.0524	-0.3605	1.0000	-0.1100	0.9892	-0.1146	0.4993	-0.2596
LOC104910218	0.1272	2.3081	0.9329	0.5802	0.7662	1.0366	1.0000	-0.2073	1.0000	1.5669	1.0000	-0.1486	1.0000	0.3292
LOC104910219	1.0000	-0.8591	1.0000	0.2177	0.9361	-1.5509	0.9096	-1.3717	1.0000	-0.1232	1.0000	0.9730	1.0000	0.0649
LOC104910220	1.0000	-2.4776	1.0000	-1.0017	1.0000	0.6823	0.9786	0.9248	1.0000	0.7365	1.0000	2.3455	1.0000	0.9917
LOC104910221	0.4175	-0.8476	1.0000	-0.1804	0.2568	0.7938	0.1600	0.7496	1.0000	0.2384	0.6588	0.9201	1.0000	0.1998
LOC104910222	0.0000	-1.3687	0.0000	-1.1307	0.2162	0.4033	0.0000	0.8103	1.0000	0.2116	0.2735	0.4653	0.0000	0.6244
LOC104910223	0.0000	-1.4438	0.0000	-1.7327	0.0001	1.0934	0.1263	0.3147	1.0000	0.2835	1.0000	0.0069	0.0238	-0.4907
LOC104910224	0.0028	2.3932	0.0007	1.8304	0.0009	2.5112	0.1152	1.1258	1.0000	0.8339	1.0000	0.2880	0.7218	-0.5420
LOC104910225	0.8226	3.0199	0.7674	3.0956	1.0000	0.0000	1.0000	0.0000	1.0000	0.0000	1.0000	0.0559	1.0000	0.0000
LOC104910228	1.0000	0.0000	1.0000	0.0000	1.0000	0.0000	1.0000	0.0000	1.0000	0.0000	1.0000	0.0000	1.0000	0.0000
LOC104910229	0.0000	2.3772	0.0001	1.8684	0.6313	-0.6973	0.6890	-0.5653	1.0000	0.4426	1.0000	-0.0519	1.0000	0.5804
LOC104910231	1.0000	0.0000	1.0000	0.0000	0.8011	3.0840	1.0000	0.0000	1.0000	0.0000	1.0000	0.0000	1.0000	-3.1344
LOC104910232	0.0317	-0.5494	0.4381	-0.1941	0.8033	-0.1352	0.4180	0.1932	1.0000	-0.2730	1.0000	0.0955	1.0000	0.0605
LOC104910234	0.1875	1.0245	0.0325	1.3843	0.1018	-2.1196	0.0885	-2.1801	1.0000	-0.8005	0.9688	-0.4234	1.0000	-0.8468
LOC104910235	1.0000	0.0000	1.0000	0.0000	1.0000	0.0000	1.0000	0.0000	1.0000	0.0000	1.0000	0.0000	1.0000	0.0000
LOC104910236	1.0000	0.0000	1.0000	0.0000	1.0000	0.0000	1.0000	0.0000	1.0000	0.0000	1.0000	0.0000	1.0000	0.0000
LOC104910237	1.0000	0.0000	1.0000	2.2534	1.0000	0.0000	1.0000	0.0000	1.0000	0.0000	1.0000	2.3480	1.0000	0.0000
LOC104910238	1.0000	0.0000	1.0000	0.0000	1.0000	0.0000	1.0000	0.0000	1.0000	0.0000	1.0000	0.0000	1.0000	0.0000
LOC104910239	1.0000	0.0000	1.0000	0.0000	1.0000	0.0000	1.0000	0.0000	1.0000	0.0000	1.0000	0.0000	1.0000	0.0000
LOC104910240	1.0000	0.0000	1.0000	0.0000	1.0000	0.0000	1.0000	0.0000	1.0000	0.0000	1.0000	0.0000	1.0000	0.0000
LOC104910241	0.3780	1.2923	0.1157	1.5708	0.0997	1.7327	1.0000	0.3120	1.0000	-0.1253	1.0000	0.1648	0.2994	-1.5472
LOC104910242	0.0009	3.7629	0.0000	4.3842	0.0750	2.8588	0.1528	2.5431	1.0000	-0.1045	0.8875	0.5207	1.0000	-0.4207
LOC104910243	0.0252	2.5410	0.0000	4.8026	0.8176	0.7522	0.1705	2.5373	1.0000	-1.4918	0.6637	0.7576	1.0000	0.2917
LOC104910244	1.0000	0.0000	1.0000	-0.1452	1.0000	0.0000	1.0000	-2.3200	1.0000	2.2735	1.0000	2.3480	1.0000	0.0000
LOC104910245	1.0000	0.0000	1.0000	0.0000	1.0000	0.0000	1.0000	0.0000	1.0000	0.0000	1.0000	0.0000	1.0000	0.0000
LOC104910246	0.2069	0.3337	0.0002	0.4439	0.0822	0.4153	0.3232	-0.1740	1.0000	0.0159	0.7407	0.1374	0.0001	-0.5683
LOC104910247	0.0023	1.8766	0.0000	2.8908	0.0000	2.6534	0.0000	2.6769	1.0000	-0.4249	0.3504	0.5966	0.4476	-0.3958
LOC104910248	0.0001	-1.3602	0.0339	-0.8540	0.0000	2.1558	0.0000	1.9077	1.0000	0.0446	0.4066	0.5615	0.7027	-0.1962
LOC104910249	0.0000	-0.9021	0.0000	-1.1302	0.0000	-0.9850	0.0000	-1.1387	1.0000	0.0604	0.4476	-0.1554	0.7161	-0.0879
LOC104910250	0.5754	-0.3928	0.3714	-0.3651	0.1981	-0.6323	0.0496	-0.7002	1.0000	0.4625	0.5784	0.5032	0.7529	0.4014
LOC104910251	0.0078	-2.5773	0.1009	-1.8223	0.0527	-2.0356	0.9761	-0.3643	1.0000	-0.3292	1.0000	0.4416	0.6285	1.3476
LOC104910252	1.0000	0.0000	1.0000	0.0000	1.0000	2.2471	1.0000	0.0000	1.0000	0.0000	1.0000	0.0000	1.0000	-2.2908
LOC104910256	0.0849	1.5502	0.0444	2.0657	0.0264	1.7641	0.0040	2.3283	1.0000	-0.8984	1.0000	-0.3713	1.0000	-0.3306
LOC104910257	0.0138	1.4606	0.0000	2.2896	0.0002	1.7650	0.0000	2.0944	1.0000	-0.5106	0.9108	0.3277	1.0000	-0.1764
LOC104910258	0.2714	0.8603	0.1714	0.7780	0.3315	0.7028	0.9582	0.2462	1.0000	0.2334	1.0000	0.1642	1.0000	-0.2162
LOC104910259	1.0000	0.0000	1.0000	0.0000	1.0000	0.0000	1.0000	0.0000	1.0000	0.0000	1.0000	0.0000	1.0000	0.0000
LOC104910260	0.0013	-0.8810	0.0000	-0.7738	0.0000	-1.6068	0.0000	-1.1600	1.0000	-0.0737	1.0000	0.0452	0.4503	0.3799
LOC104910261	0.0034	-0.4742	0.0001	-0.3592	0.3435	0.1931	0.0001	0.3507	1.0000	0.0775	0.1801	0.2045	0.0441	0.2402
LOC104910262	1.0000	0.0000	1.0000	0.0000	1.0000	0.0000	1.0000	0.0000	1.0000	0.0000	1.0000	0.0000	1.0000	0.0000
LOC104910263	1.0000	0.0000	1.0000	0.0000	1.0000	0.0000	1.0000	0.0000	1.0000	0.0000	1.0000	0.0000	1.0000	0.0000
LOC104910264	0.8226	-3.3425	1.0000	-0.1598	0.0790	2.2557	0.0873	2.1944	1.0000	-0.1179	1.0000	3.2002	1.0000	-0.1776
LOC104910265	1.0000	0.0000	1.0000	0.0000	1.0000	2.2425	1.0000	0.0000	1.0000	0.0000	1.0000	0.0000	1.0000	-2.2889
LOC104910266	0.4039	0.4418	0.8620	-0.1602	0.0206	0.8357	0.0010	0.8144	1.0000	0.0554	0.2745	-0.5298	1.0000	0.0411
LOC104910267	0.3494	0.2371	0.0127	0.2980	0.0382	-0.4870	0.0000	-0.5626	1.0000	0.0810	0.6450	0.1542	1.0000	0.0089
LOC104910268	0.0009	0.8884	0.0000	0.9744	0.0008	0.8554	0.0019	0.5920	1.0000	-0.0414	1.0000	0.0554	0.3549	-0.3012
LOC104910269	0.7938	1.1369	1.0000	-0.0117	1.0000	-0.1785	0.0203	-5.0408	1.0000	1.7520	1.0000	0.6250	1.0000	-3.1344
LOC104910270	1.0000	-1.1562	1.0000	0.0000	1.0000	0.3474	0.7710	3.1709	1.0000	-3.2304	1.0000	-2.2956	1.0000	-0.4570
LOC104910271	1.0000	0.1953	1.0000	0.0400	0.5528	1.2884	1.0000	0.0074	1.0000	1.3498	0.9773	1.2168	1.0000	0.0780
LOC104910272	1.0000	0.0000	1.0000	0.0000	1.0000	0.0000	1.0000	0.0000	1.0000	0.0000	1.0000	0.0000	1.0000	0.0000
LOC104910273	0.0000	-1.4297	0.0000	-1.7365	0.7852	-0.1956	0.2958	-0.2736	1.0000	0.0025	0.9043	-0.2942	1.0000	-0.0707
LOC104910274	0.2921	0.2560	0.0047	0.3315	0.0137	0.5003	0.0000	0.4942	1.0000	0.1311	0.3106	0.2197	0.6894	0.1296
LOC104910275	1.0000	0.0000	1.0000	0.0000	1.0000	0.0000	1.0000	0.0000	1.0000	0.0000	1.0000	0.0000	1.0000	0.0000
LOC104910276	0.0018	-1.0663	0.0022	-0.8032	0.8694	0.1605	0.0732	0.4305	1.0000	-0.0937	1.0000	0.1820	0.8937	0.1794
LOC104910277	0.0003	2.0467	0.0000	2.0112	0.0000	3.4695	0.0000	3.3451	1.0000	-0.0450	1.0000	-0.0660	1.0000	-0.1614
LOC104910279	1.0000	0.0000	1.0000	0.0000	1.0000	0.0000	1.0000	0.0000	1.0000	0.0000	1.0000	0.0000	1.0000	0.0000
LOC104910280	0.0750	0.5087	0.0453	0.4529	0.2202	-0.3987	1.0000	-0.0713	1.0000	-0.3645	0.1517	-0.4058	1.0000	-0.0295
LOC104910281	0.6363	-0.3615	0.1285	-0.6795	0.1193	0.7102	0.8633	0.1667	1.0000	0.2986	1.0000	-0.0046	0.9333	-0.2384
LOC104910282	0.0023	0.9079	0.0000	0.8557	0.0000	1.5383	0.0000	1.4270	1.0000	0.1226	1.0000	0.0842	1.0000	0.0175
LOC104910283	0.0535	-3.3582	0.0569	-2.0861	0.3300	0.9190	0.6093	0.5832	1.0000	0.1274	1.0000	1.4325	1.0000	-0.2046
LOC104910284	0.0029	1.3037	0.0097	0.8902	0.0030	1.2473	0.0003	1.1503	1.0000	0.3183	1.0000	-0.0810	0.9274	0.2283
LOC104910285	1.0000	-0.2931	1.0000	0.0000	1.0000	-0.1597	1.0000	0.0000	1.0000	-2.3771	1.0000	-2.2956	1.0000	-2.2908
LOC104910286	0.0000	-2.8604	0.0000	-2.8646	1.0000	0.0156	1.0000	-0.0972	1.0000	-0.1507	1.0000	-0.1423	0.7908	-0.2576
LOC104910287	1.0000	0.0000	1.0000	0.0000	1.0000	0.0000	1.0000	0.0000	1.0000	0.0000	1.0000	0.0000	1.0000	0.0000
LOC104910288	1.0000	0.0000	1.0000	0.0000	1.0000	0.0000	1.0000	0.0000	1.0000	0.0000	1.0000	0.0000	1.0000	0.0000
LOC104910289	1.0000	-2.4788	1.0000	0.0000	1.0000	-2.4061	1.0000	0.0000	1.0000	-2.3771	1.0000	0.0000	1.0000	0.0000

LOC104910291	1.0000	0.3033	1.0000	0.4767	0.7628	-1.4078	0.3945	-2.0639	1.0000	-0.1273	1.0000	0.0584	1.0000	-0.7795
LOC104910292	0.8249	3.0143	0.4662	1.9029	1.0000	0.0000	1.0000	-2.3177	1.0000	2.2675	1.0000	1.2750	1.0000	0.0000
LOC104910294	1.0000	2.1850	1.0000	2.2507	1.0000	2.2425	1.0000	2.3242	1.0000	0.0000	1.0000	0.0495	1.0000	0.0652
LOC104910295	1.0000	0.0000	1.0000	0.0000	0.8033	3.0892	1.0000	0.0000	1.0000	0.0000	1.0000	0.0000	1.0000	-3.1369
LOC104910296	1.0000	0.0000	1.0000	0.0000	1.0000	0.0000	1.0000	0.0000	1.0000	0.0000	1.0000	0.0000	1.0000	0.0000
LOC104910297	1.0000	0.0000	1.0000	0.0000	1.0000	0.0000	1.0000	0.0000	1.0000	0.0000	1.0000	0.0000	1.0000	0.0000
LOC104910298	0.0992	2.0582	0.6654	0.5021	1.0000	-0.7130	0.6311	-0.6757	1.0000	1.8990	1.0000	0.3614	0.4809	1.9503
LOC104910299	0.1000	1.0873	0.8439	0.2086	0.8011	-0.4897	0.0006	-1.6649	1.0000	1.2449	0.8732	0.3820	1.0000	0.0791
LOC104910300	1.0000	0.0000	1.0000	0.0000	1.0000	0.0000	1.0000	0.0000	1.0000	0.0000	1.0000	0.0000	1.0000	0.0000
LOC104910301	0.0233	-1.2536	0.0000	-2.2759	1.0000	-0.0647	0.0001	-1.1263	1.0000	0.5916	0.9729	-0.4192	0.5060	-0.4685
LOC104910302	0.0025	-2.1803	0.0004	-2.5518	1.0000	0.1835	1.0000	0.1607	1.0000	-0.2328	1.0000	-0.5929	1.0000	-0.2504
LOC104910303	1.0000	0.0000	1.0000	0.0000	1.0000	0.0000	1.0000	0.0000	1.0000	0.0000	1.0000	0.0000	1.0000	0.0000
LOC104910304	1.0000	0.0000	1.0000	0.0000	1.0000	0.0000	1.0000	0.0000	1.0000	0.0000	1.0000	0.0000	1.0000	0.0000
LOC104910305	1.0000	2.1850	1.0000	0.0000	1.0000	0.0000	1.0000	0.0000	1.0000	0.0000	1.0000	-2.2956	1.0000	0.0000
LOC104910306	0.2012	4.2368	1.0000	0.0000	1.0000	2.2471	1.0000	0.0000	1.0000	0.0000	0.4711	-4.3669	1.0000	-2.2908
LOC104910307	1.0000	0.1035	1.0000	0.1889	0.4809	0.5693	0.0195	1.1560	1.0000	-0.4287	1.0000	-0.3329	1.0000	0.1588
LOC104910308	1.0000	0.0000	1.0000	0.0000	1.0000	0.0000	1.0000	0.0000	1.0000	0.0000	1.0000	0.0000	1.0000	0.0000
LOC104910309	1.0000	0.0000	1.0000	0.0000	1.0000	0.0000	1.0000	0.0000	1.0000	0.0000	1.0000	0.0000	1.0000	0.0000
LOC104910310	1.0000	0.0000	1.0000	0.0000	1.0000	0.0000	1.0000	0.0000	1.0000	0.0000	1.0000	0.0000	1.0000	0.0000
LOC104910311	1.0000	0.0000	1.0000	0.0000	1.0000	0.0000	1.0000	0.0000	1.0000	0.0000	1.0000	0.0000	1.0000	0.0000
LOC104910313	1.0000	0.0000	1.0000	0.0000	1.0000	0.0000	1.0000	0.0000	1.0000	0.0000	1.0000	0.0000	1.0000	0.0000
LOC104910314	1.0000	-1.2537	1.0000	0.2237	0.2836	-4.1861	0.4370	-3.6968	1.0000	-0.5124	1.0000	0.9853	1.0000	0.0000
LOC104910315	1.0000	-0.7234	1.0000	0.2237	1.0000	-1.1039	0.7799	0.9494	1.0000	-0.5112	1.0000	0.4471	0.8047	1.5487
LOC104910316	0.0401	0.6717	0.4182	0.3444	0.0000	1.4860	0.0000	1.2181	1.0000	-0.1027	0.5720	-0.4166	0.1522	-0.3650
LOC104910318	0.8383	0.2705	0.0119	0.9470	0.3350	-0.6080	0.3862	0.4769	1.0000	-0.6357	1.0000	0.0563	0.7085	0.4591
LOC104910319	1.0000	2.1902	1.0000	0.0000	1.0000	2.2425	1.0000	0.0000	1.0000	0.0000	1.0000	-2.2992	1.0000	-2.2889
LOC104910320	0.0001	-2.4518	0.0000	-2.0717	0.0005	1.3198	0.0012	1.0067	1.0000	0.3475	1.0000	0.7435	1.0000	0.0399
LOC104910321	0.9724	-0.5465	0.6224	0.9851	0.6263	0.7844	1.0000	0.3973	1.0000	-1.4762	1.0000	0.0609	0.1202	-1.8575
LOC104910322	0.4876	0.2960	1.0000	-0.0381	0.0002	-1.2129	0.0006	-0.9292	1.0000	0.0464	0.6774	-0.2780	0.7287	0.3317
LOC104910323	1.0000	0.0000	1.0000	0.0000	1.0000	0.0000	1.0000	2.3242	1.0000	0.0000	1.0000	0.0000	1.0000	2.3543
LOC104910324	0.8226	3.0199	1.0000	0.0000	1.0000	2.2425	0.2785	4.0986	1.0000	0.0000	1.0000	-3.1429	0.9124	1.8420
LOC104910325	1.0000	0.0000	1.0000	0.0000	1.0000	2.2471	0.7701	3.1732	1.0000	0.0000	1.0000	0.0000	1.0000	0.9164
LOC104910326	1.0000	0.0000	1.0000	0.0000	1.0000	2.2471	1.0000	2.3242	1.0000	0.0000	1.0000	0.0000	1.0000	0.0640
LOC104910327	0.0000	2.0225	0.0000	1.5058	0.0143	1.1609	0.2925	0.4524	1.0000	0.5669	1.0000	0.0628	1.0000	-0.1391
LOC104910328	0.0438	1.0433	0.0261	0.8592	0.6331	-0.5273	0.0661	-1.0281	1.0000	0.2342	1.0000	0.0616	1.0000	-0.2599
LOC104910329	1.0000	0.0000	1.0000	0.0000	1.0000	0.0000	1.0000	0.0000	1.0000	0.0000	1.0000	0.0000	1.0000	0.0000
LOC104910330	1.0000	2.1850	1.0000	0.0000	1.0000	0.0000	1.0000	0.0000	1.0000	0.0000	1.0000	-2.2956	1.0000	0.0000
LOC104910331	1.0000	0.0000	1.0000	0.0000	1.0000	0.0000	1.0000	0.0000	1.0000	0.0000	1.0000	0.0000	1.0000	0.0000
LOC104910332	1.0000	2.1902	1.0000	0.0000	1.0000	0.0000	1.0000	0.0000	1.0000	0.0000	1.0000	-2.2992	1.0000	0.0000
LOC104910333	1.0000	0.0000	1.0000	-2.3960	1.0000	0.0000	1.0000	0.0075	1.0000	2.2676	1.0000	0.0000	1.0000	2.3554
LOC104910334	1.0000	0.0000	1.0000	0.0000	1.0000	2.2471	1.0000	0.0000	1.0000	0.0000	1.0000	0.0000	1.0000	-2.2908
LOC104910335	0.8985	0.1107	0.0156	-0.3514	0.7690	0.1477	0.7701	-0.0848	1.0000	-0.0939	0.0006	-0.5441	0.0793	-0.3206
LOC104910336	0.6855	0.3374	0.0000	1.3725	0.8295	0.2486	0.2260	0.5152	1.0000	-0.7366	0.8975	0.3057	0.5130	-0.4633
LOC104910337	0.0288	-2.8956	0.0108	-2.2772	0.0000	2.3342	0.0017	1.3800	1.0000	0.3295	1.0000	0.9628	0.3577	-0.6213
LOC104910338	1.0000	0.5453	0.9762	0.7508	0.2858	2.1449	1.0000	0.5371	1.0000	0.7357	1.0000	0.9709	1.0000	-0.8670
LOC104910339	1.0000	-0.0382	0.6522	0.7855	1.0000	-0.0346	0.5035	1.1298	1.0000	-1.0884	1.0000	-0.2545	1.0000	0.0800
LOC104910340	1.0000	0.0000	1.0000	0.0000	1.0000	0.0000	1.0000	0.0000	1.0000	0.0000	1.0000	0.0000	1.0000	0.0000
LOC104910341	0.6953	0.5503	0.2868	0.9534	0.0803	-2.3736	0.1389	-2.0327	1.0000	-0.2765	1.0000	0.1361	1.0000	0.0716
LOC104910342	1.0000	-0.8561	1.0000	0.0000	0.4809	-3.7967	1.0000	0.0000	1.0000	-3.7629	1.0000	-3.1468	1.0000	0.0000
LOC104910343	1.0000	0.0000	1.0000	0.0000	1.0000	0.0000	1.0000	0.0000	1.0000	0.0000	1.0000	0.0000	1.0000	0.0000
LOC104910344	0.1938	0.6535	0.8260	0.1913	0.3252	-0.6374	0.0038	-1.1547	1.0000	0.2320	1.0000	-0.2188	1.0000	-0.2844
LOC104910345	1.0000	0.1446	0.1448	-1.0251	0.2484	1.2079	0.2406	-0.7572	1.0000	1.8886	0.9525	0.7397	1.0000	-0.0772
LOC104910346	0.2102	1.8484	0.1045	-2.8859	0.7663	1.0386	0.8740	-0.6481	1.0000	1.7587	0.1779	-2.9802	1.0000	0.0765
LOC104910347	1.0000	2.1850	1.0000	-2.3985	1.0000	0.0000	1.0000	-2.3200	1.0000	2.2734	1.0000	-2.2957	1.0000	0.0000
LOC104910348	1.0000	0.0000	1.0000	0.0000	1.0000	0.0000	1.0000	0.0000	1.0000	0.0000	1.0000	0.0000	1.0000	0.0000
LOC104910349	1.0000	0.0000	1.0000	0.0000	1.0000	0.0000	1.0000	0.0000	1.0000	0.0000	1.0000	0.0000	1.0000	0.0000
LOC104910350	0.0065	1.3889	0.0490	0.9008	0.0142	1.3416	0.0154	1.0298	1.0000	0.1261	0.8884	-0.3496	1.0000	-0.1822
LOC104910351	0.0713	0.8823	0.0228	0.6620	1.0000	0.0281	0.0198	-0.8701	1.0000	0.6586	0.4748	0.4497	1.0000	-0.2372
LOC104910352	1.0000	-0.1217	0.9362	0.1348	0.1110	-0.6198	0.0098	-0.7846	1.0000	-0.4769	1.0000	-0.2096	0.2005	-0.6392
LOC104910353	0.0415	1.6057	0.1738	1.0880	0.0122	1.8616	0.1874	1.0654	1.0000	0.2209	1.0000	-0.2839	0.7580	-0.5705
LOC104910354	1.0000	0.0000	1.0000	0.0000	1.0000	0.0000	1.0000	0.0000	1.0000	0.0000	1.0000	0.0000	1.0000	0.0000
LOC104910355	0.4260	0.4683	0.0000	1.3589	0.0000	1.8106	0.0000	1.7746	1.0000	-0.3194	0.1664	0.5877	0.3153	-0.3438
LOC104910356	1.0000	-2.4788	0.9062	1.2235	1.0000	-2.4061	1.0000	-2.3177	1.0000	-0.1089	0.8607	3.7297	1.0000	0.0000
LOC104910357	1.0000	-0.1574	0.2367	0.3062	0.0132	-1.2113	0.0120	-0.6236	1.0000	0.2849	0.0069	0.7609	0.0261	0.8757
LOC104910358	1.0000	0.0000	1.0000	0.0000	1.0000	0.0000	1.0000	0.0000	1.0000	0.0000	1.0000	0.0000	1.0000	0.0000
LOC104910359	1.0000	0.6001	0.6224	1.6101	0.1247	2.1322	0.0065	3.4118	1.0000	-0.9614	1.0000	0.0546	1.0000	0.3156
LOC104910360	0.9545	1.0652	0.2552	4.0066	0.1203	2.7069	0.0490	4.8569	1.0000	-2.3757	1.0000	0.4459	1.0000	-0.2729
LOC104910361	1.0000	0.0603	0.6844	-0.0859	0.6168	-0.1523	0.4340	-0.1135	1.0000	0.0174	0.8899	-0.1164	1.0000	0.0601
LOC104910362	1.0000	2.1850	1.0000	0.0000	0.4809	3.6122	1.0000	2.3256	1.0000	0.0000	1.0000	-2.2957	1.0000	-1.3086
LOC104910363	1.0000	0.0000	1.0000	0.0000	1.0000	0.0000	1.0000	0.0000	1.0000	0.0000	1.0000	0.0000	1.0000	0.0000
LOC104910364	1.0000	-0.3163	0.7674	-3.2498	1.0000	-1.0156	0.7710	-3.1633	1.0000	-0.1218	1.0000	-3.1467	1.0000	-2.2908
LOC104910365	0.8249	3.0143	0.4991	1.3064	0.0144	5.7104	0.0121	2.6701	1.0000	3.1126	0.9075	1.5318	1.0000	0.0961
LOC104910366	0.8249	-3.3439	1.0000	2.2507	1.0000	-1.0167	1.0000	0.0000	1.0000	-3.2319	1.0000	2.3456	1.0000	-2.2907
LOC104910367	1.0000	0.0000	1.0000	0.0000	1.0000	0.0000	1.0000	0.0000	1.0000	0.0000	1.0000	0.0000	1.0000	0.0000
LOC104910368	0.3667	-0.5264	0.0901	-0.5805	0.1793	0.5433	0.0001	0.9700	1.0000	0.4111	0.8620	0.3709	0.0024	0.8457
LOC104910369	1.0000	0.0000	1.0000	2.2508	0.8033	3.0789	0.7701	3.1732	1.0000	0.0000	1.0000	2.3456	1.0000	0.0730
LOC104910370	1.0000	0.0000	1.0000	-2.3986	1.0000	0.0000	1.0000	0.0057	1.0000	2.2734	1.0000	0.0000	1.0000	2.3554
LOC104910371	1.0000	0.1021	1.0000	-0.1090	0.0031	1.0135	0.0050	0.9747	1.0000	-0.1196	0.8607	-0.3154	1.0000	-0.1510
LOC104910372	1.0000	0.0000	1.0000	0.0000	1.0000	0.0000	1.0000	0.0000	1.0000	0.0000	1.0000	0.0000	1.0000	0.0000
LOC104910373	1.0000	0.1611	1.0000	0.3353	0.0057	1.6202	0.0002	2.0172	1.0000	-0.5346	1.0000	-0.3615	1.0000	-0.1342
LOC104910374	0.2705	-1.4673	1.0000	0.0780	0.2384	-1.7005	0.6924	0.8233	1.0000	-0.9453	1.0000	0.6132	0.5737	1.5850
LOC104910375	1.0000	0.0000	1.0000	-2.3986	1.0000	0.0000	1.0000	-2.3200	1.0000	2.2734	1.0000	0.0000	1.0000	0.0000
LOC104910377	1.0000	-2.4788	1.0000	0.0000	1.0000	-0.1597	0.7701	3.1732	1.0000	-2.3771	1.0000	0.0000	1.0000	0.9164
LOC104910378	1.0000	2.1902	1.0000	0.0000	1.0000	0.0000	1.0000	2.3242	1.0000	0.0000	1.0000	-2.2992	1.0000	2.3543
LOC104910379	0.0810	-4.8315	0.9062	-1.5339	0.0000	3.5647	0.0000	3.7740	1.0000	-1.0714	1.0000	2.3480	0.0667	-0.8617
LOC104910381	1.0000	0.0000	0.9943	0.7489	0.8033	3.0892	1.0000	-0.8404	1.0000	3.1056	0.6950	4.1075	1.0000	-0.7815
LOC104910382	0.1793	-0.5652	0.1296	-0.5302	1.0000	-0.0510	0.9120	-0.1355	1.0000	-0.2984	0.9974	-0.2499	0.5383	-0.3760
LOC104910383	1.0000	0.0000	1.0000	0.0000	1.0000	0.0000	1.0000	0.0000	1.0000	0.0000	1.0000	0.0000	1.0000	0.0000
LOC104910384	0.0075	2.9626	0.5077	1.3087	1.0000	0.7306	0.5168	1.4855	1.0000	-0.1245	0.0944	-1.7706	1.0000	0.6363
LOC104910385	0.0632	-2.2670	0.0004	-3.9483	0.0003	1.9491	0.6972	0.4377	1.0000	0.3618	1.0000	-1.3189	0.0278	-1.1425
LOC104910387	0.0442	0.9990	0.0000	1.7871	0.0000	1.8478	0.0000	2.2155	1.0000	-0.5197	0.9428	0.2800	0.9195	-0.1486
LOC104910388	0.1256	0.7217	0.0012	1.1177	0.0032	1.1855	0.0001	1.3061	1.0000	-0.3959	1.0000	0.0105	0.8266	-0.2749
LOC104910389	1.0000	0.0000	1.0000	0.0000	1.0000	0.0000	1.0000	0.0000	1.0000	0.0000	1.0000	0.0000	1.0000	0.0000

LOC104910390	1.0000	0.0000	1.0000	0.0000	1.0000	0.0000	1.0000	0.0000	1.0000	0.0000	1.0000	0.0000	1.0000	0.0000
LOC104910391	0.5221	1.1680	0.1057	2.7242	0.6553	0.9713	0.9096	1.3850	1.0000	-1.4982	1.0000	0.0555	0.8664	-1.0820
LOC104910392	1.0000	0.0000	1.0000	0.0000	1.0000	0.0000	1.0000	0.0000	1.0000	0.0000	1.0000	0.0000	1.0000	0.0000
LOC104910393	0.2548	1.1895	0.1659	1.0879	0.0451	1.8029	0.0176	1.5117	1.0000	0.2256	1.0000	0.1291	1.0000	-0.0718
LOC104910394	1.0000	0.2116	0.7763	0.7745	0.0236	1.9851	0.0342	2.1405	1.0000	-0.5150	1.0000	0.0590	1.0000	-0.3536
LOC104910396	1.0000	0.0000	1.0000	0.0000	1.0000	0.0000	1.0000	0.0000	1.0000	0.0000	1.0000	0.0000	1.0000	0.0000
LOC104910397	0.0054	0.8104	0.0000	0.6804	0.0002	0.8601	0.0019	0.5139	1.0000	0.1914	1.0000	0.0748	0.7792	-0.1482
LOC104910398	1.0000	0.0000	1.0000	0.0000	1.0000	0.0000	1.0000	0.0000	1.0000	0.0000	1.0000	0.0000	1.0000	0.0000
LOC104910399	0.0013	1.0828	0.0000	1.3289	0.0000	1.4663	0.0000	1.0452	1.0000	0.1697	0.1139	0.4318	0.6783	-0.2440
LOC104910400	1.0000	2.1902	1.0000	0.0000	1.0000	2.2425	1.0000	0.0000	1.0000	0.0000	1.0000	-2.2992	1.0000	-2.2889
LOC104910401	0.8226	-3.3425	0.2284	-2.0424	0.7694	1.0409	1.0000	-0.3929	1.0000	1.7497	1.0000	3.1976	1.0000	0.3286
LOC104910402	1.0000	0.0000	1.0000	-2.3960	1.0000	0.0000	1.0000	0.0062	1.0000	2.2676	1.0000	0.0000	1.0000	2.3543
LOC104910403	0.9545	-1.6935	0.9062	-1.5349	0.4809	-3.7970	0.4370	-3.6973	1.0000	-0.1216	1.0000	0.0514	1.0000	0.0000
LOC104910404	0.5086	3.5456	0.7666	-3.2534	0.0938	4.5491	1.0000	0.5371	1.0000	3.1126	0.8607	-3.6732	1.0000	-0.8666
LOC104910405	0.8249	3.0255	0.2542	4.0037	1.0000	2.2471	1.0000	0.0000	1.0000	0.0000	1.0000	0.9709	1.0000	-2.2907
LOC104910406	1.0000	-0.0336	0.2687	0.3266	0.5405	0.2712	0.2991	0.3105	1.0000	-0.2347	1.0000	0.1341	0.8695	-0.1904
LOC104910407	0.8249	-3.3412	0.6224	-1.9244	1.0000	-1.0194	0.2461	-4.0849	1.0000	0.8006	1.0000	2.3480	1.0000	-2.2889
LOC104910408	0.0445	-0.3836	0.0000	-0.4916	0.0000	-0.7519	0.0000	-0.9107	1.0000	-0.0699	0.3322	-0.1656	0.0567	-0.2234
LOC104910409	0.0036	1.0672	0.0001	1.1933	0.0000	-2.3846	0.0101	-1.1604	1.0000	-0.3768	0.9052	-0.2393	0.6749	0.8544
LOC104910410	0.8925	-0.2292	0.0061	-0.7953	1.0000	-0.1362	0.4822	-0.2585	1.0000	0.4382	1.0000	-0.1148	0.7287	0.3181
LOC104910411	1.0000	0.0000	1.0000	2.2534	1.0000	2.2426	1.0000	0.0000	1.0000	0.0000	1.0000	2.3480	1.0000	-2.2889
LOC104910415	1.0000	0.0000	1.0000	0.0000	1.0000	0.0000	1.0000	0.0000	1.0000	0.0000	1.0000	0.0000	1.0000	0.0000
LOC104910416	1.0000	0.0000	1.0000	-0.1452	1.0000	0.0000	1.0000	-2.3200	1.0000	2.2735	1.0000	2.3480	1.0000	0.0000
LOC104910419	1.0000	0.0000	1.0000	2.2534	1.0000	0.0000	1.0000	0.0000	1.0000	0.0000	1.0000	2.3480	1.0000	0.0000
LOC104910420	1.0000	0.0000	1.0000	0.0000	1.0000	0.0000	1.0000	0.0000	1.0000	0.0000	1.0000	0.0000	1.0000	0.0000
LOC104910421	1.0000	0.0000	1.0000	0.0000	1.0000	0.0000	1.0000	0.0000	1.0000	0.0000	1.0000	0.0000	1.0000	0.0000
LOC104910422	1.0000	0.0000	1.0000	2.2533	1.0000	0.0000	1.0000	0.0000	1.0000	0.0000	1.0000	2.3480	1.0000	0.0000
LOC104910423	1.0000	-0.0384	0.1496	1.8720	1.0000	-1.1065	0.3714	1.7021	1.0000	-1.0431	1.0000	0.8769	0.6583	1.7692
LOC104910424	1.0000	0.0000	1.0000	0.0000	1.0000	0.0000	1.0000	2.3242	1.0000	0.0000	1.0000	0.0000	1.0000	2.3543
LOC104910425	0.2507	0.4148	0.0347	0.4977	0.1277	0.4751	0.2833	0.3028	1.0000	-0.1621	1.0000	-0.0653	0.3615	-0.3258
LOC104910426	0.0062	1.8593	0.1756	1.4665	0.3756	0.9132	1.0000	0.0066	1.0000	-1.5300	0.0007	-1.9198	0.0051	-2.4349
LOC104910427	0.4026	0.9409	0.1713	1.2318	0.0361	1.6266	0.0068	1.8072	1.0000	-0.3152	1.0000	-0.0127	1.0000	-0.1316
LOC104910428	1.0000	2.1902	1.0000	2.2507	1.0000	2.2471	1.0000	2.3256	1.0000	0.0000	1.0000	0.0471	1.0000	0.0649
LOC104910429	1.0000	0.0000	1.0000	0.0000	1.0000	0.0000	1.0000	0.0000	1.0000	0.0000	1.0000	0.0000	1.0000	0.0000
LOC104910430	1.0000	0.0000	1.0000	0.0000	1.0000	0.0000	1.0000	0.0000	1.0000	0.0000	1.0000	0.0000	1.0000	0.0000
LOC104910431	1.0000	-0.1193	0.9362	-0.7357	0.0026	1.7766	1.0000	-0.3399	1.0000	-0.4254	0.7554	-1.0261	0.0000	-2.5301
LOC104910432	1.0000	0.0000	1.0000	0.0000	1.0000	0.0000	1.0000	0.0000	1.0000	0.0000	1.0000	0.0000	1.0000	0.0000
LOC104910433	1.0000	0.0000	1.0000	0.0000	1.0000	0.0000	1.0000	0.0000	1.0000	0.0000	1.0000	0.0000	1.0000	0.0000
LOC104910434	1.0000	0.0000	1.0000	0.0000	1.0000	0.0000	1.0000	0.0000	1.0000	0.0000	1.0000	0.0000	1.0000	0.0000
LOC104910436	1.0000	2.1850	1.0000	0.0000	1.0000	0.0000	1.0000	2.3256	1.0000	0.0000	1.0000	-2.2957	1.0000	2.3555
LOC104910437	1.0000	0.0000	0.7674	3.0888	1.0000	0.0000	1.0000	0.0000	1.0000	0.0000	1.0000	3.1946	1.0000	0.0000
LOC104910438	1.0000	0.0000	1.0000	0.0000	1.0000	0.0000	1.0000	0.0000	1.0000	0.0000	1.0000	0.0000	1.0000	0.0000
LOC104910439	1.0000	0.0000	1.0000	0.0000	1.0000	0.0000	1.0000	0.0000	1.0000	0.0000	1.0000	0.0000	1.0000	0.0000
LOC104910440	1.0000	0.0000	1.0000	0.0000	1.0000	0.0000	1.0000	0.0000	1.0000	0.0000	1.0000	0.0000	1.0000	0.0000
LOC104910441	1.0000	0.0000	1.0000	0.0000	1.0000	0.0000	1.0000	0.0000	1.0000	0.0000	1.0000	0.0000	1.0000	0.0000
LOC104910442	1.0000	0.0000	1.0000	0.0000	1.0000	0.0000	1.0000	0.0000	1.0000	0.0000	1.0000	0.0000	1.0000	0.0000
LOC104910443	0.1851	4.4812	0.0758	4.5597	1.0000	2.2471	1.0000	2.3242	1.0000	0.0000	1.0000	0.0590	1.0000	0.0640
LOC104910444	0.8249	-3.3439	0.2495	-4.1757	1.0000	-0.1754	0.9786	-0.9107	1.0000	0.7956	1.0000	0.0000	1.0000	0.0716
LOC104910445	0.1161	0.7023	0.1684	0.5146	1.0000	-0.1652	0.0087	-1.1528	1.0000	-0.0658	1.0000	-0.2405	0.0986	-1.0478
LOC104910446	0.3247	2.2123	0.6215	1.6060	1.0000	-0.1597	1.0000	-2.3199	1.0000	-0.1045	1.0000	-0.7112	1.0000	-2.2907
LOC104910447	1.0000	0.0000	1.0000	0.0000	1.0000	0.0000	1.0000	0.0000	1.0000	0.0000	1.0000	0.0000	1.0000	0.0000
LOC104910448	0.7079	0.7303	1.0000	-0.0516	0.5705	-1.6609	1.0000	-0.3516	1.0000	0.4513	1.0000	-0.3120	0.6247	1.7634
LOC104910449	1.0000	-2.4776	0.7666	-3.2534	1.0000	-0.1625	1.0000	-0.8416	1.0000	0.7365	1.0000	0.0000	1.0000	0.0661
LOC104910450	0.0087	0.9702	0.0000	1.2321	0.0000	2.0238	0.0000	1.7025	1.0000	-0.2434	1.0000	0.0331	0.0311	-0.5566
LOC104910451	0.3837	-0.3111	0.0239	-0.4437	0.5170	0.2266	0.6187	0.1430	1.0000	0.0630	1.0000	-0.0575	1.0000	-0.0161
LOC104910453	1.0000	0.0000	1.0000	0.0000	1.0000	0.0000	1.0000	0.0000	1.0000	0.0000	1.0000	0.0000	1.0000	0.0000
LOC104910454	1.0000	0.0000	1.0000	0.0000	1.0000	0.0000	1.0000	0.0000	1.0000	0.0000	1.0000	0.0000	1.0000	0.0000
LOC104910455	1.0000	0.0000	1.0000	0.0000	1.0000	0.0000	1.0000	0.0000	1.0000	0.0000	1.0000	0.0000	1.0000	0.0000
LOC104910456	0.1383	-0.4645	0.0158	-0.4827	0.0006	0.8031	0.0000	0.8622	1.0000	0.0758	1.0000	0.0695	0.8234	0.1398
LOC104910457	1.0000	0.3586	1.0000	0.3841	0.0808	1.5681	0.0134	2.1101	1.0000	-0.6829	1.0000	-0.6527	1.0000	-0.1385
LOC104910458	1.0000	0.0000	1.0000	0.0000	1.0000	0.0000	1.0000	0.0000	1.0000	0.0000	1.0000	0.0000	1.0000	0.0000
LOC104910459	0.1298	0.8461	0.1341	0.5599	0.0000	1.6876	0.0000	1.3744	1.0000	0.2176	1.0000	-0.0567	1.0000	-0.0894
LOC104910460	1.0000	0.0000	0.9062	-1.5349	0.0535	4.7653	1.0000	0.6976	1.0000	3.6423	1.0000	2.3480	1.0000	-0.3886
LOC104910461	1.0000	0.0000	1.0000	0.0000	1.0000	0.0000	1.0000	2.3242	1.0000	0.0000	1.0000	0.0000	1.0000	2.3543
LOC104910463	0.1347	-3.0507	0.0400	-2.4937	0.7813	-0.8449	0.3772	0.7815	1.0000	0.3205	1.0000	0.8992	0.0767	1.9528
LOC104910464	1.0000	-1.1537	0.7674	3.0888	1.0000	-0.1789	1.0000	2.3256	1.0000	-3.2304	1.0000	0.8962	1.0000	-0.7793
LOC104910465	1.0000	-0.3458	0.5019	-1.6409	1.0000	-0.8812	1.0000	-0.5490	1.0000	0.1235	1.0000	-1.1654	1.0000	0.4629
LOC104910466	0.0331	1.9685	0.8669	0.4855	0.0154	2.1394	0.0116	1.5202	1.0000	0.8339	0.8605	-0.6323	1.0000	0.2321
LOC104910467	0.8034	0.3218	0.6488	0.3017	0.0000	1.9256	0.0000	1.3400	1.0000	0.0635	1.0000	0.0602	0.1306	-0.5125
LOC104910468	1.0000	0.0000	1.0000	0.0000	1.0000	0.0000	1.0000	0.0000	1.0000	0.0000	1.0000	0.0000	1.0000	0.0000
LOC104910469	0.0041	0.7070	0.0000	1.0647	0.0408	0.5242	0.0000	0.7878	1.0000	-0.1458	0.5145	0.2253	0.9194	0.1269
LOC104910470	0.0822	1.3368	0.6952	0.4670	0.2605	0.9672	0.4357	0.6502	1.0000	0.2111	0.7635	-0.6452	1.0000	-0.1018
LOC104910471	1.0000	2.1902	1.0000	0.0000	1.0000	0.0000	1.0000	0.0000	1.0000	0.0000	1.0000	-2.2992	1.0000	0.0000
LOC104910472	0.4119	0.3990	0.0022	0.7292	0.0000	1.6401	0.0000	1.8998	1.0000	0.2127	0.1014	0.5568	0.0257	0.4787
LOC104910473	0.9464	-0.5683	0.1077	-1.2412	0.4704	1.0416	0.6950	0.4380	1.0000	1.1178	1.0000	0.4600	0.7940	0.5129
LOC104910474	0.0430	0.7824	0.0014	0.8773	0.0000	1.3779	0.0000	1.1451	1.0000	0.2674	0.5924	0.3749	1.0000	0.0398
LOC104910476	0.5780	-1.5569	0.2517	-2.4819	0.0000	3.1998	0.1185	1.3853	1.0000	0.1246	1.0000	-0.7966	0.0007	-1.6854
LOC104910477	1.0000	0.0000	1.0000	0.0000	1.0000	0.0000	1.0000	0.0000	1.0000	0.0000	1.0000	0.0000	1.0000	0.0000
LOC104910479	0.0349	2.6767	0.3694	1.0094	0.0038	2.9742	0.0399	1.6299	1.0000	1.3485	1.0000	-0.2978	1.0000	0.0084
LOC104910481	0.0288	1.1464	0.0005	1.5511	0.0238	1.1116	0.0000	1.8587	1.0000	-0.4959	1.0000	-0.0817	0.8941	0.2534
LOC104910482	1.0000	0.0184	0.7304	0.0792	0.9951	-0.0659	0.7771	-0.0684	1.0000	0.0003	1.0000	0.0731	1.0000	0.0031
LOC104910483	1.0000	0.0000	1.0000	0.0000	1.0000	0.0000	1.0000	0.0000	1.0000	0.0000	1.0000	0.0000	1.0000	0.0000
LOC104910484	0.5086	3.5431	1.0000	2.2507	1.0000	0.0000	0.7710	3.1709	1.0000	0.0000	1.0000	-1.3254	1.0000	3.2049
LOC104910485	1.0000	0.0000	1.0000	0.0000	1.0000	0.0000	1.0000	0.0000	1.0000	0.0000	1.0000	0.0000	1.0000	0.0000
LOC104910488	0.0010	2.2072	0.0000	2.4776	0.0000	3.2415	0.0000	2.7017	1.0000	-0.0176	1.0000	0.2689	0.1223	-0.5439
LOC104910489	1.0000	0.0000	1.0000	0.0000	1.0000	0.0000	1.0000	0.0000	1.0000	0.0000	1.0000	0.0000	1.0000	0.0000
LOC104910490	1.0000	0.0000	1.0000	0.0000	1.0000	0.0000	1.0000	0.0000	1.0000	0.0000	1.0000	0.0000	1.0000	0.0000
LOC104910491	1.0000	0.0000	1.0000	0.0000	1.0000	0.0000	1.0000	0.0000	1.0000	0.0000	1.0000	0.0000	1.0000	0.0000
LOC104910492	1.0000	0.0000	1.0000	0.0000	1.0000	0.0000	1.0000	0.0000	1.0000	0.0000	1.0000	0.0000	1.0000	0.0000
LOC104910493	1.0000	0.0117	0.0057	1.0372	0.0014	1.2313	0.0000	2.3297	1.0000	-1.3095	0.9640	-0.2675	0.7526	-0.2032
LOC104910494	1.0000	-0.1211	0.0162	0.5945	0.0027	0.9919	0.0000	1.7938	1.0000	-0.6249	1.0000	0.1043	0.6687	0.1837
LOC104910495	1.0000	0.0000	1.0000	2.2533	1.0000	2.2471	1.0000	0.0000	1.0000	0.0000	1.0000	2.3480	1.0000	-2.2907
LOC104910496	0.6994	-1.2896	0.0143	2.2348	0.8297	-1.1335	0.2794	1.5159	1.0000	-1.0650	0.0698	2.4676	0.5565	1.5862

LOC104910497	0.0010	-6.0222	0.0066	-3.4535	0.7515	-0.5385	0.6012	-0.7488	1.0000	-0.3490	1.0000	2.3480	1.0000	-0.5543
LOC104910498	1.0000	2.1850	0.1467	1.8706	1.0000	2.2471	0.9786	0.9243	1.0000	3.1126	0.2582	2.9422	0.8937	1.8362
LOC104910500	1.0000	0.0000	1.0000	0.0000	1.0000	0.0000	1.0000	0.0000	1.0000	0.0000	1.0000	0.0000	1.0000	0.0000
LOC104910501	1.0000	0.0000	0.7674	3.0888	1.0000	0.0000	1.0000	0.0000	1.0000	0.0000	1.0000	3.1946	1.0000	0.0000
LOC104910503	0.6048	-0.5774	0.4979	-0.6685	0.4174	-0.7755	1.0000	-0.1694	1.0000	-0.4360	1.0000	-0.5144	1.0000	0.1777
LOC104910506	1.0000	0.0000	1.0000	0.0000	1.0000	0.0000	1.0000	0.0000	1.0000	0.0000	1.0000	0.0000	1.0000	0.0000
LOC104910507	1.0000	0.0000	1.0000	0.0000	1.0000	0.0000	1.0000	0.0000	1.0000	0.0000	1.0000	0.0000	1.0000	0.0000
LOC104910508	0.8226	3.0199	1.0000	2.2534	1.0000	2.2425	1.0000	2.3242	1.0000	0.0000	1.0000	-0.7956	1.0000	0.0652
LOC104910509	1.0000	0.0000	1.0000	0.0000	1.0000	0.0000	1.0000	0.0000	1.0000	0.0000	1.0000	0.0000	1.0000	0.0000
LOC104910510	1.0000	0.0000	1.0000	0.0000	1.0000	0.0000	1.0000	0.0000	1.0000	0.0000	1.0000	0.0000	1.0000	0.0000
LOC104910511	0.0212	2.1668	1.0000	-0.0282	0.5246	1.0774	0.1350	1.1380	1.0000	0.9928	0.3216	-1.1849	0.4563	1.0621
LOC104910512	1.0000	0.0000	1.0000	0.0000	1.0000	0.0000	1.0000	0.0000	1.0000	0.0000	1.0000	0.0000	1.0000	0.0000
LOC104910513	1.0000	0.0000	1.0000	0.0000	1.0000	0.0000	1.0000	0.0000	1.0000	0.0000	1.0000	0.0000	1.0000	0.0000
LOC104910514	1.0000	0.0000	1.0000	0.0000	1.0000	0.0000	1.0000	0.0000	1.0000	0.0000	1.0000	0.0000	1.0000	0.0000
LOC104910515	0.5086	3.5431	0.2495	4.0050	1.0000	0.0000	1.0000	0.0000	1.0000	0.0000	1.0000	0.4448	1.0000	0.0000
LOC104910516	1.0000	0.0000	1.0000	0.0000	1.0000	0.0000	1.0000	0.0000	1.0000	0.0000	1.0000	0.0000	1.0000	0.0000
LOC104910517	1.0000	0.0000	1.0000	0.0000	1.0000	0.0000	1.0000	0.0000	1.0000	0.0000	1.0000	0.0000	1.0000	0.0000
LOC104910518	1.0000	0.0000	1.0000	0.0000	1.0000	0.0000	1.0000	0.0000	1.0000	0.0000	1.0000	0.0000	1.0000	0.0000
LOC104910519	1.0000	0.0000	1.0000	0.0000	1.0000	0.0000	1.0000	0.0000	1.0000	0.0000	1.0000	0.0000	1.0000	0.0000
LOC104910520	1.0000	0.0000	1.0000	0.0000	1.0000	0.0000	1.0000	0.0000	1.0000	0.0000	1.0000	0.0000	1.0000	0.0000
LOC104910521	1.0000	0.0000	1.0000	2.2507	1.0000	2.2425	1.0000	0.0000	1.0000	0.0000	1.0000	2.3456	1.0000	-2.2889
LOC104910522	1.0000	-0.2892	1.0000	0.0000	1.0000	-0.1597	1.0000	0.0000	1.0000	-2.3771	1.0000	-2.2992	1.0000	-2.2907
LOC104910523	1.0000	0.0000	1.0000	0.0000	1.0000	0.0000	1.0000	0.0000	1.0000	0.0000	1.0000	0.0000	1.0000	0.0000
LOC104910524	1.0000	0.0000	1.0000	0.0000	1.0000	0.0000	1.0000	0.0000	1.0000	0.0000	1.0000	0.0000	1.0000	0.0000
LOC104910525	0.0001	-2.5933	0.0016	-1.8388	0.1013	-1.0297	0.3713	-0.5960	1.0000	-0.3585	1.0000	0.4107	1.0000	0.0806
LOC104910526	1.0000	0.5815	1.0000	-0.1682	1.0000	-1.0167	1.0000	-0.5236	1.0000	0.4112	1.0000	-0.3291	1.0000	0.9169
LOC104910527	1.0000	0.0000	1.0000	0.0000	1.0000	0.0000	1.0000	0.0000	1.0000	0.0000	1.0000	0.0000	1.0000	0.0000
LOC104910529	0.5391	-0.8327	1.0000	-0.4239	0.7485	0.5063	0.7228	0.7210	1.0000	-1.3203	0.9238	-0.9052	0.3073	-1.1016
LOC104910530	0.1995	-1.3302	0.0629	-2.0835	1.0000	0.2357	1.0000	-0.1152	1.0000	-0.3505	0.9847	-1.0954	0.6707	-0.6951
LOC104910531	1.0000	-0.2920	1.0000	-0.1472	1.0000	-2.4055	1.0000	0.0057	1.0000	-0.1034	1.0000	0.0495	1.0000	2.3554
LOC104910532	0.3181	-2.3898	0.4431	-3.7875	1.0000	-0.8839	0.4370	-3.6973	1.0000	-0.8150	1.0000	-2.2957	0.7477	-3.6583
LOC104910533	0.0153	-2.3723	0.0076	-2.5783	0.7062	-0.5782	0.5946	-0.6075	1.0000	-0.1388	1.0000	-0.3300	1.0000	-0.1565
LOC104910534	1.0000	-0.3452	0.4437	-1.0227	0.0009	2.4670	0.0095	1.4814	1.0000	0.9719	1.0000	0.3117	1.0000	-0.0086
LOC104910535	1.0000	0.0000	0.1388	-4.4811	1.0000	2.2426	0.3949	-2.0636	1.0000	4.3298	1.0000	0.0000	1.0000	0.0661
LOC104910536	1.0000	0.0000	1.0000	-2.3985	1.0000	0.0000	1.0000	-2.3199	1.0000	2.2734	1.0000	0.0000	1.0000	0.0000
LOC104910537	0.7616	-0.5398	0.2786	-0.8762	1.0000	-0.2839	0.3526	-0.8246	1.0000	-0.0487	1.0000	-0.3759	0.8521	-0.5846
LOC104910538	1.0000	-0.0669	0.0062	-0.4574	0.7980	-0.1437	1.0000	0.0299	1.0000	0.0925	0.3958	-0.2857	0.2488	0.2694
LOC104910539	1.0000	0.5414	1.0000	0.5210	1.0000	-2.4061	0.9096	-1.3717	1.0000	1.2628	1.0000	1.2774	1.0000	2.3554
LOC104910540	0.8249	3.0255	0.7674	-3.2498	1.0000	0.0000	0.7710	-3.1633	1.0000	3.1056	1.0000	-3.1467	1.0000	0.0000
LOC104910541	1.0000	-0.7252	1.0000	-0.8597	0.3227	-4.1836	0.1318	-4.3887	1.0000	0.1796	1.0000	0.0603	1.0000	0.0000
LOC104910542	1.0000	-0.0938	1.0000	-0.5543	1.0000	0.0567	1.0000	0.0073	1.0000	-0.4312	1.0000	-0.8853	1.0000	-0.4783
LOC104910543	1.0000	2.1902	0.4431	3.6191	1.0000	0.0000	1.0000	2.3242	1.0000	0.0000	1.0000	1.4306	1.0000	2.3543
LOC104910544	1.0000	0.0000	1.0000	0.0000	1.0000	2.2426	1.0000	0.0000	1.0000	0.0000	1.0000	0.0000	1.0000	-2.2889
LOC104910545	1.0000	0.0000	1.0000	2.2507	1.0000	2.2471	1.0000	0.0000	1.0000	0.0000	1.0000	2.3456	1.0000	-2.2907
LOC104910546	1.0000	0.0000	1.0000	0.0000	1.0000	0.0000	1.0000	0.0000	1.0000	0.0000	1.0000	0.0000	1.0000	0.0000
LOC104910547	0.0049	-0.5996	0.0000	-0.8738	0.0000	-1.0576	0.0000	-1.0858	1.0000	0.1681	1.0000	-0.0936	0.8937	0.1463
LOC104910548	0.0191	-5.3955	1.0000	-0.7173	0.0230	-5.3098	0.5078	-1.4647	1.0000	-0.6982	0.6861	4.1255	1.0000	3.2066
LOC104910550	0.6881	1.4496	1.0000	-2.3985	1.0000	-0.1630	1.0000	-2.3199	1.0000	-0.1045	0.6427	-4.0589	1.0000	-2.2889
LOC104910551	0.0019	-0.8737	0.0000	-0.9622	0.0790	-0.5201	0.2256	-0.1908	1.0000	0.0853	1.0000	0.0100	0.0350	0.4186
LOC104910552	0.0000	-2.9790	0.0000	-2.3578	0.8322	0.1372	0.2424	-0.2455	1.0000	-0.3246	1.0000	0.3094	0.0007	-0.7027
LOC104910553	1.0000	-0.2021	1.0000	0.1496	0.1817	-0.9790	0.1013	-0.9356	1.0000	0.1166	0.8029	0.4843	1.0000	0.1696
LOC104910555	0.5391	-3.8767	1.0000	2.2534	0.9468	-1.5484	1.0000	0.0000	1.0000	-3.7606	1.0000	2.3480	1.0000	-2.2907
LOC104910556	1.0000	0.0000	1.0000	2.2507	1.0000	0.0000	0.1321	4.3960	1.0000	0.0000	1.0000	2.3455	0.3369	4.4317
LOC104910557	1.0000	-2.4776	1.0000	-0.1429	1.0000	-2.4055	1.0000	-2.3177	1.0000	-0.1078	1.0000	2.3480	1.0000	0.0000
LOC104910558	0.0604	-0.5423	0.0891	-0.3239	0.0000	-1.4114	0.0000	-1.1534	1.0000	-0.2963	1.0000	-0.0639	1.0000	-0.0313
LOC104910559	1.0000	0.5381	1.0000	-1.0008	0.4426	1.8953	0.0413	2.4546	1.0000	0.7365	1.0000	-0.7919	0.5802	1.3024
LOC104910560	0.6881	1.7520	0.4649	1.3304	0.4424	1.8936	0.1877	1.6643	1.0000	1.2635	1.0000	0.8712	0.8110	1.0438
LOC104910561	0.2268	0.9284	0.8722	0.3287	0.5719	-0.7140	0.0008	-2.3650	1.0000	0.9048	1.0000	0.3133	0.9571	-0.7387
LOC104910563	0.1699	1.9514	0.0007	2.3515	0.7408	0.9199	1.0000	0.4756	1.0000	0.1759	0.8528	0.5813	1.0000	-0.2660
LOC104910564	1.0000	-0.7243	1.0000	-0.1429	1.0000	-0.5754	1.0000	0.8545	1.0000	-1.8822	1.0000	-1.3257	1.0000	-0.4568
LOC104910565	0.0208	1.2397	0.0061	1.1581	0.0764	-1.6137	0.0433	-1.4237	1.0000	0.0790	1.0000	0.0102	1.0000	0.2800
LOC104910566	0.0278	0.9790	0.0006	1.1112	0.2773	-0.7151	0.0279	-1.1039	1.0000	0.0343	1.0000	0.1766	1.0000	-0.3504
LOC104910568	0.5086	0.3681	0.1230	0.5244	0.0189	-1.1140	0.0315	-0.8206	1.0000	-0.0266	1.0000	0.1419	1.0000	0.2718
LOC104910571	0.1435	0.9481	0.0156	1.0260	0.2407	0.7497	0.7302	-0.3833	1.0000	0.3334	0.8022	0.4254	0.4015	-0.7921
LOC104910573	0.1421	0.6382	0.8580	0.1954	1.0000	-0.1463	0.4541	-0.3631	1.0000	-0.0583	0.4531	-0.4857	0.9124	-0.2661
LOC104910574	0.3405	-0.3608	0.3971	-0.2870	0.1339	-0.4879	0.9837	0.1022	1.0000	-0.4006	0.6728	-0.3156	0.9264	0.1913
LOC104910575	0.8249	-3.3439	1.0000	0.0000	1.0000	-1.0206	1.0000	0.0000	1.0000	-3.2319	1.0000	0.0000	1.0000	-2.2889
LOC104910576	0.2814	0.8830	0.0034	1.5743	0.0000	2.3604	0.0000	2.3073	1.0000	-0.2519	0.8269	0.4603	0.8110	-0.2947
LOC104910577	0.2072	-2.2114	0.9062	-1.5359	0.0727	1.5193	0.0080	2.4148	1.0000	-1.4681	1.0000	-0.7966	0.7380	-0.5767
LOC104910578	1.0000	-2.4788	1.0000	0.0000	0.1945	2.3621	1.0000	0.0000	1.0000	-2.3771	1.0000	0.0000	0.1366	-4.8207
LOC104910579	0.6923	-2.0829	0.4148	-2.2334	0.3991	1.2125	1.0000	0.0059	1.0000	0.1845	1.0000	0.0495	0.6819	-1.0214
LOC104910580	1.0000	2.1902	1.0000	-0.1449	1.0000	2.2471	1.0000	-2.3177	1.0000	2.2676	1.0000	0.0471	1.0000	-2.2907
LOC104910581	1.0000	0.0000	0.0781	4.5608	1.0000	2.2426	0.0325	5.0558	1.0000	0.0000	0.3216	4.6747	0.2994	2.8014
LOC104910582	1.0000	2.1850	1.0000	0.0000	1.0000	0.0000	1.0000	0.0000	1.0000	0.0000	1.0000	-2.2957	1.0000	0.0000
LOC104910583	1.0000	-2.4776	1.0000	0.0000	1.0000	-2.4056	1.0000	2.3256	1.0000	-2.3757	1.0000	0.0000	1.0000	2.3554
LOC104910584	0.3179	-0.7624	0.0000	-2.2518	0.6508	-0.4365	0.2367	-0.6044	1.0000	0.2413	0.3260	-1.2369	1.0000	0.0812
LOC104910585	1.0000	0.0000	1.0000	0.0000	1.0000	0.0000	1.0000	0.0000	1.0000	0.0000	1.0000	0.0000	1.0000	0.0000
LOC104910586	1.0000	0.0000	1.0000	0.0000	1.0000	0.0000	1.0000	0.0000	1.0000	0.0000	1.0000	0.0000	1.0000	0.0000
LOC104910587	1.0000	0.0000	1.0000	-0.9973	0.8033	3.0789	1.0000	0.5377	1.0000	3.1056	1.0000	2.3480	1.0000	0.6046
LOC104910588	0.3736	0.2611	0.0000	0.4619	0.8917	0.1090	0.0476	-0.2533	1.0000	0.0336	0.1473	0.2461	0.0836	-0.3222
LOC104910590	0.0000	-2.3110	0.0000	-2.4689	0.0000	-0.7979	0.0000	-0.8662	1.0000	-0.1206	0.5247	-0.2663	0.4580	-0.1833
LOC104910593	1.0000	-2.4788	0.7666	-3.2534	1.0000	-0.1597	0.9786	0.9243	1.0000	0.7357	1.0000	0.0000	0.8937	1.8362
LOC104910594	1.0000	0.0215	0.2972	-0.5913	0.0056	-1.8671	0.0004	-2.1463	1.0000	0.1648	0.9320	-0.4367	1.0000	-0.1091
LOC104910596	1.0000	0.5856	0.4431	-3.7876	0.0328	2.4749	0.1877	1.6641	1.0000	0.4115	0.6427	-4.0589	1.0000	-0.3958
LOC104910597	1.0000	0.0000	1.0000	0.0000	1.0000	0.0000	1.0000	0.0000	1.0000	0.0000	1.0000	0.0000	1.0000	0.0000
LOC104910598	1.0000	0.0000	1.0000	0.0000	1.0000	0.0000	1.0000	0.0000	1.0000	0.0000	1.0000	0.0000	1.0000	0.0000
LOC104910599	0.6927	1.4475	0.2495	4.0050	0.9361	1.2064	0.7701	3.1732	1.0000	-2.3757	1.0000	0.0588	1.0000	-0.4570
LOC104910600	1.0000	-2.4776	1.0000	0.0000	1.0000	-2.4056	1.0000	0.0000	1.0000	-2.3757	1.0000	0.0000	1.0000	0.0000
LOC104910601	0.0000	1.5522	0.0000	0.8544	0.4967	0.1997	0.5487	-0.1074	1.0000	0.0677	0.0000	-0.6179	0.2314	-0.2338
LOC104910602	0.3089	3.9281	0.1388	4.3092	0.0060	5.5215	0.0711	4.6474	1.0000	0.0000	1.0000	0.3634	0.7256	-0.8924
LOC104910603	1.0000	0.0000	1.0000	0.0000	1.0000	0.0000	1.0000	0.0000	1.0000	0.0000	1.0000	0.0000	1.0000	0.0000
LOC104910604	0.0497	-1.2608	0.0002	-1.8950	0.4618	0.5152	1.0000	-0.1043	1.0000	0.1884	1.0000	-0.4333	0.7644	-0.4287
LOC104910605	1.0000	0.5414	1.0000	0.2177	0.0786	2.8533	1.0000	-0.5236	1.0000	1.2628	1.0000	0.9730	0.2549	-2.1107

LOC104910606	0.0000	-1.1799	0.0000	-0.7985	0.0000	0.8872	0.0000	0.9496	1.0000	-0.1915	0.8052	0.2036	0.7323	-0.1230
LOC104910607	1.0000	0.0265	0.2445	0.2523	0.0000	1.1885	0.0000	1.1931	1.0000	-0.0462	0.8149	0.1928	1.0000	-0.0357
LOC104910608	0.0126	1.5437	0.3080	0.4113	0.0000	3.3566	0.0000	1.9105	1.0000	1.1307	1.0000	0.0094	0.5406	-0.3151
LOC104910609	0.0145	1.5514	0.0271	1.0269	0.7380	-0.5556	0.0899	-1.2110	1.0000	0.3806	1.0000	-0.1274	1.0000	-0.2622
LOC104910610	0.8366	0.9078	0.1542	2.3762	1.0000	-1.0194	1.0000	-2.3177	1.0000	-0.9614	1.0000	0.5196	1.0000	-2.2889
LOC104910611	0.9893	-0.5022	0.4746	-1.2947	0.1664	-1.8474	0.6831	-0.8102	1.0000	-0.2778	0.8840	-1.0624	1.0000	0.7671
LOC104910612	1.0000	0.0000	1.0000	0.0000	1.0000	2.2426	1.0000	0.0000	1.0000	0.0000	1.0000	0.0000	1.0000	-2.2889
LOC104910613	1.0000	0.0000	1.0000	2.2507	1.0000	0.0000	1.0000	0.0000	1.0000	0.0000	1.0000	2.3456	1.0000	0.0000
LOC104910615	1.0000	-1.1561	0.1541	-2.6962	1.0000	0.7338	0.1477	-2.5295	1.0000	1.5649	1.0000	0.0495	0.8937	-1.6949
LOC104910616	0.0046	-1.7053	0.5478	-0.4844	0.0004	-2.1697	0.2392	-0.7764	1.0000	-0.5969	0.9421	0.6370	0.8238	0.8010
LOC104910617	0.1418	1.2999	0.0052	1.5009	0.0000	3.4747	0.0000	2.8727	1.0000	0.2734	0.8269	0.4864	0.5941	-0.3235
LOC104910618	0.6927	1.4466	1.0000	-2.3960	1.0000	-0.1597	1.0000	0.0075	1.0000	-0.1089	0.6450	-4.0575	1.0000	0.0649
LOC104910619	1.0000	-0.3358	1.0000	0.3659	0.8184	0.7561	0.2178	1.8814	1.0000	-0.6504	1.0000	0.0569	1.0000	0.4786
LOC104910620	0.8137	0.3927	0.0462	0.9001	0.0000	1.9570	0.0000	1.9171	1.0000	-0.3992	1.0000	0.1194	0.3114	-0.4342
LOC104910621	0.0359	0.6181	0.0034	0.4944	0.0946	0.4556	0.0138	0.4370	1.0000	0.1070	1.0000	-0.0018	1.0000	0.0953
LOC104910622	0.5086	3.5456	1.0000	0.0000	1.0000	0.0000	1.0000	0.0000	1.0000	0.0000	0.8607	-3.6732	1.0000	0.0000
LOC104910623	0.8516	0.0834	0.8523	0.0443	0.0001	-0.6289	0.0000	-0.6261	1.0000	-0.1068	0.5138	-0.1336	0.7864	-0.0982
LOC104910624	1.0000	0.0000	1.0000	0.0000	1.0000	0.0000	1.0000	0.0000	1.0000	0.0000	1.0000	0.0000	1.0000	0.0000
LOC104910625	0.6998	0.2042	0.1432	0.3541	0.0042	-0.8313	0.0002	-0.9887	1.0000	-0.4534	0.5536	-0.2905	0.1458	-0.6052
LOC104910626	1.0000	0.0000	1.0000	0.0000	1.0000	0.0000	1.0000	0.0000	1.0000	0.0000	1.0000	0.0000	1.0000	0.0000
LOC104910627	1.0000	0.0000	1.0000	0.0000	1.0000	0.0000	1.0000	0.0000	1.0000	0.0000	1.0000	0.0000	1.0000	0.0000
LOC104910628	1.0000	0.0000	1.0000	0.0000	1.0000	0.0000	1.0000	0.0000	1.0000	0.0000	1.0000	0.0000	1.0000	0.0000
LOC104910629	1.0000	0.0000	1.0000	0.0000	1.0000	0.0000	1.0000	0.0000	1.0000	0.0000	1.0000	0.0000	1.0000	0.0000
LOC104910630	1.0000	-2.4776	1.0000	0.0000	1.0000	-2.4056	1.0000	0.0000	1.0000	-2.3757	1.0000	0.0000	1.0000	0.0000
LOC104910631	1.0000	0.0000	1.0000	0.0000	1.0000	0.0000	1.0000	0.0000	1.0000	0.0000	1.0000	0.0000	1.0000	0.0000
LOC104910632	1.0000	0.0000	0.7666	-3.2534	1.0000	0.0000	0.7701	-3.1666	1.0000	3.1126	1.0000	0.0000	1.0000	0.0000
LOC104910633	0.0227	-5.2317	0.4431	-3.7865	0.8480	0.5912	0.0797	1.9214	1.0000	-1.4703	1.0000	0.0000	1.0000	-0.1382
LOC104910634	1.0000	2.1850	0.2495	4.0050	1.0000	0.0000	1.0000	0.0000	1.0000	0.0000	1.0000	1.8196	1.0000	0.0000
LOC104910635	1.0000	0.0000	1.0000	0.0000	1.0000	0.0000	1.0000	0.0000	1.0000	0.0000	1.0000	0.0000	1.0000	0.0000
LOC104910636	0.4391	0.6296	0.1546	0.6991	0.0000	2.0315	0.0000	1.6379	1.0000	0.3151	0.8785	0.3975	1.0000	-0.0708
LOC104910637	1.0000	0.0000	1.0000	0.0000	1.0000	0.0000	1.0000	0.0000	1.0000	0.0000	1.0000	0.0000	1.0000	0.0000
LOC104910638	1.0000	0.0000	1.0000	0.0000	1.0000	0.0000	1.0000	0.0000	1.0000	0.0000	1.0000	0.0000	1.0000	0.0000
LOC104910639	0.0239	1.6405	0.0002	1.4364	0.0000	2.9221	0.0000	2.7829	1.0000	0.5157	0.9442	0.3183	0.3574	0.3768
LOC104910640	1.0000	2.1850	1.0000	-0.1449	0.8033	3.0789	1.0000	0.0075	1.0000	2.2676	1.0000	0.0495	1.0000	-0.7769
LOC104910641	0.2601	0.9500	0.1785	0.9108	0.0166	1.4450	1.0000	0.0806	1.0000	0.5208	0.9376	0.4948	0.3065	-0.8371
LOC104910642	0.0177	1.8738	0.0011	1.9580	0.7775	0.6541	0.8636	-0.6451	1.0000	0.2750	0.9744	0.3709	0.6819	-1.0212
LOC104910643	0.0212	2.7563	1.0000	0.0789	0.1867	1.9967	0.1430	1.4478	1.0000	1.0964	0.2142	-1.5633	1.0000	0.5553
LOC104910644	1.0000	0.0858	0.5489	-0.2910	0.0071	1.0395	0.0000	1.4176	1.0000	-0.8891	0.0000	-1.2542	0.0458	-0.5054
LOC104910646	0.1721	0.9476	0.0941	0.8462	0.0001	1.8711	0.0005	1.4383	1.0000	0.2560	1.0000	0.1662	1.0000	-0.1718
LOC104910647	0.0284	2.0971	0.0758	1.5195	0.0056	2.3784	0.4306	0.9775	1.0000	0.4304	1.0000	-0.1367	0.3665	-0.9645
LOC104910649	1.0000	-2.4775	1.0000	0.0000	1.0000	-2.4056	1.0000	0.0000	1.0000	-2.3757	1.0000	0.0000	1.0000	0.0000
LOC104910650	0.0150	2.5474	0.0347	1.8417	1.0000	-0.7128	0.8662	0.6582	1.0000	0.5722	1.0000	-0.1251	0.4805	1.9498
LOC104910651	0.3159	3.9314	0.7186	1.0553	1.0000	0.0000	0.7701	-3.1666	1.0000	3.1126	1.0000	0.3618	1.0000	0.0000
LOC104910652	0.1417	0.9677	0.6049	0.4706	0.6615	0.5270	0.7701	-0.4262	1.0000	0.2028	1.0000	-0.2802	0.5643	-0.7447
LOC104910653	0.0025	1.5337	0.0014	1.1177	0.0001	1.6963	0.0098	0.9559	1.0000	0.4704	1.0000	0.0657	0.8709	-0.2667
LOC104910654	0.0384	1.0508	0.0016	1.2316	0.9093	-0.3019	0.9732	-0.2454	1.0000	-0.3866	1.0000	-0.1955	1.0000	-0.3300
LOC104910655	0.0001	1.8379	0.4543	0.4019	1.0000	-0.1980	0.0128	-1.3605	1.0000	0.6965	0.1440	-0.7238	0.9114	-0.4584
LOC104910656	0.0107	2.2119	0.0158	2.2334	0.9056	0.6244	0.2794	1.5159	1.0000	-0.8150	0.6410	-0.7922	1.0000	0.0788
LOC104910657	0.7270	0.2606	0.6049	-0.2126	0.5879	0.2992	0.0803	-0.5214	1.0000	0.2037	0.8841	-0.2567	0.1232	-0.6118
LOC104910658	0.5391	-3.8767	1.0000	-1.0027	1.0000	-0.1865	1.0000	0.5319	1.0000	-0.6421	1.0000	2.3480	1.0000	0.0745
LOC104910659	1.0000	0.1480	0.1048	-1.3657	0.5047	-1.8710	0.4947	-0.7809	1.0000	0.8466	1.0000	-0.6548	0.4809	1.9503
LOC104910660	0.8808	0.0883	1.0000	-0.0036	0.3669	-0.2110	0.1062	-0.1784	1.0000	0.0530	1.0000	-0.0262	0.8631	0.0908
LOC104910661	1.0000	0.0000	1.0000	0.0000	1.0000	0.0000	1.0000	0.0000	1.0000	0.0000	1.0000	0.0000	1.0000	0.0000
LOC104910662	1.0000	-2.4788	1.0000	0.0000	1.0000	-2.4061	1.0000	0.0000	1.0000	-2.3771	1.0000	0.0000	1.0000	0.0000
LOC104910663	0.0000	-3.2590	0.0000	-4.0454	0.0832	0.6339	0.0019	0.7078	1.0000	0.2649	1.0000	-0.5162	0.4174	0.3466
LOC104910664	1.0000	0.0000	1.0000	0.0000	1.0000	0.0000	1.0000	0.0000	1.0000	0.0000	1.0000	0.0000	1.0000	0.0000
LOC104910665	0.2433	-1.7414	0.0193	-1.7340	1.0000	-0.0779	0.2682	0.6530	1.0000	0.7943	1.0000	0.8094	0.0169	1.5212
LOC104910666	1.0000	0.0000	1.0000	0.0000	1.0000	0.0000	1.0000	0.0000	1.0000	0.0000	1.0000	0.0000	1.0000	0.0000
LOC104910668	0.0444	1.0485	0.0005	1.2439	0.0000	2.0179	0.0000	1.6034	1.0000	0.1089	0.8605	0.3187	0.7287	-0.3007
LOC104910669	1.0000	0.0000	1.0000	0.0000	1.0000	0.0000	1.0000	0.0000	1.0000	0.0000	1.0000	0.0000	1.0000	0.0000
LOC104910671	0.1027	1.4764	0.3442	1.2303	1.0000	-0.4446	0.6142	-1.7592	1.0000	-0.6824	0.5459	-0.9228	0.6865	-1.9991
LOC104910672	1.0000	0.0000	1.0000	0.0000	1.0000	0.0000	1.0000	0.0000	1.0000	0.0000	1.0000	0.0000	1.0000	0.0000
LOC104910673	0.0084	-0.8469	0.0001	-0.8576	0.0000	1.3180	0.0000	0.8743	1.0000	0.1564	1.0000	0.1577	0.3232	-0.2839
LOC104910674	1.0000	-0.3288	1.0000	-2.3959	1.0000	0.7306	0.9167	1.3833	1.0000	-0.9638	1.0000	-3.1382	1.0000	-0.3113
LOC104910675	0.1697	-0.6632	0.0000	-1.0235	0.0000	-2.8190	0.0000	-2.4648	1.0000	0.8793	0.1777	0.5345	0.0232	1.2349
LOC104910677	0.2144	-2.6412	0.0803	-2.3640	1.0000	-0.4450	1.0000	0.1395	1.0000	0.5932	1.0000	0.8994	0.6819	1.1782
LOC104910678	0.0625	1.7294	0.0058	1.7879	0.0079	2.1794	0.0005	2.0182	1.0000	0.5234	0.7200	0.5959	0.8958	0.3678
LOC104910679	0.0163	1.9162	0.0100	1.7842	0.0001	2.9085	0.0000	2.5495	1.0000	0.0936	1.0000	-0.0347	1.0000	-0.2731
LOC104910680	1.0000	0.2673	0.1085	0.9460	0.0000	2.0452	0.0000	2.6809	1.0000	-0.5424	1.0000	0.1502	1.0000	0.0980
LOC104910681	1.0000	-0.4891	1.0000	0.1865	0.1702	1.0789	0.6205	0.9019	1.0000	-0.7766	1.0000	-0.0880	0.4341	-0.9593
LOC104910682	0.5173	0.2192	0.7128	0.1140	0.0105	0.5628	0.0001	0.5671	1.0000	-0.1181	0.6368	-0.2099	0.8937	-0.1064
LOC104910683	1.0000	-2.4788	1.0000	2.2534	1.0000	-2.4061	1.0000	2.3256	1.0000	-2.3771	1.0000	2.3480	1.0000	2.3554
LOC104910685	1.0000	2.1902	1.0000	-2.3986	1.0000	0.0000	1.0000	-2.3199	1.0000	2.2734	1.0000	-2.2992	1.0000	0.0000
LOC104910686	0.0000	0.8785	0.0000	0.6827	0.0000	1.0617	0.0000	0.9528	1.0000	0.1072	1.0000	-0.0754	1.0000	0.0044
LOC104910688	1.0000	0.0000	1.0000	0.0000	1.0000	0.0000	1.0000	0.0000	1.0000	0.0000	1.0000	0.0000	1.0000	0.0000
LOC104910689	1.0000	2.1902	1.0000	0.0000	1.0000	0.0000	1.0000	0.0000	1.0000	0.0000	1.0000	-2.2992	1.0000	0.0000
LOC104910690	0.1380	2.5675	1.0000	-1.0008	0.6601	1.5906	1.0000	0.5372	1.0000	0.7365	0.2593	-2.8332	1.0000	-0.3113
LOC104910691	0.6927	1.4475	1.0000	-2.3986	0.2858	2.1452	0.2460	2.3279	1.0000	-0.1034	0.6450	-4.0575	1.0000	0.0778
LOC104910692	0.0142	5.3234	0.7268	1.0575	0.0080	5.5129	0.0041	2.8594	1.0000	3.1126	0.8269	-1.0380	1.0000	0.4889
LOC104910693	0.3352	0.2834	0.0141	0.3247	0.0000	0.9312	0.0000	1.0210	1.0000	0.2085	0.2872	0.2638	0.0460	0.3037
LOC104910694	0.0000	1.9341	0.0000	2.0638	0.0000	2.3024	0.0000	1.7364	1.0000	0.2808	0.3240	0.4229	0.4036	-0.2814
LOC104910695	1.0000	0.0000	1.0000	0.0000	1.0000	0.0000	1.0000	0.0000	1.0000	0.0000	1.0000	0.0000	1.0000	0.0000
LOC104910696	1.0000	0.0000	1.0000	0.0000	1.0000	2.2426	1.0000	0.0000	1.0000	0.0000	1.0000	0.0000	1.0000	-2.2889
LOC104910697	1.0000	-2.4788	1.0000	0.0000	1.0000	-2.4061	1.0000	0.0000	1.0000	-2.3771	1.0000	0.0000	1.0000	0.0000
LOC104910698	1.0000	-0.2920	1.0000	0.0000	1.0000	-2.4056	1.0000	0.0000	1.0000	-2.3757	1.0000	-2.2957	1.0000	0.0000
LOC104910699	1.0000	0.6087	0.0564	1.9597	0.2785	-4.1857	0.9096	-1.3717	1.0000	-0.5115	0.9086	0.8487	1.0000	2.3554
LOC104910700	1.0000	0.0000	1.0000	0.0000	0.8033	3.0789	1.0000	0.0000	1.0000	0.0000	1.0000	0.0000	1.0000	-3.1317
LOC104910701	1.0000	0.1168	0.7666	-3.2534	1.0000	-0.8798	1.0000	0.5372	1.0000	-1.3447	0.2100	-4.8283	1.0000	0.0749
LOC104910702	1.0000	-0.1446	0.1749	-1.3656	0.4219	0.9121	0.0121	-2.6563	1.0000	-0.7764	0.0630	-2.0031	0.0000	-4.3459
LOC104910703	1.0000	-2.4788	1.0000	0.0000	1.0000	-2.4061	1.0000	0.0000	1.0000	-2.3771	1.0000	0.0000	1.0000	0.0000
LOC104910704	1.0000	-2.4788	0.7666	3.0922	1.0000	-2.4061	1.0000	0.0000	1.0000	-2.3771	1.0000	3.1976	1.0000	0.0000
LOC104910705	0.0700	1.0108	0.8190	0.2313	0.5851	0.5068	0.1855	0.6590	1.0000	0.1605	0.4861	-0.6081	0.9237	0.3188
LOC104910706	0.0814	0.7177	0.0018	0.9370	0.0020	-1.4697	0.2463	-0.5585	1.0000	-0.2814	1.0000	-0.0489	0.5521	0.6358

LOC104910708	1.0000	0.1678	1.0000	-0.2460	0.4906	-0.7716	0.5522	-0.5379	1.0000	0.1569	1.0000	-0.2446	1.0000	0.3955
LOC104910709	1.0000	0.0296	0.6813	0.4665	1.0000	0.0371	0.5451	0.5854	1.0000	-0.7771	1.0000	-0.3291	1.0000	-0.2232
LOC104910710	1.0000	-0.0781	0.0847	-0.3276	0.0036	-0.8165	0.2532	-0.2432	1.0000	0.0496	0.9048	-0.1866	0.0094	0.6269
LOC104910711	0.2872	-0.2086	0.0000	-0.3831	0.0000	-1.0621	0.0000	-1.0532	1.0000	0.0351	0.5234	-0.1272	1.0000	0.0499
LOC104910712	1.0000	0.0000	1.0000	0.0000	1.0000	0.0000	1.0000	0.0000	1.0000	0.0000	1.0000	0.0000	1.0000	0.0000
LOC104910713	1.0000	0.0000	1.0000	0.0000	1.0000	0.0000	1.0000	0.0000	1.0000	0.0000	1.0000	0.0000	1.0000	0.0000
LOC104910714	1.0000	0.0000	1.0000	0.0000	1.0000	0.0000	1.0000	0.0000	1.0000	0.0000	1.0000	0.0000	1.0000	0.0000
LOC104910715	1.0000	0.0000	1.0000	0.0000	1.0000	0.0000	1.0000	0.0000	1.0000	0.0000	1.0000	0.0000	1.0000	0.0000
LOC104910716	1.0000	0.0000	1.0000	0.0000	1.0000	0.0000	1.0000	0.0000	1.0000	0.0000	1.0000	0.0000	1.0000	0.0000
LOC104910717	1.0000	0.0000	1.0000	0.0000	1.0000	0.0000	1.0000	0.0000	1.0000	0.0000	1.0000	0.0000	1.0000	0.0000
LOC104910718	1.0000	0.0000	1.0000	0.0000	1.0000	0.0000	1.0000	0.0000	1.0000	0.0000	1.0000	0.0000	1.0000	0.0000
LOC104910719	0.0047	0.8002	0.0000	1.1166	0.0354	-0.8213	0.1915	-0.3407	1.0000	0.0134	0.1936	0.3441	0.2811	0.5046
LOC104910720	1.0000	0.0000	1.0000	-2.3986	0.4809	3.6122	1.0000	-2.3199	1.0000	2.2734	1.0000	0.0000	0.7287	-3.6637
LOC104910721	1.0000	0.0000	0.7666	3.0922	1.0000	0.0000	1.0000	0.0000	1.0000	0.0000	1.0000	3.1976	1.0000	0.0000
LOC104910722	1.0000	0.5414	1.0000	0.0000	1.0000	0.6740	1.0000	2.3242	1.0000	-2.3771	1.0000	-3.1429	1.0000	-0.7782
LOC104910723	0.3698	4.2134	1.0000	0.0000	0.4809	3.6101	1.0000	2.3242	1.0000	0.0000	0.5389	-4.3475	1.0000	-1.3084
LOC104910724	1.0000	-0.8591	0.7735	0.7731	0.4809	-3.7967	0.9096	-1.3722	1.0000	-0.1210	0.9082	1.5316	1.0000	2.3554
LOC104910725	0.0146	2.2746	0.0209	1.5299	1.0000	-0.5736	0.0516	-2.8809	1.0000	0.9929	1.0000	0.2644	1.0000	-1.3089
LOC104910726	0.2293	2.4073	1.0000	0.5134	0.9361	1.2061	0.9096	-1.3726	1.0000	1.2654	1.0000	-0.6045	1.0000	-1.3089
LOC104910727	0.8249	3.0255	1.0000	2.2507	1.0000	2.2471	0.7701	3.1732	1.0000	0.0000	1.0000	-0.8001	1.0000	0.9164
LOC104910728	1.0000	-2.4776	0.7304	-1.3883	0.9361	1.2045	0.4035	-2.0617	1.0000	1.9501	1.0000	3.1946	1.0000	-1.3081
LOC104910729	0.4620	0.8094	0.4135	-0.6995	0.6773	0.5781	1.0000	0.0669	1.0000	0.9206	0.9043	-0.5763	0.8932	0.4130
LOC104910731	0.8474	0.6233	0.2898	-1.2735	0.1846	-2.7129	0.0553	-2.3148	1.0000	0.5032	0.4742	-1.3815	1.0000	0.9149
LOC104910733	0.5384	3.5543	1.0000	2.2534	1.0000	2.2471	1.0000	0.0000	1.0000	0.0000	1.0000	-1.3292	1.0000	-2.2906
LOC104910734	0.0087	0.9640	0.0000	1.1048	0.1088	0.6076	0.2646	0.3610	1.0000	-0.1863	1.0000	-0.0361	0.2511	-0.4288
LOC104910736	1.0000	0.0000	1.0000	0.0000	1.0000	0.0000	1.0000	0.0000	1.0000	0.0000	1.0000	0.0000	1.0000	0.0000
LOC104910737	0.8464	-0.3619	0.0065	-1.3763	0.0000	1.7412	0.0701	0.7015	1.0000	0.4878	0.9819	-0.5158	0.2571	-0.5478
LOC104910738	0.1052	0.7518	1.0000	-0.0204	1.0000	0.1417	1.0000	-0.0340	1.0000	-0.4015	0.0042	-1.1613	0.4653	-0.5707
LOC104910740	0.8249	-3.3439	1.0000	0.6944	1.0000	-1.0167	0.9096	1.3849	1.0000	-0.9587	1.0000	3.1976	1.0000	1.4485
LOC104910741	1.0000	0.0476	1.0000	0.6944	0.5035	-3.7981	1.0000	0.8518	1.0000	-1.4917	1.0000	-0.8613	1.0000	3.2049
LOC104910742	1.0000	0.1924	0.7674	3.0956	1.0000	0.3547	1.0000	0.0000	1.0000	-3.2319	1.0000	-0.4672	0.7455	-3.6684
LOC104910743	1.0000	-0.3496	1.0000	0.1327	0.2898	-2.4979	1.0000	-0.3805	1.0000	-0.6796	1.0000	-0.1918	1.0000	1.4485
LOC104910744	0.4398	0.8859	1.0000	-0.0551	0.2712	0.9890	0.2966	0.8556	1.0000	0.0053	0.4868	-0.9251	1.0000	-0.1202
LOC104910745	0.1793	1.8828	0.3475	1.2297	0.2602	1.6062	1.0000	0.0090	1.0000	0.2637	1.0000	-0.3785	0.5410	-1.3282
LOC104910746	1.0000	2.1850	0.7674	-3.2570	1.0000	0.0000	0.7710	-3.1693	1.0000	3.1191	1.0000	-2.2957	1.0000	0.0000
LOC104910747	1.0000	-2.4776	1.0000	0.0000	0.9361	1.2064	0.7710	3.1753	1.0000	-2.3757	1.0000	0.0000	1.0000	-0.4568
LOC104910748	1.0000	0.0000	1.0000	0.0000	1.0000	0.0000	1.0000	0.0000	1.0000	0.0000	1.0000	0.0000	1.0000	0.0000
LOC104910749	0.3012	1.0004	1.0000	0.2292	0.0002	2.0642	0.0030	1.6129	1.0000	0.1291	0.8328	-0.6312	0.8914	-0.3198
LOC104910752	0.6348	0.7103	0.8250	0.5436	1.0000	-0.0296	0.9546	0.4127	1.0000	0.0341	1.0000	-0.1187	1.0000	0.4843
LOC104910753	1.0000	-0.0696	0.2627	-0.3210	0.5168	-0.2984	0.0880	-0.4331	1.0000	0.2252	1.0000	-0.0141	1.0000	0.0963
LOC104910754	1.0000	0.0000	1.0000	0.0000	1.0000	0.0000	1.0000	2.3256	1.0000	0.0000	1.0000	0.0000	1.0000	2.3554
LOC104910755	1.0000	2.1902	1.0000	0.0000	0.4809	3.6122	1.0000	0.0000	1.0000	0.0000	1.0000	-2.2992	0.7287	-3.6637
LOC104910756	0.0089	3.2849	0.2920	1.2112	1.0000	-2.4061	0.0501	-4.8619	1.0000	2.4298	1.0000	0.3913	1.0000	0.0000
LOC104910757	1.0000	0.0000	1.0000	0.0000	1.0000	0.0000	1.0000	0.0000	1.0000	0.0000	1.0000	0.0000	1.0000	0.0000
LOC104910758	0.1455	-0.2765	0.0563	-0.1965	0.8564	-0.0877	1.0000	-0.0307	1.0000	-0.0728	1.0000	0.0202	1.0000	-0.0102
LOC104910759	1.0000	0.1207	1.0000	0.5225	1.0000	-0.8758	1.0000	0.3951	1.0000	-0.8238	1.0000	-0.4101	1.0000	0.4605
LOC104910760	1.0000	0.2135	0.2268	1.7061	0.2098	1.4479	0.4962	1.4811	1.0000	-1.0389	1.0000	0.4592	0.6093	-1.0051
LOC104910761	0.0087	1.6401	1.0000	0.0960	0.0039	1.6027	1.0000	-0.1093	1.0000	1.3978	1.0000	-0.1330	0.9329	-0.3089
LOC104910762	1.0000	0.2173	0.2061	1.2647	0.8522	0.5938	0.8636	0.6593	1.0000	-0.7782	1.0000	0.2782	0.7938	-0.7093
LOC104910763	1.0000	0.0000	1.0000	0.0000	1.0000	0.0000	1.0000	0.0000	1.0000	0.0000	1.0000	0.0000	1.0000	0.0000
LOC104910765	1.0000	0.4322	0.3609	1.1106	0.2798	-2.0608	0.4962	-1.4668	1.0000	-0.5273	1.0000	0.1591	1.0000	0.0727
LOC104910766	0.2582	1.6787	0.0027	3.5369	1.0000	0.5015	0.9164	1.3891	1.0000	-1.4982	1.0000	0.3543	1.0000	-0.6146
LOC104910767	0.0052	1.9621	0.0000	2.2459	0.0000	2.8287	0.0000	2.4971	1.0000	-0.1294	1.0000	0.1643	0.6093	-0.4575
LOC104910768	0.2213	0.7687	0.0000	2.0006	0.0010	1.3718	0.0000	1.8965	1.0000	-0.9189	0.9188	0.3219	0.5853	-0.3916
LOC104910769	0.6207	-0.2954	0.6739	-0.2177	0.0479	0.7103	0.7530	0.1893	1.0000	-0.1666	1.0000	-0.0776	0.0512	-0.6858
LOC104910770	1.0000	0.5381	1.0000	0.0000	1.0000	0.6746	0.4622	3.6989	1.0000	-2.3757	1.0000	-3.1383	1.0000	0.6042
LOC104910771	0.0443	1.1904	0.6016	0.4578	0.8206	0.3519	0.8593	-0.4002	1.0000	-0.3794	0.0820	-1.0964	0.1912	-1.1253
LOC104910772	1.0000	0.0000	1.0000	0.0000	1.0000	0.0000	1.0000	0.0000	1.0000	0.0000	1.0000	0.0000	1.0000	0.0000
LOC104910773	0.0479	-0.5152	0.0000	-0.7717	0.0479	-0.4835	0.0000	-0.6375	1.0000	0.1552	1.0000	-0.0882	1.0000	0.0074
LOC104910774	1.0000	0.3241	1.0000	-0.1640	1.0000	0.4575	0.5745	1.1648	1.0000	-0.8169	0.9323	-1.3003	1.0000	-0.1073
LOC104910775	0.0390	2.1101	0.0588	1.3786	0.0000	3.8244	0.0000	2.4945	1.0000	0.8264	1.0000	0.1169	0.4901	-0.4986
LOC104910776	0.8226	3.0199	1.0000	-2.3986	1.0000	2.2426	1.0000	-2.3199	1.0000	2.2735	1.0000	-3.1429	1.0000	-2.2889
LOC104910777	0.0010	1.4632	0.0052	1.0578	0.9064	0.2752	0.4514	0.4945	1.0000	-0.1838	0.3199	-0.5781	1.0000	0.0412
LOC104910780	0.0396	0.5395	0.0019	0.7234	1.0000	0.0730	0.7190	0.1686	1.0000	0.0363	0.5692	0.2346	0.8937	0.1386
LOC104910781	0.0421	0.3694	0.0396	0.2336	0.0000	-1.5436	0.0000	-1.4250	1.0000	-0.0599	0.3027	-0.1837	1.0000	0.0644
LOC104910782	1.0000	0.1169	0.9525	-0.3032	0.1186	1.0266	0.1359	0.7386	1.0000	0.1241	1.0000	-0.2832	1.0000	-0.1516
LOC104910783	0.0029	1.6838	0.0048	1.1659	0.0005	1.8754	0.0164	1.0004	1.0000	0.5016	1.0000	-0.0043	0.7613	-0.3624
LOC104910784	1.0000	0.0000	1.0000	0.0000	1.0000	0.0000	1.0000	0.0000	1.0000	0.0000	1.0000	0.0000	1.0000	0.0000
LOC104910785	0.0994	1.8318	1.0000	0.2841	0.0002	3.1423	0.0145	1.3353	1.0000	1.5090	1.0000	-0.0173	1.0000	-0.2932
LOC104910786	0.0195	0.7305	0.7862	0.1346	0.1889	-0.5056	0.4069	-0.2570	1.0000	0.2625	0.3972	-0.3191	0.1082	0.5195
LOC104910787	1.0000	0.0000	1.0000	0.0000	1.0000	0.0000	1.0000	0.0000	1.0000	0.0000	1.0000	0.0000	1.0000	0.0000
LOC104910788	1.0000	0.0000	1.0000	0.0000	1.0000	0.0000	1.0000	0.0000	1.0000	0.0000	1.0000	0.0000	1.0000	0.0000
LOC104910789	1.0000	0.0000	1.0000	0.0000	1.0000	0.0000	1.0000	0.0000	1.0000	0.0000	1.0000	0.0000	1.0000	0.0000
LOC104910790	1.0000	0.0000	1.0000	2.2534	1.0000	0.0000	1.0000	0.0000	1.0000	0.0000	1.0000	2.3480	1.0000	0.0000
LOC104910792	1.0000	2.1850	1.0000	0.0000	1.0000	0.0000	1.0000	0.0000	1.0000	0.0000	1.0000	-2.2957	1.0000	0.0000
LOC104910793	1.0000	-0.3395	0.9062	1.2214	1.0000	-0.5739	1.0000	0.0045	1.0000	-1.8802	1.0000	-0.3314	1.0000	-1.3089
LOC104910794	0.0742	2.1390	0.0226	2.1489	0.9361	-1.5509	0.4370	-3.6972	1.0000	-0.1210	1.0000	-0.1052	1.0000	-2.2906
LOC104910796	1.0000	-0.2882	1.0000	0.3659	1.0000	-2.4056	0.7701	-3.1666	1.0000	0.7365	1.0000	1.4306	1.0000	0.0000
LOC104910797	1.0000	0.0000	1.0000	0.0000	1.0000	0.0000	1.0000	0.0000	1.0000	0.0000	1.0000	0.0000	1.0000	0.0000
LOC104910798	1.0000	0.0000	1.0000	0.0000	1.0000	0.0000	1.0000	0.0000	1.0000	0.0000	1.0000	0.0000	1.0000	0.0000
LOC104910799	1.0000	0.0771	0.6218	-0.2894	1.0000	0.1070	1.0000	-0.0743	1.0000	0.0072	0.8607	-0.3477	1.0000	-0.1699
LOC104910800	1.0000	0.0000	1.0000	0.0000	1.0000	0.0000	1.0000	0.0000	1.0000	0.0000	1.0000	0.0000	1.0000	0.0000
LOC104910801	1.0000	-0.2931	1.0000	0.0000	1.0000	-2.4061	1.0000	0.0000	1.0000	-2.3771	1.0000	-2.2957	1.0000	0.0000
LOC104910802	1.0000	0.0000	1.0000	2.2534	1.0000	0.0000	1.0000	2.3242	1.0000	0.0000	1.0000	2.3480	1.0000	2.3543
LOC104910803	0.8249	3.0143	1.0000	-2.3986	1.0000	2.2471	1.0000	-2.3199	1.0000	2.2735	1.0000	-3.1383	1.0000	-2.2906
LOC104910804	1.0000	0.0000	1.0000	0.0000	1.0000	2.2471	1.0000	0.0000	1.0000	0.0000	1.0000	0.0000	1.0000	-2.2906
LOC104910805	1.0000	0.0000	1.0000	0.0000	1.0000	0.0000	1.0000	0.0000	1.0000	0.0000	1.0000	0.0000	1.0000	0.0000
LOC104910806	1.0000	0.0000	1.0000	-2.3986	1.0000	0.0000	1.0000	-2.3199	1.0000	2.2735	1.0000	0.0000	1.0000	0.0000
LOC104910807	0.6936	1.4536	1.0000	-2.3986	0.1167	2.7088	1.0000	0.0045	1.0000	-0.1034	0.6450	-4.0608	0.1658	-2.8155
LOC104910808	0.4801	2.0051	0.7338	1.0561	1.0000	-2.4061	0.7710	-3.1693	1.0000	0.7402	1.0000	-0.1926	1.0000	0.0000
LOC104910809	0.0018	1.4612	0.0235	0.9900	0.5098	0.5555	1.0000	0.1089	1.0000	0.1001	0.8603	-0.3615	0.9323	-0.3436
LOC104910810	1.0000	0.0000	1.0000	2.2507	1.0000	2.2426	0.7701	3.1732	1.0000	0.0000	1.0000	2.3456	1.0000	0.9168
LOC104910811	1.0000	0.0000	1.0000	-0.1688	0.8011	3.0840	0.4370	-3.6968	1.0000	3.6396	0.8785	3.7234	1.0000	-3.1344

LOC104910812	1.0000	-0.2882	1.0000	2.2507	1.0000	0.6746	1.0000	0.0000	1.0000	-2.3757	1.0000	0.0471	1.0000	-3.1317
LOC104910813	0.1551	-1.7537	1.0000	-0.1703	0.0222	-3.3496	1.0000	-0.2445	1.0000	-0.9773	1.0000	0.6195	0.6937	2.1413
LOC104910814	0.6619	0.3891	0.8528	-0.2115	0.8564	0.2586	1.0000	0.0067	1.0000	0.5120	1.0000	-0.0784	1.0000	0.2641
LOC104910815	0.6936	1.4536	0.4583	0.9046	0.9361	1.2045	1.0000	-0.2449	1.0000	2.2057	0.4618	1.6934	1.0000	0.7678
LOC104910816	0.8226	3.0199	1.0000	0.0000	1.0000	0.0000	1.0000	0.0000	1.0000	0.0000	1.0000	-3.1429	1.0000	0.0000
LOC104910818	0.0072	-0.9049	0.0000	-1.3185	0.4369	-0.3309	0.0672	-0.5370	1.0000	-0.2573	0.2482	-0.6605	0.3373	-0.4607
LOC104910819	0.0044	-2.7565	0.1232	-1.7021	0.1645	-1.2648	0.1605	-1.5200	1.0000	-0.6263	1.0000	0.4416	0.9145	-0.8781
LOC104910820	0.8226	-3.3425	0.0445	-4.9507	0.1797	1.9988	1.0000	0.1933	1.0000	1.5688	1.0000	0.0000	1.0000	-0.2335
LOC104910821	1.0000	0.0000	1.0000	0.0000	1.0000	0.0000	1.0000	0.0000	1.0000	0.0000	1.0000	0.0000	1.0000	0.0000
LOC104910823	0.0000	1.8068	0.0000	1.4194	0.0005	1.2349	0.0000	1.2376	1.0000	0.0599	0.5603	-0.3163	1.0000	0.0677
LOC104910824	0.3159	3.9314	0.4691	3.6126	1.0000	2.2426	1.0000	0.0000	1.0000	0.0000	1.0000	-0.3347	1.0000	-2.2889
LOC104910825	1.0000	-0.3535	0.3104	1.0754	0.4518	-1.1582	0.5856	-1.1503	1.0000	-0.6303	0.8732	0.8119	1.0000	-0.6168
LOC104910826	0.0000	1.3210	0.0000	1.7011	0.0000	2.0598	0.0000	1.9041	1.0000	0.1731	0.0132	0.5673	1.0000	0.0230
LOC104910827	0.0022	-1.1458	0.0000	-1.3454	0.0569	-0.7308	0.0089	-0.6050	1.0000	0.2911	1.0000	0.1058	0.4263	0.4205
LOC104910828	1.0000	0.0000	1.0000	0.0000	1.0000	0.0000	1.0000	0.0000	1.0000	0.0000	1.0000	0.0000	1.0000	0.0000
LOC104910829	1.0000	0.0000	1.0000	0.0000	1.0000	0.0000	1.0000	0.0000	1.0000	0.0000	1.0000	0.0000	1.0000	0.0000
LOC104910831	1.0000	0.0000	1.0000	0.0000	1.0000	0.0000	1.0000	0.0000	1.0000	0.0000	1.0000	0.0000	1.0000	0.0000
LOC104910832	1.0000	0.0000	1.0000	0.0000	1.0000	0.0000	1.0000	0.0000	1.0000	0.0000	1.0000	0.0000	1.0000	0.0000
LOC104910833	0.0000	1.0915	0.0000	1.2340	0.0000	1.4215	0.0000	1.0766	1.0000	0.1310	0.3114	0.2879	0.2127	-0.2081
LOC104910834	0.1380	2.5670	0.3981	1.0478	0.0107	3.3306	0.5557	0.9787	1.0000	1.9491	1.0000	0.4647	1.0000	-0.3937
LOC104910835	1.0000	2.1901	0.7666	3.0922	0.8011	3.0840	1.0000	0.0000	1.0000	0.0000	1.0000	0.8994	1.0000	-3.1344
LOC104910836	1.0000	0.0000	1.0000	0.0000	1.0000	0.0000	1.0000	0.0000	1.0000	0.0000	1.0000	0.0000	1.0000	0.0000
LOC104910837	1.0000	0.0000	1.0000	0.0000	1.0000	0.0000	1.0000	0.0000	1.0000	0.0000	1.0000	0.0000	1.0000	0.0000
LOC104910838	1.0000	0.0000	1.0000	0.0000	1.0000	0.0000	1.0000	0.0000	1.0000	0.0000	1.0000	0.0000	1.0000	0.0000
LOC104910839	1.0000	-2.4788	0.2552	4.0066	1.0000	-2.4061	1.0000	0.0000	1.0000	-2.3771	0.6450	4.1180	1.0000	0.0000
LOC104910840	0.9025	-0.8957	0.2430	-2.0462	0.8033	0.7726	1.0000	0.0039	1.0000	0.2768	1.0000	-0.8675	1.0000	-0.4908
LOC104910841	0.4738	0.2549	1.0000	-0.0625	0.5717	0.2397	0.2362	0.2598	1.0000	0.0762	0.6957	-0.2290	1.0000	0.0990
LOC104910842	0.7594	0.1533	0.0191	0.3643	0.0045	-0.7025	0.0012	-0.5498	1.0000	-0.0406	0.7224	0.1828	1.0000	0.1178
LOC104910843	0.0447	0.6676	0.0238	0.5769	0.5979	0.2675	1.0000	0.0593	1.0000	0.0952	1.0000	0.0210	1.0000	-0.1073
LOC104910844	1.0000	0.0000	1.0000	0.0000	1.0000	0.0000	1.0000	0.0000	1.0000	0.0000	1.0000	0.0000	1.0000	0.0000
LOC104910846	1.0000	0.0000	1.0000	0.0000	1.0000	0.0000	1.0000	0.0000	1.0000	0.0000	1.0000	0.0000	1.0000	0.0000
LOC104910848	0.0006	0.9634	0.0001	0.8555	0.7934	-0.1894	0.9855	0.1065	1.0000	-0.4816	0.0192	-0.5775	0.9270	-0.1788
LOC104910849	1.0000	0.0000	1.0000	0.0000	1.0000	0.0000	1.0000	0.0000	1.0000	0.0000	1.0000	0.0000	1.0000	0.0000
LOC104910850	0.0851	1.0420	0.0046	1.1206	0.0724	1.0770	0.2225	0.6431	1.0000	0.3414	0.7567	0.4291	1.0000	-0.0918
LOC104910851	0.1589	0.5256	0.0034	0.6703	0.0009	1.0111	0.0000	0.9382	1.0000	-0.0279	1.0000	0.1291	1.0000	-0.0964
LOC104910852	0.6927	0.5086	0.0051	1.2829	0.0140	1.2906	0.0048	1.3195	1.0000	-0.1360	0.4527	0.6559	1.0000	-0.0984
LOC104910853	0.5927	-1.5556	0.4431	-3.7865	0.7749	-1.4101	1.0000	0.7001	1.0000	-0.8165	1.0000	-3.1429	0.9865	1.2995
LOC104910855	1.0000	-0.1834	0.0022	-0.9267	0.6783	0.3287	0.8182	0.1597	1.0000	0.7856	1.0000	0.0554	0.1426	0.6208
LOC104910857	0.1360	0.4103	1.0000	0.0444	0.3801	-0.2832	0.0002	-0.5171	1.0000	0.2681	1.0000	-0.0847	1.0000	0.0394
LOC104910858	0.3259	-0.2659	0.0004	-0.4687	0.0001	-0.8626	0.0000	-0.9694	1.0000	0.1010	1.0000	-0.0905	1.0000	0.0006
LOC104910859	0.0004	1.0391	0.5315	0.2697	0.8202	-0.2054	0.1603	-0.5093	1.0000	0.5186	0.7224	-0.2401	0.9317	0.2167
LOC104910860	1.0000	0.2029	0.0635	2.2773	1.0000	0.7335	0.7186	1.2292	1.0000	-0.1179	0.3571	1.9738	1.0000	0.3817
LOC104910861	1.0000	0.0405	0.9633	0.1117	0.3980	0.4225	0.0796	0.5041	1.0000	-0.4000	0.7035	-0.3131	0.5365	-0.3089
LOC104910862	0.6338	-0.1333	0.0768	-0.1756	0.0000	-0.7563	0.0000	-0.6399	1.0000	0.1092	0.9553	0.0791	0.1594	0.2317
LOC104910863	1.0000	0.0000	1.0000	0.0000	1.0000	0.0000	1.0000	0.0000	1.0000	0.0000	1.0000	0.0000	1.0000	0.0000
LOC104910864	0.0265	-0.7043	0.0000	-1.3427	0.7267	0.1915	0.1518	0.2847	1.0000	0.3382	0.8417	-0.2875	0.0716	0.4375
LOC104910865	0.2435	0.3333	0.0437	0.3537	0.0052	-0.6880	0.0000	-0.8385	1.0000	0.0385	1.0000	0.0707	1.0000	-0.1092
LOC104910866	0.2303	-2.2166	0.0113	-3.3308	0.9417	-0.3848	1.0000	0.1304	1.0000	0.3168	1.0000	-0.7919	0.8877	0.8368
LOC104910867	0.0275	2.1053	0.0025	1.6243	0.0001	3.0055	0.0014	1.6958	1.0000	1.2751	0.4324	0.8148	1.0000	-0.0309
LOC104910868	0.4111	0.7414	0.0044	1.4557	0.0001	2.0251	0.0000	1.9938	1.0000	-0.0565	0.5128	0.6714	1.0000	-0.0870
LOC104910869	0.0102	1.6186	0.7302	-0.4399	0.0128	1.5308	0.4656	0.5033	1.0000	0.9781	0.1311	-1.0676	1.0000	-0.0434
LOC104910870	0.0091	0.6906	0.0000	1.0032	0.0000	1.1113	0.0000	1.1661	1.0000	-0.2689	1.0000	0.0561	0.5546	-0.2093
LOC104910871	1.0000	0.0000	1.0000	0.0000	1.0000	0.0000	1.0000	2.3256	1.0000	0.0000	1.0000	0.0000	1.0000	2.3555
LOC104910872	0.0000	2.4808	0.0000	1.4582	0.0021	1.5589	0.6269	0.3317	1.0000	0.8972	1.0000	-0.1151	0.8198	-0.3259
LOC104910873	0.3503	0.8985	0.0050	1.3357	0.0001	2.3741	0.0000	2.0937	1.0000	0.3549	0.2666	0.8127	1.0000	0.0902
LOC104910874	1.0000	-2.4788	0.2495	-4.1757	0.4428	1.8950	0.1112	1.6480	1.0000	1.6499	1.0000	0.0000	0.4513	1.4144
LOC104910875	0.0267	0.9702	0.0001	1.0435	0.0000	3.7777	0.0000	3.3138	1.0000	0.2441	0.6911	0.3300	0.4437	-0.2134
LOC104910876	0.5515	0.8906	0.5563	0.3717	0.0000	3.1463	0.0000	1.5396	1.0000	1.6675	0.1185	1.1544	1.0000	0.0670
LOC104910877	1.0000	-0.7296	0.9754	0.7517	0.2833	-4.1854	0.7701	-3.1666	1.0000	-1.0383	1.0000	0.4467	1.0000	0.0000
LOC104910878	1.0000	0.0000	1.0000	0.0000	1.0000	0.0000	1.0000	0.0000	1.0000	0.0000	1.0000	0.0000	1.0000	0.0000
LOC104910879	0.9074	0.4610	0.9524	-0.3120	1.0000	-0.0732	1.0000	-0.1271	1.0000	0.1555	0.9615	-0.6055	1.0000	0.0943
LOC104910880	1.0000	0.0000	1.0000	0.0000	1.0000	0.0000	1.0000	0.0000	1.0000	0.0000	1.0000	0.0000	1.0000	0.0000
LOC104910882	0.0000	-2.2864	0.0000	-1.8932	0.7009	0.3262	0.0000	0.9260	1.0000	-0.1831	1.0000	0.2234	0.0624	0.4211
LOC104910883	1.0000	0.0000	1.0000	0.0000	1.0000	2.2425	1.0000	0.0000	1.0000	0.0000	1.0000	0.0000	1.0000	-2.2889
LOC104910884	0.3089	3.9281	1.0000	0.0000	1.0000	2.2471	1.0000	2.3242	1.0000	0.0000	0.6427	-4.0589	1.0000	0.0640
LOC104910885	1.0000	0.0468	1.0000	-0.1674	0.8175	0.7525	0.9096	-1.3722	1.0000	-0.1210	1.0000	-0.3282	0.5105	-2.2499
LOC104910887	1.0000	-0.2920	0.9148	1.2162	1.0000	-2.4056	1.0000	0.8547	1.0000	-0.1034	1.0000	1.4294	1.0000	3.2081
LOC104910888	0.7377	0.8285	0.2422	1.7087	0.0793	1.5695	0.0553	2.3293	1.0000	-1.5960	0.9591	-0.7179	0.5742	-0.8373
LOC104910889	0.7400	-0.3633	0.4082	0.5361	0.0981	0.8188	0.0000	1.8422	1.0000	-0.6113	1.0000	0.2982	0.5219	0.4166
LOC104910890	0.8249	3.0255	0.4431	3.6191	1.0000	2.2425	0.1317	4.3958	1.0000	0.0000	1.0000	0.5843	0.6865	2.1417
LOC104910891	1.0000	0.0000	1.0000	0.0000	1.0000	0.0000	1.0000	0.0000	1.0000	0.0000	1.0000	0.0000	1.0000	0.0000
LOC104910892	1.0000	0.0000	1.0000	0.0000	1.0000	0.0000	1.0000	0.0000	1.0000	0.0000	1.0000	0.0000	1.0000	0.0000
LOC104910893	1.0000	0.0000	1.0000	0.0000	1.0000	0.0000	1.0000	0.0000	1.0000	0.0000	1.0000	0.0000	1.0000	0.0000
LOC104910894	1.0000	0.0000	1.0000	0.0000	1.0000	0.0000	1.0000	0.0000	1.0000	0.0000	1.0000	0.0000	1.0000	0.0000
LOC104910895	0.9545	-1.6907	1.0000	0.1337	1.0000	-0.1814	0.3358	1.2773	1.0000	0.2609	0.8269	2.1235	0.4384	1.7332
LOC104910896	0.0736	1.5001	0.5653	0.5686	0.6373	-1.3491	0.0011	-5.7269	1.0000	0.7454	1.0000	-0.1766	0.7287	-3.6634
LOC104910897	1.0000	0.0000	1.0000	0.0000	1.0000	0.0000	1.0000	0.0000	1.0000	0.0000	1.0000	0.0000	1.0000	0.0000
LOC104910898	1.0000	0.0000	1.0000	0.0000	1.0000	0.0000	1.0000	0.0000	1.0000	0.0000	1.0000	0.0000	1.0000	0.0000
LOC104910899	1.0000	0.0000	1.0000	0.0000	1.0000	0.0000	1.0000	0.0000	1.0000	0.0000	1.0000	0.0000	1.0000	0.0000
LOC104910900	1.0000	0.0000	1.0000	0.0000	1.0000	0.0000	1.0000	0.0000	1.0000	0.0000	1.0000	0.0000	1.0000	0.0000
LOC104910901	1.0000	0.0000	1.0000	0.0000	1.0000	0.0000	1.0000	0.0000	1.0000	0.0000	1.0000	0.0000	1.0000	0.0000
LOC104910902	1.0000	-0.2882	0.2535	-4.1743	1.0000	-0.1625	1.0000	0.0071	1.0000	1.6478	1.0000	-2.2992	0.8939	1.8363
LOC104910903	1.0000	-2.4776	1.0000	0.0000	1.0000	-0.1591	0.4370	3.7041	1.0000	-2.3757	1.0000	0.0000	1.0000	1.4485
LOC104910904	0.6663	1.3328	1.0000	0.6944	1.0000	-0.1811	1.0000	0.0057	1.0000	-0.9587	0.8607	-1.6120	1.0000	-0.7769
LOC104910905	1.0000	0.0000	1.0000	0.0000	1.0000	0.0000	1.0000	0.0000	1.0000	0.0000	1.0000	0.0000	1.0000	0.0000
LOC104910906	1.0000	0.1489	0.1542	2.3762	0.8695	-0.9607	0.9096	1.3850	1.0000	-2.6545	1.0000	-0.4407	1.0000	-0.3119
LOC104910907	1.0000	-0.3436	1.0000	-0.5755	0.5708	1.0844	1.0000	0.0072	1.0000	0.8319	1.0000	0.6156	1.0000	-0.2352
LOC104910908	0.0000	1.5400	0.0000	1.8655	0.0000	1.3450	0.0000	0.7773	1.0000	-0.1307	0.8269	0.2094	0.0000	-0.6912
LOC104910909	0.2325	-1.2516	0.0136	-1.3697	0.0002	1.8068	0.0139	0.9531	1.0000	0.6809	1.0000	0.5785	1.0000	-0.1682
LOC104910910	1.0000	0.2067	1.0000	0.5289	0.0360	1.9099	0.1260	1.7989	1.0000	-0.5150	1.0000	-0.1869	0.7633	-0.6186
LOC104910911	1.0000	0.0000	1.0000	0.0000	1.0000	0.0000	1.0000	0.0000	1.0000	0.0000	1.0000	0.0000	1.0000	0.0000
LOC104910912	1.0000	0.0000	1.0000	0.0000	1.0000	0.0000	1.0000	0.0000	1.0000	0.0000	1.0000	0.0000	1.0000	0.0000
LOC104910913	1.0000	0.0000	1.0000	0.0000	0.8033	3.0789	1.0000	2.3256	1.0000	0.0000	1.0000	0.0000	1.0000	-0.7769

LOC104910914	1.0000	0.0000	1.0000	-2.3986	1.0000	2.2470	1.0000	0.0045	1.0000	2.2734	1.0000	0.0000	1.0000	0.0640
LOC104910915	1.0000	0.0000	1.0000	0.0000	1.0000	0.0000	1.0000	0.0000	1.0000	0.0000	1.0000	0.0000	1.0000	0.0000
LOC104910916	1.0000	0.0000	1.0000	0.0000	1.0000	0.0000	1.0000	0.0000	1.0000	0.0000	1.0000	0.0000	1.0000	0.0000
LOC104910917	0.0033	1.2967	0.0004	1.0559	0.0000	2.1787	0.0000	2.0727	1.0000	0.2786	1.0000	0.0499	0.8686	0.1780
LOC104910921	1.0000	0.2186	1.0000	0.2538	1.0000	-0.2037	0.6432	0.5142	1.0000	0.0884	1.0000	0.1360	0.6087	0.8060
LOC104910922	1.0000	-0.2423	0.9762	-0.2994	1.0000	-0.0836	1.0000	0.1204	1.0000	-0.1289	1.0000	-0.1747	1.0000	0.0797
LOC104910926	1.0000	0.0000	1.0000	0.0000	1.0000	0.0000	1.0000	0.0000	1.0000	0.0000	1.0000	0.0000	1.0000	0.0000
LOC104910927	1.0000	0.0000	1.0000	-2.3986	1.0000	2.2425	1.0000	0.8518	1.0000	2.2734	1.0000	0.0000	1.0000	0.9164
LOC104910928	0.6957	1.7476	1.0000	0.2660	0.6654	1.5927	0.8866	-0.6509	1.0000	2.6190	0.8260	1.1597	1.0000	0.3812
LOC104910929	1.0000	2.1901	0.4431	-3.7875	1.0000	2.2426	0.9096	-1.3726	1.0000	3.6423	1.0000	-2.2992	1.0000	0.0652
LOC104910930	1.0000	-0.0377	0.7186	1.0553	1.0000	-0.1894	1.0000	-0.8419	1.0000	-1.0389	1.0000	0.0580	0.8937	-1.6949
LOC104910931	1.0000	0.0000	0.7666	3.0922	0.2781	3.9963	1.0000	0.0000	1.0000	0.0000	1.0000	3.1976	0.5093	-4.0498
LOC104910932	0.0292	1.9737	0.8915	0.3584	0.1458	1.5325	0.5112	0.6189	1.0000	1.2097	1.0000	-0.3903	1.0000	0.3079
LOC104910934	1.0000	0.0000	1.0000	0.0000	1.0000	0.0000	1.0000	0.0000	1.0000	0.0000	1.0000	0.0000	1.0000	0.0000
LOC104910936	1.0000	0.0000	1.0000	0.0000	1.0000	0.0000	1.0000	0.0000	1.0000	0.0000	1.0000	0.0000	1.0000	0.0000
LOC104910938	0.0000	0.8002	0.0000	0.9584	0.0000	-0.9928	0.0000	-1.0598	1.0000	-0.2012	1.0000	-0.0313	0.4925	-0.2619
LOC104910939	0.0000	1.8118	0.0000	2.1732	0.3739	0.3244	0.6618	0.1876	1.0000	-0.1943	0.7128	0.1782	0.4509	-0.3264
LOC104910940	0.7910	0.5468	0.0751	1.1525	0.2461	-1.6984	0.0198	-3.1616	1.0000	0.1494	0.6381	0.7724	1.0000	-1.3088
LOC104910942	1.0000	0.5420	0.6180	-1.9232	1.0000	-2.4056	0.2426	-4.0840	1.0000	1.6505	1.0000	-0.7956	1.0000	0.0000
LOC104910946	0.0753	-1.7299	0.0000	-3.0415	0.0705	1.1726	0.1274	0.6143	1.0000	1.1024	1.0000	-0.1967	0.4492	0.5461
LOC104910947	0.9534	-0.5378	0.6185	0.6765	0.1993	1.1658	1.0000	-0.2444	1.0000	-0.5262	1.0000	0.6996	0.0574	-1.9367
LOC104910950	1.0000	-0.1768	0.2646	-0.6332	0.0950	-1.1808	0.0226	-1.1916	1.0000	0.4928	1.0000	0.0490	1.0000	0.4867
LOC104910953	0.0000	0.8836	0.0000	0.7334	0.0000	1.0384	0.0000	0.7080	1.0000	0.1645	1.0000	0.0265	0.3915	-0.1603
LOC104910954	0.0000	3.0442	0.0000	2.5815	0.0000	2.8671	0.0001	1.2137	1.0000	0.8623	0.5389	0.4122	0.0031	-0.7881
LOC104910956	0.0027	1.8753	0.0000	3.1680	0.0000	1.6454	0.0001	1.9491	1.0000	-0.9152	0.8915	0.3907	0.2618	-0.6101
LOC104910957	0.4447	0.3262	0.7110	0.1672	1.0000	-0.0211	0.5533	-0.2078	1.0000	-0.0326	1.0000	-0.1797	0.8304	-0.2142
LOC104910961	0.0489	-5.6892	0.4431	-3.7875	0.1122	-3.3623	0.4370	-3.6972	1.0000	-1.9197	1.0000	0.0000	1.0000	-2.2889
LOC104910962	0.0020	0.6273	0.0003	0.4806	0.0000	-1.2662	0.0000	-1.1091	1.0000	-0.1781	0.0714	-0.3122	1.0000	-0.0169
LOC104910963	0.0062	1.1051	0.0000	1.6305	0.6071	-0.4306	0.2021	-0.7370	1.0000	-0.4488	1.0000	0.0868	0.4814	-0.7507
LOC104910964	0.2703	-0.2496	0.0093	-0.3287	0.0058	-0.5258	0.0000	-0.5484	1.0000	-0.0284	0.9920	-0.0957	1.0000	-0.0459
LOC104910965	0.9545	1.0644	0.4437	3.6202	1.0000	0.6780	0.7701	3.1732	1.0000	-2.3771	1.0000	0.0576	1.0000	0.0723
LOC104910966	1.0000	-0.0401	0.9126	0.2320	0.7762	-0.3580	0.2038	-0.8320	1.0000	-0.1811	1.0000	0.1021	0.6968	-0.6504
LOC104910968	1.0000	0.0000	1.0000	0.0000	1.0000	0.0000	1.0000	0.0000	1.0000	0.0000	1.0000	0.0000	1.0000	0.0000
LOC104910969	1.0000	0.0000	1.0000	0.0000	1.0000	0.0000	1.0000	0.0000	1.0000	0.0000	1.0000	0.0000	1.0000	0.0000
LOC104910970	1.0000	-0.0661	0.1455	-0.2538	0.0006	-0.7137	0.0003	-0.6027	1.0000	0.0237	0.7644	-0.1531	0.8612	0.1382
LOC104910971	1.0000	0.0000	1.0000	0.0000	1.0000	0.0000	1.0000	0.0000	1.0000	0.0000	1.0000	0.0000	1.0000	0.0000
LOC104910972	1.0000	2.1850	1.0000	2.2507	1.0000	0.0000	1.0000	0.0000	1.0000	0.0000	1.0000	0.0495	1.0000	0.0000
LOC104910973	1.0000	-2.4788	1.0000	0.0000	1.0000	-2.4061	1.0000	0.0000	1.0000	-2.3771	1.0000	0.0000	1.0000	0.0000
LOC104910974	0.0497	0.7280	0.0007	0.8851	0.2257	-0.5886	0.1079	-0.5867	1.0000	-0.3709	0.8899	-0.2034	0.7287	-0.3636
LOC104910975	0.0019	-0.5284	0.0000	-0.5640	0.1499	0.2729	0.0001	0.3020	1.0000	-0.0634	0.9086	-0.0863	1.0000	-0.0286
LOC104910976	0.9650	1.0580	1.0000	-0.1594	0.0526	2.9859	0.7296	1.2292	1.0000	0.7304	1.0000	-0.4715	0.6944	-1.0200
LOC104910980	0.0000	1.6951	0.0000	1.7367	0.0000	2.5643	0.0000	1.8683	1.0000	0.3494	0.3980	0.4054	0.1781	-0.3389
LOC104910981	0.0681	-0.3039	0.0000	-0.4506	0.0021	-0.4604	0.0000	-0.5121	1.0000	0.0563	0.9158	-0.0783	1.0000	0.0101
LOC104910982	0.0000	2.8243	0.0000	2.3347	0.3286	0.7888	0.2722	0.7402	1.0000	0.2233	0.8607	-0.2502	1.0000	0.1809
LOC104910983	0.2861	0.2631	0.0014	0.3061	0.9092	-0.0853	1.0000	-0.0341	1.0000	-0.0541	1.0000	0.0017	1.0000	0.0028
LOC104910984	0.6601	0.3544	0.0112	0.9166	0.0184	1.0375	0.0094	0.9828	1.0000	-0.2538	0.9094	0.3201	0.8402	-0.3042
LOC104910985	0.0155	1.9167	0.0017	1.7563	0.0072	2.0748	0.0835	1.1885	1.0000	0.5870	0.8831	0.4372	1.0000	-0.2982
LOC104910986	0.0110	1.5090	0.2804	0.7120	0.0556	-2.2217	0.0203	-2.2232	1.0000	0.0720	0.4891	-0.7117	1.0000	0.0762
LOC104910987	0.8544	0.2049	0.8746	-0.1407	0.0000	1.6840	0.0000	1.2520	1.0000	0.1063	1.0000	-0.2259	0.3351	-0.3189
LOC104910989	1.0000	-0.3341	0.2741	2.1685	1.0000	-0.5711	0.6142	1.7721	1.0000	-1.8818	1.0000	0.6158	1.0000	0.4627
LOC104910990	0.0142	3.1884	0.2481	1.3521	0.0010	3.8319	0.0016	2.4570	1.0000	1.6538	1.0000	-0.1582	1.0000	0.2807
LOC104910991	1.0000	0.0000	1.0000	0.6940	0.8033	3.0789	1.0000	-2.3177	1.0000	2.2676	1.0000	3.1946	1.0000	-3.1317
LOC104910992	1.0000	0.1181	0.2805	-1.6830	0.6150	0.9110	0.7258	-0.7280	1.0000	1.0713	1.0000	-0.7122	1.0000	-0.5563
LOC104910993	0.7824	0.9263	0.8617	-0.8266	0.5285	1.0772	0.9393	0.4538	1.0000	0.8300	0.9318	-0.9084	1.0000	0.2128
LOC104910994	1.0000	0.0000	1.0000	0.0000	1.0000	0.0000	1.0000	0.0000	1.0000	0.0000	1.0000	0.0000	1.0000	0.0000
LOC104910995	1.0000	-0.1661	0.0413	-4.9488	1.0000	0.4346	0.3362	-1.6817	1.0000	-0.1282	0.1340	-5.0150	0.1763	-2.2457
LOC104910996	1.0000	0.8767	1.0000	-2.3986	0.8011	-3.2629	1.0000	-2.3199	1.0000	-0.9587	0.5389	-4.3475	1.0000	0.0000
LOC104910997	1.0000	0.0000	1.0000	0.0000	1.0000	0.0000	1.0000	0.0000	1.0000	0.0000	1.0000	0.0000	1.0000	0.0000
LOC104910998	1.0000	0.0000	1.0000	0.0000	1.0000	0.0000	1.0000	0.0000	1.0000	0.0000	1.0000	0.0000	1.0000	0.0000
LOC104910999	0.0175	0.8882	0.1142	0.4780	0.9511	0.1979	1.0000	-0.0910	1.0000	0.4140	1.0000	0.0158	1.0000	0.1260
LOC104911001	0.1947	-0.3215	0.0008	-0.3803	0.0000	-1.0278	0.0000	-0.8529	1.0000	-0.0963	0.8808	-0.1427	1.0000	0.0844
LOC104911002	0.0000	-2.5130	0.0001	-1.4964	0.0000	1.6259	0.0000	1.7971	1.0000	0.0672	0.3131	1.0987	0.4632	0.2466
LOC104911003	0.9613	-0.1674	0.6723	0.1691	0.0000	1.9566	0.0000	1.8144	1.0000	0.1596	0.1687	0.5099	1.0000	0.0231
LOC104911005	0.3827	-0.1946	0.0002	-0.3580	0.0063	-0.4747	0.0000	-0.4535	1.0000	0.0719	0.9755	-0.0786	0.7455	0.0995
LOC104911006	1.0000	0.3510	0.7763	0.7745	1.0000	-0.1810	0.1291	1.7987	1.0000	-0.1273	1.0000	0.3106	0.3359	1.8672
LOC104911008	0.0082	-0.4437	0.0000	-0.5117	0.0000	-1.1760	0.0000	-1.2172	1.0000	-0.0405	0.9005	-0.0966	1.0000	-0.0758
LOC104911009	0.0802	-4.8275	0.6847	-0.9917	1.0000	-0.4418	1.0000	-0.3426	1.0000	0.4378	0.4563	4.4217	1.0000	0.5450
LOC104911010	1.0000	-0.1888	0.1241	-0.6310	0.4684	-0.5538	0.0841	-0.7336	1.0000	0.5652	1.0000	0.1365	0.8984	0.3902
LOC104911011	0.0091	0.5187	0.0000	0.8540	0.0018	0.5859	0.0000	0.7080	1.0000	-0.2897	1.0000	0.0562	0.4598	-0.1635
LOC104911012	1.0000	0.1082	0.1517	-0.7391	0.0665	-1.2706	0.0002	-1.9646	1.0000	0.0116	0.3151	-0.8209	0.7373	-0.6714
LOC104911013	0.9903	0.4029	0.5432	-0.6524	1.0000	-0.1934	0.2320	-1.1821	1.0000	0.8475	1.0000	-0.1953	1.0000	-0.1360
LOC104911014	0.4695	-0.1688	0.7468	-0.0483	0.0521	-0.3415	0.0697	-0.1408	1.0000	0.1814	0.0024	0.3144	0.0000	0.3873
LOC104911015	0.8161	1.1481	0.8241	0.5966	0.3910	1.5011	0.2388	1.4108	1.0000	0.8006	1.0000	0.2706	1.0000	0.7127
LOC104911016	1.0000	0.0000	1.0000	0.0000	1.0000	0.0000	1.0000	0.0000	1.0000	0.0000	1.0000	0.0000	1.0000	0.0000
LOC104911017	0.9517	0.2276	0.4002	-0.4283	0.0042	1.1335	0.0000	1.6904	1.0000	0.0801	0.5859	-0.5630	0.0191	0.6437
LOC104911019	0.0413	0.9943	0.0003	1.2580	0.0000	1.8780	0.0000	1.2645	1.0000	-0.0428	1.0000	0.2356	0.0299	-0.6485
LOC104911020	1.0000	0.2998	0.7708	0.7730	0.1614	-4.4916	0.9096	-1.3721	1.0000	-0.8169	1.0000	-0.3395	1.0000	2.3543
LOC104911021	0.0048	0.9763	0.0000	1.8998	0.0135	0.7759	0.0000	1.3274	1.0000	-0.6854	0.8009	0.2526	1.0000	-0.1260
LOC104911022	0.0010	-1.3252	0.0003	-1.0731	0.9279	0.1552	0.5365	0.2357	1.0000	-0.0522	1.0000	0.2122	1.0000	0.0308
LOC104911023	0.0001	-1.1725	0.0000	-1.1171	0.0001	-1.1248	0.0015	-0.6530	1.0000	-0.1414	1.0000	-0.0724	0.6214	0.3389
LOC104911024	0.0000	-1.2516	0.0000	-1.4819	0.9060	-0.0728	0.1850	-0.1137	1.0000	-0.0407	0.1228	-0.2586	0.7908	-0.0762
LOC104911025	1.0000	0.0000	1.0000	0.0000	1.0000	0.0000	1.0000	0.0000	1.0000	0.0000	1.0000	0.0000	1.0000	0.0000
LOC104911026	1.0000	0.0000	1.0000	0.0000	1.0000	0.0000	1.0000	0.0000	1.0000	0.0000	1.0000	0.0000	1.0000	0.0000
LOC104911027	1.0000	2.1901	1.0000	0.0000	1.0000	0.0000	1.0000	0.0000	1.0000	0.0000	1.0000	-2.2992	1.0000	0.0000
LOC104911028	0.0000	1.2737	0.0000	1.7687	0.0000	1.7517	0.0000	1.7609	1.0000	0.1108	0.0062	0.6178	0.8176	0.1259
LOC104911029	0.0011	1.5743	0.0000	1.4435	0.0000	3.1637	0.0000	2.5174	1.0000	0.3764	0.9238	0.2596	0.5491	-0.2649
LOC104911030	0.0012	1.4107	0.0009	0.8930	0.0000	2.4839	0.0000	1.6030	1.0000	0.6435	1.0000	0.1397	0.5474	-0.2266
LOC104911031	1.0000	0.0000	1.0000	0.0000	1.0000	0.0000	1.0000	0.0000	1.0000	0.0000	1.0000	0.0000	1.0000	0.0000
LOC104911033	1.0000	0.0000	0.4691	3.6126	1.0000	0.0000	0.7710	3.1753	1.0000	0.0000	0.8785	3.7234	1.0000	3.2081
LOC104911034	1.0000	0.0000	1.0000	0.0000	1.0000	2.2470	1.0000	0.0000	1.0000	0.0000	1.0000	0.0000	1.0000	-2.2906
LOC104911035	1.0000	0.0000	1.0000	0.0000	1.0000	0.0000	1.0000	0.0000	1.0000	0.0000	1.0000	0.0000	1.0000	0.0000
LOC104911036	1.0000	0.0000	1.0000	0.0000	1.0000	0.0000	1.0000	0.0000	1.0000	0.0000	1.0000	0.0000	1.0000	0.0000
LOC104911037	1.0000	0.0000	1.0000	-0.1449	1.0000	2.2426	1.0000	-2.3177	1.0000	2.2676	1.0000	2.3456	1.0000	-2.2889

LOC104911040	1.0000	0.0000	1.0000	0.0000	1.0000	0.0000	1.0000	0.0000	1.0000	0.0000	1.0000	0.0000	1.0000	0.0000
LOC104911041	1.0000	0.0000	1.0000	0.0000	1.0000	0.0000	1.0000	0.0000	1.0000	0.0000	1.0000	0.0000	1.0000	0.0000
LOC104911042	1.0000	0.0000	1.0000	0.0000	1.0000	0.0000	1.0000	0.0000	1.0000	0.0000	1.0000	0.0000	1.0000	0.0000
LOC104911043	1.0000	0.0000	0.7666	-3.2534	1.0000	2.2426	1.0000	-0.8419	1.0000	3.1126	1.0000	0.0000	1.0000	0.0652
LOC104911044	1.0000	0.0000	1.0000	-2.3959	0.8033	3.0789	1.0000	0.0062	1.0000	2.2676	1.0000	0.0000	1.0000	-0.7782
LOC104911045	1.0000	-2.4776	1.0000	-0.1452	1.0000	-0.1624	1.0000	0.8541	1.0000	-0.1034	1.0000	2.3480	1.0000	0.9168
LOC104911046	1.0000	2.1901	1.0000	-2.3959	0.4809	3.6122	1.0000	-2.3177	1.0000	2.2676	1.0000	-2.2992	0.7287	-3.6637
LOC104911047	0.7950	0.4269	0.5895	-0.3915	1.0000	-0.0582	0.3634	-0.5960	1.0000	0.4718	1.0000	-0.3348	1.0000	-0.0616
LOC104911048	1.0000	-0.3236	1.0000	-0.1666	1.0000	0.3405	0.9096	-1.3717	1.0000	0.4081	1.0000	0.5866	1.0000	-1.3039
LOC104911049	0.6700	0.9998	1.0000	-0.1725	0.8273	0.7510	1.0000	0.4092	1.0000	0.8148	1.0000	-0.3408	1.0000	0.4807
LOC104911050	0.8226	3.0199	1.0000	0.1335	0.4809	3.6101	1.0000	-0.3800	1.0000	4.0267	1.0000	1.2794	1.0000	0.0752
LOC104911052	1.0000	-0.3476	0.0758	4.5597	0.7692	-1.4082	1.0000	2.3256	1.0000	-4.4584	1.0000	0.3106	1.0000	-0.7793
LOC104911053	1.0000	0.1255	0.7315	1.0510	1.0000	0.0577	1.0000	-0.8429	1.0000	-1.3418	1.0000	-0.4155	0.5105	-2.2499
LOC104911054	0.8501	0.1179	0.6851	0.0966	0.7446	-0.1523	0.0915	-0.2801	1.0000	0.0170	1.0000	0.0086	1.0000	-0.1064
LOC104911055	1.0000	0.0000	1.0000	0.0000	1.0000	0.0000	1.0000	0.0000	1.0000	0.0000	1.0000	0.0000	1.0000	0.0000
LOC104911056	1.0000	0.0000	1.0000	0.0000	1.0000	0.0000	1.0000	0.0000	1.0000	0.0000	1.0000	0.0000	1.0000	0.0000
LOC104911060	1.0000	0.0000	1.0000	0.0000	1.0000	0.0000	1.0000	0.0000	1.0000	0.0000	1.0000	0.0000	1.0000	0.0000
LOC104911061	1.0000	-0.3348	0.7189	1.0558	1.0000	-1.1017	0.9786	0.9243	1.0000	-1.0389	1.0000	0.3601	1.0000	0.9919
LOC104911062	1.0000	0.0000	1.0000	0.0000	1.0000	0.0000	1.0000	0.0000	1.0000	0.0000	1.0000	0.0000	1.0000	0.0000
LOC104911063	1.0000	2.1850	1.0000	0.0000	1.0000	0.0000	1.0000	0.0000	1.0000	0.0000	1.0000	-2.2957	1.0000	0.0000
LOC104911064	1.0000	0.0000	1.0000	0.0000	1.0000	0.0000	1.0000	0.0000	1.0000	0.0000	1.0000	0.0000	1.0000	0.0000
LOC104911065	0.0000	-2.2687	0.0000	-2.1889	0.0000	-0.7920	0.0000	-1.2944	1.0000	0.2448	0.2530	0.3371	0.0899	-0.2528
LOC104911066	1.0000	0.0000	1.0000	0.0000	1.0000	0.0000	1.0000	0.0000	1.0000	0.0000	1.0000	0.0000	1.0000	0.0000
LOC104911067	1.0000	0.0000	1.0000	0.0000	1.0000	0.0000	1.0000	0.0000	1.0000	0.0000	1.0000	0.0000	1.0000	0.0000
LOC104911068	0.8249	-3.3439	1.0000	0.0000	0.8033	-3.2636	0.7701	3.1732	1.0000	-3.2319	1.0000	0.0000	1.0000	3.2066
LOC104911069	1.0000	0.0000	1.0000	0.0000	1.0000	0.0000	1.0000	0.0000	1.0000	0.0000	1.0000	0.0000	1.0000	0.0000
LOC104911070	1.0000	0.0560	1.0000	-0.0561	0.8033	-0.6047	0.2524	-1.3990	1.0000	-0.2364	1.0000	-0.3470	0.7633	-1.0431
LOC104911071	0.7303	0.7213	0.1189	-1.7026	0.0285	1.7735	1.0000	0.2276	1.0000	0.6292	0.2582	-1.7859	0.5095	-0.9099
LOC104911072	1.0000	0.0000	1.0000	0.0000	1.0000	0.0000	1.0000	0.0000	1.0000	0.0000	1.0000	0.0000	1.0000	0.0000
LOC104911073	0.1238	-1.7076	0.6097	0.3805	0.3201	-1.1029	1.0000	-0.0558	1.0000	-0.0022	0.0048	2.1134	0.3458	1.0626
LOC104911074	1.0000	-0.2920	1.0000	0.0000	1.0000	-2.4056	1.0000	0.0000	1.0000	-2.3757	1.0000	-2.2958	1.0000	0.0000
LOC104911075	0.5407	0.7513	1.0000	0.1384	0.5932	0.6544	0.6483	-0.5001	1.0000	0.7203	1.0000	0.1193	0.9318	-0.4281
LOC104911076	0.0423	-5.0436	0.1546	-2.6963	0.8717	-0.9619	1.0000	-0.2069	1.0000	-0.1263	1.0000	2.3480	1.0000	0.6340
LOC104911077	0.0865	-0.3693	0.0003	-0.4136	0.1287	0.3258	0.0331	0.2333	1.0000	0.0716	1.0000	0.0402	1.0000	-0.0144
LOC104911079	1.0000	0.0000	1.0000	2.2507	1.0000	0.0000	1.0000	0.0000	1.0000	0.0000	1.0000	2.3456	1.0000	0.0000
LOC104911080	0.0565	-3.2106	0.0953	-2.8836	0.5804	-1.0100	0.2202	-1.8680	1.0000	-0.2920	1.0000	0.0514	0.9835	-1.1477
LOC104911081	1.0000	-1.1562	0.6180	1.6082	1.0000	0.3490	1.0000	0.8545	1.0000	-0.9614	1.0000	1.8196	1.0000	-0.4570
LOC104911082	0.2299	-0.3856	0.0281	-0.4879	0.0005	-0.9474	0.0361	-0.4357	1.0000	-0.0404	1.0000	-0.1323	0.1464	0.4766
LOC104911083	0.8359	0.0853	0.6215	-0.0689	0.0573	-0.3186	0.0000	-0.3852	1.0000	0.0646	0.9436	-0.0774	1.0000	0.0029
LOC104911084	1.0000	0.0000	1.0000	0.0000	1.0000	0.0000	1.0000	0.0000	1.0000	0.0000	1.0000	0.0000	1.0000	0.0000
LOC104911085	1.0000	0.0000	1.0000	0.0000	1.0000	0.0000	1.0000	0.0000	1.0000	0.0000	1.0000	0.0000	1.0000	0.0000
LOC104911086	0.3722	-0.6885	0.9476	-0.3079	1.0000	-0.0798	1.0000	0.1821	1.0000	-0.6912	1.0000	-0.2978	0.8426	-0.4245
LOC104911087	0.6881	1.7543	0.9779	-1.0833	0.6654	1.5931	1.0000	-0.3798	1.0000	1.6478	1.0000	-1.1674	1.0000	-0.3119
LOC104911089	1.0000	0.0000	0.7666	3.0922	1.0000	0.0000	1.0000	0.0000	1.0000	0.0000	1.0000	3.1976	1.0000	0.0000
LOC104911090	0.9700	-0.0980	1.0000	-0.0028	0.0005	0.8479	0.0000	1.0403	1.0000	-0.0304	1.0000	0.0784	0.6815	0.1675
LOC104911091	0.4065	0.5050	0.1443	0.5278	0.0000	2.3495	0.0000	1.9489	1.0000	0.0905	1.0000	0.1271	0.3840	-0.3037
LOC104911092	0.0024	2.2168	0.0038	1.4928	0.0000	3.1399	0.0000	2.4125	1.0000	0.9536	1.0000	0.2431	0.9710	0.2320
LOC104911093	0.9545	1.0679	0.5867	0.9872	0.9361	1.2064	0.9096	-1.3722	1.0000	1.2661	0.9773	1.2164	1.0000	-1.3086
LOC104911094	0.7763	0.1116	1.0000	0.0055	0.1593	-0.2860	0.0000	-0.3727	1.0000	-0.0075	0.7284	-0.1013	0.7450	-0.0889
LOC104911095	0.0005	0.9862	0.0000	1.2533	0.0000	2.9414	0.0000	2.7441	1.0000	-0.1267	1.0000	0.1539	0.2424	-0.3169
LOC104911096	0.8249	-3.3439	1.0000	-2.3986	0.8033	-3.2636	1.0000	-2.3199	1.0000	-0.9587	1.0000	0.0000	1.0000	0.0000
LOC104911097	1.0000	0.0000	0.7674	-3.2570	0.0179	5.2616	0.3417	1.6933	1.0000	3.1191	1.0000	0.0000	1.0000	-0.4207
LOC104911099	0.0678	1.0150	0.0072	1.1435	0.1515	0.8592	0.0072	1.1193	1.0000	-0.0017	1.0000	0.1378	1.0000	0.2641
LOC104911100	0.8226	3.0199	0.9787	0.7514	0.2829	3.9942	1.0000	0.0058	1.0000	3.1191	1.0000	0.9748	1.0000	-0.8428
LOC104911101	0.0459	0.6861	0.0000	1.0550	0.0000	1.4696	0.0000	1.4471	1.0000	-0.1508	0.7971	0.2309	0.7629	-0.1666
LOC104911102	0.0147	0.4367	0.0000	0.6184	0.0001	0.6292	0.0005	0.3240	1.0000	0.0377	0.1585	0.2323	0.0170	-0.2614
LOC104911103	0.6877	-2.0832	1.0000	0.3687	1.0000	0.3669	0.9801	0.9246	1.0000	-1.0414	1.0000	1.4325	1.0000	-0.4800
LOC104911104	0.0000	0.9317	0.0000	1.3440	0.0000	0.9377	0.0000	1.4153	1.0000	-0.3506	1.0000	0.0750	0.6891	0.1323
LOC104911105	0.0008	1.7489	0.0001	1.7062	0.0008	1.7119	0.0008	1.5770	1.0000	-0.2270	1.0000	-0.2565	0.7625	-0.3551
LOC104911106	0.0000	0.8900	0.0012	0.4034	0.0404	0.5007	0.7978	0.0722	1.0000	-0.0968	0.0000	-0.5708	0.0001	-0.5207
LOC104911107	0.0000	-4.2691	0.0000	-3.8791	0.1755	-0.5983	0.0001	-0.7575	1.0000	0.4584	0.8759	0.8675	0.5803	0.3066
LOC104911108	0.0000	-3.6735	0.0000	-3.1615	0.2301	-0.4954	0.0733	-0.5966	1.0000	0.1562	1.0000	0.6831	1.0000	0.0619
LOC104911110	0.0032	-1.4441	0.0000	-1.4997	0.0205	-1.1125	0.0000	-1.4166	1.0000	0.5406	0.7627	0.4959	1.0000	0.2369
LOC104911111	1.0000	-0.0560	0.8907	-0.1247	0.2908	0.4621	0.8866	-0.1251	1.0000	0.2024	1.0000	0.1464	0.4805	-0.3803
LOC104911112	1.0000	2.1901	0.7674	-3.2499	1.0000	0.0000	0.7710	-3.1633	1.0000	3.1056	1.0000	-2.2992	1.0000	0.0000
LOC104911114	0.0001	1.5484	0.0000	2.3450	0.0002	1.2667	0.0000	1.4681	1.0000	-0.0757	0.0088	0.7336	1.0000	0.1340
LOC104911116	0.0000	2.5418	0.0000	2.4541	0.0000	3.1745	0.0000	2.3711	1.0000	0.5241	0.3370	0.4512	0.6062	-0.2720
LOC104911118	1.0000	-0.0485	0.0920	-0.1764	0.0051	-0.4680	0.0000	-0.4098	1.0000	0.0865	1.0000	-0.0292	0.4513	0.1497
LOC104911120	0.0000	1.1047	0.0001	0.7152	0.4668	-0.2623	0.0045	-0.5359	1.0000	0.0981	0.4712	-0.2789	0.8793	-0.1699
LOC104911121	0.6447	0.1433	0.1189	-0.1878	0.1390	0.2960	0.7146	-0.0693	1.0000	0.3109	1.0000	-0.0077	1.0000	-0.0492
LOC104911122	1.0000	0.0000	1.0000	0.0000	1.0000	0.0000	1.0000	0.0000	1.0000	0.0000	1.0000	0.0000	1.0000	0.0000
LOC104911123	1.0000	0.8875	1.0000	0.2184	0.0048	2.9031	0.1900	1.6645	1.0000	0.4094	1.0000	-0.2463	0.6600	-0.8272
LOC104911124	1.0000	2.1901	1.0000	2.2534	0.8011	3.0840	1.0000	0.0000	1.0000	0.0000	1.0000	0.0489	1.0000	-3.1344
LOC104911126	1.0000	-2.4776	1.0000	0.0000	1.0000	-2.4056	0.7701	3.1732	1.0000	-2.3757	1.0000	0.0000	1.0000	3.2066
LOC104911127	0.8226	3.0199	1.0000	0.6912	1.0000	0.0000	1.0000	-2.3199	1.0000	2.2734	1.0000	0.0533	1.0000	0.0000
LOC104911128	1.0000	0.0000	1.0000	-2.3959	1.0000	0.0000	1.0000	-2.3178	1.0000	2.2677	1.0000	0.0000	1.0000	0.0000
LOC104911129	1.0000	0.0974	0.0537	0.4373	0.0000	1.3693	0.0000	1.0531	1.0000	0.1392	0.0658	0.4918	0.6740	-0.1700
LOC104911130	0.3089	3.9281	1.0000	-2.3960	1.0000	0.0000	1.0000	-2.3178	1.0000	2.2676	0.6427	-4.0589	1.0000	0.0000
LOC104911131	0.0511	-0.4819	0.0003	-0.4452	0.0056	-0.6517	0.0025	-0.3811	1.0000	-0.1333	1.0000	-0.0840	0.7772	0.1435
LOC104911132	1.0000	0.5815	1.0000	0.6987	0.4062	1.6823	1.0000	-2.3178	1.0000	-0.9638	1.0000	-0.8584	0.0855	-5.0038
LOC104911133	0.4802	0.2244	0.8786	-0.0594	0.0361	0.4712	0.0002	0.4181	1.0000	0.1209	0.7487	-0.1498	1.0000	0.0727
LOC104911135	0.1686	0.7735	0.5703	0.2751	0.1058	-0.9454	0.0002	-1.5632	1.0000	0.3274	1.0000	-0.1558	1.0000	-0.2856
LOC104911136	1.0000	0.0000	1.0000	0.0000	1.0000	0.0000	1.0000	0.0000	1.0000	0.0000	1.0000	0.0000	1.0000	0.0000
LOC104911138	0.0043	-1.2686	0.0263	-0.5652	0.2849	-0.5400	0.0068	-0.6792	1.0000	0.2704	0.0119	0.9842	1.0000	0.1368
LOC104911140	1.0000	0.0000	1.0000	2.2507	1.0000	0.0000	1.0000	0.0000	1.0000	0.0000	1.0000	2.3456	1.0000	0.0000
LOC104911143	0.0006	0.7705	0.0074	0.4784	1.0000	0.0151	0.3807	0.1725	1.0000	-0.3045	0.0019	-0.5834	0.8110	-0.1407
LOC104911144	0.4834	0.5975	0.2016	0.6312	0.0484	1.0722	1.0000	0.1047	1.0000	0.3239	0.8875	0.3744	0.3579	-0.6332
LOC104911145	1.0000	0.0000	1.0000	-2.3960	1.0000	0.0000	1.0000	-2.3178	1.0000	2.2676	1.0000	0.0000	1.0000	0.0000
LOC104911146	0.0713	1.4197	0.0157	1.9145	0.0223	1.7185	0.0000	3.0499	1.0000	-0.7849	1.0000	-0.2815	0.5617	0.5510
LOC104911147	0.0392	2.9665	0.9062	-1.5359	0.1954	2.5399	1.0000	0.3941	1.0000	1.2654	0.0844	-3.2368	0.9181	-0.8772
LOC104911148	0.0251	1.8341	0.0003	2.0706	0.0001	2.5446	0.0000	2.5549	1.0000	-0.4362	1.0000	-0.1966	0.7466	-0.4308
LOC104911149	0.1380	1.3100	0.0327	1.2609	1.0000	-0.1944	0.7731	-0.5639	1.0000	0.4465	0.9479	0.4038	1.0000	0.0788
LOC104911150	0.0856	1.4611	0.0171	1.2450	0.7298	0.6885	1.0000	0.1054	1.0000	0.6597	0.8885	0.4519	1.0000	0.0808

LOC104911151	1.0000	0.0000	1.0000	0.0000	1.0000	0.0000	1.0000	0.0000	1.0000	0.0000	1.0000	0.0000	1.0000	0.0000
LOC104911152	1.0000	0.3945	0.1565	1.5784	0.3865	-1.8746	1.0000	0.5632	1.0000	-0.8967	1.0000	0.2998	0.7908	1.5489
LOC104911153	0.9545	1.0644	0.4431	3.6191	1.0000	-0.1630	0.4622	3.6989	1.0000	-2.3771	1.0000	0.0569	1.0000	1.4474
LOC104911154	0.7972	0.8927	0.2517	-2.4819	0.8011	-3.2629	0.7713	-0.9365	1.0000	1.3515	0.8372	-2.0207	0.7287	3.7384
LOC104911155	1.0000	0.2067	0.6909	0.7887	0.2917	1.3331	1.0000	0.0074	1.0000	0.1810	1.0000	0.7739	0.5584	-1.1430
LOC104911156	1.0000	0.0000	1.0000	0.0000	1.0000	0.0000	1.0000	0.0000	1.0000	0.0000	1.0000	0.0000	1.0000	0.0000
LOC104911157	1.0000	0.0000	1.0000	0.0000	1.0000	0.0000	1.0000	0.0000	1.0000	0.0000	1.0000	0.0000	1.0000	0.0000
LOC104911158	0.1005	0.4932	0.0821	0.3685	0.0224	-0.6804	0.1766	-0.3215	1.0000	-0.0164	1.0000	-0.1304	0.4805	0.3461
LOC104911159	0.0003	1.6112	0.0000	1.5536	1.0000	-0.0978	0.7701	-0.3232	1.0000	-0.1328	1.0000	-0.1788	1.0000	-0.3482
LOC104911160	1.0000	-0.3449	0.8772	0.4830	0.0890	1.6779	0.6835	0.8249	1.0000	-0.1298	1.0000	0.7123	0.5130	-0.9772
LOC104911161	0.0216	1.2509	0.0262	0.8800	0.0000	1.9662	0.0000	1.6303	1.0000	0.3780	1.0000	0.0220	1.0000	0.0532
LOC104911162	1.0000	-0.3217	1.0000	0.0780	0.0002	3.3004	0.0290	1.8045	1.0000	1.0999	0.9075	1.5302	0.9820	-0.3886
LOC104911163	0.1394	1.9828	1.0000	0.0569	0.2694	1.8499	1.0000	-0.2753	1.0000	2.1991	1.0000	0.2953	1.0000	0.0795
LOC104911164	0.3351	0.7860	0.6242	0.5186	0.1544	0.9456	0.0044	1.3648	1.0000	-0.1190	1.0000	-0.3758	0.9934	0.2927
LOC104911165	1.0000	-0.8627	0.8681	0.4802	0.2376	1.5996	0.0465	1.7233	1.0000	0.5635	0.6178	1.9334	0.8749	0.6960
LOC104911166	0.8092	0.3457	0.0394	0.9756	0.1045	-1.1879	1.0000	-0.2104	1.0000	-0.6462	1.0000	0.0012	1.0000	0.3394
LOC104911167	0.0000	1.2735	0.0000	0.9372	0.0206	-0.6025	0.0004	-0.7076	1.0000	0.1398	0.5505	-0.1855	1.0000	0.0384
LOC104911168	0.1177	-0.4832	0.3472	-0.2564	0.5877	-0.2283	0.7421	0.1495	1.0000	-0.0477	0.8875	0.1938	0.3446	0.3358
LOC104911169	0.5867	1.3553	0.2101	1.2640	0.7628	1.0365	1.0000	0.4728	1.0000	1.1014	0.7982	1.0324	1.0000	0.5439
LOC104911170	1.0000	0.0000	0.7666	-3.2534	1.0000	0.0000	1.0000	0.0059	1.0000	3.1126	1.0000	0.0000	1.0000	3.2049
LOC104911171	0.0086	-1.0030	0.0049	-0.7766	0.1571	-0.5520	0.0002	-1.0163	1.0000	0.0715	0.8840	0.3101	0.6647	-0.3867
LOC104911172	0.0221	1.5205	0.0001	2.0793	0.2171	1.0216	0.6607	0.6313	1.0000	-0.4140	1.0000	0.1591	0.4964	-0.8001
LOC104911173	0.6814	-0.7270	0.0037	5.5295	0.4863	-0.8865	0.0059	5.3610	1.0000	-5.7971	1.0000	0.3177	1.0000	0.3929
LOC104911174	0.0079	1.1663	0.0518	0.7357	0.0000	2.1148	0.0000	2.1128	1.0000	0.1171	0.8733	-0.2995	1.0000	0.1208
LOC104911175	0.8249	-3.3412	0.9062	-1.5339	0.8033	-3.2622	0.4370	-3.6968	1.0000	0.4098	1.0000	2.3480	1.0000	0.0000
LOC104911176	1.0000	-0.3195	1.0000	2.2534	0.8011	-3.2629	1.0000	0.0000	1.0000	-3.2304	1.0000	-0.7977	1.0000	0.0000
LOC104911177	1.0000	0.0000	1.0000	0.0000	1.0000	0.0000	1.0000	0.0000	1.0000	0.0000	1.0000	0.0000	1.0000	0.0000
LOC104911178	1.0000	0.0000	1.0000	0.0000	1.0000	0.0000	1.0000	0.0000	1.0000	0.0000	1.0000	0.0000	1.0000	0.0000
LOC104911179	0.6961	0.8728	1.0000	0.0594	0.0403	1.8148	1.0000	-0.6858	1.0000	-0.1210	0.8788	-0.9169	0.0122	-2.6291
LOC104911181	1.0000	0.0000	1.0000	0.0000	1.0000	0.0000	1.0000	0.0000	1.0000	0.0000	1.0000	0.0000	1.0000	0.0000
LOC104911182	1.0000	-0.3436	1.0000	-0.9997	1.0000	-0.5709	1.0000	0.0074	1.0000	-1.0414	1.0000	-1.7112	1.0000	-0.4587
LOC104911183	1.0000	0.0000	1.0000	0.0000	1.0000	2.2426	1.0000	0.0000	1.0000	0.0000	1.0000	0.0000	1.0000	-2.2889
LOC104911184	1.0000	0.0000	1.0000	0.0000	1.0000	0.0000	0.7710	3.1753	1.0000	0.0000	1.0000	0.0000	1.0000	3.2081
LOC104911185	0.4622	-1.8159	0.7934	-1.1128	0.1071	-4.7451	0.0806	-4.6365	1.0000	-0.1360	1.0000	0.5858	1.0000	0.0000
LOC104911186	1.0000	0.0000	1.0000	0.0000	1.0000	2.2426	0.7701	3.1732	1.0000	0.0000	1.0000	0.0000	1.0000	0.9168
LOC104911187	0.8249	-3.3412	1.0000	-0.9997	0.0220	2.5723	0.0008	3.1041	1.0000	-0.1218	1.0000	2.3456	1.0000	0.4163
LOC104911188	1.0000	0.0000	1.0000	0.0000	1.0000	0.0000	1.0000	0.0000	1.0000	0.0000	1.0000	0.0000	1.0000	0.0000
LOC104911190	1.0000	0.0000	1.0000	0.0000	1.0000	0.0000	1.0000	0.0000	1.0000	0.0000	1.0000	0.0000	1.0000	0.0000
LOC104911191	1.0000	0.0000	1.0000	0.0000	1.0000	0.0000	1.0000	0.0000	1.0000	0.0000	1.0000	0.0000	1.0000	0.0000
LOC104911194	1.0000	0.0000	1.0000	0.0000	1.0000	0.0000	1.0000	0.0000	1.0000	0.0000	1.0000	0.0000	1.0000	0.0000
LOC104911195	1.0000	0.0000	1.0000	2.2507	1.0000	0.0000	1.0000	0.0000	1.0000	0.0000	1.0000	2.3456	1.0000	0.0000
LOC104911196	1.0000	0.0000	1.0000	0.0000	1.0000	0.0000	1.0000	0.0000	1.0000	0.0000	1.0000	0.0000	1.0000	0.0000
LOC104911197	1.0000	0.0000	1.0000	2.2507	1.0000	0.0000	1.0000	0.0000	1.0000	0.0000	1.0000	2.3456	1.0000	0.0000
LOC104911201	0.0018	-0.5198	0.0000	-0.6469	0.0000	-1.2517	0.0000	-1.2634	1.0000	0.1009	1.0000	-0.0138	0.8397	0.0955
LOC104911202	1.0000	-1.1561	1.0000	2.2507	0.8033	-3.2622	1.0000	0.0000	1.0000	-3.2289	1.0000	0.0495	1.0000	0.0000
LOC104911204	1.0000	0.2836	0.1130	0.9434	0.0763	-1.9402	0.0213	-2.2237	1.0000	-0.0300	0.6450	0.6402	1.0000	-0.3106
LOC104911205	0.7435	0.3632	0.3068	0.4340	0.0036	-1.5762	0.0705	-0.8006	1.0000	0.2880	0.8627	0.3677	0.1355	1.0683
LOC104911207	0.0814	1.6661	0.0006	2.8401	0.9164	0.4632	0.4106	1.3503	1.0000	-0.8115	1.0000	0.3658	1.0000	0.0777
LOC104911209	0.3159	3.9314	1.0000	0.0000	0.4809	3.6101	1.0000	2.3256	1.0000	0.0000	0.6450	-4.0608	1.0000	-1.3081
LOC104911210	1.0000	2.1850	1.0000	-2.3986	1.0000	0.0000	1.0000	-2.3199	1.0000	2.2734	1.0000	-2.2958	1.0000	0.0000
LOC104911211	1.0000	2.1901	1.0000	0.0000	1.0000	0.0000	1.0000	0.0000	1.0000	0.0000	1.0000	-2.2992	1.0000	0.0000
LOC104911212	1.0000	0.0000	1.0000	0.0000	1.0000	0.0000	1.0000	0.0000	1.0000	0.0000	1.0000	0.0000	1.0000	0.0000
LOC104911213	1.0000	0.0000	1.0000	-2.3960	1.0000	0.0000	1.0000	-2.3178	1.0000	2.2676	1.0000	0.0000	1.0000	0.0000
LOC104911215	1.0000	0.6037	0.4194	-1.5167	1.0000	-0.1850	0.2230	-1.8678	1.0000	1.2202	1.0000	-0.8868	1.0000	-0.4568
LOC104911217	0.9522	0.6158	0.6436	0.9464	0.5799	0.9063	1.0000	0.5631	1.0000	-0.4280	1.0000	-0.0889	0.8496	-0.7694
LOC104911218	1.0000	0.0000	1.0000	0.0000	1.0000	0.0000	1.0000	0.0000	1.0000	0.0000	1.0000	0.0000	1.0000	0.0000
LOC104911219	1.0000	0.0000	1.0000	-2.3960	0.2840	3.9988	1.0000	-2.3178	1.0000	2.2676	1.0000	0.0000	0.5105	-4.0506
LOC104911221	1.0000	0.1189	1.0000	0.2919	1.0000	-0.1907	0.2819	1.2292	1.0000	-0.1269	1.0000	0.0589	0.5603	1.3001
LOC104911222	1.0000	0.0000	1.0000	0.0000	1.0000	0.0000	1.0000	0.0000	1.0000	0.0000	1.0000	0.0000	1.0000	0.0000
LOC104911223	0.6936	1.4536	0.6224	-1.9258	1.0000	-2.4056	0.6172	-1.7596	1.0000	1.6538	1.0000	-1.7141	1.0000	2.3555
LOC104911224	1.0000	-0.2882	0.9784	0.7485	1.0000	-0.1624	0.9786	0.9238	1.0000	0.7416	1.0000	1.8175	0.8937	1.8364
LOC104911225	0.4597	0.6274	0.0957	0.8867	0.7464	-0.5388	1.0000	0.0843	1.0000	-0.2082	1.0000	0.0617	1.0000	0.4173
LOC104911227	0.0613	-0.3228	0.0000	-0.6092	0.0000	-0.8609	0.0000	-0.6319	1.0000	0.1189	0.4627	-0.1548	0.0009	0.3535
LOC104911228	1.0000	-2.4788	0.9062	1.2225	1.0000	-2.4061	1.0000	-2.3178	1.0000	-0.1089	0.8607	3.7288	1.0000	0.0000
LOC104911229	0.5646	-0.9979	1.0000	-0.1652	0.8480	0.5912	0.4743	1.3642	1.0000	-1.4726	1.0000	-0.6369	0.8120	-0.7068
LOC104911230	1.0000	-2.4776	1.0000	2.2534	1.0000	-2.4056	1.0000	2.3242	1.0000	-2.3757	1.0000	2.3480	1.0000	2.3543
LOC104911232	0.0611	1.0448	0.7189	0.2443	0.0022	1.2663	1.0000	-0.0425	1.0000	0.7644	1.0000	-0.0242	0.2797	-0.5362
LOC104911233	1.0000	0.0941	0.7447	0.1898	0.1639	0.6341	0.7722	0.1868	1.0000	0.0938	1.0000	0.2021	0.6531	-0.3510
LOC104911235	0.0200	2.2163	0.0003	2.2255	0.7033	0.7691	0.5962	-1.1462	1.0000	0.6371	0.5879	0.6635	0.7146	-1.2685
LOC104911236	0.0086	-0.5270	0.0000	-0.5158	0.2582	-0.2484	0.0165	-0.2616	1.0000	0.0939	0.9595	0.1187	0.9270	0.0859
LOC104911237	1.0000	0.0000	1.0000	0.0000	1.0000	0.0000	1.0000	0.0000	1.0000	0.0000	1.0000	0.0000	1.0000	0.0000
LOC104911238	1.0000	0.0000	1.0000	0.0000	1.0000	0.0000	1.0000	0.0000	1.0000	0.0000	1.0000	0.0000	1.0000	0.0000
LOC104911239	1.0000	0.0000	1.0000	0.0000	1.0000	0.0000	1.0000	0.0000	1.0000	0.0000	1.0000	0.0000	1.0000	0.0000
LOC104911240	0.4619	0.4871	1.0000	0.1501	0.0345	-1.2301	0.0605	-1.1210	1.0000	-0.3927	0.3227	-0.7153	1.0000	-0.2762
LOC104911241	0.3265	-1.0233	0.2604	-0.5000	0.1130	-1.5854	0.1371	-0.6325	1.0000	0.5152	0.1483	1.0527	0.0386	1.4765
LOC104911242	0.0000	-2.6182	0.0000	-2.9512	0.3707	0.3500	0.0024	0.7099	1.0000	-0.2283	0.8799	-0.5478	1.0000	0.1365
LOC104911243	0.0645	1.9024	0.3048	1.0743	1.0000	-0.5739	1.0000	-0.2065	1.0000	0.6404	1.0000	-0.1709	1.0000	1.0198
LOC104911245	0.7092	0.1730	0.0275	0.2644	1.0000	-0.0116	1.0000	-0.3664	1.0000	0.1628	0.1624	0.2682	0.6481	-0.1886
LOC104911246	1.0000	-0.2920	1.0000	-0.1557	1.0000	-2.4056	0.7710	-3.1633	1.0000	0.7318	1.0000	0.9035	1.0000	0.0000
LOC104911248	1.0000	0.8905	0.9062	-1.5349	1.0000	-1.0194	0.4370	-3.6972	1.0000	0.4129	0.8269	-2.0167	1.0000	-2.2889
LOC104911249	1.0000	-2.4776	0.9062	-1.5349	1.0000	-2.4056	0.4370	-3.6972	1.0000	1.2661	1.0000	2.3480	1.0000	0.0000
LOC104911250	0.7162	-0.3109	0.0212	-0.7591	0.5538	0.3240	0.1542	0.4119	1.0000	0.5198	1.0000	0.0858	0.0794	0.6168
LOC104911252	1.0000	-0.3554	1.0000	-0.0186	0.3997	-1.3163	0.2242	-1.0569	1.0000	0.7675	0.5842	1.1203	0.9181	1.0344
LOC104911253	0.9274	-0.3520	0.3690	-0.7234	0.1227	-1.5326	0.0112	-1.8246	1.0000	0.6185	1.0000	0.2630	1.0000	0.3306
LOC104911254	0.4316	1.4478	0.0933	2.1526	0.4837	1.3130	1.0000	0.0066	1.0000	-0.6504	1.0000	0.0597	0.3644	-1.9615
LOC104911256	0.4083	0.3653	0.0202	0.6732	0.2259	0.4395	0.7606	0.2379	1.0000	-0.5143	0.9927	-0.1926	0.0510	-0.7092
LOC104911257	1.0000	0.5420	1.0000	0.0000	1.0000	-2.4056	1.0000	0.0000	1.0000	-2.3757	1.0000	-3.1429	1.0000	0.0000
LOC104911258	1.0000	0.0000	1.0000	0.0000	1.0000	0.0000	1.0000	0.0000	1.0000	0.0000	1.0000	0.0000	1.0000	0.0000
LOC104911259	1.0000	0.0000	1.0000	0.0000	1.0000	2.2470	1.0000	0.0000	1.0000	0.0000	1.0000	0.0000	1.0000	-2.2906
LOC104911263	0.6909	-0.2191	0.4817	-0.2187	0.4430	-0.3065	1.0000	-0.0376	1.0000	-0.0763	1.0000	-0.0615	0.9185	0.1979
LOC104911264	0.6229	1.0597	0.1515	1.3619	1.0000	-0.5753	0.3945	-2.0639	1.0000	0.1789	1.0000	0.4965	1.0000	-1.3084
LOC104911265	1.0000	0.0000	1.0000	2.2507	1.0000	0.0000	1.0000	2.3242	1.0000	0.0000	1.0000	2.3456	1.0000	2.3543
LOC104911267	0.5965	-0.9938	0.7231	1.0549	0.2633	-1.5988	0.9786	0.9238	1.0000	-2.4436	1.0000	-0.4053	1.0000	0.0765

LOC104911269	1.0000	0.5381	0.0774	4.5589	0.9361	1.2064	1.0000	2.3256	1.0000	-2.3757	0.9094	1.5312	1.0000	-1.3086
LOC104911270	0.6795	1.0689	0.8181	-0.4128	0.0004	3.1400	0.0000	2.0144	1.0000	1.5114	1.0000	0.0555	0.9032	0.4016
LOC104911271	0.4587	0.5753	0.7345	0.3248	0.8611	-0.3712	0.9579	-0.2309	1.0000	-0.1332	0.9278	-0.3712	1.0000	0.0163
LOC104911272	0.0034	-0.4574	0.0000	-0.6830	0.0000	-1.0817	0.0000	-1.0235	1.0000	0.0222	0.2761	-0.1911	0.8381	0.0859
LOC104911273	1.0000	0.0000	1.0000	0.0000	1.0000	0.0000	1.0000	0.0000	1.0000	0.0000	1.0000	0.0000	1.0000	0.0000
LOC104911274	1.0000	-0.2892	1.0000	-1.0008	0.1203	2.7066	0.4962	1.4811	1.0000	0.7357	1.0000	0.0489	1.0000	-0.4860
LOC104911275	1.0000	0.5370	1.0000	2.2507	1.0000	-0.1630	1.0000	0.0000	1.0000	-2.3771	1.0000	-0.7943	1.0000	-2.2889
LOC104911276	0.0291	-0.6024	0.1634	-0.2743	0.0033	-0.7345	0.0022	-0.5359	1.0000	-0.0564	0.5684	0.2846	0.9382	0.1475
LOC104911277	0.2226	0.4929	0.0004	0.8426	0.7436	0.2249	0.1340	0.4472	1.0000	-0.3868	1.0000	-0.0288	0.9863	-0.1602
LOC104911278	1.0000	0.0744	0.7666	0.1216	0.0065	-0.8205	0.0000	-0.9318	1.0000	-0.2181	0.9277	-0.1588	0.5781	-0.3264
LOC104911281	1.0000	0.0000	1.0000	0.0000	1.0000	0.0000	1.0000	0.0000	1.0000	0.0000	1.0000	0.0000	1.0000	0.0000
LOC104911282	1.0000	0.0000	1.0000	0.0000	1.0000	0.0000	1.0000	2.3256	1.0000	0.0000	1.0000	0.0000	1.0000	2.3555
LOC104911284	1.0000	0.0717	1.0000	0.3207	1.0000	-0.3022	0.6172	-1.1607	1.0000	-1.0979	0.6921	-0.8477	0.1717	-1.9509
LOC104911285	1.0000	0.2135	0.0128	-5.2993	1.0000	0.1152	0.4680	-1.1139	1.0000	0.9928	0.3216	-4.6160	1.0000	-0.2278
LOC104911288	1.0000	0.0000	1.0000	0.0000	1.0000	0.0000	1.0000	0.0000	1.0000	0.0000	1.0000	0.0000	1.0000	0.0000
LOC104911290	1.0000	0.0000	1.0000	0.0000	1.0000	0.0000	1.0000	0.0000	1.0000	0.0000	1.0000	0.0000	1.0000	0.0000
LOC104911291	0.0000	-1.4690	0.0000	-1.5063	0.0000	-0.8029	0.0000	-0.4857	1.0000	-0.2318	0.4784	-0.2575	0.9248	0.0903
LOC104911292	0.0000	2.2532	0.0000	1.6870	0.3468	-0.6205	0.0000	-1.8109	1.0000	-0.1635	0.0016	-0.7180	0.0510	-1.3451
LOC104911293	0.0044	-1.3800	0.0000	-2.0197	0.0004	1.1778	0.0007	0.9150	1.0000	-0.0050	0.7623	-0.6310	0.6944	-0.2595
LOC104911296	0.2497	0.9632	0.0500	1.1481	0.5227	0.6137	1.0000	0.3015	1.0000	-0.1280	1.0000	0.0643	1.0000	-0.4366
LOC104911297	1.0000	0.0201	0.0002	3.2571	1.0000	-0.6707	0.9786	0.9240	1.0000	-1.8087	0.3051	1.4401	1.0000	-0.2243
LOC104911299	1.0000	0.0000	1.0000	0.0000	1.0000	0.0000	1.0000	0.0000	1.0000	0.0000	1.0000	0.0000	1.0000	0.0000
LOC104911300	1.0000	0.0000	1.0000	0.0000	1.0000	0.0000	1.0000	0.0000	1.0000	0.0000	1.0000	0.0000	1.0000	0.0000
LOC104911303	1.0000	0.0000	1.0000	0.0000	1.0000	0.0000	1.0000	0.0000	1.0000	0.0000	1.0000	0.0000	1.0000	0.0000
LOC104911304	1.0000	0.0000	1.0000	0.0000	1.0000	0.0000	1.0000	0.0000	1.0000	0.0000	1.0000	0.0000	1.0000	0.0000
LOC104911305	0.9719	-0.1079	0.2281	-0.2871	1.0000	0.0807	0.5081	0.1717	1.0000	-0.0449	0.7610	-0.2128	1.0000	0.0488
LOC104911306	0.9197	0.1477	0.5344	0.2002	0.0711	-0.6970	0.1433	-0.3883	1.0000	-0.0972	1.0000	-0.0330	1.0000	0.2177
LOC104911307	1.0000	0.0000	1.0000	0.0000	1.0000	0.0000	1.0000	0.0000	1.0000	0.0000	1.0000	0.0000	1.0000	0.0000
LOC104911308	0.0627	-0.4911	0.0000	-0.6777	1.0000	-0.0552	0.8251	0.0730	1.0000	0.0437	1.0000	-0.1290	0.6442	0.1797
LOC104911310	0.0000	1.2614	0.0000	1.3712	0.1186	0.3809	0.0000	0.6576	1.0000	-0.3468	0.1614	-0.2256	1.0000	-0.0675
LOC104911311	1.0000	0.0000	1.0000	0.0000	1.0000	0.0000	1.0000	0.0000	1.0000	0.0000	1.0000	0.0000	1.0000	0.0000
LOC104911312	1.0000	0.0000	1.0000	0.0000	1.0000	0.0000	1.0000	0.0000	1.0000	0.0000	1.0000	0.0000	1.0000	0.0000
LOC104911313	1.0000	0.4278	0.6089	-0.8107	1.0000	-0.1894	0.9367	-0.4411	1.0000	1.2799	1.0000	0.0584	0.9116	1.0344
LOC104911316	0.7902	1.1381	0.7226	1.0540	0.8033	-3.2636	0.7710	-3.1693	1.0000	-0.1140	1.0000	-0.1930	1.0000	0.0000
LOC104911318	0.1014	1.4059	0.0002	2.4009	0.0000	3.4888	0.0000	3.2279	1.0000	-0.6962	1.0000	0.3127	0.0021	-0.9521
LOC104911319	1.0000	-2.4788	1.0000	-2.3985	1.0000	-2.4061	1.0000	-2.3199	1.0000	-0.1045	1.0000	0.0000	1.0000	0.0000
LOC104911320	1.0000	0.0000	1.0000	0.0000	1.0000	0.0000	1.0000	0.0000	1.0000	0.0000	1.0000	0.0000	1.0000	0.0000
LOC104911321	0.0233	-1.0213	0.0002	-1.2982	1.0000	0.1171	0.0058	0.7705	1.0000	0.0119	1.0000	-0.2504	0.0703	0.6718
LOC104911322	1.0000	0.0000	1.0000	-2.3960	1.0000	0.0000	1.0000	-2.3178	1.0000	2.2676	1.0000	0.0000	1.0000	0.0000
LOC104911323	1.0000	0.0000	1.0000	0.0000	1.0000	0.0000	1.0000	0.0000	1.0000	0.0000	1.0000	0.0000	1.0000	0.0000
LOC104911324	0.5086	-3.8790	1.0000	-2.3960	0.9361	-1.5509	0.9167	1.3833	1.0000	-1.4938	1.0000	0.0000	1.0000	1.4453
LOC104911326	0.4354	-2.0188	0.6224	-1.9244	0.3028	-2.7160	0.9802	-0.9111	1.0000	-0.8839	1.0000	-0.7956	1.0000	0.9186
LOC104911327	0.2156	-0.2952	0.0027	-0.3402	0.0000	-1.0480	0.0000	-0.7642	1.0000	0.1868	0.5817	0.1536	0.0009	0.4746
LOC104911328	1.0000	0.0000	1.0000	0.0000	1.0000	0.0000	1.0000	0.0000	1.0000	0.0000	1.0000	0.0000	1.0000	0.0000
LOC104911329	0.3092	4.2299	1.0000	-1.0008	0.4809	3.6101	0.7701	-3.1666	1.0000	3.1126	0.8269	-2.0158	0.7287	-3.6634
LOC104911330	0.4359	1.4446	1.0000	-0.5567	1.0000	0.1996	1.0000	0.3121	1.0000	0.2615	0.4575	-1.7329	1.0000	0.3817
LOC104911331	0.0000	-1.6331	0.0000	-1.2488	0.0210	-0.7963	0.0000	-1.4954	1.0000	0.2796	0.2884	0.6769	0.6093	-0.4143
LOC104911332	1.0000	0.0000	1.0000	-2.3985	1.0000	0.0000	1.0000	-2.3199	1.0000	2.2734	1.0000	0.0000	1.0000	0.0000
LOC104911333	1.0000	2.1850	1.0000	0.0000	1.0000	0.0000	0.7701	3.1732	1.0000	0.0000	1.0000	-2.2958	1.0000	3.2066
LOC104911334	0.0087	1.7633	0.0191	1.2872	1.0000	0.3239	0.2603	-1.0655	1.0000	0.5910	1.0000	0.1289	0.7604	-0.7916
LOC104911335	1.0000	0.0000	1.0000	0.0000	1.0000	0.0000	1.0000	0.0000	1.0000	0.0000	1.0000	0.0000	1.0000	0.0000
LOC104911336	1.0000	2.1850	1.0000	2.2507	1.0000	0.0000	1.0000	0.0000	1.0000	0.0000	1.0000	0.0495	1.0000	0.0000
LOC104911338	0.5096	1.1826	0.1699	1.4666	0.0008	2.5878	0.0000	3.3217	1.0000	-0.1268	1.0000	0.1728	0.4812	0.6122
LOC104911339	0.0114	-0.8265	0.0014	-0.6272	0.0891	0.5363	1.0000	0.0637	1.0000	0.2557	0.2189	0.4685	0.6753	-0.2127
LOC104911340	0.4958	0.3643	0.5427	-0.2708	0.1712	-0.5531	0.0163	-0.7387	1.0000	-0.1138	0.1112	-0.7386	0.8440	-0.2959
LOC104911341	1.0000	-0.3433	0.8221	0.5980	0.1547	1.5538	0.1620	1.5346	1.0000	-0.1242	1.0000	0.8301	1.0000	-0.1386
LOC104911342	1.0000	2.1901	0.4691	3.6126	1.0000	0.0000	1.0000	0.0000	1.0000	0.0000	1.0000	1.4259	1.0000	0.0000
LOC104911344	1.0000	0.1920	0.1223	1.0446	0.8341	-0.4446	1.0000	0.0071	1.0000	-0.9043	1.0000	-0.0427	0.9814	-0.4502
LOC104911346	1.0000	0.0000	1.0000	0.0000	1.0000	0.0000	1.0000	0.0000	1.0000	0.0000	1.0000	0.0000	1.0000	0.0000
LOC104911347	1.0000	0.0000	1.0000	0.0000	1.0000	0.0000	1.0000	0.0000	1.0000	0.0000	1.0000	0.0000	1.0000	0.0000
LOC104911348	0.8819	-0.0810	0.2611	-0.1208	0.9999	-0.0573	0.0186	-0.2143	1.0000	0.1119	0.9498	0.0845	1.0000	-0.0402
LOC104911349	1.0000	0.0000	1.0000	0.0000	1.0000	0.0000	1.0000	0.0000	1.0000	0.0000	1.0000	0.0000	1.0000	0.0000
LOC104911350	1.0000	-2.4788	1.0000	0.0000	1.0000	-2.4061	0.7710	3.1753	1.0000	-2.3771	1.0000	0.0000	1.0000	3.2081
LOC104911351	0.0127	1.2108	0.0000	2.2981	0.7787	0.3419	0.0401	1.0829	1.0000	-0.9288	1.0000	0.1686	1.0000	-0.1861
LOC104911352	1.0000	-0.0288	1.0000	-0.1772	0.2978	0.7174	0.4996	-0.6461	1.0000	-0.1988	1.0000	-0.3344	0.0109	-1.5623
LOC104911353	1.0000	0.0000	1.0000	-2.3960	1.0000	0.0000	1.0000	0.0075	1.0000	2.2676	1.0000	0.0000	1.0000	2.3554
LOC104911354	1.0000	-0.1512	0.8390	0.5467	0.1280	-1.2560	0.5231	0.7285	1.0000	-1.1864	1.0000	-0.4804	0.8227	0.8015
LOC104911355	0.3381	0.3258	0.9854	-0.0719	0.0609	-0.5914	0.0051	-0.5507	1.0000	0.1692	0.7958	-0.2144	0.8694	0.2172
LOC104911356	0.1346	0.8872	0.0719	0.7115	0.0563	0.8839	0.3737	0.4497	1.0000	0.1269	1.0000	-0.0379	0.8381	-0.3021
LOC104911357	0.4629	-1.4626	0.6205	-0.5806	1.0000	-0.0475	0.1392	-1.3683	1.0000	0.6764	0.5375	1.5850	0.9853	-0.6353
LOC104911358	0.0005	0.8456	0.0000	0.9520	0.1994	0.3726	0.2224	-0.2663	1.0000	-0.0015	0.9407	0.1155	0.0021	-0.6347
LOC104911359	1.0000	0.0000	1.0000	0.0000	1.0000	0.0000	1.0000	0.0000	1.0000	0.0000	1.0000	0.0000	1.0000	0.0000
LOC104911360	1.0000	0.0000	1.0000	0.0000	1.0000	2.2425	1.0000	0.0000	1.0000	0.0000	1.0000	0.0000	1.0000	-2.2889
LOC104911361	1.0000	0.0000	1.0000	0.0000	1.0000	0.0000	1.0000	0.0000	1.0000	0.0000	1.0000	0.0000	1.0000	0.0000
LOC104911362	0.1380	2.5675	1.0000	-0.6953	0.6654	1.5931	1.0000	0.6984	1.0000	1.2635	0.4748	-1.9834	1.0000	0.3802
LOC104911363	0.0403	-0.4267	0.0000	-0.5085	0.3606	-0.2198	0.0036	-0.3200	1.0000	0.0933	1.0000	0.0244	1.0000	-0.0015
LOC104911364	1.0000	2.1850	1.0000	-2.3985	1.0000	0.0000	1.0000	-2.3199	1.0000	2.2734	1.0000	-2.2958	1.0000	0.0000
LOC104911365	0.5928	-1.5556	0.1552	2.0180	1.0000	-0.4859	1.0000	0.5384	1.0000	-1.3469	0.3684	2.2436	1.0000	-0.3138
LOC104911366	0.0002	-0.7693	0.0000	-0.8433	0.7033	-0.1339	0.1921	-0.1687	1.0000	0.0192	1.0000	-0.0411	1.0000	-0.0101
LOC104911367	0.0000	-1.0831	0.0000	-1.4989	0.0000	-0.7153	0.0000	-0.7605	1.0000	0.0885	0.0311	-0.3154	1.0000	0.0485
LOC104911368	1.0000	-0.2892	0.7674	-3.2570	0.5084	1.8779	1.0000	0.0031	1.0000	0.7402	1.0000	-2.2992	1.0000	-1.1412
LOC104911369	1.0000	-1.1585	0.7666	-3.2534	1.0000	-1.0167	0.9786	0.9240	1.0000	-0.1187	1.0000	-2.2958	0.8939	1.8357
LOC104911370	1.0000	0.0918	1.0000	0.0572	0.9468	-0.2490	0.4057	-0.4504	1.0000	0.1985	1.0000	0.1750	1.0000	-0.0042
LOC104911371	0.0138	0.9250	0.3488	0.3248	0.6313	-0.3172	0.0282	-0.7475	1.0000	0.4157	1.0000	-0.1723	1.0000	-0.0118
LOC104911372	0.7373	0.2786	0.2969	-0.3958	0.5525	0.3266	0.1712	-0.4865	1.0000	0.1944	0.6123	-0.4668	0.2088	-0.6148
LOC104911373	0.3029	0.2324	0.8747	0.0544	0.1402	-0.3186	0.0000	-0.5078	1.0000	0.1257	1.0000	-0.0387	1.0000	-0.0578
LOC104911374	0.0576	0.9848	0.1484	0.5212	0.0002	1.8850	0.0001	1.2632	1.0000	0.7425	0.9218	0.2897	1.0000	0.1256
LOC104911375	1.0000	-0.0646	0.5429	-0.0881	0.0000	-0.7972	0.0000	-0.9573	1.0000	-0.0068	1.0000	-0.0177	0.5723	-0.1621
LOC104911377	0.6881	1.7514	0.9763	0.7505	0.2263	2.5323	0.3362	1.6944	1.0000	0.7402	1.0000	-0.2463	1.0000	-0.1050
LOC104911378	1.0000	-0.1351	1.0000	-0.1767	1.0000	-0.4433	1.0000	-0.2076	1.0000	0.0895	1.0000	0.0589	1.0000	0.3291
LOC104911379	0.6881	1.7537	0.0758	4.5597	1.0000	0.6816	0.7701	3.1732	1.0000	-2.3771	1.0000	0.3101	1.0000	0.0716
LOC104911380	0.4976	1.9967	0.7186	1.0553	1.0000	-0.1624	0.7701	-3.1666	1.0000	0.7365	1.0000	-0.1908	1.0000	-2.2889
LOC104911381	0.4135	-0.9781	0.9952	0.3516	0.7579	-0.5384	1.0000	-0.1267	1.0000	-0.4694	0.8565	0.8726	1.0000	-0.0541

LOC104911382	1.0000	-0.3294	0.5050	-1.6397	1.0000	-0.7149	1.0000	-0.5492	1.0000	0.8170	1.0000	-0.4772	1.0000	0.9922
LOC104911384	1.0000	0.2121	0.0226	2.1489	0.0001	2.9333	0.0000	3.3619	1.0000	-0.5088	0.4309	1.4384	1.0000	-0.0821
LOC104911385	0.9899	0.4042	1.0000	-0.1764	0.0542	1.5437	0.0089	1.6176	1.0000	0.2226	1.0000	-0.3454	1.0000	0.3007
LOC104911387	1.0000	0.0000	1.0000	0.0000	1.0000	0.0000	1.0000	0.0000	1.0000	0.0000	1.0000	0.0000	1.0000	0.0000
LOC104911388	1.0000	0.5414	0.7674	3.0888	1.0000	0.6780	0.2460	4.0916	1.0000	-2.3771	1.0000	0.0533	1.0000	0.9923
LOC104911389	0.0000	-3.7761	0.0000	-2.6333	0.0004	-1.6716	0.0591	-0.8340	1.0000	-0.4470	1.0000	0.7115	0.9054	0.3984
LOC104911391	1.0000	-2.4788	1.0000	0.0000	1.0000	-2.4061	1.0000	0.0000	1.0000	-2.3771	1.0000	0.0000	1.0000	0.0000
LOC104911392	0.1994	0.6718	0.0001	1.4511	1.0000	-0.1584	0.4537	0.5037	1.0000	-0.7857	1.0000	0.0038	1.0000	-0.1186
LOC104911393	1.0000	2.1850	1.0000	0.0000	1.0000	0.0000	1.0000	0.0000	1.0000	0.0000	1.0000	-2.2958	1.0000	0.0000
LOC104911394	0.0001	1.0957	0.0000	1.1666	0.0000	2.3502	0.0000	1.7080	1.0000	0.2030	0.5392	0.2876	0.0027	-0.4325
LOC104911395	0.4986	0.4452	0.0141	0.9735	0.0041	1.2843	0.0000	1.4212	1.0000	-0.1078	0.5980	0.4330	1.0000	0.0374
LOC104911396	0.9925	0.2556	0.1066	0.9960	0.0002	1.4810	0.0183	1.0963	1.0000	-0.3789	0.9688	0.3766	0.0638	-0.7531
LOC104911397	0.9917	0.1348	0.0301	0.4573	0.0107	0.7169	0.0000	0.8161	1.0000	-0.1603	1.0000	0.1726	1.0000	-0.0595
LOC104911398	0.3856	0.1774	0.3696	0.1053	0.0014	0.5069	0.0000	0.4120	1.0000	0.0638	1.0000	0.0041	1.0000	-0.0257
LOC104911401	0.1950	-0.6869	0.0524	-0.6958	0.3384	-0.5272	0.0031	-1.0080	1.0000	0.1759	1.0000	0.1791	0.9203	-0.3012
LOC104911402	0.8226	3.0199	1.0000	0.0000	1.0000	0.0000	1.0000	0.0000	1.0000	0.0000	1.0000	-3.1429	1.0000	0.0000
LOC104911403	0.0089	1.0636	0.0002	1.0333	0.5761	0.3768	0.3894	0.3711	1.0000	-0.1313	1.0000	-0.1497	1.0000	-0.1364
LOC104911404	0.6989	0.5091	0.0148	1.1651	0.0004	1.7000	0.0867	0.9157	1.0000	-0.1319	0.7188	0.5378	0.0547	-0.9126
LOC104911405	1.0000	0.0061	1.0000	-0.0168	0.0004	-0.5896	0.0000	-0.5705	1.0000	-0.0432	1.0000	-0.0535	1.0000	-0.0188
LOC104911406	0.0055	1.0075	0.0000	0.9877	0.0000	1.3080	0.0000	1.0246	1.0000	0.1814	0.9533	0.1759	1.0000	-0.0947
LOC104911407	0.3151	0.7061	0.0515	0.9346	0.1579	0.8397	0.3218	0.6104	1.0000	-0.3652	1.0000	-0.1235	0.4738	-0.5891
LOC104911408	0.0000	1.5080	0.0001	0.8602	0.9361	0.1619	0.0157	-0.6742	1.0000	0.5967	1.0000	-0.0397	0.9428	-0.2345
LOC104911409	1.0000	0.0304	1.0000	-0.1801	0.9201	-0.3363	0.6098	-0.4186	1.0000	-0.2242	0.9306	-0.4153	1.0000	-0.2994
LOC104911410	0.1216	-1.1187	0.0977	-0.8436	0.2671	-0.8029	1.0000	-0.1205	1.0000	0.1359	1.0000	0.4256	0.3096	0.8251
LOC104911411	0.8249	3.0143	1.0000	-0.1452	0.8011	3.0840	1.0000	-2.3199	1.0000	2.2734	1.0000	-0.7919	1.0000	-3.1344
LOC104911412	0.2666	-0.3072	0.0000	-0.5420	0.3399	-0.2704	0.0000	-0.5935	1.0000	0.1933	1.0000	-0.0293	0.8441	-0.1257
LOC104911413	1.0000	0.0976	0.9716	0.1030	0.0081	0.8155	0.0587	0.4600	1.0000	0.0987	1.0000	0.1184	0.6425	-0.2471
LOC104911414	0.1413	0.6481	0.0715	0.6004	0.0000	1.3298	0.0069	0.7863	1.0000	0.1531	1.0000	0.1197	0.3503	-0.3813
LOC104911415	1.0000	0.3491	0.9062	1.2225	1.0000	-0.1781	0.9096	1.3850	1.0000	-1.4982	1.0000	-0.6341	1.0000	0.0732
LOC104911416	1.0000	0.0000	1.0000	0.0000	1.0000	0.0000	1.0000	0.0000	1.0000	0.0000	1.0000	0.0000	1.0000	0.0000
LOC104911418	0.6380	0.3632	1.0000	0.1322	0.6530	0.4424	0.4622	0.4203	1.0000	-0.3384	0.5089	-0.5552	0.8564	-0.3566
LOC104911419	1.0000	0.5420	1.0000	0.0000	1.0000	-2.4056	1.0000	2.3242	1.0000	-2.3757	1.0000	-3.1429	1.0000	2.3543
LOC104911420	0.8760	-0.6443	1.0000	-0.1792	0.0159	1.4903	0.5873	0.6238	1.0000	-0.1314	1.0000	0.3457	0.2147	-0.9915
LOC104911421	0.0493	0.3247	0.0000	0.3162	0.8381	0.0834	1.0000	0.0027	1.0000	-0.0151	1.0000	-0.0111	0.7026	-0.0903
LOC104911422	0.0013	0.7552	0.0000	0.6826	0.0357	0.5202	0.1294	0.2979	1.0000	0.0413	1.0000	-0.0174	0.7106	-0.1736
LOC104911423	1.0000	0.0000	1.0000	0.0000	1.0000	0.0000	1.0000	2.3242	1.0000	0.0000	1.0000	0.0000	1.0000	2.3543
LOC104911424	0.5972	1.3553	0.7675	1.0614	0.5567	1.2856	0.9801	0.9225	1.0000	-0.1140	1.0000	-0.4055	1.0000	-0.4790
LOC104911425	0.0002	-0.9760	0.0000	-0.8822	0.4561	-0.2550	0.0000	-0.7572	1.0000	0.2831	0.3109	0.3894	0.6476	-0.2144
LOC104911426	1.0000	-2.4788	1.0000	-0.1625	1.0000	-0.1597	1.0000	-0.8416	1.0000	0.7357	1.0000	3.1946	1.0000	0.0649
LOC104911427	1.0000	2.1850	1.0000	2.2534	1.0000	0.0000	1.0000	2.3256	1.0000	0.0000	1.0000	0.0514	1.0000	2.3555
LOC104911428	1.0000	-1.2491	0.2688	-2.4764	1.0000	-0.5733	1.0000	0.0094	1.0000	0.4254	1.0000	-0.7956	1.0000	1.0201
LOC104911429	0.1052	0.3663	0.0009	0.4171	0.0728	0.3886	0.0026	0.3816	1.0000	-0.1728	0.9060	-0.1098	0.4856	-0.1752
LOC104911430	0.8501	-0.2128	1.0000	-0.0770	0.0002	1.2805	0.0000	1.6554	1.0000	-1.3977	0.0000	-1.2523	0.0000	-1.0213
LOC104911431	1.0000	0.5864	1.0000	0.0000	0.0139	2.8198	0.4370	3.7037	1.0000	-3.2289	0.6427	-4.0589	0.0602	-2.4052
LOC104911432	0.2632	1.8696	0.2338	1.7080	0.0000	4.4635	0.0000	3.9355	1.0000	-0.6592	0.8646	-0.8174	0.0032	-1.1808
LOC104911433	1.0000	0.0000	1.0000	0.0000	1.0000	0.0000	1.0000	0.0000	1.0000	0.0000	1.0000	0.0000	1.0000	0.0000
LOC104911434	0.5086	3.5456	0.7674	3.0889	0.8033	3.0789	1.0000	0.0000	1.0000	0.0000	1.0000	-0.4766	1.0000	-3.1317
LOC104911435	0.5780	-1.5564	0.9148	1.2198	0.4426	-2.2477	0.9096	1.3850	1.0000	-2.1875	1.0000	0.5836	1.0000	1.4486
LOC104911436	0.1856	-4.5762	0.2495	-4.1757	0.4426	-2.2459	0.9786	-0.9111	1.0000	-0.4301	1.0000	0.0000	1.0000	0.9149
LOC104911437	1.0000	0.0000	1.0000	0.0000	1.0000	0.0000	1.0000	2.3242	1.0000	0.0000	1.0000	0.0000	1.0000	2.3543
LOC104911438	1.0000	2.1901	1.0000	2.2508	0.5076	3.6021	0.4370	3.7037	1.0000	0.0000	1.0000	0.0471	1.0000	0.0762
LOC104911439	0.0796	1.2888	0.0033	1.9305	0.0094	1.6010	0.0001	2.3667	1.0000	-0.7628	1.0000	-0.1120	1.0000	0.0055
LOC104911440	1.0000	0.0000	1.0000	0.0000	1.0000	0.0000	1.0000	0.0000	1.0000	0.0000	1.0000	0.0000	1.0000	0.0000
LOC104911441	1.0000	2.1901	1.0000	0.0000	1.0000	0.0000	1.0000	0.0000	1.0000	0.0000	1.0000	-2.2992	1.0000	0.0000
LOC104911442	0.8226	3.0199	1.0000	0.0000	1.0000	0.0000	1.0000	0.0000	1.0000	0.0000	1.0000	-3.1429	1.0000	0.0000
LOC104911443	0.0001	1.4117	0.0000	1.7024	0.0034	1.1444	0.0008	1.0036	1.0000	-0.0373	0.7914	0.2659	1.0000	-0.1740
LOC104911444	1.0000	0.0000	1.0000	0.0000	1.0000	0.0000	1.0000	0.0000	1.0000	0.0000	1.0000	0.0000	1.0000	0.0000
LOC104911445	1.0000	-2.4776	1.0000	0.0000	1.0000	-2.4056	1.0000	0.0000	1.0000	-2.3757	1.0000	0.0000	1.0000	0.0000
LOC104911446	1.0000	0.0000	1.0000	0.0000	1.0000	0.0000	1.0000	0.0000	1.0000	0.0000	1.0000	0.0000	1.0000	0.0000
LOC104911447	1.0000	0.0000	1.0000	0.0000	1.0000	0.0000	1.0000	0.0000	1.0000	0.0000	1.0000	0.0000	1.0000	0.0000
LOC104911448	0.0058	3.4610	0.0002	6.0206	0.4584	1.8985	0.7701	3.1732	1.0000	-2.3771	1.0000	0.0603	0.9852	-1.1488
LOC104911449	0.0180	5.3329	0.7186	1.0553	1.0000	0.0000	0.7701	-3.1666	1.0000	3.1126	0.8354	-1.0437	1.0000	0.0000
LOC104911450	0.0269	5.1972	0.4029	1.9116	1.0000	2.2470	1.0000	-2.3199	1.0000	2.2734	0.9320	-0.9076	1.0000	-2.2906
LOC104911451	1.0000	-0.1966	0.2903	1.1614	0.9561	-0.3616	0.8685	0.6605	1.0000	-0.9450	1.0000	0.4280	1.0000	0.0802
LOC104911452	0.2530	0.3285	0.9484	-0.0808	0.6365	-0.1941	1.0000	-0.0095	1.0000	0.2078	0.7458	-0.1891	0.0973	0.3984
LOC104911453	0.3582	0.3765	0.2231	0.2730	0.0927	0.5239	1.0000	0.0472	1.0000	0.4144	0.4903	0.3203	1.0000	-0.0572
LOC104911454	0.5416	0.3879	0.0118	0.8299	0.1023	0.7047	0.0040	0.9033	1.0000	-0.3101	1.0000	0.1422	1.0000	-0.1104
LOC104911455	0.0678	-0.3583	0.0001	-0.4630	0.0000	-1.1240	0.0000	-0.8924	1.0000	-0.0843	0.6116	-0.1763	0.6682	0.1533
LOC104911456	0.0833	1.2974	0.8995	0.3632	1.0000	-0.0625	1.0000	0.1122	1.0000	0.2405	0.7721	-0.6821	1.0000	0.4207
LOC104911457	1.0000	-2.4776	1.0000	2.2508	1.0000	-2.4056	0.2460	4.0916	1.0000	-2.3757	1.0000	2.3456	0.5105	4.1268
LOC104911458	0.0618	0.8733	0.1299	0.6888	0.0116	1.1201	0.0000	1.3788	1.0000	-0.4467	0.3216	-0.6187	1.0000	-0.1836
LOC104911459	0.0694	1.2084	0.0121	1.4691	0.0000	2.7144	0.0000	2.7860	1.0000	-0.3695	1.0000	-0.0946	0.8110	-0.2930
LOC104911460	0.9269	-0.0982	0.0038	0.3985	1.0000	0.0044	0.1128	0.2475	1.0000	-0.2203	0.1939	0.2893	1.0000	0.0293
LOC104911462	0.0070	2.9632	0.6963	0.6457	0.1867	1.9967	1.0000	0.2591	1.0000	1.0964	0.3313	-1.2044	0.9885	-0.6344
LOC104911463	0.9175	0.3027	0.0448	0.9546	0.6918	-0.5818	0.9046	-0.3277	1.0000	0.0256	0.4709	0.6926	1.0000	0.2854
LOC104911464	0.2770	0.3653	0.0000	0.6683	0.0000	2.1747	0.0000	2.5103	1.0000	0.0372	0.4065	0.3541	0.0000	0.3771
LOC104911466	0.0000	-2.2404	0.0000	-2.8475	0.0008	-1.5001	0.0000	-2.2733	1.0000	0.4879	1.0000	-0.1066	1.0000	-0.2790
LOC104911468	1.0000	-0.0424	1.0000	-0.1280	0.0311	1.0775	0.1186	0.7191	1.0000	-0.0868	1.0000	-0.1616	0.5628	-0.4369
LOC104911469	1.0000	-0.8577	0.4156	1.9155	0.5041	-3.7956	0.6483	1.7684	1.0000	-1.4938	1.0000	1.2809	0.5410	4.1211
LOC104911470	0.1245	0.4990	0.1287	0.3573	0.8082	-0.1766	0.0211	-0.5396	1.0000	0.2935	0.9920	0.1635	1.0000	-0.0663
LOC104911471	0.5384	3.5543	1.0000	2.2534	1.0000	2.2470	1.0000	2.3256	1.0000	0.0000	1.0000	-1.3292	1.0000	0.0649
LOC104911472	0.8858	1.0028	1.0000	0.6940	1.0000	0.2017	1.0000	0.0062	1.0000	-1.4938	0.6191	-1.8193	0.8939	-1.6957
LOC104911473	1.0000	-0.2931	1.0000	-0.1452	1.0000	-2.4061	1.0000	0.0045	1.0000	-0.1045	1.0000	0.0514	1.0000	2.3543
LOC104911474	1.0000	0.0000	0.7674	-3.2499	1.0000	0.0000	0.7710	-3.1633	1.0000	3.1056	1.0000	0.0000	1.0000	0.0000
LOC104911475	1.0000	0.0000	1.0000	2.2534	1.0000	0.0000	1.0000	2.3242	1.0000	0.0000	1.0000	2.3480	1.0000	2.3543
LOC104911476	0.5780	-1.5569	1.0000	2.2508	0.4426	-2.2479	1.0000	0.0000	1.0000	-4.4573	1.0000	-0.7966	1.0000	-2.2889
LOC104911477	0.8226	3.0199	0.7666	3.0922	1.0000	0.0000	1.0000	0.0000	1.0000	0.0000	1.0000	0.0547	1.0000	0.0000
LOC104911478	0.4782	2.0004	1.0000	-0.1630	1.0000	0.6780	0.9956	0.9147	1.0000	0.7402	0.9075	-1.4162	1.0000	0.9893
LOC104911479	1.0000	0.0000	1.0000	0.0000	1.0000	0.0000	1.0000	0.0000	1.0000	0.0000	1.0000	0.0000	1.0000	0.0000
LOC104911480	1.0000	0.0000	1.0000	0.0000	1.0000	0.0000	1.0000	0.0000	1.0000	0.0000	1.0000	0.0000	1.0000	0.0000
LOC104911481	1.0000	0.0000	1.0000	0.0000	1.0000	0.0000	1.0000	0.0000	1.0000	0.0000	1.0000	0.0000	1.0000	0.0000
LOC104911482	1.0000	0.0000	1.0000	0.0000	1.0000	0.0000	1.0000	0.0000	1.0000	0.0000	1.0000	0.0000	1.0000	0.0000
LOC104911483	1.0000	0.3477	0.2907	-1.6810	0.9468	-1.5484	0.8994	-0.5594	1.0000	1.3854	1.0000	-0.6318	0.5223	2.3911

LOC104911484	0.0000	-1.0243	0.0000	-0.9789	0.0000	-1.2520	0.0000	-1.1588	1.0000	-0.0115	1.0000	0.0462	0.7952	0.0873
LOC104911485	0.1212	-0.4369	0.0064	-0.3980	0.0002	-0.9753	0.0000	-0.7460	1.0000	0.2962	0.1949	0.3483	0.0274	0.5286
LOC104911486	0.0000	1.0958	0.0006	0.6232	0.0794	0.5290	0.3640	0.2296	1.0000	0.4180	1.0000	-0.0393	0.9907	0.1258
LOC104911487	1.0000	0.0000	1.0000	-2.3986	1.0000	0.0000	1.0000	-2.3199	1.0000	2.2734	1.0000	0.0000	1.0000	0.0000
LOC104911488	1.0000	-0.3503	0.0151	2.2362	0.7753	-0.5470	1.0000	0.6984	1.0000	-1.6354	0.7609	0.9591	1.0000	-0.3880
LOC104911490	1.0000	0.5453	0.1388	4.3092	1.0000	-2.4061	0.7710	3.1753	1.0000	-2.3771	1.0000	1.2783	1.0000	3.2081
LOC104911491	1.0000	0.0000	0.7674	-3.2570	1.0000	0.0000	0.7710	-3.1693	1.0000	3.1191	1.0000	0.0000	1.0000	0.0000
LOC104911494	1.0000	2.1850	1.0000	-1.0017	1.0000	0.0000	0.7701	-3.1666	1.0000	3.1126	1.0000	0.0495	1.0000	0.0000
LOC104911495	1.0000	0.0000	1.0000	0.0000	1.0000	0.0000	1.0000	0.0000	1.0000	0.0000	1.0000	0.0000	1.0000	0.0000
LOC104911497	0.0071	2.3786	0.1083	1.1211	0.5629	1.0694	0.6918	0.5794	1.0000	1.1445	1.0000	-0.1006	0.9778	0.6540
LOC104911498	0.0107	0.8666	0.0394	0.4975	0.8879	0.1700	0.2455	-0.3584	1.0000	0.3313	1.0000	-0.0263	0.9966	-0.1931
LOC104911499	0.9390	-0.5696	1.0000	-0.5543	1.0000	-0.6580	1.0000	-0.3811	1.0000	-0.8976	1.0000	-0.8773	1.0000	-0.6168
LOC104911500	0.8226	-3.3425	1.0000	-1.0017	1.0000	0.3490	0.4962	1.4811	1.0000	-0.1179	1.0000	2.3456	1.0000	1.0201
LOC104911501	0.0231	2.7544	0.3144	0.8739	0.0000	3.7373	0.4462	0.7586	1.0000	2.0592	1.0000	0.2031	0.3110	-0.9090
LOC104911502	0.1175	1.2932	0.1100	1.2526	0.0006	1.9748	0.0013	1.9280	1.0000	-0.4401	0.9176	-0.4734	0.6362	-0.4849
LOC104911503	1.0000	0.0064	0.0318	-0.2197	0.5750	-0.1526	0.0000	-0.4243	1.0000	0.1722	1.0000	-0.0407	0.8315	-0.0941
LOC104911504	0.8463	0.3278	0.9876	0.1514	0.0003	1.5448	0.0048	0.9473	1.0000	0.4465	1.0000	0.2839	1.0000	-0.1462
LOC104911505	0.0011	-2.1220	0.0562	-0.9520	0.0011	-2.0984	0.0015	-1.6813	1.0000	-0.2049	0.6379	0.9773	1.0000	0.2139
LOC104911506	0.8249	-3.3412	0.2535	-4.1743	1.0000	-0.1742	1.0000	0.5636	1.0000	0.7940	1.0000	0.0000	0.7915	1.5482
LOC104911507	0.0291	0.3889	0.0000	0.4124	0.0048	0.4728	0.0541	0.1913	1.0000	-0.0474	1.0000	-0.0117	0.0077	-0.3234
LOC104911508	0.1670	1.2548	0.1298	1.5390	0.6183	0.7793	0.2826	1.2293	1.0000	-0.7737	1.0000	-0.4878	1.0000	-0.3269
LOC104911509	0.5384	3.5542	0.7674	3.0889	1.0000	2.2426	1.0000	0.0000	1.0000	0.0000	1.0000	-0.4823	1.0000	-2.2889
LOC104911510	0.8249	-3.3412	1.0000	-0.1449	0.8033	-3.2622	1.0000	-2.3178	1.0000	-0.9614	1.0000	2.3456	1.0000	0.0000
LOC104911511	1.0000	0.0000	1.0000	0.0000	1.0000	0.0000	1.0000	0.0000	1.0000	0.0000	1.0000	0.0000	1.0000	0.0000
LOC104911512	1.0000	0.0000	1.0000	2.2534	1.0000	0.0000	1.0000	0.0000	1.0000	0.0000	1.0000	2.3480	1.0000	0.0000
LOC104911513	0.9430	0.2555	0.4624	0.4365	1.0000	-0.0440	1.0000	-0.0444	1.0000	-0.1345	1.0000	0.0612	1.0000	-0.1248
LOC104911514	0.4743	2.0033	0.9062	1.2235	1.0000	-2.4056	1.0000	0.0075	1.0000	-0.1078	1.0000	-0.8853	1.0000	2.3555
LOC104911515	0.1142	0.5574	0.0064	0.6221	0.9715	-0.1188	0.6322	-0.2211	1.0000	-0.0357	1.0000	0.0462	1.0000	-0.1287
LOC104911516	1.0000	-2.4788	0.7666	-3.2534	1.0000	-0.1597	1.0000	0.0073	1.0000	0.7357	1.0000	0.0000	1.0000	0.9169
LOC104911517	1.0000	0.0000	1.0000	2.2534	1.0000	0.0000	1.0000	0.0000	1.0000	0.0000	1.0000	2.3480	1.0000	0.0000
LOC104911518	1.0000	0.0000	1.0000	0.0000	1.0000	0.0000	1.0000	0.0000	1.0000	0.0000	1.0000	0.0000	1.0000	0.0000
LOC104911519	0.0020	-1.2313	0.0000	-1.7729	0.6231	0.2728	0.6618	-0.1871	1.0000	0.2872	1.0000	-0.2421	1.0000	-0.1681
LOC104911520	0.9539	0.4148	0.6893	0.4294	0.0000	2.5904	0.0000	1.7839	1.0000	0.3065	1.0000	0.3335	0.5046	-0.4974
LOC104911521	0.0053	1.6471	0.0129	1.0920	0.0000	2.5615	0.0000	1.7209	1.0000	0.7954	1.0000	0.2520	1.0000	-0.0438
LOC104911522	0.8109	1.1304	0.7674	-3.2499	1.0000	-0.1793	0.4992	1.4810	1.0000	-0.1245	0.3380	-4.6100	0.7908	1.5489
LOC104911525	1.0000	0.0000	1.0000	0.0000	1.0000	0.0000	1.0000	0.0000	1.0000	0.0000	1.0000	0.0000	1.0000	0.0000
LOC104911526	1.0000	0.0000	1.0000	0.0000	1.0000	0.0000	1.0000	0.0000	1.0000	0.0000	1.0000	0.0000	1.0000	0.0000
LOC104911527	1.0000	0.0000	1.0000	0.0000	1.0000	0.0000	1.0000	0.0000	1.0000	0.0000	1.0000	0.0000	1.0000	0.0000
LOC104911528	1.0000	0.0000	1.0000	0.0000	1.0000	0.0000	1.0000	0.0000	1.0000	0.0000	1.0000	0.0000	1.0000	0.0000
LOC104911529	0.8249	-3.3439	1.0000	-2.3959	1.0000	0.7446	1.0000	-2.3178	1.0000	-0.9638	1.0000	0.0000	0.5413	-4.0582
LOC104911530	1.0000	0.5414	1.0000	2.2533	0.9361	1.2042	1.0000	0.0000	1.0000	-2.3771	1.0000	-0.7956	0.7287	-3.6634
LOC104911532	1.0000	-2.4788	0.7674	3.0889	1.0000	-0.1597	1.0000	2.3256	1.0000	-2.3771	1.0000	3.1946	1.0000	0.0649
LOC104911533	1.0000	0.0000	1.0000	0.0000	1.0000	0.0000	1.0000	0.0000	1.0000	0.0000	1.0000	0.0000	1.0000	0.0000
LOC104911534	1.0000	0.0000	1.0000	0.0000	1.0000	0.0000	1.0000	0.0000	1.0000	0.0000	1.0000	0.0000	1.0000	0.0000
LOC104911535	1.0000	0.0000	1.0000	0.0000	1.0000	0.0000	1.0000	0.0000	1.0000	0.0000	1.0000	0.0000	1.0000	0.0000
LOC104911536	0.6881	1.7543	0.1478	4.3119	0.4424	1.8936	1.0000	2.3256	1.0000	-2.3757	1.0000	0.0593	0.6865	-1.9990
LOC104911537	1.0000	0.0118	1.0000	-0.0779	0.0000	-1.5087	0.0395	-0.7435	1.0000	-0.4949	0.1832	-0.5734	1.0000	0.2754
LOC104911538	1.0000	0.1003	1.0000	-0.0575	0.0023	-1.1130	0.0001	-0.9535	1.0000	0.4567	0.5692	0.3138	0.2524	0.6246
LOC104911540	1.0000	0.0000	1.0000	0.0000	1.0000	0.0000	1.0000	0.0000	1.0000	0.0000	1.0000	0.0000	1.0000	0.0000
LOC104911541	1.0000	2.1850	0.7674	3.0956	1.0000	2.2426	1.0000	2.3256	1.0000	0.0000	1.0000	0.9035	1.0000	0.0661
LOC104911542	0.8249	-3.3412	1.0000	0.0000	0.8033	-3.2622	0.7701	3.1732	1.0000	-3.2289	1.0000	0.0000	1.0000	3.2066
LOC104911543	1.0000	2.1851	1.0000	-2.3960	0.8011	3.0840	1.0000	0.0062	1.0000	2.2676	1.0000	-2.2958	1.0000	-0.7795
LOC104911545	1.0000	0.0000	1.0000	0.0000	1.0000	0.0000	1.0000	0.0000	1.0000	0.0000	1.0000	0.0000	1.0000	0.0000
LOC104911546	1.0000	0.0596	0.4950	0.8999	0.9361	0.5161	1.0000	0.0069	1.0000	-0.1272	1.0000	0.7169	0.9885	-0.6344
LOC104911547	0.0002	1.5282	0.0014	1.1026	0.0004	-2.4245	0.1176	-0.8964	1.0000	0.0891	0.7740	-0.3186	0.1846	1.6262
LOC104911548	1.0000	-0.0028	1.0000	0.0951	1.0000	0.0497	0.8065	-0.1423	1.0000	0.1574	0.8314	0.2657	1.0000	-0.0334
LOC104911549	1.0000	2.1901	1.0000	-2.3986	1.0000	0.0000	1.0000	-2.3199	1.0000	2.2735	1.0000	-2.2992	1.0000	0.0000
LOC104911554	0.2714	2.4169	0.7666	-3.2534	1.0000	-2.4056	1.0000	-0.8416	1.0000	0.7365	0.1882	-5.0277	1.0000	2.3555
LOC104911555	1.0000	0.0000	1.0000	0.0000	1.0000	0.0000	1.0000	0.0000	1.0000	0.0000	1.0000	0.0000	1.0000	0.0000
LOC104911556	1.0000	0.0000	1.0000	0.0000	1.0000	0.0000	1.0000	0.0000	1.0000	0.0000	1.0000	0.0000	1.0000	0.0000
LOC104911557	1.0000	0.0000	1.0000	0.0000	1.0000	0.0000	1.0000	0.0000	1.0000	0.0000	1.0000	0.0000	1.0000	0.0000
LOC104911558	1.0000	-1.2504	0.0113	-3.3308	0.6932	0.9301	0.8345	0.4462	1.0000	1.2762	1.0000	-0.7956	0.8198	0.8006
LOC104911559	0.3325	-1.2204	1.0000	0.0754	0.0555	-2.2214	1.0000	-0.2977	1.0000	-1.4629	1.0000	-0.1594	1.0000	0.4653
LOC104911560	0.3365	-1.3170	1.0000	-0.1761	0.6417	-0.9069	0.7948	-0.5108	1.0000	0.2393	0.6050	1.3976	1.0000	0.6442
LOC104911561	0.8473	0.3504	0.9168	0.2440	0.0085	-1.8100	0.1234	-0.9784	1.0000	-0.0772	1.0000	-0.1700	0.9124	0.7628
LOC104911562	1.0000	0.5464	0.4431	-3.7876	1.0000	-2.4056	1.0000	-0.5247	1.0000	1.2661	1.0000	-3.1467	1.0000	3.2049
LOC104911564	1.0000	0.0000	1.0000	2.2508	1.0000	0.0000	1.0000	0.0000	1.0000	0.0000	1.0000	2.3456	1.0000	0.0000
LOC104911565	1.0000	0.0000	1.0000	0.0000	1.0000	0.0000	1.0000	0.0000	1.0000	0.0000	1.0000	0.0000	1.0000	0.0000
LOC104911566	1.0000	2.1851	1.0000	0.0000	1.0000	2.2426	1.0000	2.3256	1.0000	0.0000	1.0000	-2.2958	1.0000	0.0661
LOC104911567	1.0000	0.0000	1.0000	0.0000	1.0000	0.0000	1.0000	0.0000	1.0000	0.0000	1.0000	0.0000	1.0000	0.0000
LOC104911568	1.0000	0.0000	1.0000	0.0000	1.0000	0.0000	1.0000	2.3241	1.0000	0.0000	1.0000	0.0000	1.0000	2.3543
LOC104911569	0.1812	0.9843	0.0233	1.6235	1.0000	0.2351	0.1956	1.1723	1.0000	-1.1075	0.9572	-0.4592	1.0000	-0.1640
LOC104911570	1.0000	2.1850	1.0000	0.0000	1.0000	2.2425	1.0000	0.0000	1.0000	0.0000	1.0000	-2.2958	1.0000	-2.2889
LOC104911571	1.0000	-0.1628	0.7263	-0.7406	0.6618	-1.3481	0.4641	-1.1152	1.0000	0.2259	1.0000	-0.3407	1.0000	0.4627
LOC104911572	1.0000	0.0000	1.0000	0.0000	1.0000	0.0000	1.0000	0.0000	1.0000	0.0000	1.0000	0.0000	1.0000	0.0000
LOC104911573	1.0000	0.0000	1.0000	0.0000	1.0000	0.0000	1.0000	0.0000	1.0000	0.0000	1.0000	0.0000	1.0000	0.0000
LOC104911574	1.0000	-2.4776	1.0000	0.0000	1.0000	-2.4056	1.0000	0.0000	1.0000	-2.3757	1.0000	0.0000	1.0000	0.0000
LOC104911575	1.0000	0.0000	1.0000	0.0000	1.0000	0.0000	1.0000	0.0000	1.0000	0.0000	1.0000	0.0000	1.0000	0.0000
LOC104911576	1.0000	0.0000	1.0000	0.0000	1.0000	0.0000	1.0000	0.0000	1.0000	0.0000	1.0000	0.0000	1.0000	0.0000
LOC104911578	1.0000	-2.4776	1.0000	-2.3985	1.0000	0.6783	0.9096	1.3845	1.0000	-0.1034	1.0000	0.0000	1.0000	0.6041
LOC104911579	1.0000	0.1646	0.4190	-0.7967	1.0000	-0.3470	0.6745	-0.6136	1.0000	0.4892	1.0000	-0.4575	1.0000	0.2412
LOC104911580	0.9684	-0.5176	0.0221	1.9277	1.0000	0.2077	0.0123	2.1095	1.0000	-1.2491	0.5203	1.2056	0.8898	0.6553
LOC104911581	1.0000	0.0455	0.7473	0.1099	0.1442	-0.4333	0.0036	-0.5114	1.0000	-0.1210	1.0000	-0.0460	0.7874	-0.1927
LOC104911584	1.0000	-0.0403	0.0002	-0.3610	0.6394	-0.1692	0.0003	-0.3418	1.0000	-0.0694	0.0034	-0.3773	0.0946	-0.2362
LOC104911585	1.0000	0.0000	1.0000	0.0000	1.0000	0.0000	0.0402	4.8608	1.0000	0.0000	1.0000	0.0000	0.1347	4.8972
LOC104911586	0.1689	1.1312	0.2249	0.5801	0.4232	-0.9548	0.0140	-1.4204	1.0000	1.1777	0.4742	0.6415	1.0000	0.7133
LOC104911589	0.9537	-0.3550	0.7913	0.3181	0.0579	-1.7334	0.0950	-1.1010	1.0000	0.5407	0.1427	1.2279	0.6818	1.1781
LOC104911590	0.0000	1.3192	0.0000	1.1031	0.0000	-1.9403	0.0000	-2.0724	1.0000	0.3823	0.8785	0.1772	1.0000	0.2543
LOC104911591	0.5712	0.7550	0.5889	0.6548	0.0545	-2.5102	0.1877	-1.6501	1.0000	-0.2642	1.0000	-0.3509	1.0000	0.6041
LOC104911592	1.0000	0.6043	1.0000	0.0386	1.0000	-0.1865	0.7715	-0.9364	1.0000	0.8189	1.0000	0.2714	1.0000	0.0752
LOC104911593	0.8226	3.0199	1.0000	0.1281	1.0000	2.2425	0.6431	-1.7680	1.0000	4.0426	1.0000	1.2787	1.0000	0.0652
LOC104911594	1.0000	-0.2882	1.0000	-0.1452	1.0000	0.6783	1.0000	-2.3199	1.0000	-0.1034	1.0000	0.0489	1.0000	-3.1344
LOC104911595	1.0000	0.0000	1.0000	0.0000	1.0000	0.0000	1.0000	2.3256	1.0000	0.0000	1.0000	0.0000	1.0000	2.3555

LOC104911600	0.1249	0.8441	0.0007	1.0623	0.0003	1.5498	0.0001	1.2046	1.0000	0.3780	0.2404	0.6081	1.0000	0.0369
LOC104911601	1.0000	0.2130	0.6523	0.7855	0.0341	1.7596	0.0001	2.8844	1.0000	-0.6901	1.0000	-0.1049	0.8210	0.4450
LOC104911602	0.0664	1.6560	0.0006	2.1450	0.0014	2.2571	0.0745	1.4147	1.0000	0.0892	0.7085	0.5861	0.4738	-0.7511
LOC104911603	1.0000	2.1851	0.7666	3.0922	1.0000	0.0000	1.0000	0.0000	1.0000	0.0000	1.0000	0.9006	1.0000	0.0000
LOC104911604	1.0000	0.0000	1.0000	2.2508	1.0000	2.2425	1.0000	0.0000	1.0000	0.0000	1.0000	2.3456	1.0000	-2.2889
LOC104911605	0.0719	4.8747	0.1384	4.3086	0.8033	3.0892	1.0000	0.0000	1.0000	0.0000	1.0000	-0.5915	1.0000	-3.1369
LOC104911606	0.5751	-1.2988	1.0000	-0.8619	0.1163	-2.9016	1.0000	0.0073	1.0000	-0.7802	1.0000	-0.3361	0.6865	2.1419
LOC104911607	1.0000	0.0000	1.0000	0.0000	1.0000	0.0000	1.0000	0.0000	1.0000	0.0000	1.0000	0.0000	1.0000	0.0000
LOC104911609	1.0000	0.0000	1.0000	0.0000	1.0000	0.0000	1.0000	0.0000	1.0000	0.0000	1.0000	0.0000	1.0000	0.0000
LOC104911610	0.2105	0.5119	0.0286	0.5280	0.4692	-0.3812	0.0783	-0.4936	1.0000	0.2722	0.6820	0.3020	1.0000	0.1638
LOC104911611	0.0002	-6.2124	0.0004	-2.4434	1.0000	0.0468	0.8399	-0.3123	1.0000	0.5081	0.4563	4.4223	1.0000	0.1528
LOC104911613	1.0000	0.2219	0.2474	1.3533	0.2894	-2.4999	0.9786	-0.9103	1.0000	-0.6840	1.0000	0.4622	1.0000	0.9168
LOC104911615	0.0000	-3.4371	0.0000	-2.3812	1.0000	0.0271	0.2846	0.4440	1.0000	-0.1990	0.8565	0.8726	0.9759	0.2221
LOC104911616	1.0000	0.5420	1.0000	-2.3960	1.0000	-2.4056	1.0000	-2.3178	1.0000	-0.1078	1.0000	-3.1429	1.0000	0.0000
LOC104911617	1.0000	0.0000	1.0000	0.0000	1.0000	0.0000	1.0000	0.0000	1.0000	0.0000	1.0000	0.0000	1.0000	0.0000
LOC104911619	1.0000	0.0000	1.0000	-0.1429	1.0000	0.0000	0.9164	1.3891	1.0000	2.2676	1.0000	2.3480	0.7453	3.7416
LOC104911620	1.0000	0.0000	1.0000	0.0000	1.0000	0.0000	1.0000	2.3241	1.0000	0.0000	1.0000	0.0000	1.0000	2.3543
LOC104911621	1.0000	0.0000	1.0000	0.0000	1.0000	2.2425	1.0000	0.0000	1.0000	0.0000	1.0000	0.0000	1.0000	-2.2889
LOC104911622	1.0000	0.0000	1.0000	0.0000	1.0000	0.0000	1.0000	0.0000	1.0000	0.0000	1.0000	0.0000	1.0000	0.0000
LOC104911623	1.0000	0.0000	1.0000	0.0000	1.0000	0.0000	1.0000	0.0000	1.0000	0.0000	1.0000	0.0000	1.0000	0.0000
LOC104911624	1.0000	0.0000	1.0000	0.0000	1.0000	0.0000	1.0000	0.0000	1.0000	0.0000	1.0000	0.0000	1.0000	0.0000
LOC104911625	1.0000	0.5464	1.0000	-0.1608	1.0000	-0.1591	1.0000	0.0072	1.0000	0.7318	1.0000	0.0496	1.0000	0.9149
LOC104911626	1.0000	0.3037	0.0974	2.1527	0.9100	0.6262	0.3361	1.6955	1.0000	-1.3466	1.0000	0.5067	1.0000	-0.2732
LOC104911627	1.0000	-0.2931	0.6215	-1.9216	1.0000	-0.1597	0.6172	-1.7581	1.0000	1.6470	1.0000	0.0514	1.0000	0.0649
LOC104911628	0.5086	3.5431	1.0000	-0.1688	1.0000	0.0000	0.9096	-1.3717	1.0000	3.6395	1.0000	0.0554	1.0000	2.3555
LOC104911629	1.0000	0.5864	1.0000	0.3833	0.2745	1.8499	1.0000	0.5626	1.0000	0.8006	1.0000	0.6149	1.0000	-0.4867
LOC104911630	0.5844	0.4113	0.0389	0.7944	0.0487	0.8739	0.0372	0.7715	1.0000	-0.0552	0.8875	0.3417	1.0000	-0.1504
LOC104911632	1.0000	-0.0706	0.9743	-0.0552	0.0117	-0.6266	0.0000	-0.7042	1.0000	0.0417	1.0000	0.0694	1.0000	-0.0300
LOC104911633	0.7810	-0.7540	0.8672	0.4785	0.7629	-0.8500	1.0000	-0.2973	1.0000	-0.7849	1.0000	0.4600	1.0000	-0.2273
LOC104911635	0.4166	0.9759	1.0000	-0.1772	1.0000	0.1635	0.1624	-1.5204	1.0000	0.6303	1.0000	-0.5125	0.7758	-1.0468
LOC104911636	0.7250	0.3310	0.0186	0.7612	0.0073	1.0502	0.0000	1.5143	1.0000	-0.2895	1.0000	0.1533	0.9429	0.1866
LOC104911637	0.3635	0.7177	0.2545	0.8092	0.6783	0.4918	0.1122	0.9963	1.0000	-0.3220	1.0000	-0.2169	1.0000	0.1878
LOC104911638	0.3123	1.0446	0.4914	0.5222	0.0028	1.8704	0.0227	1.1085	1.0000	0.5139	1.0000	0.0150	1.0000	-0.2327
LOC104911639	0.9545	1.0672	1.0000	-0.1449	0.2858	2.1449	0.6142	1.7721	1.0000	-0.1089	1.0000	-1.3266	1.0000	-0.4791
LOC104911640	1.0000	0.5420	1.0000	-0.1472	1.0000	0.6783	1.0000	-2.3199	1.0000	-0.1034	1.0000	-0.7966	1.0000	-3.1344
LOC104911641	0.7902	1.1393	0.5865	0.9876	0.3922	1.5029	1.0000	0.0094	1.0000	0.4032	1.0000	0.2731	0.8620	-1.0809
LOC104911642	1.0000	0.0000	1.0000	-2.3960	1.0000	0.0000	1.0000	0.0062	1.0000	2.2676	1.0000	0.0000	1.0000	2.3543
LOC104911644	0.0366	1.5607	0.9322	0.3180	1.0000	0.2562	1.0000	-0.1357	1.0000	1.2373	1.0000	0.0112	0.6818	0.8535
LOC104911645	0.0129	0.9707	0.0000	1.5154	0.0033	-1.5985	1.0000	-0.1211	1.0000	-0.5819	1.0000	-0.0256	0.2428	0.9014
LOC104911646	0.8982	-0.6011	1.0000	-0.1630	0.8309	-1.1322	1.0000	0.5367	1.0000	-1.5939	1.0000	-1.1654	1.0000	0.0750
LOC104911647	0.0005	-0.5683	0.0000	-0.7785	0.0048	-0.4776	0.0001	-0.3322	1.0000	0.1976	1.0000	0.0000	0.0031	0.3479
LOC104911649	0.1811	-0.3718	0.0017	-0.4620	0.0000	-1.0442	0.0000	-1.0188	1.0000	-0.0433	1.0000	-0.1218	1.0000	-0.0141
LOC104911650	0.0000	1.9438	0.0000	2.0701	0.0000	3.0926	0.0000	2.4553	1.0000	-0.0171	1.0000	0.1269	0.0502	-0.6440
LOC104911651	0.0956	0.4864	0.0000	0.6285	0.6435	0.2090	1.0000	0.0400	1.0000	0.0273	0.6524	0.1823	0.8877	-0.1339
LOC104911652	1.0000	-0.3294	0.3421	1.5203	0.4809	-3.7967	1.0000	-0.8416	1.0000	-0.6504	0.9773	1.2144	1.0000	2.3555
LOC104911655	0.7583	-0.8239	0.4670	3.6266	0.6373	-1.3491	0.4622	3.6989	1.0000	-4.9245	1.0000	-0.6112	1.0000	0.0745
LOC104911656	0.5384	3.5542	1.0000	2.2533	1.0000	0.0000	1.0000	0.0000	1.0000	0.0000	1.0000	-1.3292	1.0000	0.0000
LOC104911657	0.7717	0.1259	0.6436	-0.0848	0.1412	-0.3465	0.0000	-0.5113	1.0000	0.1780	1.0000	-0.0211	1.0000	0.0182
LOC104911658	1.0000	0.0491	0.9524	-0.3120	0.5879	-1.0103	0.0544	-2.3154	1.0000	0.1534	1.0000	-0.1970	0.9835	-1.1482
LOC104911659	1.0000	-0.3163	1.0000	-2.3960	0.8033	-3.2622	1.0000	0.0075	1.0000	-0.9614	1.0000	-3.1467	1.0000	2.3555
LOC104911660	1.0000	0.0000	1.0000	0.0000	1.0000	0.0000	1.0000	0.0000	1.0000	0.0000	1.0000	0.0000	1.0000	0.0000
LOC104911661	0.1856	-4.5762	0.0011	-3.1885	0.7469	0.7683	0.0562	-1.5383	1.0000	1.6669	1.0000	3.2002	0.9875	-0.6341
LOC104911662	1.0000	-0.1627	0.8594	0.4373	0.2243	-1.3832	0.0173	-3.1636	1.0000	-0.4705	1.0000	0.1420	0.5109	-2.2511
LOC104911663	1.0000	0.0000	1.0000	0.0000	1.0000	2.2425	1.0000	2.3256	1.0000	0.0000	1.0000	0.0000	1.0000	0.0661
LOC104911664	1.0000	0.0000	1.0000	2.2508	1.0000	2.2470	1.0000	0.0000	1.0000	0.0000	1.0000	2.3456	1.0000	-2.2906
LOC104911665	0.0479	0.4692	0.8863	-0.0662	0.2888	-0.3115	0.0001	-0.5688	1.0000	0.3883	0.8795	-0.1352	0.9162	0.1340
LOC104911666	1.0000	0.0000	1.0000	0.0000	1.0000	0.0000	1.0000	0.0000	1.0000	0.0000	1.0000	0.0000	1.0000	0.0000
LOC104911667	0.0000	-0.6207	0.0000	-0.7426	0.7845	-0.0917	0.0055	-0.2081	1.0000	0.1097	1.0000	0.0001	1.0000	-0.0014
LOC104911668	1.0000	0.0000	1.0000	0.0000	1.0000	0.0000	1.0000	0.0000	1.0000	0.0000	1.0000	0.0000	1.0000	0.0000
LOC104911670	1.0000	0.0000	1.0000	-2.3960	1.0000	0.0000	1.0000	-2.3178	1.0000	2.2676	1.0000	0.0000	1.0000	0.0000
LOC104911671	0.9872	-0.0708	0.4872	-0.1666	0.0000	1.3721	0.0000	1.1376	1.0000	0.2927	0.2266	0.2086	1.0000	0.0629
LOC104911672	1.0000	0.0000	1.0000	0.0000	1.0000	0.0000	1.0000	0.0000	1.0000	0.0000	1.0000	0.0000	1.0000	0.0000
LOC104911673	1.0000	0.0000	1.0000	0.0000	1.0000	0.0000	1.0000	0.0000	1.0000	0.0000	1.0000	0.0000	1.0000	0.0000
LOC104911675	1.0000	0.0000	1.0000	0.0000	1.0000	0.0000	1.0000	0.0000	1.0000	0.0000	1.0000	0.0000	1.0000	0.0000
LOC104911676	0.8425	0.8181	1.0000	0.6912	0.9361	-1.5532	1.0000	-2.3199	1.0000	-1.4918	0.7743	-1.6347	1.0000	-2.2889
LOC104911677	0.0417	-2.2603	1.0000	-0.1598	0.5329	-0.7651	0.0873	2.1944	1.0000	-2.5687	1.0000	-0.4731	1.0000	0.3916
LOC104911679	0.0089	-0.7338	0.1967	-0.2836	0.0919	-0.5454	1.0000	-0.0133	1.0000	-0.2126	0.7598	0.2482	0.5421	0.3264
LOC104911681	0.0548	-0.4459	0.0024	-0.4502	0.0018	-0.6495	0.0003	-0.5306	1.0000	-0.1972	0.7086	-0.1893	1.0000	-0.0739
LOC104911682	1.0000	0.0000	1.0000	-2.3960	1.0000	0.0000	1.0000	-2.3178	1.0000	2.2676	1.0000	0.0000	1.0000	0.0000
LOC104911683	1.0000	-0.1296	0.0509	-0.7210	1.0000	-0.0199	1.0000	-0.1044	1.0000	0.0284	0.6190	-0.5535	1.0000	-0.0517
LOC104911685	0.5220	1.1729	0.7800	0.7737	0.7004	-1.9462	1.0000	-0.5176	1.0000	-0.5259	0.8607	-0.9104	1.0000	0.9186
LOC104911686	1.0000	0.0000	1.0000	0.0000	1.0000	0.0000	1.0000	0.0000	1.0000	0.0000	1.0000	0.0000	1.0000	0.0000
LOC104911687	0.2370	-0.2954	0.0002	-0.5004	0.0000	-1.5630	0.0000	-0.8100	1.0000	-0.0983	0.2506	-0.2908	0.0003	0.6602
LOC104911688	0.1012	-0.4008	0.0308	-0.4276	0.0055	-0.6214	0.0152	-0.4884	1.0000	-0.2890	0.2660	-0.3036	0.8306	-0.1512
LOC104911689	1.0000	0.0000	1.0000	0.0000	1.0000	0.0000	1.0000	0.0000	1.0000	0.0000	1.0000	0.0000	1.0000	0.0000
LOC104911690	0.0000	-1.1867	0.0000	-1.2298	0.0107	-0.5355	0.3394	-0.0998	1.0000	-0.2284	0.2872	-0.2594	0.1208	0.2125
LOC104911691	0.0000	-7.0594	0.0000	-4.4992	0.4073	-0.6624	0.3940	-0.6290	1.0000	-0.3407	1.0000	2.3456	1.0000	-0.3065
LOC104911692	0.0565	-3.2106	0.0066	-5.4489	0.0176	-5.3108	0.1877	-1.6500	1.0000	0.0192	1.0000	-2.2958	0.7287	3.7387
LOC104911693	0.7109	0.5270	1.0000	-0.1286	0.0000	2.4190	0.0000	1.5842	1.0000	0.8542	1.0000	0.2159	1.0000	0.0294
LOC104911694	0.0364	0.8552	0.0000	1.4192	0.0000	1.6889	0.0000	1.5987	1.0000	-0.1012	0.2715	0.4752	0.9032	-0.1874
LOC104911695	0.3577	0.3755	0.0182	0.4884	0.0021	0.9342	0.0001	0.7254	1.0000	0.1470	0.6116	0.2729	1.0000	-0.0581
LOC104911696	0.4265	0.2125	0.0276	0.2639	0.5213	-0.1821	0.1951	-0.1836	1.0000	0.1815	0.2142	0.2451	0.4084	0.1852
LOC104911697	0.0000	2.3204	0.0000	2.7370	0.0000	3.0097	0.0000	2.4665	1.0000	0.1715	0.2061	0.6009	0.2705	-0.3634
LOC104911698	1.0000	-2.4788	1.0000	0.6987	1.0000	-2.4061	1.0000	-2.3178	1.0000	-0.1089	1.0000	3.2002	1.0000	0.0000
LOC104911699	0.0053	-0.6187	0.0000	-0.9077	0.4809	-0.1983	0.3572	-0.1298	1.0000	0.1111	0.8603	-0.1657	0.4273	0.1828
LOC104911700	0.0015	2.2572	0.0144	1.5974	0.0001	2.9093	0.0008	1.9740	1.0000	0.2750	1.0000	-0.3755	0.4685	-0.6618
LOC104911701	0.0079	1.6824	0.0000	3.0250	0.0000	2.7137	0.0000	2.9944	1.0000	-0.4117	0.0267	0.9426	1.0000	-0.1293
LOC104911702	0.2360	-0.8029	0.3193	-0.5780	0.0107	-1.6233	0.6132	-0.3901	1.0000	0.0077	1.0000	0.2435	0.1727	1.2462
LOC104911703	0.8276	-0.1825	0.7407	0.1689	0.8114	-0.1765	0.0427	-0.5162	1.0000	-0.1695	1.0000	0.1944	0.1866	-0.5028
LOC104911704	0.2585	1.2556	0.0006	1.8308	0.0041	2.0789	0.2839	0.9827	1.0000	0.3717	0.2467	0.9557	0.5448	-0.7189
LOC104911705	1.0000	2.1901	1.0000	2.2508	1.0000	0.0000	1.0000	0.0000	1.0000	0.0000	1.0000	0.0471	1.0000	0.0000
LOC104911706	1.0000	0.0000	1.0000	0.0000	1.0000	2.2426	1.0000	0.0000	1.0000	0.0000	1.0000	0.0000	1.0000	-2.2889
LOC104911707	0.0604	0.5825	0.0091	0.5584	0.0000	1.6258	0.0000	0.9113	1.0000	-0.0198	1.0000	-0.0304	0.0007	-0.7299

LOC104911708	1.0000	0.0000	1.0000	0.0000	1.0000	0.0000	1.0000	0.0000	1.0000	0.0000	1.0000	0.0000	1.0000	0.0000
LOC104911709	1.0000	-2.4776	1.0000	0.0000	1.0000	-2.4056	1.0000	0.0000	1.0000	-2.3757	1.0000	0.0000	1.0000	0.0000
LOC104911710	0.9545	1.0672	1.0000	-2.3985	1.0000	0.6816	1.0000	-2.3200	1.0000	-0.1045	0.8607	-3.6732	1.0000	-3.1369
LOC104911711	0.5779	1.3527	0.6224	-1.9258	1.0000	-0.1789	0.6172	-1.7602	1.0000	0.7988	0.4861	-2.4826	1.0000	-0.7795
LOC104911712	1.0000	2.1850	1.0000	0.0000	1.0000	0.0000	1.0000	0.0000	1.0000	0.0000	1.0000	-2.2958	1.0000	0.0000
LOC104911713	1.0000	-0.3562	0.4766	-1.2945	1.0000	-0.1935	0.4652	-1.1144	1.0000	-0.1321	0.8841	-1.0622	0.7629	-1.0450
LOC104911714	1.0000	0.0000	1.0000	0.0000	1.0000	0.0000	1.0000	0.0000	1.0000	0.0000	1.0000	0.0000	1.0000	0.0000
LOC104911715	0.0001	-1.2455	0.0000	-1.4046	0.0000	-2.9148	0.0000	-1.1527	1.0000	0.3174	1.0000	0.1727	0.0000	2.0843
LOC104911716	1.0000	-1.1537	1.0000	0.1327	1.0000	0.3467	0.9786	-0.9114	1.0000	0.7988	0.8269	2.1242	1.0000	-0.4568
LOC104911718	1.0000	0.0000	1.0000	0.0000	1.0000	0.0000	1.0000	0.0000	1.0000	0.0000	1.0000	0.0000	1.0000	0.0000
LOC104911719	1.0000	0.0000	1.0000	0.0000	1.0000	0.0000	1.0000	0.0000	1.0000	0.0000	1.0000	0.0000	1.0000	0.0000
LOC104911720	0.0486	1.8406	0.0428	1.4414	0.0000	2.7959	0.0000	2.7125	1.0000	-0.3208	0.7567	-0.7070	0.7554	-0.3975
LOC104911721	0.8815	0.1664	0.7023	0.1460	0.0006	0.9543	0.0005	0.6560	1.0000	0.1214	1.0000	0.1126	0.8112	-0.1746
LOC104911722	1.0000	0.0000	1.0000	-0.1449	1.0000	0.0000	1.0000	-2.3178	1.0000	2.2676	1.0000	2.3456	1.0000	0.0000
LOC104911723	0.0000	-0.7713	0.0000	-1.0690	0.4674	-0.1692	0.0011	-0.3081	1.0000	0.1619	0.8553	-0.1226	1.0000	0.0294
LOC104911724	0.8226	3.0199	1.0000	0.0000	1.0000	2.2426	1.0000	0.0000	1.0000	0.0000	1.0000	-3.1429	1.0000	-2.2889
LOC104911725	1.0000	2.1850	0.7674	3.0889	1.0000	2.2426	0.7710	3.1709	1.0000	0.0000	1.0000	0.8992	1.0000	0.9164
LOC104911726	1.0000	0.0000	1.0000	0.0000	1.0000	0.0000	1.0000	0.0000	1.0000	0.0000	1.0000	0.0000	1.0000	0.0000
LOC104911727	1.0000	-0.2892	0.9762	0.7528	0.9361	1.2042	1.0000	-0.8388	1.0000	0.7304	1.0000	1.8187	1.0000	-1.3081
LOC104911728	0.8199	0.7517	0.1783	1.2019	1.0000	-0.8798	0.4992	-1.4660	1.0000	0.1217	1.0000	0.5889	1.0000	-0.4568
LOC104911729	0.0048	-0.5342	0.0000	-0.5928	0.0018	-0.5191	0.0000	-0.3646	1.0000	-0.0206	1.0000	-0.0665	0.5087	0.1395
LOC104911730	0.0000	1.6651	0.0000	1.0557	0.0000	2.3409	0.0000	1.0317	1.0000	0.6929	1.0000	0.0947	0.0099	-0.6121
LOC104911731	0.0125	0.5024	0.0012	0.3580	0.0005	-0.7016	0.0000	-0.8194	1.0000	0.0031	0.8269	-0.1287	0.9380	-0.1086
LOC104911732	0.5551	0.2154	0.1509	0.2084	0.0000	-1.1756	0.0000	-0.9715	1.0000	0.0117	1.0000	0.0181	0.6033	0.2213
LOC104911733	1.0000	2.1850	0.7674	-3.2570	1.0000	0.0000	1.0000	-0.8442	1.0000	3.1191	1.0000	-2.2958	1.0000	2.3543
LOC104911734	1.0000	0.0000	0.7666	-3.2534	1.0000	0.0000	1.0000	-0.8419	1.0000	3.1126	1.0000	0.0000	1.0000	2.3543
LOC104911735	1.0000	0.0000	1.0000	0.0000	1.0000	0.0000	1.0000	0.0000	1.0000	0.0000	1.0000	0.0000	1.0000	0.0000
LOC104911737	0.2448	0.5316	0.0004	0.9962	0.0026	1.0213	0.0000	1.1828	1.0000	-0.0514	0.4563	0.4282	1.0000	0.1172
LOC104911738	1.0000	-0.1315	1.0000	0.2853	1.0000	-0.4428	0.5346	0.9706	1.0000	-0.3674	1.0000	0.0578	0.8120	1.0437
LOC104911739	1.0000	0.0000	1.0000	0.0000	1.0000	0.0000	1.0000	0.0000	1.0000	0.0000	1.0000	0.0000	1.0000	0.0000
LOC104911740	1.0000	-0.0737	0.8362	-0.3957	0.0596	1.4187	0.0574	1.0936	1.0000	0.4926	1.0000	0.1838	1.0000	0.1778
LOC104911741	1.0000	0.0000	1.0000	0.0000	0.8011	3.0840	1.0000	0.0000	1.0000	0.0000	1.0000	0.0000	1.0000	-3.1344
LOC104911742	1.0000	0.0000	1.0000	0.0000	1.0000	0.0000	1.0000	0.0000	1.0000	0.0000	1.0000	0.0000	1.0000	0.0000
LOC104911743	1.0000	-1.1561	0.7674	-3.2499	1.0000	-1.0194	1.0000	-0.8388	1.0000	-0.1218	1.0000	-2.2958	1.0000	0.0661
LOC104911744	1.0000	0.5414	1.0000	0.0000	1.0000	0.6816	1.0000	0.0000	1.0000	-2.3771	1.0000	-3.1429	1.0000	-3.1369
LOC104911745	1.0000	-1.1537	1.0000	0.0000	1.0000	-1.0191	1.0000	2.3256	1.0000	-3.2304	1.0000	-2.2992	1.0000	0.0661
LOC104911746	0.7185	0.1539	0.1214	0.2701	0.1755	0.3623	0.0007	0.4981	1.0000	-0.0114	0.9819	0.1193	0.8810	0.1297
LOC104911747	1.0000	-0.0064	0.1293	-0.9140	0.0023	-2.9010	0.0013	-1.9271	1.0000	0.6054	1.0000	-0.2950	0.5565	1.5860
LOC104911748	0.0000	-1.1950	0.0000	-1.0765	0.0000	-1.3004	0.0000	-1.0877	1.0000	-0.0479	1.0000	0.0833	0.4983	0.1713
LOC104911749	0.0148	-0.5011	0.0110	-0.3375	0.0000	-1.0253	0.0000	-0.6426	1.0000	-0.1833	1.0000	-0.0064	0.5183	0.2051
LOC104911750	0.1031	0.3883	0.0017	0.4692	0.3166	0.2658	0.2441	0.2241	1.0000	-0.0747	1.0000	0.0202	0.9122	-0.1100
LOC104911751	0.0000	2.2756	0.0000	2.1086	0.0004	1.7224	0.0000	1.6403	1.0000	0.0021	1.0000	-0.1533	1.0000	-0.0799
LOC104911752	0.0014	0.5032	0.0000	0.3983	0.9272	-0.0672	0.1130	-0.1489	1.0000	-0.0546	0.5045	-0.1476	0.4541	-0.1312
LOC104911753	1.0000	0.0000	1.0000	0.0000	1.0000	0.0000	1.0000	0.0000	1.0000	0.0000	1.0000	0.0000	1.0000	0.0000
LOC104911754	0.6753	-0.8537	0.2307	1.0196	0.1551	1.1596	0.8435	0.5254	1.0000	-0.2696	0.1995	1.6118	0.5093	-0.9052
LOC104911756	1.0000	0.0000	1.0000	0.0000	1.0000	0.0000	1.0000	2.3256	1.0000	0.0000	1.0000	0.0000	1.0000	2.3555
LOC104911757	1.0000	-2.4788	1.0000	0.0000	1.0000	-2.4061	1.0000	0.0000	1.0000	-2.3771	1.0000	0.0000	1.0000	0.0000
LOC104911758	1.0000	0.0000	1.0000	0.0000	1.0000	0.0000	1.0000	0.0000	1.0000	0.0000	1.0000	0.0000	1.0000	0.0000
LOC104911759	1.0000	-0.1774	0.8856	0.2914	0.5674	0.4315	0.0802	0.8343	1.0000	-0.4614	1.0000	0.0174	1.0000	-0.0533
LOC104911761	0.0004	0.6934	0.0000	0.9849	0.0000	-1.2936	0.0000	-0.9199	1.0000	-0.2035	0.9986	0.1001	0.7287	0.1763
LOC104911762	0.0005	-1.8976	0.0000	-1.6984	1.0000	-0.1516	0.0035	0.7214	1.0000	-0.5191	1.0000	-0.3068	0.4150	0.3654
LOC104911763	0.0082	-5.5464	0.0413	-4.9488	1.0000	0.3756	0.7554	0.6409	1.0000	-0.6278	1.0000	0.0000	1.0000	-0.3591
LOC104911764	0.9500	-0.0868	1.0000	0.0057	1.0000	-0.0552	1.0000	0.0176	1.0000	0.1227	0.2845	0.2290	0.3963	0.2000
LOC104911765	0.0000	1.3044	0.0000	1.4998	0.0000	1.3521	0.0000	1.1085	1.0000	0.2984	0.0002	0.5062	1.0000	0.0593
LOC104911766	1.0000	-1.2491	1.0000	-0.4762	0.2132	1.4482	1.0000	0.0070	1.0000	0.1810	1.0000	0.9748	0.4468	-1.2571
LOC104911767	0.0031	-0.5050	0.0000	-0.4908	0.0000	-1.2661	0.0000	-1.0628	1.0000	-0.0365	1.0000	-0.0098	0.3206	0.1724
LOC104911768	1.0000	-0.3195	0.4431	3.6192	1.0000	-0.1811	1.0000	2.3256	1.0000	-3.2304	1.0000	0.5843	1.0000	-0.7769
LOC104911769	1.0000	0.0000	1.0000	0.0000	1.0000	0.0000	1.0000	0.0000	1.0000	0.0000	1.0000	0.0000	1.0000	0.0000
LOC104911770	0.9845	0.1223	0.5189	0.1399	0.0002	1.0317	0.0387	0.3396	1.0000	0.4651	0.0176	0.4955	0.2888	-0.2232
LOC104911771	1.0000	-2.4776	1.0000	0.0000	1.0000	-2.4056	1.0000	0.0000	1.0000	-2.3757	1.0000	0.0000	1.0000	0.0000
LOC104911772	1.0000	2.1850	1.0000	2.2508	1.0000	0.0000	1.0000	0.0000	1.0000	0.0000	1.0000	0.0495	1.0000	0.0000
LOC104911774	0.8849	-0.5989	1.0000	-0.3414	1.0000	-0.1877	0.2826	-1.5016	1.0000	0.4355	1.0000	0.7102	1.0000	-0.8694
LOC104911775	1.0000	-0.3195	1.0000	-0.1718	0.8011	-3.2629	0.1321	-4.3889	1.0000	1.1014	1.0000	1.2793	1.0000	0.0000
LOC104911776	0.0562	0.6135	0.1011	0.4253	0.0000	1.1362	0.0000	0.9669	1.0000	0.0723	1.0000	-0.1020	1.0000	-0.0882
LOC104911777	1.0000	0.0000	1.0000	0.0000	1.0000	2.2471	1.0000	0.0000	1.0000	0.0000	1.0000	0.0000	1.0000	-2.2906
LOC104911778	1.0000	0.0220	0.2134	-0.3978	1.0000	0.1059	0.8434	-0.1394	1.0000	0.1594	0.9755	-0.2470	1.0000	-0.0811
LOC104911780	0.3199	-0.2501	0.0001	-0.3960	0.0080	-0.5179	0.0000	-0.5281	1.0000	0.0275	0.9606	-0.1052	1.0000	0.0230
LOC104911781	0.0757	1.1501	0.6180	0.3977	0.0003	1.7002	0.0050	1.1541	1.0000	0.4011	0.9994	-0.3450	1.0000	-0.1469
LOC104911785	0.8517	-0.3556	1.0000	-0.0690	0.0158	-1.9609	0.0065	-1.3797	1.0000	0.2718	0.6537	0.5711	0.8175	0.8634
LOC104911786	1.0000	-2.4788	1.0000	2.2533	1.0000	-2.4061	1.0000	0.0000	1.0000	-2.3771	1.0000	2.3480	1.0000	0.0000
LOC104911787	0.0000	-0.8539	0.0000	-0.8475	0.0248	-0.4848	0.7782	-0.0648	1.0000	0.2224	0.3764	0.2418	0.0000	0.6477
LOC104911788	1.0000	0.0000	1.0000	0.0000	1.0000	0.0000	1.0000	0.0000	1.0000	0.0000	1.0000	0.0000	1.0000	0.0000
LOC104911789	1.0000	0.1609	1.0000	-0.1128	0.4279	0.5679	1.0000	-0.1395	1.0000	0.4660	1.0000	0.2028	1.0000	-0.2324
LOC104911790	1.0000	-0.3384	1.0000	-0.1429	0.9468	-1.5506	1.0000	0.8545	1.0000	-1.4982	1.0000	-1.3246	1.0000	0.9164
LOC104911791	1.0000	0.0000	1.0000	-0.1429	1.0000	2.2426	0.9096	1.3850	1.0000	2.2676	1.0000	2.3480	1.0000	1.4486
LOC104911793	1.0000	0.5370	1.0000	2.2533	1.0000	-0.1597	1.0000	0.0000	1.0000	-2.3771	1.0000	-0.7919	1.0000	-2.2906
LOC104911794	0.8226	3.0199	1.0000	2.2508	1.0000	2.2426	0.4370	3.7037	1.0000	0.0000	1.0000	-0.7966	1.0000	1.4486
LOC104911795	1.0000	0.0498	1.0000	-0.0274	1.0000	-0.0890	1.0000	0.0071	1.0000	-0.0749	1.0000	-0.1422	1.0000	0.0270
LOC104911796	0.0424	0.5587	0.0008	0.6095	0.3396	0.3165	0.0501	0.4195	1.0000	-0.1484	1.0000	-0.0858	1.0000	-0.0401
LOC104911797	0.0000	-1.2463	0.0000	-1.4899	0.0000	-1.3092	0.0000	-1.3636	1.0000	0.0146	0.6450	-0.2161	1.0000	-0.0358
LOC104911798	0.5126	-0.7101	0.5034	-0.4502	1.0000	0.0368	1.0000	-0.0426	1.0000	-0.0820	1.0000	0.1876	1.0000	-0.1546
LOC104911799	0.0000	-1.0716	0.0000	-1.1771	0.0005	0.5554	1.0000	0.0181	1.0000	0.0406	1.0000	-0.0526	0.0000	-0.4917
LOC104911800	1.0000	0.0000	1.0000	0.0000	1.0000	0.0000	1.0000	0.0000	1.0000	0.0000	1.0000	0.0000	1.0000	0.0000
LOC104911801	0.0444	1.0541	0.0001	1.4125	0.0000	3.1895	0.0000	2.8583	1.0000	-0.0784	0.9643	0.2905	0.2728	-0.4031
LOC104911803	0.3192	4.2259	0.4991	-1.6416	0.0018	5.8282	0.1652	1.2939	1.0000	4.5819	1.0000	-1.1637	1.0000	0.0814
LOC104911804	0.0000	1.4282	0.0000	1.5291	0.0000	0.9617	0.0000	0.7105	1.0000	0.1998	0.2582	0.3126	1.0000	-0.0469
LOC104911805	0.4209	1.5357	0.1464	4.3063	0.0828	2.2539	0.0109	5.2130	1.0000	-3.2304	1.0000	-0.5932	1.0000	-0.3253
LOC104911806	0.0032	2.2658	0.3326	0.8062	0.0000	3.1120	0.0004	1.8102	1.0000	0.9727	0.9940	-0.4736	0.9145	-0.3280
LOC104911807	0.0002	-1.1983	0.0000	-1.0562	0.1428	-0.4578	0.0011	-0.6389	1.0000	0.0980	0.9289	0.2544	1.0000	-0.0776
LOC104911808	1.0000	-0.2032	1.0000	-0.0977	0.0597	0.9455	0.0763	0.8630	1.0000	0.2353	0.9881	0.3601	1.0000	0.1558
LOC104911809	1.0000	2.1901	1.0000	-0.1449	1.0000	0.0000	1.0000	-2.3178	1.0000	2.2676	1.0000	0.0471	1.0000	0.0000
LOC104911810	0.1851	4.5039	0.7674	-3.2499	1.0000	2.2471	1.0000	0.0074	1.0000	3.1056	0.4062	-4.6342	1.0000	0.9164
LOC104911811	0.3357	-2.3895	1.0000	-2.3960	0.1702	-4.4910	1.0000	-2.3178	1.0000	-2.1873	1.0000	-2.2958	1.0000	0.0000

LOC104911812	0.0269	1.2736	0.0044	0.9454	0.0000	2.0437	0.0000	1.6840	1.0000	0.3498	1.0000	0.0368	1.0000	-0.0031
LOC104911813	0.2327	0.8187	0.0018	1.4874	0.6253	0.4809	0.5304	0.5836	1.0000	-0.4853	1.0000	0.1943	1.0000	-0.3803
LOC104911815	1.0000	0.0000	1.0000	0.0000	1.0000	0.0000	1.0000	0.0000	1.0000	0.0000	1.0000	0.0000	1.0000	0.0000
LOC104911816	0.0000	1.5374	0.0000	1.9931	0.0000	1.3226	0.0000	1.5543	1.0000	-0.3650	1.0000	0.1025	1.0000	-0.1273
LOC104911817	0.3159	3.9314	0.4024	-2.2295	1.0000	0.0000	0.1321	-4.3889	1.0000	4.3319	1.0000	-1.7130	1.0000	0.0000
LOC104911818	1.0000	0.3418	1.0000	-0.1664	1.0000	0.1978	0.8310	0.7796	1.0000	0.2556	1.0000	-0.2404	1.0000	0.8486
LOC104911819	0.8108	1.1326	0.8984	0.3906	0.0252	2.5689	0.1177	1.3857	1.0000	1.3548	1.0000	0.6289	1.0000	0.1728
LOC104911820	1.0000	-0.2931	1.0000	0.0000	1.0000	-2.4061	1.0000	2.3241	1.0000	-2.3771	1.0000	-2.2958	1.0000	2.3543
LOC104911821	0.0000	-1.7971	0.0000	-2.3337	0.0000	-1.2714	0.0000	-1.2225	1.0000	0.0112	0.0002	-0.5130	0.9877	0.0655
LOC104911823	0.7853	-0.5196	0.0049	-1.6411	1.0000	0.0112	0.3521	-0.6235	1.0000	0.4232	0.8770	-0.6887	1.0000	-0.2063
LOC104911824	1.0000	0.2007	1.0000	-0.4768	1.0000	-0.1789	1.0000	-0.6849	1.0000	1.0995	1.0000	0.4441	1.0000	0.6041
LOC104911825	1.0000	0.0000	1.0000	0.0000	1.0000	0.0000	1.0000	0.0000	1.0000	0.0000	1.0000	0.0000	1.0000	0.0000
LOC104911826	0.0098	1.2112	0.0064	1.0395	1.0000	0.1378	0.7602	0.2909	1.0000	-0.0398	1.0000	-0.2008	1.0000	0.1184
LOC104911827	1.0000	0.0000	1.0000	0.0000	1.0000	0.0000	1.0000	0.0000	1.0000	0.0000	1.0000	0.0000	1.0000	0.0000
LOC104911828	1.0000	0.0000	1.0000	2.2508	1.0000	0.0000	1.0000	2.3256	1.0000	0.0000	1.0000	2.3456	1.0000	2.3555
LOC104911829	0.0633	-2.1354	1.0000	0.2918	0.2120	-1.5988	0.7210	-1.2163	1.0000	-1.2265	0.9744	1.2156	1.0000	-0.8429
LOC104911830	0.7695	-0.7492	0.7666	-0.6764	0.4791	-1.5313	0.0952	-2.1819	1.0000	0.1877	1.0000	0.2735	1.0000	-0.4586
LOC104911831	0.8249	-3.3439	1.0000	2.2508	0.8033	-3.2636	1.0000	0.0000	1.0000	-3.2319	1.0000	2.3456	1.0000	0.0000
LOC104911832	0.2950	1.0117	0.3975	0.8795	0.9361	-0.3820	1.0000	0.2905	1.0000	0.0307	1.0000	-0.0850	1.0000	0.7127
LOC104911833	1.0000	0.0000	1.0000	0.0000	1.0000	0.0000	1.0000	0.0000	1.0000	0.0000	1.0000	0.0000	1.0000	0.0000
LOC104911834	1.0000	0.0000	1.0000	-0.1452	1.0000	2.2426	1.0000	-2.3200	1.0000	2.2735	1.0000	2.3480	1.0000	-2.2889
LOC104911835	0.1046	0.3051	0.0004	0.3698	0.0000	0.7402	0.0000	0.4645	1.0000	0.0428	0.7902	0.1203	0.0535	-0.2273
LOC104911836	1.0000	0.0000	1.0000	0.0000	1.0000	0.0000	1.0000	0.0000	1.0000	0.0000	1.0000	0.0000	1.0000	0.0000
LOC104911837	0.0389	0.6712	0.1209	0.4080	0.0893	0.5120	0.0728	0.4242	1.0000	-0.2036	0.2459	-0.4564	0.4605	-0.2865
LOC104911838	0.9303	0.1378	0.3516	-0.2399	0.8117	0.1648	0.0000	1.1904	1.0000	-0.0036	0.3383	-0.3691	0.0000	1.0276
LOC104911839	1.0000	-0.2920	1.0000	-2.3985	0.0000	5.0004	0.0000	5.3067	1.0000	-0.1034	1.0000	-2.2958	1.0000	0.2024
LOC104911840	1.0000	0.0000	1.0000	0.0000	1.0000	2.2426	1.0000	2.3257	1.0000	0.0000	1.0000	0.0000	1.0000	0.0661
LOC104911841	1.0000	2.1851	1.0000	2.2507	1.0000	0.0000	1.0000	0.0000	1.0000	0.0000	1.0000	0.0495	1.0000	0.0000
LOC104911842	0.6359	-0.2054	0.0176	-0.4547	0.0000	-1.1711	0.0000	-1.3946	1.0000	0.1112	1.0000	-0.1248	1.0000	-0.1054
LOC104911843	0.7561	0.2308	0.0597	0.4646	0.1290	0.5326	0.1150	0.4107	1.0000	-0.1159	1.0000	0.1290	0.6897	-0.2334
LOC104911844	0.3934	0.8638	1.0000	0.0485	1.0000	0.0411	0.0150	-1.6739	1.0000	0.9169	1.0000	0.1144	0.7604	-0.7916
LOC104911845	0.0001	1.5669	0.0000	1.8664	0.0291	0.9770	0.1114	0.7603	1.0000	-0.2891	1.0000	0.0231	0.4815	-0.5011
LOC104911846	0.1995	0.8736	0.0370	0.9852	0.7518	0.4061	1.0000	-0.1915	1.0000	0.1412	1.0000	0.2658	0.9362	-0.4500
LOC104911848	0.0961	2.0646	0.0902	1.8555	0.6300	0.9670	1.0000	0.6976	1.0000	-0.1210	1.0000	-0.3204	1.0000	-0.3880
LOC104911849	1.0000	2.1901	0.7666	3.0922	1.0000	0.0000	1.0000	2.3257	1.0000	0.0000	1.0000	0.8994	1.0000	2.3555
LOC104911850	0.1190	-0.7554	0.0004	-0.9939	0.2033	-0.5155	0.0018	-0.8621	1.0000	0.2558	1.0000	0.0324	1.0000	-0.0853
LOC104911851	1.0000	-0.0476	1.0000	0.2194	0.0139	2.2535	0.0000	3.4467	1.0000	-0.5150	1.0000	-0.2414	0.4462	0.6821
LOC104911852	1.0000	0.0000	1.0000	-2.3985	1.0000	2.2426	1.0000	-2.3200	1.0000	2.2734	1.0000	0.0000	1.0000	-2.2889
LOC104911853	0.3181	-2.3898	1.0000	-0.1598	0.1614	-4.4916	1.0000	0.0066	1.0000	-1.3443	1.0000	0.9035	1.0000	3.2066
LOC104911854	0.7328	-2.0827	0.6182	1.6074	1.0000	-1.1065	1.0000	0.8541	1.0000	-1.8737	1.0000	1.8196	1.0000	0.0730
LOC104911855	1.0000	0.0000	1.0000	0.0000	1.0000	0.0000	1.0000	2.3241	1.0000	0.0000	1.0000	0.0000	1.0000	2.3543
LOC104911857	1.0000	0.0000	1.0000	2.2533	0.4809	3.6101	0.7710	3.1709	1.0000	0.0000	1.0000	2.3480	1.0000	-0.4570
LOC104911858	1.0000	0.0000	1.0000	-0.1429	1.0000	2.2426	1.0000	-2.3178	1.0000	2.2676	1.0000	2.3480	1.0000	-2.2889
LOC104911859	0.0000	-2.0762	0.0000	-1.9980	0.0291	-0.6714	0.0001	0.4579	1.0000	0.2816	0.8377	0.3720	0.0000	1.4173
LOC104911860	1.0000	0.0000	0.7666	3.0922	1.0000	2.2470	0.7701	3.1732	1.0000	0.0000	1.0000	3.1976	1.0000	0.9164
LOC104911862	0.3843	0.4549	0.5218	0.2645	0.3550	0.4338	0.3945	0.3200	1.0000	0.2715	1.0000	0.0908	1.0000	0.1615
LOC104911863	1.0000	0.0000	1.0000	0.0000	1.0000	0.0000	1.0000	0.0000	1.0000	0.0000	1.0000	0.0000	1.0000	0.0000
LOC104911864	0.6410	-0.1774	0.0000	-0.6587	0.5431	0.1883	1.0000	-0.0426	1.0000	0.1931	0.3505	-0.2749	1.0000	-0.0314
LOC104911865	0.4241	-1.8074	0.0003	-5.9234	1.0000	0.2065	1.0000	-0.0936	1.0000	1.0577	1.0000	-3.1468	0.9295	0.7651
LOC104911866	1.0000	0.0000	1.0000	0.0000	1.0000	0.0000	1.0000	0.0000	1.0000	0.0000	1.0000	0.0000	1.0000	0.0000
LOC104911867	1.0000	0.0000	1.0000	0.0000	1.0000	2.2470	1.0000	0.0000	1.0000	0.0000	1.0000	0.0000	1.0000	-2.2906
LOC104911868	1.0000	0.0000	1.0000	0.0000	1.0000	0.0000	1.0000	0.0000	1.0000	0.0000	1.0000	0.0000	1.0000	0.0000
LOC104911870	1.0000	-0.2882	1.0000	-0.1452	1.0000	-2.4056	1.0000	-2.3200	1.0000	-0.1034	1.0000	0.0489	1.0000	0.0000
LOC104911871	0.0122	-2.3636	0.0019	-3.1091	0.8033	0.3960	0.6961	0.4377	1.0000	-0.4304	1.0000	-1.1747	0.9748	-0.3894
LOC104911872	0.0003	6.1755	0.0000	4.0549	1.0000	0.0000	1.0000	-2.3200	1.0000	2.2734	1.0000	0.2589	1.0000	0.0000
LOC104911873	0.5086	3.5456	1.0000	2.2533	1.0000	0.0000	1.0000	0.0000	1.0000	0.0000	1.0000	-1.3257	1.0000	0.0000
LOC104911874	1.0000	0.0000	1.0000	0.0000	1.0000	0.0000	0.7710	3.1709	1.0000	0.0000	1.0000	0.0000	1.0000	3.2049
LOC104911875	1.0000	0.0000	1.0000	0.0000	1.0000	0.0000	1.0000	0.0000	1.0000	0.0000	1.0000	0.0000	1.0000	0.0000
LOC104911876	1.0000	0.0000	1.0000	0.0000	1.0000	0.0000	1.0000	0.0000	1.0000	0.0000	1.0000	0.0000	1.0000	0.0000
LOC104911877	1.0000	0.0000	1.0000	0.0000	1.0000	0.0000	1.0000	0.0000	1.0000	0.0000	1.0000	0.0000	1.0000	0.0000
LOC104911878	1.0000	-1.1585	0.2941	-1.6794	0.2908	1.6929	1.0000	0.0075	1.0000	1.9125	1.0000	1.4326	1.0000	0.2427
LOC104911879	0.8249	3.0143	1.0000	-0.1452	1.0000	0.0000	1.0000	-2.3200	1.0000	2.2734	1.0000	-0.7919	1.0000	0.0000
LOC104911880	1.0000	0.1953	1.0000	-1.0008	1.0000	-0.1785	0.7701	-3.1666	1.0000	-0.1169	1.0000	-1.3189	1.0000	-3.1344
LOC104911881	1.0000	-0.2882	1.0000	-2.3985	1.0000	-2.4056	1.0000	-2.3200	1.0000	-0.1034	1.0000	-2.2992	1.0000	0.0000
LOC104911882	1.0000	0.0000	1.0000	0.0000	1.0000	0.0000	1.0000	0.0000	1.0000	0.0000	1.0000	0.0000	1.0000	0.0000
LOC104911885	0.8249	-3.3412	1.0000	-0.1472	0.8033	-3.2622	1.0000	-2.3200	1.0000	-0.9563	1.0000	2.3456	1.0000	0.0000
LOC104911886	1.0000	-0.2920	1.0000	2.2507	1.0000	-2.4056	1.0000	2.3241	1.0000	-2.3757	1.0000	0.0495	1.0000	2.3543
LOC104911887	0.1171	0.9381	0.4075	0.5151	0.0051	1.3934	0.0551	0.8753	1.0000	0.0609	0.9401	-0.3510	0.5601	-0.4553
LOC104911891	0.1374	2.5657	0.0226	2.1493	0.0188	3.2298	0.2124	1.6620	1.0000	1.2635	0.8521	0.8734	1.0000	-0.2939
LOC104911892	1.0000	0.0000	1.0000	0.0000	1.0000	0.0000	1.0000	0.0000	1.0000	0.0000	1.0000	0.0000	1.0000	0.0000
LOC104911893	1.0000	2.1851	1.0000	0.0000	1.0000	0.0000	1.0000	0.0000	1.0000	0.0000	1.0000	-2.2959	1.0000	0.0000
LOC104911894	0.8226	-3.3425	1.0000	-0.1452	0.8011	-3.2629	1.0000	0.0045	1.0000	-0.9587	1.0000	2.3480	1.0000	2.3543
LOC104911895	1.0000	0.0000	1.0000	0.0000	1.0000	0.0000	1.0000	0.0000	1.0000	0.0000	1.0000	0.0000	1.0000	0.0000
LOC104911896	1.0000	-2.4788	1.0000	-2.3985	1.0000	-2.4061	1.0000	-2.3200	1.0000	-0.1045	1.0000	0.0000	1.0000	0.0000
LOC104911897	1.0000	2.1901	1.0000	-0.1625	0.2840	3.9988	1.0000	0.5372	1.0000	3.1126	1.0000	0.8962	1.0000	-0.3119
LOC104911898	0.0004	1.9356	0.0000	2.3324	0.0000	2.3204	0.0000	2.0365	1.0000	-0.0220	0.7549	0.3824	0.8475	-0.3059
LOC104911899	0.9531	0.0987	0.2394	-0.2097	0.0000	0.8722	0.0000	0.6111	1.0000	0.1791	1.0000	-0.1180	1.0000	-0.0783
LOC104911900	1.0000	-0.3217	0.7674	3.0889	1.0000	-1.0156	0.7701	3.1732	1.0000	-3.2289	1.0000	0.0533	1.0000	0.9164
LOC104911901	1.0000	0.0000	0.7674	-3.2570	0.0938	4.5491	1.0000	0.0061	1.0000	3.1191	1.0000	0.0000	0.7915	-1.3979
LOC104911902	0.4710	-0.6373	0.0081	-1.6913	0.0015	1.3240	0.0007	1.2127	1.0000	0.0513	0.4746	-0.9888	1.0000	-0.0489
LOC104911903	1.0000	0.0000	1.0000	0.0000	1.0000	0.0000	1.0000	0.0000	1.0000	0.0000	1.0000	0.0000	1.0000	0.0000
LOC104911904	1.0000	0.0000	1.0000	0.0000	1.0000	2.2470	1.0000	2.3257	1.0000	0.0000	1.0000	0.0000	1.0000	0.0649
LOC104911905	1.0000	0.5453	1.0000	2.2533	0.1908	2.3554	0.4370	3.7041	1.0000	-2.3771	1.0000	-0.7977	0.8648	-1.0799
LOC104911906	0.1006	-1.4999	0.0584	-1.9021	0.0164	-2.2961	0.0071	-2.9392	1.0000	-0.2025	1.0000	-0.5932	1.0000	-0.8429
LOC104911907	0.7477	-0.3613	0.8890	-0.2145	0.2220	-0.7300	0.9120	0.2007	1.0000	-0.1665	1.0000	-0.0076	0.2881	0.7696
LOC104911908	1.0000	-0.2920	0.7711	0.7724	0.9468	1.2126	1.0000	0.0068	1.0000	1.2661	0.6450	2.3766	1.0000	0.0738
LOC104911909	0.0001	0.9823	0.0000	0.8673	1.0000	0.0399	1.0000	0.0568	1.0000	-0.1036	0.6537	-0.2053	1.0000	-0.0814
LOC104911910	0.1072	0.4268	0.3661	0.1734	0.9168	-0.1108	0.0141	-0.3842	1.0000	0.0979	0.8899	-0.1427	0.7287	-0.1702
LOC104911911	0.9899	0.4885	0.0313	1.6988	0.0002	2.5313	0.0000	2.8339	1.0000	-0.3791	0.8306	0.8380	1.0000	-0.0690
LOC104911912	0.4782	2.0004	1.0000	-0.1783	1.0000	-0.1597	0.4562	-2.0752	1.0000	1.9711	1.0000	-0.1905	1.0000	0.0649
LOC104911914	1.0000	0.4767	1.0000	-0.1759	1.0000	0.4593	1.0000	-0.3068	1.0000	0.8434	1.0000	0.2073	1.0000	0.0788
LOC104911915	0.0882	-1.6381	0.5884	-0.5857	0.0000	2.0234	0.0046	1.3517	1.0000	-0.0399	0.8607	1.0295	0.1473	-0.7069
LOC104911916	0.6665	-0.4073	0.0275	-1.3830	0.0302	1.1761	0.0009	1.2373	1.0000	-0.2335	0.2482	-1.1962	1.0000	-0.1689

LOC104911917	0.8249	3.0143	1.0000	0.0000	1.0000	0.0000	1.0000	0.0000	1.0000	0.0000	1.0000	-3.1383	1.0000	0.0000
LOC104911918	0.5317	-0.5350	0.1062	0.8478	0.0011	1.4135	0.0000	1.9737	1.0000	-0.7622	0.5226	0.6405	1.0000	-0.1971
LOC104911919	0.0063	-0.6182	0.0000	-1.1112	0.0147	0.5123	0.0008	0.5434	1.0000	-0.0819	0.0076	-0.5620	1.0000	-0.0459
LOC104911920	0.0000	-1.4647	0.0000	-1.9027	0.0000	-0.6756	0.0000	-0.7848	1.0000	0.0448	0.0787	-0.3815	1.0000	-0.0590
LOC104911921	0.2092	-2.6409	1.0000	-0.1612	0.9350	0.5178	0.0007	3.2509	1.0000	-1.5965	1.0000	0.8994	0.3577	1.1355
LOC104911922	1.0000	0.0000	1.0000	0.0000	1.0000	0.0000	1.0000	0.0000	1.0000	0.0000	1.0000	0.0000	1.0000	0.0000
LOC104911923	1.0000	0.0000	1.0000	0.0000	1.0000	0.0000	1.0000	0.0000	1.0000	0.0000	1.0000	0.0000	1.0000	0.0000
LOC104911924	1.0000	0.0000	1.0000	2.2533	1.0000	0.0000	1.0000	0.0000	1.0000	0.0000	1.0000	2.3480	1.0000	0.0000
LOC104911925	1.0000	0.5945	1.0000	0.6956	0.8011	-3.2629	1.0000	-2.3178	1.0000	-0.9614	1.0000	-0.8675	1.0000	0.0000
LOC104911926	1.0000	0.0589	0.9062	0.1394	0.1308	0.7749	0.0000	1.0706	1.0000	0.0948	1.0000	0.1874	0.2630	0.3944
LOC104911927	0.2781	-1.4706	0.6400	-1.1309	0.0005	2.0913	0.0417	1.4383	1.0000	-0.2913	1.0000	0.0588	0.1393	-0.9432
LOC104911928	0.0145	3.1865	0.0000	6.3226	0.0173	3.2264	0.1321	4.3960	1.0000	-2.3771	0.9425	0.6374	0.4457	-1.2568
LOC104911933	1.0000	0.0462	1.0000	-0.1682	0.6301	0.9668	1.0000	0.6976	1.0000	-0.1216	1.0000	-0.3291	1.0000	-0.3880
LOC104911934	1.0000	-0.8510	1.0000	0.6956	1.0000	0.5100	0.9096	1.3850	1.0000	-1.4938	1.0000	0.0532	1.0000	-0.6170
LOC104911937	1.0000	-0.7243	0.2688	-2.4764	0.8888	0.5863	1.0000	0.4083	1.0000	0.4254	1.0000	-1.3257	1.0000	0.2596
LOC104911938	1.0000	-0.1206	0.0503	-0.6364	0.4390	0.3396	0.3036	0.3384	1.0000	-0.1658	0.2072	-0.6693	1.0000	-0.1643
LOC104911939	0.0000	-2.6132	0.0000	-3.0654	0.0009	-0.8644	0.0000	-0.7723	1.0000	0.2252	1.0000	-0.2165	0.5082	0.3226
LOC104911940	0.0292	-1.8459	0.0414	-1.2815	0.0345	-1.6926	1.0000	0.0049	1.0000	0.0526	1.0000	0.6297	0.0313	1.7495
LOC104911941	1.0000	0.5773	0.6180	1.6082	1.0000	-1.0172	1.0000	0.0075	1.0000	-0.9614	1.0000	0.0619	1.0000	0.0649
LOC104911942	0.1212	1.3276	0.0817	1.5199	0.8695	-0.9605	1.0000	-0.5495	1.0000	-0.3398	1.0000	-0.1381	1.0000	0.0763
LOC104911943	0.5255	1.0900	1.0000	-0.5737	1.0000	-0.8796	0.4160	-1.3358	1.0000	0.5211	0.6331	-1.1294	1.0000	0.0749
LOC104911944	0.5086	3.5431	1.0000	0.0000	1.0000	2.2470	1.0000	0.0000	1.0000	0.0000	0.8607	-3.6719	1.0000	-2.2906
LOC104911945	0.1652	1.7582	0.1484	2.0180	0.6794	0.9480	0.9946	0.9258	1.0000	-1.0344	0.8047	-0.7745	0.8649	-1.0523
LOC104911946	1.0000	0.5420	1.0000	-0.1616	1.0000	-2.4056	1.0000	0.0058	1.0000	0.7416	1.0000	0.0559	1.0000	3.2066
LOC104911947	1.0000	0.0000	1.0000	0.0000	1.0000	0.0000	1.0000	2.3241	1.0000	0.0000	1.0000	0.0000	1.0000	2.3543
LOC104911948	1.0000	0.0011	0.9828	0.2485	0.7203	-0.4582	0.5762	-0.5088	1.0000	-0.1924	1.0000	0.0664	1.0000	-0.2370
LOC104911949	1.0000	0.0715	0.8268	-0.4108	0.1876	0.8322	0.0031	1.5368	1.0000	-0.5139	0.4669	-0.9856	1.0000	0.1979
LOC104911950	0.0028	-2.0566	0.0000	-2.3539	0.6412	0.4183	0.4170	-0.4266	1.0000	0.3494	1.0000	0.0612	0.6500	-0.4978
LOC104911951	0.0000	-2.4238	0.0002	-1.8435	0.5016	-0.4143	0.1126	-0.7237	1.0000	-0.3143	1.0000	0.2801	0.4794	-0.6181
LOC104911952	0.0034	-2.3596	0.0000	-2.1331	0.9761	-0.2534	0.4794	-0.3456	1.0000	0.7864	0.8437	1.0338	0.3615	0.6967
LOC104911953	0.8208	0.1823	0.1615	0.3770	1.0000	-0.0143	1.0000	0.0314	1.0000	-0.0773	1.0000	0.1318	1.0000	-0.0261
LOC104911954	0.2548	-0.6826	0.3931	-0.5102	0.2257	0.6054	0.5083	0.3637	1.0000	-0.4895	1.0000	-0.3054	0.1262	-0.7291
LOC104911955	0.6145	1.0577	1.0000	-0.1682	1.0000	-0.1811	1.0000	0.0065	1.0000	-0.5088	0.4473	-1.7335	1.0000	-0.3148
LOC104911956	1.0000	-1.2522	0.4156	1.9155	1.0000	-0.1894	1.0000	-2.3179	1.0000	-1.8822	1.0000	1.2837	0.5093	-4.0498
LOC104911957	0.2841	1.0096	1.0000	-0.1753	0.3910	0.9489	0.5080	0.7951	1.0000	-0.1299	0.2884	-1.3047	1.0000	-0.2779
LOC104911958	0.0004	-3.1230	0.0000	-4.6081	0.7788	-0.4876	1.0000	-0.1609	1.0000	0.1602	1.0000	-1.3246	0.7977	0.4910
LOC104911959	1.0000	0.0000	1.0000	0.0000	1.0000	0.0000	1.0000	0.0000	1.0000	0.0000	1.0000	0.0000	1.0000	0.0000
LOC104911963	0.2600	2.3889	1.0000	0.3782	0.0001	4.1500	1.0000	0.5636	1.0000	1.6470	1.0000	-0.3391	0.0323	-1.9328
LOC104911964	0.3711	-0.5890	0.5901	-0.2720	0.0035	1.0947	0.7446	0.2126	1.0000	0.5295	0.1529	0.8586	0.5939	-0.3475
LOC104911965	1.0000	2.1851	1.0000	0.0000	0.8033	3.0892	1.0000	0.0000	1.0000	0.0000	1.0000	-2.2959	1.0000	-3.1369
LOC104911966	1.0000	0.0000	1.0000	0.0000	1.0000	0.0000	1.0000	0.0000	1.0000	0.0000	1.0000	0.0000	1.0000	0.0000
LOC104911967	1.0000	0.0000	1.0000	0.0000	1.0000	0.0000	1.0000	0.0000	1.0000	0.0000	1.0000	0.0000	1.0000	0.0000
LOC104911968	1.0000	0.0000	1.0000	0.0000	1.0000	0.0000	1.0000	0.0000	1.0000	0.0000	1.0000	0.0000	1.0000	0.0000
LOC104911969	0.8736	0.7203	0.2347	1.8684	0.8257	-1.1286	1.0000	-0.8442	1.0000	-1.5933	1.0000	-0.4518	1.0000	-1.3127
LOC104911970	0.8226	3.0199	1.0000	0.0000	0.8033	3.0789	1.0000	0.0000	1.0000	0.0000	1.0000	-3.1429	1.0000	-3.1317
LOC104911971	0.4422	0.3690	0.0155	0.5695	0.4089	-0.4247	0.0296	-0.6279	1.0000	-0.1766	1.0000	0.0353	0.6298	-0.3728
LOC104911972	1.0000	-2.4788	1.0000	0.0000	1.0000	-0.1597	1.0000	0.0000	1.0000	-2.3771	1.0000	0.0000	1.0000	-2.2906
LOC104911974	1.0000	0.0000	1.0000	0.0000	1.0000	0.0000	1.0000	0.0000	1.0000	0.0000	1.0000	0.0000	1.0000	0.0000
LOC104911975	1.0000	0.0000	1.0000	0.0000	1.0000	0.0000	1.0000	0.0000	1.0000	0.0000	1.0000	0.0000	1.0000	0.0000
LOC104911976	0.6881	1.7520	0.9062	1.2235	1.0000	-0.1624	1.0000	-2.3179	1.0000	-0.1078	1.0000	-0.6335	1.0000	-2.2889
LOC104911977	1.0000	-0.2892	1.0000	-0.1449	1.0000	-2.4061	1.0000	-2.3179	1.0000	-0.1089	1.0000	0.0471	1.0000	0.0000
LOC104911978	0.8226	3.0199	0.7666	3.0922	1.0000	0.0000	1.0000	0.0000	1.0000	0.0000	1.0000	0.0547	1.0000	0.0000
LOC104911979	0.0960	0.4017	0.2450	0.2068	0.1337	-0.3662	0.0366	-0.3505	1.0000	-0.0685	0.4199	-0.2493	1.0000	-0.0469
LOC104911981	1.0000	0.0000	1.0000	0.0000	1.0000	0.0000	1.0000	0.0000	1.0000	0.0000	1.0000	0.0000	1.0000	0.0000
LOC104911982	1.0000	0.0000	1.0000	0.0000	1.0000	0.0000	1.0000	0.0000	1.0000	0.0000	1.0000	0.0000	1.0000	0.0000
LOC104911983	1.0000	-0.2920	1.0000	-0.1452	1.0000	-2.4056	1.0000	-2.3200	1.0000	-0.1034	1.0000	0.0514	1.0000	0.0000
LOC104911984	0.8226	1.0250	0.9148	1.2162	0.5036	-3.7981	1.0000	0.0057	1.0000	-1.4917	0.9323	-1.3175	1.0000	2.3555
LOC104911985	1.0000	2.1851	1.0000	0.0000	1.0000	0.0000	1.0000	0.0000	1.0000	0.0000	1.0000	-2.2959	1.0000	0.0000
LOC104911986	0.5836	-0.1859	0.0855	-0.2664	0.0000	-0.9373	0.0000	-0.5422	1.0000	0.0605	1.0000	-0.0076	0.0211	0.4588
LOC104911987	0.8226	-0.3544	0.0284	-1.1698	0.1359	-0.8890	0.1711	-0.7517	1.0000	-0.1296	0.3622	-0.9334	1.0000	0.0108
LOC104911988	1.0000	0.0000	1.0000	0.0000	1.0000	0.0000	1.0000	0.0000	1.0000	0.0000	1.0000	0.0000	1.0000	0.0000
LOC104911990	0.7877	1.1367	0.6855	0.6432	1.0000	-0.1789	1.0000	0.0067	1.0000	1.0995	1.0000	0.6259	0.9851	1.2965
LOC104911993	1.0000	-0.3262	0.7666	-3.2534	0.8033	-3.2622	1.0000	-0.8419	1.0000	-0.1170	1.0000	-3.1383	1.0000	2.3543
LOC104911994	1.0000	2.1901	0.4690	3.6126	1.0000	0.0000	1.0000	0.0000	1.0000	0.0000	1.0000	1.4259	1.0000	0.0000
LOC104911995	0.0000	-1.8522	0.0000	-1.2593	0.1149	-0.3062	0.2498	-0.1376	1.0000	-0.0580	0.0022	0.5472	0.7135	0.1157
LOC104911996	1.0000	-0.0726	0.4757	0.7315	0.6743	-1.0008	0.0865	-2.1807	1.0000	0.0218	0.8171	0.8363	0.9852	-1.1488
LOC104911997	0.0000	-4.8241	0.0000	-3.3085	0.0147	-1.1942	0.0015	-1.0693	1.0000	0.3905	0.6320	1.9311	0.6707	0.5207
LOC104911999	0.5086	-3.8790	0.7666	-3.2534	1.0000	-0.7149	1.0000	0.5373	1.0000	-0.6504	1.0000	0.0000	1.0000	0.6050
LOC104912000	1.0000	-0.0513	0.9062	-0.1598	1.0000	-0.1531	0.2081	-0.4721	1.0000	-0.0198	1.0000	-0.1171	0.7781	-0.3333
LOC104912001	1.0000	0.1783	1.0000	-0.0618	0.7050	-0.4597	0.2192	-0.8634	1.0000	-0.1354	1.0000	-0.3626	0.9017	-0.5318
LOC104912002	1.0000	-0.3226	0.9762	0.7528	0.4758	1.3180	1.0000	0.5427	1.0000	-0.6546	1.0000	0.4420	0.5617	-1.4312
LOC104912003	1.0000	2.1851	1.0000	0.0000	1.0000	2.2470	1.0000	0.0000	1.0000	0.0000	1.0000	-2.2959	1.0000	-2.2906
LOC104912004	1.0000	-2.4788	0.2920	-4.1632	1.0000	-0.1597	0.2831	-4.0731	1.0000	1.6362	1.0000	0.0000	1.0000	-2.2906
LOC104912005	0.6192	0.3205	0.8917	-0.1358	1.0000	0.0255	0.0005	-1.1467	1.0000	0.5885	1.0000	0.1480	0.3879	-0.5778
LOC104912006	0.0013	-1.7180	0.0024	-1.1737	1.0000	0.0642	0.9579	0.1485	1.0000	0.2629	0.5523	0.8220	0.7148	0.3519
LOC104912007	1.0000	-2.4776	1.0000	0.6956	1.0000	0.6783	1.0000	0.0062	1.0000	-0.1078	1.0000	3.1976	1.0000	-0.7795
LOC104912008	1.0000	0.0408	0.0954	2.5625	1.0000	-0.1902	0.6172	1.7712	1.0000	-1.4918	1.0000	1.0194	1.0000	0.4637
LOC104912009	0.8496	-0.4409	0.9784	-0.3134	0.6865	-0.5814	1.0000	0.0202	1.0000	0.1571	1.0000	0.3032	0.5325	0.7636
LOC104912011	1.0000	0.0000	1.0000	2.2533	1.0000	0.0000	1.0000	0.0000	1.0000	0.0000	1.0000	2.3480	1.0000	0.0000
LOC104912012	0.8226	-3.3425	0.7666	3.0922	0.8011	-3.2629	1.0000	2.3241	1.0000	-3.2304	1.0000	3.1976	1.0000	2.3543
LOC104912013	1.0000	0.0000	1.0000	0.0000	1.0000	0.0000	1.0000	0.0000	1.0000	0.0000	1.0000	0.0000	1.0000	0.0000
LOC104912014	1.0000	0.0000	1.0000	0.0000	1.0000	0.0000	1.0000	0.0000	1.0000	0.0000	1.0000	0.0000	1.0000	0.0000
LOC104912015	0.0005	1.4271	0.0000	2.2503	0.0003	1.3936	0.0000	1.7278	1.0000	-0.5247	0.7310	0.3051	0.9779	-0.1876
LOC104912017	1.0000	0.0000	1.0000	0.0000	1.0000	0.0000	1.0000	0.0000	1.0000	0.0000	1.0000	0.0000	1.0000	0.0000
LOC104912020	1.0000	0.0000	1.0000	0.0000	1.0000	0.0000	1.0000	0.0000	1.0000	0.0000	1.0000	0.0000	1.0000	0.0000
LOC104912022	1.0000	0.0000	1.0000	0.0000	1.0000	0.0000	1.0000	0.0000	1.0000	0.0000	1.0000	0.0000	1.0000	0.0000
LOC104912027	0.6129	0.3271	1.0000	-0.1143	0.5838	0.3268	0.2436	0.4393	1.0000	0.2737	1.0000	-0.1528	0.5186	0.3944
LOC104912028	0.8035	1.1451	0.0193	3.1320	1.0000	0.3474	0.6483	1.7684	1.0000	-0.9614	0.8607	1.0288	1.0000	0.4614
LOC104912029	0.9580	-0.0896	0.0177	-0.3196	0.0561	-0.4473	0.0086	-0.3482	1.0000	0.1999	1.0000	-0.0170	0.1797	0.3041
LOC104912030	0.5620	1.3143	1.0000	0.2184	0.1135	1.8354	0.0804	1.9211	1.0000	-0.1216	0.7681	-1.2114	1.0000	-0.0336
LOC104912031	1.0000	0.0000	1.0000	0.0000	1.0000	0.0000	1.0000	0.0000	1.0000	0.0000	1.0000	0.0000	1.0000	0.0000
LOC104912032	0.8035	1.1451	0.1491	1.8713	0.8011	-3.2629	1.0000	0.0074	1.0000	-0.1210	1.0000	0.6227	1.0000	3.2066
LOC104912034	1.0000	2.1901	1.0000	-1.0027	1.0000	0.0000	1.0000	0.0058	1.0000	3.1191	1.0000	0.0489	1.0000	3.2066

LOC104912035	1.0000	0.0000	1.0000	0.0000	1.0000	0.0000	1.0000	0.0000	1.0000	0.0000	1.0000	0.0000	1.0000	0.0000
LOC104912037	1.0000	-2.4788	1.0000	-0.1452	0.2263	2.5324	0.9162	1.3864	1.0000	-0.1045	1.0000	2.3480	0.7474	-1.2567
LOC104912038	1.0000	0.0000	1.0000	2.2533	1.0000	0.0000	1.0000	2.3257	1.0000	0.0000	1.0000	2.3480	1.0000	2.3555
LOC104912039	1.0000	-0.3195	0.5159	-1.6382	1.0000	-0.1789	0.7796	-0.9349	1.0000	1.3455	1.0000	0.0532	1.0000	0.6041
LOC104912040	1.0000	0.3529	0.0228	4.9588	0.6394	1.1498	0.1321	4.3960	1.0000	-3.7629	1.0000	0.7104	1.0000	-0.5726
LOC104912041	0.2290	1.0749	0.0061	2.1948	0.0965	1.4128	0.2432	1.3443	1.0000	-0.7767	1.0000	0.3517	0.6093	-0.8460
LOC104912042	1.0000	-0.2920	0.4642	-3.7944	1.0000	-0.1591	1.0000	-0.5262	1.0000	1.2738	1.0000	-2.2959	1.0000	0.9164
LOC104912044	0.2043	0.9114	0.1538	0.9877	0.1862	0.9107	0.3788	0.7815	1.0000	-0.6601	0.7731	-0.5748	0.4730	-0.7894
LOC104912045	0.3367	-2.3921	1.0000	-0.5493	1.0000	0.0567	1.0000	0.3137	1.0000	-0.4412	1.0000	1.4326	1.0000	-0.1736
LOC104912046	1.0000	0.0000	1.0000	0.0000	1.0000	0.0000	1.0000	0.0000	1.0000	0.0000	1.0000	0.0000	1.0000	0.0000
LOC104912047	1.0000	0.0000	1.0000	0.0000	1.0000	0.0000	1.0000	0.0000	1.0000	0.0000	1.0000	0.0000	1.0000	0.0000
LOC104912048	0.0044	0.7474	0.0000	0.6846	0.0000	1.3069	0.0000	1.0693	1.0000	0.1509	1.0000	0.1003	1.0000	-0.0834
LOC104912049	1.0000	0.0000	1.0000	0.0000	1.0000	0.0000	1.0000	0.0000	1.0000	0.0000	1.0000	0.0000	1.0000	0.0000
LOC104912050	1.0000	0.0000	1.0000	0.0000	1.0000	0.0000	1.0000	0.0000	1.0000	0.0000	1.0000	0.0000	1.0000	0.0000
LOC104912051	1.0000	-0.2931	1.0000	0.0000	0.2892	2.1431	0.4370	3.7037	1.0000	-2.3771	1.0000	-2.2959	1.0000	-0.8666
LOC104912052	0.0694	-1.9868	0.6503	-1.1266	1.0000	-0.1975	0.0001	2.2209	1.0000	-0.8157	1.0000	0.0576	0.0056	1.6100
LOC104912053	0.0000	1.3681	0.0000	1.7324	0.0000	1.3337	0.0000	0.7873	1.0000	-0.1949	0.2468	0.1809	0.0000	-0.7364
LOC104912055	1.0000	-2.4788	1.0000	-0.1449	0.6654	1.5927	0.9096	1.3850	1.0000	-0.1089	1.0000	2.3456	1.0000	-0.3119
LOC104912056	0.5086	3.5456	1.0000	2.2508	1.0000	0.0000	1.0000	0.0000	1.0000	0.0000	1.0000	-1.3266	1.0000	0.0000
LOC104912058	1.0000	0.0000	1.0000	0.0000	1.0000	0.0000	1.0000	2.3257	1.0000	0.0000	1.0000	0.0000	1.0000	2.3555
LOC104912059	0.0037	1.1418	0.0000	1.0208	0.0000	1.9652	0.0000	1.8395	1.0000	0.1384	1.0000	0.0299	1.0000	0.0196
LOC104912060	0.2261	-2.3747	0.3451	-0.9307	0.0459	1.5619	0.0338	1.2638	1.0000	0.2630	0.7635	1.7439	1.0000	-0.0156
LOC104912061	0.7000	0.3989	0.0003	1.6116	0.0000	1.7456	0.0000	2.5281	1.0000	-0.8759	0.9381	0.3453	1.0000	-0.0917
LOC104912063	1.0000	0.0000	1.0000	2.2508	1.0000	0.0000	0.7710	3.1709	1.0000	0.0000	1.0000	2.3456	1.0000	3.2049
LOC104912064	0.0357	-1.2309	0.0002	-1.4475	0.0000	1.7394	0.0000	1.5731	1.0000	0.3832	1.0000	0.1801	0.7284	0.2247
LOC104912065	0.0766	0.5495	0.1593	0.3374	0.2669	-0.4199	0.2976	-0.3023	1.0000	-0.2279	0.1158	-0.4255	1.0000	-0.1008
LOC104912067	0.0251	0.8532	0.0019	0.9845	0.0006	1.1285	0.0015	0.9633	1.0000	-0.1669	1.0000	-0.0190	0.4959	-0.3229
LOC104912068	0.0664	-0.4748	0.0000	-0.4918	0.0042	0.6385	0.0000	0.8092	1.0000	0.1635	0.6427	0.1589	0.0024	0.3400
LOC104912069	1.0000	0.0000	1.0000	0.0000	1.0000	0.0000	1.0000	0.0000	1.0000	0.0000	1.0000	0.0000	1.0000	0.0000
LOC104912070	1.0000	0.0000	1.0000	0.0000	1.0000	0.0000	1.0000	2.3241	1.0000	0.0000	1.0000	0.0000	1.0000	2.3543
LOC104912072	1.0000	0.0000	1.0000	0.0000	1.0000	0.0000	1.0000	0.0000	1.0000	0.0000	1.0000	0.0000	1.0000	0.0000
LOC104912073	0.4158	-0.4070	0.4625	-0.2767	0.0000	1.3564	0.0000	0.9759	1.0000	0.0753	1.0000	0.2181	0.3060	-0.2982
LOC104912074	1.0000	-0.2931	0.7666	-3.2534	0.9361	1.2042	1.0000	0.0066	1.0000	0.7357	1.0000	-2.2959	1.0000	-0.4568
LOC104912075	0.0233	-1.3122	0.8613	-0.2611	0.1732	-0.8178	0.7214	-0.3121	1.0000	-0.2116	0.5347	0.8536	1.0000	0.3004
LOC104912076	1.0000	0.0095	0.0000	-0.8759	1.0000	-0.0141	0.9310	-0.0770	1.0000	0.5157	0.3550	-0.3581	0.0728	0.4584
LOC104912077	1.0000	0.2133	1.0000	-0.8635	1.0000	-0.1946	0.7246	-1.2170	1.0000	0.1843	1.0000	-0.8861	1.0000	-0.8428
LOC104912078	0.0323	0.9998	0.0013	1.1860	0.1978	0.6634	0.2477	0.6051	1.0000	-0.4619	0.9572	-0.2653	0.4856	-0.5140
LOC104912079	1.0000	0.0000	1.0000	-2.3985	1.0000	0.0000	1.0000	0.0045	1.0000	2.2734	1.0000	0.0000	1.0000	2.3543
LOC104912080	1.0000	0.2511	1.0000	-0.0550	0.5196	-0.8350	1.0000	-0.2754	1.0000	-0.1276	1.0000	-0.4272	1.0000	0.4336
LOC104912081	0.4422	0.2843	0.4318	0.2049	0.0032	0.7264	0.0000	0.8136	1.0000	0.1588	1.0000	0.0948	0.3402	0.2554
LOC104912082	1.0000	-0.1361	1.0000	-0.1746	0.0006	2.4169	0.0000	2.6950	1.0000	-0.1303	1.0000	-0.1557	1.0000	0.1547
LOC104912083	0.1377	1.0787	0.0617	0.9424	0.0000	2.3495	0.0000	2.0288	1.0000	0.2528	1.0000	0.1274	1.0000	-0.0614
LOC104912084	0.0325	-0.4043	0.0012	-0.4771	0.0032	0.5261	0.0033	0.4240	1.0000	0.0997	1.0000	0.0391	1.0000	0.0017
LOC104912085	0.0000	-1.8050	0.0000	-1.7914	0.0000	1.7953	0.0000	1.2703	1.0000	-0.0831	1.0000	-0.0570	0.0003	-0.6046
LOC104912086	0.0236	0.8656	0.0000	1.1608	0.0000	1.5675	0.0000	1.9336	1.0000	-0.2247	1.0000	0.0811	0.8955	0.1460
LOC104912087	0.0016	0.7276	0.0004	0.4999	0.0601	0.4682	0.0114	0.3668	1.0000	0.1632	1.0000	-0.0519	1.0000	0.0648
LOC104912088	1.0000	0.0000	1.0000	0.0000	1.0000	0.0000	1.0000	0.0000	1.0000	0.0000	1.0000	0.0000	1.0000	0.0000
LOC104912089	1.0000	0.0000	1.0000	0.0000	1.0000	0.0000	1.0000	0.0000	1.0000	0.0000	1.0000	0.0000	1.0000	0.0000
LOC104912090	1.0000	0.0000	1.0000	0.0000	1.0000	0.0000	1.0000	0.0000	1.0000	0.0000	1.0000	0.0000	1.0000	0.0000
LOC104912091	0.0502	-0.3520	0.0000	-0.4136	0.0315	-0.3524	0.0000	-0.5166	1.0000	0.1142	1.0000	0.0649	1.0000	-0.0445
LOC104912092	0.6927	1.4466	1.0000	-0.5557	1.0000	-0.1597	1.0000	0.5635	1.0000	1.6499	1.0000	-0.3282	0.5105	2.3934
LOC104912093	1.0000	-0.0545	1.0000	0.0253	0.7289	0.2417	0.0259	0.6032	1.0000	-0.0775	1.0000	0.0148	0.6529	0.2904
LOC104912094	1.0000	-1.1561	1.0000	0.0000	0.8033	-3.2622	1.0000	2.3257	1.0000	-3.2289	1.0000	-2.2958	1.0000	2.3555
LOC104912095	0.1876	4.2322	1.0000	-2.3985	1.0000	2.2426	1.0000	-2.3200	1.0000	2.2734	0.4563	-4.3639	1.0000	-2.2888
LOC104912096	1.0000	-2.4776	1.0000	0.3672	1.0000	0.6746	0.7210	1.2300	1.0000	0.7318	0.8607	3.7288	0.9835	1.2979
LOC104912097	0.0000	1.1540	0.0000	1.0654	0.9361	-0.0776	0.1671	0.1293	1.0000	-0.1929	0.0031	-0.2693	1.0000	0.0195
LOC104912098	0.2284	1.0775	0.6487	0.7858	0.7628	-0.8517	1.0000	-0.3800	1.0000	-1.0836	0.2436	-1.3709	1.0000	-0.6097
LOC104912099	1.0000	0.5464	1.0000	0.6944	1.0000	-2.4056	1.0000	-2.3200	1.0000	-0.1034	1.0000	0.0532	1.0000	0.0000
LOC104912101	0.3919	-0.8636	0.0473	-1.2762	1.0000	-0.0027	1.0000	-0.1676	1.0000	0.2416	1.0000	-0.1566	1.0000	0.0811
LOC104912102	1.0000	0.0000	1.0000	0.0000	1.0000	0.0000	1.0000	0.0000	1.0000	0.0000	1.0000	0.0000	1.0000	0.0000
LOC104912103	0.7233	-0.1839	0.5116	-0.1821	1.0000	0.0146	0.2512	0.2516	1.0000	-0.1315	1.0000	-0.1175	1.0000	0.1092
LOC104912106	1.0000	-0.8620	1.0000	-0.1612	0.4809	-3.7967	1.0000	0.0059	1.0000	-0.6504	1.0000	0.0563	1.0000	3.2049
LOC104912108	0.0061	0.8231	0.0000	0.6664	0.0000	1.1774	0.0000	0.9398	1.0000	0.1848	1.0000	0.0406	1.0000	-0.0466
LOC104912109	0.0000	2.6621	0.0000	1.8029	0.1662	-1.8489	0.0205	-2.0144	1.0000	0.6231	1.0000	-0.2254	1.0000	0.4629
LOC104912110	0.8226	-3.3425	0.2920	-4.1891	1.0000	-1.0191	0.9875	-0.9156	1.0000	0.8060	1.0000	0.0000	1.0000	0.9168
LOC104912112	0.4973	-1.1167	1.0000	0.3635	1.0000	-0.6594	1.0000	0.5372	1.0000	-1.8112	1.0000	-0.3316	1.0000	-0.6156
LOC104912114	0.0000	-0.9916	0.0000	-0.9804	0.2231	-0.3164	0.7710	0.0640	1.0000	-0.0419	1.0000	-0.0193	0.0086	0.3431
LOC104912115	0.3797	0.8472	0.1782	0.8102	0.8695	-0.4547	1.0000	-0.2691	1.0000	0.3702	1.0000	0.3409	1.0000	0.5555
LOC104912116	0.0087	2.3022	0.2888	0.9542	0.0000	3.5850	0.0000	2.7340	1.0000	0.6842	0.7392	-0.6493	1.0000	-0.1576
LOC104912117	1.0000	-0.0240	0.3602	0.1458	0.1954	-0.3120	0.6124	-0.1057	1.0000	-0.1349	1.0000	0.0484	1.0000	0.0779
LOC104912118	1.0000	-0.3524	0.3476	0.9656	0.0828	1.7406	0.0023	1.8822	1.0000	0.8349	0.1188	2.1618	0.2130	0.9779
LOC104912120	0.0022	-0.9893	0.0000	-1.2439	0.4631	-0.3118	0.0309	-0.5503	1.0000	0.0452	1.0000	-0.1967	0.9139	-0.1889
LOC104912121	0.5996	-0.5523	0.2480	0.6305	0.0152	1.1913	0.0000	2.2297	1.0000	-0.6874	0.7745	0.5112	0.6429	0.3530
LOC104912123	1.0000	0.0000	1.0000	0.0000	1.0000	0.0000	1.0000	2.3257	1.0000	0.0000	1.0000	0.0000	1.0000	2.3555
LOC104912124	1.0000	0.0000	1.0000	0.0000	1.0000	0.0000	1.0000	2.3240	1.0000	0.0000	1.0000	0.0000	1.0000	2.3543
LOC104912125	0.1279	1.3454	0.0001	2.3980	0.0000	2.5519	0.0000	2.9567	1.0000	-0.6890	1.0000	0.3663	0.9561	-0.2852
LOC104912126	0.5934	-1.5577	1.0000	-0.8620	1.0000	-0.4984	1.0000	0.2587	1.0000	-0.1259	1.0000	0.5866	1.0000	0.6346
LOC104912127	0.1612	0.7413	0.0082	1.3466	0.0000	1.9835	0.0000	2.0844	1.0000	-0.4899	1.0000	0.1301	0.5093	-0.3788
LOC104912128	0.6209	-0.3265	0.6461	-0.2424	0.0000	1.3014	0.0000	1.1564	1.0000	0.1610	0.9533	0.2580	1.0000	0.0243
LOC104912129	1.0000	0.0000	1.0000	-2.3960	1.0000	0.0000	1.0000	-2.3179	1.0000	2.2676	1.0000	0.0000	1.0000	0.0000
LOC104912130	1.0000	0.0000	1.0000	0.0000	1.0000	0.0000	1.0000	0.0000	1.0000	0.0000	1.0000	0.0000	1.0000	0.0000
LOC104912131	0.9860	-0.3498	0.9651	-0.3069	0.1085	1.0659	0.2966	0.8561	1.0000	-0.6656	1.0000	-0.6090	0.3019	-0.8712
LOC104912132	1.0000	0.2496	1.0000	0.1289	0.0023	1.6633	0.0153	1.0299	1.0000	0.7975	0.6156	0.6894	1.0000	0.1697
LOC104912133	1.0000	-2.4788	1.0000	0.6987	1.0000	-0.1630	1.0000	-2.3179	1.0000	-0.1089	1.0000	3.2002	1.0000	-2.2888
LOC104912134	1.0000	0.0942	0.1745	0.5799	0.0732	-0.8050	0.2034	-0.6068	1.0000	-0.5341	1.0000	-0.0374	0.9439	-0.3320
LOC104912135	1.0000	-0.0755	0.0622	-1.8079	1.0000	0.2081	0.7609	0.4611	1.0000	0.6917	0.8372	-1.0356	0.5018	0.9454
LOC104912136	1.0000	0.0000	1.0000	0.0000	1.0000	0.0000	1.0000	0.0000	1.0000	0.0000	1.0000	0.0000	1.0000	0.0000
LOC104912137	1.0000	0.5420	1.0000	0.0000	0.4584	1.8990	1.0000	2.3257	1.0000	-2.3757	1.0000	-3.1429	0.6944	-2.0004
LOC104912138	0.7605	-0.2626	0.2014	-0.4929	0.0584	-0.7752	0.0879	-0.6304	1.0000	-0.2525	0.6050	-0.4709	1.0000	-0.1057
LOC104912139	0.0728	-0.3013	0.0002	-0.3370	0.0000	-1.3322	0.0000	-1.1674	1.0000	-0.1080	0.3730	-0.1312	0.9095	0.0626
LOC104912140	0.4851	-0.3730	0.0001	1.0487	0.0101	0.8252	0.0000	1.8672	1.0000	-1.1129	0.6178	0.3239	1.0000	-0.0620
LOC104912141	0.0097	-0.4908	0.1275	-0.2282	0.4213	0.2238	0.0000	0.5407	1.0000	-0.2963	1.0000	-0.0204	1.0000	0.0281
LOC104912142	0.4135	-0.8177	0.6424	-0.5838	0.4509	-0.7726	0.1914	0.9313	1.0000	-0.7050	1.0000	-0.4604	0.3635	1.0040

LOC104912145	1.0000	-0.7243	0.8238	0.5988	1.0000	0.1177	0.4634	1.1293	1.0000	-0.1268	0.9744	1.2156	0.9562	0.8945
LOC104912147	1.0000	0.0000	1.0000	0.0000	1.0000	0.0000	1.0000	0.0000	1.0000	0.0000	1.0000	0.0000	1.0000	0.0000
LOC104912148	1.0000	0.0000	1.0000	0.0000	1.0000	2.2426	1.0000	0.0000	1.0000	0.0000	1.0000	0.0000	1.0000	-2.2888
LOC104912150	0.0001	1.0137	0.0000	1.2636	0.0000	2.0037	0.0000	1.9124	1.0000	-0.1057	0.8932	0.1593	0.4269	-0.1877
LOC104912151	0.5993	0.2313	0.2083	0.2978	0.0275	0.5868	0.0051	0.5681	1.0000	-0.0629	1.0000	0.0176	1.0000	-0.0742
LOC104912152	1.0000	0.4349	0.5422	0.8025	1.0000	-0.3801	0.6432	-0.9503	1.0000	-0.1296	1.0000	0.2508	1.0000	-0.6947
LOC104912153	1.0000	0.0000	1.0000	0.0000	1.0000	0.0000	1.0000	0.0000	1.0000	0.0000	1.0000	0.0000	1.0000	0.0000
LOC104912154	1.0000	0.4219	0.1330	2.5751	0.1656	1.5471	0.2439	2.3286	1.0000	-1.8822	1.0000	0.2547	0.5105	-1.1083
LOC104912155	0.8249	-3.3412	0.7674	3.0889	0.8033	-3.2622	0.7710	3.1753	1.0000	-3.2289	1.0000	3.1946	1.0000	3.2081
LOC104912156	0.0279	1.6164	0.0000	2.0656	0.2388	-1.6989	0.7701	-0.6270	1.0000	0.1513	0.5013	0.6158	0.8768	1.2314
LOC104912157	0.0020	0.9720	0.0000	0.8929	0.7630	-0.2333	0.6487	-0.2082	1.0000	-0.0039	1.0000	-0.0711	1.0000	0.0283
LOC104912158	0.0646	1.0367	0.2925	0.5635	1.0000	-0.1441	1.0000	0.1421	1.0000	0.2849	1.0000	-0.1693	0.6062	0.5795
LOC104912159	0.0146	1.2345	0.2251	0.8073	0.0001	1.7187	0.3749	0.6762	1.0000	-0.6730	0.0332	-1.0914	0.0000	-1.7149
LOC104912161	1.0000	2.1901	1.0000	0.0000	1.0000	0.0000	1.0000	0.0000	1.0000	0.0000	1.0000	-2.2992	1.0000	0.0000
LOC104912162	1.0000	0.0000	1.0000	0.0000	1.0000	0.0000	1.0000	0.0000	1.0000	0.0000	1.0000	0.0000	1.0000	0.0000
LOC104912163	0.3148	3.9252	0.4431	3.6192	0.5076	3.6021	0.7710	3.1709	1.0000	0.0000	1.0000	-0.3291	1.0000	-0.4550
LOC104912164	1.0000	0.0000	1.0000	0.0000	1.0000	2.2470	1.0000	0.0000	1.0000	0.0000	1.0000	0.0000	1.0000	-2.2906
LOC104912165	1.0000	-0.0402	0.6180	-1.9239	1.0000	0.3596	0.9786	-0.9111	1.0000	-0.1253	0.8269	-2.0165	0.8047	-1.3971
LOC104912166	1.0000	0.0000	1.0000	-2.3985	1.0000	0.0000	1.0000	-2.3200	1.0000	2.2734	1.0000	0.0000	1.0000	0.0000
LOC104912167	1.0000	0.1850	0.0324	0.9821	0.5661	-0.5697	1.0000	0.1437	1.0000	-0.5094	1.0000	0.3032	1.0000	0.2142
LOC104912168	1.0000	0.0000	1.0000	0.0000	1.0000	0.0000	1.0000	0.0000	1.0000	0.0000	1.0000	0.0000	1.0000	0.0000
LOC104912169	0.1369	-1.7544	0.0359	-2.2002	0.0033	-5.5939	0.0008	-5.7242	1.0000	0.1056	1.0000	-0.3291	1.0000	0.0000
LOC104912170	1.0000	0.0000	1.0000	0.0000	1.0000	0.0000	1.0000	0.0000	1.0000	0.0000	1.0000	0.0000	1.0000	0.0000
LOC104912171	1.0000	0.0000	1.0000	0.0000	1.0000	0.0000	1.0000	0.0000	1.0000	0.0000	1.0000	0.0000	1.0000	0.0000
LOC104912172	1.0000	0.0000	0.4431	3.6191	1.0000	0.0000	1.0000	0.0000	1.0000	0.0000	0.8607	3.7288	1.0000	0.0000
LOC104912173	1.0000	-0.2892	1.0000	-0.1429	0.9468	1.2118	0.9096	1.3854	1.0000	-0.1089	1.0000	0.0489	1.0000	0.0738
LOC104912174	0.4950	1.7587	1.0000	-0.1472	1.0000	-2.4056	0.9162	1.3864	1.0000	-0.1035	0.8372	-2.0207	0.7453	3.7416
LOC104912175	1.0000	-0.1262	1.0000	0.0157	0.0504	-0.7921	0.7121	-0.1986	1.0000	-0.2030	1.0000	-0.0458	0.5656	0.3970
LOC104912176	0.9650	1.0594	0.4437	3.6202	1.0000	-2.4056	1.0000	0.0000	1.0000	-2.3757	1.0000	0.0604	1.0000	0.0000
LOC104912177	1.0000	0.0000	1.0000	-2.3960	1.0000	0.0000	1.0000	-2.3179	1.0000	2.2676	1.0000	0.0000	1.0000	0.0000
LOC104912178	1.0000	0.0000	1.0000	0.0000	1.0000	0.0000	1.0000	0.0000	1.0000	0.0000	1.0000	0.0000	1.0000	0.0000
LOC104912179	0.0000	-1.5714	0.0000	-2.2419	0.0000	-0.9890	0.0000	-0.9237	1.0000	-0.0626	0.0000	-0.7213	1.0000	0.0083
LOC104912180	0.3757	-0.5262	0.0508	-0.6995	0.9543	-0.1764	0.1002	-0.6810	1.0000	0.1052	1.0000	-0.0577	0.7946	-0.3944
LOC104912181	0.0460	2.9629	1.0000	0.2802	1.0000	0.6780	0.3597	-1.0808	1.0000	3.2984	1.0000	0.6376	0.7908	1.5494
LOC104912182	1.0000	-2.4788	0.4431	-3.7876	0.9361	1.2042	1.0000	0.3938	1.0000	1.2654	1.0000	0.0000	1.0000	0.4629
LOC104912183	1.0000	-1.1514	1.0000	2.2533	1.0000	-0.1785	1.0000	0.0000	1.0000	-3.2289	1.0000	0.0489	1.0000	-3.1344
LOC104912184	0.0000	-3.0485	0.0000	-2.1343	0.8011	0.2802	0.0107	0.6251	1.0000	0.2465	0.4865	1.1755	0.0574	0.6005
LOC104912185	1.0000	-2.4788	0.4635	-3.7803	0.6601	1.5904	0.1748	1.9159	1.0000	1.2548	1.0000	0.0000	0.5187	1.5948
LOC104912186	1.0000	0.0792	0.1495	0.2588	0.0696	-0.4634	0.1683	0.2480	1.0000	-0.1405	1.0000	0.0537	0.0003	0.5785
LOC104912188	0.1416	-0.3441	0.0013	-0.2610	0.2237	0.2904	0.0000	0.5240	1.0000	0.2391	0.0004	0.3350	0.0000	0.4777
LOC104912189	1.0000	-0.2882	1.0000	2.2533	1.0000	-2.4056	1.0000	0.0000	1.0000	-2.3757	1.0000	0.0489	1.0000	0.0000
LOC104912190	0.0381	-0.6225	0.0000	-1.0066	0.0000	-1.5224	0.0000	-1.9619	1.0000	0.2399	1.0000	-0.1323	1.0000	-0.1956
LOC104912191	1.0000	-1.1514	0.9154	-0.5280	0.8033	-3.2622	0.4670	-1.1155	1.0000	1.9206	0.4857	2.5903	0.5093	4.1263
LOC104912192	1.0000	0.0000	1.0000	0.0000	1.0000	0.0000	1.0000	0.0000	1.0000	0.0000	1.0000	0.0000	1.0000	0.0000
LOC104912194	1.0000	-0.2931	1.0000	0.0000	1.0000	-0.1597	1.0000	0.0000	1.0000	-2.3771	1.0000	-2.2958	1.0000	-2.2906
LOC104912195	0.0001	-2.0962	0.0001	-2.0003	1.0000	-0.0967	1.0000	0.1346	1.0000	-0.4551	1.0000	-0.3481	1.0000	-0.2211
LOC104912196	1.0000	-2.4788	1.0000	0.0000	1.0000	-0.1597	1.0000	2.3240	1.0000	-2.3771	1.0000	0.0000	1.0000	0.0640
LOC104912198	1.0000	0.3564	1.0000	-1.0017	0.6409	0.9683	0.0874	2.1944	1.0000	-0.6522	0.8372	-2.0207	1.0000	0.5781
LOC104912200	1.0000	-1.1537	0.4431	-3.7876	1.0000	-0.1765	1.0000	0.0046	1.0000	0.4115	1.0000	-2.2992	1.0000	0.5998
LOC104912201	1.0000	0.0000	1.0000	-2.3985	1.0000	0.0000	1.0000	-2.3200	1.0000	2.2734	1.0000	0.0000	1.0000	0.0000
LOC104912202	0.5780	-1.5569	0.1546	2.3755	1.0000	-0.8842	1.0000	0.8519	1.0000	-2.1858	0.7608	1.7449	1.0000	-0.4550
LOC104912203	1.0000	0.0000	1.0000	0.0000	0.8033	3.0892	0.4370	3.7037	1.0000	0.0000	1.0000	0.0000	1.0000	0.6034
LOC104912204	1.0000	0.0000	1.0000	0.0000	1.0000	0.0000	1.0000	0.0000	1.0000	0.0000	1.0000	0.0000	1.0000	0.0000
LOC104912205	1.0000	0.0000	1.0000	0.0000	1.0000	0.0000	1.0000	0.0000	1.0000	0.0000	1.0000	0.0000	1.0000	0.0000
LOC104912208	1.0000	0.0000	1.0000	0.0000	1.0000	0.0000	1.0000	0.0000	1.0000	0.0000	1.0000	0.0000	1.0000	0.0000
LOC104912209	1.0000	0.0000	1.0000	0.0000	1.0000	2.2470	1.0000	2.3257	1.0000	0.0000	1.0000	0.0000	1.0000	0.0649
LOC104912210	1.0000	0.5453	1.0000	-0.1429	1.0000	-2.4061	1.0000	0.0074	1.0000	-0.1089	1.0000	-0.7977	1.0000	2.3555
LOC104912211	1.0000	0.5414	1.0000	0.1330	1.0000	-0.1597	0.9786	-0.9107	1.0000	1.6499	1.0000	1.2787	1.0000	0.9164
LOC104912212	0.5328	-0.9877	0.9163	0.4599	0.5162	-1.0391	1.0000	-0.2065	1.0000	-0.7660	1.0000	0.6948	1.0000	0.0765
LOC104912214	0.0289	0.4966	0.0282	0.3776	0.0245	0.5276	0.0007	0.5673	1.0000	-0.0073	0.9443	-0.1130	1.0000	0.0394
LOC104912217	0.0001	1.1591	0.0000	1.0455	0.0000	1.2688	0.0001	0.8124	1.0000	0.0883	1.0000	-0.0121	0.2055	-0.3620
LOC104912219	0.2959	-2.0245	0.9967	-1.0889	0.6301	-1.3466	0.6429	-1.7650	1.0000	-0.8910	1.0000	0.0559	1.0000	-1.3086
LOC104912220	1.0000	-2.4788	0.7227	1.0571	0.5134	1.9109	1.0000	0.5377	1.0000	0.7305	0.4563	4.4223	1.0000	-0.6219
LOC104912221	0.8457	-1.0284	0.9062	-1.5359	0.1614	-4.4918	0.9096	-1.3722	1.0000	-0.8154	1.0000	-1.3320	1.0000	2.3555
LOC104912222	1.0000	0.0491	1.0000	-0.1535	0.1142	1.8369	0.0996	1.4236	1.0000	1.0139	1.0000	0.8302	0.8163	0.6193
LOC104912223	0.0296	-0.4447	0.0000	-0.7411	0.7704	-0.1181	0.0416	-0.2357	1.0000	0.3272	1.0000	0.0443	0.2172	0.2163
LOC104912224	0.0021	0.8329	0.0000	1.1553	0.0000	1.3092	0.0000	1.3567	1.0000	0.1151	0.0126	0.4508	0.5536	0.1682
LOC104912226	1.0000	0.0000	1.0000	2.2533	1.0000	2.2426	1.0000	2.3240	1.0000	0.0000	1.0000	2.3480	1.0000	0.0652
LOC104912227	0.0861	0.7040	0.5079	0.2607	0.1032	0.6594	1.0000	0.0449	1.0000	0.4510	1.0000	0.0194	1.0000	-0.1592
LOC104912228	1.0000	0.1601	1.0000	0.0236	0.0000	2.2215	0.0000	1.7142	1.0000	0.3229	1.0000	0.2009	0.7704	-0.1760
LOC104912229	1.0000	0.0000	1.0000	-2.3985	1.0000	0.0000	1.0000	-2.3200	1.0000	2.2734	1.0000	0.0000	1.0000	0.0000
LOC104912230	1.0000	2.1850	1.0000	0.0000	1.0000	0.0000	1.0000	0.0000	1.0000	0.0000	1.0000	-2.2958	1.0000	0.0000
LOC104912231	0.8226	3.0199	1.0000	2.2508	1.0000	0.0000	1.0000	0.0000	1.0000	0.0000	1.0000	-0.7966	1.0000	0.0000
LOC104912233	1.0000	-0.2179	1.0000	-0.1769	0.8596	-0.5100	1.0000	-0.1409	1.0000	-0.1258	1.0000	-0.0740	1.0000	0.2442
LOC104912234	0.5086	3.5431	1.0000	0.0000	1.0000	0.0000	1.0000	0.0000	1.0000	0.0000	0.8607	-3.6719	1.0000	0.0000
LOC104912236	0.6936	1.4527	1.0000	0.6944	1.0000	-0.1597	0.6142	1.7719	1.0000	-0.1045	1.0000	-0.8625	0.8937	1.8362
LOC104912238	1.0000	0.0000	1.0000	0.0000	1.0000	0.0000	1.0000	0.0000	1.0000	0.0000	1.0000	0.0000	1.0000	0.0000
LOC104912240	1.0000	0.0000	1.0000	0.0000	1.0000	0.0000	1.0000	0.0000	1.0000	0.0000	1.0000	0.0000	1.0000	0.0000
LOC104912241	1.0000	2.1850	1.0000	0.0000	1.0000	2.2426	1.0000	0.0000	1.0000	0.0000	1.0000	-2.2958	1.0000	-2.2888
LOC104912242	1.0000	0.3529	0.6751	0.7933	0.9361	-1.5509	0.2831	-4.0731	1.0000	0.2556	1.0000	0.7123	1.0000	-2.2906
LOC104912244	1.0000	0.0000	1.0000	0.0000	1.0000	2.2426	1.0000	0.0000	1.0000	0.0000	1.0000	0.0000	1.0000	-2.2888
LOC104912245	0.4601	-1.1594	0.5693	-0.6758	1.0000	0.3204	0.9154	-0.4921	1.0000	0.0179	1.0000	0.5161	0.7588	-0.7902
LOC104912246	0.0727	1.1362	0.1579	0.6734	0.4704	0.6204	0.0292	-1.2161	1.0000	0.1584	0.9332	-0.2976	0.0024	-1.6785
LOC104912247	0.0357	0.9998	0.0000	1.7526	0.0000	1.7696	0.1024	0.7851	1.0000	-0.4455	0.7777	0.3239	0.0000	-1.4188
LOC104912248	0.3777	0.2822	0.4271	0.1423	0.1303	0.3797	0.0738	0.2690	1.0000	0.2648	0.9072	0.1386	0.6865	0.1599
LOC104912249	0.1877	4.4789	1.0000	-1.0051	1.0000	0.0000	1.0000	-0.8429	1.0000	3.1190	0.6450	-2.2673	1.0000	2.3555
LOC104912250	0.6936	1.4536	1.0000	0.3653	1.0000	-0.1591	0.7710	-3.1693	1.0000	0.7416	1.0000	-0.3305	1.0000	-2.2907
LOC104912252	0.1708	1.0416	0.2592	0.8079	0.0918	1.1528	0.2479	0.8329	1.0000	0.0917	1.0000	-0.1307	1.0000	-0.2261
LOC104912253	0.0207	0.6266	1.0000	0.0551	0.0014	0.7954	0.0001	0.6127	1.0000	0.3757	0.8351	-0.1822	0.5646	0.1987
LOC104912254	0.2144	-0.2622	0.0033	-0.2740	0.5065	-0.1701	0.0006	-0.3009	1.0000	-0.0095	1.0000	-0.0091	0.5122	-0.1352
LOC104912255	0.6185	0.4626	0.1465	0.7724	1.0000	-0.1150	0.0564	0.9599	1.0000	-0.9209	0.6442	-0.6029	1.0000	0.1603
LOC104912256	0.2288	-0.5509	0.7509	-0.1404	0.7336	-0.2549	0.9267	-0.1106	1.0000	-0.1650	0.8064	0.2566	1.0000	-0.0153
LOC104912257	0.2699	0.8342	0.3688	0.4394	0.4612	0.5708	1.0000	0.1188	1.0000	0.6783	1.0000	0.2985	1.0000	0.2315

LOC104912258	0.0003	-0.7007	0.0000	-0.5441	0.0000	-0.9028	0.0000	-0.7898	1.0000	-0.1371	1.0000	0.0325	1.0000	-0.0186
LOC104912259	0.2484	1.0165	0.4870	-0.7496	1.0000	-0.1990	0.5961	-0.7495	1.0000	-0.2447	0.0139	-2.0009	0.7557	-0.7906
LOC104912260	0.6089	1.0557	0.1533	1.0508	0.0102	2.3225	0.9911	0.3506	1.0000	1.2680	0.2366	1.2848	0.5763	-0.6882
LOC104912261	0.4638	1.0251	1.0000	0.3415	1.0000	0.2067	0.8975	-0.5577	1.0000	0.4355	1.0000	-0.2341	1.0000	-0.3219
LOC104912262	0.2959	1.7028	0.5029	1.3073	1.0000	-1.0171	1.0000	0.5384	1.0000	-0.1210	1.0000	-0.5065	1.0000	1.4485
LOC104912263	1.0000	2.1851	1.0000	2.2533	0.5076	3.6021	1.0000	2.3240	1.0000	0.0000	1.0000	0.0514	1.0000	-1.3057
LOC104912264	1.0000	0.0000	1.0000	0.0000	1.0000	0.0000	1.0000	0.0000	1.0000	0.0000	1.0000	0.0000	1.0000	0.0000
LOC104912265	1.0000	0.0000	1.0000	0.0000	1.0000	0.0000	1.0000	0.0000	1.0000	0.0000	1.0000	0.0000	1.0000	0.0000
LOC104912266	1.0000	0.0000	1.0000	0.0000	1.0000	0.0000	1.0000	0.0000	1.0000	0.0000	1.0000	0.0000	1.0000	0.0000
LOC104912267	1.0000	-2.4788	1.0000	2.2533	1.0000	-2.4061	1.0000	0.0000	1.0000	-2.3771	1.0000	2.3480	1.0000	0.0000
LOC104912268	1.0000	-0.2882	0.4431	-3.7865	1.0000	-0.1624	1.0000	0.3938	1.0000	1.2635	1.0000	-2.2992	0.8939	1.8363
LOC104912269	1.0000	-0.3485	1.0000	-0.1728	0.7628	-1.4076	0.2461	-4.0849	1.0000	-0.4280	1.0000	-0.2457	1.0000	-3.1344
LOC104912270	0.0000	1.3995	0.0000	1.2303	0.0001	-1.3353	0.0000	-1.1163	1.0000	-0.0879	0.3195	-0.2454	1.0000	0.1379
LOC104912271	1.0000	0.1953	0.0768	-4.7329	0.7663	1.0386	0.4992	-1.4660	1.0000	1.3498	0.8785	-3.6647	0.9837	-1.1475
LOC104912272	1.0000	2.1851	1.0000	0.0000	1.0000	0.0000	0.2461	4.0903	1.0000	0.0000	1.0000	-2.2958	0.5105	4.1259
LOC104912273	0.2119	-0.8456	0.0619	-0.8979	0.8824	0.2557	0.0307	0.7777	1.0000	-0.1322	1.0000	-0.1702	0.6829	0.3986
LOC104912274	0.8784	-0.2303	0.2072	-0.4686	1.0000	-0.0009	1.0000	0.0623	1.0000	0.3760	1.0000	0.1533	0.4602	0.4462
LOC104912275	0.5086	3.5431	1.0000	0.0000	0.1614	4.2993	1.0000	2.3240	1.0000	0.0000	0.8607	-3.6719	0.6865	-1.9991
LOC104912276	0.0000	-1.1367	0.0000	-1.5054	0.0007	-0.5432	0.0000	-0.8598	1.0000	0.1739	0.4857	-0.1822	0.5217	-0.1373
LOC104912277	1.0000	0.0000	1.0000	0.0000	1.0000	0.0000	1.0000	0.0000	1.0000	0.0000	1.0000	0.0000	1.0000	0.0000
LOC104912278	1.0000	0.0000	0.7666	-3.2534	1.0000	0.0000	0.7701	-3.1666	1.0000	3.1126	1.0000	0.0000	1.0000	0.0000
LOC104912279	0.6606	-0.9008	0.6057	-1.3227	0.2898	-2.4979	0.1701	-2.5251	1.0000	0.0805	1.0000	-0.3294	1.0000	0.0640
LOC104912280	0.0482	1.1142	0.0016	1.2715	0.0432	1.0697	0.9801	0.2189	1.0000	0.1203	0.9874	0.2866	0.2468	-0.7283
LOC104912282	0.8227	0.1336	0.8771	-0.0889	0.2985	-0.2942	0.1090	-0.2920	1.0000	0.0965	1.0000	-0.1122	0.9756	0.1053
LOC104912283	0.9854	-0.0576	1.0000	0.0230	0.8641	-0.0826	1.0000	-0.0084	1.0000	0.0787	0.2580	0.1718	0.3580	0.1576
LOC104912284	0.0001	0.9159	0.0000	0.8882	0.2173	-0.3822	0.0229	-0.4980	1.0000	-0.1180	0.9304	-0.1323	0.7532	-0.2277
LOC104912285	1.0000	0.0000	1.0000	0.0000	0.2840	3.9988	1.0000	2.3240	1.0000	0.0000	1.0000	0.0000	0.8939	-1.6957
LOC104912286	1.0000	0.0000	1.0000	-2.3985	1.0000	0.0000	1.0000	-2.3200	1.0000	2.2734	1.0000	0.0000	1.0000	0.0000
LOC104912287	0.0560	-0.5284	0.0029	-0.4985	0.1015	-0.4384	0.0001	-0.6821	1.0000	-0.0079	1.0000	0.0344	0.6275	-0.2455
LOC104912288	0.8249	3.0143	1.0000	0.0000	1.0000	0.0000	1.0000	0.0000	1.0000	0.0000	1.0000	-3.1383	1.0000	0.0000
LOC104912289	1.0000	0.0000	1.0000	0.0000	1.0000	0.0000	1.0000	0.0000	1.0000	0.0000	1.0000	0.0000	1.0000	0.0000
LOC104912290	0.5086	-3.8796	1.0000	-1.0027	0.9361	-1.5532	1.0000	0.0058	1.0000	-0.6479	1.0000	2.3480	1.0000	0.9168
LOC104912291	1.0000	0.0000	1.0000	0.0000	1.0000	0.0000	1.0000	0.0000	1.0000	0.0000	1.0000	0.0000	1.0000	0.0000
LOC104912292	1.0000	0.0000	1.0000	0.0000	1.0000	0.0000	1.0000	0.0000	1.0000	0.0000	1.0000	0.0000	1.0000	0.0000
LOC104912293	1.0000	0.5464	1.0000	2.2508	1.0000	-0.1624	0.7710	3.1753	1.0000	-2.3757	1.0000	-0.8001	1.0000	0.9186
LOC104912294	1.0000	0.5370	1.0000	2.2533	1.0000	0.6780	0.4370	3.7037	1.0000	-2.3771	1.0000	-0.7919	1.0000	0.6041
LOC104912295	0.6926	-2.0812	0.2920	-4.1891	1.0000	-1.1020	1.0000	-0.3848	1.0000	-0.1169	1.0000	-2.2992	1.0000	0.6041
LOC104912297	1.0000	0.0000	1.0000	2.2508	1.0000	2.2426	1.0000	0.0000	1.0000	0.0000	1.0000	2.3456	1.0000	-2.2888
LOC104912298	1.0000	-2.4788	1.0000	0.0000	1.0000	-2.4061	1.0000	0.0000	1.0000	-2.3771	1.0000	0.0000	1.0000	0.0000
LOC104912299	0.2365	0.6772	0.5141	0.3718	0.0207	-1.5265	0.2590	-0.5928	1.0000	0.0887	1.0000	-0.2054	0.1823	1.0203
LOC104912301	1.0000	0.0000	0.4634	-3.7803	1.0000	0.0000	0.4632	-3.6900	1.0000	3.6299	1.0000	0.0000	1.0000	0.0000
LOC104912302	1.0000	0.5968	0.9754	0.7517	0.6360	0.9650	0.7186	1.2292	1.0000	-0.6504	1.0000	-0.4954	1.0000	-0.3872
LOC104912303	1.0000	2.1901	1.0000	0.0000	1.0000	2.2426	0.2461	4.0903	1.0000	0.0000	1.0000	-2.2992	0.8939	1.8363
LOC104912304	0.0193	1.9615	0.0000	2.1715	1.0000	-0.4443	0.1940	-1.6493	1.0000	0.5823	0.2853	0.8047	1.0000	-0.6156
LOC104912305	0.6348	1.0520	0.7185	0.6437	1.0000	0.3655	1.0000	-0.6864	1.0000	0.1845	1.0000	-0.2159	1.0000	-0.8670
LOC104912306	1.0000	0.0000	1.0000	0.0000	1.0000	0.0000	1.0000	0.0000	1.0000	0.0000	1.0000	0.0000	1.0000	0.0000
LOC104912307	1.0000	-0.2892	1.0000	0.0000	1.0000	-2.4061	1.0000	0.0000	1.0000	-2.3771	1.0000	-2.2992	1.0000	0.0000
LOC104912308	0.0024	-2.3604	0.0002	-2.5534	0.8333	0.2986	1.0000	0.0055	1.0000	-0.2310	1.0000	-0.4190	0.6738	-0.5194
LOC104912309	0.0656	1.2121	0.8142	-0.2736	0.3246	0.7480	0.0067	-1.5121	1.0000	0.7716	0.4491	-0.7012	0.0325	-1.4833
LOC104912310	0.0187	3.1817	0.5793	0.9864	0.0136	3.3260	0.4085	1.3514	1.0000	1.2628	0.8044	-0.9129	0.8058	-0.7069
LOC104912312	1.0000	0.0000	1.0000	0.0000	1.0000	0.0000	1.0000	0.0000	1.0000	0.0000	1.0000	0.0000	1.0000	0.0000
LOC104912313	0.6881	1.7520	0.9062	-1.5349	0.6966	1.5803	1.0000	-0.5247	1.0000	1.2661	0.8269	-2.0158	1.0000	-0.8395
LOC104912314	0.1128	0.5853	0.0000	0.8904	0.0000	2.0063	0.0000	1.9298	1.0000	0.1327	0.0594	0.4511	1.0000	0.0614
LOC104912315	0.9730	-0.1596	1.0000	-0.0507	0.4685	-0.4023	0.0135	0.6291	1.0000	0.0481	1.0000	0.1717	0.0004	1.0863
LOC104912316	0.0000	-1.9962	0.0000	-1.5104	1.0000	0.0692	0.0000	1.1599	1.0000	0.0875	0.6238	0.5902	0.0000	1.1811
LOC104912317	1.0000	0.0000	1.0000	0.0000	1.0000	0.0000	1.0000	0.0000	1.0000	0.0000	1.0000	0.0000	1.0000	0.0000
LOC104912318	1.0000	0.0000	1.0000	0.0000	1.0000	0.0000	1.0000	2.3240	1.0000	0.0000	1.0000	0.0000	1.0000	2.3543
LOC104912319	0.1974	-4.5752	1.0000	2.2533	0.4561	-2.2447	0.7710	3.1710	1.0000	-4.4557	1.0000	2.3480	1.0000	0.9149
LOC104912320	0.0786	-0.3486	0.0000	-0.6100	0.0799	-0.3462	0.0000	-0.4531	1.0000	0.2132	1.0000	-0.0353	0.7121	0.1123
LOC104912321	1.0000	-0.3384	0.6180	-1.9239	0.9468	-1.5505	1.0000	0.3118	1.0000	0.2623	1.0000	-1.3254	0.6865	2.1414
LOC104912322	0.4241	-1.8074	0.5540	-0.7463	1.0000	0.3737	1.0000	0.0071	1.0000	0.4383	0.9846	1.5086	1.0000	0.0783
LOC104912323	0.6157	-0.9156	1.0000	-0.0440	0.4080	-1.3161	1.0000	0.0074	1.0000	0.0213	0.9640	0.9069	0.6384	1.3498
LOC104912324	0.0960	-2.0004	0.0563	-3.0485	0.1690	-1.8471	0.2800	-1.5020	1.0000	-0.2761	1.0000	-1.3257	1.0000	0.0750
LOC104912327	0.0000	-2.9553	0.0000	-2.5846	0.7079	-0.1717	0.0000	0.6582	1.0000	0.1685	0.5226	0.5532	0.0000	1.0037
LOC104912328	0.2237	0.4561	0.0028	0.8658	0.1891	-0.5385	1.0000	-0.0900	1.0000	-0.6918	0.8059	-0.2706	0.9886	-0.2362
LOC104912329	0.0000	-1.0675	0.0000	-1.2566	0.0000	-0.8437	0.0000	-0.7996	1.0000	0.0835	0.7721	-0.0932	0.6059	0.1330
LOC104912330	1.0000	0.0047	0.5522	-0.1100	0.0507	0.3834	0.1063	0.2034	1.0000	-0.1159	0.3460	-0.2178	0.0455	-0.2904
LOC104912332	0.3122	0.3951	0.0022	0.5153	0.0055	0.7525	0.0000	1.3682	1.0000	-0.4936	0.3322	-0.3605	0.7485	0.1278
LOC104912334	1.0000	0.0000	1.0000	0.0000	1.0000	0.0000	1.0000	0.0000	1.0000	0.0000	1.0000	0.0000	1.0000	0.0000
LOC104912335	1.0000	0.0489	0.2571	0.2487	0.9419	0.1156	0.7710	0.1234	1.0000	-0.1240	1.0000	0.0866	1.0000	-0.1099
LOC104912336	0.5093	-0.1457	0.0873	-0.1409	0.2679	0.2035	0.0088	0.2019	1.0000	0.0919	0.6659	0.1090	0.6544	0.0953
LOC104912337	0.3362	0.6636	0.0046	1.2325	0.0000	2.2253	0.0000	1.6400	1.0000	0.0467	0.4311	0.6310	0.1620	-0.5277
LOC104912338	1.0000	0.0522	0.8235	-0.9444	0.5513	-1.6581	0.5798	-1.1502	1.0000	0.0873	1.0000	-0.8996	1.0000	0.6041
LOC104912339	1.0000	0.5420	1.0000	-2.3985	1.0000	-2.4056	1.0000	-2.3200	1.0000	-0.1035	1.0000	-3.1429	1.0000	0.0000
LOC104912341	1.0000	0.0000	1.0000	0.0000	1.0000	0.0000	0.7710	3.1753	1.0000	0.0000	1.0000	0.0000	1.0000	3.2081
LOC104912342	1.0000	0.0000	1.0000	-0.1449	0.8033	3.0789	1.0000	-2.3179	1.0000	2.2676	1.0000	2.3456	1.0000	-3.1317
LOC104912343	1.0000	0.0742	0.7715	-0.0987	0.0123	-0.6242	0.0015	-0.5353	1.0000	-0.0450	0.6920	-0.2049	1.0000	0.0496
LOC104912346	0.2882	0.4289	0.5985	0.2171	0.7131	0.2322	0.0012	0.7274	1.0000	-0.3349	0.1375	-0.5329	0.9222	0.1671
LOC104912348	0.3711	0.9370	0.0479	1.5049	0.0003	2.2845	0.0060	1.8629	1.0000	-0.3169	1.0000	0.2619	0.3521	-0.7352
LOC104912349	0.0096	-3.7282	0.0045	-3.5647	0.4261	-0.8794	1.0000	0.1200	1.0000	-0.1332	1.0000	0.0489	0.8278	0.8712
LOC104912350	1.0000	-0.0499	1.0000	0.0109	1.0000	-0.0504	0.2242	0.3434	1.0000	0.2101	0.8607	0.2833	0.1162	0.6062
LOC104912351	1.0000	0.0000	1.0000	0.0000	1.0000	2.2470	1.0000	0.0000	1.0000	0.0000	1.0000	0.0000	1.0000	-2.2907
LOC104912352	1.0000	2.1901	0.7674	3.0956	1.0000	2.2470	1.0000	2.3257	1.0000	0.0000	1.0000	0.9006	1.0000	0.0649
LOC104912353	0.0250	-1.3266	0.1141	-0.7763	0.1737	0.6506	0.0002	1.1684	1.0000	-0.3339	1.0000	0.2301	1.0000	0.1937
LOC104912354	0.5086	-3.8790	0.7674	-3.2499	0.4809	-3.7967	1.0000	0.0074	1.0000	-0.6533	1.0000	0.0000	1.0000	3.2066
LOC104912355	0.1360	0.6983	0.0109	0.7891	0.0893	0.7725	0.3903	0.3995	1.0000	0.3040	0.6071	0.4076	1.0000	-0.0644
LOC104912356	0.3856	0.1761	0.3298	-0.1026	0.4476	0.1628	0.8607	0.0399	1.0000	0.0837	0.1948	-0.1827	1.0000	-0.0342
LOC104912358	0.0004	-0.9591	0.0000	-1.2350	0.1886	-0.3816	1.0000	0.0250	1.0000	0.2121	1.0000	-0.0526	0.0006	0.6227
LOC104912359	0.0020	1.1907	0.0047	0.8958	1.0000	0.0752	1.0000	-0.1343	1.0000	0.0457	0.9311	-0.2376	1.0000	-0.1585
LOC104912360	1.0000	0.4623	1.0000	0.2935	1.0000	0.0547	0.6835	0.8249	1.0000	-0.1298	1.0000	-0.2890	1.0000	0.6455
LOC104912361	0.0152	0.7519	0.0000	1.2878	0.5151	-0.3271	0.0444	-0.6583	1.0000	-0.2599	0.4465	0.2851	0.2532	-0.5855
LOC104912362	0.2182	-0.2896	0.0000	-0.4987	0.2138	-0.2898	0.0000	-0.5714	1.0000	0.1804	1.0000	-0.0164	0.9628	-0.0958
LOC104912363	0.3781	0.1866	0.3754	0.1012	0.0000	0.6422	0.0000	0.6220	1.0000	0.1441	0.9944	0.0712	0.4564	0.1296

LOC104912364	0.0000	2.3188	0.0000	1.8735	1.0000	-0.1974	0.2244	-0.9103	1.0000	0.4470	1.0000	0.0130	1.0000	-0.2613
LOC104912365	0.0007	2.0197	0.0000	3.0263	0.6363	-0.6934	0.8620	0.6591	1.0000	-1.0910	1.0000	-0.0789	1.0000	0.2649
LOC104912366	1.0000	-0.2920	1.0000	2.2533	1.0000	0.6746	1.0000	0.0000	1.0000	-2.3757	1.0000	0.0514	1.0000	-3.1317
LOC104912367	0.0039	1.4225	0.0000	2.5247	1.0000	-0.2701	0.8364	0.4466	1.0000	-1.0498	1.0000	0.0627	1.0000	-0.3284
LOC104912368	0.8457	-0.3530	0.0540	-0.9460	0.0000	1.9822	0.0021	0.9858	1.0000	0.7564	1.0000	0.1794	0.8672	-0.2349
LOC104912369	0.1096	4.6934	0.1543	2.3762	0.0000	6.3133	0.0018	3.6153	1.0000	2.2676	1.0000	0.0594	1.0000	-0.3987
LOC104912370	1.0000	0.0000	0.7674	3.0889	0.0033	5.6323	0.0235	5.0500	1.0000	0.0000	1.0000	3.1946	0.9124	-0.6034
LOC104912371	1.0000	0.2193	0.9541	-0.3102	0.4682	-0.8211	0.9355	-0.4400	1.0000	-0.4739	0.5089	-0.9914	1.0000	-0.0862
LOC104912372	0.0124	1.3685	0.1830	0.6238	0.0000	2.5384	0.0000	1.5709	1.0000	0.7646	1.0000	0.0359	0.9307	-0.1933
LOC104912373	1.0000	0.0000	1.0000	0.0000	1.0000	0.0000	1.0000	0.0000	1.0000	0.0000	1.0000	0.0000	1.0000	0.0000
LOC104912374	0.1585	0.7420	0.1117	0.6683	0.6836	-0.4070	1.0000	0.0075	1.0000	-0.3895	0.6096	-0.4505	1.0000	0.0347
LOC104912375	0.1578	0.5723	0.0338	0.5088	1.0000	0.1159	0.0000	1.1377	1.0000	0.0630	1.0000	0.0141	0.0000	1.0943
LOC104912376	1.0000	0.0000	1.0000	0.0000	1.0000	0.0000	1.0000	0.0000	1.0000	0.0000	1.0000	0.0000	1.0000	0.0000
LOC104912377	0.8121	0.3017	0.0671	0.8413	0.2107	0.7395	0.0362	0.9117	1.0000	-0.4867	1.0000	0.0635	0.8937	-0.3113
LOC104912381	0.0000	-1.1213	0.0000	-1.5526	0.1535	-0.3312	0.4700	0.0689	1.0000	-0.1304	0.0000	-0.5492	0.0044	0.2745
LOC104912382	0.2364	0.7119	0.1779	0.6947	1.0000	0.0041	1.0000	0.1626	1.0000	-0.1271	1.0000	-0.1346	1.0000	0.0324
LOC104912384	0.2029	-0.6484	0.0322	-0.8950	1.0000	-0.1536	1.0000	-0.0502	1.0000	-0.3891	0.5657	-0.6247	0.8837	-0.2803
LOC104912385	1.0000	0.0000	1.0000	2.2533	1.0000	0.0000	1.0000	0.0000	1.0000	0.0000	1.0000	2.3480	1.0000	0.0000
LOC104912386	1.0000	0.0000	0.4689	3.6127	1.0000	0.0000	1.0000	0.0000	1.0000	0.0000	0.8785	3.7233	1.0000	0.0000
LOC104912388	0.8226	-3.3425	1.0000	-1.0017	1.0000	-1.0191	1.0000	-0.8419	1.0000	-0.1179	1.0000	2.3456	1.0000	0.0652
LOC104912389	0.0535	0.9553	0.0079	0.9268	0.2068	0.7611	0.0036	0.9830	1.0000	0.1679	1.0000	0.1534	0.6830	0.3909
LOC104912390	1.0000	0.0000	1.0000	0.0000	1.0000	0.0000	1.0000	2.3257	1.0000	0.0000	1.0000	0.0000	1.0000	2.3555
LOC104912391	0.0394	0.6916	0.0006	0.7431	0.0000	1.5218	0.0000	1.4862	1.0000	0.0672	1.0000	0.1303	1.0000	0.0358
LOC104912393	1.0000	2.1851	1.0000	0.0000	1.0000	0.0000	1.0000	0.0000	1.0000	0.0000	1.0000	-2.2957	1.0000	0.0000
LOC104912394	1.0000	-0.2920	0.9062	-1.5348	1.0000	-2.4056	0.4370	-3.6968	1.0000	1.2635	1.0000	0.0495	1.0000	0.0000
LOC104912395	1.0000	-0.2892	1.0000	-0.9997	1.0000	-2.4061	1.0000	-0.8388	1.0000	0.7305	1.0000	0.0471	1.0000	2.3555
LOC104912396	1.0000	0.0000	1.0000	0.0000	1.0000	0.0000	1.0000	0.0000	1.0000	0.0000	1.0000	0.0000	1.0000	0.0000
LOC104912397	1.0000	-2.4776	1.0000	0.0000	1.0000	-0.1591	1.0000	2.3240	1.0000	-2.3757	1.0000	0.0000	1.0000	0.0640
LOC104912398	1.0000	0.2121	1.0000	0.0798	1.0000	-0.1959	1.0000	-0.6850	1.0000	0.1810	1.0000	0.0608	1.0000	-0.3077
LOC104912399	1.0000	0.0000	1.0000	0.0000	1.0000	0.0000	0.2426	4.0910	1.0000	0.0000	1.0000	0.0000	0.5093	4.1263
LOC104912401	0.3902	0.3597	1.0000	-0.0587	0.0600	0.5980	0.7318	0.1698	1.0000	0.3387	1.0000	-0.0655	1.0000	-0.0865
LOC104912402	0.7322	-2.0746	0.2541	-2.4812	0.4154	1.2133	1.0000	-0.2440	1.0000	0.4291	1.0000	0.0471	0.6819	-1.0212
LOC104912403	0.8249	3.0143	1.0000	0.0000	1.0000	0.0000	1.0000	0.0000	1.0000	0.0000	1.0000	-3.1383	1.0000	0.0000
LOC104912404	0.8226	3.0199	0.7674	-3.2499	1.0000	0.0000	0.7710	-3.1633	1.0000	3.1056	1.0000	-3.1429	1.0000	0.0000
LOC104912405	1.0000	0.0000	1.0000	0.0000	1.0000	0.0000	1.0000	0.0000	1.0000	0.0000	1.0000	0.0000	1.0000	0.0000
LOC104912406	0.8249	3.0255	1.0000	2.2507	1.0000	0.0000	1.0000	0.0000	1.0000	0.0000	1.0000	-0.8001	1.0000	0.0000
LOC104912410	1.0000	0.0000	1.0000	0.0000	1.0000	2.2426	1.0000	2.3240	1.0000	0.0000	1.0000	0.0000	1.0000	0.0652
LOC104912411	1.0000	0.0000	1.0000	0.0000	1.0000	0.0000	1.0000	0.0000	1.0000	0.0000	1.0000	0.0000	1.0000	0.0000
LOC104912412	0.0000	-1.5355	0.0000	-1.4721	1.0000	-0.0315	1.0000	0.0078	1.0000	-0.0691	1.0000	0.0063	1.0000	-0.0244
LOC104912413	0.6991	-0.7984	0.7726	0.7717	1.0000	0.0415	0.0379	2.1375	1.0000	-1.9158	1.0000	-0.3425	1.0000	0.1825
LOC104912415	0.0000	-1.8372	0.0000	-3.3483	0.3192	0.3822	0.0017	0.6684	1.0000	0.2550	0.2049	-1.2434	0.0436	0.5467
LOC104912416	1.0000	0.0000	1.0000	0.0000	1.0000	0.0000	1.0000	2.3257	1.0000	0.0000	1.0000	0.0000	1.0000	2.3555
LOC104912417	0.0000	1.8945	0.0000	1.7693	0.0000	3.3905	0.0000	2.4744	1.0000	0.5674	0.4857	0.4576	0.2118	-0.3398
LOC104912418	0.9650	1.0733	0.0852	-4.7292	1.0000	-0.1597	0.7922	-0.9350	1.0000	2.1976	0.8764	-3.6794	1.0000	1.4453
LOC104912421	0.0034	-0.5266	0.0001	-0.3874	0.2085	-0.2491	0.8421	-0.0499	1.0000	-0.1036	1.0000	0.0484	0.7566	0.1003
LOC104912422	0.1995	-0.6607	0.0032	-0.8988	0.1808	-0.6000	0.5222	0.2386	1.0000	0.3399	1.0000	0.1117	0.0001	1.1818
LOC104912423	0.8448	1.1167	1.0000	0.5219	0.5567	1.2856	0.4057	1.3511	1.0000	0.4081	1.0000	-0.1854	1.0000	0.4793
LOC104912424	1.0000	0.0000	1.0000	0.0000	1.0000	0.0000	1.0000	0.0000	1.0000	0.0000	1.0000	0.0000	1.0000	0.0000
LOC104912425	0.8249	-3.3412	1.0000	-2.3985	1.0000	-1.0156	1.0000	-2.3200	1.0000	-0.9563	1.0000	0.0000	1.0000	-2.2906
LOC104912426	1.0000	-2.4776	1.0000	0.0000	1.0000	-2.4056	0.7710	3.1709	1.0000	-2.3757	1.0000	0.0000	1.0000	3.2049
LOC104912427	0.0000	-1.2027	0.0000	-1.3264	0.0028	-0.5371	0.0000	-1.0816	1.0000	-0.0751	0.6956	-0.1854	0.0000	-0.6134
LOC104912428	0.0000	-0.7026	0.0000	-1.2033	0.0000	-0.9302	0.0000	-1.3542	1.0000	-0.1403	0.0000	-0.6283	0.0002	-0.5584
LOC104912429	1.0000	-1.1562	1.0000	2.2508	0.8011	-3.2629	0.1321	4.3960	1.0000	-3.2304	1.0000	0.0495	0.3369	4.4317
LOC104912430	1.0000	-0.1106	0.8790	-0.1440	0.0009	-1.3144	0.0001	-1.2981	1.0000	-0.0745	1.0000	-0.0948	1.0000	-0.0518
LOC104912431	1.0000	-0.2215	1.0000	-0.3110	0.9966	-0.3386	1.0000	-0.1157	1.0000	0.1286	1.0000	0.0532	1.0000	0.3603
LOC104912432	0.8249	3.0254	1.0000	0.0000	1.0000	2.2470	1.0000	2.3257	1.0000	0.0000	1.0000	-3.1468	1.0000	0.0649
LOC104912434	0.6936	1.4527	0.9762	-1.0826	1.0000	-2.4061	0.2426	-4.0840	1.0000	1.6499	1.0000	-0.8617	1.0000	0.0000
LOC104912435	0.8226	3.0199	1.0000	-0.1616	1.0000	0.0000	1.0000	0.0058	1.0000	3.1190	1.0000	0.0559	1.0000	3.2066
LOC104912436	0.0044	0.4689	0.0023	0.2848	0.0640	0.3235	0.0252	0.2065	1.0000	0.1074	1.0000	-0.0639	1.0000	-0.0042
LOC104912440	0.8087	-0.1705	1.0000	0.0221	0.9520	-0.1167	1.0000	0.0446	1.0000	-0.2367	1.0000	-0.0320	1.0000	-0.0689
LOC104912442	0.4873	0.5139	0.0132	1.1321	0.0121	-1.8074	0.0605	-1.4708	1.0000	-0.7671	1.0000	-0.1291	1.0000	-0.4203
LOC104912443	0.5557	-0.4934	0.0037	-1.5121	0.0551	-1.1869	0.0406	-0.9507	1.0000	0.2047	0.6957	-0.8036	0.9854	0.4481
LOC104912445	1.0000	0.0000	1.0000	0.0000	1.0000	0.0000	1.0000	0.0000	1.0000	0.0000	1.0000	0.0000	1.0000	0.0000
LOC104912446	0.0173	0.9682	0.0000	1.2663	0.0000	2.3889	0.0000	1.9964	1.0000	0.2114	0.1286	0.5219	0.7389	-0.1761
LOC104912447	0.5519	-0.2126	0.1085	0.3224	0.0006	-0.8010	0.0011	-0.6882	1.0000	-0.5119	1.0000	0.0345	0.2579	-0.3958
LOC104912448	0.8318	-0.0924	0.1754	-0.1458	1.0000	0.0581	1.0000	0.0138	1.0000	0.0272	1.0000	-0.0136	1.0000	-0.0120
LOC104912449	0.0034	2.0653	0.0166	1.3899	0.0000	2.5673	0.0041	1.6965	1.0000	0.4230	1.0000	-0.2261	0.7059	-0.4346
LOC104912450	0.3175	0.2581	0.1881	0.2275	0.0002	-0.9535	0.0000	-1.0018	1.0000	0.0503	1.0000	0.0309	1.0000	0.0075
LOC104912451	0.1022	0.8844	0.0079	0.9912	0.0000	1.7525	0.0000	1.6418	1.0000	-0.0553	1.0000	0.0661	1.0000	-0.1578
LOC104912452	0.0083	1.1778	0.0137	0.8427	0.0000	1.7296	0.0000	1.2213	1.0000	0.3094	1.0000	-0.0111	1.0000	-0.1914
LOC104912453	0.9545	1.0644	0.7674	3.0956	1.0000	-2.4061	1.0000	0.0000	1.0000	-2.3771	1.0000	-0.4730	1.0000	0.0000
LOC104912454	1.0000	0.0000	1.0000	0.0000	1.0000	0.0000	1.0000	0.0000	1.0000	0.0000	1.0000	0.0000	1.0000	0.0000
LOC104912455	0.0098	-5.5472	0.0003	-5.9256	0.3071	-1.4656	0.3526	-0.9680	1.0000	0.3465	1.0000	0.0000	1.0000	0.8483
LOC104912457	0.6881	1.7537	0.1388	4.3092	1.0000	-2.4061	1.0000	0.0000	1.0000	-2.3771	1.0000	0.0585	1.0000	0.0000
LOC104912458	0.4962	0.8734	0.2066	1.4421	1.0000	0.3724	0.7888	0.7248	1.0000	-0.5447	1.0000	0.0304	1.0000	-0.1785
LOC104912459	0.0024	-1.5635	0.0000	-1.8377	0.0265	0.8373	0.0000	1.4022	1.0000	0.2563	1.0000	-0.0040	0.0024	0.8273
LOC104912460	0.2197	0.4463	0.0381	0.4777	0.9018	0.1653	0.9067	-0.1194	1.0000	0.0879	1.0000	0.1299	0.9225	-0.1918
LOC104912462	1.0000	-0.2892	1.0000	0.6959	1.0000	-2.4061	1.0000	0.8541	1.0000	-0.1045	1.0000	0.9006	1.0000	3.2066
LOC104912464	1.0000	0.0000	1.0000	0.6944	1.0000	0.0000	1.0000	-2.3200	1.0000	2.2734	1.0000	3.1976	1.0000	0.0000
LOC104912465	0.4704	-0.4816	0.9125	-0.1806	1.0000	0.0763	1.0000	-0.0508	1.0000	-0.1603	1.0000	0.1514	0.9660	-0.2837
LOC104912467	0.0000	-2.1637	0.0000	-2.5576	0.0002	-0.8359	0.0010	-0.4737	1.0000	0.2473	1.0000	-0.1344	0.0028	0.6140
LOC104912468	0.0170	0.7758	0.0002	0.8430	0.0000	1.6799	0.0000	1.4732	1.0000	0.0355	1.0000	0.1178	0.7629	-0.1645
LOC104912469	0.1927	-0.2805	0.0000	-0.4477	0.1610	-0.2853	0.0453	-0.2268	1.0000	0.0293	0.8723	-0.1248	0.8920	0.0935
LOC104912471	0.0100	0.7772	0.0000	0.9992	0.2460	0.4118	0.5674	0.2043	1.0000	-0.0562	0.8759	0.1778	0.6285	-0.2567
LOC104912473	0.0002	-1.8198	0.0000	-1.5817	0.4795	-0.4034	1.0000	0.0415	1.0000	0.2278	0.8028	0.4744	0.0665	0.6730
LOC104912474	0.4743	0.3324	0.1395	0.3606	1.0000	0.1066	0.9647	-0.1160	1.0000	0.1219	0.9907	0.1652	1.0000	-0.0953
LOC104912475	1.0000	0.0000	1.0000	0.0000	1.0000	0.0000	1.0000	0.0000	1.0000	0.0000	1.0000	0.0000	1.0000	0.0000
LOC104912476	0.0372	-3.3578	0.0022	-5.5849	0.7716	0.5532	0.9830	0.3477	1.0000	0.0069	1.0000	-2.2992	1.0000	-0.1957
LOC104912477	0.0001	-0.6586	0.0000	-0.5929	0.9278	-0.0765	0.8297	0.0545	1.0000	-0.3228	0.2218	-0.2440	0.2488	-0.1865
LOC104912481	0.3900	-1.3050	0.1359	-1.8304	1.0000	0.3749	1.0000	-0.1410	1.0000	0.1873	1.0000	-0.3291	1.0000	-0.3258
LOC104912482	0.0038	-0.6892	0.0000	-1.0447	1.0000	-0.0830	1.0000	-0.0331	1.0000	0.0110	0.3988	-0.3318	1.0000	0.0666
LOC104912483	0.0839	0.3132	0.0435	0.1743	0.6228	-0.1350	0.0259	-0.1900	1.0000	-0.0090	0.4330	-0.1356	0.9880	-0.0584
LOC104912484	0.0000	-2.3846	0.0000	-2.6549	0.0007	-0.4983	0.0000	-0.7219	1.0000	0.1409	0.8785	-0.1174	0.8109	-0.0778

LOC104912485	0.2255	1.0207	1.0000	0.1941	0.0344	1.4041	1.0000	0.1422	1.0000	0.7187	1.0000	-0.0957	0.7370	-0.5412
LOC104912486	0.1121	-0.4741	0.1612	-0.2799	0.0000	-1.1325	0.0000	-0.8001	1.0000	-0.1011	1.0000	0.1051	0.7122	0.2392
LOC104912488	1.0000	0.2011	0.9062	1.2223	0.7662	1.0366	0.6142	1.7719	1.0000	-0.9563	1.0000	0.0576	1.0000	-0.2278
LOC104912491	0.0000	1.9949	0.0000	1.9677	0.0000	3.6852	0.0000	2.6983	1.0000	0.5811	0.0829	0.5709	0.1389	-0.3944
LOC104912492	0.5086	3.5431	0.7666	3.0922	1.0000	0.0000	1.0000	0.0000	1.0000	0.0000	1.0000	-0.4744	1.0000	0.0000
LOC104912493	1.0000	0.0000	1.0000	0.0000	1.0000	0.0000	1.0000	0.0000	1.0000	0.0000	1.0000	0.0000	1.0000	0.0000
LOC104912494	0.0001	-0.9650	0.0000	-1.4601	0.0042	-0.6089	0.0000	-1.5756	1.0000	0.2372	0.8995	-0.2451	0.0000	-0.7248
LOC104912495	0.4350	0.5028	0.0081	0.9253	0.0000	1.5227	0.0000	1.7027	1.0000	-0.2302	1.0000	0.2056	1.0000	-0.0434
LOC104912496	1.0000	2.1900	1.0000	0.0000	1.0000	0.0000	1.0000	0.0000	1.0000	0.0000	1.0000	-2.2992	1.0000	0.0000
LOC104912497	0.8745	-0.1532	0.0850	0.3192	0.0000	1.4007	0.0000	1.6701	1.0000	-0.1436	0.3054	0.3390	0.7485	0.1281
LOC104912498	1.0000	0.0032	0.6483	0.1096	0.7809	0.1313	0.0329	0.3447	1.0000	-0.0558	1.0000	0.0636	0.7148	0.1632
LOC104912499	0.6677	0.2936	0.0018	0.9739	0.6976	-0.2882	0.1788	-0.6386	1.0000	-0.2732	0.5289	0.4172	0.4154	-0.6219
LOC104912500	0.6048	0.2967	0.8573	-0.1604	1.0000	-0.1165	1.0000	-0.1194	1.0000	0.1541	0.8507	-0.2894	1.0000	0.1597
LOC104912501	0.1843	-1.1310	0.2674	-0.8803	0.4812	-0.7386	1.0000	0.1288	1.0000	0.1324	1.0000	0.4007	0.2665	1.0096
LOC104912504	1.0000	0.1965	1.0000	0.3667	0.0510	2.3695	0.9786	0.9243	1.0000	-0.1179	1.0000	0.0604	0.2944	-1.5622
LOC104912505	0.3144	1.0680	0.4106	0.5353	0.2415	0.9576	1.0000	-0.1426	1.0000	0.5970	1.0000	0.0723	0.9054	-0.4981
LOC104912507	0.1694	-0.6020	0.0537	-0.8618	0.3684	0.3819	0.6693	0.2869	1.0000	-0.2774	0.5666	-0.5223	0.5483	-0.3640
LOC104912508	1.0000	0.3510	1.0000	-0.6954	1.0000	0.1929	1.0000	0.6989	1.0000	-0.1232	1.0000	-1.1662	1.0000	0.3834
LOC104912509	0.0024	-1.0996	0.0007	-1.0818	0.0652	-0.7167	0.4519	-0.2742	1.0000	-0.2405	1.0000	-0.2093	1.0000	0.2048
LOC104912510	0.9625	-0.5439	1.0000	0.1344	0.9945	0.4879	1.0000	0.0071	1.0000	-1.0859	1.0000	-0.4025	0.2974	-1.5628
LOC104912511	0.2027	-0.2797	0.2972	-0.1423	0.0605	-0.3685	0.7559	0.0706	1.0000	-0.3009	0.6857	-0.1510	0.5861	0.1424
LOC104912512	0.0000	-0.7429	0.0000	-0.8616	0.0041	-0.4767	0.0000	-0.3704	1.0000	0.1468	1.0000	0.0406	0.0472	0.2582
LOC104912514	1.0000	0.1130	0.3144	0.5731	0.0028	1.1936	0.0010	1.2299	1.0000	-0.4732	1.0000	-0.0021	0.4626	-0.4300
LOC104912515	0.9545	1.0672	0.6215	1.6074	1.0000	-0.1597	1.0000	0.8545	1.0000	-0.1089	1.0000	0.4429	1.0000	0.9164
LOC104912516	0.4101	0.9774	1.0000	-0.1704	0.7016	0.7782	1.0000	0.2022	1.0000	0.8323	1.0000	-0.2964	1.0000	0.2689
LOC104912517	0.0631	-0.4745	0.0004	-0.5291	0.0055	-0.6331	0.0002	-0.5297	1.0000	0.1231	1.0000	0.0819	0.5007	0.2329
LOC104912520	1.0000	0.0678	1.0000	-0.0100	0.0000	-1.9949	0.0000	-2.2910	1.0000	-0.6118	0.1392	-0.6815	0.4959	-0.9040
LOC104912521	1.0000	2.1851	1.0000	0.0000	1.0000	0.0000	1.0000	2.3240	1.0000	0.0000	1.0000	-2.2957	1.0000	2.3543
LOC104912522	1.0000	0.0000	1.0000	0.0000	1.0000	0.0000	1.0000	0.0000	1.0000	0.0000	1.0000	0.0000	1.0000	0.0000
LOC104912523	1.0000	0.0000	1.0000	0.0000	1.0000	0.0000	1.0000	0.0000	1.0000	0.0000	1.0000	0.0000	1.0000	0.0000
LOC104912524	1.0000	-0.3462	0.4437	3.6202	0.7660	-1.4055	0.4547	3.7078	1.0000	-4.4568	1.0000	-0.6335	1.0000	0.6041
LOC104912525	0.9950	0.5050	0.0520	1.3278	1.0000	-0.4418	0.9046	-0.5560	1.0000	0.4321	0.2818	1.2810	1.0000	0.3293
LOC104912526	0.0000	-2.0879	0.0000	-2.1937	0.0968	-0.5580	0.4019	-0.2591	1.0000	-0.0543	1.0000	-0.1470	0.7521	0.2506
LOC104912527	1.0000	0.0000	0.7674	-3.2570	0.8011	3.0840	1.0000	0.5362	1.0000	3.1190	1.0000	0.0000	1.0000	0.6041
LOC104912528	0.0294	-2.3716	0.3422	-1.8566	0.8731	-0.4321	0.5798	-1.1502	1.0000	-0.9976	1.0000	-0.4752	0.3331	-1.7139
LOC104912529	1.0000	0.5381	0.4634	-3.7803	0.2858	2.1452	1.0000	0.3973	1.0000	1.2565	1.0000	-3.1383	1.0000	-0.4789
LOC104912530	0.0193	-0.5062	0.0000	-0.5633	0.0022	-0.6395	0.0000	-0.6498	1.0000	-0.0197	1.0000	-0.0638	1.0000	-0.0242
LOC104912531	1.0000	0.0000	1.0000	0.0000	1.0000	2.2470	1.0000	0.0000	1.0000	0.0000	1.0000	0.0000	1.0000	-2.2906
LOC104912532	1.0000	-2.4776	0.7666	-3.2534	1.0000	-0.1591	1.0000	-0.8416	1.0000	0.7365	1.0000	0.0000	1.0000	0.0649
LOC104912533	0.0016	1.1620	0.0000	1.6209	0.9510	-0.1984	0.5601	0.3212	1.0000	-0.3083	1.0000	0.1608	1.0000	0.2152
LOC104912534	1.0000	0.5381	1.0000	-0.6969	1.0000	-0.1591	1.0000	-0.5239	1.0000	1.2661	1.0000	0.0554	1.0000	0.9165
LOC104912536	1.0000	2.1901	0.7674	3.0889	1.0000	2.2470	1.0000	0.0000	1.0000	0.0000	1.0000	0.8962	1.0000	-2.2906
LOC104912537	1.0000	0.0000	0.7666	3.0922	1.0000	2.2470	1.0000	0.0000	1.0000	0.0000	1.0000	3.1976	1.0000	-2.2906
LOC104912538	1.0000	0.0000	1.0000	-2.3985	1.0000	0.0000	1.0000	0.0057	1.0000	2.2734	1.0000	0.0000	1.0000	2.3554
LOC104912539	0.8249	-3.3412	1.0000	-2.3960	0.8033	-3.2622	1.0000	0.0062	1.0000	-0.9614	1.0000	0.0000	1.0000	2.3543
LOC104912540	1.0000	-2.4776	1.0000	-2.3985	1.0000	-0.1624	1.0000	0.0057	1.0000	-0.1034	1.0000	0.0000	1.0000	0.0661
LOC104912541	0.8226	3.0199	1.0000	0.0000	1.0000	0.0000	1.0000	0.0000	1.0000	0.0000	1.0000	-3.1429	1.0000	0.0000
LOC104912542	1.0000	0.0000	1.0000	2.2508	1.0000	0.0000	1.0000	0.0000	1.0000	0.0000	1.0000	2.3456	1.0000	0.0000
LOC104912543	0.0105	1.4880	0.0126	0.9889	0.1143	1.0229	0.8513	-0.2853	1.0000	0.8053	0.9569	0.3190	0.7916	-0.4989
LOC104912544	0.0000	1.3321	0.0000	1.3036	0.0005	-1.5498	0.0000	-1.9629	1.0000	0.2440	0.7952	0.2256	1.0000	-0.1662
LOC104912545	0.0088	2.5557	0.0004	3.1342	0.0043	2.5176	0.0195	2.6652	1.0000	-0.6504	1.0000	-0.0661	0.9380	-0.4985
LOC104912546	1.0000	-0.0489	0.2558	-0.2016	0.0035	-0.6954	0.0000	-0.7510	1.0000	0.1398	1.0000	-0.0003	1.0000	0.0899
LOC104912547	0.1115	-0.6798	0.0675	-0.6346	0.0578	-0.7587	0.0124	-0.8691	1.0000	-0.3452	0.9657	-0.2869	0.6531	-0.4495
LOC104912548	0.0230	-0.5917	0.1242	-0.3028	0.4685	-0.2464	0.0237	-0.4181	1.0000	-0.1935	1.0000	0.1094	0.1351	-0.3587
LOC104912549	0.1424	-0.9376	0.1528	-0.7413	0.6918	-0.4036	0.7210	0.2872	1.0000	0.1714	1.0000	0.3797	0.1607	0.8659
LOC104912550	0.9888	-0.0931	0.1733	0.2586	0.6815	-0.1895	0.6210	0.1243	1.0000	-0.1733	0.7610	0.1905	0.8965	0.1442
LOC104912551	1.0000	-0.2367	1.0000	-0.0147	0.8916	0.2439	0.1978	-0.6720	1.0000	0.7889	0.0430	1.0176	1.0000	-0.1245
LOC104912553	0.7849	0.5440	0.6771	0.7118	0.9570	-0.3584	1.0000	0.1731	1.0000	-0.2948	1.0000	-0.1158	1.0000	0.2440
LOC104912554	1.0000	2.1850	1.0000	-2.3960	1.0000	0.0000	1.0000	-2.3179	1.0000	2.2676	1.0000	-2.2958	1.0000	0.0000
LOC104912555	0.4947	0.4152	0.9559	0.1129	0.0000	1.4124	0.0217	0.6212	1.0000	0.2918	1.0000	0.0005	0.1048	-0.4982
LOC104912556	0.0014	-0.4946	0.0000	-0.6743	0.0011	-0.4841	0.0000	-0.4486	1.0000	0.0911	0.9931	-0.0764	0.3625	0.1318
LOC104912557	0.0031	2.0024	0.0000	1.7190	0.2590	0.9682	0.9495	0.2562	1.0000	0.7868	0.6053	0.5130	1.0000	0.0805
LOC104912558	0.3089	3.9281	1.0000	-2.3960	1.0000	2.2470	1.0000	0.8545	1.0000	2.2676	0.6427	-4.0589	1.0000	0.9165
LOC104912559	1.0000	0.0000	1.0000	0.0000	1.0000	0.0000	1.0000	2.3257	1.0000	0.0000	1.0000	0.0000	1.0000	2.3555
LOC104912560	1.0000	-2.4776	0.4670	3.6266	1.0000	-2.4056	1.0000	2.3240	1.0000	-2.3757	0.8759	3.7343	1.0000	2.3543
LOC104912561	0.0000	-2.5625	0.0000	-1.7812	0.9369	-0.1408	1.0000	0.0425	1.0000	-0.0279	0.3838	0.7682	0.9809	0.1621
LOC104912562	0.5203	-0.1897	0.0000	-0.4221	0.0001	-0.7936	0.0000	-0.8377	1.0000	0.0739	0.6277	-0.1459	1.0000	0.0345
LOC104912563	1.0000	-2.4788	1.0000	2.2533	1.0000	-2.4061	1.0000	0.0000	1.0000	-2.3771	1.0000	2.3480	1.0000	0.0000
LOC104912564	0.7251	1.4375	1.0000	-0.9997	1.0000	0.6816	1.0000	-0.8388	1.0000	0.7305	1.0000	-1.7054	1.0000	-0.7804
LOC104912565	1.0000	0.8825	0.1126	4.5751	1.0000	-1.0206	1.0000	2.3257	1.0000	-3.2319	1.0000	0.3274	1.0000	0.0661
LOC104912566	0.0588	2.8450	0.5159	0.8982	0.1322	2.7027	1.0000	0.1936	1.0000	2.4180	1.0000	0.5020	1.0000	-0.0849
LOC104912567	0.1851	4.4812	0.6180	1.6082	0.8011	3.0840	1.0000	-2.3179	1.0000	2.2676	1.0000	-0.4979	1.0000	-3.1344
LOC104912568	0.0454	1.2794	0.4006	0.5995	1.0000	-0.3123	0.7602	-0.4529	1.0000	0.3258	1.0000	-0.3387	1.0000	0.1936
LOC104912569	1.0000	0.0000	1.0000	0.0000	1.0000	0.0000	1.0000	0.0000	1.0000	0.0000	1.0000	0.0000	1.0000	0.0000
LOC104912570	1.0000	-1.1561	1.0000	2.2508	1.0000	-1.0194	1.0000	0.0000	1.0000	-3.2289	1.0000	0.0495	1.0000	-2.2888
LOC104912571	0.0018	0.6613	0.0006	0.5445	0.2177	0.3190	0.3402	0.1927	1.0000	0.0440	1.0000	-0.0583	1.0000	-0.0749
LOC104912572	1.0000	0.1172	0.8390	-0.3961	1.0000	-0.4933	0.0032	-3.5046	1.0000	1.3141	0.9953	0.8173	0.8939	-1.6953
LOC104912573	0.0000	0.9669	0.0000	1.0185	0.0000	1.0911	0.0000	0.9503	1.0000	0.0519	0.9994	0.1172	1.0000	-0.0826
LOC104912574	1.0000	-0.0381	1.0000	2.2508	1.0000	0.3649	1.0000	2.3240	1.0000	-4.1522	0.8269	-2.0174	0.5105	-2.2505
LOC104912575	1.0000	0.0000	1.0000	0.0000	1.0000	2.2470	1.0000	0.0000	1.0000	0.0000	1.0000	0.0000	1.0000	-2.2906
LOC104912576	0.6927	1.4466	0.3421	1.5203	1.0000	-2.4061	0.3583	1.6919	1.0000	0.7357	1.0000	0.8301	0.1506	4.8945
LOC104912577	0.9545	1.0644	1.0000	0.0000	1.0000	-2.4061	1.0000	0.0000	1.0000	-2.3771	0.8607	-3.6719	1.0000	0.0000
LOC104912578	1.0000	0.0000	1.0000	0.0000	1.0000	0.0000	1.0000	0.0000	1.0000	0.0000	1.0000	0.0000	1.0000	0.0000
LOC104912579	1.0000	0.0000	1.0000	0.0000	1.0000	0.0000	1.0000	0.0000	1.0000	0.0000	1.0000	0.0000	1.0000	0.0000
LOC104912581	0.0000	1.2992	0.0000	1.7248	0.0000	2.0392	0.0000	2.2803	1.0000	-0.0073	0.0925	0.4329	0.3817	0.2416
LOC104912582	0.3365	1.5667	1.0000	0.3841	1.0000	-0.1848	1.0000	0.5632	1.0000	0.2623	0.8607	-0.9108	1.0000	1.0195
LOC104912583	1.0000	-0.0575	0.2577	-0.1133	0.4627	-0.1674	1.0000	0.0171	1.0000	0.1780	0.6187	0.1346	0.0003	0.3673
LOC104912584	0.0026	0.6846	0.0021	0.5373	0.0000	0.9795	0.0000	0.6851	1.0000	0.1632	1.0000	0.0297	0.8665	-0.1255
LOC104912585	0.0017	-0.6001	0.0000	-0.9700	0.0000	-1.1426	0.0000	-0.8283	1.0000	0.2029	0.7275	-0.1535	0.0001	0.5228
LOC104912586	0.0134	0.9282	0.0000	1.5508	0.0001	1.2432	0.0000	1.2181	1.0000	-0.2546	0.4604	0.3821	0.6583	-0.2696
LOC104912587	1.0000	0.0000	1.0000	0.0000	1.0000	0.0000	1.0000	0.0000	1.0000	0.0000	1.0000	0.0000	1.0000	0.0000
LOC104912588	1.0000	0.0000	1.0000	2.2508	1.0000	0.0000	1.0000	0.0000	1.0000	0.0000	1.0000	2.3456	1.0000	0.0000
LOC104912589	1.0000	2.1850	1.0000	2.2533	0.8033	3.0892	1.0000	0.0000	1.0000	0.0000	1.0000	0.0514	1.0000	-3.1369

LOC104912590	0.0044	1.2231	0.0000	2.5214	0.0000	1.7033	0.0000	2.6841	1.0000	-0.4791	0.0007	0.8348	0.0215	0.5127
LOC104912591	0.8226	0.7431	0.0051	2.7695	0.7757	-1.4051	1.0000	0.5372	1.0000	-1.3455	0.9929	0.6797	1.0000	0.6034
LOC104912593	1.0000	-0.1252	1.0000	0.2711	0.0089	1.5670	0.0000	2.6702	1.0000	-0.5763	1.0000	-0.1720	0.5109	0.5308
LOC104912594	1.0000	0.0000	1.0000	0.0000	1.0000	0.0000	1.0000	0.0000	1.0000	0.0000	1.0000	0.0000	1.0000	0.0000
LOC104912596	0.0096	0.4771	0.0000	0.5467	0.8033	0.1046	0.9632	0.0299	1.0000	-0.1065	1.0000	-0.0245	0.1442	-0.1752
LOC104912598	1.0000	2.1850	0.6180	-1.9232	0.4809	3.6101	0.2427	-4.0840	1.0000	4.0267	1.0000	0.0514	0.7287	-3.6634
LOC104912599	1.0000	0.0000	1.0000	-2.3985	1.0000	0.0000	1.0000	-2.3200	1.0000	2.2734	1.0000	0.0000	1.0000	0.0000
LOC104912600	1.0000	0.0000	1.0000	0.0000	1.0000	0.0000	1.0000	0.0000	1.0000	0.0000	1.0000	0.0000	1.0000	0.0000
LOC104912601	1.0000	2.1901	0.7674	-3.2499	1.0000	2.2426	1.0000	0.0074	1.0000	3.1056	1.0000	-2.2992	1.0000	0.9168
LOC104912602	1.0000	-0.2020	0.6381	-0.5835	1.0000	-0.0466	0.3140	0.8016	1.0000	0.2772	1.0000	-0.0913	0.3787	1.1311
LOC104912603	1.0000	0.0000	1.0000	0.0000	1.0000	0.0000	1.0000	0.0000	1.0000	0.0000	1.0000	0.0000	1.0000	0.0000
LOC104912605	0.0140	0.9524	0.0511	0.7380	1.0000	0.1034	0.3834	-0.3647	1.0000	-0.0062	1.0000	-0.2070	0.4530	-0.4664
LOC104912607	1.0000	-0.7235	0.6547	0.9470	0.0361	1.9078	1.0000	0.3137	1.0000	-0.1266	0.7186	1.5639	0.1087	-1.7180
LOC104912609	0.0000	1.6359	0.0000	1.4210	0.0000	1.0500	0.0000	0.7393	1.0000	0.2939	1.0000	0.0928	1.0000	-0.0111
LOC104912610	0.0406	-0.3307	0.0000	-0.3256	0.0332	0.3486	0.0003	0.2486	1.0000	0.1101	0.3855	0.1278	1.0000	0.0152
LOC104912612	0.0998	1.0509	0.3105	0.7420	0.0005	1.6706	0.0014	1.5356	1.0000	0.0309	1.0000	-0.2638	1.0000	-0.0887
LOC104912614	1.0000	0.0000	0.7666	3.0922	1.0000	0.0000	1.0000	0.0000	1.0000	0.0000	1.0000	3.1976	1.0000	0.0000
LOC104912615	0.0648	-4.8294	0.9148	-1.5280	1.0000	-0.4466	0.5792	1.1652	1.0000	-1.0719	1.0000	2.3480	1.0000	0.5442
LOC104912616	0.0268	-0.7144	0.0032	-0.6297	0.0000	-1.5146	0.0003	-0.7601	1.0000	-0.2363	1.0000	-0.1396	0.1971	0.5262
LOC104912617	0.0034	0.9701	0.0000	1.5328	0.0000	2.5796	0.0000	2.4686	1.0000	-0.2578	0.4639	0.3203	0.1509	-0.3618
LOC104912619	0.9545	1.0672	1.0000	-0.1452	1.0000	-2.4061	1.0000	-2.3200	1.0000	-0.1045	1.0000	-1.3257	1.0000	0.0000
LOC104912620	0.5086	3.5456	1.0000	0.0000	0.8011	3.0840	1.0000	0.0000	1.0000	0.0000	0.8607	-3.6732	1.0000	-3.1344
LOC104912621	1.0000	0.0000	1.0000	-1.0017	1.0000	2.2426	1.0000	-0.8416	1.0000	3.1126	1.0000	2.3456	1.0000	0.0661
LOC104912622	1.0000	0.0000	1.0000	0.0000	1.0000	2.2470	1.0000	0.0000	1.0000	0.0000	1.0000	0.0000	1.0000	-2.2906
LOC104912625	0.1406	-0.3148	0.0000	-0.4737	0.0008	0.5793	0.0000	0.4702	1.0000	0.1759	1.0000	0.0290	0.9489	0.0715
LOC104912627	1.0000	2.1901	0.4689	3.6126	1.0000	0.0000	1.0000	0.0000	1.0000	0.0000	1.0000	1.4259	1.0000	0.0000
LOC104912628	0.0000	2.5528	0.0000	2.2400	0.1130	1.2973	0.0742	1.1730	1.0000	0.3231	1.0000	0.0161	1.0000	0.1996
LOC104912630	1.0000	0.0000	1.0000	2.2533	1.0000	2.2426	1.0000	0.0000	1.0000	0.0000	1.0000	2.3480	1.0000	-2.2888
LOC104912631	0.8249	3.0254	0.7674	-3.2499	1.0000	2.2426	0.7710	-3.1633	1.0000	3.1056	1.0000	-3.1468	1.0000	-2.2888
LOC104912632	0.0004	1.5456	0.0000	1.3985	0.0000	2.2961	0.0000	2.1448	1.0000	0.3062	1.0000	0.1694	0.9237	0.1605
LOC104912634	0.5391	-3.8819	0.2495	4.0050	1.0000	0.1989	0.2461	4.0903	1.0000	-3.7658	0.6427	4.1168	1.0000	0.0763
LOC104912635	1.0000	0.3545	0.1545	2.3755	0.6503	0.9646	0.1715	2.5457	1.0000	-1.4875	1.0000	0.5242	1.0000	0.0824
LOC104912637	0.4620	0.2725	0.1699	0.2619	0.1622	0.3955	0.0007	0.5301	1.0000	-0.0037	1.0000	-0.0031	0.8381	0.1366
LOC104912638	1.0000	2.1850	0.7674	3.0889	1.0000	0.0000	0.7701	3.1732	1.0000	0.0000	1.0000	0.8992	1.0000	3.2066
LOC104912639	1.0000	0.0000	0.7666	3.0922	1.0000	2.2470	1.0000	2.3257	1.0000	0.0000	1.0000	3.1976	1.0000	0.0649
LOC104912647	1.0000	0.0059	0.0035	0.7352	0.0689	1.0370	0.0000	1.5755	1.0000	-0.0849	0.0688	0.6591	0.0879	0.4512
LOC104912648	0.0849	0.8124	0.0000	1.2859	0.1540	0.6867	0.2176	0.4874	1.0000	-0.1020	0.5182	0.3872	0.7455	-0.2944
LOC104912649	0.0050	1.1177	0.0000	1.6817	0.0000	1.4331	0.0000	2.0239	1.0000	-0.3961	1.0000	0.1821	0.6265	0.2020
LOC104912650	0.1729	0.8840	0.0073	0.9547	0.6120	-0.4598	1.0000	-0.1254	1.0000	0.0496	1.0000	0.1294	0.8832	0.3878
LOC104912651	0.7596	0.2662	0.0023	0.8468	0.0000	1.4027	0.0000	1.4089	1.0000	0.0462	0.1375	0.6394	1.0000	0.0579
LOC104912652	1.0000	0.0827	0.4779	-0.2792	0.0696	0.6249	0.0218	0.5867	1.0000	0.1712	1.0000	-0.1776	1.0000	0.1393
LOC104912653	1.0000	2.1901	1.0000	2.2533	1.0000	2.2426	1.0000	0.0000	1.0000	0.0000	1.0000	0.0489	1.0000	-2.2888
LOC104912655	1.0000	0.0000	1.0000	0.0000	1.0000	0.0000	1.0000	2.3257	1.0000	0.0000	1.0000	0.0000	1.0000	2.3554
LOC104912656	0.9108	-0.6241	0.2290	-0.9808	0.0035	1.7788	0.1502	0.8556	1.0000	0.7974	1.0000	0.4568	1.0000	-0.1277
LOC104912657	0.0565	-1.1905	0.0029	-1.5925	0.0051	1.2674	0.0062	0.9800	1.0000	-0.0882	1.0000	-0.4792	0.6524	-0.3759
LOC104912658	0.1726	0.9623	1.0000	-0.0233	0.4356	-0.8173	0.0027	-1.7366	1.0000	0.8629	1.0000	-0.1145	1.0000	-0.0542
LOC104912660	1.0000	0.0000	1.0000	0.0000	1.0000	0.0000	1.0000	0.0000	1.0000	0.0000	1.0000	0.0000	1.0000	0.0000
LOC104912662	0.6936	1.4536	1.0000	0.3847	1.0000	-2.4056	1.0000	0.3118	1.0000	1.6505	1.0000	0.6146	0.3369	4.4316
LOC104912664	0.0034	-2.2670	0.0261	-1.7216	0.2347	-0.9518	0.1982	0.7835	1.0000	-0.7146	1.0000	-0.1560	0.1915	1.0248
LOC104912665	0.0010	0.5827	0.0000	0.5018	0.4979	0.1830	0.0000	0.4569	1.0000	-0.1060	0.3978	-0.1748	0.4139	0.1744
LOC104912666	1.0000	-0.3395	0.0587	-2.3568	1.0000	-0.1847	0.5159	-0.9577	1.0000	1.5316	1.0000	-0.4715	1.0000	0.7685
LOC104912668	0.0000	-1.4619	0.0000	-1.3751	0.0899	-0.5040	0.7411	-0.1454	1.0000	-0.0402	1.0000	0.0602	0.3475	0.3234
LOC104912669	0.5842	-0.4891	0.3111	-0.5554	1.0000	-0.1169	0.7615	-0.2763	1.0000	0.0242	1.0000	-0.0304	1.0000	-0.1288
LOC104912671	1.0000	0.0000	1.0000	0.0000	1.0000	0.0000	1.0000	0.0000	1.0000	0.0000	1.0000	0.0000	1.0000	0.0000
LOC104912672	0.3089	3.9281	1.0000	-1.0051	1.0000	2.2470	1.0000	-0.8442	1.0000	3.1190	1.0000	-1.7124	1.0000	0.0640
LOC104912674	1.0000	0.0000	1.0000	0.6959	1.0000	0.0000	1.0000	-2.3200	1.0000	2.2734	1.0000	3.2002	1.0000	0.0000
LOC104912675	1.0000	-0.8577	0.0450	4.7755	0.5041	-3.7956	1.0000	2.3240	1.0000	-3.7607	0.7672	1.7465	1.0000	2.3543
LOC104912676	1.0000	0.0000	1.0000	0.0000	1.0000	0.0000	1.0000	0.0000	1.0000	0.0000	1.0000	0.0000	1.0000	0.0000
LOC104912679	1.0000	0.0000	0.4431	-3.7865	1.0000	0.0000	0.4370	-3.6968	1.0000	3.6396	1.0000	0.0000	1.0000	0.0000
LOC104912680	1.0000	-2.4776	1.0000	0.0000	1.0000	-2.4056	1.0000	0.0000	1.0000	-2.3757	1.0000	0.0000	1.0000	0.0000
LOC104912681	0.3234	1.5681	0.0001	3.9255	1.0000	0.1978	0.0515	2.8939	1.0000	-1.4912	0.7464	0.8558	0.7601	1.2001
LOC104912684	1.0000	0.0000	1.0000	0.0000	1.0000	0.0000	1.0000	0.0000	1.0000	0.0000	1.0000	0.0000	1.0000	0.0000
LOC104912685	1.0000	0.0000	1.0000	0.0000	1.0000	0.0000	1.0000	0.0000	1.0000	0.0000	1.0000	0.0000	1.0000	0.0000
LOC104912686	1.0000	-0.2931	1.0000	2.2533	1.0000	-2.4061	1.0000	0.0000	1.0000	-2.3771	1.0000	0.0514	1.0000	0.0000
LOC104912687	1.0000	0.1474	1.0000	0.3848	1.0000	0.3013	1.0000	0.3121	1.0000	-0.8989	1.0000	-0.6542	0.8145	-0.8861
LOC104912688	1.0000	0.2011	0.4642	-3.7944	0.7780	1.0427	0.5794	1.1635	1.0000	0.4210	0.8607	-3.6719	1.0000	0.5426
LOC104912689	0.4238	-0.8221	0.0091	-1.9735	0.8140	-0.4576	1.0000	-0.2517	1.0000	-0.1305	0.5963	-1.2745	1.0000	0.0802
LOC104912690	1.0000	0.0000	1.0000	2.2508	1.0000	0.0000	1.0000	0.0000	1.0000	0.0000	1.0000	2.3456	1.0000	0.0000
LOC104912691	0.0000	-0.7737	0.0000	-0.7611	0.4294	0.1830	0.3394	0.1125	1.0000	0.1384	0.6091	0.1639	0.9631	0.0740
LOC104912692	1.0000	0.5881	0.4026	1.9109	1.0000	0.3474	0.6172	1.7712	1.0000	-0.9587	1.0000	0.3618	1.0000	0.4627
LOC104912693	1.0000	0.0000	1.0000	0.0000	1.0000	0.0000	1.0000	0.0000	1.0000	0.0000	1.0000	0.0000	1.0000	0.0000
LOC104912694	1.0000	0.5420	1.0000	2.2508	1.0000	-2.4056	1.0000	0.0000	1.0000	-2.3757	1.0000	-0.7966	1.0000	0.0000
LOC104912695	0.0081	0.5532	0.0005	0.3966	0.0439	0.4447	0.3860	0.1312	1.0000	0.2691	0.7914	0.1248	1.0000	-0.0402
LOC104912696	1.0000	0.1859	0.5831	0.3708	0.0059	1.2623	0.0037	1.0658	1.0000	-0.0315	1.0000	0.1594	0.9248	-0.2277
LOC104912699	0.0404	-1.4747	0.4377	-1.0229	0.9026	-0.3310	0.0000	2.4753	1.0000	-1.1164	1.0000	-0.6542	0.0002	1.6990
LOC104912700	0.0000	2.4550	0.0000	1.6788	0.0653	1.1727	0.0121	1.0272	1.0000	0.8558	1.0000	0.0920	0.2562	0.7164
LOC104912701	0.5696	-0.1757	0.5341	-0.0975	0.5559	-0.1775	1.0000	0.0129	1.0000	0.0529	0.6861	0.1442	0.1175	0.2485
LOC104912702	1.0000	-2.4788	1.0000	-2.3960	0.9361	1.2042	1.0000	0.0074	1.0000	-0.1089	1.0000	0.0000	1.0000	-1.3081
LOC104912703	1.0000	0.0000	1.0000	0.0000	1.0000	0.0000	1.0000	0.0000	1.0000	0.0000	1.0000	0.0000	1.0000	0.0000
LOC104912704	1.0000	2.1850	1.0000	0.0000	1.0000	0.0000	1.0000	2.3257	1.0000	0.0000	1.0000	-2.2957	1.0000	2.3554
LOC104912705	1.0000	0.0000	1.0000	0.0000	1.0000	0.0000	1.0000	0.0000	1.0000	0.0000	1.0000	0.0000	1.0000	0.0000
LOC104912707	1.0000	0.0000	1.0000	0.0000	1.0000	0.0000	1.0000	0.0000	1.0000	0.0000	1.0000	0.0000	1.0000	0.0000
LOC104912708	1.0000	0.0000	1.0000	0.0000	1.0000	0.0000	1.0000	0.0000	1.0000	0.0000	1.0000	0.0000	1.0000	0.0000
LOC104912709	1.0000	-2.4776	1.0000	0.0000	0.6654	1.5931	0.7710	3.1710	1.0000	-2.3757	1.0000	0.0000	1.0000	-0.8445
LOC104912710	1.0000	2.1850	1.0000	0.0000	1.0000	0.0000	1.0000	0.0000	1.0000	0.0000	1.0000	-2.2957	1.0000	0.0000
LOC104912711	1.0000	0.0000	1.0000	0.0000	0.8011	3.0840	1.0000	0.0000	1.0000	0.0000	1.0000	0.0000	1.0000	-3.1344
LOC104912712	1.0000	-2.4776	1.0000	0.0000	1.0000	-0.1624	1.0000	0.0000	1.0000	-2.3757	1.0000	0.0000	1.0000	-2.2888
LOC104912713	1.0000	0.0000	1.0000	0.0000	1.0000	0.0000	1.0000	0.0000	1.0000	0.0000	1.0000	0.0000	1.0000	0.0000
LOC104912714	1.0000	0.0000	1.0000	0.0000	1.0000	0.0000	1.0000	0.0000	1.0000	0.0000	1.0000	0.0000	1.0000	0.0000
LOC104912715	1.0000	2.1900	1.0000	0.0000	0.4809	3.6101	0.2785	4.0986	1.0000	0.0000	1.0000	-2.2992	1.0000	0.4658
LOC104912716	1.0000	0.0000	1.0000	0.0000	1.0000	0.0000	1.0000	2.3257	1.0000	0.0000	1.0000	0.0000	1.0000	2.3554
LOC104912717	1.0000	0.0000	1.0000	0.0000	1.0000	0.0000	1.0000	0.0000	1.0000	0.0000	1.0000	0.0000	1.0000	0.0000
LOC104912718	1.0000	2.1900	1.0000	-2.3960	1.0000	2.2426	1.0000	-2.3179	1.0000	2.2676	1.0000	-2.2992	1.0000	-2.2888

LOC104912719	0.1404	0.7724	0.5636	-0.3250	0.0000	2.2975	0.0535	0.6775	1.0000	0.7047	0.8269	-0.3807	0.0010	-0.9093
LOC104912720	0.1203	0.7481	0.2195	0.5101	0.0834	0.7946	0.1439	0.6304	1.0000	0.0891	1.0000	-0.1369	1.0000	-0.0691
LOC104912721	1.0000	0.0000	1.0000	-2.3960	0.5080	3.6208	1.0000	0.0074	1.0000	2.2677	1.0000	0.0000	1.0000	-1.3110
LOC104912722	0.1740	-0.4745	0.0282	-0.5419	0.0000	1.4141	0.0121	0.5240	1.0000	0.2105	1.0000	0.1541	0.0000	-0.6753
LOC104912723	1.0000	0.0000	1.0000	2.2508	1.0000	0.0000	1.0000	0.0000	1.0000	0.0000	1.0000	2.3456	1.0000	0.0000
LOC104912724	0.0005	-0.9155	0.0000	-0.8365	0.0004	-0.8860	0.1444	-0.2450	1.0000	-0.0811	1.0000	0.0126	0.0023	0.5670
LOC104912726	1.0000	0.0000	1.0000	-2.3960	1.0000	0.0000	1.0000	-2.3179	1.0000	2.2677	1.0000	0.0000	1.0000	0.0000
LOC104912727	1.0000	0.0000	1.0000	0.0000	1.0000	0.0000	1.0000	0.0000	1.0000	0.0000	1.0000	0.0000	1.0000	0.0000
LOC104912728	0.5021	-1.1148	1.0000	-0.5493	0.6375	-1.3445	0.9877	-0.9060	1.0000	-0.9026	1.0000	-0.3305	1.0000	-0.4587
LOC104912729	1.0000	0.0000	1.0000	0.0000	1.0000	0.0000	1.0000	0.0000	1.0000	0.0000	1.0000	0.0000	1.0000	0.0000
LOC104912730	1.0000	0.0000	1.0000	0.0000	1.0000	0.0000	1.0000	0.0000	1.0000	0.0000	1.0000	0.0000	1.0000	0.0000
LOC104912731	0.0000	-1.0165	0.0000	-1.0810	0.0000	-1.4016	0.0000	-1.3529	1.0000	0.0540	1.0000	0.0018	0.7781	0.1085
LOC104912732	1.0000	-1.1562	1.0000	2.2508	0.8011	-3.2629	1.0000	0.0000	1.0000	-3.2304	1.0000	0.0495	1.0000	0.0000
LOC104912738	1.0000	0.3596	0.1056	1.2133	0.2894	-2.4999	0.2846	-1.5026	1.0000	0.4410	0.2713	1.3064	1.0000	1.4489
LOC104912739	0.8589	-1.0248	1.0000	-0.4783	1.0000	-0.8850	1.0000	-0.6858	1.0000	-0.1202	1.0000	0.4420	1.0000	0.0768
LOC104912740	1.0000	2.1850	1.0000	0.0000	1.0000	0.0000	1.0000	0.0000	1.0000	0.0000	1.0000	-2.2957	1.0000	0.0000
LOC104912741	0.0026	-0.9498	0.0001	-1.2490	0.0464	-0.6188	0.0005	-0.6584	1.0000	0.3520	1.0000	0.0646	0.5509	0.3165
LOC104912746	1.0000	0.0000	1.0000	0.0000	1.0000	0.0000	1.0000	0.0000	1.0000	0.0000	1.0000	0.0000	1.0000	0.0000
LOC104912747	0.0111	0.4258	0.0016	0.3133	0.0207	-0.4144	0.0002	-0.3651	1.0000	0.0044	0.8605	-0.0962	1.0000	0.0583
LOC104912748	0.5008	-1.1181	0.8221	0.5980	0.8276	0.6764	1.0000	-0.3791	1.0000	-0.8967	1.0000	0.8301	0.1765	-1.9505
LOC104912749	0.0005	-0.7602	0.0000	-0.9463	0.0009	-0.7014	0.0000	-0.8558	1.0000	0.0639	1.0000	-0.1097	1.0000	-0.0842
LOC104912750	1.0000	0.0000	1.0000	0.0000	1.0000	0.0000	1.0000	0.0000	1.0000	0.0000	1.0000	0.0000	1.0000	0.0000
LOC104912752	0.2054	1.8480	0.0160	2.0026	1.0000	0.3438	0.9801	-0.9127	1.0000	0.7988	0.6951	0.9691	1.0000	-0.4550
LOC104912760	0.9521	-0.1858	0.7119	-0.1990	0.1889	-0.5722	0.8351	0.1699	1.0000	-0.1904	1.0000	-0.1885	0.2415	0.5587
LOC104912761	0.4139	0.2737	0.0285	0.4026	0.7396	-0.1762	0.5361	0.1638	1.0000	-0.1797	1.0000	-0.0398	0.8815	0.1661
LOC104912762	0.8249	3.0254	1.0000	2.2508	1.0000	2.2425	1.0000	2.3240	1.0000	0.0000	1.0000	-0.8001	1.0000	0.0652
LOC104912763	0.0163	-0.3903	0.0000	-0.3911	1.0000	-0.0313	0.0009	0.2792	1.0000	0.0219	1.0000	0.0335	0.0005	0.3376
LOC104912764	0.5126	0.6328	0.0000	2.1588	0.0096	1.6766	0.0000	2.1366	1.0000	-0.3217	0.0233	1.2188	1.0000	0.1432
LOC104912766	0.8941	0.1029	1.0000	0.0348	0.1956	-0.3394	0.0083	-0.3550	1.0000	0.0818	1.0000	0.0265	1.0000	0.0707
LOC104912768	0.0414	1.6403	0.0000	2.9307	0.2028	-1.8511	0.3952	-2.0640	1.0000	-1.0934	1.0000	0.1984	1.0000	-1.3081
LOC104912769	0.1630	1.0072	0.4179	0.5903	0.0000	2.4301	0.0000	1.9515	1.0000	0.4624	1.0000	0.0619	1.0000	0.0023
LOC104912770	0.2563	0.5321	0.0249	0.6016	0.0000	2.2281	0.0000	1.9986	1.0000	0.1544	0.8840	0.2353	1.0000	-0.0708
LOC104912771	1.0000	0.1164	0.7888	-0.2855	0.0250	1.0303	0.0397	0.7205	1.0000	0.4480	1.0000	0.0610	1.0000	0.1448
LOC104912772	1.0000	0.0000	1.0000	0.0000	1.0000	0.0000	1.0000	0.0000	1.0000	0.0000	1.0000	0.0000	1.0000	0.0000
LOC104912773	0.8378	-0.6501	0.1655	-2.2058	1.0000	0.2737	0.1512	-2.0301	1.0000	0.6844	1.0000	-0.8610	0.6246	-1.6128
LOC104912774	1.0000	-1.2458	1.0000	0.5219	1.0000	0.1138	0.9096	-1.3717	1.0000	-0.5112	1.0000	1.2775	0.6865	-1.9990
LOC104912775	1.0000	2.1900	1.0000	2.2533	1.0000	0.0000	1.0000	0.0000	1.0000	0.0000	1.0000	0.0489	1.0000	0.0000
LOC104912776	0.7777	0.1019	0.4991	-0.0771	0.9468	0.0651	0.9039	0.0346	1.0000	0.0316	0.5902	-0.1352	1.0000	0.0064
LOC104912777	1.0000	0.0000	1.0000	0.0000	1.0000	0.0000	1.0000	0.0000	1.0000	0.0000	1.0000	0.0000	1.0000	0.0000
LOC104912779	0.5441	0.7660	0.6117	-0.5589	0.6306	-0.9070	0.0436	-1.7089	1.0000	0.6215	0.7902	-0.6893	1.0000	-0.1738
LOC104912780	0.0001	0.7936	0.0000	0.4699	0.0000	1.7075	0.0000	1.3109	1.0000	0.3833	1.0000	0.0711	1.0000	-0.0086
LOC104912781	0.5400	0.3910	0.0056	0.7797	0.0898	-0.8587	1.0000	-0.0416	1.0000	-0.0543	0.6693	0.3460	0.1658	0.7653
LOC104912782	0.0014	-0.5332	0.0000	-0.7234	0.0000	-0.8513	0.0000	-0.8678	1.0000	0.1886	1.0000	0.0106	0.3502	0.1768
LOC104912783	1.0000	0.2664	1.0000	0.0585	0.5224	-0.8887	0.0233	-1.6111	1.0000	0.6207	1.0000	0.4314	1.0000	-0.1051
LOC104912784	0.8249	3.0143	1.0000	0.0000	0.0938	4.5491	0.2461	4.0903	1.0000	0.0000	1.0000	-3.1383	1.0000	-0.4790
LOC104912785	0.4639	0.3100	1.0000	0.0648	0.4035	-0.3622	0.0142	-0.5906	1.0000	0.1455	1.0000	-0.0858	1.0000	-0.0779
LOC104912786	0.9471	0.0999	1.0000	0.0433	0.4061	-0.2795	0.0036	-0.4250	1.0000	0.1712	0.9314	0.1279	1.0000	0.0295
LOC104912787	0.6757	-0.1443	0.0074	-0.3387	0.9631	0.0763	0.8052	-0.0641	1.0000	0.1775	1.0000	-0.0039	1.0000	0.0421
LOC104912788	1.0000	-1.1561	1.0000	0.6912	1.0000	0.7314	0.9096	1.3849	1.0000	-0.9563	1.0000	0.8992	1.0000	-0.3106
LOC104912791	0.2901	0.8661	0.0164	0.7985	0.0000	2.8314	0.0000	2.3214	1.0000	0.3243	1.0000	0.2686	0.9412	-0.1824
LOC104912792	0.0164	1.7957	0.0038	1.4592	0.0000	4.0184	0.0000	3.1402	1.0000	0.3913	1.0000	0.0633	0.3273	-0.4868
LOC104912793	0.0000	-2.8128	0.0000	-2.8059	0.8712	0.2116	0.2095	0.4594	1.0000	0.2035	1.0000	0.2251	0.4180	0.4586
LOC104912794	0.2972	1.0074	0.0123	1.8374	0.0376	1.5772	0.0170	1.7727	1.0000	-0.5262	1.0000	0.3110	1.0000	-0.3299
LOC104912795	0.6881	1.7537	1.0000	0.5219	1.0000	-0.1630	0.9096	-1.3721	1.0000	1.2628	1.0000	0.0580	1.0000	0.0652
LOC104912796	1.0000	0.0000	1.0000	0.0000	1.0000	0.0000	1.0000	0.0000	1.0000	0.0000	1.0000	0.0000	1.0000	0.0000
LOC104912797	0.0624	-0.5770	0.0021	-0.5576	0.0000	-1.7729	0.0000	-1.6414	1.0000	0.0811	1.0000	0.1148	0.9768	0.2198
LOC104912799	0.5411	1.1558	0.0083	3.3456	1.0000	-0.1781	1.0000	0.8519	1.0000	-1.4918	1.0000	0.6840	1.0000	-0.4617
LOC104912800	1.0000	-2.4788	1.0000	0.6956	0.9361	1.2042	1.0000	0.0074	1.0000	-0.1089	1.0000	3.1976	1.0000	-1.3081
LOC104912801	1.0000	-0.3257	1.0000	-0.6927	0.8011	-3.2629	1.0000	0.4074	1.0000	0.4046	1.0000	0.0554	0.5406	4.1317
LOC104912802	0.0002	0.8849	0.0000	1.0014	0.4963	-0.2621	0.8376	0.0976	1.0000	-0.2385	1.0000	-0.1097	1.0000	0.1287
LOC104912803	0.6796	-0.1147	0.0000	-0.4168	0.0000	-1.2144	0.0000	-1.2548	1.0000	-0.0160	0.0035	-0.3060	1.0000	-0.0511
LOC104912804	1.0000	0.0000	1.0000	0.0000	1.0000	0.0000	1.0000	2.3240	1.0000	0.0000	1.0000	0.0000	1.0000	2.3543
LOC104912805	1.0000	0.0000	1.0000	2.2533	1.0000	0.0000	1.0000	0.0000	1.0000	0.0000	1.0000	2.3480	1.0000	0.0000
LOC104912806	0.9810	-0.3547	1.0000	-0.1119	1.0000	-0.0514	0.8432	0.3126	1.0000	0.0865	1.0000	0.3456	0.8232	0.4556
LOC104912807	0.9666	0.3913	0.0001	2.5723	0.0030	1.6375	0.0000	2.8732	1.0000	-1.4659	0.5013	0.7252	1.0000	-0.2242
LOC104912808	0.6957	1.7486	1.0000	-0.7037	0.1182	2.5432	1.0000	0.6980	1.0000	1.2738	1.0000	-1.1645	1.0000	-0.5735
LOC104912809	1.0000	0.0000	1.0000	0.0000	1.0000	0.0000	1.0000	0.0000	1.0000	0.0000	1.0000	0.0000	1.0000	0.0000
LOC104912811	0.8549	0.8070	0.6215	1.6060	1.0000	0.5040	1.0000	0.0045	1.0000	-1.4918	1.0000	-0.7087	0.6865	-1.9994
LOC104912812	1.0000	2.1901	0.4334	1.3350	0.0019	5.7365	0.5741	1.1646	1.0000	3.6396	0.2854	2.9377	0.6477	-0.8956
LOC104912815	0.2424	0.7475	0.0051	1.1005	0.0000	2.8740	0.0000	2.2522	1.0000	0.2390	0.3660	0.6075	0.2800	-0.3718
LOC104912816	0.0164	5.4352	0.0501	1.5224	0.0000	7.8634	0.0001	2.4625	1.0000	4.5593	0.8607	0.7896	0.1398	-0.7811
LOC104912818	1.0000	0.0000	1.0000	2.2533	1.0000	0.0000	1.0000	0.0000	1.0000	0.0000	1.0000	2.3480	1.0000	0.0000
LOC104912820	1.0000	-0.1683	1.0000	0.1665	0.7527	-0.5398	1.0000	0.1299	1.0000	-0.4687	1.0000	-0.1241	1.0000	0.2032
LOC104912821	1.0000	0.0000	1.0000	-2.3960	1.0000	0.0000	1.0000	0.0074	1.0000	2.2677	1.0000	0.0000	1.0000	2.3554
LOC104912822	0.0868	0.5356	0.9621	0.0838	1.0000	-0.0115	0.3700	-0.2420	1.0000	0.2666	0.9272	-0.1748	1.0000	0.0388
LOC104912823	0.9073	0.2401	0.0000	1.9188	0.0229	1.0772	0.0003	1.6704	1.0000	-0.8654	0.1517	0.8307	0.9315	-0.2629
LOC104912824	0.0027	-0.8937	0.0000	-1.3385	0.0014	-0.9181	0.0000	-1.1484	1.0000	0.2335	1.0000	-0.1986	1.0000	0.0078
LOC104912825	0.9545	1.0644	1.0000	0.6940	0.2858	2.1449	0.4562	2.0902	1.0000	-0.1089	1.0000	-0.4752	1.0000	-0.1716
LOC104912826	1.0000	0.0000	1.0000	-0.1449	1.0000	0.0000	1.0000	-2.3179	1.0000	2.2677	1.0000	2.3456	1.0000	0.0000
LOC104912827	0.1380	2.5670	1.0000	-0.1449	1.0000	-0.1630	1.0000	-2.3179	1.0000	-0.1089	0.2592	-2.8337	1.0000	-2.2888
LOC104912828	0.8249	3.0254	0.7674	3.0889	1.0000	2.2425	1.0000	0.0000	1.0000	0.0000	1.0000	0.0496	1.0000	-2.2888
LOC104912829	1.0000	-0.2882	0.2920	4.0165	1.0000	-2.4056	1.0000	0.0000	1.0000	-2.3757	1.0000	1.8233	1.0000	0.0000
LOC104912830	1.0000	-2.4776	1.0000	-0.1452	1.0000	-2.4056	1.0000	-2.3200	1.0000	-0.1034	1.0000	2.3480	1.0000	0.0000
LOC104912831	0.8226	3.0199	0.7666	3.0922	1.0000	0.0000	1.0000	2.3257	1.0000	0.0000	1.0000	0.0547	1.0000	2.3554
LOC104912832	1.0000	0.0000	1.0000	0.0000	1.0000	0.0000	0.7701	3.1732	1.0000	0.0000	1.0000	0.0000	1.0000	3.2066
LOC104912833	1.0000	0.8846	0.4971	1.0924	0.3860	1.5009	0.8257	0.7781	1.0000	0.7963	0.9553	1.0233	1.0000	0.0792
LOC104912834	1.0000	0.0034	0.0674	-0.1498	0.0000	-0.7692	0.0000	-0.6345	1.0000	-0.0764	0.0213	-0.2173	0.9791	0.0645
LOC104912835	0.0001	1.0246	0.0000	1.0151	0.0179	-0.7598	0.4310	0.2316	1.0000	-0.1552	0.9437	-0.1530	0.0026	0.8429
LOC104912836	0.0071	1.9619	0.0003	2.6061	0.0000	2.7592	0.0000	3.3765	1.0000	-0.5934	1.0000	0.0605	1.0000	0.0285
LOC104912838	1.0000	2.1901	1.0000	-0.1472	1.0000	2.2425	0.4037	2.0749	1.0000	2.2734	1.0000	0.0470	0.6937	2.1413
LOC104912839	0.7485	0.1675	1.0000	-0.0140	0.5363	-0.2345	0.0440	-0.3351	1.0000	-0.0592	0.5382	-0.2278	0.8393	-0.1541
LOC104912841	1.0000	0.3737	0.4339	-0.8100	1.0000	0.3227	0.5594	-0.8409	1.0000	0.1528	0.7187	-1.0182	0.6089	-1.0056
LOC104912842	0.2607	1.1256	0.0552	1.5880	0.4707	0.9713	0.9167	0.5655	1.0000	-0.3398	1.0000	0.1306	0.7477	-0.7385

LOC104912843	1.0000	0.0000	1.0000	2.2508	1.0000	0.0000	1.0000	0.0000	1.0000	0.0000	1.0000	2.3457	1.0000	0.0000
LOC104912844	1.0000	0.0000	1.0000	0.0000	1.0000	0.0000	1.0000	0.0000	1.0000	0.0000	1.0000	0.0000	1.0000	0.0000
LOC104912845	1.0000	2.1850	1.0000	0.0000	1.0000	2.2470	1.0000	0.0000	1.0000	0.0000	1.0000	-2.2957	1.0000	-2.2907
LOC104912846	1.0000	2.1901	1.0000	0.0000	1.0000	0.0000	1.0000	0.0000	1.0000	0.0000	1.0000	-2.2992	1.0000	0.0000
LOC104912847	0.3148	-4.2689	0.9062	-1.5339	0.2833	-4.1854	0.4370	-3.6968	1.0000	-0.5112	1.0000	2.3480	1.0000	0.0000
LOC104912848	1.0000	0.0000	1.0000	-2.3960	1.0000	2.2425	1.0000	-2.3179	1.0000	2.2677	1.0000	0.0000	1.0000	-2.2888
LOC104912849	1.0000	0.0000	1.0000	0.0000	1.0000	0.0000	1.0000	0.0000	1.0000	0.0000	1.0000	0.0000	1.0000	0.0000
LOC104912851	1.0000	0.1874	1.0000	0.0966	0.9969	0.1796	1.0000	0.0914	1.0000	0.0048	1.0000	-0.0740	1.0000	-0.0762
LOC104912852	1.0000	-0.2931	0.2947	-1.6794	1.0000	-2.4061	0.0516	-2.8809	1.0000	2.7691	1.0000	1.4371	1.0000	2.3543
LOC104912853	0.2293	0.4157	1.0000	-0.0168	0.1699	-0.5086	0.0000	-1.0104	1.0000	0.2483	1.0000	-0.1715	0.8388	-0.2479
LOC104912854	0.8249	3.0254	1.0000	0.0000	1.0000	2.2470	1.0000	0.0000	1.0000	0.0000	1.0000	-3.1468	1.0000	-2.2907
LOC104912855	0.0050	0.8759	0.0000	1.0318	0.2057	0.4936	0.0001	0.7315	1.0000	0.2002	0.2761	0.3686	0.1509	0.4414
LOC104912856	0.9545	0.0641	0.1506	-0.1324	0.0000	-1.1365	0.0000	-1.3101	1.0000	0.0481	0.5136	-0.1358	0.6724	-0.1201
LOC104912857	1.0000	2.1901	1.0000	-0.1557	1.0000	0.0000	0.7710	-3.1633	1.0000	3.1056	1.0000	0.9006	1.0000	0.0000
LOC104912858	0.0000	-1.7774	0.0000	-1.3485	0.6283	-0.2445	0.0079	0.6163	1.0000	-0.2747	1.0000	0.1668	0.0554	0.5907
LOC104912859	0.0004	-1.6851	0.0000	-1.7908	0.8811	0.2116	1.0000	0.0757	1.0000	0.2646	1.0000	0.1720	1.0000	0.1328
LOC104912860	1.0000	0.0000	1.0000	0.0000	1.0000	0.0000	1.0000	0.0000	1.0000	0.0000	1.0000	0.0000	1.0000	0.0000
LOC104912861	1.0000	-2.4788	1.0000	0.6912	1.0000	-2.4061	1.0000	0.0057	1.0000	-0.1045	1.0000	3.1946	1.0000	2.3554
LOC104912862	1.0000	0.0000	1.0000	0.0000	1.0000	0.0000	1.0000	0.0000	1.0000	0.0000	1.0000	0.0000	1.0000	0.0000
LOC104912863	0.8249	3.0254	0.7186	1.0553	1.0000	0.0000	0.7701	-3.1666	1.0000	3.1126	1.0000	1.2775	1.0000	0.0000
LOC104912864	1.0000	0.0000	1.0000	0.0000	1.0000	0.0000	1.0000	0.0000	1.0000	0.0000	1.0000	0.0000	1.0000	0.0000
LOC104912865	1.0000	0.6037	1.0000	-0.7281	0.4809	-3.7970	0.2439	-2.3155	1.0000	0.8185	1.0000	-0.4982	1.0000	2.3543
LOC104912866	0.0126	1.0393	0.0249	0.9596	0.8974	0.2577	1.0000	-0.0428	1.0000	-0.4804	0.4411	-0.5493	0.2093	-0.7776
LOC104912867	1.0000	0.0000	1.0000	0.0000	1.0000	0.0000	1.0000	0.0000	1.0000	0.0000	1.0000	0.0000	1.0000	0.0000
LOC104912869	1.0000	-2.4776	1.0000	-2.3985	1.0000	-0.1624	1.0000	0.0045	1.0000	-0.1034	1.0000	0.0000	1.0000	0.0652
LOC104912871	0.7001	-0.2660	0.8696	-0.1471	1.0000	0.1009	0.6924	-0.1488	1.0000	0.1652	0.8385	0.2963	1.0000	-0.0831
LOC104912872	0.6936	1.4527	0.2495	4.0050	0.6654	1.5927	0.4622	3.6989	1.0000	-2.3771	1.0000	0.0569	1.0000	-0.3137
LOC104912873	1.0000	-0.7294	1.0000	0.2194	1.0000	-0.1875	1.0000	0.0094	1.0000	-0.5150	1.0000	0.4459	1.0000	-0.3116
LOC104912874	1.0000	0.0000	1.0000	-2.3985	0.4809	3.6122	1.0000	0.0057	1.0000	2.2734	1.0000	0.0000	1.0000	-1.3086
LOC104912875	1.0000	2.1850	1.0000	2.2533	1.0000	0.0000	1.0000	0.0000	1.0000	0.0000	1.0000	0.0513	1.0000	0.0000
LOC104912876	0.0000	1.4888	0.0000	1.0910	1.0000	0.0006	0.5594	0.2640	1.0000	-0.6768	0.0000	-1.0650	0.3938	-0.4101
LOC104912877	0.0566	2.8469	0.7268	1.0575	1.0000	0.6780	1.0000	-0.8419	1.0000	0.7357	0.8269	-1.0391	1.0000	-0.7795
LOC104912879	0.4664	-1.6749	0.0327	-3.1965	0.0241	1.7209	0.4421	0.7585	1.0000	0.1865	1.0000	-1.3292	0.5105	-0.7755
LOC104912881	1.0000	0.0000	1.0000	0.0000	1.0000	2.2470	1.0000	0.0000	1.0000	0.0000	1.0000	0.0000	1.0000	-2.2906
LOC104912882	1.0000	0.0000	1.0000	-2.3985	1.0000	0.0000	1.0000	-2.3200	1.0000	2.2734	1.0000	0.0000	1.0000	0.0000
LOC104912883	0.7827	0.1007	0.1197	-0.1584	0.0010	-0.5448	0.0000	-0.3759	1.0000	0.0269	0.1156	-0.2196	0.1950	0.2015
LOC104912884	0.8819	0.1822	0.4268	0.2537	1.0000	0.1188	1.0000	-0.0506	1.0000	0.1308	0.9107	0.2146	1.0000	-0.0326
LOC104912885	0.3843	1.1159	0.3845	1.0611	0.0218	1.8190	0.1830	1.2497	1.0000	0.1028	1.0000	0.0570	0.8814	-0.4701
LOC104912886	0.1216	-1.1187	0.0057	-1.4876	1.0000	-0.2004	0.2346	-0.6569	1.0000	0.1375	1.0000	-0.2206	1.0000	-0.3144
LOC104912887	1.0000	0.0000	1.0000	0.0000	1.0000	0.0000	1.0000	0.0000	1.0000	0.0000	1.0000	0.0000	1.0000	0.0000
LOC104912888	0.1325	0.4452	1.0000	0.0059	0.0508	0.5213	0.0052	0.5049	1.0000	-0.0223	0.0766	-0.4505	1.0000	-0.0343
LOC104912892	1.0000	0.0532	0.4597	0.2354	0.0012	0.8849	0.0000	1.0308	1.0000	0.0410	0.8915	0.2355	0.7142	0.1915
LOC104912895	0.0280	-0.4515	0.6834	-0.0713	0.5707	-0.1751	0.0006	0.3069	1.0000	-0.2241	0.4348	0.1686	0.0567	0.2635
LOC104912896	1.0000	-0.1673	0.9486	-0.3230	0.9361	0.3064	0.4814	0.5156	1.0000	-0.1263	1.0000	-0.2719	1.0000	0.0817
LOC104912897	0.0000	-0.7412	0.0000	-0.9561	0.0000	-0.8623	0.0000	-0.8822	1.0000	0.0947	0.9687	-0.1085	0.8988	0.0793
LOC104912898	1.0000	2.1901	1.0000	2.2533	1.0000	0.0000	1.0000	0.0000	1.0000	0.0000	1.0000	0.0489	1.0000	0.0000
LOC104912900	0.0005	1.8043	0.0000	1.7997	0.0000	2.1170	0.0000	1.6453	1.0000	0.1760	1.0000	0.1831	0.6865	-0.2931
LOC104912904	0.4889	-0.8818	0.2026	-1.0636	0.0158	-2.5130	0.0211	-2.2228	1.0000	-0.2222	1.0000	-0.3911	1.0000	0.0756
LOC104912905	0.1708	-1.2077	0.8619	-0.8261	0.3144	-0.9237	0.6267	0.6907	1.0000	-1.4240	0.8309	-1.0375	1.0000	0.1931
LOC104912906	0.1101	4.6954	0.2299	1.7069	1.0000	2.2425	0.7710	-3.1633	1.0000	3.1056	1.0000	0.2457	1.0000	-2.2888
LOC104912907	1.0000	2.1850	1.0000	0.0000	1.0000	2.2470	1.0000	0.0000	1.0000	0.0000	1.0000	-2.2957	1.0000	-2.2906
LOC104912908	1.0000	0.0000	1.0000	0.0000	1.0000	0.0000	1.0000	0.0000	1.0000	0.0000	1.0000	0.0000	1.0000	0.0000
LOC104912909	1.0000	-0.2410	1.0000	0.1270	0.6680	-0.5187	0.0723	-1.1598	1.0000	-0.6131	1.0000	-0.2326	0.1323	-1.2489
LOC104912910	0.0209	3.1958	0.0121	5.1239	0.9468	1.1991	0.7710	3.1752	1.0000	-2.3757	1.0000	-0.5669	1.0000	-0.4518
LOC104912911	1.0000	-0.0177	0.7438	0.2557	0.6734	0.3377	0.0495	0.6826	1.0000	-0.2279	1.0000	0.0595	1.0000	0.1221
LOC104912912	0.0000	1.1825	0.0000	1.3940	0.0000	1.6344	0.0000	1.6637	1.0000	-0.1805	1.0000	0.0436	0.7516	-0.1457
LOC104912913	0.4304	-1.1658	0.6939	-0.9890	0.5914	-0.7637	1.0000	0.2901	1.0000	-0.1313	1.0000	0.0593	0.8550	0.9279
LOC104912914	1.0000	-2.4776	1.0000	0.0000	1.0000	-2.4056	1.0000	0.0000	1.0000	-2.3757	1.0000	0.0000	1.0000	0.0000
LOC104912915	0.7954	0.6287	0.0065	3.4520	0.5441	-1.1498	0.9096	1.3849	1.0000	-2.8396	1.0000	-0.0318	1.0000	-0.3112
LOC104912916	0.3769	0.7518	0.0026	2.0969	0.2075	-1.1772	0.5673	0.8541	1.0000	-1.5023	1.0000	-0.1559	1.0000	0.5274
LOC104912917	0.0007	1.0518	0.0000	1.3094	0.0000	1.6185	0.0000	1.4581	1.0000	0.0381	0.4639	0.3104	0.9095	-0.1135
LOC104912918	0.3290	0.3972	0.0679	0.3851	0.0007	0.9577	0.0000	0.9147	1.0000	0.3146	0.4823	0.3161	0.3483	0.2779
LOC104912919	1.0000	0.0142	0.6279	-0.0619	0.1445	-0.3008	0.0000	-0.3266	1.0000	0.0016	1.0000	-0.0620	1.0000	-0.0190
LOC104912920	0.2523	1.6807	0.1550	1.7454	0.2519	1.5971	0.0281	2.3201	1.0000	-0.1218	1.0000	-0.0503	1.0000	0.6077
LOC104912921	0.7625	0.9225	0.9082	0.5438	0.0440	1.9137	0.1842	1.2958	1.0000	0.4167	1.0000	0.0600	1.0000	-0.1812
LOC104912922	1.0000	-0.1326	0.0587	-2.3568	0.9375	0.5180	0.5599	0.6726	1.0000	0.5867	0.7610	-1.6314	0.8528	0.7455
LOC104912923	0.0588	-1.0672	0.0000	-2.2393	0.5081	-0.4685	1.0000	-0.0197	1.0000	0.2893	0.5247	-0.8706	0.2335	0.7433
LOC104912924	0.2143	-0.9656	1.0000	-0.0667	0.0395	1.0033	1.0000	-0.1837	1.0000	-0.2433	0.8553	0.6665	0.0036	-1.4252
LOC104912925	1.0000	0.0000	1.0000	-2.3960	1.0000	0.0000	1.0000	-2.3179	1.0000	2.2677	1.0000	0.0000	1.0000	0.0000
LOC104912926	0.2298	-0.2477	0.0000	-0.3703	1.0000	-0.0534	0.0002	-0.3281	1.0000	0.2381	0.6781	0.1280	1.0000	-0.0325
LOC104912929	0.0749	1.4196	0.0001	2.7932	0.0456	1.4506	0.0027	2.3936	1.0000	-1.3962	1.0000	-0.0195	0.9035	-0.4538
LOC104912930	1.0000	0.2029	0.6401	-1.1309	1.0000	0.3474	0.0891	-2.7166	1.0000	1.7533	1.0000	0.4439	1.0000	-1.3084
LOC104912931	0.2334	0.2341	0.2984	0.1171	0.2229	-0.2425	0.6671	-0.0723	1.0000	0.0924	1.0000	-0.0123	0.0610	0.2673
LOC104912932	0.2376	0.8755	0.7064	0.3786	0.2524	0.7902	0.4343	0.5243	1.0000	0.3855	1.0000	-0.0989	1.0000	0.1254
LOC104912933	1.0000	0.0000	1.0000	0.0000	1.0000	0.0000	1.0000	0.0000	1.0000	0.0000	1.0000	0.0000	1.0000	0.0000
LOC104912934	1.0000	2.1850	1.0000	0.6912	1.0000	0.0000	1.0000	-2.3200	1.0000	2.2734	1.0000	0.8992	1.0000	0.0000
LOC104912935	1.0000	2.1850	0.7674	-3.2499	1.0000	0.0000	1.0000	0.0074	1.0000	3.1056	1.0000	-2.2957	1.0000	3.2066
LOC104912936	1.0000	-0.3434	1.0000	-0.1716	1.0000	-0.8850	1.0000	0.0043	1.0000	-0.8084	1.0000	-0.6350	1.0000	0.0762
LOC104912937	1.0000	0.0000	1.0000	0.0000	1.0000	0.0000	1.0000	0.0000	1.0000	0.0000	1.0000	0.0000	1.0000	0.0000
LOC104912938	1.0000	2.1850	1.0000	-1.0017	0.0691	4.7549	0.2178	1.8814	1.0000	3.1126	1.0000	0.0495	1.0000	0.2669
LOC104912939	0.9923	0.0817	0.5223	0.1262	0.2434	-0.3125	0.8168	-0.0801	1.0000	0.0243	1.0000	0.0816	0.2681	0.2642
LOC104912940	0.0006	-0.6272	0.0000	-0.7715	0.9474	-0.0703	1.0000	-0.0229	1.0000	0.1222	1.0000	-0.0093	0.3019	0.1752
LOC104912941	1.0000	0.1732	0.1193	0.8193	0.2141	0.7202	0.0007	1.2533	1.0000	-0.2861	0.9671	0.3719	0.8941	0.2529
LOC104912942	0.0594	0.6409	0.0037	0.6539	0.0000	1.7870	0.0000	1.4837	1.0000	0.1523	0.9736	0.1786	0.8110	-0.1438
LOC104912943	0.0000	1.5300	0.0000	1.4836	0.0000	2.2530	0.0000	1.9371	1.0000	0.0481	1.0000	0.0130	0.4678	-0.2643
LOC104912944	1.0000	0.0000	1.0000	0.0000	1.0000	0.0000	1.0000	0.0000	1.0000	0.0000	1.0000	0.0000	1.0000	0.0000
LOC104912945	0.0000	1.9199	0.0000	1.9238	0.0000	1.9398	0.0000	1.4189	1.0000	0.1608	0.9323	0.1794	0.3529	-0.3521
LOC104912946	0.1618	-1.0508	1.0000	-0.1061	0.1417	-1.0852	0.7326	0.5332	1.0000	-1.1003	1.0000	-0.1300	0.9181	0.5161
LOC104912947	1.0000	0.0000	1.0000	0.0000	1.0000	0.0000	1.0000	0.0000	1.0000	0.0000	1.0000	0.0000	1.0000	0.0000
LOC104912948	1.0000	0.0468	0.9062	1.2223	0.9361	-1.5509	1.0000	0.8519	1.0000	-1.4912	1.0000	-0.3282	1.0000	0.9149
LOC104912949	1.0000	-2.4788	1.0000	0.0000	1.0000	-0.1597	1.0000	0.0000	1.0000	-2.3771	1.0000	0.0000	1.0000	-2.2907
LOC104912950	1.0000	-0.3288	1.0000	0.0000	0.8033	-3.2635	1.0000	2.3257	1.0000	-3.2319	1.0000	-3.1383	1.0000	2.3554
LOC104912951	0.0016	-0.5923	0.0000	-0.6064	0.0000	-0.7801	0.0000	-0.7692	1.0000	-0.0479	1.0000	-0.0497	1.0000	-0.0327

LOC104912952	0.0029	-0.7373	0.0000	-1.1155	0.0002	-0.8622	0.0000	-0.9515	1.0000	0.3022	1.0000	-0.0636	0.6191	0.2183
LOC104912953	0.4801	2.0051	0.7674	3.0956	0.0188	3.2295	0.4370	3.7037	1.0000	-2.3771	0.9101	-1.4174	0.1717	-1.9510
LOC104912954	0.0588	1.6589	0.3311	0.7110	0.0000	2.9730	0.0003	1.6389	1.0000	1.1558	1.0000	0.2235	1.0000	-0.1707
LOC104912955	1.0000	-0.2882	0.7674	3.0956	1.0000	-2.4056	0.7710	3.1752	1.0000	-2.3757	1.0000	0.9006	1.0000	3.2081
LOC104912956	0.1453	1.2550	0.5584	0.8887	0.0247	1.5578	0.0246	1.6241	1.0000	-0.4790	0.6218	-0.8322	0.9535	-0.4098
LOC104912957	0.0018	1.2907	0.0063	1.0031	0.0000	2.2412	0.0000	1.7426	1.0000	0.3837	1.0000	0.1121	1.0000	-0.1051
LOC104912958	0.7116	0.1636	1.0000	-0.0490	0.0417	-0.5124	0.0527	-0.3175	1.0000	-0.1287	0.2503	-0.3282	1.0000	0.0735
LOC104912959	1.0000	0.2007	1.0000	-0.1472	1.0000	0.7335	0.0518	2.8941	1.0000	-0.9587	1.0000	-1.3254	0.7588	1.1999
LOC104912960	1.0000	0.0000	1.0000	0.0000	1.0000	0.0000	1.0000	0.0000	1.0000	0.0000	1.0000	0.0000	1.0000	0.0000
LOC104912961	1.0000	0.0000	1.0000	0.0000	1.0000	0.0000	1.0000	0.0000	1.0000	0.0000	1.0000	0.0000	1.0000	0.0000
LOC104912962	0.0001	0.9525	0.0007	0.6338	0.3726	-0.3282	0.1082	-0.3663	1.0000	0.1585	0.9405	-0.1497	1.0000	0.1251
LOC104912963	0.0000	-1.5590	0.0000	-1.3395	0.5980	0.1236	0.0000	0.6147	1.0000	0.1512	0.0009	0.3829	0.0000	0.6476
LOC104912964	0.4552	0.8498	0.2225	0.8642	0.1356	1.3552	0.0796	1.1068	1.0000	0.2786	1.0000	0.3078	1.0000	0.0280
LOC104912965	0.1419	2.5630	0.0038	3.4469	1.0000	-2.4061	1.0000	-2.3180	1.0000	-0.1089	0.9375	0.7817	1.0000	0.0000
LOC104912966	0.0662	-2.1367	0.7726	-1.1137	0.6820	0.5783	0.3317	1.0932	1.0000	-0.9775	1.0000	0.0569	0.9114	-0.4584
LOC104912967	1.0000	-0.1906	0.6122	-0.5585	0.0351	-2.2114	0.0427	-1.7087	1.0000	-0.1313	1.0000	-0.4820	1.0000	0.3815
LOC104912968	1.0000	0.0502	0.7324	-1.3907	0.5041	-3.7956	0.4057	-2.0667	1.0000	0.5758	1.0000	-0.8603	1.0000	2.3543
LOC104912971	1.0000	0.0000	1.0000	0.0000	1.0000	0.0000	1.0000	0.0000	1.0000	0.0000	1.0000	0.0000	1.0000	0.0000
LOC104912973	1.0000	2.1850	1.0000	2.2508	1.0000	0.0000	1.0000	0.0000	1.0000	0.0000	1.0000	0.0495	1.0000	0.0000
LOC104912974	0.3092	4.2299	1.0000	2.2508	1.0000	0.0000	1.0000	2.3257	1.0000	0.0000	0.8269	-2.0165	1.0000	2.3554
LOC104912976	0.4383	-0.7409	0.4431	0.6556	0.4908	-0.7654	0.8877	-0.3493	1.0000	-0.3495	0.5692	1.0576	1.0000	0.0764
LOC104912977	1.0000	0.1184	0.4179	1.1722	0.5833	0.9033	1.0000	0.0060	1.0000	-0.8174	1.0000	0.2452	0.3356	-1.7140
LOC104912978	0.0057	1.2801	0.0000	1.9860	0.0000	1.7593	0.0000	1.9235	1.0000	-0.5314	1.0000	0.1867	0.4327	-0.3593
LOC104912979	0.1893	-0.9628	0.3778	-0.6136	0.9008	-0.2953	0.8205	-0.2702	1.0000	0.0936	1.0000	0.4567	1.0000	0.1289
LOC104912981	1.0000	2.1900	1.0000	0.0000	0.5080	3.6208	0.7710	3.1710	1.0000	0.0000	1.0000	-2.2992	1.0000	-0.4617
LOC104912982	0.6481	-0.1329	0.1747	-0.1539	0.0009	-0.5678	0.0000	-0.5723	1.0000	0.0348	1.0000	0.0260	1.0000	0.0351
LOC104912983	0.7342	0.1987	0.0009	-0.8549	0.5101	0.2966	0.8942	-0.1348	1.0000	-0.0611	0.0001	-1.1027	0.2837	-0.4864
LOC104912984	0.0142	-0.4738	0.0083	-0.3331	0.0001	-0.8154	0.0000	-0.5033	1.0000	-0.1485	1.0000	0.0042	0.7060	0.1698
LOC104912985	0.7496	0.3746	0.0195	1.0999	0.0006	1.3949	0.0000	1.9303	1.0000	-0.7132	1.0000	0.0277	1.0000	-0.1695
LOC104912986	0.8226	3.0199	0.7666	3.0922	0.8011	3.0840	1.0000	2.3240	1.0000	0.0000	1.0000	0.0547	1.0000	-0.7795
LOC104912988	0.0150	1.1502	0.0000	1.8410	0.0000	2.5634	0.0000	2.6986	1.0000	-0.4352	0.7500	0.2738	0.3574	-0.2893
LOC104912989	0.5928	1.3488	1.0000	-0.6953	0.7663	1.0386	1.0000	0.0046	1.0000	0.4129	0.7641	-1.6296	1.0000	-0.6168
LOC104912990	0.5079	-0.6476	0.0001	-1.8107	0.0025	-2.3166	0.0076	-1.1458	1.0000	0.6652	0.9666	-0.4844	0.0722	1.8441
LOC104912991	0.0111	-0.6622	0.0000	-1.0635	0.0000	-1.0936	0.0000	-0.9686	1.0000	0.0929	0.5737	-0.2972	0.7372	0.2228
LOC104912992	0.4532	0.6523	0.0179	0.8323	0.0001	1.6508	0.0102	0.9228	1.0000	0.3610	0.5247	0.5481	0.5935	-0.3635
LOC104912993	0.7266	0.1264	1.0000	-0.0378	0.0000	-1.0566	0.0000	-0.8522	1.0000	-0.0425	0.4723	-0.1947	0.5774	0.1675
LOC104912994	0.0023	1.1559	0.0000	1.7505	0.0004	1.0312	0.0000	1.4947	1.0000	-0.1338	0.2865	0.4731	0.2580	0.3345
LOC104912995	0.0008	1.9726	0.0000	2.7616	0.9419	-0.4004	1.0000	0.0067	1.0000	-1.6714	0.1266	-0.8766	0.4508	-1.2577
LOC104912996	1.0000	0.0000	1.0000	0.0000	1.0000	0.0000	1.0000	2.3240	1.0000	0.0000	1.0000	0.0000	1.0000	2.3543
LOC104912998	0.0302	1.0008	0.0801	0.7127	0.0002	1.3642	0.0001	1.2761	1.0000	-0.0059	0.8931	-0.2839	1.0000	-0.0947
LOC104912999	0.1699	0.4116	0.1920	0.2828	0.0001	-1.1561	0.0001	-0.8131	1.0000	0.2722	0.9217	0.1543	0.0686	0.6175
LOC104913001	1.0000	0.0000	1.0000	0.0000	1.0000	0.0000	1.0000	0.0000	1.0000	0.0000	1.0000	0.0000	1.0000	0.0000
LOC104913002	1.0000	0.0000	0.7666	-3.2534	1.0000	0.0000	0.9786	0.9240	1.0000	3.1126	1.0000	0.0000	0.5105	4.1259
LOC104913003	0.1978	0.3105	0.0003	0.4521	1.0000	0.0008	0.9912	0.0473	1.0000	-0.2025	1.0000	-0.0484	0.6818	-0.1499
LOC104913005	0.0948	1.3720	0.0012	2.1500	0.4692	-1.5333	0.8169	-0.9274	1.0000	-0.5383	1.0000	0.2581	1.0000	0.0749
LOC104913007	1.0000	-0.3262	1.0000	-2.3960	1.0000	-0.1785	1.0000	0.8545	1.0000	-0.9614	1.0000	-3.1383	1.0000	0.0723
LOC104913009	0.5243	-0.1647	0.0100	-0.2380	0.3089	0.2042	0.0130	0.2226	1.0000	-0.0081	1.0000	-0.0691	1.0000	0.0155
LOC104913010	1.0000	-0.0467	0.0201	-0.4000	1.0000	-0.0222	0.4548	-0.1627	1.0000	0.1284	0.7467	-0.2138	1.0000	-0.0041
LOC104913011	0.0043	0.8847	0.0000	1.1689	0.0000	1.8315	0.0000	1.8893	1.0000	-0.2165	1.0000	0.0808	0.7135	-0.1523
LOC104913014	0.0694	0.5854	0.4843	-0.2438	0.1506	0.4757	0.6134	-0.1884	1.0000	-0.0059	0.0019	-0.8235	0.0133	-0.6653
LOC104913015	0.0369	0.4819	0.0882	0.2580	0.0047	0.5916	0.0355	0.2982	1.0000	0.2174	1.0000	0.0076	1.0000	-0.0696
LOC104913016	1.0000	-0.1055	0.8161	0.3041	1.0000	0.0550	0.0029	1.1891	1.0000	-0.6574	1.0000	-0.2338	0.5611	0.4852
LOC104913017	1.0000	2.1850	0.4437	3.6202	1.0000	2.2470	1.0000	0.0000	1.0000	0.0000	1.0000	1.4326	1.0000	-2.2907
LOC104913018	0.5086	3.5431	0.4431	3.6192	1.0000	0.0000	1.0000	0.0000	1.0000	0.0000	1.0000	0.0569	1.0000	0.0000
LOC104913019	0.2064	0.2629	1.0000	-0.0228	0.0045	0.4944	0.0015	0.2454	1.0000	0.1603	0.6913	-0.1129	0.7488	-0.0830
LOC104913021	0.0110	2.4918	0.0148	2.3143	0.9361	-1.5529	0.9167	-1.3690	1.0000	-0.1273	1.0000	-0.2906	1.0000	0.0652
LOC104913023	1.0000	0.0000	0.7666	-3.2534	1.0000	0.0000	0.7701	-3.1666	1.0000	3.1126	1.0000	0.0000	1.0000	0.0000
LOC104913024	0.1096	4.6934	0.9062	1.2214	0.8033	3.0892	1.0000	0.0045	1.0000	2.2734	0.9741	-1.0994	1.0000	-0.7815
LOC104913025	0.2590	0.8748	1.0000	-0.2834	0.1053	0.9964	0.0473	1.2487	1.0000	-0.0251	0.1297	-1.1740	1.0000	0.2274
LOC104913026	0.1644	0.8384	0.0000	1.7120	0.0001	1.5968	0.0000	2.0325	1.0000	-0.5319	0.7930	0.3580	1.0000	-0.0807
LOC104913028	0.6936	0.1544	0.0801	0.3385	0.0005	-0.8075	0.0000	-0.8635	1.0000	-0.2058	1.0000	-0.0100	0.5119	-0.2559
LOC104913029	1.0000	0.0000	1.0000	0.0000	1.0000	0.0000	1.0000	0.0000	1.0000	0.0000	1.0000	0.0000	1.0000	0.0000
LOC104913030	0.0000	-1.5571	0.0000	-1.6610	0.0000	-1.2672	0.0000	-1.0674	1.0000	-0.0691	0.5999	-0.1603	0.6347	0.1363
LOC104913032	0.0000	-0.7455	0.0000	-0.7132	0.0000	-0.8573	0.0000	-0.5915	1.0000	-0.0806	1.0000	-0.0361	0.4210	0.1906
LOC104913033	0.5415	-0.2077	0.3990	0.1495	0.3283	0.2752	0.0000	0.6647	1.0000	-0.3128	1.0000	0.0578	0.8742	0.0834
LOC104913034	0.0000	0.9002	0.0000	1.0662	0.6454	0.1702	1.0000	0.0462	1.0000	-0.2270	1.0000	-0.0490	0.0955	-0.3444
LOC104913035	0.0002	1.4043	0.0000	2.2503	0.0032	1.1040	0.0762	0.8116	1.0000	-0.6434	0.9120	0.2137	0.0142	-0.9298
LOC104913036	0.8987	-0.5984	0.2899	-1.2750	1.0000	0.3635	0.2348	-1.3973	1.0000	0.7178	1.0000	0.0568	0.7749	-1.0426
LOC104913037	0.0000	-0.7549	0.0000	-0.8067	0.0000	-0.7074	0.0000	-0.6667	1.0000	0.1480	0.8605	0.1085	0.2554	0.1942
LOC104913038	1.0000	0.0000	0.7666	3.0922	1.0000	0.0000	1.0000	2.3240	1.0000	0.0000	1.0000	3.1976	1.0000	2.3543
LOC104913039	0.3098	-0.5919	1.0000	-0.1376	0.4809	0.4112	0.0000	1.5334	1.0000	-0.4785	1.0000	-0.0111	0.0692	0.6509
LOC104913040	1.0000	0.0000	1.0000	0.0000	1.0000	0.0000	1.0000	0.0000	1.0000	0.0000	1.0000	0.0000	1.0000	0.0000
LOC104913041	1.0000	-2.4776	0.7674	-3.2570	1.0000	0.6783	0.0631	2.3298	1.0000	0.7416	1.0000	0.0000	0.1858	2.3994
LOC104913042	1.0000	0.0000	1.0000	0.0000	1.0000	0.0000	1.0000	0.0000	1.0000	0.0000	1.0000	0.0000	1.0000	0.0000
LOC104913043	1.0000	0.0000	1.0000	0.0000	1.0000	0.0000	1.0000	0.0000	1.0000	0.0000	1.0000	0.0000	1.0000	0.0000
LOC104913045	0.0073	1.3644	0.0000	1.5095	0.0438	0.9752	0.0081	0.9574	1.0000	0.2108	0.7457	0.3658	1.0000	0.1975
LOC104913046	1.0000	0.0000	1.0000	0.0000	1.0000	0.0000	1.0000	0.0000	1.0000	0.0000	1.0000	0.0000	1.0000	0.0000
LOC104913047	1.0000	0.0000	1.0000	0.0000	0.8033	3.0892	1.0000	2.3240	1.0000	0.0000	1.0000	0.0000	1.0000	-0.7815
LOC104913048	0.9742	-0.1616	0.0365	0.5132	1.0000	0.0028	0.4342	0.2646	1.0000	0.2579	0.0009	0.9456	0.1825	0.5270
LOC104913049	0.0539	0.9329	0.0048	0.9705	0.0948	-1.0973	0.7237	-0.3120	1.0000	0.0881	1.0000	0.1379	0.3103	0.8783
LOC104913050	1.0000	0.0000	1.0000	0.0000	1.0000	0.0000	1.0000	0.0000	1.0000	0.0000	1.0000	0.0000	1.0000	0.0000
LOC104913051	1.0000	0.0901	0.3302	-1.0592	0.4867	0.8682	1.0000	0.0074	1.0000	0.7552	1.0000	-0.3772	1.0000	-0.0982
LOC104913054	1.0000	-0.0377	1.0000	0.2155	1.0000	-0.5709	1.0000	-0.0017	1.0000	-0.5061	1.0000	-0.2470	1.0000	0.0690
LOC104913055	0.1128	1.2435	0.0001	1.9042	0.0001	2.0452	0.0001	1.9036	1.0000	0.1056	0.3284	0.7770	1.0000	-0.0308
LOC104913056	1.0000	0.0000	1.0000	0.0000	1.0000	0.0000	1.0000	0.0000	1.0000	0.0000	1.0000	0.0000	1.0000	0.0000
LOC104913057	0.2251	-0.2349	0.0004	-0.3130	0.0006	-0.5366	0.0000	-0.4062	1.0000	0.0106	1.0000	-0.0553	0.3804	0.1462
LOC104913059	0.0419	5.0480	0.2555	2.1641	1.0000	2.2470	1.0000	0.0074	1.0000	2.2677	1.0000	-0.5069	1.0000	0.0649
LOC104913060	0.1063	0.6905	0.0994	0.4711	0.0000	1.4314	0.0000	1.0985	1.0000	0.3423	1.0000	0.1366	1.0000	0.0183
LOC104913062	1.0000	2.1900	1.0000	0.0000	1.0000	2.2470	1.0000	0.0000	1.0000	0.0000	1.0000	-2.2992	1.0000	-2.2907
LOC104913063	0.0007	1.3171	0.0209	0.6597	0.2936	0.5317	0.0145	-0.8776	1.0000	0.5574	1.0000	-0.0863	0.0867	-0.8484
LOC104913064	0.6008	0.2437	0.0119	0.6602	0.0684	-0.5986	0.3139	-0.3835	1.0000	-0.6041	0.9443	-0.1781	0.5000	-0.3851
LOC104913065	1.0000	0.0000	1.0000	0.0000	1.0000	2.2425	1.0000	0.0000	1.0000	0.0000	1.0000	0.0000	1.0000	-2.2888
LOC104913067	1.0000	-0.0696	1.0000	0.0110	0.8924	0.1112	0.9696	0.0566	1.0000	-0.1193	1.0000	-0.0277	0.7883	-0.1686

LOC104913068	0.4336	-0.2445	1.0000	0.0666	0.0001	-0.9434	0.0005	-0.6788	1.0000	-0.3008	1.0000	0.0219	1.0000	-0.0304
LOC104913069	0.3961	0.3600	0.3691	0.2087	0.0000	1.1376	0.0000	0.7137	1.0000	0.1224	1.0000	-0.0158	0.1519	-0.2948
LOC104913071	0.2421	0.7176	0.0925	0.6563	0.0024	1.3540	0.0032	1.0036	1.0000	0.1548	1.0000	0.1067	1.0000	-0.1900
LOC104913072	0.0000	2.0840	0.0000	2.1047	0.0003	0.8829	0.0546	0.3420	1.0000	-0.0098	1.0000	0.0240	0.0017	-0.5446
LOC104913073	0.5391	-3.8819	0.7666	-3.2534	0.9468	-1.5505	0.7701	-3.1666	1.0000	-0.6522	1.0000	0.0000	1.0000	-2.2907
LOC104913074	0.0575	2.4130	0.9762	0.7526	1.0000	-1.0206	1.0000	-0.8416	1.0000	-0.1187	0.2872	-1.7785	1.0000	0.0661
LOC104913075	0.0036	-2.8382	0.0000	-4.8958	1.0000	-0.3133	0.0224	-1.2151	1.0000	0.7351	1.0000	-1.3189	1.0000	-0.1677
LOC104913076	1.0000	0.0000	1.0000	0.0000	1.0000	0.0000	1.0000	0.0000	1.0000	0.0000	1.0000	0.0000	1.0000	0.0000
LOC104913077	1.0000	0.0000	1.0000	0.0000	1.0000	0.0000	1.0000	0.0000	1.0000	0.0000	1.0000	0.0000	1.0000	0.0000
LOC104913078	1.0000	0.0000	1.0000	0.0000	1.0000	0.0000	1.0000	0.0000	1.0000	0.0000	1.0000	0.0000	1.0000	0.0000
LOC104913080	1.0000	0.0000	1.0000	0.0000	1.0000	0.0000	1.0000	0.0000	1.0000	0.0000	1.0000	0.0000	1.0000	0.0000
LOC104913082	1.0000	0.0000	1.0000	0.0000	1.0000	0.0000	1.0000	2.3257	1.0000	0.0000	1.0000	0.0000	1.0000	2.3554
LOC104913084	0.0001	-1.2584	0.0000	-0.9529	0.7855	0.1739	0.0000	0.7890	1.0000	-0.1333	1.0000	0.1839	0.0144	0.4858
LOC104913085	0.0091	0.9226	0.0000	1.9061	0.0000	1.7691	0.0000	2.3339	1.0000	-0.8235	0.9896	0.1767	0.4272	-0.2468
LOC104913086	1.0000	0.0000	1.0000	0.0000	1.0000	0.0000	1.0000	0.0000	1.0000	0.0000	1.0000	0.0000	1.0000	0.0000
LOC104913088	0.8366	-0.3548	0.6550	-0.4176	0.3239	0.6925	0.0390	0.8858	1.0000	0.1064	1.0000	0.0594	0.9376	0.3067
LOC104913089	1.0000	-2.4788	1.0000	0.0000	1.0000	-2.4061	1.0000	0.0000	1.0000	-2.3771	1.0000	0.0000	1.0000	0.0000
LOC104913090	1.0000	0.0000	1.0000	-2.3960	1.0000	0.0000	1.0000	-2.3179	1.0000	2.2677	1.0000	0.0000	1.0000	0.0000
LOC104913091	0.5086	-3.8796	0.4431	-3.7865	1.0000	-0.1850	1.0000	0.3978	1.0000	-0.1237	1.0000	0.0000	1.0000	0.4642
LOC104913092	1.0000	0.0000	0.7666	3.0922	1.0000	0.0000	1.0000	2.3256	1.0000	0.0000	1.0000	3.1976	1.0000	2.3554
LOC104913093	1.0000	-2.4788	1.0000	0.6912	1.0000	-0.1597	1.0000	-2.3200	1.0000	-0.1045	1.0000	3.1946	1.0000	-2.2907
LOC104913094	1.0000	2.1850	1.0000	0.0000	1.0000	0.0000	1.0000	0.0000	1.0000	0.0000	1.0000	-2.2958	1.0000	0.0000
LOC104913095	1.0000	-0.2892	1.0000	0.6940	1.0000	-0.1630	1.0000	-2.3179	1.0000	-0.1089	1.0000	0.8962	1.0000	-2.2888
LOC104913096	0.2773	-1.8513	1.0000	-0.3890	0.2461	-1.6984	1.0000	0.0071	1.0000	-0.4784	1.0000	1.0010	0.8620	1.2343
LOC104913097	1.0000	2.1850	1.0000	-0.1449	0.8011	3.0840	1.0000	0.8538	1.0000	2.2677	1.0000	0.0495	1.0000	0.0717
LOC104913098	0.0189	1.0841	0.0123	0.9602	0.0000	1.7318	0.0000	1.6811	1.0000	-0.0022	1.0000	-0.1141	1.0000	-0.0454
LOC104913099	1.0000	0.0000	1.0000	0.0000	1.0000	0.0000	1.0000	0.0000	1.0000	0.0000	1.0000	0.0000	1.0000	0.0000
LOC104913100	1.0000	0.5414	0.9148	1.2162	1.0000	0.6740	1.0000	0.0057	1.0000	-0.1045	1.0000	0.5836	1.0000	-0.7769
LOC104913101	0.7549	0.3432	0.3684	0.4396	0.9361	0.2119	0.8664	0.2229	1.0000	0.0339	1.0000	0.1402	1.0000	0.0484
LOC104913102	1.0000	-0.0065	1.0000	-0.0083	0.0000	-1.1839	0.0000	-0.9699	1.0000	0.0193	1.0000	0.0297	0.1836	0.2392
LOC104913104	0.2197	-0.3886	0.0009	-0.6170	1.0000	0.0481	1.0000	0.0483	1.0000	0.1864	1.0000	-0.0298	0.5140	0.1913
LOC104913105	0.3346	-0.7006	0.0410	-1.1279	0.0122	-1.5273	0.1101	-0.8694	1.0000	0.0259	1.0000	-0.3869	0.8120	0.6920
LOC104913106	0.1297	1.0699	0.0707	0.9326	0.5776	0.5871	1.0000	0.0034	1.0000	0.4230	1.0000	0.2897	1.0000	-0.1587
LOC104913107	1.0000	0.2695	0.6722	0.7083	0.1277	-2.2243	0.8688	-0.6461	1.0000	-0.2885	1.0000	0.1584	0.9835	1.2969
LOC104913108	1.0000	-0.7243	0.1616	-2.6990	0.0115	2.1298	0.0006	2.1152	1.0000	0.6473	1.0000	-1.3266	0.5443	0.6369
LOC104913109	1.0000	2.1850	0.5285	-1.6445	0.0562	4.7614	0.7409	0.7203	1.0000	4.5910	1.0000	0.9035	1.0000	0.5804
LOC104913110	1.0000	0.1595	0.6182	0.5980	1.0000	-0.1969	0.8408	0.4472	1.0000	-0.6642	1.0000	-0.2120	1.0000	-0.0118
LOC104913111	0.6927	1.4475	0.9062	1.2223	1.0000	-0.1624	1.0000	0.0045	1.0000	-0.1034	1.0000	-0.3282	1.0000	0.0652
LOC104913112	0.0177	1.8738	0.0110	1.4699	0.0495	1.7029	0.0043	1.6146	1.0000	0.7209	1.0000	0.3308	0.5353	0.6394
LOC104913113	0.9921	0.1088	0.8795	-0.0600	0.0053	-0.8936	0.0032	-0.3953	1.0000	0.3592	0.4917	0.2027	0.0000	0.8589
LOC104913115	0.8358	0.1897	0.1443	0.4378	0.0000	1.5271	0.0000	1.6968	1.0000	-0.2008	1.0000	0.0619	1.0000	-0.0217
LOC104913116	1.0000	0.0000	1.0000	-2.3960	1.0000	0.0000	1.0000	-2.3179	1.0000	2.2677	1.0000	0.0000	1.0000	0.0000
LOC104913117	0.0784	-1.2804	0.7595	0.5808	1.0000	0.1360	0.0722	1.4169	1.0000	-1.8129	1.0000	0.0593	0.8514	-0.5244
LOC104913119	0.0020	-1.1042	0.0000	-1.3835	0.0002	-1.2823	0.0016	-0.8332	1.0000	-0.0114	1.0000	-0.2785	0.5661	0.4422
LOC104913121	0.0002	-0.9148	0.0000	-0.7433	0.0000	-1.0947	0.0081	-0.3903	1.0000	-0.3381	1.0000	-0.1539	0.0948	0.3701
LOC104913122	0.8678	-0.6314	0.1076	1.4463	0.8266	-0.4795	0.3285	1.0897	1.0000	-0.9752	0.5808	1.1161	1.0000	0.5973
LOC104913123	0.5598	-1.0012	1.0000	-0.1785	0.7690	-0.8374	1.0000	0.0047	1.0000	-0.5229	1.0000	0.3102	1.0000	0.3246
LOC104913124	0.4439	0.6669	0.1497	0.7794	1.0000	-0.1192	0.6157	0.4304	1.0000	0.2269	0.9952	0.3454	0.3706	0.7785
LOC104913126	0.0150	-0.5096	0.0005	-0.4475	0.0081	-0.5947	0.0060	-0.3497	1.0000	-0.0919	1.0000	-0.0177	0.7908	0.1594
LOC104913127	1.0000	-0.7296	0.9762	-1.0846	0.2833	-4.1854	0.6172	-1.7602	1.0000	-0.1215	1.0000	-0.4715	1.0000	2.3543
LOC104913129	1.0000	0.5849	0.4437	3.6202	0.8033	-3.2635	1.0000	2.3256	1.0000	-3.2319	1.0000	-0.3294	1.0000	2.3554
LOC104913130	0.0000	1.8793	0.0000	2.5817	0.0001	1.3056	0.0000	1.3808	1.0000	-0.6161	1.0000	0.0981	0.1139	-0.5323
LOC104913132	1.0000	0.0000	1.0000	0.0000	1.0000	0.0000	1.0000	0.0000	1.0000	0.0000	1.0000	0.0000	1.0000	0.0000
LOC104913134	0.5833	1.3502	1.0000	0.3841	0.1819	2.0000	0.4656	1.1287	1.0000	0.7956	1.0000	-0.1547	1.0000	-0.0693
LOC104913135	1.0000	0.0000	1.0000	0.0000	1.0000	0.0000	1.0000	0.0000	1.0000	0.0000	1.0000	0.0000	1.0000	0.0000
LOC104913136	0.0050	1.9623	0.0012	1.5781	0.0000	3.0760	0.0000	2.4461	1.0000	0.6630	1.0000	0.2879	1.0000	0.0321
LOC104913137	1.0000	0.0000	1.0000	0.0000	1.0000	0.0000	1.0000	0.0000	1.0000	0.0000	1.0000	0.0000	1.0000	0.0000
LOC104913138	0.0613	-0.3352	0.0232	-0.2369	0.0000	-1.1033	0.0000	-0.5861	1.0000	-0.2112	0.8797	-0.1008	0.0693	0.3127
LOC104913139	1.0000	0.0000	1.0000	0.0000	1.0000	0.0000	1.0000	0.0000	1.0000	0.0000	1.0000	0.0000	1.0000	0.0000
LOC104913140	0.0242	-0.5444	0.0006	-0.5626	1.0000	0.0605	0.0185	0.3246	1.0000	-0.1823	0.8507	-0.1889	1.0000	0.0869
LOC104913142	0.4751	-0.3217	0.7344	-0.1578	0.8581	-0.1539	1.0000	-0.0142	1.0000	-0.1352	1.0000	0.0408	1.0000	0.0091
LOC104913144	0.2959	-1.5014	0.1610	-2.2047	1.0000	0.3057	0.9018	-0.5570	1.0000	0.2177	1.0000	-0.4744	0.9860	-0.6354
LOC104913145	1.0000	0.0000	1.0000	0.0000	1.0000	0.0000	1.0000	0.0000	1.0000	0.0000	1.0000	0.0000	1.0000	0.0000
LOC104913146	1.0000	0.0000	1.0000	0.0000	1.0000	0.0000	1.0000	0.0000	1.0000	0.0000	1.0000	0.0000	1.0000	0.0000
LOC104913147	1.0000	0.0000	1.0000	0.0000	1.0000	0.0000	1.0000	0.0000	1.0000	0.0000	1.0000	0.0000	1.0000	0.0000
LOC104913148	1.0000	0.0000	1.0000	-2.3960	1.0000	0.0000	1.0000	-2.3179	1.0000	2.2677	1.0000	0.0000	1.0000	0.0000
LOC104913149	0.9545	1.0672	1.0000	-2.3960	0.3144	2.1521	1.0000	-2.3179	1.0000	-0.1089	0.8607	-3.6732	0.2306	-4.6095
LOC104913150	1.0000	0.0452	0.0009	-0.5164	1.0000	-0.0438	0.0000	-0.6184	1.0000	0.4493	1.0000	-0.0990	0.9618	-0.1202
LOC104913151	1.0000	2.1850	1.0000	0.0000	1.0000	2.2470	1.0000	0.0000	1.0000	0.0000	1.0000	-2.2958	1.0000	-2.2907
LOC104913154	1.0000	-0.3257	0.3489	-1.2953	1.0000	0.3438	0.2800	-1.5020	1.0000	1.9171	1.0000	0.9770	1.0000	0.0768
LOC104913156	1.0000	0.0000	1.0000	0.0000	1.0000	0.0000	1.0000	0.0000	1.0000	0.0000	1.0000	0.0000	1.0000	0.0000
LOC104913158	1.0000	0.0000	1.0000	-0.1472	1.0000	2.2425	1.0000	0.0045	1.0000	2.2734	1.0000	2.3457	1.0000	0.0652
LOC104913159	0.9545	1.0672	1.0000	0.0000	1.0000	-2.4061	1.0000	2.3256	1.0000	-2.3771	0.8607	-3.6732	1.0000	2.3554
LOC104913160	1.0000	-2.4776	1.0000	0.6940	1.0000	-2.4056	1.0000	0.0074	1.0000	-0.1078	1.0000	3.1946	1.0000	2.3554
LOC104913161	1.0000	-0.0627	0.8959	-0.3588	0.0551	-2.2196	0.1407	-1.2432	1.0000	0.2516	1.0000	-0.0326	0.8664	1.2353
LOC104913162	1.0000	-0.0974	0.3501	-1.8580	1.0000	-0.1925	0.5796	-1.1517	1.0000	0.3414	0.9075	-1.4162	1.0000	-0.6156
LOC104913163	1.0000	0.0000	1.0000	0.0000	1.0000	0.0000	1.0000	0.0000	1.0000	0.0000	1.0000	0.0000	1.0000	0.0000
LOC104913164	1.0000	0.0000	1.0000	0.0000	1.0000	2.2470	1.0000	0.0000	1.0000	0.0000	1.0000	0.0000	1.0000	-2.2908
LOC104913165	1.0000	0.4663	0.2555	2.1641	0.1707	-4.4923	0.3949	2.0772	1.0000	-2.1898	1.0000	-0.5054	0.3369	4.4317
LOC104913166	0.0000	-2.6814	0.0000	-2.2326	0.0094	-0.8835	0.1065	-0.5416	1.0000	-0.4038	1.0000	0.0588	1.0000	-0.0555
LOC104913167	0.0000	-0.9143	0.0000	-0.9484	0.0000	-0.8915	0.0000	-0.8575	1.0000	-0.0983	0.7972	-0.1204	1.0000	-0.0590
LOC104913168	0.7550	-0.1793	1.0000	-0.0512	0.7131	0.1846	0.1200	0.2892	1.0000	-0.0342	1.0000	0.1068	1.0000	0.0789
LOC104913169	1.0000	-0.3452	0.3422	-1.8566	0.2678	1.2426	0.1931	1.1717	1.0000	0.3389	1.0000	-1.1663	1.0000	0.2704
LOC104913175	1.0000	2.1850	1.0000	0.0000	1.0000	0.0000	1.0000	0.0000	1.0000	0.0000	1.0000	-2.2958	1.0000	0.0000
LOC104913176	1.0000	0.0000	1.0000	0.0000	1.0000	0.0000	1.0000	0.0000	1.0000	0.0000	1.0000	0.0000	1.0000	0.0000
LOC104913177	1.0000	-0.3195	0.6224	1.6084	1.0000	-1.0171	1.0000	-2.3200	1.0000	-0.9587	1.0000	0.9734	1.0000	-2.2909
LOC104913178	0.7123	-0.5458	0.0289	1.5050	1.0000	0.1162	0.0252	1.6255	1.0000	-1.2979	0.7946	0.7664	1.0000	0.2168
LOC104913181	1.0000	0.0000	1.0000	0.0000	1.0000	0.0000	1.0000	0.0000	1.0000	0.0000	1.0000	0.0000	1.0000	0.0000
LOC104913184	0.8249	-3.3412	1.0000	-0.1664	1.0000	-1.0194	1.0000	0.0124	1.0000	0.7852	0.6427	4.1168	0.8939	1.8371
LOC104913189	1.0000	0.0000	1.0000	0.0000	1.0000	0.0000	1.0000	0.0000	1.0000	0.0000	1.0000	0.0000	1.0000	0.0000
LOC104913190	0.0788	2.2256	1.0000	-0.7305	1.0000	-1.0194	1.0000	-0.5495	1.0000	1.3548	0.4428	-1.5855	0.8939	1.8371
LOC104913191	1.0000	-2.4776	0.9062	-1.5359	1.0000	-0.1591	1.0000	0.0046	1.0000	1.2661	1.0000	2.3457	1.0000	1.4453
LOC104913192	0.5086	3.5431	0.6180	-1.9232	1.0000	0.0000	0.2429	-4.0840	1.0000	4.0267	1.0000	-1.3246	1.0000	0.0000

LOC104913193	1.0000	-2.4776	1.0000	0.0000	1.0000	-2.4056	1.0000	2.3240	1.0000	-2.3757	1.0000	0.0000	1.0000	2.3543
LOC104913194	1.0000	0.0000	1.0000	0.0000	1.0000	0.0000	1.0000	0.0000	1.0000	0.0000	1.0000	0.0000	1.0000	0.0000
LOC104913195	1.0000	0.0000	1.0000	0.0000	1.0000	0.0000	1.0000	0.0000	1.0000	0.0000	1.0000	0.0000	1.0000	0.0000
LOC104913196	0.2628	1.6844	0.9691	-0.2997	0.2562	1.6030	1.0000	0.1202	1.0000	1.7858	1.0000	-0.1765	1.0000	0.3138
LOC104913197	1.0000	2.1900	1.0000	-0.1449	1.0000	0.0000	0.9167	1.3833	1.0000	2.2677	1.0000	0.0470	0.7455	3.7355
LOC104913198	1.0000	0.3561	0.6218	-1.9228	0.8309	0.7600	1.0000	-0.3798	1.0000	0.2615	0.8372	-2.0207	1.0000	-0.8694
LOC104913199	0.5280	0.8229	0.8261	0.4350	0.0823	-2.3732	0.0134	-2.4939	1.0000	0.7150	1.0000	0.3409	1.0000	0.6034
LOC104913200	1.0000	0.0000	1.0000	0.0000	1.0000	0.0000	1.0000	0.0000	1.0000	0.0000	1.0000	0.0000	1.0000	0.0000
LOC104913201	1.0000	-2.4788	1.0000	0.0000	1.0000	-2.4061	1.0000	0.0000	1.0000	-2.3771	1.0000	0.0000	1.0000	0.0000
LOC104913202	1.0000	0.0000	1.0000	0.0000	1.0000	0.0000	1.0000	0.0000	1.0000	0.0000	1.0000	0.0000	1.0000	0.0000
LOC104913204	1.0000	0.0000	1.0000	0.0000	1.0000	0.0000	1.0000	0.0000	1.0000	0.0000	1.0000	0.0000	1.0000	0.0000
LOC104913205	0.5384	3.5542	1.0000	-2.3960	1.0000	2.2425	1.0000	-2.3179	1.0000	2.2676	0.8764	-3.6794	1.0000	-2.2888
LOC104913206	1.0000	0.0000	1.0000	-0.1452	1.0000	0.0000	1.0000	-2.3200	1.0000	2.2734	1.0000	2.3480	1.0000	0.0000
LOC104913207	1.0000	0.5832	1.0000	0.0000	1.0000	0.3490	1.0000	2.3256	1.0000	-3.2304	0.6450	-4.0575	1.0000	-1.3086
LOC104913209	0.1913	0.3639	0.2653	0.2318	0.1524	0.3742	0.1050	0.2928	1.0000	-0.0968	0.6054	-0.2155	0.6651	-0.1716
LOC104913210	1.0000	0.0000	0.1384	4.3086	1.0000	2.2470	1.0000	0.0000	1.0000	0.0000	0.4563	4.4217	1.0000	-2.2909
LOC104913211	1.0000	-0.2892	1.0000	-2.3960	0.9361	1.2042	0.6142	1.7721	1.0000	-0.1089	1.0000	-2.2992	1.0000	0.4629
LOC104913214	0.7734	-0.2611	1.0000	0.0301	0.0000	-2.3909	0.8597	-0.1807	1.0000	-0.3195	1.0000	-0.0179	0.0005	1.8980
LOC104913215	1.0000	0.2043	0.5021	1.3057	0.1819	2.0005	0.9786	0.9243	1.0000	-0.1170	1.0000	1.0003	0.6263	-1.1922
LOC104913216	1.0000	0.0000	1.0000	0.0000	1.0000	0.0000	0.7710	3.1752	1.0000	0.0000	1.0000	0.0000	1.0000	3.2081
LOC104913217	0.0604	1.7122	0.0002	2.0753	0.0000	2.9168	0.0001	2.2590	1.0000	0.2525	0.5877	0.6431	0.7793	-0.3848
LOC104913218	0.8441	-0.1615	0.6180	-0.1649	0.0000	1.2699	0.0000	1.3509	1.0000	0.0480	1.0000	0.0552	0.8120	0.1339
LOC104913219	1.0000	-1.1537	1.0000	0.0000	1.0000	-0.1754	1.0000	0.0000	1.0000	-3.2319	1.0000	-2.2992	1.0000	-3.1369
LOC104913220	1.0000	0.0000	1.0000	0.0000	1.0000	0.0000	1.0000	0.0000	1.0000	0.0000	1.0000	0.0000	1.0000	0.0000
LOC104913221	1.0000	2.1850	1.0000	0.0000	0.8033	3.0892	1.0000	0.0000	1.0000	0.0000	1.0000	-2.2958	1.0000	-3.1369
LOC104913222	0.8226	-3.3425	1.0000	-0.1429	0.5476	1.2880	1.0000	0.0062	1.0000	-0.9614	1.0000	2.3480	0.5105	-2.2505
LOC104913223	0.9152	0.3744	0.0002	1.5779	0.0001	1.7451	0.0000	2.1517	1.0000	-0.2934	0.2039	0.9211	1.0000	0.1198
LOC104913227	0.6362	-0.1596	0.9339	-0.0371	0.6094	0.1650	0.0028	0.2603	1.0000	-0.0931	1.0000	0.0420	1.0000	0.0075
LOC104913229	0.3437	0.6303	0.0003	1.6778	0.0000	2.1430	0.0000	2.8245	1.0000	-0.9194	1.0000	0.1416	0.8073	-0.2308
LOC104913230	1.0000	-0.2463	1.0000	0.1161	0.2014	-1.5304	0.7940	-0.5114	1.0000	-0.1296	1.0000	0.2483	0.9561	0.8952
LOC104913231	0.8249	3.0254	0.7674	-3.2499	1.0000	0.0000	0.7710	-3.1633	1.0000	3.1056	1.0000	-3.1468	1.0000	0.0000
LOC104913232	0.1464	-2.8575	0.4154	-1.5158	1.0000	-0.0078	1.0000	0.1720	1.0000	0.0589	1.0000	1.4317	1.0000	0.2436
LOC104913233	1.0000	0.0000	1.0000	0.0000	1.0000	0.0000	1.0000	0.0000	1.0000	0.0000	1.0000	0.0000	1.0000	0.0000
LOC104913234	0.4216	-1.2212	0.1586	-0.8716	1.0000	-0.1965	0.9654	-0.2309	1.0000	0.8603	0.5783	1.2243	0.5525	0.8289
LOC104913240	1.0000	2.1900	1.0000	0.0000	1.0000	0.0000	1.0000	0.0000	1.0000	0.0000	1.0000	-2.2992	1.0000	0.0000
LOC104913241	1.0000	-2.4788	0.7674	-3.2499	1.0000	-2.4061	0.7710	-3.1632	1.0000	0.7305	1.0000	0.0000	1.0000	0.0000
LOC104913242	1.0000	2.1850	1.0000	0.0000	1.0000	0.0000	1.0000	0.0000	1.0000	0.0000	1.0000	-2.2958	1.0000	0.0000
LOC104913243	1.0000	0.0000	1.0000	0.0000	1.0000	0.0000	1.0000	0.0000	1.0000	0.0000	1.0000	0.0000	1.0000	0.0000
LOC104913244	1.0000	0.0000	1.0000	0.0000	1.0000	0.0000	1.0000	0.0000	1.0000	0.0000	1.0000	0.0000	1.0000	0.0000
LOC104913246	1.0000	0.0000	1.0000	0.0000	1.0000	0.0000	1.0000	0.0000	1.0000	0.0000	1.0000	0.0000	1.0000	0.0000
LOC104913247	1.0000	0.0000	1.0000	0.0000	1.0000	2.2470	1.0000	0.0000	1.0000	0.0000	1.0000	0.0000	1.0000	-2.2909
LOC104913248	1.0000	0.0000	1.0000	0.0000	1.0000	0.0000	1.0000	0.0000	1.0000	0.0000	1.0000	0.0000	1.0000	0.0000
LOC104913250	1.0000	0.0000	1.0000	0.0000	1.0000	0.0000	1.0000	0.0000	1.0000	0.0000	1.0000	0.0000	1.0000	0.0000
LOC104913252	1.0000	0.1965	1.0000	0.5243	1.0000	-0.1811	0.4370	-3.6968	1.0000	0.4094	1.0000	0.7559	1.0000	-3.1317
LOC104913253	0.5664	-0.2451	0.6799	-0.1621	0.6168	0.2004	0.0081	0.5126	1.0000	-0.2568	1.0000	-0.1612	1.0000	0.0620
LOC104913254	0.3856	-0.7028	0.0047	-1.2044	0.2274	-0.9050	0.0028	-1.3855	1.0000	0.5493	1.0000	0.0605	1.0000	0.0705
LOC104913255	1.0000	0.0000	0.7674	-3.2499	1.0000	2.2425	0.7710	-3.1632	1.0000	3.1056	1.0000	0.0000	1.0000	-2.2888
LOC104913256	0.9098	-0.1360	0.0066	-0.5917	0.0000	-1.3038	0.0000	-1.3550	1.0000	-0.0625	0.1462	-0.5059	1.0000	-0.1079
LOC104913257	0.0732	-3.2137	0.0953	-2.8836	0.1399	-2.2279	0.0210	-5.0413	1.0000	-0.2927	1.0000	0.0513	1.0000	-3.1344
LOC104913258	1.0000	0.0000	1.0000	0.0000	1.0000	0.0000	1.0000	0.0000	1.0000	0.0000	1.0000	0.0000	1.0000	0.0000
LOC104913259	1.0000	0.0000	0.7674	3.0889	1.0000	0.0000	1.0000	0.0000	1.0000	0.0000	1.0000	3.1946	1.0000	0.0000
LOC104913261	1.0000	0.0000	1.0000	0.0000	1.0000	0.0000	1.0000	0.0000	1.0000	0.0000	1.0000	0.0000	1.0000	0.0000
LOC104913262	1.0000	0.0000	1.0000	0.0000	1.0000	0.0000	1.0000	0.0000	1.0000	0.0000	1.0000	0.0000	1.0000	0.0000
LOC104913263	0.7321	-2.0746	1.0000	-0.6388	1.0000	-0.5753	0.3366	-1.6811	1.0000	0.6473	0.8372	2.1256	1.0000	-0.4561
LOC104913264	0.6612	-0.4697	1.0000	0.1729	0.5916	-0.5401	0.7544	-0.3824	1.0000	-0.2928	1.0000	0.3596	1.0000	-0.1288
LOC104913265	1.0000	-0.2920	0.2535	-4.1743	1.0000	-2.4056	1.0000	-0.3803	1.0000	1.6478	1.0000	-2.2957	0.7455	3.7355
LOC104913266	0.9545	1.0652	1.0000	2.2508	1.0000	-2.4056	1.0000	0.0000	1.0000	-2.3757	1.0000	-1.3254	1.0000	0.0000
LOC104913268	0.3148	3.9252	1.0000	0.6956	0.5080	3.6208	0.9096	1.3850	1.0000	2.2676	1.0000	-0.8603	1.0000	0.0732
LOC104913269	0.4585	-0.6217	0.1964	-0.6827	0.2804	-0.6931	0.2035	-0.6718	1.0000	-0.2833	1.0000	-0.3274	1.0000	-0.2513
LOC104913271	1.0000	-0.3519	1.0000	0.2951	1.0000	0.5465	0.0472	1.7243	1.0000	-0.6232	1.0000	0.0581	1.0000	0.5747
LOC104913274	1.0000	0.0000	1.0000	0.0000	1.0000	0.0000	1.0000	0.0000	1.0000	0.0000	1.0000	0.0000	1.0000	0.0000
LOC104913275	0.8249	3.0254	1.0000	-0.1674	0.2840	3.9988	0.7823	0.9516	1.0000	3.6423	1.0000	0.5854	1.0000	0.6340
LOC104913276	1.0000	0.0000	1.0000	0.0000	1.0000	0.0000	1.0000	0.0000	1.0000	0.0000	1.0000	0.0000	1.0000	0.0000
LOC104913277	0.6135	1.3609	0.7231	1.0549	1.0000	-0.1789	1.0000	0.0031	1.0000	-0.1146	1.0000	-0.4105	1.0000	0.0717
LOC104913278	1.0000	0.1362	0.0074	1.7845	0.0852	-2.1052	1.0000	-0.2071	1.0000	-0.8855	0.6211	0.7724	1.0000	1.0198
LOC104913279	1.0000	-1.1561	1.0000	-1.0017	0.5852	1.2964	1.0000	-0.8416	1.0000	-0.1170	1.0000	0.0495	0.5280	-2.2532
LOC104913280	1.0000	0.0000	1.0000	0.0000	1.0000	0.0000	1.0000	0.0000	1.0000	0.0000	1.0000	0.0000	1.0000	0.0000
LOC104913281	0.0000	-3.6350	0.0000	-4.0387	0.2012	-0.6130	0.0565	-0.4807	1.0000	0.0842	1.0000	-0.3063	0.8297	0.2238
LOC104913282	1.0000	0.0000	0.0520	-4.9414	0.8011	3.0840	0.3662	-1.6778	1.0000	4.7850	1.0000	0.0000	1.0000	0.0718
LOC104913283	0.0998	0.6905	0.0041	0.7785	0.0000	2.2696	0.0000	1.8030	1.0000	0.3111	0.4206	0.4145	0.8937	-0.1488
LOC104913284	0.0648	0.8460	0.0000	1.3279	0.0000	2.8659	0.0000	2.6840	1.0000	-0.1992	0.7851	0.2952	0.1072	-0.3764
LOC104913285	1.0000	0.0000	1.0000	2.2508	1.0000	0.0000	1.0000	0.0000	1.0000	0.0000	1.0000	2.3457	1.0000	0.0000
LOC104913287	0.5391	-3.8767	0.4383	-1.5100	1.0000	-0.7166	1.0000	-0.3926	1.0000	1.2141	0.8759	3.7343	0.7908	1.5495
LOC104913288	0.2837	-0.5368	0.2653	0.3215	0.0213	-0.8879	0.0501	-0.5746	1.0000	0.0579	0.0082	0.9336	0.6865	0.3791
LOC104913289	0.0055	-3.8342	0.0019	-3.1102	0.0748	-1.9403	0.0871	-1.4575	1.0000	0.1473	1.0000	0.8963	1.0000	0.6363
LOC104913290	0.8326	-0.3585	1.0000	-0.0549	0.1187	0.8792	0.5634	0.4506	1.0000	-0.1957	1.0000	0.1214	0.4158	-0.6210
LOC104913291	0.3242	-1.2236	0.1004	-1.6159	0.4205	-0.8796	0.9802	-0.3338	1.0000	-0.0249	1.0000	-0.4062	1.0000	0.5265
LOC104913293	0.5378	3.5350	0.4025	1.9118	1.0000	2.2470	1.0000	-2.3179	1.0000	2.2676	1.0000	0.7511	1.0000	-2.2909
LOC104913294	0.5086	3.5431	0.7223	1.0562	0.0535	4.7653	0.3714	1.7021	1.0000	3.1056	1.0000	0.7497	1.0000	0.0796
LOC104913296	1.0000	0.0000	1.0000	0.0000	1.0000	2.2425	1.0000	0.0000	1.0000	0.0000	1.0000	0.0000	1.0000	-2.2888
LOC104913297	1.0000	0.0000	1.0000	-2.3960	1.0000	0.0000	1.0000	-2.3179	1.0000	2.2676	1.0000	0.0000	1.0000	0.0000
LOC104913298	0.2447	-1.2377	0.0882	-1.2285	0.9361	-0.4063	0.6624	-0.4712	1.0000	0.3497	1.0000	0.3749	1.0000	0.2859
LOC104913299	1.0000	2.1850	1.0000	0.0000	1.0000	0.0000	1.0000	0.0000	1.0000	0.0000	1.0000	-2.2957	1.0000	0.0000
LOC104913300	0.2677	0.3273	0.1014	0.3855	0.0041	-0.7973	0.0122	-0.6160	1.0000	-0.1783	1.0000	-0.1087	1.0000	0.0089
LOC104913301	0.6926	-2.0812	0.7674	-3.2499	0.6928	0.7646	1.0000	0.5384	1.0000	-1.0429	1.0000	-2.2993	0.7028	-1.2669
LOC104913302	1.0000	0.0000	1.0000	0.0000	1.0000	0.0000	1.0000	0.0000	1.0000	0.0000	1.0000	0.0000	1.0000	0.0000
LOC104913303	1.0000	0.0000	1.0000	0.0000	1.0000	2.2425	1.0000	0.0000	1.0000	0.0000	1.0000	0.0000	1.0000	-2.2888
LOC104913304	0.1348	0.2960	0.0001	0.3801	0.8564	0.0925	0.0085	0.2579	1.0000	-0.0353	1.0000	0.0611	0.5138	0.1349
LOC104913305	0.1947	-0.4242	0.0014	-0.4640	0.0063	-0.7840	0.0873	-0.2831	1.0000	-0.0085	1.0000	-0.0362	0.0206	0.4972
LOC104913308	1.0000	0.0000	0.2526	2.1629	1.0000	2.2470	1.0000	0.8545	1.0000	2.2676	0.3185	4.6738	1.0000	0.9165
LOC104913309	0.1811	1.0690	0.4511	0.5346	1.0000	-0.1947	0.0808	-1.4572	1.0000	0.4848	1.0000	-0.0362	0.8532	-0.7699
LOC104913310	0.9545	0.0966	1.0000	0.0507	0.2892	0.2822	0.0020	0.4378	1.0000	-0.0652	1.0000	-0.0982	0.9619	0.0971
LOC104913311	0.0028	1.4292	0.0000	1.2266	0.0000	2.1731	0.0000	1.9558	1.0000	0.1321	1.0000	-0.0570	1.0000	-0.0795

LOC104913312	0.0000	-4.6983	0.0000	-2.7265	0.0058	1.1036	0.0176	0.8222	1.0000	0.0906	0.5697	2.0826	0.9865	-0.1817
LOC104913313	1.0000	0.0000	1.0000	0.0000	1.0000	0.0000	0.7701	3.1732	1.0000	0.0000	1.0000	0.0000	1.0000	3.2066
LOC104913314	1.0000	-0.3482	0.1455	-1.0877	0.0314	-3.3496	0.0079	-2.1723	1.0000	0.6477	1.0000	-0.0757	0.8937	1.8364
LOC104913315	1.0000	-0.3257	1.0000	-2.3985	1.0000	-0.1765	1.0000	0.0057	1.0000	-0.9587	1.0000	-3.1383	1.0000	-0.7804
LOC104913316	0.0490	0.9371	0.0037	1.0620	0.0004	1.3912	0.0000	1.5171	1.0000	-0.1799	1.0000	-0.0384	1.0000	-0.0429
LOC104913317	0.1856	-4.5762	1.0000	-2.3985	1.0000	-0.8796	0.3950	2.0766	1.0000	-2.1852	1.0000	0.0000	1.0000	0.7679
LOC104913318	1.0000	0.0699	0.2384	-0.4496	0.1706	-0.5941	0.4447	-0.3046	1.0000	-0.0146	0.4220	-0.5228	0.8425	0.2791
LOC104913321	1.0000	-1.2521	1.0000	0.3672	0.3991	1.2125	0.3672	1.6934	1.0000	-1.0414	1.0000	0.5871	1.0000	-0.5564
LOC104913322	1.0000	0.0000	1.0000	0.0000	1.0000	0.0000	1.0000	0.0000	1.0000	0.0000	1.0000	0.0000	1.0000	0.0000
LOC104913323	1.0000	0.0000	1.0000	0.0000	1.0000	0.0000	1.0000	0.0000	1.0000	0.0000	1.0000	0.0000	1.0000	0.0000
LOC104913324	0.5153	0.1535	0.4868	-0.0794	0.0000	-0.9137	0.0000	-0.8032	1.0000	0.0187	0.1469	-0.2020	0.4370	0.1347
LOC104913325	0.3954	1.3914	0.3952	1.0475	0.2909	1.3356	0.8636	0.6593	1.0000	0.1803	1.0000	-0.1549	1.0000	-0.4913
LOC104913326	0.8226	3.0199	1.0000	0.0000	1.0000	0.0000	1.0000	0.0000	1.0000	0.0000	1.0000	-3.1429	1.0000	0.0000
LOC104913327	1.0000	0.0000	1.0000	0.0000	1.0000	0.0000	1.0000	2.3240	1.0000	0.0000	1.0000	0.0000	1.0000	2.3543
LOC104913328	0.0697	0.3599	0.0203	0.2877	0.0006	0.5903	0.0000	0.5915	1.0000	-0.1163	0.4857	-0.1756	0.6977	-0.1082
LOC104913329	0.0002	1.3690	0.0000	1.3035	0.0000	1.6358	0.0000	1.4920	1.0000	0.1001	1.0000	0.0495	1.0000	-0.0349
LOC104913330	0.0415	-3.3586	0.0000	-6.9130	0.0058	1.8382	0.2460	0.5879	1.0000	1.3333	1.0000	-2.2957	1.0000	0.0831
LOC104913331	0.0134	-5.3976	0.0002	-6.0226	0.0004	2.0910	0.0354	1.1320	1.0000	0.5910	1.0000	0.0000	0.8350	-0.3633
LOC104913333	0.1876	4.2322	0.1464	4.3063	1.0000	2.2470	0.4370	3.7037	1.0000	0.0000	1.0000	0.0568	1.0000	1.4482
LOC104913334	1.0000	0.0000	1.0000	0.0000	1.0000	0.0000	1.0000	2.3241	1.0000	0.0000	1.0000	0.0000	1.0000	2.3543
LOC104913336	0.0008	0.9928	0.0933	0.4841	0.9540	-0.1533	0.0099	-0.6686	1.0000	0.3508	1.0000	-0.1461	1.0000	-0.1613
LOC104913337	1.0000	2.1850	0.7666	3.0922	1.0000	0.0000	1.0000	2.3256	1.0000	0.0000	1.0000	0.9006	1.0000	2.3554
LOC104913338	1.0000	0.0000	1.0000	0.0000	1.0000	0.0000	1.0000	0.0000	1.0000	0.0000	1.0000	0.0000	1.0000	0.0000
LOC104913339	0.0300	1.2109	0.0032	1.1445	0.1742	0.9262	0.0000	1.8288	1.0000	0.0344	1.0000	-0.0195	0.0149	0.9393
LOC104913340	0.9120	0.1832	0.0089	0.7525	0.0047	-1.1753	0.0479	-0.7762	1.0000	0.0177	0.0953	0.5957	0.7709	0.4186
LOC104913341	1.0000	0.1965	1.0000	-0.4018	1.0000	-1.0191	0.2141	-2.5445	1.0000	1.5872	1.0000	1.0046	1.0000	0.0661
LOC104913344	1.0000	-0.1537	0.6887	0.2800	0.0138	0.8306	0.0000	1.7581	1.0000	-0.6409	1.0000	-0.1935	0.5275	0.2946
LOC104913345	0.9545	-0.1516	0.2592	0.3548	0.5494	-0.3021	0.3243	-0.3491	1.0000	-0.2338	0.8354	0.2839	0.8120	-0.2755
LOC104913346	1.0000	-0.2892	1.0000	0.0000	1.0000	-2.4061	1.0000	0.0000	1.0000	-2.3771	1.0000	-2.2993	1.0000	0.0000
LOC104913349	1.0000	-0.0056	0.2397	0.4907	0.0036	1.1346	0.0017	0.9621	1.0000	0.1888	0.2127	0.7006	1.0000	0.0235
LOC104913350	0.3149	0.6559	1.0000	-0.0231	0.5362	0.5026	0.0939	-0.7694	1.0000	0.5356	1.0000	-0.1336	0.3458	-0.7298
LOC104913351	1.0000	0.0596	0.1216	1.0437	0.5906	-0.6609	0.9419	-0.4403	1.0000	-0.8265	1.0000	0.1709	0.9124	-0.6034
LOC104913352	1.0000	0.0000	1.0000	0.0000	1.0000	0.0000	1.0000	0.0000	1.0000	0.0000	1.0000	0.0000	1.0000	0.0000
LOC104913353	0.6273	-0.2054	0.0014	-0.5880	0.0020	-0.7786	0.0000	-0.8758	1.0000	-0.0528	0.1446	-0.4235	0.9835	-0.1456
LOC104913356	1.0000	-0.0300	0.6580	-0.0539	0.7628	-0.1001	0.4542	-0.0718	1.0000	0.0050	1.0000	-0.0068	1.0000	0.0382
LOC104913357	0.7820	0.4295	0.0002	1.6771	0.0000	2.6113	0.0000	2.5359	1.0000	-0.2085	0.0619	1.0524	0.6575	-0.2795
LOC104913358	0.0477	-1.0130	0.0212	-0.9253	0.0138	-1.1914	0.1012	-0.6529	1.0000	0.0034	1.0000	0.1024	0.6107	0.5459
LOC104913359	0.4153	0.3378	0.9930	0.0746	0.0106	-0.8861	0.0000	-1.2990	1.0000	0.0844	1.0000	-0.1650	0.8110	-0.3243
LOC104913360	0.5891	-0.4606	0.4366	-0.4867	1.0000	-0.0224	0.2784	0.5413	1.0000	-0.0853	1.0000	-0.0996	0.6564	0.4827
LOC104913361	0.0001	-0.6834	0.0000	-0.8375	0.0000	-1.0547	0.0000	-0.7962	1.0000	0.0589	1.0000	-0.0825	0.0539	0.3238
LOC104913362	1.0000	0.0000	1.0000	0.0000	1.0000	0.0000	1.0000	0.0000	1.0000	0.0000	1.0000	0.0000	1.0000	0.0000
LOC104913363	1.0000	2.1850	0.4431	-3.7865	1.0000	0.0000	1.0000	0.0072	1.0000	3.6396	1.0000	-2.2957	0.7287	3.7387
LOC104913364	1.0000	0.0000	1.0000	0.0000	1.0000	0.0000	1.0000	0.0000	1.0000	0.0000	1.0000	0.0000	1.0000	0.0000
LOC104913365	0.0065	-2.6714	0.0429	-2.0140	0.2615	-1.0570	1.0000	0.0091	1.0000	-1.1814	1.0000	-0.5010	1.0000	-0.0978
LOC104913366	0.0636	1.0977	0.0465	1.1040	0.0550	1.0606	0.0000	1.6410	1.0000	0.1495	1.0000	0.1730	0.0665	0.7433
LOC104913367	1.0000	0.5945	1.0000	-1.0017	0.8011	-3.2629	0.7701	-3.1666	1.0000	-0.1179	1.0000	-1.7225	1.0000	0.0000
LOC104913368	1.0000	-2.4788	1.0000	-2.3960	1.0000	-0.1630	1.0000	0.8567	1.0000	-0.1089	1.0000	0.0000	1.0000	0.9187
LOC104913369	0.0126	0.6214	0.0000	0.6715	0.0000	1.9616	0.0000	1.6768	1.0000	0.1537	0.5568	0.2172	0.6331	-0.1243
LOC104913373	0.2611	1.1356	0.4185	0.7408	0.0011	2.0919	0.0187	1.3858	1.0000	0.3186	1.0000	-0.0651	0.8728	-0.3807
LOC104913374	1.0000	0.0029	0.6097	-0.1025	0.7261	0.1383	0.8830	0.0633	1.0000	0.0663	1.0000	-0.0255	1.0000	-0.0035
LOC104913375	0.7531	0.5043	0.0036	1.9996	0.7079	-0.6055	1.0000	0.4071	1.0000	-1.0994	1.0000	0.4053	1.0000	-0.0851
LOC104913376	0.0000	-0.9725	0.0000	-0.9841	0.0000	-1.4744	0.0000	-1.5241	1.0000	-0.0189	1.0000	-0.0178	1.0000	-0.0638
LOC104913377	0.3370	0.5498	0.0017	1.1580	0.0000	1.6053	0.0000	1.8252	1.0000	-0.2955	0.9094	0.3237	1.0000	-0.0709
LOC104913378	1.0000	0.0000	1.0000	0.0000	0.4809	3.6101	1.0000	0.0000	1.0000	0.0000	1.0000	0.0000	0.7287	-3.6634
LOC104913379	0.8553	-0.2832	0.6344	-0.2566	1.0000	-0.0479	0.3435	0.3175	1.0000	0.0591	1.0000	0.0959	0.3638	0.4266
LOC104913381	0.9545	1.0644	1.0000	2.2508	0.4424	1.8933	0.2426	4.0910	1.0000	-2.3771	1.0000	-1.3254	1.0000	-0.2278
LOC104913382	1.0000	0.0000	1.0000	2.2508	1.0000	2.2470	1.0000	0.0000	1.0000	0.0000	1.0000	2.3457	1.0000	-2.2909
LOC104913383	1.0000	0.0000	1.0000	0.0000	1.0000	0.0000	1.0000	0.0000	1.0000	0.0000	1.0000	0.0000	1.0000	0.0000
LOC104913384	0.0006	1.5940	0.0001	1.5903	0.0000	2.5819	0.0000	2.3854	1.0000	-0.1358	1.0000	-0.1258	0.5113	-0.3259
LOC104913385	0.5036	-0.2215	0.4515	-0.1612	0.3738	-0.2539	0.1999	-0.2514	1.0000	-0.0534	1.0000	0.0176	1.0000	-0.0464
LOC104913386	0.8093	0.7523	0.7695	0.6939	0.5862	0.9080	0.2820	1.0785	1.0000	0.3381	1.0000	0.2956	1.0000	0.5154
LOC104913387	0.9441	0.7827	0.0525	1.7599	1.0000	0.3655	0.9786	-0.9114	1.0000	-0.1215	0.8607	0.8712	0.7892	-1.3988
LOC104913389	0.5086	-3.8790	0.9754	-1.0835	0.6394	1.1498	1.0000	0.3118	1.0000	0.2637	1.0000	3.1976	1.0000	-0.5730
LOC104913390	0.0000	3.7308	0.0000	3.8706	0.0000	3.8318	0.0000	3.5422	1.0000	0.2615	0.8014	0.4112	1.0000	-0.0263
LOC104913391	0.0020	-0.5786	0.0000	-0.8214	0.5474	0.1602	0.1863	-0.1650	1.0000	0.1536	1.0000	-0.0762	0.4132	-0.1654
LOC104913392	1.0000	-0.2931	1.0000	2.2533	1.0000	-2.4061	1.0000	2.3241	1.0000	-2.3771	1.0000	0.0514	1.0000	2.3543
LOC104913393	0.0000	-0.6712	0.0000	-0.9624	0.0002	-0.5918	0.0000	-0.6851	1.0000	0.0738	0.3768	-0.2052	1.0000	-0.0138
LOC104913394	0.1877	4.4789	1.0000	0.0406	0.0691	4.7549	1.0000	0.0072	1.0000	4.5797	1.0000	0.2742	1.0000	-0.1341
LOC104913397	0.0547	2.3516	0.0009	1.8703	0.2518	1.5982	0.4321	0.8217	1.0000	1.3834	0.3252	0.9140	0.9874	0.6126
LOC104913398	0.2854	-0.3109	0.0116	-0.3701	0.0217	0.5042	0.0130	0.3394	1.0000	0.0898	1.0000	0.0438	1.0000	-0.0700
LOC104913399	0.0038	0.4566	0.0000	0.4635	0.0000	-0.7255	0.0000	-0.6717	1.0000	-0.1701	0.1732	-0.1507	0.5763	-0.1103
LOC104913401	1.0000	0.0000	1.0000	0.0000	1.0000	0.0000	1.0000	0.0000	1.0000	0.0000	1.0000	0.0000	1.0000	0.0000
LOC104913402	0.7256	-0.4124	0.1103	-1.1027	0.0056	-1.8086	0.0305	-1.4866	1.0000	-0.4142	0.3566	-1.0931	1.0000	-0.0857
LOC104913403	1.0000	0.0000	1.0000	0.0000	1.0000	2.2425	1.0000	0.0000	1.0000	0.0000	1.0000	0.0000	1.0000	-2.2888
LOC104913404	1.0000	0.0000	1.0000	0.0000	1.0000	0.0000	1.0000	0.0000	1.0000	0.0000	1.0000	0.0000	1.0000	0.0000
LOC104913405	1.0000	0.0000	1.0000	0.0000	1.0000	0.0000	1.0000	0.0000	1.0000	0.0000	1.0000	0.0000	1.0000	0.0000
LOC104913406	1.0000	-2.4788	0.7674	-3.2499	1.0000	-2.4061	1.0000	-0.8404	1.0000	0.7305	1.0000	0.0000	1.0000	2.3543
LOC104913409	0.6448	-0.9298	0.8072	-0.4359	1.0000	0.2944	0.1605	-1.5200	1.0000	-0.1346	1.0000	0.3713	0.0789	-1.9427
LOC104913410	1.0000	0.0000	1.0000	-2.3985	1.0000	0.0000	1.0000	-2.3200	1.0000	2.2734	1.0000	0.0000	1.0000	0.0000
LOC104913411	0.0004	-0.5666	0.0000	-0.6366	1.0000	0.0179	0.9786	0.0299	1.0000	-0.0159	0.9729	-0.0737	1.0000	0.0007
LOC104913412	0.8329	-0.1372	1.0000	0.0155	0.6690	-0.1911	0.6439	0.1304	1.0000	-0.2078	1.0000	-0.0411	1.0000	0.1214
LOC104913413	0.2251	-0.5538	0.4460	-0.2575	1.0000	0.1086	0.5813	0.2042	1.0000	0.0234	0.7323	0.3315	1.0000	0.1223
LOC104913414	1.0000	0.0040	0.7351	0.1421	0.0000	1.1039	0.0000	0.9729	1.0000	0.1186	0.6629	0.2707	1.0000	-0.0056
LOC104913417	0.2621	1.0519	0.0007	2.6070	0.0000	2.3037	0.0000	3.8349	1.0000	-1.3981	1.0000	0.1629	1.0000	0.1337
LOC104913418	0.0071	-0.8767	0.0005	-0.6293	0.1216	-0.4679	0.0086	-0.4934	1.0000	-0.0874	1.0000	0.1723	1.0000	-0.1103
LOC104913419	0.0379	-0.7819	0.0635	-0.4617	0.0237	-0.6979	0.0428	-0.4780	1.0000	-0.0995	0.9974	0.2340	1.0000	0.1231
LOC104913420	0.8249	3.0254	0.7674	3.0889	1.0000	0.0000	1.0000	2.3256	1.0000	0.0000	1.0000	0.0496	1.0000	2.3554
LOC104913421	1.0000	0.0000	1.0000	2.2533	1.0000	0.0000	1.0000	2.3256	1.0000	0.0000	1.0000	2.3480	1.0000	2.3554
LOC104913422	0.0196	1.7495	0.0003	2.3600	0.8249	0.6725	0.0932	1.4693	1.0000	-0.3398	1.0000	0.2782	1.0000	0.4600
LOC104913423	0.3148	3.9252	1.0000	0.0000	1.0000	2.2470	1.0000	0.0000	1.0000	0.0000	0.6450	-4.0575	1.0000	-2.2909
LOC104913424	0.2809	1.3587	0.2544	2.1622	0.7660	-1.4099	1.0000	-2.3179	1.0000	-2.1881	0.3764	-1.4024	1.0000	-3.1317
LOC104913425	0.2744	1.2623	0.5112	0.8944	0.8249	-1.1362	0.8238	-0.7639	1.0000	0.0878	1.0000	-0.2727	1.0000	0.4637
LOC104913426	0.3010	0.8106	0.3687	0.6935	0.0550	-2.0350	0.1084	-1.6334	1.0000	-0.3317	1.0000	-0.4374	1.0000	0.0761

LOC104913427	0.5086	-3.8790	1.0000	-2.3960	1.0000	-0.7128	1.0000	0.0062	1.0000	-1.4938	1.0000	0.0000	1.0000	-0.7795
LOC104913428	0.5086	3.5456	1.0000	0.6959	0.2231	4.3199	0.9096	1.3849	1.0000	2.2734	1.0000	-0.4744	1.0000	-0.6204
LOC104913429	1.0000	0.0000	1.0000	0.0000	1.0000	0.0000	1.0000	0.0000	1.0000	0.0000	1.0000	0.0000	1.0000	0.0000
LOC104913430	0.9671	-1.6927	0.7338	1.0561	1.0000	-0.1833	1.0000	-0.8442	1.0000	-0.6421	0.8372	2.1278	1.0000	-1.3089
LOC104913431	0.4806	0.6792	0.5655	0.5285	0.0075	1.5275	0.0000	1.8260	1.0000	-0.1304	1.0000	-0.2693	1.0000	0.1711
LOC104913432	0.0702	0.6567	0.0003	0.9802	0.0000	-1.8843	0.0336	-0.8350	1.0000	-0.6617	0.6450	-0.3244	0.9853	0.3966
LOC104913433	0.2858	1.4448	0.0222	1.9274	0.8287	0.7574	0.8219	0.7775	1.0000	0.2648	0.8875	0.7587	1.0000	0.2919
LOC104913435	1.0000	0.5833	1.0000	-0.6953	0.1834	1.8535	0.1899	1.6642	1.0000	0.4129	1.0000	-0.8603	1.0000	0.2274
LOC104913437	1.0000	0.0000	1.0000	0.0000	1.0000	2.2425	1.0000	0.0000	1.0000	0.0000	1.0000	0.0000	1.0000	-2.2889
LOC104913439	0.0000	1.7202	0.0000	2.1860	0.0000	2.0977	0.0000	1.9248	1.0000	-0.4278	1.0000	0.0520	0.0046	-0.5955
LOC104913440	1.0000	0.0547	0.7236	-0.7406	0.1685	1.1332	0.0001	2.0555	1.0000	-0.1294	0.8607	-0.9130	0.2735	0.7988
LOC104913441	0.5421	0.1506	1.0000	-0.0017	0.0009	-0.5621	0.0000	-0.4493	1.0000	0.1195	1.0000	-0.0206	0.1158	0.2378
LOC104913442	0.6941	0.5757	0.3657	-0.6085	0.6263	0.5476	0.0044	-1.5166	1.0000	1.2774	1.0000	0.1050	0.6062	-0.7803
LOC104913443	0.9298	-0.5605	0.7959	-0.4345	1.0000	-0.4017	0.6018	-0.7503	1.0000	0.6302	1.0000	0.7731	1.0000	0.2895
LOC104913444	1.0000	0.0000	1.0000	0.0000	1.0000	0.0000	1.0000	0.0000	1.0000	0.0000	1.0000	0.0000	1.0000	0.0000
LOC104913445	0.5779	1.3527	1.0000	0.0398	1.0000	0.3490	0.7713	-0.9365	1.0000	1.3515	1.0000	0.0593	1.0000	0.0749
LOC104913446	0.0028	-2.6048	0.0000	-2.2531	0.9257	-0.3107	1.0000	0.0416	1.0000	0.0056	1.0000	0.3735	0.8426	0.3684
LOC104913447	0.2384	-2.6422	0.1518	-2.2062	0.3407	1.1837	0.4160	0.8204	1.0000	0.4355	1.0000	0.9035	1.0000	0.0799
LOC104913449	0.9932	-0.2219	1.0000	0.1621	0.3951	-0.6465	0.0699	0.7687	1.0000	-0.1323	1.0000	0.2651	0.0137	1.2865
LOC104913452	0.0003	1.1426	0.0000	1.4521	0.0000	1.5123	0.0000	1.3717	1.0000	-0.1733	1.0000	0.1503	0.4453	-0.3082
LOC104913453	0.6492	-0.2722	0.0008	-0.7375	0.0002	-1.2953	0.0000	-1.1199	1.0000	0.1710	0.8297	-0.2796	0.7365	0.3556
LOC104913454	1.0000	0.0000	1.0000	0.0000	1.0000	0.0000	1.0000	0.0000	1.0000	0.0000	1.0000	0.0000	1.0000	0.0000
LOC104913455	1.0000	2.1900	1.0000	0.0000	0.8033	3.0789	1.0000	0.0000	1.0000	0.0000	1.0000	-2.2993	1.0000	-3.1316
LOC104913456	1.0000	0.0000	1.0000	0.0000	1.0000	0.0000	1.0000	0.0000	1.0000	0.0000	1.0000	0.0000	1.0000	0.0000
LOC104913457	1.0000	-1.1562	0.7674	3.0956	0.8011	-3.2629	1.0000	0.0000	1.0000	-3.2304	1.0000	0.9035	1.0000	0.0000
LOC104913458	0.4429	-0.6509	0.7977	-0.3991	0.9682	0.2112	0.0314	0.9989	1.0000	-0.4889	1.0000	-0.2255	0.9757	0.3022
LOC104913459	0.9650	1.0595	0.5064	1.3043	0.2858	2.1452	0.1125	2.1968	1.0000	0.7415	1.0000	1.0024	0.9983	0.7945
LOC104913460	0.0140	2.4895	0.2495	0.8094	0.0033	2.5813	0.6215	0.5463	1.0000	1.9015	1.0000	0.2389	1.0000	-0.1281
LOC104913461	1.0000	0.0000	1.0000	0.0000	1.0000	2.2425	1.0000	0.0000	1.0000	0.0000	1.0000	0.0000	1.0000	-2.2889
LOC104913462	0.0000	-3.6964	0.0036	-2.4273	1.0000	-0.1664	0.0000	2.3852	1.0000	-1.2255	1.0000	0.0569	0.0000	1.3310
LOC104913463	1.0000	-1.1585	1.0000	-2.3985	0.0892	2.2608	0.0002	3.9514	1.0000	-0.9587	1.0000	-2.2957	0.7915	0.7312
LOC104913466	1.0000	0.0256	0.0492	-0.3083	0.1404	-0.4319	0.0053	-0.3961	1.0000	-0.1423	0.0139	-0.4629	1.0000	-0.0984
LOC104913467	1.0000	0.0000	1.0000	0.0000	1.0000	0.0000	1.0000	0.0000	1.0000	0.0000	1.0000	0.0000	1.0000	0.0000
LOC104913468	1.0000	0.0000	1.0000	0.0000	1.0000	0.0000	1.0000	0.0000	1.0000	0.0000	1.0000	0.0000	1.0000	0.0000
LOC104913469	0.0002	2.6197	0.0001	2.5774	0.0920	1.6806	0.5659	0.8562	1.0000	0.1198	1.0000	0.0938	0.6722	-0.6952
LOC104913470	0.0000	6.4298	0.0000	6.7320	0.0006	6.1042	0.0407	4.8626	1.0000	0.0000	1.0000	0.2810	0.3341	-1.2562
LOC104913472	0.5469	1.0842	1.0000	0.2177	0.7628	-1.4078	1.0000	0.3899	1.0000	-0.8154	0.3444	-1.6835	1.0000	0.9893
LOC104913473	1.0000	-0.3364	1.0000	-1.0051	0.4809	-3.7970	1.0000	-0.8442	1.0000	-0.6479	1.0000	-1.3254	1.0000	2.3543
LOC104913477	0.1421	-0.3183	0.4631	-0.1469	0.0000	-1.4402	0.0000	-0.9709	1.0000	-0.2346	1.0000	-0.0514	0.4791	0.2396
LOC104913478	1.0000	-0.2882	0.7674	-3.2570	1.0000	0.6783	0.7710	-3.1693	1.0000	0.7415	1.0000	-2.2993	1.0000	-3.1344
LOC104913482	1.0000	0.2007	0.9062	1.2214	1.0000	-1.0191	1.0000	0.0045	1.0000	-0.9587	1.0000	0.0569	1.0000	0.0652
LOC104913486	0.2050	0.5789	0.0207	0.7666	0.8745	0.2403	0.9903	0.1519	1.0000	-0.2758	1.0000	-0.0760	0.7395	-0.3635
LOC104913488	1.0000	-0.2892	1.0000	0.0000	0.4584	1.8985	0.7710	3.1710	1.0000	-2.3771	1.0000	-2.2993	0.9877	-1.1503
LOC104913489	0.4274	1.1899	0.7934	0.6112	1.0000	0.2704	0.4106	-1.3375	1.0000	0.5250	1.0000	-0.0386	0.8717	-1.0804
LOC104913491	1.0000	-0.1008	0.5379	1.0863	0.2942	-1.4648	1.0000	0.5632	1.0000	-1.3975	1.0000	-0.1998	1.0000	0.6338
LOC104913492	1.0000	-2.4776	0.2535	-4.1743	1.0000	-2.4056	1.0000	-0.3794	1.0000	1.6478	1.0000	0.0000	0.7287	3.7387
LOC104913493	0.6927	-0.8023	0.0433	-1.5081	0.4910	-0.8288	0.0569	-1.5385	1.0000	0.5655	1.0000	-0.1266	1.0000	-0.1363
LOC104913496	0.3091	-0.4118	0.0005	0.7271	0.0000	1.5105	0.0000	2.2387	1.0000	-0.5581	0.0311	0.5938	0.5729	0.1789
LOC104913497	1.0000	0.0000	1.0000	0.0000	1.0000	0.0000	1.0000	0.0000	1.0000	0.0000	1.0000	0.0000	1.0000	0.0000
LOC104913498	0.0008	0.7952	0.0003	0.5111	0.9361	0.1052	0.6976	0.1142	1.0000	0.1309	0.8661	-0.1421	0.8422	0.1447
LOC104913499	0.0224	2.7559	0.0557	1.6229	0.0115	2.8936	0.0203	1.8829	1.0000	1.0806	1.0000	-0.0168	1.0000	0.0853
LOC104913500	1.0000	0.0000	1.0000	2.2508	1.0000	0.0000	1.0000	0.0000	1.0000	0.0000	1.0000	2.3457	1.0000	0.0000
LOC104913501	0.5696	1.3088	1.0000	-0.5563	0.0529	2.0392	0.2386	1.4119	1.0000	0.2632	0.5784	-1.5974	1.0000	-0.3590
LOC104913502	0.4597	0.4776	0.0007	1.2477	0.3071	0.5345	0.2037	0.6043	1.0000	-0.4589	0.8759	0.3223	0.7287	-0.3845
LOC104913504	0.0001	0.6504	0.0000	0.6401	0.0000	-0.9200	0.0000	-1.0476	1.0000	0.1750	0.3407	0.1768	1.0000	0.0520
LOC104913505	1.0000	0.0000	1.0000	0.0000	1.0000	0.0000	1.0000	0.0000	1.0000	0.0000	1.0000	0.0000	1.0000	0.0000
LOC104913507	1.0000	0.0000	1.0000	0.0000	1.0000	0.0000	1.0000	0.0000	1.0000	0.0000	1.0000	0.0000	1.0000	0.0000
LOC104913508	1.0000	0.0000	1.0000	2.2533	1.0000	0.0000	1.0000	2.3256	1.0000	0.0000	1.0000	2.3481	1.0000	2.3554
LOC104913510	0.0001	-1.3408	0.0000	-0.9176	0.7314	-0.2054	1.0000	-0.0048	1.0000	-0.0090	0.3807	0.4229	0.6865	0.1937
LOC104913512	0.0962	-0.9352	0.0000	-0.9344	0.0000	-2.2067	0.0000	-1.2639	1.0000	0.4729	0.4114	0.4891	0.0000	1.4114
LOC104913513	0.1050	-1.2430	0.6215	-0.2576	0.1459	-1.1491	0.7170	-0.2347	1.0000	0.3687	0.0116	1.3709	0.0117	1.2927
LOC104913514	0.3641	-4.2658	1.0000	-0.6954	0.9301	0.7520	0.4085	1.3514	1.0000	-0.5105	1.0000	3.1946	1.0000	0.0828
LOC104913515	1.0000	-0.3227	0.6182	1.6074	0.7679	1.0340	1.0000	0.8547	1.0000	-0.9587	1.0000	0.9738	0.9863	-1.1453
LOC104913516	1.0000	-2.4788	1.0000	0.6944	0.5084	1.8779	1.0000	-2.3200	1.0000	-0.1045	1.0000	3.1976	0.4068	-4.3405
LOC104913517	1.0000	-0.8591	1.0000	0.3659	0.0544	2.3181	0.0195	2.5649	1.0000	-0.6504	1.0000	0.5860	1.0000	-0.3947
LOC104913519	0.2204	1.8445	1.0000	0.1369	0.7663	1.0380	0.0881	-2.7157	1.0000	1.7488	1.0000	0.0608	0.6865	-1.9994
LOC104913520	0.7801	0.1791	0.0000	0.8463	0.0000	-2.0015	0.0000	-1.4972	1.0000	-0.0511	0.0025	0.6273	0.6536	0.4579
LOC104913521	1.0000	0.0367	0.2738	-0.2964	0.4385	0.3183	0.5066	0.2020	1.0000	0.1905	1.0000	-0.1307	1.0000	0.0801
LOC104913523	1.0000	-0.1448	0.0000	0.5810	0.0007	-1.4887	0.0000	-1.1403	1.0000	0.4507	0.0000	1.1924	0.0032	0.8030
LOC104913524	1.0000	0.1953	0.7674	-3.2570	1.0000	-1.0194	1.0000	-0.8429	1.0000	-0.1113	0.8785	-3.6647	1.0000	0.0661
LOC104913525	0.4144	-0.4159	0.1574	-0.4475	0.0443	-0.7999	0.3305	-0.3387	1.0000	-0.0113	1.0000	-0.0324	0.4563	0.4537
LOC104913526	0.9545	1.0672	1.0000	2.2508	1.0000	-0.1597	1.0000	0.0000	1.0000	-2.3771	1.0000	-1.3266	1.0000	-2.2909
LOC104913527	0.0000	-2.5795	0.0000	-2.3139	0.5460	0.3692	0.0121	0.8996	1.0000	-0.3864	1.0000	-0.1086	1.0000	0.1482
LOC104913528	0.3746	-1.3021	0.0943	-2.8827	1.0000	-0.0327	0.7710	0.5777	1.0000	-0.1304	1.0000	-1.7141	1.0000	0.4851
LOC104913529	1.0000	-0.0872	1.0000	-0.1755	0.8141	-1.4015	0.4263	-1.3333	1.0000	0.5142	1.0000	0.4538	1.0000	0.6041
LOC104913531	0.5252	1.2831	0.3026	1.1599	1.0000	0.3706	0.5238	0.9725	1.0000	0.1747	1.0000	0.0623	0.9947	0.7894
LOC104913532	1.0000	0.0000	1.0000	0.0000	1.0000	0.0000	1.0000	0.0000	1.0000	0.0000	1.0000	0.0000	1.0000	0.0000
LOC104913533	1.0000	0.5464	1.0000	-1.0017	0.0291	3.1158	0.0119	2.6698	1.0000	0.7365	1.0000	-0.8001	1.0000	0.2974
LOC104913535	0.1133	-0.8847	1.0000	-0.1328	0.7848	0.2680	0.0627	0.7884	1.0000	-0.5567	1.0000	0.2078	1.0000	-0.0298
LOC104913536	1.0000	0.3073	0.3858	0.8757	0.0109	1.6233	0.0065	1.7658	1.0000	-0.2732	1.0000	0.3045	1.0000	-0.1305
LOC104913537	0.7424	0.2001	1.0000	-0.0124	0.9140	-0.1460	1.0000	-0.0623	1.0000	0.0651	1.0000	-0.1336	0.9671	0.1550
LOC104913538	0.4629	0.4694	0.2202	0.5375	0.7881	0.2920	0.9264	-0.1994	1.0000	-0.2840	1.0000	-0.1995	0.2080	-0.7697
LOC104913539	1.0000	0.0000	1.0000	2.2533	1.0000	2.2425	1.0000	2.3256	1.0000	0.0000	1.0000	2.3481	1.0000	0.0661
LOC104913543	1.0000	0.3663	0.0362	2.3884	0.4256	-1.3154	0.7246	1.2302	1.0000	-2.1647	1.0000	-0.1428	1.0000	0.3825
LOC104913544	0.9545	1.0672	1.0000	0.2935	1.0000	0.6780	0.1319	-4.3887	1.0000	1.9531	0.9773	1.2163	1.0000	-3.1344
LOC104913548	0.8271	0.3516	0.0389	1.3792	0.9604	-0.3695	0.6704	0.6297	1.0000	-1.0244	1.0000	0.0091	1.0000	-0.0202
LOC104913549	0.8582	-0.3584	0.3971	-0.5323	0.1438	0.8670	0.0022	1.0898	1.0000	-0.1779	1.0000	-0.3369	1.0000	0.0585
LOC104913550	0.0196	-0.5883	0.0000	-0.8306	0.0391	-0.5046	0.0011	-0.3593	1.0000	0.0281	0.6728	-0.2005	0.4778	0.1787
LOC104913551	0.0000	6.5958	0.0320	1.5298	0.0058	5.5185	1.0000	0.1488	1.0000	5.1651	1.0000	0.2165	1.0000	-0.1776
LOC104913552	0.0000	-1.4041	0.0000	-1.5749	0.0000	-1.3510	0.0000	-1.2574	1.0000	0.1371	1.0000	-0.0213	0.1415	0.2362
LOC104913553	0.0000	-2.0709	0.0000	-2.5420	0.0000	-2.1871	0.0000	-3.0847	1.0000	0.1490	1.0000	-0.3148	0.1654	-0.7439
LOC104913554	1.0000	0.4657	0.7594	0.7941	0.0000	3.2856	0.0000	2.8962	1.0000	0.1269	1.0000	0.4657	1.0000	-0.2605
LOC104913555	1.0000	2.1850	1.0000	-0.6944	1.0000	2.2470	1.0000	-0.5229	1.0000	3.6396	1.0000	0.9006	1.0000	0.9169

LOC104913556	0.0000	-1.5589	0.0000	-0.9720	0.0000	-2.7541	0.0000	-2.5060	1.0000	0.3792	0.0172	0.9801	0.5670	0.6333
LOC104913558	0.9248	0.7762	0.4234	1.1739	0.4480	1.2024	0.3146	1.5223	1.0000	-0.5152	1.0000	-0.1056	1.0000	-0.1940
LOC104913559	0.3031	0.2675	0.8546	-0.0618	0.1007	0.3480	1.0000	0.0281	1.0000	0.1904	0.8874	-0.1257	0.7287	-0.1238
LOC104913560	0.3148	3.9252	0.7666	-3.2534	1.0000	0.0000	1.0000	-0.8419	1.0000	3.1126	0.6450	-4.0575	1.0000	2.3543
LOC104913561	1.0000	2.1900	0.7674	3.0889	1.0000	0.0000	1.0000	0.0000	1.0000	0.0000	1.0000	0.8963	1.0000	0.0000
LOC104913562	0.0568	1.2763	0.1046	1.0634	1.0000	0.1801	0.6957	-0.6745	1.0000	-0.1364	1.0000	-0.3364	0.6087	-0.9846
LOC104913563	0.8226	3.0199	1.0000	2.2508	1.0000	0.0000	1.0000	0.0000	1.0000	0.0000	1.0000	-0.7966	1.0000	0.0000
LOC104913564	0.0626	0.6889	1.0000	-0.0008	0.9793	-0.1350	0.0360	-0.7763	1.0000	0.0823	0.1479	-0.5983	0.3487	-0.5560
LOC104913565	0.0082	1.4474	0.4722	0.4508	1.0000	-0.0427	0.3818	-0.5949	1.0000	0.6266	0.8837	-0.3526	1.0000	0.0808
LOC104913566	1.0000	0.0000	1.0000	0.0000	1.0000	2.2425	1.0000	0.0000	1.0000	0.0000	1.0000	0.0000	1.0000	-2.2889
LOC104913567	0.0000	1.2397	0.0000	1.0287	0.0000	-1.3277	0.0000	-1.1375	1.0000	-0.0147	0.0857	-0.2132	0.6247	0.1792
LOC104913568	0.4899	0.7748	0.0016	2.3373	0.1443	1.2505	0.0007	2.5200	1.0000	-1.2501	1.0000	0.3206	1.0000	0.0226
LOC104913569	0.0000	1.9129	0.0000	1.9305	0.8038	0.3188	0.4085	-0.5286	1.0000	-0.2592	0.8617	-0.2294	0.0904	-1.1008
LOC104913570	0.7765	-0.1120	0.7047	0.0849	0.5383	-0.1694	0.1000	0.1984	1.0000	-0.4022	0.4518	-0.1925	1.0000	-0.0280
LOC104913571	0.0227	5.1922	0.0374	2.3882	0.4809	3.6101	0.7210	1.2285	1.0000	3.1190	1.0000	0.4307	1.0000	0.7682
LOC104913572	0.0004	-1.2957	0.0002	-1.6005	0.0840	0.5883	0.0005	1.0480	1.0000	-0.4936	0.1527	-0.7841	1.0000	-0.0310
LOC104913574	1.0000	-0.1635	1.0000	-0.1167	0.6657	0.4724	0.9901	0.2593	1.0000	-0.1180	1.0000	-0.0634	1.0000	-0.3308
LOC104913575	0.3540	0.9418	1.0000	0.3176	1.0000	0.2498	0.9542	-0.3982	1.0000	0.4426	1.0000	-0.1693	1.0000	-0.2025
LOC104913578	1.0000	0.0000	1.0000	-2.3985	1.0000	2.2425	1.0000	-2.3200	1.0000	2.2734	1.0000	0.0000	1.0000	-2.2889
LOC104913579	1.0000	-0.3217	1.0000	-0.6349	1.0000	-0.1785	1.0000	-0.2067	1.0000	1.5649	1.0000	1.2867	0.7908	1.5494
LOC104913580	0.2581	0.2733	0.1114	0.2205	0.0221	0.4661	0.0004	0.4362	1.0000	-0.0211	1.0000	-0.0606	1.0000	-0.0445
LOC104913581	0.0536	1.2069	0.0596	0.7692	0.0001	1.9422	0.0000	1.5052	1.0000	0.2946	1.0000	-0.1302	1.0000	-0.1403
LOC104913585	1.0000	-0.0343	1.0000	-0.0212	0.6471	0.1404	0.9919	0.0349	1.0000	-0.0599	1.0000	-0.0338	0.3615	-0.1592
LOC104913586	1.0000	0.0000	1.0000	2.2508	1.0000	2.2470	1.0000	0.0000	1.0000	0.0000	1.0000	2.3457	1.0000	-2.2909
LOC104913587	0.0043	-0.4582	0.0002	-0.4040	0.0000	-0.8002	0.0000	-0.6778	1.0000	-0.0469	1.0000	0.0196	0.8699	0.0811
LOC104913588	0.3135	0.3854	0.0978	0.4259	0.0000	1.3087	0.0000	1.0805	1.0000	-0.0787	1.0000	-0.0244	0.1730	-0.2992
LOC104913589	1.0000	0.5832	1.0000	0.2195	0.7628	1.0365	0.9096	-1.3721	1.0000	0.4094	1.0000	0.0600	0.6865	-1.9991
LOC104913591	1.0000	0.0000	1.0000	2.2509	1.0000	0.0000	0.1711	4.4060	1.0000	0.0000	1.0000	2.3457	0.4003	4.4407
LOC104913592	1.0000	-0.0392	0.0167	1.8395	0.0954	0.9960	0.0000	3.1775	1.0000	-1.7595	1.0000	0.1279	0.7287	0.4246
LOC104913593	1.0000	2.1850	1.0000	-2.3985	1.0000	2.2425	1.0000	-2.3200	1.0000	2.2734	1.0000	-2.2957	1.0000	-2.2889
LOC104913595	0.3770	-0.1730	0.1615	-0.1214	1.0000	-0.0447	0.5478	0.0659	1.0000	-0.0980	1.0000	-0.0340	1.0000	0.0180
LOC104913597	0.0004	1.6534	0.0000	1.9488	0.0000	2.9231	0.0000	2.3911	1.0000	0.1008	0.5523	0.4038	0.1820	-0.4311
LOC104913598	0.0059	1.4705	0.0000	1.7659	0.0526	1.1377	0.0077	1.2082	1.0000	-0.3395	1.0000	-0.0329	0.9728	-0.2651
LOC104913600	0.5003	-0.2629	0.0000	-0.8441	0.0002	-1.0214	0.0000	-1.0658	1.0000	0.2933	0.6424	-0.2756	0.6795	0.2523
LOC104913601	0.3749	0.5578	0.0069	1.0592	0.1779	-0.9570	0.0905	-1.0327	1.0000	-0.2341	1.0000	0.2801	1.0000	-0.3017
LOC104913602	1.0000	0.0000	1.0000	0.0000	1.0000	0.0000	1.0000	0.0000	1.0000	0.0000	1.0000	0.0000	1.0000	0.0000
LOC104913604	0.9545	-1.6928	1.0000	-0.1472	0.4809	-3.7967	1.0000	-2.3200	1.0000	-1.4912	1.0000	0.0495	1.0000	0.0000
LOC104913605	1.0000	-0.2920	1.0000	-0.1452	1.0000	-2.4056	1.0000	-2.3200	1.0000	-0.1034	1.0000	0.0513	1.0000	0.0000
LOC104913606	0.0000	-1.4272	0.0000	-0.9543	0.0000	-1.3136	0.0000	-0.9972	1.0000	-0.0083	0.0412	0.4779	0.2363	0.3140
LOC104913607	1.0000	0.0000	1.0000	0.0000	1.0000	0.0000	1.0000	0.0000	1.0000	0.0000	1.0000	0.0000	1.0000	0.0000
LOC104913608	1.0000	0.0000	1.0000	0.0000	1.0000	0.0000	1.0000	0.0000	1.0000	0.0000	1.0000	0.0000	1.0000	0.0000
LOC104913609	1.0000	0.0026	1.0000	0.0670	0.0000	1.4085	0.0012	0.8508	1.0000	0.2790	0.8269	0.3555	0.6054	-0.2722
LOC104913610	0.2478	0.4604	0.0284	0.6452	0.0947	-0.6733	1.0000	-0.1057	1.0000	-0.4451	0.8875	-0.2472	1.0000	0.1287
LOC104913611	1.0000	0.0000	1.0000	0.0000	1.0000	0.0000	1.0000	0.0000	1.0000	0.0000	1.0000	0.0000	1.0000	0.0000
LOC104913612	0.4035	0.4872	0.0050	0.9941	1.0000	-0.0642	0.1321	0.6450	1.0000	-0.4025	1.0000	0.1187	0.9059	0.3156
LOC104913614	1.0000	0.0000	1.0000	0.0000	1.0000	0.0000	1.0000	0.0000	1.0000	0.0000	1.0000	0.0000	1.0000	0.0000
LOC104913617	0.0000	1.8022	0.0001	1.5028	1.0000	0.0134	1.0000	-0.2124	1.0000	-0.6858	0.0163	-0.9745	0.2758	-0.9091
LOC104913620	0.8513	-0.1399	0.5032	0.2384	0.0421	0.5330	0.0000	1.2165	1.0000	-0.3974	1.0000	-0.0073	0.2944	0.2908
LOC104913621	0.0020	0.9255	0.0000	1.2815	0.6303	0.2659	0.0000	1.2179	1.0000	-0.3731	1.0000	-0.0045	0.0392	0.5901
LOC104913622	0.2913	1.1944	0.1792	1.4648	1.0000	0.0198	0.6452	0.9628	1.0000	-0.6764	1.0000	-0.4018	1.0000	0.2655
LOC104913623	1.0000	0.1183	0.8225	0.2161	0.5070	-0.6104	0.1542	0.6145	1.0000	0.3024	0.8406	0.4179	0.0012	1.5347
LOC104913624	0.0000	-6.6677	0.0000	-4.4412	0.6903	-0.4848	1.0000	-0.1833	1.0000	-0.0064	1.0000	2.3457	1.0000	0.2999
LOC104913625	0.1006	-0.4781	0.2913	-0.2465	0.7664	-0.1823	0.9691	-0.0725	1.0000	-0.1551	1.0000	0.0896	1.0000	-0.0373
LOC104913627	0.4124	0.9053	0.0128	1.8378	1.0000	-0.0464	0.7731	-0.9370	1.0000	-0.6918	1.0000	0.2467	0.4347	-1.5805
LOC104913628	0.0000	-2.3055	0.0000	-2.2298	0.0885	-0.6646	0.3376	-0.2756	1.0000	0.2797	0.9994	0.3678	0.0838	0.6760
LOC104913629	0.0196	-3.0013	0.1071	-1.6156	1.0000	0.0782	1.0000	0.2862	1.0000	-0.1331	1.0000	1.2747	1.0000	0.0811
LOC104913630	1.0000	0.0000	1.0000	0.0000	1.0000	2.2469	1.0000	0.0000	1.0000	0.0000	1.0000	0.0000	1.0000	-2.2909
LOC104913631	0.0000	1.0249	0.0000	0.8095	0.0000	1.2120	0.0000	1.2289	1.0000	-0.1334	0.1655	-0.3365	0.9202	-0.1115
LOC104913632	1.0000	-0.0036	1.0000	-0.1770	1.0000	0.1645	0.4992	0.6595	1.0000	-0.3504	1.0000	-0.5134	1.0000	0.1556
LOC104913633	1.0000	-0.4965	0.0913	-1.2347	0.9805	-0.5054	0.0186	-1.8675	1.0000	0.7788	1.0000	0.0575	1.0000	-0.5771
LOC104913634	0.0023	0.8286	0.0000	1.6206	0.0004	0.7971	0.0000	1.0191	1.0000	-0.5205	0.4618	0.2838	0.1798	-0.2926
LOC104913635	0.3599	-0.4391	1.0000	-0.0504	0.0000	-1.7195	0.0078	-1.0097	1.0000	-0.3802	1.0000	0.0201	0.9844	0.3339
LOC104913636	0.0017	-0.6112	0.0101	-0.2480	0.8033	0.1102	0.0000	0.5708	1.0000	-0.0726	0.0190	0.3025	0.0000	0.3926
LOC104913637	1.0000	-2.4776	1.0000	0.0000	1.0000	-2.4056	1.0000	2.3241	1.0000	-2.3757	1.0000	0.0000	1.0000	2.3543
LOC104913638	1.0000	0.0000	1.0000	0.0000	1.0000	2.2469	1.0000	0.0000	1.0000	0.0000	1.0000	0.0000	1.0000	-2.2909
LOC104913639	0.7252	0.2449	0.0023	0.8268	0.0172	0.7747	0.0000	1.3103	1.0000	-0.4962	1.0000	0.1009	1.0000	0.0477
LOC104913643	0.0036	1.5321	0.0005	1.5399	1.0000	-0.2888	0.1816	-1.0499	1.0000	0.1137	1.0000	0.1334	0.8204	-0.6418
LOC104913644	1.0000	0.0026	1.0000	0.0914	1.0000	0.0165	0.2362	0.3925	1.0000	-0.1310	1.0000	-0.0293	0.8133	0.2506
LOC104913648	1.0000	0.0000	1.0000	0.0000	1.0000	0.0000	1.0000	2.3241	1.0000	0.0000	1.0000	0.0000	1.0000	2.3543
LOC104913651	0.4238	-0.5733	1.0000	-0.1044	0.4130	-0.6087	0.0633	-0.9443	1.0000	-0.0933	0.9238	0.3894	1.0000	-0.4212
LOC104913653	0.0037	-1.1449	0.0000	-0.9891	0.3549	-0.3512	0.4235	-0.2368	1.0000	-0.0772	1.0000	0.0914	1.0000	0.0426
LOC104913658	0.0003	-1.0705	0.0000	-0.7361	0.0029	-0.9002	0.0000	-0.7735	1.0000	0.3081	0.0016	0.6555	0.0716	0.4413
LOC104913659	0.0011	2.2993	0.1226	1.1516	0.8151	-1.1321	0.2798	-1.5022	1.0000	0.4383	0.6295	-0.6960	1.0000	0.0749
LOC104913661	1.0000	0.0000	1.0000	2.2509	1.0000	0.0000	1.0000	0.0000	1.0000	0.0000	1.0000	2.3457	1.0000	0.0000
LOC104913662	0.1789	-4.5815	1.0000	-2.3960	0.2223	-4.4946	1.0000	0.0074	1.0000	-2.1959	1.0000	0.0000	1.0000	2.3554
LOC104913664	0.4678	0.6761	0.0001	2.2972	0.9419	0.3090	1.0000	0.0070	1.0000	-1.1033	0.7688	0.5255	0.1593	-1.4026
LOC104913665	0.2232	0.3916	0.9807	0.0599	0.5029	-0.2562	0.0005	-0.6550	1.0000	0.1889	1.0000	-0.1292	0.7931	-0.2033
LOC104913666	1.0000	0.0000	1.0000	0.0000	1.0000	0.0000	1.0000	0.0000	1.0000	0.0000	1.0000	0.0000	1.0000	0.0000
LOC104913668	1.0000	0.0000	1.0000	0.0000	1.0000	0.0000	1.0000	0.0000	1.0000	0.0000	1.0000	0.0000	1.0000	0.0000
LOC104913670	0.1394	-0.7859	0.2014	-0.4358	0.0000	1.5437	0.0000	1.3131	1.0000	0.5979	0.0203	0.9649	0.1283	0.3746
LOC104913673	1.0000	2.1850	0.9062	-1.5349	0.2840	3.9988	1.0000	0.6976	1.0000	3.6423	1.0000	0.0513	1.0000	0.3802
LOC104913674	0.2651	0.7245	0.0005	1.2888	1.0000	-0.1226	1.0000	-0.1287	1.0000	-0.3704	1.0000	0.2072	0.8782	-0.3672
LOC104913675	0.3300	1.0518	0.4438	1.1795	0.6301	-1.3466	0.7779	0.9523	1.0000	-1.2860	0.4569	-1.1591	1.0000	1.0201
LOC104913676	1.0000	0.0506	0.3629	-0.4443	1.0000	0.0519	1.0000	0.0618	1.0000	0.1976	1.0000	-0.2837	1.0000	0.2171
LOC104913678	0.0445	1.7957	0.0503	1.4373	0.1310	1.6898	0.7837	0.5806	1.0000	0.5163	1.0000	0.1788	0.8283	-0.5763
LOC104913681	1.0000	-2.4788	1.0000	0.0000	1.0000	-0.1597	1.0000	2.3241	1.0000	-2.3771	1.0000	0.0000	1.0000	0.0640
LOC104913683	0.1899	-0.8639	0.1296	-0.6961	0.0178	0.9916	0.0000	1.4214	1.0000	0.0858	1.0000	0.2658	0.1717	0.5173
LOC104913684	1.0000	0.5464	1.0000	2.2509	1.0000	-2.4056	0.7701	3.1732	1.0000	-2.3757	1.0000	-0.8001	1.0000	3.2066
LOC104913685	1.0000	0.0000	0.7674	3.0956	1.0000	0.0000	1.0000	0.0000	1.0000	0.0000	1.0000	3.2002	1.0000	0.0000
LOC104913686	1.0000	-0.8568	1.0000	-0.1948	1.0000	0.5067	1.0000	-0.3936	1.0000	0.2717	1.0000	0.9628	1.0000	-0.6192
LOC104913687	1.0000	-0.7263	0.5796	0.9858	1.0000	0.1240	0.1899	1.6642	1.0000	-0.5096	0.9741	1.2157	0.8254	1.0417
LOC104913688	1.0000	0.0000	1.0000	-2.3985	1.0000	0.0000	0.9096	1.3849	1.0000	2.2734	1.0000	0.0000	0.7287	3.7387
LOC104913690	0.4327	-0.2436	0.5327	-0.1459	0.0000	-1.4402	0.0000	-1.3023	1.0000	-0.1531	1.0000	-0.0438	1.0000	-0.0087

LOC104913691	1.0000	-0.2920	0.9062	1.2225	1.0000	-2.4056	1.0000	0.8538	1.0000	-0.1078	1.0000	1.4317	1.0000	3.2049
LOC104913692	0.4697	0.4823	1.0000	-0.0552	0.0430	-1.2843	0.0201	-1.0882	1.0000	0.3878	1.0000	-0.1388	0.8297	0.5874
LOC104913693	1.0000	-0.0187	1.0000	0.1765	0.6960	-0.4873	1.0000	0.0105	1.0000	0.0502	1.0000	0.2575	0.7638	0.5541
LOC104913694	0.0000	-1.3084	0.0000	-1.2715	0.0510	-0.3365	0.0039	-0.2208	1.0000	0.2884	0.0155	0.3374	0.0000	0.4090
LOC104913695	1.0000	0.0805	0.8707	-0.1814	0.0017	1.2775	0.0000	1.4965	1.0000	0.2472	1.0000	-0.0067	0.2409	0.4688
LOC104913696	0.2954	-0.6420	1.0000	0.1098	0.0000	-2.4958	0.0001	-1.4356	1.0000	0.1168	0.0906	0.8827	0.1866	1.1798
LOC104913697	1.0000	0.0000	1.0000	0.0000	1.0000	0.0000	1.0000	2.3241	1.0000	0.0000	1.0000	0.0000	1.0000	2.3543
LOC104913698	1.0000	0.0000	1.0000	0.0000	1.0000	0.0000	1.0000	2.3241	1.0000	0.0000	1.0000	0.0000	1.0000	2.3543
LOC104913700	0.0943	-0.9062	0.5895	0.4084	0.3091	-0.5818	0.2778	0.5882	1.0000	-0.9954	1.0000	0.3315	1.0000	0.1839
LOC104913701	0.5745	0.3015	0.0000	0.9548	0.0000	2.5458	0.0000	2.6390	1.0000	0.0471	0.0016	0.7137	0.6913	0.1475
LOC104913702	1.0000	-0.3164	0.4025	1.9118	1.0000	-0.1742	0.6142	1.7721	1.0000	-0.9614	1.0000	1.2775	1.0000	0.9915
LOC104913703	1.0000	-2.4788	1.0000	-0.1429	1.0000	-0.1630	1.0000	0.0074	1.0000	-0.1089	1.0000	2.3481	1.0000	0.0660
LOC104913704	0.6881	1.7537	0.5021	1.3057	1.0000	0.6816	1.0000	0.5372	1.0000	0.7357	1.0000	0.3095	1.0000	0.6034
LOC104913705	1.0000	0.0000	1.0000	-2.3960	0.8011	3.0840	1.0000	0.8545	1.0000	2.2676	1.0000	0.0000	1.0000	0.0723
LOC104913706	1.0000	-2.4788	1.0000	0.0000	1.0000	-2.4061	1.0000	0.0000	1.0000	-2.3771	1.0000	0.0000	1.0000	0.0000
LOC104913707	1.0000	0.0000	1.0000	0.0000	1.0000	0.0000	1.0000	0.0000	1.0000	0.0000	1.0000	0.0000	1.0000	0.0000
LOC104913708	1.0000	0.0000	1.0000	0.0000	1.0000	0.0000	1.0000	0.0000	1.0000	0.0000	1.0000	0.0000	1.0000	0.0000
LOC104913710	1.0000	0.0000	1.0000	0.0000	1.0000	0.0000	1.0000	0.0000	1.0000	0.0000	1.0000	0.0000	1.0000	0.0000
LOC104913712	0.2154	-1.4379	0.2553	-0.8597	0.5668	-0.7145	0.1131	-1.1908	1.0000	0.5505	0.7462	1.1445	1.0000	0.0817
LOC104913713	1.0000	0.0000	1.0000	0.0000	1.0000	0.0000	1.0000	0.0000	1.0000	0.0000	1.0000	0.0000	1.0000	0.0000
LOC104913717	1.0000	0.0000	1.0000	0.0000	1.0000	0.0000	1.0000	0.0000	1.0000	0.0000	1.0000	0.0000	1.0000	0.0000
LOC104913718	0.0050	1.9682	0.0000	2.4845	0.0001	2.4434	0.3087	0.9865	1.0000	0.0584	0.5382	0.5878	0.0192	-1.3958
LOC104913722	1.0000	0.6163	0.0026	2.2763	0.3975	1.2111	0.3358	1.2773	1.0000	-0.1266	0.1965	1.5522	1.0000	-0.0550
LOC104913723	0.0089	1.9678	0.2173	1.0085	0.0002	2.4928	0.1626	1.1339	1.0000	0.5723	1.0000	-0.3655	0.3585	-0.7747
LOC104913724	0.5833	1.3514	1.0000	-0.1598	1.0000	0.7368	0.0125	2.6694	1.0000	-0.1170	0.7635	-1.6300	0.2236	1.8225
LOC104913725	0.0000	-2.0972	0.0000	-2.3056	0.0000	0.8173	0.4344	0.1639	1.0000	0.0592	0.9920	-0.1358	0.0000	-0.5901
LOC104913726	0.0000	-5.5652	0.0000	-8.1433	0.1738	0.5343	0.0003	0.9028	1.0000	-0.4765	1.0000	-3.1468	1.0000	-0.1050
LOC104913727	0.0000	-5.9833	0.0000	-8.0799	0.1995	-0.7431	0.0081	-1.0122	1.0000	-0.1235	1.0000	-2.2993	0.8244	-0.3891
LOC104913729	0.0280	-5.2303	0.1392	-4.4820	0.7062	-1.1509	0.1321	-4.3889	1.0000	-0.7772	1.0000	0.0000	0.5105	-4.0491
LOC104913730	0.0000	-3.3757	0.0000	-3.5094	0.0000	-0.9702	0.0000	-1.2650	1.0000	0.0274	0.9987	-0.0947	0.0022	-0.2624
LOC104913731	0.3148	-4.2689	0.4431	-3.7865	0.0020	2.6260	0.1269	1.7991	1.0000	-0.5112	1.0000	0.0000	0.1325	-1.3385
LOC104913732	0.7034	-0.7973	0.3254	-0.9329	0.7919	0.5005	0.1420	0.9927	1.0000	-0.0082	1.0000	-0.1293	0.7886	0.4904
LOC104913733	1.0000	0.0000	1.0000	0.0000	0.8033	3.0789	1.0000	2.3241	1.0000	0.0000	1.0000	0.0000	1.0000	-0.7782
LOC104913734	0.8226	-3.3425	1.0000	-1.0008	0.2642	1.8517	0.9786	0.9243	1.0000	-0.1179	1.0000	2.3481	0.7580	-1.0441
LOC104913735	0.0000	1.8103	0.0000	1.9708	0.6026	0.3988	0.1797	0.6012	1.0000	0.1263	0.8171	0.2964	0.8275	0.3324
LOC104913736	0.0386	-0.6269	0.0162	-0.5441	0.5948	-0.2298	0.2156	0.2820	1.0000	-0.3855	0.7610	-0.2910	1.0000	0.1332
LOC104913737	0.3089	-4.2696	0.9148	1.2198	1.0000	0.1057	0.6483	1.7684	1.0000	-1.8822	0.8785	3.7233	1.0000	-0.2228
LOC104913738	1.0000	0.0000	1.0000	0.0000	1.0000	0.0000	1.0000	0.0000	1.0000	0.0000	1.0000	0.0000	1.0000	0.0000
LOC104913740	0.4801	2.0051	0.1621	2.3788	0.4424	1.8933	0.6439	1.7796	1.0000	-0.1089	1.0000	0.2735	1.0000	-0.2259
LOC104913741	0.0001	0.7011	0.0002	0.3990	0.0000	-0.8825	0.0000	-1.1408	1.0000	0.1868	0.8737	-0.1025	1.0000	-0.0659
LOC104913742	0.0000	-6.4332	0.0000	-6.2161	0.9857	0.1271	0.3901	0.3551	1.0000	-0.1879	1.0000	0.0470	1.0000	0.0439
LOC104913743	0.0000	1.5908	0.0000	1.9055	0.0000	1.6235	0.0000	1.5102	1.0000	-0.2658	1.0000	0.0600	0.2088	-0.3747
LOC104913745	0.4238	-1.8118	1.0000	-0.4260	0.9519	0.6538	0.8940	0.5728	1.0000	-0.1272	1.0000	1.2789	1.0000	-0.2032
LOC104913746	1.0000	0.4221	0.7865	-1.1189	1.0000	-0.5733	0.2626	-2.3201	1.0000	0.4379	0.9773	-1.0985	1.0000	-1.3088
LOC104913747	0.8450	0.8149	0.0758	4.5597	0.9468	-1.5535	1.0000	0.0000	1.0000	-3.7607	1.0000	-0.1547	1.0000	-2.2889
LOC104913748	0.7887	1.1408	1.0000	0.0000	1.0000	-1.0191	1.0000	0.0000	1.0000	-3.2304	0.3216	-4.6160	1.0000	-2.2889
LOC104913749	0.0012	1.9108	0.2200	0.8095	0.1779	1.0787	0.5676	0.5379	1.0000	0.5619	0.7290	-0.5225	1.0000	0.0279
LOC104913751	1.0000	-0.1429	0.0299	-0.7745	1.0000	0.0664	0.0951	-0.6221	1.0000	0.2067	0.8247	-0.4124	0.5522	-0.4772
LOC104913752	0.5630	0.3093	0.4501	0.2990	0.0056	-1.0741	1.0000	-0.1118	1.0000	-0.4557	0.3810	-0.4552	0.4653	0.5106
LOC104913753	0.0000	-2.6649	0.0000	-3.2364	0.0000	-1.4209	0.0000	-1.5412	1.0000	0.3965	1.0000	-0.1606	0.8235	0.2817
LOC104913755	0.6066	1.3438	0.2941	1.1583	0.0000	4.0002	0.0000	3.0145	1.0000	1.1048	0.8915	0.9312	1.0000	0.1171
LOC104913756	1.0000	0.1569	0.7674	0.3978	0.3704	-0.8081	1.0000	0.1426	1.0000	-0.3257	1.0000	-0.0756	0.6665	0.6267
LOC104913758	1.0000	0.0000	1.0000	0.0000	1.0000	0.0000	1.0000	0.0000	1.0000	0.0000	1.0000	0.0000	1.0000	0.0000
LOC104913759	0.0317	-1.2296	0.1302	-0.7090	0.5122	0.3812	0.0545	0.6428	1.0000	0.1135	0.6284	0.6440	0.5802	0.3756
LOC104913760	0.4051	0.4548	0.1005	0.5927	0.1354	0.6489	0.2903	0.4346	1.0000	-0.0374	1.0000	0.1121	0.8699	-0.2487
LOC104913763	0.2075	1.2038	0.5724	0.7946	0.0877	1.3617	0.9062	0.5753	1.0000	-0.6977	0.3097	-1.0947	0.0773	-1.4786
LOC104913764	0.9943	-0.4994	0.1226	-1.7006	0.2953	-1.4615	0.2869	-1.2135	1.0000	0.1243	0.8875	-1.0654	1.0000	0.3818
LOC104913766	0.4912	1.1811	0.4154	-1.5158	0.1583	1.5495	0.0467	1.4396	1.0000	0.8308	0.3468	-1.8568	0.5565	0.7249
LOC104913767	1.0000	-0.8577	1.0000	0.6987	1.0000	0.2002	1.0000	0.8538	1.0000	-1.4938	1.0000	0.0559	1.0000	-0.8434
LOC104913769	0.6551	0.2658	0.0043	0.7567	0.0000	2.2399	0.0000	2.3328	1.0000	0.0526	0.0406	0.5574	0.5809	0.1545
LOC104913771	0.2878	-0.2132	0.0016	-0.3021	0.0000	-1.0416	0.0000	-0.9926	1.0000	0.0170	1.0000	-0.0598	1.0000	0.0722
LOC104913773	0.4200	-0.2401	0.0004	-0.4732	0.9262	-0.1086	0.4274	0.1329	1.0000	0.0071	0.5548	-0.2140	0.4068	0.2560
LOC104913775	0.0038	-0.4750	0.0000	-0.6254	0.0000	-0.7069	0.0001	-0.3506	1.0000	-0.0027	0.7324	-0.1410	0.0043	0.3594
LOC104913777	0.0000	-1.7202	0.0000	-2.1055	0.0000	-1.1890	0.0009	-0.7392	1.0000	0.1234	1.0000	-0.2479	0.0596	0.5811
LOC104913778	0.7791	0.3069	0.1314	0.7282	0.0000	1.7929	0.0000	1.7075	1.0000	-0.4306	1.0000	0.0038	0.1692	-0.5083
LOC104913779	0.0032	-0.7690	0.0072	-0.6661	0.0000	-1.6367	0.0000	-1.2319	1.0000	-0.2354	1.0000	-0.1214	0.9787	0.1739
LOC104913781	1.0000	0.0000	0.7674	3.0889	1.0000	0.0000	1.0000	2.3256	1.0000	0.0000	1.0000	3.1946	1.0000	2.3554
LOC104913782	1.0000	0.2011	1.0000	-0.1630	1.0000	0.3421	1.0000	0.5319	1.0000	-0.1113	1.0000	-0.4744	1.0000	0.0763
LOC104913783	0.1101	4.6954	0.7189	1.0558	0.1736	4.3051	0.7186	1.2292	1.0000	3.1126	1.0000	-0.4068	1.0000	0.0763
LOC104913784	0.0000	1.5889	0.0000	2.1737	0.0000	1.8485	0.0000	1.6770	1.0000	-0.2556	0.5290	0.3447	0.0789	-0.4194
LOC104913785	1.0000	0.1841	0.0338	1.5495	0.0376	1.4026	0.0223	1.6800	1.0000	-0.8313	0.9458	0.5610	0.6584	-0.5361
LOC104913786	1.0000	-0.0273	0.3473	0.8058	0.0478	1.3207	0.0630	1.2296	1.0000	-0.1338	0.8548	0.7085	1.0000	-0.2171
LOC104913789	0.9650	1.0594	1.0000	-2.3960	1.0000	-2.4056	1.0000	-2.3179	1.0000	-0.1078	0.8785	-3.6647	1.0000	0.0000
LOC104913791	0.0007	1.1700	0.0000	1.2057	1.0000	0.0862	0.0199	-0.5374	1.0000	-0.9271	0.0000	-0.8804	0.0000	-1.5443
LOC104913792	1.0000	0.0000	1.0000	0.0000	1.0000	0.0000	1.0000	0.0000	1.0000	0.0000	1.0000	0.0000	1.0000	0.0000
LOC104913793	0.8458	0.8174	1.0000	-0.1708	1.0000	-0.1865	1.0000	-0.5239	1.0000	-0.1188	0.9773	-1.1014	1.0000	-0.4568
LOC104913795	1.0000	0.1870	1.0000	-0.2649	0.9759	0.2918	1.0000	0.2386	1.0000	-0.1315	0.9052	-0.5738	1.0000	-0.1797
LOC104913796	1.0000	-0.2892	0.0038	-5.4498	1.0000	-2.4061	0.0305	-3.0296	1.0000	2.9197	1.0000	-2.2993	1.0000	2.3543
LOC104913797	0.0015	-1.8658	0.0000	-2.0905	0.4313	-0.5440	0.8740	-0.1350	1.0000	0.2274	1.0000	0.0228	0.0439	0.6436
LOC104913798	0.7266	1.4633	0.6180	1.6082	1.0000	-0.1624	0.6142	1.7721	1.0000	-0.1078	1.0000	0.0537	0.8937	1.8364
LOC104913799	1.0000	-2.4776	1.0000	0.0000	1.0000	-2.4056	1.0000	0.0000	1.0000	-2.3757	1.0000	0.0000	1.0000	0.0000
LOC104913802	1.0000	-0.2242	0.2635	0.8688	1.0000	0.2275	0.0959	1.1158	1.0000	-0.9798	1.0000	0.1242	1.0000	-0.0870
LOC104913803	0.8795	0.2507	0.0986	0.6935	0.2785	0.5889	0.8283	0.2602	1.0000	-0.0833	0.8820	0.3702	0.6920	-0.4078
LOC104913804	0.6923	0.3183	0.7306	0.1951	0.9836	0.1661	0.5214	-0.2582	1.0000	0.1304	1.0000	0.0196	0.7471	-0.2856
LOC104913806	1.0000	0.0000	0.9062	1.2235	1.0000	0.0000	1.0000	-2.3179	1.0000	2.2676	0.8607	3.7297	1.0000	0.0000
LOC104913808	1.0000	0.2026	0.5029	1.3073	1.0000	0.3406	1.0000	0.0074	1.0000	-0.1245	1.0000	1.0010	1.0000	-0.4550
LOC104913809	0.1839	1.2666	0.5054	-0.6409	0.7276	0.6908	0.0123	1.1837	1.0000	1.0152	0.6057	-0.8781	0.0068	1.5155
LOC104913810	0.5086	3.5431	1.0000	0.6940	1.0000	0.0000	0.9164	1.3892	1.0000	2.2676	1.0000	-0.4752	0.7453	3.7416
LOC104913812	0.4676	-0.3206	0.0001	-0.7623	0.0675	-0.6108	0.0027	-0.5547	1.0000	0.1459	0.7370	-0.2816	0.7915	0.2108
LOC104913813	0.3471	-0.2667	0.0016	-0.4058	0.0001	-0.8505	0.0000	-0.8244	1.0000	0.1485	1.0000	0.0217	0.6135	0.1782
LOC104913814	0.1347	1.2018	0.0950	1.1504	0.0003	1.9606	0.0197	1.3864	1.0000	-0.1333	1.0000	-0.1727	0.2885	-0.6999
LOC104913816	1.0000	0.0243	0.0034	0.6061	0.0000	-4.6784	0.0000	-2.8538	1.0000	-0.0329	0.0399	0.5589	0.1520	1.7962
LOC104913817	0.0387	-0.3675	0.0002	-0.3248	0.0047	-0.4784	0.0000	-0.4575	1.0000	0.0077	1.0000	0.0631	1.0000	0.0346

LOC104913818	1.0000	0.0000	1.0000	0.0000	1.0000	0.0000	1.0000	0.0000	1.0000	0.0000	1.0000	0.0000	1.0000	0.0000
LOC104913819	0.0538	0.7773	0.4980	0.2907	0.0002	-1.8822	0.0000	-2.0202	1.0000	0.1169	0.6811	-0.3562	1.0000	-0.0121
LOC104913820	0.0095	5.4529	0.0016	3.7113	0.2186	4.2822	1.0000	-2.3200	1.0000	2.2734	1.0000	0.6368	0.4066	-4.3406
LOC104913821	0.0137	-0.4081	0.0000	-0.5228	0.5608	-0.1432	0.6317	-0.0641	1.0000	0.1033	1.0000	0.0014	0.1616	0.1876
LOC104913822	1.0000	-0.0956	0.6708	-1.1237	1.0000	-0.1909	0.6483	0.6923	1.0000	0.5154	1.0000	-0.4979	0.4497	1.4136
LOC104913824	0.0004	0.9987	0.0107	0.5199	1.0000	-0.0533	0.8325	-0.1413	1.0000	0.3685	1.0000	-0.0960	0.6703	0.2886
LOC104913826	0.0000	1.5146	0.0000	1.5641	0.0000	-1.1093	0.0000	-1.1044	1.0000	-0.2466	0.2453	-0.1866	0.6627	-0.2356
LOC104913827	0.6727	-0.4286	1.0000	-0.0303	1.0000	-0.0594	0.5102	0.3877	1.0000	-0.2784	1.0000	0.1323	1.0000	0.1732
LOC104913829	1.0000	0.0000	1.0000	0.0000	1.0000	0.0000	1.0000	2.3256	1.0000	0.0000	1.0000	0.0000	1.0000	2.3554
LOC104913830	1.0000	0.0000	1.0000	0.0000	1.0000	0.0000	1.0000	0.0000	1.0000	0.0000	1.0000	0.0000	1.0000	0.0000
LOC104913831	0.0000	-1.6886	0.0000	-1.8568	0.0000	-1.3700	0.0000	-1.0610	1.0000	0.0904	1.0000	-0.0643	0.1545	0.4071
LOC104913832	1.0000	0.0000	1.0000	0.0000	1.0000	0.0000	1.0000	0.0000	1.0000	0.0000	1.0000	0.0000	1.0000	0.0000
LOC104913833	0.3256	-0.2435	0.7639	-0.0802	0.3155	0.2338	0.0002	0.4317	1.0000	-0.1141	1.0000	0.0611	0.9124	0.0885
LOC104913836	1.0000	0.0000	1.0000	0.0000	1.0000	0.0000	1.0000	0.0000	1.0000	0.0000	1.0000	0.0000	1.0000	0.0000
LOC104913837	1.0000	0.1716	0.3056	0.7156	0.0073	-2.1420	0.1078	-1.5282	1.0000	-0.5445	1.0000	0.0121	1.0000	0.0774
LOC104913838	0.0000	-6.9592	0.0000	-3.9667	0.0009	-2.3179	0.0001	-2.5663	1.0000	0.0670	1.0000	3.1976	1.0000	-0.1753
LOC104913839	1.0000	0.0000	1.0000	0.0000	1.0000	0.0000	1.0000	0.0000	1.0000	0.0000	1.0000	0.0000	1.0000	0.0000
LOC104913840	1.0000	0.0000	1.0000	0.0000	1.0000	0.0000	1.0000	0.0000	1.0000	0.0000	1.0000	0.0000	1.0000	0.0000
LOC104913841	1.0000	0.0000	1.0000	0.0000	1.0000	0.0000	1.0000	0.0000	1.0000	0.0000	1.0000	0.0000	1.0000	0.0000
LOC104913842	0.0000	-6.9016	0.0106	-2.1885	0.2063	-1.0773	1.0000	-0.1306	1.0000	-0.4449	0.5389	4.4097	0.9194	0.5140
LOC104913844	1.0000	-2.4776	1.0000	-2.3985	1.0000	0.6783	1.0000	-2.3200	1.0000	-0.1034	1.0000	0.0000	1.0000	-3.1344
LOC104913846	1.0000	-0.2882	1.0000	0.0000	1.0000	-0.1591	0.7710	3.1710	1.0000	-2.3757	1.0000	-2.2993	1.0000	0.9149
LOC104913847	1.0000	-0.0370	1.0000	-0.1674	0.8694	0.5783	1.0000	0.6988	1.0000	-0.5088	1.0000	-0.6343	1.0000	-0.3874
LOC104913848	0.5258	-0.1806	0.0042	-0.4464	0.0000	-1.4202	0.0000	-1.0450	1.0000	-0.0686	0.1198	-0.3231	0.2276	0.3121
LOC104913849	1.0000	0.0519	1.0000	0.2611	0.0766	1.3920	0.0278	1.3359	1.0000	-0.2515	1.0000	-0.0310	1.0000	-0.3065
LOC104913850	0.8249	3.0143	0.9148	-1.5387	1.0000	2.2469	0.4547	-3.7024	1.0000	3.6521	1.0000	-0.7919	1.0000	-2.2909
LOC104913851	0.7768	-0.3984	1.0000	-0.1811	0.6197	0.4110	0.9566	0.1876	1.0000	-0.3410	1.0000	-0.1180	0.3844	-0.5669
LOC104913853	1.0000	0.0000	1.0000	0.0000	1.0000	0.0000	1.0000	0.0000	1.0000	0.0000	1.0000	0.0000	1.0000	0.0000
LOC104913856	0.0430	-2.2591	0.4195	-1.5160	1.0000	-0.1917	1.0000	0.0059	1.0000	-0.6983	1.0000	0.0567	1.0000	-0.4964
LOC104913857	0.0000	-1.0994	0.0000	-1.1323	0.0001	-0.6676	0.0000	-0.5397	1.0000	-0.2061	0.4057	-0.2265	1.0000	-0.0720
LOC104913858	1.0000	0.0000	1.0000	0.0000	1.0000	0.0000	1.0000	0.0000	1.0000	0.0000	1.0000	0.0000	1.0000	0.0000
LOC104913859	1.0000	0.0000	1.0000	0.0000	1.0000	2.2469	1.0000	2.3241	1.0000	0.0000	1.0000	0.0000	1.0000	0.0640
LOC104913860	0.5086	-3.8796	0.2059	-1.3978	0.4809	-3.7970	0.0097	-3.2863	1.0000	1.7881	0.4563	4.4217	1.0000	2.3543
LOC104913861	0.0423	-5.0436	0.0008	-3.9555	0.6371	-1.3481	0.0040	-2.9358	1.0000	1.1297	1.0000	2.3457	1.0000	-0.4568
LOC104913862	1.0000	-0.0318	0.0041	0.5651	0.2333	0.3847	0.0000	0.8715	1.0000	-0.2455	0.4316	0.3621	0.5114	0.2454
LOC104913863	0.8489	0.1611	0.4370	-0.1948	0.1104	0.4732	0.7755	0.1052	1.0000	0.2632	1.0000	-0.0791	1.0000	-0.0986
LOC104913864	0.5771	1.3509	0.6908	0.6433	0.5524	1.2899	1.0000	0.0068	1.0000	1.1014	1.0000	0.4123	1.0000	-0.1744
LOC104913865	0.2177	1.3513	0.6331	0.7982	1.0000	-0.1945	0.5745	-1.1506	1.0000	0.0878	1.0000	-0.4505	1.0000	-0.8664
LOC104913866	0.6600	0.1867	0.8154	-0.0789	0.8899	0.1070	1.0000	0.0349	1.0000	0.1272	0.9993	-0.1263	1.0000	0.0616
LOC104913867	1.0000	0.0000	1.0000	0.0000	1.0000	0.0000	1.0000	0.0000	1.0000	0.0000	1.0000	0.0000	1.0000	0.0000
LOC104913868	1.0000	0.0000	1.0000	0.0000	1.0000	0.0000	1.0000	0.0000	1.0000	0.0000	1.0000	0.0000	1.0000	0.0000
LOC104913869	0.8249	3.0143	1.0000	0.0000	1.0000	0.0000	1.0000	0.0000	1.0000	0.0000	1.0000	-3.1383	1.0000	0.0000
LOC104913870	1.0000	0.0000	1.0000	0.0000	1.0000	0.0000	1.0000	0.0000	1.0000	0.0000	1.0000	0.0000	1.0000	0.0000
LOC104913871	1.0000	0.0000	1.0000	0.0000	1.0000	0.0000	1.0000	0.0000	1.0000	0.0000	1.0000	0.0000	1.0000	0.0000
LOC104913872	1.0000	-0.0892	0.0240	-1.2128	0.7390	0.4175	0.0136	0.9355	1.0000	0.3266	0.6015	-0.7868	0.0937	0.8468
LOC104913873	0.0010	-0.5878	0.0000	-0.8394	0.1447	-0.2940	0.0001	-0.3993	1.0000	-0.0063	0.2762	-0.2458	0.7900	-0.1068
LOC104913875	1.0000	0.0000	1.0000	0.0000	1.0000	0.0000	1.0000	0.0000	1.0000	0.0000	1.0000	0.0000	1.0000	0.0000
LOC104913877	0.0787	-0.3279	0.0073	-0.3040	0.0633	-0.3615	0.0022	-0.3330	1.0000	-0.0056	1.0000	0.0303	1.0000	0.0284
LOC104913878	0.0001	1.7290	0.0000	1.9713	0.0000	2.6143	0.0000	2.7912	1.0000	0.1199	0.6191	0.3745	0.6819	0.2979
LOC104913879	0.2311	-0.2274	0.0586	-0.1757	0.9369	0.0680	0.8938	-0.0386	1.0000	0.0345	0.8269	0.0987	0.9499	-0.0665
LOC104913880	0.2303	0.7802	0.6780	0.2724	0.7480	-0.4637	0.0000	-2.1664	1.0000	0.8608	0.8793	0.3661	0.6246	-0.8339
LOC104913882	0.0000	-1.5090	0.0000	-1.6463	0.0000	-0.7013	0.0000	-0.4732	1.0000	-0.0845	0.4653	-0.2096	0.3764	0.1491
LOC104913884	0.0985	0.6068	0.0000	1.2993	0.0000	1.5941	0.0000	1.6315	1.0000	-0.4671	0.7598	0.2378	0.0218	-0.4232
LOC104913885	0.5086	-3.8790	1.0000	-0.6912	0.4670	1.3193	1.0000	0.3953	1.0000	-0.1273	1.0000	3.1976	0.7588	-1.0444
LOC104913886	1.0000	2.1900	1.0000	0.0000	1.0000	0.0000	1.0000	0.0000	1.0000	0.0000	1.0000	-2.2992	1.0000	0.0000
LOC104913887	1.0000	0.0000	1.0000	0.0000	1.0000	2.2425	1.0000	0.0000	1.0000	0.0000	1.0000	0.0000	1.0000	-2.2889
LOC104913889	1.0000	0.0000	1.0000	0.0000	1.0000	0.0000	1.0000	0.0000	1.0000	0.0000	1.0000	0.0000	1.0000	0.0000
LOC104913890	0.0000	2.0989	0.0000	2.1175	0.0000	1.4994	0.0000	1.7616	1.0000	-0.6355	0.1151	-0.5984	0.2100	-0.3631
LOC104913891	0.0000	2.4525	0.0000	2.9878	0.0001	1.8413	0.0000	2.4848	1.0000	-0.7028	1.0000	-0.1614	1.0000	-0.0593
LOC104913892	1.0000	0.0000	1.0000	2.2509	0.5076	3.6021	1.0000	0.0000	1.0000	0.0000	1.0000	2.3457	0.7477	-3.6583
LOC104913893	0.0838	0.6324	0.0125	0.5869	0.0749	0.5911	0.2606	0.3442	1.0000	0.0807	1.0000	0.0454	1.0000	-0.1610
LOC104913894	0.2172	0.6752	0.0674	0.7301	0.0364	0.9554	0.0127	0.8953	1.0000	-0.0463	1.0000	0.0195	1.0000	-0.1045
LOC104913895	0.6881	1.7520	0.6180	1.6082	1.0000	-2.4056	1.0000	0.0062	1.0000	-0.1078	1.0000	-0.2463	1.0000	2.3543
LOC104913898	0.0282	0.4877	0.0130	0.4197	0.0012	0.6475	0.0007	0.5225	1.0000	-0.0819	0.8607	-0.1373	0.3774	-0.2006
LOC104913899	1.0000	0.0000	1.0000	0.0000	1.0000	2.2425	1.0000	0.0000	1.0000	0.0000	1.0000	0.0000	1.0000	-2.2889
LOC104913900	1.0000	2.1900	1.0000	2.2533	1.0000	2.2469	1.0000	0.0000	1.0000	0.0000	1.0000	0.0489	1.0000	-2.2909
LOC104913901	1.0000	0.0000	1.0000	0.0000	1.0000	0.0000	1.0000	0.0000	1.0000	0.0000	1.0000	0.0000	1.0000	0.0000
LOC104913902	1.0000	0.0000	1.0000	0.0000	1.0000	0.0000	1.0000	0.0000	1.0000	0.0000	1.0000	0.0000	1.0000	0.0000
LOC104913903	0.6384	0.1685	0.1164	0.2437	0.5853	0.1786	1.0000	0.0128	1.0000	0.0379	0.9072	0.1249	0.8232	-0.1238
LOC104913904	0.0261	2.5929	0.0021	3.5387	0.3922	1.5029	0.1531	2.5439	1.0000	-0.9614	1.0000	-0.0237	1.0000	0.0787
LOC104913905	1.0000	-0.3217	0.0330	3.0089	1.0000	-1.0194	0.9096	1.3845	1.0000	-0.9563	0.2676	2.3796	1.0000	1.4486
LOC104913906	0.4471	-0.4926	0.0718	-0.7523	0.1438	-0.8241	0.0076	-1.1257	1.0000	0.1610	1.0000	-0.0871	1.0000	-0.1354
LOC104913907	0.0068	0.7097	0.0013	0.5218	0.0161	0.6345	0.0033	0.4442	1.0000	0.1537	1.0000	-0.0209	1.0000	-0.0314
LOC104913908	0.1876	-0.2725	0.0000	-0.3670	0.0000	-0.7335	0.0000	-0.7634	1.0000	0.1227	1.0000	0.0405	0.7554	0.0981
LOC104913909	0.8260	-0.2188	0.6009	-0.3106	1.0000	-0.1499	1.0000	0.0052	1.0000	-0.1021	1.0000	-0.1832	1.0000	0.0564
LOC104913910	0.8226	-0.0880	0.0000	-0.3458	0.0150	-0.3771	0.0000	-0.7555	1.0000	0.2286	1.0000	-0.0172	0.2671	-0.1446
LOC104913913	0.2298	0.9657	0.6940	0.4284	0.3276	0.7917	0.9942	-0.3085	1.0000	0.0717	0.9326	-0.4550	0.3139	-1.0206
LOC104913914	0.6048	-0.2150	0.0068	-0.4561	0.8732	0.1191	1.0000	-0.0124	1.0000	0.0628	0.9307	-0.1655	1.0000	-0.0644
LOC104913915	1.0000	0.0000	1.0000	0.0000	0.8033	3.0892	1.0000	0.0000	1.0000	0.0000	1.0000	0.0000	1.0000	-3.1369
LOC104913917	1.0000	-2.4788	1.0000	-2.3960	1.0000	-0.1597	1.0000	-2.3179	1.0000	-0.1089	1.0000	0.0000	1.0000	-2.2909
LOC104913918	1.0000	-0.8561	1.0000	-1.0008	1.0000	0.5042	0.0547	2.3285	1.0000	-0.6504	1.0000	-0.7977	0.6843	1.1774
LOC104913919	0.8580	0.3070	0.0019	1.2876	0.0001	1.5711	0.0000	2.5269	1.0000	-0.3650	0.3799	0.6317	0.0729	0.5974
LOC104913920	0.1766	-0.5057	1.0000	-0.0269	0.0010	-1.0671	0.1807	-0.4645	1.0000	-0.6364	1.0000	-0.1431	1.0000	-0.0266
LOC104913921	0.0069	1.5709	1.0000	-0.2449	1.0000	0.0772	0.0182	-1.7574	1.0000	0.4442	0.0254	-1.3598	0.2442	-1.3848
LOC104913922	1.0000	2.1850	1.0000	-0.9973	1.0000	2.2469	1.0000	0.5384	1.0000	3.1057	1.0000	0.0514	1.0000	1.4485
LOC104913924	0.0211	2.2166	0.0115	1.7325	0.0006	2.7690	0.0002	2.2044	1.0000	0.6484	1.0000	0.1728	1.0000	0.0822
LOC104913925	1.0000	-0.0968	1.0000	0.0376	0.0005	1.0506	0.0000	1.0846	1.0000	0.1217	0.9094	0.2681	0.8768	0.1609
LOC104913926	0.0095	1.3575	1.0000	0.0943	0.0041	1.4484	0.0030	0.9836	1.0000	0.8600	0.8265	-0.3903	0.5509	0.4014
LOC104913927	0.3148	0.7931	0.0054	1.4602	0.0011	1.6428	0.0000	2.4741	1.0000	-0.4216	1.0000	0.2534	0.5274	0.4117
LOC104913928	0.8249	3.0254	1.0000	0.0000	1.0000	0.0000	1.0000	0.0000	1.0000	0.0000	1.0000	-3.1468	1.0000	0.0000
LOC104913929	0.6582	-0.1738	0.0099	-0.4801	0.0005	-0.7793	0.0282	-0.4052	1.0000	-0.0572	0.1832	-0.3516	0.2120	0.3207
LOC104913930	0.0527	0.7957	0.0018	1.0810	0.0000	2.5656	0.0000	2.3607	1.0000	-0.1879	1.0000	0.1126	0.0975	-0.3839
LOC104913931	0.0960	-0.3782	0.8718	-0.0649	0.9668	0.0793	0.2092	0.1825	1.0000	-0.1498	0.6637	0.1767	1.0000	-0.0413

LOC104913932	0.1607	-0.4443	0.0006	-0.5669	0.0001	-1.0693	0.0001	-0.6739	1.0000	0.2744	0.9773	0.1650	0.0136	0.6750
LOC104913933	0.7155	0.4797	1.0000	0.1677	0.7673	0.4675	0.3018	0.6866	1.0000	0.1283	1.0000	-0.1710	0.9696	0.3509
LOC104913934	1.0000	0.0000	1.0000	-2.3985	1.0000	0.0000	1.0000	-2.3200	1.0000	2.2734	1.0000	0.0000	1.0000	0.0000
LOC104913935	1.0000	0.0000	1.0000	0.0000	0.8033	3.0892	1.0000	2.3241	1.0000	0.0000	1.0000	0.0000	1.0000	-0.7815
LOC104913936	0.0000	-1.1592	0.0000	-1.5797	0.1007	-0.3940	0.0001	-0.4430	1.0000	0.2106	0.8939	-0.1965	0.5554	0.1662
LOC104913937	0.0016	-5.9175	0.1480	-2.2075	0.1331	1.0410	0.1070	1.2063	1.0000	-0.6487	1.0000	3.2002	0.8730	-0.4775
LOC104913940	0.2956	-0.2381	0.0000	-0.6051	1.0000	0.0171	0.5124	-0.0983	1.0000	0.1041	0.2781	-0.2498	1.0000	-0.0057
LOC104913942	0.6445	0.2870	0.0545	0.5821	0.1796	-0.6111	0.8165	-0.1778	1.0000	-0.3564	1.0000	-0.0484	1.0000	0.0819
LOC104913943	1.0000	-0.1042	0.0007	2.2348	0.2847	0.7880	0.0000	2.7276	1.0000	-1.4173	0.3272	0.9285	0.6456	0.5270
LOC104913944	0.0000	-1.9877	0.0000	-1.9458	0.0028	-0.4973	0.0000	-0.6455	1.0000	-0.0067	1.0000	0.0475	0.3469	-0.1494
LOC104913945	1.0000	-0.1374	0.1546	-2.6963	0.0864	1.5701	0.0710	1.4162	1.0000	0.0866	0.4857	-2.4811	1.0000	-0.0622
LOC104913946	0.2656	-1.2410	1.0000	-0.2840	0.5246	-0.7710	1.0000	0.2132	1.0000	-0.3393	1.0000	0.6323	0.9605	0.6536
LOC104913948	1.0000	-0.3477	1.0000	0.2128	0.3214	1.3411	0.9162	-1.3749	1.0000	-0.5061	1.0000	0.0610	0.0571	-3.2246
LOC104913950	0.5968	-0.2078	0.4765	-0.1683	0.0461	-0.5673	0.0001	-0.6205	1.0000	0.0336	1.0000	0.0852	1.0000	-0.0125
LOC104913952	1.0000	-2.4788	1.0000	2.2533	1.0000	-2.4061	1.0000	0.0000	1.0000	-2.3771	1.0000	2.3481	1.0000	0.0000
LOC104913953	1.0000	-0.2892	0.0853	-4.7292	0.4424	1.8933	0.5079	-1.4647	1.0000	2.1976	1.0000	-2.2992	0.9835	-1.1475
LOC104913956	0.8808	-0.1533	1.0000	0.0638	0.4540	-0.3350	0.1378	0.3777	1.0000	-0.2816	1.0000	-0.0527	0.2360	0.4374
LOC104913957	0.5646	0.3016	0.0000	1.1722	0.0001	1.1636	0.0000	1.9777	1.0000	-0.4924	0.1803	0.3942	0.1145	0.3271
LOC104913958	0.2829	0.3975	0.0006	0.6734	0.3214	0.3502	0.0017	0.6218	1.0000	-0.2737	1.0000	0.0137	1.0000	0.0021
LOC104913960	1.0000	0.0000	1.0000	0.0000	0.8033	3.0892	1.0000	0.0000	1.0000	0.0000	1.0000	0.0000	1.0000	-3.1369
LOC104913961	0.0013	-0.4684	0.0000	-0.7179	0.0000	-0.8955	0.0000	-1.0078	1.0000	0.1907	1.0000	-0.0464	0.8306	0.0834
LOC104913962	0.0000	1.9637	0.0000	2.8350	0.0000	3.0923	0.0000	3.3989	1.0000	-0.4932	0.6072	0.3969	0.7119	-0.1788
LOC104913964	0.2579	0.8800	0.7738	-0.4466	1.0000	-0.0946	0.9248	0.3090	1.0000	0.2455	0.3123	-1.0707	0.7248	0.6567
LOC104913965	0.0000	-1.1383	0.0000	-1.3296	0.0000	-1.3745	0.0000	-1.2120	1.0000	0.0531	0.9428	-0.1258	0.5564	0.2208
LOC104913967	0.0082	-0.9183	0.0001	-1.2897	0.0014	-1.0875	0.0454	-0.7543	1.0000	-0.4666	0.0743	-0.8238	1.0000	-0.1258
LOC104913969	0.0002	-0.5837	0.0000	-0.7109	0.0000	-0.6955	0.0000	-0.5757	1.0000	0.0581	1.0000	-0.0571	0.1315	0.1833
LOC104913970	0.0667	-0.6240	0.0045	-0.6885	0.6298	-0.2277	1.0000	-0.0467	1.0000	0.0531	1.0000	0.0008	0.6836	0.2390
LOC104913973	0.0187	-2.4771	0.0803	-2.3640	0.7515	-0.5385	0.2292	0.9884	1.0000	-0.5982	1.0000	-0.4752	0.4979	0.9285
LOC104913974	1.0000	0.0000	0.7674	-3.2499	1.0000	0.0000	0.7710	-3.1632	1.0000	3.1057	1.0000	0.0000	1.0000	0.0000
LOC104913976	1.0000	0.0000	1.0000	0.0000	1.0000	0.0000	1.0000	0.0000	1.0000	0.0000	1.0000	0.0000	1.0000	0.0000
LOC104913979	0.6396	0.2750	0.1058	0.5007	0.0122	0.7931	0.0327	0.5765	1.0000	-0.0531	1.0000	0.1851	0.6711	-0.2631
LOC104913980	1.0000	-0.3364	1.0000	0.6940	1.0000	-0.1902	1.0000	-2.3180	1.0000	-1.4945	1.0000	-0.4752	0.7477	-3.6583
LOC104913981	0.8226	3.0199	1.0000	0.0000	1.0000	0.0000	1.0000	2.3256	1.0000	0.0000	1.0000	-3.1429	1.0000	2.3554
LOC104913982	1.0000	0.0000	1.0000	0.0000	1.0000	0.0000	1.0000	0.0000	1.0000	0.0000	1.0000	0.0000	1.0000	0.0000
LOC104913984	0.0000	-4.9281	0.0000	-5.6535	0.6454	0.3453	0.0127	0.7369	1.0000	-0.0803	1.0000	-0.7977	0.6944	0.3161
LOC104913985	1.0000	0.0000	0.7674	3.0956	1.0000	2.2425	0.7701	3.1732	1.0000	0.0000	1.0000	3.2002	1.0000	0.9168
LOC104913986	1.0000	0.0000	1.0000	0.0000	1.0000	0.0000	1.0000	2.3256	1.0000	0.0000	1.0000	0.0000	1.0000	2.3554
LOC104913987	0.0081	1.0411	0.0001	1.3167	0.0116	-1.3159	0.1148	-0.8019	1.0000	-0.5675	0.8579	-0.2791	1.0000	-0.0484
LOC104913988	0.0153	0.3955	0.0000	0.5326	0.1388	-0.2756	0.0038	-0.2617	1.0000	-0.0093	0.5013	0.1405	1.0000	0.0098
LOC104913990	1.0000	-2.4788	1.0000	0.0000	1.0000	-2.4061	1.0000	0.0000	1.0000	-2.3771	1.0000	0.0000	1.0000	0.0000
LOC104913992	1.0000	-2.4776	1.0000	-0.9973	1.0000	-0.1624	0.7210	1.2300	1.0000	0.7318	1.0000	2.3481	0.6865	2.1419
LOC104913993	0.0366	-0.5324	0.0000	-0.6423	1.0000	0.0539	0.0201	0.2887	1.0000	-0.0260	0.9721	-0.1237	0.3860	0.2140
LOC104913994	0.0546	0.9985	0.0001	1.1294	0.0000	2.0012	0.0000	1.9388	1.0000	-0.0869	1.0000	0.0598	0.9073	-0.1375
LOC104913995	1.0000	0.0000	1.0000	0.0000	1.0000	0.0000	1.0000	0.0000	1.0000	0.0000	1.0000	0.0000	1.0000	0.0000
LOC104913996	1.0000	0.0000	1.0000	0.0000	1.0000	0.0000	1.0000	0.0000	1.0000	0.0000	1.0000	0.0000	1.0000	0.0000
LOC104913997	1.0000	0.0000	1.0000	-2.3985	1.0000	0.0000	1.0000	-2.3200	1.0000	2.2734	1.0000	0.0000	1.0000	0.0000
LOC104913998	1.0000	0.0000	1.0000	0.0000	1.0000	0.0000	1.0000	0.0000	1.0000	0.0000	1.0000	0.0000	1.0000	0.0000
LOC104913999	1.0000	2.1850	1.0000	-0.1557	1.0000	2.2469	0.7710	-3.1632	1.0000	3.1057	1.0000	0.9035	1.0000	-2.2909
LOC104914000	1.0000	0.0000	1.0000	0.0000	1.0000	0.0000	1.0000	0.0000	1.0000	0.0000	1.0000	0.0000	1.0000	0.0000
LOC104914002	0.3315	0.6085	0.0511	0.7704	0.9287	-0.2747	0.9053	-0.2283	1.0000	-0.2572	1.0000	-0.0859	1.0000	-0.2016
LOC104914003	1.0000	0.0000	1.0000	-2.3985	0.0091	5.5287	0.0171	3.1764	1.0000	2.2734	1.0000	0.0000	1.0000	-0.0458
LOC104914004	0.3181	-2.3898	0.7250	-1.3874	0.7404	0.7702	0.0647	1.6375	1.0000	-0.1292	1.0000	0.9006	0.8476	0.7446
LOC104914005	0.8249	-3.3412	0.6884	-0.8268	0.2908	1.6939	0.9348	0.4545	1.0000	1.7543	0.4563	4.4223	1.0000	0.5257
LOC104914006	0.0000	-2.3847	0.0000	-2.6433	0.0000	2.3886	0.0000	1.8800	1.0000	0.0955	1.0000	-0.1502	0.0069	-0.4074
LOC104914007	0.2168	-3.0304	0.0002	-4.0288	0.9728	0.5791	0.5355	0.5344	1.0000	1.0149	1.0000	0.0489	0.3369	0.9684
LOC104914008	0.9532	0.6139	0.0610	1.7646	1.0000	0.4572	1.0000	0.3146	1.0000	-0.4373	0.9672	0.7267	1.0000	-0.5735
LOC104914009	0.0000	1.0157	0.0000	1.3997	0.0285	0.4822	0.7922	0.0816	1.0000	-0.3079	0.9449	0.0871	0.0000	-0.7030
LOC104914010	1.0000	0.0000	1.0000	2.2509	1.0000	0.0000	1.0000	0.0000	1.0000	0.0000	1.0000	2.3457	1.0000	0.0000
LOC104914011	1.0000	0.0000	1.0000	0.0000	1.0000	0.0000	1.0000	0.0000	1.0000	0.0000	1.0000	0.0000	1.0000	0.0000
LOC104914012	0.6877	-2.0832	0.7674	-3.2499	0.0000	2.9766	0.0000	3.5531	1.0000	-1.0414	1.0000	-2.2957	0.7783	-0.4623
LOC104914013	0.0604	-3.2079	0.0038	-5.4498	0.0000	2.6182	0.0024	1.7051	1.0000	0.0213	1.0000	-2.2992	0.0705	-0.8907
LOC104914014	0.1141	-0.4801	0.3828	-0.2327	0.0401	-0.5693	1.0000	-0.0185	1.0000	-0.4031	1.0000	-0.1442	0.9286	0.1501
LOC104914016	1.0000	0.0000	1.0000	0.0000	1.0000	0.0000	1.0000	0.0000	1.0000	0.0000	1.0000	0.0000	1.0000	0.0000
LOC104914020	1.0000	0.0000	1.0000	0.0000	1.0000	0.0000	1.0000	0.0000	1.0000	0.0000	1.0000	0.0000	1.0000	0.0000
LOC104914021	1.0000	0.0000	1.0000	0.0000	1.0000	0.0000	1.0000	0.0000	1.0000	0.0000	1.0000	0.0000	1.0000	0.0000
LOC104914022	1.0000	0.0000	1.0000	0.0000	1.0000	0.0000	1.0000	0.0000	1.0000	0.0000	1.0000	0.0000	1.0000	0.0000
LOC104914023	1.0000	0.0000	1.0000	0.0000	1.0000	0.0000	1.0000	0.0000	1.0000	0.0000	1.0000	0.0000	1.0000	0.0000
LOC104914024	1.0000	0.0000	1.0000	0.0000	1.0000	0.0000	1.0000	0.0000	1.0000	0.0000	1.0000	0.0000	1.0000	0.0000
LOC104914025	1.0000	0.0000	1.0000	0.0000	1.0000	0.0000	1.0000	0.0000	1.0000	0.0000	1.0000	0.0000	1.0000	0.0000
LOC104914026	1.0000	0.0000	1.0000	0.0000	1.0000	0.0000	1.0000	0.0000	1.0000	0.0000	1.0000	0.0000	1.0000	0.0000
LOC104914027	1.0000	-2.4776	0.4642	-3.7944	1.0000	0.6783	0.9162	-1.3767	1.0000	1.2738	1.0000	0.0000	1.0000	-0.7795
LOC104914028	0.0000	-4.8730	0.0000	-7.1555	1.0000	-0.1475	0.3914	-0.5758	1.0000	0.0656	1.0000	-2.2957	1.0000	-0.3629
LOC104914031	0.6936	1.4527	0.4634	-3.7803	1.0000	-2.4061	0.9167	-1.3690	1.0000	1.2548	0.6450	-4.0608	1.0000	2.3543
LOC104914032	0.0000	-4.1488	0.0000	-4.2161	0.0001	-1.4733	0.0000	-1.4740	1.0000	0.2614	1.0000	0.2082	0.9300	0.2633
LOC104914033	1.0000	0.0000	1.0000	0.0000	1.0000	0.0000	1.0000	0.0000	1.0000	0.0000	1.0000	0.0000	1.0000	0.0000
LOC104914035	0.2038	0.2892	0.0001	0.3803	0.7834	-0.1148	0.0069	-0.2868	1.0000	0.0183	0.8018	0.1218	0.5328	-0.1484
LOC104914036	1.0000	-0.2696	0.4051	-0.9299	0.0123	1.3414	0.1390	1.0131	1.0000	0.0910	0.9844	-0.5570	1.0000	-0.2240
LOC104914038	0.1430	1.8883	0.7223	1.0562	1.0000	-0.1865	1.0000	0.0105	1.0000	-0.6546	0.3834	-1.4802	1.0000	-0.4561
LOC104914039	0.0473	0.4324	0.0001	0.3400	0.0000	-1.0684	0.0000	-0.9572	1.0000	-0.0439	0.6242	-0.1241	1.0000	0.0740
LOC104914040	1.0000	0.0000	1.0000	0.0000	1.0000	0.0000	1.0000	0.0000	1.0000	0.0000	1.0000	0.0000	1.0000	0.0000
LOC104914041	0.9404	0.0678	0.0440	0.1943	0.2711	-0.2147	0.0060	-0.2560	1.0000	-0.0981	1.0000	0.0407	0.4066	-0.1341
LOC104914042	1.0000	-2.4776	0.0931	2.5625	1.0000	-2.4056	1.0000	0.0062	1.0000	-0.1078	0.1335	5.0746	1.0000	2.3543
LOC104914043	1.0000	0.0000	1.0000	0.0000	1.0000	0.0000	1.0000	0.0000	1.0000	0.0000	1.0000	0.0000	1.0000	0.0000
LOC104914044	0.7902	0.3186	0.0187	1.0242	0.0000	1.5654	0.0128	0.8981	1.0000	-0.0023	0.3227	0.7175	0.0621	-0.6697
LOC104914045	0.0414	0.4793	1.0000	0.0094	0.0000	1.3934	0.0000	1.1327	1.0000	0.3181	0.8899	-0.1391	1.0000	0.0622
LOC104914046	0.0079	2.9615	0.0117	2.6848	0.3868	1.5024	1.0000	0.5362	1.0000	-0.1146	1.0000	-0.3883	0.8620	-1.0811
LOC104914047	1.0000	0.0000	1.0000	0.0000	1.0000	0.0000	1.0000	0.0000	1.0000	0.0000	1.0000	0.0000	1.0000	0.0000
LOC104914048	1.0000	0.0812	0.8863	-0.1806	0.0128	1.0717	0.0723	0.6560	1.0000	-0.0238	1.0000	-0.2734	0.5057	-0.4390
LOC104914049	0.0001	-1.8018	0.0000	-1.9687	0.6897	0.2720	0.0378	0.5400	1.0000	0.1013	1.0000	-0.0502	0.4643	0.3719
LOC104914050	0.8249	3.0254	1.0000	2.2533	1.0000	0.0000	1.0000	0.0000	1.0000	0.0000	1.0000	-0.7977	1.0000	0.0000
LOC104914052	0.0000	-3.2971	0.0000	-2.9731	0.0000	-0.7354	0.0000	-1.0078	1.0000	0.0746	0.0426	0.4107	0.1773	-0.1932
LOC104914053	1.0000	-0.1659	1.0000	0.2221	0.3865	-1.8746	0.2626	-2.3201	1.0000	-0.3367	1.0000	0.0598	1.0000	-0.7795
LOC104914055	1.0000	0.0000	1.0000	0.0000	1.0000	0.0000	1.0000	0.0000	1.0000	0.0000	1.0000	0.0000	1.0000	0.0000

LOC104914056	0.9363	0.1491	0.0899	0.4229	0.0001	1.0626	0.0059	0.5871	1.0000	-0.0936	0.9569	0.1923	0.0149	-0.5655
LOC104914057	0.3102	0.6655	0.0000	1.3992	0.0003	1.4903	0.0111	0.9415	1.0000	-0.2861	0.4816	0.4607	0.0143	-0.8292
LOC104914059	1.0000	-0.0251	0.2545	-0.1314	0.0150	0.4617	0.0000	0.3850	1.0000	0.2946	0.2990	0.2019	0.0762	0.2233
LOC104914060	0.8746	0.3496	0.0000	2.0534	0.0000	2.2118	0.0000	2.1936	1.0000	-0.5141	0.0182	1.2020	0.3989	-0.5274
LOC104914061	0.6488	0.3572	1.0000	0.0348	0.0000	1.6377	0.0000	1.2455	1.0000	-0.1016	0.7715	-0.4113	0.2302	-0.4885
LOC104914063	0.2790	0.8333	0.6786	-0.3471	0.1904	0.9128	0.6380	-0.4202	1.0000	0.6120	0.7760	-0.5562	0.5109	-0.7169
LOC104914064	0.0002	1.7515	0.0000	1.9362	0.0104	1.3504	0.0010	1.2822	1.0000	-0.0889	1.0000	0.1135	1.0000	-0.1490
LOC104914065	0.8882	-0.5953	0.6182	1.6074	0.2858	-2.4984	0.9167	1.3803	1.0000	-2.4377	1.0000	-0.2484	1.0000	1.4453
LOC104914066	1.0000	0.0000	1.0000	0.0000	1.0000	0.0000	1.0000	0.0000	1.0000	0.0000	1.0000	0.0000	1.0000	0.0000
LOC104914067	1.0000	0.5453	0.9148	1.2251	0.0202	3.2224	0.4054	2.0777	1.0000	-0.1045	1.0000	0.5866	0.4643	-1.2545
LOC104914068	0.0000	-1.9631	0.0000	-1.9189	0.0000	-1.7935	0.0000	-1.3725	1.0000	0.0728	1.0000	0.1297	0.0602	0.4979
LOC104914069	1.0000	2.1900	1.0000	2.2533	1.0000	0.0000	1.0000	0.0000	1.0000	0.0000	1.0000	0.0489	1.0000	0.0000
LOC104914071	1.0000	2.1850	1.0000	2.2509	1.0000	0.0000	1.0000	0.0000	1.0000	0.0000	1.0000	0.0495	1.0000	0.0000
LOC104914072	1.0000	0.0000	0.7666	3.0922	0.8011	3.0840	1.0000	0.0000	1.0000	0.0000	1.0000	3.1976	1.0000	-3.1344
LOC104914073	1.0000	2.1850	1.0000	-0.9997	0.4809	3.6101	1.0000	0.0072	1.0000	3.1057	1.0000	0.0495	1.0000	-0.4570
LOC104914076	1.0000	0.0000	1.0000	0.0000	1.0000	0.0000	1.0000	0.0000	1.0000	0.0000	1.0000	0.0000	1.0000	0.0000
LOC104914077	1.0000	0.5884	1.0000	-0.1658	0.9361	-1.5529	0.9096	-1.3722	1.0000	-0.1210	1.0000	-0.8586	1.0000	0.0661
LOC104914079	0.1564	1.7614	0.0372	1.8453	1.0000	-1.1017	0.2781	-4.0944	1.0000	-0.1174	1.0000	-0.0244	1.0000	-3.1344
LOC104914081	0.0000	-3.6332	0.0000	-3.7822	0.0000	-4.9704	0.0000	-4.2245	1.0000	-0.0449	0.9942	-0.1816	0.0746	0.7044
LOC104914082	1.0000	0.0000	1.0000	2.2509	1.0000	0.0000	1.0000	0.0000	1.0000	0.0000	1.0000	2.3457	1.0000	0.0000
LOC104914083	0.0760	-0.6489	0.0011	-0.8550	0.0090	-1.0201	0.0194	-0.5061	1.0000	0.1371	1.0000	-0.0563	0.1307	0.6567
LOC104914085	0.3492	0.2327	0.2740	0.1385	0.2231	0.2763	0.7986	0.0581	1.0000	0.1536	1.0000	0.0726	1.0000	-0.0581
LOC104914086	1.0000	2.1900	1.0000	2.2509	1.0000	2.2469	1.0000	0.0000	1.0000	0.0000	1.0000	0.0470	1.0000	-2.2909
LOC104914088	0.5384	3.5542	1.0000	-2.3985	1.0000	2.2469	1.0000	0.0057	1.0000	2.2734	0.8764	-3.6794	1.0000	0.0649
LOC104914089	1.0000	0.0000	1.0000	2.2533	0.4809	3.6122	1.0000	0.0000	1.0000	0.0000	1.0000	2.3481	0.7287	-3.6637
LOC104914090	0.0000	1.4779	0.0000	2.0559	0.5417	-0.3154	0.0000	1.2302	1.0000	-0.6896	1.0000	-0.0999	0.0051	0.8625
LOC104914091	0.0849	-1.1361	0.4843	-0.4356	0.0785	-0.9096	0.6317	-0.3307	1.0000	-0.2239	0.9808	0.4925	0.9061	0.3583
LOC104914093	0.6362	0.2674	0.0000	1.0769	0.9396	0.1425	0.0678	0.5948	1.0000	-0.7509	1.0000	0.0702	0.6865	-0.2933
LOC104914095	0.0228	3.0829	0.0065	3.3493	0.0181	3.2251	0.0007	3.7941	1.0000	-0.1034	1.0000	0.1655	1.0000	0.4620
LOC104914102	1.0000	0.0000	1.0000	-2.3985	1.0000	2.2469	0.9162	1.3864	1.0000	2.2734	1.0000	0.0000	1.0000	1.4496
LOC104914104	0.0070	-2.0248	0.0001	-1.9515	0.0562	-1.2962	0.0045	-1.3042	1.0000	0.6561	1.0000	0.7437	0.9059	0.6523
LOC104914106	1.0000	0.0000	1.0000	0.0000	1.0000	0.0000	1.0000	0.0000	1.0000	0.0000	1.0000	0.0000	1.0000	0.0000
LOC104914108	1.0000	-0.0460	0.3909	0.3024	0.6211	0.2935	0.3894	0.3168	1.0000	-0.0111	0.6888	0.3486	1.0000	0.0190
LOC104914110	1.0000	-2.4776	0.9876	-1.0904	1.0000	-2.4056	1.0000	-0.3848	1.0000	1.6647	1.0000	3.1976	0.7287	3.7384
LOC104914112	0.9546	-0.1418	1.0000	0.0394	1.0000	0.0607	0.7710	0.1484	1.0000	-0.0238	1.0000	0.1666	1.0000	0.0698
LOC104914114	0.0634	0.6467	0.0780	0.4667	0.0241	-0.8465	0.3995	-0.2755	1.0000	0.0526	1.0000	-0.1143	0.0958	0.6293
LOC104914115	1.0000	0.0000	1.0000	0.0000	1.0000	0.0000	1.0000	0.0000	1.0000	0.0000	1.0000	0.0000	1.0000	0.0000
LOC104914119	0.3538	-0.7095	0.2331	0.7652	0.8982	-0.2909	0.1086	0.9503	1.0000	-1.1212	1.0000	0.3680	1.0000	0.1264
LOC104914121	0.0864	0.6431	0.0081	0.7280	0.0000	1.7837	0.0001	0.8938	1.0000	0.1386	0.8785	0.2373	0.0041	-0.7449
LOC104914122	1.0000	0.0000	1.0000	0.0000	1.0000	0.0000	1.0000	0.0000	1.0000	0.0000	1.0000	0.0000	1.0000	0.0000
LOC104914124	0.0373	0.6566	0.0126	0.5169	0.0000	1.1364	0.0000	1.1485	1.0000	0.0060	1.0000	-0.1206	1.0000	0.0236
LOC104914125	1.0000	0.0000	1.0000	0.0000	1.0000	0.0000	1.0000	0.0000	1.0000	0.0000	1.0000	0.0000	1.0000	0.0000
LOC104914126	1.0000	0.0000	1.0000	0.0000	1.0000	0.0000	1.0000	0.0000	1.0000	0.0000	1.0000	0.0000	1.0000	0.0000
LOC104914127	0.9671	-1.6927	0.0445	-4.9507	1.0000	0.5027	1.0000	-0.2076	1.0000	1.0387	1.0000	-2.2957	1.0000	0.3294
LOC104914128	0.0008	-2.8612	0.0000	-3.0303	0.0012	1.4564	0.0000	1.6983	1.0000	0.5194	1.0000	0.3684	0.0160	0.7736
LOC104914131	0.0000	1.3904	0.0000	1.3253	0.4556	0.2806	0.1806	0.3148	1.0000	-0.2121	0.2593	-0.2649	0.8838	-0.1718
LOC104914133	1.0000	-0.2920	1.0000	0.0000	1.0000	-0.1591	1.0000	0.0000	1.0000	-2.3757	1.0000	-2.2957	1.0000	-2.2909
LOC104914134	1.0000	0.2029	0.4688	3.6127	1.0000	-1.0191	1.0000	2.3256	1.0000	-3.2304	1.0000	0.0533	1.0000	0.0660
LOC104914136	1.0000	0.0000	1.0000	0.0000	1.0000	0.0000	1.0000	0.0000	1.0000	0.0000	1.0000	0.0000	1.0000	0.0000
LOC104914141	0.8249	3.0143	0.7674	-3.2570	0.0101	5.3980	0.3436	1.6950	1.0000	3.1190	1.0000	-3.1383	1.0000	-0.5547
LOC104914142	1.0000	-0.8620	0.9862	-1.0773	0.9361	-1.5529	0.6476	-1.7495	1.0000	0.2556	1.0000	0.0563	1.0000	0.0660
LOC104914144	0.6102	-1.1112	1.0000	-1.0008	1.0000	-0.6583	0.1072	2.1985	1.0000	-1.8053	1.0000	-1.7117	0.8306	1.0466
LOC104914145	0.2264	1.8555	0.3583	-1.1509	0.0826	2.2590	0.5032	0.6578	1.0000	2.3311	1.0000	-0.6561	0.7890	0.7317
LOC104914146	0.2043	-0.2726	0.3899	0.1231	0.0000	-1.8428	0.0000	-2.0261	1.0000	-0.1248	0.1075	0.2831	0.2476	-0.3039
LOC104914147	0.4263	-0.2372	0.0003	-0.5293	1.0000	-0.0710	0.5203	-0.1304	1.0000	0.0125	0.3642	-0.2656	1.0000	-0.0400
LOC104914148	0.1893	0.6859	0.0000	1.5323	0.6169	0.3968	0.0000	1.6705	1.0000	-0.6672	1.0000	0.1965	0.1586	0.6213
LOC104914149	0.6877	-2.0809	1.0000	-0.6362	0.2785	-4.1857	0.0489	-4.8482	1.0000	0.6371	0.8269	2.1235	1.0000	0.0000
LOC104914150	0.9770	0.4923	1.0000	0.1329	0.8012	0.7759	1.0000	-0.3772	1.0000	-0.6843	0.8371	-1.0388	0.2396	-1.8365
LOC104914152	0.9650	1.0580	1.0000	0.0000	1.0000	-2.4061	1.0000	0.0000	1.0000	-2.3771	0.8785	-3.6647	1.0000	0.0000
LOC104914153	0.0009	2.2946	0.0439	1.7125	0.8207	-1.1361	0.0708	-4.6403	1.0000	-0.1272	0.5980	-0.6964	0.7477	-3.6583
LOC104914155	1.0000	0.0455	1.0000	-0.0393	0.0526	0.4280	0.0493	0.2809	1.0000	-0.1098	0.6703	-0.1804	0.1907	-0.2504
LOC104914156	0.9009	-0.4300	1.0000	0.2544	0.8581	-0.4447	1.0000	-0.0977	1.0000	-0.5629	1.0000	0.1350	1.0000	-0.2140
LOC104914157	1.0000	-0.2051	0.6847	0.7902	0.1188	-2.2268	0.2789	1.2296	1.0000	-0.9438	1.0000	0.0600	0.1189	2.5210
LOC104914158	0.4507	-1.8112	1.0000	0.3687	1.0000	-0.4582	1.0000	-0.8404	1.0000	-1.6029	1.0000	0.5866	0.7287	-1.9910
LOC104914159	0.4371	0.7516	0.0361	0.9910	0.0000	2.7935	0.0000	2.0128	1.0000	0.2099	0.7314	0.4732	0.0801	-0.5580
LOC104914161	0.0556	-0.3349	0.0000	-0.5694	1.0000	0.0148	0.1611	-0.1444	1.0000	0.1048	0.7112	-0.1171	1.0000	-0.0492
LOC104914162	0.0386	5.0437	0.4135	1.9103	1.0000	0.0000	1.0000	0.0062	1.0000	2.2676	1.0000	-0.7585	1.0000	2.3543
LOC104914163	0.0240	0.4971	0.5273	0.1134	0.0190	0.4869	1.0000	0.0134	1.0000	0.2161	0.7345	-0.1560	0.1694	-0.2522
LOC104914164	0.6669	0.6266	0.7950	0.6107	0.0097	1.7284	0.0066	1.7304	1.0000	-0.2920	1.0000	-0.2973	1.0000	-0.2899
LOC104914165	0.0923	2.0630	1.0000	0.1383	0.0000	3.7282	0.0000	1.9854	1.0000	1.7871	1.0000	-0.1177	1.0000	0.0516
LOC104914166	0.5385	0.8231	0.7726	0.3970	0.0000	3.1190	0.0000	1.9365	1.0000	0.3466	1.0000	-0.0687	0.0227	-0.8325
LOC104914167	1.0000	0.0000	1.0000	-1.0017	0.0058	5.5185	0.0001	3.4372	1.0000	3.1126	1.0000	2.3457	0.2963	1.0657
LOC104914168	1.0000	0.0000	1.0000	-2.3960	1.0000	0.0000	1.0000	-2.3180	1.0000	2.2676	1.0000	0.0000	1.0000	0.0000
LOC104914170	0.8249	3.0254	1.0000	0.0000	0.8033	3.0789	1.0000	2.3241	1.0000	0.0000	1.0000	-3.1468	1.0000	-0.7782
LOC104914171	0.4639	0.4495	0.0232	0.8156	0.0000	2.3063	0.0000	2.0533	1.0000	-0.1357	1.0000	0.2462	0.1576	-0.3796
LOC104914173	0.0000	-0.9950	0.0000	-1.2760	0.0000	-0.8764	0.0000	-0.5239	1.0000	0.0547	0.1551	-0.2138	0.0000	0.4119
LOC104914174	0.4266	-0.1699	0.0011	-0.3045	0.0077	-0.4881	0.0069	-0.2531	1.0000	-0.0137	0.4311	-0.1355	0.2206	0.2277
LOC104914175	0.0585	-0.4586	0.0014	-0.4522	0.5681	0.1983	0.0001	0.4748	1.0000	-0.1639	0.8785	-0.1448	0.8857	0.1196
LOC104914176	0.0006	-0.5740	0.0000	-0.8529	0.1647	-0.2543	0.0764	-0.1586	1.0000	0.1806	1.0000	-0.0858	0.0090	0.2813
LOC104914177	0.0241	-1.6416	0.0067	-1.4305	1.0000	0.0190	0.7708	-0.3058	1.0000	-0.1711	1.0000	0.0498	0.7491	-0.4899
LOC104914178	1.0000	0.1077	0.9828	-0.2921	1.0000	-0.1855	0.7926	-0.5112	1.0000	-0.1294	1.0000	-0.5172	1.0000	-0.4468
LOC104914179	0.3089	3.9281	1.0000	-0.1472	1.0000	0.0000	1.0000	-2.3200	1.0000	2.2734	1.0000	-1.7124	1.0000	0.0000
LOC104914181	1.0000	0.0000	0.1388	4.3092	1.0000	0.0000	1.0000	0.0000	1.0000	0.0000	0.4563	4.4223	1.0000	0.0000
LOC104914182	1.0000	-0.0166	0.0000	-0.6920	0.0000	-1.4560	0.0000	-1.3817	1.0000	0.1220	0.0173	-0.5405	0.8939	0.2021
LOC104914184	0.0084	1.7193	0.5818	0.3831	0.0000	3.0180	0.0003	1.3106	1.0000	1.2783	1.0000	-0.0427	0.7287	-0.4261
LOC104914185	0.9036	0.1142	0.3666	-0.1836	0.0502	0.4615	0.0022	0.3953	1.0000	0.4060	1.0000	0.1207	0.0463	0.3447
LOC104914186	0.5126	0.5663	0.8221	-0.3274	0.0105	-2.2149	1.0000	-0.1331	1.0000	0.4691	1.0000	-0.4117	0.0009	2.5574
LOC104914187	1.0000	2.1900	0.7666	3.0922	1.0000	2.2425	1.0000	0.0000	1.0000	0.0000	1.0000	0.8994	1.0000	-2.2889
LOC104914188	0.4801	-0.2592	0.5387	-0.1085	0.0003	0.8004	0.0000	0.7822	1.0000	0.1905	0.0867	0.3531	0.3343	0.1770
LOC104914191	1.0000	0.0000	1.0000	-2.3985	1.0000	0.0000	1.0000	-2.3200	1.0000	2.2734	1.0000	0.0000	1.0000	0.0000
LOC104914193	0.3691	-1.0175	0.3498	1.2314	0.3963	-0.8609	1.0000	0.0070	1.0000	-2.0629	1.0000	0.1935	0.6285	-1.1926
LOC104914194	1.0000	-0.0967	0.9784	0.7520	0.1702	-4.4910	0.9786	0.9248	1.0000	-1.3469	1.0000	-0.4978	0.5093	4.1263
LOC104914197	1.0000	0.0500	0.2015	0.7002	1.0000	0.0650	0.3754	0.6889	1.0000	-0.3938	1.0000	0.2694	1.0000	0.2346

LOC104914200	0.0000	1.9089	0.0000	1.6247	1.0000	-0.0950	0.8525	0.2062	1.0000	0.1721	1.0000	-0.1007	0.6533	0.4767
LOC104914201	0.0578	-1.2365	0.0036	-1.2020	0.0227	0.9870	0.0001	1.0859	1.0000	0.2576	1.0000	0.3074	0.4766	0.3595
LOC104914202	0.6099	-0.9999	1.0000	-0.1681	0.7578	0.6921	0.0197	1.8826	1.0000	-0.7828	1.0000	0.0603	1.0000	0.4163
LOC104914203	0.0000	-0.8342	0.0000	-0.7044	0.3260	-0.2416	0.0000	0.3678	1.0000	-0.1152	1.0000	0.0268	0.0001	0.4990
LOC104914204	0.0025	-0.6913	0.0000	-0.6782	0.0000	-1.0005	0.0005	-0.4592	1.0000	-0.1777	0.9178	-0.1514	0.0866	0.3695
LOC104914206	0.0000	-0.7899	0.0000	-0.7840	0.1945	-0.2949	1.0000	0.0316	1.0000	-0.1776	0.6768	-0.1591	0.6011	0.1548
LOC104914207	0.7288	-0.3277	0.8358	0.2158	0.3740	0.5999	0.0000	1.4000	1.0000	-0.2354	0.9675	0.3228	0.3369	0.5785
LOC104914208	0.2144	-1.6904	0.0884	1.8545	0.1184	1.2956	0.0209	2.2373	1.0000	-1.4699	0.2716	2.0865	0.8493	-0.5279
LOC104914209	1.0000	0.0000	1.0000	0.0000	1.0000	0.0000	1.0000	0.0000	1.0000	0.0000	1.0000	0.0000	1.0000	0.0000
LOC104914210	0.0219	0.7049	0.0311	0.4249	0.0000	1.2178	0.0000	0.7868	1.0000	0.2080	1.0000	-0.0576	0.5522	-0.2177
LOC104914211	0.6881	1.4496	0.1388	4.3092	1.0000	-2.4061	1.0000	0.0000	1.0000	-2.3771	1.0000	0.3634	1.0000	0.0000
LOC104914212	0.1698	-0.2706	0.0000	-0.4343	1.0000	-0.0333	0.4318	-0.0956	1.0000	0.1137	1.0000	-0.0374	1.0000	0.0562
LOC104914214	0.1564	2.5599	1.0000	-0.8513	0.0359	3.1074	1.0000	0.4758	1.0000	1.9385	0.7173	-1.4469	0.9094	-0.6762
LOC104914215	0.3840	0.4495	0.0177	0.6329	0.0013	1.1482	0.0000	1.3154	1.0000	-0.1710	1.0000	0.0278	1.0000	0.0004
LOC104914218	1.0000	0.1025	0.0273	0.9422	1.0000	0.1069	0.0666	0.8861	1.0000	0.1772	0.0718	1.0286	0.1239	0.9603
LOC104914219	0.4374	1.5427	0.1471	2.0182	1.0000	0.7317	1.0000	0.5372	1.0000	-0.1179	1.0000	0.3705	1.0000	-0.3110
LOC104914220	1.0000	0.0000	1.0000	0.0000	1.0000	0.0000	1.0000	0.0000	1.0000	0.0000	1.0000	0.0000	1.0000	0.0000
LOC104914221	1.0000	0.0000	1.0000	0.0000	1.0000	0.0000	1.0000	0.0000	1.0000	0.0000	1.0000	0.0000	1.0000	0.0000
LOC104914223	1.0000	-0.0951	0.6111	0.6954	0.0002	2.6321	0.0003	2.1610	1.0000	0.3362	0.7488	1.1445	1.0000	-0.1273
LOC104914225	0.0139	-0.3896	0.0002	-0.3456	0.0000	-1.6790	0.0000	-1.4243	1.0000	-0.1684	0.6726	-0.1124	0.9035	0.0922
LOC104914226	1.0000	0.0000	1.0000	0.0000	1.0000	0.0000	1.0000	0.0000	1.0000	0.0000	1.0000	0.0000	1.0000	0.0000
LOC104914227	0.0524	1.8617	1.0000	0.0204	1.0000	0.2715	0.0581	-2.0228	1.0000	1.2123	0.8603	-0.6150	0.8616	-1.0807
LOC104914228	1.0000	0.0000	1.0000	0.0000	1.0000	2.2425	0.7710	3.1710	1.0000	0.0000	1.0000	0.0000	1.0000	0.9164
LOC104914230	0.6969	0.7344	1.0000	-0.3252	0.0000	2.8977	0.0040	1.6616	1.0000	0.5788	1.0000	-0.4656	0.3469	-0.6443
LOC104914232	0.0147	-3.6176	0.0235	-2.1106	0.3617	0.8416	1.0000	0.2516	1.0000	0.2774	1.0000	1.8149	1.0000	-0.3103
LOC104914233	0.8226	-3.3425	0.4024	-2.2295	1.0000	-0.1789	0.7190	-1.2157	1.0000	1.1014	1.0000	2.3481	1.0000	0.0723
LOC104914234	1.0000	0.0000	1.0000	0.0000	1.0000	0.0000	1.0000	0.0000	1.0000	0.0000	1.0000	0.0000	1.0000	0.0000
LOC104914235	0.0000	-1.8096	0.0000	-2.2886	1.0000	-0.0217	0.2525	-0.3911	1.0000	0.2677	1.0000	-0.1994	1.0000	-0.1010
LOC104914236	1.0000	0.0000	1.0000	-2.3985	1.0000	0.0000	1.0000	-2.3200	1.0000	2.2734	1.0000	0.0000	1.0000	0.0000
LOC104914237	1.0000	0.2026	1.0000	-0.6954	1.0000	0.3491	1.0000	-0.5236	1.0000	0.4081	1.0000	-0.4766	1.0000	-0.4570
LOC104914238	0.4302	-1.1654	0.6395	0.7830	0.4003	-1.3128	0.6355	0.9639	1.0000	-1.2469	1.0000	0.7109	0.9124	1.0344
LOC104914239	1.0000	0.5900	1.0000	-0.1630	1.0000	0.7368	1.0000	-0.8429	1.0000	-0.1113	1.0000	-0.8625	0.8939	-1.6953
LOC104914240	0.9671	-1.6927	0.7640	-1.4013	0.9468	-1.5535	0.7710	-1.2244	1.0000	0.5902	1.0000	0.9006	1.0000	0.9187
LOC104914241	0.0000	2.1421	0.0000	1.9005	0.0000	2.4903	0.0000	1.4479	1.0000	0.5693	0.5746	0.3428	0.3310	-0.4671
LOC104914242	0.0207	0.6310	0.0275	0.5279	0.3383	-0.4164	0.3675	-0.2580	1.0000	-0.0359	1.0000	-0.1284	1.0000	0.1278
LOC104914243	0.9441	0.1820	1.0000	0.1139	0.5487	-0.3668	0.6895	-0.2326	1.0000	-0.0854	1.0000	-0.1415	1.0000	0.0546
LOC104914244	0.0000	-1.7241	0.0000	-1.9943	1.0000	-0.0688	0.3087	0.3510	1.0000	-0.0753	1.0000	-0.3333	0.6145	0.3515
LOC104914245	1.0000	0.0000	1.0000	0.0000	1.0000	0.0000	1.0000	0.0000	1.0000	0.0000	1.0000	0.0000	1.0000	0.0000
LOC104914246	1.0000	0.0000	1.0000	0.0000	1.0000	0.0000	1.0000	0.0000	1.0000	0.0000	1.0000	0.0000	1.0000	0.0000
LOC104914247	1.0000	0.0000	1.0000	0.0000	1.0000	0.0000	1.0000	0.0000	1.0000	0.0000	1.0000	0.0000	1.0000	0.0000
LOC104914248	1.0000	0.0000	1.0000	0.0000	1.0000	0.0000	1.0000	0.0000	1.0000	0.0000	1.0000	0.0000	1.0000	0.0000
LOC104914250	1.0000	-2.4776	1.0000	-2.3960	1.0000	-2.4056	1.0000	-2.3180	1.0000	-0.1078	1.0000	0.0000	1.0000	0.0000
LOC104914251	1.0000	0.0000	1.0000	0.0000	1.0000	0.0000	1.0000	0.0000	1.0000	0.0000	1.0000	0.0000	1.0000	0.0000
LOC104914253	1.0000	-0.0791	0.0065	1.3606	0.3492	-0.7727	0.2771	0.7952	1.0000	-0.8074	0.6337	0.6543	0.5410	0.7719
LOC104914254	0.4972	-1.1163	1.0000	0.1354	1.0000	-0.6592	0.3409	1.2782	1.0000	-0.9019	1.0000	0.3618	0.8110	1.0438
LOC104914255	0.4694	-0.4058	0.3608	-0.3360	0.0138	-1.0692	0.0125	-0.7482	1.0000	0.3251	0.7211	0.4086	0.3638	0.6508
LOC104914256	0.1470	1.1173	0.3144	0.8758	0.0793	1.2292	0.2735	0.9163	1.0000	-0.2652	0.9846	-0.4949	0.7536	-0.5722
LOC104914260	1.0000	0.0000	1.0000	0.0000	1.0000	2.2425	1.0000	0.0000	1.0000	0.0000	1.0000	0.0000	1.0000	-2.2889
LOC104914261	0.5617	0.1309	0.0179	0.1872	0.3630	0.1744	0.1881	0.1092	1.0000	0.0067	0.8840	0.0755	0.9835	-0.0531
LOC104914262	1.0000	0.0277	0.0593	0.3670	0.3698	0.2633	0.0126	0.4156	1.0000	-0.1828	0.9314	0.1701	1.0000	-0.0231
LOC104914263	0.0000	1.8378	0.0000	2.0060	0.0000	1.9052	0.0000	1.7294	1.0000	0.0614	0.9052	0.2441	1.0000	-0.1097
LOC104914264	0.0441	0.4608	0.0000	0.8659	0.0000	1.0055	0.0000	0.9967	1.0000	-0.0587	0.0360	0.3597	1.0000	-0.0612
LOC104914265	0.9099	0.4560	0.0076	1.7854	0.1863	1.1916	0.0044	1.8638	1.0000	-0.4796	0.5330	0.8599	1.0000	0.1954
LOC104914266	1.0000	-0.2920	0.9062	1.2225	1.0000	-0.1591	1.0000	0.0062	1.0000	-0.1078	1.0000	1.4317	1.0000	0.0640
LOC104914267	1.0000	0.0000	1.0000	0.0000	1.0000	0.0000	1.0000	0.0000	1.0000	0.0000	1.0000	0.0000	1.0000	0.0000
LOC104914268	1.0000	0.0000	1.0000	0.0000	1.0000	0.0000	1.0000	0.0000	1.0000	0.0000	1.0000	0.0000	1.0000	0.0000
LOC104914270	0.4197	0.7200	0.6723	0.4456	0.0004	1.8028	0.0001	1.5800	1.0000	0.2818	1.0000	0.0165	1.0000	0.0585
LOC104914271	0.0237	-5.2312	0.0019	-5.5832	0.7438	-0.8444	0.2354	-1.3970	1.0000	0.3182	1.0000	0.0000	1.0000	-0.2278
LOC104914272	0.0047	0.9944	0.0000	1.5063	0.0000	2.0728	0.0000	1.9451	1.0000	-0.1724	0.4857	0.3527	0.1508	-0.2944
LOC104914273	0.0000	-1.0525	0.0000	-0.7797	0.0001	-0.7401	0.0000	-0.6648	1.0000	-0.1421	0.8607	0.1425	1.0000	-0.0612
LOC104914277	0.5812	-0.2856	0.0003	-0.6157	0.5392	-0.3078	0.0309	0.3362	1.0000	0.0965	0.7549	-0.2234	0.0001	0.7471
LOC104914280	0.6561	0.4013	0.0768	1.0028	0.1290	0.7694	0.0000	2.0130	1.0000	-0.9420	1.0000	-0.3274	0.7686	0.3065
LOC104914281	0.0644	0.4896	0.0111	0.4313	0.2126	-0.4193	1.0000	-0.0520	1.0000	-0.4860	0.0009	-0.5336	1.0000	-0.1119
LOC104914283	1.0000	0.0694	0.7593	0.2259	1.0000	0.0829	1.0000	-0.0572	1.0000	0.1833	0.9311	0.3505	1.0000	0.0477
LOC104914284	0.8467	-1.0326	0.8628	-0.8260	0.0022	2.3802	0.0027	1.8333	1.0000	0.5237	1.0000	0.7497	1.0000	-0.0154
LOC104914285	1.0000	-2.4788	0.4021	-2.2294	0.1908	2.3554	0.2002	1.3433	1.0000	1.9530	1.0000	2.3457	0.7629	0.9503
LOC104914286	1.0000	-0.3227	0.2332	-2.0432	0.0801	2.2585	1.0000	0.1662	1.0000	1.7533	1.0000	0.0559	1.0000	-0.3298
LOC104914287	0.0982	4.8951	1.0000	0.6956	1.0000	0.0000	1.0000	-2.3180	1.0000	2.2676	0.6615	-1.8267	1.0000	0.0000
LOC104914288	0.0264	-0.6287	0.0001	-0.6398	0.0000	-2.0700	0.0000	-2.0455	1.0000	-0.1248	1.0000	-0.1233	1.0000	-0.0917
LOC104914289	1.0000	0.0000	1.0000	0.0000	1.0000	0.0000	1.0000	0.0000	1.0000	0.0000	1.0000	0.0000	1.0000	0.0000
LOC104914290	0.9829	-0.0757	0.1103	-0.2145	0.0292	0.4335	0.0000	0.5318	1.0000	0.0924	1.0000	-0.0339	0.3095	0.1951
LOC104914291	0.0036	-0.4998	0.0001	-0.3664	0.0798	-0.3086	1.0000	0.0281	1.0000	-0.2915	0.6558	-0.1460	1.0000	0.0507
LOC104914292	1.0000	-0.0115	0.2830	-0.3953	0.0358	-0.7790	0.0011	-1.0003	1.0000	-0.0797	0.5025	-0.4497	0.8973	-0.2948
LOC104914294	0.0013	-0.7006	0.0000	-1.0852	0.0000	-1.1878	0.0000	-1.0621	1.0000	0.1201	0.5811	-0.2522	0.5105	0.2512
LOC104914295	0.0719	4.8747	1.0000	2.2509	0.3240	3.9829	1.0000	2.3241	1.0000	0.0000	0.3760	-2.6659	0.9136	-1.6898
LOC104914297	1.0000	2.1850	1.0000	2.2508	1.0000	0.0000	0.7710	3.1710	1.0000	0.0000	1.0000	0.0495	1.0000	3.2049
LOC104914298	0.5694	-0.5512	0.4228	0.7379	0.1883	-0.9656	1.0000	0.2437	1.0000	-1.2396	1.0000	0.0600	1.0000	-0.0248
LOC104914299	1.0000	0.0000	1.0000	0.0000	1.0000	0.0000	1.0000	0.0000	1.0000	0.0000	1.0000	0.0000	1.0000	0.0000
LOC104914300	0.0014	0.9525	0.0006	0.7358	0.0000	1.2693	0.0000	0.9544	1.0000	0.1635	1.0000	-0.0375	0.8768	-0.1443
LOC104914301	0.0172	-1.1432	0.0000	-1.3405	0.2632	-0.5951	0.0006	-0.6474	1.0000	0.4610	0.8888	0.2768	0.3183	0.4112
LOC104914302	0.6551	0.2984	1.0000	0.0082	0.0003	-1.6796	0.0311	-0.8205	1.0000	-0.2527	0.3809	-0.5300	0.6655	0.6144
LOC104914304	1.0000	0.0000	1.0000	0.0000	1.0000	0.0000	1.0000	0.0000	1.0000	0.0000	1.0000	0.0000	1.0000	0.0000
LOC104914305	1.0000	0.0000	1.0000	0.0000	1.0000	0.0000	1.0000	0.0000	1.0000	0.0000	1.0000	0.0000	1.0000	0.0000
LOC104914307	0.4238	0.2056	0.9054	-0.0566	0.0027	-0.6335	0.0000	-0.5831	1.0000	-0.0644	0.0579	-0.3139	1.0000	-0.0068
LOC104914309	0.6571	0.8709	0.9313	0.3057	0.6203	0.7796	0.6662	0.4859	1.0000	1.4085	0.7181	0.8606	0.4069	1.1284
LOC104914310	0.1031	-0.5345	0.0000	-0.6783	0.9138	-0.1328	0.4201	0.1597	1.0000	-0.0848	0.7010	-0.2184	0.5119	0.2124
LOC104914311	1.0000	2.1850	0.9062	1.2225	1.0000	2.2469	1.0000	-2.3180	1.0000	2.2676	1.0000	1.4317	1.0000	-2.2909
LOC104914312	1.0000	0.0482	0.1049	0.8468	0.0107	1.1227	0.0476	0.9658	1.0000	-0.4962	1.0000	0.3164	0.2265	-0.6449
LOC104914314	0.0586	1.2035	0.0001	1.8225	0.0149	1.3417	0.0000	1.7466	1.0000	-0.3757	1.0000	0.2522	1.0000	0.0307
LOC104914316	1.0000	0.0000	1.0000	0.0000	1.0000	0.0000	1.0000	0.0000	1.0000	0.0000	1.0000	0.0000	1.0000	0.0000
LOC104914317	0.1644	1.6721	0.4176	0.9905	0.0528	1.8279	1.0000	0.1932	1.0000	0.6429	1.0000	-0.0243	0.4901	-0.9855
LOC104914319	0.1906	-0.3709	0.0000	-0.8903	0.0003	-0.9203	0.0000	-1.1915	1.0000	-0.0593	0.0068	-0.5662	0.3963	-0.3234
LOC104914320	0.0000	1.0378	0.0000	1.4100	0.0000	1.5683	0.0000	1.3579	1.0000	0.0339	0.0512	0.4202	0.2841	-0.1696

LOC104914321	0.1025	-0.3738	0.1425	-0.2229	0.0464	-0.4234	0.4698	0.1287	1.0000	-0.3042	0.8875	-0.1418	0.2356	0.2520
LOC104914325	1.0000	-0.1808	0.0925	-1.1617	0.2602	-1.0854	0.1223	-1.1100	1.0000	0.4147	0.9852	-0.5549	1.0000	0.3977
LOC104914326	0.8215	0.3542	0.1043	0.6889	0.0064	-2.1552	0.0001	-2.5196	1.0000	-0.0295	0.9741	0.3176	1.0000	-0.3886
LOC104914327	1.0000	-2.4776	1.0000	-2.3985	1.0000	-0.1591	1.0000	0.8541	1.0000	-0.1034	1.0000	0.0000	1.0000	0.9165
LOC104914332	0.0000	-1.1030	0.0000	-0.8744	0.0002	0.7493	0.0000	0.8643	1.0000	-0.1516	0.8702	0.0892	1.0000	-0.0319
LOC104914333	0.0248	-0.7262	0.5910	-0.1854	0.0000	1.6722	0.0000	1.8896	1.0000	-0.2978	0.4275	0.2555	0.8717	-0.0761
LOC104914335	1.0000	0.5900	0.7823	0.7692	0.6581	1.2690	0.4160	1.3496	1.0000	0.4129	1.0000	0.6105	1.0000	0.4827
LOC104914336	1.0000	0.0000	1.0000	0.0000	1.0000	0.0000	1.0000	2.3256	1.0000	0.0000	1.0000	0.0000	1.0000	2.3554
LOC104914337	0.0000	-1.3363	0.0000	-1.1240	0.7693	0.1199	0.1412	0.2003	1.0000	-0.0557	0.9008	0.1702	1.0000	0.0319
LOC104914338	1.0000	0.0000	1.0000	0.0000	1.0000	0.0000	1.0000	0.0000	1.0000	0.0000	1.0000	0.0000	1.0000	0.0000
LOC104914340	1.0000	0.0000	1.0000	0.0000	1.0000	0.0000	1.0000	0.0000	1.0000	0.0000	1.0000	0.0000	1.0000	0.0000
LOC104914341	0.3391	0.3672	1.0000	-0.0774	0.6822	-0.2261	0.2788	-0.3314	1.0000	-0.0213	0.1211	-0.4561	1.0000	-0.1203
LOC104914342	1.0000	0.1470	0.0399	2.0573	0.0697	-4.9571	1.0000	0.0052	1.0000	-1.2717	1.0000	0.6347	0.7287	3.7387
LOC104914343	1.0000	-2.4776	0.4431	-3.7876	0.6601	1.5906	0.4370	-3.6972	1.0000	1.2661	1.0000	0.0000	0.5093	-4.0498
LOC104914344	0.0000	-1.8627	0.0000	-2.0238	0.0000	-3.1436	0.0000	-1.2665	1.0000	0.3212	1.0000	0.1717	0.0000	2.2045
LOC104914345	0.8249	3.0254	1.0000	-0.1449	1.0000	0.0000	1.0000	-2.3180	1.0000	2.2676	1.0000	-0.8001	1.0000	0.0000
LOC104914346	1.0000	0.0000	1.0000	0.0000	1.0000	0.0000	1.0000	0.0000	1.0000	0.0000	1.0000	0.0000	1.0000	0.0000
LOC104914347	0.0010	1.4065	0.0251	0.7453	1.0000	0.1912	1.0000	-0.1361	1.0000	0.3019	0.7391	-0.3449	1.0000	-0.0249
LOC104914348	0.0000	2.0248	0.0000	1.7649	0.0090	-0.8508	0.0004	-0.6499	1.0000	0.0833	0.6450	-0.1636	0.6731	0.2881
LOC104914349	1.0000	0.0000	1.0000	0.0000	1.0000	0.0000	1.0000	0.0000	1.0000	0.0000	1.0000	0.0000	1.0000	0.0000
LOC104914350	1.0000	0.0000	1.0000	-2.3985	1.0000	0.0000	1.0000	0.0045	1.0000	2.2734	1.0000	0.0000	1.0000	2.3543
LOC104914351	1.0000	-1.1514	0.6180	1.6082	0.7662	1.0366	0.3949	2.0772	1.0000	-0.9614	1.0000	1.8187	1.0000	0.0777
LOC104914353	1.0000	0.0000	1.0000	0.0000	1.0000	0.0000	1.0000	0.0000	1.0000	0.0000	1.0000	0.0000	1.0000	0.0000
LOC104914355	1.0000	-2.4776	1.0000	2.2533	1.0000	-2.4056	1.0000	0.0000	1.0000	-2.3757	1.0000	2.3481	1.0000	0.0000
LOC104914357	0.5578	1.3126	0.4431	3.6192	0.4809	-3.7970	1.0000	0.0000	1.0000	-3.7635	0.5657	-1.5990	1.0000	0.0000
LOC104914359	0.0523	-1.0929	0.0068	-1.1468	0.2962	-0.6015	0.1989	-0.5603	1.0000	0.1526	1.0000	0.1125	1.0000	0.2015
LOC104914360	0.0387	-5.0448	0.0067	-5.4483	1.0000	-0.6614	0.1887	-1.6499	1.0000	0.3694	1.0000	0.0000	1.0000	-0.6149
LOC104914363	0.4479	-0.3066	0.1135	-0.3461	0.2208	-0.3929	0.2646	-0.2518	1.0000	0.1542	1.0000	0.1275	0.4936	0.2998
LOC104914367	0.0007	2.3718	0.0488	1.5033	1.0000	-0.4473	0.1477	-2.5295	1.0000	0.0854	0.4531	-0.7695	0.6942	-1.9982
LOC104914368	0.9489	0.3174	0.1766	1.0873	0.1062	-1.3420	0.5344	0.7273	1.0000	-1.1053	1.0000	-0.3307	0.6842	0.9657
LOC104914370	1.0000	0.0000	0.7674	3.0956	1.0000	0.0000	1.0000	2.3241	1.0000	0.0000	1.0000	3.2002	1.0000	2.3543
LOC104914371	0.9545	1.0672	1.0000	-0.6943	1.0000	-0.1630	1.0000	0.3899	1.0000	1.2654	1.0000	-0.4744	0.9136	1.8339
LOC104914373	1.0000	0.0000	0.7666	-3.2534	1.0000	0.0000	0.7701	-3.1666	1.0000	3.1126	1.0000	0.0000	1.0000	0.0000
LOC104914376	0.0209	0.7710	0.0087	0.6650	0.8908	-0.1813	0.7674	-0.1807	1.0000	-0.0475	1.0000	-0.1437	1.0000	-0.0415
LOC104914377	0.0001	-3.7701	0.0191	-2.4081	0.3644	-0.7796	1.0000	-0.0900	1.0000	-0.7967	1.0000	0.5792	1.0000	-0.1049
LOC104914378	0.4231	-0.9820	0.1169	-1.7010	0.0038	1.5629	0.0026	1.5725	1.0000	-0.3478	0.8983	-1.0585	0.8631	-0.3325
LOC104914384	1.0000	-2.4776	1.0000	0.0000	1.0000	-2.4056	1.0000	0.0000	1.0000	-2.3758	1.0000	0.0000	1.0000	0.0000
LOC104914390	1.0000	0.0000	0.4431	-3.7876	1.0000	2.2425	1.0000	0.0065	1.0000	3.6423	1.0000	0.0000	1.0000	1.4486
LOC104914392	0.0022	-1.0574	0.0008	-0.8860	0.3616	-0.3710	0.4340	0.2466	1.0000	-0.2792	1.0000	-0.0972	0.5105	0.3452
LOC104914393	1.0000	0.0013	1.0000	0.1778	0.0142	1.4638	0.0092	1.4349	1.0000	-0.1288	1.0000	0.0599	1.0000	-0.1557
LOC104914394	0.0649	1.1507	0.8416	0.2326	0.1158	0.9575	0.4368	0.4544	1.0000	0.7661	1.0000	-0.1389	1.0000	0.2670
LOC104914395	1.0000	0.1939	1.0000	0.6956	1.0000	0.3662	0.3949	2.0772	1.0000	-1.8818	0.9591	-1.4034	1.0000	-0.1737
LOC104914396	0.8249	3.0143	1.0000	2.2533	0.1736	4.3051	0.7701	3.1732	1.0000	0.0000	1.0000	-0.7919	0.9852	-1.1488
LOC104914397	1.0000	-1.2521	1.0000	-2.3985	0.8698	0.5799	0.0297	3.0421	1.0000	-1.8788	1.0000	-3.1384	1.0000	0.5783
LOC104914398	0.0464	1.9721	1.0000	0.3431	0.4166	1.2070	0.4962	0.8819	1.0000	0.6265	0.5167	-0.9904	1.0000	0.3170
LOC104914402	0.4766	0.2634	0.9869	0.0626	0.0030	0.7221	0.0083	0.4175	1.0000	0.3229	1.0000	0.1356	1.0000	0.0235
LOC104914404	1.0000	0.0000	1.0000	0.0000	1.0000	0.0000	1.0000	2.3256	1.0000	0.0000	1.0000	0.0000	1.0000	2.3554
LOC104914405	1.0000	0.2011	0.9148	1.2162	0.8033	-3.2622	1.0000	0.0045	1.0000	-0.9563	1.0000	0.0554	1.0000	2.3542
LOC104914407	1.0000	0.0000	1.0000	0.0000	1.0000	0.0000	1.0000	0.0000	1.0000	0.0000	1.0000	0.0000	1.0000	0.0000
LOC104914408	1.0000	0.0000	1.0000	0.0000	1.0000	0.0000	1.0000	0.0000	1.0000	0.0000	1.0000	0.0000	1.0000	0.0000
LOC104914409	1.0000	0.0000	1.0000	0.0000	1.0000	0.0000	1.0000	0.0000	1.0000	0.0000	1.0000	0.0000	1.0000	0.0000
LOC104914410	0.0328	2.5170	0.6845	0.7908	1.0000	0.3498	0.8672	0.6587	1.0000	1.0999	0.9663	-0.6059	0.7123	1.4195
LOC104914411	0.1071	0.9764	1.0000	0.1303	0.6184	-0.6066	0.1267	-0.9310	1.0000	0.3951	0.8601	-0.4376	1.0000	0.0795
LOC104914412	1.0000	0.0000	1.0000	0.0000	1.0000	0.0000	1.0000	0.0000	1.0000	0.0000	1.0000	0.0000	1.0000	0.0000
LOC104914413	1.0000	0.0000	1.0000	0.0000	1.0000	0.0000	1.0000	0.0000	1.0000	0.0000	1.0000	0.0000	1.0000	0.0000
LOC104914414	1.0000	0.0000	1.0000	0.0000	1.0000	0.0000	1.0000	0.0000	1.0000	0.0000	1.0000	0.0000	1.0000	0.0000
LOC104914415	1.0000	0.0000	1.0000	0.0000	1.0000	0.0000	1.0000	0.0000	1.0000	0.0000	1.0000	0.0000	1.0000	0.0000
LOC104914416	1.0000	0.0000	1.0000	0.0000	1.0000	0.0000	1.0000	0.0000	1.0000	0.0000	1.0000	0.0000	1.0000	0.0000
LOC104914417	1.0000	0.0000	1.0000	0.0000	1.0000	2.2425	1.0000	0.0000	1.0000	0.0000	1.0000	0.0000	1.0000	-2.2889
LOC104914418	1.0000	0.0000	1.0000	0.0000	1.0000	0.0000	1.0000	0.0000	1.0000	0.0000	1.0000	0.0000	1.0000	0.0000
LOC104914419	1.0000	0.0000	1.0000	0.0000	1.0000	0.0000	1.0000	0.0000	1.0000	0.0000	1.0000	0.0000	1.0000	0.0000
LOC104914420	1.0000	-2.4776	1.0000	0.0000	1.0000	-2.4056	1.0000	0.0000	1.0000	-2.3758	1.0000	0.0000	1.0000	0.0000
LOC104914422	1.0000	0.0000	1.0000	0.0000	1.0000	0.0000	1.0000	0.0000	1.0000	0.0000	1.0000	0.0000	1.0000	0.0000
LOC104914423	1.0000	-0.0239	0.6872	-0.2287	0.0898	-0.8587	0.6716	-0.2551	1.0000	-0.0575	0.9957	-0.2492	0.5218	0.5509
LOC104914425	0.9407	-0.1767	0.9266	-0.1835	0.0001	-1.7244	0.5236	-0.3586	1.0000	-0.3701	0.8327	-0.3667	0.1215	0.9983
LOC104914426	0.5309	-0.3308	0.4571	-0.2366	1.0000	-0.0512	1.0000	0.0071	1.0000	-0.1224	1.0000	-0.0159	1.0000	-0.0565
LOC104914427	1.0000	-2.4776	1.0000	-1.0027	1.0000	-2.4056	0.7710	-3.1693	1.0000	0.7415	1.0000	2.3481	1.0000	0.0000
LOC104914428	0.5519	0.4720	1.0000	0.0893	0.0131	-1.4171	0.2180	-0.6287	1.0000	-0.1695	0.6803	-0.5375	0.7262	0.6284
LOC104914429	1.0000	0.0000	1.0000	0.0000	1.0000	0.0000	1.0000	0.0000	1.0000	0.0000	1.0000	0.0000	1.0000	0.0000
LOC104914431	0.6936	1.4527	1.0000	-2.3985	1.0000	0.6780	1.0000	-2.3200	1.0000	-0.1045	0.6450	-4.0608	1.0000	-3.1344
LOC104914439	1.0000	2.1900	1.0000	0.0000	1.0000	0.0000	1.0000	0.0000	1.0000	0.0000	1.0000	-2.2992	1.0000	0.0000
LOC104914441	1.0000	0.0000	1.0000	0.0000	1.0000	0.0000	1.0000	0.0000	1.0000	0.0000	1.0000	0.0000	1.0000	0.0000
LOC104914447	0.8226	3.0199	1.0000	0.0000	0.8011	3.0840	0.7701	3.1732	1.0000	0.0000	1.0000	-3.1429	1.0000	0.0723
LOC104914448	1.0000	0.0000	1.0000	0.0000	1.0000	0.0000	1.0000	0.0000	1.0000	0.0000	1.0000	0.0000	1.0000	0.0000
LOC104914449	1.0000	0.0000	1.0000	0.0000	1.0000	2.2425	1.0000	0.0000	1.0000	0.0000	1.0000	0.0000	1.0000	-2.2889
LOC104914452	0.0024	-0.7591	0.0059	-0.3319	1.0000	-0.0171	0.7471	-0.0744	1.0000	-0.0877	0.0593	0.3533	0.7287	-0.1385
LOC104914453	1.0000	0.3033	0.7189	-1.3897	0.0269	1.9548	0.0632	1.6371	1.0000	-0.1255	0.6102	-1.8174	0.9590	-0.4378
LOC104914454	0.0000	2.0931	0.0000	2.4841	0.0000	2.0032	0.0000	2.3188	1.0000	-0.4759	1.0000	-0.0711	0.9759	-0.1531
LOC104914455	0.0004	-0.8444	0.0040	-0.4907	0.0000	-1.1221	0.0039	-0.4857	1.0000	-0.2796	1.0000	0.0863	0.2953	0.3635
LOC104914456	0.2200	0.3957	0.7666	0.1588	0.0000	1.7411	0.0000	1.7134	1.0000	0.0985	1.0000	-0.1246	0.9863	0.0768
LOC104914458	1.0000	0.5464	1.0000	-2.3960	1.0000	-2.4056	1.0000	-2.3180	1.0000	-0.1078	1.0000	-3.1468	1.0000	0.0000
LOC104914459	1.0000	-2.4776	1.0000	2.2508	0.9361	1.2045	1.0000	0.0000	1.0000	-2.3758	1.0000	2.3457	0.7287	-3.6634
LOC104914461	1.0000	-0.0281	0.0005	-0.2532	0.0031	-0.4416	0.0000	-0.4327	1.0000	0.0908	0.4311	-0.1220	0.5196	0.1051
LOC104914462	0.2627	-0.2271	0.0477	-0.2148	0.0321	-0.3632	0.0000	-0.3847	1.0000	0.0229	1.0000	0.0475	1.0000	0.0066
LOC104914463	0.3067	0.8468	0.7695	0.3981	0.1863	0.9243	0.1733	0.8890	1.0000	-0.2281	0.6450	-0.6673	1.0000	-0.2598
LOC104914464	1.0000	-0.2920	1.0000	0.0000	1.0000	-2.4056	1.0000	2.3241	1.0000	-2.3758	1.0000	-2.2957	1.0000	2.3542
LOC104914465	0.0000	-1.7642	0.0000	-1.7493	0.0216	-0.3805	0.0000	-0.3969	1.0000	-0.0257	1.0000	0.0010	1.0000	-0.0373
LOC104914466	1.0000	0.0816	0.1933	0.5800	0.3856	0.4337	0.0875	0.6817	1.0000	-0.7205	1.0000	-0.2093	0.3514	-0.4651
LOC104914467	0.6155	-0.9271	0.0257	1.4357	0.4542	0.7296	0.8502	0.4815	1.0000	-0.3894	0.0224	1.9878	0.7588	-0.6290
LOC104914470	0.0000	-2.3555	0.0000	-2.0686	0.0579	-0.5767	0.1065	-0.3315	1.0000	0.0271	0.9514	0.3269	0.5017	0.2777
LOC104914473	0.0480	-1.8899	0.1751	-1.1522	1.0000	-0.1082	0.3541	0.7135	1.0000	-0.2262	1.0000	0.5247	0.6746	0.5976
LOC104914474	1.0000	0.0000	1.0000	0.0000	1.0000	0.0000	1.0000	0.0000	1.0000	0.0000	1.0000	0.0000	1.0000	0.0000
LOC104914475	1.0000	0.0000	1.0000	2.2508	1.0000	2.2425	1.0000	0.0000	1.0000	0.0000	1.0000	2.3457	1.0000	-2.2889

LOC104914478	1.0000	0.0000	1.0000	0.0000	1.0000	0.0000	1.0000	0.0000	1.0000	0.0000	1.0000	0.0000	1.0000	0.0000
LOC104914479	1.0000	0.0039	0.0108	-0.6042	1.0000	0.1062	0.0298	-0.5234	1.0000	-0.2208	0.0015	-0.8153	0.0005	-0.8445
LOC104914480	1.0000	-0.2920	1.0000	0.0000	1.0000	-0.1591	1.0000	0.0000	1.0000	-2.3758	1.0000	-2.2957	1.0000	-2.2909
LOC104914481	1.0000	0.0000	1.0000	0.0000	1.0000	0.0000	1.0000	0.0000	1.0000	0.0000	1.0000	0.0000	1.0000	0.0000
LOC104914482	0.3168	2.2149	0.2544	2.1622	0.9361	1.2061	1.0000	-2.3180	1.0000	-0.1089	1.0000	-0.1551	0.7287	-3.6637
LOC104914483	1.0000	0.0000	1.0000	-0.1452	1.0000	0.0000	1.0000	-2.3200	1.0000	2.2734	1.0000	2.3481	1.0000	0.0000
LOC104914484	0.0002	-2.0652	0.0000	-2.4183	0.2488	-0.6701	0.4349	-0.4976	1.0000	0.0335	1.0000	-0.3089	1.0000	0.2120
LOC104914487	0.0365	2.2962	0.1593	1.0431	0.6383	0.9709	0.1262	-1.7846	1.0000	1.6651	1.0000	0.4326	0.8734	-1.0836
LOC104914488	1.0000	-2.4788	1.0000	0.0000	1.0000	-2.4061	1.0000	0.0000	1.0000	-2.3771	1.0000	0.0000	1.0000	0.0000
LOC104914489	1.0000	0.0000	1.0000	0.0000	1.0000	0.0000	1.0000	0.0000	1.0000	0.0000	1.0000	0.0000	1.0000	0.0000
LOC104914490	0.8249	-3.3439	0.7666	-3.2534	1.0000	-0.1793	0.7701	-3.1666	1.0000	-0.1187	1.0000	0.0000	1.0000	-3.1344
LOC104914491	0.6918	-0.4087	0.9136	0.1723	0.1920	-0.8255	1.0000	-0.1324	1.0000	0.3370	0.1383	0.9301	0.0974	1.0356
LOC104914493	0.0013	1.4183	0.0001	1.5162	0.0072	-1.8689	0.0021	-2.3200	1.0000	-0.1244	1.0000	-0.0205	1.0000	-0.5735
LOC104914494	0.8249	3.0143	0.4687	3.6127	0.4809	3.6101	1.0000	2.3241	1.0000	0.0000	1.0000	0.5858	1.0000	-1.3084
LOC104914495	0.3651	0.9428	0.1302	1.2529	0.3687	0.9303	0.0867	1.4356	1.0000	-0.4454	1.0000	-0.1228	1.0000	0.0627
LOC104914496	0.2214	-2.2106	1.0000	-0.3884	0.4048	0.9482	0.0102	1.7490	1.0000	-0.3125	0.9082	1.5316	0.7892	0.4905
LOC104914497	0.5391	-3.8819	0.2545	-4.1774	0.0007	2.8238	0.0084	2.1865	1.0000	0.2660	1.0000	0.0000	1.0000	-0.3675
LOC104914498	1.0000	-0.2920	1.0000	0.6956	0.9361	1.2064	1.0000	-2.3180	1.0000	-0.1077	1.0000	0.9006	0.7287	-3.6637
LOC104914499	1.0000	-0.8610	0.7797	-1.1174	1.0000	0.2118	0.4996	-1.4673	1.0000	0.8205	1.0000	0.5836	1.0000	-0.8468
LOC104914500	1.0000	-2.4788	1.0000	0.0000	1.0000	-2.4061	1.0000	0.0000	1.0000	-2.3771	1.0000	0.0000	1.0000	0.0000
LOC104914501	1.0000	2.1850	1.0000	-2.3985	0.4809	3.6101	0.9162	1.3864	1.0000	2.2734	1.0000	-2.2957	1.0000	0.0765
LOC104914502	1.0000	0.0000	0.4431	-3.7865	0.8011	3.0840	0.9096	-1.3721	1.0000	3.6396	1.0000	0.0000	1.0000	-0.7795
LOC104914503	1.0000	0.0000	1.0000	0.0000	1.0000	0.0000	1.0000	0.0000	1.0000	0.0000	1.0000	0.0000	1.0000	0.0000
LOC104914507	0.0033	3.0905	0.0901	1.8562	1.0000	-0.1793	1.0000	-0.5236	1.0000	0.4081	0.6105	-0.8114	1.0000	0.0723
LOC104914508	1.0000	2.1850	1.0000	0.0000	1.0000	0.0000	1.0000	0.0000	1.0000	0.0000	1.0000	-2.2957	1.0000	0.0000
LOC104914509	1.0000	2.1900	1.0000	0.0000	1.0000	0.0000	1.0000	0.0000	1.0000	0.0000	1.0000	-2.2992	1.0000	0.0000
LOC104914510	0.0006	1.6635	0.0000	1.8871	1.0000	-0.1154	0.2193	0.7691	1.0000	-0.2130	1.0000	0.0225	0.6150	0.6815
LOC104914513	1.0000	0.0000	1.0000	0.0000	1.0000	0.0000	1.0000	0.0000	1.0000	0.0000	1.0000	0.0000	1.0000	0.0000
LOC104914514	0.8249	-3.3439	1.0000	0.0000	0.5786	1.2809	0.0000	6.2686	1.0000	-3.2319	1.0000	0.0000	0.1554	1.7020
LOC104914515	0.4423	1.2705	0.5627	0.7220	0.0144	1.9520	0.0171	1.5121	1.0000	0.6904	1.0000	0.1505	1.0000	0.2555
LOC104914519	1.0000	0.0000	1.0000	0.0000	1.0000	0.0000	1.0000	0.0000	1.0000	0.0000	1.0000	0.0000	1.0000	0.0000
LOC104914521	0.0000	-0.9043	0.0000	-0.7516	0.0000	-0.9548	0.0000	-0.6801	1.0000	-0.1692	1.0000	-0.0045	0.9747	0.1116
LOC104914523	0.0648	-4.8294	0.9062	-1.5348	0.0111	1.9804	0.0203	2.2374	1.0000	-1.0691	1.0000	2.3456	0.4319	-0.8093
LOC104914524	0.0000	-0.9544	0.0000	-0.5859	0.0004	-0.7326	0.0000	-0.5185	1.0000	-0.2015	0.7635	0.1804	1.0000	0.0192
LOC104914527	1.0000	0.0438	0.9414	-0.0740	0.0271	0.5246	0.0046	0.4361	1.0000	0.1266	1.0000	0.0221	1.0000	0.0435
LOC104914528	0.0013	0.6279	0.0084	0.2904	0.0001	0.7624	0.0000	0.7416	1.0000	-0.1281	0.0000	-0.4543	0.3501	-0.1438
LOC104914530	0.0188	-1.3759	0.0005	-1.2927	0.0619	0.7951	0.0000	1.9150	1.0000	0.2897	1.0000	0.3897	0.0000	1.4136
LOC104914531	0.9246	0.7736	1.0000	-0.8596	1.0000	0.3596	0.7296	-1.2146	1.0000	0.1747	0.6954	-1.4503	0.8047	-1.3971
LOC104914533	0.0600	0.3933	0.0145	0.3183	0.0074	-0.5657	0.0000	-0.6292	1.0000	-0.0414	0.9486	-0.1045	0.9980	-0.0998
LOC104914535	1.0000	2.1850	1.0000	0.0000	1.0000	0.0000	1.0000	0.0000	1.0000	0.0000	1.0000	-2.2958	1.0000	0.0000
LOC104914536	0.0499	-1.0388	0.0196	-0.7539	0.0000	1.4620	0.0067	0.7554	1.0000	-0.0513	1.0000	0.2464	0.0075	-0.7521
LOC104914538	1.0000	0.0000	0.6224	-1.9244	0.0001	6.2432	0.1127	1.6477	1.0000	4.0300	1.0000	2.3481	0.9049	-0.5314
LOC104914539	1.0000	0.0000	1.0000	0.0000	1.0000	0.0000	1.0000	0.0000	1.0000	0.0000	1.0000	0.0000	1.0000	0.0000
LOC104914540	0.3092	4.2299	0.0412	4.7731	1.0000	0.0000	1.0000	2.3241	1.0000	0.0000	1.0000	0.5247	1.0000	2.3542
LOC104914541	0.6585	-1.2842	1.0000	-0.1557	0.0919	-4.7440	1.0000	-0.8388	1.0000	-1.5987	1.0000	-0.4744	1.0000	2.3554
LOC104914543	1.0000	0.0000	0.9062	1.2223	1.0000	2.2425	1.0000	-2.3200	1.0000	2.2734	0.8607	3.7297	1.0000	-2.2889
LOC104914544	1.0000	-2.4788	0.9148	-1.5387	1.0000	-0.1597	0.4547	-3.7024	1.0000	1.2722	1.0000	2.3481	1.0000	-2.2909
LOC104914545	1.0000	0.0000	1.0000	0.0000	1.0000	0.0000	1.0000	0.0000	1.0000	0.0000	1.0000	0.0000	1.0000	0.0000
LOC104914549	0.6085	0.8115	0.0144	2.0048	1.0000	-0.4017	1.0000	0.0070	1.0000	-0.8961	1.0000	0.3052	1.0000	-0.4810
LOC104914551	1.0000	0.0000	1.0000	0.0000	1.0000	0.0000	1.0000	0.0000	1.0000	0.0000	1.0000	0.0000	1.0000	0.0000
LOC104914552	0.8489	0.2114	0.2393	0.4516	0.0131	0.8921	0.0000	1.3072	1.0000	-0.4601	1.0000	-0.2078	1.0000	-0.0379
LOC104914553	1.0000	0.0000	1.0000	-2.3960	1.0000	0.0000	1.0000	-2.3180	1.0000	2.2676	1.0000	0.0000	1.0000	0.0000
LOC104914555	1.0000	0.0000	1.0000	0.0000	1.0000	0.0000	1.0000	0.0000	1.0000	0.0000	1.0000	0.0000	1.0000	0.0000
LOC104914556	0.0649	-0.6839	1.0000	-0.0164	0.1154	-0.5949	1.0000	-0.0648	1.0000	-0.3936	0.8401	0.2858	1.0000	0.1431
LOC104914557	0.6090	-0.9164	1.0000	-0.8626	0.3962	-1.3147	0.3952	-2.0640	1.0000	-0.9441	1.0000	-0.8858	0.8937	-1.6947
LOC104914561	0.0000	-0.7882	0.0000	-0.6440	0.0000	-0.9170	0.0000	-0.3886	1.0000	-0.2314	0.9569	-0.0750	0.0123	0.3027
LOC104914563	0.0661	0.7159	0.0000	0.8712	0.0000	1.3470	0.0000	1.2397	1.0000	-0.0327	1.0000	0.1339	0.8815	-0.1349
LOC104914564	1.0000	0.0000	0.7674	3.0889	0.8033	3.0789	1.0000	2.3256	1.0000	0.0000	1.0000	3.1946	1.0000	-0.7769
LOC104914565	0.0099	2.0076	0.0694	1.1999	0.0000	2.8118	0.0000	2.2509	1.0000	0.7175	1.0000	-0.0781	1.0000	0.1646
LOC104914567	1.0000	-0.7296	0.1616	-2.6978	0.6927	0.7651	1.0000	0.1927	1.0000	0.6484	1.0000	-1.3189	1.0000	0.0786
LOC104914568	0.8295	-0.3132	0.8146	0.3873	0.0458	0.9280	0.0005	1.5357	1.0000	-0.7114	1.0000	0.0022	1.0000	-0.1001
LOC104914570	1.0000	-0.0791	0.0011	-0.8743	0.8322	-0.1867	0.8196	-0.1345	1.0000	0.2172	0.1710	-0.5665	0.6801	0.2716
LOC104914571	1.0000	-2.4776	1.0000	0.0000	1.0000	-2.4056	1.0000	2.3241	1.0000	-2.3758	1.0000	0.0000	1.0000	2.3543
LOC104914572	1.0000	0.0000	1.0000	0.0000	1.0000	0.0000	1.0000	0.0000	1.0000	0.0000	1.0000	0.0000	1.0000	0.0000
LOC104914574	0.0000	1.8907	0.0000	2.1838	0.0000	1.8804	0.0000	1.6610	1.0000	0.1729	0.1984	0.4754	1.0000	-0.0469
LOC104914577	0.5059	-0.3636	0.2302	0.4374	0.0537	-0.7718	0.3212	-0.4325	1.0000	-0.4376	0.7337	0.3759	1.0000	-0.0913
LOC104914579	0.8249	-3.3412	1.0000	-0.1429	0.7662	1.0366	1.0000	0.8567	1.0000	-0.9613	1.0000	2.3481	0.9835	-1.1470
LOC104914580	1.0000	-2.4788	1.0000	0.0000	1.0000	-2.4061	1.0000	0.0000	1.0000	-2.3771	1.0000	0.0000	1.0000	0.0000
LOC104914581	0.1139	1.8359	0.0945	2.5620	0.2836	-4.1861	1.0000	0.8545	1.0000	-1.8834	0.4743	-1.1671	1.0000	3.2066
LOC104914582	0.7500	-0.5148	0.1734	-1.1514	1.0000	-0.1961	1.0000	-0.2108	1.0000	-0.6388	0.4090	-1.2695	0.7011	-0.6458
LOC104914583	1.0000	0.0000	1.0000	-2.3985	1.0000	0.0000	1.0000	-2.3200	1.0000	2.2734	1.0000	0.0000	1.0000	0.0000
LOC104914585	1.0000	0.1133	1.0000	-0.8629	0.0294	1.9549	0.5124	0.9724	1.0000	-0.1282	0.9844	-1.0992	0.2532	-1.1032
LOC104914586	1.0000	0.0000	1.0000	0.0000	1.0000	0.0000	1.0000	0.0000	1.0000	0.0000	1.0000	0.0000	1.0000	0.0000
LOC104914587	1.0000	2.1900	1.0000	0.0000	1.0000	2.2425	0.1396	4.3976	1.0000	0.0000	1.0000	-2.2992	0.6944	2.1432
LOC104914588	0.0047	-0.6979	0.0000	-0.7165	0.8995	-0.1146	0.3903	0.1599	1.0000	-0.0602	1.0000	-0.0667	0.5078	0.2198
LOC104914589	0.9316	-0.1215	0.2237	0.2768	0.7768	-0.1607	0.0001	0.6499	1.0000	-0.5601	0.9798	-0.1523	0.3841	0.2543
LOC104914590	1.0000	0.0039	0.8717	-0.1100	0.4876	0.2697	0.5194	0.2041	1.0000	-0.0876	1.0000	-0.1913	0.9286	-0.1492
LOC104914591	1.0000	0.0000	1.0000	0.0000	1.0000	0.0000	1.0000	0.0000	1.0000	0.0000	1.0000	0.0000	1.0000	0.0000
LOC104914592	1.0000	0.0000	1.0000	0.0000	1.0000	0.0000	1.0000	0.0000	1.0000	0.0000	1.0000	0.0000	1.0000	0.0000
LOC104914596	1.0000	0.0000	1.0000	0.0000	1.0000	0.0000	1.0000	0.0000	1.0000	0.0000	1.0000	0.0000	1.0000	0.0000
LOC104914598	0.0020	-0.7310	0.0038	-0.4866	0.0000	-1.1658	0.1541	-0.2716	1.0000	-0.4559	0.8501	-0.1987	0.0936	0.4443
LOC104914600	0.0000	-1.3490	0.0002	-1.0043	0.0011	0.8385	0.0000	1.2280	1.0000	-0.4001	1.0000	-0.0411	1.0000	-0.0028
LOC104914601	0.0000	-1.3559	0.0000	-1.3020	0.0205	0.5588	0.0000	0.7379	1.0000	-0.1578	1.0000	-0.0901	1.0000	0.0279
LOC104914602	0.9341	-0.2975	0.5492	-0.6154	1.0000	0.1810	0.0533	1.0348	1.0000	-0.3975	0.7064	-0.7065	0.7451	0.4575
LOC104914604	0.0764	0.4477	0.0096	0.4408	0.0000	1.3972	0.0000	1.2413	1.0000	-0.1293	1.0000	-0.1231	0.0457	-0.2791
LOC104914605	0.0010	0.7628	0.0000	1.4305	0.0000	1.3829	0.0000	1.4083	1.0000	-0.3862	0.1355	0.2955	0.0017	-0.3532
LOC104914606	1.0000	-0.3466	0.4195	1.1735	0.7628	-1.4078	1.0000	0.3978	1.0000	-0.8174	1.0000	0.7120	1.0000	0.9946
LOC104914608	1.0000	0.0000	1.0000	0.0000	1.0000	0.0000	1.0000	0.0000	1.0000	0.0000	1.0000	0.0000	1.0000	0.0000
LOC104914610	1.0000	0.0000	1.0000	0.0000	1.0000	0.0000	1.0000	0.0000	1.0000	0.0000	1.0000	0.0000	1.0000	0.0000
LOC104914612	0.1877	-0.3507	0.0000	-0.7187	0.9343	-0.0932	1.0000	-0.0239	1.0000	0.0426	0.2713	-0.3123	0.8749	0.1179
LOC104914614	0.0000	-1.3501	0.0000	-1.1353	0.0000	-1.4006	0.0000	-0.7073	1.0000	-0.2581	1.0000	-0.0298	0.0226	0.4424
LOC104914617	0.0072	-0.5078	0.0067	-0.3416	0.3907	-0.2042	0.0904	-0.2064	1.0000	-0.0651	0.9313	0.1135	1.0000	-0.0617
LOC104914618	0.8520	0.8121	1.0000	0.3692	0.5041	-3.7956	1.0000	0.5373	1.0000	-0.6490	0.9847	-1.0954	0.7287	3.7387

LOC104914619	1.0000	0.0060	0.4932	0.2136	0.1386	-0.5319	0.7170	-0.1503	1.0000	-0.3668	1.0000	-0.1468	1.0000	0.0225
LOC104914622	0.0001	-6.2986	0.1399	-1.8316	0.0101	-2.6007	0.9160	-0.4927	1.0000	-0.8794	0.8785	3.7233	0.8620	1.2344
LOC104914623	1.0000	2.1900	1.0000	2.2533	1.0000	2.2469	1.0000	0.0000	1.0000	0.0000	1.0000	0.0489	1.0000	-2.2909
LOC104914624	0.4318	0.4346	0.0555	0.6503	0.4433	0.4221	0.1925	0.5039	1.0000	-0.1314	1.0000	0.0969	1.0000	-0.0456
LOC104914628	0.9545	-1.6928	1.0000	-2.3985	1.0000	0.2073	1.0000	0.0045	1.0000	-1.4912	1.0000	-2.2958	0.9124	-1.7021
LOC104914629	1.0000	-1.1585	0.4687	3.6127	1.0000	-0.1793	1.0000	2.3256	1.0000	-3.2319	1.0000	1.4294	1.0000	-0.7793
LOC104914631	0.9948	0.1198	0.5491	0.1701	0.0000	1.2206	0.0000	1.2647	1.0000	0.3620	0.1678	0.4273	0.0261	0.4136
LOC104914633	0.2333	-0.2945	0.5374	-0.1019	0.0782	0.3938	0.0000	0.7969	1.0000	-0.2913	0.9251	-0.0857	0.6285	0.1183
LOC104914634	0.0292	0.6260	0.0114	0.5675	0.0860	-0.5817	0.6343	-0.1885	1.0000	-0.4499	0.0539	-0.4951	1.0000	-0.0492
LOC104914635	1.0000	2.1850	1.0000	0.0000	1.0000	0.0000	1.0000	0.0000	1.0000	0.0000	1.0000	-2.2958	1.0000	0.0000
LOC104914636	1.0000	0.0000	1.0000	0.0000	1.0000	0.0000	1.0000	0.0000	1.0000	0.0000	1.0000	0.0000	1.0000	0.0000
LOC104914637	1.0000	0.0000	1.0000	0.0000	1.0000	0.0000	1.0000	0.0000	1.0000	0.0000	1.0000	0.0000	1.0000	0.0000
LOC104914639	0.0804	1.0516	0.0001	1.6869	0.0007	1.7594	0.0000	1.8496	1.0000	-0.3754	1.0000	0.2711	0.9488	-0.2828
LOC104914640	1.0000	0.0000	1.0000	0.0000	1.0000	2.2425	1.0000	0.0000	1.0000	0.0000	1.0000	0.0000	1.0000	-2.2889
LOC104914641	1.0000	0.0000	1.0000	-2.3960	1.0000	0.0000	1.0000	-2.3180	1.0000	2.2676	1.0000	0.0000	1.0000	0.0000
LOC104914642	0.3357	-2.3859	0.7666	-3.2534	0.3449	1.1424	0.3333	1.6949	1.0000	-1.3434	1.0000	-2.2992	0.7562	-0.7911
LOC104914643	0.9219	0.7756	0.7854	-0.8502	1.0000	0.1136	0.5139	-1.3581	1.0000	0.8526	1.0000	-0.7585	1.0000	-0.6156
LOC104914644	1.0000	0.0000	1.0000	0.0000	0.8033	3.0892	1.0000	0.0000	1.0000	0.0000	1.0000	0.0000	1.0000	-3.1369
LOC104914645	1.0000	0.4679	1.0000	0.3659	1.0000	-0.8812	1.0000	-0.8419	1.0000	-1.3434	0.6954	-1.4507	1.0000	-1.3084
LOC104914647	0.0784	-0.9592	0.0048	-0.9830	1.0000	-0.1721	1.0000	-0.0390	1.0000	-0.0848	1.0000	-0.0926	1.0000	0.0563
LOC104914649	1.0000	-0.0020	0.0159	0.4349	0.1417	-0.4269	1.0000	0.0282	1.0000	-0.4613	1.0000	-0.0138	1.0000	-0.0010
LOC104914651	0.5086	3.5456	1.0000	-0.1472	0.8033	3.0891	1.0000	-2.3200	1.0000	2.2734	1.0000	-1.3266	1.0000	-3.1368
LOC104914652	0.0336	-2.3707	0.0128	-5.2993	0.7662	0.4903	0.1999	1.0641	1.0000	-0.6495	0.8607	-3.6732	1.0000	-0.0670
LOC104914654	1.0000	0.0000	1.0000	2.2508	1.0000	0.0000	1.0000	0.0000	1.0000	0.0000	1.0000	2.3456	1.0000	0.0000
LOC104914656	0.1847	0.6829	1.0000	0.0912	0.0079	-1.5066	0.0000	-1.7978	1.0000	0.6099	1.0000	0.0280	1.0000	0.3240
LOC104914657	1.0000	0.0000	1.0000	-2.3985	1.0000	0.0000	1.0000	0.0057	1.0000	2.2734	1.0000	0.0000	1.0000	2.3554
LOC104914658	1.0000	0.5414	1.0000	-2.3985	1.0000	-2.4061	1.0000	0.0057	1.0000	-0.1045	1.0000	-3.1429	1.0000	2.3554
LOC104914659	0.6881	1.7543	1.0000	0.3653	1.0000	0.6783	1.0000	-0.8429	1.0000	0.7415	1.0000	-0.6343	1.0000	-0.7793
LOC104914660	1.0000	0.0204	0.2495	0.7632	0.0000	-2.7319	1.0000	-0.2885	1.0000	-1.2828	0.7313	-0.5338	0.6072	1.1631
LOC104914661	1.0000	0.0000	1.0000	0.0000	1.0000	0.0000	1.0000	2.3241	1.0000	0.0000	1.0000	0.0000	1.0000	2.3543
LOC104914662	0.9545	1.0672	1.0000	0.6944	1.0000	-2.4061	1.0000	-2.3199	1.0000	-0.1045	1.0000	-0.4752	1.0000	0.0000
LOC104914663	0.8926	-0.2475	0.7447	0.3357	0.0275	-1.1023	0.1733	0.6516	1.0000	-0.7799	1.0000	-0.1838	0.0879	0.9837
LOC104914664	0.0010	-0.5524	0.0000	-0.4411	0.0001	-0.6874	0.0044	-0.2889	1.0000	-0.2423	0.6442	-0.1184	0.3369	0.1622
LOC104914665	0.0251	-0.7841	1.0000	-0.0061	0.0170	-0.7979	1.0000	0.0579	1.0000	-0.6971	1.0000	0.0934	1.0000	0.1640
LOC104914666	0.6306	0.5013	0.5869	0.5878	0.0421	1.2091	0.0000	2.1637	1.0000	-0.5946	0.9007	-0.4999	0.6745	0.3700
LOC104914670	0.0000	-1.8973	0.0000	-1.6225	0.3270	-0.2619	1.0000	-0.0483	1.0000	-0.1440	0.9992	0.1445	1.0000	0.0767
LOC104914671	1.0000	0.0000	1.0000	0.0000	1.0000	0.0000	1.0000	0.0000	1.0000	0.0000	1.0000	0.0000	1.0000	0.0000
LOC104914673	1.0000	0.0000	1.0000	0.0000	1.0000	0.0000	1.0000	0.0000	1.0000	0.0000	1.0000	0.0000	1.0000	0.0000
LOC104914674	1.0000	-2.4788	1.0000	-2.3960	1.0000	-2.4061	1.0000	0.0074	1.0000	-0.1089	1.0000	0.0000	1.0000	2.3554
LOC104914676	0.0286	-0.5236	0.0003	-0.6202	0.0002	-0.8327	0.0000	-0.8926	1.0000	-0.0414	1.0000	-0.1270	1.0000	-0.0957
LOC104914677	1.0000	-0.0107	1.0000	-0.0312	0.0121	-0.4281	0.0003	-0.3277	1.0000	-0.1284	0.6450	-0.1367	1.0000	-0.0224
LOC104914678	0.1877	4.4789	1.0000	-2.3960	1.0000	0.0000	1.0000	0.0074	1.0000	2.2676	0.3199	-4.6138	1.0000	2.3554
LOC104914681	1.0000	0.0000	0.7674	-3.2499	1.0000	0.0000	0.7710	-3.1632	1.0000	3.1056	1.0000	0.0000	1.0000	0.0000
LOC104914682	0.0000	-1.5104	0.0000	-1.7108	0.0076	-0.4894	1.0000	-0.0062	1.0000	-0.1598	0.2273	-0.3478	0.0215	0.3287
LOC104914683	1.0000	0.0000	1.0000	0.0000	1.0000	0.0000	1.0000	0.0000	1.0000	0.0000	1.0000	0.0000	1.0000	0.0000
LOC104914684	0.5212	0.8242	0.0080	1.5628	0.0050	1.8180	0.0104	1.5202	1.0000	-0.1302	0.6450	0.6207	0.8336	-0.4184
LOC104914686	0.0000	-1.3235	0.0000	-1.2392	0.0000	-0.8451	0.0000	-0.8606	1.0000	0.0899	0.6770	0.1871	1.0000	0.0789
LOC104914687	0.0000	-2.7231	0.0000	-3.8396	0.0000	-1.2282	0.0000	-1.2436	1.0000	0.3676	0.6857	-0.7380	0.6093	0.3567
LOC104914688	0.2069	-1.6879	0.1467	-2.2087	0.7438	-0.8444	1.0000	0.2892	1.0000	0.0375	1.0000	-0.4744	0.6843	1.1774
LOC104914690	0.0002	1.5124	0.0000	1.9516	0.0000	1.9943	0.0000	1.7917	1.0000	-0.0570	0.4348	0.3990	0.6865	-0.2472
LOC104914691	0.9545	1.0652	0.8265	-0.9448	0.6647	1.5885	0.5796	-1.1517	1.0000	2.4237	1.0000	0.4448	1.0000	-0.3110
LOC104914697	0.4920	-0.5276	0.3717	0.5249	0.0000	1.5139	0.0000	2.1201	1.0000	-0.8761	1.0000	0.1873	0.7180	-0.2643
LOC104914698	1.0000	-0.2341	0.5678	0.7961	0.6910	0.5743	0.1212	1.3867	1.0000	-0.9788	1.0000	0.0611	1.0000	-0.1628
LOC104914699	0.6769	0.2028	0.3224	0.2795	0.0000	1.0745	0.0000	1.1586	1.0000	-0.0376	1.0000	0.0527	1.0000	0.0553
LOC104914701	0.8793	-0.1903	0.9176	-0.1462	0.5684	0.3158	0.1811	0.3927	1.0000	0.0030	1.0000	0.0614	1.0000	0.0837
LOC104914702	1.0000	0.0000	1.0000	0.0000	1.0000	0.0000	1.0000	0.0000	1.0000	0.0000	1.0000	0.0000	1.0000	0.0000
LOC104914703	0.8249	-3.3439	1.0000	-2.3960	0.8033	-3.2635	1.0000	0.0075	1.0000	-0.9638	1.0000	0.0000	1.0000	2.3554
LOC104914705	1.0000	0.0000	1.0000	-0.1429	0.4809	3.6101	1.0000	0.0062	1.0000	2.2677	1.0000	2.3481	1.0000	-1.3084
LOC104914706	1.0000	-0.0307	0.3784	0.0978	0.0193	-0.4067	0.0514	-0.1774	1.0000	-0.1394	1.0000	0.0015	0.7947	0.0958
LOC104914707	0.0015	-0.9051	0.0000	-1.1212	0.0000	-1.2722	0.0000	-0.9316	1.0000	-0.0471	0.8785	-0.2504	0.6567	0.2988
LOC104914708	0.0000	-3.0410	0.0000	-2.4906	0.0001	1.4139	0.0000	1.5426	1.0000	0.0531	0.8202	0.6151	0.6277	0.1867
LOC104914710	1.0000	-0.0740	1.0000	-0.1318	0.2845	0.7097	0.1457	0.6811	1.0000	0.5945	0.7704	0.5513	0.4951	0.5742
LOC104914711	0.0000	0.9678	0.0000	0.6275	0.0447	-0.5106	0.0030	-0.4914	1.0000	0.0054	0.0925	-0.3219	1.0000	0.0315
LOC104914713	0.0185	-0.5940	0.0118	-0.4972	0.0000	-1.2612	0.0000	-0.9519	1.0000	-0.3777	0.6156	-0.2685	1.0000	-0.0630
LOC104914716	1.0000	-2.4776	1.0000	-2.3985	1.0000	-2.4056	1.0000	0.8519	1.0000	-0.1034	1.0000	0.0000	1.0000	3.2049
LOC104914717	1.0000	0.0000	1.0000	0.0000	1.0000	0.0000	1.0000	2.3256	1.0000	0.0000	1.0000	0.0000	1.0000	2.3554
LOC104914718	0.0604	2.8512	0.0586	2.2755	1.0000	-0.1625	0.7701	-3.1666	1.0000	0.7365	1.0000	0.1822	1.0000	-2.2889
LOC104914720	1.0000	0.0000	1.0000	0.0000	1.0000	0.0000	1.0000	0.0000	1.0000	0.0000	1.0000	0.0000	1.0000	0.0000
LOC104914722	0.7861	-0.2570	0.5511	0.2333	0.0001	1.1777	0.0000	1.0544	1.0000	-0.3737	1.0000	0.1270	0.0255	-0.4922
LOC104914728	0.0001	1.2022	0.0236	0.6462	0.0001	-1.7707	0.0000	-1.8965	1.0000	0.0500	0.1852	-0.4951	1.0000	-0.0707
LOC104914729	0.0000	1.5606	0.0000	1.5656	0.0000	1.9598	0.0000	1.6240	1.0000	0.1951	0.7428	0.2125	0.9245	-0.1367
LOC104914731	1.0000	0.6002	0.4991	1.3064	1.0000	0.1978	0.9786	0.9248	1.0000	-0.6504	1.0000	0.0596	1.0000	0.0774
LOC104914732	0.1374	2.5657	0.1288	1.5717	0.0107	3.3308	0.0821	1.7555	1.0000	1.6383	1.0000	0.6830	1.0000	0.0808
LOC104914733	0.0098	1.6714	0.0034	1.4223	0.4046	-0.9568	0.8150	-0.4681	1.0000	0.0886	1.0000	-0.1517	1.0000	0.5800
LOC104914734	0.0000	1.1813	0.0000	1.2720	0.0000	1.7723	0.0000	1.6256	1.0000	0.2283	0.2339	0.3286	1.0000	0.0844
LOC104914735	1.0000	0.0000	1.0000	0.0000	1.0000	0.0000	1.0000	0.0000	1.0000	0.0000	1.0000	0.0000	1.0000	0.0000
LOC104914736	1.0000	0.0000	1.0000	0.0000	1.0000	0.0000	1.0000	0.0000	1.0000	0.0000	1.0000	0.0000	1.0000	0.0000
LOC104914738	0.6926	-2.0844	0.2535	-4.1743	0.2836	-4.1861	0.6172	-1.7581	1.0000	-0.1279	1.0000	-2.2958	1.0000	2.3554
LOC104914742	0.5258	0.4454	0.0000	1.7507	0.0041	-1.5465	0.0005	-1.8540	1.0000	-0.4448	0.0178	0.8703	0.7701	-0.7478
LOC104914743	0.6723	0.2920	0.9611	0.1260	0.0254	0.7638	0.0002	0.9012	1.0000	0.0371	1.0000	-0.1170	0.9145	0.1806
LOC104914744	0.4523	0.3762	0.4431	0.2750	0.0001	1.2076	0.0000	1.0772	1.0000	0.1513	1.0000	0.0626	1.0000	0.0282
LOC104914746	1.0000	0.0373	0.6688	0.0926	1.0000	-0.8556	1.0000	-0.5097	1.0000	-0.0586	1.0000	0.0092	0.1961	0.2933
LOC104914750	0.0008	1.3203	0.0000	1.2677	0.0000	2.2655	0.0000	1.9046	1.0000	0.3168	0.8194	0.2798	1.0000	-0.0359
LOC104914751	0.0839	0.7938	0.3388	0.4593	0.0054	1.1118	0.0167	0.8247	1.0000	0.4725	1.0000	0.1503	0.9151	0.1963
LOC104914752	0.6111	0.5479	0.0050	1.3126	0.0071	1.4917	0.0096	1.2092	1.0000	-0.0541	0.3733	0.7230	0.8809	-0.3333
LOC104914753	0.2534	0.3654	1.0000	-0.0628	0.1438	0.4309	1.0000	0.0243	1.0000	0.5739	0.9052	0.1590	0.7609	0.1724
LOC104914755	1.0000	0.0000	1.0000	0.0000	1.0000	0.0000	1.0000	0.0000	1.0000	0.0000	1.0000	0.0000	1.0000	0.0000
LOC104914759	1.0000	0.0000	1.0000	0.0000	1.0000	0.0000	1.0000	0.0000	1.0000	0.0000	1.0000	0.0000	1.0000	0.0000
LOC104914760	0.6703	-0.2981	0.8077	0.1441	1.0000	-0.1136	1.0000	-0.0279	1.0000	-0.1848	0.8785	0.2699	1.0000	-0.0904
LOC104914761	0.9586	-0.1517	0.0378	0.5252	0.5917	0.2853	0.1356	0.4092	1.0000	-0.2966	0.4114	0.3897	0.9418	-0.1690
LOC104914762	0.0000	-1.0162	0.0000	-1.1231	0.3650	-0.2500	1.0000	-0.0326	1.0000	-0.0043	1.0000	-0.0973	0.2678	0.2179
LOC104914763	1.0000	2.1900	1.0000	2.2508	1.0000	2.2469	1.0000	2.3241	1.0000	0.0000	1.0000	0.0470	1.0000	0.0640
LOC104914767	0.4636	0.6716	0.0862	1.0592	0.0107	-2.6006	0.6483	-0.6770	1.0000	-0.5123	1.0000	-0.1127	0.7287	1.4183

LOC104914768	0.9406	0.0733	0.6723	0.0682	0.0826	0.3217	0.5791	0.0818	1.0000	0.0507	1.0000	0.0578	0.2580	-0.1846
LOC104914769	0.1380	0.7847	0.7302	-0.2506	0.0000	1.6890	0.0170	0.7062	1.0000	0.7884	1.0000	-0.2303	0.9454	-0.1842
LOC104914770	0.1012	-0.6367	0.0006	-0.6968	0.0626	-0.5532	0.0000	-1.0353	1.0000	0.1856	1.0000	0.1380	0.6506	-0.2909
LOC104914772	0.0767	0.7542	0.0154	0.8335	0.0007	-1.6232	0.0432	-0.9833	1.0000	-0.6362	0.3206	-0.5438	1.0000	0.0078
LOC104914773	0.5146	1.1732	0.0296	1.7181	1.0000	0.3605	0.2626	-2.3201	1.0000	0.4379	0.6002	0.9881	0.5236	-2.2483
LOC104914775	1.0000	-0.0402	0.6128	-1.3340	1.0000	-0.1874	0.8678	-0.7769	1.0000	0.6523	1.0000	-0.6335	1.0000	0.0727
LOC104914776	1.0000	0.0000	1.0000	0.0000	1.0000	0.0000	1.0000	0.0000	1.0000	0.0000	1.0000	0.0000	1.0000	0.0000
LOC104914777	1.0000	0.0000	1.0000	0.0000	1.0000	0.0000	1.0000	0.0000	1.0000	0.0000	1.0000	0.0000	1.0000	0.0000
LOC104914779	1.0000	0.2164	0.2053	1.2650	0.0117	1.6212	0.0027	2.2129	1.0000	-1.0918	1.0000	-0.0368	0.7687	-0.4991
LOC104914780	0.0000	-1.3192	0.0000	-0.9795	0.0024	-0.5887	0.0000	-0.7471	1.0000	-0.0956	0.6123	0.2579	0.2585	-0.2485
LOC104914781	1.0000	-0.3449	0.2429	-2.0462	1.0000	0.2720	0.4316	-1.3382	1.0000	0.5290	1.0000	-1.1663	0.8664	-1.0799
LOC104914782	0.2497	-1.0094	0.1945	-0.7795	0.5821	0.5088	0.5906	0.3624	1.0000	0.1123	1.0000	0.3602	1.0000	-0.0240
LOC104914783	1.0000	-0.0514	1.0000	0.0049	0.2204	0.3525	0.3412	0.2052	1.0000	-0.0861	1.0000	-0.0170	0.4877	-0.2270
LOC104914786	0.0206	1.2563	0.0721	1.2617	0.0164	1.2210	0.0020	1.4537	1.0000	-0.4538	0.9052	-0.4347	1.0000	-0.2126
LOC104914787	0.0000	-0.7639	0.0000	-0.8776	0.0000	-1.2470	0.0000	-1.1133	1.0000	-0.0019	0.7211	-0.1034	0.2963	0.1373
LOC104914788	0.3827	-0.7343	0.4427	-0.6661	0.0002	1.6091	0.0012	1.2971	1.0000	-0.2513	1.0000	-0.1696	0.2802	-0.5563
LOC104914789	0.6016	-1.5517	0.3472	-1.8549	0.0167	2.1726	0.1940	1.1718	1.0000	0.3361	1.0000	0.0532	0.6470	-0.6627
LOC104914790	1.0000	0.0000	1.0000	0.0000	1.0000	0.0000	1.0000	0.0000	1.0000	0.0000	1.0000	0.0000	1.0000	0.0000
LOC104914791	1.0000	-0.2882	0.6504	-1.9122	0.1203	2.7069	1.0000	0.3146	1.0000	1.6383	1.0000	0.0489	0.9558	-0.7381
LOC104914793	0.5815	-0.1299	0.6880	-0.0560	0.1186	-0.2617	0.0001	-0.3033	1.0000	-0.0457	1.0000	0.0407	0.7533	-0.0817
LOC104914794	0.0000	-1.5508	0.0000	-1.6180	0.0000	1.5094	0.0000	1.3096	1.0000	-0.0196	1.0000	-0.0736	0.1453	-0.2153
LOC104914795	1.0000	2.1850	1.0000	0.0000	1.0000	0.0000	1.0000	0.0000	1.0000	0.0000	1.0000	-2.2958	1.0000	0.0000
LOC104914796	1.0000	-2.4788	0.1392	-4.4820	1.0000	-2.4061	0.3949	-2.0643	1.0000	1.9552	1.0000	0.0000	1.0000	2.3543
LOC104914797	1.0000	0.0000	1.0000	0.0000	1.0000	0.0000	1.0000	0.0000	1.0000	0.0000	1.0000	0.0000	1.0000	0.0000
LOC104914799	1.0000	0.0000	1.0000	0.0000	1.0000	0.0000	1.0000	0.0000	1.0000	0.0000	1.0000	0.0000	1.0000	0.0000
LOC104914800	0.8262	-0.2497	0.4843	-0.2652	1.0000	0.0151	1.0000	-0.0195	1.0000	0.1735	1.0000	0.1703	1.0000	0.1507
LOC104914801	0.8002	0.4309	0.6470	0.4003	0.9269	-0.3371	1.0000	0.0828	1.0000	-0.4167	1.0000	-0.4333	1.0000	0.0066
LOC104914802	0.9538	0.1372	0.4159	-0.2768	0.0000	2.1724	0.0000	1.2680	1.0000	0.4405	1.0000	0.0399	0.0412	-0.4571
LOC104914803	0.1951	-1.4958	1.0000	0.1654	0.6650	0.5366	0.0078	1.5281	1.0000	-0.8285	0.9139	0.8496	1.0000	0.1729
LOC104914804	0.7513	-0.2847	0.3729	0.3373	0.3946	0.3828	0.0243	0.6416	1.0000	-0.1566	0.6072	0.4783	1.0000	0.1096
LOC104914806	0.1763	0.9448	0.0439	0.9050	0.0000	2.2021	0.0000	1.7875	1.0000	0.0492	1.0000	0.0253	0.4078	-0.3550
LOC104914807	0.0256	1.0205	0.0000	1.6501	0.0000	2.5208	0.0000	2.6418	1.0000	-0.4096	1.0000	0.2357	0.0522	-0.2819
LOC104914810	0.0000	-1.3633	0.0000	-1.0790	0.0005	-0.6077	0.2038	-0.1569	1.0000	-0.1603	0.8788	0.1367	0.0455	0.2957
LOC104914811	0.0001	-1.0684	0.3221	-0.2771	0.8899	0.1222	0.0002	0.6615	1.0000	-0.4296	0.4709	0.3721	0.9852	0.1133
LOC104914812	0.8074	-0.2538	0.4114	-0.4133	0.0321	-1.0363	0.0020	-1.3661	1.0000	-0.2750	0.8207	-0.4229	0.7377	-0.6007
LOC104914813	0.6318	0.3065	0.0001	0.9296	0.1992	0.5066	0.9946	0.1202	1.0000	-0.0318	0.0756	0.6028	0.4248	-0.4140
LOC104914814	0.7225	0.2374	0.0449	0.5639	0.0024	-1.1528	0.0005	-1.1135	1.0000	-0.6412	0.6766	-0.3017	0.3841	-0.5962
LOC104914815	0.1057	-0.5021	0.2093	-0.1978	0.0425	-0.5978	0.0003	-0.4967	1.0000	0.2000	0.0044	0.5171	0.2080	0.3061
LOC104914816	0.2948	-0.2688	1.0000	-0.0192	0.0000	-0.8781	0.0000	-0.8079	1.0000	-0.1856	1.0000	0.0765	0.9835	-0.1094
LOC104914818	0.0693	-1.7287	0.0790	-1.0614	0.6843	0.4919	1.0000	0.1147	1.0000	0.5630	0.6427	1.2470	1.0000	0.1896
LOC104914821	0.0000	-2.4246	0.0001	-1.3410	0.0021	-1.2378	0.0003	-1.2426	1.0000	-0.0907	0.3185	1.0060	1.0000	-0.0928
LOC104914822	1.0000	0.0000	1.0000	-2.3960	1.0000	0.0000	1.0000	-2.3180	1.0000	2.2677	1.0000	0.0000	1.0000	0.0000
LOC104914825	0.0000	-0.8952	0.0000	-1.1229	0.0000	-1.6578	0.0000	-1.2574	1.0000	0.4631	0.4999	0.2482	0.0000	0.8717
LOC104914826	1.0000	-0.8620	0.4670	1.9290	0.8151	0.7542	0.0883	2.7290	1.0000	-1.4938	1.0000	1.2962	1.0000	0.4787
LOC104914828	0.1767	0.3890	0.0084	0.4637	0.0000	1.1326	0.0000	1.0504	1.0000	0.0522	0.9187	0.1373	1.0000	-0.0281
LOC104914829	0.0069	0.8993	0.0000	1.2503	0.0000	1.7600	0.0000	1.4994	1.0000	0.0685	0.2100	0.4354	0.7059	-0.1845
LOC104914830	0.0000	1.2702	0.0000	1.9575	0.0000	1.8316	0.0000	2.1399	1.0000	-0.4946	0.7081	0.2045	0.5872	-0.1812
LOC104914834	1.0000	0.0000	1.0000	2.2533	1.0000	0.0000	1.0000	0.0000	1.0000	0.0000	1.0000	2.3481	1.0000	0.0000
LOC104914835	0.6290	0.6189	0.0119	1.3865	0.0008	1.8012	0.0000	2.3299	1.0000	-0.4465	1.0000	0.3279	1.0000	0.0844
LOC104914838	0.8249	3.0143	1.0000	0.3687	0.1054	4.5443	0.7210	1.2296	1.0000	3.1057	1.0000	0.5881	1.0000	-0.1726
LOC104914839	1.0000	0.0000	1.0000	0.0000	1.0000	0.0000	1.0000	0.0000	1.0000	0.0000	1.0000	0.0000	1.0000	0.0000
LOC104914840	1.0000	2.1900	1.0000	-2.3960	1.0000	0.0000	1.0000	-2.3180	1.0000	2.2676	1.0000	-2.2992	1.0000	0.0000
LOC104914841	1.0000	0.0268	1.0000	-0.0598	0.9357	-0.1812	0.0585	-0.5275	1.0000	0.2298	1.0000	0.1552	1.0000	-0.1088
LOC104914842	0.0016	1.4016	0.0000	1.7355	0.0000	2.7201	0.0000	2.4299	1.0000	-0.2414	1.0000	0.1062	0.1367	-0.5279
LOC104914843	1.0000	0.0000	1.0000	0.0000	1.0000	0.0000	1.0000	0.0000	1.0000	0.0000	1.0000	0.0000	1.0000	0.0000
LOC104914844	1.0000	0.0000	1.0000	0.0000	1.0000	0.0000	1.0000	0.0000	1.0000	0.0000	1.0000	0.0000	1.0000	0.0000
LOC104914847	1.0000	-0.8577	0.5066	-1.6418	1.0000	0.2038	0.7731	-0.9370	1.0000	0.8230	1.0000	0.0559	1.0000	-0.3119
LOC104914849	0.9545	-1.6935	1.0000	0.0000	0.4809	-3.7970	1.0000	0.0000	1.0000	-3.7635	1.0000	-2.2958	1.0000	0.0000
LOC104914851	1.0000	0.0000	1.0000	0.0000	1.0000	0.0000	1.0000	0.0000	1.0000	0.0000	1.0000	0.0000	1.0000	0.0000
LOC104914852	1.0000	0.0000	1.0000	0.0000	1.0000	0.0000	1.0000	0.0000	1.0000	0.0000	1.0000	0.0000	1.0000	0.0000
LOC104914853	0.7908	0.2499	0.6734	-0.2212	0.2453	0.4949	0.1919	0.4197	1.0000	0.1003	0.7251	-0.3613	1.0000	0.0256
LOC104914856	0.1827	0.6261	0.0004	0.8172	0.0000	1.1680	0.0000	1.2348	1.0000	-0.0787	1.0000	0.1247	1.0000	-0.0069
LOC104914857	0.8582	0.2211	0.2070	0.4865	0.0235	0.8507	0.0884	0.5423	1.0000	0.1203	0.7487	0.3986	0.9639	-0.1824
LOC104914859	0.0587	-1.5060	0.0039	-1.3910	0.5847	-0.5322	0.9466	-0.1746	1.0000	0.3985	1.0000	0.5231	0.3220	0.7606
LOC104914861	0.1473	1.2185	0.2672	1.0111	0.1916	1.0098	0.0588	1.0929	1.0000	0.0917	1.0000	-0.1056	1.0000	0.1787
LOC104914865	0.3548	0.2271	0.2389	0.1810	0.0855	-0.3728	0.7027	-0.0917	1.0000	0.0276	1.0000	-0.0066	0.0730	0.3127
LOC104914866	0.0000	1.8512	0.0000	2.0395	0.0000	1.5895	0.0000	1.7585	1.0000	-0.5749	0.3704	-0.3734	0.1460	-0.3999
LOC104914870	1.0000	-0.0020	1.0000	0.0100	0.8276	0.6753	0.4620	0.8770	1.0000	-0.1281	1.0000	-0.1054	1.0000	0.0792
LOC104914872	1.0000	0.0000	1.0000	0.0000	1.0000	0.0000	1.0000	0.0000	1.0000	0.0000	1.0000	0.0000	1.0000	0.0000
LOC104914873	0.4996	-1.4996	0.8299	-0.9449	0.1846	-2.7149	0.3330	-1.6811	1.0000	-0.1289	1.0000	0.4388	1.0000	0.9168
LOC104914875	0.0161	0.9657	0.0004	0.8548	0.1577	-0.8448	0.0001	-1.2606	1.0000	0.1168	1.0000	0.0167	1.0000	-0.2887
LOC104914876	0.8523	0.8095	0.7315	1.0551	1.0000	-0.1848	0.3415	1.6949	1.0000	-0.6592	1.0000	-0.4064	0.8652	1.2335
LOC104914877	0.5384	3.5542	1.0000	2.2508	0.8011	3.0840	1.0000	0.0000	1.0000	0.0000	1.0000	-1.3320	1.0000	-3.1344
LOC104914878	0.9545	1.0644	1.0000	2.2533	0.6966	1.5793	0.7701	3.1732	1.0000	-2.3771	1.0000	-1.3246	1.0000	-0.8394
LOC104914879	1.0000	0.0000	1.0000	0.0000	1.0000	0.0000	1.0000	0.0000	1.0000	0.0000	1.0000	0.0000	1.0000	0.0000
LOC104914880	0.6099	1.0572	1.0000	0.2296	0.2888	1.3329	1.0000	0.1561	1.0000	0.9928	1.0000	0.1832	1.0000	-0.1771
LOC104914881	0.3089	-4.2696	1.0000	0.3659	1.0000	-0.1911	0.1422	2.0470	1.0000	-1.0389	0.8607	3.7288	0.7640	1.2010
LOC104914883	0.2895	1.3963	0.0223	2.4930	1.0000	0.1132	1.0000	0.5372	1.0000	-1.0395	1.0000	0.0601	1.0000	-0.6156
LOC104914886	0.0000	1.3034	0.0000	1.2543	0.0000	0.6997	0.0000	0.4391	1.0000	-0.1019	0.5785	-0.1385	0.0012	-0.3568
LOC104914888	0.0000	-0.9790	0.0000	-1.3435	0.0000	-0.9095	0.0000	-1.1005	1.0000	0.2767	1.0000	-0.0754	1.0000	0.0913
LOC104914889	0.0750	1.3815	0.0001	2.2583	0.0000	2.6133	0.0000	2.8809	1.0000	-0.6263	1.0000	0.2580	0.7533	-0.3579
LOC104914890	0.0010	1.2634	0.0000	1.0896	0.0000	2.4069	0.0000	1.8380	1.0000	0.1591	1.0000	-0.0015	0.0297	-0.4038
LOC104914891	0.0000	1.2845	0.0000	1.5139	0.0000	1.6552	0.0000	1.2666	1.0000	0.0664	0.3217	0.3091	0.2488	-0.3169
LOC104914894	0.2034	4.4738	1.0000	0.3635	0.5076	3.6021	1.0000	-0.8416	1.0000	3.1126	1.0000	-0.8837	1.0000	-1.3039
LOC104914895	0.3192	4.2259	0.0255	4.9567	1.0000	2.2469	0.4370	3.7037	1.0000	0.0000	1.0000	0.7112	1.0000	1.4482
LOC104914896	0.0343	1.8306	0.0038	1.7655	0.2648	1.2165	1.0000	0.1602	1.0000	0.3372	1.0000	0.3049	0.6825	-0.6961
LOC104914898	0.1096	4.6934	0.4234	1.1739	0.0001	6.1719	0.7948	0.9568	1.0000	3.6299	1.0000	0.2462	0.1388	-1.5435
LOC104914900	0.2599	-0.3979	0.6876	-0.1416	0.0020	-0.9079	0.0008	-0.6723	1.0000	-0.0287	0.8009	0.2398	0.8785	0.2119
LOC104914902	0.2385	0.4534	0.0032	0.7946	0.0401	0.6545	0.6852	0.2026	1.0000	0.0779	0.2917	0.4335	0.3502	-0.3688
LOC104914903	0.0004	-1.5654	0.0000	-1.4813	0.2936	0.4469	0.5772	0.2204	1.0000	0.0807	1.0000	0.1786	1.0000	-0.1393
LOC104914905	1.0000	0.0000	1.0000	0.0000	1.0000	0.0000	1.0000	0.0000	1.0000	0.0000	1.0000	0.0000	1.0000	0.0000
LOC104914906	0.4369	-1.1667	1.0000	-0.1762	0.0238	1.6082	0.0318	1.3647	1.0000	0.0162	0.9238	1.0244	1.0000	-0.2173
LOC104914909	0.1303	0.3249	0.3358	0.1299	0.0001	-0.7313	0.0000	-0.9374	1.0000	0.1704	1.0000	-0.0112	1.0000	-0.0295

LOC104914911	0.7902	1.1381	0.7057	0.7932	0.0001	3.4636	0.0000	2.7989	1.0000	1.0806	1.0000	0.7728	0.7915	0.4406
LOC104914913	1.0000	0.0000	1.0000	0.0000	1.0000	0.0000	1.0000	0.0000	1.0000	0.0000	1.0000	0.0000	1.0000	0.0000
LOC104914914	1.0000	0.0000	1.0000	0.0000	1.0000	0.0000	1.0000	0.0000	1.0000	0.0000	1.0000	0.0000	1.0000	0.0000
LOC104914915	1.0000	0.0000	1.0000	0.0000	1.0000	0.0000	1.0000	0.0000	1.0000	0.0000	1.0000	0.0000	1.0000	0.0000
LOC104914916	0.1867	1.1604	0.0392	1.2944	0.0202	1.7281	0.0013	1.6954	1.0000	0.0050	1.0000	0.1534	1.0000	-0.0287
LOC104914917	0.4841	0.8089	0.5737	-0.6160	0.0048	1.7099	0.1788	0.8961	1.0000	0.3060	0.3912	-1.1059	0.6865	-0.5008
LOC104914919	0.0124	0.9118	0.0000	1.8148	0.0001	1.2594	0.0000	1.4737	1.0000	-0.4294	0.1586	0.4875	0.7947	-0.2073
LOC104914920	0.0007	1.0869	0.0000	1.3078	0.0000	1.8403	0.0000	1.4604	1.0000	-0.1586	1.0000	0.0771	0.0147	-0.5303
LOC104914921	0.7508	0.1118	0.0250	0.2289	0.0848	-0.3321	0.0014	-0.3226	1.0000	0.0033	0.6195	0.1321	1.0000	0.0183
LOC104914926	0.0631	0.9345	0.0262	0.7951	0.0002	1.4908	0.0051	0.9124	1.0000	0.1398	1.0000	0.0096	0.3660	-0.4333
LOC104914928	0.0246	0.4765	0.0000	0.5776	0.2248	-0.3089	0.0000	-0.7768	1.0000	-0.1448	1.0000	-0.0316	0.0000	-0.6062
LOC104914929	0.3698	4.2134	0.0372	2.3895	0.0101	5.3980	0.0198	2.5651	1.0000	3.1056	0.6040	1.3982	1.0000	0.3154
LOC104914930	0.1039	2.0661	1.0000	-0.1782	1.0000	-0.7130	0.4086	-1.3372	1.0000	1.2202	0.6450	-1.0083	1.0000	0.6043
LOC104914931	0.1940	2.5524	0.4424	1.6884	0.1182	2.5432	0.1384	2.0464	1.0000	0.7365	1.0000	-0.1042	1.0000	0.2442
LOC104914932	0.0000	2.2530	0.0000	2.6141	0.0000	2.9783	0.0000	2.5049	1.0000	0.0648	0.3309	0.4390	0.3665	-0.4031
LOC104914933	0.0000	0.8669	0.0000	0.7368	0.0022	0.6154	0.0107	0.3680	1.0000	0.2556	0.6650	0.1393	1.0000	0.0137
LOC104914934	1.0000	0.0000	1.0000	2.2508	1.0000	0.0000	1.0000	0.0000	1.0000	0.0000	1.0000	2.3456	1.0000	0.0000
LOC104914935	1.0000	0.0000	1.0000	0.0000	1.0000	0.0000	1.0000	0.0000	1.0000	0.0000	1.0000	0.0000	1.0000	0.0000
LOC104914937	1.0000	0.0000	1.0000	0.0000	1.0000	0.0000	1.0000	0.0000	1.0000	0.0000	1.0000	0.0000	1.0000	0.0000
LOC104914938	1.0000	0.0000	1.0000	0.0000	1.0000	0.0000	1.0000	0.0000	1.0000	0.0000	1.0000	0.0000	1.0000	0.0000
LOC104914939	1.0000	0.0000	1.0000	0.0000	1.0000	0.0000	1.0000	0.0000	1.0000	0.0000	1.0000	0.0000	1.0000	0.0000
LOC104914940	1.0000	0.0000	1.0000	0.0000	1.0000	0.0000	1.0000	0.0000	1.0000	0.0000	1.0000	0.0000	1.0000	0.0000
LOC104914941	1.0000	0.0000	0.7666	-3.2534	1.0000	0.0000	0.7701	-3.1666	1.0000	3.1126	1.0000	0.0000	1.0000	0.0000
LOC104914942	0.8051	0.6304	0.6128	0.5973	0.0003	2.2888	0.0189	1.3862	1.0000	0.3170	1.0000	0.3037	0.4936	-0.5799
LOC104914943	0.7126	-0.1420	1.0000	-0.0333	0.0000	-0.9930	0.0000	-0.7742	1.0000	-0.1488	1.0000	-0.0273	1.0000	0.0768
LOC104914944	0.0002	0.9595	0.0000	0.9574	0.0004	0.8740	0.0000	0.8228	1.0000	0.0069	1.0000	0.0174	1.0000	-0.0383
LOC104914945	0.0015	0.7620	0.0003	0.6039	0.0534	-0.5679	0.1944	-0.2792	1.0000	0.2040	1.0000	0.0572	0.0703	0.4974
LOC104914946	0.0000	-1.1400	0.0000	-0.8461	0.0397	0.5200	0.6276	0.1194	1.0000	0.0040	0.5783	0.3103	0.0510	-0.3925
LOC104914947	0.0595	2.4999	0.9674	0.3951	0.2687	1.8519	0.6965	0.6922	1.0000	1.7401	1.0000	-0.3419	1.0000	0.5963
LOC104914948	0.0228	3.0829	0.0553	1.7616	0.0105	3.3321	0.8250	0.7771	1.0000	1.6505	1.0000	0.3560	0.6506	-0.8961
LOC104914949	1.0000	0.0000	1.0000	0.0000	1.0000	0.0000	1.0000	0.0000	1.0000	0.0000	1.0000	0.0000	1.0000	0.0000
LOC104914952	0.8249	3.0254	1.0000	-1.0017	1.0000	2.2469	0.7701	-3.1666	1.0000	3.1126	1.0000	-0.8001	1.0000	-2.2908
LOC104914955	0.6619	0.1690	0.9253	0.0765	0.0000	-1.3622	0.0003	-0.6996	1.0000	0.0819	1.0000	0.0004	0.0008	0.7491
LOC104914956	0.0000	-1.6121	0.0000	-1.6814	0.1791	-0.2913	0.0001	-0.3798	1.0000	-0.1696	0.4639	-0.2256	0.0723	-0.2525
LOC104914959	0.0016	0.8389	0.0021	0.5377	0.0000	2.0121	0.0000	1.6550	1.0000	0.1807	1.0000	-0.1082	0.6819	-0.1720
LOC104914961	0.0015	2.3374	0.0000	1.8012	0.0000	2.9955	0.0000	2.1045	1.0000	0.9417	0.8775	0.4148	1.0000	0.0586
LOC104914962	1.0000	0.0000	1.0000	0.0000	1.0000	0.0000	1.0000	0.0000	1.0000	0.0000	1.0000	0.0000	1.0000	0.0000
LOC104914965	1.0000	0.3141	0.4344	0.8863	0.3101	0.9173	0.0201	1.5632	1.0000	-0.4439	1.0000	0.1420	1.0000	0.2057
LOC104914968	0.2308	0.4762	0.1198	0.5126	0.0444	0.7232	0.0122	0.7012	1.0000	0.0381	1.0000	0.0904	1.0000	0.0225
LOC104914969	1.0000	-2.4776	0.7666	3.0922	1.0000	-2.4056	0.7710	3.1710	1.0000	-2.3758	1.0000	3.1976	1.0000	3.2049
LOC104914972	0.0003	1.4352	0.0013	0.9663	0.0001	1.4458	0.0004	1.0191	1.0000	0.4461	1.0000	-0.0097	1.0000	0.0268
LOC104914973	0.3953	1.2880	0.7708	0.6932	0.3990	1.2125	0.4620	0.8770	1.0000	0.6438	1.0000	0.0612	1.0000	0.3151
LOC104914976	0.0789	0.9469	0.2315	0.6203	1.0000	-0.1027	0.9122	0.2113	1.0000	0.1318	1.0000	-0.1802	0.7260	0.4566
LOC104914977	0.1727	0.4475	0.4527	0.2678	1.0000	0.0074	0.4983	-0.2479	1.0000	-0.0817	0.7590	-0.2487	0.5898	-0.3318
LOC104914979	0.1201	1.3878	0.0599	1.2834	0.5667	0.8764	0.9696	0.3777	1.0000	0.3712	1.0000	0.2801	1.0000	-0.1228
LOC104914980	0.8784	0.2885	0.7708	0.2645	0.9320	0.2226	0.0022	1.0661	1.0000	-0.4438	0.8338	-0.4584	0.5582	0.4066
LOC104914981	1.0000	0.3510	0.6224	1.6084	0.6305	1.1523	0.2463	2.3286	1.0000	-1.4912	1.0000	-0.2455	1.0000	-0.3214
LOC104914982	0.4950	1.7587	0.1384	4.3086	0.2892	2.1434	0.0208	5.0478	1.0000	-2.3758	1.0000	0.0565	1.0000	0.4791
LOC104914983	0.0212	2.7563	0.0330	2.8752	0.0009	3.2905	0.0000	4.2264	1.0000	-0.9614	0.6836	-0.8488	1.0000	-0.0314
LOC104914985	0.8249	3.0144	1.0000	0.0000	1.0000	0.0000	1.0000	0.0000	1.0000	0.0000	1.0000	-3.1384	1.0000	0.0000
LOC104914986	0.1888	0.8265	0.0000	1.5387	0.0374	1.1110	0.0236	0.8465	1.0000	-0.1391	0.2939	0.5866	0.5353	-0.3953
LOC104914987	0.2437	-0.9590	0.1401	-1.0402	0.3605	-0.7221	0.6817	-0.4467	1.0000	-0.3685	1.0000	-0.4378	1.0000	-0.0881
LOC104914990	1.0000	0.1136	0.8876	-0.1310	0.3921	-0.3768	0.0139	-0.7286	1.0000	0.0976	1.0000	-0.1373	0.9039	-0.2491
LOC104914991	1.0000	0.0000	1.0000	0.0000	1.0000	0.0000	1.0000	0.0000	1.0000	0.0000	1.0000	0.0000	1.0000	0.0000
LOC104914992	1.0000	0.8875	0.2542	4.0037	1.0000	-0.1789	0.7710	3.1752	1.0000	-3.2304	1.0000	-0.2469	1.0000	0.0727
LOC104914993	1.0000	0.0000	1.0000	0.0000	1.0000	0.0000	1.0000	0.0000	1.0000	0.0000	1.0000	0.0000	1.0000	0.0000
LOC104914994	0.0926	1.4465	0.5658	0.7224	0.3905	-1.8727	0.8938	-0.5584	1.0000	0.2226	0.9111	-0.4887	0.7908	1.5487
LOC104914995	0.0022	0.9552	0.0020	0.8492	0.0001	1.1090	0.0252	0.6419	1.0000	-0.4520	0.1011	-0.5433	0.0001	-0.9106
LOC104914997	0.4623	-0.2150	0.0343	-0.2488	0.0000	-0.8724	0.0000	-0.8998	1.0000	-0.0242	1.0000	-0.0463	1.0000	-0.0466
LOC104914998	1.0000	-0.1086	0.2836	-0.3058	1.0000	0.1131	0.0699	-0.4510	1.0000	0.1977	1.0000	0.0137	0.4993	-0.3636
LOC104915000	0.1630	0.9750	0.0088	1.2681	0.0000	2.8451	0.0000	2.5278	1.0000	-0.3000	1.0000	0.0044	0.1014	-0.6137
LOC104915001	1.0000	2.1900	1.0000	2.2508	0.4809	3.6122	1.0000	2.3241	1.0000	0.0000	1.0000	0.0470	1.0000	-1.3088
LOC104915002	0.1936	1.2026	1.0000	0.2251	1.0000	-0.3769	1.0000	0.2217	1.0000	-0.5273	0.1406	-1.4963	1.0000	0.0781
LOC104915003	0.1096	-0.3476	0.0000	-0.4375	0.0000	-0.7928	0.0000	-0.8880	1.0000	0.0497	1.0000	-0.0281	1.0000	-0.0393
LOC104915004	0.0053	1.3097	0.0052	1.0811	0.0054	1.3490	0.1440	0.6682	1.0000	0.1392	1.0000	-0.0773	0.3816	-0.5402
LOC104915005	1.0000	2.1850	0.4030	1.9126	0.8011	3.0840	0.9096	1.3854	1.0000	2.2676	0.8269	2.1253	1.0000	0.6043
LOC104915006	0.4866	1.0238	0.0089	2.6808	0.5089	0.8871	0.4992	1.4798	1.0000	-1.5901	1.0000	0.0608	0.6100	-1.0049
LOC104915007	1.0000	0.0022	0.0186	-0.2195	0.0142	0.4211	0.0007	0.3039	1.0000	0.1464	1.0000	-0.0626	1.0000	0.0345
LOC104915009	1.0000	0.0000	0.7674	3.0956	1.0000	0.0000	0.7710	3.1710	1.0000	0.0000	1.0000	3.2002	1.0000	3.2049
LOC104915011	0.4427	1.5330	0.0225	2.4940	0.8033	-3.2622	0.7710	-3.1632	1.0000	-0.1218	0.9107	0.8498	1.0000	0.0000
LOC104915012	1.0000	0.0000	1.0000	2.2533	1.0000	2.2424	1.0000	2.3241	1.0000	0.0000	1.0000	2.3481	1.0000	0.0652
LOC104915014	1.0000	-0.2931	1.0000	2.2508	1.0000	-2.4061	0.7701	3.1732	1.0000	-2.3771	1.0000	0.0495	1.0000	3.2066
LOC104915015	1.0000	0.0000	1.0000	0.0000	0.8033	3.0789	1.0000	0.0000	1.0000	0.0000	1.0000	0.0000	1.0000	-3.1317
LOC104915016	0.3358	1.0564	0.0904	1.7539	0.9794	0.5624	0.8238	0.7778	1.0000	-0.9000	1.0000	-0.1918	0.8856	-0.6782
LOC104915017	0.2579	1.6464	0.6957	0.6493	0.0665	1.7519	0.5967	-0.9686	1.0000	0.8581	1.0000	-0.1332	0.0534	-1.8684
LOC104915018	0.1329	1.2045	0.1261	1.3047	0.9748	-0.3436	0.9155	0.5065	1.0000	-0.6278	0.8820	-0.5183	1.0000	0.2269
LOC104915019	0.9786	-0.2250	0.9785	-0.1494	1.0000	-0.0436	0.2855	0.4527	1.0000	-0.0309	1.0000	0.0577	0.5617	0.4665
LOC104915020	0.1471	0.9657	0.0795	0.8854	0.0198	1.3937	0.0008	1.4311	1.0000	0.2748	1.0000	0.2075	0.8608	0.3169
LOC104915021	1.0000	-0.0937	0.8250	-0.9422	0.5862	0.9077	1.0000	0.0075	1.0000	0.3356	1.0000	-0.4988	1.0000	-0.5557
LOC104915022	1.0000	-0.3344	0.7666	-3.2534	1.0000	-0.7136	1.0000	0.0066	1.0000	-0.6522	0.8607	-3.6732	1.0000	0.0723
LOC104915023	1.0000	-0.3217	1.0000	0.2184	0.7663	1.0386	1.0000	0.3946	1.0000	0.4098	1.0000	0.9738	1.0000	-0.2278
LOC104915024	0.4767	1.0270	1.0000	0.3896	1.0000	0.0255	0.3939	0.8933	1.0000	0.2718	1.0000	-0.3511	0.5517	1.1500
LOC104915025	0.9545	1.0652	1.0000	-0.6379	0.0742	2.7104	1.0000	-0.4581	1.0000	2.4160	1.0000	0.7503	0.9536	-0.7392
LOC104915026	1.0000	-0.0332	0.3316	-0.1779	0.1852	-0.3285	0.3094	-0.1804	1.0000	0.0281	1.0000	-0.1043	0.6277	0.1807
LOC104915027	0.5086	3.5431	0.6180	1.6082	1.0000	2.2424	0.0323	3.0416	1.0000	2.2676	1.0000	0.4448	0.1145	3.1073
LOC104915028	0.8226	3.0199	1.0000	0.6956	0.8033	3.0891	1.0000	0.8545	1.0000	2.2676	1.0000	0.0547	1.0000	0.0716
LOC104915034	1.0000	0.4233	1.0000	0.6956	0.6647	-1.9401	1.0000	0.0062	1.0000	-1.8834	0.7608	-1.6308	1.0000	0.0640
LOC104915035	0.6394	0.5785	0.0263	1.7186	1.0000	-0.1981	1.0000	0.0072	1.0000	-1.3215	1.0000	-0.1694	0.4993	-1.1103
LOC104915036	1.0000	0.3609	1.0000	0.3912	0.9613	-0.3636	0.4992	-1.4660	1.0000	-0.6948	0.9520	-0.6575	0.4924	-1.7965
LOC104915037	1.0000	-0.1247	0.0628	0.8434	0.0155	-1.3895	0.0240	-1.4026	1.0000	-0.6634	0.9928	0.3190	0.8523	-0.6706
LOC104915038	0.8226	3.0199	1.0000	0.0000	1.0000	0.0000	0.7710	3.1710	1.0000	0.0000	1.0000	-3.1429	1.0000	3.2049
LOC104915039	0.0341	1.7722	1.0000	0.1164	0.9538	0.6537	0.0513	-2.0203	1.0000	0.9545	0.6767	-0.6859	0.3270	-1.7142
LOC104915040	1.0000	0.0000	1.0000	0.0000	1.0000	0.0000	1.0000	0.0000	1.0000	0.0000	1.0000	0.0000	1.0000	0.0000

LOC104915043	0.6969	-0.1700	0.2650	-0.1782	0.7595	0.1616	0.3253	0.1664	1.0000	-0.0559	1.0000	-0.0518	1.0000	-0.0466
LOC104915044	0.0000	2.1848	0.0000	1.6443	0.0009	-3.9671	0.0076	-2.0098	1.0000	0.1699	0.7971	-0.3563	0.6865	2.1419
LOC104915045	0.0119	-3.7297	1.0000	-0.5760	0.0259	-2.7469	0.2199	-1.8673	1.0000	-0.8138	0.6450	2.3747	1.0000	0.0727
LOC104915046	1.0000	-2.4788	1.0000	0.0000	1.0000	-0.1630	1.0000	0.0000	1.0000	-2.3771	1.0000	0.0000	1.0000	-2.2889
LOC104915047	1.0000	0.2167	0.1018	1.4547	0.0290	-2.8575	0.3872	1.1062	1.0000	-1.5696	1.0000	-0.3259	0.1763	2.3979
LOC104915049	0.5079	-0.1694	0.0002	-0.3915	0.0816	-0.3251	0.0000	-0.5791	1.0000	0.1079	0.9091	-0.1020	0.5406	-0.1419
LOC104915050	0.0545	1.1548	0.0067	1.3645	0.0000	2.4289	0.0000	1.7707	1.0000	0.0599	1.0000	0.2846	0.1053	-0.5824
LOC104915051	0.2731	1.6781	0.2299	1.7069	0.0014	2.7055	0.0000	3.8029	1.0000	-0.6546	1.0000	-0.6218	0.7931	0.4466
LOC104915052	0.5690	-0.9257	0.6518	-1.1341	0.1549	-1.7191	0.8636	-0.6454	1.0000	-0.6983	1.0000	-0.8990	1.0000	0.3812
LOC104915053	0.0006	1.7696	0.0000	1.5397	0.0000	2.6687	0.0000	1.8410	1.0000	0.1515	1.0000	-0.0664	0.0359	-0.6683
LOC104915054	0.0037	1.3131	0.0000	1.7003	0.0000	2.5928	0.0000	2.3413	1.0000	-0.1893	1.0000	0.2135	0.1738	-0.4326
LOC104915057	0.0006	2.0383	0.0000	2.1913	0.0001	1.9321	0.0005	1.5263	1.0000	0.0281	1.0000	0.1992	0.7059	-0.3663
LOC104915058	0.3461	-0.1776	0.0000	-0.3395	0.0001	-0.5547	0.0000	-0.4270	1.0000	0.1593	1.0000	0.0097	0.0005	0.2920
LOC104915059	1.0000	0.3021	1.0000	-0.0401	0.9086	0.4606	0.9163	-0.4916	1.0000	0.8363	1.0000	0.5104	1.0000	-0.1078
LOC104915061	1.0000	0.0000	1.0000	0.0000	1.0000	0.0000	1.0000	0.0000	1.0000	0.0000	1.0000	0.0000	1.0000	0.0000
LOC104915062	0.0008	0.6594	0.0062	0.3529	0.0000	-1.1398	0.0000	-1.4454	1.0000	0.1443	0.7015	-0.1500	0.9239	-0.1558
LOC104915063	1.0000	0.0000	1.0000	0.0000	1.0000	0.0000	1.0000	0.0000	1.0000	0.0000	1.0000	0.0000	1.0000	0.0000
LOC104915064	1.0000	0.0000	1.0000	0.0000	1.0000	2.2469	1.0000	0.0000	1.0000	0.0000	1.0000	0.0000	1.0000	-2.2909
LOC104915065	1.0000	0.0000	1.0000	0.0000	1.0000	0.0000	1.0000	0.0000	1.0000	0.0000	1.0000	0.0000	1.0000	0.0000
LOC104915066	0.0000	-0.7720	0.0000	-0.7516	0.0053	0.4363	1.0000	0.0030	1.0000	0.0890	0.6020	0.1220	0.0003	-0.3391
LOC104915067	0.3247	2.2123	1.0000	0.1840	0.0184	3.3212	1.0000	0.3702	1.0000	2.3937	1.0000	0.4118	1.0000	-0.5383
LOC104915068	0.3467	0.9160	0.0088	1.1508	0.0000	3.3087	0.0000	1.7051	1.0000	0.9053	0.0311	1.1588	0.0436	-0.6862
LOC104915069	0.0012	1.1592	0.0026	0.8290	0.0000	2.8290	0.0000	1.8070	1.0000	0.2604	1.0000	-0.0549	0.0006	-0.7527
LOC104915070	0.0001	1.6710	0.0000	1.8929	0.0000	3.6351	0.0000	2.6102	1.0000	-0.0170	1.0000	0.2193	0.0002	-1.0337
LOC104915071	0.0036	1.2097	0.0000	1.5126	0.0000	3.3097	0.0000	2.3954	1.0000	0.1856	0.3348	0.5014	0.0001	-0.7228
LOC104915072	0.5243	0.7630	0.4883	-0.6736	1.0000	0.3728	0.0880	-1.4592	1.0000	0.6246	0.6524	-0.8010	0.4068	-1.2071
LOC104915073	0.8226	-3.3425	0.9062	-1.5349	0.8011	-3.2629	0.9096	-1.3726	1.0000	0.4115	1.0000	2.3481	1.0000	2.3543
LOC104915074	0.5745	1.0139	0.1107	1.4562	1.0000	0.0202	1.0000	-0.2895	1.0000	-0.3892	1.0000	0.0662	1.0000	-0.6926
LOC104915076	1.0000	-0.0761	0.9587	0.2307	0.0189	-1.4830	0.2377	-0.8242	1.0000	-0.3785	1.0000	-0.0572	1.0000	0.2857
LOC104915077	0.8249	-3.3439	1.0000	0.0000	0.8033	-3.2636	1.0000	0.0000	1.0000	-3.2319	1.0000	0.0000	1.0000	0.0000
LOC104915078	0.0315	1.0842	0.0004	1.3746	0.0714	0.8794	0.0000	1.5622	1.0000	-0.5479	1.0000	-0.2455	1.0000	0.1428
LOC104915080	0.6352	-0.2379	0.5941	-0.1716	0.0955	-0.5356	0.0038	-0.6279	1.0000	-0.0187	1.0000	0.0609	1.0000	-0.1058
LOC104915082	0.0049	-0.6613	0.0000	-0.7648	0.0626	0.4291	0.0315	0.2438	1.0000	0.0926	1.0000	0.0013	0.9260	-0.0876
LOC104915083	0.3214	1.5691	0.6403	0.7839	1.0000	-0.7130	1.0000	0.3118	1.0000	0.2609	1.0000	-0.5108	0.9851	1.2966
LOC104915084	1.0000	0.0000	1.0000	0.0000	1.0000	0.0000	1.0000	0.0000	1.0000	0.0000	1.0000	0.0000	1.0000	0.0000
LOC104915085	1.0000	2.1850	1.0000	0.0000	1.0000	0.0000	1.0000	0.0000	1.0000	0.0000	1.0000	-2.2958	1.0000	0.0000
LOC104915088	0.0042	0.5008	0.0067	0.3415	0.0000	0.7066	0.0000	0.7080	1.0000	0.0876	1.0000	-0.0585	0.7940	0.0943
LOC104915089	0.1245	0.7924	0.4034	0.3648	0.0000	2.1735	0.0000	1.5398	1.0000	0.5954	1.0000	0.1840	1.0000	-0.0315
LOC104915091	0.0933	1.3684	0.0499	1.2490	0.0018	2.0139	0.1811	0.9923	1.0000	0.3168	1.0000	0.2117	0.4667	-0.7000
LOC104915092	0.1346	-0.4961	0.9535	0.0814	1.0000	-0.0366	0.6678	-0.1646	1.0000	-0.1149	0.2147	0.4754	0.7073	-0.2371
LOC104915093	0.0028	2.1567	0.1212	0.8136	0.0005	2.4351	0.1084	0.8279	1.0000	1.4798	1.0000	0.1591	1.0000	-0.1201
LOC104915094	0.1612	-0.3442	0.0000	-0.5614	0.3741	-0.2232	0.5005	-0.1035	1.0000	0.1437	1.0000	-0.0611	0.1278	0.2694
LOC104915095	1.0000	0.0000	1.0000	2.2533	1.0000	0.0000	1.0000	0.0000	1.0000	0.0000	1.0000	2.3480	1.0000	0.0000
LOC104915100	1.0000	-2.4788	1.0000	2.2533	1.0000	-2.4061	0.2460	4.0916	1.0000	-2.3771	1.0000	2.3480	0.5105	4.1268
LOC104915101	1.0000	0.0000	1.0000	0.0000	1.0000	0.0000	1.0000	0.0000	1.0000	0.0000	1.0000	0.0000	1.0000	0.0000
LOC104915102	1.0000	0.0000	1.0000	-2.3960	1.0000	2.2425	1.0000	0.0062	1.0000	2.2676	1.0000	0.0000	1.0000	0.0652
LOC104915104	1.0000	-0.2285	0.1551	2.3761	1.0000	0.3764	0.2607	2.3250	1.0000	-3.1536	1.0000	-0.5717	0.4051	-1.2088
LOC104915106	1.0000	-0.2073	0.8617	-0.2388	0.9357	0.2732	0.0034	1.1864	1.0000	-0.1815	1.0000	-0.2070	0.2399	0.7369
LOC104915109	1.0000	0.5756	0.1384	4.3086	1.0000	0.7338	0.7701	3.1732	1.0000	-3.2289	1.0000	0.3659	1.0000	-0.8431
LOC104915110	0.8249	0.4749	1.0000	0.1164	0.0147	1.8338	0.0157	1.3793	1.0000	0.2435	1.0000	-0.1055	1.0000	-0.2094
LOC104915116	0.0268	1.1880	0.0035	1.2720	0.0039	1.4215	0.1657	0.7772	1.0000	0.0169	1.0000	0.1106	0.3374	-0.6271
LOC104915117	0.7009	-0.1652	0.0240	-0.2912	0.0000	-1.2081	0.0000	-0.8995	1.0000	0.0798	1.0000	-0.0325	0.0880	0.3931
LOC104915118	0.3339	0.6640	1.0000	-0.0286	0.7006	0.4144	1.0000	-0.0718	1.0000	0.3348	0.9575	-0.3434	1.0000	-0.1471
LOC104915119	1.0000	0.0121	0.3713	0.8814	0.8032	-0.5086	0.8938	-0.5584	1.0000	-0.2775	1.0000	0.6031	1.0000	-0.3221
LOC104915120	0.0254	5.1879	0.2526	2.1629	0.0531	4.7637	0.9096	1.3850	1.0000	2.2676	1.0000	-0.6527	0.8616	-1.0807
LOC104915121	0.0412	2.9750	0.6635	0.7099	0.1954	2.5403	1.0000	0.3206	1.0000	2.6038	1.0000	0.3754	1.0000	0.3931
LOC104915122	0.0001	2.9934	0.0000	1.9399	0.0000	3.3397	0.2375	0.8437	1.0000	1.7095	0.3137	0.6765	0.2593	-0.7748
LOC104915123	1.0000	-0.2204	1.0000	-0.0568	0.4887	-0.9122	0.2402	-1.3982	1.0000	0.0068	1.0000	0.1818	1.0000	-0.4790
LOC104915124	0.0001	0.8924	0.0000	0.8138	0.3980	-0.2797	0.0061	-0.4786	1.0000	0.0627	1.0000	-0.0016	0.9549	-0.1304
LOC104915125	1.0000	0.0000	1.0000	2.2533	1.0000	2.2468	1.0000	0.0000	1.0000	0.0000	1.0000	2.3480	1.0000	-2.2909
LOC104915127	0.8249	3.0254	0.7674	3.0955	1.0000	0.0000	0.7701	3.1732	1.0000	0.0000	1.0000	0.0540	1.0000	3.2066
LOC104915129	1.0000	0.0000	1.0000	0.0000	1.0000	0.0000	1.0000	0.0000	1.0000	0.0000	1.0000	0.0000	1.0000	0.0000
LOC104915130	0.0854	-2.7932	0.0144	-2.4033	0.0249	-3.4713	0.0205	-2.2233	1.0000	0.1850	1.0000	0.5880	1.0000	1.4485
LOC104915131	1.0000	0.0000	1.0000	0.0000	1.0000	0.0000	1.0000	0.0000	1.0000	0.0000	1.0000	0.0000	1.0000	0.0000
LOC104915132	1.0000	0.0000	1.0000	0.0000	1.0000	0.0000	1.0000	0.0000	1.0000	0.0000	1.0000	0.0000	1.0000	0.0000
LOC104915134	0.0237	0.7807	0.0007	0.7354	0.9942	0.1354	1.0000	0.0793	1.0000	-0.0128	1.0000	-0.0466	1.0000	-0.0628
LOC104915138	0.0081	-0.5933	0.0001	-0.6031	0.0679	-0.4242	0.0001	-0.4879	1.0000	0.0775	1.0000	0.0800	1.0000	0.0188
LOC104915139	1.0000	0.0000	1.0000	0.0000	1.0000	0.0000	1.0000	0.0000	1.0000	0.0000	1.0000	0.0000	1.0000	0.0000
LOC104915140	0.0268	-0.8547	0.0010	-0.8581	0.0106	0.7738	0.0275	0.4673	1.0000	0.0341	1.0000	0.0426	0.4904	-0.2660
LOC104915141	0.0415	1.2839	0.0000	2.4537	0.0000	2.6577	0.0000	2.9349	1.0000	-0.7025	0.7020	0.4757	0.3200	-0.4233
LOC104915142	0.0004	1.4133	0.0000	1.2767	0.0000	1.9793	0.0001	1.1029	1.0000	0.4799	0.5666	0.3560	0.3164	-0.3930
LOC104915143	0.0139	0.9536	0.0286	0.6753	0.0018	1.0821	0.0049	0.7980	1.0000	0.1355	1.0000	-0.1271	1.0000	-0.1387
LOC104915145	1.0000	0.0000	1.0000	0.0000	1.0000	0.0000	1.0000	0.0000	1.0000	0.0000	1.0000	0.0000	1.0000	0.0000
LOC104915146	1.0000	0.0000	1.0000	0.0000	1.0000	0.0000	1.0000	0.0000	1.0000	0.0000	1.0000	0.0000	1.0000	0.0000
LOC104915148	1.0000	-0.1232	0.3950	-0.5296	0.0207	1.0579	0.2899	0.5623	1.0000	-0.3847	0.4336	-0.7784	0.0494	-0.8694
LOC104915150	0.5086	3.5456	1.0000	2.2508	0.2231	4.3199	1.0000	0.0000	1.0000	0.0000	1.0000	-1.3266	0.4034	-4.3683
LOC104915152	0.0437	0.6979	0.0000	0.9267	0.0000	1.3813	0.0000	1.2455	1.0000	-0.0246	0.8607	0.2172	0.7609	-0.1536
LOC104915153	0.0653	1.5559	0.0004	2.6059	0.0000	2.8460	0.0000	3.5422	1.0000	-1.0859	1.0000	-0.0314	0.7114	-0.3877
LOC104915154	0.4106	0.9424	0.7544	0.5200	0.0001	2.3286	0.0000	2.3038	1.0000	0.0007	1.0000	-0.4077	1.0000	-0.0184
LOC104915155	0.1219	2.0562	0.0309	1.3834	0.0000	4.4997	0.0000	2.7636	1.0000	1.5323	0.4147	0.8737	1.0000	-0.2005
LOC104915156	0.0110	1.2153	0.0568	0.7354	0.0000	2.7243	0.0000	1.9102	1.0000	0.7267	0.9123	0.2652	1.0000	-0.0755
LOC104915157	1.0000	0.0000	1.0000	0.0000	1.0000	0.0000	1.0000	2.3241	1.0000	0.0000	1.0000	0.0000	1.0000	2.3543
LOC104915158	0.3091	-2.3844	1.0000	0.0000	0.0560	-5.3159	0.7710	3.1752	1.0000	-5.2833	1.0000	-3.1429	1.0000	3.2081
LOC104915160	1.0000	-0.0054	0.7929	-0.0604	1.0000	0.0029	0.5037	-0.0981	1.0000	0.0423	1.0000	0.0007	1.0000	-0.0523
LOC104915161	0.1976	0.4516	0.0021	0.6240	0.0000	1.2192	0.0000	1.1416	1.0000	0.0624	0.7047	0.2478	1.0000	-0.0118
LOC104915162	0.3223	1.0533	0.0146	2.0040	0.0031	2.0409	0.0000	3.0176	1.0000	-0.8948	1.0000	0.0611	1.0000	0.0813
LOC104915163	0.3986	-0.5470	0.0823	-0.6748	0.2825	0.5266	1.0000	-0.0178	1.0000	0.6202	0.6957	0.5034	1.0000	0.0799
LOC104915164	1.0000	-0.1522	0.6779	-0.3460	0.1125	0.9967	0.7207	0.3381	1.0000	0.3673	1.0000	0.1869	0.9869	-0.2865
LOC104915166	0.0002	2.0082	0.2256	0.6637	0.0000	2.9101	0.0000	1.8657	1.0000	0.9771	0.8607	-0.3593	1.0000	-0.0718
LOC104915167	1.0000	-0.0377	0.2429	-0.9887	0.6933	0.9272	0.2020	-1.1557	1.0000	1.8027	1.0000	0.8759	1.0000	-0.2720
LOC104915169	1.0000	2.1900	1.0000	2.2509	0.8011	3.0840	0.7701	3.1732	1.0000	0.0000	1.0000	0.0470	1.0000	0.0723
LOC104915170	0.0006	-1.8157	0.0075	-1.3149	0.3818	-0.6358	0.0923	-0.7943	1.0000	-0.2907	1.0000	0.2204	0.9223	-0.4483
LOC104915171	1.0000	-0.0640	0.4663	0.3252	0.0000	1.3840	0.0000	1.4461	1.0000	0.0223	0.3698	0.4256	0.9875	0.0916

LOC104915172	1.0000	0.0000	1.0000	0.0000	1.0000	0.0000	1.0000	0.0000	1.0000	0.0000	1.0000	0.0000	1.0000	0.0000
LOC104915176	1.0000	0.0000	1.0000	0.0000	1.0000	0.0000	1.0000	0.0000	1.0000	0.0000	1.0000	0.0000	1.0000	0.0000
LOC104915177	1.0000	0.5464	1.0000	2.2509	1.0000	-0.1591	0.7710	3.1752	1.0000	-2.3758	1.0000	-0.8001	1.0000	0.9169
LOC104915179	1.0000	-0.3195	0.9148	1.2289	1.0000	-0.1789	1.0000	-2.3180	1.0000	-0.9614	1.0000	0.5866	1.0000	-3.1344
LOC104915180	0.3355	-0.2969	0.0323	-0.4246	0.7053	0.1674	0.2767	0.2264	1.0000	0.0982	1.0000	-0.0155	0.7287	0.1626
LOC104915181	0.0002	1.0908	0.0014	0.7762	0.0000	1.7836	0.0000	1.2324	1.0000	0.3659	1.0000	0.0660	0.7200	-0.1787
LOC104915182	0.0000	1.3971	0.0000	1.5423	0.0000	2.0762	0.0000	1.8857	1.0000	-0.1969	1.0000	-0.0375	0.0577	-0.3764
LOC104915183	0.6957	1.7476	0.2265	-2.0430	1.0000	0.6816	0.4057	-1.3370	1.0000	2.6051	1.0000	-1.1638	1.0000	0.6034
LOC104915184	0.0049	1.6192	0.0000	1.9110	0.0000	2.9650	0.0000	2.2815	1.0000	0.4425	0.0849	0.7480	0.8022	-0.2348
LOC104915185	0.0000	2.0286	0.0000	2.1941	0.0000	3.1244	0.0000	2.5041	1.0000	0.0779	0.6450	0.2603	0.0000	-0.5344
LOC104915187	1.0000	-0.0859	1.0000	-0.0017	0.0367	-1.8815	0.0001	-2.5199	1.0000	0.2493	1.0000	0.3398	1.0000	-0.3874
LOC104915188	1.0000	0.0000	1.0000	0.0000	1.0000	0.0000	1.0000	0.0000	1.0000	0.0000	1.0000	0.0000	1.0000	0.0000
LOC104915189	1.0000	-0.0675	0.1449	0.7733	0.0000	1.8167	0.0000	1.7712	1.0000	-0.3410	0.7704	0.5124	0.6389	-0.3828
LOC104915190	1.0000	0.0000	1.0000	0.0000	1.0000	0.0000	1.0000	0.0000	1.0000	0.0000	1.0000	0.0000	1.0000	0.0000
LOC104915191	0.0224	-0.4191	0.0000	-0.7714	0.0647	-0.3512	0.0000	-0.5131	1.0000	0.1990	0.7224	-0.1406	1.0000	0.0414
LOC104915195	1.0000	-0.1648	0.4632	1.1783	1.0000	0.3030	0.1234	1.7984	1.0000	-1.2820	1.0000	0.0634	1.0000	0.2139
LOC104915196	1.0000	0.8866	0.2542	4.0037	1.0000	-1.0167	0.7701	3.1732	1.0000	-3.2319	1.0000	-0.2469	1.0000	0.9165
LOC104915197	0.0786	-0.3702	0.0000	-0.6191	0.0000	-0.8838	0.0000	-0.8432	1.0000	-0.0047	0.3052	-0.2415	1.0000	0.0411
LOC104915198	0.2917	0.6113	0.1038	0.6045	0.0000	2.1613	0.0000	1.6411	1.0000	0.5655	0.4062	0.5714	1.0000	0.0473
LOC104915199	0.0000	2.5932	0.0000	2.0601	1.0000	-0.1071	0.0738	-1.7384	1.0000	-0.2266	0.0716	-0.7506	0.1142	-1.8561
LOC104915200	1.0000	0.0000	1.0000	0.0000	1.0000	0.0000	1.0000	0.0000	1.0000	0.0000	1.0000	0.0000	1.0000	0.0000
LOC104915201	1.0000	0.0000	1.0000	0.0000	1.0000	0.0000	1.0000	0.0000	1.0000	0.0000	1.0000	0.0000	1.0000	0.0000
LOC104915202	0.0000	2.7840	0.0000	2.8276	0.0023	2.4985	0.0025	1.7481	1.0000	0.1704	1.0000	0.2338	0.7138	-0.5714
LOC104915204	1.0000	0.0000	1.0000	2.2509	1.0000	2.2468	1.0000	0.0000	1.0000	0.0000	1.0000	2.3456	1.0000	-2.2909
LOC104915205	0.0296	5.4262	0.0954	2.1539	0.0528	4.9659	0.0885	2.1947	1.0000	3.1057	1.0000	-0.0491	1.0000	0.3884
LOC104915207	0.9893	-0.1279	0.7407	-0.1800	1.0000	-0.0618	1.0000	-0.0195	1.0000	-0.1313	1.0000	-0.1711	1.0000	-0.0849
LOC104915208	0.0018	1.2712	0.0000	1.9280	0.0056	1.1135	0.0906	0.7574	1.0000	-0.3580	0.7549	0.3096	0.0964	-0.7107
LOC104915209	0.0040	2.7179	0.0024	2.0983	0.0005	2.9698	0.0020	2.0776	1.0000	0.8165	1.0000	0.2123	1.0000	-0.0735
LOC104915210	1.0000	0.0000	1.0000	0.0000	1.0000	0.0000	1.0000	0.0000	1.0000	0.0000	1.0000	0.0000	1.0000	0.0000
LOC104915211	1.0000	0.0000	1.0000	0.0000	1.0000	0.0000	1.0000	0.0000	1.0000	0.0000	1.0000	0.0000	1.0000	0.0000
LOC104915212	1.0000	0.0000	1.0000	0.0000	1.0000	0.0000	1.0000	0.0000	1.0000	0.0000	1.0000	0.0000	1.0000	0.0000
LOC104915213	1.0000	0.0000	1.0000	0.0000	1.0000	0.0000	1.0000	0.0000	1.0000	0.0000	1.0000	0.0000	1.0000	0.0000
LOC104915214	0.8667	-0.1252	0.5110	0.1558	1.0000	0.0731	0.1252	0.2787	1.0000	-0.0567	0.6096	0.2373	0.8088	0.1540
LOC104915216	0.4915	0.5460	1.0000	0.1663	0.0860	0.9200	0.7701	0.2834	1.0000	0.2073	1.0000	-0.1593	0.6975	-0.4254
LOC104915218	1.0000	0.0000	1.0000	0.0000	1.0000	0.0000	1.0000	0.0000	1.0000	0.0000	1.0000	0.0000	1.0000	0.0000
LOC104915219	1.0000	2.1850	0.7666	-3.2534	0.0938	4.5491	1.0000	0.0066	1.0000	3.1126	1.0000	-2.2958	0.7908	-1.3984
LOC104915220	1.0000	0.0000	1.0000	0.0000	1.0000	0.0000	1.0000	0.0000	1.0000	0.0000	1.0000	0.0000	1.0000	0.0000
LOC104915221	0.3864	-0.6751	0.0736	-1.0004	0.7072	0.3484	0.2130	0.5898	1.0000	-0.0419	1.0000	-0.3526	1.0000	0.2063
LOC104915223	0.5086	3.5456	0.4431	3.6192	1.0000	2.2468	1.0000	0.0000	1.0000	0.0000	1.0000	0.0559	1.0000	-2.2908
LOC104915224	1.0000	-0.3335	0.2920	-4.1891	0.4657	1.1538	1.0000	0.0045	1.0000	0.2728	0.8607	-3.6732	0.9122	-0.8790
LOC104915225	0.0049	0.6473	0.1172	0.2449	0.3532	0.2740	0.1048	0.2717	1.0000	0.0430	0.1226	-0.3469	1.0000	0.0457
LOC104915226	1.0000	0.0000	1.0000	0.0000	1.0000	0.0000	1.0000	0.0000	1.0000	0.0000	1.0000	0.0000	1.0000	0.0000
LOC104915227	0.0002	0.8395	0.0000	0.7310	0.0001	0.9102	0.0000	1.0840	1.0000	-0.1447	0.4799	-0.2405	1.0000	0.0326
LOC104915229	0.7565	0.2789	0.3155	0.3185	0.0000	1.6308	0.0003	0.8570	1.0000	0.2688	0.7293	0.3201	0.2857	-0.5017
LOC104915231	0.9545	-1.6928	0.2535	-4.1743	0.9361	-1.5529	0.9801	-0.9105	1.0000	0.2615	1.0000	-2.2958	1.0000	0.9164
LOC104915232	1.0000	-0.2920	1.0000	-2.3960	1.0000	-0.1625	1.0000	0.0075	1.0000	-0.1077	1.0000	-2.2958	1.0000	0.0660
LOC104915233	0.7577	-0.1987	0.5154	-0.1929	0.3807	-0.3140	0.0082	-0.5897	1.0000	0.2217	0.8328	0.2400	1.0000	-0.0511
LOC104915234	0.4330	1.1838	0.0001	2.4739	0.0068	2.1837	0.0613	1.5550	1.0000	0.1203	0.0311	1.4252	0.9111	-0.4985
LOC104915235	0.3659	0.6313	0.9428	0.2681	0.0531	-1.4629	0.0189	-1.8677	1.0000	-0.3389	0.5388	-0.6918	0.9562	-0.7396
LOC104915237	1.0000	2.1900	1.0000	2.2533	1.0000	2.2468	1.0000	0.0000	1.0000	0.0000	1.0000	0.0489	1.0000	-2.2908
LOC104915238	1.0000	0.0000	1.0000	2.2509	1.0000	0.0000	1.0000	0.0000	1.0000	0.0000	1.0000	2.3456	1.0000	0.0000
LOC104915239	0.0046	2.4233	0.0000	4.3860	0.0000	3.1724	0.0000	4.9793	1.0000	-2.1881	1.0000	-0.2424	0.7073	-0.3860
LOC104915240	0.0083	2.3312	0.0000	3.3734	0.0034	2.5813	0.0002	3.3120	1.0000	-1.0389	1.0000	0.0060	1.0000	-0.3121
LOC104915241	0.5391	0.2518	0.7437	-0.1294	0.3188	0.3107	0.4473	0.1823	1.0000	0.0237	0.3704	-0.3438	1.0000	-0.0984
LOC104915242	1.0000	0.0000	1.0000	0.0000	1.0000	0.0000	1.0000	2.3256	1.0000	0.0000	1.0000	0.0000	1.0000	2.3554
LOC104915243	1.0000	0.0000	1.0000	0.0000	1.0000	0.0000	1.0000	0.0000	1.0000	0.0000	1.0000	0.0000	1.0000	0.0000
LOC104915244	0.6758	0.3873	0.0216	0.9315	0.0004	1.3387	0.0009	1.2681	1.0000	-0.4011	1.0000	0.1568	0.3442	-0.4615
LOC104915247	1.0000	0.0000	1.0000	0.0000	1.0000	0.0000	1.0000	0.0000	1.0000	0.0000	1.0000	0.0000	1.0000	0.0000
LOC104915248	1.0000	0.0000	1.0000	-2.3985	0.0531	4.7637	0.9096	1.3845	1.0000	2.2734	1.0000	0.0000	0.8616	-1.0807
LOC104915249	1.0000	-2.4788	1.0000	-0.1472	0.1908	2.3554	0.9162	1.3864	1.0000	-0.1045	1.0000	2.3456	0.8720	-1.0785
LOC104915251	0.0000	-4.4092	0.0000	-3.6477	1.0000	-0.0993	1.0000	-0.1353	1.0000	-0.0320	1.0000	0.7489	1.0000	-0.0641
LOC104915253	0.0115	1.3726	0.0124	0.9875	0.0000	2.4623	0.0000	1.8893	1.0000	0.5289	1.0000	0.1574	1.0000	-0.0402
LOC104915255	0.0207	1.1912	0.0448	1.0111	0.0243	-1.6941	0.0834	-1.2882	1.0000	-0.3308	0.6342	-0.4998	1.0000	0.0795
LOC104915256	0.3425	0.4346	0.0243	-0.5477	0.0000	1.5632	0.0008	0.6943	1.0000	0.7681	1.0000	-0.2015	1.0000	-0.0979
LOC104915257	1.0000	0.0000	1.0000	0.0000	1.0000	0.0000	1.0000	0.0000	1.0000	0.0000	1.0000	0.0000	1.0000	0.0000
LOC104915258	1.0000	0.0000	1.0000	0.0000	1.0000	0.0000	1.0000	0.0000	1.0000	0.0000	1.0000	0.0000	1.0000	0.0000
LOC104915259	1.0000	0.0000	1.0000	0.0000	1.0000	0.0000	1.0000	0.0000	1.0000	0.0000	1.0000	0.0000	1.0000	0.0000
LOC104915260	1.0000	0.0782	0.0131	-0.4428	0.0208	0.5840	0.0931	0.2861	1.0000	0.4641	1.0000	-0.0432	0.6865	0.1720
LOC104915261	1.0000	0.0000	1.0000	0.0000	1.0000	0.0000	1.0000	0.0000	1.0000	0.0000	1.0000	0.0000	1.0000	0.0000
LOC104915262	1.0000	0.0000	1.0000	0.0000	1.0000	0.0000	1.0000	0.0000	1.0000	0.0000	1.0000	0.0000	1.0000	0.0000
LOC104915264	0.0000	-2.4016	0.0000	-1.9525	1.0000	-0.1138	0.6517	-0.2018	1.0000	0.2514	0.6462	0.7126	1.0000	0.1670
LOC104915265	0.0458	-1.8902	0.0098	-2.1233	0.0054	1.4633	0.0365	0.9983	1.0000	0.2800	1.0000	0.0603	1.0000	-0.1808
LOC104915266	1.0000	0.0000	1.0000	0.0000	1.0000	0.0000	1.0000	0.0000	1.0000	0.0000	1.0000	0.0000	1.0000	0.0000
LOC104915267	1.0000	0.0000	1.0000	0.0000	1.0000	0.0000	1.0000	0.0000	1.0000	0.0000	1.0000	0.0000	1.0000	0.0000
LOC104915269	0.3884	0.5061	0.3140	0.4028	0.0000	2.1248	0.0000	2.1569	1.0000	0.0586	1.0000	-0.0328	1.0000	0.0952
LOC104915270	1.0000	0.5414	0.9062	-1.5359	1.0000	-0.1597	0.9096	-1.3722	1.0000	1.2654	1.0000	-0.7966	1.0000	0.0649
LOC104915271	1.0000	0.0000	1.0000	0.0000	1.0000	0.0000	1.0000	0.0000	1.0000	0.0000	1.0000	0.0000	1.0000	0.0000
LOC104915274	1.0000	0.0000	1.0000	2.2509	1.0000	0.0000	1.0000	0.0000	1.0000	0.0000	1.0000	2.3456	1.0000	0.0000
LOC104915275	0.0000	2.1175	0.0000	2.5078	0.0000	4.2834	0.0000	3.3218	1.0000	0.2129	0.0720	0.6145	0.0024	-0.7444
LOC104915277	0.0673	0.7763	0.3207	0.4048	0.4800	-0.4925	0.1216	-0.6657	1.0000	0.2428	1.0000	-0.1147	1.0000	0.0784
LOC104915278	0.1510	0.3698	0.0850	0.3200	0.0790	-0.4752	0.0046	-0.5282	1.0000	-0.1328	0.8377	-0.1698	0.8763	-0.1804
LOC104915286	1.0000	0.0000	1.0000	0.0000	1.0000	0.0000	0.7701	3.1732	1.0000	0.0000	1.0000	0.0000	1.0000	3.2066
LOC104915287	1.0000	0.2067	1.0000	0.2251	0.5329	1.0793	1.0000	0.2212	1.0000	0.4310	1.0000	0.4612	1.0000	-0.4211
LOC104915288	0.8249	3.0254	0.7674	-3.2570	1.0000	0.0000	0.7710	-3.1693	1.0000	3.1190	1.0000	-3.1468	1.0000	0.0000
LOC104915289	1.0000	0.2900	0.4291	0.5995	0.1147	0.9700	0.3177	0.6909	1.0000	-0.2172	1.0000	0.1083	0.6779	-0.4839
LOC104915290	0.0014	0.6909	0.0000	0.7642	1.0000	0.0653	1.0000	-0.0422	1.0000	0.0432	0.9091	0.1288	1.0000	-0.0581
LOC104915292	0.9210	-0.0762	0.1883	-0.1576	0.0000	-1.1199	0.0000	-1.1037	1.0000	0.0114	1.0000	-0.0577	1.0000	0.0341
LOC104915295	1.0000	0.0000	1.0000	0.0000	1.0000	0.0000	1.0000	0.0000	1.0000	0.0000	1.0000	0.0000	1.0000	0.0000
LOC104915298	1.0000	2.1900	0.2495	-4.1757	1.0000	2.2425	0.2429	-4.0840	1.0000	4.0267	1.0000	-2.2992	1.0000	-2.2889
LOC104915299	1.0000	-0.1001	0.9045	-0.1140	0.9099	0.1296	0.5238	0.2070	1.0000	-0.2641	0.7623	-0.2644	0.8727	-0.1795
LOC104915300	0.5352	1.0892	0.2335	1.0795	0.0914	1.6788	0.6615	0.6301	1.0000	0.6899	0.9225	0.6968	1.0000	-0.3541
LOC104915303	0.0000	2.3719	0.0000	3.0047	0.0000	3.3952	0.0000	2.4184	1.0000	-0.2655	0.4709	0.3793	0.0000	-1.2375
LOC104915305	1.0000	2.1900	1.0000	0.0000	1.0000	0.0000	1.0000	0.0000	1.0000	0.0000	1.0000	-2.2992	1.0000	0.0000

LOC104915307	0.4963	-0.2181	0.0300	-0.3141	1.0000	0.0308	0.7246	-0.0933	1.0000	0.0559	1.0000	-0.0274	1.0000	-0.0637
LOC104915308	1.0000	0.0000	1.0000	0.0000	1.0000	0.0000	1.0000	0.0000	1.0000	0.0000	1.0000	0.0000	1.0000	0.0000
LOC104915310	1.0000	0.0000	1.0000	0.0000	1.0000	0.0000	1.0000	0.0000	1.0000	0.0000	1.0000	0.0000	1.0000	0.0000
LOC104915311	1.0000	0.0000	1.0000	0.0000	1.0000	0.0000	1.0000	0.0000	1.0000	0.0000	1.0000	0.0000	1.0000	0.0000
LOC104915312	0.2800	-0.2545	0.0002	-0.3970	0.0001	-0.7162	0.0000	-0.7837	1.0000	0.0199	0.8052	-0.1103	1.0000	-0.0429
LOC104915313	0.7897	0.2636	0.9855	0.1247	0.0112	0.8958	1.0000	0.0516	1.0000	-0.1191	1.0000	-0.2443	0.0010	-0.9556
LOC104915314	1.0000	-0.8632	1.0000	-1.0017	1.0000	0.4991	1.0000	0.0066	1.0000	-0.6490	1.0000	-0.7943	0.9847	-1.1466
LOC104915316	0.1214	1.2795	0.2552	0.6983	0.2919	1.0464	0.7503	0.3803	1.0000	0.5940	1.0000	0.0225	1.0000	-0.0747
LOC104915317	0.0046	0.9934	0.0209	0.8547	0.8251	0.2070	1.0000	-0.0211	1.0000	0.1241	1.0000	0.0018	1.0000	-0.0975
LOC104915318	0.5189	0.7427	0.1234	0.8518	0.1072	1.1532	0.7281	-0.4866	1.0000	0.3618	0.8269	0.4857	0.0893	-1.2713
LOC104915319	1.0000	0.0927	0.3889	0.1965	0.2463	-0.3822	0.0000	-0.8417	1.0000	0.0023	1.0000	0.1198	0.1785	-0.4497
LOC104915320	0.7849	1.1386	1.0000	0.3580	1.0000	-0.1789	1.0000	0.0031	1.0000	-0.1146	1.0000	-0.8870	1.0000	0.0718
LOC104915321	1.0000	0.0000	1.0000	0.0000	1.0000	0.0000	1.0000	0.0000	1.0000	0.0000	1.0000	0.0000	1.0000	0.0000
LOC104915322	1.0000	0.0000	1.0000	0.0000	1.0000	0.0000	1.0000	0.0000	1.0000	0.0000	1.0000	0.0000	1.0000	0.0000
LOC104915323	1.0000	0.0000	1.0000	0.0000	1.0000	0.0000	1.0000	0.0000	1.0000	0.0000	1.0000	0.0000	1.0000	0.0000
LOC104915324	1.0000	0.0000	1.0000	0.0000	1.0000	0.0000	1.0000	0.0000	1.0000	0.0000	1.0000	0.0000	1.0000	0.0000
LOC104915325	1.0000	0.0000	1.0000	0.0000	1.0000	0.0000	1.0000	0.0000	1.0000	0.0000	1.0000	0.0000	1.0000	0.0000
LOC104915326	0.4940	-0.3363	0.4770	-0.3350	1.0000	0.0183	0.9367	0.1358	1.0000	-0.3269	0.7740	-0.3163	0.9013	-0.2048
LOC104915327	0.0456	1.8536	0.0936	1.2103	0.0000	3.4549	0.0080	1.4479	1.0000	1.0933	0.9998	0.4672	0.0837	-0.9108
LOC104915330	0.3640	0.3508	1.0000	0.0469	0.9361	-0.1327	0.9220	-0.0884	1.0000	0.0649	0.8607	-0.2267	1.0000	0.1142
LOC104915331	1.0000	-2.4788	1.0000	0.0000	0.0489	2.9940	0.0235	5.0500	1.0000	-2.3771	1.0000	0.0000	1.0000	-0.3685
LOC104915335	1.0000	2.1900	1.0000	-2.3960	1.0000	0.0000	1.0000	-2.3180	1.0000	2.2676	1.0000	-2.2992	1.0000	0.0000
LOC104915336	0.1842	0.5743	0.1143	0.4831	0.9583	0.1527	0.3312	0.3529	1.0000	-0.0248	1.0000	-0.1053	1.0000	0.1813
LOC104915338	0.7248	-0.1448	0.2868	-0.1871	0.0095	-0.5724	0.0000	-0.8197	1.0000	-0.1310	0.7874	-0.1621	0.1161	-0.3725
LOC104915340	0.0003	2.2745	0.0000	1.6844	0.0000	2.9296	0.0000	1.8288	1.0000	0.8030	1.0000	0.2256	0.7402	-0.2935
LOC104915341	1.0000	0.0107	1.0000	0.0199	0.4164	-0.2146	0.8571	-0.0706	1.0000	0.0146	1.0000	0.0364	0.6498	0.1641
LOC104915342	0.2510	0.8483	1.0000	0.1259	0.0012	1.6817	0.3221	0.5681	1.0000	0.5953	1.0000	-0.1141	0.5384	-0.5135
LOC104915343	0.0003	-0.7048	0.0000	-0.9748	0.5405	0.1747	0.4597	-0.0901	1.0000	-0.0626	0.0194	-0.3204	0.0036	-0.3213
LOC104915344	0.0131	1.8798	0.0001	2.3264	0.1360	1.4757	0.1531	1.2559	1.0000	-0.1269	1.0000	0.3294	1.0000	-0.3472
LOC104915345	0.7098	-0.1255	0.0489	-0.1859	0.0003	-0.6417	0.0000	-0.6055	1.0000	0.0161	1.0000	-0.0320	1.0000	0.0579
LOC104915347	0.0209	2.3400	1.0000	0.2975	0.8700	0.7629	0.5120	-0.9580	1.0000	1.1411	0.5859	-0.8847	1.0000	-0.5737
LOC104915348	0.7832	0.1249	0.1300	0.2329	0.0010	-0.7136	0.0000	-0.6646	1.0000	-0.2461	0.8875	-0.1259	0.6584	-0.1934
LOC104915349	0.3986	0.3059	0.0125	0.4521	0.2886	-0.3612	0.1699	-0.2739	1.0000	-0.1459	1.0000	0.0125	1.0000	-0.0546
LOC104915350	0.0001	1.2239	0.0000	1.0008	0.2227	0.4872	0.2138	0.3812	1.0000	0.1392	1.0000	-0.0718	1.0000	0.0403
LOC104915351	0.8760	-0.1413	0.1892	0.2931	1.0000	0.0347	0.7579	0.1184	1.0000	-0.2584	0.8607	0.1893	0.8937	-0.1702
LOC104915352	0.0145	0.9491	0.0000	1.0942	0.0000	1.7163	0.0000	1.5256	1.0000	-0.1050	1.0000	0.0516	0.5007	-0.2916
LOC104915354	1.0000	-0.0951	0.0774	1.4384	0.0656	1.7506	0.0326	1.5587	1.0000	0.3381	0.1091	1.8892	1.0000	0.1502
LOC104915355	1.0000	-0.3444	0.9148	-1.5280	1.0000	-0.8796	1.0000	0.0094	1.0000	-0.8200	0.8269	-2.0167	1.0000	0.0750
LOC104915356	0.0432	1.7180	0.0014	1.6425	0.8033	0.7772	1.0000	0.1198	1.0000	0.8425	0.3718	0.7822	1.0000	0.1915
LOC104915357	0.1986	-1.3291	1.0000	-0.1769	1.0000	-0.2894	0.2689	0.9243	1.0000	-0.6640	1.0000	0.5045	0.9562	0.5572
LOC104915358	1.0000	-0.0262	0.1097	-0.6702	1.0000	0.0531	0.8196	-0.2318	1.0000	0.6138	1.0000	-0.0170	0.9619	0.3327
LOC104915359	0.0825	0.5342	0.0001	0.7252	0.0041	0.7614	0.0001	0.7449	1.0000	-0.0131	0.8354	0.1923	1.0000	-0.0230
LOC104915360	1.0000	-0.2892	1.0000	0.0000	1.0000	-2.4061	1.0000	0.0000	1.0000	-2.3771	1.0000	-2.2992	1.0000	0.0000
LOC104915361	0.8468	0.7239	0.8390	0.4863	0.0012	2.2962	1.0000	0.0072	1.0000	0.5851	1.0000	0.3568	0.0100	-1.7011
LOC104915362	1.0000	-0.0862	1.0000	0.3405	0.1606	1.2476	0.3243	0.9065	1.0000	-0.1265	1.0000	0.3034	0.9853	-0.4677
LOC104915365	0.0000	1.6888	0.0000	1.1252	0.0000	1.7062	0.0000	1.1608	1.0000	0.6610	1.0000	0.1114	1.0000	0.1236
LOC104915366	0.8880	-0.6170	0.2201	-0.9518	1.0000	0.2082	0.6245	-0.5315	1.0000	0.5206	1.0000	0.1994	1.0000	-0.2142
LOC104915367	0.8249	-3.3412	0.4633	-3.7804	1.0000	0.7338	0.6172	1.1738	1.0000	0.4032	1.0000	0.0000	1.0000	0.8501
LOC104915368	0.1817	-0.3483	0.0279	-0.3735	0.8761	-0.1053	0.1634	-0.2464	1.0000	0.1760	0.8400	0.1643	1.0000	0.0410
LOC104915371	1.0000	0.0000	1.0000	0.0000	1.0000	0.0000	1.0000	0.0000	1.0000	0.0000	1.0000	0.0000	1.0000	0.0000
LOC104915373	1.0000	0.0000	1.0000	2.2533	1.0000	2.2425	1.0000	0.0000	1.0000	0.0000	1.0000	2.3480	1.0000	-2.2889
LOC104915374	1.0000	0.0000	1.0000	0.0000	1.0000	0.0000	1.0000	0.0000	1.0000	0.0000	1.0000	0.0000	1.0000	0.0000
LOC104915375	1.0000	0.0000	1.0000	0.0000	1.0000	0.0000	1.0000	0.0000	1.0000	0.0000	1.0000	0.0000	1.0000	0.0000
LOC104915377	0.0116	0.4494	0.0000	0.5170	0.0000	-1.3178	0.0000	-1.0970	1.0000	-0.2091	0.7224	-0.1295	1.0000	0.0182
LOC104915382	0.0367	2.6754	0.1070	1.4460	1.0000	-0.1811	1.0000	0.2209	1.0000	1.3515	1.0000	0.1387	0.6308	1.7635
LOC104915384	1.0000	2.1850	0.7666	3.0922	0.8033	3.0789	0.7710	3.1752	1.0000	0.0000	1.0000	0.9006	1.0000	0.0752
LOC104915385	1.0000	0.0000	1.0000	0.0000	1.0000	0.0000	1.0000	0.0000	1.0000	0.0000	1.0000	0.0000	1.0000	0.0000
LOC104915386	1.0000	0.0000	1.0000	0.0000	1.0000	0.0000	1.0000	0.0000	1.0000	0.0000	1.0000	0.0000	1.0000	0.0000
LOC104915387	1.0000	0.0000	1.0000	0.0000	1.0000	0.0000	1.0000	0.0000	1.0000	0.0000	1.0000	0.0000	1.0000	0.0000
LOC104915388	0.3926	0.1940	0.0080	0.1954	0.0043	-0.4940	0.0000	-0.4324	1.0000	-0.1279	0.4753	-0.1141	0.9356	-0.0604
LOC104915389	1.0000	0.0000	1.0000	0.0000	1.0000	0.0000	1.0000	0.0000	1.0000	0.0000	1.0000	0.0000	1.0000	0.0000
LOC104915390	1.0000	0.0000	1.0000	0.0000	1.0000	0.0000	1.0000	0.0000	1.0000	0.0000	1.0000	0.0000	1.0000	0.0000
LOC104915391	1.0000	0.0000	1.0000	0.0000	1.0000	0.0000	1.0000	0.0000	1.0000	0.0000	1.0000	0.0000	1.0000	0.0000
LOC104915392	0.0025	1.4393	0.0000	1.1935	0.0000	1.8101	0.0000	1.2824	1.0000	0.3250	1.0000	0.0944	0.8830	-0.1935
LOC104915393	1.0000	0.5464	0.7674	3.0955	0.9361	1.2045	0.4370	3.7041	1.0000	-2.3758	1.0000	0.0540	1.0000	0.0752
LOC104915394	1.0000	0.0000	1.0000	0.0000	1.0000	0.0000	1.0000	0.0000	1.0000	0.0000	1.0000	0.0000	1.0000	0.0000
LOC104915395	1.0000	0.0000	1.0000	-2.3985	1.0000	0.0000	1.0000	0.0057	1.0000	2.2734	1.0000	0.0000	1.0000	2.3554
LOC104915397	0.2900	-1.1256	0.4883	-0.4737	0.1995	-1.3005	0.0214	-1.2750	1.0000	0.4856	0.3168	1.1529	1.0000	0.5218
LOC104915398	1.0000	0.0123	0.3482	-0.5167	0.0005	-2.7066	0.0655	-0.7601	1.0000	0.9999	0.9974	0.4813	0.0001	2.9572
LOC104915399	0.5086	3.5456	0.0415	4.7737	0.3267	4.0111	0.7701	3.1732	1.0000	0.0000	0.9744	1.2156	1.0000	-0.8470
LOC104915400	0.3717	1.1104	0.0011	2.3981	0.0320	1.7615	0.0020	2.3959	1.0000	-0.6882	0.8565	0.6117	1.0000	-0.0442
LOC104915401	0.8639	0.2599	0.7674	-0.2965	0.2322	-0.7573	0.0262	-1.1387	1.0000	-0.0959	0.4475	-0.6384	0.9966	-0.4696
LOC104915402	1.0000	0.5414	1.0000	2.2533	1.0000	-2.4061	1.0000	0.0000	1.0000	-2.3771	1.0000	-0.7956	1.0000	0.0000
LOC104915403	1.0000	0.0000	1.0000	2.2509	1.0000	0.0000	1.0000	0.0000	1.0000	0.0000	1.0000	2.3456	1.0000	0.0000
LOC104915404	1.0000	-2.4788	1.0000	2.2509	0.9361	1.2061	1.0000	0.0000	1.0000	-2.3771	1.0000	2.3456	0.7287	-3.6637
LOC104915405	1.0000	0.0000	1.0000	0.0000	1.0000	0.0000	1.0000	0.0000	1.0000	0.0000	1.0000	0.0000	1.0000	0.0000
LOC104915406	0.0144	-0.7784	0.0773	-0.3792	0.0000	-1.4099	0.0345	-0.4625	1.0000	-0.1702	0.8265	0.2426	0.0106	0.7820
LOC104915407	1.0000	-0.3436	0.5842	-1.3278	0.0079	2.3166	0.4013	0.9804	1.0000	0.6414	1.0000	-0.3291	0.6780	-0.6875
LOC104915408	1.0000	0.0000	1.0000	0.0000	1.0000	0.0000	1.0000	0.0000	1.0000	0.0000	1.0000	0.0000	1.0000	0.0000
LOC104915409	1.0000	-2.4776	0.4431	3.6192	1.0000	-0.1591	1.0000	0.0000	1.0000	-2.3758	0.8607	3.7288	1.0000	-2.2908
LOC104915410	1.0000	0.0000	1.0000	0.0000	1.0000	0.0000	1.0000	2.3241	1.0000	0.0000	1.0000	0.0000	1.0000	2.3543
LOC104915411	0.1183	1.1180	0.0265	1.0782	0.1445	0.9151	1.0000	0.2389	1.0000	-0.1289	1.0000	-0.1608	0.3776	-0.7993
LOC104915412	0.2249	0.6037	0.6827	0.2348	1.0000	-0.1066	0.2286	-0.5004	1.0000	0.2045	1.0000	-0.1497	1.0000	-0.1811
LOC104915413	0.0000	-1.5863	0.0000	-1.8320	0.0000	-1.0783	0.0000	-0.7548	1.0000	0.0612	0.6646	-0.1722	0.0002	0.3899
LOC104915414	1.0000	0.0000	1.0000	0.0000	1.0000	0.0000	1.0000	0.0000	1.0000	0.0000	1.0000	0.0000	1.0000	0.0000
LOC104915415	1.0000	0.0000	0.7674	-3.2499	1.0000	2.2468	0.7710	-3.1632	1.0000	3.1057	1.0000	0.0000	1.0000	-2.2908
LOC104915416	0.6877	0.6200	1.0000	-0.0995	0.0001	2.2728	0.0703	0.9578	1.0000	0.8456	1.0000	0.1403	0.6175	-0.4593
LOC104915417	1.0000	0.0000	1.0000	0.0000	1.0000	0.0000	1.0000	0.0000	1.0000	0.0000	1.0000	0.0000	1.0000	0.0000
LOC104915418	0.0144	2.8299	0.0138	2.5920	0.0007	3.1762	0.0048	2.8607	1.0000	-0.1210	1.0000	-0.3484	1.0000	-0.4344
LOC104915419	0.0011	-0.5683	0.0000	-0.5054	0.0342	-0.3881	0.0000	-0.3749	1.0000	0.0551	0.6450	0.1307	0.8752	0.0739
LOC104915420	0.8448	1.1167	0.4437	3.6202	1.0000	0.7306	1.0000	2.3241	1.0000	-3.2319	1.0000	-0.8718	0.8939	-1.6944
LOC104915421	1.0000	0.0000	1.0000	-1.0051	1.0000	0.0000	0.7710	-3.1693	1.0000	3.1190	1.0000	2.3456	1.0000	0.0000
LOC104915422	0.8226	3.0199	1.0000	-2.3960	1.0000	0.0000	1.0000	-2.3180	1.0000	2.2676	1.0000	-3.1429	1.0000	0.0000

LOC104915423	0.0800	0.4506	0.1731	0.2106	1.0000	-0.0185	0.0273	-0.3243	1.0000	0.2542	1.0000	0.0268	1.0000	-0.0490
LOC104915425	0.0802	0.8450	0.0000	1.2719	0.2057	0.6734	0.0432	0.6706	1.0000	0.0636	0.3116	0.5044	1.0000	0.0670
LOC104915426	1.0000	0.0000	1.0000	0.0000	1.0000	0.0000	1.0000	0.0000	1.0000	0.0000	1.0000	0.0000	1.0000	0.0000
LOC104915427	0.4488	-0.5458	0.1484	-0.6016	0.0392	0.8961	0.0135	0.7588	1.0000	0.1805	1.0000	0.1388	1.0000	0.0485
LOC104915428	0.0095	2.6063	0.5267	0.9233	0.1230	1.8319	1.0000	-0.2988	1.0000	0.5714	0.4034	-1.1042	0.3056	-1.5611
LOC104915429	1.0000	0.0000	1.0000	0.0000	1.0000	0.0000	1.0000	0.0000	1.0000	0.0000	1.0000	0.0000	1.0000	0.0000
LOC104915430	1.0000	0.0347	0.3526	-0.3115	0.0192	-0.8948	0.0002	-1.0170	1.0000	0.1426	1.0000	-0.1902	1.0000	0.0257
LOC104915431	0.0000	-1.2453	0.0000	-1.4397	0.0001	-0.6585	0.0000	-0.4599	1.0000	-0.2198	0.0018	-0.4014	1.0000	-0.0163
LOC104915432	1.0000	0.0000	1.0000	0.0000	1.0000	0.0000	1.0000	0.0000	1.0000	0.0000	1.0000	0.0000	1.0000	0.0000
LOC104915433	1.0000	0.0000	1.0000	0.0000	1.0000	0.0000	1.0000	0.0000	1.0000	0.0000	1.0000	0.0000	1.0000	0.0000
LOC104915434	0.0659	-0.5085	0.4146	-0.1988	0.2836	-0.3429	1.0000	0.0568	1.0000	-0.1884	1.0000	0.1342	0.6800	0.2187
LOC104915435	1.0000	0.0000	1.0000	0.0000	1.0000	0.0000	1.0000	0.0000	1.0000	0.0000	1.0000	0.0000	1.0000	0.0000
LOC104915436	1.0000	0.0000	1.0000	0.0000	1.0000	0.0000	1.0000	0.0000	1.0000	0.0000	1.0000	0.0000	1.0000	0.0000
LOC104915438	1.0000	0.0000	1.0000	0.0000	1.0000	0.0000	1.0000	0.0000	1.0000	0.0000	1.0000	0.0000	1.0000	0.0000
LOC104915439	1.0000	0.1286	0.5132	0.2546	1.0000	-0.0203	0.3888	0.3286	1.0000	-0.6457	0.3224	-0.5080	0.6579	-0.2899
LOC104915440	1.0000	-0.0283	1.0000	-0.0301	0.9023	-0.1986	1.0000	-0.0971	1.0000	0.1792	1.0000	0.1905	0.8381	0.2862
LOC104915441	1.0000	0.0000	1.0000	0.0000	1.0000	0.0000	1.0000	0.0000	1.0000	0.0000	1.0000	0.0000	1.0000	0.0000
LOC104915442	1.0000	0.0000	1.0000	0.0000	1.0000	2.2425	1.0000	0.0000	1.0000	0.0000	1.0000	0.0000	1.0000	-2.2889
LOC104915443	0.0259	0.8310	0.0067	0.7131	0.6927	0.2779	1.0000	0.0454	1.0000	0.1217	1.0000	0.0218	1.0000	-0.1045
LOC104915444	1.0000	0.0840	0.2309	0.6940	0.0044	-1.8929	1.0000	-0.2369	1.0000	-0.9200	1.0000	-0.2988	0.8592	0.7441
LOC104915445	1.0000	0.0000	1.0000	0.0000	1.0000	2.2468	1.0000	0.0000	1.0000	0.0000	1.0000	0.0000	1.0000	-2.2908
LOC104915446	0.5086	3.5431	1.0000	2.2509	1.0000	0.0000	1.0000	0.0000	1.0000	0.0000	1.0000	-1.3254	1.0000	0.0000
LOC104915448	1.0000	0.0000	1.0000	0.0000	1.0000	0.0000	1.0000	0.0000	1.0000	0.0000	1.0000	0.0000	1.0000	0.0000
LOC104915449	1.0000	0.0000	1.0000	0.0000	1.0000	0.0000	1.0000	0.0000	1.0000	0.0000	1.0000	0.0000	1.0000	0.0000
LOC104915450	1.0000	0.0000	1.0000	0.0000	1.0000	0.0000	1.0000	0.0000	1.0000	0.0000	1.0000	0.0000	1.0000	0.0000
LOC104915451	1.0000	0.0000	1.0000	-2.3985	0.8011	3.0840	1.0000	-2.3199	1.0000	2.2734	1.0000	0.0000	1.0000	-3.1344
LOC104915452	1.0000	0.0000	1.0000	0.0000	1.0000	0.0000	1.0000	0.0000	1.0000	0.0000	1.0000	0.0000	1.0000	0.0000
LOC104915453	1.0000	0.0000	1.0000	0.0000	1.0000	0.0000	1.0000	0.0000	1.0000	0.0000	1.0000	0.0000	1.0000	0.0000
LOC104915454	1.0000	0.0000	1.0000	0.0000	1.0000	0.0000	1.0000	0.0000	1.0000	0.0000	1.0000	0.0000	1.0000	0.0000
LOC104915455	1.0000	0.0000	1.0000	0.0000	1.0000	0.0000	1.0000	0.0000	1.0000	0.0000	1.0000	0.0000	1.0000	0.0000
LOC104915456	1.0000	0.0000	1.0000	0.0000	1.0000	0.0000	1.0000	0.0000	1.0000	0.0000	1.0000	0.0000	1.0000	0.0000
LOC104915457	0.5667	0.4715	0.0454	0.9734	0.8624	0.3187	1.0000	0.1372	1.0000	-0.2607	1.0000	0.2557	0.8833	-0.4359
LOC104915458	1.0000	2.1850	1.0000	-2.3985	1.0000	0.0000	1.0000	-2.3199	1.0000	2.2734	1.0000	-2.2958	1.0000	0.0000
LOC104915459	1.0000	0.0000	1.0000	0.0000	1.0000	0.0000	1.0000	0.0000	1.0000	0.0000	1.0000	0.0000	1.0000	0.0000
LOC104915461	0.5086	3.5431	1.0000	-0.1594	1.0000	0.0000	1.0000	-0.8404	1.0000	3.1057	1.0000	-0.4744	1.0000	2.3543
LOC104915462	0.8834	0.4551	0.6881	0.6354	0.0000	3.4977	0.0000	3.8773	1.0000	-0.6471	0.9773	-0.4563	0.6246	-0.2661
LOC104915463	0.0000	-1.2428	0.0000	-0.8534	0.5474	-0.2242	0.9919	-0.0354	1.0000	-0.0264	0.0030	0.3758	0.2781	0.1676
LOC104915464	1.0000	0.0000	1.0000	0.0000	1.0000	0.0000	1.0000	0.0000	1.0000	0.0000	1.0000	0.0000	1.0000	0.0000
LOC104915465	1.0000	0.0000	1.0000	0.0000	1.0000	0.0000	1.0000	0.0000	1.0000	0.0000	1.0000	0.0000	1.0000	0.0000
LOC104915466	1.0000	0.0000	1.0000	0.6959	1.0000	0.0000	1.0000	0.8519	1.0000	2.2734	1.0000	3.2002	1.0000	3.2049
LOC104915467	1.0000	0.0000	1.0000	0.0000	1.0000	0.0000	1.0000	0.0000	1.0000	0.0000	1.0000	0.0000	1.0000	0.0000
LOC104915468	1.0000	0.0000	1.0000	0.0000	1.0000	0.0000	1.0000	0.0000	1.0000	0.0000	1.0000	0.0000	1.0000	0.0000
LOC104915469	1.0000	-0.3227	1.0000	2.2509	0.8011	-3.2629	1.0000	0.0000	1.0000	-3.2304	1.0000	-0.7966	1.0000	0.0000
LOC104915470	1.0000	0.0000	1.0000	-2.3985	0.8011	3.0840	1.0000	-2.3199	1.0000	2.2734	1.0000	0.0000	1.0000	-3.1344
LOC104915471	1.0000	2.1851	1.0000	0.0000	0.8033	3.0789	1.0000	0.0000	1.0000	0.0000	1.0000	-2.2958	1.0000	-3.1316
LOC104915472	1.0000	0.0000	1.0000	0.0000	1.0000	0.0000	1.0000	0.0000	1.0000	0.0000	1.0000	0.0000	1.0000	0.0000
LOC104915473	1.0000	2.1900	1.0000	0.0000	1.0000	0.0000	1.0000	2.3241	1.0000	0.0000	1.0000	-2.2992	1.0000	2.3543
LOC104915474	1.0000	0.0000	1.0000	2.2533	1.0000	2.2425	1.0000	0.0000	1.0000	0.0000	1.0000	2.3480	1.0000	-2.2889
LOC104915475	0.5378	3.5350	1.0000	0.0000	1.0000	0.0000	1.0000	0.0000	1.0000	0.0000	0.8785	-3.6648	1.0000	0.0000
LOC104915476	1.0000	0.0000	1.0000	0.0000	1.0000	0.0000	1.0000	0.0000	1.0000	0.0000	1.0000	0.0000	1.0000	0.0000
LOC104915477	1.0000	0.0000	1.0000	-2.3985	1.0000	0.0000	1.0000	-2.3199	1.0000	2.2734	1.0000	0.0000	1.0000	0.0000
LOC104915478	1.0000	0.0000	1.0000	0.0000	1.0000	0.0000	1.0000	0.0000	1.0000	0.0000	1.0000	0.0000	1.0000	0.0000
LOC104915479	1.0000	0.0000	1.0000	0.0000	1.0000	0.0000	1.0000	0.0000	1.0000	0.0000	1.0000	0.0000	1.0000	0.0000
LOC104915480	1.0000	0.0000	1.0000	0.0000	1.0000	0.0000	1.0000	0.0000	1.0000	0.0000	1.0000	0.0000	1.0000	0.0000
LOC104915481	1.0000	0.0000	1.0000	0.0000	1.0000	0.0000	1.0000	0.0000	1.0000	0.0000	1.0000	0.0000	1.0000	0.0000
LOC104915482	1.0000	0.0000	1.0000	0.0000	1.0000	0.0000	1.0000	0.0000	1.0000	0.0000	1.0000	0.0000	1.0000	0.0000
LOC104915483	1.0000	0.0000	1.0000	0.0000	1.0000	0.0000	1.0000	0.0000	1.0000	0.0000	1.0000	0.0000	1.0000	0.0000
LOC104915484	1.0000	0.0000	1.0000	0.0000	1.0000	0.0000	1.0000	0.0000	1.0000	0.0000	1.0000	0.0000	1.0000	0.0000
LOC104915485	1.0000	-0.8510	0.4633	-3.7804	0.3656	1.4722	0.0001	3.0210	1.0000	-0.1299	1.0000	-3.1468	0.0907	1.4354
LOC104915486	0.0810	0.4931	1.0000	-0.0655	0.2370	-0.3880	0.0020	-0.6079	1.0000	0.2058	0.3303	-0.3386	1.0000	-0.0056
LOC104915487	0.6993	0.2599	0.2438	0.4329	0.0012	0.9984	0.0000	1.6401	1.0000	-0.4530	0.8311	-0.2659	0.7239	0.1988
LOC104915488	1.0000	0.0000	1.0000	0.0000	1.0000	0.0000	1.0000	0.0000	1.0000	0.0000	1.0000	0.0000	1.0000	0.0000
LOC104915489	1.0000	0.6080	1.0000	-0.1708	0.1290	1.6427	0.1113	1.6469	1.0000	-0.1228	1.0000	-0.8969	1.0000	-0.1214
LOC104915490	1.0000	-0.7263	0.7439	-0.8853	0.2156	1.4495	1.0000	0.1449	1.0000	1.1358	1.0000	1.0017	1.0000	-0.1567
LOC104915491	0.8249	3.0254	1.0000	-0.1449	1.0000	0.0000	1.0000	0.0075	1.0000	2.2676	1.0000	-0.8001	1.0000	2.3554
LOC104915493	0.4685	0.3819	0.7293	-0.1980	0.0001	-1.8180	0.0000	-1.8153	1.0000	0.1998	0.6450	-0.3663	1.0000	0.2098
LOC104915494	1.0000	0.1483	1.0000	0.0218	0.0451	-2.3245	0.0582	-2.0228	1.0000	-0.2280	1.0000	-0.3482	1.0000	0.0751
LOC104915495	0.0434	1.1820	0.2168	0.7284	0.9449	-0.3643	0.0053	-2.4833	1.0000	-0.1277	0.6126	-0.5732	0.0563	-2.2450
LOC104915496	0.4427	1.5330	0.1492	2.0189	0.3910	1.5011	1.0000	0.5384	1.0000	-0.1218	1.0000	0.3739	0.8620	-1.0805
LOC104915497	0.0068	0.9264	0.0014	0.5792	0.0000	-1.7443	0.0000	-2.0488	1.0000	0.5248	0.9146	0.1913	1.0000	0.2250
LOC104915498	0.0648	0.7411	0.0010	0.8064	0.0000	1.4720	0.0000	1.1316	1.0000	0.0553	1.0000	0.1326	0.6223	-0.2806
LOC104915499	0.3049	-0.4759	0.0165	-0.7474	1.0000	-0.0739	0.4905	0.2460	1.0000	-0.2228	0.4257	-0.4793	1.0000	0.1059
LOC104915500	0.9650	1.0748	1.0000	0.1280	1.0000	0.6783	0.9786	-0.9107	1.0000	1.6505	1.0000	0.7284	1.0000	0.0723
LOC104915501	0.0007	-0.5065	0.0000	-0.5815	0.0000	-0.8735	0.0000	-0.8137	1.0000	0.0434	1.0000	-0.0194	0.5322	0.1088
LOC104915502	0.0101	-0.6813	0.0000	-0.8341	0.0000	-1.1790	0.0000	-1.1564	1.0000	0.1112	1.0000	-0.0276	1.0000	0.1412
LOC104915504	1.0000	-1.1514	1.0000	0.0000	0.8033	-3.2622	1.0000	2.3256	1.0000	-3.2289	1.0000	-2.2992	1.0000	2.3554
LOC104915505	1.0000	-2.4776	1.0000	-0.1429	1.0000	-0.1591	1.0000	0.0062	1.0000	-0.1077	1.0000	2.3480	1.0000	0.0640
LOC104915506	0.4876	0.6768	0.0130	1.1469	0.0000	2.3114	0.0000	2.8616	1.0000	0.0235	0.7057	0.5118	0.0832	0.5827
LOC104915507	1.0000	-2.4776	1.0000	0.0000	1.0000	-2.4056	1.0000	0.0000	1.0000	-2.3758	1.0000	0.0000	1.0000	0.0000
LOC104915508	1.0000	0.0000	1.0000	2.2533	1.0000	0.0000	1.0000	2.3241	1.0000	0.0000	1.0000	2.3480	1.0000	2.3543
LOC104915509	1.0000	-0.2920	1.0000	-2.3960	0.0729	2.8574	1.0000	0.0062	1.0000	-0.1077	1.0000	-2.2958	0.1085	-2.9636
LOC104915510	0.2619	-0.2264	0.4431	-0.0970	0.8011	-0.0972	1.0000	-0.0248	1.0000	-0.0470	0.8785	0.0951	1.0000	0.0307
LOC104915511	0.5008	0.3419	1.0000	0.0643	0.0023	0.9017	0.3369	0.2726	1.0000	0.0906	1.0000	-0.1748	0.0838	-0.5348
LOC104915512	1.0000	0.0000	1.0000	0.0000	0.8033	3.0789	0.7701	3.1732	1.0000	0.0000	1.0000	0.0000	1.0000	0.0730
LOC104915513	1.0000	0.0987	1.0000	2.2509	0.2628	1.1645	1.0000	2.3256	1.0000	-5.8036	0.0051	-3.8133	0.0000	-4.7028
LOC104915514	0.9650	1.0580	1.0000	0.6944	1.0000	-0.1597	1.0000	-2.3199	1.0000	-0.1045	1.0000	-0.4715	1.0000	-2.2908
LOC104915515	0.2570	0.7577	0.4633	-0.6803	1.0000	-0.2671	0.1764	0.7665	1.0000	-0.2054	0.0187	-1.6330	0.2740	0.8383
LOC104915516	1.0000	0.0000	1.0000	0.0000	1.0000	0.0000	1.0000	0.0000	1.0000	0.0000	1.0000	0.0000	1.0000	0.0000
LOC104915517	1.0000	0.0000	1.0000	-0.1429	1.0000	2.2468	1.0000	-2.3180	1.0000	2.2676	1.0000	2.3480	1.0000	-2.2908
LOC104915518	1.0000	-1.2470	0.5137	-1.2944	1.0000	-0.1882	0.9018	-0.5570	1.0000	0.9888	1.0000	0.9693	1.0000	0.6337
LOC104915519	1.0000	0.0127	0.8071	0.3385	1.0000	0.1322	0.0067	1.3153	1.0000	-0.8760	0.8788	-0.5394	0.9662	0.3125
LOC104915520	0.5780	-1.5564	1.0000	-0.1557	0.7660	-1.4095	0.0123	2.6705	1.0000	-1.3466	1.0000	0.0559	0.0535	2.7398
LOC104915521	0.8249	3.0254	1.0000	-2.3985	1.0000	0.0000	0.9167	1.3803	1.0000	2.2734	1.0000	-3.1468	0.7455	3.7355

LOC104915522	0.0005	2.4072	1.0000	-0.1779	1.0000	-0.4446	0.0195	-2.5510	1.0000	0.9549	0.0183	-1.6153	0.9835	-1.1475
LOC104915523	1.0000	0.0000	1.0000	-2.3960	1.0000	0.0000	1.0000	-2.3180	1.0000	2.2676	1.0000	0.0000	1.0000	0.0000
LOC104915524	1.0000	0.1512	1.0000	0.0819	0.3136	-0.6360	0.0905	-0.6329	1.0000	0.2555	1.0000	0.1973	1.0000	0.2649
LOC104915525	1.0000	0.0000	1.0000	0.0000	1.0000	0.0000	1.0000	0.0000	1.0000	0.0000	1.0000	0.0000	1.0000	0.0000
LOC104915526	0.0000	-6.1730	0.0000	-6.7110	1.0000	0.0591	0.1632	0.3687	1.0000	-0.2652	1.0000	-0.7977	1.0000	0.0525
LOC104915527	0.0000	-7.4194	0.0000	-7.8328	0.0000	-1.1370	0.0000	-1.1543	1.0000	-0.0727	1.0000	-0.4752	1.0000	-0.0841
LOC104915528	1.0000	0.0000	1.0000	0.0000	1.0000	0.0000	1.0000	0.0000	1.0000	0.0000	1.0000	0.0000	1.0000	0.0000
LOC104915529	1.0000	2.1900	1.0000	2.2533	1.0000	0.0000	1.0000	0.0000	1.0000	0.0000	1.0000	0.0489	1.0000	0.0000
LOC104915530	1.0000	0.0000	1.0000	0.0000	1.0000	0.0000	1.0000	0.0000	1.0000	0.0000	1.0000	0.0000	1.0000	0.0000
LOC104915532	1.0000	0.0000	1.0000	0.0000	1.0000	0.0000	1.0000	0.0000	1.0000	0.0000	1.0000	0.0000	1.0000	0.0000
LOC104915533	0.1036	-0.3329	0.0136	-0.2116	0.0081	-0.4974	0.0000	-0.4963	1.0000	-0.0110	0.5544	0.1223	1.0000	-0.0040
LOC104915534	1.0000	0.0000	1.0000	0.0000	1.0000	0.0000	1.0000	0.0000	1.0000	0.0000	1.0000	0.0000	1.0000	0.0000
LOC104915535	1.0000	0.0000	1.0000	0.0000	1.0000	0.0000	1.0000	0.0000	1.0000	0.0000	1.0000	0.0000	1.0000	0.0000
LOC104915536	1.0000	0.0000	1.0000	0.0000	1.0000	0.0000	1.0000	0.0000	1.0000	0.0000	1.0000	0.0000	1.0000	0.0000
LOC104915537	1.0000	0.0000	1.0000	0.0000	1.0000	0.0000	1.0000	0.0000	1.0000	0.0000	1.0000	0.0000	1.0000	0.0000
LOC104915538	0.9968	-0.1362	0.9943	-0.1165	0.0241	-0.8585	0.0000	-1.4783	1.0000	-0.0412	1.0000	-0.0086	0.2974	-0.6523
LOC104915539	1.0000	2.1850	1.0000	0.0000	1.0000	2.2468	1.0000	0.0000	1.0000	0.0000	1.0000	-2.2958	1.0000	-2.2907
LOC104915540	1.0000	0.0000	1.0000	0.0000	1.0000	0.0000	1.0000	0.0000	1.0000	0.0000	1.0000	0.0000	1.0000	0.0000
LOC104915541	0.9264	0.2606	1.0000	-0.1268	0.8141	-0.3342	0.0261	-0.9040	1.0000	0.4941	1.0000	0.1169	1.0000	-0.0703
LOC104915542	0.1853	0.4207	0.1484	0.2977	0.5631	-0.2499	0.0389	-0.4471	1.0000	0.0665	1.0000	-0.0439	1.0000	-0.1247
LOC104915543	1.0000	0.0000	1.0000	0.0000	1.0000	0.0000	1.0000	0.0000	1.0000	0.0000	1.0000	0.0000	1.0000	0.0000
LOC104915544	1.0000	0.2915	0.0783	-0.8758	0.0758	1.0353	1.0000	-0.1504	1.0000	0.9001	1.0000	-0.2569	0.9886	-0.2812
LOC104915545	1.0000	-0.0414	0.1170	0.3926	0.2402	0.3884	0.0307	0.5776	1.0000	-0.4127	1.0000	0.0332	0.7741	-0.2180
LOC104915546	0.3684	1.0473	0.0195	1.5658	0.0011	2.1980	0.0009	2.0689	1.0000	-0.1337	1.0000	0.3968	1.0000	-0.2563
LOC104915547	0.6926	0.7328	1.0000	0.4664	0.0919	-4.7440	1.0000	-0.6856	1.0000	-0.3751	1.0000	-0.6269	0.7453	3.7416
LOC104915548	0.4588	0.3231	0.0671	0.3446	1.0000	0.0267	0.0005	0.5738	1.0000	-0.1361	1.0000	-0.1034	0.0575	0.4157
LOC104915549	0.3159	3.9314	1.0000	0.0000	1.0000	0.0000	1.0000	0.0000	1.0000	0.0000	0.6450	-4.0608	1.0000	0.0000
LOC104915550	0.0864	-0.4325	0.0039	-0.4387	0.0091	-0.6033	0.0396	-0.3139	1.0000	-0.1204	1.0000	-0.1139	0.6942	0.1745
LOC104915551	1.0000	0.0000	1.0000	0.0000	1.0000	0.0000	1.0000	0.0000	1.0000	0.0000	1.0000	0.0000	1.0000	0.0000
LOC104915552	1.0000	-1.1562	0.7674	-3.2570	0.8011	-3.2629	0.7710	-3.1693	1.0000	-0.1146	1.0000	-2.2958	1.0000	0.0000
LOC104915553	0.9671	-1.6927	1.0000	-2.3985	0.9468	-1.5485	0.9096	1.3845	1.0000	-1.4875	1.0000	-2.2958	1.0000	1.4482
LOC104915554	0.0485	0.6741	0.0126	0.5640	0.8033	0.1799	0.2230	0.3263	1.0000	0.1530	1.0000	0.0554	0.5694	0.3064
LOC104915555	0.6392	0.5333	0.4327	0.7514	0.0990	-1.4827	1.0000	-0.2490	1.0000	-0.4451	1.0000	-0.2176	0.9983	0.7945
LOC104915556	0.5255	-0.3599	0.9084	-0.1325	1.0000	-0.0360	0.4737	-0.2613	1.0000	0.1742	0.6611	0.4147	1.0000	-0.0444
LOC104915557	1.0000	0.0000	1.0000	0.0000	1.0000	0.0000	1.0000	0.0000	1.0000	0.0000	1.0000	0.0000	1.0000	0.0000
LOC104915558	0.2406	0.3613	0.0001	0.6747	0.0007	0.7892	0.0000	0.8903	1.0000	-0.1909	0.9681	0.1361	1.0000	-0.0839
LOC104915559	1.0000	0.0000	1.0000	0.0000	1.0000	0.0000	1.0000	0.0000	1.0000	0.0000	1.0000	0.0000	1.0000	0.0000
LOC104915560	0.0170	-0.5953	0.0564	-0.3181	0.5455	-0.2015	0.1622	0.2186	1.0000	-0.2886	1.0000	0.0014	0.8297	0.1371
LOC104915561	0.7849	0.2318	0.5248	0.3105	0.0760	-0.8189	0.1061	-0.7309	1.0000	-0.6411	0.3313	-0.5509	0.6362	-0.5456
LOC104915562	1.0000	0.0000	1.0000	0.0000	1.0000	0.0000	1.0000	0.0000	1.0000	0.0000	1.0000	0.0000	1.0000	0.0000
LOC104915563	1.0000	0.0000	1.0000	0.0000	1.0000	0.0000	1.0000	0.0000	1.0000	0.0000	1.0000	0.0000	1.0000	0.0000
LOC104915564	1.0000	0.0000	1.0000	0.0000	1.0000	0.0000	1.0000	0.0000	1.0000	0.0000	1.0000	0.0000	1.0000	0.0000
LOC104915566	1.0000	0.0119	0.7699	0.0818	0.6880	-0.1663	0.0071	-0.4626	1.0000	-0.0135	1.0000	0.0692	0.2530	-0.3052
LOC104915567	0.0001	1.5631	0.0111	0.6578	0.0000	1.9462	0.0001	0.9409	1.0000	0.5786	0.8572	-0.3160	0.1626	-0.4205
LOC104915568	1.0000	0.0000	1.0000	-2.3960	1.0000	0.0000	1.0000	-2.3180	1.0000	2.2676	1.0000	0.0000	1.0000	0.0000
LOC104915569	1.0000	-0.0456	0.2220	1.2671	1.0000	0.1173	0.2154	1.3460	1.0000	-1.3582	1.0000	-0.0352	1.0000	-0.1221
LOC104915570	1.0000	0.0000	1.0000	0.0000	1.0000	0.0000	1.0000	0.0000	1.0000	0.0000	1.0000	0.0000	1.0000	0.0000
LOC104915571	1.0000	0.0000	1.0000	0.0000	1.0000	2.2469	1.0000	0.0000	1.0000	0.0000	1.0000	0.0000	1.0000	-2.2907
LOC104915572	1.0000	-0.0081	0.0473	0.3698	0.8322	-0.1350	1.0000	0.0141	1.0000	-0.1850	0.7635	0.2061	1.0000	-0.0293
LOC104915573	1.0000	0.0000	1.0000	0.0000	1.0000	0.0000	1.0000	0.0000	1.0000	0.0000	1.0000	0.0000	1.0000	0.0000
LOC104915575	1.0000	0.0000	1.0000	0.0000	1.0000	0.0000	1.0000	0.0000	1.0000	0.0000	1.0000	0.0000	1.0000	0.0000
LOC104915578	1.0000	0.0000	1.0000	0.0000	1.0000	0.0000	1.0000	0.0000	1.0000	0.0000	1.0000	0.0000	1.0000	0.0000
LOC104915579	1.0000	0.5420	1.0000	-0.1608	1.0000	-2.4056	0.7710	-3.1632	1.0000	0.7318	1.0000	0.0533	1.0000	0.0000
LOC104915580	0.5086	3.5456	1.0000	-2.3985	1.0000	0.0000	1.0000	0.0045	1.0000	2.2734	0.8607	-3.6732	1.0000	2.3543
LOC104915581	1.0000	0.0000	1.0000	0.0000	1.0000	0.0000	1.0000	0.0000	1.0000	0.0000	1.0000	0.0000	1.0000	0.0000
LOC104915582	1.0000	0.0000	1.0000	0.0000	1.0000	0.0000	1.0000	0.0000	1.0000	0.0000	1.0000	0.0000	1.0000	0.0000
LOC104915583	1.0000	0.0000	1.0000	0.0000	1.0000	0.0000	1.0000	0.0000	1.0000	0.0000	1.0000	0.0000	1.0000	0.0000
LOC104915584	1.0000	0.0000	1.0000	0.0000	1.0000	0.0000	1.0000	0.0000	1.0000	0.0000	1.0000	0.0000	1.0000	0.0000
LOC104915585	0.0944	0.7749	1.0000	0.1052	0.1323	-0.8182	0.0001	-1.5432	1.0000	0.2988	0.8676	-0.3596	0.9863	-0.4216
LOC104915586	1.0000	0.0000	1.0000	0.0000	1.0000	0.0000	1.0000	0.0000	1.0000	0.0000	1.0000	0.0000	1.0000	0.0000
LOC104915588	1.0000	0.0000	1.0000	0.0000	1.0000	0.0000	1.0000	0.0000	1.0000	0.0000	1.0000	0.0000	1.0000	0.0000
LOC104915589	1.0000	0.0000	1.0000	0.0000	1.0000	0.0000	1.0000	0.0000	1.0000	0.0000	1.0000	0.0000	1.0000	0.0000
LOC104915590	1.0000	0.0000	1.0000	0.0000	1.0000	0.0000	0.2461	4.0903	1.0000	0.0000	1.0000	0.0000	0.5105	4.1259
LOC104915591	1.0000	0.0000	1.0000	0.0000	1.0000	0.0000	1.0000	0.0000	1.0000	0.0000	1.0000	0.0000	1.0000	0.0000
LOC104915593	1.0000	-2.4776	1.0000	0.0000	1.0000	-2.4056	1.0000	0.0000	1.0000	-2.3758	1.0000	0.0000	1.0000	0.0000
LOC104915594	0.1843	1.2501	0.1833	1.2297	1.0000	-0.0253	0.2542	1.1618	1.0000	-0.3083	1.0000	-0.3262	0.7629	0.8850
LOC104915595	0.8249	3.0143	1.0000	0.0000	1.0000	0.0000	1.0000	0.0000	1.0000	0.0000	1.0000	-3.1384	1.0000	0.0000
LOC104915596	1.0000	0.0000	1.0000	0.0000	1.0000	0.0000	1.0000	0.0000	1.0000	0.0000	1.0000	0.0000	1.0000	0.0000
LOC104915597	0.9545	-1.6907	0.9062	1.2225	0.9361	-1.5512	0.3949	2.0772	1.0000	-1.4945	1.0000	1.4306	0.6867	2.1416
LOC104915598	0.0672	-0.3633	0.0000	-0.5318	0.2890	-0.2275	0.0259	-0.2550	1.0000	0.0216	0.8405	-0.1347	1.0000	-0.0011
LOC104915599	0.1995	-0.2864	0.1677	-0.2037	0.0000	-0.8501	0.0000	-0.6591	1.0000	-0.1964	0.9741	-0.1025	1.0000	0.0003
LOC104915600	1.0000	0.0000	1.0000	0.0000	1.0000	0.0000	1.0000	0.0000	1.0000	0.0000	1.0000	0.0000	1.0000	0.0000
LOC104915601	1.0000	0.0000	1.0000	0.0000	1.0000	0.0000	1.0000	0.0000	1.0000	0.0000	1.0000	0.0000	1.0000	0.0000
LOC104915602	1.0000	0.0000	1.0000	0.0000	1.0000	0.0000	1.0000	0.0000	1.0000	0.0000	1.0000	0.0000	1.0000	0.0000
LOC104915603	1.0000	0.0000	1.0000	0.0000	1.0000	0.0000	1.0000	0.0000	1.0000	0.0000	1.0000	0.0000	1.0000	0.0000
LOC104915604	1.0000	0.0000	1.0000	0.0000	1.0000	0.0000	1.0000	0.0000	1.0000	0.0000	1.0000	0.0000	1.0000	0.0000
LOC104915605	1.0000	0.0000	1.0000	0.0000	1.0000	0.0000	1.0000	0.0000	1.0000	0.0000	1.0000	0.0000	1.0000	0.0000
LOC104915606	1.0000	0.0000	1.0000	0.0000	1.0000	0.0000	1.0000	0.0000	1.0000	0.0000	1.0000	0.0000	1.0000	0.0000
LOC104915607	1.0000	0.0000	1.0000	0.0000	1.0000	0.0000	1.0000	0.0000	1.0000	0.0000	1.0000	0.0000	1.0000	0.0000
LOC104915608	1.0000	0.0000	1.0000	0.0000	1.0000	0.0000	1.0000	0.0000	1.0000	0.0000	1.0000	0.0000	1.0000	0.0000
LOC104915609	1.0000	0.0000	1.0000	0.0000	1.0000	0.0000	1.0000	0.0000	1.0000	0.0000	1.0000	0.0000	1.0000	0.0000
LOC104915610	0.0409	-3.4926	1.0000	0.2128	0.0781	-2.5081	0.1292	1.7972	1.0000	-1.9064	1.0000	1.8188	0.1747	2.3974
LOC104915611	1.0000	0.0000	1.0000	0.0000	1.0000	0.0000	1.0000	0.0000	1.0000	0.0000	1.0000	0.0000	1.0000	0.0000
LOC104915613	1.0000	0.0000	1.0000	0.0000	1.0000	0.0000	1.0000	0.0000	1.0000	0.0000	1.0000	0.0000	1.0000	0.0000
LOC104915614	0.3641	-4.2658	1.0000	-0.1452	0.3227	-4.1836	1.0000	-2.3199	1.0000	-1.8737	1.0000	2.3480	1.0000	0.0000
LOC104915615	1.0000	0.0000	1.0000	0.0000	1.0000	0.0000	1.0000	0.0000	1.0000	0.0000	1.0000	0.0000	1.0000	0.0000
LOC104915616	1.0000	-1.1562	1.0000	-0.5555	0.8011	-3.2629	0.9786	-0.9103	1.0000	0.7940	1.0000	1.4317	1.0000	3.2066
LOC104915617	1.0000	0.0000	1.0000	0.0000	1.0000	0.0000	1.0000	0.0000	1.0000	0.0000	1.0000	0.0000	1.0000	0.0000
LOC104915618	1.0000	0.0000	1.0000	0.0000	1.0000	0.0000	1.0000	0.0000	1.0000	0.0000	1.0000	0.0000	1.0000	0.0000
LOC104915619	1.0000	0.0000	1.0000	-2.3960	1.0000	0.0000	1.0000	0.0075	1.0000	2.2676	1.0000	0.0000	1.0000	2.3554
LOC104915620	1.0000	0.0000	0.7674	-3.2499	0.0175	5.1145	0.3358	1.6952	1.0000	3.1057	1.0000	0.0000	1.0000	-0.2726
LOC104915621	0.0000	1.5118	0.0000	1.5038	0.0015	1.0531	0.0014	0.8408	1.0000	-0.0025	1.0000	0.0053	0.8110	-0.2079
LOC104915622	1.0000	0.0000	1.0000	0.0000	1.0000	0.0000	1.0000	0.0000	1.0000	0.0000	1.0000	0.0000	1.0000	0.0000

LOC104915623	1.0000	0.0000	1.0000	0.0000	1.0000	0.0000	1.0000	0.0000	1.0000	0.0000	1.0000	0.0000	1.0000	0.0000
LOC104915624	1.0000	0.0000	1.0000	0.0000	1.0000	0.0000	1.0000	0.0000	1.0000	0.0000	1.0000	0.0000	1.0000	0.0000
LOC104915625	1.0000	0.0000	1.0000	0.0000	1.0000	0.0000	1.0000	0.0000	1.0000	0.0000	1.0000	0.0000	1.0000	0.0000
LOC104915626	1.0000	0.0000	1.0000	-2.3960	1.0000	0.0000	1.0000	-2.3180	1.0000	2.2676	1.0000	0.0000	1.0000	0.0000
LOC104915627	0.2202	1.8459	0.1484	2.0180	1.0000	0.3475	0.5159	1.4816	1.0000	-0.1113	1.0000	0.0612	1.0000	1.0220
LOC104915628	1.0000	0.0000	1.0000	0.0000	1.0000	2.2425	1.0000	0.0000	1.0000	0.0000	1.0000	0.0000	1.0000	-2.2889
LOC104915629	1.0000	0.0000	1.0000	-2.3985	1.0000	0.0000	1.0000	0.0057	1.0000	2.2734	1.0000	0.0000	1.0000	2.3554
LOC104915630	1.0000	0.0000	1.0000	2.2533	1.0000	0.0000	1.0000	0.0000	1.0000	0.0000	1.0000	2.3480	1.0000	0.0000
LOC104915631	1.0000	0.0000	1.0000	0.0000	0.8011	3.0840	1.0000	2.3241	1.0000	0.0000	1.0000	0.0000	1.0000	-0.7795
LOC104915632	1.0000	0.0000	1.0000	0.0000	1.0000	0.0000	1.0000	0.0000	1.0000	0.0000	1.0000	0.0000	1.0000	0.0000
LOC104915633	1.0000	-0.0400	1.0000	0.0301	0.6813	-0.2844	0.0172	-0.6773	1.0000	0.0437	1.0000	0.1274	0.7373	-0.3408
LOC104915634	1.0000	0.0000	1.0000	2.2509	1.0000	0.0000	1.0000	0.0000	1.0000	0.0000	1.0000	2.3456	1.0000	0.0000
LOC104915635	1.0000	0.0000	1.0000	0.0000	1.0000	0.0000	1.0000	0.0000	1.0000	0.0000	1.0000	0.0000	1.0000	0.0000
LOC104915637	1.0000	-0.2892	0.9062	-1.5359	1.0000	0.6740	1.0000	0.3938	1.0000	1.2654	1.0000	0.0470	1.0000	0.9923
LOC104915638	1.0000	-2.4788	1.0000	0.0000	1.0000	-0.1630	1.0000	2.3241	1.0000	-2.3771	1.0000	0.0000	1.0000	0.0652
LOC104915639	1.0000	0.0000	1.0000	0.0000	1.0000	0.0000	1.0000	0.0000	1.0000	0.0000	1.0000	0.0000	1.0000	0.0000
LOC104915640	0.5086	-3.8790	0.1456	-4.4788	0.4809	-3.7967	0.7238	-1.2144	1.0000	0.5642	1.0000	0.0000	1.0000	3.2066
LOC104915641	1.0000	0.0000	1.0000	0.6959	1.0000	0.0000	1.0000	0.8541	1.0000	2.2734	1.0000	3.2002	1.0000	3.2066
LOC104915642	1.0000	0.0000	1.0000	0.0000	1.0000	0.0000	1.0000	0.0000	1.0000	0.0000	1.0000	0.0000	1.0000	0.0000
LOC104915643	1.0000	-0.2892	1.0000	0.0000	1.0000	-0.1630	1.0000	0.0000	1.0000	-2.3771	1.0000	-2.2992	1.0000	-2.2889
LOC104915644	1.0000	0.0000	1.0000	0.0000	1.0000	0.0000	1.0000	0.0000	1.0000	0.0000	1.0000	0.0000	1.0000	0.0000
LOC104915647	1.0000	0.5881	1.0000	-0.4694	0.0352	2.4721	0.0216	1.8838	1.0000	1.0869	1.0000	0.0569	0.8133	0.5150
LOC104915648	0.8249	-3.3412	0.4670	3.6266	0.5782	1.2821	0.2461	4.0903	1.0000	-3.2289	0.8759	3.7343	1.0000	-0.4779
LOC104915649	1.0000	0.0000	1.0000	0.0000	1.0000	0.0000	1.0000	0.0000	1.0000	0.0000	1.0000	0.0000	1.0000	0.0000
LOC104915650	1.0000	0.2011	1.0000	0.2195	1.0000	-1.0156	0.5741	1.1646	1.0000	0.4098	1.0000	0.4459	0.3618	2.6076
LOC104915651	1.0000	0.0000	1.0000	0.0000	1.0000	0.0000	0.7710	3.1710	1.0000	0.0000	1.0000	0.0000	1.0000	3.2049
LOC104915652	1.0000	0.0000	1.0000	0.0000	1.0000	0.0000	1.0000	0.0000	1.0000	0.0000	1.0000	0.0000	1.0000	0.0000
LOC104915653	1.0000	2.1851	1.0000	0.0000	1.0000	0.0000	1.0000	0.0000	1.0000	0.0000	1.0000	-2.2959	1.0000	0.0000
LOC104915654	1.0000	0.0000	1.0000	0.0000	1.0000	0.0000	1.0000	0.0000	1.0000	0.0000	1.0000	0.0000	1.0000	0.0000
LOC104915655	1.0000	0.0000	1.0000	0.0000	1.0000	0.0000	1.0000	0.0000	1.0000	0.0000	1.0000	0.0000	1.0000	0.0000
LOC104915656	0.9671	-1.6927	0.2584	-1.4471	0.8288	0.7575	0.9460	-0.4891	1.0000	1.5345	1.0000	1.8149	1.0000	0.2963
LOC104915657	1.0000	0.4690	0.2495	4.0050	0.1614	-4.4916	1.0000	0.0000	1.0000	-4.4568	0.8825	-1.0634	1.0000	0.0000
LOC104915658	0.2862	-1.3242	0.1772	-1.3642	0.1495	-1.7205	0.0033	-3.5036	1.0000	0.0857	1.0000	0.0596	0.8937	-1.6947
LOC104915659	1.0000	-2.4776	1.0000	-0.1472	1.0000	-2.4056	1.0000	-2.3199	1.0000	-0.1034	1.0000	2.3456	1.0000	0.0000
LOC104915660	0.5086	-3.8790	1.0000	-0.1472	1.0000	-0.7128	1.0000	0.0045	1.0000	-1.4912	1.0000	2.3456	1.0000	-0.7795
LOC104915661	0.0000	1.5664	0.1663	0.7198	0.0000	1.8439	0.0000	1.9192	1.0000	-0.6297	0.0000	-1.4647	0.0956	-0.5493
LOC104915662	1.0000	0.0000	1.0000	0.0000	1.0000	0.0000	1.0000	0.0000	1.0000	0.0000	1.0000	0.0000	1.0000	0.0000
LOC104915663	1.0000	0.0000	1.0000	0.0000	1.0000	0.0000	1.0000	0.0000	1.0000	0.0000	1.0000	0.0000	1.0000	0.0000
LOC104915664	0.4318	-0.3626	0.5573	0.2094	0.0379	-0.7938	0.0291	-0.5648	1.0000	-0.3415	0.8601	0.2407	1.0000	-0.1063
LOC104915665	1.0000	0.0000	1.0000	0.0000	1.0000	0.0000	1.0000	0.0000	1.0000	0.0000	1.0000	0.0000	1.0000	0.0000
LOC104915666	0.1190	-1.5429	0.0691	-1.4633	1.0000	-0.1997	0.7725	0.4156	1.0000	-0.0317	1.0000	0.0596	0.8181	0.5870
LOC104915667	1.0000	0.0000	1.0000	0.0000	1.0000	0.0000	1.0000	0.0000	1.0000	0.0000	1.0000	0.0000	1.0000	0.0000
LOC104915668	0.4912	1.1811	0.0774	4.5589	1.0000	0.1138	0.4370	3.7041	1.0000	-4.1507	0.8607	-0.9124	1.0000	-0.6154
LOC104915669	0.9545	1.0652	1.0000	0.0000	1.0000	-2.4056	1.0000	0.0000	1.0000	-2.3758	0.8607	-3.6719	1.0000	0.0000
LOC104915670	0.0000	-1.1912	0.0000	-0.9820	0.0000	-1.2048	0.0000	-1.0818	1.0000	-0.1975	1.0000	0.0242	1.0000	-0.0698
LOC104915672	1.0000	0.2774	1.0000	0.3902	1.0000	0.1577	0.2441	-2.3153	1.0000	-0.3410	1.0000	-0.2208	0.1668	-2.8156
LOC104915673	0.9650	1.0580	1.0000	0.0000	1.0000	-0.1597	1.0000	0.0000	1.0000	-2.3771	0.8785	-3.6648	1.0000	-2.2908
LOC104915674	1.0000	0.0000	1.0000	0.0000	1.0000	0.0000	1.0000	0.0000	1.0000	0.0000	1.0000	0.0000	1.0000	0.0000
LOC104915675	1.0000	0.0000	1.0000	0.0000	1.0000	0.0000	1.0000	0.0000	1.0000	0.0000	1.0000	0.0000	1.0000	0.0000
LOC104915676	0.9303	-0.5620	1.0000	-0.0552	0.6373	-1.3491	0.1263	-1.7839	1.0000	0.5038	0.8607	1.0284	1.0000	0.0765
LOC104915677	1.0000	-2.4776	1.0000	2.2533	1.0000	-2.4056	1.0000	0.0000	1.0000	-2.3758	1.0000	2.3480	1.0000	0.0000
LOC104915678	0.6012	0.3678	0.5738	-0.3732	0.0059	0.9239	0.1017	0.5797	1.0000	-0.2721	0.0985	-1.0008	0.0862	-0.6106
LOC104915679	1.0000	0.0000	1.0000	0.0000	1.0000	0.0000	1.0000	0.0000	1.0000	0.0000	1.0000	0.0000	1.0000	0.0000
LOC104915680	1.0000	0.0000	1.0000	0.0000	1.0000	0.0000	1.0000	0.0000	1.0000	0.0000	1.0000	0.0000	1.0000	0.0000
LOC104915681	0.0295	0.8603	0.2368	0.4360	0.0004	1.1876	0.0003	0.9118	1.0000	0.1398	0.9234	-0.2730	1.0000	-0.1306
LOC104915682	1.0000	0.0000	1.0000	0.0000	0.8011	3.0840	1.0000	0.0000	1.0000	0.0000	1.0000	0.0000	1.0000	-3.1344
LOC104915683	1.0000	-2.4776	0.7674	-3.2499	1.0000	-2.4056	0.7710	-3.1632	1.0000	0.7318	1.0000	0.0000	1.0000	0.0000
LOC104915684	1.0000	0.0000	1.0000	0.0000	1.0000	0.0000	1.0000	0.0000	1.0000	0.0000	1.0000	0.0000	1.0000	0.0000
LOC104915685	1.0000	0.0000	1.0000	0.0000	1.0000	0.0000	1.0000	0.0000	1.0000	0.0000	1.0000	0.0000	1.0000	0.0000
LOC104915686	0.8859	0.6166	0.9807	0.3444	0.0000	2.9059	0.0000	2.4502	1.0000	0.2109	1.0000	-0.0436	1.0000	-0.2280
LOC104915687	1.0000	-1.1514	1.0000	-1.0017	0.0127	2.7443	1.0000	0.5372	1.0000	-0.1170	1.0000	0.0470	0.0449	-2.3282
LOC104915688	1.0000	0.0000	1.0000	0.0000	1.0000	0.0000	1.0000	2.3241	1.0000	0.0000	1.0000	0.0000	1.0000	2.3543
LOC104915689	0.5391	-3.8819	1.0000	-2.3985	0.9468	-1.5556	1.0000	-2.3199	1.0000	-1.4918	1.0000	0.0000	1.0000	-2.2889
LOC104915690	1.0000	0.0000	1.0000	0.0000	1.0000	0.0000	1.0000	0.0000	1.0000	0.0000	1.0000	0.0000	1.0000	0.0000
LOC104915692	1.0000	0.0000	1.0000	0.0000	1.0000	0.0000	1.0000	0.0000	1.0000	0.0000	1.0000	0.0000	1.0000	0.0000
LOC104915693	0.8520	0.1519	0.0000	0.7934	0.2870	0.3390	0.0000	1.0776	1.0000	-0.6265	1.0000	0.0283	0.8744	0.1187
LOC104915694	1.0000	0.0000	1.0000	0.0000	1.0000	0.0000	1.0000	0.0000	1.0000	0.0000	1.0000	0.0000	1.0000	0.0000
LOC104915695	0.3148	3.9252	1.0000	-0.1625	1.0000	2.2469	1.0000	-0.8419	1.0000	3.1126	1.0000	-0.8610	1.0000	0.0640
LOC104915697	0.7023	1.0401	0.0247	3.0138	0.0282	1.9892	0.0018	3.6153	1.0000	-1.8822	1.0000	0.0811	1.0000	-0.2575
LOC104915698	1.0000	0.5414	1.0000	0.6940	1.0000	-0.1630	1.0000	-2.3179	1.0000	-0.1089	1.0000	0.0533	1.0000	-2.2889
LOC104915699	0.5351	0.2131	0.0004	0.5121	0.3496	0.2776	0.0167	0.4102	1.0000	-0.4591	0.8122	-0.1498	0.2072	-0.3216
LOC104915701	1.0000	0.0000	1.0000	0.0000	1.0000	0.0000	1.0000	0.0000	1.0000	0.0000	1.0000	0.0000	1.0000	0.0000
LOC104915702	0.0000	-3.7066	0.0000	-3.9908	0.0268	-0.7887	0.0000	-1.4832	1.0000	0.5297	1.0000	0.2612	1.0000	-0.1613
LOC104915703	0.4379	0.6768	0.2244	0.8607	0.2074	-1.1772	0.7701	-0.5975	1.0000	-0.3266	1.0000	-0.1251	1.0000	0.2536
LOC104915704	1.0000	0.0000	1.0000	0.0000	1.0000	2.2469	1.0000	0.0000	1.0000	0.0000	1.0000	0.0000	1.0000	-2.2908
LOC104915705	1.0000	0.0000	1.0000	0.0000	1.0000	0.0000	1.0000	0.0000	1.0000	0.0000	1.0000	0.0000	1.0000	0.0000
LOC104915706	0.1920	0.8763	0.2813	0.5265	0.3212	0.7418	1.0000	0.1586	1.0000	1.0247	0.4006	0.6896	0.7389	0.4464
LOC104915707	1.0000	-0.0764	0.1227	-0.3460	0.0693	-0.5543	0.0287	-0.4634	1.0000	0.0332	0.8395	-0.2233	1.0000	0.1275
LOC104915708	1.0000	-0.0963	0.1021	1.5372	0.1378	1.1261	0.0000	3.0496	1.0000	-1.6696	1.0000	-0.0233	1.0000	0.2583
LOC104915709	0.8249	-3.3412	0.7674	-3.2499	0.8033	-3.2622	0.7710	-3.1632	1.0000	-0.1218	1.0000	0.0000	1.0000	0.0000
LOC104915710	1.0000	0.0000	1.0000	0.0000	1.0000	0.0000	1.0000	0.0000	1.0000	0.0000	1.0000	0.0000	1.0000	0.0000
LOC104915712	0.1174	1.5967	0.5077	1.3087	1.0000	-0.4932	1.0000	0.0072	1.0000	-1.3495	0.1664	-1.6363	1.0000	-0.8445
LOC104915714	1.0000	0.0000	1.0000	-2.3985	1.0000	0.0000	1.0000	-2.3199	1.0000	2.2734	1.0000	0.0000	1.0000	0.0000
LOC104915716	0.0001	-0.8937	0.0000	-0.8247	0.0004	-0.7554	0.0001	-0.5271	1.0000	-0.2786	0.8600	-0.1966	1.0000	-0.0451
LOC104915717	1.0000	0.0000	1.0000	0.0000	1.0000	0.0000	1.0000	0.0000	1.0000	0.0000	1.0000	0.0000	1.0000	0.0000
LOC104915718	1.0000	0.0000	1.0000	0.0000	1.0000	0.0000	1.0000	0.0000	1.0000	0.0000	1.0000	0.0000	1.0000	0.0000
LOC104915721	0.5086	-0.5632	0.0267	-1.0605	0.0227	-1.4984	0.0052	-1.3113	1.0000	0.7458	1.0000	0.2633	0.4508	0.9396
LOC104915722	0.0003	-0.5638	0.0000	-0.6117	0.0000	-0.9765	0.0000	-0.7497	1.0000	-0.0826	0.7213	-0.1180	0.5612	0.1501
LOC104915724	1.0000	0.0000	1.0000	0.0000	1.0000	0.0000	1.0000	0.0000	1.0000	0.0000	1.0000	0.0000	1.0000	0.0000
LOC104915725	1.0000	-0.1179	1.0000	0.0402	0.1184	-1.9832	0.4701	-0.6914	1.0000	0.5714	0.8607	0.7423	0.3258	1.8688
LOC104915726	1.0000	2.1900	1.0000	-2.3985	1.0000	2.2469	1.0000	0.0045	1.0000	2.2734	1.0000	-2.2992	1.0000	0.0640
LOC104915727	0.0141	2.4876	0.0375	2.0587	0.0529	2.0394	0.0512	2.0346	1.0000	-0.1232	0.9755	-0.5448	1.0000	-0.1232
LOC104915728	1.0000	0.0000	1.0000	0.0000	1.0000	0.0000	1.0000	0.0000	1.0000	0.0000	1.0000	0.0000	1.0000	0.0000

LOC104915729	1.0000	0.1838	0.5449	0.5684	0.0007	1.8681	0.0000	2.4740	1.0000	-0.5039	1.0000	-0.1182	1.0000	0.1008
LOC104915730	1.0000	0.0000	1.0000	0.0000	1.0000	0.0000	1.0000	0.0000	1.0000	0.0000	1.0000	0.0000	1.0000	0.0000
LOC104915731	1.0000	0.0000	1.0000	0.0000	1.0000	0.0000	1.0000	0.0000	1.0000	0.0000	1.0000	0.0000	1.0000	0.0000
LOC104915732	1.0000	0.0000	1.0000	0.0000	1.0000	0.0000	1.0000	0.0000	1.0000	0.0000	1.0000	0.0000	1.0000	0.0000
LOC104915733	1.0000	0.0000	1.0000	0.0000	1.0000	0.0000	1.0000	0.0000	1.0000	0.0000	1.0000	0.0000	1.0000	0.0000
LOC104915734	0.7938	1.1370	0.0040	5.4075	0.8099	1.0224	0.2426	4.0910	1.0000	-3.2289	0.9681	0.9092	1.0000	-0.2241
LOC104915735	1.0000	0.0000	1.0000	0.0000	1.0000	2.2469	1.0000	0.0000	1.0000	0.0000	1.0000	0.0000	1.0000	-2.2908
LOC104915736	0.2338	1.0180	1.0000	0.0810	0.3926	0.7864	0.4528	0.6158	1.0000	0.3086	0.8059	-0.6176	1.0000	0.1437
LOC104915737	1.0000	0.0000	1.0000	0.0000	1.0000	0.0000	1.0000	0.0000	1.0000	0.0000	1.0000	0.0000	1.0000	0.0000
LOC104915738	0.9545	1.0652	1.0000	0.0000	1.0000	-2.4055	0.4370	3.7041	1.0000	-2.3758	0.8607	-3.6719	0.7287	3.7387
LOC104915739	1.0000	0.0035	0.7398	-0.2822	0.0527	-1.1194	0.0000	-2.3814	1.0000	-0.0349	1.0000	-0.3088	0.1915	-1.2863
LOC104915740	0.9545	1.0679	0.9762	0.7508	1.0000	0.6746	0.7701	-3.1666	1.0000	0.7365	1.0000	0.4429	1.0000	-3.1316
LOC104915742	1.0000	0.0000	1.0000	0.0000	1.0000	0.0000	1.0000	0.0000	1.0000	0.0000	1.0000	0.0000	1.0000	0.0000
LOC104915743	1.0000	-0.1031	0.0010	0.5513	0.0008	0.8762	0.0000	1.3194	1.0000	-0.4679	0.7628	0.1993	1.0000	-0.0165
LOC104915744	0.0673	-4.8289	1.0000	-2.3985	0.2898	-2.4979	1.0000	-2.3199	1.0000	-2.4369	1.0000	0.0000	1.0000	-2.2907
LOC104915745	1.0000	0.0000	1.0000	0.0000	1.0000	0.0000	1.0000	0.0000	1.0000	0.0000	1.0000	0.0000	1.0000	0.0000
LOC104915746	1.0000	0.0000	1.0000	0.0000	1.0000	0.0000	1.0000	0.0000	1.0000	0.0000	1.0000	0.0000	1.0000	0.0000
LOC104915747	1.0000	0.0000	1.0000	0.0000	1.0000	0.0000	1.0000	0.0000	1.0000	0.0000	1.0000	0.0000	1.0000	0.0000
LOC104915748	0.8977	-0.1357	0.7717	0.1313	0.0458	-0.6072	1.0000	0.0378	1.0000	-0.1523	1.0000	0.1275	0.1232	0.4983
LOC104915749	1.0000	0.0000	1.0000	0.0000	1.0000	0.0000	1.0000	0.0000	1.0000	0.0000	1.0000	0.0000	1.0000	0.0000
LOC104915750	1.0000	0.0000	1.0000	0.0000	1.0000	0.0000	1.0000	0.0000	1.0000	0.0000	1.0000	0.0000	1.0000	0.0000
LOC104915751	1.0000	0.0000	1.0000	-2.3960	1.0000	0.0000	1.0000	-2.3179	1.0000	2.2676	1.0000	0.0000	1.0000	0.0000
LOC104915752	1.0000	0.0000	1.0000	0.0000	1.0000	0.0000	1.0000	0.0000	1.0000	0.0000	1.0000	0.0000	1.0000	0.0000
LOC104915753	1.0000	0.0000	1.0000	0.0000	1.0000	0.0000	1.0000	0.0000	1.0000	0.0000	1.0000	0.0000	1.0000	0.0000
LOC104915754	1.0000	0.0000	1.0000	0.0000	1.0000	0.0000	1.0000	0.0000	1.0000	0.0000	1.0000	0.0000	1.0000	0.0000
LOC104915755	1.0000	0.0000	1.0000	0.0000	1.0000	0.0000	1.0000	0.0000	1.0000	0.0000	1.0000	0.0000	1.0000	0.0000
LOC104915756	1.0000	0.0000	1.0000	0.0000	1.0000	0.0000	1.0000	0.0000	1.0000	0.0000	1.0000	0.0000	1.0000	0.0000
LOC104915757	1.0000	0.0000	1.0000	0.0000	1.0000	0.0000	1.0000	0.0000	1.0000	0.0000	1.0000	0.0000	1.0000	0.0000
LOC104915759	0.0313	-0.8307	0.0484	-0.5744	0.0716	-0.7480	0.0475	-0.5834	1.0000	-0.1629	1.0000	0.1039	1.0000	0.0094
LOC104915760	0.9447	0.2191	0.0161	-0.8397	0.1201	-0.8989	0.0237	-0.7915	1.0000	0.3410	0.3181	-0.7047	0.7425	0.4545
LOC104915761	0.5007	-0.1690	0.0159	-0.2619	0.0000	-1.2126	0.0000	-0.5818	1.0000	-0.2201	0.0199	-0.3003	0.0034	0.4178
LOC104915762	0.6877	-2.0809	1.0000	0.5243	0.2785	-1.4857	1.0000	-0.5236	1.0000	-0.5115	0.8372	2.1256	1.0000	3.2066
LOC104915763	0.0005	-0.7118	0.0000	-1.0211	0.0002	-0.7671	0.0089	-0.3292	1.0000	-0.0840	0.1637	-0.3811	0.0647	0.3593
LOC104915764	0.0907	-1.6062	0.0655	-3.0450	0.0558	-1.6625	0.4821	-1.1117	1.0000	-1.0349	0.4857	-2.4818	1.0000	-0.4783
LOC104915765	0.0278	-2.8973	0.2545	-4.1774	0.5729	-0.7129	0.0042	2.3898	1.0000	-1.7661	1.0000	-3.1429	0.2715	1.3409
LOC104915766	1.0000	0.0000	1.0000	0.0000	1.0000	0.0000	1.0000	0.0000	1.0000	0.0000	1.0000	0.0000	1.0000	0.0000
LOC104915767	1.0000	0.0000	1.0000	0.0000	1.0000	0.0000	1.0000	0.0000	1.0000	0.0000	1.0000	0.0000	1.0000	0.0000
LOC104915769	1.0000	-0.2882	1.0000	-1.0008	1.0000	-2.4055	1.0000	-0.8416	1.0000	0.7365	1.0000	0.0489	1.0000	2.3554
LOC104915770	1.0000	0.1249	0.2555	0.4054	0.0390	0.6916	0.0000	1.0475	1.0000	-0.1986	1.0000	0.0988	0.8725	0.1660
LOC104915771	1.0000	-2.4788	1.0000	0.0000	1.0000	-2.4061	1.0000	0.0000	1.0000	-2.3771	1.0000	0.0000	1.0000	0.0000
LOC104915772	1.0000	0.0000	1.0000	0.0000	1.0000	0.0000	0.2461	4.0903	1.0000	0.0000	1.0000	0.0000	0.5105	4.1259
LOC104915773	1.0000	0.0000	1.0000	0.0000	1.0000	0.0000	1.0000	0.0000	1.0000	0.0000	1.0000	0.0000	1.0000	0.0000
LOC104915774	0.3827	-0.2118	0.9645	0.0417	0.1074	0.3231	0.7550	-0.0606	1.0000	-0.1786	1.0000	0.0869	0.0000	-0.5564
LOC104915776	0.7197	-0.1151	0.8087	0.0515	0.0000	-0.7574	0.0000	-0.6448	1.0000	-0.1758	1.0000	0.0027	1.0000	-0.0571
LOC104915777	1.0000	0.0000	1.0000	0.0000	1.0000	0.0000	1.0000	0.0000	1.0000	0.0000	1.0000	0.0000	1.0000	0.0000
LOC104915778	1.0000	2.1900	1.0000	-0.1472	1.0000	2.2424	1.0000	-2.3199	1.0000	2.2734	1.0000	0.0470	1.0000	-2.2889
LOC104915779	0.5375	0.6311	0.2393	0.7651	0.0179	1.4295	0.0277	1.1669	1.0000	-0.0427	1.0000	0.1078	0.9351	-0.2995
LOC104915780	1.0000	0.0000	1.0000	0.0000	1.0000	0.0000	1.0000	0.0000	1.0000	0.0000	1.0000	0.0000	1.0000	0.0000
LOC104915781	0.5026	0.4107	0.0000	1.3236	0.7498	0.2851	0.0000	1.0170	1.0000	-0.0681	0.0000	0.8595	0.0035	0.6746
LOC104915782	0.1062	-0.4627	0.0152	-0.4190	0.3880	0.2554	0.0032	0.4047	1.0000	-0.0286	1.0000	0.0283	0.8271	0.1256
LOC104915783	1.0000	-0.2602	0.2567	-0.8603	0.3886	0.7377	0.4686	0.5165	1.0000	0.4459	1.0000	-0.1423	1.0000	0.2305
LOC104915785	1.0000	0.0000	1.0000	0.0000	1.0000	0.0000	1.0000	0.0000	1.0000	0.0000	1.0000	0.0000	1.0000	0.0000
LOC104915786	0.0719	-0.8698	0.7338	-0.2588	0.9205	0.1815	0.3376	0.3895	1.0000	-0.1591	0.7364	0.4654	1.0000	0.0588
LOC104915787	0.0338	-0.6341	0.3090	-0.3066	0.0000	-1.2608	0.3544	-0.2670	1.0000	-0.4868	1.0000	-0.1487	0.1558	0.5108
LOC104915788	0.0104	1.2311	0.0075	1.0167	0.0055	1.2255	0.0002	1.3035	1.0000	-0.1360	0.8269	-0.3354	1.0000	-0.0491
LOC104915789	1.0000	0.5414	0.4135	1.9103	1.0000	-2.4061	1.0000	-2.3179	1.0000	-0.1089	1.0000	1.2774	1.0000	0.9000
LOC104915790	0.1016	-3.2093	0.0520	-4.9414	0.8094	-0.5462	0.4948	0.8822	1.0000	-0.4879	1.0000	-2.2959	0.7683	0.9504
LOC104915791	0.3820	0.6665	0.5179	0.5327	0.0001	1.7292	0.0611	0.9972	1.0000	-0.2910	0.9773	-0.4129	0.0169	-1.0160
LOC104915794	0.3234	-1.3162	1.0000	-0.1708	1.0000	-0.1950	1.0000	0.2586	1.0000	-1.0942	1.0000	0.0603	0.9863	-0.6357
LOC104915795	0.5438	-0.1730	0.1182	-0.2043	0.4863	-0.1917	0.7065	0.0761	1.0000	-0.1302	0.6968	-0.1496	0.6579	0.1439
LOC104915796	1.0000	0.0000	1.0000	0.0000	1.0000	0.0000	1.0000	2.3241	1.0000	0.0000	1.0000	0.0000	1.0000	2.3543
LOC104915797	0.0001	-1.0729	0.0561	-0.4509	0.0093	-0.7213	0.9875	0.0741	1.0000	-0.4728	1.0000	0.1627	0.3246	0.3294
LOC104915798	1.0000	-0.2031	0.2636	0.4990	0.0006	1.6734	0.0000	2.4885	1.0000	-0.7599	1.0000	-0.0416	1.0000	0.0655
LOC104915799	0.0000	-7.4067	0.0000	-4.5933	0.3378	-0.8240	0.2069	-0.5734	1.0000	0.2444	1.0000	3.1976	0.7451	0.4947
LOC104915800	1.0000	-0.0914	0.0103	0.4412	0.3869	0.2989	0.0000	0.7735	1.0000	-0.4724	1.0000	0.0730	1.0000	0.0108
LOC104915801	1.0000	0.1208	1.0000	0.0253	0.4838	-0.4142	1.0000	-0.0291	1.0000	-0.1759	0.8607	-0.2611	0.9777	0.2108
LOC104915802	1.0000	0.0000	1.0000	0.0000	1.0000	0.0000	1.0000	0.0000	1.0000	0.0000	1.0000	0.0000	1.0000	0.0000
LOC104915803	1.0000	0.0000	1.0000	0.0000	1.0000	0.0000	1.0000	0.0000	1.0000	0.0000	1.0000	0.0000	1.0000	0.0000
LOC104915804	1.0000	0.0000	1.0000	0.0000	1.0000	0.0000	1.0000	0.0000	1.0000	0.0000	1.0000	0.0000	1.0000	0.0000
LOC104915805	1.0000	0.0000	1.0000	0.0000	1.0000	0.0000	1.0000	0.0000	1.0000	0.0000	1.0000	0.0000	1.0000	0.0000
LOC104915806	1.0000	0.0000	1.0000	0.0000	1.0000	0.0000	1.0000	0.0000	1.0000	0.0000	1.0000	0.0000	1.0000	0.0000
LOC104915807	0.0216	-0.4473	0.0821	-0.2060	0.0000	-0.8459	0.0000	-0.5107	1.0000	-0.3202	1.0000	-0.0663	1.0000	0.0208
LOC104915808	0.8249	-3.3412	1.0000	-1.0051	1.0000	-0.1821	0.7210	1.2285	1.0000	-0.1113	1.0000	2.3456	0.9835	1.2976
LOC104915809	1.0000	0.1064	0.1379	0.4068	0.9020	0.1889	0.2785	0.4001	1.0000	-0.2063	1.0000	0.1043	1.0000	0.0086
LOC104915810	1.0000	0.0104	0.2207	0.1918	0.0003	0.6744	0.0000	0.9413	1.0000	-0.3513	0.7635	-0.1573	0.9788	-0.0775
LOC104915811	0.5745	-0.1815	0.0013	-0.3315	0.0000	-1.0054	0.0000	-0.7613	1.0000	-0.0424	0.3934	-0.1801	0.3592	0.2070
LOC104915813	0.9671	-1.6927	1.0000	0.5192	0.0262	2.2901	0.0006	2.9048	1.0000	-0.1299	0.8741	2.1171	0.9248	0.4912
LOC104915814	0.0369	0.4847	0.0000	0.7023	0.0316	-0.5446	0.0007	-0.5248	1.0000	-0.1870	1.0000	0.0408	0.7915	-0.1619
LOC104915815	1.0000	2.1851	1.0000	-2.3985	0.4809	3.6101	1.0000	0.8541	1.0000	2.2734	1.0000	-2.2959	1.0000	-0.4568
LOC104915816	1.0000	0.0000	1.0000	0.0000	1.0000	0.0000	1.0000	0.0000	1.0000	0.0000	1.0000	0.0000	1.0000	0.0000
LOC104915817	1.0000	0.0000	1.0000	0.0000	1.0000	0.0000	1.0000	0.0000	1.0000	0.0000	1.0000	0.0000	1.0000	0.0000
LOC104915818	0.8249	3.0143	1.0000	-2.3985	1.0000	0.0000	1.0000	-2.3199	1.0000	2.2734	1.0000	-3.1384	1.0000	0.0000
LOC104915820	1.0000	0.0000	1.0000	2.2509	1.0000	0.0000	1.0000	0.0000	1.0000	0.0000	1.0000	2.3456	1.0000	0.0000
LOC104915821	1.0000	0.0000	1.0000	0.0000	1.0000	0.0000	1.0000	0.0000	1.0000	0.0000	1.0000	0.0000	1.0000	0.0000
LOC104915822	1.0000	0.0000	1.0000	0.0000	1.0000	0.0000	1.0000	0.0000	1.0000	0.0000	1.0000	0.0000	1.0000	0.0000
LOC104915823	0.0228	-0.3569	0.1818	-0.1415	0.0000	-1.3069	0.0000	-0.9620	1.0000	-0.3110	0.8875	-0.0834	1.0000	0.0398
LOC104915824	1.0000	0.0000	1.0000	0.0000	1.0000	2.2469	1.0000	0.0000	1.0000	0.0000	1.0000	0.0000	1.0000	-2.2907
LOC104915825	1.0000	0.0000	1.0000	0.0000	1.0000	0.0000	1.0000	0.0000	1.0000	0.0000	1.0000	0.0000	1.0000	0.0000
LOC104915827	0.1082	-0.4371	0.0176	0.3853	0.0000	1.3431	0.0000	2.1071	1.0000	-0.8312	1.0000	0.0047	1.0000	-0.0612
LOC104915828	0.5895	-0.7181	1.0000	0.3912	0.1401	0.9485	0.0169	1.7731	1.0000	-1.6754	1.0000	-0.5606	0.2997	-0.8426
LOC104915830	0.5611	0.6353	0.1828	0.8100	0.2245	-1.1727	0.0126	-2.3148	1.0000	0.0584	1.0000	0.2495	0.8648	-1.0802
LOC104915831	0.2456	-0.4463	0.5573	-0.1123	0.8070	0.1724	0.0004	0.4247	1.0000	0.1697	0.0036	0.5173	0.0083	0.4273
LOC104915832	0.0469	0.8257	0.0000	1.6306	0.0002	1.1668	0.0000	2.1614	1.0000	-0.7394	1.0000	0.0760	0.6077	0.2611

LOC104915833	0.6737	0.9974	1.0000	-0.1716	1.0000	-0.1848	0.4547	-3.7024	1.0000	-0.1146	0.8390	-1.2854	0.7287	-3.6637
LOC104915834	1.0000	0.0000	1.0000	0.0000	1.0000	0.0000	1.0000	0.0000	1.0000	0.0000	1.0000	0.0000	1.0000	0.0000
LOC104915835	1.0000	-0.0570	0.3651	-0.1904	0.0004	-0.7846	0.0000	-1.0208	1.0000	-0.0521	0.6478	-0.1745	0.4047	-0.2818
LOC104915836	1.0000	0.0000	1.0000	0.0000	1.0000	0.0000	1.0000	0.0000	1.0000	0.0000	1.0000	0.0000	1.0000	0.0000
LOC104915838	0.0153	-1.5987	0.1727	-0.8737	0.0189	-1.5469	0.2525	-0.7763	1.0000	-0.3813	1.0000	0.3543	1.0000	0.3955
LOC104915840	0.7172	0.1472	0.1415	0.2077	1.0000	-0.0127	0.7926	0.0776	1.0000	-0.1215	1.0000	-0.0480	1.0000	-0.0238
LOC104915841	0.4339	-0.3389	0.9747	0.1167	0.5463	-0.2890	0.9096	0.1227	1.0000	-0.3898	1.0000	0.0802	1.0000	0.0302
LOC104915842	0.0003	-0.8341	0.0007	-0.6760	0.0000	-1.4871	0.0000	-1.2074	1.0000	-0.2557	1.0000	-0.0848	1.0000	0.0293
LOC104915843	1.0000	-2.4776	0.7666	3.0922	0.6647	1.5885	1.0000	2.3256	1.0000	-2.3758	1.0000	3.1976	0.8939	-1.6940
LOC104915844	0.7284	-0.5543	0.2111	0.9992	0.0013	1.6582	0.0000	2.2119	1.0000	-0.6990	0.7284	0.8670	1.0000	-0.1447
LOC104915845	0.8374	-1.0310	1.0000	-0.6944	0.7660	-1.4095	1.0000	-0.5236	1.0000	-0.8169	1.0000	-0.4752	1.0000	0.0730
LOC104915846	0.9091	0.2585	0.4404	0.5497	0.0240	1.1115	0.0000	1.6475	1.0000	-0.3186	1.0000	-0.0182	0.9796	0.2183
LOC104915848	0.0001	-0.8138	0.0000	-0.8874	0.0001	-0.9289	0.0000	-0.6508	1.0000	0.0009	1.0000	-0.0610	0.4601	0.2856
LOC104915850	1.0000	0.0000	1.0000	0.0000	1.0000	0.0000	1.0000	0.0000	1.0000	0.0000	1.0000	0.0000	1.0000	0.0000
LOC104915851	1.0000	0.0000	1.0000	0.0000	1.0000	0.0000	1.0000	0.0000	1.0000	0.0000	1.0000	0.0000	1.0000	0.0000
LOC104915852	1.0000	0.0000	1.0000	0.0000	1.0000	0.0000	1.0000	0.0000	1.0000	0.0000	1.0000	0.0000	1.0000	0.0000
LOC104915853	0.0546	-0.4071	0.8335	-0.0687	0.0000	-1.2081	0.0000	-0.8445	1.0000	-0.3318	1.0000	0.0190	1.0000	0.0351
LOC104915856	0.0003	-0.7153	0.0001	-0.5055	0.0000	-1.7277	0.0000	-0.9428	1.0000	-0.2750	1.0000	-0.0528	0.0495	0.5151
LOC104915857	0.1148	0.5177	0.0000	1.0257	0.0000	1.2262	0.0000	1.6583	1.0000	-0.3550	0.9233	0.1658	1.0000	0.0835
LOC104915858	0.0599	-1.2979	0.0861	-0.8368	0.8276	0.2972	0.6435	0.3126	1.0000	0.2546	0.8175	0.7301	1.0000	0.2768
LOC104915860	1.0000	0.0000	1.0000	0.0000	1.0000	0.0000	1.0000	0.0000	1.0000	0.0000	1.0000	0.0000	1.0000	0.0000
LOC104915861	0.0538	-0.5268	0.0456	-0.2753	0.4095	-0.2834	0.0005	0.4032	1.0000	-0.1535	0.9249	0.1117	0.0000	0.5408
LOC104915862	0.0010	-0.5100	0.0000	-0.4593	0.0000	-1.1433	0.0000	-0.6309	1.0000	-0.4294	0.0002	-0.3664	0.8744	0.0892
LOC104915863	0.1099	-0.4635	0.0436	-0.3694	0.1103	0.4493	0.0000	0.8113	1.0000	-0.1721	1.0000	-0.0662	0.6285	0.1964
LOC104915864	1.0000	0.0000	1.0000	0.0000	1.0000	0.0000	1.0000	0.0000	1.0000	0.0000	1.0000	0.0000	1.0000	0.0000
LOC104915867	0.8328	-0.3544	0.0019	-1.6629	0.8059	0.3880	0.8515	0.2397	1.0000	0.4915	0.6872	-0.8046	0.9287	0.3510
LOC104915868	1.0000	-0.0098	1.0000	-0.0630	0.0131	1.0087	0.0114	0.7305	1.0000	0.5443	0.6424	0.5020	0.7121	0.2659
LOC104915869	0.0003	-0.7504	0.0001	-0.4966	0.0000	-1.0477	0.0000	-0.6248	1.0000	-0.2273	1.0000	0.0401	0.3535	0.2022
LOC104915870	1.0000	2.1900	1.0000	0.0000	1.0000	2.2424	0.7710	3.1710	1.0000	0.0000	1.0000	-2.2992	1.0000	0.9164
LOC104915871	1.0000	-0.0554	0.0936	-0.3015	0.0128	-0.5515	0.0013	-0.5332	1.0000	-0.2047	0.0152	-0.4389	0.7172	-0.1805
LOC104915872	0.0010	-0.5965	0.0000	-0.7343	0.0000	-1.3961	0.0000	-1.1824	1.0000	-0.1858	0.1069	-0.3116	1.0000	0.0346
LOC104915873	0.0000	-0.7187	0.0000	-0.8297	0.0000	-1.0080	0.0000	-0.6732	1.0000	0.0609	1.0000	-0.0378	0.0011	0.4016
LOC104915874	1.0000	-2.4776	1.0000	0.0000	1.0000	-2.4055	1.0000	0.0000	1.0000	-2.3758	1.0000	0.0000	1.0000	0.0000
LOC104915875	0.0000	-0.6684	0.0000	-0.7796	0.0000	-0.6871	0.0000	-0.4288	1.0000	-0.1762	0.0275	-0.2752	0.8822	0.0881
LOC104915876	1.0000	0.0000	1.0000	0.0000	1.0000	0.0000	1.0000	0.0000	1.0000	0.0000	1.0000	0.0000	1.0000	0.0000
LOC104915877	0.2561	-0.4128	0.0672	-0.3180	0.0308	0.6006	0.0017	0.4363	1.0000	0.0978	0.7505	0.2073	1.0000	-0.0602
LOC104915878	1.0000	0.8846	0.7189	-1.3897	1.0000	-1.0171	0.7190	-1.2157	1.0000	1.1014	1.0000	-1.1638	1.0000	0.9165
LOC104915879	1.0000	0.0000	1.0000	0.0000	1.0000	0.0000	1.0000	0.0000	1.0000	0.0000	1.0000	0.0000	1.0000	0.0000
LOC104915880	1.0000	0.0000	1.0000	0.0000	1.0000	0.0000	1.0000	0.0000	1.0000	0.0000	1.0000	0.0000	1.0000	0.0000
LOC104915881	1.0000	0.0000	1.0000	0.0000	1.0000	0.0000	1.0000	0.0000	1.0000	0.0000	1.0000	0.0000	1.0000	0.0000
LOC104915882	1.0000	0.0000	1.0000	0.0000	1.0000	0.0000	1.0000	0.0000	1.0000	0.0000	1.0000	0.0000	1.0000	0.0000
LOC104915883	1.0000	0.0000	1.0000	0.0000	1.0000	0.0000	1.0000	0.0000	1.0000	0.0000	1.0000	0.0000	1.0000	0.0000
LOC104915884	0.6936	1.4527	0.1478	4.3119	0.2910	2.1469	1.0000	2.3241	1.0000	-2.3771	1.0000	0.3633	0.5109	-2.2511
LOC104915885	1.0000	-0.8677	1.0000	0.0000	1.0000	-0.1880	1.0000	0.0000	1.0000	-3.7658	1.0000	-3.1384	0.7287	-3.6634
LOC104915888	1.0000	0.0000	1.0000	0.0000	1.0000	0.0000	1.0000	0.0000	1.0000	0.0000	1.0000	0.0000	1.0000	0.0000
LOC104915889	1.0000	2.1851	1.0000	0.0000	1.0000	0.0000	1.0000	2.3256	1.0000	0.0000	1.0000	-2.2959	1.0000	2.3554
LOC104915890	1.0000	0.0000	1.0000	2.2509	1.0000	0.0000	1.0000	0.0000	1.0000	0.0000	1.0000	2.3456	1.0000	0.0000
LOC104915891	1.0000	0.0000	1.0000	0.0000	1.0000	2.2425	1.0000	0.0000	1.0000	0.0000	1.0000	0.0000	1.0000	-2.2889
LOC104915892	0.0257	-0.3827	0.2219	-0.1765	0.1492	-0.2938	0.0368	0.2500	1.0000	-0.3870	0.4138	-0.1681	0.5000	0.1638
LOC104915894	0.0993	-1.0225	0.0007	-1.7844	0.1998	0.7182	0.0000	1.3768	1.0000	-0.0515	0.6681	-0.8022	0.1818	0.6162
LOC104915895	0.8226	-3.3425	0.1476	-4.4851	0.8011	-3.2629	0.4057	-2.0656	1.0000	1.1048	1.0000	0.0000	1.0000	2.3554
LOC104915897	0.0403	-0.4626	0.1298	-0.2702	0.1687	0.3441	0.0000	0.6699	1.0000	-0.2854	1.0000	-0.0800	1.0000	0.0478
LOC104915898	0.0002	-1.1673	0.0266	-0.5268	0.0002	-1.2451	0.1951	-0.3157	1.0000	-0.2568	0.5474	0.3965	0.0613	0.6810
LOC104915899	0.0003	-2.2178	0.0848	-1.4221	0.0521	0.9326	0.0000	2.3967	1.0000	-1.2483	1.0000	-0.4407	0.9428	0.2291
LOC104915901	1.0000	0.0000	1.0000	0.0000	1.0000	0.0000	1.0000	0.0000	1.0000	0.0000	1.0000	0.0000	1.0000	0.0000
LOC104915902	1.0000	0.1181	1.0000	-0.1787	0.7756	-1.4115	0.1555	-2.0361	1.0000	0.6961	1.0000	0.4110	1.0000	0.0730
LOC104915905	0.8940	0.2494	0.3787	0.3916	0.1018	0.7995	0.0046	0.9033	1.0000	-0.1609	1.0000	-0.0033	1.0000	-0.0515
LOC104915908	0.0000	-0.9553	0.0000	-0.6487	0.0000	-1.1526	0.0000	-0.8174	1.0000	-0.1939	0.9718	0.1250	0.7916	0.1470
LOC104915909	0.0007	-1.9943	0.0000	-2.6715	1.0000	-0.0985	0.0357	0.8274	1.0000	-0.4639	0.6331	-1.1294	0.6626	0.4716
LOC104915912	0.3274	-0.4149	0.5405	-0.2448	0.0562	-0.6235	0.3878	-0.2987	1.0000	-0.2338	1.0000	-0.0511	1.0000	0.0974
LOC104915913	1.0000	0.0000	1.0000	0.0000	1.0000	0.0000	1.0000	0.0000	1.0000	0.0000	1.0000	0.0000	1.0000	0.0000
LOC104915914	1.0000	0.0000	1.0000	0.0000	1.0000	0.0000	1.0000	0.0000	1.0000	0.0000	1.0000	0.0000	1.0000	0.0000
LOC104915915	1.0000	0.0000	1.0000	0.0000	1.0000	0.0000	1.0000	0.0000	1.0000	0.0000	1.0000	0.0000	1.0000	0.0000
LOC104915916	1.0000	0.0000	1.0000	0.0000	1.0000	0.0000	1.0000	2.3256	1.0000	0.0000	1.0000	0.0000	1.0000	2.3554
LOC104915917	1.0000	0.0000	1.0000	0.0000	1.0000	0.0000	1.0000	0.0000	1.0000	0.0000	1.0000	0.0000	1.0000	0.0000
LOC104915918	1.0000	-2.4776	0.7674	-3.2499	0.9468	1.1991	0.9801	0.9246	1.0000	0.7318	1.0000	0.0000	1.0000	0.4637
LOC104915919	1.0000	2.1851	1.0000	-2.3960	1.0000	0.0000	1.0000	0.0062	1.0000	2.2676	1.0000	-2.2959	1.0000	2.3543
LOC104915920	1.0000	0.0000	1.0000	0.0000	1.0000	0.0000	1.0000	0.0000	1.0000	0.0000	1.0000	0.0000	1.0000	0.0000
LOC104915921	0.0083	0.7220	0.0000	1.4071	0.0000	1.7228	0.0000	1.7717	1.0000	-0.4438	0.5526	0.2545	0.0014	-0.3896
LOC104915922	1.0000	0.0000	1.0000	0.0000	1.0000	0.0000	1.0000	0.0000	1.0000	0.0000	1.0000	0.0000	1.0000	0.0000
LOC104915923	0.1779	-1.5740	1.0000	0.3781	0.0020	-5.7170	1.0000	0.0043	1.0000	-1.6504	1.0000	0.3102	0.5093	4.1263
LOC104915924	0.0028	-0.7289	0.0966	-0.3649	0.3823	0.2520	0.5548	0.1579	1.0000	-0.1973	0.9073	0.1809	0.1937	-0.2849
LOC104915925	1.0000	0.0000	1.0000	0.0000	1.0000	0.0000	1.0000	0.0000	1.0000	0.0000	1.0000	0.0000	1.0000	0.0000
LOC104915926	0.0062	-1.0694	0.1585	-0.1770	0.2184	-0.5425	0.0001	0.3850	1.0000	0.1263	0.0000	1.0297	0.0000	1.0600
LOC104915931	1.0000	0.0404	1.0000	-0.0171	0.0002	-1.0290	0.0000	-1.2984	1.0000	-0.1410	0.8915	-0.1865	0.4901	-0.4057
LOC104915932	0.0000	1.0640	0.0000	1.3008	0.1324	-0.4445	0.0021	-0.5352	1.0000	-0.1759	1.0000	0.0710	0.6076	-0.2605
LOC104915935	1.0000	0.0000	1.0000	-0.1449	1.0000	0.0000	0.9096	1.3854	1.0000	2.2676	1.0000	2.3456	0.7287	3.7387
LOC104915936	0.0000	-0.8395	0.0000	-0.7608	0.0000	-1.6781	0.0000	-0.8487	1.0000	-0.2231	0.9042	-0.1311	0.0053	0.6140
LOC104915937	1.0000	0.0000	0.7674	3.0955	1.0000	0.0000	1.0000	0.0000	1.0000	0.0000	1.0000	3.2002	1.0000	0.0000
LOC104915938	0.5021	-1.1148	1.0000	-0.4768	0.1846	-2.7131	0.7190	-1.2157	1.0000	-0.5921	1.0000	0.0569	1.0000	0.9165
LOC104915939	1.0000	0.0000	1.0000	0.0000	1.0000	0.0000	1.0000	0.0000	1.0000	0.0000	1.0000	0.0000	1.0000	0.0000
LOC104915940	1.0000	0.0000	1.0000	0.0000	1.0000	0.0000	1.0000	0.0000	1.0000	0.0000	1.0000	0.0000	1.0000	0.0000
LOC104915941	0.0381	-0.4412	0.0000	-0.5450	0.0000	-1.0777	0.0000	-1.0328	1.0000	-0.1139	0.5336	-0.2057	1.0000	-0.0643
LOC104915944	0.9008	-0.0882	0.2694	0.1820	0.0014	-0.6654	0.0000	-0.6756	1.0000	-0.3310	1.0000	-0.0486	0.2734	-0.3359
LOC104915946	0.2182	-1.1449	0.2354	-0.9515	0.0012	1.8421	0.0000	1.9150	1.0000	-0.2775	1.0000	-0.0652	1.0000	-0.2007
LOC104915947	1.0000	0.4344	0.8621	0.4776	1.0000	-1.1039	1.0000	-0.2980	1.0000	0.1810	1.0000	0.2427	1.0000	0.9923
LOC104915949	1.0000	0.0000	1.0000	0.0000	1.0000	0.0000	1.0000	0.0000	1.0000	0.0000	1.0000	0.0000	1.0000	0.0000
LOC104915950	0.0000	-3.6373	0.0000	-2.6098	0.0693	-0.7609	0.1353	0.5096	1.0000	-0.6707	1.0000	0.3684	0.2413	0.6078
LOC104915951	0.1131	0.6326	0.0030	0.7887	0.9488	0.1420	0.4180	0.3169	1.0000	0.0029	1.0000	0.1669	0.9691	0.1818
LOC104915952	1.0000	0.0000	1.0000	0.0000	1.0000	0.0000	1.0000	0.0000	1.0000	0.0000	1.0000	0.0000	1.0000	0.0000
LOC104915953	0.1717	0.3478	0.0000	0.7412	0.0000	1.7849	0.0000	2.0454	1.0000	-0.0279	0.0240	0.3790	0.2432	0.2373
LOC104915954	0.0167	-0.4982	0.0000	-0.8178	0.0000	-1.2560	0.0000	-1.0176	1.0000	-0.3732	0.0000	-0.6814	0.8717	-0.1289
LOC104915955	1.0000	0.0000	1.0000	0.0000	1.0000	0.0000	1.0000	0.0000	1.0000	0.0000	1.0000	0.0000	1.0000	0.0000

LOC104915956	1.0000	0.0000	1.0000	0.0000	1.0000	0.0000	1.0000	0.0000	1.0000	0.0000	1.0000	0.0000	1.0000	0.0000
LOC104915957	1.0000	0.0000	1.0000	0.0000	1.0000	0.0000	1.0000	0.0000	1.0000	0.0000	1.0000	0.0000	1.0000	0.0000
LOC104915958	0.9671	-1.6863	1.0000	0.6944	1.0000	-0.1833	0.3950	2.0766	1.0000	-1.4875	1.0000	0.8994	1.0000	0.7679
LOC104915959	1.0000	0.0000	1.0000	0.0000	1.0000	0.0000	1.0000	0.0000	1.0000	0.0000	1.0000	0.0000	1.0000	0.0000
LOC104915960	0.0107	-0.6437	0.1298	-0.2884	0.0001	-0.9706	0.5881	-0.1520	1.0000	-0.3602	1.0000	0.0072	0.1184	0.4641
LOC104915965	0.0003	0.6777	0.0000	0.5268	0.5800	-0.1817	1.0000	0.0263	1.0000	0.0658	1.0000	-0.0732	0.1381	0.2795
LOC104915967	0.0332	-1.0311	0.0054	-0.8301	0.9847	0.1667	0.0676	0.4778	1.0000	0.2605	0.6987	0.4744	0.1367	0.5818
LOC104915970	0.5086	-0.2110	1.0000	-0.0058	1.0000	-0.0175	0.0000	0.3913	1.0000	-0.2350	1.0000	-0.0175	0.2784	0.1794
LOC104915971	0.4782	-0.2763	0.9409	0.0685	1.0000	-0.0533	0.0000	0.7313	1.0000	-0.4722	0.9942	-0.1120	0.0665	0.3210
LOC104915972	0.8226	3.0199	0.6224	-1.9258	1.0000	2.2425	0.9786	-0.9114	1.0000	4.0300	1.0000	-0.7966	1.0000	0.9168
LOC104915974	1.0000	0.0000	1.0000	0.0000	1.0000	0.0000	1.0000	0.0000	1.0000	0.0000	1.0000	0.0000	1.0000	0.0000
LOC104915975	0.5123	-0.4341	0.6771	-0.2909	1.0000	-0.0538	0.0270	0.7051	1.0000	-0.4093	1.0000	-0.2498	0.5189	0.3603
LOC104915976	1.0000	-0.0968	0.4156	0.2967	1.0000	-0.0343	0.0202	0.6097	1.0000	-0.3060	1.0000	0.1014	0.4972	0.3441
LOC104915977	0.2391	0.6535	0.0237	0.9775	0.4595	0.4724	0.0528	0.8820	1.0000	-0.4894	1.0000	-0.1518	1.0000	-0.0686
LOC104915978	0.5166	-0.2960	0.0125	-0.3429	0.0001	-1.2523	0.0000	-1.1404	1.0000	-0.3110	0.0585	-0.3475	0.6285	-0.1945
LOC104915979	0.0344	-1.8362	0.8429	-0.3978	0.1786	-1.1737	0.7701	0.4408	1.0000	-0.6338	0.9945	0.8164	0.5617	0.9874
LOC104915980	0.8686	0.2775	0.0164	0.8961	0.0571	0.9653	0.0000	1.7710	1.0000	-1.0123	0.6537	-0.3777	0.8832	-0.1970
LOC104915981	0.8351	0.1862	0.2531	0.2904	0.2087	0.4649	0.0000	0.7199	1.0000	-0.0924	1.0000	0.0256	0.7287	0.1711
LOC104915983	0.5391	-3.8767	1.0000	0.0000	1.0000	-0.1754	1.0000	0.0000	1.0000	-3.7607	1.0000	0.0000	0.7455	-3.6684
LOC104915984	1.0000	0.0000	1.0000	2.2509	0.4809	3.6101	1.0000	0.0000	1.0000	0.0000	1.0000	2.3456	0.7287	-3.6634
LOC104915985	0.4132	-0.5885	0.2833	-0.5302	0.0009	-1.9133	0.0126	-1.1144	1.0000	0.0665	1.0000	0.1411	0.6214	0.8730
LOC104915986	0.3193	-1.3158	0.1262	-1.7043	0.4483	-1.1585	0.5067	0.6593	1.0000	0.1309	1.0000	-0.2469	0.0821	1.9539
LOC104915987	1.0000	-0.0510	1.0000	0.0657	0.0000	1.3226	0.0000	1.5389	1.0000	-0.4005	0.9242	-0.2691	0.8512	-0.1752
LOC104915988	0.0000	-0.7509	0.0000	-0.6886	0.0000	-0.9693	0.0000	-0.8713	1.0000	-0.1979	0.6660	-0.1237	0.9049	-0.0941
LOC104915989	1.0000	0.0000	1.0000	0.0000	1.0000	0.0000	1.0000	0.0000	1.0000	0.0000	1.0000	0.0000	1.0000	0.0000
LOC104915990	1.0000	0.0000	1.0000	0.0000	1.0000	0.0000	1.0000	0.0000	1.0000	0.0000	1.0000	0.0000	1.0000	0.0000
LOC104915991	0.1374	2.5657	0.8218	0.5975	0.0742	2.7104	0.6312	0.9640	1.0000	1.6505	1.0000	-0.2918	1.0000	-0.0862
LOC104915992	0.0399	-0.6315	0.0134	-0.3612	0.5464	-0.2574	0.0471	-0.2945	1.0000	0.0491	0.2327	0.3339	1.0000	0.0195
LOC104915993	0.0000	-1.1351	0.0000	-0.8241	0.1673	-0.2952	0.0031	0.3595	1.0000	-0.3932	1.0000	-0.0686	0.0998	0.2681
LOC104915995	1.0000	-0.1714	0.0680	0.9939	0.6597	0.4267	0.0003	1.5768	1.0000	-0.8022	0.9437	0.3709	0.8721	0.3429
LOC104915996	1.0000	0.0000	1.0000	0.0000	0.8033	3.0789	1.0000	2.3256	1.0000	0.0000	1.0000	0.0000	1.0000	-0.7769
LOC104915997	1.0000	0.0000	1.0000	0.0000	0.4809	3.6101	1.0000	0.0000	1.0000	0.0000	1.0000	0.0000	0.7287	-3.6634
LOC104915999	1.0000	0.0000	1.0000	0.0000	1.0000	0.0000	1.0000	0.0000	1.0000	0.0000	1.0000	0.0000	1.0000	0.0000
LOC104916000	1.0000	0.0000	1.0000	0.0000	1.0000	0.0000	1.0000	0.0000	1.0000	0.0000	1.0000	0.0000	1.0000	0.0000
LOC104916001	1.0000	0.0000	1.0000	0.0000	1.0000	0.0000	1.0000	0.0000	1.0000	0.0000	1.0000	0.0000	1.0000	0.0000
LOC104916002	1.0000	0.0000	1.0000	0.0000	1.0000	0.0000	1.0000	0.0000	1.0000	0.0000	1.0000	0.0000	1.0000	0.0000
LOC104916003	0.5780	-1.5564	0.9779	-1.0833	1.0000	-0.4952	0.0053	2.2595	1.0000	-0.4321	1.0000	0.0533	0.0263	2.3304
LOC104916004	1.0000	0.0000	0.7674	3.0955	1.0000	2.2424	1.0000	0.0000	1.0000	0.0000	1.0000	3.2002	1.0000	-2.2889
LOC104916005	1.0000	0.0000	1.0000	0.0000	1.0000	0.0000	1.0000	0.0000	1.0000	0.0000	1.0000	0.0000	1.0000	0.0000
LOC104916006	1.0000	0.0000	1.0000	0.0000	1.0000	0.0000	1.0000	0.0000	1.0000	0.0000	1.0000	0.0000	1.0000	0.0000
LOC104916007	1.0000	0.0000	1.0000	0.0000	1.0000	0.0000	1.0000	0.0000	1.0000	0.0000	1.0000	0.0000	1.0000	0.0000
LOC104916008	0.3089	3.9281	1.0000	2.2533	1.0000	0.0000	1.0000	0.0000	1.0000	0.0000	1.0000	-1.7117	1.0000	0.0000
LOC104916009	1.0000	0.0000	1.0000	0.0000	1.0000	0.0000	1.0000	0.0000	1.0000	0.0000	1.0000	0.0000	1.0000	0.0000
LOC104916010	1.0000	0.0000	1.0000	0.0000	1.0000	0.0000	1.0000	0.0000	1.0000	0.0000	1.0000	0.0000	1.0000	0.0000
LOC104916013	0.1464	-2.8575	0.1045	-2.8877	1.0000	0.3048	1.0000	0.0075	1.0000	0.0641	1.0000	0.0495	1.0000	-0.2322
LOC104916014	1.0000	0.0000	1.0000	0.0000	1.0000	0.0000	1.0000	0.0000	1.0000	0.0000	1.0000	0.0000	1.0000	0.0000
LOC104916015	0.0000	0.9208	0.0000	1.2867	0.0000	1.1419	0.0000	1.1248	1.0000	-0.0508	0.2267	0.3275	1.0000	-0.0643
LOC104916016	0.0120	-0.6231	0.0000	-0.9585	0.0000	-1.4734	0.0000	-0.7782	1.0000	-0.0528	0.1863	-0.3771	0.0071	0.6473
LOC104916018	0.0036	-0.7165	0.0000	-0.8177	0.0000	-1.1892	0.0000	-1.0001	1.0000	0.0027	1.0000	-0.0866	0.7588	0.1970
LOC104916019	0.0283	-0.5802	0.5616	-0.1961	0.0015	-0.8133	0.6172	-0.1960	1.0000	-0.2372	0.9888	0.1579	0.3893	0.3847
LOC104916022	0.0000	-1.0525	0.0000	-1.2531	0.0000	-1.3446	0.0001	-0.8218	1.0000	-0.1648	0.4754	-0.3542	0.3684	0.3638
LOC104916023	0.0180	-0.9158	0.0219	-0.5935	0.0286	-0.9402	0.1296	-0.4792	1.0000	-0.1910	1.0000	0.1432	0.9856	0.2794
LOC104916024	0.9671	-1.6927	1.0000	0.3858	1.0000	-0.7166	1.0000	0.5636	1.0000	0.2613	0.6465	2.3777	0.7915	1.5493
LOC104916025	0.0300	-0.5134	0.0000	-1.4664	0.0648	0.4180	0.0251	0.2525	1.0000	-0.3681	0.0000	-1.3084	0.0000	-0.5266
LOC104916026	0.0005	-1.6884	0.0002	-1.8946	0.0065	-1.2935	0.0417	-0.9824	1.0000	-0.6681	0.6565	-0.8632	1.0000	-0.3483
LOC104916027	1.0000	0.0000	1.0000	-2.3960	1.0000	0.0000	1.0000	-2.3179	1.0000	2.2677	1.0000	0.0000	1.0000	0.0000
LOC104916028	0.8249	-3.3439	1.0000	0.0000	0.8033	-3.2636	1.0000	0.0000	1.0000	-3.2319	1.0000	0.0000	1.0000	0.0000
LOC104916029	0.5629	-0.3509	1.0000	0.0112	0.0164	-0.9526	0.5640	-0.2584	1.0000	-0.3235	1.0000	0.0483	0.7257	0.3780
LOC104916030	1.0000	0.0000	0.4021	-2.2287	0.0531	4.7637	0.1421	1.4486	1.0000	4.3298	1.0000	2.3480	0.6557	1.0550
LOC104916031	1.0000	-0.0212	0.1114	0.5960	1.0000	0.1550	0.0000	1.1889	1.0000	-0.5038	1.0000	0.1284	0.1461	0.5375
LOC104916032	0.0133	-5.3972	0.0931	-2.8833	0.1702	1.1915	0.1602	1.1526	1.0000	-0.2936	1.0000	2.3456	1.0000	-0.3295
LOC104916034	0.8759	-0.1295	0.6806	-0.1276	0.5290	-0.2482	0.3905	-0.2130	1.0000	-0.0414	1.0000	-0.0252	1.0000	0.0006
LOC104916035	0.8187	-0.1469	0.2884	-0.2302	1.0000	-0.0080	0.2552	0.2160	1.0000	-0.1696	0.5666	-0.2425	1.0000	0.0592
LOC104916036	0.3089	3.9281	0.6413	0.7853	0.0000	6.3813	0.0331	1.9432	1.0000	4.0238	1.0000	1.0162	1.0000	-0.3731
LOC104916038	0.6680	-1.2815	0.9062	-1.5339	1.0000	-0.1886	1.0000	0.6991	1.0000	-1.0691	1.0000	-1.3292	1.0000	-0.1753
LOC104916039	0.5099	-0.5039	1.0000	-0.0860	0.4641	0.4588	0.0567	0.6736	1.0000	-0.0284	0.8690	0.4033	1.0000	0.1879
LOC104916040	0.2331	0.2288	0.0030	0.3067	0.0715	-0.3425	0.0231	-0.2374	1.0000	-0.1967	0.7806	-0.1070	0.9237	-0.0861
LOC104916041	0.0087	-0.5595	0.0001	-0.3824	0.0825	-0.4391	0.5030	0.0895	1.0000	-0.1897	1.0000	0.0003	0.0560	0.3452
LOC104916042	0.4232	0.2858	0.0610	0.4031	0.0537	-0.5698	0.1430	-0.3494	1.0000	-0.3703	0.6857	-0.2425	1.0000	-0.1444
LOC104916043	1.0000	-0.0422	0.0473	0.1689	0.0000	-1.1424	0.0000	-0.7856	1.0000	-0.2380	1.0000	-0.0146	0.7700	0.1246
LOC104916044	0.0002	-0.6180	0.0000	-0.6952	0.0000	-0.7976	0.0000	-0.6701	1.0000	-0.0442	0.6565	-0.1087	0.8297	0.0894
LOC104916045	1.0000	0.0000	1.0000	2.2509	1.0000	0.0000	1.0000	0.0000	1.0000	0.0000	1.0000	2.3456	1.0000	0.0000
LOC104916047	0.0001	-0.8191	0.0067	-0.3376	0.0000	-1.4658	0.0000	-1.0052	1.0000	-0.5200	1.0000	-0.0269	1.0000	-0.0555
LOC104916048	1.0000	0.0000	1.0000	0.0000	1.0000	0.0000	1.0000	0.0000	1.0000	0.0000	1.0000	0.0000	1.0000	0.0000
LOC104916049	1.0000	0.0000	1.0000	0.0000	1.0000	0.0000	1.0000	0.0000	1.0000	0.0000	1.0000	0.0000	1.0000	0.0000
LOC104916050	1.0000	0.0000	1.0000	0.0000	1.0000	0.0000	1.0000	0.0000	1.0000	0.0000	1.0000	0.0000	1.0000	0.0000
LOC104916051	0.0107	2.2113	0.0000	4.7393	0.0000	3.2743	0.0000	5.1843	1.0000	-2.1858	1.0000	0.3270	0.9157	-0.2833
LOC104916052	1.0000	0.0000	1.0000	0.0000	1.0000	0.0000	1.0000	0.0000	1.0000	0.0000	1.0000	0.0000	1.0000	0.0000
LOC104916053	1.0000	0.0000	1.0000	0.0000	1.0000	0.0000	1.0000	0.0000	1.0000	0.0000	1.0000	0.0000	1.0000	0.0000
LOC104916054	1.0000	0.0000	1.0000	0.0000	1.0000	0.0000	1.0000	0.0000	1.0000	0.0000	1.0000	0.0000	1.0000	0.0000
LOC104916055	0.8249	-3.3412	0.7666	-3.2534	0.8033	-3.2622	0.7701	-3.1666	1.0000	-0.1170	1.0000	0.0000	1.0000	0.0000
LOC104916056	1.0000	0.0000	1.0000	0.0000	1.0000	0.0000	1.0000	0.0000	1.0000	0.0000	1.0000	0.0000	1.0000	0.0000
LOC104916057	0.8249	3.0254	1.0000	0.0000	1.0000	0.0000	1.0000	0.0000	1.0000	0.0000	1.0000	-3.1468	1.0000	0.0000
LOC104916058	0.0868	-0.8722	0.7369	-0.3237	0.5023	-0.4405	1.0000	-0.0276	1.0000	-0.6064	1.0000	-0.0452	1.0000	-0.1839
LOC104916059	1.0000	0.0000	1.0000	0.0000	1.0000	0.0000	1.0000	0.0000	1.0000	0.0000	1.0000	0.0000	1.0000	0.0000
LOC104916060	0.8373	-1.0330	0.6359	0.7837	1.0000	-0.8767	1.0000	0.0070	1.0000	-0.4301	0.8372	1.4025	1.0000	0.4616
LOC104916061	1.0000	0.0000	1.0000	0.0000	1.0000	0.0000	1.0000	0.0000	1.0000	0.0000	1.0000	0.0000	1.0000	0.0000
LOC104916062	1.0000	0.0000	1.0000	0.0000	1.0000	0.0000	1.0000	0.0000	1.0000	0.0000	1.0000	0.0000	1.0000	0.0000
LOC104916063	1.0000	0.0000	1.0000	0.0000	1.0000	0.0000	1.0000	0.0000	1.0000	0.0000	1.0000	0.0000	1.0000	0.0000
LOC104916064	1.0000	0.0000	1.0000	0.0000	1.0000	0.0000	1.0000	0.0000	1.0000	0.0000	1.0000	0.0000	1.0000	0.0000
LOC104916066	1.0000	0.0971	0.5641	0.2748	0.0764	0.7581	0.1183	0.5696	1.0000	0.4249	0.2840	0.6168	0.9287	0.2415
LOC104916067	1.0000	-0.1359	0.4280	1.1754	0.1961	1.4369	0.0540	2.0348	1.0000	-1.0719	1.0000	0.2467	0.9398	-0.4646
LOC104916068	1.0000	-0.1189	0.0909	0.3682	0.3575	0.3862	0.0000	0.8183	1.0000	-0.2622	0.5955	0.2415	0.6942	0.1803

LOC104916069	1.0000	-2.4788	1.0000	2.2509	1.0000	-2.4061	1.0000	0.0000	1.0000	-2.3771	1.0000	2.3456	1.0000	0.0000
LOC104916070	0.2040	-0.2617	0.0221	-0.2677	0.0000	-1.3240	0.0000	-1.1277	1.0000	-0.4029	0.0025	-0.3967	0.5342	-0.2003
LOC104916071	0.0406	0.6728	0.0000	1.0889	0.0000	2.4112	0.0000	2.4139	1.0000	0.0917	0.1595	0.5237	0.7181	0.1019
LOC104916072	0.0081	-1.5832	0.4190	-0.4888	0.0602	0.9094	0.0088	0.9208	1.0000	-0.2828	0.4639	0.8241	0.8937	-0.2689
LOC104916073	1.0000	0.0000	1.0000	0.0000	1.0000	0.0000	1.0000	0.0000	1.0000	0.0000	1.0000	0.0000	1.0000	0.0000
LOC104916074	0.0014	1.3882	0.0000	1.7486	0.0128	1.1615	0.0030	1.1955	1.0000	-0.2479	1.0000	0.1260	1.0000	-0.2048
LOC104916079	0.2261	-1.2623	0.1616	-0.9237	0.1492	-1.2560	0.0152	-1.5495	1.0000	0.3678	0.9755	0.7246	1.0000	0.0830
LOC104916080	0.0902	-3.0458	0.0562	-3.0491	0.1974	1.1698	0.3201	0.9062	1.0000	0.0368	1.0000	0.0495	1.0000	-0.2212
LOC104916081	0.4329	-0.4398	1.0000	0.1100	1.0000	-0.0673	0.7377	-0.2181	1.0000	-0.3651	1.0000	0.1973	0.3601	-0.5111
LOC104916082	1.0000	0.0000	1.0000	-2.3960	1.0000	0.0000	1.0000	-2.3179	1.0000	2.2677	1.0000	0.0000	1.0000	0.0000
LOC104916083	0.0167	-0.9480	0.0018	-0.8477	1.0000	0.0003	0.0000	1.1437	1.0000	-0.3244	1.0000	-0.2126	0.0023	0.8295
LOC104916084	0.3150	-4.2704	0.5021	-1.6423	1.0000	-1.1020	1.0000	-0.5480	1.0000	0.4300	1.0000	3.1947	1.0000	0.9946
LOC104916085	1.0000	0.0000	1.0000	0.0000	1.0000	0.0000	1.0000	0.0000	1.0000	0.0000	1.0000	0.0000	1.0000	0.0000
LOC104916086	0.2617	-0.5843	0.8851	0.1889	1.0000	0.0160	0.0016	0.9755	1.0000	-0.5039	1.0000	0.2857	0.3252	0.4647
LOC104916088	0.1797	-0.3972	0.0349	-0.3822	0.0000	-1.3695	0.0000	-1.4550	1.0000	-0.0447	1.0000	-0.0167	1.0000	-0.1235
LOC104916090	0.3147	-0.3774	0.9944	0.0691	0.0058	0.6762	0.0000	1.0336	1.0000	-0.4539	1.0000	0.0049	0.9980	-0.0890
LOC104916092	1.0000	0.0000	1.0000	0.0000	1.0000	0.0000	1.0000	0.0000	1.0000	0.0000	1.0000	0.0000	1.0000	0.0000
LOC104916093	0.9734	-0.1666	0.0383	0.6428	1.0000	0.0123	0.0002	0.9573	1.0000	-0.7464	1.0000	0.0749	0.8864	0.2056
LOC104916095	0.3654	-0.5785	0.4431	-0.4789	0.0331	-1.0970	0.8491	-0.2457	1.0000	-0.3507	1.0000	-0.2383	0.7287	0.5069
LOC104916099	1.0000	0.0000	1.0000	0.0000	1.0000	0.0000	1.0000	0.0000	1.0000	0.0000	1.0000	0.0000	1.0000	0.0000
LOC104916100	1.0000	0.3491	1.0000	0.0779	1.0000	0.7298	1.0000	0.2583	1.0000	0.5680	1.0000	0.3106	1.0000	0.0824
LOC104916101	0.2415	0.6341	0.0003	1.3175	0.0000	1.5581	0.0004	1.3803	1.0000	-0.3603	0.7567	0.3406	0.1769	-0.5277
LOC104916103	0.3006	-2.0266	1.0000	-0.1688	0.3865	-1.8746	1.0000	0.0069	1.0000	-1.2834	1.0000	0.5858	1.0000	0.6041
LOC104916105	1.0000	0.0000	1.0000	0.0000	1.0000	0.0000	1.0000	0.0000	1.0000	0.0000	1.0000	0.0000	1.0000	0.0000
LOC104916107	0.4597	-0.2915	0.2809	-0.2627	0.0000	-1.3611	0.0000	-1.1618	1.0000	-0.1423	1.0000	-0.0999	1.0000	0.0641
LOC104916108	0.3729	-0.3182	0.0075	-0.5626	0.0000	-1.0804	0.0000	-1.1273	1.0000	0.2450	1.0000	0.0123	0.8120	0.2025
LOC104916109	1.0000	-0.0540	1.0000	-0.1788	0.0552	-1.9098	0.9598	-0.3985	1.0000	-0.6195	0.7489	-0.7350	0.9568	0.8956
LOC104916110	1.0000	0.0000	1.0000	0.0000	1.0000	2.2469	1.0000	0.0000	1.0000	0.0000	1.0000	0.0000	1.0000	-2.2907
LOC104916111	1.0000	0.0000	1.0000	0.0000	1.0000	0.0000	1.0000	0.0000	1.0000	0.0000	1.0000	0.0000	1.0000	0.0000
LOC104916112	0.1765	-0.5772	0.3076	-0.2917	0.0000	1.3653	0.0000	0.7454	1.0000	0.3122	0.1291	0.6103	0.2781	-0.3022
LOC104916113	0.0000	-1.2905	0.0000	-2.4857	0.0008	0.6068	0.0001	0.4282	1.0000	-0.2505	0.0000	-1.4339	0.0003	-0.4225
LOC104916114	0.9545	-1.6907	0.9947	-1.0788	1.0000	-0.1868	0.8310	0.7796	1.0000	0.2544	1.0000	0.8963	0.8620	1.2346
LOC104916115	0.8249	0.1073	0.0019	0.3508	0.2936	0.2373	0.0000	0.6491	1.0000	-0.2558	1.0000	-0.0010	0.4629	0.1611
LOC104916116	0.0000	-0.9872	0.0000	-0.7058	0.0058	-0.5182	0.4088	-0.1413	1.0000	-0.3700	1.0000	-0.0766	1.0000	0.0124
LOC104916117	1.0000	0.0000	1.0000	0.0000	1.0000	0.0000	1.0000	0.0000	1.0000	0.0000	1.0000	0.0000	1.0000	0.0000
LOC104916118	0.2812	-0.7847	0.7525	-0.3718	0.2149	-0.7982	0.4781	-0.5710	1.0000	-0.6672	1.0000	-0.2445	0.9515	-0.4369
LOC104916119	1.0000	0.0000	1.0000	0.0000	1.0000	0.0000	1.0000	0.0000	1.0000	0.0000	1.0000	0.0000	1.0000	0.0000
LOC104916121	1.0000	-2.4776	0.4431	-3.7865	1.0000	0.6746	0.4370	-3.6968	1.0000	1.2635	1.0000	0.0000	1.0000	-3.1316
LOC104916122	0.7225	-0.8102	1.0000	0.0846	1.0000	-0.4077	0.8654	0.6595	1.0000	-0.5954	1.0000	0.3103	1.0000	0.4787
LOC104916123	1.0000	0.0000	1.0000	0.0000	1.0000	2.2425	1.0000	0.0000	1.0000	0.0000	1.0000	0.0000	1.0000	-2.2889
LOC104916126	1.0000	0.0000	1.0000	0.0000	1.0000	2.2425	1.0000	0.0000	1.0000	0.0000	1.0000	0.0000	1.0000	-2.2889
LOC104916127	1.0000	0.0000	1.0000	0.0000	1.0000	0.0000	1.0000	0.0000	1.0000	0.0000	1.0000	0.0000	1.0000	0.0000
LOC104916128	1.0000	0.0000	1.0000	0.0000	1.0000	0.0000	1.0000	0.0000	1.0000	0.0000	1.0000	0.0000	1.0000	0.0000
LOC104916129	1.0000	0.0000	1.0000	0.0000	1.0000	0.0000	1.0000	0.0000	1.0000	0.0000	1.0000	0.0000	1.0000	0.0000
LOC104916130	1.0000	0.0000	1.0000	0.0000	1.0000	0.0000	1.0000	0.0000	1.0000	0.0000	1.0000	0.0000	1.0000	0.0000
LOC104916131	1.0000	0.0000	1.0000	0.0000	1.0000	0.0000	1.0000	0.0000	1.0000	0.0000	1.0000	0.0000	1.0000	0.0000
LOC104916132	1.0000	-0.7243	0.6465	-1.1281	0.4106	1.2071	1.0000	-0.1782	1.0000	0.8264	1.0000	0.4439	1.0000	-0.5533
LOC104916133	1.0000	0.0000	1.0000	0.0000	1.0000	0.0000	1.0000	0.0000	1.0000	0.0000	1.0000	0.0000	1.0000	0.0000
LOC104916134	0.3370	-1.0759	0.4636	-0.7542	1.0000	-0.0202	1.0000	0.1821	1.0000	-0.1215	1.0000	0.2087	1.0000	0.0800
LOC104916135	1.0000	0.0000	1.0000	0.0000	1.0000	0.0000	1.0000	0.0000	1.0000	0.0000	1.0000	0.0000	1.0000	0.0000
LOC104916136	1.0000	0.0000	1.0000	0.0000	1.0000	0.0000	1.0000	0.0000	1.0000	0.0000	1.0000	0.0000	1.0000	0.0000
LOC104916137	1.0000	0.0000	1.0000	0.0000	1.0000	0.0000	1.0000	0.0000	1.0000	0.0000	1.0000	0.0000	1.0000	0.0000
LOC104916138	1.0000	0.0000	1.0000	0.0000	1.0000	0.0000	1.0000	0.0000	1.0000	0.0000	1.0000	0.0000	1.0000	0.0000
LOC104916139	1.0000	0.0000	1.0000	0.0000	1.0000	0.0000	1.0000	0.0000	1.0000	0.0000	1.0000	0.0000	1.0000	0.0000
LOC104916140	1.0000	0.0000	1.0000	2.2509	1.0000	0.0000	1.0000	0.0000	1.0000	0.0000	1.0000	2.3456	1.0000	0.0000
LOC104916142	1.0000	0.5414	1.0000	2.2509	1.0000	-2.4061	1.0000	0.0000	1.0000	-2.3771	1.0000	-0.7966	1.0000	0.0000
LOC104916146	0.0846	0.5459	0.0000	1.5152	0.6392	0.2221	0.0000	1.1416	1.0000	-0.6231	0.3051	0.3580	0.3551	0.3021
LOC104916148	1.0000	0.0000	1.0000	0.0000	1.0000	0.0000	1.0000	0.0000	1.0000	0.0000	1.0000	0.0000	1.0000	0.0000
LOC104916149	0.1381	0.4337	0.0023	0.4849	0.0208	0.5792	0.0506	0.3407	1.0000	-0.0685	1.0000	-0.0041	0.2852	-0.3008
LOC104916151	1.0000	0.0000	1.0000	0.0000	1.0000	0.0000	1.0000	0.0000	1.0000	0.0000	1.0000	0.0000	1.0000	0.0000
LOC104916152	0.3272	-0.8505	0.4516	-0.7082	0.9188	-0.2606	1.0000	-0.1748	1.0000	-0.7086	0.9816	-0.5559	0.8939	-0.6209
LOC104916153	0.0685	0.9070	0.0034	0.9369	0.0499	0.8130	0.0017	1.0230	1.0000	-0.1071	1.0000	-0.0628	1.0000	0.1119
LOC104916154	0.7306	0.3723	0.0157	1.2286	0.0002	1.5411	0.0000	2.4228	1.0000	-1.0082	1.0000	-0.1337	1.0000	-0.1125
LOC104916155	0.0000	-0.9087	0.0000	-0.5591	0.0000	-1.2224	0.0000	-0.8640	1.0000	-0.2284	0.5915	0.1340	0.7200	0.1368
LOC104916157	1.0000	0.0824	0.0594	0.5216	0.6696	0.2417	0.0338	0.5588	1.0000	-0.3757	1.0000	0.0785	1.0000	-0.0514
LOC104916158	0.1416	-1.7538	1.0000	-0.1794	1.0000	-0.1956	0.2097	-1.3299	1.0000	0.1116	0.4132	1.7004	0.6819	-1.0212
LOC104916159	0.0083	-0.7194	0.0161	-0.5707	0.0011	-0.8532	0.0057	-0.6029	1.0000	-0.0968	1.0000	0.0634	0.9536	0.1569
LOC104916160	0.0005	-0.5756	0.0000	-0.7954	0.0306	-0.3759	0.0000	-0.3547	1.0000	0.0765	0.6987	-0.1307	0.7263	0.1034
LOC104916161	1.0000	0.2064	0.7186	-1.3890	0.7156	0.7667	1.0000	-0.2979	1.0000	0.1759	0.9075	-1.4163	0.9124	-0.8801
LOC104916163	0.1212	0.5226	0.0851	0.3500	0.0602	-0.6311	0.0120	-0.5539	1.0000	0.3654	0.8607	0.2045	0.2835	0.4487
LOC104916164	0.2421	1.6576	0.5032	1.3055	0.6597	-1.9401	1.0000	-0.8442	1.0000	-1.0363	0.5434	-1.3934	1.0000	0.0640
LOC104916165	0.1228	0.8546	0.2946	0.7186	1.0000	0.1873	0.8559	-0.4008	1.0000	-1.3685	0.0004	-1.4964	0.0002	-1.9551
LOC104916166	0.5384	3.5542	1.0000	0.6940	1.0000	0.0000	1.0000	-2.3179	1.0000	2.2677	1.0000	-0.4823	1.0000	0.0000
LOC104916167	1.0000	-0.1221	0.4271	1.1697	0.4542	0.7238	1.0000	-0.5262	1.0000	-2.8305	0.1520	-1.5521	0.0000	-4.0853
LOC104916168	1.0000	0.0000	1.0000	0.0000	1.0000	0.0000	1.0000	0.0000	1.0000	0.0000	1.0000	0.0000	1.0000	0.0000
LOC104916169	1.0000	0.0000	1.0000	0.0000	1.0000	0.0000	1.0000	0.0000	1.0000	0.0000	1.0000	0.0000	1.0000	0.0000
LOC104916170	1.0000	0.0000	1.0000	0.0000	1.0000	0.0000	1.0000	0.0000	1.0000	0.0000	1.0000	0.0000	1.0000	0.0000
LOC104916171	1.0000	0.0408	0.4633	-3.7804	0.1161	1.8340	1.0000	0.3973	1.0000	-0.1282	0.6983	-4.0460	0.2997	-1.5615
LOC104916172	1.0000	0.0000	1.0000	-2.3960	1.0000	0.0000	1.0000	-2.3179	1.0000	2.2677	1.0000	0.0000	1.0000	0.0000
LOC104916173	1.0000	0.0000	1.0000	0.0000	1.0000	0.0000	1.0000	0.0000	1.0000	0.0000	1.0000	0.0000	1.0000	0.0000
LOC104916174	1.0000	0.0000	1.0000	0.0000	1.0000	0.0000	1.0000	0.0000	1.0000	0.0000	1.0000	0.0000	1.0000	0.0000
LOC104916175	0.0180	0.7640	0.2205	0.3152	0.0069	-1.0106	0.0012	-0.8123	1.0000	0.3368	1.0000	-0.0977	0.3615	0.5427
LOC104916179	1.0000	0.0000	1.0000	0.0000	1.0000	0.0000	1.0000	0.0000	1.0000	0.0000	1.0000	0.0000	1.0000	0.0000
LOC104916180	0.0210	0.6274	0.0000	1.0448	0.0104	0.6509	0.0001	0.7230	1.0000	-0.4110	1.0000	0.0177	0.1887	-0.3343
LOC104916181	0.0616	0.4945	0.0000	0.9284	0.0000	1.1736	0.0000	1.1519	1.0000	-0.3171	0.9533	0.1294	0.0155	-0.3337
LOC104916182	0.3129	-0.3251	0.8291	-0.0996	0.0001	-1.1028	0.0007	-0.6403	1.0000	-0.2224	1.0000	0.0161	0.7215	0.2481
LOC104916183	0.0139	1.6474	0.0013	1.7593	0.4581	0.7828	0.2072	0.9899	1.0000	-0.1329	1.0000	-0.0091	1.0000	0.0806
LOC104916184	1.0000	0.0000	1.0000	0.0000	1.0000	0.0000	1.0000	0.0000	1.0000	0.0000	1.0000	0.0000	1.0000	0.0000
LOC104916185	1.0000	0.0000	1.0000	0.0000	1.0000	0.0000	1.0000	0.0000	1.0000	0.0000	1.0000	0.0000	1.0000	0.0000
LOC104916187	0.0245	-0.5439	0.0001	-0.5954	0.0000	-1.2189	0.0000	-1.5111	1.0000	-0.0355	1.0000	-0.0745	0.4210	-0.3202
LOC104916188	0.0009	-0.8261	0.0014	-0.5600	0.3276	0.2751	0.0001	0.5570	1.0000	-0.1036	0.9007	0.1766	0.5105	0.1853
LOC104916191	0.6881	1.4496	0.0328	3.0090	0.9361	1.2061	0.4038	2.0762	1.0000	-0.1089	0.6375	1.4633	1.0000	0.7671
LOC104916192	1.0000	2.1851	1.0000	0.0000	1.0000	2.2469	0.7710	3.1709	1.0000	0.0000	1.0000	-2.2959	1.0000	0.9149

LOC104916195	1.0000	-0.0835	0.0796	0.4656	0.1588	0.5204	0.0069	0.6444	1.0000	-0.0801	0.2839	0.4812	1.0000	0.0493
LOC104916196	0.0000	-2.6505	0.0000	-3.1864	0.0000	-0.8185	0.0000	-0.6493	1.0000	-0.0370	0.0684	-0.5615	0.5351	0.1367
LOC104916197	0.0000	-2.6955	0.0000	-2.6781	0.0000	-1.9681	0.0000	-2.5006	1.0000	-0.1197	1.0000	-0.0900	0.0107	-0.6470
LOC104916198	0.4866	-0.3883	0.0383	-0.5020	0.1954	0.5165	0.0821	0.3666	1.0000	0.3596	0.8840	0.2599	0.6952	0.2182
LOC104916199	1.0000	0.0000	1.0000	0.0000	0.8011	3.0840	1.0000	0.0000	1.0000	0.0000	1.0000	0.0000	1.0000	-3.1344
LOC104916200	0.5354	-0.5880	0.4341	-0.4807	0.6820	0.3982	0.1976	0.5778	1.0000	0.3181	0.9945	0.4393	0.5306	0.5038
LOC104916203	1.0000	0.8139	0.3471	1.2314	0.2384	1.6009	0.6343	0.9641	1.0000	0.2609	1.0000	0.6941	1.0000	-0.3687
LOC104916204	0.0116	-0.5354	0.2226	-0.2017	0.0000	-1.0429	0.0012	-0.5317	1.0000	-0.3377	1.0000	0.0079	0.8381	0.1787
LOC104916205	0.1179	-0.3859	0.0000	-0.4364	0.0000	-1.3881	0.0000	-1.2037	1.0000	-0.2508	0.0020	-0.2889	1.0000	-0.0597
LOC104916207	0.0001	1.2835	0.0000	1.2147	0.0067	-1.3035	0.0005	-1.5367	1.0000	-0.2926	0.4816	-0.3520	0.8093	-0.5244
LOC104916208	1.0000	0.0000	1.0000	0.0000	1.0000	0.0000	1.0000	2.3241	1.0000	0.0000	1.0000	0.0000	1.0000	2.3543
LOC104916209	1.0000	2.1900	1.0000	-0.1598	1.0000	0.0000	1.0000	-0.8419	1.0000	3.1126	1.0000	0.9006	1.0000	2.3543
LOC104916210	0.0134	-0.5820	0.0415	-0.3827	0.0005	-0.7687	0.3188	-0.2048	1.0000	-0.2066	1.0000	0.0044	0.1127	0.3614
LOC104916211	0.5369	0.7296	1.0000	-0.1010	0.1837	1.0119	0.0392	1.0370	1.0000	0.1279	0.6647	-0.6845	1.0000	0.1603
LOC104916212	0.0039	-0.5736	0.0000	-0.6640	0.0001	-0.7444	0.0000	-0.5686	1.0000	-0.2691	0.0462	-0.3479	1.0000	-0.0874
LOC104916214	0.9545	-1.6907	1.0000	0.6987	0.2376	1.5996	1.0000	0.8545	1.0000	-1.4945	1.0000	0.9006	0.1717	-2.2460
LOC104916215	0.2469	-0.3494	0.1551	-0.2973	0.9661	-0.0961	0.0116	0.4392	1.0000	-0.2274	0.9162	-0.1650	0.1761	0.3119
LOC104916217	1.0000	0.0000	1.0000	0.0000	1.0000	0.0000	1.0000	0.0000	1.0000	0.0000	1.0000	0.0000	1.0000	0.0000
LOC104916218	1.0000	2.1851	1.0000	0.6940	1.0000	0.0000	1.0000	-2.3179	1.0000	2.2677	1.0000	0.8992	1.0000	0.0000
LOC104916219	0.8249	-3.3412	0.4125	-2.2277	0.8033	-3.2622	0.7296	-1.2146	1.0000	1.0963	1.0000	2.3456	1.0000	3.2049
LOC104916221	0.0085	-1.5808	0.0010	-1.3022	0.0026	-1.8314	0.0116	-0.9635	1.0000	0.2006	0.8732	0.4894	0.1460	1.0686
LOC104916222	0.0360	-1.0270	0.7335	-0.2339	0.0208	-1.1660	0.3043	-0.4500	1.0000	-0.4126	0.8621	0.3940	0.9831	0.3111
LOC104916223	1.0000	0.2206	1.0000	-0.0108	0.6688	0.8867	0.0887	1.3019	1.0000	0.2723	1.0000	0.0575	0.8175	0.6917
LOC104916224	1.0000	0.0000	1.0000	0.0000	1.0000	0.0000	1.0000	0.0000	1.0000	0.0000	1.0000	0.0000	1.0000	0.0000
LOC104916225	0.6007	0.9286	0.7708	0.7730	0.9363	0.5186	0.0133	2.3287	1.0000	-1.0714	0.5382	-1.2255	0.8501	0.7440
LOC104916228	0.0264	-0.5335	0.8222	0.0960	0.0387	0.4498	0.0000	0.9177	1.0000	-0.4543	0.7635	0.1883	1.0000	0.0212
LOC104916229	0.9457	0.2724	0.0263	1.3843	1.0000	-0.1263	0.2989	0.8564	1.0000	-0.8316	1.0000	0.2948	1.0000	0.1550
LOC104916231	1.0000	0.0000	1.0000	0.0000	1.0000	0.0000	1.0000	0.0000	1.0000	0.0000	1.0000	0.0000	1.0000	0.0000
LOC104916232	0.6878	-2.0809	1.0000	2.2509	0.2785	-4.1857	1.0000	2.3256	1.0000	-4.1514	1.0000	0.0470	1.0000	2.3554
LOC104916233	1.0000	0.0000	1.0000	0.0000	1.0000	0.0000	1.0000	0.0000	1.0000	0.0000	1.0000	0.0000	1.0000	0.0000
LOC104916234	1.0000	0.0000	1.0000	0.0000	1.0000	0.0000	1.0000	0.0000	1.0000	0.0000	1.0000	0.0000	1.0000	0.0000
LOC104916235	1.0000	0.0000	1.0000	0.0000	1.0000	0.0000	1.0000	0.0000	1.0000	0.0000	1.0000	0.0000	1.0000	0.0000
LOC104916237	0.2228	-0.4607	0.0444	-0.5908	0.1251	-0.5498	0.0705	-0.4355	1.0000	0.0483	1.0000	-0.0691	0.9940	0.1690
LOC104916241	0.3295	-0.1869	0.0061	-0.2310	0.0000	-0.8612	0.0000	-0.6328	1.0000	-0.1211	0.3241	-0.1529	0.7602	0.1130
LOC104916242	1.0000	0.0000	1.0000	0.0000	1.0000	0.0000	1.0000	0.0000	1.0000	0.0000	1.0000	0.0000	1.0000	0.0000
LOC104916243	0.8226	-3.3425	1.0000	0.0000	0.8011	-3.2629	1.0000	0.0000	1.0000	-3.2304	1.0000	0.0000	1.0000	0.0000
LOC104916244	0.3150	-4.2704	0.2920	-4.1633	1.0000	-0.5759	0.4727	1.1306	1.0000	-0.1343	1.0000	0.0000	0.5565	1.5864
LOC104916245	1.0000	-2.4788	1.0000	-2.3985	1.0000	-2.4061	1.0000	0.0045	1.0000	-0.1045	1.0000	0.0000	1.0000	2.3543
LOC104916246	0.0003	-0.9414	0.0000	-0.5645	0.0000	-1.0896	0.0000	-0.9066	1.0000	0.0420	0.1743	0.4325	0.4307	0.2289
LOC104916247	0.0003	-0.5859	0.0000	-0.4991	0.0114	-0.4356	0.2882	-0.1184	1.0000	-0.1366	1.0000	-0.0373	0.2314	0.1860
LOC104916248	0.9917	0.3319	0.5916	0.7266	1.0000	-0.3267	0.6922	-0.8085	1.0000	-0.6522	1.0000	-0.2419	0.7687	-1.1311
LOC104916249	1.0000	0.0000	1.0000	0.0000	1.0000	0.0000	1.0000	0.0000	1.0000	0.0000	1.0000	0.0000	1.0000	0.0000
LOC104916251	1.0000	-0.1652	1.0000	-0.4768	1.0000	-0.6599	1.0000	0.0046	1.0000	-0.5945	1.0000	-0.8987	1.0000	0.0756
LOC104916253	1.0000	0.0000	1.0000	0.0000	0.8033	3.0891	1.0000	0.0000	1.0000	0.0000	1.0000	0.0000	1.0000	-3.1368
LOC104916254	0.0468	-0.6144	0.0086	-0.5471	0.0011	-0.8825	0.0045	-0.5400	1.0000	-0.1654	1.0000	-0.0847	0.9284	0.1832
LOC104916255	0.1086	-0.3943	1.0000	-0.0069	0.0004	-0.7913	0.1779	-0.2344	1.0000	-0.4741	1.0000	-0.0748	1.0000	0.0895
LOC104916258	0.0581	0.4498	0.0000	0.8617	0.1930	0.3049	0.0000	0.6134	1.0000	-0.2443	0.6357	0.1810	1.0000	0.0710
LOC104916259	0.3203	-0.3466	0.7614	-0.1131	0.0028	0.6776	0.0000	0.6844	1.0000	-0.1800	1.0000	0.0652	0.6649	-0.1683
LOC104916260	0.7482	0.1991	0.0001	0.9402	0.0072	0.7161	0.0000	1.1365	1.0000	-0.6241	1.0000	0.1305	0.5880	-0.1948
LOC104916261	1.0000	0.0000	1.0000	0.0000	1.0000	0.0000	1.0000	0.0000	1.0000	0.0000	1.0000	0.0000	1.0000	0.0000
LOC104916263	0.0049	-0.5443	0.0000	-0.6024	0.0000	-1.0867	0.0000	-0.7745	1.0000	-0.0657	0.9932	-0.1116	0.3943	0.2525
LOC104916264	1.0000	0.0000	1.0000	0.0000	1.0000	0.0000	1.0000	0.0000	1.0000	0.0000	1.0000	0.0000	1.0000	0.0000
LOC104916265	1.0000	2.1900	1.0000	-2.3960	1.0000	2.2425	1.0000	0.0075	1.0000	2.2677	1.0000	-2.2992	1.0000	0.0660
LOC104916266	0.0014	0.9752	0.0000	1.2213	0.0009	1.0319	0.0000	1.0849	1.0000	-0.1671	1.0000	0.0908	1.0000	-0.1071
LOC104916267	0.7471	-0.3977	1.0000	0.0165	0.2237	0.7083	0.0003	1.1048	1.0000	-0.0971	0.9892	0.3298	0.7287	0.3098
LOC104916268	0.0800	-0.5232	0.2175	-0.2917	0.0407	-0.5695	0.3497	-0.2023	1.0000	-0.1204	1.0000	0.1242	0.5195	0.2532
LOC104916269	1.0000	0.0000	1.0000	0.0000	1.0000	0.0000	1.0000	0.0000	1.0000	0.0000	1.0000	0.0000	1.0000	0.0000
LOC104916270	1.0000	0.3646	0.6044	0.9075	0.8850	0.5249	1.0000	-0.5497	1.0000	-0.6924	1.0000	-0.1424	0.1577	-1.7653
LOC104916271	0.3377	-2.0224	0.2561	-1.5760	0.9865	0.5621	0.5688	-0.8396	1.0000	0.5006	1.0000	0.9738	0.7233	-0.8920
LOC104916272	0.4995	0.8948	0.0021	5.6431	0.1846	-2.7131	1.0000	0.0000	1.0000	-4.9238	1.0000	-0.3188	1.0000	-2.2907
LOC104916274	0.1361	-1.4958	0.0002	-4.0288	0.0857	-1.5259	0.4922	-0.6913	1.0000	-0.1320	0.3633	-2.6687	1.0000	0.7096
LOC104916275	0.1064	1.0570	0.0158	1.1867	0.2007	0.7393	0.0006	1.4296	1.0000	-0.5641	0.8904	-0.4214	1.0000	0.1350
LOC104916276	0.0744	0.4397	0.0000	0.6502	0.0000	0.8858	0.0000	1.0358	1.0000	-0.2668	1.0000	-0.0429	0.7929	-0.1090
LOC104916277	0.0016	-3.1918	0.0277	-2.3028	0.2914	0.7957	0.0007	1.6096	1.0000	-0.3237	1.0000	0.5860	0.6422	0.5026
LOC104916278	1.0000	0.0000	1.0000	-1.0051	1.0000	2.2425	0.7710	-3.1693	1.0000	3.1190	1.0000	2.3456	1.0000	-2.2889
LOC104916280	1.0000	-0.1723	1.0000	-0.1305	1.0000	0.0528	0.3498	0.5182	1.0000	-0.4925	0.9402	-0.4377	1.0000	-0.0225
LOC104916281	1.0000	0.0000	1.0000	0.0000	1.0000	0.0000	1.0000	0.0000	1.0000	0.0000	1.0000	0.0000	1.0000	0.0000
LOC104916282	0.9545	1.0644	1.0000	2.2509	1.0000	0.6740	1.0000	2.3241	1.0000	-2.3771	1.0000	-1.3254	1.0000	-0.7782
LOC104916283	0.1923	1.1971	0.5463	0.7991	1.0000	0.1118	1.0000	-0.3934	1.0000	-0.1281	0.9942	-0.5166	0.9949	-0.6325
LOC104916284	1.0000	0.0000	1.0000	0.0000	1.0000	0.0000	1.0000	0.0000	1.0000	0.0000	1.0000	0.0000	1.0000	0.0000
LOC104916285	0.6111	-0.4417	0.8513	-0.2578	1.0000	0.1214	0.9245	-0.1948	1.0000	-0.0537	1.0000	0.1427	0.8346	-0.3642
LOC104916287	0.0051	-1.0643	0.0001	-0.9492	0.3728	0.4508	0.7049	0.1630	1.0000	0.4542	0.3216	0.5839	1.0000	0.1732
LOC104916289	0.0000	-1.1612	0.0000	-1.1624	0.0000	-1.0873	0.0000	-0.6306	1.0000	-0.1765	0.8553	-0.1659	0.2442	0.2858
LOC104916290	0.1388	1.4043	0.0016	1.5126	0.0000	3.9480	0.0000	4.0898	1.0000	0.0662	1.0000	0.1889	0.6093	0.2173
LOC104916291	0.7651	-0.7502	1.0000	-0.1726	0.8503	0.5913	1.0000	0.2591	1.0000	-0.7799	1.0000	-0.1930	0.5007	-1.1095
LOC104916292	0.0000	-2.1722	0.0000	-2.2812	0.0014	-0.7401	0.0000	-0.7154	1.0000	-0.0555	1.0000	-0.1536	1.0000	-0.0261
LOC104916293	0.5780	-1.5569	1.0000	-0.1429	1.0000	-0.4955	0.1709	2.5398	1.0000	-2.1881	1.0000	-0.7956	1.0000	0.8462
LOC104916294	1.0000	-0.0030	0.0150	0.6409	0.0366	-0.5694	0.9555	0.1055	1.0000	-0.3304	0.3755	0.3248	0.2593	0.3520
LOC104916295	0.7266	1.4633	1.0000	-0.6969	0.0002	4.0332	0.0129	2.3291	1.0000	1.2661	1.0000	-0.8736	1.0000	-0.4350
LOC104916296	1.0000	0.0000	1.0000	0.0000	1.0000	0.0000	1.0000	0.0000	1.0000	0.0000	1.0000	0.0000	1.0000	0.0000
LOC104916298	0.6793	0.2860	1.0000	0.1062	0.0021	0.9688	0.0000	1.3949	1.0000	0.1874	1.0000	0.0219	0.0102	0.6220
LOC104916299	0.0099	1.5399	0.0093	1.1612	0.0090	1.4945	0.3979	0.5923	1.0000	0.5042	1.0000	0.1419	0.8120	-0.3916
LOC104916300	1.0000	0.0000	1.0000	0.0000	1.0000	0.0000	1.0000	0.0000	1.0000	0.0000	1.0000	0.0000	1.0000	0.0000
LOC104916301	0.0000	-1.0679	0.0002	-0.8415	0.0000	-1.3917	0.0000	-0.7302	1.0000	-0.2847	1.0000	-0.0458	0.3459	0.3846
LOC104916302	0.3530	0.2871	0.0000	0.7645	0.0000	1.6973	0.0000	1.9743	1.0000	-0.0278	0.0014	0.4623	0.1712	0.2548
LOC104916303	0.4622	0.3890	0.0282	0.5902	0.0229	0.8318	0.0000	1.4860	1.0000	-0.5324	0.5720	-0.3176	0.9380	0.1313
LOC104916305	0.8608	-0.5784	0.7409	0.5209	0.0904	1.2482	0.0007	1.7736	1.0000	-0.3702	0.8946	0.7561	1.0000	0.1600
LOC104916307	0.0000	-0.7750	0.0000	-0.4821	0.0000	-0.8840	0.0000	-0.4400	1.0000	-0.2777	1.0000	0.0273	0.3680	0.1715
LOC104916308	0.0076	-0.5922	0.0000	-0.5570	0.0068	-0.6687	0.0001	-0.4464	1.0000	-0.0607	1.0000	-0.0124	0.7729	0.1687
LOC104916309	0.1991	-0.5169	1.0000	0.0005	0.5703	0.2774	0.0000	0.7756	1.0000	-0.2982	0.7149	0.2341	0.5410	0.2089
LOC104916310	1.0000	0.0000	1.0000	0.0000	1.0000	0.0000	1.0000	2.3241	1.0000	0.0000	1.0000	0.0000	1.0000	2.3543
LOC104916311	1.0000	0.0000	1.0000	-2.3985	1.0000	2.2425	1.0000	0.0057	1.0000	2.2734	1.0000	0.0000	1.0000	0.0661
LOC104916312	0.6881	1.4496	1.0000	0.0000	1.0000	-0.1597	1.0000	0.0000	1.0000	-2.3771	0.6427	-4.0589	1.0000	-2.2907

LOC104916313	0.0002	-0.7321	0.0000	-0.9522	0.0000	-1.3563	0.0000	-0.9868	1.0000	-0.2838	0.0113	-0.4906	1.0000	0.0929
LOC104916314	0.5251	0.2915	1.0000	0.0912	0.8905	-0.1931	0.5022	-0.2687	1.0000	-0.1943	0.4527	-0.3803	0.8586	-0.2600
LOC104916315	1.0000	0.0000	1.0000	0.0000	1.0000	0.0000	1.0000	0.0000	1.0000	0.0000	1.0000	0.0000	1.0000	0.0000
LOC104916316	1.0000	0.0000	1.0000	0.0000	1.0000	0.0000	1.0000	0.0000	1.0000	0.0000	1.0000	0.0000	1.0000	0.0000
LOC104916317	1.0000	0.0264	0.3620	0.2541	0.0001	-1.0603	0.0567	-0.4017	1.0000	-0.4870	0.7015	-0.2467	0.9515	0.1780
LOC104916319	1.0000	0.0000	1.0000	0.0000	1.0000	0.0000	1.0000	0.0000	1.0000	0.0000	1.0000	0.0000	1.0000	0.0000
LOC104916320	1.0000	0.0000	1.0000	0.0000	1.0000	0.0000	1.0000	0.0000	1.0000	0.0000	1.0000	0.0000	1.0000	0.0000
LOC104916321	1.0000	0.0000	1.0000	0.0000	1.0000	0.0000	1.0000	0.0000	1.0000	0.0000	1.0000	0.0000	1.0000	0.0000
LOC104916322	1.0000	0.0000	1.0000	0.0000	1.0000	0.0000	1.0000	0.0000	1.0000	0.0000	1.0000	0.0000	1.0000	0.0000
LOC104916323	1.0000	0.0000	1.0000	0.0000	1.0000	0.0000	1.0000	0.0000	1.0000	0.0000	1.0000	0.0000	1.0000	0.0000
LOC104916324	1.0000	-0.0432	0.0563	-2.0870	0.0295	1.9892	0.0000	2.0434	1.0000	1.3997	1.0000	-0.6328	0.0039	1.4635
LOC104916326	1.0000	0.0000	1.0000	0.0000	1.0000	0.0000	1.0000	0.0000	1.0000	0.0000	1.0000	0.0000	1.0000	0.0000
LOC104916327	0.2245	1.5833	0.0680	-2.3611	0.1095	1.6482	1.0000	-0.1446	1.0000	1.1484	0.0445	-2.7942	0.8297	-0.6420
LOC104916328	0.3576	0.1972	0.0001	0.3417	1.0000	-0.0139	0.5260	0.0781	1.0000	-0.0147	0.5166	0.1420	0.8908	0.0819
LOC104916329	1.0000	0.0000	1.0000	0.0000	1.0000	0.0000	1.0000	0.0000	1.0000	0.0000	1.0000	0.0000	1.0000	0.0000
LOC104916330	1.0000	0.0000	1.0000	0.0000	1.0000	0.0000	1.0000	0.0000	1.0000	0.0000	1.0000	0.0000	1.0000	0.0000
LOC104916331	1.0000	0.0000	1.0000	0.0000	1.0000	0.0000	1.0000	0.0000	1.0000	0.0000	1.0000	0.0000	1.0000	0.0000
LOC104916332	1.0000	0.0000	1.0000	0.0000	1.0000	0.0000	1.0000	0.0000	1.0000	0.0000	1.0000	0.0000	1.0000	0.0000
LOC104916334	1.0000	0.0000	1.0000	0.0000	1.0000	0.0000	1.0000	0.0000	1.0000	0.0000	1.0000	0.0000	1.0000	0.0000
LOC104916335	0.8293	0.1448	0.0120	0.4712	0.2946	-0.3461	0.2687	0.2495	1.0000	-0.3861	1.0000	-0.0490	0.6739	0.2149
LOC104916336	0.0000	-0.9109	0.0000	-0.8747	0.0001	0.6582	0.0000	0.7192	1.0000	-0.1073	1.0000	-0.0590	1.0000	-0.0415
LOC104916337	0.1223	-0.4266	0.6879	-0.1209	0.0000	-1.1921	0.0194	-0.4368	1.0000	-0.2065	1.0000	0.1114	0.0360	0.5540
LOC104916338	1.0000	0.0120	0.0325	0.5886	0.0090	0.7598	0.0000	1.4628	1.0000	-0.4590	1.0000	0.1306	0.5336	0.2516
LOC104916339	0.0034	-1.1641	0.0006	-1.1719	0.9805	0.1362	0.0017	0.7734	1.0000	-0.4520	0.6954	-0.4448	0.8515	0.1967
LOC104916340	0.0866	-0.8219	1.0000	-0.1950	0.2993	0.4718	0.4088	0.4596	1.0000	-0.5465	1.0000	0.0921	0.2180	-0.5467
LOC104916343	0.0654	2.8419	1.0000	0.5210	0.2858	2.1449	0.9096	-1.3717	1.0000	1.2628	0.8371	-1.0388	0.5105	-2.2503
LOC104916345	1.0000	0.0000	1.0000	2.2508	1.0000	2.2425	1.0000	0.0000	1.0000	0.0000	1.0000	2.3456	1.0000	-2.2889
LOC104916346	0.7651	0.2511	0.4709	0.2414	0.0430	0.6943	0.0000	1.1263	1.0000	-0.3133	0.7394	-0.3110	1.0000	0.1266
LOC104916348	1.0000	2.1851	1.0000	2.2508	1.0000	2.2469	1.0000	0.0000	1.0000	0.0000	1.0000	0.0495	1.0000	-2.2907
LOC104916349	1.0000	2.1851	1.0000	-0.1449	1.0000	2.2425	1.0000	0.8545	1.0000	2.2677	1.0000	0.0495	1.0000	0.9168
LOC104916350	0.0000	-2.4884	0.0000	-2.4137	0.0191	0.8542	0.0000	1.2758	1.0000	-0.1603	1.0000	-0.0736	0.7064	0.2659
LOC104916351	0.1746	1.0855	0.0058	2.0903	0.2948	0.8478	0.0015	2.2723	1.0000	-1.3505	1.0000	-0.3367	1.0000	0.0790
LOC104916352	1.0000	0.5464	1.0000	2.2509	1.0000	-2.4055	1.0000	2.3241	1.0000	-2.3758	1.0000	-0.8001	1.0000	2.3543
LOC104916353	1.0000	0.1527	1.0000	0.0919	1.0000	0.0775	0.5784	0.5785	1.0000	-0.1207	1.0000	-0.1720	0.9553	0.3807
LOC104916356	0.8081	-0.1883	0.0950	0.3802	0.0003	0.9194	0.0000	1.4212	1.0000	-0.3940	0.9623	0.1875	0.8944	0.1173
LOC104916357	0.0127	-1.5570	0.0053	-1.1738	1.0000	0.1452	0.1564	0.5280	1.0000	0.2443	0.9005	0.6385	0.2301	0.6316
LOC104916358	0.9464	0.2594	0.5839	-0.6141	0.1649	-1.0563	0.7018	-0.5643	1.0000	-1.1763	0.0007	-2.0381	0.7383	-0.6717
LOC104916359	1.0000	0.0000	1.0000	0.0000	1.0000	2.2424	1.0000	0.0000	1.0000	0.0000	1.0000	0.0000	1.0000	-2.2889
LOC104916360	1.0000	-2.4788	1.0000	0.0000	1.0000	-0.1597	1.0000	2.3241	1.0000	-2.3771	1.0000	0.0000	1.0000	0.0640
LOC104916362	1.0000	0.0000	1.0000	0.0000	1.0000	0.0000	1.0000	0.0000	1.0000	0.0000	1.0000	0.0000	1.0000	0.0000
LOC104916363	0.5675	-0.6961	0.8854	0.3609	0.0788	1.0335	0.0023	1.5369	1.0000	-0.6704	1.0000	0.3978	1.0000	-0.1616
LOC104916364	1.0000	0.0000	1.0000	0.0000	1.0000	2.2469	1.0000	0.0000	1.0000	0.0000	1.0000	0.0000	1.0000	-2.2907
LOC104916365	0.5141	0.7414	0.2436	0.8050	1.0000	-0.1994	0.9980	-0.3098	1.0000	0.1898	1.0000	0.2639	1.0000	0.0799
LOC104916367	0.0000	-1.0812	0.0000	-1.0666	0.0000	-1.3505	0.0000	-0.9050	1.0000	-0.1928	0.6878	-0.1660	0.5109	0.2580
LOC104916369	0.9256	0.1448	0.0012	0.8081	0.0319	0.6542	0.0000	1.0994	1.0000	-0.6583	1.0000	0.0180	0.7293	-0.2041
LOC104916371	0.0017	1.2890	0.0000	1.6459	0.0000	2.2505	0.0000	2.1264	1.0000	-0.0600	0.8372	0.3145	0.5421	-0.1742
LOC104916372	0.5416	0.5253	0.0000	1.6758	0.0000	2.2221	0.0000	2.8277	1.0000	-0.3983	0.1670	0.7661	0.6865	0.2172
LOC104916374	0.0005	-0.8994	0.0045	-0.5244	0.0000	-1.2274	0.0000	-0.9752	1.0000	-0.3423	1.0000	0.0452	1.0000	-0.0841
LOC104916375	1.0000	-0.8610	1.0000	0.0000	1.0000	-0.7088	1.0000	0.0000	1.0000	-3.7658	1.0000	-3.1429	1.0000	-3.1368
LOC104916377	0.0000	-1.6870	0.0008	-0.7261	0.0000	-1.8413	0.0014	-0.7089	1.0000	-0.6980	0.8171	0.2753	0.3780	0.4398
LOC104916378	0.9949	-0.1065	0.1647	-0.3452	1.0000	0.0213	0.9238	-0.0944	1.0000	-0.1599	0.3137	-0.3881	0.5027	-0.2727
LOC104916379	0.1034	1.2512	0.0245	1.6245	0.0688	1.3140	1.0000	0.3590	1.0000	-0.6306	1.0000	-0.2460	0.0386	-1.5809
LOC104916380	1.0000	0.4652	0.4632	1.1783	0.7628	-1.4078	0.9096	-1.3726	1.0000	-0.8154	1.0000	-0.0983	1.0000	-0.7795
LOC104916381	0.5924	-0.1399	0.9413	0.0410	0.0000	-0.9013	0.0000	-0.6386	1.0000	-0.1912	1.0000	0.0017	0.9980	0.0775
LOC104916382	0.0000	-1.8031	0.0000	-0.9694	0.3788	-0.3843	0.5209	0.1689	1.0000	-0.2196	0.1595	0.6254	0.4557	0.3407
LOC104916383	0.7346	0.1312	0.0003	0.3876	0.0000	0.8571	0.0000	1.0630	1.0000	-0.2711	1.0000	-0.0034	1.0000	-0.0605
LOC104916384	0.9303	0.1349	0.0374	0.5107	0.0000	1.0964	0.0000	0.9960	1.0000	-0.0158	0.4650	0.3730	0.9853	-0.1096
LOC104916385	1.0000	0.1216	0.0013	2.0607	1.0000	-0.1973	0.0689	1.4159	1.0000	-1.2014	0.5855	0.7477	1.0000	0.4160
LOC104916386	0.5946	0.1462	0.0000	0.5250	0.0045	0.5179	0.0000	0.5773	1.0000	-0.3048	0.8915	0.0860	0.1139	-0.2398
LOC104916387	0.5086	-3.8796	1.0000	-2.3960	1.0000	-0.7107	0.4037	2.0762	1.0000	-1.4945	1.0000	0.0000	0.9863	1.2952
LOC104916388	1.0000	-0.0932	1.0000	-0.0104	0.0230	1.9524	0.2228	1.0721	1.0000	0.5211	1.0000	0.6233	1.0000	-0.3504
LOC104916390	0.3544	-0.7733	0.7465	-0.2150	0.0327	1.2287	0.0000	1.9083	1.0000	-0.6007	1.0000	-0.0271	1.0000	0.0866
LOC104916391	1.0000	0.0000	1.0000	0.0000	1.0000	0.0000	1.0000	0.0000	1.0000	0.0000	1.0000	0.0000	1.0000	0.0000
LOC104916392	1.0000	0.0000	1.0000	0.0000	1.0000	0.0000	1.0000	0.0000	1.0000	0.0000	1.0000	0.0000	1.0000	0.0000
LOC104916393	1.0000	0.0577	0.7674	-0.2026	0.6331	-0.3232	0.0346	0.6209	1.0000	-0.3263	0.3409	-0.5745	0.1352	0.6252
LOC104916394	0.0063	-2.1837	0.0001	-2.8010	1.0000	0.1608	0.9096	0.2583	1.0000	-0.2932	1.0000	-0.8987	1.0000	-0.1870
LOC104916395	0.3374	0.5152	0.2920	0.3702	0.7587	0.3178	0.6582	-0.2770	1.0000	0.3953	0.9155	0.2631	1.0000	-0.1972
LOC104916396	1.0000	-0.2432	0.8204	-0.3859	0.2747	-0.8321	1.0000	0.0711	1.0000	-0.3164	0.9769	-0.4438	0.7824	0.5956
LOC104916399	1.0000	0.0000	1.0000	0.0000	1.0000	0.0000	1.0000	0.0000	1.0000	0.0000	1.0000	0.0000	1.0000	0.0000
LOC104916400	0.6317	-0.4020	0.0238	-1.0646	0.0013	1.2140	0.2626	0.5252	1.0000	-0.0572	0.5831	-0.7084	0.0767	-0.7397
LOC104916401	1.0000	-0.0756	0.4459	0.4253	0.5460	-0.5076	0.6645	-0.3546	1.0000	0.0877	0.5435	0.5942	1.0000	0.2426
LOC104916402	0.7546	0.3272	0.7045	0.3096	0.0016	1.2436	0.0000	1.5393	1.0000	-0.2201	1.0000	-0.2254	1.0000	0.0813
LOC104916403	0.8226	3.0199	1.0000	-2.3960	1.0000	2.2424	1.0000	-2.3178	1.0000	2.2677	1.0000	-3.1429	1.0000	-2.2889
LOC104916404	0.3080	-0.2870	0.5025	-0.1497	1.0000	0.0655	0.0001	0.5546	1.0000	-0.2401	1.0000	-0.0894	0.3299	0.2561
LOC104916405	1.0000	-0.1193	0.0004	0.7554	0.6751	-0.2501	0.0031	0.6601	1.0000	-0.8003	1.0000	0.0863	1.0000	0.1169
LOC104916408	1.0000	0.0100	0.0171	1.2150	0.0325	0.8496	0.0000	1.6385	1.0000	-1.0748	1.0000	0.1401	0.7617	-0.2781
LOC104916409	0.0747	-0.3492	0.6685	-0.0810	1.0000	-0.0020	0.0008	0.3366	1.0000	-0.2646	1.0000	0.0161	0.9202	0.0785
LOC104916410	1.0000	-0.0938	1.0000	-0.6944	1.0000	-0.8798	0.4370	-3.6968	1.0000	-0.8186	0.9083	-1.4181	0.7287	-3.6637
LOC104916411	1.0000	0.0000	1.0000	0.0000	1.0000	0.0000	1.0000	0.0000	1.0000	0.0000	1.0000	0.0000	1.0000	0.0000
LOC104916413	1.0000	0.0000	1.0000	0.0000	1.0000	0.0000	1.0000	0.0000	1.0000	0.0000	1.0000	0.0000	1.0000	0.0000
LOC104916414	0.9545	1.0672	1.0000	-0.6943	0.1183	2.5429	0.0324	2.1395	1.0000	1.2654	1.0000	-0.4744	0.7915	0.8682
LOC104916416	1.0000	2.1900	1.0000	0.0000	1.0000	0.0000	1.0000	0.0000	1.0000	0.0000	1.0000	-2.2992	1.0000	0.0000
LOC104916417	1.0000	-0.2920	1.0000	0.0000	1.0000	-2.4055	1.0000	0.0000	1.0000	-2.3758	1.0000	-2.2959	1.0000	0.0000
LOC104916418	0.6853	-0.3606	0.2209	0.6934	0.0738	0.7765	0.0000	1.7459	1.0000	-1.1220	1.0000	-0.0566	1.0000	-0.1475
LOC104916420	0.5086	-3.8790	1.0000	-0.8607	0.8151	0.7542	1.0000	0.2593	1.0000	0.5642	0.8607	3.7288	1.0000	0.0780
LOC104916421	0.0140	1.0460	0.0018	1.1461	0.0029	1.1480	0.0000	1.5370	1.0000	-0.4439	0.8161	-0.3311	1.0000	-0.0488
LOC104916422	1.0000	0.0000	1.0000	2.2509	1.0000	0.0000	1.0000	2.3241	1.0000	0.0000	1.0000	2.3456	1.0000	2.3543
LOC104916423	1.0000	0.0000	1.0000	0.0000	1.0000	0.0000	1.0000	0.0000	1.0000	0.0000	1.0000	0.0000	1.0000	0.0000
LOC104916424	1.0000	0.0509	1.0000	0.2287	1.0000	0.5040	1.0000	0.2247	1.0000	0.8165	1.0000	1.0164	1.0000	0.5450
LOC104916425	0.8366	-0.3084	0.2495	0.7664	0.3104	0.6746	0.0020	1.4535	1.0000	-0.9844	1.0000	0.1073	1.0000	-0.1909
LOC104916426	0.3192	4.2259	1.0000	-0.1616	0.0045	5.6386	0.5794	1.4858	1.0000	3.1190	1.0000	-1.1612	0.6747	-1.0059
LOC104916427	1.0000	0.0000	1.0000	0.0000	1.0000	0.0000	1.0000	0.0000	1.0000	0.0000	1.0000	0.0000	1.0000	0.0000
LOC104916428	1.0000	0.0000	1.0000	0.0000	1.0000	0.0000	1.0000	0.0000	1.0000	0.0000	1.0000	0.0000	1.0000	0.0000

LOC104916429	1.0000	0.0000	1.0000	0.0000	1.0000	0.0000	1.0000	0.0000	1.0000	0.0000	1.0000	0.0000	1.0000	0.0000
LOC104916430	1.0000	0.0000	1.0000	0.0000	1.0000	0.0000	1.0000	0.0000	1.0000	0.0000	1.0000	0.0000	1.0000	0.0000
LOC104916431	1.0000	0.0000	1.0000	0.0000	1.0000	0.0000	1.0000	0.0000	1.0000	0.0000	1.0000	0.0000	1.0000	0.0000
LOC104916432	1.0000	0.0000	1.0000	0.0000	1.0000	0.0000	1.0000	0.0000	1.0000	0.0000	1.0000	0.0000	1.0000	0.0000
LOC104916434	1.0000	2.1851	1.0000	0.0000	1.0000	0.0000	1.0000	0.0000	1.0000	0.0000	1.0000	-2.2959	1.0000	0.0000
LOC104916435	0.0018	-0.8485	0.0629	-0.2913	0.0051	0.6851	0.0000	1.3197	1.0000	-0.4505	1.0000	0.1190	0.2488	0.1896
LOC104916438	0.0000	-0.9709	0.0000	-1.0926	0.0000	-1.3861	0.0000	-1.2644	1.0000	-0.1350	0.1701	-0.2442	1.0000	-0.0075
LOC104916440	0.0583	-0.4619	0.0000	-0.7237	1.0000	-0.0507	0.0442	-0.2996	1.0000	0.1552	1.0000	-0.0942	1.0000	-0.0884
LOC104916443	1.0000	-0.6461	0.5029	-1.6425	1.0000	0.4589	0.9049	0.5705	1.0000	0.1269	1.0000	-0.8625	1.0000	0.2429
LOC104916444	1.0000	0.0000	1.0000	2.2533	1.0000	2.2424	1.0000	0.0000	1.0000	0.0000	1.0000	2.3480	1.0000	-2.2889
LOC104916445	1.0000	0.0000	1.0000	0.0000	1.0000	0.0000	1.0000	0.0000	1.0000	0.0000	1.0000	0.0000	1.0000	0.0000
LOC104916447	1.0000	0.0000	1.0000	0.0000	1.0000	0.0000	1.0000	0.0000	1.0000	0.0000	1.0000	0.0000	1.0000	0.0000
LOC104916448	0.5308	-0.2951	0.8606	-0.1185	0.0008	-1.0045	0.0006	-0.7688	1.0000	-0.3239	1.0000	-0.1344	1.0000	-0.0828
LOC104916449	1.0000	0.0000	1.0000	0.0000	1.0000	2.2469	1.0000	2.3241	1.0000	0.0000	1.0000	0.0000	1.0000	0.0640
LOC104916451	0.6149	-0.3755	0.7149	0.1858	1.0000	-0.0102	0.0343	0.5384	1.0000	-0.2608	0.9017	0.3131	0.5805	0.2963
LOC104916452	0.0001	-0.6123	0.0000	-0.4815	0.0000	-0.6969	0.0000	-0.4655	1.0000	-0.2034	1.0000	-0.0603	1.0000	0.0338
LOC104916453	0.1166	-0.9938	0.0981	-0.6179	0.3913	0.4733	0.0088	0.7324	1.0000	0.2444	0.6442	0.6368	0.2215	0.5088
LOC104916454	1.0000	0.5381	0.7666	-3.2534	1.0000	-0.1591	0.7701	-3.1666	1.0000	0.7365	1.0000	-3.1384	1.0000	-2.2907
LOC104916455	0.0508	-0.7623	0.0039	-0.6381	0.0122	0.7046	0.0000	0.8060	1.0000	0.3383	0.4307	0.4773	0.0435	0.4435
LOC104916456	1.0000	0.0000	1.0000	0.0000	1.0000	0.0000	1.0000	0.0000	1.0000	0.0000	1.0000	0.0000	1.0000	0.0000
LOC104916458	0.2097	2.4021	0.0450	4.7755	0.0787	2.7127	0.1396	4.3976	1.0000	-2.3758	1.0000	-0.1261	0.9617	-0.7393
LOC104916459	0.2992	0.4334	0.5378	0.2574	0.6317	-0.2628	0.0006	0.8378	1.0000	-0.5411	0.0402	-0.7059	0.0923	0.5636
LOC104916460	0.8202	0.3436	0.7378	0.3972	0.8788	-0.4058	0.3532	-0.8257	1.0000	-0.4139	1.0000	-0.3508	0.5482	-0.8300
LOC104916462	0.0083	-2.2980	0.0000	-6.3578	0.4160	0.7317	0.0442	1.0379	1.0000	-0.2062	0.4563	-4.3639	1.0000	0.1120
LOC104916463	0.0000	-2.2137	0.0000	-1.9405	0.7534	0.1849	0.0000	0.7693	1.0000	-0.4509	1.0000	-0.1617	0.7412	0.1413
LOC104916465	1.0000	0.0002	0.0481	0.5368	0.4901	-0.3736	0.9873	-0.1064	1.0000	0.0418	0.1327	0.5913	0.7393	0.3151
LOC104916466	1.0000	0.1559	0.6080	0.6577	0.3441	0.9321	0.3589	0.8408	1.0000	0.0112	1.0000	0.5260	1.0000	-0.0696
LOC104916468	0.5126	0.6727	0.1851	0.8619	0.3713	0.7380	0.0679	1.0998	1.0000	-0.3327	1.0000	-0.1341	1.0000	0.0316
LOC104916469	1.0000	0.0000	1.0000	0.0000	1.0000	0.0000	1.0000	0.0000	1.0000	0.0000	1.0000	0.0000	1.0000	0.0000
LOC104916470	0.0088	5.4449	0.1621	2.3788	0.0312	4.9489	0.9096	1.3850	1.0000	2.2677	1.0000	-0.6942	0.7040	-1.2668
LOC104916471	1.0000	0.0000	1.0000	2.2509	1.0000	0.0000	1.0000	0.0000	1.0000	0.0000	1.0000	2.3456	1.0000	0.0000
LOC104916472	0.4430	0.5842	0.1086	0.7626	0.3276	0.6430	0.0672	0.9165	1.0000	-0.2844	1.0000	-0.0930	1.0000	-0.0040
LOC104916474	0.8319	-0.3153	1.0000	-0.0737	0.0005	-2.3031	1.0000	-0.1070	1.0000	-0.3830	1.0000	-0.1298	0.0337	1.8210
LOC104916475	0.0029	-0.7405	0.0035	-0.4393	0.0019	-0.7589	0.0006	-0.4991	1.0000	-0.3281	1.0000	-0.0134	1.0000	-0.0620
LOC104916478	1.0000	0.0000	1.0000	2.2533	1.0000	0.0000	1.0000	0.0000	1.0000	0.0000	1.0000	2.3480	1.0000	0.0000
LOC104916481	0.1617	0.5928	0.0269	0.7121	0.0000	2.1338	0.0000	2.1743	1.0000	-0.1196	1.0000	0.0162	1.0000	-0.0676
LOC104916482	1.0000	2.1900	1.0000	-0.1429	1.0000	2.2469	1.0000	-2.3178	1.0000	2.2677	1.0000	0.0489	1.0000	-2.2907
LOC104916483	0.9545	-1.6935	1.0000	0.0000	1.0000	-0.7130	1.0000	2.3241	1.0000	-3.7635	1.0000	-2.2959	1.0000	-0.7795
LOC104916484	1.0000	0.0000	1.0000	0.0000	1.0000	0.0000	1.0000	0.0000	1.0000	0.0000	1.0000	0.0000	1.0000	0.0000
LOC104916485	0.6881	1.4496	1.0000	-0.1625	0.6654	1.5927	0.7190	1.2294	1.0000	0.7357	1.0000	-0.8622	1.0000	0.3815
LOC104916486	0.0000	-1.0933	0.0000	-1.0001	0.3801	-0.2965	0.3161	0.1918	1.0000	-0.3716	0.7364	-0.2658	1.0000	0.1236
LOC104916487	1.0000	0.0000	1.0000	0.0000	1.0000	0.0000	1.0000	0.0000	1.0000	0.0000	1.0000	0.0000	1.0000	0.0000
LOC104916488	1.0000	0.0000	1.0000	0.0000	1.0000	0.0000	1.0000	0.0000	1.0000	0.0000	1.0000	0.0000	1.0000	0.0000
LOC104916489	1.0000	0.0000	1.0000	0.0000	1.0000	0.0000	1.0000	0.0000	1.0000	0.0000	1.0000	0.0000	1.0000	0.0000
LOC104916490	0.1926	1.3411	0.0856	1.1784	0.0356	1.7584	0.4462	0.7586	1.0000	0.5823	1.0000	0.4351	0.9684	-0.4109
LOC104916491	1.0000	-2.4776	1.0000	0.0000	1.0000	-0.1591	1.0000	2.3256	1.0000	-2.3758	1.0000	0.0000	1.0000	0.0649
LOC104916492	1.0000	0.0000	1.0000	0.0000	1.0000	0.0000	1.0000	0.0000	1.0000	0.0000	1.0000	0.0000	1.0000	0.0000
LOC104916493	0.8226	3.0199	1.0000	0.0000	1.0000	0.0000	1.0000	0.0000	1.0000	0.0000	1.0000	-3.1429	1.0000	0.0000
LOC104916494	1.0000	0.0000	1.0000	0.0000	1.0000	0.0000	1.0000	0.0000	1.0000	0.0000	1.0000	0.0000	1.0000	0.0000
LOC104916496	0.5086	1.1758	1.0000	0.3654	1.0000	0.1151	1.0000	0.5370	1.0000	-1.0498	0.3524	-1.8561	1.0000	-0.6168
LOC104916497	1.0000	0.0000	1.0000	0.0000	1.0000	0.0000	1.0000	0.0000	1.0000	0.0000	1.0000	0.0000	1.0000	0.0000
LOC104916498	0.6585	-1.2842	0.0098	-3.0135	0.1132	1.5027	0.9297	0.3300	1.0000	1.2378	1.0000	-0.4766	1.0000	0.0798
LOC104916499	0.4654	-0.2314	0.7614	0.1222	0.0351	0.4828	0.0000	0.8159	1.0000	-0.1061	0.1761	0.2604	0.1485	0.2314
LOC104916500	0.0804	-0.7808	0.6958	-0.2922	1.0000	0.0003	0.2216	0.5060	1.0000	-0.7807	1.0000	-0.2782	0.8133	-0.2698
LOC104916501	1.0000	0.0000	1.0000	0.0000	1.0000	0.0000	1.0000	0.0000	1.0000	0.0000	1.0000	0.0000	1.0000	0.0000
LOC104916502	1.0000	-0.0913	1.0000	-0.0919	0.7266	-0.2386	0.3945	0.2506	1.0000	-0.2972	0.7577	-0.2830	0.8138	0.2014
LOC104916504	1.0000	0.0000	1.0000	0.0000	1.0000	0.0000	1.0000	0.0000	1.0000	0.0000	1.0000	0.0000	1.0000	0.0000
LOC104916505	0.4583	0.4135	0.0049	1.0045	1.0000	0.0182	0.1288	0.5128	1.0000	-0.4077	1.0000	0.1947	1.0000	0.0972
LOC104916506	1.0000	-0.3418	1.0000	-0.1706	0.4809	1.3159	0.0523	1.8515	1.0000	0.2648	1.0000	0.4467	0.8315	0.8027
LOC104916507	1.0000	0.0000	1.0000	0.0000	1.0000	0.0000	1.0000	0.0000	1.0000	0.0000	1.0000	0.0000	1.0000	0.0000
LOC104916508	0.0000	-1.2060	0.1239	-0.3732	0.3078	-0.3906	0.8904	0.0960	1.0000	-0.4413	0.4062	0.4057	1.0000	0.0527
LOC104916510	1.0000	0.0000	1.0000	0.0000	1.0000	0.0000	1.0000	0.0000	1.0000	0.0000	1.0000	0.0000	1.0000	0.0000
LOC104916511	0.2624	-0.2326	0.0011	-0.3399	0.0000	-1.4658	0.0000	-1.3349	1.0000	-0.1533	0.1234	-0.2484	1.0000	-0.0159
LOC104916512	1.0000	2.1900	1.0000	-2.3960	0.2829	3.9942	1.0000	0.8538	1.0000	2.2677	1.0000	-2.2992	1.0000	-0.8429
LOC104916513	1.0000	0.0537	0.8993	0.0868	0.7728	0.1607	0.2046	0.2802	1.0000	-0.2191	0.9094	-0.1727	1.0000	-0.0919
LOC104916514	1.0000	0.0000	1.0000	0.0000	1.0000	0.0000	1.0000	0.0000	1.0000	0.0000	1.0000	0.0000	1.0000	0.0000
LOC104916515	0.3945	0.6851	0.0347	0.9248	1.0000	0.2076	1.0000	0.0076	1.0000	-0.0779	1.0000	0.1740	1.0000	-0.2720
LOC104916516	0.3379	0.4135	0.1724	0.4253	0.4646	-0.3687	0.0019	-1.0268	1.0000	-0.0341	1.0000	-0.0107	0.2056	-0.6895
LOC104916517	0.0431	1.3228	0.0055	1.1456	0.0003	1.8938	0.7710	0.3398	1.0000	0.7927	0.4230	0.6306	0.2258	-0.7555
LOC104916518	1.0000	0.3412	0.9326	0.5806	0.1032	1.2236	0.0686	1.4161	1.0000	-0.7628	0.9998	-0.5143	0.7715	-0.5697
LOC104916519	1.0000	0.0000	1.0000	0.0000	1.0000	0.0000	1.0000	0.0000	1.0000	0.0000	1.0000	0.0000	1.0000	0.0000
LOC104916520	1.0000	0.0000	1.0000	0.0000	1.0000	2.2469	0.4548	3.7078	1.0000	0.0000	1.0000	0.0000	1.0000	1.4496
LOC104916521	0.0226	-0.9469	0.0038	-0.9029	0.2840	-0.4917	1.0000	-0.0212	1.0000	-0.4349	0.7874	-0.3826	1.0000	0.0393
LOC104916522	1.0000	0.0000	1.0000	0.0000	1.0000	0.0000	1.0000	0.0000	1.0000	0.0000	1.0000	0.0000	1.0000	0.0000
LOC104916523	0.0000	-0.6345	0.0000	-0.6453	0.0001	-0.6078	0.0000	-0.3734	1.0000	0.0098	1.0000	0.0114	0.0075	0.2493
LOC104916524	1.0000	-0.0225	0.5143	0.1930	0.0000	0.9662	0.0000	1.1844	1.0000	-0.2522	1.0000	-0.0236	1.0000	-0.0280
LOC104916525	1.0000	-0.0353	0.0492	-0.2441	0.0033	0.5177	0.0258	0.2456	1.0000	0.1456	1.0000	-0.0498	0.6326	-0.1199
LOC104916526	1.0000	0.0000	1.0000	0.0000	1.0000	0.0000	1.0000	0.0000	1.0000	0.0000	1.0000	0.0000	1.0000	0.0000
LOC104916527	0.0081	-0.8810	0.0593	-0.5394	0.0404	-0.6912	0.4046	-0.3053	1.0000	-0.4602	1.0000	-0.1046	1.0000	-0.0677
LOC104916528	0.2167	0.2399	0.0000	0.6888	0.2632	-0.2504	0.0001	0.3631	1.0000	-0.2981	0.3604	0.1636	0.0551	0.3219
LOC104916529	1.0000	-0.2920	0.7674	3.0955	1.0000	-0.1624	1.0000	2.3256	1.0000	-2.3758	1.0000	0.9035	1.0000	0.0661
LOC104916532	1.0000	0.2716	0.0062	1.8373	0.0003	1.7051	0.0000	2.8291	1.0000	-1.4629	1.0000	0.1124	0.7955	-0.3326
LOC104916533	0.5086	3.5431	1.0000	-0.1429	1.0000	0.0000	1.0000	0.0062	1.0000	2.2677	1.0000	-1.3246	1.0000	2.3543
LOC104916534	0.0535	0.8393	0.0020	1.0488	0.4655	0.4441	0.0002	1.1847	1.0000	-0.2071	1.0000	0.0142	0.2599	0.5401
LOC104916535	0.0837	0.7844	0.0481	0.6607	0.0000	1.6288	0.0000	1.4584	1.0000	0.0062	1.0000	-0.1055	0.9206	-0.1591
LOC104916536	0.6011	-0.6787	0.3620	0.7223	0.3417	0.6707	0.4838	0.6183	1.0000	-0.8082	0.8841	0.6115	0.2150	-0.8482
LOC104916537	1.0000	0.0000	1.0000	0.0000	1.0000	0.0000	1.0000	0.0000	1.0000	0.0000	1.0000	0.0000	1.0000	0.0000
LOC104916538	0.0304	0.8424	0.0000	1.1505	0.0026	1.0390	0.0000	1.3483	1.0000	-0.1352	0.9994	0.1879	0.9103	0.1827
LOC104916540	0.0373	2.2954	0.0191	1.7392	0.0079	2.4453	0.0108	1.7493	1.0000	1.0295	0.9846	0.4911	0.9886	0.3406
LOC104916541	1.0000	0.0000	1.0000	0.0000	1.0000	0.0000	1.0000	0.0000	1.0000	0.0000	1.0000	0.0000	1.0000	0.0000
LOC104916542	1.0000	0.0000	1.0000	0.0000	1.0000	0.0000	1.0000	0.0000	1.0000	0.0000	1.0000	0.0000	1.0000	0.0000
LOC104916543	0.7938	1.1355	0.5050	-1.6397	0.0001	3.4581	0.0560	1.5536	1.0000	1.3485	0.9094	-1.4150	0.8234	-0.5490
LOC104916544	1.0000	0.0000	1.0000	0.0000	1.0000	0.0000	1.0000	0.0000	1.0000	0.0000	1.0000	0.0000	1.0000	0.0000

LOC104916545	0.2012	4.2368	1.0000	0.6940	1.0000	0.0000	1.0000	0.0075	1.0000	2.2677	1.0000	-1.1710	1.0000	2.3554
LOC104916546	1.0000	0.0000	1.0000	0.0000	1.0000	0.0000	1.0000	0.0000	1.0000	0.0000	1.0000	0.0000	1.0000	0.0000
LOC104916547	0.0000	-3.1705	0.0000	-3.1930	0.4389	0.2994	0.0494	0.4403	1.0000	-0.1922	1.0000	-0.2006	1.0000	-0.0436
LOC104916548	1.0000	0.0000	1.0000	0.0000	1.0000	0.0000	1.0000	0.0000	1.0000	0.0000	1.0000	0.0000	1.0000	0.0000
LOC104916549	0.0903	2.7161	0.0563	2.7269	0.0188	3.2298	0.9096	1.3845	1.0000	-0.1034	1.0000	-0.0886	0.1717	-1.9510
LOC104916550	1.0000	0.0000	1.0000	0.0000	1.0000	0.0000	1.0000	2.3256	1.0000	0.0000	1.0000	0.0000	1.0000	2.3554
LOC104916551	1.0000	0.0000	1.0000	0.0000	1.0000	0.0000	1.0000	0.0000	1.0000	0.0000	1.0000	0.0000	1.0000	0.0000
LOC104916552	1.0000	0.0000	1.0000	0.0000	1.0000	0.0000	1.0000	0.0000	1.0000	0.0000	1.0000	0.0000	1.0000	0.0000
LOC104916553	1.0000	0.0000	1.0000	0.0000	1.0000	0.0000	1.0000	0.0000	1.0000	0.0000	1.0000	0.0000	1.0000	0.0000
LOC104916554	1.0000	0.0000	1.0000	0.0000	1.0000	0.0000	1.0000	0.0000	1.0000	0.0000	1.0000	0.0000	1.0000	0.0000
LOC104916555	1.0000	0.0000	1.0000	0.0000	1.0000	0.0000	1.0000	0.0000	1.0000	0.0000	1.0000	0.0000	1.0000	0.0000
LOC104916556	1.0000	0.0000	1.0000	0.0000	1.0000	0.0000	1.0000	0.0000	1.0000	0.0000	1.0000	0.0000	1.0000	0.0000
LOC104916557	1.0000	-2.4788	1.0000	0.0000	1.0000	-0.1630	1.0000	0.0000	1.0000	-2.3771	1.0000	0.0000	1.0000	-2.2889
LOC104916558	0.4688	0.7050	0.0005	1.6855	0.4731	0.6949	0.0033	1.5381	1.0000	-1.9739	0.0126	-0.9801	0.0025	-1.1242
LOC104916559	1.0000	0.0000	1.0000	0.0000	1.0000	0.0000	1.0000	0.0000	1.0000	0.0000	1.0000	0.0000	1.0000	0.0000
LOC104916560	1.0000	0.0000	1.0000	0.0000	1.0000	0.0000	1.0000	0.0000	1.0000	0.0000	1.0000	0.0000	1.0000	0.0000
LOC104916561	1.0000	0.0000	1.0000	0.0000	1.0000	0.0000	1.0000	0.0000	1.0000	0.0000	1.0000	0.0000	1.0000	0.0000
LOC104916562	1.0000	0.0000	0.7666	3.0922	1.0000	0.0000	1.0000	0.0000	1.0000	0.0000	1.0000	3.1976	1.0000	0.0000
LOC104916563	0.3075	-0.5726	0.0335	-0.5543	0.0180	-1.1542	0.0044	-0.8258	1.0000	0.0749	1.0000	0.1038	0.6256	0.4075
LOC104916564	1.0000	0.0000	1.0000	0.0000	1.0000	0.0000	1.0000	0.0000	1.0000	0.0000	1.0000	0.0000	1.0000	0.0000
LOC104916565	1.0000	0.0000	1.0000	0.0000	1.0000	0.0000	1.0000	0.0000	1.0000	0.0000	1.0000	0.0000	1.0000	0.0000
LOC104916566	1.0000	0.0000	1.0000	0.0000	1.0000	0.0000	1.0000	0.0000	1.0000	0.0000	1.0000	0.0000	1.0000	0.0000
LOC104916567	1.0000	0.0000	1.0000	0.0000	1.0000	0.0000	1.0000	0.0000	1.0000	0.0000	1.0000	0.0000	1.0000	0.0000
LOC104916568	1.0000	0.0000	1.0000	0.0000	1.0000	0.0000	1.0000	0.0000	1.0000	0.0000	1.0000	0.0000	1.0000	0.0000
LOC104916569	0.8249	-3.3412	1.0000	0.0000	0.8033	-3.2622	1.0000	2.3256	1.0000	-3.2289	1.0000	0.0000	1.0000	2.3554
LOC104916570	1.0000	2.1851	1.0000	0.0000	1.0000	0.0000	1.0000	0.0000	1.0000	0.0000	1.0000	-2.2959	1.0000	0.0000
LOC104916571	1.0000	0.0000	1.0000	0.0000	1.0000	0.0000	1.0000	0.0000	1.0000	0.0000	1.0000	0.0000	1.0000	0.0000
LOC104916572	1.0000	-0.0967	0.0635	2.2773	1.0000	-0.8812	0.7701	-3.1666	1.0000	-1.3434	0.8617	1.0328	0.7287	-3.6634
LOC104916573	1.0000	0.0000	1.0000	-2.3960	1.0000	0.0000	1.0000	-2.3178	1.0000	2.2677	1.0000	0.0000	1.0000	0.0000
LOC104916574	1.0000	0.0000	1.0000	0.0000	1.0000	0.0000	1.0000	0.0000	1.0000	0.0000	1.0000	0.0000	1.0000	0.0000
LOC104916575	1.0000	0.0000	1.0000	0.0000	1.0000	0.0000	1.0000	0.0000	1.0000	0.0000	1.0000	0.0000	1.0000	0.0000
LOC104916576	0.9996	-0.3558	0.5627	-0.5906	1.0000	0.1919	1.0000	-0.0659	1.0000	-0.0549	1.0000	-0.2780	1.0000	-0.3107
LOC104916577	1.0000	2.1900	1.0000	0.0000	1.0000	2.2424	1.0000	0.0000	1.0000	0.0000	1.0000	-2.2992	1.0000	-2.2889
LOC104916578	0.2107	1.1977	0.0757	1.1259	0.1240	-2.1014	1.0000	0.3291	1.0000	-0.1294	1.0000	-0.1929	0.1306	2.3127
LOC104916579	1.0000	0.0000	1.0000	0.0000	1.0000	0.0000	1.0000	0.0000	1.0000	0.0000	1.0000	0.0000	1.0000	0.0000
LOC104916580	0.1877	4.4789	1.0000	2.2509	1.0000	2.2469	0.2426	4.0910	1.0000	0.0000	0.6450	-2.2673	0.8937	1.8362
LOC104916581	0.8042	0.2867	0.0014	0.8748	0.1447	-0.8237	0.7418	0.2295	1.0000	0.1918	0.0432	0.7942	0.0041	1.2503
LOC104916582	1.0000	0.0000	1.0000	0.0000	1.0000	0.0000	1.0000	0.0000	1.0000	0.0000	1.0000	0.0000	1.0000	0.0000
LOC104916584	1.0000	0.0000	1.0000	0.0000	1.0000	0.0000	1.0000	0.0000	1.0000	0.0000	1.0000	0.0000	1.0000	0.0000
LOC104916585	1.0000	0.0000	1.0000	0.0000	1.0000	0.0000	1.0000	0.0000	1.0000	0.0000	1.0000	0.0000	1.0000	0.0000
LOC104916586	1.0000	0.0000	1.0000	0.0000	1.0000	0.0000	1.0000	0.0000	1.0000	0.0000	1.0000	0.0000	1.0000	0.0000
LOC104916587	1.0000	0.0000	1.0000	0.0000	1.0000	0.0000	1.0000	0.0000	1.0000	0.0000	1.0000	0.0000	1.0000	0.0000
LOC104916588	0.8226	-3.3425	1.0000	0.0000	0.8011	-3.2629	1.0000	0.0000	1.0000	-3.2304	1.0000	0.0000	1.0000	0.0000
LOC104916589	1.0000	0.0000	1.0000	0.0000	1.0000	0.0000	1.0000	0.0000	1.0000	0.0000	1.0000	0.0000	1.0000	0.0000
LOC104916590	1.0000	0.0000	1.0000	0.0000	1.0000	0.0000	1.0000	0.0000	1.0000	0.0000	1.0000	0.0000	1.0000	0.0000
LOC104916591	1.0000	0.0000	1.0000	0.0000	1.0000	0.0000	1.0000	0.0000	1.0000	0.0000	1.0000	0.0000	1.0000	0.0000
LOC104916592	1.0000	0.0000	1.0000	0.0000	1.0000	0.0000	1.0000	0.0000	1.0000	0.0000	1.0000	0.0000	1.0000	0.0000
LOC104916593	1.0000	0.0000	1.0000	0.0000	1.0000	0.0000	1.0000	0.0000	1.0000	0.0000	1.0000	0.0000	1.0000	0.0000
LOC104916594	1.0000	0.0000	1.0000	0.0000	1.0000	0.0000	1.0000	0.0000	1.0000	0.0000	1.0000	0.0000	1.0000	0.0000
LOC104916595	1.0000	0.0000	1.0000	-2.3960	1.0000	0.0000	1.0000	-2.3178	1.0000	2.2677	1.0000	0.0000	1.0000	0.0000
LOC104916596	1.0000	0.0000	1.0000	0.0000	1.0000	0.0000	1.0000	0.0000	1.0000	0.0000	1.0000	0.0000	1.0000	0.0000
LOC104916597	1.0000	0.0000	1.0000	0.0000	1.0000	0.0000	1.0000	0.0000	1.0000	0.0000	1.0000	0.0000	1.0000	0.0000
LOC104916598	1.0000	0.0000	1.0000	0.0000	1.0000	0.0000	1.0000	0.0000	1.0000	0.0000	1.0000	0.0000	1.0000	0.0000
LOC104916599	1.0000	0.0000	1.0000	0.0000	1.0000	0.0000	1.0000	0.0000	1.0000	0.0000	1.0000	0.0000	1.0000	0.0000
LOC104916600	1.0000	0.0000	1.0000	0.0000	1.0000	0.0000	1.0000	0.0000	1.0000	0.0000	1.0000	0.0000	1.0000	0.0000
LOC104916601	1.0000	2.1851	1.0000	0.0000	1.0000	0.0000	1.0000	0.0000	1.0000	0.0000	1.0000	-2.2959	1.0000	0.0000
LOC104916602	0.1612	0.5790	0.0001	1.0275	0.0000	1.2788	0.0000	1.5629	1.0000	-0.3662	1.0000	0.0951	1.0000	-0.0742
LOC104916603	0.5086	3.5456	0.7674	3.0889	1.0000	2.2469	1.0000	0.0000	1.0000	0.0000	1.0000	-0.4766	1.0000	-2.2907
LOC104916604	1.0000	0.0000	1.0000	0.0000	1.0000	0.0000	1.0000	0.0000	1.0000	0.0000	1.0000	0.0000	1.0000	0.0000
LOC104916606	1.0000	0.5864	1.0000	0.3746	0.8033	-3.2622	0.9801	0.9262	1.0000	-0.1218	1.0000	-0.3278	0.5105	4.1268
LOC104916607	0.9087	-0.6078	1.0000	0.2297	0.5514	-1.6603	0.4634	-1.1150	1.0000	0.4364	0.7353	1.2887	1.0000	0.9917
LOC104916608	0.0079	0.9813	0.0000	1.1625	0.0520	0.7673	0.0868	0.6103	1.0000	-0.1939	1.0000	0.0009	0.6001	-0.3440
LOC104916609	1.0000	-2.4788	0.7674	-3.2570	0.1196	2.5464	0.7210	1.2289	1.0000	0.7403	1.0000	0.0000	1.0000	-0.5742
LOC104916610	1.0000	0.0000	1.0000	2.2509	1.0000	0.0000	1.0000	0.0000	1.0000	0.0000	1.0000	2.3456	1.0000	0.0000
LOC104916611	1.0000	0.0000	1.0000	0.0000	1.0000	0.0000	1.0000	0.0000	1.0000	0.0000	1.0000	0.0000	1.0000	0.0000
LOC104916612	1.0000	0.0000	1.0000	-2.3985	1.0000	0.0000	1.0000	-2.3199	1.0000	2.2734	1.0000	0.0000	1.0000	0.0000
LOC104916614	1.0000	0.0000	1.0000	0.0000	1.0000	0.0000	1.0000	0.0000	1.0000	0.0000	1.0000	0.0000	1.0000	0.0000
LOC104916615	1.0000	0.0000	0.7666	-3.2534	0.2840	3.9988	0.7701	-3.1666	1.0000	3.1126	1.0000	0.0000	0.5105	-4.0506
LOC104916616	1.0000	2.1900	1.0000	0.0000	1.0000	2.2469	1.0000	0.0000	1.0000	0.0000	1.0000	-2.2992	1.0000	-2.2907
LOC104916617	1.0000	0.0000	1.0000	0.0000	1.0000	0.0000	1.0000	0.0000	1.0000	0.0000	1.0000	0.0000	1.0000	0.0000
LOC104916618	0.0028	-5.8035	1.0000	0.2184	0.7277	0.5223	0.0043	2.5762	1.0000	-2.0409	0.6427	4.1168	1.0000	0.0125
LOC104916619	0.0000	-8.1210	0.0000	-7.4185	0.0000	-4.5920	0.0000	-1.1947	1.0000	0.4601	0.8820	1.1807	0.0000	3.8598
LOC104916620	1.0000	0.0000	1.0000	0.0000	1.0000	0.0000	1.0000	0.0000	1.0000	0.0000	1.0000	0.0000	1.0000	0.0000
LOC104916621	0.8595	0.5218	0.0050	2.7685	0.6374	-1.3442	1.0000	0.0061	1.0000	-1.8086	1.0000	0.4396	1.0000	-0.4586
LOC104916622	1.0000	-2.4776	1.0000	0.0000	1.0000	0.6822	1.0000	0.0000	1.0000	-2.3758	1.0000	0.0000	1.0000	-3.1368
LOC104916624	1.0000	0.5420	0.4431	-3.7865	0.6601	1.5906	0.1236	1.7986	1.0000	1.2635	1.0000	-3.1429	0.5018	1.4825
LOC104916625	1.0000	0.0000	1.0000	0.0000	1.0000	0.0000	1.0000	0.0000	1.0000	0.0000	1.0000	0.0000	1.0000	0.0000
LOC104916626	1.0000	-2.4776	1.0000	0.0000	1.0000	-2.4055	1.0000	0.0000	1.0000	-2.3758	1.0000	0.0000	1.0000	0.0000
LOC104916627	1.0000	-2.4776	1.0000	2.2509	1.0000	-0.1591	0.7710	3.1709	1.0000	-2.3758	1.0000	2.3456	1.0000	0.9149
LOC104916629	1.0000	-0.3478	1.0000	0.6956	0.2873	1.3342	0.1709	2.5399	1.0000	-1.8822	1.0000	-0.8556	0.8937	-0.6788
LOC104916630	1.0000	0.0000	1.0000	0.0000	1.0000	0.0000	1.0000	0.0000	1.0000	0.0000	1.0000	0.0000	1.0000	0.0000
LOC104916631	1.0000	2.1900	1.0000	0.0000	1.0000	0.0000	1.0000	0.0000	1.0000	0.0000	1.0000	-2.2992	1.0000	0.0000
LOC104916632	1.0000	0.0000	1.0000	0.0000	1.0000	0.0000	1.0000	0.0000	1.0000	0.0000	1.0000	0.0000	1.0000	0.0000
LOC104916633	1.0000	-2.4776	1.0000	2.2533	1.0000	-2.4055	1.0000	0.0000	1.0000	-2.3758	1.0000	2.3480	1.0000	0.0000
LOC104916634	1.0000	0.0000	1.0000	0.0000	1.0000	0.0000	1.0000	0.0000	1.0000	0.0000	1.0000	0.0000	1.0000	0.0000
LOC104916635	1.0000	0.0000	1.0000	0.0000	1.0000	0.0000	1.0000	0.0000	1.0000	0.0000	1.0000	0.0000	1.0000	0.0000
LOC104916636	1.0000	0.0000	1.0000	0.0000	1.0000	0.0000	1.0000	0.0000	1.0000	0.0000	1.0000	0.0000	1.0000	0.0000
LOC104916637	0.0094	1.9671	0.0354	1.7007	0.0942	-4.7437	1.0000	-0.2979	1.0000	-0.3787	0.8350	-0.6366	0.5105	4.1259
LOC104916638	1.0000	2.1900	1.0000	0.0000	1.0000	0.0000	1.0000	0.0000	1.0000	0.0000	1.0000	-2.2992	1.0000	0.0000
LOC104916639	1.0000	0.0000	1.0000	0.0000	1.0000	0.0000	1.0000	0.0000	1.0000	0.0000	1.0000	0.0000	1.0000	0.0000
LOC104916640	1.0000	0.0000	1.0000	0.0000	1.0000	0.0000	1.0000	0.0000	1.0000	0.0000	1.0000	0.0000	1.0000	0.0000
LOC104916641	1.0000	0.0000	1.0000	0.0000	1.0000	0.0000	1.0000	0.0000	1.0000	0.0000	1.0000	0.0000	1.0000	0.0000
LOC104916642	0.0000	-0.7358	0.0000	-0.5856	0.0000	-1.2502	0.0000	-1.0194	1.0000	-0.2029	1.0000	-0.0402	1.0000	0.0341

LOC104916643	1.0000	0.0000	1.0000	0.0000	1.0000	0.0000	1.0000	0.0000	1.0000	0.0000	1.0000	0.0000	1.0000	0.0000
LOC104916644	1.0000	0.0000	1.0000	0.0000	0.4809	3.6122	1.0000	0.0000	1.0000	0.0000	1.0000	0.0000	0.7287	-3.6637
LOC104916645	1.0000	0.0000	1.0000	-2.3960	1.0000	0.0000	1.0000	-2.3178	1.0000	2.2677	1.0000	0.0000	1.0000	0.0000
LOC104916646	0.5848	-1.0035	0.6885	-0.5182	1.0000	0.3734	1.0000	0.0073	1.0000	0.6587	0.8265	1.1602	1.0000	0.2980
LOC104916647	0.9958	-0.3578	0.0597	-1.2207	0.0084	-3.1255	0.0712	-1.1587	1.0000	0.4174	1.0000	-0.4324	0.1717	2.3977
LOC104916648	1.0000	0.0000	1.0000	-0.1612	1.0000	2.2469	1.0000	0.0059	1.0000	3.1126	1.0000	3.1976	1.0000	0.9149
LOC104916649	1.0000	-0.3195	0.2495	4.0050	1.0000	-0.1789	0.2426	4.0910	1.0000	-3.2304	1.0000	0.9726	1.0000	0.9919
LOC104916650	0.0227	5.1922	1.0000	-0.1760	0.8033	3.0891	0.3402	-1.6822	1.0000	4.8000	1.0000	-0.4397	1.0000	0.0716
LOC104916651	0.0753	0.9269	0.0045	1.1557	0.0002	1.5305	0.0029	1.2596	1.0000	-0.3735	1.0000	-0.1283	0.1141	-0.6338
LOC104916652	1.0000	0.0000	1.0000	0.0000	1.0000	0.0000	1.0000	0.0000	1.0000	0.0000	1.0000	0.0000	1.0000	0.0000
LOC104916653	1.0000	0.0000	0.7666	3.0922	1.0000	0.0000	1.0000	0.0000	1.0000	0.0000	1.0000	3.1976	1.0000	0.0000
LOC104916654	1.0000	0.1641	0.6504	0.9458	1.0000	-0.1927	0.3330	1.2772	1.0000	-1.2482	1.0000	-0.4596	1.0000	0.2267
LOC104916655	0.1011	-1.2324	0.0234	-0.9606	0.9257	0.2603	0.0043	0.8693	1.0000	0.2996	0.8899	0.5894	0.0119	0.9142
LOC104916656	0.9983	0.3599	1.0000	-0.6986	0.6092	-0.7646	0.4547	-3.7024	1.0000	-2.0353	0.0088	-3.1102	0.0812	-5.0060
[truncated: 1,010,574 more chars]
