# Supplementary material for: Response of Turkey Muscle Satellite Cells to Thermal Challenge. II. Transcriptome Effects in Differentiating Cells
Source: Front Physiol. 2017 Nov 30;8:948. doi: 10.3389/fphys.2017.00948 (PMC5714890; doi:10.3389/fphys.2017.00948)
Supplement: Supplementary file 10 [file Table4.PDF]

Table S4. Fifty genes showing the greatest differential expression in each pairwise comparison of treatment groups. Genes highlighted red are up-regulated in the comparison whereas genes highlighted in green are down-regulated.

Temperature comparisons by line

	Feature ID	Description	FDR p-value correction	Log_2FC
**RBC2 33° vs 38°**	LOC104913551	uncharacterized LOC104913551	1.48E-05	6.596
	LOC104913470	periplakin-like	2.91E-05	6.430
	LOC104911872	uncharacterized LOC104911872	2.55E-04	6.175
	NTRK1	neurotrophic tyrosine kinase, receptor, type 1	2.76E-04	6.168
	TDRP	testis development related protein	9.63E-04	5.942
	LOC104909892	uncharacterized LOC104909892	3.82E-13	-5.765
	LOC104916618	adenylate cyclase type 10-like	2.82E-03	-5.803
	LEF1	lymphoid enhancer-binding factor 1	2.69E-03	-5.804
	LOC104910064	uncharacterized LOC104910064	3.39E-03	-5.805
	CCDC36	coiled-coil domain containing 36	4.83E-03	-5.806
	LOC104913937	double C2-like domain-containing protein beta	1.60E-03	-5.918
	LOC104913727	myosin-8-like	6.91E-12	-5.983
	LOC104910497	uncharacterized LOC104910497	9.82E-04	-6.022
	STMN4	stathmin-like 4	1.65E-25	-6.095
	SLC25A48	solute carrier family 25, member 48	5.53E-04	-6.120
	LOC104915526	myosin heavy chain, skeletal muscle-like	1.13E-25	-6.173
	KBTBD12	kelch repeat and BTB (POZ) domain containing 12	6.02E-04	-6.210
	LOC100550580	proto-oncogene tyrosine-protein kinase ROS	2.04E-04	-6.212
	LOC104911611	uncharacterized LOC104911611	1.94E-04	-6.212
	LDB3	LIM domain binding 3	1.41E-124	-6.214
	EGF	epidermal growth factor	1.10E-65	-6.258
	MYL3	myosin, light chain 3, alkali; ventricular, skeletal, slow	6.61E-130	-6.283
	LOC104914622	uncharacterized LOC104914622	1.17E-04	-6.299
	LOC104913742	myosin-6-like	2.64E-18	-6.433
	KIF26A	kinesin family member 26A	1.16E-31	-6.485
	MLANA	melan-A	1.45E-04	-6.528
	LOC104909805	triadin-like	7.47E-05	-6.529
	CLIC5	chloride intracellular channel 5	4.86E-05	-6.532
	LOC104916992	uncharacterized LOC104916992	2.46E-78	-6.565
	MYL1	myosin, light chain 1, alkali; skeletal, fast	2.45E-277	-6.586
	LOC104909252	myosin-3-like	1.56E-05	-6.600
	MYH7B	myosin, heavy chain 7B, cardiac muscle, beta	1.57E-82	-6.628
	LOC104913624	zinc finger protein RFP-like	1.76E-05	-6.668
	ITGB3	integrin, beta 3 (platelet glycoprotein IIIa, antigen CD61)	1.20E-62	-6.725
	ADCY8	adenylate cyclase 8 (brain)	1.67E-04	-6.796
	LOC104913842	myosin-7-like	2.23E-05	-6.902
	LOC104913838	uncharacterized LOC104913838	4.68E-07	-6.959
	LOC104911691	rac GTPase-activating protein 1-like	1.44E-06	-7.059
	TRDN	triadin	5.64E-08	-7.203
	LOC104915799	uncharacterized LOC104915799	6.10E-07	-7.407
	LOC104915527	myosin-8-like	8.15E-67	-7.419
	SV2B	synaptic vesicle glycoprotein 2B	3.54E-06	-7.443
	LOC100544198	myosin heavy chain, skeletal muscle, adult	1.98E-79	-7.480
	LOC104917219	myosin-7-like	1.15E-22	-7.725
	LOC100542552	myosin heavy chain, skeletal muscle-like	8.34E-09	-7.781
	MMP7	matrix metallopeptidase 7 (matrilysin, uterine)	3.35E-12	-7.872
	LOC104916619	myosin light chain 3, skeletal muscle isoform-like	1.42E-09	-8.121
	LOC100543330	myosin heavy chain, skeletal muscle-like	1.47E-57	-8.381
	LOC100543400	matrix metalloproteinase-27-like	1.20E-24	-9.514
	NPY	neuropeptide Y	7.79E-17	-10.039
**F 33° vs 38°**	TECRL	trans-2,3-enoyl-CoA reductase-like	4.77E-07	7.694
	LOC104913470	periplakin-like	2.38E-07	6.732
	SLC13A1	solute carrier family 13 (sodium/sulfate symporter), member 1	1.87E-05	6.616
	LOC100549208	limbin-like	1.13E-06	6.571
	LOC104911928	DNA replication ATP-dependent helicase/nuclease DNA2-like	1.27E-05	6.323
	LOC100544125	ectonucleotide pyrophosphatase/phosphodiesterase family member 1-like	8.87E-05	6.104
	LOC104911448	uncharacterized LOC104911448	1.92E-04	6.021
	LOC100542876	keratin, type I cytoskeletal 9-like	3.85E-04	-5.927
	SV2B	synaptic vesicle glycoprotein 2B	1.35E-18	-5.957
	LOC104909718	uncharacterized LOC104909718	2.03E-04	-6.021

	C7H21orf58	chromosome 7 open reading frame, human C21orf58	1.57E-04	-6.023
	LOC104913331	putative E3 ubiquitin-protein ligase SH3RF2	1.57E-04	-6.023
	LOC104909291	myosin heavy chain, skeletal muscle, adult-like	1.61E-04	-6.023
	LDB3	LIM domain binding 3	2.59E-51	-6.061
	LRRC14B	leucine rich repeat containing 14B	2.11E-21	-6.075
	KMO	kynurenine 3-monooxygenase (kynurenine 3-hydroxylase)	9.56E-05	-6.113
	FBN2	fibrillin 2	8.37E-05	-6.115
	INHA	inhibin, alpha	4.21E-20	-6.115
	LOC104913742	myosin-6-like	2.23E-20	-6.216
	GUCA1B	guanylate cyclase activator 1B (retina)	2.68E-05	-6.281
	ITGB3	integrin, beta 3 (platelet glycoprotein IIIa, antigen CD61)	9.18E-75	-6.283
	LOC104916992	uncharacterized LOC104916992	4.03E-112	-6.353
	LOC100540751	dual specificity protein phosphatase 13 isoform B-like	1.60E-05	-6.358
	LOC104916462	CCAAT/enhancer-binding protein alpha-like	1.59E-05	-6.358
	EEF1A2	eukaryotic translation elongation factor 1 alpha 2	0.00E+00	-6.371
	LOC100549331	myosin-7-like	0.00E+00	-6.412
	LOC100550580	proto-oncogene tyrosine-protein kinase ROS	9.13E-06	-6.434
	SLN	sarcolipin	7.03E-80	-6.440
	MYH7B	myosin, heavy chain 7B, cardiac muscle, beta	0.00E+00	-6.459
	LOC104909805	triadin-like	2.00E-06	-6.568
	SLC25A48	solute carrier family 25, member 48	1.05E-06	-6.632
	LOC104909252	myosin-3-like	6.28E-07	-6.693
	LOC104915526	myosin heavy chain, skeletal muscle-like	3.60E-29	-6.711
	MYL1	myosin, light chain 1, alkali; skeletal, fast	0.00E+00	-6.722
	MNX1	motor neuron and pancreas homeobox 1	9.97E-77	-6.794
	LOC100542552	myosin heavy chain, skeletal muscle-like	2.67E-07	-6.864
	LOC104913330	putative E3 ubiquitin-protein ligase SH3RF2	4.96E-08	-6.913
	GUCA1C	guanylate cyclase activator 1C	3.05E-08	-7.009
	LOC104917219	myosin-7-like	2.38E-69	-7.031
	LOC104914028	collagen alpha-1(XX) chain-like	2.41E-08	-7.155
	MYL3	myosin, light chain 3, alkali; ventricular, skeletal, slow	0.00E+00	-7.377
	LOC104916619	myosin light chain 3, skeletal muscle isoform-like	1.68E-154	-7.418
	VGLL2	vestigial-like family member 2	1.54E-11	-7.424
	NPY	neuropeptide Y	4.52E-79	-7.529
	LOC104915527	myosin-8-like	1.17E-75	-7.833
	LOC104913727	myosin-8-like	2.90E-16	-8.080
	RAB38	RAB38, member RAS oncogene family	2.71E-17	-8.124
	LOC104913726	myosin-7-like	9.75E-19	-8.143
	LOC100543330	myosin heavy chain, skeletal muscle-like	3.30E-54	-10.316
	LOC100544198	myosin heavy chain, skeletal muscle, adult	2.33E-94	-11.218
**RBC2 43° vs 38°**	LOC104912816	thrombospondin type-1 domain-containing protein 4-like	1.43E-13	7.863
	LOC104916036	zinc finger and BTB domain-containing protein 7B-like	2.49E-05	6.381
	LOC104912369	collagen alpha-1(XI) chain-like	3.95E-05	6.313
	LOC104914538	metabotropic glutamate receptor 4-like	6.92E-05	6.243
	LOC100544134	thrombospondin type-1 domain-containing protein 7A	1.38E-04	6.237
	LOC104914898	mitogen-activated protein kinase kinase kinase 1-like	1.21E-04	6.172
	LOC100541413	delta and Notch-like epidermal growth factor-related receptor	1.25E-04	6.170
	LOC104913470	periplakin-like	5.67E-04	6.104
	LOC100540961	heparan sulfate glucosamine 3-O-sulfotransferase 4	2.06E-04	6.093
	LOC100549706	semaphorin-3A-like	3.77E-04	6.011
	LOC104911803	kalirin-like	1.77E-03	5.828
	LOC104912812	uncharacterized LOC104912812	1.93E-03	5.737
	LOC104910365	uncharacterized LOC104910365	1.44E-02	5.710
	LOC104916426	uncharacterized LOC104916426	4.45E-03	5.639
	LOC104912370	collagen alpha-1(XI) chain-like	3.27E-03	5.632
	TRIM2	tripartite motif containing 2	3.27E-03	5.632
	LOC104909995	phosphatidate phosphatase LPIN3-like	3.27E-03	5.632
	LOC104916797	kinesin-like protein KIF20B	3.27E-03	5.632
	NOX4	NADPH oxidase 4	3.47E-03	5.631
	LOC104914003	uncharacterized LOC104914003	9.11E-03	5.529
	LOC104910602	uncharacterized LOC104910602	5.99E-03	5.521
	LOC104917369	junctophilin-1-like	5.99E-03	5.521
	LOC100547657	cadherin EGF LAG seven-pass G-type receptor 2	5.81E-03	5.519
	LOC104913551	uncharacterized LOC104913551	5.82E-03	5.519
	LOC104914167	agrin-like	5.82E-03	5.518
	KAL1	Kallmann syndrome 1 sequence	7.95E-03	5.513

	LOC104910692	teneurin-3-like	7.96E-03	5.513
	ADCY3	adenylate cyclase 3	1.10E-02	5.400
	LOC104914141	uncharacterized LOC104914141	1.01E-02	5.398
	LOC104914929	A disintegrin and metalloproteinase with thrombospondin motifs 6-like	1.01E-02	5.398
	LOC104909322	elastin-like	1.21E-02	5.392
	LOC104911097	uncharacterized LOC104911097	1.79E-02	5.262
	LOC104909526	uncharacterized LOC104909526	1.95E-02	5.259
	LOC100541159	neural-cadherin-like	1.95E-02	5.259
	LOC104909653	disheveled-associated activator of morphogenesis 2-like	2.03E-08	5.194
	GIF	gastric intrinsic factor (vitamin B synthesis)	3.56E-02	-5.146
	LOC104917139	germin-like protein subfamily 2 member 2	4.91E-09	-5.294
	LOC104910189	uncharacterized LOC104910189	3.51E-02	-5.308
	LOC104910548	uncharacterized LOC104910548	2.30E-02	-5.310
	LOC104911692	uncharacterized LOC104911692	1.76E-02	-5.311
	LOC100543786	heat shock protein beta-7-like	1.76E-02	-5.311
	CSF3	colony stimulating factor 3 (granulocyte)	1.76E-02	-5.311
	ADARB2	adenosine deaminase, RNA-specific, B2 (non-functional)	1.90E-02	-5.311
	IKZF3	IKAROS family zinc finger 3 (Aiolos)	4.53E-03	-5.593
	LOC104912169	uncharacterized LOC104912169	3.30E-03	-5.594
	LOC104915923	uncharacterized LOC104915923	1.99E-03	-5.717
	CFD	complement factor D (adipsin)	5.24E-14	-5.796
	BAI2	brain-specific angiogenesis inhibitor 2	3.45E-04	-6.124
	LOC100544139	gamma-aminobutyric acid receptor subunit pi-like	3.12E-06	-6.703
	RBP4	retinol binding protein 4, plasma	1.44E-06	-6.761
**F 43° vs 38°**	ZP4	zona pellucida glycoprotein 4	1.07E-05	6.343
	LOC104914514	plexin-A2-like	3.10E-05	6.269
	EXPH5	exophilin 5	5.66E-05	6.195
	LOC100548679	uncharacterized protein KIAA0825-like	1.24E-04	6.026
	FAM46D	family with sequence similarity 46, member D	4.59E-04	5.837
	LOC104917369	junctophilin-1-like	1.61E-03	5.618
	LOC100550586	tyrosine-protein kinase SgK223-like	1.65E-03	5.618
	LOC100547657	cadherin EGF LAG seven-pass G-type receptor 2	3.81E-03	5.494
	GDF11	growth differentiation factor 11	5.93E-03	5.361
	LOC104911173	ryanodine receptor 3-like	5.93E-03	5.361
	TECRL	trans-2,3-enoyl-CoA reductase-like	8.63E-03	5.357
	LOC104911839	uncharacterized LOC104911839	1.56E-12	5.307
	CALCA	calcitonin-related polypeptide alpha	2.45E-02	5.224
	LOC104911805	semaphorin-5B-like	1.09E-02	5.213
	LOC104909322	elastin-like	1.14E-02	5.213
	LOC104916051	uncharacterized LOC104916051	2.35E-11	5.184
	LOC104910581	uncharacterized LOC104910581	3.25E-02	5.056
	LOC104912370	collagen alpha-1(XI) chain-like	2.35E-02	5.050
	LOC104915331	connector enhancer of kinase suppressor of ras 2-like	2.35E-02	5.050
	NWD2	NACHT and WD repeat domain containing 2	2.35E-02	5.050
	LOC100544125	ectonucleotide pyrophosphatase/phosphodiesterase family member 1-like	2.03E-02	5.048
	PCDH17	protocadherin 17	2.07E-02	5.048
	LOC104914982	rho guanine nucleotide exchange factor 28-like	2.08E-02	5.048
	LOC104915239	solute carrier family 12 member 2-like	8.76E-10	4.979
	LOC104913470	periplakin-like	4.07E-02	4.863
	LOC100540476	tau-tubulin kinase 1-like	4.06E-02	4.863
	RNF222	ring finger protein 222	3.84E-02	4.862
	LOC104911585	uncharacterized LOC104911585	4.02E-02	4.861
	LOC100550557	caskin-1-like	4.02E-02	4.861
	LOC104910360	carboxypeptidase A6-like	4.90E-02	4.857
	BMP15	bone morphogenetic protein 15	4.01E-02	-4.853
	TMEM247	transmembrane protein 247	4.01E-02	-4.853
	LOC100543786	heat shock protein beta-7-like	4.08E-02	-4.856
	LOC104917171	very long-chain acyl-CoA synthetase-like	2.07E-02	-5.041
	LOC104909815	proto-oncogene tyrosine-protein kinase ROS-like	2.07E-02	-5.041
	LOC104910269	uncharacterized LOC104910269	2.03E-02	-5.041
	LOC104913257	uncharacterized LOC104913257	2.10E-02	-5.041
	CALN1	calneuron 1	1.14E-02	-5.205
	LOC100547944	riboflavin-binding protein	1.14E-02	-5.205
	LOC104909649	uncharacterized LOC104909649	1.14E-02	-5.205
	LOC100541465	cytochrome P450 2J2-like	1.09E-02	-5.206
	PLSCR5	phospholipid scramblase family, member 5	6.01E-03	-5.354

	PKHD1	polycystic kidney and hepatic disease 1 (autosomal recessive)	3.11E-03	-5.488
	FAM19A1	family with sequence similarity 19 (chemokine (C-C motif)-like), member A1	1.52E-03	-5.719
	LOC104912169	uncharacterized LOC104912169	8.46E-04	-5.724
	REC8	REC8 meiotic recombination protein	8.53E-04	-5.724
	LOC104910896	uncharacterized LOC104910896	1.14E-03	-5.727
	LOC100545776	vesicular inhibitory amino acid transporter-like	3.21E-04	-5.929
	GOLPH3L	golgi phosphoprotein 3-like	1.25E-04	-6.019
	RBP4	retinol binding protein 4, plasma	1.53E-08	-6.915

Line comparisons by temperature treatment

**33° F vs RBC2**	LOC100539697	integrin beta-like protein 1	3.19E-02	2.998
	CNGA3	cyclic nucleotide gated channel alpha 3	3.08E-03	2.740
	LOC104911073	uncharacterized LOC104911073	4.84E-03	2.113
	LOC104914467	uncharacterized LOC104914467	2.24E-02	1.988
	SPON1	spondin 1, extracellular matrix protein	3.18E-22	1.960
	LOC104909548	uncharacterized LOC104909548	4.18E-03	1.900
	LOC100544508	transmembrane protein 2-like	1.45E-03	1.808
	CAPN8	calpain 8	5.11E-03	1.779
	IL13RA2	interleukin 13 receptor, alpha 2	5.49E-04	1.774
	CCDC69	coiled-coil domain containing 69	5.54E-04	1.746
	BMP3	bone morphogenetic protein 3	1.02E-05	1.669
	CEMIP	cell migration inducing protein, hyaluronan binding	6.19E-03	1.633
	AJAP1	adherens junctions associated protein 1	7.86E-06	1.536
	METRN	meteorin, glial cell differentiation regulator	7.89E-04	-1.580
	LOC104915522	uncharacterized LOC104915522	1.83E-02	-1.615
	LOC104915515	myelin-oligodendrocyte glycoprotein-like	1.87E-02	-1.633
	LOC100547159	uncharacterized LOC100547159	2.38E-02	-1.666
	EPHA7	EPH receptor A7	1.87E-02	-1.688
	TMCC3	transmembrane and coiled-coil domain family 3	3.64E-08	-1.692
	LOC104910060	fibrocystin-like	7.66E-05	-1.716
	MGP	matrix Gla protein	9.09E-65	-1.840
	SOX10	SRY (sex determining region Y)-box 10	4.61E-02	-1.849
	CBX7	chromobox homolog 7	2.98E-02	-1.858
	GIPC3	GIPC PDZ domain containing family, member 3	2.37E-02	-1.861
	LOC104909502	uncharacterized LOC104909502	4.13E-05	-1.903
	LOC104910426	uncharacterized LOC104910426	7.20E-04	-1.920
	CDH6	cadherin 6, type 2, K-cadherin (fetal kidney)	3.79E-15	-1.929
	FXYD2	FXYD domain containing ion transport regulator 2	1.95E-02	-1.954
	LOC100544245	nebulin	1.38E-03	-1.962
	MYOM2	myomesin 2	1.99E-02	-1.998
	LOC104912259	uncharacterized LOC104912259	1.39E-02	-2.001
	TTPA	tocopherol (alpha) transfer protein	7.73E-04	-2.010
	LOC104916358	arf-GAP with coiled-coil, ANK repeat and PH domain-containing protein 1-like	6.99E-04	-2.038
	CPS1	carbamoyl-phosphate synthase 1, mitochondrial	1.32E-02	-2.047
	LOC104910046	uncharacterized LOC104910046	6.67E-08	-2.083
	ARHGEF16	Rho guanine nucleotide exchange factor (GEF) 16	2.75E-03	-2.152
	NCF1	neutrophil cytosolic factor 1	2.84E-07	-2.301
	LOC104910133	uncharacterized LOC104910133	6.24E-09	-2.521
	LOC104909501	uncharacterized LOC104909501	2.13E-06	-2.789
	LOC104916327	semaphorin-6C-like	4.45E-02	-2.794
	COL24A1	collagen, type XXIV, alpha 1	1.98E-06	-2.815
	LOC100546071	receptor tyrosine-protein kinase erbB-3-like	3.26E-02	-2.884
	LOC104916656	uncharacterized LOC104916656	8.79E-03	-3.110
	LOC104910058	fibrocystin-like	4.73E-06	-3.194
	ROBO2	roundabout, axon guidance receptor, homolog 2 (Drosophila)	3.47E-05	-3.456
	LOC104915513	histone deacetylase 7-like	5.11E-03	-3.813
	LOC104917072	zinc finger protein 502-like	2.29E-06	-3.963
	TECRL	trans-2,3-enoyl-CoA reductase-like	1.00E-07	-4.141
	LOC100542432	histamine N-methyltransferase-like	1.20E-02	-5.697
	MUC3A	mucin 3A, cell surface associated	6.59E-03	-6.218
**38° F vs RBC2**	TECRL	trans-2,3-enoyl-CoA reductase-like	5.33E-03	-10.580
**43° F vs RBC2**	LOC104909548	uncharacterized LOC104909548	4.21E-14	5.664
	CNGA3	cyclic nucleotide gated channel alpha 3	7.88E-04	4.090
	LOC104916619	myosin light chain 3, skeletal muscle isoform-like	6.95E-116	3.860
	LOC100544938	cytochrome P450 26B1	6.75E-03	3.774
	RASD2	RASD family, member 2	3.33E-20	3.766

SLC7A14	solute carrier family 7, member 14	2.73E-02	3.479
WASF3	WAS protein family, member 3	3.21E-03	3.246
LOC100549696	peroxidasin homolog	6.68E-12	3.178
LOC100539697	integrin beta-like protein 1	8.83E-03	3.096
LOC104917080	uncharacterized LOC104917080	9.73E-04	2.977
LOC104915398	histone H2A-IV-like	1.15E-04	2.957
SGPP2	sphingosine-1-phosphate phosphatase 2	6.11E-13	2.805
ENPP2	ectonucleotide pyrophosphatase/phosphodiesterase 2	4.22E-05	2.693
NPY	neuropeptide Y	4.06E-56	2.629
ALDH1A3	aldehyde dehydrogenase 1 family, member A3	1.65E-13	2.572
LOC104914186	uncharacterized LOC104914186	8.57E-04	2.557
RSAD2	radical S-adenosyl methionine domain containing 2	8.77E-03	2.507
CYGB	cytoglobin	4.90E-45	2.409
LOC104916003	adenylate cyclase type 10-like	2.63E-02	2.330
RARB	retinoic acid receptor, beta	4.05E-02	2.257
NRTN	neurturin	1.37E-06	2.255
SPON1	spondin 1, extracellular matrix protein	7.05E-61	2.232
LOC104914344	uncharacterized LOC104914344	4.22E-09	2.204
SBK2	SH3 domain binding kinase family, member 2	1.11E-02	2.094
RPS4X	ribosomal protein S4, X-linked	1.29E-24	-2.133
SLIT3	slit homolog 3 (Drosophila)	6.22E-51	-2.233
LOC104915687	fibrous sheath CABYR-binding protein-like	4.49E-02	-2.328
RAC2	ras-related C3 botulinum toxin substrate 2 (rho family, small GTP binding protein Rac2)	4.73E-03	-2.354
CAPN9	calpain 9	6.23E-04	-2.361
LOC100549208	limbin-like	7.95E-03	-2.433
ANKS4B	ankyrin repeat and sterile alpha motif domain containing 4B	7.25E-03	-2.434
LOC104910426	uncharacterized LOC104910426	5.13E-03	-2.435
LOC104910431	regulating synaptic membrane exocytosis protein 2-like	1.89E-05	-2.530
B4GALT6	UDP-Gal:betaGlcNAc beta 1,4- galactosyltransferase, polypeptide 6	1.31E-02	-2.564
LOC104911179	uncharacterized LOC104911179	1.22E-02	-2.629
ROBO2	roundabout, axon guidance receptor, homolog 2 (Drosophila)	1.17E-03	-2.654
LOC100547159	uncharacterized LOC100547159	9.19E-04	-2.706
LOC100550045	toll-like receptor 13	2.22E-02	-2.776
COL24A1	collagen, type XXIV, alpha 1	8.81E-10	-2.851
LOC104909502	uncharacterized LOC104909502	7.93E-07	-2.900
CD55	CD55 molecule, decay accelerating factor for complement (Cromer blood group)	3.71E-03	-3.095
GALNT3	polypeptide N-acetylgalactosaminyltransferase 3	8.55E-07	-3.268
LOC104916167	butyrophilin subfamily 1 member A1-like	1.55E-07	-4.085
LOC104910702	uncharacterized LOC104910702	1.23E-09	-4.346
LOC104915513	histone deacetylase 7-like	4.93E-07	-4.703
LOC104909649	uncharacterized LOC104909649	4.00E-02	-5.325
LOC104917072	zinc finger protein 502-like	1.02E-03	-5.982
TECRL	trans-2,3-enoyl-CoA reductase-like	6.44E-114	-6.170
PKHD1	polycystic kidney and hepatic disease 1 (autosomal recessive)	9.92E-05	-6.301
MUC3A	mucin 3A, cell surface associated	1.05E-23	-6.338
